# Genome-wide characterization of circulating metabolic biomarkers

**DOI:** 10.1038/s41586-024-07148-y

**Published:** 2024-03-06

**Authors:** Minna K. Karjalainen, Savita Karthikeyan, Clare Oliver-Williams, Eeva Sliz, Elias Allara, Wing Tung Fung, Praveen Surendran, Weihua Zhang, Pekka Jousilahti, Kati Kristiansson, Veikko Salomaa, Matt Goodwin, David A. Hughes, Michael Boehnke, Lilian Fernandes Silva, Xianyong Yin, Anubha Mahajan, Matt J. Neville, Natalie R. van Zuydam, Renée de Mutsert, Ruifang Li-Gao, Dennis O. Mook-Kanamori, Ayse Demirkan, Jun Liu, Raymond Noordam, Stella Trompet, Zhengming Chen, Christiana Kartsonaki, Liming Li, Kuang Lin, Fiona A. Hagenbeek, Jouke Jan Hottenga, René Pool, M. Arfan Ikram, Joyce van Meurs, Toomas Haller, Yuri Milaneschi, Mika Kähönen, Pashupati P. Mishra, Peter K. Joshi, Erin Macdonald-Dunlop, Massimo Mangino, Jonas Zierer, Ilhan E. Acar, Carel B. Hoyng, Yara T. E. Lechanteur, Lude Franke, Alexander Kurilshikov, Alexandra Zhernakova, Marian Beekman, Erik B. van den Akker, Ivana Kolcic, Ozren Polasek, Igor Rudan, Christian Gieger, Melanie Waldenberger, Folkert W. Asselbergs, China Kadoorie Biobank Collaborative Group, Estonian Biobank Research Team, FinnGen, Caroline Hayward, Jingyuan Fu, Anneke I. den Hollander, Cristina Menni, Tim D. Spector, James F. Wilson, Terho Lehtimäki, Olli T. Raitakari, Brenda W. J. H. Penninx, Tonu Esko, Robin G. Walters, J. Wouter Jukema, Naveed Sattar, Mohsen Ghanbari, Ko Willems van Dijk, Fredrik Karpe, Mark I. McCarthy, Markku Laakso, Marjo-Riitta Järvelin, Nicholas J. Timpson, Markus Perola, Jaspal S. Kooner, John C. Chambers, Cornelia van Duijn, P. Eline Slagboom, Dorret I. Boomsma, John Danesh, Mika Ala-Korpela, Adam S. Butterworth, Johannes Kettunen

**Affiliations:** 1 Systems Epidemiology, Faculty of Medicine, University of Oulu and Biocenter Oulu, Oulu, Finland; 2 Research Unit of Population Health, Faculty of Medicine, University of Oulu, Oulu, Finland; 3 Northern Finland Birth Cohorts, Arctic Biobank, Infrastructure for Population Studies, Faculty of Medicine, University of Oulu, Oulu, Finland; 4 British Heart Foundation Cardiovascular Epidemiology Unit, Department of Public Health and Primary Care, University of Cambridge, Cambridge, UK; 5 Public Health Specialty Training Programme, Cambridge, UK; 6 National Institute for Health and Care Research Blood and Transplant Research Unit in Donor Health and Behaviour, University of Cambridge, Cambridge, UK; 7 Victor Phillip Dahdaleh Heart and Lung Research Institute, University of Cambridge, Cambridge, UK; 8 Rutherford Fund Fellow, Department of Public Health and Primary Care, University of Cambridge, Cambridge, UK; 9 British Heart Foundation Centre of Research Excellence, University of Cambridge, Cambridge, UK; 10 Health Data Research UK Cambridge, Wellcome Genome Campus and University of Cambridge, Cambridge, UK; 11 Department of Epidemiology and Biostatistics, School of Public Health, Imperial College London, London, UK; 12 Department of Cardiology, Ealing Hospital, London North West University Healthcare NHS Trust, London, UK; 13 Department of Public Health and Welfare, Finnish Institute for Health and Welfare, Helsinki, Finland; 14 MRC Integrative Epidemiology Unit, University of Bristol, Bristol, UK; 15 Population Health Science, Bristol Medical School, University of Bristol, Bristol, UK; 16 Department of Biostatistics and Center for Statistical Genetics, University of Michigan, Ann Arbor, MI USA; 17 Institute of Clinical Medicine, Internal Medicine, University of Eastern Finland, Kuopio, Finland; 18 Department of Epidemiology, School of Public Health, Nanjing Medical University, Jiangsu, China; 19 Wellcome Centre for Human Genetics, Nuffield Department of Medicine, University of Oxford, Oxford, UK; 20 NIHR Oxford Biomedical Research Centre, OUHFT Oxford, Oxford, UK; 21 Oxford Centre for Diabetes, Endocrinology and Metabolism, Radcliffe Department of Medicine, University of Oxford, Oxford, UK; 22 Department of Clinical Epidemiology, Leiden University Medical Center, Leiden, The Netherlands; 23 Department of Public Health and Primary Care, Leiden University Medical Center, Leiden, The Netherlands; 24 Surrey Institute for People-Centred AI, University of Surrey, Guildford, UK; 25 Section of Statistical Multi-Omics, Department of Clinical and Experimental Medicine, University of Surrey, Guildford, UK; 26 Nuffield Department of Population Health, University of Oxford, Oxford, UK; 27 Department of Epidemiology, Erasmus MC, University Medical Center Rotterdam, Rotterdam, The Netherlands; 28 Department of Internal Medicine, Section of Gerontology and Geriatrics, Leiden University Medical Center, Leiden, The Netherlands; 29 Department of Cardiology, Leiden University Medical Center, Leiden, The Netherlands; 30 MRC Population Health Research Unit, University of Oxford, Oxford, UK; 31 Department of Epidemiology and Biostatistics, School of Public Health, Peking University, Beijing, China; 32 Peking University Center for Public Health and Epidemic Preparedness and Response, Beijing, China; 33 Key Laboratory of Epidemiology of Major Diseases, Peking University, Ministry of Education, Beijing, China; 34 Department of Biological Psychology, Vrije Universiteit Amsterdam, Amsterdam, The Netherlands; 35 Amsterdam Public Health Research Institute, Amsterdam, The Netherlands; 36 Institute for Molecular Medicine Finland (FIMM), HiLIFE, University of Helsinki, Helsinki, Finland; 37 Department of Internal Medicine, Erasmus MC, University Medical Center Rotterdam, Rotterdam, The Netherlands; 38 Institute of Genomics, University of Tartu, Tartu, Estonia; 39 Department of Psychiatry, Amsterdam Neuroscience and Amsterdam Public Health, Amsterdam UMC, Vrije Universiteit Amsterdam, Amsterdam, The Netherlands; 40 Finnish Cardiovascular Research Center Tampere, Faculty of Medicine and Health Technology, Tampere University, Tampere, Finland; 41 Department of Clinical Physiology, Tampere University Hospital, Tampere, Finland; 42 Department of Clinical Chemistry, Faculty of Medicine and Health Technology, Tampere University, Tampere, Finland; 43 Department of Clinical Chemistry, Fimlab Laboratories, Tampere, Finland; 44 Centre for Global Health, Usher Institute, University of Edinburgh, Edinburgh, Scotland; 45 Department of Twin Research and Genetic Epidemiology, King’s College London, London, UK; 46 NIHR Biomedical Research Centre at Guy’s and St Thomas’ Foundation Trust, London, UK; 47 Department of Biosystems Science and Engineering, ETH Zurich, Basel, Switzerland; 48 Department of Ophthalmology, Radboud University Medical Center, Nijmegen, The Netherlands; 49 Department of Genetics, University Medical Center Groningen, University of Groningen, Groningen, The Netherlands; 50 Section of Molecular Epidemiology, Department of Biomedical Data Sciences, Leiden University Medical Center, Leiden, The Netherlands; 51 Center for Computational Biology, Leiden University Medical Center, Leiden, The Netherlands; 52 The Delft Bioinformatics Lab, Delft University of Technology, Delft, The Netherlands; 53 Department of Public Health, School of Medicine, University of Split, Split, Croatia; 54 Research Unit Molecular Epidemiology, Institute of Epidemiology, Helmholtz Zentrum München, German Research Center for Environmental Health, Neuherberg, Germany; 55 German Center for Cardiovascular Research (DZHK), Partner Site Munich Heart Alliance, Munich, Germany; 56 Amsterdam University Medical Centers, Department of Cardiology, University of Amsterdam, Amsterdam, The Netherlands; 57 Health Data Research UK and Institute of Health Informatics, University College London, London, UK; 58 Medical Research Council Human Genetics Unit, Institute of Genetics and Cancer, University of Edinburgh, Edinburgh, UK; 59 Department of Pediatrics, University Medical Center Groningen, University of Groningen, Groningen, The Netherlands; 60 Genomics Research Center, Abbvie, Cambridge, MA USA; 61 Research Centre of Applied and Preventive Cardiovascular Medicine, University of Turku, Turku, Finland; 62 Department of Clinical Physiology and Nuclear Medicine, Turku University Hospital, Turku, Finland; 63 Centre for Population Health Research, University of Turku and Turku University Hospital, Turku, Finland; 64 InFLAMES Research Flagship, University of Turku, Turku, Finland; 65 Netherlands Heart Institute, Utrecht, The Netherlands; 66 School of Cardiovascular and Metabolic Health, University of Glasgow, Glasgow, UK; 67 Department of Human Genetics, Leiden University Medical Center, Leiden, The Netherlands; 68 Department of Internal Medicine, Division Endocrinology, Leiden University Medical Center, Leiden, The Netherlands; 69 Leiden Laboratory for Experimental Vascular Medicine, Leiden University Medical Center, Leiden, The Netherlands; 70 Kuopio University Hospital, Kuopio, Finland; 71 Department of Life Sciences, College of Health and Life Sciences, Brunel University London, Uxbridge, UK; 72 Unit of Primary Health Care, Oulu University Hospital, OYS, Oulu, Finland; 73 Diabetes and Obesity Research Program, University of Helsinki, Helsinki, Finland; 74 Estonian Genome Center, University of Tartu, Tartu, Estonia; 75 Imperial College Healthcare NHS Trust, Imperial College London, London, UK; 76 MRC-PHE Centre for Environment and Health, Imperial College London, London, UK; 77 National Heart and Lung Institute, Imperial College London, London, UK; 78 Lee Kong Chian School of Medicine, Nanyang Technological University, Singapore, Singapore; 79 Amsterdam Reproduction and Development (AR&D) Research Institute, Amsterdam, The Netherlands; 80 Department of Human Genetics, Wellcome Sanger Institute, Hinxton, UK; 81 NMR Metabolomics Laboratory, School of Pharmacy, University of Eastern Finland, Kuopio, Finland; 82 Present Address: Genentech, South San Francisco, CA USA

**Keywords:** Genetics research, Predictive markers, Genome-wide association studies, Metabolomics

## Abstract

Genome-wide association analyses using high-throughput metabolomics platforms have led to novel insights into the biology of human metabolism^1–7^. This detailed knowledge of the genetic determinants of systemic metabolism has been pivotal for uncovering how genetic pathways influence biological mechanisms and complex diseases^8–11^. Here we present a genome-wide association study for 233 circulating metabolic traits quantified by nuclear magnetic resonance spectroscopy in up to 136,016 participants from 33 cohorts. We identify more than 400 independent loci and assign probable causal genes at two-thirds of these using manual curation of plausible biological candidates. We highlight the importance of sample and participant characteristics that can have significant effects on genetic associations. We use detailed metabolic profiling of lipoprotein- and lipid-associated variants to better characterize how known lipid loci and novel loci affect lipoprotein metabolism at a granular level. We demonstrate the translational utility of comprehensively phenotyped molecular data, characterizing the metabolic associations of intrahepatic cholestasis of pregnancy. Finally, we observe substantial genetic pleiotropy for multiple metabolic pathways and illustrate the importance of careful instrument selection in Mendelian randomization analysis, revealing a putative causal relationship between acetone and hypertension. Our publicly available results provide a foundational resource for the community to examine the role of metabolism across diverse diseases.

## Abstract (web summary)

A meta-analysis of genome-wide association studies for 233 circulating metabolites from 33 cohorts reveals more than 400 loci and suggests probable causal genes, providing insights into metabolic pathways and disease aetiology.

## Main

Large genome-wide association studies (GWASs) coupled with metabolic profiling platforms have successfully identified many loci associated with circulating metabolic traits^1–7,12–16^. For example, studies combining genomics with detailed metabolic profiling from a high-throughput metabolomics platform based on nuclear magnetic resonance spectroscopy^17^ have enabled the identification of dozens of loci for traits associated with circulating lipid, lipoprotein and fatty acid and small molecules such as amino acids^2,4,5,9,18,19^. These studies have provided novel insights into the biology of human metabolism and have guided large-scale epidemiological studies, such as Mendelian randomization analyses to infer causal relationships^17^. Here, using the same NMR metabolomics platform from Nightingale Health with an updated quantification version, we considerably extend our previous GWAS^4^ of 123 circulating metabolic traits in up to around 25,000 participants to study 233 traits in more than 135,000 participants.

## Genetic discovery

GWAS was performed under the additive model separately in each of 33 cohorts (Supplementary Table 1). Subsequent meta-analysis involved 233 metabolic traits (Supplementary Table 2), including 213 lipid and lipoprotein parameters or fatty acids and 20 non-lipid traits (amino acids, ketone bodies and glycolysis/gluconeogenesis, fluid balance and inflammation-related metabolites). After variant filtering and quality control, up to 13,389,637 imputed autosomal single-nucleotide polymorphisms (SNPs) were included in the meta-analysis for up to 136,016 participants.

**Fig. 1:**
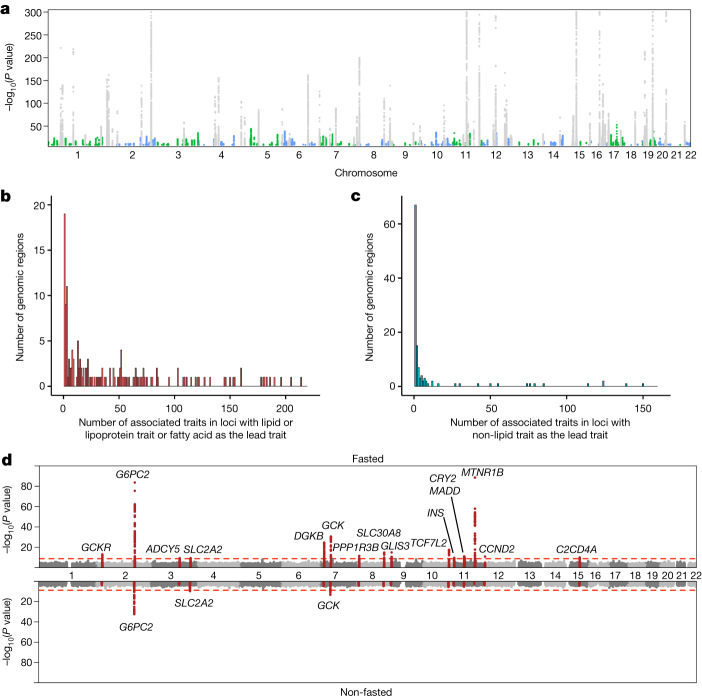
Results of the GWAS meta-analysis of 233 metabolic traits. **a**, Manhattan plot summarizing the metabolic trait associations from inverse variance-weighted GWAS meta-analysis. Loci that do not overlap with those identified in the previous large-scale NMR metabolomics GWAS^4,5^ are shown in blue and green. Only genome-wide significant SNPs (two-sided *P* < 1.8 × 10^−9^) are shown and −log_10_(*P* values) were capped at 300. **b**,**c**, Numbers of associated metabolic traits at the 276 associated genomic regions are shown separately for genomic regions in which the lead trait was a lipid, lipoprotein or fatty acid trait (**b**; 155 loci; median 24 traits per locus) and for those in which the lead trait was a non-lipid trait (**c**; 121 loci; median one trait per locus). **d**, Results of the GWAS for glucose for the fasted (top; total *n* = 68,559) and non-fasted (bottom; total *n* = 58,112) cohorts. The red line indicates the threshold for genome-wide significance. The 500-kb regions around lead SNPs in the fasted cohorts are highlighted.

In the meta-analysis, we detected genome-wide significant associations for all 233 metabolic traits (Supplementary Data Figs. 1–3 and Supplementary Tables 4 and 5) with extensive pleiotropy and polygenicity. We detected 276 broad regions (defined as a ±500-kb region around the set of genome-wide significant SNPs) associated with at least one metabolic trait (Fig. 1a and Supplementary Table 4). Eighty-six of these regions were associated with just a single metabolic trait, whereas most regions harboured associations with multiple traits (Fig. 1b,c) up to a maximum of 214 associated traits at the well-characterized lipid-associated *APOE* region. The lipid, lipoprotein and fatty acid traits were mostly demonstrably polygenic, with 60 traits having associations at more than 50 loci, 137 traits (64.3%) having associations at 20–50 loci, and 16 traits (7.5%) having associations at fewer than 20 loci (Supplementary Tables 5 and 6). Most non-lipid traits had substantially fewer associated loci (13 with fewer than 20 associated traits; 65% of all 20 non-lipid traits), including 3 glucose metabolism-related traits (lactate, pyruvate and glycerol) having fewer than 5 associated loci, whereas glycoprotein A and some amino acids had associations at 20–33 loci and creatine had associations at 49 loci (Supplementary Tables 5 and 6). The non-lipid traits accounted for most of the regions with a single associated trait (*n* = 67; 78%), and the majority (*n* = 163; 57%) of the regions with non-lipid trait associations had fewer than 5 associated metabolic traits in total. By contrast, the lipid, lipoprotein and fatty acid trait-associated regions (*n* = 186) were generally more pleiotropic with 75% (*n* = 140) of the regions being associated with 5 or more traits. The pleiotropy difference is owing to the fact that lipoprotein metabolism is a continuum, with genes often affecting several particle categories, whereas the non-lipid traits are often affected by more distinct processes and enzymatic modifications, thus leading to less pleiotropy^3,9,20^. Within the 276 regions, we found 8,795 lead SNP–lead trait associations corresponding to 1,447 unique lead SNPs (Supplementary Table 5). After resolving independent signals on the basis of pairwise linkage disequilibrium (LD), we concluded that the 276 broad regions involved at least 443 independent loci. We estimated the genome-wide common variant heritability for 223 traits also available in UK Biobank (Supplementary Table 6). Median trait heritability was 0.29, of which only around a quarter was explained by the lead SNPs, supporting the high polygenicity of many of the traits.

## Ancestry-stratified analyses

**Extended Data Fig. 1:**
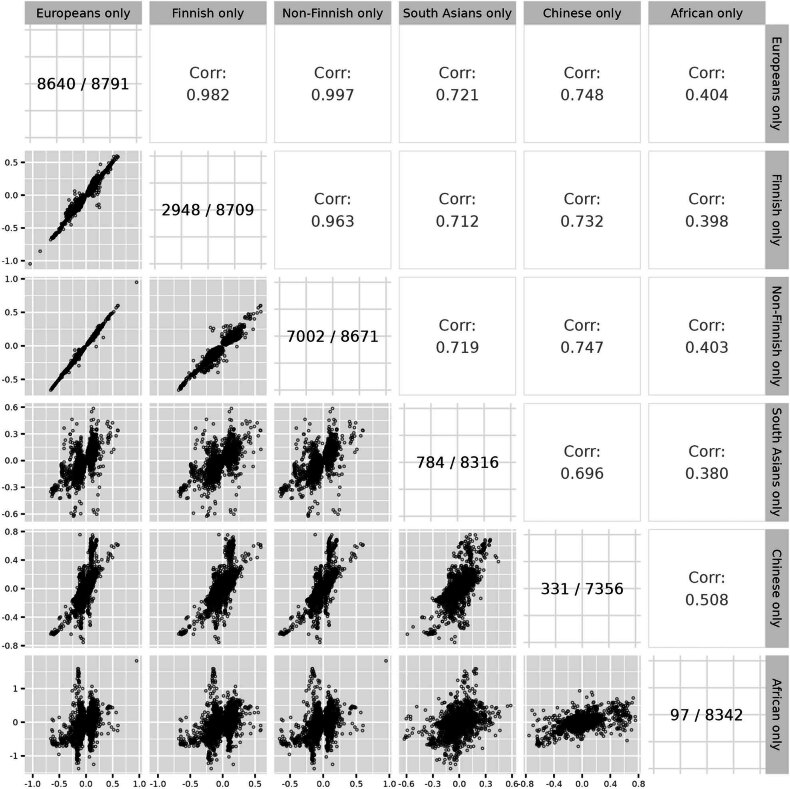
Comparisons of effect sizes across ancestries. Scatter plots (lower left) show SNP effect sizes of the lead SNP – metabolic trait pairs within the 276 associated genomic regions across different ancestries (Europeans, *n* = 120,251; Finnish, *n* = 27,577; non-Finnish Europeans, *n* = 92,664; South Asians, *n* = 11,340; Han Chinese, *n* = 4,435; Africans, *n* = 1,405). Pearson correlation (Corr) values (*r*) from each comparison are shown (upper right). The diagonal squares show the number of the 8,795 associations that could be tested in each ancestry group (denominator) and the number that reached the traditional level of genome-wide significance (*p* < 5 ×10^−8^) (numerator).

To investigate the generalizability of our associations across ethnic groups and search for additional ancestry-specific association signals, we conducted ancestry-stratified analyses of South Asian (five cohorts, 11,340 participants), East Asian (one cohort, 4,435 participants), Finnish (six cohorts, 27,577 participants) and non-Finnish European (21 cohorts, 92,664 participants) cohorts (Supplementary Table 7). To investigate the generalizability further, we additionally performed a post hoc comparison of the associations to a small population with African ancestry (*n* = 1,405). Associations were strongly positively correlated across ancestry groups (Extended Data Fig. 1), suggesting that associations are broadly transferable across ancestries. Stronger correlations were seen between Finnish and non-Finnish Europeans (*r* = 0.96) compared with East Asians and South Asians (*r* ≈ 0.7) and Africans (*r* ≈ 0.4). For some loci, effect estimates were notably stronger in one ancestry than another. The number of genome-wide significant associations was strongly related to sample size, ranging from 7,002 associations in the non-Finnish Europeans to 331 in the East Asian and 97 in the African participants. We did not detect any novel genomic regions in any of the ancestry groups beyond the 276 discovered in the ancestry-combined meta-analysis, suggesting that substantially larger sample sizes of participants with non-European ancestry will be required in future studies.

## Associations in UK Biobank

**Extended Data Fig. 2:**
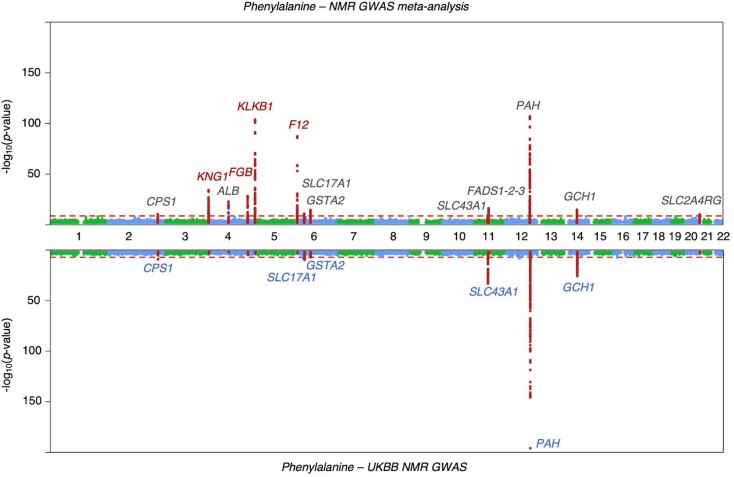
Mirrored Manhattan plot showing the results of genome-wide association study of phenylalanine in the NMR GWAS meta-analysis and UK Biobank. The top panel of the mirrored Manhattan plot shows the NMR inverse variance weighted GWAS meta-analysis results (*n* = 136,016) and the bottom panel the UKBB results (*n* = 115,025). The red lines indicate the thresholds for genome-wide significance (top panel *p* < 1.8 × 10^−9^; bottom panel *p* < 5 × 10^−8^). 500-kb regions around lead SNPs in the NMR GWAS are highlighted. Loci indicated in red have roles in coagulation-related pathways. Loci indicated in blue were genome-wide significant in both NMR GWAS meta-analysis and UK Biobank.

**Extended Data Fig. 3:**
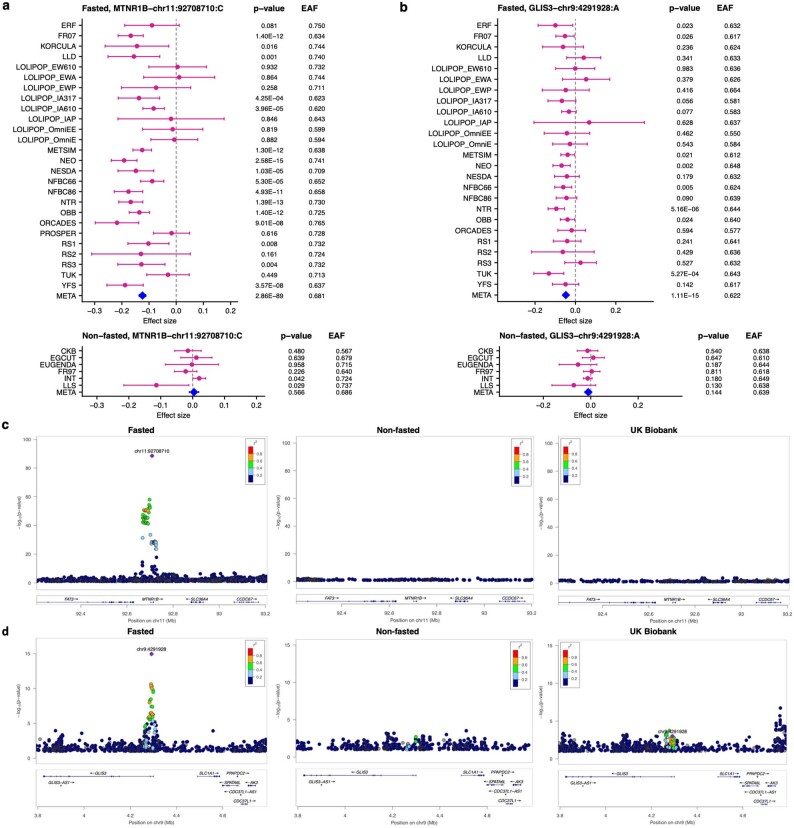
Examples of glucose associations for fasted and non-fasted cohorts. The forest plots in panels a and b show examples of two lead SNPs in which glucose associations were significant in the fasted cohorts (top; *n* = 68,559) and non-significant in the non-fasted cohorts (bottom; *n* = 58,112). The associations were analyzed by inverse variance weighted GWAS meta-analysis. These associations were absent in the UK Biobank. Effect sizes (betas and 95% confidence intervals), effect allele frequencies (EAF) and p-values are indicated for each cohort. Cohort acronyms can be found in Supplementary Table 1. Panels c and d show regional association plots of the *MTNR1B* (c) and *GLIS3* (d) loci in the fasted (left) and non-fasted (center) cohorts and in UK Biobank (right). SNPs with *p* < 0.1 are shown. 500-kb flanking regions around each lead SNP are shown. The linkage disequilibrium values (*r*^2^) are based on the 1000Genomes European population.

The availability of NMR data from the UK Biobank resource^21^ (March 2021 release) enabled us to check for associations of the lead variants in an independent population and to assess the effects of participant characteristics and sample-related factors on our associations. Of the 8,502 lead SNP–metabolic trait pairs that could be tested in up to 115,078 UK Biobank participants with European ancestry, 5,442 (64.0%) associated at *P* < 5 × 10^−8^, and a further 772 (9.1%; 328 unique SNPs) associated at *P* < 1 × 10^−5^ (Supplementary Table 8). When we performed further stratified analyses in cohorts with different sample types (serum, *n* = 90,223; plasma, *n* = 45,793), and in fasted (*n* = 68,559) and non-fasted (*n* = 58,112) cohorts, we detected that, in addition to subtle differences in population ancestry between the studies, sample type and fasting status were probably the major drivers of non-replication. The UK Biobank NMR measurements were performed on EDTA plasma samples, whereas the current meta-analysis involved predominantly serum samples. For example, several of the non-replicating associations with phenylalanine were in coagulation-related loci (for example, *KLKB1*, *F12*, *KNG1* and *FGB*) but these signals were absent in UK Biobank (Extended Data Fig. 2 and Supplementary Table 8)—therefore we speculate that the removal of clotting factors in the preparation of serum could reveal associations with phenylalanine via coagulation. Two loci (*NHLRC1*, lead SNP rs73726535; *TXNRD1*, lead SNP rs191631370) also had associations for phenylalanine in UK Biobank that were absent in the current meta-analysis. Similarly, we found associations with glucose that did not replicate in the UK Biobank, including a well-known association at the melatonin receptor 1B gene^22^ (*MTNR1B*), a key regulator in glucose metabolism (rs10830963; meta-analysis *P* value = 1.5 × 10^−60^; UK Biobank *P* value = 0.60). The UK Biobank predominantly includes non-fasted samples, but the current meta-analysis mainly consists of cohorts (26 cohorts) with fasted samples (Supplementary Table 1), and our fasting-stratified meta-analysis suggested that some of the glucose associations were driven by cohorts with predominantly fasted samples (Fig. 1d and Extended Data Fig. 3) and are thus absent in UK Biobank. In addition to *MTNR1B* rs10830963 (*P* values 2.9 × 10^−89^ and 0.57 in meta-analysis of fasted and non-fasted cohorts, respectively), the association of which was also previously shown to be absent in non-fasting samples^23^, GLIS family zinc finger 3 (*GLIS3*; a known diabetes risk gene^24^ with a role in pancreatic beta cell biology) rs10974438 represents another example of an association that was not robustly replicated in UK Biobank (meta-analysis *P* value = 4.0 × 10^−14^; UK Biobank *P* value = 0.001) and was characterized by the absence of signals in the non-fasted cohorts (*P* values 1.1 × 10^−15^ and 0.14 in meta-analysis of fasted and non-fasted cohorts; Extended Data Fig. 3).

Many of the metabolic trait associations differed by sample type and fasting status, although comparisons with the overall associations are complicated by the reduced power of the stratified analyses. For example, associations of several lipoprotein subclass measures were substantially affected by fasting status at loci with central roles in lipid biology, such as *LPA* and *ANGPTL3* (Supplementary Table 9). Similarly, multiple loci had greater than twofold higher or lower effect estimates in cohorts using serum compared to those using plasma (Supplementary Table 9). These differences were detected both for lipid and non-lipid traits, with some associations being notably augmented by removal of plasma samples. However, only 7 additional loci (beyond the 276 initially associated genomic regions) were detected in analyses stratified by sample type and 10 were detected in fasting-stratified analyses (Supplementary Tables 10 and 11)—for example, *C2CD4A* rs10083587 for glucose and *KAT5* rs12787843 for creatinine, both of which showed associations only in fasting cohorts (*P* values 1.3 × 10^−10^ and 7.6 × 10^−10^, respectively). We note that the effects of the sample type and fasting status require careful consideration when interpreting the results of GWAS of metabolic traits and conducting downstream analyses, such as Mendelian randomization studies using trait-associated variants as instruments.

## Novel loci and candidate genes

We conducted extensive manual curation to prioritize 231 likely causal genes with clear biological relevance to the associated traits at 297 (67.0%) of the 443 loci (Methods). As some regions were extremely complex and pleiotropic owing to overlapping genetic associations of up to 11 independent lead variants with heterogeneous associations across the metabolic traits, we characterized these loci in detail to pinpoint potential multiple probable causal genes within each locus (Supplementary Table 5). For example, in a 7.6-Mb region on chromosome 16 with 139 associated metabolic traits, we identified 6 distinct biologically relevant potential causal genes: lecithin-cholesterol acyltransferase (*LCAT*; associated with multiple lipoprotein subclass measures), solute carrier family 7 member 6 (*SLC7A6*; associated with acetate and creatinine), pyruvate dehydrogenase phosphatase regulatory subunit (*PDPR*; associated with pyruvate and amino acids), alanyl-tRNA synthetase 1 (*AARS*; associated with pyruvate and amino acids), tyrosine aminotransferase (*TAT*; associated with tyrosine) and haptoglobin (*HP*; associated with a range of lipoprotein subclass measures, fatty acids, cholesterol, apolipoprotein B (apoB) and glycoprotein acetylation). This locus exemplifies the complexity of the metabolic trait-associated loci. For additional loci without an obvious biological candidate, we assigned a further 39 probable causal genes on the basis of SNP function or the presence of probable functional (missense, stop gained or splice region) variants in strong LD (*r*^2^ ≥ 0.8) with the lead variant (Supplementary Table 5).

We performed an extensive comparison of the discovered associations to previously reported genetic associations of metabolic traits and traditional clinical lipids (high density lipoprotein C (HDL-C), low density lipoprotein C (LDL-C), triglycerides and total cholesterol; Supplementary Table 5). In comparison to previous large-scale NMR metabolomics GWASs^4,5^, we identified 212 additional associated genomic regions (Supplementary Table 4). These included 138 novel genomic regions for the lipoprotein, lipid and fatty acid traits, and 113 novel regions associated with the non-lipid traits. New associations for several lipoprotein subclass measures were detected in loci previously associated with clinical lipids, such as the locus containing low density lipoprotein receptor adapter protein 1 gene (*LDLRAP1*), which is involved in cholesterol metabolism. This locus was previously known to be associated with LDL-C, triglycerides and total cholesterol^25–27^, and we found associations at this locus with several lipoprotein subclass measures, lipids and fatty acids (Supplementary Table 5). Locus containing the sterol *O*-acyltransferase 2 gene (*SOAT2*; which functions in cholesterol metabolism) represents another example of a novel biologically plausible locus associated with the lipoprotein and lipid traits. Our analyses also identified genetic associations with detailed lipoprotein subclass measures in loci that have not previously been reported to be associated with traditional clinical lipids. Compared with the largest trans-ancestry study of clinical lipids to date^27^, we detected associations at twelve additional loci (Supplementary Table 5) for the lipid and lipoprotein traits (corresponding to 6.5% of all lipoprotein and lipid trait-associated regions): gene encoding type 2 lactosamine α-2,3-sialyltransferase (*ST3GAL6*; which functions in glycolipid metabolism) represents an example of a biologically plausible gene associated with multiple lipoprotein subclass measures and lipids.

Novel loci were also detected for small molecules such as phenylalanine and glutamine. For phenylalanine, we detected associations at 13 loci. Novel phenylalanine-associated loci include both a well-known metabolic trait-associated locus (*FADS1–FADS2*) and two novel, biologically plausible loci (*GSTA2* and *SLC2A4RG*). For example, *SLC2A4RG* encodes SLC2A4 regulator, a transcription factor involved in the activation of solute carrier family 2 member 4 (SLC2A4, also known as GLUT4), a key regulator of glucose transport. For glutamine, we detected associations at 26 loci. Of note, seven of the loci were associated only with glutamine (*GLS*, *PLCL1*, *SFXN1*, *KCNK16*, *MED23*, *SLC25A29* and *PCK1*). Thus, these associations are likely to represent biology local to glutamine, most of the loci having biologically plausible candidate genes with roles in glutamine metabolism (*GLS*), amino acid transport (*SFXN1* and *SLC25A29*) or glucose and gluconeogenesis-related pathways (*PCK1* and *KCNK16*). *KCNK16*, a known type 2 diabetes susceptibility gene that encodes the potassium channel subfamily K member 16, a pancreatic potassium channel, represents an example of a novel glutamine-associated locus with a role in glucose biology^28,29^.

## Effects of apoB variants

**Fig. 2:**
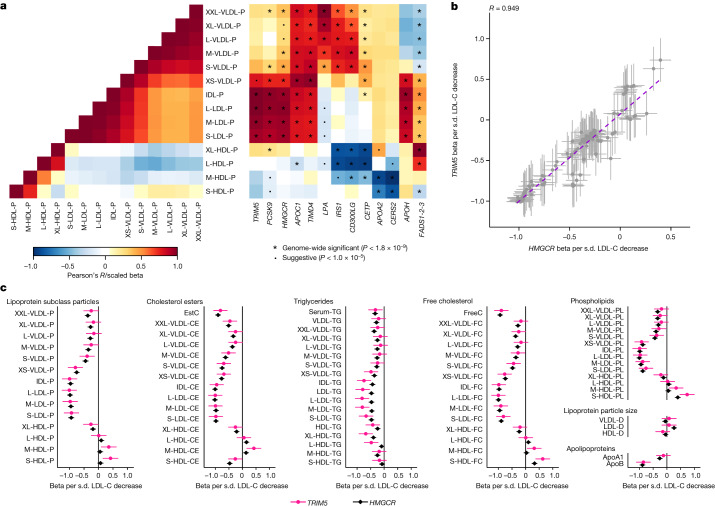
Effects of SNPs across lipoprotein and lipid traits. **a**, Heat maps of the correlation structure of lipoprotein subclass particle concentrations (left) and the association landscapes of exemplar SNPs (right). In the heat maps, pairwise correlations of lipoprotein subclass particle concentrations (calculated in FINRISK 1997; left) and effect estimates for the SNP–metabolic trait associations (right) are represented as a colour range. The SNP effect sizes were scaled relative to the absolute maximum effect size in each locus. Each column represents a single SNP, and each row corresponds to a single metabolic measure. Two-sided tests were used. **b**,**c**, Scatter plot (**b**) and forest plots (**c**) of the effect estimates (betas and 95% confidence intervals) for *TRIM5* and *HMGCR* lead SNPs (rs11601507 and rs12916, respectively; *n* = 136,016 individuals) across the lipoprotein and lipid traits. **b**, A best fit regression line (purple dashed line) and an estimate of Pearson’s correlation coefficient *R* (for betas of 116 SNP–trait pairs) are shown. The effect estimates (s.d. units) were scaled relative to a one-s.d. decrease in LDL cholesterol. VLDL, LDL, IDL and HDL are classified into different particle sizes (in order of decreasing size: XXL, XL, L, M, S and XS). Detailed descriptions of the metabolic traits and abbreviations are shown in Supplementary Table 2. ApoA1, apolipoprotein A1. Source Data

**Extended Data Fig. 4:**
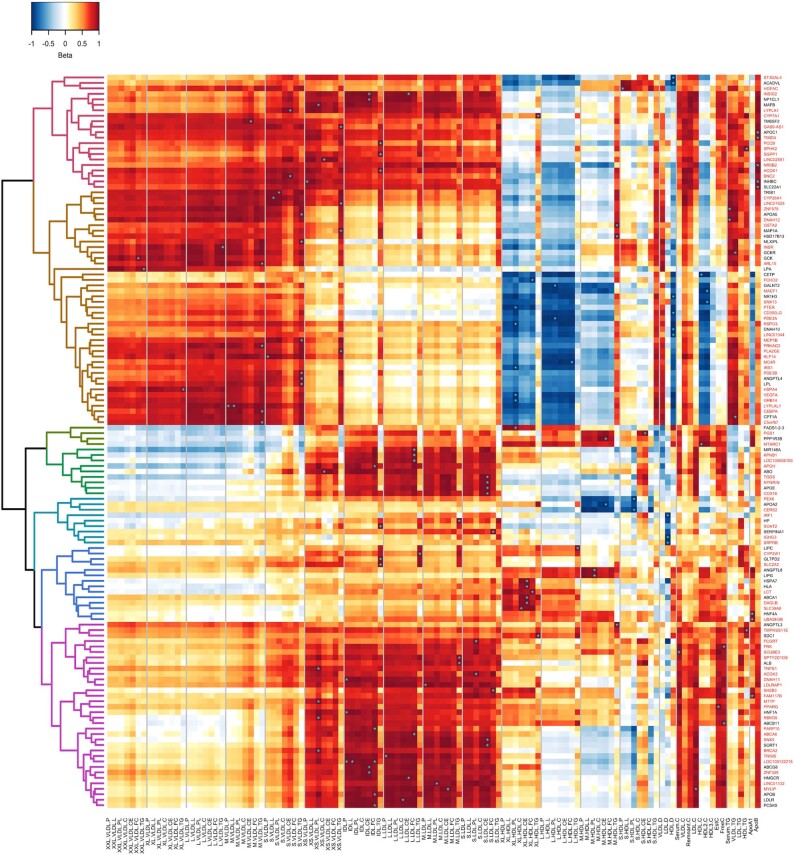
Heat map of lipoprotein and lipid associations. Lipoprotein and lipid associated loci with similar association patterns across the lipoprotein measures were grouped together in a dendrogram based on hierarchical clustering of the SNP effects. The apolipoprotein B associated loci (*n* = 134, *p* < 0.05) were included since apolipoprotein B represents a causal part of the lipoprotein metabolism for cardiovascular disease. The heat map illustrates the resemblances of the association landscapes; each row represents a single SNP, each column corresponds to a single metabolic measure, and the scaled effect estimates from inverse variance weighted GWAS meta-analysis for the SNP-metabolite associations are visualized with a colour range. Effect sizes were scaled relative to the absolute maximum effect size (beta) in each locus. Loci that were not identified in the previous large-scale NMR metabolomics GWAS are indicated by red. Traits with absolute maximum effects in each locus are indicated by asterisks. For clarity, two of the clusters (brown and purple) are highlighted in Extended Data Fig. 5.

**Extended Data Fig. 5:**
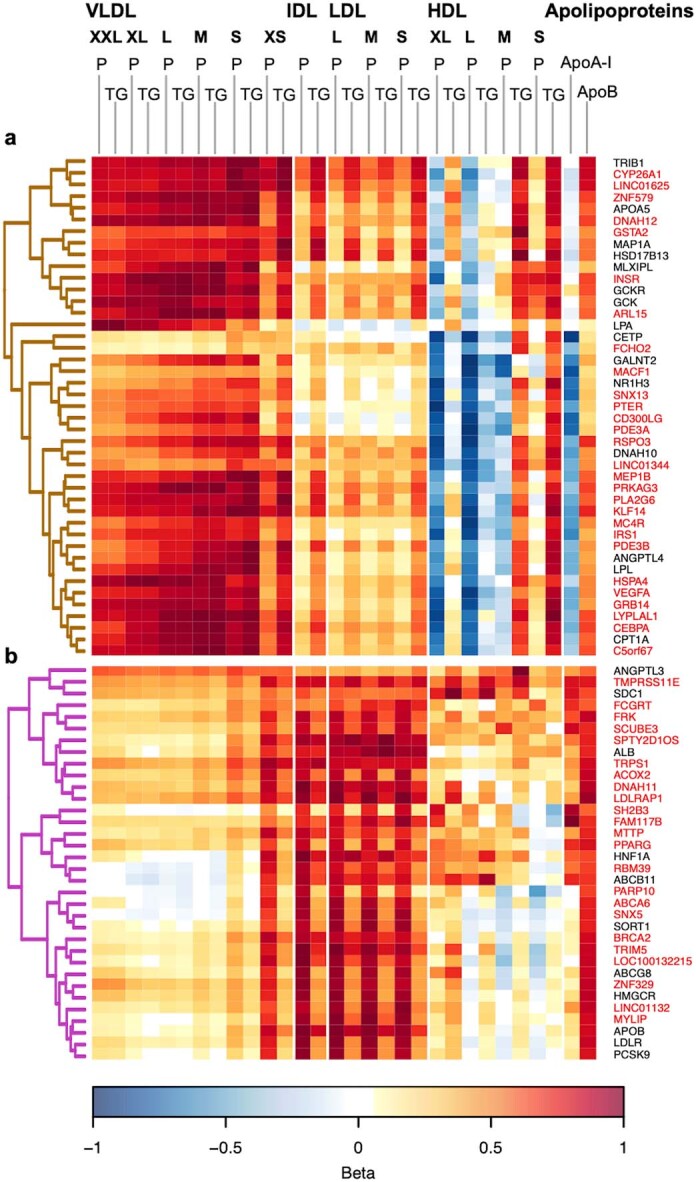
A zoomed heat map of lipoprotein and lipid associations. The full heat map including all the loci and a full set of lipoprotein traits is shown in Extended Data Fig. 4. For clarity, two of the clusters are highlighted here. For details, see legend for Extended Data Fig. 4. Panels a and b corresponding to the brown and purple branches of the dendrogram shown in the full-sized heat map, respectively. Effect sizes were scaled relative to the absolute maximum effect size (beta) in each locus. In the heat map, each row represents a single SNP, each column corresponds to a single metabolic measure, and the effect estimates for the SNP-metabolite associations are visualized with a colour range. Loci highlighted in red were not identified in the previous NMR metabolomics GWAS.

To provide insights into the distinct ways in which lipid loci can affect the continuum of lipoprotein metabolism, we characterized clusters of genes with similar metabolic association profiles. The effect estimates were scaled by dividing all effect estimates of a given SNP using the strongest association effect estimate across all metabolic associations in each locus. In this way, the scaled effect estimates for all SNPs were between −1 and 1, and the statistical strength of an association affects the clustering less, and more emphasis is given to the association landscape in guiding the clustering. We concentrated on 134 loci with nominal evidence (*P* < 0.05) of an association with apoB, as recent studies have highlighted the predominant role of apoB in coronary artery disease aetiology^30–32^. The clustering of the loci produced at least seven major clusters of loci (Extended Data Figs. 4 and 5). The top cluster in Extended Data Fig. 4 is very similar to the previously observed epidemiological association profile with type 2 diabetes risk^33^ and adiposity^34^. The second cluster of loci (Extended Data Figs. 4 and 5) primarily shows increasing triglyceride-rich very low density lipoprotein (VLDL) particles and decreasing large HDL particles. The genes in this cluster, such as *LPL*, *MLXIPL* and *ANGPTL4*, relate to triglyceride metabolism, and glucose metabolism, exemplified by *GCK*, *GCKR* and *INSR*. The other clusters associate primarily with LDL particles and generally less with other lipoproteins. The lowest cluster includes biologically relevant genes that are known to affect LDL cholesterol in circulation, including *APOB*, *LDLR*, *PCSK9*, *SORT1* and *HMGCR*. Despite the strong correlation structure within the lipid and apolipoprotein traits, we identified several loci with association patterns that do not follow the between-trait correlation structure (Fig. 2a and Extended Data Figs. 4 and 5). For example, some loci (*APOC1* and *TIMD4*) are strongly associated with all the apoB-containing particles (VLDL, intermediate density lipoprotein (IDL) and LDL), whereas other loci are predominantly associated with IDL and LDL particles (*PCSK9*, *HMGCR* and *TRIM5*), with VLDL and the largest HDL particles (*IRS1* and *CD300LG*), or with medium and small HDL particles (*APOA2* and *CERS2*). Several SNPs also exhibit discordant associations within highly correlated metabolic traits (for example, *LPA* and *APOH* SNPs within apoB-containing particles and *FADS* cluster SNP within both apoB-containing and HDL particles; Fig. 2a).

Metabolic profiles of 84 novel loci that were not identified in the previous NMR GWASs^2,4,5^ were characterized here using the clustering approach (Extended Data Figs. 4 and 5). As the approach we have taken uses scaled effect estimates, our results are not directly comparable to previous studies which have used unscaled effect estimates^9^ or numbers of associations per lipoprotein type^5^ in clustering. Even though many loci, such as the master regulator genes *PCSK9* and *LDLR*, clustered mostly similarly as reported previously^5,9^, the genetically calibrated approach applied here can specifically add to the understanding of the detailed metabolic effects of less well-known lipid-associated loci as their metabolic association patterns have not been previously characterized. The tripartite motif-containing protein 5 gene (*TRIM5*) is an example of a poorly characterized locus associated with 42 lipoprotein and lipid traits (Supplementary Table 5). *TRIM5* is best known for its role in antiviral host defence^35^, but variants near *TRIM5* have also been associated with several traits related to liver biology, such as levels of liver enzymes^36^, and have recently been reported to associate with risk of coronary artery disease^37^. Notably, the metabolic effects on the lipoprotein and lipid traits of the lead *TRIM5* variant (rs11601507, p.Val112Ile) appear similar to those of the *HMGCR* variant rs12916 (Fig. 2b,c), the metabolic effects of which are concordant with those of statin therapy^38–40^. The mechanism by which *TRIM5* affects lipid and lipoprotein levels and predisposes to coronary artery disease is unclear and it has been speculated to be related to innate immunity^41^. A recent study using a mouse model of nonalcoholic fatty liver disease suggested that TRIM5 may mediate degradation of DEAD-box protein 5, which could affect mTORC1 signalling and the LDL receptor pathway, consequently affecting lipid accumulation and inflammation^42^. Irrespective of the pathophysiological mechanism, our findings raise the possibility that inhibition of TRIM5 could provide an alternative therapeutic pathway for reducing the risk of cardiovascular disease via lowering the concentrations of circulating atherosclerotic apoB-containing lipoprotein particles similar to PCSK9-inhibition therapies that are useful for statin intolerant individuals or for statin users requiring further risk reduction. Although we specifically chose the *TRIM5* association for further investigation, our clustering analysis suggests there are several other novel loci worthy of further in-depth investigation.

## Metabolic trait variants and diseases

To investigate the roles of the metabolic trait-associated variants in disease, we scanned all the disease and trait associations of the 1,447 lead SNPs in the (1) FinnGen study (data freeze 7, up to 309,154 participants, 3,095 phenotypes), a dataset linking genomic information from Finnish participants to digital health care data^43^, and (2) curated collections of published GWASs, including PhenoScanner^44,45^ and GWAS catalog^46^ (Supplementary Table 5). In addition, we scanned the SNPs for association with gene expression and protein levels.

**Fig. 3:**
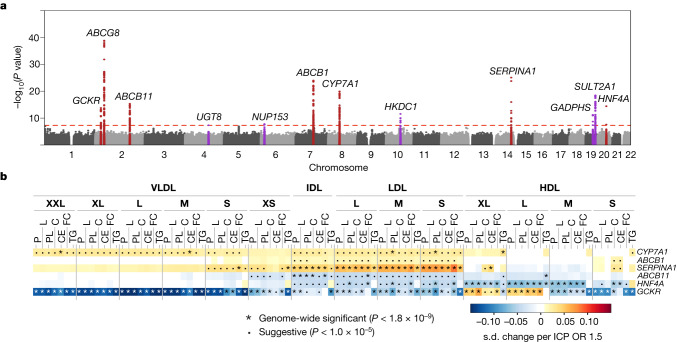
Metabolic trait-associated variants are associated with ICP. **a**,**b**, Manhattan plot of the GWAS of intrahepatic cholestasis of pregnancy (ICP) (**a**) and heat map of loci associated with metabolic traits and ICP (**b**). Twelve loci were associated with ICP in the FinnGen study (1,460 cases, 172,286 controls). **a**, The 500-kb regions flanking the lead SNPs are highlighted, and the nearest gene is indicated for each signal. The ICP GWAS was performed with scalable and accurate implementation of generalized mixed model (SAIGE). Loci that overlap with the loci identified in the NMR meta-analysis are indicated in red. **b**, Loci that are likely to have shared causal variants with the metabolic traits are included. The heat map illustrates the resemblances of the association landscapes. Each row represents a single SNP, each column corresponds to a single metabolic measure, and the scaled effect estimates for the SNP–metabolite associations from inverse variance-weighted GWAS meta-analysis are represented as a colour range. The associations were scaled with respect to their associations with ICP (s.d. change per ICP odds ratio (OR) 1.5). Detailed descriptions of the metabolic traits and abbreviations are shown in Supplementary Table 2.

Most (*n* = 1,279) of the 1,447 lead SNPs had previously reported associations (*P* < 5 × 10^−8^) with traits or diseases, including directly relevant outcomes such as use of statin medication and hypercholesterolaemia (Supplementary Table 5). Most of the SNPs (*n* = 1,270) were also associated with messenger RNA (mRNA) or protein levels (Supplementary Table 5), indicating that at least some of the associations are likely mediated by direct or indirect effects of SNPs on mRNA or protein levels. Seven metabolic trait-associated loci (*GCKR*, *ABCG8*, *ABCB11*, *ABCB1*, *CYP7A1*, *SERPINA1* and *HNF4A*) were associated (*P* < 5 × 10^−8^) with risk of intrahepatic cholestasis of pregnancy (ICP) in FinnGen (Fig. 3a and Supplementary Table 12), of which all except *ABCG8* showed robust evidence of colocalization or shared regional associations with the metabolic trait associations (Supplementary Table 13). ICP is a cholestatic disorder with onset in the second or third trimester of pregnancy, that is characterized by pruritus and elevated concentrations of serum aminotransferases and bile acids. ICP increases the risk of meconium staining of amniotic fluid, preterm delivery, fetal bradycardia, fetal distress and fetal loss^47^. The genetic background of ICP is poorly characterized with few published GWASs^7,48^ and the metabolic effect of the ICP loci has not been characterized. Compared with results of a recent ICP GWAS that included data from meta-analysis of an earlier FinnGen release (data freeze 4) and two other cohorts^48^, associations at nine loci (*GCKR*, *ABCG8*, *ABCB11*, *ABCB1–ABCB4*, *CYP7A1*, *SERPINA1*, *GAPDHS*–*TMEM147*, *SULT2A1* and *HNF4A*) were replicated here and three novel loci (*UGT8*, *NUP153* and *HKDC1*) were additionally identified. Rare coding variants at two of the loci, within the *ABCB11* and *ABCB4* genes, have additionally been previously reported in ICP^49,50^. A pathway analysis of the ICP-associated loci showed that biological processes related to bile acid, glucose and lipid metabolism were enriched for ICP (Supplementary Table 14), consistent with the metabolic trait associations. For some loci (*CYP7A1*, *ABCB1* and *SERPINA1*), the most profound associations were detected for IDL and LDL particles, whereas two loci (*HNF4A* and *GCKR*) were more pleiotropic, with effects across both apoB-containing and HDL particles (Fig. 3b). At three of the loci (*CYP7A1*, *ABCB1* and *SERPINA1*) the ICP-predisposing alleles were associated with higher concentrations of IDL and LDL subclass measures, whereas the direction of the association was reversed for others (*GCKR*, *ABCB11* and *HNF4A*). This information may be useful when considering these genes as therapeutic targets, as targets that adversely influence atherosclerotic lipids in pregnant women may be undesirable, despite the relatively short treatment period. By characterizing the associations of ICP-associated loci with metabolic traits in detail, we exemplify the value of combining the metabolic association information with disease associations to clarify the metabolic underpinnings of poorly understood conditions.

## Mendelian randomization

Finally, we exploited the absence of UK Biobank from our GWAS meta-analysis to perform a two-sample Mendelian randomization analysis to investigate associations of genetically predicted levels of the 20 non-lipid traits with 460 Phecodes and 52 quantitative traits from the UK Biobank. Initial Mendelian randomization analyses using all lead variants for each trait as genetic instruments identified 503 significant associations (*P* < 4.88 × 10^−6^) under the inverse variance-weighted model, including positive associations between glucose and diabetes, creatinine and renal failure, and amino acids with diabetes (Supplementary Tables 15 and 16), all of which represent well-known causal relationships. Less well-characterized relationships included a positive association between genetically predicted lactate levels and benign neoplasm of uterus. This potentially causal association is concordant with a recent GWAS that linked genetic tendency to gain muscle mass with uterine fibroids^51^. We also found an inverse association between genetically predicted circulating glycine levels and blood pressure, which is supported by a strong observational association with hypertension^52^ and by genetic data^53^. This finding suggests a potential mediator for the previously reported inverse association of glycine levels with myocardial infarction^54^. These examples highlight the value of linking data on genetics, metabolic traits and disease outcomes at scale to identify novel causal relationships between metabolic traits and disease.

**Extended Data Fig. 6:**
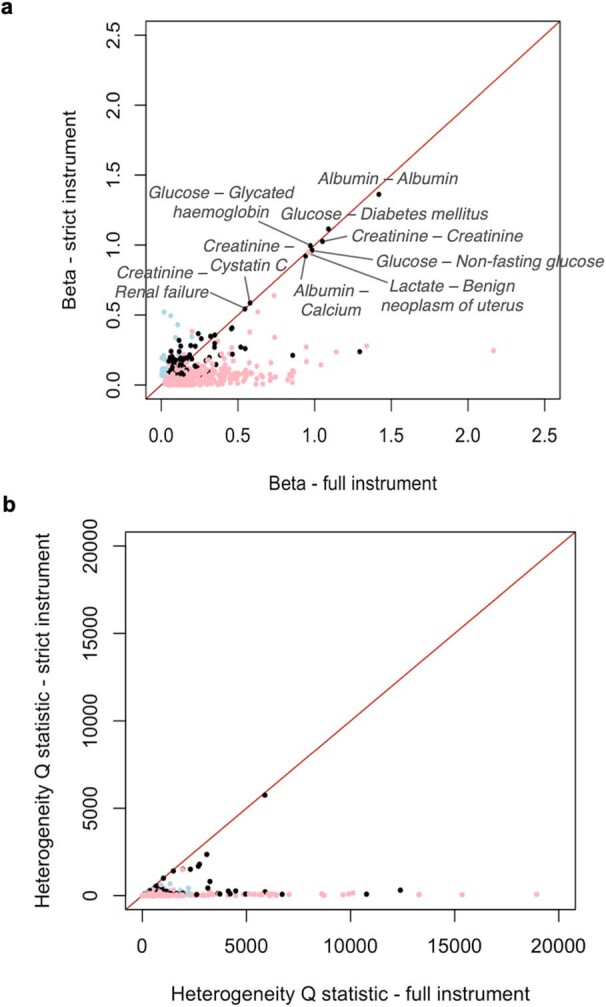
Influence of pleiotropy on Mendelian randomization estimates. The effect estimates (absolute betas) (panel a) and heterogeneity Q statistics (panel b) from the Mendelian randomization (MR) analyses using the full (pleiotropic) and strict (non-pleiotropic) MR instruments are shown. MR was performed using a fixed-effects inverse-variance weighted method. Associations that were significant (*p* < 4.88 × 10^−6^) using either the full or strict instrument or both were included; some of the significant exposure-outcome associations are indicated. Estimates indicated in light pink and light blue were not significant with the strict and full instruments, respectively. For clarity, very large beta (>5) and Q values (>60,000) were excluded from the plots.

Restricting the analyses to less pleiotropic variants (associated with fewer than 5 metabolic traits), the association estimates were on average considerably weaker with less between-variant heterogeneity (median absolute beta, 0.058 versus 0.152; Q-statistic, 34.2 versus 385.6, Extended Data Fig. 6), suggesting that pleiotropy was driving many of the initial Mendelian randomization associations. Results using two alternative thresholds for variant pleiotropy (fewer than three metabolic trait associations and fewer than seven metabolic traits associations) were very similar (Supplementary Table 17), suggesting that the findings are not sensitive to the choice of threshold. This clearly emphasizes that pleiotropy should be carefully considered when selecting instrument SNPs for Mendelian randomization to avoid false interpretations about potential causal relationships.

**Fig. 4:**
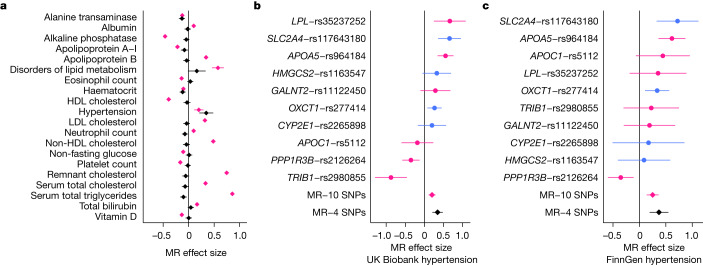
Mendelian randomization suggests a causal association between acetone and hypertension. **a**, Effect estimates (betas per s.d. increase in acetone) from Mendelian randomization (MR) analysis performed under the inverse variance-weighted model are shown for the UK Biobank outcomes that were significant (*P* < 4.88 × 10^−6^) with the full (pleiotropic, *n* = 10 instrument SNPs, pink) or strict (non-pleiotropic, *n* = 4 instrument SNPs, black) set of instruments. Betas and *P* values are shown in Supplementary Table 15. **b**,**c**, Effect estimates (betas per s.d. increase in acetone) in Mendelian randomization analysis with hypertension as the outcome in the UK Biobank (**b**; 104,824 cases with hypertension, 367,542 controls) and FinnGen (**c**; 70,651 cases with hypertension, 223,663 controls) datasets. Single-SNP Mendelian randomization effect estimates and 95% confidence intervals are shown, with the SNPs in the strict instrument coloured blue and the other SNPs coloured pink. Mendelian randomization effect estimates are shown with pink and black diamonds for the full instrument (all ten SNPs) and strict instrument (four non-pleiotropic SNPs), respectively. Source Data

As an example, the Mendelian randomization results for acetone were substantially affected by the inclusion of more pleiotropic SNPs in the instrument (Fig. 4). Acetone is a ketone body that is produced primarily in the liver during fasting and which has been associated with several cardiometabolic conditions including heart failure^55^ and diabetes^56^ in biochemical and epidemiological studies. In the GWAS, we identified associations for acetone at ten loci (only one associated locus—*APOA5*—was identified in the previous NMR GWAS meta-analysis^4^), and Mendelian randomization yielded 20 robust associations (Fig. 4a). These included associations with triglycerides, HDL cholesterol and remnant cholesterol, probably reflecting the inclusion of well-known lipid loci (*LPL*, *APOA5*, *TRIB1*, *APOC1*, *GALNT2* and *PPP1R3B*) in the instrument. The less pleiotropic instrument for acetone included only four loci: 3-hydroxy-3-methylglutaryl-CoA synthase 2 (*HMGCS2*), 3-oxoacid CoA-transferase 1 (*OXCT1*), cytochrome P450 family 2 subfamily E member 1 (*CYP2E1*) and *SLC2A4*, all of which have direct roles in ketone body or glycaemic-related pathways. Using these 4 variants only, the positive association with hypertension (OR per s.d. higher genetically predicted acetone level = 1.41, *P* = 6.9 × 10^−7^) was robust (Fig. 4a,b) and was also replicated in FinnGen (OR 1.45, *P* = 4.5 × 10^−5^) (Fig. 4c). Consistent with these results, acetone has recently been suggested as a biomarker for hypertension^57^. It should be noted that previous studies using the NMR metabolomics platform had incorrectly labelled acetone as acetoacetate, which was detected and corrected in 2020 and later versions of the platform. The discovery regarding this potential causal relationship between acetone and hypertension is noteworthy, since the data on the role of ketogenic diets in hypertension are suggestive but inconclusive^58,59^ and ketone bodies have also emerged as potential therapeutic agents for coronary disease^60^. This finding concords with preclinical and human studies that link interventions that alter levels of ketone bodies, such as ketogenic diets and ketone salt supplementation, with changes in blood pressure^61,62^, leading to suggestions that ketone bodies could be a promising potential therapeutic strategy for hypertension and other cardiovascular diseases^60,63^. The mechanisms by which ketone bodies influence risk of hypertension are currently unclear, with both indirect (for example, obesity and diabetes) and direct (for example, sympathetic nervous system activity, vasodilation and cardiac endothelial cell proliferation) pathways being suggested^64–66^. A recent study in the UK Biobank demonstrated that some loci and pathways associated with the non-lipid NMR traits are highly pleiotropic, with the less pleiotropic variants often reflecting biology more proximal to the traits^67^. This is also in line with our findings as demonstrated by the identification of several pleiotropic triglyceride-related genes that are associated with acetone levels, as well as four less pleiotropic acetone-associated loci with direct links to pathways related to ketone biology. These results accentuate that genetic pleiotropy can be common for metabolic measures, even for some non-lipid traits, and that careful selection of variants for Mendelian randomization is crucial to avoid bias due to pervasive pleiotropy.

## Limitations

The predominance of participants of European ancestries (27 out of 33 cohorts) meant that we had limited power to detect associations in other ancestry groups. However, our ancestry-stratified comparisons suggested that the associations discovered were broadly transferable across ancestries. Future larger studies of diverse ancestries, including African ancestries, will be required to better understand genetic regulation of metabolism on a global scale. Our NMR-based study was also limited in the number of metabolic traits analysed compared with studies using mass spectrometry, a complementary method that can simultaneously measure thousands of metabolites. Although mass spectrometry is more sensitive, NMR is analytically more robust, high-throughput and low cost, thus our study includes more than sixfold more participants than the largest GWAS of mass spectrometry-based circulating metabolites^68^ enabling much deeper characterization of the genetic regulation. Furthermore, mass spectrometry cannot provide the detailed analysis of lipoprotein subclasses that is available from NMR platforms. Another limitation is that although we identified differences in genetic associations according to fasting status and sample type, the mechanisms explaining these differences remain suggestive and require further investigations. These differences suggest that caution should be used when interpreting heritability estimates across different studies, such as UK Biobank. Furthermore, we have described the detailed metabolic associations of genetic loci associated with ICP, and it should be noted that many of the ICP-associated loci are known to be associated with liver function enzymes or bilirubin, increased levels of which are included in diagnostic criteria for ICP. However, the presence of pruritus (itching) is required for ICP to be diagnosed, and the ICP cases defined through hospital discharge registries included in the GWAS should therefore represent true symptomatic cases.

## Conclusion

Through this large-scale, genome-wide meta-analysis including more than 136,000 participants, we identified more than 8,000 genetic associations of circulating metabolic biomarkers involving over 400 loci. The fivefold increase in sample size and doubling of the number of metabolic traits compared to our previous GWAS meta-analysis of NMR metabolic traits led to a marked increase in the number of significant associations (62 associated loci previously^4^), leading to a substantial improvement in understanding of genetic regulation of systemic metabolism. Key features of our meta-analysis are the inclusion of participants from 33 cohorts, which enables the discovery of many new robust associations with evidence from independent datasets. Through internal comparisons across these datasets and external comparison with UK Biobank, we have highlighted the important role that sample and participant characteristics, such as sample type and fasting status, can have in revealing or masking genetic associations, with significant consequences for biological interpretation and downstream analyses. Our extensive manual curation to identify highly probable causal genes at nearly 300 associated loci provides a useful resource to further biological understanding of the associations and allows high-confidence identification of causal genes for disease associations that colocalize. For the remaining loci, our results provide a starting point for identification of genes that have so far not been known to be involved in metabolic regulation. Our comparison of the fine-grained metabolic associations across the lipoprotein measures enables the identification of clusters of genes with similar metabolic profiles, suggesting TRIM5 as a potential therapeutic target for lowering pro-atherogenic lipid levels, and therefore cardiovascular diseases, as the metabolic profile of *TRIM5* aligns well with genes that affect LDL cholesterol intake to hepatocytes through the LDL receptor. By making the summary statistics publicly available, we provide a valuable resource for Mendelian randomization studies and have illustrated the potential pitfalls of using pleiotropic variants as genetic instrumental variables. Finally, we have illustrated the potential to use these findings to shed light on inadequately characterized diseases by examining the metabolic effects of genetic variants associated with ICP, a disease with a largely unknown genetic background.

## Methods

### NMR metabolomics

In this work, we expand our previous GWAS of 123 human metabolic traits in ~25,000 individuals^4^ to include additional cohorts and a more comprehensive panel of metabolic traits. Up to 233 serum or plasma metabolic traits were quantified in 33 cohorts (total sample size up to 136,016) using an updated quantification version of the same NMR metabolomics platform^17^ as in the previous study. The NMR metabolomics platform provides data of lipoprotein subclasses and their lipid concentrations and compositions, apoAI and apoB, cholesterol and triglyceride measures, albumin, various fatty acids and low-molecular-weight metabolites—for example, amino acids, glycolysis-related measures and ketone bodies. In this work, the metabolic traits were quantified in the following cohorts (described in detail in Supplementary Notes and Supplementary Table 1): Avon Longitudinal Study of Parents and Children (ALSPAC), China Kadoorie Biobank (CKB), Estonian Genome Center of University of Tartu Cohort (EGCUT), The Erasmus Rucphen Family study (ERF), European Genetic Database (EUGENDA), FINRISK 1997 (FR97), FINRISK 2007 (FR07, that is, DILGOM), The INTERVAL Bioresource (INTERVAL), CROATIA-Korcula Study (KORCULA), LifeLines-DEEP (LLD), Leiden Longevity Study (LLS), eight subcohorts from the London Life Sciences Prospective Population Study (LOLIPOP), The Metabolic Syndrome in Men study (METSIM), The Netherlands Epidemiology of Obesity Study (NEO), The Netherlands Study of Depression and Anxiety (NESDA), Northern Finland Birth Cohort 1966 (NFBC1966), NFBC1986, The Netherlands Twin Register (NTR), Oxford Biobank (OBB), Orkney Complex Disease Study (ORCADES), PROspective Study of Pravastatin in the Elderly at Risk (PROSPER), three subcohorts from the Rotterdam Study (RS), TwinsUK (TUK), and The Cardiovascular Risk in Young Finns Study (YFS). Most of the cohorts consisted of individuals of European ancestry (six Finnish and 21 non-Finnish), and six cohorts had individuals of Asian ancestry (one Han Chinese and five South Asian). All participants gave informed consent and all studies were approved by the ethical committees of the participating centres.

Detailed description of the NMR method is given in the Supplementary Notes.

### Genome-wide association study

A GWAS was performed for 233 metabolic traits (Supplementary Table 2) in each of 33 cohorts (Supplementary Table 1), leading to inclusion of up to 136,016 individuals with both NMR metabolic trait measurements and genome-wide SNP data available. Pregnant individuals or those using lipid-lowering medication were excluded from the study. SNPs were imputed using the Haplotype Reference Consortium release 1.1 or the 1000 Genomes Project phase 3 release, and GWAS was performed under the additive model separately in each cohort (details in Supplementary Table 3). Before analyses, the metabolic trait distributions were adjusted for age, sex, principal components and relevant study-specific covariates (see Supplementary Table 3), and inverse rank normal transformation of trait residuals was performed. The cohorts were combined in fixed-effect meta-analysis with METAL^69^, and the SNPs were filtered to those present in at least seven cohorts. The NMR metabolic traits are highly correlated and using the Bonferroni correction to account for multiple testing would result in an overconservative threshold for genome-wide significance. We therefore used the number of principal components (28) explaining >95% variation in the metabolic traits defined in the largest cohort, INTERVAL, to correct for multiple testing, and our genome-wide significance threshold was set to *P* < 1.8 × 10^−9^ (standard genome-wide significance level, *P* < 5 × 10^−8^, divided by 28). After the primary GWAS, fasting- and sample type-stratified analyses were performed for the 233 metabolic traits. In these analyses 26 of the cohorts were classified as fasted (*n* = 68,559), six cohorts were classified as non-fasted (*n* = 58,112), seventeen cohorts were classified as having serum samples (*n* = 90,223) and sixteen cohorts had plasma samples (*n* = 45,793; see Supplementary Table 1). To define associated loci across the metabolic traits, we defined a 500-kb window flanking each SNP meeting the significance threshold, pooled together these windows from all metabolic traits for each chromosome, and iteratively merged the windows. As this approach can lead to inclusion of multiple independent signals within these loci, we further defined potential independent signals that reside within the defined loci based on pairwise LD (*r*^2^ cut-off of 0.3, defined in INTERVAL and FINRISK97) of all the lead SNPs within each locus. Regional association plots were created in LocusZoom, v. 1.4. We assigned the associated lead SNPs to the most likely causal genes based on two criteria: (1) we prioritized genes with clear biological relevance to the associated metabolic traits; and (2) if no biologically plausible causal gene was detected and the lead SNP was a functional variant (missense, splice region or stop gained) or in high LD (*r*^2^ > 0.8 in INTERVAL) with such a variant, the gene with the functional variant was assigned as the most likely candidate gene. If criteria 1 and 2 were not fulfilled, the nearest gene was indicated as the candidate gene.

### Ancestry-specific analyses

We conducted ancestry-stratified analyses within our primary discovery meta-analysis for South Asian (five cohorts, 11,340 participants), East Asian (one cohort, 4,435 participants), all European (27 cohorts, 120,241 participants), Finnish (six cohorts, 27,577 participants) and non-Finnish European (21 cohorts, 92,664 participants) participants. For these ancestry-specific analyses, we used the standard threshold for genome-wide significance (*P* < 5 × 10^−8^). To also compare to participants with African ancestry, we conducted an African-specific subgroup analysis using the UK Biobank dataset (March 2021 release). Using self-reported ethnicity information (Field 21000: Ethnicity background) from the baseline questionnaire, 1,405 participants with African ancestry were identified as having Caribbean (code 4001), African (code 4002), or any other Black background (code 4003). Variant QC was performed by excluding SNPs with minor allele frequency <1%, INFO score <0.4, and variants in complex LD regions. LD thinning was performed with *r*^2^ < 0.1, a window size of 1,000 and a step size of 80. Related individuals were identified and excluded using relatedness data provided by the UK Biobank (Field 22021: Genetic kinship to other participants). Outliers of the first 6 genetic principal components computed on the unrelated samples were removed from the analysis. NMR metabolic traits were adjusted for age, sex, fasting status and 10 genetic principal components, and trait residuals were inverse rank normal-transformed. Associations between SNPs and metabolic traits were tested using PLINK 2.0.

### Replication in publicly available data

UK Biobank SNP–metabolic trait summary statistics were downloaded (https://gwas.mrcieu.ac.uk/datasets/?gwas_id__icontains=met-d) from the IEU Open GWAS Project^70^. These summary statistics were derived from the publicly available March 2021 release of the UK Biobank data in which the metabolic traits were measured with a similar NMR technology (newer version of the Nightingale Health platform) as in our study. The data were used to compare the association of our lead SNP–metabolic trait pairs within the 276 associated regions. Two thresholds were used to define an association in the UK Biobank data: the standard genome-wide significance level (*P* < 5 × 10^−8^) and the suggestive level of significance (*p* < 1 × 10^−5^).

### Heritability and variance explained

We used GCTA-GREML^71^ v. 1.94 to estimate common variant heritability for each trait using an independent dataset, specifically the UK Biobank phase 1 NMR release. This research was conducted using the UKBB Resource under application number 7439. We randomly selected 10,000 unrelated UK Biobank participants of European ancestry with available NMR data and filtered imputed variants to minor allele frequency >0.005, missingness <0.1 and Hardy–Weinberg equilibrium *P* value <10^−6^. We removed technical variation from the traits using methods described previously^72^, and adjusted the traits for age, sex, lipid-lowering medication usage and the first 10 genetic principal components of ancestry. Traits were rank inverse normal-transformed prior to GREML analysis. Variance explained by the lead SNPs for each trait was estimated as described before^73^.

### Comparing to previous associations

We performed an extensive comparison of our metabolic trait associations to previous GWASs of metabolic traits. Our comparisons were divided into three groups: (1) comparison to results of previously published large GWAS of circulating NMR traits^4,5^; (2) comparison with loci associated with clinical lipids (including those from the UK Biobank September 2019 version 3 release)^21,25–27,74^; and (3) comparison with an extensive list of associations from previous metabolite and metabolomic studies^11,13,53,75–87^. The comparisons were performed by indicating: (1) co-located known variants; (2) any known associations within a 500-kb flank of a lead SNP; or (3) known associations in LD (*r*^2^ > 0.3, defined in INTERVAL) with a lead SNP. Since we used the UK Biobank for replication, we did not compare the associations to those from studies that used UK Biobank NMR metabolomics as a single cohort without validation cohorts^67,88^.

In addition to comparing to previous metabolic trait associations, we screened previous disease and trait associations (*P* value cut-off 5 × 10^−8^) of the lead SNPs using PhenoScanner, v2^44,45^, and NHGRI-EBI GWAS Catalog^46^ (associations downloaded on 30 March 2023 using the gwasrapidd R package, v. 0.99.14^89^). In addition, we screened the FinnGen^43^ data freeze 7 summary statistics of 3,095 disease endpoints for overlapping associations (*P* value cut-off 5 × 10^−8^). Associations with gene expression and protein levels were screened using PhenoScanner, v2^44,45^.

### Metabolic effects of lipoprotein loci

To compare the metabolic effects of lipoprotein, lipid and apolipoprotein-associated variants, the effect estimates were visualized as colour-coded heat maps. To allow comparison of SNP effects, the estimates were scaled relative to the highest absolute value of the estimate for each SNP. In this analysis, we included lead SNPs at the 276 initially defined regions that were associated with any of the lipoprotein lipids or apolipoproteins at genome-wide significance and nominally associated (*P* < 0.05) with apoB. We used these criteria to restrict the analysis to SNPs associated with apoB, because apoB is known to be a causal part of lipoprotein metabolism for cardiovascular disease^30–32^. To exclude signals with similar effects across the metabolic traits due to the same causal gene, we included only a single SNP from the initially defined genomic regions that had multiple independent signals if the patterns of metabolic traits associations were similar (*R* > 0.5). In the heat maps each line represents a single SNP, each column corresponds to a single metabolic measure, and the scaled effect estimates for the SNP-metabolite associations are visualized with a colour range. Directions of effects are shown in relation to the allele associated with increased apoB. To group SNPs with similar effects together, dendrograms were constructed based on hierarchical clustering of the scaled SNP effects. Heat maps were constructed using the heatmap.2 function of the gplots v. 3.0.3 R package. Pearson correlations were assessed in R, v. 4.0.0.

### Intrahepatic cholestasis of pregnancy

We assessed overlap of our metabolic trait associations with ICP using summary statistics from the FinnGen study^43^ data freeze 7 (O15_ICP; 1,460 cases, 172,286 controls). ICP cases were defined through hospital discharge registry, ICD10 code O26.6 and ICD9 codes 6467A and 6467X. Using the nearest genes at each associated locus, we performed gene ontology (GO) enrichment analysis to search for enriched biological process and molecular function GO terms^90,91^. We assessed colocalizations of association signals using the hypothesis prioritization for multi-trait colocalization (HyPrColoc) R library, v. 1.0, in which an efficient deterministic Bayesian algorithm is used to detect colocalization across vast numbers of traits simultaneously^92^. We searched for colocalization at single causal variants and shared regional associations. To visualize SNP effects across lipid and lipoprotein traits, heat maps were constructed using the heatmap.2 function of the gplots v. 3.0.3 R package. The following SNPs were included in the heat maps: *GCKR*-rs1260326, *ABCB11*-rs10184673, *ABCB1*-rs17209837, *CYP7A1*-rs9297994, *SERPINA1*-rs28929474 and *HNF4A*-rs1800961. Effects of the metabolic trait-associated SNPs were scaled relative to an odds ratio of 1.5 for ICP.

### Mendelian randomization

Two-sample Mendelian randomization was performed using 20 NMR non-lipid metabolic traits (including amino acids (alanine, glutamine, glycine, histidine, isoleucine, leucine, valine, phenylalanine and tyrosine), ketone bodies (acetate, acetone and 3-hydroxybutyrate), and glycolysis/gluconeogenesis (glucose, lactate, pyruvate, glycerol and citrate), fluid balance (albumin and creatinine) or inflammation-related (glycoprotein acetylation) metabolic traits) as exposures and 460 Phecodes and 52 quantitative traits from the UK Biobank^21^ as outcomes. We defined two sets of instruments for the analyses that are referred to as full and strict instruments. As initial instruments we used the 334 lead variants (a single instrument SNP per each defined associated locus) associated with these traits (‘full instruments’). To avoid potential bias due to pleiotropy, we also selected a subset of 193 variants (‘strict instruments’) that had fewer than 5 associations across all 233 metabolic traits. Our threshold of 5 associations was based on empirical assessment of the distribution of per-variant trait associations. To investigate the sensitivity of the Mendelian randomization analyses to the choice of threshold, we also tested using fewer than 3 associations and fewer than 7 associations. We defined disease outcomes in UK Biobank using a curated list of major Phecodes available in the PheWAS R package^93,94^. To restrict our analysis to major disease outcomes, we discarded any sub-categories (that is, Phecodes with 4 or more characters) and removed outcomes with fewer than 100 events across up to 367,542 unrelated UK Biobank participants with European ancestry. The resulting 460 diseases were grouped into 15 broad domains: circulatory system, dermatologic, digestive, endocrine/metabolic, genitourinary, haematopoietic, infectious diseases, mental disorders, musculoskeletal, neoplasms, neurological, pregnancy complications, respiratory, sense organs and symptoms. We also analysed 52 quantitative traits available in UK Biobank, including blood pressure, lung function measures, blood cell traits and clinical chemistry biomarkers. In our replication analysis (acetone as the exposure and hypertension as the outcome), we used essential hypertension from the FinnGen study^43^ data freeze 7 as the outcome (hypertension essential, I9_HYPTENSESS; 70,651 cases, 223,663 controls). Cases were defined through hospital discharge registry, ICD10 code I10, ICD9 codes 4019X and 4039A, ICD8 codes 40199, 40299, 40399, 40499, 40209, 40100, 40291, 40191 and 40290.

We performed univariable Mendelian randomization using the inverse variance-weighted method for each instrument^95^. We also performed sensitivity analyses using Mendelian randomization–Egger regression to account for unmeasured pleiotropy^96^ and weighted median regression to assess robustness to invalid genetic instruments^97^. Our primary analyses were based on fixed-effect models, but as sensitivity analyses we used random-effect models to account for between-variant heterogeneity, which we quantified using the I-squared statistic. The Mendelian randomization analyses were performed using the MendelianRandomization package v. 0.5.1^98^ or the TwoSampleMR package v. 0.5.3^99^. Single-SNP Mendelian randomization estimates were based on the Wald ratio. We considered the fixed-effects inverse variance-weighted method as the main Mendelian randomization model but report the results of all models in Supplementary Table 15. To account for multiple testing, associations with *P* < 4.88 × 10^−6^ were considered significant (Bonferroni correction to account for testing of 20 metabolic traits with 512 outcomes).

### FinnGen study

In the present study, we used GWAS summary statistics of 3,095 disease endpoints from FinnGen data freeze 7. Full description of the FinnGen study^43^ and data analysis steps is provided in the Supplementary Notes. FinnGen contributors are listed in Supplementary Table 18.

### Statistics and reproducibility

The meta-analyses were conducted independently by two investigators in two different centres (University of Oulu, Finland and University of Cambridge, UK), and the summary statistics were compared to verify consistency of results.

### Reporting summary

Further information on research design is available in the Nature Portfolio Reporting Summary linked to this article.

## Online content

Any methods, additional references, Nature Portfolio reporting summaries, source data, extended data, supplementary information, acknowledgements, peer review information; details of author contributions and competing interests; and statements of data and code availability are available at 10.1038/s41586-024-07148-y.

### Supplementary information

Supplementary Notes This file contains study descriptions, acknowledgements and funding information, and details of NMR metabolomics.

Reporting Summary

Supplementary Data 1 Supplementary Fig. 1. Manhattan plots showing the NMR GWAS meta-analysis results of 233 metabolic traits.

Supplementary Data 2 Supplementary Fig. 2. Regional associations plots for the most significantly associated metabolic traits in each genomic region.

Supplementary Data 3 Supplementary Fig. 3. Forest plots showing the associations of the lead SNPs in each cohort.

Supplementary Tables This file contains Supplementary Tables 1–18.

### Source data

Source Data Figs. 2 and 4

## Data Availability

Full GWAS summary statistics are publicly available through the NHGRI-EBI GWAS catalogue (GCST90301941–GCST90302173) and https://www.phpc.cam.ac.uk/ceu/lipids-and-metabolites/. Individual-level raw metabolic data from the INTERVAL study can be requested as instructed in https://www.phpc.cam.ac.uk/ceu/lipids-and-metabolites/. For access to individual-level genotype and phenotype data for the other studies included in this meta-analysis, please see Supplementary Table 1 for details of websites or references of the individual studies. The NMR metabolomics platform, including the proprietary analysis software, is protected by the intellectual property rights of Nightingale Health, therefore the NMR spectra are not in the possession of the authors and cannot be made publicly available. Source data are provided with this paper.

## References

[1] Suhre K (2011). Human metabolic individuality in biomedical and pharmaceutical research. Nature.

[2] Kettunen J (2012). Genome-wide association study identifies multiple loci influencing human serum metabolite levels. Nat. Genet..

[3] Shin SY (2014). An atlas of genetic influences on human blood metabolites. Nat. Genet..

[4] Kettunen J (2016). Genome-wide study for circulating metabolites identifies 62 loci and reveals novel systemic effects of LPA. Nat. Commun..

[5] Gallois A (2019). A comprehensive study of metabolite genetics reveals strong pleiotropy and heterogeneity across time and context. Nat. Commun..

[6] Lotta LA (2021). A cross-platform approach identifies genetic regulators of human metabolism and health. Nat. Genet..

[7] Yin X (2022). Genome-wide association studies of metabolites in Finnish men identify disease-relevant loci. Nat. Commun..

[8] Chambers JC (2011). Genome-wide association study identifies loci influencing concentrations of liver enzymes in plasma. Nat. Genet..

[9] Tukiainen T (2012). Detailed metabolic and genetic characterization reveals new associations for 30 known lipid loci. Hum. Mol. Genet..

[10] Visscher PM (2017). 10 years of GWAS discovery: biology, function, and translation. Am. J. Hum. Gen..

[11] Locke AE (2019). Exome sequencing of Finnish isolates enhances rare-variant association power. Nature.

[12] Illig T (2010). A genome-wide perspective of genetic variation in human metabolism. Nat. Genet..

[13] Draisma HHM (2015). Genome-wide association study identifies novel genetic variants contributing to variation in blood metabolite levels. Nat. Commun..

[14] Long T (2017). Whole-genome sequencing identifies common-to-rare variants associated with human blood metabolites. Nat. Genet..

[15] Tabassum R (2019). Genetic architecture of human plasma lipidome and its link to cardiovascular disease. Nat. Commun..

[16] Hagenbeek FA (2020). Heritability estimates for 361 blood metabolites across 40 genome-wide association studies. Nat. Commun..

[17] Wurtz P (2017). Quantitative serum nuclear magnetic resonance metabolomics in large-scale epidemiology: a primer on -omic technologies. Am. J. Epidemiol..

[18] Inouye M (2012). Novel loci for metabolic networks and multi-tissue expression studies reveal genes for atherosclerosis. PLoS Genet..

[19] Teslovich TM (2018). Identification of seven novel loci associated with amino acid levels using single-variant and gene-based tests in 8545 Finnish men from the METSIM study. Hum. Mol. Genet..

[20] Würtz P (2013). Lipoprotein subclass profiling reveals pleiotropy in the genetic variants of lipid risk factors for coronary heart disease: A note on mendelian randomization studies. J. Am. Coll. Cardiol..

[21] Sudlow C (2015). UK Biobank: an open access resource for identifying the causes of a wide range of complex diseases of middle and old age. PLoS Med..

[22] Lyssenko V (2009). Common variant in *MTNR1B* associated with increased risk of type 2 diabetes and impaired early insulin secretion. Nat. Genet..

[23] Li-Gao R (2021). Genetic studies of metabolomics change after a liquid meal illuminate novel pathways for glucose and lipid metabolism. Diabetes.

[24] Barrett JC (2009). Genome-wide association study and meta-analysis find that over 40 loci affect risk of type 1 diabetes. Nat. Genet..

[25] Willer CJ (2013). Discovery and refinement of loci associated with lipid levels. Nat. Genet..

[26] Klarin D (2018). Genetics of blood lipids among ~300,000 multi-ethnic participants of the Million Veteran Program. Nat. Genet..

[27] Graham SE (2021). The power of genetic diversity in genome-wide association studies of lipids. Nature.

[28] Dickerson MT, Vierra NC, Milian SC, Dadi PK, Jacobson DA (2017). Osteopontin activates the diabetes-associated potassium channel TALK-1 in pancreatic β- cells. PLoS ONE.

[29] Graff SM (2021). A *KCNK16* mutation causing TALK-1 gain of function is associated with maturity-onset diabetes of the young. JCI Insight.

[30] Ference BA (2019). Association of triglyceride-lowering LPL variants and LDL-C-lowering LDLR variants with risk of coronary heart disease. JAMA.

[31] Sniderman AD (2019). Apolipoprotein B particles and cardiovascular disease: a narrative review. JAMA Cardiol..

[32] Ala-Korpela M (2019). The culprit is the carrier, not the loads: cholesterol, triglycerides and apolipoprotein B in atherosclerosis and coronary heart disease. Int. J. Epidemiol..

[33] Ahola-Olli AV (2019). Circulating metabolites and the risk of type 2 diabetes: a prospective study of 11,896 young adults from four Finnish cohorts. Diabetologia.

[34] Würtz P (2014). Metabolic signatures of adiposity in young adults: Mendelian randomization analysis and effects of weight change. PLoS Med..

[35] Rahm N, Telenti A (2012). The role of tripartite motif family members in mediating susceptibility to HIV-1 infection. Curr. Opin. HIV AIDS.

[36] Pazoki R (2021). Genetic analysis in European ancestry individuals identifies 517 loci associated with liver enzymes. Nat. Commun..

[37] van der Harst P, Verweij N (2018). Identification of 64 novel genetic loci provides an expanded view on the genetic architecture of coronary artery disease. Circ. Res..

[38] Wurtz P (2016). Metabolomic profiling of statin use and genetic inhibition of HMG-CoA reductase. J. Am. Coll. Cardiol..

[39] Sliz E (2018). Metabolomic consequences of genetic inhibition of PCSK9 compared with statin treatment. Circulation.

[40] Holmes MV, Ala-Korpela M (2019). What is ‘LDL cholesterol’?. Nat. Rev. Cardiol..

[41] Hughes MF (2018). Exploring coronary artery disease GWAs targets with functional links to immunometabolism. Front. Cardiovasc. Med..

[42] Zhang Y (2023). RNA helicase DEAD-box protein 5 alleviates nonalcoholic steatohepatitis progression via tethering TSC complex and suppressing mTORC1 signaling. Hepatology.

[43] Kurki MI (2023). FinnGen provides genetic insights from a well-phenotyped isolated population. Nature.

[44] Staley JR (2016). PhenoScanner: a database of human genotype-phenotype associations. Bioinformatics.

[45] Kamat MA (2019). PhenoScanner V2: an expanded tool for searching human genotype–phenotype associations. Bioinformatics.

[46] Sollis E (2023). The NHGRI-EBI GWAS catalog: knowledgebase and deposition resource. Nucleic Acids Res..

[47] Pusl T, Beuers U (2007). Intrahepatic cholestasis of pregnancy. Orphanet J. Rare Dis..

[48] Dixon PH (2022). GWAS meta-analysis of intrahepatic cholestasis of pregnancy implicates multiple hepatic genes and regulatory elements. Nat. Commun..

[49] Strautnieks SS (2008). Severe bile salt export pump deficiency: 82 different *ABCB11* mutations in 109 families. Gastroenterology.

[50] Turro E (2020). Whole-genome sequencing of patients with rare diseases in a national health system. Nature.

[51] Sliz E (2023). Evidence of a causal effect of genetic tendency to gain muscle mass on uterine leiomyomata. Nat. Commun..

[52] Julkunen H (2023). Atlas of plasma NMR biomarkers for health and disease in 118,461 individuals from the UK Biobank. Nat. Commun..

[53] Wittemans LBL (2019). Assessing the causal association of glycine with risk of cardio-metabolic diseases. Nat. Commun..

[54] Ding Y (2016). Plasma glycine and risk of acute myocardial infarction in patients with suspected stable angina pectoris. J. Am. Heart Assoc..

[55] Gladding PA (2022). Metabolomics and a breath sensor identify acetone as a biomarker for heart failure. Biomolecules.

[56] Mahendran Y (2013). Association of ketone body levels with hyperglycemia and type 2 diabetes in 9,398 Finnish men. Diabetes.

[57] Palmu J (2022). Comprehensive biomarker profiling of hypertension in 36985 Finnish individuals. J. Hypertens..

[58] Barrea L (2023). Very low-calorie ketogenic diet (VLCKD): an antihypertensive nutritional approach. J. Transl. Med..

[59] di Raimondo D (2021). Ketogenic diet, physical activity, and hypertension–a narrative review. Nutrients.

[60] Yurista SR (2021). Therapeutic potential of ketone bodies for patients with cardiovascular disease: JACC state-of-the-art review. J. Am. Coll. Card..

[61] Holland AM, Qazi AS, Beasley KN, Bennett HR (2019). Blood and cardiovascular health parameters after supplementing with ketone salts for six weeks. J. Insul. Resist..

[62] Myette-Côté É, Caldwell HG, Ainslie PN, Clarke K, Little JP (2019). A ketone monoester drink reduces the glycemic response to an oral glucose challenge in individuals with obesity: a randomized trial. Am. J. Clin. Nutr..

[63] Costa TJ (2022). The janus face of ketone bodies in hypertension. J. Hypertens..

[64] Kimura I (2011). Short-chain fatty acids and ketones directly regulate sympathetic nervous system via G protein-coupled receptor 41 (GPR41). Proc. Natl Acad. Sci. USA.

[65] McCarthy CG (2021). Ketone body β-hydroxybutyrate is an autophagy-dependent vasodilator. JCI Insight.

[66] Weis E (2022). Ketone body oxidation increases cardiac endothelial cell proliferation. EMBO Mol. Med..

[67] Smith CJ (2022). Integrative analysis of metabolite GWAS illuminates the molecular basis of pleiotropy and genetic correlation. eLife.

[68] Surendran P (2022). Rare and common genetic determinants of metabolic individuality and their effects on human health. Nat. Med..

[69] Willer CJ, Li Y, Abecasis GR (2010). METAL: fast and efficient meta-analysis of genomewide association scans. Bioinformatics.

[70] Elsworth, B. et al. The MRC IEU OpenGWAS data infrastructure. Preprint at *bioRxiv*10.1101/2020.08.10.244293 (2020).

[71] Yang J (2010). Common SNPs explain a large proportion of the heritability for human height. Nat. Genet..

[72] Ritchie SC (2023). Quality control and removal of technical variation of NMR metabolic biomarker 1 data in ~120,000 UK Biobank participants. Sci. Data.

[73] Ahola-Olli AV (2017). Genome-wide association study identifies 27 loci influencing concentrations of circulating cytokines and growth factors. Am. J. Hum. Genet..

[74] Hindy G (2022). Rare coding variants in 35 genes associate with circulating lipid levels—a multi-ancestry analysis of 170,000 exomes. Am. J. Hum. Genet..

[75] Chen, J. et al. The trans-ancestral genomic architecture of glycemic traits. *Nat. Genet.* **53**, 840–860 (2021). 10.1038/s41588-021-00852-9 PMC7610958 34059833

[76] Davis JP (2017). Common, low-frequency, and rare genetic variants associated with lipoprotein subclasses and triglyceride measures in Finnish men from the METSIM study. PLoS Genet..

[77] de Oliveira Otto MC (2018). Genome-wide association meta-analysis of circulating odd-numbered chain saturated fatty acids: results from the CHARGE Consortium. PLoS ONE.

[78] Demirkan A (2012). Genome-wide association study identifies novel loci associated with circulating phospho- and sphingolipid concentrations. PLoS Genet..

[79] Franceschini N (2012). Discovery and fine mapping of serum protein loci through transethnic meta-analysis. Am. J. Hum. Genet..

[80] Guan W (2014). Genome-wide association study of plasma n6 polyunsaturated fatty acids within the cohorts for heart and aging research in genomic epidemiology consortium. Circ. Cardiovasc. Genet..

[81] Kanai M (2018). Genetic analysis of quantitative traits in the Japanese population links cell types to complex human diseases. Nat. Genet..

[82] Lemaitre RN (2015). Genetic loci associated with circulating levels of very long-chain saturated fatty acids. J. Lipid Res..

[83] Lemaitre RN (2011). Genetic loci associated with plasma phospholipid N-3 fatty acids: a meta-analysis of genome-wide association studies from the CHARGE Consortium. PLoS Genet..

[84] Sinnott-Armstrong N (2021). Genetics of 35 blood and urine biomarkers in the UK Biobank. Nat. Genet..

[85] Tin A (2016). *GCKR* and *PPP1R3B* identified as genome-wide significant loci for plasma lactate: the Atherosclerosis Risk in Communities (ARIC) study. Diabet. Med..

[86] Wu JHY (2013). Genome-wide association study identifies novel loci associated with concentrations of four plasma phospholipid fatty acids in the de novo lipogenesis pathway: results from the Cohorts for Heart and Aging Research in Genomic Epidemiology (CHARGE) consortium. Circ. Cardiovasc. Genet..

[87] Wuttke M (2019). A catalog of genetic loci associated with kidney function from analyses of a million individuals. Nat. Genet..

[88] Richardson TG (2022). Characterising metabolomic signatures of lipid-modifying therapies through drug target mendelian randomisation. PLoS Biol..

[89] Magno R, Maia AT (2020). Gwasrapidd: an R package to query, download and wrangle GWAS catalog data. Bioinformatics.

[90] Carbon S (2021). The Gene Ontology resource: enriching a GOld mine. Nucleic Acids Res..

[91] Mi H, Muruganujan A, Casagrande JT, Thomas PD (2013). Large-scale gene function analysis with the panther classification system. Nat. Protoc..

[92] Foley CN (2021). A fast and efficient colocalization algorithm for identifying shared genetic risk factors across multiple traits. Nat. Commun..

[93] Carroll RJ, Bastarache L, Denny JC (2014). R PheWAS: data analysis and plotting tools for phenome-wide association studies in the R environment. Bioinformatics.

[94] Denny JC (2013). Systematic comparison of phenome-wide association study of electronic medical record data and genome-wide association study data. Nat. Biotechnol..

[95] Burgess S, Butterworth A, Thompson SG (2013). Mendelian randomization analysis with multiple genetic variants using summarized data. Genet. Epidemiol..

[96] Bowden J, Davey Smith G, Burgess S (2015). Mendelian randomization with invalid instruments: effect estimation and bias detection through Egger regression. Int. J. Epidemiol..

[97] Bowden J, Davey Smith G, Haycock PC, Burgess S (2016). Consistent estimation in Mendelian randomization with some invalid instruments using a weighted median estimator. Genet. Epidemiol..

[98] Yavorska OO, Burgess S (2017). MendelianRandomization: an R package for performing Mendelian randomization analyses using summarized data. Int. J. Epidemiol..

[99] Hemani G (2018). The MR-Base platform supports systematic causal inference across the human phenome. eLife.

