# Supplementary material for: Genome-wide characterization of circulating metabolic biomarkers
Source: Nature. 2024 Mar 6;628(8006):130–8. doi: 10.1038/s41586-024-07148-y (PMC10990933; doi:10.1038/s41586-024-07148-y)
Supplement: Supplementary file 2 — Reporting Summary [file 41586_2024_7148_MOESM2_ESM.pdf]

nature portfolio

Corresponding author(s): Minna K. Karjalainen

Last updated by author(s): Feb 14, 2024

Reporting Summary

Nature Portfolio wishes to improve the reproducibility of the work that we publish. This form provides structure for consistency and transparency in reporting. For further information on Nature Portfolio policies, see our Editorial Policies and the Editorial Policy Checklist.

Statistics

For all statistical analyses, confirm that the following items are present in the figure legend, table legend, main text, or Methods section.

n/a	Confirmed	
☐	☒	The exact sample size (*n*) for each experimental group/condition, given as a discrete number and unit of measurement
☒	☐	A statement on whether measurements were taken from distinct samples or whether the same sample was measured repeatedly
☐	☒	The statistical test(s) used AND whether they are one- or two-sided<br>*Only common tests should be described solely by name; describe more complex techniques in the Methods section.*
☐	☒	A description of all covariates tested
☐	☒	A description of any assumptions or corrections, such as tests of normality and adjustment for multiple comparisons
☐	☒	A full description of the statistical parameters including central tendency (e.g. means) or other basic estimates (e.g. regression coefficient) AND variation (e.g. standard deviation) or associated estimates of uncertainty (e.g. confidence intervals)
☐	☒	For null hypothesis testing, the test statistic (e.g. *F*, *t*, *r*) with confidence intervals, effect sizes, degrees of freedom and *P* value noted<br>*Give P values as exact values whenever suitable.*
☒	☐	For Bayesian analysis, information on the choice of priors and Markov chain Monte Carlo settings
☒	☐	For hierarchical and complex designs, identification of the appropriate level for tests and full reporting of outcomes
☐	☒	Estimates of effect sizes (e.g. Cohen's *d*, Pearson's *r*), indicating how they were calculated

Our web collection on statistics for biologists contains articles on many of the points above.

Software and code

Policy information about availability of computer code

Data collection	The nuclear magnetic resonance spectroscopy (NMR) data were acquired using commercial software by Nightingale Health Ltd., quantification version 2016.
Data analysis	BOLT-LMM v. 2.3.2; GCTA v. 1.91.1 and 1.94; GEMMA 0.97; Plink v. 2.0; RegScan v. 0.5; Rvtest v. 2.0.6; SNPTEST v. 2.5.1, 2.5.2 and 2.5.4; METAL v. 2011; LocusZoom v. 1.4; R v. 3.4.3 and 4.0.0; R packages gplots 3.0.3, HyPrColoc 1.0, MendelianRandomization 0.5.199 and TwoSampleMR 0.5.3.

For manuscripts utilizing custom algorithms or software that are central to the research but not yet described in published literature, software must be made available to editors and reviewers. We strongly encourage code deposition in a community repository (e.g. GitHub). See the Nature Portfolio guidelines for submitting code & software for further information.

Data

Policy information about availability of data

All manuscripts must include a data availability statement. This statement should provide the following information, where applicable:

- Accession codes, unique identifiers, or web links for publicly available datasets
- A description of any restrictions on data availability
- For clinical datasets or third party data, please ensure that the statement adheres to our policy

Full GWAS summary statistics are publicly available through the NHGRI-EBI GWAS Catalog (GCST90301941-GCST90302173) and https://www.phpc.cam.ac.uk/ceu/

lipids-and-metabolites/. Individual-level raw metabolic data from the INTERVAL study can be requested as instructed in https://www.phpc.cam.ac.uk/ceu/lipids-and-metabolites/. For the access to individual-level genotype and phenotype data for the other studies included in this meta-analysis, please see Supplementary Table 1 for details of websites or references of the individual studies. The NMR metabolomics platform, including the proprietary analysis software, is protected by the intellectual property rights of the Nightingale Health Plc, Therefore the NMR spectra are not in the possession of the authors of this paper and cannot be made publicly available.

Human research participants

Policy information about studies involving human research participants and Sex and Gender in Research.

Reporting on sex and gender	We utilized data from studies that comprised both men and women. We did not perform any sex-specific analyses. Biological sex was used as a covariate in the analyses as indicated in the manuscript.
Population characteristics	In the principal analyses, we utilized data from 33 cohorts/subcohorts. In addition, we used data from two biobanks/biobank-related projects. Details of the participants are summarized in the Supplementary Tables and Supplementary Notes. We used age, sex, population stratification and other relevant study-specific factors as covariates as indicated in the manuscript and Supplementary Tables.
Recruitment	The studies are predominantly population-based cohorts recruited from a variety of settings, including primary care registries, household surveys and blood donors.
Ethics oversight	The studies were approved by appropriate local ethics committees; the committees are indicated in the Supplementary Notes. The following committees approved the studies: Avon Longitudinal Study of Parents and Children: ALSPAC Law and Ethics committee; China Kadoorie Biobank: Oxford Tropical Research Ethics Committee, the Ethical Review Committees of the Chinese Centre for Disease Control and Prevention, Chinese Academy of Medical Sciences, and the Institutional Review Board (IRB) at Peking University; Estonian Genome Center, Institute of Genomics, University of Tartu: Research Ethics Committee of the University of Tartu; Erasmus Rucphen Family study: Medical ethics committee of the Erasmus Medical Center, Rotterdam, the Netherlands; European Genetic Database: Institutional review board in Radboud UMC (Commissie Mensgebonden Onderzoek Radboudum), ethics committees in Cologne and Nijmegen; FINRISK: The Coordinating Ethics Committee of the Helsinki and Uusimaa Hospital District; The INTERVAL Bioresource: the National Research Ethics Service Committee East of England - Cambridge East; CROATIA-Korcula: Ethics committees of the Medical School of the University of Zagreb, the Medical School of the University of Split and the National Health Service, Lothian, Scotland; LifeLines-DEEP: The University Medical Center Groningen review board; Leiden Longevity Study: Ethical committee of the Leiden University Medical Center; London Life Sciences Prospective Population Study: National Research Ethics Service; The Metabolic Syndrome in Men study: Ethics Committee of the University of Eastern Finland and Kuopio University Hospital in Kuopio, Finland; The Netherlands Study of Depression and Anxiety: Ethical review boards of the participating research centers in Amsterdam, Leiden, and Groningen; Northern Finland Birth Cohort: Northern Ostrobothnia Hospital District Ethical Committee; The Netherlands Twin Register: Central Ethics Committee on Research Involving Human Subjects of the VU University Medical Center, Amsterdam; Oxford Biobank: South Central - Oxford C Research Ethics Committee; Orkney Complex Disease Study: Research Ethics Committees in Orkney, Aberdeen (North of Scotland REC), and South East Scotland REC, NHS Lothian; PROspective Study of Pravastatin in the Elderly at Risk: The institutional ethics review boards of centers of Cork University (Ireland), Glasgow University, Scotland, and Leiden University Medical Center, the Netherlands; Rotterdam Study: Medical Ethics Committee of the Erasmus MC and the Dutch Ministry of Health, Welfare and SportTwinsUK: St. Thomas' Hospital Research Ethics Committee; The Cardiovascular Risk in Young Finns Study: Ethics Committee of the wellbeing services county of Southwest Finland.

Note that full information on the approval of the study protocol must also be provided in the manuscript.

Field-specific reporting

Please select the one below that is the best fit for your research. If you are not sure, read the appropriate sections before making your selection.

☒ Life sciences ☐ Behavioural & social sciences ☐ Ecological, evolutionary & environmental sciences

For a reference copy of the document with all sections, see nature.com/documents/nr-reporting-summary-flat.pdf

Life sciences study design

All studies must disclose on these points even when the disclosure is negative.

Sample size	All participants with available genotype and metabolite data were included. Total sample size was up to 136,016.
Data exclusions	The quality control steps including participant/data exclusions are described in the manuscript. Pregnant individuals, statin users and data outliers were excluded in relevant cohorts. Genetic variants that did not meet quality control thresholds were excluded.
Replication	This is a meta-analysis, and as such includes internal replication. Replication of the findings was further investigated in the UK Biobank for those genetic variants and metabolic traits that were available in the UK Biobank data (8,502 of 8,795 lead SNP - metabolic trait pairs).

Randomization	This is a genome-wide association study and thus the randomization was due to genetic variants.
Blinding	This was not a clinical trial so there was no requirement for blinding. Due to the sheer size of the genetic data, the investigators were blinded to the genotype group allocation.

Reporting for specific materials, systems and methods

We require information from authors about some types of materials, experimental systems and methods used in many studies. Here, indicate whether each material, system or method listed is relevant to your study. If you are not sure if a list item applies to your research, read the appropriate section before selecting a response.

Materials & experimental systems

n/a	Involved in the study
☒	☐ Antibodies
☒	☐ Eukaryotic cell lines
☒	☐ Palaeontology and archaeology
☒	☐ Animals and other organisms
☒	☐ Clinical data
☒	☐ Dual use research of concern

Methods

n/a	Involved in the study
☒	☐ ChIP-seq
☒	☐ Flow cytometry
☒	☐ MRI-based neuroimaging
