# Supplementary material for: Genome-wide characterization of circulating metabolic biomarkers
Source: Nature. 2024 Mar 6;628(8006):130–8. doi: 10.1038/s41586-024-07148-y (PMC10990933; doi:10.1038/s41586-024-07148-y)
Supplement: Supplementary file 3 — Supplementary Fig. 1. Manhattan plots showing the NMR GWAS meta-analysis results of 233 metabolic traits. [file 41586_2024_7148_MOESM3_ESM.pdf]

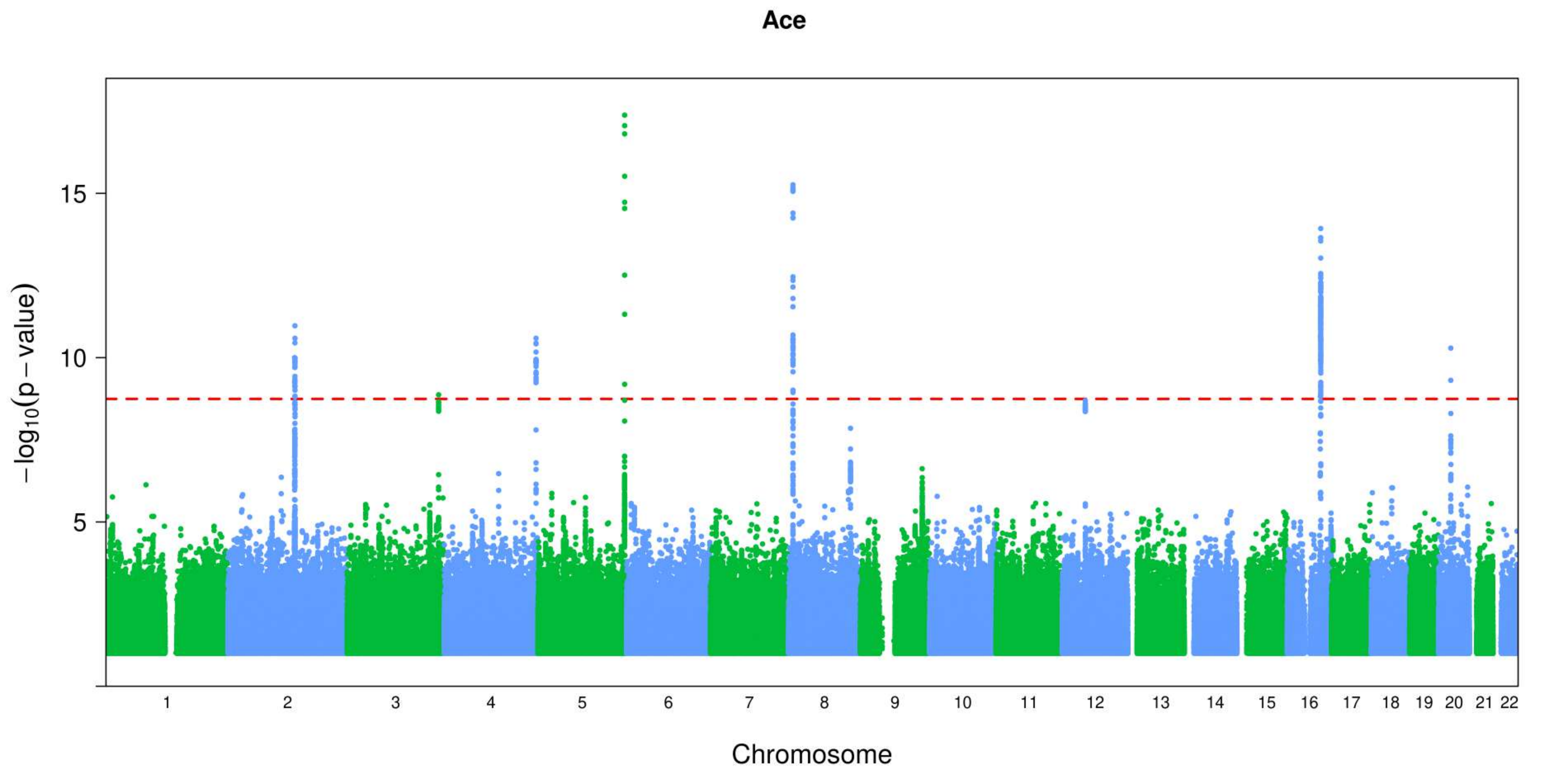

Ace
−log10(p−value)
15
10
5
1
2
3
4
5
6
7
8
9
10
11
12
13
14
15
16
17
18
19
20
21
22
Chromosome

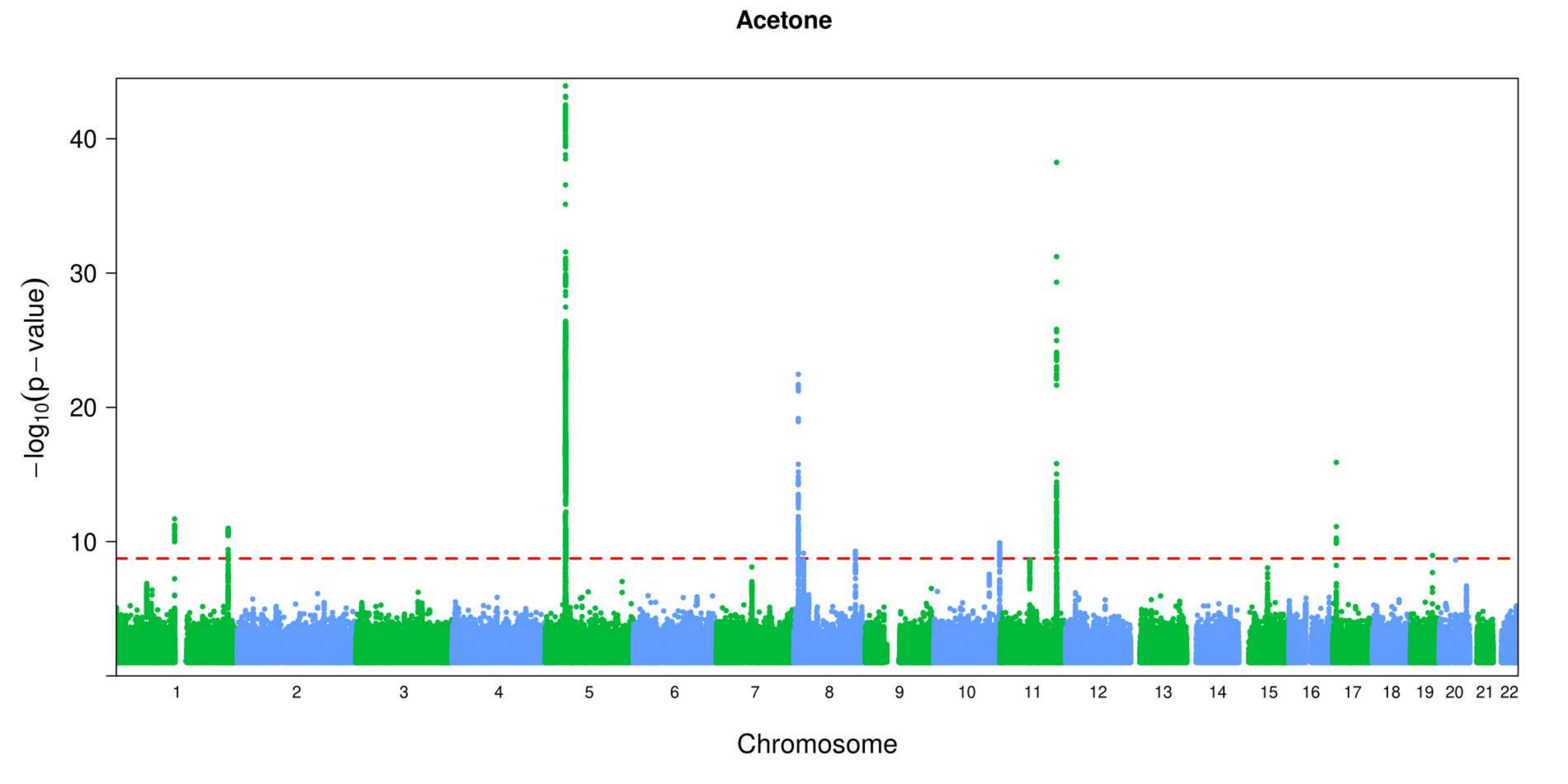

Acetone
40
30
20
10
−log10(p − value)
1
2
3
4
5
6
7
8
9
10
11
12
13
14
15
16
17
18
19
20
21
22
Chromosome

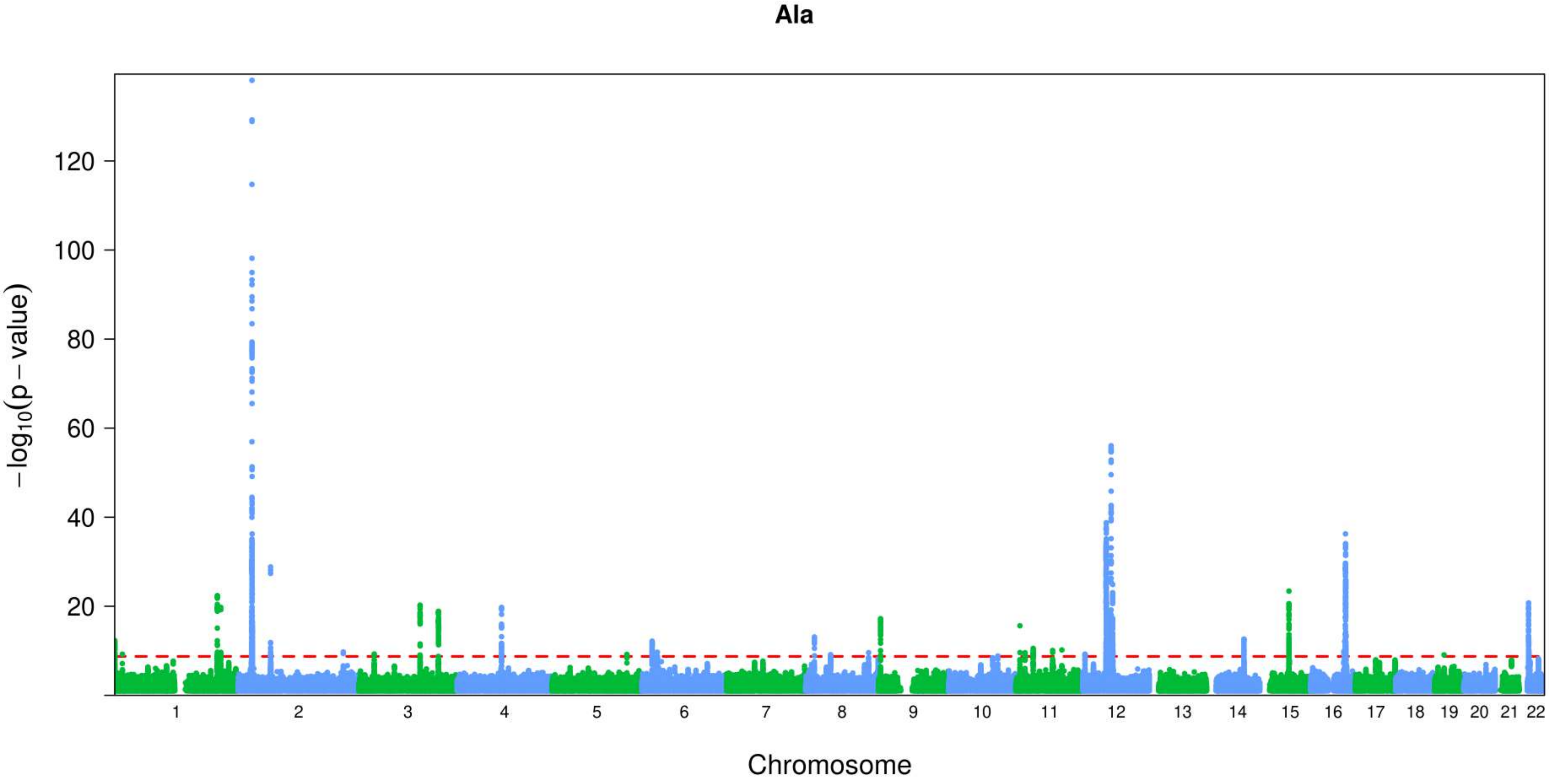
Ala
120
100
80
60
40
20
$-\log_{10}(p-\text{value})$
1
2
3
4
5
6
7
8
9
10
11
12
13
14
15
16
17
18
19
20
21
22
Chromosome

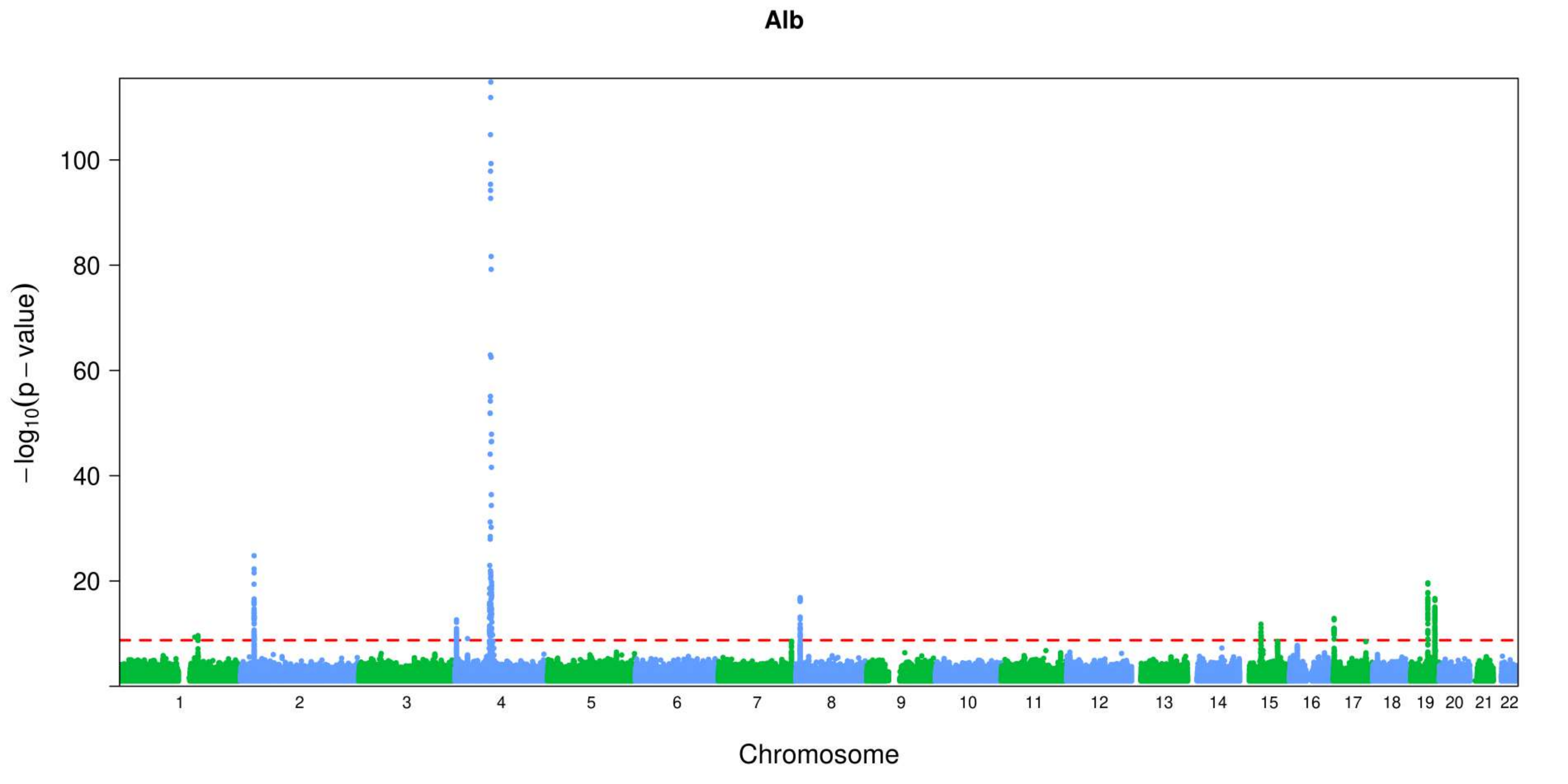

Alb
100
80
60
40
20
−log10(p − value)
1
2
3
4
5
6
7
8
9
10
11
12
13
14
15
16
17
18
19
20
21
22
Chromosome

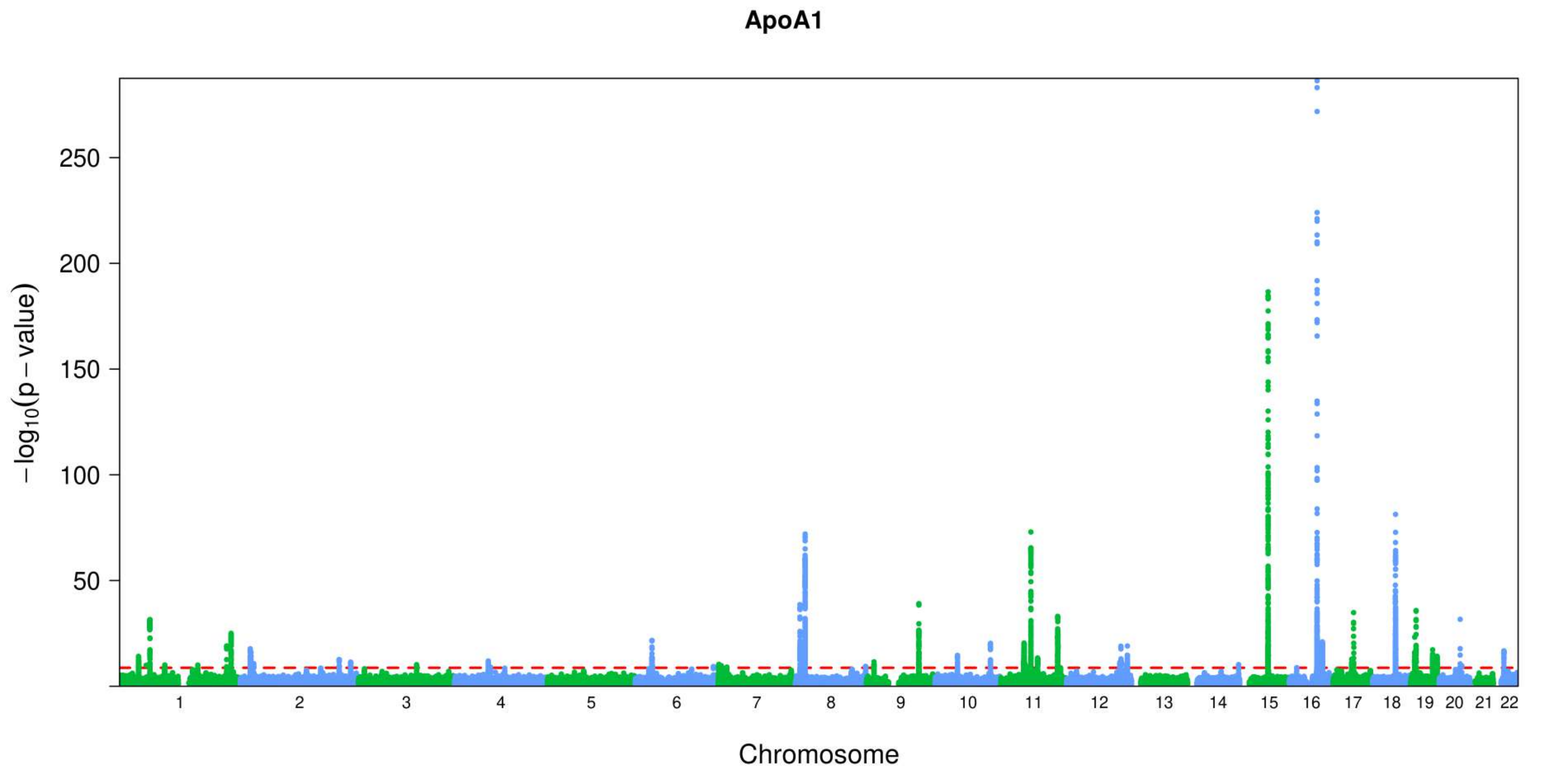
ApoA1
250
200
150
100
50
$-\log_{10}(p-\text{value})$
1
2
3
4
5
6
7
8
9
10
11
12
13
14
15
16
17
18
19
20
21
22
Chromosome

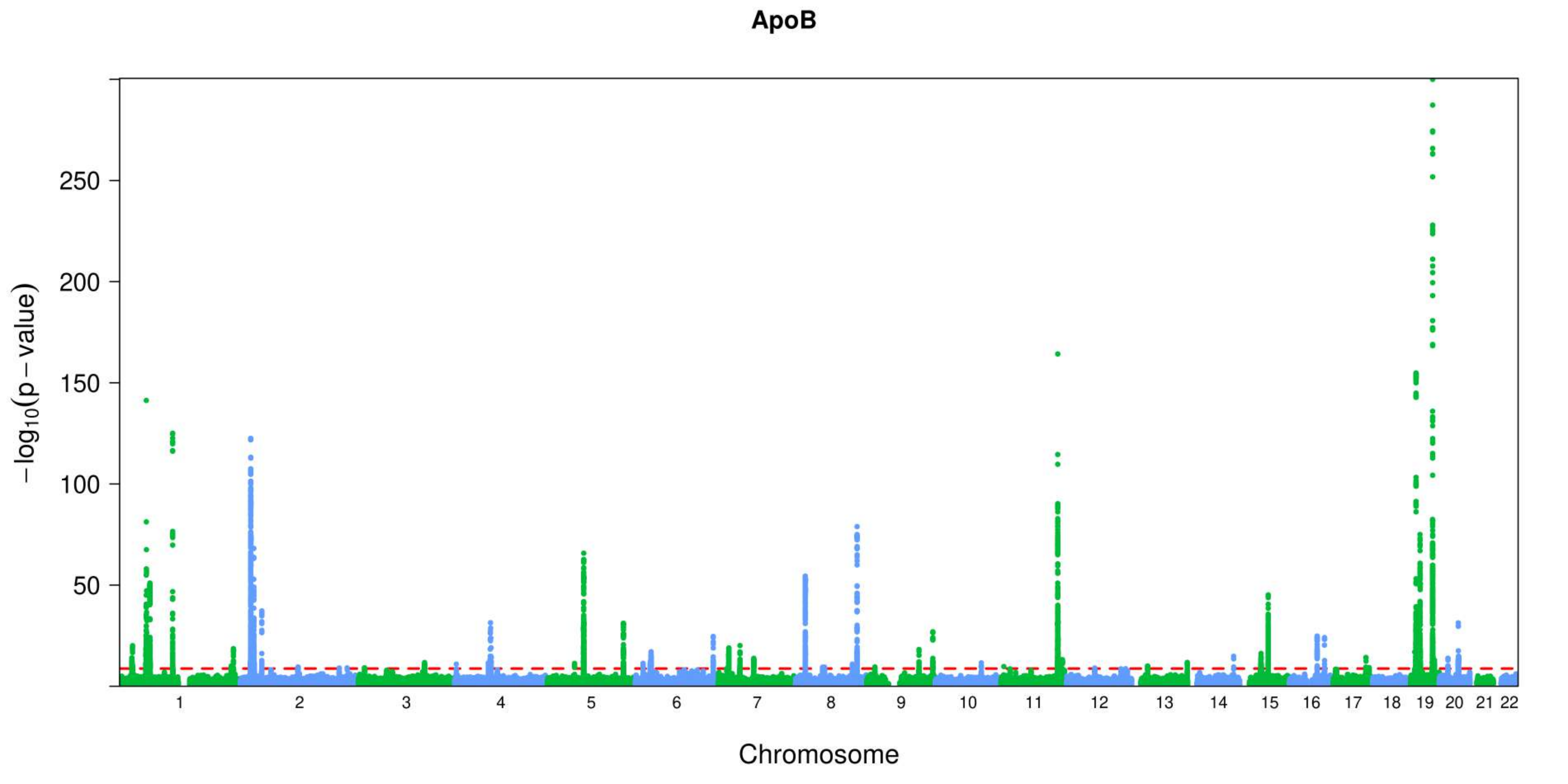
ApoB
−log10(p − value)
250
200
150
100
50
Chromosome
1
2
3
4
5
6
7
8
9
10
11
12
13
14
15
16
17
18
19
20
21
22

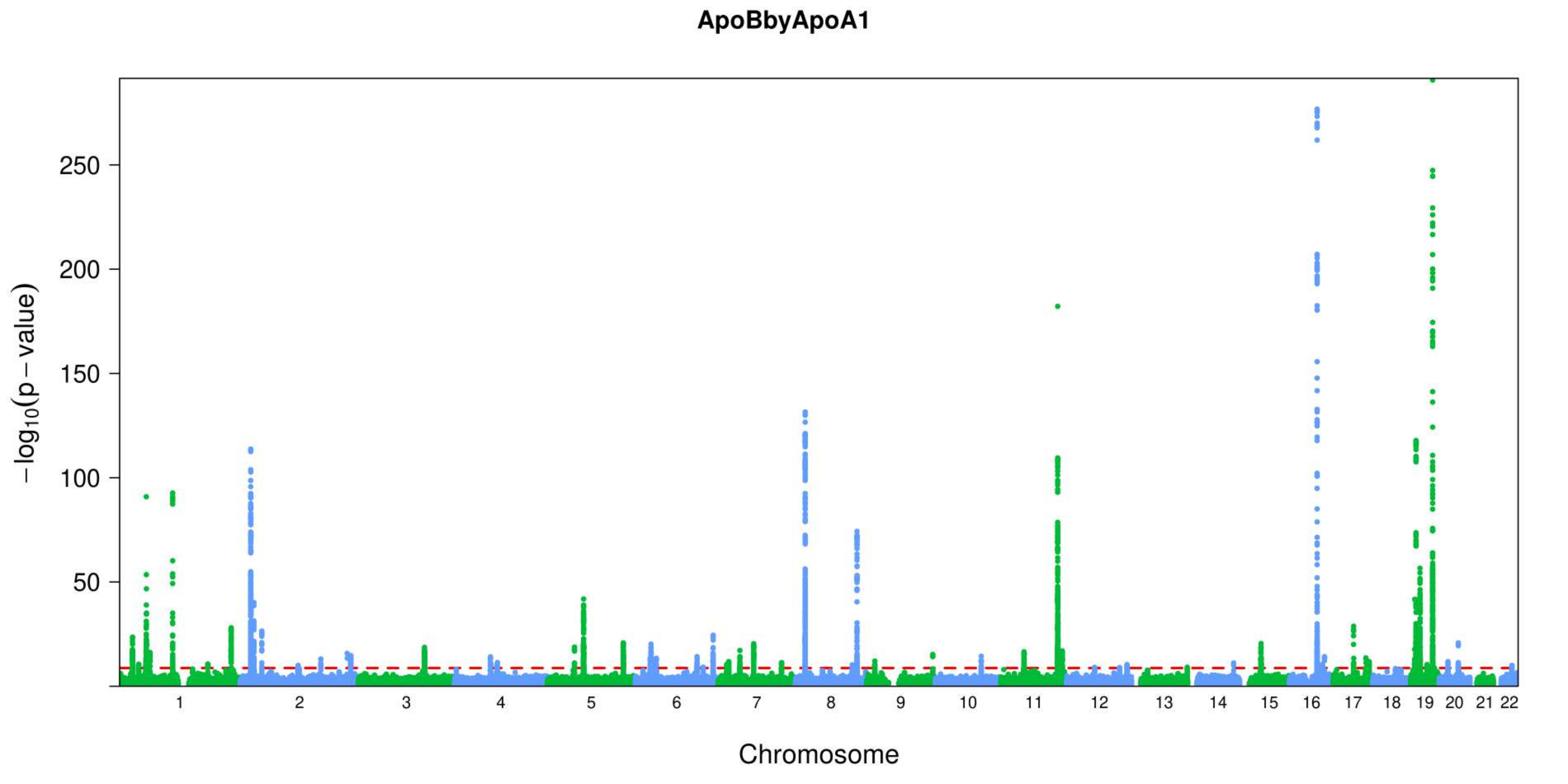
ApoBbyApoA1
-log10(p - value)
250
200
150
100
50
Chromosome
1
2
3
4
5
6
7
8
9
10
11
12
13
14
15
16
17
18
19
20
21
22

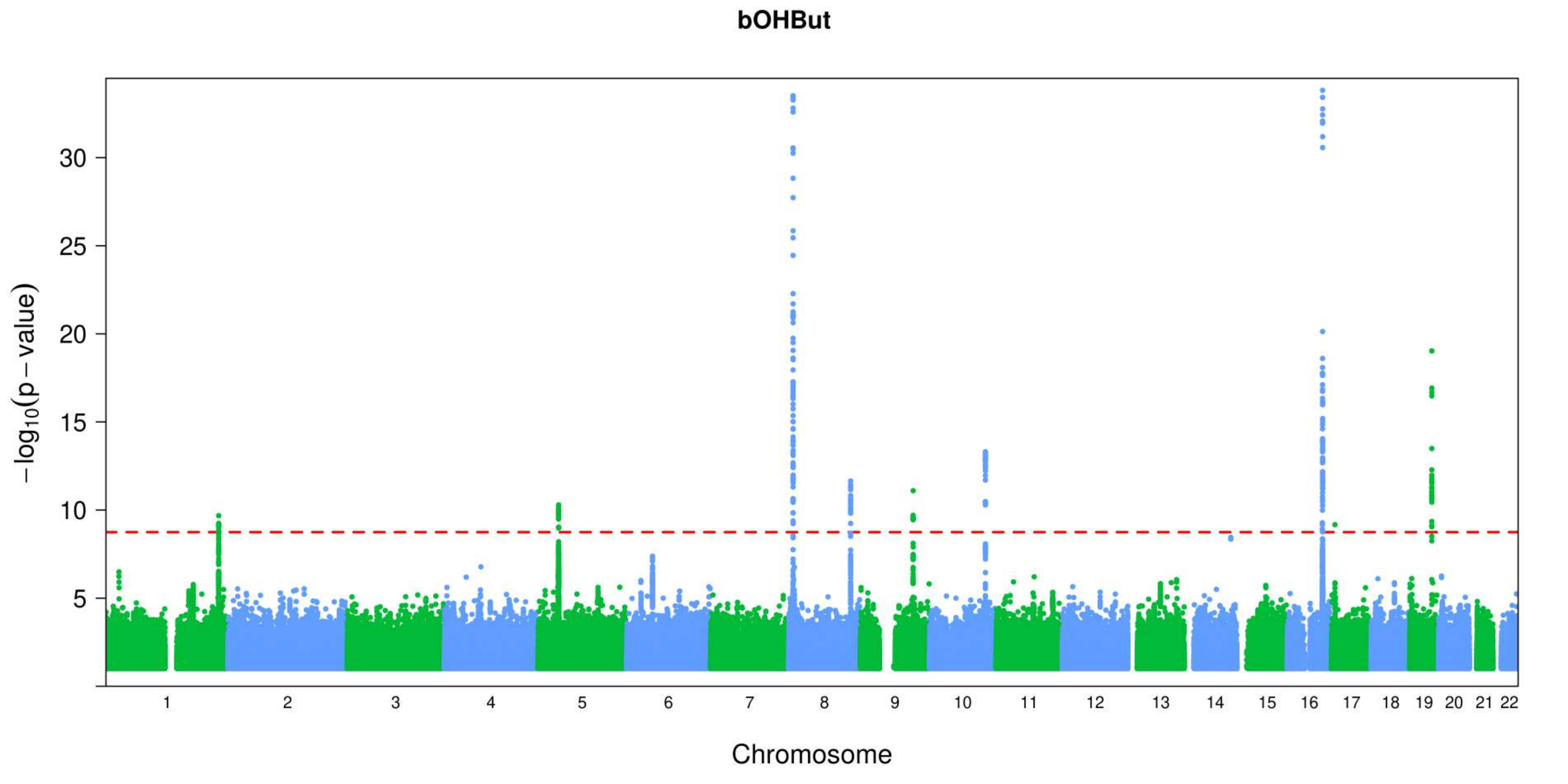

bOHBut
$-\log_{10}(p-\text{value})$
30
25
20
15
10
5
1
2
3
4
5
6
7
8
9
10
11
12
13
14
15
16
17
18
19
20
21
22
Chromosome

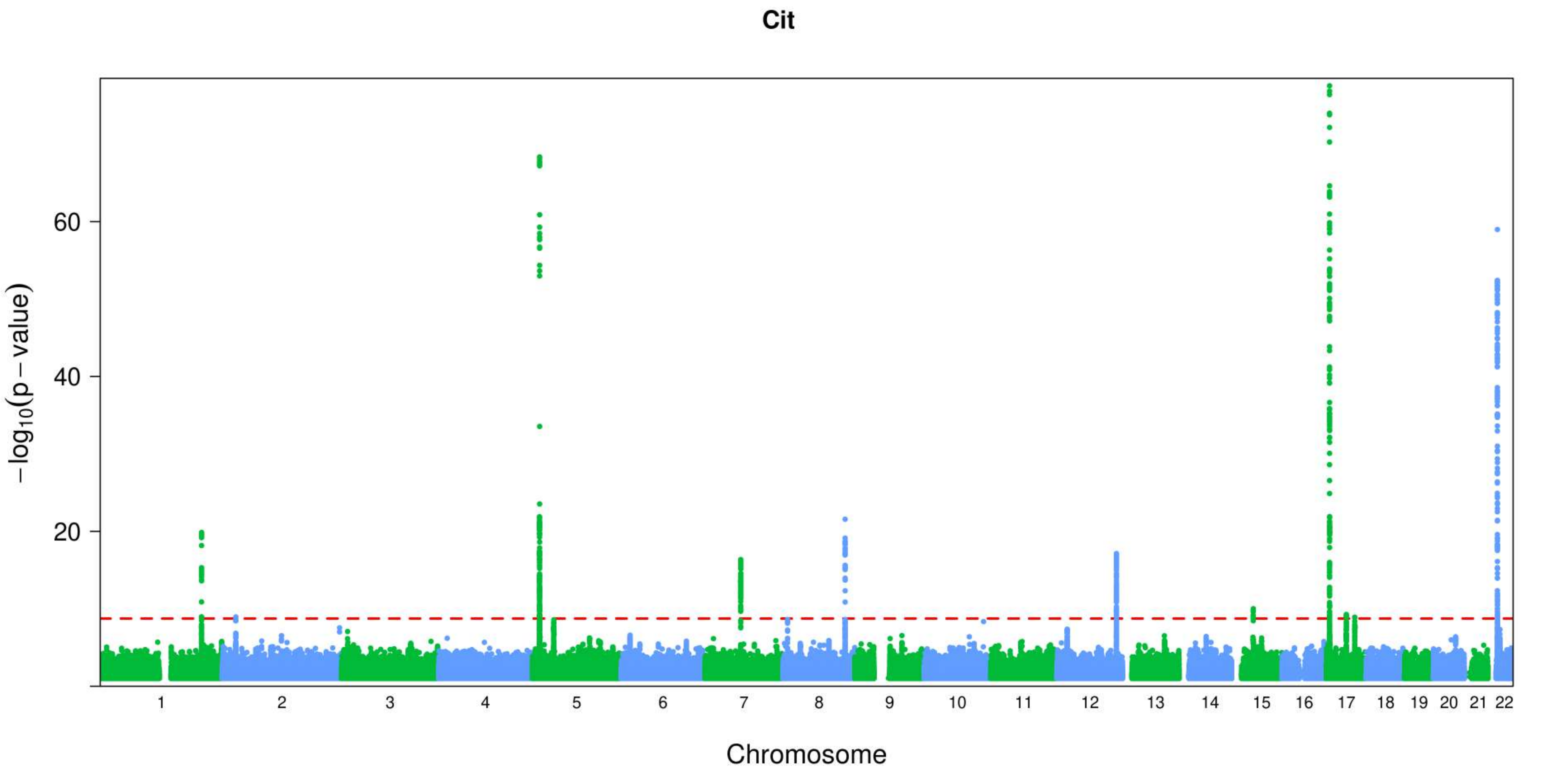
Cit
60
40
20
−log10(p − value)
1
2
3
4
5
6
7
8
9
10
11
12
13
14
15
16
17
18
19
20
21
22
Chromosome

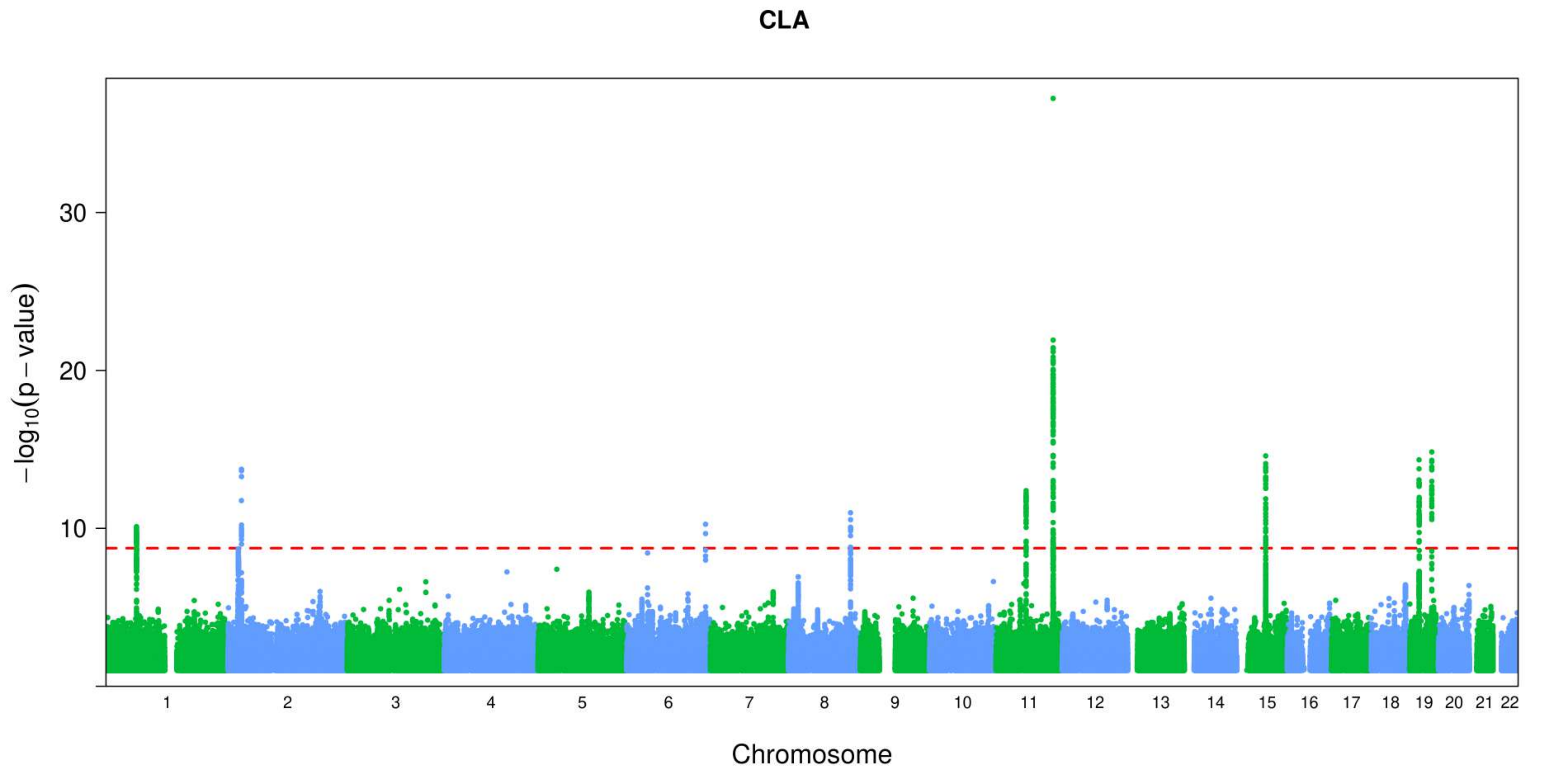
CLA
−log10(p − value)
30
20
10
1
2
3
4
5
6
7
8
9
10
11
12
13
14
15
16
17
18
19
20
21
22
Chromosome

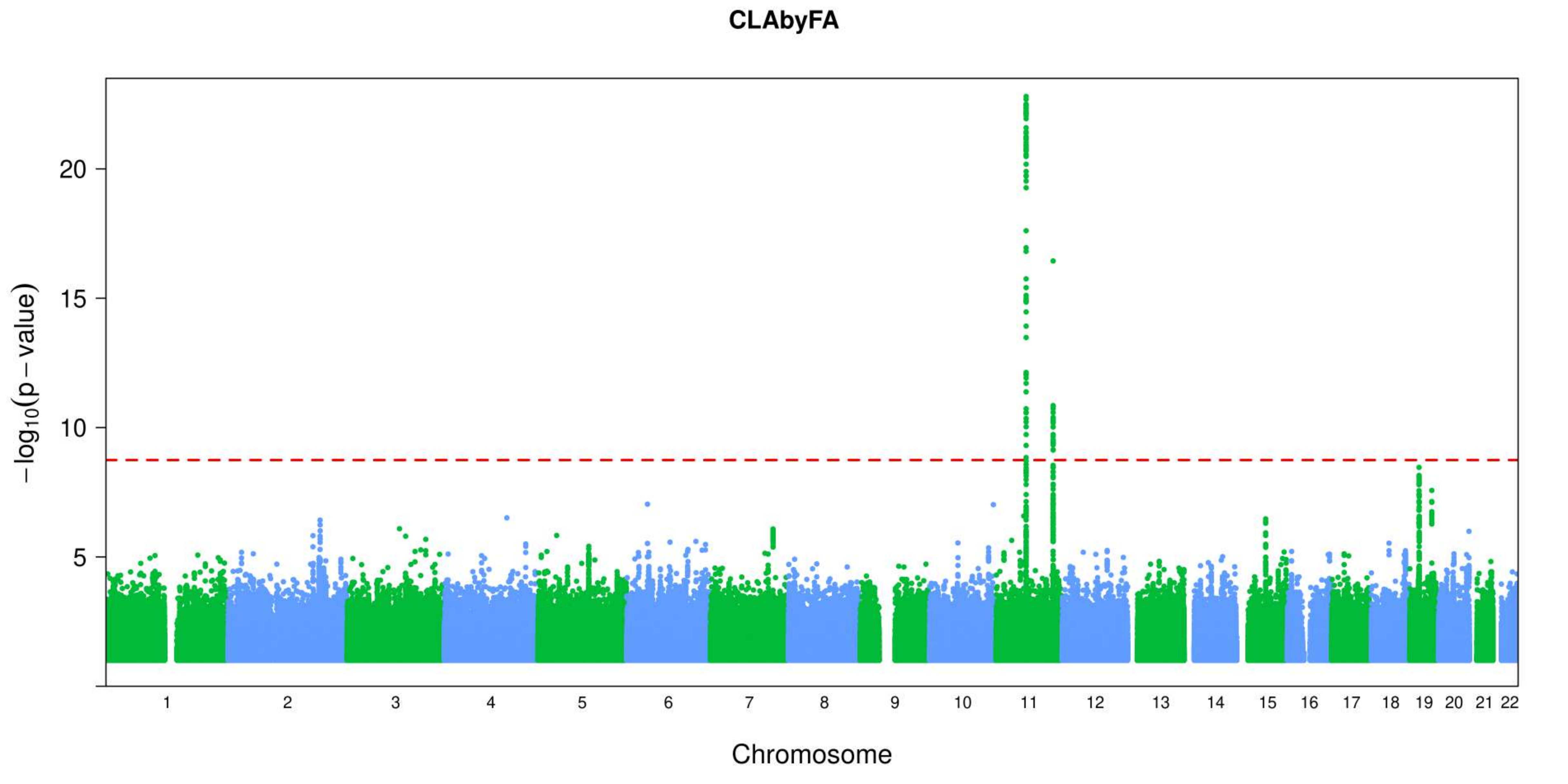

CLAbyFA
20
15
10
5
$-\log_{10}(p-\text{value})$
1
2
3
4
5
6
7
8
9
10
11
12
13
14
15
16
17
18
19
20
21
22
Chromosome

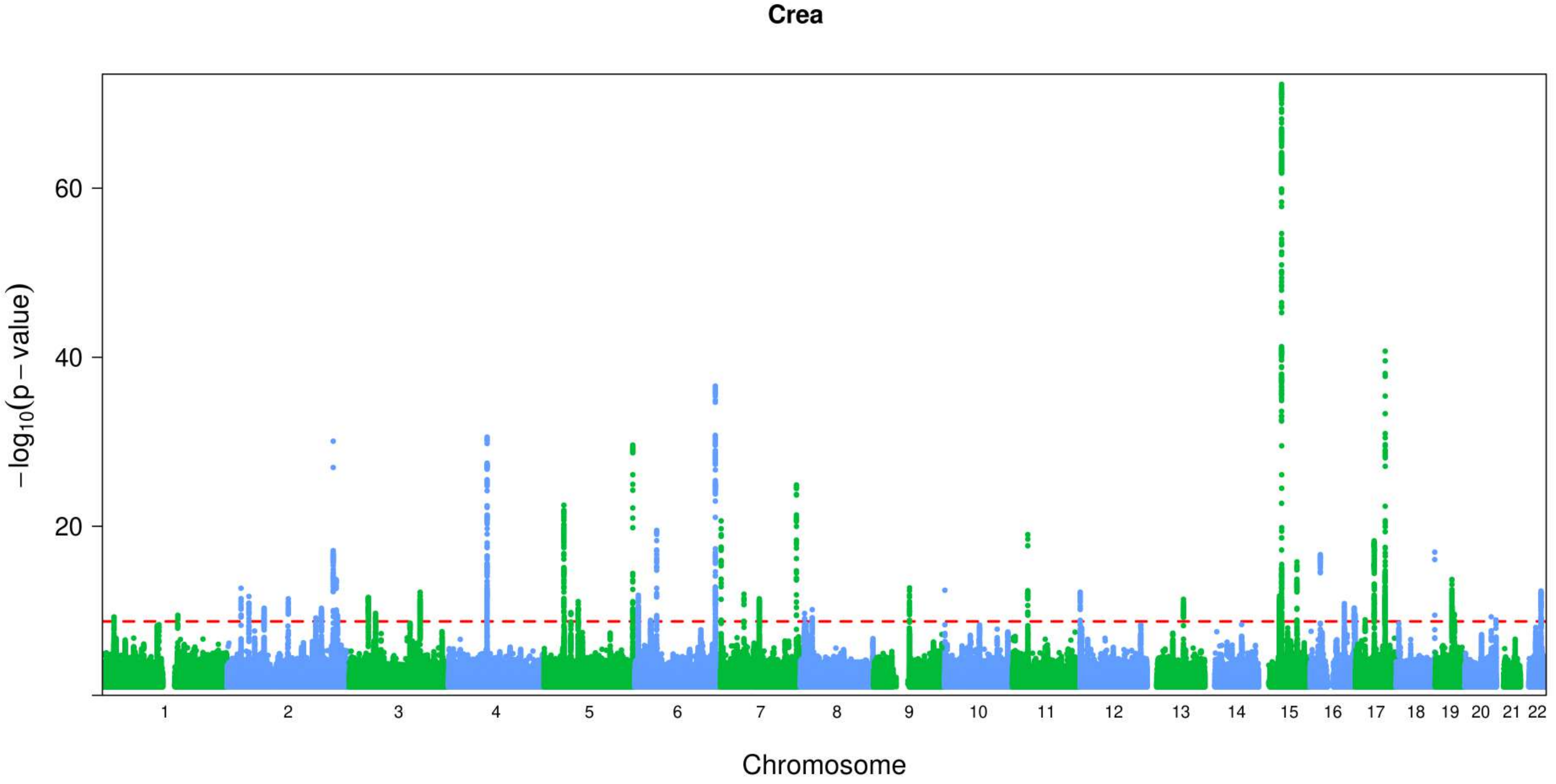
Crea
60
40
20
−log10(p − value)
1
2
3
4
5
6
7
8
9
10
11
12
13
14
15
16
17
18
19
20
21
22
Chromosome

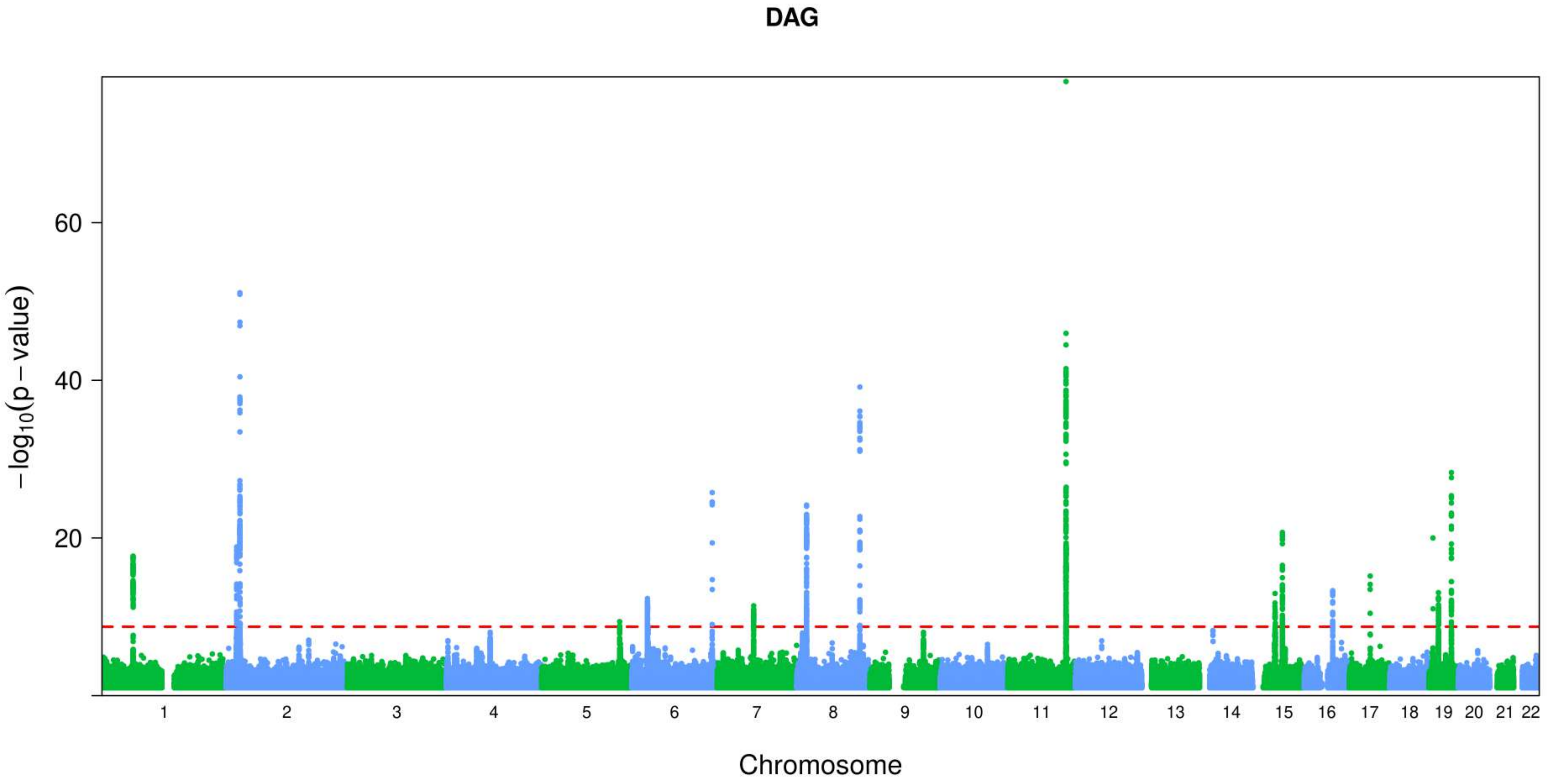
DAG
60
40
20
−log10(p − value)
1
2
3
4
5
6
7
8
9
10
11
12
13
14
15
16
17
18
19
20
21
22
Chromosome

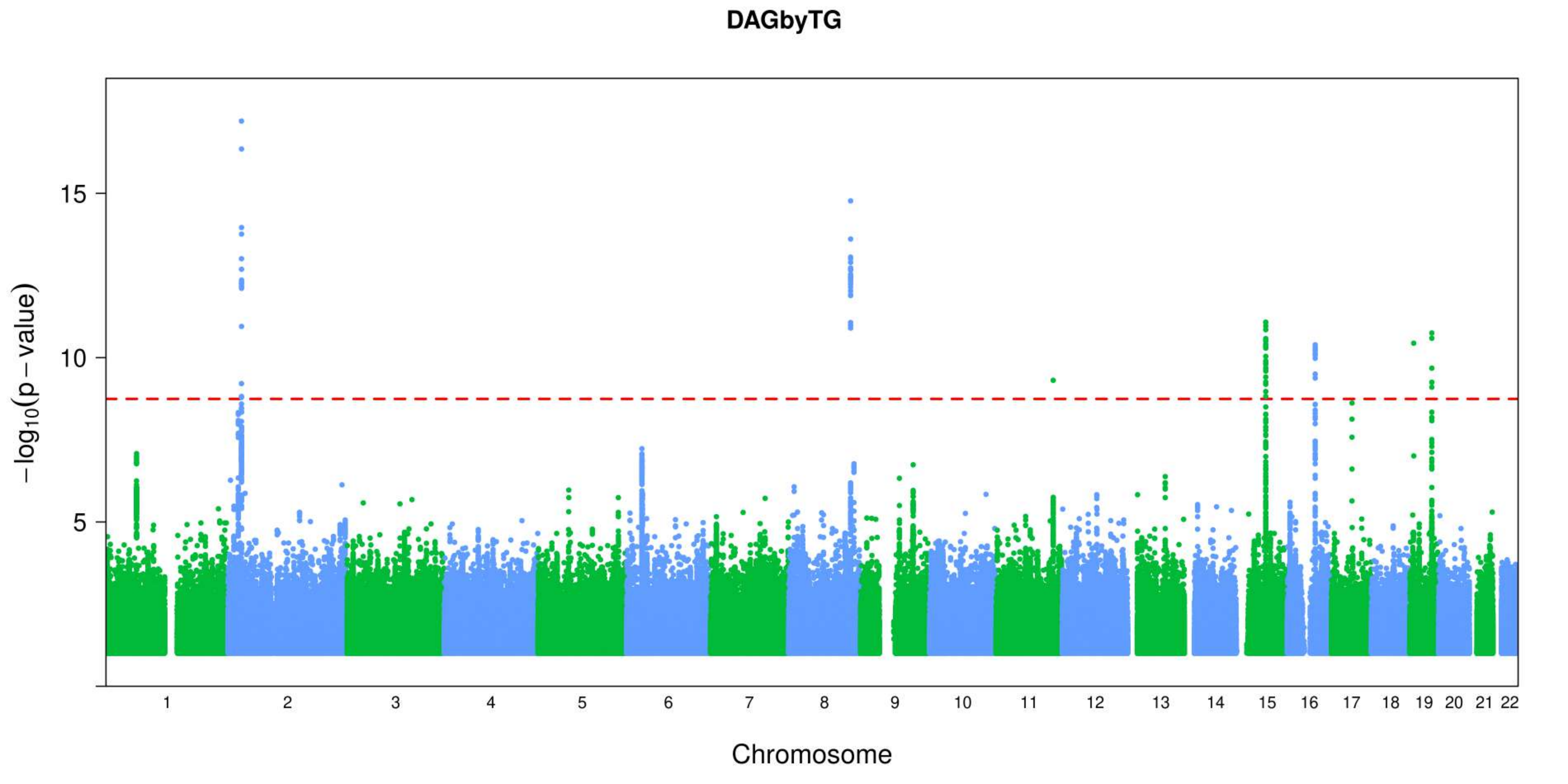
DAGbyTG
15
10
5
$-\log_{10}(p-\text{value})$
1
2
3
4
5
6
7
8
9
10
11
12
13
14
15
16
17
18
19
20
21
22
Chromosome

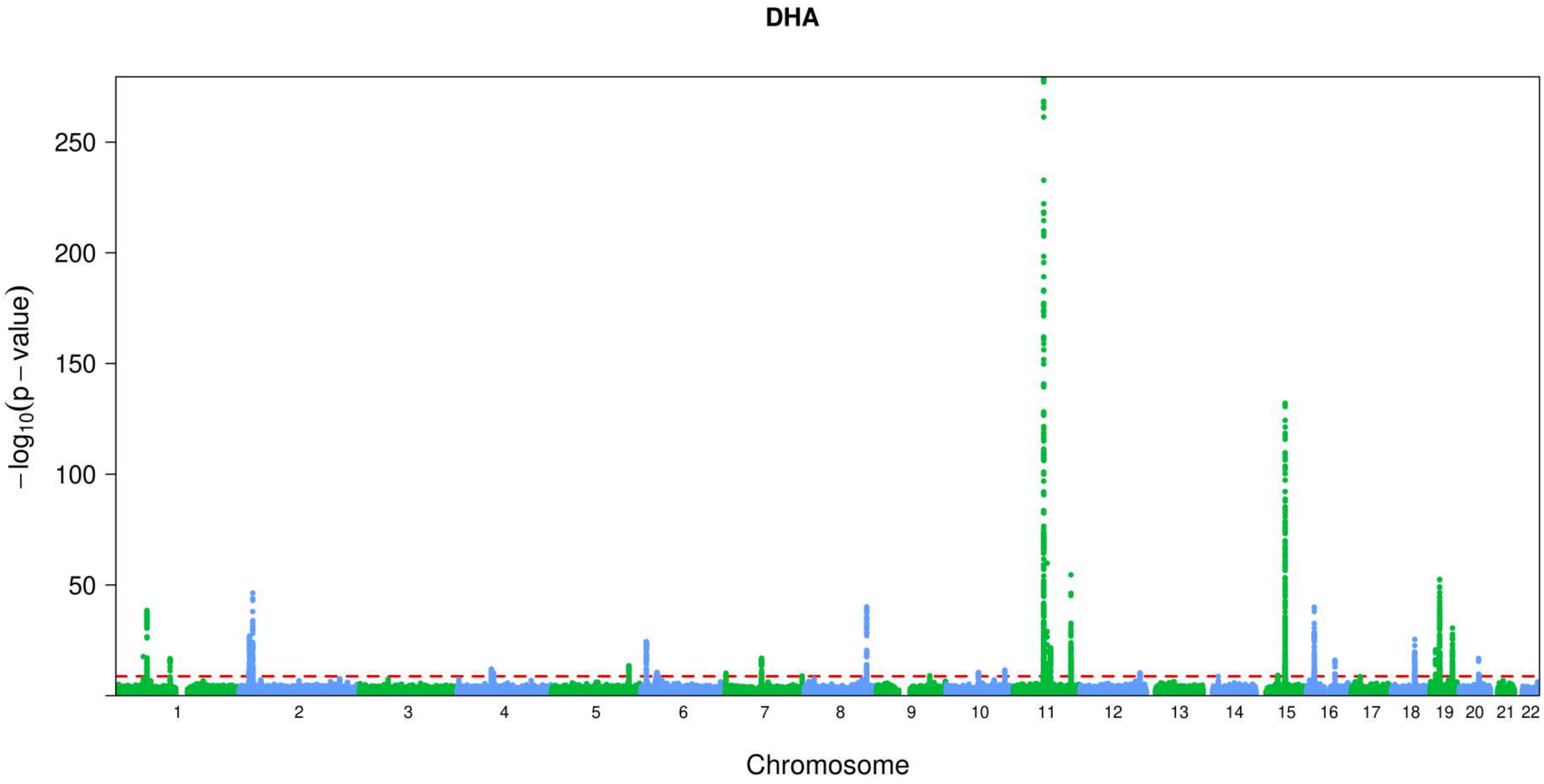
DHA
250
200
150
100
50
−log10(p − value)
1
2
3
4
5
6
7
8
9
10
11
12
13
14
15
16
17
18
19
20
21
22
Chromosome

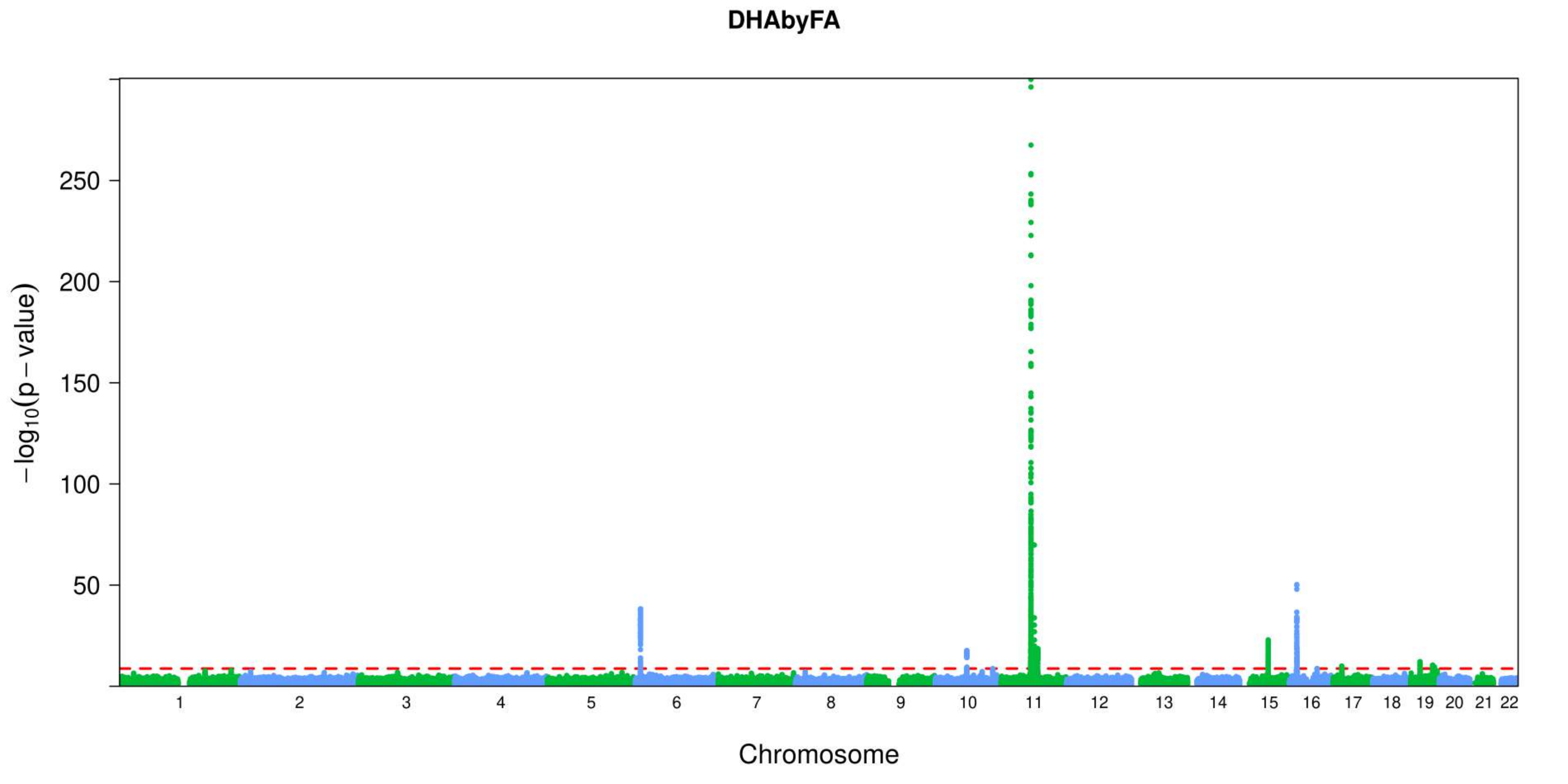
DHAbyFA
250
200
150
100
50
−log10(p − value)
1
2
3
4
5
6
7
8
9
10
11
12
13
14
15
16
17
18
19
20
21
22
Chromosome

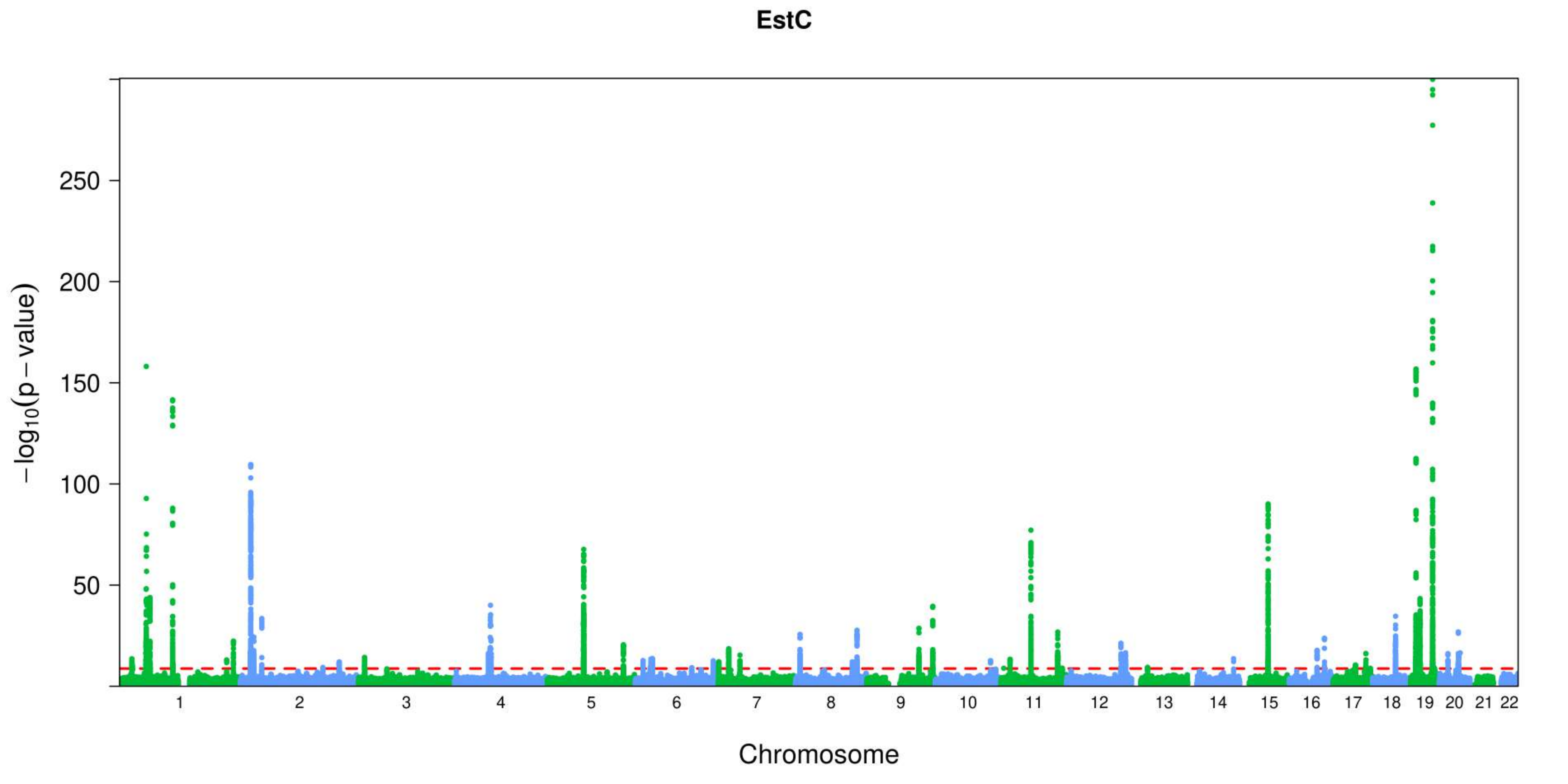

EstC
250
200
150
100
50
$-\log_{10}(p-\text{value})$
1
2
3
4
5
6
7
8
9
10
11
12
13
14
15
16
17
18
19
20
21
22
Chromosome

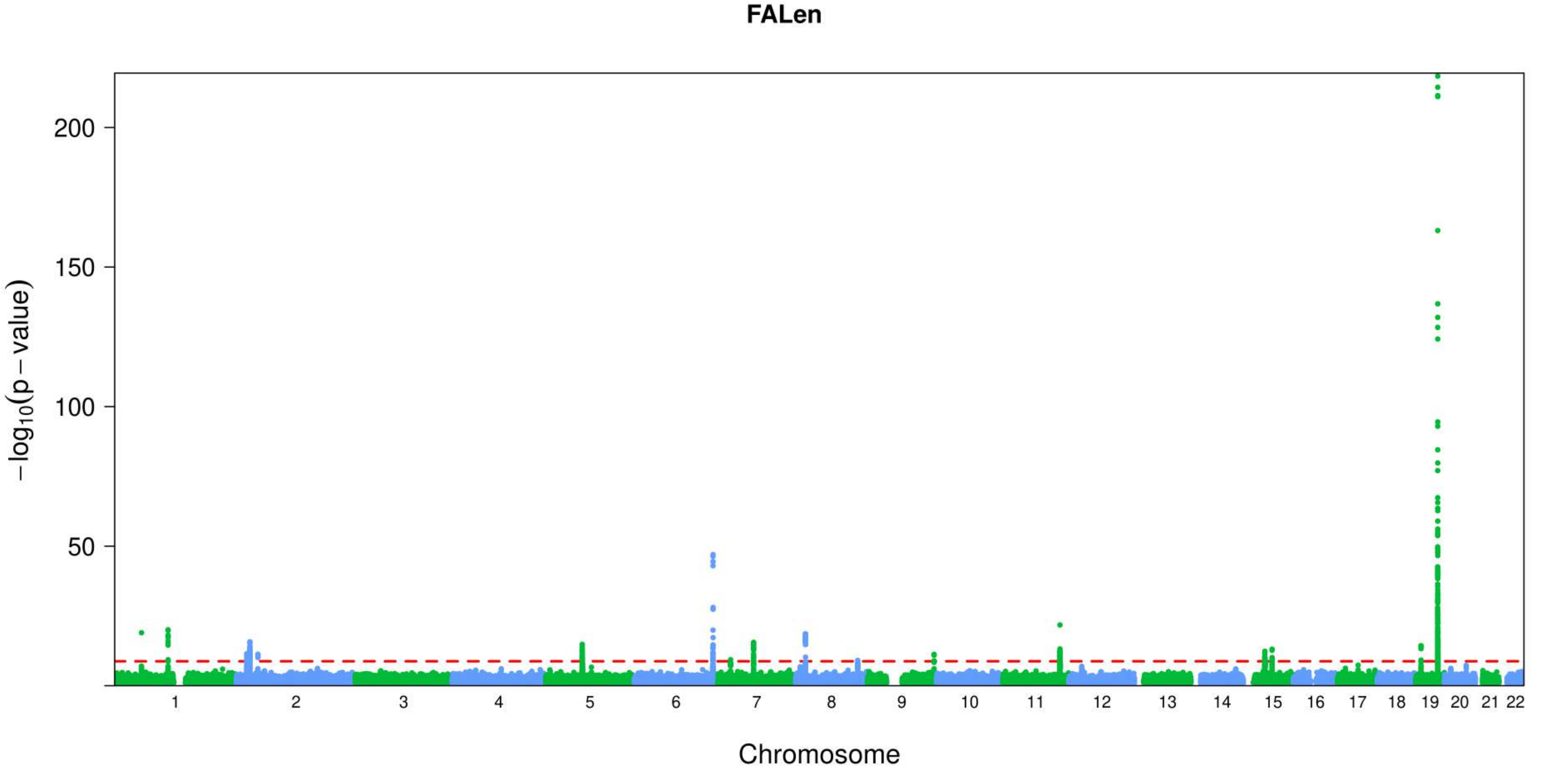
FALen
200
150
100
50
$-\log_{10}(p-\text{value})$
1
2
3
4
5
6
7
8
9
10
11
12
13
14
15
16
17
18
19
20
21
22
Chromosome

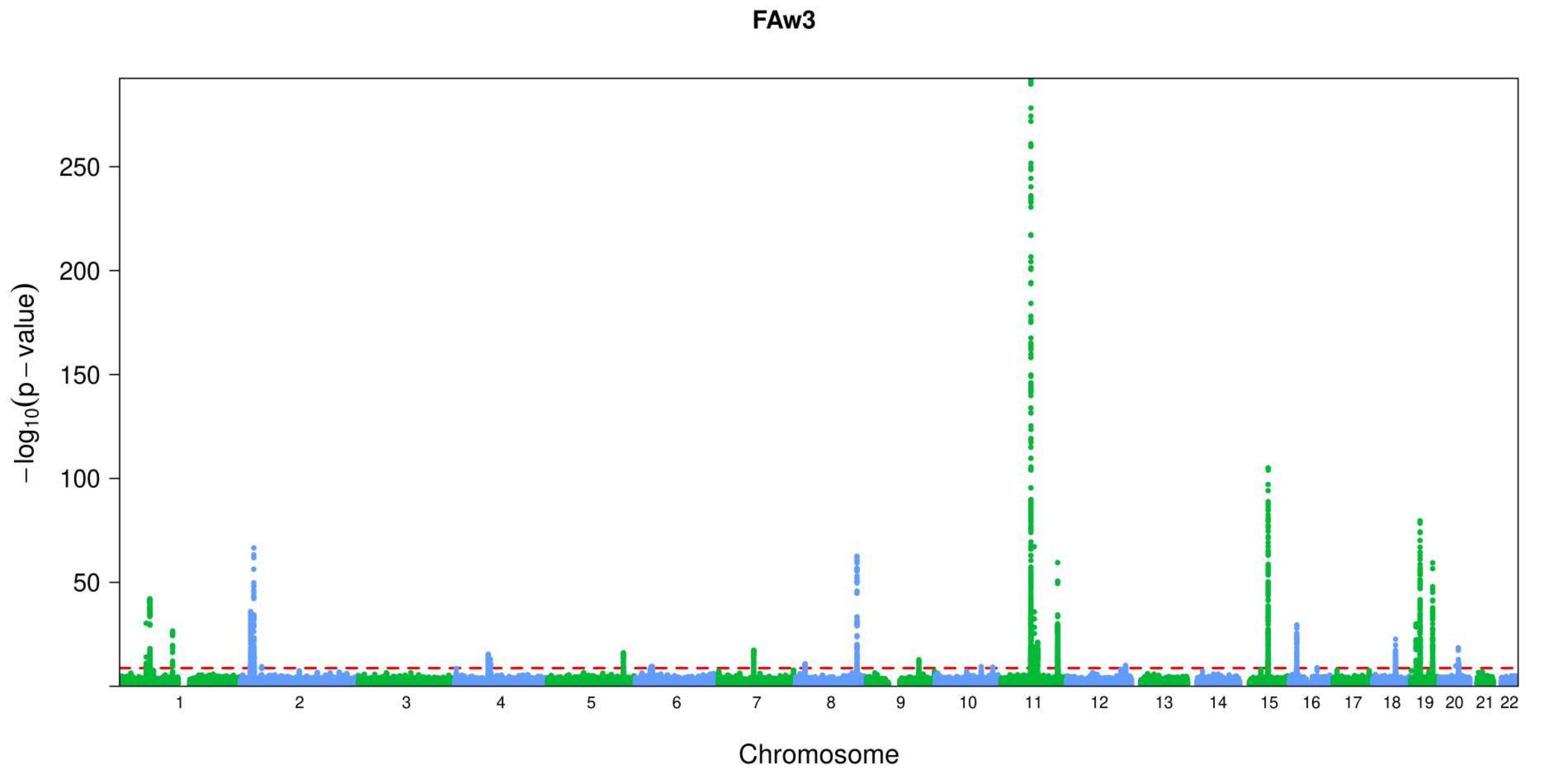

FAw3
250
200
150
100
50
$-\log_{10}(p-\text{value})$
1
2
3
4
5
6
7
8
9
10
11
12
13
14
15
16
17
18
19
20
21
22
Chromosome

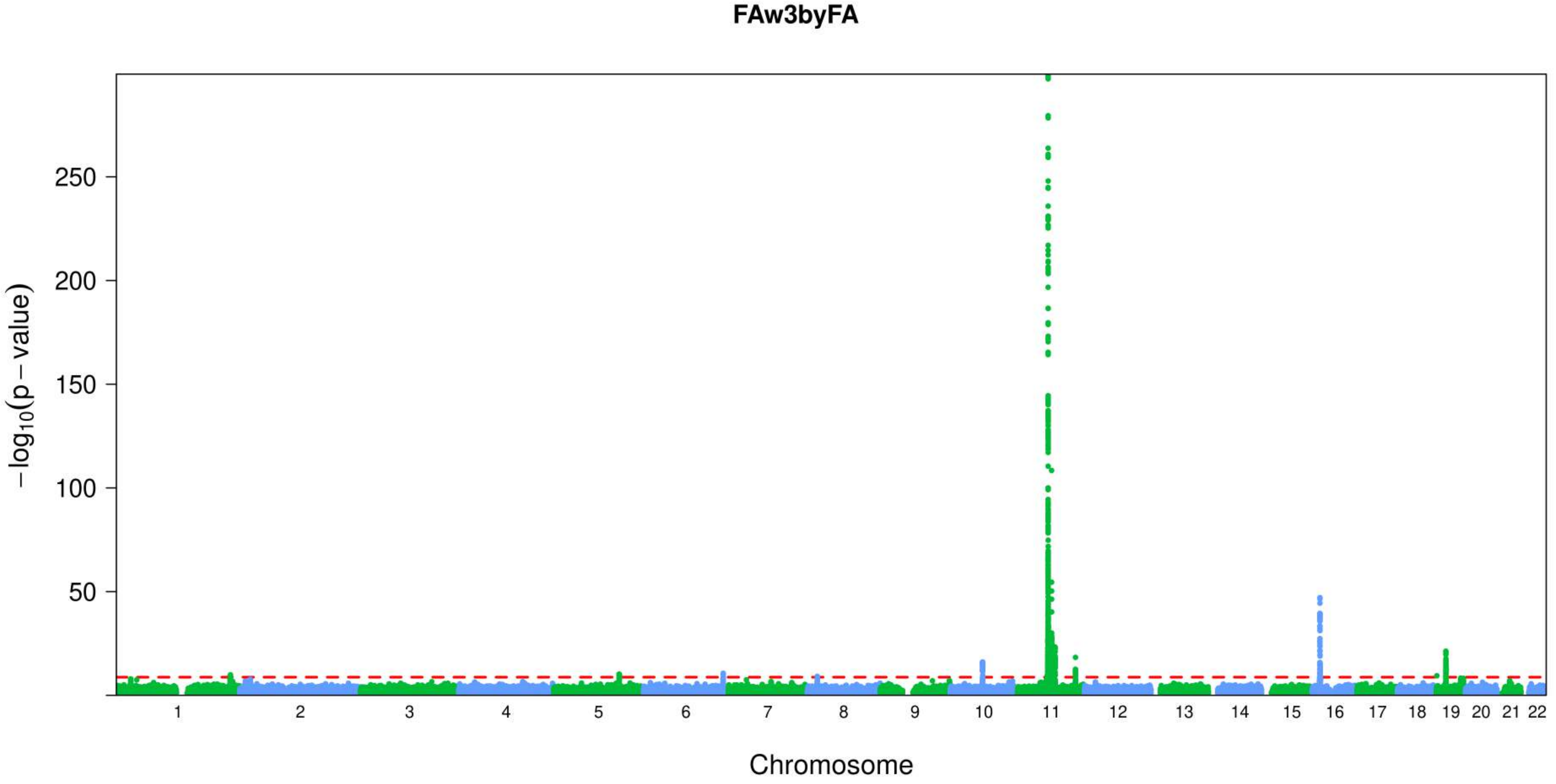

FAw3byFA
250
200
150
100
50
−log10(p − value)
1
2
3
4
5
6
7
8
9
10
11
12
13
14
15
16
17
18
19
20
21
22
Chromosome

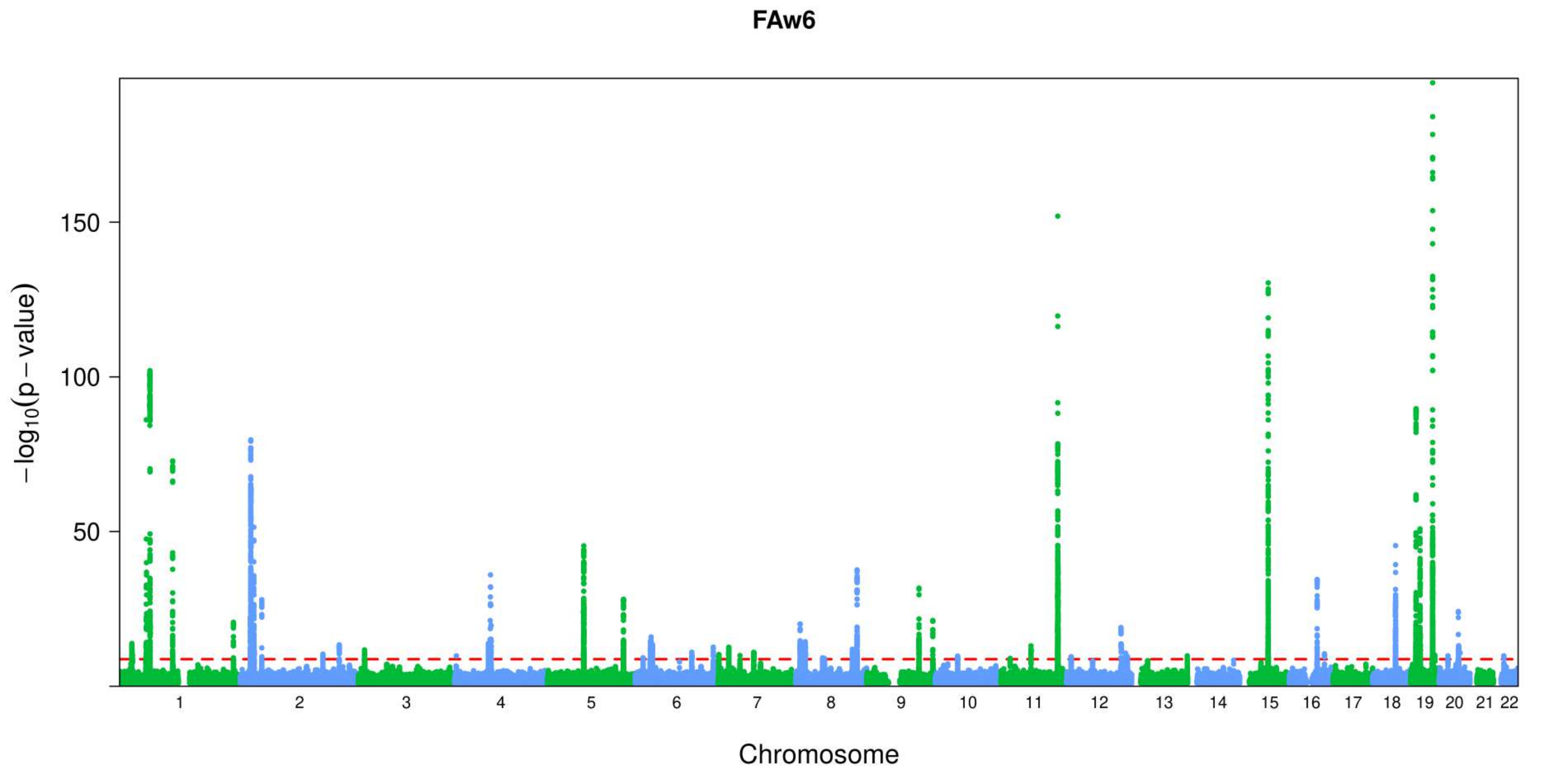

FAw6
150
100
50
−log10(p − value)
1
2
3
4
5
6
7
8
9
10
11
12
13
14
15
16
17
18
19
20
21
22
Chromosome

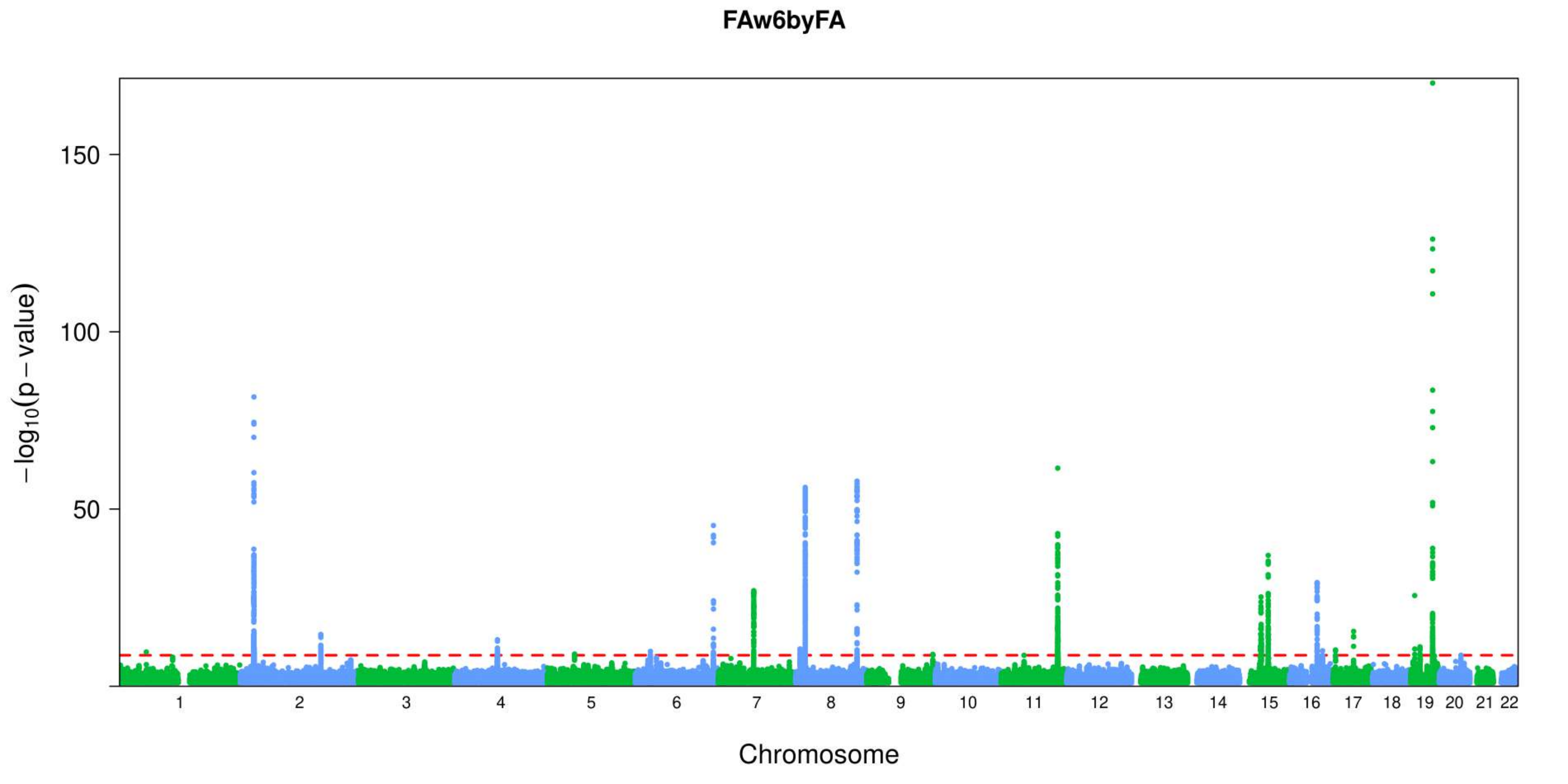

FAw6byFA
150
100
50
−log10(p − value)
1
2
3
4
5
6
7
8
9
10
11
12
13
14
15
16
17
18
19
20
21
22
Chromosome

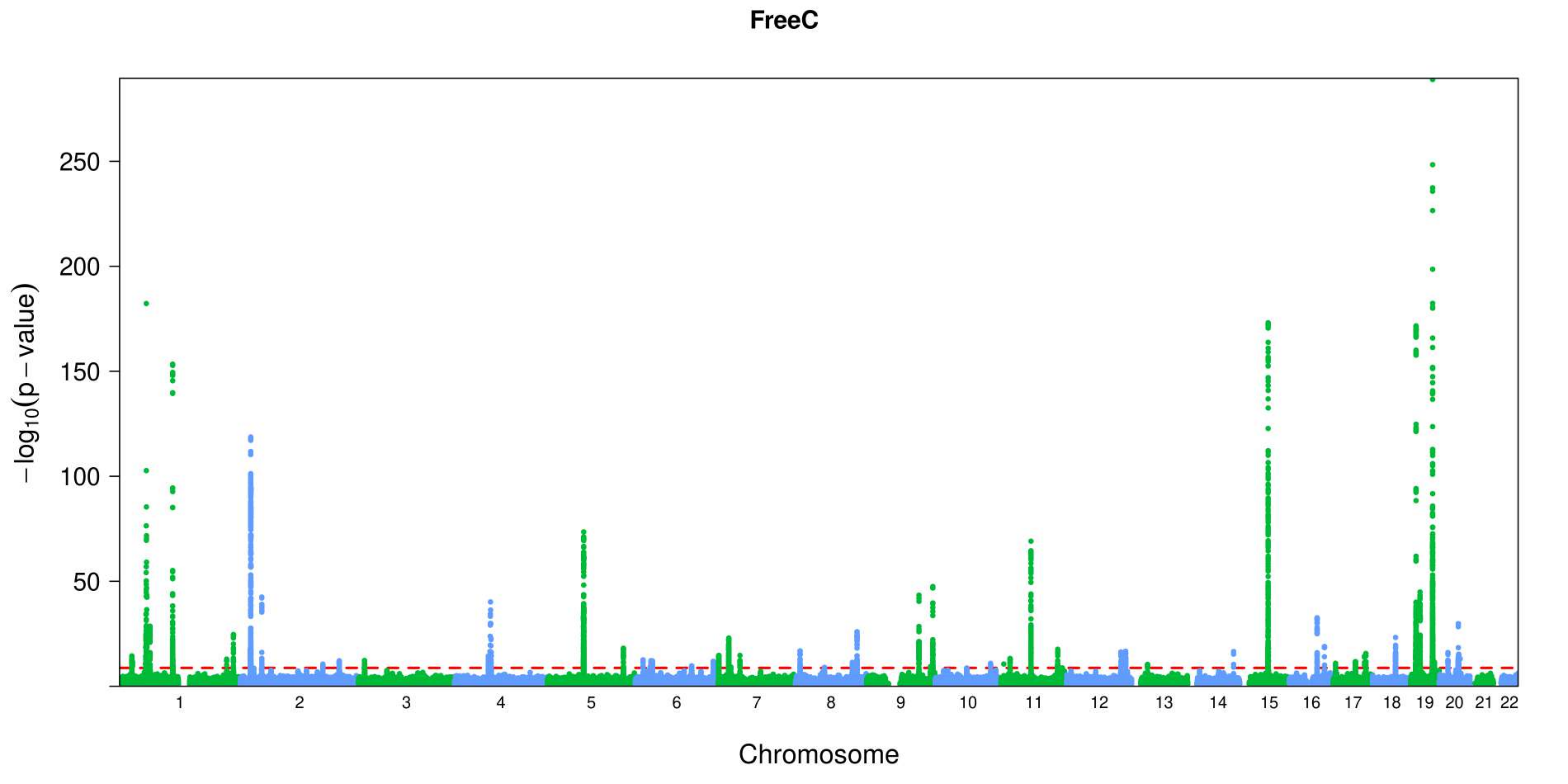
FreeC
250
200
150
100
50
$-\log_{10}(p-value)$
1
2
3
4
5
6
7
8
9
10
11
12
13
14
15
16
17
18
19
20
21
22
Chromosome

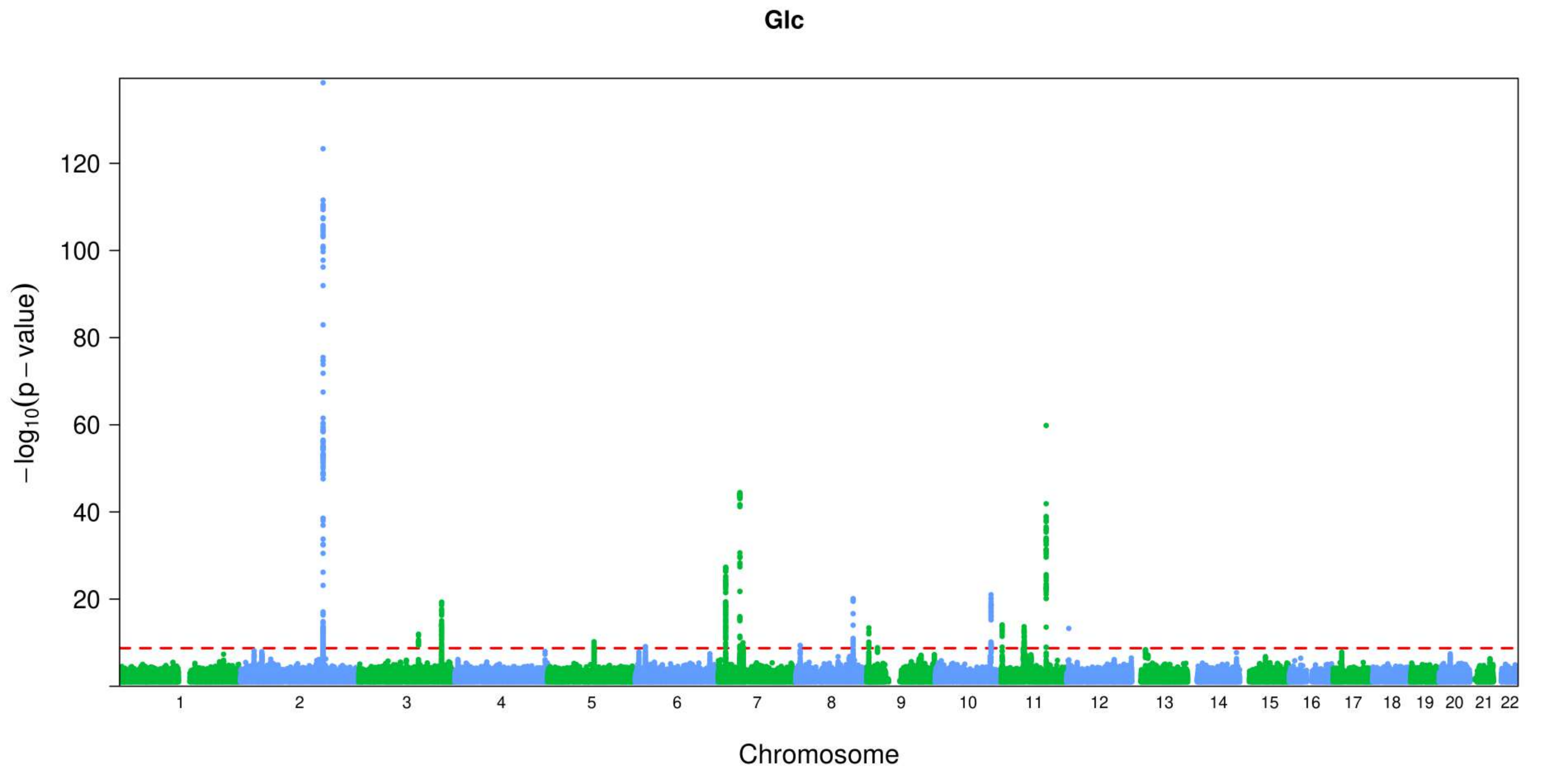
Glc
−log10(p − value)
120
100
80
60
40
20
1
2
3
4
5
6
7
8
9
10
11
12
13
14
15
16
17
18
19
20
21
22
Chromosome

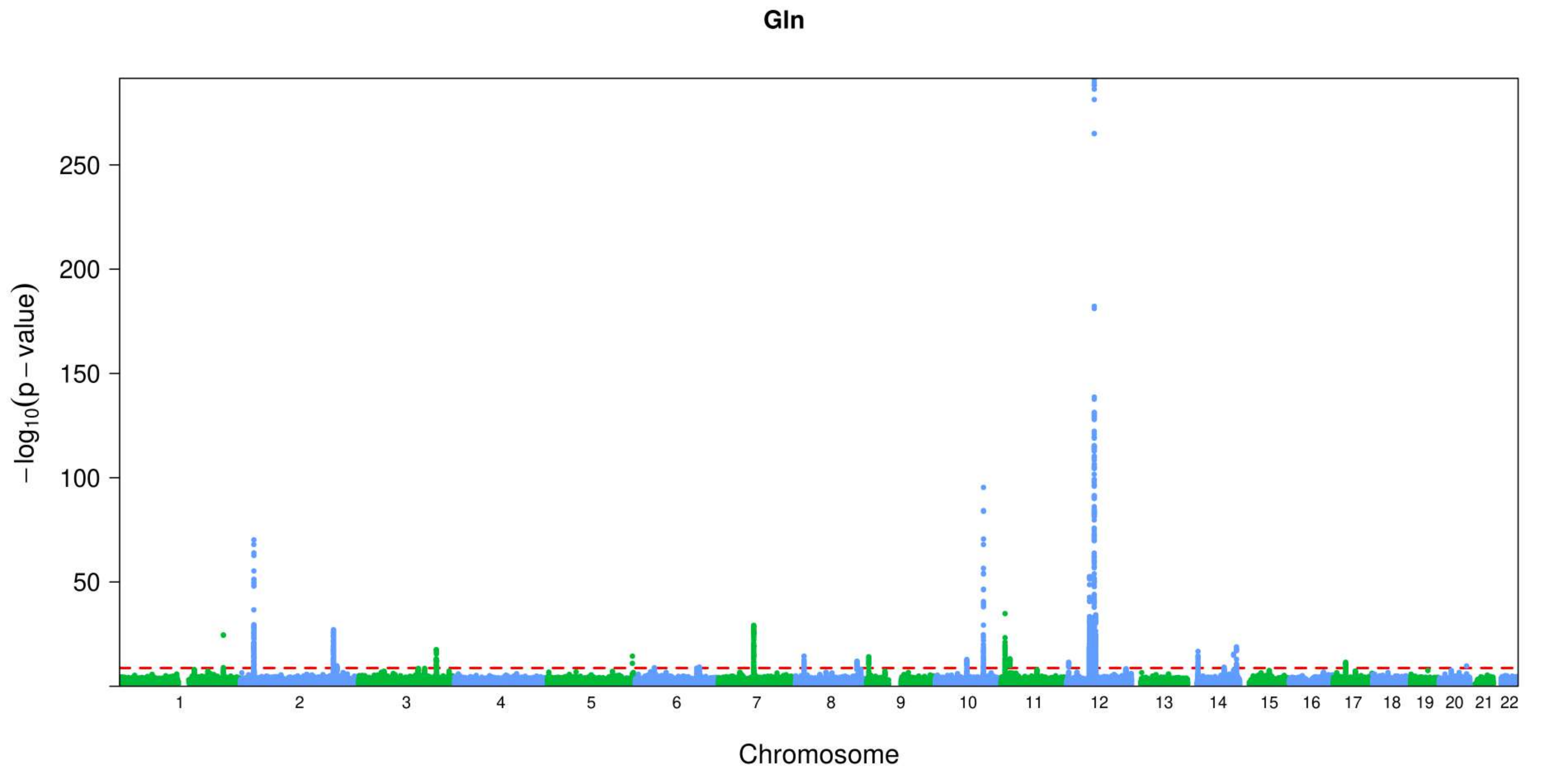

Gln
250
200
150
100
50
−log10(p − value)
1
2
3
4
5
6
7
8
9
10
11
12
13
14
15
16
17
18
19
20
21
22
Chromosome

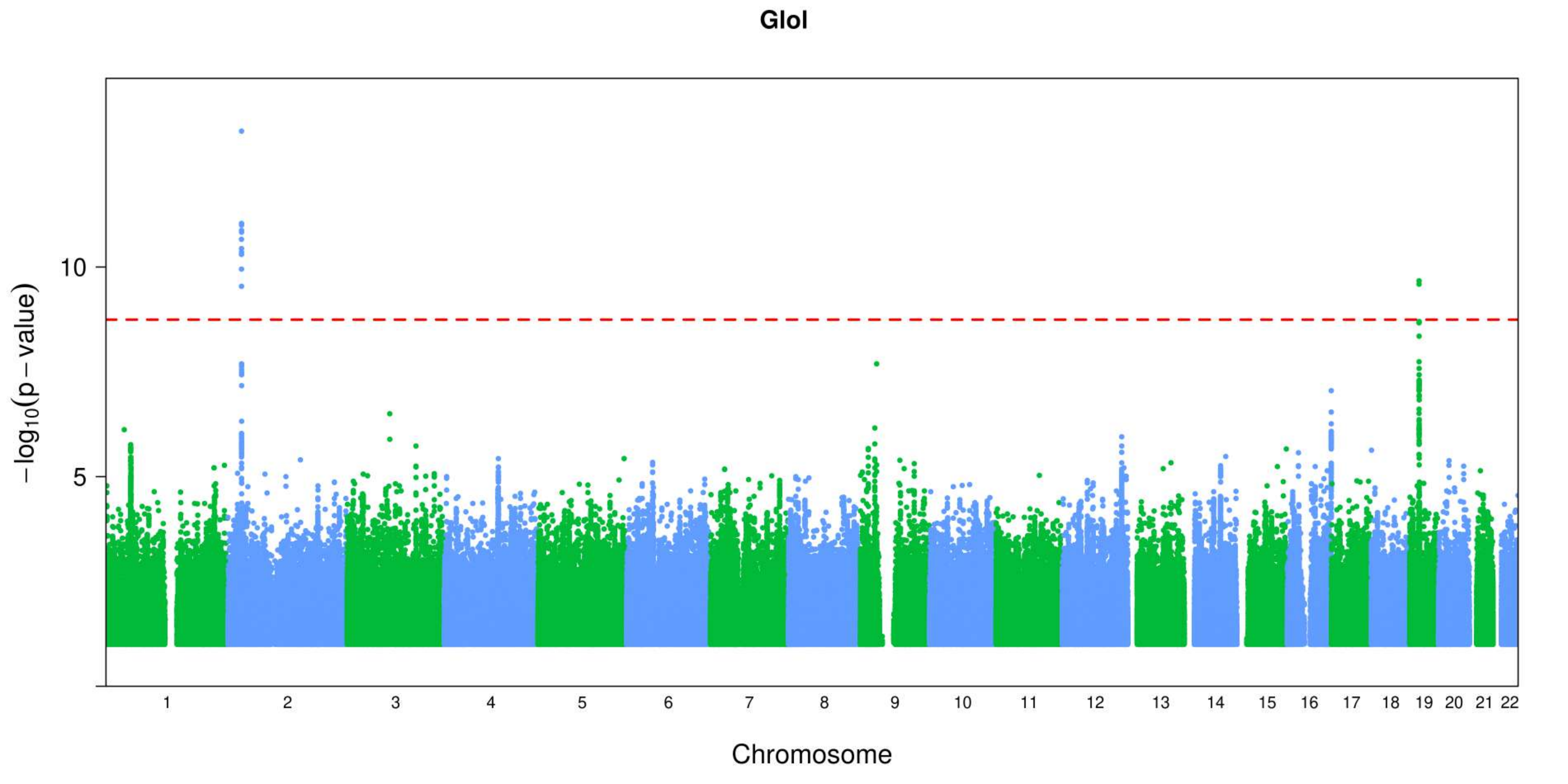
GloI
10
5
−log10(p − value)
1
2
3
4
5
6
7
8
9
10
11
12
13
14
15
16
17
18
19
20
21
22
Chromosome

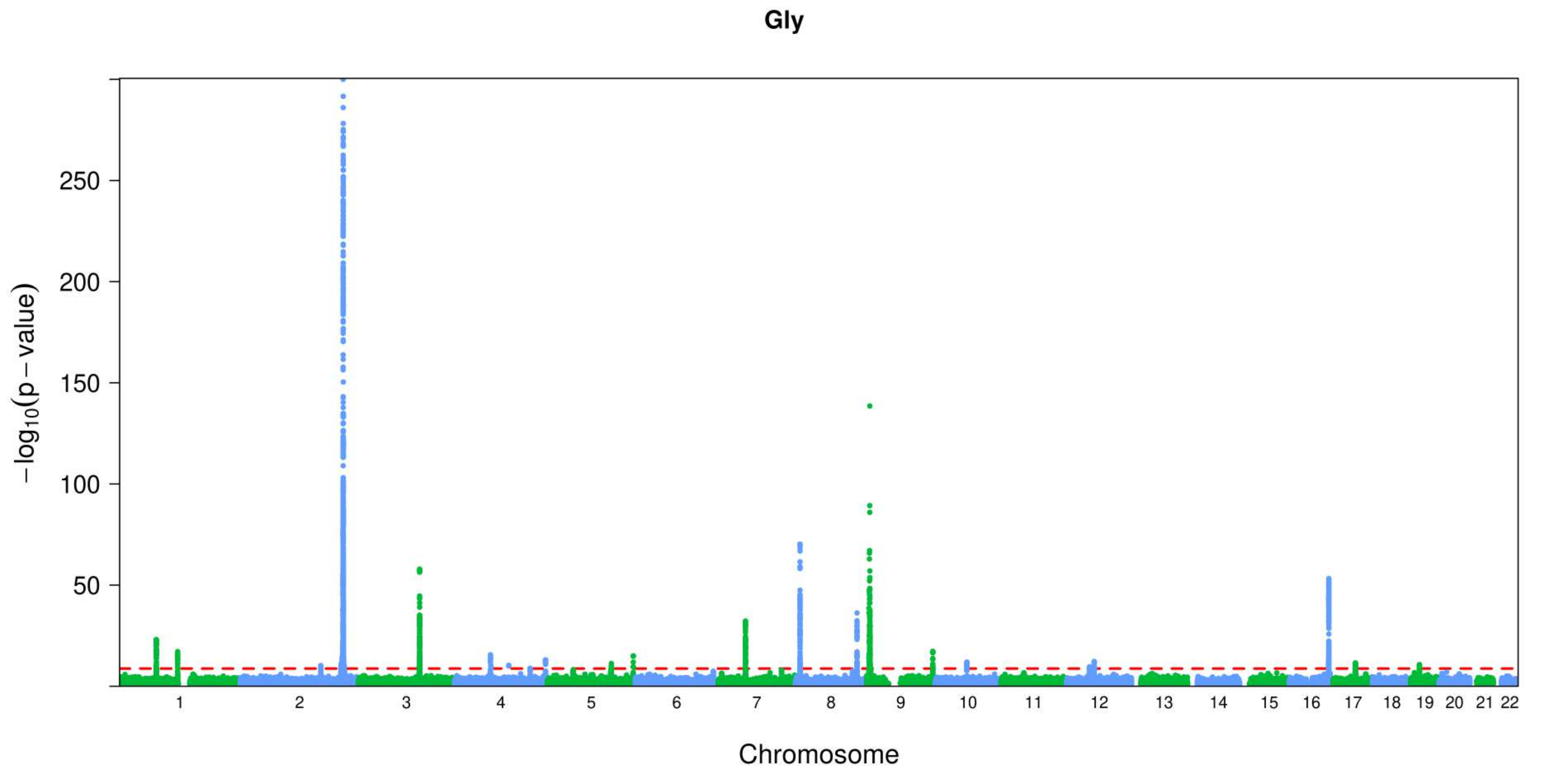
Gly
250
200
150
100
50
$-\log_{10}(p-\text{value})$
1
2
3
4
5
6
7
8
9
10
11
12
13
14
15
16
17
18
19
20
21
22
Chromosome

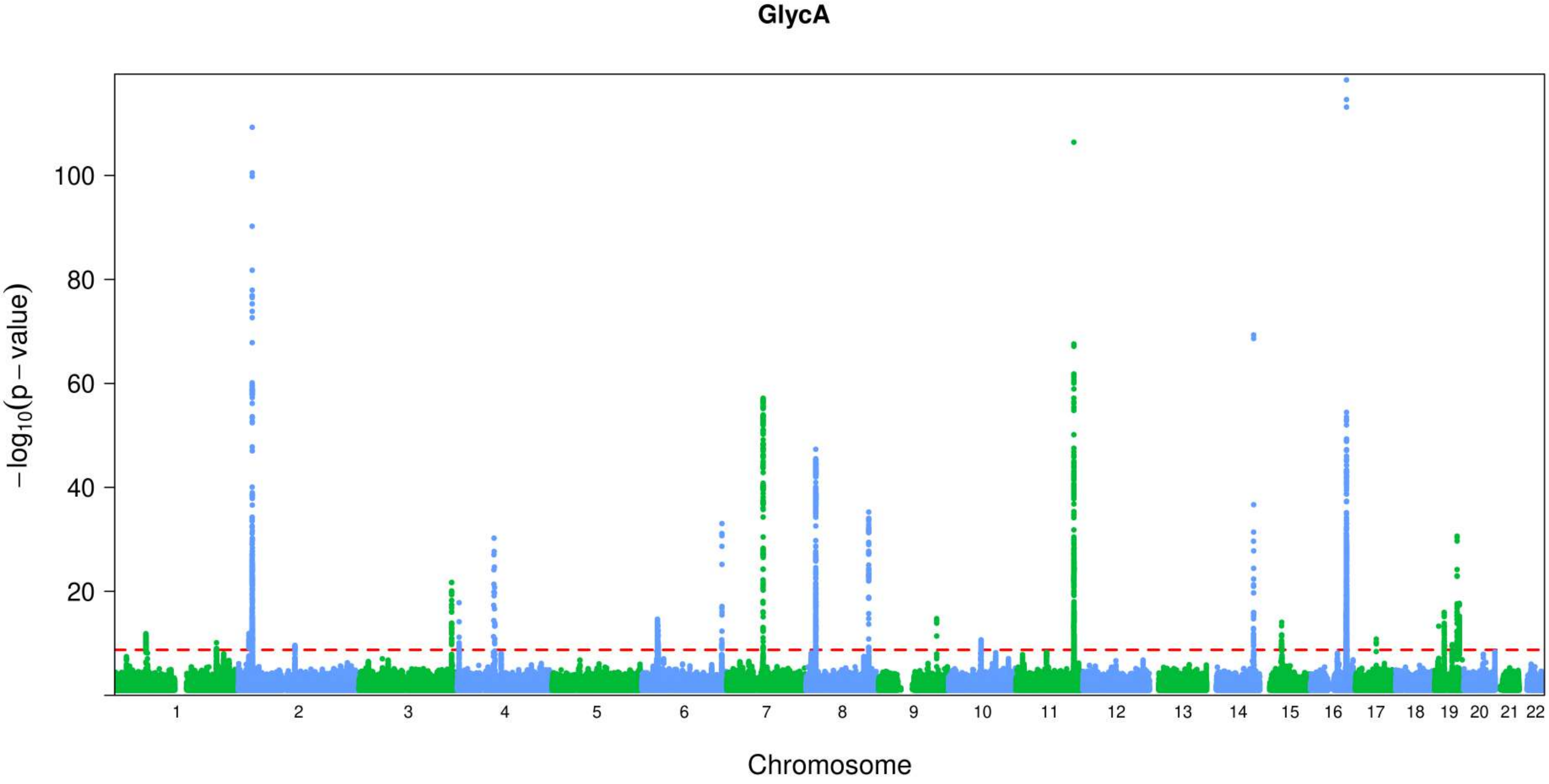
GlycA
-log10(p-value)
100
80
60
40
20
Chromosome
1
2
3
4
5
6
7
8
9
10
11
12
13
14
15
16
17
18
19
20
21
22

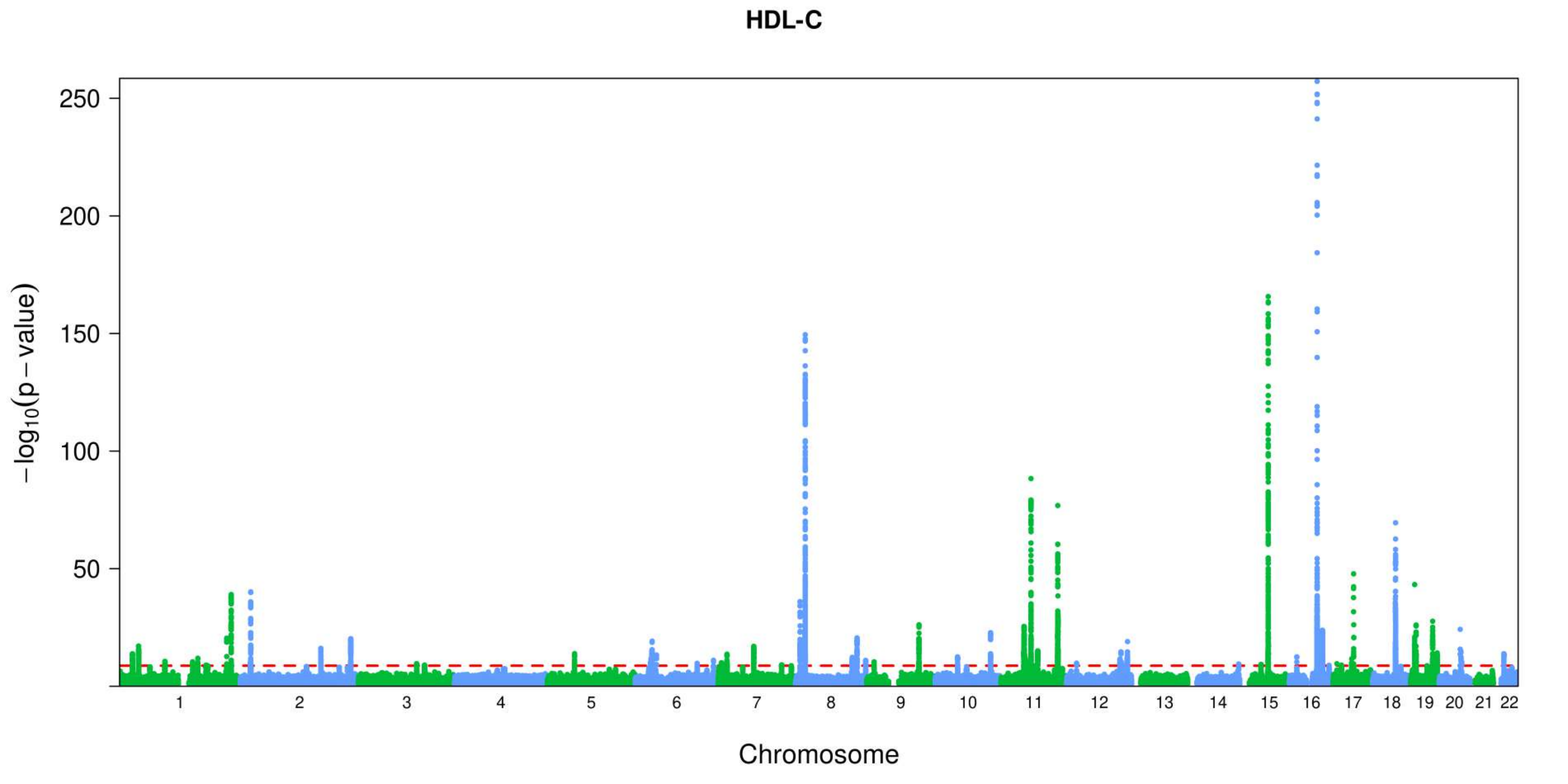
HDL-C
250
200
150
100
50
−log10(p − value)
1
2
3
4
5
6
7
8
9
10
11
12
13
14
15
16
17
18
19
20
21
22
Chromosome

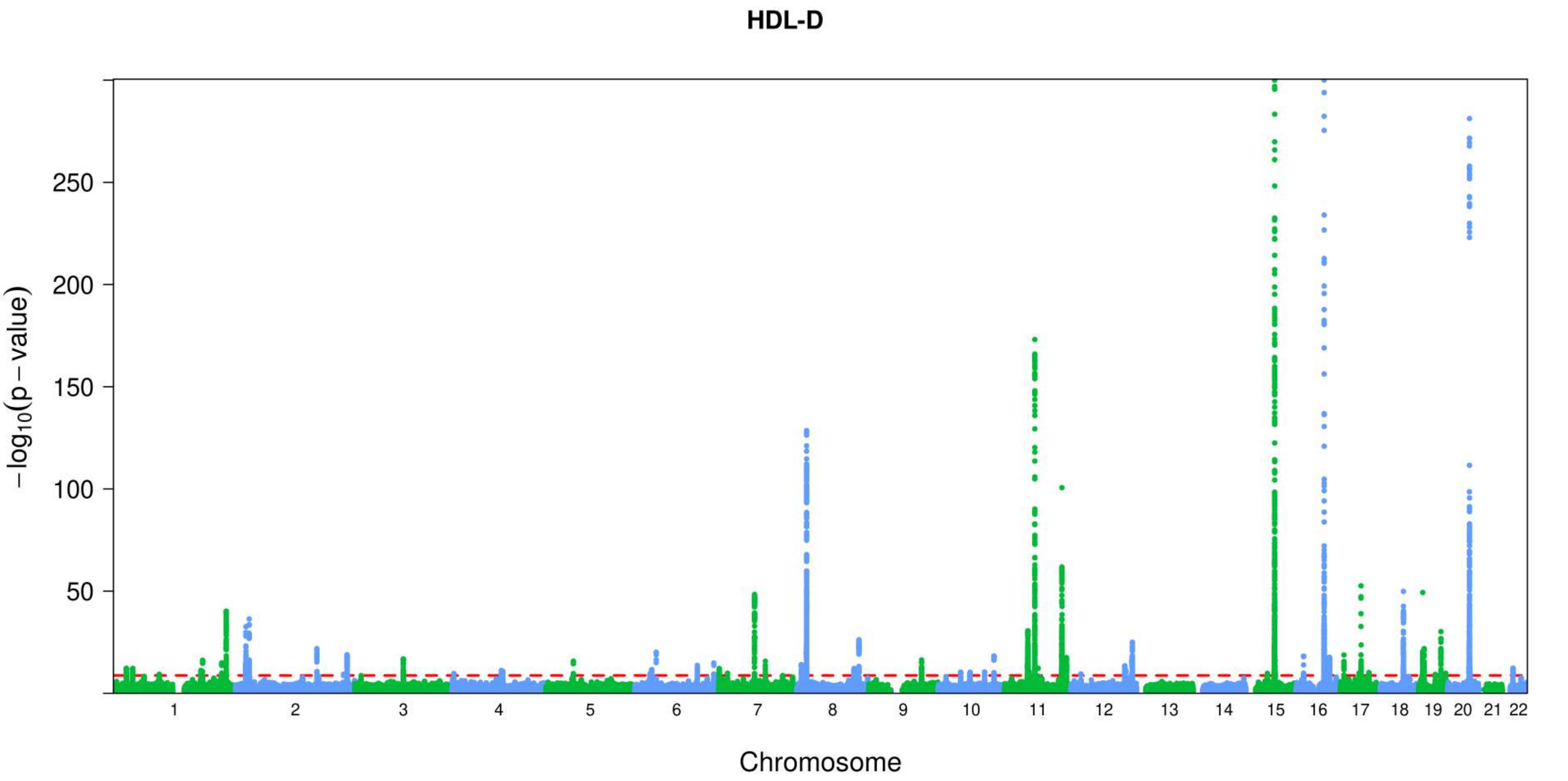
HDL-D
250
200
150
100
50
−log10(p − value)
1
2
3
4
5
6
7
8
9
10
11
12
13
14
15
16
17
18
19
20
21
22
Chromosome

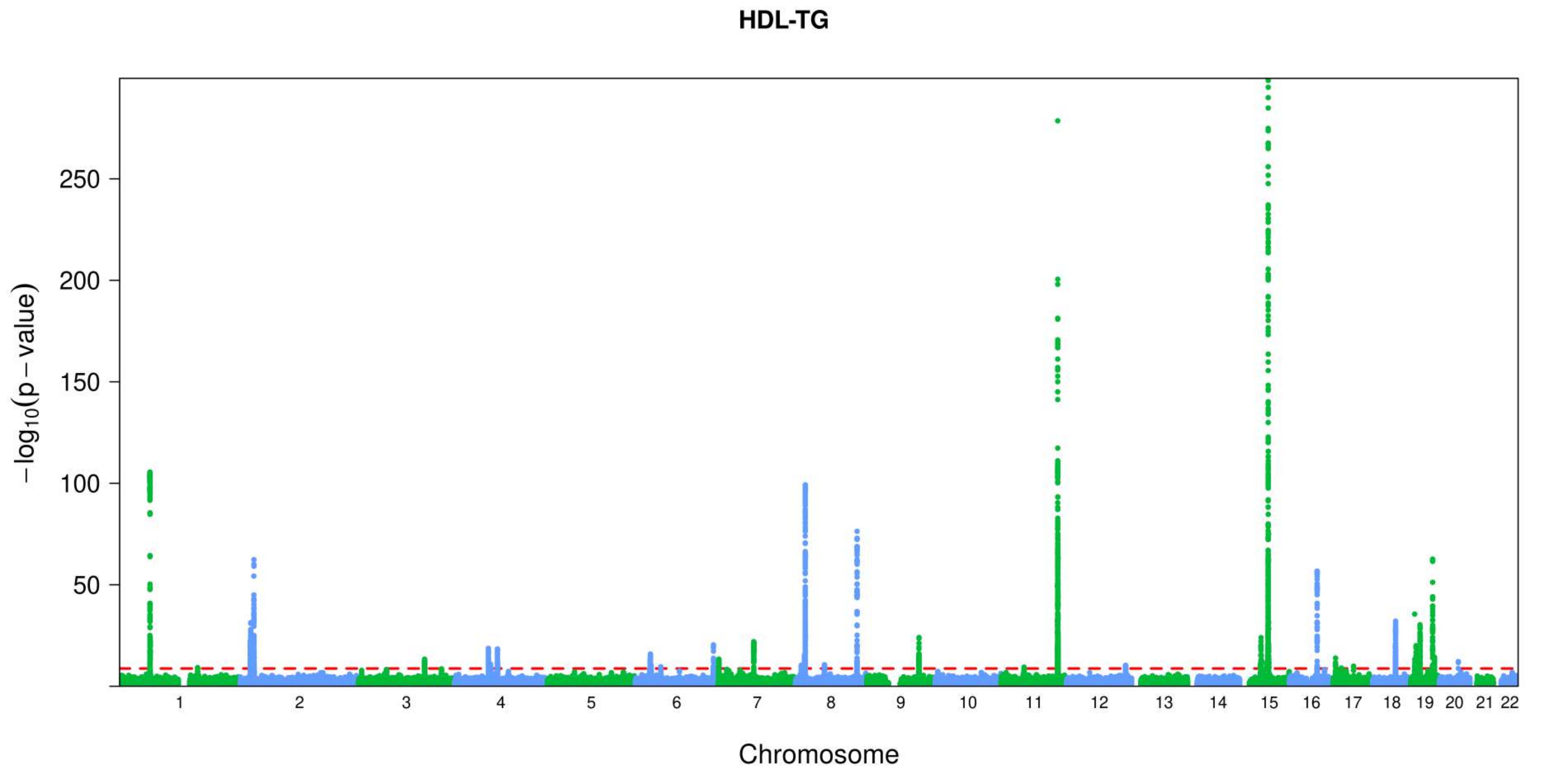
HDL-TG
$-\log_{10}(p-value)$
250
200
150
100
50
Chromosome
1
2
3
4
5
6
7
8
9
10
11
12
13
14
15
16
17
18
19
20
21
22

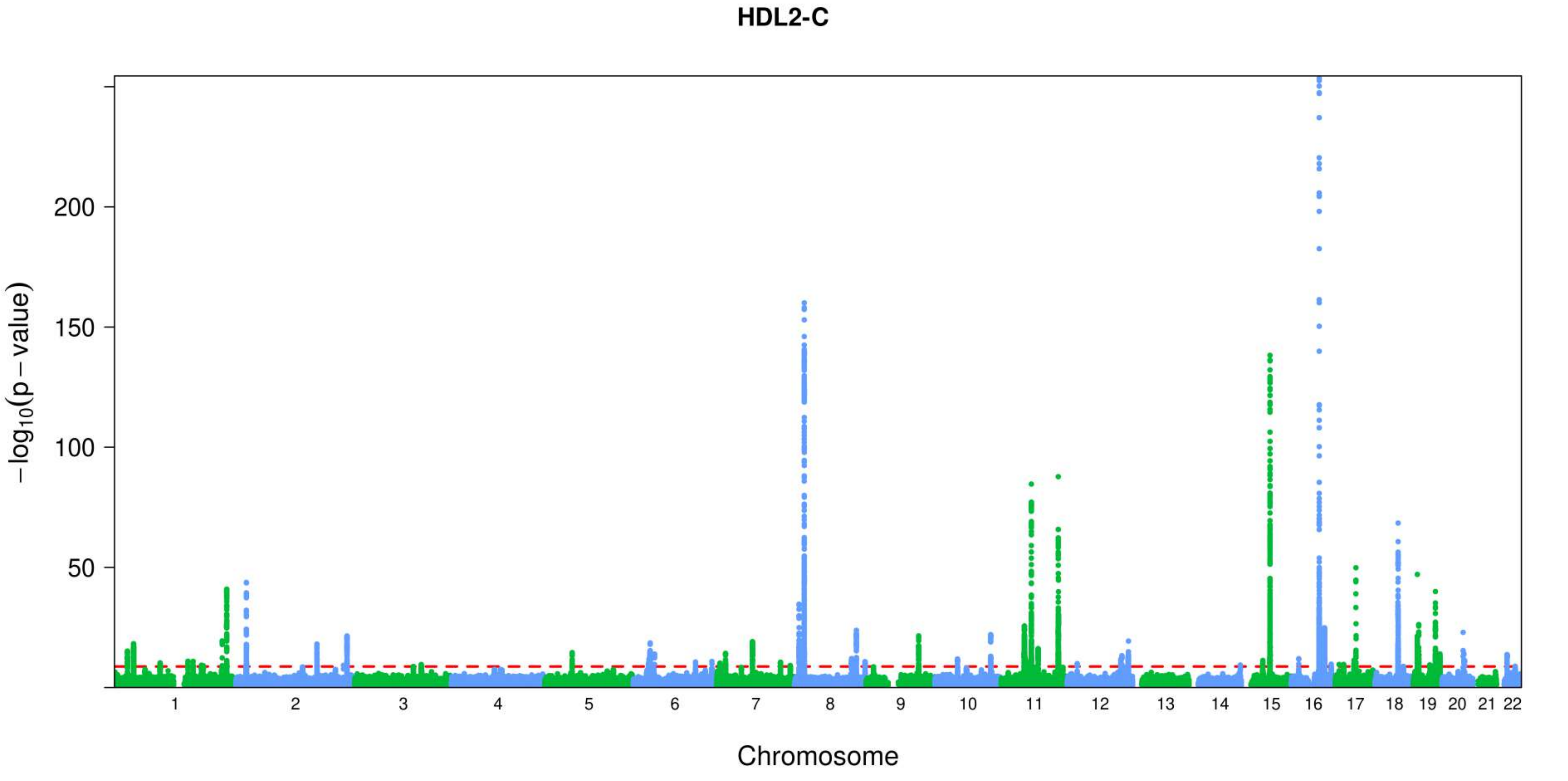

HDL2-C
200
150
100
50
−log10(p − value)
1
2
3
4
5
6
7
8
9
10
11
12
13
14
15
16
17
18
19
20
21
22
Chromosome

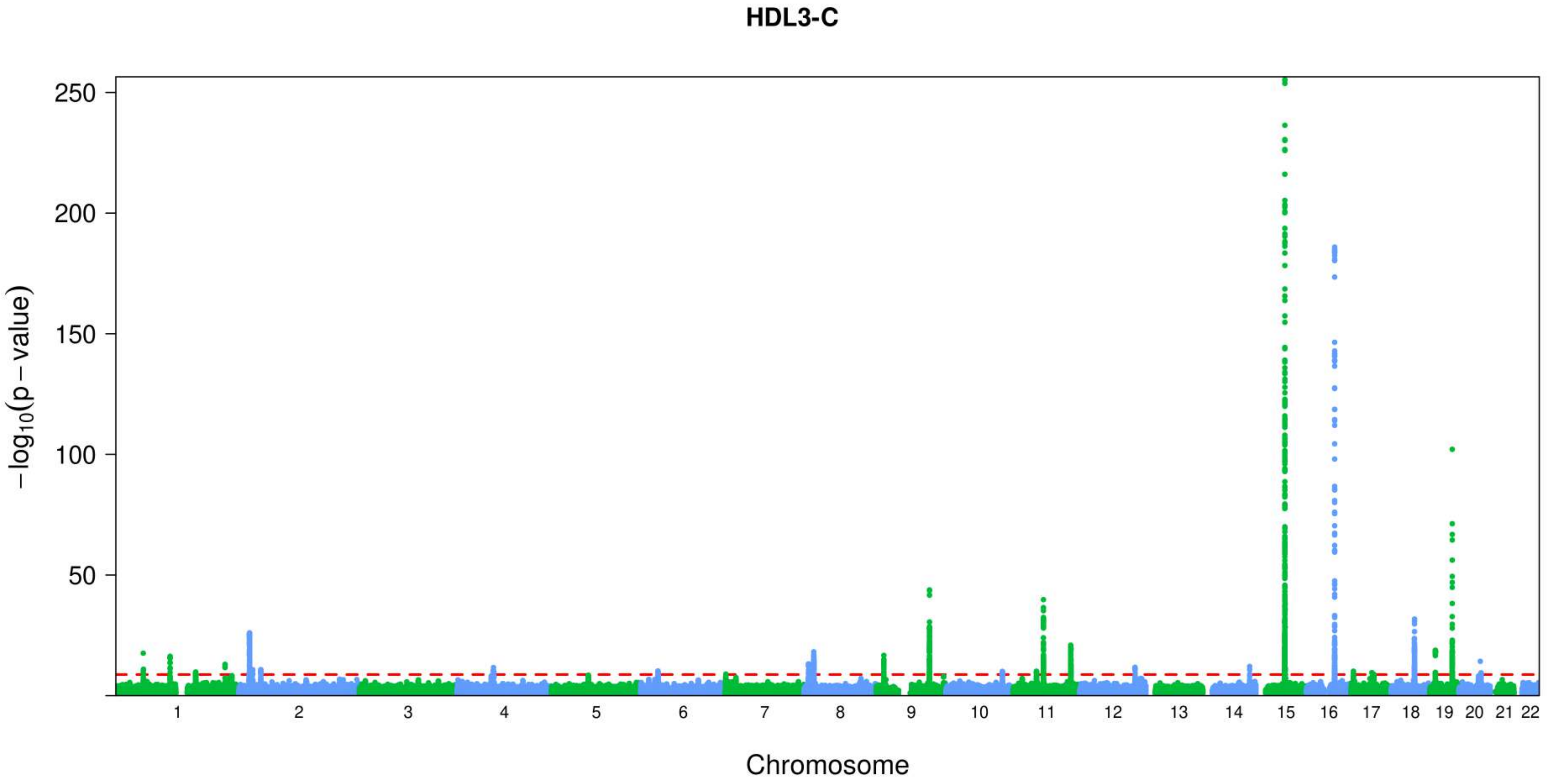
HDL3-C
250
200
150
100
50
$-\log_{10}(p-\text{value})$
1
2
3
4
5
6
7
8
9
10
11
12
13
14
15
16
17
18
19
20
21
22
Chromosome

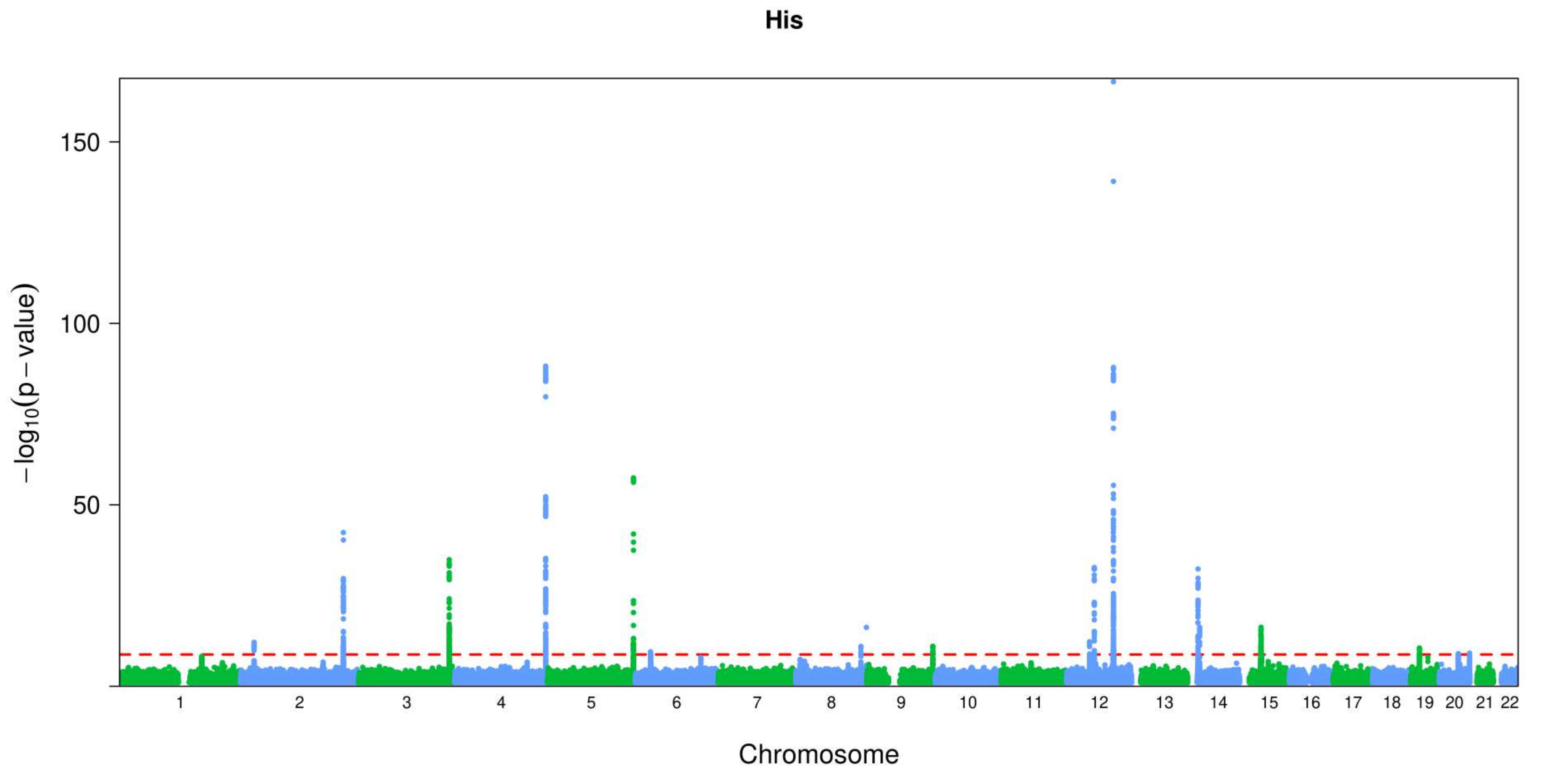
His
150
100
50
−log10(p − value)
1
2
3
4
5
6
7
8
9
10
11
12
13
14
15
16
17
18
19
20
21
22
Chromosome

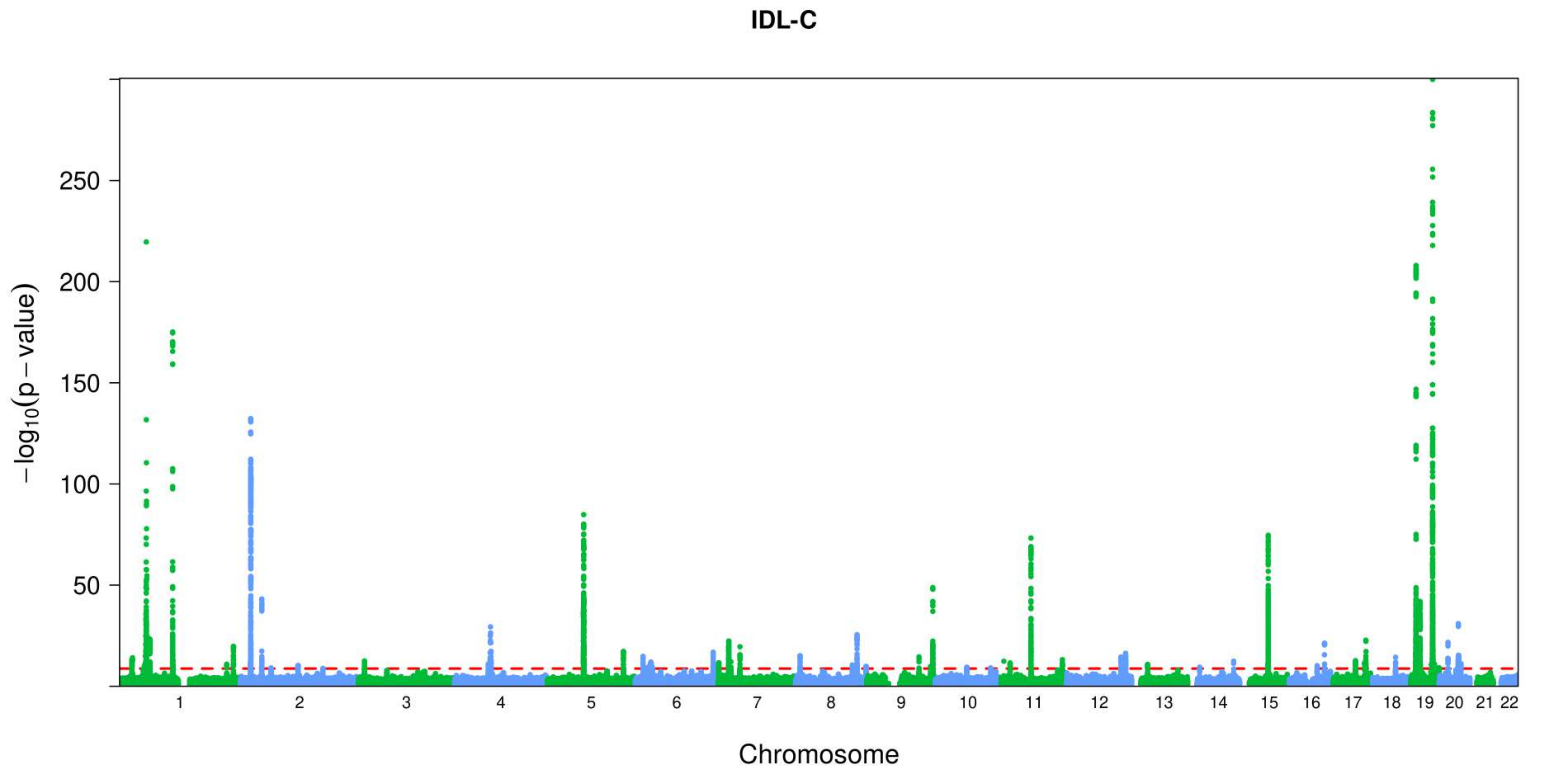
IDL-C
250
200
150
100
50
$-\log_{10}(p-value)$
1
2
3
4
5
6
7
8
9
10
11
12
13
14
15
16
17
18
19
20
21
22
Chromosome

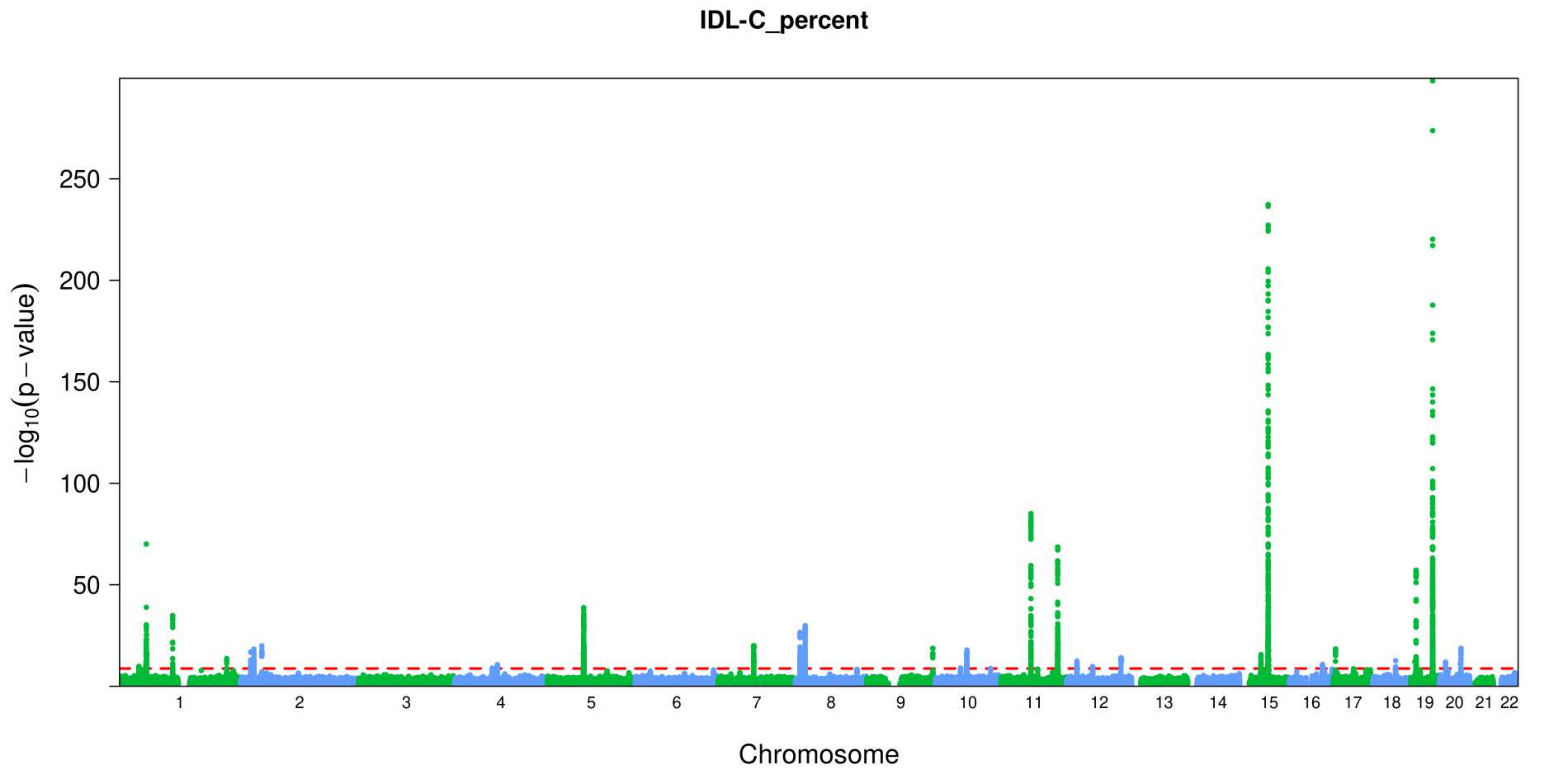

IDL-C_percent
250
200
150
100
50
−log10(p − value)
1
2
3
4
5
6
7
8
9
10
11
12
13
14
15
16
17
18
19
20
21
22
Chromosome

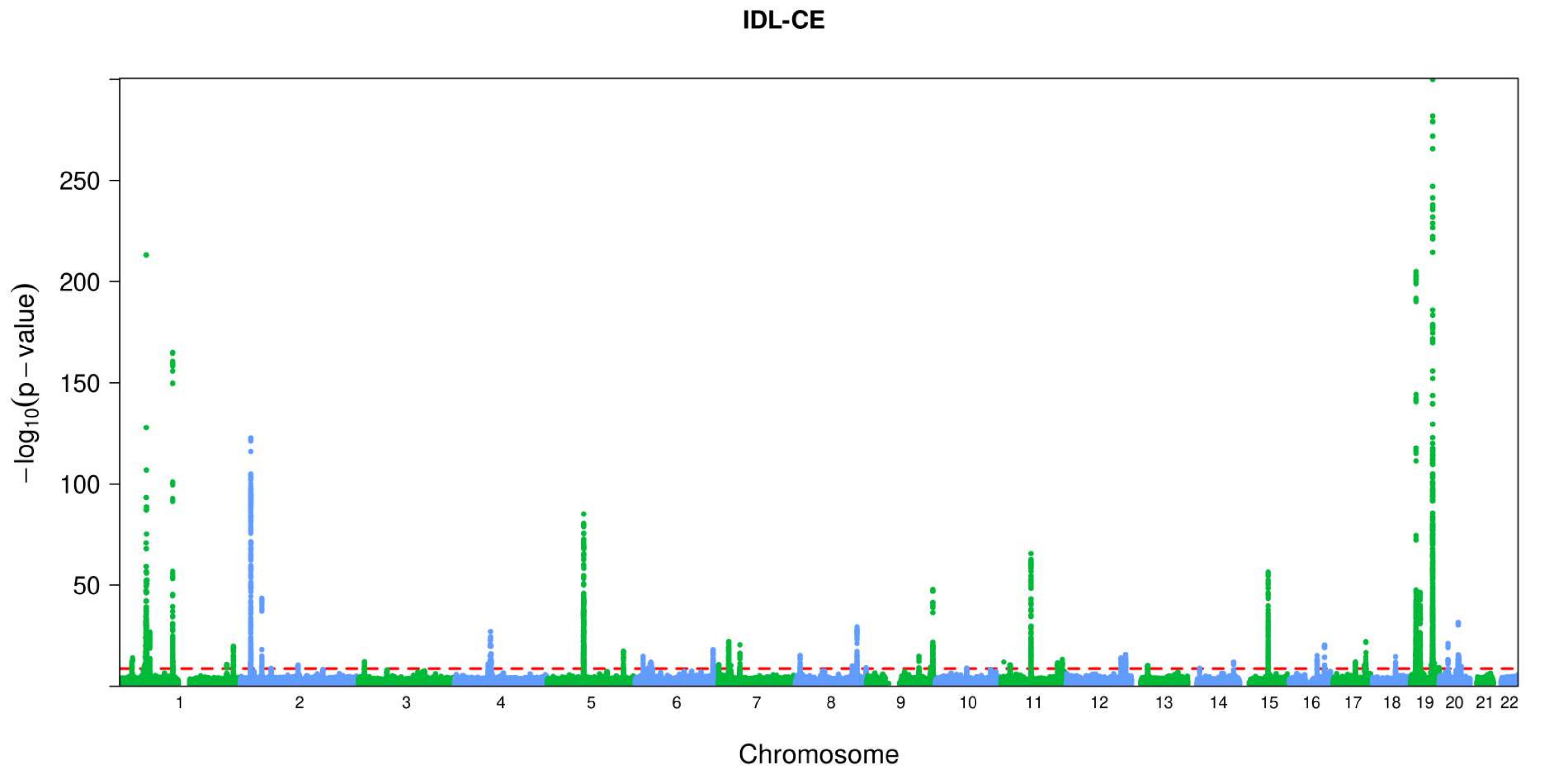
IDL-CE
250
200
150
100
50
−log10(p − value)
1
2
3
4
5
6
7
8
9
10
11
12
13
14
15
16
17
18
19
20
21
22
Chromosome

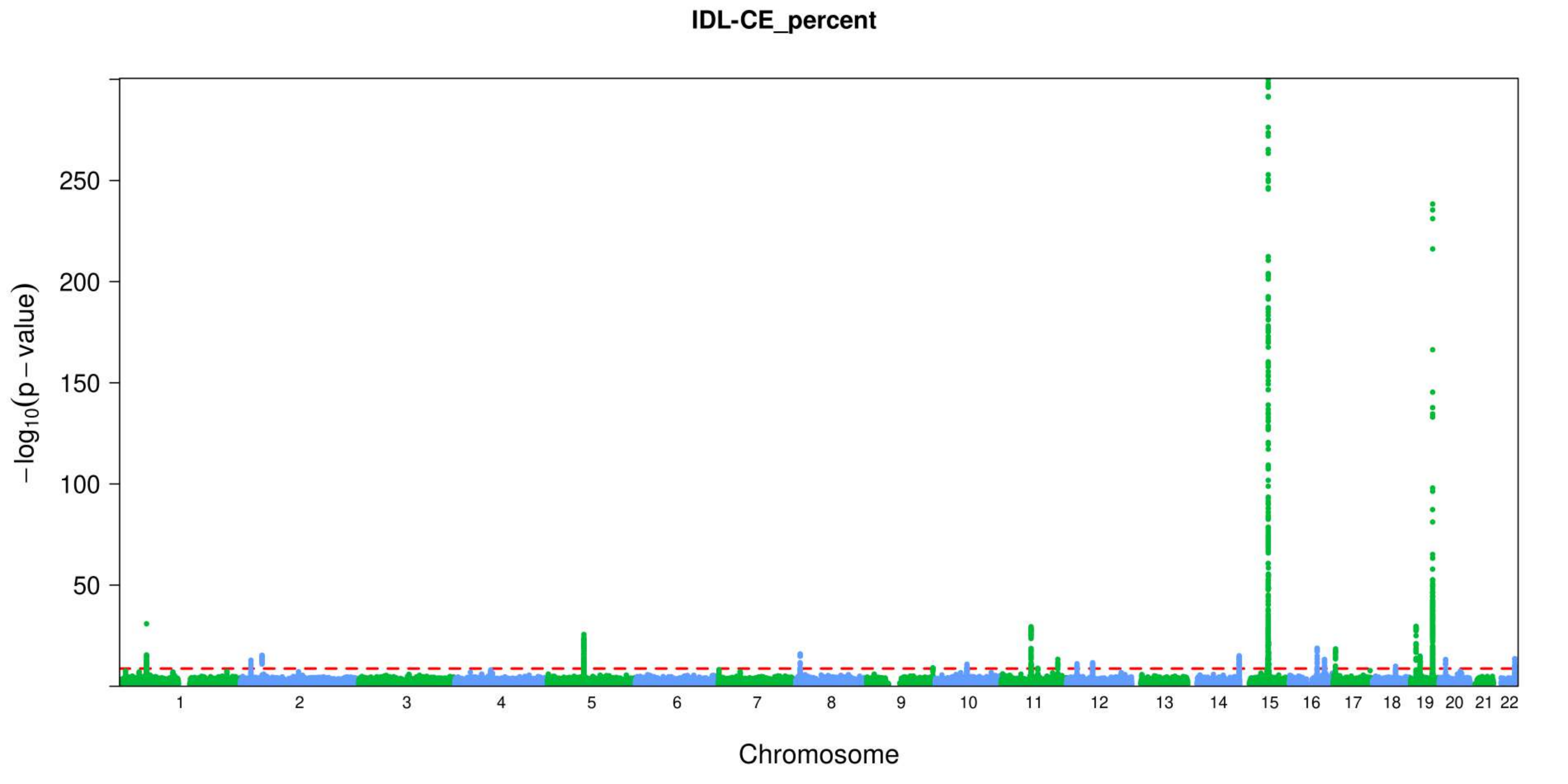
IDL-CE_percent
250
200
150
100
50
-log10(p - value)
1
2
3
4
5
6
7
8
9
10
11
12
13
14
15
16
17
18
19
20
21
22
Chromosome

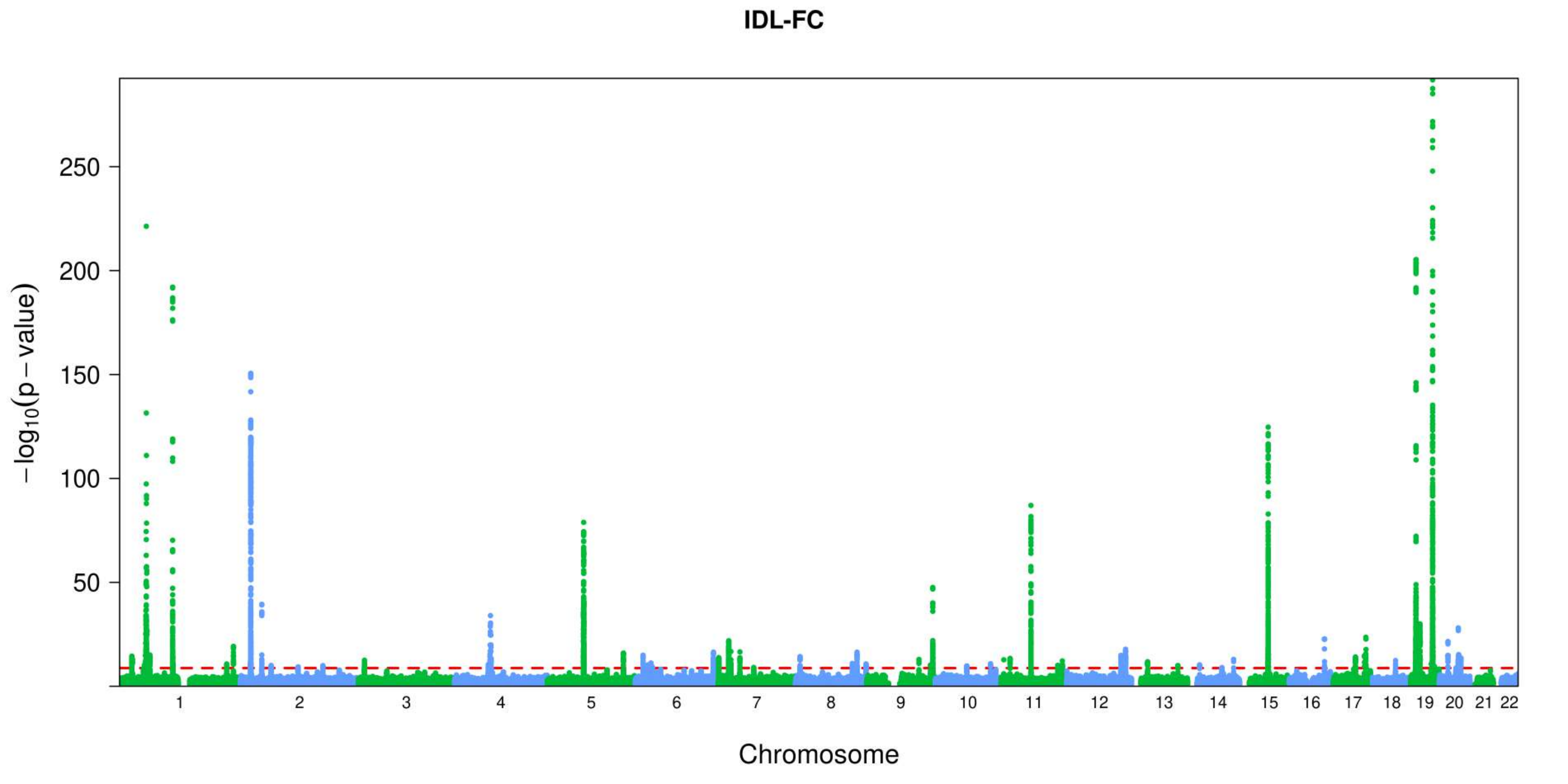
IDL-FC
250
200
150
100
50
−log10(p − value)
1
2
3
4
5
6
7
8
9
10
11
12
13
14
15
16
17
18
19
20
21
22
Chromosome

IDL-FC_percent

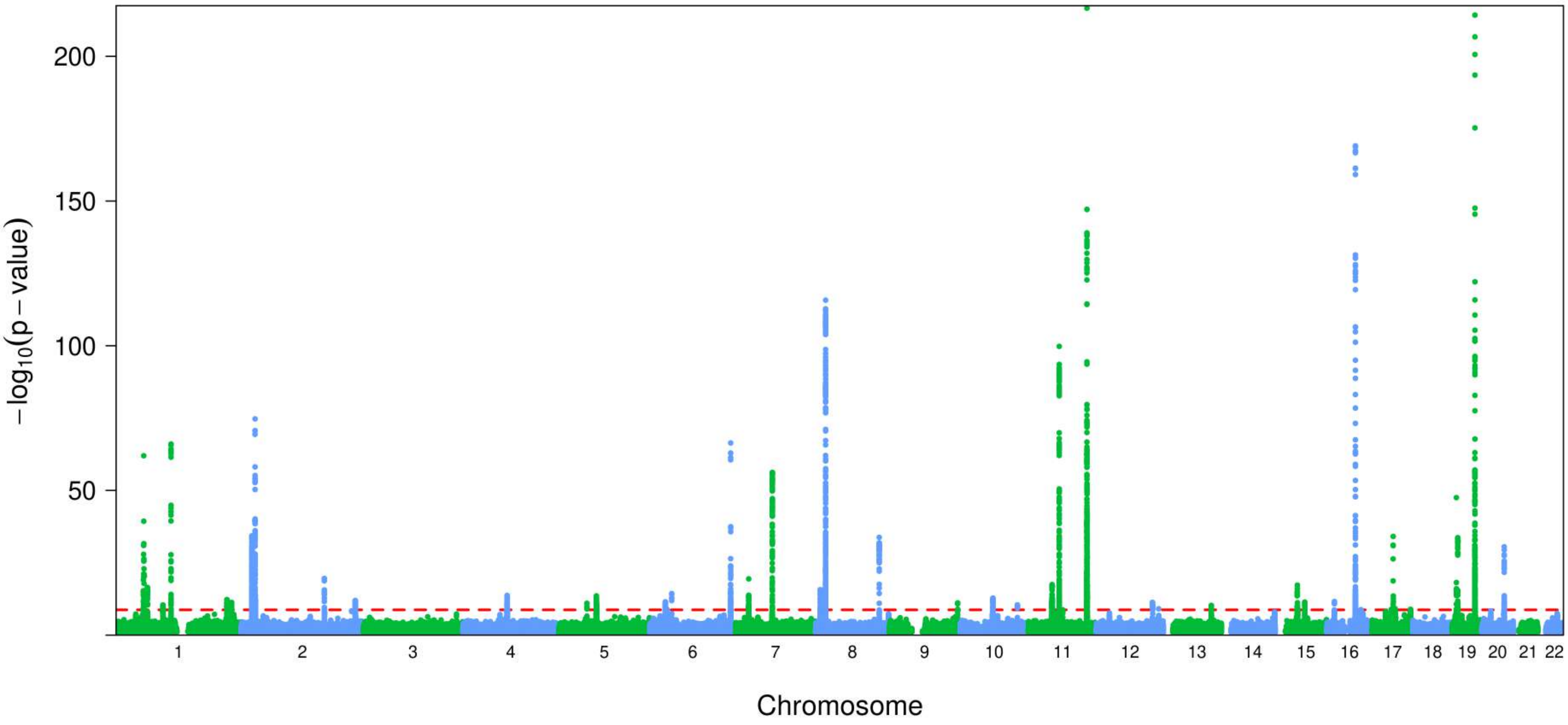

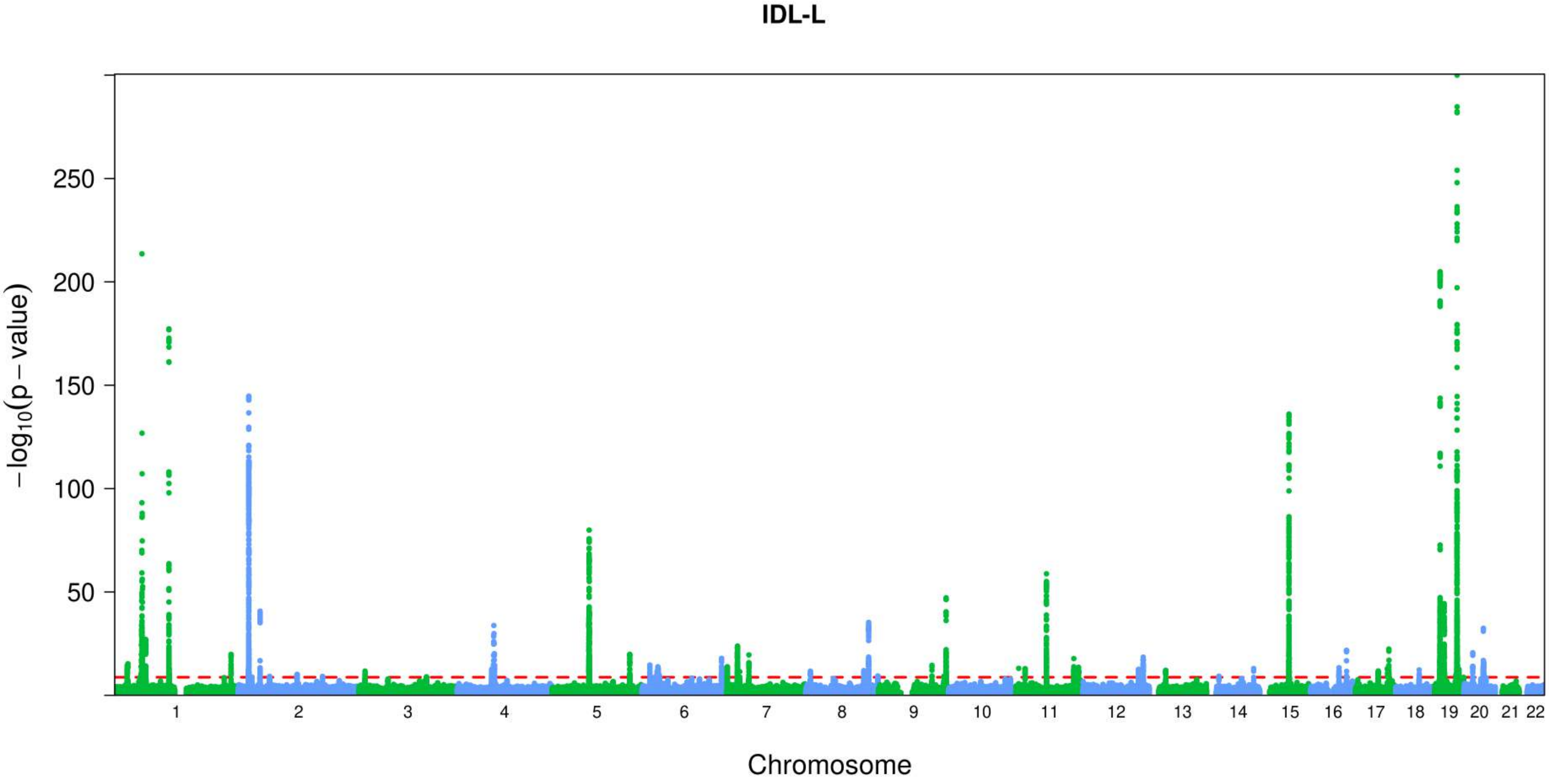
IDL-L
250
200
150
100
50
$-\log_{10}(p-\text{value})$
1
2
3
4
5
6
7
8
9
10
11
12
13
14
15
16
17
18
19
20
21
22
Chromosome

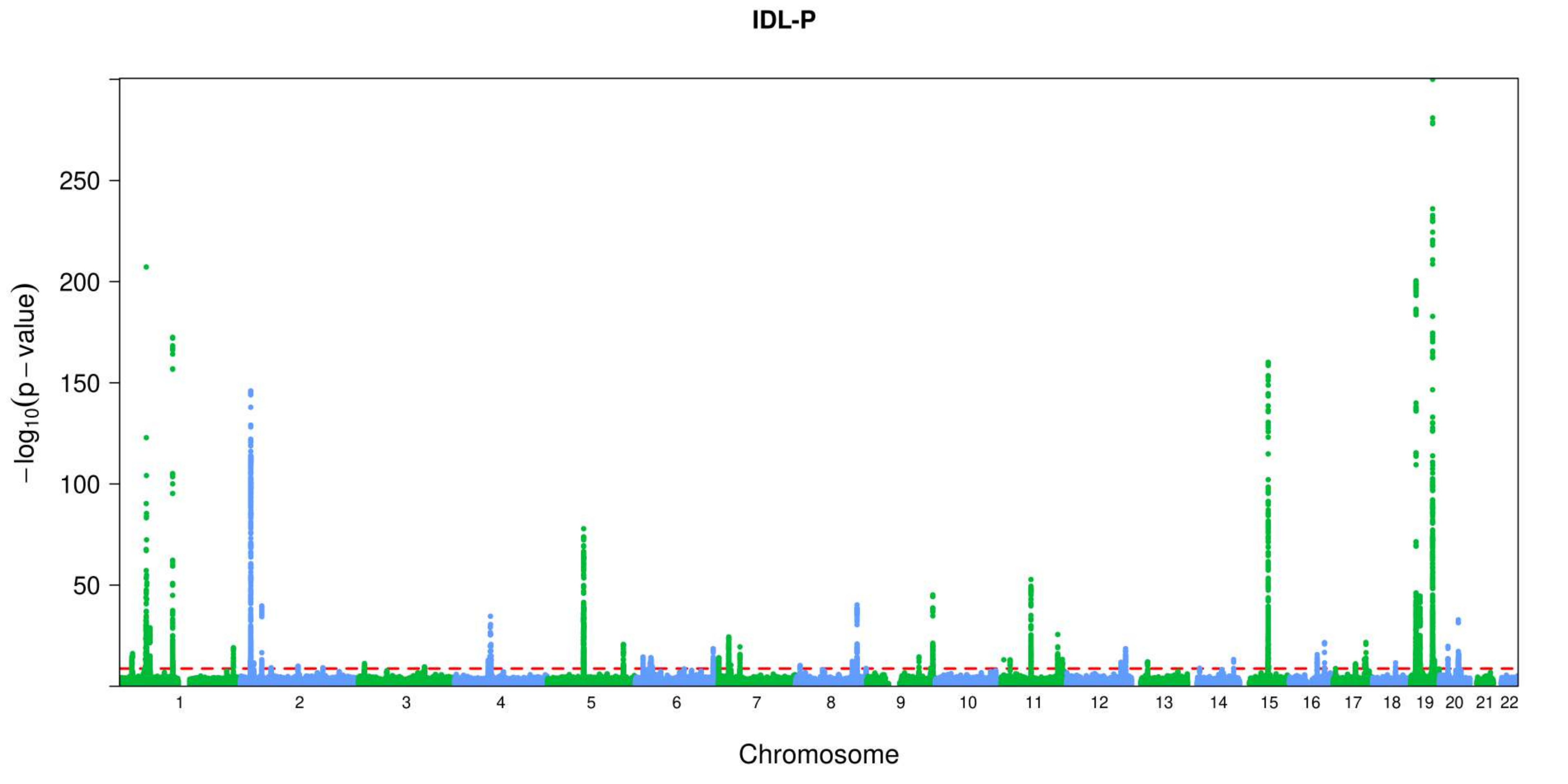
IDL-P
250
200
150
100
50
−log10(p − value)
1
2
3
4
5
6
7
8
9
10
11
12
13
14
15
16
17
18
19
20
21
22
Chromosome

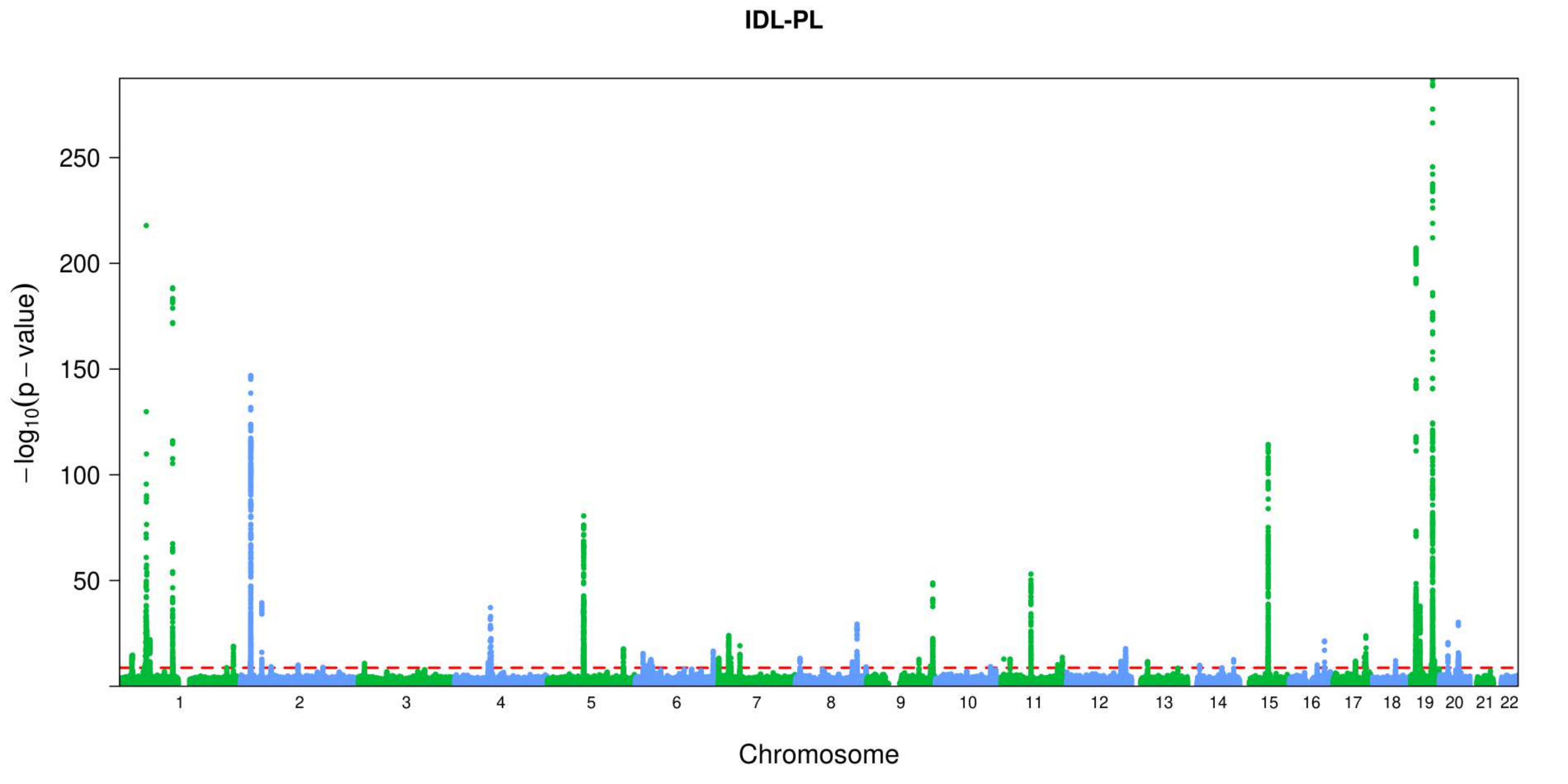

IDL-PL
250
200
150
100
50
−log10(p − value)
1
2
3
4
5
6
7
8
9
10
11
12
13
14
15
16
17
18
19
20
21
22
Chromosome

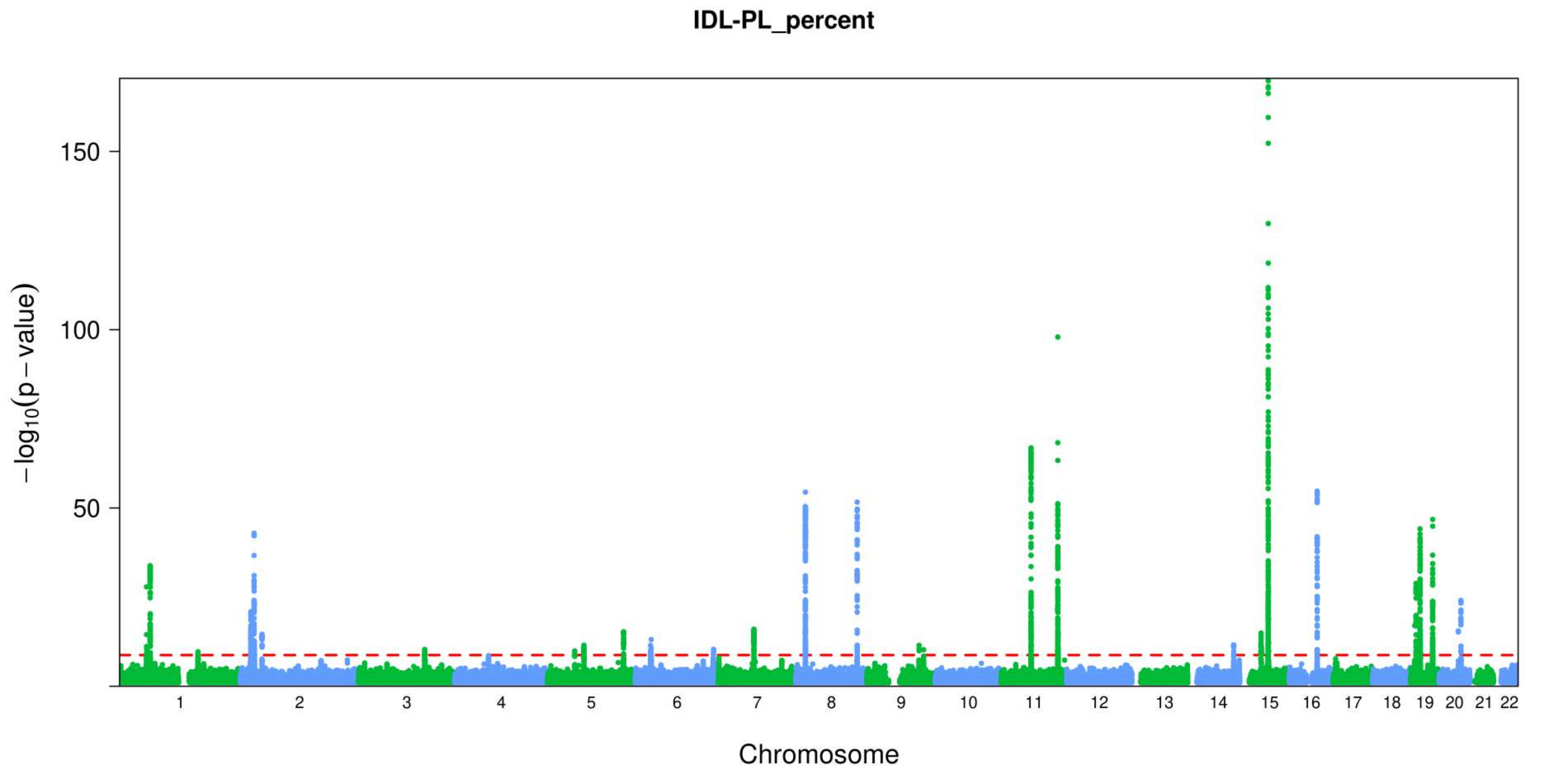
IDL-PL_percent
$-\log_{10}(p-\text{value})$
150
100
50
1
2
3
4
5
6
7
8
9
10
11
12
13
14
15
16
17
18
19
20
21
22
Chromosome

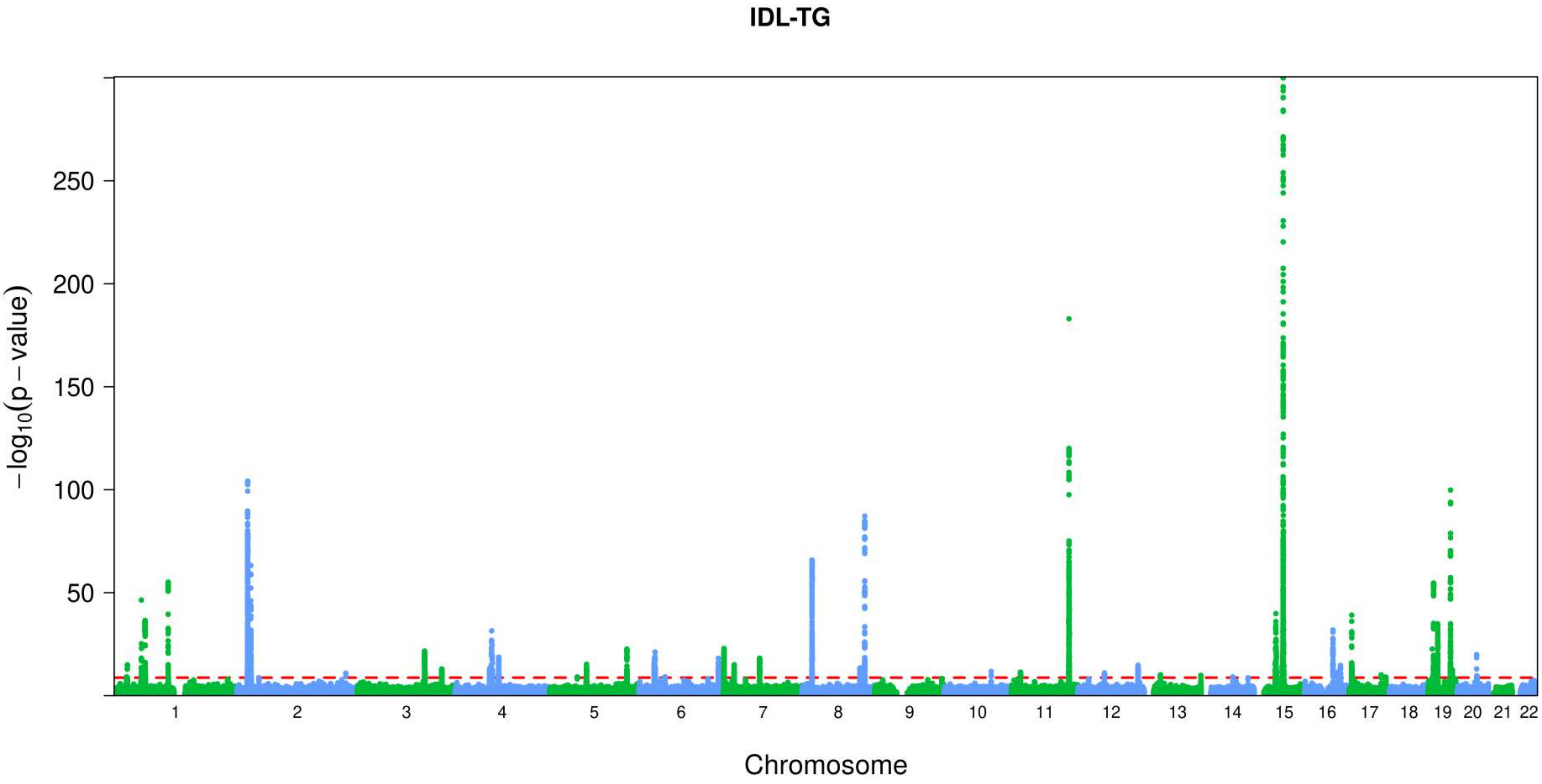
IDL-TG
250
200
150
100
50
$-\log_{10}(p-\text{value})$
1
2
3
4
5
6
7
8
9
10
11
12
13
14
15
16
17
18
19
20
21
22
Chromosome

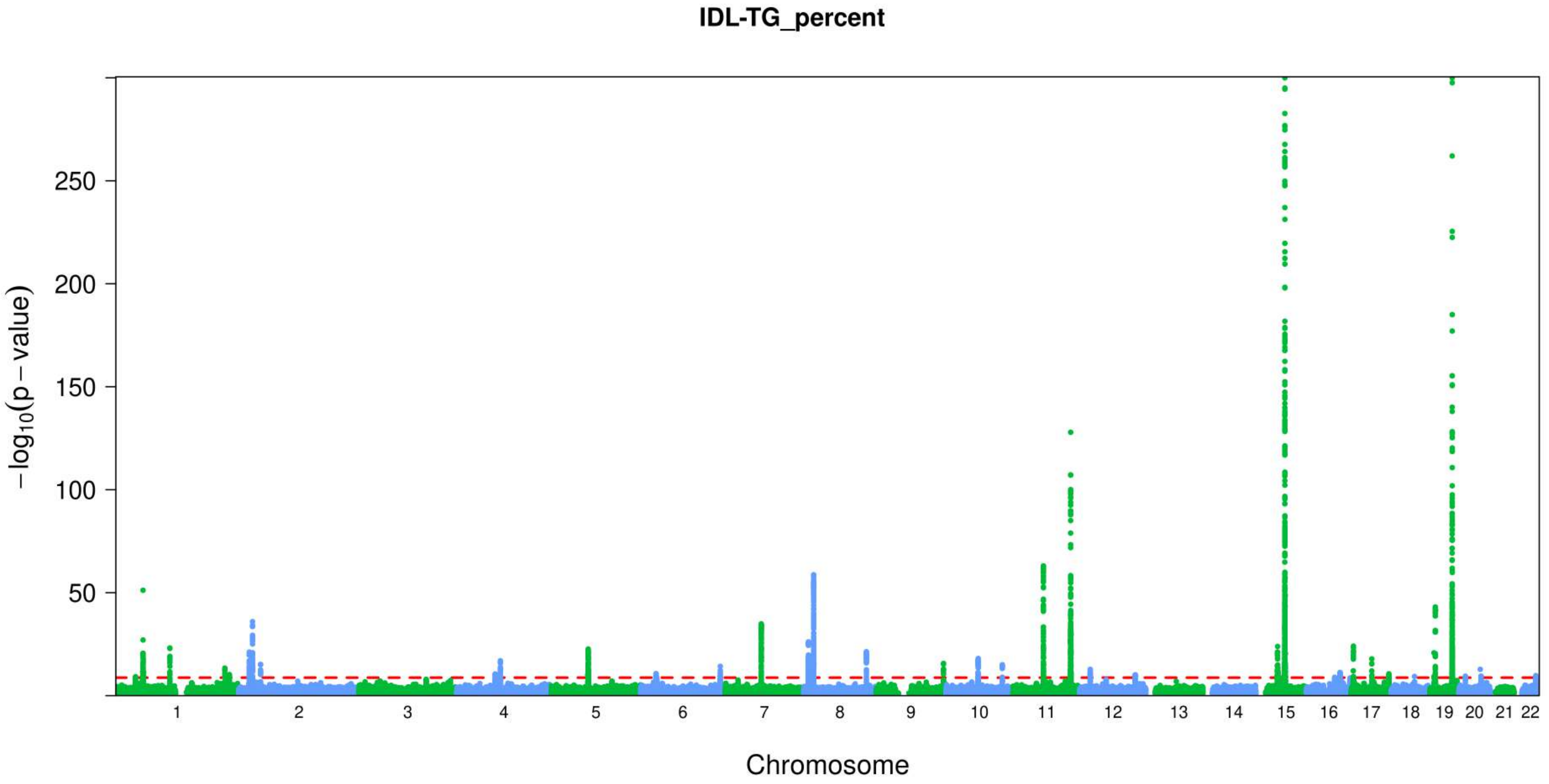
IDL-TG_percent
250
200
150
100
50
$-\log_{10}(p-\text{value})$
1
2
3
4
5
6
7
8
9
10
11
12
13
14
15
16
17
18
19
20
21
22
Chromosome

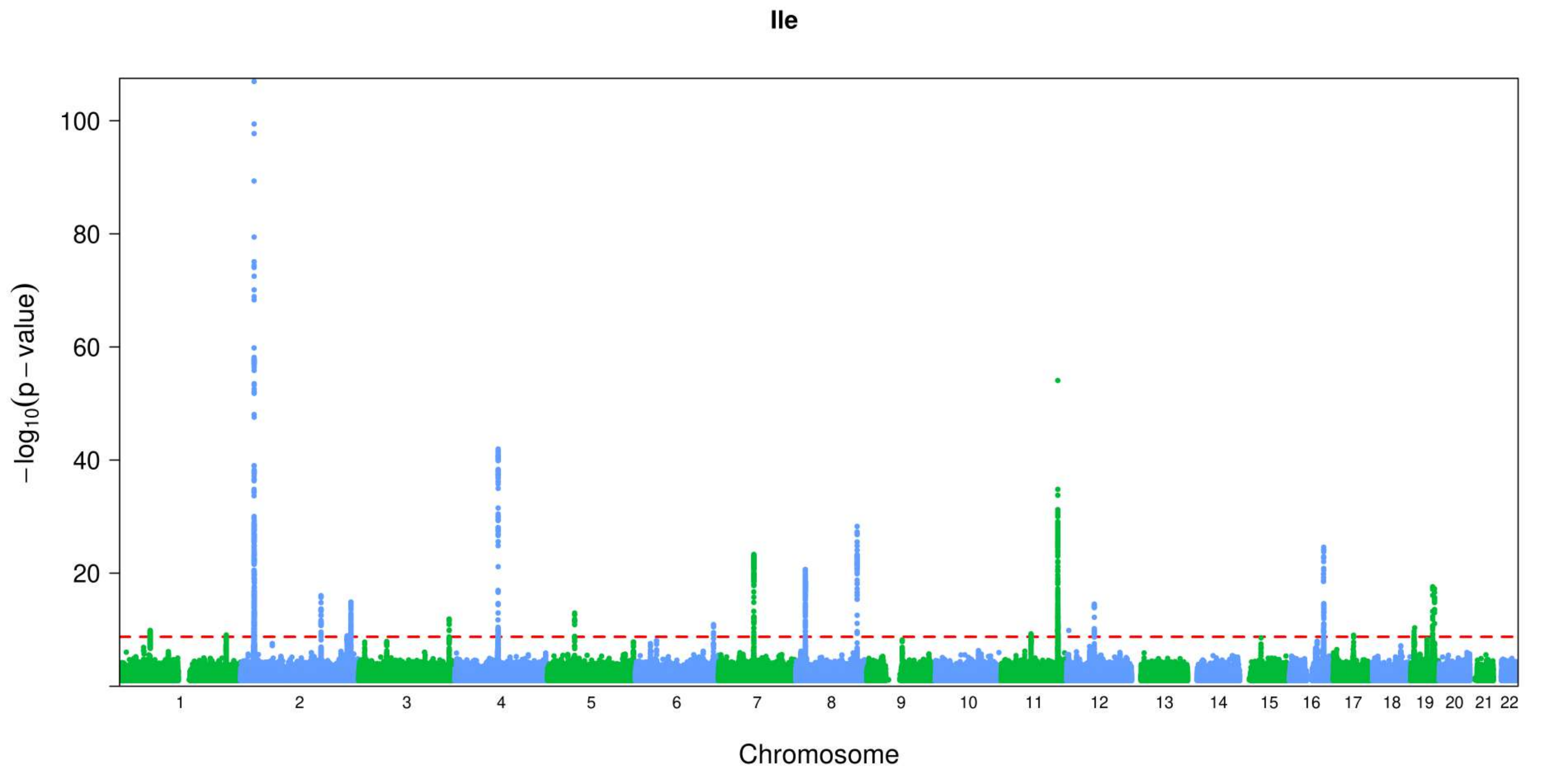

Ile
100
80
60
40
20
−log10(p − value)
1
2
3
4
5
6
7
8
9
10
11
12
13
14
15
16
17
18
19
20
21
22
Chromosome

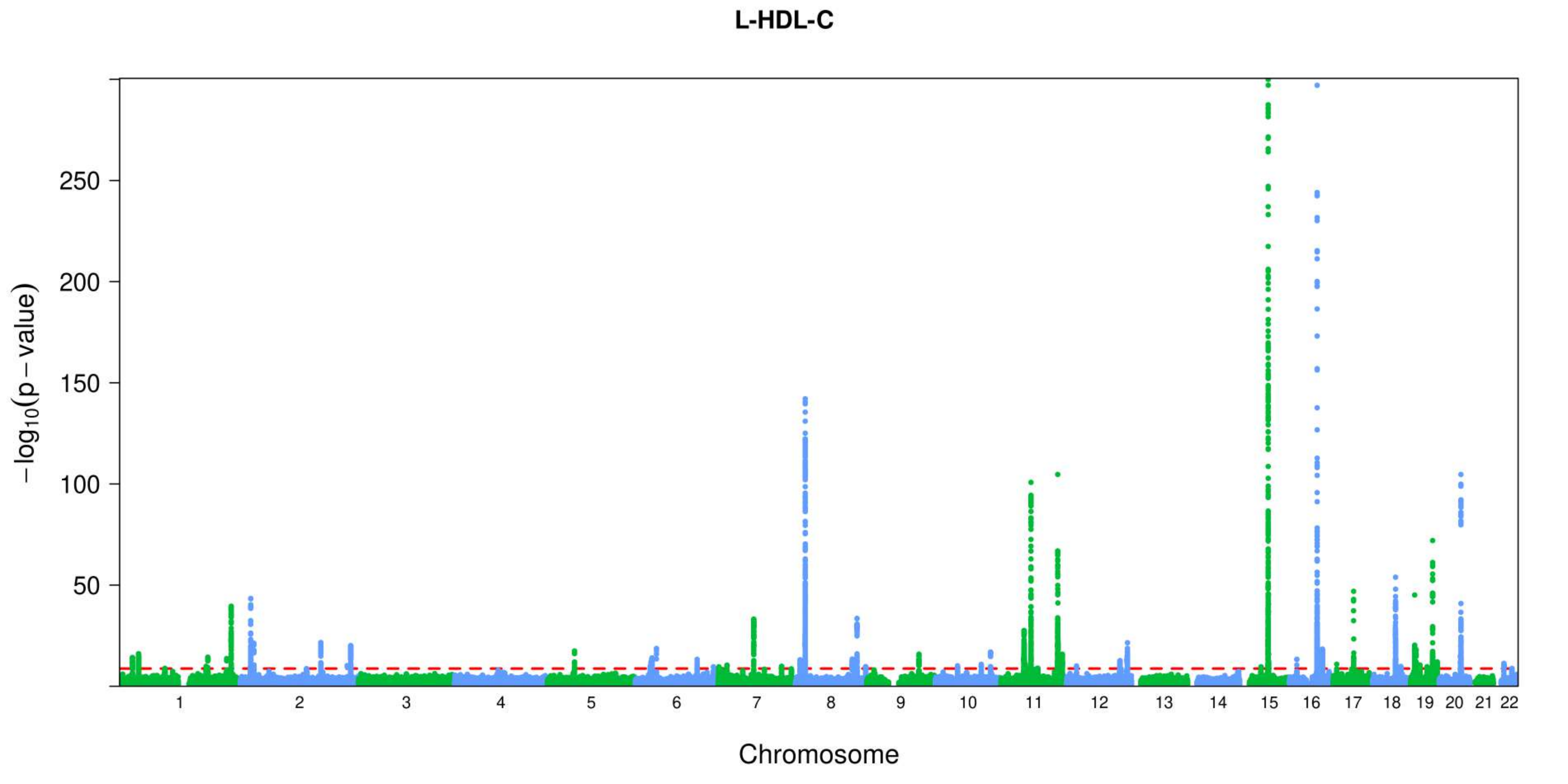

L-HDL-C
250
200
150
100
50
−log10(p − value)
1
2
3
4
5
6
7
8
9
10
11
12
13
14
15
16
17
18
19
20
21
22
Chromosome

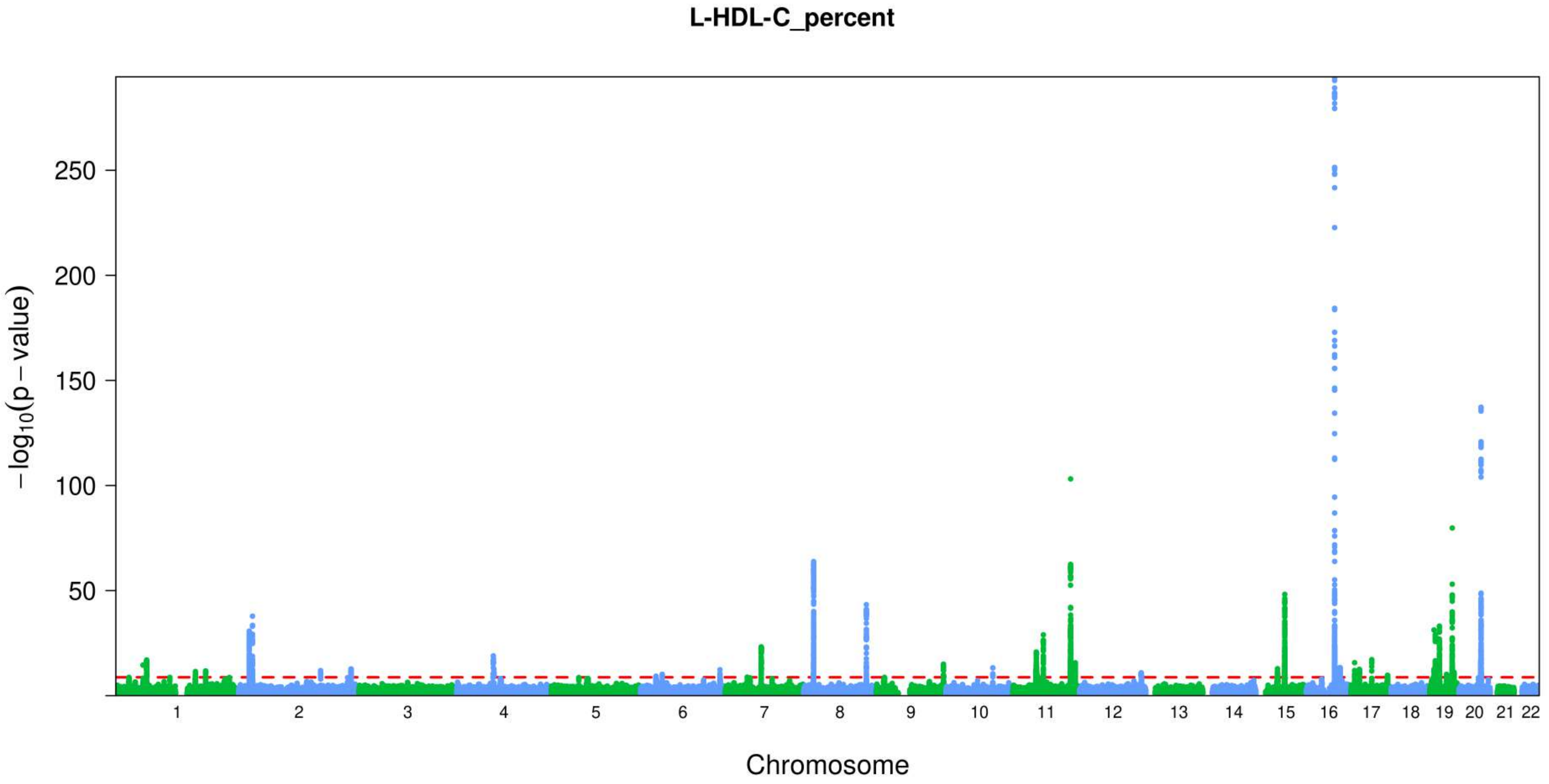
L-HDL-C_percent
250
200
150
100
50
-log10(p - value)
1
2
3
4
5
6
7
8
9
10
11
12
13
14
15
16
17
18
19
20
21
22
Chromosome

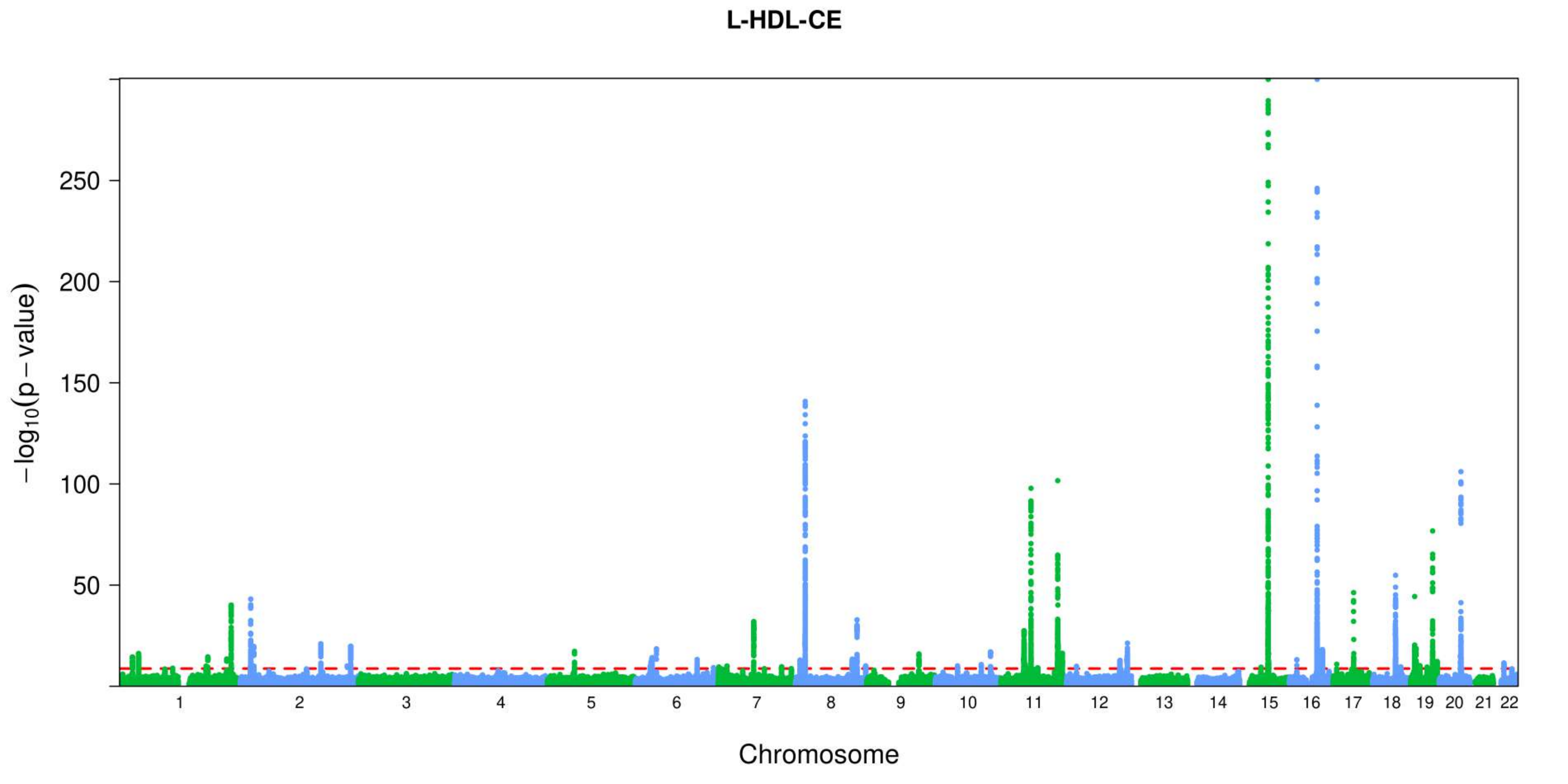

L-HDL-CE
250
200
150
100
50
−log10(p − value)
1
2
3
4
5
6
7
8
9
10
11
12
13
14
15
16
17
18
19
20
21
22
Chromosome

L-HDL-CE_percent

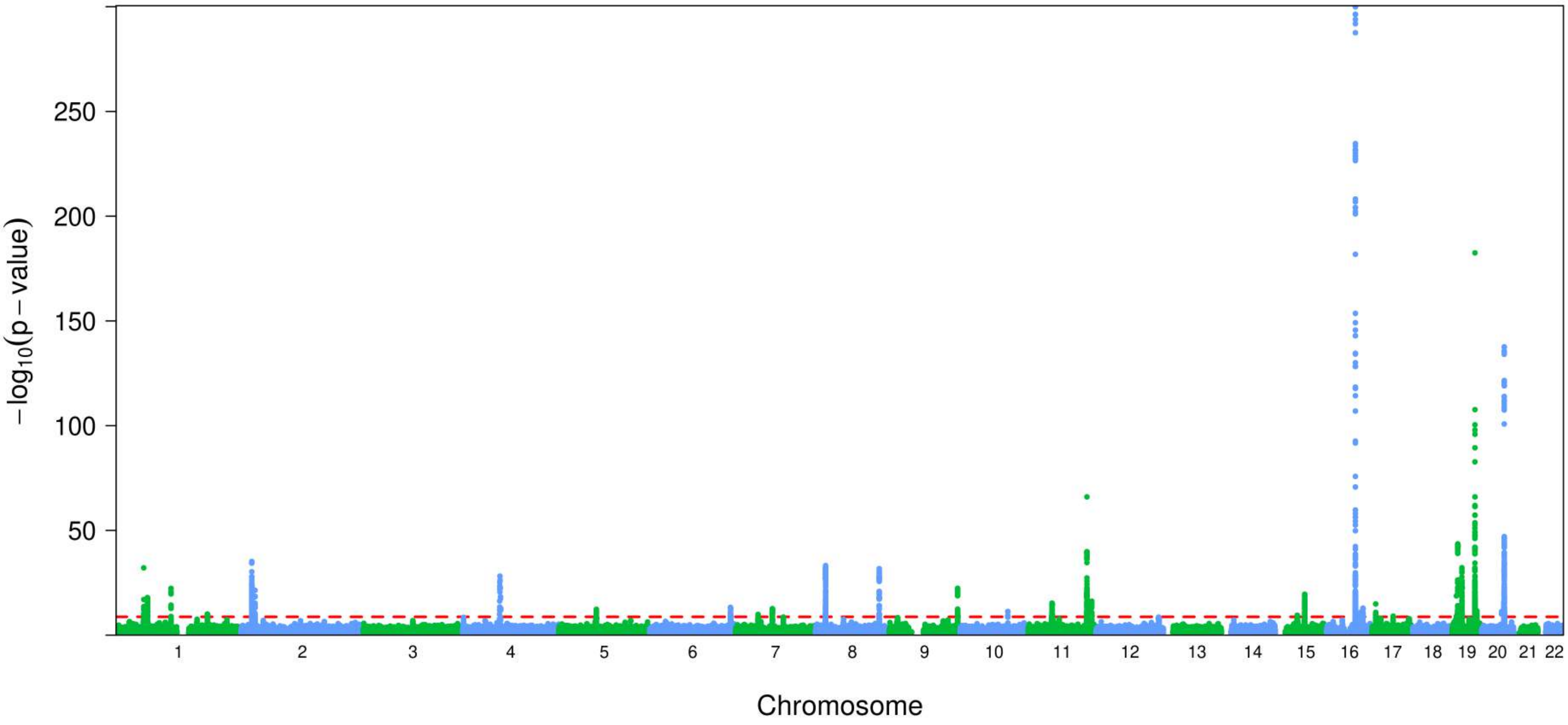

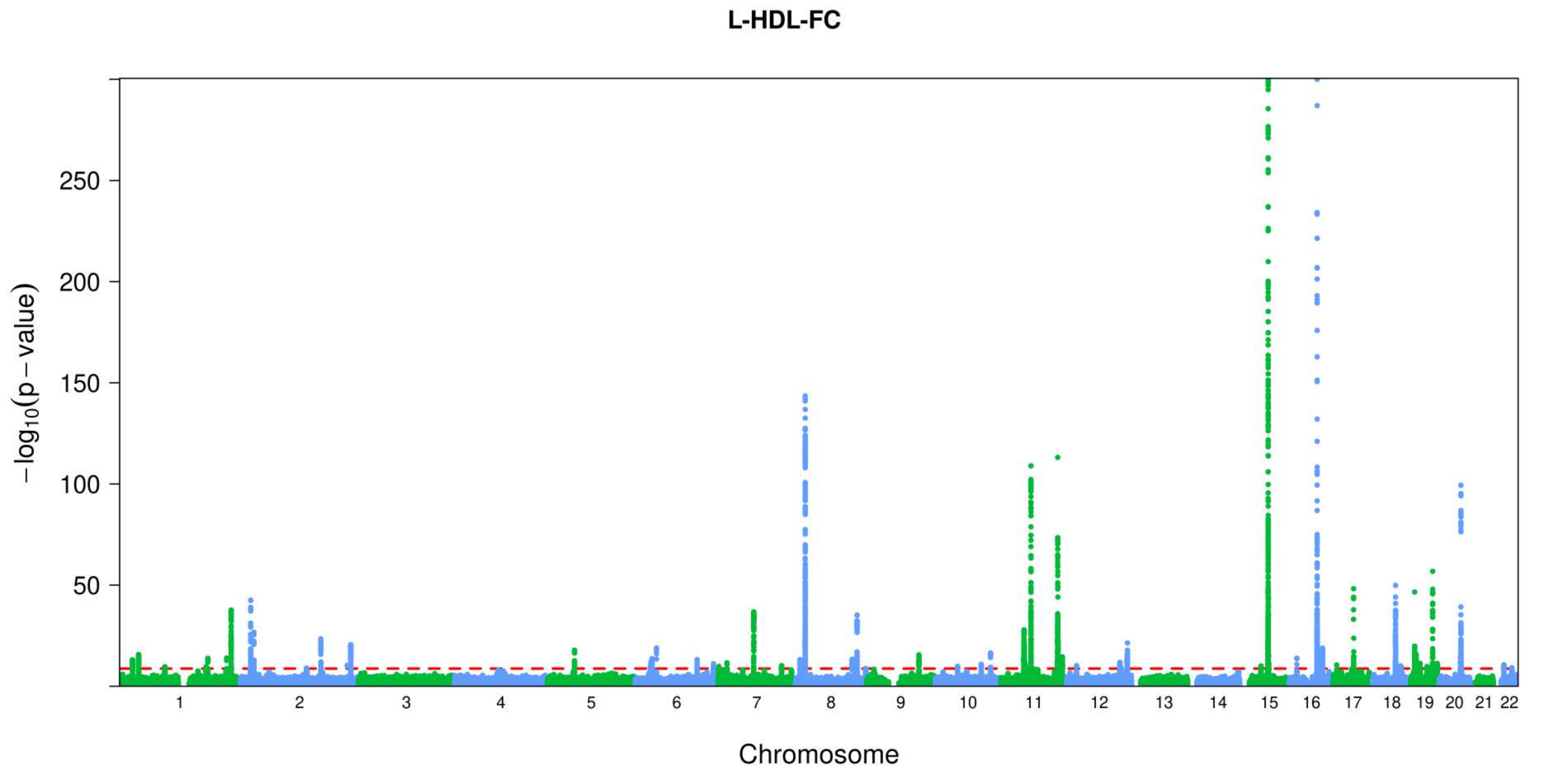

L-HDL-FC
250
200
150
100
50
$-\log_{10}(p-\text{value})$
1
2
3
4
5
6
7
8
9
10
11
12
13
14
15
16
17
18
19
20
21
22
Chromosome

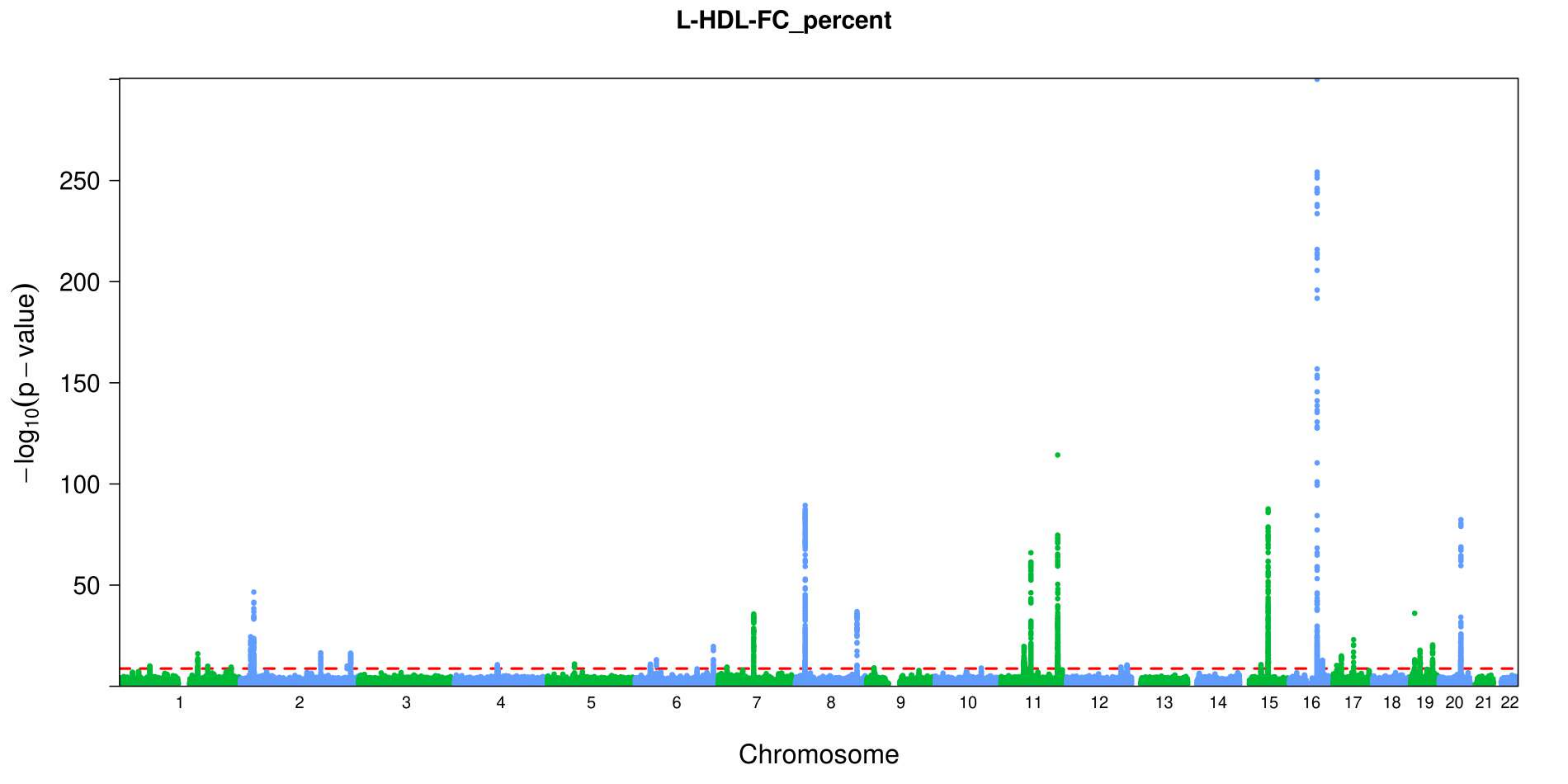
L-HDL-FC_percent
−log10(p − value)
250
200
150
100
50
Chromosome
1
2
3
4
5
6
7
8
9
10
11
12
13
14
15
16
17
18
19
20
21
22

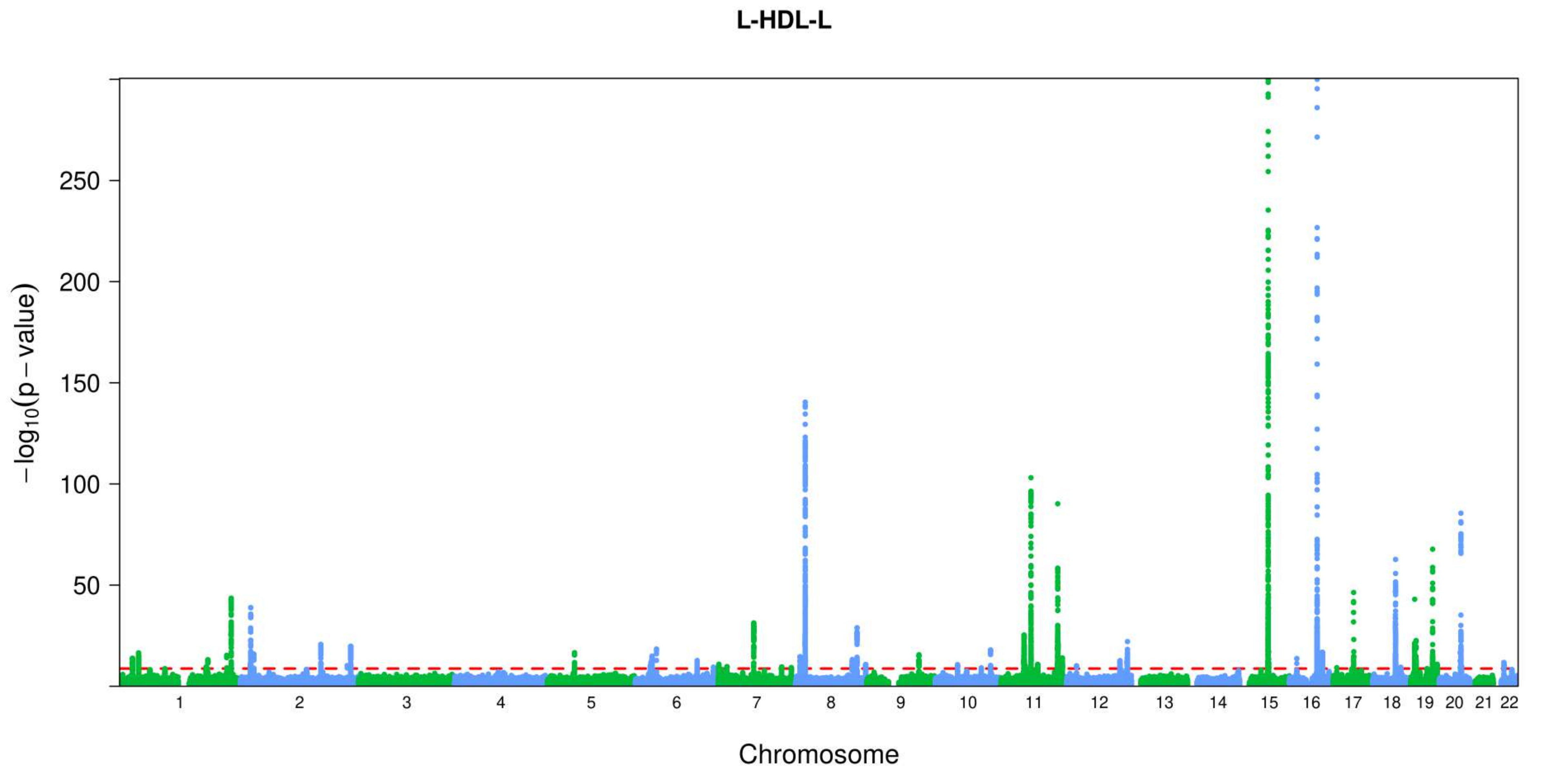
L-HDL-L
−log10(p − value)
250
200
150
100
50
1 2 3 4 5 6 7 8 9 10 11 12 13 14 15 16 17 18 19 20 21 22
Chromosome

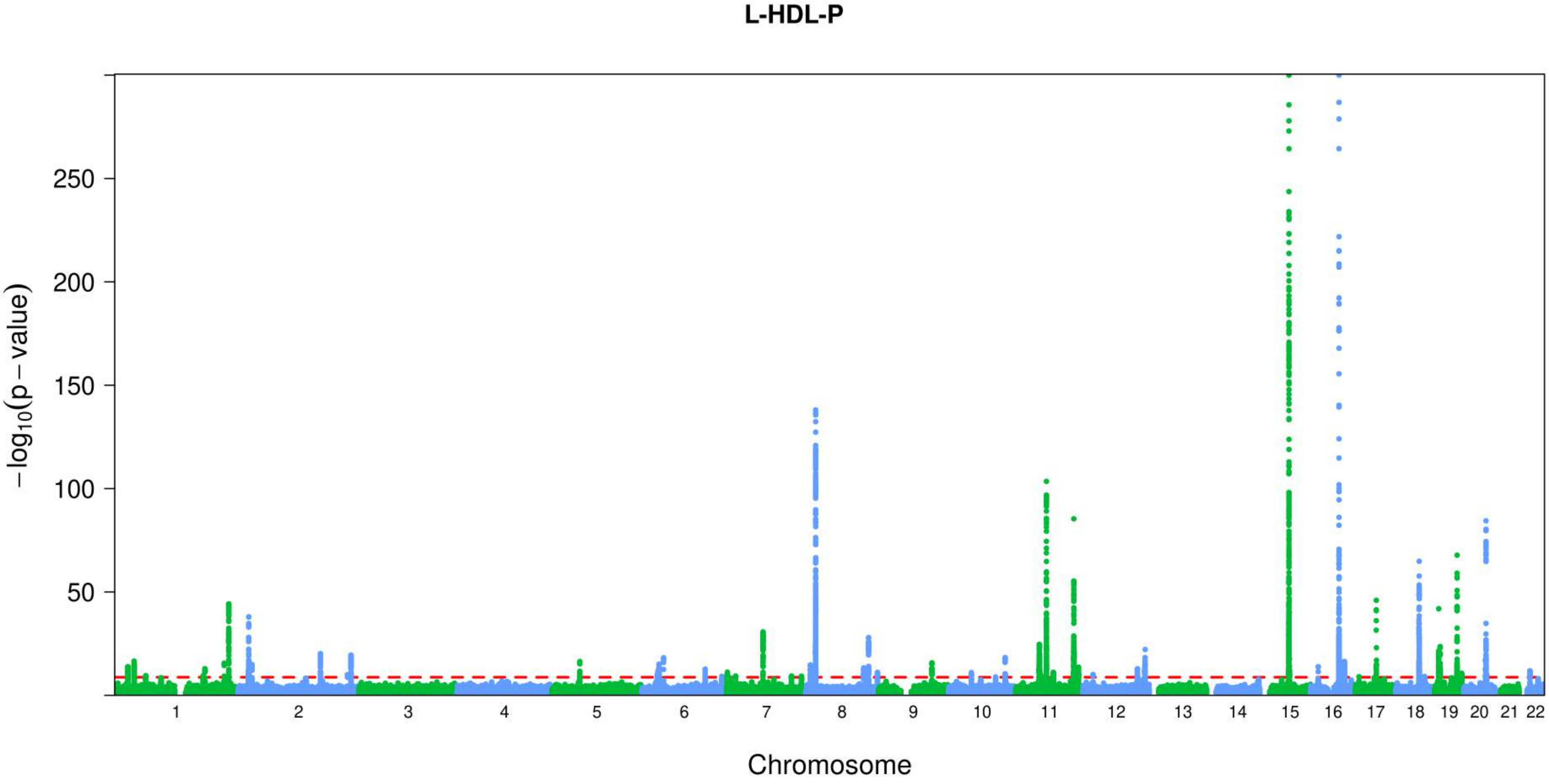
L-HDL-P
−log10(p − value)
250
200
150
100
50
1
2
3
4
5
6
7
8
9
10
11
12
13
14
15
16
17
18
19
20
21
22
Chromosome

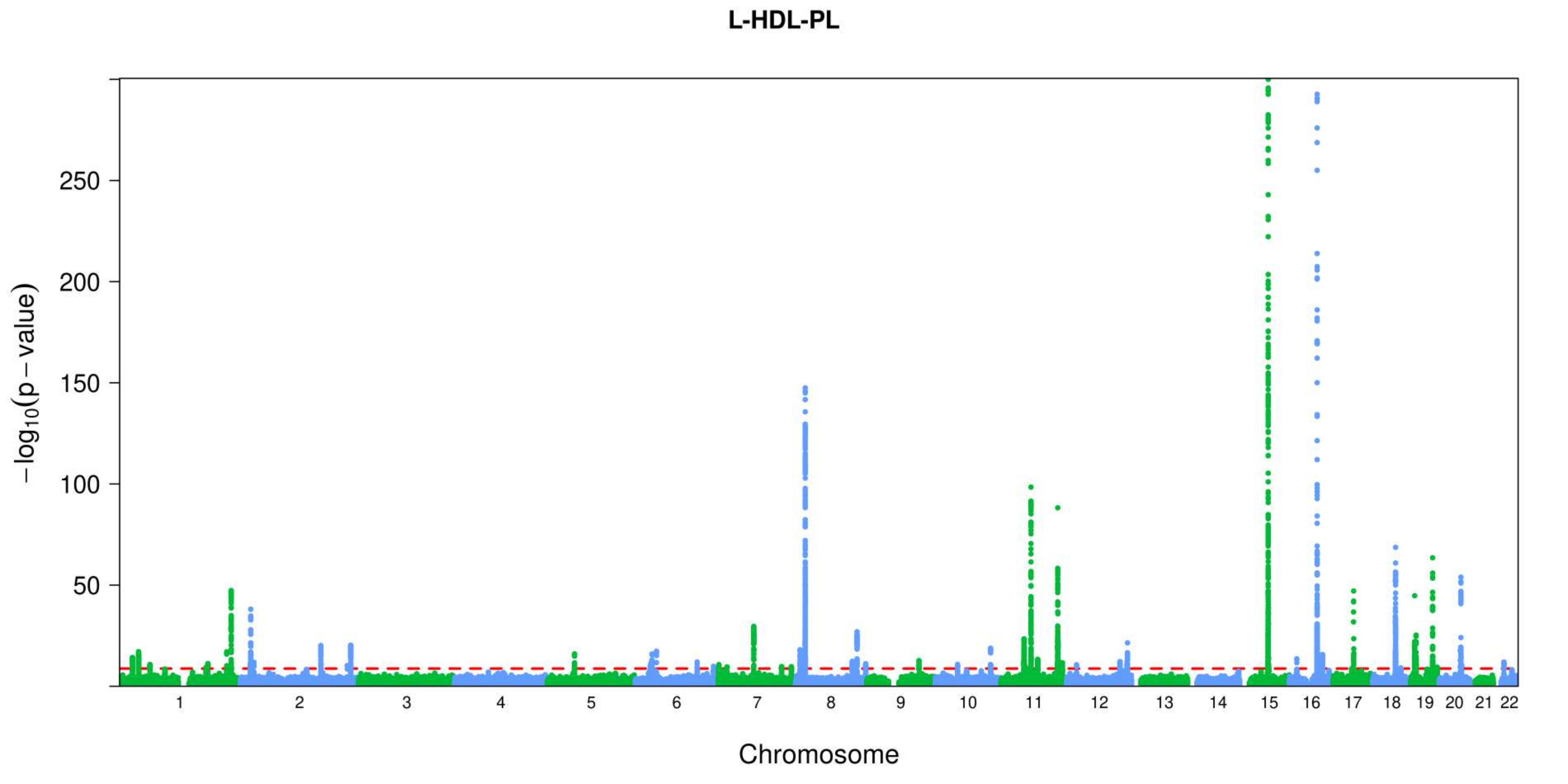
L-HDL-PL
−log10(p − value)
250
200
150
100
50
Chromosome
1 2 3 4 5 6 7 8 9 10 11 12 13 14 15 16 17 18 19 20 21 22

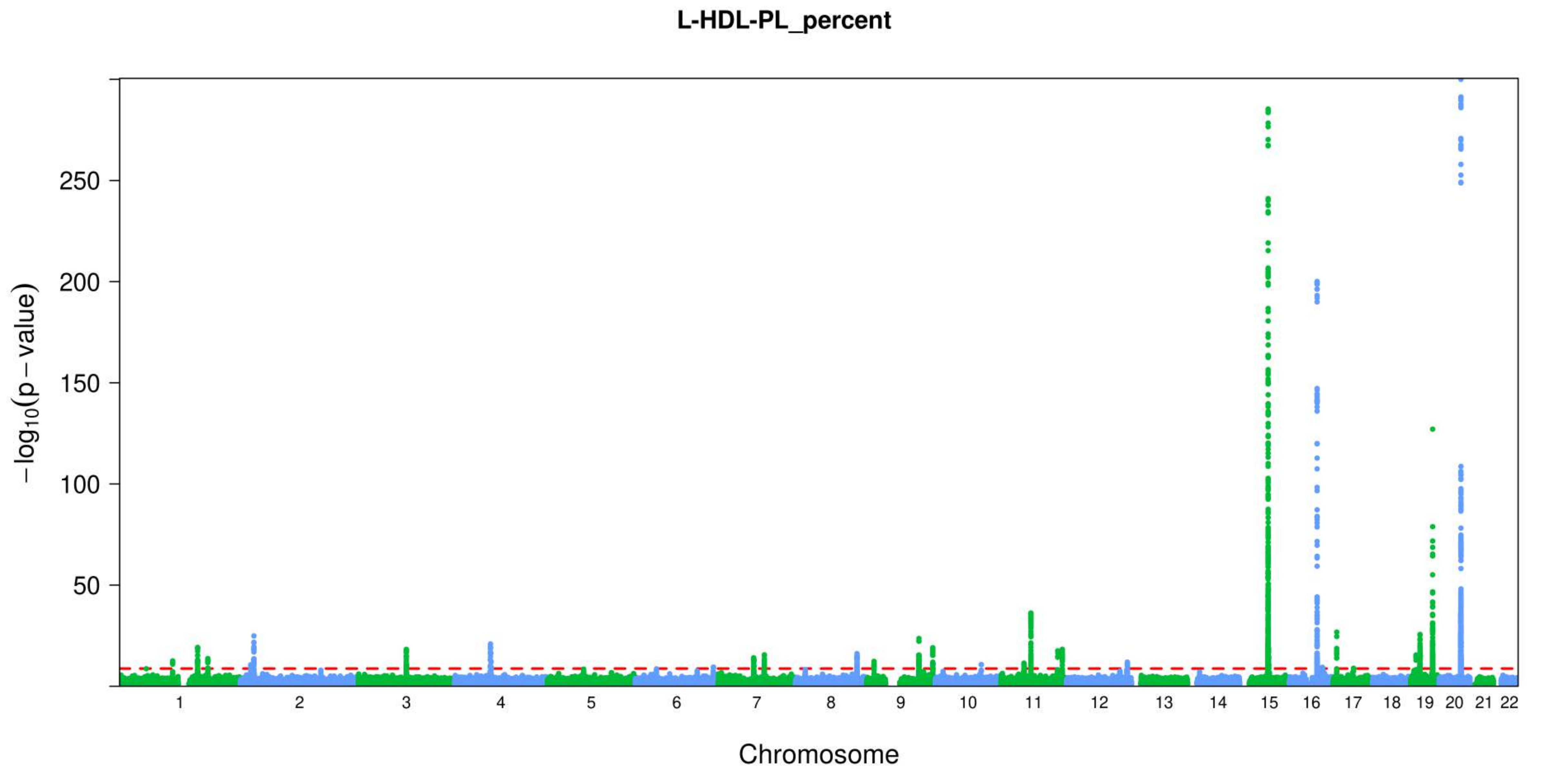

L-HDL-PL_percent
250
200
150
100
50
−log10(p − value)
1
2
3
4
5
6
7
8
9
10
11
12
13
14
15
16
17
18
19
20
21
22
Chromosome

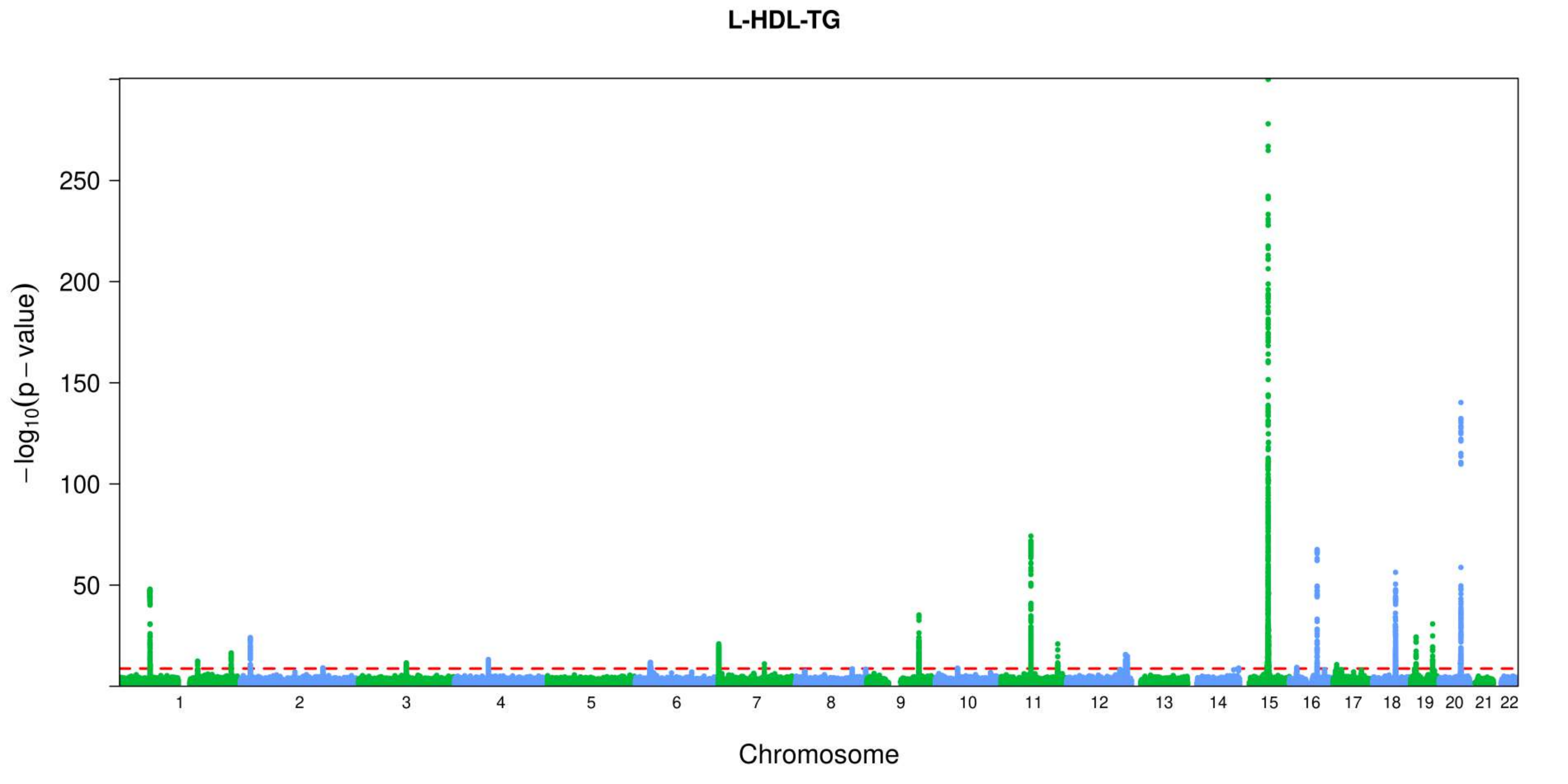
L-HDL-TG
250
200
150
100
50
−log10(p − value)
1
2
3
4
5
6
7
8
9
10
11
12
13
14
15
16
17
18
19
20
21
22
Chromosome

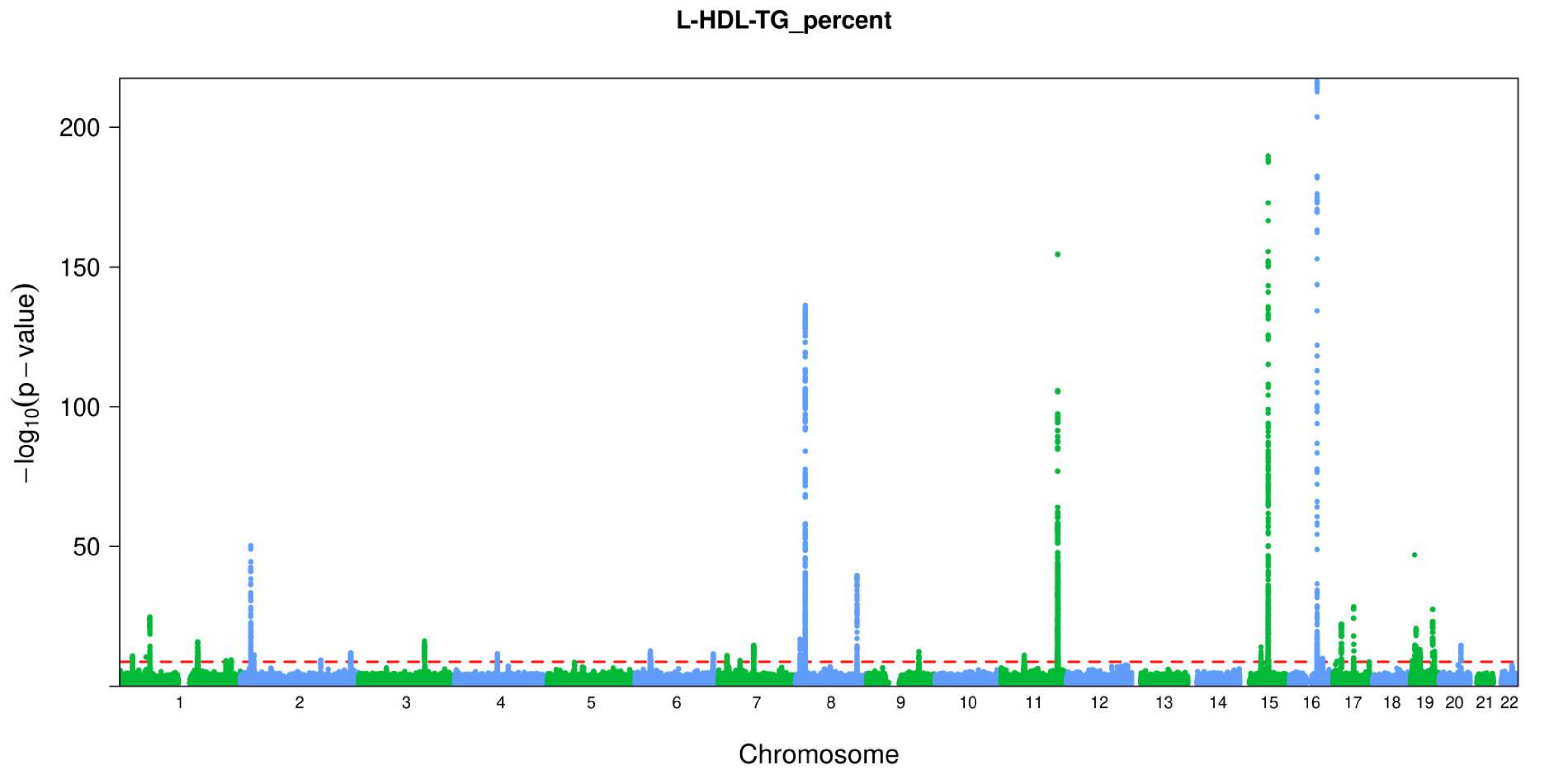
L-HDL-TG_percent
200
150
100
50
$-\log_{10}(p-\text{value})$
1
2
3
4
5
6
7
8
9
10
11
12
13
14
15
16
17
18
19
20
21
22
Chromosome

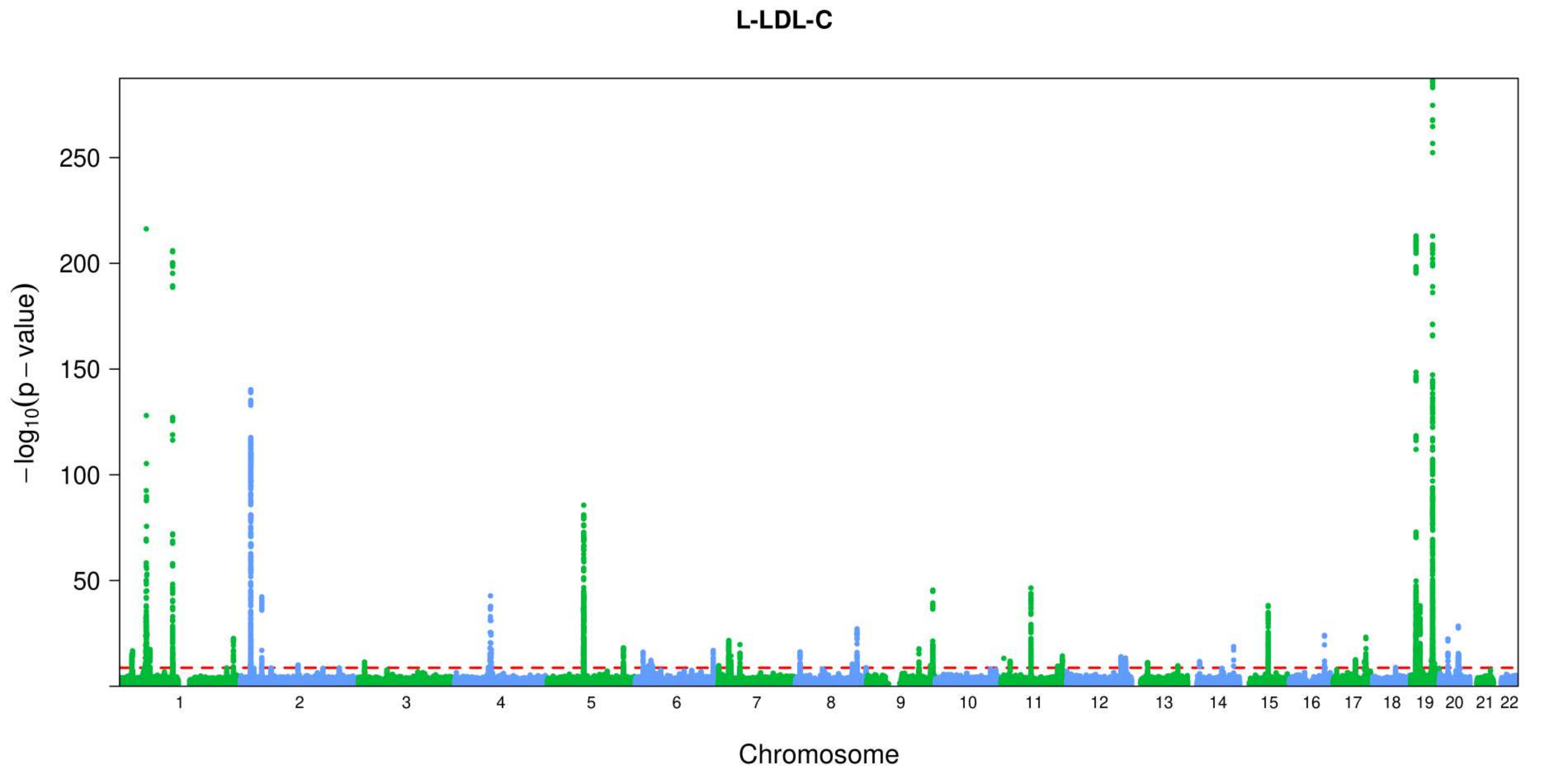

L-LDL-C
250
200
150
100
50
−log10(p − value)
1
2
3
4
5
6
7
8
9
10
11
12
13
14
15
16
17
18
19
20
21
22
Chromosome

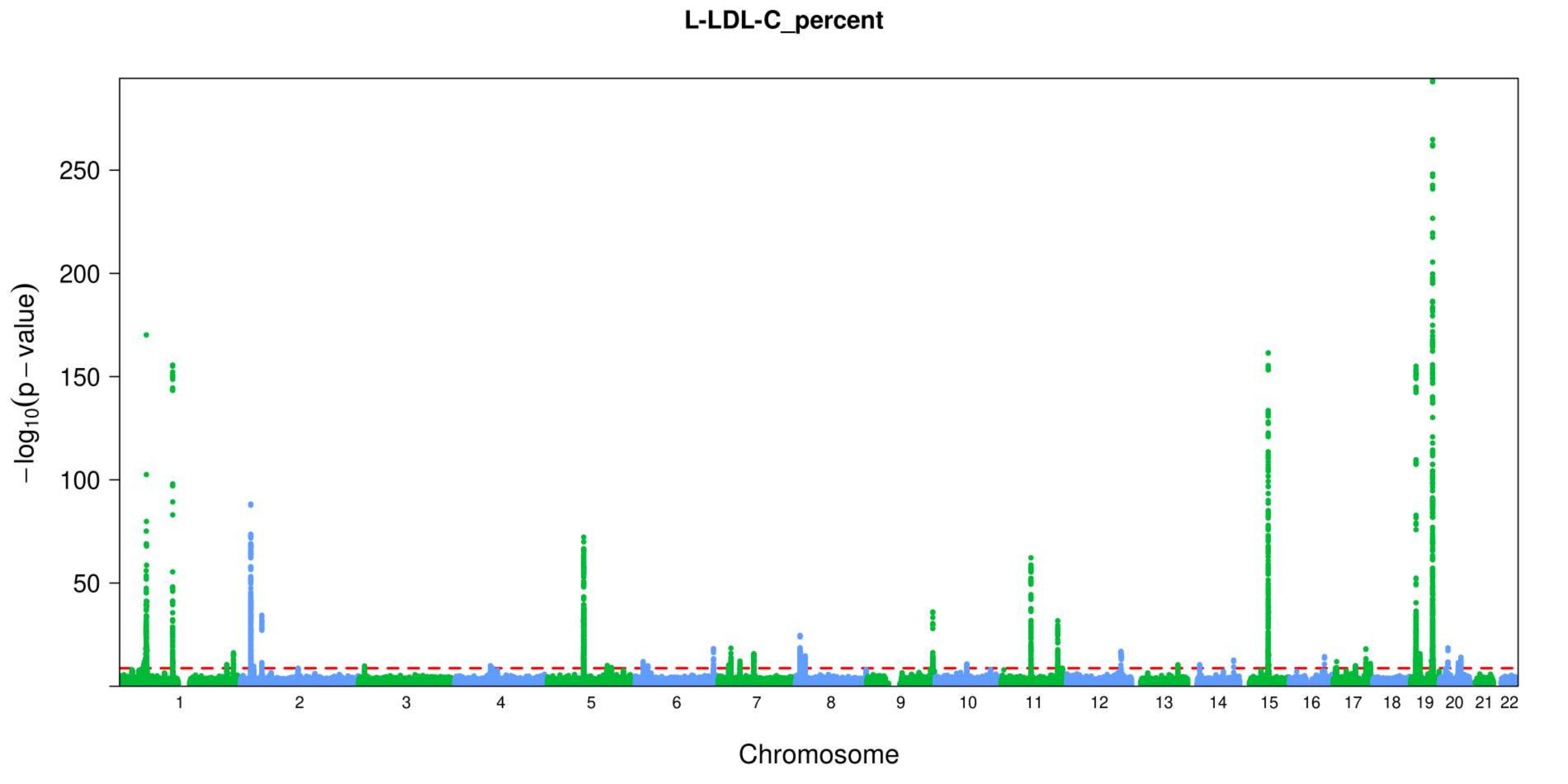
L-LDL-C_percent
−log10(p − value)
250
200
150
100
50
Chromosome
1
2
3
4
5
6
7
8
9
10
11
12
13
14
15
16
17
18
19
20
21
22

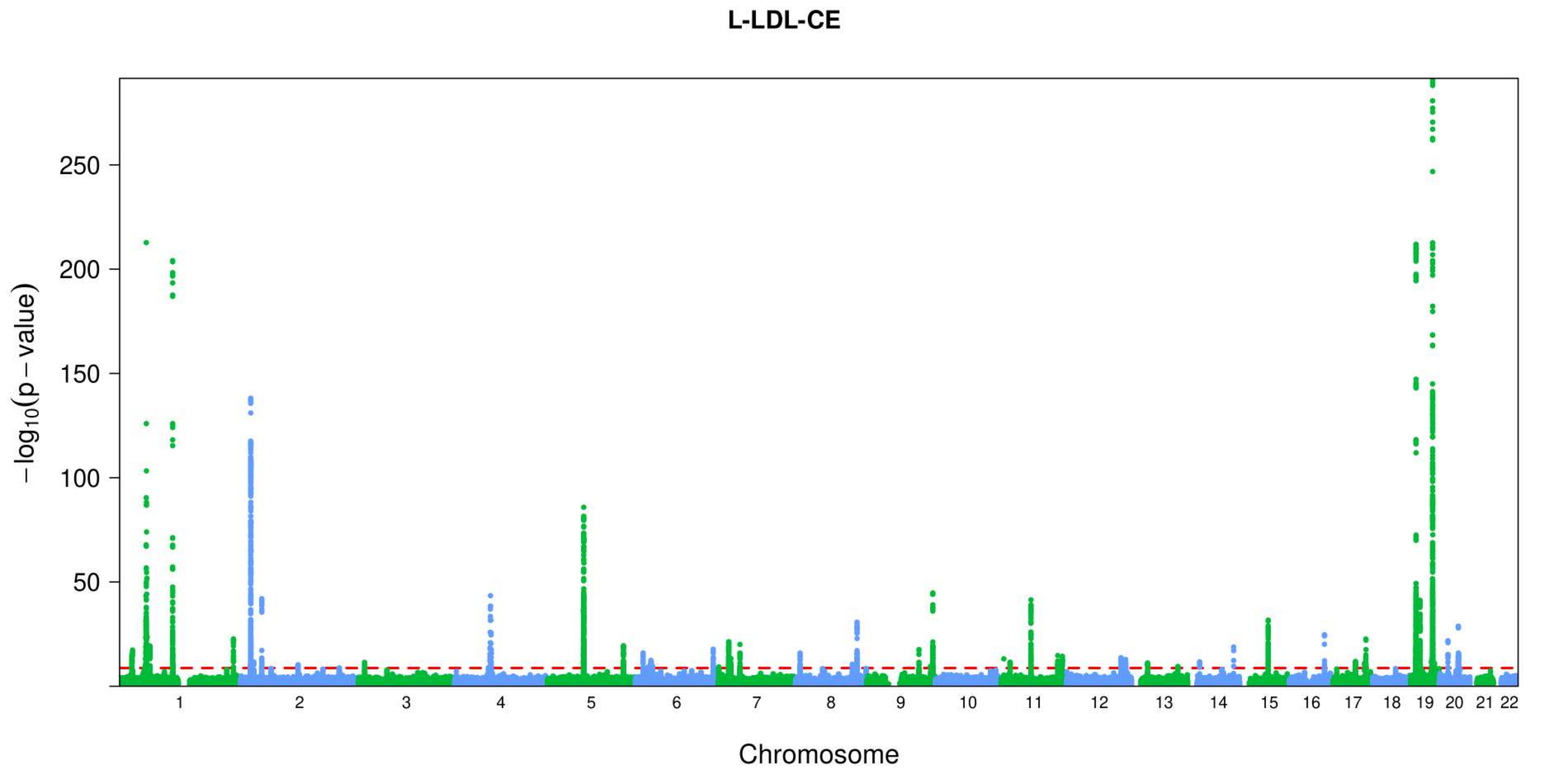
L-LDL-CE
-log10(p - value)
250
200
150
100
50
1
2
3
4
5
6
7
8
9
10
11
12
13
14
15
16
17
18
19
20
21
22
Chromosome

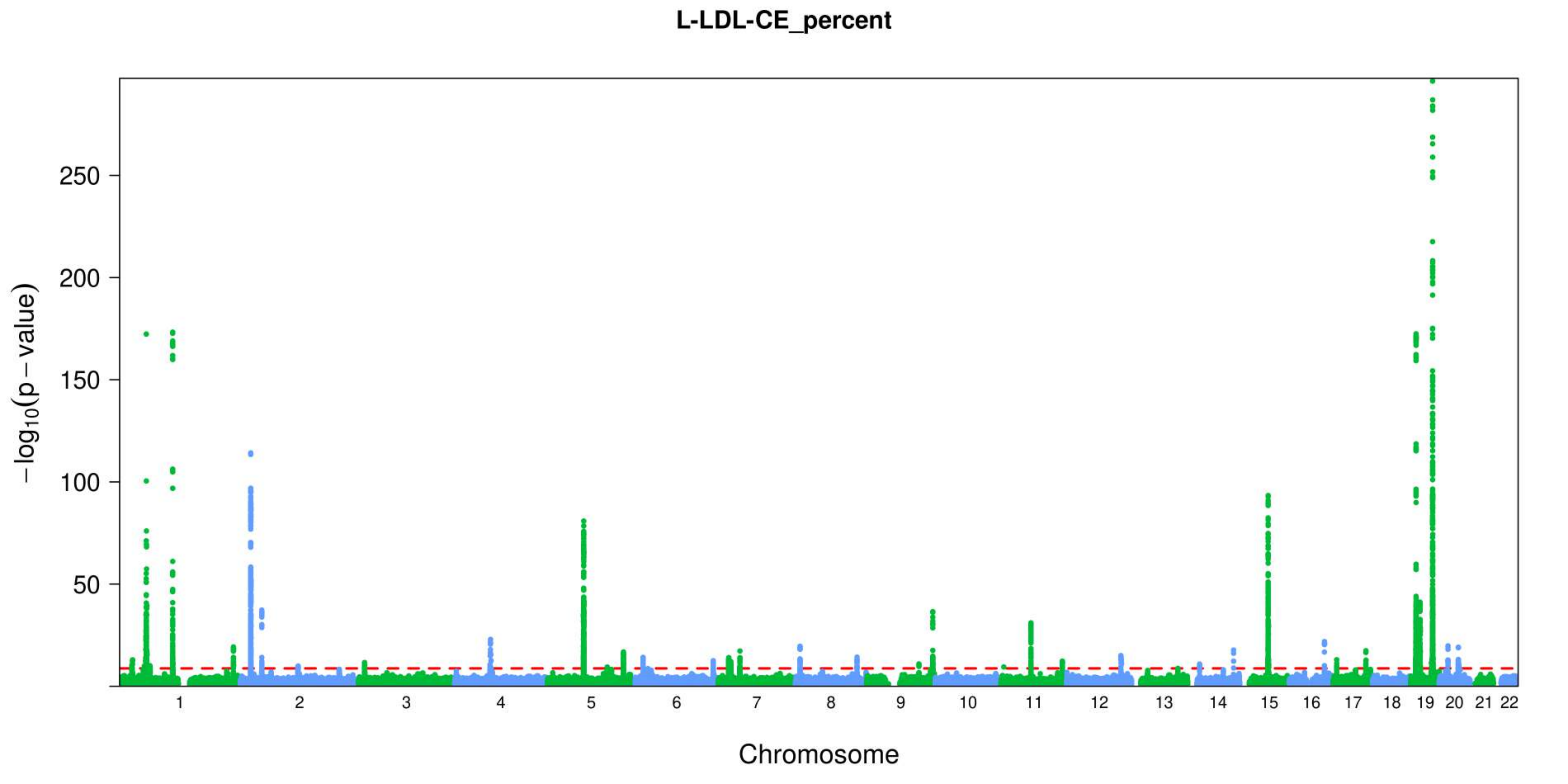
L-LDL-CE_percent
−log10(p − value)
250
200
150
100
50
Chromosome
1
2
3
4
5
6
7
8
9
10
11
12
13
14
15
16
17
18
19
20
21
22

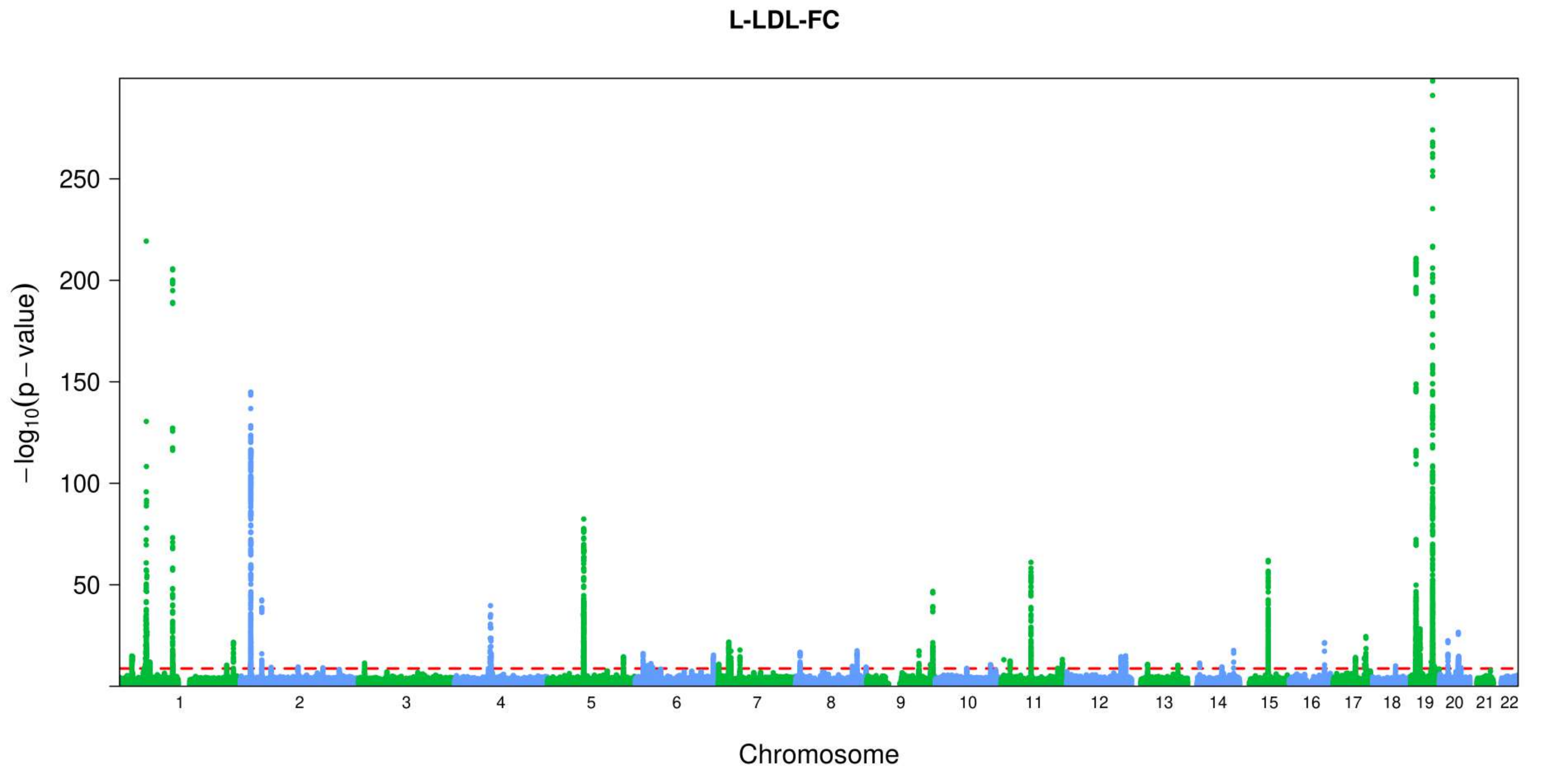

L-LDL-FC
250
200
150
100
50
-log10(p - value)
1
2
3
4
5
6
7
8
9
10
11
12
13
14
15
16
17
18
19
20
21
22
Chromosome

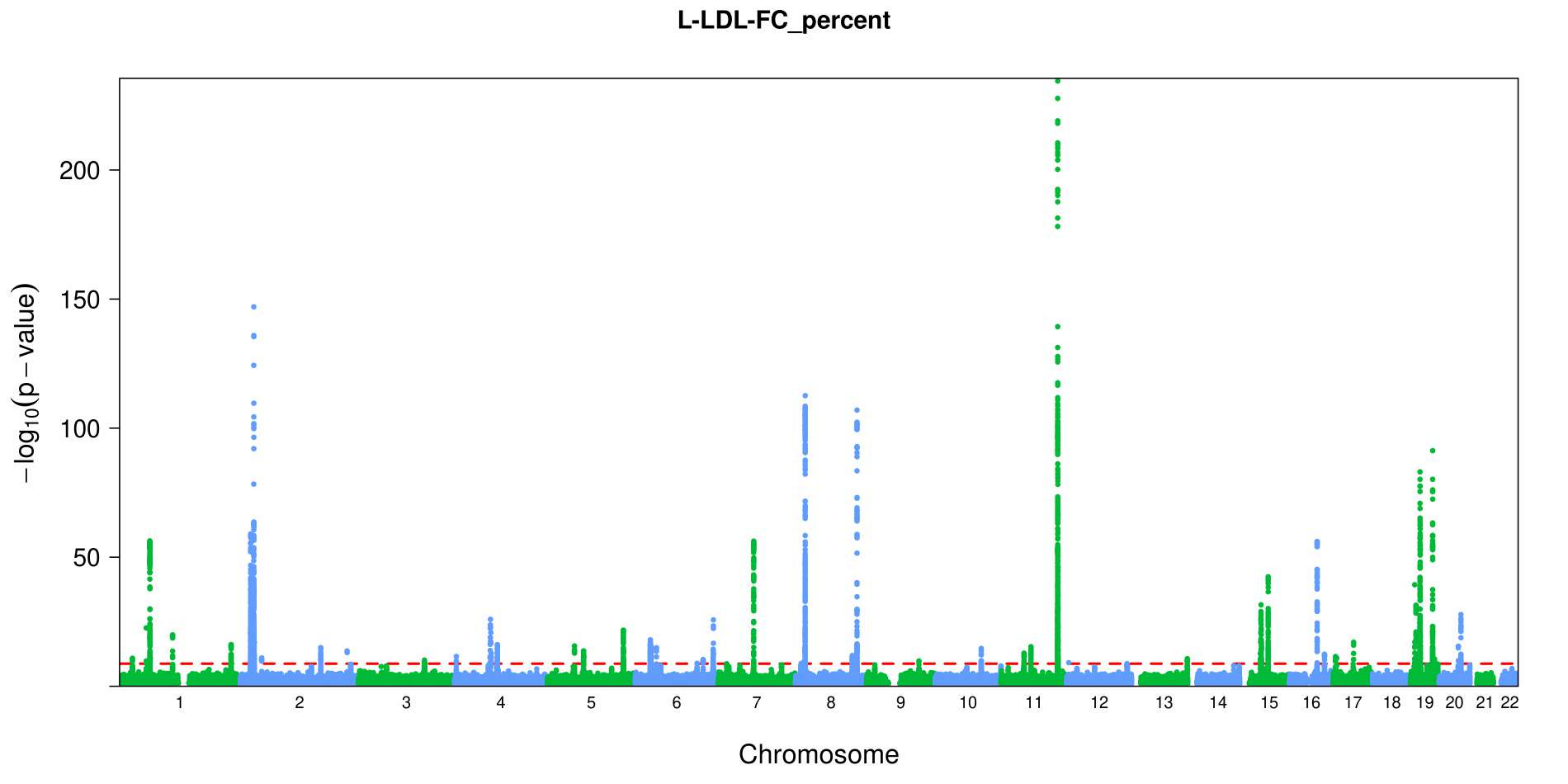

L-LDL-FC_percent
$-\log_{10}(p - \text{value})$
200
150
100
50
Chromosome
1
2
3
4
5
6
7
8
9
10
11
12
13
14
15
16
17
18
19
20
21
22

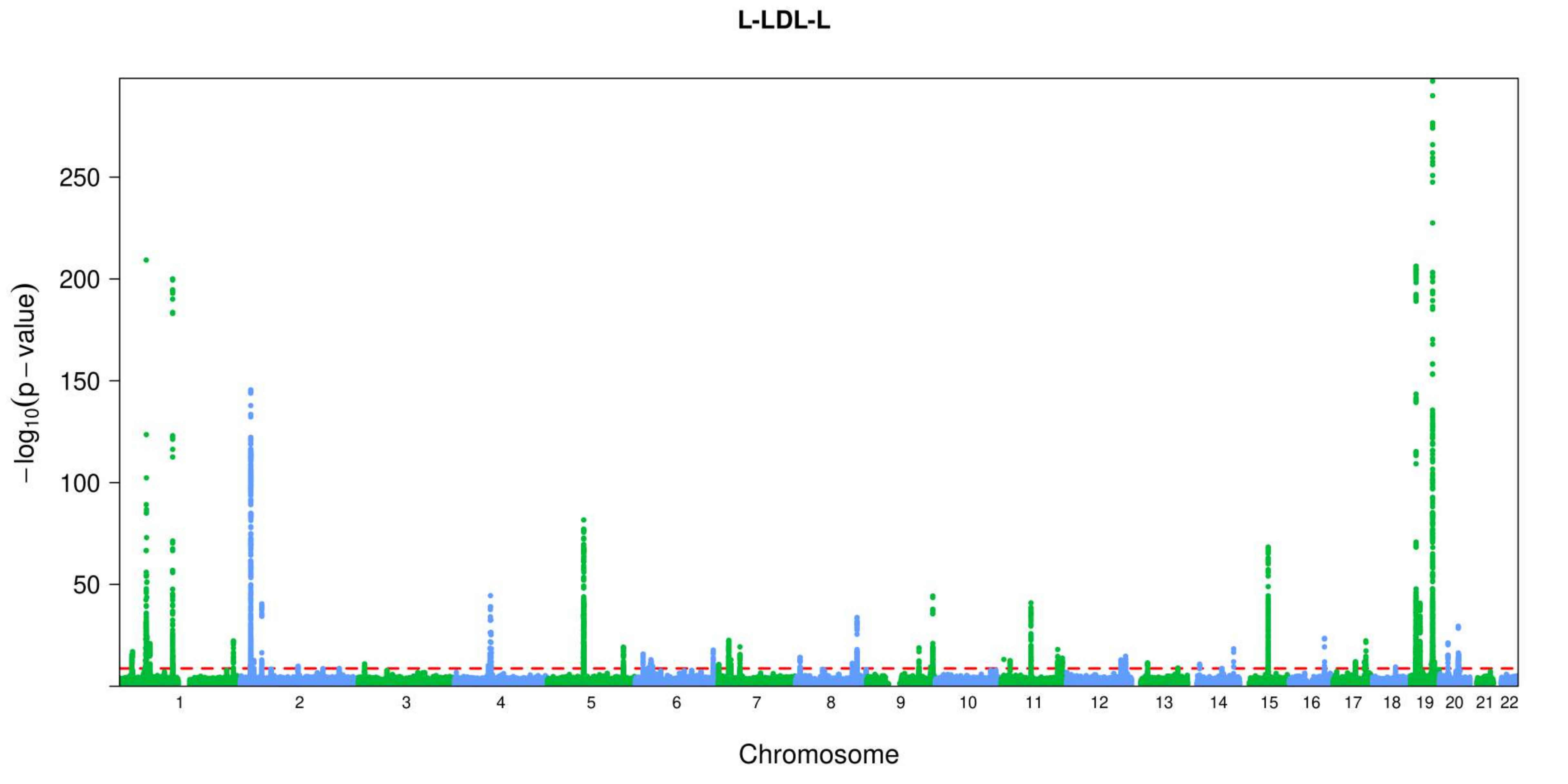
L-LDL-L
250
200
150
100
50
$-\log_{10}(p-\text{value})$
1
2
3
4
5
6
7
8
9
10
11
12
13
14
15
16
17
18
19
20
21
22
Chromosome

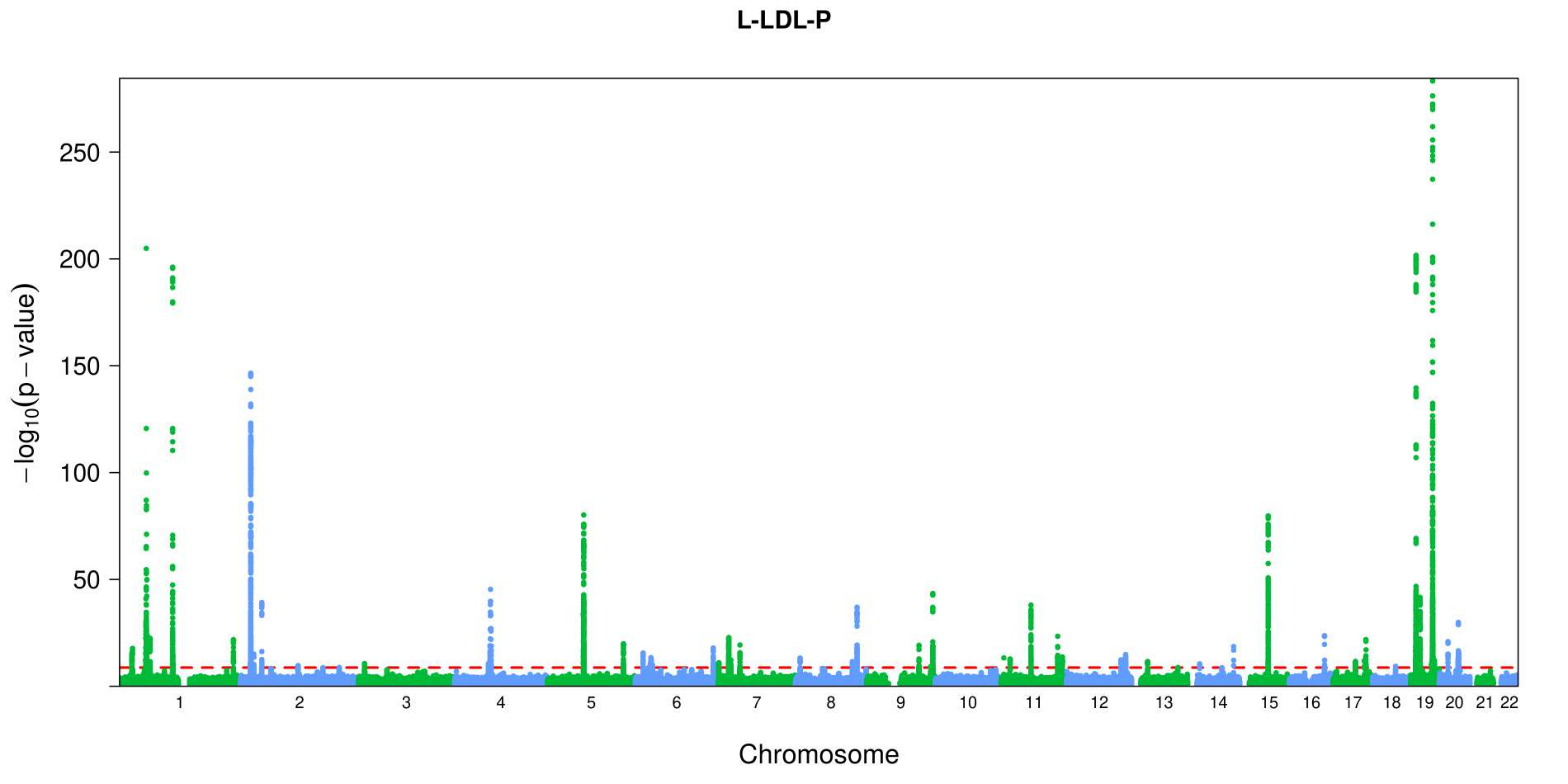

L-LDL-P
250
200
150
100
50
−log10(p − value)
1
2
3
4
5
6
7
8
9
10
11
12
13
14
15
16
17
18
19
20
21
22
Chromosome

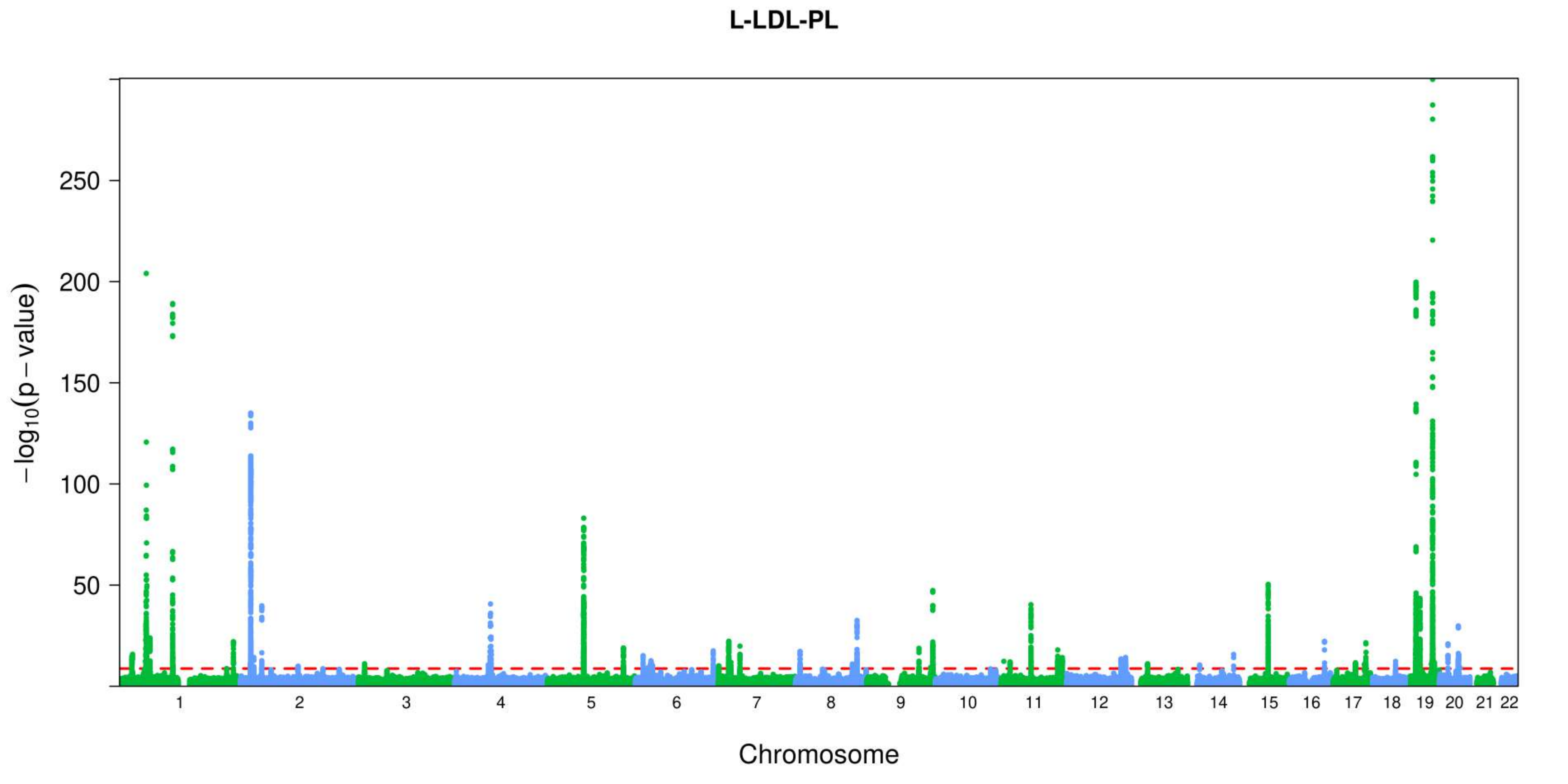

L-LDL-PL
250
200
150
100
50
−log10(p − value)
1
2
3
4
5
6
7
8
9
10
11
12
13
14
15
16
17
18
19
20
21
22
Chromosome

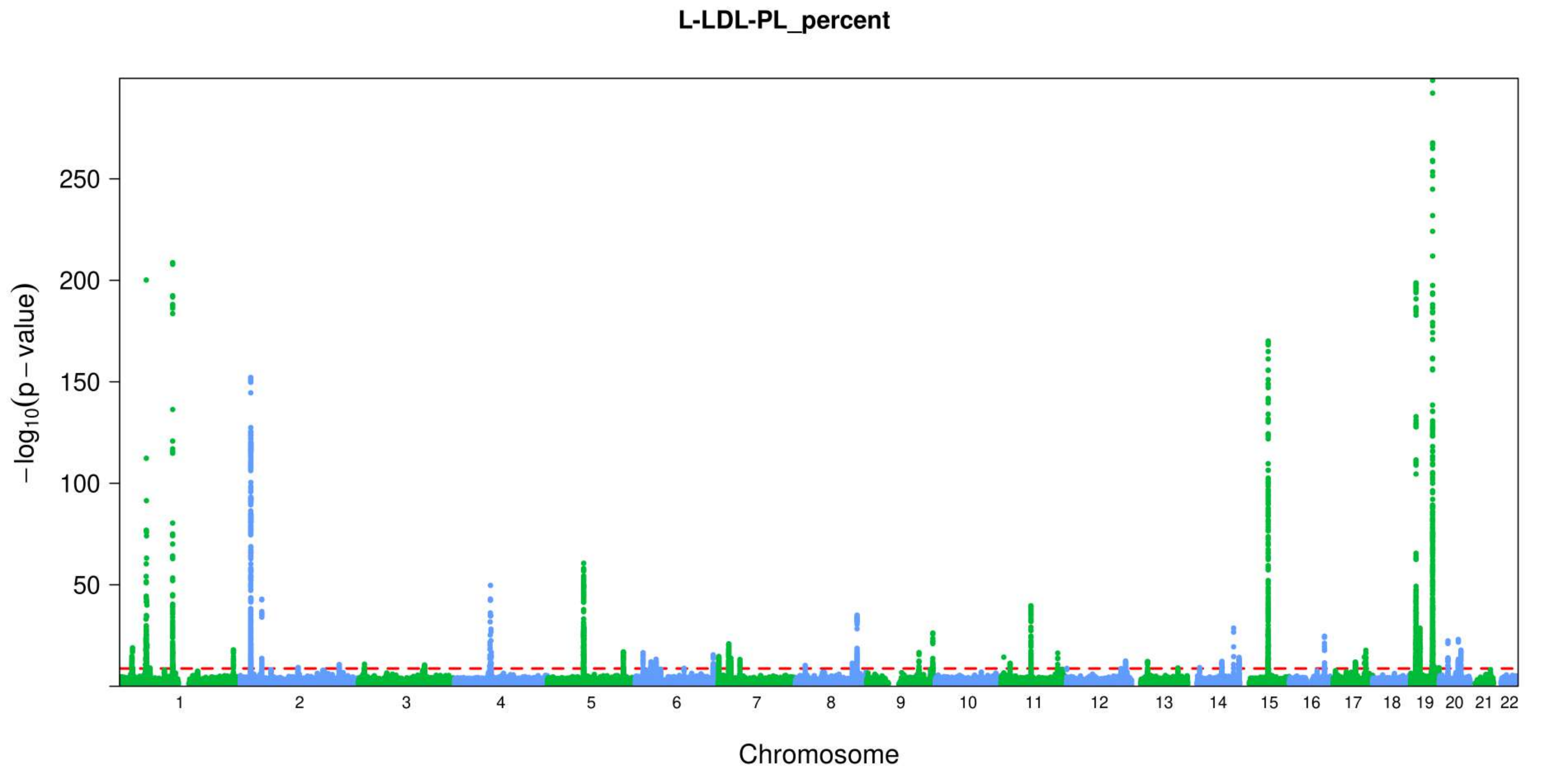
L-LDL-PL_percent
$-\log_{10}(p-value)$
250
200
150
100
50
1
2
3
4
5
6
7
8
9
10
11
12
13
14
15
16
17
18
19
20
21
22
Chromosome

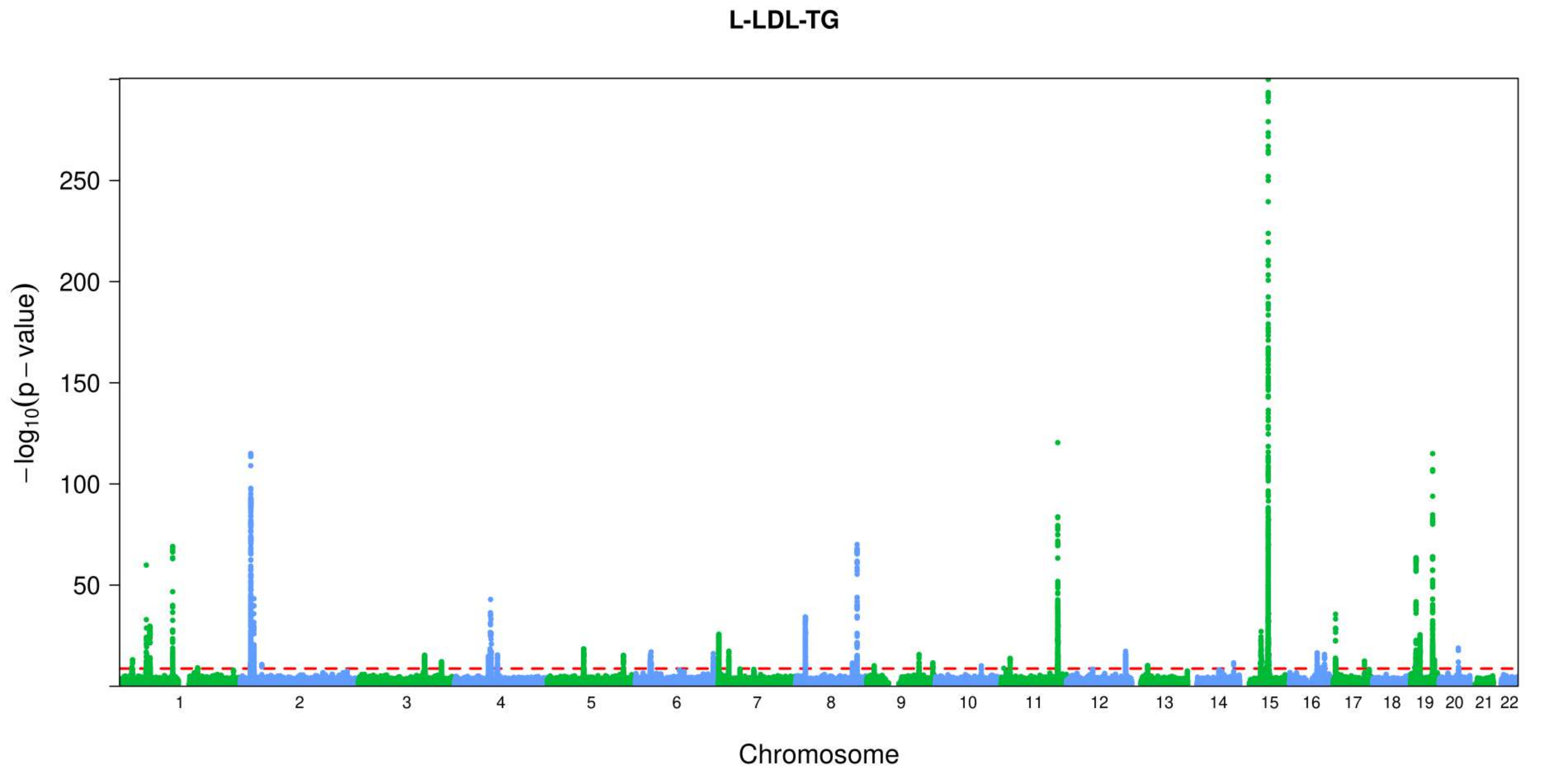

L-LDL-TG
−log10(p − value)
250
200
150
100
50
Chromosome
1
2
3
4
5
6
7
8
9
10
11
12
13
14
15
16
17
18
19
20
21
22

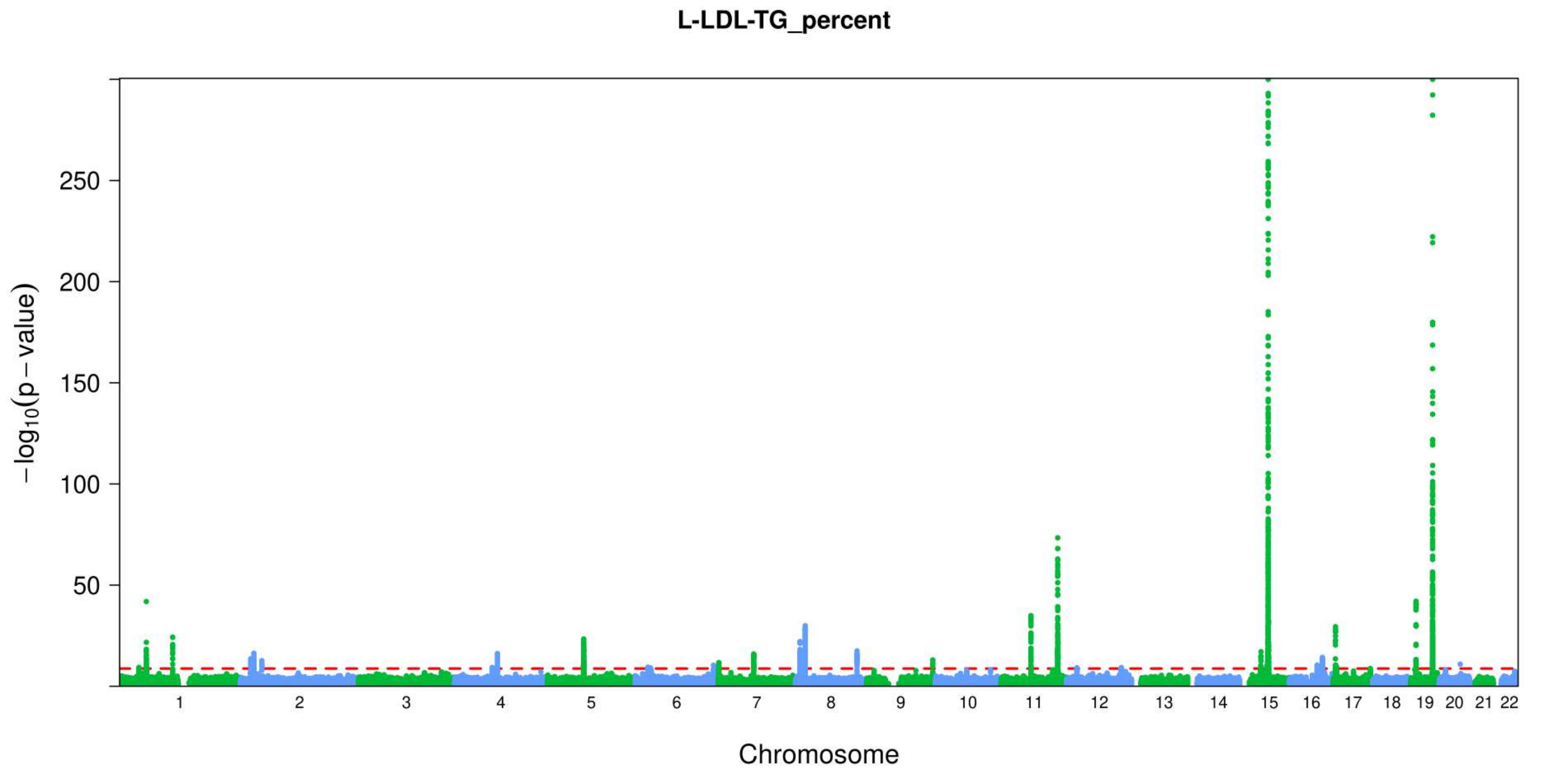
L-LDL-TG_percent
250
200
150
100
50
−log10(p − value)
1
2
3
4
5
6
7
8
9
10
11
12
13
14
15
16
17
18
19
20
21
22
Chromosome

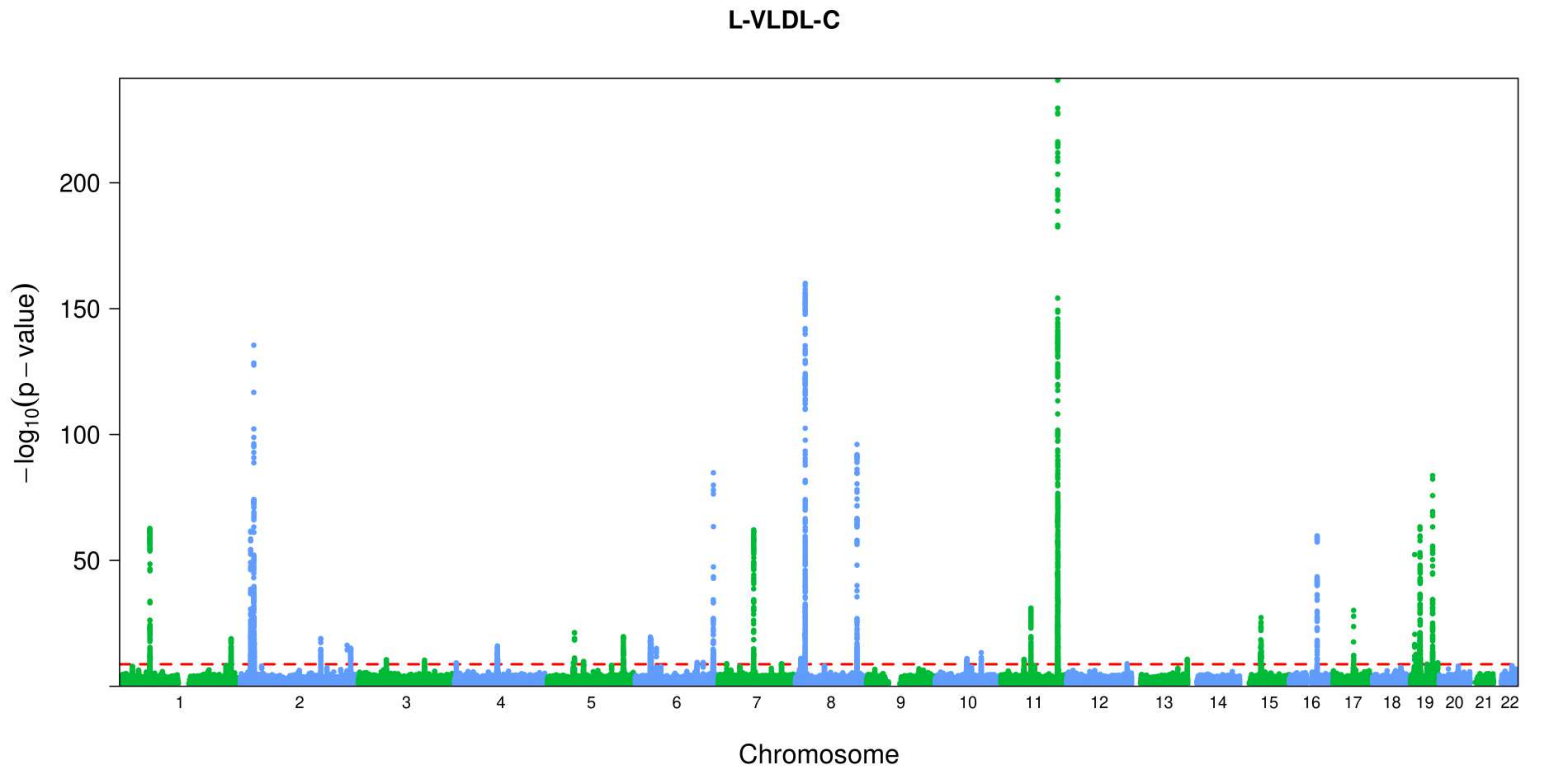
L-VLDL-C
200
150
100
50
$-\log_{10}(p-\text{value})$
1
2
3
4
5
6
7
8
9
10
11
12
13
14
15
16
17
18
19
20
21
22
Chromosome

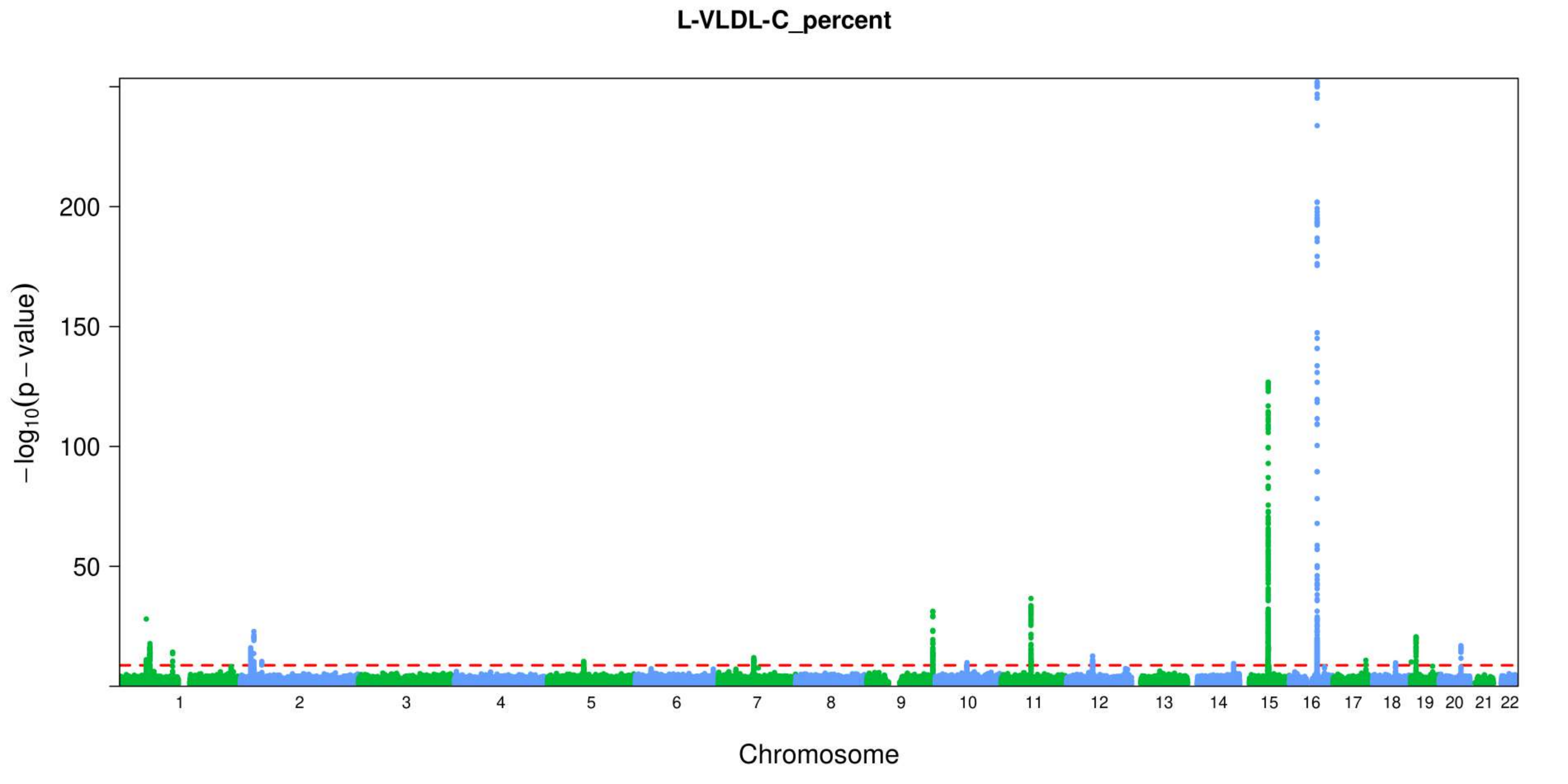

L-VLDL-C_percent
200
150
100
50
$-\log_{10}(p-\text{value})$
1
2
3
4
5
6
7
8
9
10
11
12
13
14
15
16
17
18
19
20
21
22
Chromosome

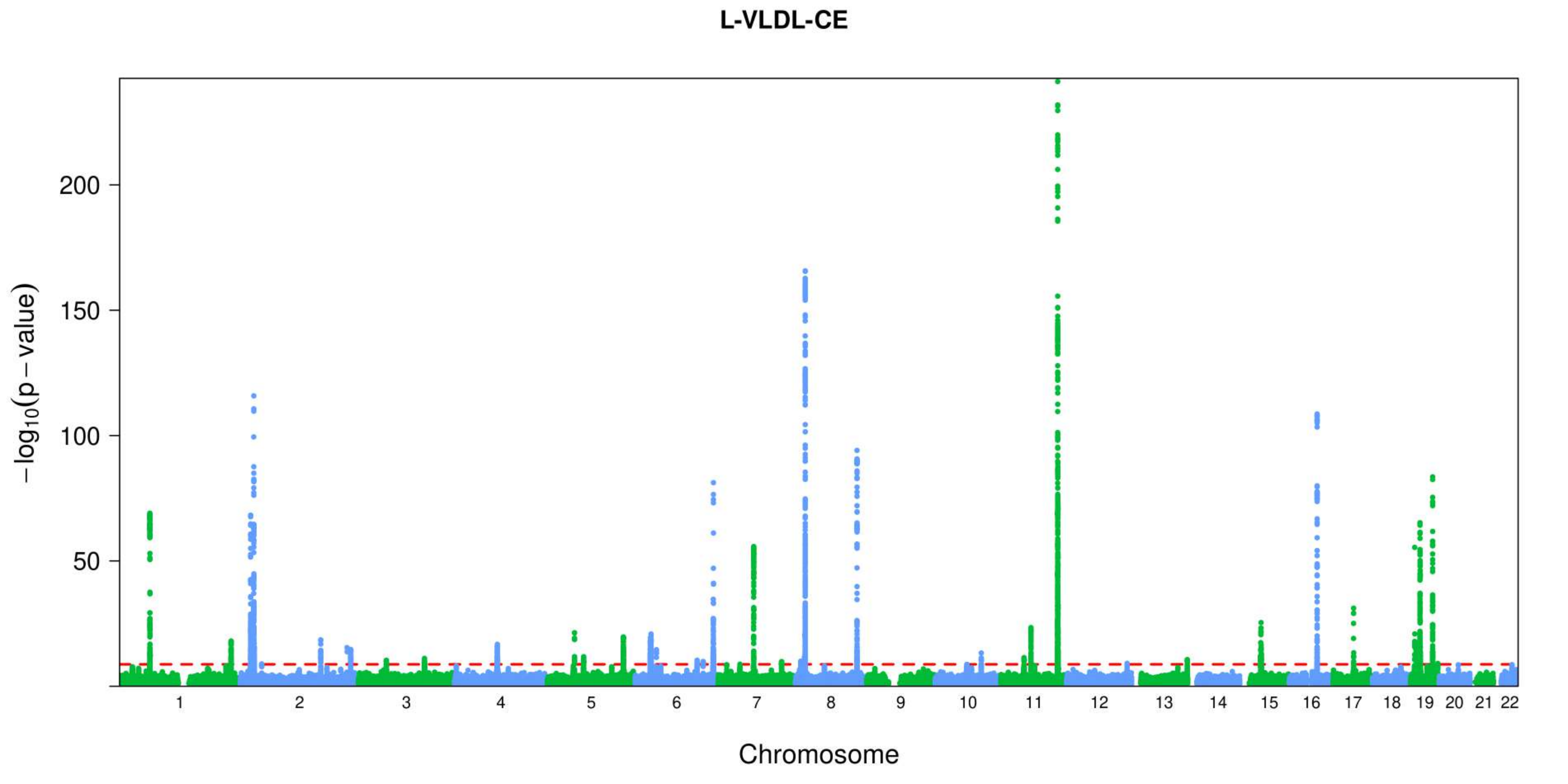
L-VLDL-CE
200
150
100
50
−log10(p − value)
1
2
3
4
5
6
7
8
9
10
11
12
13
14
15
16
17
18
19
20
21
22
Chromosome

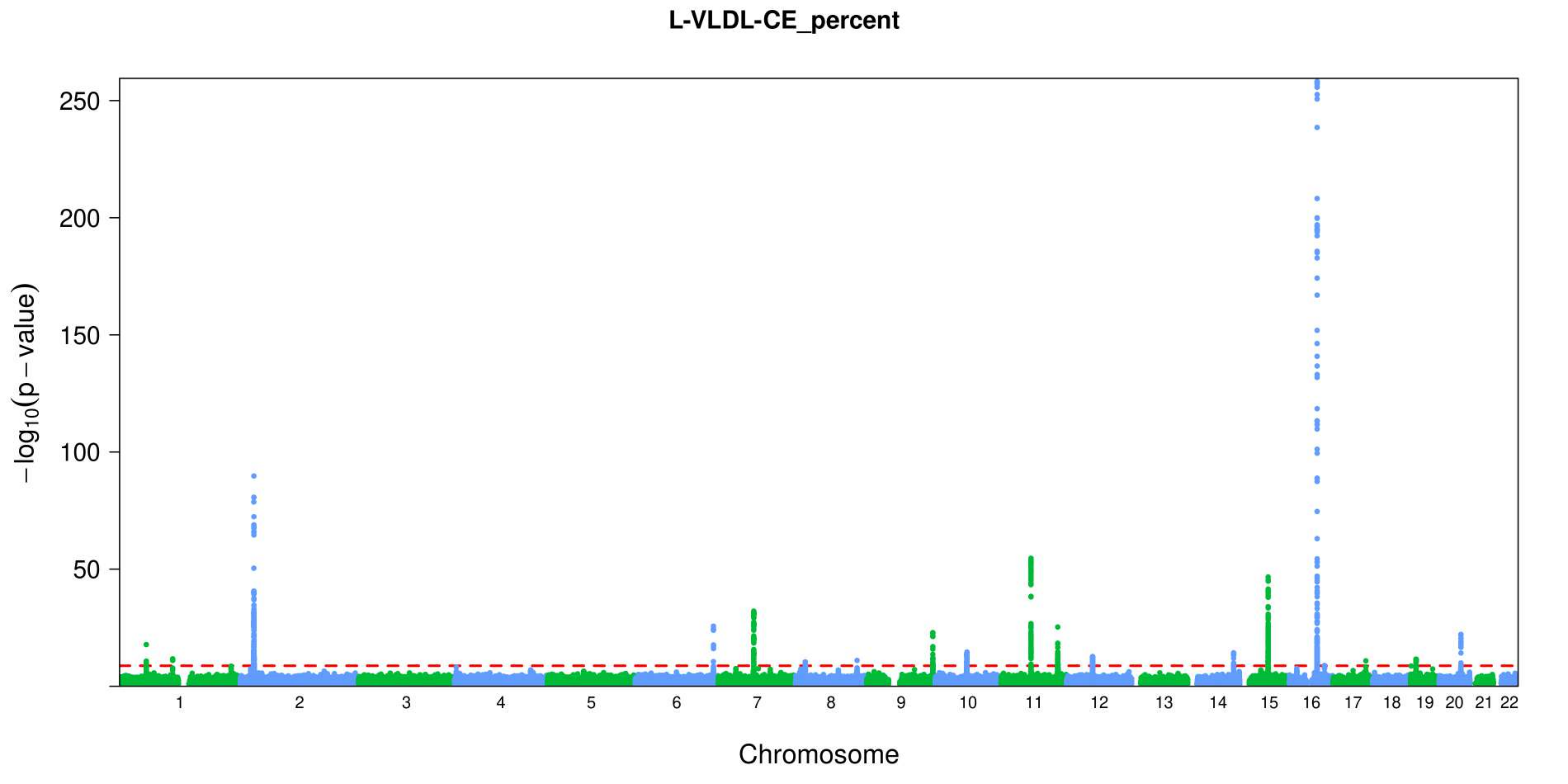

L-VLDL-CE_percent
250
200
150
100
50
−log10(p − value)
1
2
3
4
5
6
7
8
9
10
11
12
13
14
15
16
17
18
19
20
21
22
Chromosome

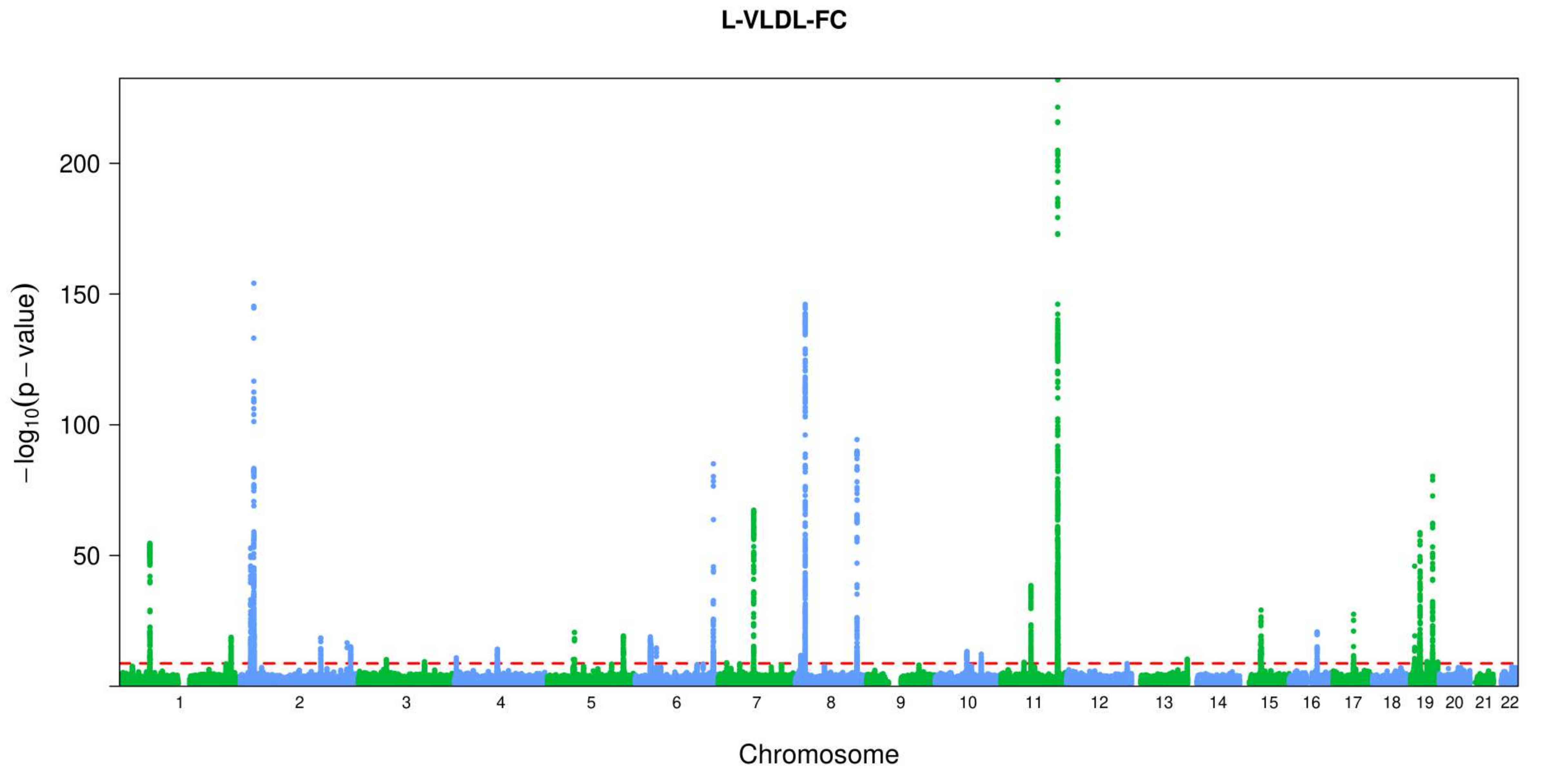
L-VLDL-FC
−log10(p − value)
200
150
100
50
Chromosome
1
2
3
4
5
6
7
8
9
10
11
12
13
14
15
16
17
18
19
20
21
22

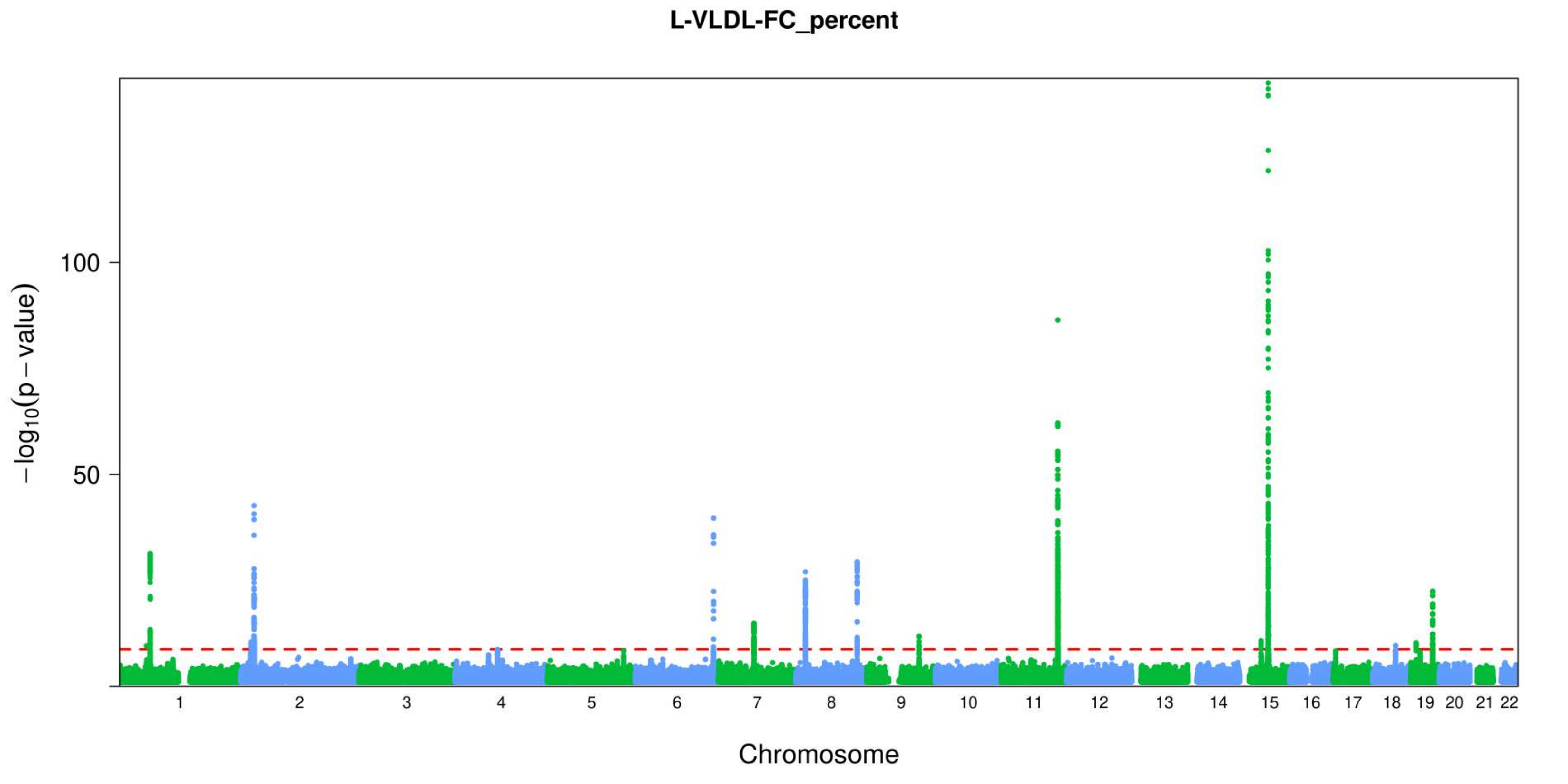

L-VLDL-FC_percent
$-\log_{10}(p-\text{value})$
100
50
Chromosome
1
2
3
4
5
6
7
8
9
10
11
12
13
14
15
16
17
18
19
20
21
22

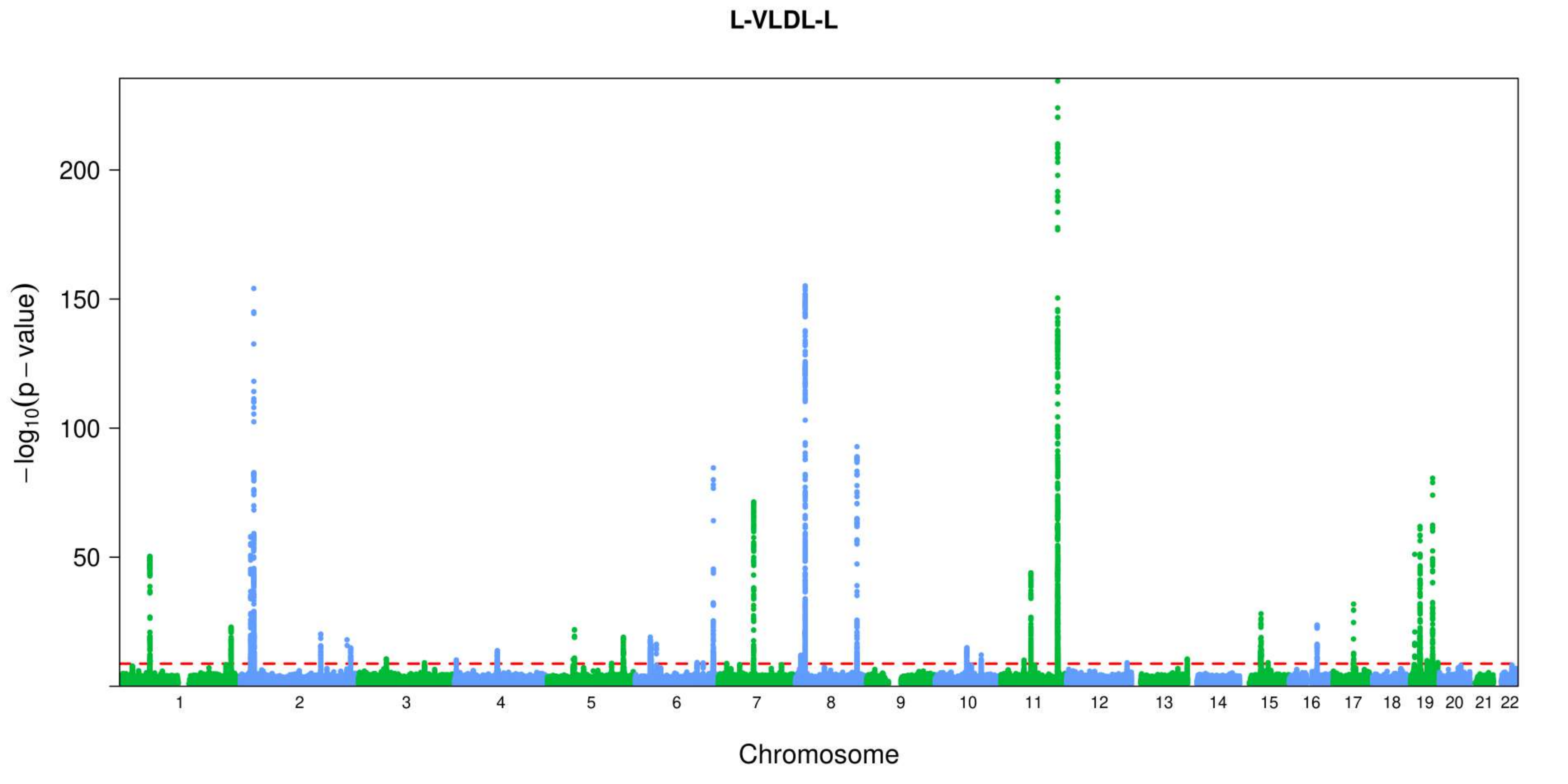

L-VLDL-L
200
150
100
50
$-\log_{10}(p-\text{value})$
1
2
3
4
5
6
7
8
9
10
11
12
13
14
15
16
17
18
19
20
21
22
Chromosome

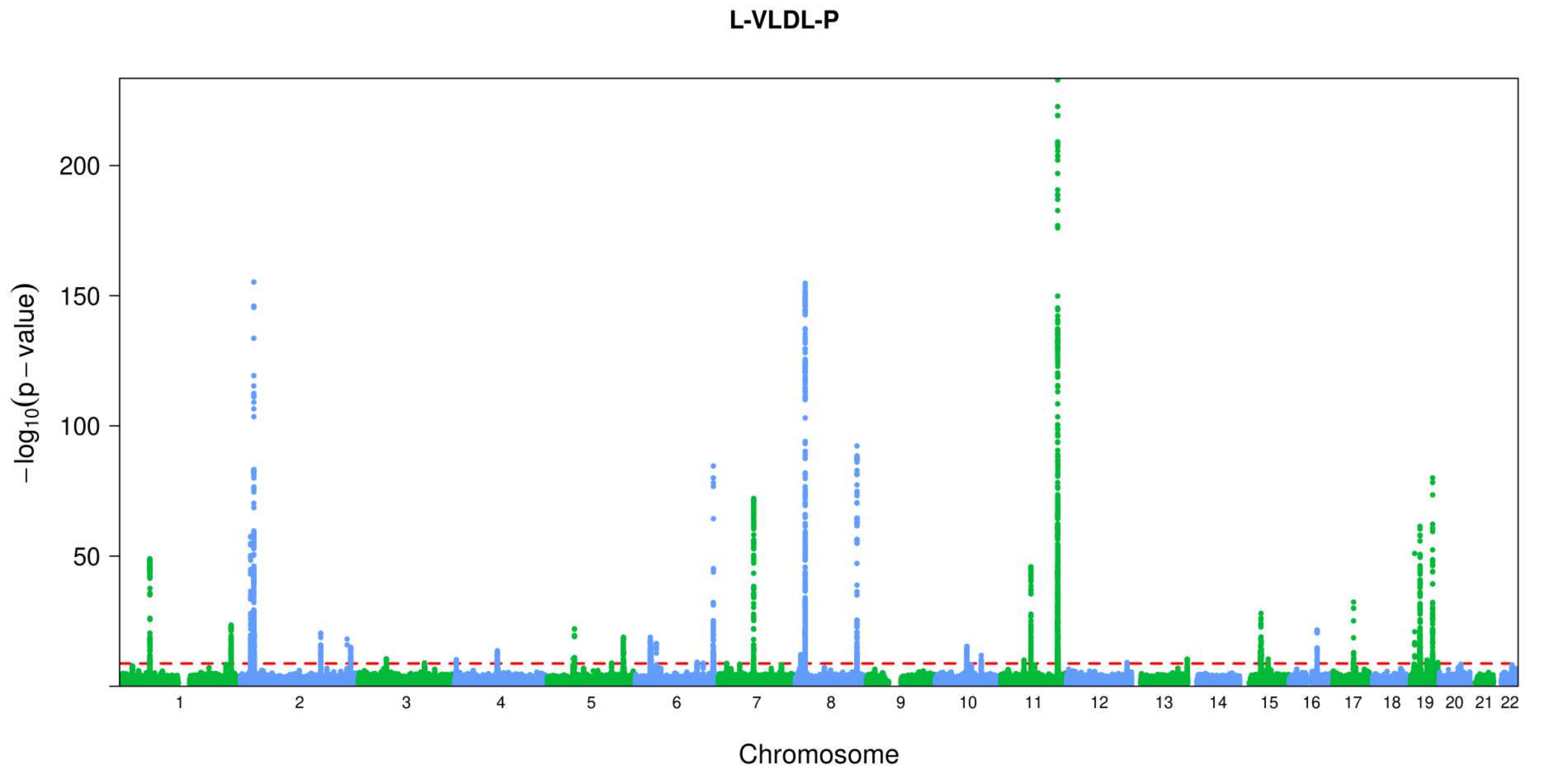

L-VLDL-P
200
150
100
50
−log10(p − value)
1
2
3
4
5
6
7
8
9
10
11
12
13
14
15
16
17
18
19
20
21
22
Chromosome

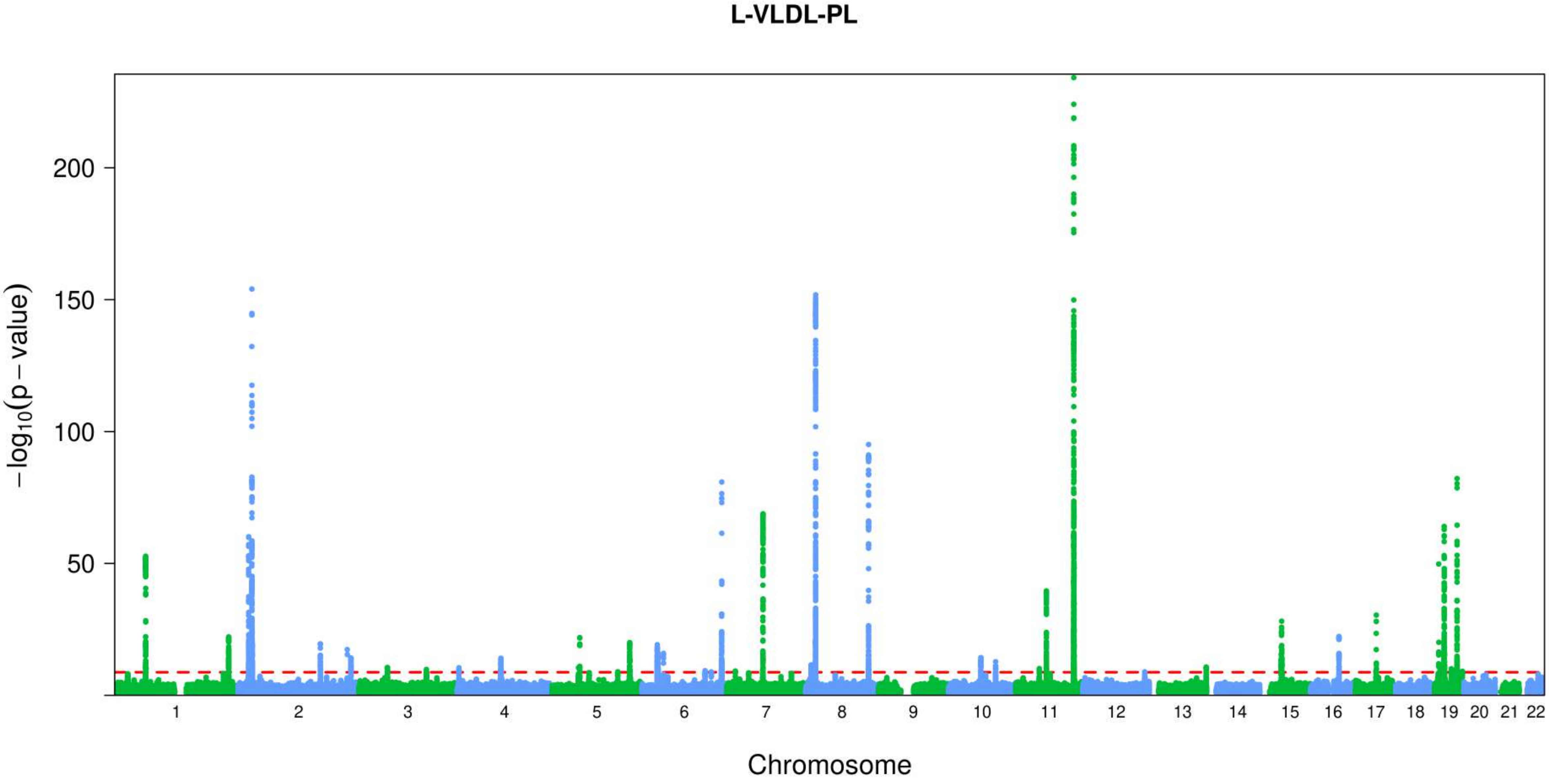
L-VLDL-PL
−log10(p − value)
200
150
100
50
1
2
3
4
5
6
7
8
9
10
11
12
13
14
15
16
17
18
19
20
21
22
Chromosome

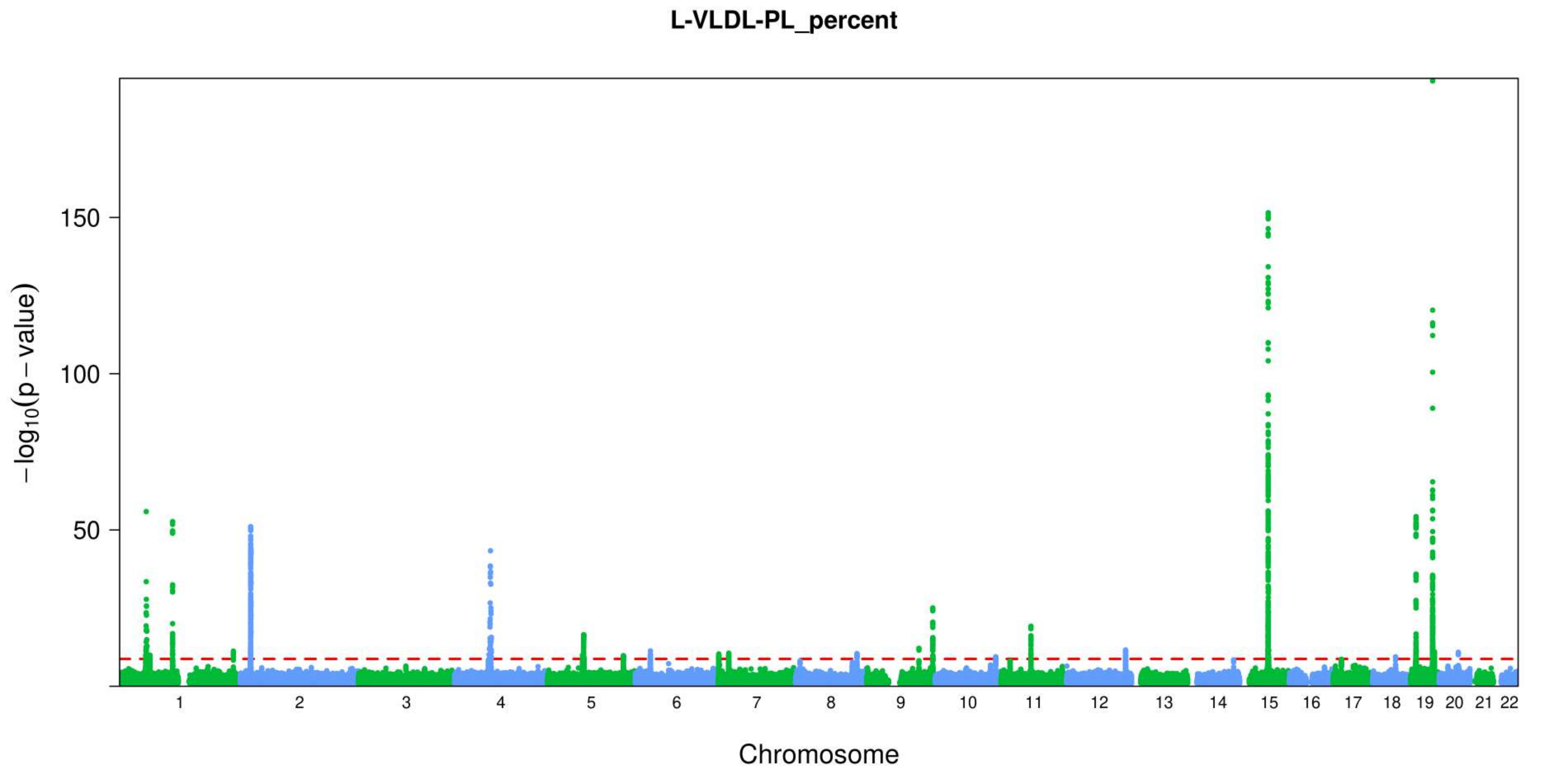
L-VLDL-PL_percent
150
100
50
−log10(p − value)
1
2
3
4
5
6
7
8
9
10
11
12
13
14
15
16
17
18
19
20
21
22
Chromosome

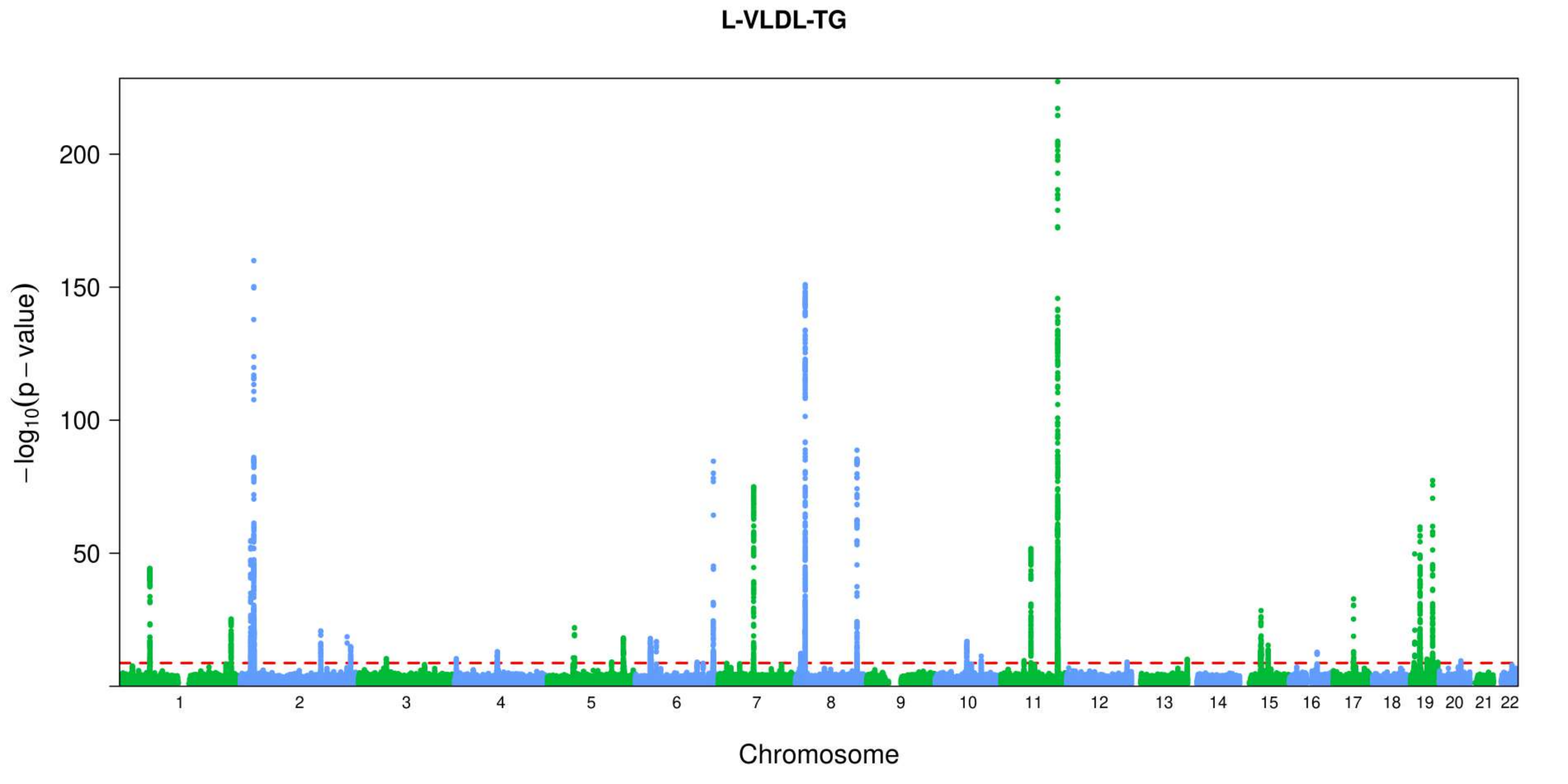

L-VLDL-TG
200
150
100
50
$-\log_{10}(p-\text{value})$
1
2
3
4
5
6
7
8
9
10
11
12
13
14
15
16
17
18
19
20
21
22
Chromosome

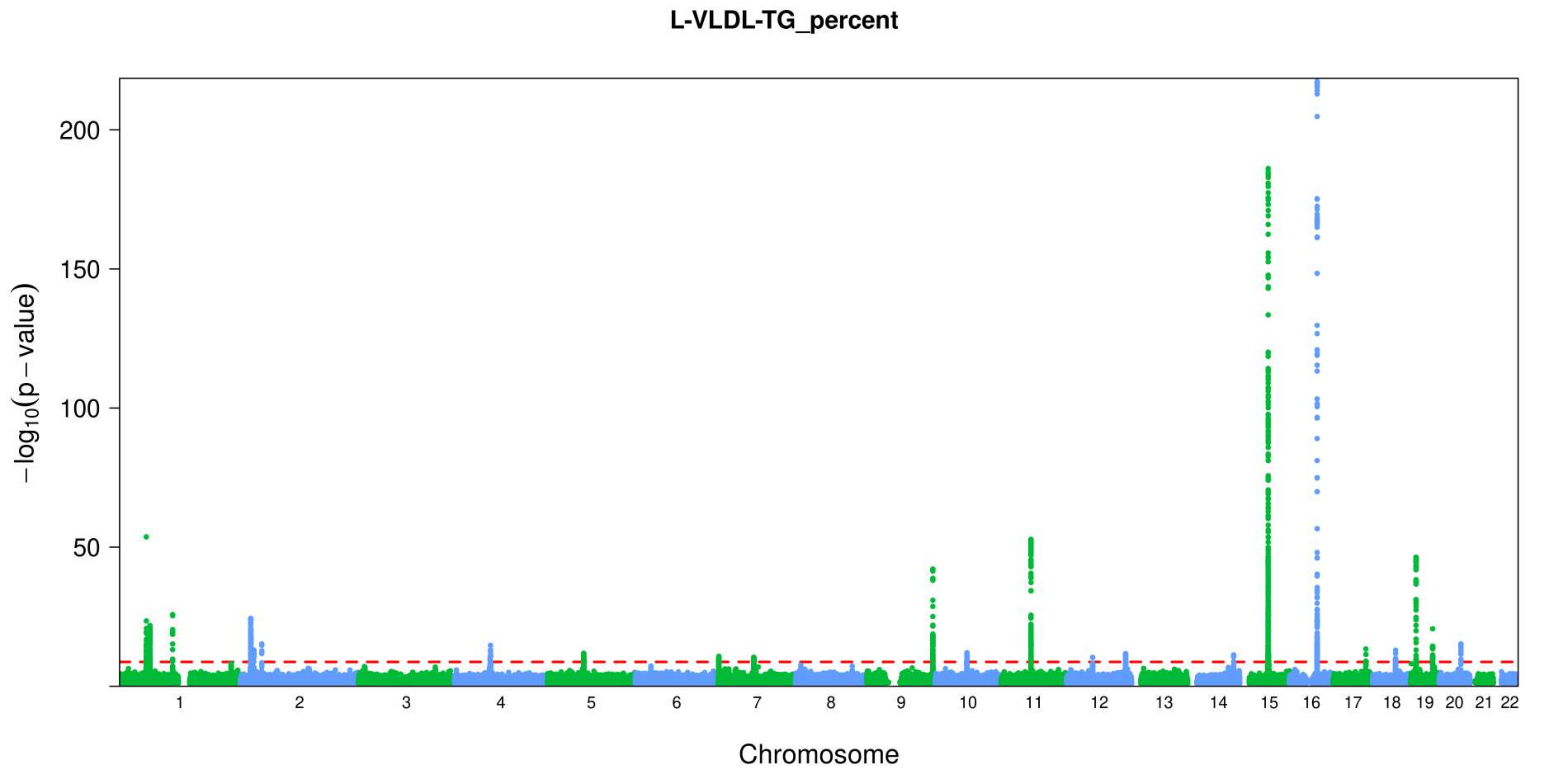
L-VLDL-TG_percent
−log10(p − value)
200
150
100
50
Chromosome
1
2
3
4
5
6
7
8
9
10
11
12
13
14
15
16
17
18
19
20
21
22

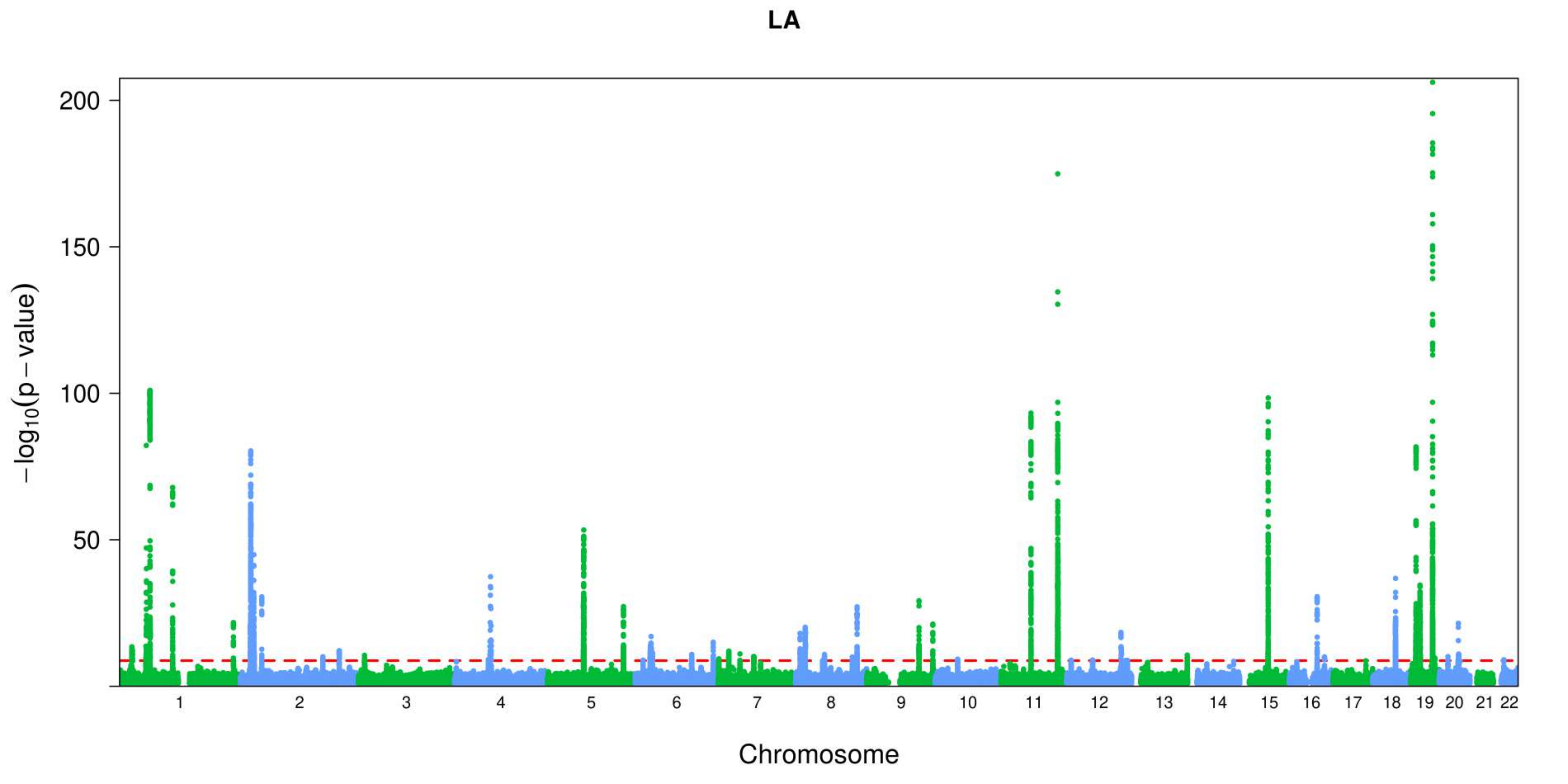
LA
200
150
100
50
−log10(p − value)
1
2
3
4
5
6
7
8
9
10
11
12
13
14
15
16
17
18
19
20
21
22
Chromosome

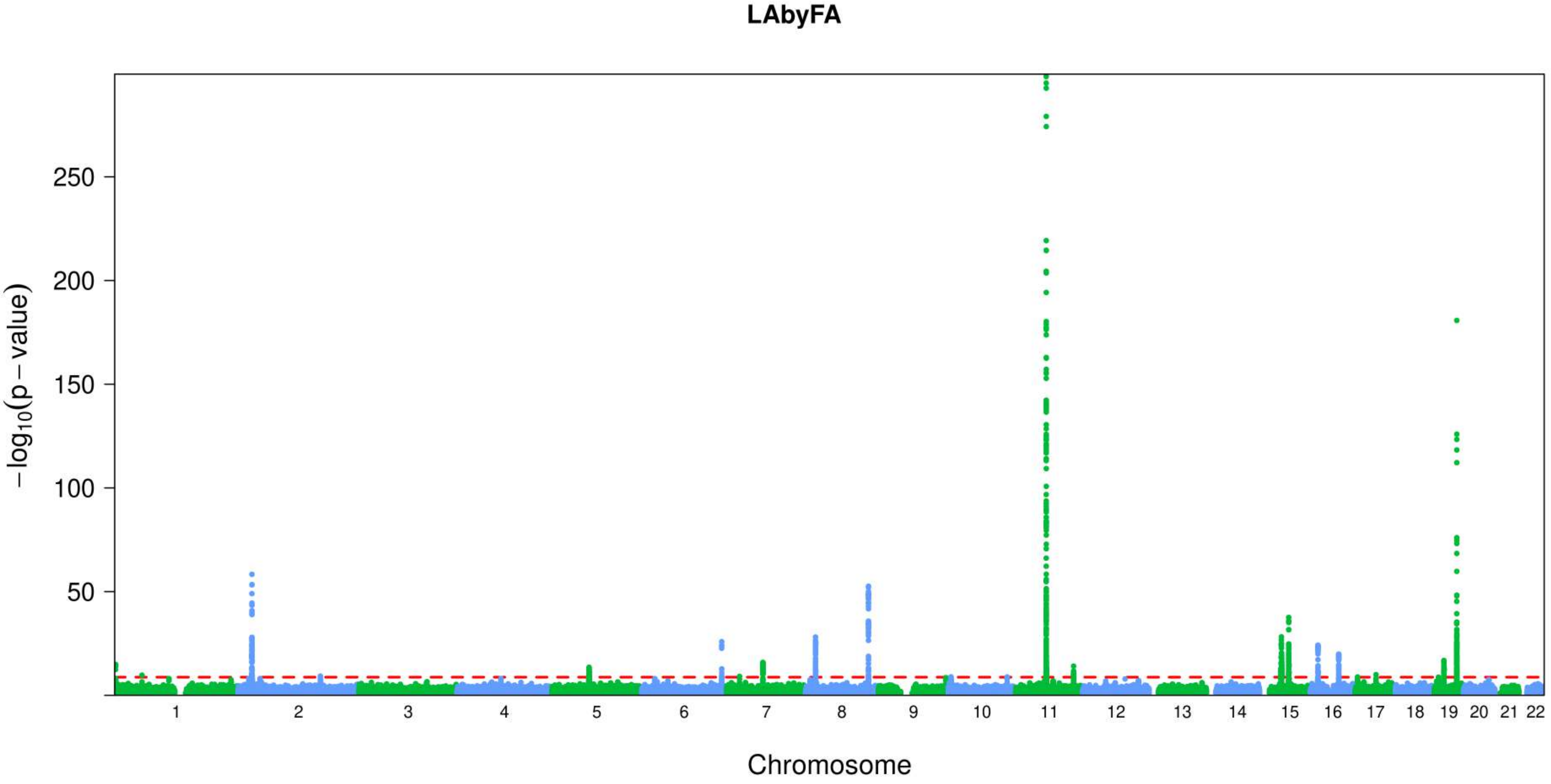
LAbyFA
250
200
150
100
50
$-\log_{10}(p-\text{value})$
1
2
3
4
5
6
7
8
9
10
11
12
13
14
15
16
17
18
19
20
21
22
Chromosome

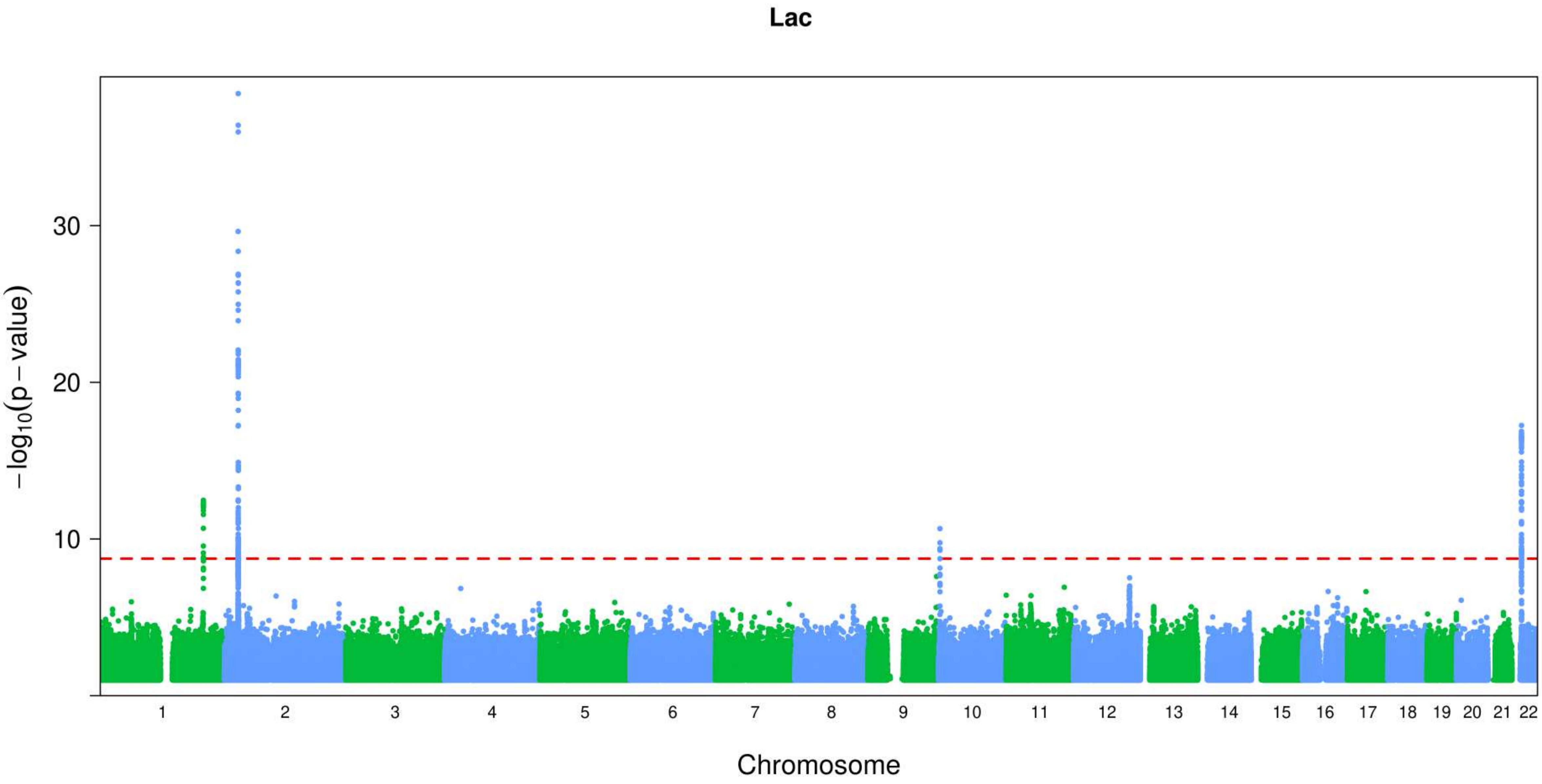

Lac
30
20
10
−log10(p − value)
1
2
3
4
5
6
7
8
9
10
11
12
13
14
15
16
17
18
19
20
21
22
Chromosome

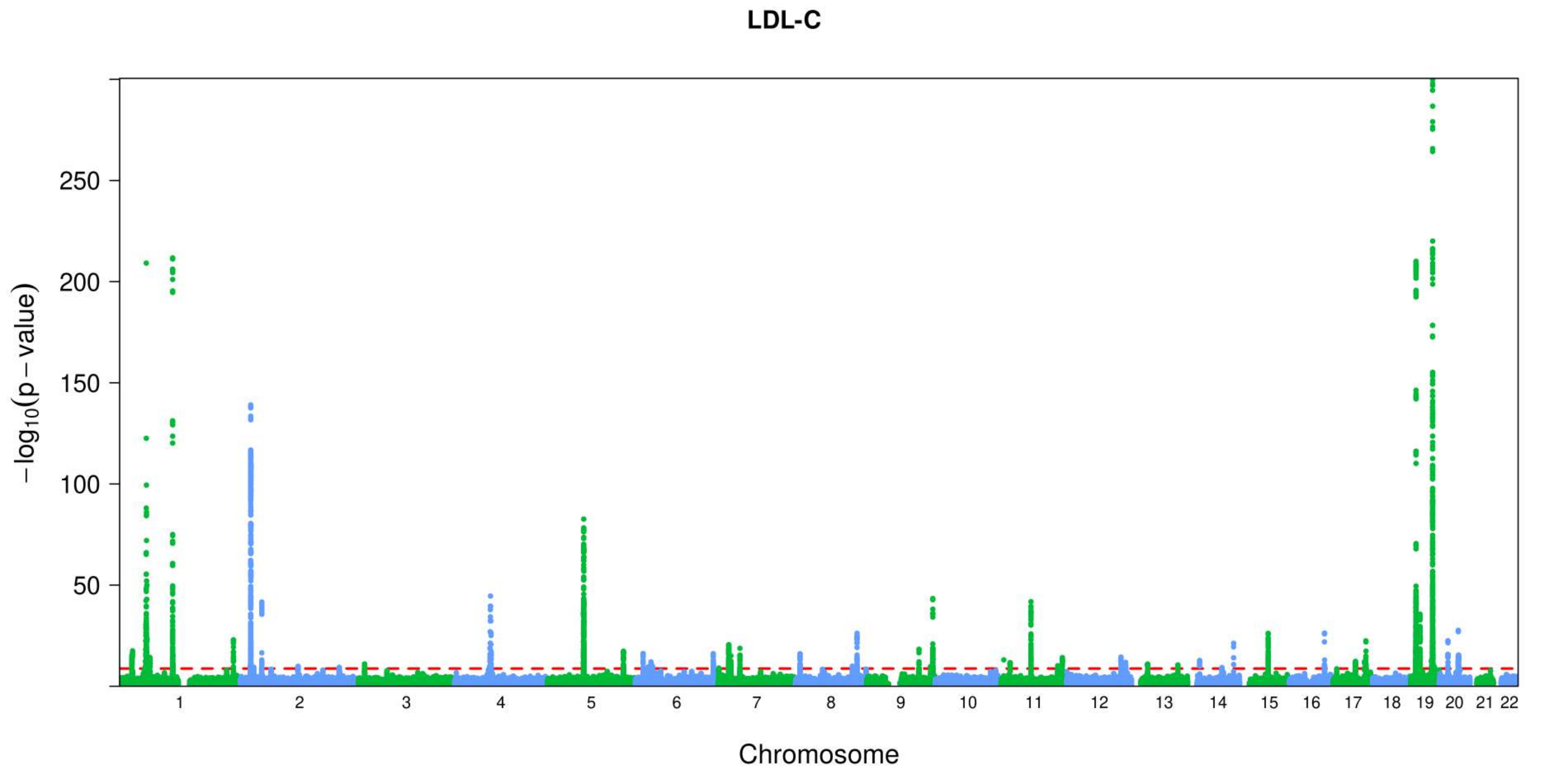
LDL-C
−log10(p − value)
250
200
150
100
50
Chromosome
1
2
3
4
5
6
7
8
9
10
11
12
13
14
15
16
17
18
19
20
21
22

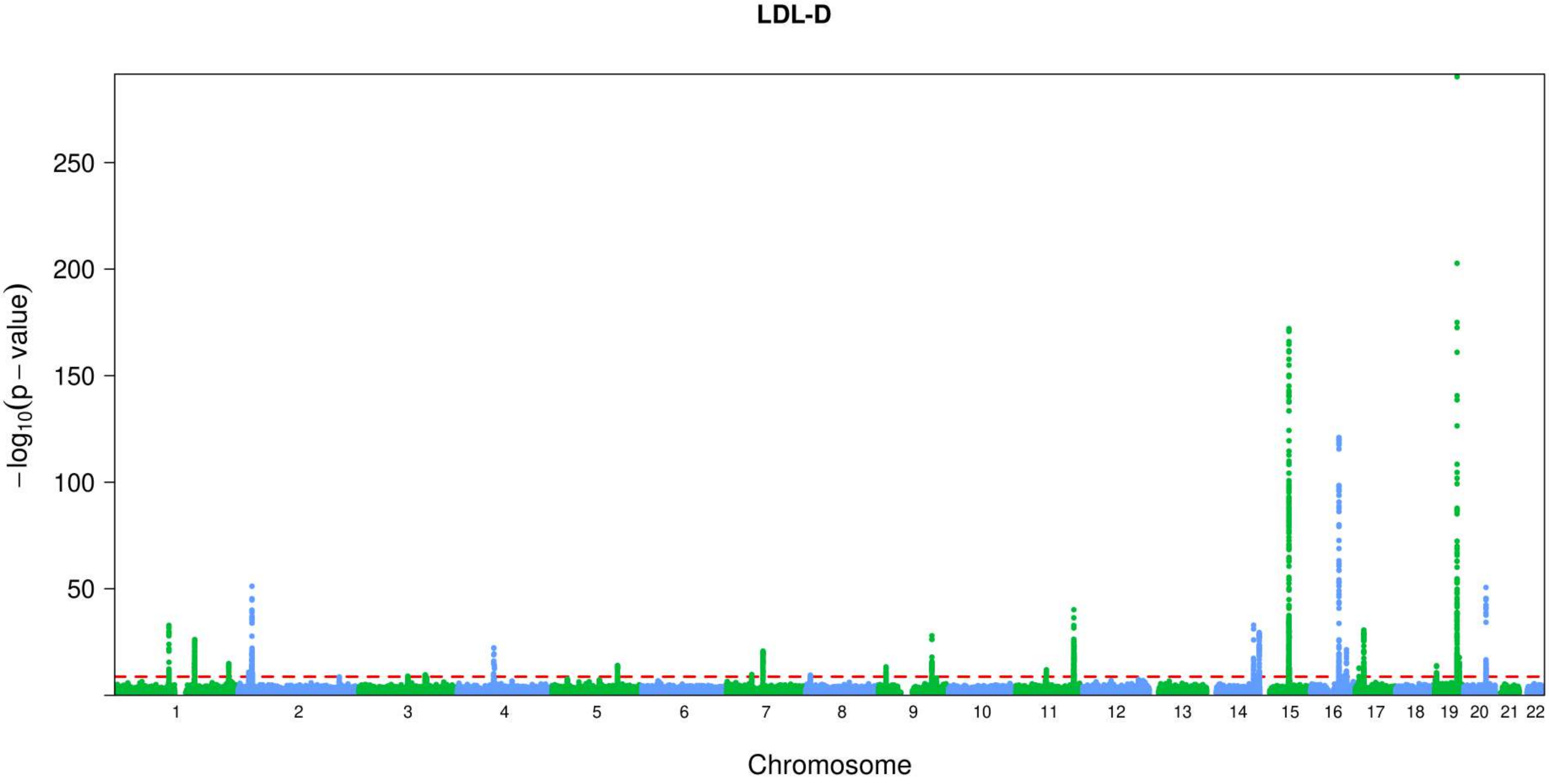

LDL-D
250
200
150
100
50
-log10(p-value)
1
2
3
4
5
6
7
8
9
10
11
12
13
14
15
16
17
18
19
20
21
22
Chromosome

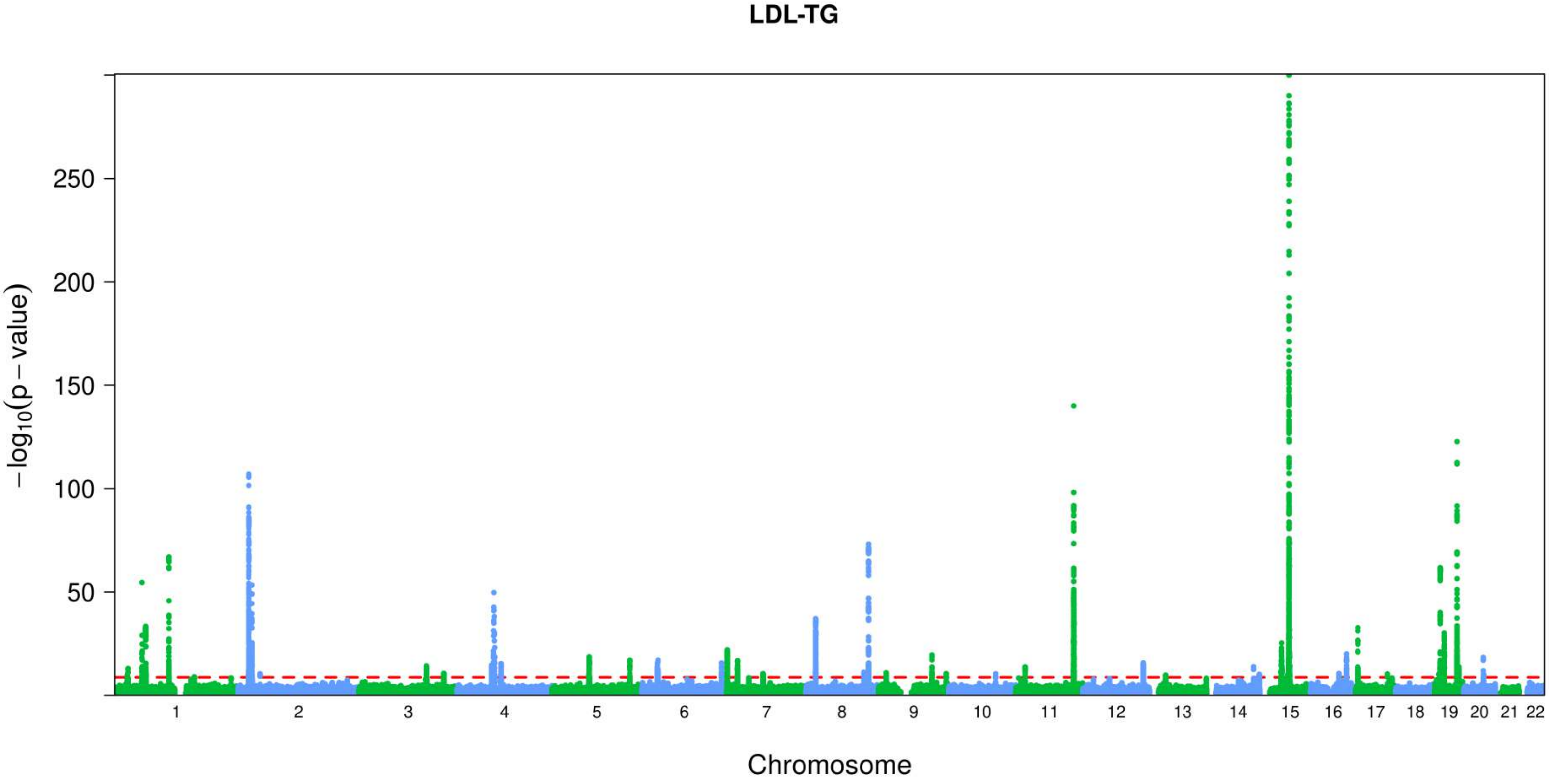
LDL-TG
250
200
150
100
50
$-\log_{10}(p-\text{value})$
1
2
3
4
5
6
7
8
9
10
11
12
13
14
15
16
17
18
19
20
21
22
Chromosome

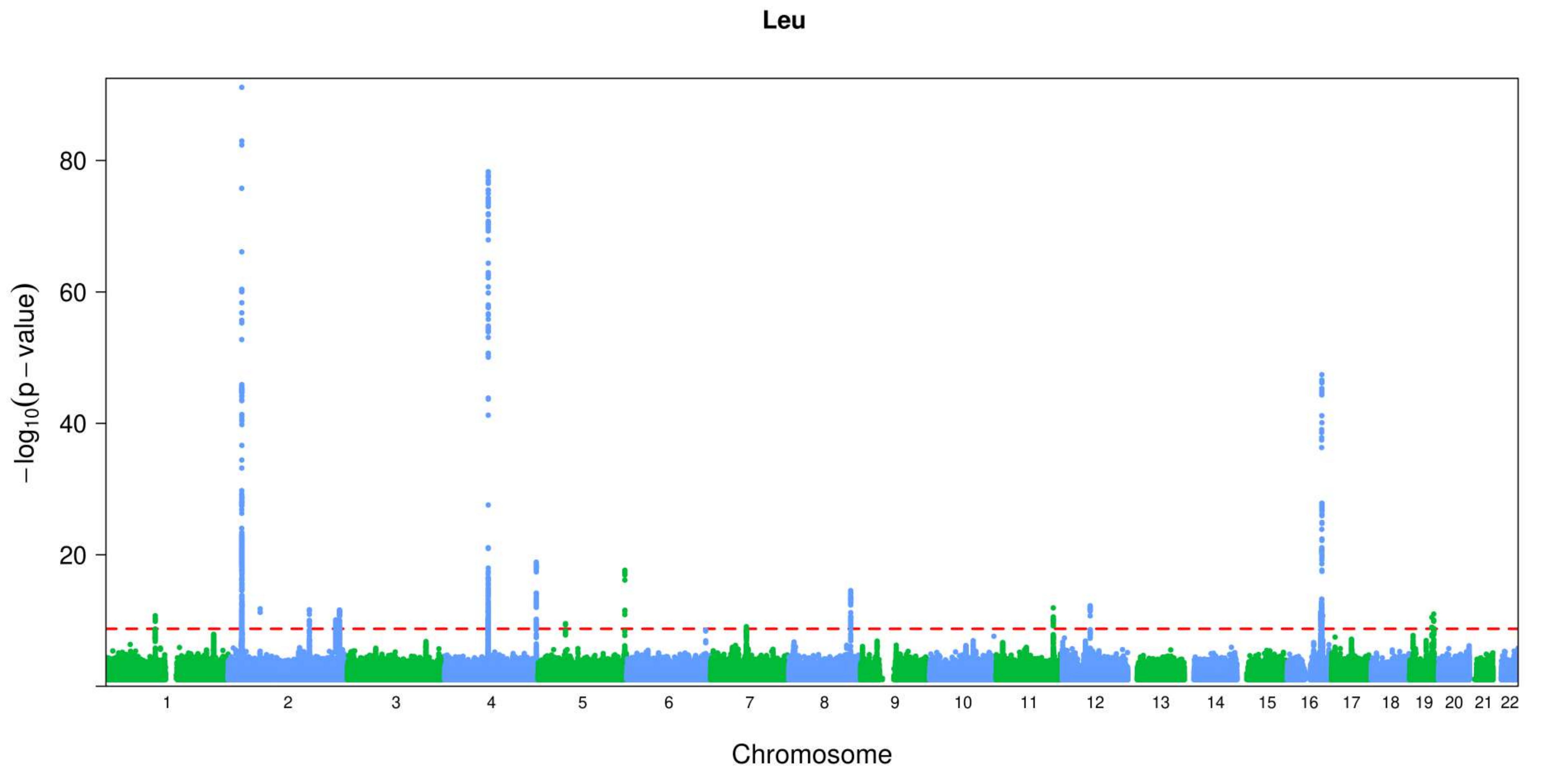
Leu
−log10(p − value)
80
60
40
20
1 2 3 4 5 6 7 8 9 10 11 12 13 14 15 16 17 18 19 20 21 22
Chromosome

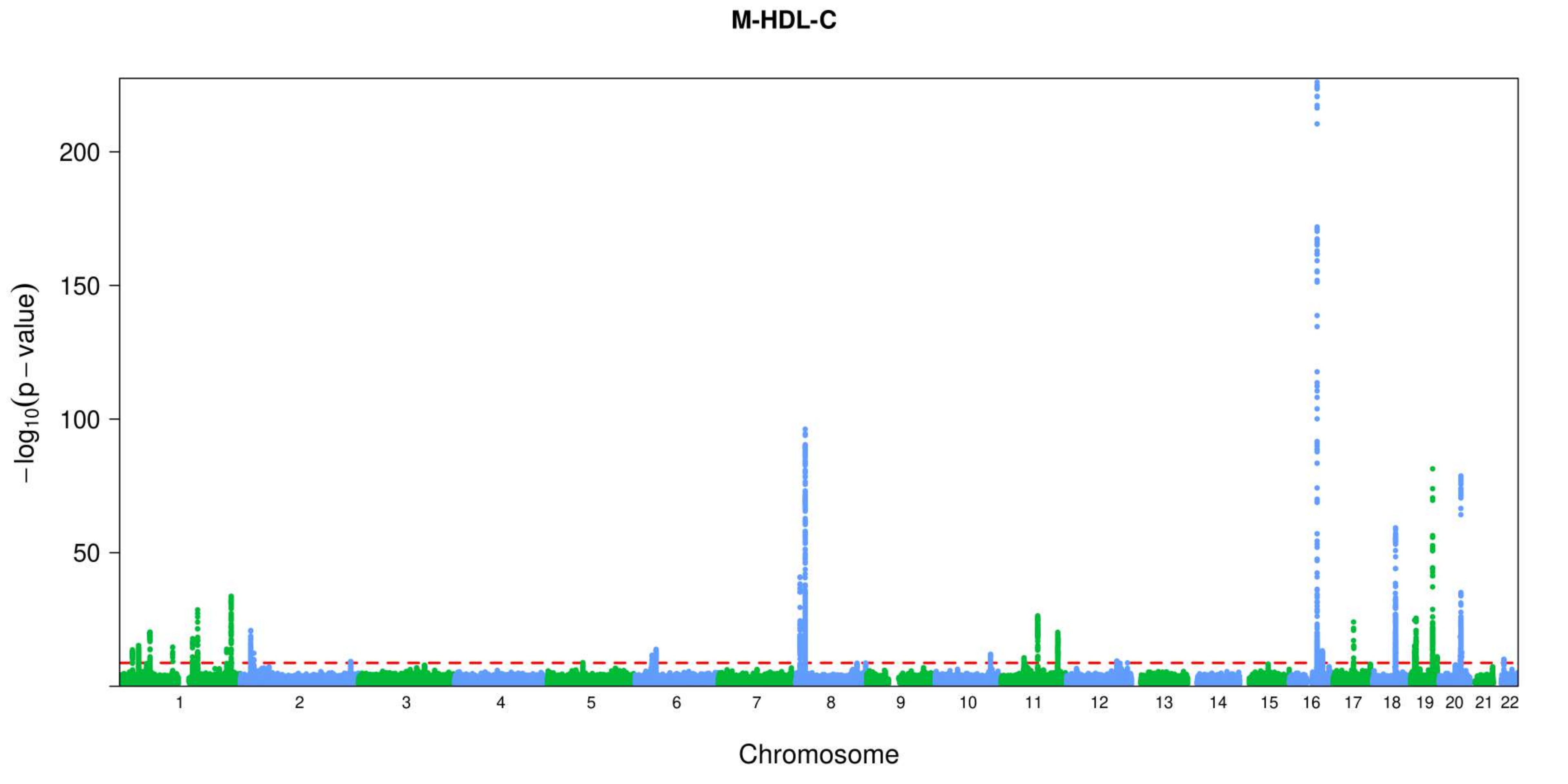

M-HDL-C
200
150
100
50
−log10(p − value)
1
2
3
4
5
6
7
8
9
10
11
12
13
14
15
16
17
18
19
20
21
22
Chromosome

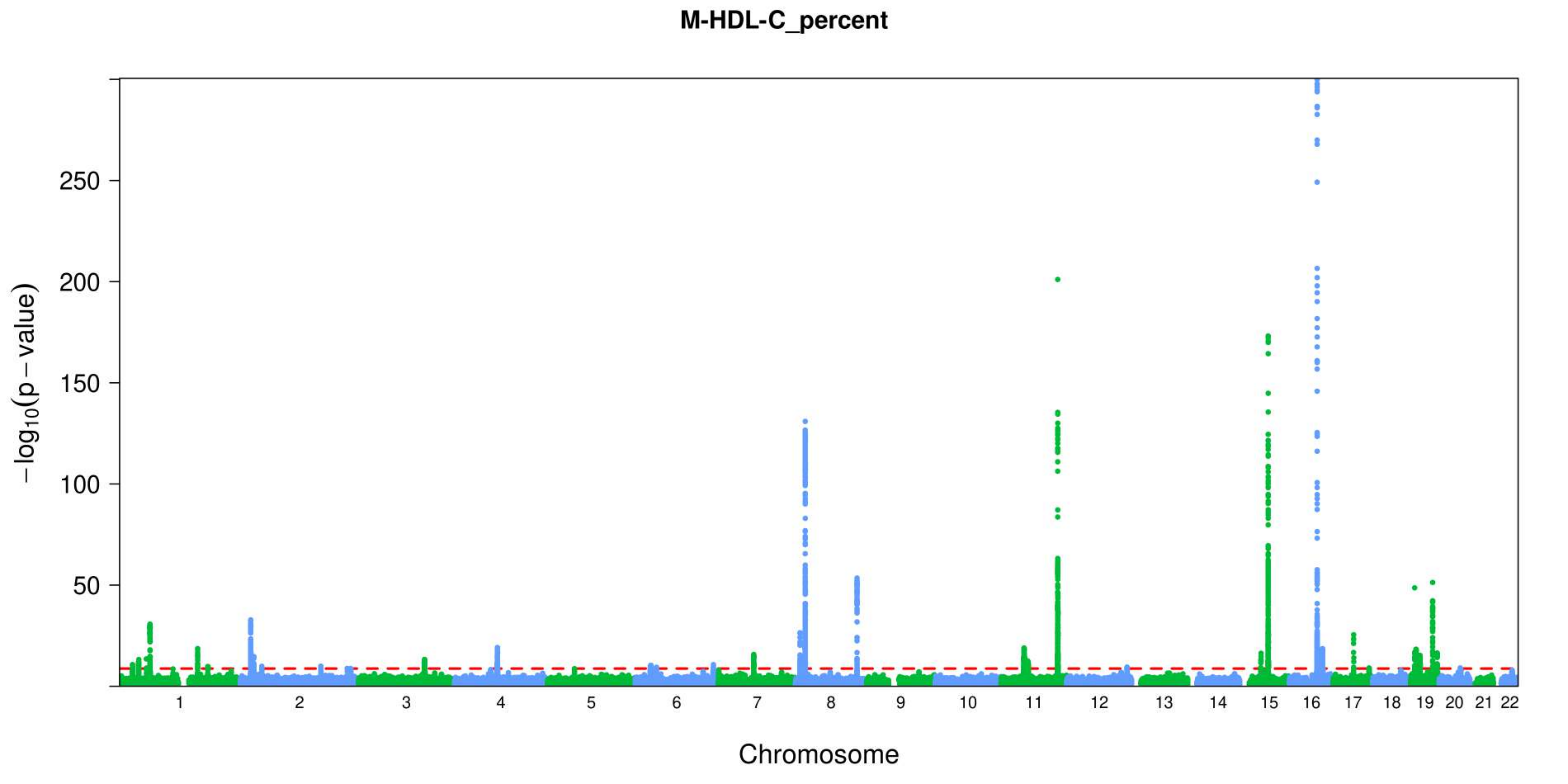
M-HDL-C_percent
250
200
150
100
50
−log10(p − value)
1
2
3
4
5
6
7
8
9
10
11
12
13
14
15
16
17
18
19
20
21
22
Chromosome

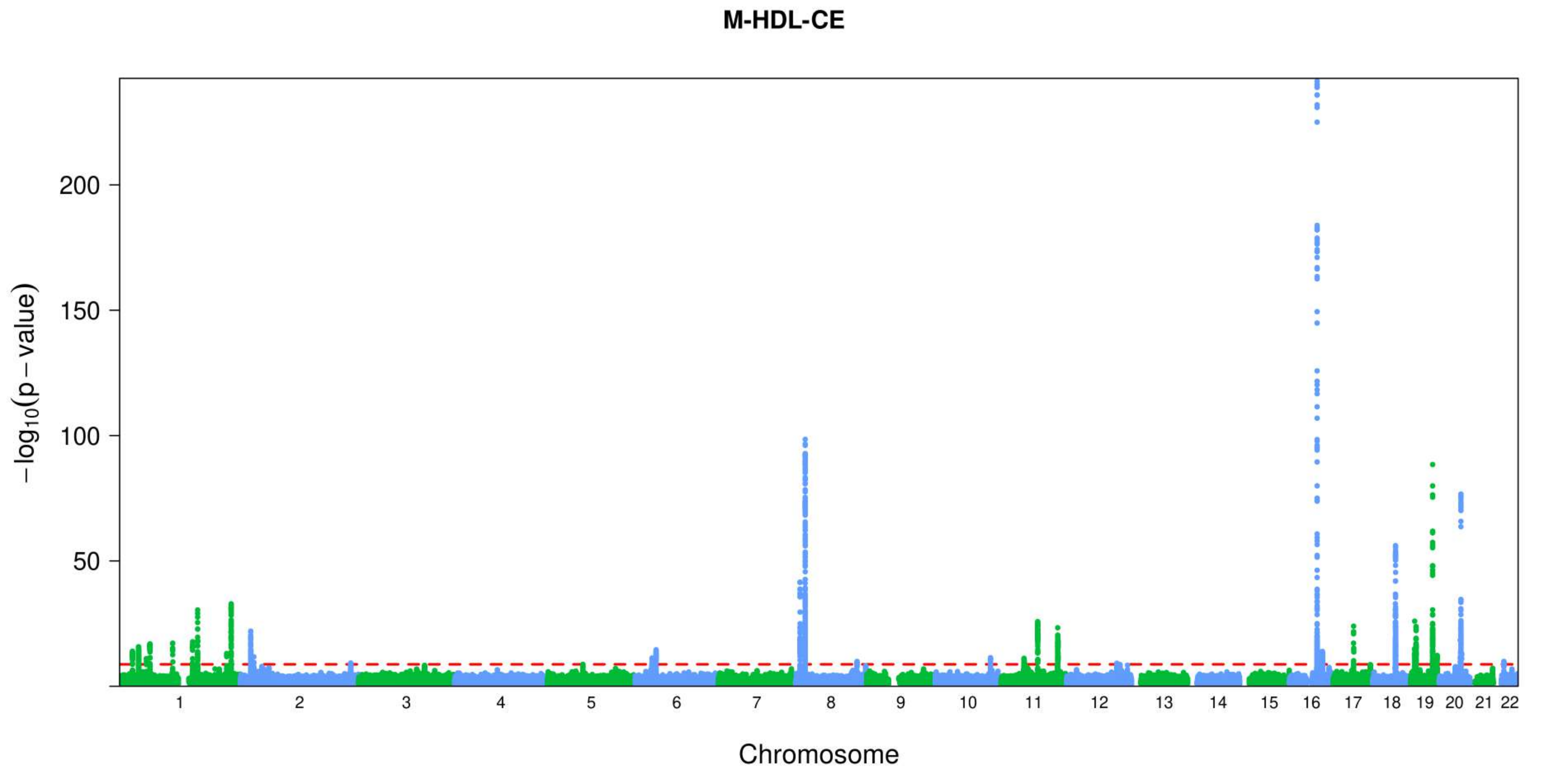

M-HDL-CE
−log10(p − value)
200
150
100
50
Chromosome
1
2
3
4
5
6
7
8
9
10
11
12
13
14
15
16
17
18
19
20
21
22

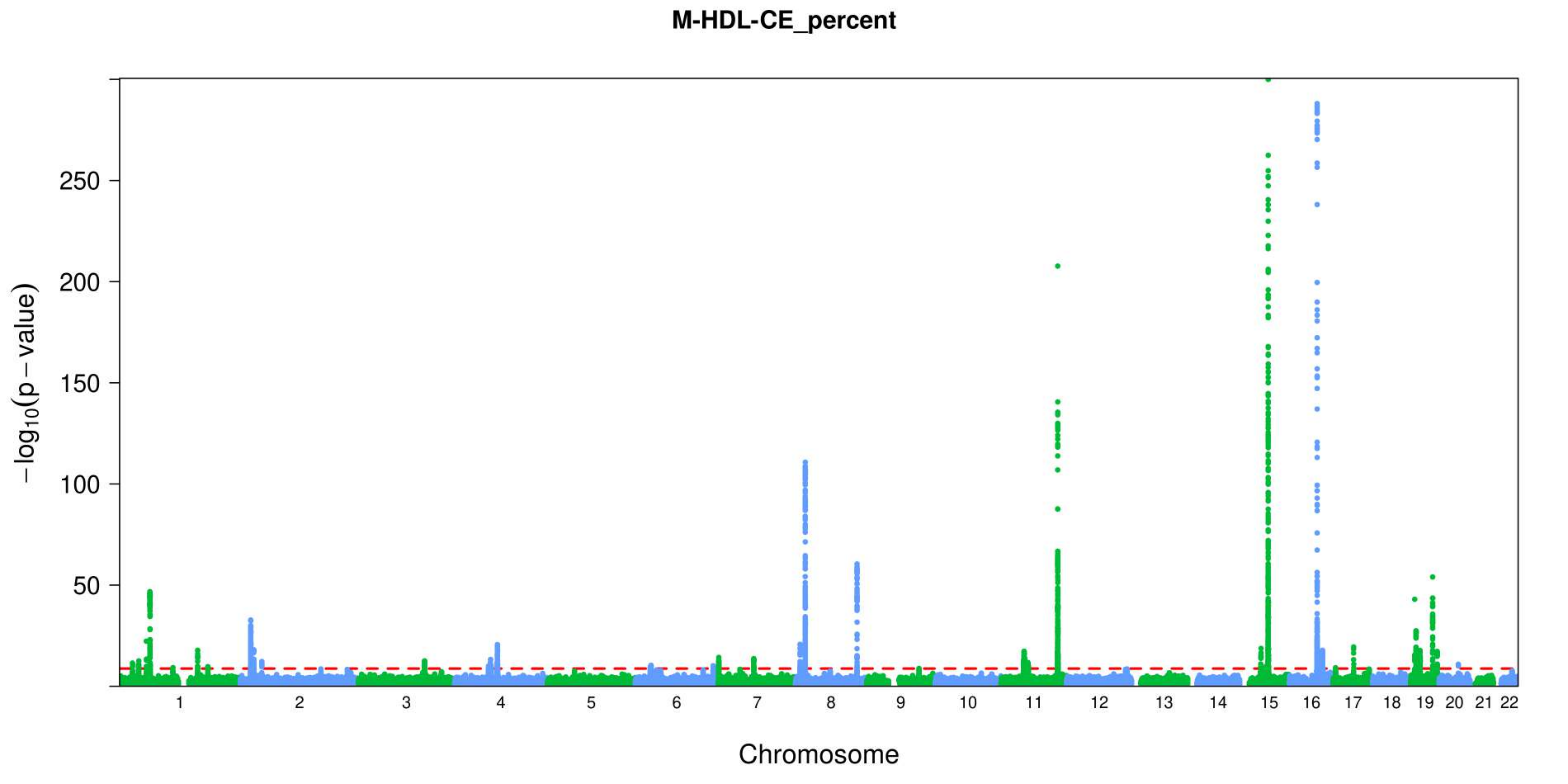

M-HDL-CE_percent
250
200
150
100
50
−log10(p − value)
1
2
3
4
5
6
7
8
9
10
11
12
13
14
15
16
17
18
19
20
21
22
Chromosome

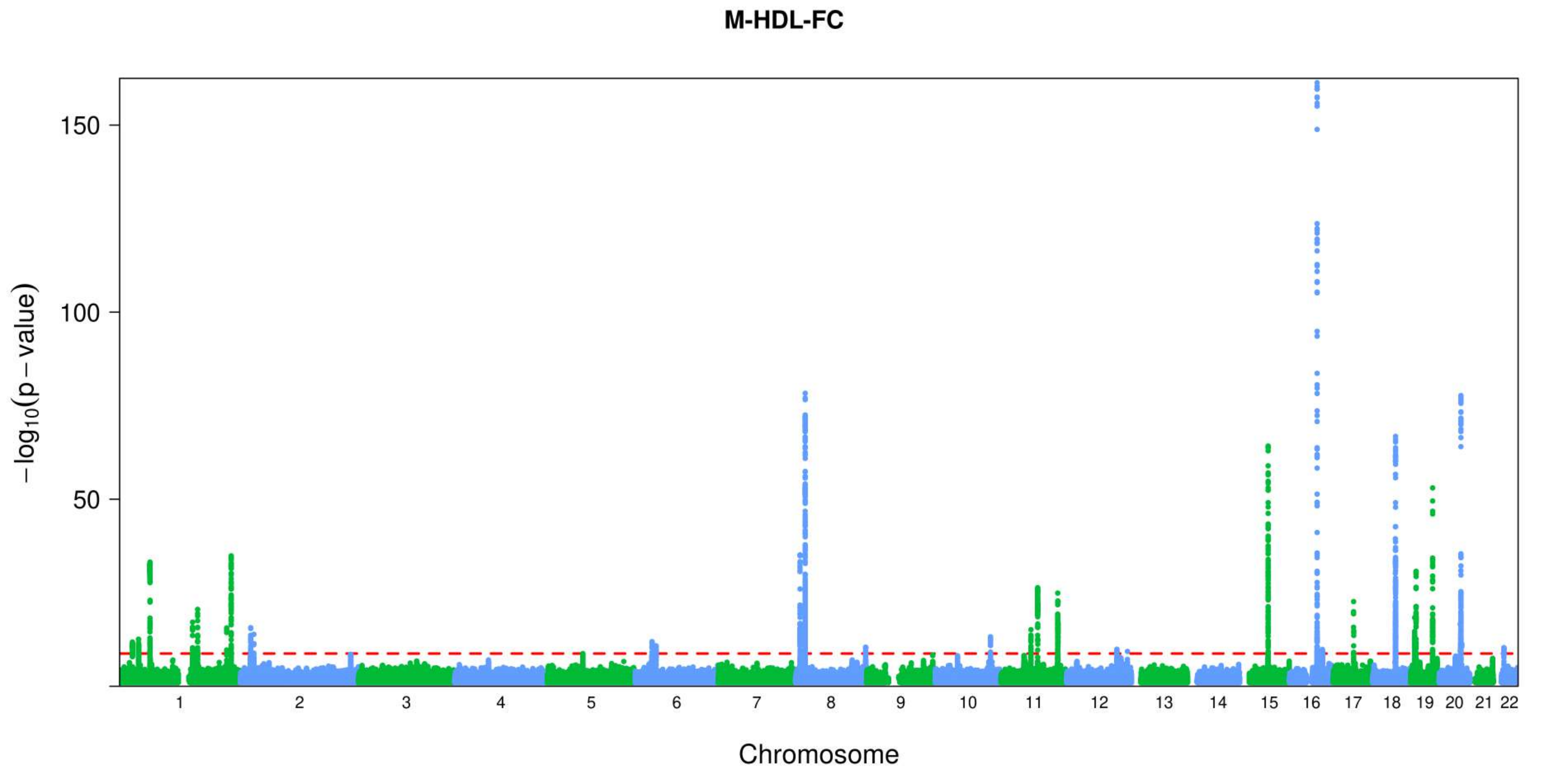

M-HDL-FC
−log10(p − value)
150
100
50
1
2
3
4
5
6
7
8
9
10
11
12
13
14
15
16
17
18
19
20
21
22
Chromosome

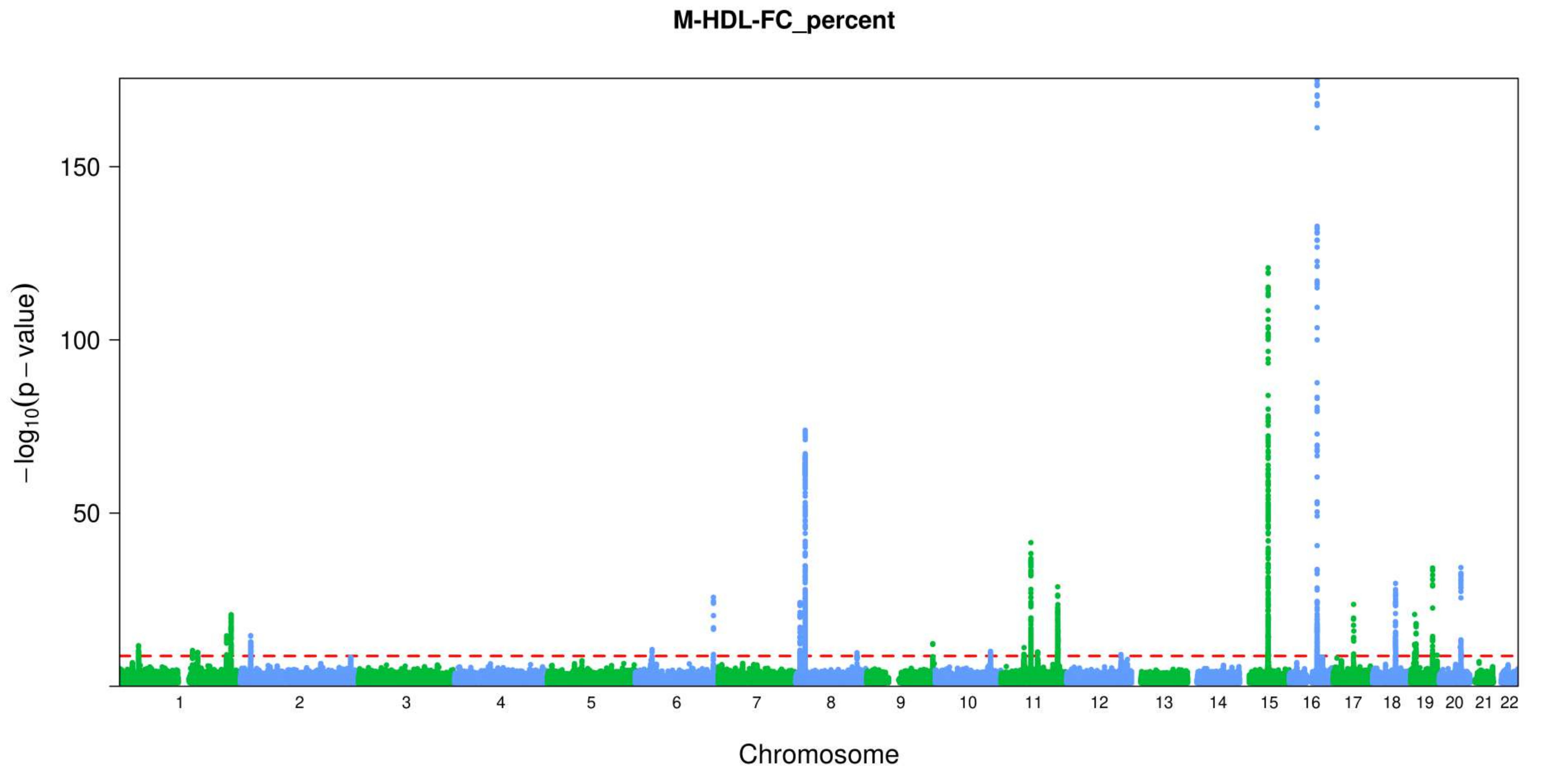
M-HDL-FC_percent
150
100
50
−log10(p − value)
1
2
3
4
5
6
7
8
9
10
11
12
13
14
15
16
17
18
19
20
21
22
Chromosome

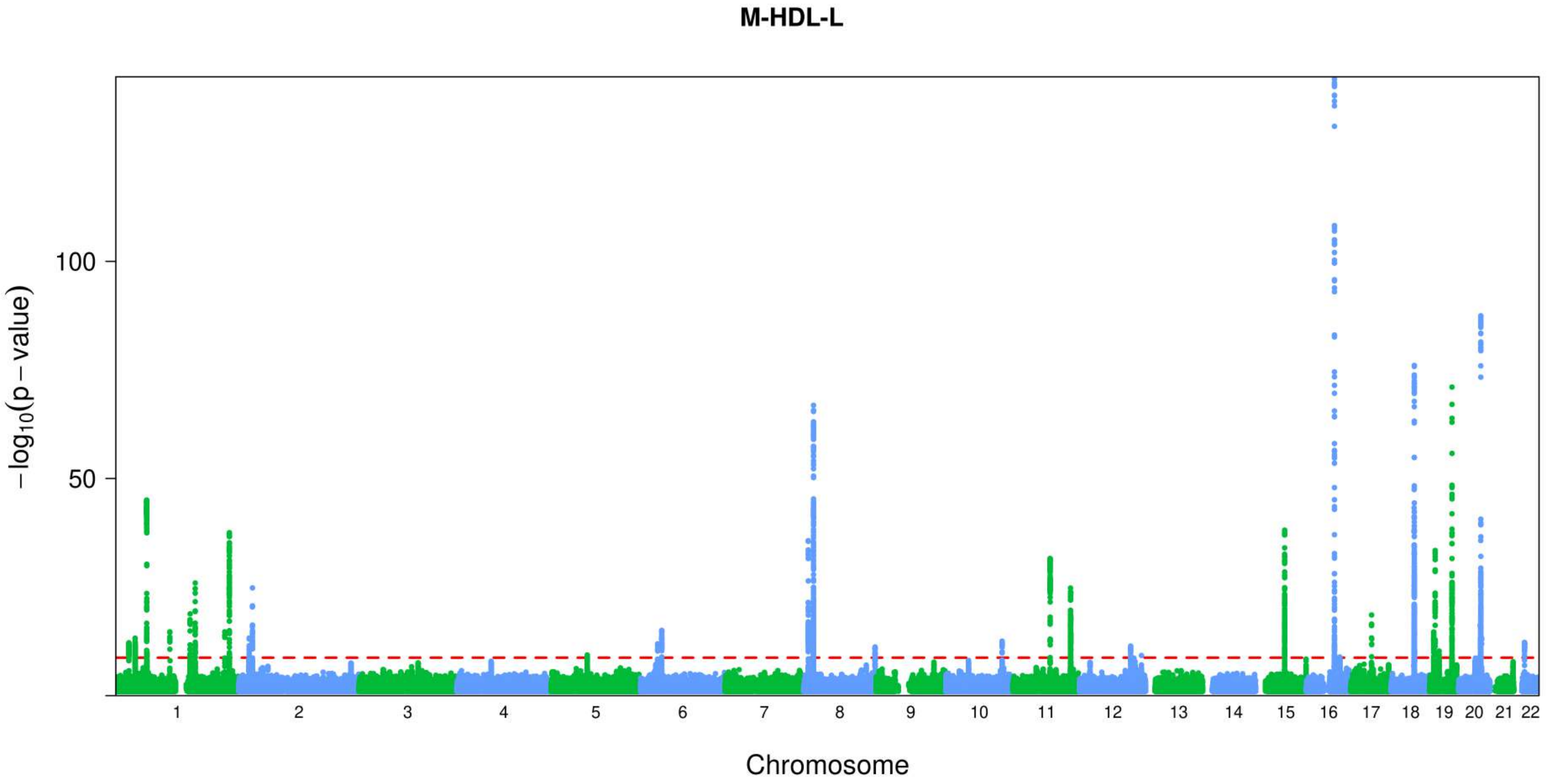
M-HDL-L
100
50
−log10(p − value)
1
2
3
4
5
6
7
8
9
10
11
12
13
14
15
16
17
18
19
20
21
22
Chromosome

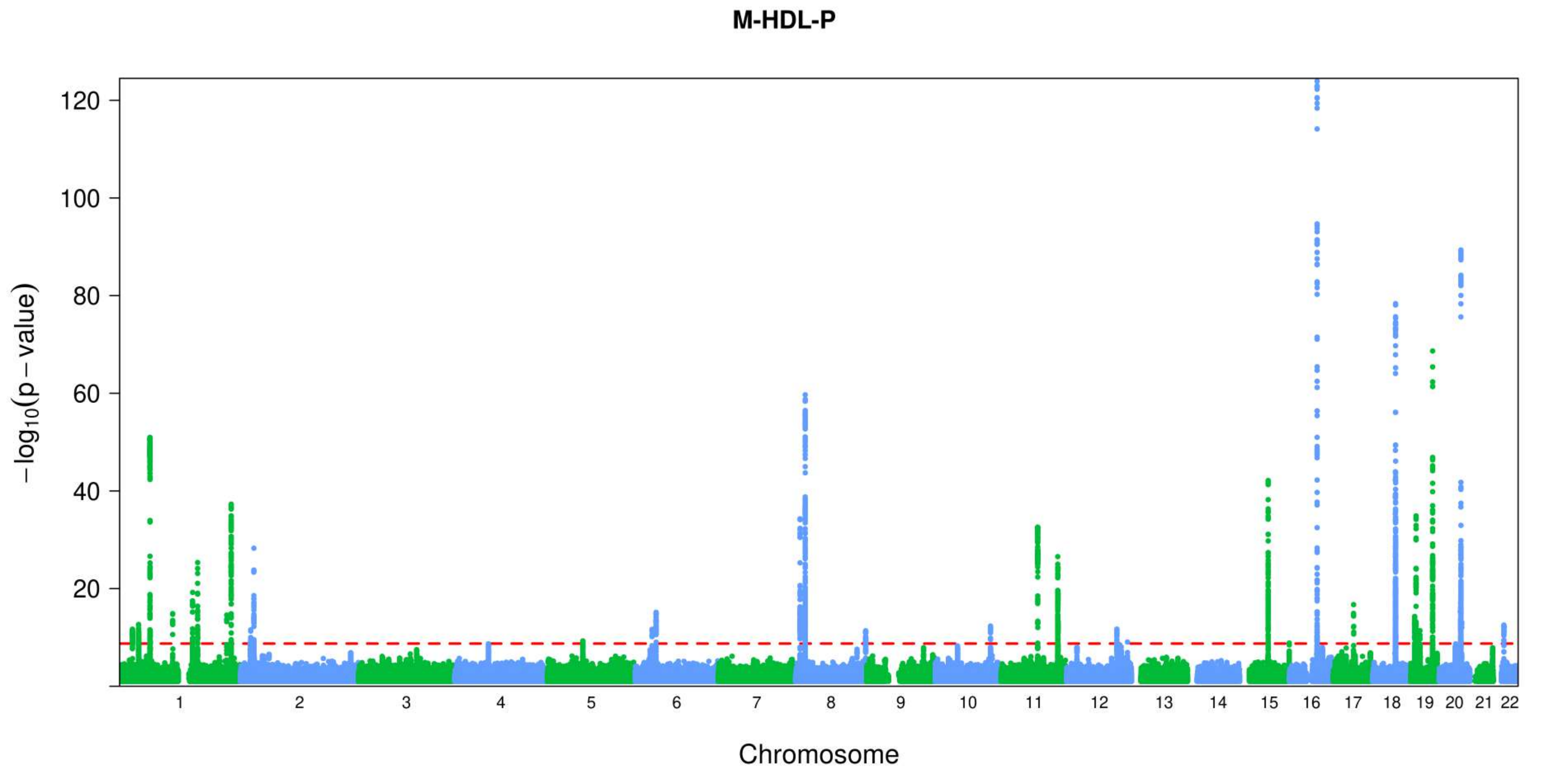
M-HDL-P
−log10(p − value)
120
100
80
60
40
20
Chromosome
1
2
3
4
5
6
7
8
9
10
11
12
13
14
15
16
17
18
19
20
21
22

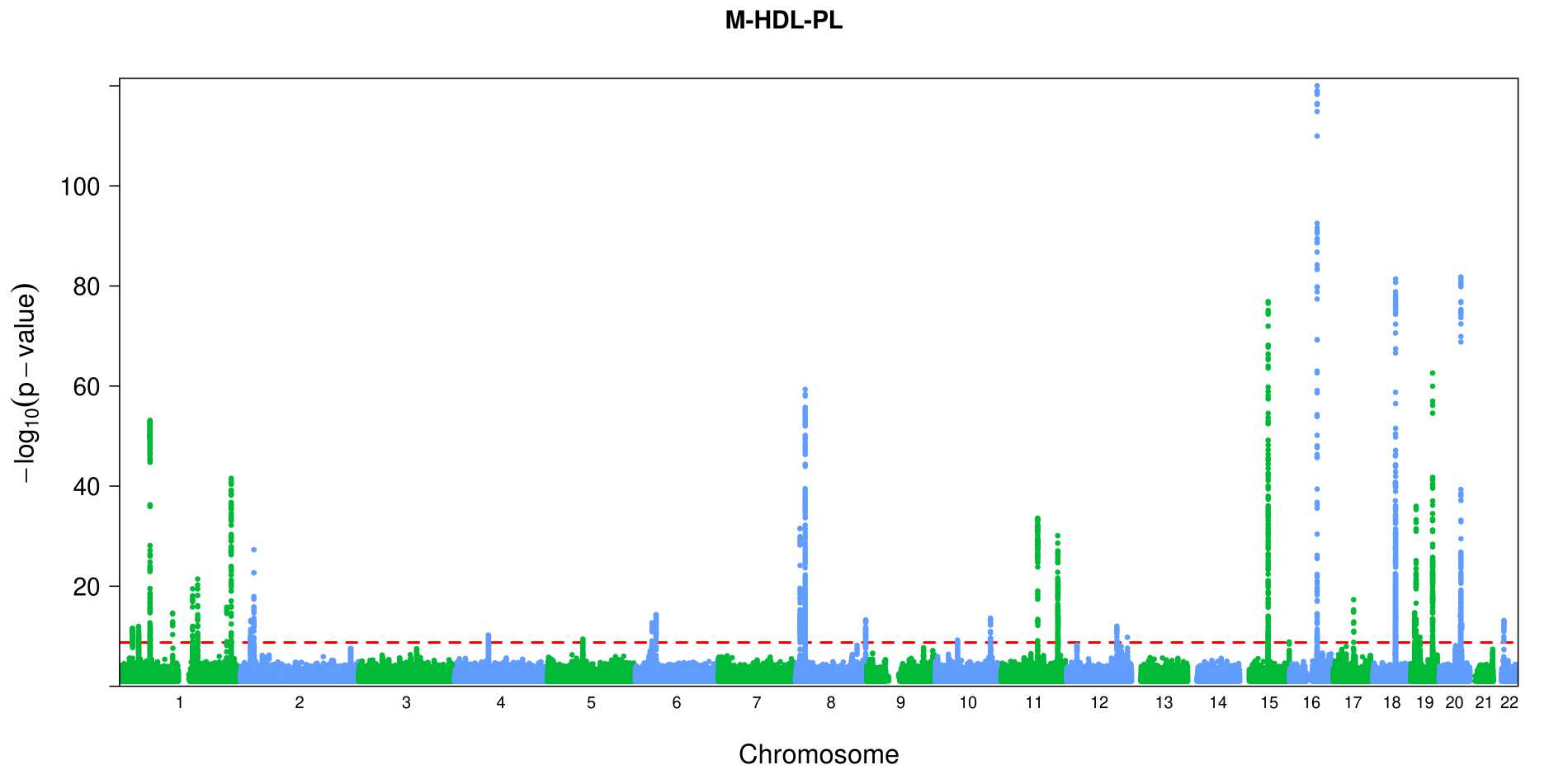

M-HDL-PL
−log10(p − value)
100
80
60
40
20
1
2
3
4
5
6
7
8
9
10
11
12
13
14
15
16
17
18
19
20
21
22
Chromosome

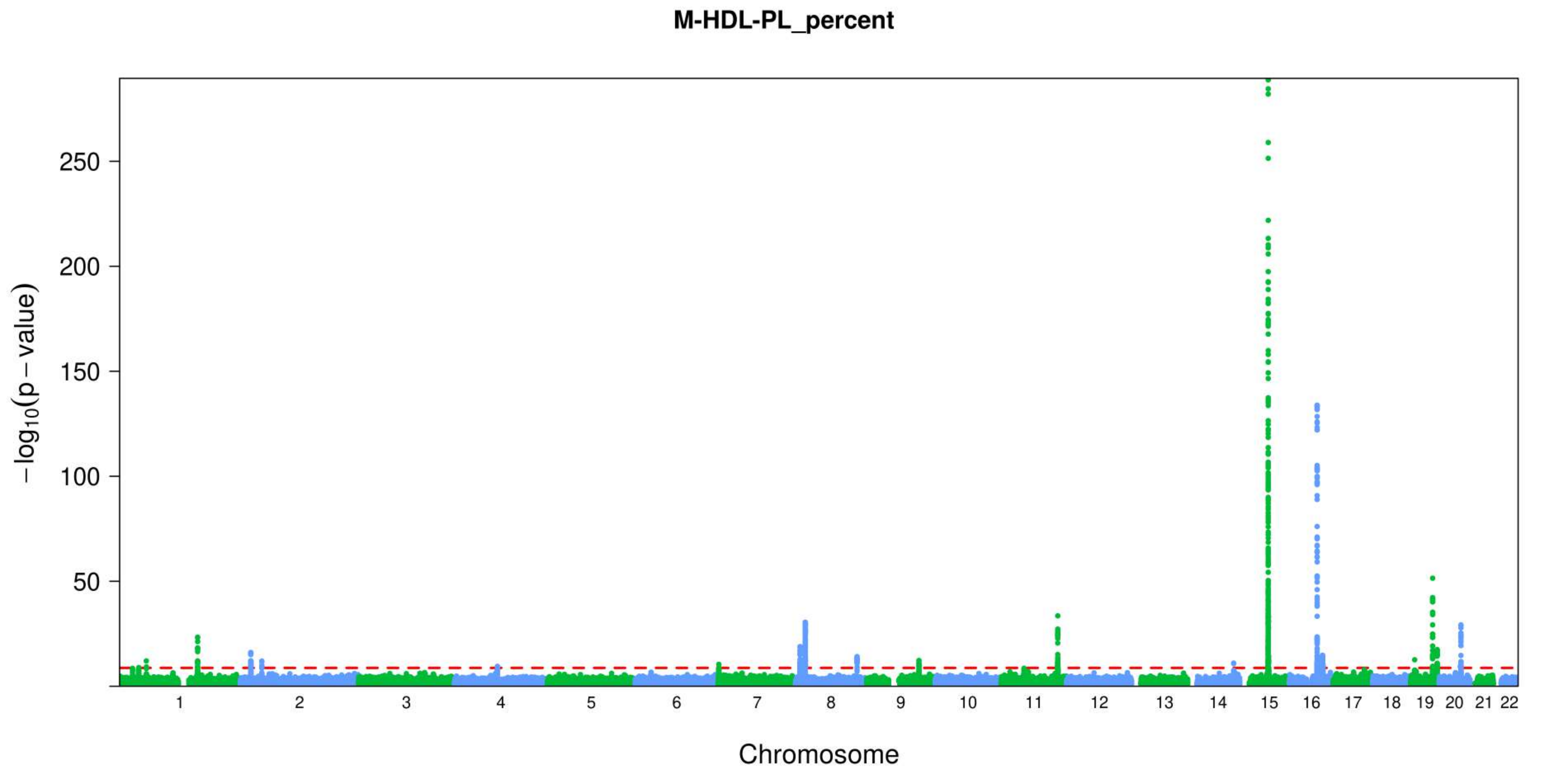

M-HDL-PL_percent
−log10(p − value)
250
200
150
100
50
Chromosome
1
2
3
4
5
6
7
8
9
10
11
12
13
14
15
16
17
18
19
20
21
22

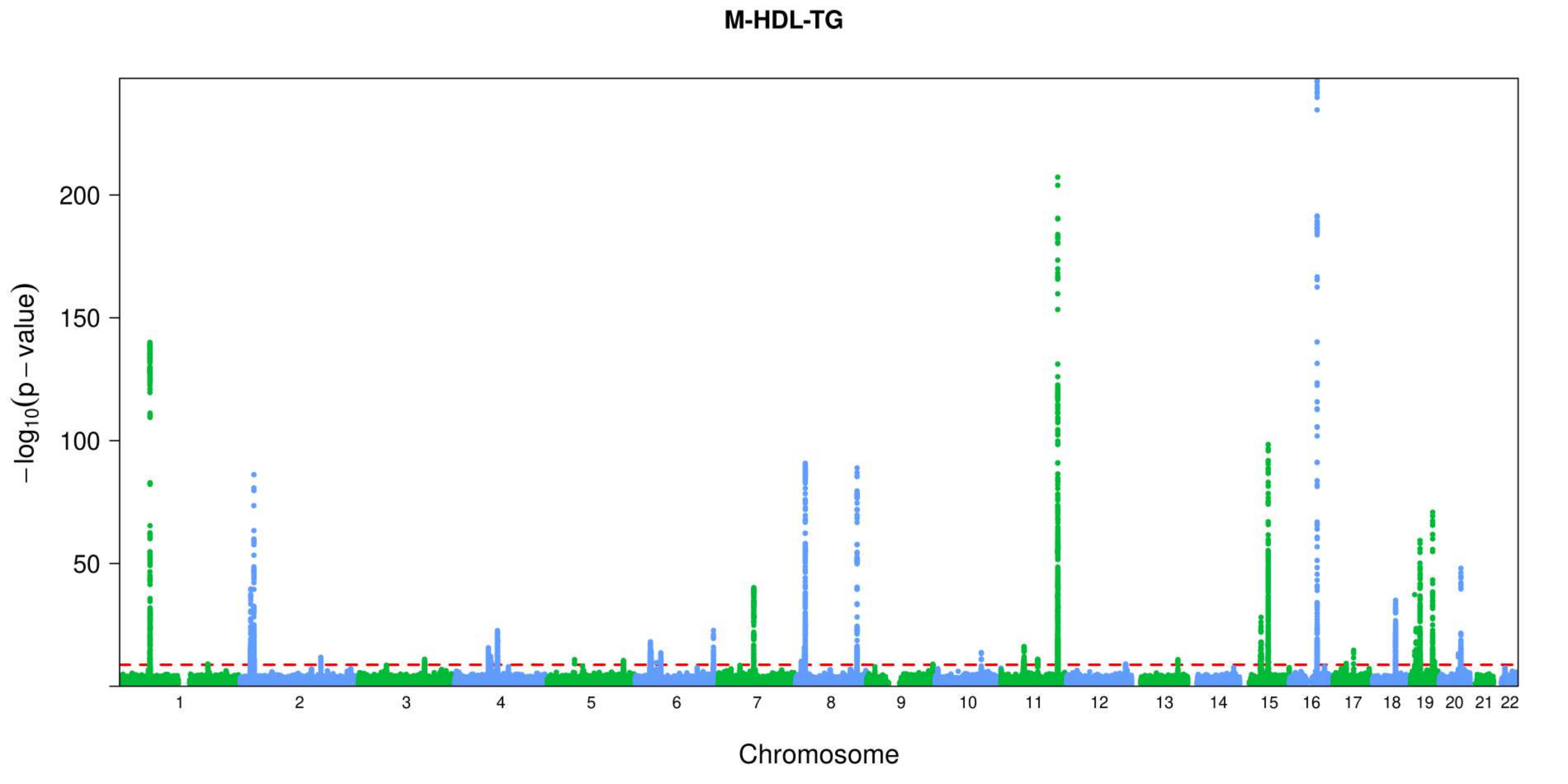
M-HDL-TG
200
150
100
50
−log10(p − value)
1
2
3
4
5
6
7
8
9
10
11
12
13
14
15
16
17
18
19
20
21
22
Chromosome

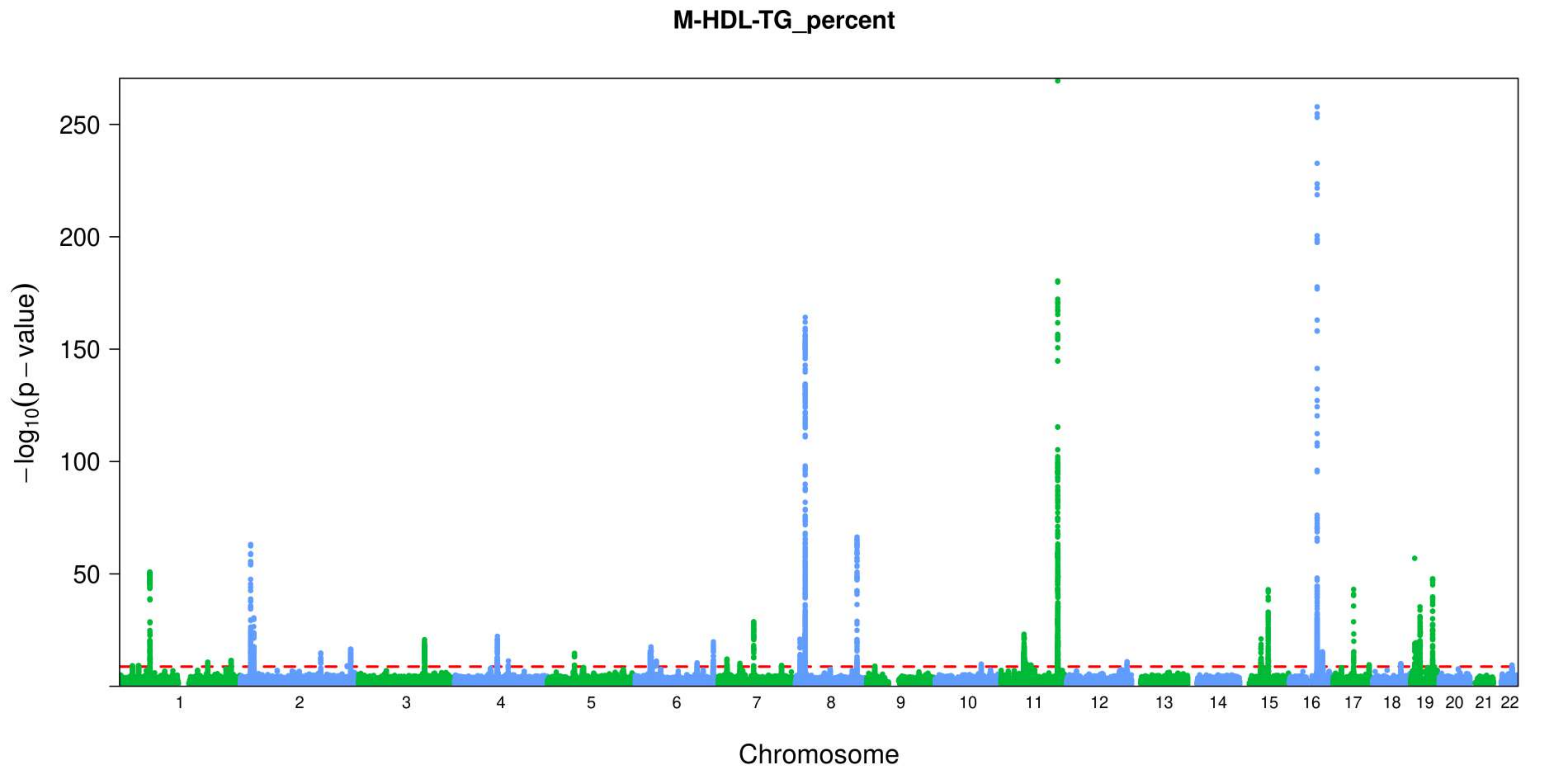
M-HDL-TG_percent
-log10(p-value)
250
200
150
100
50
1
2
3
4
5
6
7
8
9
10
11
12
13
14
15
16
17
18
19
20
21
22
Chromosome

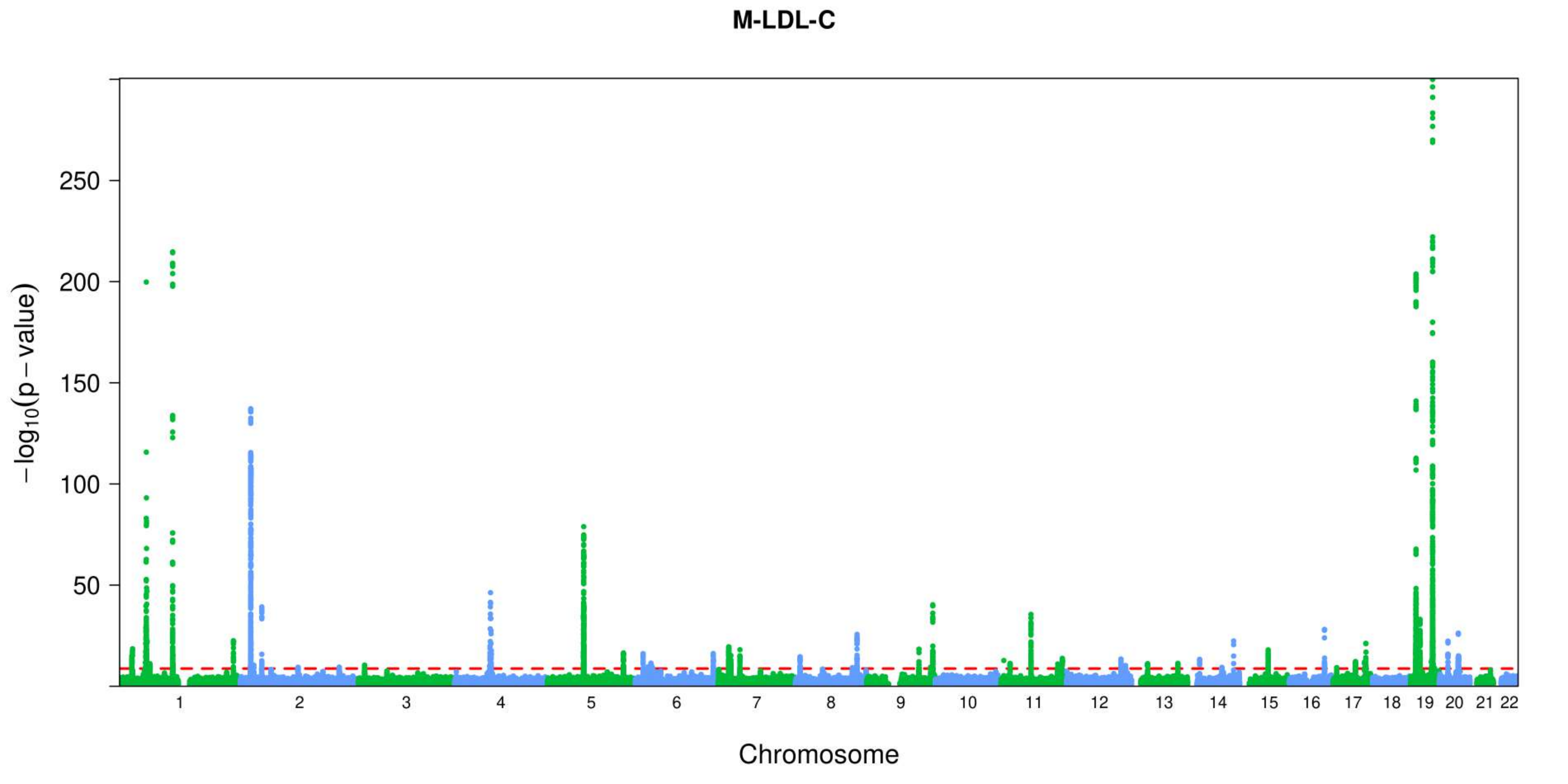
M-LDL-C
250
200
150
100
50
−log10(p − value)
1
2
3
4
5
6
7
8
9
10
11
12
13
14
15
16
17
18
19
20
21
22
Chromosome

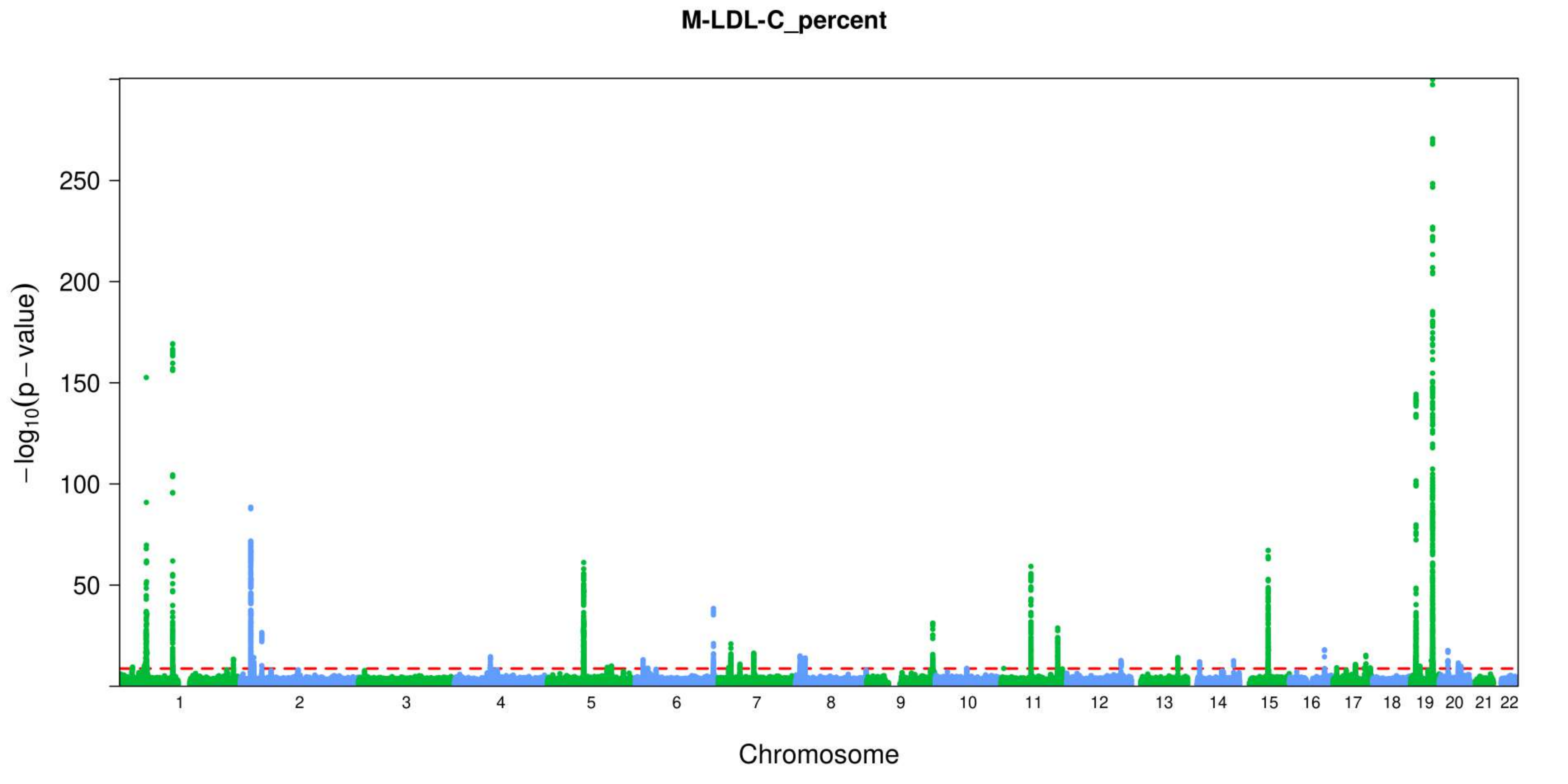
M-LDL-C_percent
$-\log_{10}(p-\text{value})$
250
200
150
100
50
Chromosome
1
2
3
4
5
6
7
8
9
10
11
12
13
14
15
16
17
18
19
20
21
22

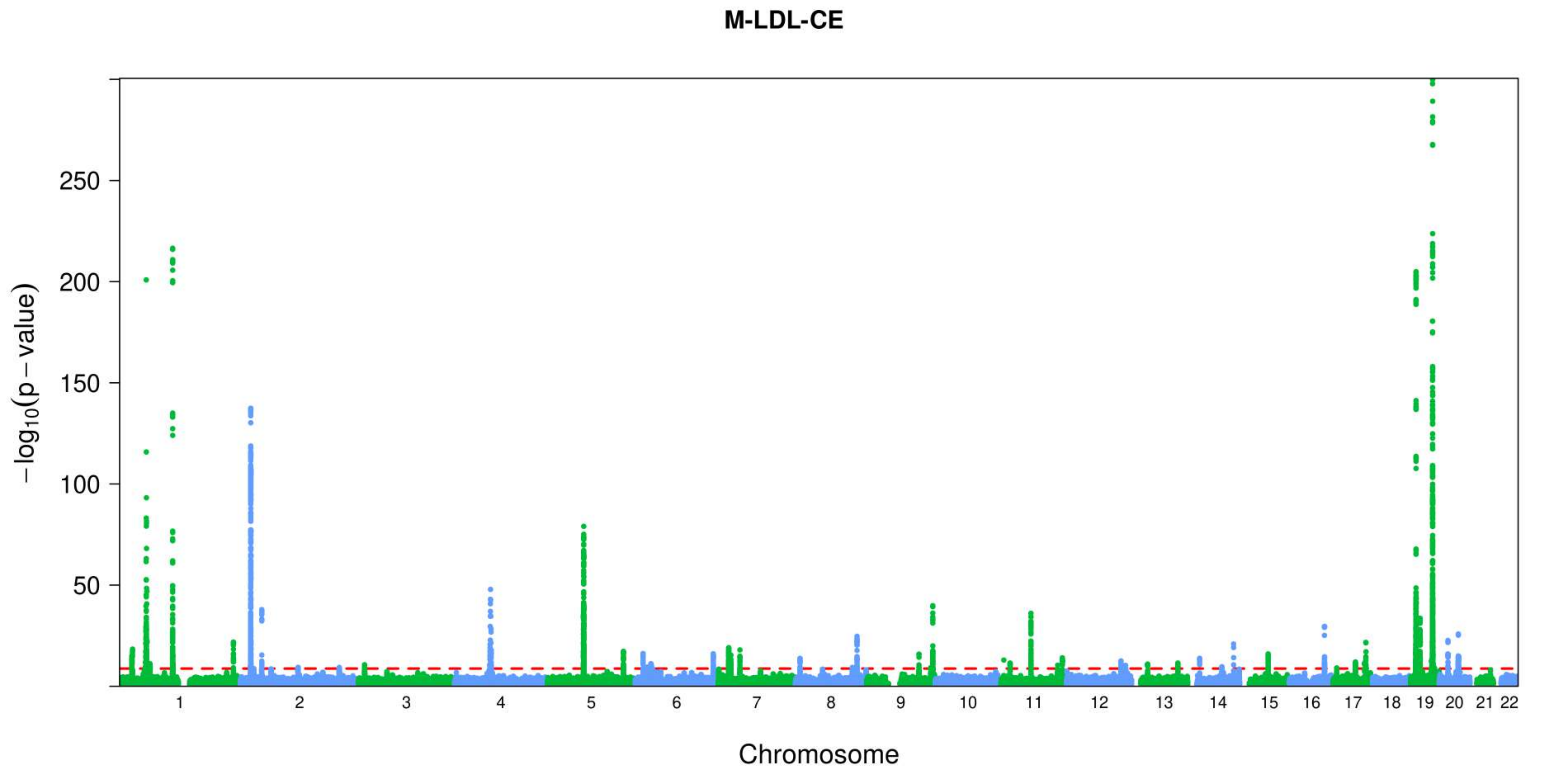
M-LDL-CE
-log10(p-value)
250
200
150
100
50
Chromosome
1
2
3
4
5
6
7
8
9
10
11
12
13
14
15
16
17
18
19
20
21
22

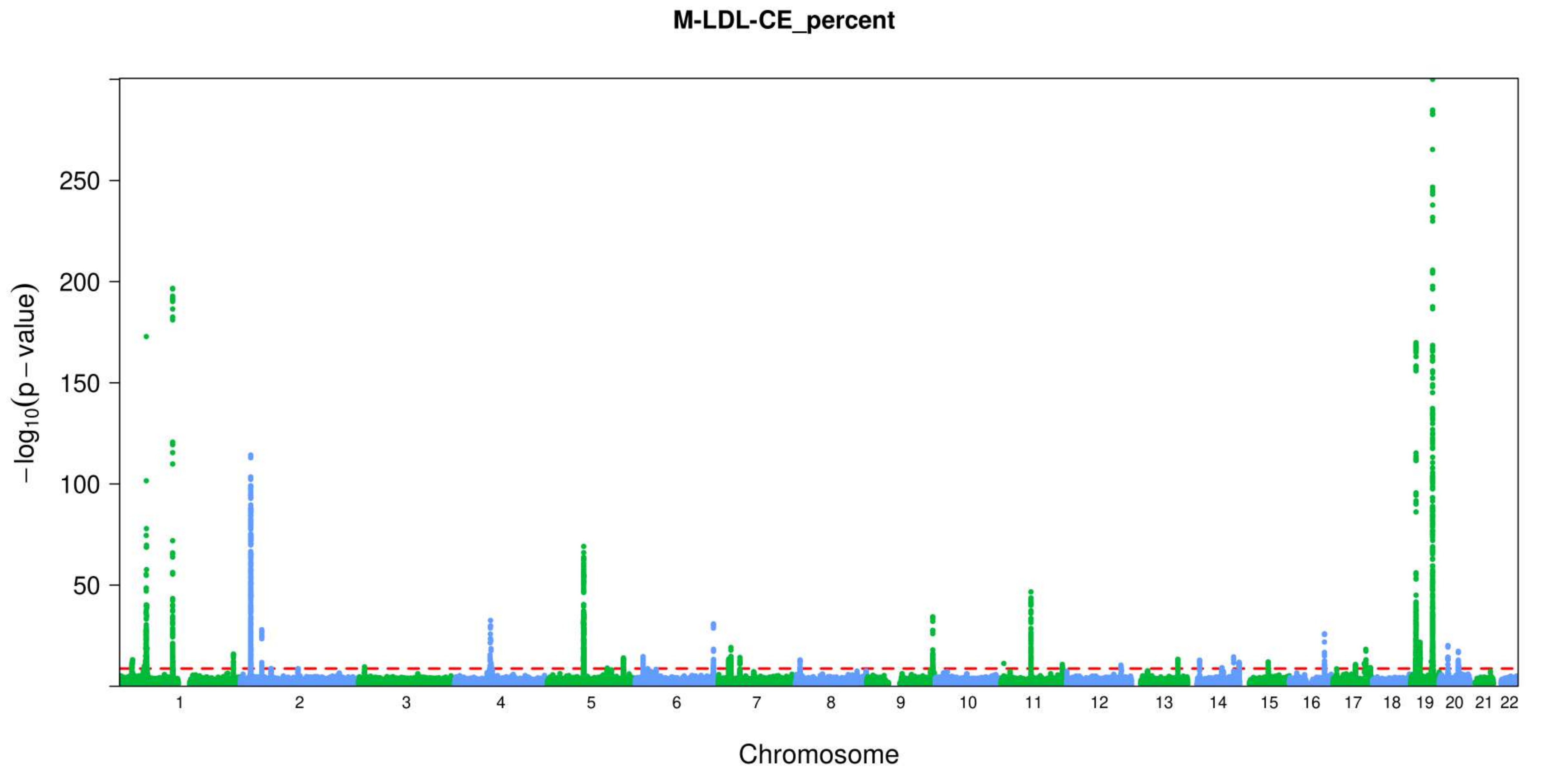

M-LDL-CE_percent
−log10(p − value)
250
200
150
100
50
1
2
3
4
5
6
7
8
9
10
11
12
13
14
15
16
17
18
19
20
21
22
Chromosome

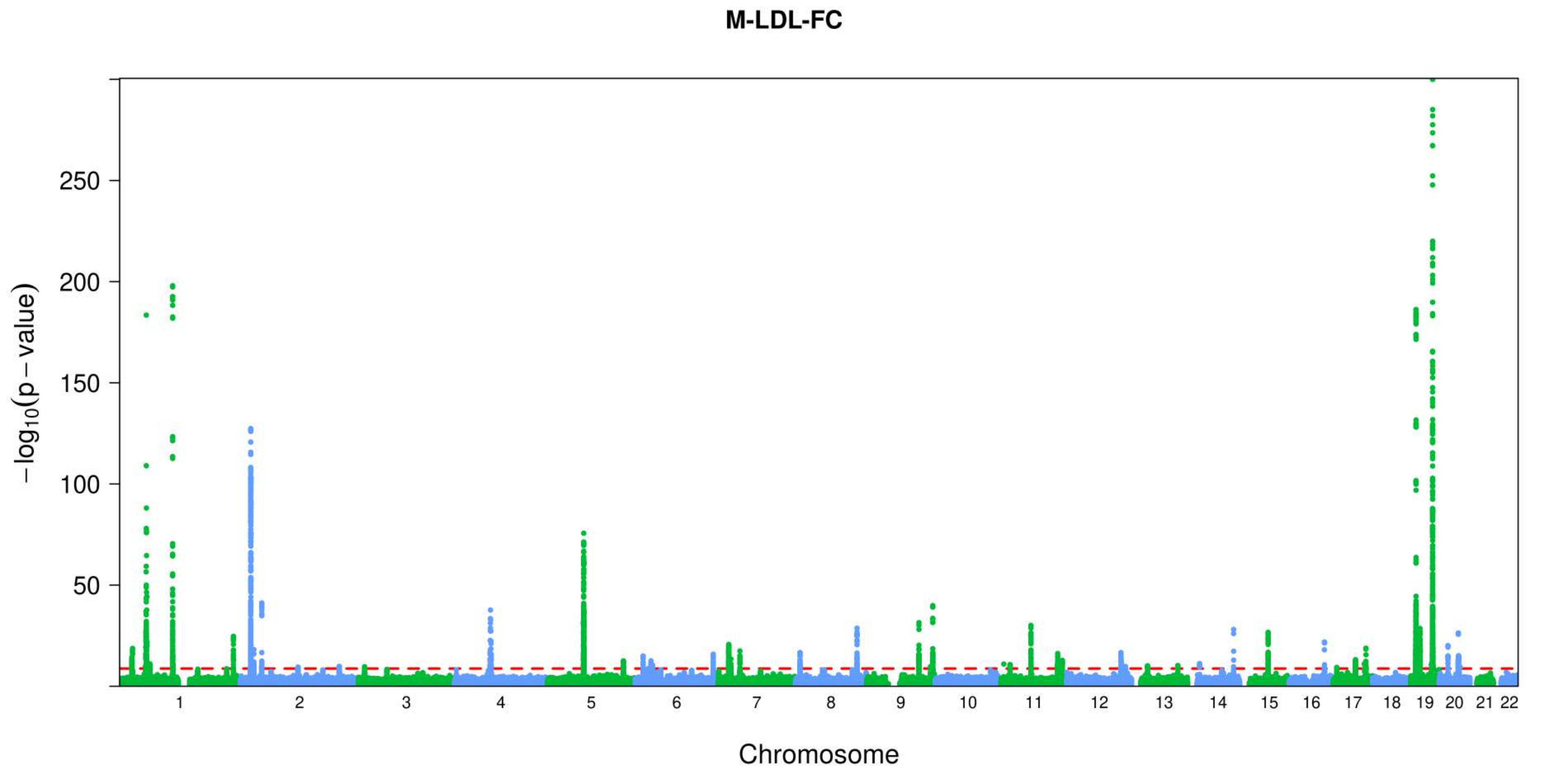
M-LDL-FC
−log10(p − value)
250
200
150
100
50
Chromosome
1
2
3
4
5
6
7
8
9
10
11
12
13
14
15
16
17
18
19
20
21
22

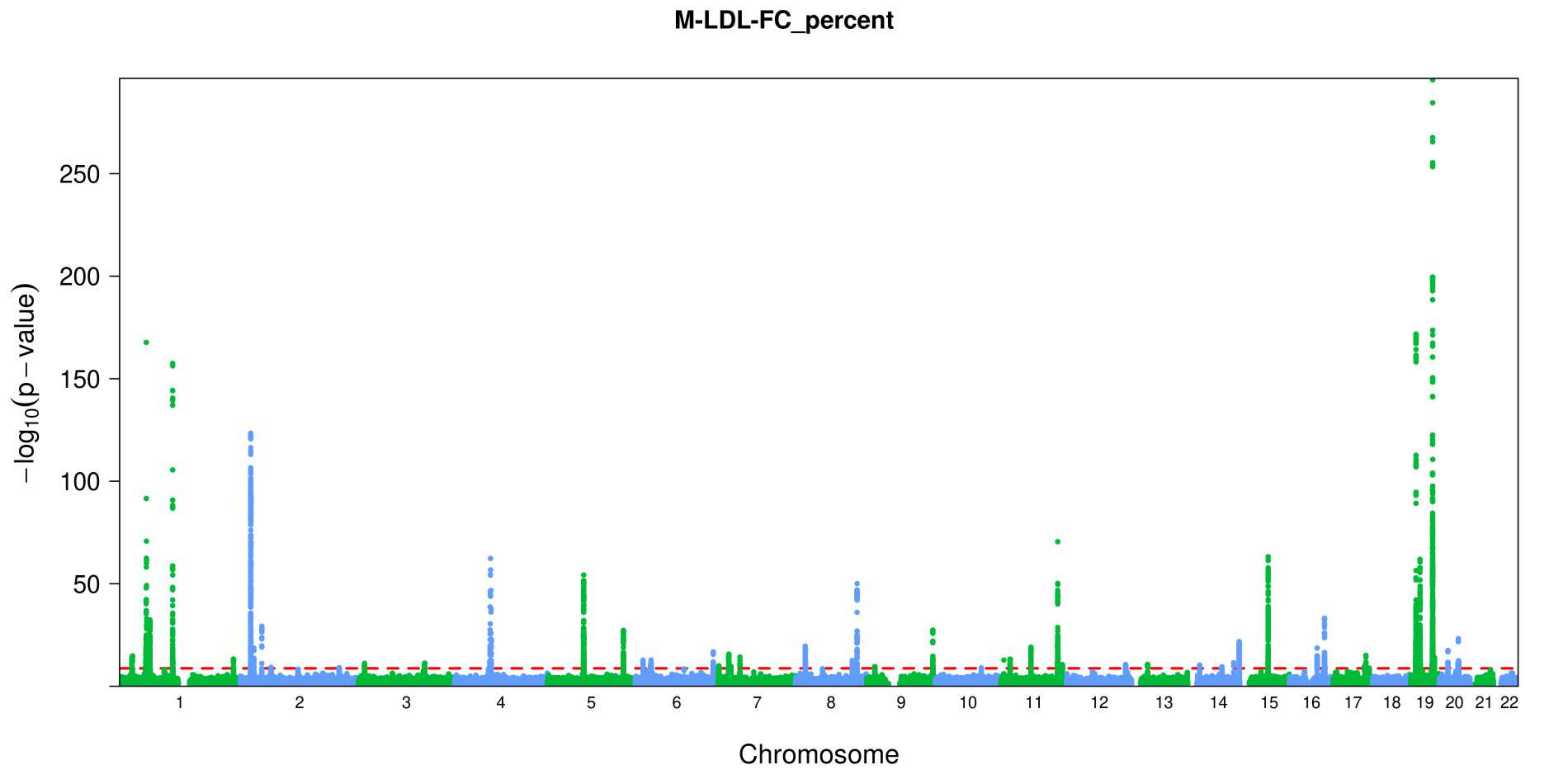

M-LDL-FC_percent
250
200
150
100
50
$-\log_{10}(p-\text{value})$
1
2
3
4
5
6
7
8
9
10
11
12
13
14
15
16
17
18
19
20
21
22
Chromosome

M-LDL-L

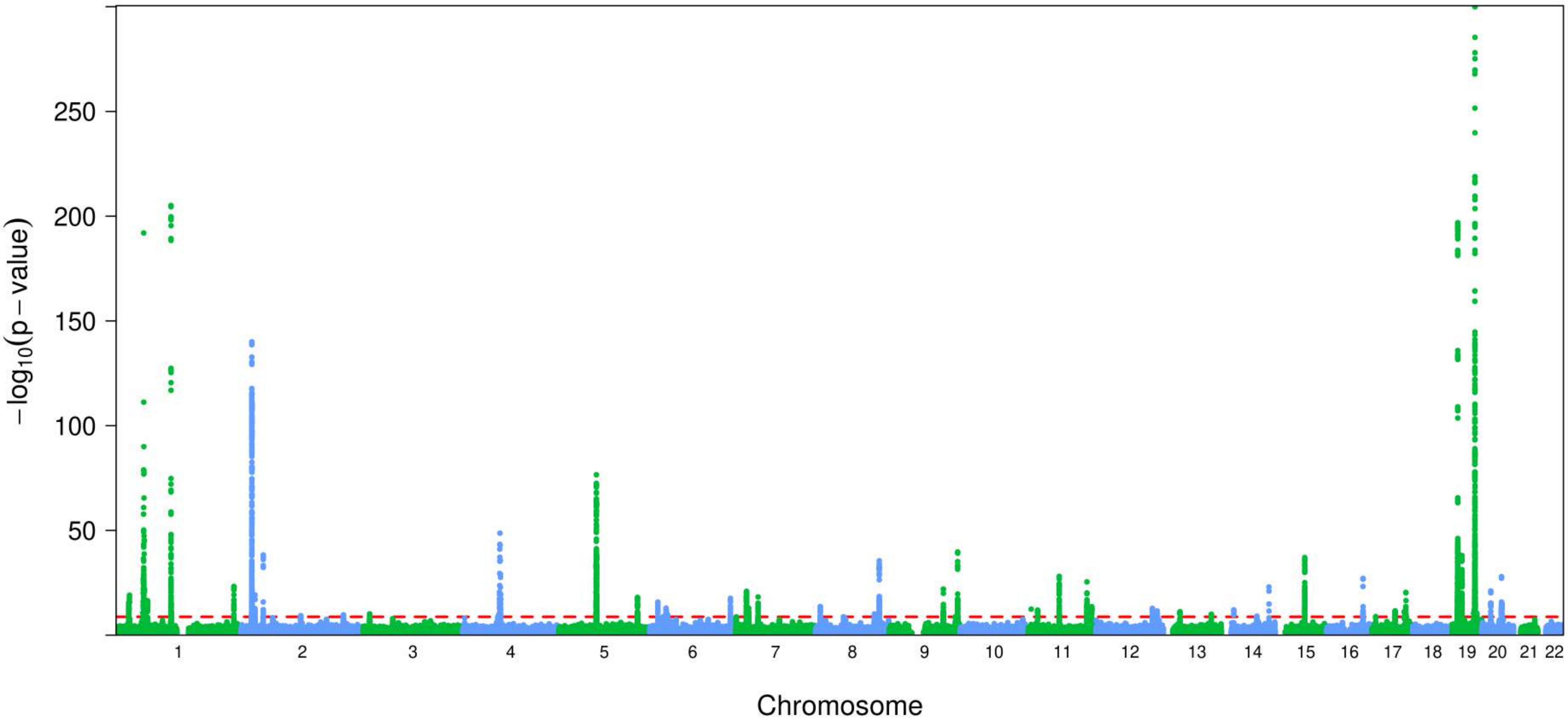

250
200
150
100
50
−log10(p−value)
1
2
3
4
5
6
7
8
9
10
11
12
13
14
15
16
17
18
19
20
21
22
Chromosome

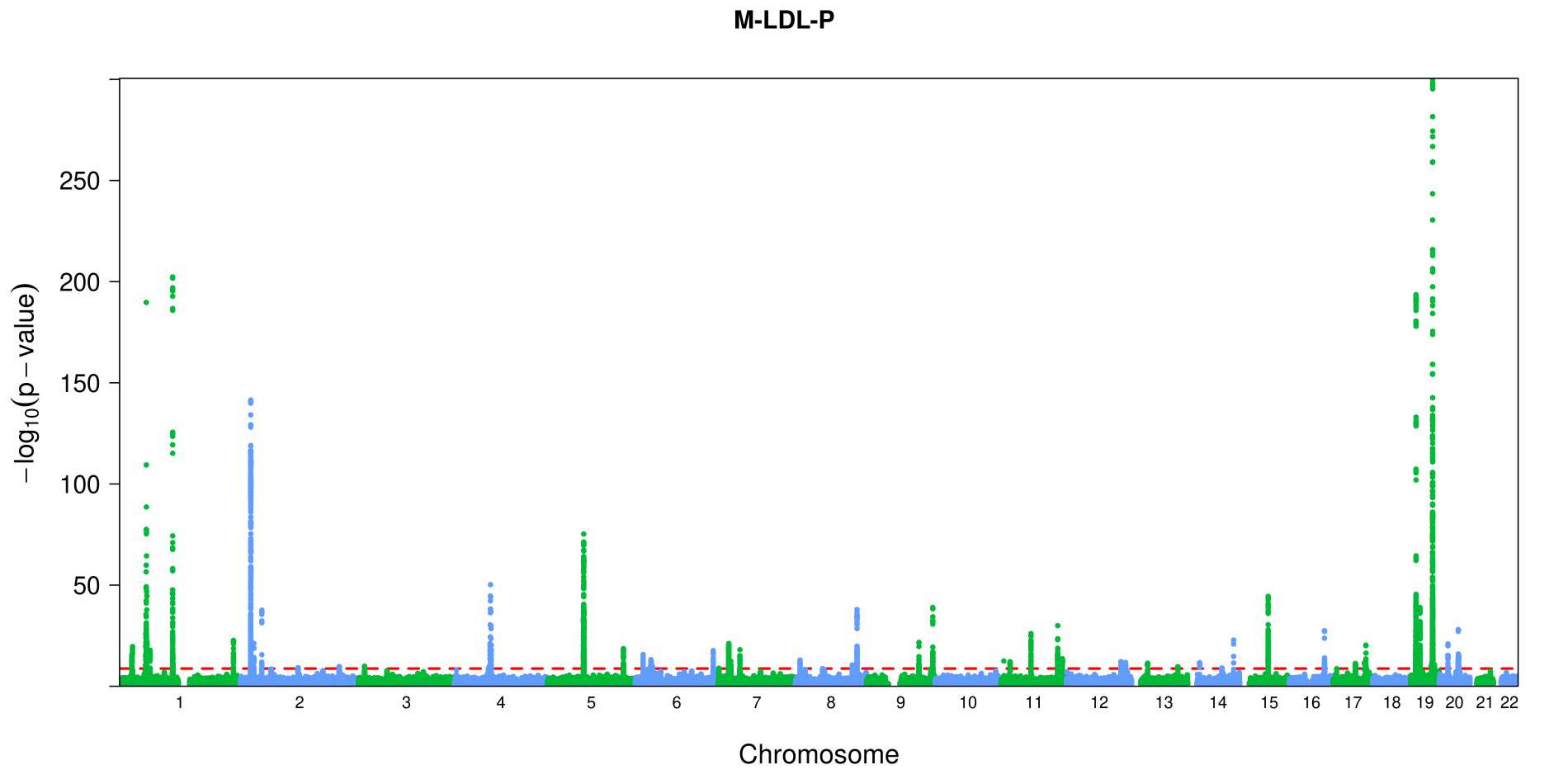
M-LDL-P
$-\log_{10}(p-\text{value})$
250
200
150
100
50
Chromosome
1
2
3
4
5
6
7
8
9
10
11
12
13
14
15
16
17
18
19
20
21
22

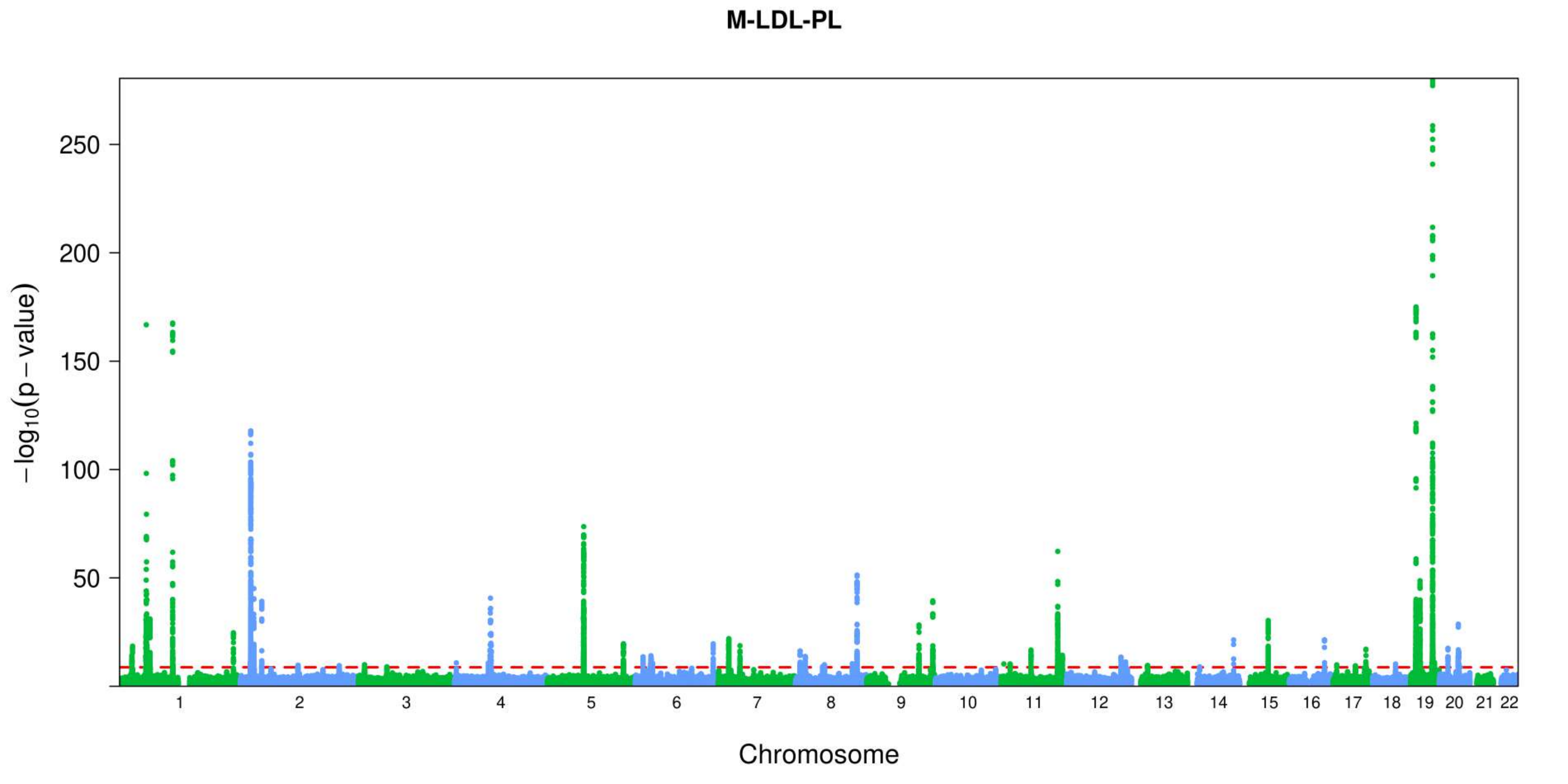
M-LDL-PL
250
200
150
100
50
$-\log_{10}(p-\text{value})$
1
2
3
4
5
6
7
8
9
10
11
12
13
14
15
16
17
18
19
20
21
22
Chromosome

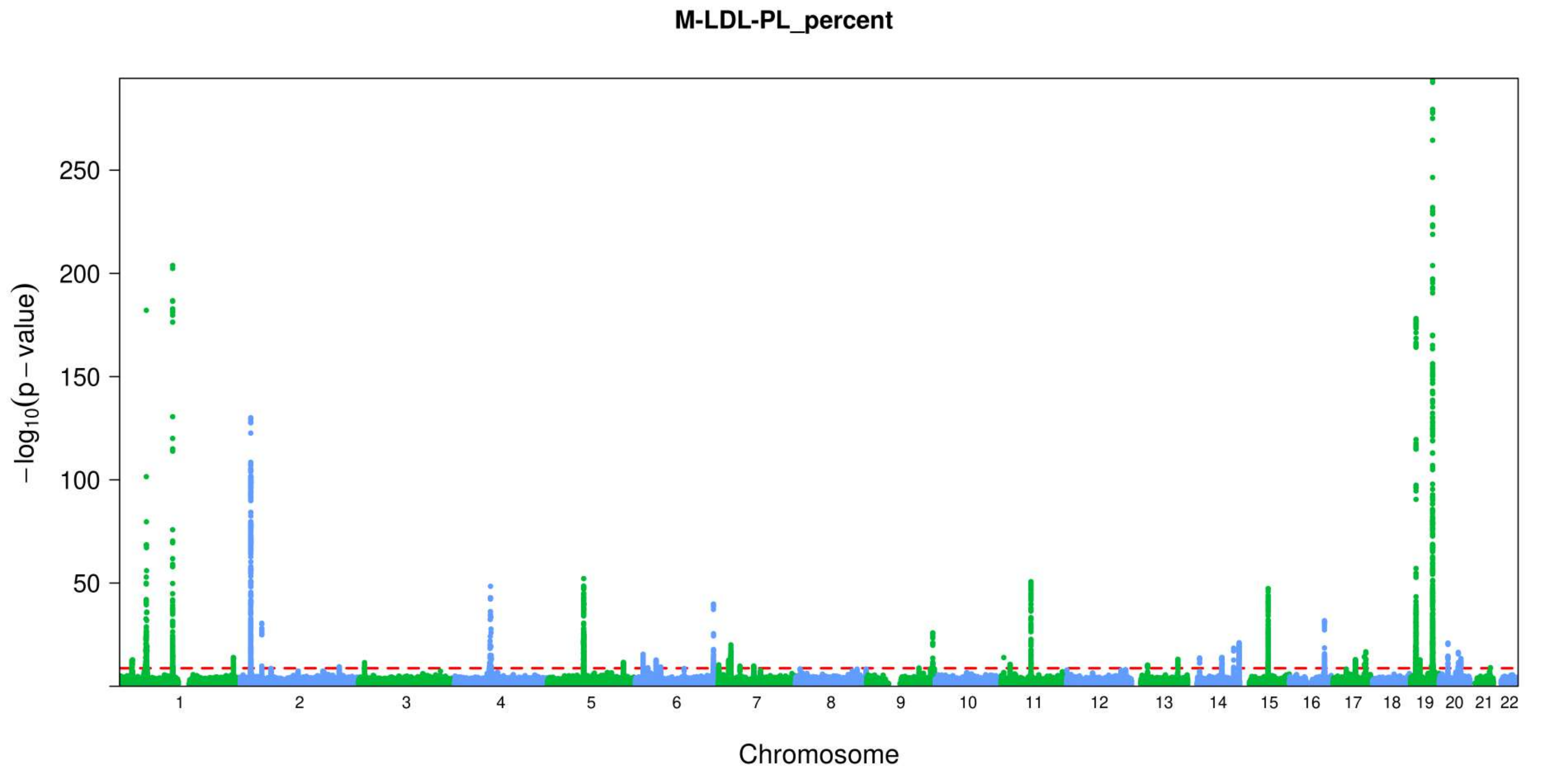
M-LDL-PL_percent
250
200
150
100
50
−log10(p − value)
1
2
3
4
5
6
7
8
9
10
11
12
13
14
15
16
17
18
19
20
21
22
Chromosome

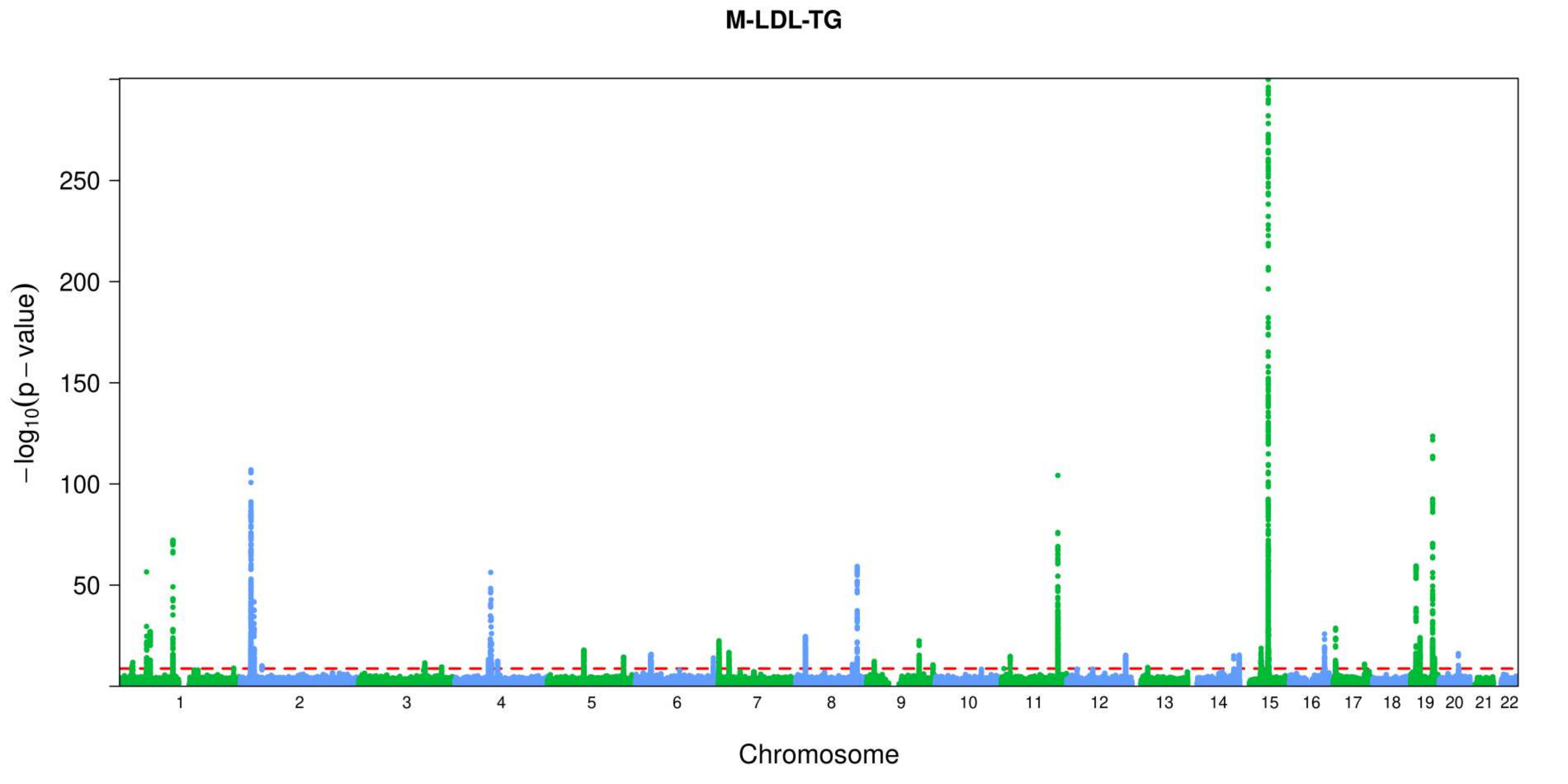
M-LDL-TG
250
200
150
100
50
−log10(p − value)
1
2
3
4
5
6
7
8
9
10
11
12
13
14
15
16
17
18
19
20
21
22
Chromosome

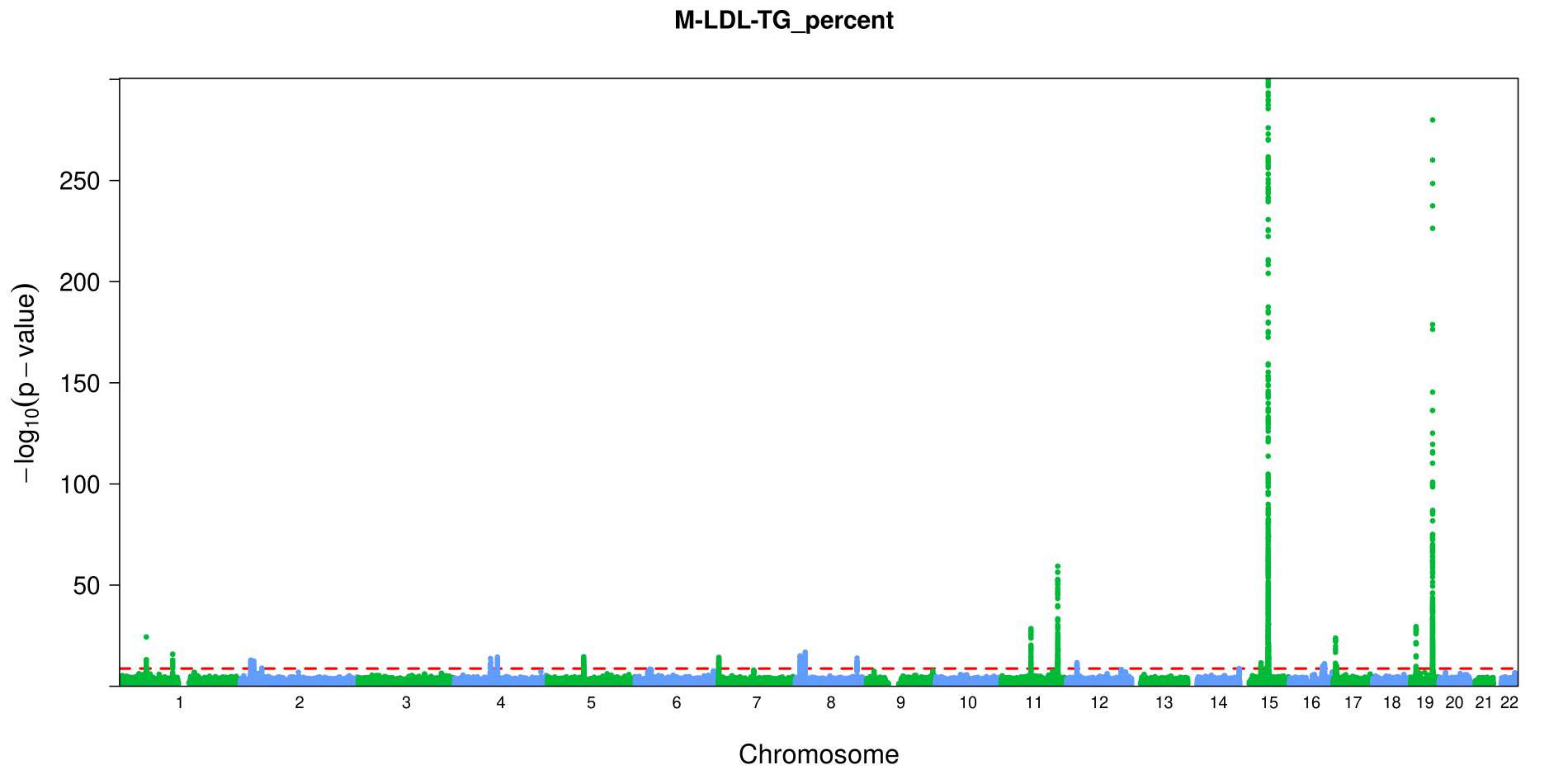

M-LDL-TG_percent
−log10(p − value)
250
200
150
100
50
1
2
3
4
5
6
7
8
9
10
11
12
13
14
15
16
17
18
19
20
21
22
Chromosome

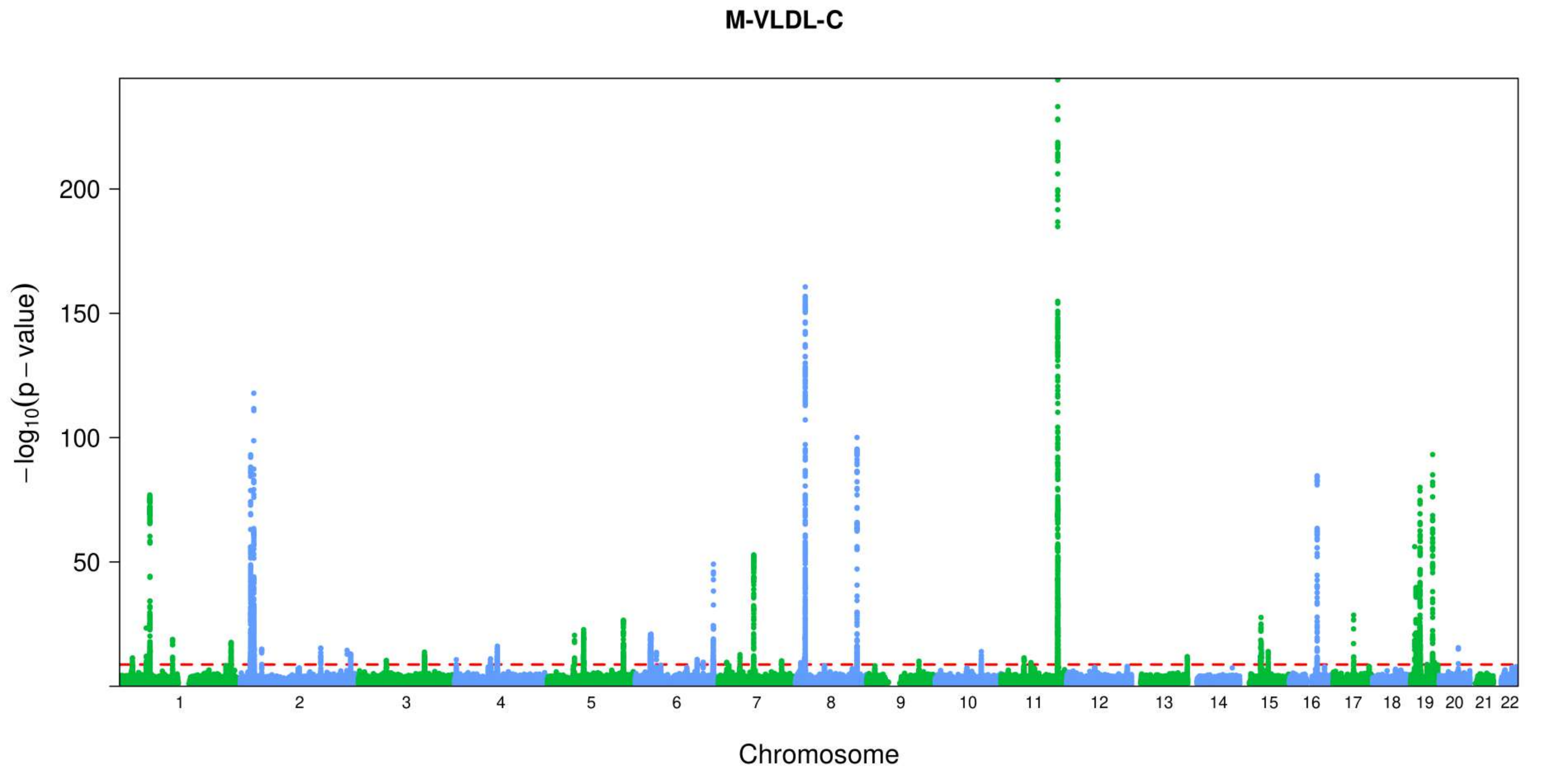
M-VLDL-C
200
150
100
50
$-\log_{10}(p-value)$
1
2
3
4
5
6
7
8
9
10
11
12
13
14
15
16
17
18
19
20
21
22
Chromosome

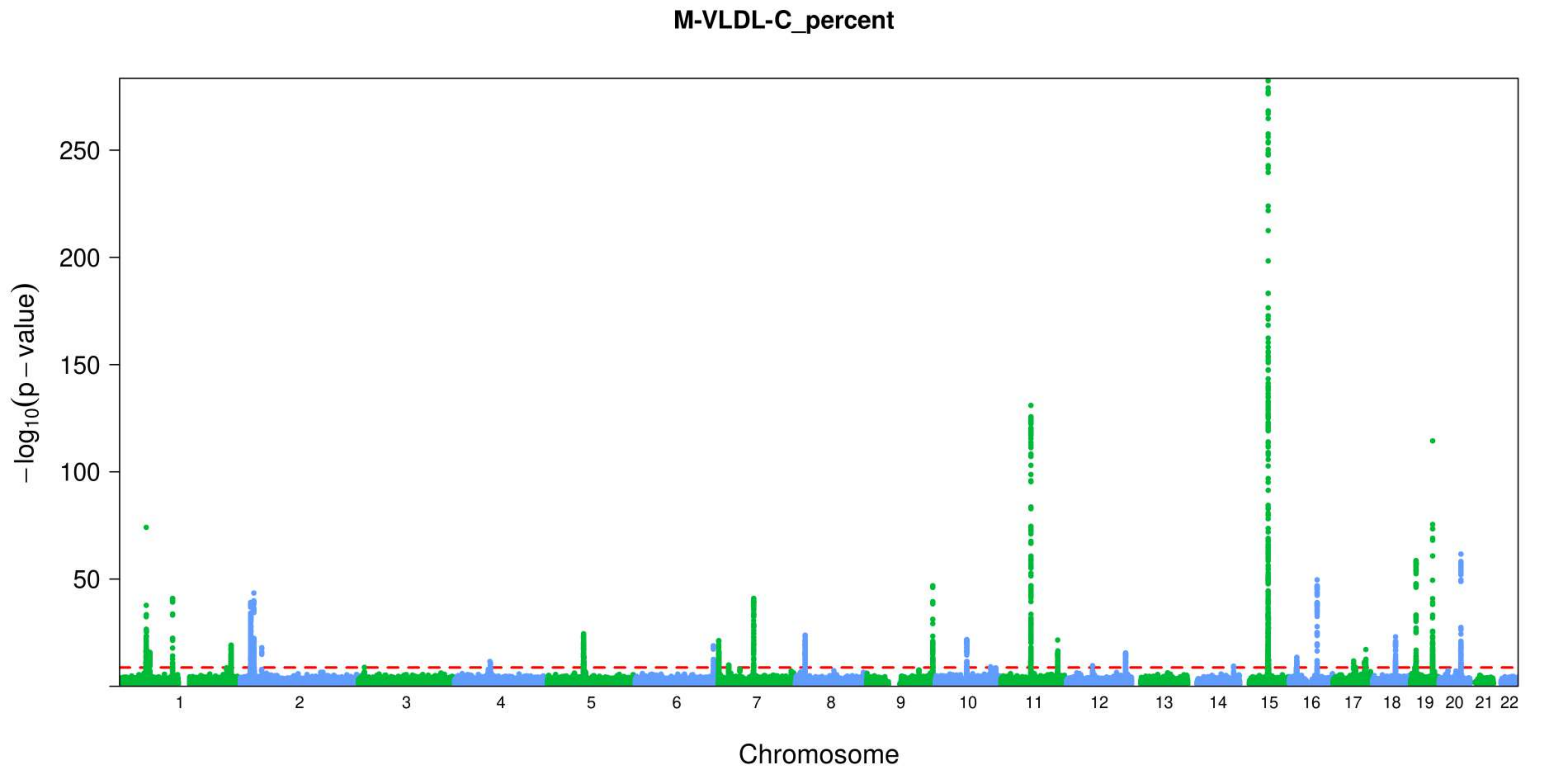
M-VLDL-C_percent
250
200
150
100
50
−log10(p − value)
1
2
3
4
5
6
7
8
9
10
11
12
13
14
15
16
17
18
19
20
21
22
Chromosome

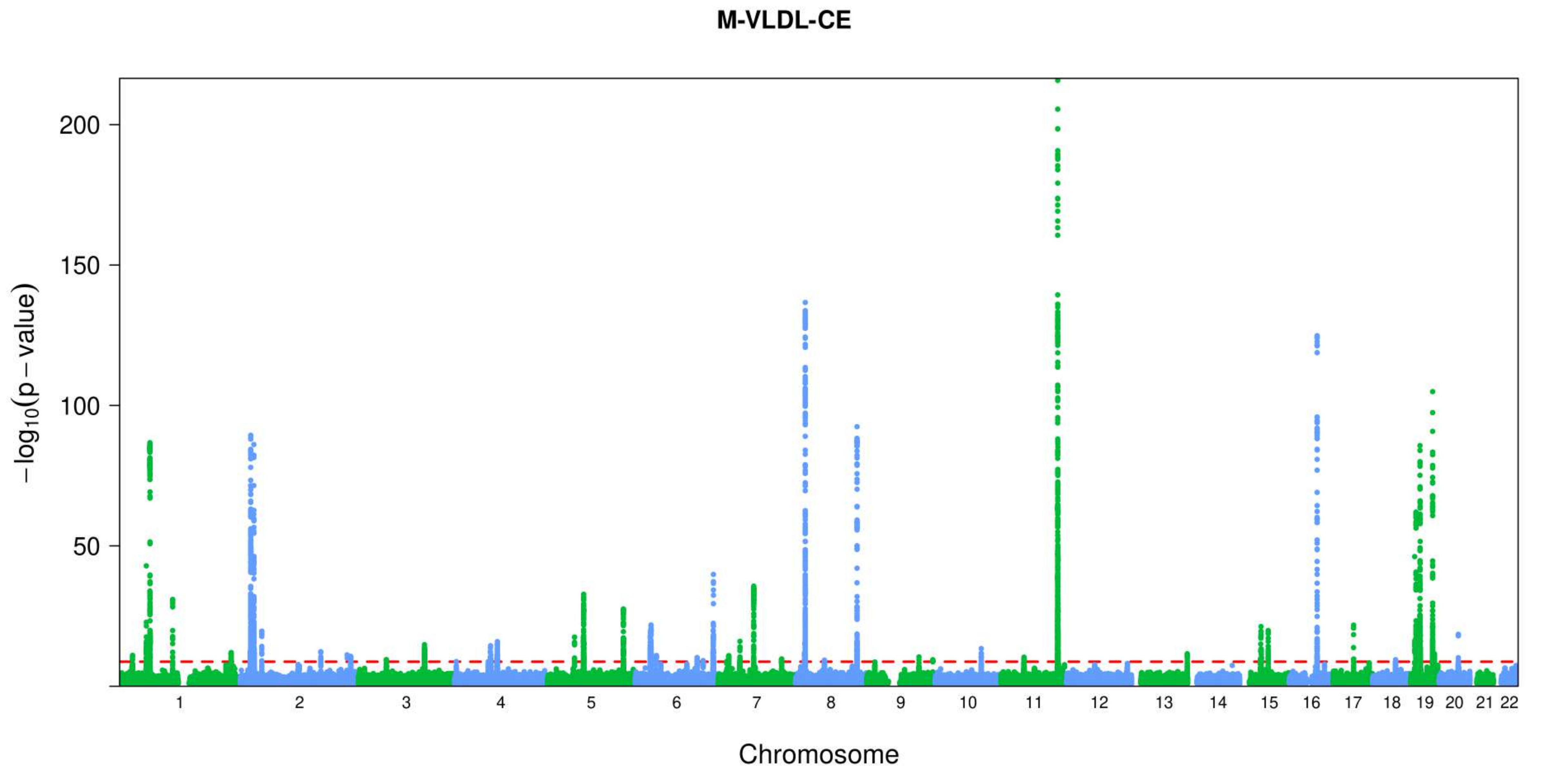
M-VLDL-CE
200
150
100
50
−log10(p − value)
1
2
3
4
5
6
7
8
9
10
11
12
13
14
15
16
17
18
19
20
21
22
Chromosome

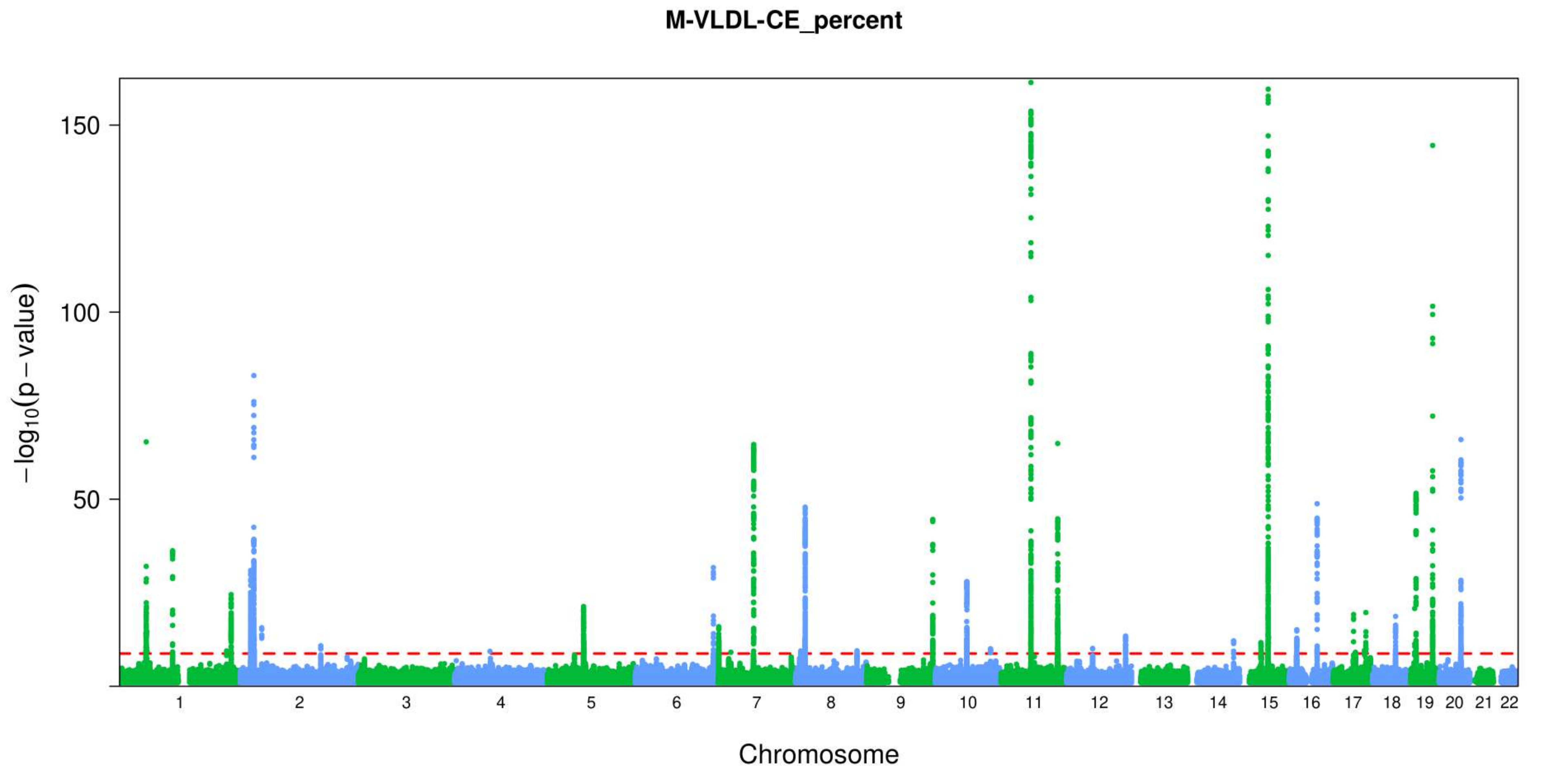
M-VLDL-CE_percent
150
100
50
−log10(p − value)
1
2
3
4
5
6
7
8
9
10
11
12
13
14
15
16
17
18
19
20
21
22
Chromosome

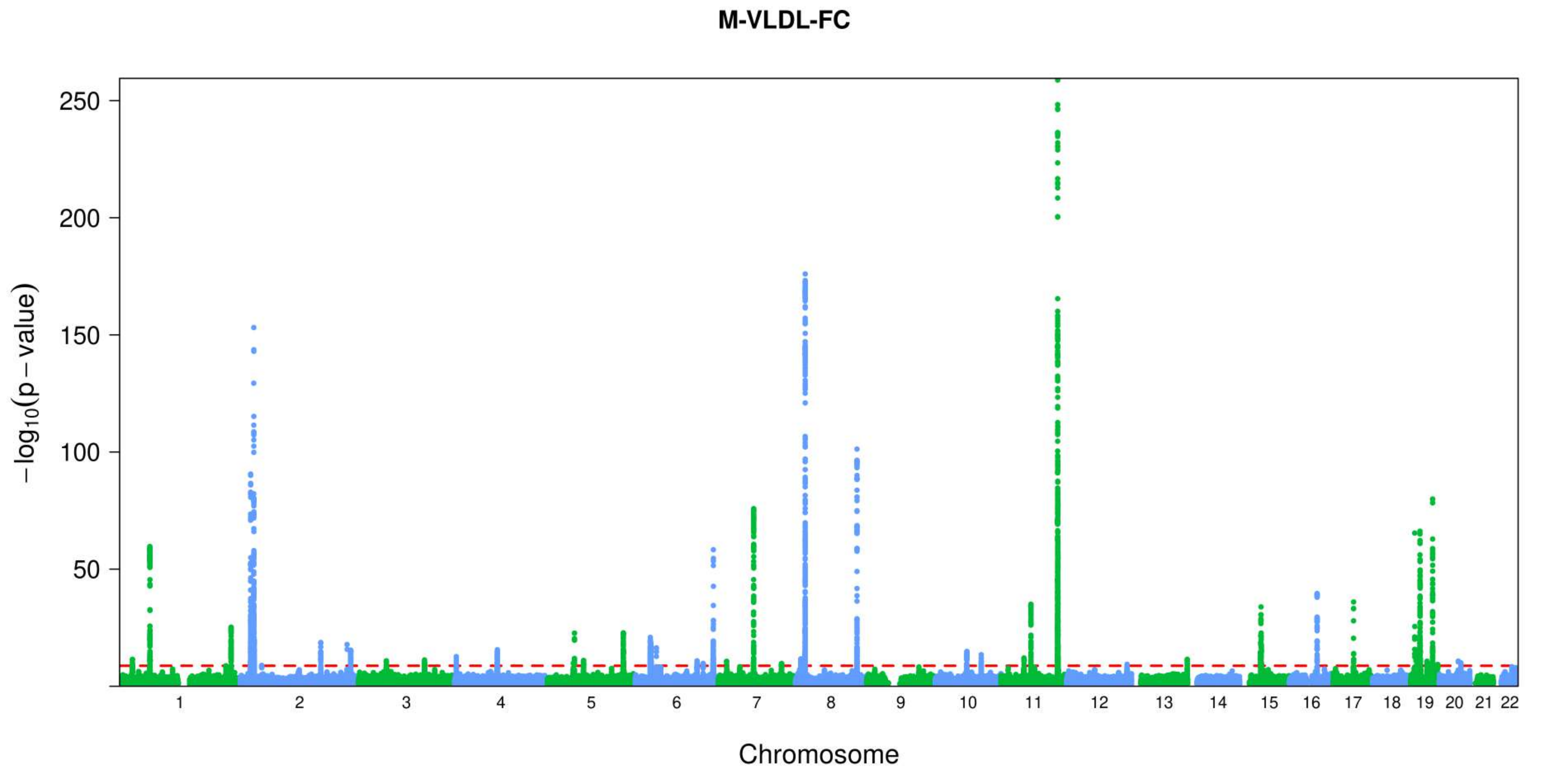
M-VLDL-FC
250
200
150
100
50
$-\log_{10}(p-\text{value})$
1
2
3
4
5
6
7
8
9
10
11
12
13
14
15
16
17
18
19
20
21
22
Chromosome

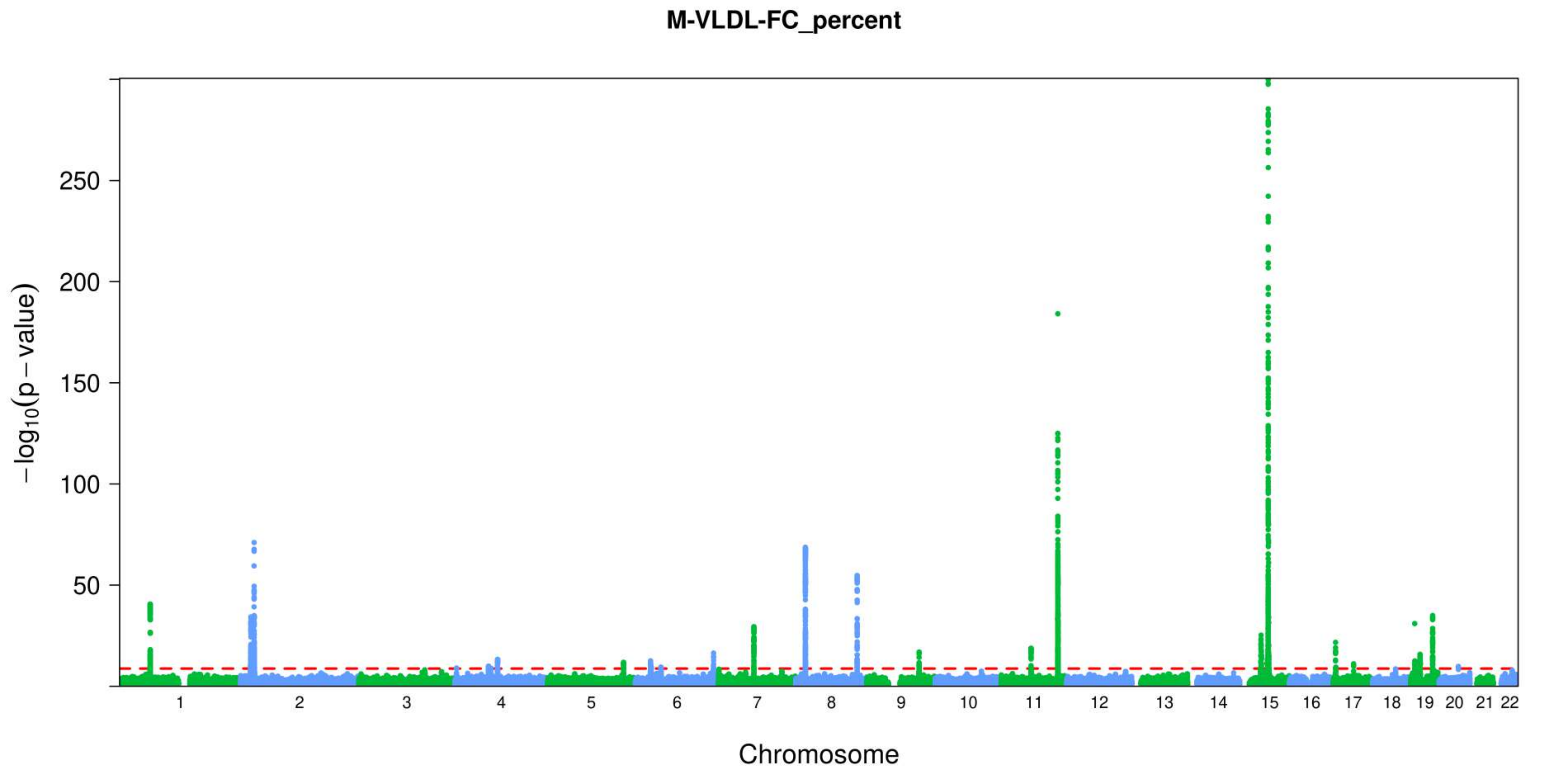

M-VLDL-FC_percent
−log10(p − value)
250
200
150
100
50
Chromosome
1
2
3
4
5
6
7
8
9
10
11
12
13
14
15
16
17
18
19
20
21
22

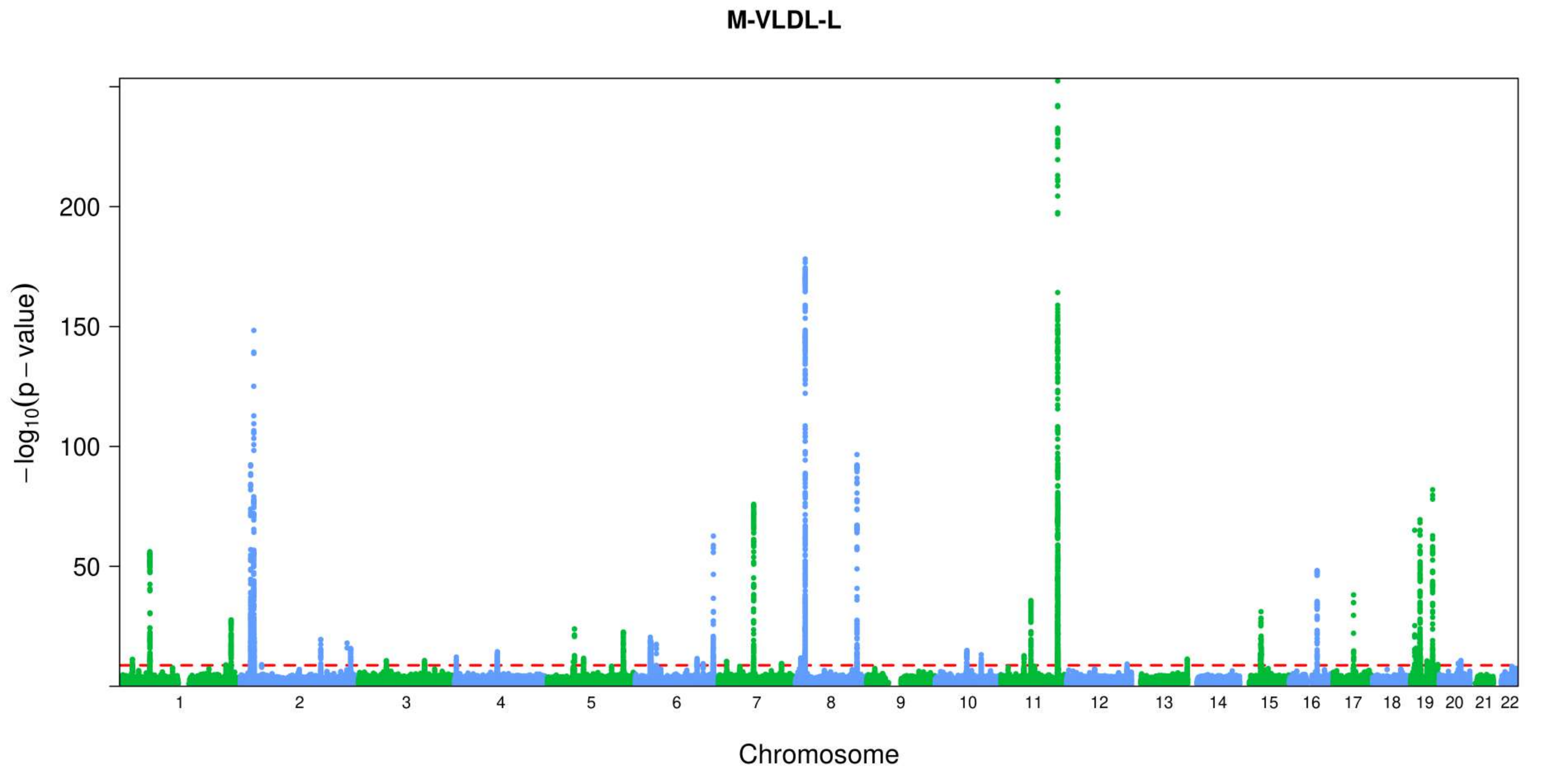
M-VLDL-L
200
150
100
50
$-\log_{10}(p-\text{value})$
1
2
3
4
5
6
7
8
9
10
11
12
13
14
15
16
17
18
19
20
21
22
Chromosome

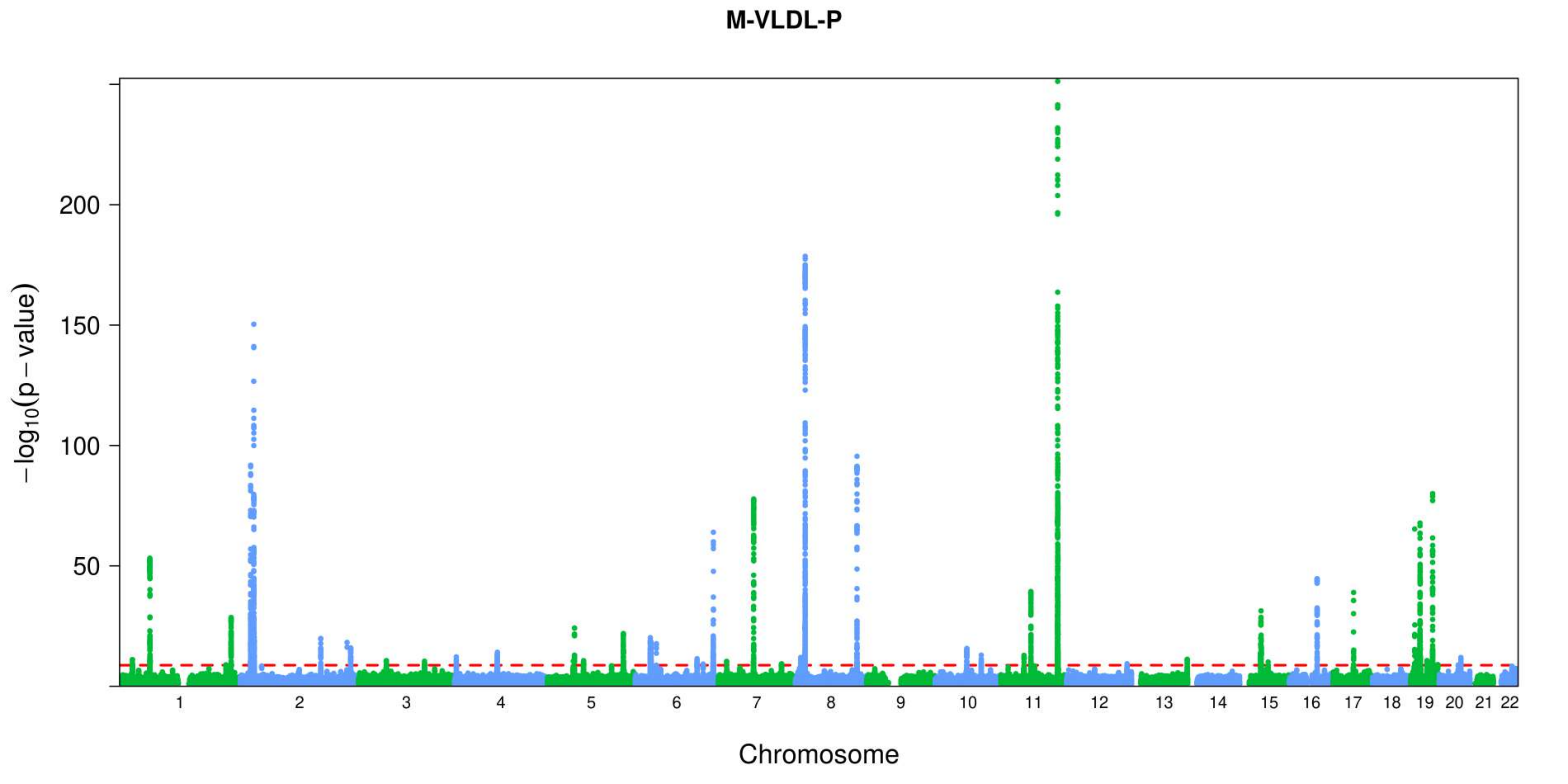
M-VLDL-P
200
150
100
50
$-\log_{10}(p-\text{value})$
1
2
3
4
5
6
7
8
9
10
11
12
13
14
15
16
17
18
19
20
21
22
Chromosome

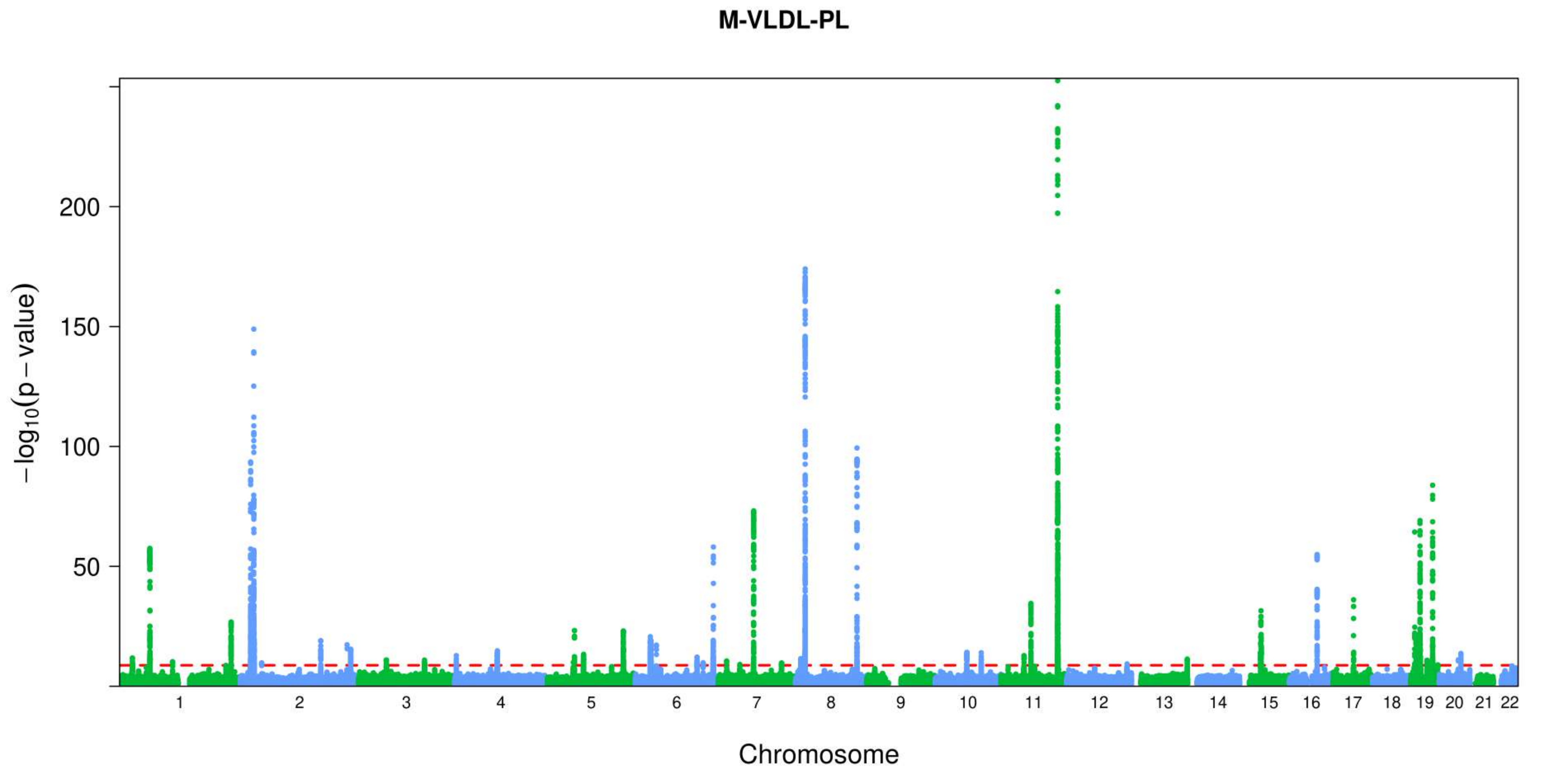
M-VLDL-PL
200
150
100
50
$-\log_{10}(p-\text{value})$
1
2
3
4
5
6
7
8
9
10
11
12
13
14
15
16
17
18
19
20
21
22
Chromosome

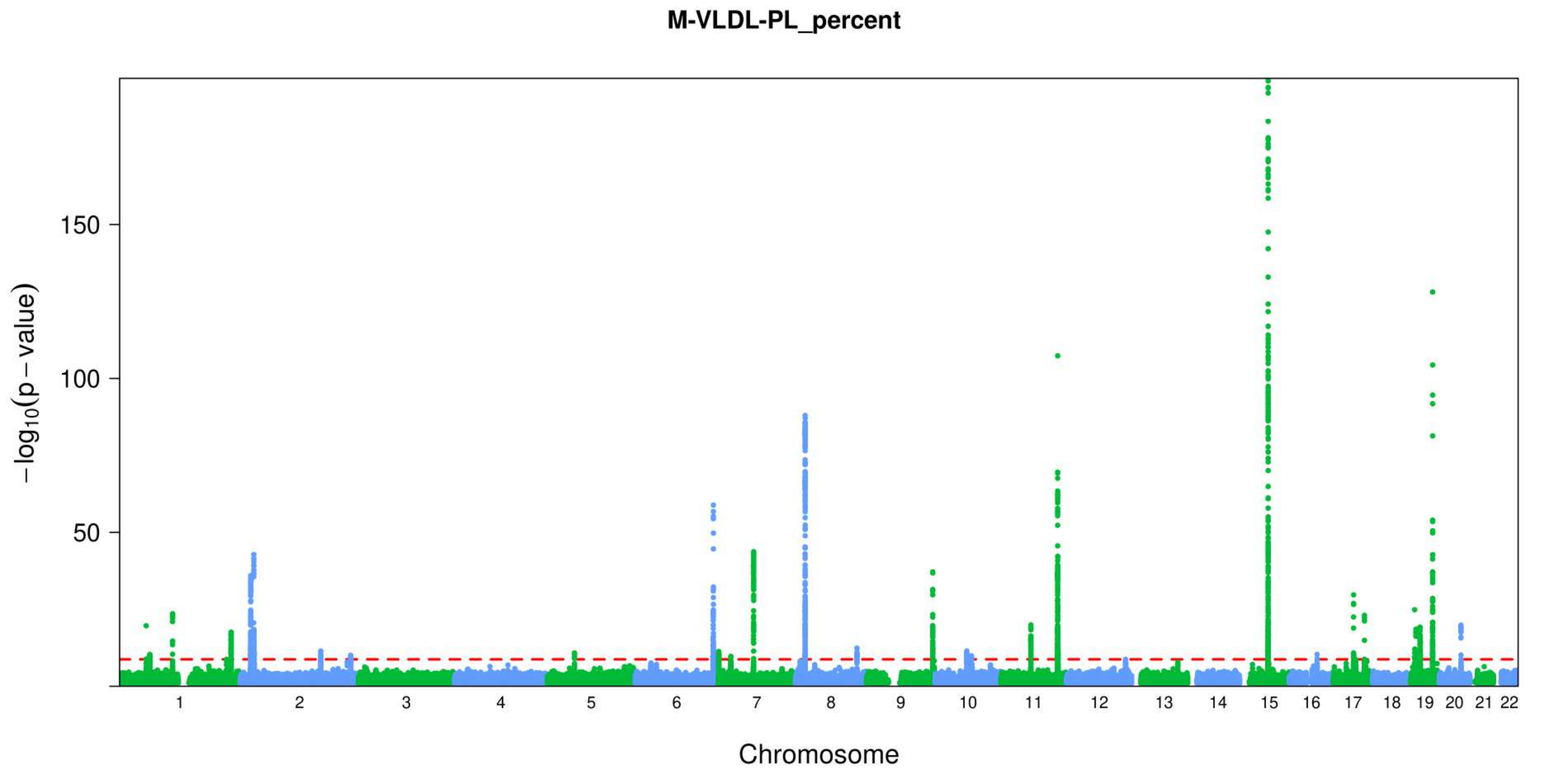
M-VLDL-PL_percent
−log10(p − value)
150
100
50
Chromosome
1
2
3
4
5
6
7
8
9
10
11
12
13
14
15
16
17
18
19
20
21
22

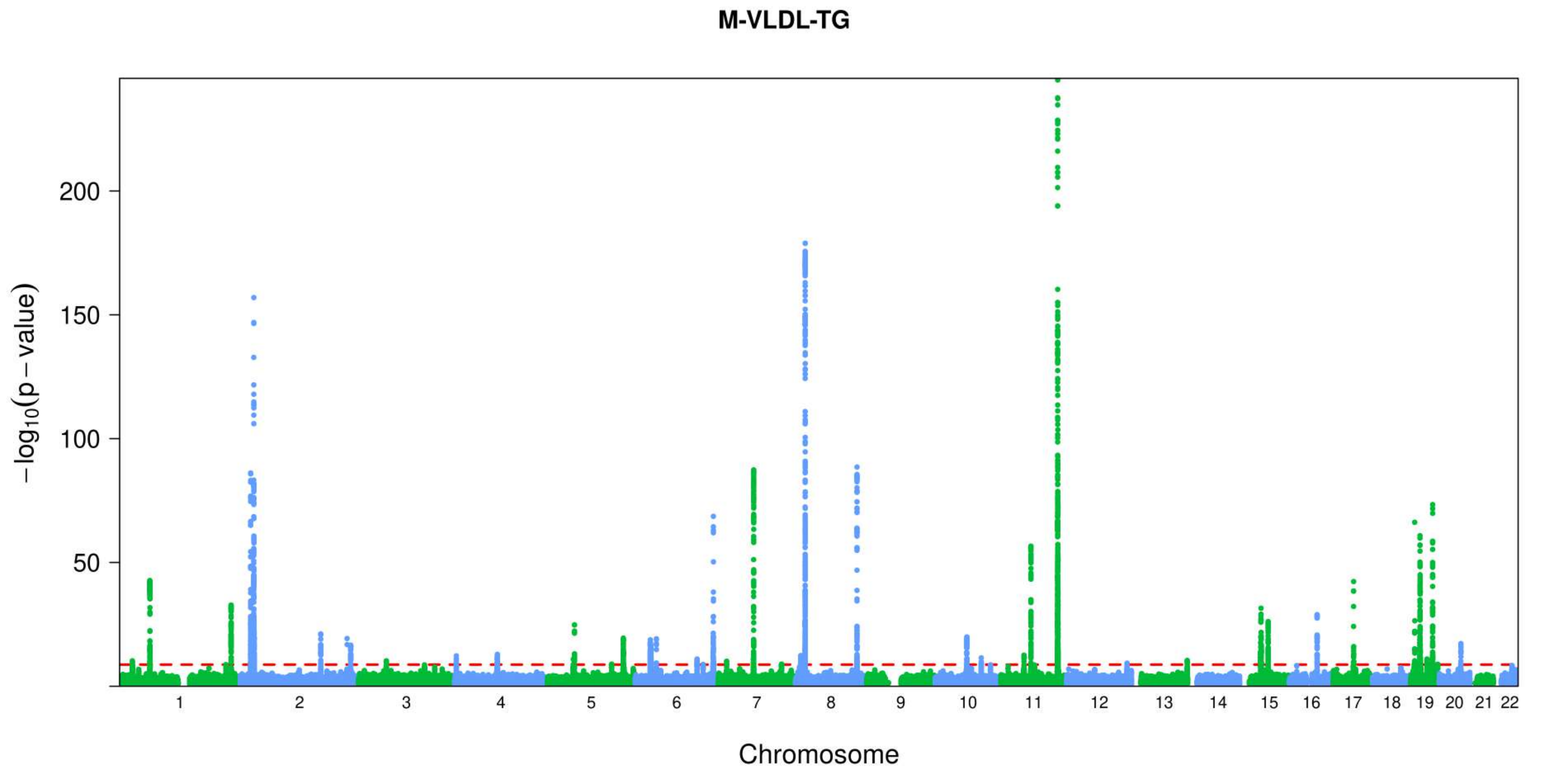
M-VLDL-TG
200
150
100
50
$-\log_{10}(p-\text{value})$
1 2 3 4 5 6 7 8 9 10 11 12 13 14 15 16 17 18 19 20 21 22
Chromosome

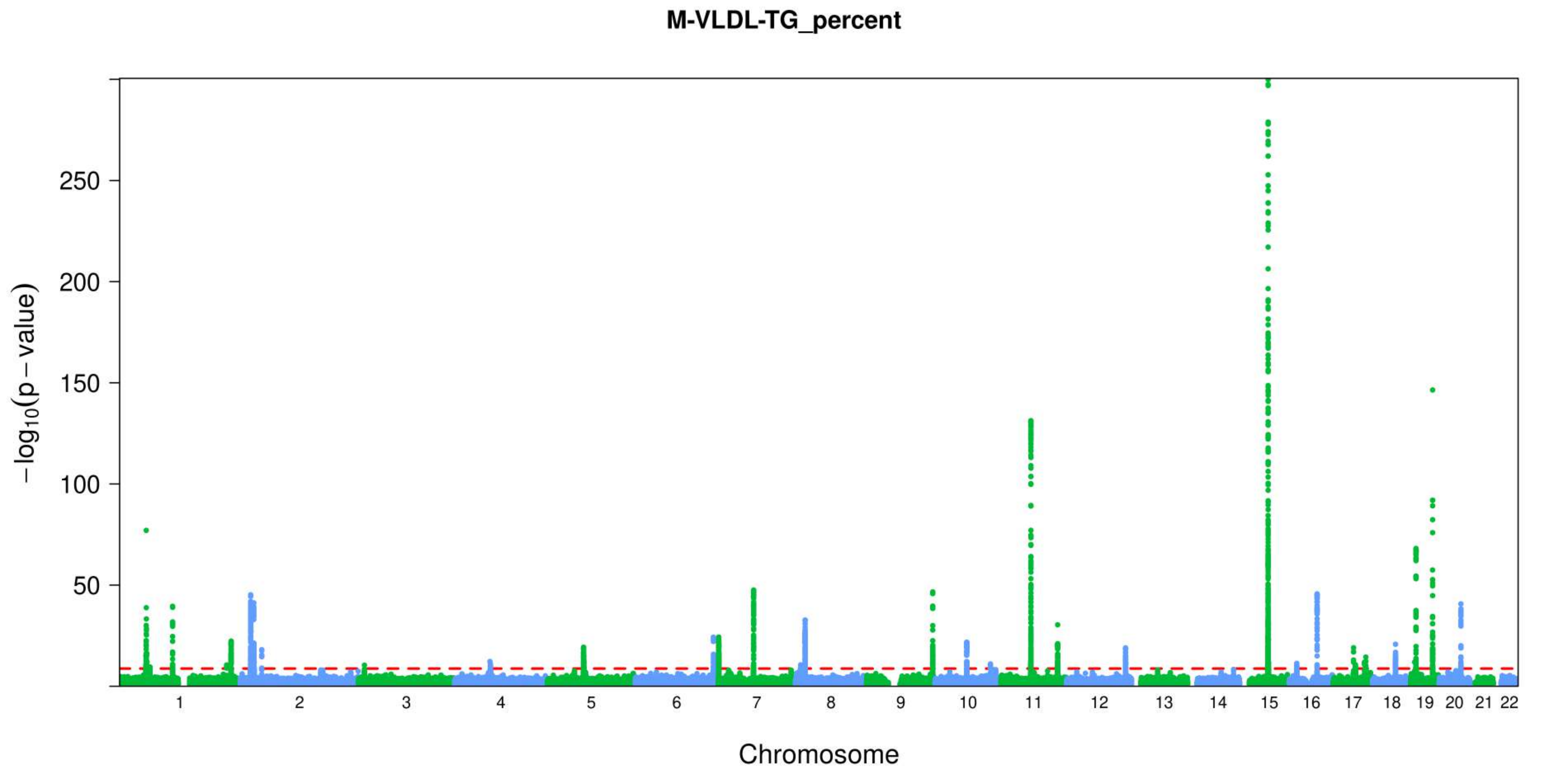
M-VLDL-TG_percent
−log10(p − value)
250
200
150
100
50
Chromosome
1
2
3
4
5
6
7
8
9
10
11
12
13
14
15
16
17
18
19
20
21
22

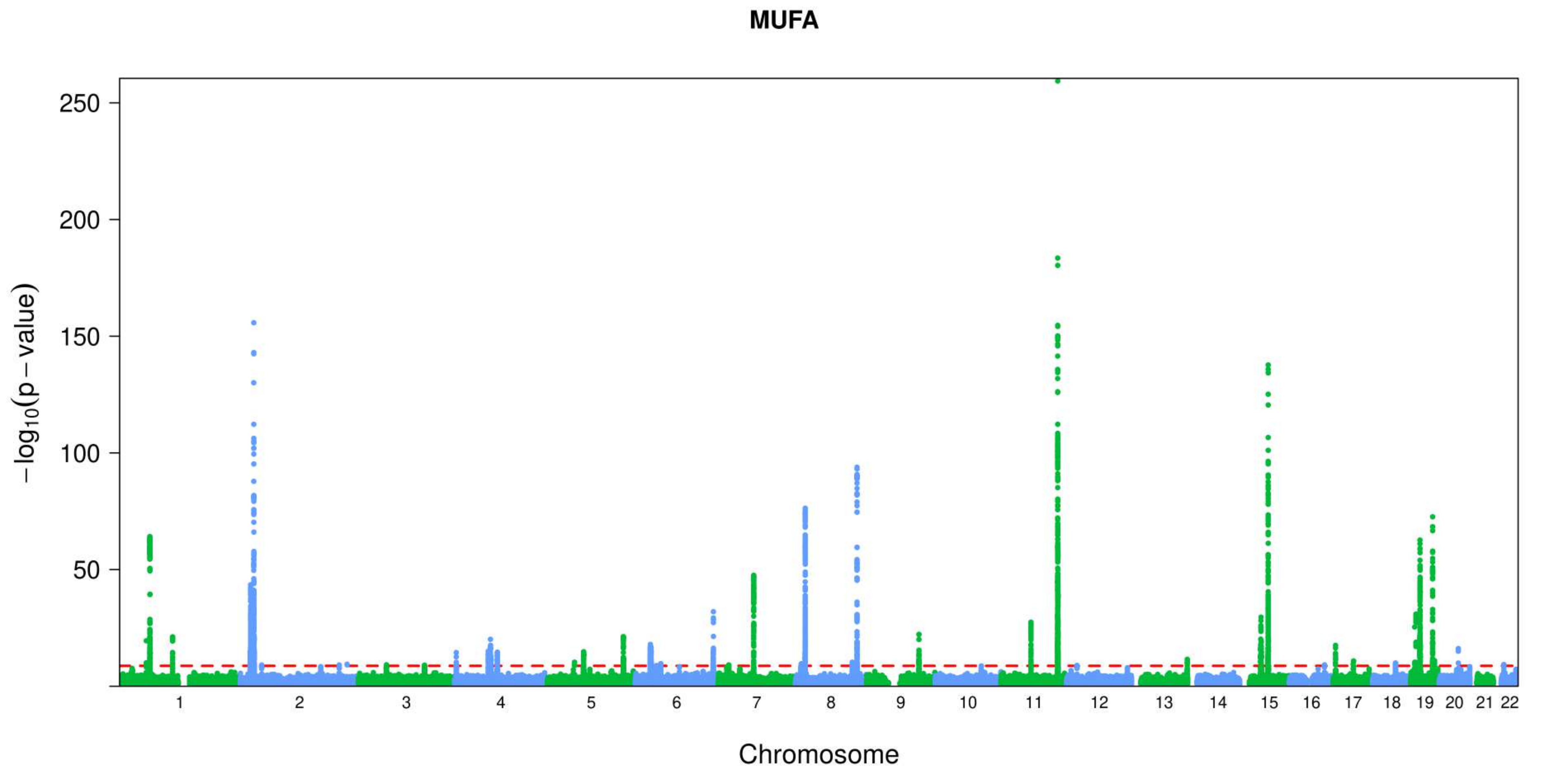

MUFA
250
200
150
100
50
−log10(p − value)
1
2
3
4
5
6
7
8
9
10
11
12
13
14
15
16
17
18
19
20
21
22
Chromosome

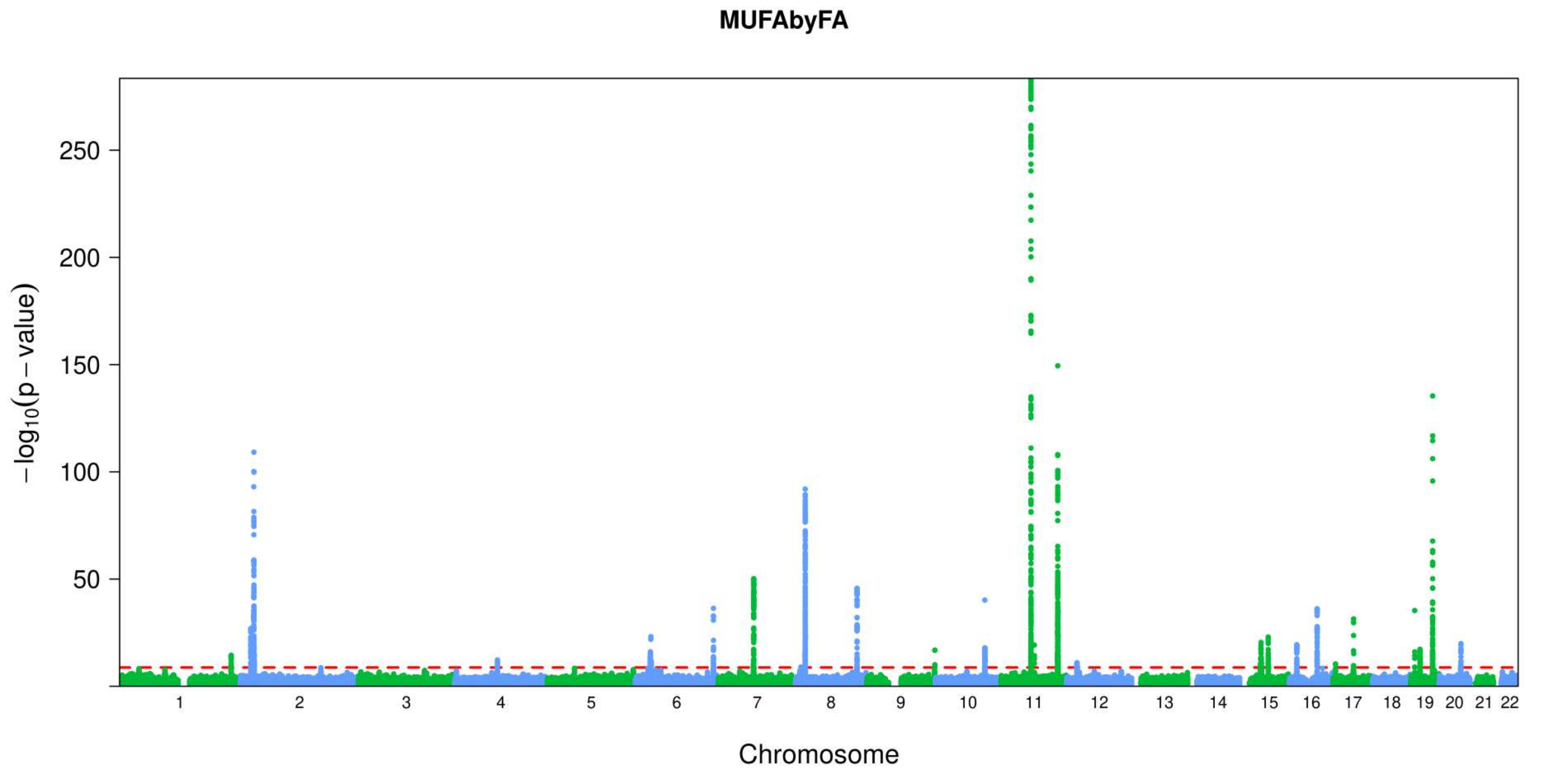

MUFAbyFA
250
200
150
100
50
$-\log_{10}(p-value)$
1
2
3
4
5
6
7
8
9
10
11
12
13
14
15
16
17
18
19
20
21
22
Chromosome

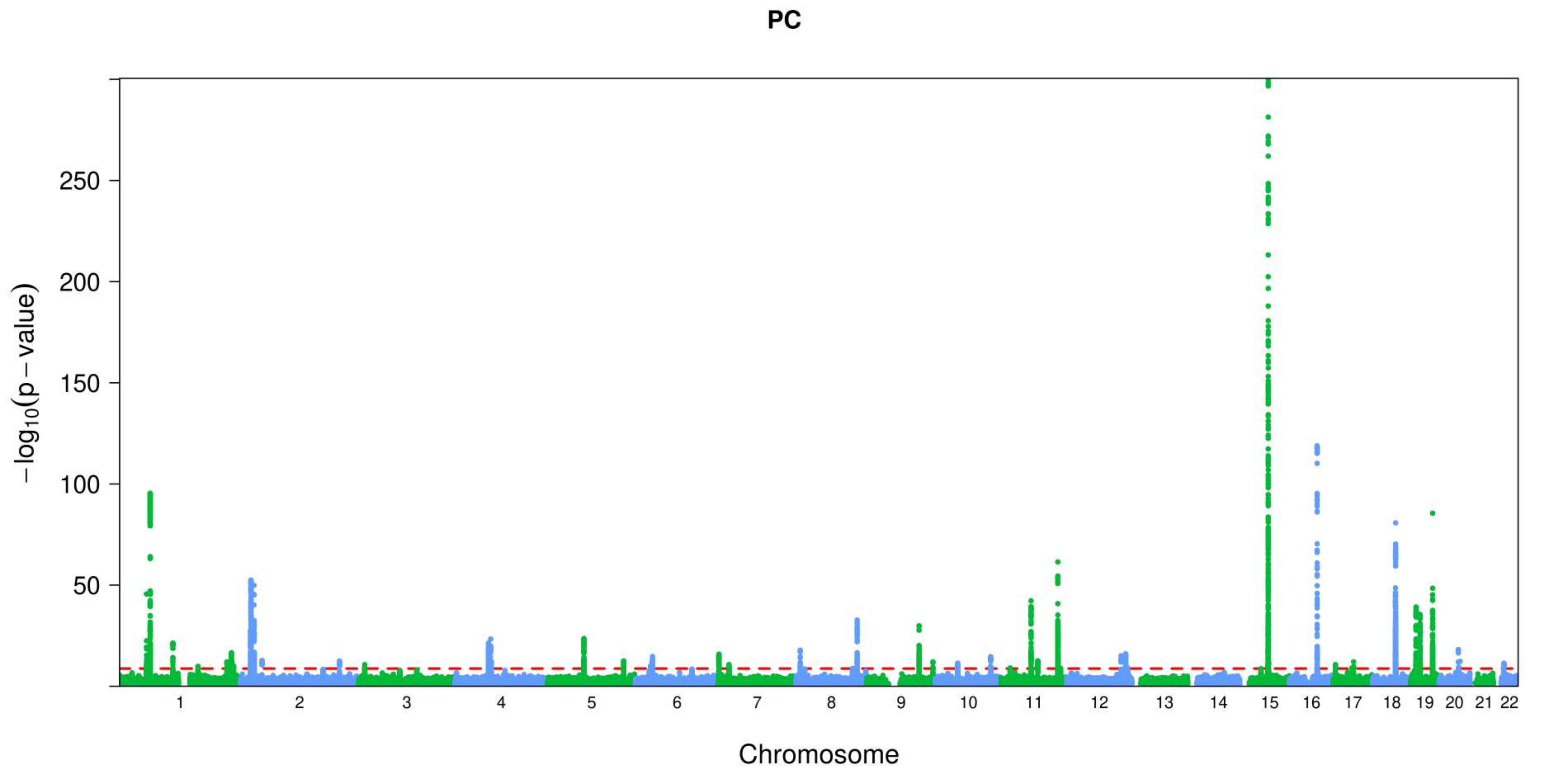
PC
-log10(p - value)
250
200
150
100
50
1
2
3
4
5
6
7
8
9
10
11
12
13
14
15
16
17
18
19
20
21
22
Chromosome

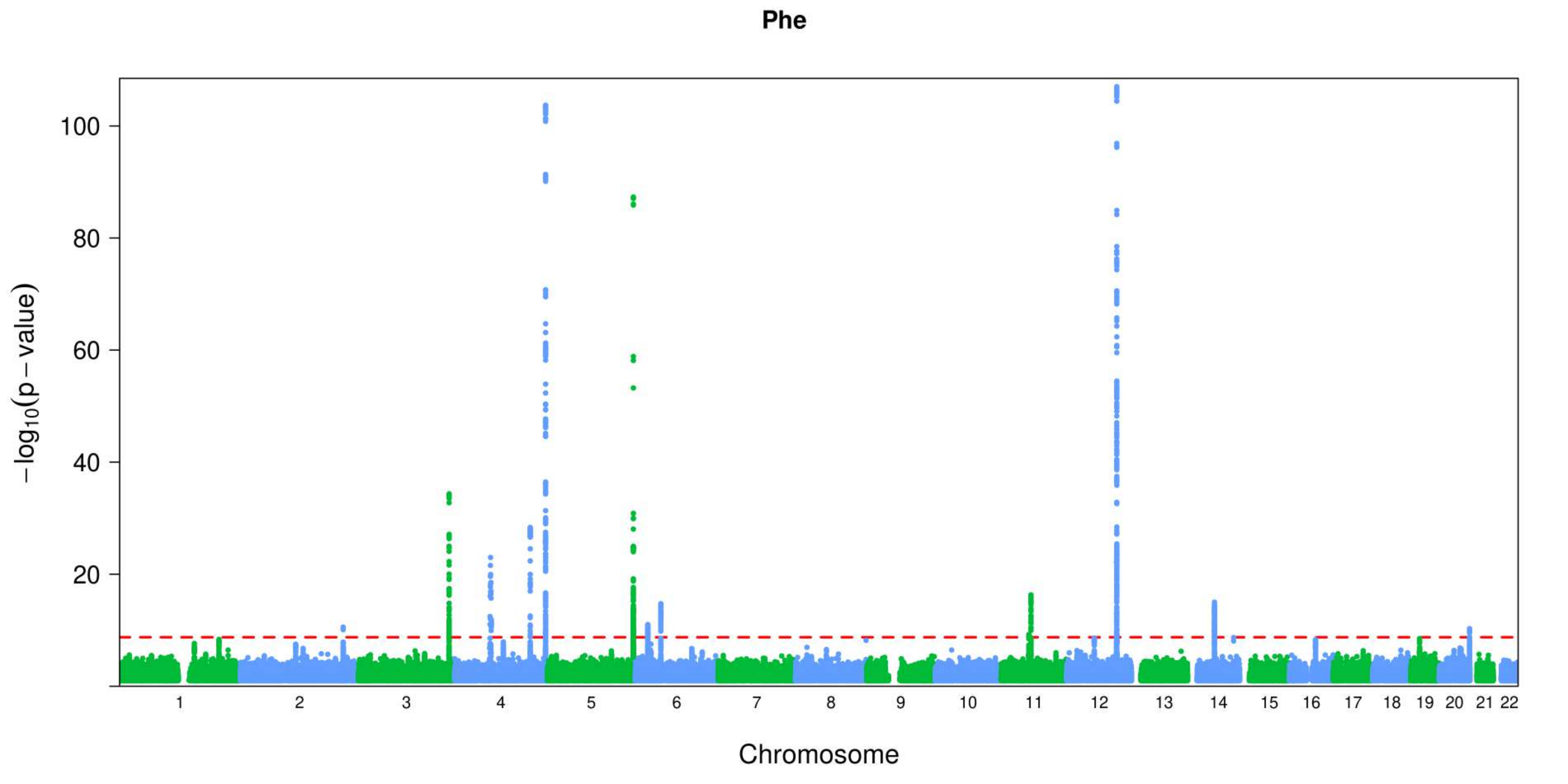

Phe
100
80
60
40
20
−log10(p − value)
1
2
3
4
5
6
7
8
9
10
11
12
13
14
15
16
17
18
19
20
21
22
Chromosome

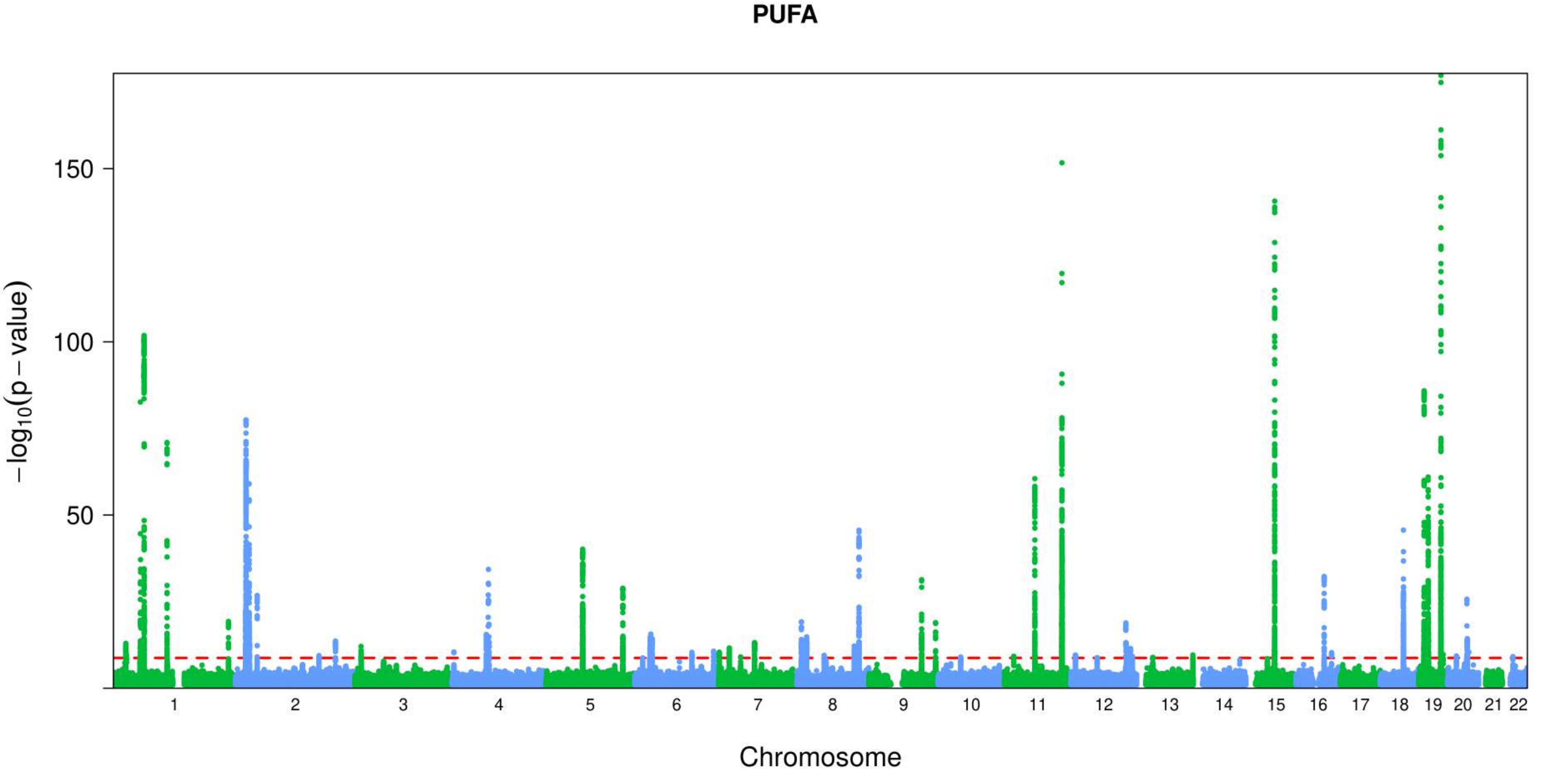
PUFA
150
100
50
−log10(p − value)
1
2
3
4
5
6
7
8
9
10
11
12
13
14
15
16
17
18
19
20
21
22
Chromosome

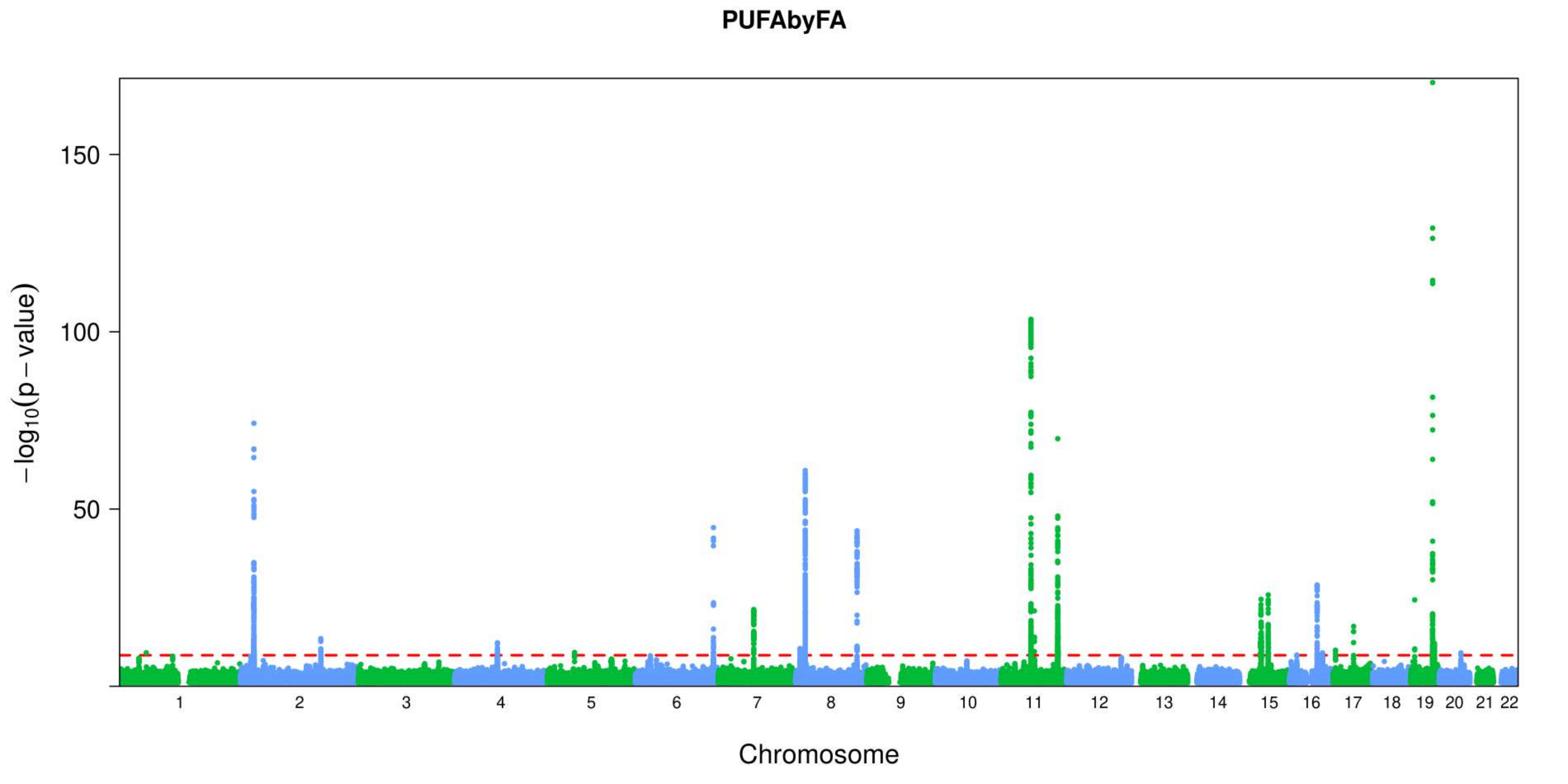

PUFAbyFA
−log10(p − value)
150
100
50
1
2
3
4
5
6
7
8
9
10
11
12
13
14
15
16
17
18
19
20
21
22
Chromosome

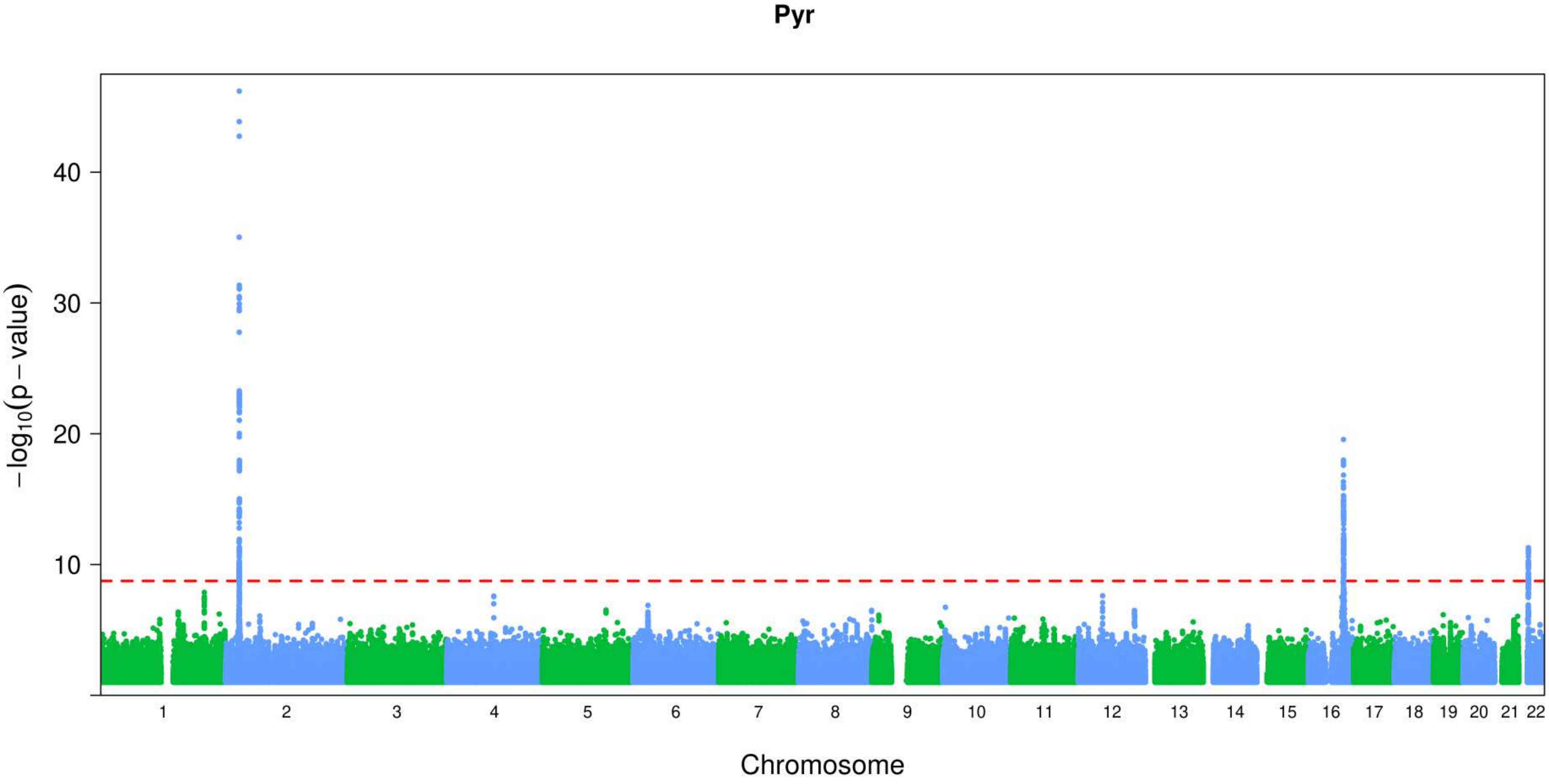

Pyr
40
30
20
10
−log10(p − value)
1
2
3
4
5
6
7
8
9
10
11
12
13
14
15
16
17
18
19
20
21
22
Chromosome

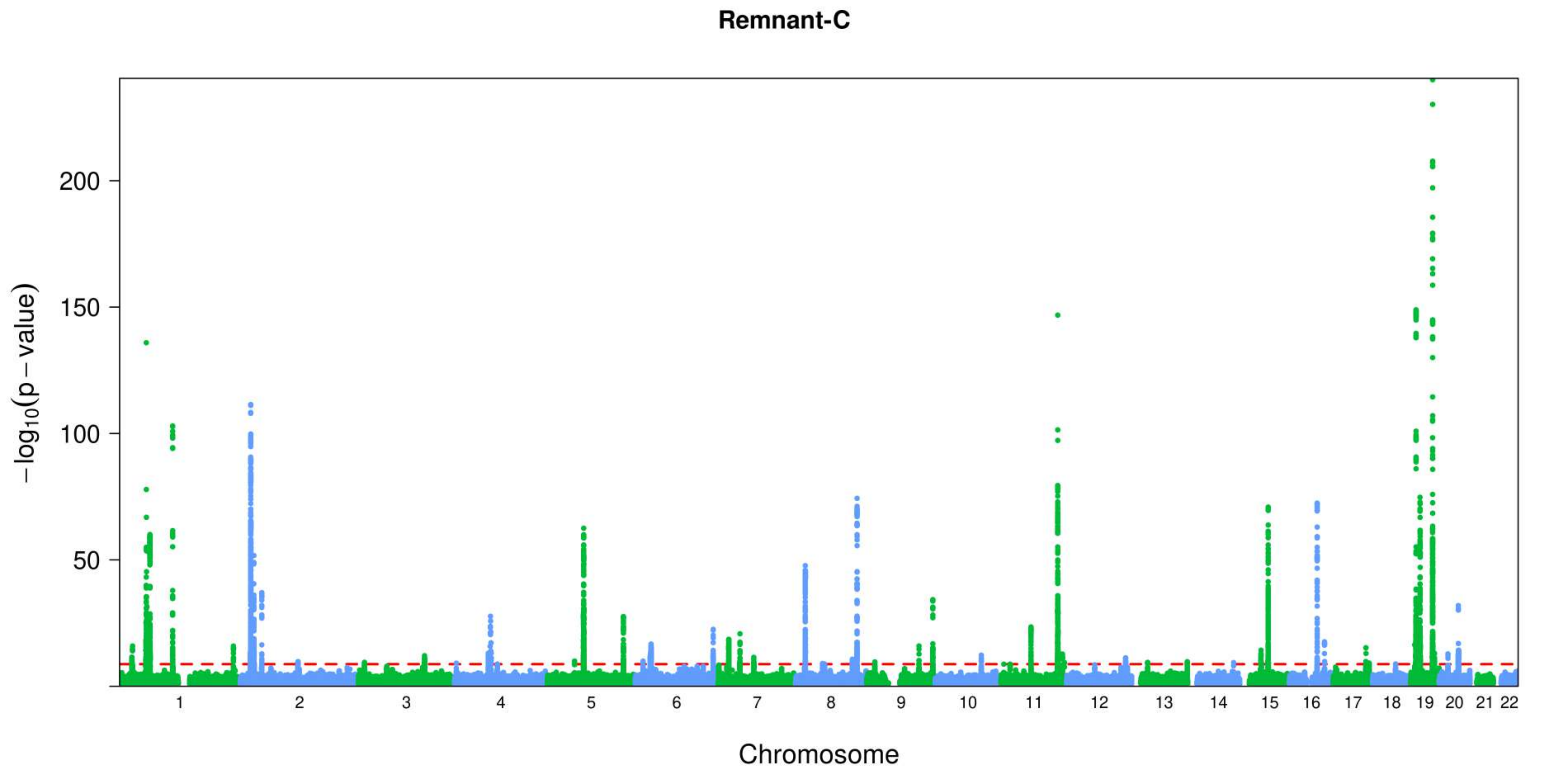

Remnant-C
−log10(p − value)
200
150
100
50
1
2
3
4
5
6
7
8
9
10
11
12
13
14
15
16
17
18
19
20
21
22
Chromosome

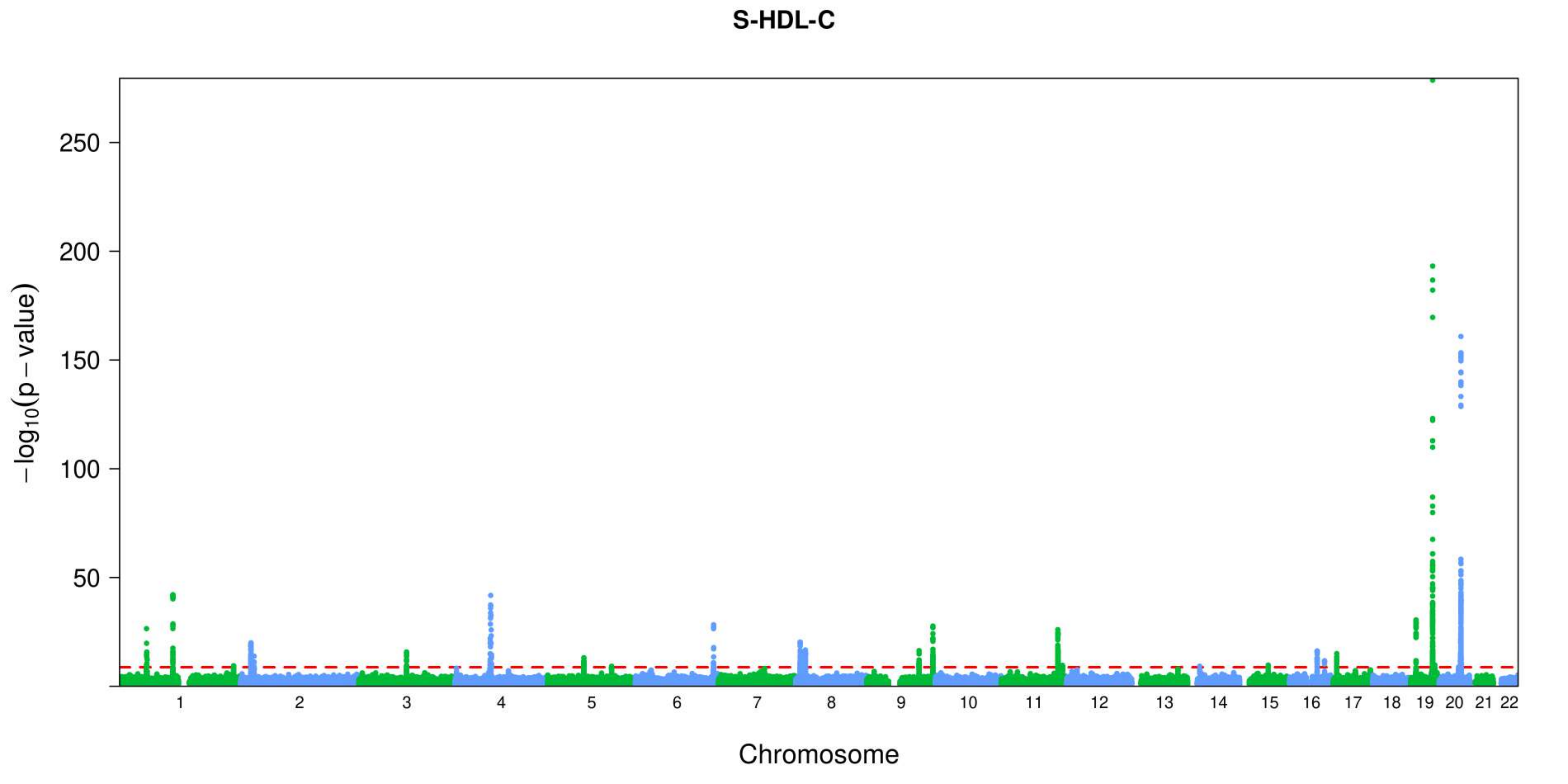
S-HDL-C
−log10(p − value)
250
200
150
100
50
Chromosome
1
2
3
4
5
6
7
8
9
10
11
12
13
14
15
16
17
18
19
20
21
22

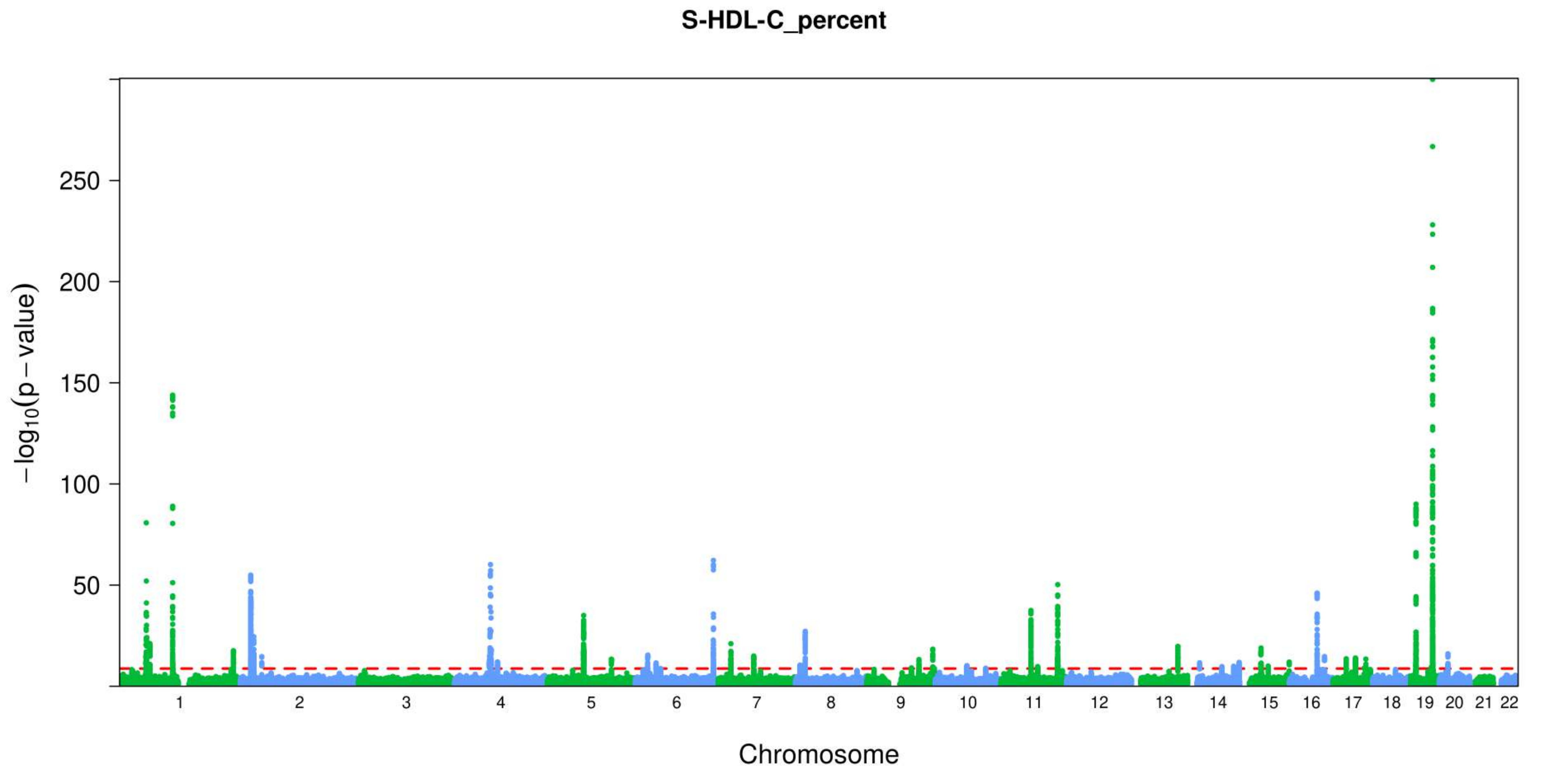
S-HDL-C_percent
250
200
150
100
50
−log10(p − value)
1
2
3
4
5
6
7
8
9
10
11
12
13
14
15
16
17
18
19
20
21
22
Chromosome

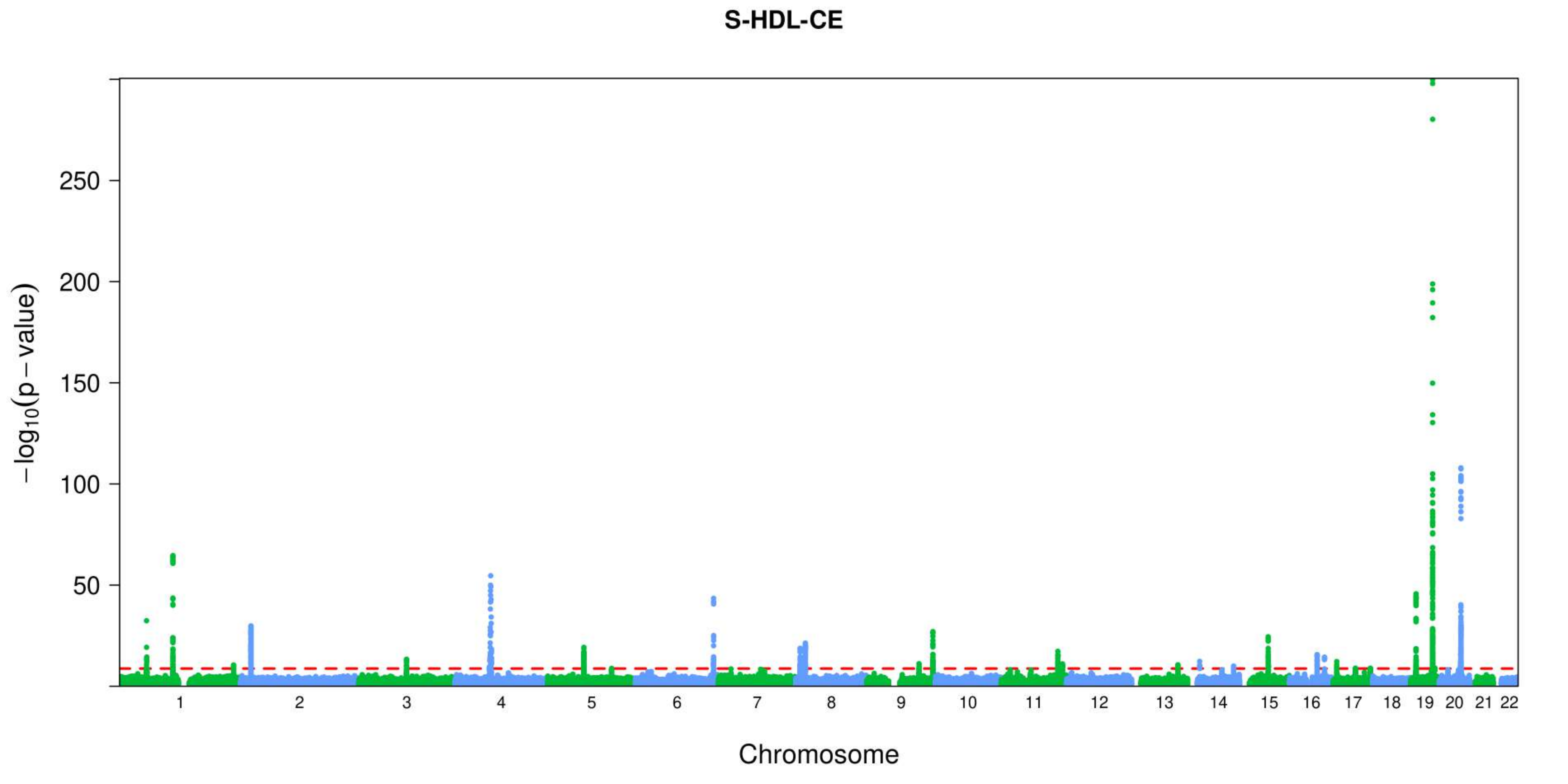
S-HDL-CE
-log10(p – value)
250
200
150
100
50
1
2
3
4
5
6
7
8
9
10
11
12
13
14
15
16
17
18
19
20
21
22
Chromosome

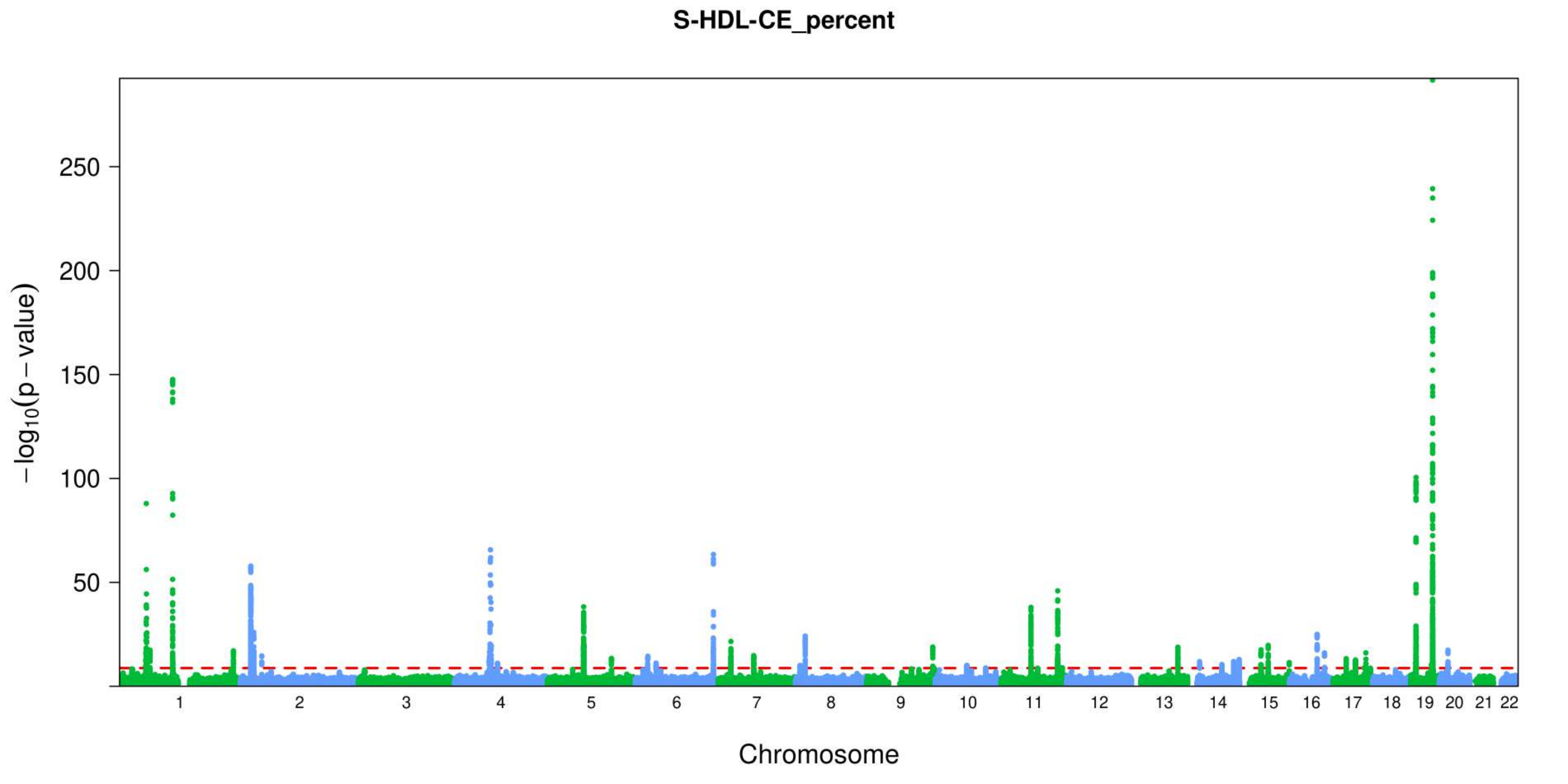

S-HDL-CE_percent
250
200
150
100
50
$-\log_{10}(p-\text{value})$
1
2
3
4
5
6
7
8
9
10
11
12
13
14
15
16
17
18
19
20
21
22
Chromosome

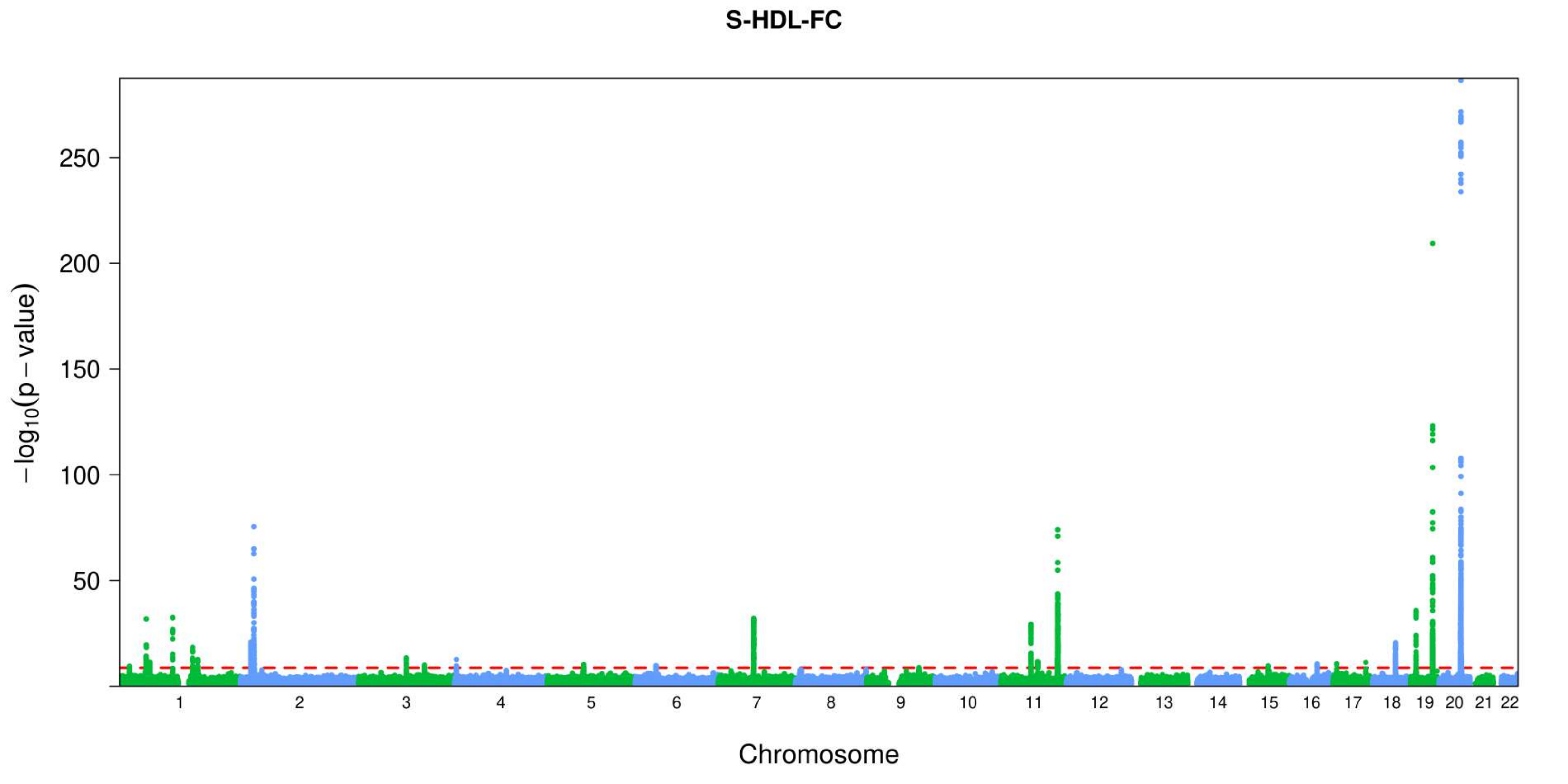
S-HDL-FC
−log10(p − value)
250
200
150
100
50
Chromosome
1
2
3
4
5
6
7
8
9
10
11
12
13
14
15
16
17
18
19
20
21
22

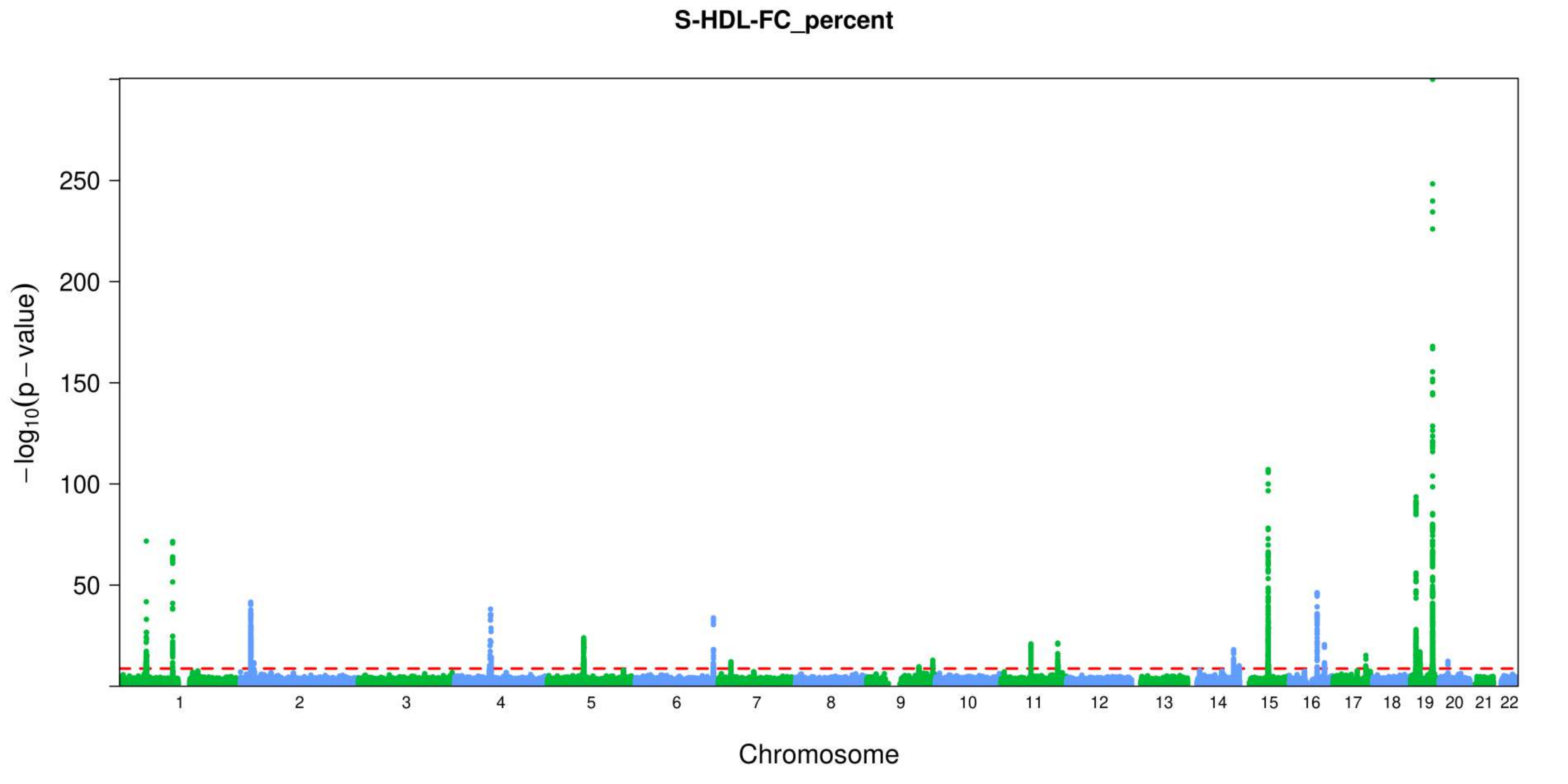

S-HDL-FC_percent
250
200
150
100
50
$-\log_{10}(p-\text{value})$
1
2
3
4
5
6
7
8
9
10
11
12
13
14
15
16
17
18
19
20
21
22
Chromosome

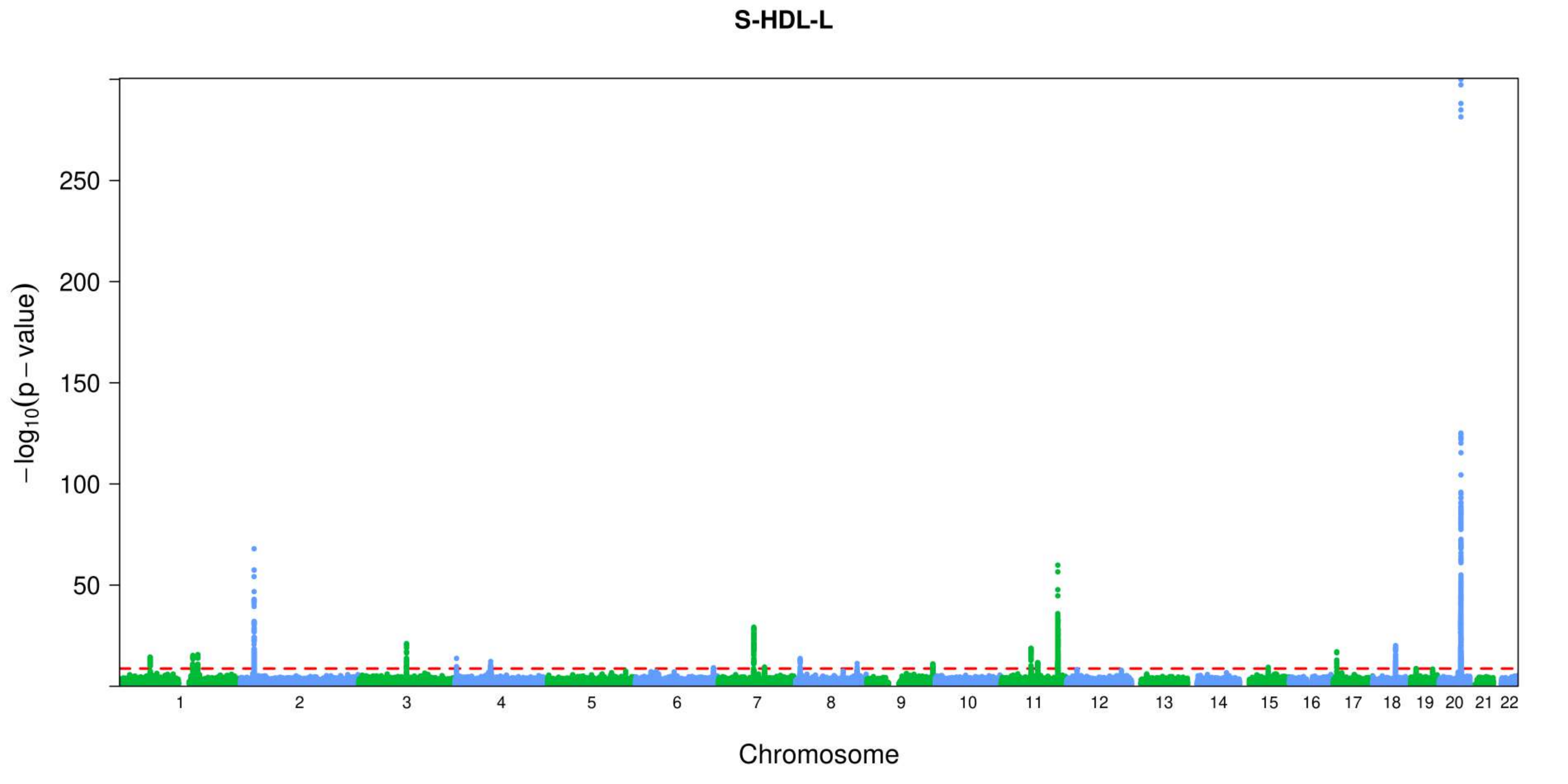
S-HDL-L
250
200
150
100
50
−log10(p − value)
1
2
3
4
5
6
7
8
9
10
11
12
13
14
15
16
17
18
19
20
21
22
Chromosome

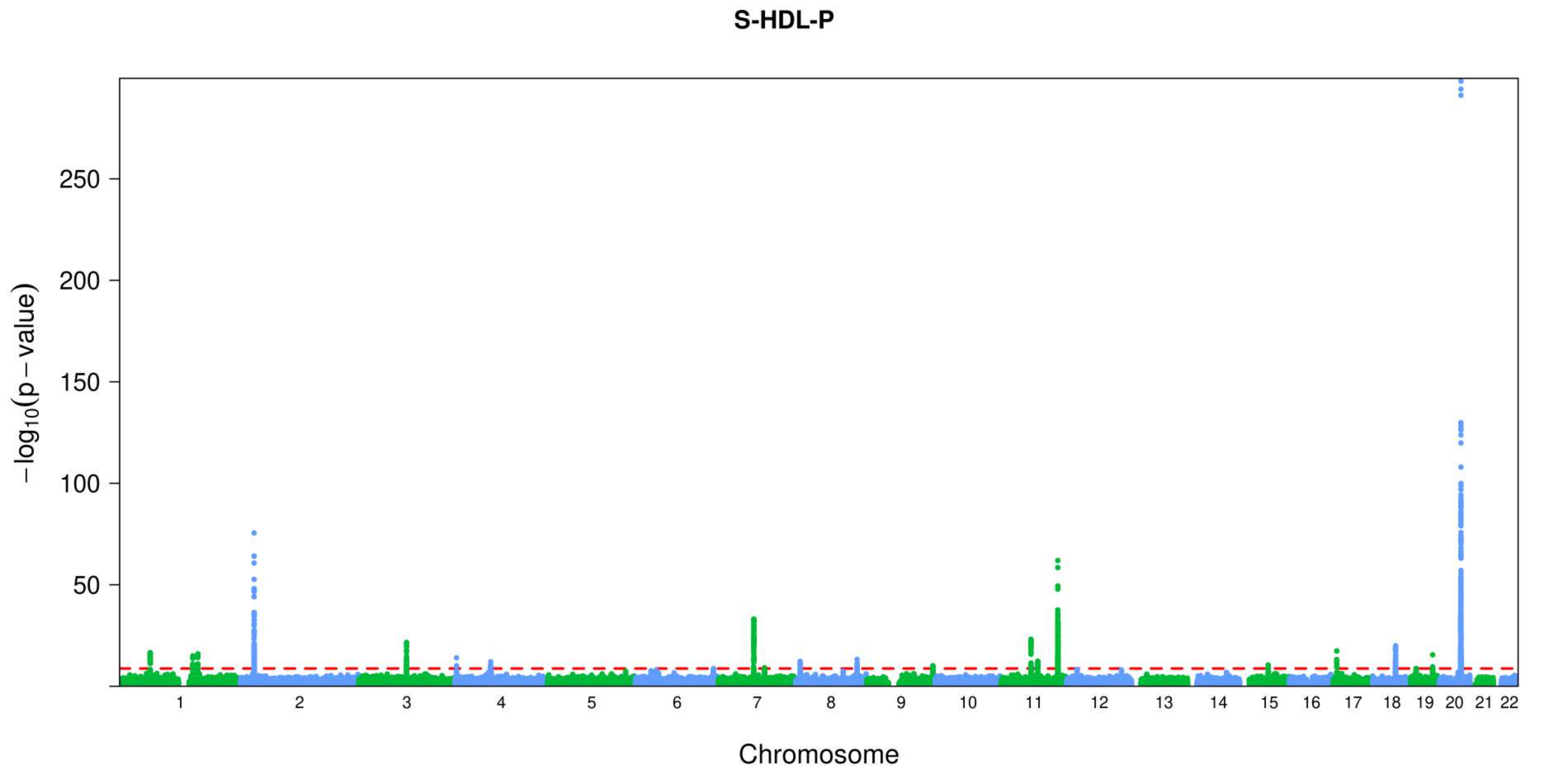
S-HDL-P
−log10(p − value)
250
200
150
100
50
1
2
3
4
5
6
7
8
9
10
11
12
13
14
15
16
17
18
19
20
21
22
Chromosome

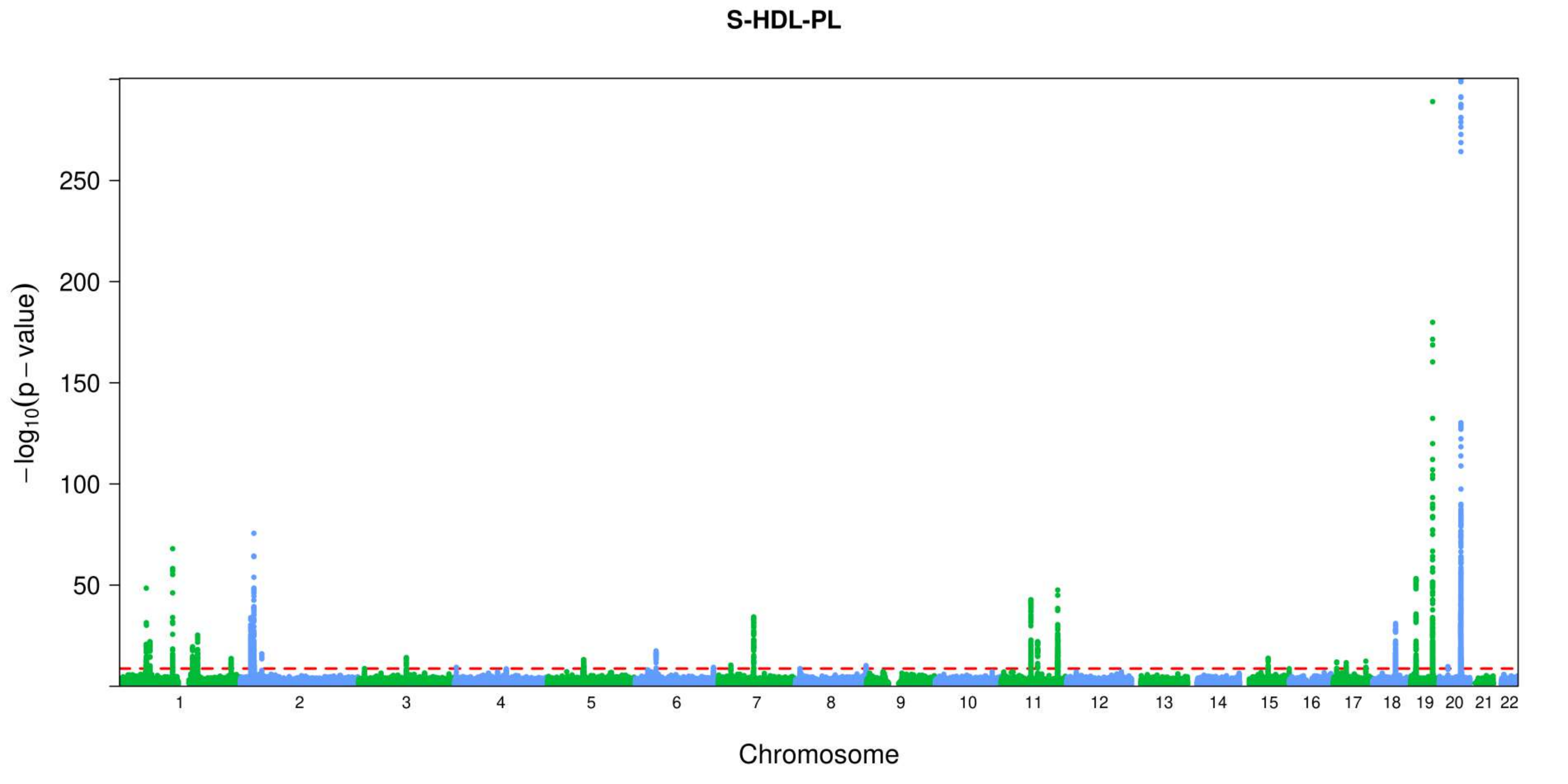
S-HDL-PL
−log10(p − value)
250
200
150
100
50
1
2
3
4
5
6
7
8
9
10
11
12
13
14
15
16
17
18
19
20
21
22
Chromosome

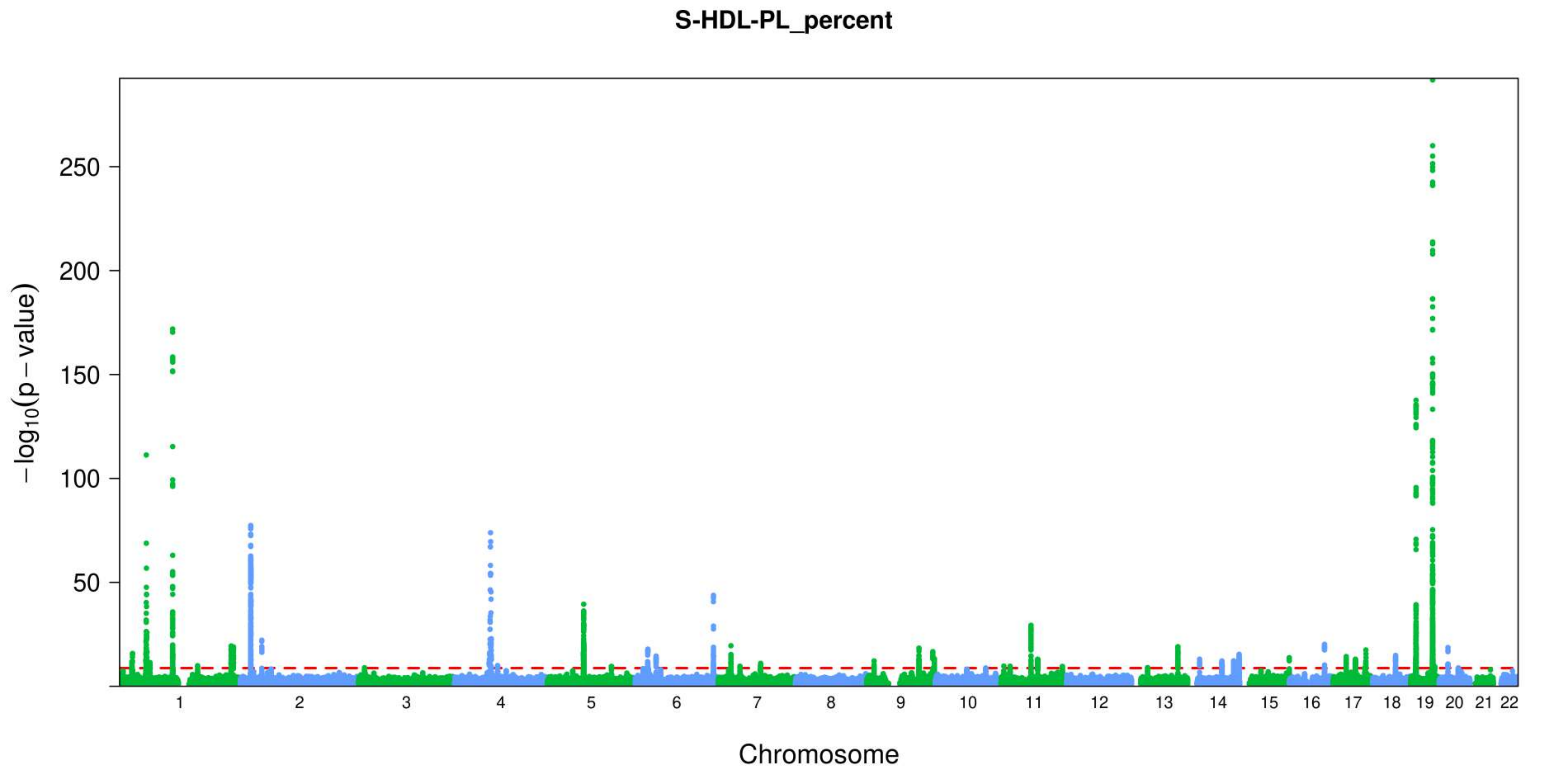
S-HDL-PL_percent
250
200
150
100
50
-log10(p - value)
1
2
3
4
5
6
7
8
9
10
11
12
13
14
15
16
17
18
19
20
21
22
Chromosome

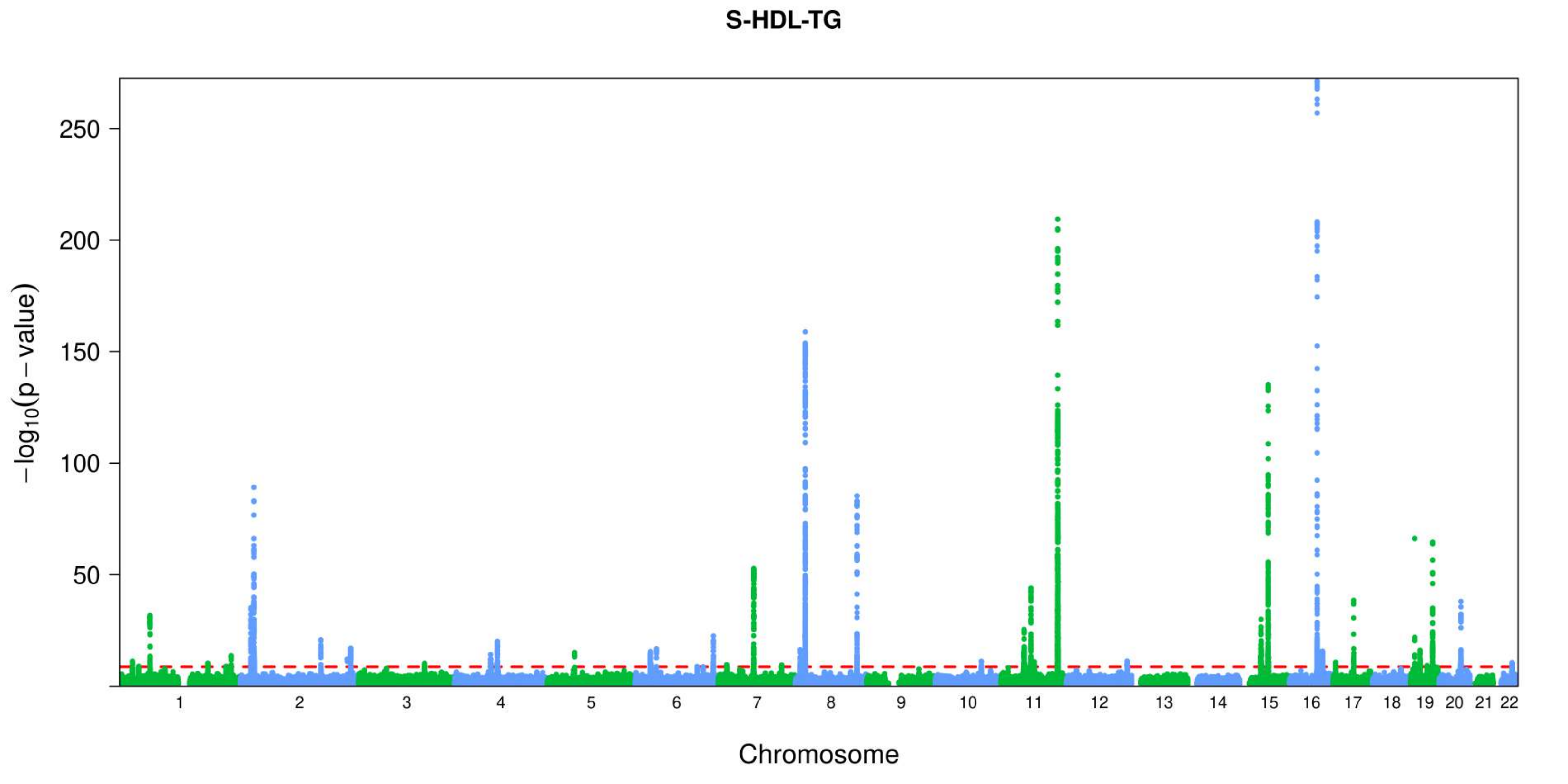
S-HDL-TG
−log10(p − value)
250
200
150
100
50
1
2
3
4
5
6
7
8
9
10
11
12
13
14
15
16
17
18
19
20
21
22
Chromosome

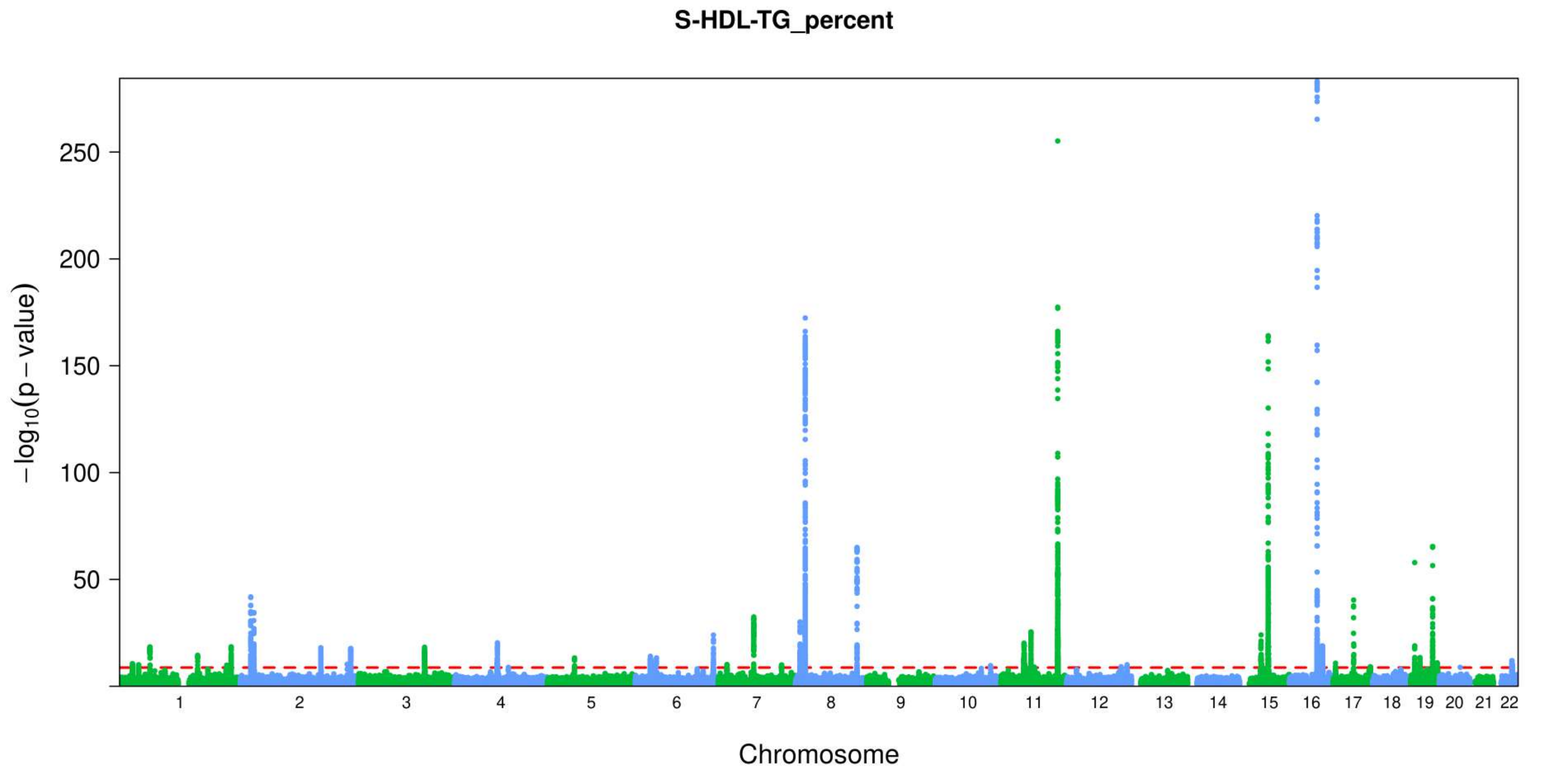
S-HDL-TG_percent
−log10(p − value)
250
200
150
100
50
1
2
3
4
5
6
7
8
9
10
11
12
13
14
15
16
17
18
19
20
21
22
Chromosome

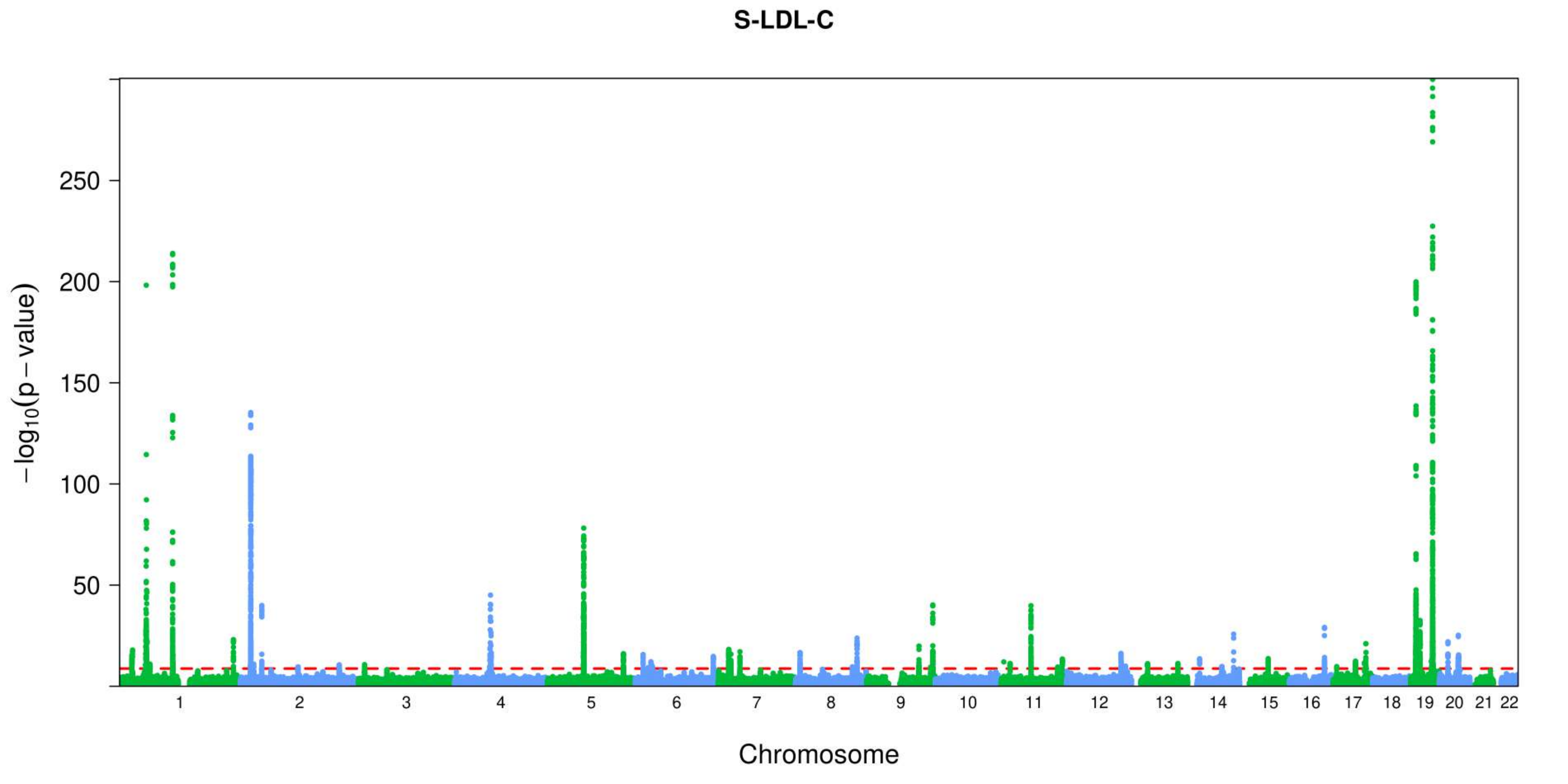
S-LDL-C
250
200
150
100
50
−log10(p − value)
1
2
3
4
5
6
7
8
9
10
11
12
13
14
15
16
17
18
19
20
21
22
Chromosome

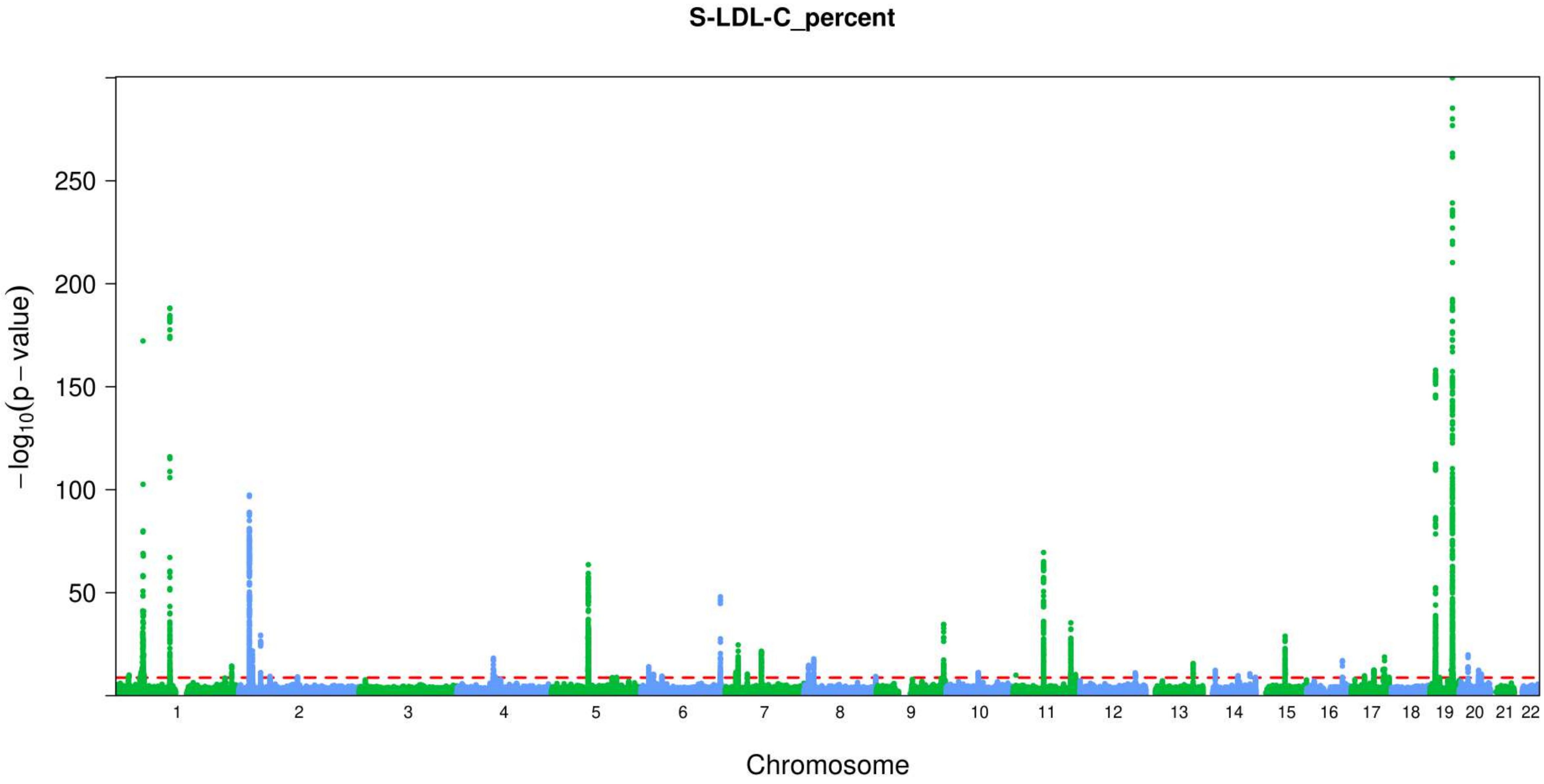
S-LDL-C_percent
−log10(p − value)
250
200
150
100
50
Chromosome
1
2
3
4
5
6
7
8
9
10
11
12
13
14
15
16
17
18
19
20
21
22

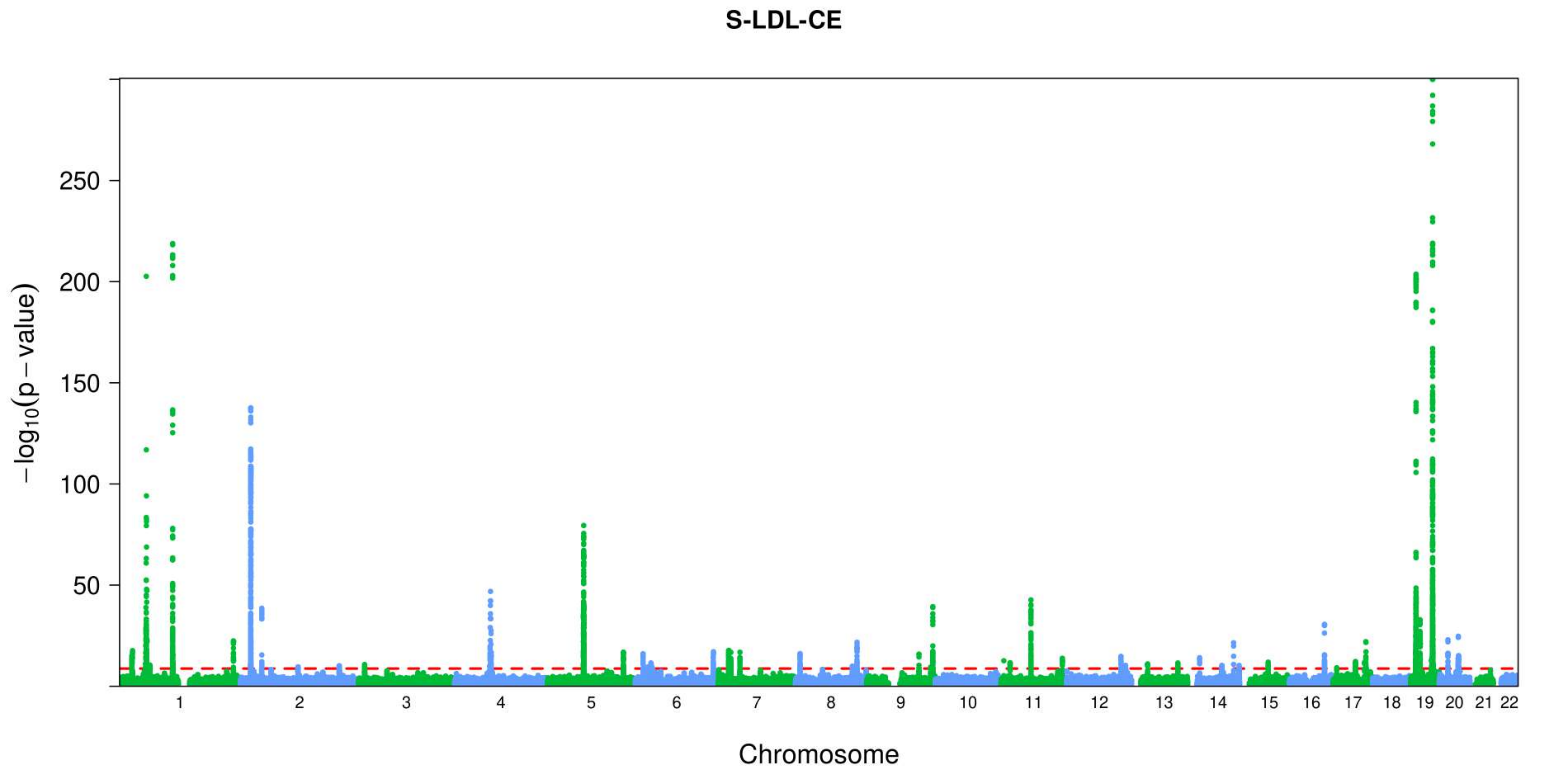
S-LDL-CE
−log10(p − value)
250
200
150
100
50
Chromosome
1
2
3
4
5
6
7
8
9
10
11
12
13
14
15
16
17
18
19
20
21
22

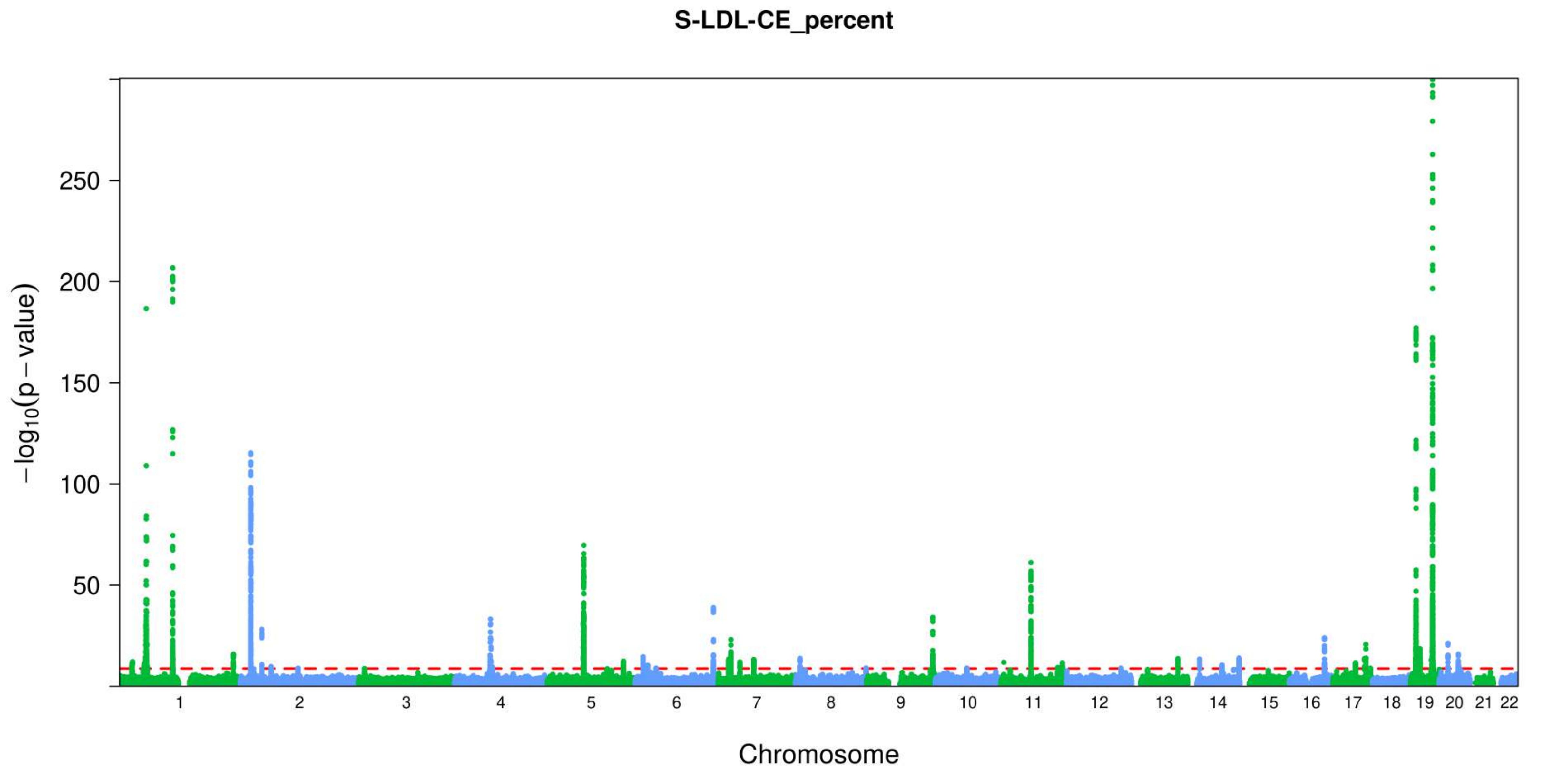
S-LDL-CE_percent
250
200
150
100
50
−log10(p − value)
1
2
3
4
5
6
7
8
9
10
11
12
13
14
15
16
17
18
19
20
21
22
Chromosome

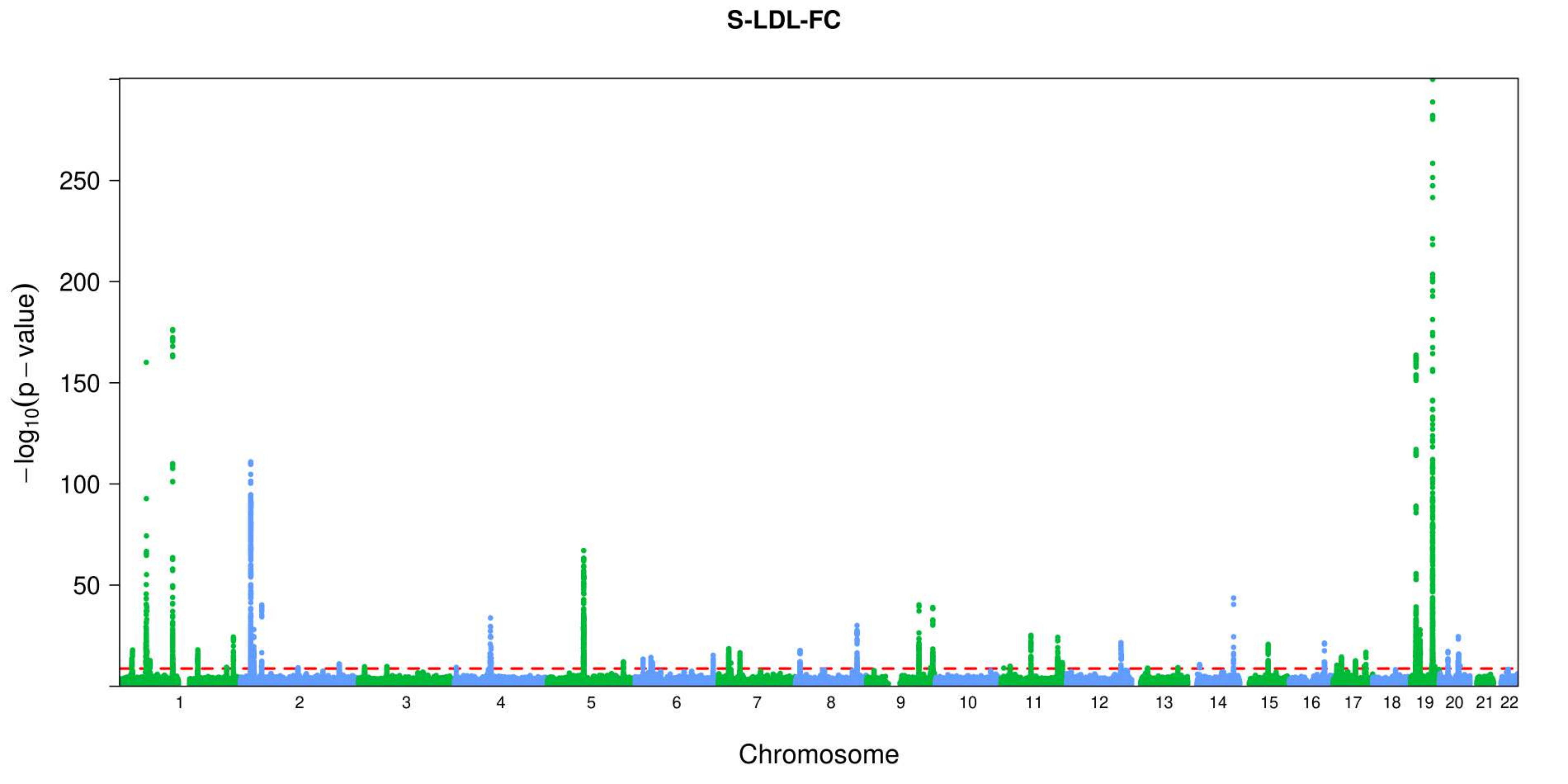
S-LDL-FC
-log10(p - value)
250
200
150
100
50
Chromosome
1
2
3
4
5
6
7
8
9
10
11
12
13
14
15
16
17
18
19
20
21
22

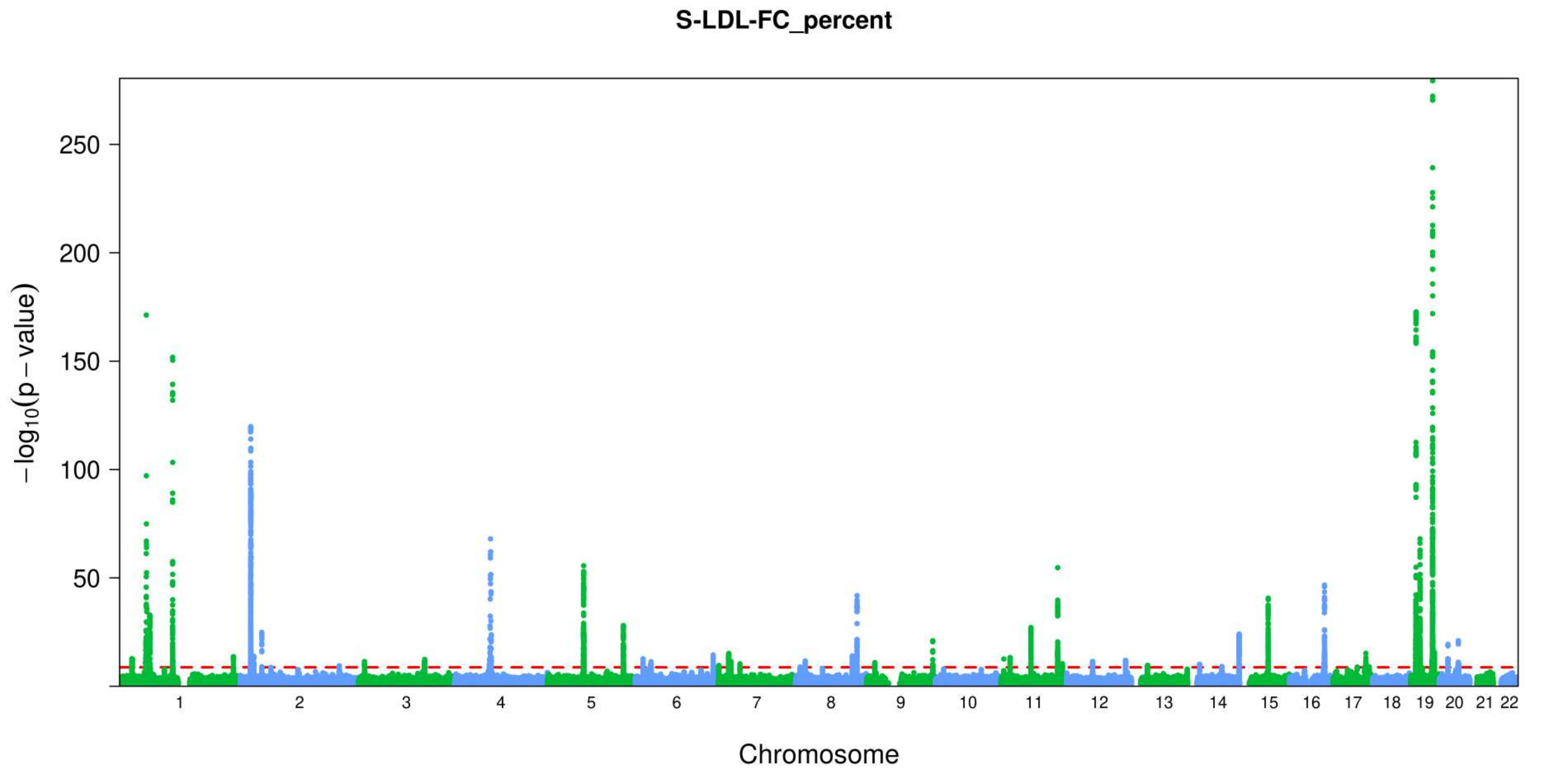
S-LDL-FC_percent
$-\log_{10}(p-\text{value})$
250
200
150
100
50
1 2 3 4 5 6 7 8 9 10 11 12 13 14 15 16 17 18 19 20 21 22
Chromosome

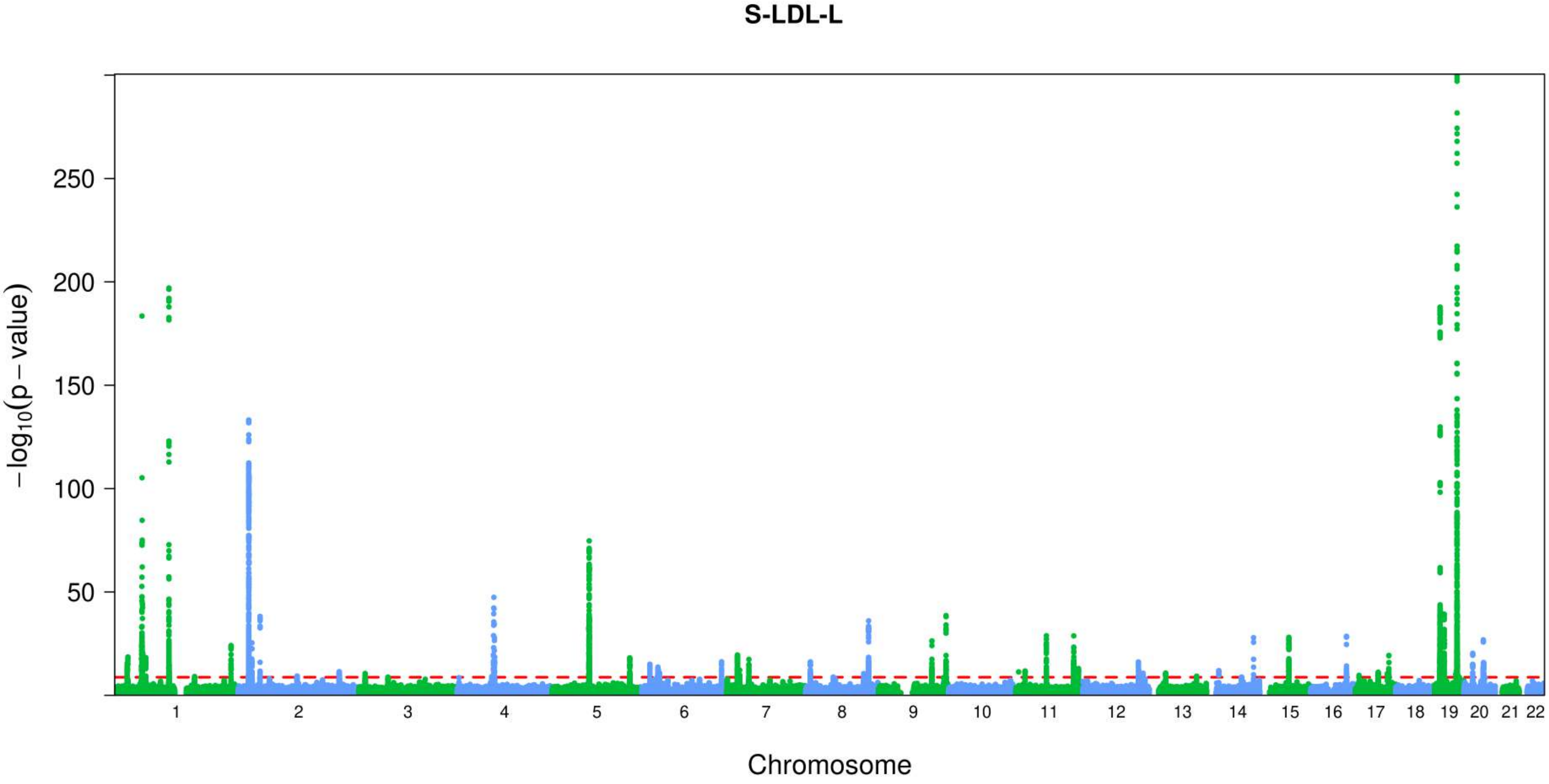
S-LDL-L
$-\log_{10}(p-\text{value})$
250
200
150
100
50
1
2
3
4
5
6
7
8
9
10
11
12
13
14
15
16
17
18
19
20
21
22
Chromosome

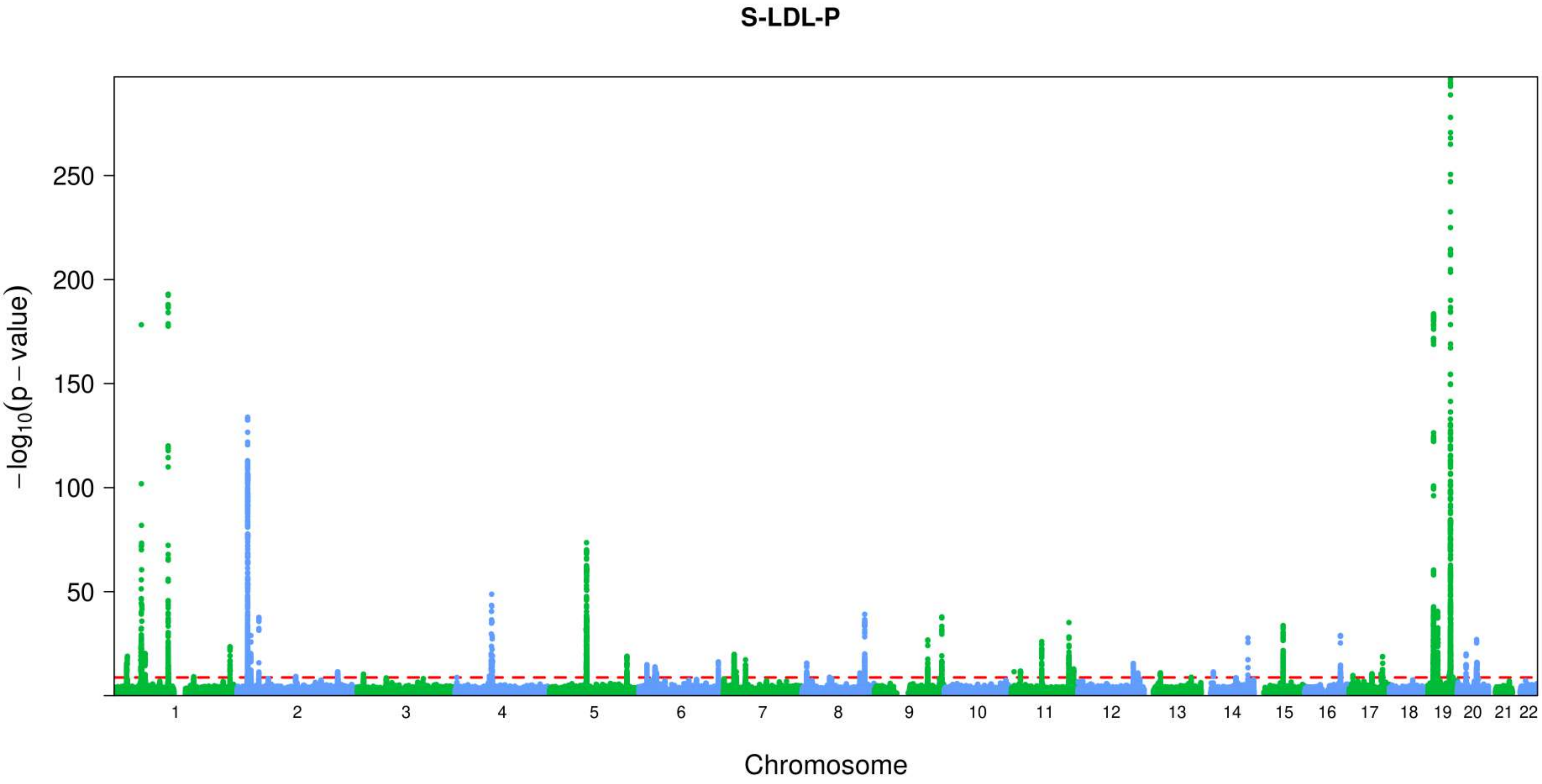
S-LDL-P
250
200
150
100
50
$-\log_{10}(p-\text{value})$
1
2
3
4
5
6
7
8
9
10
11
12
13
14
15
16
17
18
19
20
21
22
Chromosome

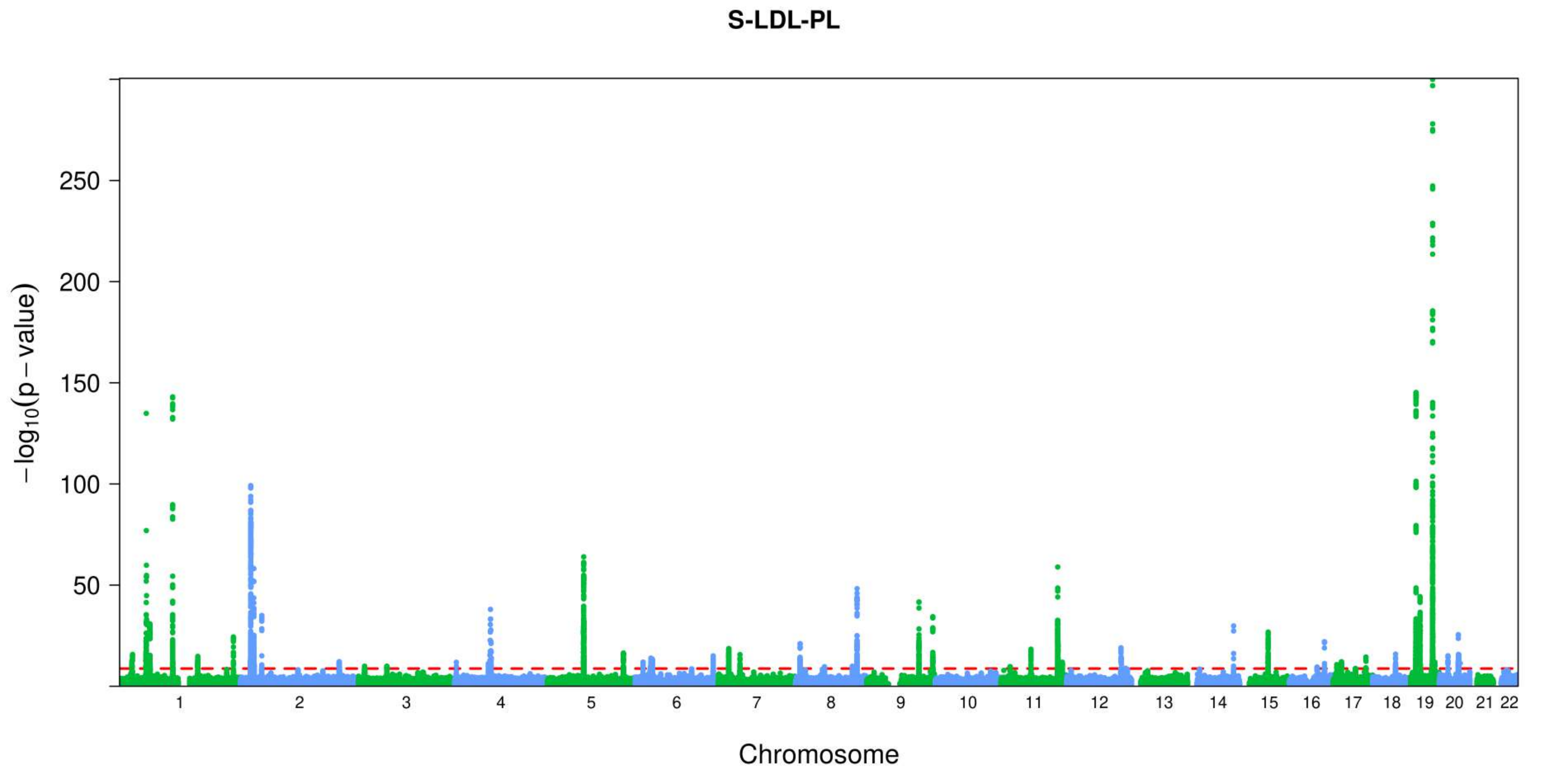
S-LDL-PL
−log10(p − value)
250
200
150
100
50
Chromosome
1
2
3
4
5
6
7
8
9
10
11
12
13
14
15
16
17
18
19
20
21
22

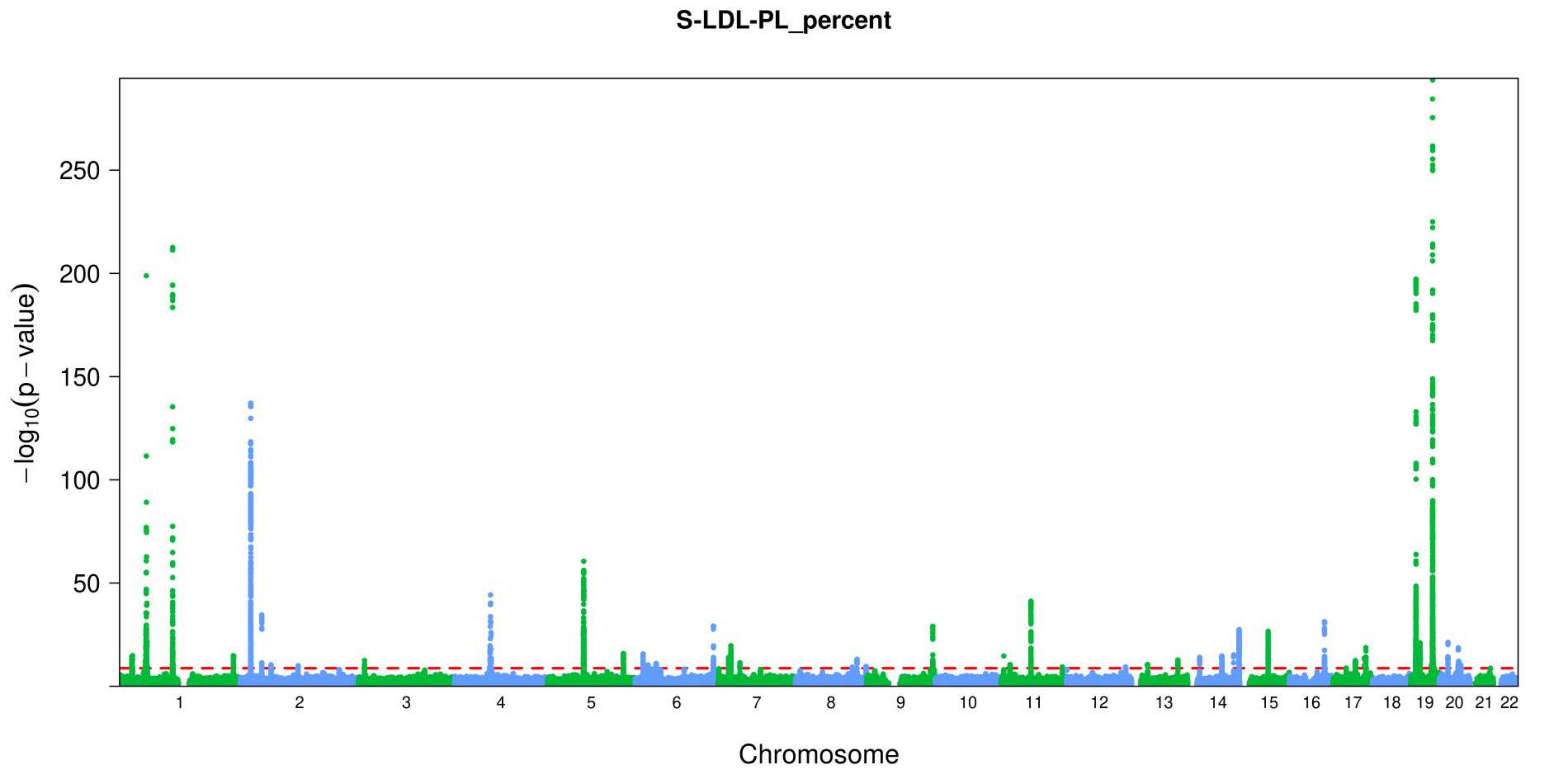
S-LDL-PL_percent
250
200
150
100
50
−log10(p − value)
1
2
3
4
5
6
7
8
9
10
11
12
13
14
15
16
17
18
19
20
21
22
Chromosome

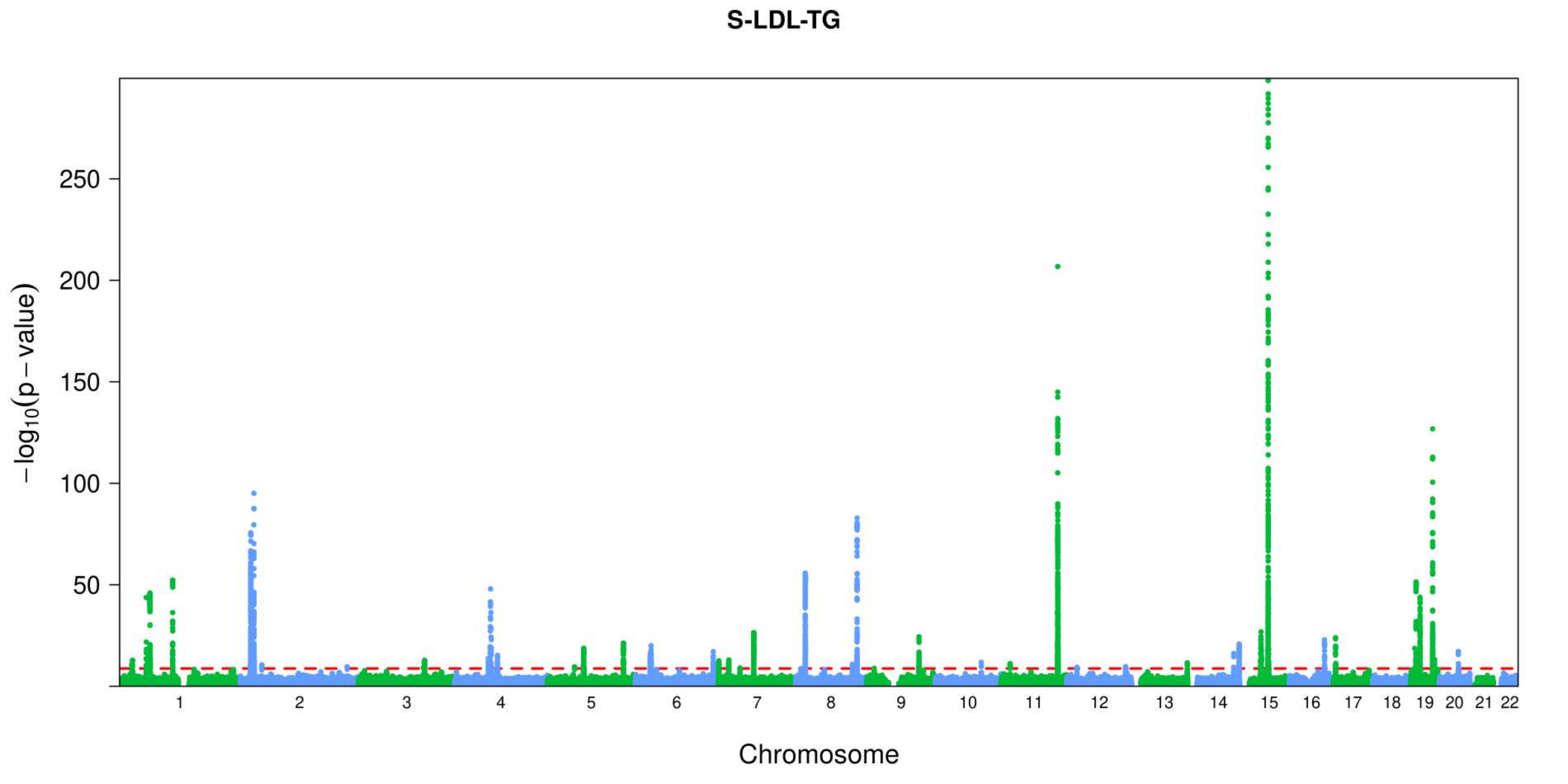
S-LDL-TG
250
200
150
100
50
−log10(p − value)
1
2
3
4
5
6
7
8
9
10
11
12
13
14
15
16
17
18
19
20
21
22
Chromosome

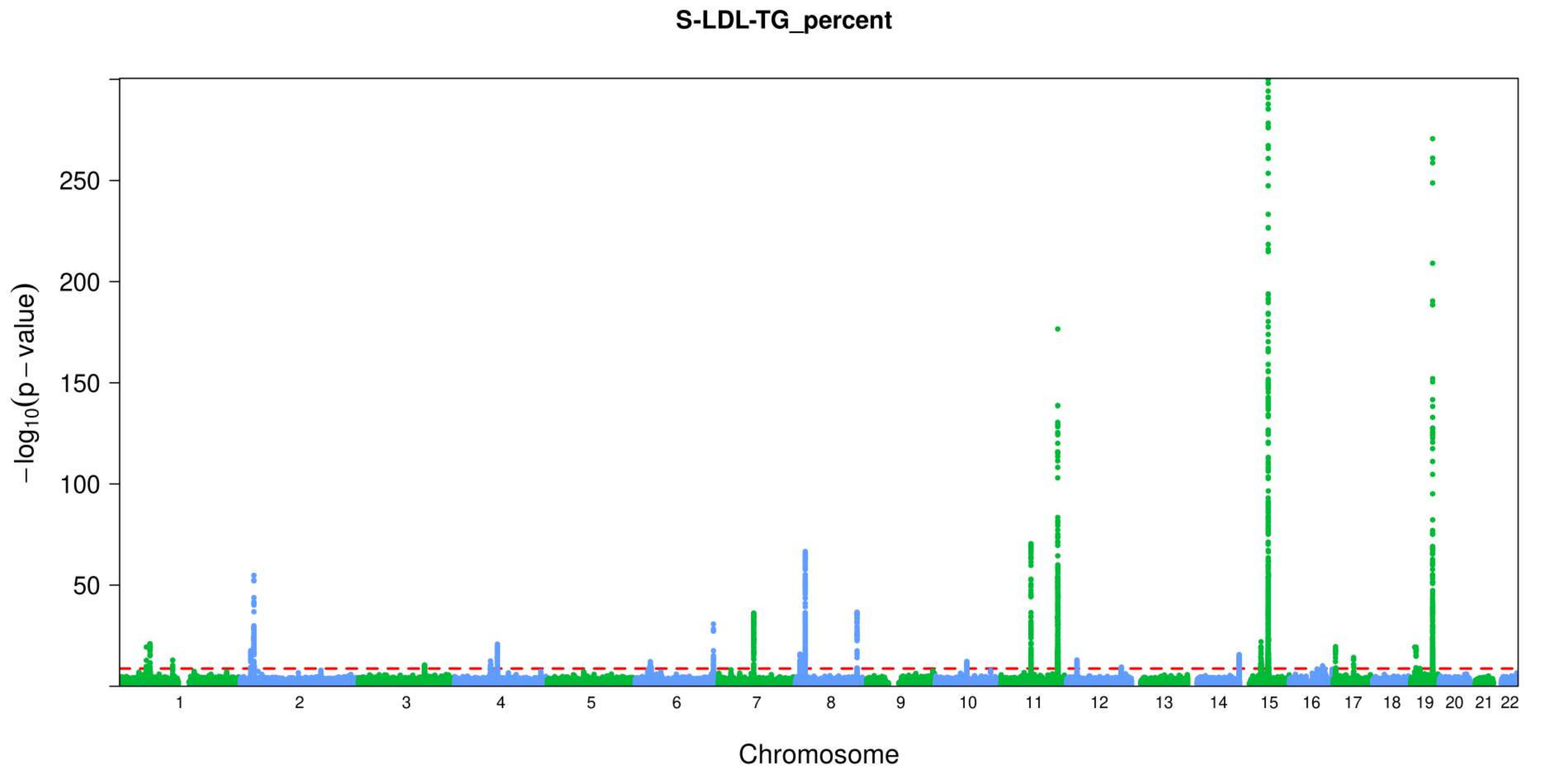

S-LDL-TG_percent
250
200
150
100
50
−log10(p − value)
1
2
3
4
5
6
7
8
9
10
11
12
13
14
15
16
17
18
19
20
21
22
Chromosome

S-VLDL-C

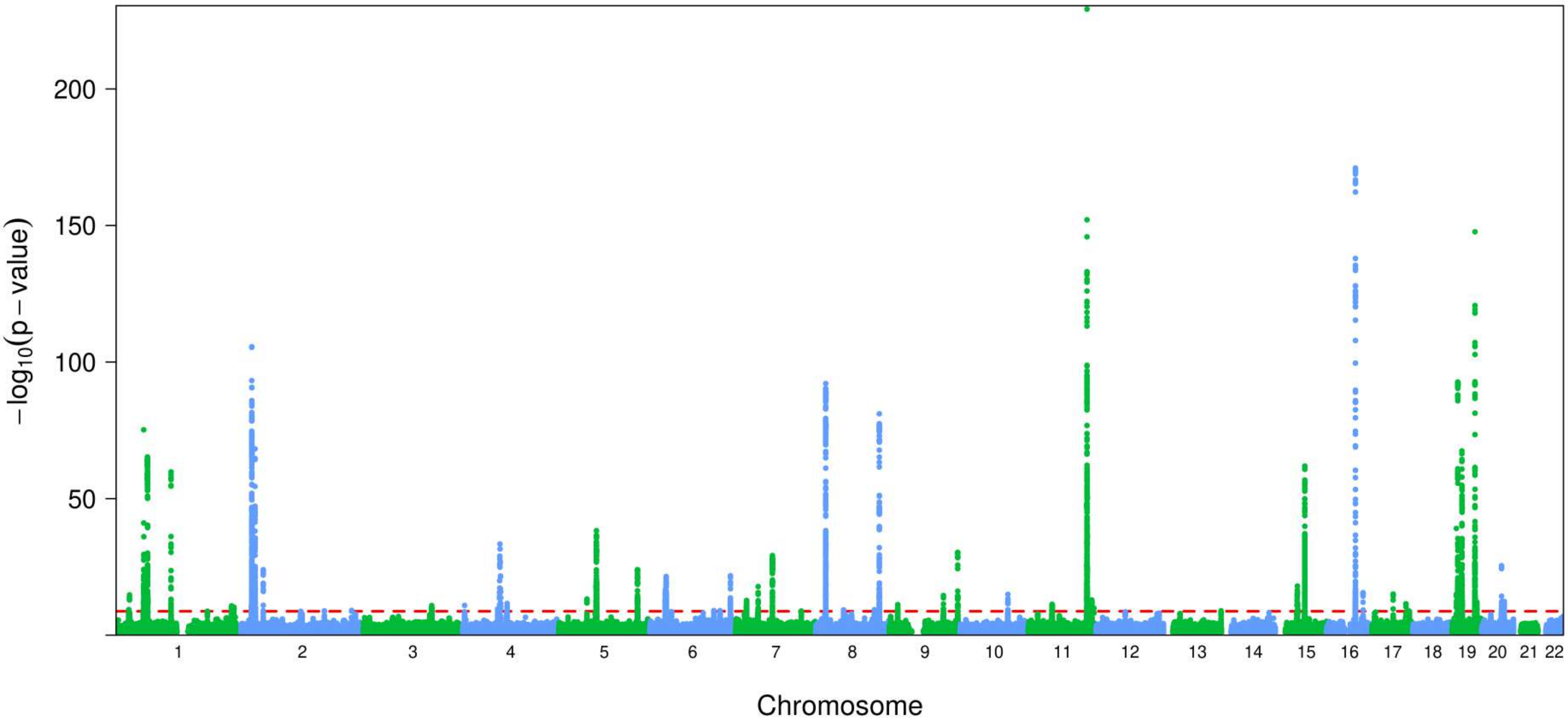

200
150
100
50
−log10(p − value)
1
2
3
4
5
6
7
8
9
10
11
12
13
14
15
16
17
18
19
20
21
22
Chromosome

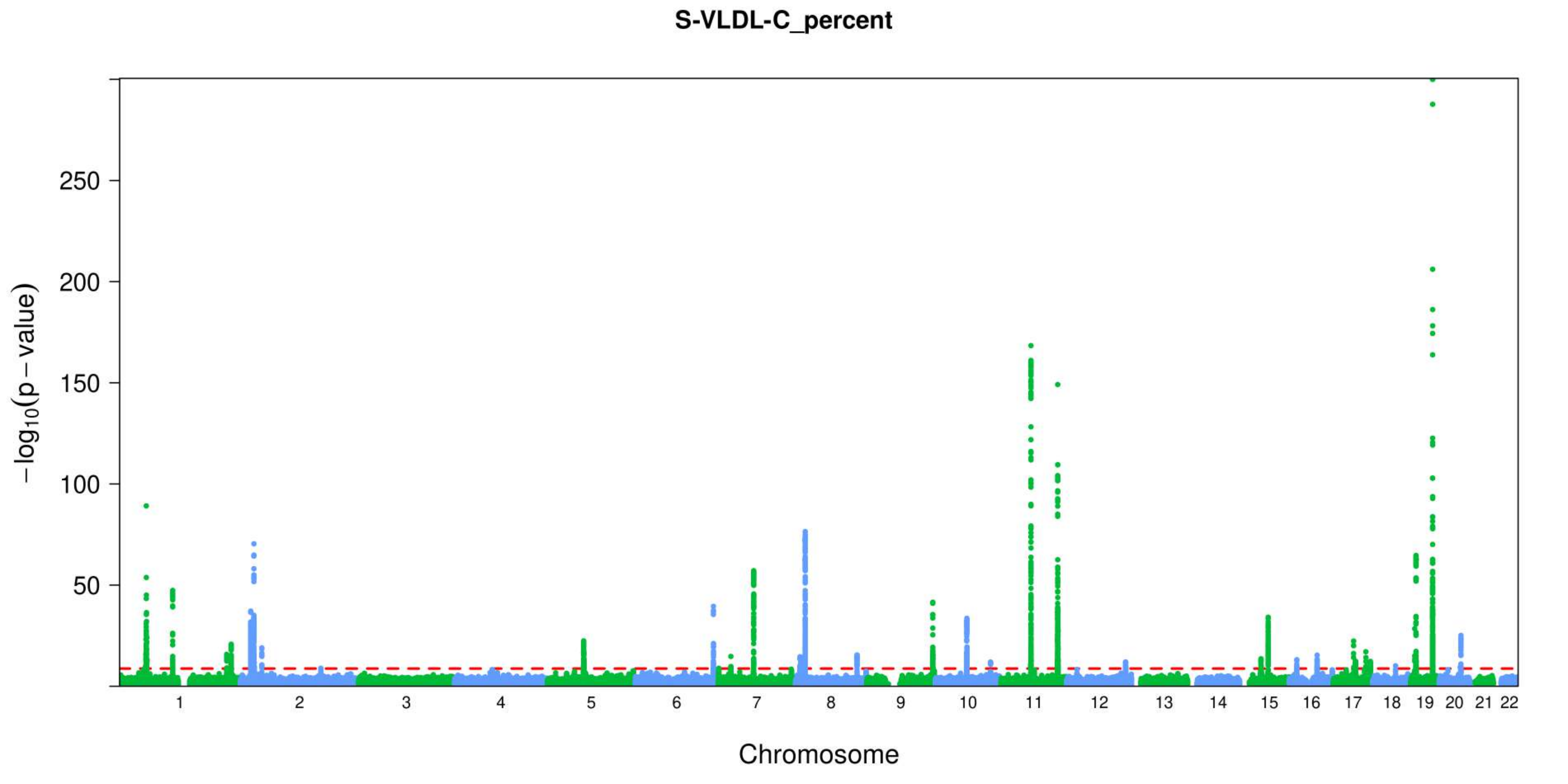
S-VLDL-C_percent
−log10(p − value)
250
200
150
100
50
1
2
3
4
5
6
7
8
9
10
11
12
13
14
15
16
17
18
19
20
21
22
Chromosome

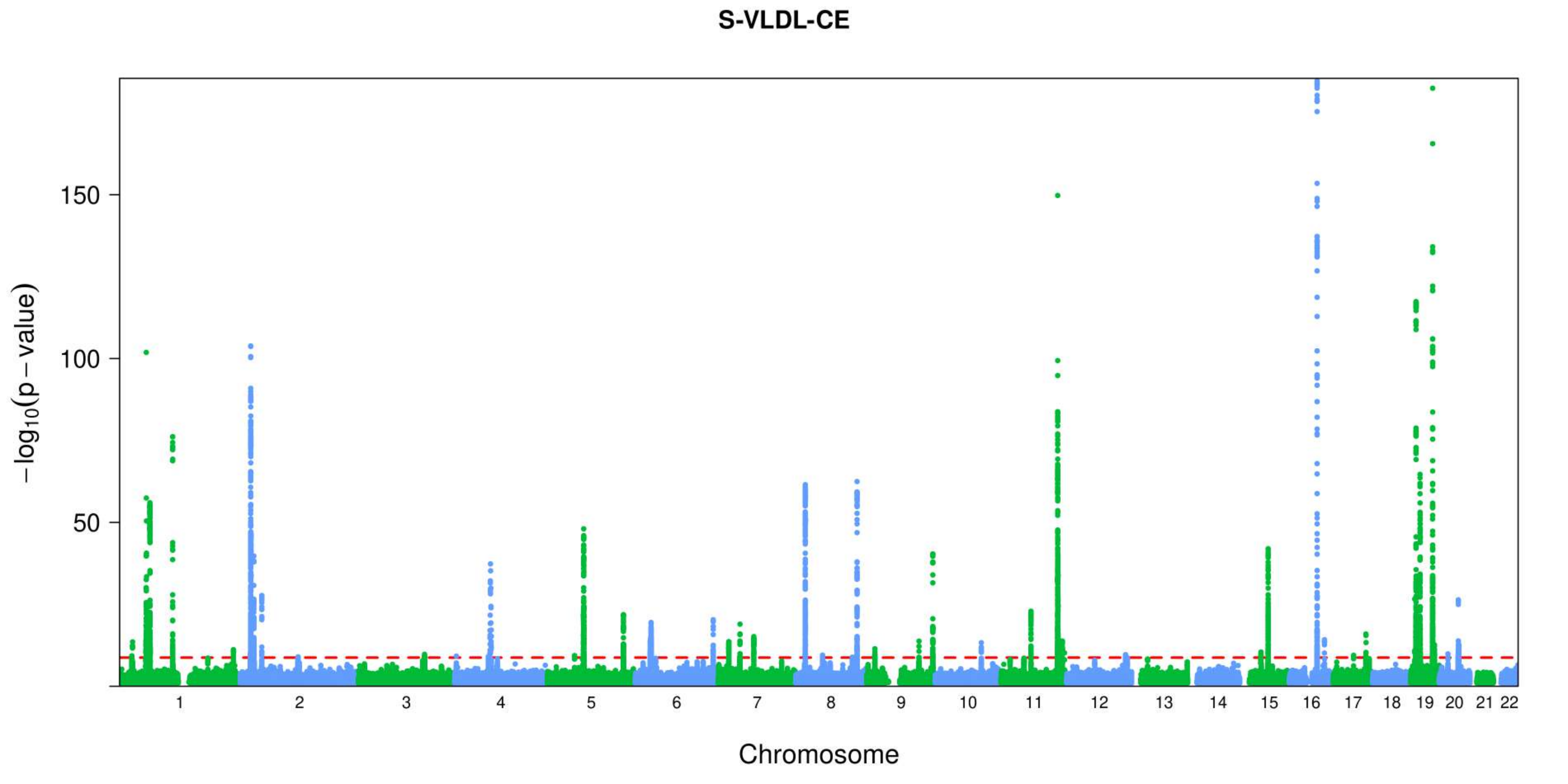
S-VLDL-CE
−log10(p − value)
150
100
50
1
2
3
4
5
6
7
8
9
10
11
12
13
14
15
16
17
18
19
20
21
22
Chromosome

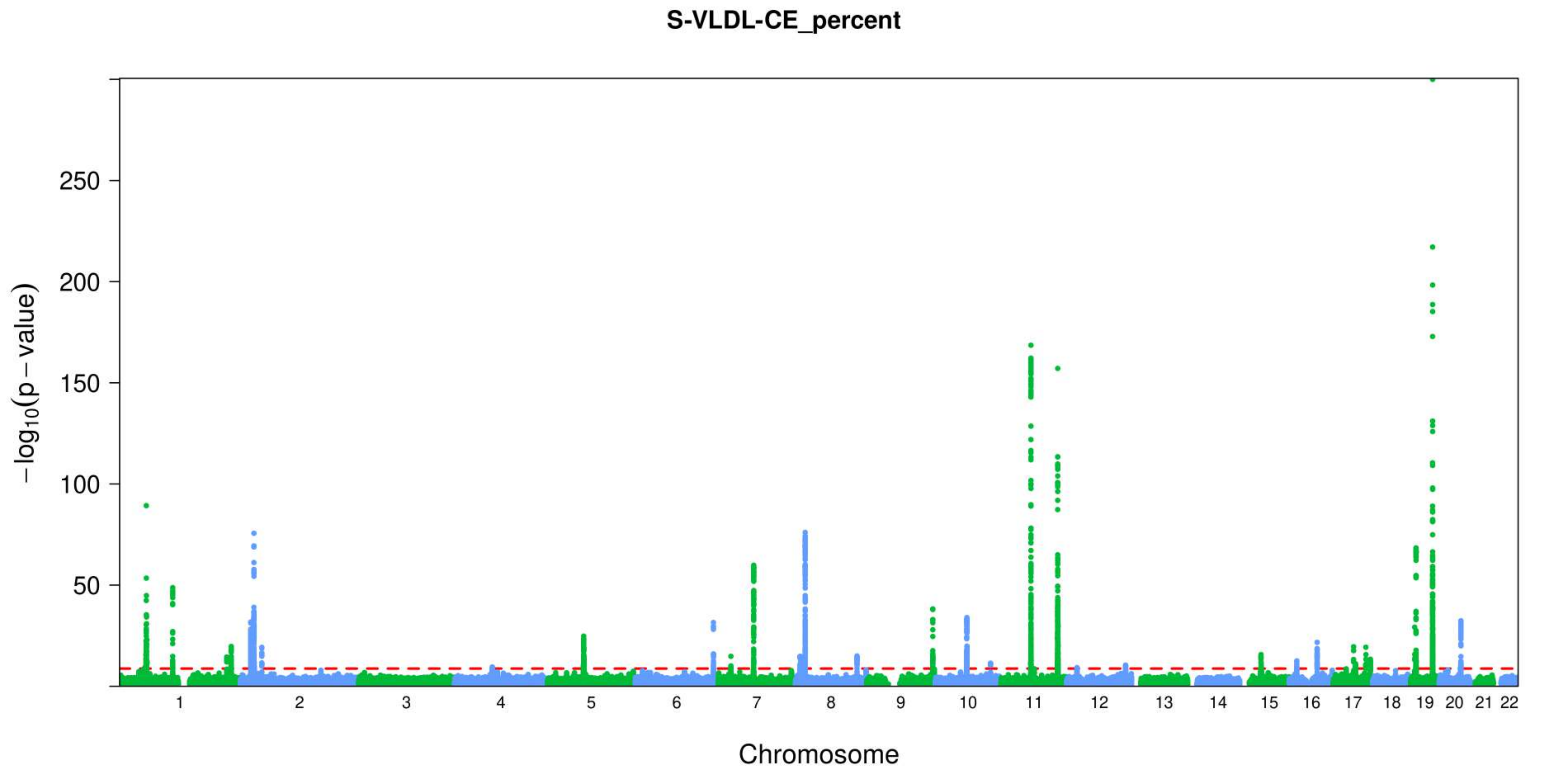
S-VLDL-CE_percent
−log10(p − value)
250
200
150
100
50
1
2
3
4
5
6
7
8
9
10
11
12
13
14
15
16
17
18
19
20
21
22
Chromosome

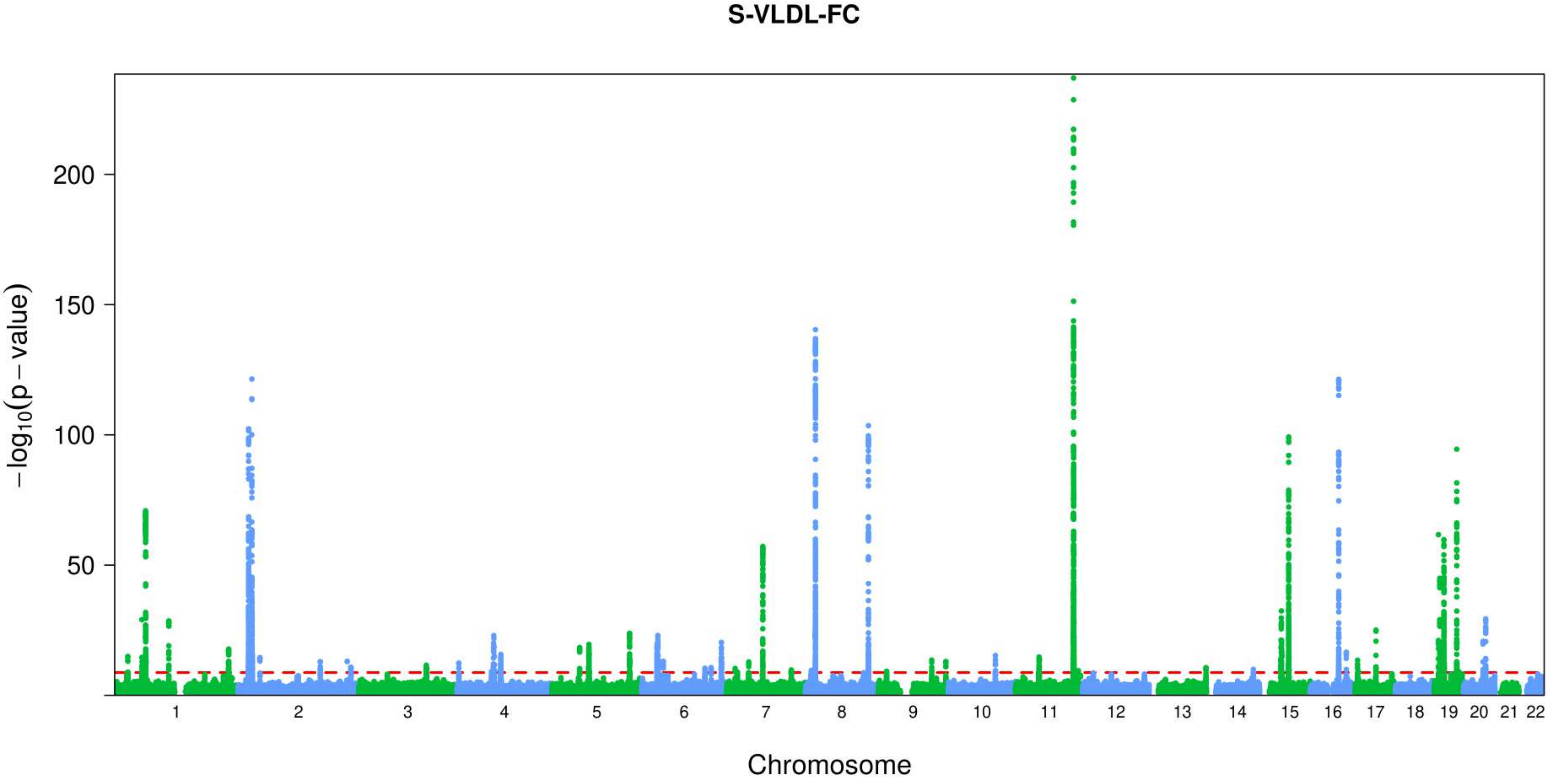

S-VLDL-FC
200
150
100
50
$-\log_{10}(p-\text{value})$
1
2
3
4
5
6
7
8
9
10
11
12
13
14
15
16
17
18
19
20
21
22
Chromosome

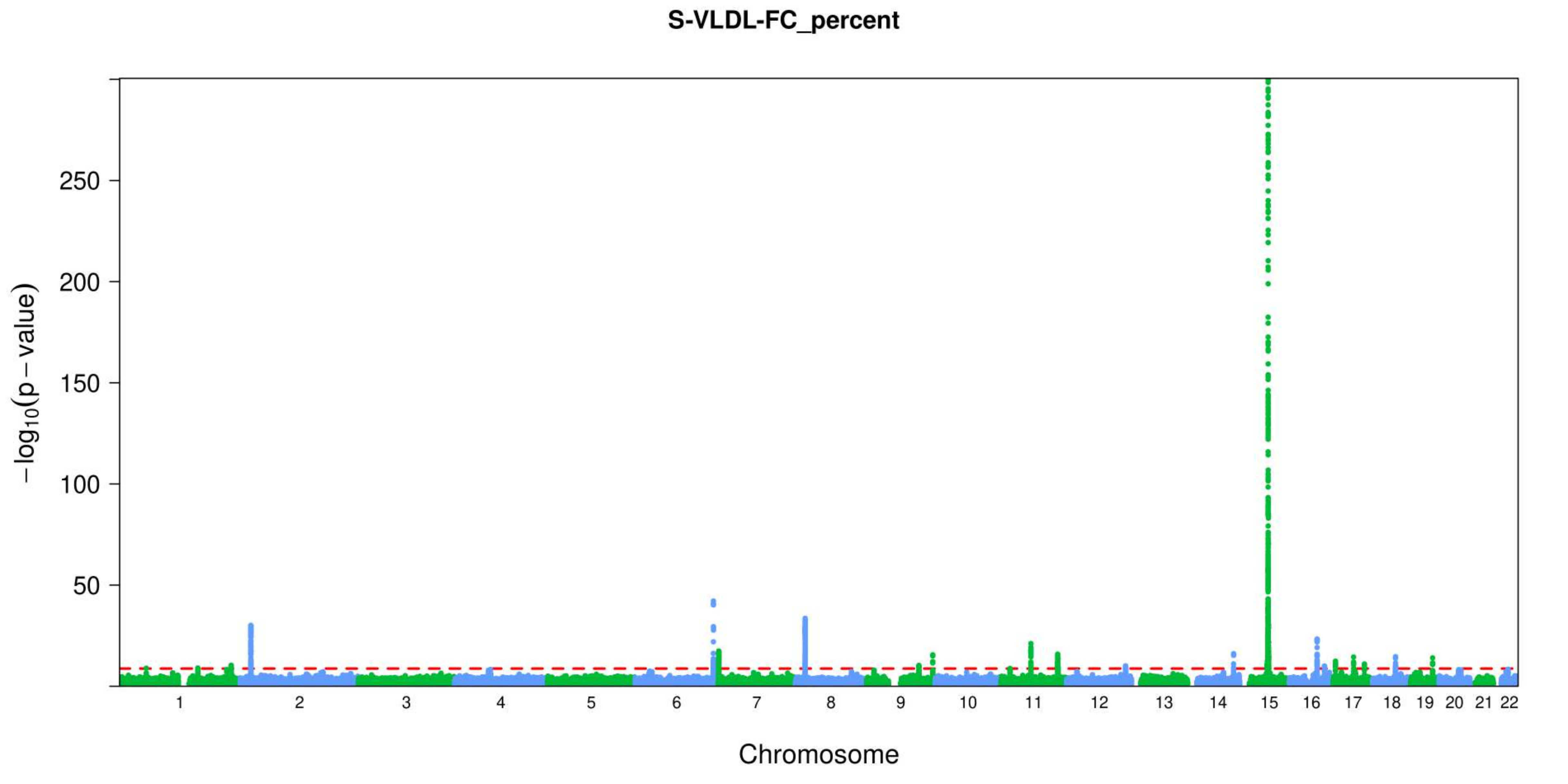
S-VLDL-FC_percent
$-\log_{10}(p-\text{value})$
250
200
150
100
50
1
2
3
4
5
6
7
8
9
10
11
12
13
14
15
16
17
18
19
20
21
22
Chromosome

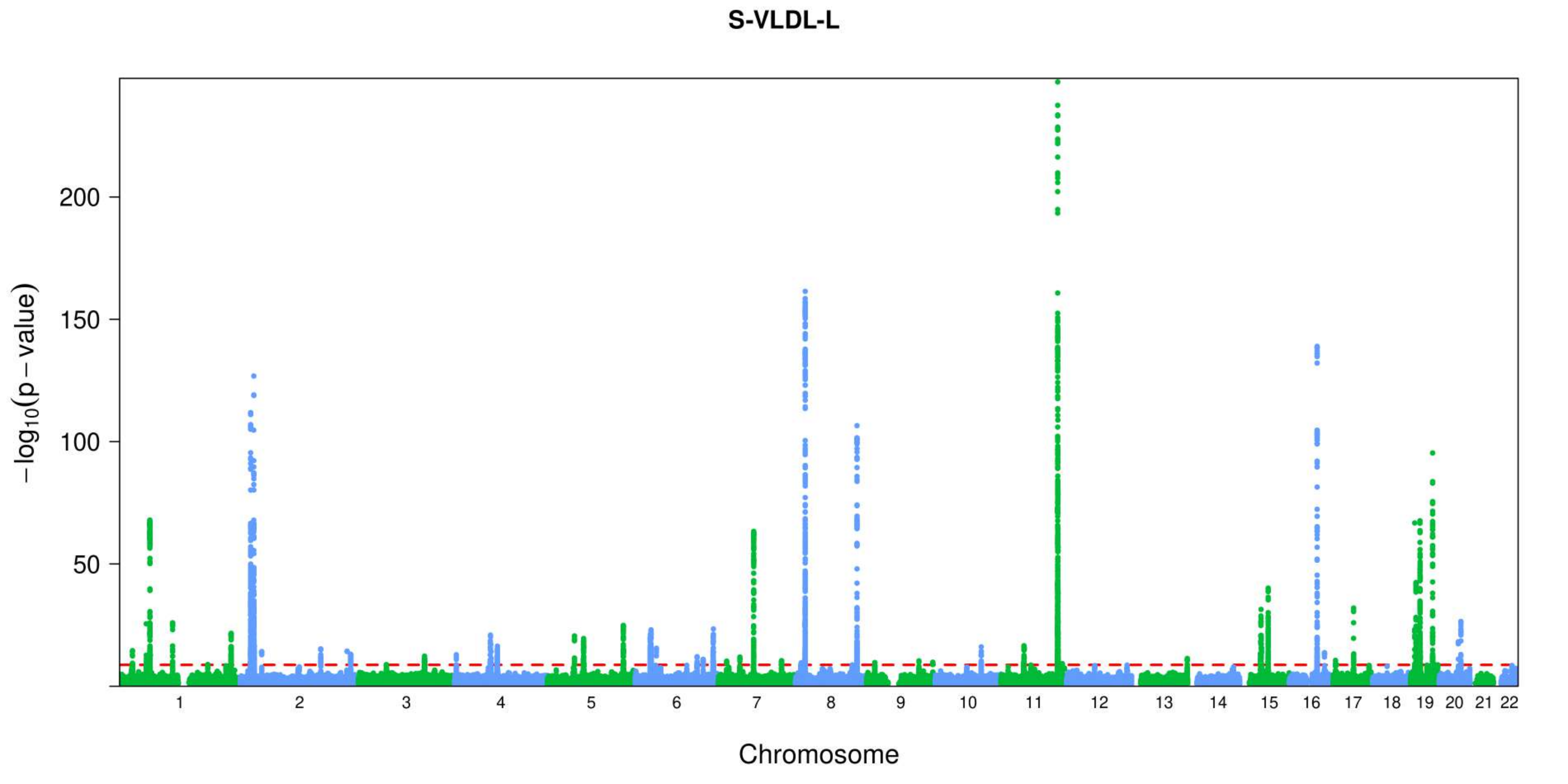

S-VLDL-L
200
150
100
50
$-\log_{10}(p-\text{value})$
1
2
3
4
5
6
7
8
9
10
11
12
13
14
15
16
17
18
19
20
21
22
Chromosome

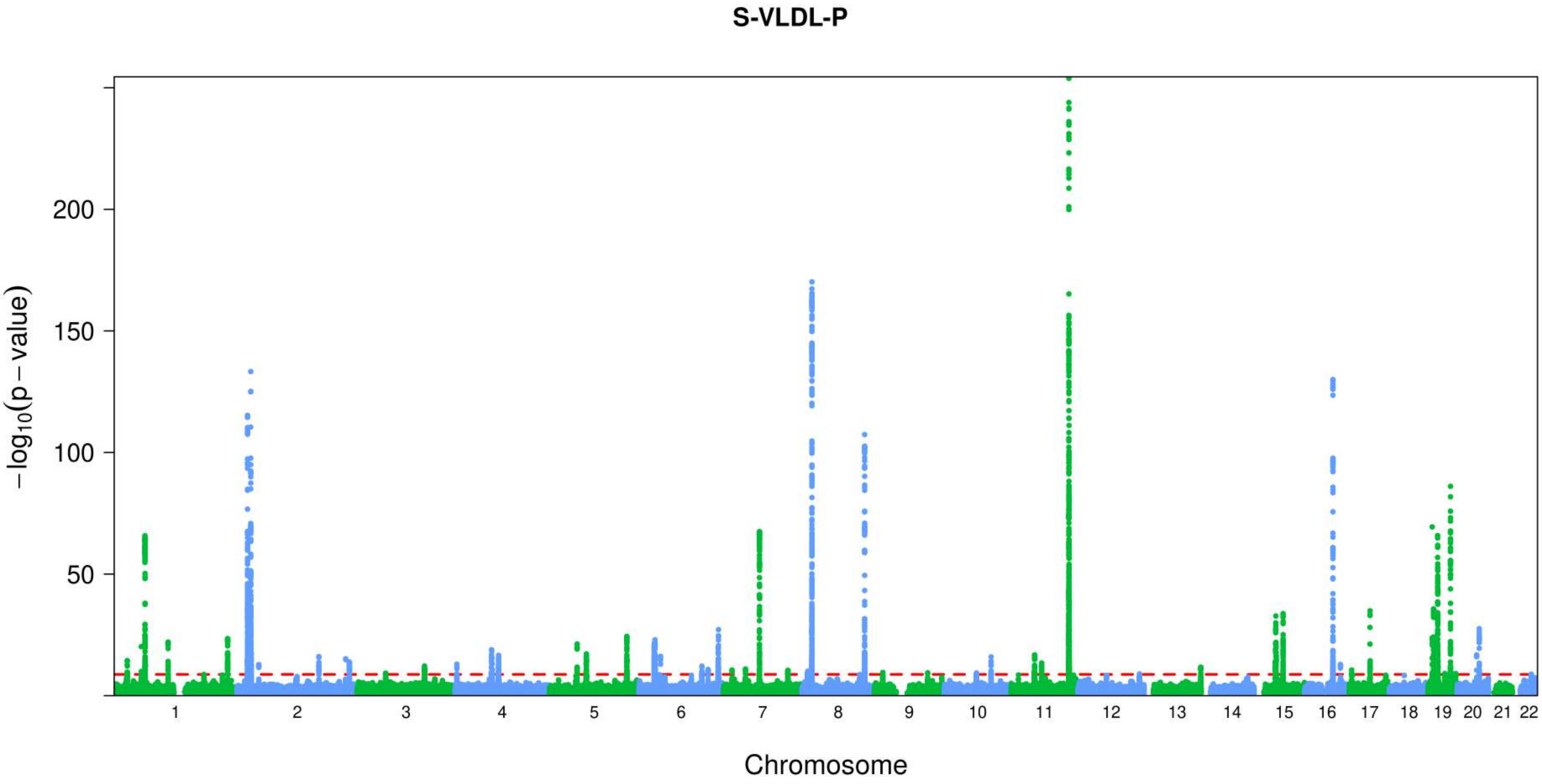
S-VLDL-P
−log10(p − value)
200
150
100
50
1
2
3
4
5
6
7
8
9
10
11
12
13
14
15
16
17
18
19
20
21
22
Chromosome

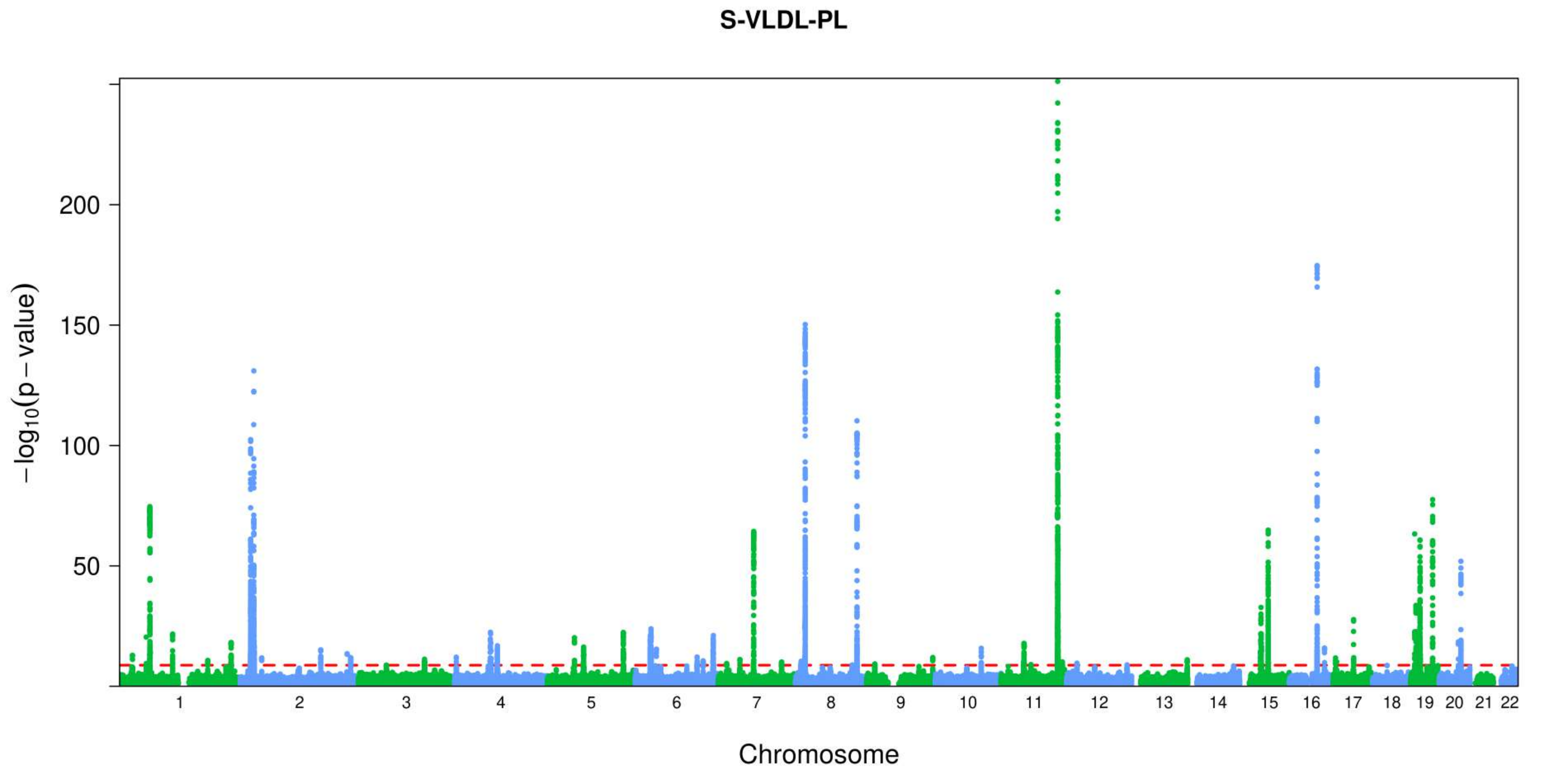

S-VLDL-PL
−log10(p − value)
200
150
100
50
Chromosome
1
2
3
4
5
6
7
8
9
10
11
12
13
14
15
16
17
18
19
20
21
22

S-VLDL-PL_percent

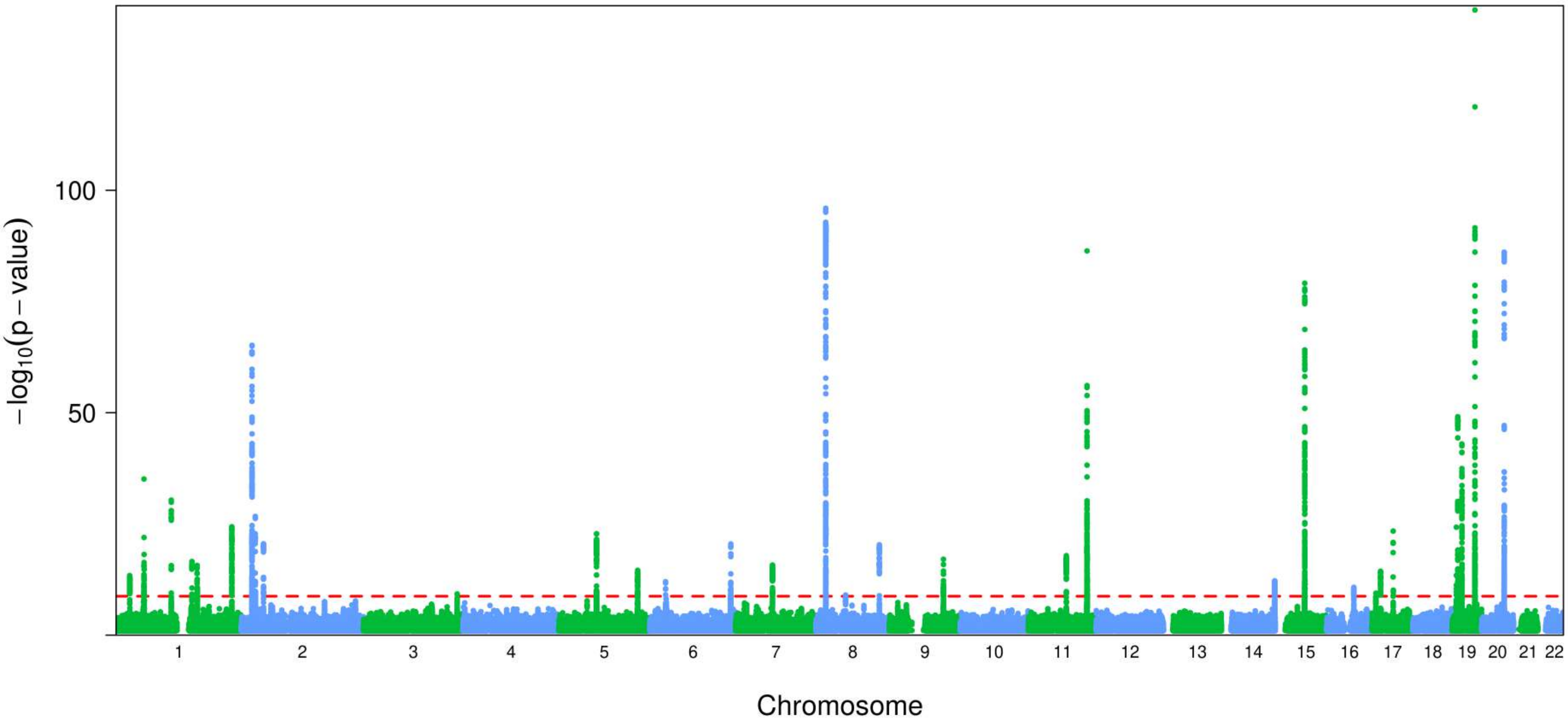

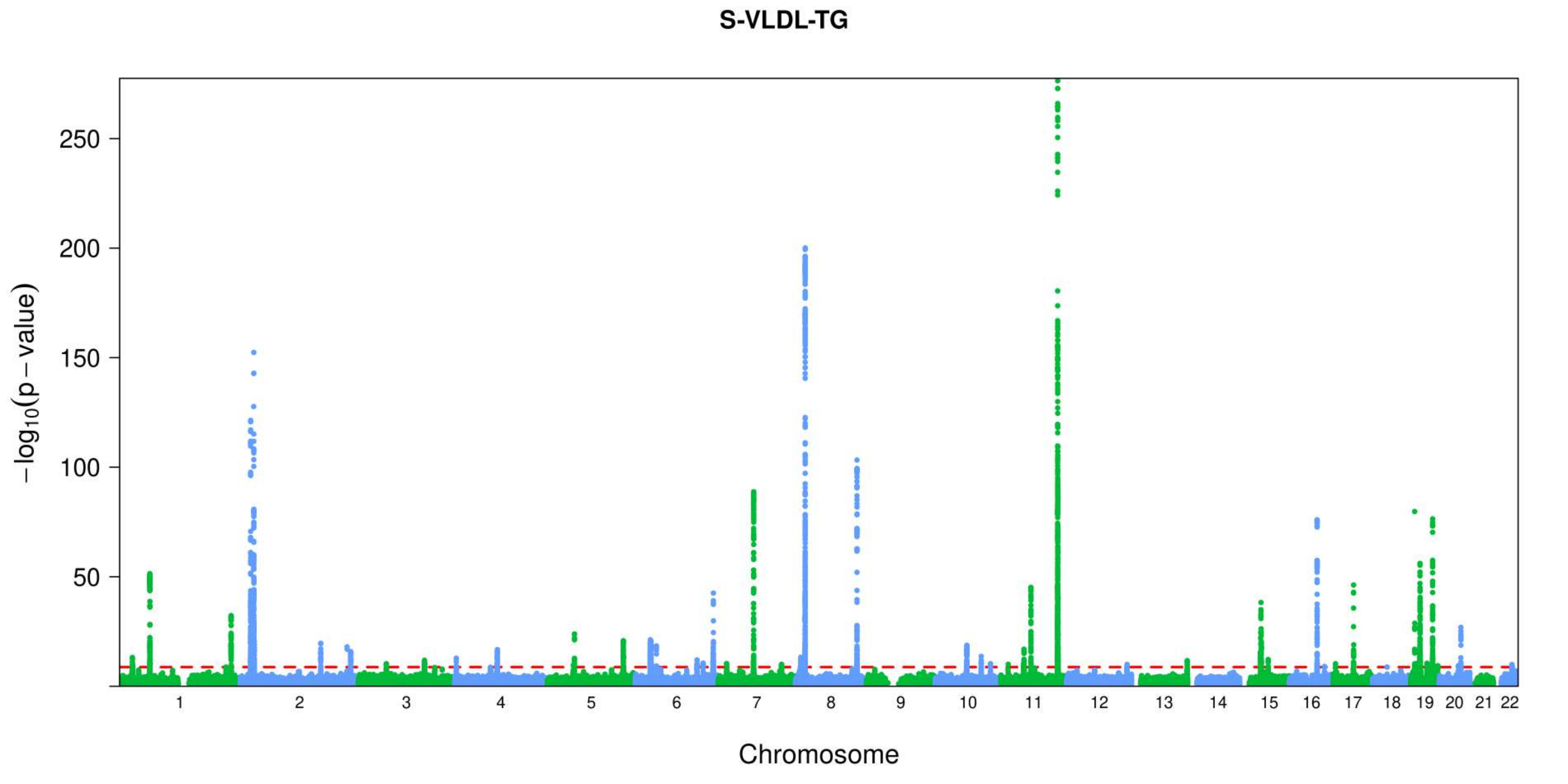
S-VLDL-TG
−log10(p − value)
250
200
150
100
50
Chromosome
1
2
3
4
5
6
7
8
9
10
11
12
13
14
15
16
17
18
19
20
21
22

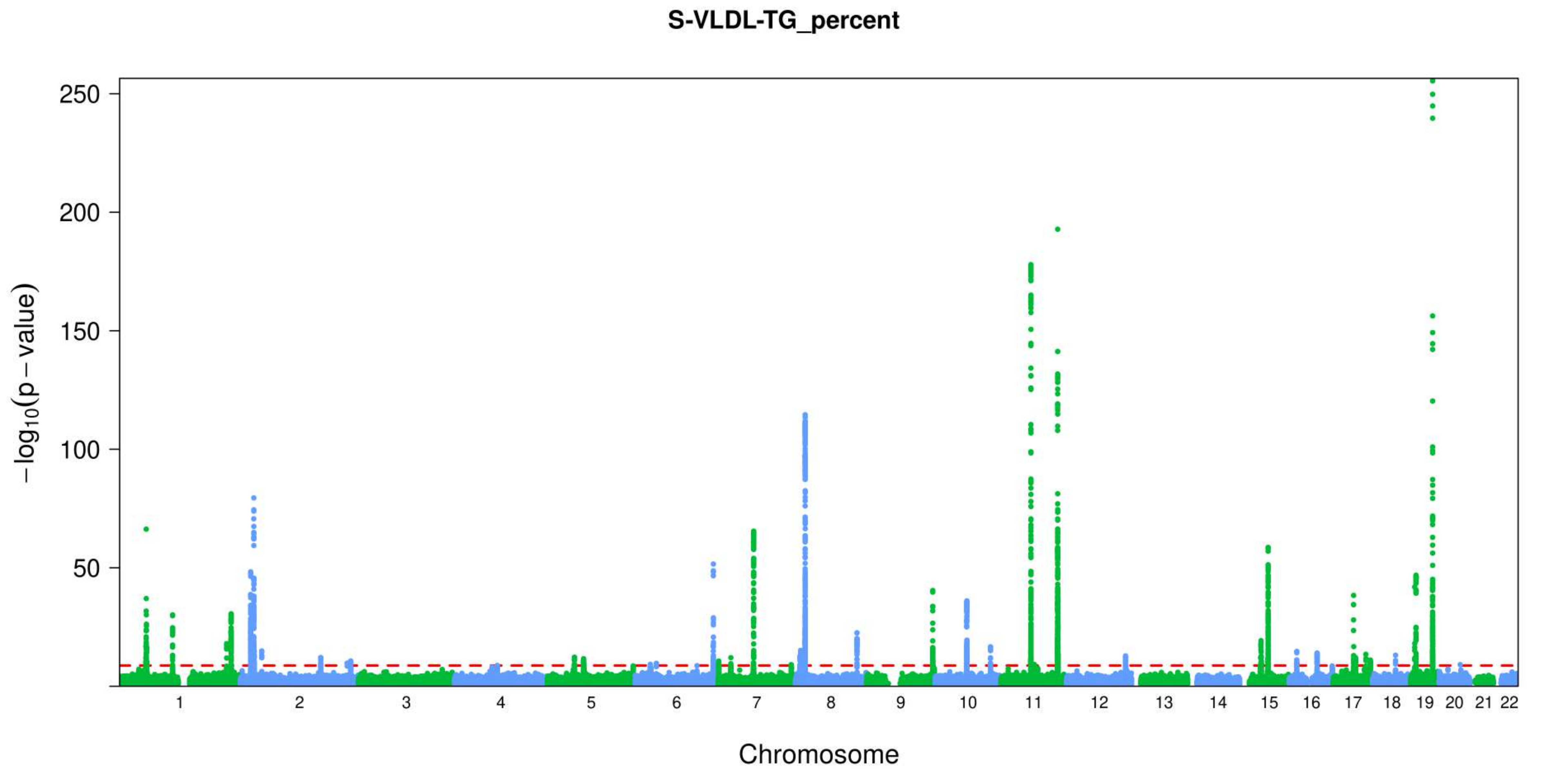
S-VLDL-TG_percent
250
200
150
100
50
-log10(p - value)
1
2
3
4
5
6
7
8
9
10
11
12
13
14
15
16
17
18
19
20
21
22
Chromosome

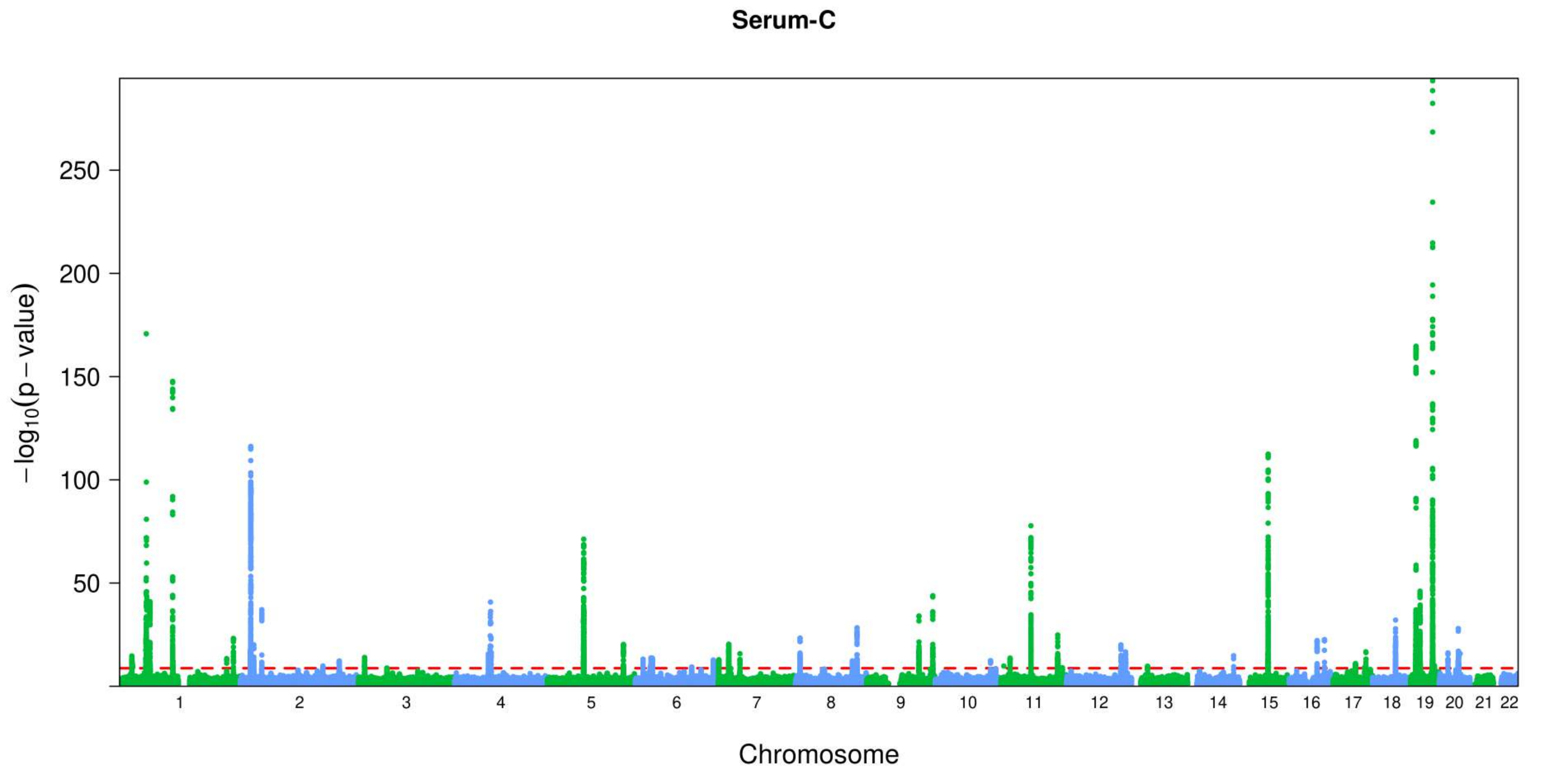
Serum-C
250
200
150
100
50
−log10(p − value)
1
2
3
4
5
6
7
8
9
10
11
12
13
14
15
16
17
18
19
20
21
22
Chromosome

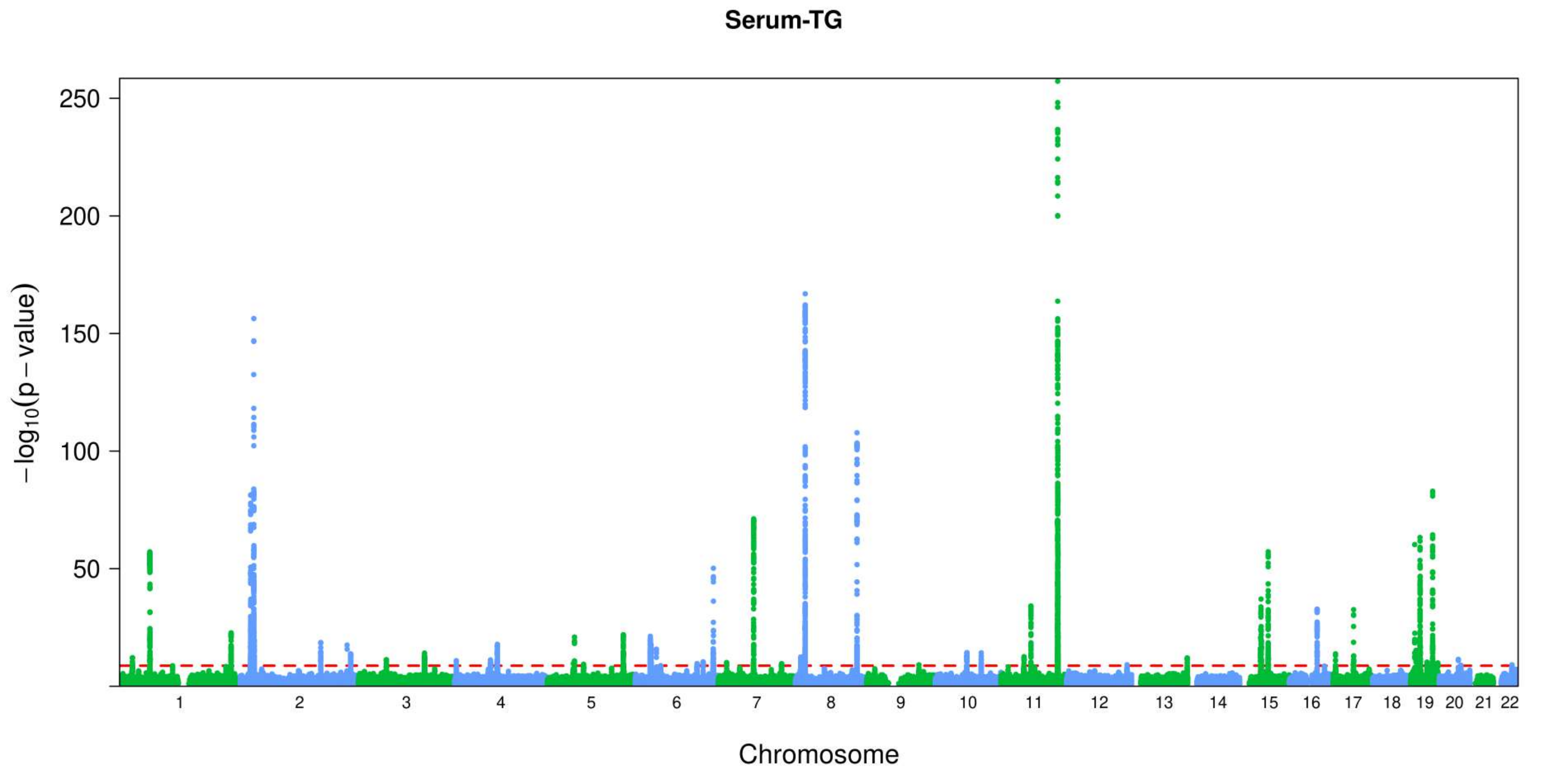

Serum-TG
250
200
150
100
50
−log10(p − value)
1
2
3
4
5
6
7
8
9
10
11
12
13
14
15
16
17
18
19
20
21
22
Chromosome

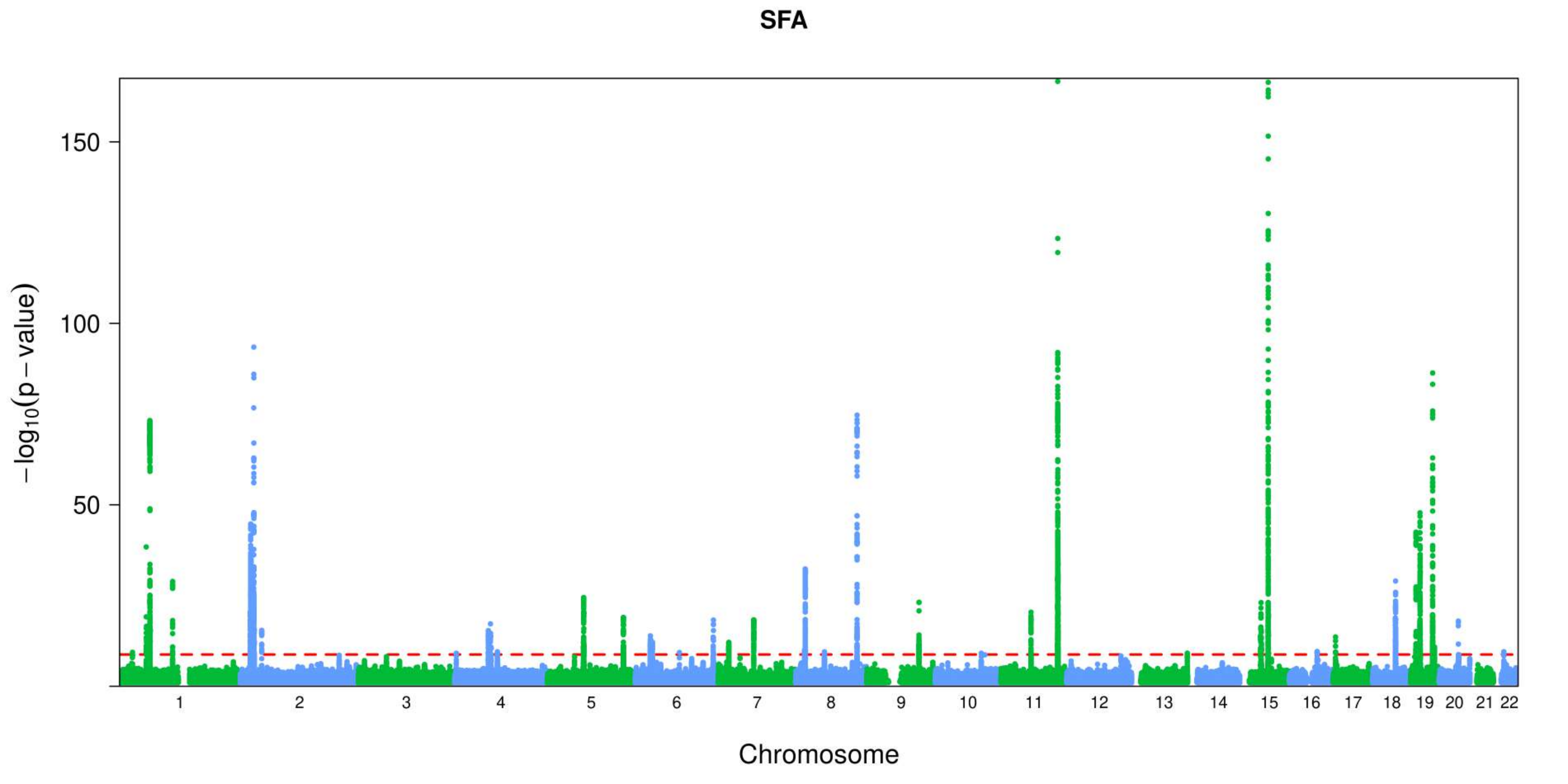
SFA
150
100
50
−log10(p − value)
1
2
3
4
5
6
7
8
9
10
11
12
13
14
15
16
17
18
19
20
21
22
Chromosome

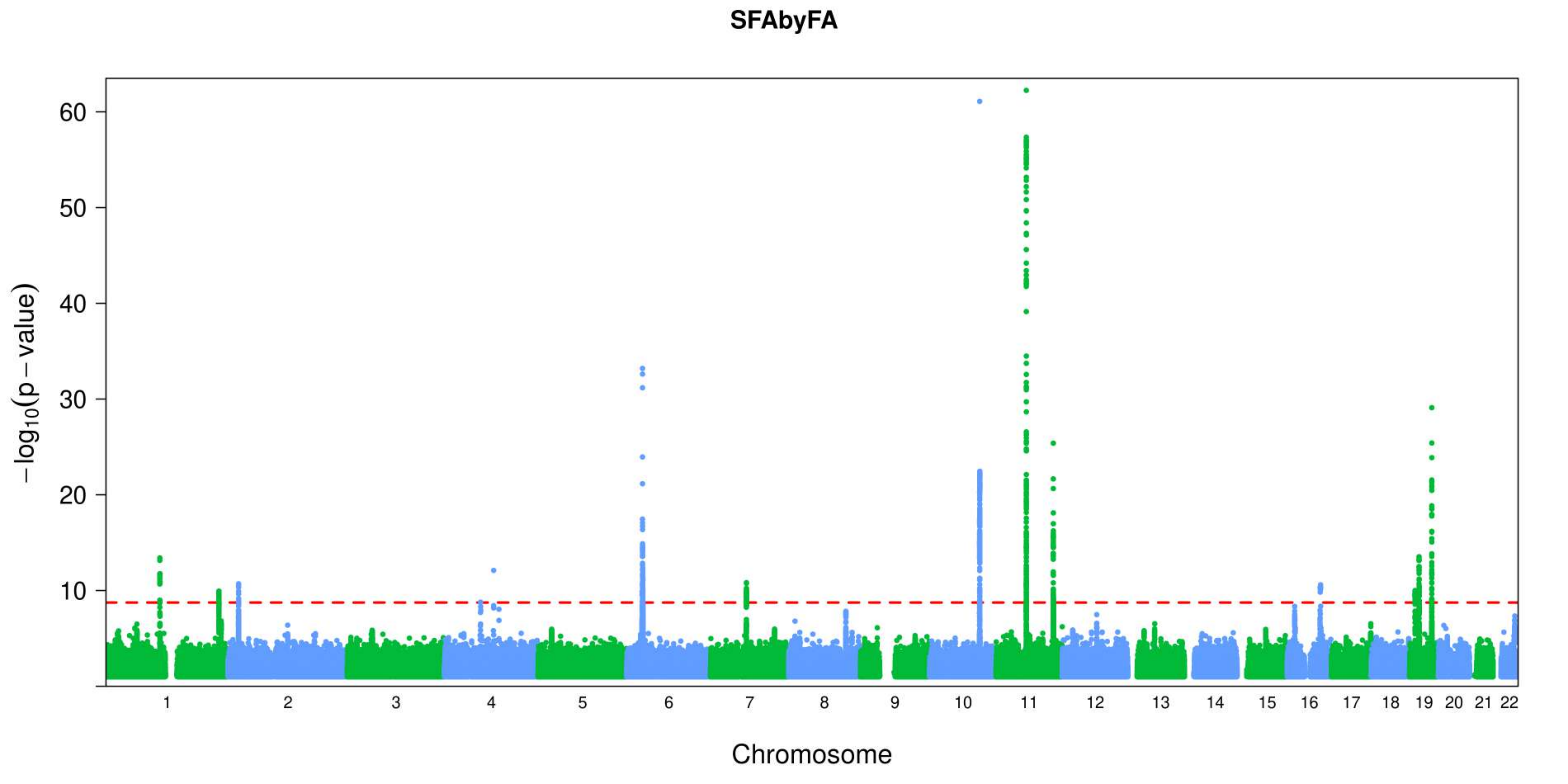
SFAbyFA
−log10(p − value)
60
50
40
30
20
10
1
2
3
4
5
6
7
8
9
10
11
12
13
14
15
16
17
18
19
20
21
22
Chromosome

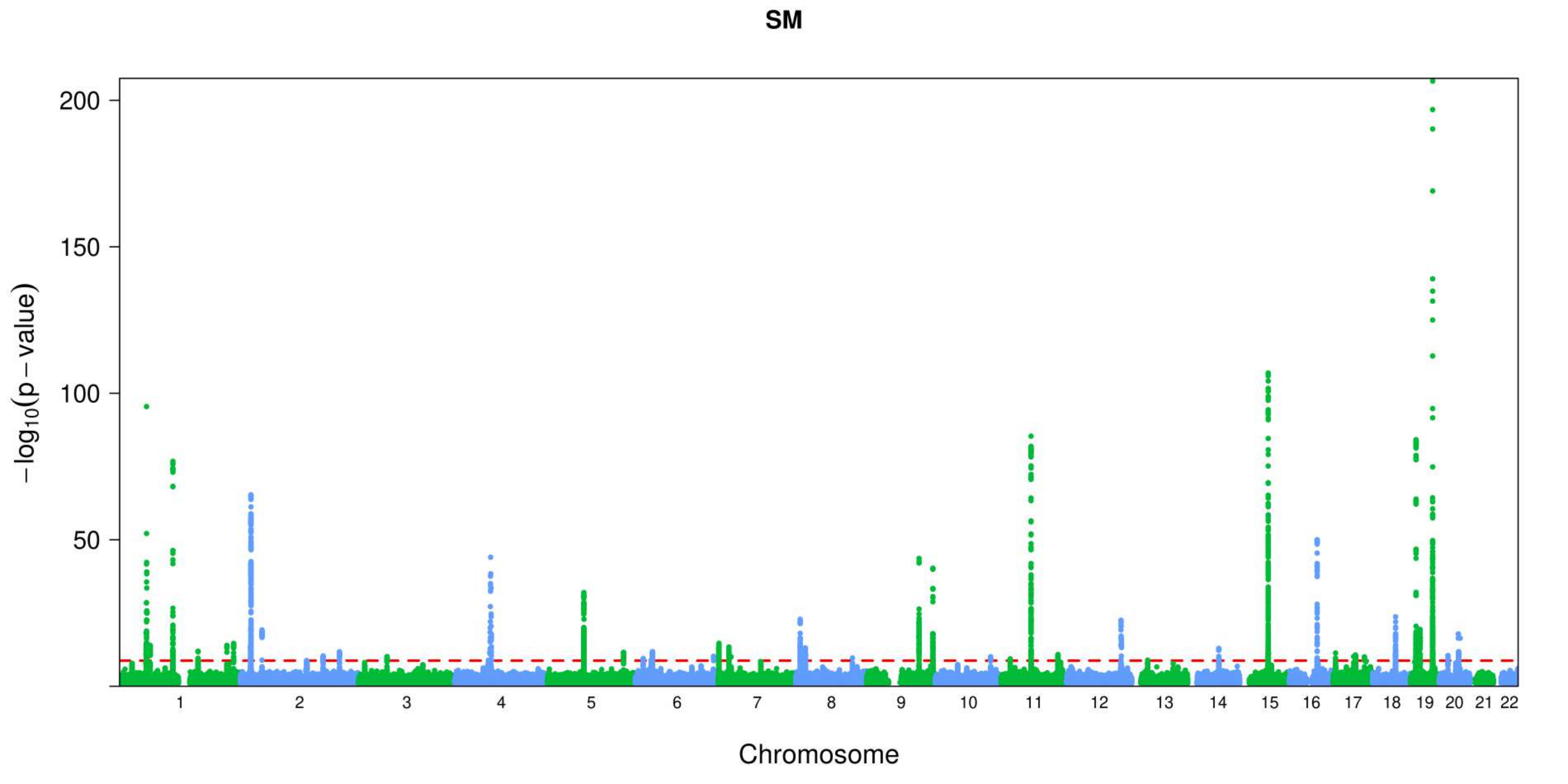
SM
200
150
100
50
−log10(p − value)
1
2
3
4
5
6
7
8
9
10
11
12
13
14
15
16
17
18
19
20
21
22
Chromosome

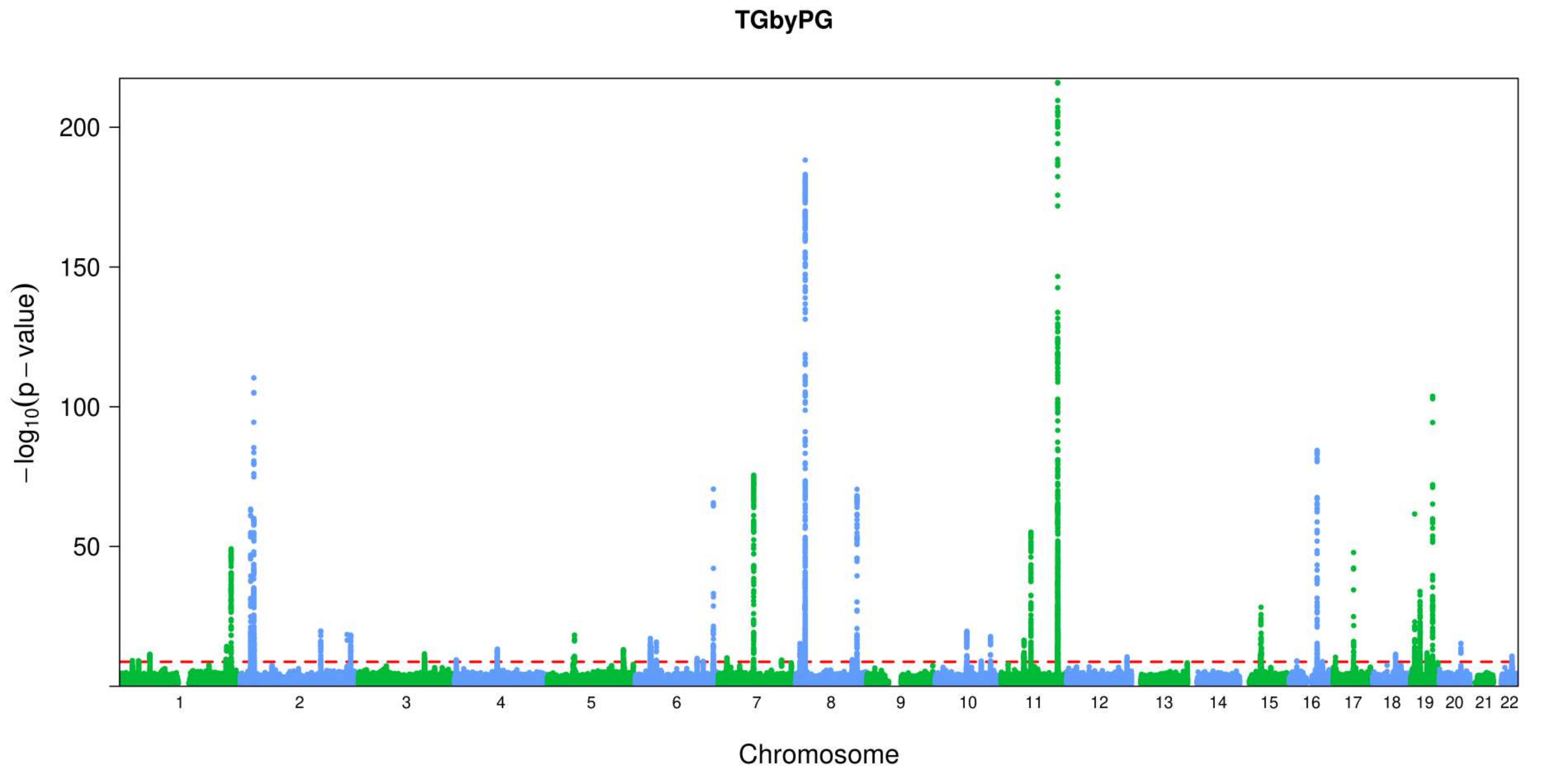
TGbyPG
−log10(p − value)
200
150
100
50
1 2 3 4 5 6 7 8 9 10 11 12 13 14 15 16 17 18 19 20 21 22
Chromosome

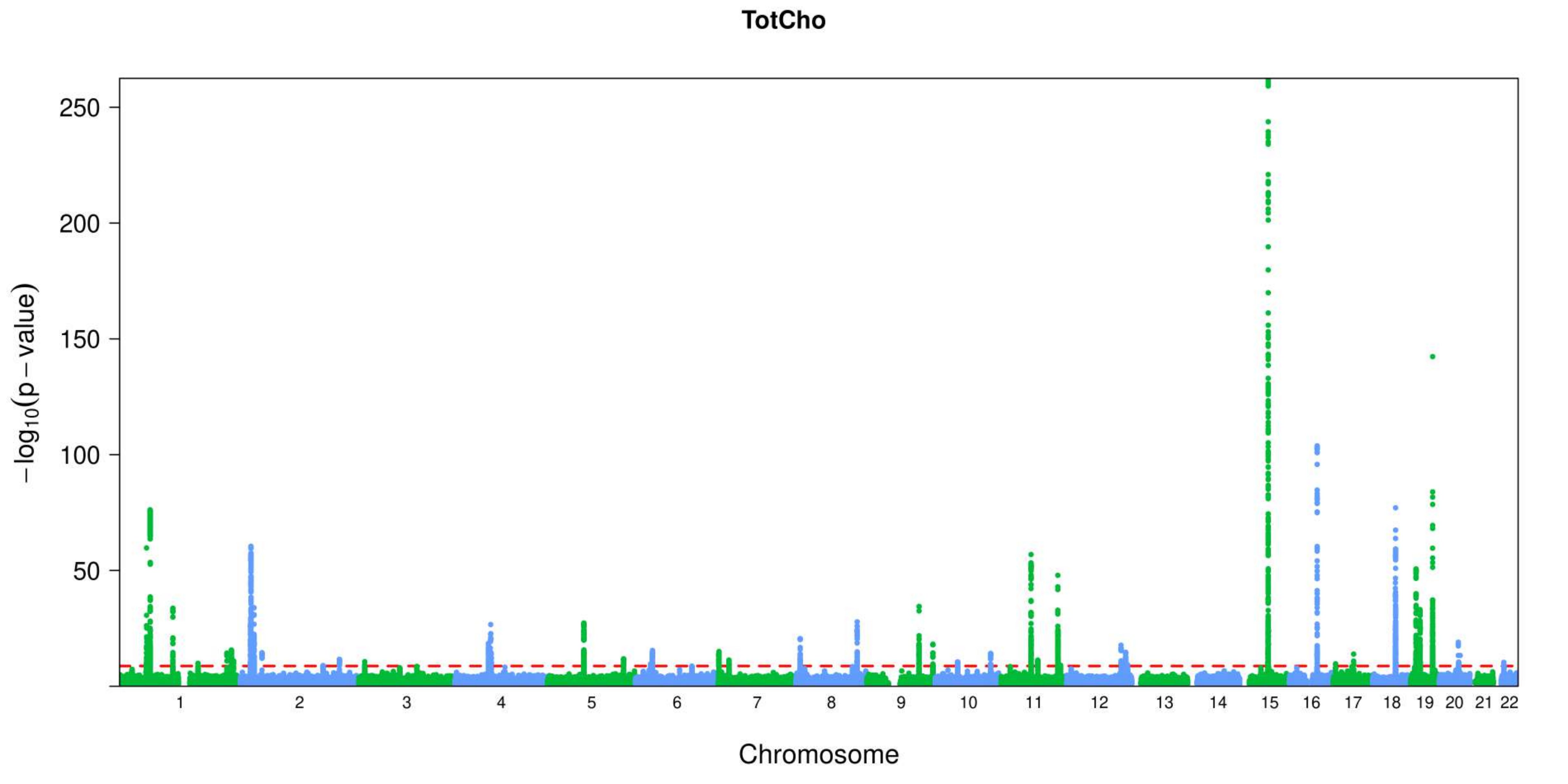
TotCho
$-\log_{10}$(p − value)
250
200
150
100
50
1
2
3
4
5
6
7
8
9
10
11
12
13
14
15
16
17
18
19
20
21
22
Chromosome

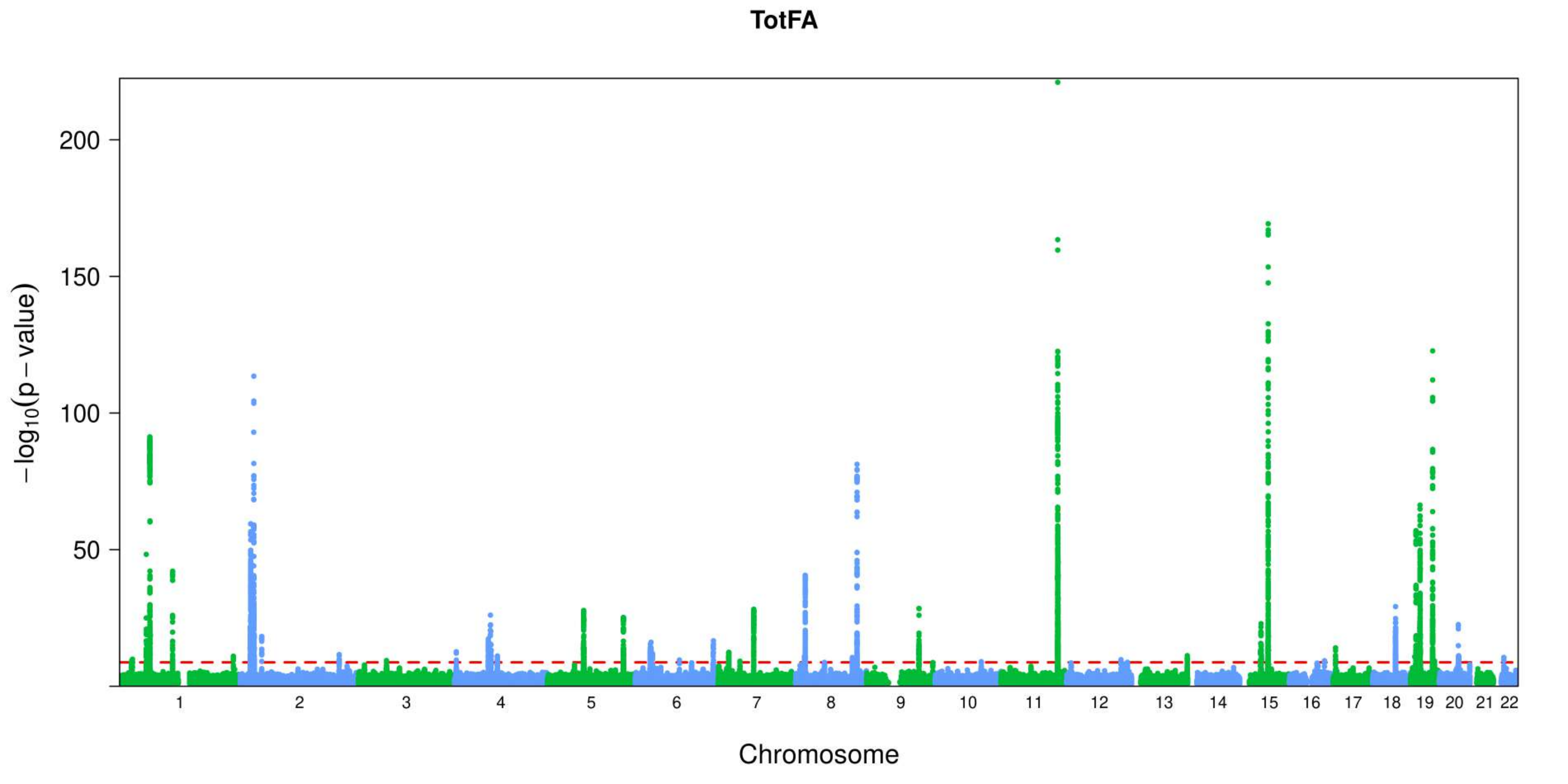
TotFA
−log10(p − value)
200
150
100
50
Chromosome
1
2
3
4
5
6
7
8
9
10
11
12
13
14
15
16
17
18
19
20
21
22

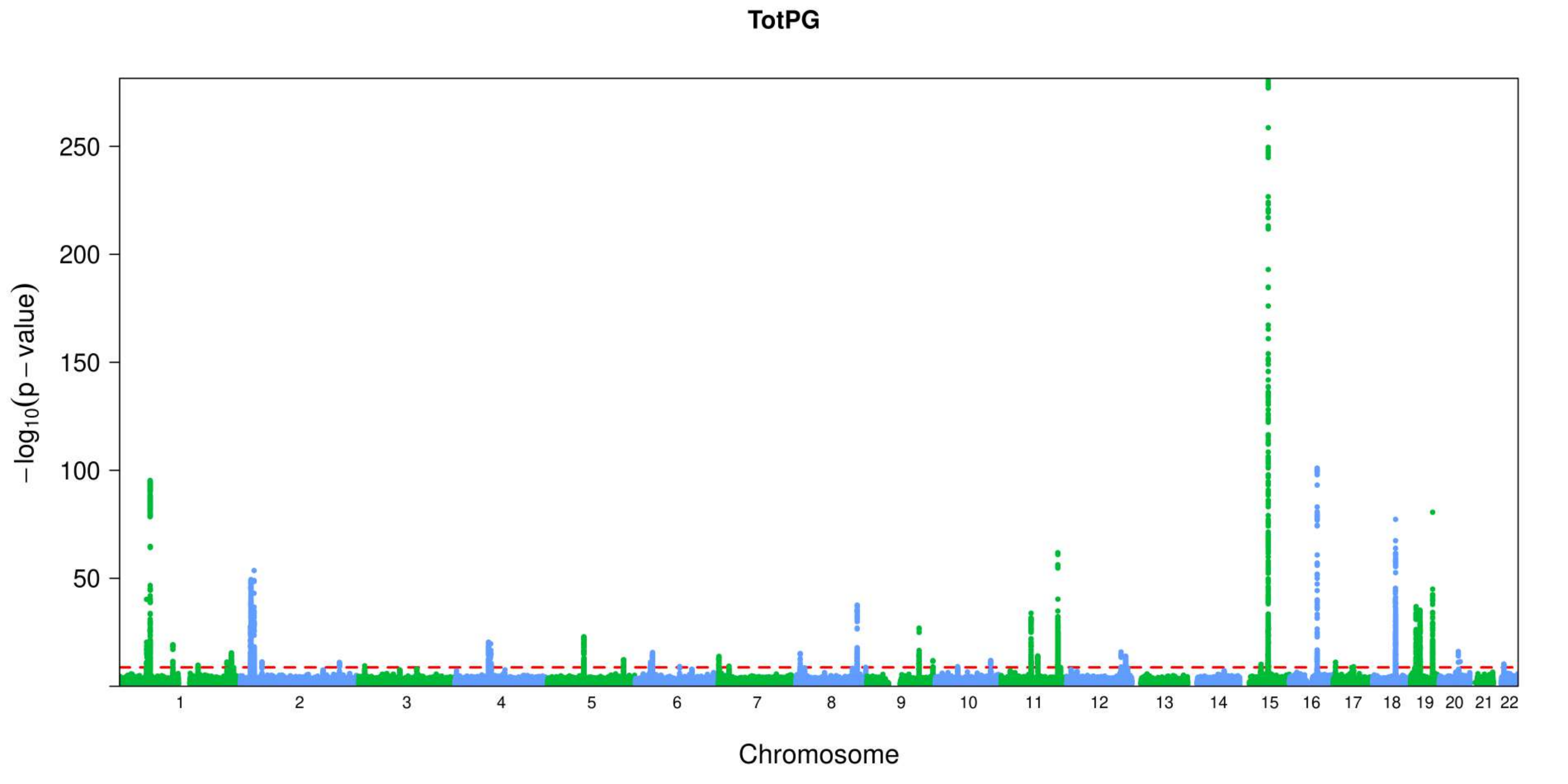
TotPG
−log10(p−value)
250
200
150
100
50
Chromosome
1
2
3
4
5
6
7
8
9
10
11
12
13
14
15
16
17
18
19
20
21
22

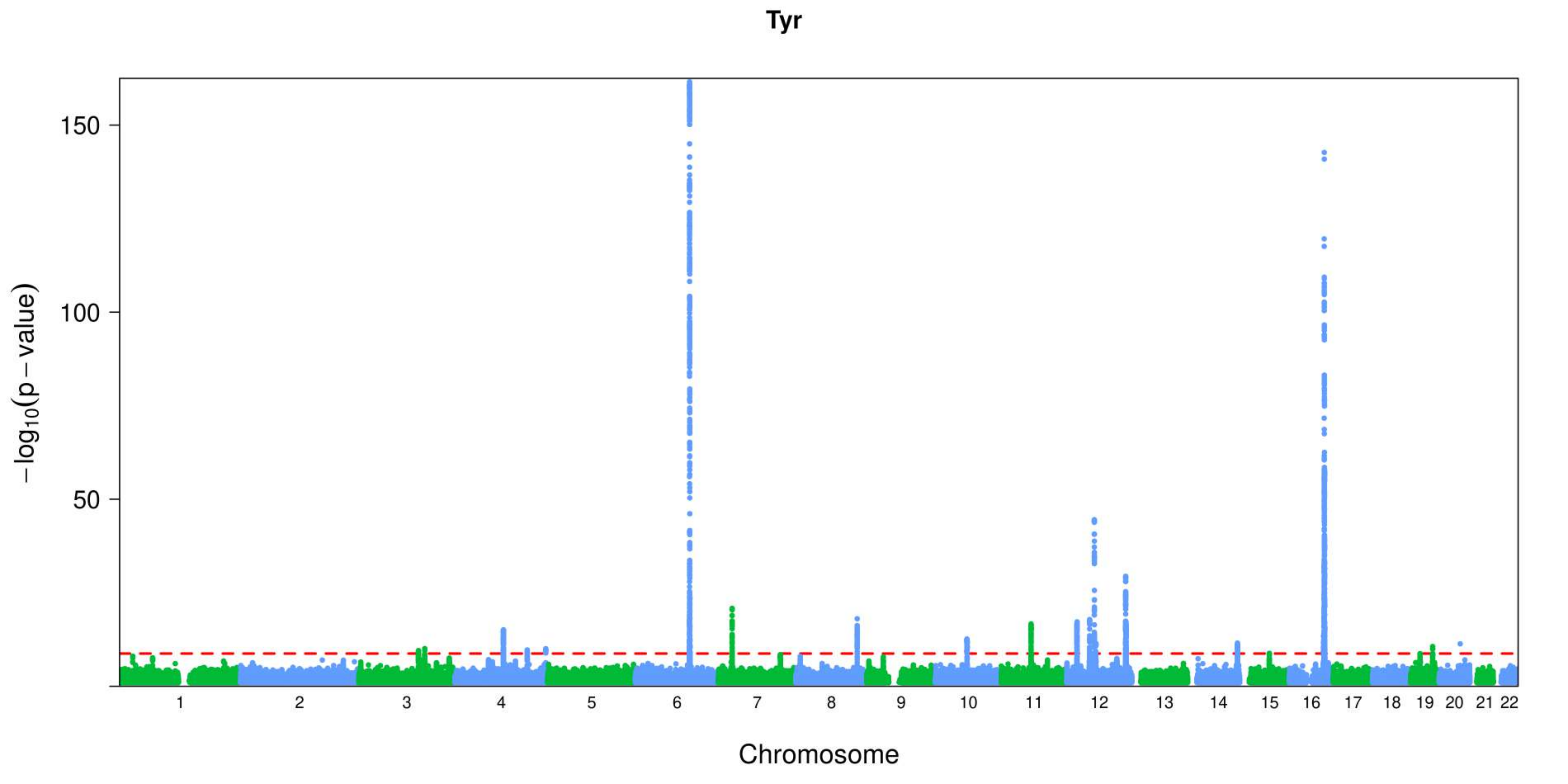
Tyr
150
100
50
−log10(p − value)
1
2
3
4
5
6
7
8
9
10
11
12
13
14
15
16
17
18
19
20
21
22
Chromosome

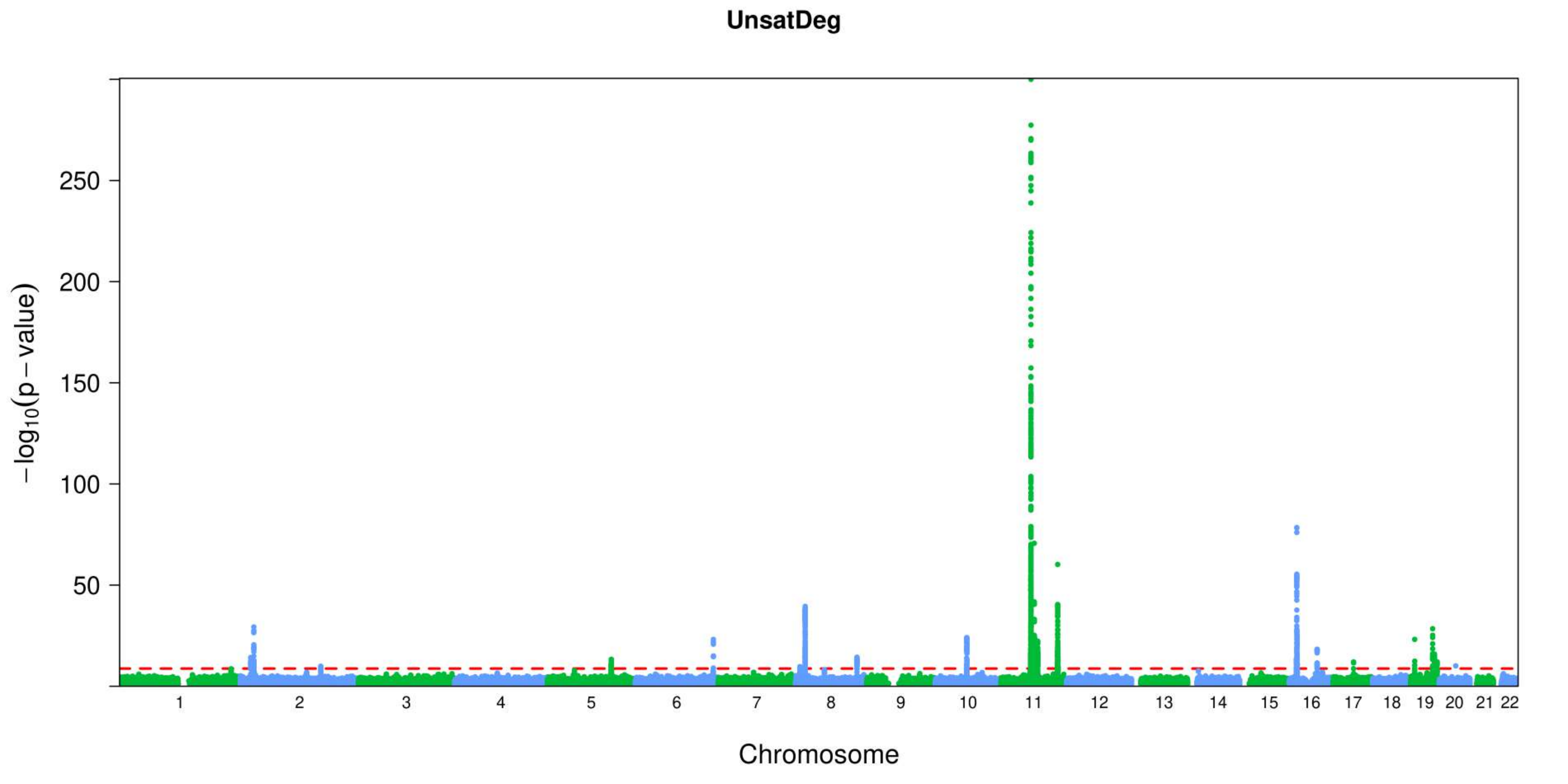
UnsatDeg
250
200
150
100
50
$-\log_{10}(p-value)$
1
2
3
4
5
6
7
8
9
10
11
12
13
14
15
16
17
18
19
20
21
22
Chromosome

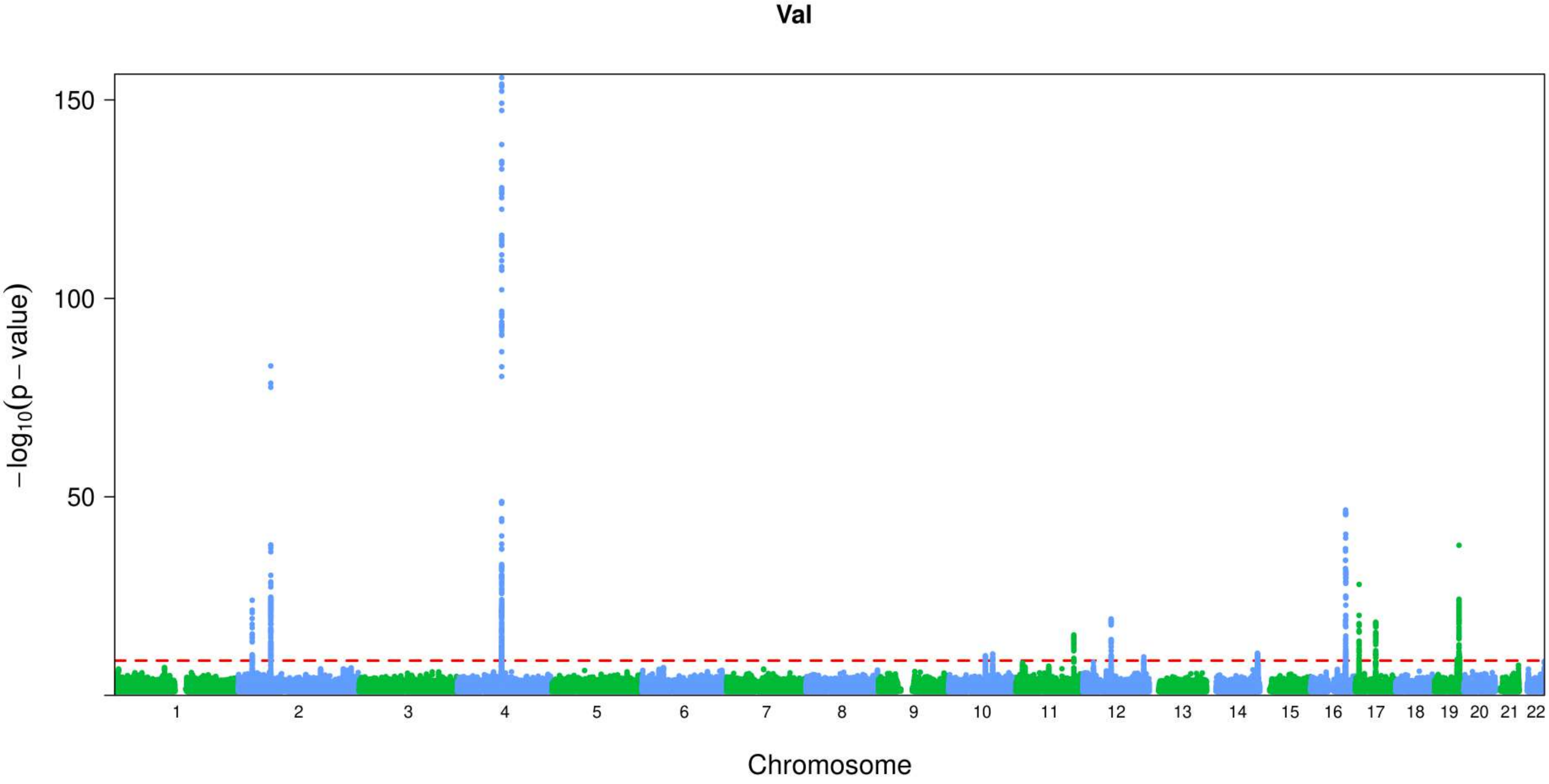

Val
150
100
50
−log10(p − value)
1
2
3
4
5
6
7
8
9
10
11
12
13
14
15
16
17
18
19
20
21
22
Chromosome

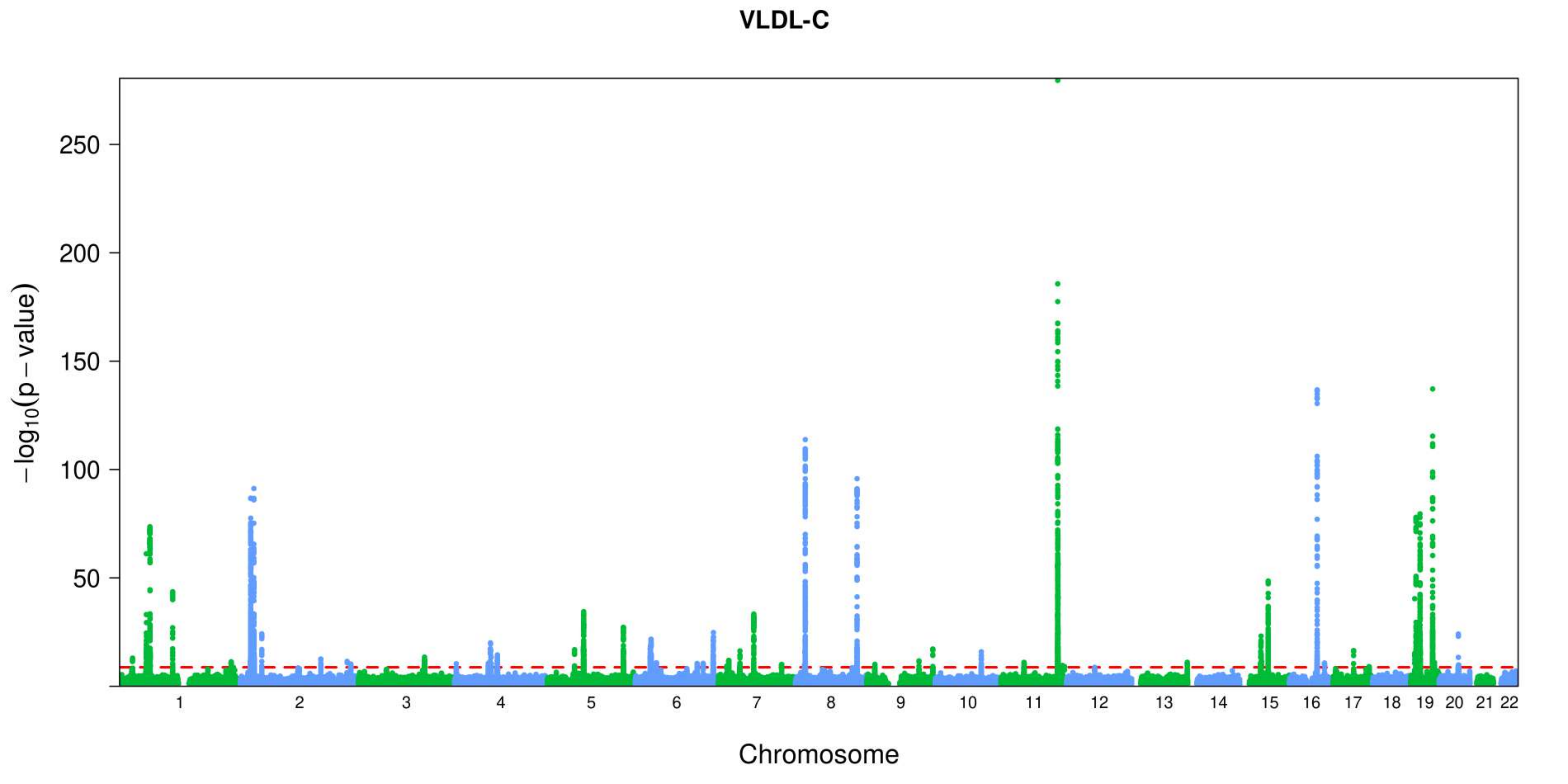
VLDL-C
250
200
150
100
50
−log10(p − value)
1
2
3
4
5
6
7
8
9
10
11
12
13
14
15
16
17
18
19
20
21
22
Chromosome

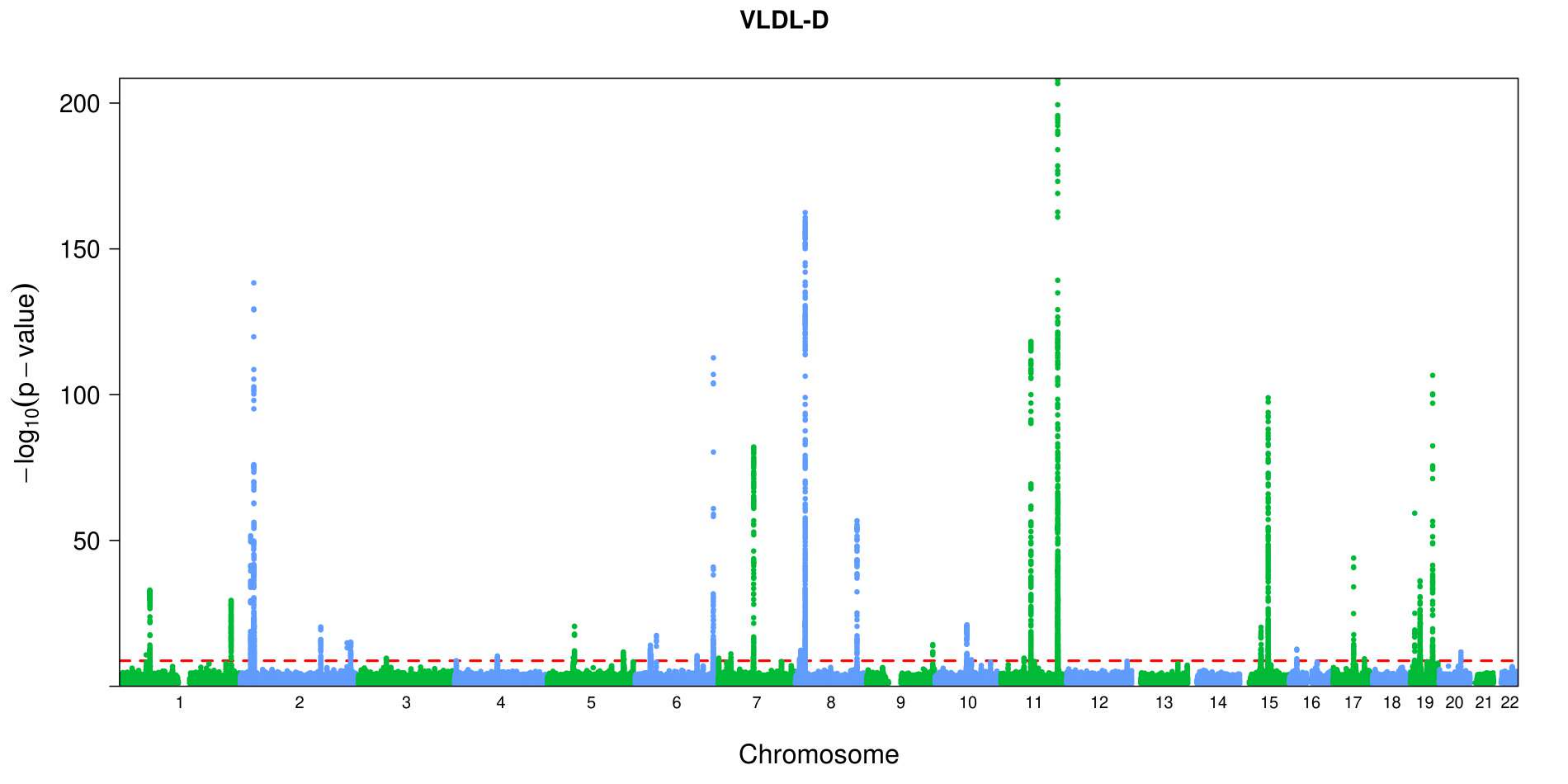
VLDL-D
200
150
100
50
$-\log_{10}(p-\text{value})$
1
2
3
4
5
6
7
8
9
10
11
12
13
14
15
16
17
18
19
20
21
22
Chromosome

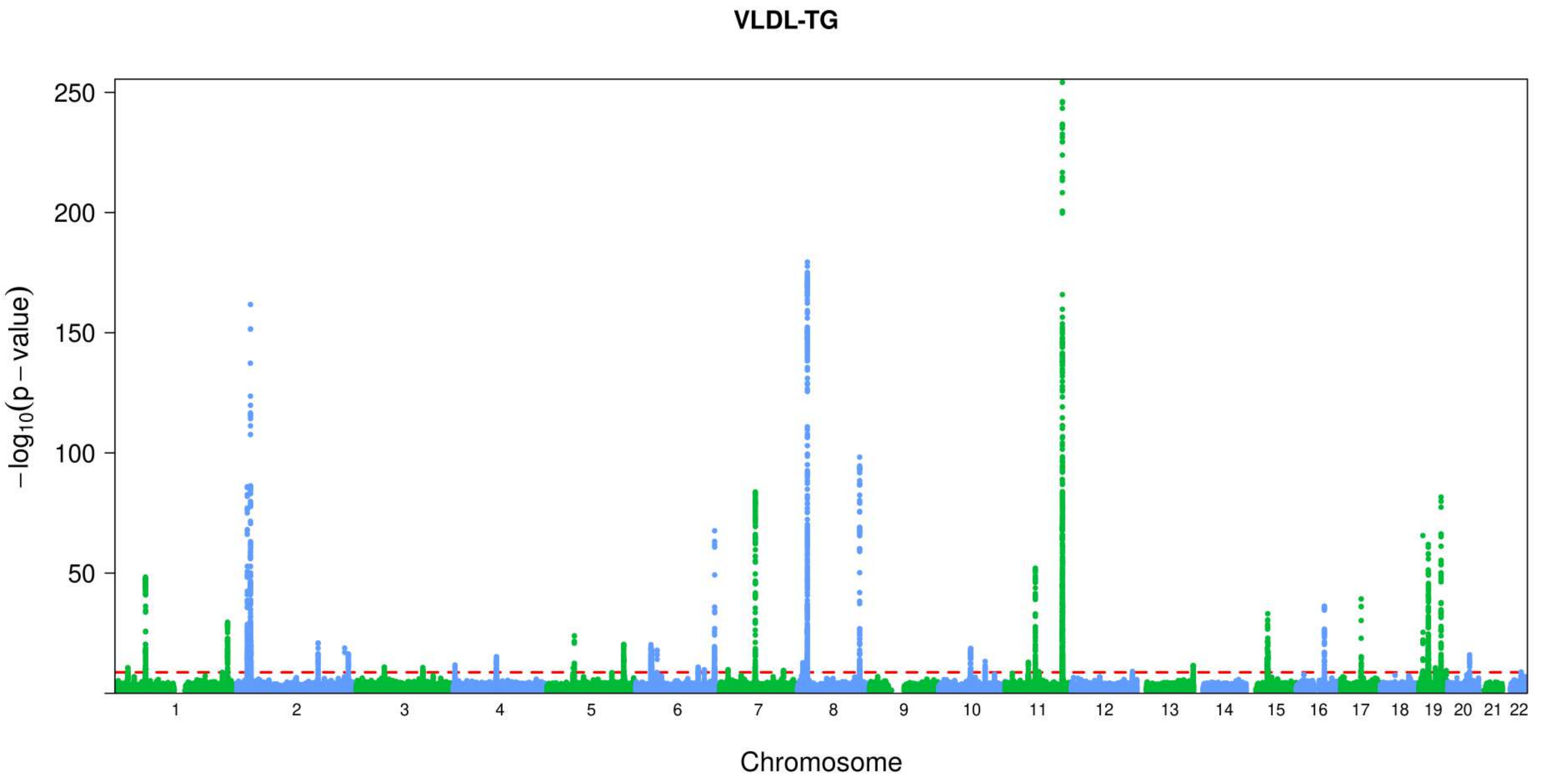
VLDL-TG
−log10(p − value)
250
200
150
100
50
Chromosome
1
2
3
4
5
6
7
8
9
10
11
12
13
14
15
16
17
18
19
20
21
22

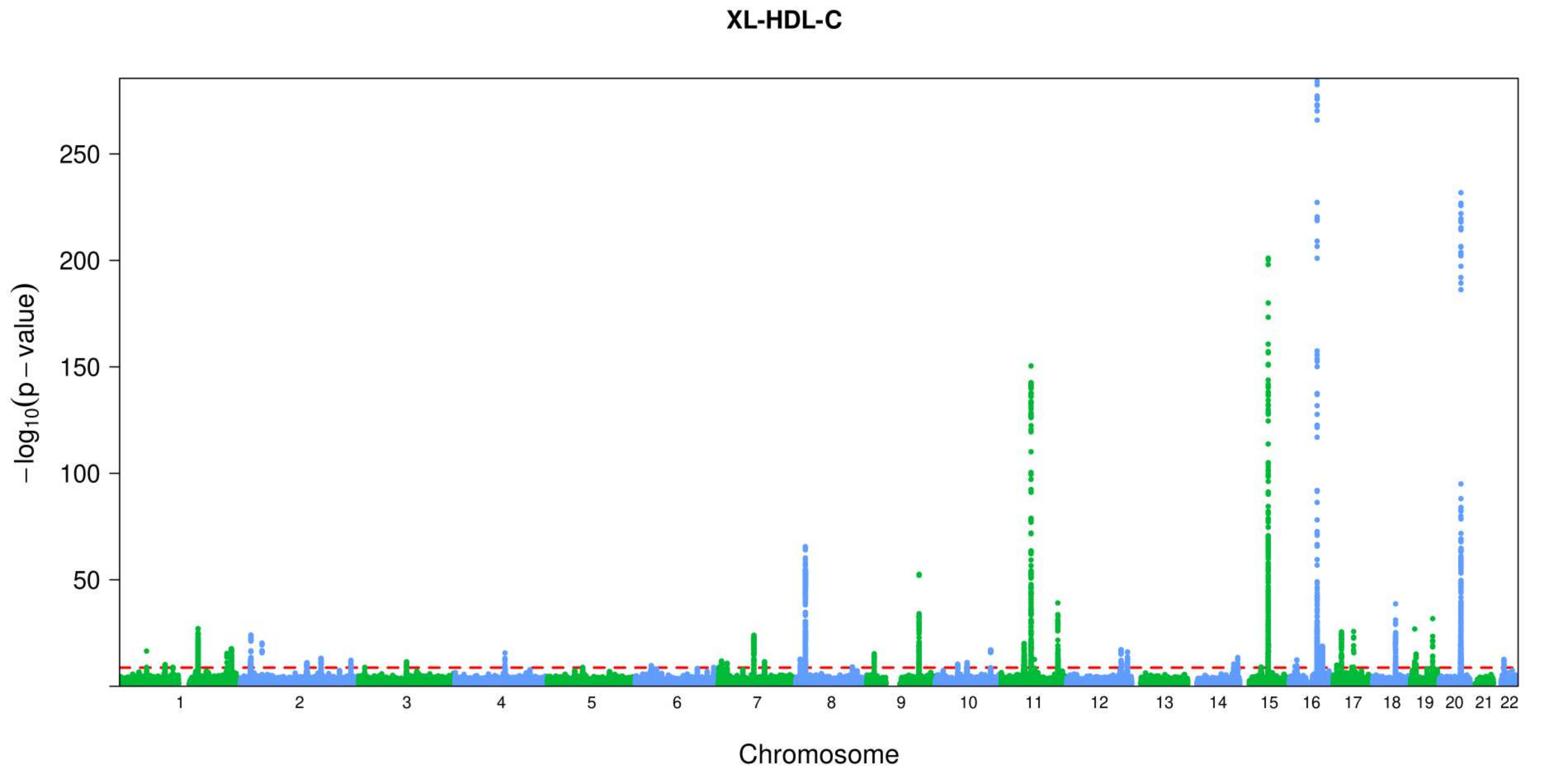
XL-HDL-C
250
200
150
100
50
−log10(p − value)
1
2
3
4
5
6
7
8
9
10
11
12
13
14
15
16
17
18
19
20
21
22
Chromosome

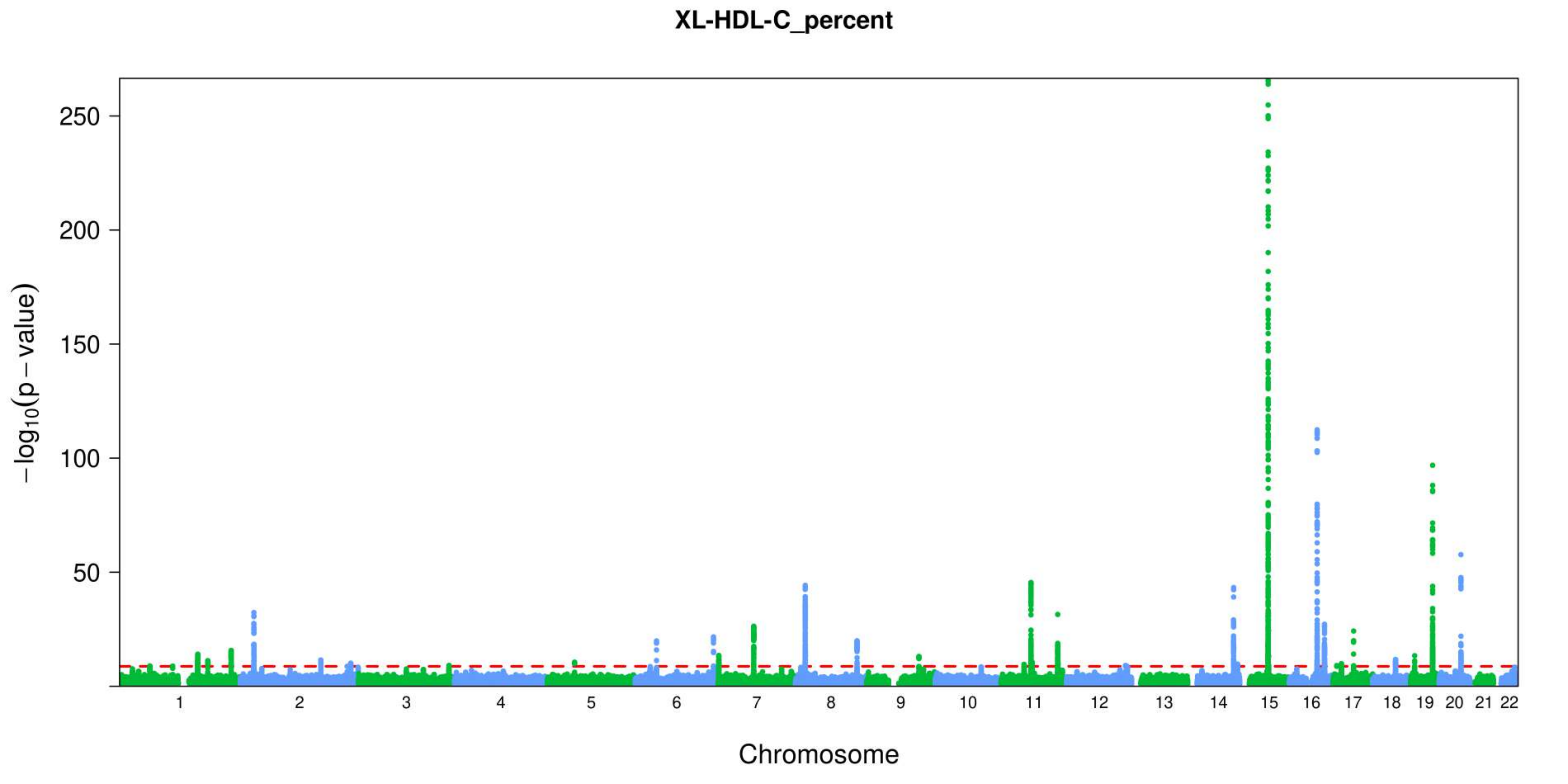
XL-HDL-C_percent
$-\log_{10}(p-\text{value})$
250
200
150
100
50
1
2
3
4
5
6
7
8
9
10
11
12
13
14
15
16
17
18
19
20
21
22
Chromosome

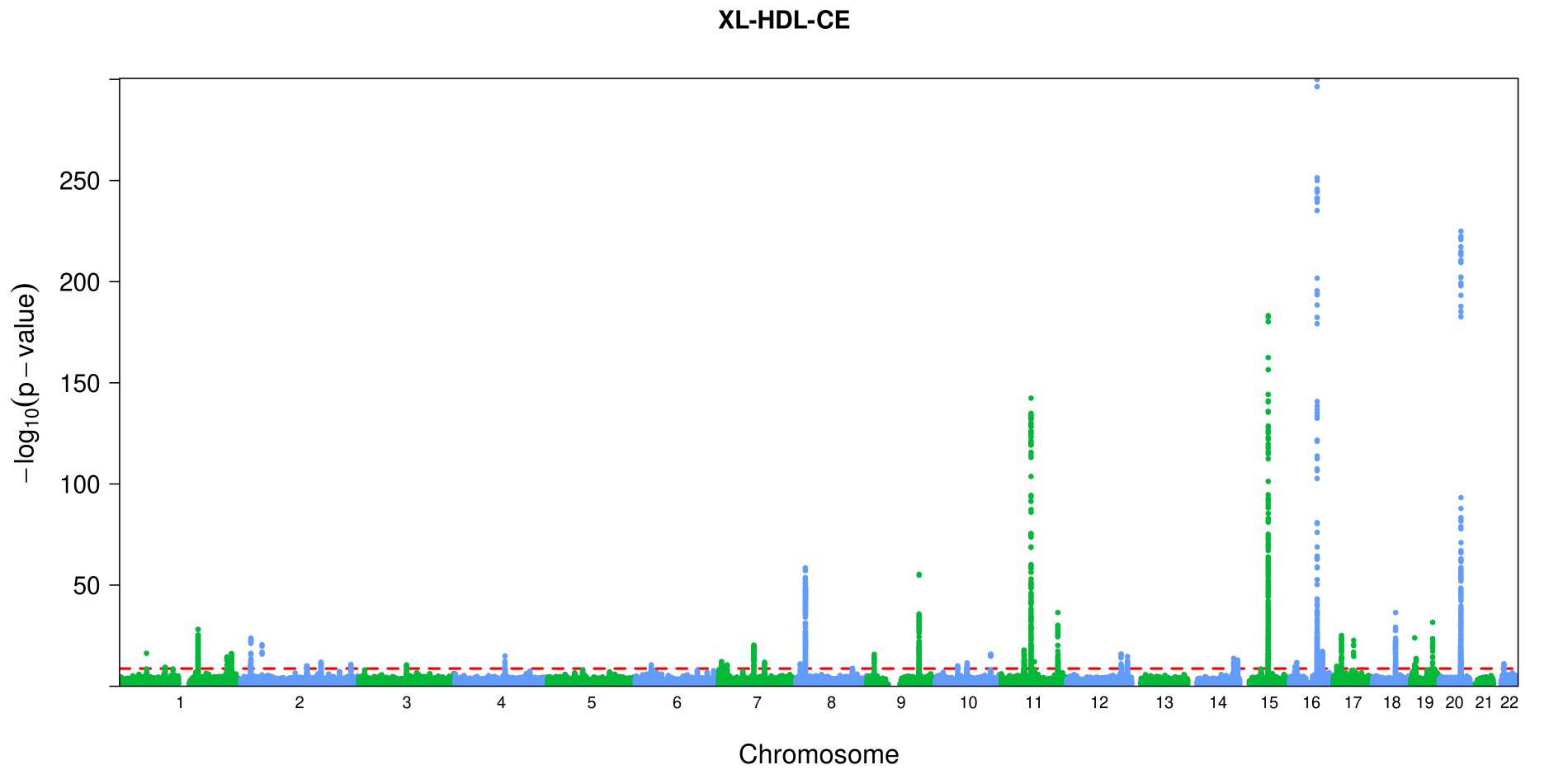
XL-HDL-CE
−log10(p − value)
250
200
150
100
50
Chromosome
1
2
3
4
5
6
7
8
9
10
11
12
13
14
15
16
17
18
19
20
21
22

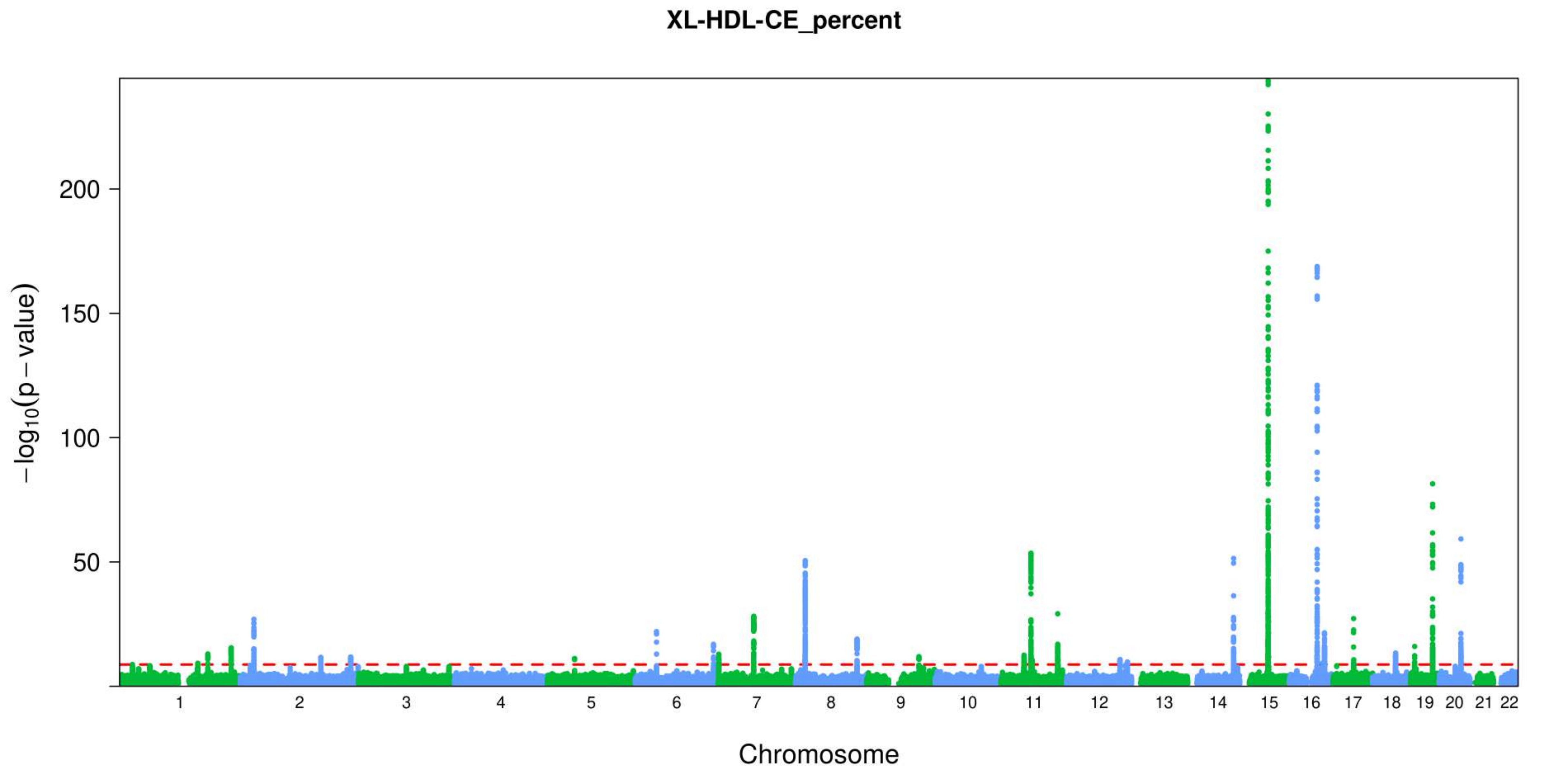

XL-HDL-CE_percent
200
150
100
50
$-\log_{10}(p-\text{value})$
1
2
3
4
5
6
7
8
9
10
11
12
13
14
15
16
17
18
19
20
21
22
Chromosome

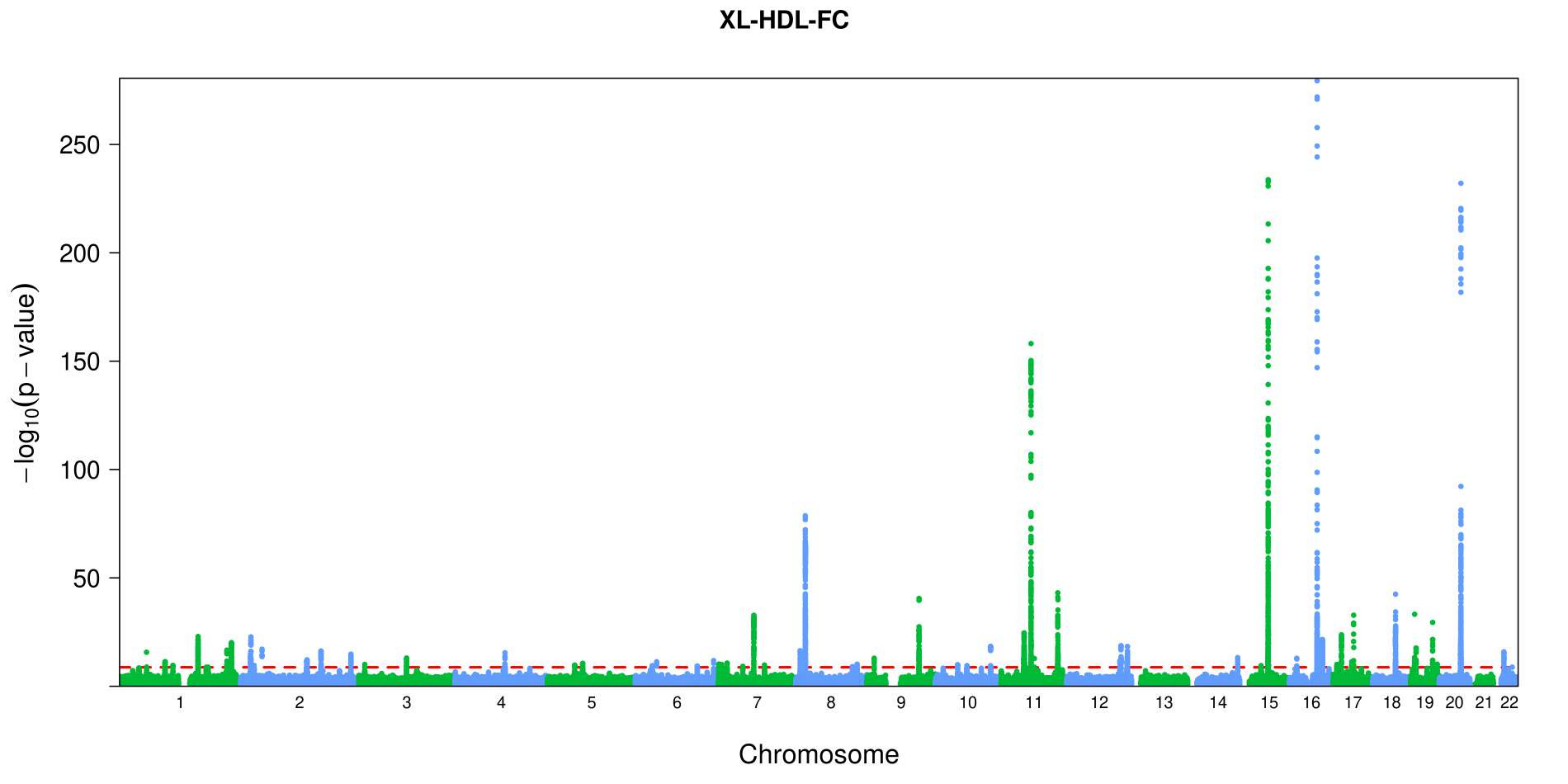
XL-HDL-FC
-log10(p - value)
250
200
150
100
50
1
2
3
4
5
6
7
8
9
10
11
12
13
14
15
16
17
18
19
20
21
22
Chromosome

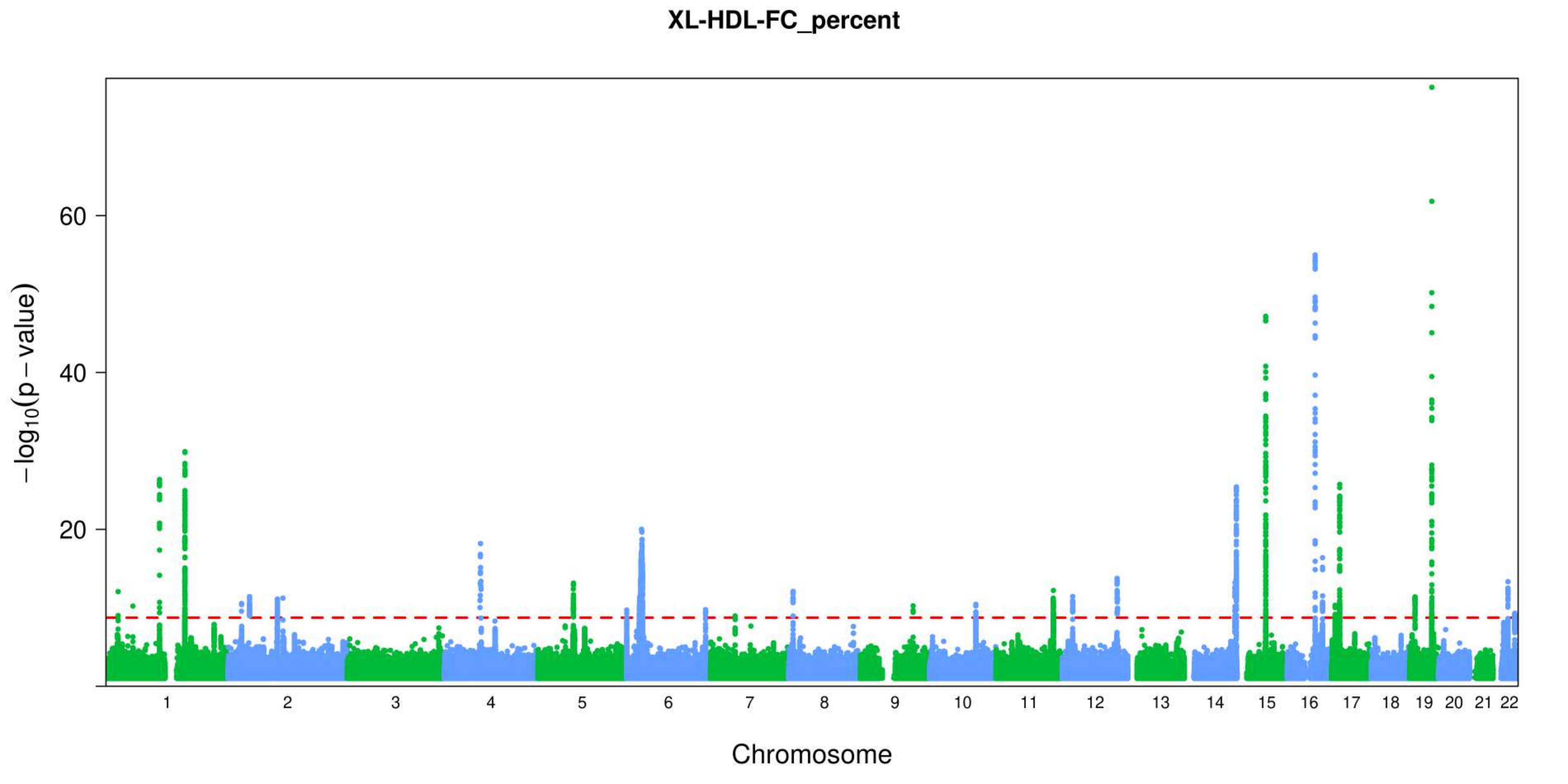
XL-HDL-FC_percent
-log10(p - value)
60
40
20
Chromosome
1
2
3
4
5
6
7
8
9
10
11
12
13
14
15
16
17
18
19
20
21
22

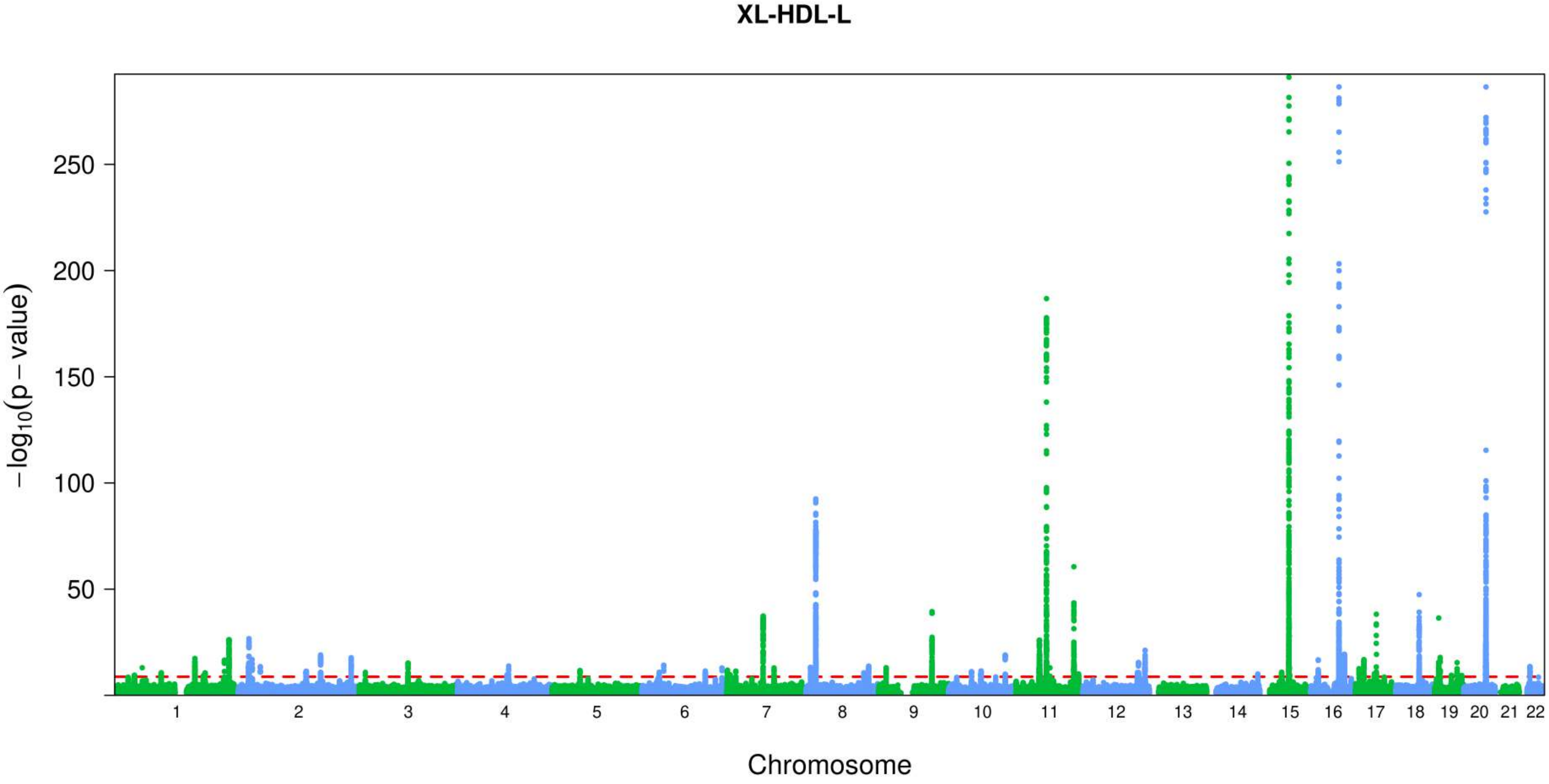
XL-HDL-L
−log10(p − value)
250
200
150
100
50
1
2
3
4
5
6
7
8
9
10
11
12
13
14
15
16
17
18
19
20
21
22
Chromosome

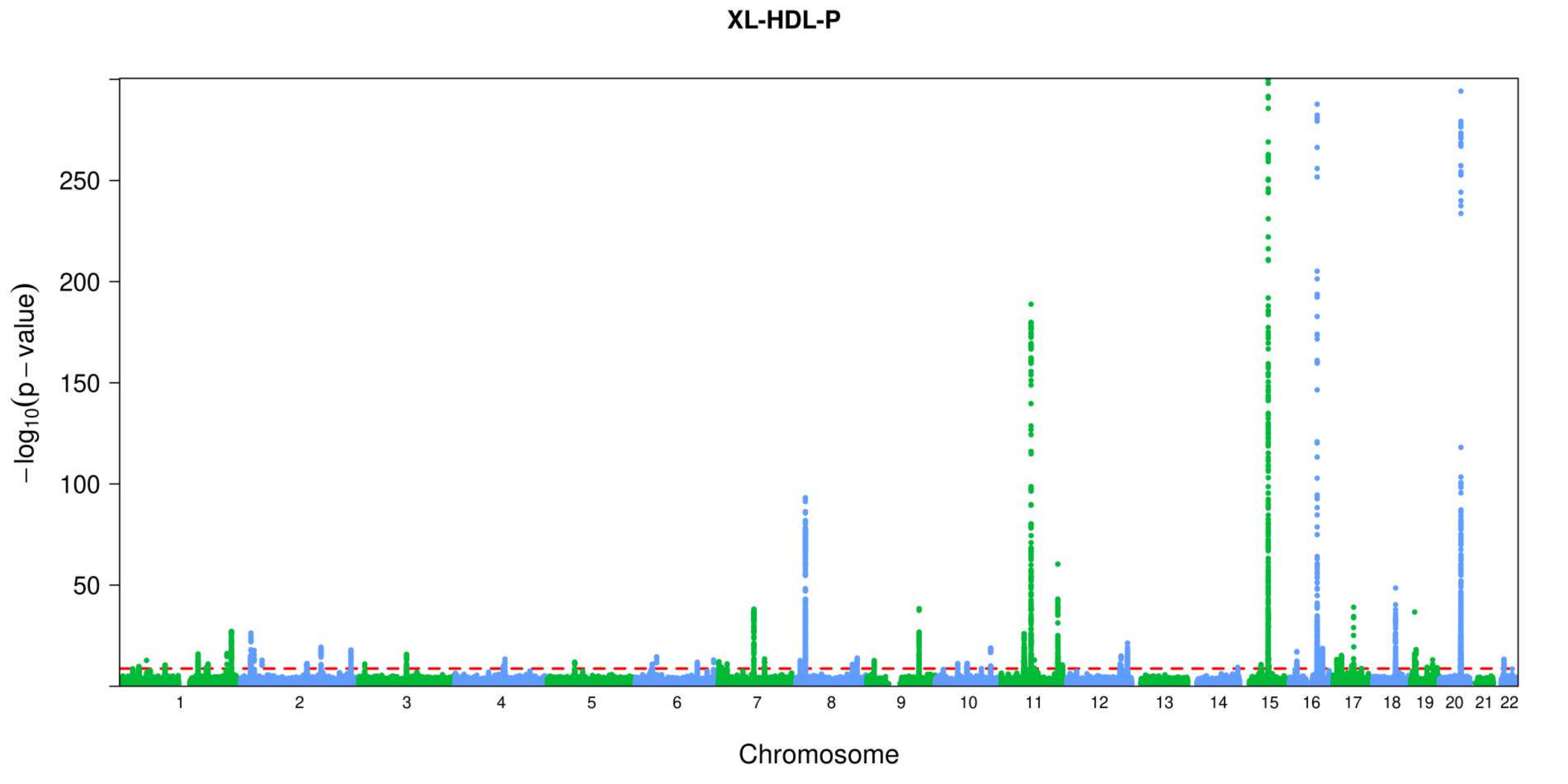
XL-HDL-P
250
200
150
100
50
-log10(p-value)
1
2
3
4
5
6
7
8
9
10
11
12
13
14
15
16
17
18
19
20
21
22
Chromosome

XL-HDL-PL

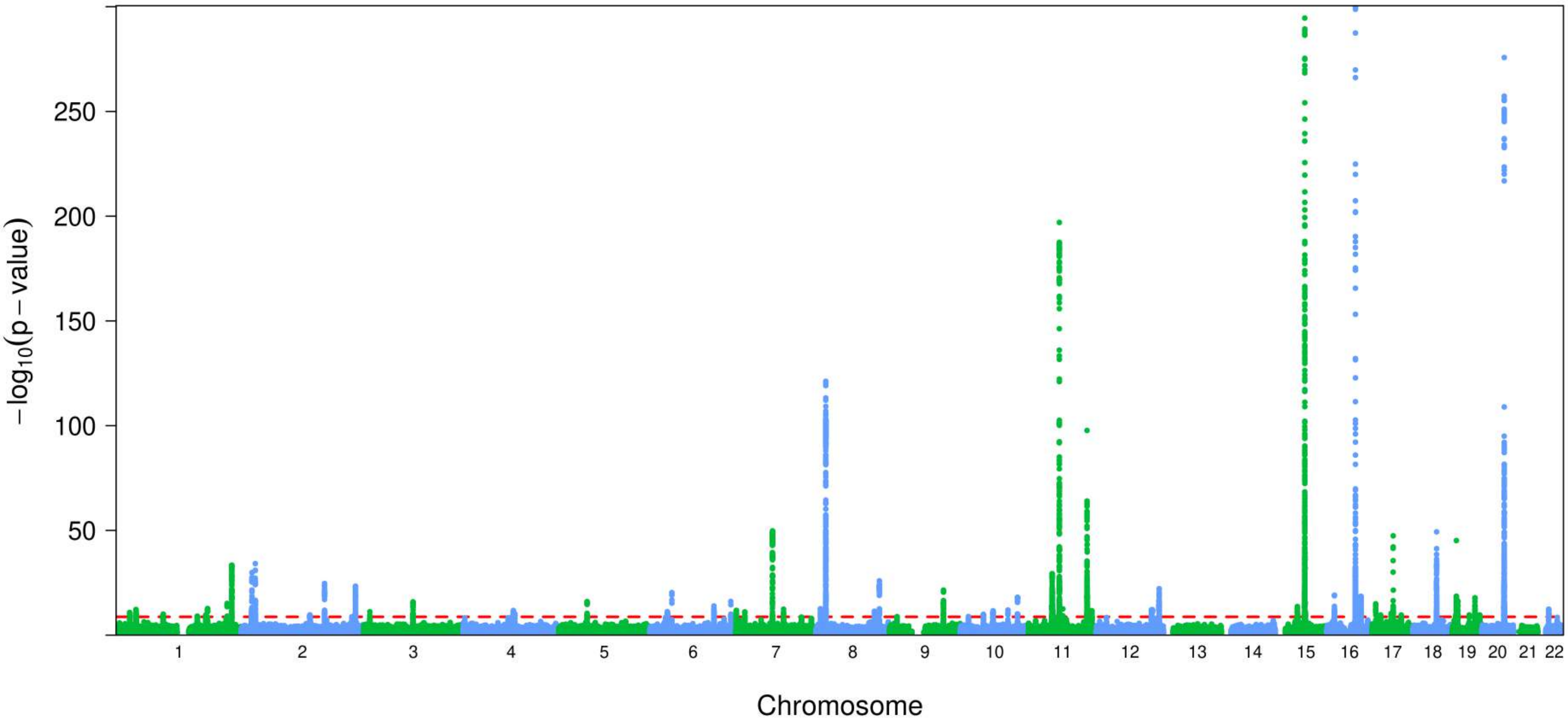

-log10(p - value)
250
200
150
100
50
1
2
3
4
5
6
7
8
9
10
11
12
13
14
15
16
17
18
19
20
21
22
Chromosome

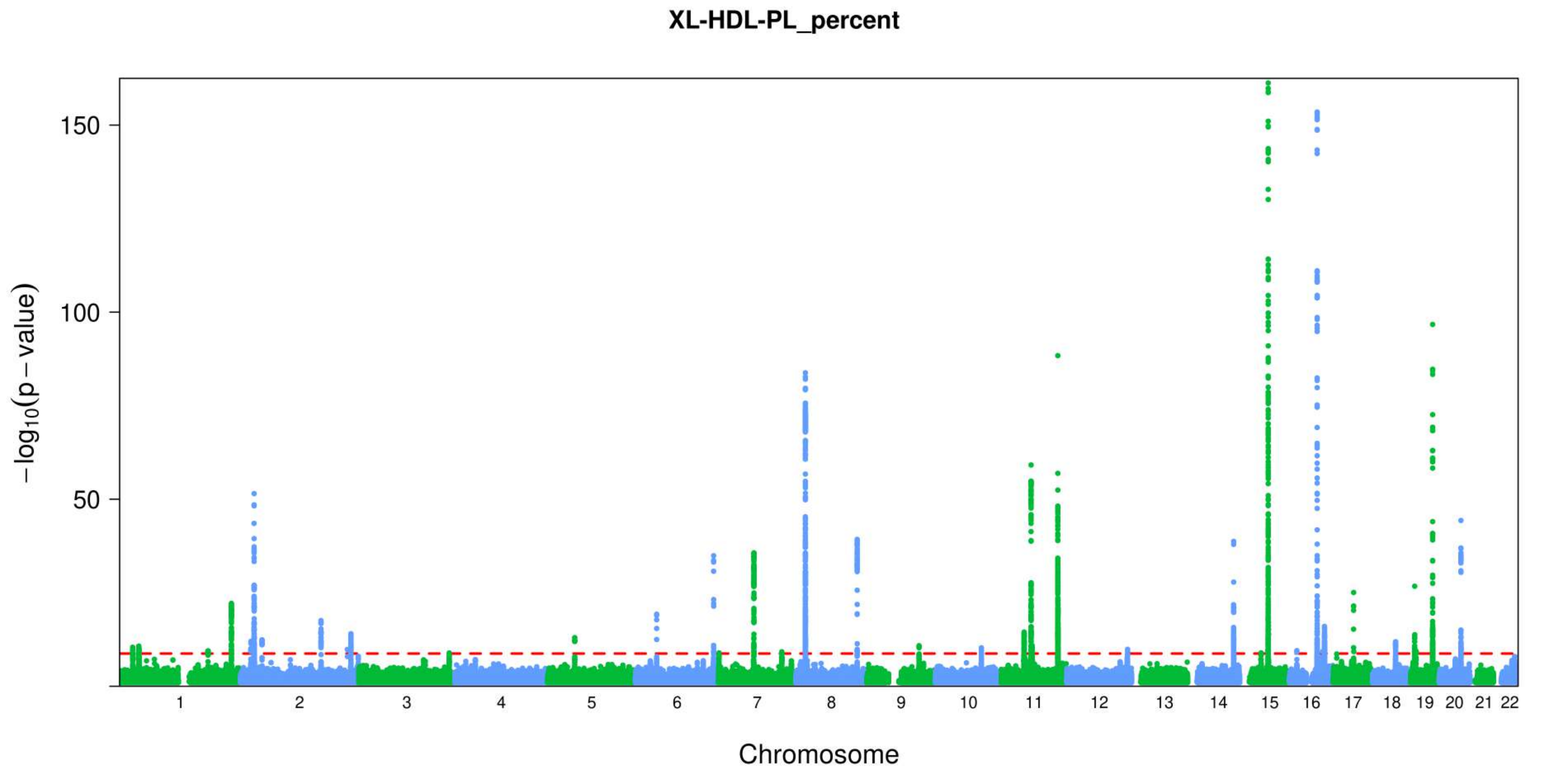

XL-HDL-PL_percent
150
100
50
−log10(p − value)
1
2
3
4
5
6
7
8
9
10
11
12
13
14
15
16
17
18
19
20
21
22
Chromosome

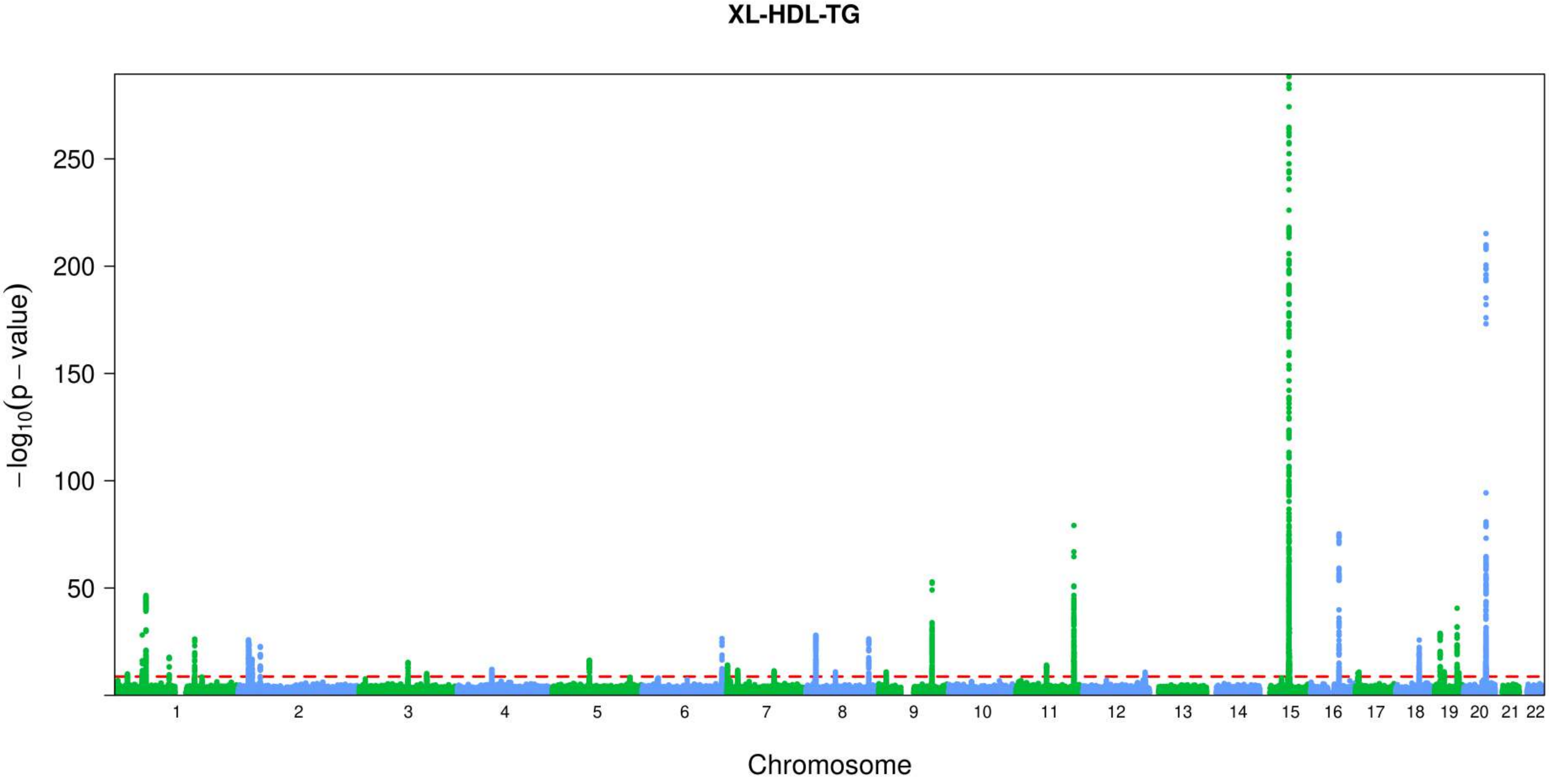

XL-HDL-TG
250
200
150
100
50
−log10(p − value)
1
2
3
4
5
6
7
8
9
10
11
12
13
14
15
16
17
18
19
20
21
22
Chromosome

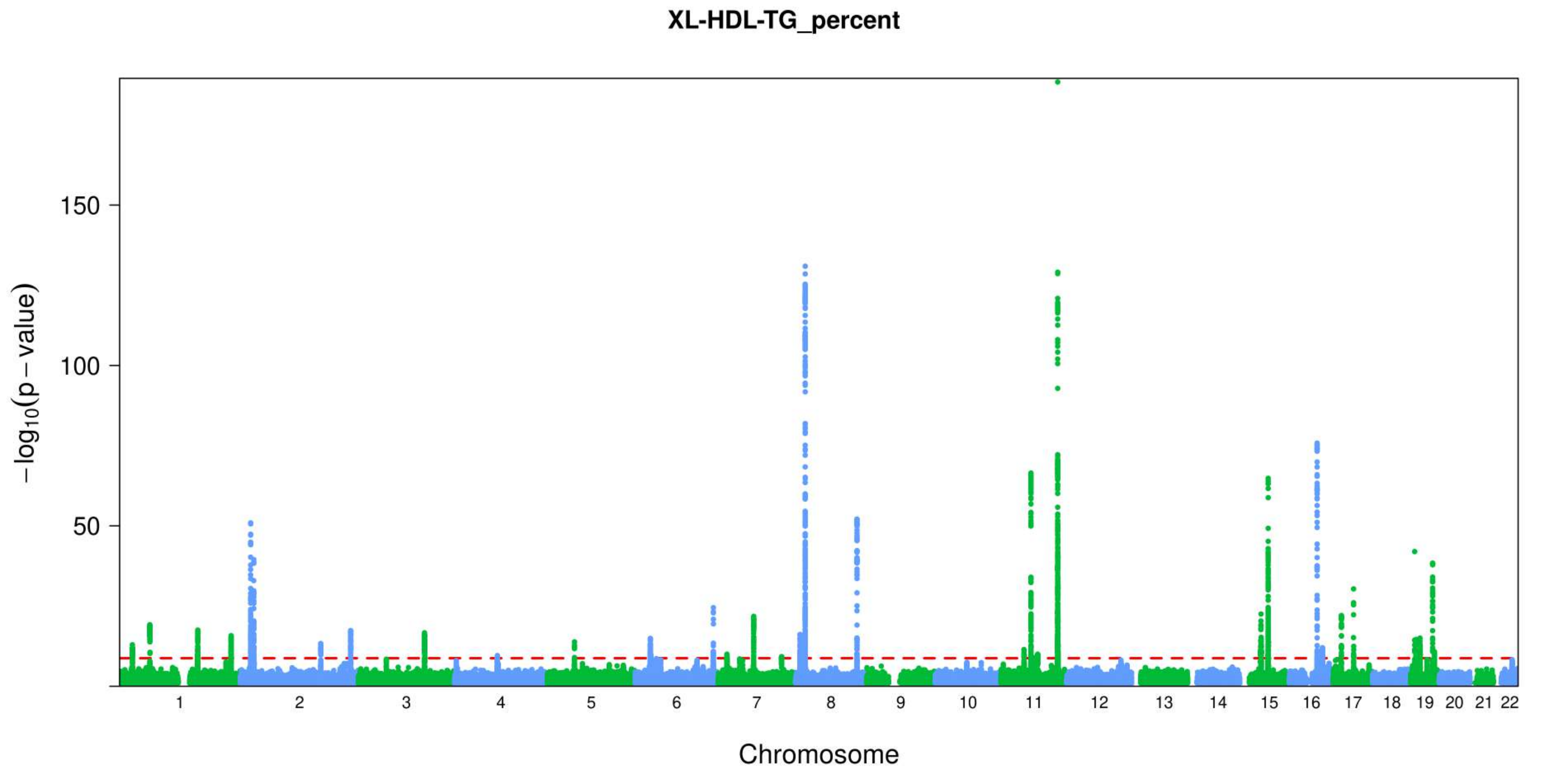
XL-HDL-TG_percent
−log10(p − value)
150
100
50
Chromosome
1
2
3
4
5
6
7
8
9
10
11
12
13
14
15
16
17
18
19
20
21
22

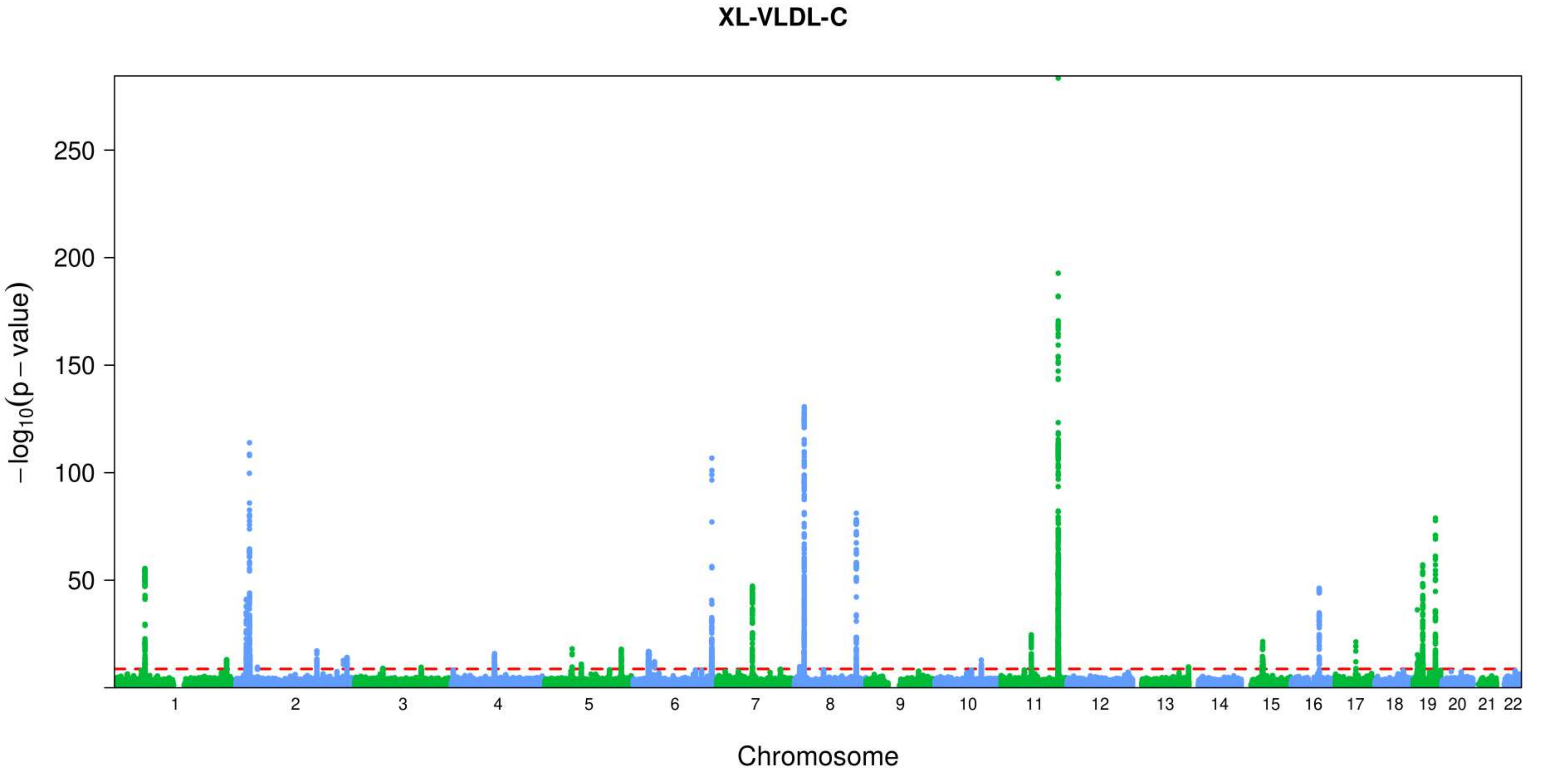
XL-VLDL-C
-log10(p - value)
250
200
150
100
50
Chromosome
1
2
3
4
5
6
7
8
9
10
11
12
13
14
15
16
17
18
19
20
21
22

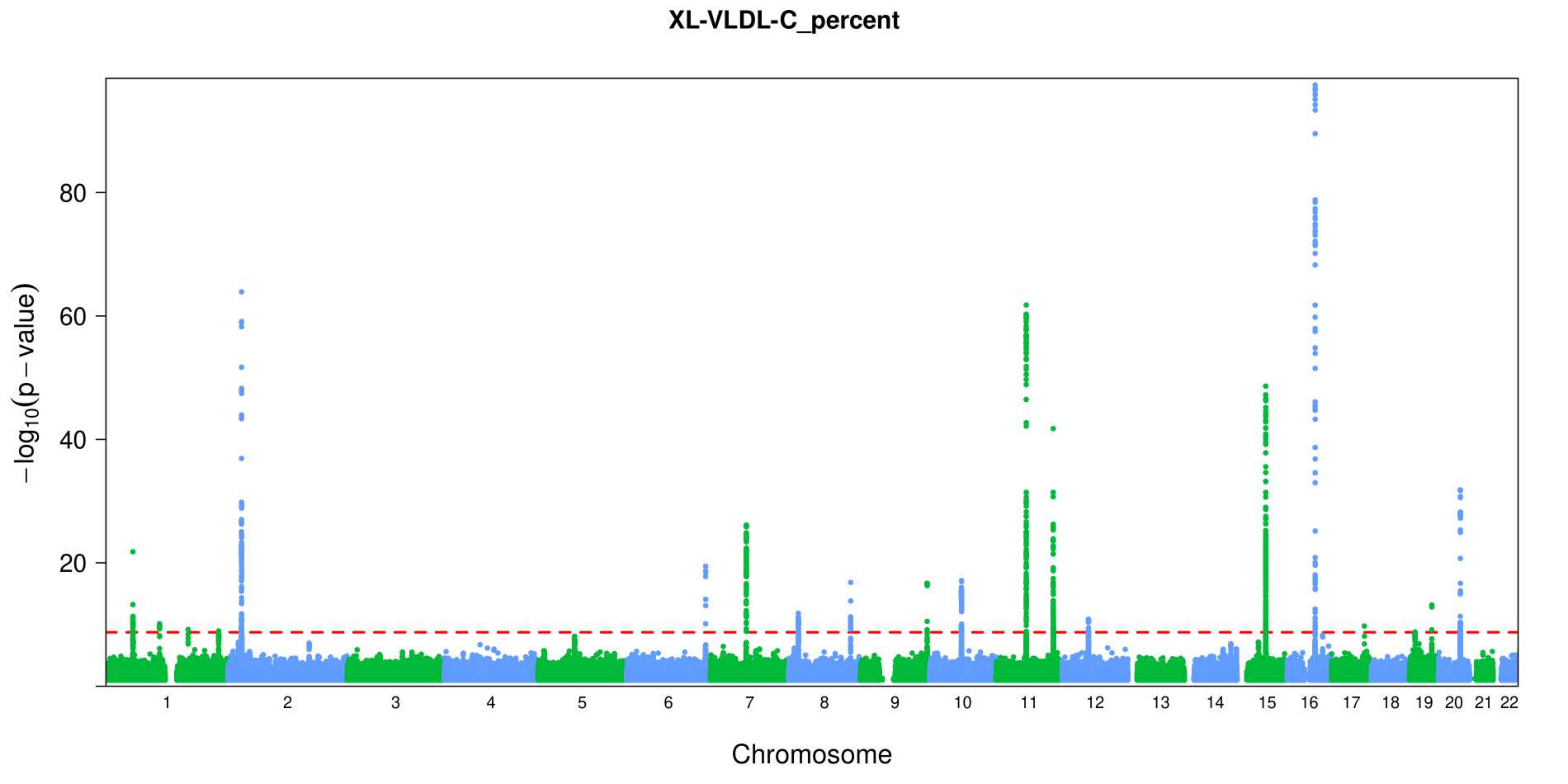
XL-VLDL-C_percent
−log10(p − value)
80
60
40
20
1
2
3
4
5
6
7
8
9
10
11
12
13
14
15
16
17
18
19
20
21
22
Chromosome

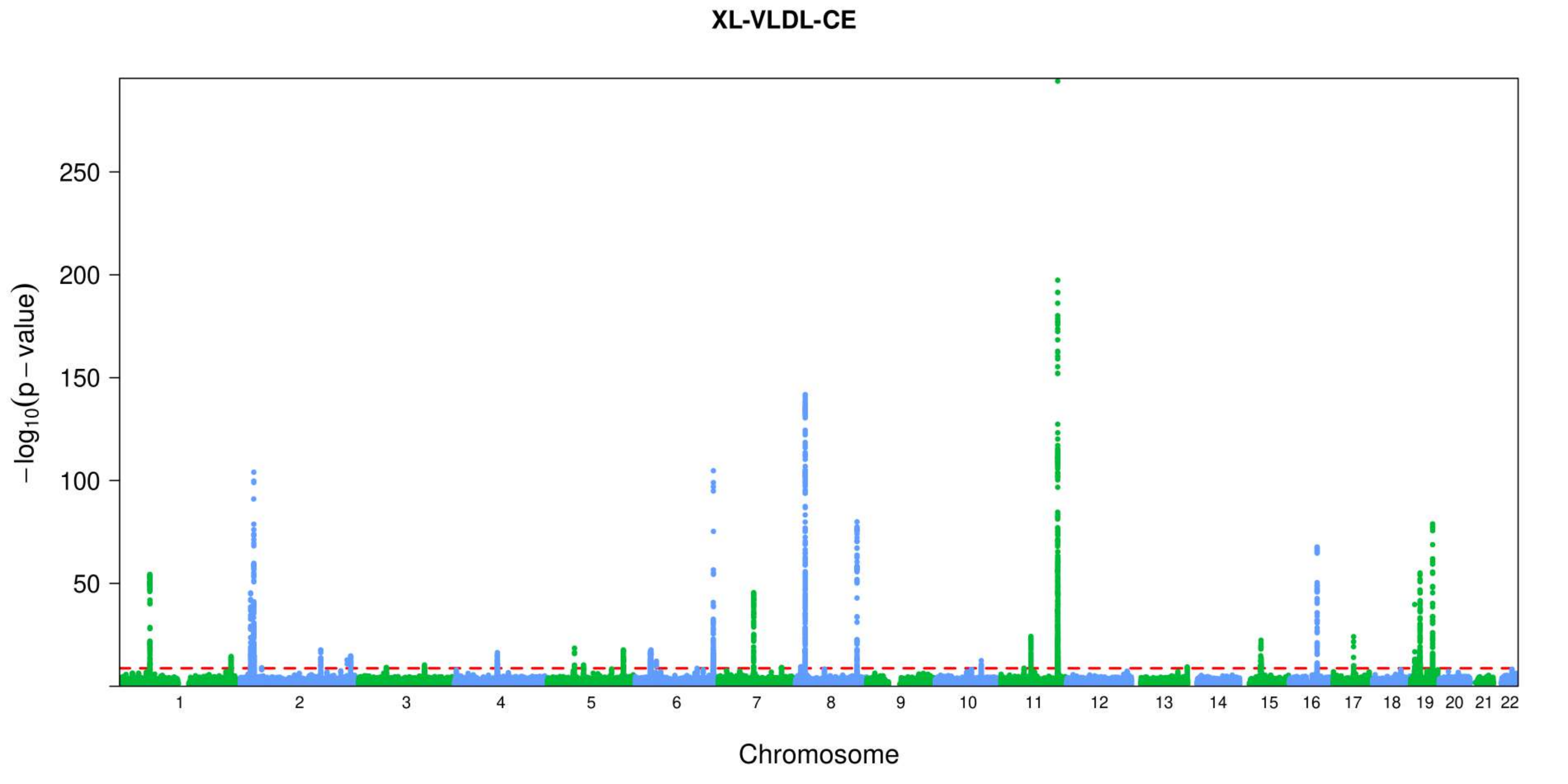

XL-VLDL-CE
250
200
150
100
50
$-\log_{10}(p-\text{value})$
1
2
3
4
5
6
7
8
9
10
11
12
13
14
15
16
17
18
19
20
21
22
Chromosome

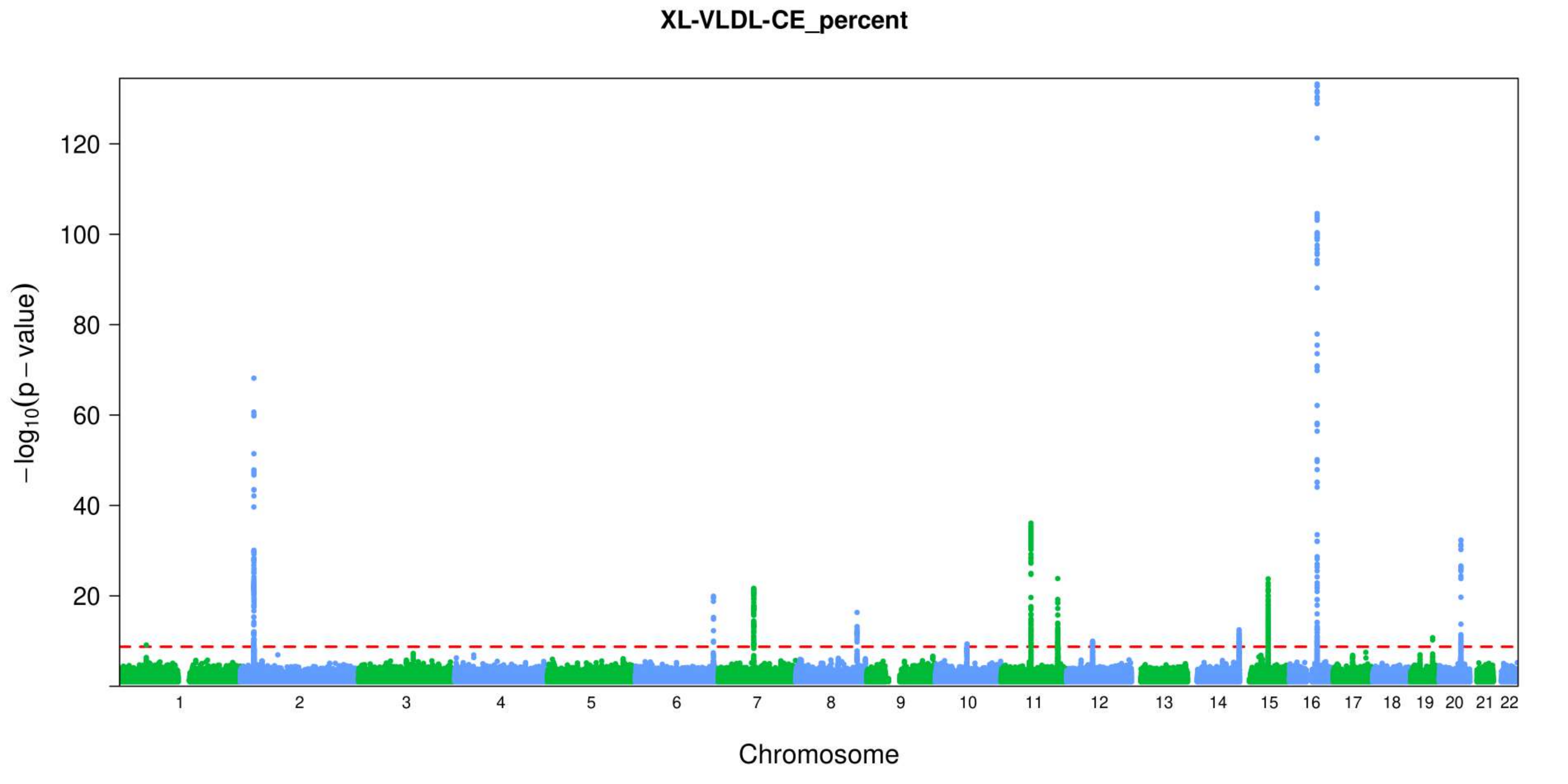
XL-VLDL-CE_percent
-log10(p-value)
120
100
80
60
40
20
1
2
3
4
5
6
7
8
9
10
11
12
13
14
15
16
17
18
19
20
21
22
Chromosome

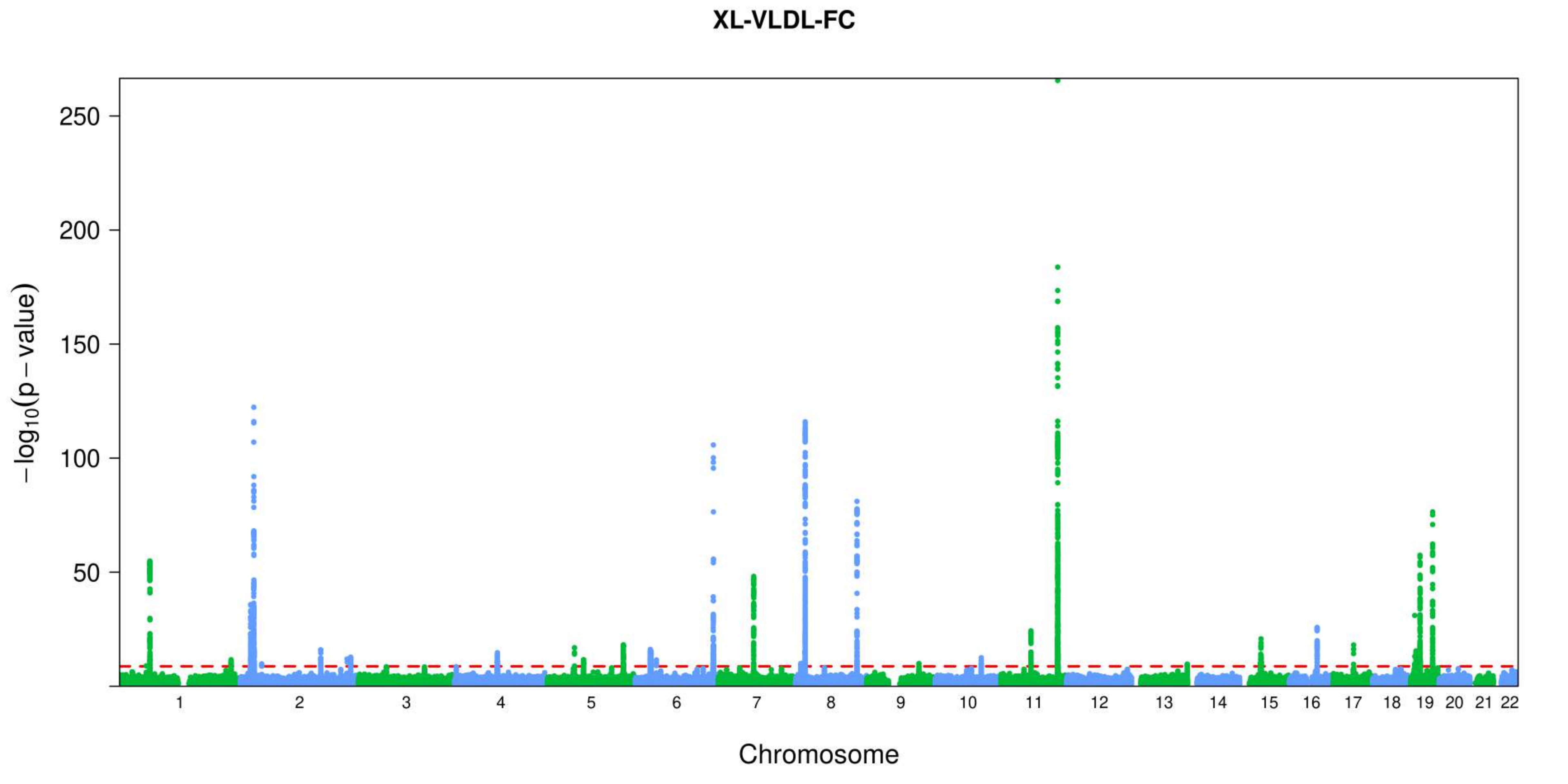

XL-VLDL-FC
250
200
150
100
50
$-\log_{10}(p-\text{value})$
1
2
3
4
5
6
7
8
9
10
11
12
13
14
15
16
17
18
19
20
21
22
Chromosome

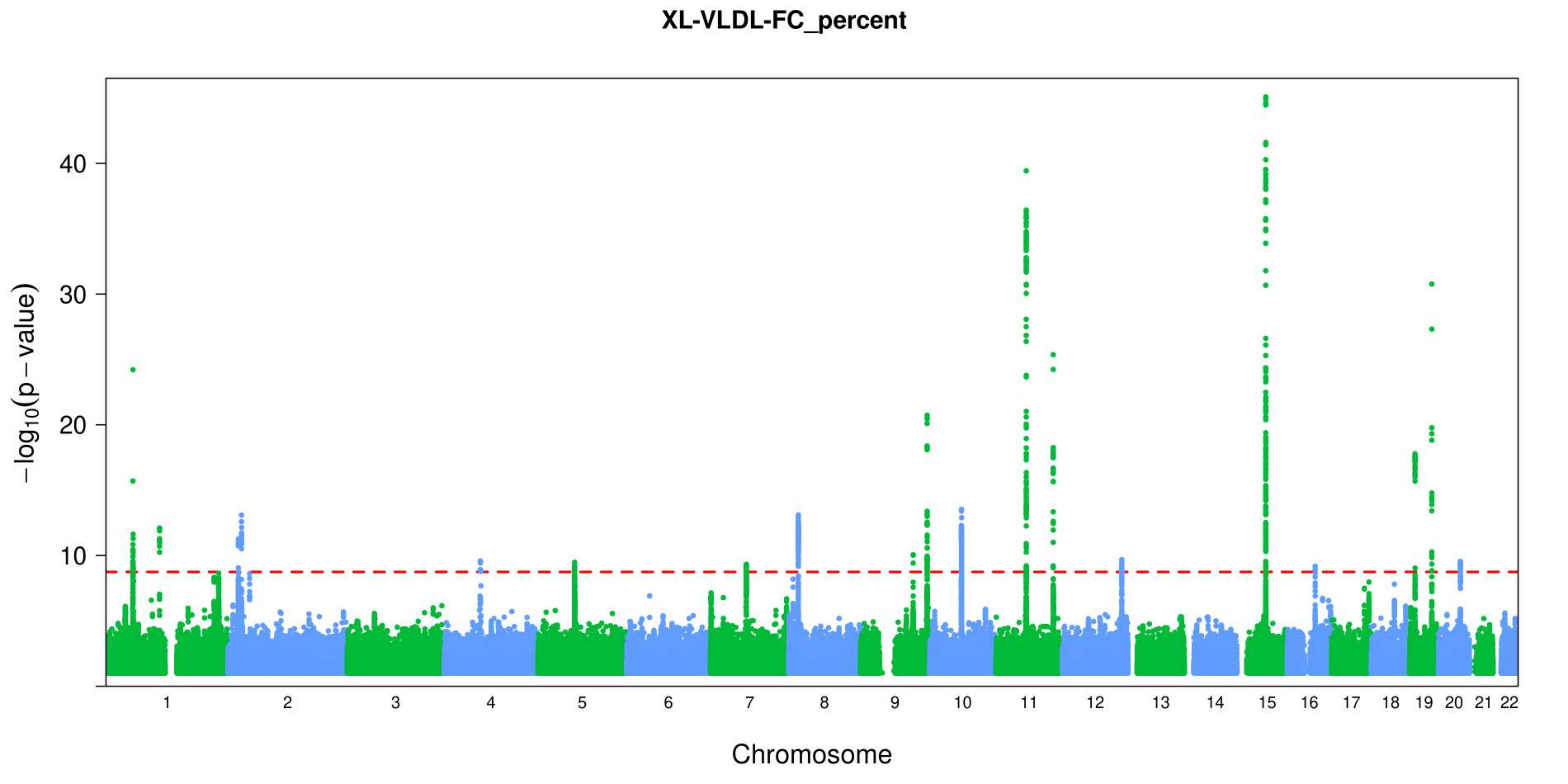

XL-VLDL-FC_percent
$-\log_{10}(p-\text{value})$
10
20
30
40
1
2
3
4
5
6
7
8
9
10
11
12
13
14
15
16
17
18
19
20
21
22
Chromosome

XL-VLDL-L

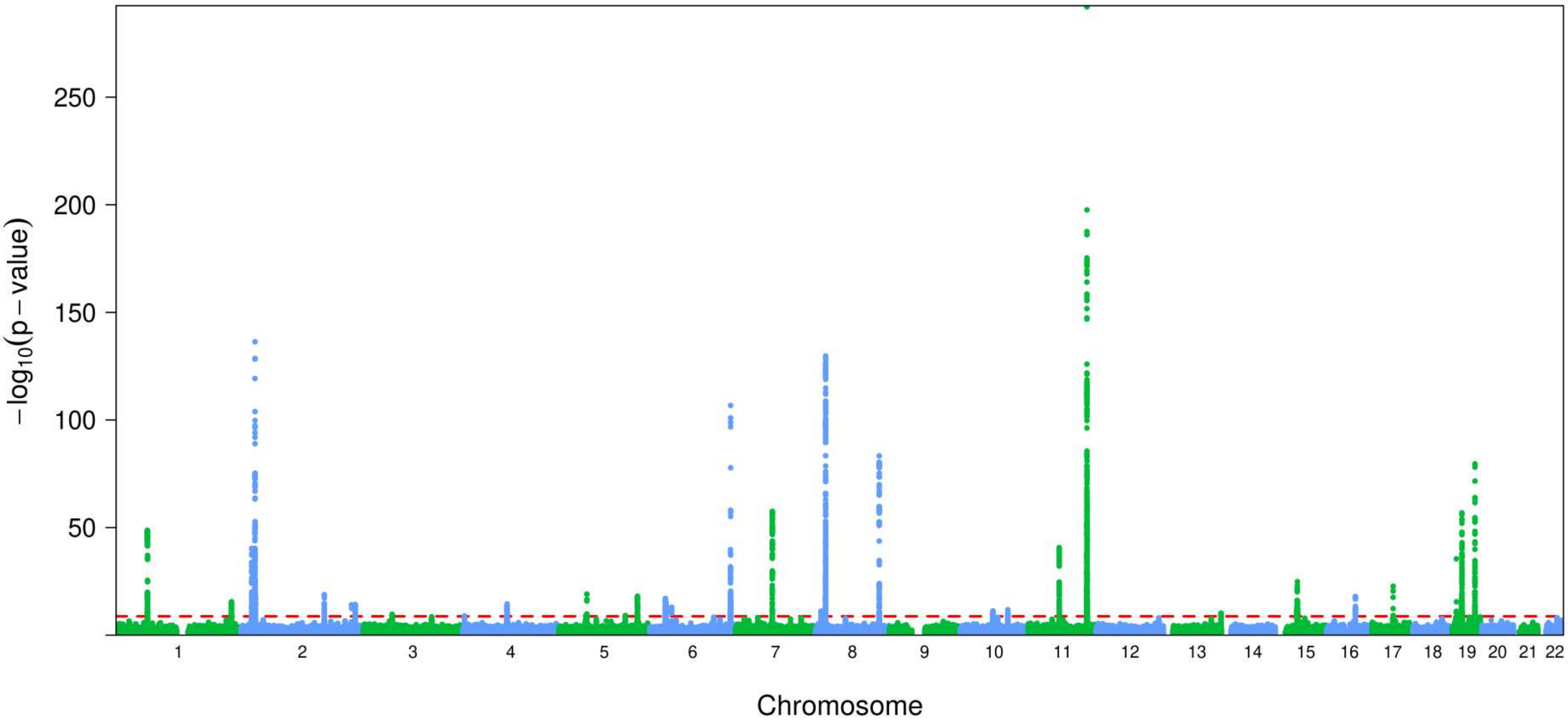

250
200
150
100
50
$-\log_{10}(p-\text{value})$
1
2
3
4
5
6
7
8
9
10
11
12
13
14
15
16
17
18
19
20
21
22
Chromosome

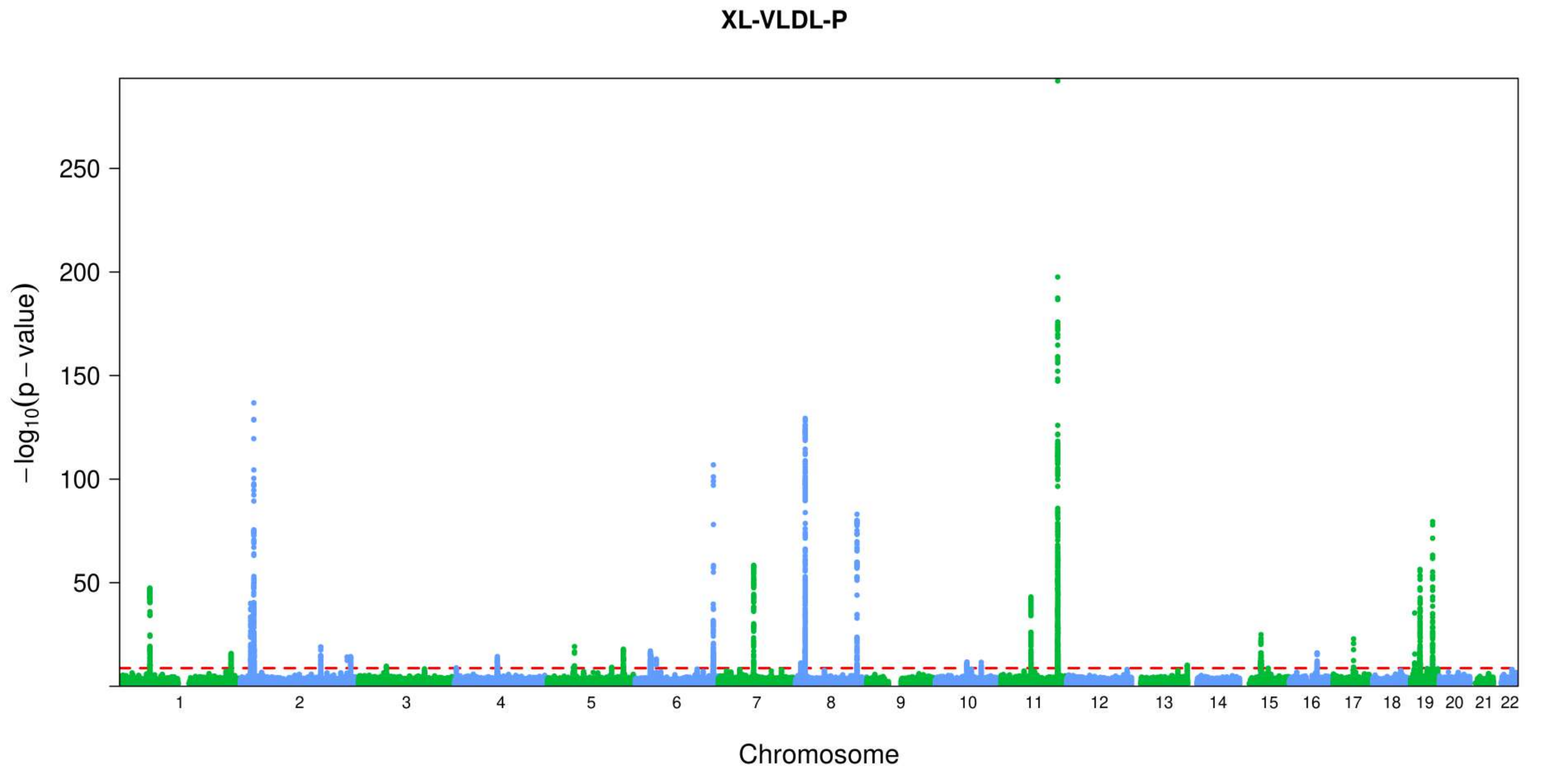
XL-VLDL-P
250
200
150
100
50
$-\log_{10}(p-\text{value})$
1
2
3
4
5
6
7
8
9
10
11
12
13
14
15
16
17
18
19
20
21
22
Chromosome

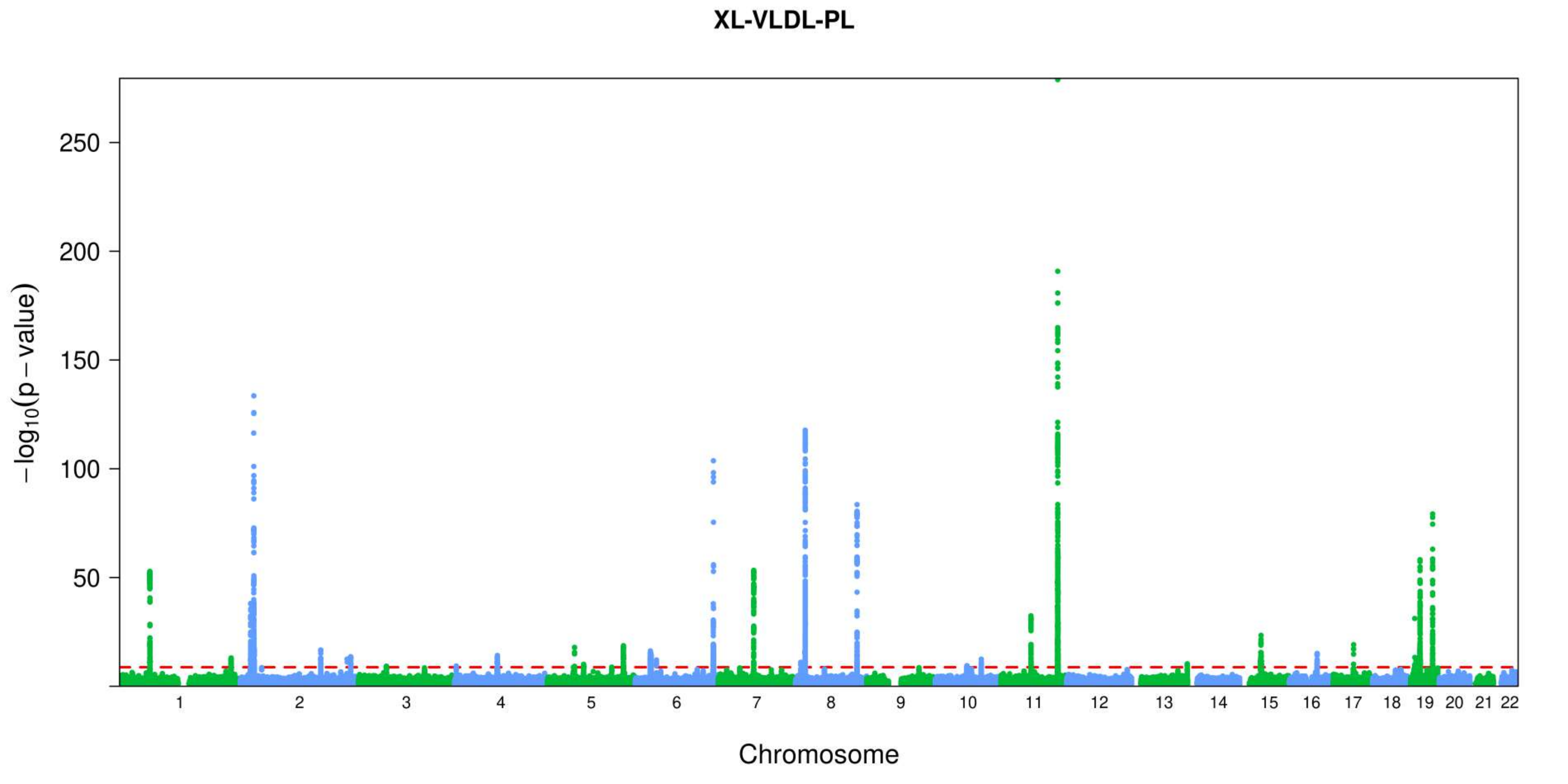
XL-VLDL-PL
250
200
150
100
50
$-\log_{10}(p-\text{value})$
1
2
3
4
5
6
7
8
9
10
11
12
13
14
15
16
17
18
19
20
21
22
Chromosome

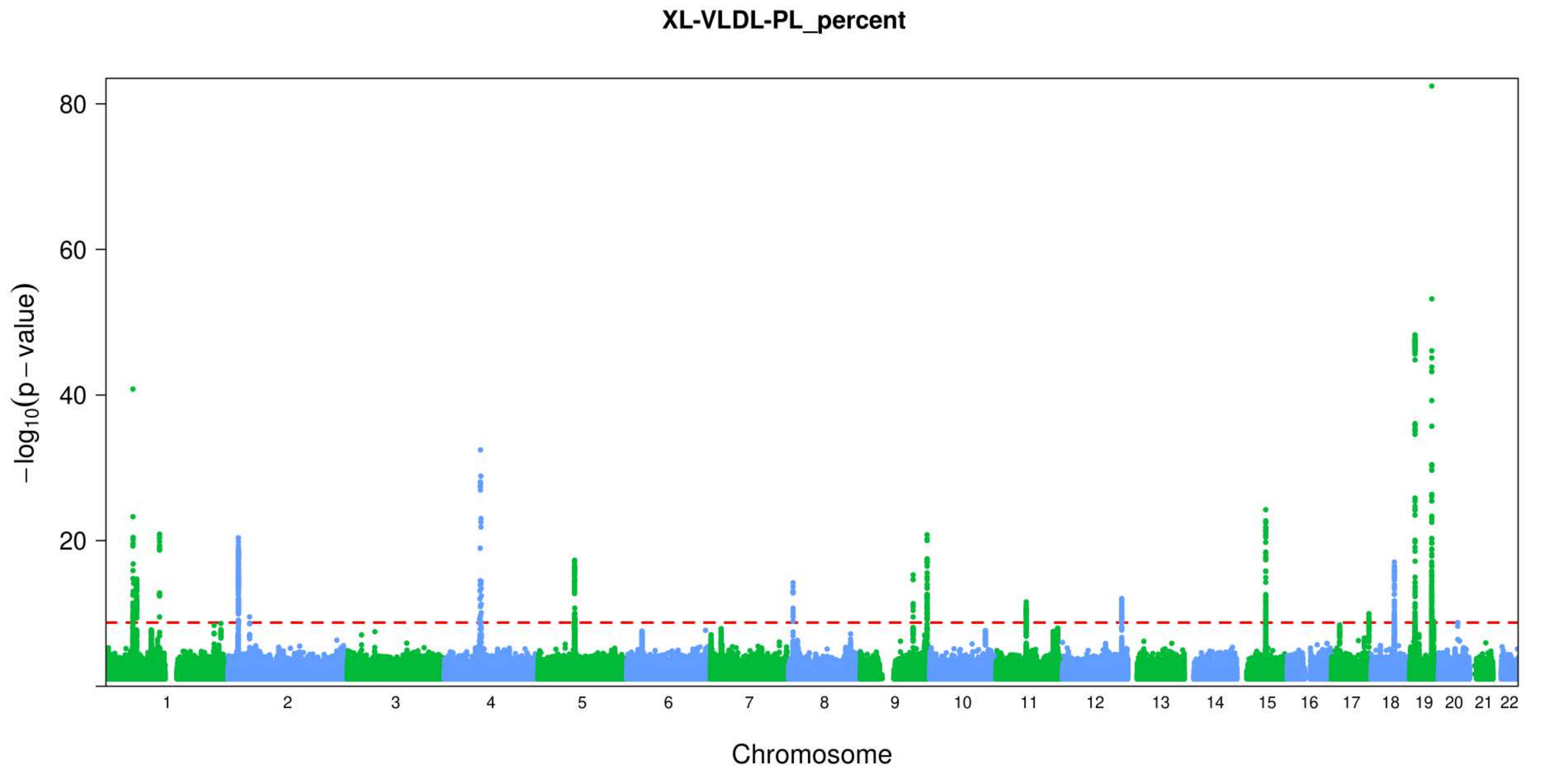

XL-VLDL-PL_percent
80
60
40
20
−log10(p − value)
1
2
3
4
5
6
7
8
9
10
11
12
13
14
15
16
17
18
19
20
21
22
Chromosome

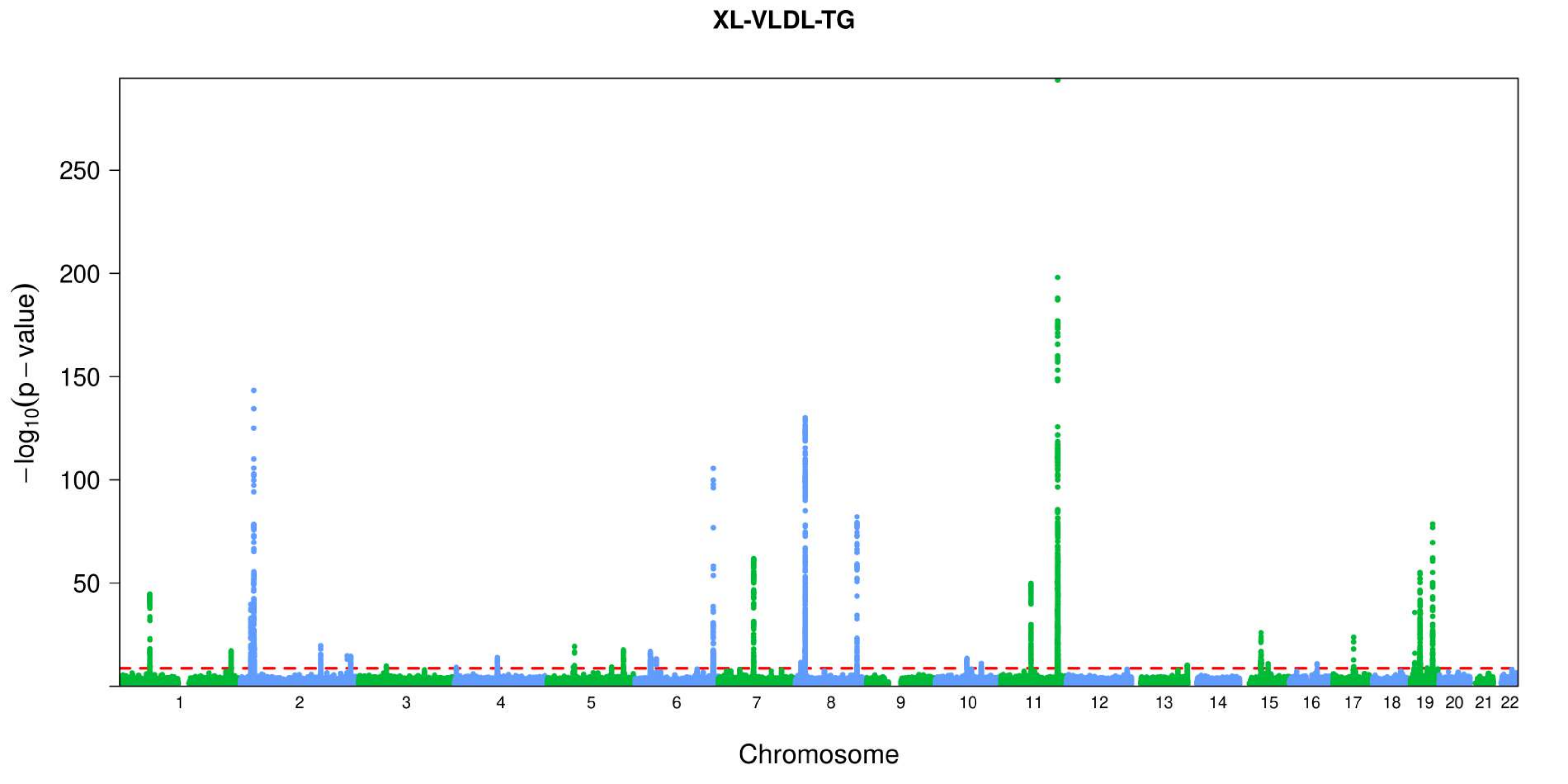

XL-VLDL-TG
250
200
150
100
50
−log10(p − value)
1
2
3
4
5
6
7
8
9
10
11
12
13
14
15
16
17
18
19
20
21
22
Chromosome

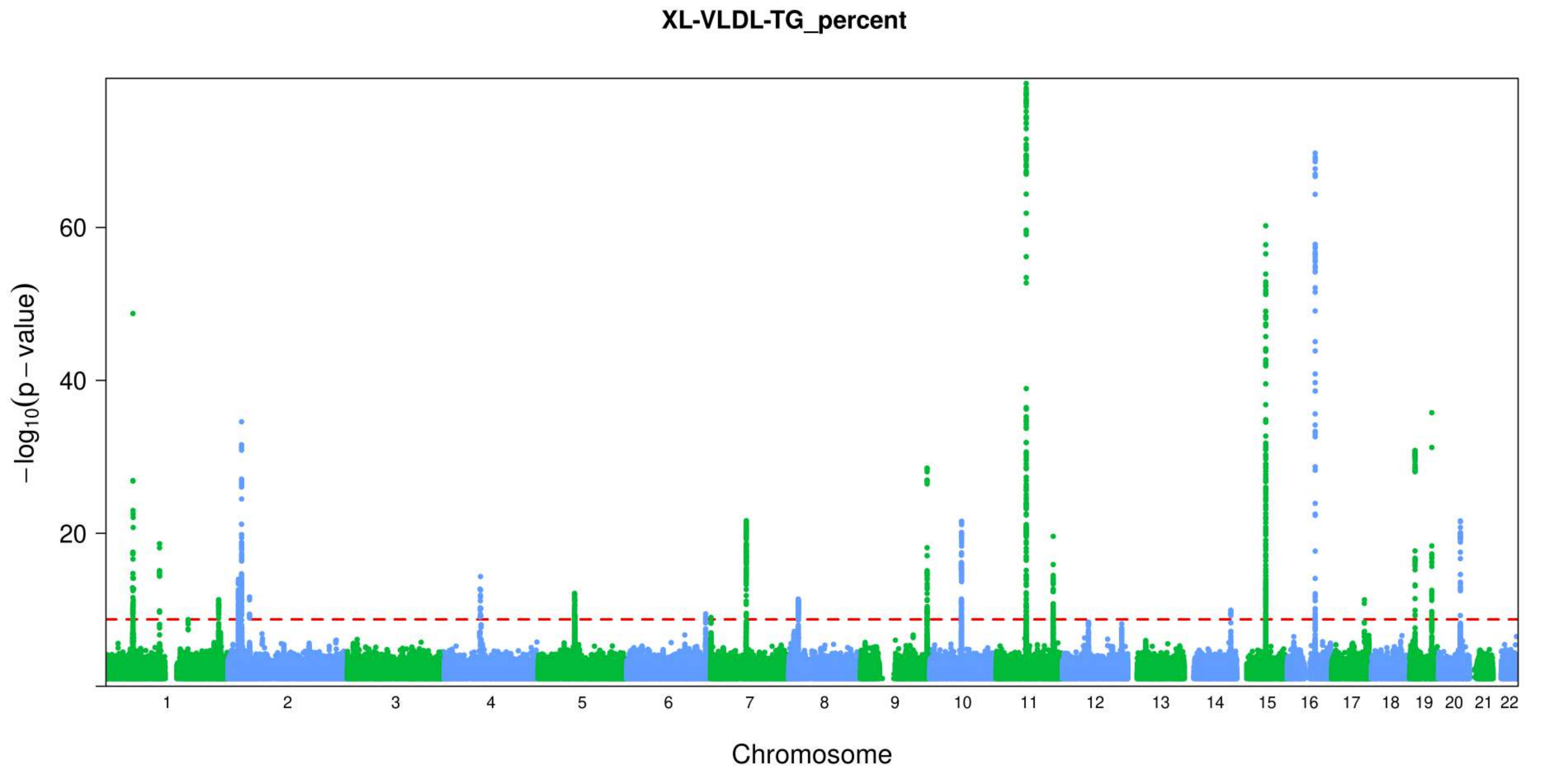
XL-VLDL-TG_percent
$-\log_{10}(p-\text{value})$
20
40
60
Chromosome
1
2
3
4
5
6
7
8
9
10
11
12
13
14
15
16
17
18
19
20
21
22

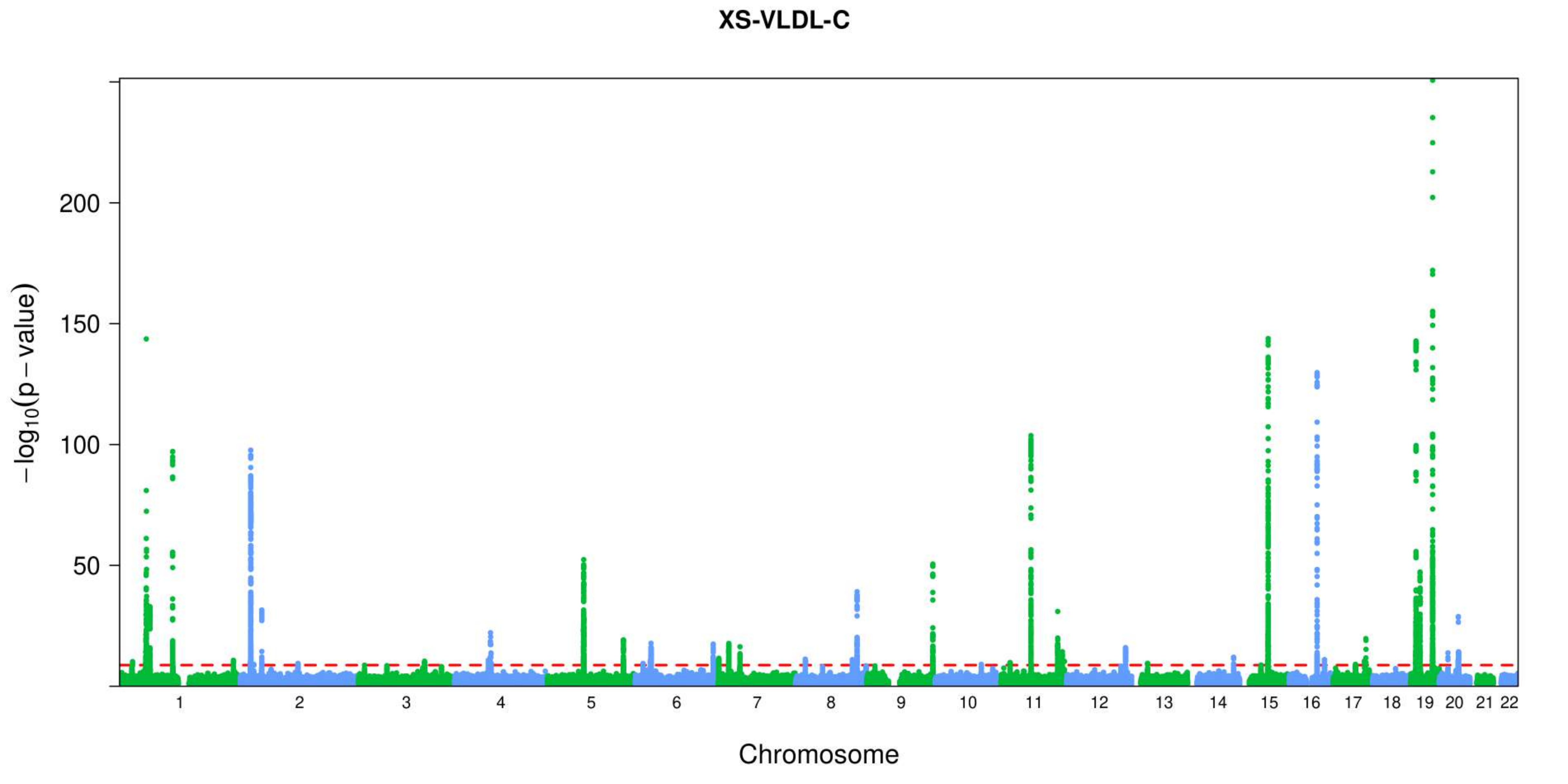
XS-VLDL-C
−log10(p − value)
200
150
100
50
1
2
3
4
5
6
7
8
9
10
11
12
13
14
15
16
17
18
19
20
21
22
Chromosome

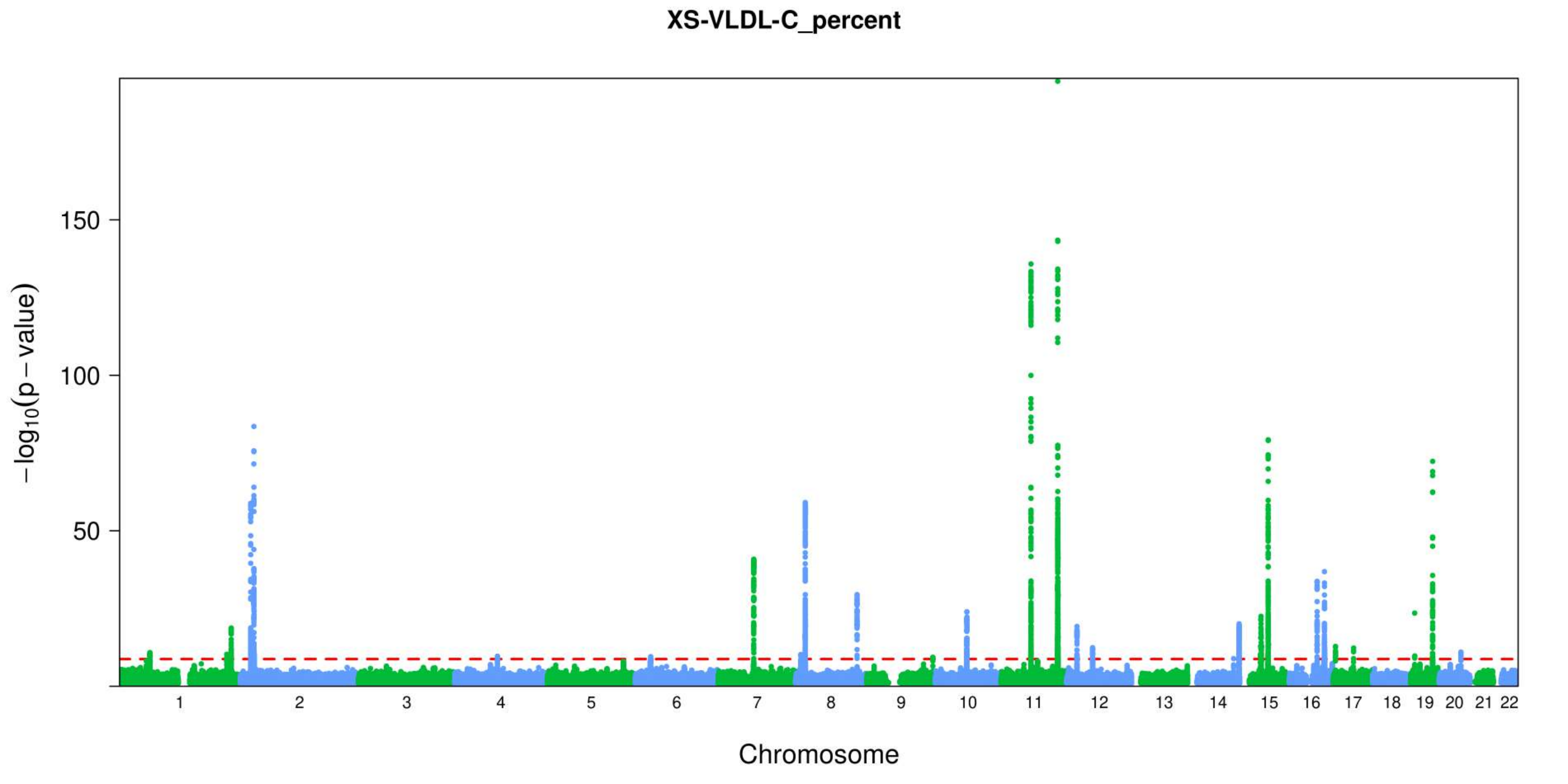
XS-VLDL-C_percent
150
100
50
−log10(p − value)
1
2
3
4
5
6
7
8
9
10
11
12
13
14
15
16
17
18
19
20
21
22
Chromosome

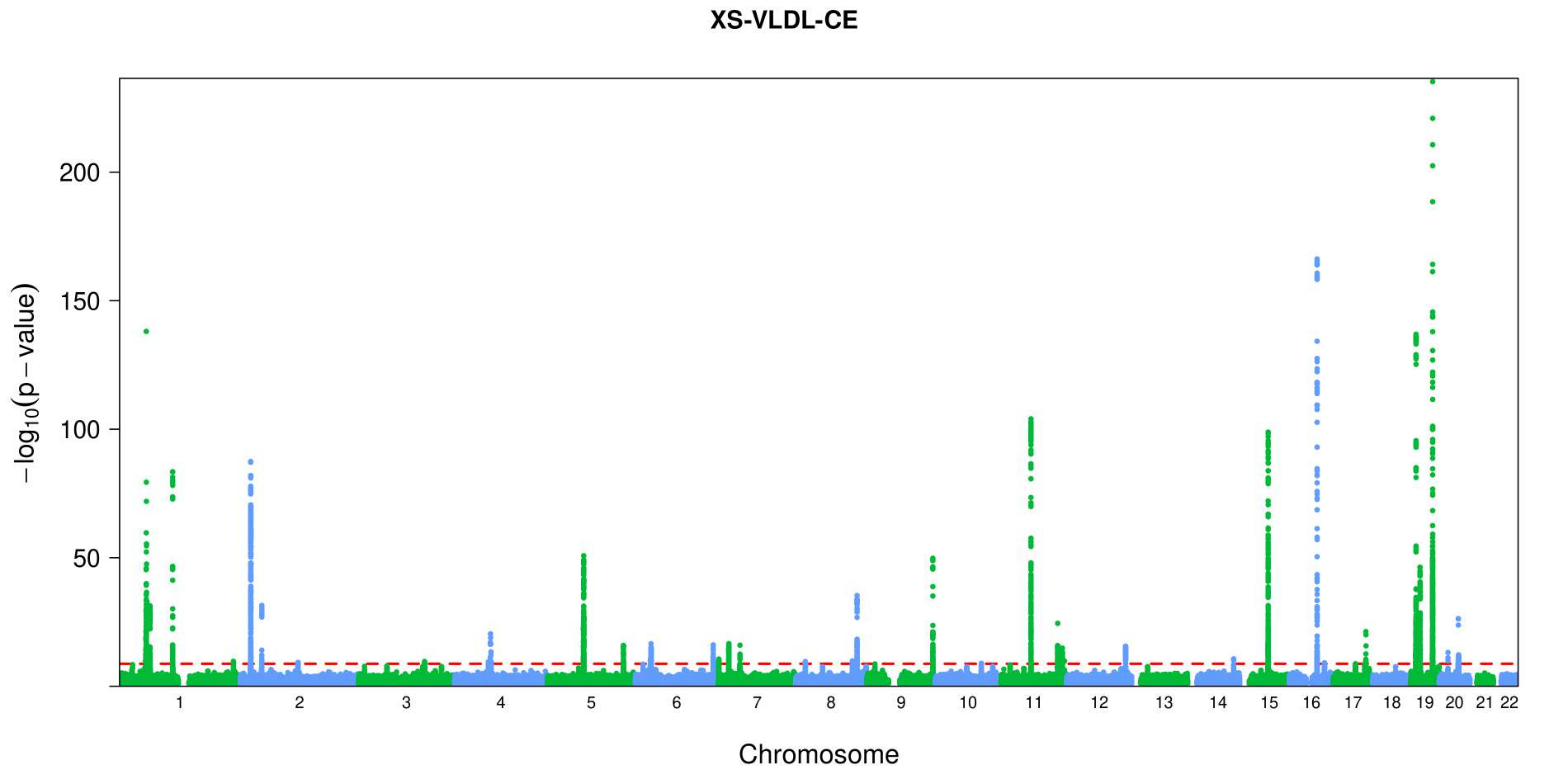
XS-VLDL-CE
200
150
100
50
$-\log_{10}(p-\text{value})$
1
2
3
4
5
6
7
8
9
10
11
12
13
14
15
16
17
18
19
20
21
22
Chromosome

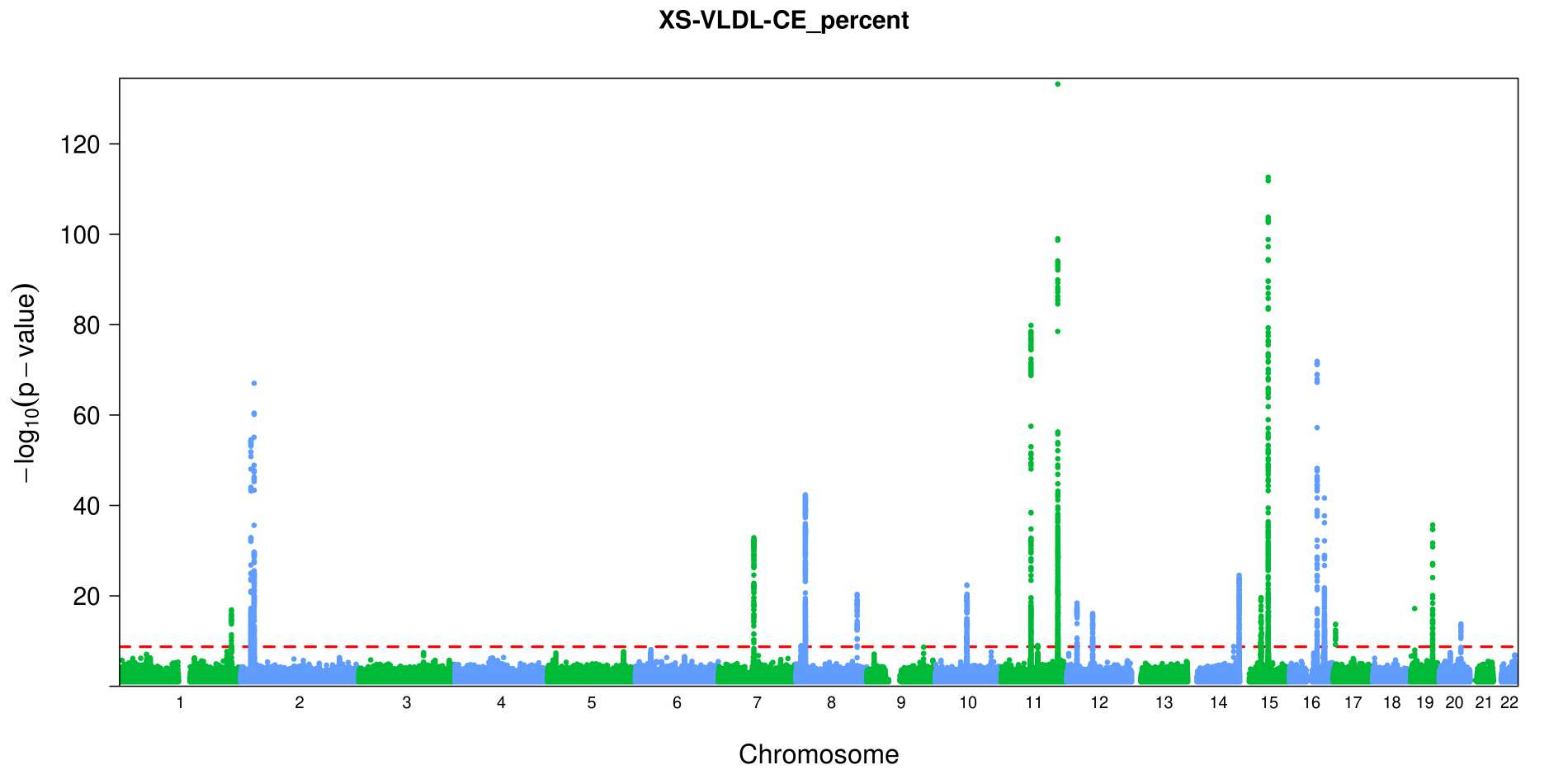
XS-VLDL-CE_percent
−log10(p − value)
120
100
80
60
40
20
Chromosome
1
2
3
4
5
6
7
8
9
10
11
12
13
14
15
16
17
18
19
20
21
22

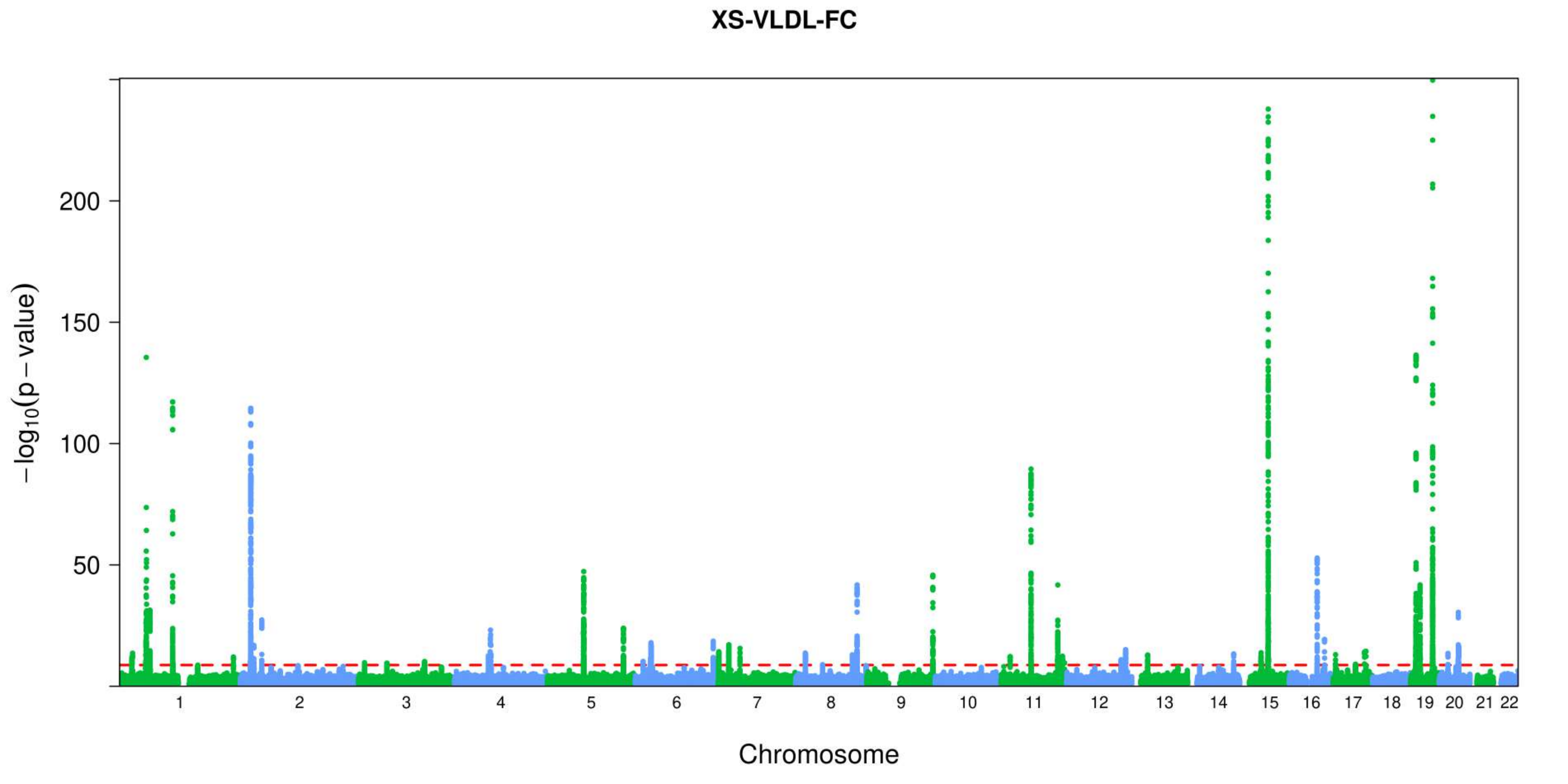
XS-VLDL-FC
−log10(p − value)
200
150
100
50
1
2
3
4
5
6
7
8
9
10
11
12
13
14
15
16
17
18
19
20
21
22
Chromosome

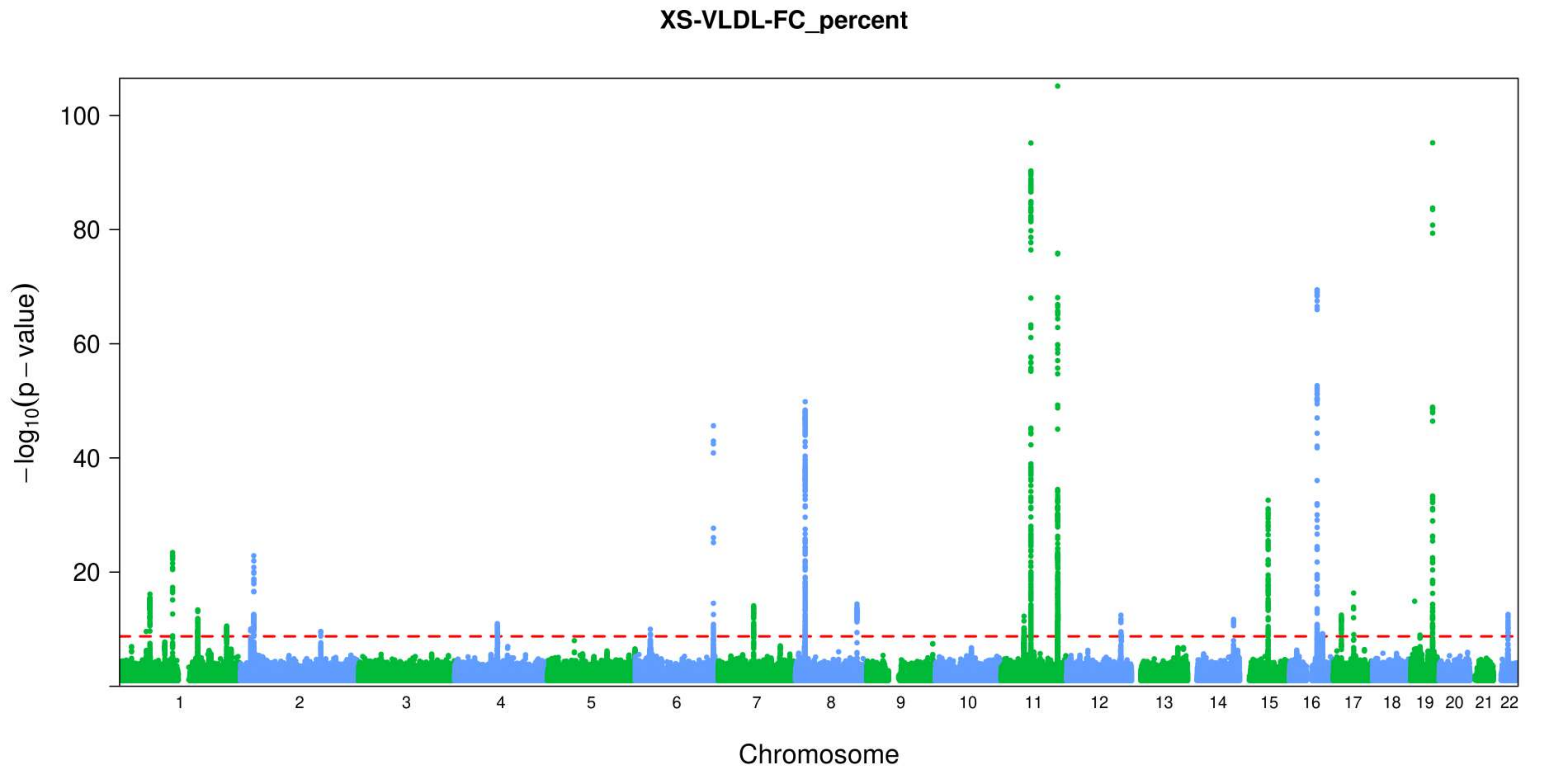
XS-VLDL-FC_percent
−log10(p−value)
100
80
60
40
20
Chromosome
1
2
3
4
5
6
7
8
9
10
11
12
13
14
15
16
17
18
19
20
21
22

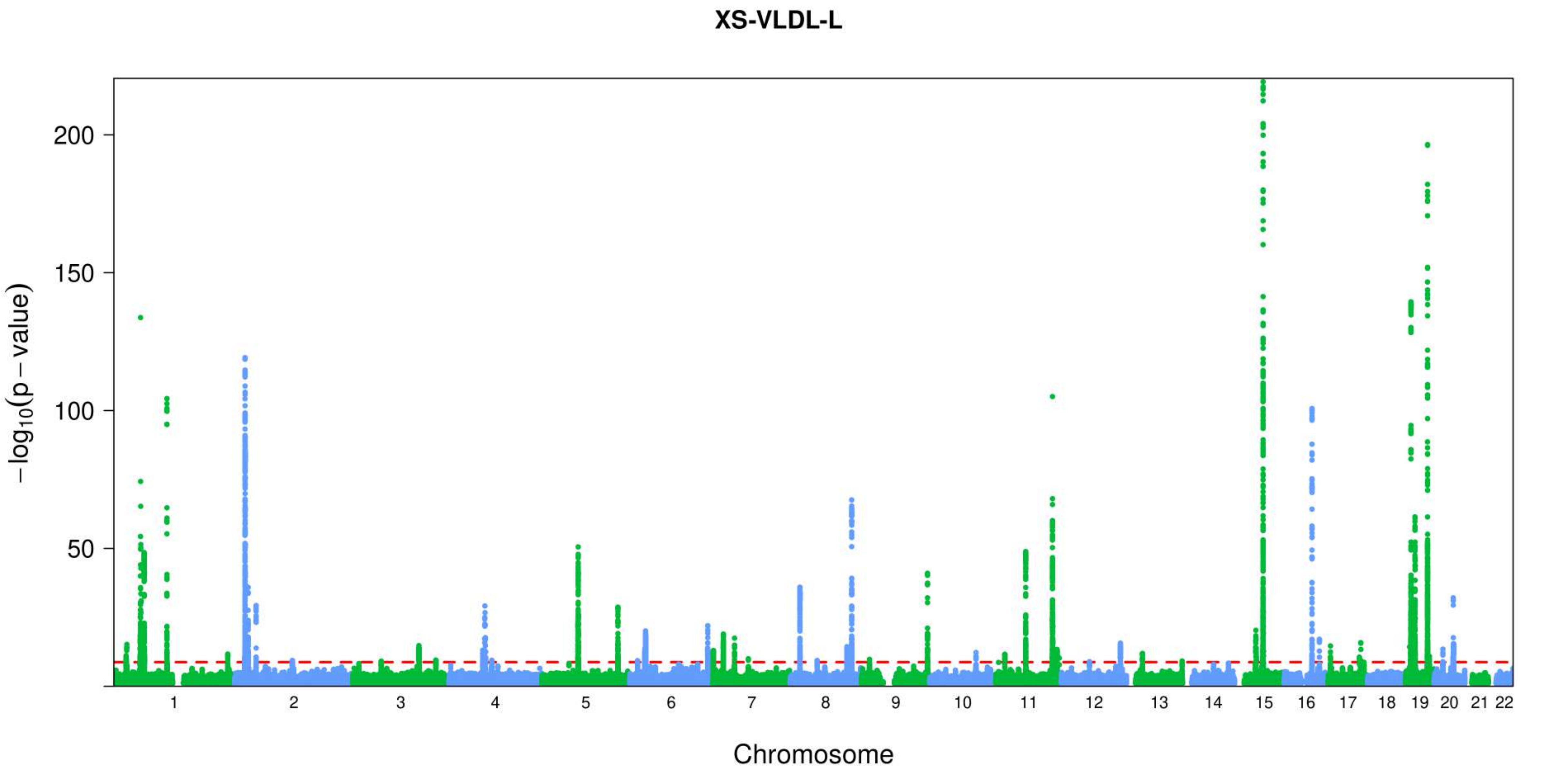
XS-VLDL-L
200
150
100
50
−log10(p − value)
1
2
3
4
5
6
7
8
9
10
11
12
13
14
15
16
17
18
19
20
21
22
Chromosome

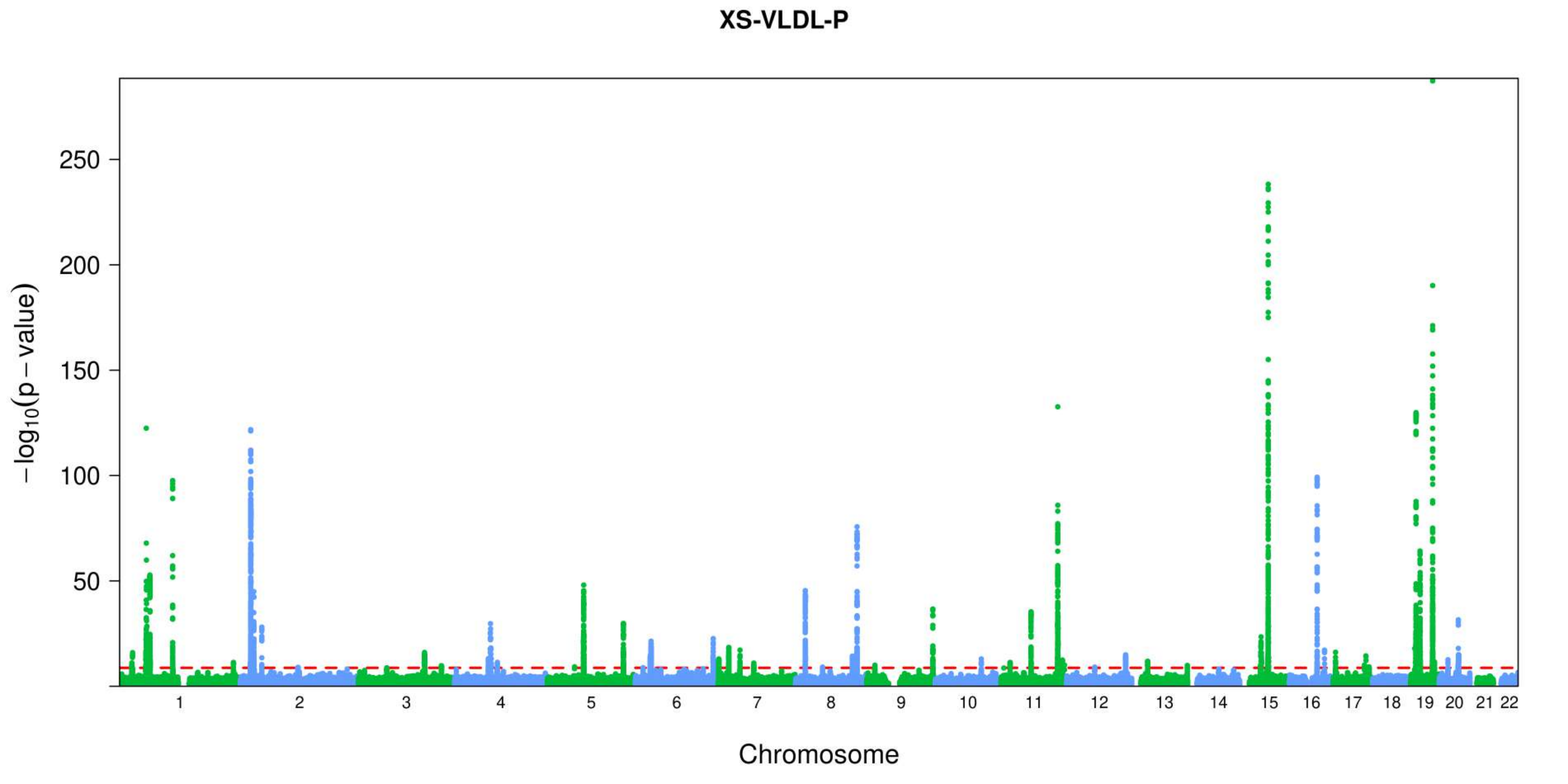
XS-VLDL-P
250
200
150
100
50
$-\log_{10}(p-\text{value})$
1
2
3
4
5
6
7
8
9
10
11
12
13
14
15
16
17
18
19
20
21
22
Chromosome

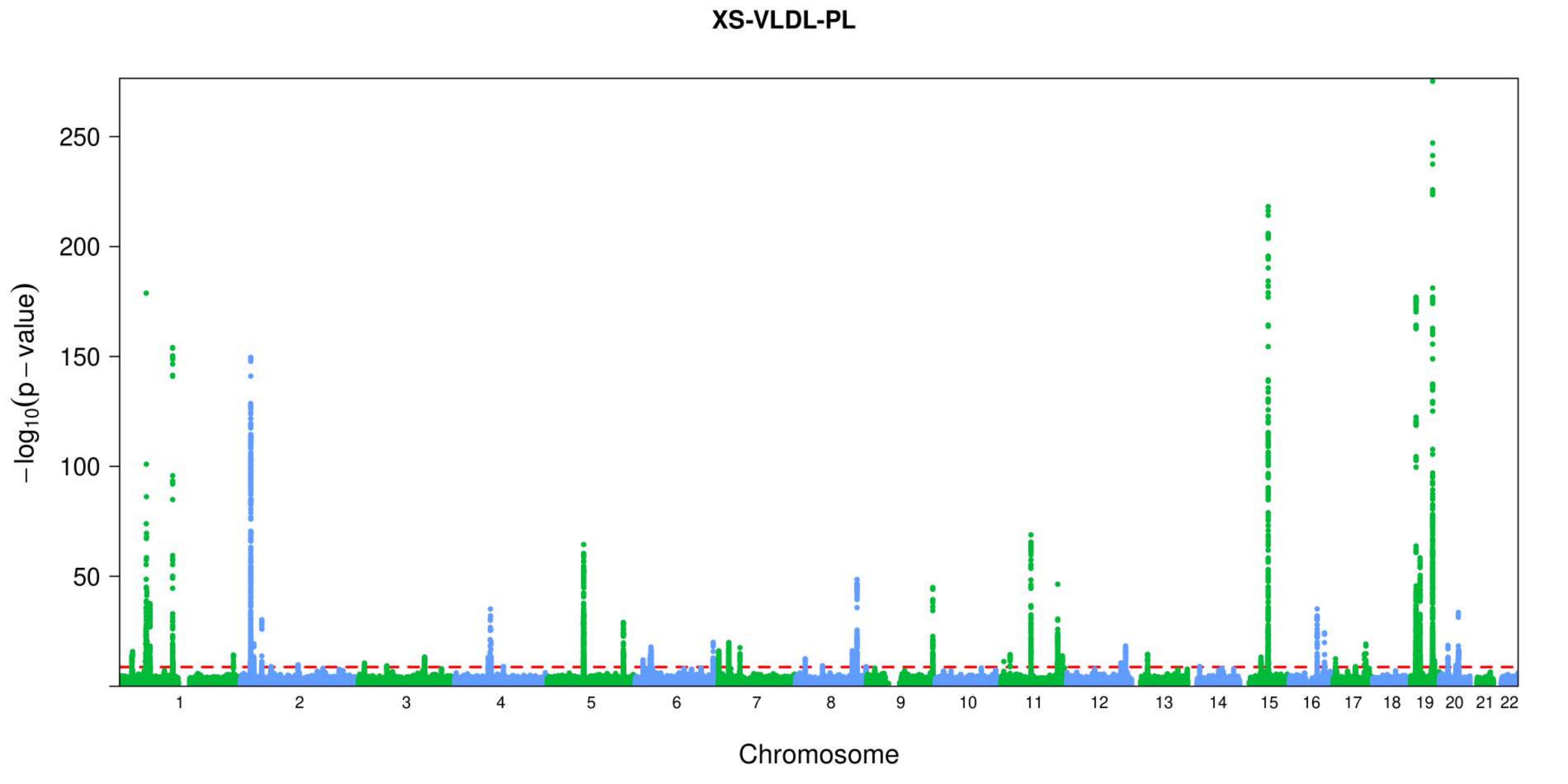
XS-VLDL-PL
$-\log_{10}(p-\text{value})$
250
200
150
100
50
Chromosome
1
2
3
4
5
6
7
8
9
10
11
12
13
14
15
16
17
18
19
20
21
22

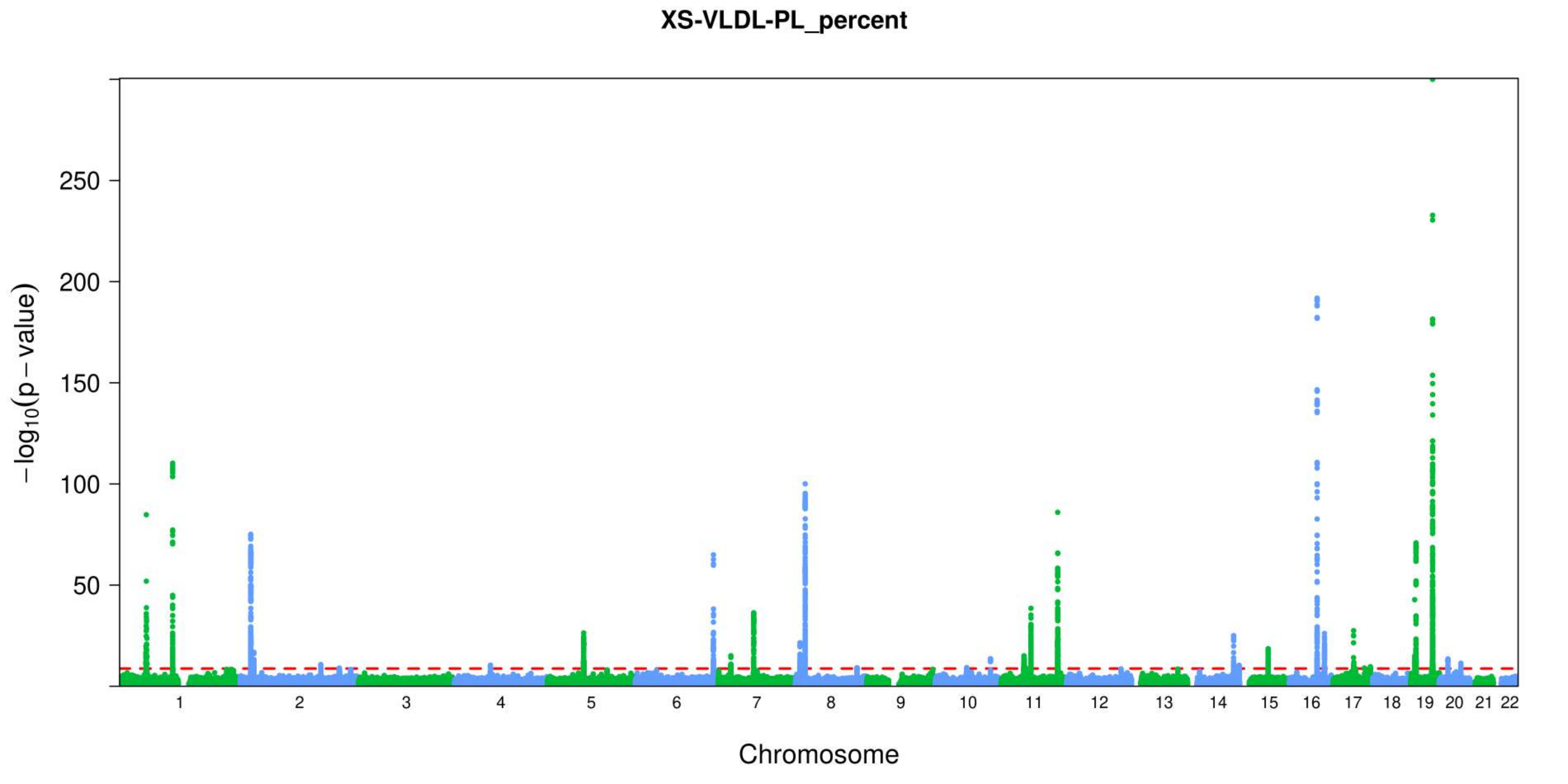

XS-VLDL-PL_percent
250
200
150
100
50
−log10(p − value)
1
2
3
4
5
6
7
8
9
10
11
12
13
14
15
16
17
18
19
20
21
22
Chromosome

XS-VLDL-TG

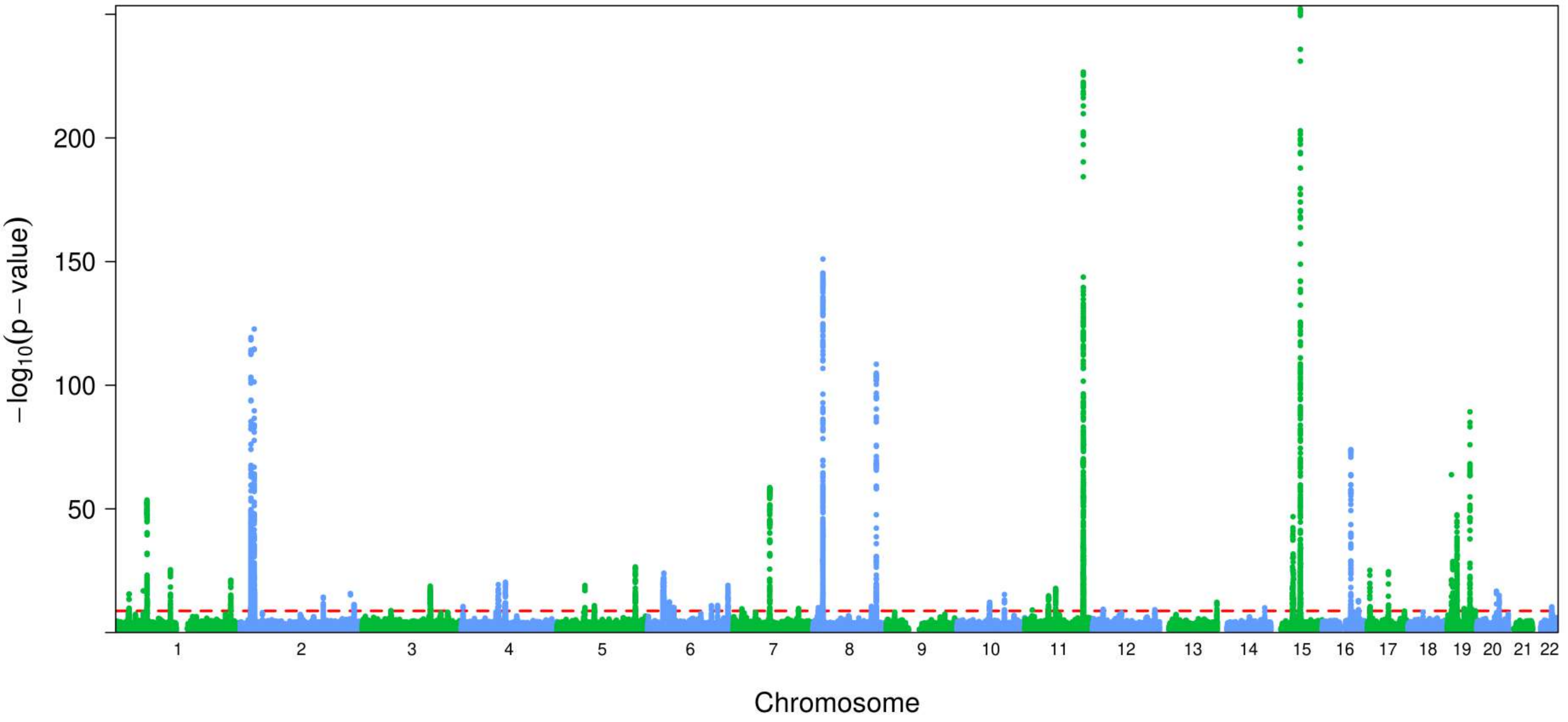
200
150
100
50
-log10(p-value)
1
2
3
4
5
6
7
8
9
10
11
12
13
14
15
16
17
18
19
20
21
22
Chromosome

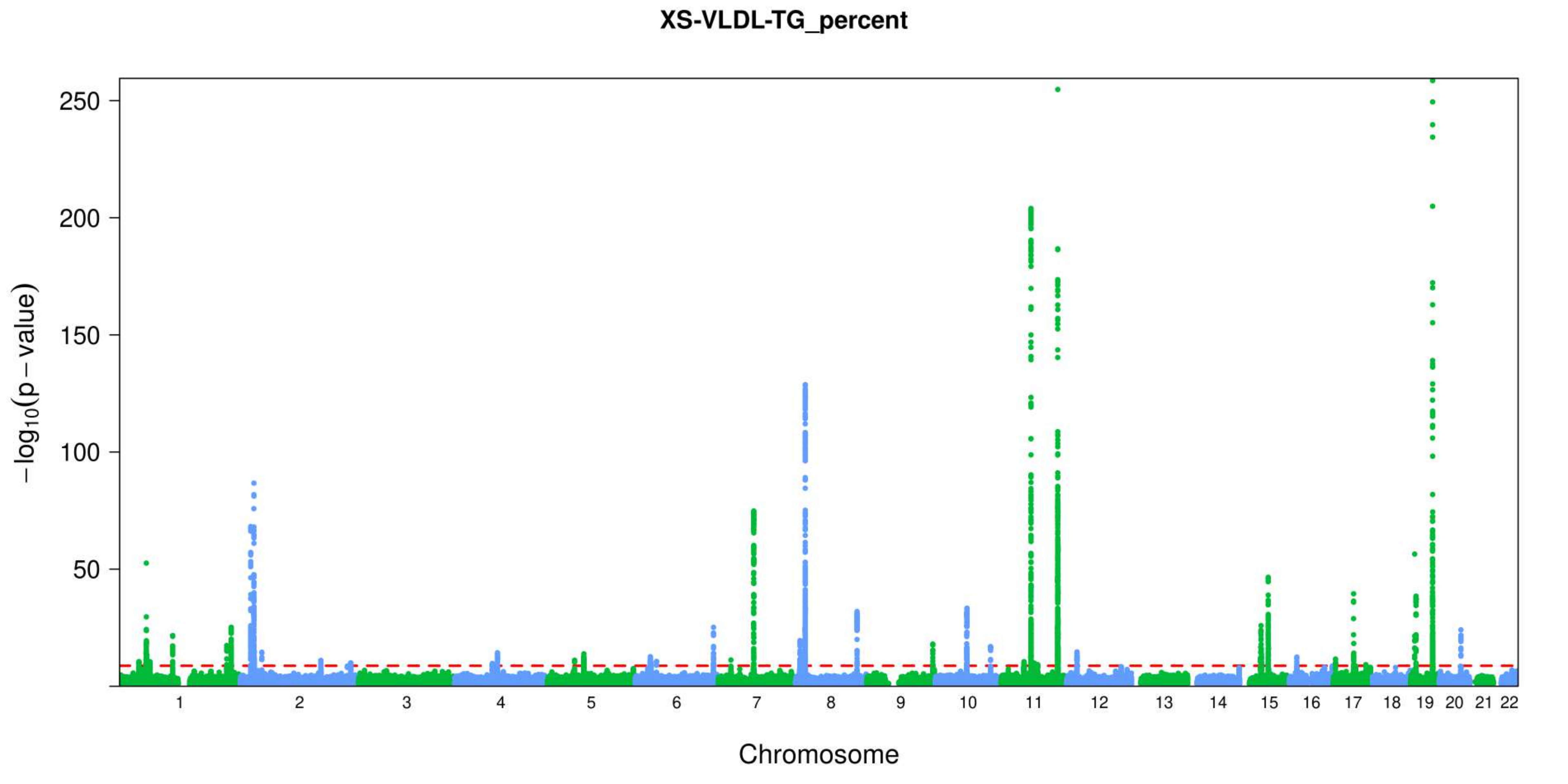
XS-VLDL-TG_percent
−log10(p − value)
250
200
150
100
50
Chromosome
1
2
3
4
5
6
7
8
9
10
11
12
13
14
15
16
17
18
19
20
21
22

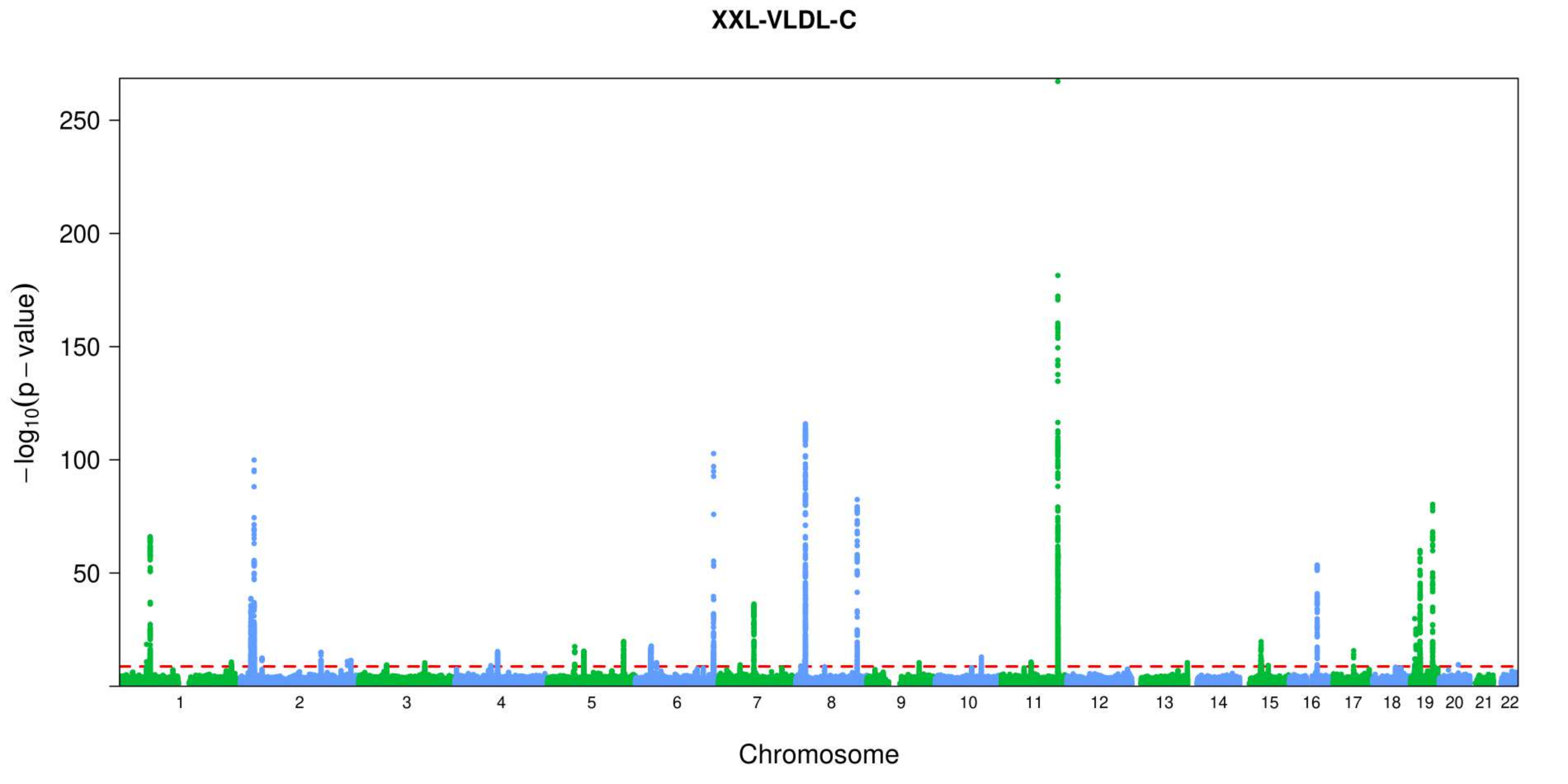
XXL-VLDL-C
250
200
150
100
50
−log10(p − value)
1
2
3
4
5
6
7
8
9
10
11
12
13
14
15
16
17
18
19
20
21
22
Chromosome

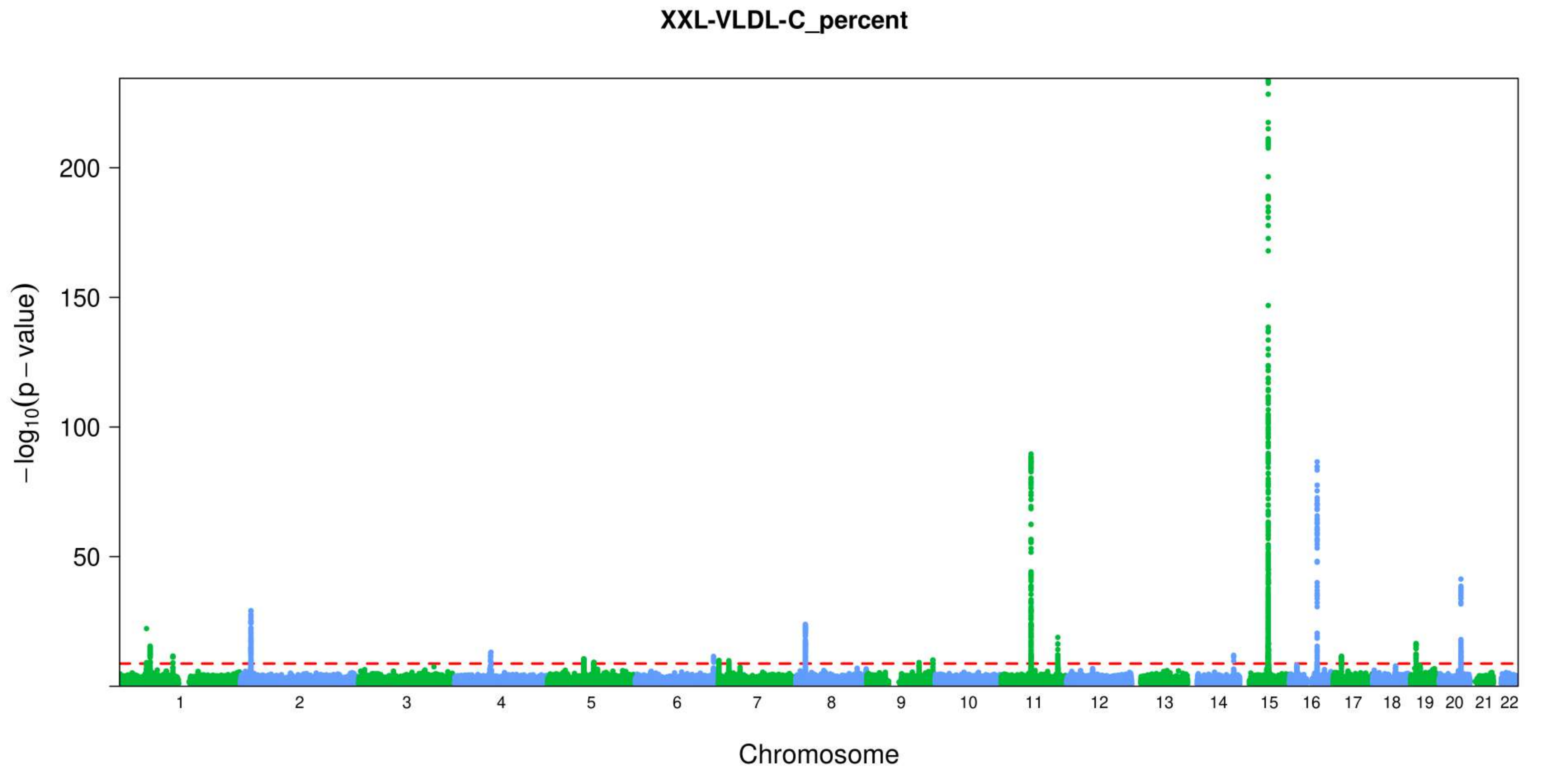
XXL-VLDL-C_percent
200
150
100
50
$-\log_{10}(p-value)$
1
2
3
4
5
6
7
8
9
10
11
12
13
14
15
16
17
18
19
20
21
22
Chromosome

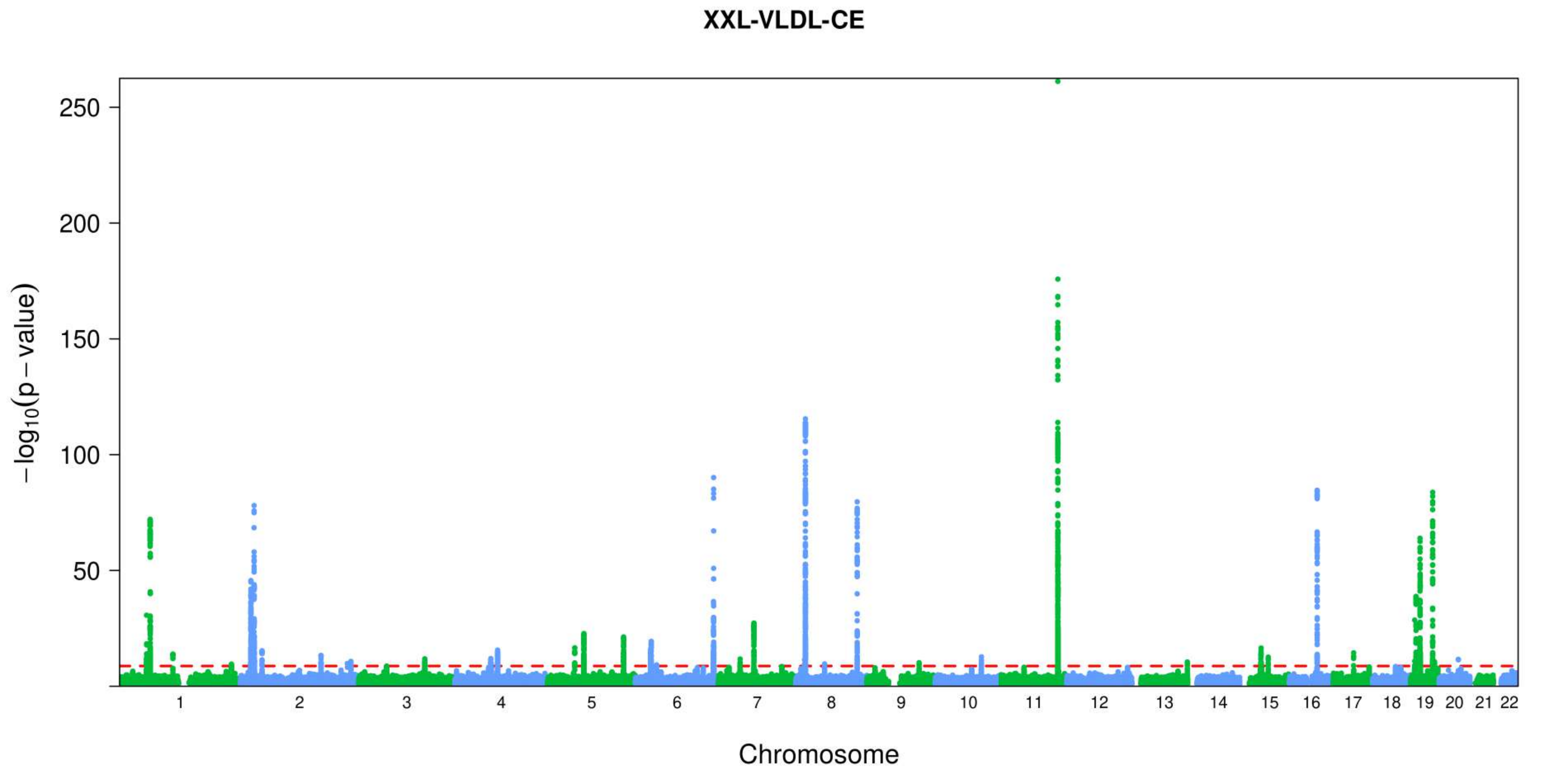
XXL-VLDL-CE
250
200
150
100
50
$-\log_{10}(p-\text{value})$
1
2
3
4
5
6
7
8
9
10
11
12
13
14
15
16
17
18
19
20
21
22
Chromosome

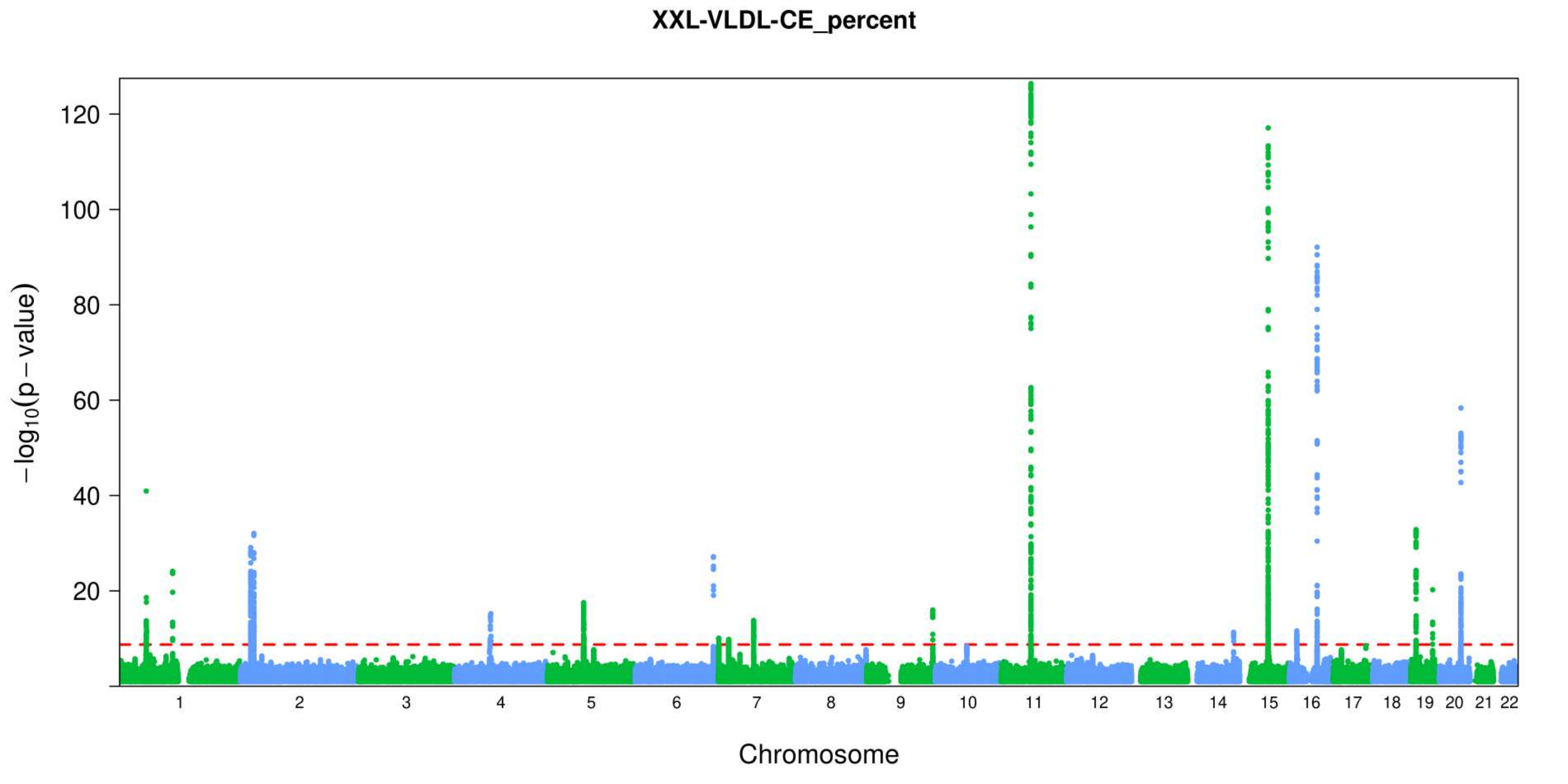
XXL-VLDL-CE_percent
−log10(p − value)
120
100
80
60
40
20
1
2
3
4
5
6
7
8
9
10
11
12
13
14
15
16
17
18
19
20
21
22
Chromosome

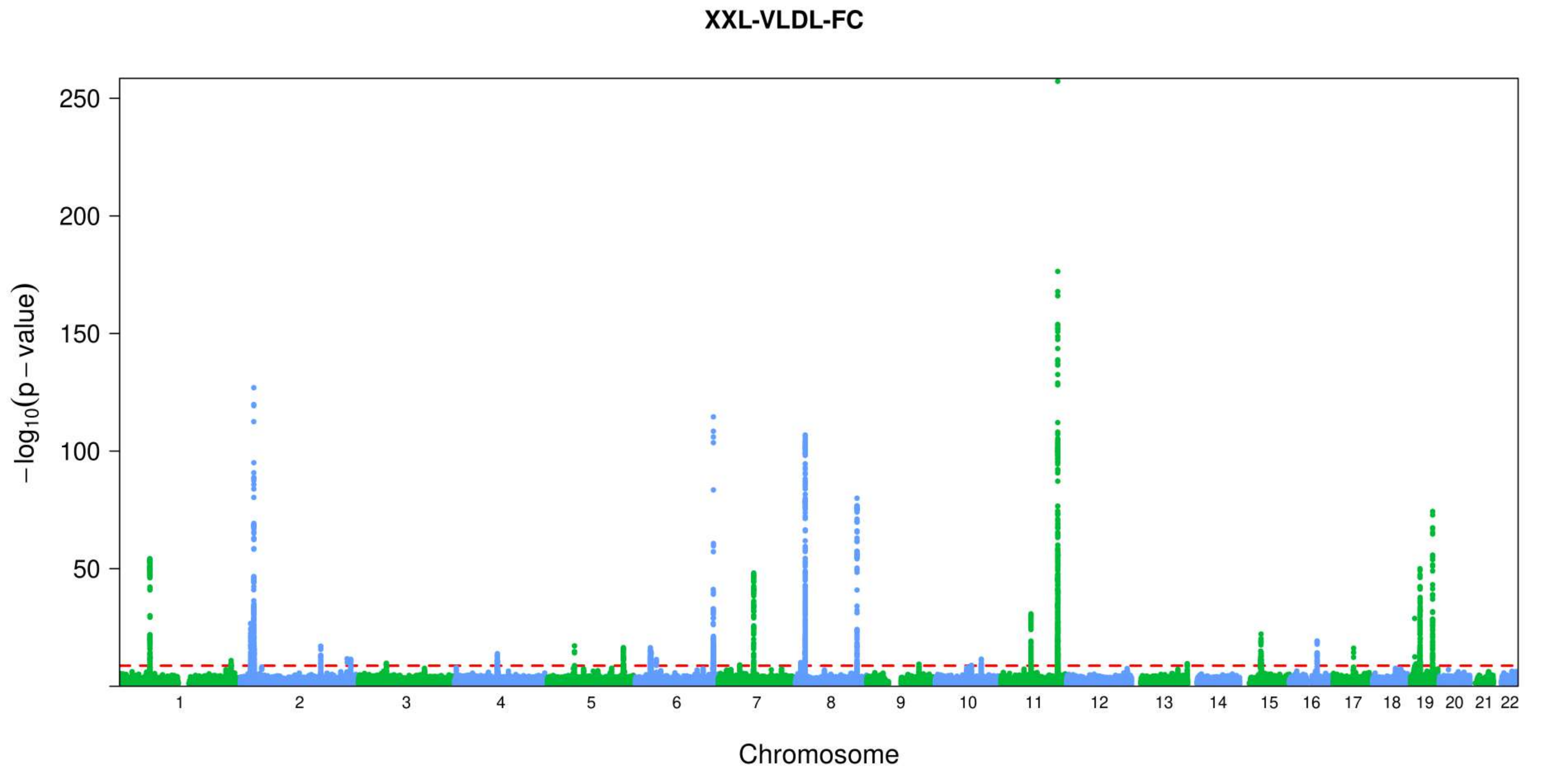
XXL-VLDL-FC
250
200
150
100
50
$-\log_{10}(p-\text{value})$
1
2
3
4
5
6
7
8
9
10
11
12
13
14
15
16
17
18
19
20
21
22
Chromosome

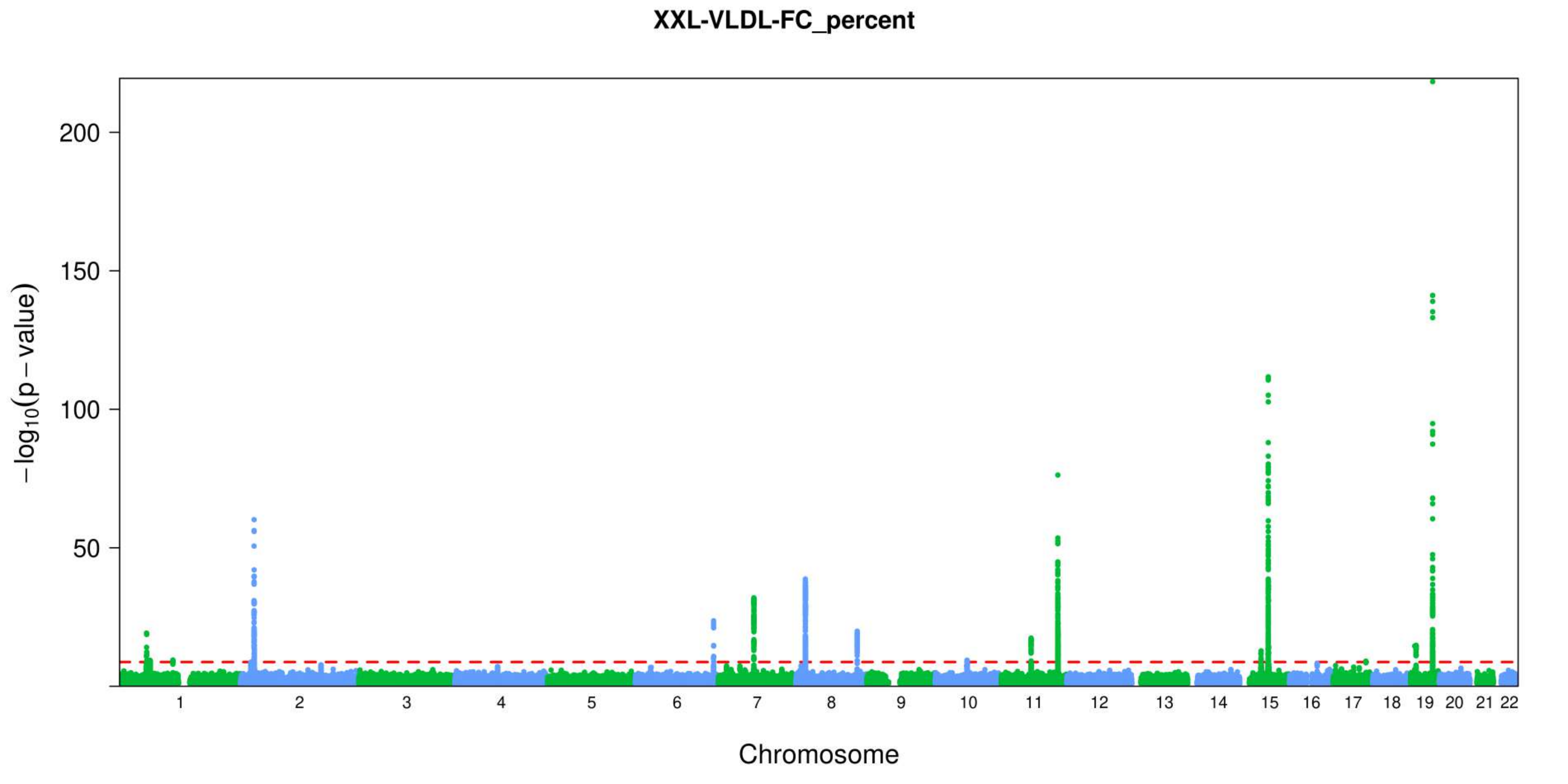
XXL-VLDL-FC_percent
200
150
100
50
−log10(p − value)
1
2
3
4
5
6
7
8
9
10
11
12
13
14
15
16
17
18
19
20
21
22
Chromosome

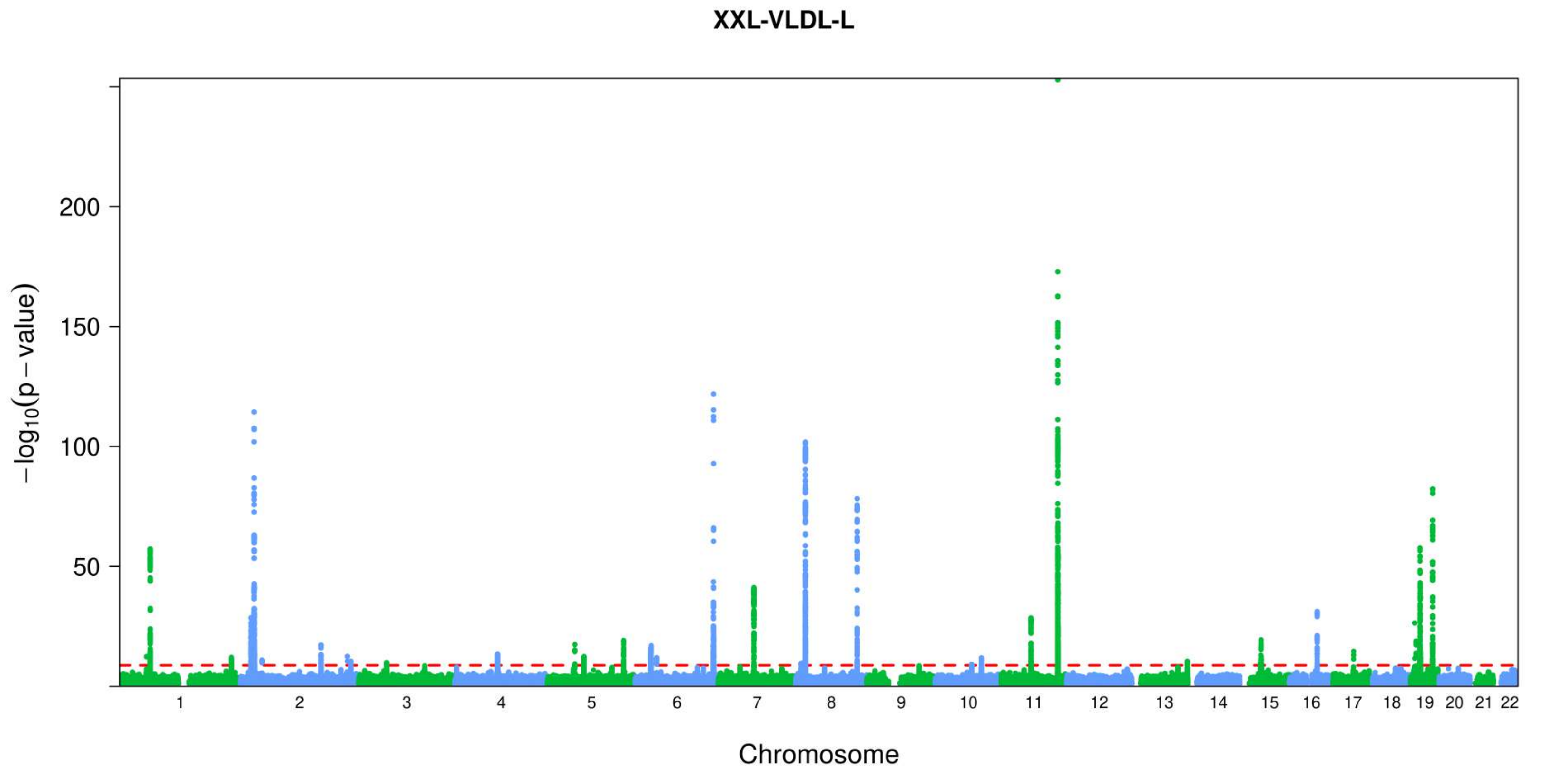
XXL-VLDL-L
−log10(p − value)
200
150
100
50
Chromosome
1
2
3
4
5
6
7
8
9
10
11
12
13
14
15
16
17
18
19
20
21
22

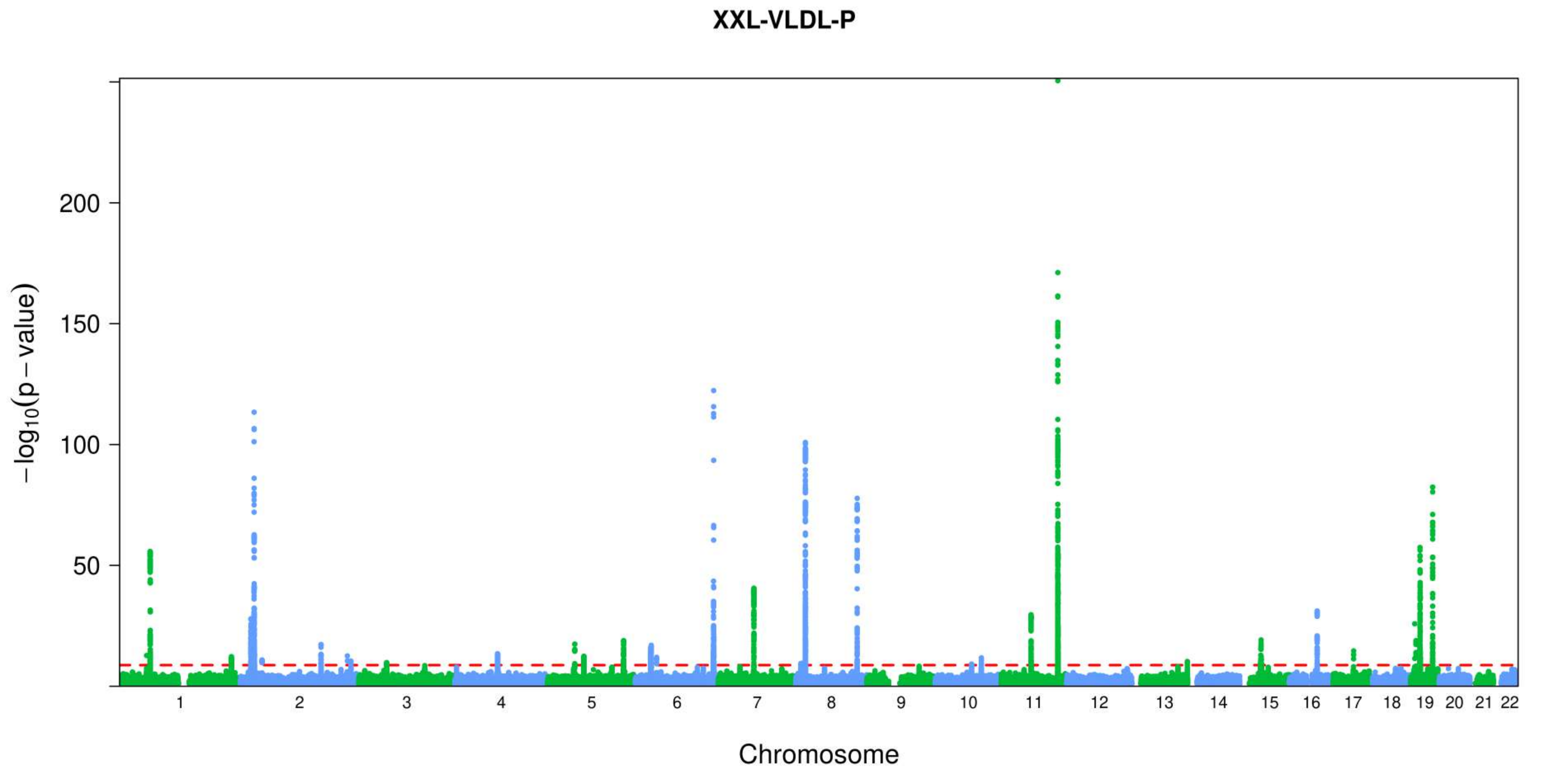
XXL-VLDL-P
200
150
100
50
$-\log_{10}(p-\text{value})$
1
2
3
4
5
6
7
8
9
10
11
12
13
14
15
16
17
18
19
20
21
22
Chromosome

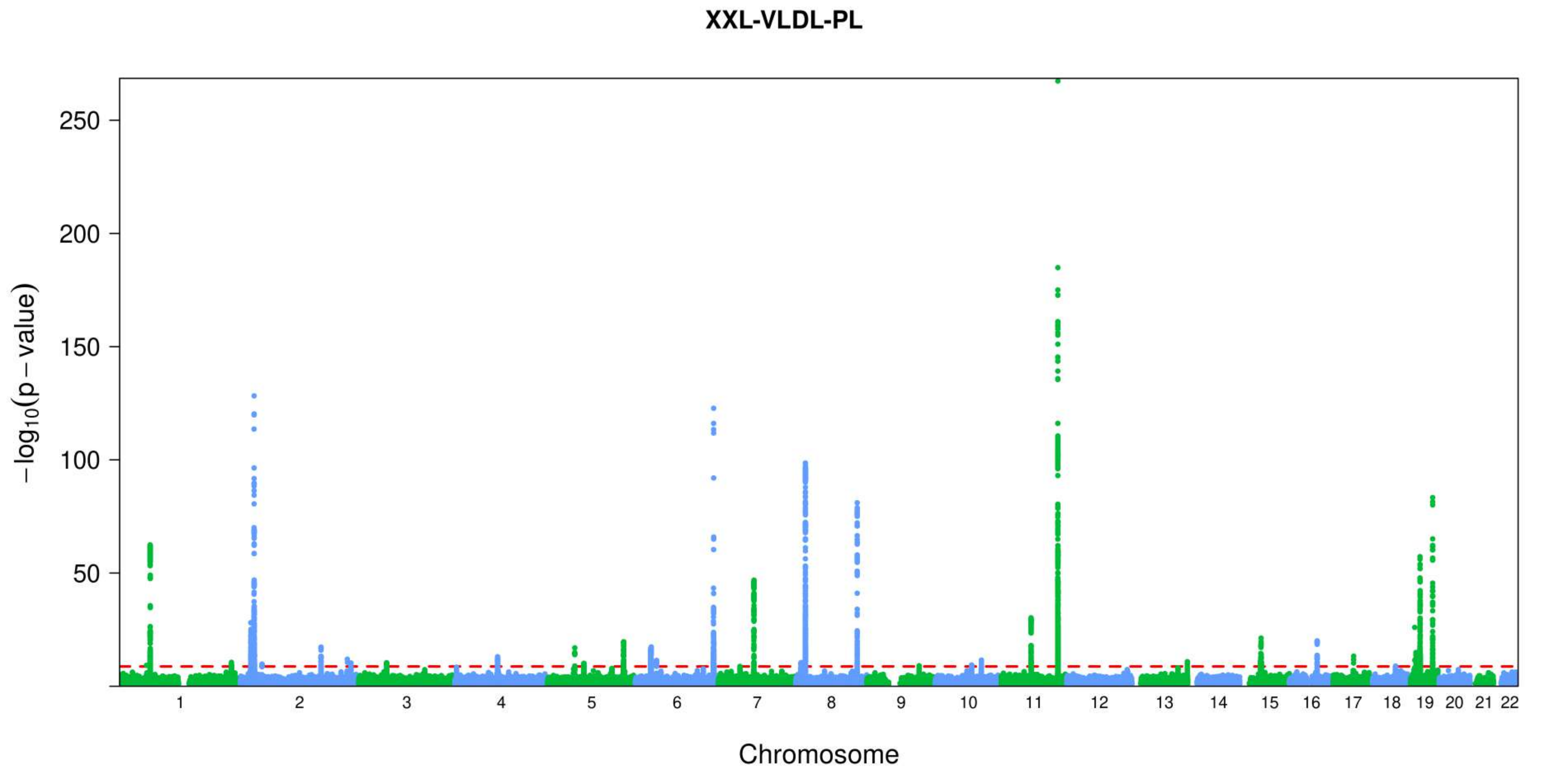
XXL-VLDL-PL
250
200
150
100
50
−log10(p − value)
1
2
3
4
5
6
7
8
9
10
11
12
13
14
15
16
17
18
19
20
21
22
Chromosome

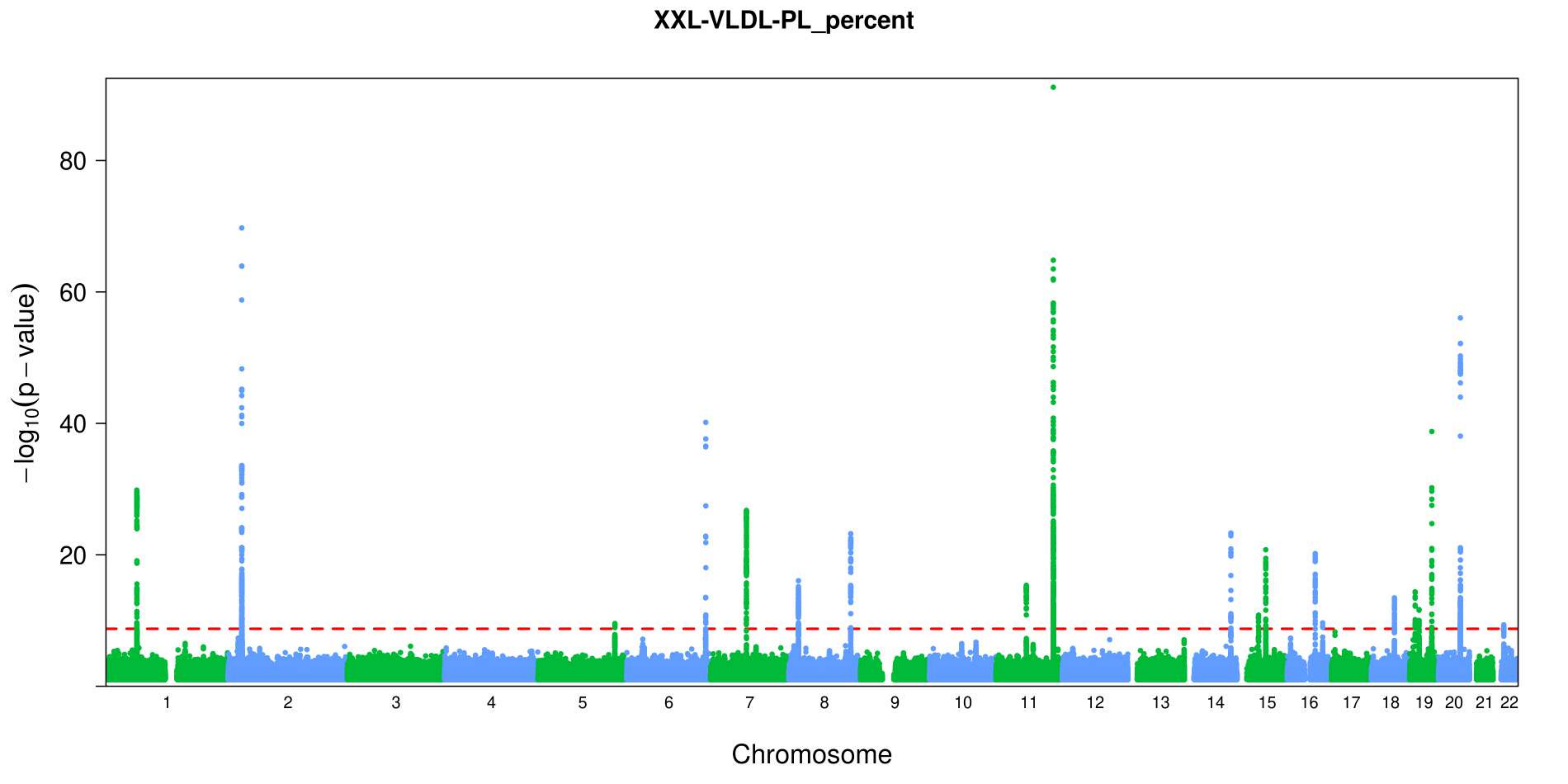

XXL-VLDL-PL_percent
80
60
40
20
−log10(p − value)
1
2
3
4
5
6
7
8
9
10
11
12
13
14
15
16
17
18
19
20
21
22
Chromosome

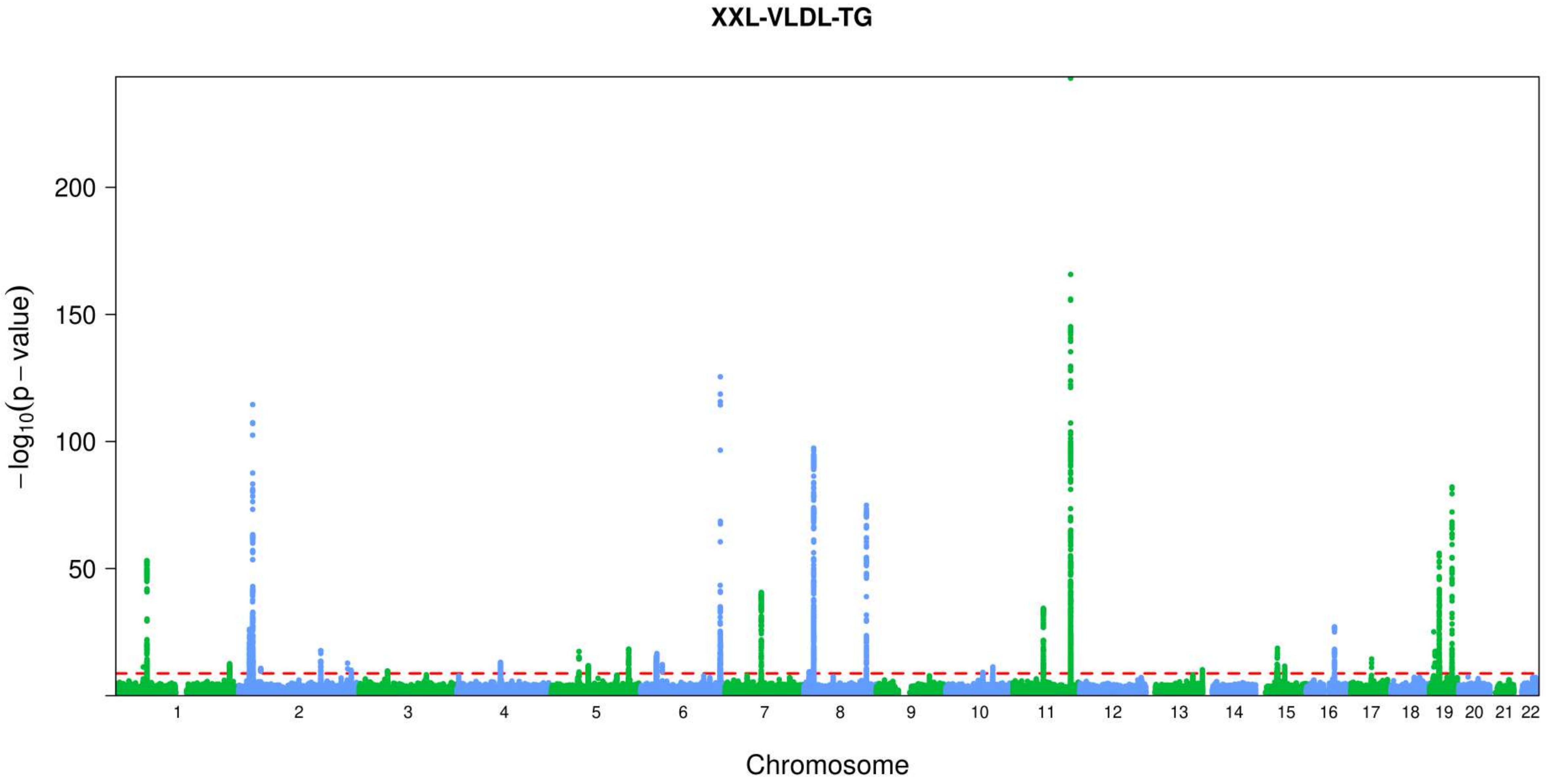

XXL-VLDL-TG
200
150
100
50
$-\log_{10}(p-\text{value})$
1
2
3
4
5
6
7
8
9
10
11
12
13
14
15
16
17
18
19
20
21
22
Chromosome

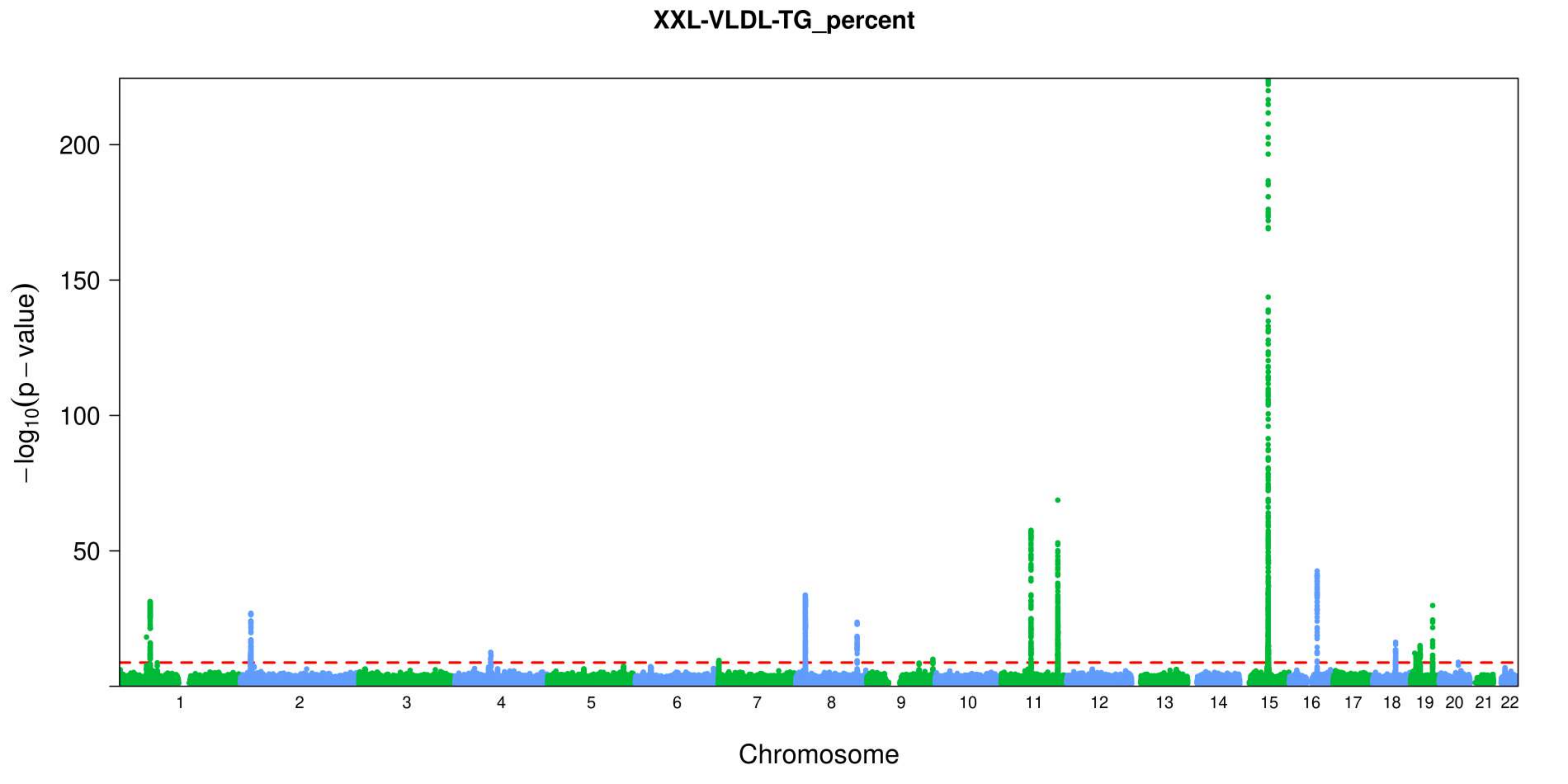
XXL-VLDL-TG_percent
200
150
100
50
−log10(p − value)
1
2
3
4
5
6
7
8
9
10
11
12
13
14
15
16
17
18
19
20
21
22
Chromosome
