# Supplementary material for: Genome-wide characterization of circulating metabolic biomarkers
Source: Nature. 2024 Mar 6;628(8006):130–8. doi: 10.1038/s41586-024-07148-y (PMC10990933; doi:10.1038/s41586-024-07148-y)
Supplement: Supplementary file 4 — Supplementary Fig. 2. Regional associations plots for the most significantly associated metabolic traits in each genomic region. [file 41586_2024_7148_MOESM4_ESM.pdf]

1_1:Ala

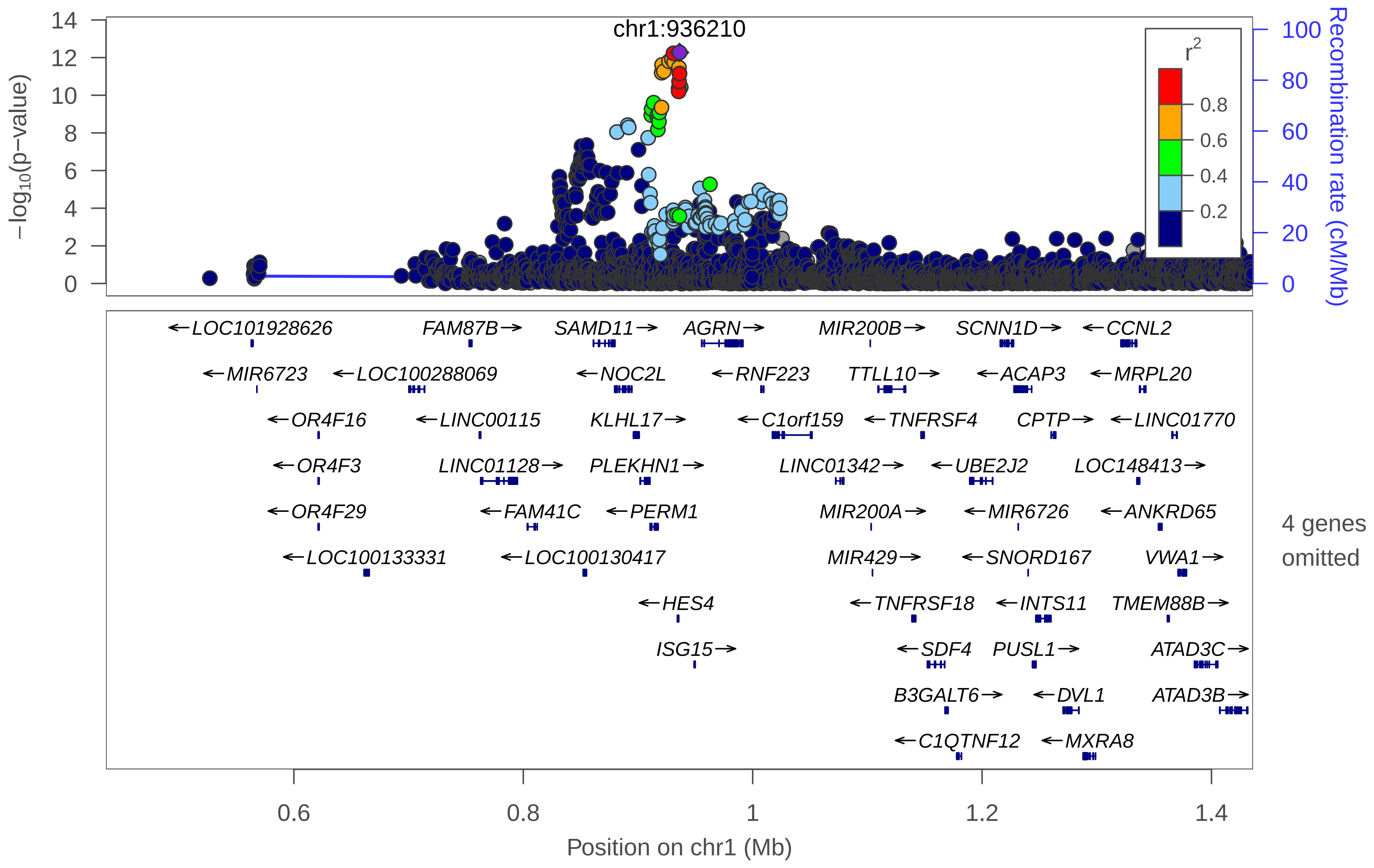

chr1:936210
−log10(p−value)
Recombination rate (cM/Mb)
r2
0.8
0.6
0.4
0.2
0
2
4
6
8
10
12
14
0
20
40
60
80
100
LOC101928626
FAM87B
SAMD11
AGRN
MIR200B
SCNN1D
CCNL2
MIR6723
LOC100288069
NOC2L
RNF223
TTLL10
ACAP3
MRPL20
OR4F16
LINC00115
KLHL17
C1orf159
TNFRSF4
CPTP
LINC01770
OR4F3
LINC01128
PLEKHN1
LINC01342
UBE2J2
LOC148413
OR4F29
FAM41C
PERM1
MIR200A
MIR6726
ANKRD65
LOC100133331
LOC100130417
MIR429
SNORD167
VWA1
HES4
TNFRSF18
INTS11
TMEM88B
ISG15
SDF4
PUSL1
ATAD3C
B3GALT6
DVL1
ATAD3B
C1QTNF12
MXRA8
4 genes omitted
0.6
0.8
1
1.2
1.4
Position on chr1 (Mb)

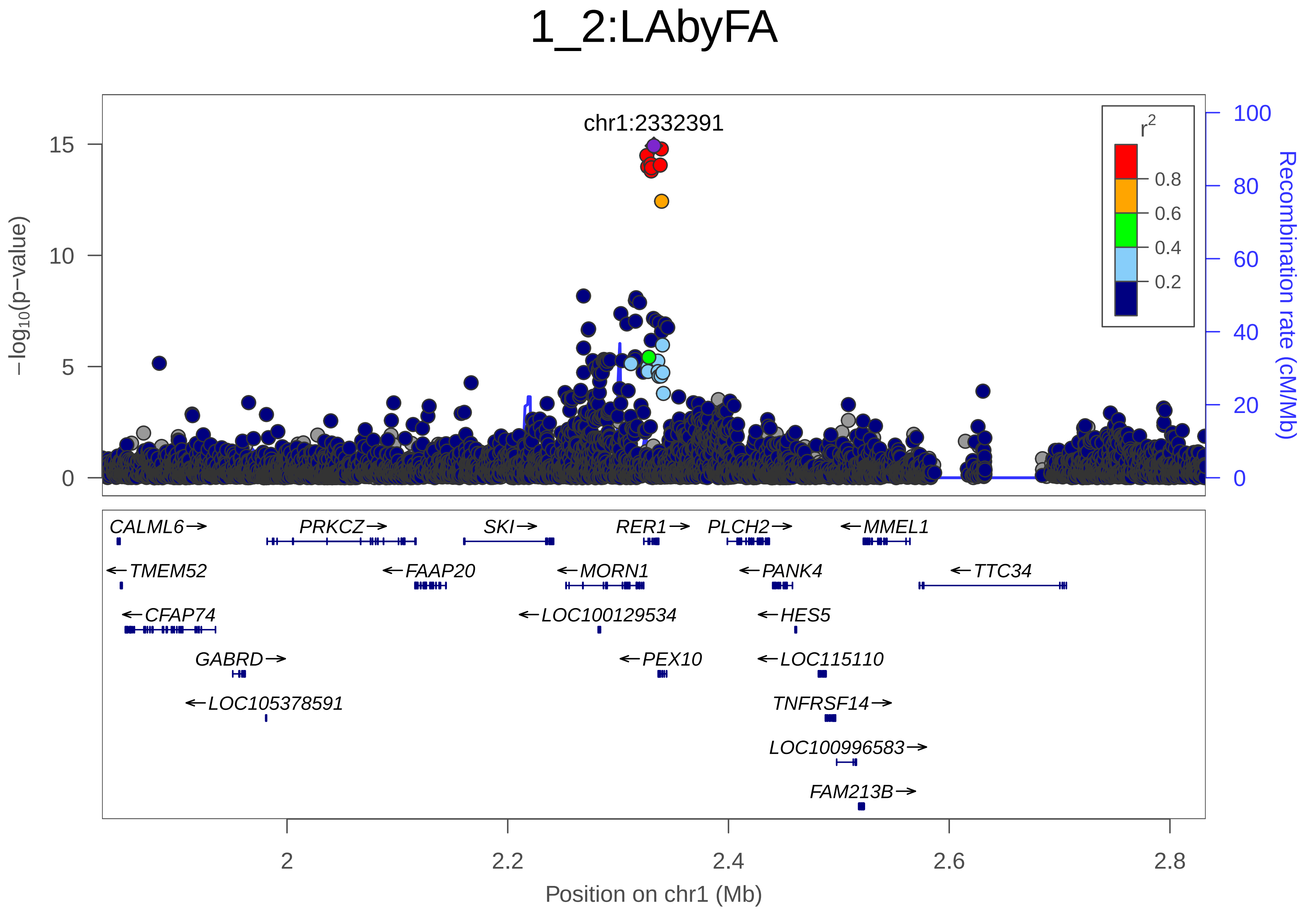
1_2:LAbyFA
chr1:2332391
r2
0.8
0.6
0.4
0.2
−log10(p−value)
Recombination rate (cM/Mb)
CALML6→
PRKCZ→
SKI→
RER1→
PLCH2→
←MMEL1
←TMEM52
←FAAP20
←MORN1
←PANK4
←TTC34
←CFAP74
←LOC100129534
←HES5
GABRD→
←PEX10
←LOC115110
←LOC105378591
TNFRSF14→
LOC100996583→
FAM213B→
Position on chr1 (Mb)

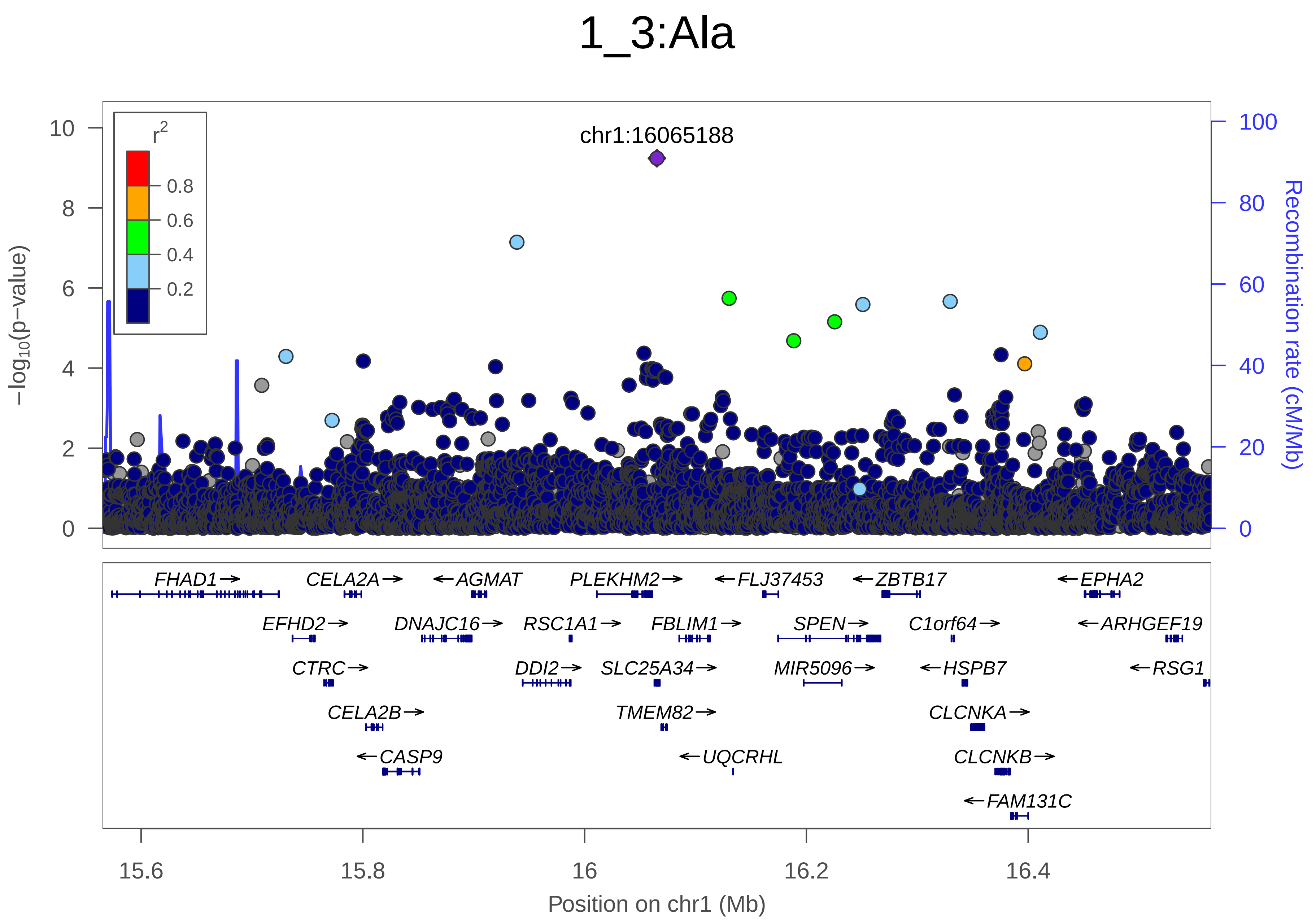

1_3:Ala
chr1:16065188
r2
0.8
0.6
0.4
0.2
−log10(p−value)
Recombination rate (cM/Mb)
FHAD1→
CELA2A→
←AGMAT
PLEKHM2→
←FLJ37453
←ZBTB17
←EPHA2
EFHD2→
DNAJC16→
RSC1A1→
FBLIM1→
SPEN→
C1orf64→
←ARHGEF19
CTRC→
DDI2→
SLC25A34→
MIR5096→
←HSPB7
←RSG1
CELA2B→
TMEM82→
CLCNKA→
←CASP9
←UQCRHL
CLCNKB→
←FAM131C
15.6
15.8
16
16.2
16.4
Position on chr1 (Mb)

1_4:S−HDL−FC

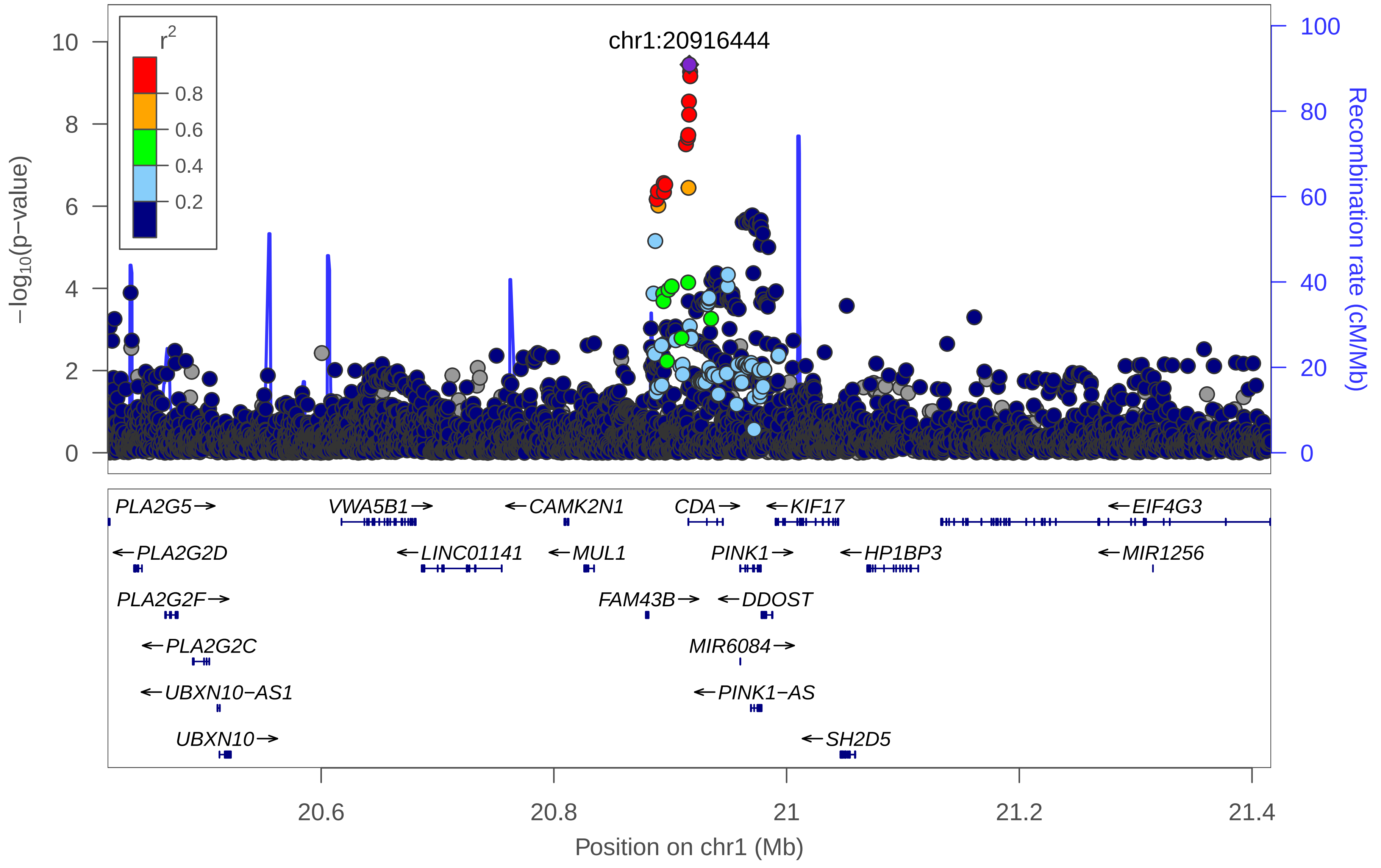

chr1:20916444
r2
0.8
0.6
0.4
0.2
10
8
6
4
2
0
−log10(p−value)
100
80
60
40
20
0
Recombination rate (cM/Mb)
PLA2G5→
VWA5B1→
←CAMK2N1
CDA→
←KIF17
←EIF4G3
←PLA2G2D
←LINC01141
←MUL1
PINK1→
←HP1BP3
←MIR1256
PLA2G2F→
FAM43B→
←DDOST
←PLA2G2C
MIR6084→
←UBXN10−AS1
←PINK1−AS
UBXN10→
←SH2D5
20.6
20.8
21
21.2
21.4
Position on chr1 (Mb)

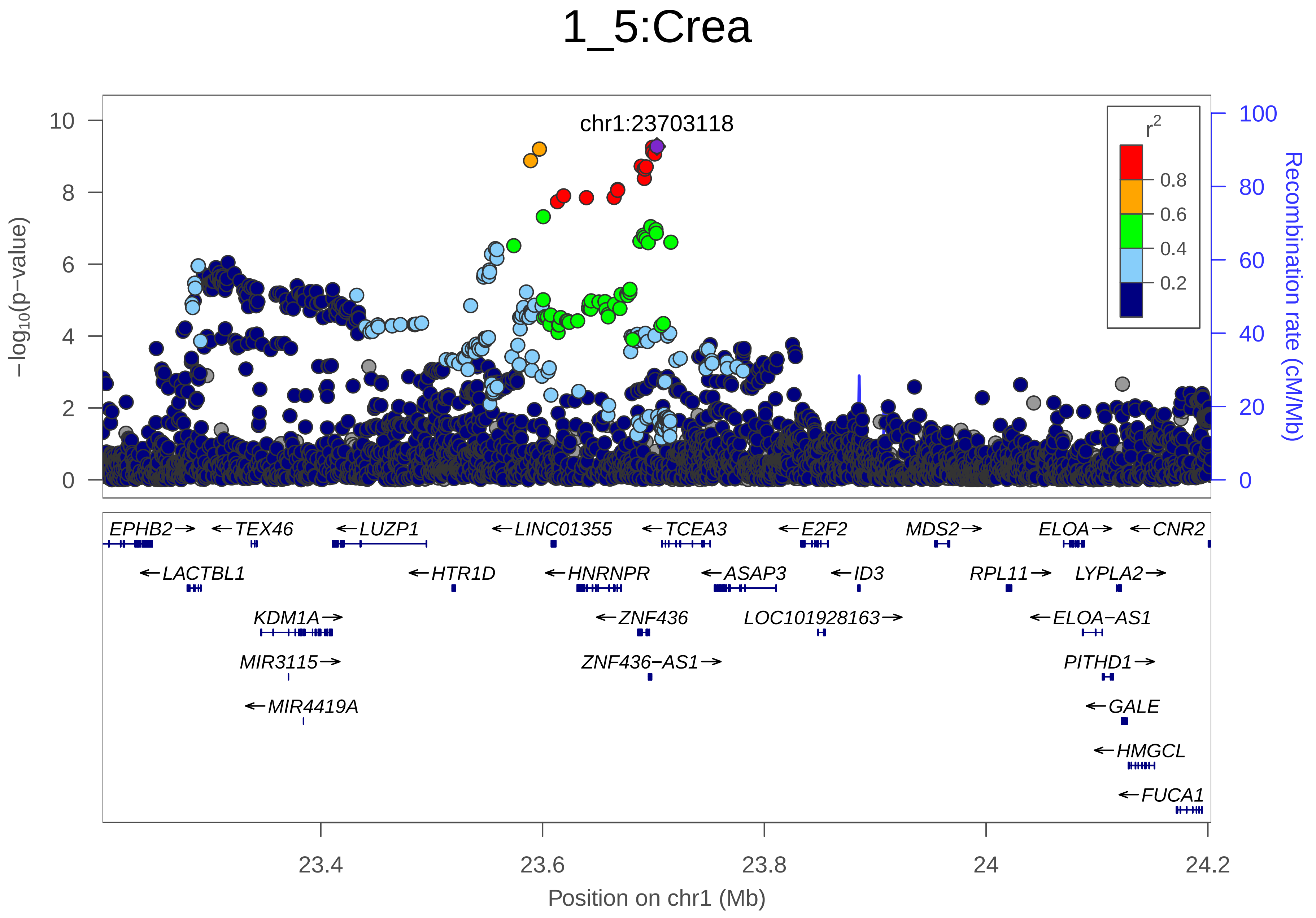

1_5:Crea
chr1:23703118
r²
0.8
0.6
0.4
0.2
−log10(p−value)
Recombination rate (cM/Mb)
EPHB2→
←TEX46
←LUZP1
←LINC01355
←TCEA3
←E2F2
MDS2→
ELOA→
←CNR2
←LACTBL1
←HTR1D
←HNRNPR
←ASAP3
←ID3
RPL11→
LYPLA2→
KDM1A→
←ZNF436
LOC101928163→
←ELOA−AS1
MIR3115→
ZNF436−AS1→
PITHD1→
←MIR4419A
←GALE
←HMGCL
←FUCA1
23.4
23.6
23.8
24
24.2
Position on chr1 (Mb)

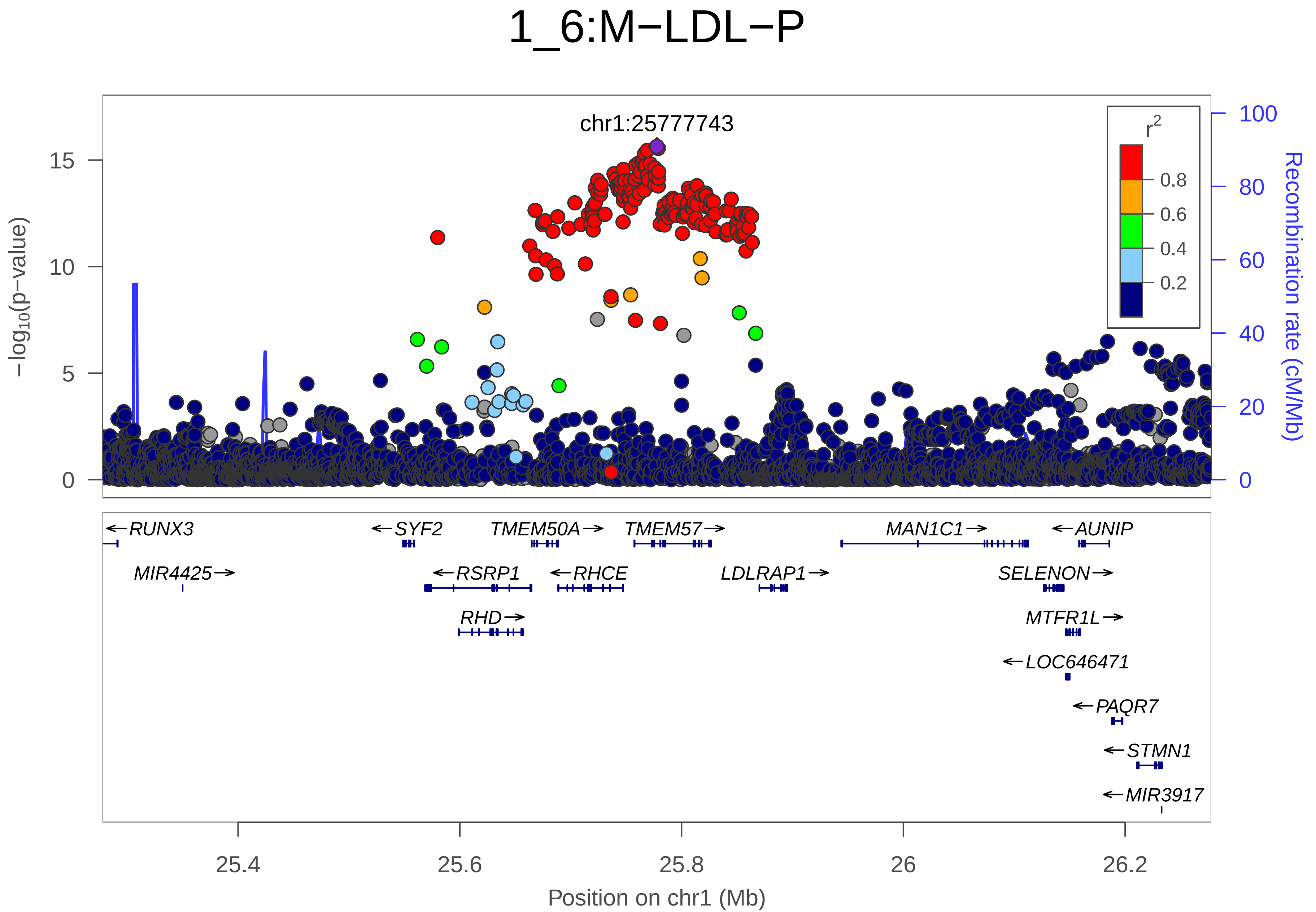

1_6:M−LDL−P
chr1:25777743
r2
0.8
0.6
0.4
0.2
−log10(p−value)
Recombination rate (cM/Mb)
15
10
5
0
100
80
60
40
20
0
←RUNX3
MIR4425→
←SYF2
←RSRP1
RHD→
TMEM50A→
←RHCE
TMEM57→
LDLRAP1→
MAN1C1→
←AUNIP
SELENON→
MTFR1L→
←LOC646471
←PAQR7
←STMN1
←MIR3917
25.4
25.6
25.8
26
26.2
Position on chr1 (Mb)

1_7:ApoBbyApoA1

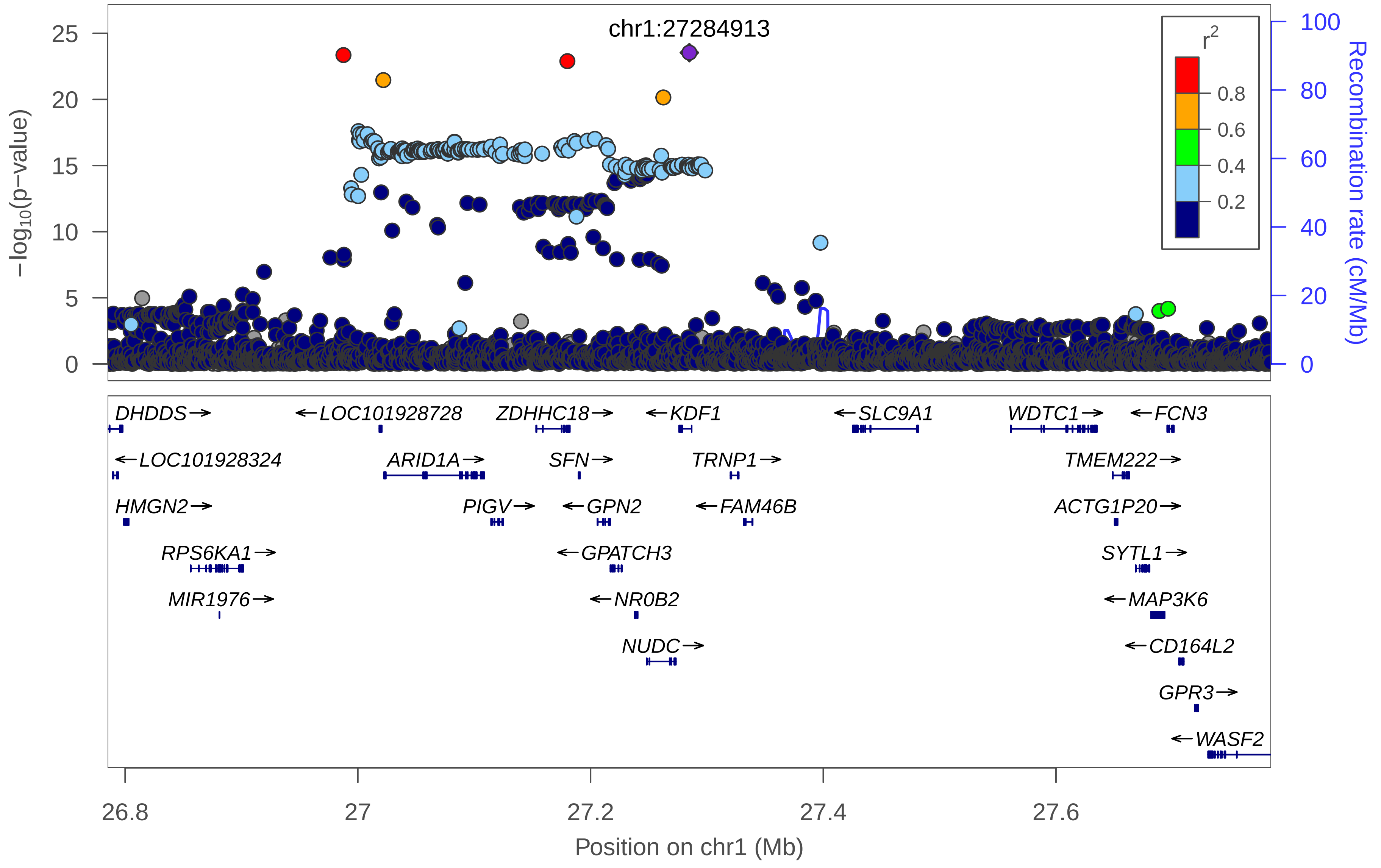

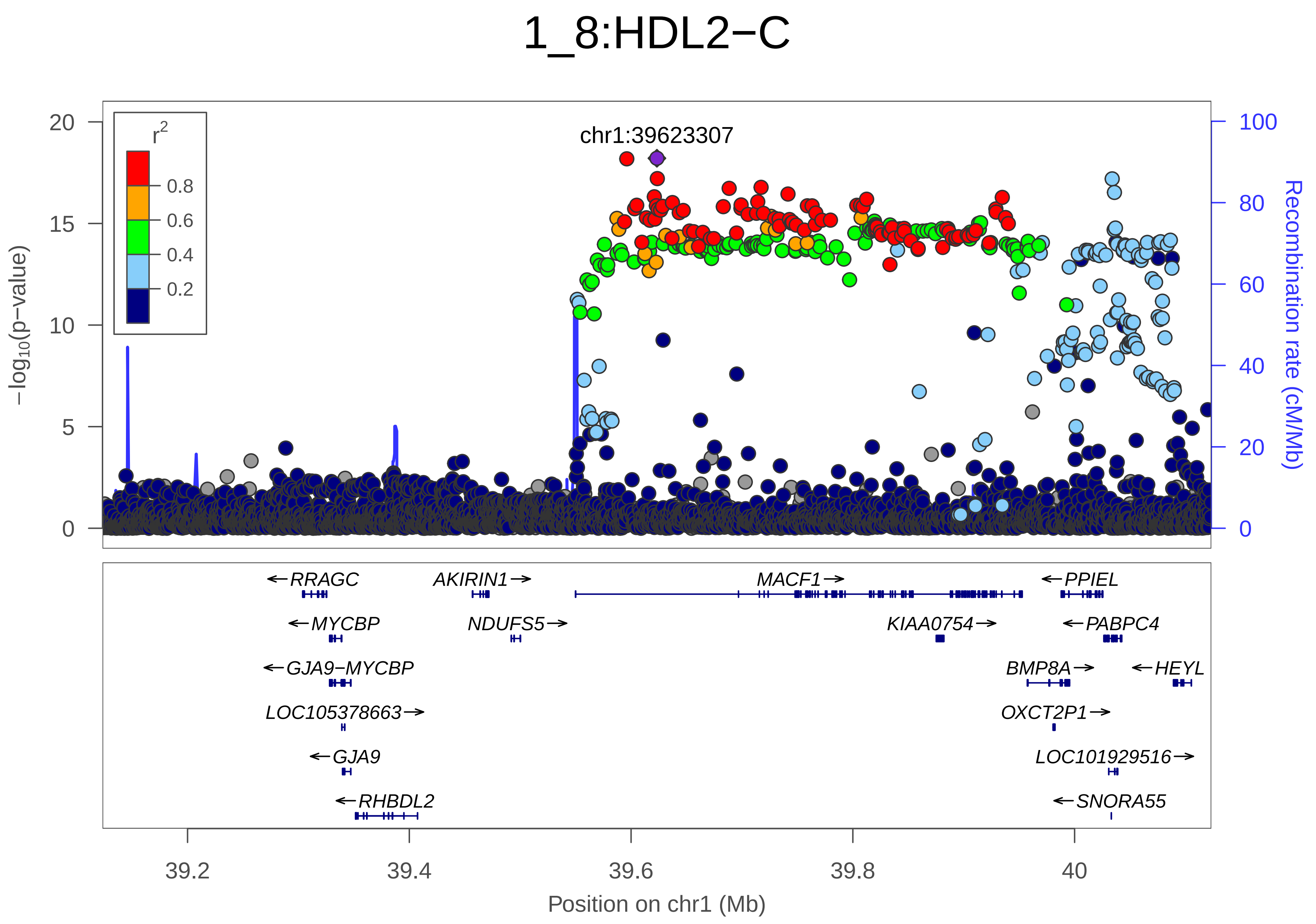

1_8:HDL2−C
chr1:39623307
r2
0.8
0.6
0.4
0.2
−log10(p−value)
Recombination rate (cM/Mb)
RRAGC
AKIRIN1
MACF1
PPIEL
MYCBP
NDUFS5
KIAA0754
PABPC4
GJA9−MYCBP
BMP8A
HEYL
LOC105378663
OXCT2P1
GJA9
LOC101929516
RHBDL2
SNORA55
Position on chr1 (Mb)

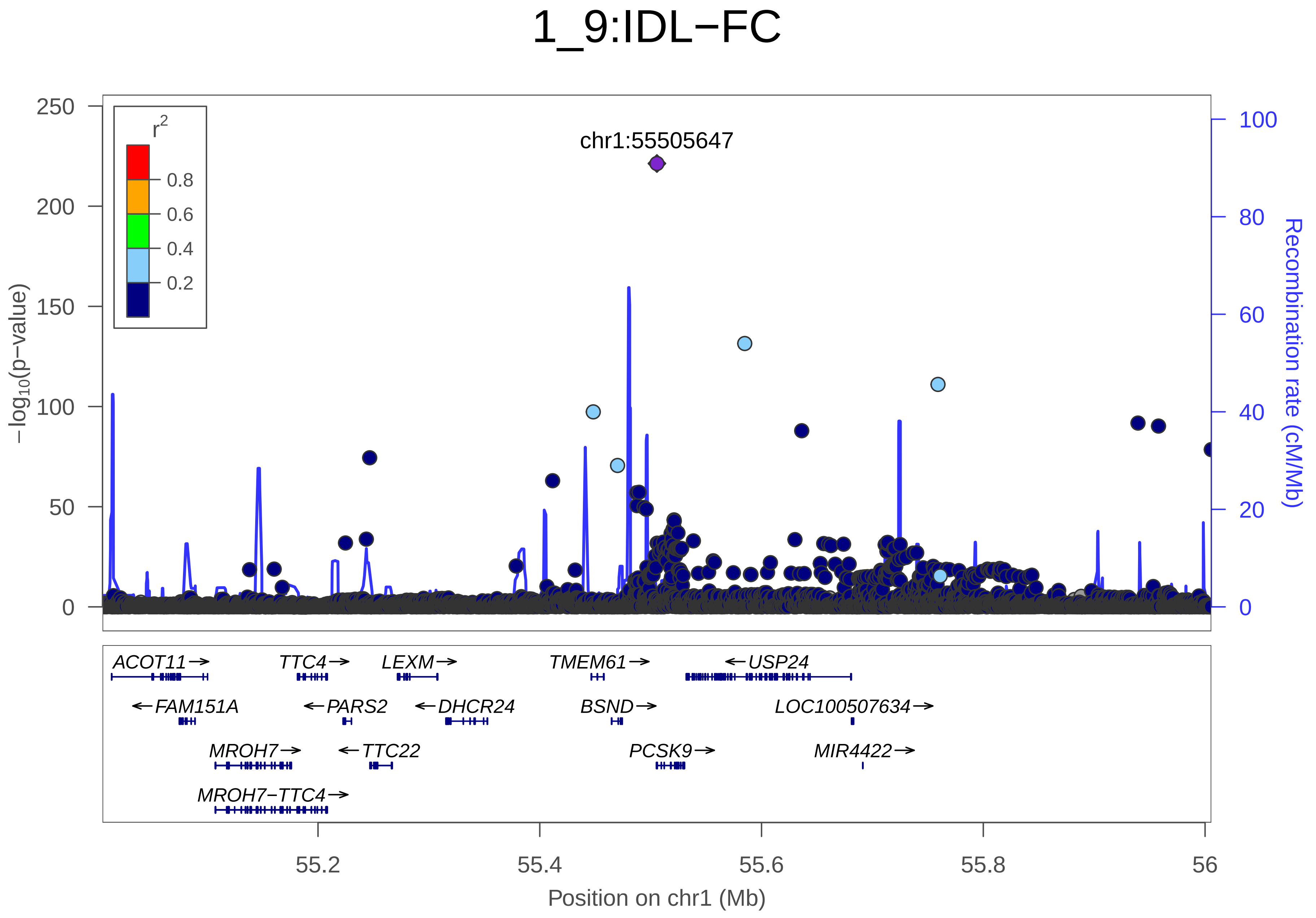

1_9:IDL−FC
chr1:55505647
r2
0.8
0.6
0.4
0.2
250
200
150
100
50
0
−log10(p−value)
100
80
60
40
20
0
Recombination rate (cM/Mb)
ACOT11→
TTC4→
LEXM→
TMEM61→
←USP24
←FAM151A
←PARS2
←DHCR24
BSND→
LOC100507634→
MROH7→
←TTC22
PCSK9→
MIR4422→
MROH7−TTC4→
55.2
55.4
55.6
55.8
56
Position on chr1 (Mb)

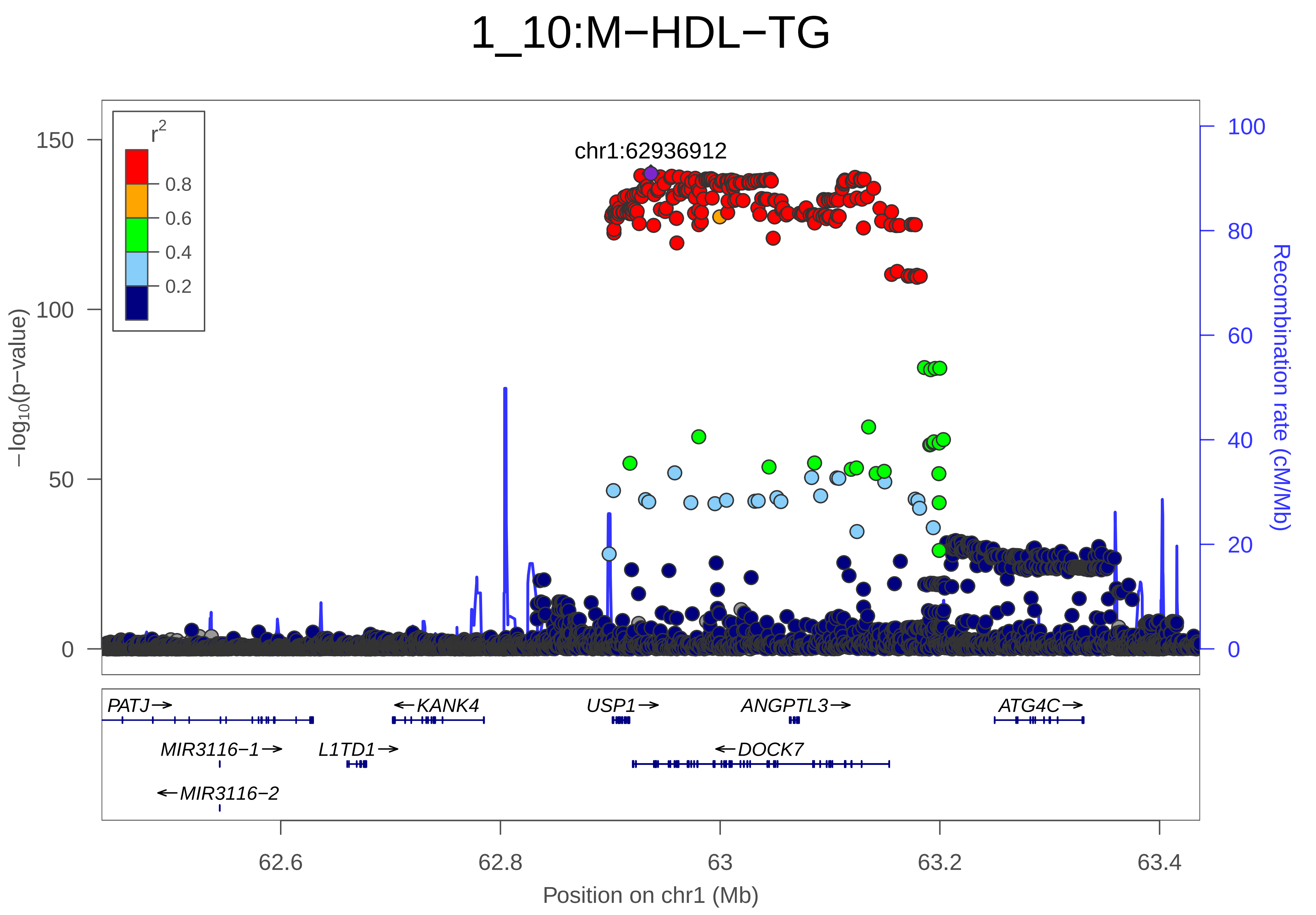
1_10:M−HDL−TG
chr1:62936912
r2
0.8
0.6
0.4
0.2
−log10(p−value)
150
100
50
0
Recombination rate (cM/Mb)
100
80
60
40
20
0
PATJ→
←KANK4
USP1→
ANGPTL3→
ATG4C→
MIR3116−1→
L1TD1→
←DOCK7
←MIR3116−2
62.6
62.8
63
63.2
63.4
Position on chr1 (Mb)

1_11:Gly

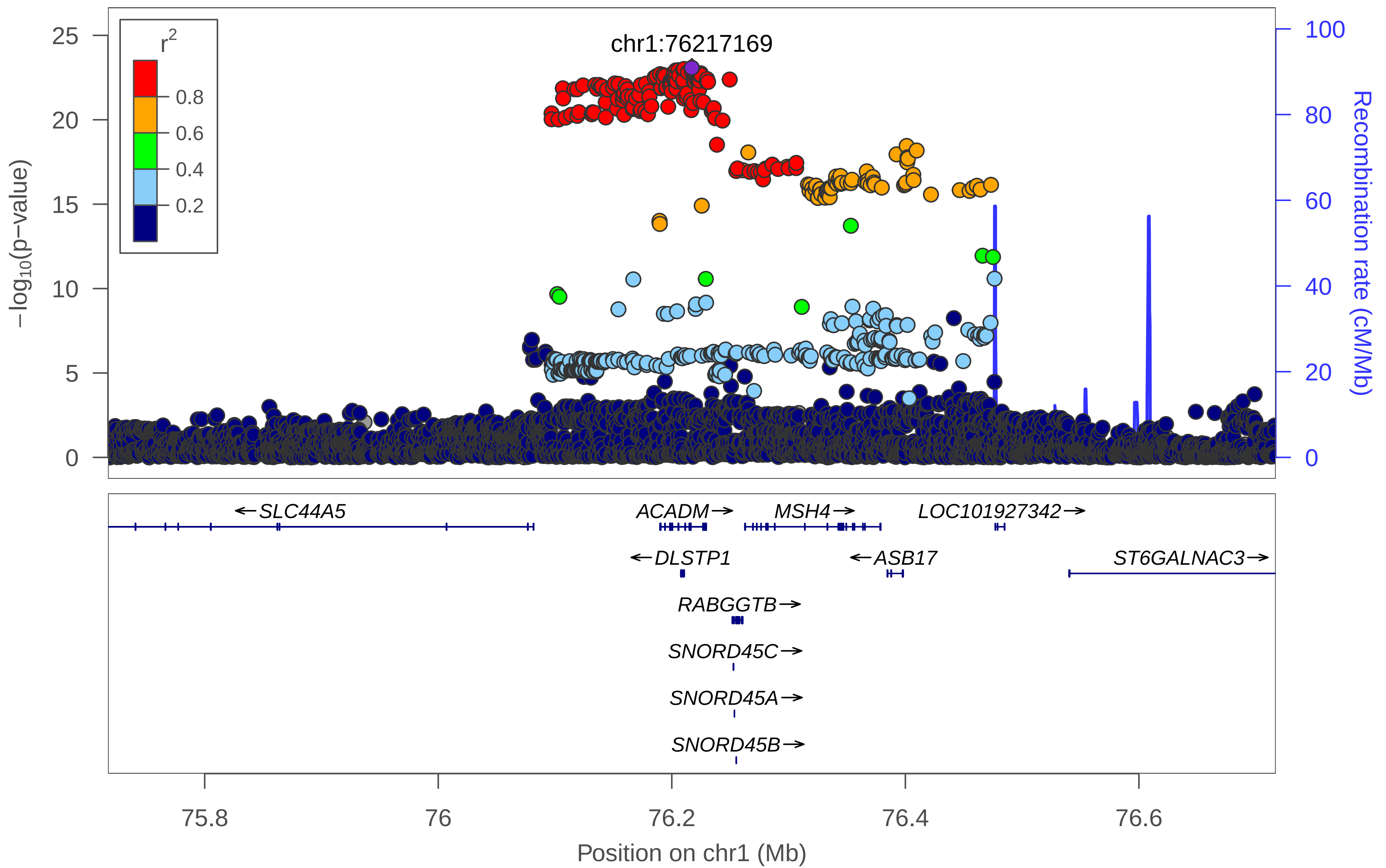
chr1:76217169
r2
0.8
0.6
0.4
0.2
25
20
15
10
5
0
−log10(p−value)
100
80
60
40
20
0
Recombination rate (cM/Mb)
←SLC44A5
ACADM→
MSH4→
LOC101927342→
←DLSTP1
←ASB17
ST6GALNAC3→
RABGGTB→
SNORD45C→
SNORD45A→
SNORD45B→
75.8
76
76.2
76.4
76.6
Position on chr1 (Mb)

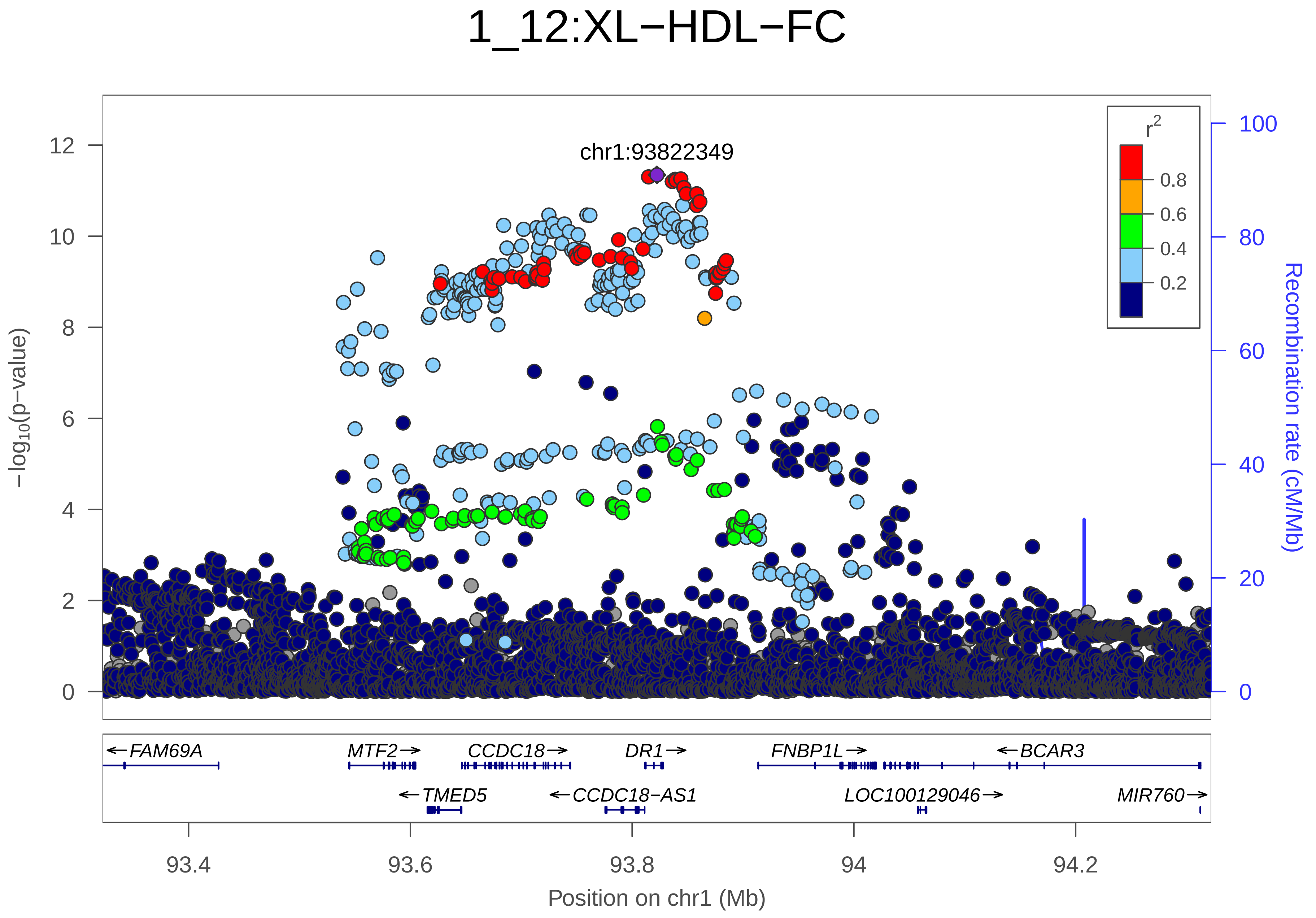
1_12:XL−HDL−FC
chr1:93822349
r2
0.8
0.6
0.4
0.2
−log10(p−value)
Recombination rate (cM/Mb)
12
10
8
6
4
2
0
100
80
60
40
20
0
←FAM69A
MTF2→
CCDC18→
DR1→
FNBP1L→
←BCAR3
←TMED5
←CCDC18−AS1
LOC100129046→
MIR760→
93.4
93.6
93.8
94
94.2
Position on chr1 (Mb)

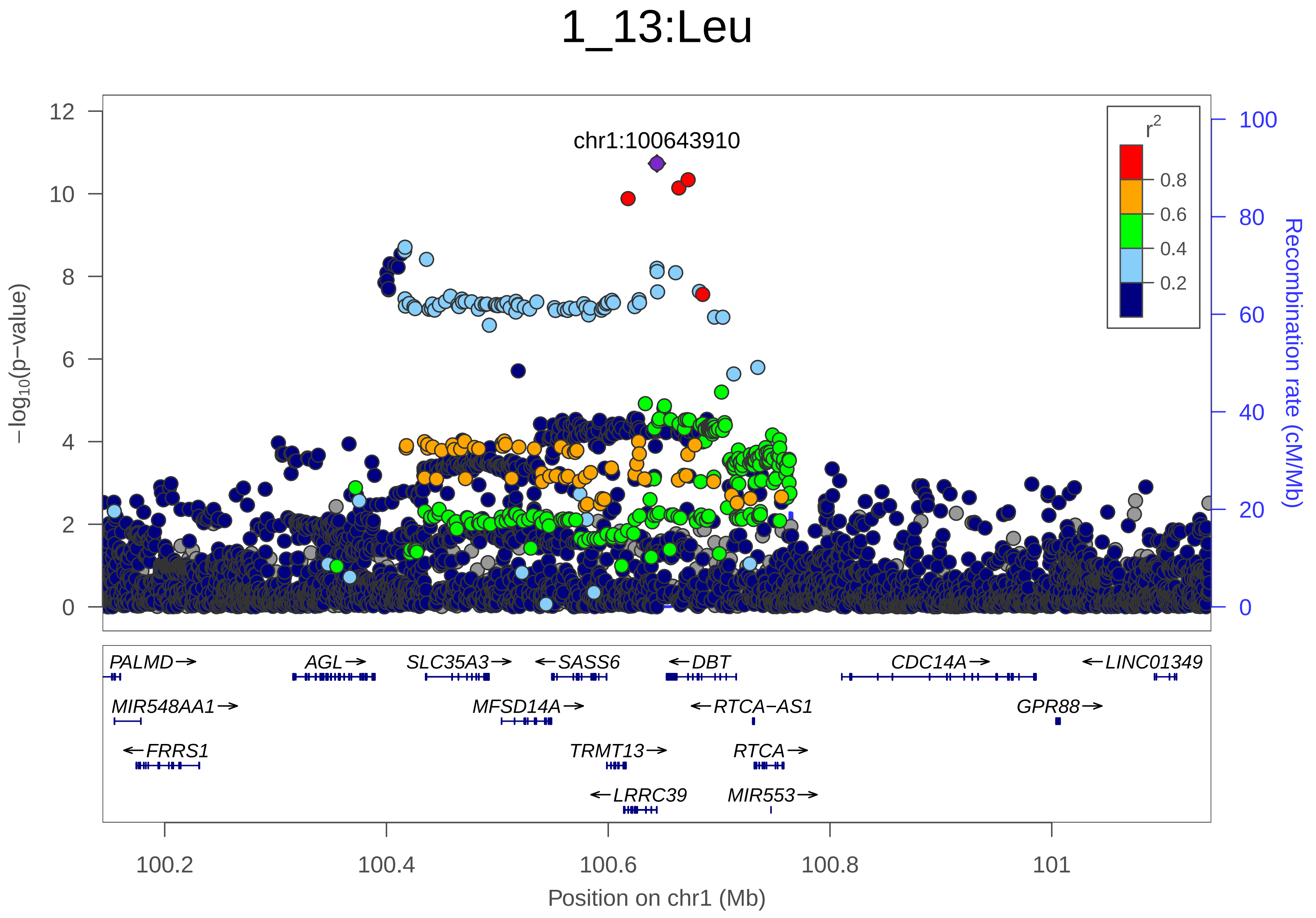

1_13:Leu
chr1:100643910
−log10(p−value)
Recombination rate (cM/Mb)
r2
0.8
0.6
0.4
0.2
0
2
4
6
8
10
12
0
20
40
60
80
100
PALMD
AGL
SLC35A3
SASS6
DBT
CDC14A
LINC01349
MIR548AA1
MFSD14A
RTCA−AS1
GPR88
FRRS1
TRMT13
RTCA
LRRC39
MIR553
100.2
100.4
100.6
100.8
101
Position on chr1 (Mb)

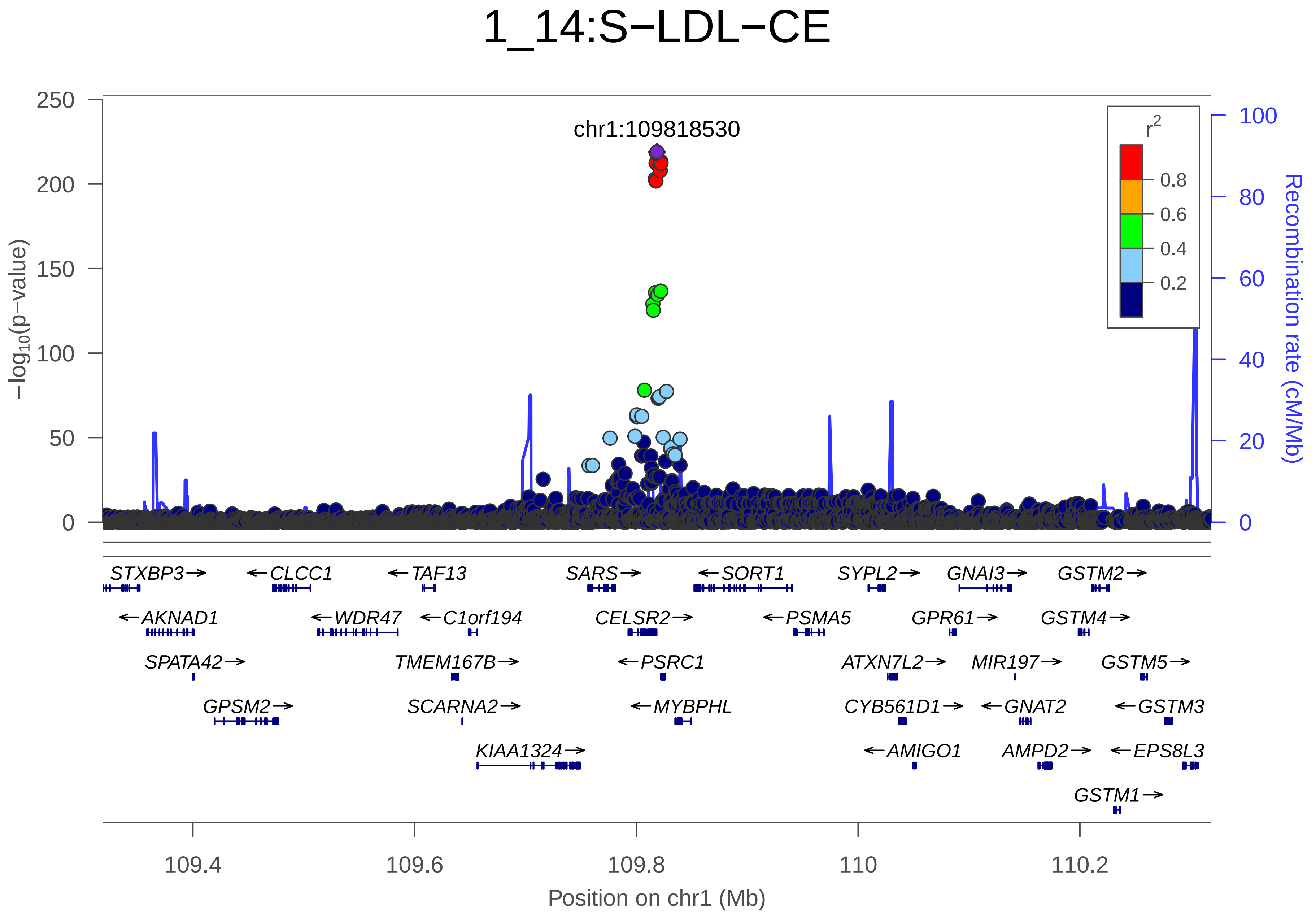

1_14:S−LDL−CE
chr1:109818530
r2
0.8
0.6
0.4
0.2
250
200
150
100
50
0
−log10(p−value)
100
80
60
40
20
0
Recombination rate (cM/Mb)
STXBP3→
←CLCC1
←TAF13
SARS→
←SORT1
SYPL2→
GNAI3→
GSTM2→
←AKNAD1
←WDR47
←C1orf194
CELSR2→
←PSMA5
GPR61→
GSTM4→
SPATA42→
TMEM167B→
←PSRC1
ATXN7L2→
MIR197→
GSTM5→
GPSM2→
SCARNA2→
←MYBPHL
CYB561D1→
←GNAT2
←GSTM3
KIAA1324→
←AMIGO1
AMPD2→
←EPS8L3
GSTM1→
109.4
109.6
109.8
110
110.2
Position on chr1 (Mb)

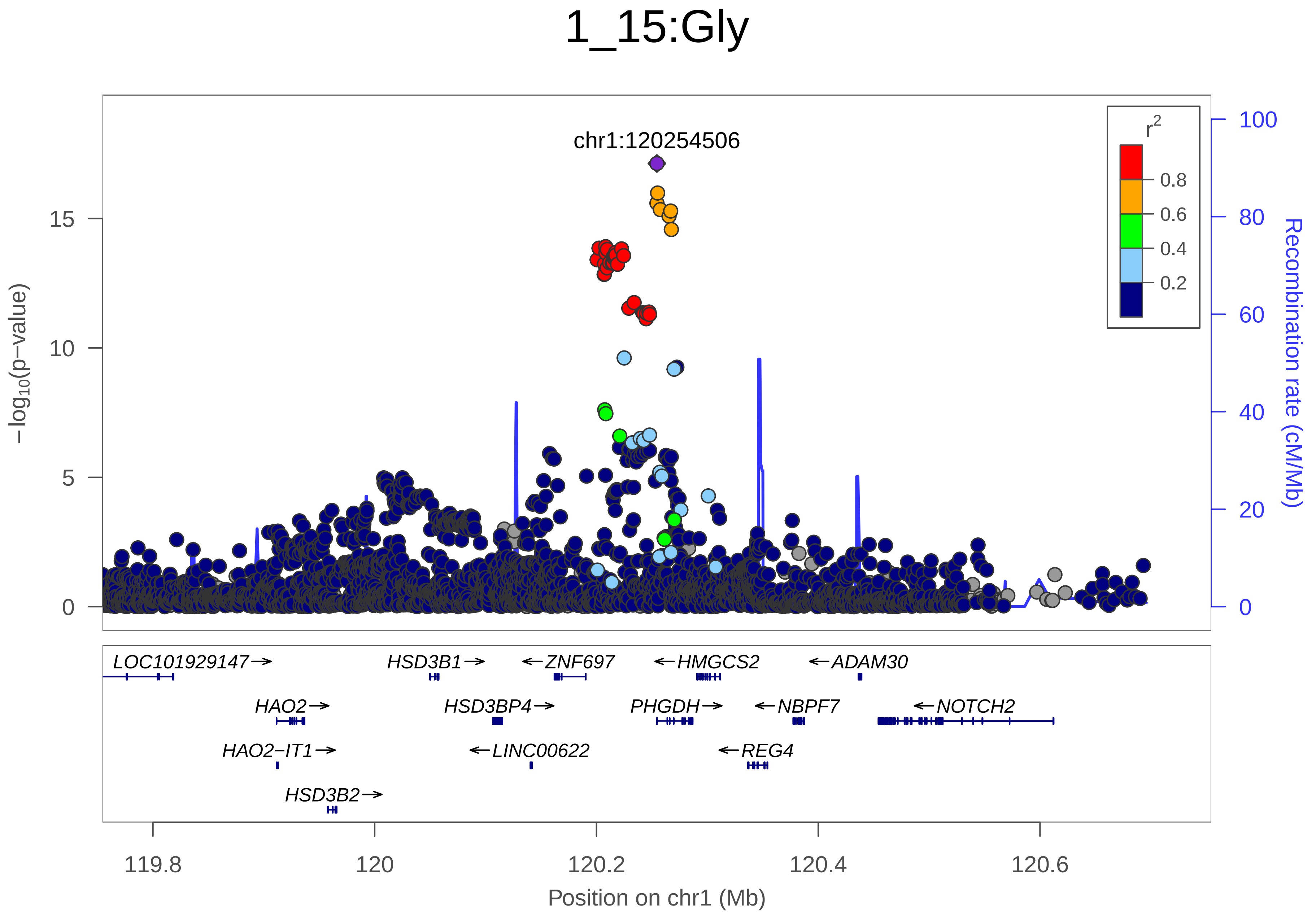

1_15:Gly
chr1:120254506
r2
0.8
0.6
0.4
0.2
−log10(p−value)
0
5
10
15
Recombination rate (cM/Mb)
0
20
40
60
80
100
LOC101929147→
HSD3B1→
←ZNF697
←HMGCS2
←ADAM30
HAO2→
HSD3BP4→
PHGDH→
←NBPF7
←NOTCH2
HAO2−IT1→
←LINC00622
←REG4
HSD3B2→
119.8
120
120.2
120.4
120.6
Position on chr1 (Mb)

1_16:S−HDL−PL

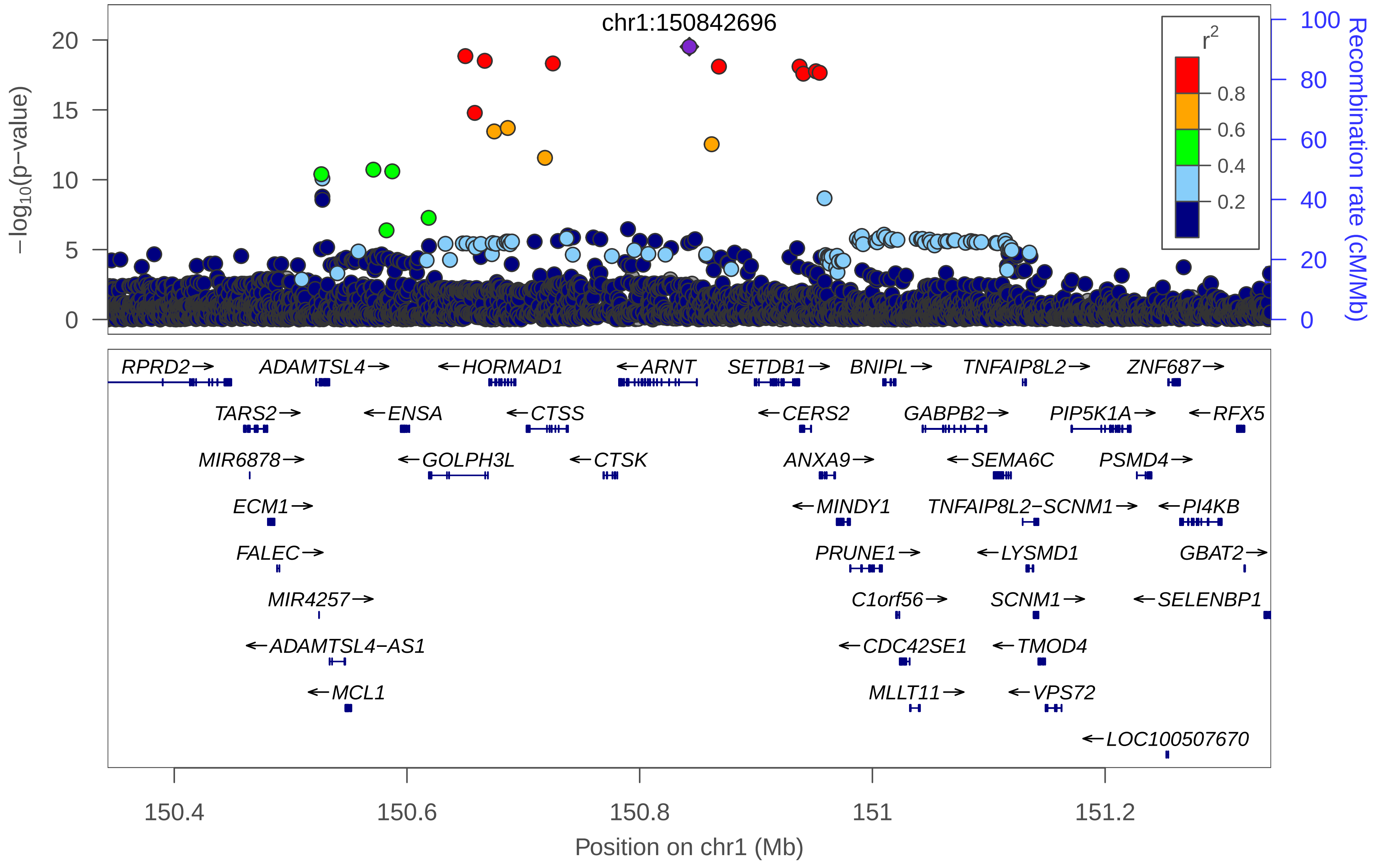

chr1:150842696
−log10(p−value)
Recombination rate (cM/Mb)
r2
0.8
0.6
0.4
0.2
0
5
10
15
20
0
20
40
60
80
100
RPRD2→
ADAMTSL4→
←HORMAD1
←ARNT
SETDB1→
BNIPL→
TNFAIP8L2→
ZNF687→
TARS2→
←ENSA
←CTSS
←CERS2
GABPB2→
PIP5K1A→
←RFX5
MIR6878→
←GOLPH3L
←CTSK
ANXA9→
←SEMA6C
PSMD4→
ECM1→
←MINDY1
TNFAIP8L2−SCNM1→
←PI4KB
FALEC→
PRUNE1→
←LYSMD1
GBAT2→
MIR4257→
C1orf56→
SCNM1→
←SELENBP1
←ADAMTSL4−AS1
←CDC42SE1
←TMOD4
←MCL1
MLLT11→
←VPS72
←LOC100507670
150.4
150.6
150.8
151
151.2
Position on chr1 (Mb)

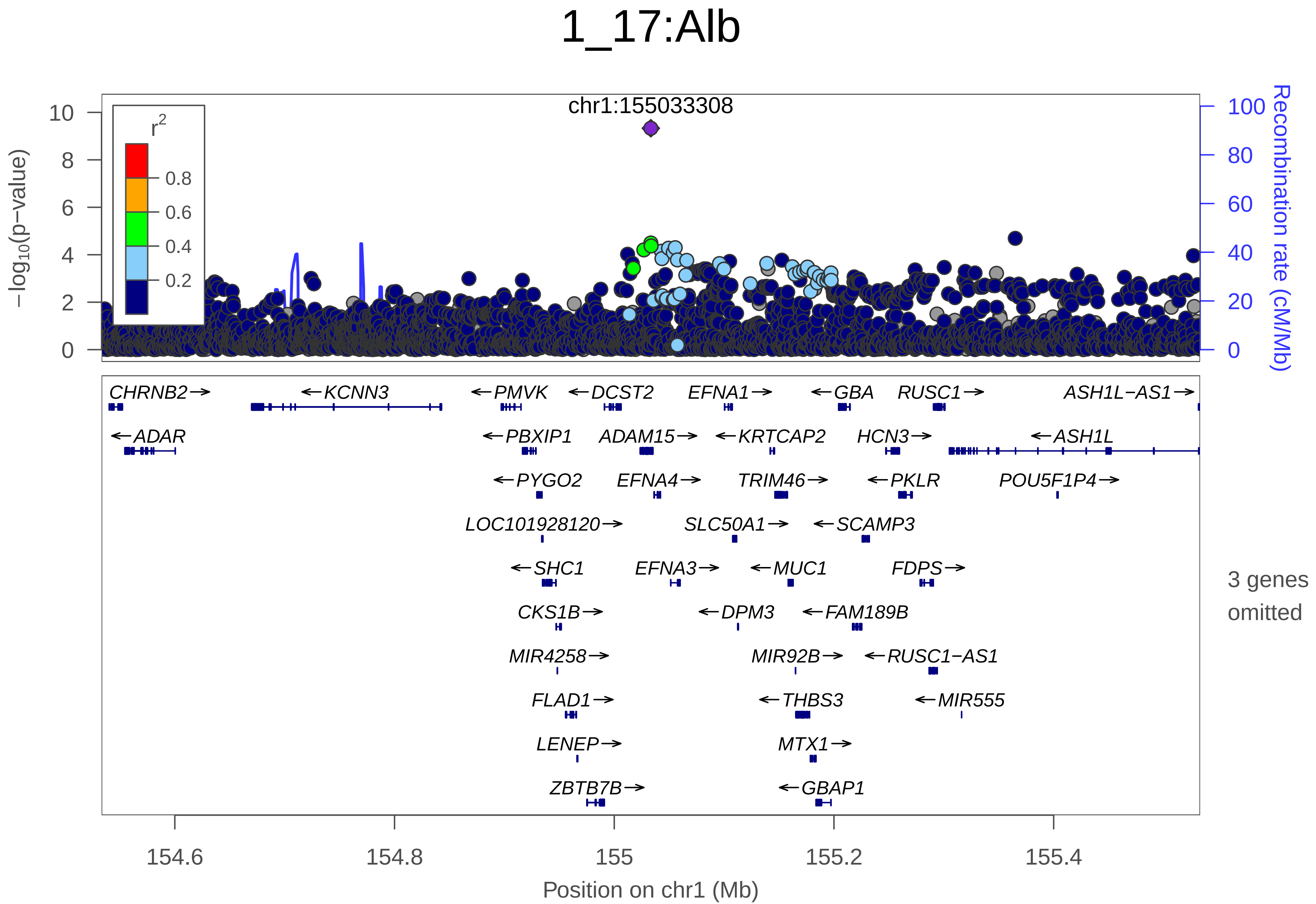

1_17:Alb
chr1:155033308
r2
0.8
0.6
0.4
0.2
−log10(p−value)
Recombination rate (cM/Mb)
CHRNB2→
←KCNN3
←PMVK
←DCST2
EFNA1→
←GBA
RUSC1→
ASH1L−AS1→
←ADAR
←PBXIP1
ADAM15→
←KRTCAP2
HCN3→
←ASH1L
←PYGO2
EFNA4→
TRIM46→
←PKLR
POU5F1P4→
LOC101928120→
SLC50A1→
←SCAMP3
←SHC1
EFNA3→
←MUC1
FDPS→
CKS1B→
←DPM3
←FAM189B
MIR4258→
MIR92B→
←RUSC1−AS1
FLAD1→
←THBS3
←MIR555
LENEP→
MTX1→
ZBTB7B→
←GBAP1
3 genes omitted
154.6
154.8
155
155.2
155.4
Position on chr1 (Mb)

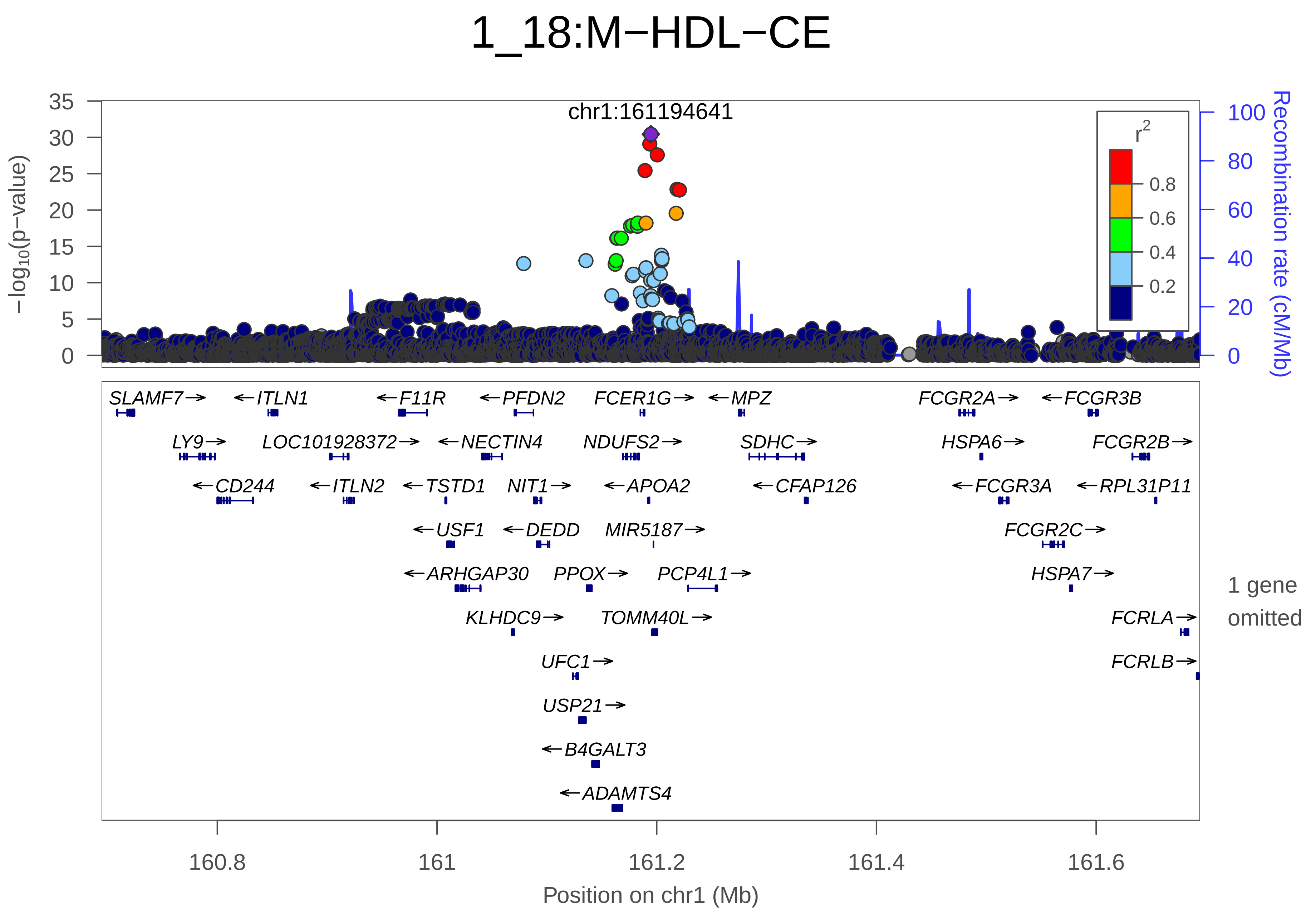

1_18:M−HDL−CE
chr1:161194641
−log10(p−value)
Recombination rate (cM/Mb)
r2
0.8
0.6
0.4
0.2
35
30
25
20
15
10
5
0
100
80
60
40
20
0
SLAMF7→
←ITLN1
←F11R
←PFDN2
FCER1G→
←MPZ
FCGR2A→
←FCGR3B
LY9→
LOC101928372→
←NECTIN4
NDUFS2→
SDHC→
HSPA6→
FCGR2B→
←CD244
←ITLN2
←TSTD1
NIT1→
←APOA2
←CFAP126
←FCGR3A
←RPL31P11
←USF1
←DEDD
MIR5187→
FCGR2C→
←ARHGAP30
PPOX→
PCP4L1→
HSPA7→
KLHDC9→
TOMM40L→
FCRLA→
UFC1→
FCRLB→
USP21→
←B4GALT3
←ADAMTS4
1 gene omitted
160.8
161
161.2
161.4
161.6
Position on chr1 (Mb)

1_19:XL−VLDL−C_percent

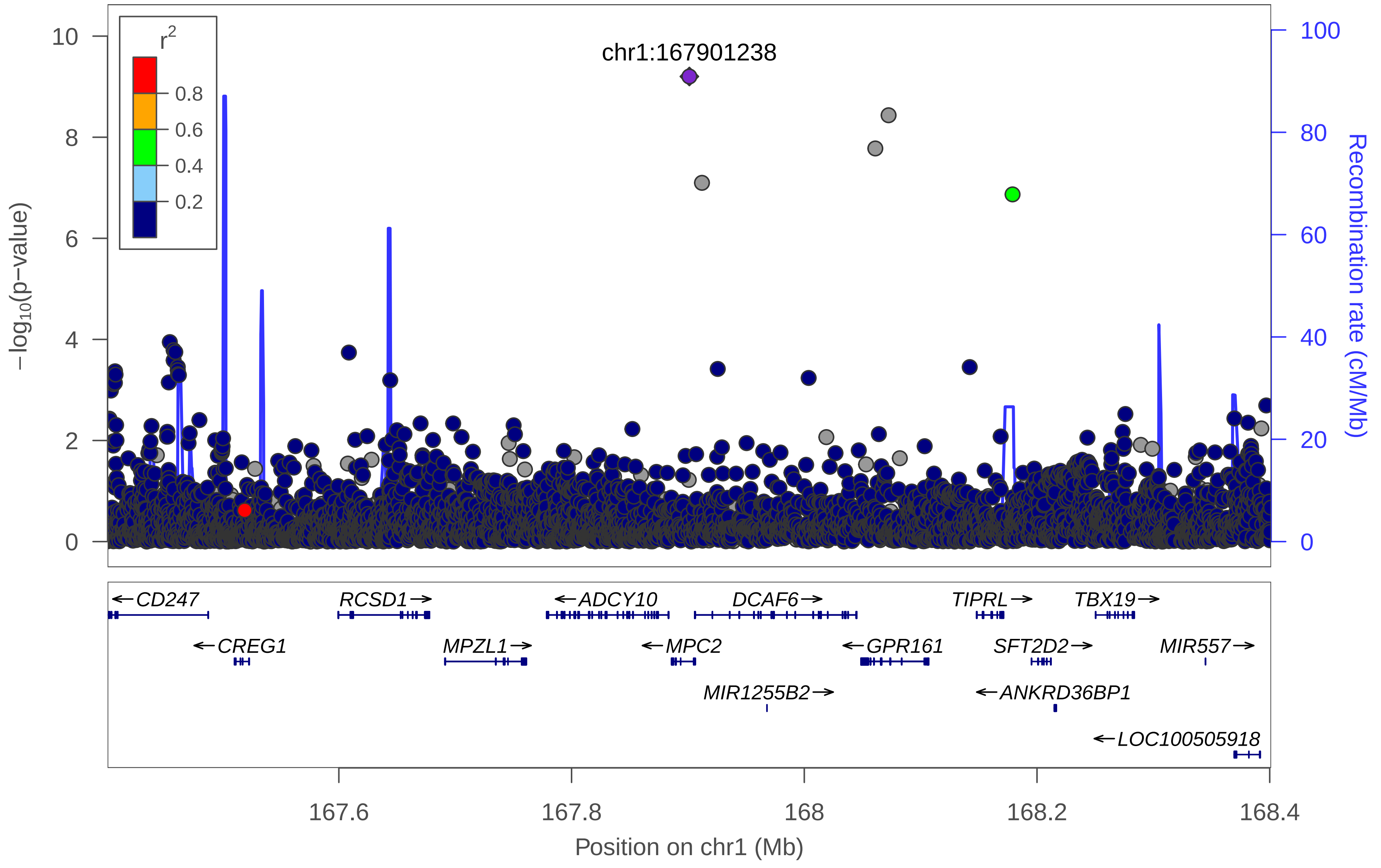

1_20:HDL−D

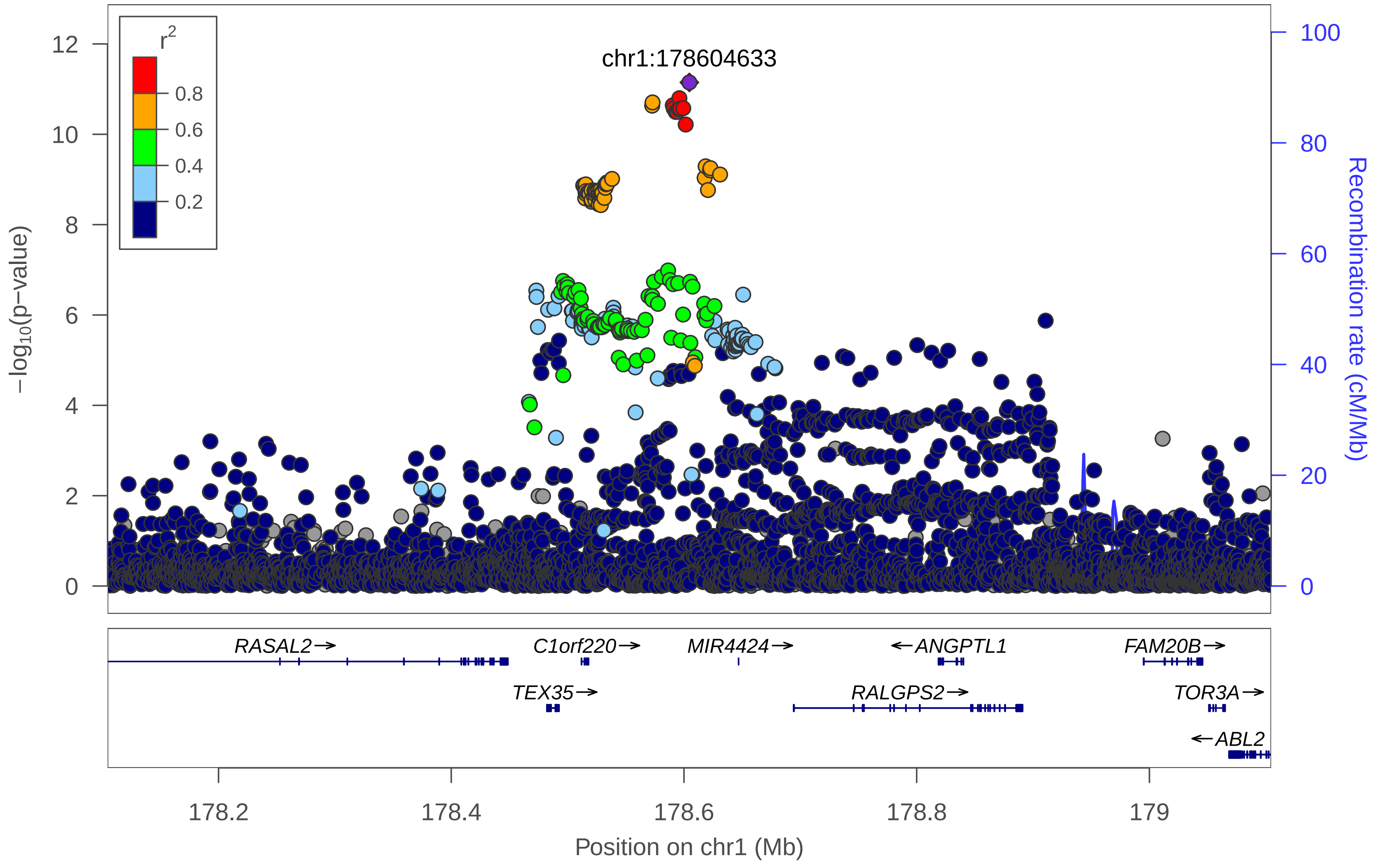

r2
0.8
0.6
0.4
0.2
chr1:178604633
−log10(p−value)
0
2
4
6
8
10
12
Recombination rate (cM/Mb)
0
20
40
60
80
100
RASAL2→
C1orf220→
MIR4424→
←ANGPTL1
FAM20B→
TEX35→
RALGPS2→
TOR3A→
←ABL2
178.2
178.4
178.6
178.8
179
Position on chr1 (Mb)

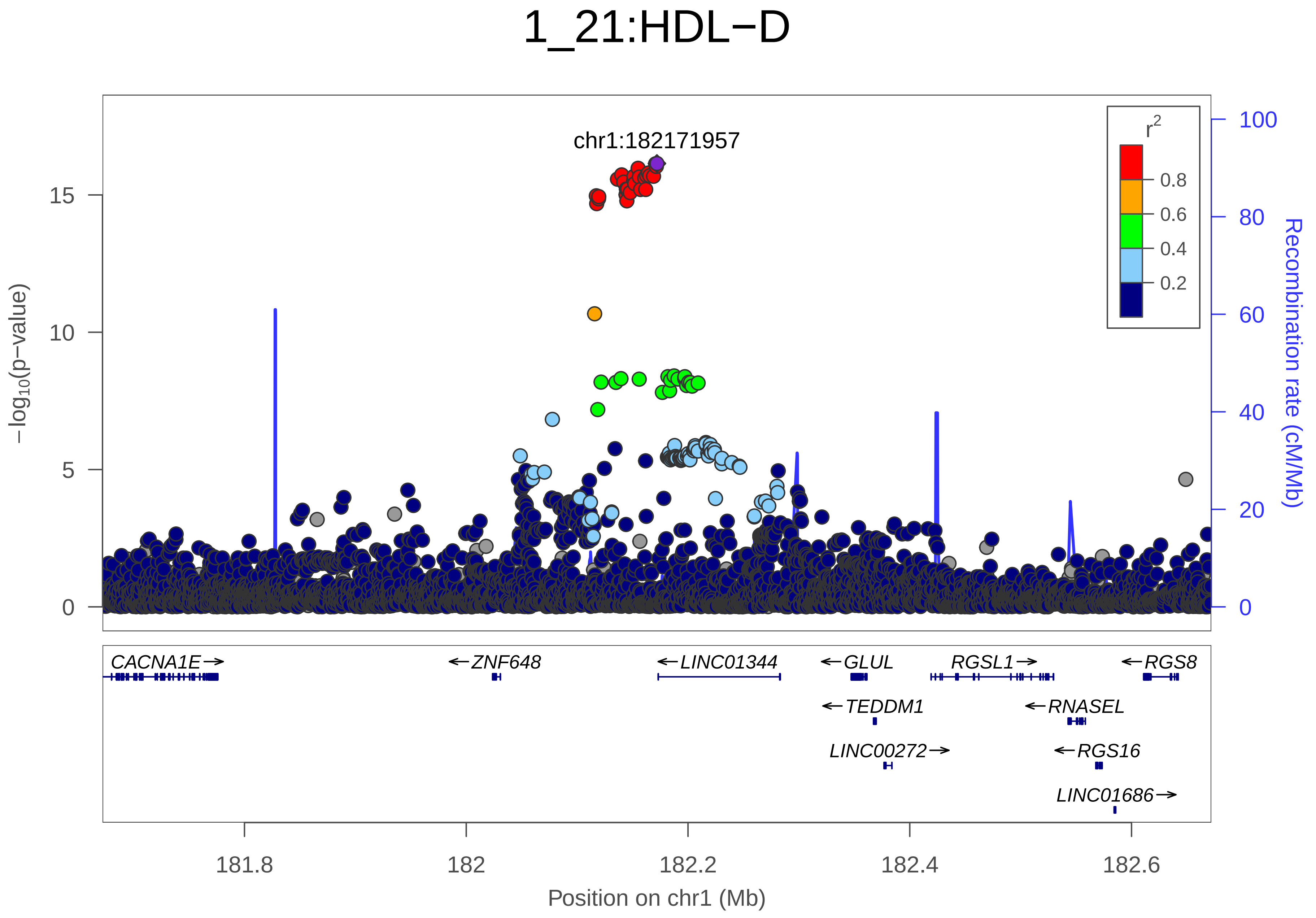
1_21:HDL−D
chr1:182171957
r2
0.8
0.6
0.4
0.2
−log10(p−value)
15
10
5
0
100
80
60
40
20
0
Recombination rate (cM/Mb)
CACNA1E→
←ZNF648
←LINC01344
←GLUL
RGSL1→
←RGS8
←TEDDM1
←RNASEL
LINC00272→
←RGS16
LINC01686→
181.8
182
182.2
182.4
182.6
Position on chr1 (Mb)

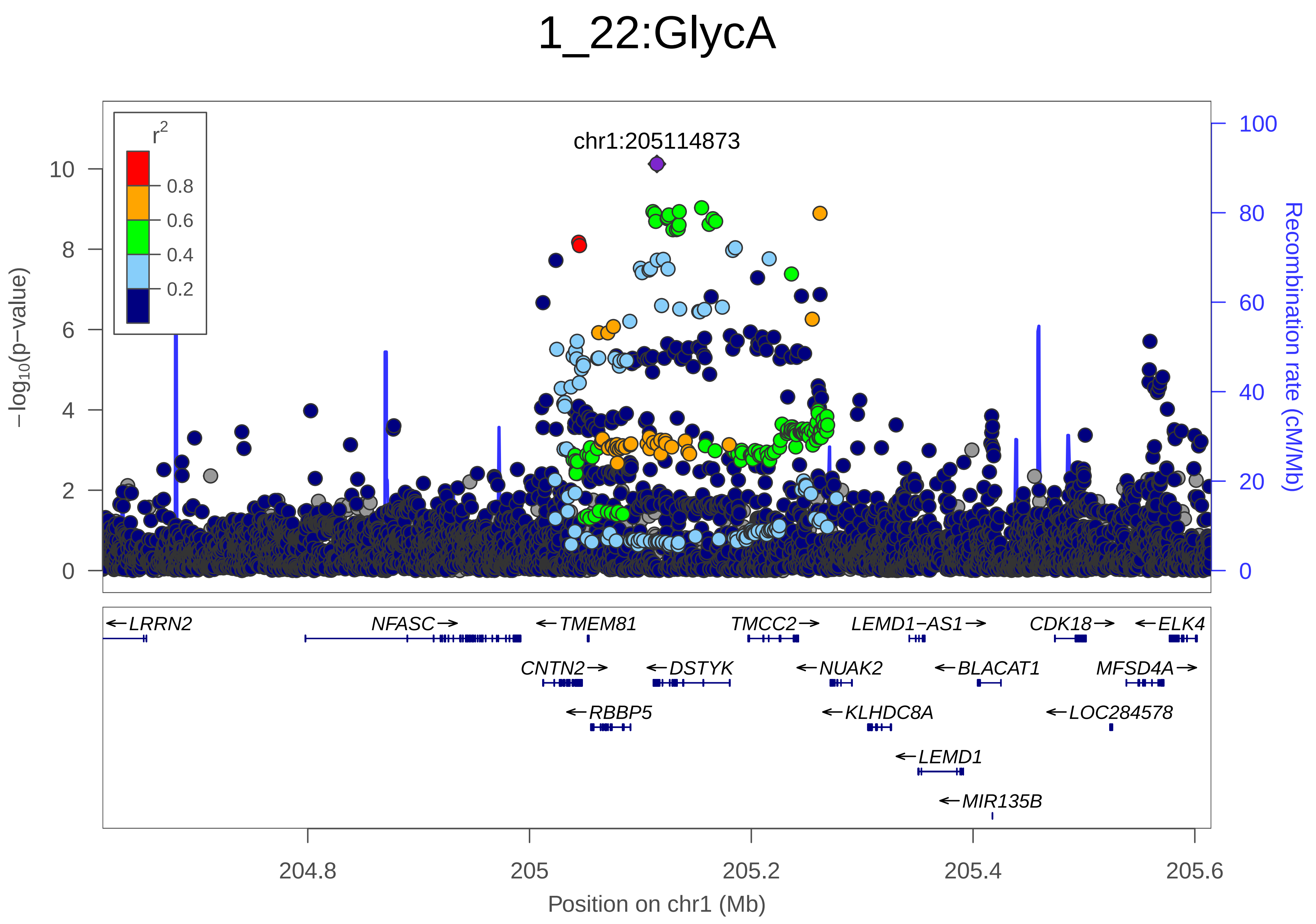

1_22:GlycA
chr1:205114873
r2
0.8
0.6
0.4
0.2
−log10(p−value)
10
8
6
4
2
0
Recombination rate (cM/Mb)
100
80
60
40
20
0
LRRN2
NFASC
TMEM81
TMCC2
LEMD1−AS1
CDK18
ELK4
CNTN2
DSTYK
NUAK2
BLACAT1
MFSD4A
RBBP5
KLHDC8A
LOC284578
LEMD1
MIR135B
204.8
205
205.2
205.4
205.6
Position on chr1 (Mb)

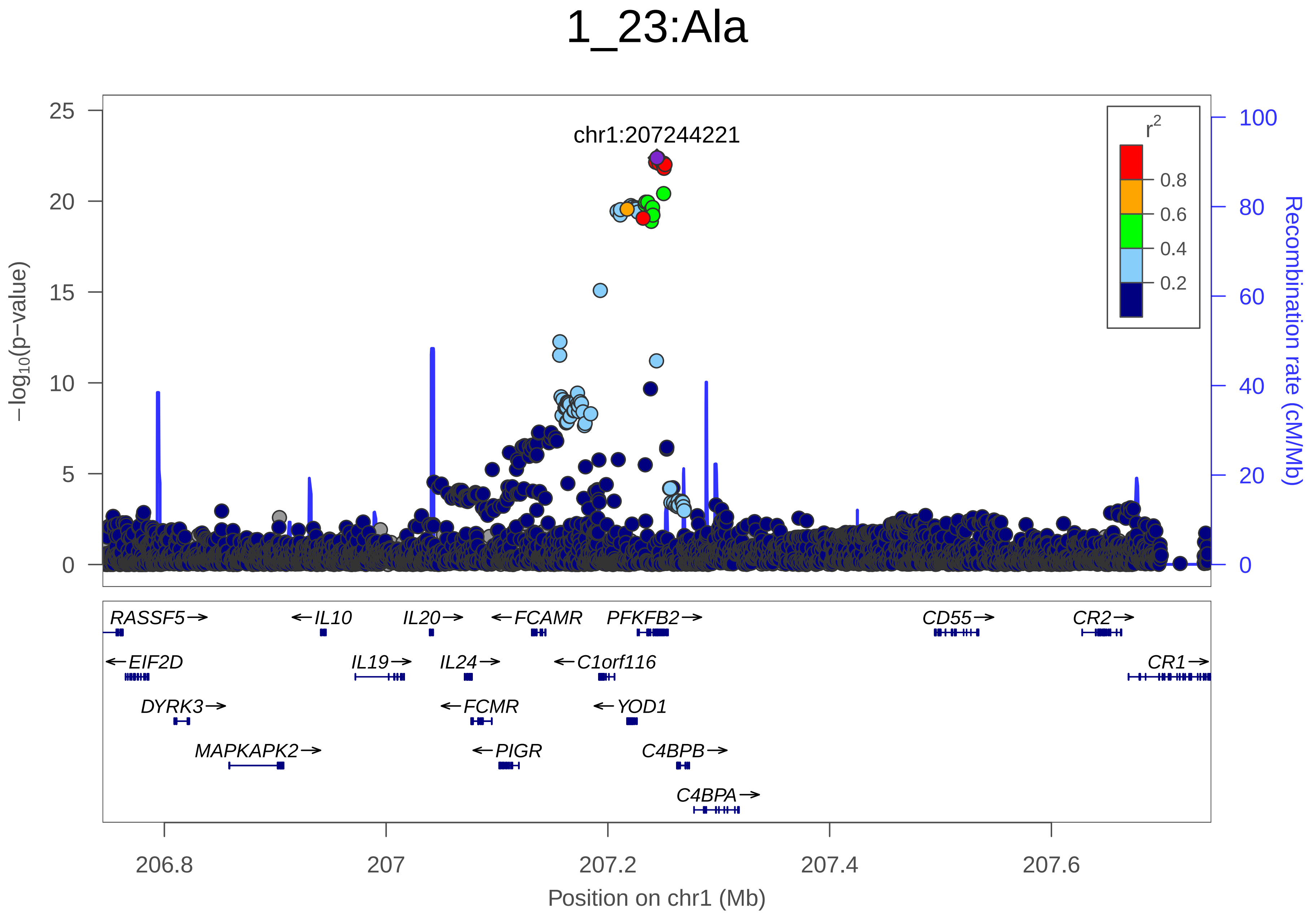

1_23:Ala
chr1:207244221
r2
0.8
0.6
0.4
0.2
-log10(p-value)
Recombination rate (cM/Mb)
RASSF5→
←IL10
IL20→
←FCAMR
PFKFB2→
CD55→
CR2→
←EIF2D
IL19→
IL24→
←C1orf116
CR1→
DYRK3→
←FCMR
←YOD1
MAPKAPK2→
←PIGR
C4BPB→
C4BPA→
206.8
207
207.2
207.4
207.6
Position on chr1 (Mb)

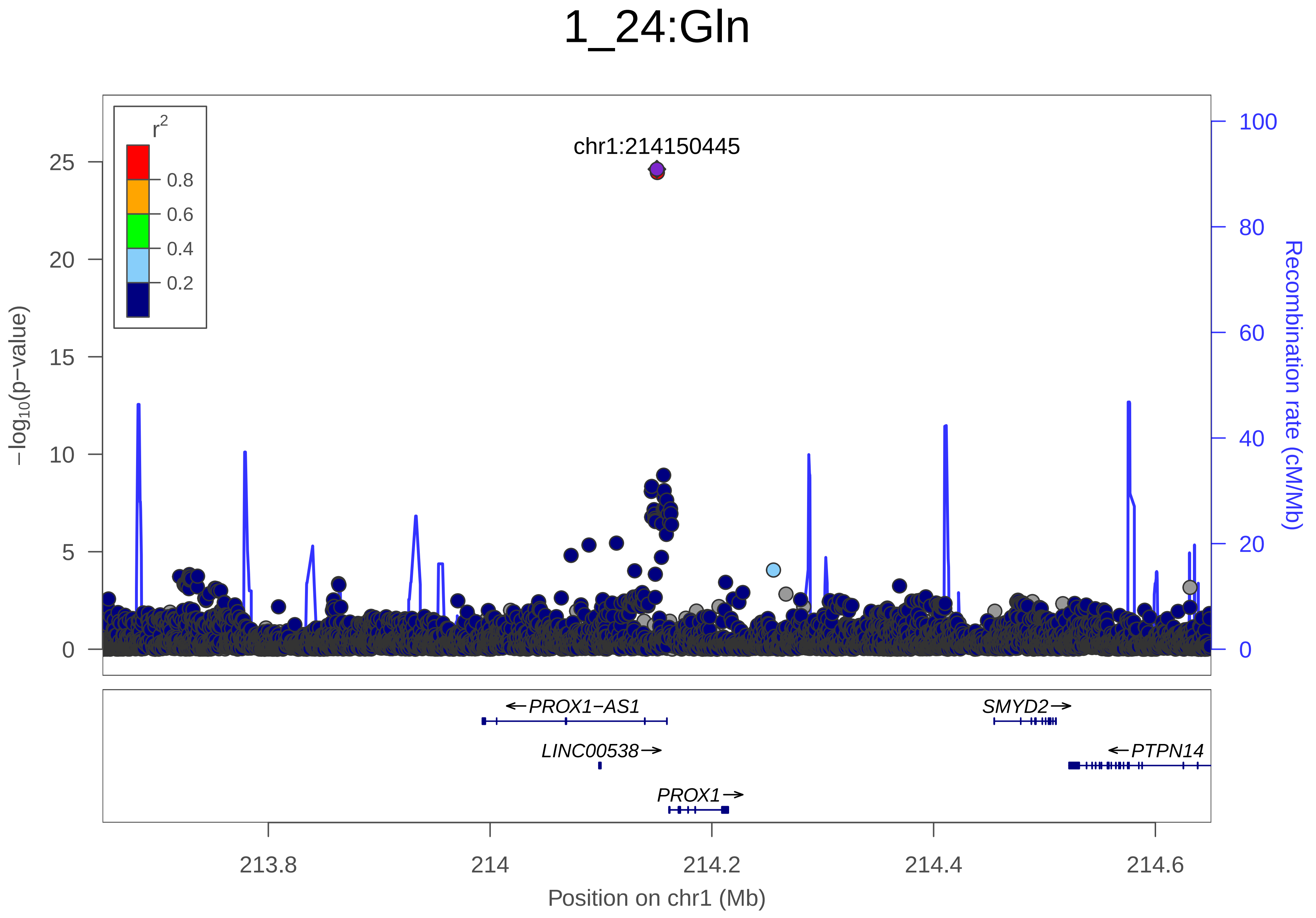

1_24:Gln
chr1:214150445
r2
0.8
0.6
0.4
0.2
−log10(p−value)
Recombination rate (cM/Mb)
0
5
10
15
20
25
0
20
40
60
80
100
←PROX1−AS1
SMYD2→
LINC00538→
←PTPN14
PROX1→
213.8
214
214.2
214.4
214.6
Position on chr1 (Mb)

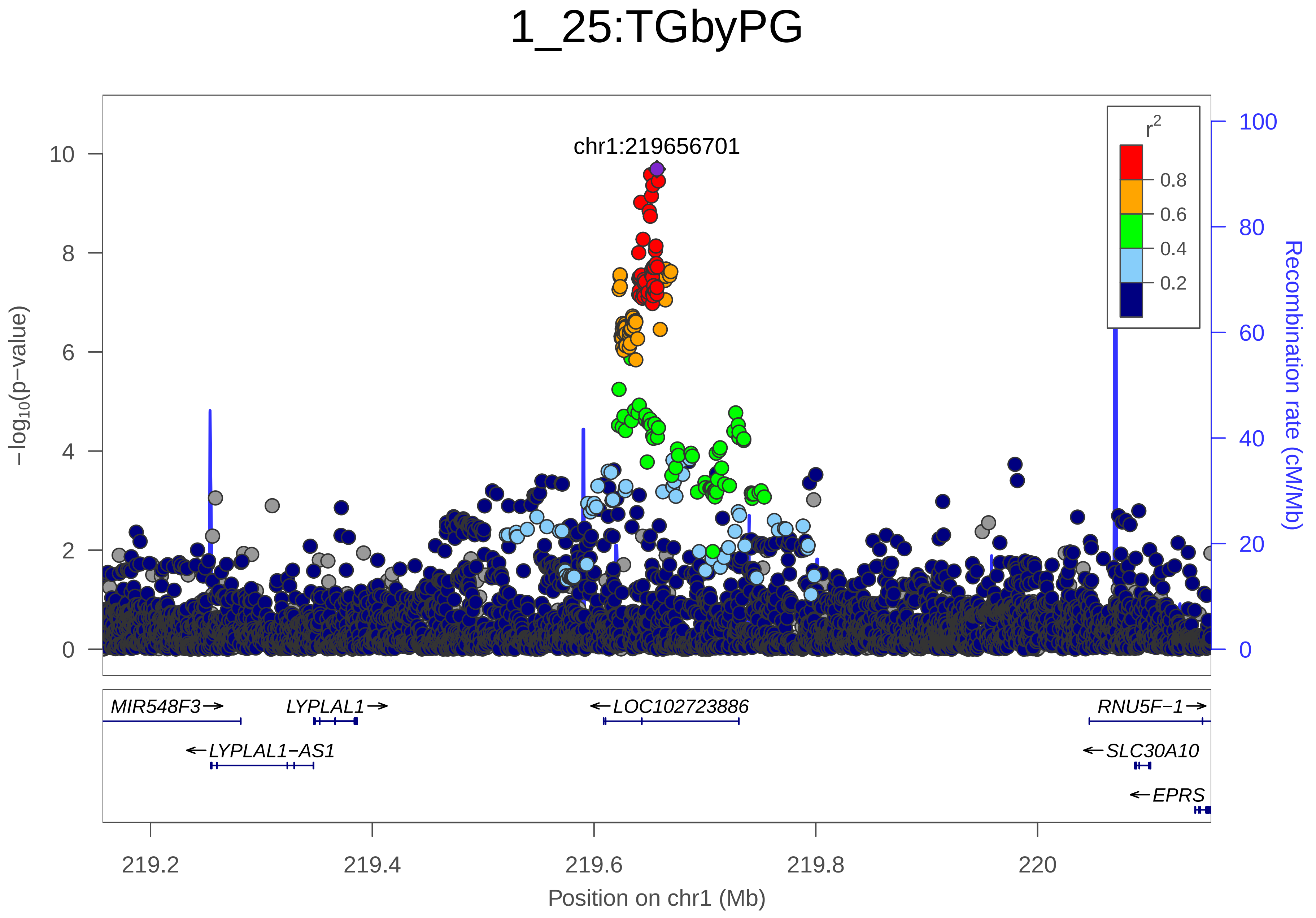

1_25:TGbyPG
chr1:219656701
r2
0.8
0.6
0.4
0.2
−log10(p−value)
Recombination rate (cM/Mb)
0
2
4
6
8
10
0
20
40
60
80
100
MIR548F3→
LYPLAL1→
←LOC102723886
RNU5F−1→
←LYPLAL1−AS1
←SLC30A10
←EPRS
219.2
219.4
219.6
219.8
220
Position on chr1 (Mb)

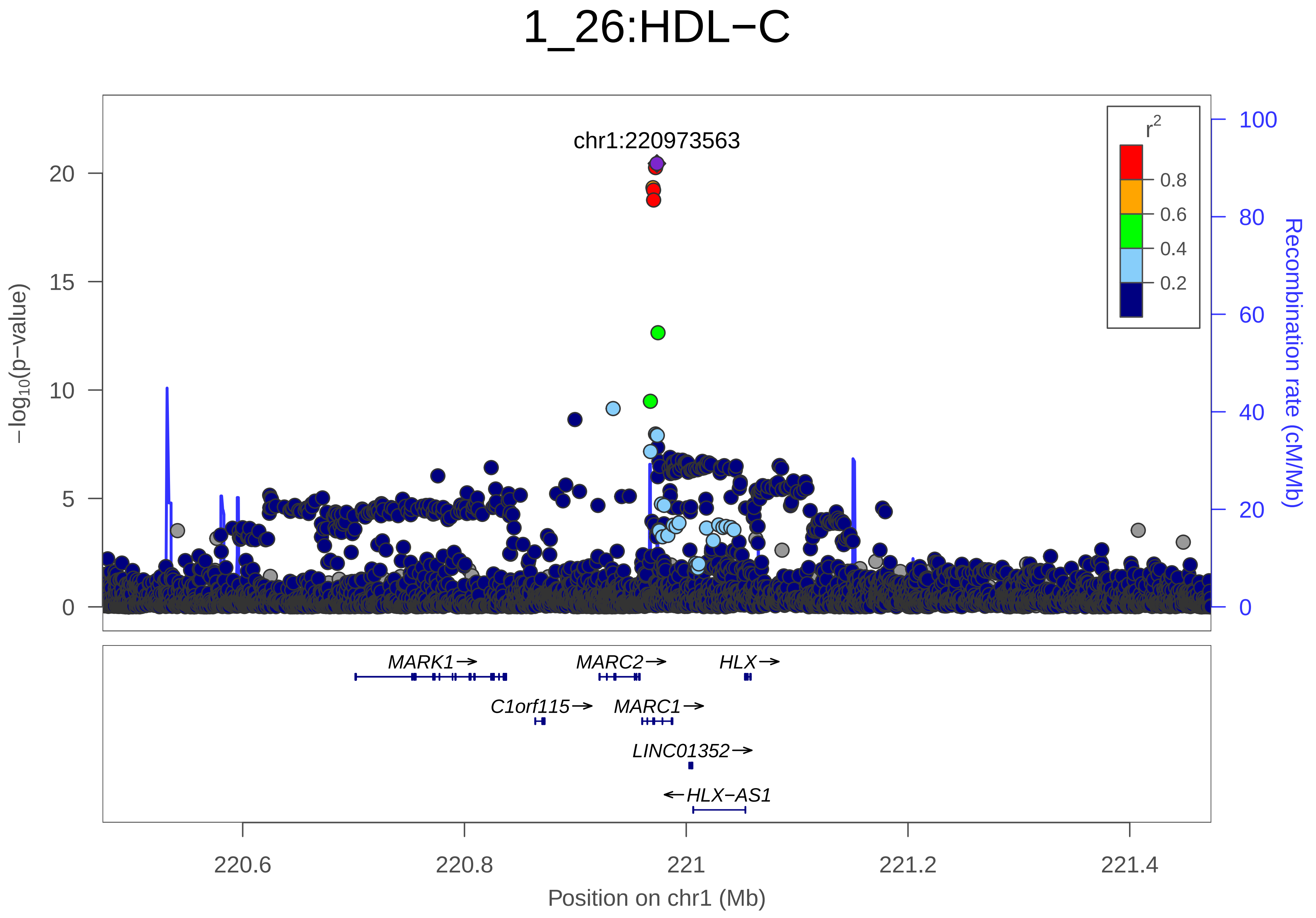

1_26:HDL−C
chr1:220973563
r2
0.8
0.6
0.4
0.2
−log10(p−value)
20
15
10
5
0
100
80
60
40
20
0
Recombination rate (cM/Mb)
MARK1→
MARC2→
HLX→
C1orf115→
MARC1→
LINC01352→
←HLX−AS1
220.6
220.8
221
221.2
221.4
Position on chr1 (Mb)

1_27:TGbyPG

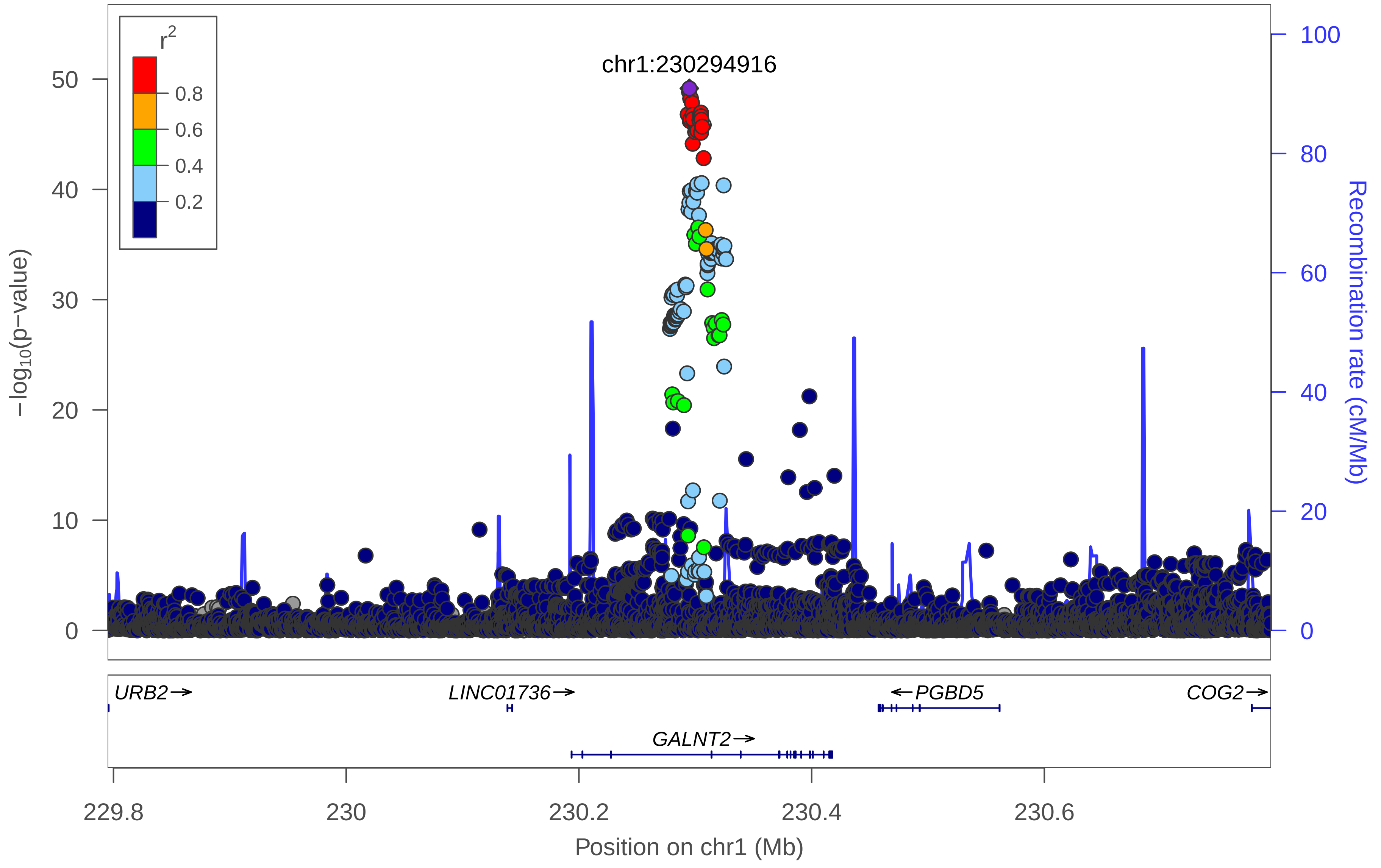

r²
0.8
0.6
0.4
0.2
chr1:230294916
50
40
30
20
10
0
−log10(p−value)
100
80
60
40
20
0
Recombination rate (cM/Mb)
URB2→
LINC01736→
←PGBD5
COG2→
GALNT2→
229.8
230
230.2
230.4
230.6
Position on chr1 (Mb)

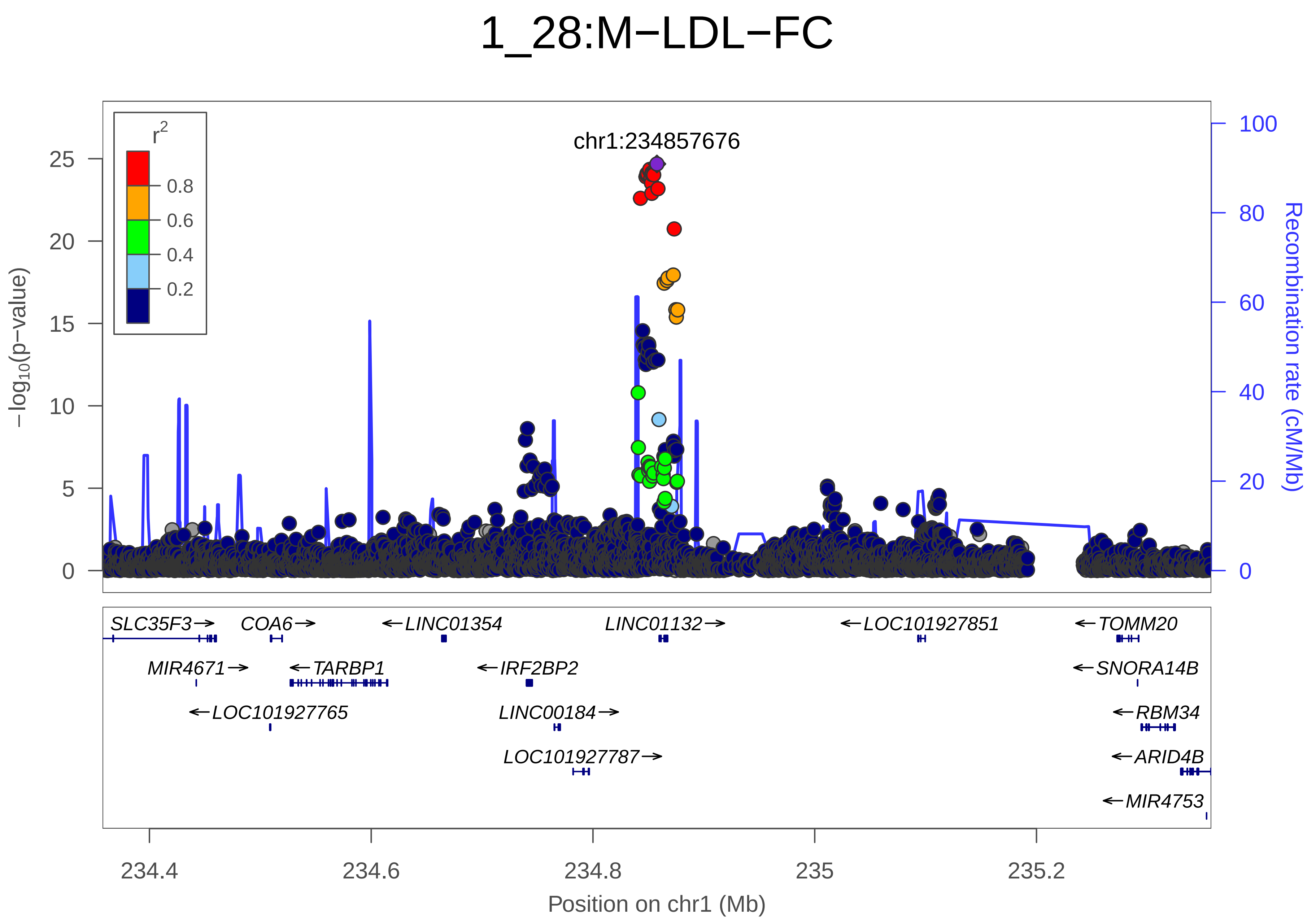

1_28:M−LDL−FC
chr1:234857676
r2
0.8
0.6
0.4
0.2
−log10(p−value)
Recombination rate (cM/Mb)
SLC35F3
COA6
LINC01354
LINC01132
LOC101927851
TOMM20
MIR4671
TARBP1
IRF2BP2
SNORA14B
LOC101927765
LINC00184
RBM34
LOC101927787
ARID4B
MIR4753
234.4
234.6
234.8
235
235.2
Position on chr1 (Mb)

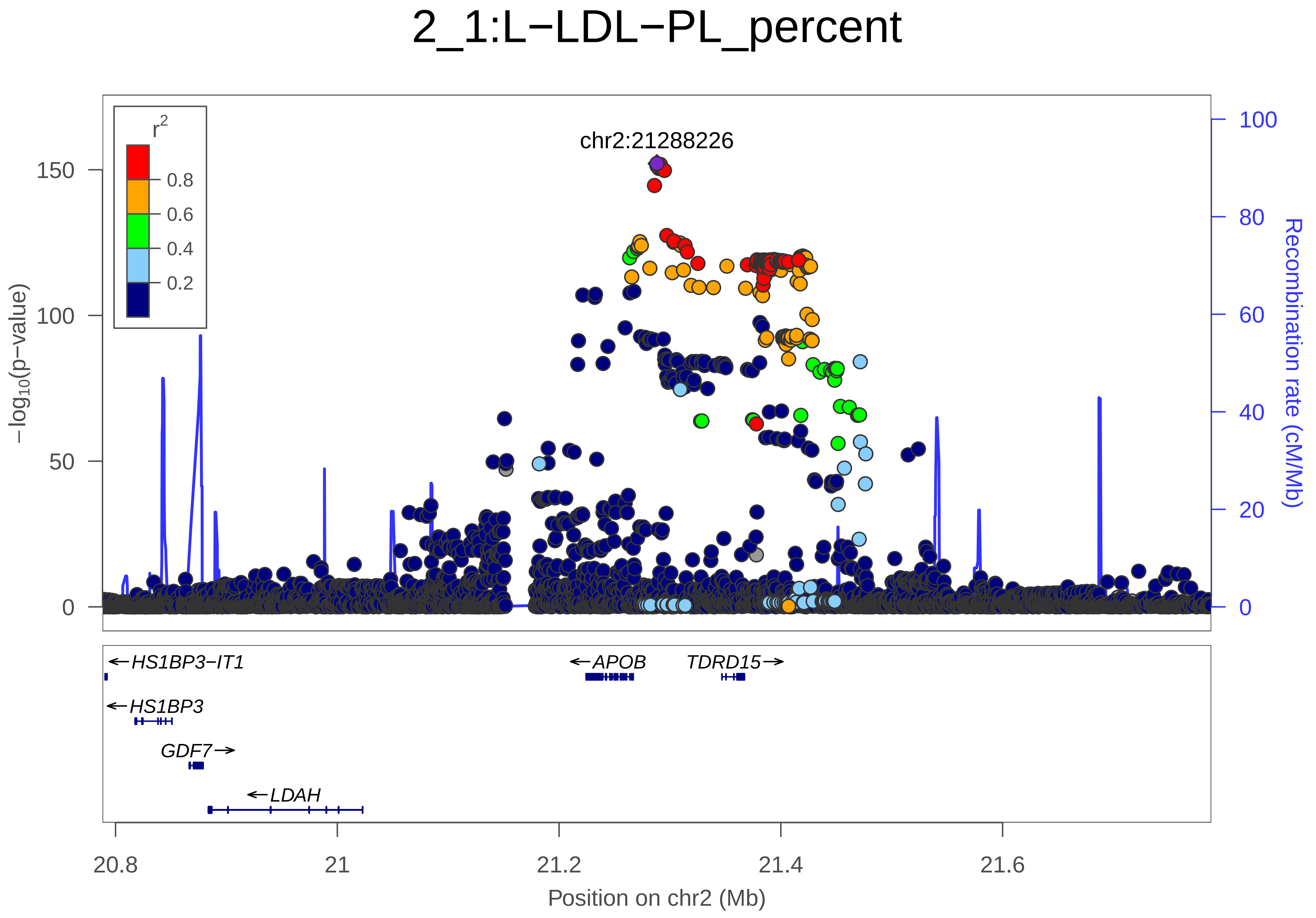

2_1:L−LDL−PL_percent
chr2:21288226
r2
0.8
0.6
0.4
0.2
−log10(p−value)
150
100
50
0
Recombination rate (cM/Mb)
100
80
60
40
20
0
←HS1BP3−IT1
←HS1BP3
GDF7→
←LDAH
←APOB
TDRD15→
20.8
21
21.2
21.4
21.6
Position on chr2 (Mb)

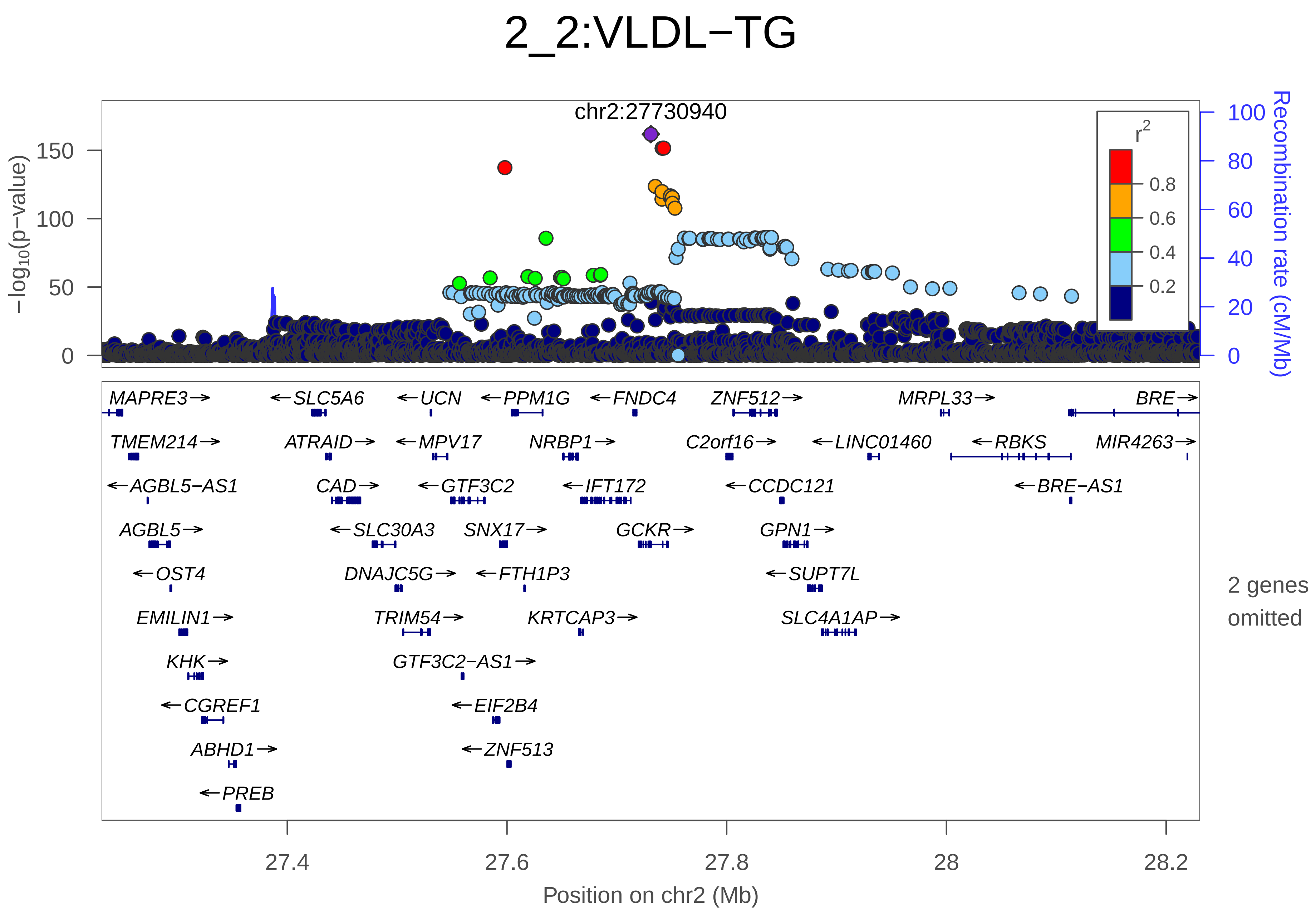

2_2:VLDL−TG
chr2:27730940
−log10(p−value)
Recombination rate (cM/Mb)
r2
0.8
0.6
0.4
0.2
0
50
100
150
0
20
40
60
80
100
MAPRE3→ ←SLC5A6 ←UCN ←PPM1G ←FNDC4 ZNF512→ MRPL33→ BRE→
TMEM214→ ATRAID→ ←MPV17 NRBP1→ C2orf16→ ←LINC01460 ←RBKS MIR4263→
←AGBL5−AS1 CAD→ ←GTF3C2 ←IFT172 ←CCDC121 ←BRE−AS1
AGBL5→ ←SLC30A3 SNX17→ GCKR→ GPN1→
←OST4 DNAJC5G→ ←FTH1P3 ←SUPT7L
EMILIN1→ TRIM54→ KRTCAP3→ SLC4A1AP→
KHK→ GTF3C2−AS1→
←CGREF1 ←EIF2B4
ABHD1→ ←ZNF513
←PREB
2 genes omitted
27.4
27.6
27.8
28
28.2
Position on chr2 (Mb)

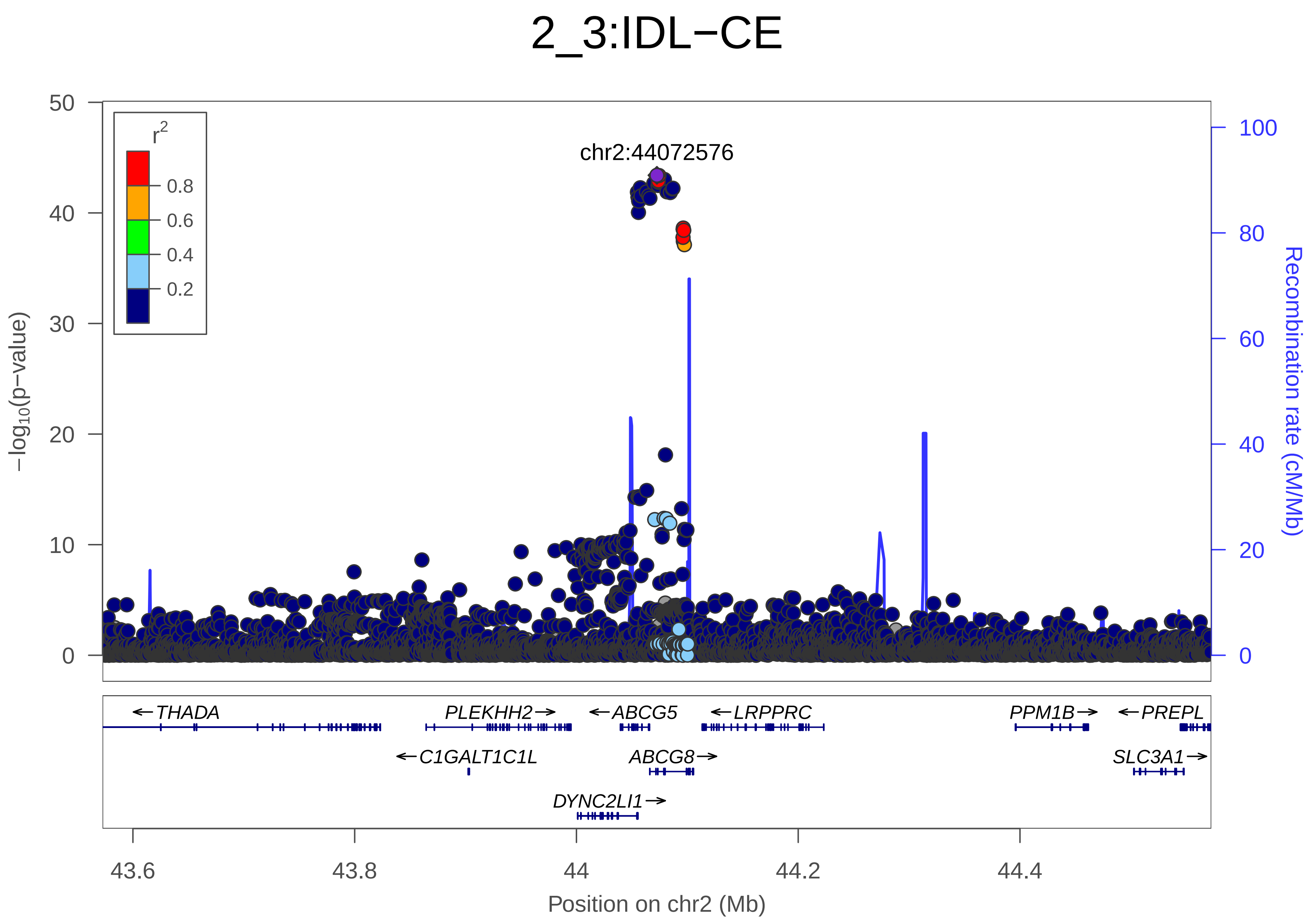

2_3:IDL−CE
chr2:44072576
r2
0.8
0.6
0.4
0.2
−log10(p−value)
Recombination rate (cM/Mb)
50
40
30
20
10
0
100
80
60
40
20
0
THADA
PLEKHH2
ABCG5
LRPPRC
PPM1B
PREPL
C1GALT1C1L
ABCG8
SLC3A1
DYNC2LI1
43.6
43.8
44
44.2
44.4
Position on chr2 (Mb)

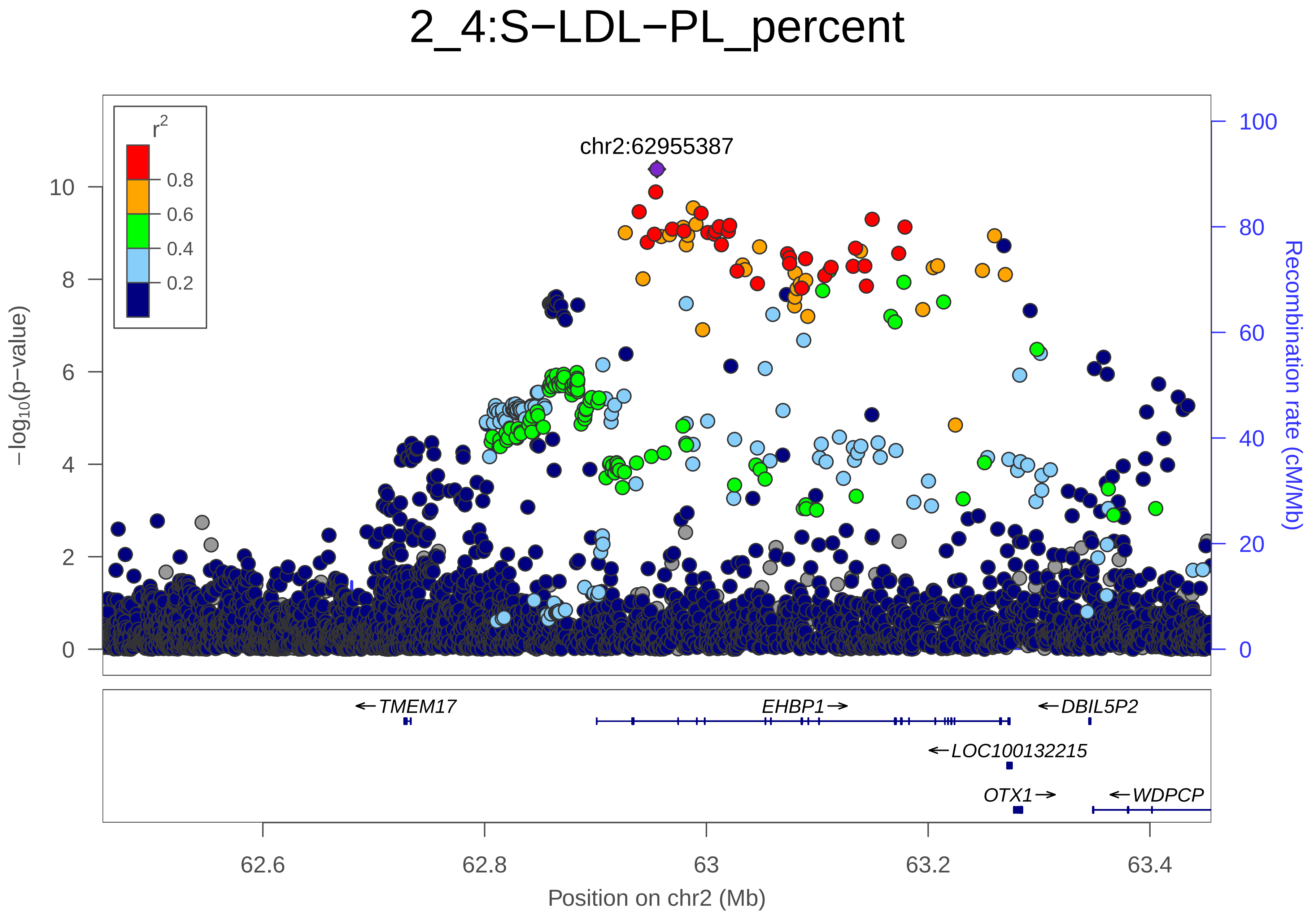

2_4:S−LDL−PL_percent
r2
0.8
0.6
0.4
0.2
chr2:62955387
−log10(p−value)
Recombination rate (cM/Mb)
0
2
4
6
8
10
0
20
40
60
80
100
←TMEM17
EHBP1→
←DBIL5P2
←LOC100132215
OTX1→
←WDPCP
62.6
62.8
63
63.2
63.4
Position on chr2 (Mb)

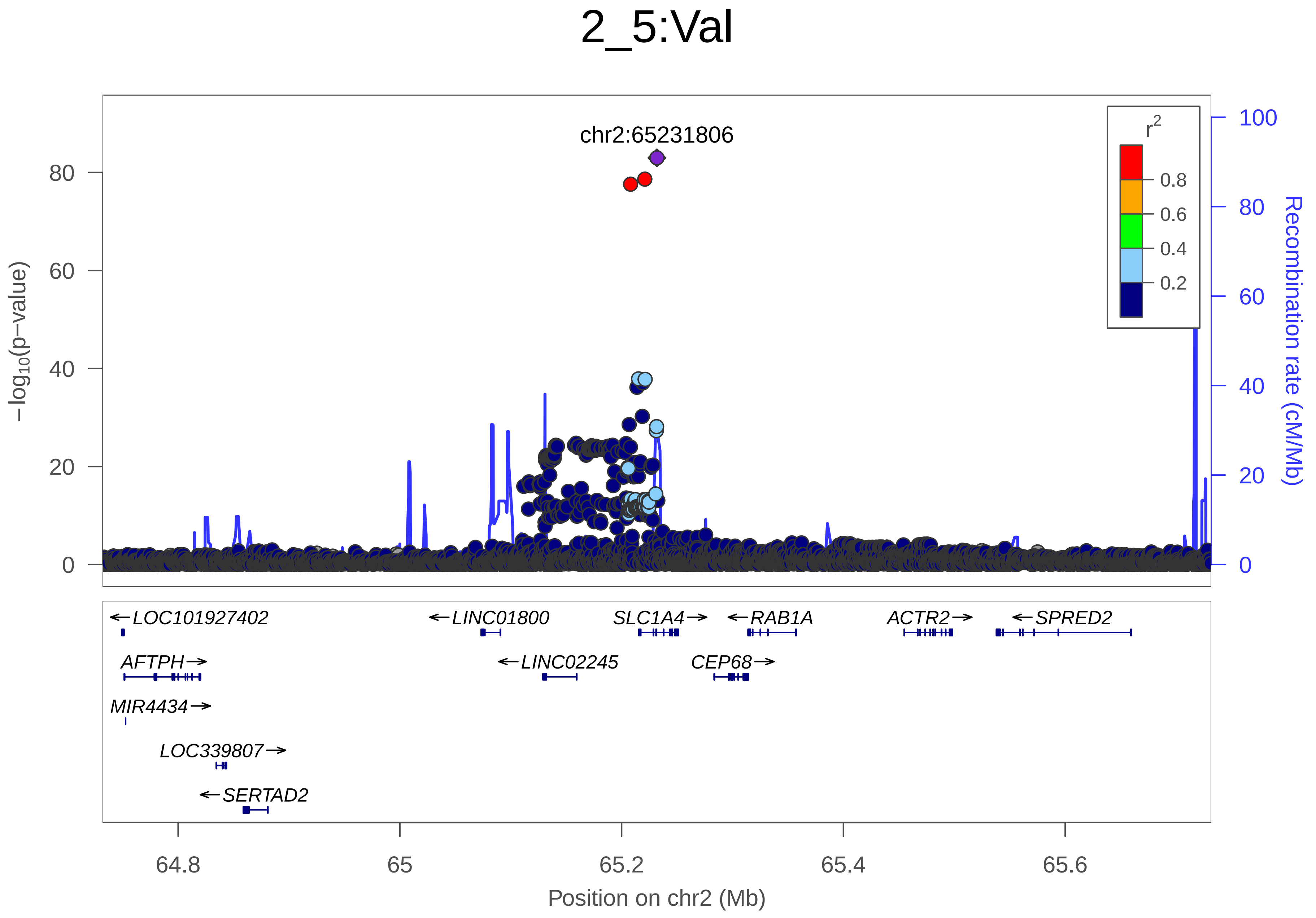

2_5:Val
chr2:65231806
−log10(p−value)
Recombination rate (cM/Mb)
r2
0.8
0.6
0.4
0.2
LOC101927402
AFTPH
MIR4434
LOC339807
SERTAD2
LINC01800
LINC02245
SLC1A4
CEP68
RAB1A
ACTR2
SPRED2
64.8
65
65.2
65.4
65.6
Position on chr2 (Mb)

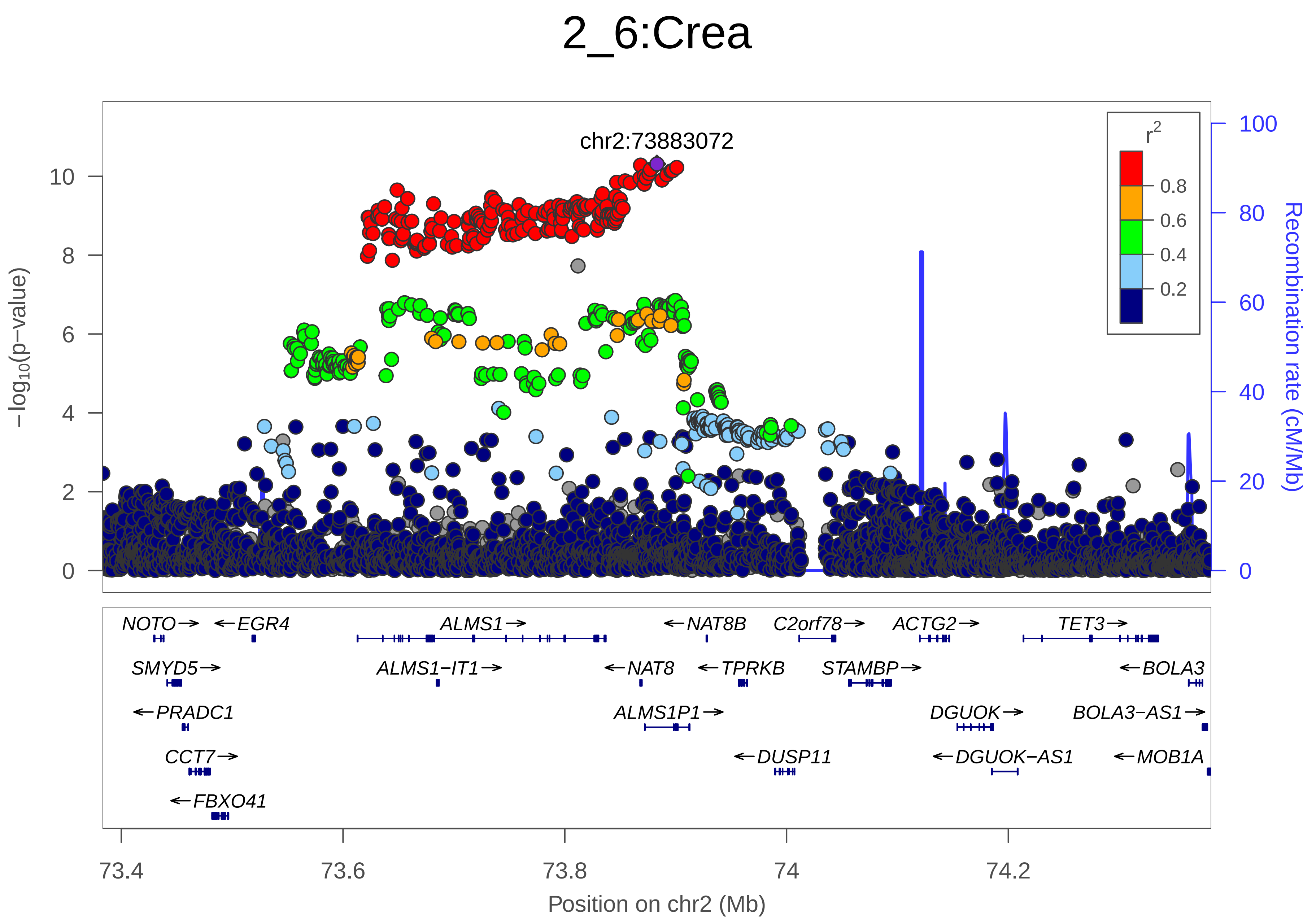

2_6:Crea
chr2:73883072
r2
0.8
0.6
0.4
0.2
−log10(p−value)
Recombination rate (cM/Mb)
NOTO→
←EGR4
ALMS1→
←NAT8B
C2orf78→
ACTG2→
TET3→
SMYD5→
ALMS1−IT1→
←NAT8
←TPRKB
STAMBP→
←BOLA3
←PRADC1
ALMS1P1→
DGUOK→
BOLA3−AS1→
CCT7→
←DUSP11
←DGUOK−AS1
←MOB1A
←FBXO41
73.4
73.6
73.8
74
74.2
Position on chr2 (Mb)

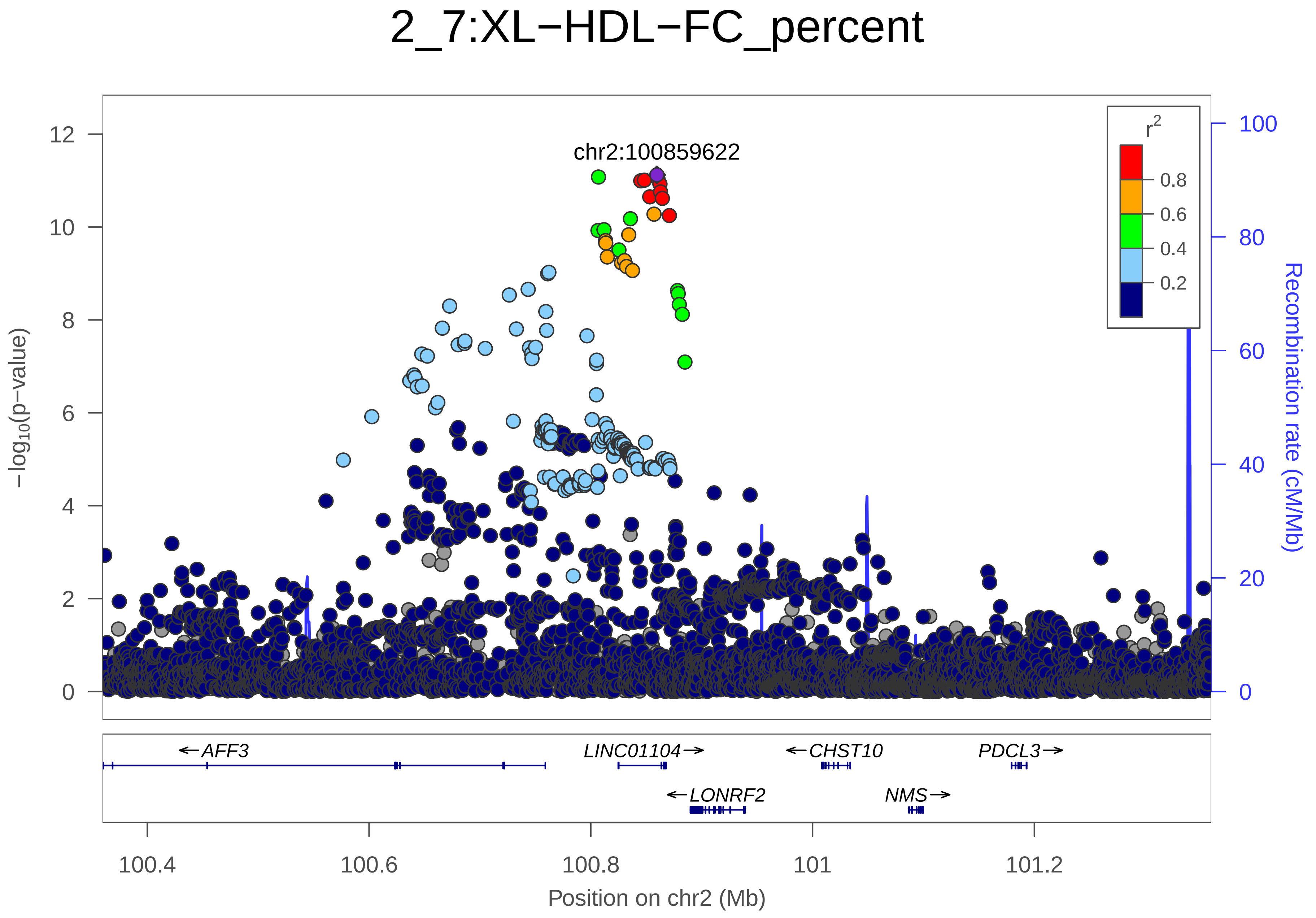

2_7:XL−HDL−FC_percent
chr2:100859622
r2
0.8
0.6
0.4
0.2
12
10
8
6
4
2
0
100
80
60
40
20
0
−log10(p−value)
Recombination rate (cM/Mb)
←AFF3
LINC01104→
←CHST10
PDCL3→
←LONRF2
NMS→
100.4
100.6
100.8
101
101.2
Position on chr2 (Mb)

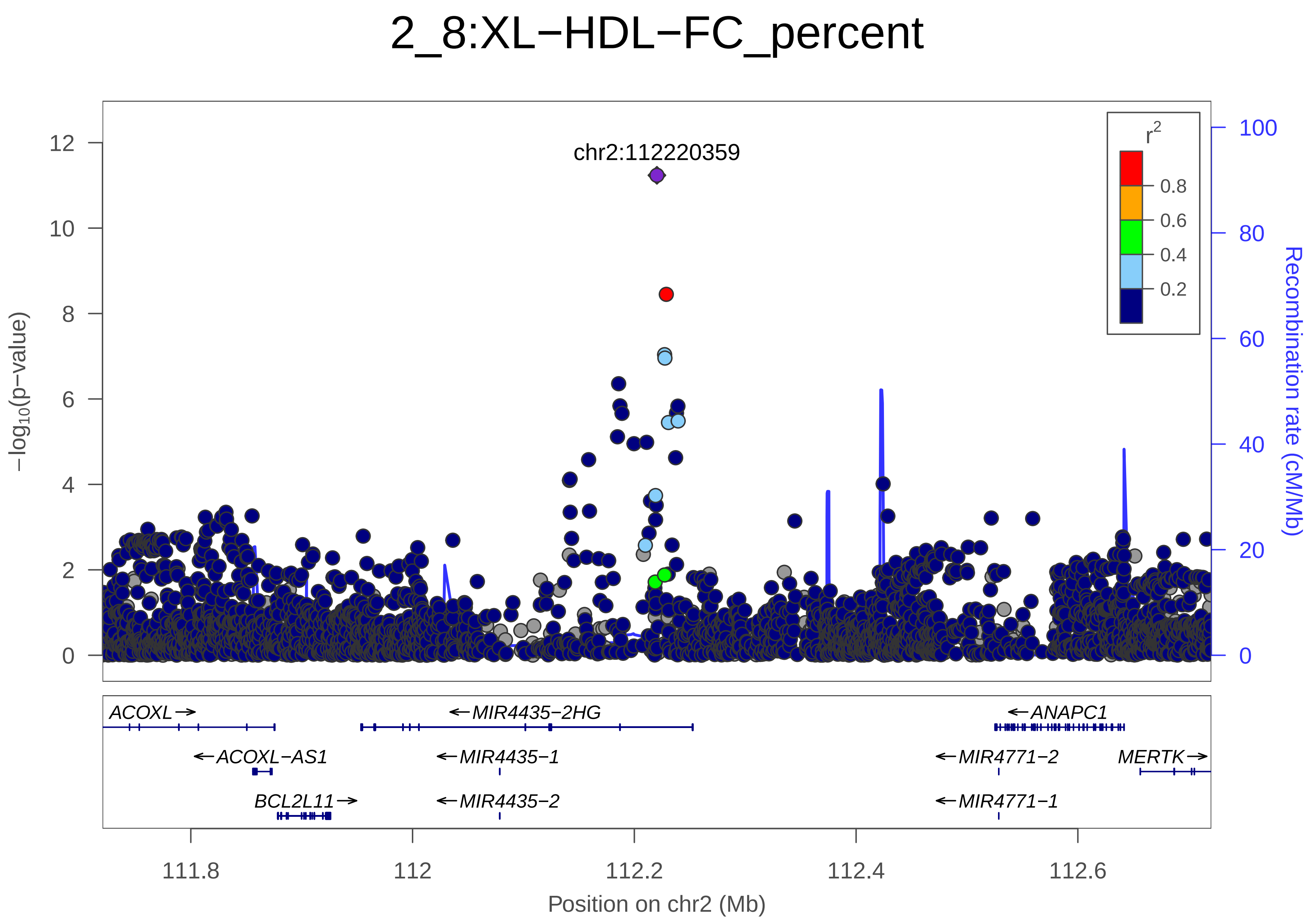
2_8:XL−HDL−FC_percent
chr2:112220359
r2
0.8
0.6
0.4
0.2
−log10(p−value)
Recombination rate (cM/Mb)
ACOXL→
←MIR4435−2HG
←ANAPC1
←ACOXL−AS1
←MIR4435−1
←MIR4771−2
MERTK→
BCL2L11→
←MIR4435−2
←MIR4771−1
111.8
112
112.2
112.4
112.6
Position on chr2 (Mb)

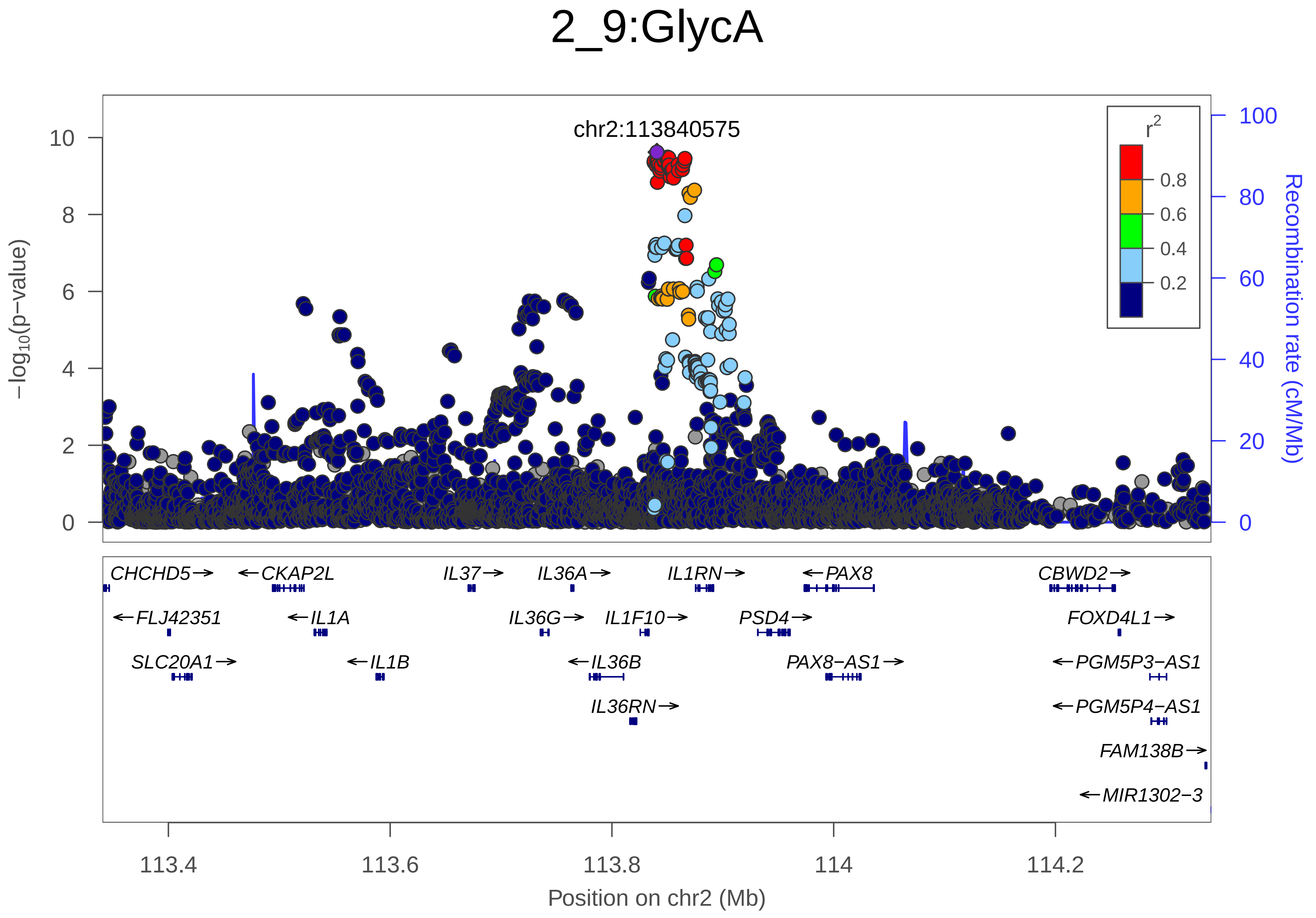
2_9:GlycA
chr2:113840575
−log10(p−value)
Recombination rate (cM/Mb)
r2
0.8
0.6
0.4
0.2
0
2
4
6
8
10
0
20
40
60
80
100
CHCHD5→
←CKAP2L
IL37→
IL36A→
IL1RN→
←PAX8
CBWD2→
←FLJ42351
←IL1A
IL36G→
IL1F10→
PSD4→
FOXD4L1→
SLC20A1→
←IL1B
←IL36B
PAX8−AS1→
←PGM5P3−AS1
IL36RN→
←PGM5P4−AS1
FAM138B→
←MIR1302−3
113.4
113.6
113.8
114
114.2
Position on chr2 (Mb)

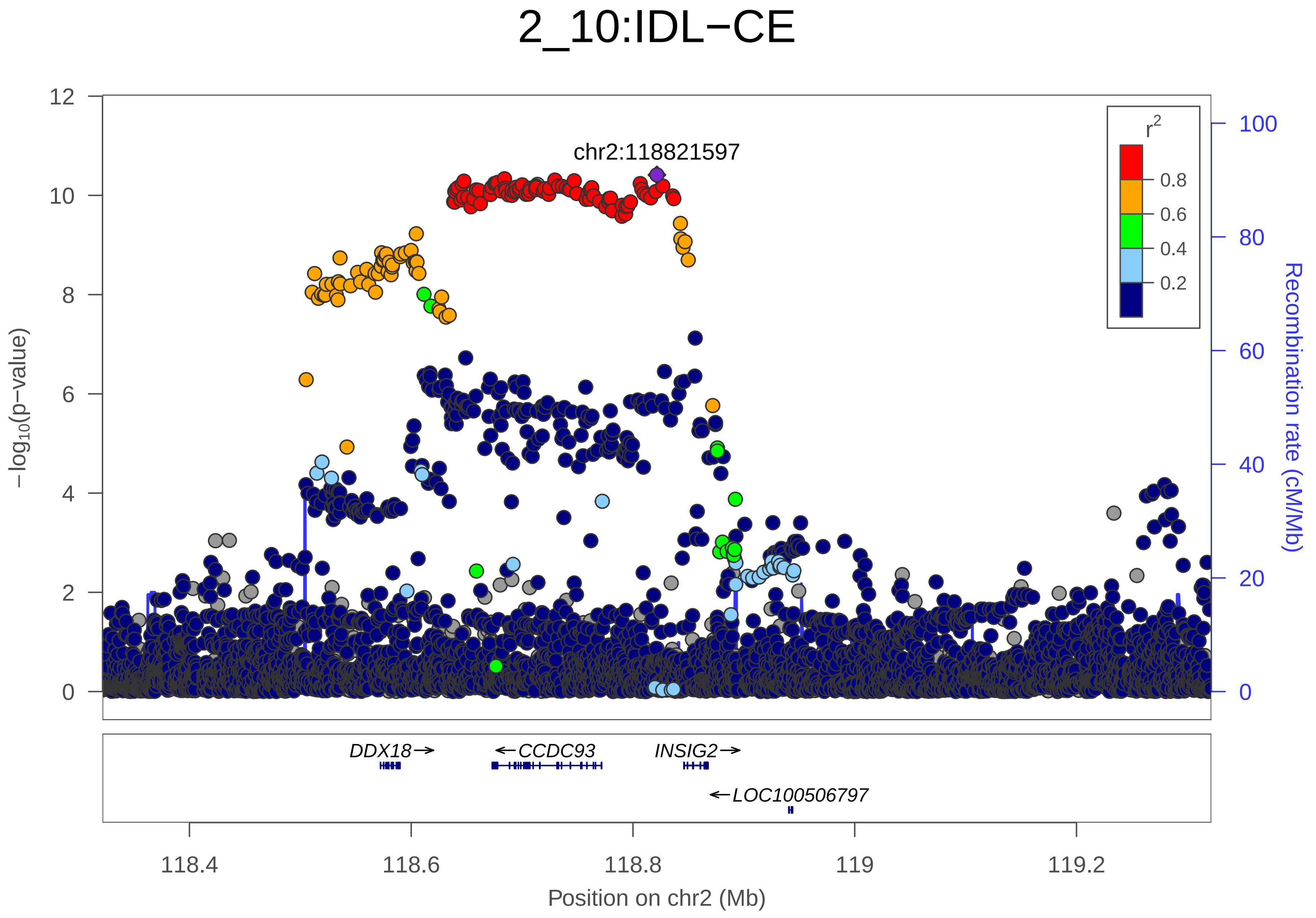

2_10:IDL−CE
chr2:118821597
r2
0.8
0.6
0.4
0.2
12
10
8
6
4
2
0
−log10(p−value)
100
80
60
40
20
0
Recombination rate (cM/Mb)
DDX18→
←CCDC93
INSIG2→
←LOC100506797
118.4
118.6
118.8
119
119.2
Position on chr2 (Mb)

2_11:Crea

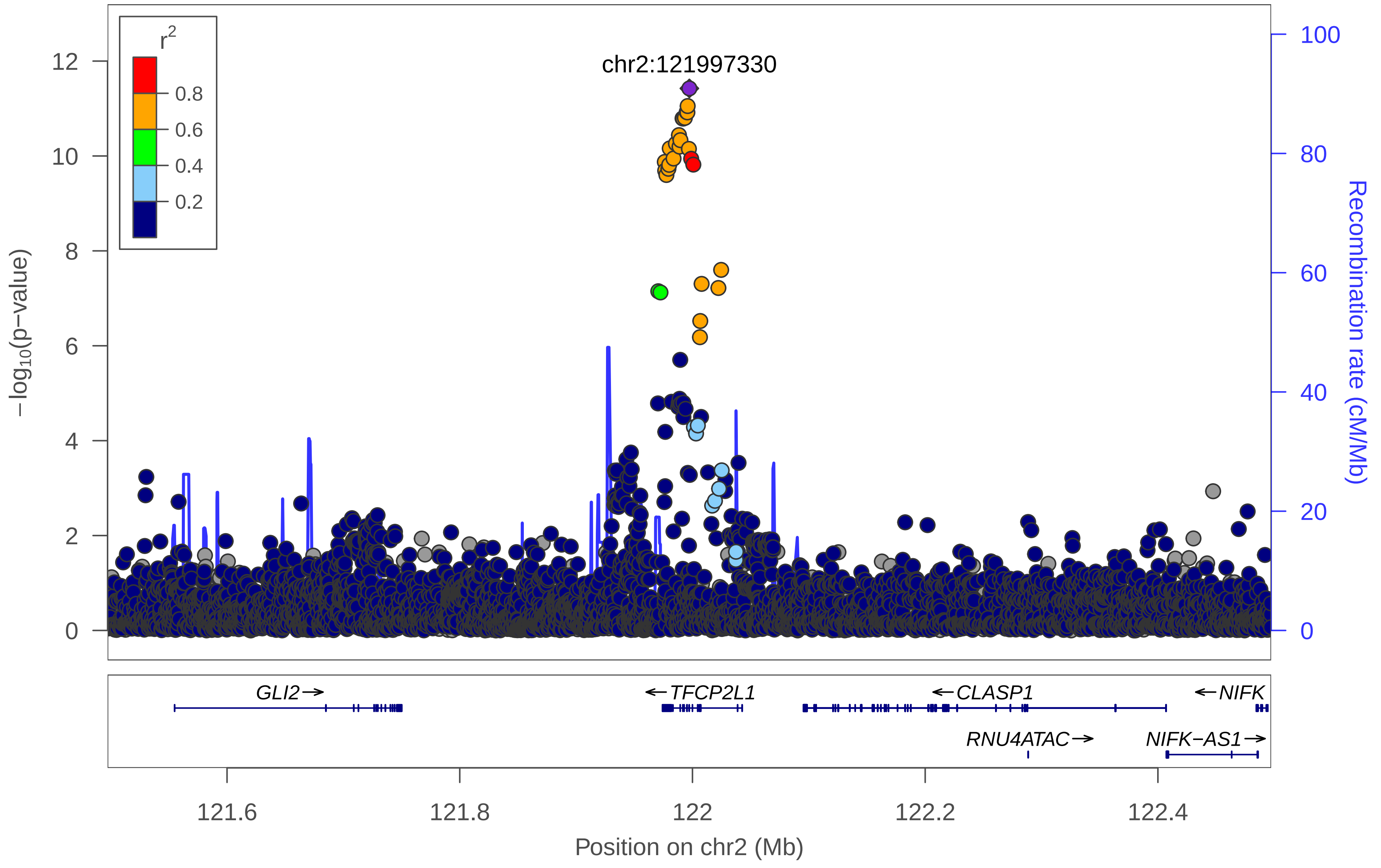

r2
0.8
0.6
0.4
0.2
chr2:121997330
−log10(p−value)
0
2
4
6
8
10
12
Recombination rate (cM/Mb)
0
20
40
60
80
100
GLI2→
←TFCP2L1
←CLASP1
←NIFK
RNU4ATAC→
NIFK−AS1→
121.6
121.8
122
122.2
122.4
Position on chr2 (Mb)

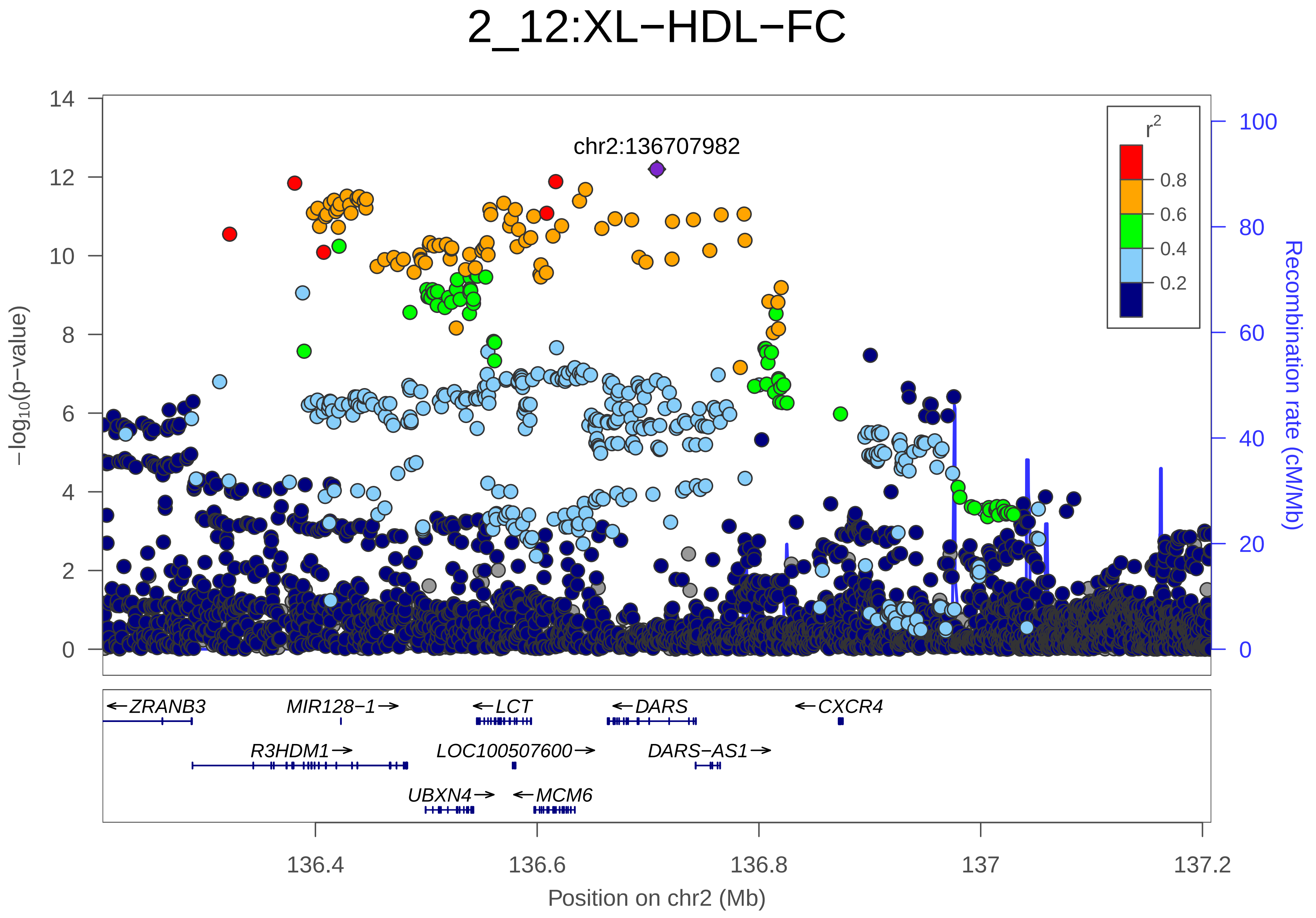

2_12:XL−HDL−FC
chr2:136707982
r2
0.8
0.6
0.4
0.2
−log10(p−value)
Recombination rate (cM/Mb)
ZRANB3
MIR128−1
LCT
DARS
CXCR4
R3HDM1
LOC100507600
DARS−AS1
UBXN4
MCM6
136.4
136.6
136.8
137
137.2
Position on chr2 (Mb)

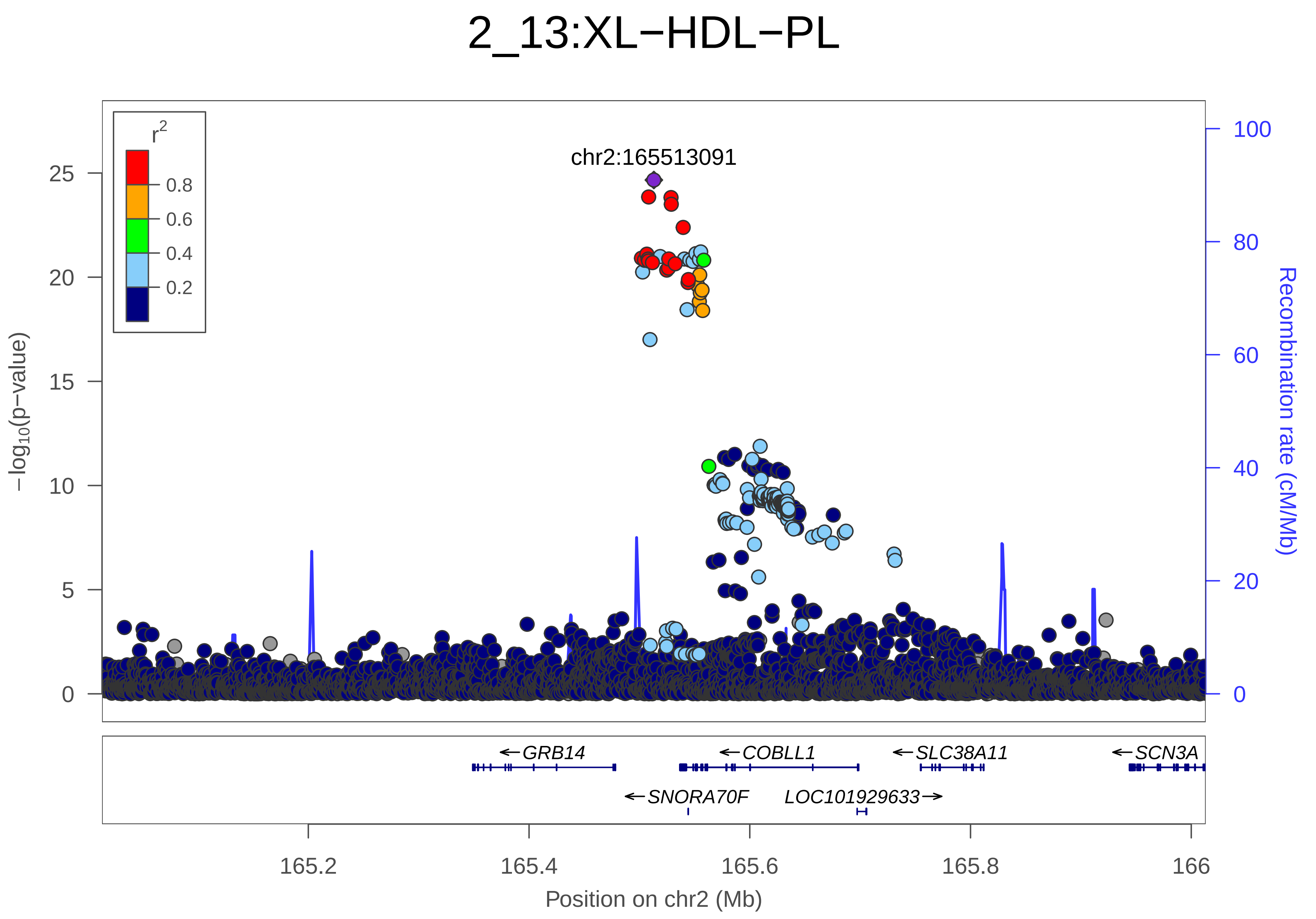

2_13:XL−HDL−PL
r2
0.8
0.6
0.4
0.2
chr2:165513091
−log10(p−value)
Recombination rate (cM/Mb)
GRB14
COBLL1
SLC38A11
SCN3A
SNORA70F
LOC101929633
165.2
165.4
165.6
165.8
166
Position on chr2 (Mb)

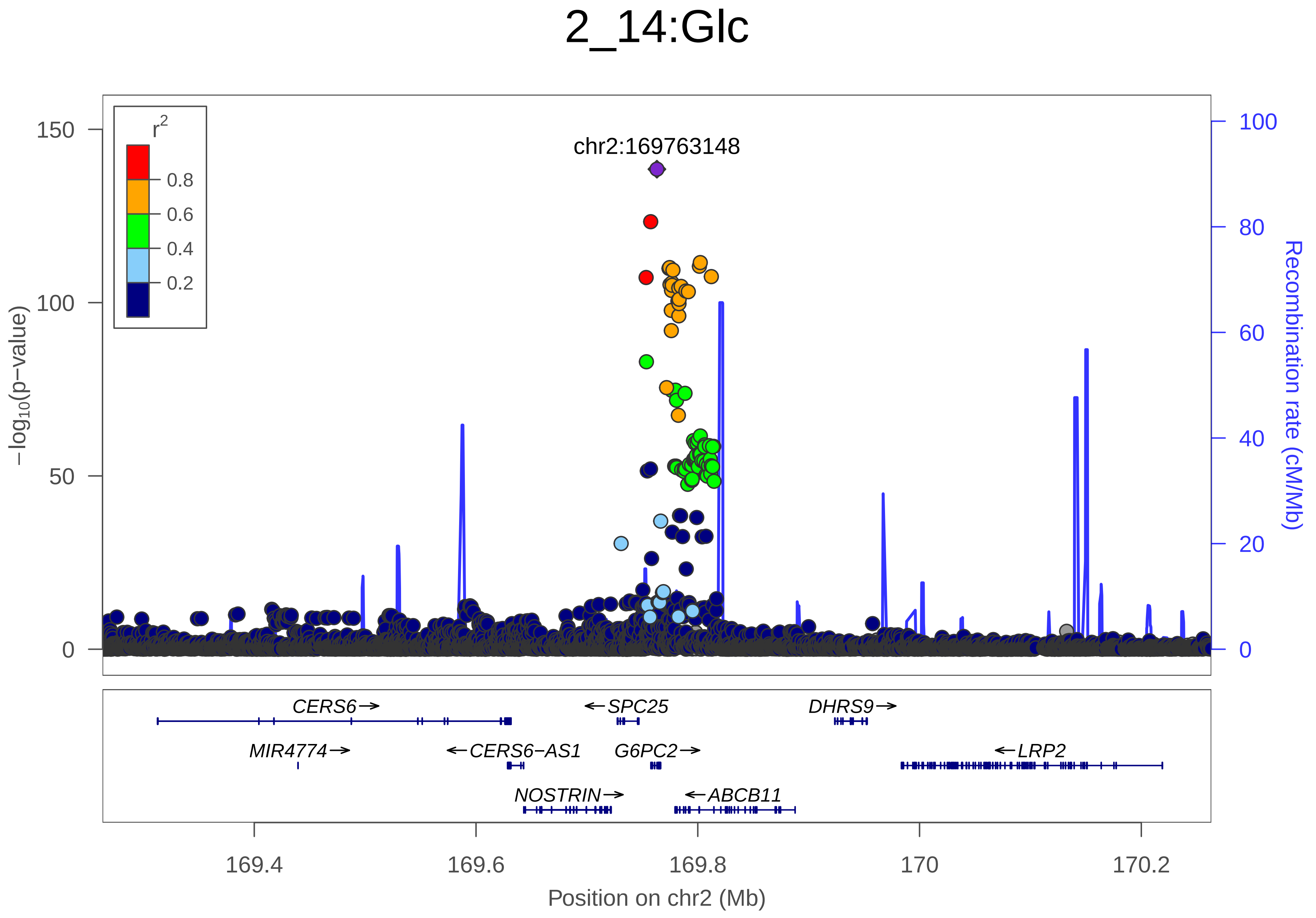

2_14:Glc
chr2:169763148
r2
0.8
0.6
0.4
0.2
−log10(p−value)
150
100
50
0
100
80
60
40
20
0
Recombination rate (cM/Mb)
CERS6→
←SPC25
DHRS9→
MIR4774→
←CERS6−AS1
G6PC2→
←LRP2
NOSTRIN→
←ABCB11
169.4
169.6
169.8
170
170.2
Position on chr2 (Mb)

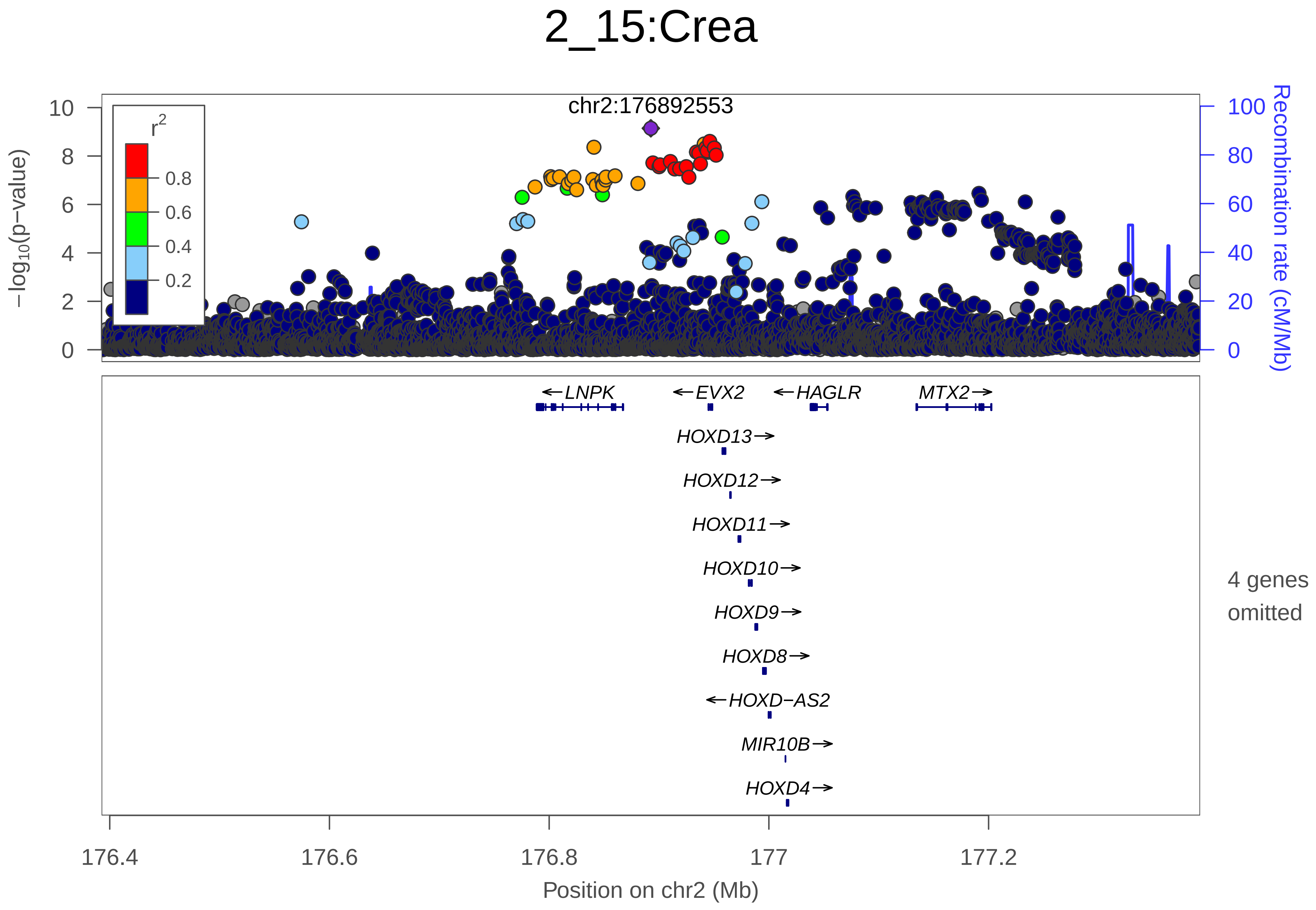
2_15:Crea
chr2:176892553
r2
0.8
0.6
0.4
0.2
−log10(p−value)
Recombination rate (cM/Mb)
←LNPK
←EVX2
←HAGLR
MTX2→
HOXD13→
HOXD12→
HOXD11→
HOXD10→
HOXD9→
HOXD8→
←HOXD−AS2
MIR10B→
HOXD4→
4 genes omitted
176.4
176.6
176.8
177
177.2
Position on chr2 (Mb)

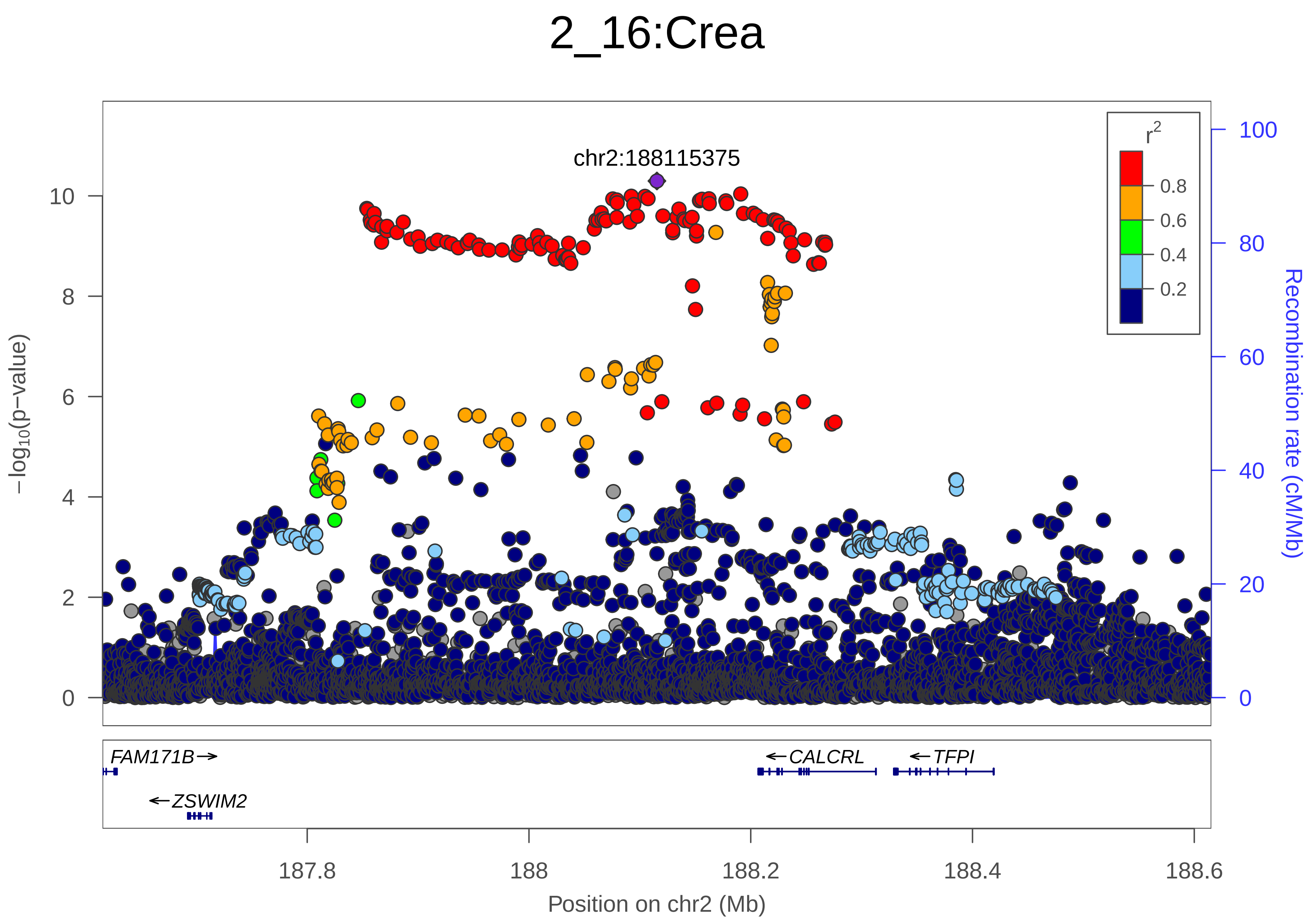

2_16:Crea
chr2:188115375
r2
0.8
0.6
0.4
0.2
10
8
6
4
2
0
−log10(p−value)
100
80
60
40
20
0
Recombination rate (cM/Mb)
FAM171B→
←ZSWIM2
←CALCRL
←TFPI
187.8
188
188.2
188.4
188.6
Position on chr2 (Mb)

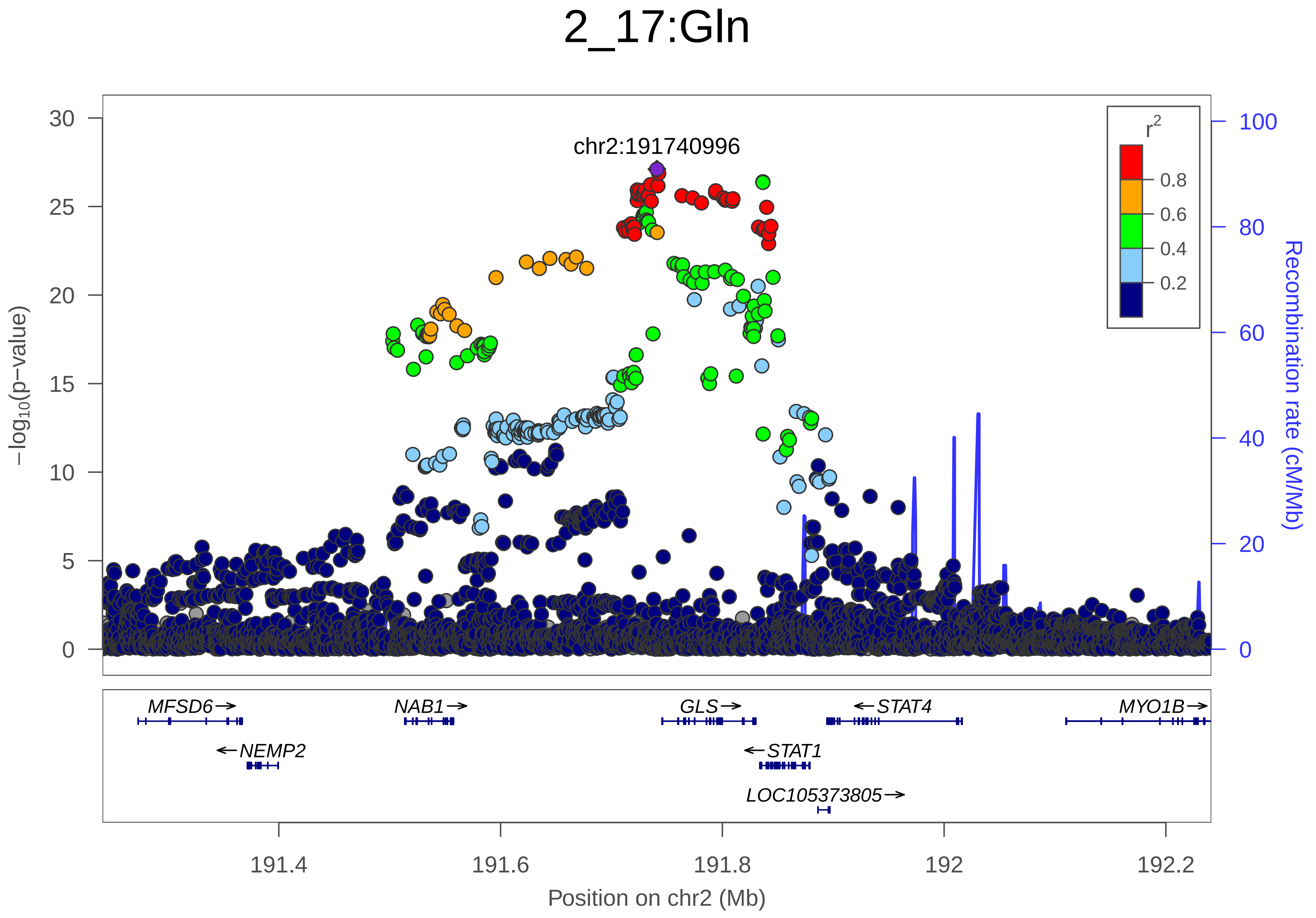

2_17:Gln
chr2:191740996
r2
0.8
0.6
0.4
0.2
−log10(p−value)
Recombination rate (cM/Mb)
MFSD6→
←NEMP2
NAB1→
GLS→
←STAT1
LOC105373805→
←STAT4
MYO1B→
191.4
191.6
191.8
192
192.2
Position on chr2 (Mb)

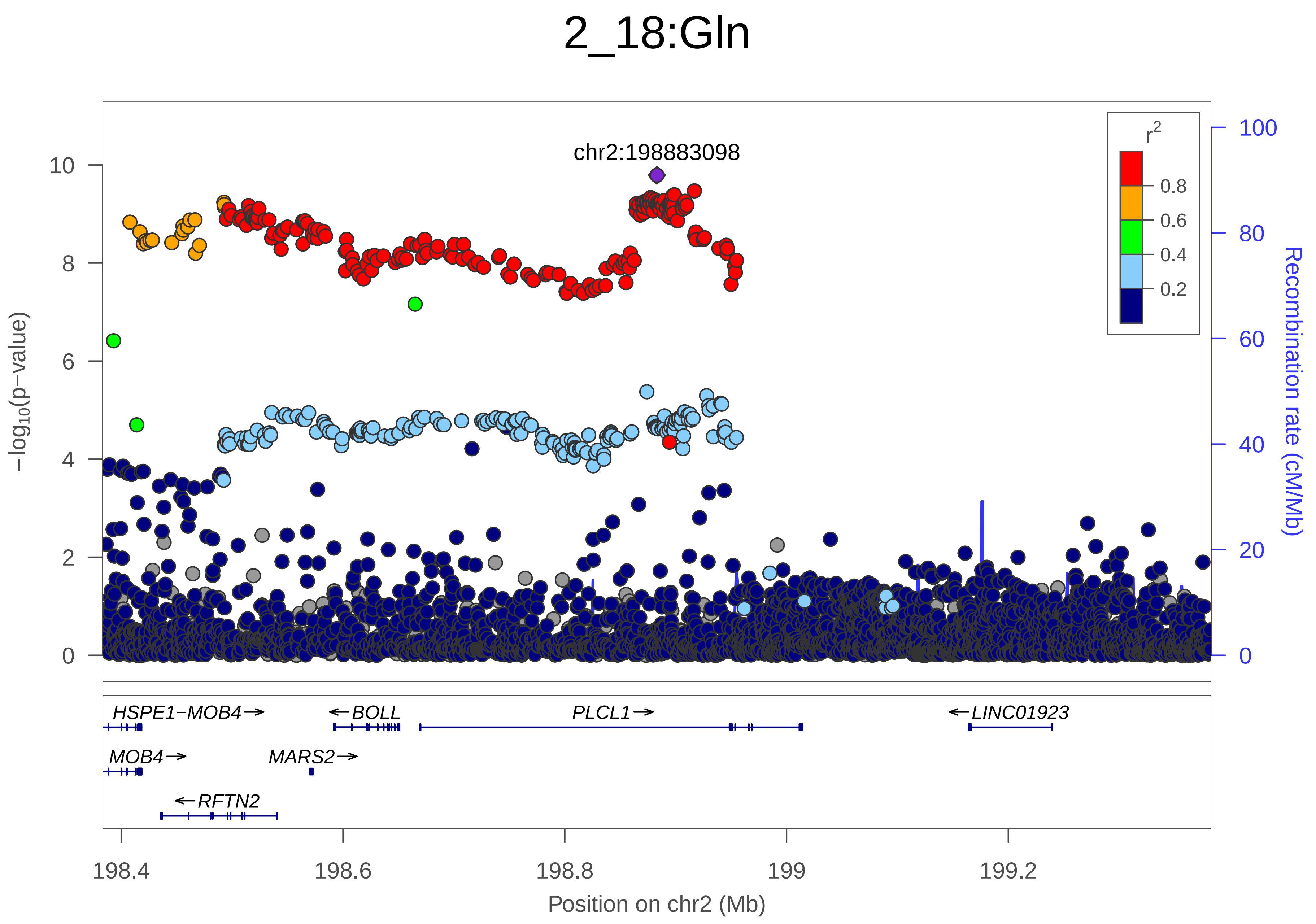

2_18:Gln
chr2:198883098
r2
0.8
0.6
0.4
0.2
−log10(p−value)
Recombination rate (cM/Mb)
HSPE1−MOB4→
←BOLL
PLCL1→
←LINC01923
MOB4→
MARS2→
←RFTN2
198.4
198.6
198.8
199
199.2
Position on chr2 (Mb)

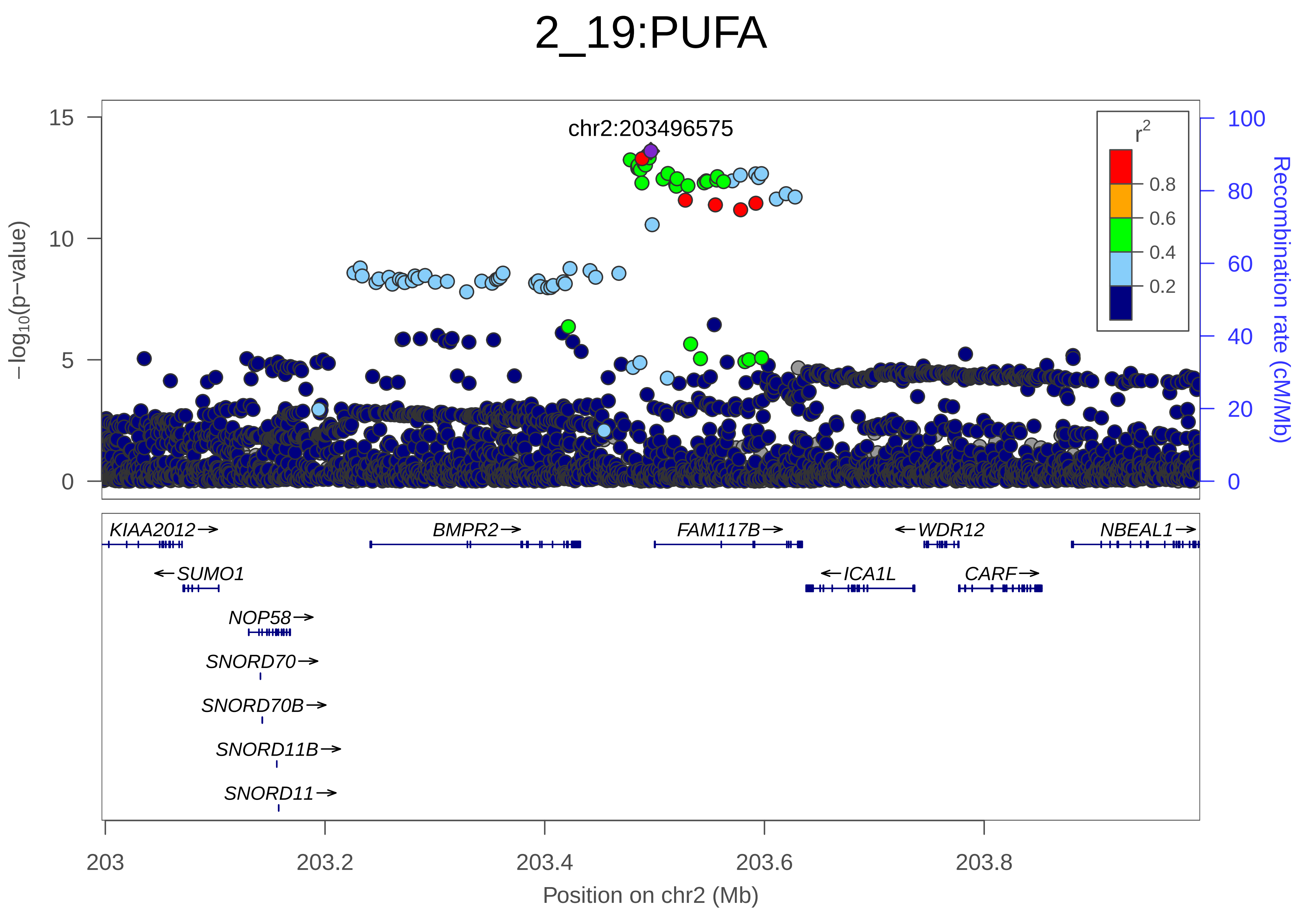

2_19:PUFA
chr2:203496575
r2
0.8
0.6
0.4
0.2
−log10(p−value)
Recombination rate (cM/Mb)
KIAA2012→
BMPR2→
FAM117B→
←WDR12
NBEAL1→
←SUMO1
←ICA1L
CARF→
NOP58→
SNORD70→
SNORD70B→
SNORD11B→
SNORD11→
203
203.2
203.4
203.6
203.8
Position on chr2 (Mb)

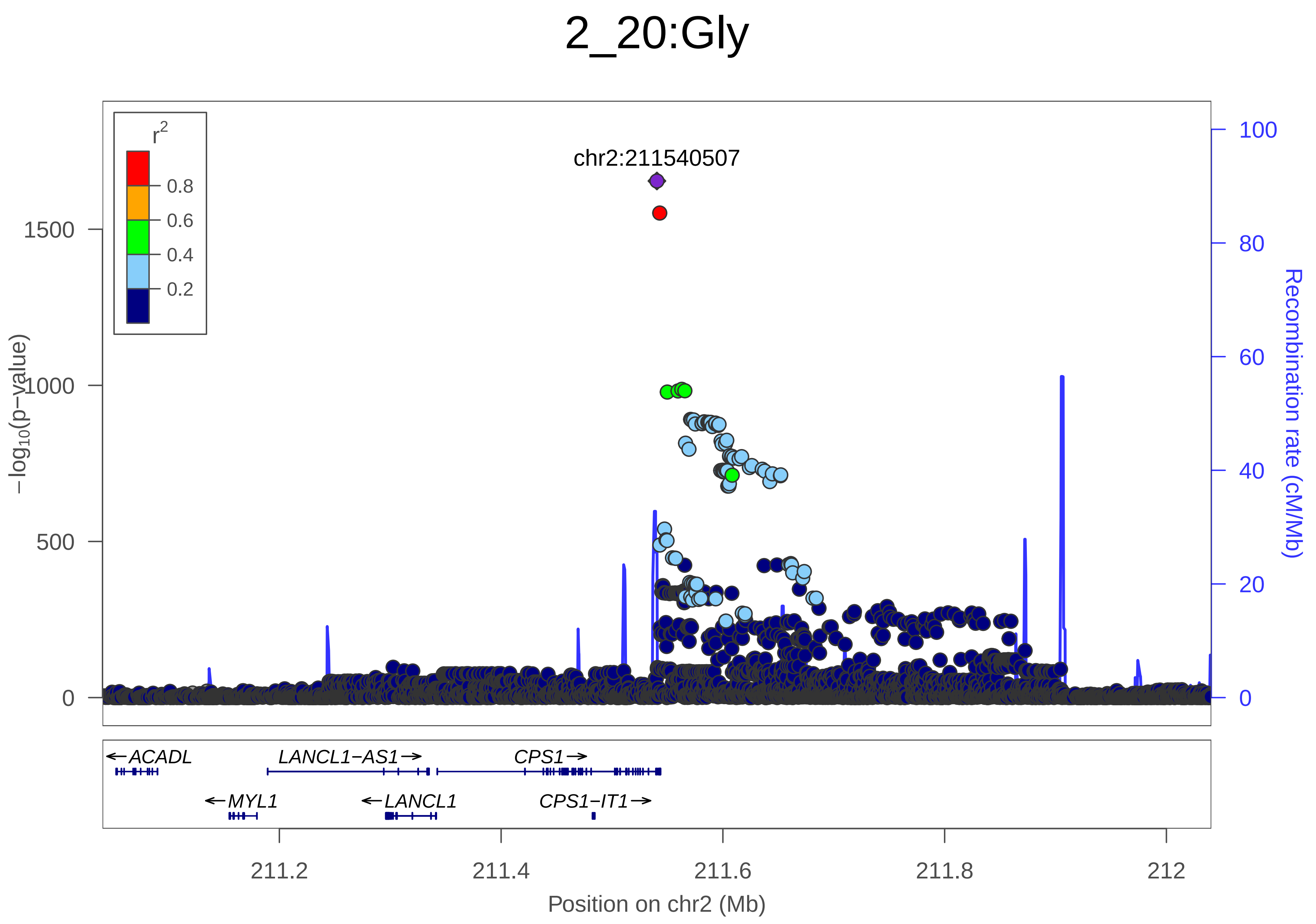
2_20:Gly
chr2:211540507
r2
0.8
0.6
0.4
0.2
1500
1000
500
0
−log10(p−value)
100
80
60
40
20
0
Recombination rate (cM/Mb)
←ACADL
LANCL1−AS1→
CPS1→
←MYL1
←LANCL1
CPS1−IT1→
211.2
211.4
211.6
211.8
212
Position on chr2 (Mb)

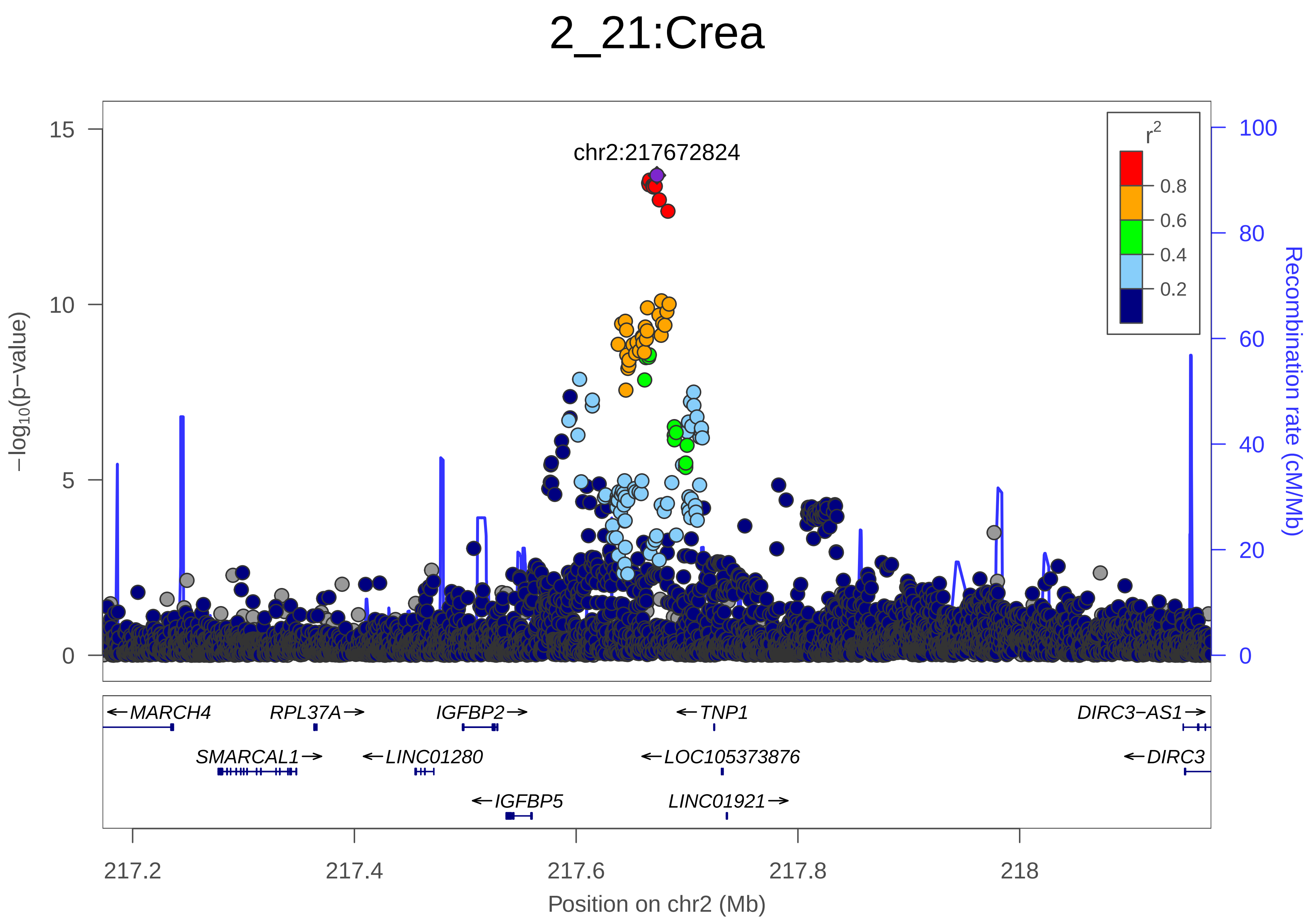

2_21:Crea
chr2:217672824
r2
0.8
0.6
0.4
0.2
15
10
5
0
100
80
60
40
20
0
−log10(p−value)
Recombination rate (cM/Mb)
MARCH4
RPL37A
IGFBP2
TNP1
DIRC3−AS1
SMARCAL1
LINC01280
LOC105373876
DIRC3
IGFBP5
LINC01921
217.2
217.4
217.6
217.8
218
Position on chr2 (Mb)

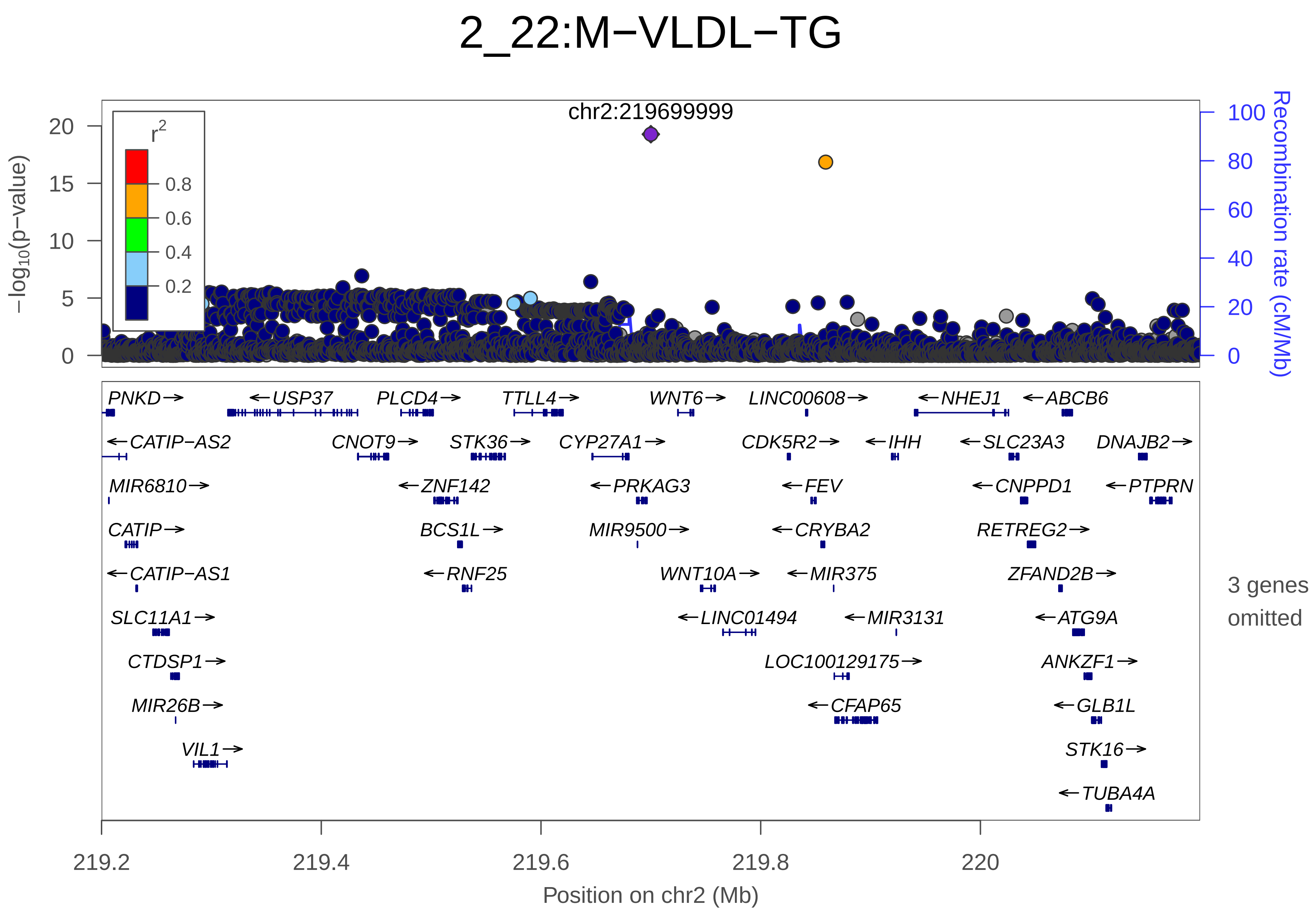

2_22:M−VLDL−TG
chr2:219699999
r2
0.8
0.6
0.4
0.2
−log10(p−value)
Recombination rate (cM/Mb)
0
5
10
15
20
0
20
40
60
80
100
PNKD→
←USP37
PLCD4→
TTLL4→
WNT6→
LINC00608→
←NHEJ1
←ABCB6
←CATIP−AS2
CNOT9→
STK36→
CYP27A1→
CDK5R2→
←IHH
←SLC23A3
DNAJB2→
MIR6810→
←ZNF142
←PRKAG3
←FEV
←CNPPD1
←PTPRN
CATIP→
BCS1L→
MIR9500→
←CRYBA2
RETREG2→
←CATIP−AS1
←RNF25
WNT10A→
←MIR375
ZFAND2B→
SLC11A1→
←LINC01494
←MIR3131
←ATG9A
CTDSP1→
LOC100129175→
ANKZF1→
MIR26B→
←CFAP65
←GLB1L
VIL1→
STK16→
←TUBA4A
3 genes omitted
219.2
219.4
219.6
219.8
220
Position on chr2 (Mb)

2_23:XL−HDL−PL

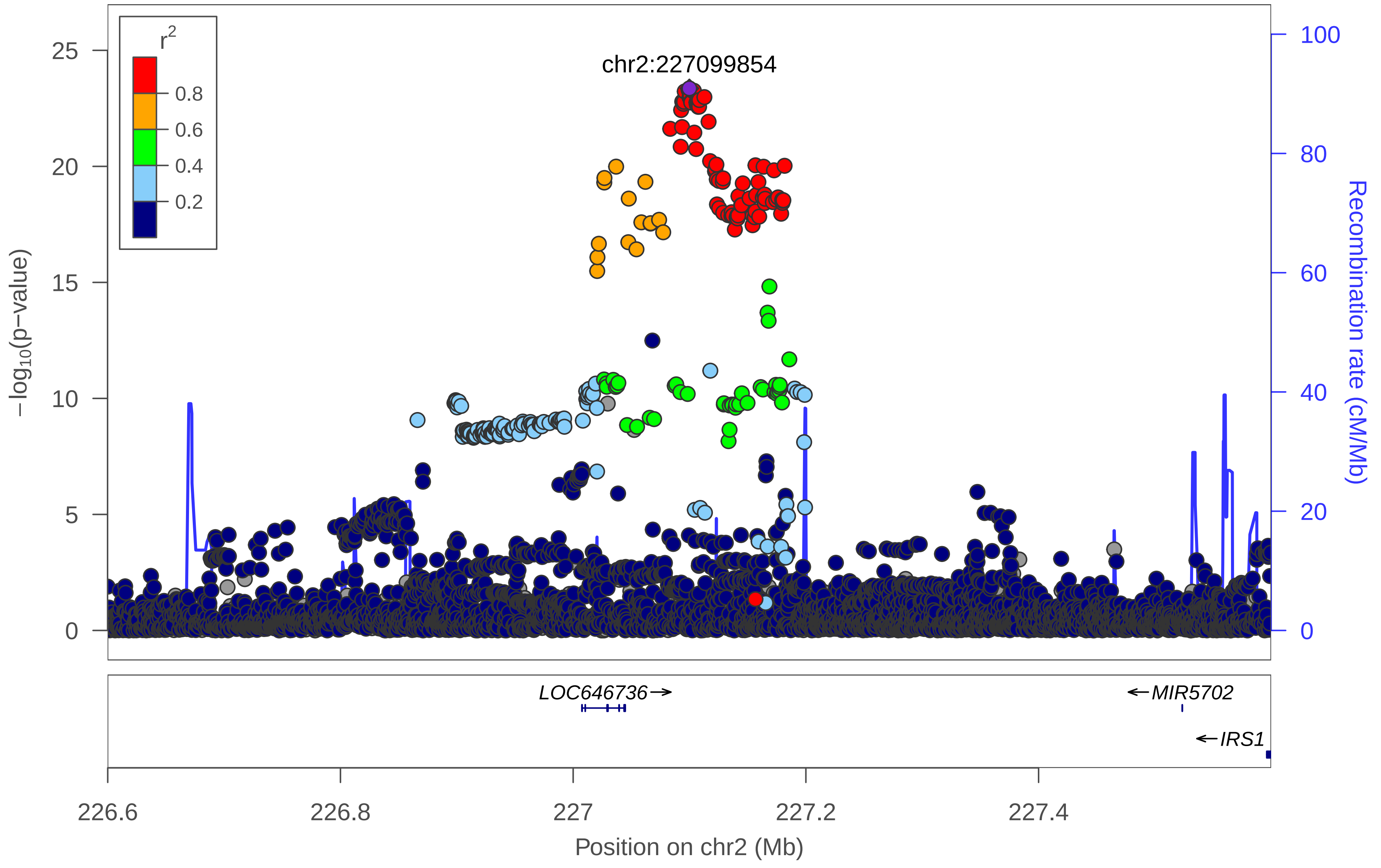

r2
0.8
0.6
0.4
0.2
chr2:227099854
−log10(p−value)
Recombination rate (cM/Mb)
LOC646736→
←MIR5702
←IRS1
226.6
226.8
227
227.2
227.4
Position on chr2 (Mb)

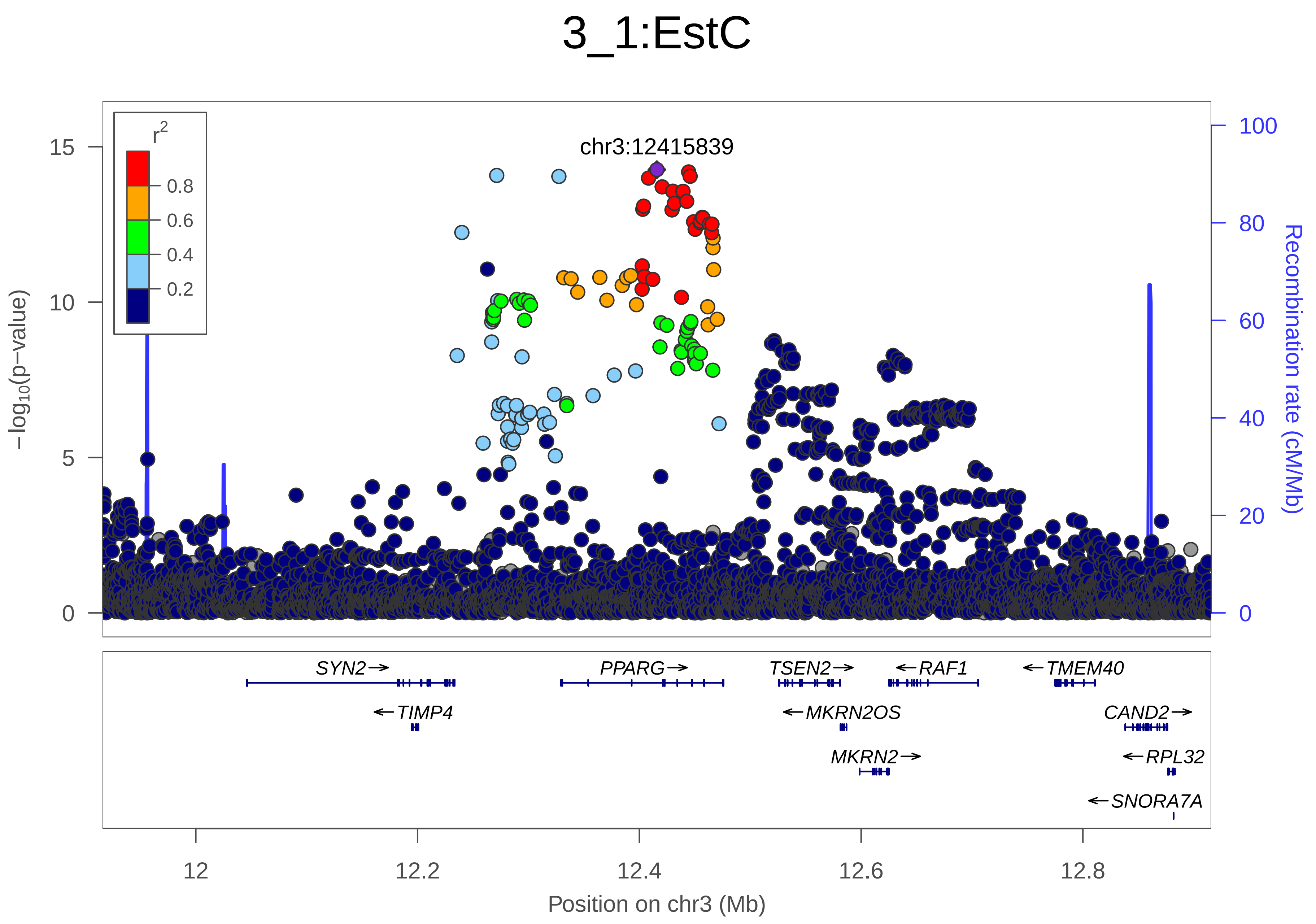

3_1:EstC
chr3:12415839
r2
0.8
0.6
0.4
0.2
15
10
5
0
−log10(p−value)
100
80
60
40
20
0
Recombination rate (cM/Mb)
SYN2→
←TIMP4
PPARG→
TSEN2→
←MKRN2OS
MKRN2→
←RAF1
←TMEM40
CAND2→
←RPL32
←SNORA7A
12
12.2
12.4
12.6
12.8
Position on chr3 (Mb)

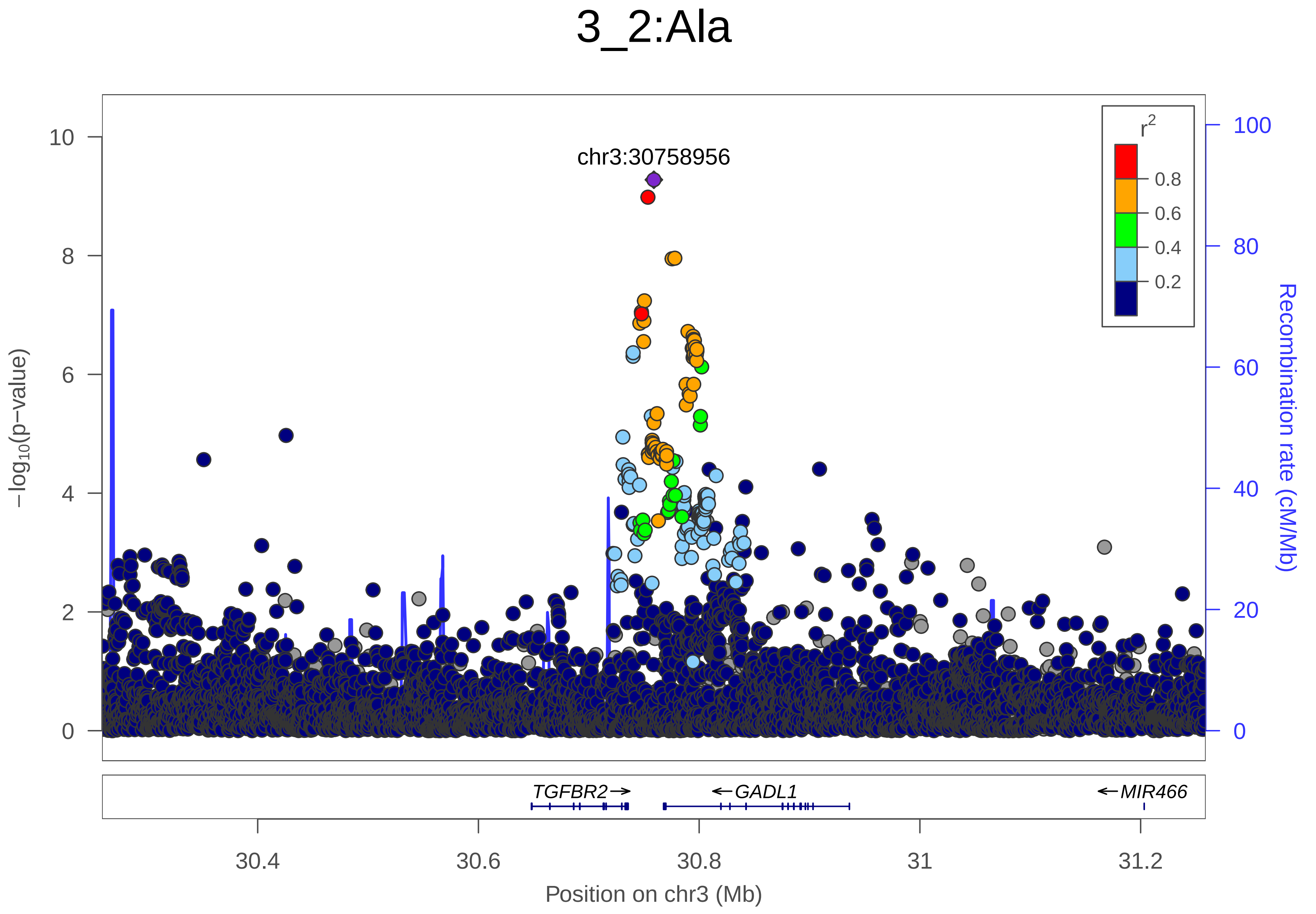

3_2:Ala
chr3:30758956
r2
0.8
0.6
0.4
0.2
−log10(p−value)
Recombination rate (cM/Mb)
TGFBR2
GADL1
MIR466
Position on chr3 (Mb)

3_3:Crea

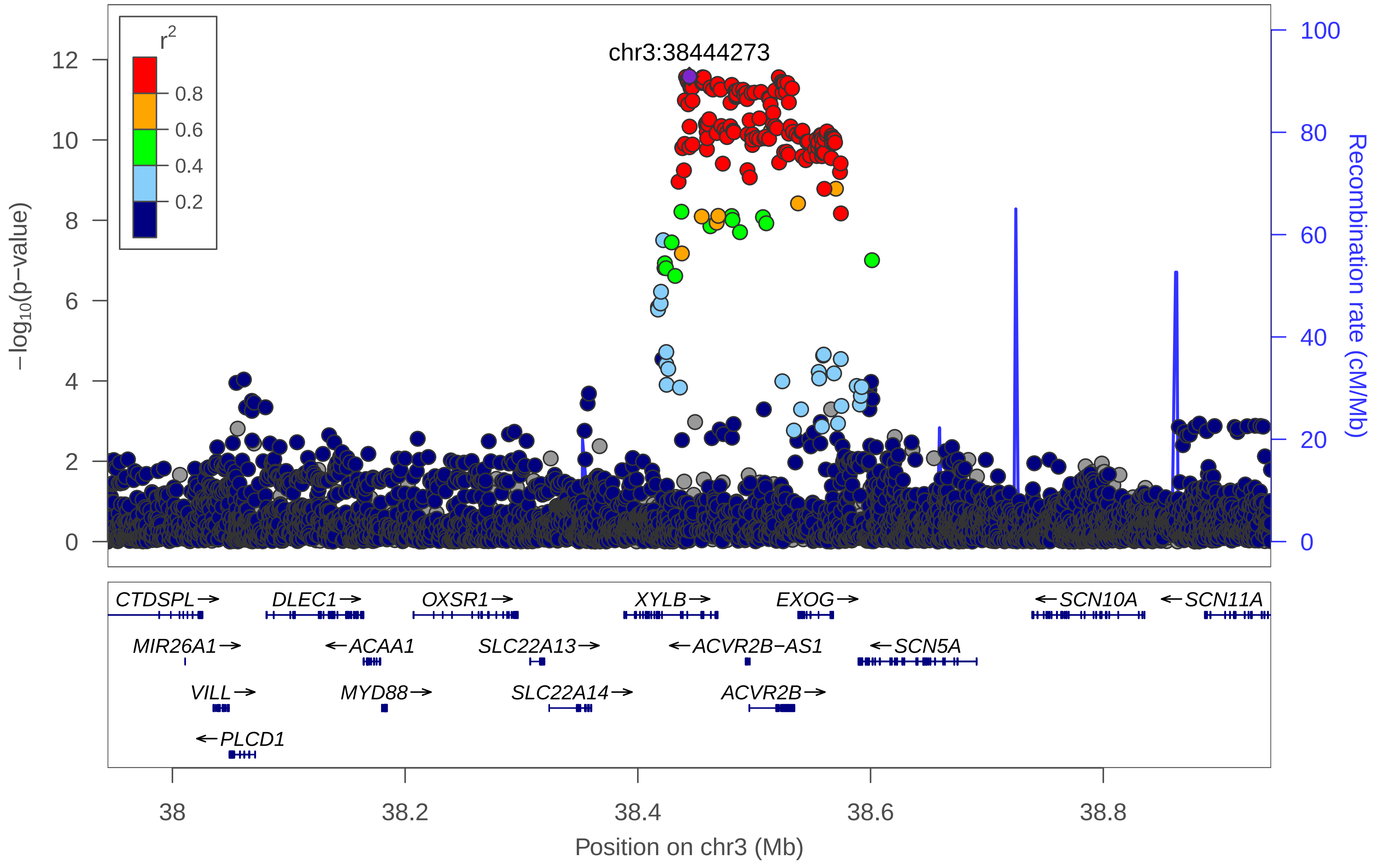
chr3:38444273
r2
0.8
0.6
0.4
0.2
12
10
8
6
4
2
0
−log10(p−value)
100
80
60
40
20
0
Recombination rate (cM/Mb)
CTDSPL→
DLEC1→
OXSR1→
XYLB→
EXOG→
←SCN10A
←SCN11A
MIR26A1→
←ACAA1
SLC22A13→
←ACVR2B−AS1
←SCN5A
VILL→
MYD88→
SLC22A14→
ACVR2B→
←PLCD1
38
38.2
38.4
38.6
38.8
Position on chr3 (Mb)

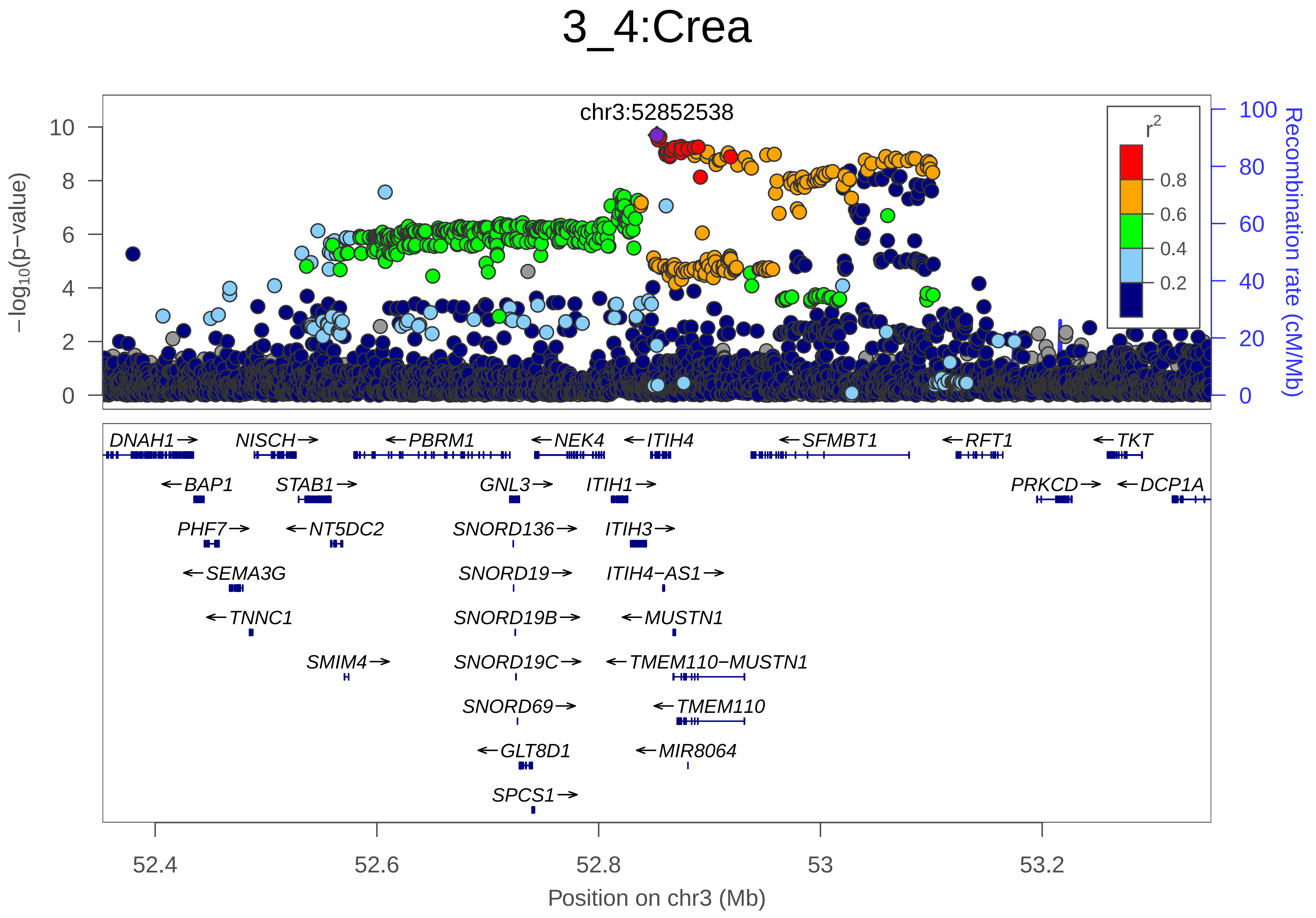

3_4:Crea
chr3:52852538
−log10(p−value)
Recombination rate (cM/Mb)
r2
0.8
0.6
0.4
0.2
DNAH1
NISCH
PBRM1
NEK4
ITIH4
SFMBT1
RFT1
TKT
BAP1
STAB1
GNL3
ITIH1
PRKCD
DCP1A
PHF7
NT5DC2
SNORD136
ITIH3
SEMA3G
SNORD19
ITIH4−AS1
TNNC1
SNORD19B
MUSTN1
SMIM4
SNORD19C
TMEM110−MUSTN1
SNORD69
TMEM110
GLT8D1
MIR8064
SPCS1
52.4
52.6
52.8
53
53.2
Position on chr3 (Mb)

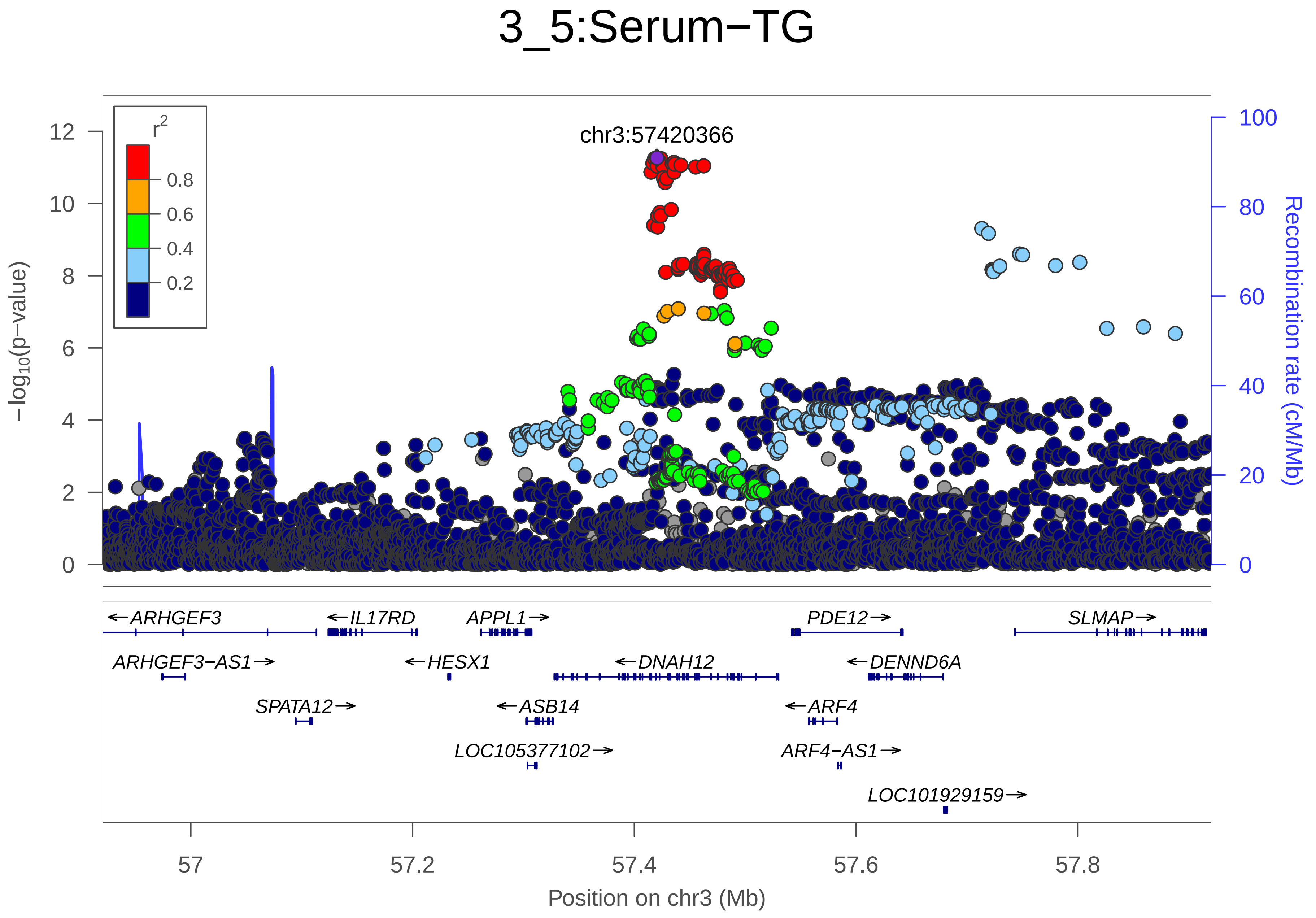

3_5:Serum−TG
chr3:57420366
r2
0.8
0.6
0.4
0.2
−log10(p−value)
Recombination rate (cM/Mb)
←ARHGEF3
←IL17RD
APPL1→
PDE12→
SLMAP→
ARHGEF3−AS1→
←HESX1
←DNAH12
←DENND6A
SPATA12→
←ASB14
←ARF4
LOC105377102→
ARF4−AS1→
LOC101929159→
Position on chr3 (Mb)

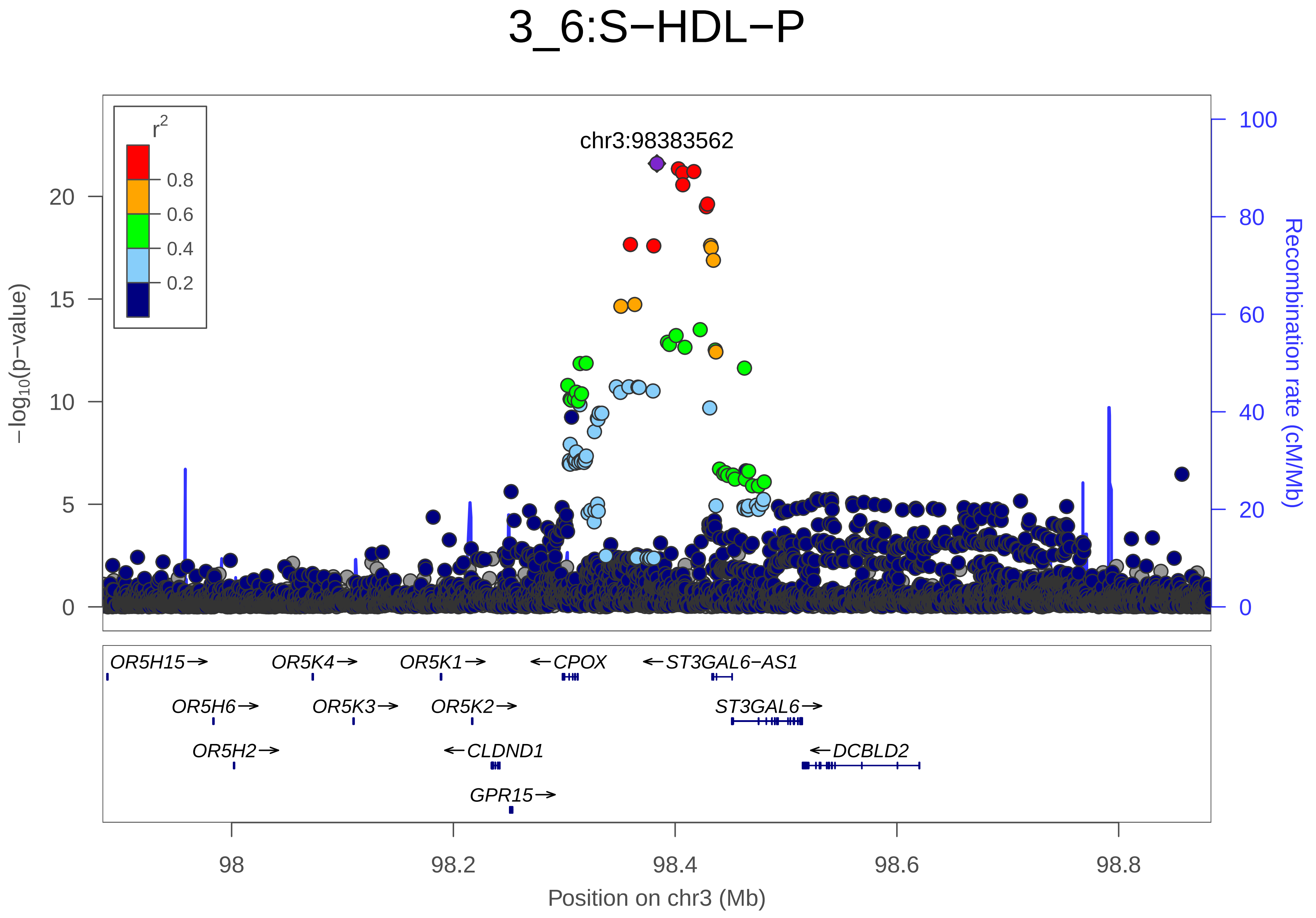
3_6:S−HDL−P
r2
0.8
0.6
0.4
0.2
chr3:98383562
−log10(p−value)
Recombination rate (cM/Mb)
0
5
10
15
20
0
20
40
60
80
100
OR5H15
OR5K4
OR5K1
CPOX
ST3GAL6−AS1
OR5H6
OR5K3
OR5K2
ST3GAL6
OR5H2
CLDND1
DCBLD2
GPR15
98
98.2
98.4
98.6
98.8
Position on chr3 (Mb)

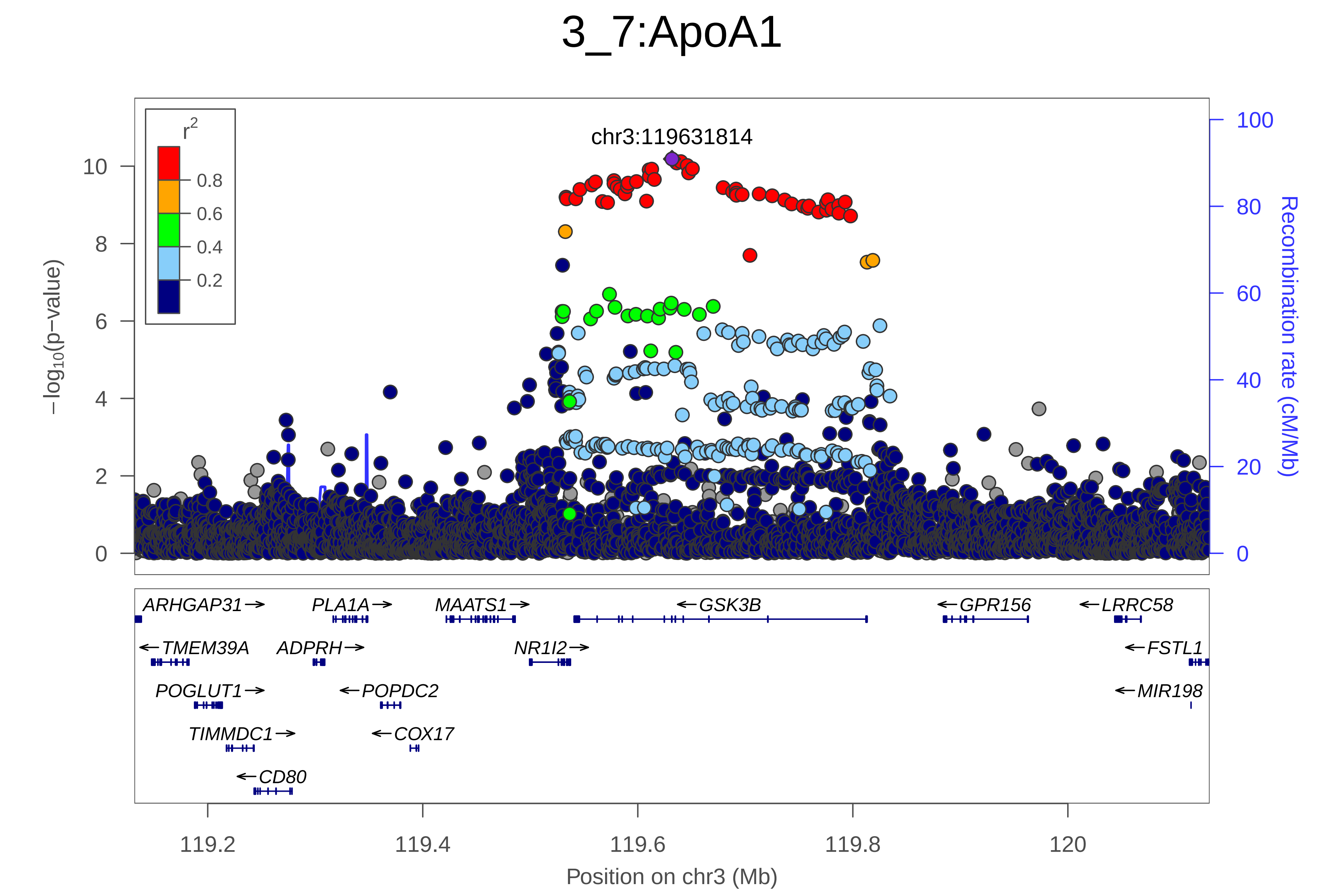
3_7:ApoA1
r2
0.8
0.6
0.4
0.2
chr3:119631814
−log10(p−value)
0
2
4
6
8
10
Recombination rate (cM/Mb)
0
20
40
60
80
100
ARHGAP31→
PLA1A→
MAATS1→
←GSK3B
←GPR156
←LRRC58
←TMEM39A
ADPRH→
NR1I2→
←FSTL1
POGLUT1→
←POPDC2
←MIR198
TIMMDC1→
←COX17
←CD80
119.2
119.4
119.6
119.8
120
Position on chr3 (Mb)

3_8:Ala

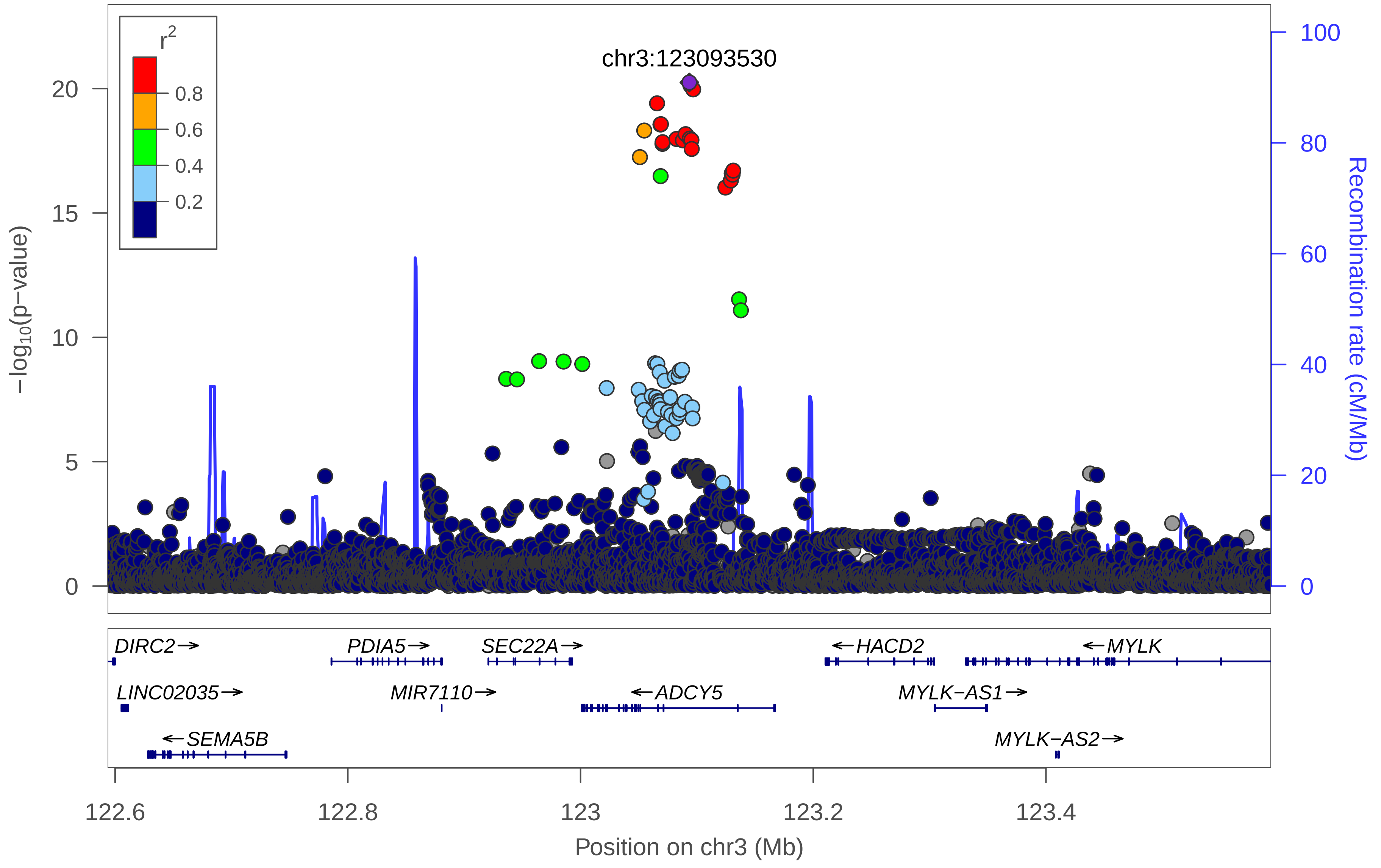

chr3:123093530
r2
0.8
0.6
0.4
0.2
−log10(p−value)
20
15
10
5
0
Recombination rate (cM/Mb)
100
80
60
40
20
0
DIRC2→
PDIA5→
SEC22A→
←HACD2
←MYLK
LINC02035→
MIR7110→
←ADCY5
MYLK−AS1→
←SEMA5B
MYLK−AS2→
122.6
122.8
123
123.2
123.4
Position on chr3 (Mb)

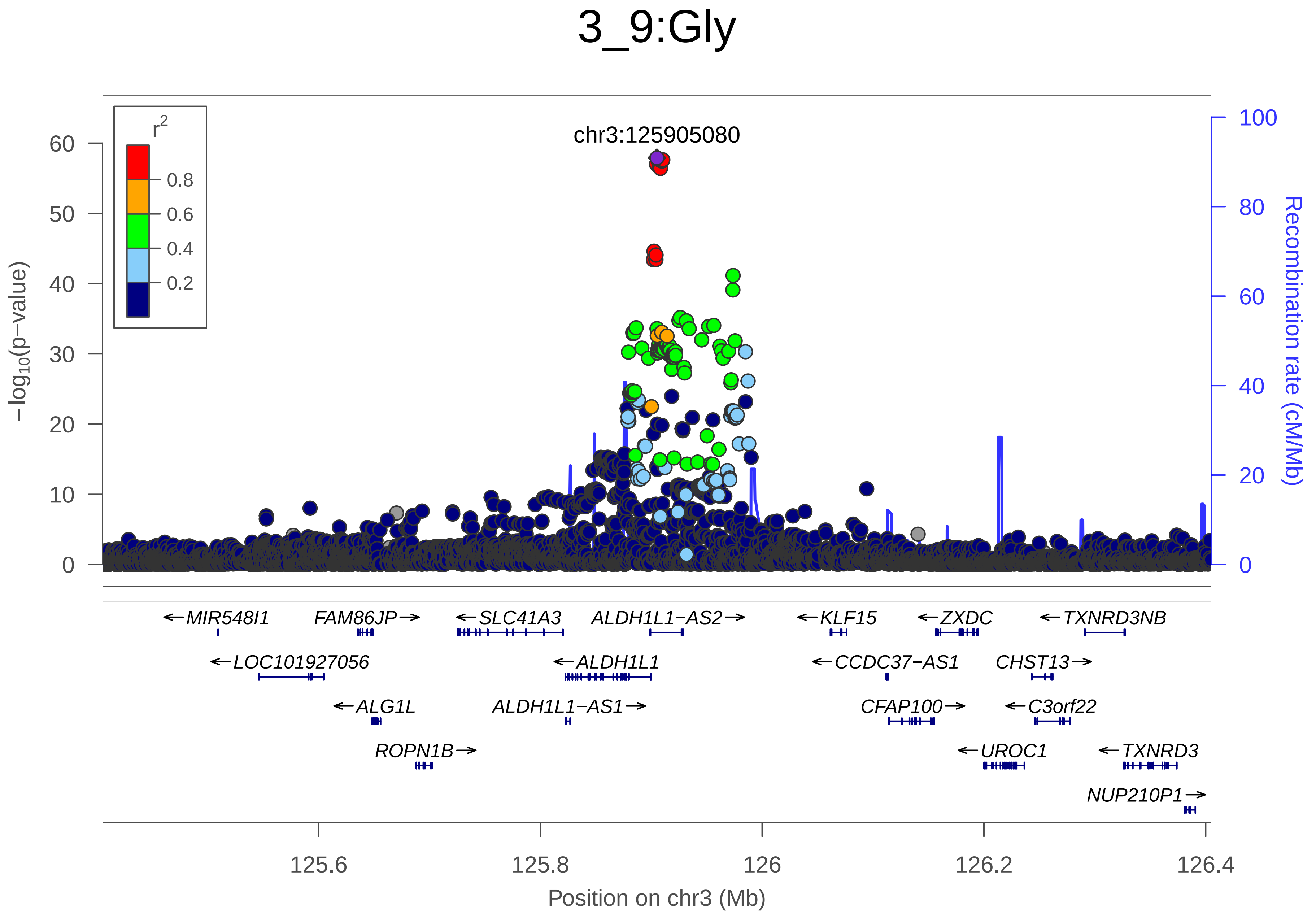
3_9:Gly
chr3:125905080
r2
0.8
0.6
0.4
0.2
−log10(p−value)
Recombination rate (cM/Mb)
MIR548I1
FAM86JP
SLC41A3
ALDH1L1−AS2
KLF15
ZXDC
TXNRD3NB
LOC101927056
ALDH1L1
CCDC37−AS1
CHST13
ALG1L
ALDH1L1−AS1
CFAP100
C3orf22
ROPN1B
UROC1
TXNRD3
NUP210P1
125.6
125.8
126
126.2
126.4
Position on chr3 (Mb)

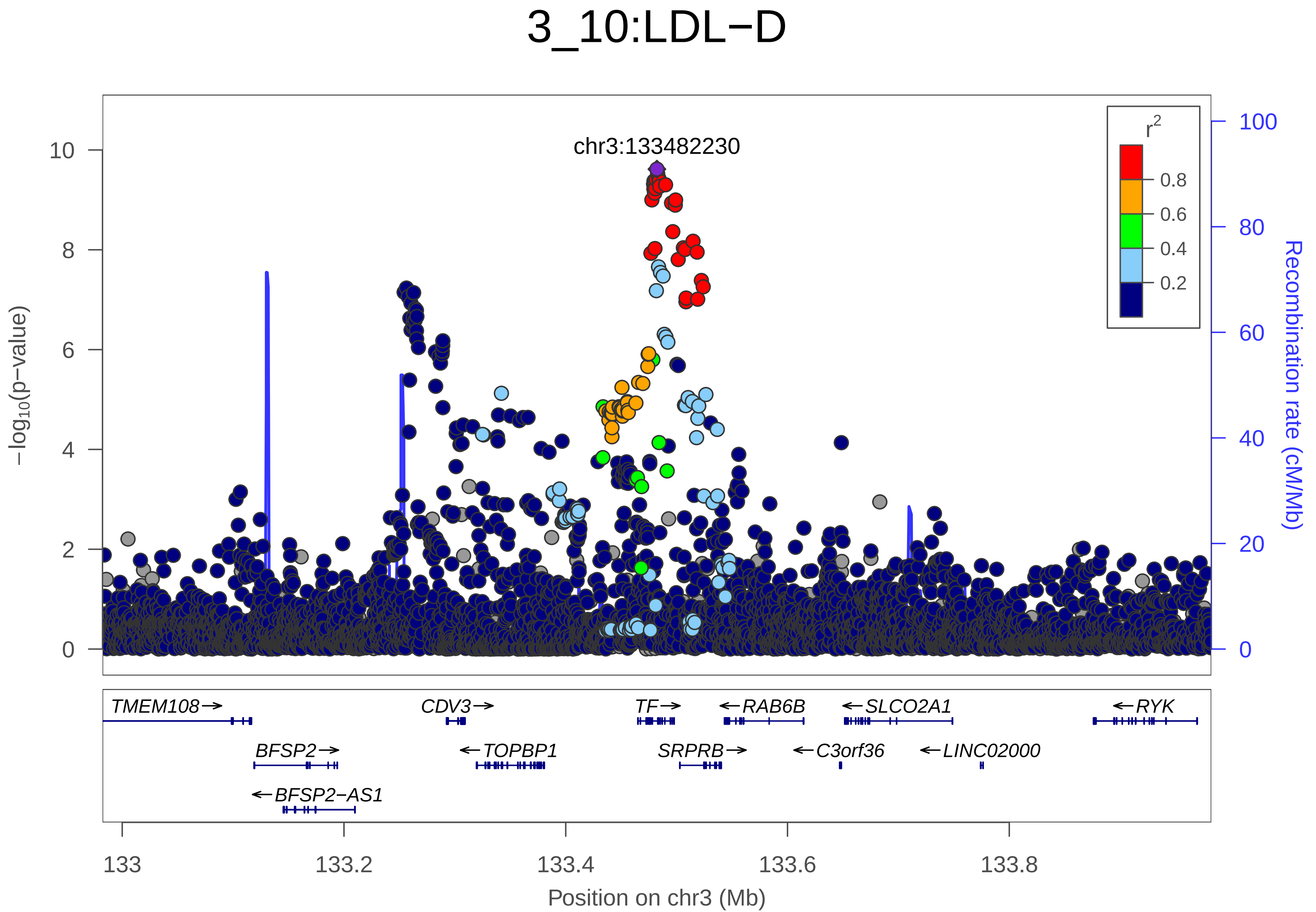

3_10:LDL−D
chr3:133482230
−log10(p−value)
Recombination rate (cM/Mb)
r2
0.8
0.6
0.4
0.2
0
2
4
6
8
10
0
20
40
60
80
100
TMEM108→
CDV3→
TF→
←RAB6B
←SLCO2A1
←RYK
BFSP2→
←TOPBP1
SRPRB→
←C3orf36
←LINC02000
←BFSP2−AS1
133
133.2
133.4
133.6
133.8
Position on chr3 (Mb)

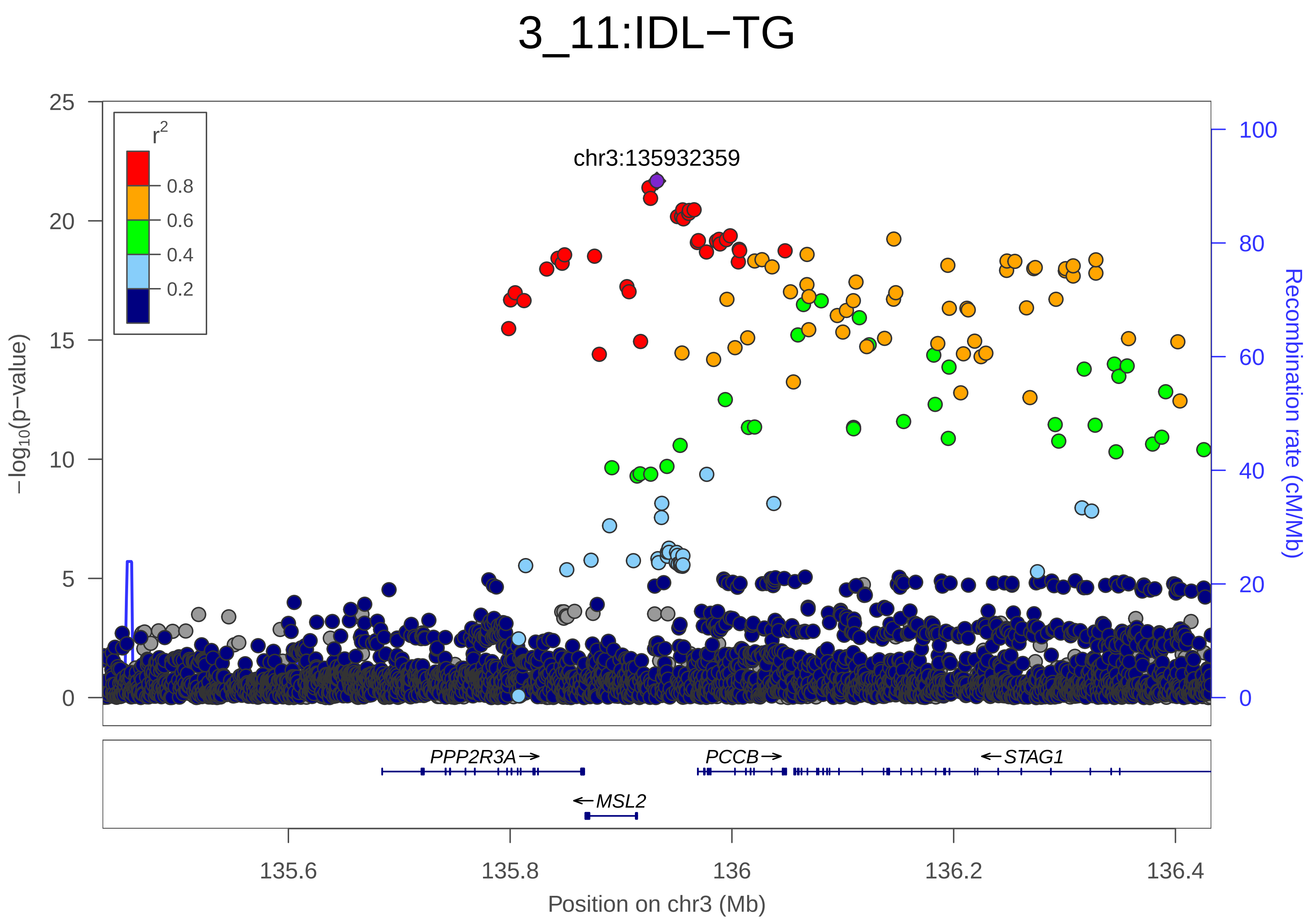

3_11:IDL−TG
r2
0.8
0.6
0.4
0.2
chr3:135932359
25
20
15
10
5
0
−log10(p−value)
100
80
60
40
20
0
Recombination rate (cM/Mb)
PPP2R3A→
PCCB→
←STAG1
←MSL2
135.6
135.8
136
136.2
136.4
Position on chr3 (Mb)

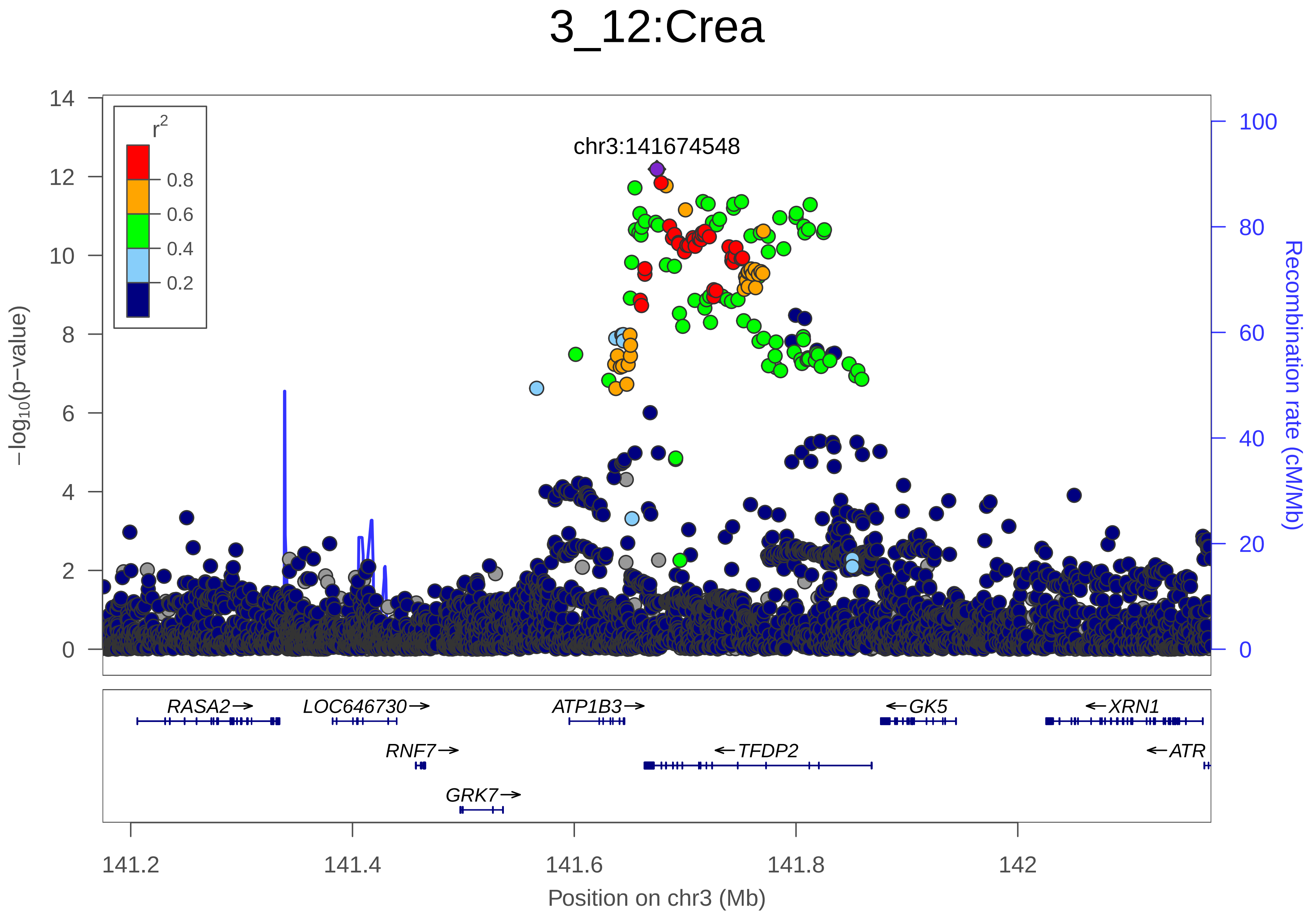
3_12:Crea
chr3:141674548
r2
0.8
0.6
0.4
0.2
−log10(p−value)
Recombination rate (cM/Mb)
0
2
4
6
8
10
12
14
0
20
40
60
80
100
RASA2→
LOC646730→
ATP1B3→
←GK5
←XRN1
RNF7→
←TFDP2
←ATR
GRK7→
141.2
141.4
141.6
141.8
142
Position on chr3 (Mb)

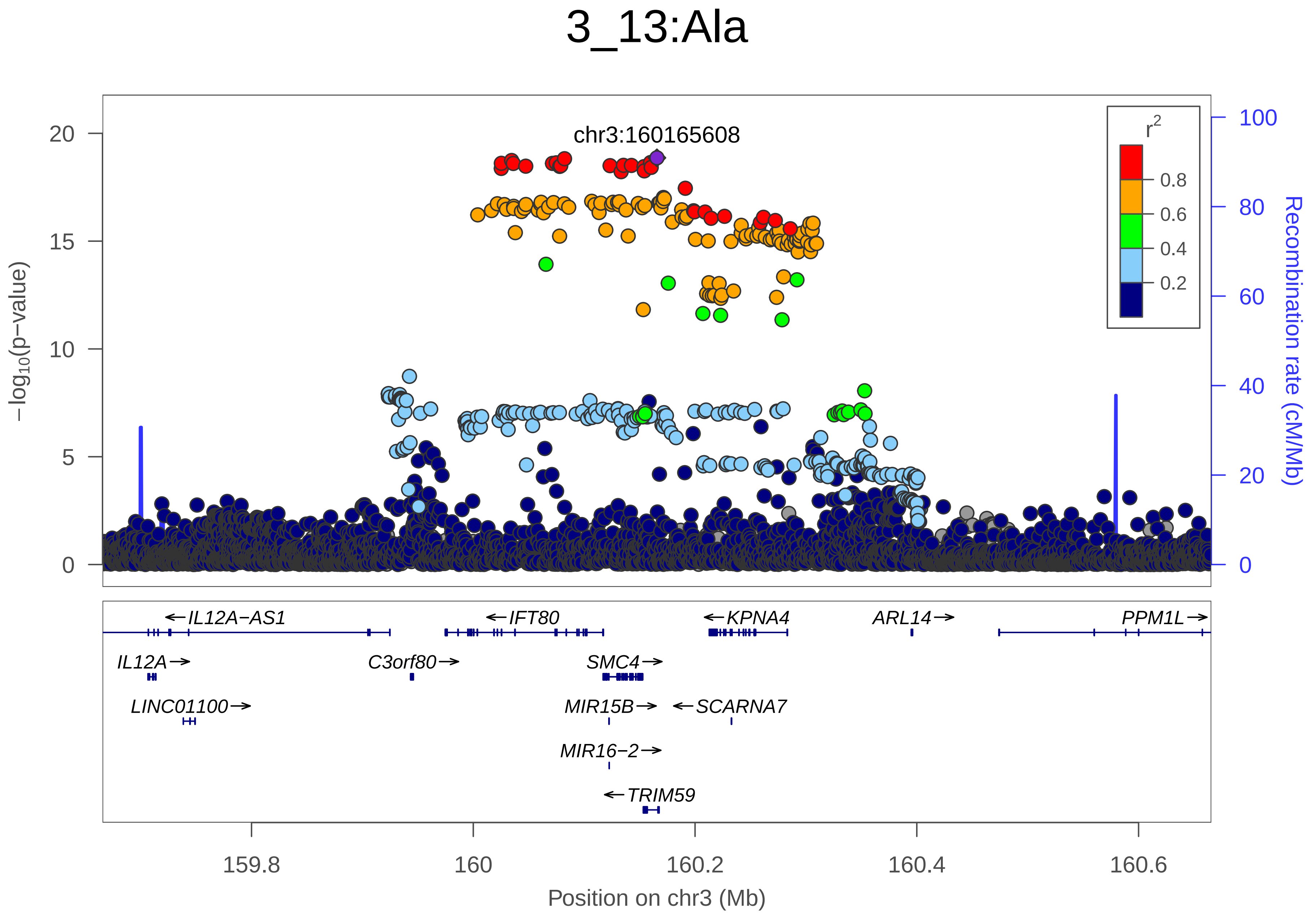
3_13:Ala
chr3:160165608
r2
0.8
0.6
0.4
0.2
−log10(p−value)
Recombination rate (cM/Mb)
0
5
10
15
20
0
20
40
60
80
100
←IL12A−AS1
←IFT80
←KPNA4
ARL14→
PPM1L→
IL12A→
C3orf80→
SMC4→
LINC01100→
MIR15B→
←SCARNA7
MIR16−2→
←TRIM59
159.8
160
160.2
160.4
160.6
Position on chr3 (Mb)

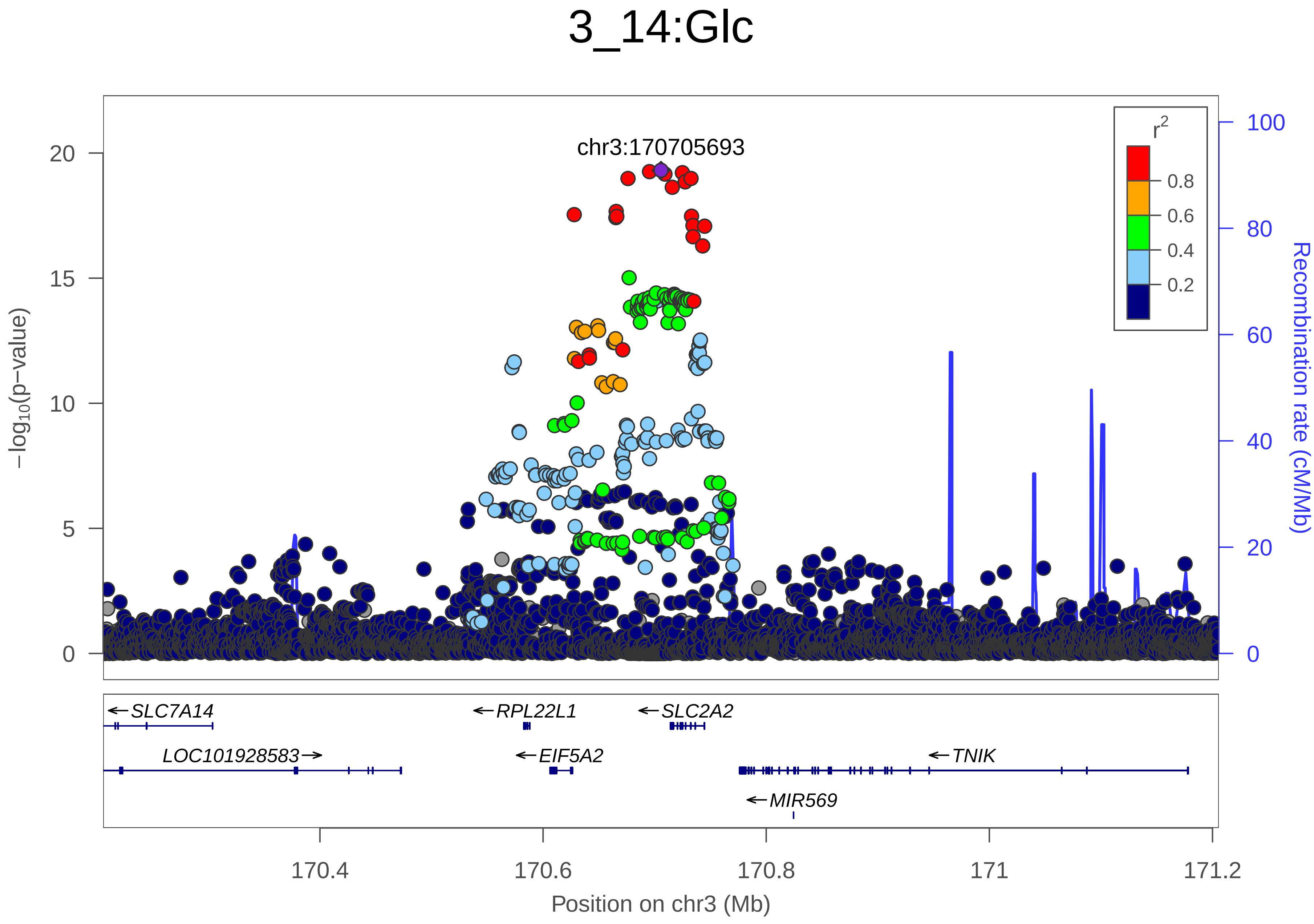
3_14:Glc
chr3:170705693
r^2
0.8
0.6
0.4
0.2
$-\log_{10}$(p−value)
0
5
10
15
20
Recombination rate (cM/Mb)
0
20
40
60
80
100
SLC7A14
RPL22L1
SLC2A2
LOC101928583
EIF5A2
TNIK
MIR569
170.4
170.6
170.8
171
171.2
Position on chr3 (Mb)

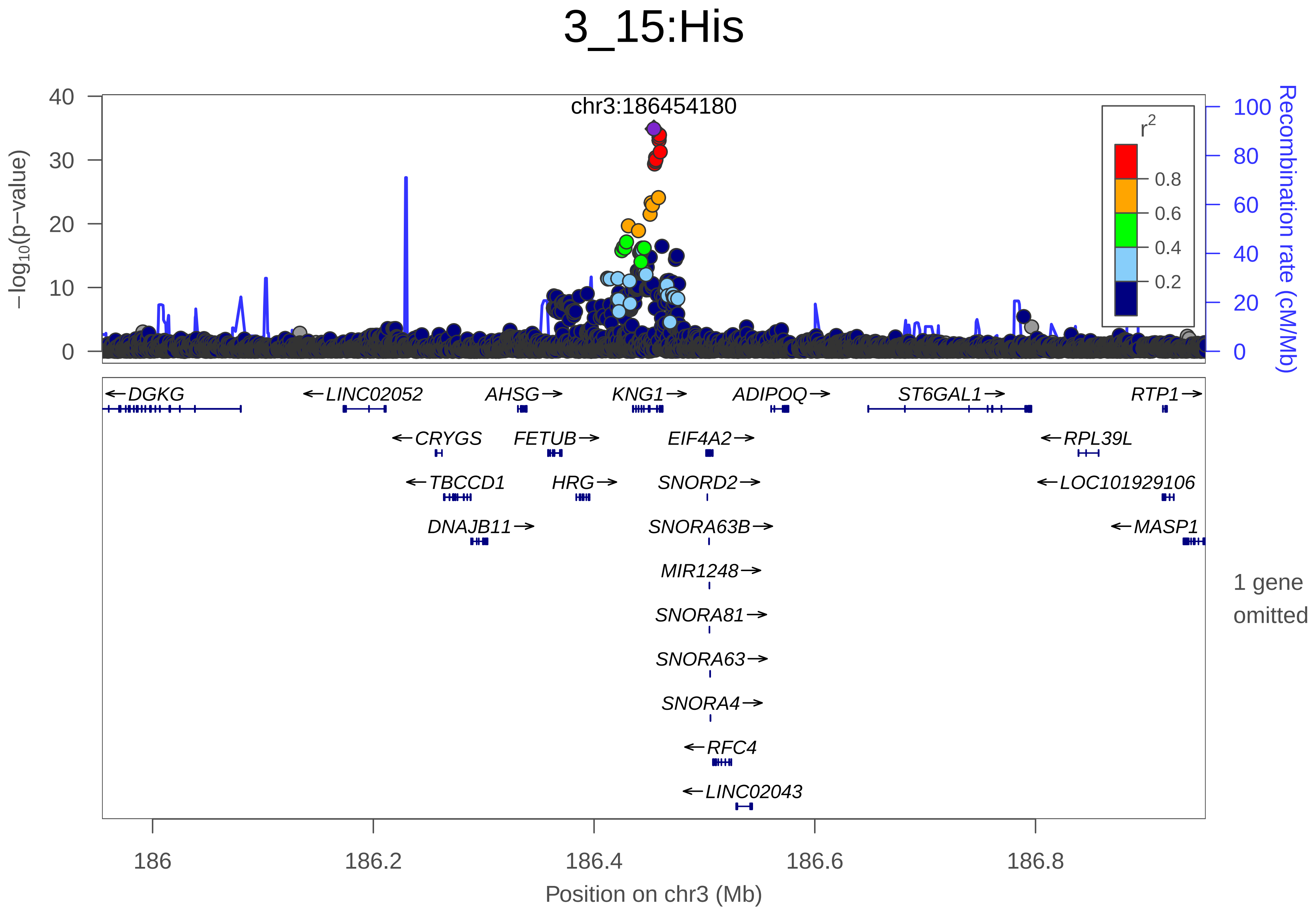
3_15:His
chr3:186454180
−log10(p−value)
Recombination rate (cM/Mb)
r2
0.8
0.6
0.4
0.2
DGKG
LINC02052
AHSG
KNG1
ADIPOQ
ST6GAL1
RTP1
CRYGS
FETUB
EIF4A2
RPL39L
TBCCD1
HRG
SNORD2
LOC101929106
DNAJB11
SNORA63B
MASP1
MIR1248
SNORA81
SNORA63
SNORA4
RFC4
LINC02043
1 gene omitted
186
186.2
186.4
186.6
186.8
Position on chr3 (Mb)

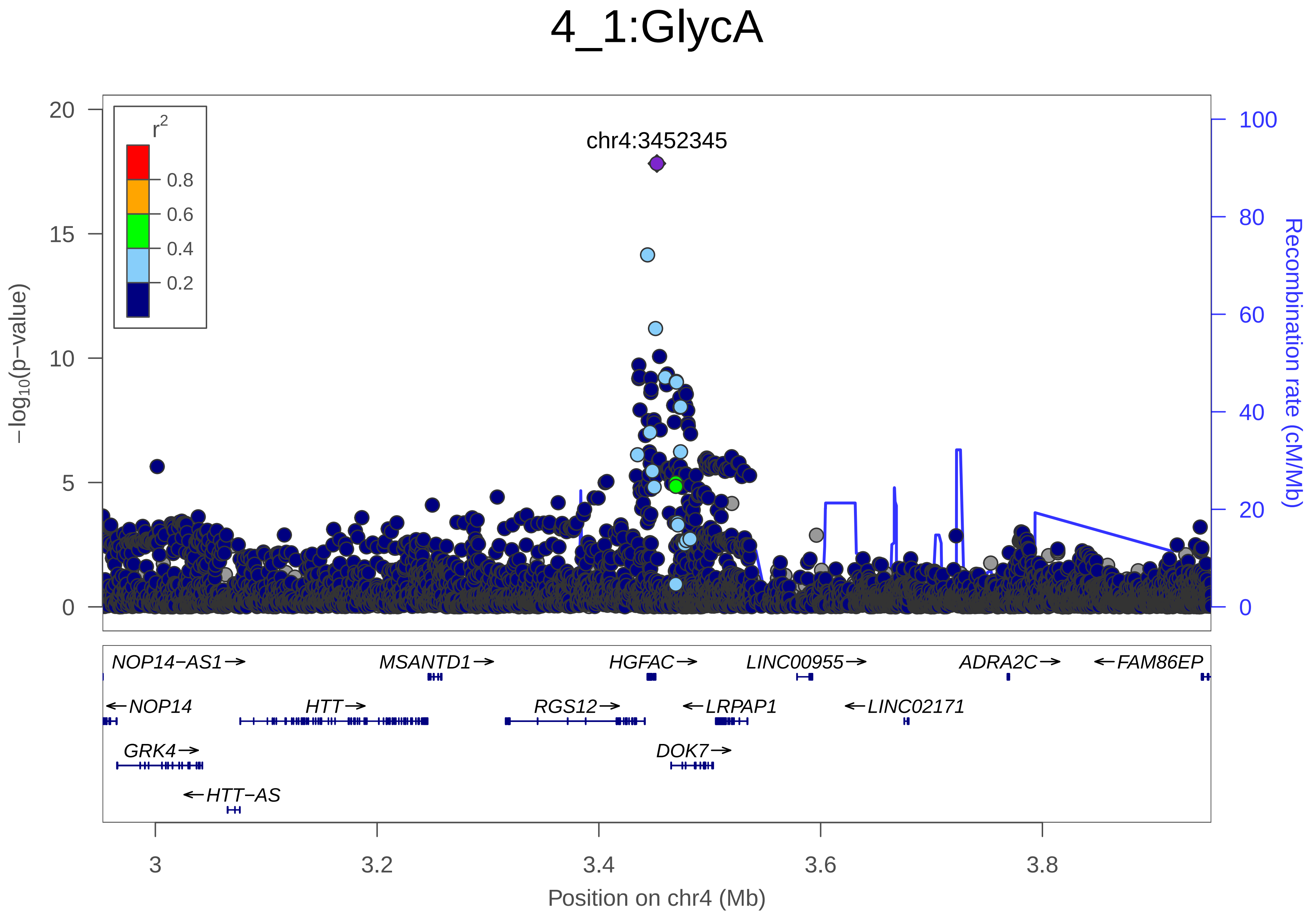

4_1:GlycA
chr4:3452345
r2
0.8
0.6
0.4
0.2
−log10(p−value)
0
5
10
15
20
Recombination rate (cM/Mb)
0
20
40
60
80
100
NOP14−AS1→
MSANTD1→
HGFAC→
LINC00955→
ADRA2C→
←FAM86EP
←NOP14
HTT→
RGS12→
←LRPAP1
←LINC02171
GRK4→
DOK7→
←HTT−AS
3
3.2
3.4
3.6
3.8
Position on chr4 (Mb)

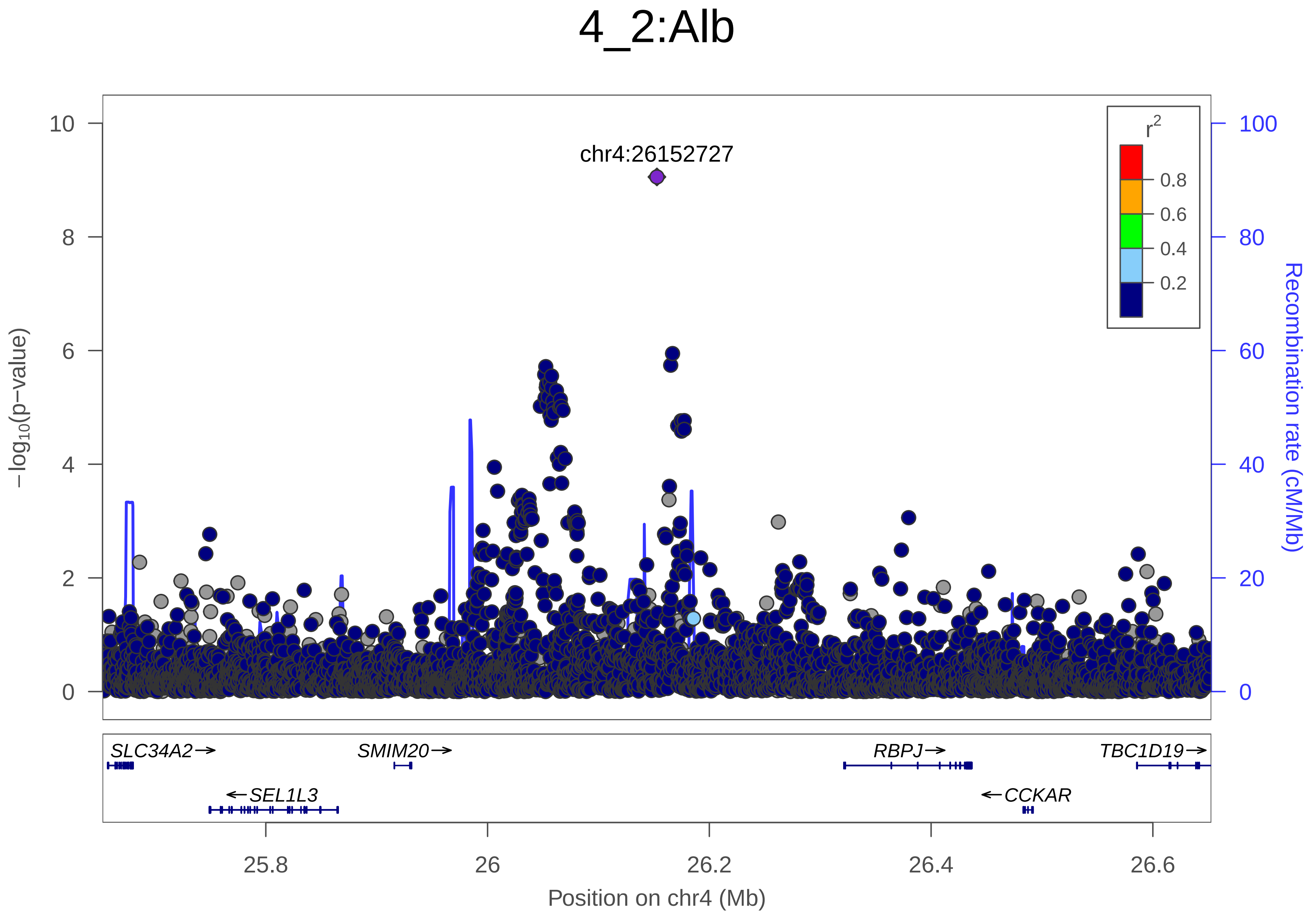

4_2:Alb
chr4:26152727
r2
0.8
0.6
0.4
0.2
−log10(p−value)
Recombination rate (cM/Mb)
10
8
6
4
2
0
100
80
60
40
20
0
SLC34A2→
SMIM20→
RBPJ→
TBC1D19→
←SEL1L3
←CCKAR
25.8
26
26.2
26.4
26.6
Position on chr4 (Mb)

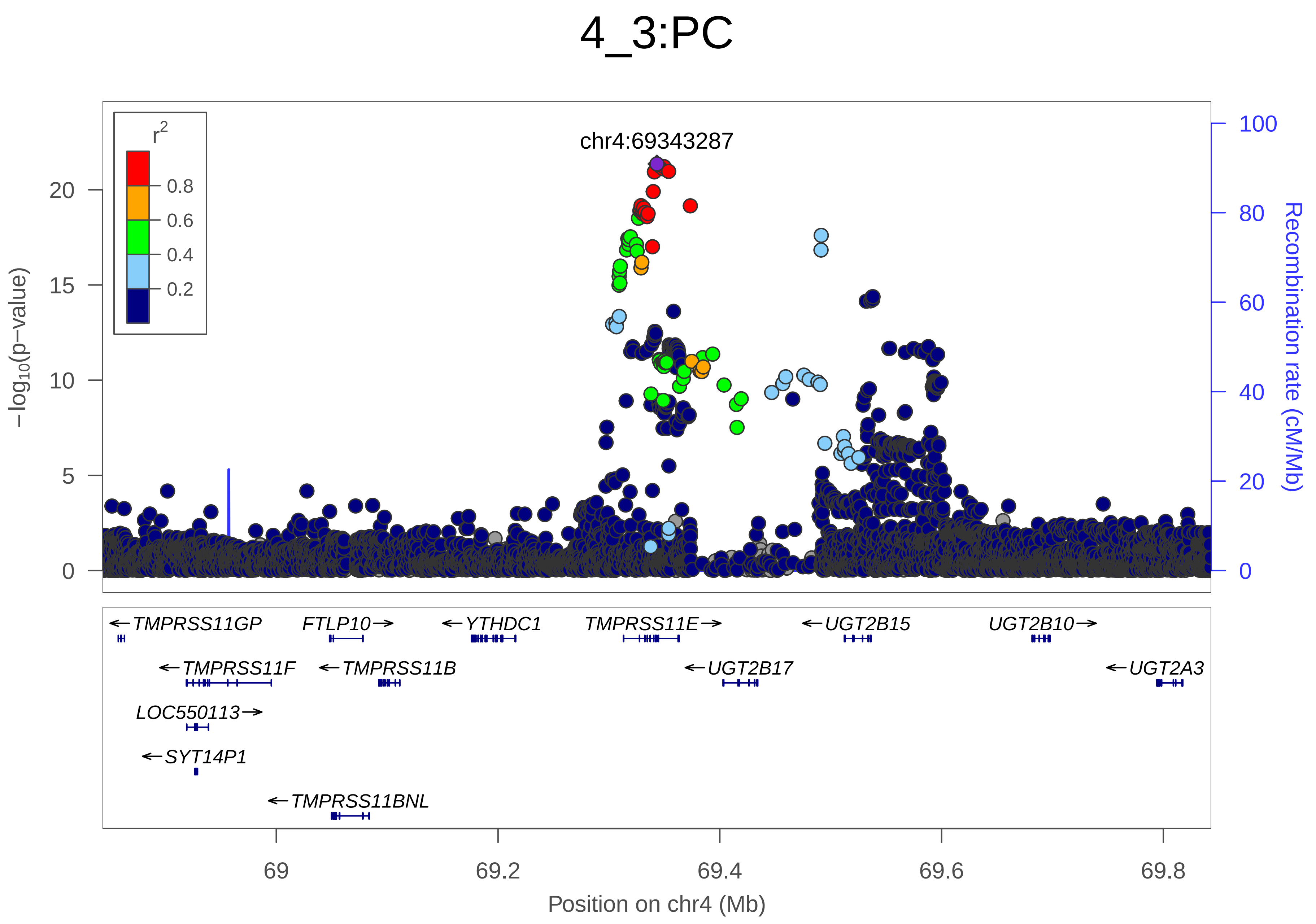

4_3:PC
chr4:69343287
r2
0.8
0.6
0.4
0.2
−log10(p−value)
Recombination rate (cM/Mb)
0
5
10
15
20
0
20
40
60
80
100
←TMPRSS11GP
FTLP10→
←YTHDC1
TMPRSS11E→
←UGT2B15
UGT2B10→
←TMPRSS11F
←TMPRSS11B
←UGT2B17
←UGT2A3
LOC550113→
←SYT14P1
←TMPRSS11BNL
69
69.2
69.4
69.6
69.8
Position on chr4 (Mb)

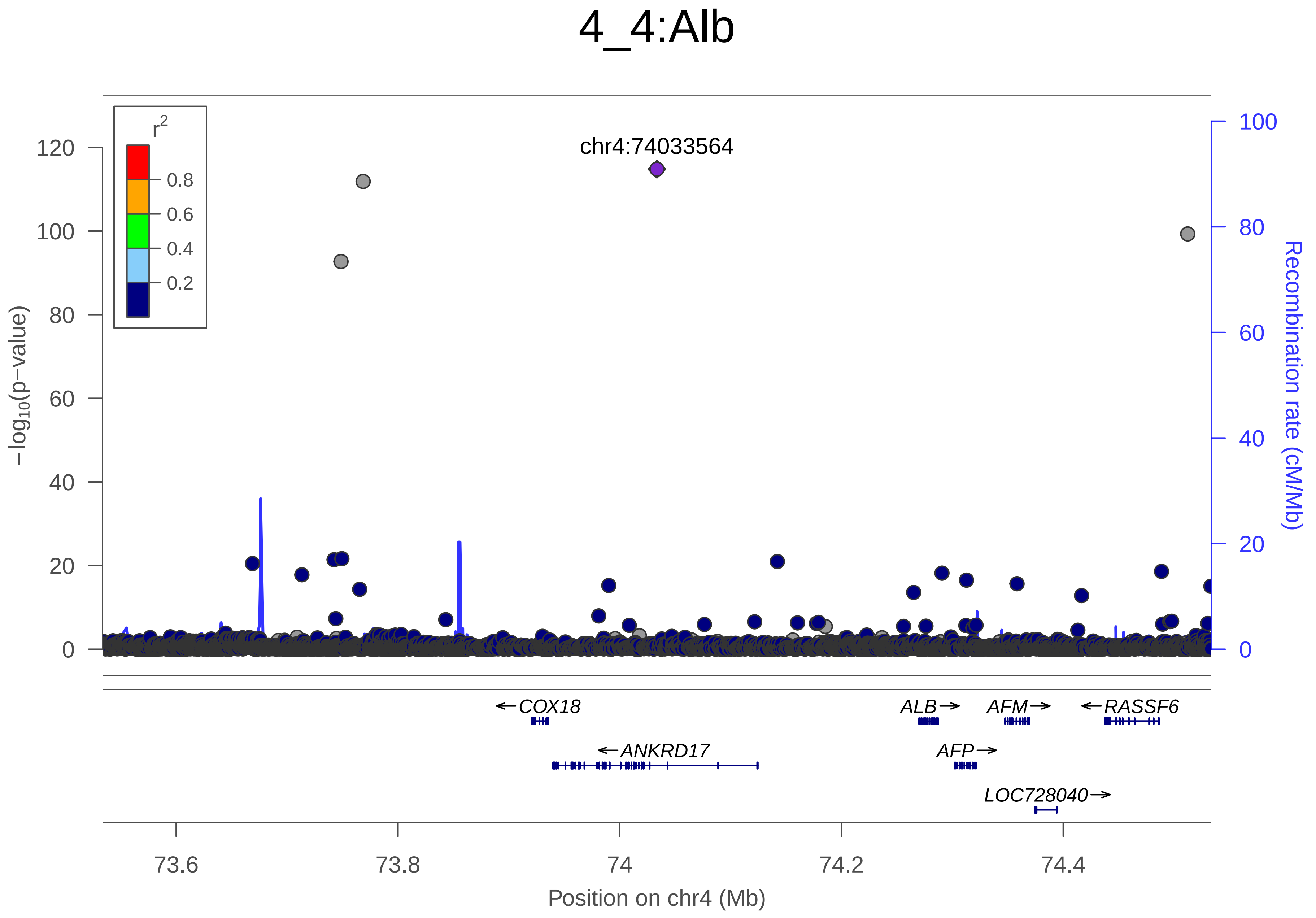

4_4:Alb
chr4:74033564
r2
0.8
0.6
0.4
0.2
0
20
40
60
80
100
120
−log10(p−value)
Recombination rate (cM/Mb)
COX18
ANKRD17
ALB
AFP
AFM
RASSF6
LOC728040
73.6
73.8
74
74.2
74.4
Position on chr4 (Mb)

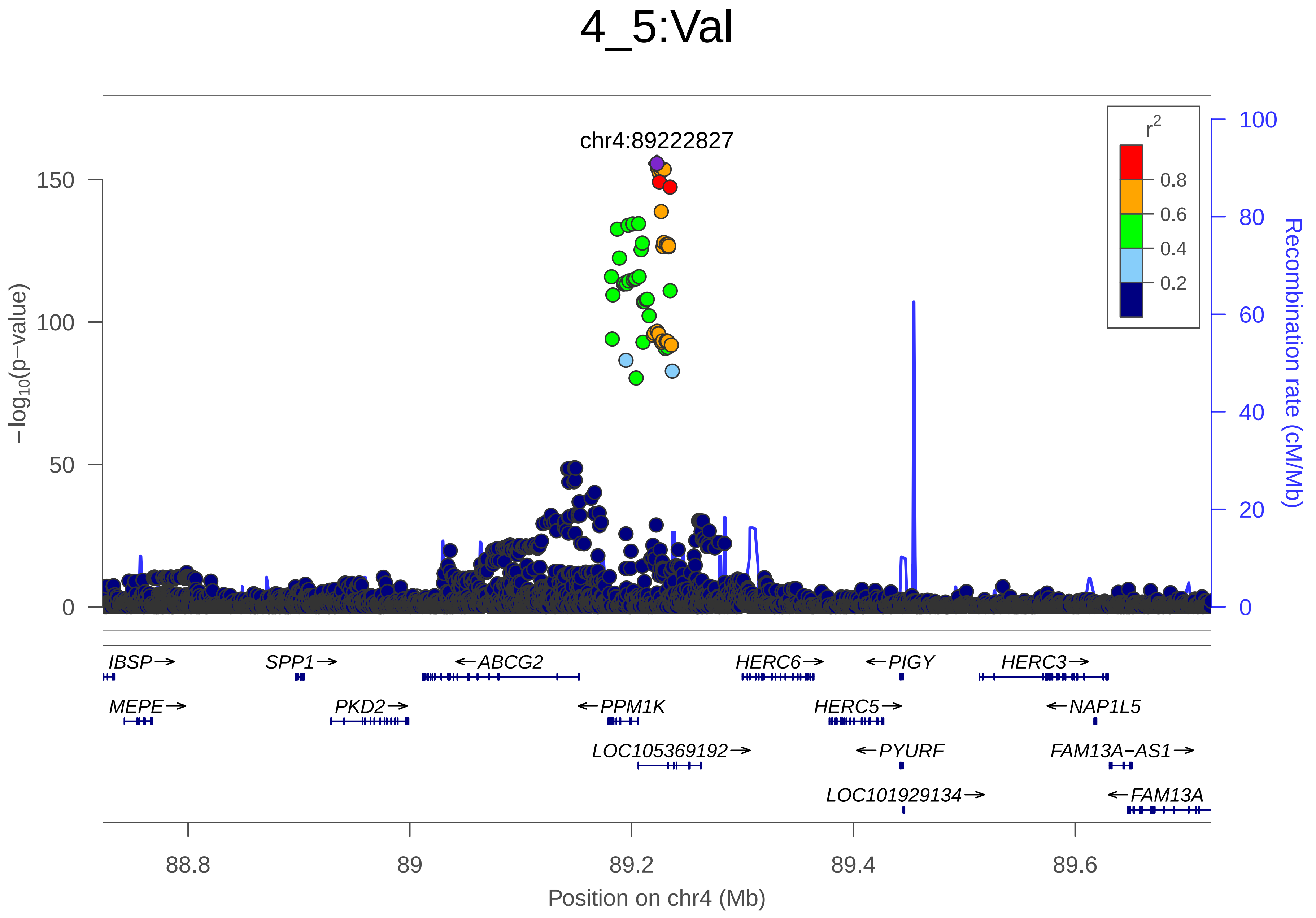

4_5:Val
chr4:89222827
−log10(p−value)
150
100
50
0
r2
0.8
0.6
0.4
0.2
100
80
60
40
20
0
Recombination rate (cM/Mb)
IBSP→
SPP1→
←ABCG2
HERC6→
←PIGY
HERC3→
MEPE→
PKD2→
←PPM1K
HERC5→
←NAP1L5
LOC105369192→
←PYURF
FAM13A−AS1→
LOC101929134→
←FAM13A
88.8
89
89.2
89.4
89.6
Position on chr4 (Mb)

4_6:Tyr

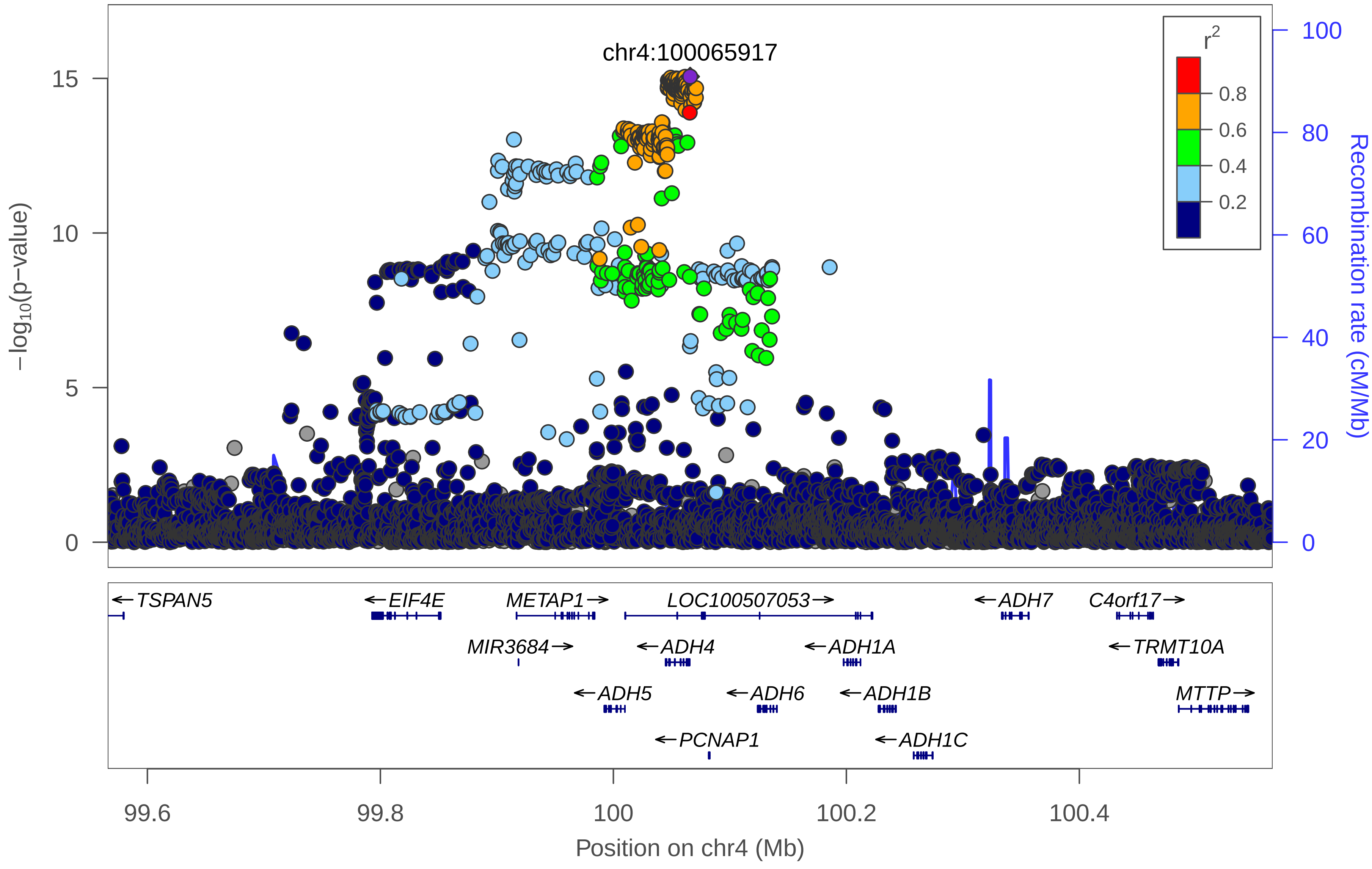
chr4:100065917
r2
0.8
0.6
0.4
0.2
−log10(p−value)
Recombination rate (cM/Mb)
0
5
10
15
0
20
40
60
80
100
TSPAN5
EIF4E
METAP1
LOC100507053
ADH7
C4orf17
MIR3684
ADH4
ADH1A
TRMT10A
ADH5
ADH6
ADH1B
MTTP
PCNAP1
ADH1C
99.6
99.8
100
100.2
100.4
Position on chr4 (Mb)

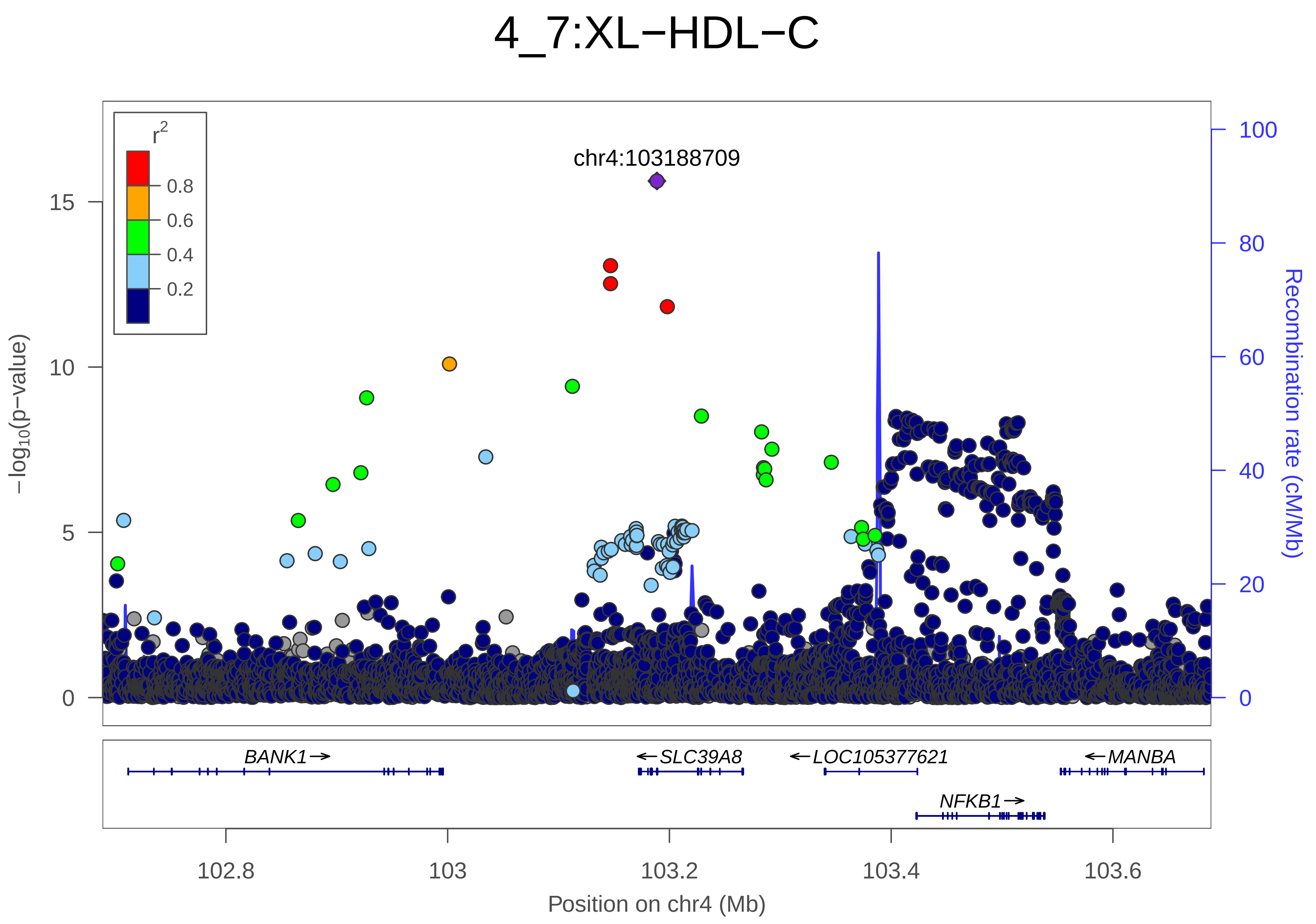

4_7:XL−HDL−C
r2
0.8
0.6
0.4
0.2
chr4:103188709
−log10(p−value)
15
10
5
0
100
80
60
40
20
0
Recombination rate (cM/Mb)
BANK1→
←SLC39A8
←LOC105377621
←MANBA
NFKB1→
102.8
103
103.2
103.4
103.6
Position on chr4 (Mb)

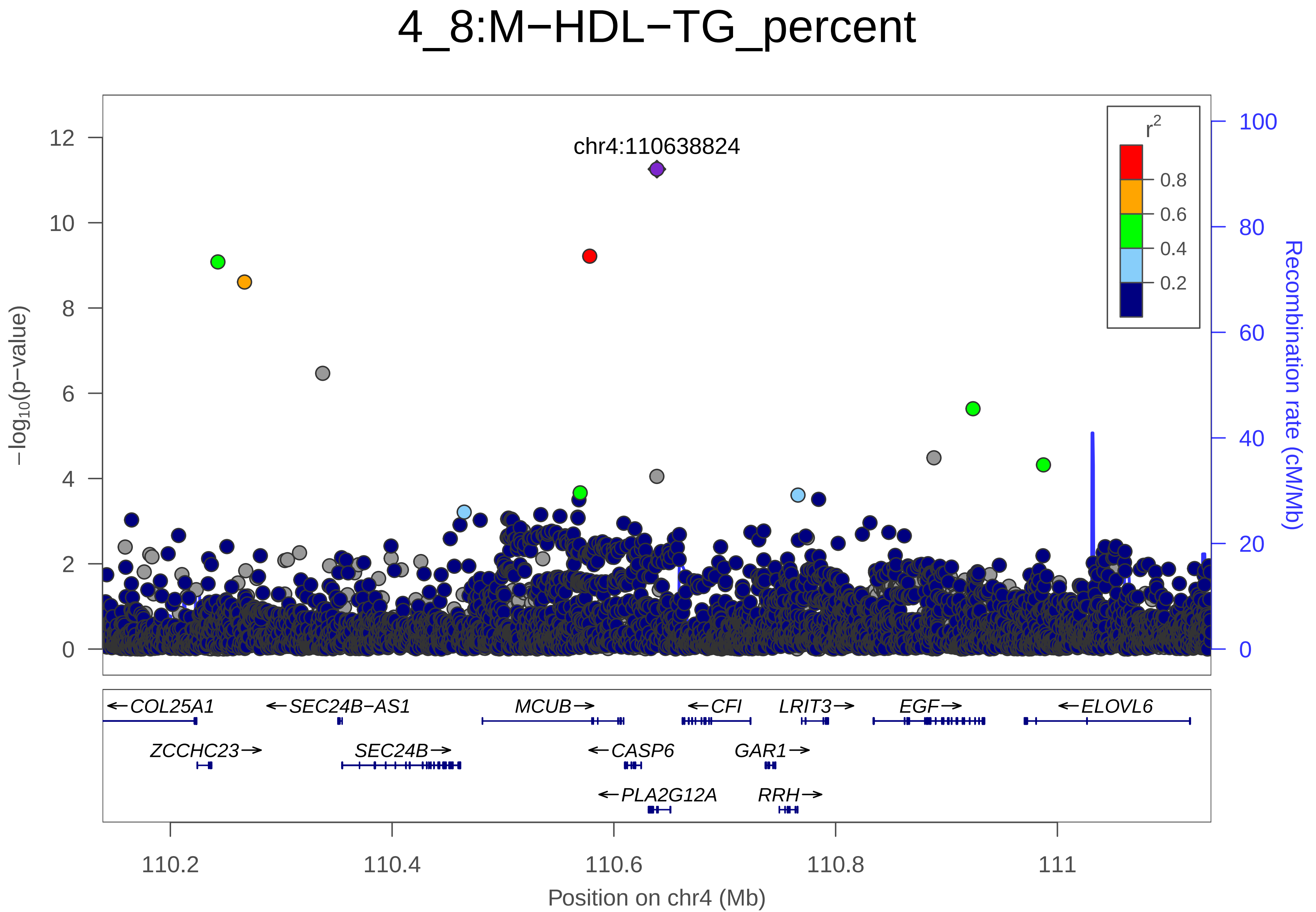

4_8:M−HDL−TG_percent
chr4:110638824
−log10(p−value)
Recombination rate (cM/Mb)
r2
0.8
0.6
0.4
0.2
0
2
4
6
8
10
12
0
20
40
60
80
100
←COL25A1
←SEC24B−AS1
MCUB→
←CFI
LRIT3→
EGF→
←ELOVL6
ZCCHC23→
SEC24B→
←CASP6
GAR1→
←PLA2G12A
RRH→
110.2
110.4
110.6
110.8
111
Position on chr4 (Mb)

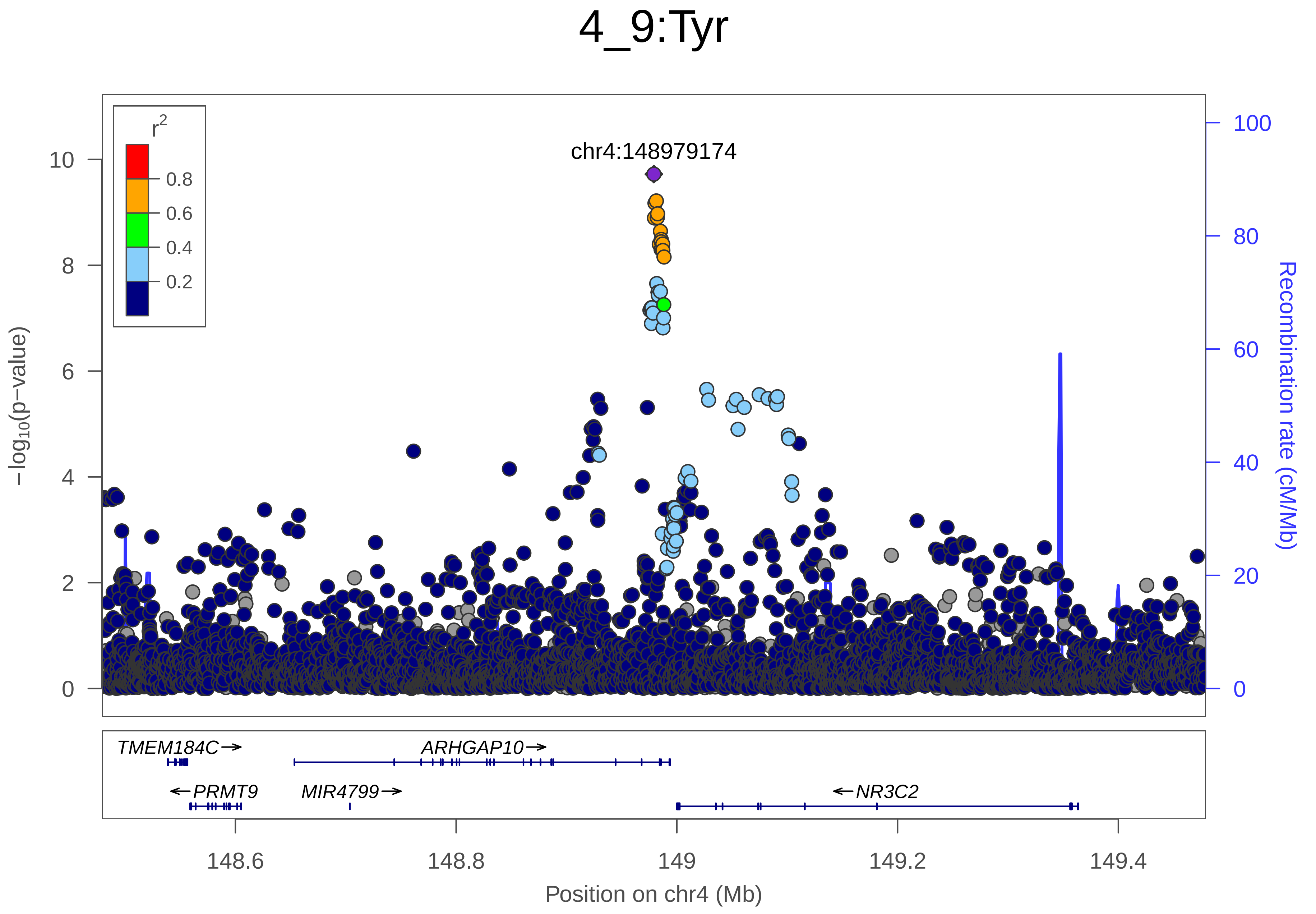

4_9:Tyr
r^2
0.8
0.6
0.4
0.2
chr4:148979174
−log10(p−value)
Recombination rate (cM/Mb)
0
2
4
6
8
10
0
20
40
60
80
100
TMEM184C→
ARHGAP10→
←PRMT9
MIR4799→
←NR3C2
148.6
148.8
149
149.2
149.4
Position on chr4 (Mb)

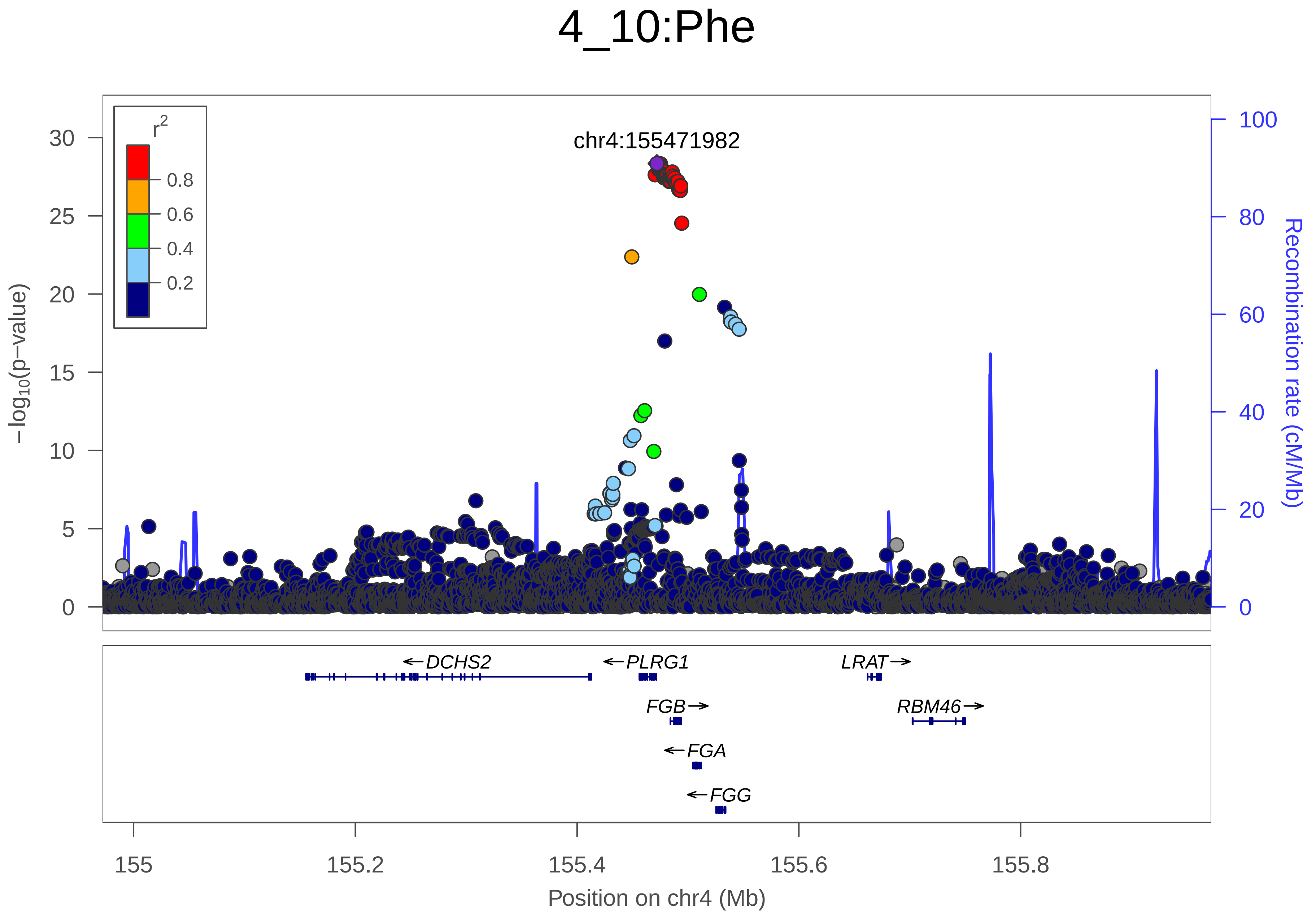
4_10:Phe
chr4:155471982
r2
0.8
0.6
0.4
0.2
−log10(p−value)
Recombination rate (cM/Mb)
DCHS2
PLRG1
LRAT
FGB
RBM46
FGA
FGG
Position on chr4 (Mb)

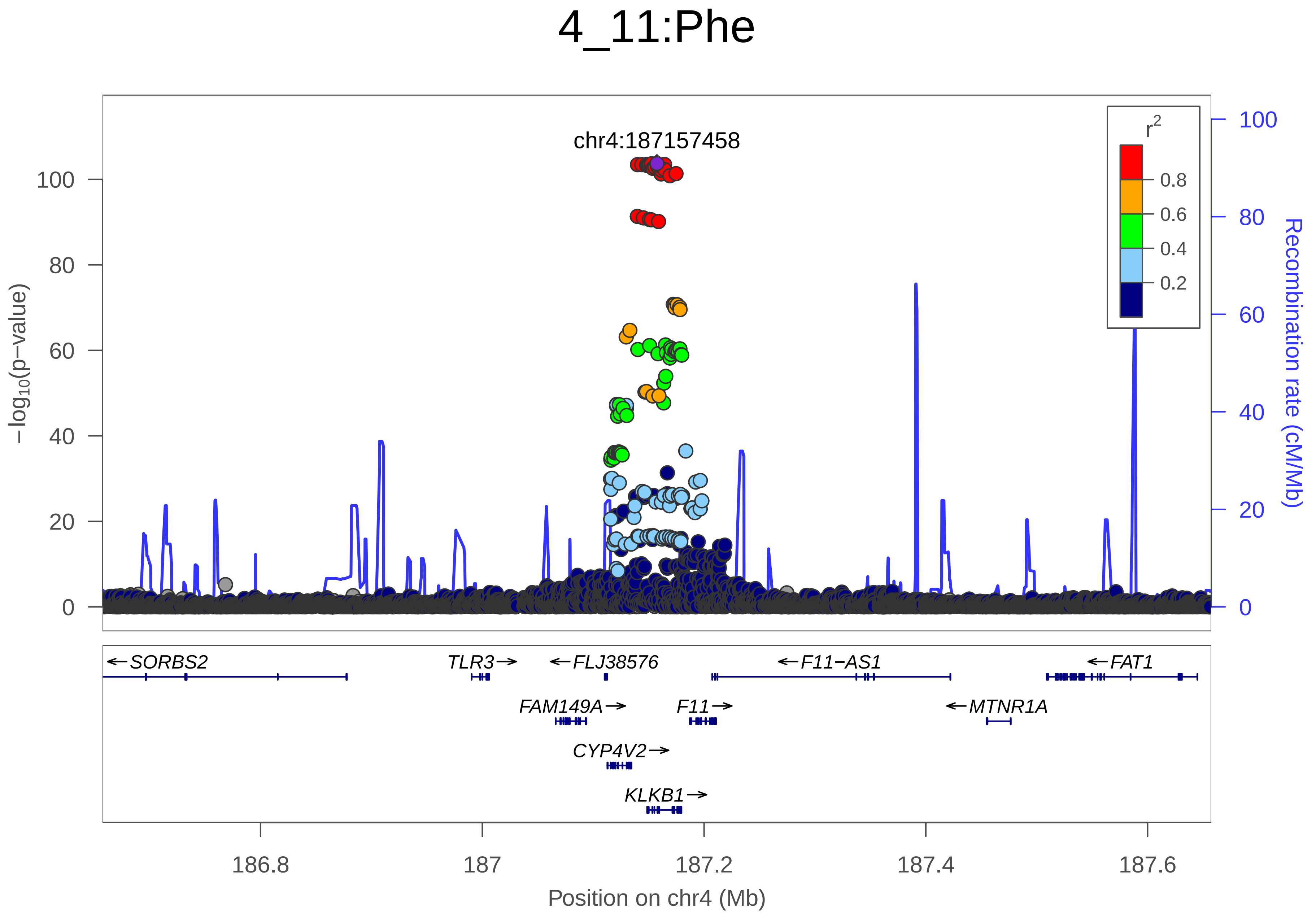

4_11:Phe
chr4:187157458
r2
0.8
0.6
0.4
0.2
−log10(p−value)
Recombination rate (cM/Mb)
100
80
60
40
20
0
SORBS2
TLR3
FLJ38576
F11−AS1
FAT1
FAM149A
F11
MTNR1A
CYP4V2
KLKB1
186.8
187
187.2
187.4
187.6
Position on chr4 (Mb)

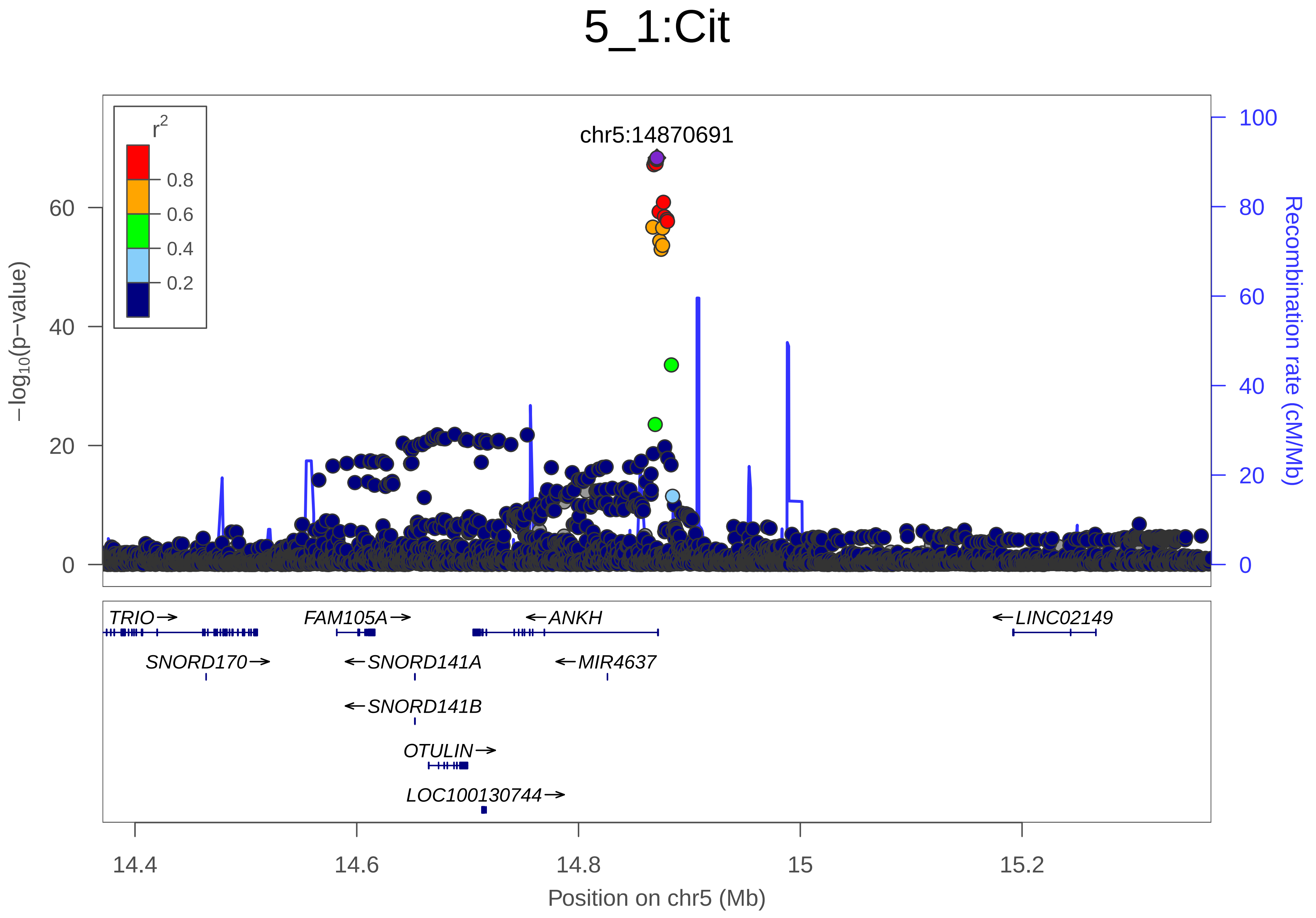
5_1:Cit
chr5:14870691
r2
0.8
0.6
0.4
0.2
−log10(p−value)
Recombination rate (cM/Mb)
0
20
40
60
0
20
40
60
80
100
TRIO→
FAM105A→
←ANKH
←LINC02149
SNORD170→
←SNORD141A
←MIR4637
←SNORD141B
OTULIN→
LOC100130744→
14.4
14.6
14.8
15
15.2
Position on chr5 (Mb)

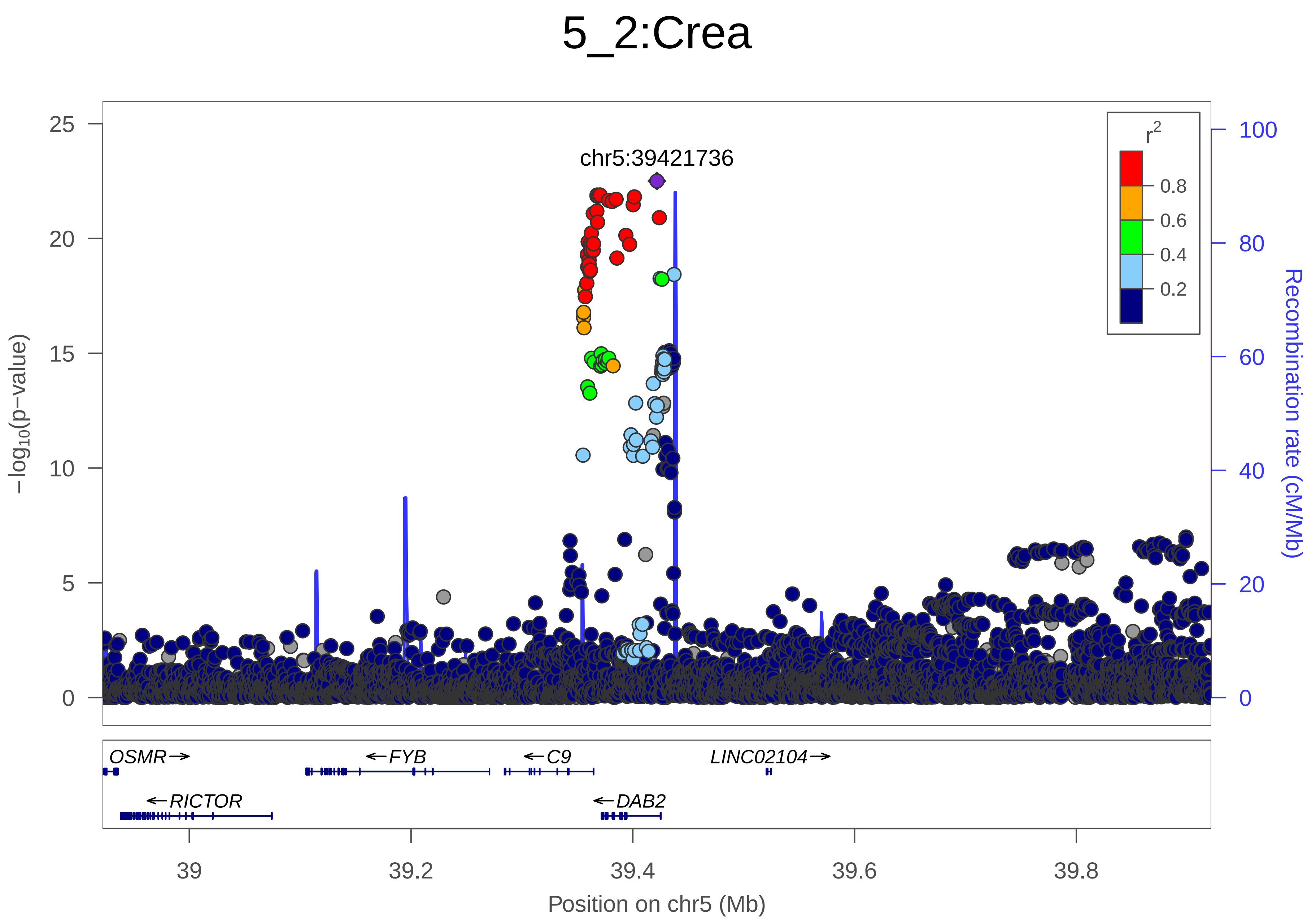
5_2:Crea
chr5:39421736
r2
0.8
0.6
0.4
0.2
25
20
15
10
5
0
100
80
60
40
20
0
−log10(p−value)
Recombination rate (cM/Mb)
OSMR
FYB
C9
LINC02104
RICTOR
DAB2
39
39.2
39.4
39.6
39.8
Position on chr5 (Mb)

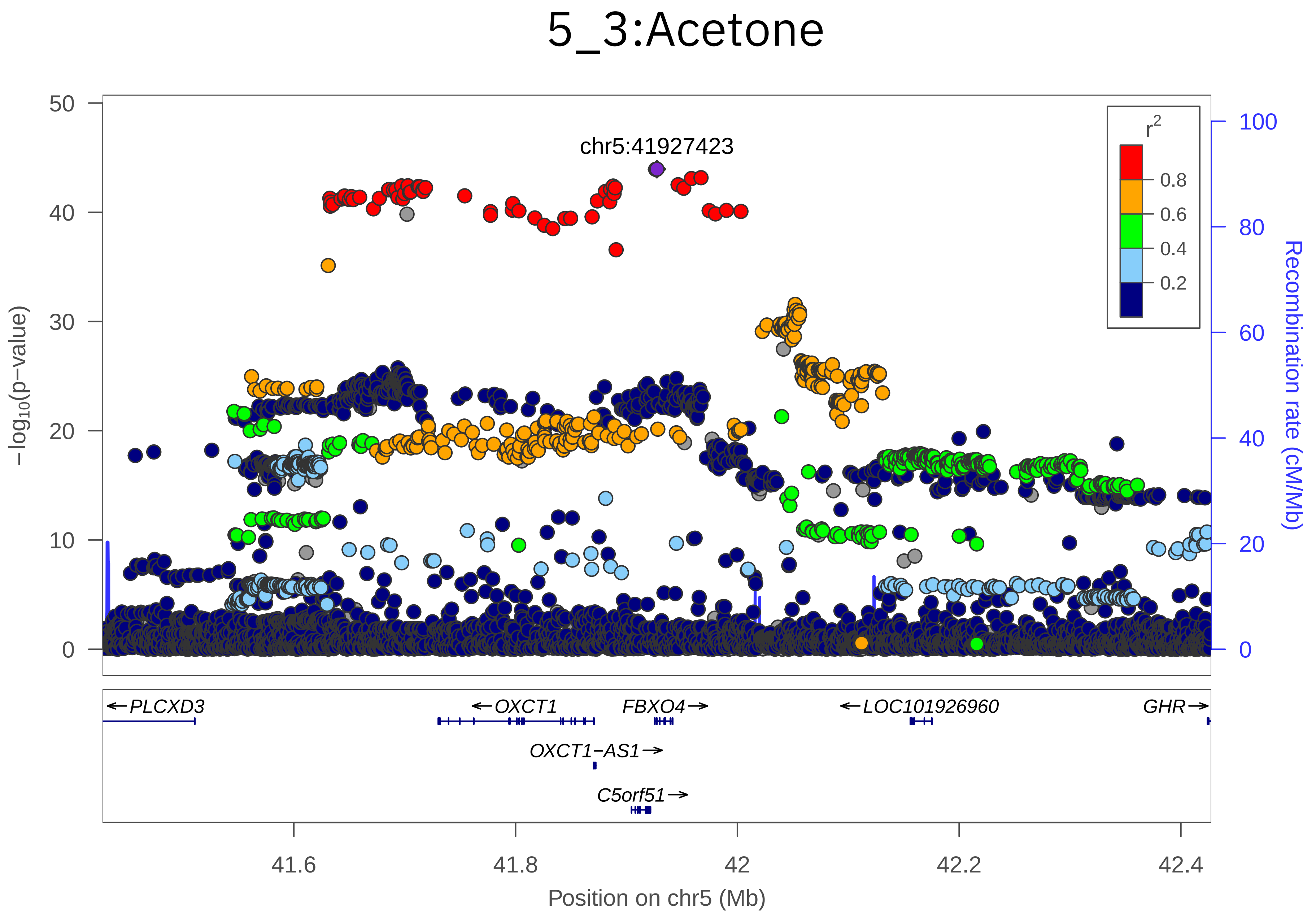

5_3:Acetone
chr5:41927423
r2
0.8
0.6
0.4
0.2
50
40
30
20
10
0
100
80
60
40
20
0
−log10(p−value)
Recombination rate (cM/Mb)
←PLCXD3
←OXCT1
FBXO4→
←LOC101926960
GHR→
OXCT1−AS1→
C5orf51→
41.6
41.8
42
42.2
42.4
Position on chr5 (Mb)

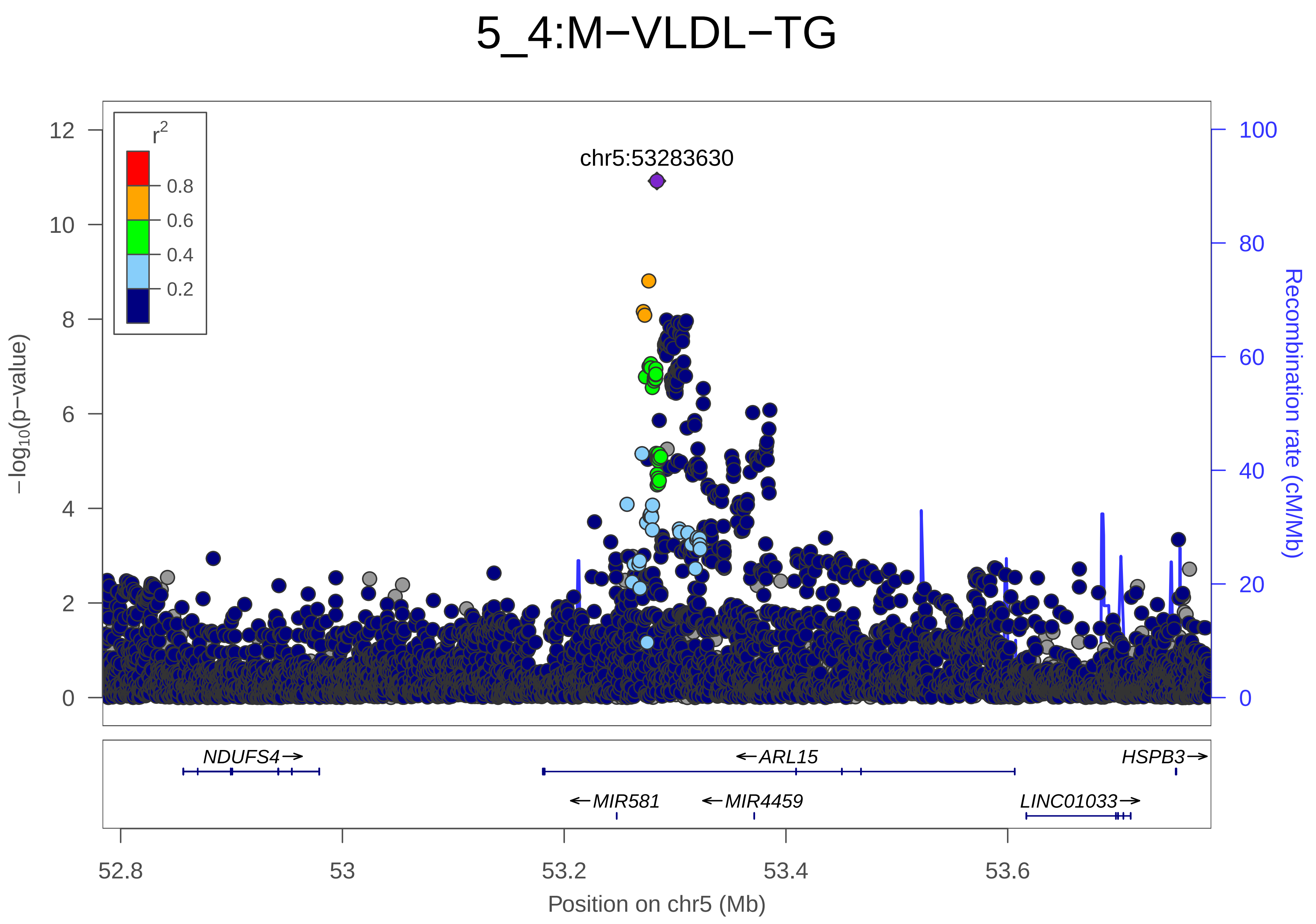
5_4:M−VLDL−TG
chr5:53283630
r2
0.8
0.6
0.4
0.2
−log10(p−value)
Recombination rate (cM/Mb)
NDUFS4→
←ARL15
HSPB3→
←MIR581
←MIR4459
LINC01033→
52.8
53
53.2
53.4
53.6
Position on chr5 (Mb)

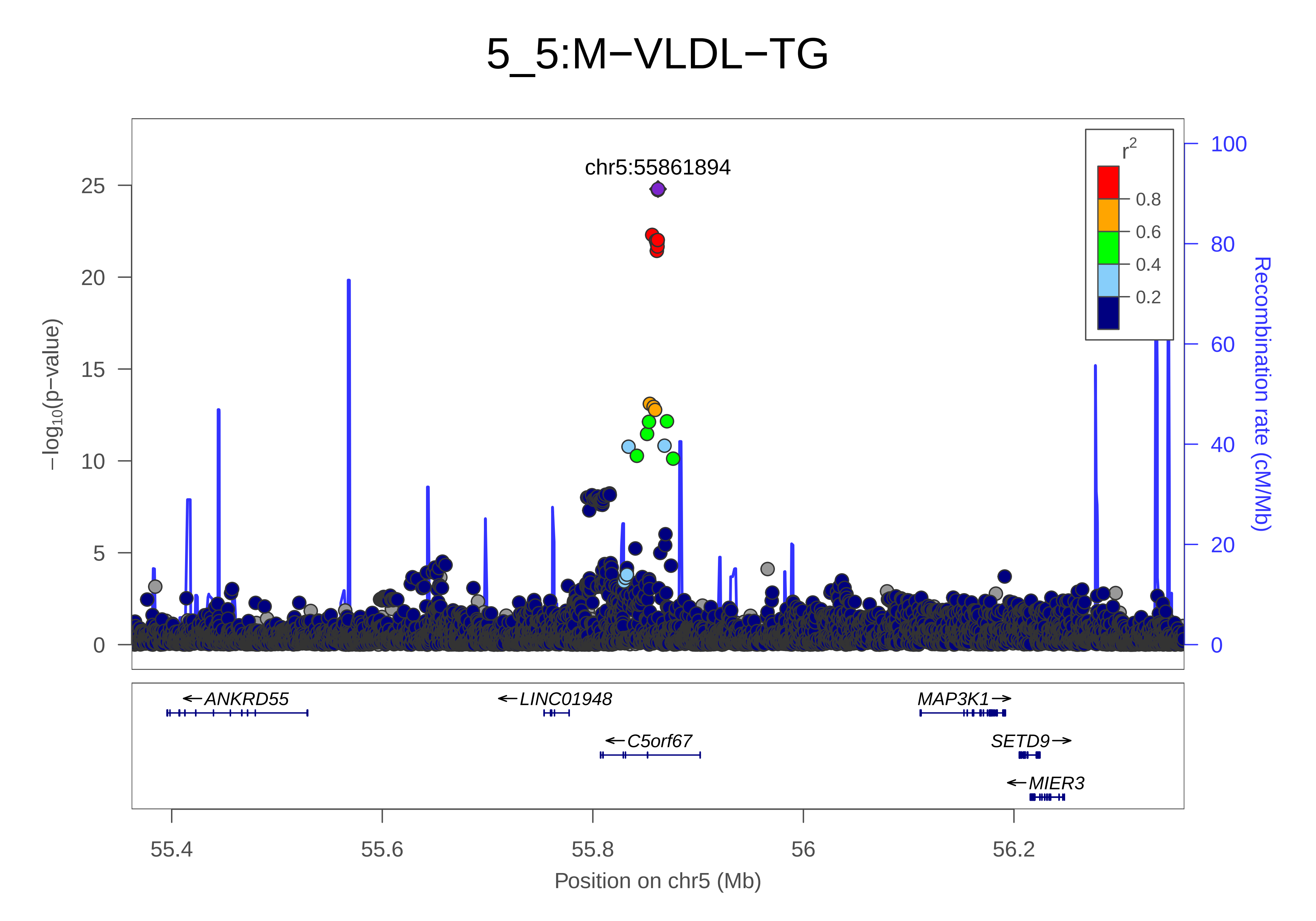
5_5:M−VLDL−TG
chr5:55861894
−log10(p−value)
Recombination rate (cM/Mb)
r2
0.8
0.6
0.4
0.2
ANKRD55
LINC01948
C5orf67
MAP3K1
SETD9
MIER3
55.4
55.6
55.8
56
56.2
Position on chr5 (Mb)

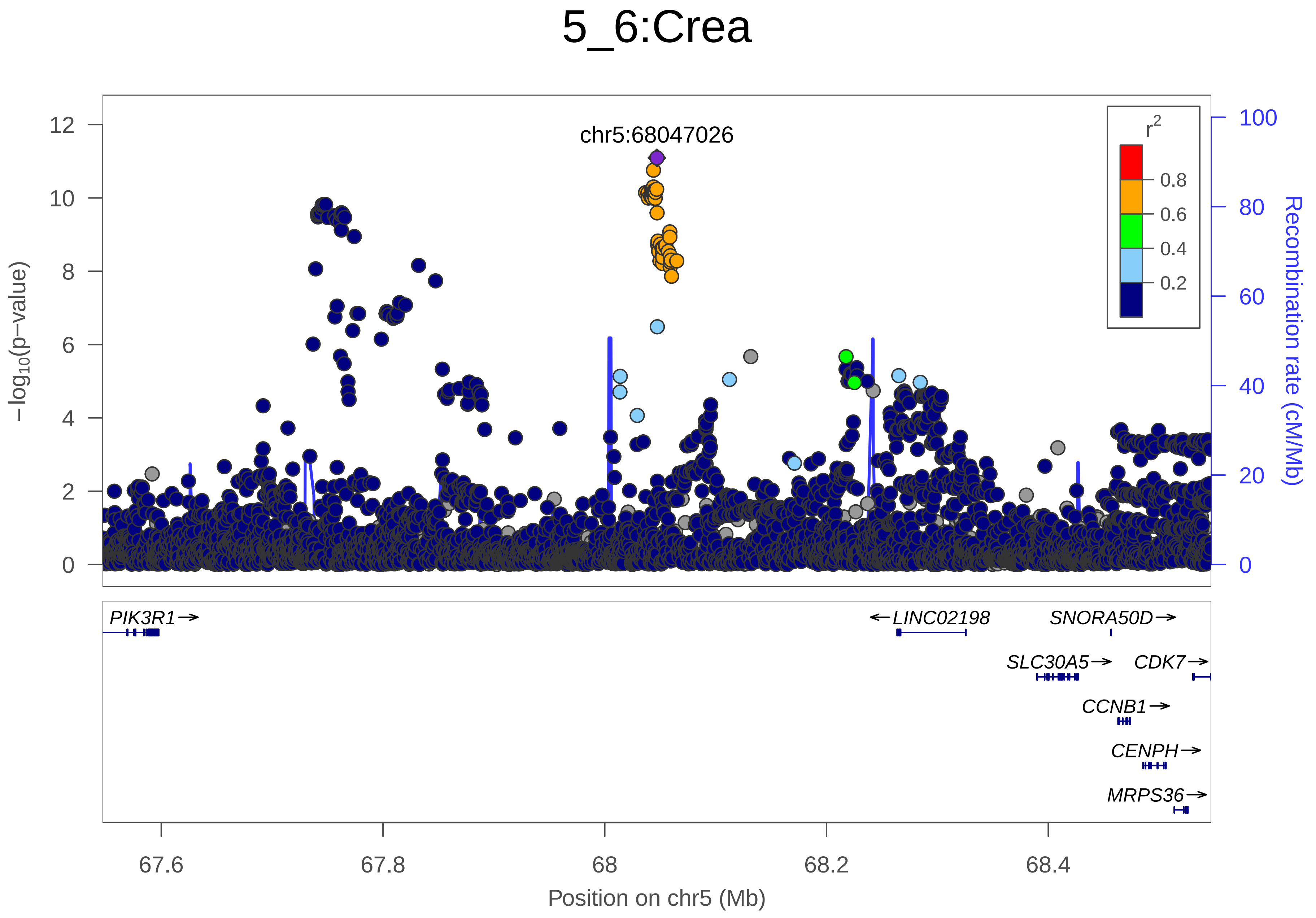

5_6:Crea
chr5:68047026
r2
0.8
0.6
0.4
0.2
−log10(p−value)
Recombination rate (cM/Mb)
PIK3R1
LINC02198
SNORA50D
SLC30A5
CDK7
CCNB1
CENPH
MRPS36
67.6
67.8
68
68.2
68.4
Position on chr5 (Mb)

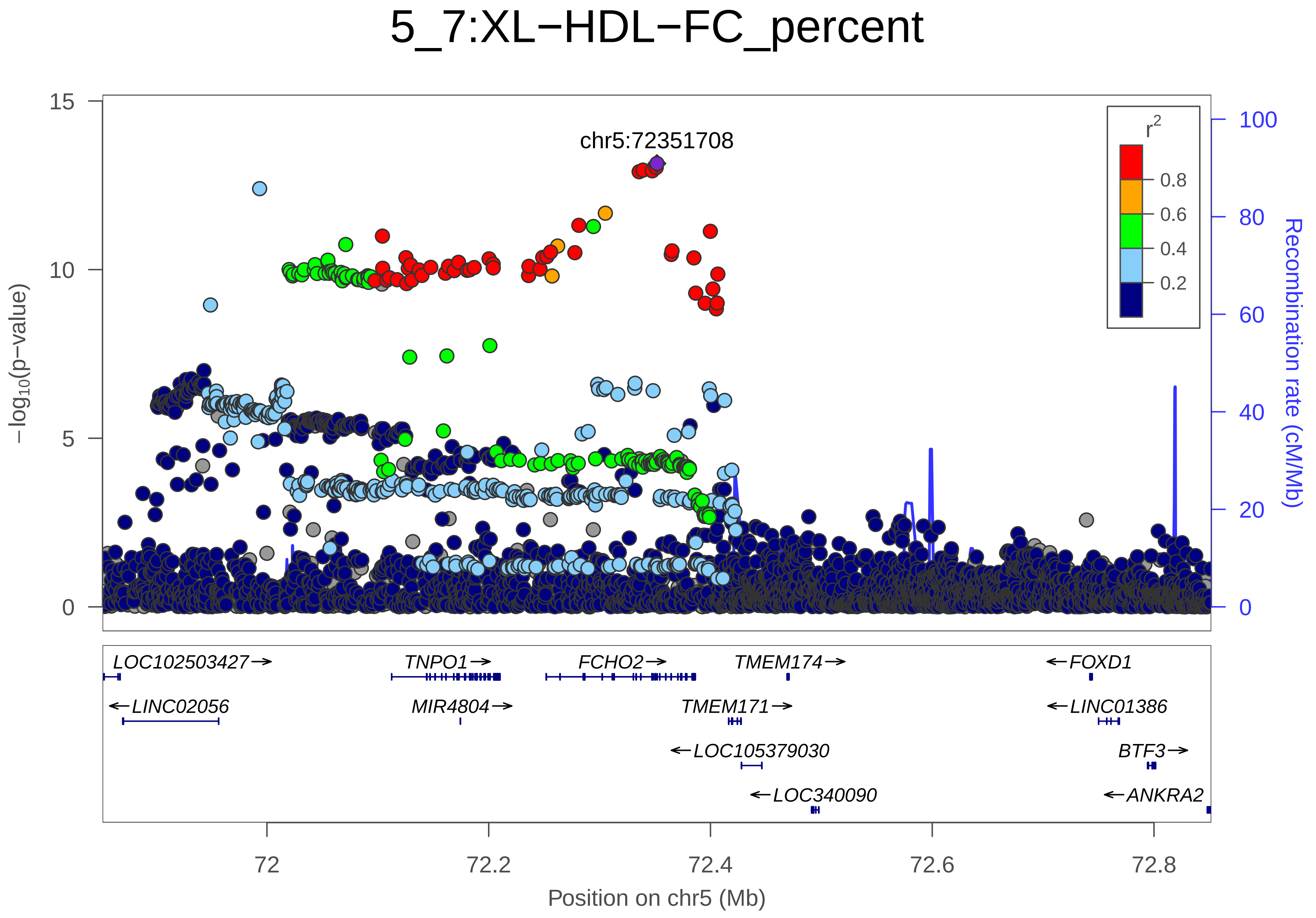
5_7:XL−HDL−FC_percent
chr5:72351708
r²
0.8
0.6
0.4
0.2
−log10(p−value)
15
10
5
0
100
80
60
40
20
0
Recombination rate (cM/Mb)
LOC102503427→
TNPO1→
FCHO2→
TMEM174→
←FOXD1
←LINC02056
MIR4804→
TMEM171→
←LINC01386
←LOC105379030
BTF3→
←LOC340090
←ANKRA2
72
72.2
72.4
72.6
72.8
Position on chr5 (Mb)

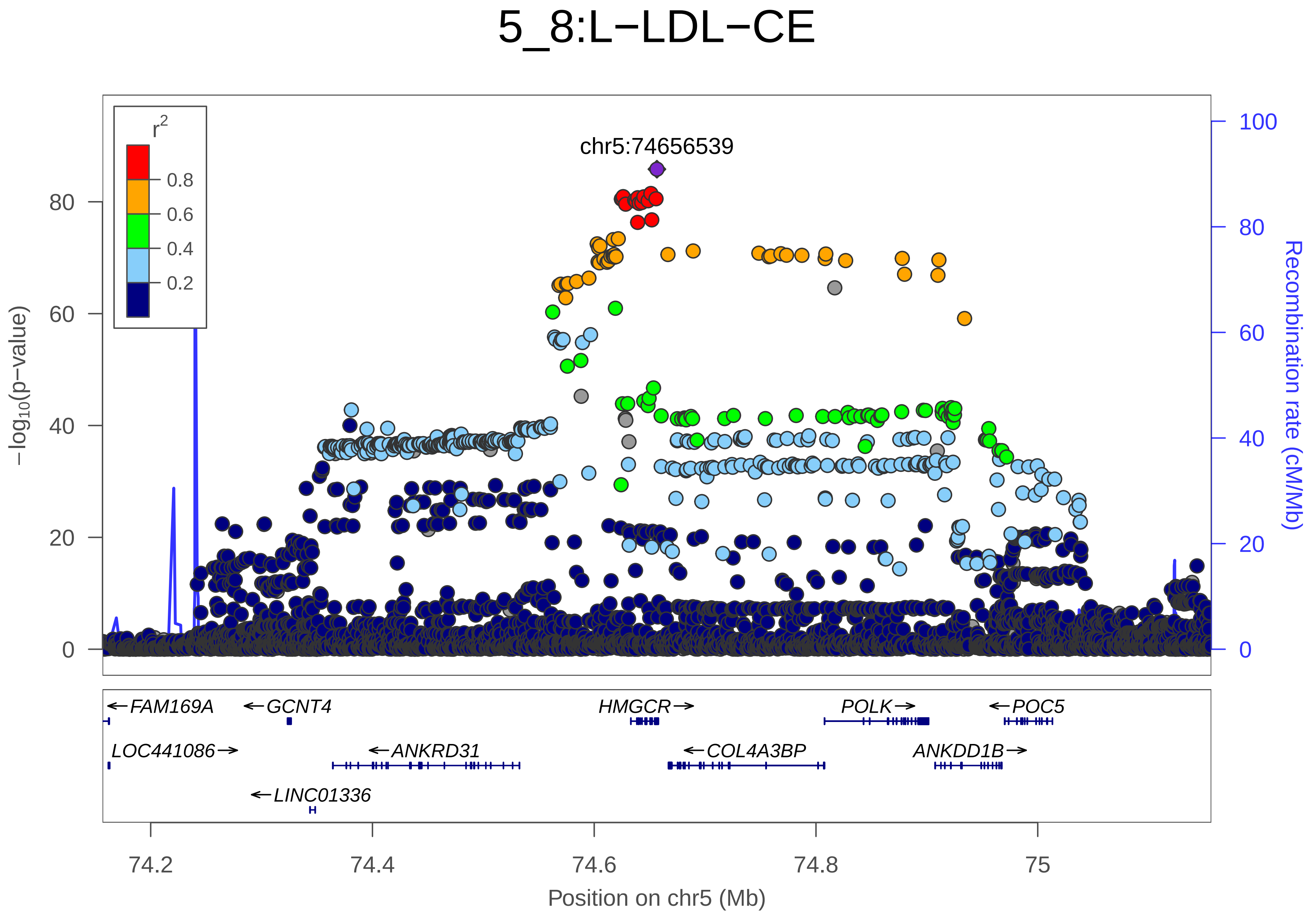

5_8:L−LDL−CE
chr5:74656539
r2
0.8
0.6
0.4
0.2
−log10(p−value)
Recombination rate (cM/Mb)
FAM169A
GCNT4
HMGCR
POLK
POC5
LOC441086
ANKRD31
COL4A3BP
ANKDD1B
LINC01336
Position on chr5 (Mb)

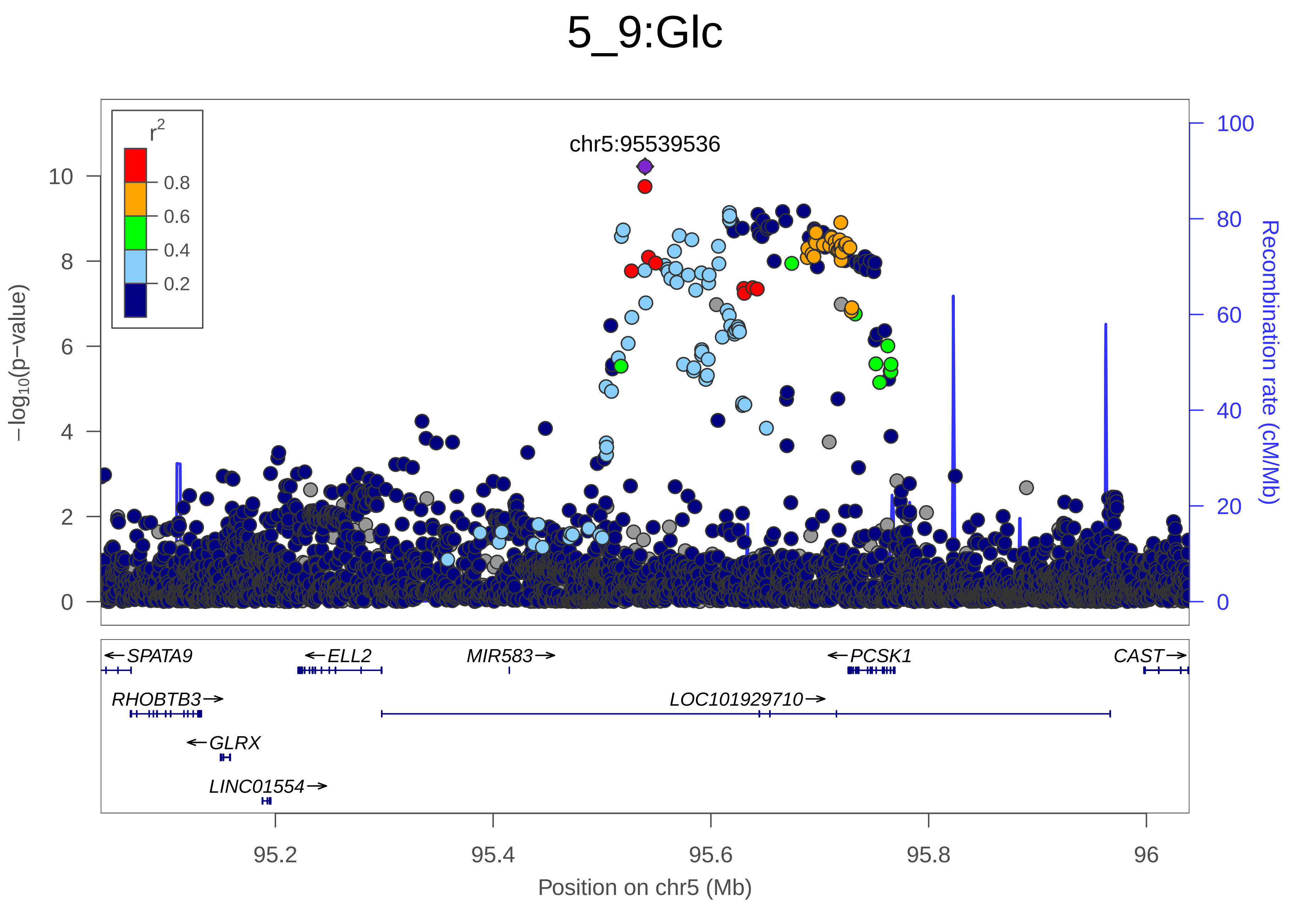

5_9:Glc
chr5:95539536
r2
0.8
0.6
0.4
0.2
−log10(p−value)
Recombination rate (cM/Mb)
0
2
4
6
8
10
0
20
40
60
80
100
←SPATA9
←ELL2
MIR583→
←PCSK1
CAST→
RHOBTB3→
LOC101929710→
←GLRX
LINC01554→
95.2
95.4
95.6
95.8
96
Position on chr5 (Mb)

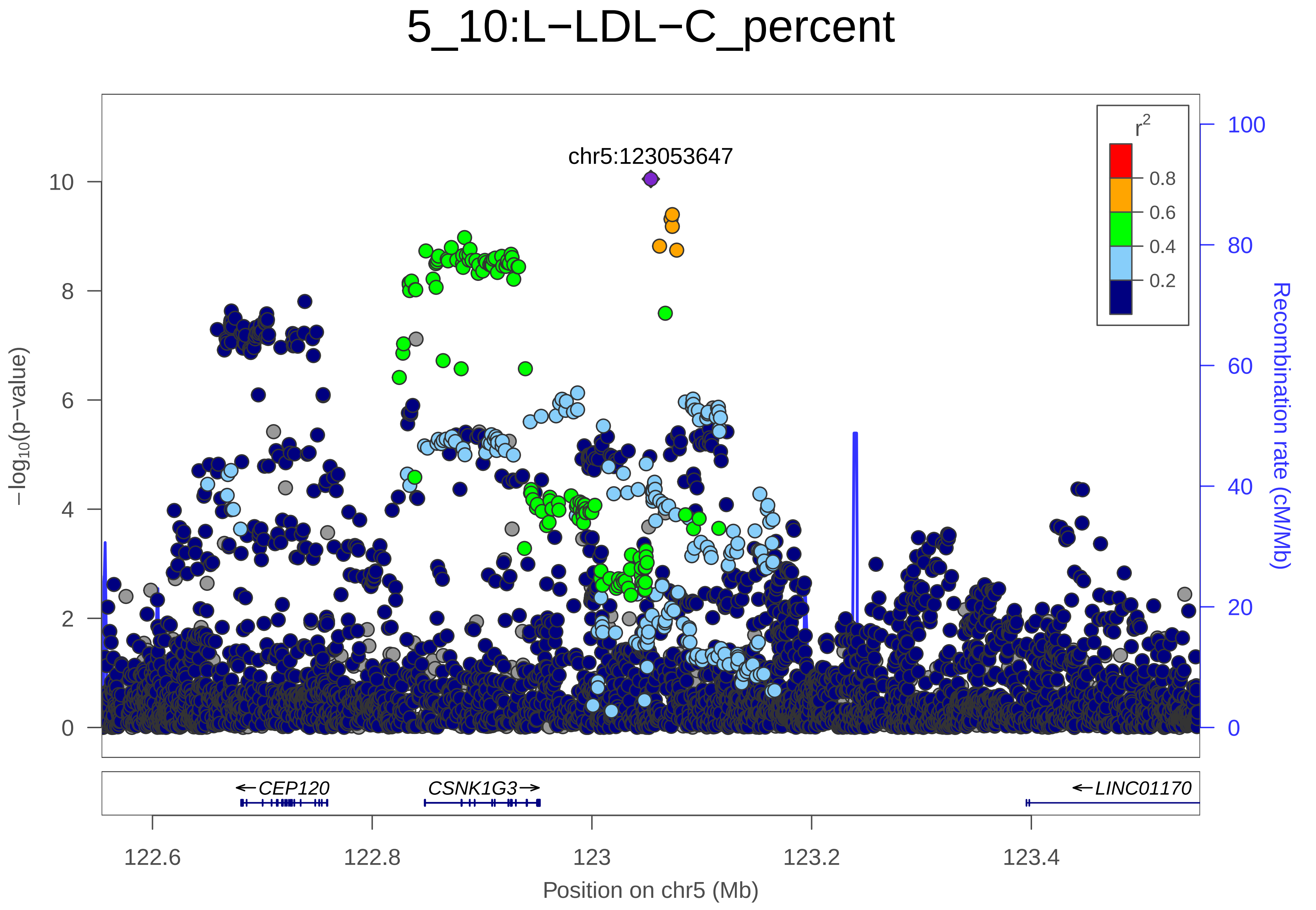
5_10:L−LDL−C_percent
chr5:123053647
r²
0.8
0.6
0.4
0.2
−log10(p−value)
Recombination rate (cM/Mb)
0
2
4
6
8
10
0
20
40
60
80
100
CEP120
CSNK1G3
LINC01170
122.6
122.8
123
123.2
123.4
Position on chr5 (Mb)

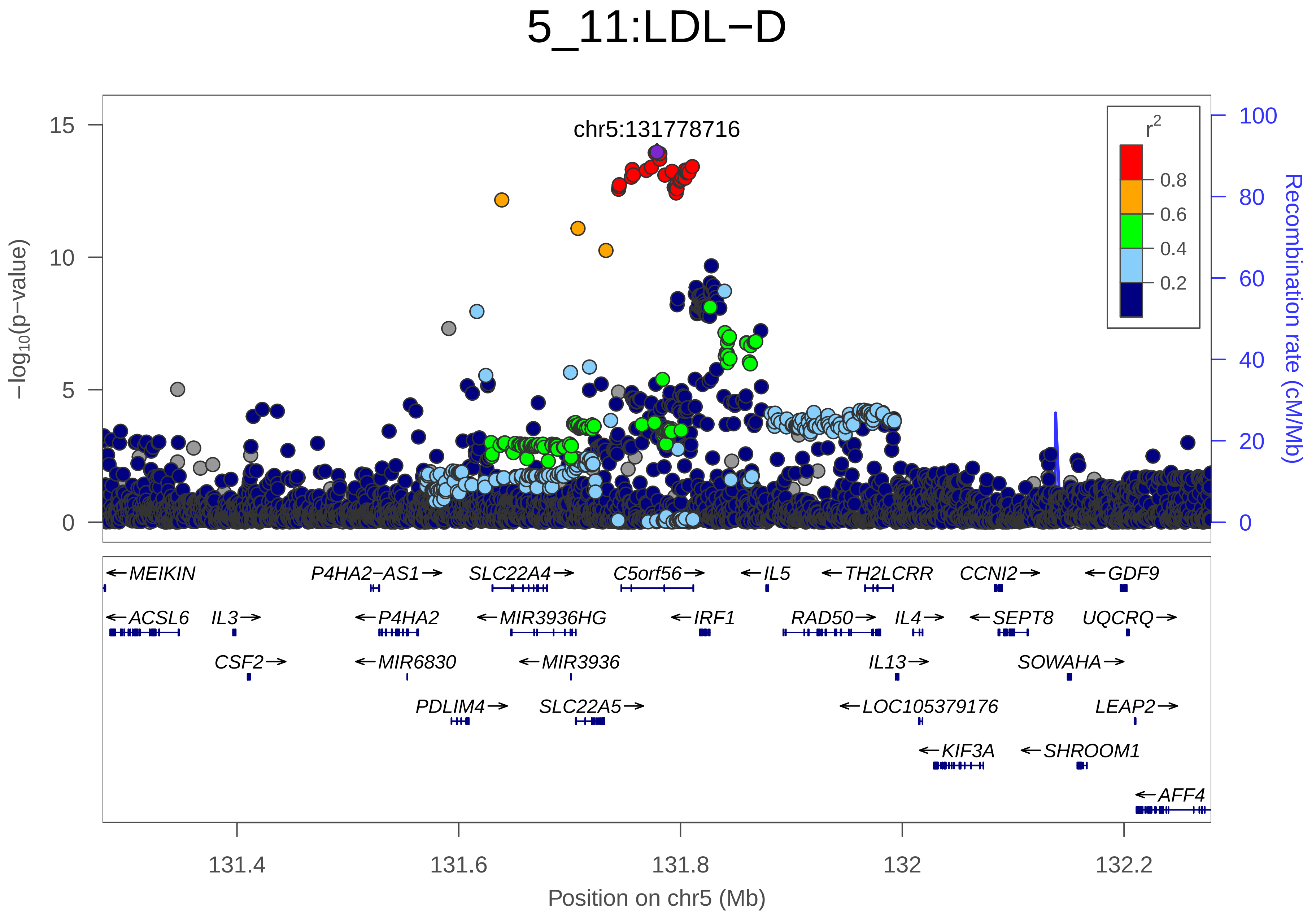

5_11:LDL−D
chr5:131778716
r2
0.8
0.6
0.4
0.2
−log10(p−value)
Recombination rate (cM/Mb)
MEIKIN
P4HA2−AS1
SLC22A4
C5orf56
IL5
TH2LCRR
CCNI2
GDF9
ACSL6
IL3
P4HA2
MIR3936HG
IRF1
RAD50
IL4
SEPT8
UQCRQ
CSF2
MIR6830
MIR3936
IL13
SOWAHA
PDLIM4
SLC22A5
LOC105379176
LEAP2
KIF3A
SHROOM1
AFF4
131.4
131.6
131.8
132
132.2
Position on chr5 (Mb)

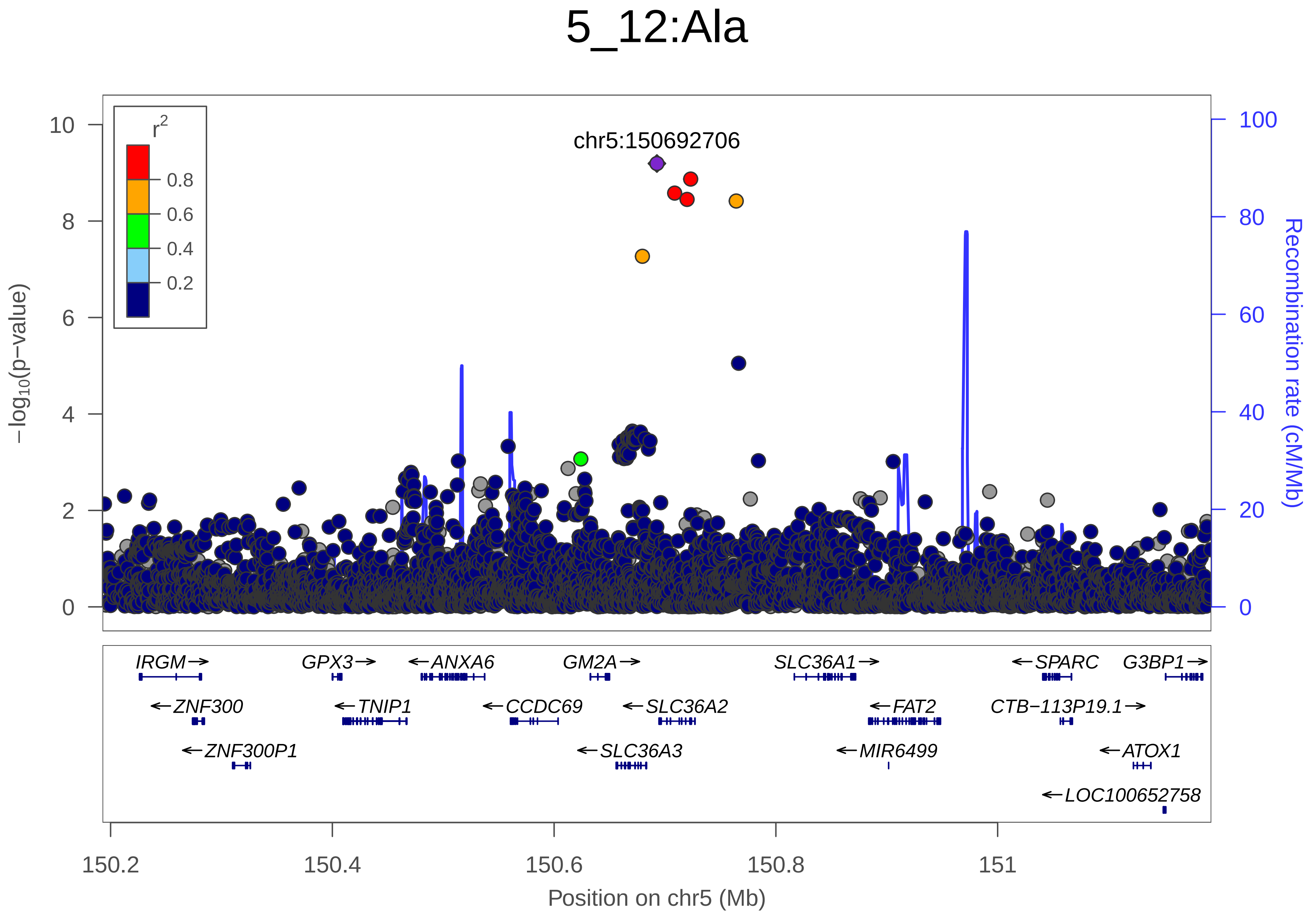

5_12:Ala
chr5:150692706
r2
0.8
0.6
0.4
0.2
−log10(p−value)
Recombination rate (cM/Mb)
10
8
6
4
2
0
100
80
60
40
20
0
IRGM
GPX3
ANXA6
GM2A
SLC36A1
SPARC
G3BP1
ZNF300
TNIP1
CCDC69
SLC36A2
FAT2
CTB−113P19.1
ZNF300P1
SLC36A3
MIR6499
ATOX1
LOC100652758
150.2
150.4
150.6
150.8
151
Position on chr5 (Mb)

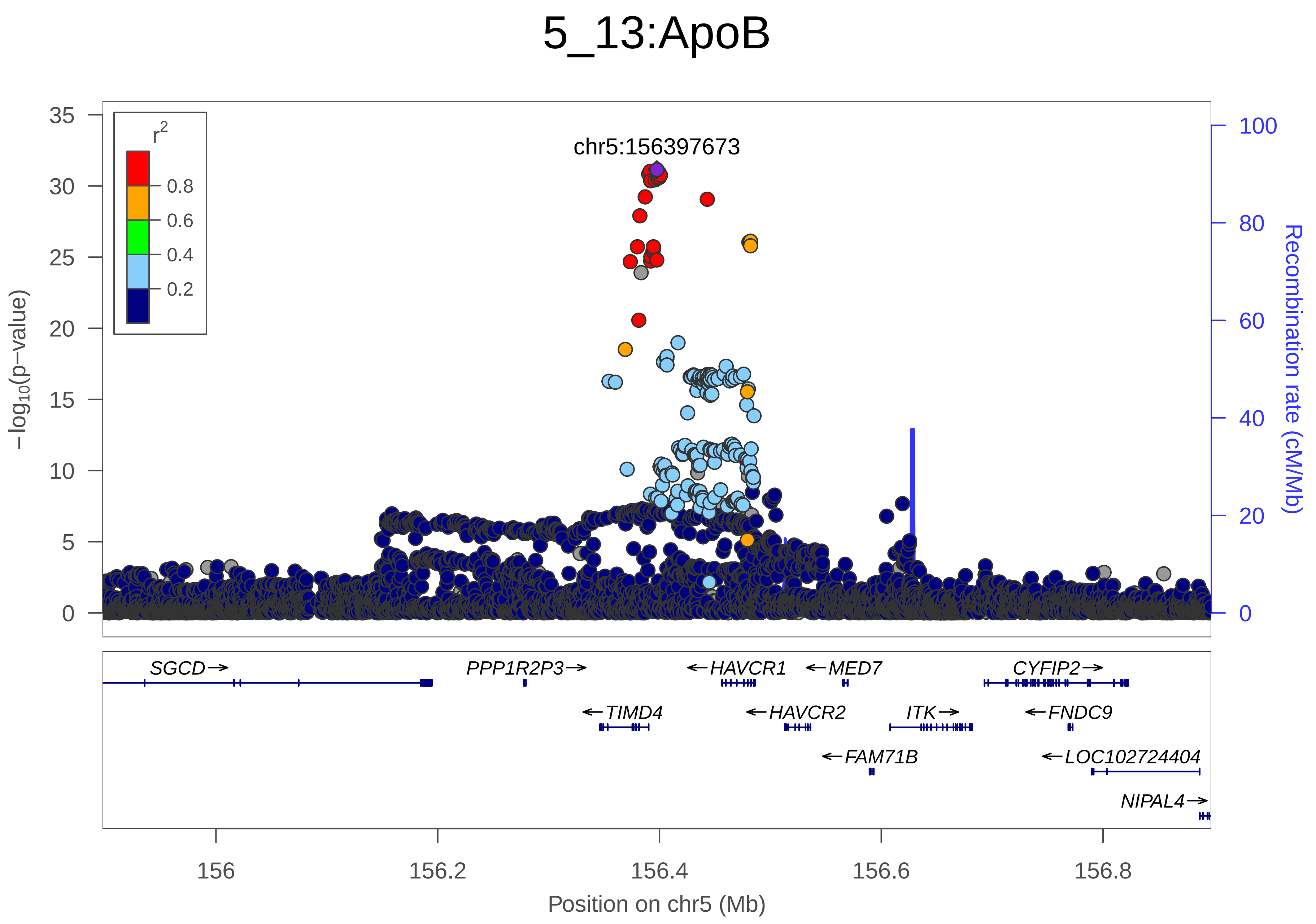

5_13:ApoB
chr5:156397673
r2
0.8
0.6
0.4
0.2
35
30
25
20
15
10
5
0
−log10(p−value)
100
80
60
40
20
0
Recombination rate (cM/Mb)
SGCD→
PPP1R2P3→
←HAVCR1
←MED7
CYFIP2→
←TIMD4
←HAVCR2
ITK→
←FNDC9
←FAM71B
←LOC102724404
NIPAL4→
156
156.2
156.4
156.6
156.8
Position on chr5 (Mb)

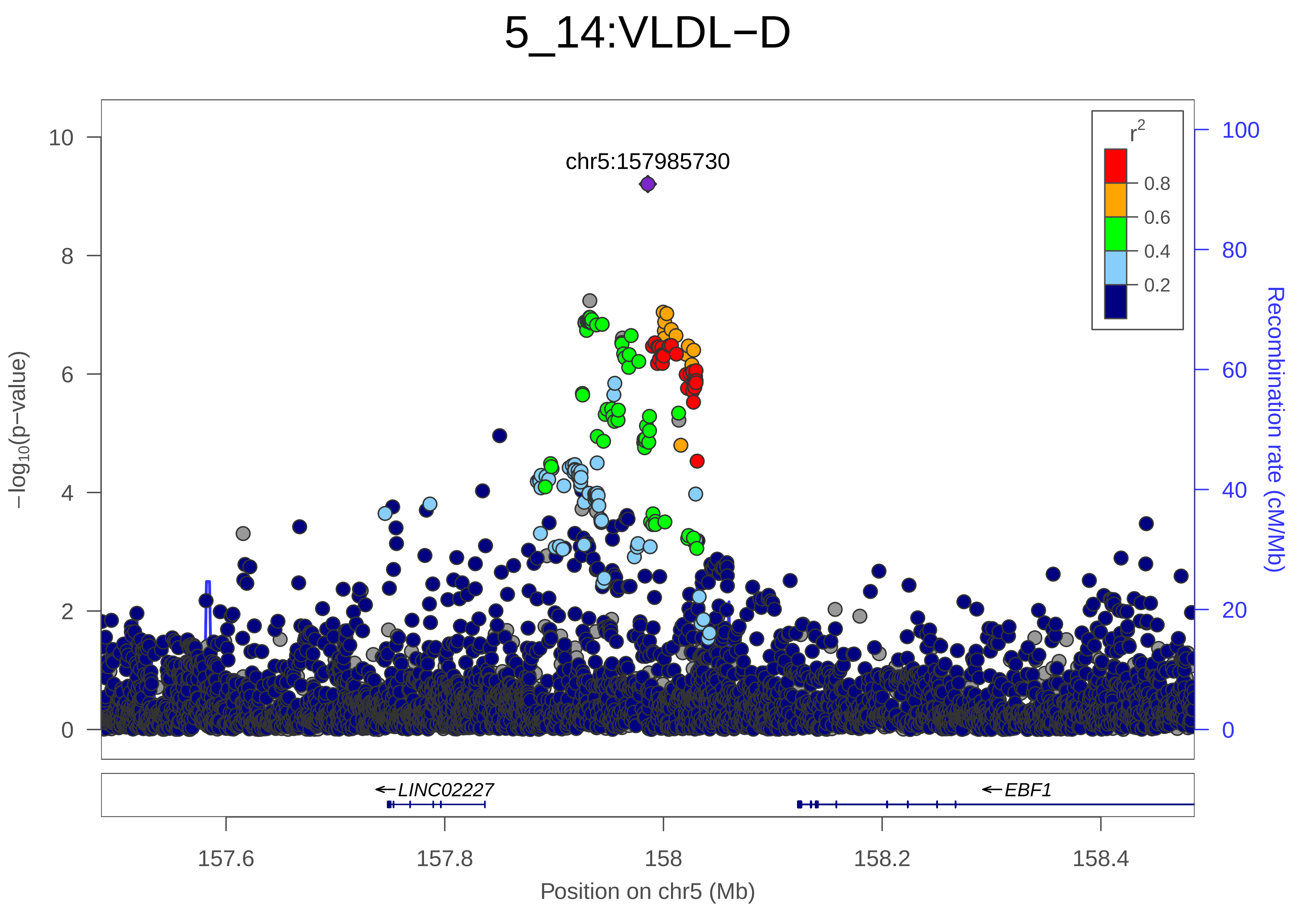
5_14:VLDL−D
chr5:157985730
r2
0.8
0.6
0.4
0.2
−log10(p−value)
Recombination rate (cM/Mb)
10
8
6
4
2
0
100
80
60
40
20
0
←LINC02227
←EBF1
157.6
157.8
158
158.2
158.4
Position on chr5 (Mb)

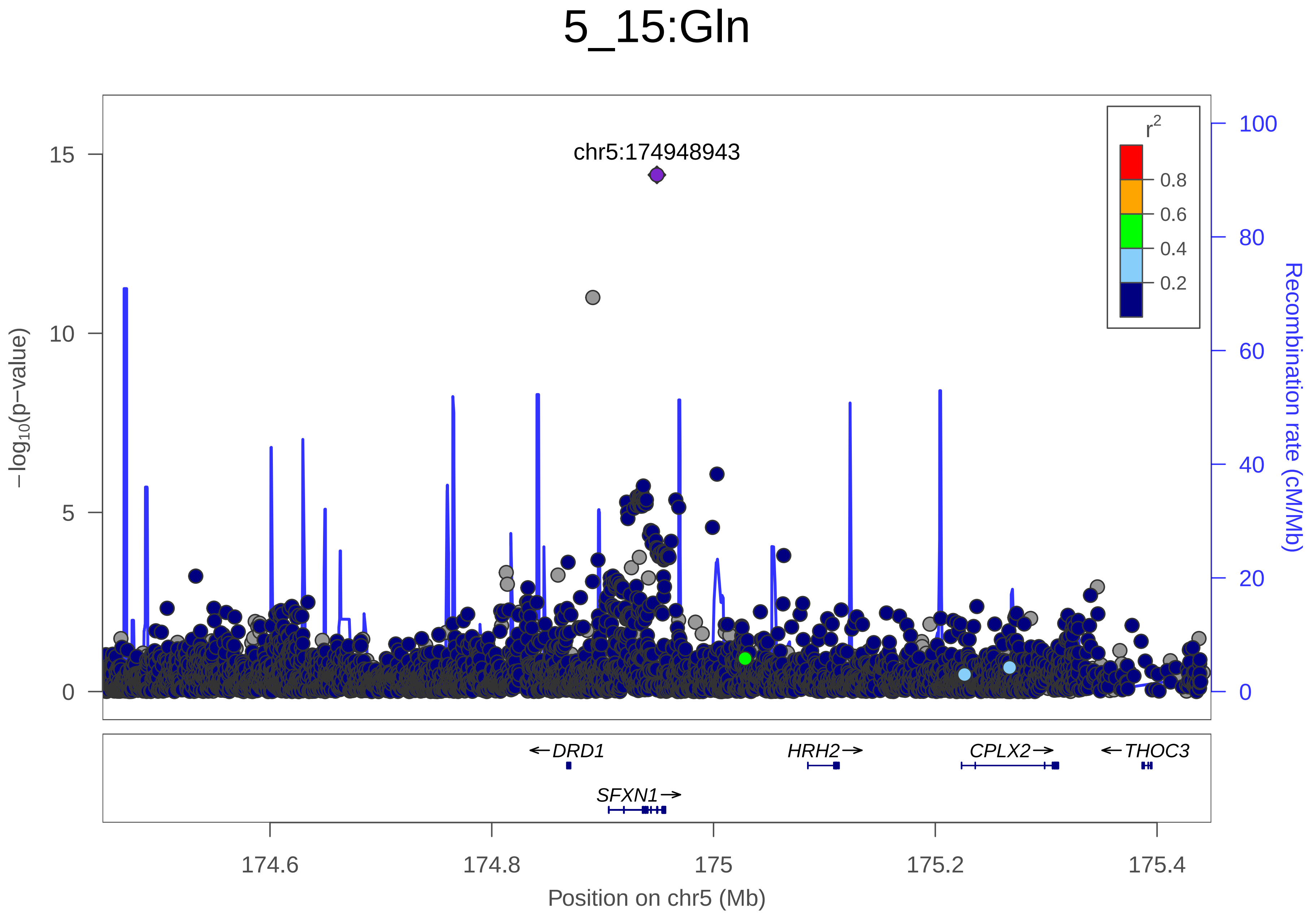
5_15:Gln
chr5:174948943
r2
0.8
0.6
0.4
0.2
15
10
5
0
−log10(p−value)
100
80
60
40
20
0
Recombination rate (cM/Mb)
←DRD1
HRH2→
CPLX2→
←THOC3
SFXN1→
174.6
174.8
175
175.2
175.4
Position on chr5 (Mb)

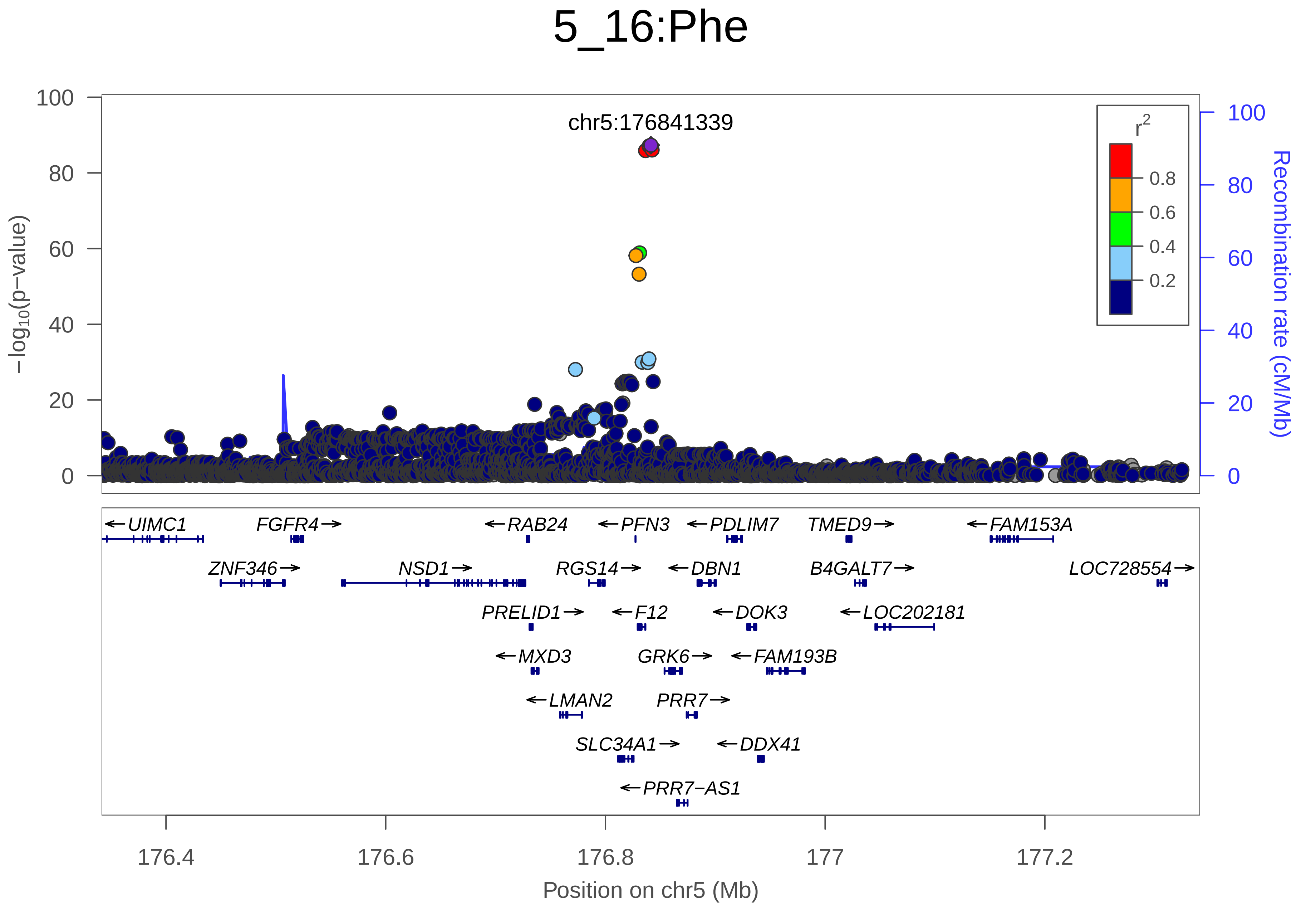
5_16:Phe
chr5:176841339
r²
0.8
0.6
0.4
0.2
−log10(p−value)
Recombination rate (cM/Mb)
0
20
40
60
80
100
UIMC1
FGFR4
RAB24
PFN3
PDLIM7
TMED9
FAM153A
ZNF346
NSD1
RGS14
DBN1
B4GALT7
LOC728554
PRELID1
F12
DOK3
LOC202181
MXD3
GRK6
FAM193B
LMAN2
PRR7
SLC34A1
DDX41
PRR7−AS1
176.4
176.6
176.8
177
177.2
Position on chr5 (Mb)

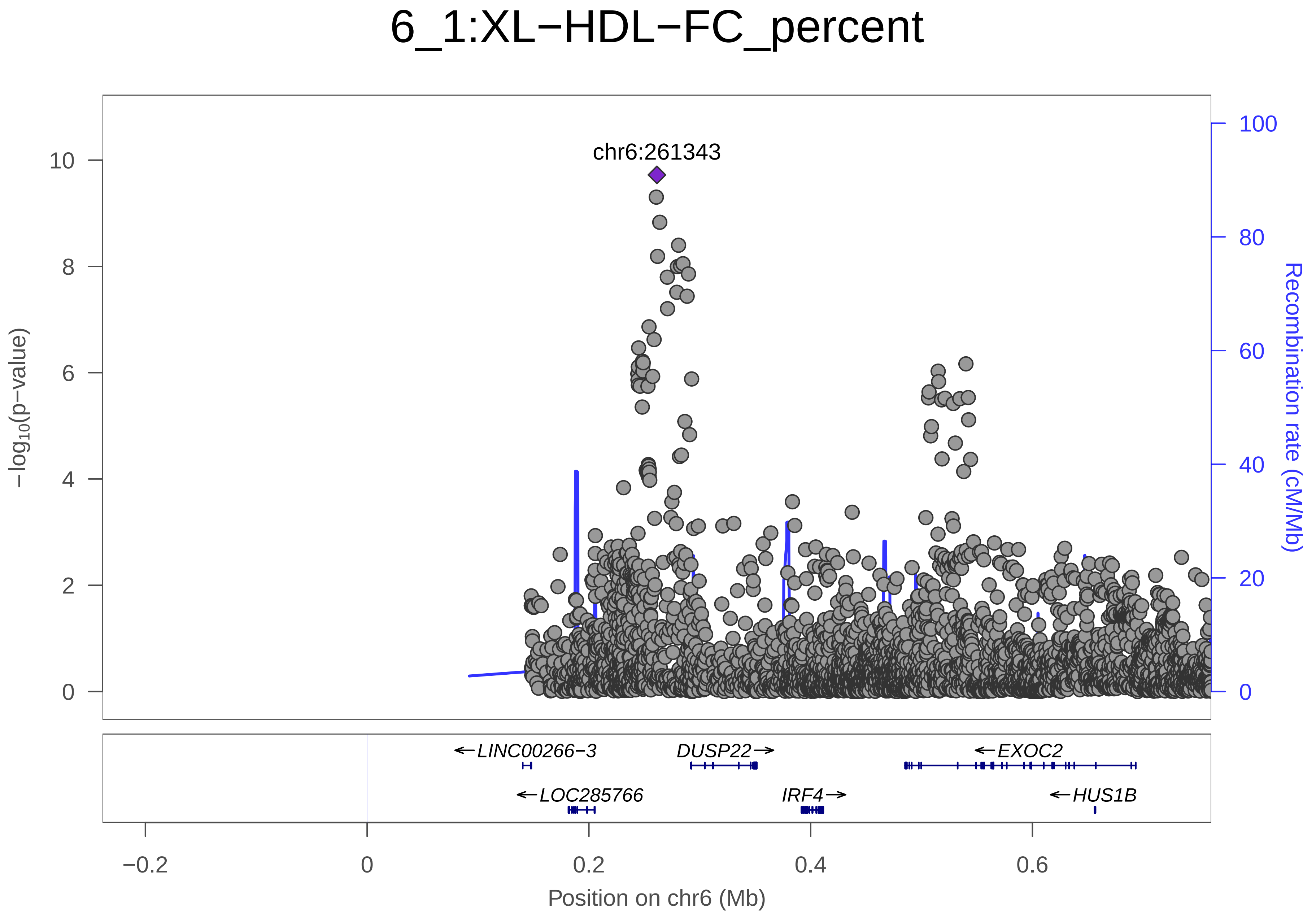

6_1:XL−HDL−FC_percent
chr6:261343
−log10(p−value)
Recombination rate (cM/Mb)
10
8
6
4
2
0
100
80
60
40
20
0
←LINC00266−3
DUSP22→
←EXOC2
←LOC285766
IRF4→
←HUS1B
−0.2
0
0.2
0.4
0.6
Position on chr6 (Mb)

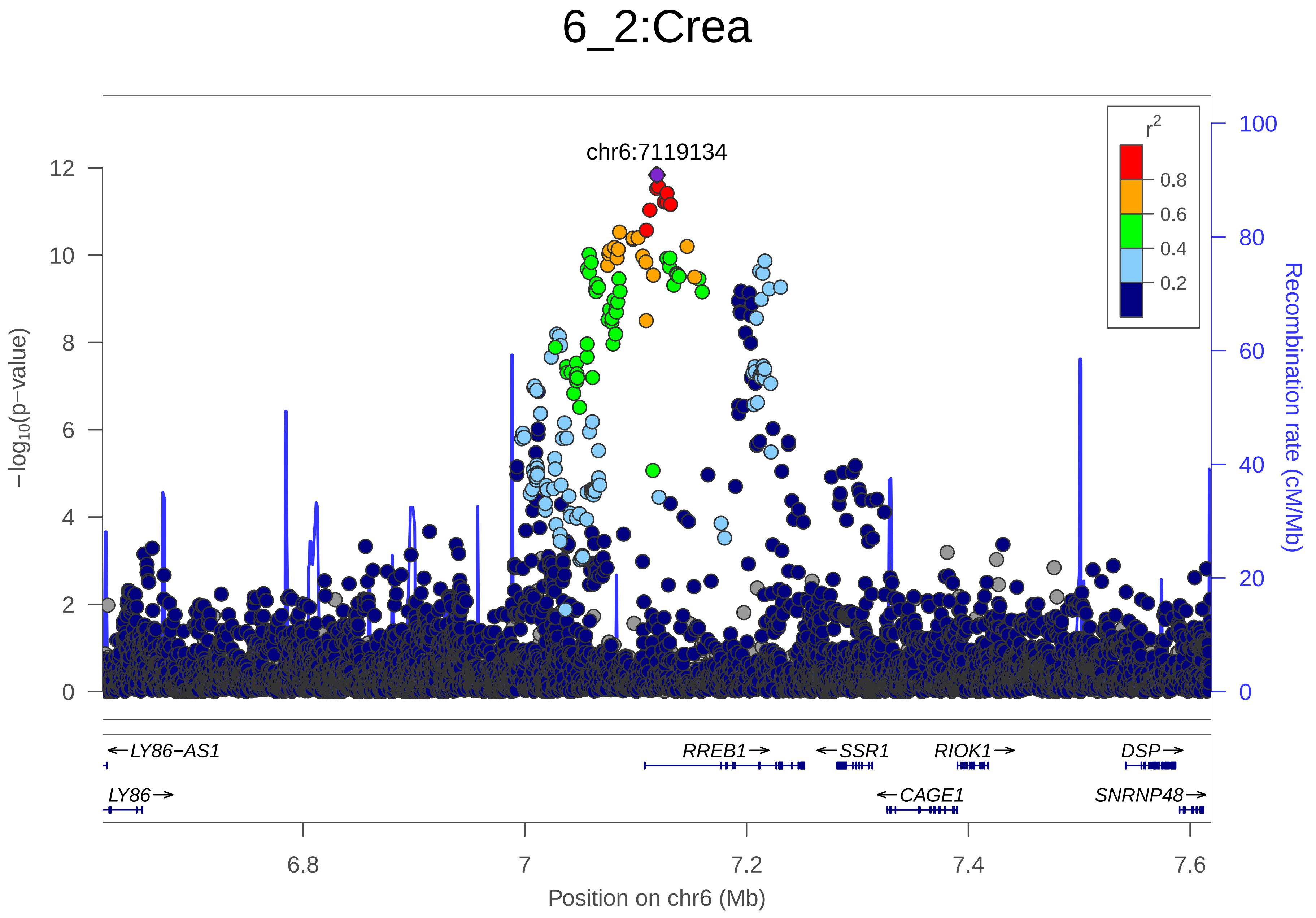

6_2:Crea
chr6:7119134
r2
0.8
0.6
0.4
0.2
12
10
8
6
4
2
0
100
80
60
40
20
0
−log10(p−value)
Recombination rate (cM/Mb)
LY86−AS1
RREB1
SSR1
RIOK1
DSP
LY86
CAGE1
SNRNP48
6.8
7
7.2
7.4
7.6
Position on chr6 (Mb)

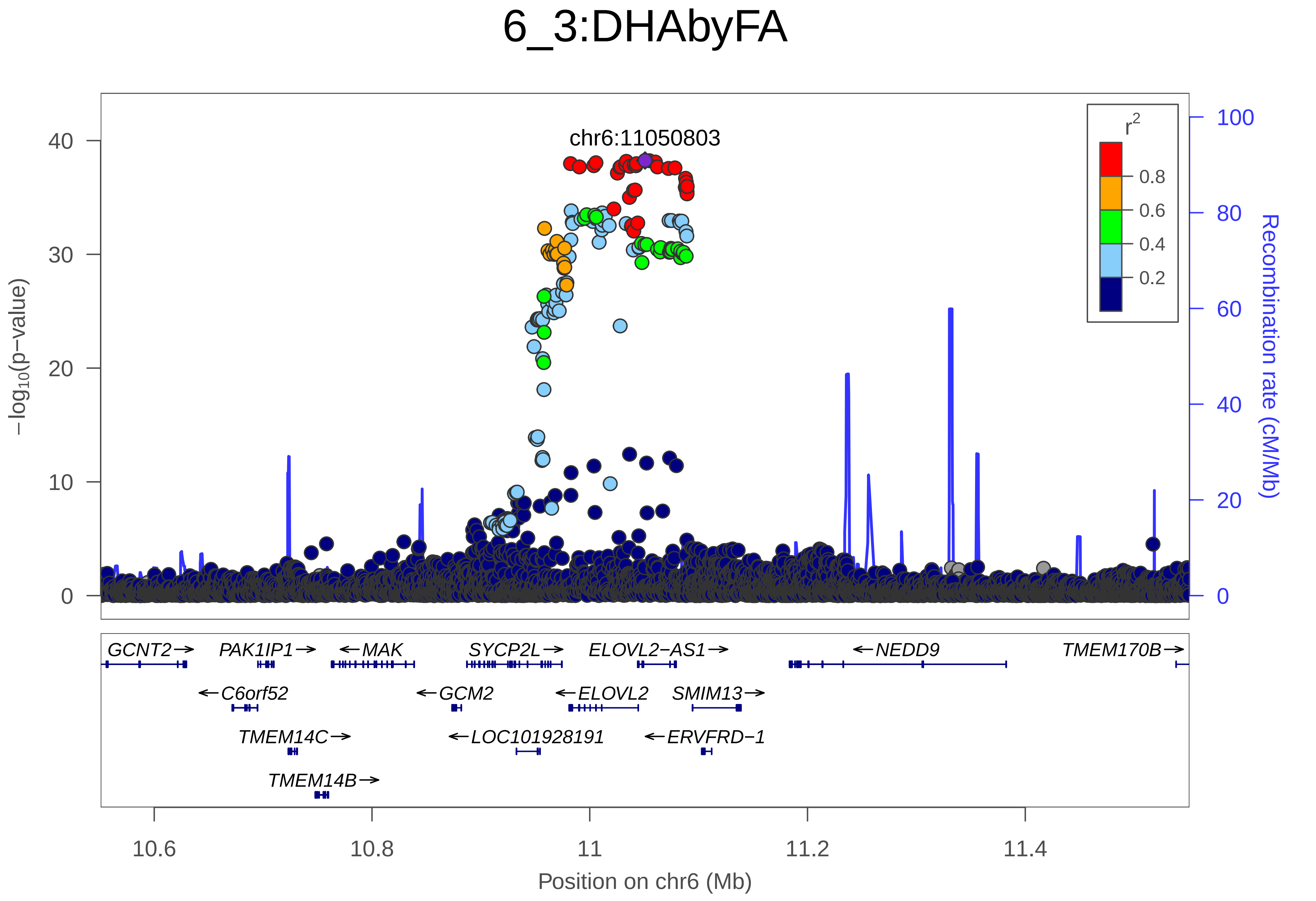

6_3:DHAbyFA
chr6:11050803
−log10(p−value)
Recombination rate (cM/Mb)
r2
0.8
0.6
0.4
0.2
0
10
20
30
40
60
80
100
GCNT2→
PAK1IP1→
←MAK
SYCP2L→
ELOVL2−AS1→
←NEDD9
TMEM170B→
←C6orf52
←GCM2
←ELOVL2
SMIM13→
TMEM14C→
←LOC101928191
←ERVFRD−1
TMEM14B→
10.6
10.8
11
11.2
11.4
Position on chr6 (Mb)

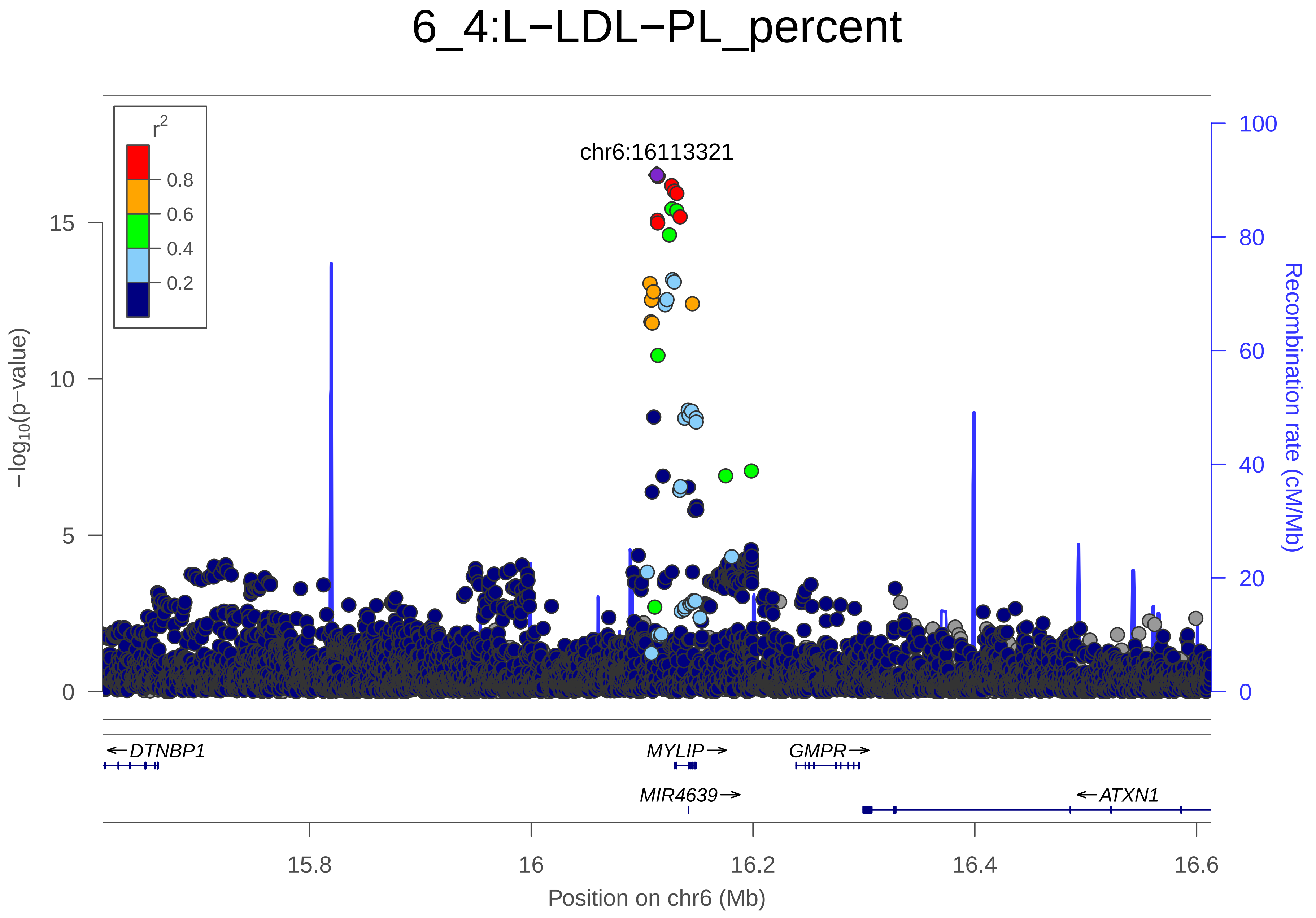
6_4:L−LDL−PL_percent
r2
0.8
0.6
0.4
0.2
chr6:16113321
15
10
5
0
−log10(p−value)
100
80
60
40
20
0
Recombination rate (cM/Mb)
←DTNBP1
MYLIP→
GMPR→
MIR4639→
←ATXN1
15.8
16
16.2
16.4
16.6
Position on chr6 (Mb)

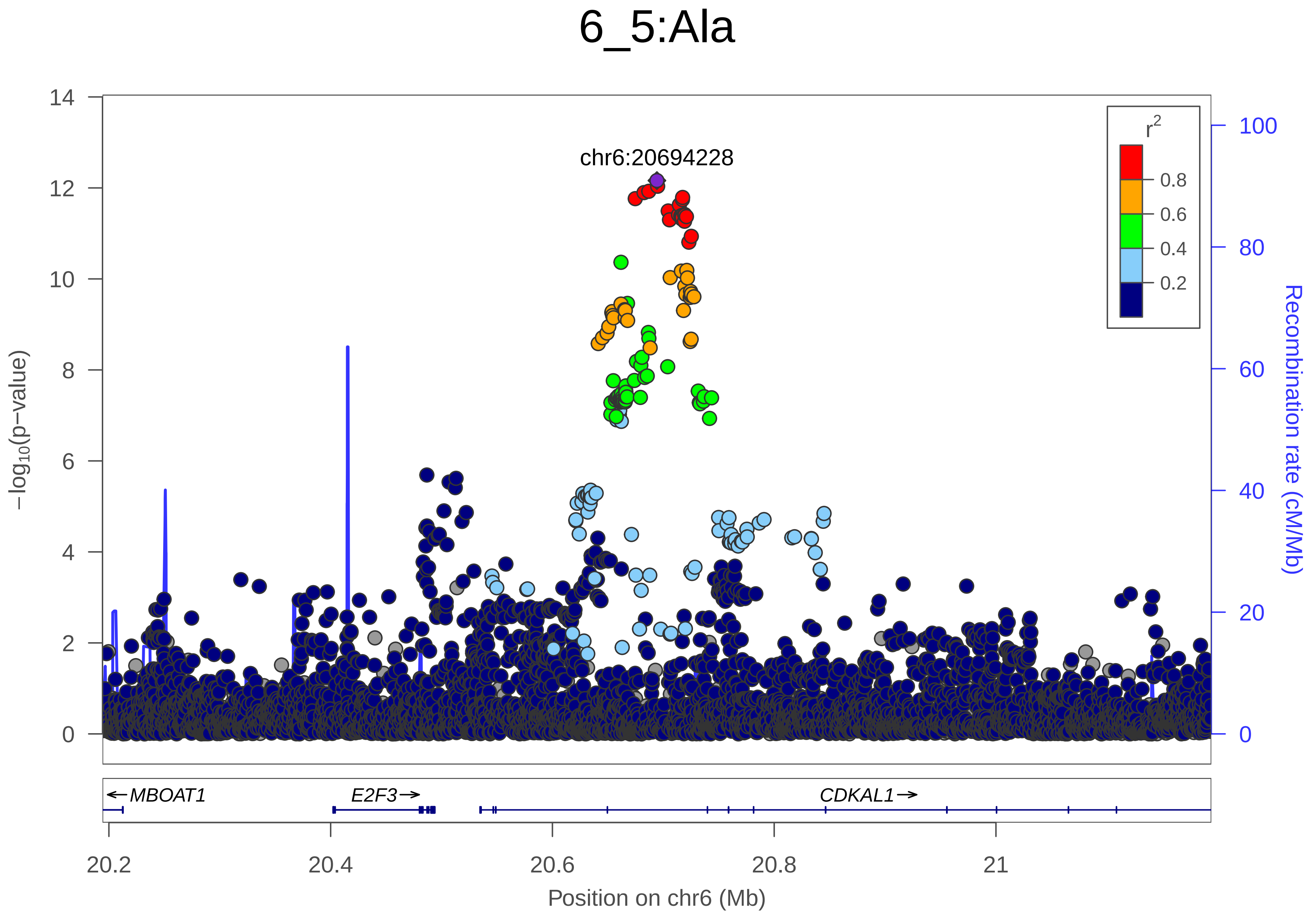
6_5:Ala
chr6:20694228
r2
0.8
0.6
0.4
0.2
14
12
10
8
6
4
2
0
−log10(p−value)
100
80
60
40
20
0
Recombination rate (cM/Mb)
MBOAT1
E2F3
CDKAL1
20.2
20.4
20.6
20.8
21
Position on chr6 (Mb)

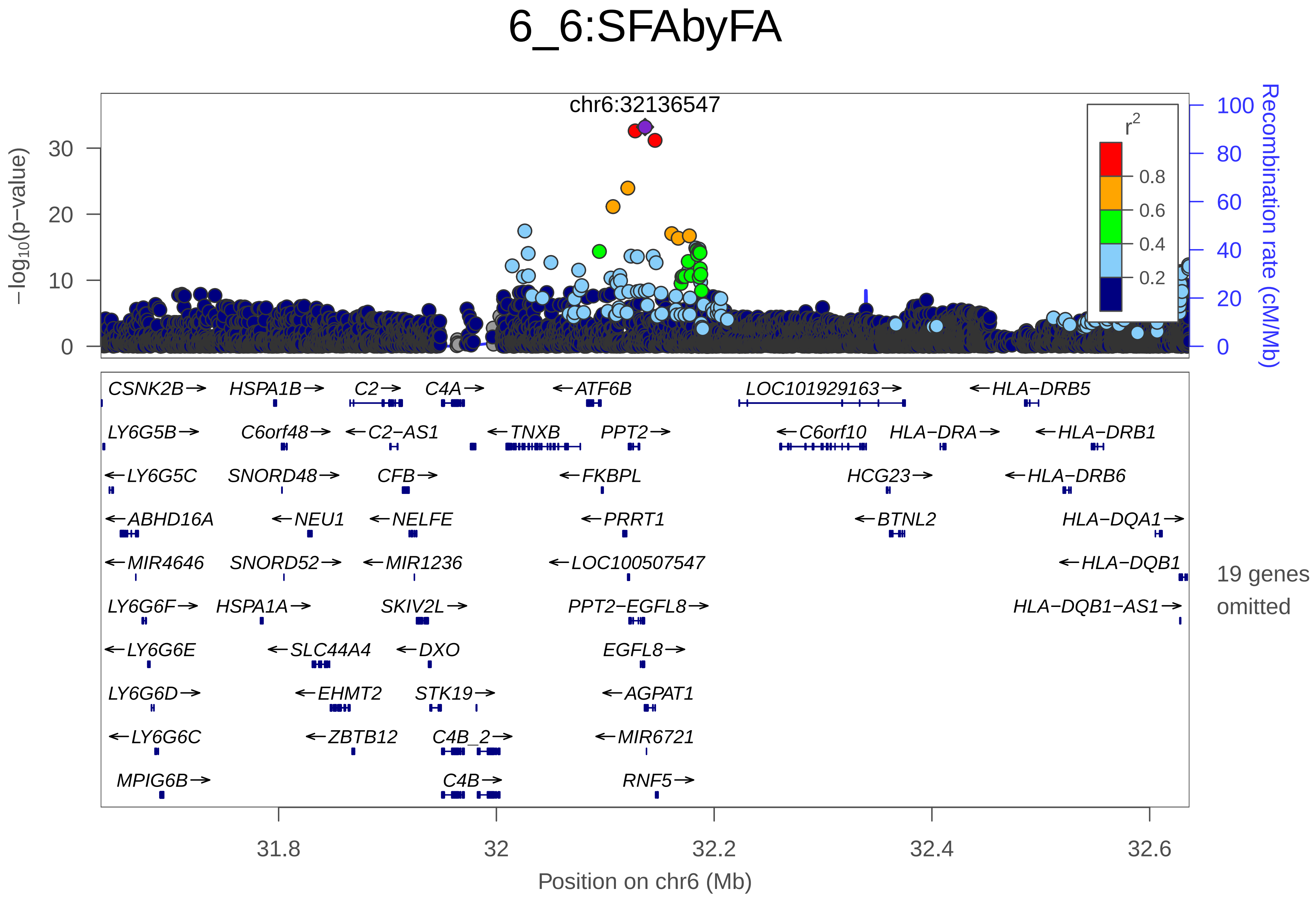
6_6:SFAbyFA
chr6:32136547
−log10(p−value)
Recombination rate (cM/Mb)
r2
0.8
0.6
0.4
0.2
0
10
20
30
0
20
40
60
80
100
CSNK2B→
HSPA1B→
C2→
C4A→
←ATF6B
LOC101929163→
←HLA−DRB5
LY6G5B→
C6orf48→
←C2−AS1
←TNXB
PPT2→
←C6orf10
HLA−DRA→
←HLA−DRB1
←LY6G5C
SNORD48→
CFB→
←FKBPL
HCG23→
←HLA−DRB6
←ABHD16A
←NEU1
←NELFE
←PRRT1
←BTNL2
HLA−DQA1→
←MIR4646
SNORD52→
←MIR1236
←LOC100507547
←HLA−DQB1
19 genes omitted
LY6G6F→
HSPA1A→
SKIV2L→
PPT2−EGFL8→
HLA−DQB1−AS1→
←LY6G6E
←SLC44A4
←DXO
EGFL8→
LY6G6D→
←EHMT2
STK19→
←AGPAT1
←LY6G6C
←ZBTB12
C4B_2→
←MIR6721
MPIG6B→
C4B→
RNF5→
31.8
32
32.2
32.4
32.6
Position on chr6 (Mb)

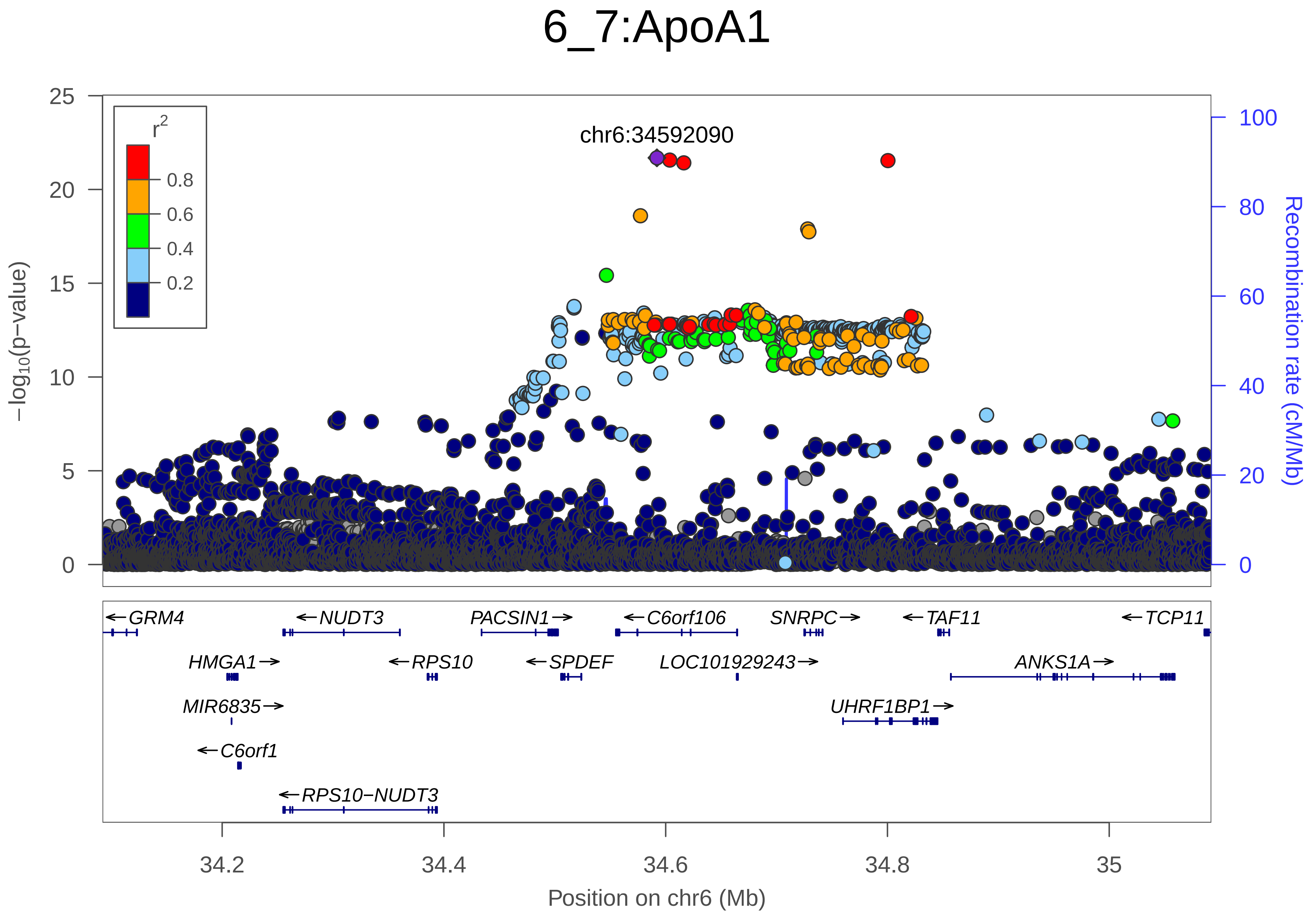
6_7:ApoA1
chr6:34592090
r2
0.8
0.6
0.4
0.2
−log10(p−value)
Recombination rate (cM/Mb)
0
5
10
15
20
25
0
20
40
60
80
100
GRM4
NUDT3
PACSIN1
C6orf106
SNRPC
TAF11
TCP11
HMGA1
RPS10
SPDEF
LOC101929243
ANKS1A
MIR6835
UHRF1BP1
C6orf1
RPS10−NUDT3
34.2
34.4
34.6
34.8
35
Position on chr6 (Mb)

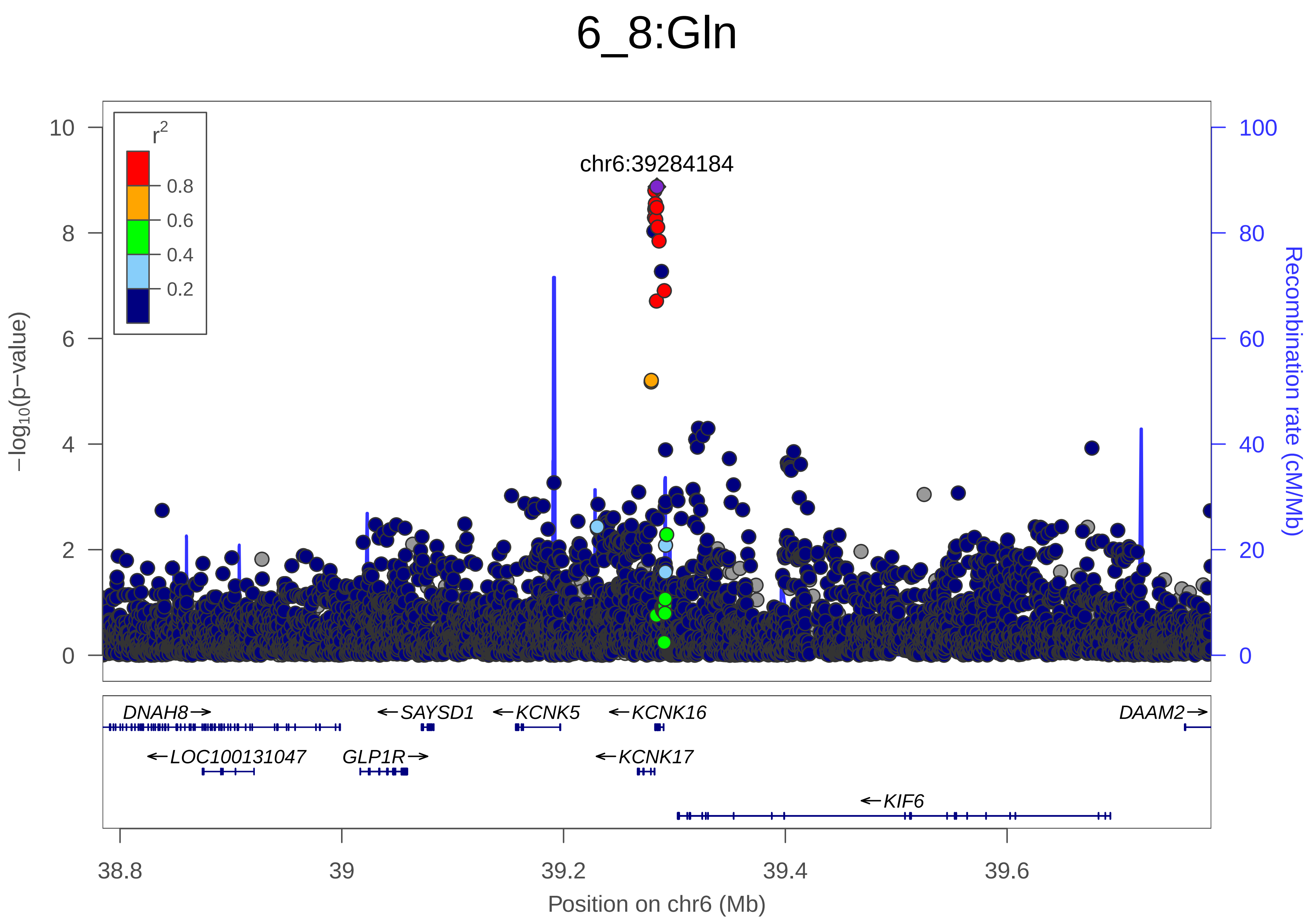

6_8:Gln
chr6:39284184
r2
0.8
0.6
0.4
0.2
−log10(p−value)
Recombination rate (cM/Mb)
0
2
4
6
8
10
0
20
40
60
80
100
DNAH8→
←SAYSD1
←KCNK5
←KCNK16
DAAM2→
←LOC100131047
GLP1R→
←KCNK17
←KIF6
38.8
39
39.2
39.4
39.6
Position on chr6 (Mb)

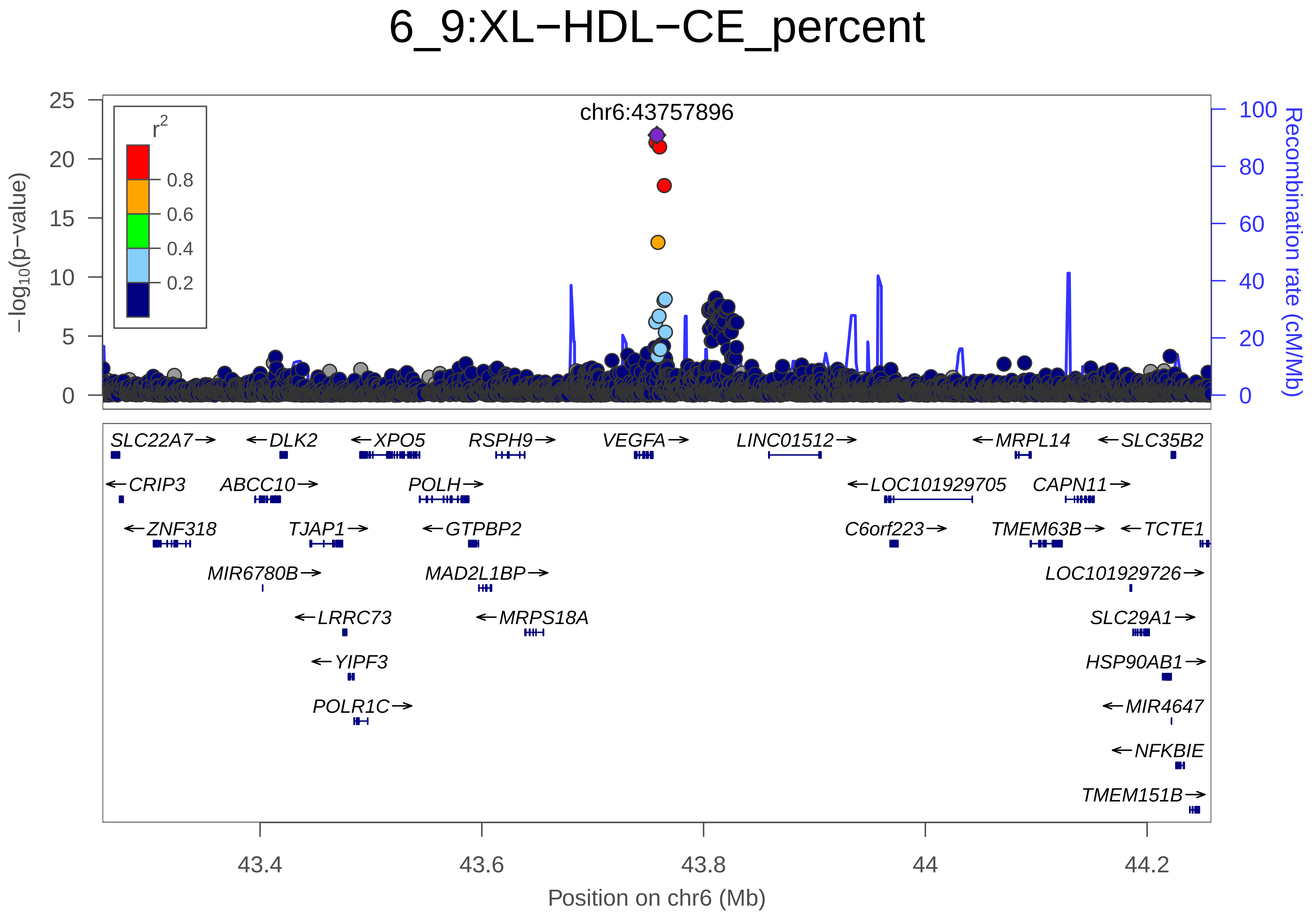

6_9:XL−HDL−CE_percent
chr6:43757896
r2
0.8
0.6
0.4
0.2
−log10(p−value)
Recombination rate (cM/Mb)
SLC22A7
DLK2
XPO5
RSPH9
VEGFA
LINC01512
MRPL14
SLC35B2
CRIP3
ABCC10
POLH
LOC101929705
CAPN11
ZNF318
TJAP1
GTPBP2
C6orf223
TMEM63B
TCTE1
MIR6780B
MAD2L1BP
LOC101929726
LRRC73
MRPS18A
SLC29A1
YIPF3
HSP90AB1
POLR1C
MIR4647
NFKBIE
TMEM151B
43.4
43.6
43.8
44
44.2
Position on chr6 (Mb)

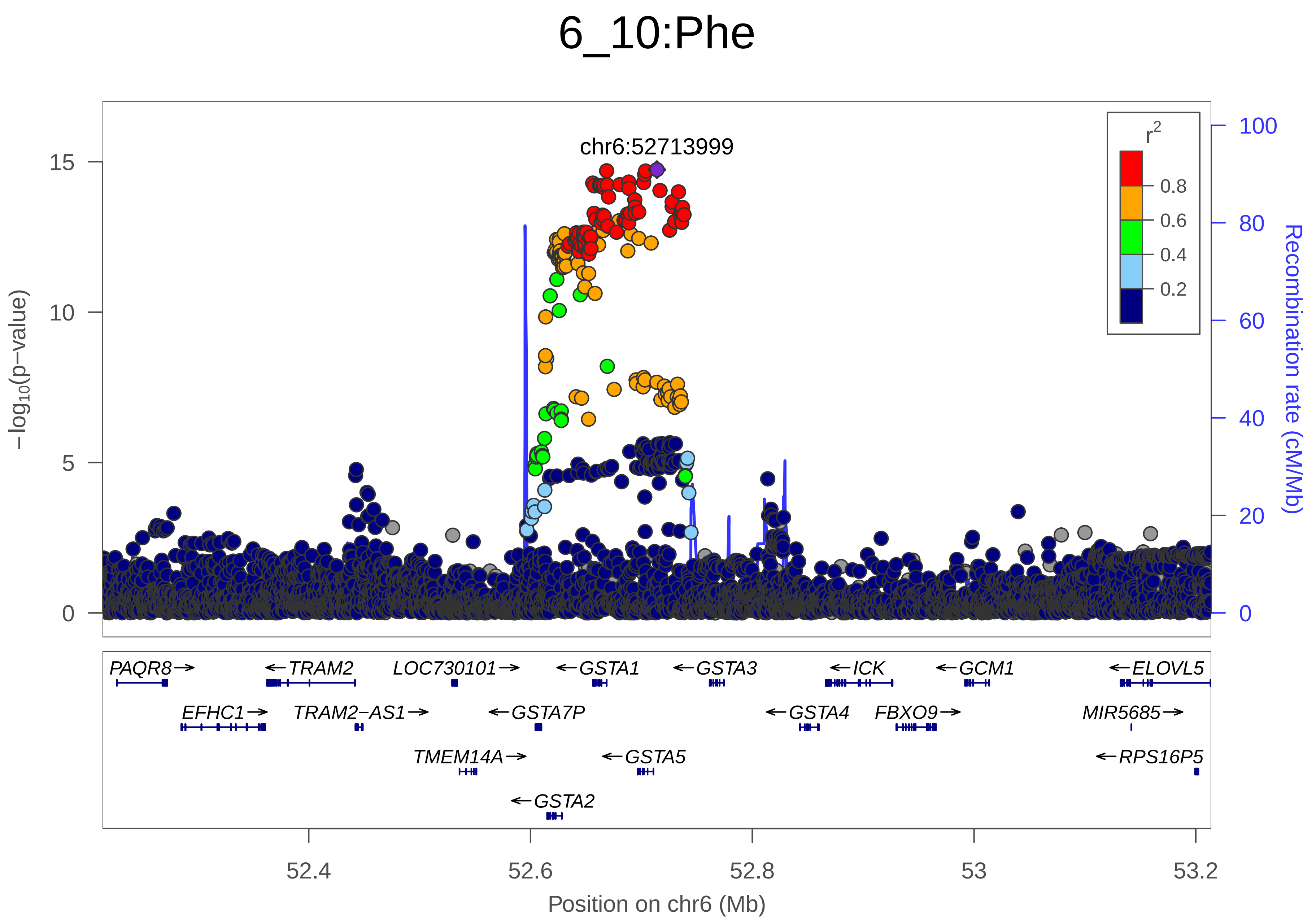
6_10:Phe
chr6:52713999
r2
0.8
0.6
0.4
0.2
−log10(p−value)
15
10
5
0
100
80
60
40
20
0
Recombination rate (cM/Mb)
PAQR8→
←TRAM2
LOC730101→
←GSTA1
←GSTA3
←ICK
←GCM1
←ELOVL5
EFHC1→
TRAM2−AS1→
←GSTA7P
←GSTA4
FBXO9→
MIR5685→
TMEM14A→
←GSTA5
←RPS16P5
←GSTA2
52.4
52.6
52.8
53
53.2
Position on chr6 (Mb)

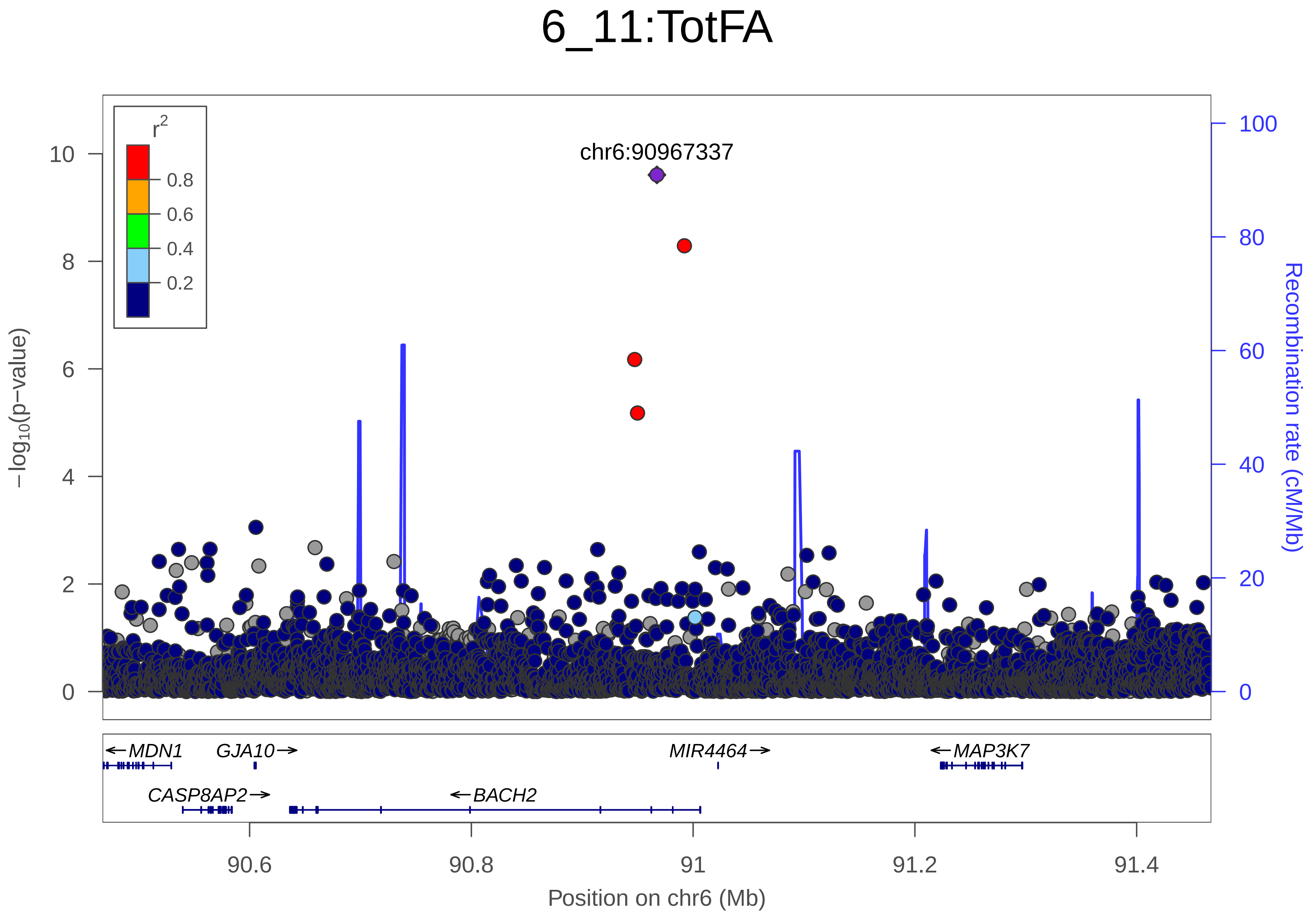

6_11:TotFA
chr6:90967337
r2
0.8
0.6
0.4
0.2
−log10(p−value)
Recombination rate (cM/Mb)
0
2
4
6
8
10
0
20
40
60
80
100
MDN1
GJA10
MIR4464
MAP3K7
CASP8AP2
BACH2
90.6
90.8
91
91.2
91.4
Position on chr6 (Mb)

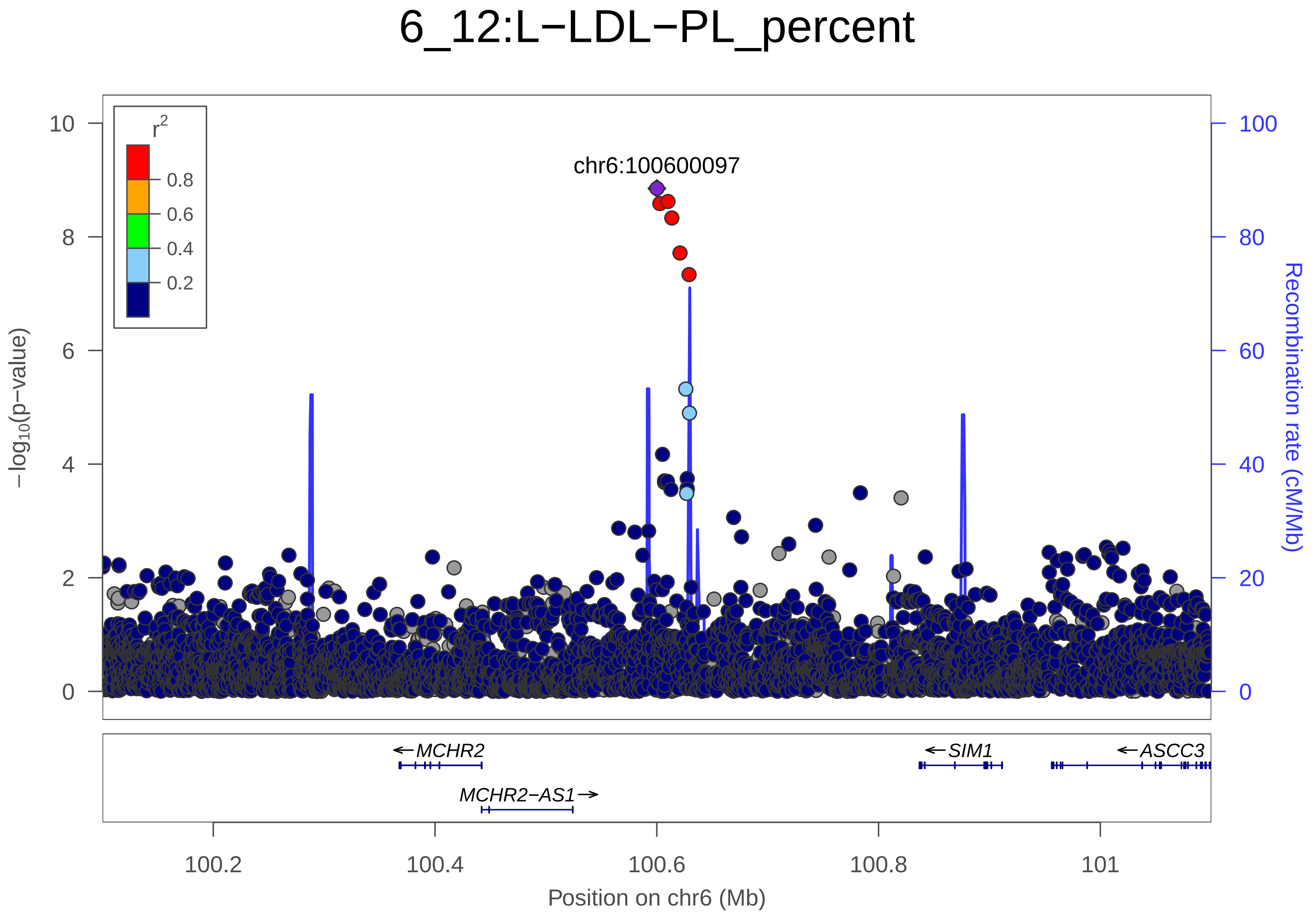

6_12:L−LDL−PL_percent
r2
0.8
0.6
0.4
0.2
chr6:100600097
−log10(p−value)
Recombination rate (cM/Mb)
0
2
4
6
8
10
0
20
40
60
80
100
←MCHR2
MCHR2−AS1→
←SIM1
←ASCC3
100.2
100.4
100.6
100.8
101
Position on chr6 (Mb)

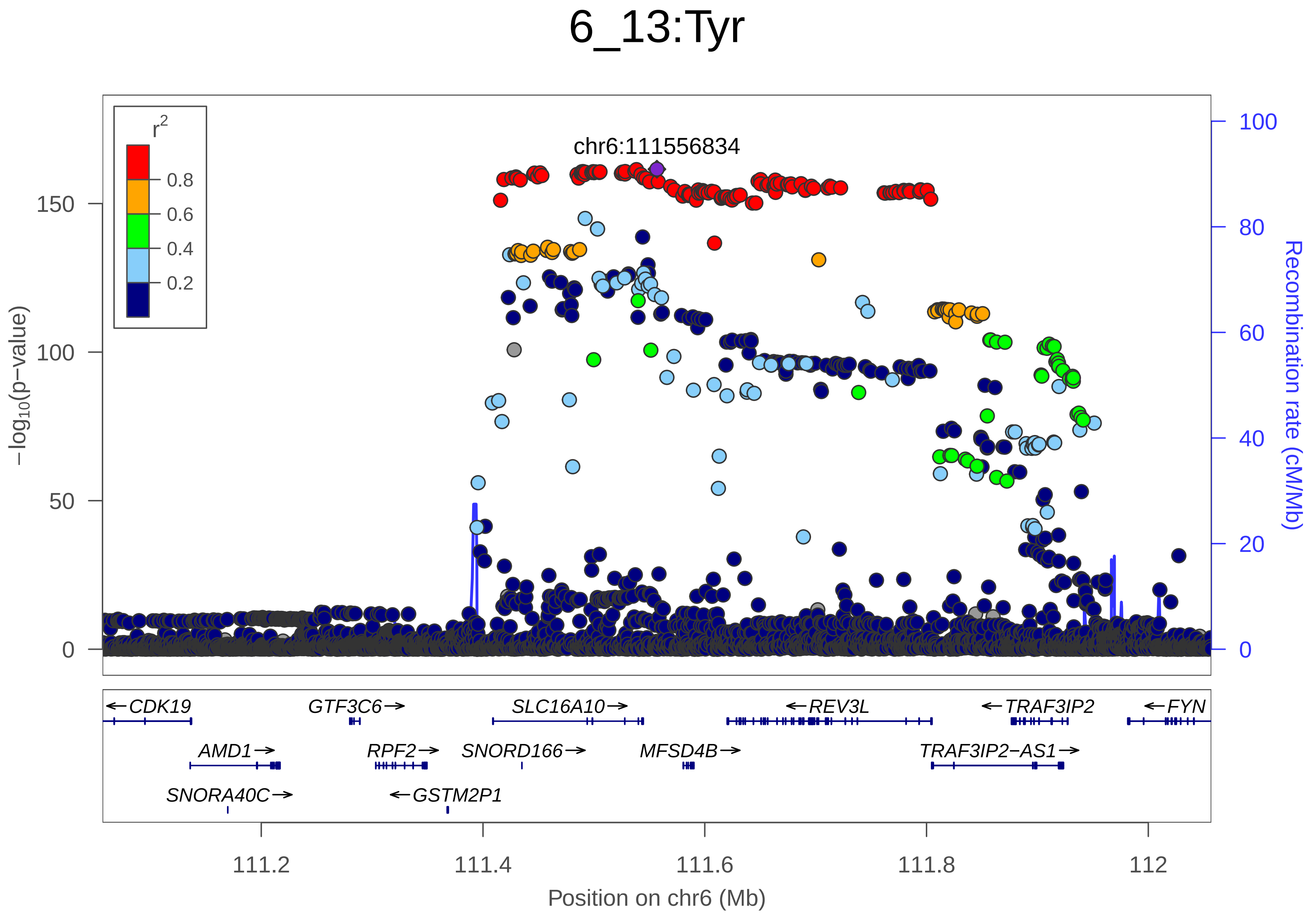

6_13:Tyr
r2
0.8
0.6
0.4
0.2
chr6:111556834
150
100
50
0
−log10(p−value)
100
80
60
40
20
0
Recombination rate (cM/Mb)
CDK19
GTF3C6
SLC16A10
REV3L
TRAF3IP2
FYN
AMD1
RPF2
SNORD166
MFSD4B
TRAF3IP2−AS1
SNORA40C
GSTM2P1
111.2
111.4
111.6
111.8
112
Position on chr6 (Mb)

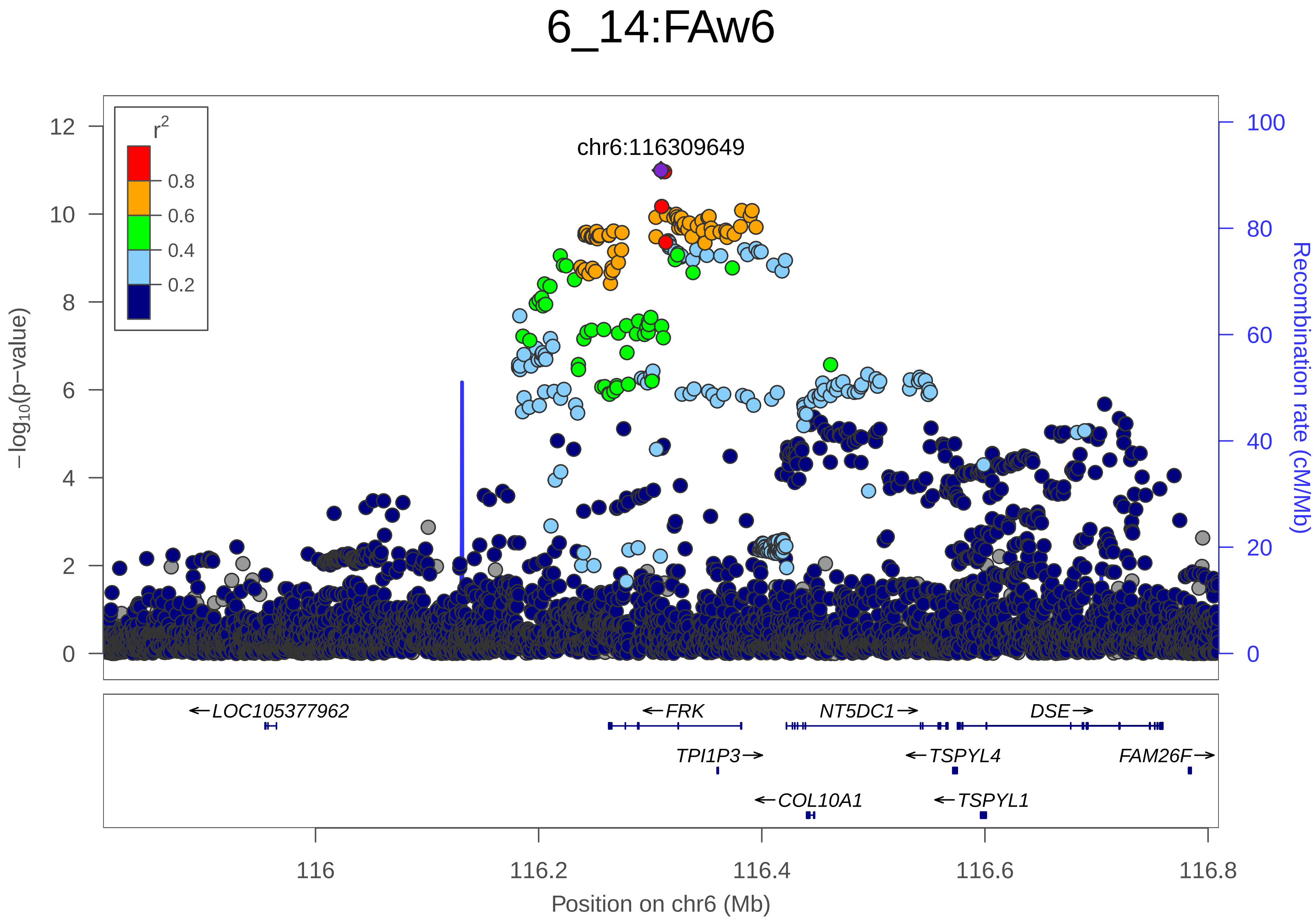

6_14:FAw6
chr6:116309649
r2
0.8
0.6
0.4
0.2
−log10(p−value)
0
2
4
6
8
10
12
Recombination rate (cM/Mb)
0
20
40
60
80
100
←LOC105377962
←FRK
NT5DC1→
DSE→
TPI1P3→
←TSPYL4
FAM26F→
←COL10A1
←TSPYL1
116
116.2
116.4
116.6
116.8
Position on chr6 (Mb)

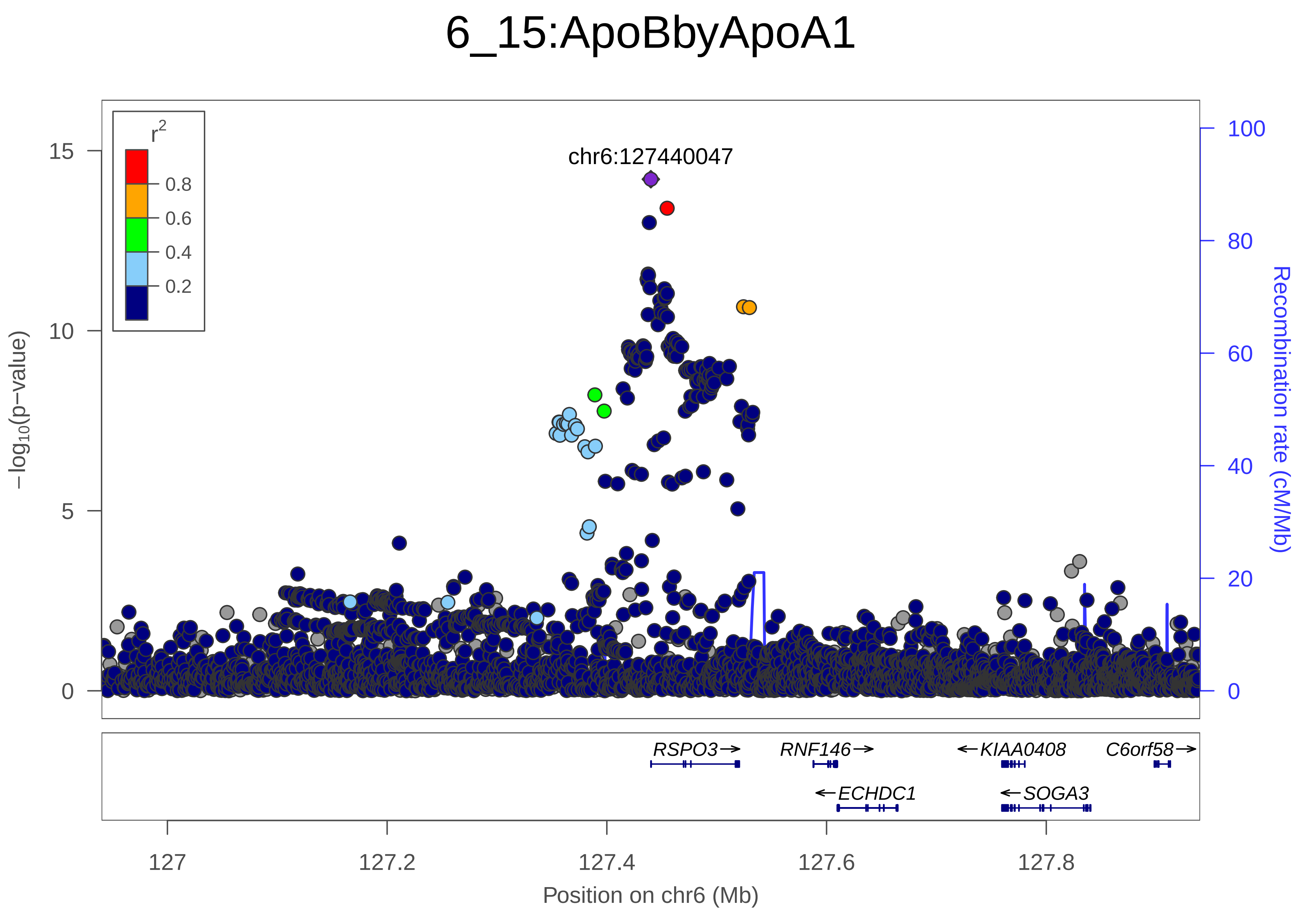

6_15:ApoBbyApoA1
chr6:127440047
r2
0.8
0.6
0.4
0.2
15
10
5
0
−log10(p−value)
100
80
60
40
20
0
Recombination rate (cM/Mb)
RSPO3→
RNF146→
←KIAA0408
C6orf58→
←ECHDC1
←SOGA3
127
127.2
127.4
127.6
127.8
Position on chr6 (Mb)

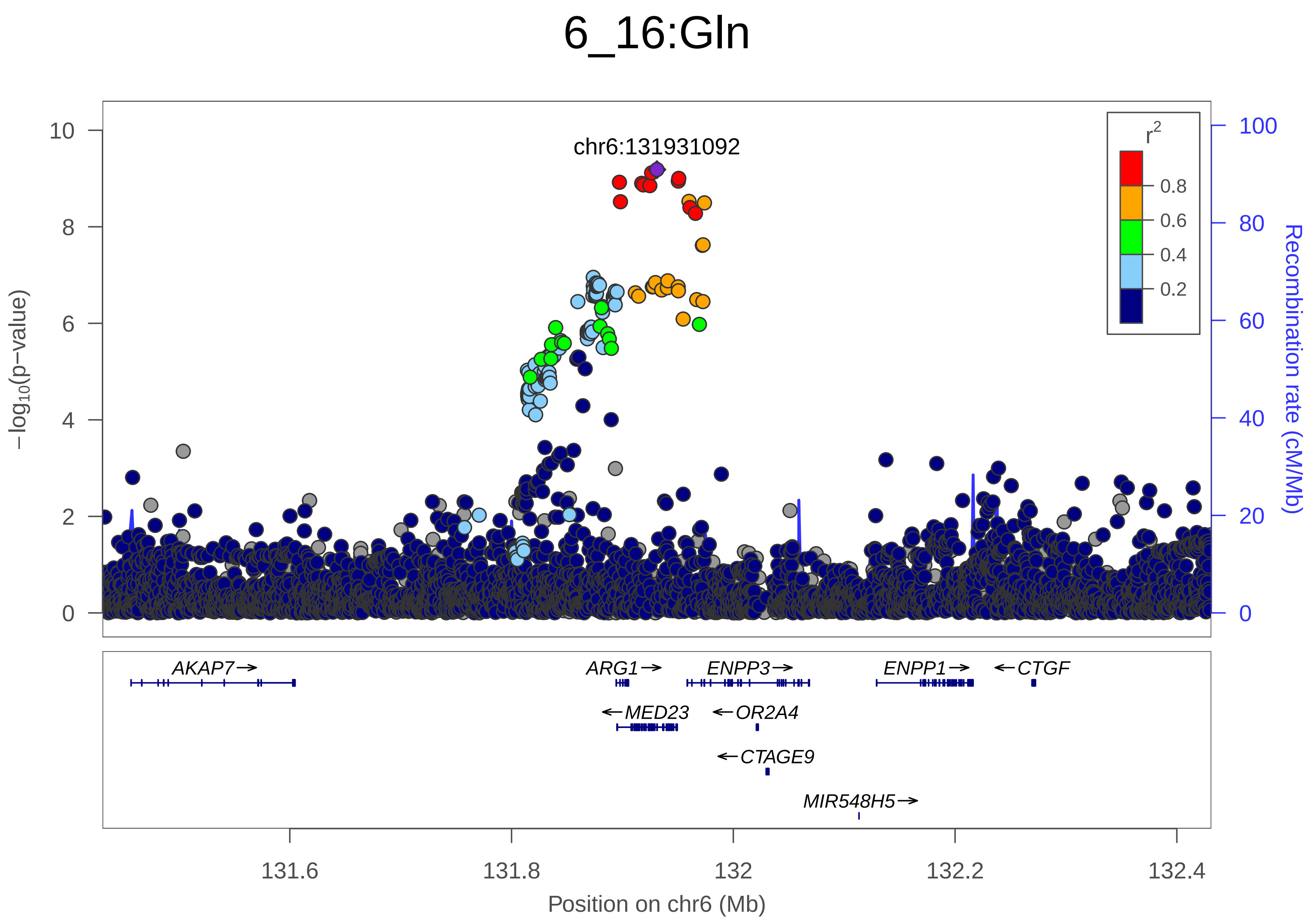

6_16:Gln
chr6:131931092
r2
0.8
0.6
0.4
0.2
−log10(p−value)
Recombination rate (cM/Mb)
AKAP7
ARG1
ENPP3
ENPP1
CTGF
MED23
OR2A4
CTAGE9
MIR548H5
131.6
131.8
132
132.2
132.4
Position on chr6 (Mb)

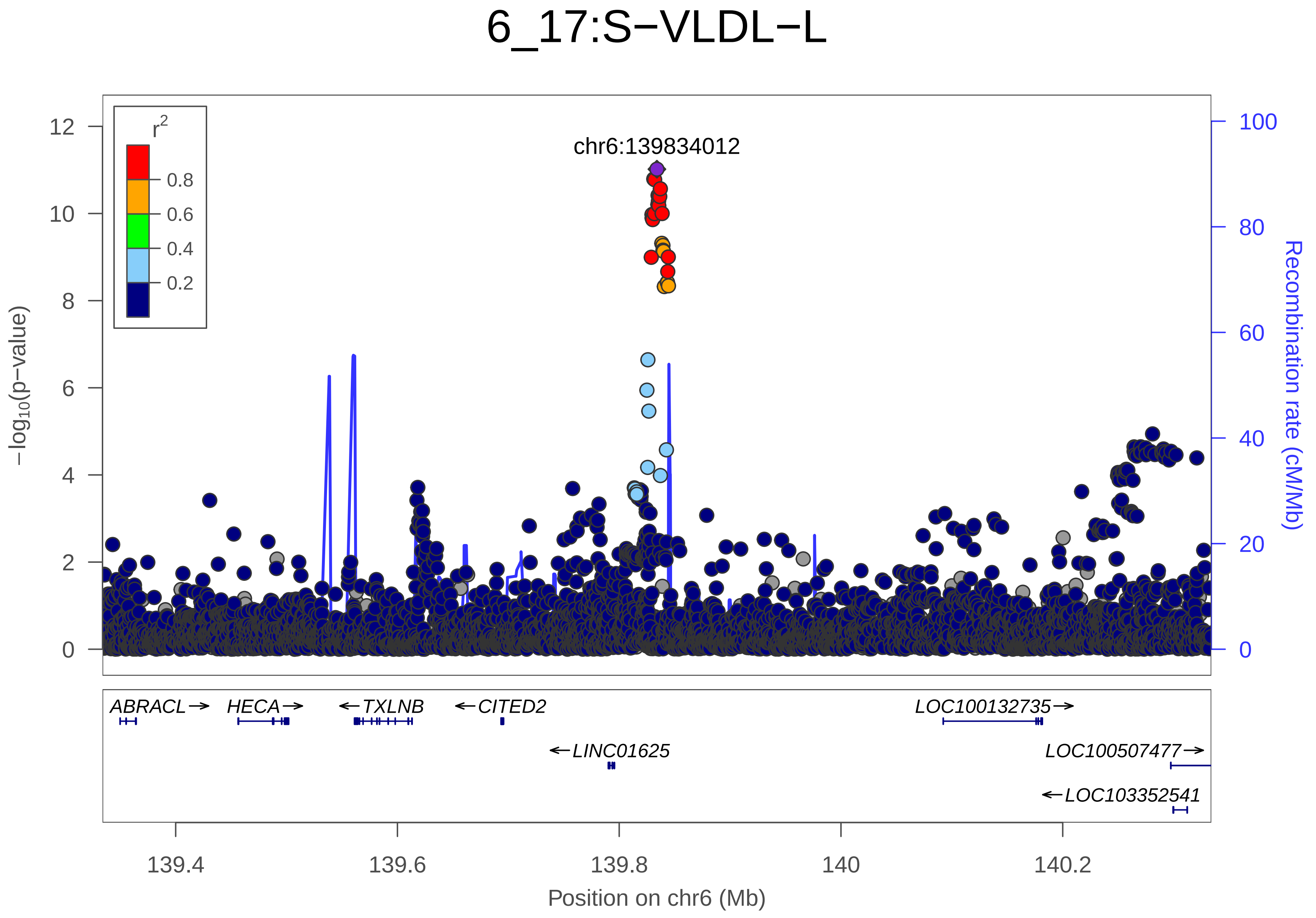

6_17:S−VLDL−L
chr6:139834012
r²
0.8
0.6
0.4
0.2
−log10(p−value)
Recombination rate (cM/Mb)
0
2
4
6
8
10
12
20
40
60
80
100
ABRACL→
HECA→
←TXLNB
←CITED2
←LINC01625
LOC100132735→
LOC100507477→
←LOC103352541
139.4
139.6
139.8
140
140.2
Position on chr6 (Mb)

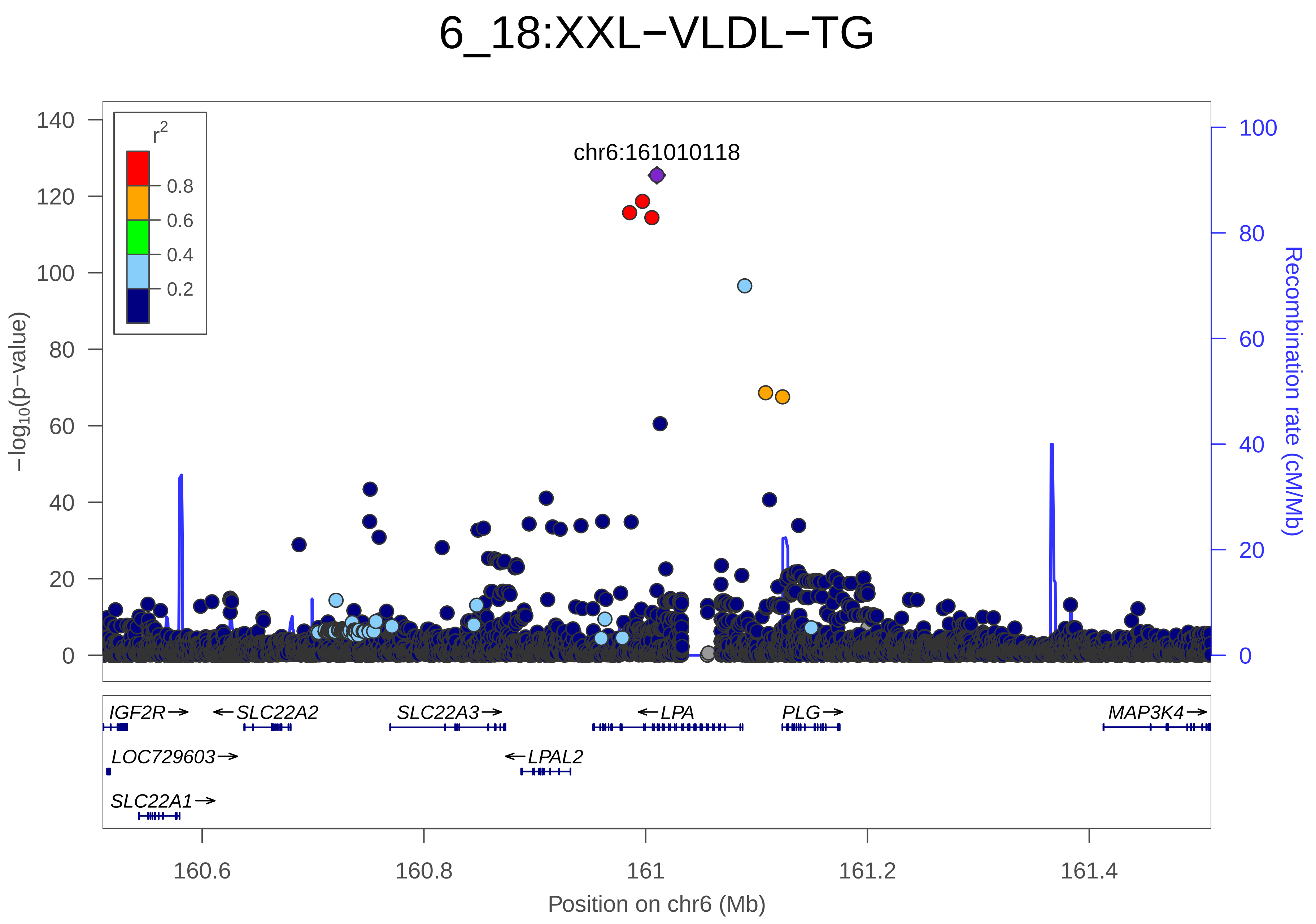

6_18:XXL−VLDL−TG
r²
0.8
0.6
0.4
0.2
chr6:161010118
−log10(p−value)
140
120
100
80
60
40
20
0
Recombination rate (cM/Mb)
100
80
60
40
20
0
IGF2R→
←SLC22A2
SLC22A3→
←LPA
PLG→
MAP3K4→
LOC729603→
←LPAL2
SLC22A1→
160.6
160.8
161
161.2
161.4
Position on chr6 (Mb)

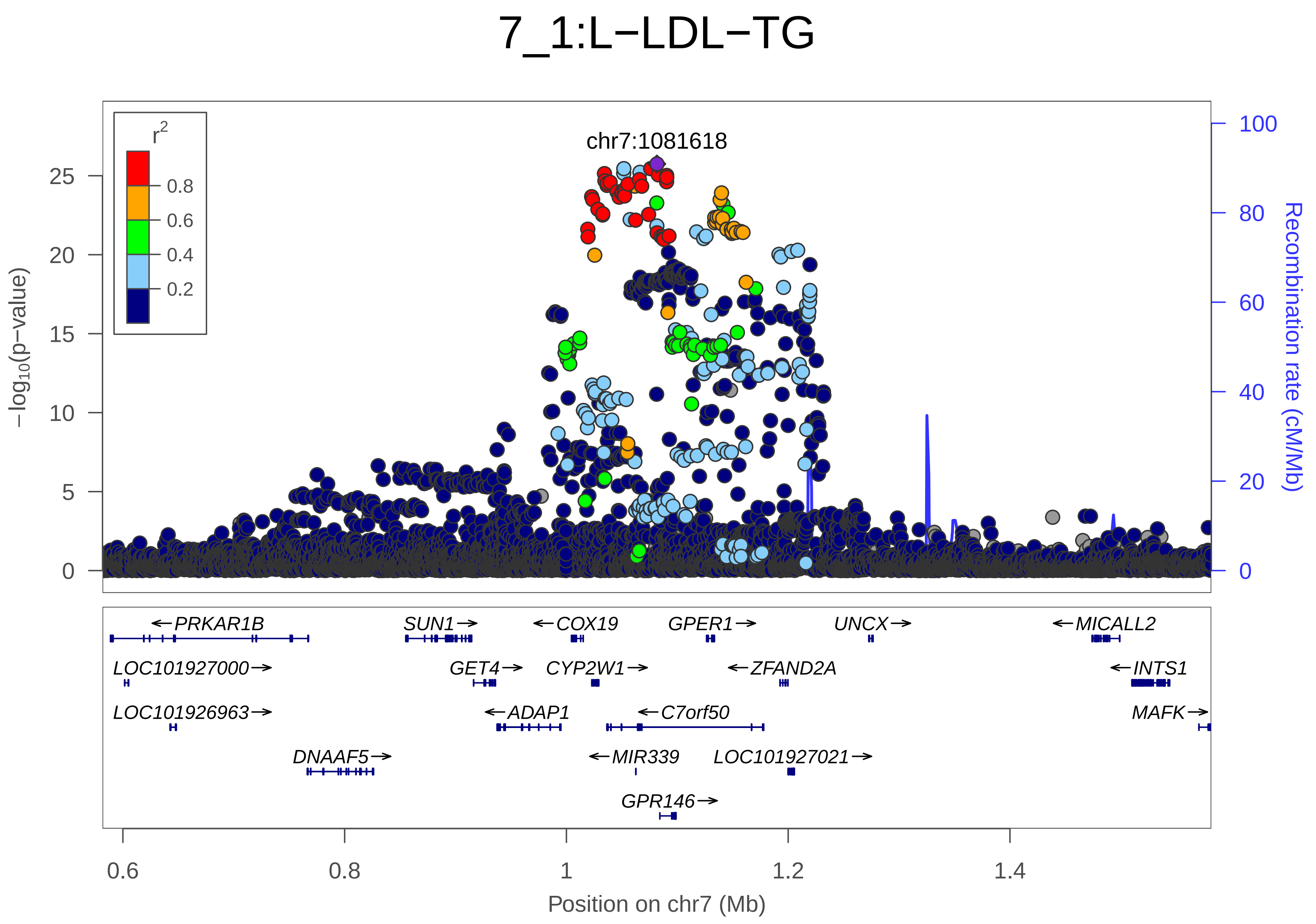

7_1:L−LDL−TG
chr7:1081618
r2
0.8
0.6
0.4
0.2
−log10(p−value)
Recombination rate (cM/Mb)
0
5
10
15
20
25
0
20
40
60
80
100
←PRKAR1B
SUN1→
←COX19
GPER1→
UNCX→
←MICALL2
LOC101927000→
GET4→
CYP2W1→
←ZFAND2A
←INTS1
LOC101926963→
←ADAP1
←C7orf50
MAFK→
DNAAF5→
←MIR339
LOC101927021→
GPR146→
0.6
0.8
1
1.2
1.4
Position on chr7 (Mb)

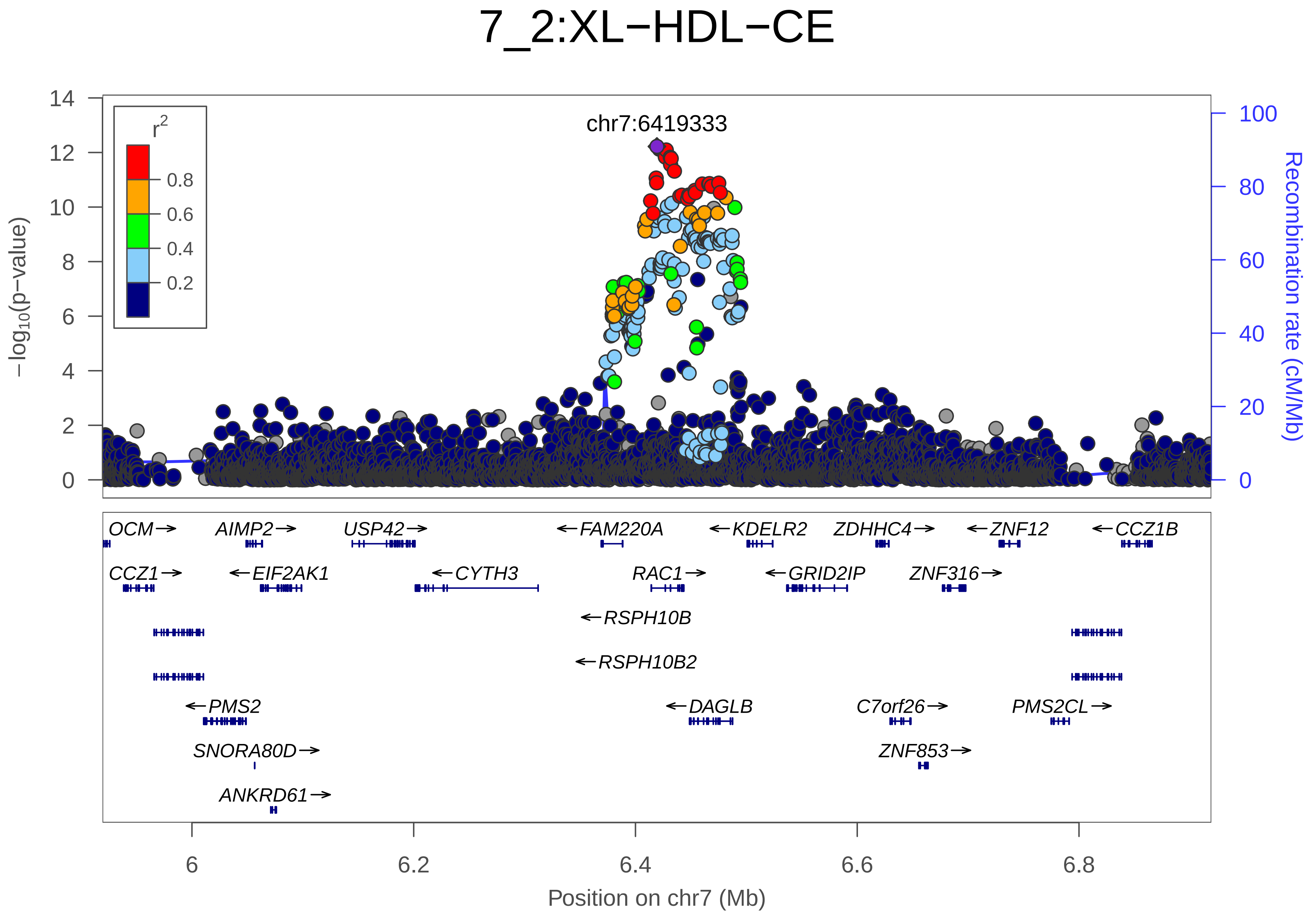
7_2:XL−HDL−CE
chr7:6419333
r²
0.8
0.6
0.4
0.2
−log10(p−value)
Recombination rate (cM/Mb)
OCM→
AIMP2→
USP42→
←FAM220A
←KDELR2
ZDHHC4→
←ZNF12
←CCZ1B
CCZ1→
←EIF2AK1
←CYTH3
RAC1→
←GRID2IP
ZNF316→
←RSPH10B
←RSPH10B2
←PMS2
←DAGLB
C7orf26→
PMS2CL→
SNORA80D→
ZNF853→
ANKRD61→
Position on chr7 (Mb)

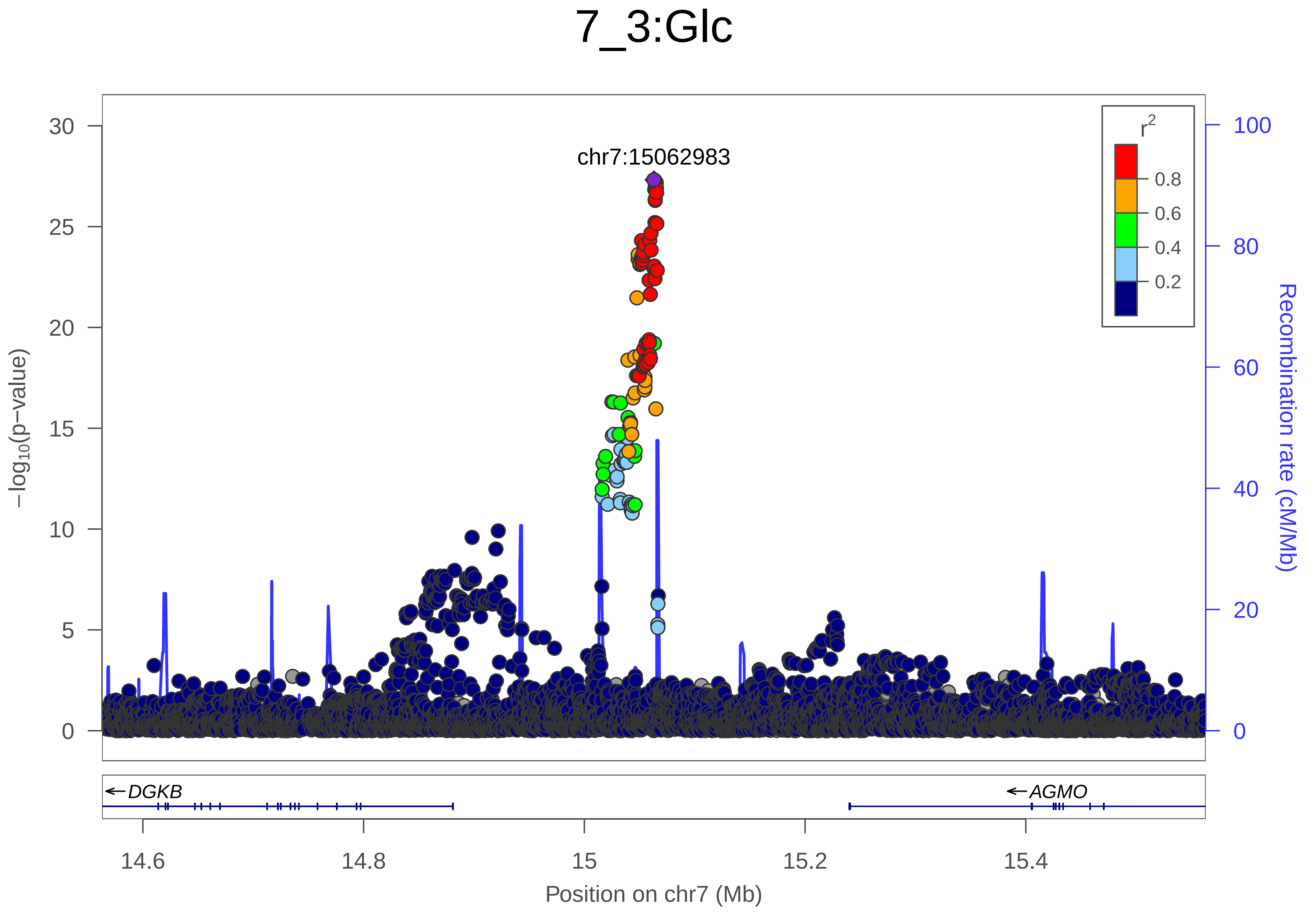

7_3:Glc
chr7:15062983
r2
0.8
0.6
0.4
0.2
−log10(p−value)
Recombination rate (cM/Mb)
30
25
20
15
10
5
0
100
80
60
40
20
0
DGKB
AGMO
14.6
14.8
15
15.2
15.4
Position on chr7 (Mb)

7_4:HDL2−C

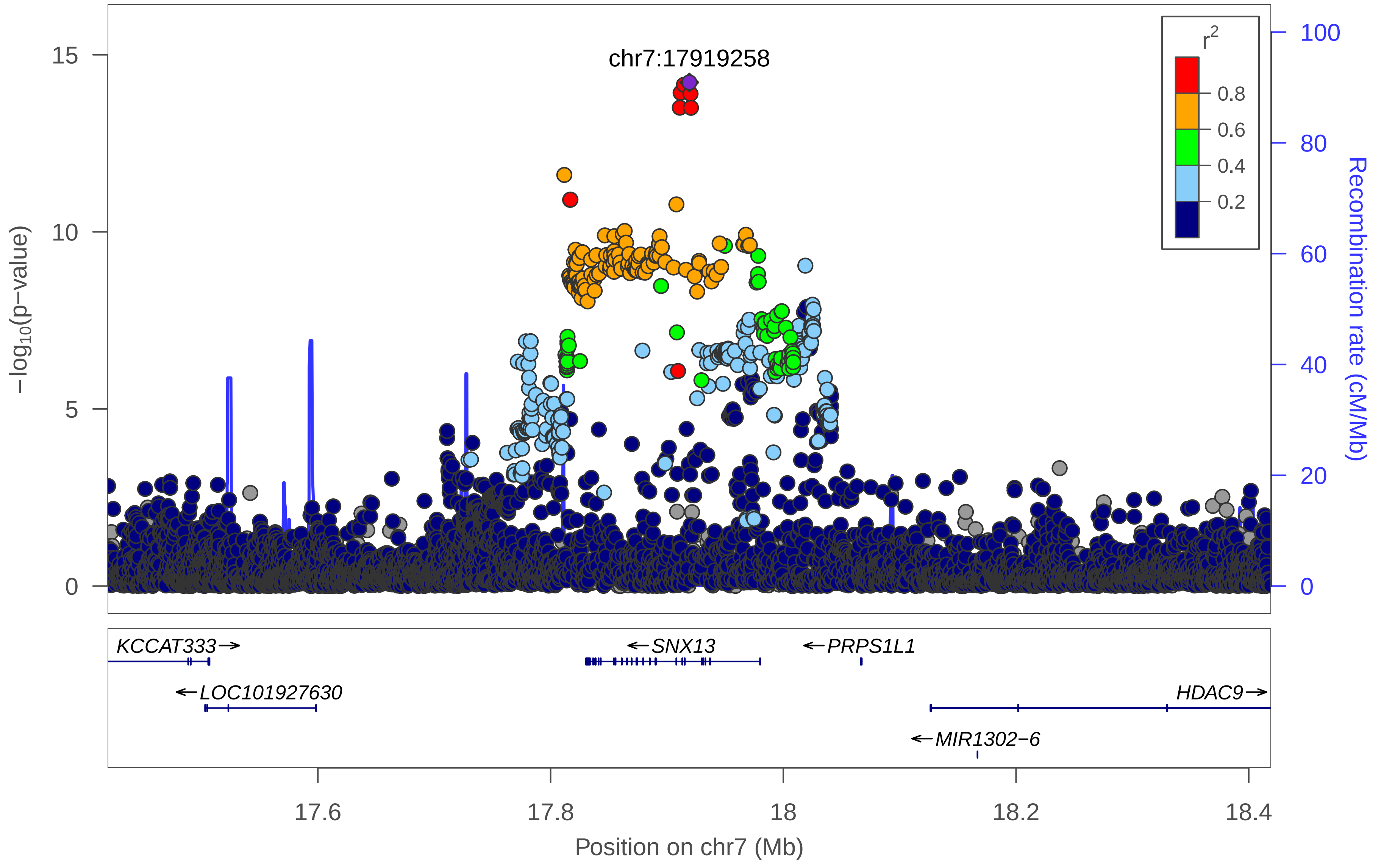
chr7:17919258
−log10(p−value)
15
10
5
0
r2
0.8
0.6
0.4
0.2
100
80
60
40
20
0
Recombination rate (cM/Mb)
KCCAT333→
←LOC101927630
←SNX13
←PRPS1L1
HDAC9→
←MIR1302−6
17.6
17.8
18
18.2
18.4
Position on chr7 (Mb)

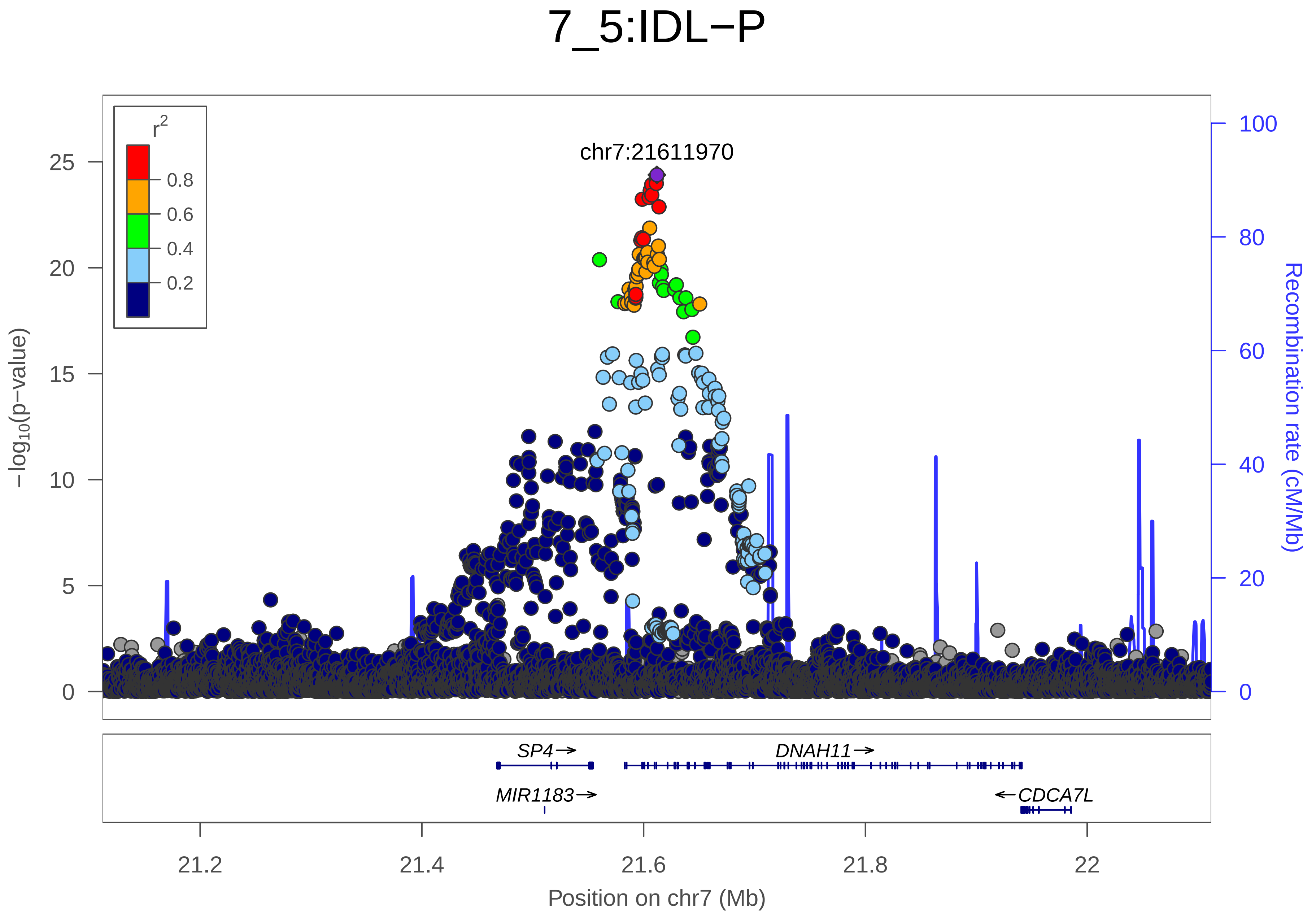

7_5:IDL−P
chr7:21611970
r2
0.8
0.6
0.4
0.2
−log10(p−value)
25
20
15
10
5
0
Recombination rate (cM/Mb)
100
80
60
40
20
0
SP4→
DNAH11→
MIR1183→
←CDCA7L
21.2
21.4
21.6
21.8
22
Position on chr7 (Mb)

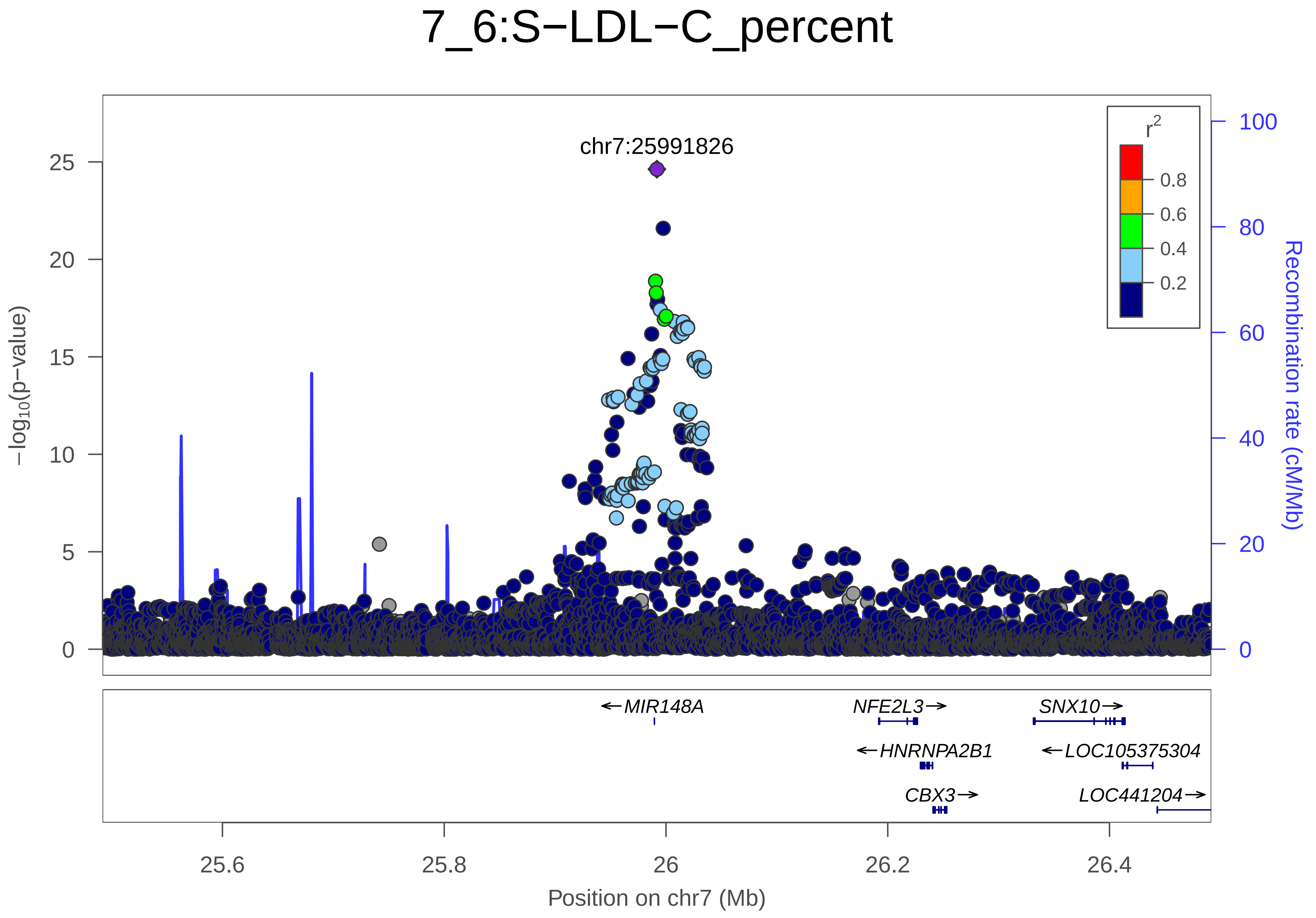

7_6:S−LDL−C_percent
chr7:25991826
r2
0.8
0.6
0.4
0.2
−log10(p−value)
0
5
10
15
20
25
Recombination rate (cM/Mb)
0
20
40
60
80
100
←MIR148A
NFE2L3→
SNX10→
←HNRNPA2B1
←LOC105375304
CBX3→
LOC441204→
25.6
25.8
26
26.2
26.4
Position on chr7 (Mb)

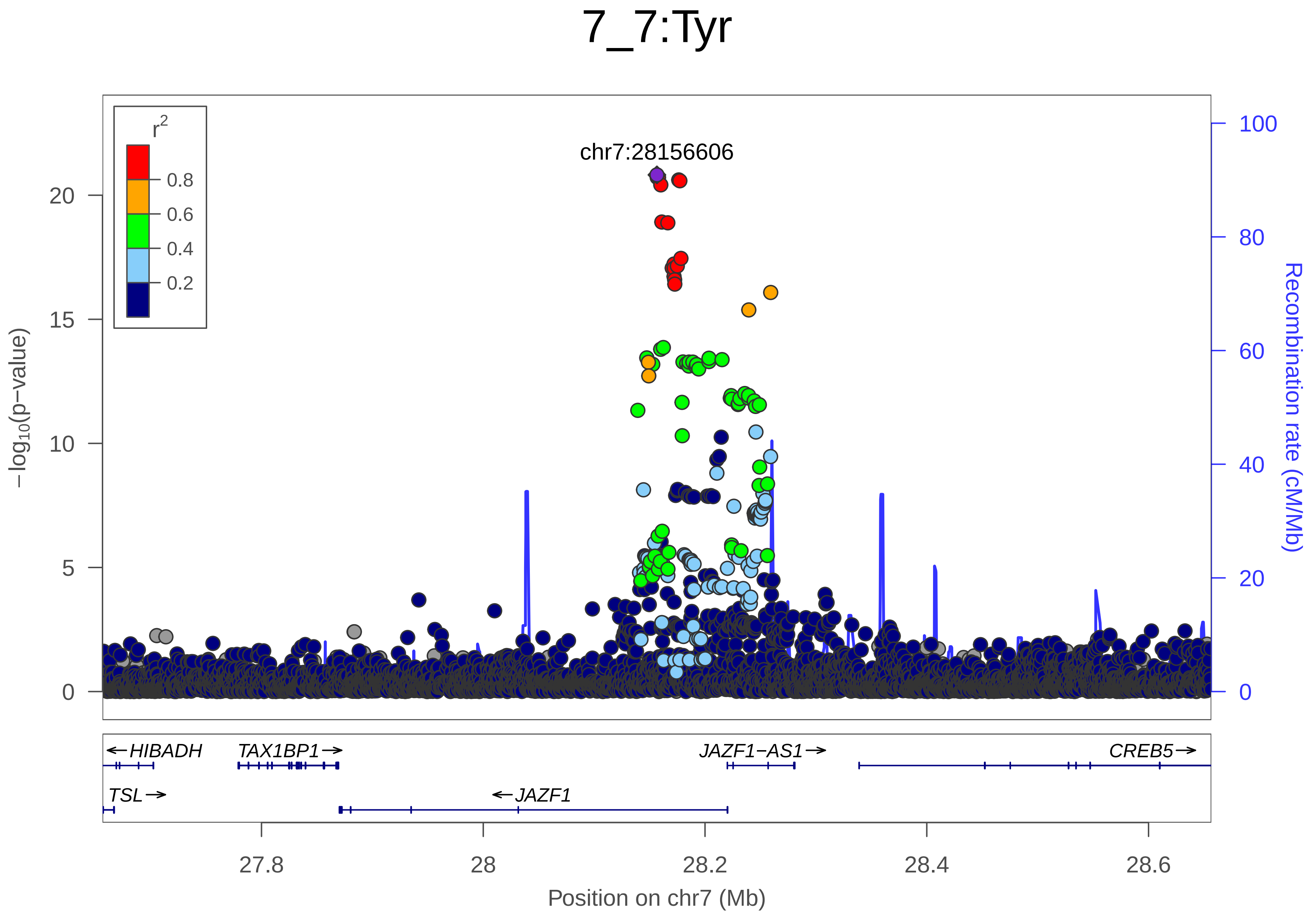
7_7:Tyr
r2
0.8
0.6
0.4
0.2
chr7:28156606
−log10(p−value)
0
5
10
15
20
Recombination rate (cM/Mb)
0
20
40
60
80
100
←HIBADH
TAX1BP1→
JAZF1−AS1→
CREB5→
TSL→
←JAZF1
27.8
28
28.2
28.4
28.6
Position on chr7 (Mb)

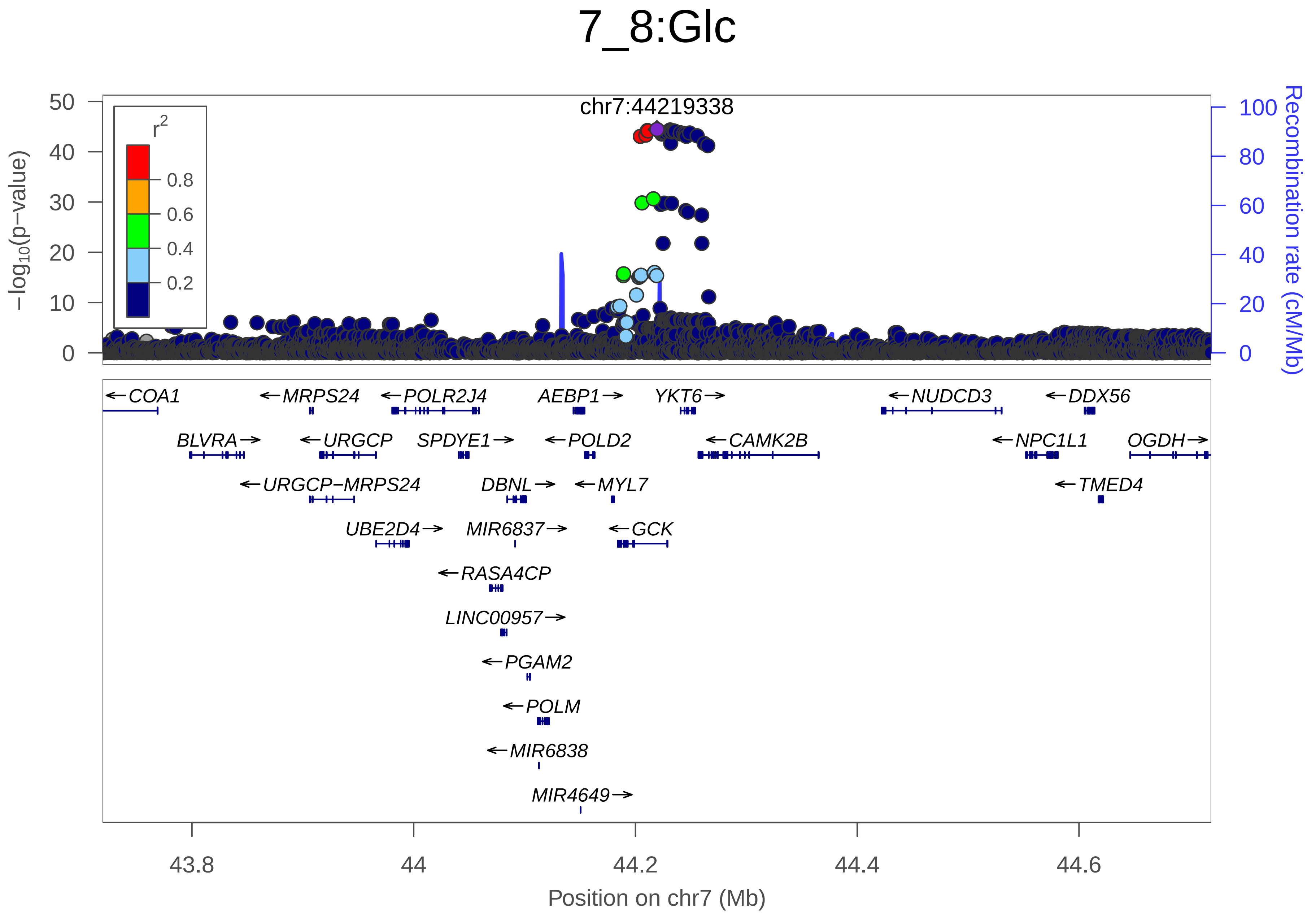

7_8:Glc
chr7:44219338
r2
0.8
0.6
0.4
0.2
−log10(p−value)
Recombination rate (cM/Mb)
0
10
20
30
40
50
0
20
40
60
80
100
←COA1
←MRPS24
←POLR2J4
AEBP1→
YKT6→
←NUDCD3
←DDX56
BLVRA→
←URGCP
SPDYE1→
←POLD2
←CAMK2B
←NPC1L1
OGDH→
←URGCP−MRPS24
DBNL→
←MYL7
←TMED4
UBE2D4→
MIR6837→
←GCK
←RASA4CP
LINC00957→
←PGAM2
←POLM
←MIR6838
MIR4649→
43.8
44
44.2
44.4
44.6
Position on chr7 (Mb)

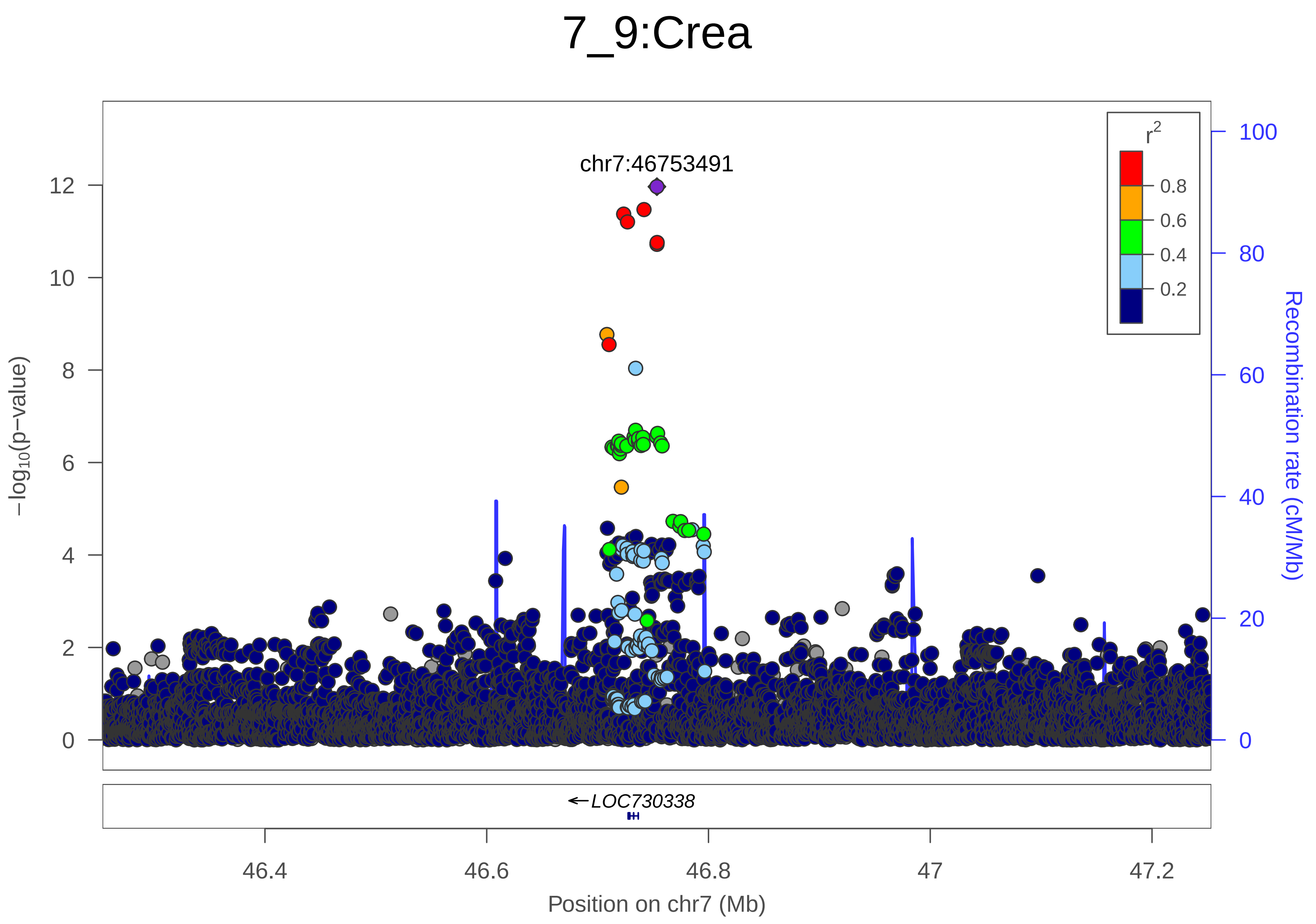
7_9:Crea
chr7:46753491
r2
0.8
0.6
0.4
0.2
−log10(p−value)
Recombination rate (cM/Mb)
0
2
4
6
8
10
12
0
20
40
60
80
100
LOC730338
46.4
46.6
46.8
47
47.2
Position on chr7 (Mb)

7_10:Glc

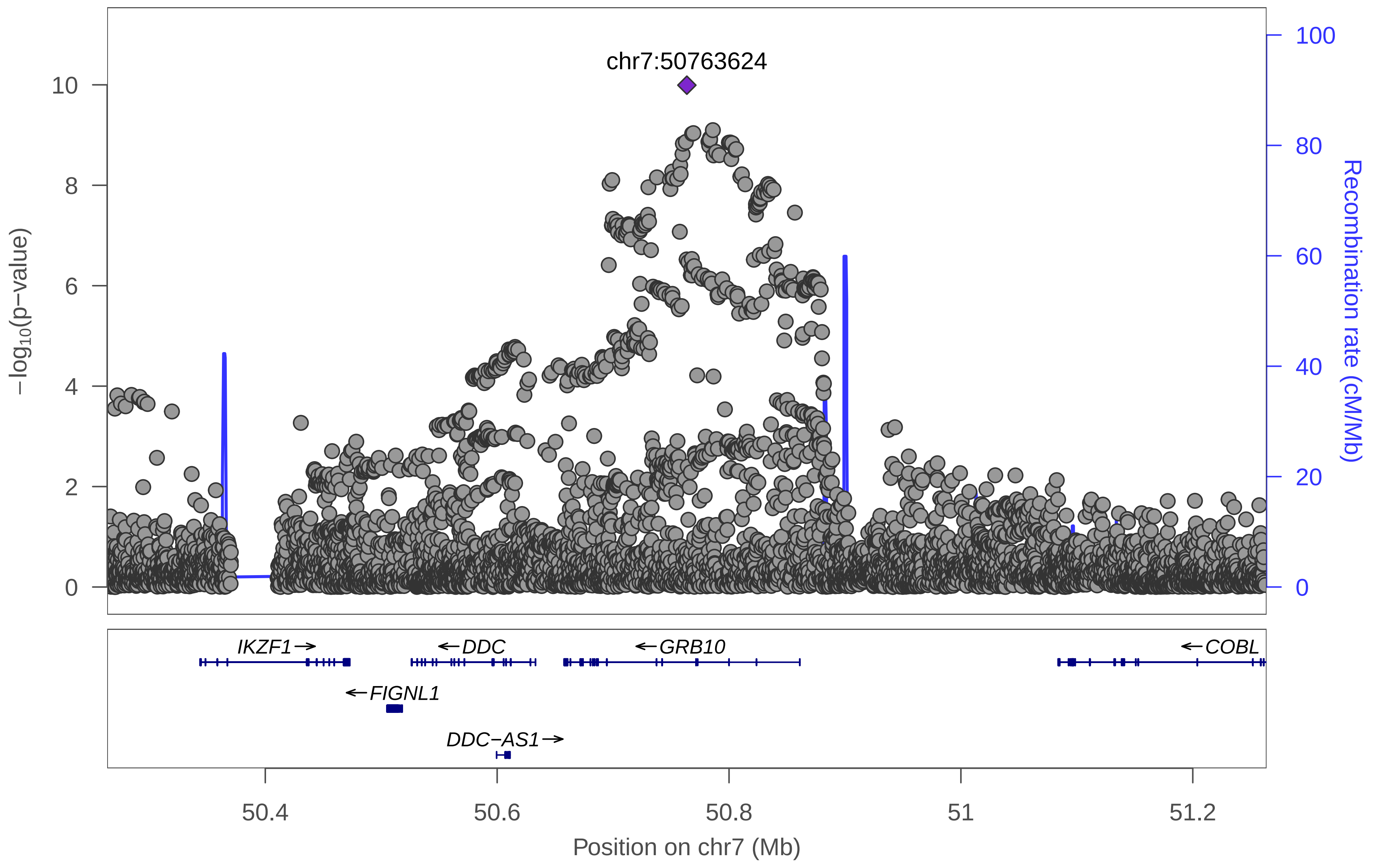

chr7:50763624
−log10(p−value)
Recombination rate (cM/Mb)
0
2
4
6
8
10
0
20
40
60
80
100
IKZF1→
←DDC
←GRB10
←COBL
←FIGNL1
DDC−AS1→
50.4
50.6
50.8
51
51.2
Position on chr7 (Mb)

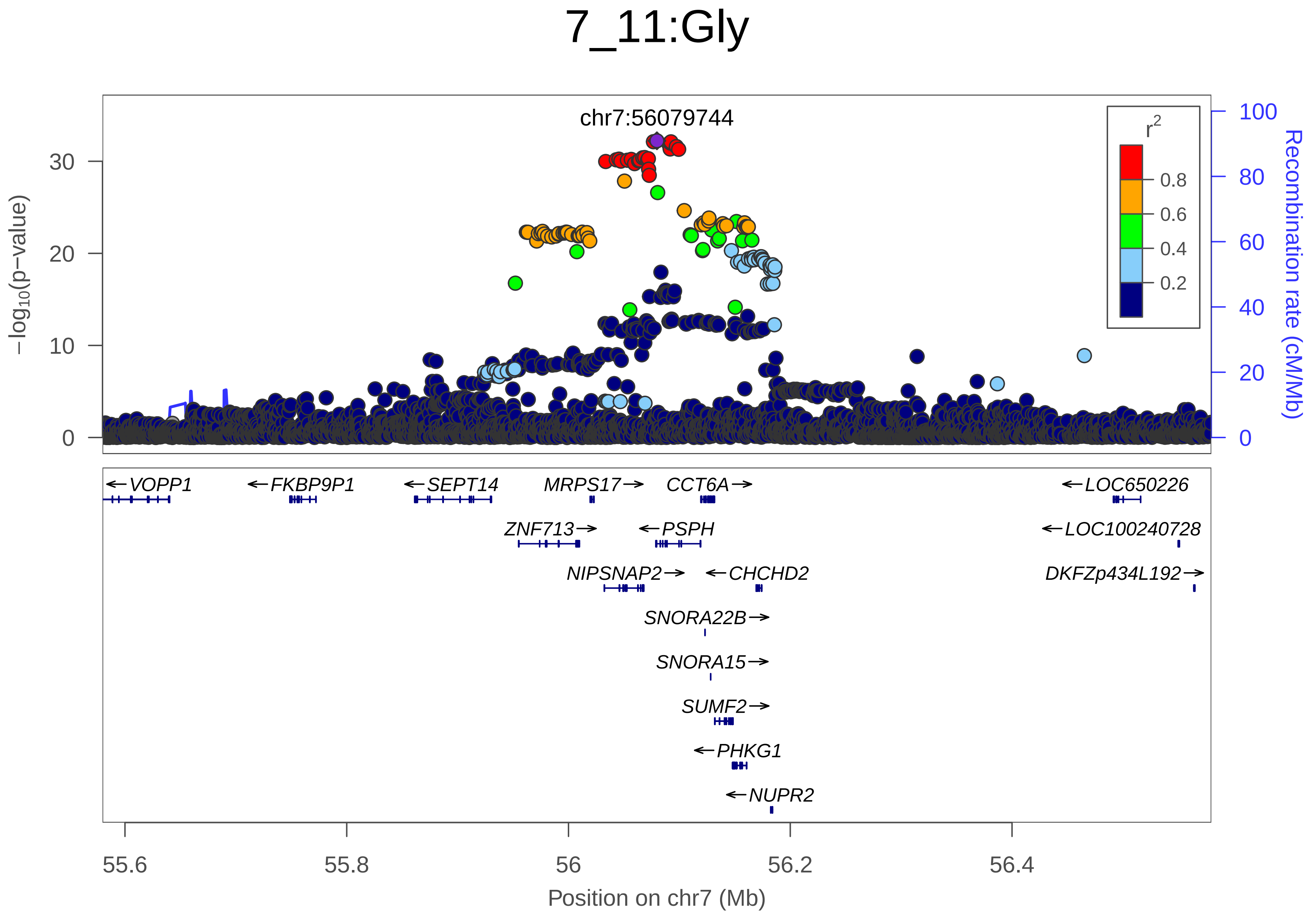

7_11:Gly
chr7:56079744
−log10(p−value)
Recombination rate (cM/Mb)
r2
0.8
0.6
0.4
0.2
0
10
20
30
0
20
40
60
80
100
VOPP1
FKBP9P1
SEPT14
MRPS17
CCT6A
LOC650226
ZNF713
PSPH
LOC100240728
NIPSNAP2
CHCHD2
DKFZp434L192
SNORA22B
SNORA15
SUMF2
PHKG1
NUPR2
55.6
55.8
56
56.2
56.4
Position on chr7 (Mb)

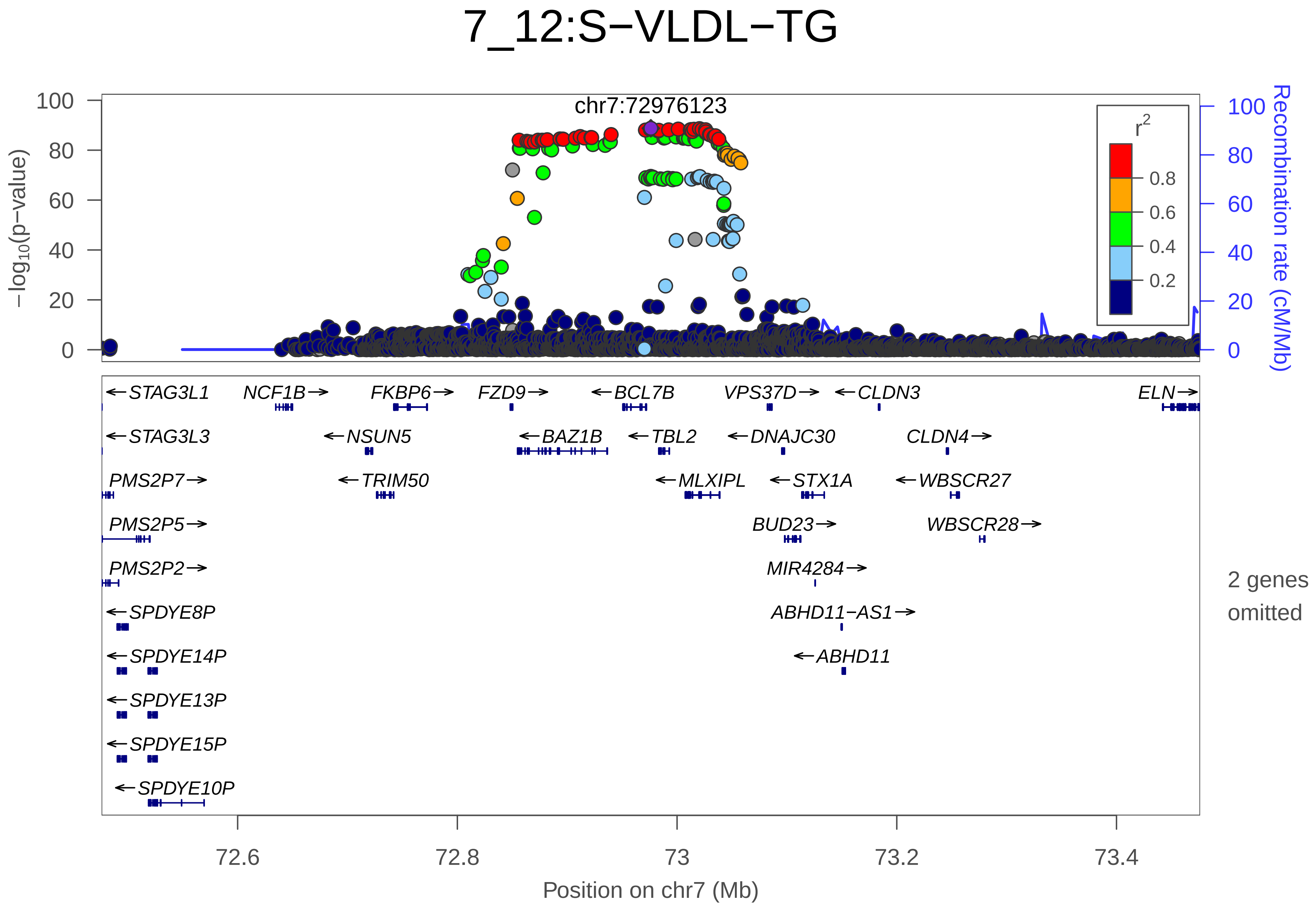
7_12:S−VLDL−TG
chr7:72976123
−log10(p−value)
Recombination rate (cM/Mb)
r2
0.8
0.6
0.4
0.2
STAG3L1
NCF1B
FKBP6
FZD9
BCL7B
VPS37D
CLDN3
ELN
STAG3L3
NSUN5
BAZ1B
TBL2
DNAJC30
CLDN4
PMS2P7
TRIM50
MLXIPL
STX1A
WBSCR27
PMS2P5
BUD23
WBSCR28
PMS2P2
MIR4284
SPDYE8P
ABHD11−AS1
SPDYE14P
ABHD11
SPDYE13P
SPDYE15P
SPDYE10P
2 genes omitted
72.6
72.8
73
73.2
73.4
Position on chr7 (Mb)

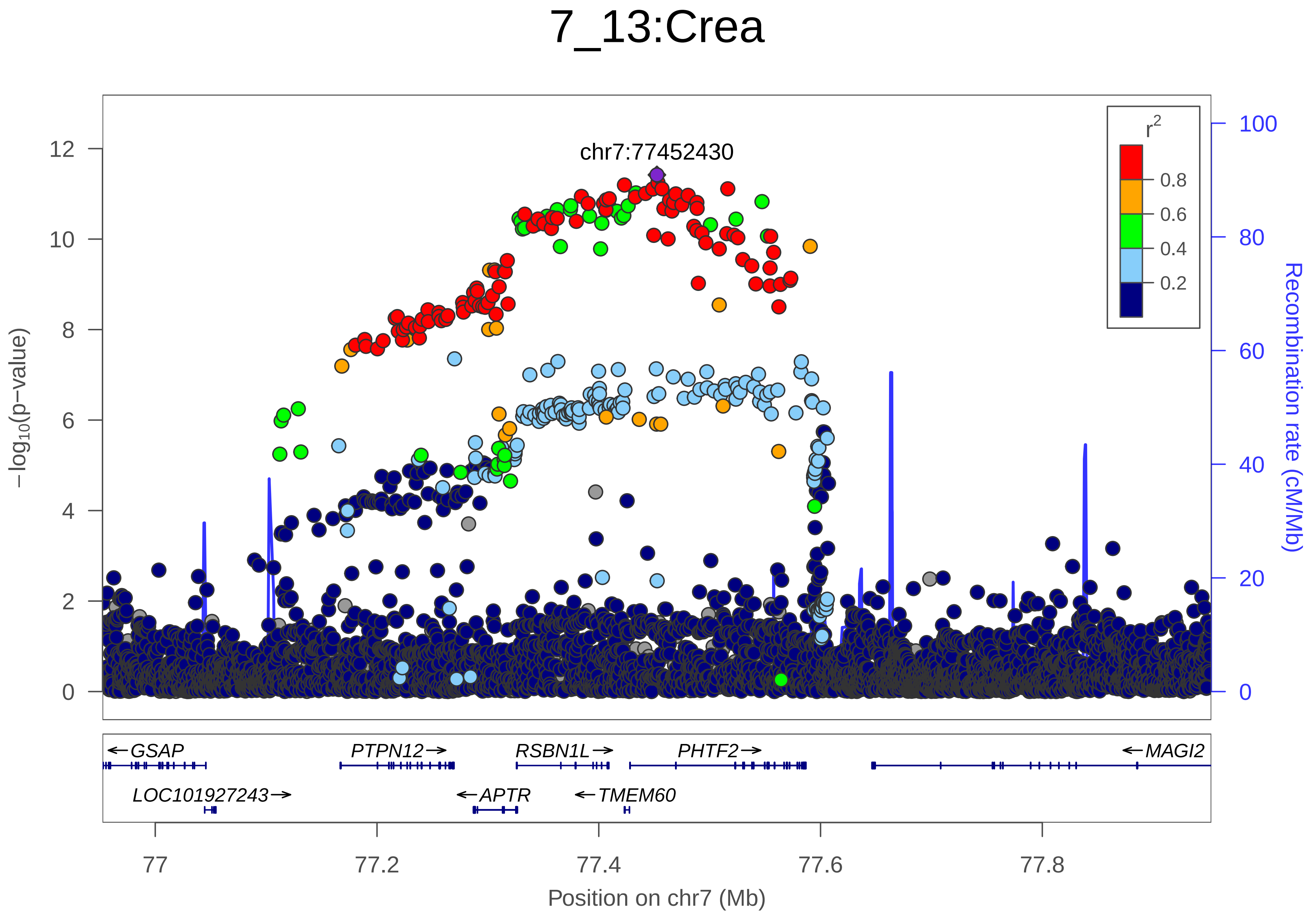

7_13:Crea
chr7:77452430
r2
0.8
0.6
0.4
0.2
−log10(p−value)
Recombination rate (cM/Mb)
12
10
8
6
4
2
0
100
80
60
40
20
0
GSAP
PTPN12
RSBN1L
PHTF2
MAGI2
LOC101927243
APTR
TMEM60
77
77.2
77.4
77.6
77.8
Position on chr7 (Mb)

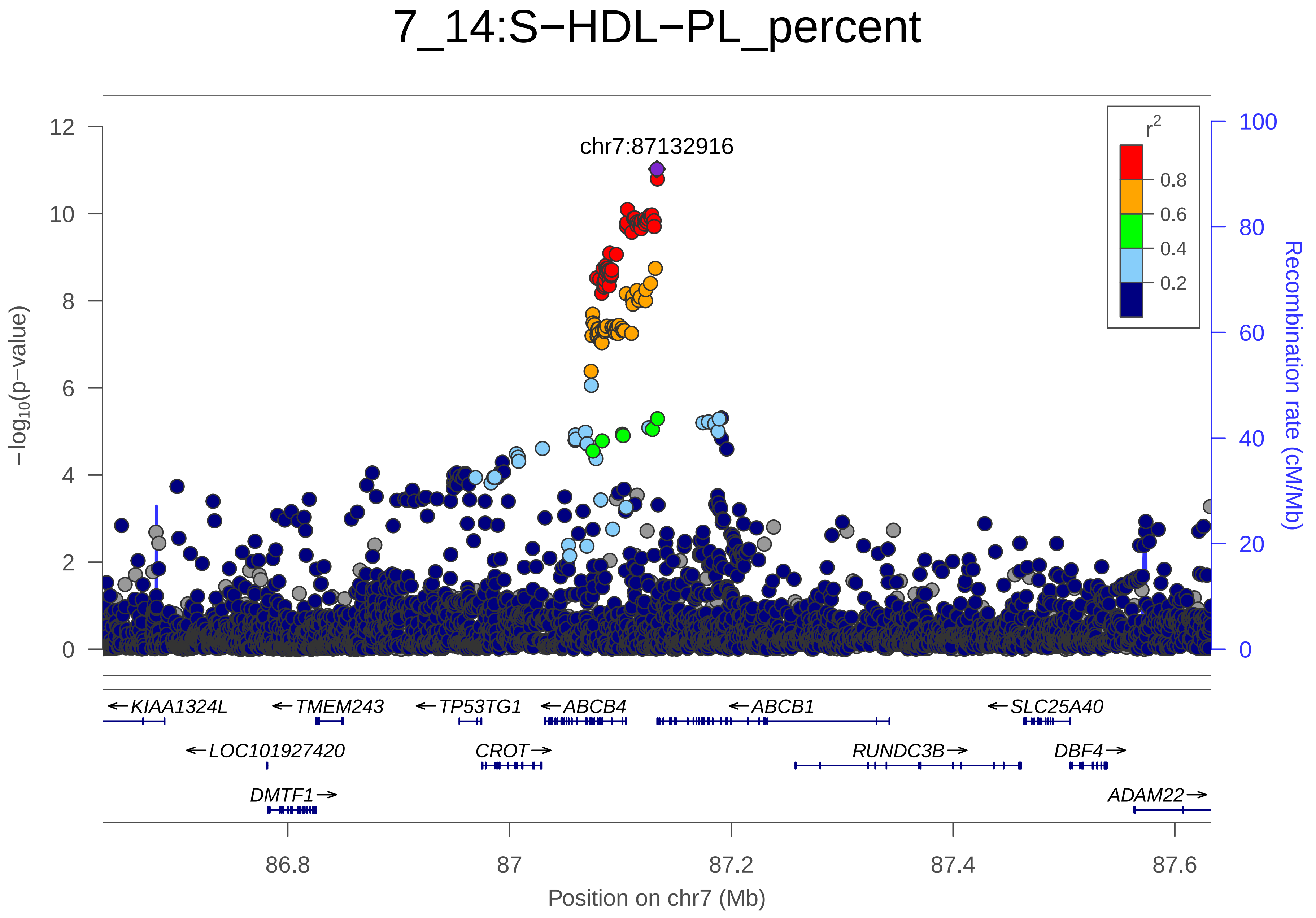

7_14:S−HDL−PL_percent
chr7:87132916
r2
0.8
0.6
0.4
0.2
12
10
8
6
4
2
0
100
80
60
40
20
0
−log10(p−value)
Recombination rate (cM/Mb)
KIAA1324L
TMEM243
TP53TG1
ABCB4
ABCB1
SLC25A40
LOC101927420
CROT
RUNDC3B
DBF4
DMTF1
ADAM22
86.8
87
87.2
87.4
87.6
Position on chr7 (Mb)

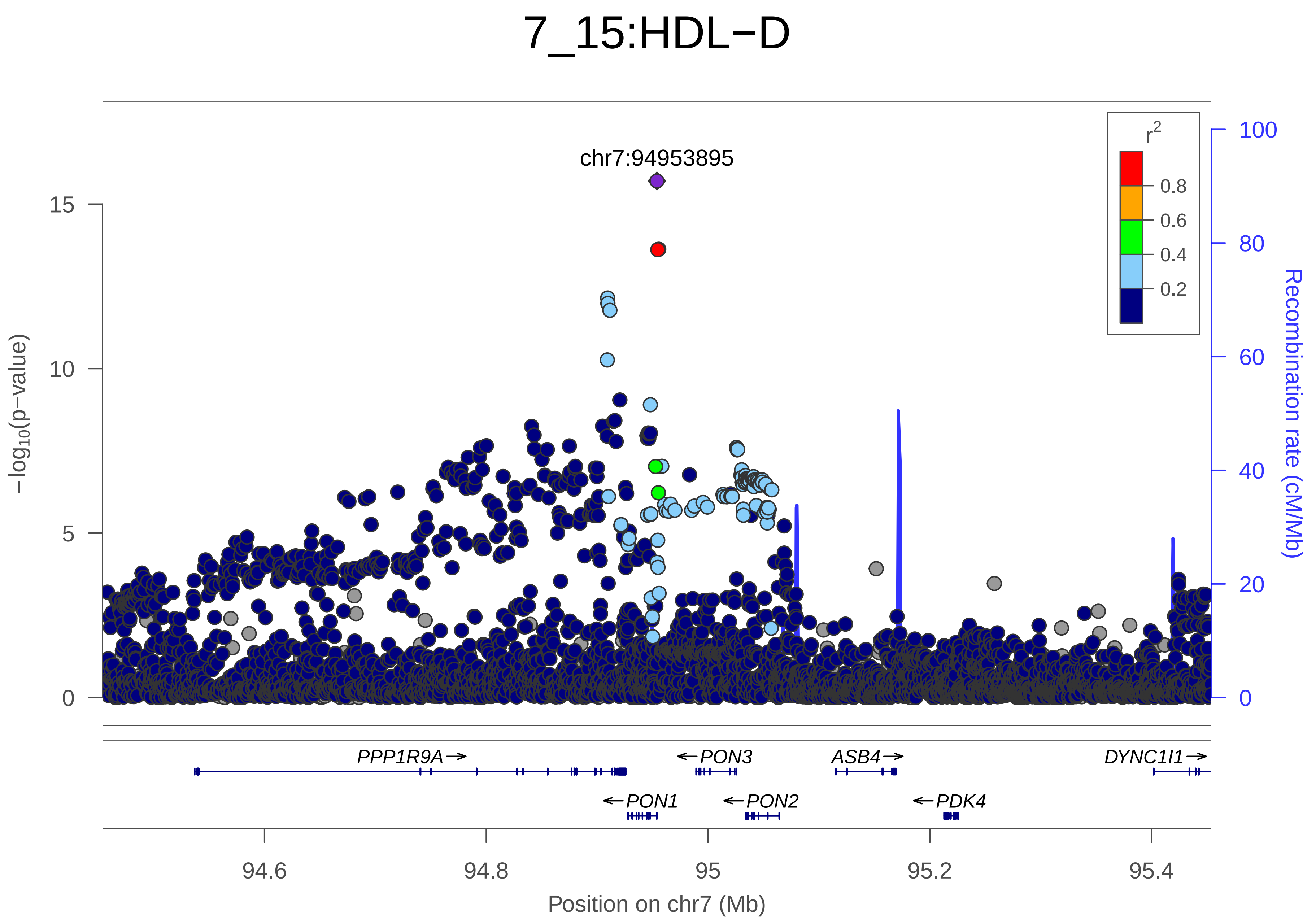

7_15:HDL−D
chr7:94953895
r2
0.8
0.6
0.4
0.2
−log10(p−value)
0
5
10
15
Recombination rate (cM/Mb)
0
20
40
60
80
100
PPP1R9A→
←PON3
ASB4→
DYNC1I1→
←PON1
←PON2
←PDK4
94.6
94.8
95
95.2
95.4
Position on chr7 (Mb)

7_16:ApoBbyApoA1

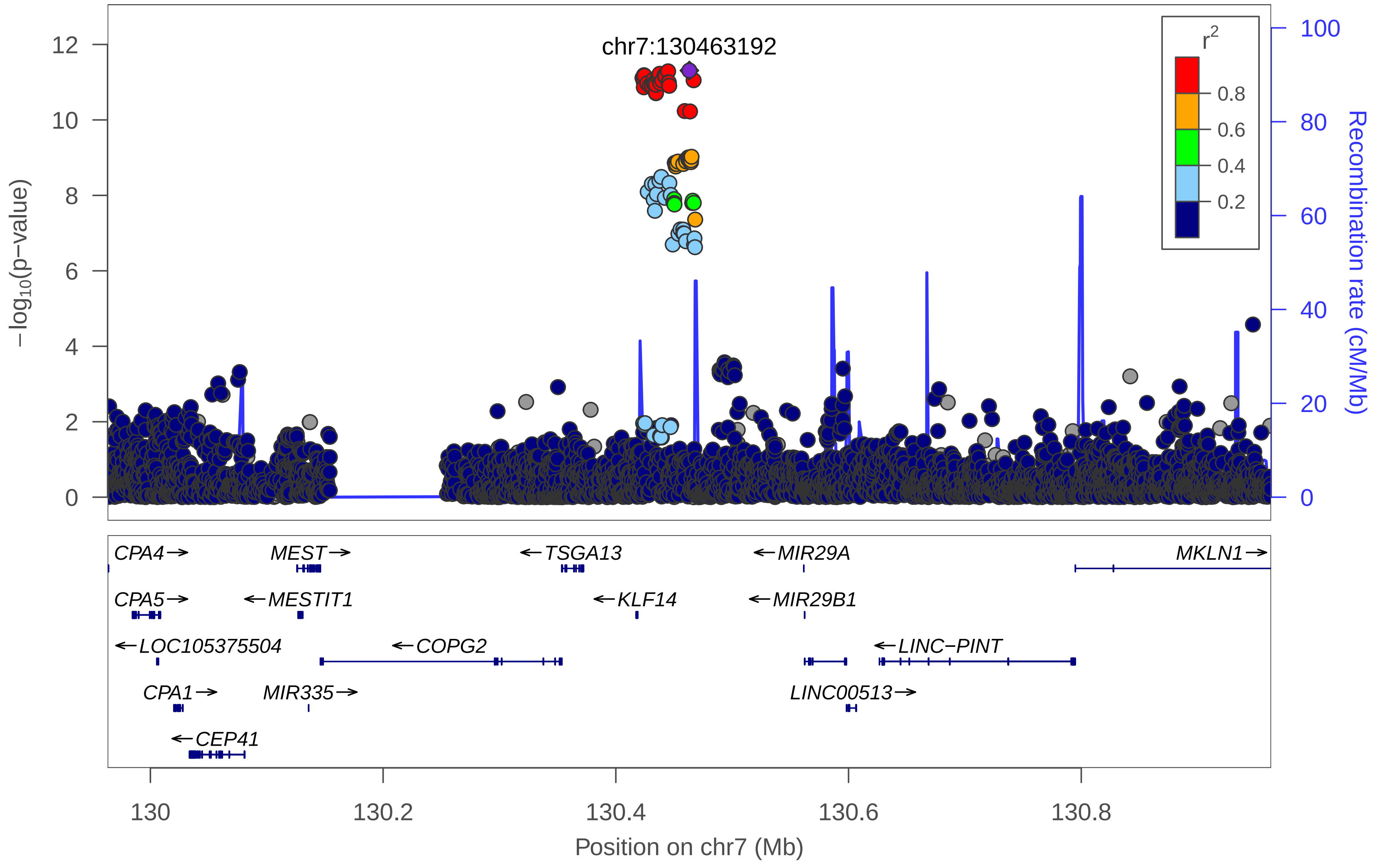

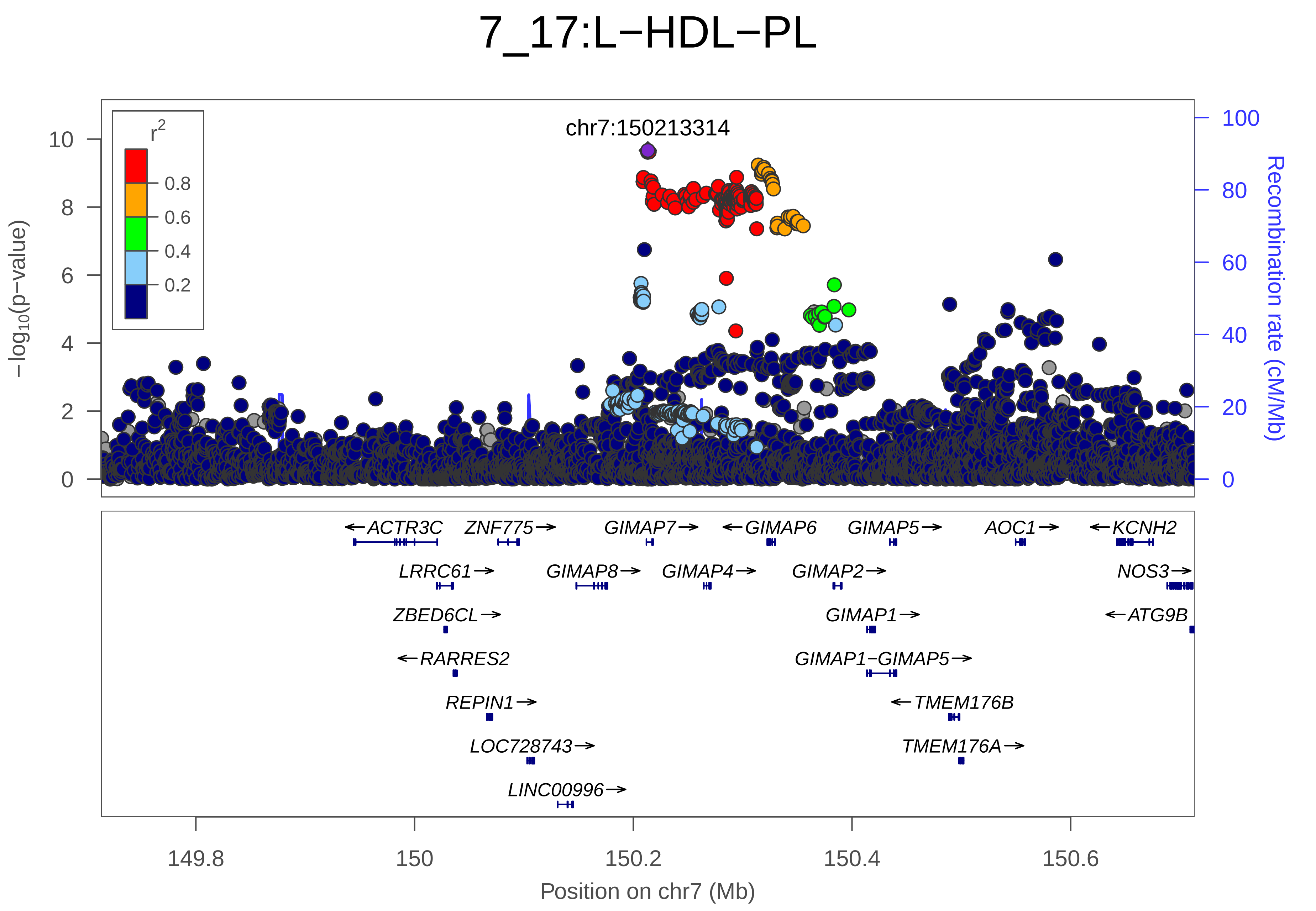
7_17:L−HDL−PL
chr7:150213314
r2
0.8
0.6
0.4
0.2
−log10(p−value)
Recombination rate (cM/Mb)
0
2
4
6
8
10
20
40
60
80
100
←ACTR3C
ZNF775→
GIMAP7→
←GIMAP6
GIMAP5→
AOC1→
←KCNH2
LRRC61→
GIMAP8→
GIMAP4→
GIMAP2→
NOS3→
ZBED6CL→
GIMAP1→
←ATG9B
←RARRES2
GIMAP1−GIMAP5→
REPIN1→
←TMEM176B
LOC728743→
TMEM176A→
LINC00996→
149.8
150
150.2
150.4
150.6
Position on chr7 (Mb)

7_18:Crea

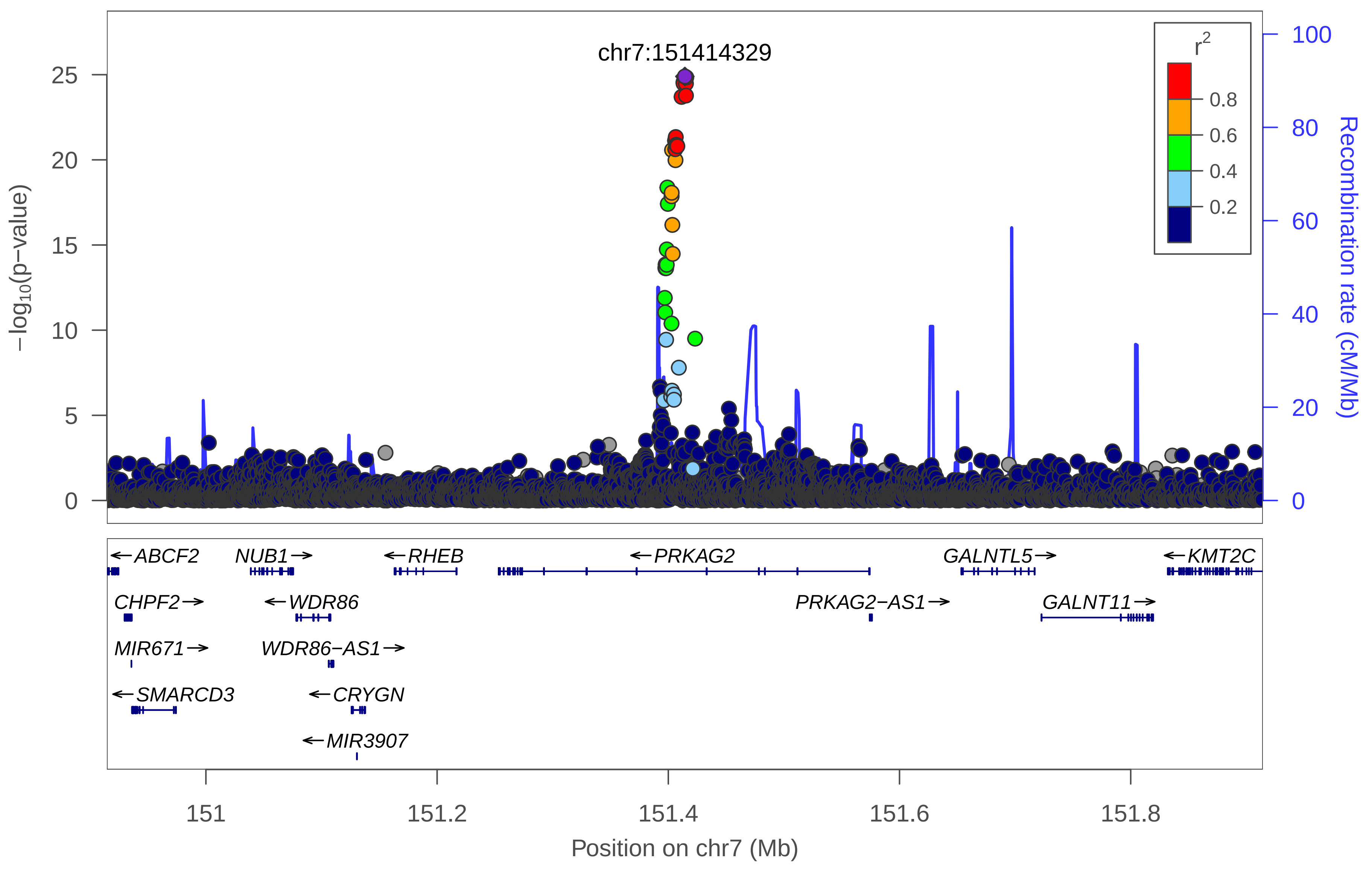

chr7:151414329
−log10(p−value)
Recombination rate (cM/Mb)
r2
0.8
0.6
0.4
0.2
25
20
15
10
5
0
100
80
60
40
20
0
←ABCF2
NUB1→
←RHEB
←PRKAG2
GALNTL5→
←KMT2C
CHPF2→
←WDR86
PRKAG2−AS1→
GALNT11→
MIR671→
WDR86−AS1→
←SMARCD3
←CRYGN
←MIR3907
151
151.2
151.4
151.6
151.8
Position on chr7 (Mb)

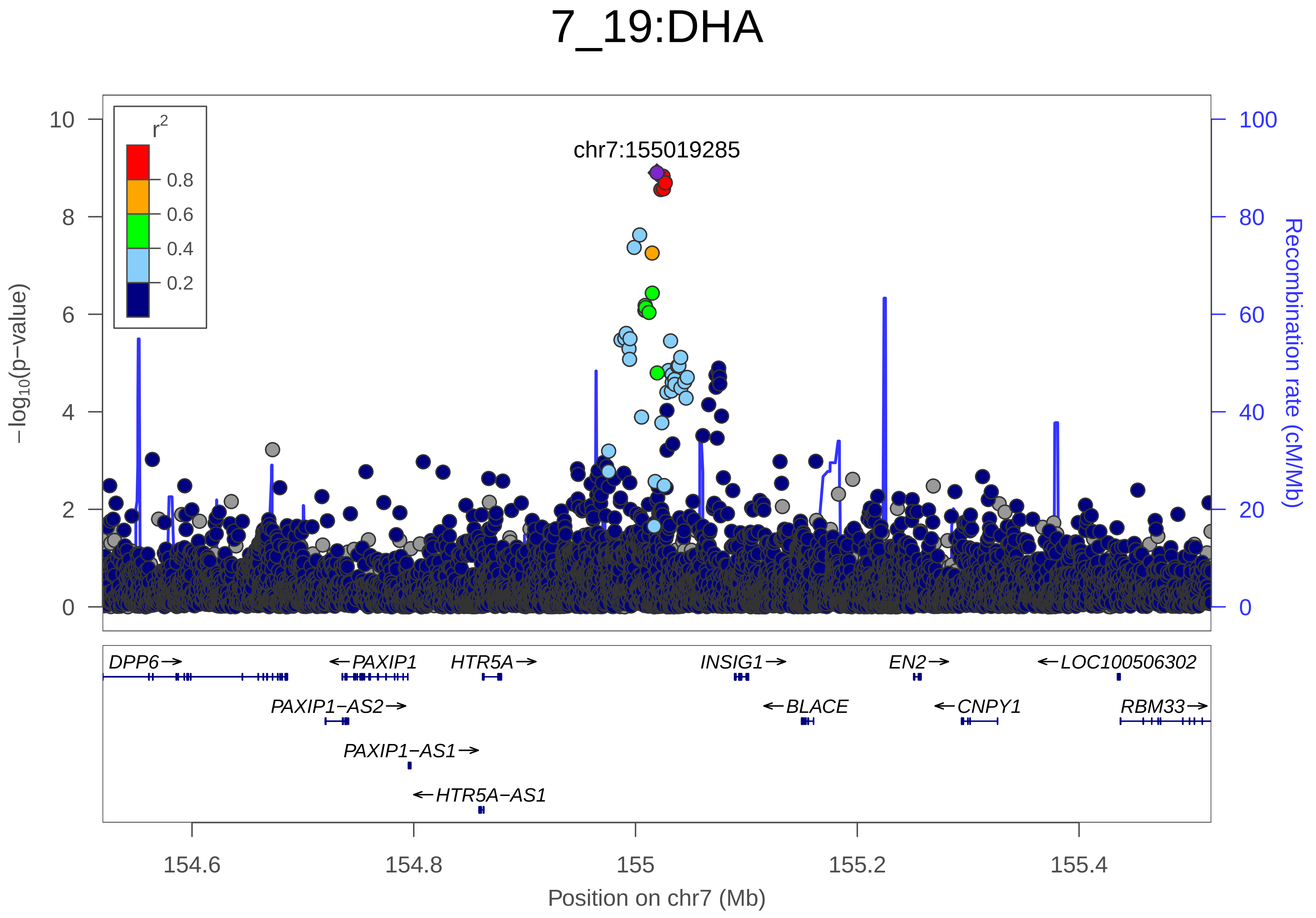
7_19:DHA
r2
0.8
0.6
0.4
0.2
chr7:155019285
−log10(p−value)
Recombination rate (cM/Mb)
0
2
4
6
8
10
0
20
40
60
80
100
DPP6→
←PAXIP1
HTR5A→
INSIG1→
EN2→
←LOC100506302
PAXIP1−AS2→
←BLACE
←CNPY1
RBM33→
PAXIP1−AS1→
←HTR5A−AS1
154.6
154.8
155
155.2
155.4
Position on chr7 (Mb)

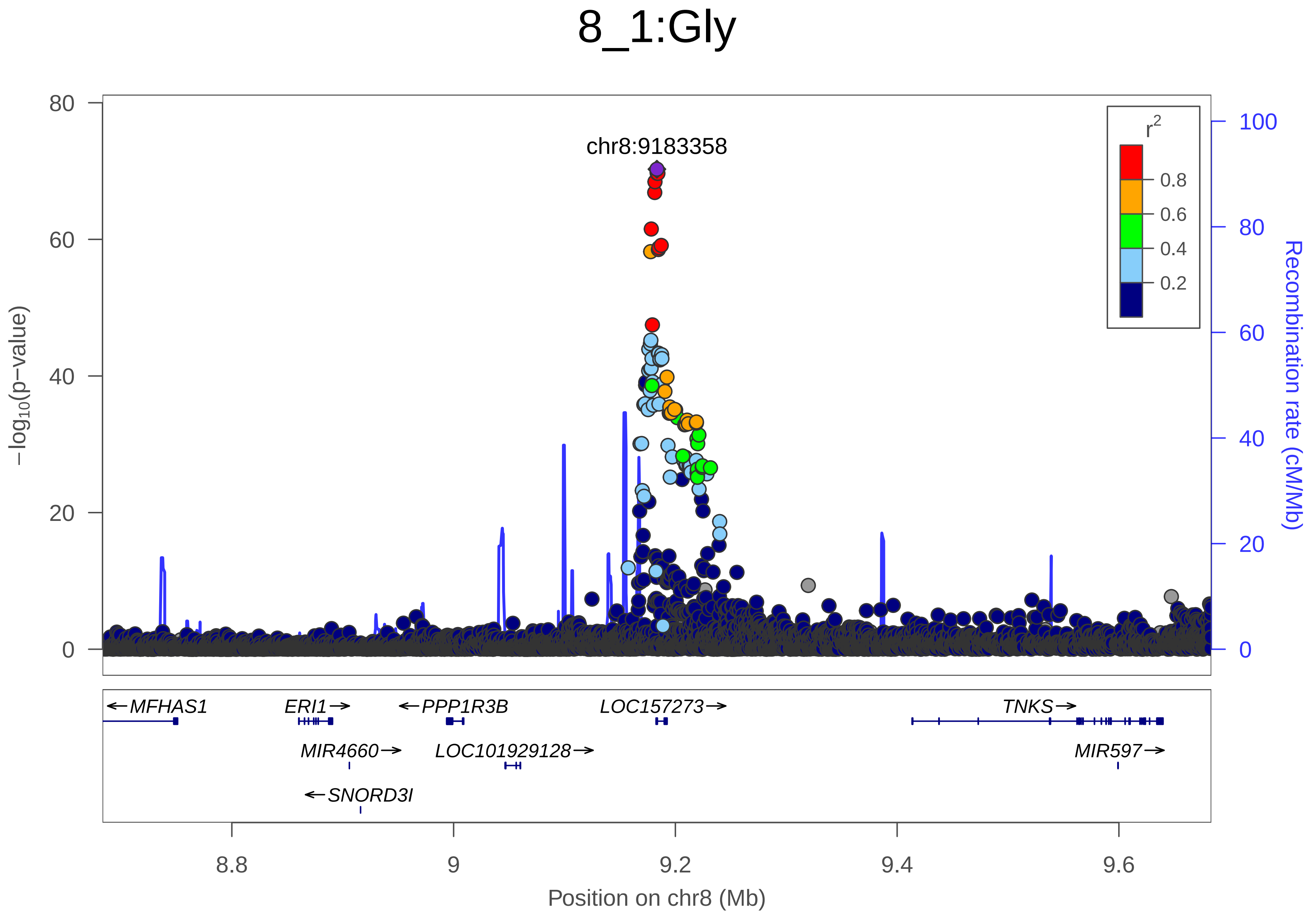
8_1:Gly
chr8:9183358
r^2
0.8
0.6
0.4
0.2
−log10(p−value)
Recombination rate (cM/Mb)
0
20
40
60
80
100
←MFHAS1
ERI1→
←PPP1R3B
LOC157273→
TNKS→
MIR4660→
LOC101929128→
MIR597→
←SNORD3I
8.8
9
9.2
9.4
9.6
Position on chr8 (Mb)

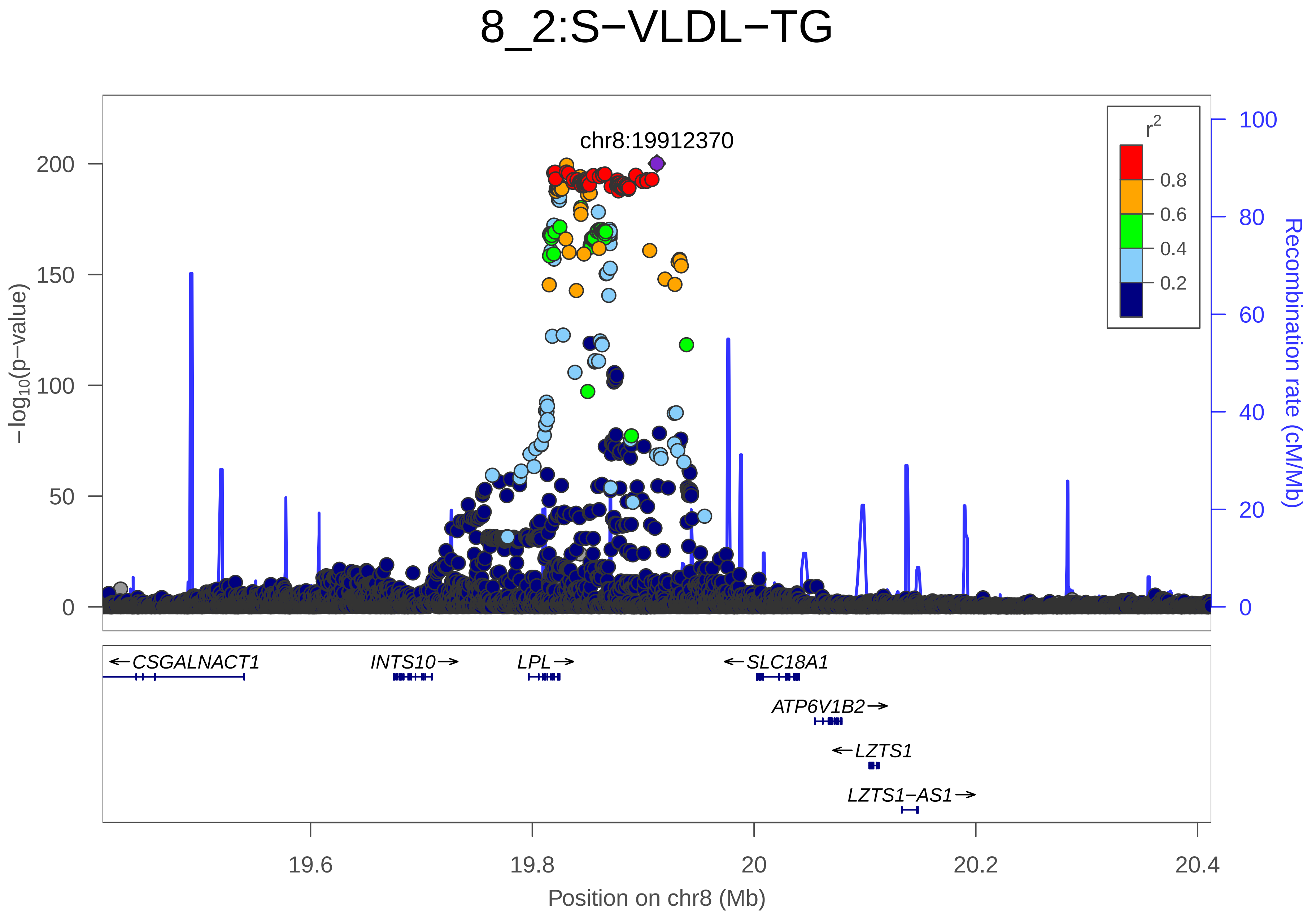

8_2:S−VLDL−TG
chr8:19912370
r²
0.8
0.6
0.4
0.2
200
150
100
50
0
−log10(p−value)
100
80
60
40
20
0
Recombination rate (cM/Mb)
←CSGALNACT1
INTS10→
LPL→
←SLC18A1
ATP6V1B2→
←LZTS1
LZTS1−AS1→
19.6
19.8
20
20.2
20.4
Position on chr8 (Mb)

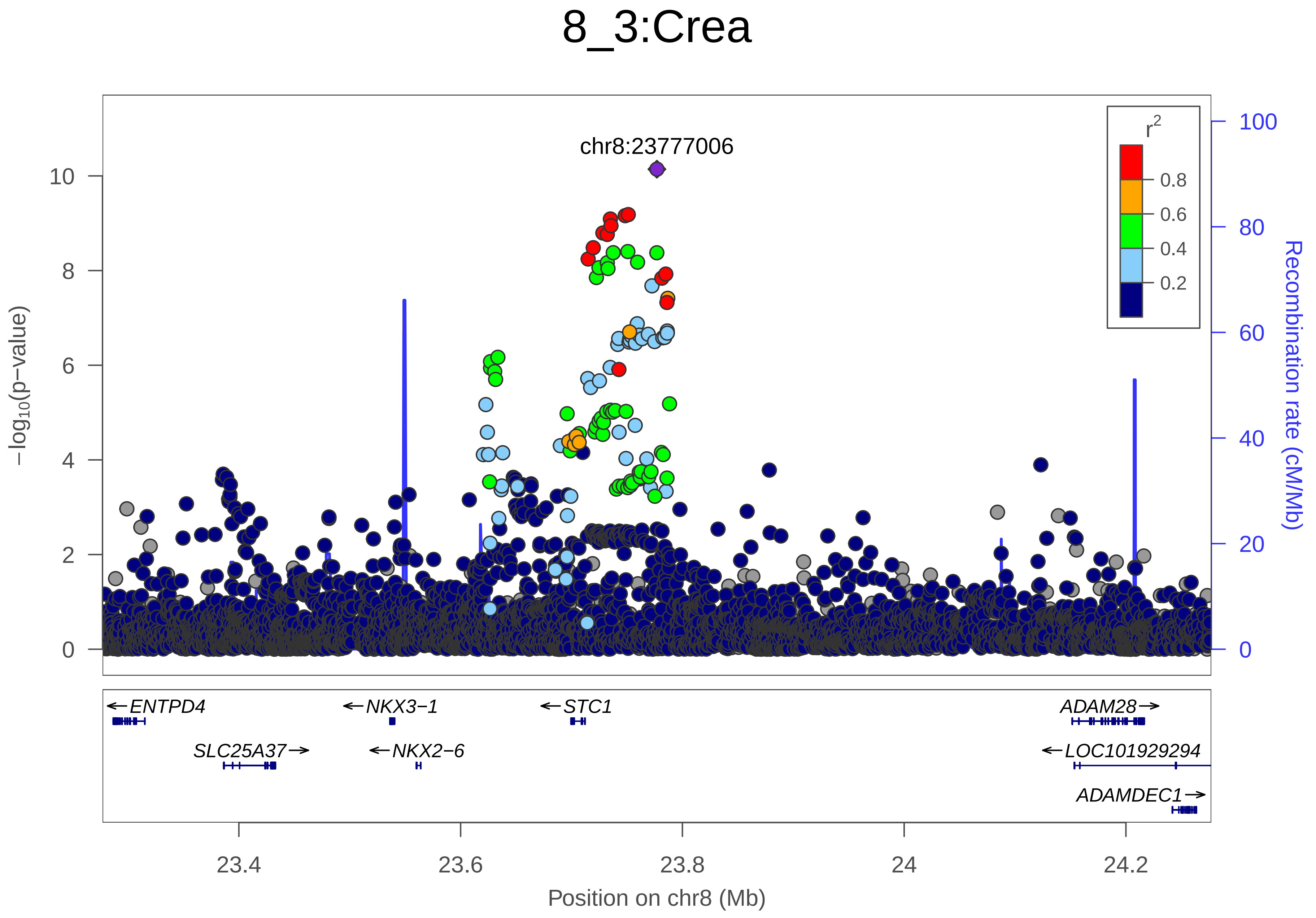

8_3:Crea
chr8:23777006
r2
0.8
0.6
0.4
0.2
−log10(p−value)
Recombination rate (cM/Mb)
ENTPD4
NKX3−1
STC1
ADAM28
SLC25A37
NKX2−6
LOC101929294
ADAMDEC1
23.4
23.6
23.8
24
24.2
Position on chr8 (Mb)

8_4:Ala

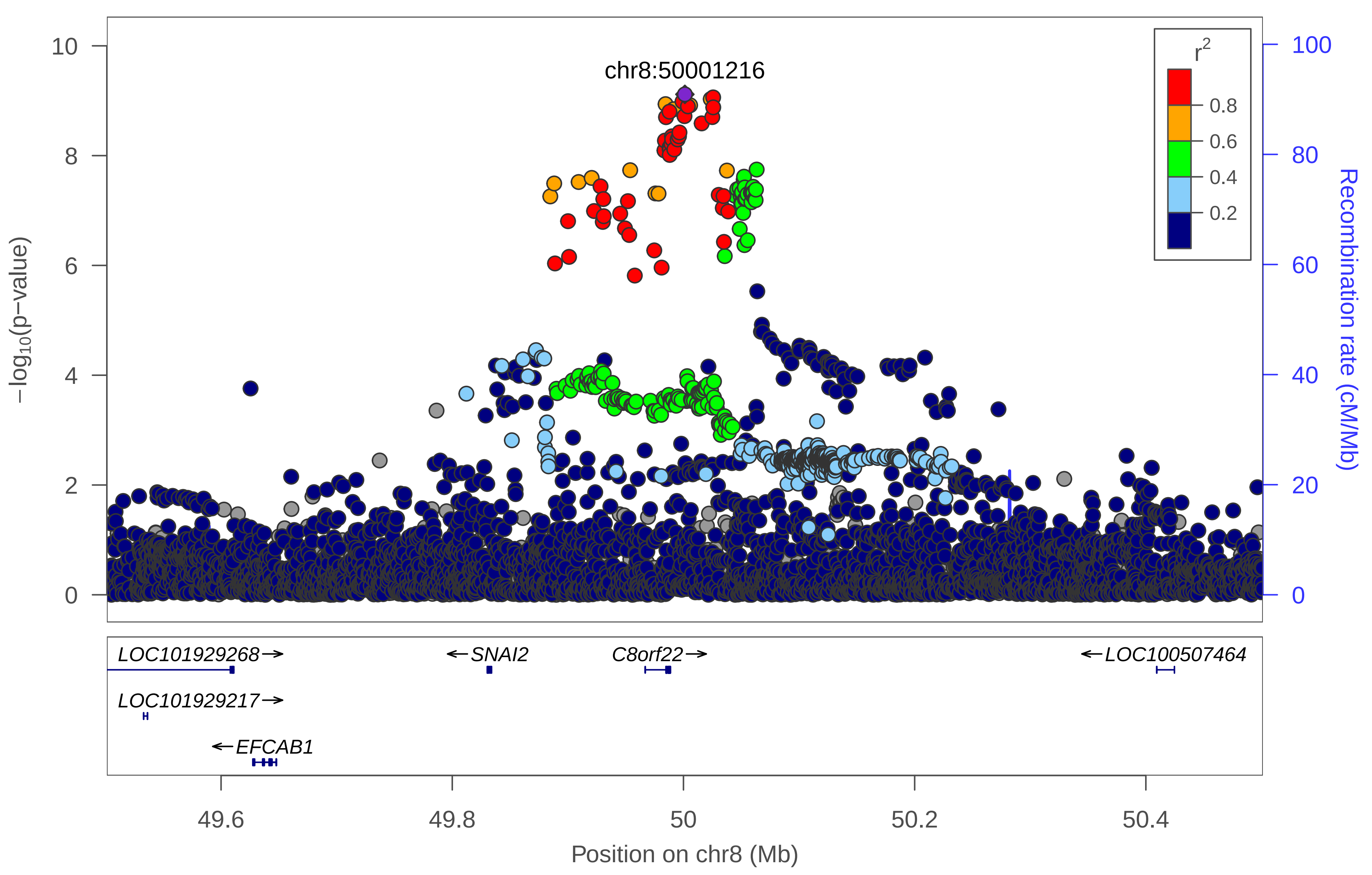

chr8:50001216
r2
0.8
0.6
0.4
0.2
10
8
6
4
2
0
−log10(p−value)
100
80
60
40
20
0
Recombination rate (cM/Mb)
LOC101929268→
←SNAI2
C8orf22→
←LOC100507464
LOC101929217→
←EFCAB1
49.6
49.8
50
50.2
50.4
Position on chr8 (Mb)

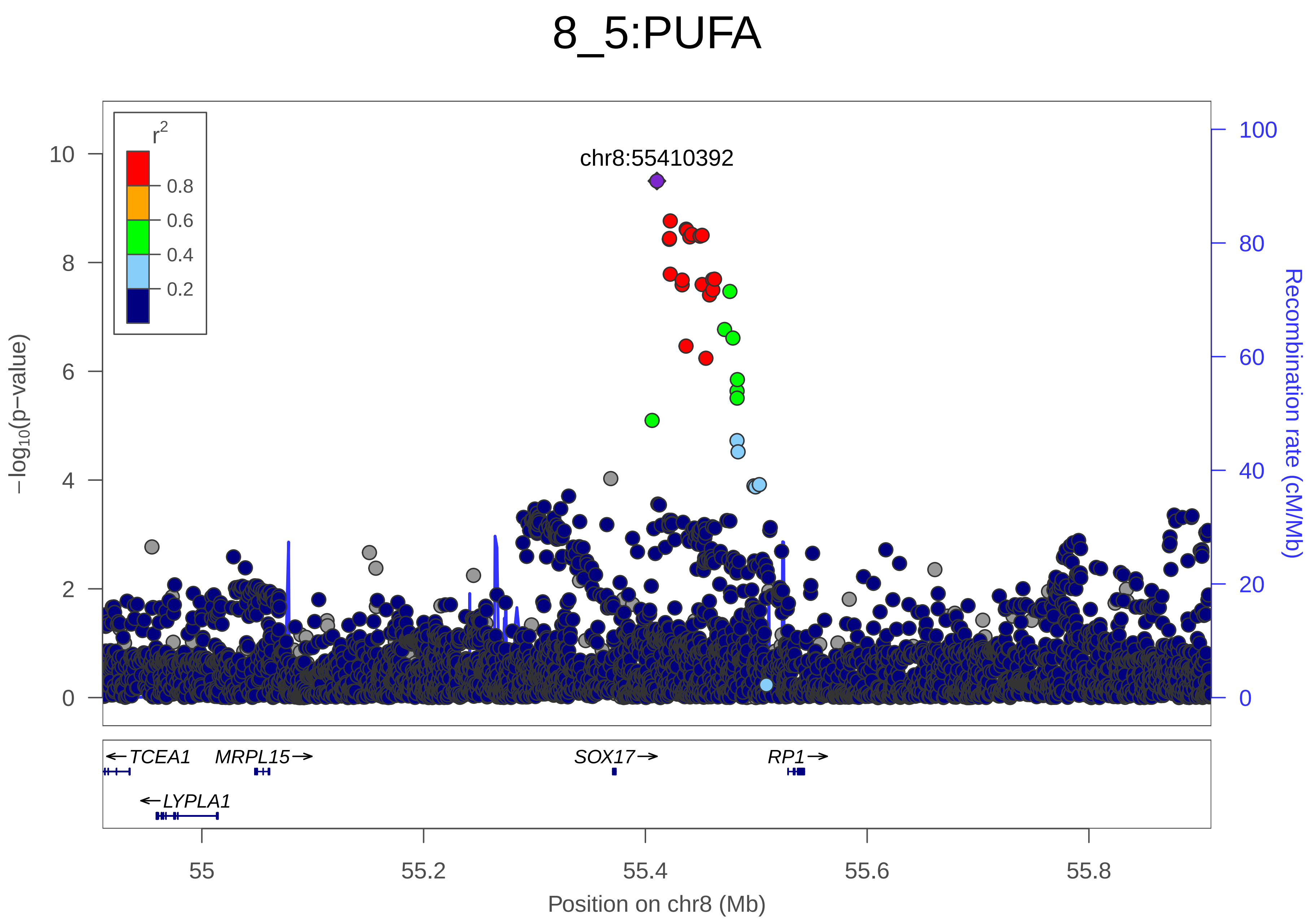
8_5:PUFA
r2
0.8
0.6
0.4
0.2
chr8:55410392
−log10(p−value)
Recombination rate (cM/Mb)
10
8
6
4
2
0
100
80
60
40
20
0
TCEA1
MRPL15
SOX17
RP1
LYPLA1
55
55.2
55.4
55.6
55.8
Position on chr8 (Mb)

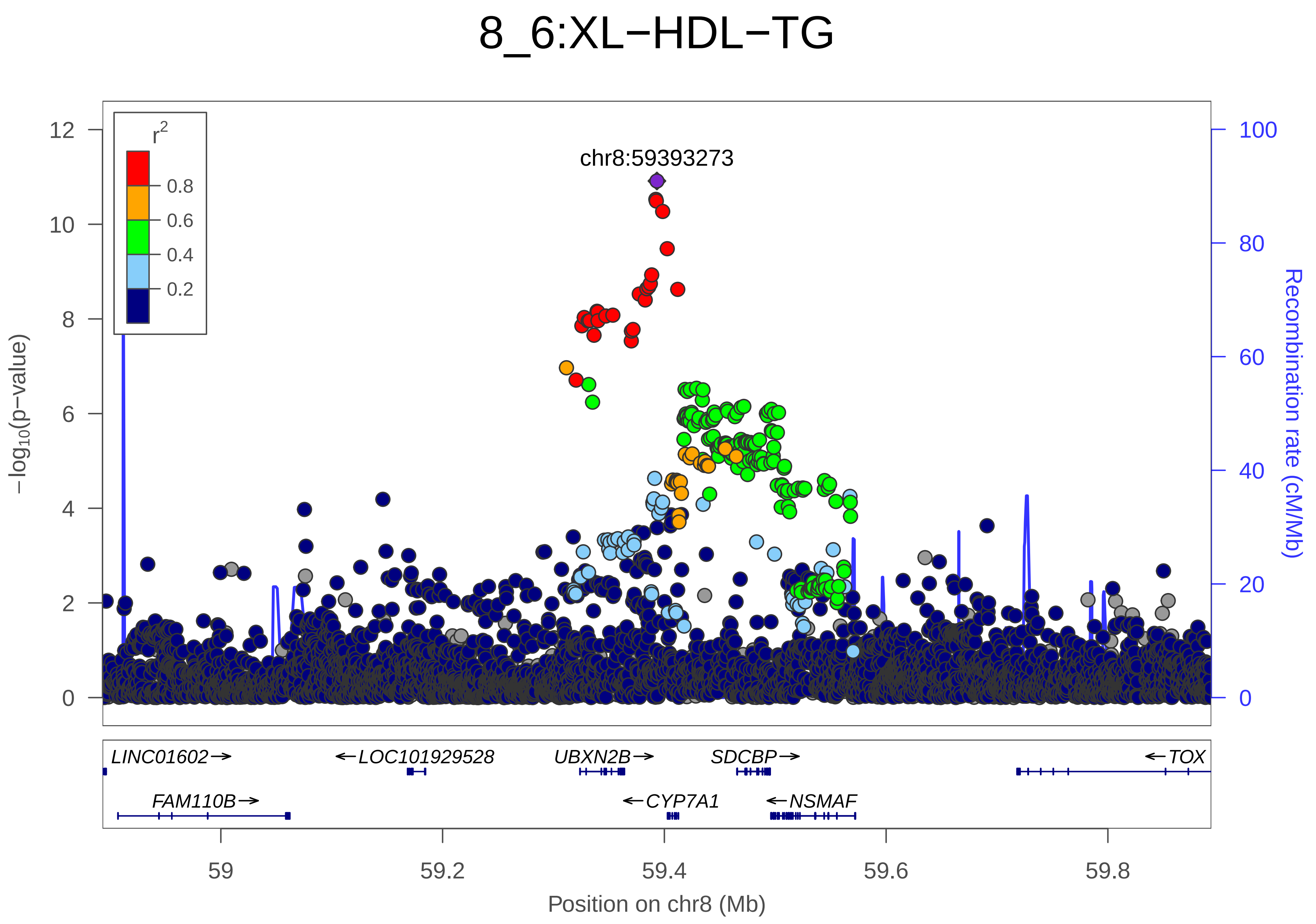

8_6:XL−HDL−TG
chr8:59393273
r2
0.8
0.6
0.4
0.2
−log10(p−value)
Recombination rate (cM/Mb)
12
10
8
6
4
2
0
100
80
60
40
20
0
LINC01602→
←LOC101929528
UBXN2B→
SDCBP→
←TOX
FAM110B→
←CYP7A1
←NSMAF
59
59.2
59.4
59.6
59.8
Position on chr8 (Mb)

8_7:XS−VLDL−PL

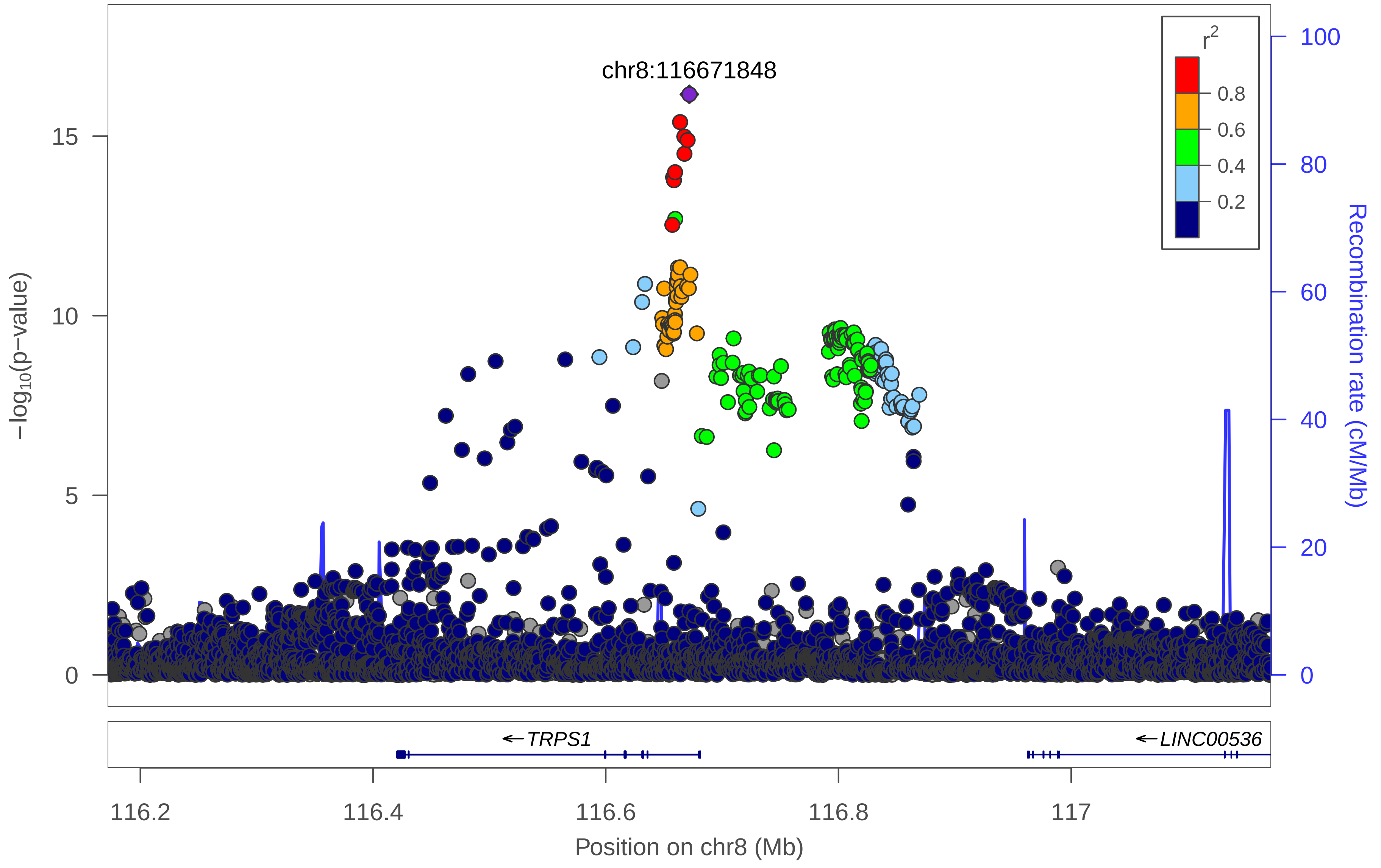
chr8:116671848
r2
0.8
0.6
0.4
0.2
15
10
5
0
100
80
60
40
20
0
−log10(p−value)
Recombination rate (cM/Mb)
TRPS1
LINC00536
116.2
116.4
116.6
116.8
117
Position on chr8 (Mb)

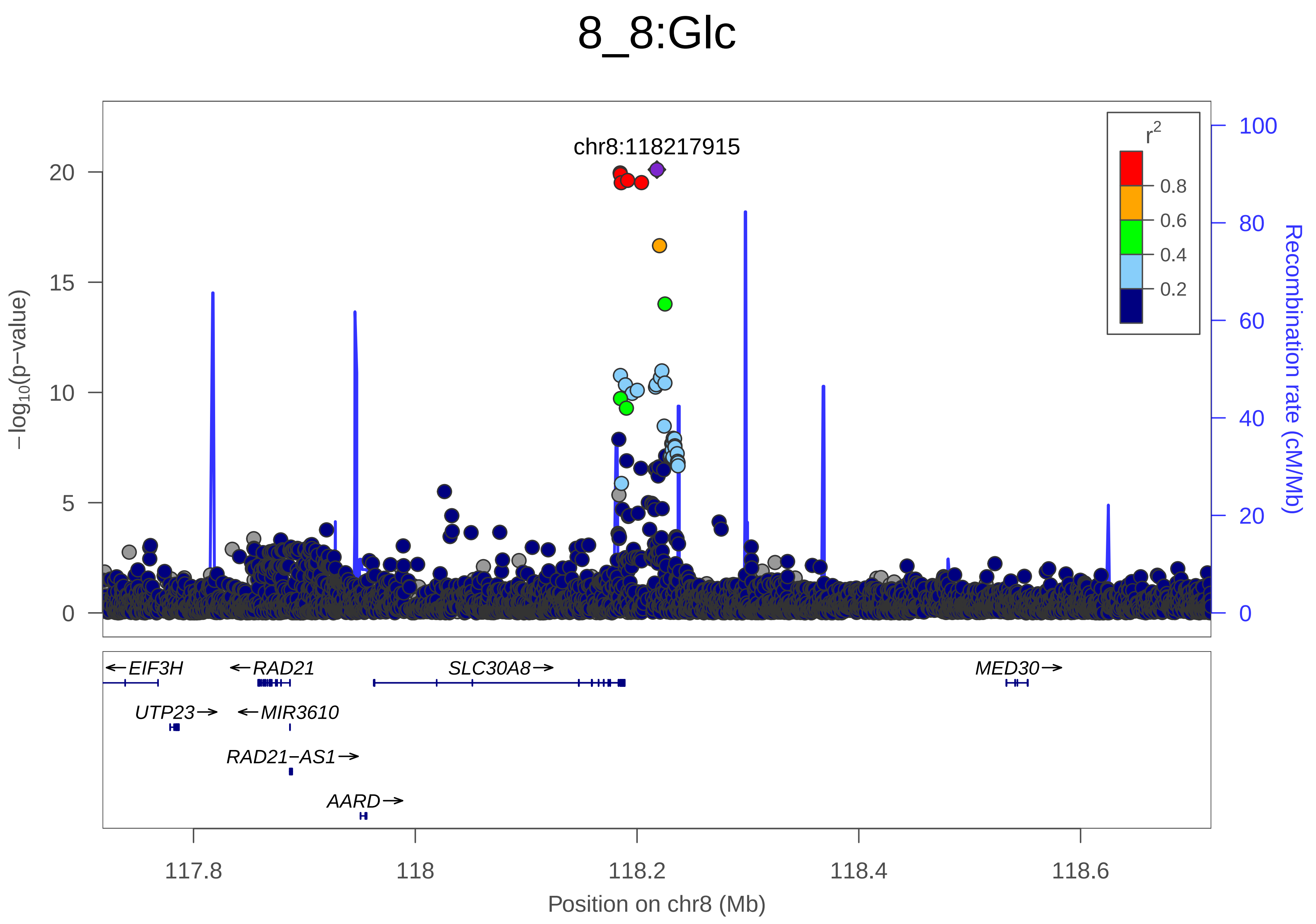
8_8:Glc
chr8:118217915
r2
0.8
0.6
0.4
0.2
−log10(p−value)
20
15
10
5
0
100
80
60
40
20
0
Recombination rate (cM/Mb)
←EIF3H
←RAD21
SLC30A8→
MED30→
UTP23→
←MIR3610
RAD21−AS1→
AARD→
117.8
118
118.2
118.4
118.6
Position on chr8 (Mb)

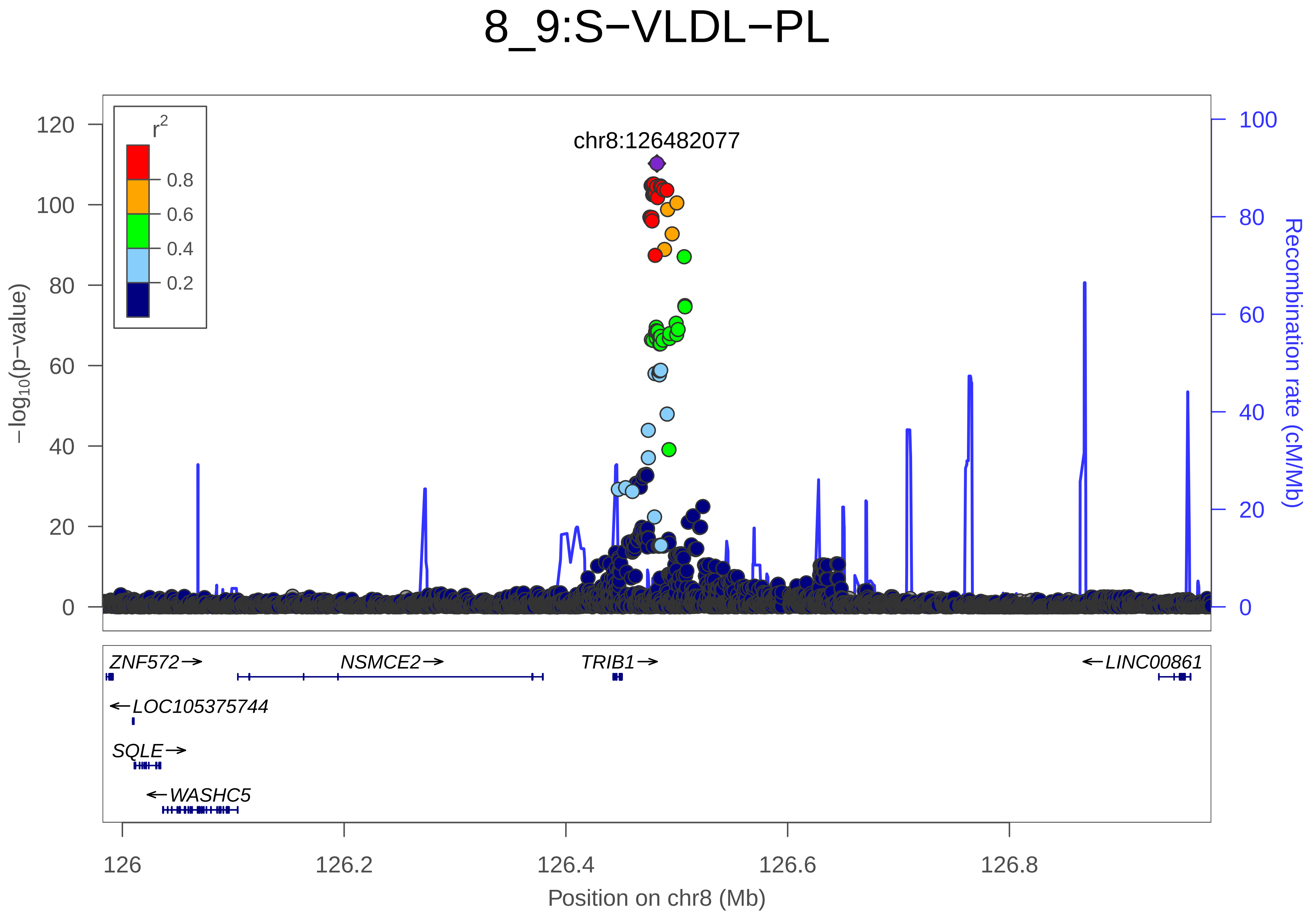
8_9:S−VLDL−PL
chr8:126482077
r2
0.8
0.6
0.4
0.2
120
100
80
60
40
20
0
−log10(p−value)
Recombination rate (cM/Mb)
ZNF572→
NSMCE2→
TRIB1→
←LINC00861
←LOC105375744
SQLE→
←WASHC5
126
126.2
126.4
126.6
126.8
Position on chr8 (Mb)

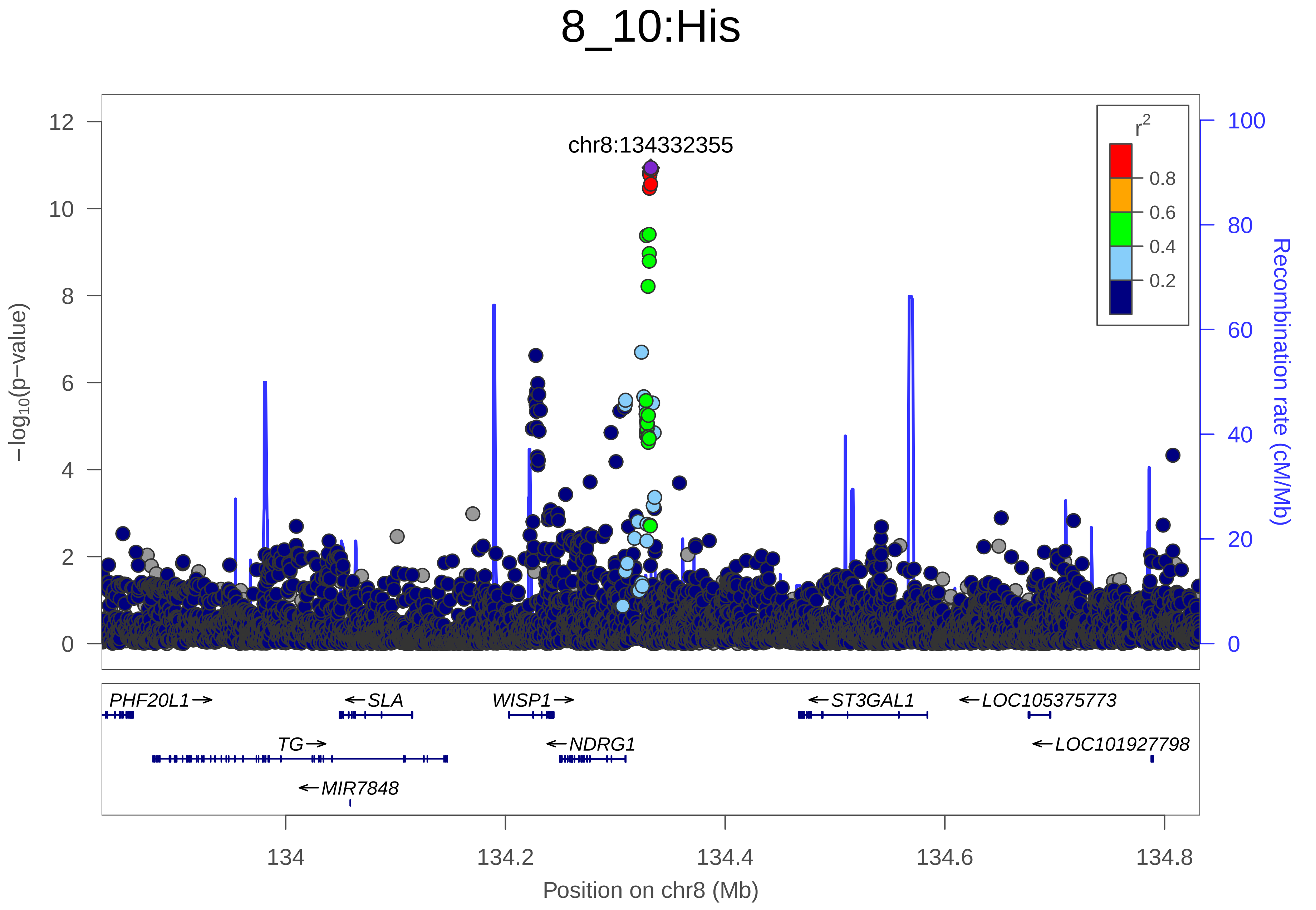
8_10:His
chr8:134332355
r²
0.8
0.6
0.4
0.2
−log10(p−value)
Recombination rate (cM/Mb)
PHF20L1→
←SLA
WISP1→
←ST3GAL1
←LOC105375773
TG→
←NDRG1
←LOC101927798
←MIR7848
134
134.2
134.4
134.6
134.8
Position on chr8 (Mb)

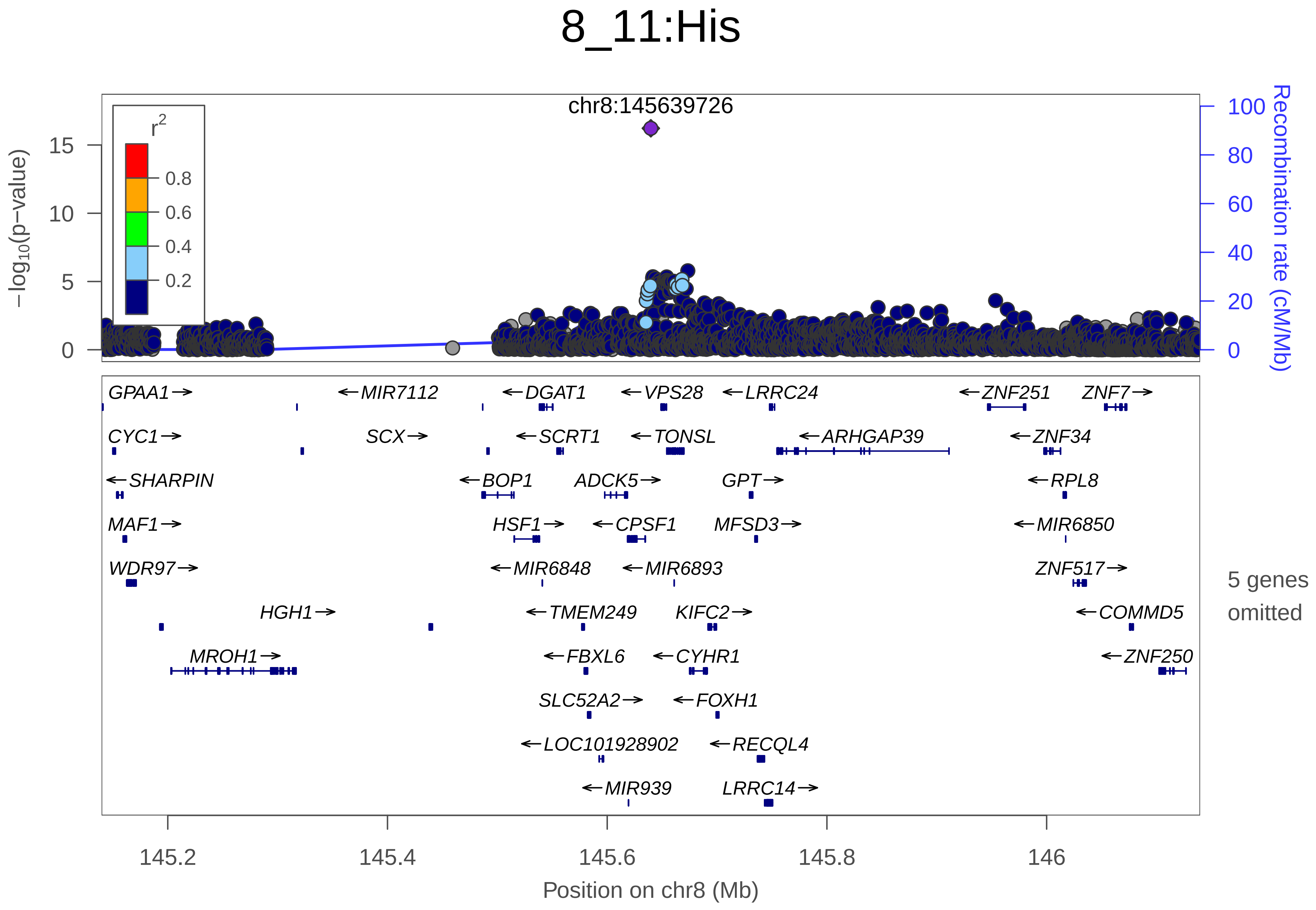

8_11:His
chr8:145639726
r2
0.8
0.6
0.4
0.2
−log10(p−value)
15
10
5
0
Recombination rate (cM/Mb)
100
80
60
40
20
0
GPAA1→
←MIR7112
←DGAT1
←VPS28
←LRRC24
←ZNF251
ZNF7→
CYC1→
SCX→
←SCRT1
←TONSL
←ARHGAP39
←ZNF34
←SHARPIN
←BOP1
ADCK5→
GPT→
←RPL8
MAF1→
HSF1→
←CPSF1
MFSD3→
←MIR6850
WDR97→
←MIR6848
←MIR6893
ZNF517→
HGH1→
←TMEM249
KIFC2→
←COMMD5
MROH1→
←FBXL6
←CYHR1
←ZNF250
SLC52A2→
←FOXH1
←LOC101928902
←RECQL4
←MIR939
LRRC14→
5 genes omitted
145.2
145.4
145.6
145.8
146
Position on chr8 (Mb)

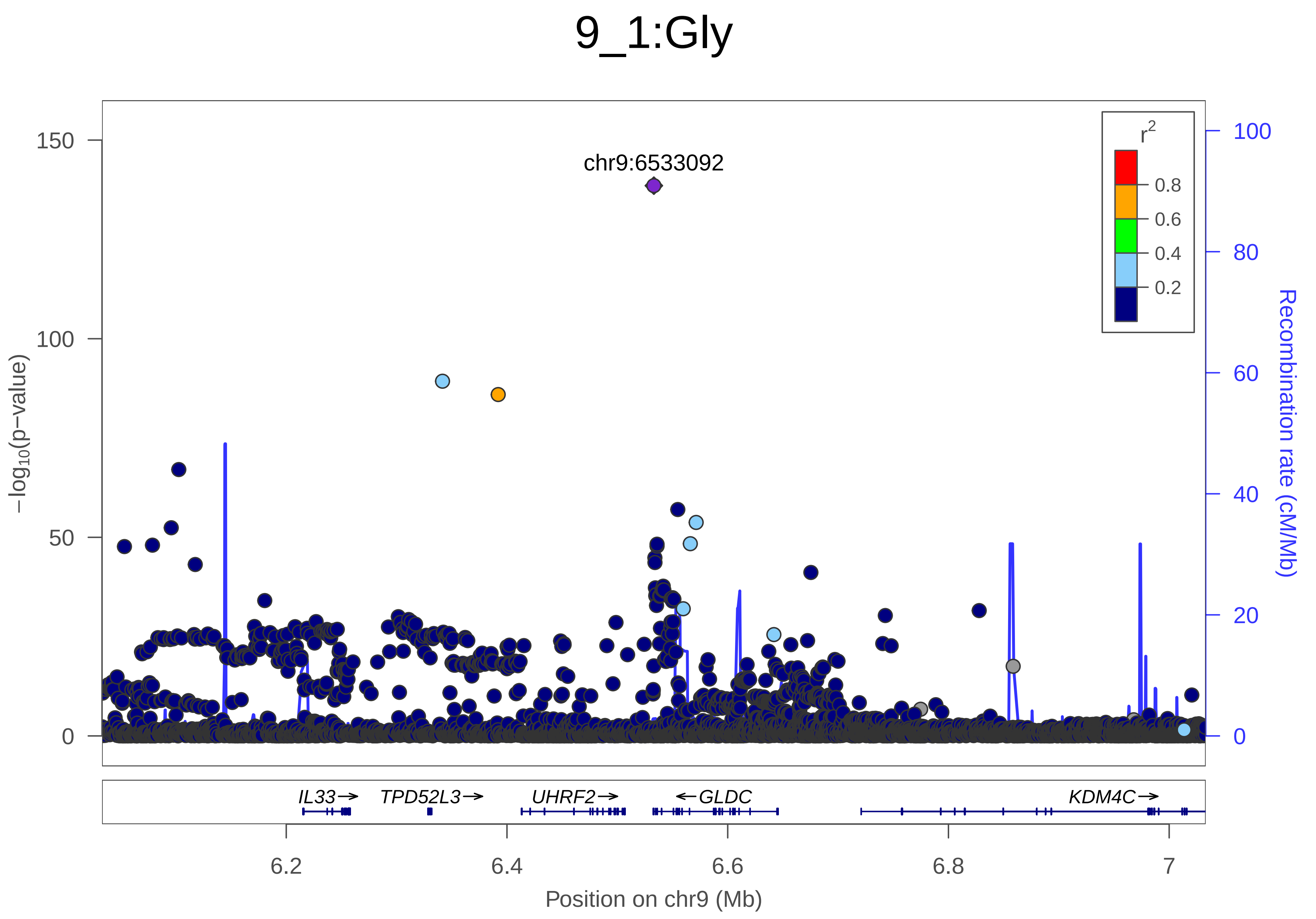

9_1:Gly
chr9:6533092
r2
0.8
0.6
0.4
0.2
−log10(p−value)
150
100
50
0
Recombination rate (cM/Mb)
100
80
60
40
20
0
IL33→
TPD52L3→
UHRF2→
←GLDC
KDM4C→
6.2
6.4
6.6
6.8
7
Position on chr9 (Mb)

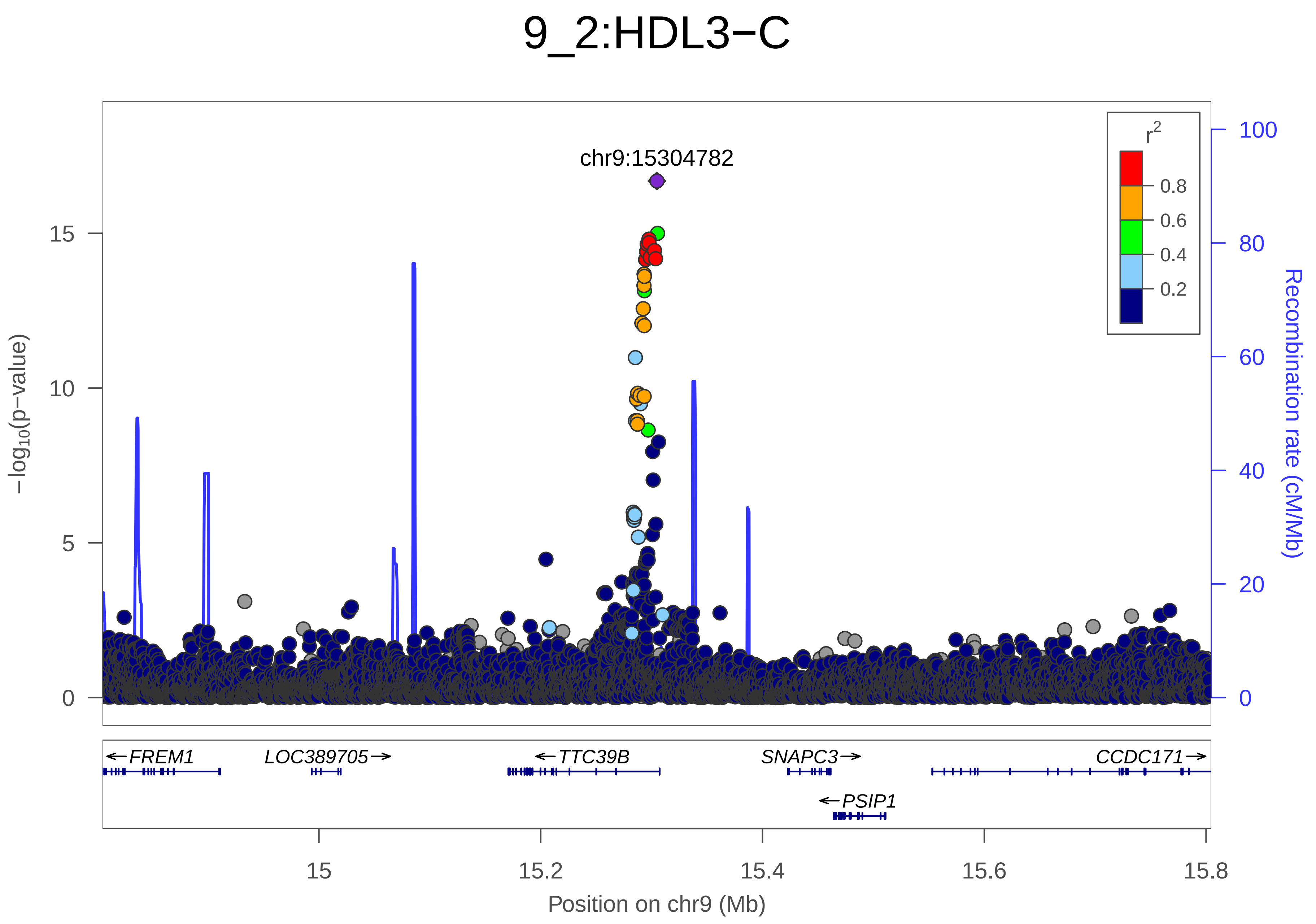
9_2:HDL3−C
chr9:15304782
−log10(p−value)
0
5
10
15
Recombination rate (cM/Mb)
0
20
40
60
80
100
r2
0.2
0.4
0.6
0.8
FREM1
LOC389705
TTC39B
SNAPC3
CCDC171
PSIP1
15
15.2
15.4
15.6
15.8
Position on chr9 (Mb)

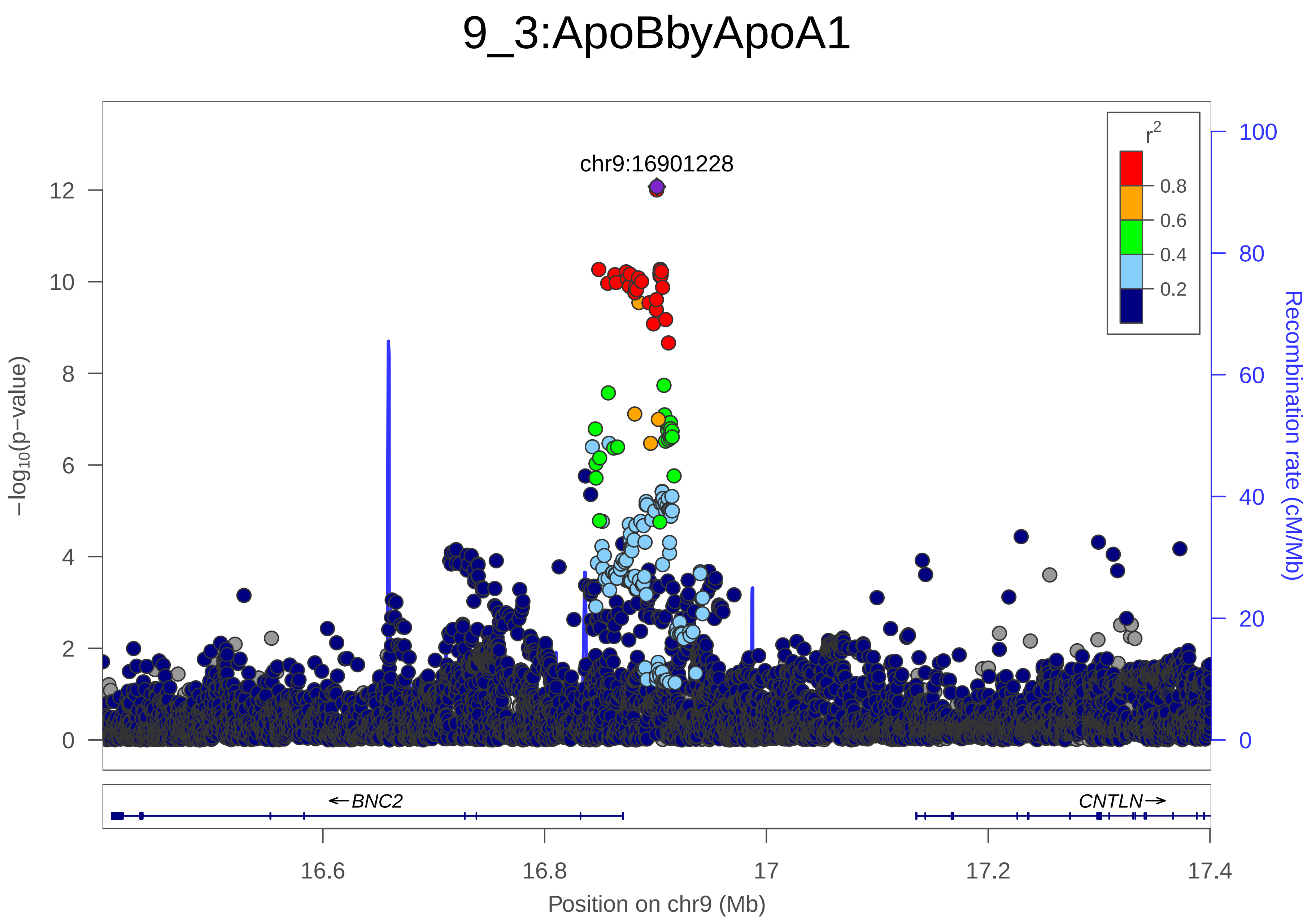

9_3:ApoBbyApoA1
chr9:16901228
r2
0.8
0.6
0.4
0.2
−log10(p−value)
Recombination rate (cM/Mb)
0
2
4
6
8
10
12
20
40
60
80
100
BNC2
CNTLN
16.6
16.8
17
17.2
17.4
Position on chr9 (Mb)

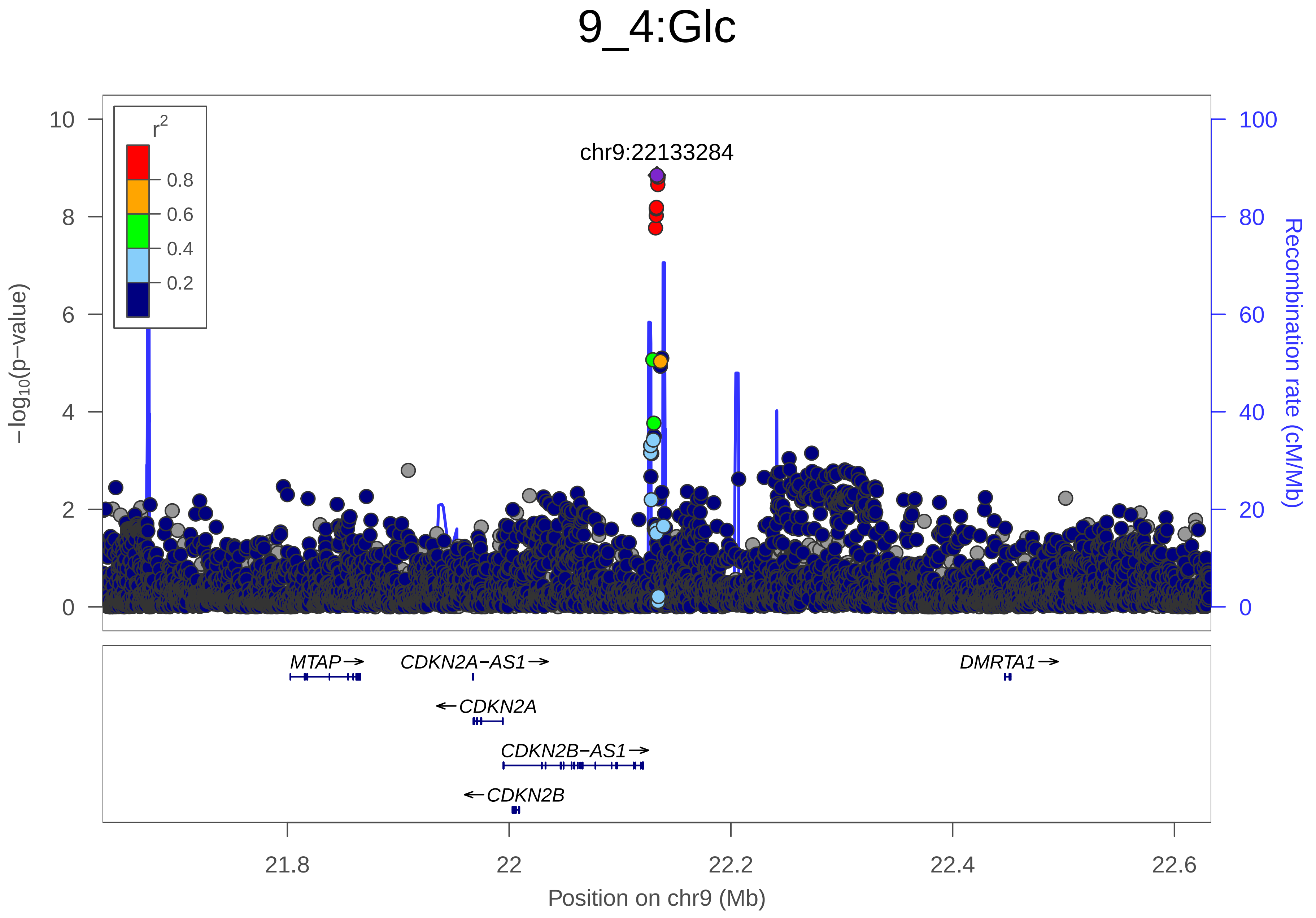

9_4:Glc
chr9:22133284
r2
0.8
0.6
0.4
0.2
−log10(p−value)
Recombination rate (cM/Mb)
0
2
4
6
8
10
0
20
40
60
80
100
MTAP→
CDKN2A−AS1→
←CDKN2A
CDKN2B−AS1→
←CDKN2B
DMRTA1→
21.8
22
22.2
22.4
22.6
Position on chr9 (Mb)

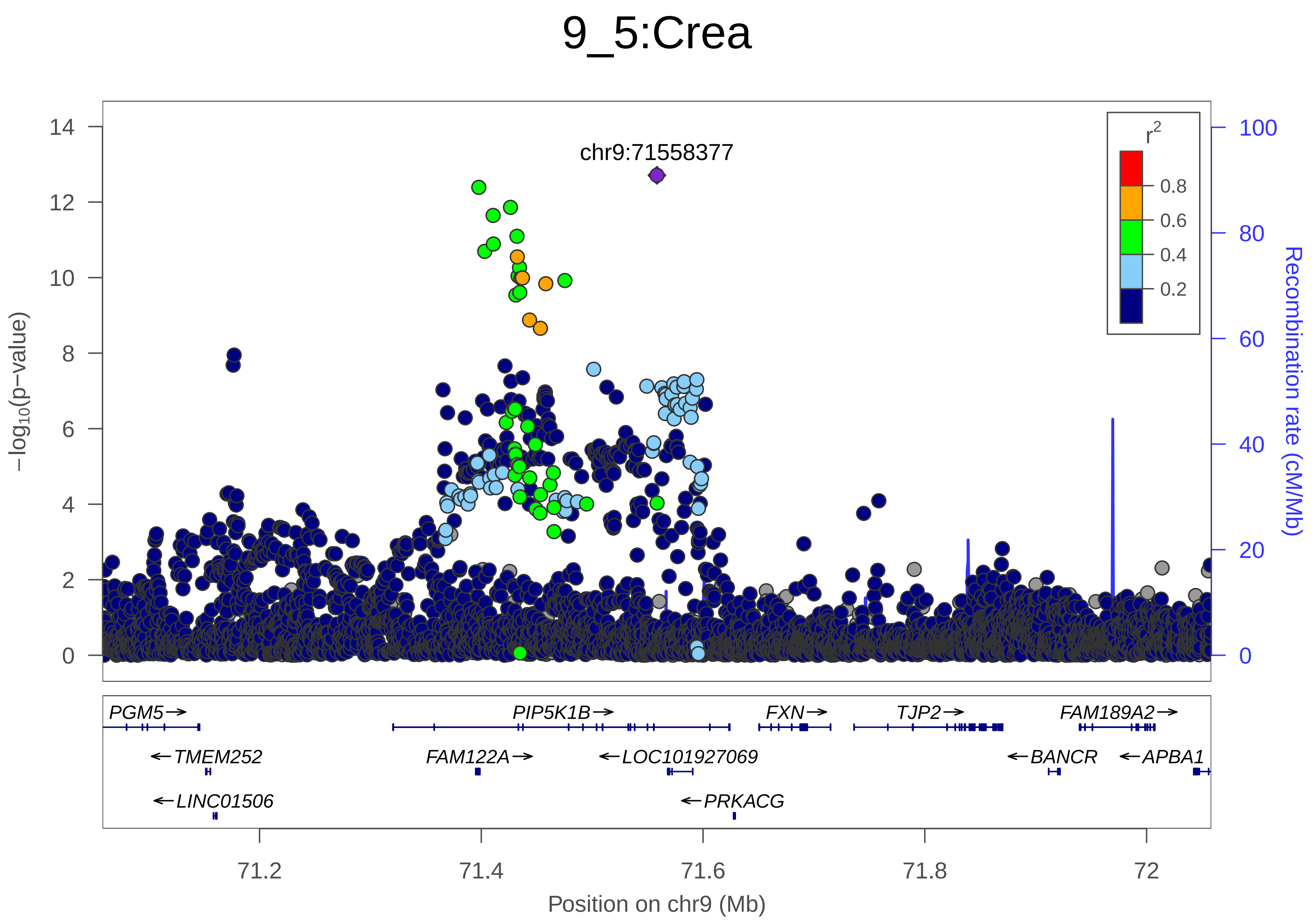
9_5:Crea
chr9:71558377
−log10(p−value)
Recombination rate (cM/Mb)
r2
0.8
0.6
0.4
0.2
0
2
4
6
8
10
12
14
0
20
40
60
80
100
PGM5→
PIP5K1B→
FXN→
TJP2→
FAM189A2→
←TMEM252
FAM122A→
←LOC101927069
←BANCR
←APBA1
←LINC01506
←PRKACG
71.2
71.4
71.6
71.8
72
Position on chr9 (Mb)

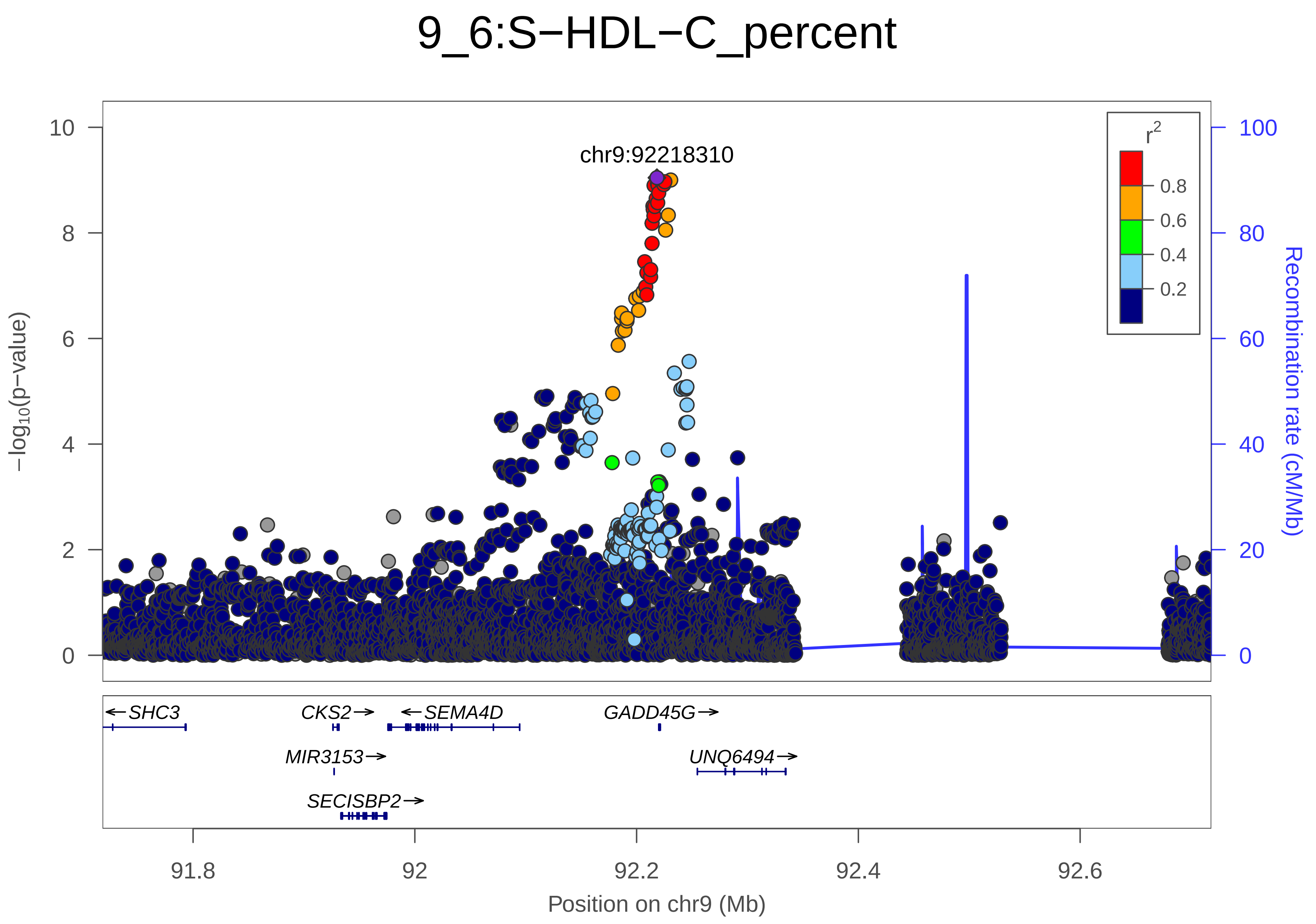
9_6:S−HDL−C_percent
chr9:92218310
r2
0.8
0.6
0.4
0.2
−log10(p−value)
Recombination rate (cM/Mb)
0
2
4
6
8
10
20
40
60
80
100
SHC3
CKS2
SEMA4D
GADD45G
MIR3153
UNQ6494
SECISBP2
91.8
92
92.2
92.4
92.6
Position on chr9 (Mb)

9_7:XL−HDL−CE

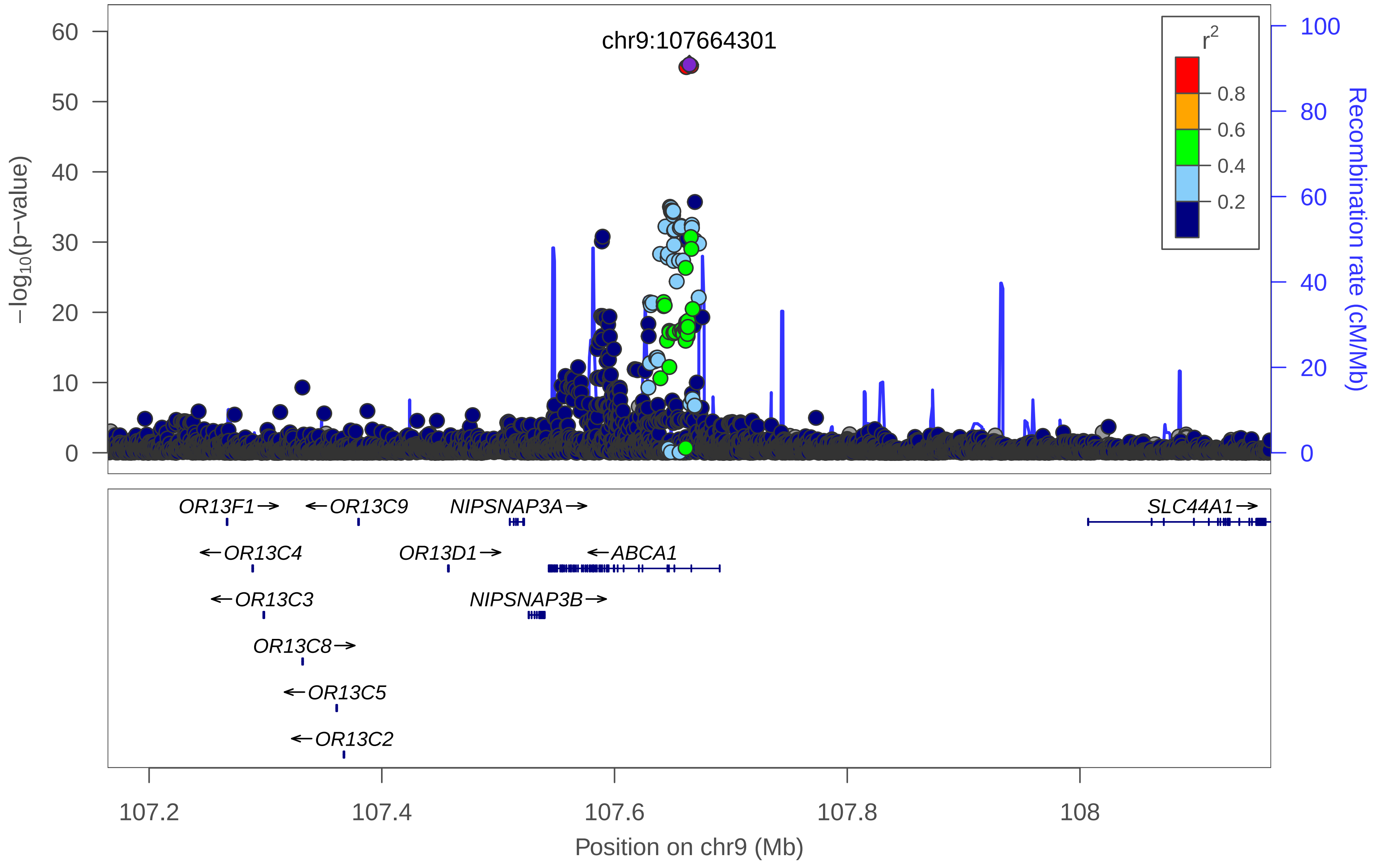
chr9:107664301
−log10(p−value)
0
10
20
30
40
50
60
Recombination rate (cM/Mb)
0
20
40
60
80
100
r2
0.2
0.4
0.6
0.8
OR13F1→
←OR13C9
NIPSNAP3A→
SLC44A1→
←OR13C4
OR13D1→
←ABCA1
←OR13C3
NIPSNAP3B→
OR13C8→
←OR13C5
←OR13C2
107.2
107.4
107.6
107.8
108
Position on chr9 (Mb)

9_8:GlycA

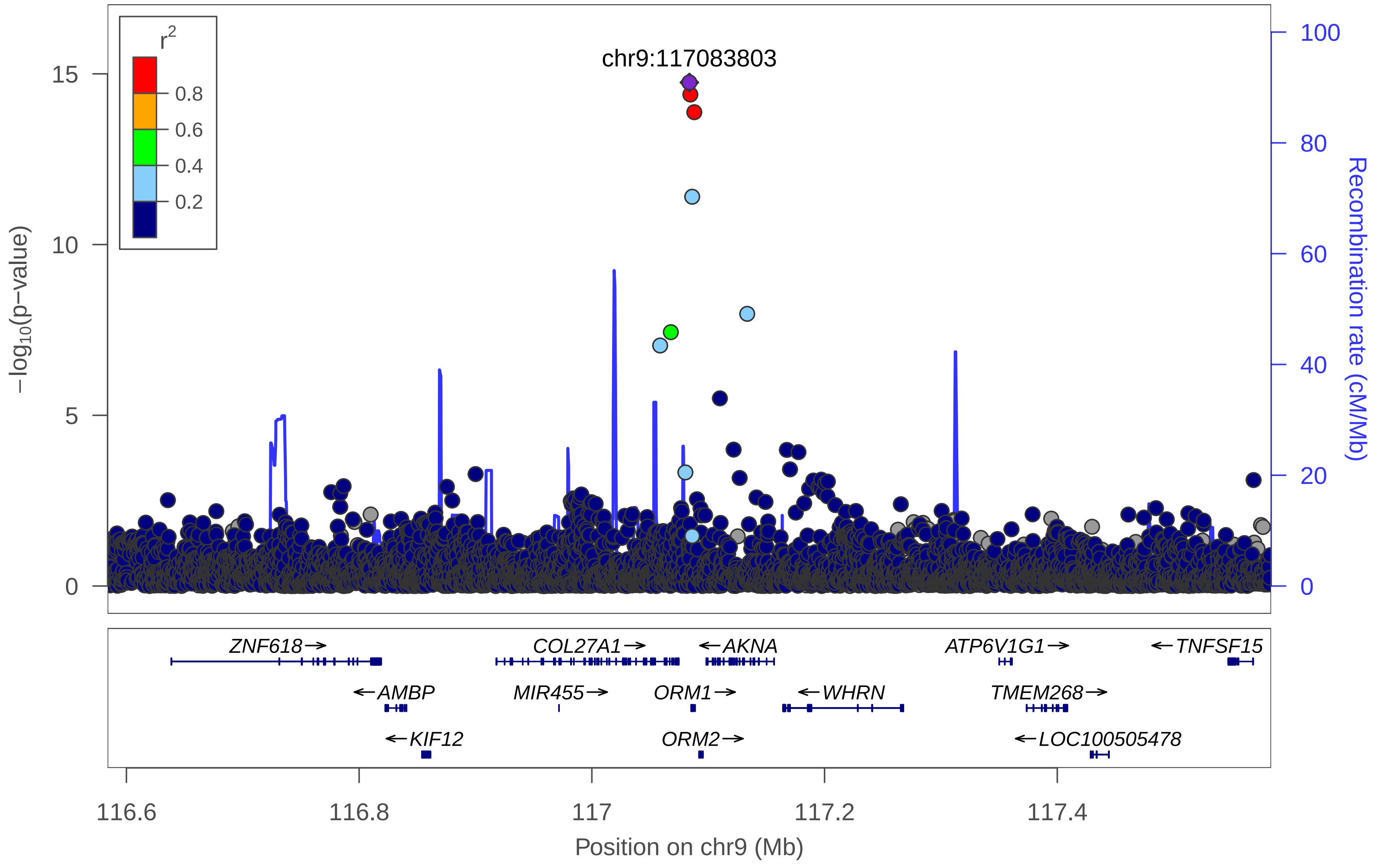

chr9:117083803
r2
0.8
0.6
0.4
0.2
−log10(p−value)
15
10
5
0
Recombination rate (cM/Mb)
100
80
60
40
20
0
ZNF618→
COL27A1→
←AKNA
ATP6V1G1→
←TNFSF15
←AMBP
MIR455→
ORM1→
←WHRN
TMEM268→
←KIF12
ORM2→
←LOC100505478
116.6
116.8
117
117.2
117.4
Position on chr9 (Mb)

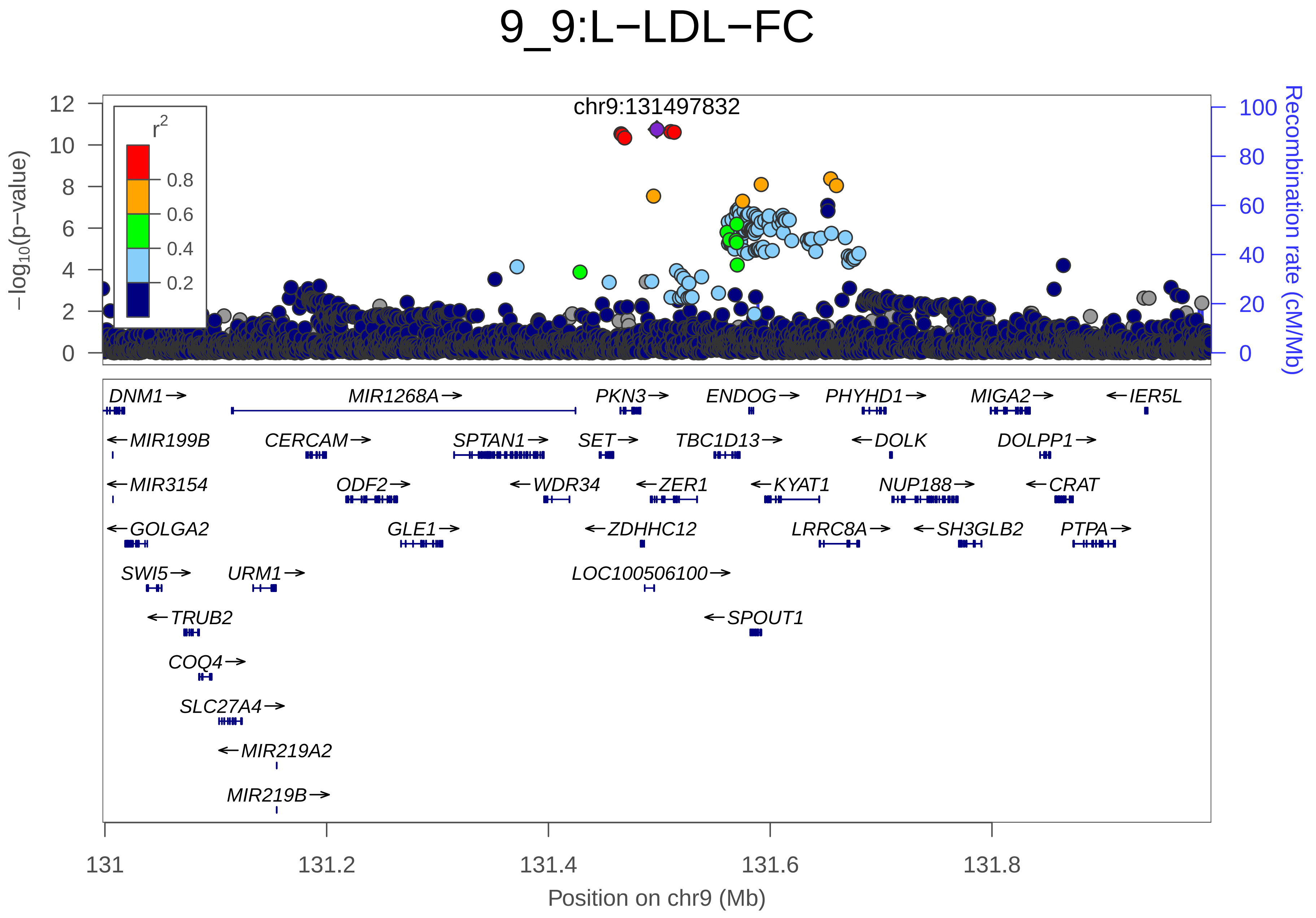
9_9:L−LDL−FC
chr9:131497832
r2
0.8
0.6
0.4
0.2
−log10(p−value)
Recombination rate (cM/Mb)
DNM1
MIR1268A
PKN3
ENDOG
PHYHD1
MIGA2
IER5L
MIR199B
CERCAM
SPTAN1
SET
TBC1D13
DOLK
DOLPP1
MIR3154
ODF2
WDR34
ZER1
KYAT1
NUP188
CRAT
GOLGA2
GLE1
ZDHHC12
LRRC8A
SH3GLB2
PTPA
SWI5
URM1
LOC100506100
TRUB2
SPOUT1
COQ4
SLC27A4
MIR219A2
MIR219B
131
131.2
131.4
131.6
131.8
Position on chr9 (Mb)

9_10:XS−VLDL−C

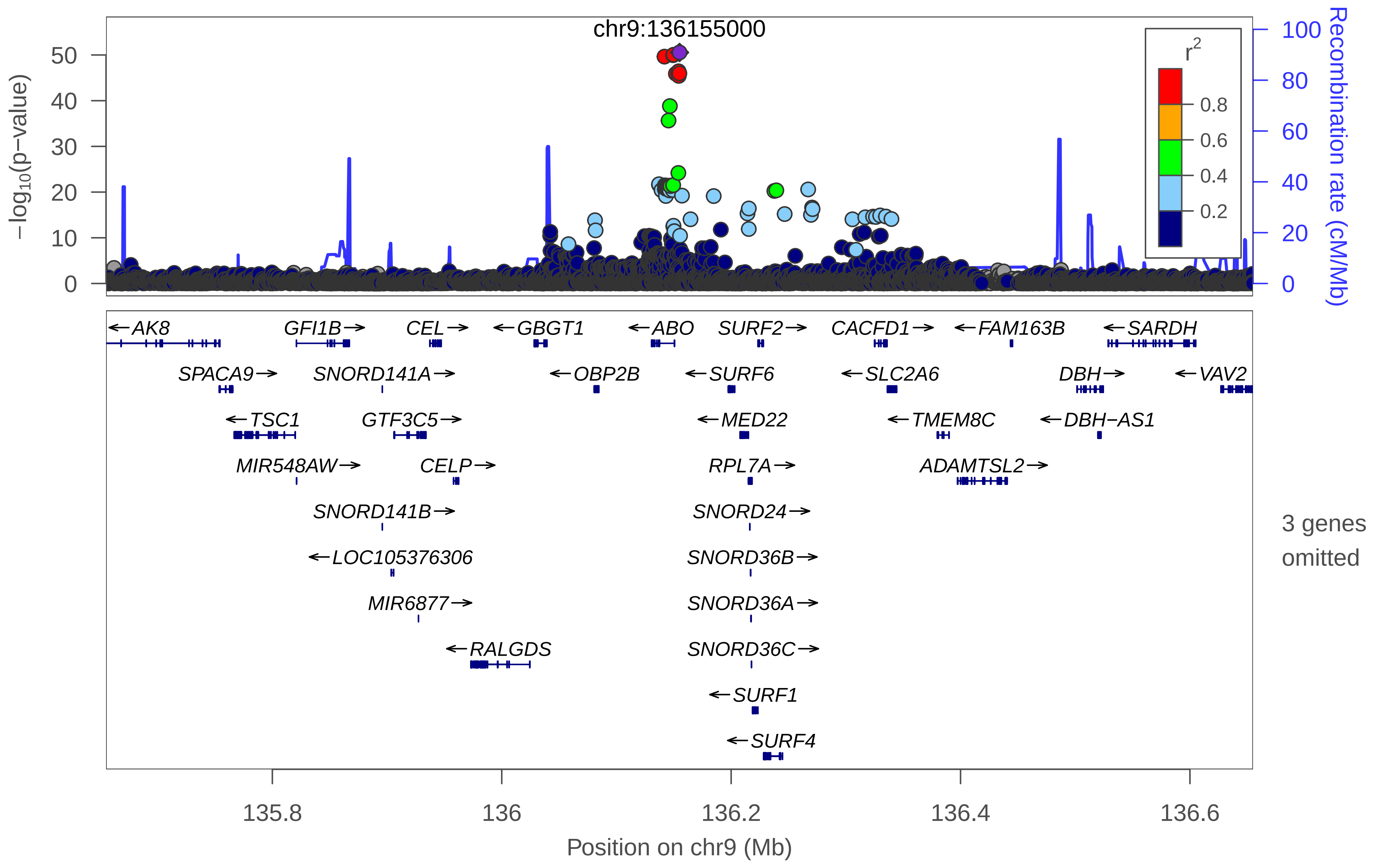

chr9:136155000
−log10(p−value)
0
10
20
30
40
50
Recombination rate (cM/Mb)
0
20
40
60
80
100
r2
0.8
0.6
0.4
0.2
AK8
GFI1B
CEL
GBGT1
ABO
SURF2
CACFD1
FAM163B
SARDH
SPACA9
SNORD141A
OBP2B
SURF6
SLC2A6
DBH
VAV2
TSC1
GTF3C5
MED22
TMEM8C
DBH−AS1
MIR548AW
CELP
RPL7A
ADAMTSL2
SNORD141B
SNORD24
LOC105376306
SNORD36B
MIR6877
SNORD36A
RALGDS
SNORD36C
SURF1
SURF4
3 genes omitted
135.8
136
136.2
136.4
136.6
Position on chr9 (Mb)

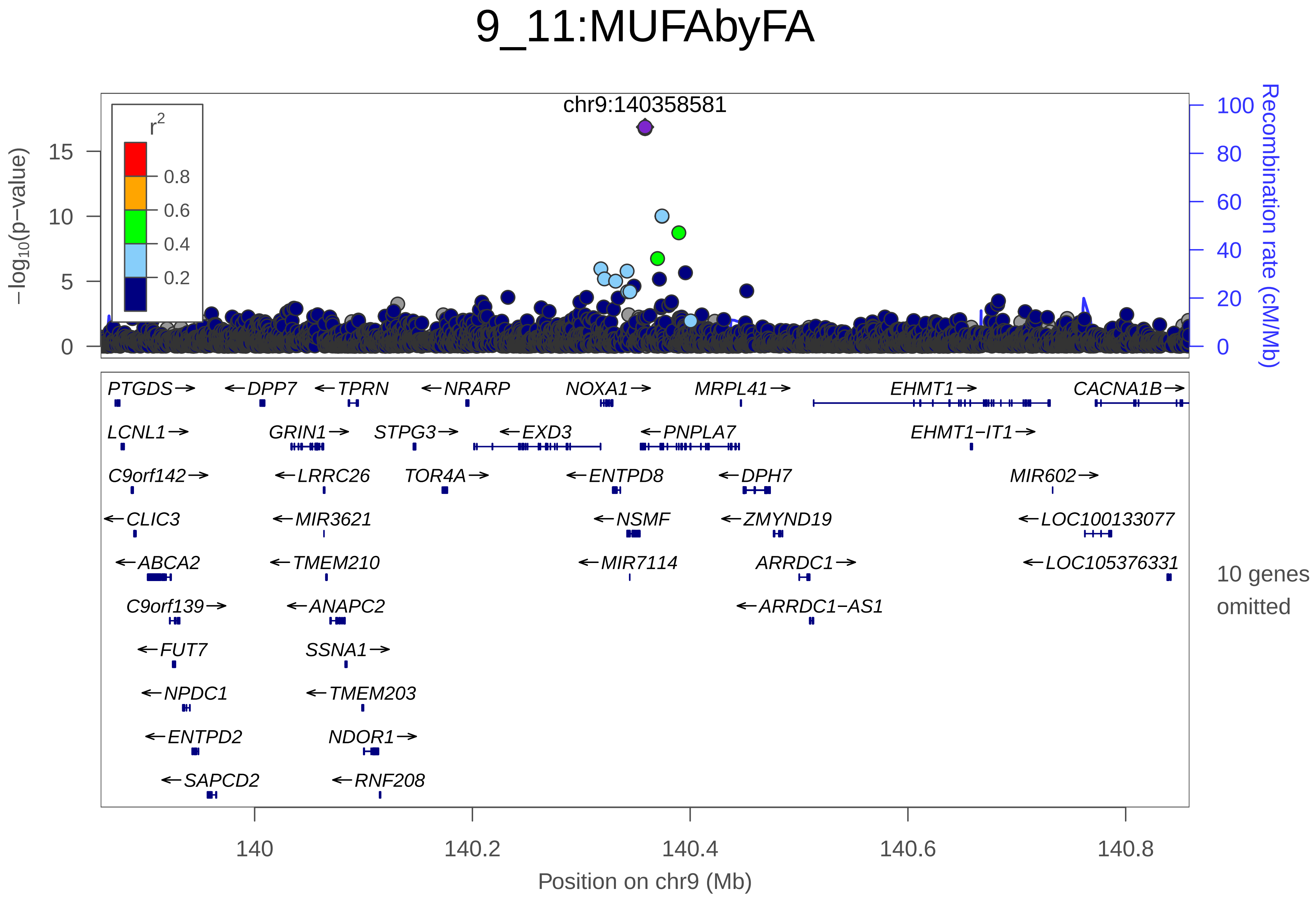
9_11:MUFAbyFA
chr9:140358581
r2
0.8
0.6
0.4
0.2
−log10(p−value)
15
10
5
0
100
80
60
40
20
0
Recombination rate (cM/Mb)
PTGDS→
←DPP7
←TPRN
←NRARP
NOXA1→
MRPL41→
EHMT1→
CACNA1B→
LCNL1→
GRIN1→
STPG3→
←EXD3
←PNPLA7
EHMT1−IT1→
C9orf142→
←LRRC26
TOR4A→
←ENTPD8
←DPH7
MIR602→
←CLIC3
←MIR3621
←NSMF
←ZMYND19
←LOC100133077
←ABCA2
←TMEM210
←MIR7114
ARRDC1→
←LOC105376331
C9orf139→
←ANAPC2
←ARRDC1−AS1
←FUT7
SSNA1→
←NPDC1
←TMEM203
←ENTPD2
NDOR1→
←SAPCD2
←RNF208
10 genes omitted
140
140.2
140.4
140.6
140.8
Position on chr9 (Mb)

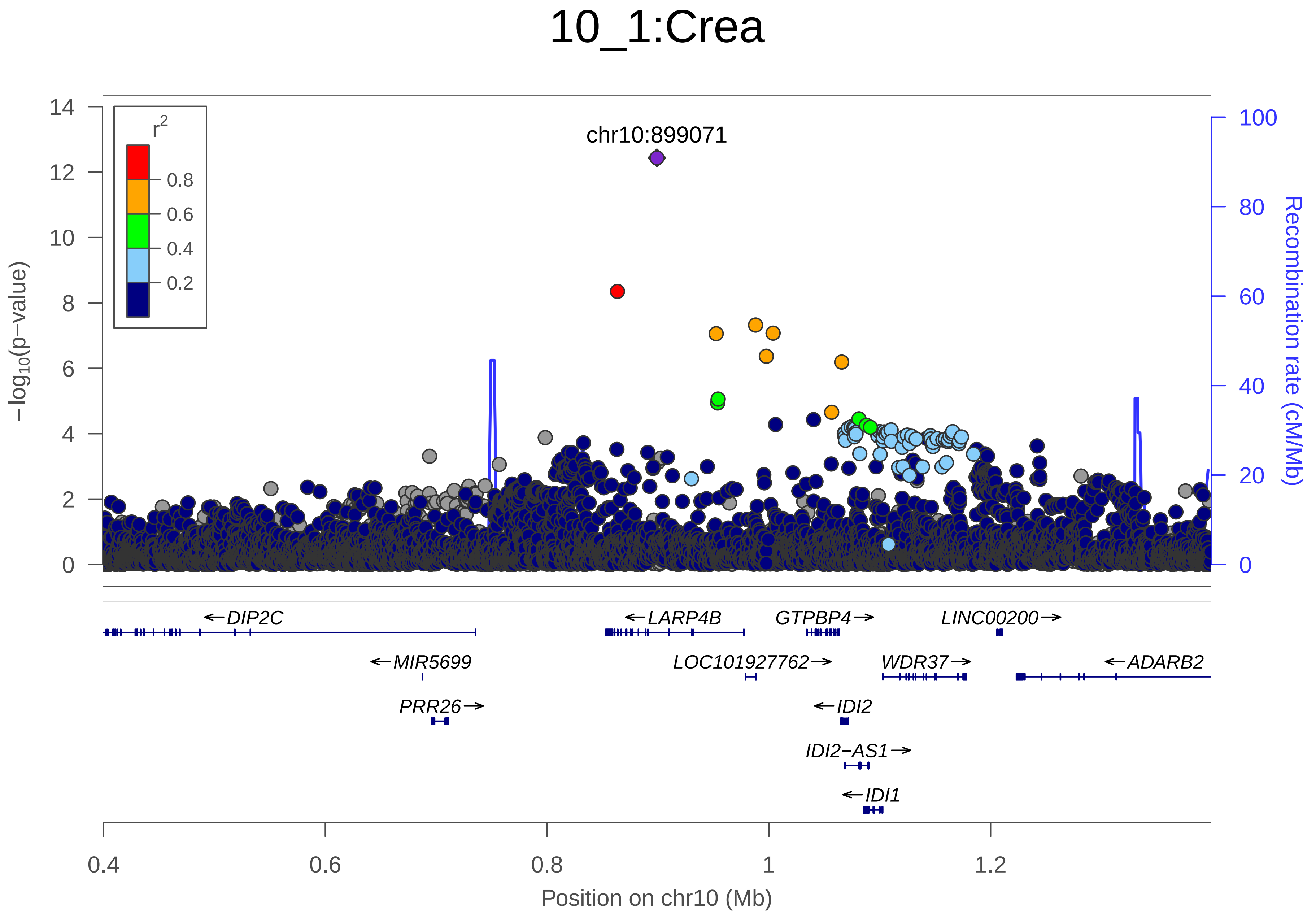

10_1:Crea
chr10:899071
r2
0.8
0.6
0.4
0.2
−log10(p−value)
Recombination rate (cM/Mb)
DIP2C
LARP4B
GTPBP4
LINC00200
MIR5699
LOC101927762
WDR37
ADARB2
PRR26
IDI2
IDI2−AS1
IDI1
Position on chr10 (Mb)

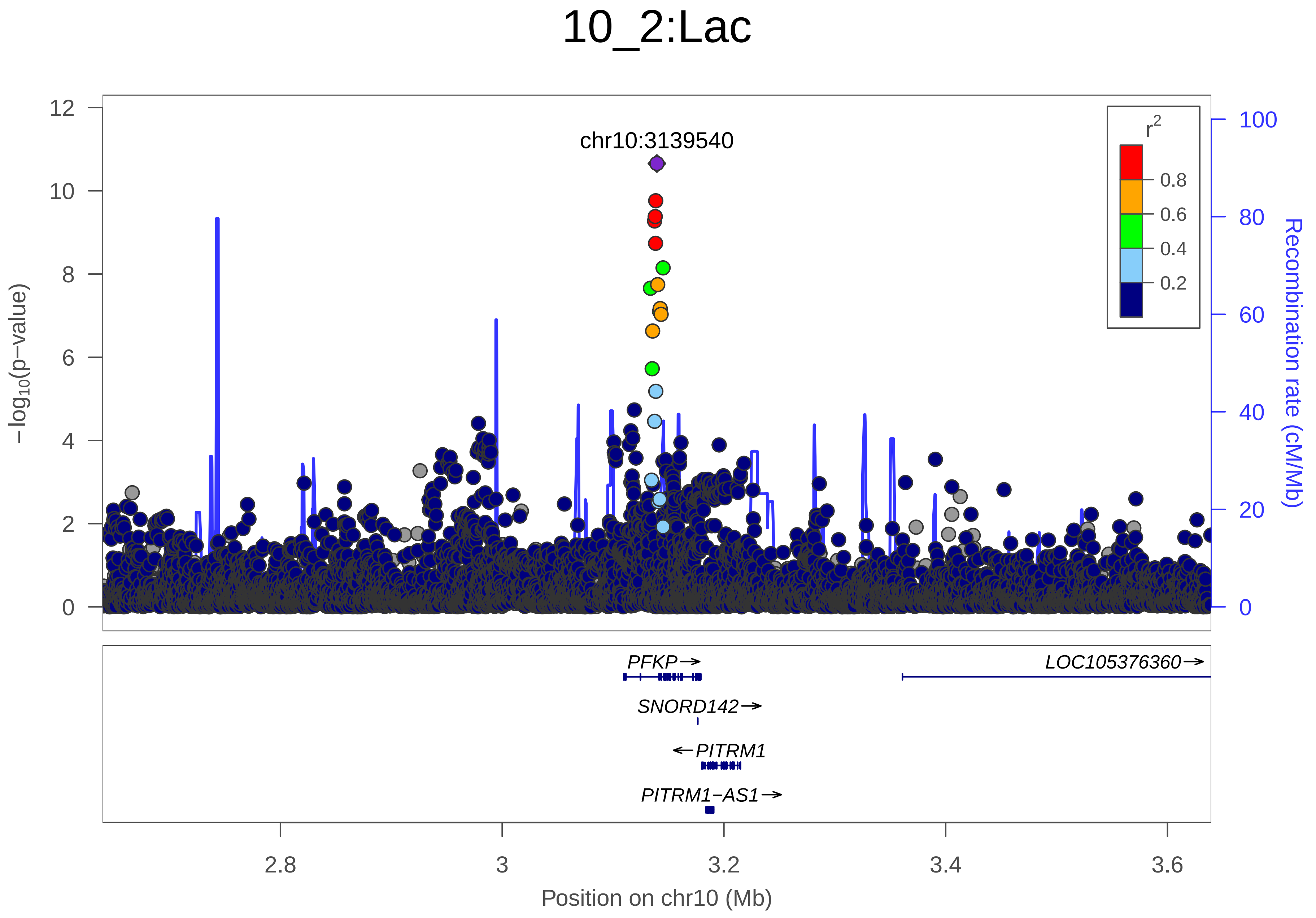
10_2:Lac
chr10:3139540
−log10(p−value)
Recombination rate (cM/Mb)
r2
0.8
0.6
0.4
0.2
12
10
8
6
4
2
0
100
80
60
40
20
0
PFKP→
LOC105376360→
SNORD142→
←PITRM1
PITRM1−AS1→
2.8
3
3.2
3.4
3.6
Position on chr10 (Mb)

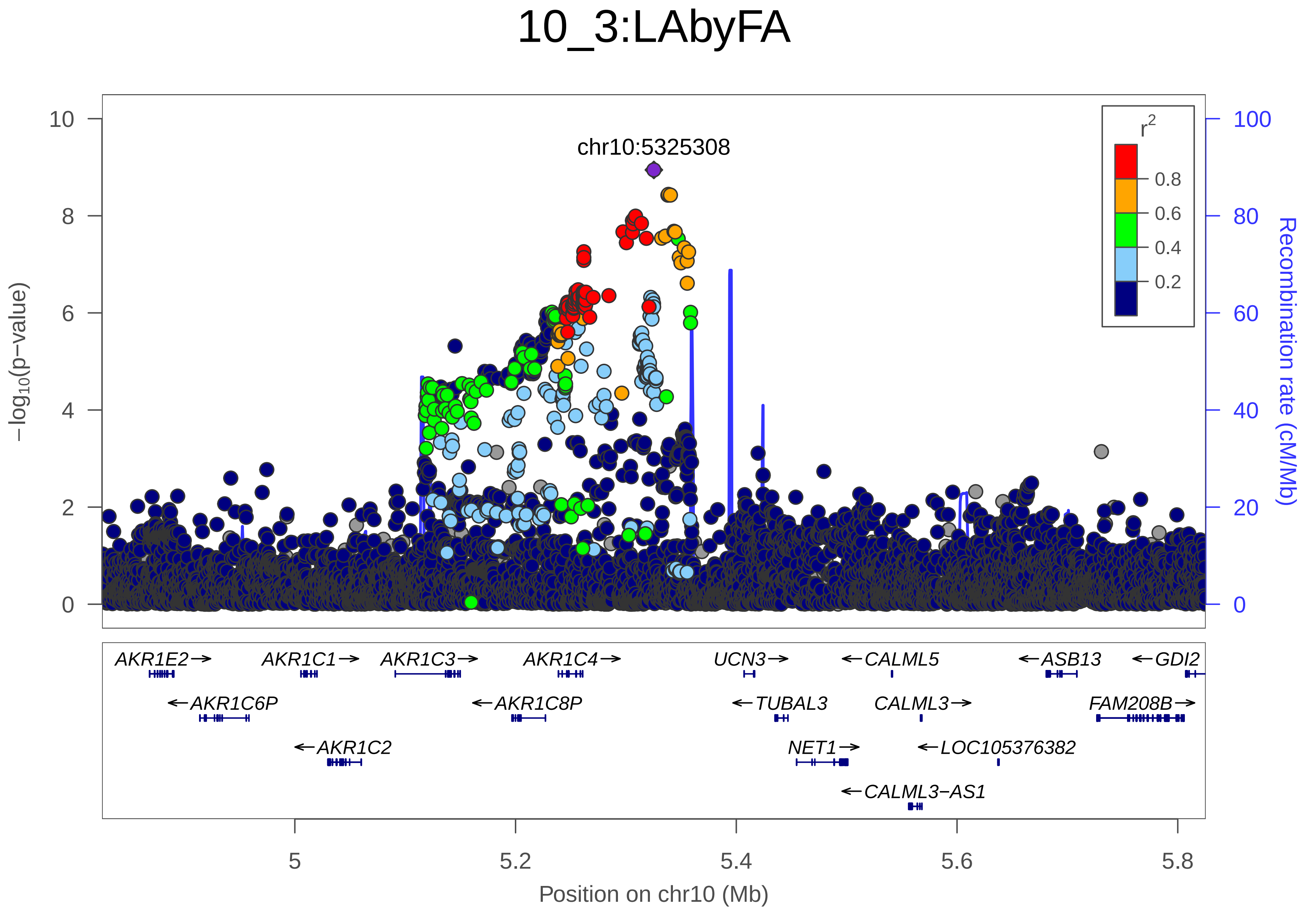
10_3:LAbyFA
chr10:5325308
−log10(p−value)
Recombination rate (cM/Mb)
r2
0.8
0.6
0.4
0.2
0
2
4
6
8
10
0
20
40
60
80
100
AKR1E2→
AKR1C1→
AKR1C3→
AKR1C4→
UCN3→
←CALML5
←ASB13
←GDI2
←AKR1C6P
←AKR1C8P
←TUBAL3
CALML3→
FAM208B→
←AKR1C2
NET1→
←LOC105376382
←CALML3−AS1
5
5.2
5.4
5.6
5.8
Position on chr10 (Mb)

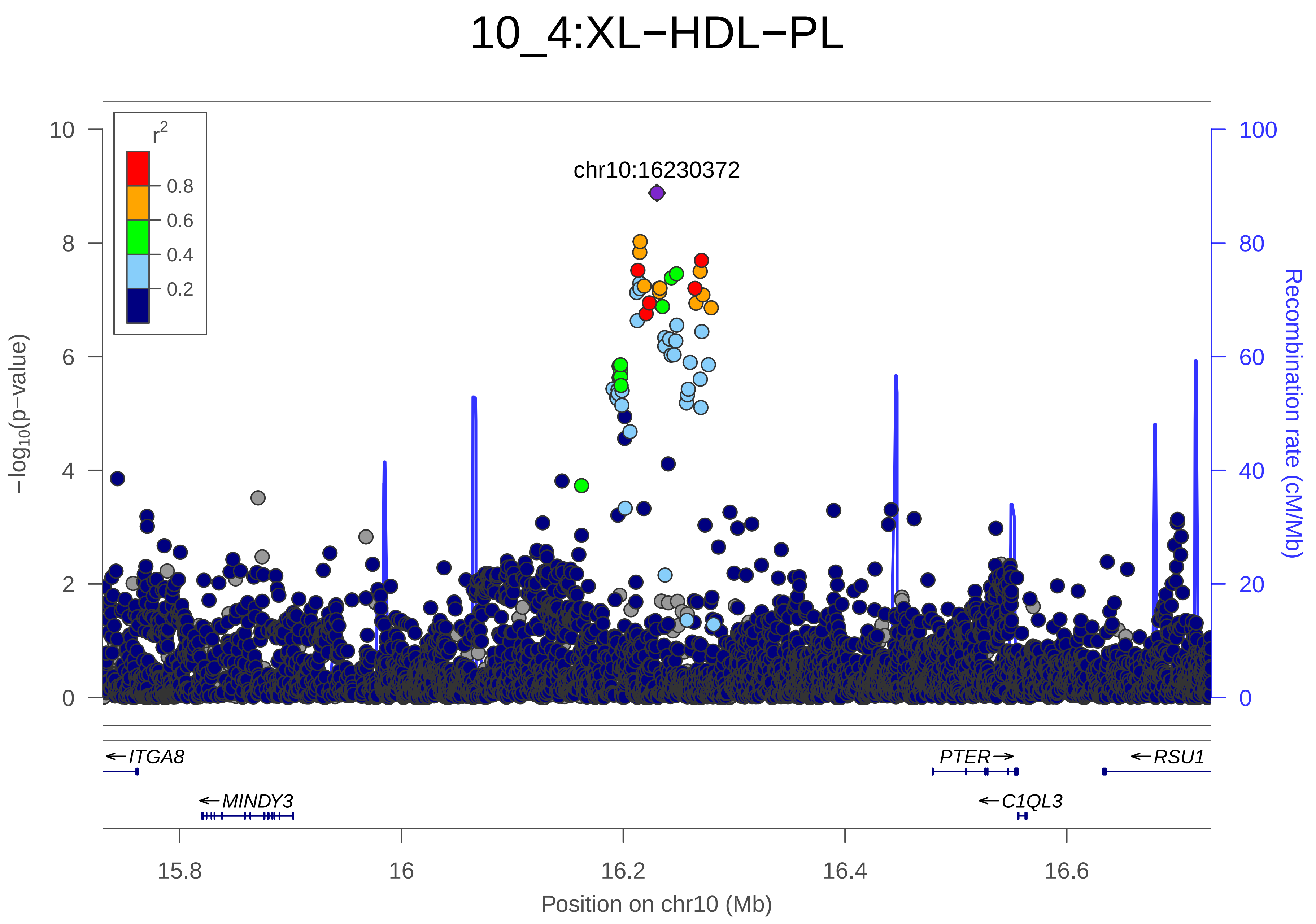

10_4:XL−HDL−PL
r2
0.8
0.6
0.4
0.2
chr10:16230372
−log10(p−value)
Recombination rate (cM/Mb)
0
2
4
6
8
10
0
20
40
60
80
100
ITGA8
MINDY3
PTER
C1QL3
RSU1
15.8
16
16.2
16.4
16.6
Position on chr10 (Mb)

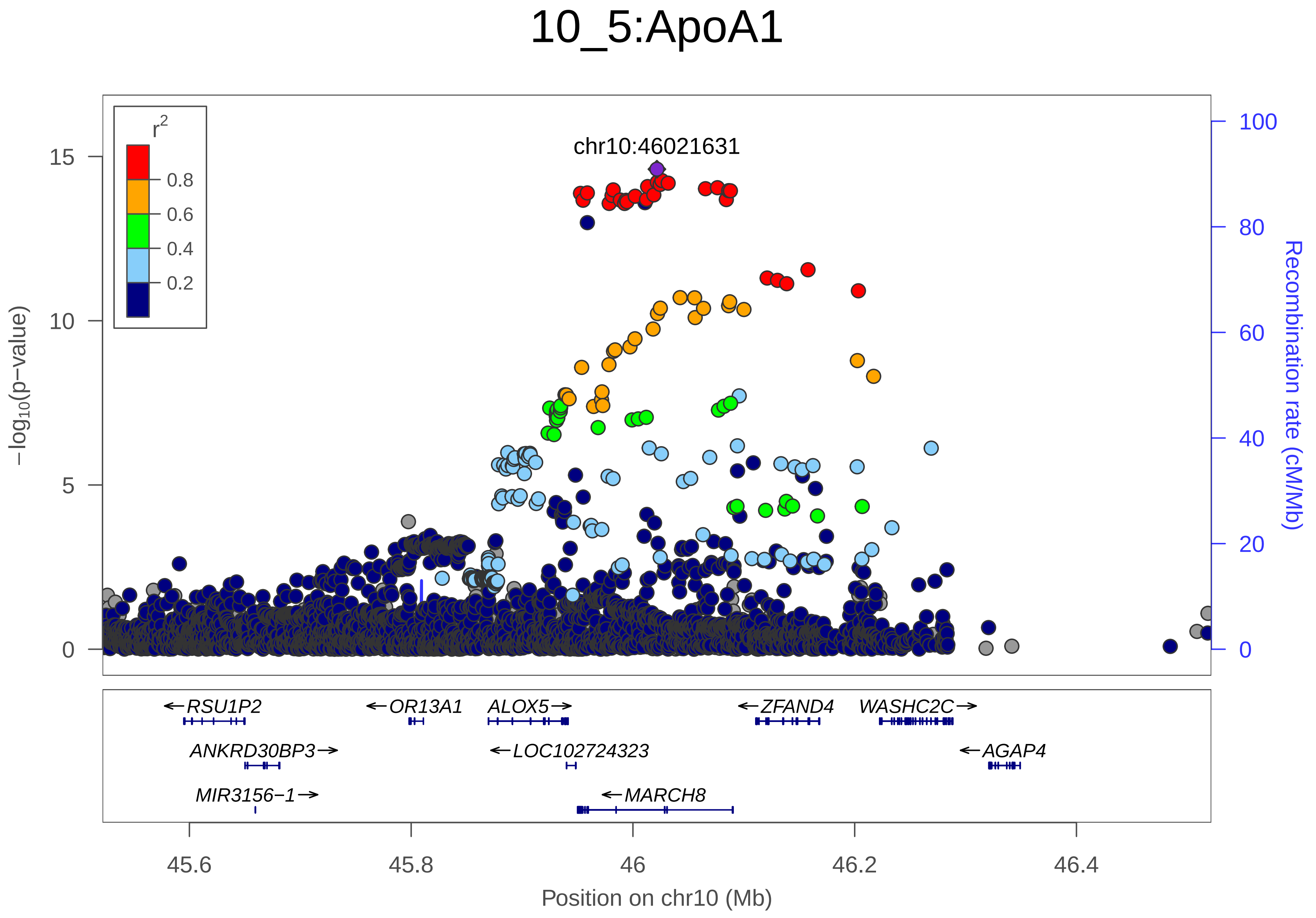

10_5:ApoA1
chr10:46021631
r2
0.8
0.6
0.4
0.2
−log10(p−value)
Recombination rate (cM/Mb)
15
10
5
0
100
80
60
40
20
0
RSU1P2
OR13A1
ALOX5
ZFAND4
WASHC2C
ANKRD30BP3
LOC102724323
AGAP4
MIR3156−1
MARCH8
45.6
45.8
46
46.2
46.4
Position on chr10 (Mb)

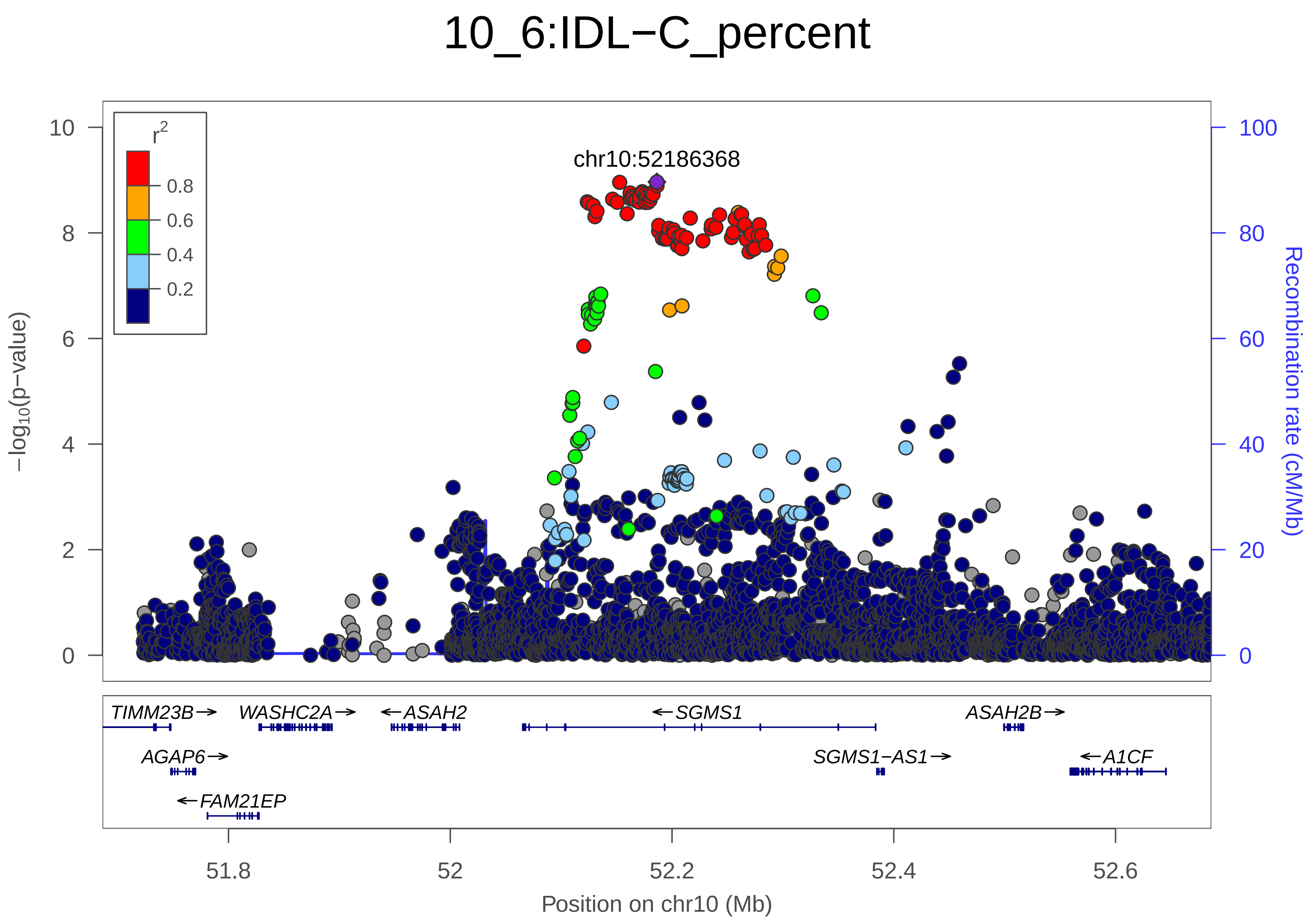
10_6:IDL−C_percent
chr10:52186368
r2
0.8
0.6
0.4
0.2
−log10(p−value)
Recombination rate (cM/Mb)
TIMM23B→
WASHC2A→
←ASAH2
←SGMS1
ASAH2B→
AGAP6→
SGMS1−AS1→
←A1CF
←FAM21EP
51.8
52
52.2
52.4
52.6
Position on chr10 (Mb)

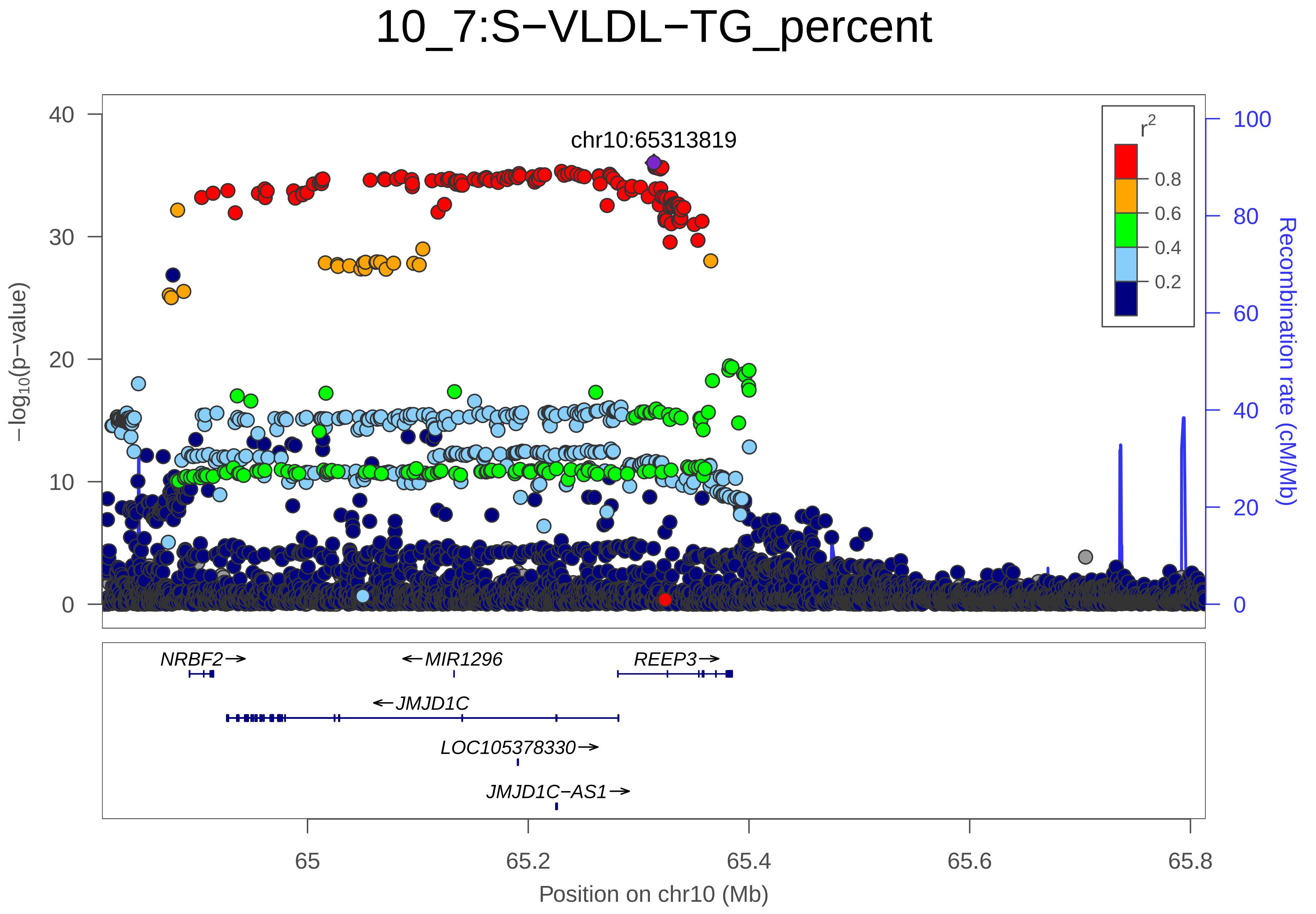
10_7:S−VLDL−TG_percent
chr10:65313819
r2
0.8
0.6
0.4
0.2
−log10(p−value)
Recombination rate (cM/Mb)
NRBF2→
←MIR1296
REEP3→
←JMJD1C
LOC105378330→
JMJD1C−AS1→
Position on chr10 (Mb)

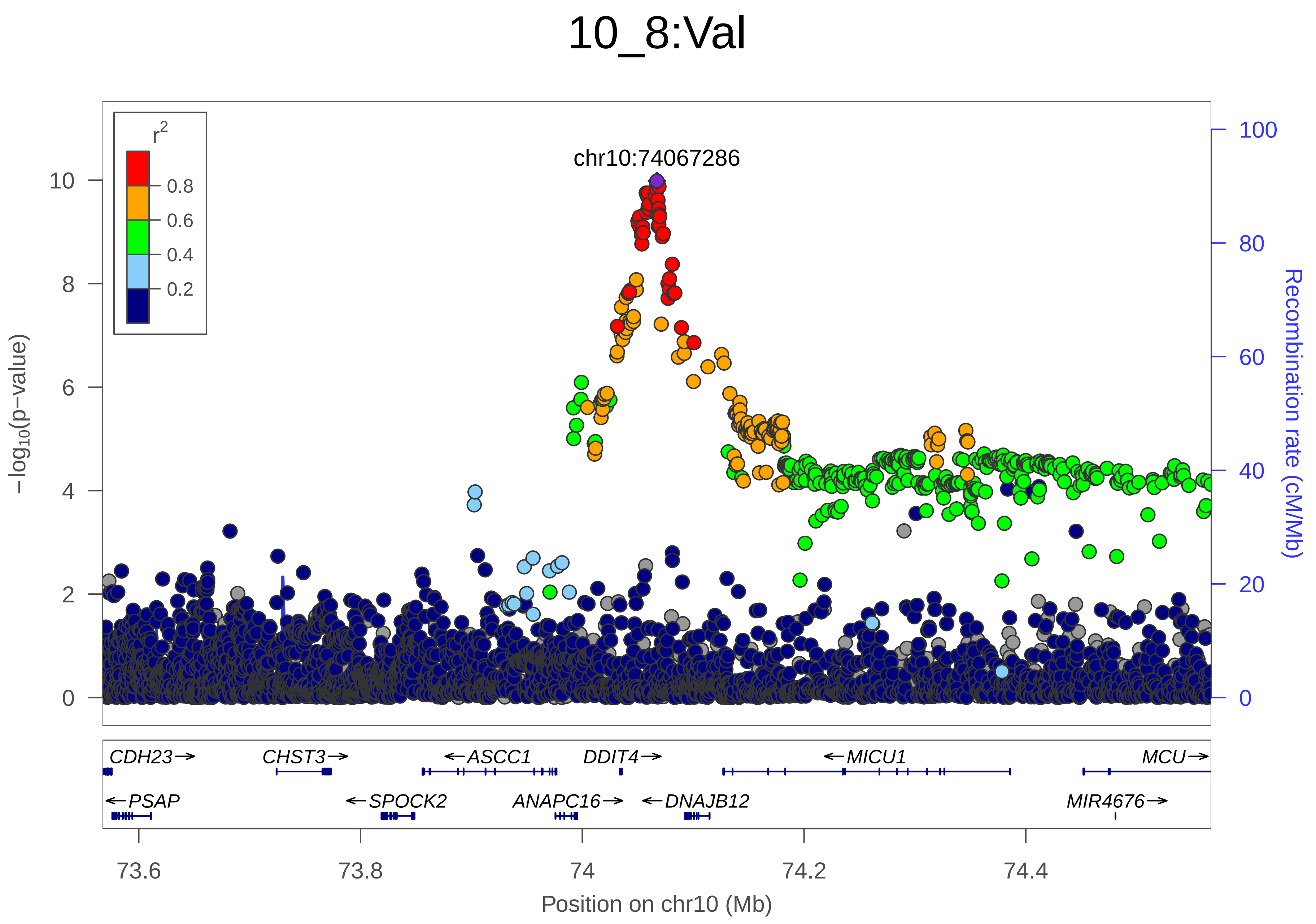

10_8:Val
r2
0.8
0.6
0.4
0.2
chr10:74067286
−log10(p−value)
Recombination rate (cM/Mb)
10
8
6
4
2
0
100
80
60
40
20
0
CDH23→
CHST3→
←ASCC1
DDIT4→
←MICU1
MCU→
←PSAP
←SPOCK2
ANAPC16→
←DNAJB12
MIR4676→
73.6
73.8
74
74.2
74.4
Position on chr10 (Mb)

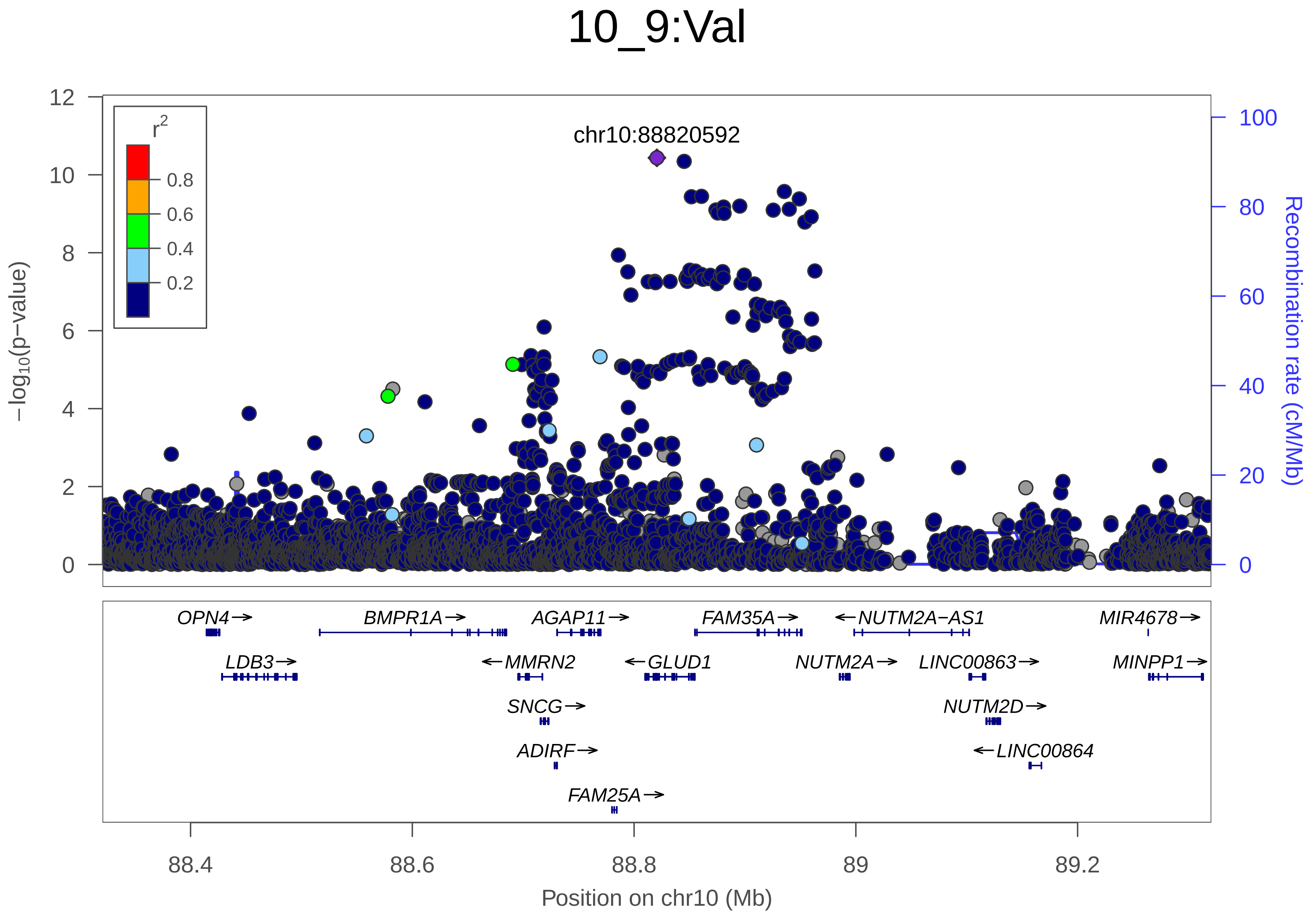

10_9:Val
chr10:88820592
r2
0.8
0.6
0.4
0.2
−log10(p−value)
Recombination rate (cM/Mb)
0
2
4
6
8
10
12
0
20
40
60
80
100
OPN4→
BMPR1A→
AGAP11→
FAM35A→
←NUTM2A−AS1
MIR4678→
LDB3→
←MMRN2
←GLUD1
NUTM2A→
LINC00863→
MINPP1→
SNCG→
NUTM2D→
ADIRF→
←LINC00864
FAM25A→
88.4
88.6
88.8
89
89.2
Position on chr10 (Mb)

10_10:S−VLDL−L

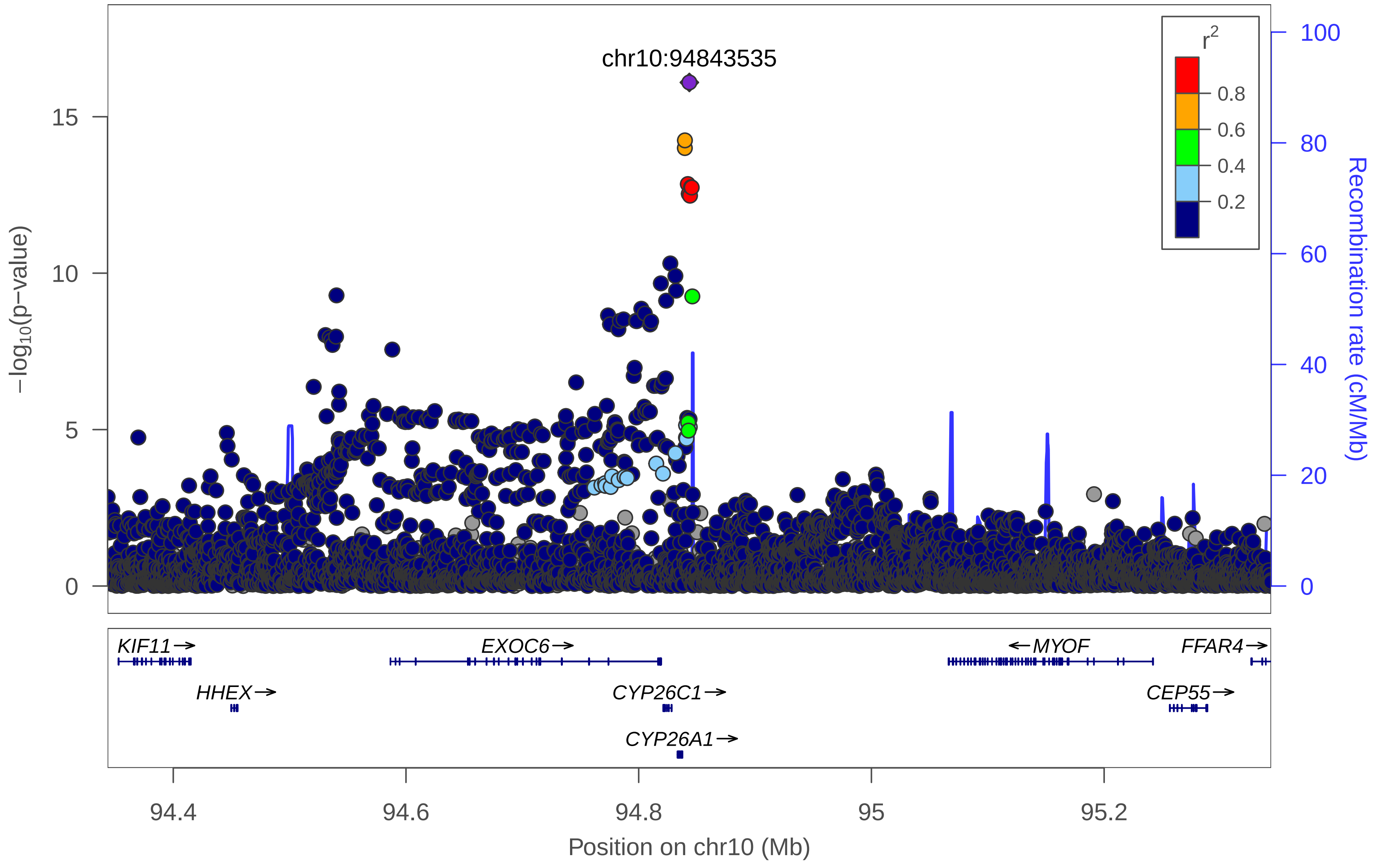

chr10:94843535
r2
0.8
0.6
0.4
0.2
15
10
5
0
100
80
60
40
20
0
−log10(p−value)
Recombination rate (cM/Mb)
KIF11→
HHEX→
EXOC6→
CYP26C1→
CYP26A1→
←MYOF
FFAR4→
CEP55→
94.4
94.6
94.8
95
95.2
Position on chr10 (Mb)

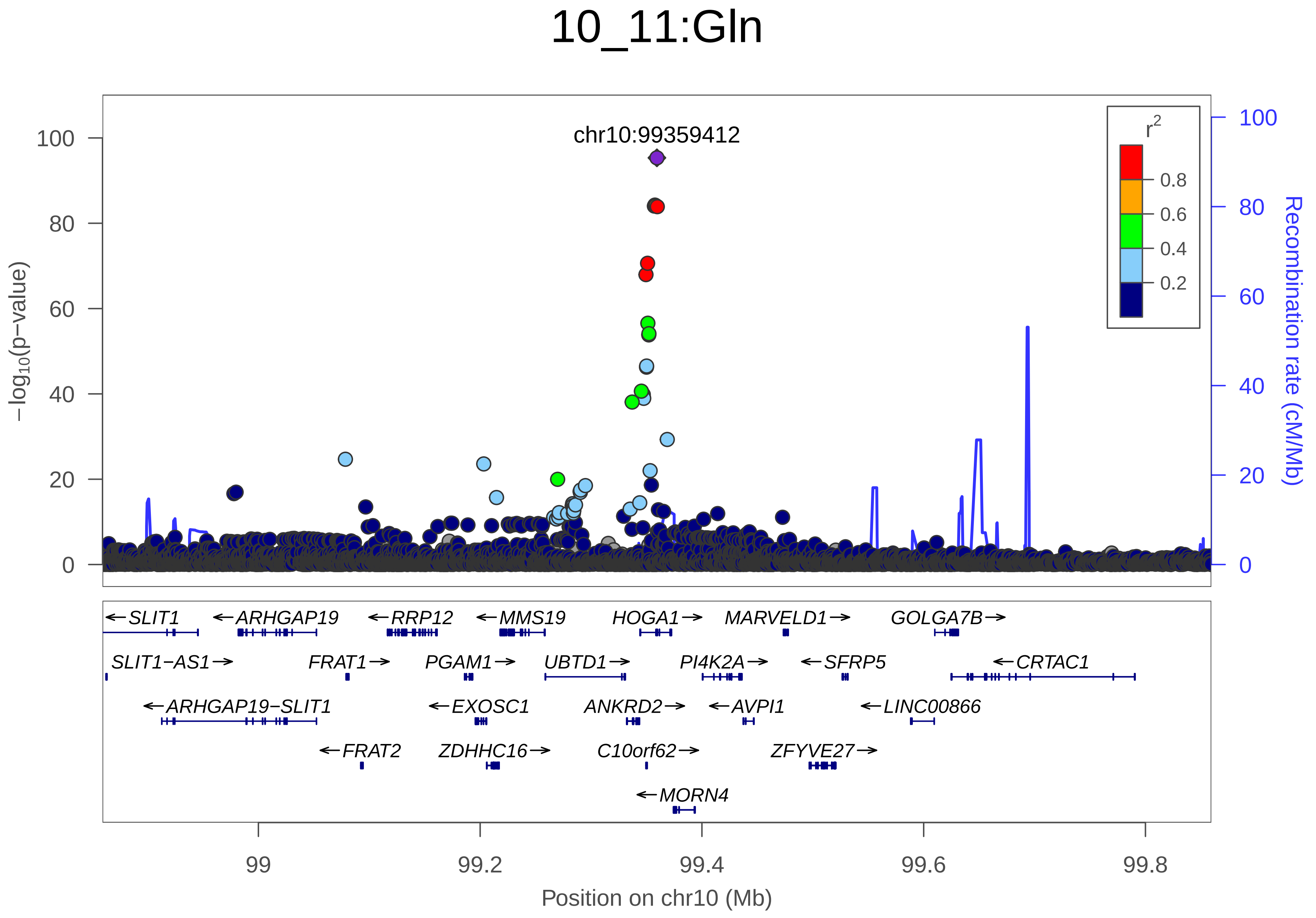
10_11:Gln
chr10:99359412
r2
0.8
0.6
0.4
0.2
100
80
60
40
20
0
−log10(p−value)
Recombination rate (cM/Mb)
SLIT1
ARHGAP19
RRP12
MMS19
HOGA1
MARVELD1
GOLGA7B
SLIT1−AS1
FRAT1
PGAM1
UBTD1
PI4K2A
SFRP5
CRTAC1
ARHGAP19−SLIT1
EXOSC1
ANKRD2
AVPI1
LINC00866
FRAT2
ZDHHC16
C10orf62
ZFYVE27
MORN4
99
99.2
99.4
99.6
99.8
Position on chr10 (Mb)

10_12:SFAbyFA

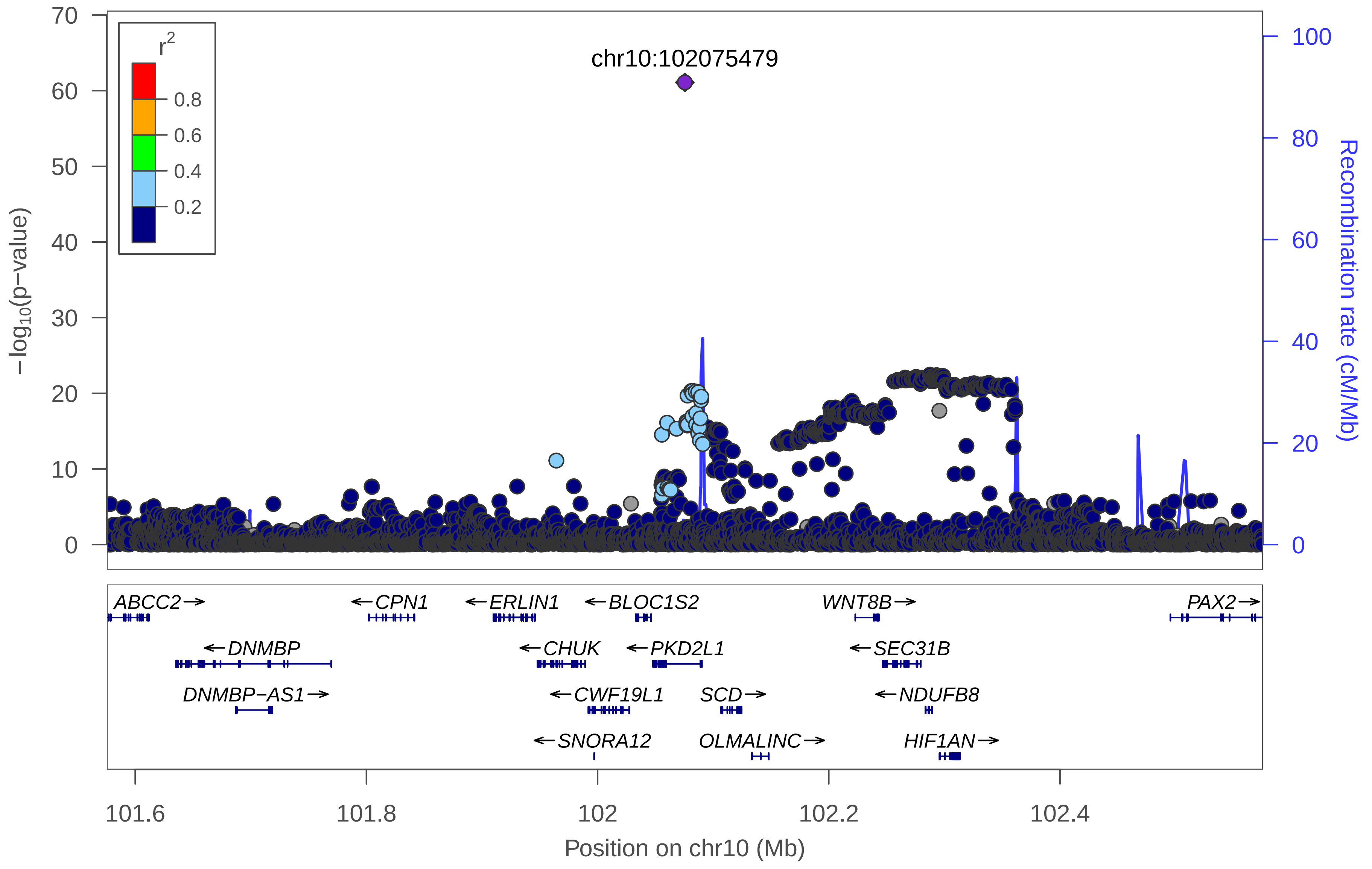

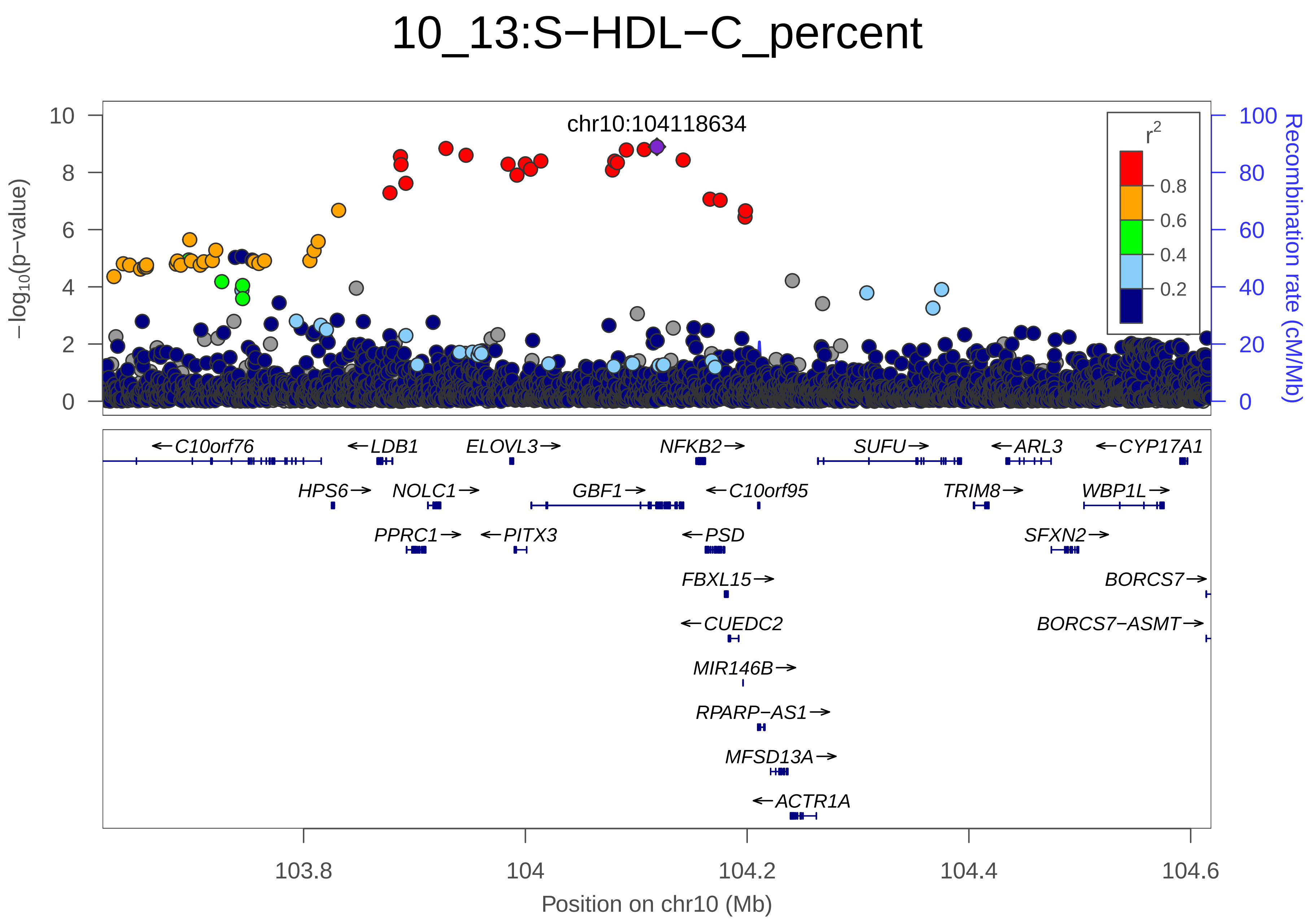
10_13:S−HDL−C_percent
chr10:104118634
−log10(p−value)
Recombination rate (cM/Mb)
r2
0.8
0.6
0.4
0.2
10
8
6
4
2
0
100
80
60
40
20
0
←C10orf76
←LDB1
ELOVL3→
NFKB2→
SUFU→
←ARL3
←CYP17A1
HPS6→
NOLC1→
GBF1→
←C10orf95
TRIM8→
WBP1L→
PPRC1→
←PITX3
←PSD
SFXN2→
FBXL15→
BORCS7→
←CUEDC2
BORCS7−ASMT→
MIR146B→
RPARP−AS1→
MFSD13A→
←ACTR1A
103.8
104
104.2
104.4
104.6
Position on chr10 (Mb)

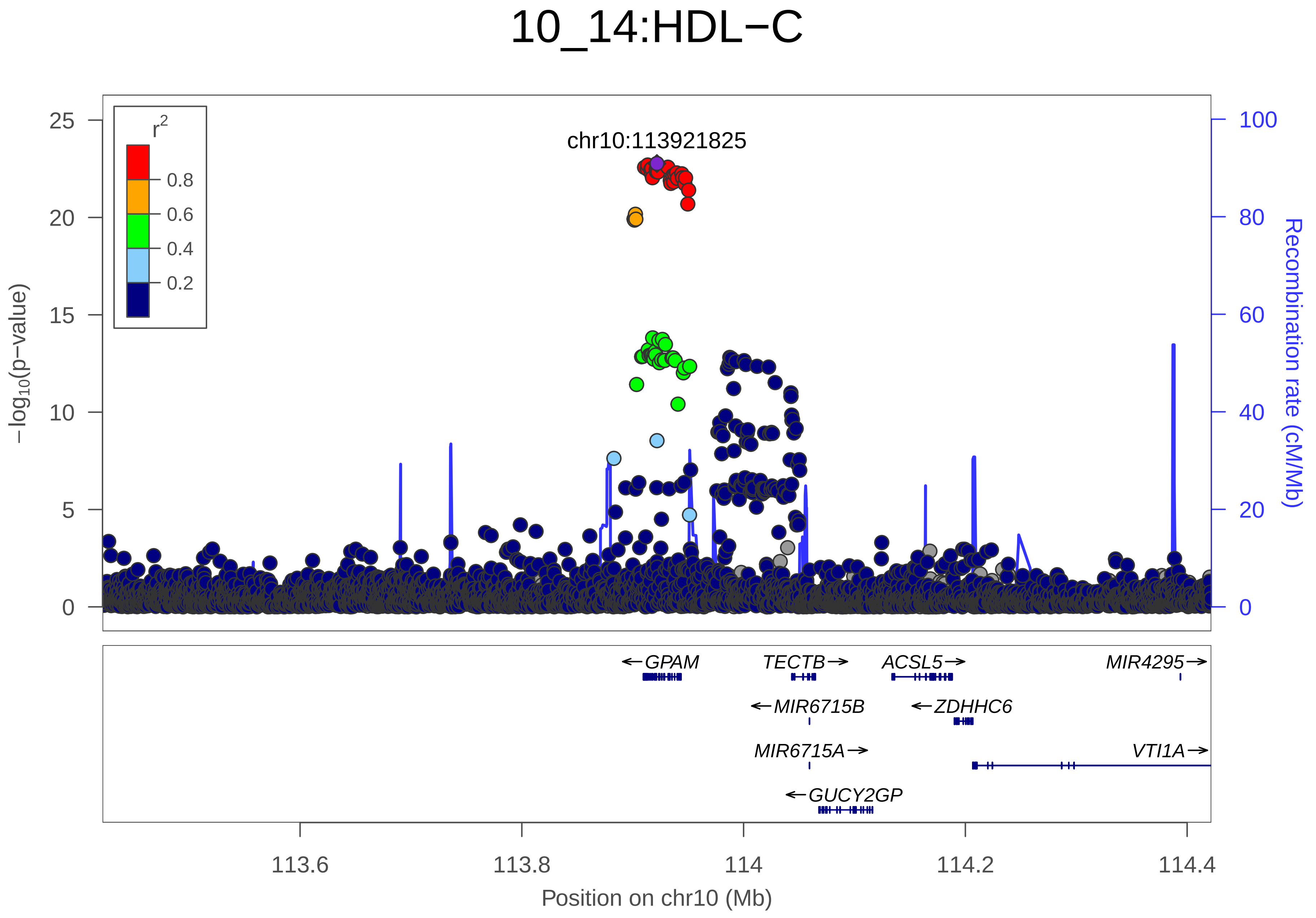

10_14:HDL−C
chr10:113921825
r2
0.8
0.6
0.4
0.2
−log10(p−value)
Recombination rate (cM/Mb)
25
20
15
10
5
0
100
80
60
40
20
0
GPAM
TECTB
ACSL5
MIR4295
MIR6715B
ZDHHC6
MIR6715A
VTI1A
GUCY2GP
113.6
113.8
114
114.2
114.4
Position on chr10 (Mb)

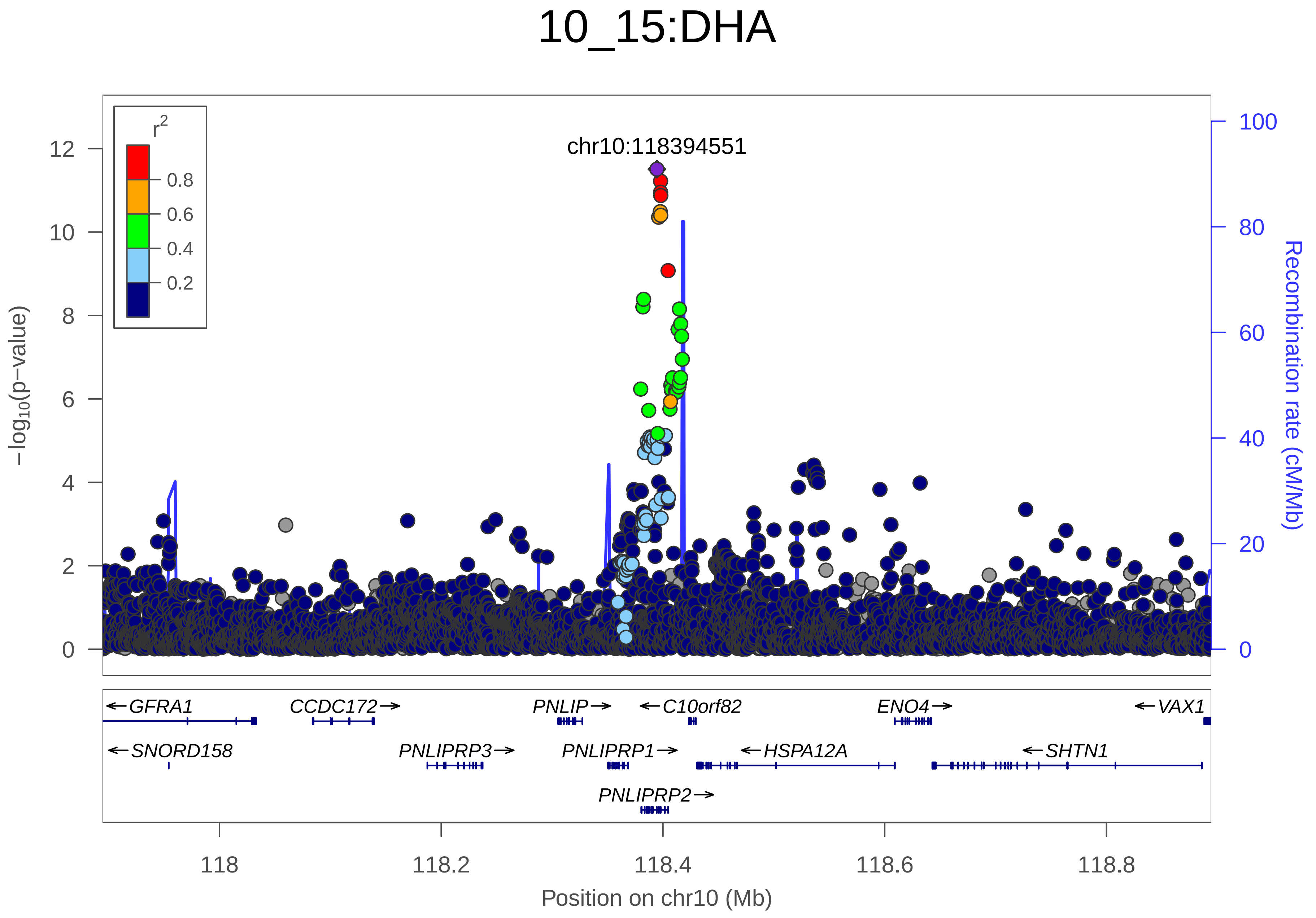
10_15:DHA
chr10:118394551
r2
0.8
0.6
0.4
0.2
12
10
8
6
4
2
0
−log10(p−value)
100
80
60
40
20
0
Recombination rate (cM/Mb)
GFRA1
CCDC172
PNLIP
C10orf82
ENO4
VAX1
SNORD158
PNLIPRP3
PNLIPRP1
HSPA12A
SHTN1
PNLIPRP2
118
118.2
118.4
118.6
118.8
Position on chr10 (Mb)

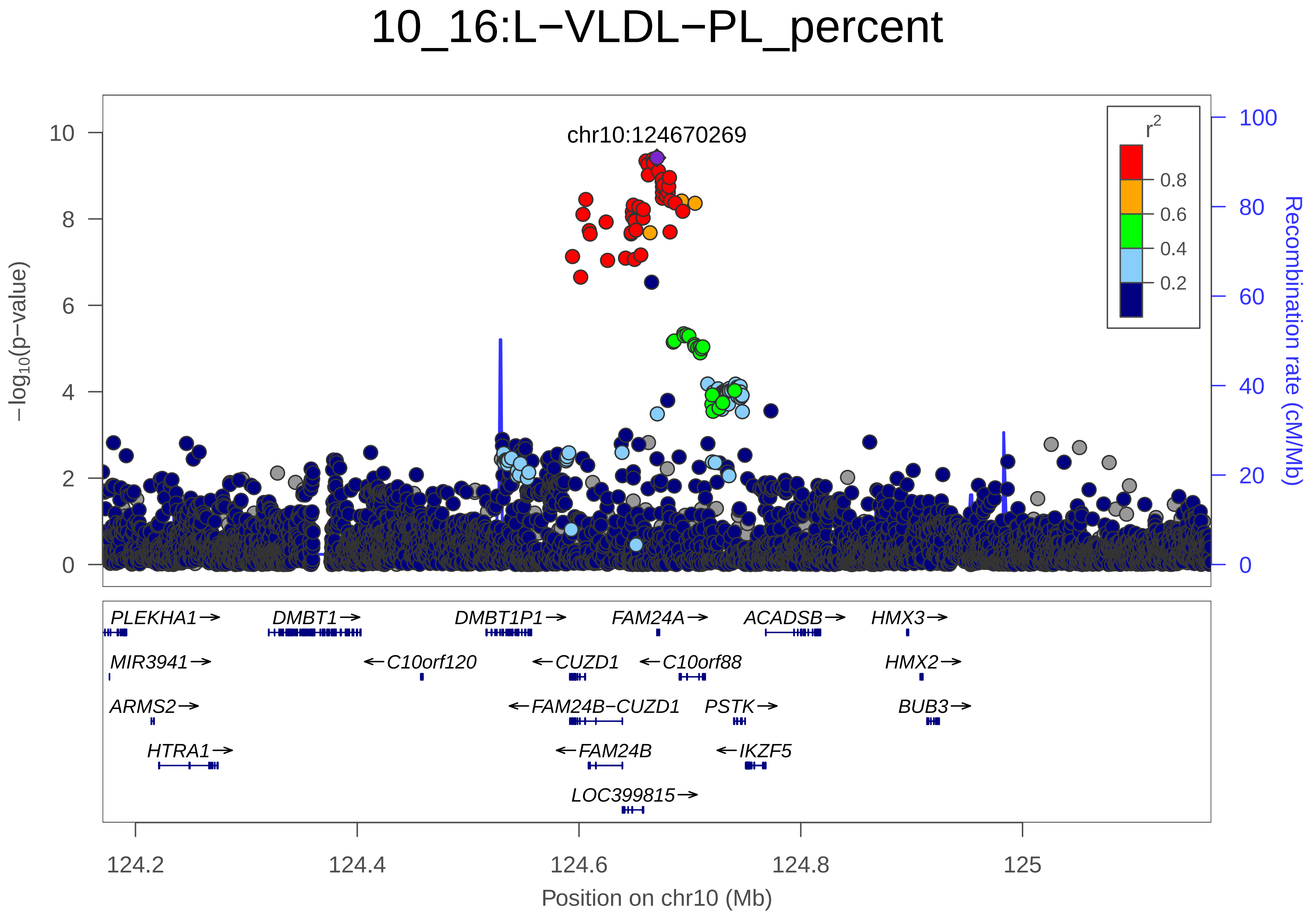

10_16:L−VLDL−PL_percent
chr10:124670269
r2
0.8
0.6
0.4
0.2
−log10(p−value)
Recombination rate (cM/Mb)
0
2
4
6
8
10
0
20
40
60
80
100
PLEKHA1→
DMBT1→
DMBT1P1→
FAM24A→
ACADSB→
HMX3→
MIR3941→
←C10orf120
←CUZD1
←C10orf88
HMX2→
ARMS2→
←FAM24B−CUZD1
PSTK→
BUB3→
HTRA1→
←FAM24B
←IKZF5
LOC399815→
124.2
124.4
124.6
124.8
125
Position on chr10 (Mb)

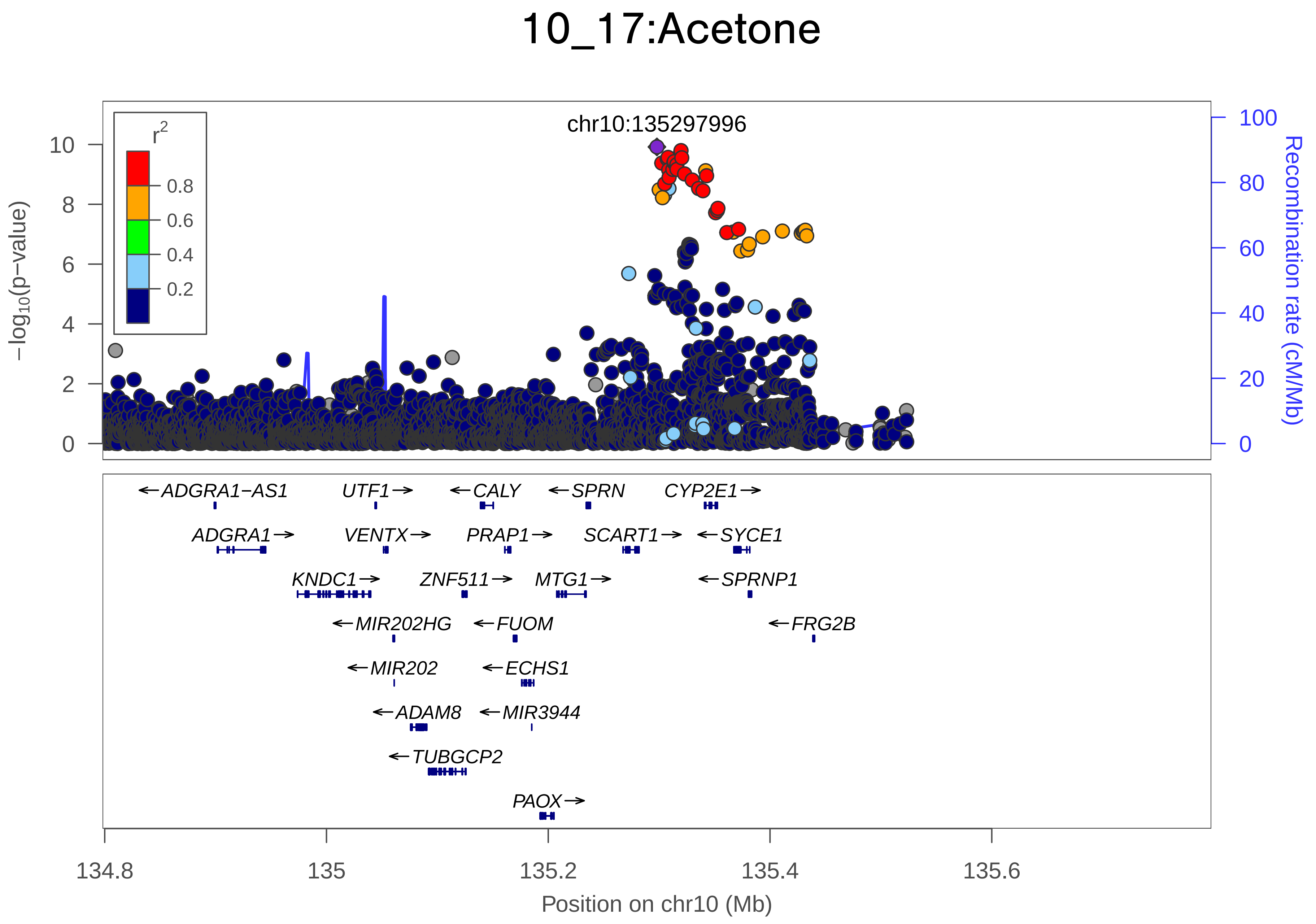
10_17:Acetone
chr10:135297996
r2
0.8
0.6
0.4
0.2
−log10(p−value)
Recombination rate (cM/Mb)
0
2
4
6
8
10
0
20
40
60
80
100
←ADGRA1−AS1
UTF1→
←CALY
←SPRN
CYP2E1→
ADGRA1→
VENTX→
PRAP1→
SCART1→
←SYCE1
KNDC1→
ZNF511→
MTG1→
←SPRNP1
←MIR202HG
←FUOM
←FRG2B
←MIR202
←ECHS1
←ADAM8
←MIR3944
←TUBGCP2
PAOX→
134.8
135
135.2
135.4
135.6
Position on chr10 (Mb)

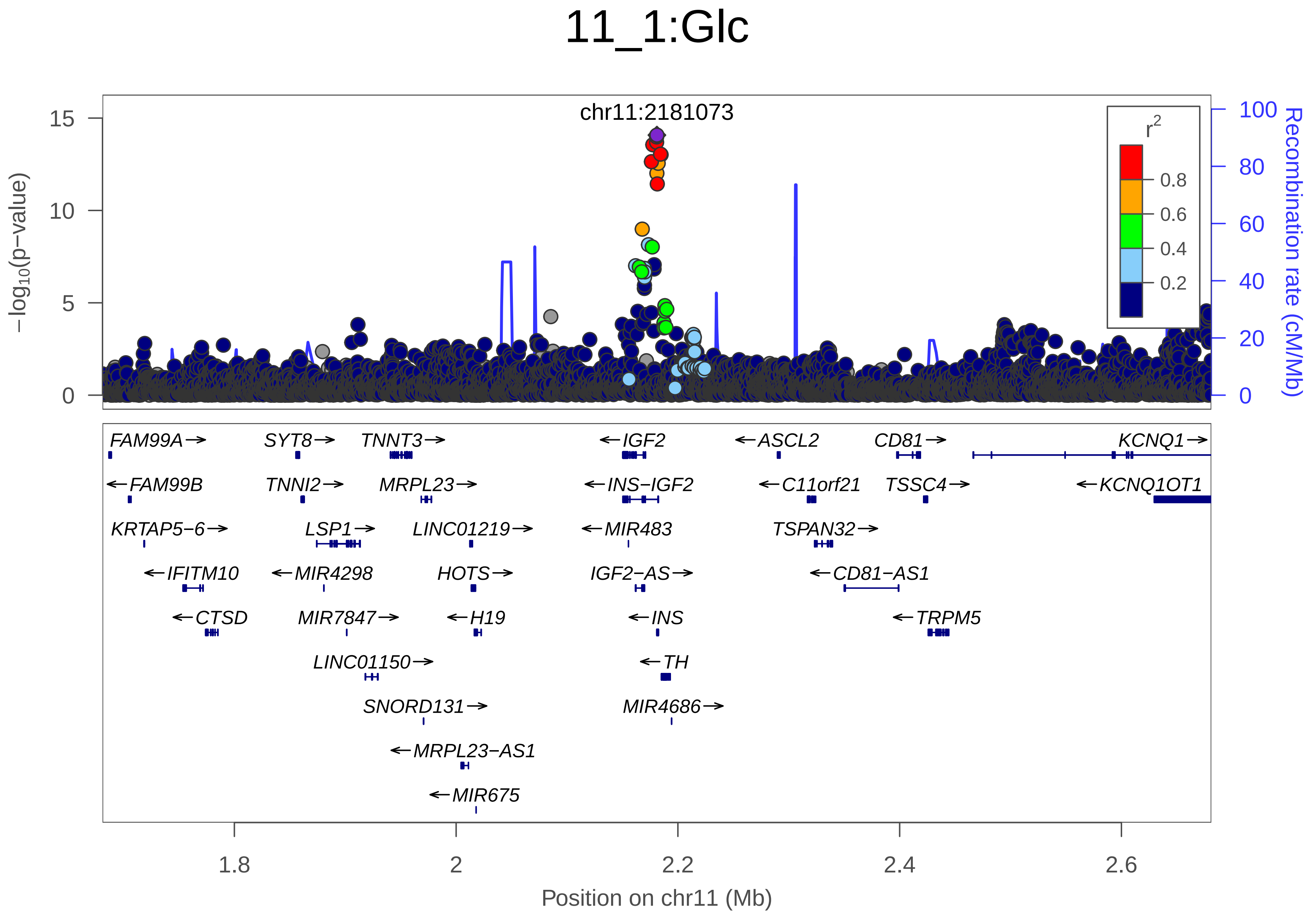

11_1:Glc
chr11:2181073
−log10(p−value)
Recombination rate (cM/Mb)
r2
0.8
0.6
0.4
0.2
FAM99A→
SYT8→
TNNT3→
←IGF2
←ASCL2
CD81→
KCNQ1→
←FAM99B
TNNI2→
MRPL23→
←INS−IGF2
←C11orf21
TSSC4→
←KCNQ1OT1
KRTAP5−6→
LSP1→
LINC01219→
←MIR483
TSPAN32→
←IFITM10
←MIR4298
HOTS→
IGF2−AS→
←CD81−AS1
←CTSD
MIR7847→
←H19
←INS
←TRPM5
LINC01150→
←TH
SNORD131→
MIR4686→
←MRPL23−AS1
←MIR675
Position on chr11 (Mb)

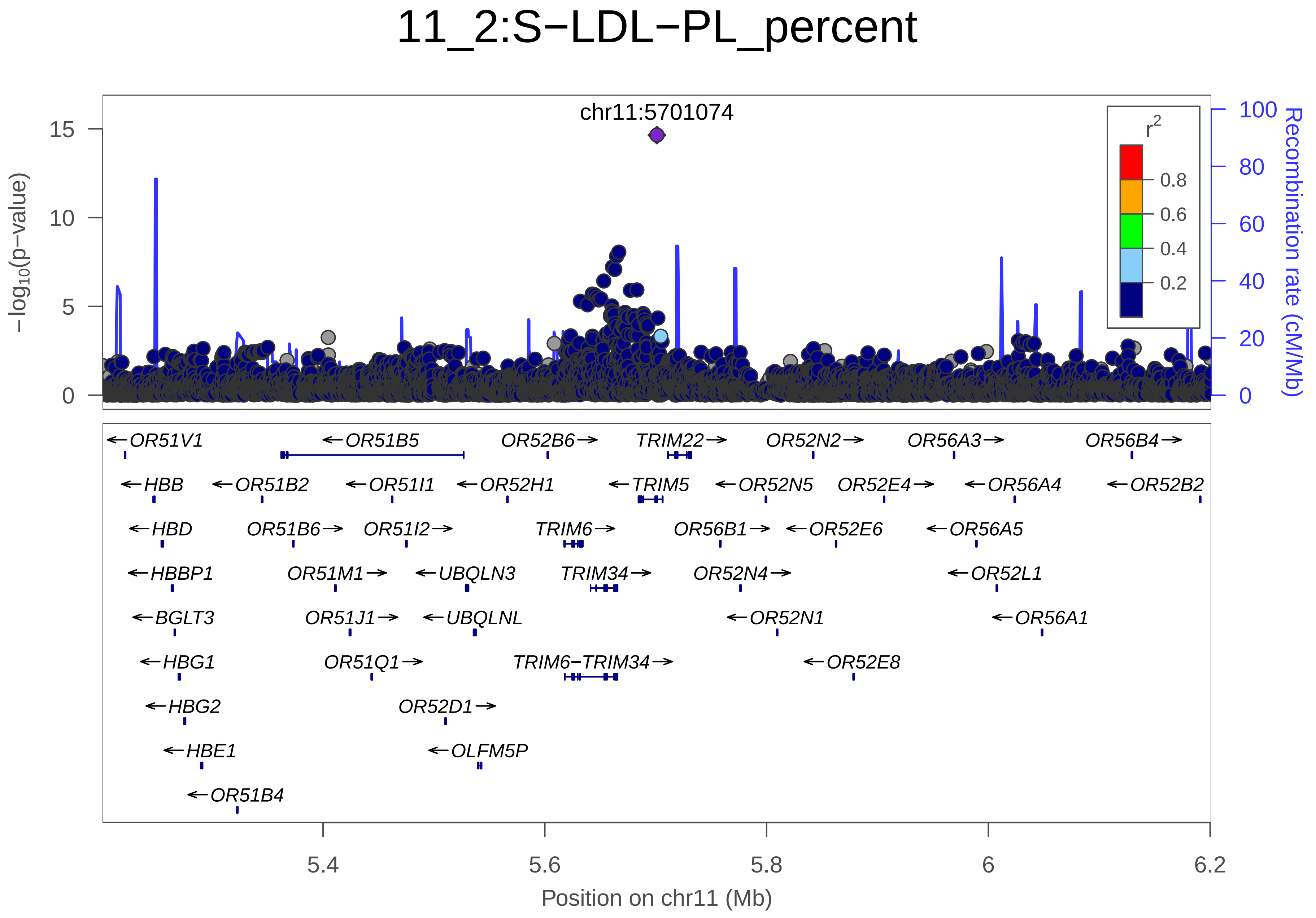

11_2:S−LDL−PL_percent
chr11:5701074
−log10(p−value)
Recombination rate (cM/Mb)
r2
0.8
0.6
0.4
0.2
0
5
10
15
0
20
40
60
80
100
OR51V1
OR51B5
OR52B6
TRIM22
OR52N2
OR56A3
OR56B4
HBB
OR51B2
OR51I1
OR52H1
TRIM5
OR52N5
OR52E4
OR56A4
OR52B2
HBD
OR51B6
OR51I2
TRIM6
OR56B1
OR52E6
OR56A5
HBBP1
OR51M1
UBQLN3
TRIM34
OR52N4
OR52L1
BGLT3
OR51J1
UBQLNL
OR52N1
OR56A1
HBG1
OR51Q1
TRIM6−TRIM34
OR52E8
HBG2
OR52D1
HBE1
OLFM5P
OR51B4
5.4
5.6
5.8
6
6.2
Position on chr11 (Mb)

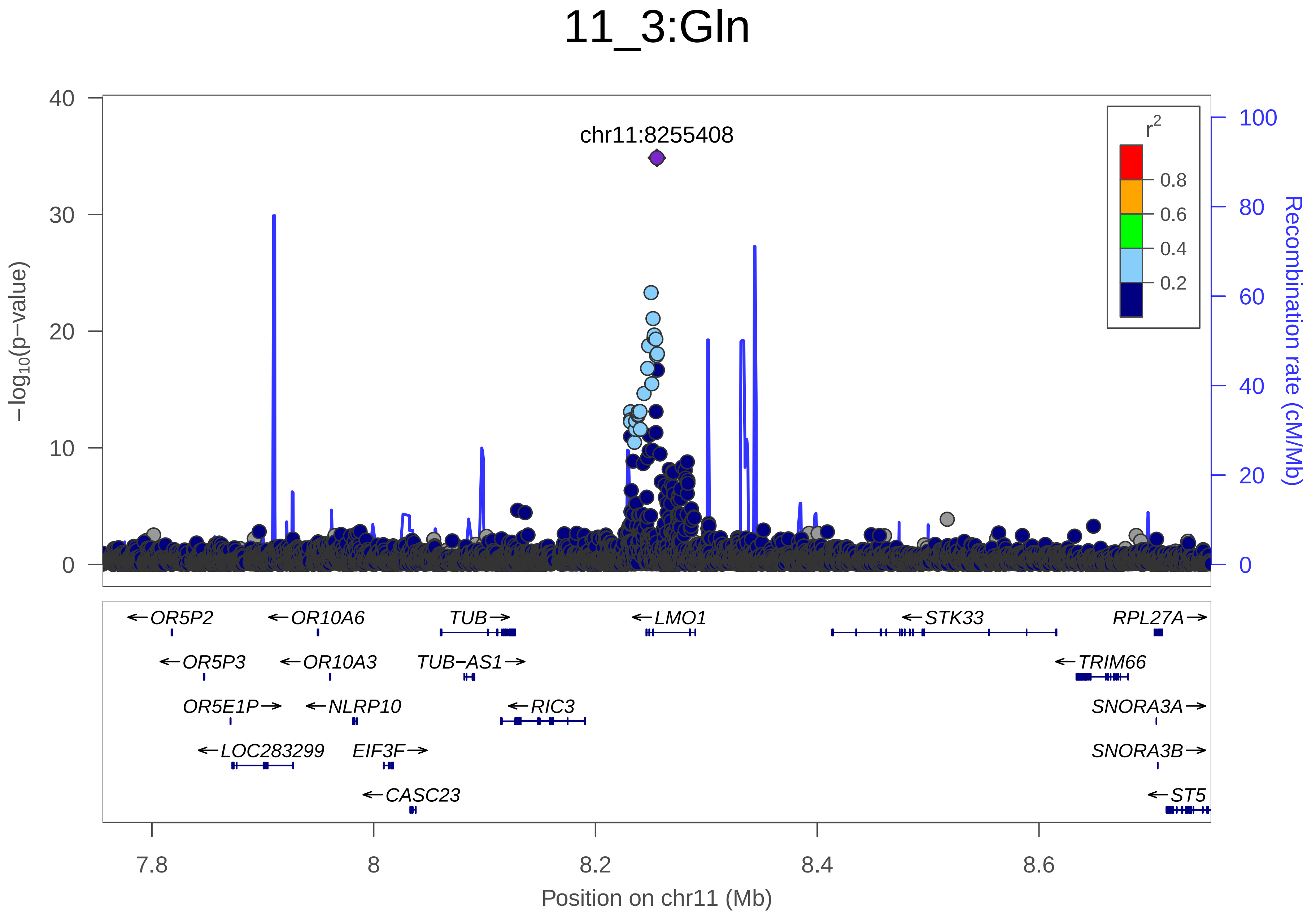

11_3:Gln
chr11:8255408
−log10(p−value)
Recombination rate (cM/Mb)
r2
0.8
0.6
0.4
0.2
OR5P2
OR10A6
TUB
LMO1
STK33
RPL27A
OR5P3
OR10A3
TUB−AS1
TRIM66
OR5E1P
NLRP10
RIC3
SNORA3A
LOC283299
EIF3F
SNORA3B
CASC23
ST5
7.8
8
8.2
8.4
8.6
Position on chr11 (Mb)

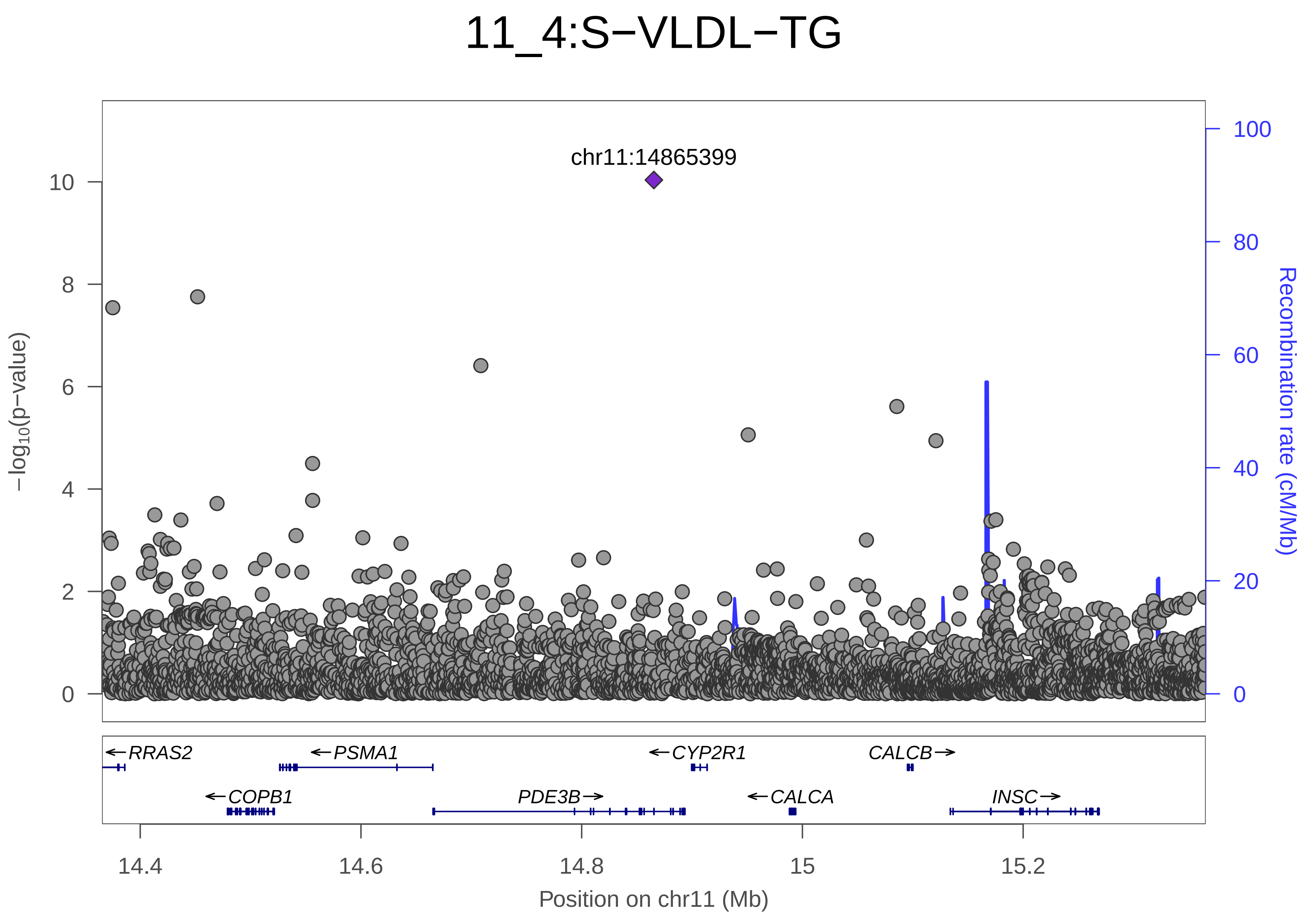
11_4:S−VLDL−TG
chr11:14865399
−log10(p−value)
Recombination rate (cM/Mb)
Position on chr11 (Mb)
RRAS2
PSMA1
CYP2R1
CALCB
COPB1
PDE3B
CALCA
INSC
0
2
4
6
8
10
20
40
60
80
100
14.4
14.6
14.8
15
15.2

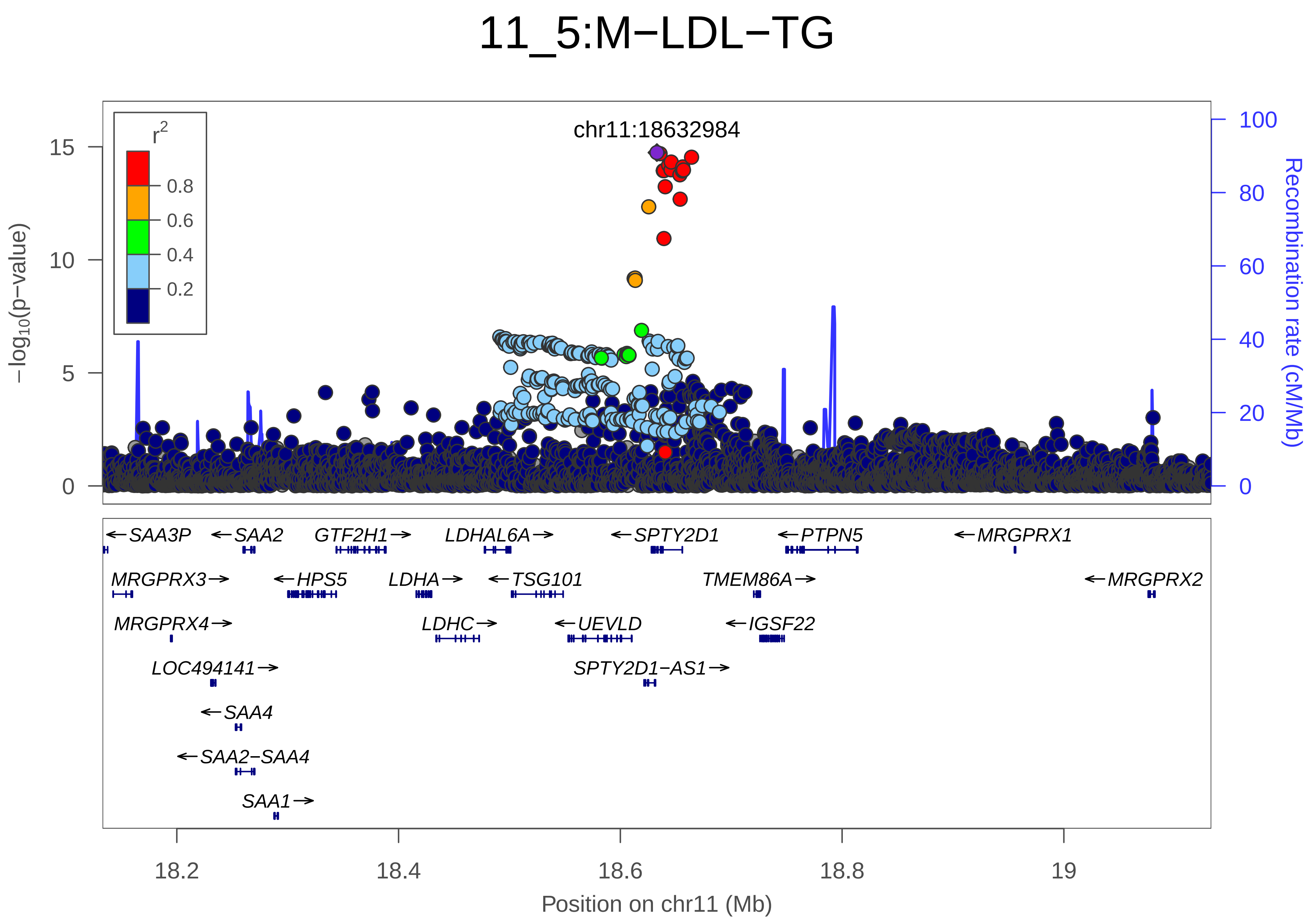

11_5:M−LDL−TG
chr11:18632984
r2
0.8
0.6
0.4
0.2
−log10(p−value)
Recombination rate (cM/Mb)
SAA3P
SAA2
GTF2H1
LDHAL6A
SPTY2D1
PTPN5
MRGPRX1
MRGPRX3
HPS5
LDHA
TSG101
TMEM86A
MRGPRX2
MRGPRX4
LDHC
UEVLD
IGSF22
LOC494141
SPTY2D1−AS1
SAA4
SAA2−SAA4
SAA1
Position on chr11 (Mb)

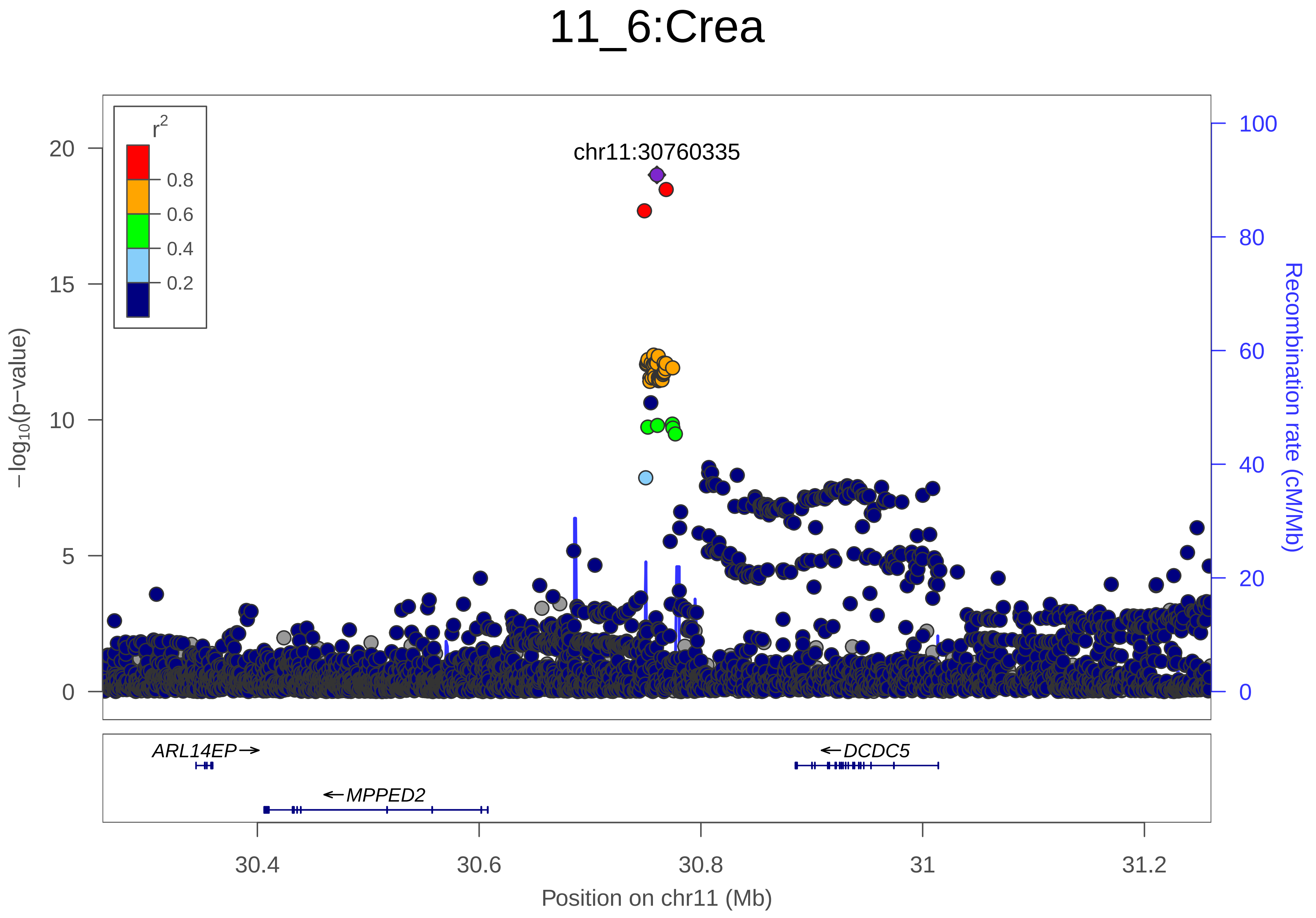

11_6:Crea
chr11:30760335
r2
0.8
0.6
0.4
0.2
−log10(p−value)
Recombination rate (cM/Mb)
ARL14EP
DCDC5
MPPED2
30.4
30.6
30.8
31
31.2
Position on chr11 (Mb)

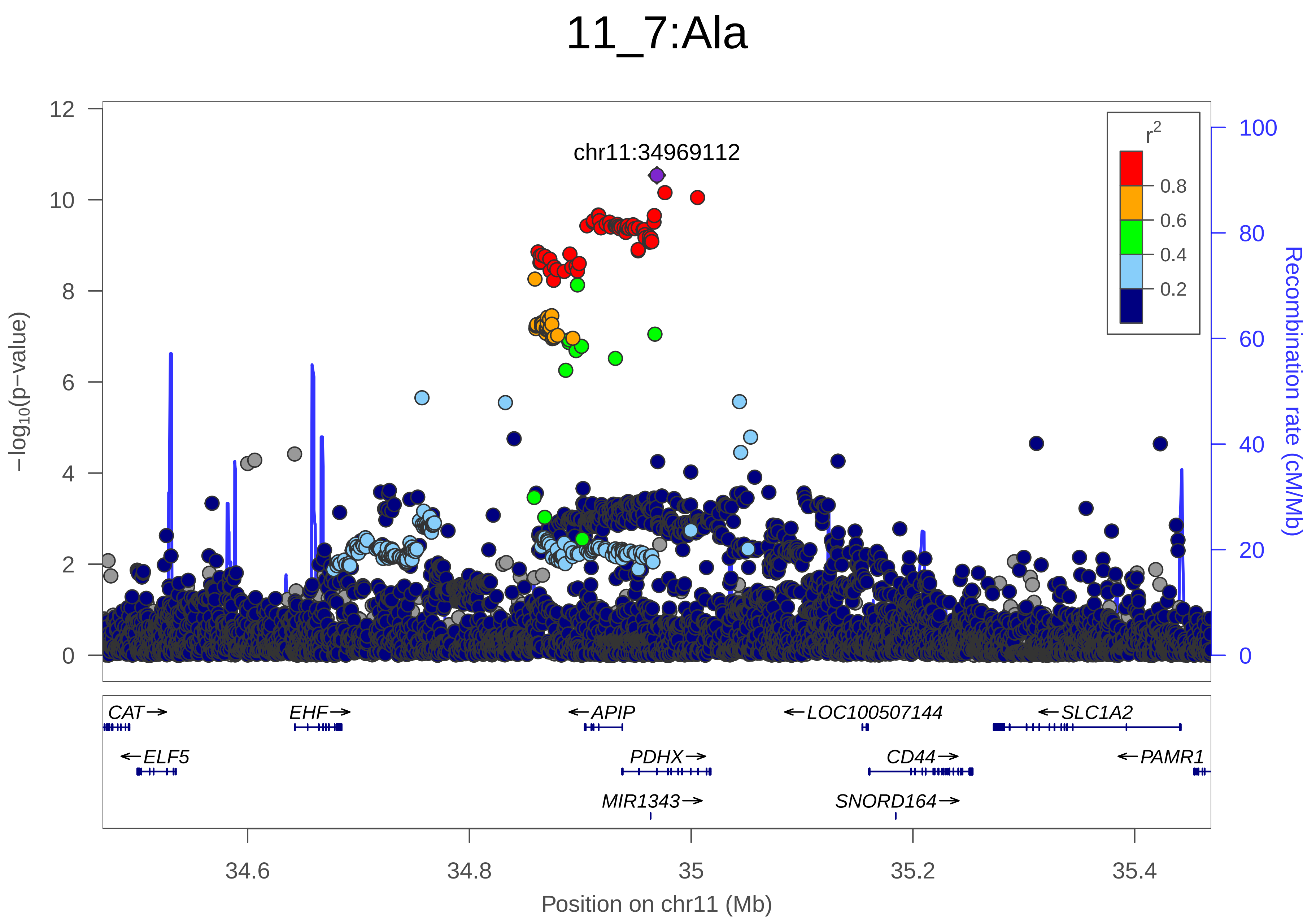

11_7:Ala
chr11:34969112
r2
0.8
0.6
0.4
0.2
−log10(p−value)
Recombination rate (cM/Mb)
0
2
4
6
8
10
12
0
20
40
60
80
100
CAT
EHF
APIP
LOC100507144
SLC1A2
ELF5
PDHX
CD44
PAMR1
MIR1343
SNORD164
34.6
34.8
35
35.2
35.4
Position on chr11 (Mb)

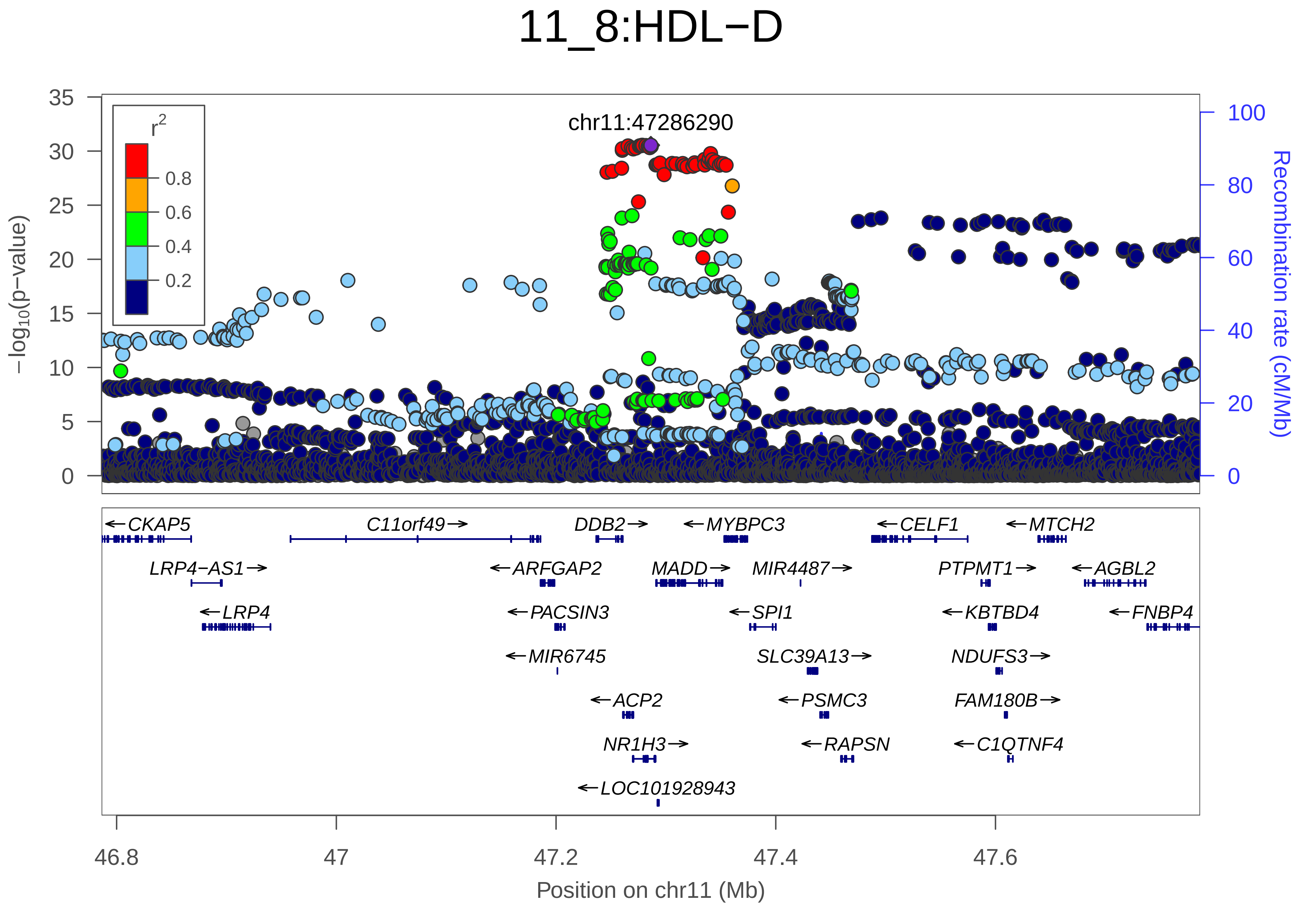

11_8:HDL−D
chr11:47286290
r2
0.8
0.6
0.4
0.2
−log10(p−value)
Recombination rate (cM/Mb)
CKAP5
C11orf49
DDB2
MYBPC3
CELF1
MTCH2
LRP4−AS1
ARFGAP2
MADD
MIR4487
PTPMT1
AGBL2
LRP4
PACSIN3
SPI1
KBTBD4
FNBP4
MIR6745
SLC39A13
NDUFS3
ACP2
PSMC3
FAM180B
NR1H3
RAPSN
C1QTNF4
LOC101928943
46.8
47
47.2
47.4
47.6
Position on chr11 (Mb)

11_9:M−HDL−C_percent

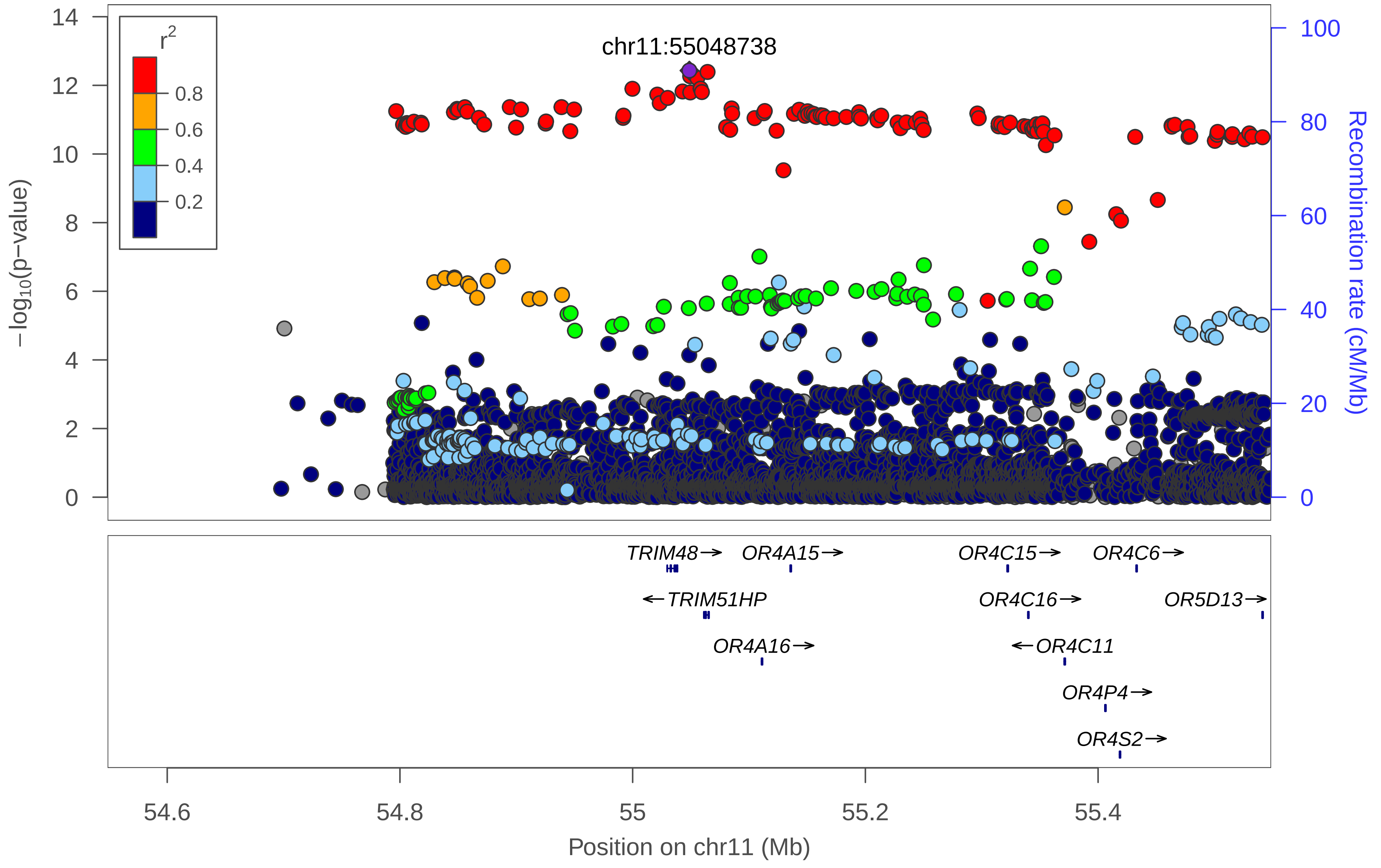

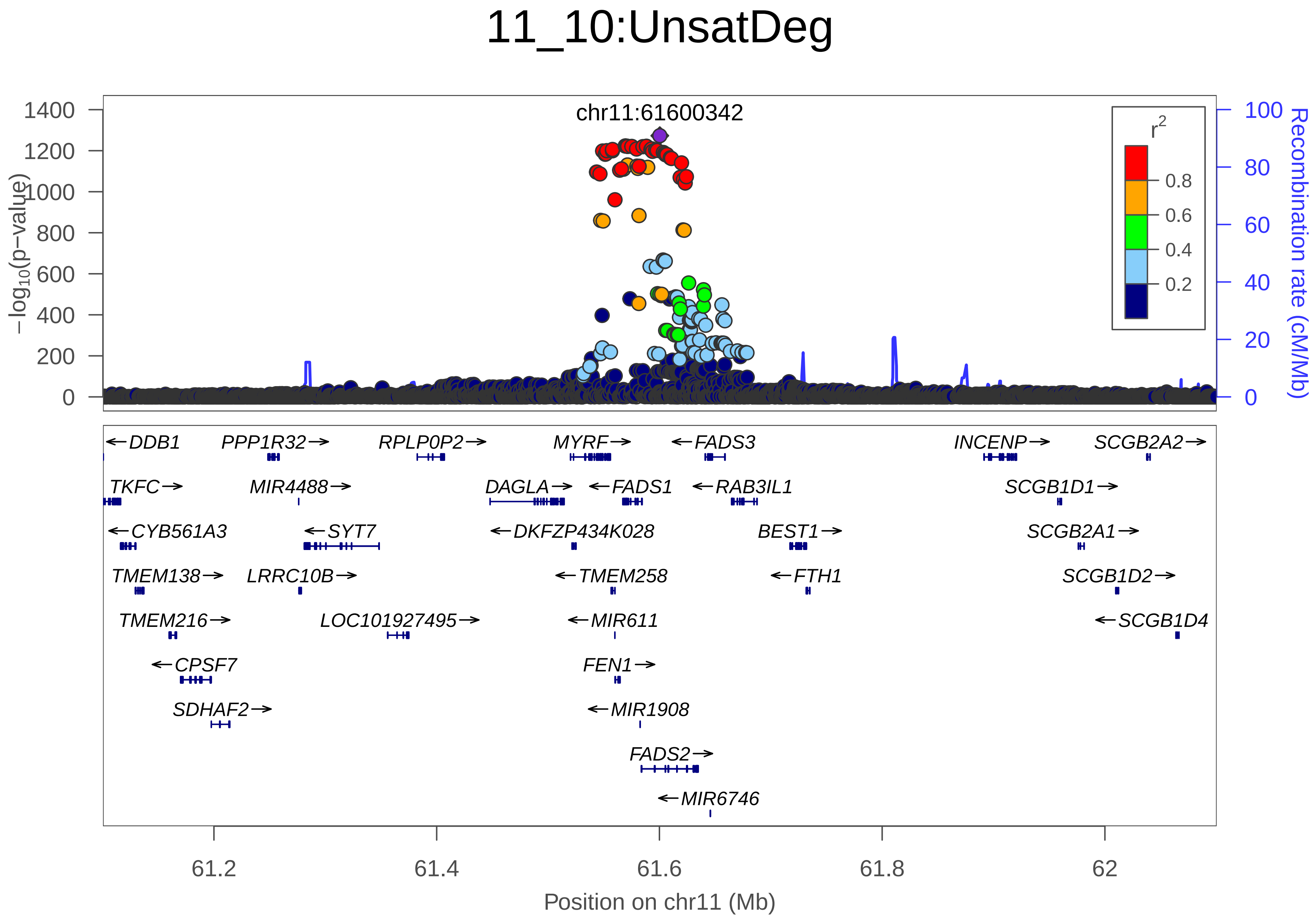
11_10:UnsatDeg
chr11:61600342
−log10(p−value)
Recombination rate (cM/Mb)
r2
0.8
0.6
0.4
0.2
DDB1
PPP1R32
RPLP0P2
MYRF
FADS3
INCENP
SCGB2A2
TKFC
MIR4488
DAGLA
FADS1
RAB3IL1
SCGB1D1
CYB561A3
SYT7
DKFZP434K028
BEST1
SCGB2A1
TMEM138
LRRC10B
TMEM258
FTH1
SCGB1D2
TMEM216
LOC101927495
MIR611
SCGB1D4
CPSF7
FEN1
SDHAF2
MIR1908
FADS2
MIR6746
Position on chr11 (Mb)

11_11:FAw3byFA

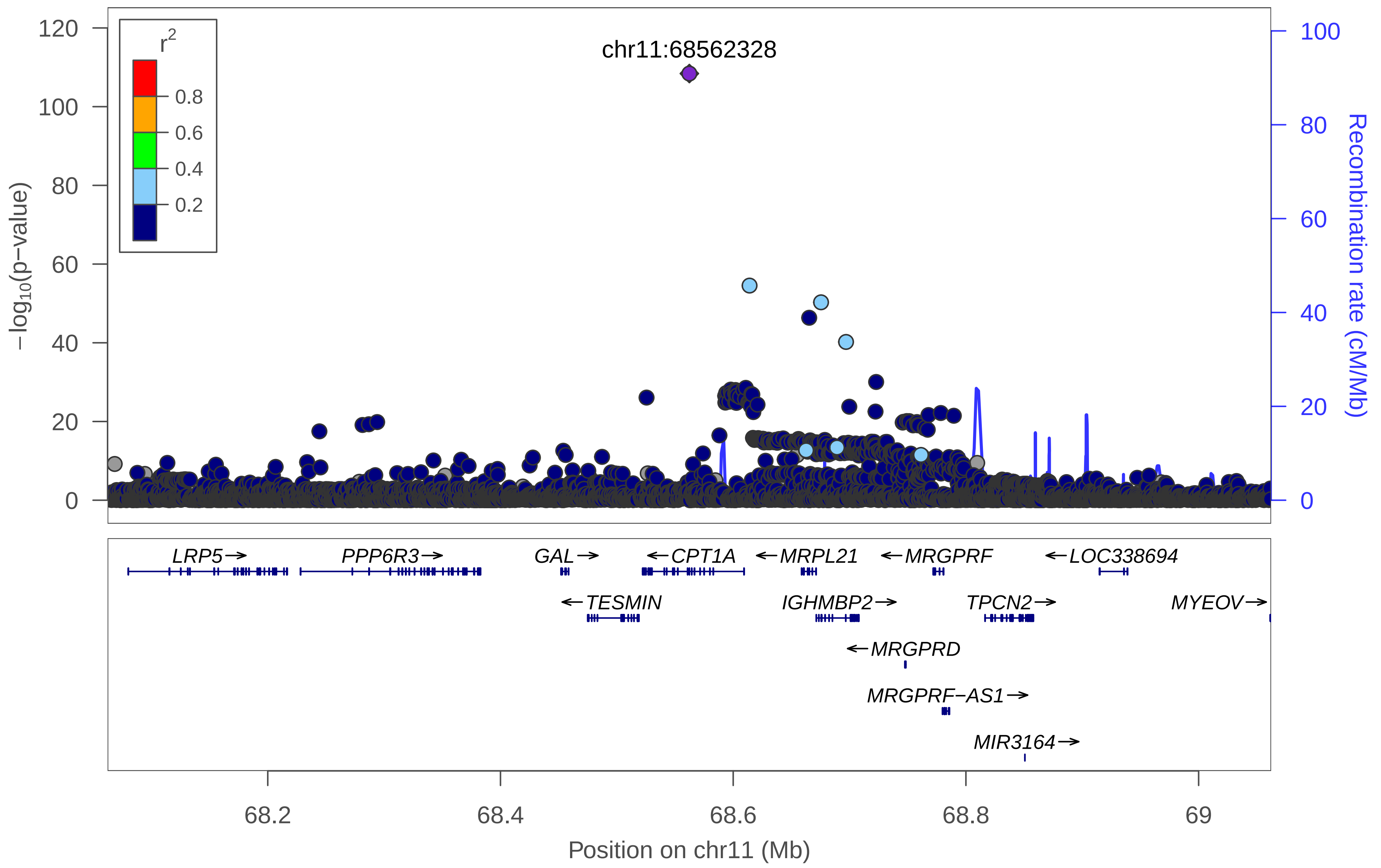

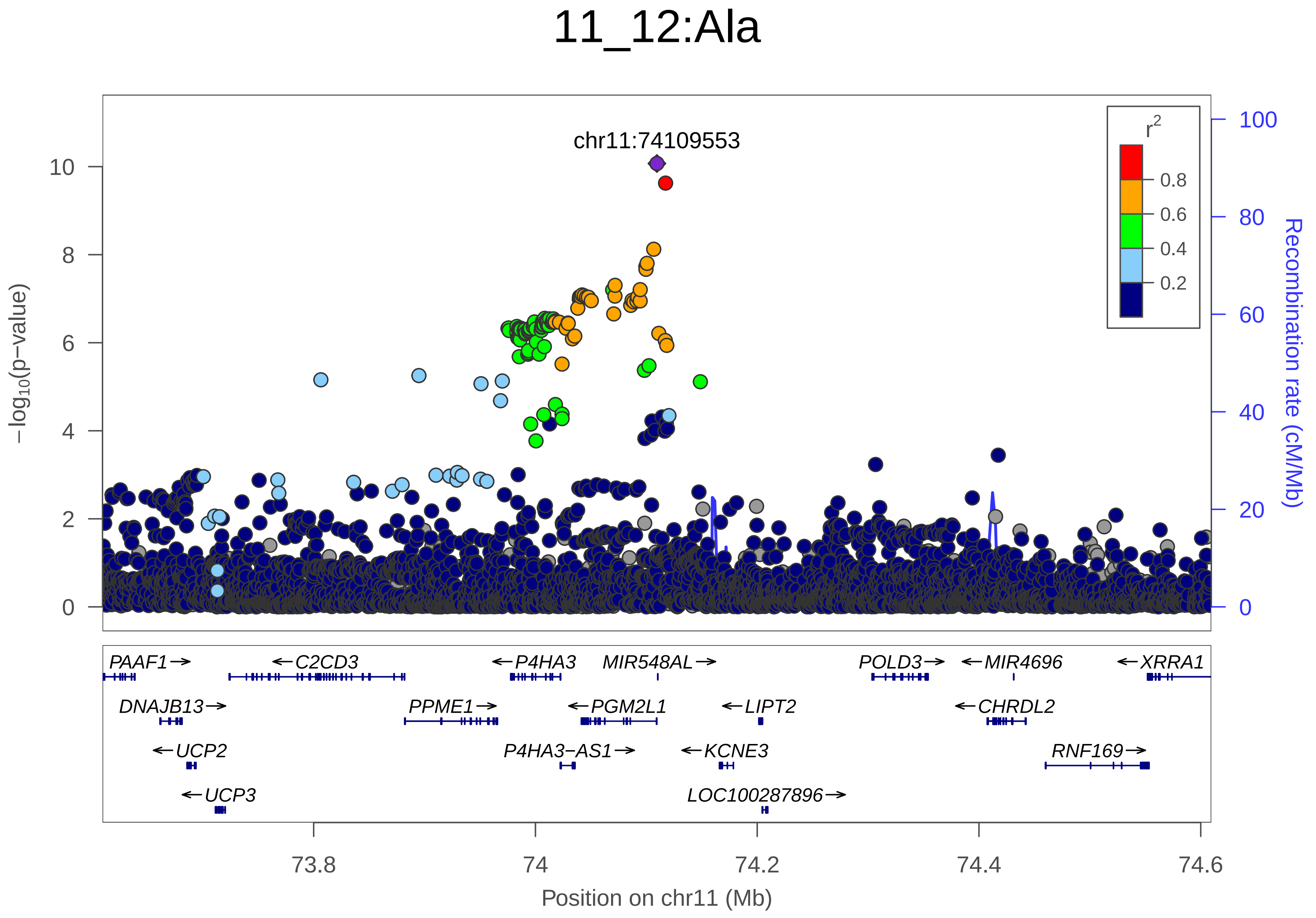

11_12:Ala
chr11:74109553
r2
0.8
0.6
0.4
0.2
−log10(p−value)
Recombination rate (cM/Mb)
PAAF1→
←C2CD3
←P4HA3
MIR548AL→
POLD3→
←MIR4696
←XRRA1
DNAJB13→
PPME1→
←PGM2L1
←LIPT2
←CHRDL2
←UCP2
P4HA3−AS1→
←KCNE3
RNF169→
←UCP3
LOC100287896→
73.8
74
74.2
74.4
74.6
Position on chr11 (Mb)

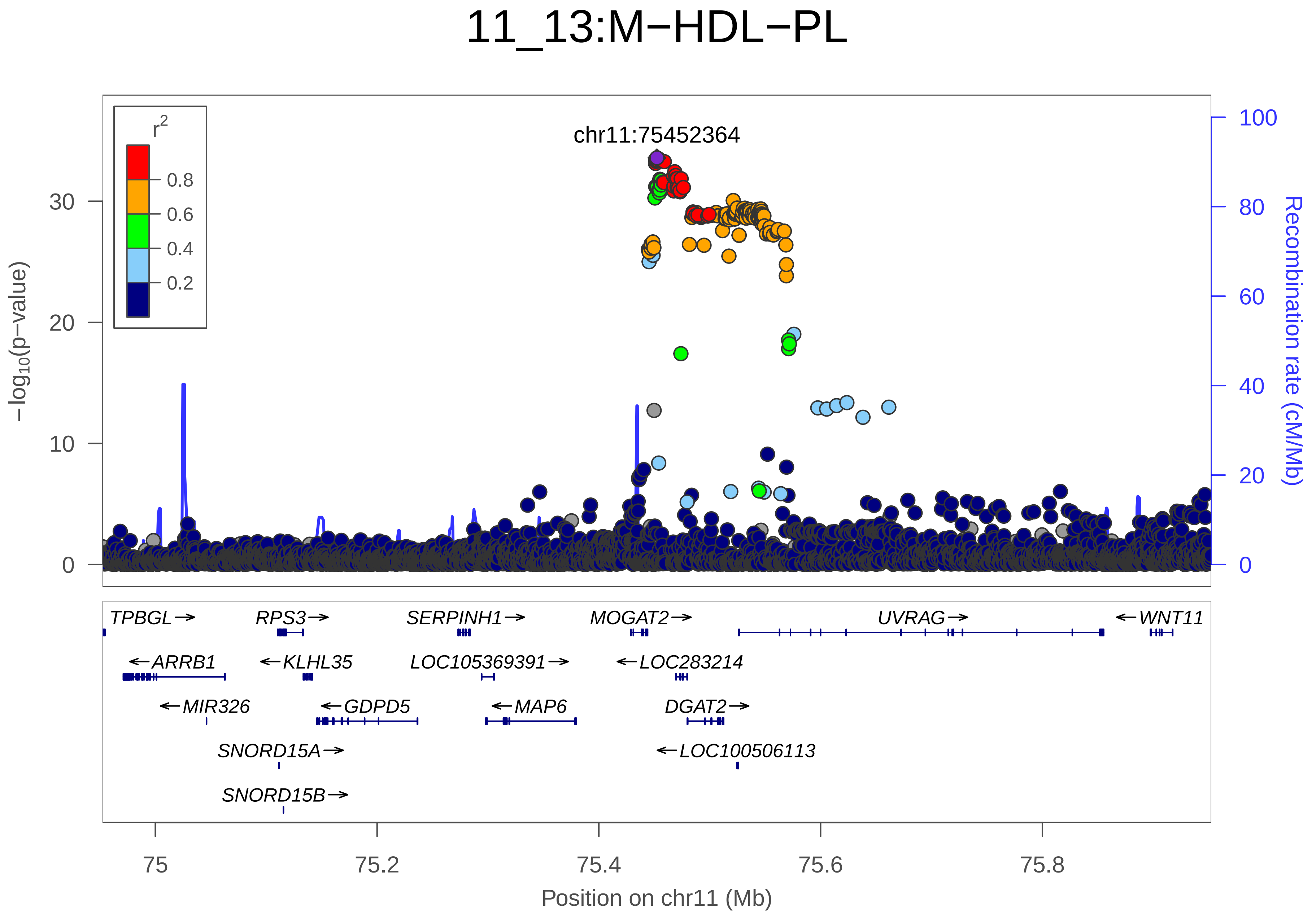

11_13:M−HDL−PL
r2
0.8
0.6
0.4
0.2
chr11:75452364
−log10(p−value)
0
10
20
30
Recombination rate (cM/Mb)
0
20
40
60
80
100
TPBGL→
←ARRB1
←MIR326
RPS3→
←KLHL35
SNORD15A→
SNORD15B→
←GDPD5
SERPINH1→
LOC105369391→
←MAP6
MOGAT2→
←LOC283214
DGAT2→
←LOC100506113
UVRAG→
←WNT11
75
75.2
75.4
75.6
75.8
Position on chr11 (Mb)

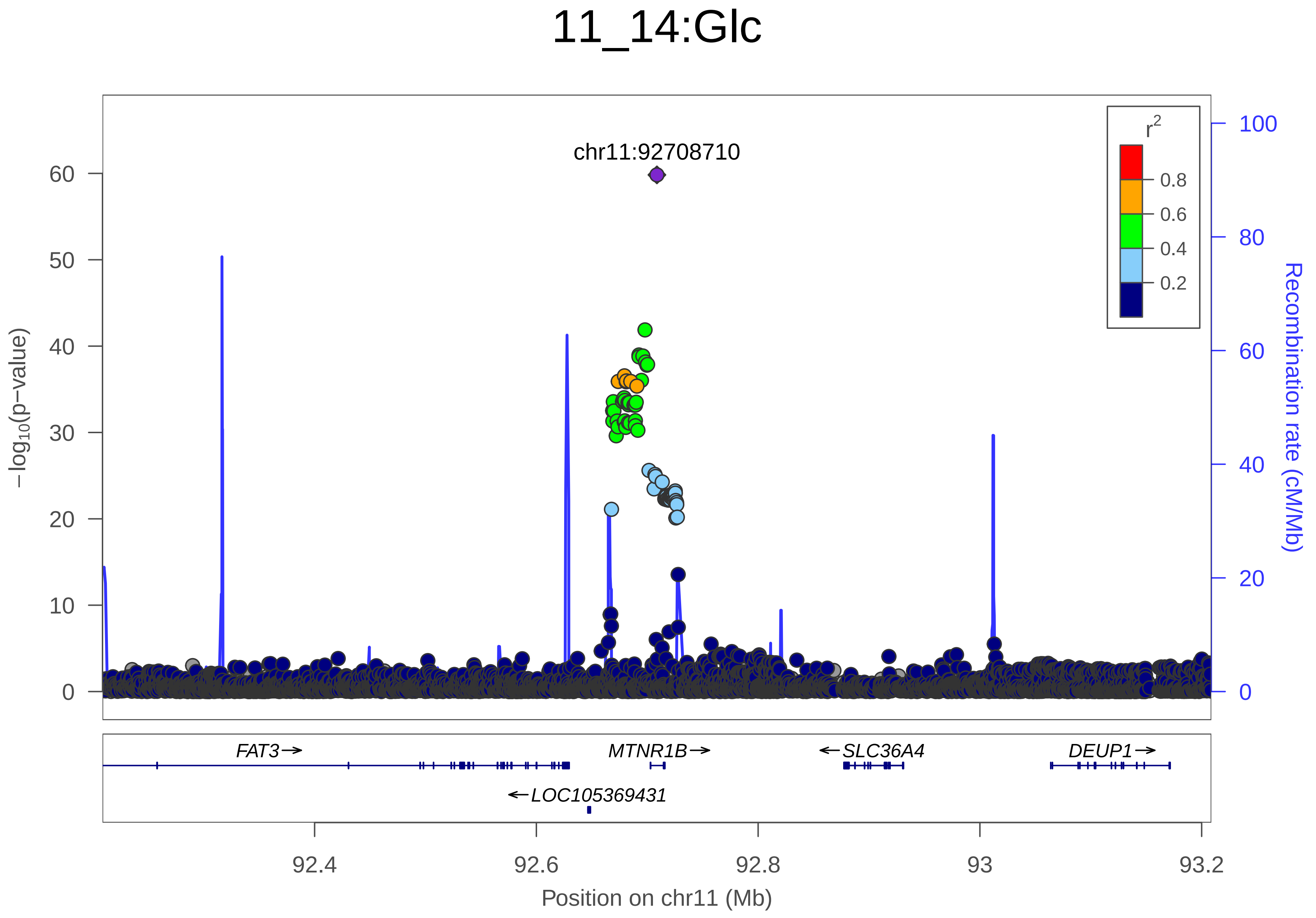

11_14:Glc
chr11:92708710
−log10(p−value)
0
10
20
30
40
50
60
Recombination rate (cM/Mb)
0
20
40
60
80
100
r2
0.8
0.6
0.4
0.2
FAT3→
MTNR1B→
←SLC36A4
DEUP1→
←LOC105369431
92.4
92.6
92.8
93
93.2
Position on chr11 (Mb)

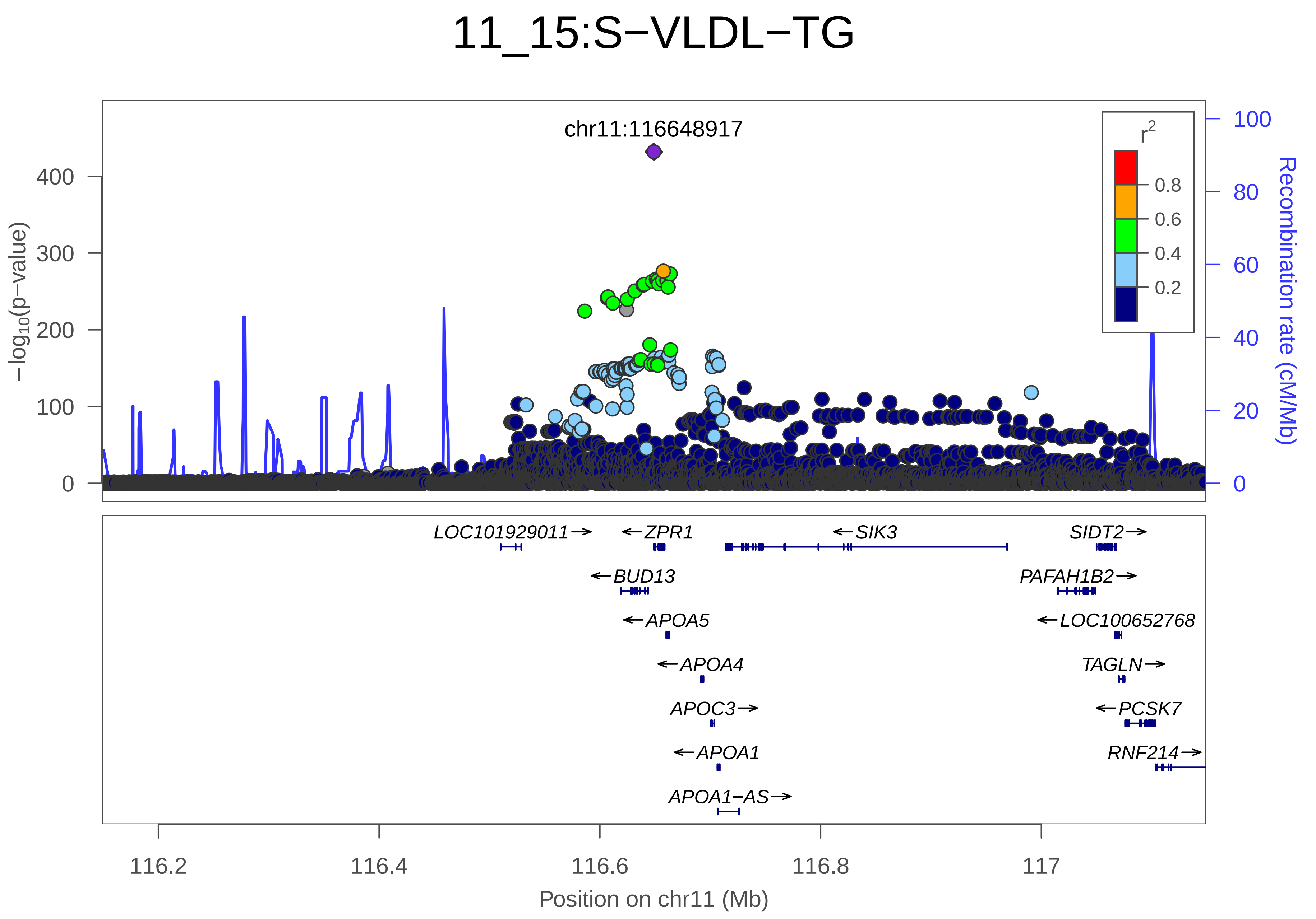

11_15:S−VLDL−TG
chr11:116648917
−log10(p−value)
Recombination rate (cM/Mb)
r2
0.8
0.6
0.4
0.2
LOC101929011
ZPR1
SIK3
SIDT2
BUD13
PAFAH1B2
APOA5
LOC100652768
APOA4
TAGLN
APOC3
PCSK7
APOA1
RNF214
APOA1−AS
Position on chr11 (Mb)

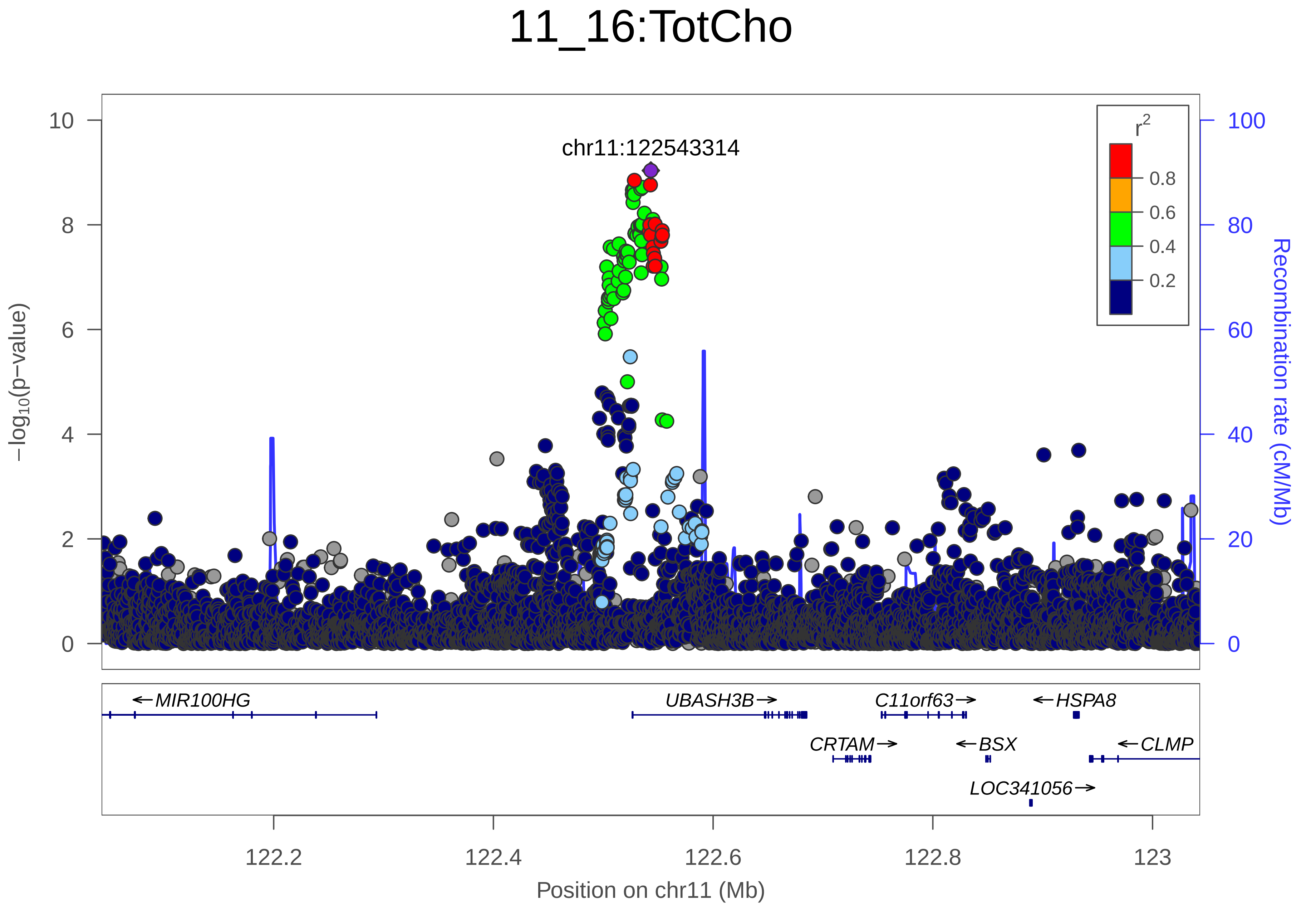

11_16:TotCho
chr11:122543314
−log10(p−value)
Recombination rate (cM/Mb)
r2
0.8
0.6
0.4
0.2
0
2
4
6
8
10
0
20
40
60
80
100
←MIR100HG
UBASH3B→
C11orf63→
←HSPA8
CRTAM→
←BSX
←CLMP
LOC341056→
122.2
122.4
122.6
122.8
123
Position on chr11 (Mb)

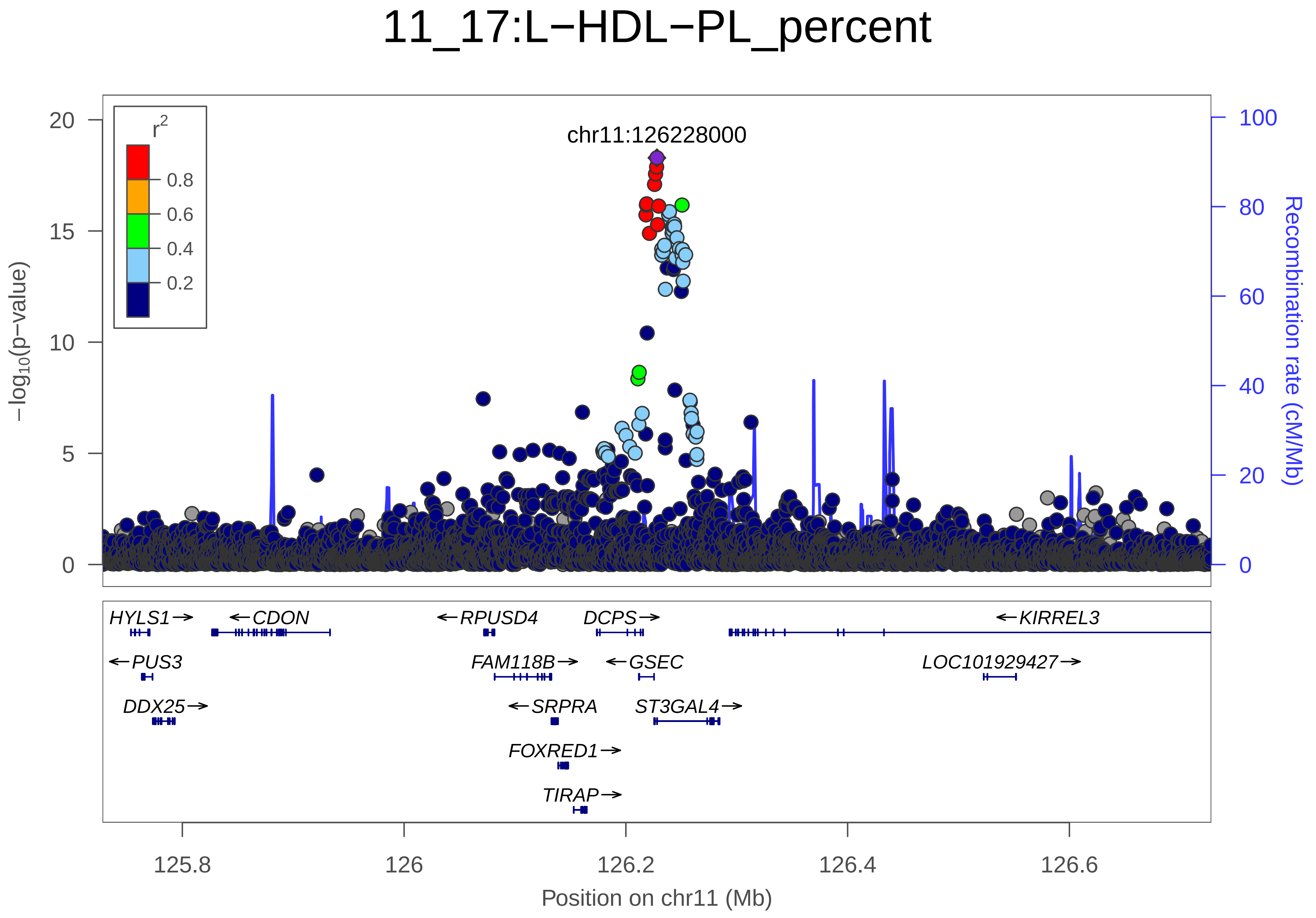

11_17:L−HDL−PL_percent
chr11:126228000
r2
0.8
0.6
0.4
0.2
−log10(p−value)
Recombination rate (cM/Mb)
0
5
10
15
20
0
20
40
60
80
100
HYLS1→
←CDON
←RPUSD4
DCPS→
←KIRREL3
←PUS3
FAM118B→
←GSEC
LOC101929427→
DDX25→
←SRPRA
ST3GAL4→
FOXRED1→
TIRAP→
125.8
126
126.2
126.4
126.6
Position on chr11 (Mb)

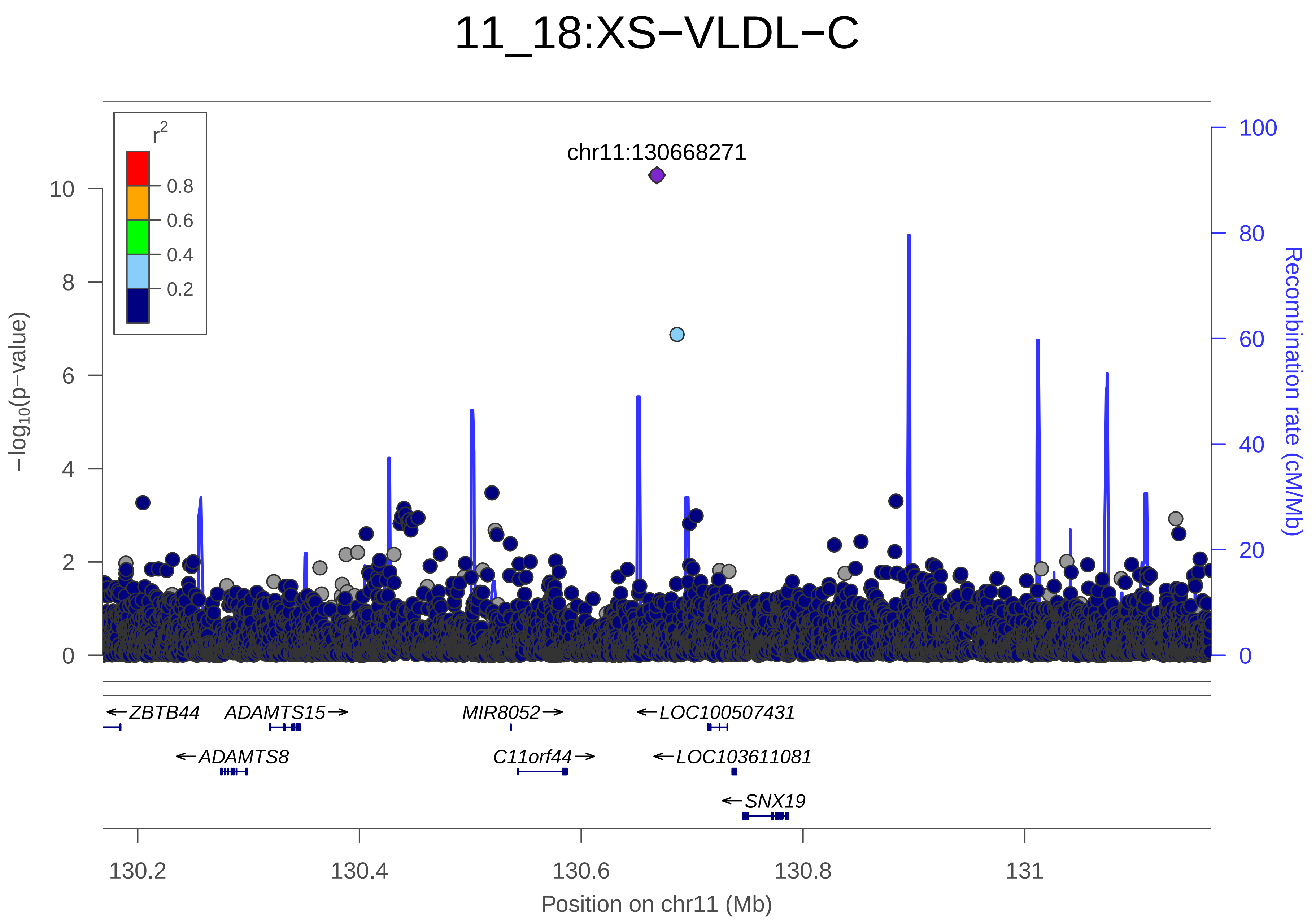

11_18:XS−VLDL−C
chr11:130668271
r2
0.8
0.6
0.4
0.2
−log10(p−value)
Recombination rate (cM/Mb)
0
2
4
6
8
10
0
20
40
60
80
100
ZBTB44
ADAMTS15
MIR8052
LOC100507431
ADAMTS8
C11orf44
LOC103611081
SNX19
130.2
130.4
130.6
130.8
131
Position on chr11 (Mb)

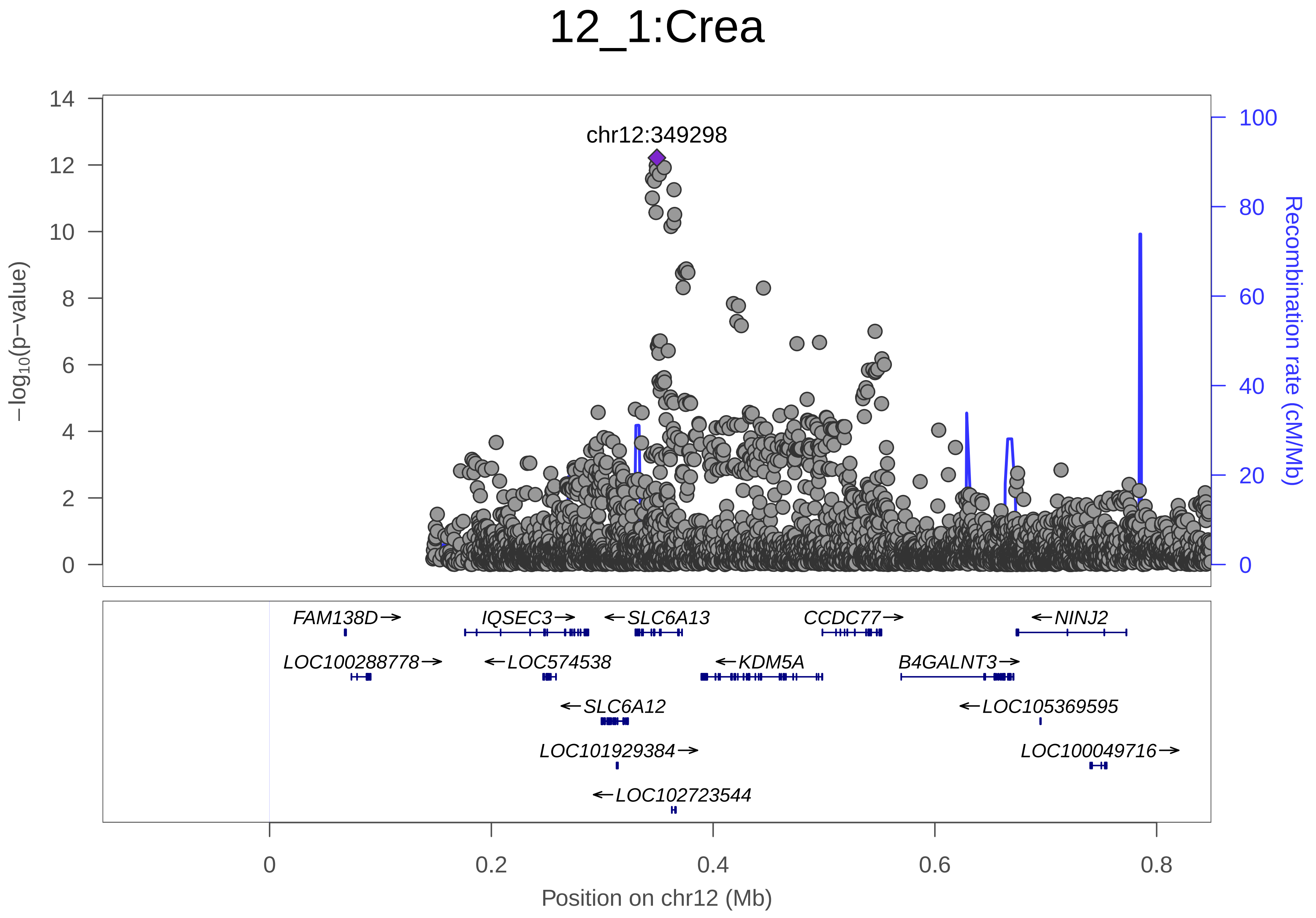

12_1:Crea
chr12:349298
−log10(p−value)
Recombination rate (cM/Mb)
14
12
10
8
6
4
2
0
100
80
60
40
20
0
FAM138D→
IQSEC3→
←SLC6A13
CCDC77→
←NINJ2
LOC100288778→
←LOC574538
←KDM5A
B4GALNT3→
←SLC6A12
←LOC105369595
LOC101929384→
LOC100049716→
←LOC102723544
0
0.2
0.4
0.6
0.8
Position on chr12 (Mb)

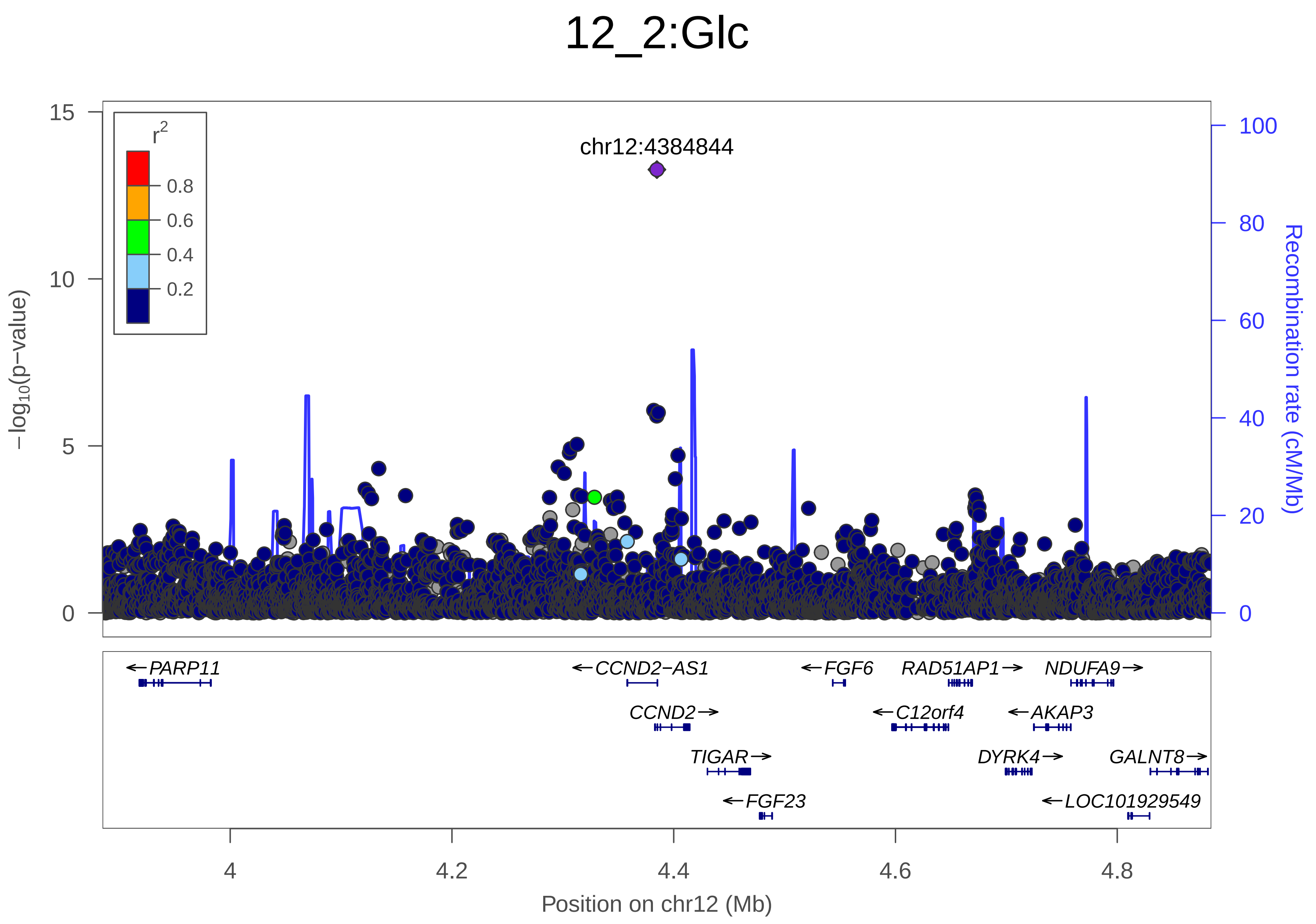
12_2:Glc
chr12:4384844
r2
0.8
0.6
0.4
0.2
15
10
5
0
−log10(p−value)
100
80
60
40
20
0
Recombination rate (cM/Mb)
←PARP11
←CCND2−AS1
CCND2→
TIGAR→
←FGF23
←FGF6
RAD51AP1→
←C12orf4
NDUFA9→
←AKAP3
DYRK4→
GALNT8→
←LOC101929549
4
4.2
4.4
4.6
4.8
Position on chr12 (Mb)

12_3:PUFA

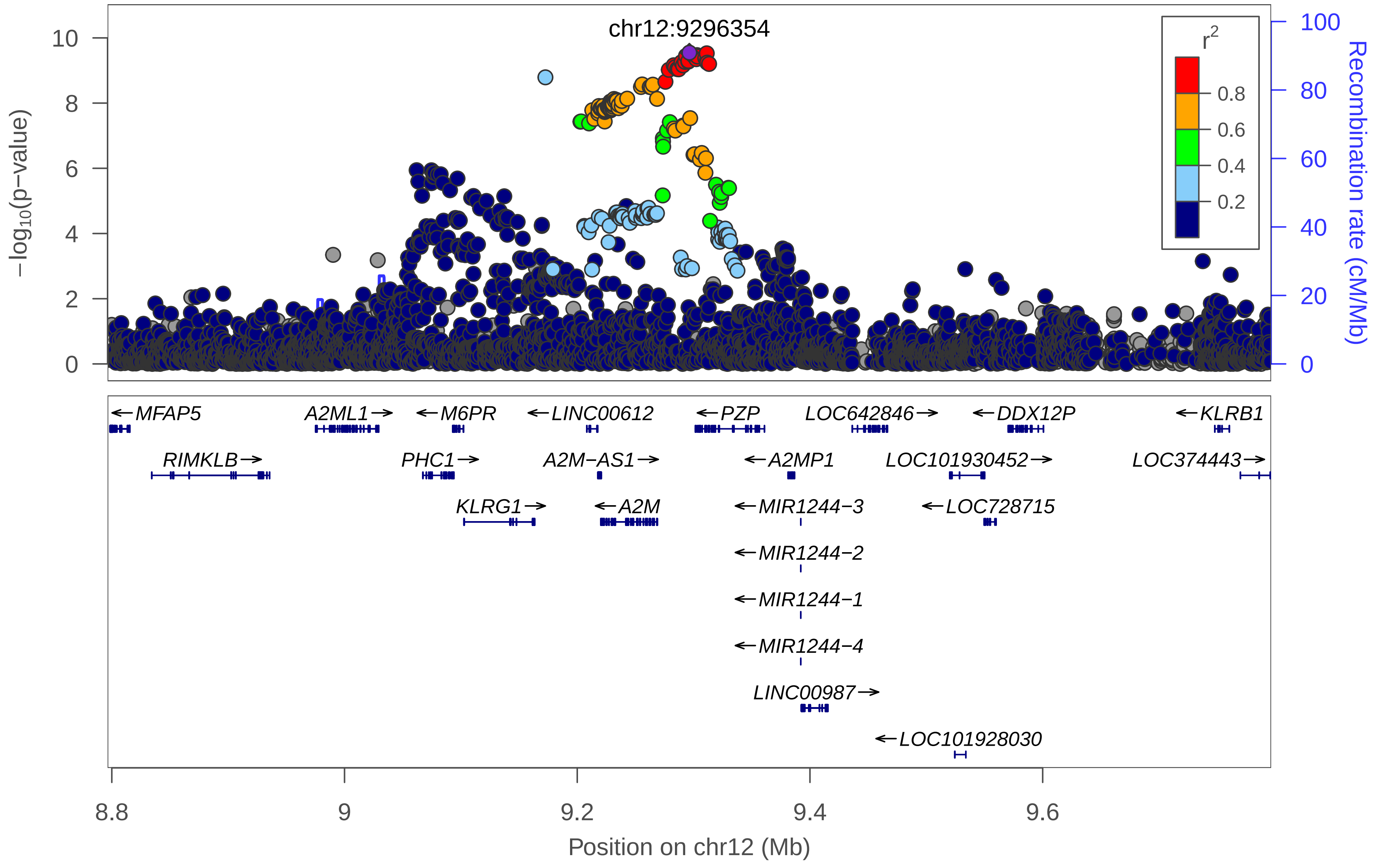

chr12:9296354
−log10(p−value)
Recombination rate (cM/Mb)
r2
0.8
0.6
0.4
0.2
0
2
4
6
8
10
0
20
40
60
80
100
←MFAP5
A2ML1→
←M6PR
←LINC00612
←PZP
LOC642846→
←DDX12P
←KLRB1
RIMKLB→
PHC1→
A2M−AS1→
←A2MP1
LOC101930452→
LOC374443→
KLRG1→
←A2M
←MIR1244−3
←LOC728715
←MIR1244−2
←MIR1244−1
←MIR1244−4
LINC00987→
←LOC101928030
8.8
9
9.2
9.4
9.6
Position on chr12 (Mb)

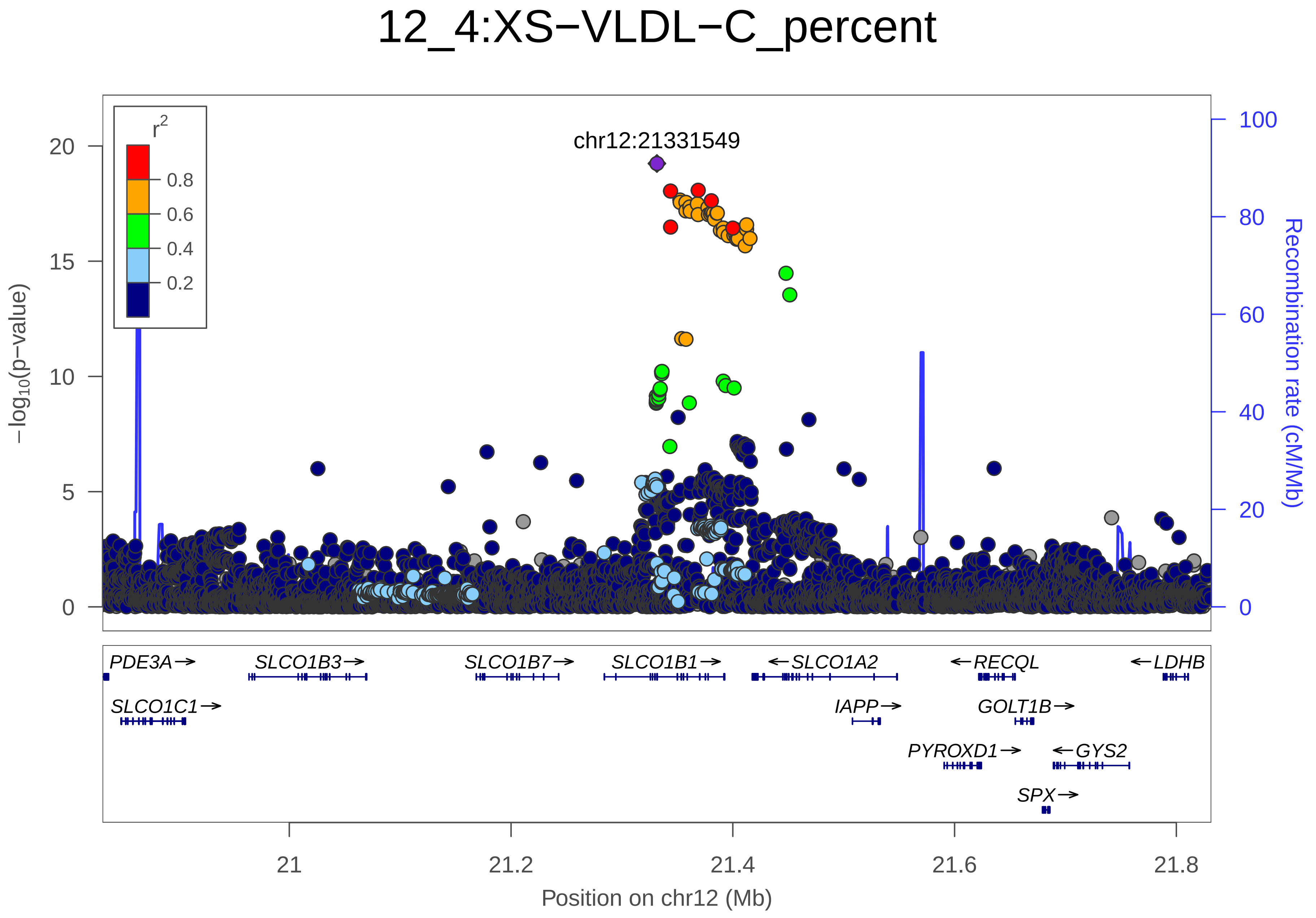
12_4:XS−VLDL−C_percent
chr12:21331549
r2
0.8
0.6
0.4
0.2
−log10(p−value)
Recombination rate (cM/Mb)
0
5
10
15
20
0
20
40
60
80
100
PDE3A→
SLCO1B3→
SLCO1B7→
SLCO1B1→
←SLCO1A2
←RECQL
←LDHB
SLCO1C1→
IAPP→
GOLT1B→
PYROXD1→
←GYS2
SPX→
21
21.2
21.4
21.6
21.8
Position on chr12 (Mb)

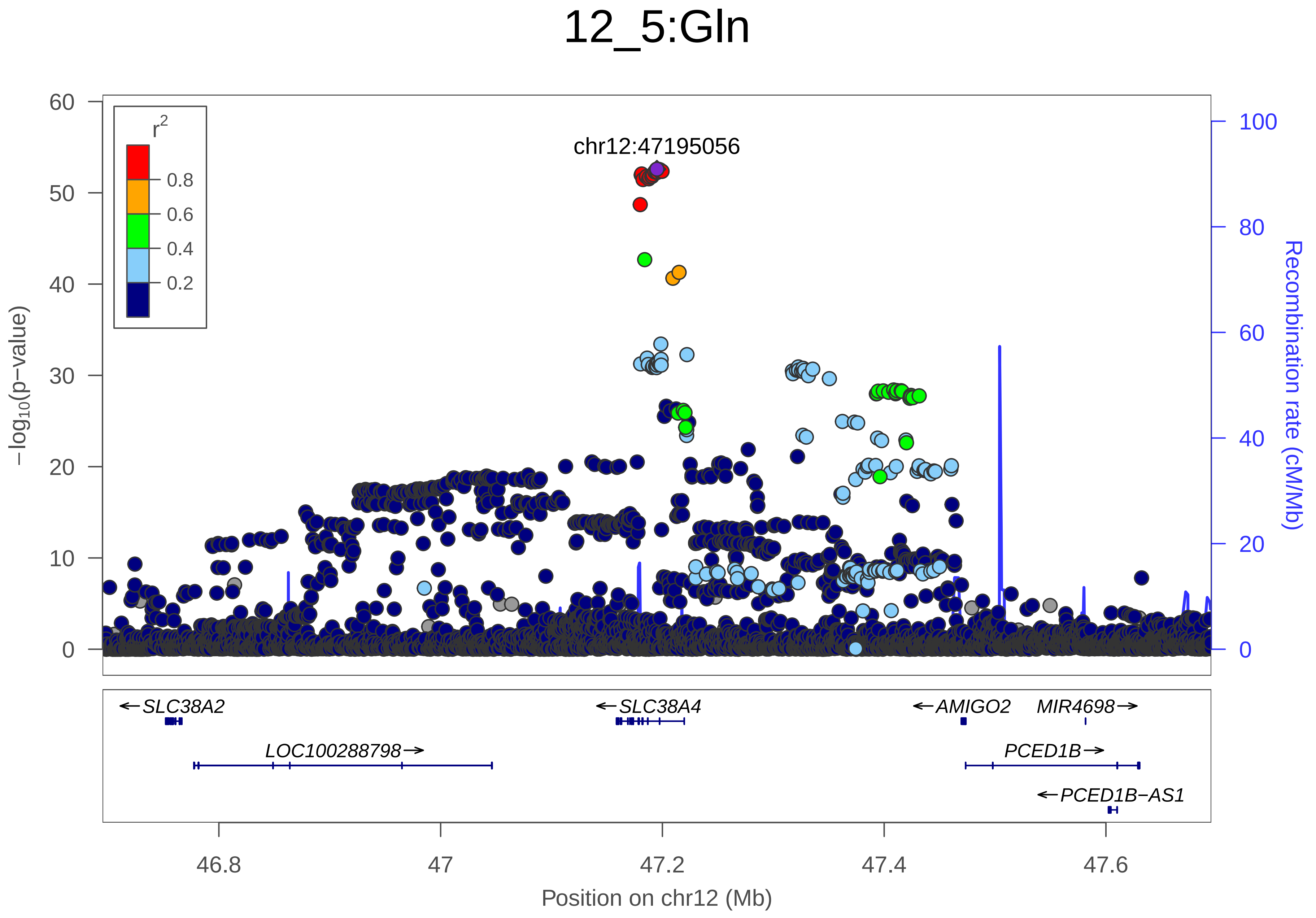

12_5:Gln
chr12:47195056
r2
0.8
0.6
0.4
0.2
−log10(p−value)
Recombination rate (cM/Mb)
60
50
40
30
20
10
0
100
80
60
40
20
0
←SLC38A2
LOC100288798→
←SLC38A4
←AMIGO2
MIR4698→
PCED1B→
←PCED1B−AS1
46.8
47
47.2
47.4
47.6
Position on chr12 (Mb)

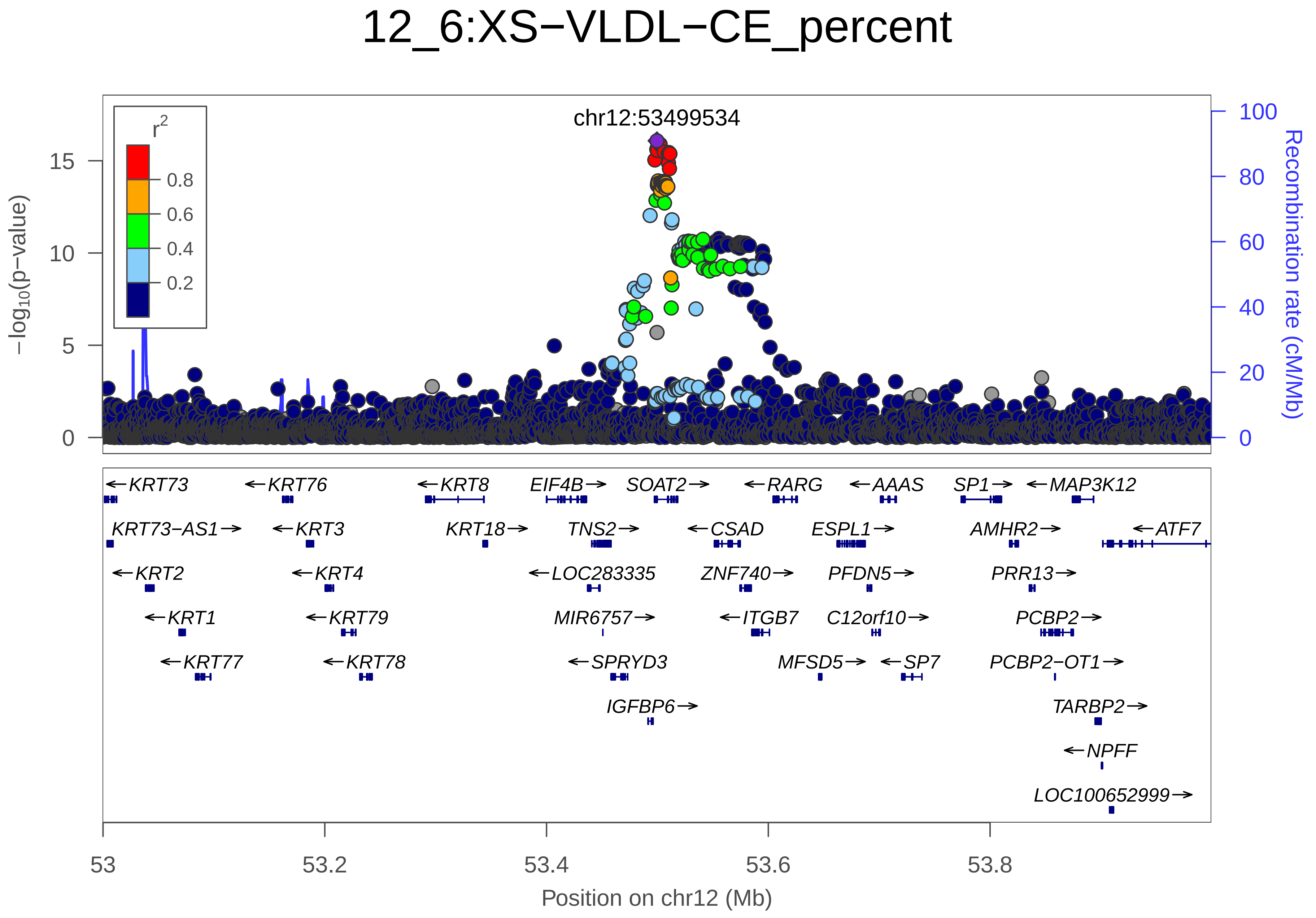

12_6:XS−VLDL−CE_percent
chr12:53499534
r2
0.8
0.6
0.4
0.2
−log10(p−value)
Recombination rate (cM/Mb)
0
5
10
15
20
40
60
80
100
KRT73
KRT76
KRT8
EIF4B
SOAT2
RARG
AAAS
SP1
MAP3K12
KRT73−AS1
KRT3
KRT18
TNS2
CSAD
ESPL1
AMHR2
ATF7
KRT2
KRT4
LOC283335
ZNF740
PFDN5
PRR13
KRT1
KRT79
MIR6757
ITGB7
C12orf10
PCBP2
KRT77
KRT78
SPRYD3
MFSD5
SP7
PCBP2−OT1
IGFBP6
TARBP2
NPFF
LOC100652999
53
53.2
53.4
53.6
53.8
Position on chr12 (Mb)

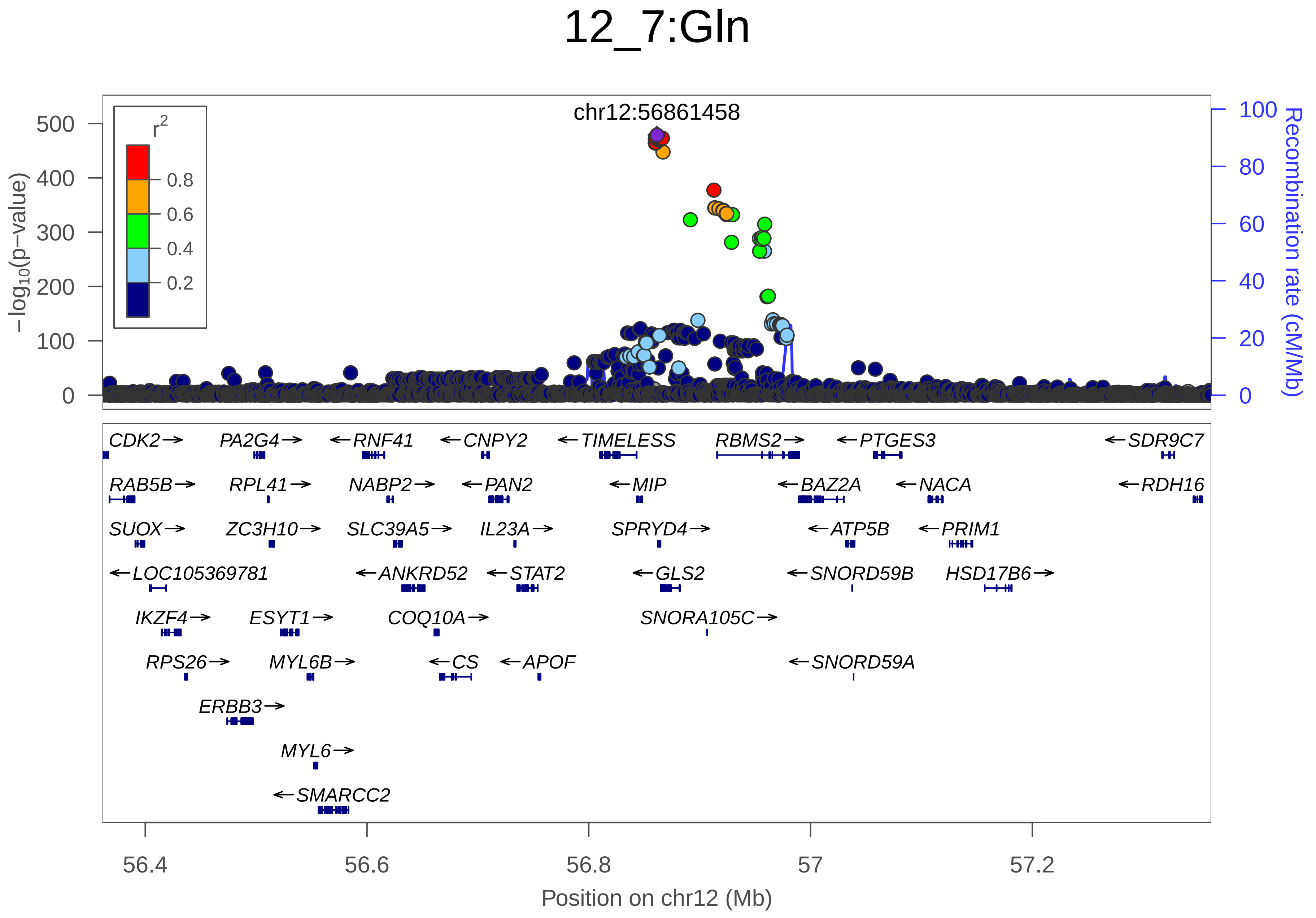
12_7:Gln
chr12:56861458
r2
0.8
0.6
0.4
0.2
−log10(p−value)
500
400
300
200
100
0
Recombination rate (cM/Mb)
100
80
60
40
20
0
CDK2→
PA2G4→
←RNF41
←CNPY2
←TIMELESS
RBMS2→
←PTGES3
←SDR9C7
RAB5B→
RPL41→
NABP2→
←PAN2
←MIP
←BAZ2A
←NACA
←RDH16
SUOX→
ZC3H10→
SLC39A5→
IL23A→
SPRYD4→
←ATP5B
←PRIM1
←LOC105369781
←ANKRD52
←STAT2
←GLS2
←SNORD59B
HSD17B6→
IKZF4→
ESYT1→
COQ10A→
SNORA105C→
RPS26→
MYL6B→
←CS
←APOF
←SNORD59A
ERBB3→
MYL6→
←SMARCC2
56.4
56.6
56.8
57
57.2
Position on chr12 (Mb)

12_8:Gln

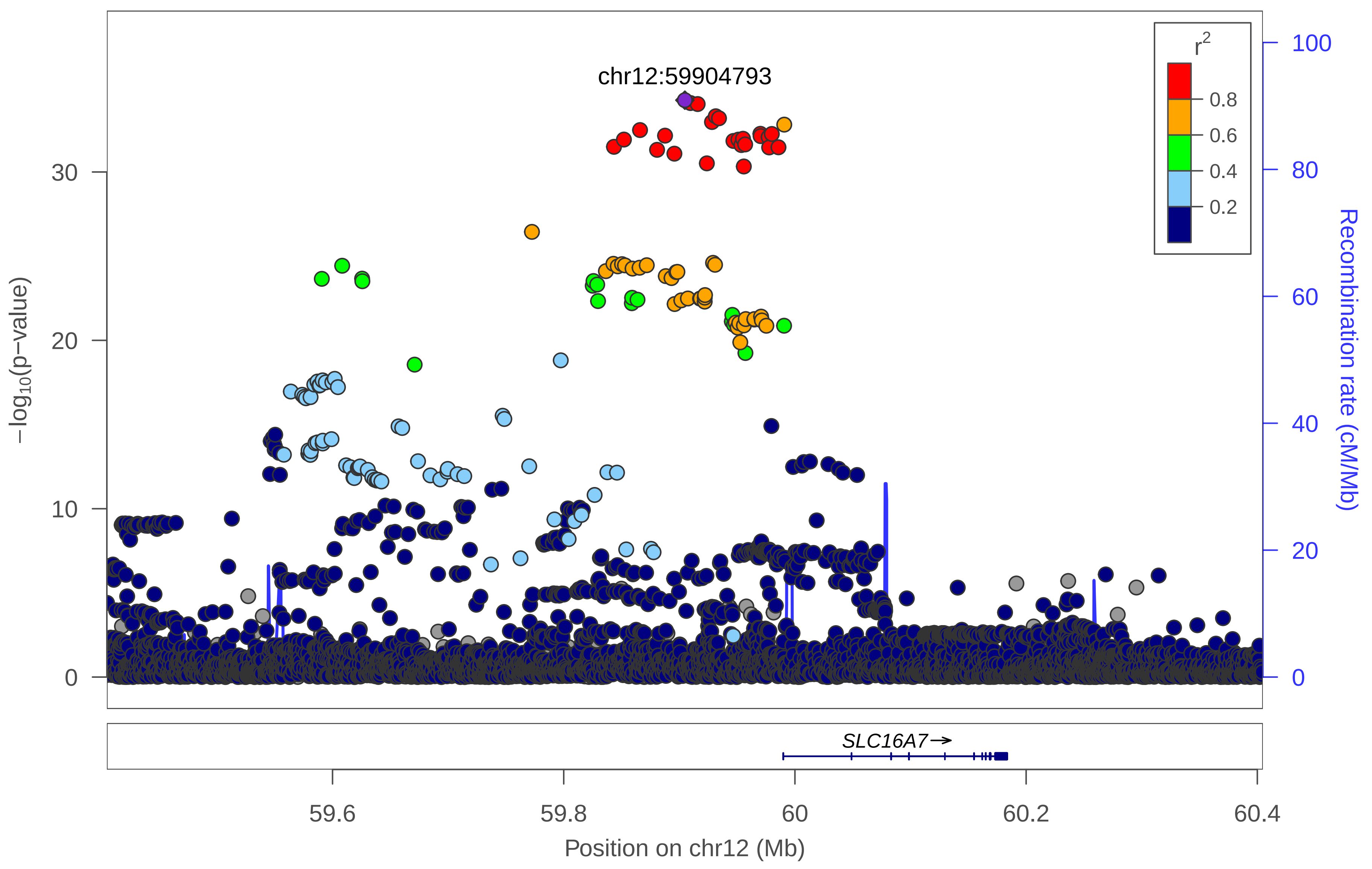

chr12:59904793
r2
0.8
0.6
0.4
0.2
−log10(p−value)
0
10
20
30
Recombination rate (cM/Mb)
0
20
40
60
80
100
SLC16A7→
59.6
59.8
60
60.2
60.4
Position on chr12 (Mb)

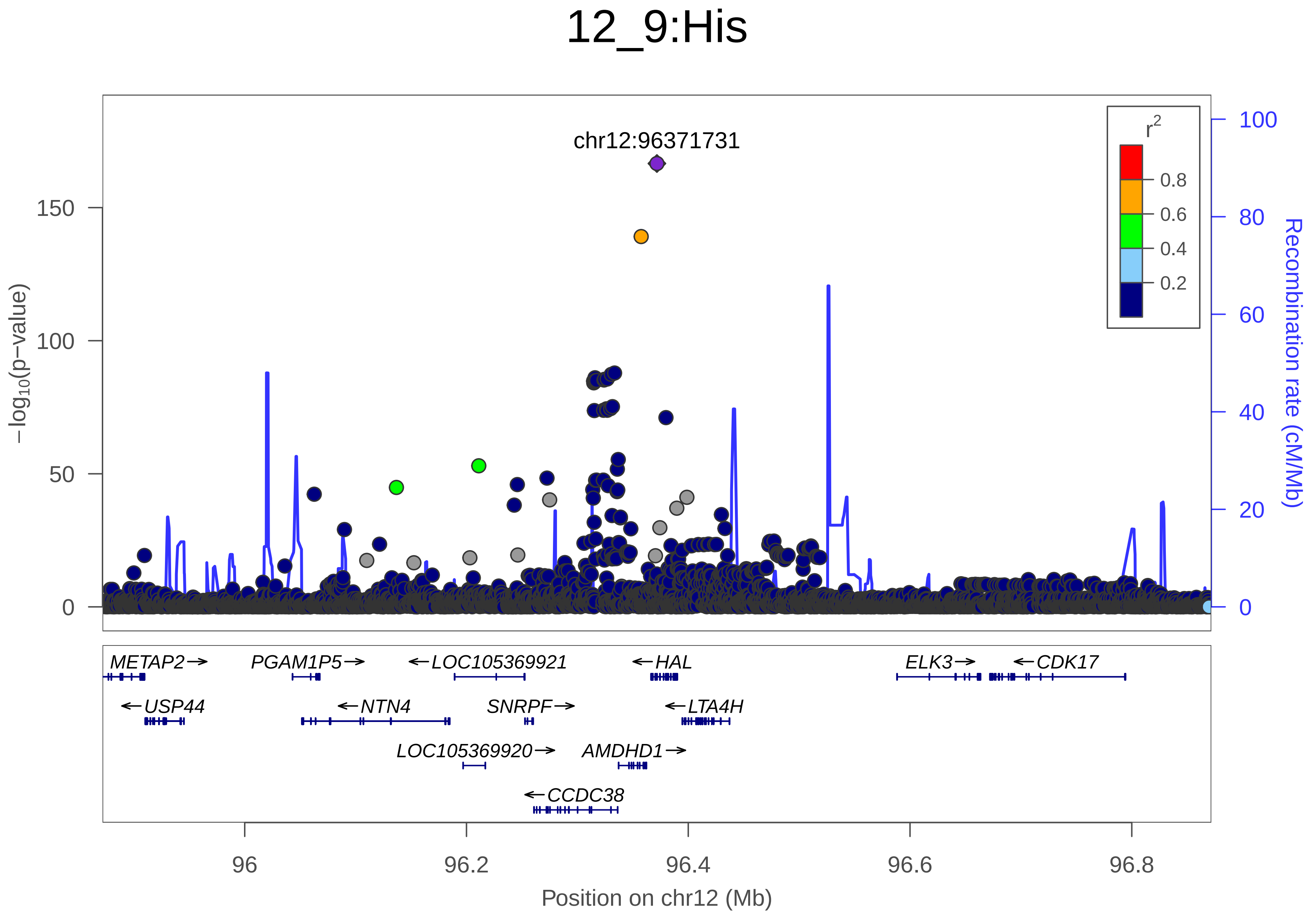
12_9:His
chr12:96371731
r2
0.8
0.6
0.4
0.2
150
100
50
0
−log10(p−value)
100
80
60
40
20
0
Recombination rate (cM/Mb)
METAP2→
PGAM1P5→
←LOC105369921
←HAL
ELK3→
←CDK17
←USP44
←NTN4
SNRPF→
←LTA4H
LOC105369920→
AMDHD1→
←CCDC38
96
96.2
96.4
96.6
96.8
Position on chr12 (Mb)

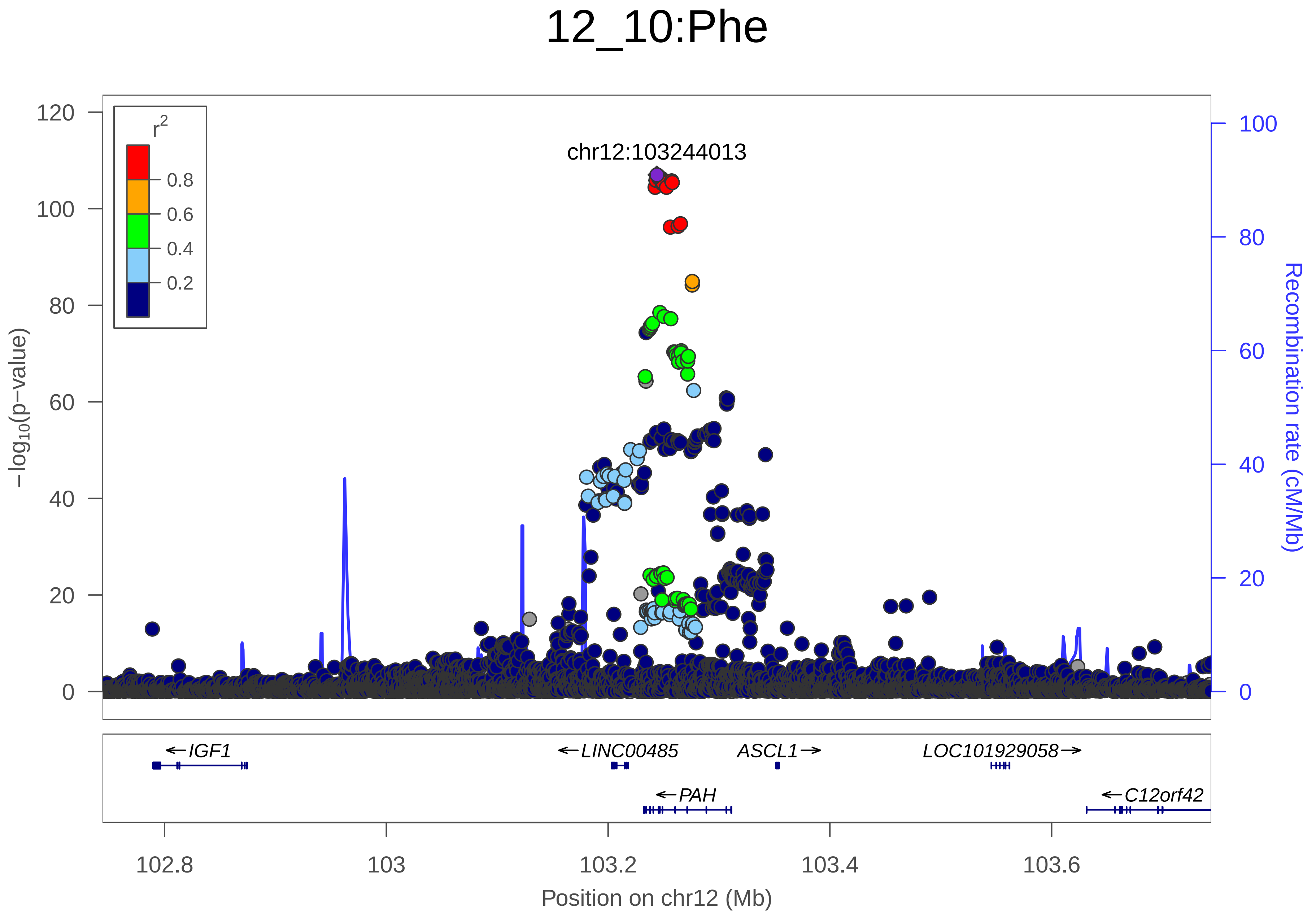

12_10:Phe
r2
0.8
0.6
0.4
0.2
chr12:103244013
120
100
80
60
40
20
0
−log10(p−value)
Recombination rate (cM/Mb)
IGF1
LINC00485
ASCL1
LOC101929058
PAH
C12orf42
102.8
103
103.2
103.4
103.6
Position on chr12 (Mb)

12_11:HDL−D

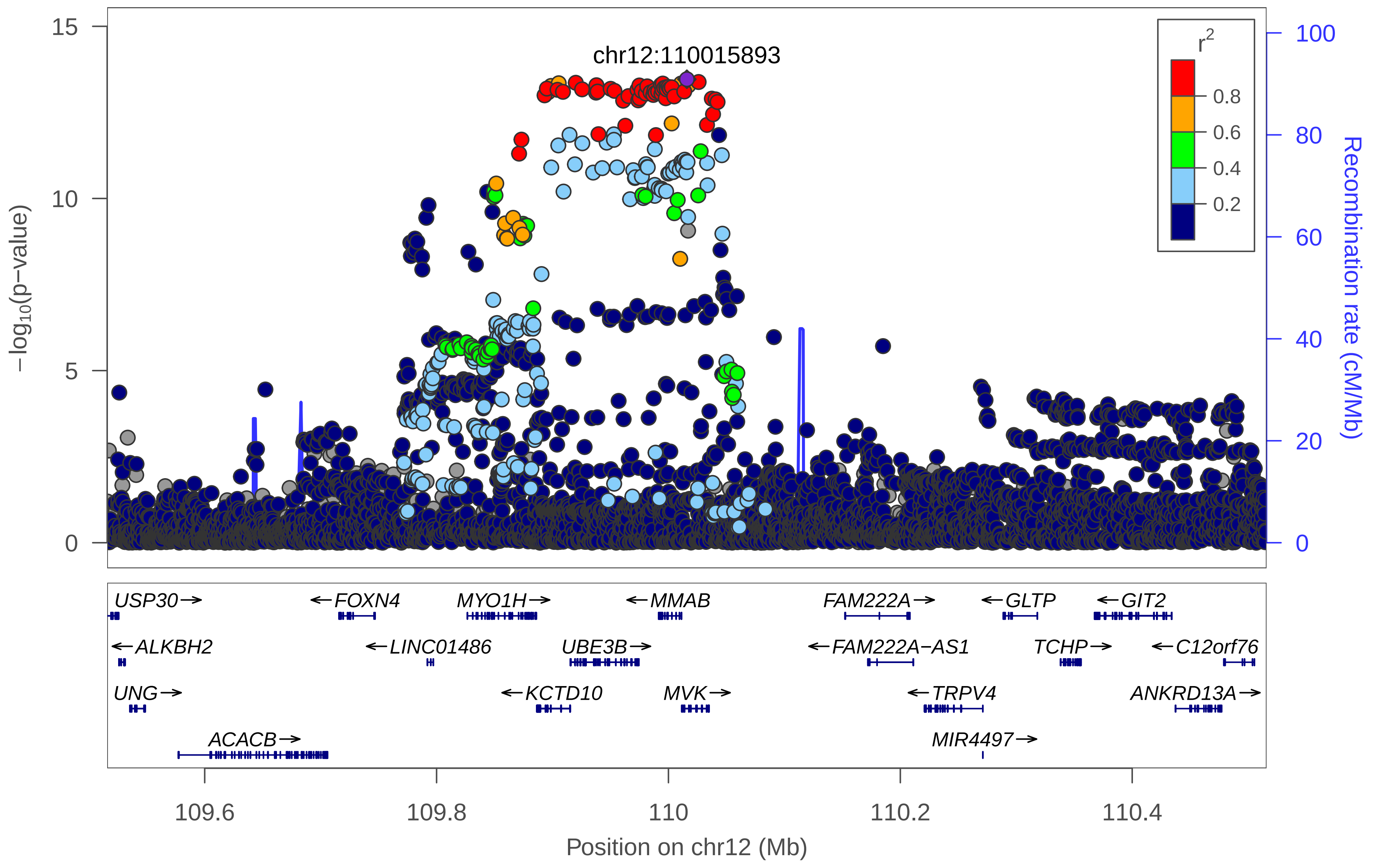

chr12:110015893
−log10(p−value)
Recombination rate (cM/Mb)
r²
0.8
0.6
0.4
0.2
15
10
5
0
100
80
60
40
20
0
USP30→
←FOXN4
MYO1H→
←MMAB
FAM222A→
←GLTP
←GIT2
←ALKBH2
←LINC01486
UBE3B→
←FAM222A−AS1
TCHP→
←C12orf76
UNG→
←KCTD10
MVK→
←TRPV4
ANKRD13A→
ACACB→
MIR4497→
109.6
109.8
110
110.2
110.4
Position on chr12 (Mb)

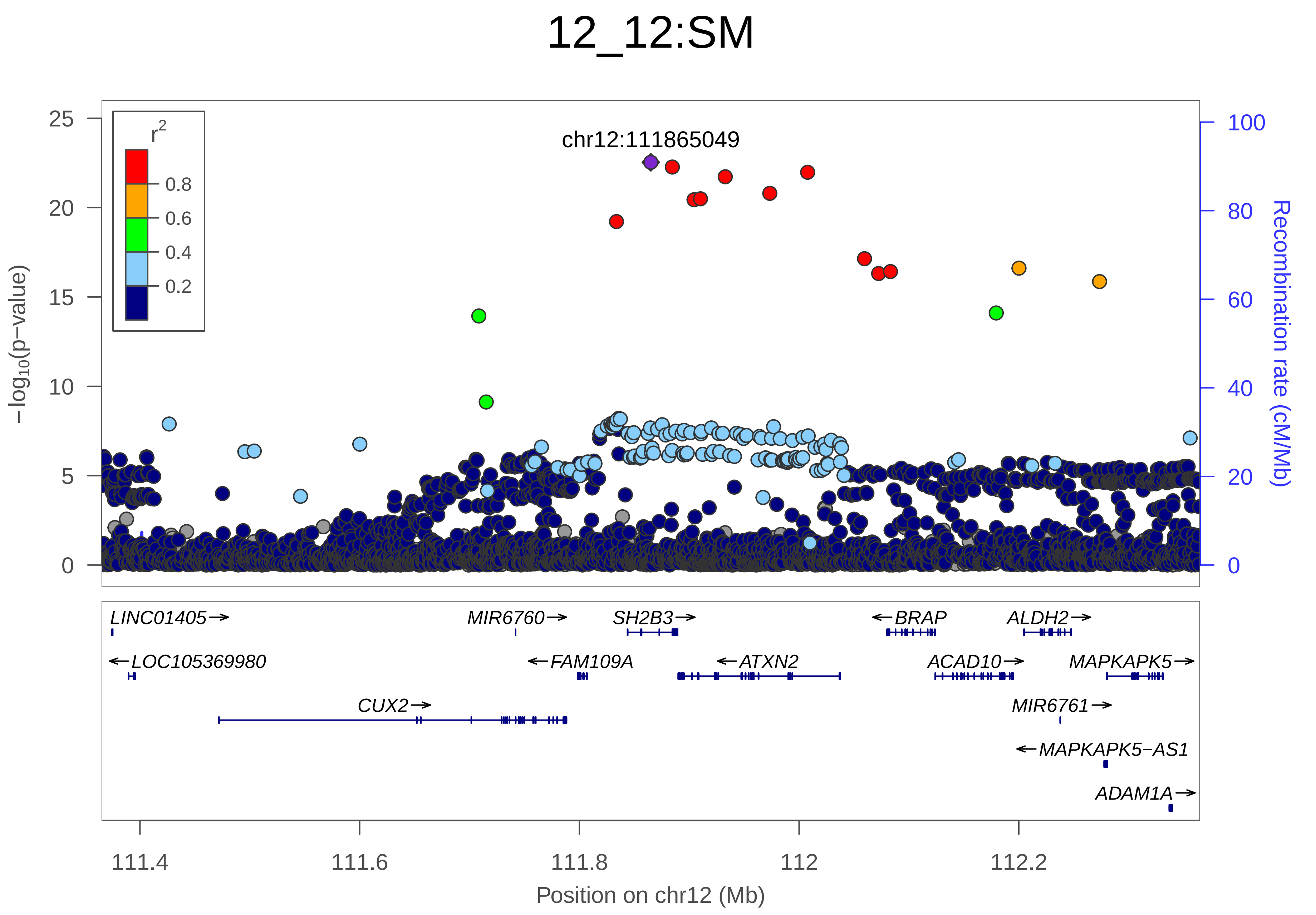
12_12:SM
chr12:111865049
r2
0.8
0.6
0.4
0.2
−log10(p−value)
Recombination rate (cM/Mb)
LINC01405→
←LOC105369980
CUX2→
MIR6760→
←FAM109A
SH2B3→
←ATXN2
←BRAP
ACAD10→
ALDH2→
MAPKAPK5→
MIR6761→
←MAPKAPK5−AS1
ADAM1A→
111.4
111.6
111.8
112
112.2
Position on chr12 (Mb)

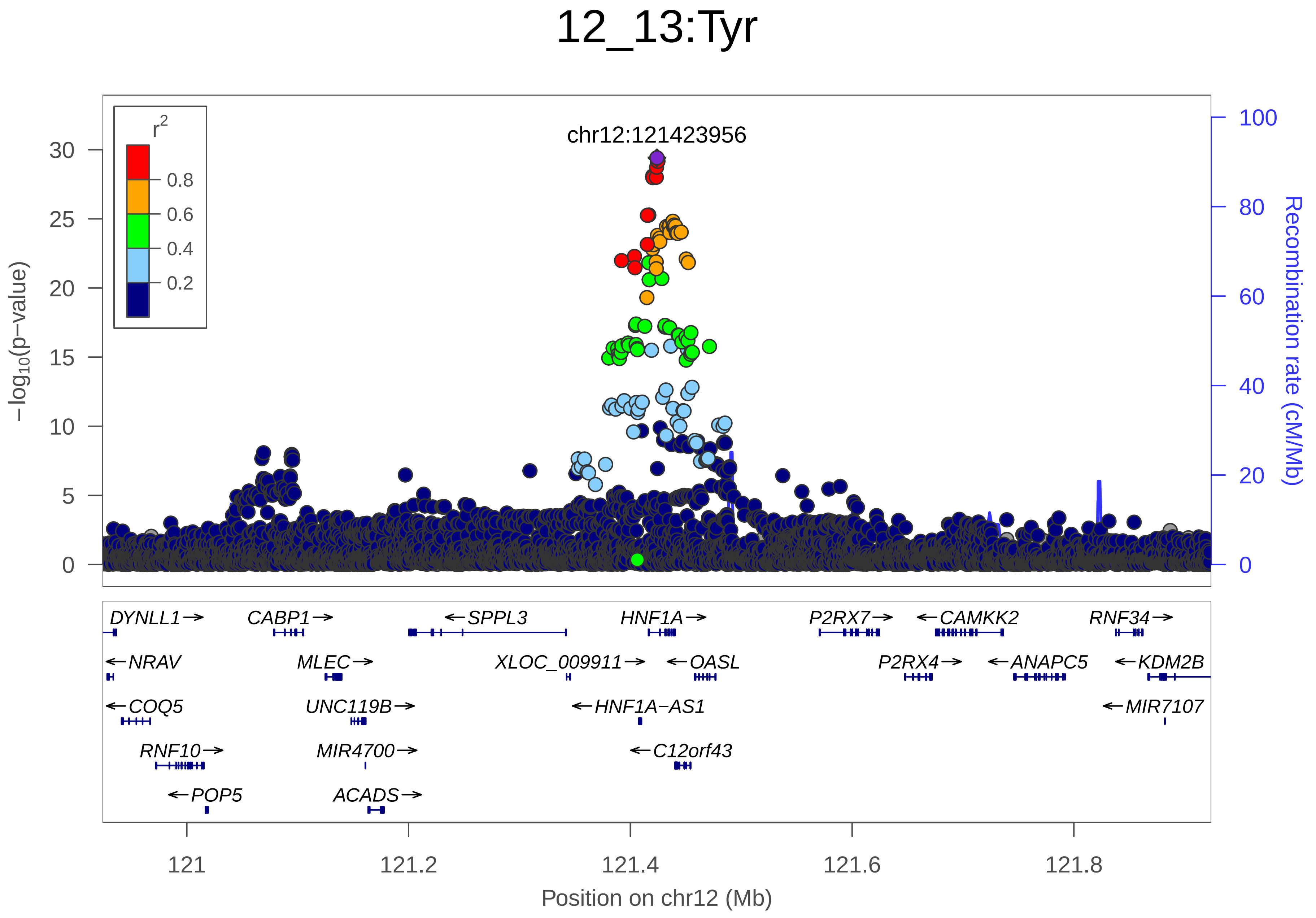

12_13:Tyr
chr12:121423956
r2
0.8
0.6
0.4
0.2
−log10(p−value)
Recombination rate (cM/Mb)
DYNLL1→
CABP1→
←SPPL3
HNF1A→
P2RX7→
←CAMKK2
RNF34→
←NRAV
MLEC→
XLOC_009911→
←OASL
P2RX4→
←ANAPC5
←KDM2B
←COQ5
UNC119B→
←HNF1A−AS1
←MIR7107
RNF10→
MIR4700→
←C12orf43
←POP5
ACADS→
121
121.2
121.4
121.6
121.8
Position on chr12 (Mb)

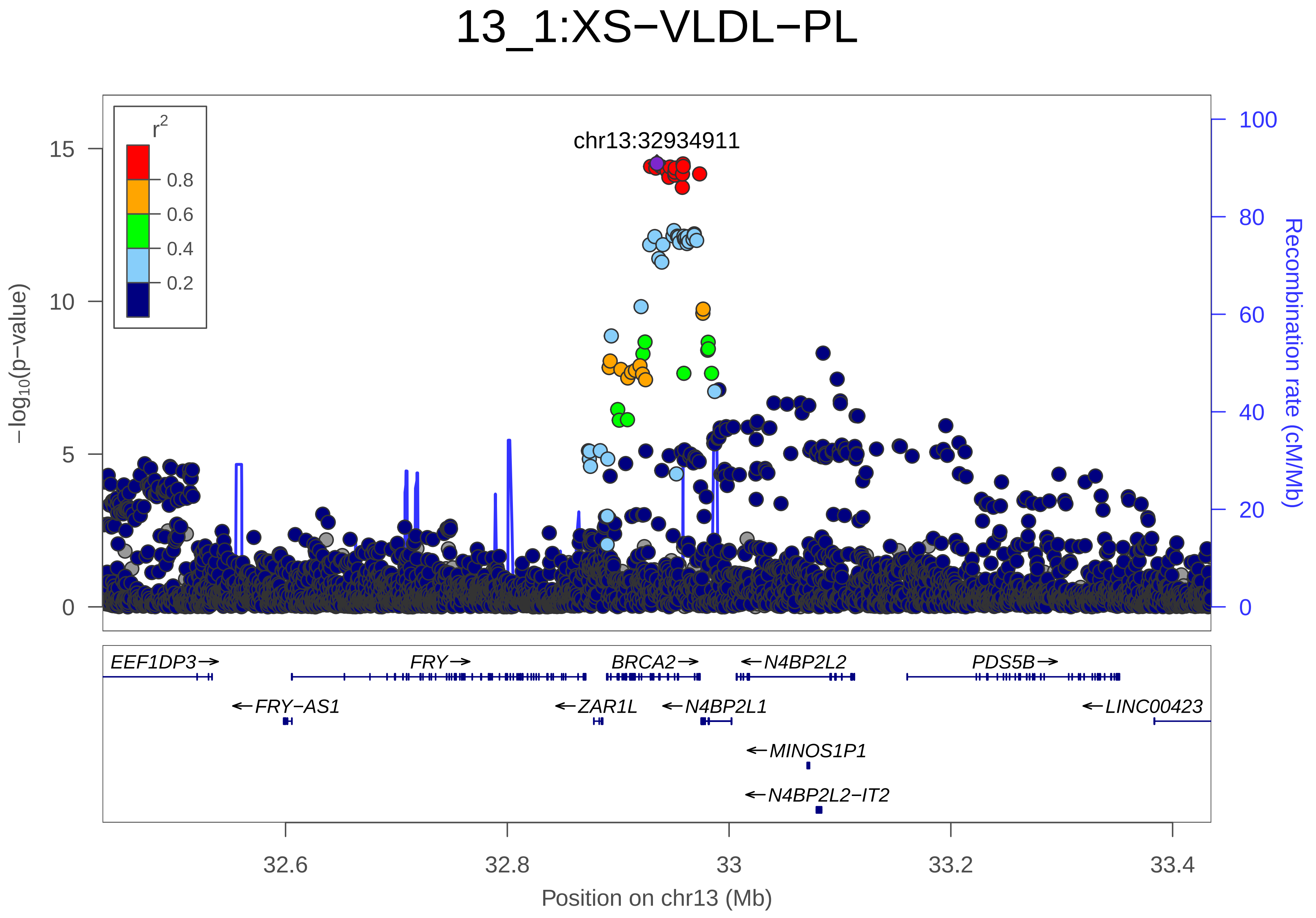

13_1:XS−VLDL−PL
chr13:32934911
r2
0.8
0.6
0.4
0.2
−log10(p−value)
Recombination rate (cM/Mb)
0
5
10
15
0
20
40
60
80
100
EEF1DP3
FRY
BRCA2
N4BP2L2
PDS5B
FRY−AS1
ZAR1L
N4BP2L1
LINC00423
MINOS1P1
N4BP2L2−IT2
32.6
32.8
33
33.2
33.4
Position on chr13 (Mb)

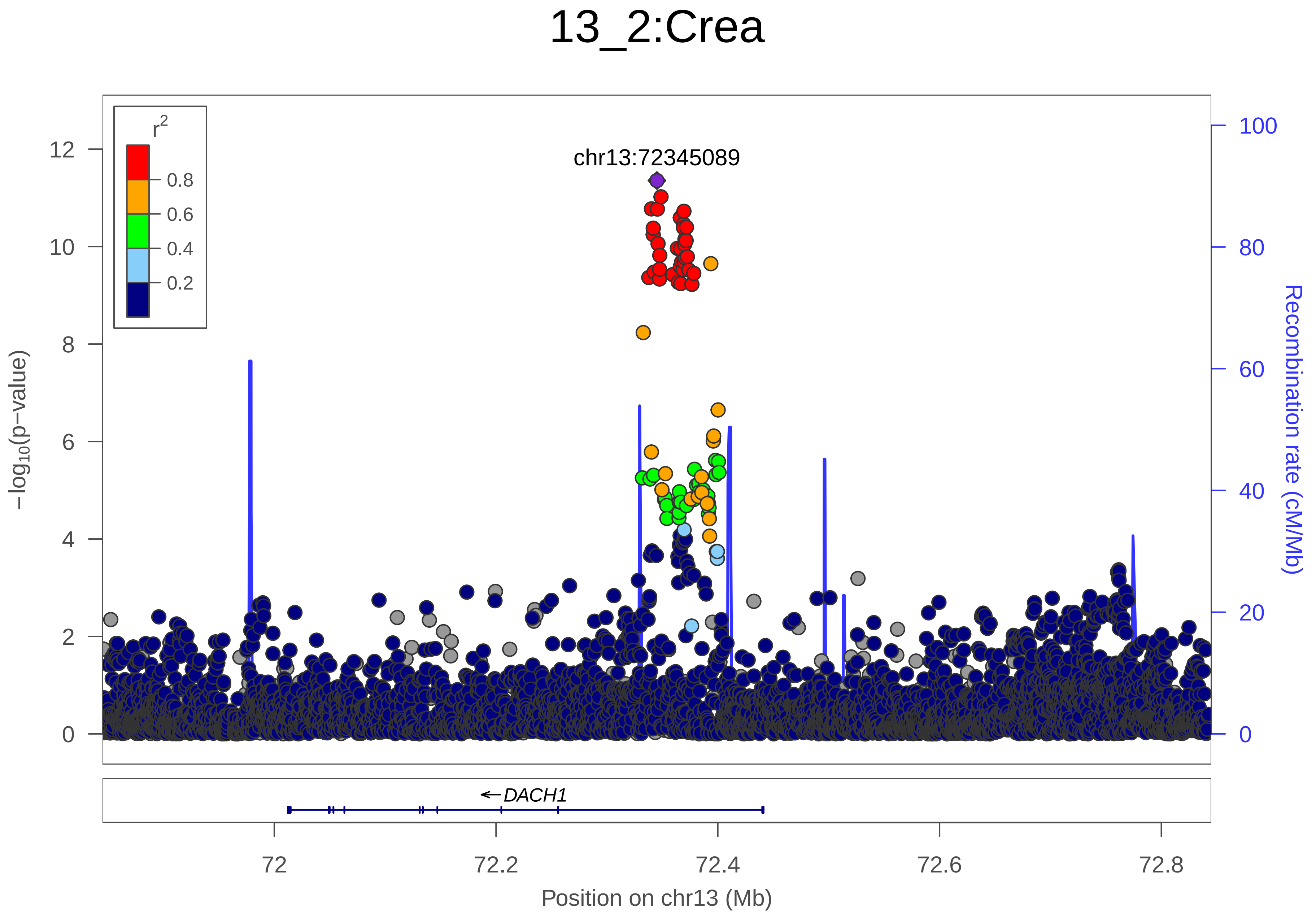

13_2:Crea
r2
0.8
0.6
0.4
0.2
chr13:72345089
12
10
8
6
4
2
0
−log10(p−value)
100
80
60
40
20
0
Recombination rate (cM/Mb)
DACH1
72
72.2
72.4
72.6
72.8
Position on chr13 (Mb)

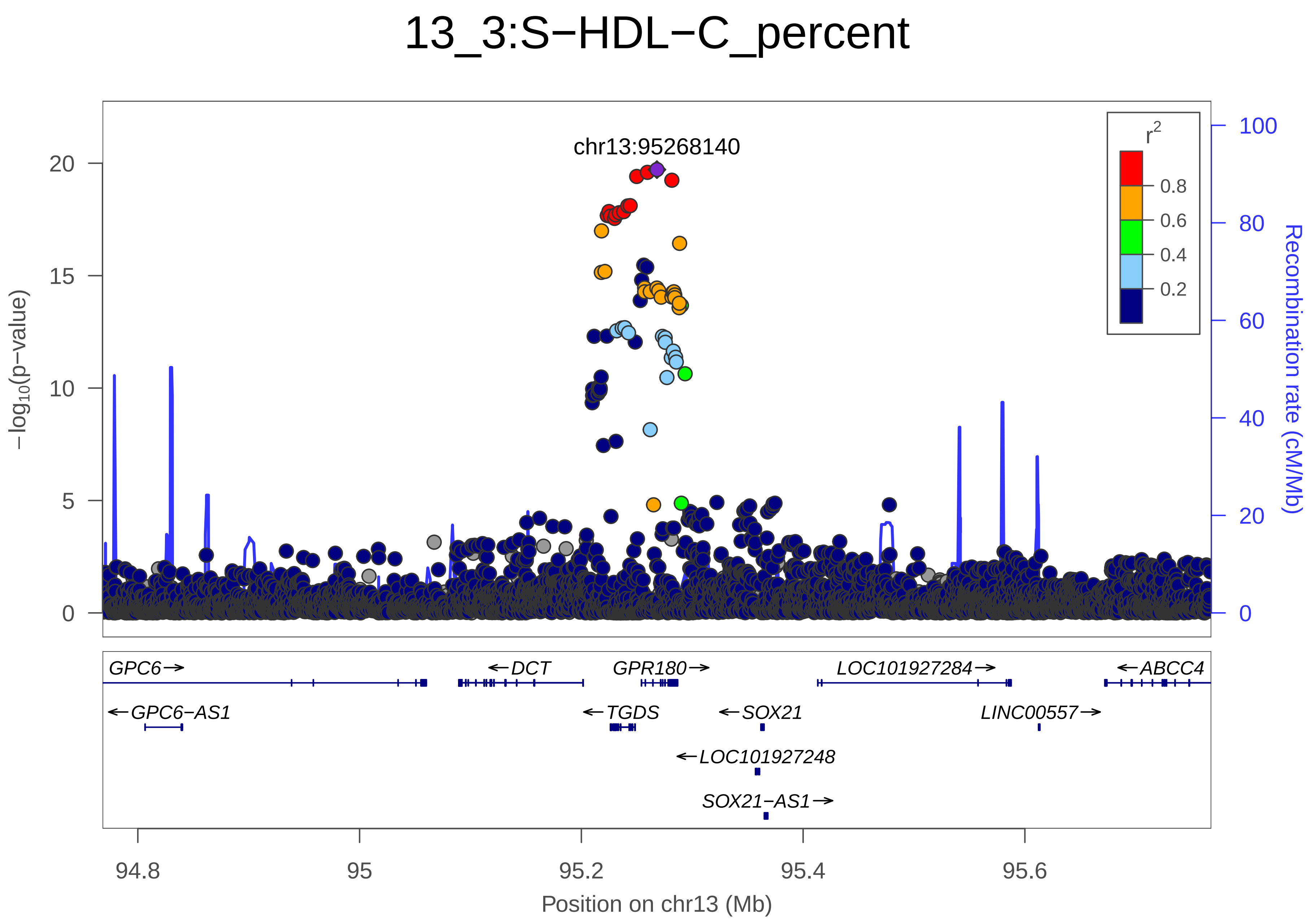

13_3:S−HDL−C_percent
chr13:95268140
−log10(p−value)
20
15
10
5
0
r2
0.8
0.6
0.4
0.2
100
80
60
40
20
0
Recombination rate (cM/Mb)
GPC6→
←DCT
GPR180→
LOC101927284→
←ABCC4
←GPC6−AS1
←TGDS
←SOX21
LINC00557→
←LOC101927248
SOX21−AS1→
94.8
95
95.2
95.4
95.6
Position on chr13 (Mb)

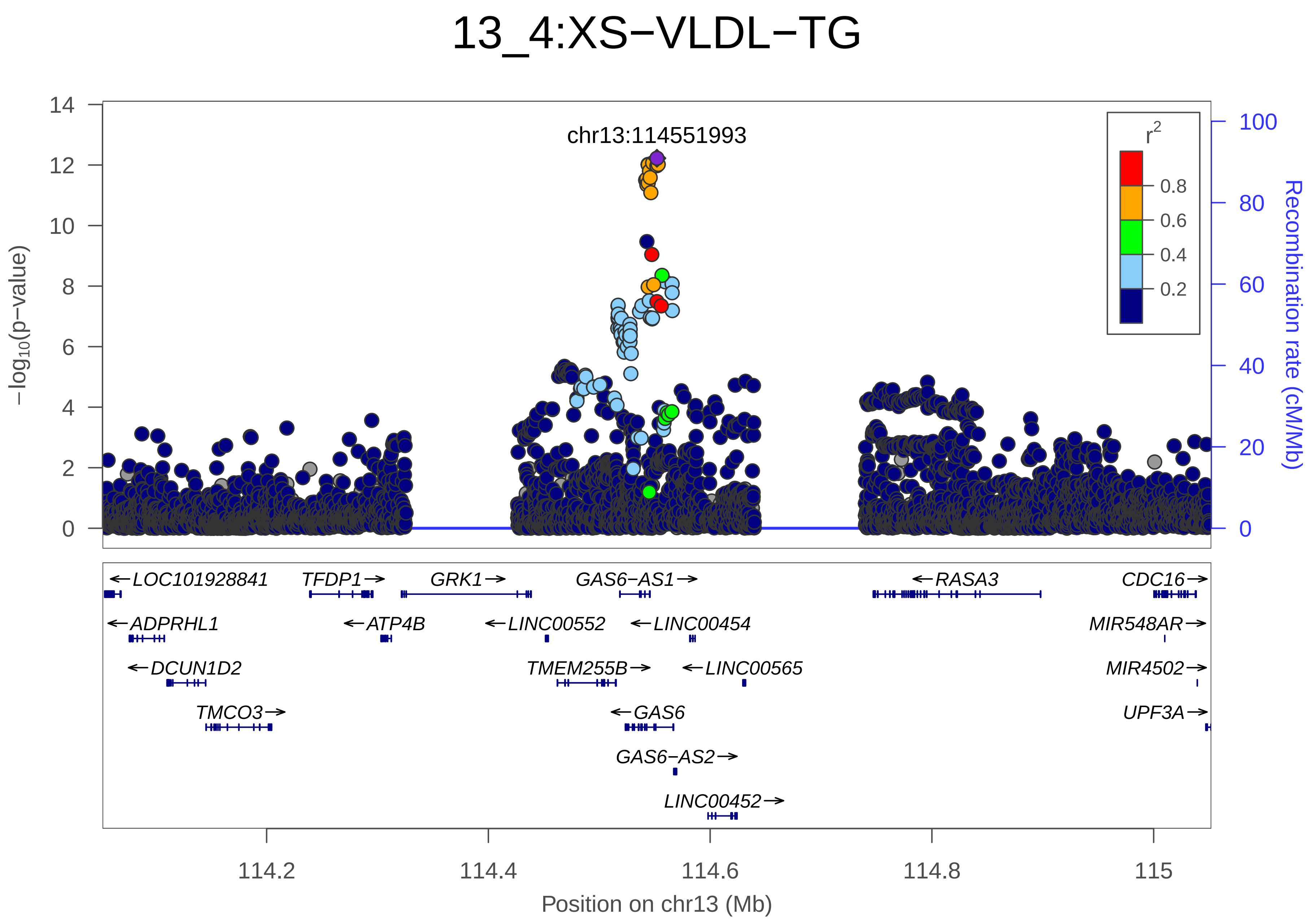
13_4:XS−VLDL−TG
chr13:114551993
−log10(p−value)
Recombination rate (cM/Mb)
r2
0.8
0.6
0.4
0.2
14
12
10
8
6
4
2
0
100
80
60
40
20
0
←LOC101928841
TFDP1→
GRK1→
GAS6−AS1→
←RASA3
CDC16→
←ADPRHL1
←ATP4B
←LINC00552
←LINC00454
MIR548AR→
←DCUN1D2
TMEM255B→
←LINC00565
MIR4502→
TMCO3→
←GAS6
UPF3A→
GAS6−AS2→
LINC00452→
114.2
114.4
114.6
114.8
115
Position on chr13 (Mb)

14_1:His

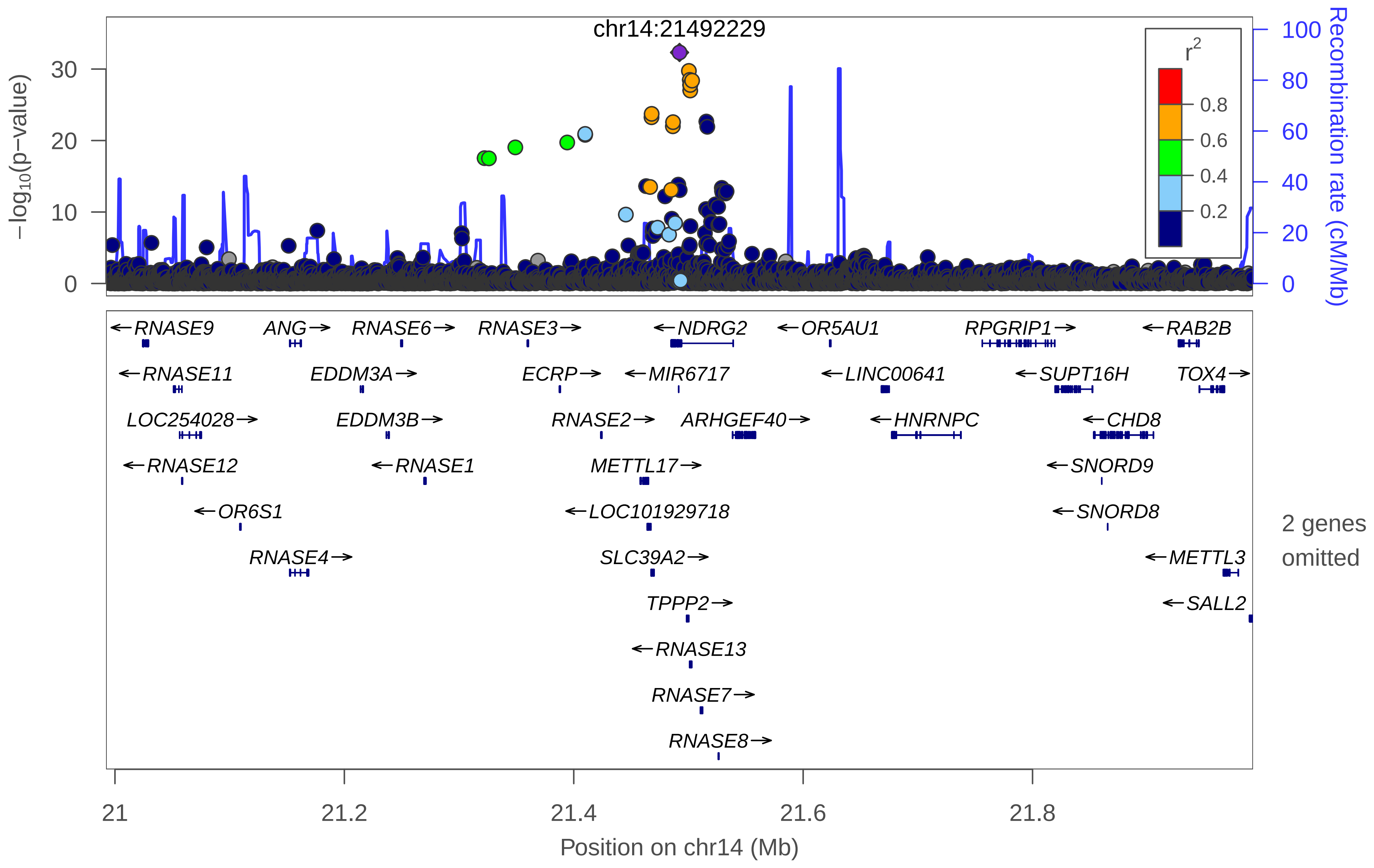

chr14:21492229
−log10(p−value)
Recombination rate (cM/Mb)
r2
0.8
0.6
0.4
0.2
RNASE9
ANG
RNASE6
RNASE3
NDRG2
OR5AU1
RPGRIP1
RAB2B
RNASE11
EDDM3A
ECRP
MIR6717
LINC00641
SUPT16H
TOX4
LOC254028
EDDM3B
RNASE2
ARHGEF40
HNRNPC
CHD8
RNASE12
RNASE1
METTL17
SNORD9
OR6S1
LOC101929718
SNORD8
RNASE4
SLC39A2
METTL3
TPPP2
SALL2
RNASE13
RNASE7
RNASE8
2 genes omitted
Position on chr14 (Mb)

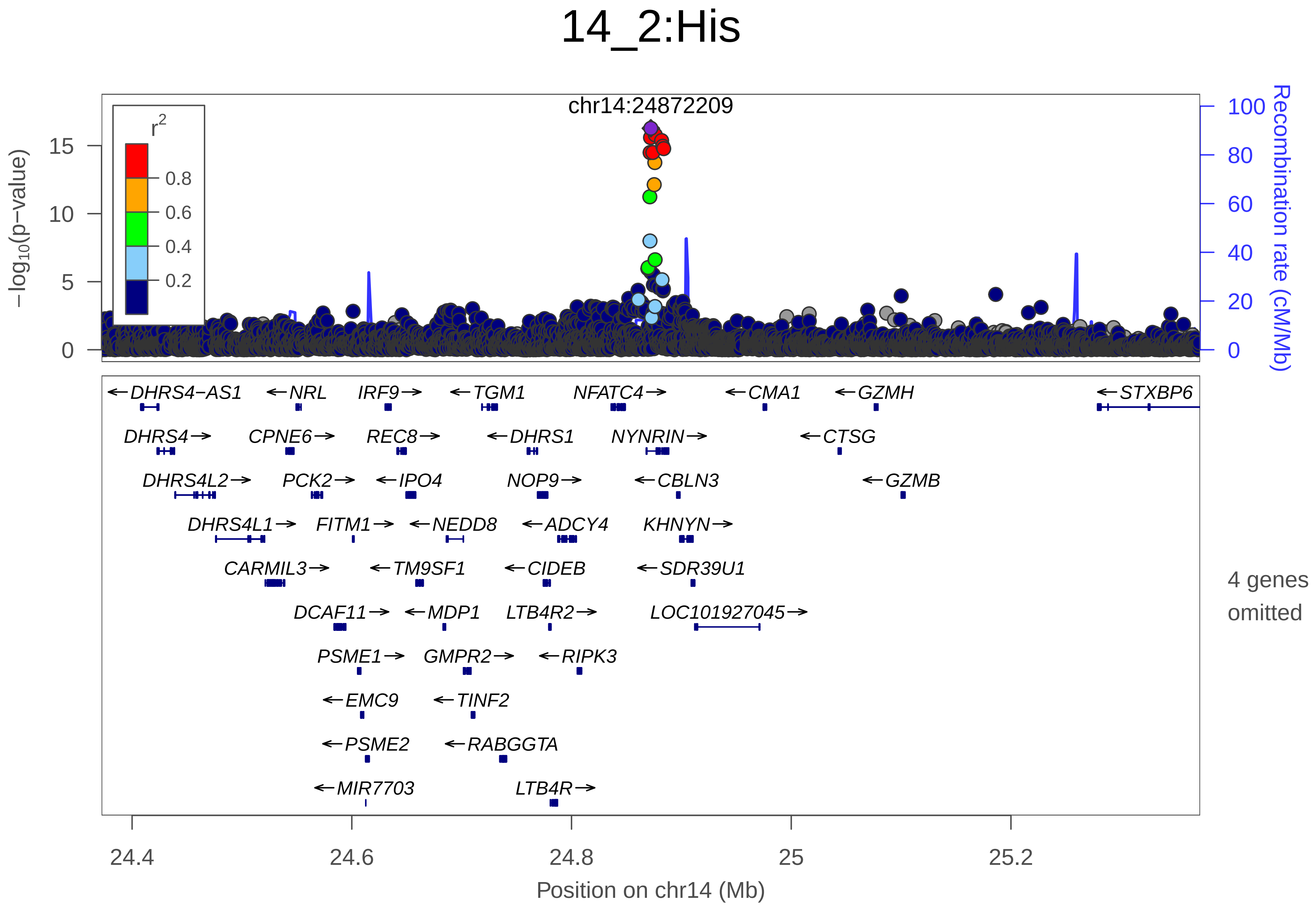

14_2:His
chr14:24872209
r2
0.8
0.6
0.4
0.2
−log10(p−value)
15
10
5
0
100
80
60
40
20
0
Recombination rate (cM/Mb)
←DHRS4−AS1
←NRL
IRF9→
←TGM1
NFATC4→
←CMA1
←GZMH
←STXBP6
DHRS4→
CPNE6→
REC8→
←DHRS1
NYNRIN→
←CTSG
DHRS4L2→
PCK2→
←IPO4
NOP9→
←CBLN3
←GZMB
DHRS4L1→
FITM1→
←NEDD8
←ADCY4
KHNYN→
CARMIL3→
←TM9SF1
←CIDEB
←SDR39U1
DCAF11→
←MDP1
LTB4R2→
LOC101927045→
PSME1→
GMPR2→
←RIPK3
←EMC9
←TINF2
←PSME2
←RABGGTA
←MIR7703
LTB4R→
4 genes omitted
24.4
24.6
24.8
25
25.2
Position on chr14 (Mb)

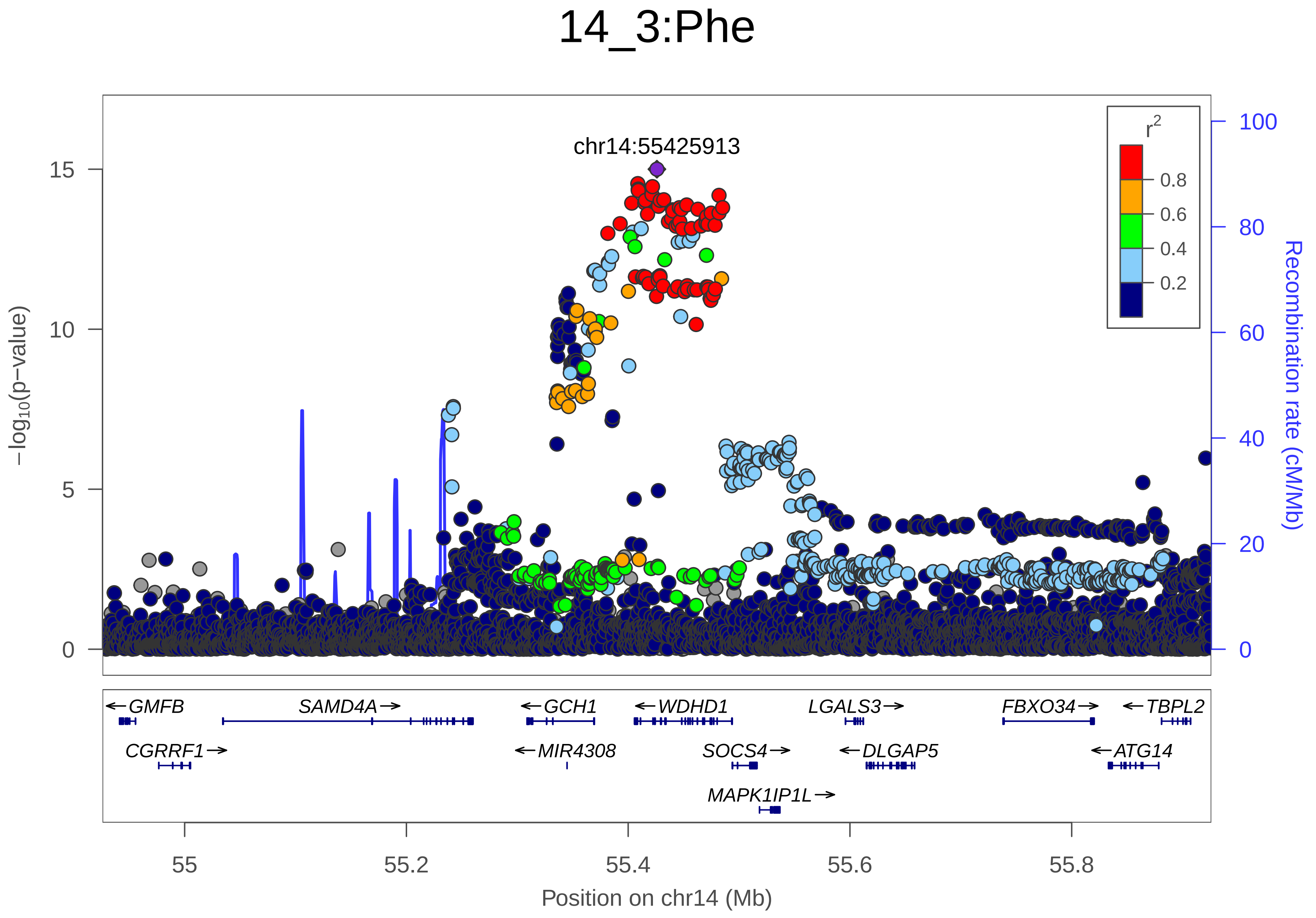
14_3:Phe
chr14:55425913
−log10(p−value)
Recombination rate (cM/Mb)
r2
0.8
0.6
0.4
0.2
0
5
10
15
20
40
60
80
100
GMFB
SAMD4A
GCH1
WDHD1
LGALS3
FBXO34
TBPL2
CGRRF1
MIR4308
SOCS4
DLGAP5
ATG14
MAPK1IP1L
55
55.2
55.4
55.6
55.8
Position on chr14 (Mb)

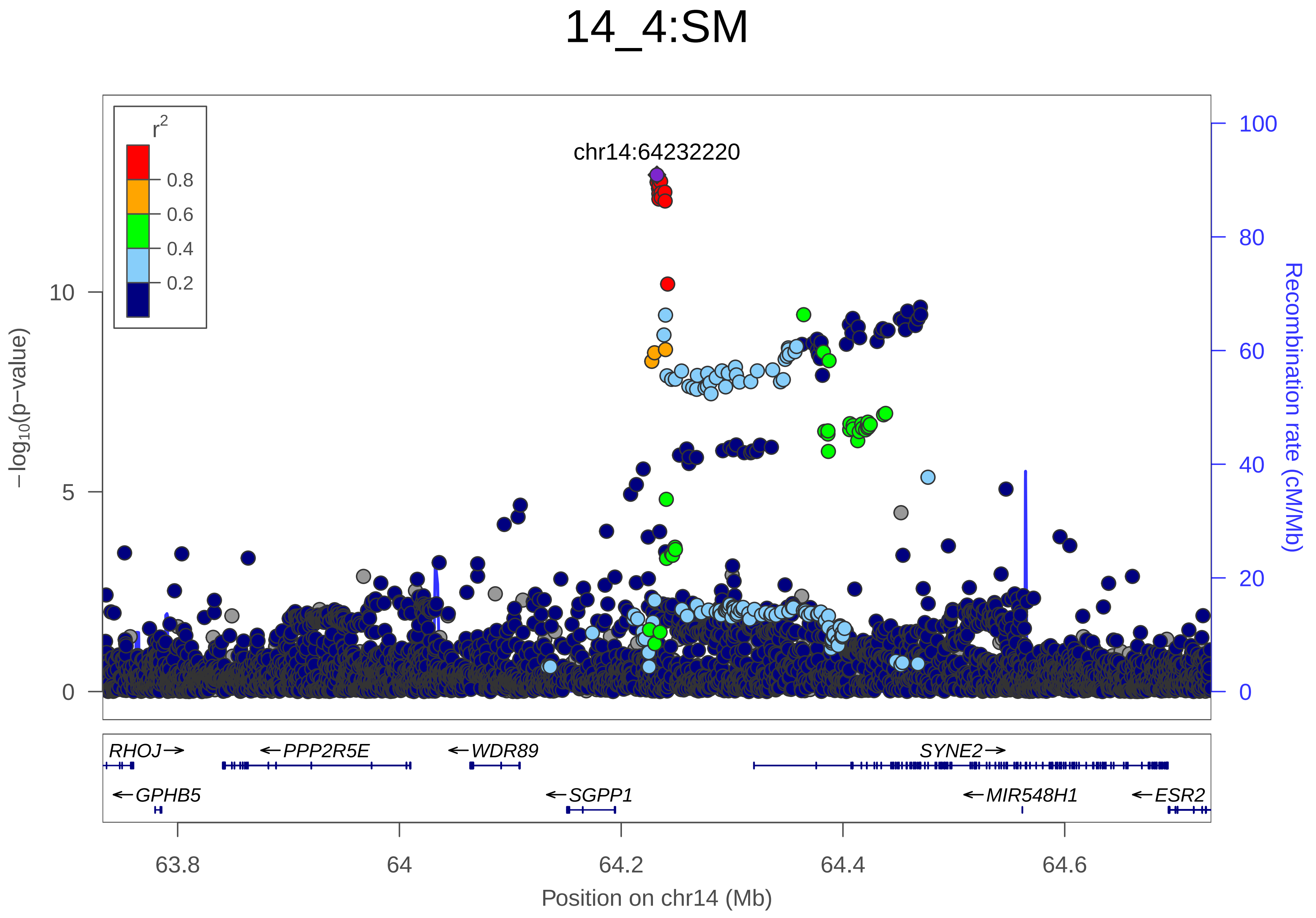

14_4:SM
chr14:64232220
r2
0.8
0.6
0.4
0.2
−log10(p−value)
10
5
0
Recombination rate (cM/Mb)
100
80
60
40
20
0
RHOJ→
←PPP2R5E
←WDR89
SYNE2→
←GPHB5
←SGPP1
←MIR548H1
←ESR2
63.8
64
64.2
64.4
64.6
Position on chr14 (Mb)

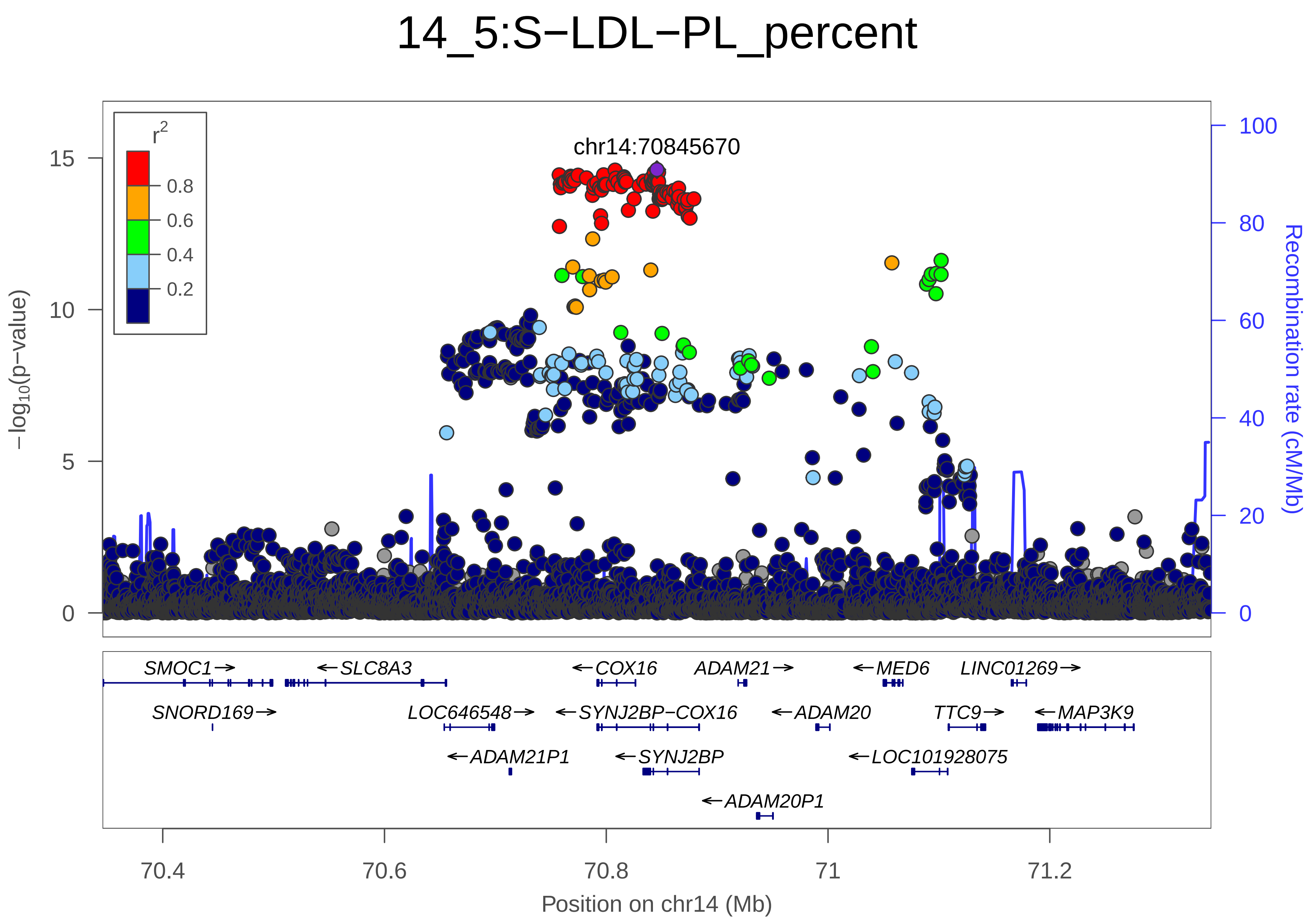

14_5:S−LDL−PL_percent
chr14:70845670
r2
0.8
0.6
0.4
0.2
15
10
5
0
−log10(p−value)
100
80
60
40
20
0
Recombination rate (cM/Mb)
SMOC1→
←SLC8A3
←COX16
ADAM21→
←MED6
LINC01269→
SNORD169→
LOC646548→
←SYNJ2BP−COX16
←ADAM20
TTC9→
←MAP3K9
←ADAM21P1
←SYNJ2BP
←LOC101928075
←ADAM20P1
70.4
70.6
70.8
71
71.2
Position on chr14 (Mb)

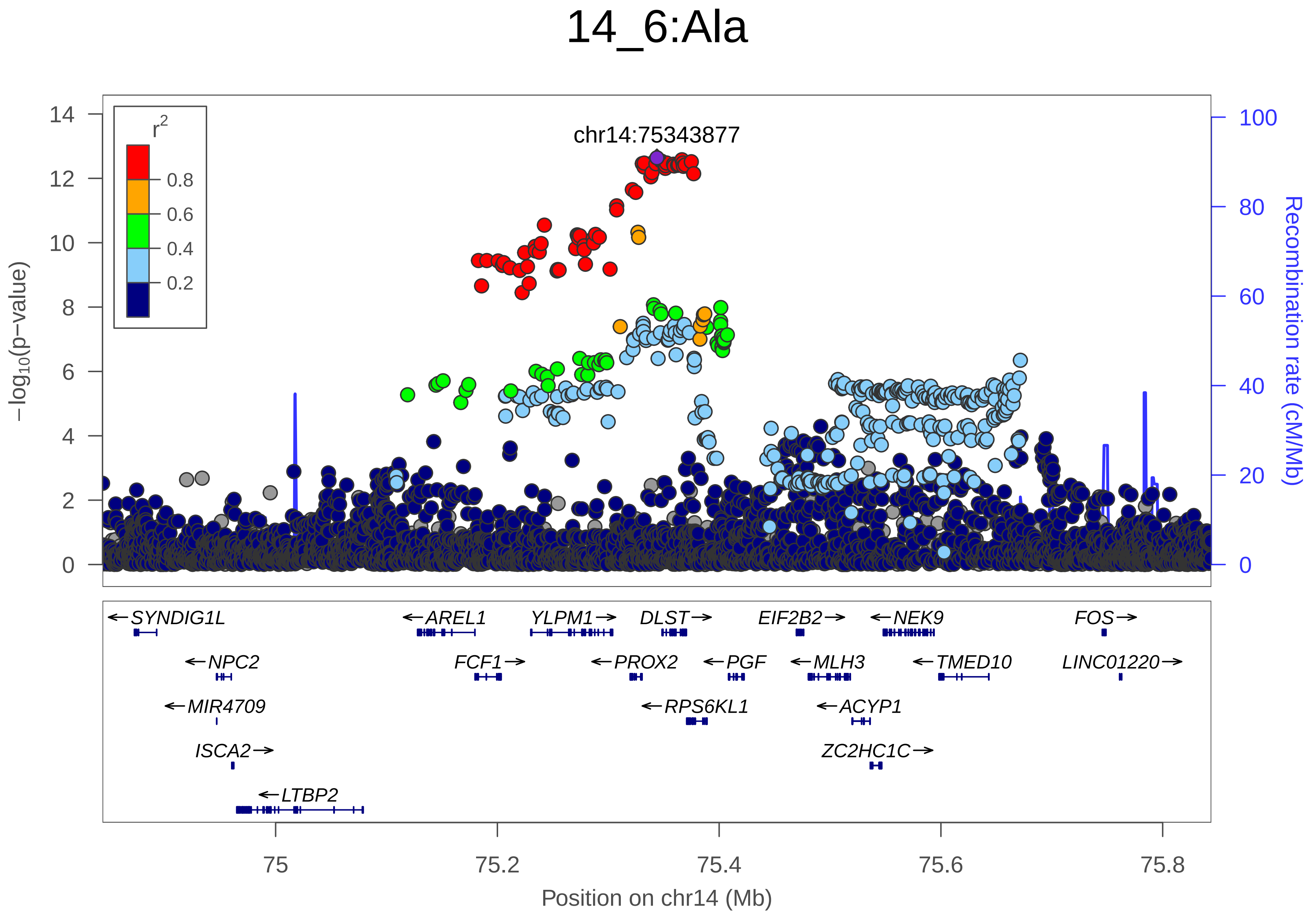
14_6:Ala
chr14:75343877
r2
0.8
0.6
0.4
0.2
−log10(p−value)
Recombination rate (cM/Mb)
0
2
4
6
8
10
12
14
0
20
40
60
80
100
SYNDIG1L
NPC2
MIR4709
ISCA2
LTBP2
AREL1
FCF1
YLPM1
PROX2
DLST
RPS6KL1
PGF
EIF2B2
MLH3
ACYP1
ZC2HC1C
NEK9
TMED10
FOS
LINC01220
75
75.2
75.4
75.6
75.8
Position on chr14 (Mb)

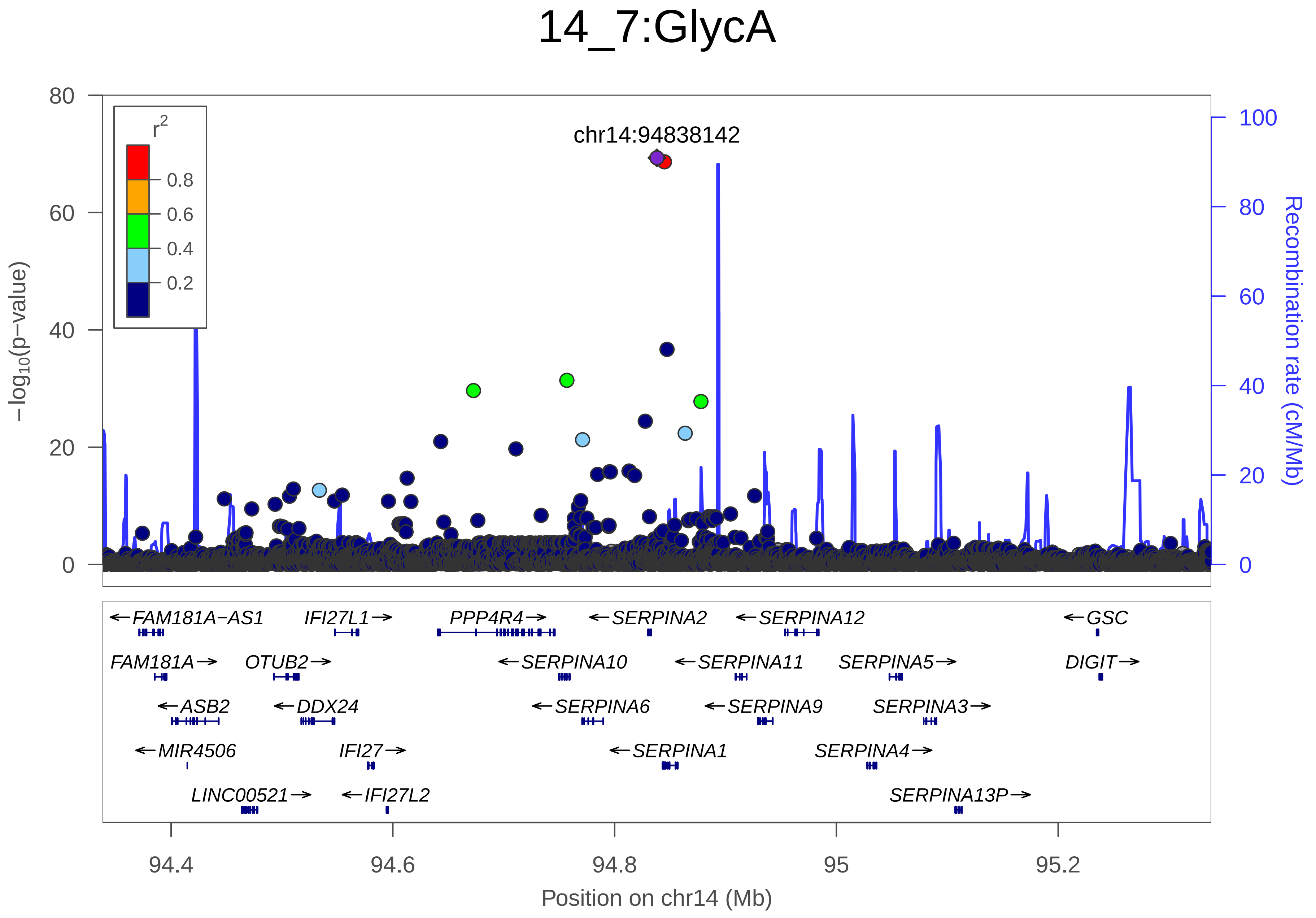
14_7:GlycA
chr14:94838142
r2
0.8
0.6
0.4
0.2
−log10(p−value)
Recombination rate (cM/Mb)
80
60
40
20
0
100
80
60
40
20
0
←FAM181A−AS1
IFI27L1→
PPP4R4→
←SERPINA2
←SERPINA12
←GSC
FAM181A→
OTUB2→
←SERPINA10
←SERPINA11
SERPINA5→
DIGIT→
←ASB2
←DDX24
←SERPINA6
←SERPINA9
SERPINA3→
←MIR4506
IFI27→
←SERPINA1
SERPINA4→
LINC00521→
←IFI27L2
SERPINA13P→
94.4
94.6
94.8
95
95.2
Position on chr14 (Mb)

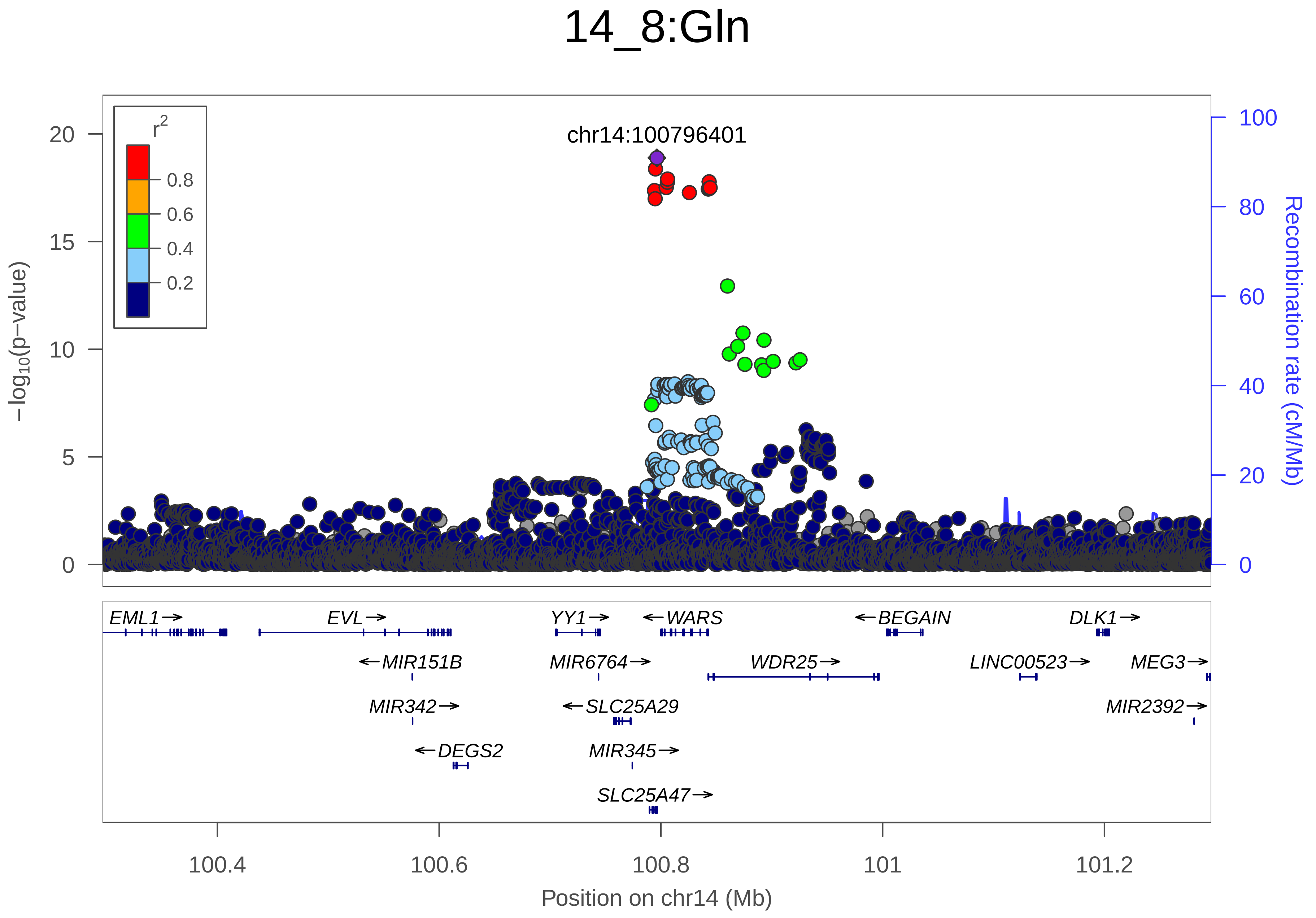

14_8:Gln
chr14:100796401
r2
0.8
0.6
0.4
0.2
−log10(p−value)
Recombination rate (cM/Mb)
0
5
10
15
20
0
20
40
60
80
100
EML1→
EVL→
YY1→
←WARS
←BEGAIN
DLK1→
←MIR151B
MIR6764→
WDR25→
LINC00523→
MEG3→
MIR342→
←SLC25A29
MIR2392→
←DEGS2
MIR345→
SLC25A47→
100.4
100.6
100.8
101
101.2
Position on chr14 (Mb)

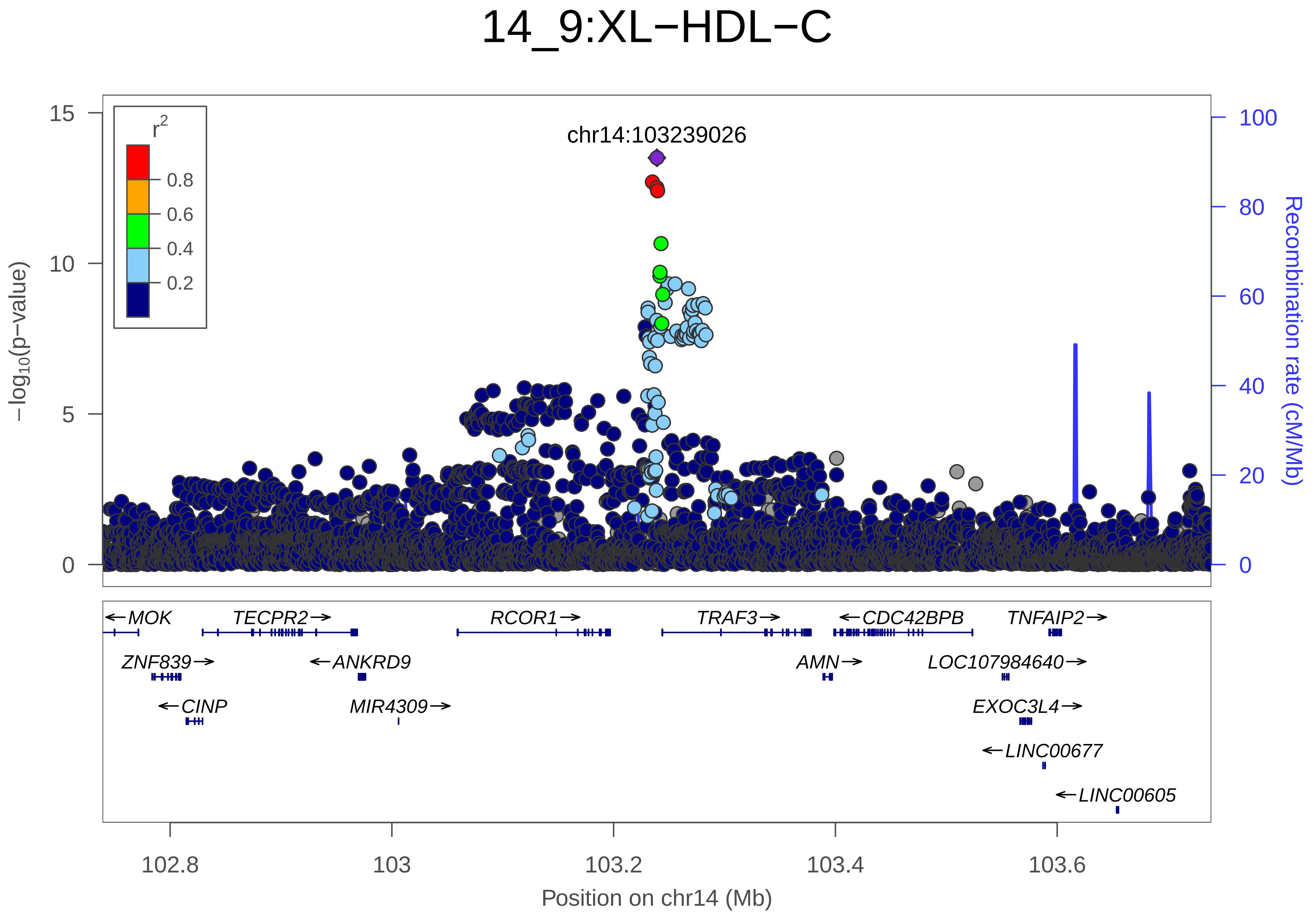

14_9:XL−HDL−C
chr14:103239026
r2
0.8
0.6
0.4
0.2
−log10(p−value)
Recombination rate (cM/Mb)
MOK
TECPR2
RCOR1
TRAF3
CDC42BPB
TNFAIP2
ZNF839
ANKRD9
AMN
LOC107984640
CINP
MIR4309
EXOC3L4
LINC00677
LINC00605
102.8
103
103.2
103.4
103.6
Position on chr14 (Mb)

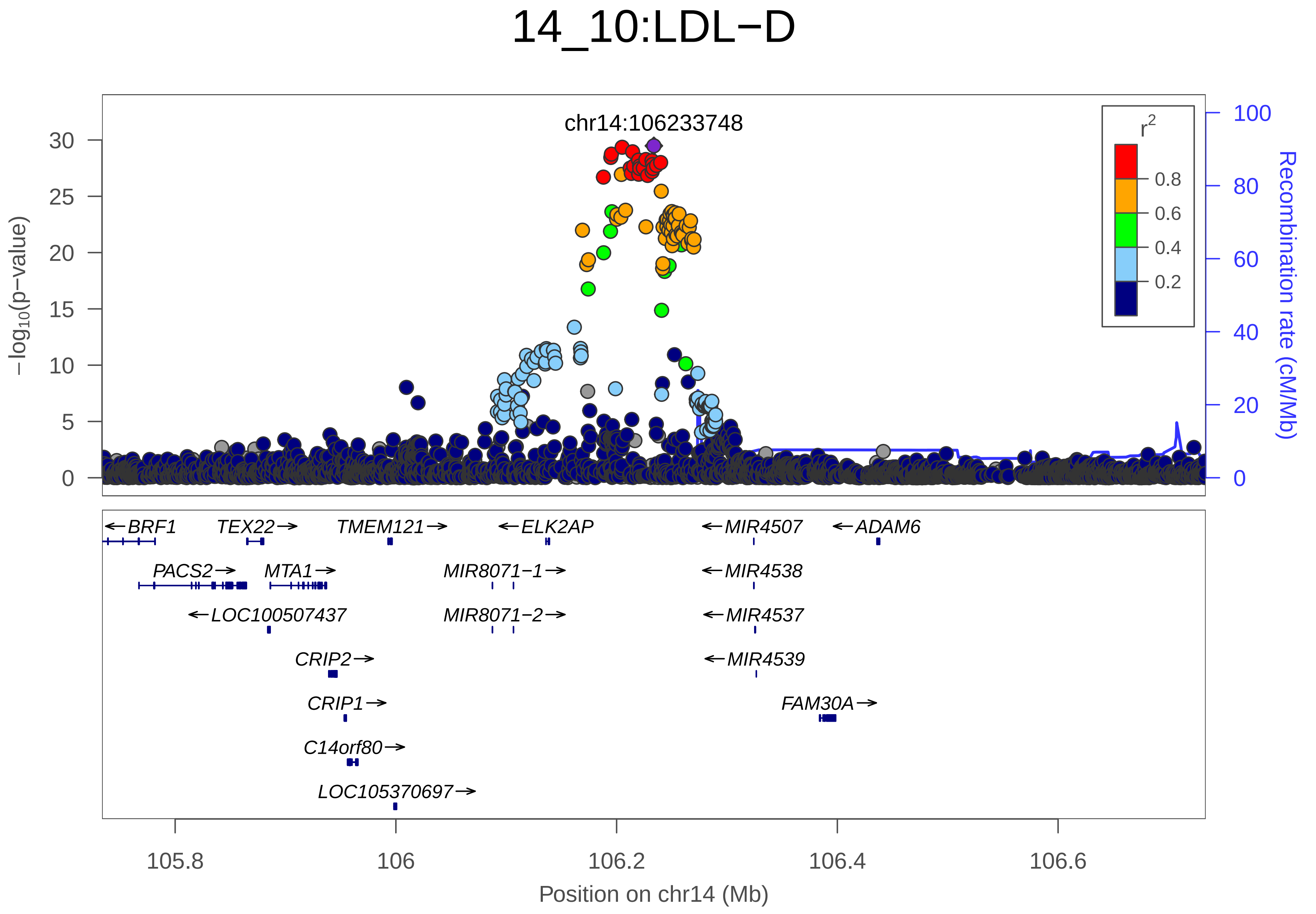

14_10:LDL−D
chr14:106233748
r2
0.8
0.6
0.4
0.2
−log10(p−value)
Recombination rate (cM/Mb)
BRF1
TEX22
TMEM121
ELK2AP
MIR4507
ADAM6
PACS2
MTA1
MIR8071−1
MIR4538
LOC100507437
MIR8071−2
MIR4537
CRIP2
MIR4539
CRIP1
FAM30A
C14orf80
LOC105370697
105.8
106
106.2
106.4
106.6
Position on chr14 (Mb)

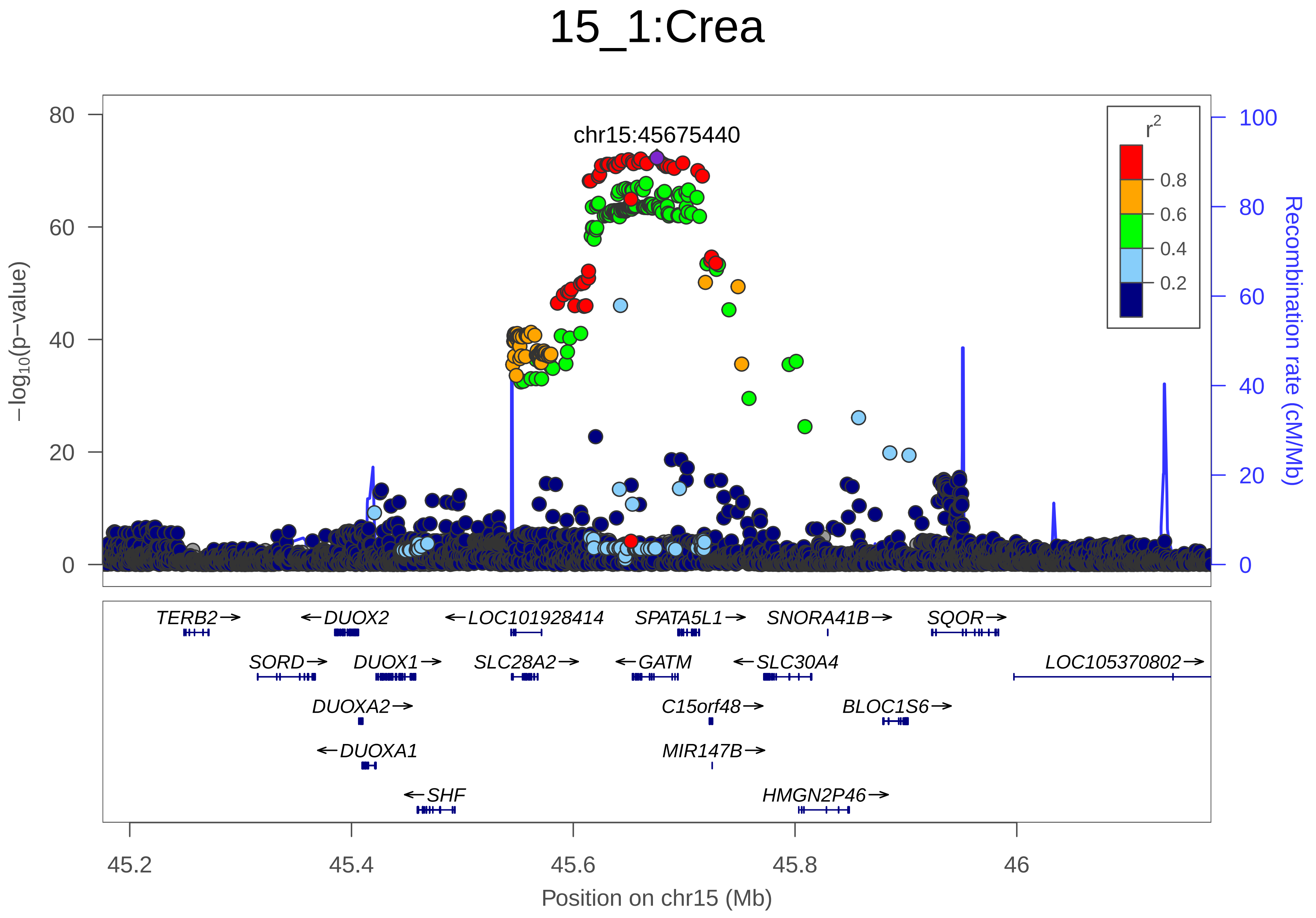

15_1:Crea
chr15:45675440
r2
0.8
0.6
0.4
0.2
−log10(p−value)
Recombination rate (cM/Mb)
0
20
40
60
80
100
TERB2→
←DUOX2
←LOC101928414
SPATA5L1→
SNORA41B→
SQOR→
SORD→
DUOX1→
SLC28A2→
←GATM
←SLC30A4
LOC105370802→
DUOXA2→
C15orf48→
BLOC1S6→
←DUOXA1
MIR147B→
←SHF
HMGN2P46→
45.2
45.4
45.6
45.8
46
Position on chr15 (Mb)

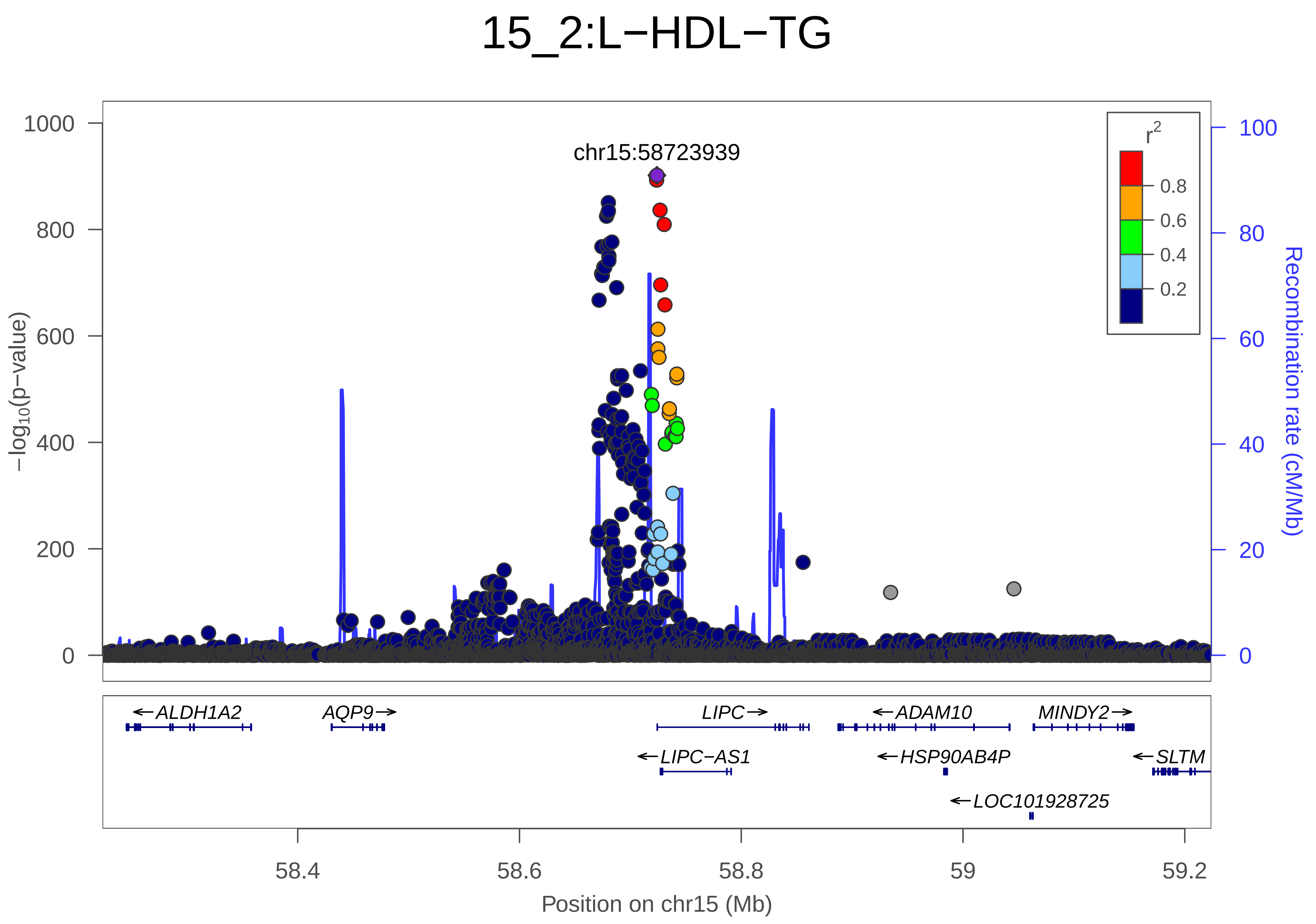
15_2:L−HDL−TG
chr15:58723939
−log10(p−value)
Recombination rate (cM/Mb)
r2
0.8
0.6
0.4
0.2
ALDH1A2
AQP9
LIPC
LIPC−AS1
ADAM10
HSP90AB4P
LOC101928725
MINDY2
SLTM
58.4
58.6
58.8
59
59.2
Position on chr15 (Mb)

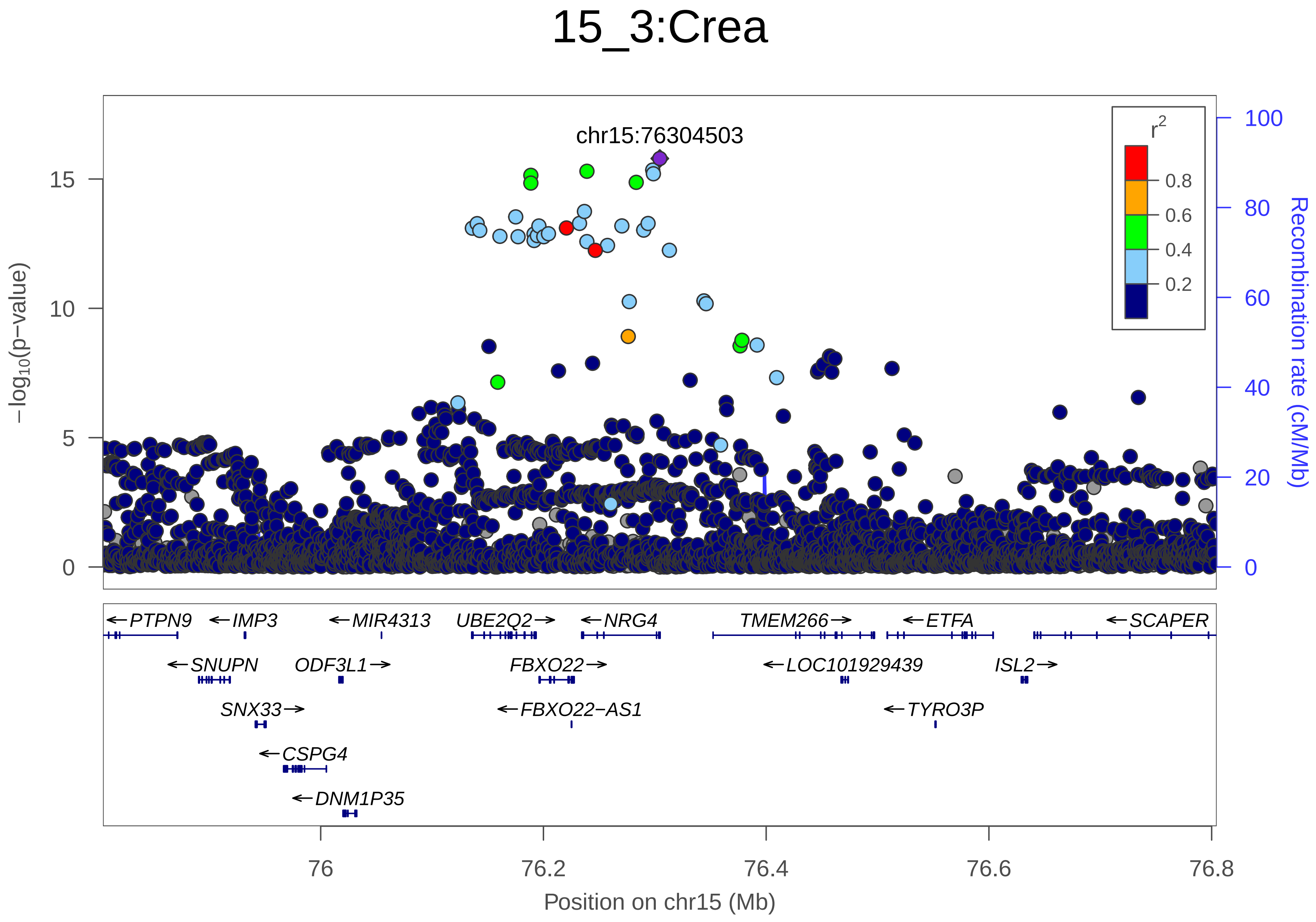

15_3:Crea
chr15:76304503
−log10(p−value)
Recombination rate (cM/Mb)
r2
0.8
0.6
0.4
0.2
0
5
10
15
0
20
40
60
80
100
←PTPN9
←IMP3
←MIR4313
UBE2Q2→
←NRG4
TMEM266→
←ETFA
←SCAPER
←SNUPN
ODF3L1→
FBXO22→
←LOC101929439
ISL2→
SNX33→
←FBXO22−AS1
←TYRO3P
←CSPG4
←DNM1P35
76
76.2
76.4
76.6
76.8
Position on chr15 (Mb)

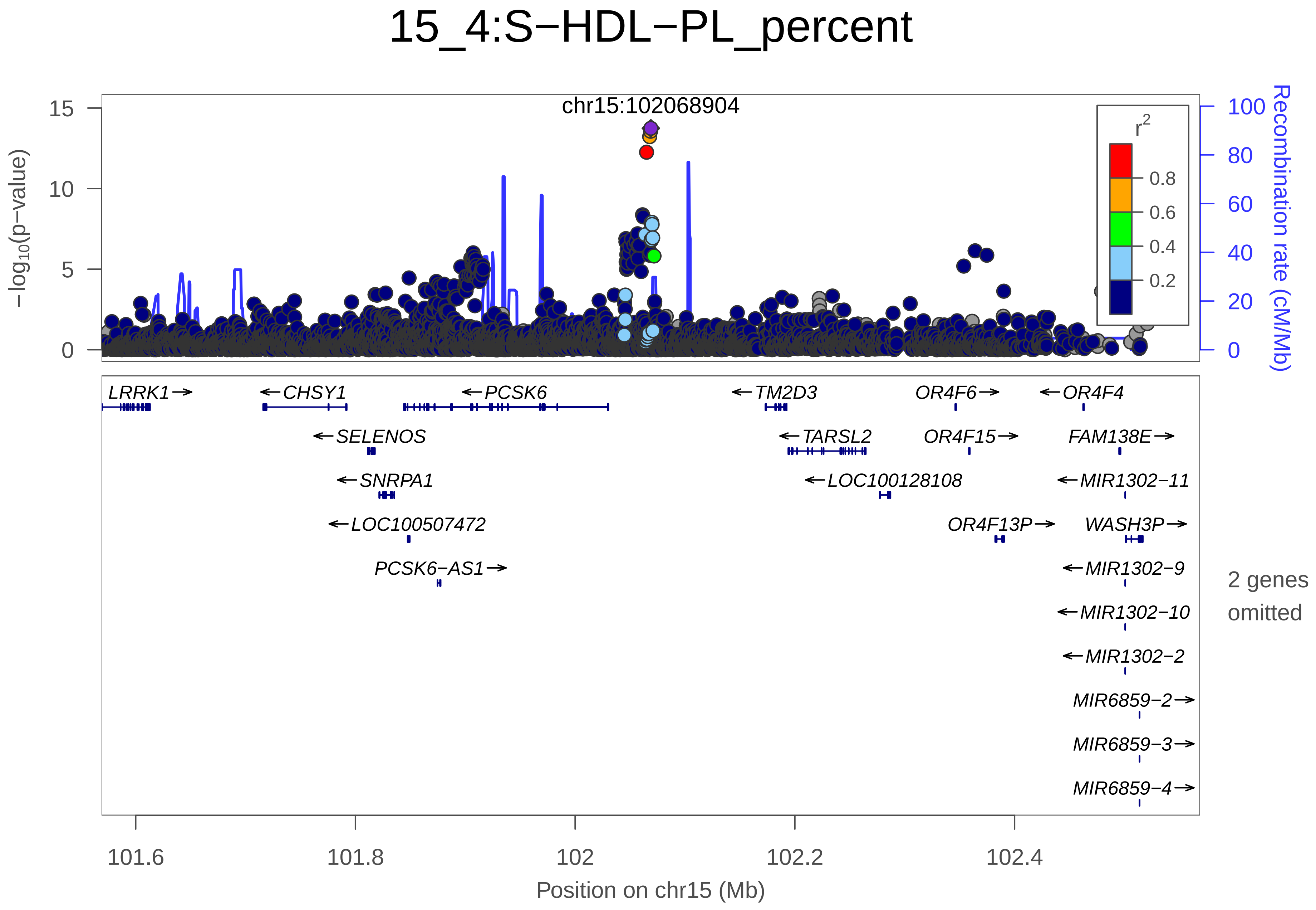

15_4:S−HDL−PL_percent
chr15:102068904
−log10(p−value)
Recombination rate (cM/Mb)
r2
0.8
0.6
0.4
0.2
LRRK1
CHSY1
PCSK6
TM2D3
OR4F6
OR4F4
SELENOS
TARSL2
OR4F15
FAM138E
SNRPA1
LOC100128108
MIR1302−11
LOC100507472
OR4F13P
WASH3P
PCSK6−AS1
MIR1302−9
MIR1302−10
MIR1302−2
MIR6859−2
MIR6859−3
MIR6859−4
2 genes omitted
101.6
101.8
102
102.2
102.4
Position on chr15 (Mb)

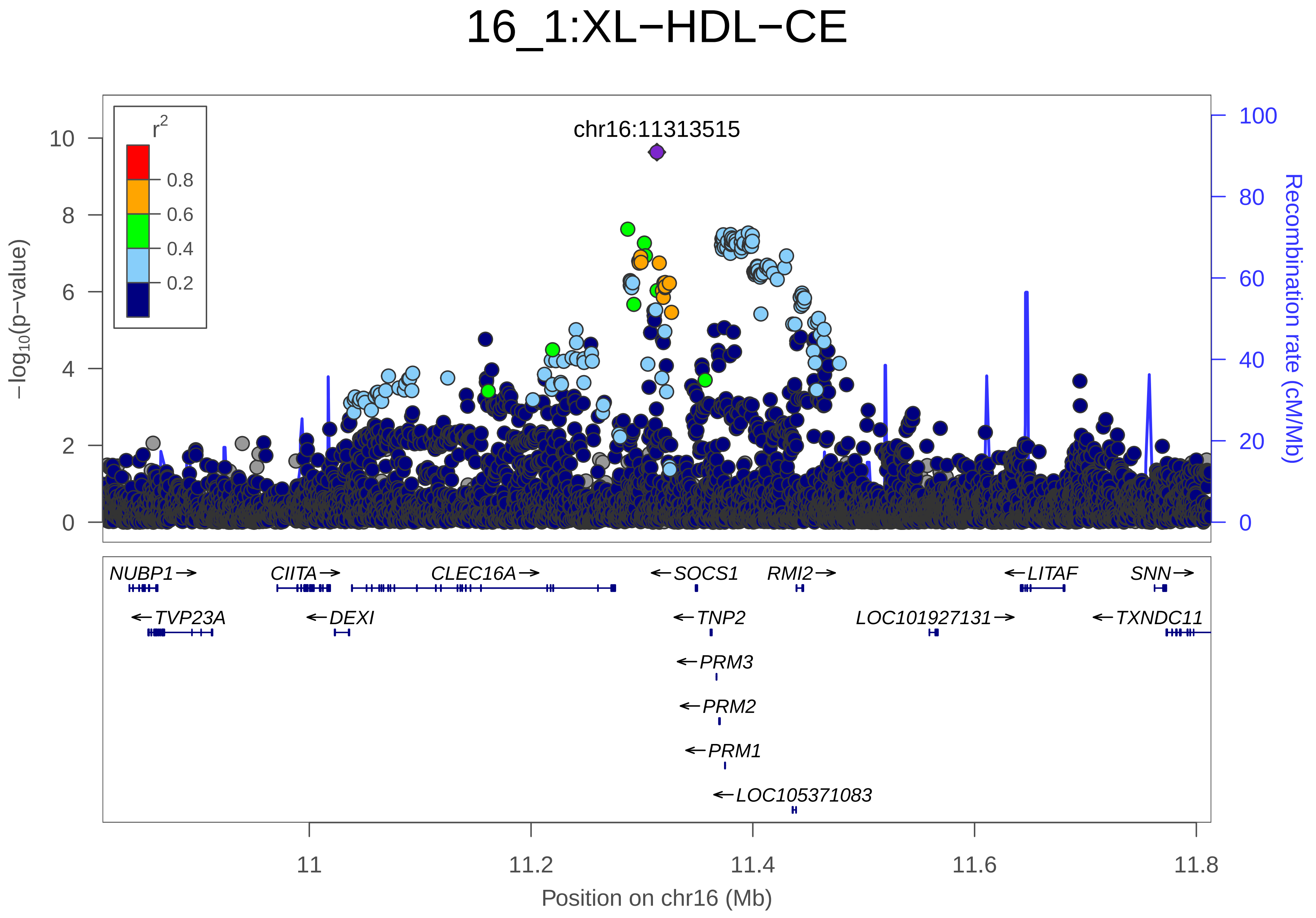

16_1:XL−HDL−CE
chr16:11313515
r2
0.8
0.6
0.4
0.2
−log10(p−value)
Recombination rate (cM/Mb)
0
2
4
6
8
10
0
20
40
60
80
100
NUBP1→
CIITA→
CLEC16A→
←SOCS1
RMI2→
←LITAF
SNN→
←TVP23A
←DEXI
←TNP2
LOC101927131→
←TXNDC11
←PRM3
←PRM2
←PRM1
←LOC105371083
11
11.2
11.4
11.6
11.8
Position on chr16 (Mb)

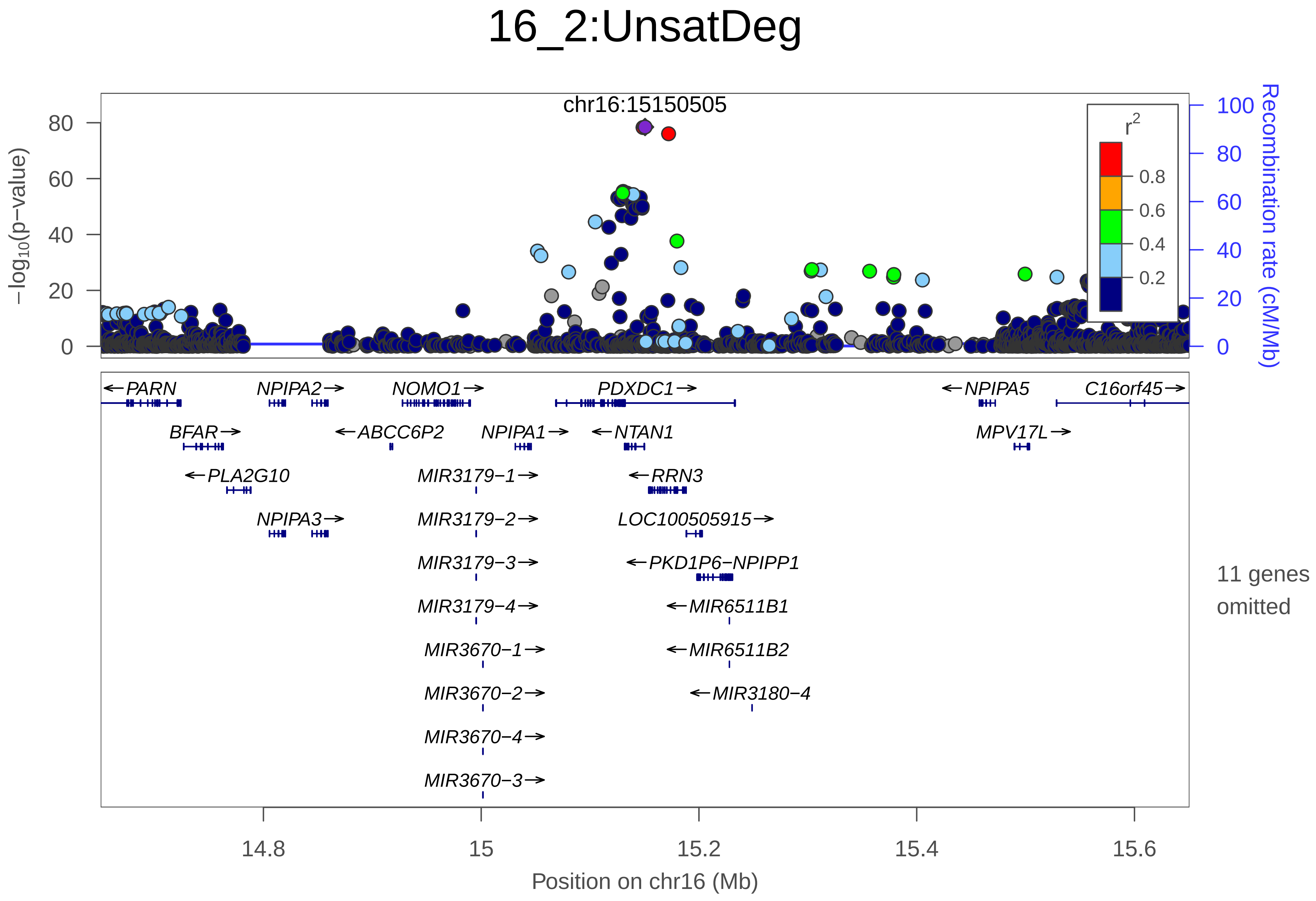
16_2:UnsatDeg
chr16:15150505
−log10(p−value)
Recombination rate (cM/Mb)
r2
0.8
0.6
0.4
0.2
PARN
NPIPA2
NOMO1
PDXDC1
NPIPA5
C16orf45
BFAR
ABCC6P2
NPIPA1
NTAN1
MPV17L
PLA2G10
MIR3179−1
RRN3
NPIPA3
MIR3179−2
LOC100505915
MIR3179−3
PKD1P6−NPIPP1
MIR3179−4
MIR6511B1
MIR3670−1
MIR6511B2
MIR3670−2
MIR3180−4
MIR3670−4
MIR3670−3
11 genes omitted
14.8
15
15.2
15.4
15.6
Position on chr16 (Mb)

16_3:Crea

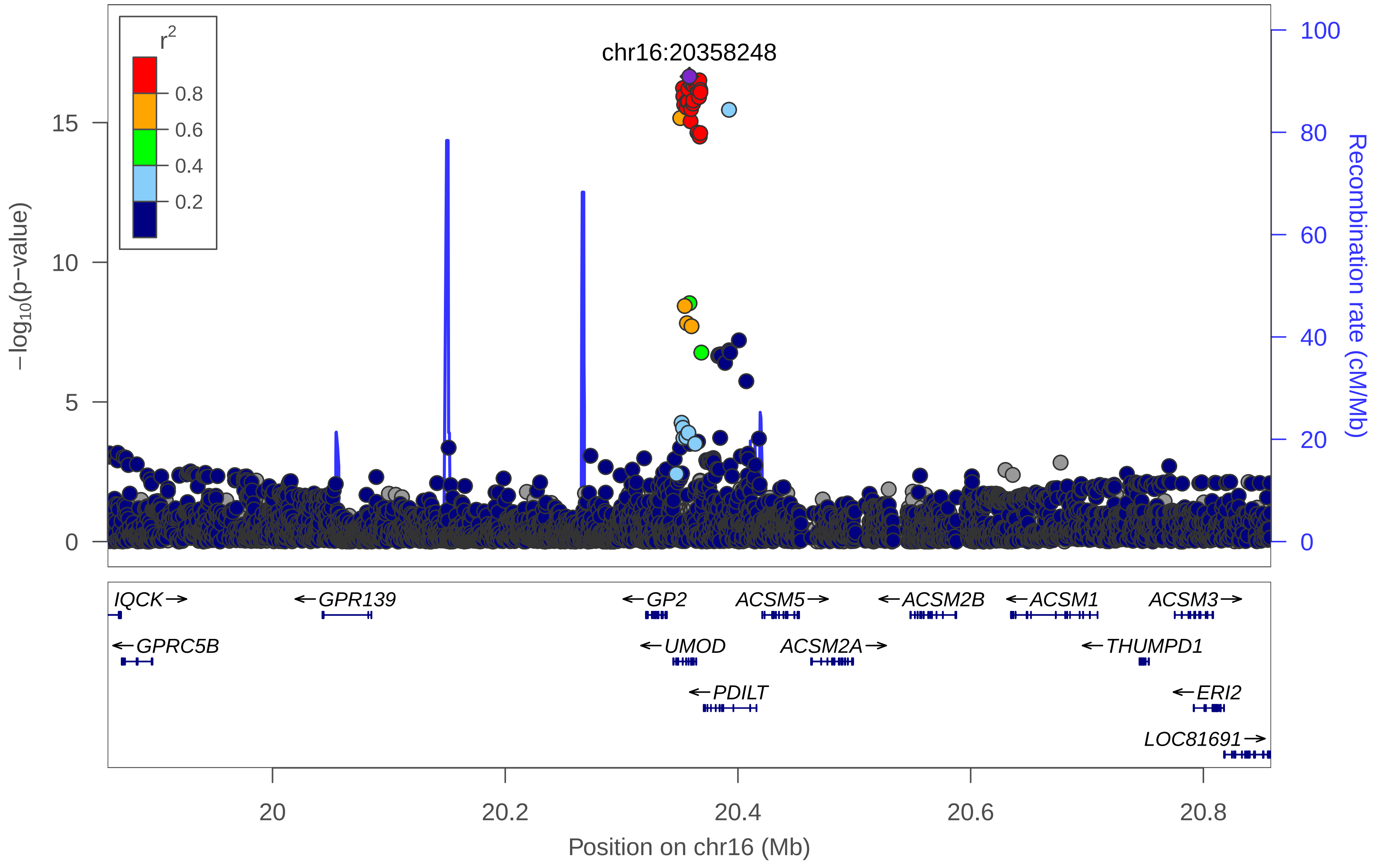

r²
0.8
0.6
0.4
0.2
chr16:20358248
−log10(p−value)
15
10
5
0
100
80
60
40
20
0
Recombination rate (cM/Mb)
IQCK→
←GPR139
←GP2
ACSM5→
←ACSM2B
←ACSM1
ACSM3→
←GPRC5B
←UMOD
ACSM2A→
←THUMPD1
←PDILT
←ERI2
LOC81691→
20
20.2
20.4
20.6
20.8
Position on chr16 (Mb)

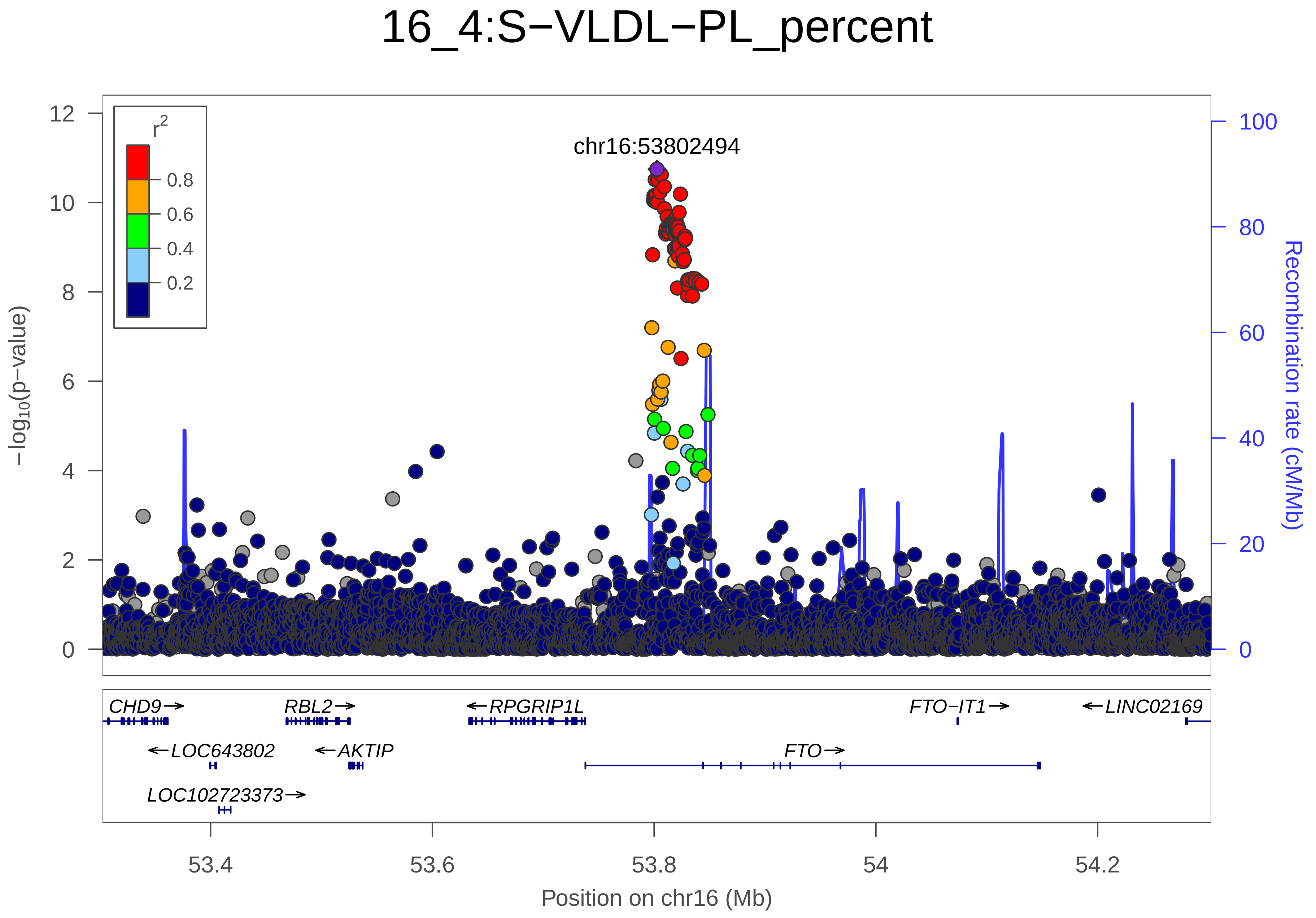

16_4:S−VLDL−PL_percent
r2
0.8
0.6
0.4
0.2
chr16:53802494
−log10(p−value)
Recombination rate (cM/Mb)
0
2
4
6
8
10
12
0
20
40
60
80
100
CHD9→
RBL2→
←RPGRIP1L
FTO−IT1→
←LINC02169
←LOC643802
←AKTIP
FTO→
LOC102723373→
53.4
53.6
53.8
54
54.2
Position on chr16 (Mb)

16_5:HDL−C

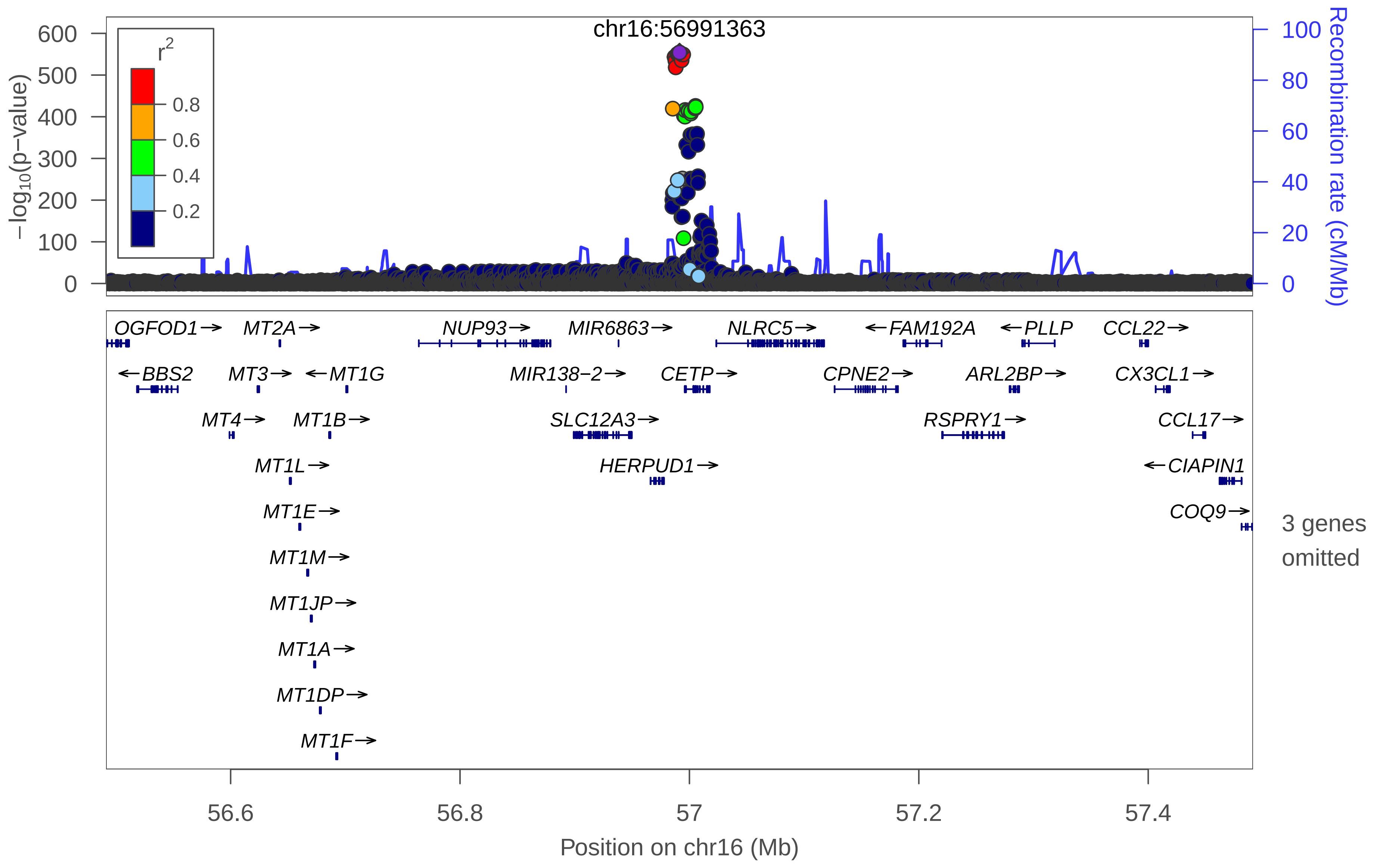

chr16:56991363
r2
0.8
0.6
0.4
0.2
600
500
400
300
200
100
0
−log10(p−value)
100
80
60
40
20
0
Recombination rate (cM/Mb)
OGFOD1→
MT2A→
NUP93→
MIR6863→
NLRC5→
←FAM192A
←PLLP
CCL22→
←BBS2
MT3→
←MT1G
MIR138−2→
CETP→
CPNE2→
ARL2BP→
CX3CL1→
MT4→
MT1B→
SLC12A3→
RSPRY1→
CCL17→
MT1L→
HERPUD1→
←CIAPIN1
MT1E→
COQ9→
3 genes omitted
MT1M→
MT1JP→
MT1A→
MT1DP→
MT1F→
56.6
56.8
57
57.2
57.4
Position on chr16 (Mb)

16_6:Tyr

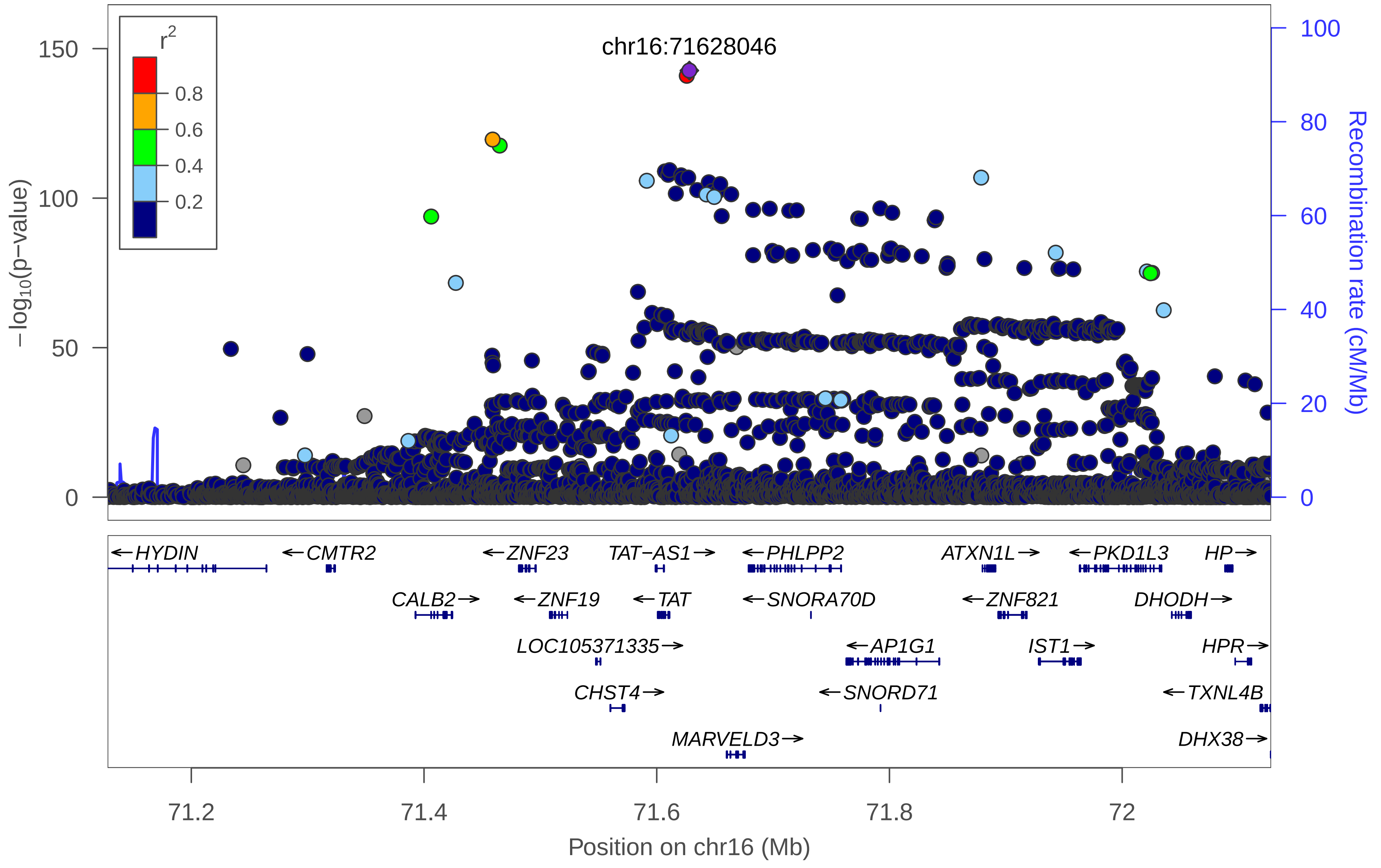

chr16:71628046
r2
0.8
0.6
0.4
0.2
150
100
50
0
−log10(p−value)
100
80
60
40
20
0
Recombination rate (cM/Mb)
←HYDIN
←CMTR2
←ZNF23
TAT−AS1→
←PHLPP2
ATXN1L→
←PKD1L3
HP→
CALB2→
←ZNF19
←TAT
←SNORA70D
←ZNF821
DHODH→
LOC105371335→
←AP1G1
IST1→
HPR→
CHST4→
←SNORD71
←TXNL4B
MARVELD3→
DHX38→
71.2
71.4
71.6
71.8
72
Position on chr16 (Mb)

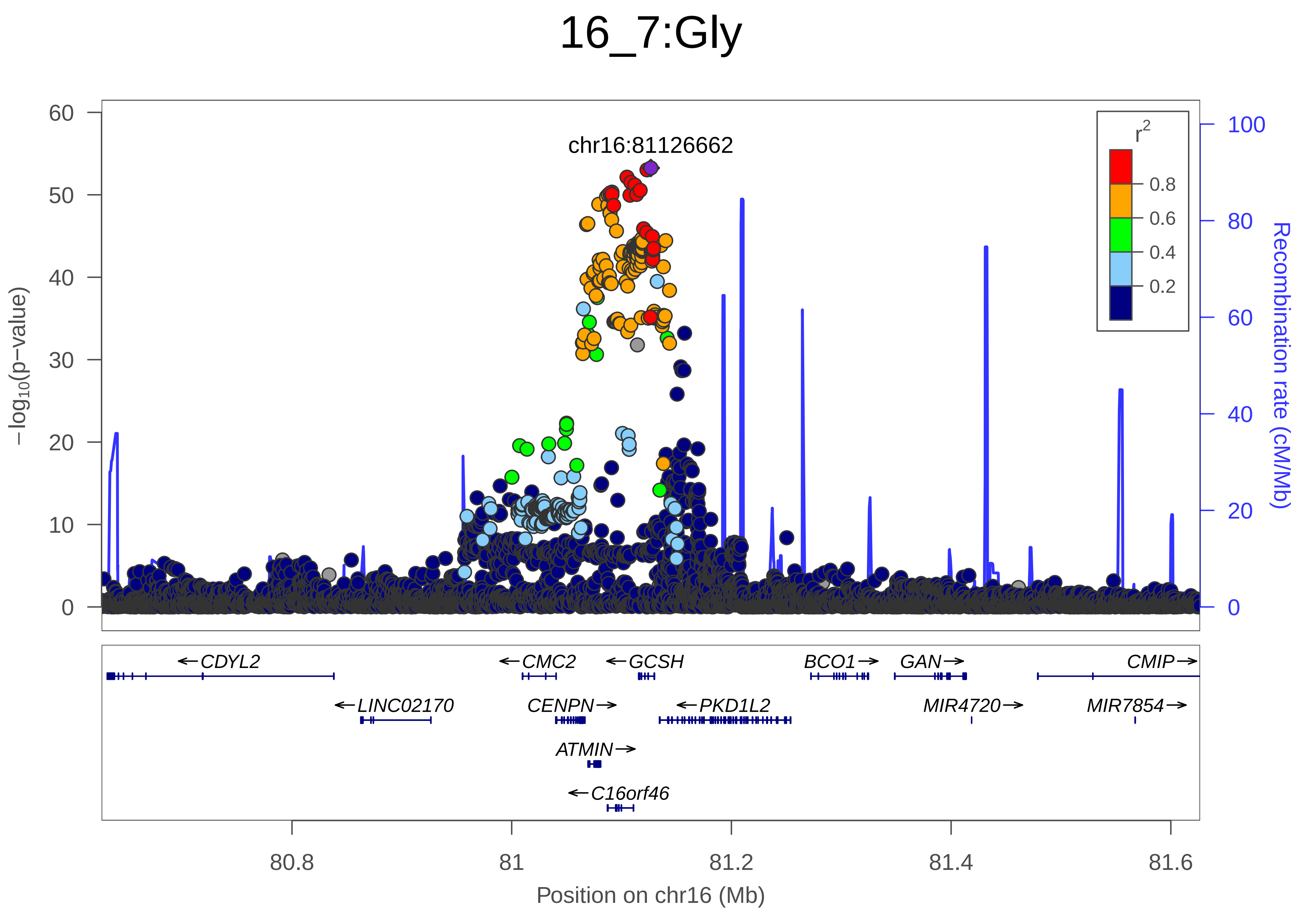

16_7:Gly
chr16:81126662
−log10(p−value)
Recombination rate (cM/Mb)
r2
0.8
0.6
0.4
0.2
60
50
40
30
20
10
0
100
80
60
40
20
0
←CDYL2
←CMC2
←GCSH
BCO1→
GAN→
CMIP→
←LINC02170
CENPN→
←PKD1L2
MIR4720→
MIR7854→
ATMIN→
←C16orf46
80.8
81
81.2
81.4
81.6
Position on chr16 (Mb)

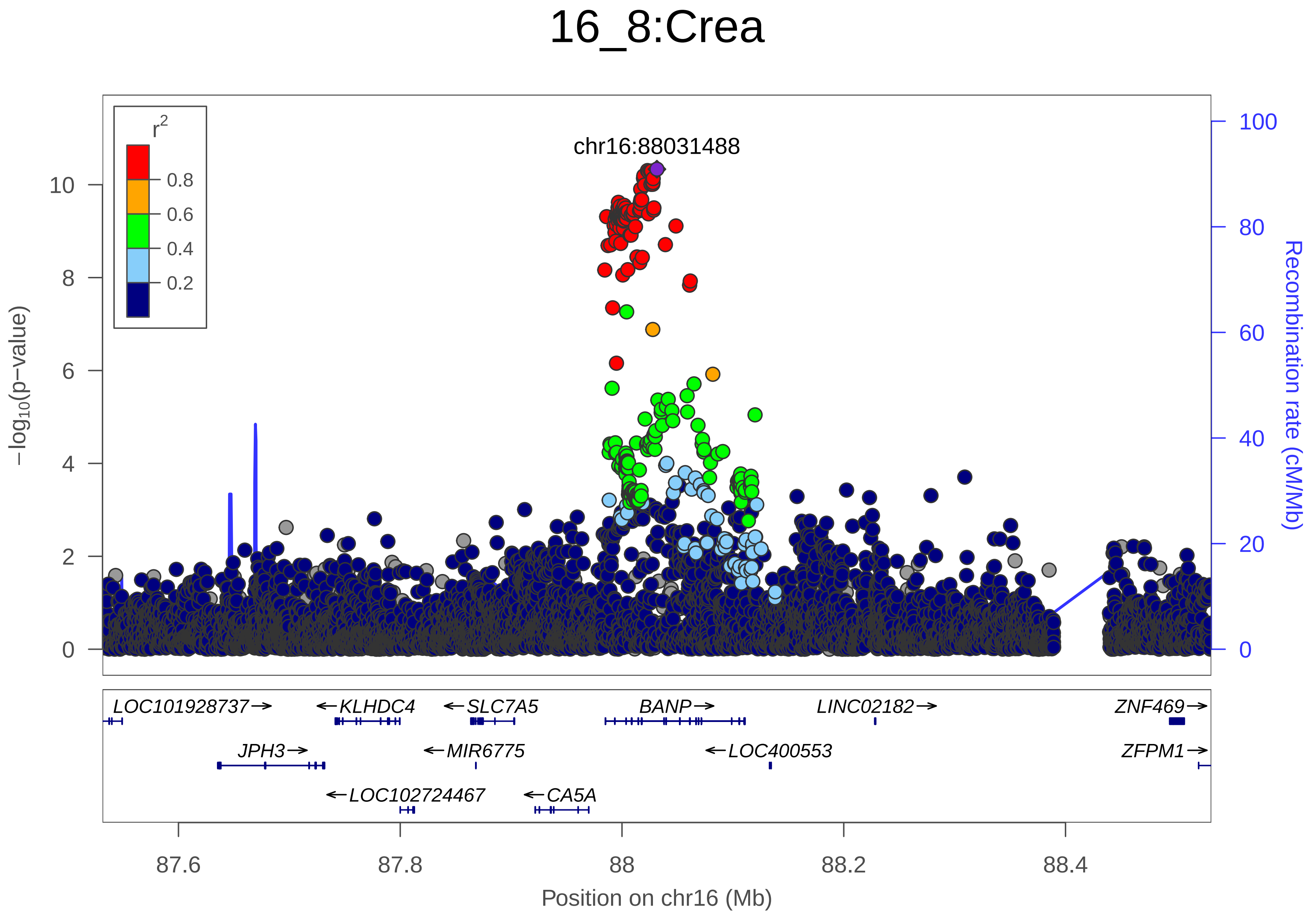

16_8:Crea
chr16:88031488
r2
0.8
0.6
0.4
0.2
10
8
6
4
2
0
−log10(p−value)
100
80
60
40
20
0
Recombination rate (cM/Mb)
LOC101928737→
←KLHDC4
←SLC7A5
BANP→
LINC02182→
ZNF469→
JPH3→
←MIR6775
←LOC400553
ZFPM1→
←LOC102724467
←CA5A
87.6
87.8
88
88.2
88.4
Position on chr16 (Mb)

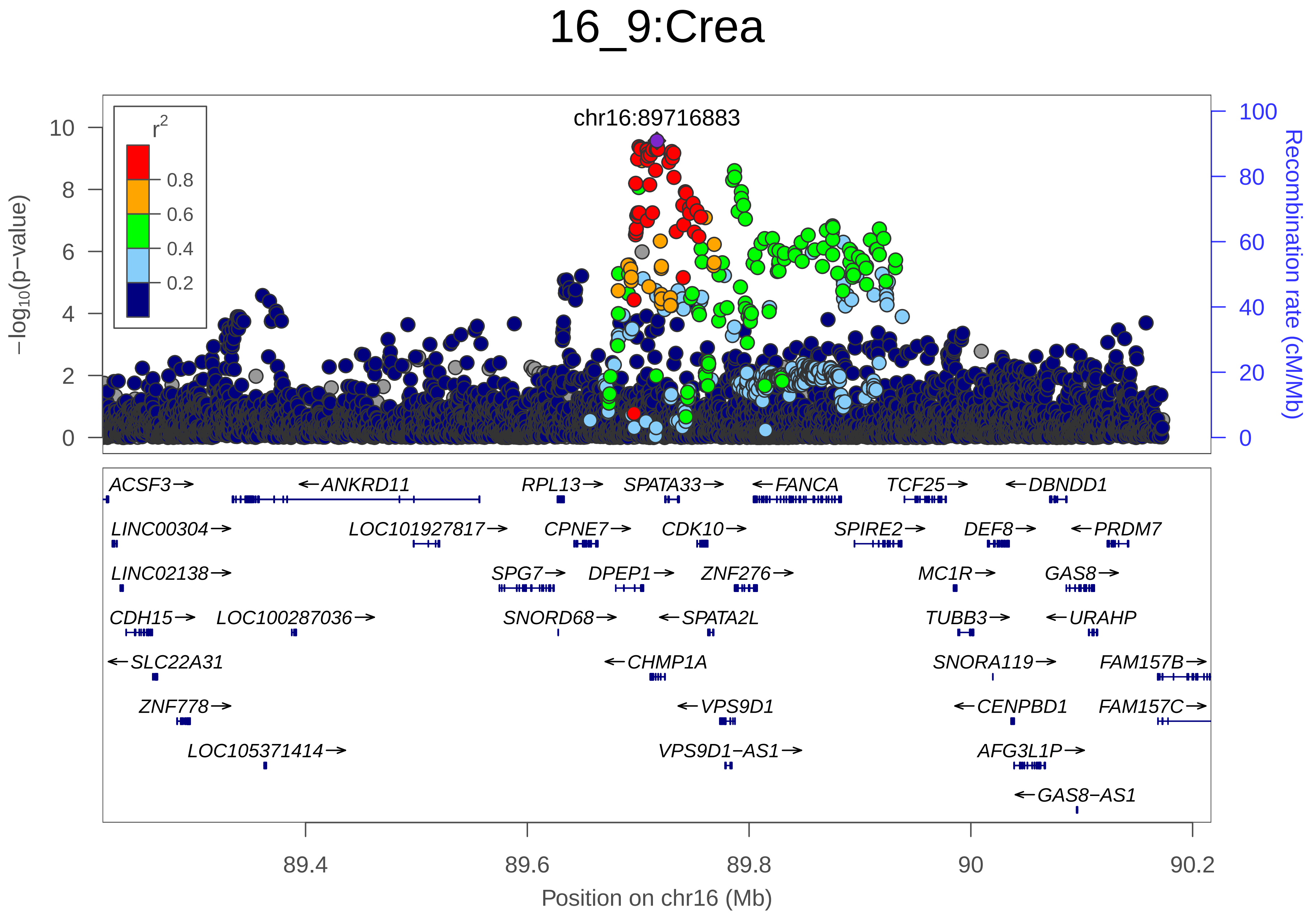

16_9:Crea
chr16:89716883
r2
0.8
0.6
0.4
0.2
−log10(p−value)
Recombination rate (cM/Mb)
Position on chr16 (Mb)
ACSF3→
←ANKRD11
RPL13→
SPATA33→
←FANCA
TCF25→
←DBNDD1
LINC00304→
LOC101927817→
CPNE7→
CDK10→
SPIRE2→
DEF8→
←PRDM7
LINC02138→
SPG7→
DPEP1→
ZNF276→
MC1R→
GAS8→
CDH15→
LOC100287036→
SNORD68→
←SPATA2L
TUBB3→
←URAHP
←SLC22A31
←CHMP1A
SNORA119→
FAM157B→
ZNF778→
←VPS9D1
←CENPBD1
FAM157C→
LOC105371414→
VPS9D1−AS1→
AFG3L1P→
←GAS8−AS1

17_1:Alb

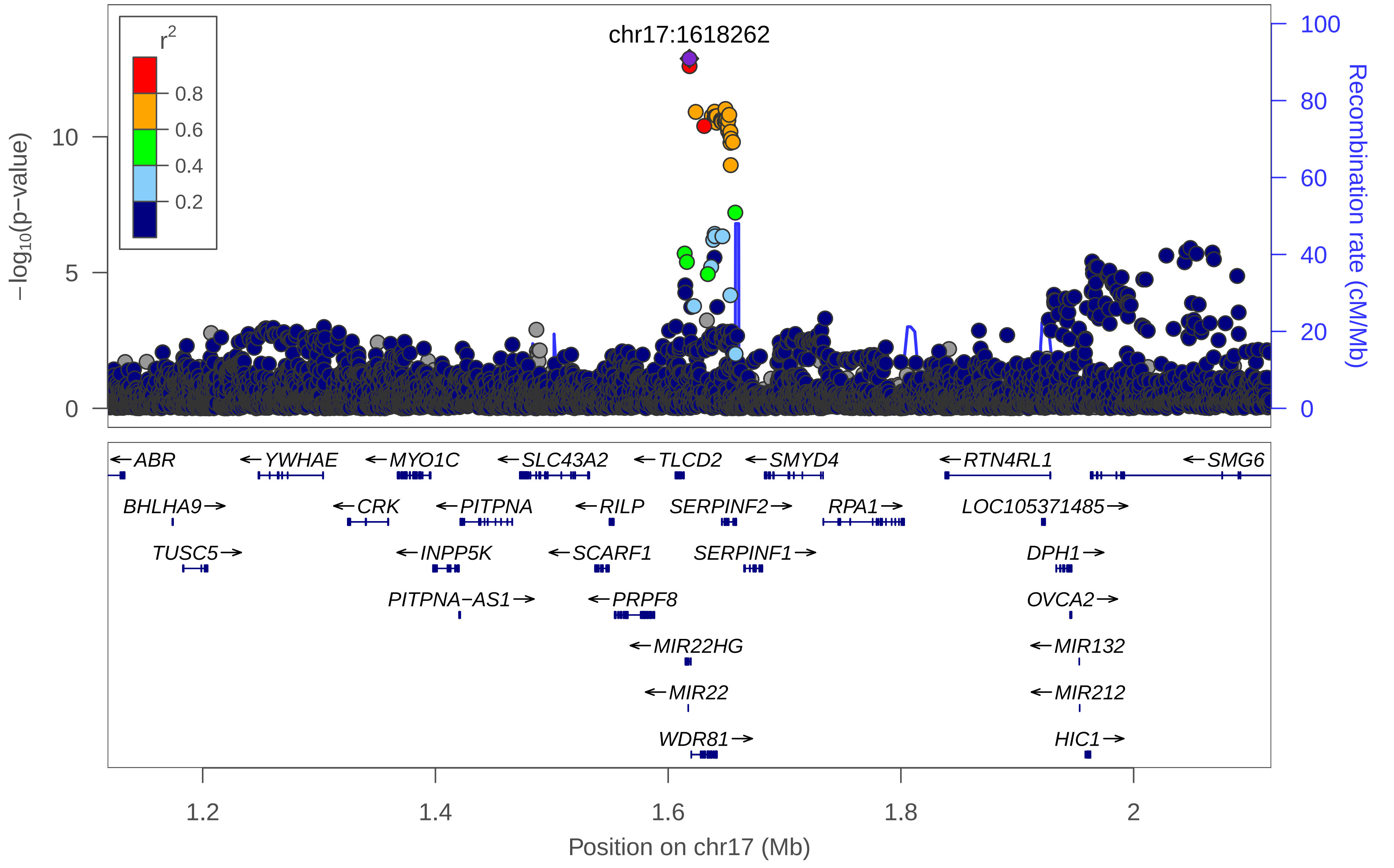

chr17:1618262
r2
0.8
0.6
0.4
0.2
−log10(p−value)
0
5
10
Recombination rate (cM/Mb)
0
20
40
60
80
100
ABR
YWHAE
MYO1C
SLC43A2
TLCD2
SMYD4
RTN4RL1
SMG6
BHLHA9
CRK
PITPNA
RILP
SERPINF2
RPA1
LOC105371485
TUSC5
INPP5K
SCARF1
SERPINF1
DPH1
PITPNA−AS1
PRPF8
OVCA2
MIR22HG
MIR132
MIR22
MIR212
WDR81
HIC1
1.2
1.4
1.6
1.8
2
Position on chr17 (Mb)

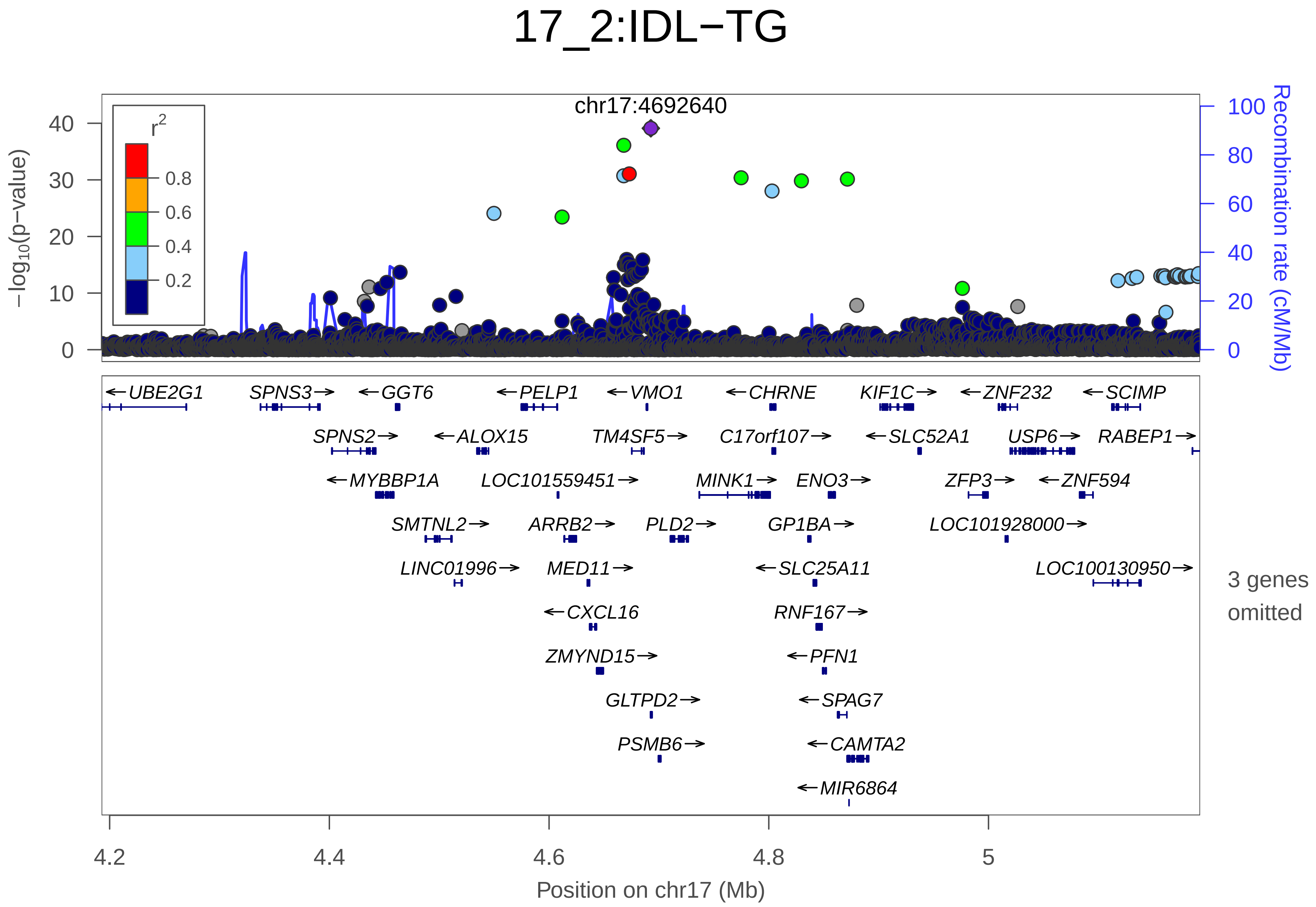
17_2:IDL−TG
chr17:4692640
r2
0.8
0.6
0.4
0.2
−log10(p−value)
0
10
20
30
40
Recombination rate (cM/Mb)
0
20
40
60
80
100
UBE2G1
SPNS3
GGT6
PELP1
VMO1
CHRNE
KIF1C
ZNF232
SCIMP
SPNS2
ALOX15
TM4SF5
C17orf107
SLC52A1
USP6
RABEP1
MYBBP1A
LOC101559451
MINK1
ENO3
ZFP3
ZNF594
SMTNL2
ARRB2
PLD2
GP1BA
LOC101928000
LINC01996
MED11
SLC25A11
LOC100130950
CXCL16
RNF167
ZMYND15
PFN1
GLTPD2
SPAG7
PSMB6
CAMTA2
MIR6864
3 genes omitted
4.2
4.4
4.6
4.8
5
Position on chr17 (Mb)

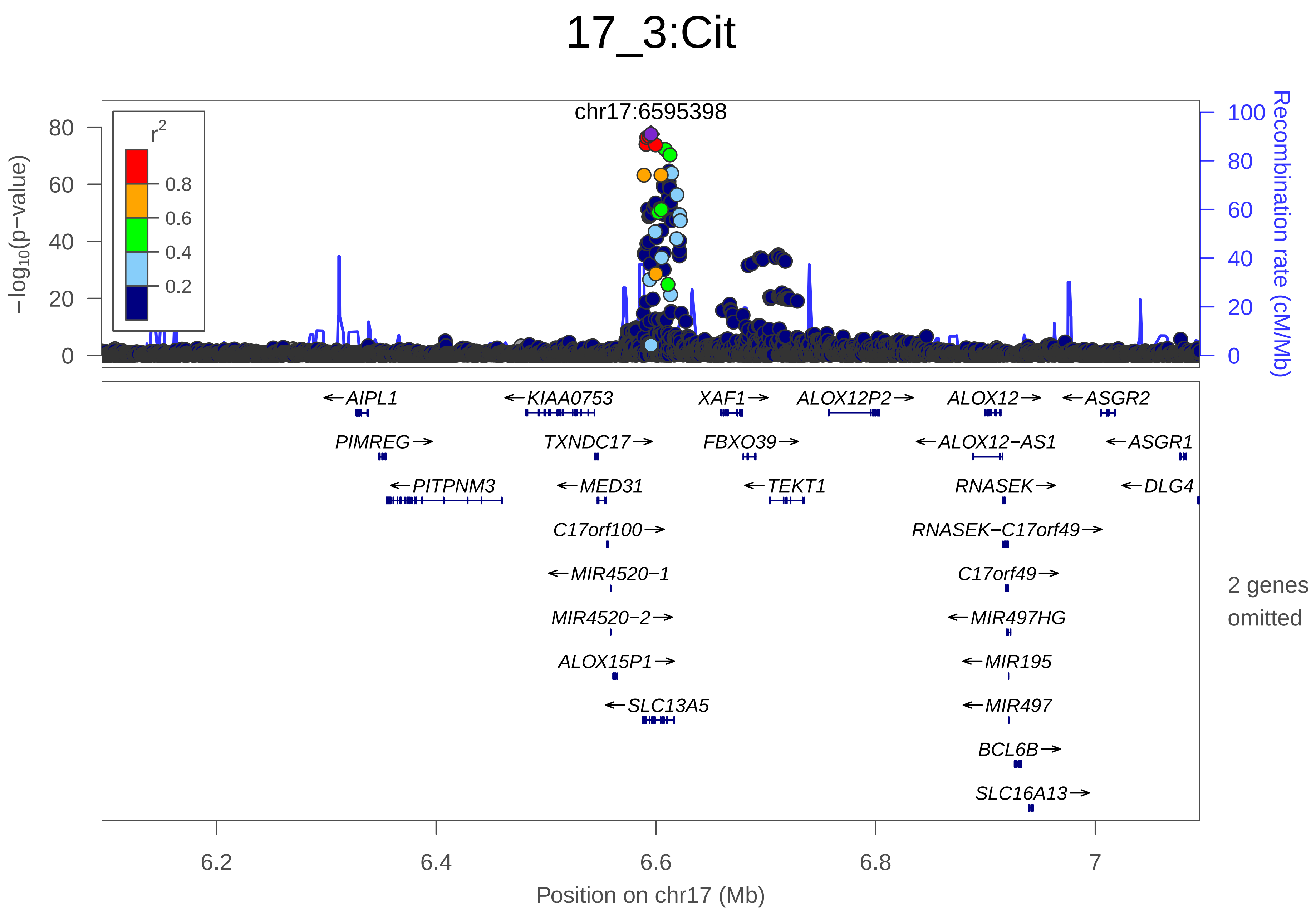

17_3:Cit
chr17:6595398
r2
0.8
0.6
0.4
0.2
−log10(p−value)
Recombination rate (cM/Mb)
AIPL1
KIAA0753
XAF1
ALOX12P2
ALOX12
ASGR2
PIMREG
TXNDC17
FBXO39
ALOX12−AS1
ASGR1
PITPNM3
MED31
TEKT1
RNASEK
DLG4
C17orf100
RNASEK−C17orf49
MIR4520−1
C17orf49
MIR4520−2
MIR497HG
ALOX15P1
MIR195
SLC13A5
MIR497
BCL6B
SLC16A13
2 genes omitted
Position on chr17 (Mb)

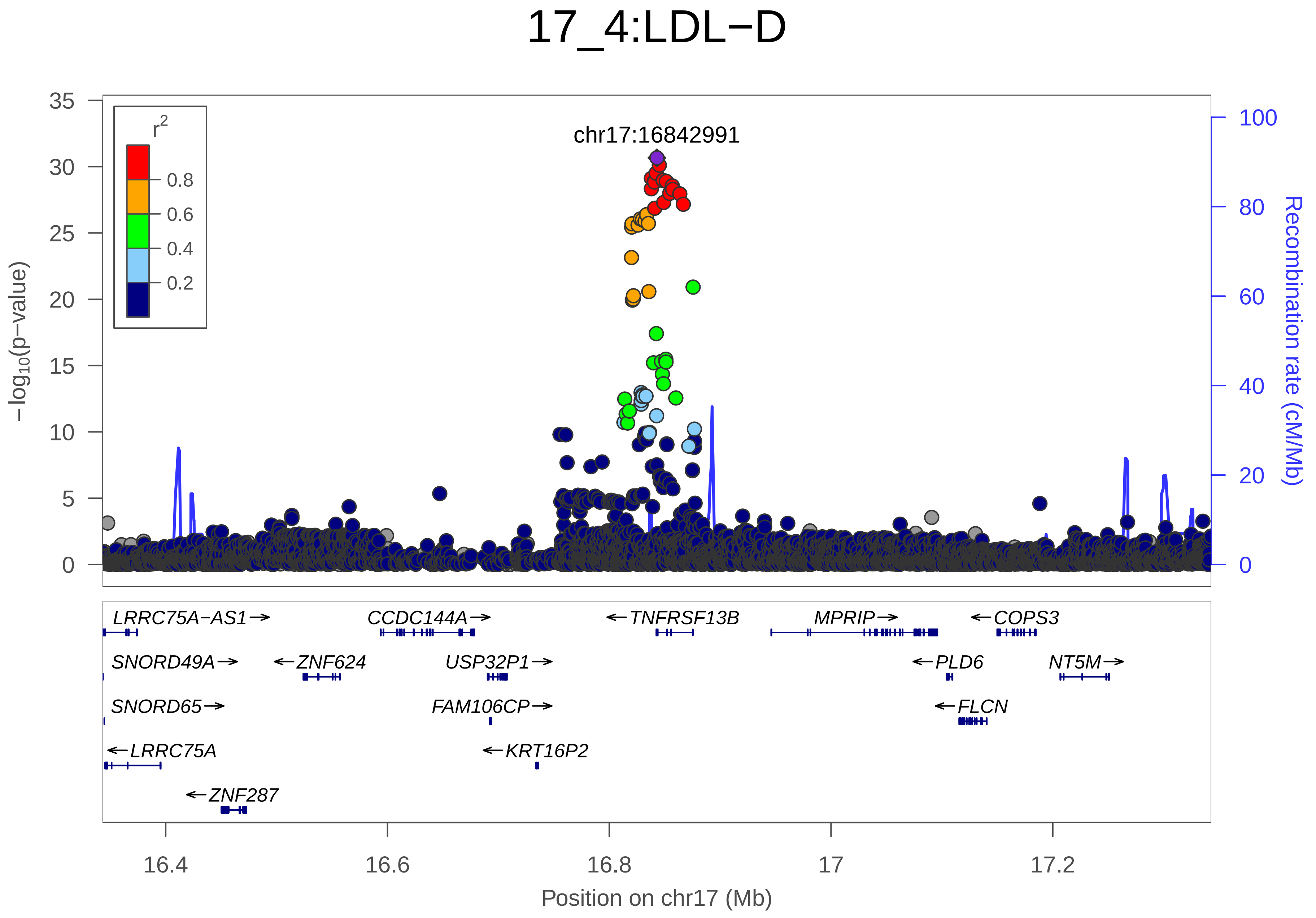

17_4:LDL−D
chr17:16842991
r2
0.8
0.6
0.4
0.2
−log10(p−value)
Recombination rate (cM/Mb)
35
30
25
20
15
10
5
0
100
80
60
40
20
0
LRRC75A−AS1→
CCDC144A→
←TNFRSF13B
MPRIP→
←COPS3
SNORD49A→
←ZNF624
USP32P1→
←PLD6
NT5M→
SNORD65→
FAM106CP→
←FLCN
←LRRC75A
←KRT16P2
←ZNF287
16.4
16.6
16.8
17
17.2
Position on chr17 (Mb)

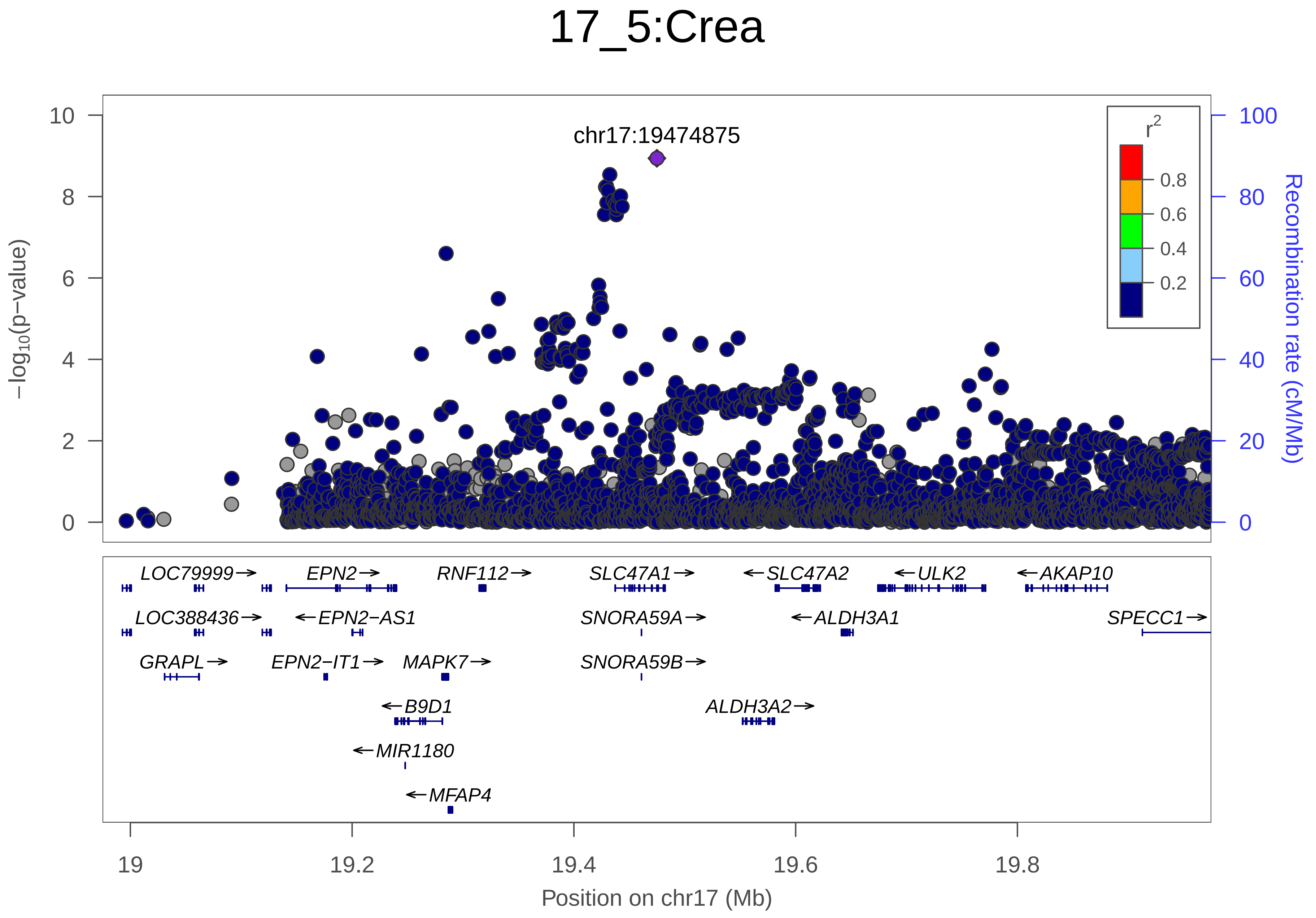

17_5:Crea
chr17:19474875
−log10(p−value)
Recombination rate (cM/Mb)
r2
0.8
0.6
0.4
0.2
LOC79999→
EPN2→
RNF112→
SLC47A1→
←SLC47A2
←ULK2
←AKAP10
LOC388436→
←EPN2−AS1
SNORA59A→
←ALDH3A1
SPECC1→
GRAPL→
EPN2−IT1→
MAPK7→
SNORA59B→
←B9D1
ALDH3A2→
←MIR1180
←MFAP4
Position on chr17 (Mb)

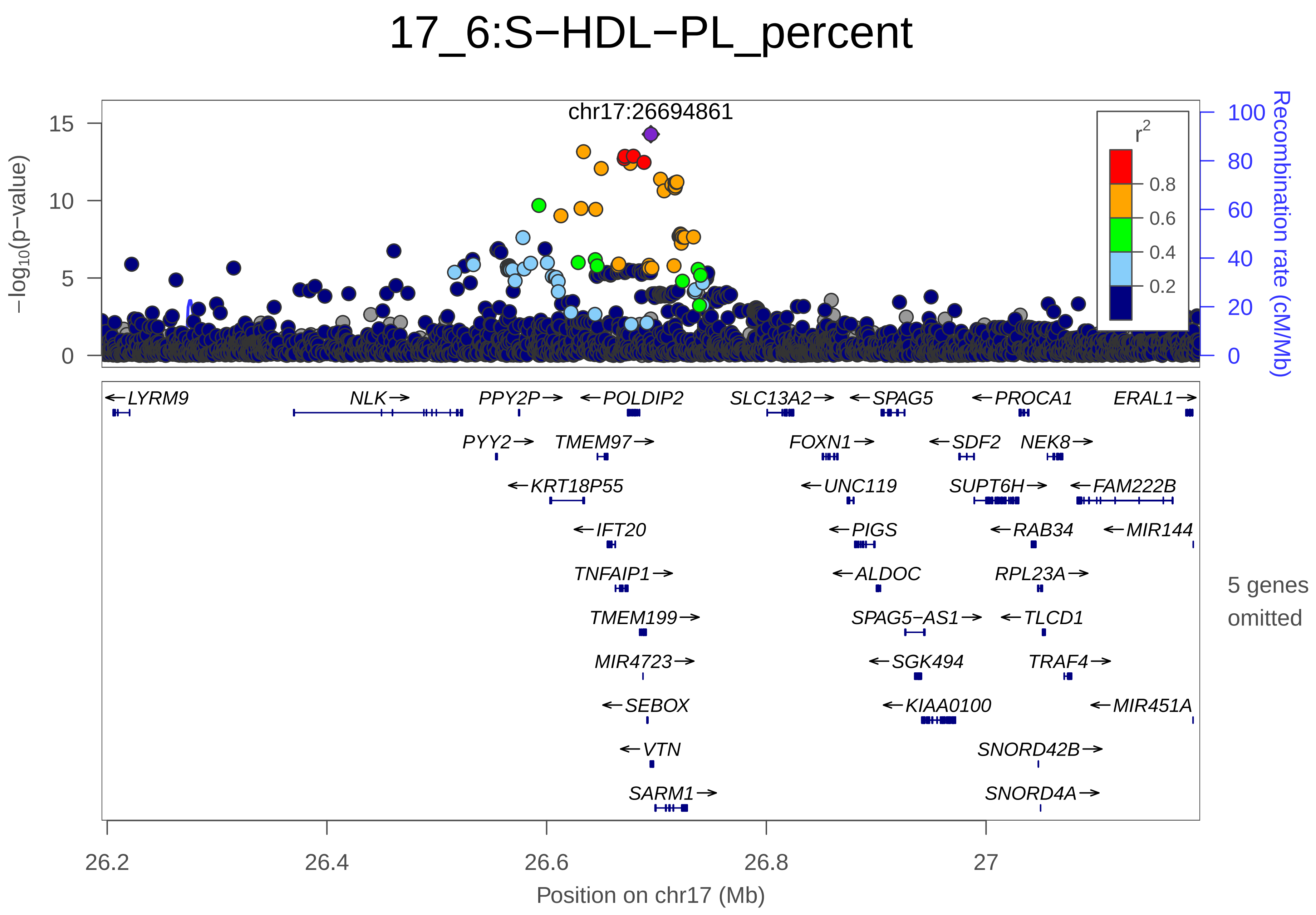
17_6:S−HDL−PL_percent
chr17:26694861
−log10(p−value)
Recombination rate (cM/Mb)
r2
0.8
0.6
0.4
0.2
LYRM9
NLK
PPY2P
POLDIP2
SLC13A2
SPAG5
PROCA1
ERAL1
PYY2
TMEM97
FOXN1
SDF2
NEK8
KRT18P55
UNC119
SUPT6H
FAM222B
IFT20
PIGS
RAB34
MIR144
TNFAIP1
ALDOC
RPL23A
TMEM199
SPAG5−AS1
TLCD1
MIR4723
SGK494
TRAF4
SEBOX
KIAA0100
MIR451A
VTN
SNORD42B
SARM1
SNORD4A
5 genes omitted
26.2
26.4
26.6
26.8
27
Position on chr17 (Mb)

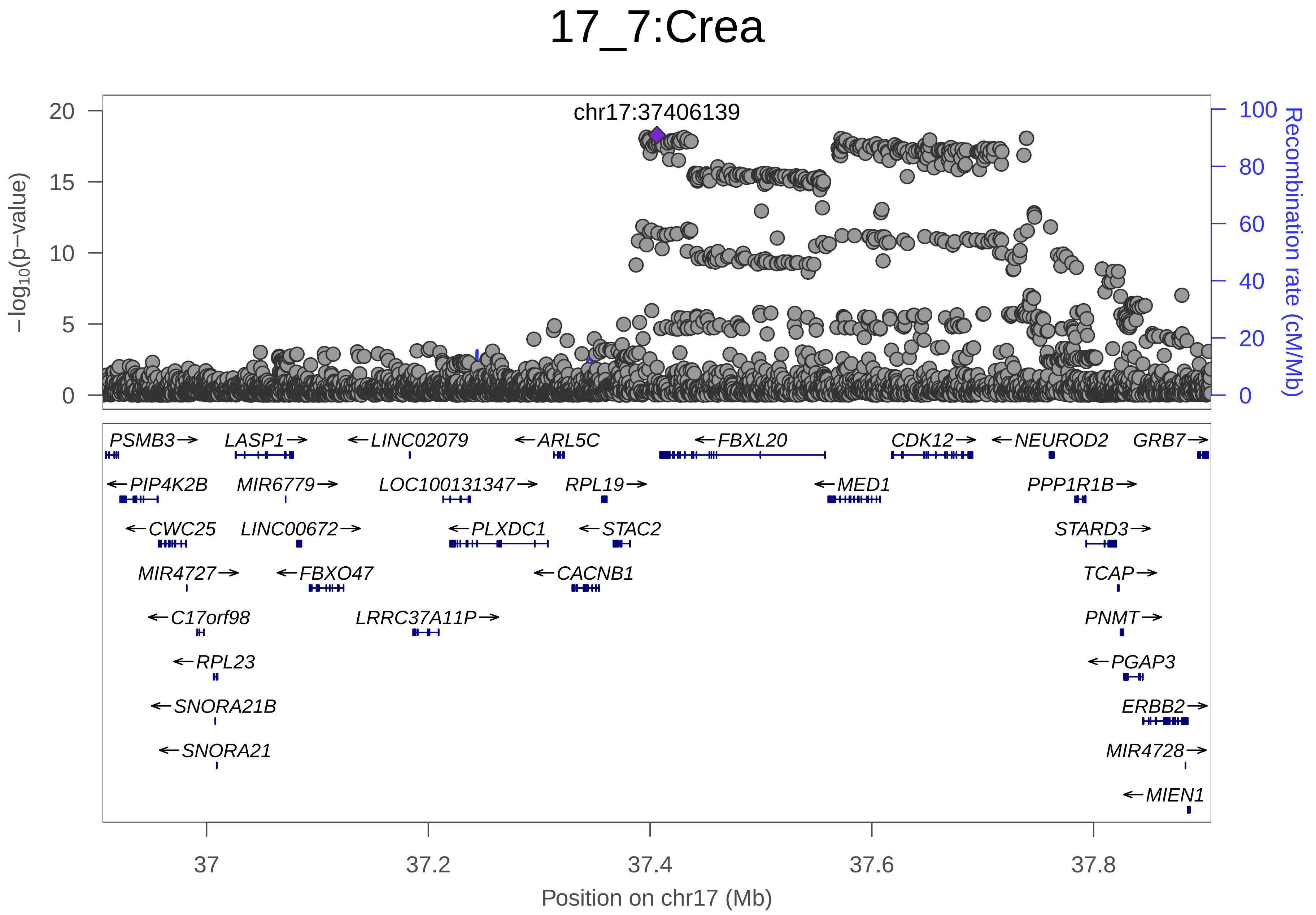

17_7:Crea
chr17:37406139
−log10(p−value)
Recombination rate (cM/Mb)
0
5
10
15
20
0
20
40
60
80
100
PSMB3→
LASP1→
←LINC02079
←ARL5C
←FBXL20
CDK12→
←NEUROD2
GRB7→
←PIP4K2B
MIR6779→
LOC100131347→
RPL19→
←MED1
PPP1R1B→
←CWC25
LINC00672→
←PLXDC1
←STAC2
STARD3→
MIR4727→
←FBXO47
←CACNB1
TCAP→
←C17orf98
LRRC37A11P→
PNMT→
←RPL23
←PGAP3
←SNORA21B
ERBB2→
←SNORA21
MIR4728→
←MIEN1
37
37.2
37.4
37.6
37.8
Position on chr17 (Mb)

17_8:HDL−D

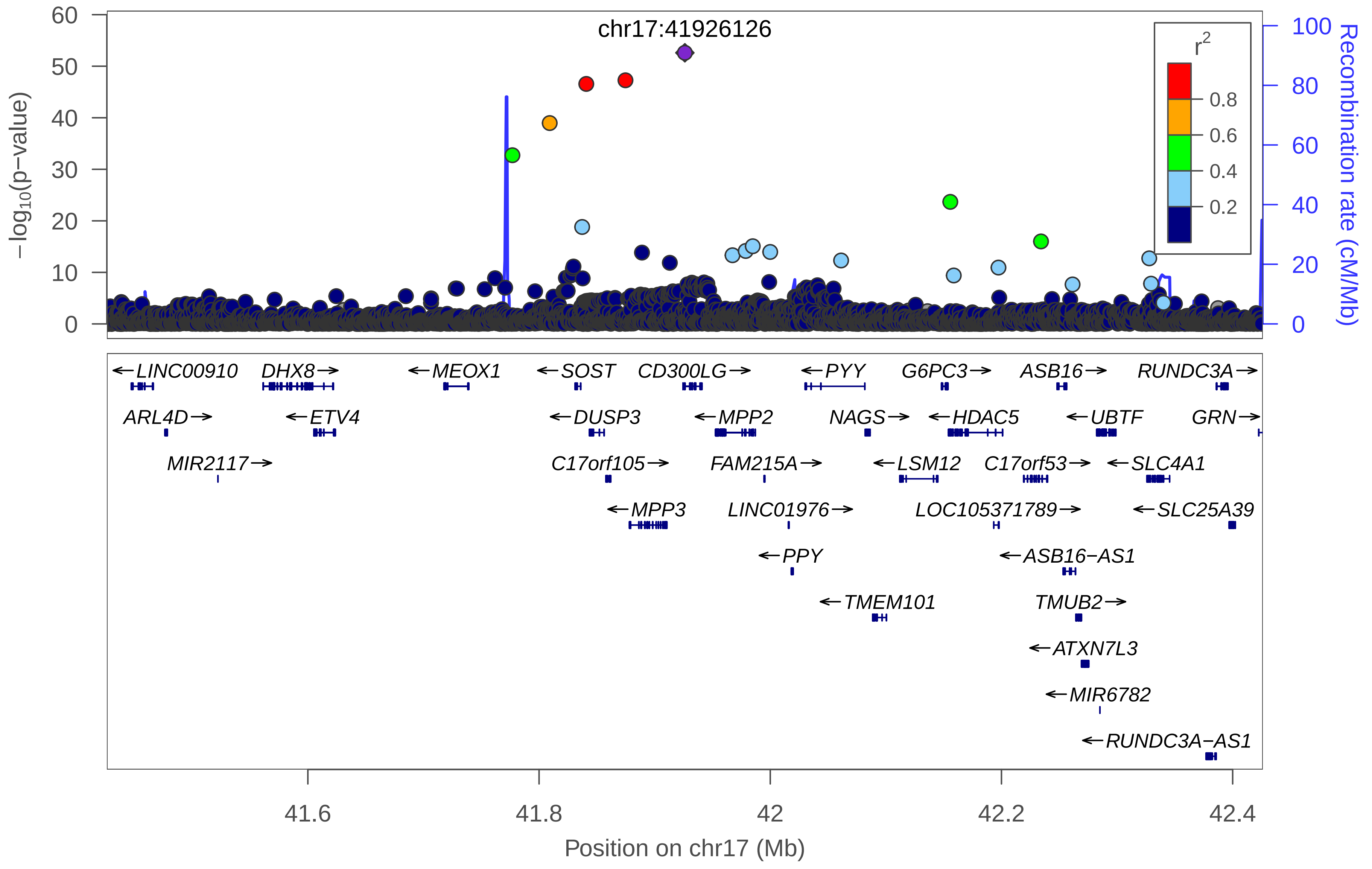

chr17:41926126
−log10(p−value)
0
10
20
30
40
50
60
Recombination rate (cM/Mb)
0
20
40
60
80
100
r2
0.8
0.6
0.4
0.2
←LINC00910
DHX8→
←MEOX1
←SOST
CD300LG→
←PYY
G6PC3→
ASB16→
RUNDC3A→
ARL4D→
←ETV4
←DUSP3
←MPP2
NAGS→
←HDAC5
←UBTF
GRN→
MIR2117→
C17orf105→
FAM215A→
←LSM12
C17orf53→
←SLC4A1
←MPP3
LINC01976→
LOC105371789→
←SLC25A39
←PPY
←ASB16−AS1
←TMEM101
TMUB2→
←ATXN7L3
←MIR6782
←RUNDC3A−AS1
41.6
41.8
42
42.2
42.4
Position on chr17 (Mb)

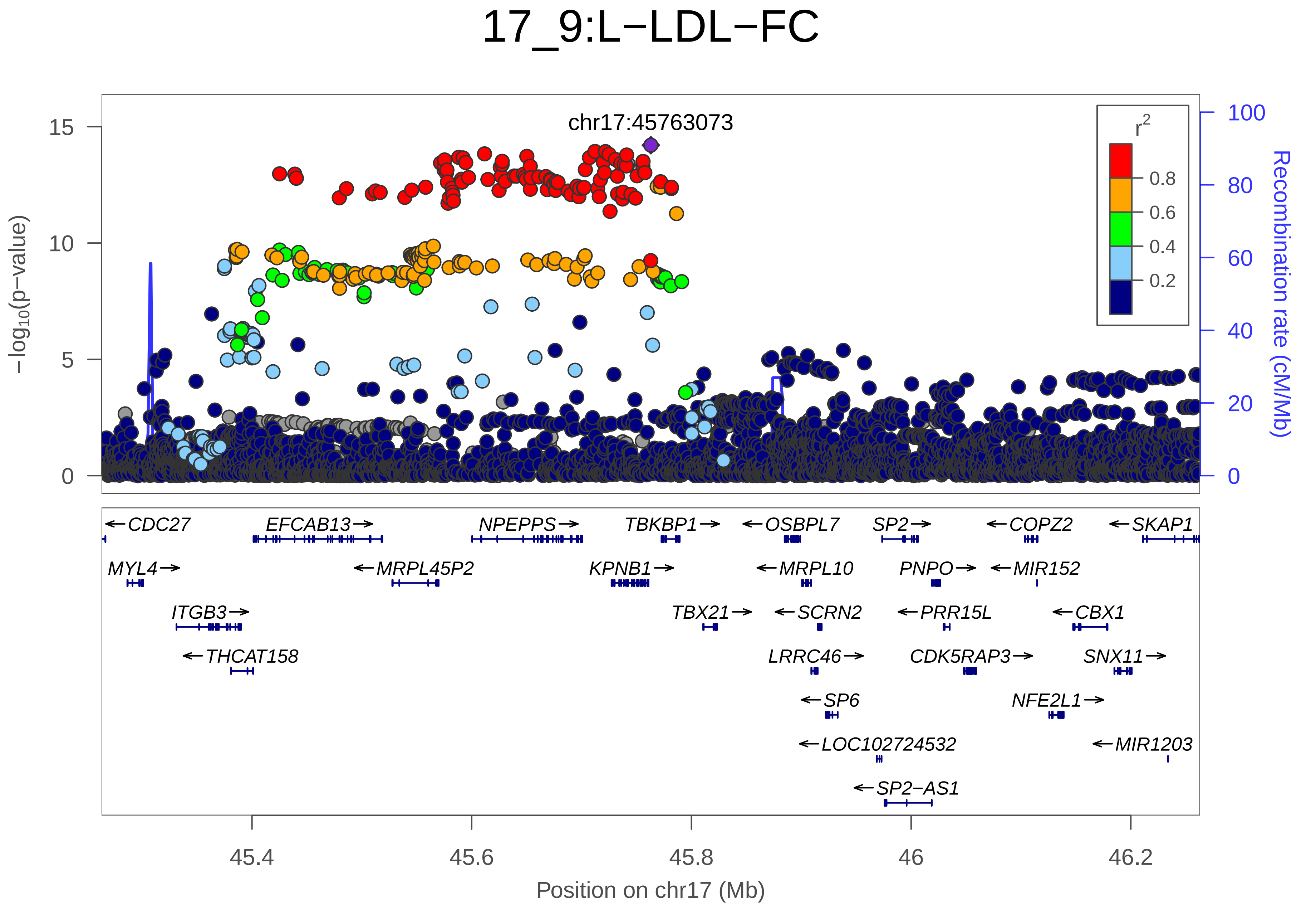

17_9:L−LDL−FC
chr17:45763073
r2
0.8
0.6
0.4
0.2
−log10(p−value)
Recombination rate (cM/Mb)
15
10
5
0
100
80
60
40
20
0
CDC27
EFCAB13
NPEPPS
TBKBP1
OSBPL7
SP2
COPZ2
SKAP1
MYL4
MRPL45P2
KPNB1
MRPL10
PNPO
MIR152
ITGB3
TBX21
SCRN2
PRR15L
CBX1
THCAT158
LRRC46
CDK5RAP3
SNX11
SP6
NFE2L1
LOC102724532
MIR1203
SP2−AS1
45.4
45.6
45.8
46
46.2
Position on chr17 (Mb)

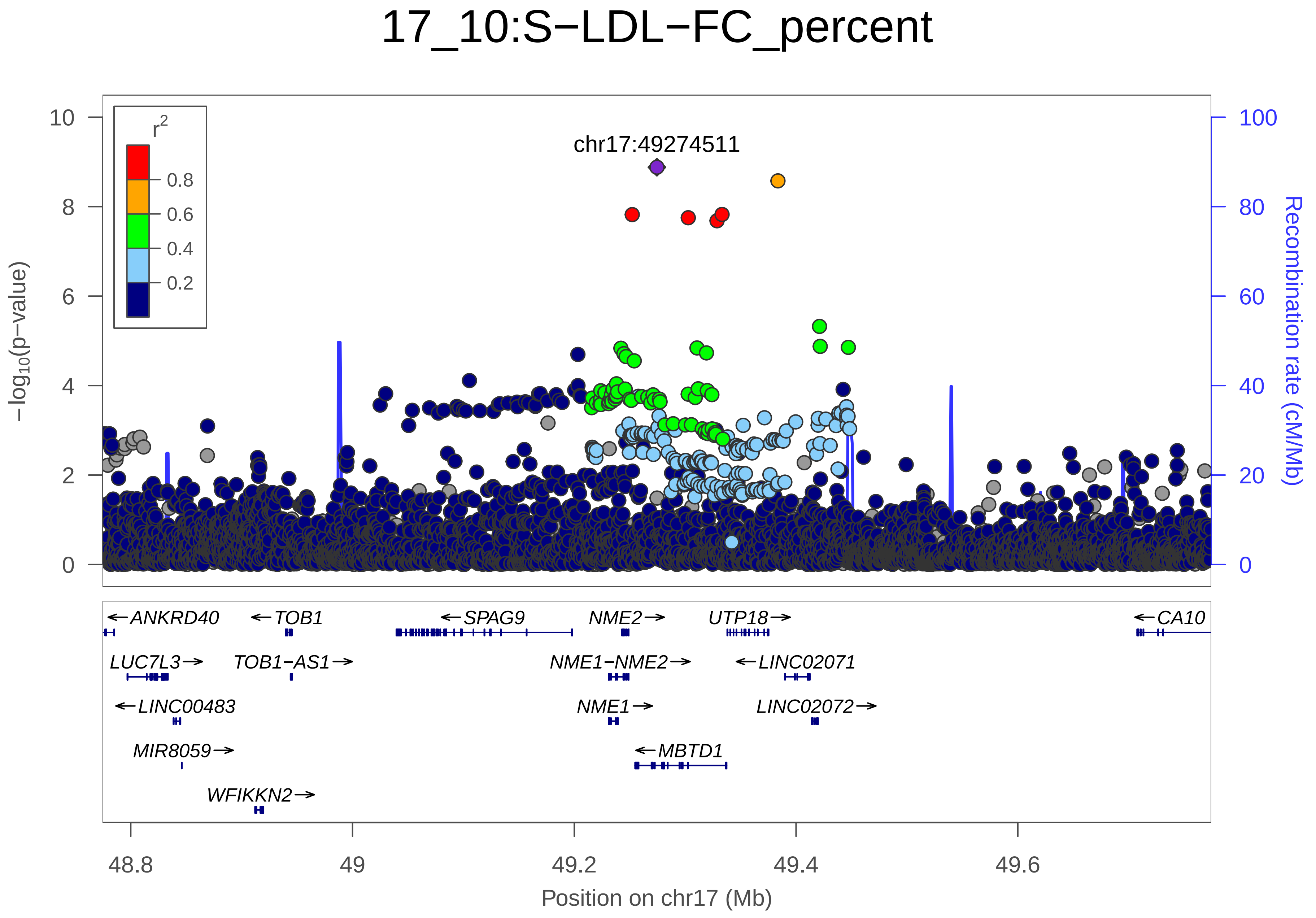
17_10:S−LDL−FC_percent
chr17:49274511
r2
0.8
0.6
0.4
0.2
−log10(p−value)
Recombination rate (cM/Mb)
ANKRD40
TOB1
SPAG9
NME2
UTP18
CA10
LUC7L3
TOB1−AS1
NME1−NME2
LINC02071
LINC00483
NME1
LINC02072
MIR8059
MBTD1
WFIKKN2
48.8
49
49.2
49.4
49.6
Position on chr17 (Mb)

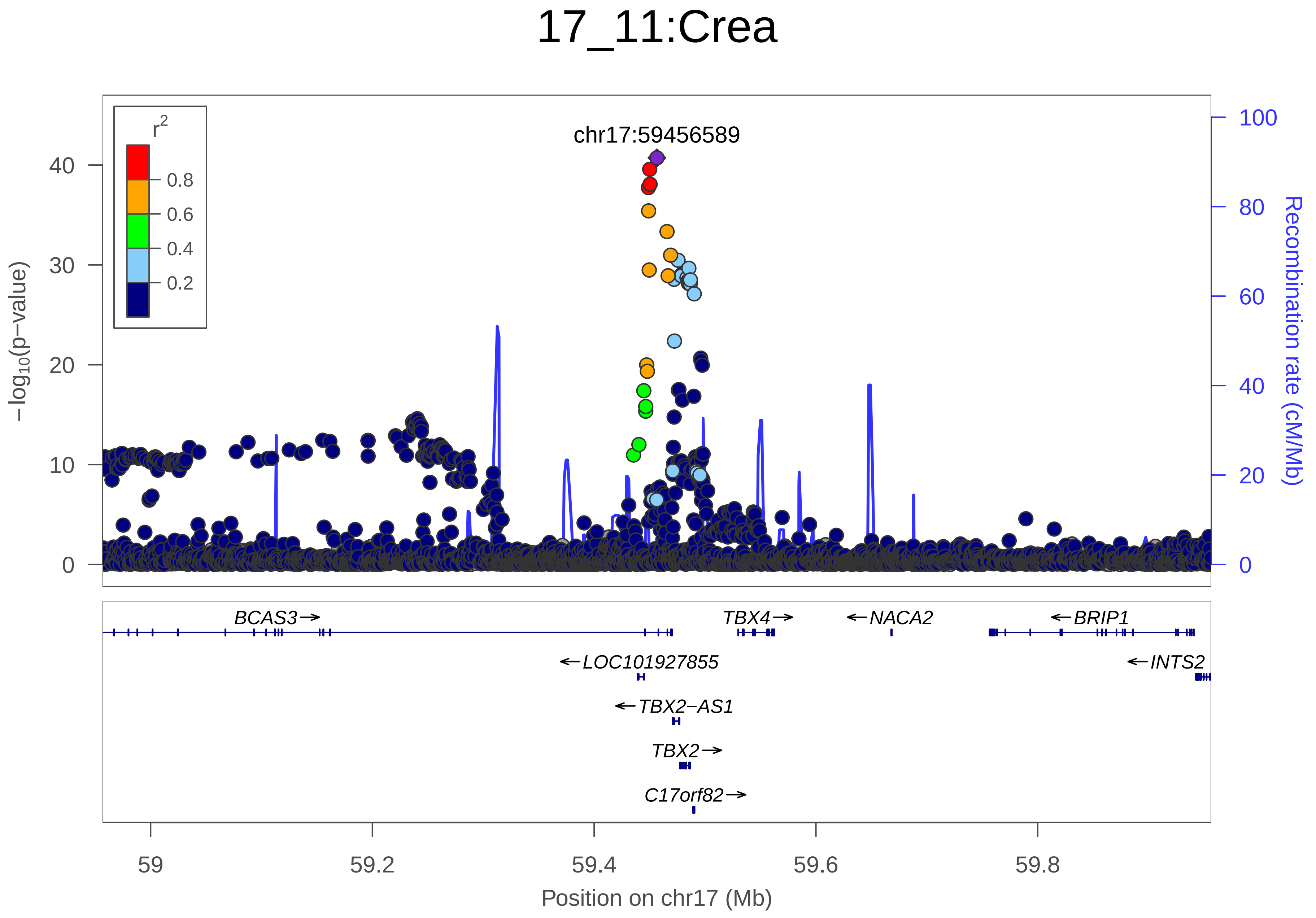
17_11:Crea
chr17:59456589
r2
0.8
0.6
0.4
0.2
−log10(p−value)
0
10
20
30
40
Recombination rate (cM/Mb)
0
20
40
60
80
100
BCAS3→
TBX4→
←NACA2
←BRIP1
←LOC101927855
←INTS2
←TBX2−AS1
TBX2→
C17orf82→
59
59.2
59.4
59.6
59.8
Position on chr17 (Mb)

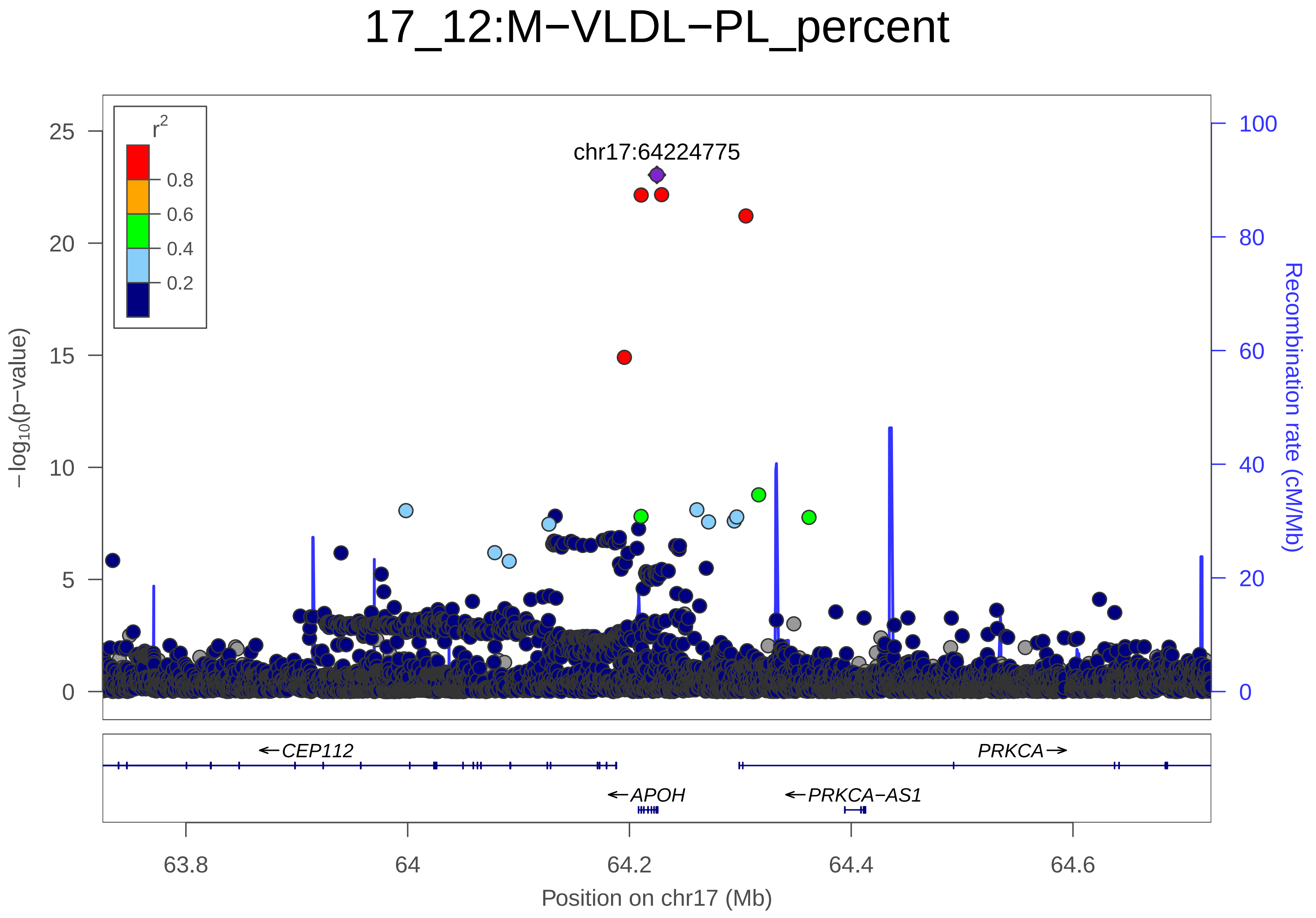
17_12:M−VLDL−PL_percent
chr17:64224775
r2
0.8
0.6
0.4
0.2
−log10(p−value)
Recombination rate (cM/Mb)
25
20
15
10
5
0
100
80
60
40
20
0
←CEP112
PRKCA→
←APOH
←PRKCA−AS1
63.8
64
64.2
64.4
64.6
Position on chr17 (Mb)

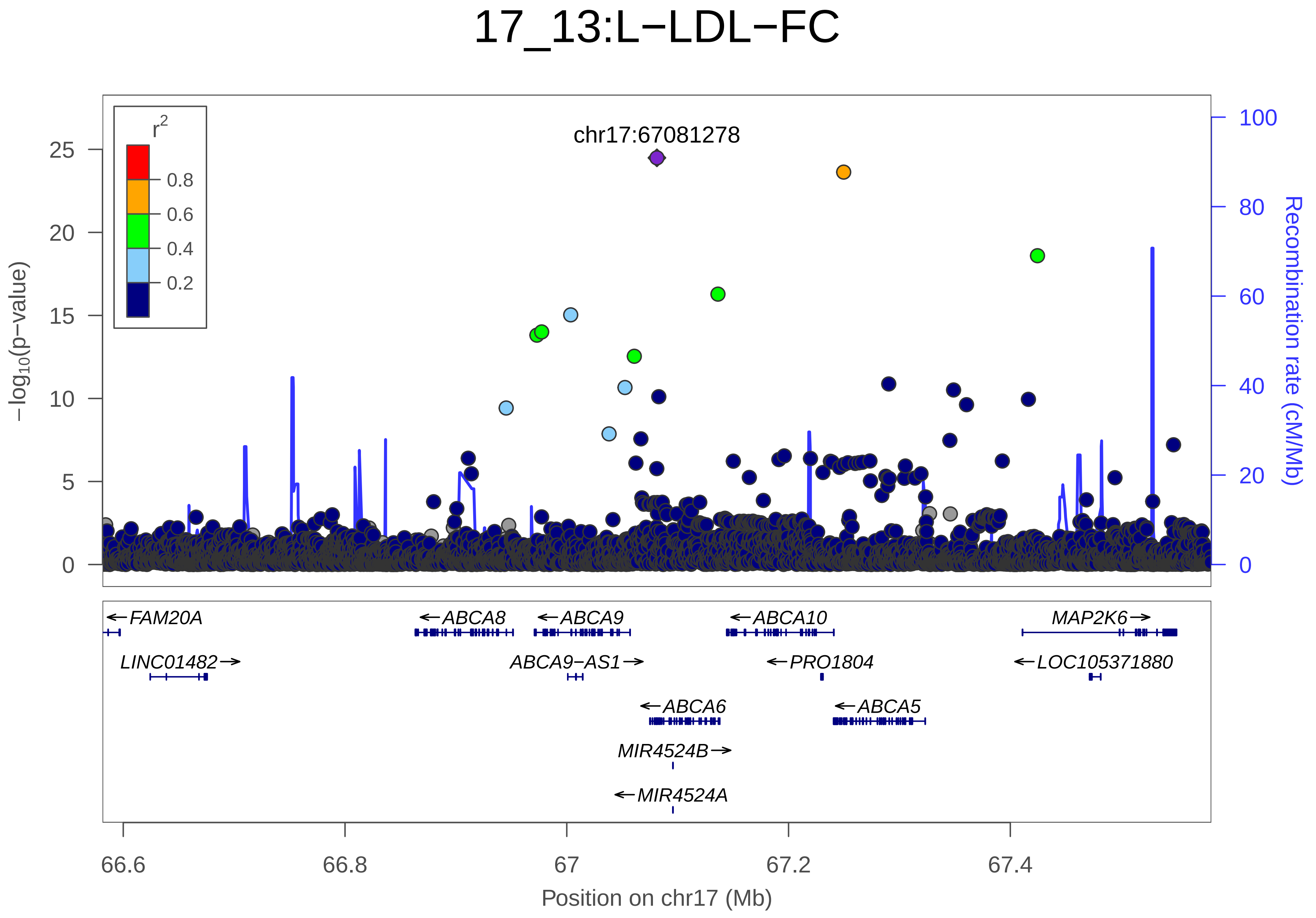

17_13:L−LDL−FC
chr17:67081278
r2
0.8
0.6
0.4
0.2
−log10(p−value)
Recombination rate (cM/Mb)
25
20
15
10
5
0
100
80
60
40
20
0
FAM20A
ABCA8
ABCA9
ABCA10
MAP2K6
LINC01482
ABCA9−AS1
PRO1804
LOC105371880
ABCA6
ABCA5
MIR4524B
MIR4524A
66.6
66.8
67
67.2
67.4
Position on chr17 (Mb)

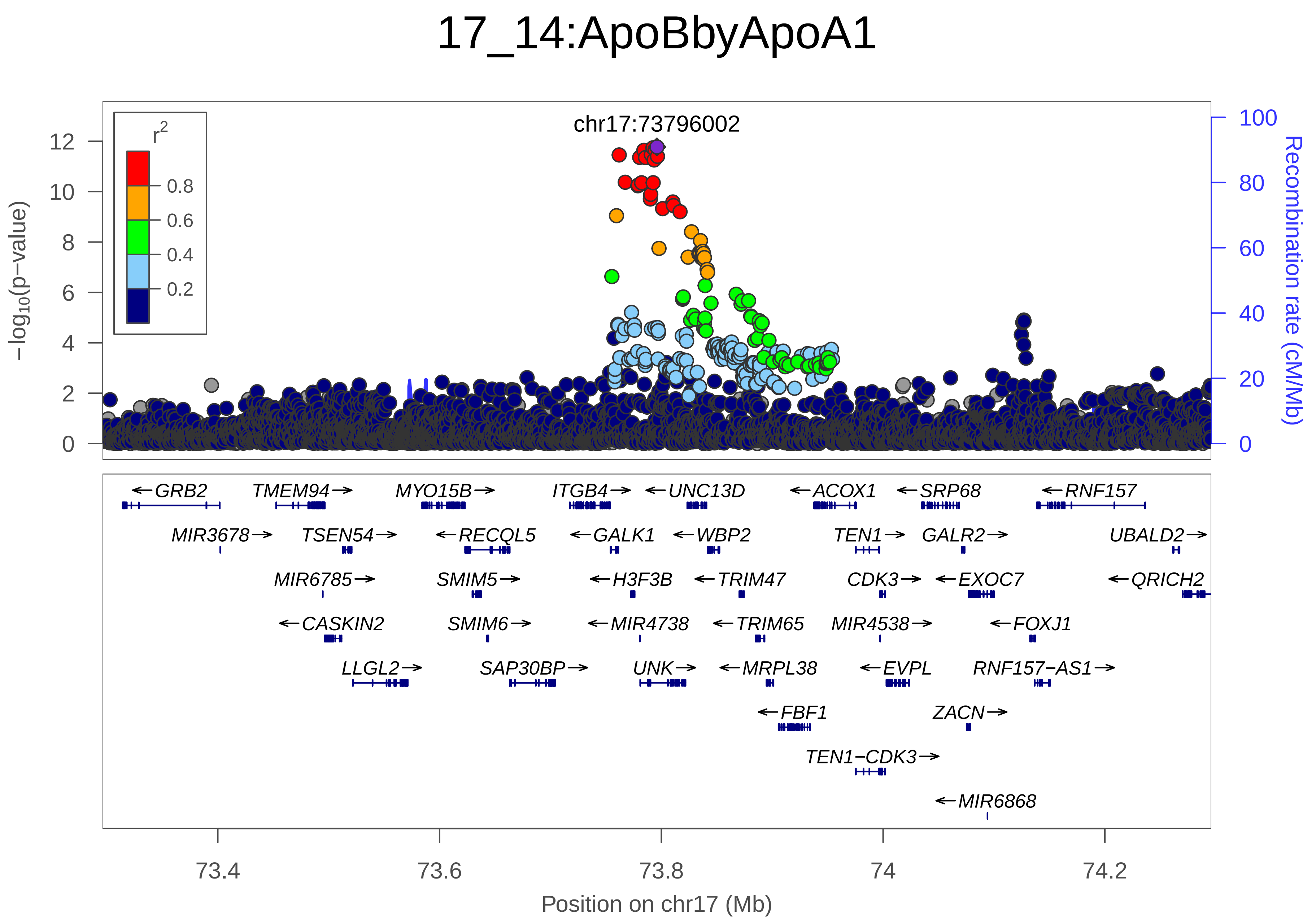

17_14:ApoBbyApoA1
chr17:73796002
r2
0.8
0.6
0.4
0.2
−log10(p−value)
Recombination rate (cM/Mb)
0
2
4
6
8
10
12
20
40
60
80
100
←GRB2
TMEM94→
MYO15B→
ITGB4→
←UNC13D
←ACOX1
←SRP68
←RNF157
MIR3678→
TSEN54→
←RECQL5
←GALK1
←WBP2
TEN1→
GALR2→
UBALD2→
MIR6785→
SMIM5→
←H3F3B
←TRIM47
CDK3→
←EXOC7
←QRICH2
←CASKIN2
SMIM6→
←MIR4738
←TRIM65
MIR4538→
←FOXJ1
LLGL2→
SAP30BP→
UNK→
←MRPL38
←EVPL
RNF157−AS1→
←FBF1
ZACN→
TEN1−CDK3→
←MIR6868
73.4
73.6
73.8
74
74.2
Position on chr17 (Mb)

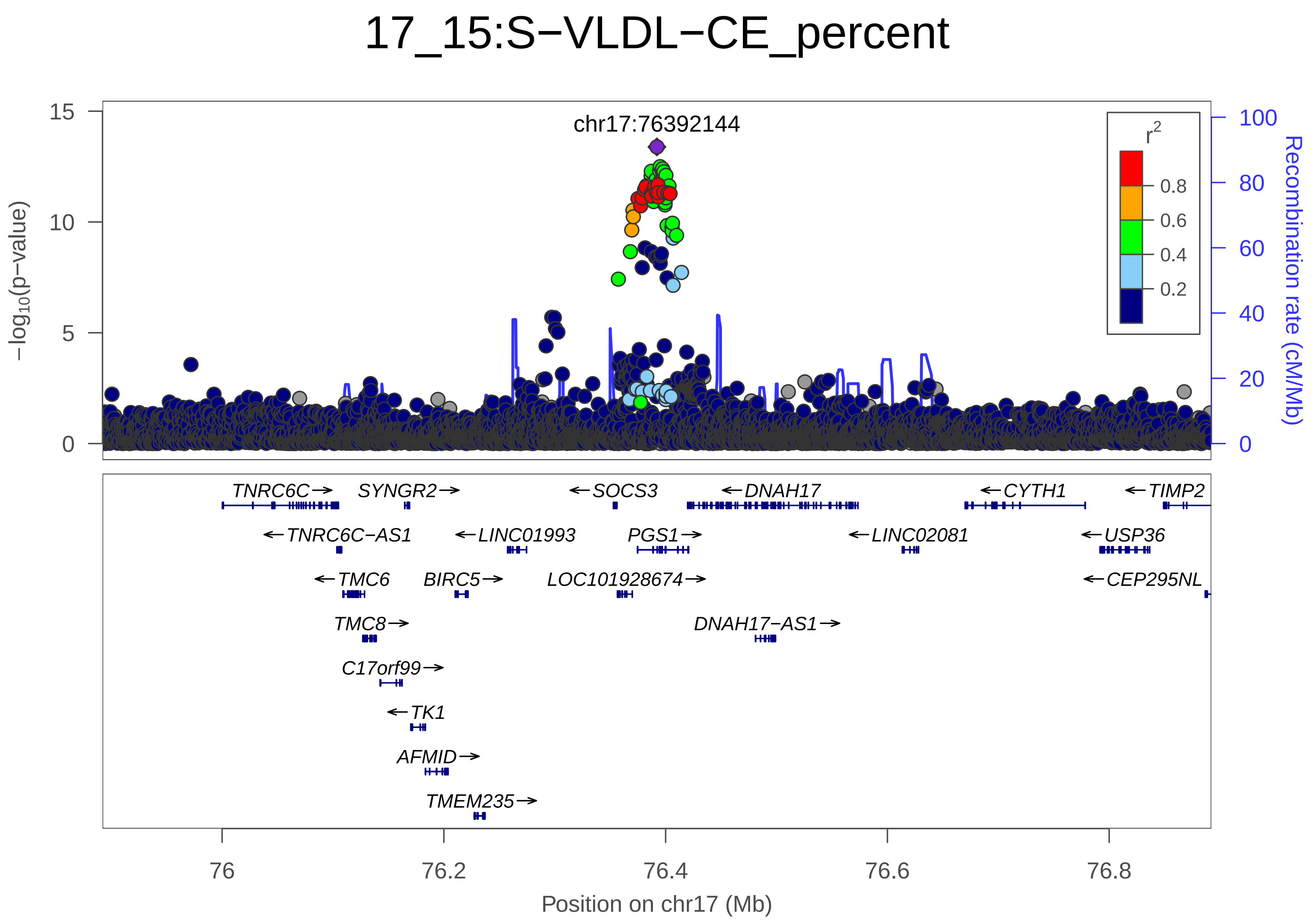

17_15:S−VLDL−CE_percent
chr17:76392144
r²
0.8
0.6
0.4
0.2
−log10(p−value)
Recombination rate (cM/Mb)
15
10
5
0
100
80
60
40
20
0
TNRC6C→
SYNGR2→
←SOCS3
←DNAH17
←CYTH1
←TIMP2
←TNRC6C−AS1
←LINC01993
PGS1→
←LINC02081
←USP36
←TMC6
BIRC5→
LOC101928674→
←CEP295NL
TMC8→
DNAH17−AS1→
C17orf99→
←TK1
AFMID→
TMEM235→
76
76.2
76.4
76.6
76.8
Position on chr17 (Mb)

18_1:S−VLDL−TG

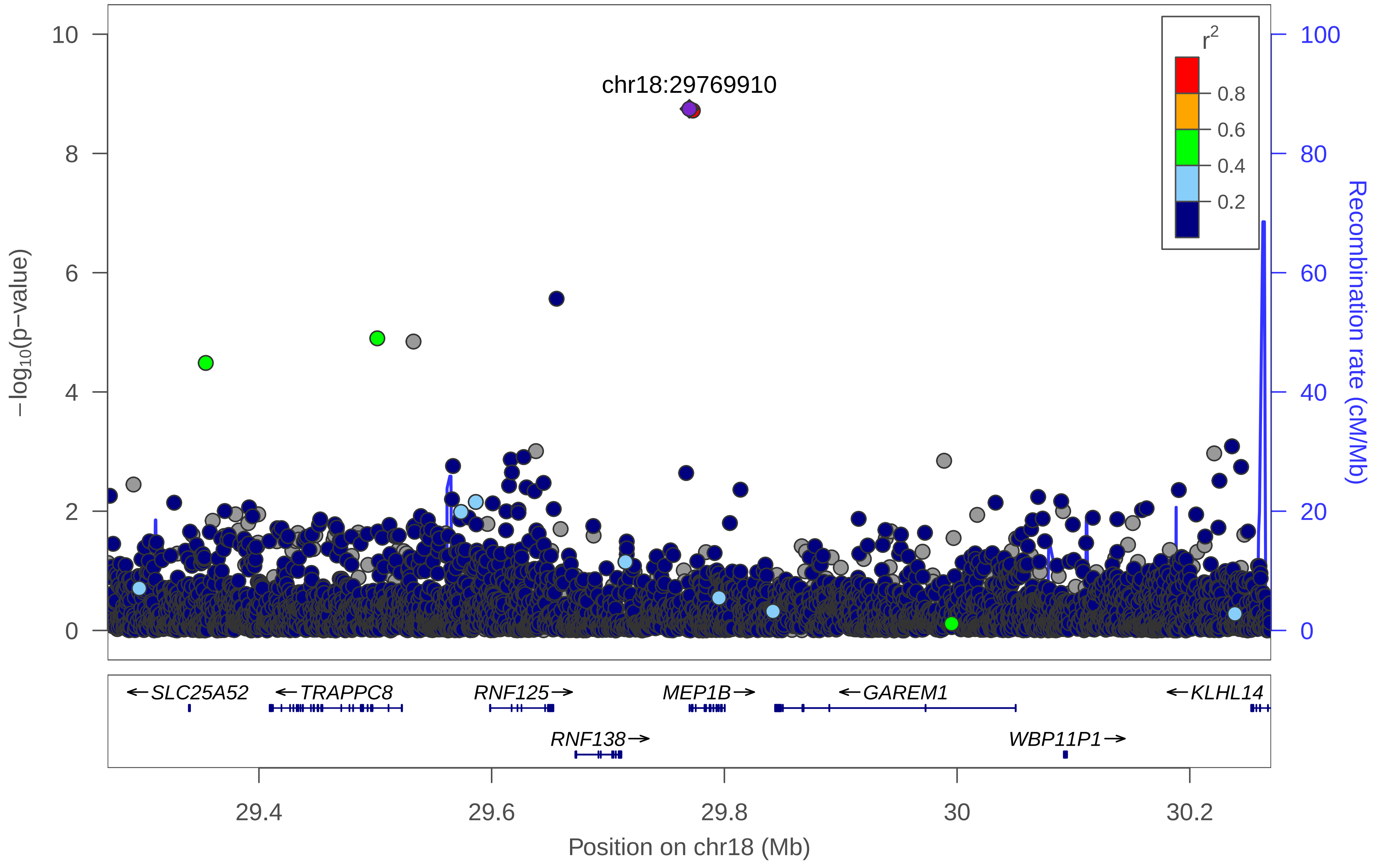

chr18:29769910
−log10(p−value)
10
8
6
4
2
0
Recombination rate (cM/Mb)
100
80
60
40
20
0
r2
0.8
0.6
0.4
0.2
←SLC25A52
←TRAPPC8
RNF125→
MEP1B→
←GAREM1
←KLHL14
RNF138→
WBP11P1→
29.4
29.6
29.8
30
30.2
Position on chr18 (Mb)

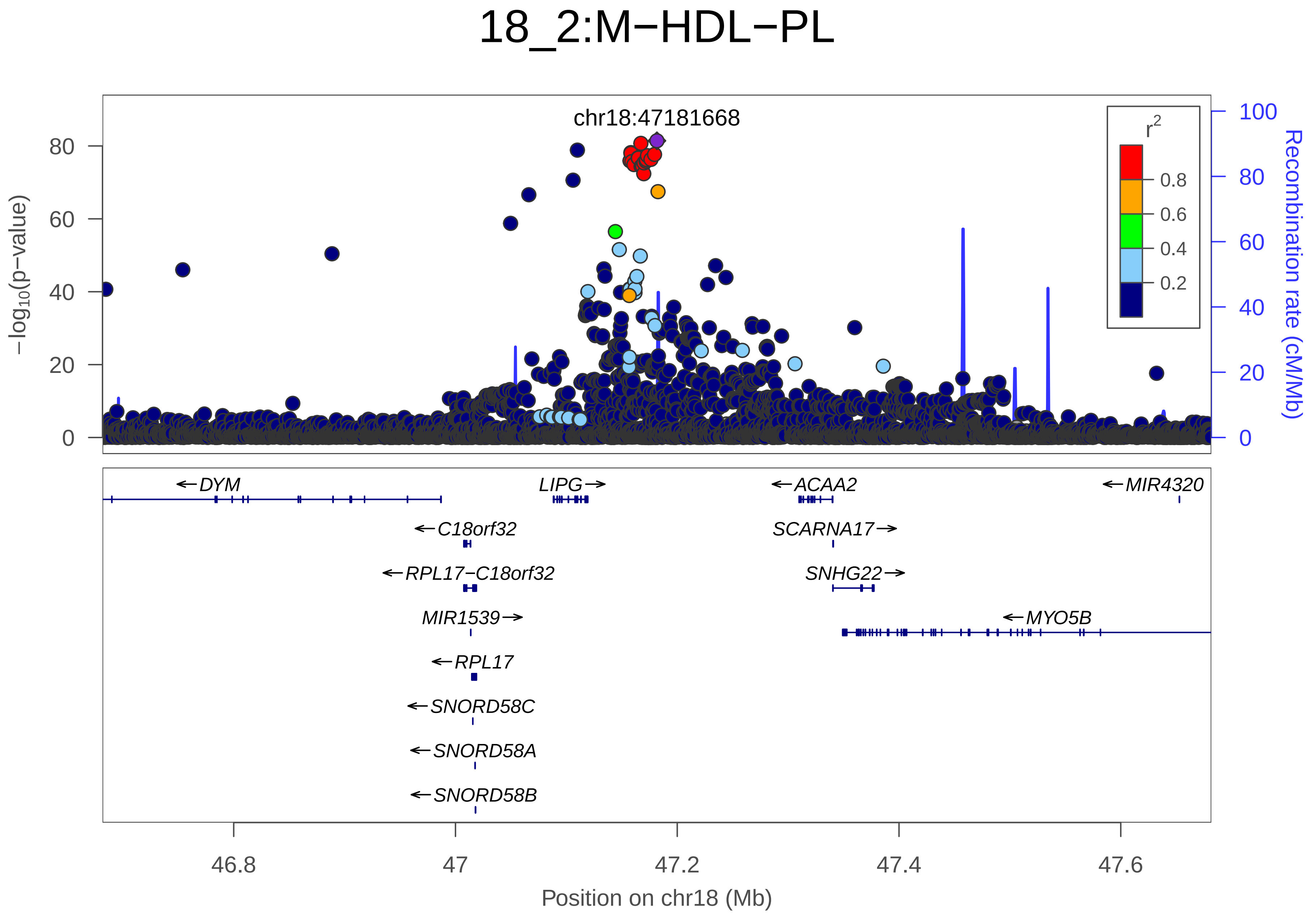
18_2:M−HDL−PL
chr18:47181668
−log10(p−value)
Recombination rate (cM/Mb)
r2
0.8
0.6
0.4
0.2
80
60
40
20
0
100
DYM
LIPG
ACAA2
MIR4320
C18orf32
SCARNA17
RPL17−C18orf32
SNHG22
MIR1539
MYO5B
RPL17
SNORD58C
SNORD58A
SNORD58B
46.8
47
47.2
47.4
47.6
Position on chr18 (Mb)

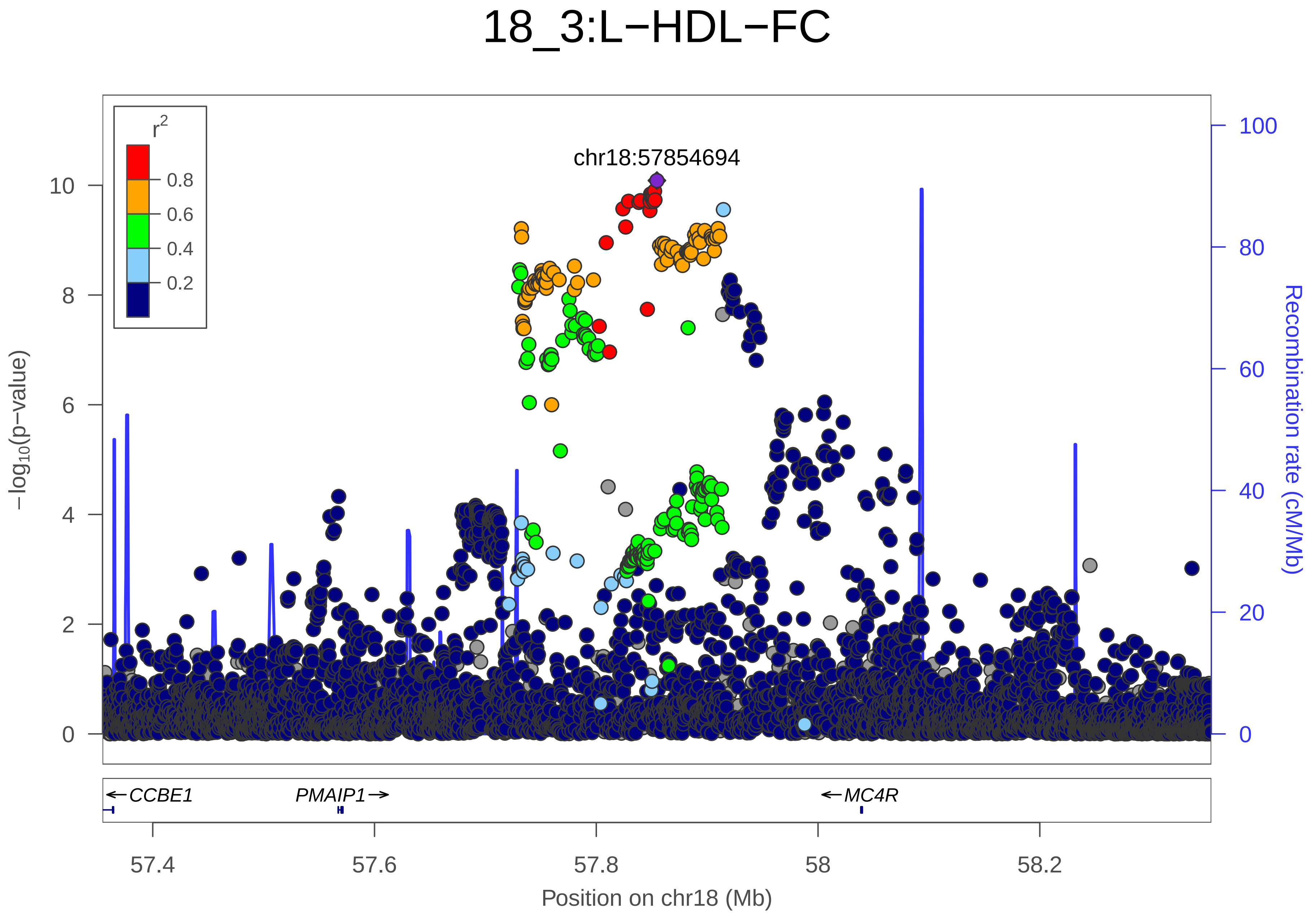

18_3:L−HDL−FC
chr18:57854694
r2
0.8
0.6
0.4
0.2
−log10(p−value)
Recombination rate (cM/Mb)
CCBE1
PMAIP1
MC4R
57.4
57.6
57.8
58
58.2
Position on chr18 (Mb)

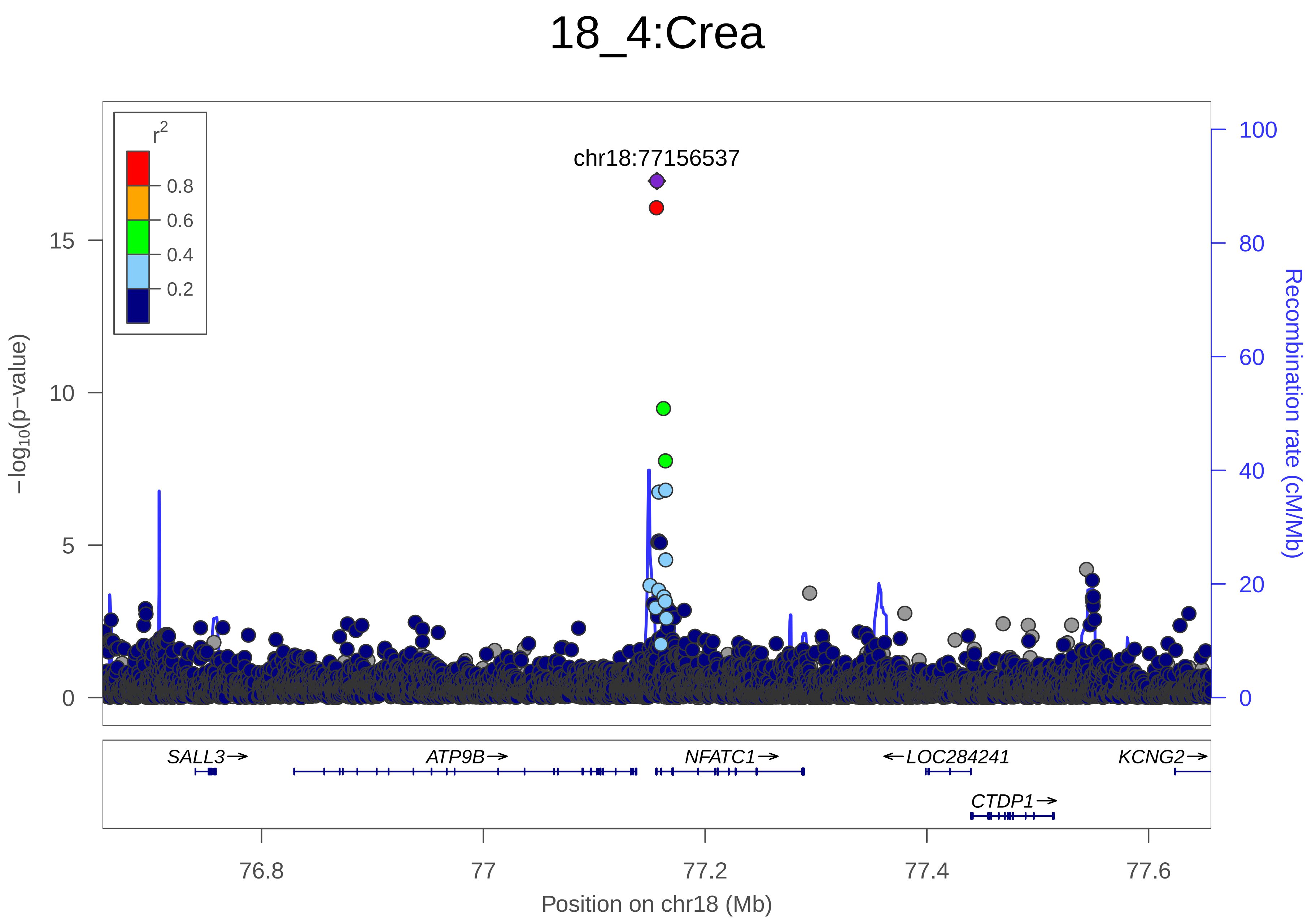

18_4:Crea
r2
0.8
0.6
0.4
0.2
chr18:77156537
−log10(p−value)
0
5
10
15
Recombination rate (cM/Mb)
0
20
40
60
80
100
SALL3→
ATP9B→
NFATC1→
←LOC284241
KCNG2→
CTDP1→
76.8
77
77.2
77.4
77.6
Position on chr18 (Mb)

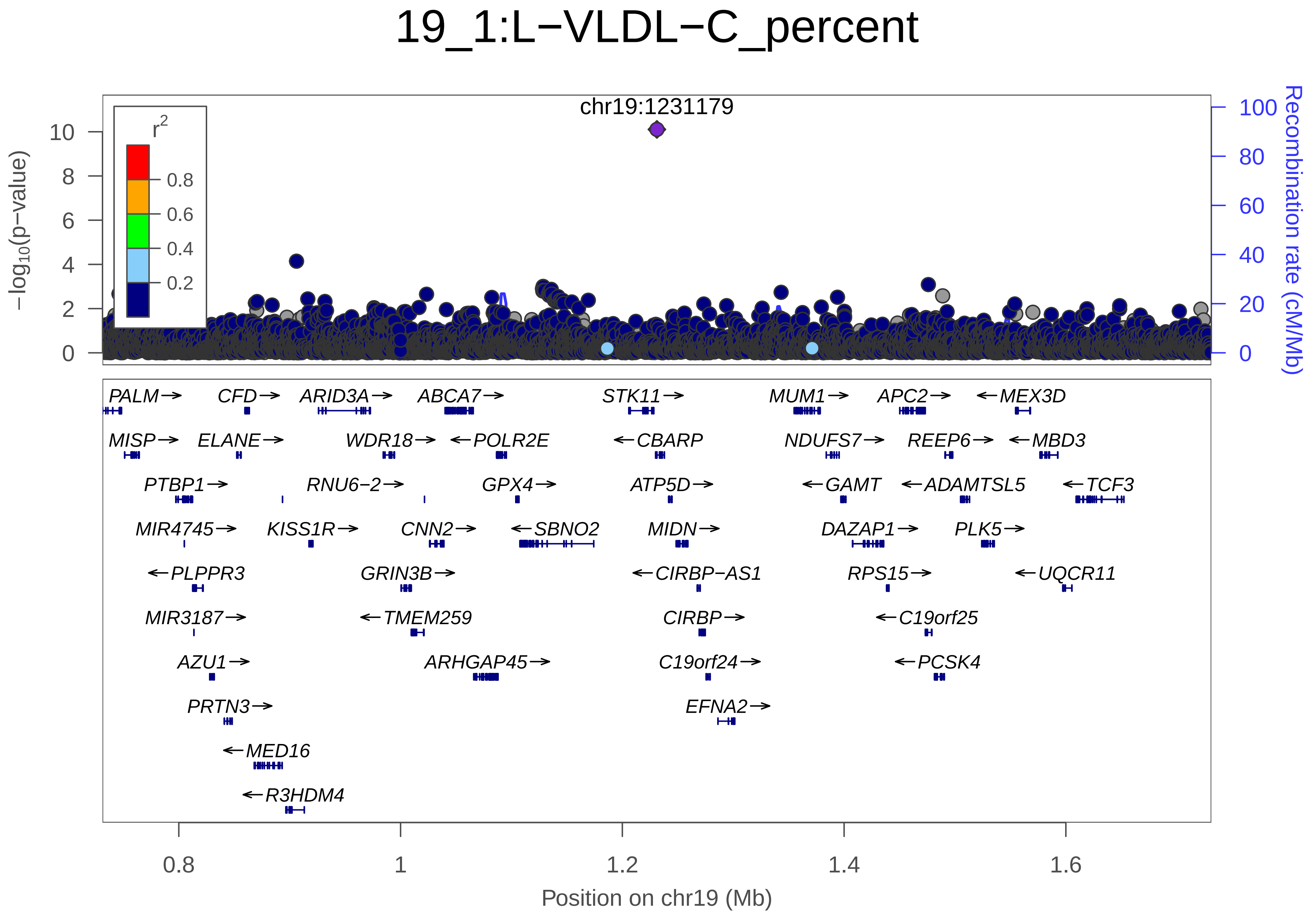
19_1:L−VLDL−C_percent
chr19:1231179
r2
0.8
0.6
0.4
0.2
−log10(p−value)
Recombination rate (cM/Mb)
PALM→
CFD→
ARID3A→
ABCA7→
STK11→
MUM1→
APC2→
←MEX3D
MISP→
ELANE→
WDR18→
←POLR2E
←CBARP
NDUFS7→
REEP6→
←MBD3
PTBP1→
RNU6−2→
GPX4→
ATP5D→
←GAMT
←ADAMTSL5
←TCF3
MIR4745→
KISS1R→
CNN2→
←SBNO2
MIDN→
DAZAP1→
PLK5→
←PLPPR3
GRIN3B→
←CIRBP−AS1
RPS15→
←UQCR11
MIR3187→
←TMEM259
CIRBP→
←C19orf25
AZU1→
ARHGAP45→
C19orf24→
←PCSK4
PRTN3→
EFNA2→
←MED16
←R3HDM4
Position on chr19 (Mb)

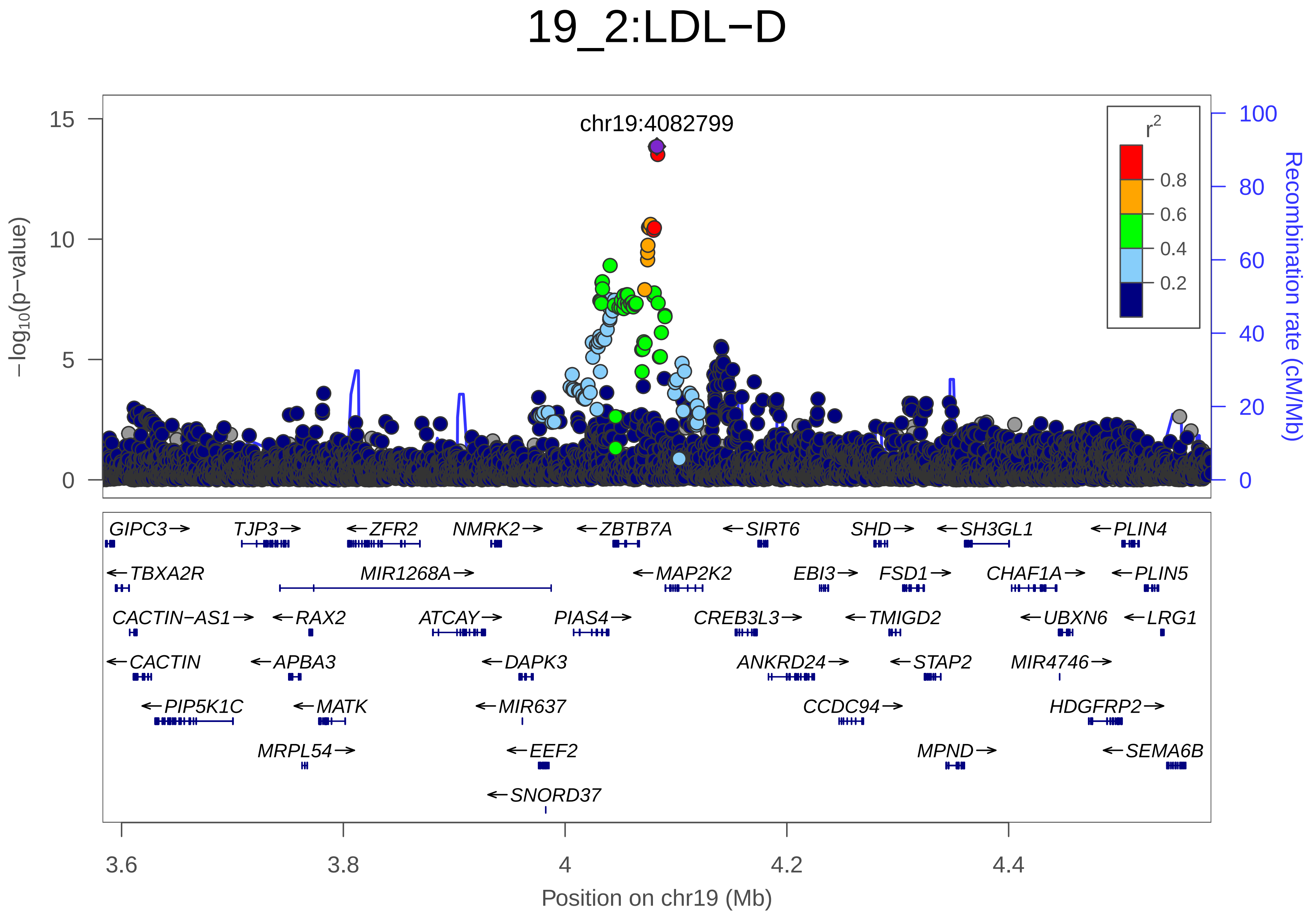

19_2:LDL−D
chr19:4082799
r²
0.8
0.6
0.4
0.2
−log10(p−value)
Recombination rate (cM/Mb)
Position on chr19 (Mb)
GIPC3→
TJP3→
←ZFR2
NMRK2→
←ZBTB7A
←SIRT6
SHD→
←SH3GL1
←PLIN4
←TBXA2R
MIR1268A→
←MAP2K2
EBI3→
FSD1→
CHAF1A→
←PLIN5
CACTIN−AS1→
←RAX2
ATCAY→
PIAS4→
CREB3L3→
←TMIGD2
←UBXN6
←LRG1
←CACTIN
←APBA3
←DAPK3
ANKRD24→
←STAP2
MIR4746→
←PIP5K1C
←MATK
←MIR637
CCDC94→
HDGFRP2→
MRPL54→
←EEF2
MPND→
←SEMA6B
←SNORD37

19_3:Ile

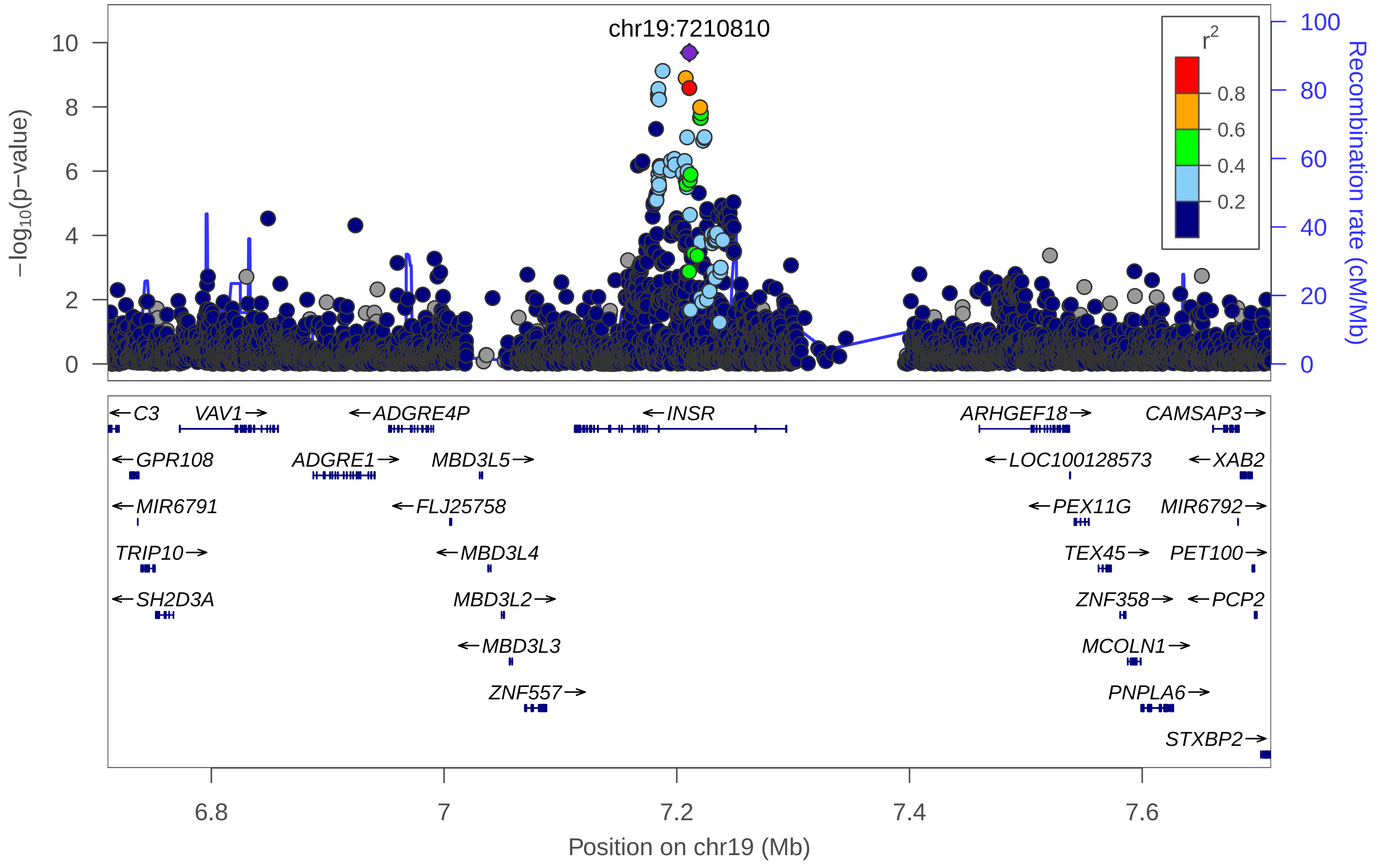

chr19:7210810
−log10(p−value)
Recombination rate (cM/Mb)
r2
0.8
0.6
0.4
0.2
0
2
4
6
8
10
20
40
60
80
100
C3
VAV1
ADGRE4P
INSR
ARHGEF18
CAMSAP3
GPR108
ADGRE1
MBD3L5
LOC100128573
XAB2
MIR6791
FLJ25758
PEX11G
MIR6792
TRIP10
MBD3L4
TEX45
PET100
SH2D3A
MBD3L2
ZNF358
PCP2
MBD3L3
MCOLN1
ZNF557
PNPLA6
STXBP2
6.8
7
7.2
7.4
7.6
Position on chr19 (Mb)

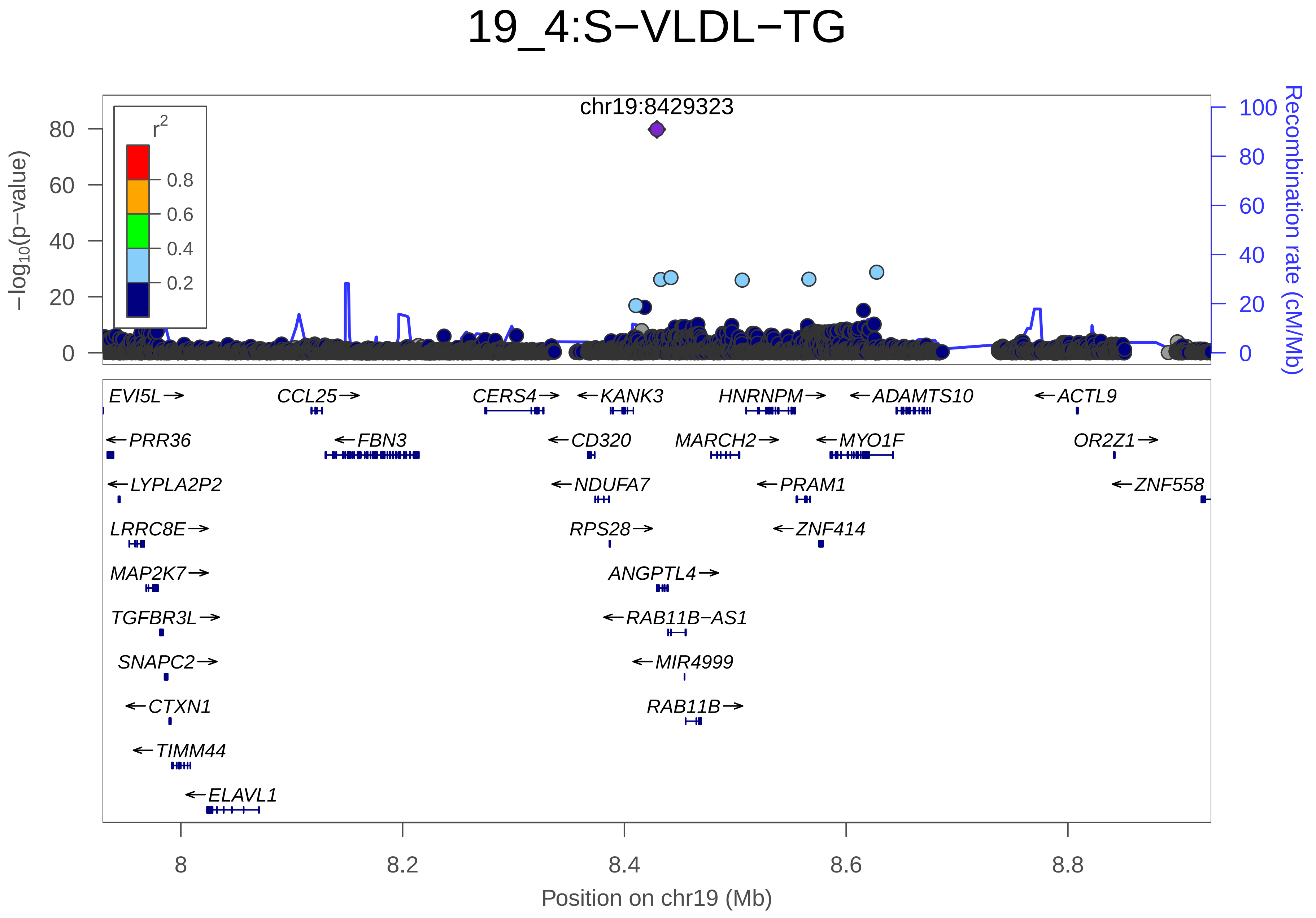

19_4:S−VLDL−TG
chr19:8429323
r2
0.8
0.6
0.4
0.2
−log10(p−value)
0
20
40
60
80
Recombination rate (cM/Mb)
0
20
40
60
80
100
EVI5L→
CCL25→
CERS4→
←KANK3
HNRNPM→
←ADAMTS10
←ACTL9
←PRR36
←FBN3
←CD320
MARCH2→
←MYO1F
OR2Z1→
←LYPLA2P2
←NDUFA7
←PRAM1
←ZNF558
LRRC8E→
RPS28→
←ZNF414
MAP2K7→
ANGPTL4→
TGFBR3L→
←RAB11B−AS1
SNAPC2→
←MIR4999
←CTXN1
RAB11B→
←TIMM44
←ELAVL1
8
8.2
8.4
8.6
8.8
Position on chr19 (Mb)

19_5:L−LDL−C

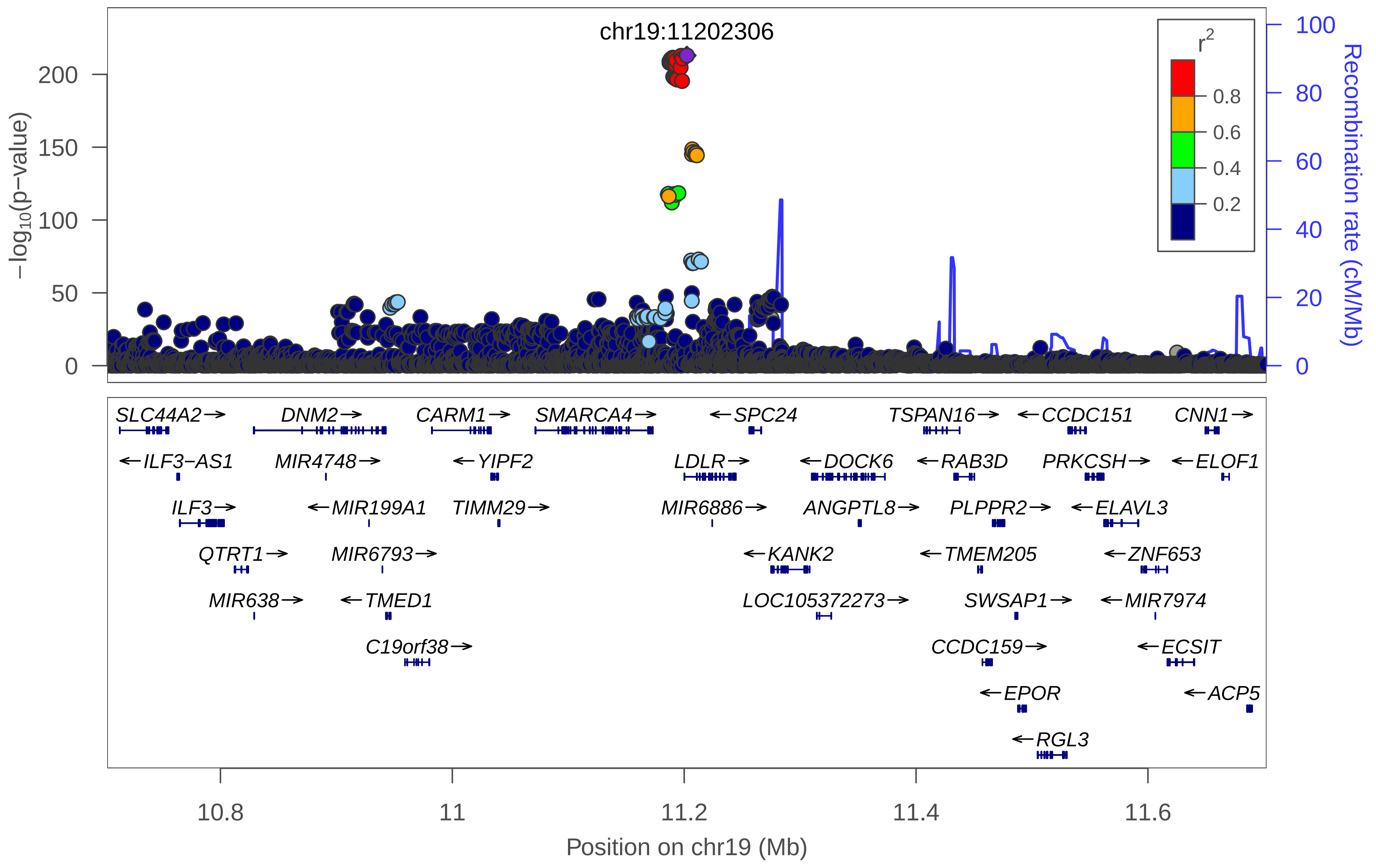
chr19:11202306
−log10(p−value)
200
150
100
50
0
r2
0.8
0.6
0.4
0.2
100
80
60
40
20
0
Recombination rate (cM/Mb)
SLC44A2→
DNM2→
CARM1→
SMARCA4→
←SPC24
TSPAN16→
←CCDC151
CNN1→
←ILF3−AS1
MIR4748→
←YIPF2
LDLR→
←DOCK6
←RAB3D
PRKCSH→
←ELOF1
ILF3→
←MIR199A1
TIMM29→
MIR6886→
ANGPTL8→
PLPPR2→
←ELAVL3
QTRT1→
MIR6793→
←KANK2
←TMEM205
←ZNF653
MIR638→
←TMED1
LOC105372273→
SWSAP1→
←MIR7974
C19orf38→
CCDC159→
←ECSIT
←EPOR
←ACP5
←RGL3
10.8
11
11.2
11.4
11.6
Position on chr19 (Mb)

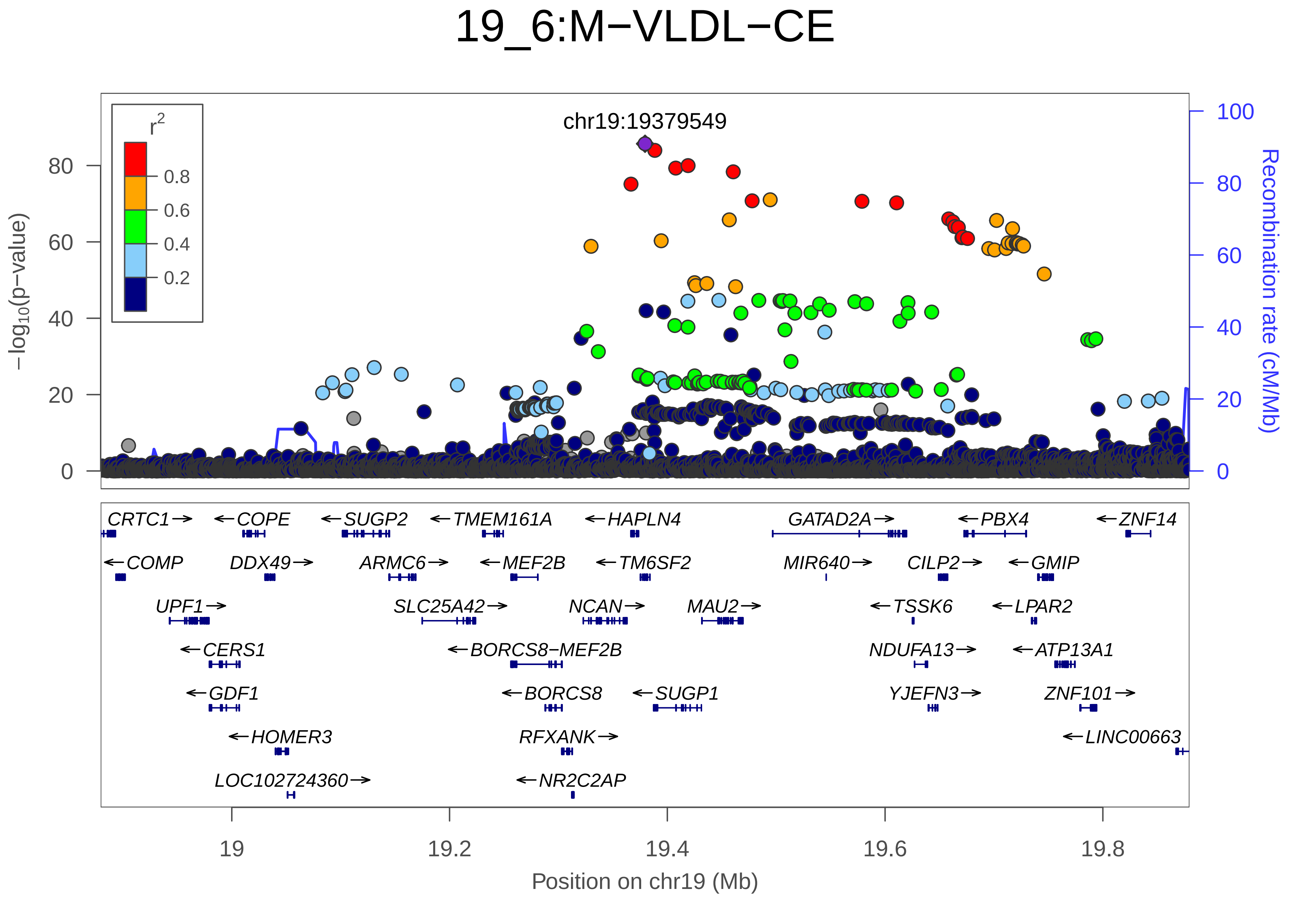
19_6:M−VLDL−CE
r2
0.8
0.6
0.4
0.2
chr19:19379549
−log10(p−value)
Recombination rate (cM/Mb)
0
20
40
60
80
100
CRTC1→
←COPE
←SUGP2
←TMEM161A
←HAPLN4
GATAD2A→
←PBX4
←ZNF14
←COMP
DDX49→
ARMC6→
←MEF2B
←TM6SF2
MIR640→
CILP2→
←GMIP
UPF1→
SLC25A42→
NCAN→
MAU2→
←TSSK6
←LPAR2
←CERS1
←BORCS8−MEF2B
NDUFA13→
←ATP13A1
←GDF1
←BORCS8
←SUGP1
YJEFN3→
ZNF101→
←HOMER3
RFXANK→
←LINC00663
LOC102724360→
←NR2C2AP
19
19.2
19.4
19.6
19.8
Position on chr19 (Mb)

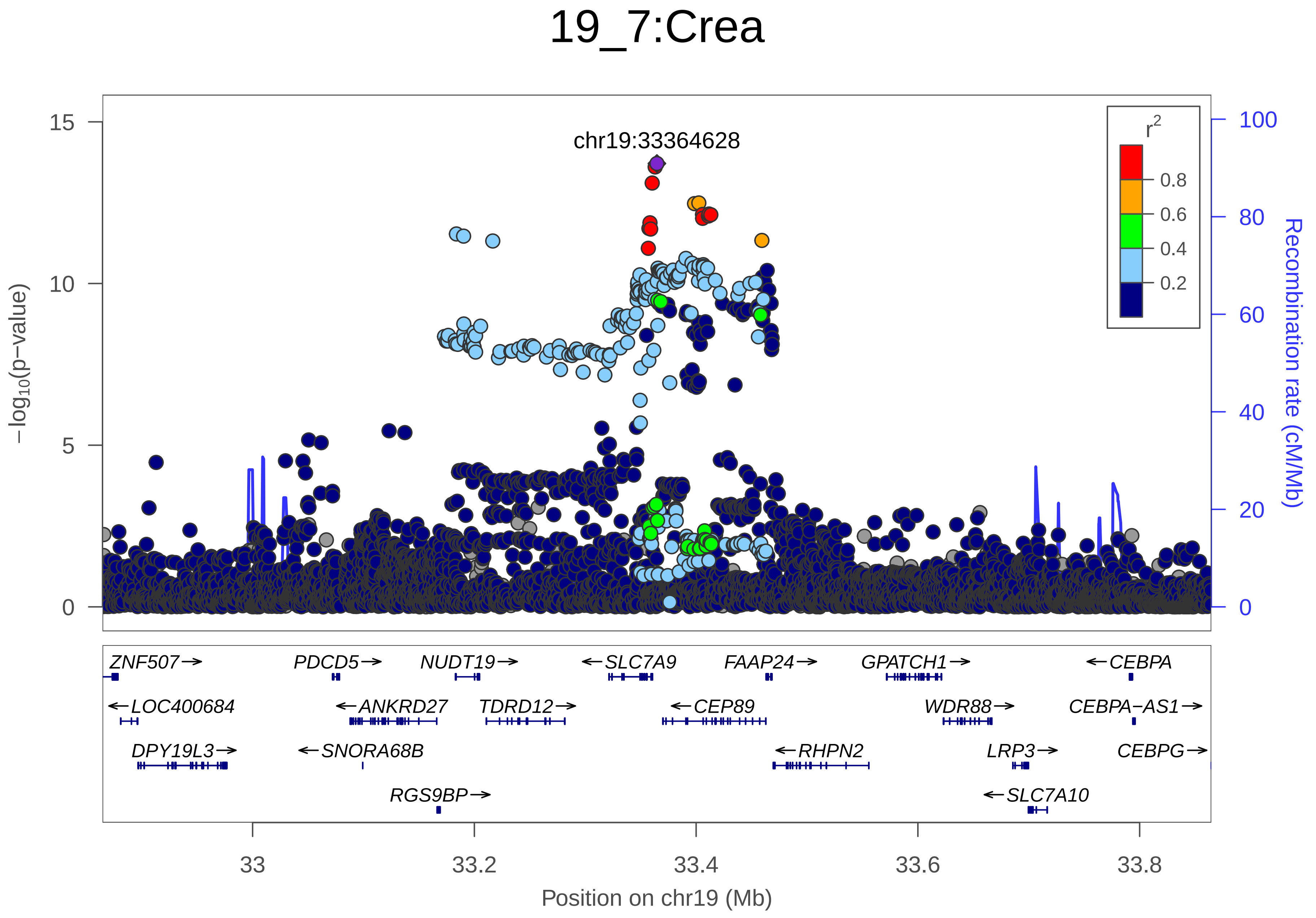

19_7:Crea
chr19:33364628
r2
0.8
0.6
0.4
0.2
15
10
5
0
−log10(p−value)
100
80
60
40
20
0
Recombination rate (cM/Mb)
ZNF507→
PDCD5→
NUDT19→
←SLC7A9
FAAP24→
GPATCH1→
←CEBPA
←LOC400684
←ANKRD27
TDRD12→
←CEP89
WDR88→
CEBPA−AS1→
DPY19L3→
←SNORA68B
←RHPN2
LRP3→
CEBPG→
RGS9BP→
←SLC7A10
33
33.2
33.4
33.6
33.8
Position on chr19 (Mb)

19_8:Alb

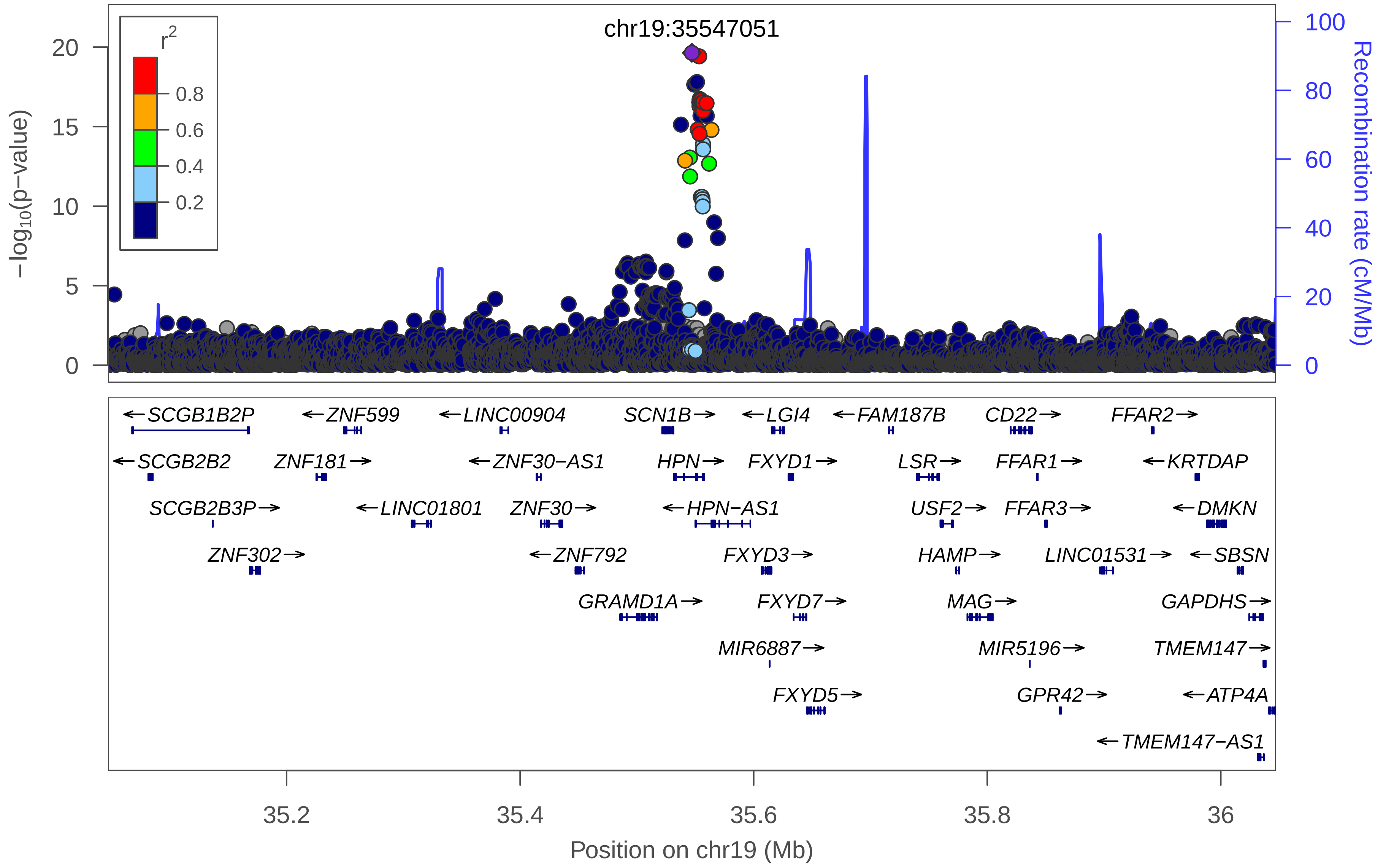
chr19:35547051
r2
0.8
0.6
0.4
0.2
−log10(p−value)
20
15
10
5
0
100
80
60
40
20
0
Recombination rate (cM/Mb)
←SCGB1B2P
←ZNF599
←LINC00904
SCN1B→
←LGI4
←FAM187B
CD22→
FFAR2→
←SCGB2B2
ZNF181→
←ZNF30−AS1
HPN→
FXYD1→
LSR→
FFAR1→
←KRTDAP
SCGB2B3P→
←LINC01801
ZNF30→
←HPN−AS1
USF2→
FFAR3→
←DMKN
ZNF302→
←ZNF792
FXYD3→
HAMP→
LINC01531→
←SBSN
GRAMD1A→
FXYD7→
MAG→
GAPDHS→
MIR6887→
MIR5196→
TMEM147→
FXYD5→
GPR42→
←ATP4A
←TMEM147−AS1
35.2
35.4
35.6
35.8
36
Position on chr19 (Mb)

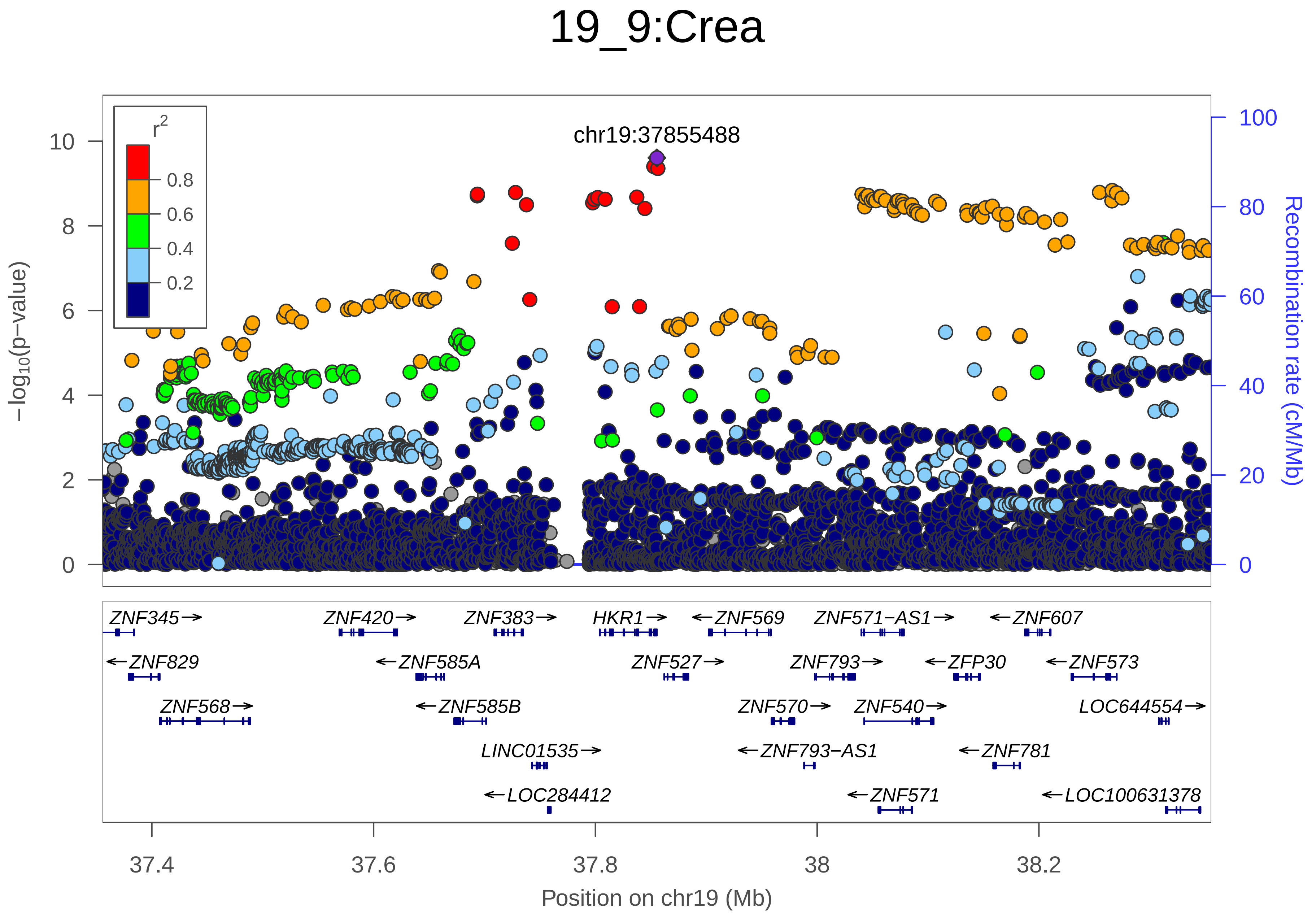

19_9:Crea
chr19:37855488
r2
0.8
0.6
0.4
0.2
−log10(p−value)
Recombination rate (cM/Mb)
0
2
4
6
8
10
0
20
40
60
80
100
ZNF345→
ZNF420→
ZNF383→
HKR1→
←ZNF569
ZNF571−AS1→
←ZNF607
←ZNF829
←ZNF585A
ZNF527→
ZNF793→
←ZFP30
←ZNF573
ZNF568→
←ZNF585B
ZNF570→
ZNF540→
LOC644554→
LINC01535→
←ZNF793−AS1
←ZNF781
←LOC284412
←ZNF571
←LOC100631378
37.4
37.6
37.8
38
38.2
Position on chr19 (Mb)

19_10:L−LDL−C_percent

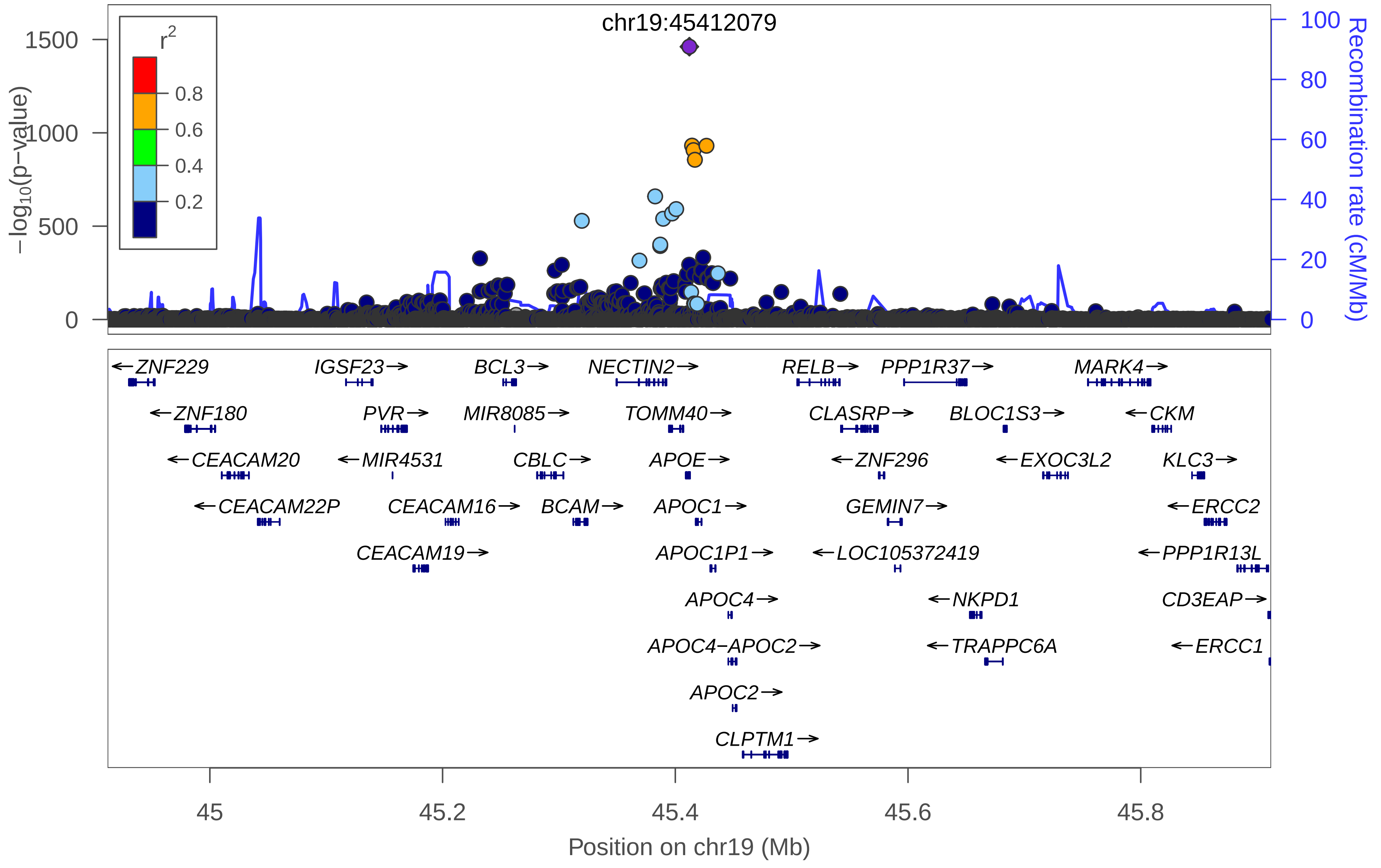

19_11:Val

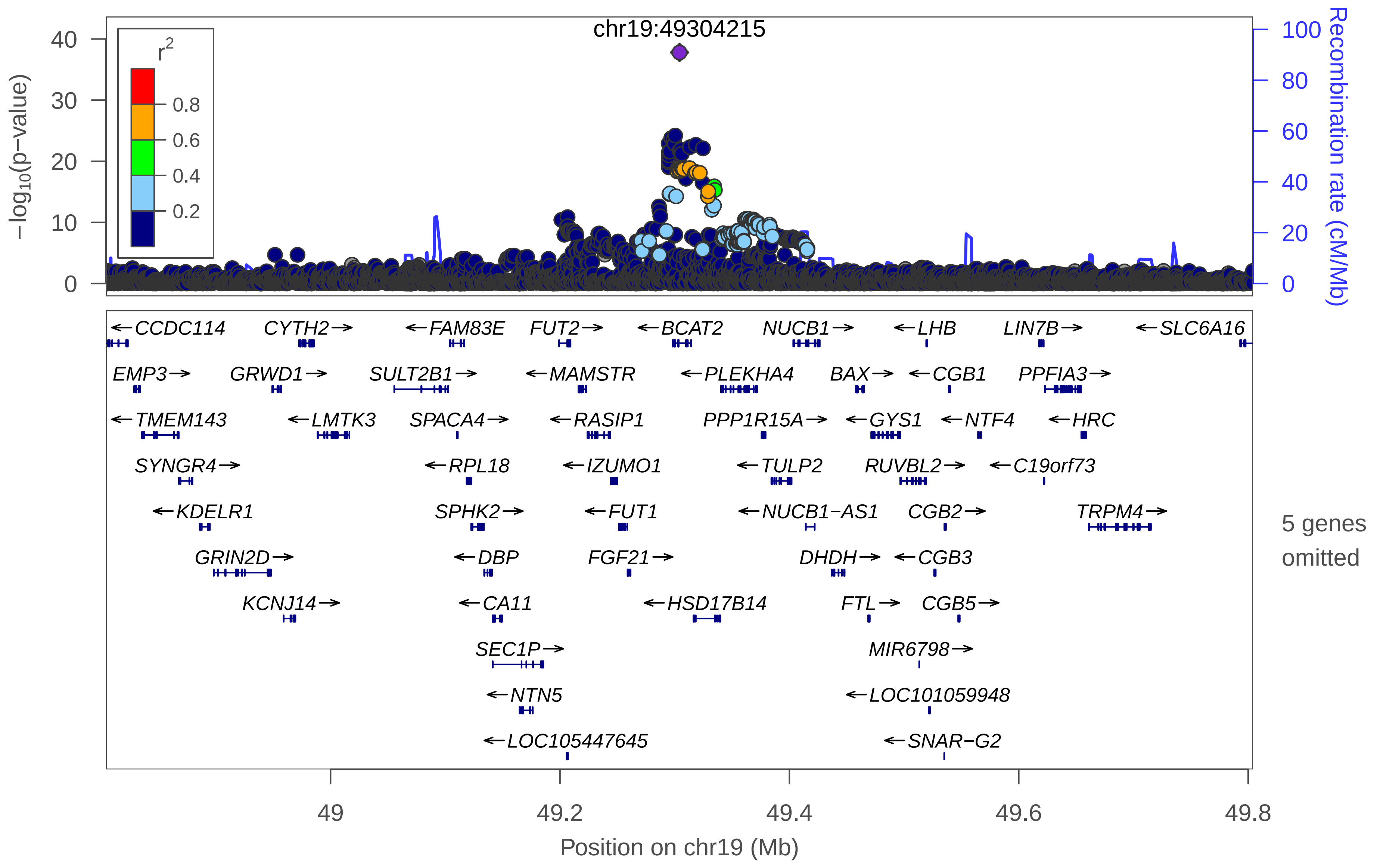
chr19:49304215
r2
0.8
0.6
0.4
0.2
−log10(p−value)
Recombination rate (cM/Mb)
0
10
20
30
40
0
20
40
60
80
100
←CCDC114
CYTH2→
←FAM83E
FUT2→
←BCAT2
NUCB1→
←LHB
LIN7B→
←SLC6A16
EMP3→
GRWD1→
SULT2B1→
←MAMSTR
←PLEKHA4
BAX→
←CGB1
PPFIA3→
←TMEM143
←LMTK3
SPACA4→
←RASIP1
PPP1R15A→
←GYS1
←NTF4
←HRC
SYNGR4→
←RPL18
←IZUMO1
←TULP2
RUVBL2→
←C19orf73
←KDELR1
SPHK2→
←FUT1
←NUCB1−AS1
CGB2→
TRPM4→
5 genes omitted
GRIN2D→
←DBP
FGF21→
DHDH→
←CGB3
KCNJ14→
←CA11
←HSD17B14
FTL→
CGB5→
SEC1P→
MIR6798→
←NTN5
←LOC101059948
←LOC105447645
←SNAR−G2
49
49.2
49.4
49.6
49.8
Position on chr19 (Mb)

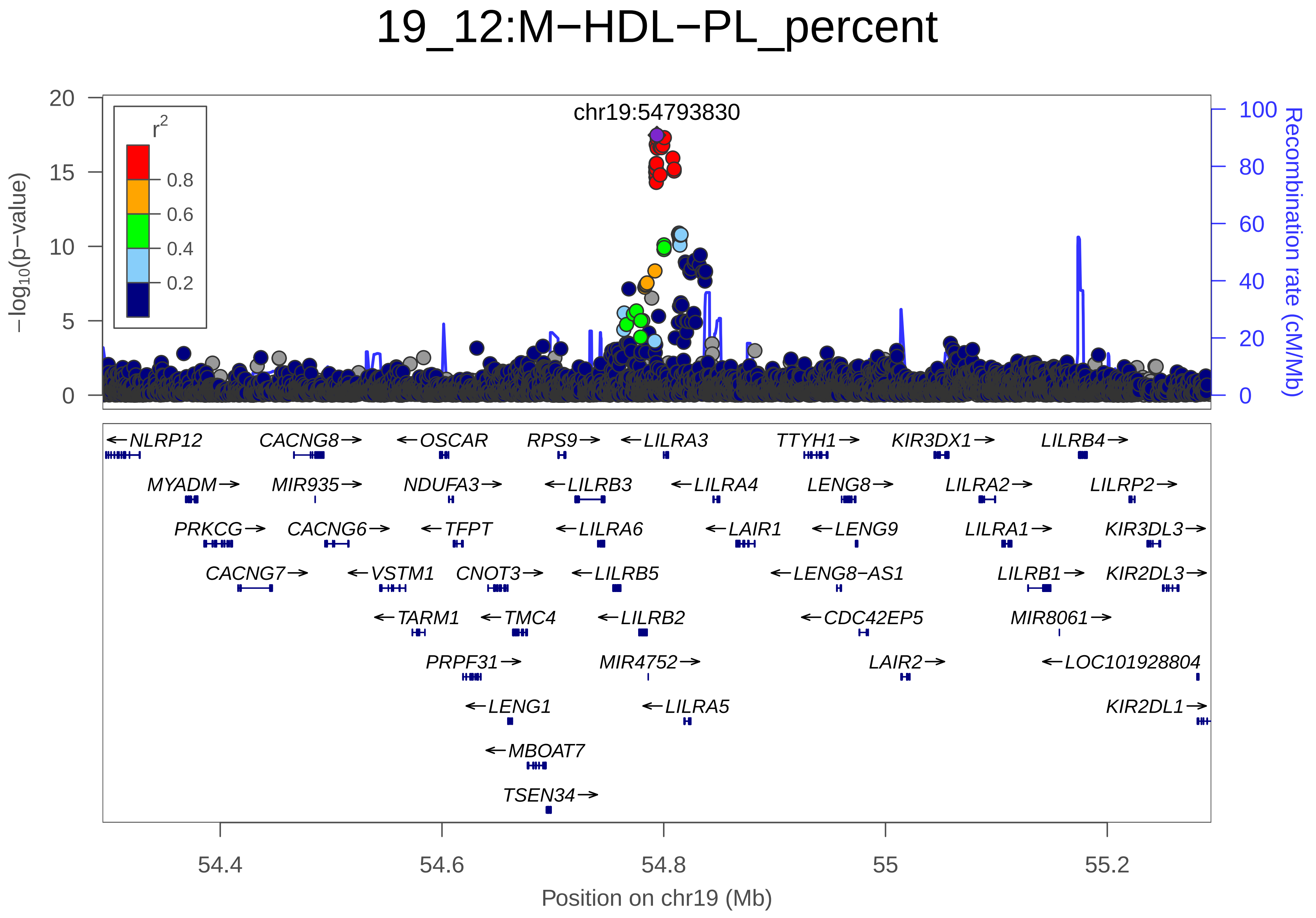
19_12:M−HDL−PL_percent
chr19:54793830
r2
0.8
0.6
0.4
0.2
−log10(p−value)
Recombination rate (cM/Mb)
NLRP12
CACNG8
OSCAR
RPS9
LILRA3
TTYH1
KIR3DX1
LILRB4
MYADM
MIR935
NDUFA3
LILRB3
LILRA4
LENG8
LILRA2
LILRP2
PRKCG
CACNG6
TFPT
LILRA6
LAIR1
LENG9
LILRA1
KIR3DL3
CACNG7
VSTM1
CNOT3
LILRB5
LENG8−AS1
LILRB1
KIR2DL3
TARM1
TMC4
LILRB2
CDC42EP5
MIR8061
PRPF31
MIR4752
LAIR2
LOC101928804
LENG1
LILRA5
KIR2DL1
MBOAT7
TSEN34
54.4
54.6
54.8
55
55.2
Position on chr19 (Mb)

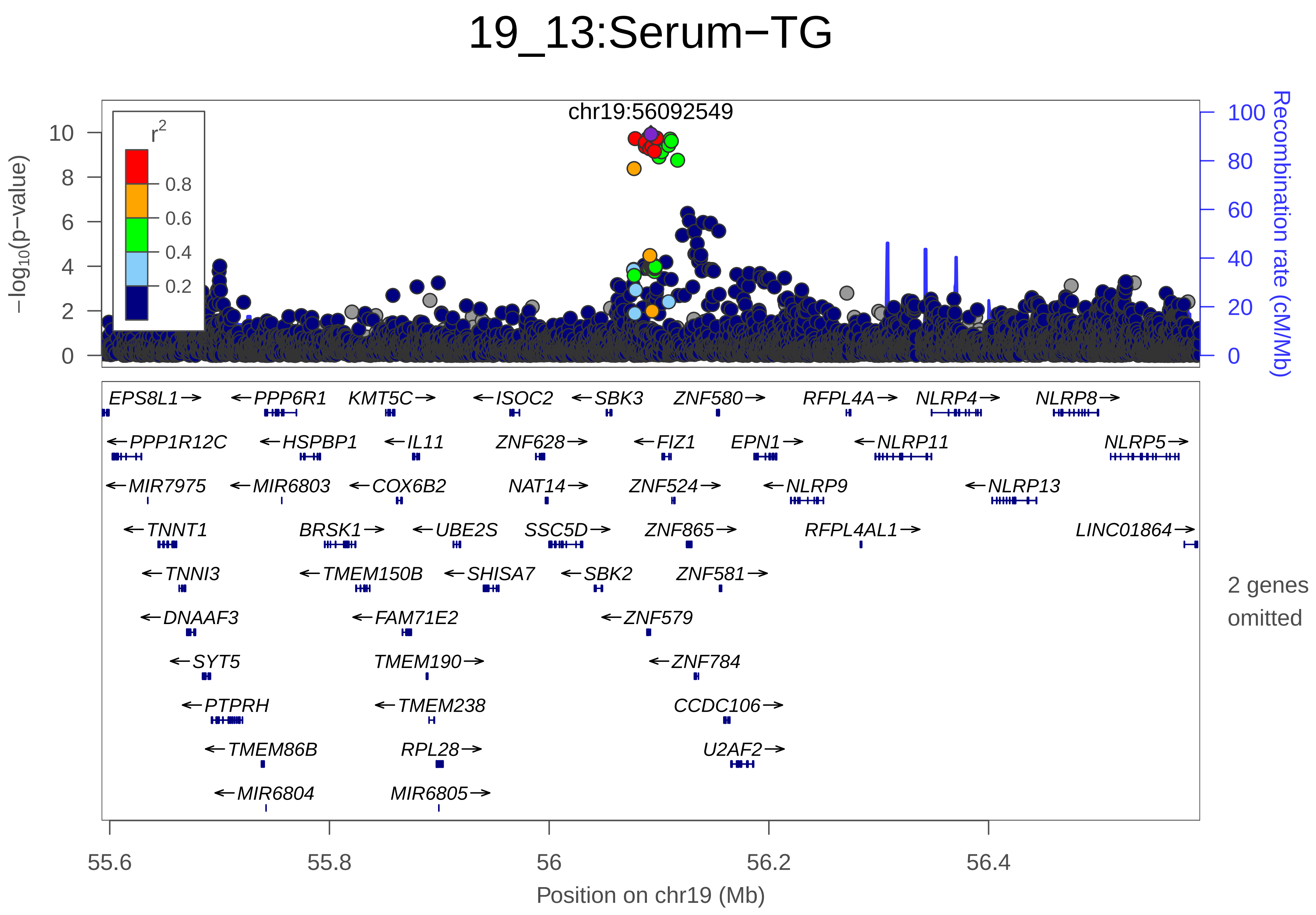
19_13:Serum−TG
chr19:56092549
r2
0.8
0.6
0.4
0.2
−log10(p−value)
Recombination rate (cM/Mb)
EPS8L1→
←PPP6R1
KMT5C→
←ISOC2
←SBK3
ZNF580→
RFPL4A→
NLRP4→
NLRP8→
←PPP1R12C
←HSPBP1
←IL11
ZNF628→
←FIZ1
EPN1→
←NLRP11
NLRP5→
←MIR7975
←MIR6803
←COX6B2
NAT14→
ZNF524→
←NLRP9
←NLRP13
←TNNT1
BRSK1→
←UBE2S
SSC5D→
ZNF865→
RFPL4AL1→
LINC01864→
←TNNI3
←TMEM150B
←SHISA7
←SBK2
ZNF581→
←DNAAF3
←FAM71E2
←ZNF579
←SYT5
TMEM190→
←ZNF784
←PTPRH
←TMEM238
CCDC106→
←TMEM86B
RPL28→
U2AF2→
←MIR6804
MIR6805→
2 genes omitted
55.6
55.8
56
56.2
56.4
Position on chr19 (Mb)

19_14:IDL−CE

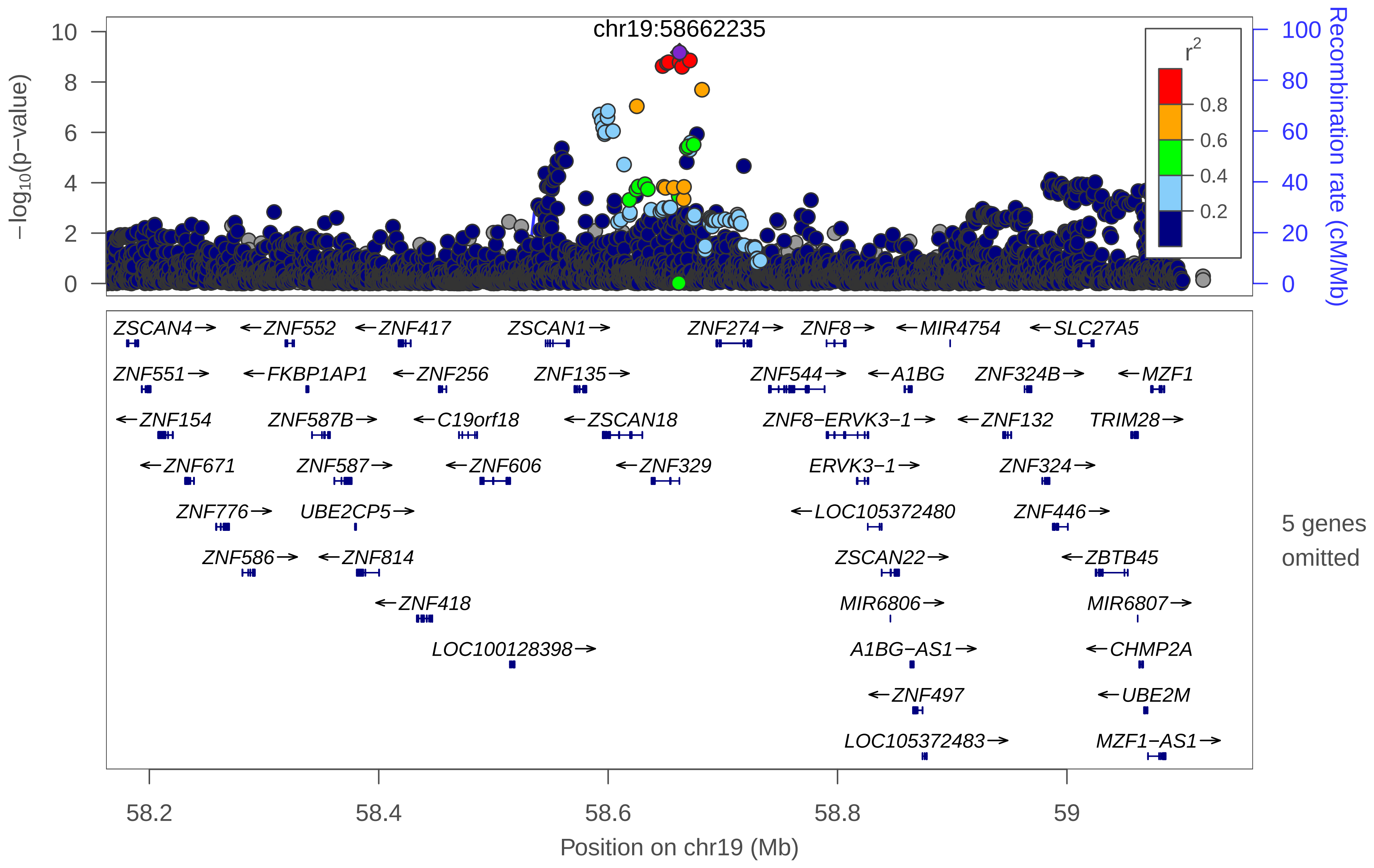

chr19:58662235
−log10(p−value)
Recombination rate (cM/Mb)
r2
0.8
0.6
0.4
0.2
ZSCAN4→
←ZNF552
←ZNF417
ZSCAN1→
ZNF274→
ZNF8→
←MIR4754
←SLC27A5
ZNF551→
←FKBP1AP1
←ZNF256
ZNF135→
ZNF544→
←A1BG
ZNF324B→
←MZF1
←ZNF154
ZNF587B→
←C19orf18
←ZSCAN18
ZNF8−ERVK3−1→
←ZNF132
TRIM28→
←ZNF671
ZNF587→
←ZNF606
←ZNF329
ERVK3−1→
ZNF324→
ZNF776→
UBE2CP5→
←LOC105372480
ZNF446→
ZNF586→
←ZNF814
ZSCAN22→
←ZBTB45
←ZNF418
MIR6806→
MIR6807→
LOC100128398→
A1BG−AS1→
←CHMP2A
←ZNF497
←UBE2M
LOC105372483→
MZF1−AS1→
5 genes omitted
58.2
58.4
58.6
58.8
59
Position on chr19 (Mb)

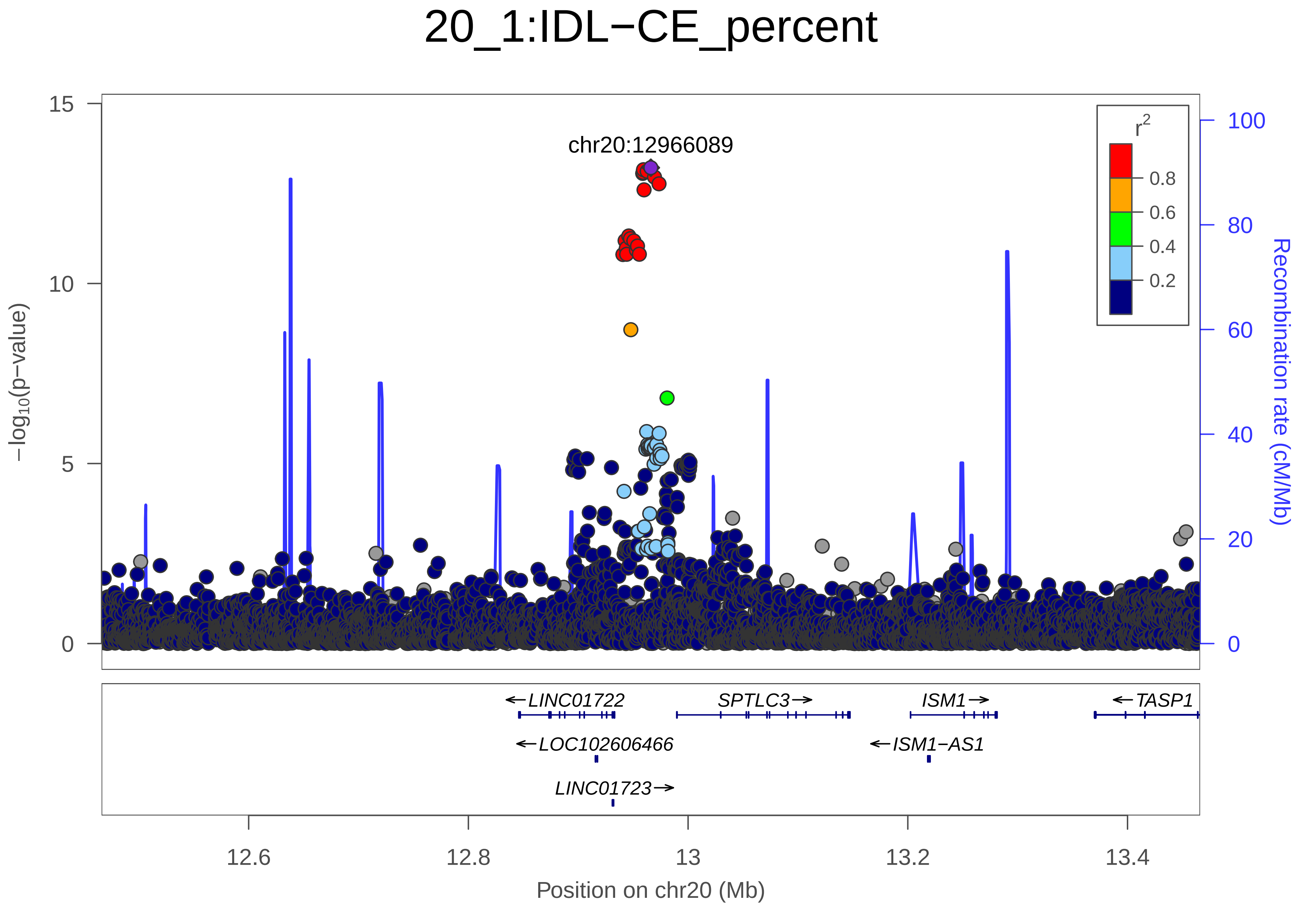
20_1:IDL−CE_percent
chr20:12966089
r2
0.8
0.6
0.4
0.2
15
10
5
0
−log10(p−value)
100
80
60
40
20
0
Recombination rate (cM/Mb)
←LINC01722
SPTLC3→
ISM1→
←TASP1
←LOC102606466
←ISM1−AS1
LINC01723→
12.6
12.8
13
13.2
13.4
Position on chr20 (Mb)

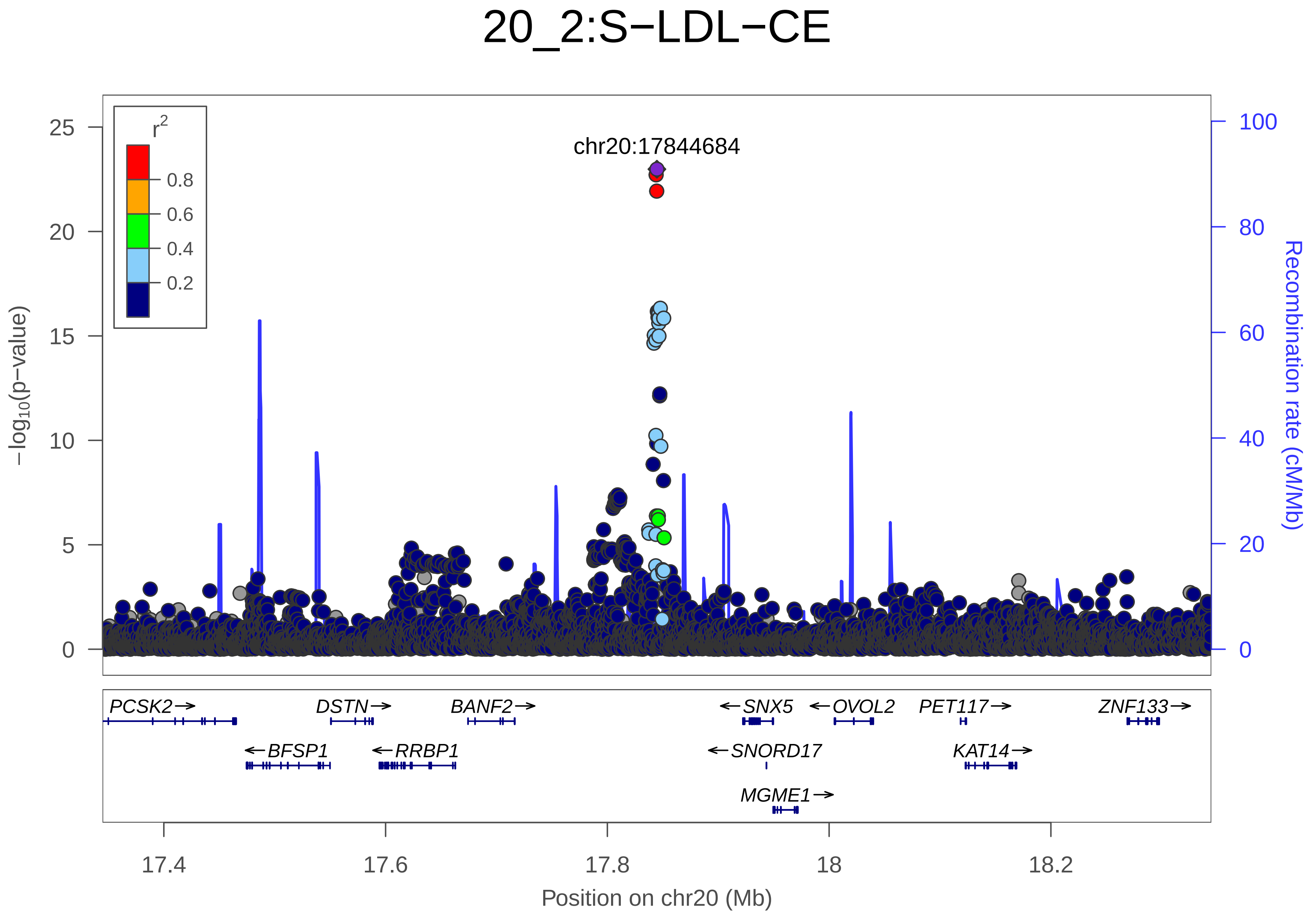

20_2:S−LDL−CE
r2
0.8
0.6
0.4
0.2
chr20:17844684
−log10(p−value)
Recombination rate (cM/Mb)
0
5
10
15
20
25
0
20
40
60
80
100
PCSK2→
DSTN→
BANF2→
←SNX5
←OVOL2
PET117→
ZNF133→
←BFSP1
←RRBP1
←SNORD17
KAT14→
MGME1→
17.4
17.6
17.8
18
18.2
Position on chr20 (Mb)

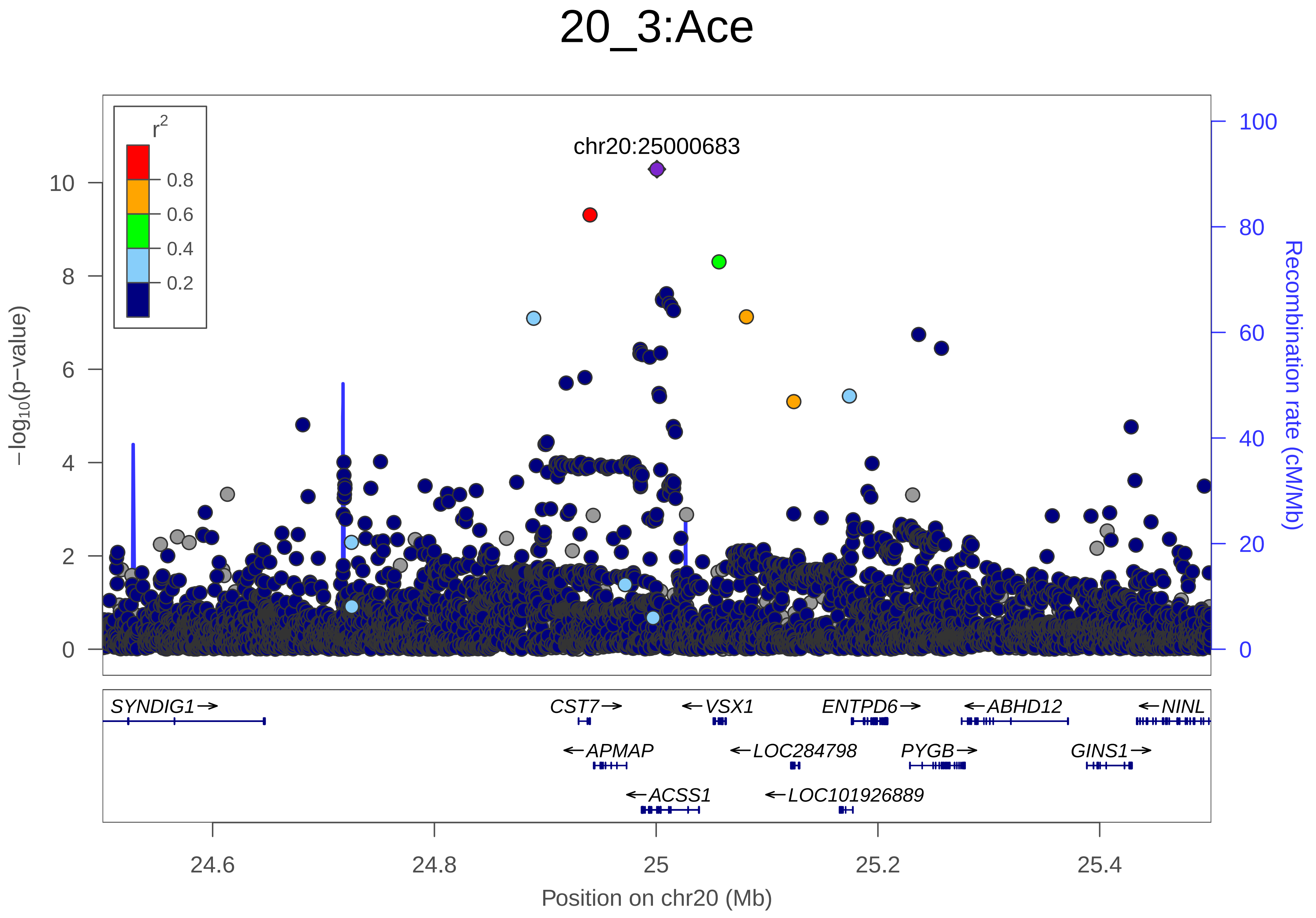

20_3:Ace
r2
0.8
0.6
0.4
0.2
chr20:25000683
−log10(p−value)
Recombination rate (cM/Mb)
0
2
4
6
8
10
0
20
40
60
80
100
SYNDIG1→
CST7→
←VSX1
ENTPD6→
←ABHD12
←NINL
←APMAP
←LOC284798
PYGB→
GINS1→
←ACSS1
←LOC101926889
24.6
24.8
25
25.2
25.4
Position on chr20 (Mb)

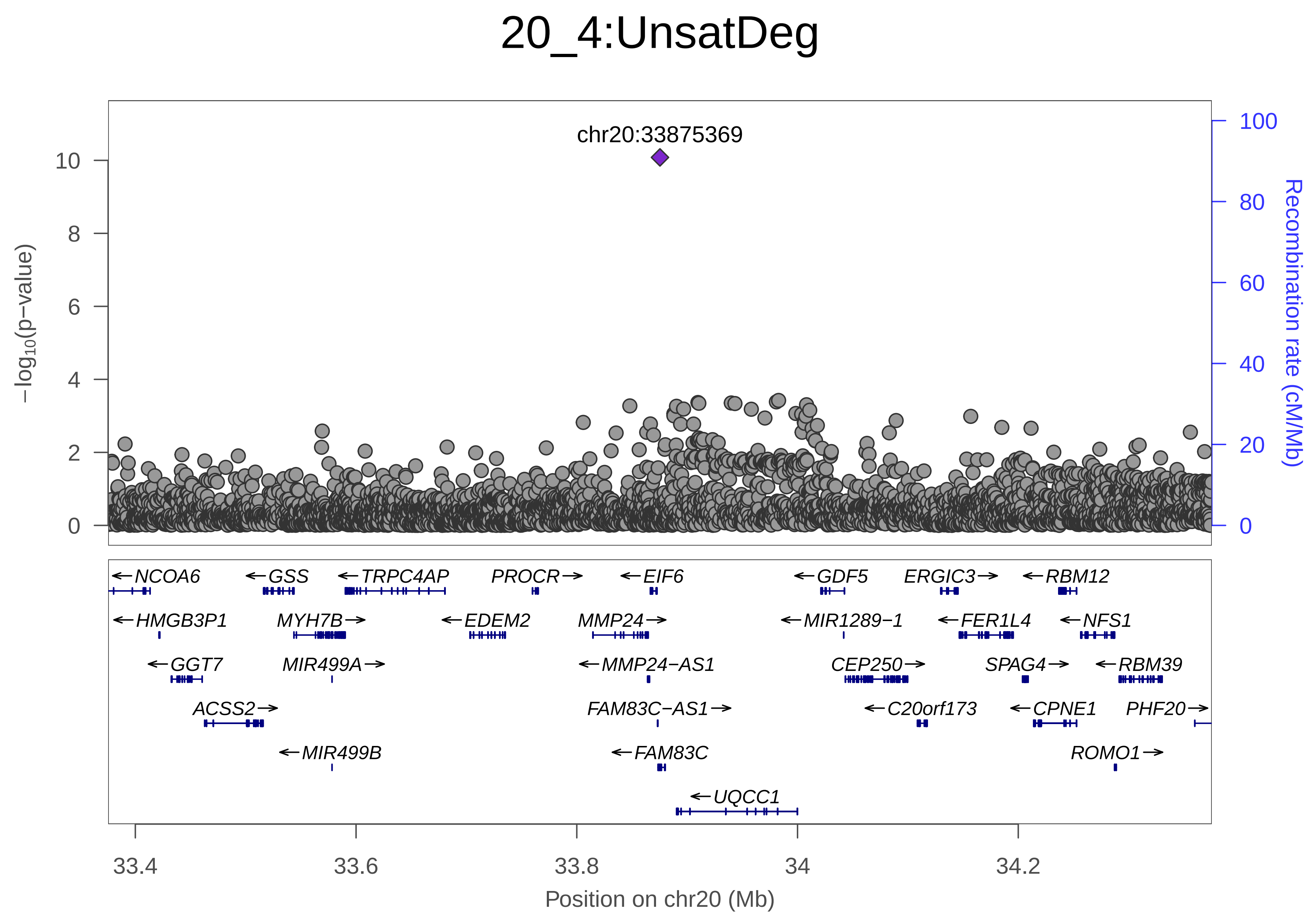
20_4:UnsatDeg
chr20:33875369
−log10(p−value)
Recombination rate (cM/Mb)
0
2
4
6
8
10
20
40
60
80
100
NCOA6
GSS
TRPC4AP
PROCR
EIF6
GDF5
ERGIC3
RBM12
HMGB3P1
MYH7B
EDEM2
MMP24
MIR1289−1
FER1L4
NFS1
GGT7
MIR499A
MMP24−AS1
CEP250
SPAG4
RBM39
ACSS2
FAM83C−AS1
C20orf173
CPNE1
PHF20
MIR499B
FAM83C
ROMO1
UQCC1
33.4
33.6
33.8
34
34.2
Position on chr20 (Mb)

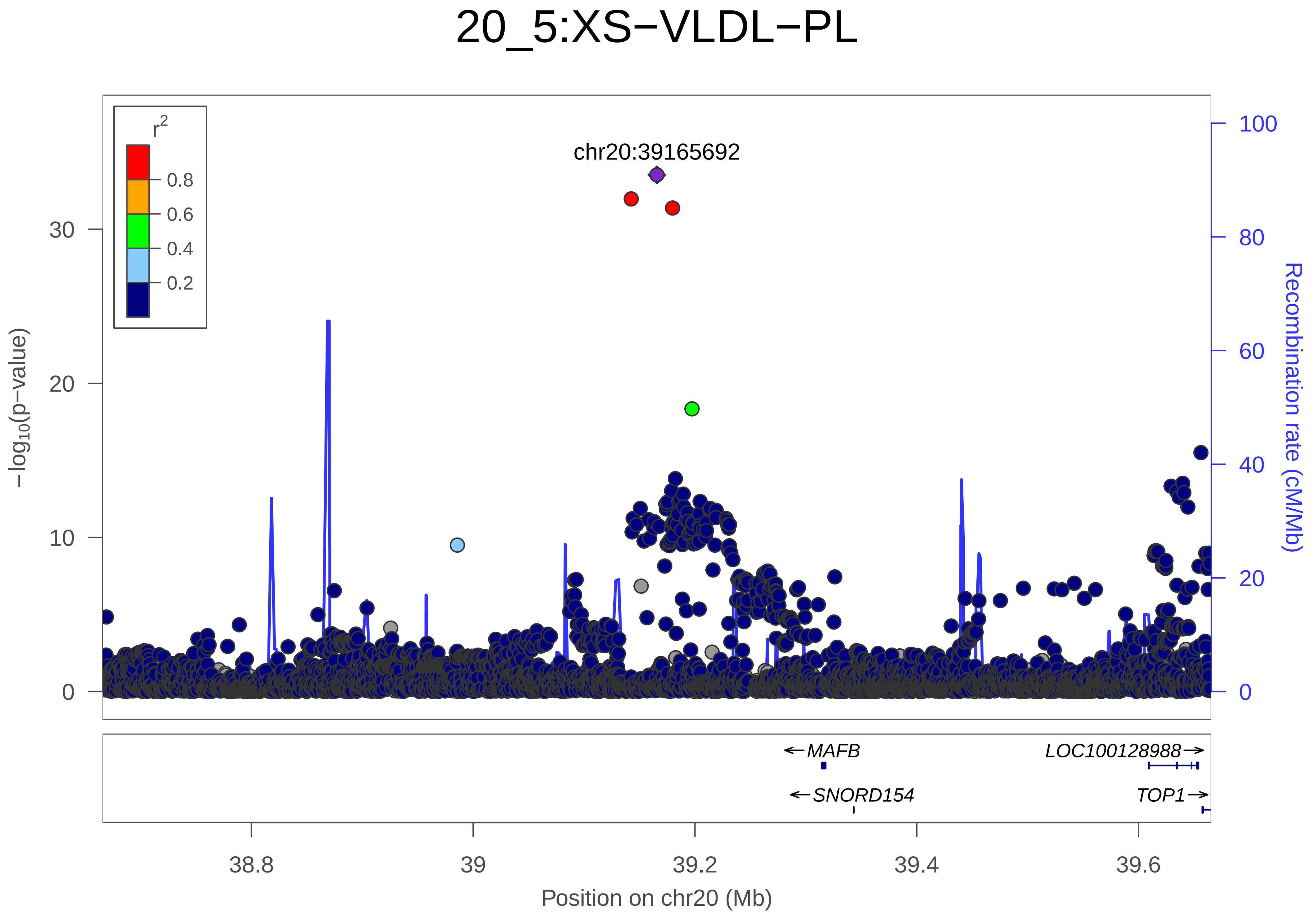
20_5:XS−VLDL−PL
r2
0.8
0.6
0.4
0.2
chr20:39165692
−log10(p−value)
0
10
20
30
Recombination rate (cM/Mb)
0
20
40
60
80
100
←MAFB
LOC100128988→
←SNORD154
TOP1→
38.8
39
39.2
39.4
39.6
Position on chr20 (Mb)

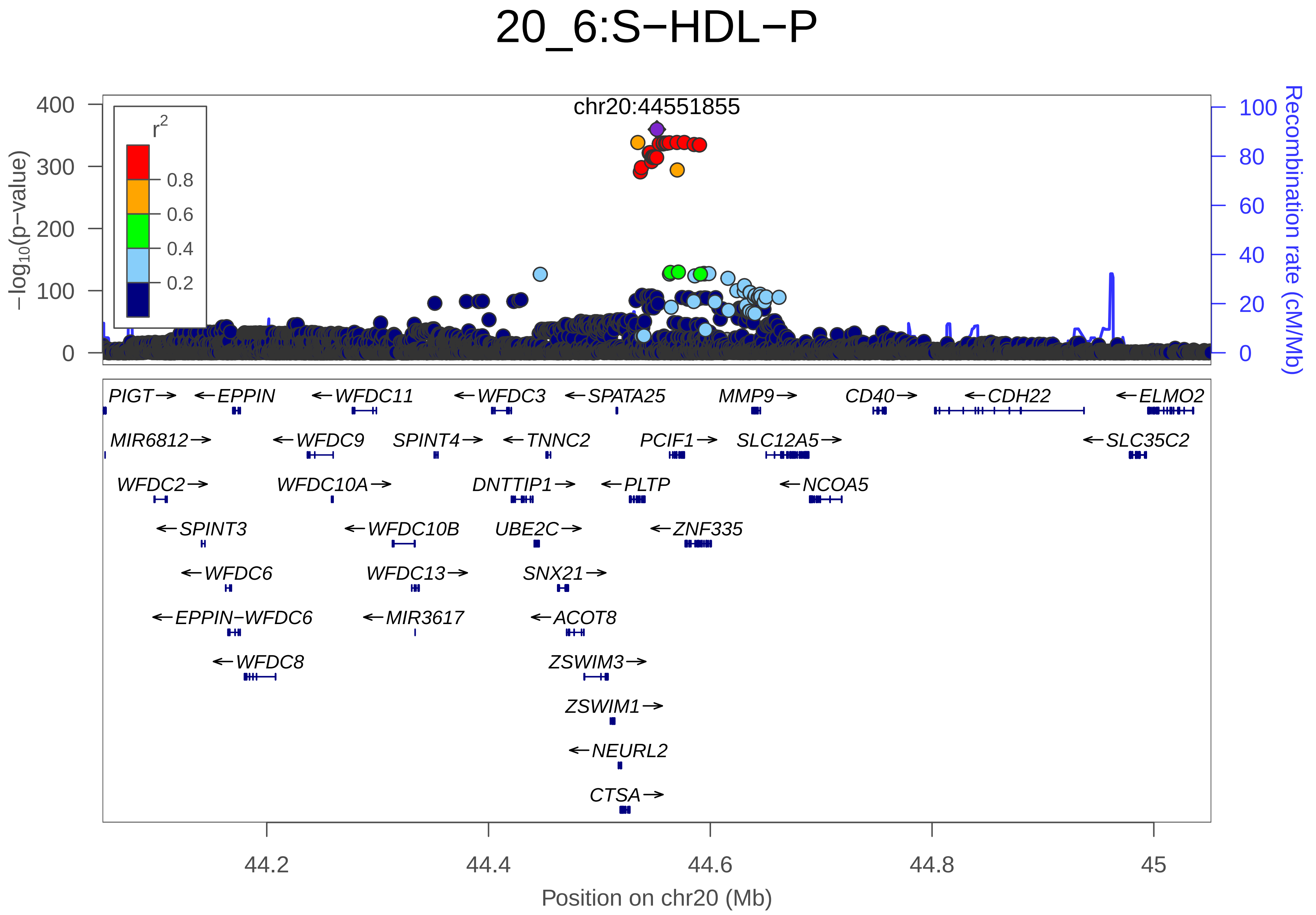

20_6:S−HDL−P
chr20:44551855
r2
0.8
0.6
0.4
0.2
−log10(p−value)
Recombination rate (cM/Mb)
400
300
200
100
0
100
80
60
40
20
0
PIGT→
←EPPIN
←WFDC11
←WFDC3
←SPATA25
MMP9→
CD40→
←CDH22
←ELMO2
MIR6812→
←WFDC9
SPINT4→
←TNNC2
PCIF1→
SLC12A5→
←SLC35C2
WFDC2→
WFDC10A→
DNTTIP1→
←PLTP
←NCOA5
←SPINT3
←WFDC10B
UBE2C→
←ZNF335
←WFDC6
WFDC13→
SNX21→
←EPPIN−WFDC6
←MIR3617
←ACOT8
←WFDC8
ZSWIM3→
ZSWIM1→
←NEURL2
CTSA→
44.2
44.4
44.6
44.8
45
Position on chr20 (Mb)

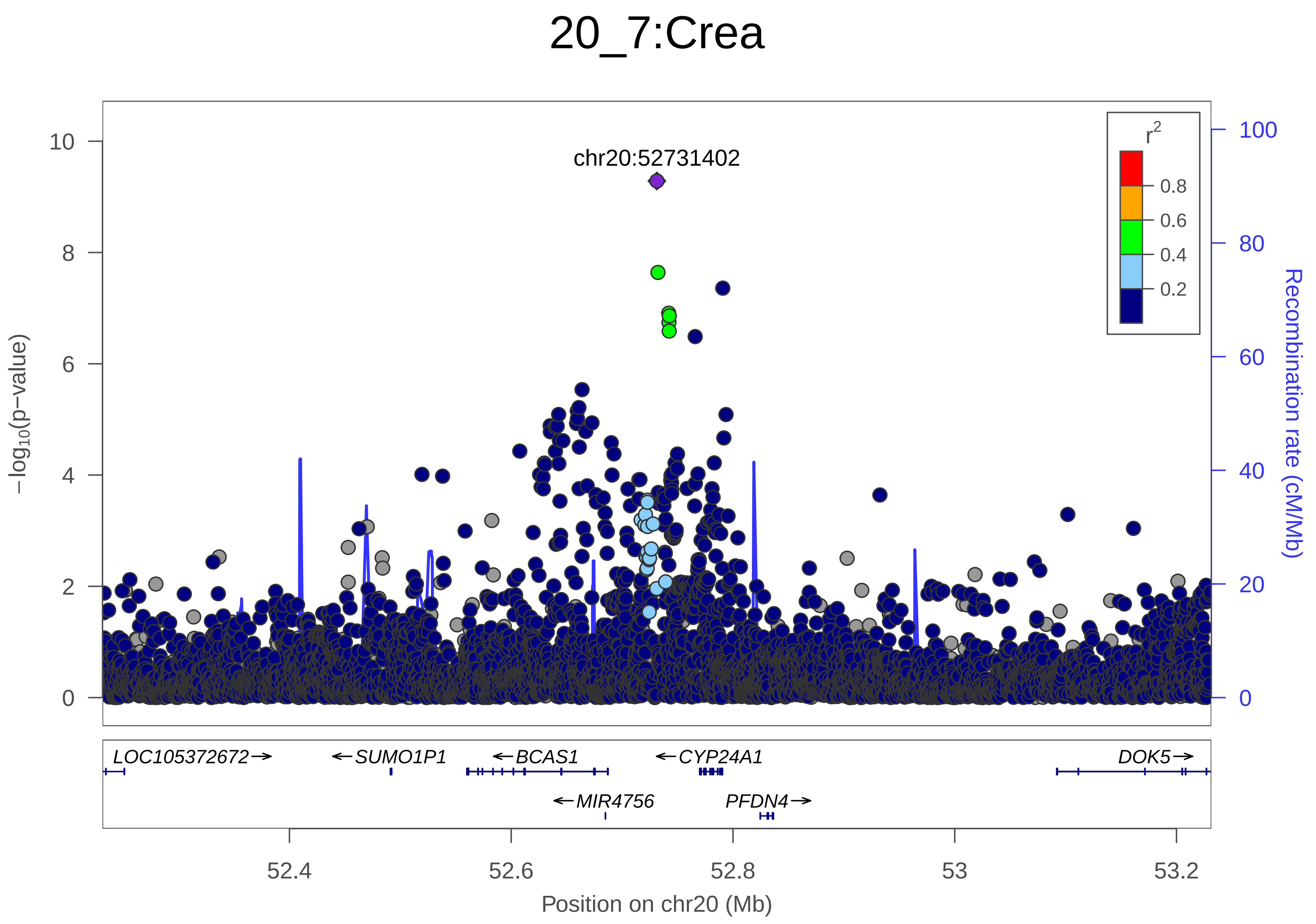
20_7:Crea
chr20:52731402
r2
0.8
0.6
0.4
0.2
10
8
6
4
2
0
−log10(p−value)
100
80
60
40
20
0
Recombination rate (cM/Mb)
LOC105372672→
←SUMO1P1
←BCAS1
←CYP24A1
DOK5→
←MIR4756
PFDN4→
52.4
52.6
52.8
53
53.2
Position on chr20 (Mb)

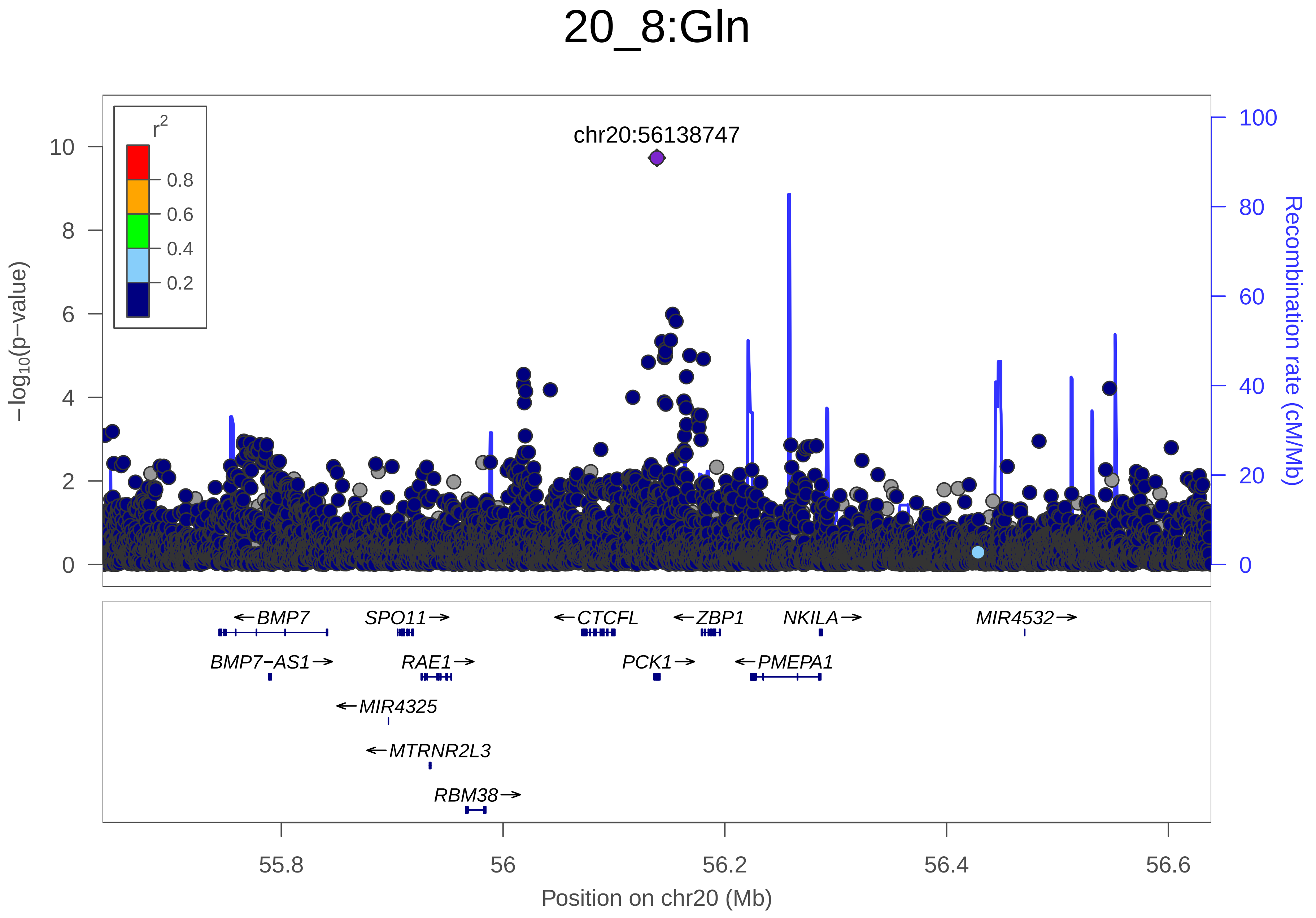

20_8:Gln
chr20:56138747
r2
0.8
0.6
0.4
0.2
−log10(p−value)
Recombination rate (cM/Mb)
BMP7
SPO11
CTCFL
ZBP1
NKILA
MIR4532
BMP7−AS1
RAE1
PCK1
PMEPA1
MIR4325
MTRNR2L3
RBM38
55.8
56
56.2
56.4
56.6
Position on chr20 (Mb)

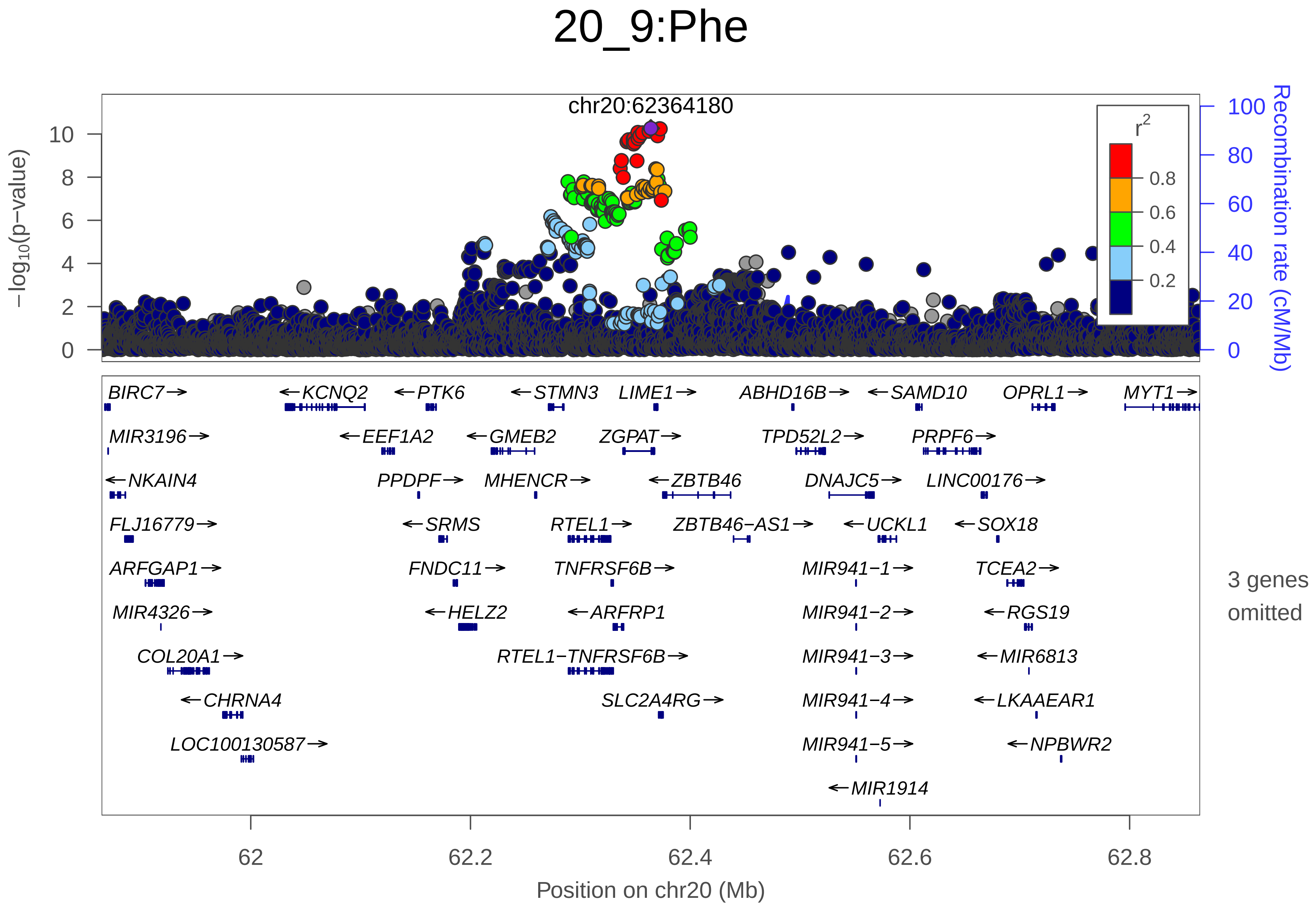

20_9:Phe
chr20:62364180
−log10(p−value)
Recombination rate (cM/Mb)
r2
0.8
0.6
0.4
0.2
0
2
4
6
8
10
0
20
40
60
80
100
BIRC7→
←KCNQ2
←PTK6
←STMN3
LIME1→
ABHD16B→
←SAMD10
OPRL1→
MYT1→
MIR3196→
←EEF1A2
←GMEB2
ZGPAT→
TPD52L2→
PRPF6→
←NKAIN4
PPDPF→
MHENCR→
←ZBTB46
DNAJC5→
LINC00176→
FLJ16779→
←SRMS
RTEL1→
ZBTB46−AS1→
←UCKL1
←SOX18
ARFGAP1→
FNDC11→
TNFRSF6B→
MIR941−1→
TCEA2→
MIR4326→
←HELZ2
←ARFRP1
MIR941−2→
←RGS19
COL20A1→
RTEL1−TNFRSF6B→
MIR941−3→
←MIR6813
←CHRNA4
SLC2A4RG→
MIR941−4→
←LKAAEAR1
LOC100130587→
MIR941−5→
←NPBWR2
←MIR1914
3 genes omitted
62
62.2
62.4
62.6
62.8
Position on chr20 (Mb)

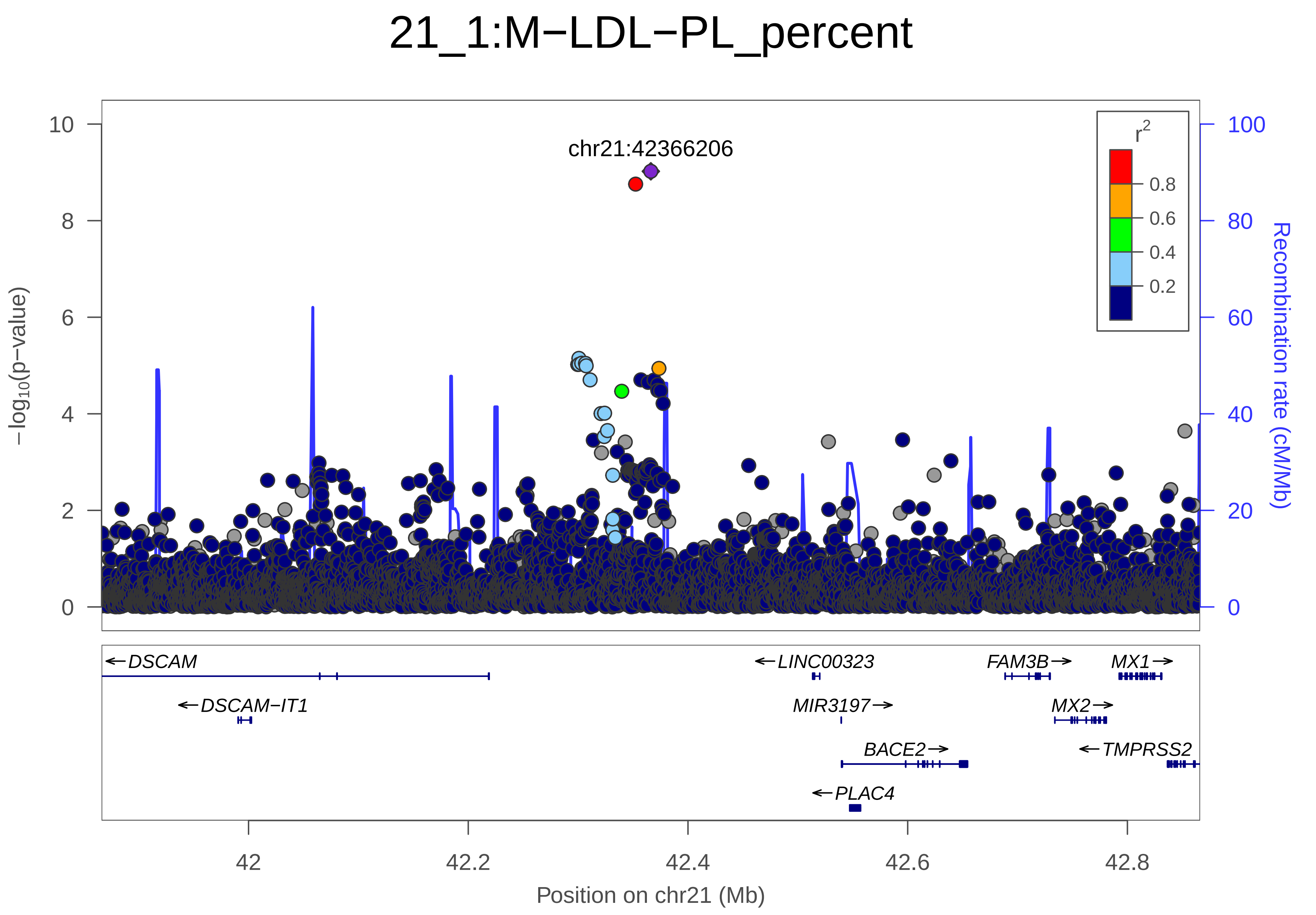

21_1:M−LDL−PL_percent
chr21:42366206
r²
0.8
0.6
0.4
0.2
−log10(p−value)
Recombination rate (cM/Mb)
10
8
6
4
2
0
100
80
60
40
20
0
←DSCAM
←LINC00323
FAM3B→
MX1→
←DSCAM−IT1
MIR3197→
MX2→
BACE2→
←TMPRSS2
←PLAC4
42
42.2
42.4
42.6
42.8
Position on chr21 (Mb)

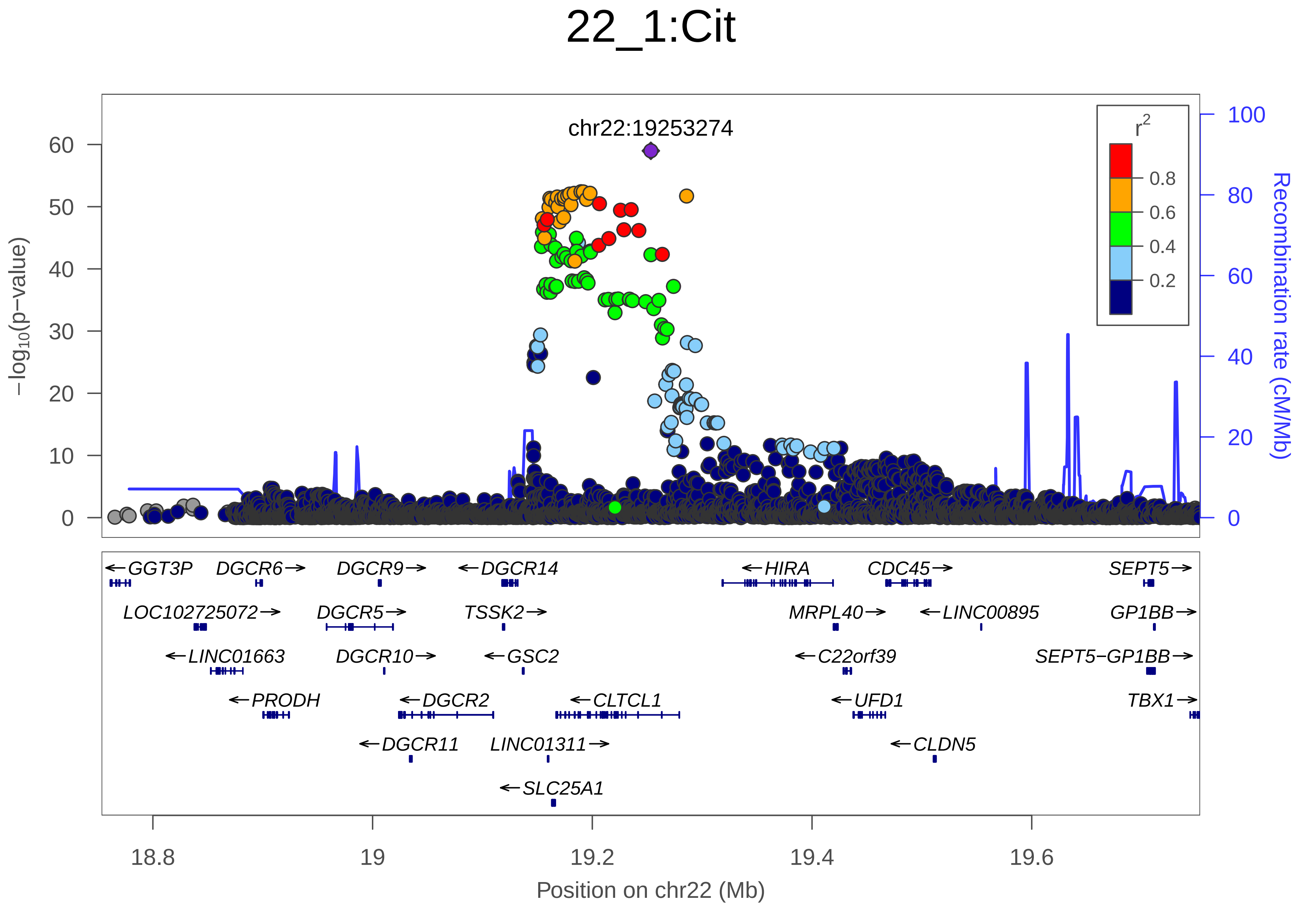

22_1:Cit
chr22:19253274
−log10(p−value)
Recombination rate (cM/Mb)
r2
0.8
0.6
0.4
0.2
0
10
20
30
40
50
60
100
80
60
40
20
0
GGT3P
DGCR6
DGCR9
DGCR14
HIRA
CDC45
SEPT5
LOC102725072
DGCR5
TSSK2
MRPL40
LINC00895
GP1BB
LINC01663
DGCR10
GSC2
C22orf39
SEPT5−GP1BB
PRODH
DGCR2
CLTCL1
UFD1
TBX1
DGCR11
LINC01311
CLDN5
SLC25A1
18.8
19
19.2
19.4
19.6
Position on chr22 (Mb)

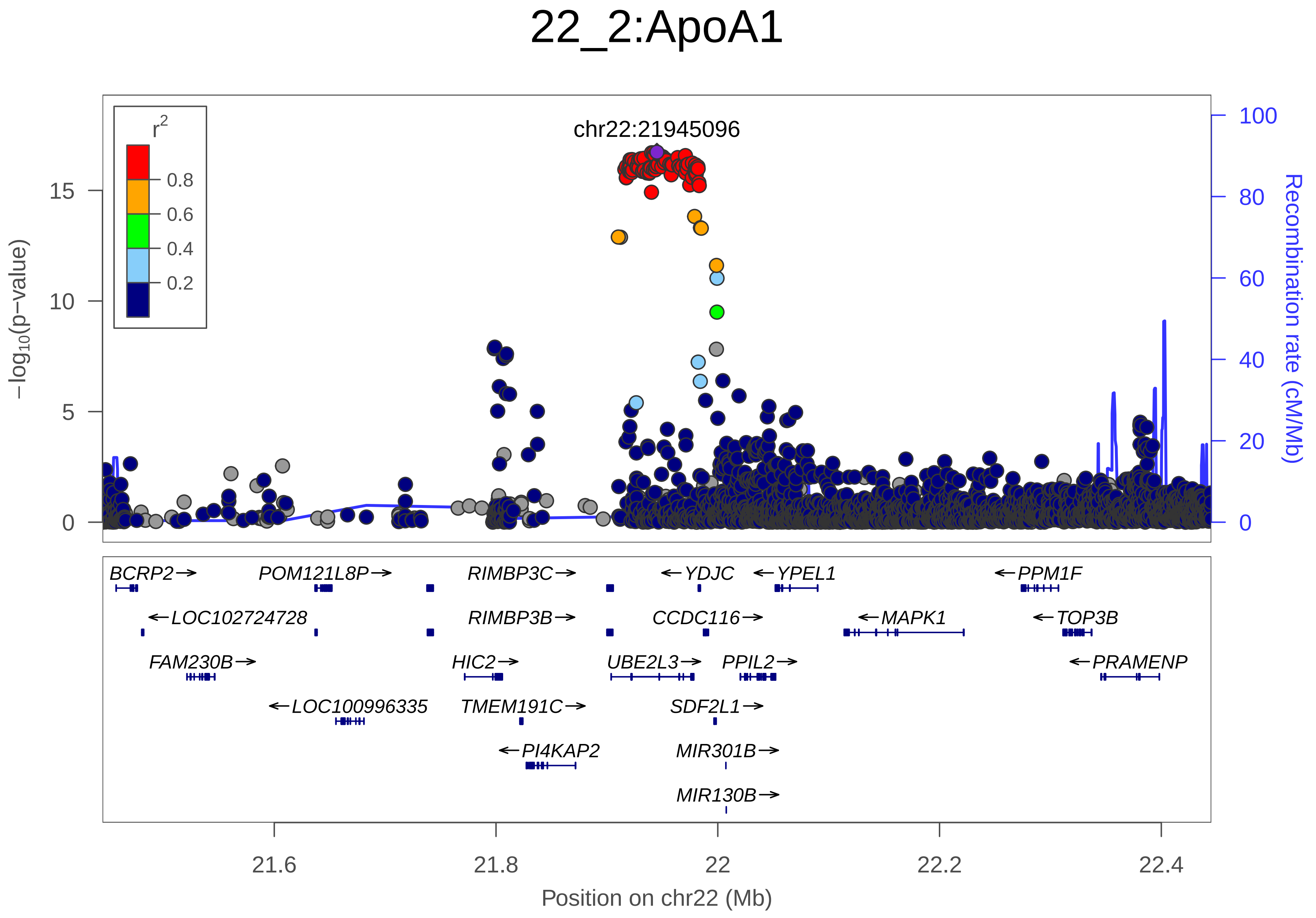
22_2:ApoA1
chr22:21945096
r2
0.8
0.6
0.4
0.2
−log10(p−value)
0
5
10
15
Recombination rate (cM/Mb)
0
20
40
60
80
100
BCRP2→
POM121L8P→
RIMBP3C→
←YDJC
←YPEL1
←PPM1F
←LOC102724728
RIMBP3B→
CCDC116→
←MAPK1
←TOP3B
FAM230B→
HIC2→
UBE2L3→
PPIL2→
←PRAMENP
←LOC100996335
TMEM191C→
SDF2L1→
←PI4KAP2
MIR301B→
MIR130B→
21.6
21.8
22
22.2
22.4
Position on chr22 (Mb)

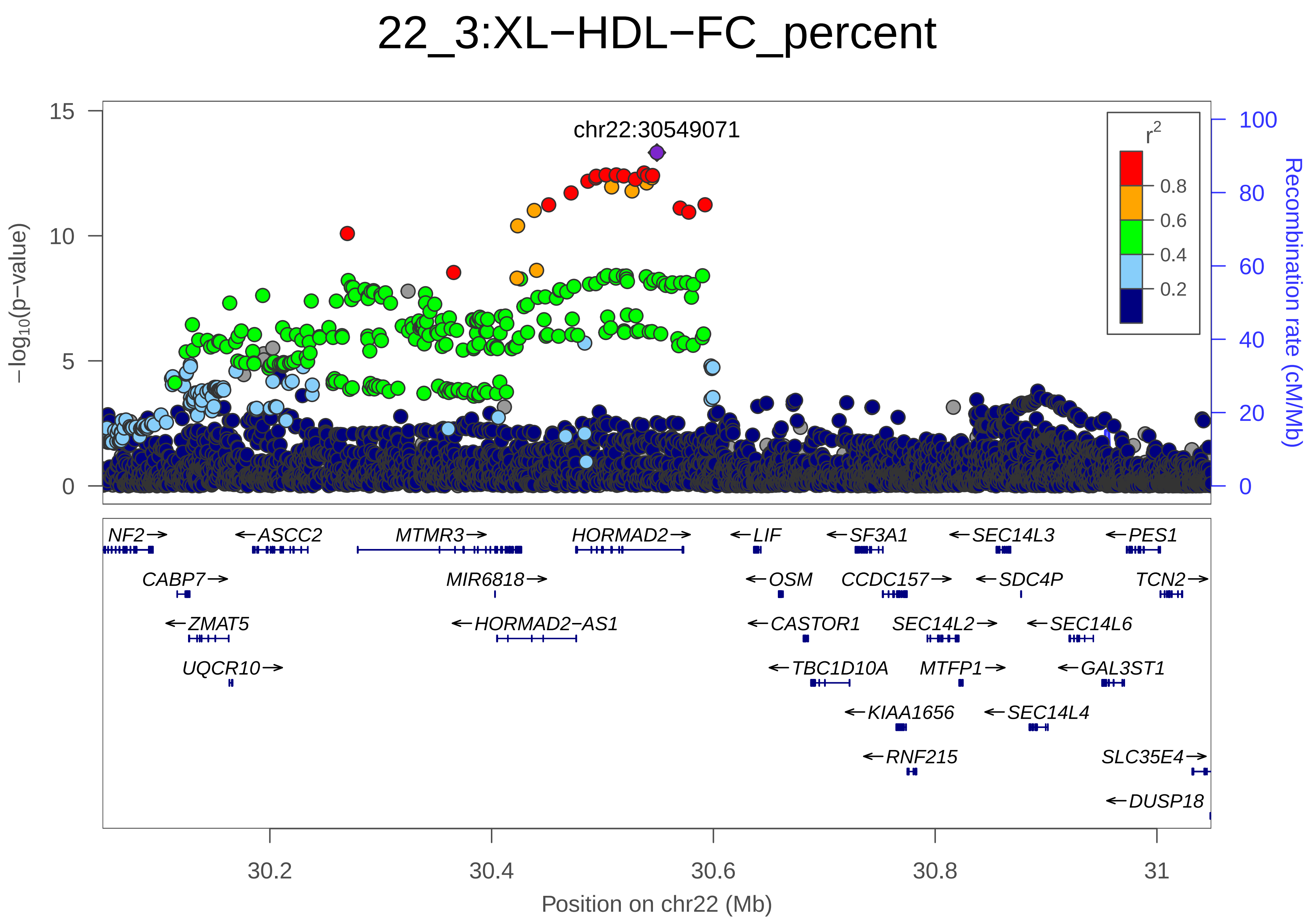

22_3:XL−HDL−FC_percent
chr22:30549071
−log10(p−value)
Recombination rate (cM/Mb)
r2
0.8
0.6
0.4
0.2
NF2→
←ASCC2
MTMR3→
HORMAD2→
←LIF
←SF3A1
←SEC14L3
←PES1
CABP7→
MIR6818→
←OSM
CCDC157→
←SDC4P
TCN2→
←ZMAT5
←HORMAD2−AS1
←CASTOR1
SEC14L2→
←SEC14L6
UQCR10→
←TBC1D10A
MTFP1→
←GAL3ST1
←KIAA1656
←SEC14L4
←RNF215
SLC35E4→
←DUSP18
Position on chr22 (Mb)

22_4:S−HDL−TG_percent

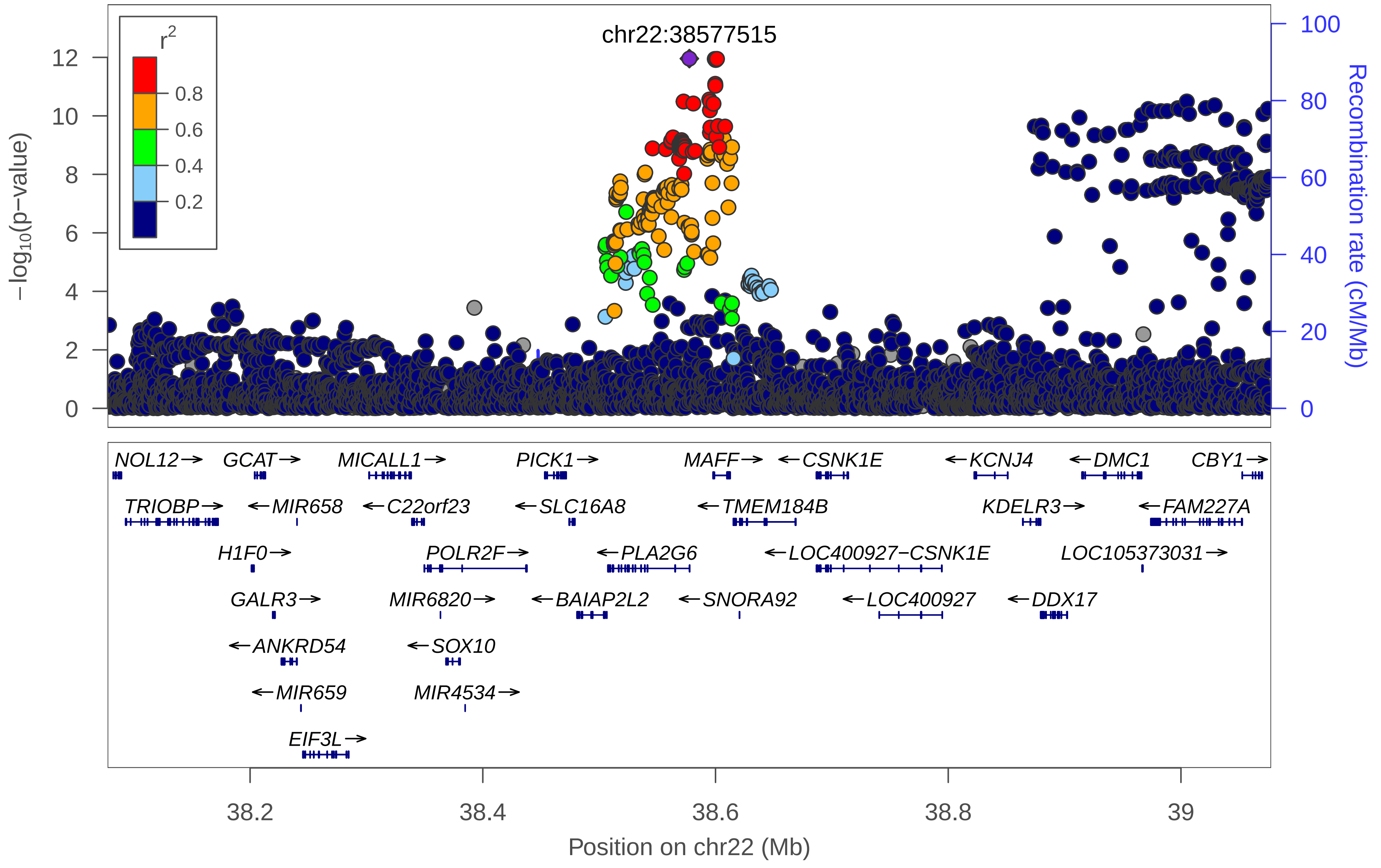

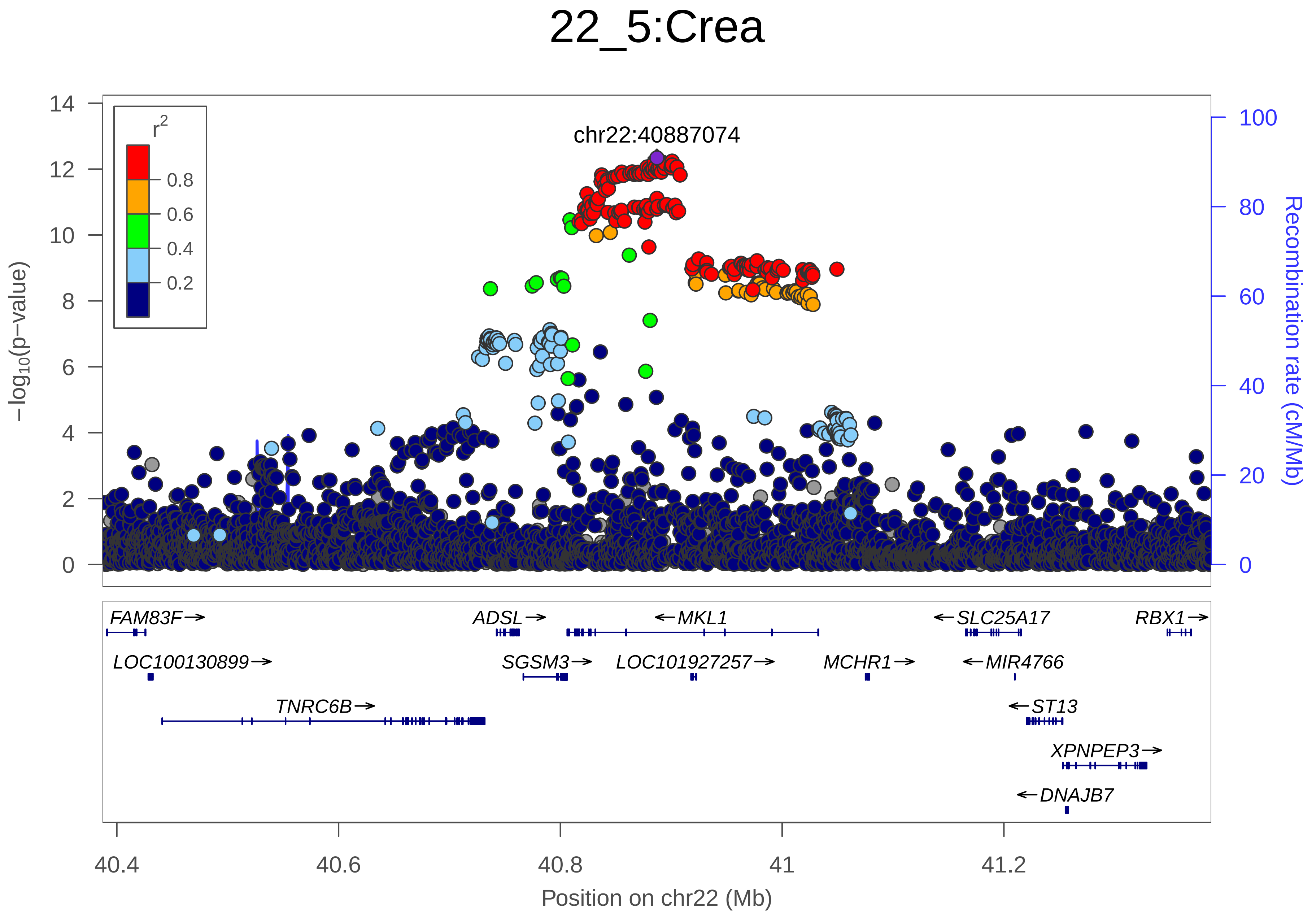

22_5:Crea
chr22:40887074
r2
0.8
0.6
0.4
0.2
−log10(p−value)
14
12
10
8
6
4
2
0
100
80
60
40
20
0
Recombination rate (cM/Mb)
FAM83F→
LOC100130899→
TNRC6B→
ADSL→
SGSM3→
←MKL1
LOC101927257→
MCHR1→
←SLC25A17
←MIR4766
←ST13
XPNPEP3→
←DNAJB7
RBX1→
40.4
40.6
40.8
41
41.2
Position on chr22 (Mb)

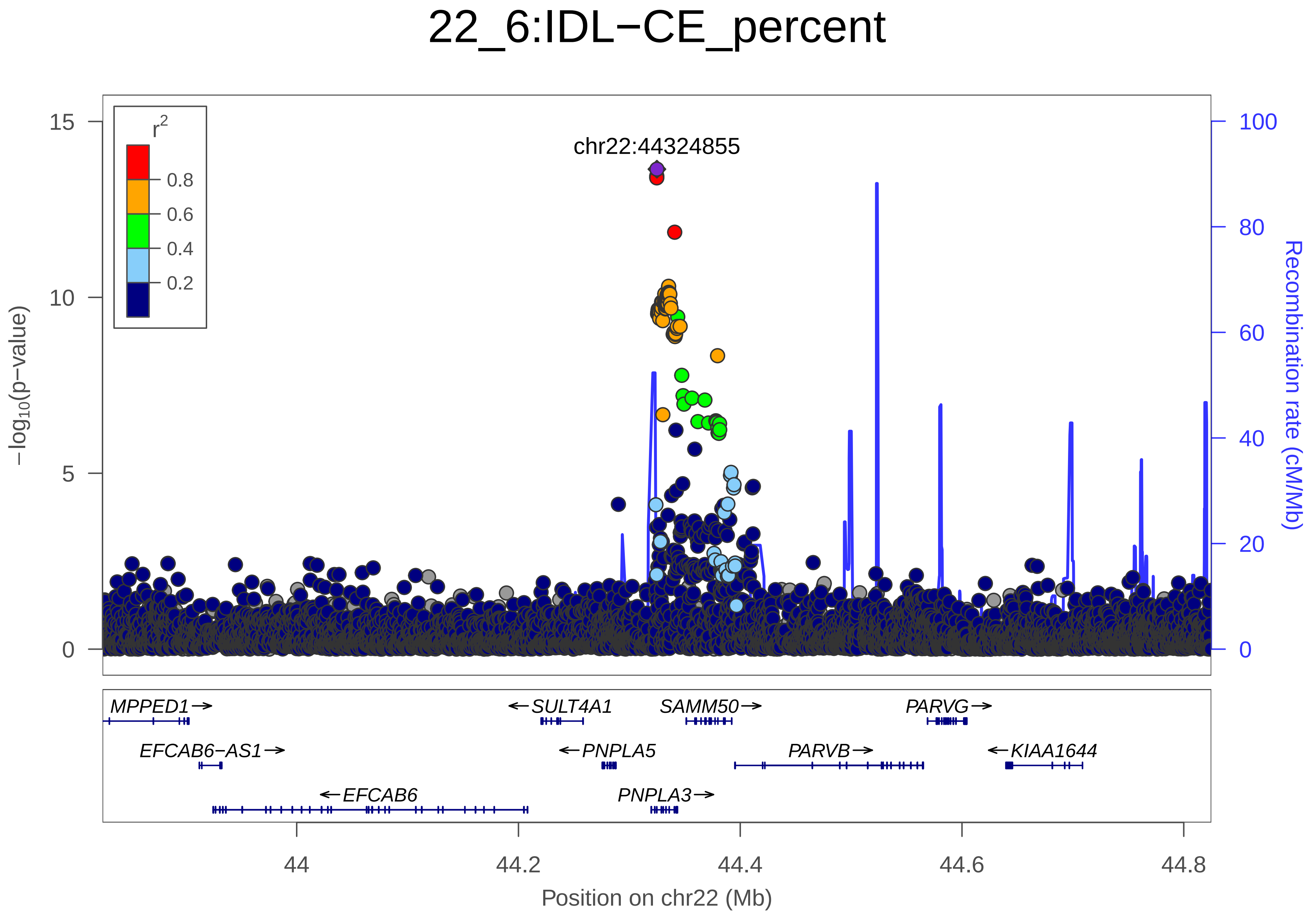

22_6:IDL−CE_percent
chr22:44324855
r2
0.8
0.6
0.4
0.2
15
10
5
0
100
80
60
40
20
0
−log10(p−value)
Recombination rate (cM/Mb)
MPPED1→
←SULT4A1
SAMM50→
PARVG→
EFCAB6−AS1→
←PNPLA5
PARVB→
←KIAA1644
←EFCAB6
PNPLA3→
44
44.2
44.4
44.6
44.8
Position on chr22 (Mb)
