# Supplementary material for: Genome-wide characterization of circulating metabolic biomarkers
Source: Nature. 2024 Mar 6;628(8006):130–8. doi: 10.1038/s41586-024-07148-y (PMC10990933; doi:10.1038/s41586-024-07148-y)
Supplement: Supplementary file 5 — Supplementary Fig. 3. Forest plots showing the associations of the lead SNPs in each cohort. [file 41586_2024_7148_MOESM5_ESM.pdf]

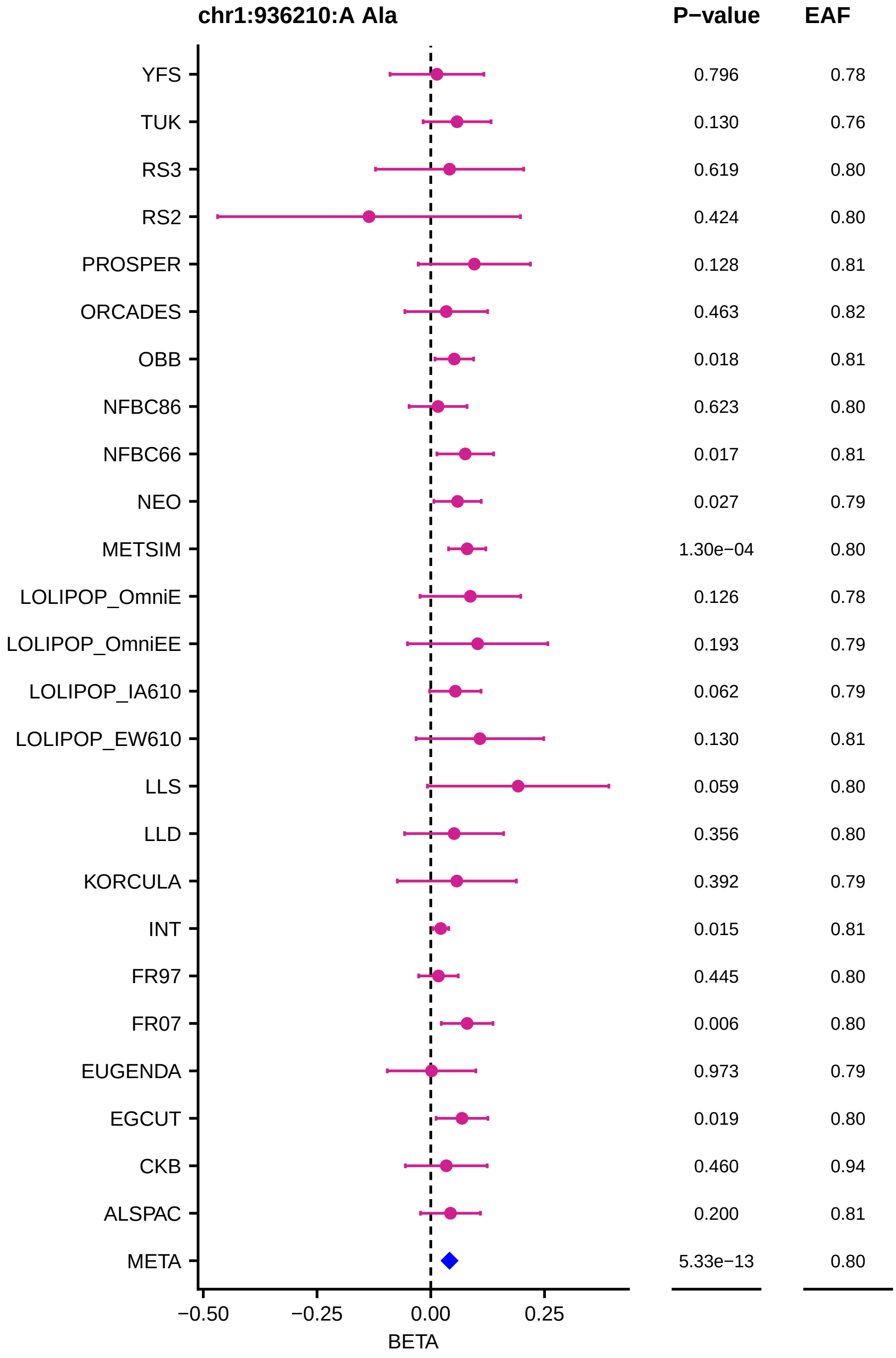
chr1:936210:A Ala
P−value
EAF
YFS 0.796 0.78
TUK 0.130 0.76
RS3 0.619 0.80
RS2 0.424 0.80
PROSPER 0.128 0.81
ORCADES 0.463 0.82
OBB 0.018 0.81
NFBC86 0.623 0.80
NFBC66 0.017 0.81
NEO 0.027 0.79
METSIM 1.30e−04 0.80
LOLIPOP_OmniE 0.126 0.78
LOLIPOP_OmniEE 0.193 0.79
LOLIPOP_IA610 0.062 0.79
LOLIPOP_EW610 0.130 0.81
LLS 0.059 0.80
LLD 0.356 0.80
KORCULA 0.392 0.79
INT 0.015 0.81
FR97 0.445 0.80
FR07 0.006 0.80
EUGENDA 0.973 0.79
EGCUT 0.019 0.80
CKB 0.460 0.94
ALSPAC 0.200 0.81
META 5.33e−13 0.80
−0.50
−0.25
0.00
0.25
BETA

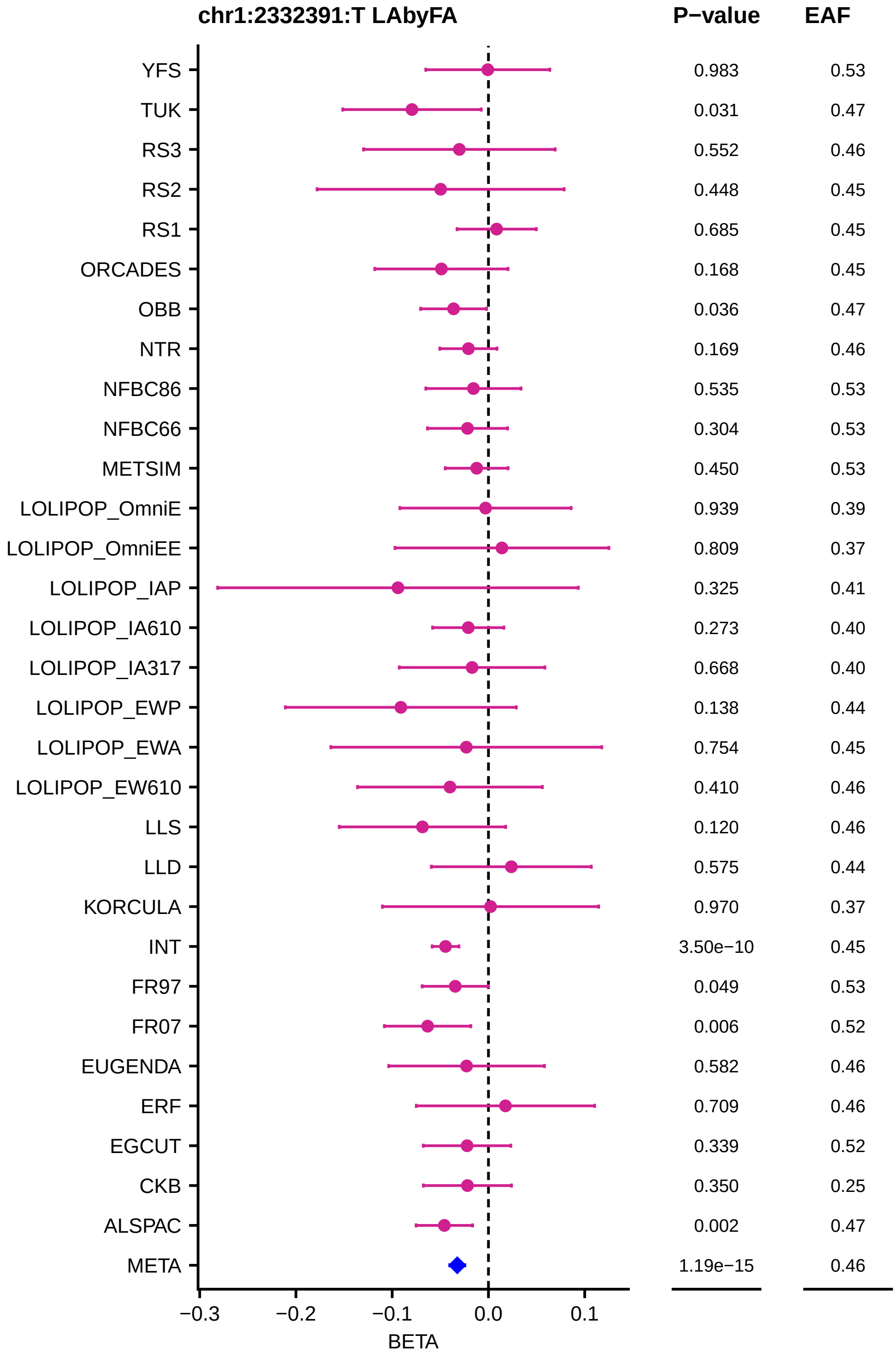
chr1:2332391:T LAbyFA
P−value
EAF
YFS 0.983 0.53
TUK 0.031 0.47
RS3 0.552 0.46
RS2 0.448 0.45
RS1 0.685 0.45
ORCADES 0.168 0.45
OBB 0.036 0.47
NTR 0.169 0.46
NFBC86 0.535 0.53
NFBC66 0.304 0.53
METSIM 0.450 0.53
LOLIPOP_OmniE 0.939 0.39
LOLIPOP_OmniEE 0.809 0.37
LOLIPOP_IAP 0.325 0.41
LOLIPOP_IA610 0.273 0.40
LOLIPOP_IA317 0.668 0.40
LOLIPOP_EWP 0.138 0.44
LOLIPOP_EWA 0.754 0.45
LOLIPOP_EW610 0.410 0.46
LLS 0.120 0.46
LLD 0.575 0.44
KORCULA 0.970 0.37
INT 3.50e−10 0.45
FR97 0.049 0.53
FR07 0.006 0.52
EUGENDA 0.582 0.46
ERF 0.709 0.46
EGCUT 0.339 0.52
CKB 0.350 0.25
ALSPAC 0.002 0.47
META 1.19e−15 0.46
−0.3
−0.2
−0.1
0.0
0.1
BETA

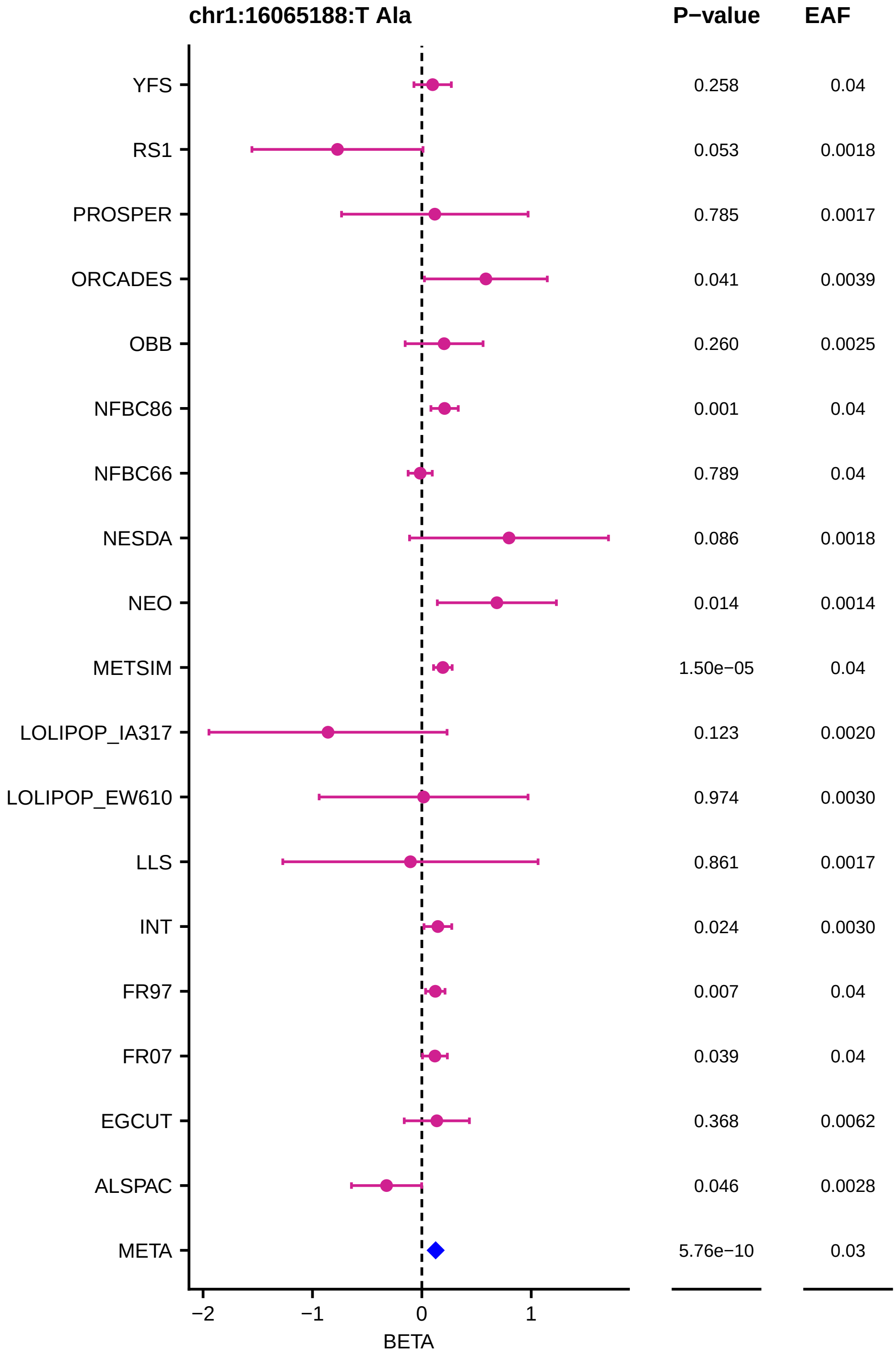
chr1:16065188:T Ala
P−value
EAF
YFS
RS1
PROSPER
ORCADES
OBB
NFBC86
NFBC66
NESDA
NEO
METSIM
LOLIPOP_IA317
LOLIPOP_EW610
LLS
INT
FR97
FR07
EGCUT
ALSPAC
META
0.258
0.053
0.785
0.041
0.260
0.001
0.789
0.086
0.014
1.50e−05
0.123
0.974
0.861
0.024
0.007
0.039
0.368
0.046
5.76e−10
0.04
0.0018
0.0017
0.0039
0.0025
0.04
0.04
0.0018
0.0014
0.04
0.0020
0.0030
0.0017
0.0030
0.04
0.04
0.0062
0.0028
0.03
−2
−1
0
1
BETA

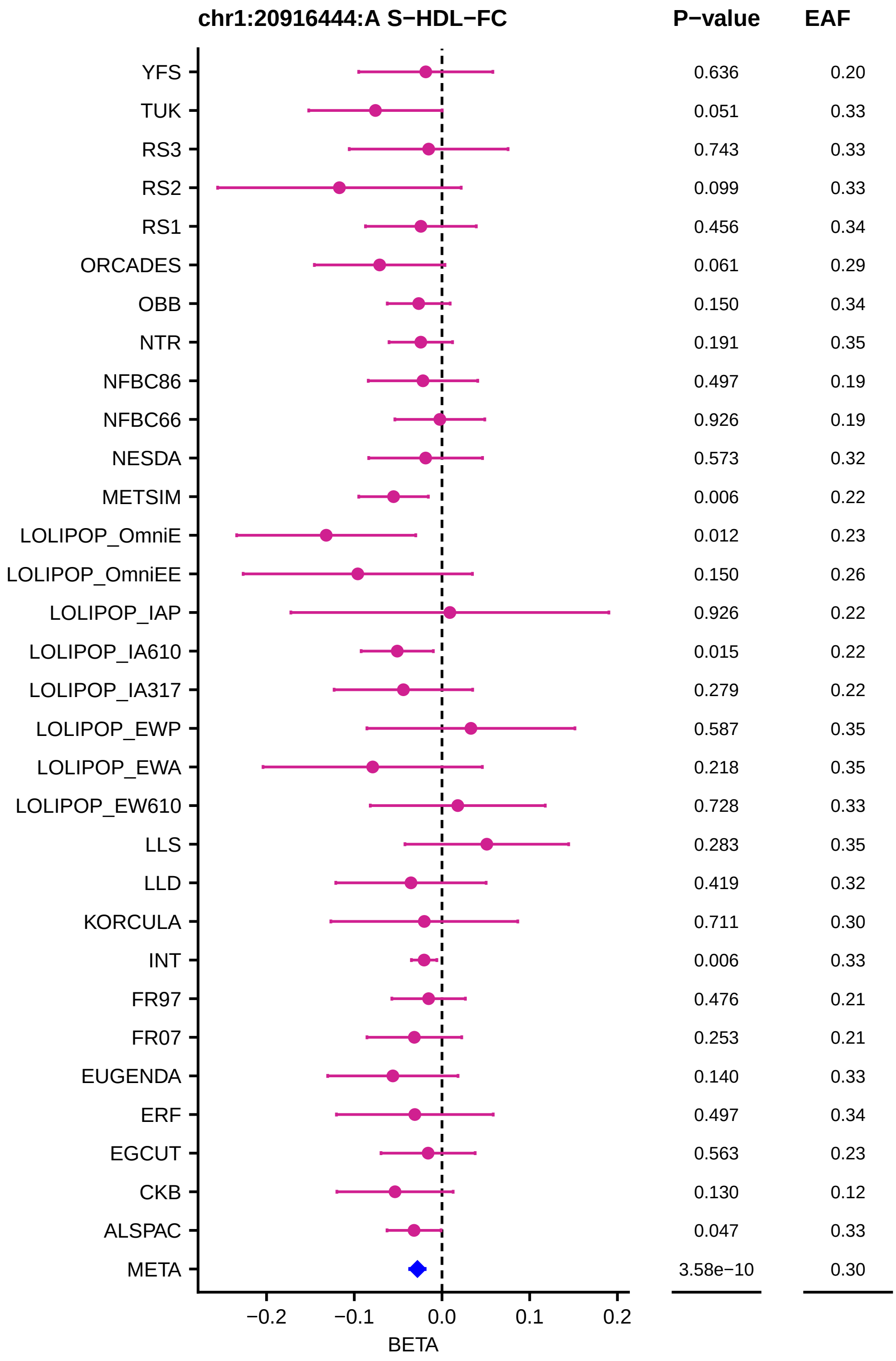
chr1:20916444:A S−HDL−FC
P−value
EAF
YFS 0.636 0.20
TUK 0.051 0.33
RS3 0.743 0.33
RS2 0.099 0.33
RS1 0.456 0.34
ORCADES 0.061 0.29
OBB 0.150 0.34
NTR 0.191 0.35
NFBC86 0.497 0.19
NFBC66 0.926 0.19
NESDA 0.573 0.32
METSIM 0.006 0.22
LOLIPOP_OmniE 0.012 0.23
LOLIPOP_OmniEE 0.150 0.26
LOLIPOP_IAP 0.926 0.22
LOLIPOP_IA610 0.015 0.22
LOLIPOP_IA317 0.279 0.22
LOLIPOP_EWP 0.587 0.35
LOLIPOP_EWA 0.218 0.35
LOLIPOP_EW610 0.728 0.33
LLS 0.283 0.35
LLD 0.419 0.32
KORCULA 0.711 0.30
INT 0.006 0.33
FR97 0.476 0.21
FR07 0.253 0.21
EUGENDA 0.140 0.33
ERF 0.497 0.34
EGCUT 0.563 0.23
CKB 0.130 0.12
ALSPAC 0.047 0.33
META 3.58e−10 0.30
−0.2
−0.1
0.0
0.1
0.2
BETA

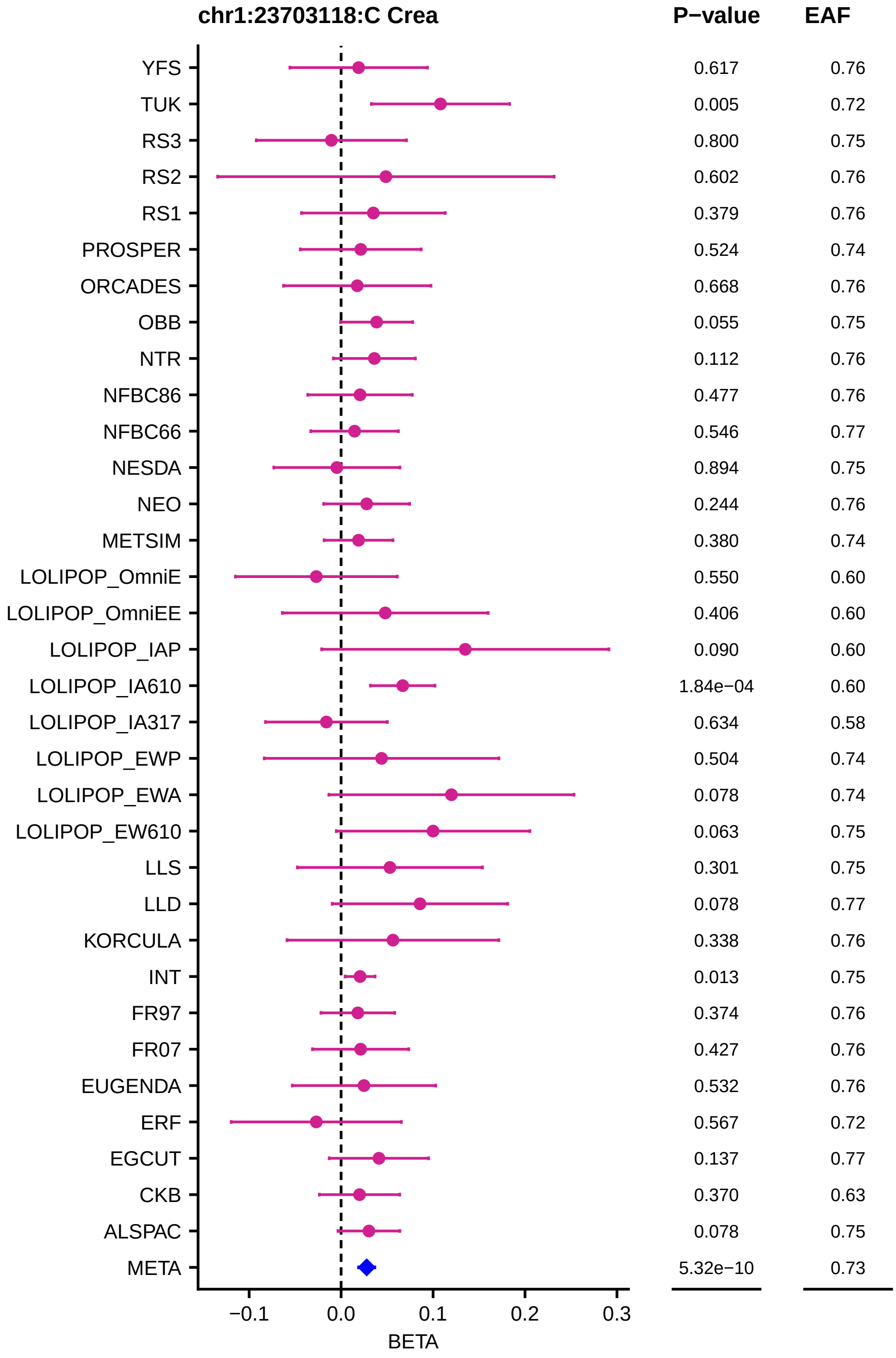
chr1:23703118:C Crea
P−value
EAF
YFS 0.617 0.76
TUK 0.005 0.72
RS3 0.800 0.75
RS2 0.602 0.76
RS1 0.379 0.76
PROSPER 0.524 0.74
ORCADES 0.668 0.76
OBB 0.055 0.75
NTR 0.112 0.76
NFBC86 0.477 0.76
NFBC66 0.546 0.77
NESDA 0.894 0.75
NEO 0.244 0.76
METSIM 0.380 0.74
LOLIPOP_OmniE 0.550 0.60
LOLIPOP_OmniEE 0.406 0.60
LOLIPOP_IAP 0.090 0.60
LOLIPOP_IA610 1.84e−04 0.60
LOLIPOP_IA317 0.634 0.58
LOLIPOP_EWP 0.504 0.74
LOLIPOP_EWA 0.078 0.74
LOLIPOP_EW610 0.063 0.75
LLS 0.301 0.75
LLD 0.078 0.77
KORCULA 0.338 0.76
INT 0.013 0.75
FR97 0.374 0.76
FR07 0.427 0.76
EUGENDA 0.532 0.76
ERF 0.567 0.72
EGCUT 0.137 0.77
CKB 0.370 0.63
ALSPAC 0.078 0.75
META 5.32e−10 0.73
−0.1
0.0
0.1
0.2
0.3
BETA

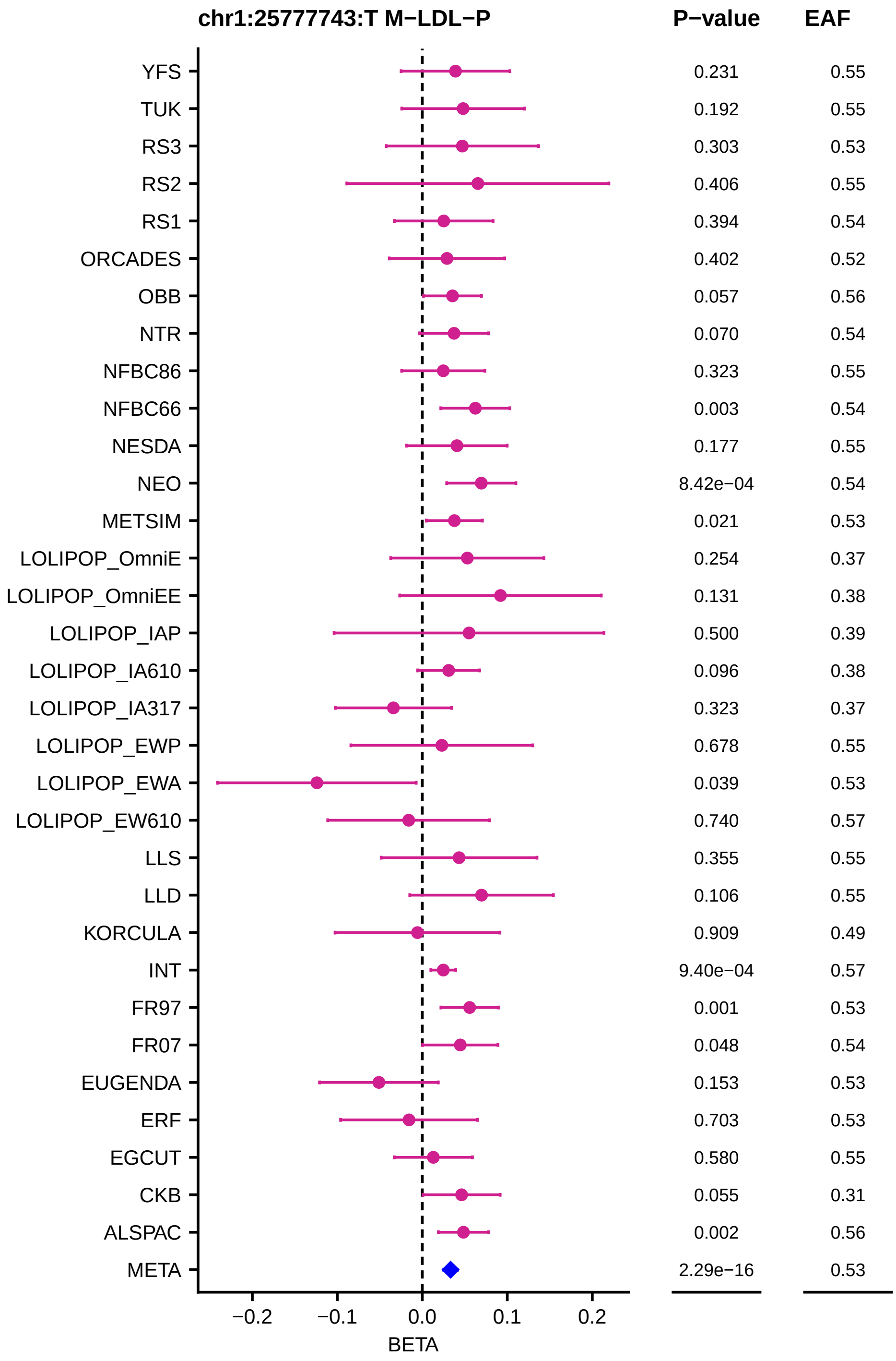

chr1:25777743:T M−LDL−P
P−value
EAF
YFS 0.231 0.55
TUK 0.192 0.55
RS3 0.303 0.53
RS2 0.406 0.55
RS1 0.394 0.54
ORCADES 0.402 0.52
OBB 0.057 0.56
NTR 0.070 0.54
NFBC86 0.323 0.55
NFBC66 0.003 0.54
NESDA 0.177 0.55
NEO 8.42e−04 0.54
METSIM 0.021 0.53
LOLIPOP_OmniE 0.254 0.37
LOLIPOP_OmniEE 0.131 0.38
LOLIPOP_IAP 0.500 0.39
LOLIPOP_IA610 0.096 0.38
LOLIPOP_IA317 0.323 0.37
LOLIPOP_EWP 0.678 0.55
LOLIPOP_EWA 0.039 0.53
LOLIPOP_EW610 0.740 0.57
LLS 0.355 0.55
LLD 0.106 0.55
KORCULA 0.909 0.49
INT 9.40e−04 0.57
FR97 0.001 0.53
FR07 0.048 0.54
EUGENDA 0.153 0.53
ERF 0.703 0.53
EGCUT 0.580 0.55
CKB 0.055 0.31
ALSPAC 0.002 0.56
META 2.29e−16 0.53
−0.2
−0.1
0.0
0.1
0.2
BETA

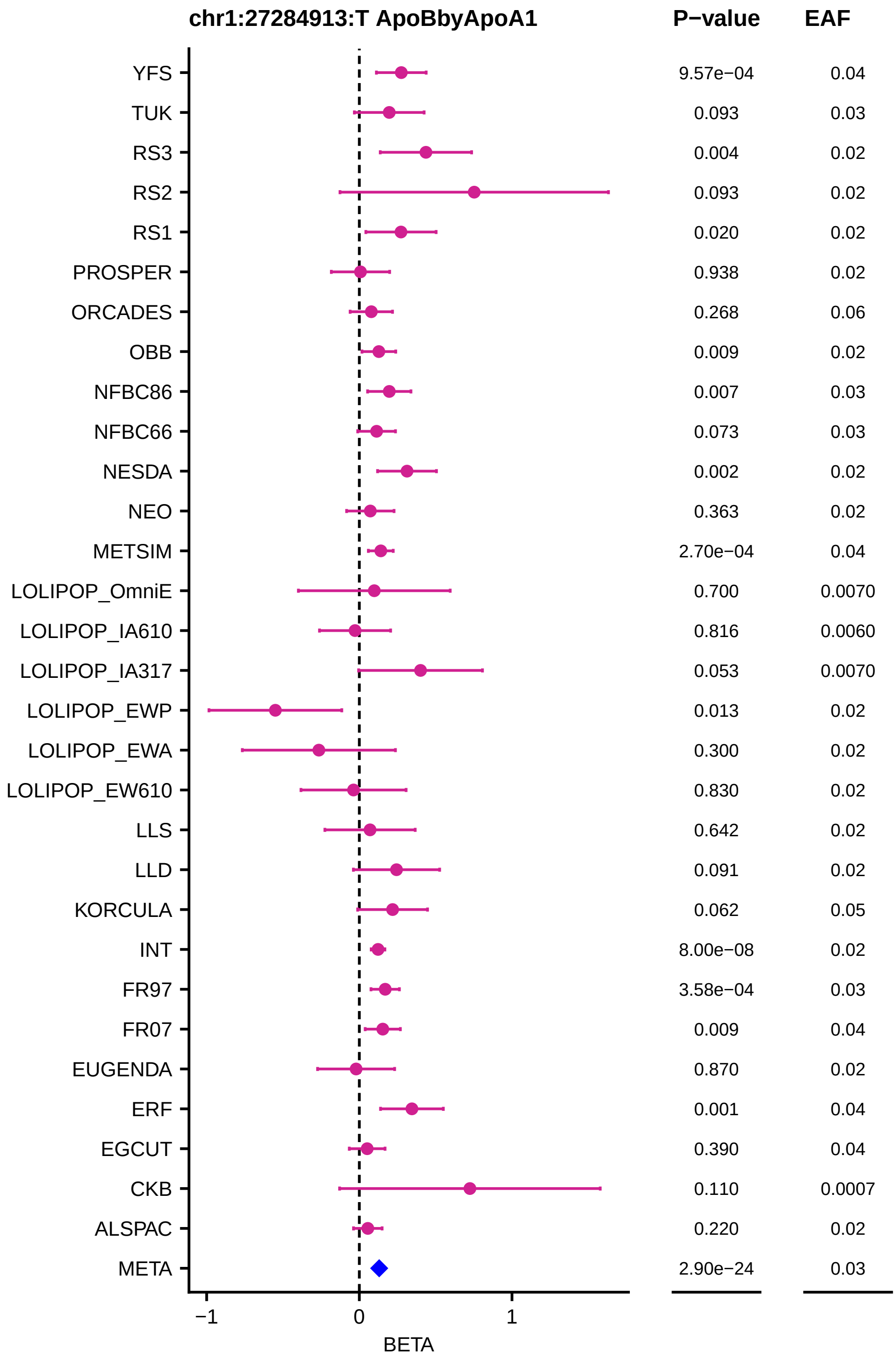
chr1:27284913:T ApoBbyApoA1
P−value
EAF
YFS
TUK
RS3
RS2
RS1
PROSPER
ORCADES
OBB
NFBC86
NFBC66
NESDA
NEO
METSIM
LOLIPOP_OmniE
LOLIPOP_IA610
LOLIPOP_IA317
LOLIPOP_EWP
LOLIPOP_EWA
LOLIPOP_EW610
LLS
LLD
KORCULA
INT
FR97
FR07
EUGENDA
ERF
EGCUT
CKB
ALSPAC
META
9.57e−04
0.093
0.004
0.093
0.020
0.938
0.268
0.009
0.007
0.073
0.002
0.363
2.70e−04
0.700
0.816
0.053
0.013
0.300
0.830
0.642
0.091
0.062
8.00e−08
3.58e−04
0.009
0.870
0.001
0.390
0.110
0.220
2.90e−24
0.04
0.03
0.02
0.02
0.02
0.02
0.06
0.02
0.03
0.03
0.02
0.02
0.04
0.0070
0.0060
0.0070
0.02
0.02
0.02
0.02
0.02
0.05
0.02
0.03
0.04
0.02
0.04
0.04
0.0007
0.02
0.03
−1
0
1
BETA

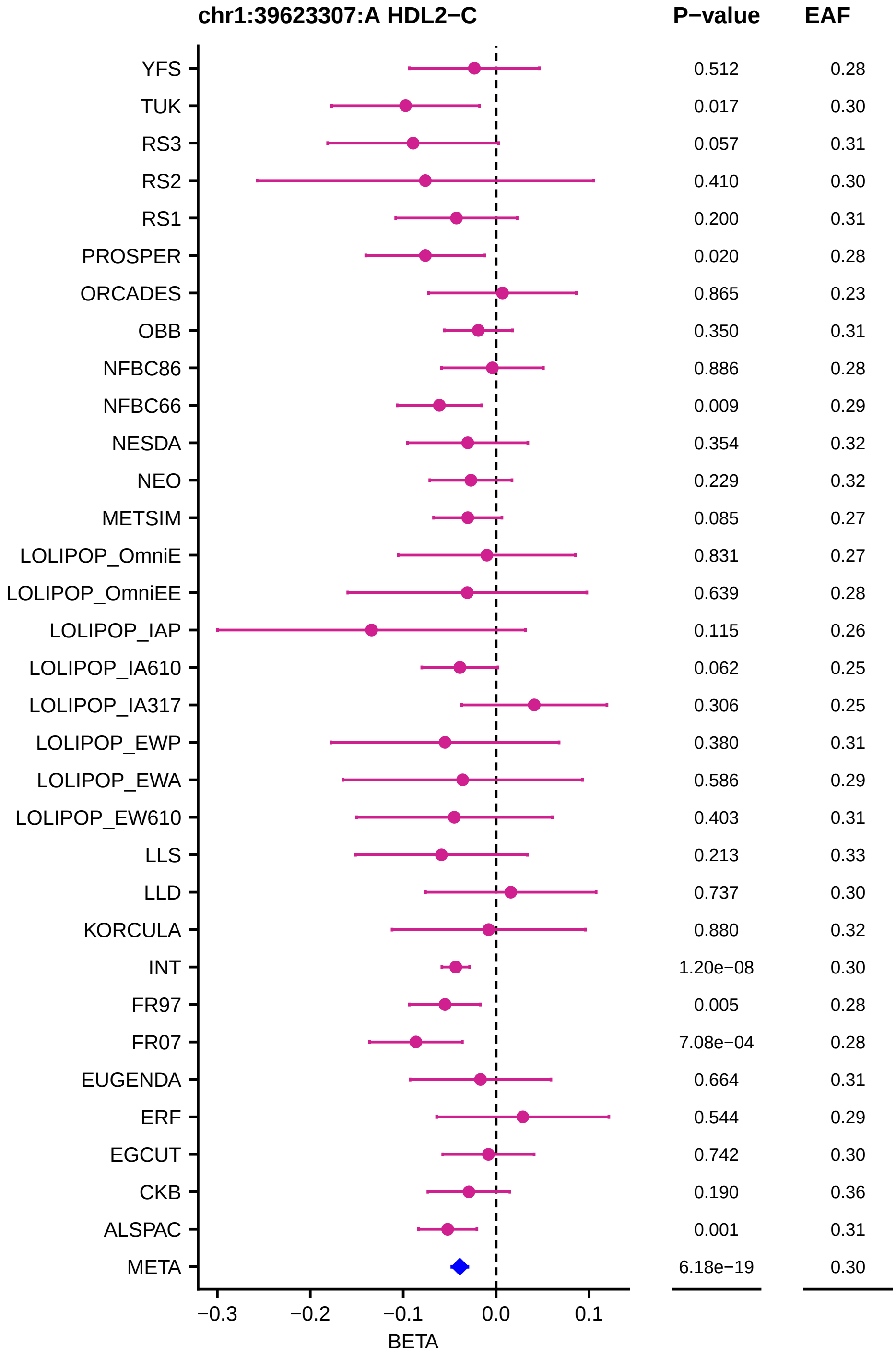
chr1:39623307:A HDL2−C
P−value
EAF
YFS 0.512 0.28
TUK 0.017 0.30
RS3 0.057 0.31
RS2 0.410 0.30
RS1 0.200 0.31
PROSPER 0.020 0.28
ORCADES 0.865 0.23
OBB 0.350 0.31
NFBC86 0.886 0.28
NFBC66 0.009 0.29
NESDA 0.354 0.32
NEO 0.229 0.32
METSIM 0.085 0.27
LOLIPOP_OmniE 0.831 0.27
LOLIPOP_OmniEE 0.639 0.28
LOLIPOP_IAP 0.115 0.26
LOLIPOP_IA610 0.062 0.25
LOLIPOP_IA317 0.306 0.25
LOLIPOP_EWP 0.380 0.31
LOLIPOP_EWA 0.586 0.29
LOLIPOP_EW610 0.403 0.31
LLS 0.213 0.33
LLD 0.737 0.30
KORCULA 0.880 0.32
INT 1.20e−08 0.30
FR97 0.005 0.28
FR07 7.08e−04 0.28
EUGENDA 0.664 0.31
ERF 0.544 0.29
EGCUT 0.742 0.30
CKB 0.190 0.36
ALSPAC 0.001 0.31
META 6.18e−19 0.30
−0.3
−0.2
−0.1
0.0
0.1
BETA

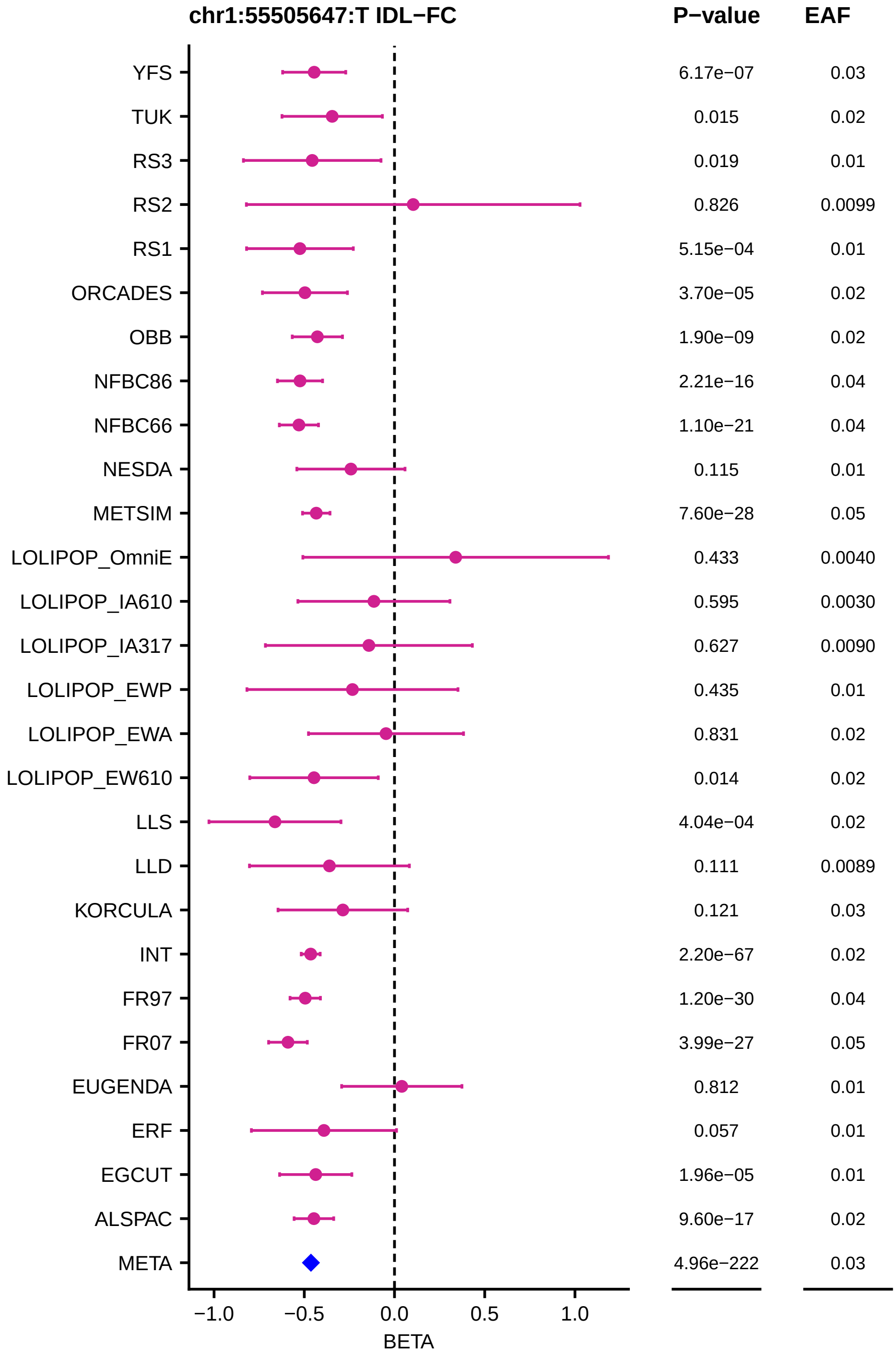

chr1:55505647:T IDL−FC
P−value
EAF
YFS
TUK
RS3
RS2
RS1
ORCADES
OBB
NFBC86
NFBC66
NESDA
METSIM
LOLIPOP_OmniE
LOLIPOP_IA610
LOLIPOP_IA317
LOLIPOP_EWP
LOLIPOP_EWA
LOLIPOP_EW610
LLS
LLD
KORCULA
INT
FR97
FR07
EUGENDA
ERF
EGCUT
ALSPAC
META
6.17e−07 0.03
0.015 0.02
0.019 0.01
0.826 0.0099
5.15e−04 0.01
3.70e−05 0.02
1.90e−09 0.02
2.21e−16 0.04
1.10e−21 0.04
0.115 0.01
7.60e−28 0.05
0.433 0.0040
0.595 0.0030
0.627 0.0090
0.435 0.01
0.831 0.02
0.014 0.02
4.04e−04 0.02
0.111 0.0089
0.121 0.03
2.20e−67 0.02
1.20e−30 0.04
3.99e−27 0.05
0.812 0.01
0.057 0.01
1.96e−05 0.01
9.60e−17 0.02
4.96e−222 0.03
−1.0
−0.5
0.0
0.5
1.0
BETA

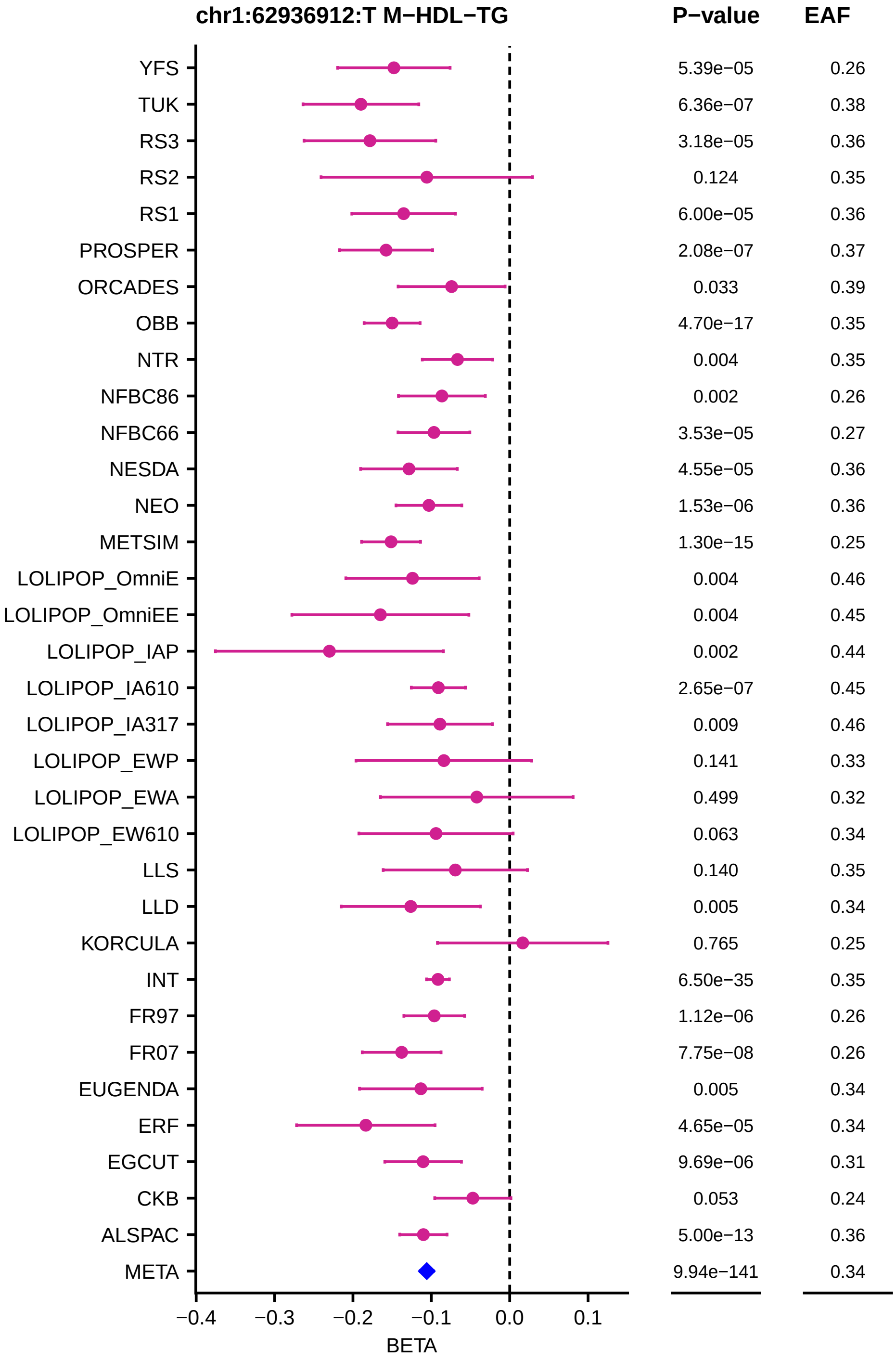

chr1:62936912:T M−HDL−TG
P−value
EAF
YFS 5.39e−05 0.26
TUK 6.36e−07 0.38
RS3 3.18e−05 0.36
RS2 0.124 0.35
RS1 6.00e−05 0.36
PROSPER 2.08e−07 0.37
ORCADES 0.033 0.39
OBB 4.70e−17 0.35
NTR 0.004 0.35
NFBC86 0.002 0.26
NFBC66 3.53e−05 0.27
NESDA 4.55e−05 0.36
NEO 1.53e−06 0.36
METSIM 1.30e−15 0.25
LOLIPOP_OmniE 0.004 0.46
LOLIPOP_OmniEE 0.004 0.45
LOLIPOP_IAP 0.002 0.44
LOLIPOP_IA610 2.65e−07 0.45
LOLIPOP_IA317 0.009 0.46
LOLIPOP_EWP 0.141 0.33
LOLIPOP_EWA 0.499 0.32
LOLIPOP_EW610 0.063 0.34
LLS 0.140 0.35
LLD 0.005 0.34
KORCULA 0.765 0.25
INT 6.50e−35 0.35
FR97 1.12e−06 0.26
FR07 7.75e−08 0.26
EUGENDA 0.005 0.34
ERF 4.65e−05 0.34
EGCUT 9.69e−06 0.31
CKB 0.053 0.24
ALSPAC 5.00e−13 0.36
META 9.94e−141 0.34
−0.4
−0.3
−0.2
−0.1
0.0
0.1
BETA

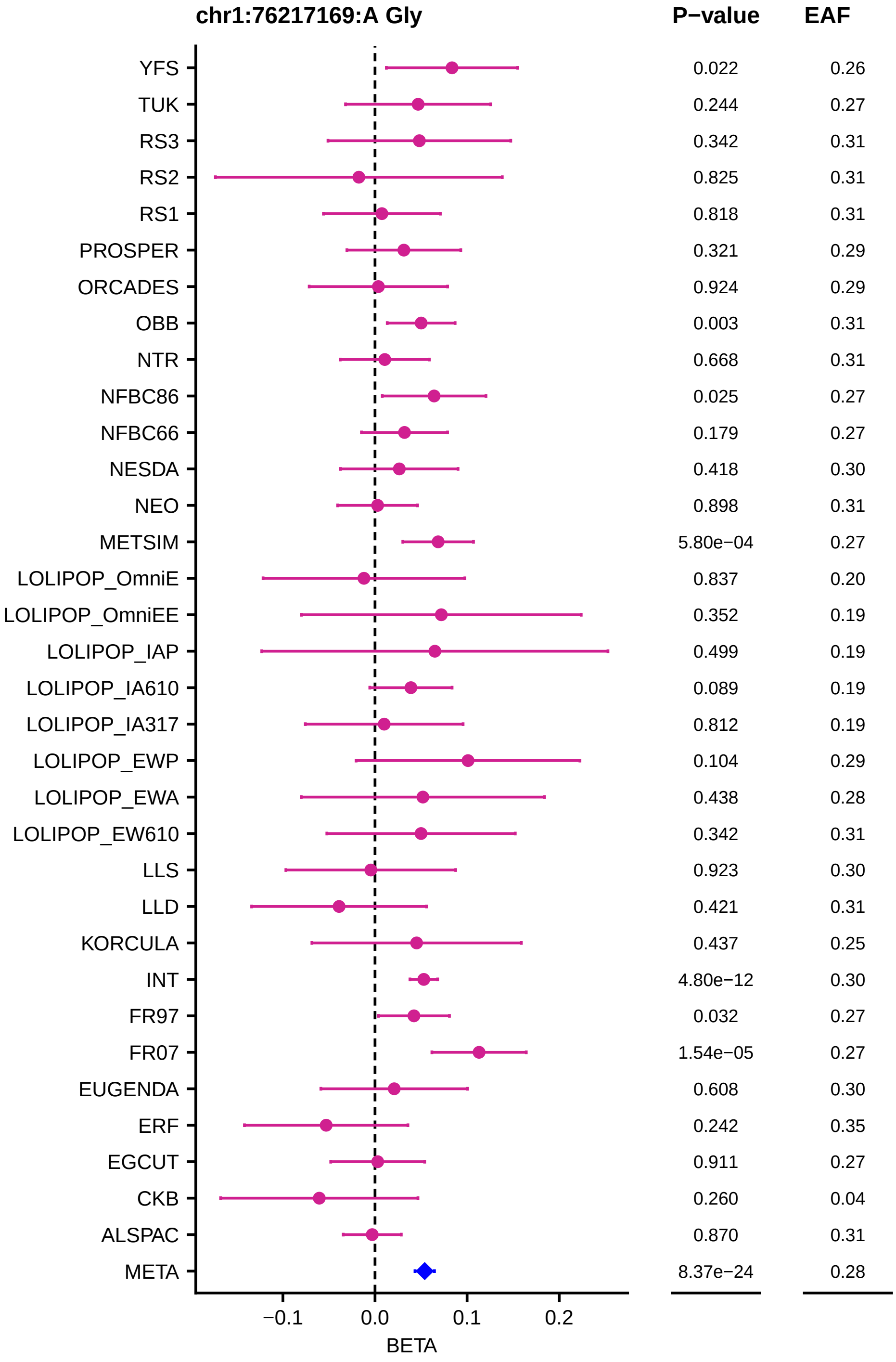
chr1:76217169:A Gly
P−value
EAF
YFS 0.022 0.26
TUK 0.244 0.27
RS3 0.342 0.31
RS2 0.825 0.31
RS1 0.818 0.31
PROSPER 0.321 0.29
ORCADES 0.924 0.29
OBB 0.003 0.31
NTR 0.668 0.31
NFBC86 0.025 0.27
NFBC66 0.179 0.27
NESDA 0.418 0.30
NEO 0.898 0.31
METSIM 5.80e−04 0.27
LOLIPOP_OmniE 0.837 0.20
LOLIPOP_OmniEE 0.352 0.19
LOLIPOP_IAP 0.499 0.19
LOLIPOP_IA610 0.089 0.19
LOLIPOP_IA317 0.812 0.19
LOLIPOP_EWP 0.104 0.29
LOLIPOP_EWA 0.438 0.28
LOLIPOP_EW610 0.342 0.31
LLS 0.923 0.30
LLD 0.421 0.31
KORCULA 0.437 0.25
INT 4.80e−12 0.30
FR97 0.032 0.27
FR07 1.54e−05 0.27
EUGENDA 0.608 0.30
ERF 0.242 0.35
EGCUT 0.911 0.27
CKB 0.260 0.04
ALSPAC 0.870 0.31
META 8.37e−24 0.28
−0.1
0.0
0.1
0.2
BETA

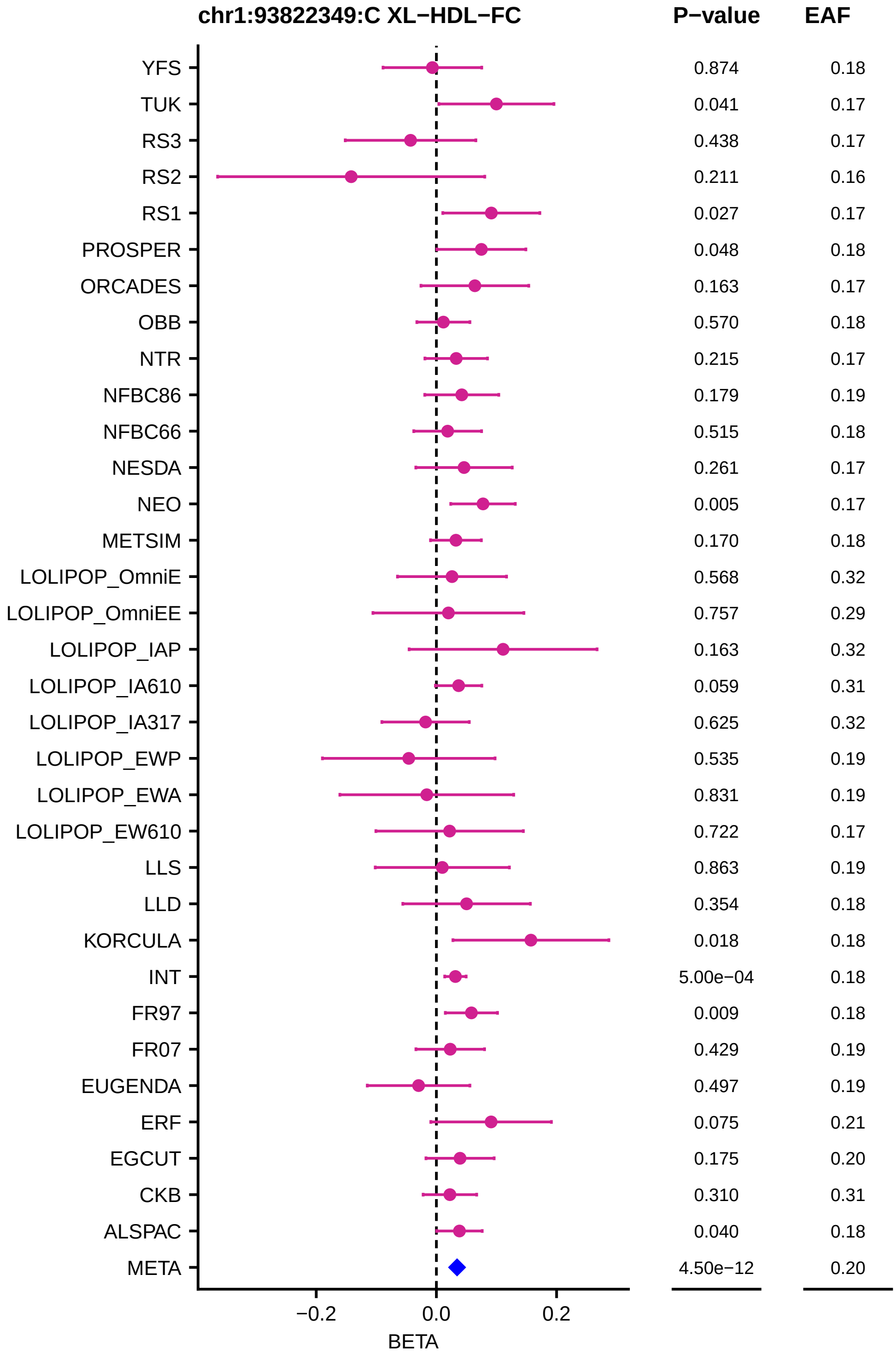

chr1:93822349:C XL−HDL−FC
P−value
EAF
YFS 0.874 0.18
TUK 0.041 0.17
RS3 0.438 0.17
RS2 0.211 0.16
RS1 0.027 0.17
PROSPER 0.048 0.18
ORCADES 0.163 0.17
OBB 0.570 0.18
NTR 0.215 0.17
NFBC86 0.179 0.19
NFBC66 0.515 0.18
NESDA 0.261 0.17
NEO 0.005 0.17
METSIM 0.170 0.18
LOLIPOP_OmniE 0.568 0.32
LOLIPOP_OmniEE 0.757 0.29
LOLIPOP_IAP 0.163 0.32
LOLIPOP_IA610 0.059 0.31
LOLIPOP_IA317 0.625 0.32
LOLIPOP_EWP 0.535 0.19
LOLIPOP_EWA 0.831 0.19
LOLIPOP_EW610 0.722 0.17
LLS 0.863 0.19
LLD 0.354 0.18
KORCULA 0.018 0.18
INT 5.00e−04 0.18
FR97 0.009 0.18
FR07 0.429 0.19
EUGENDA 0.497 0.19
ERF 0.075 0.21
EGCUT 0.175 0.20
CKB 0.310 0.31
ALSPAC 0.040 0.18
META 4.50e−12 0.20
−0.2
0.0
0.2
BETA

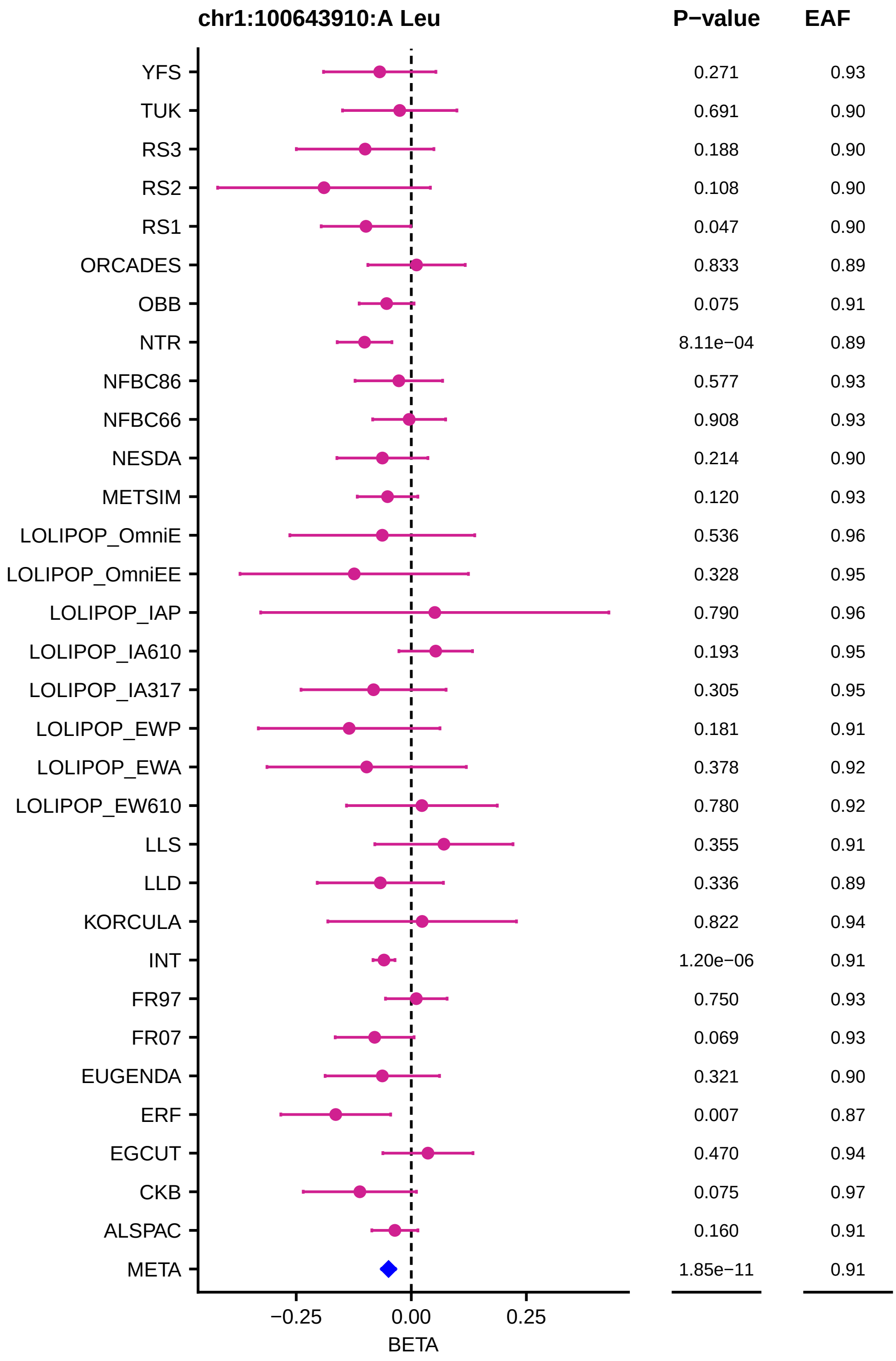
chr1:100643910:A Leu
P−value
EAF
YFS 0.271 0.93
TUK 0.691 0.90
RS3 0.188 0.90
RS2 0.108 0.90
RS1 0.047 0.90
ORCADES 0.833 0.89
OBB 0.075 0.91
NTR 8.11e−04 0.89
NFBC86 0.577 0.93
NFBC66 0.908 0.93
NESDA 0.214 0.90
METSIM 0.120 0.93
LOLIPOP_OmniE 0.536 0.96
LOLIPOP_OmniEE 0.328 0.95
LOLIPOP_IAP 0.790 0.96
LOLIPOP_IA610 0.193 0.95
LOLIPOP_IA317 0.305 0.95
LOLIPOP_EWP 0.181 0.91
LOLIPOP_EWA 0.378 0.92
LOLIPOP_EW610 0.780 0.92
LLS 0.355 0.91
LLD 0.336 0.89
KORCULA 0.822 0.94
INT 1.20e−06 0.91
FR97 0.750 0.93
FR07 0.069 0.93
EUGENDA 0.321 0.90
ERF 0.007 0.87
EGCUT 0.470 0.94
CKB 0.075 0.97
ALSPAC 0.160 0.91
META 1.85e−11 0.91
−0.25
0.00
0.25
BETA

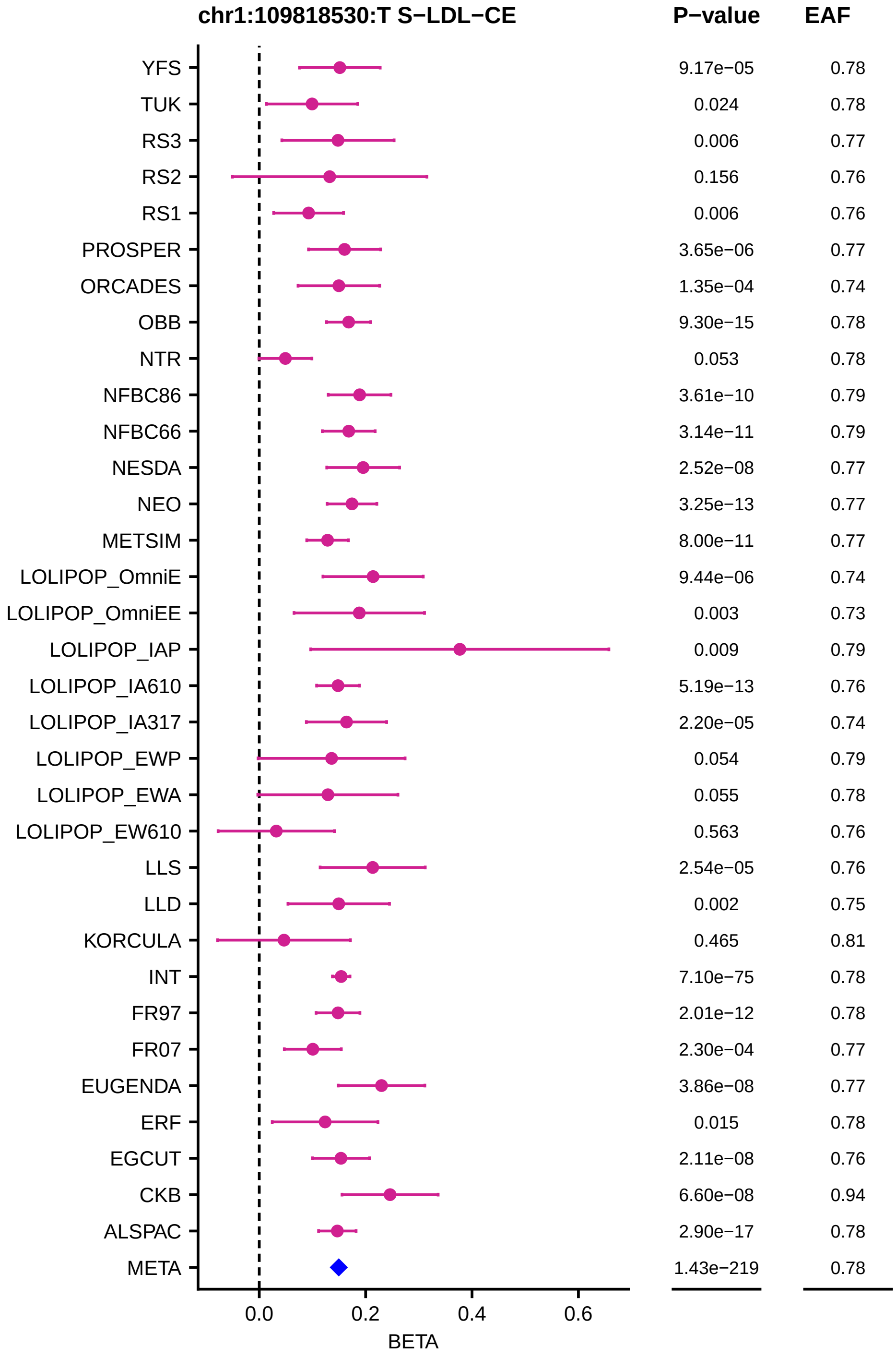
chr1:109818530:T S−LDL−CE
P−value
EAF
YFS 9.17e−05 0.78
TUK 0.024 0.78
RS3 0.006 0.77
RS2 0.156 0.76
RS1 0.006 0.76
PROSPER 3.65e−06 0.77
ORCADES 1.35e−04 0.74
OBB 9.30e−15 0.78
NTR 0.053 0.78
NFBC86 3.61e−10 0.79
NFBC66 3.14e−11 0.79
NESDA 2.52e−08 0.77
NEO 3.25e−13 0.77
METSIM 8.00e−11 0.77
LOLIPOP_OmniE 9.44e−06 0.74
LOLIPOP_OmniEE 0.003 0.73
LOLIPOP_IAP 0.009 0.79
LOLIPOP_IA610 5.19e−13 0.76
LOLIPOP_IA317 2.20e−05 0.74
LOLIPOP_EWP 0.054 0.79
LOLIPOP_EWA 0.055 0.78
LOLIPOP_EW610 0.563 0.76
LLS 2.54e−05 0.76
LLD 0.002 0.75
KORCULA 0.465 0.81
INT 7.10e−75 0.78
FR97 2.01e−12 0.78
FR07 2.30e−04 0.77
EUGENDA 3.86e−08 0.77
ERF 0.015 0.78
EGCUT 2.11e−08 0.76
CKB 6.60e−08 0.94
ALSPAC 2.90e−17 0.78
META 1.43e−219 0.78
0.0
0.2
0.4
0.6
BETA

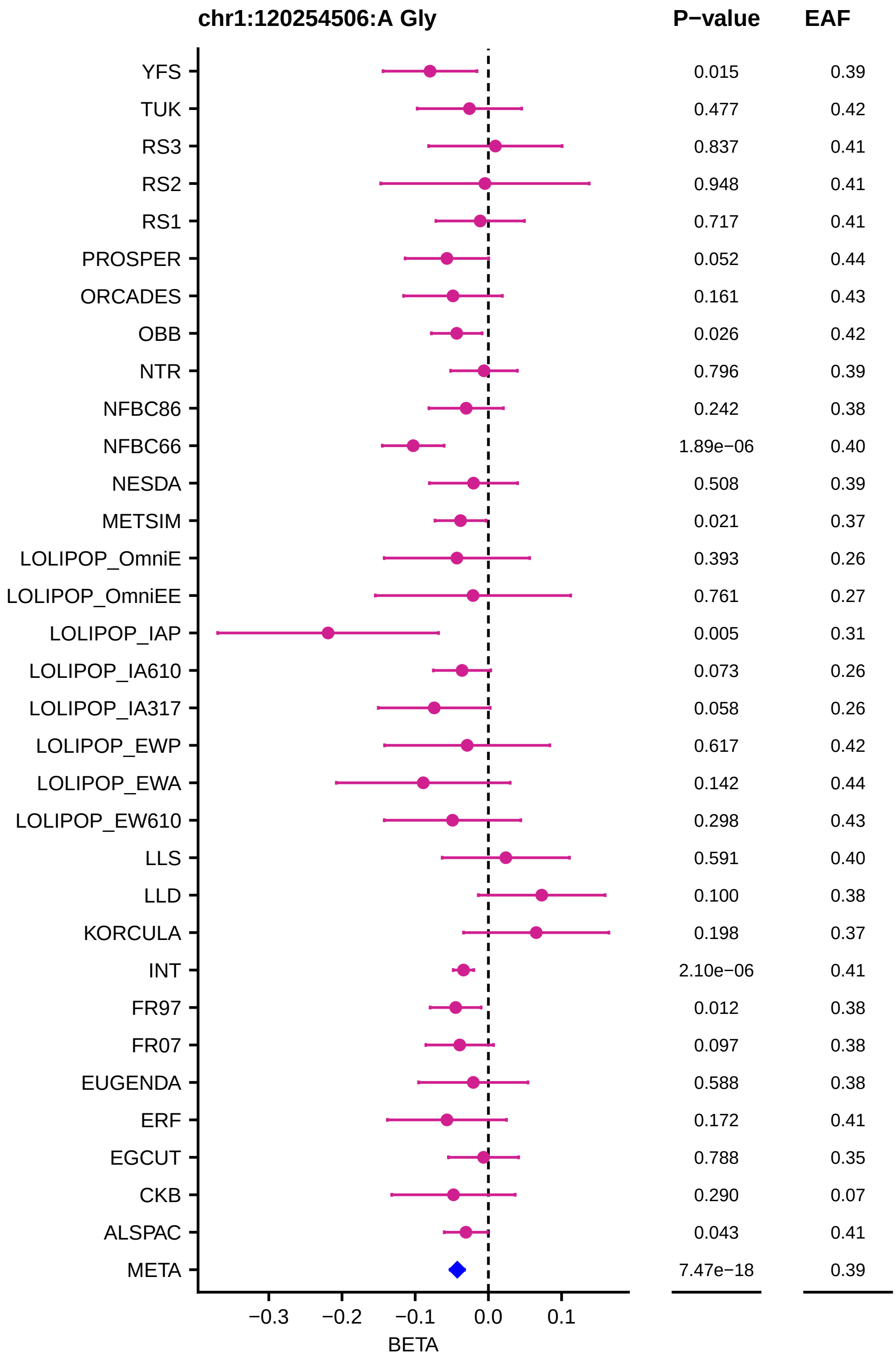
chr1:120254506:A Gly
P−value
EAF
YFS 0.015 0.39
TUK 0.477 0.42
RS3 0.837 0.41
RS2 0.948 0.41
RS1 0.717 0.41
PROSPER 0.052 0.44
ORCADES 0.161 0.43
OBB 0.026 0.42
NTR 0.796 0.39
NFBC86 0.242 0.38
NFBC66 1.89e−06 0.40
NESDA 0.508 0.39
METSIM 0.021 0.37
LOLIPOP_OmniE 0.393 0.26
LOLIPOP_OmniEE 0.761 0.27
LOLIPOP_IAP 0.005 0.31
LOLIPOP_IA610 0.073 0.26
LOLIPOP_IA317 0.058 0.26
LOLIPOP_EWP 0.617 0.42
LOLIPOP_EWA 0.142 0.44
LOLIPOP_EW610 0.298 0.43
LLS 0.591 0.40
LLD 0.100 0.38
KORCULA 0.198 0.37
INT 2.10e−06 0.41
FR97 0.012 0.38
FR07 0.097 0.38
EUGENDA 0.588 0.38
ERF 0.172 0.41
EGCUT 0.788 0.35
CKB 0.290 0.07
ALSPAC 0.043 0.41
META 7.47e−18 0.39
−0.3
−0.2
−0.1
0.0
0.1
BETA

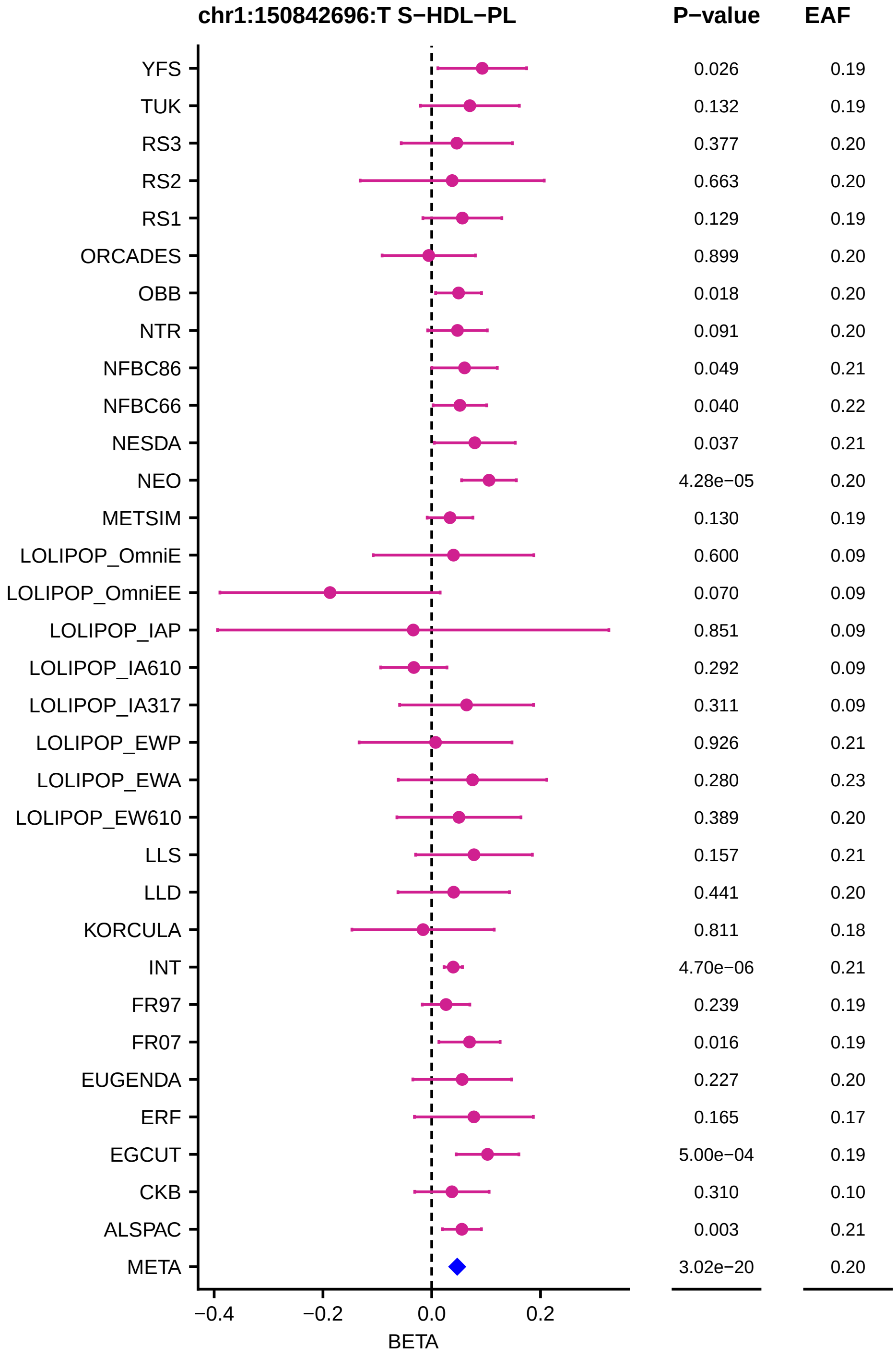
chr1:150842696:T S−HDL−PL
P−value
EAF
YFS 0.026 0.19
TUK 0.132 0.19
RS3 0.377 0.20
RS2 0.663 0.20
RS1 0.129 0.19
ORCADES 0.899 0.20
OBB 0.018 0.20
NTR 0.091 0.20
NFBC86 0.049 0.21
NFBC66 0.040 0.22
NESDA 0.037 0.21
NEO 4.28e−05 0.20
METSIM 0.130 0.19
LOLIPOP_OmniE 0.600 0.09
LOLIPOP_OmniEE 0.070 0.09
LOLIPOP_IAP 0.851 0.09
LOLIPOP_IA610 0.292 0.09
LOLIPOP_IA317 0.311 0.09
LOLIPOP_EWP 0.926 0.21
LOLIPOP_EWA 0.280 0.23
LOLIPOP_EW610 0.389 0.20
LLS 0.157 0.21
LLD 0.441 0.20
KORCULA 0.811 0.18
INT 4.70e−06 0.21
FR97 0.239 0.19
FR07 0.016 0.19
EUGENDA 0.227 0.20
ERF 0.165 0.17
EGCUT 5.00e−04 0.19
CKB 0.310 0.10
ALSPAC 0.003 0.21
META 3.02e−20 0.20
−0.4
−0.2
0.0
0.2
BETA

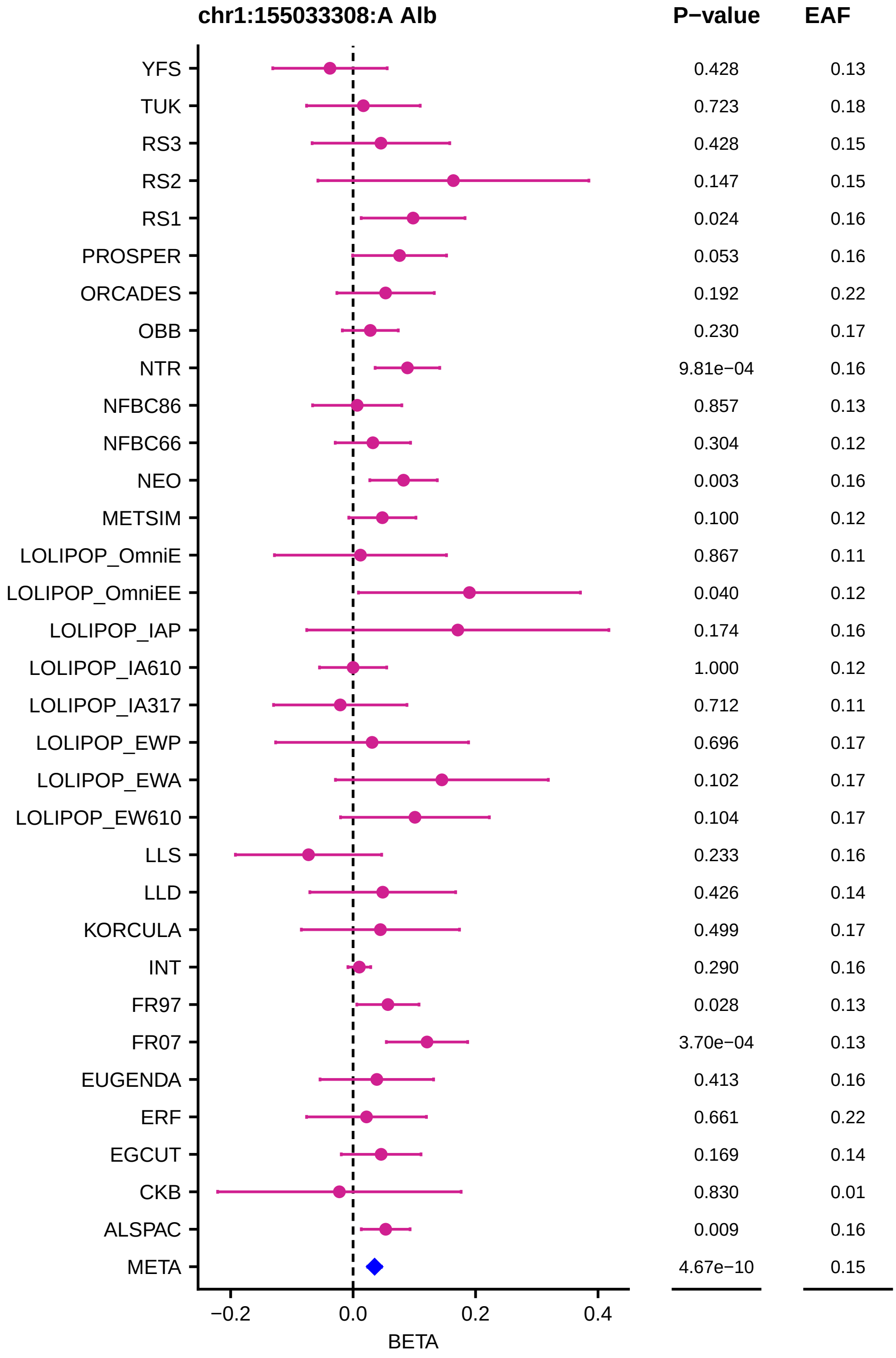
chr1:155033308:A Alb
P−value
EAF
YFS 0.428 0.13
TUK 0.723 0.18
RS3 0.428 0.15
RS2 0.147 0.15
RS1 0.024 0.16
PROSPER 0.053 0.16
ORCADES 0.192 0.22
OBB 0.230 0.17
NTR 9.81e−04 0.16
NFBC86 0.857 0.13
NFBC66 0.304 0.12
NEO 0.003 0.16
METSIM 0.100 0.12
LOLIPOP_OmniE 0.867 0.11
LOLIPOP_OmniEE 0.040 0.12
LOLIPOP_IAP 0.174 0.16
LOLIPOP_IA610 1.000 0.12
LOLIPOP_IA317 0.712 0.11
LOLIPOP_EWP 0.696 0.17
LOLIPOP_EWA 0.102 0.17
LOLIPOP_EW610 0.104 0.17
LLS 0.233 0.16
LLD 0.426 0.14
KORCULA 0.499 0.17
INT 0.290 0.16
FR97 0.028 0.13
FR07 3.70e−04 0.13
EUGENDA 0.413 0.16
ERF 0.661 0.22
EGCUT 0.169 0.14
CKB 0.830 0.01
ALSPAC 0.009 0.16
META 4.67e−10 0.15
−0.2
0.0
0.2
0.4
BETA

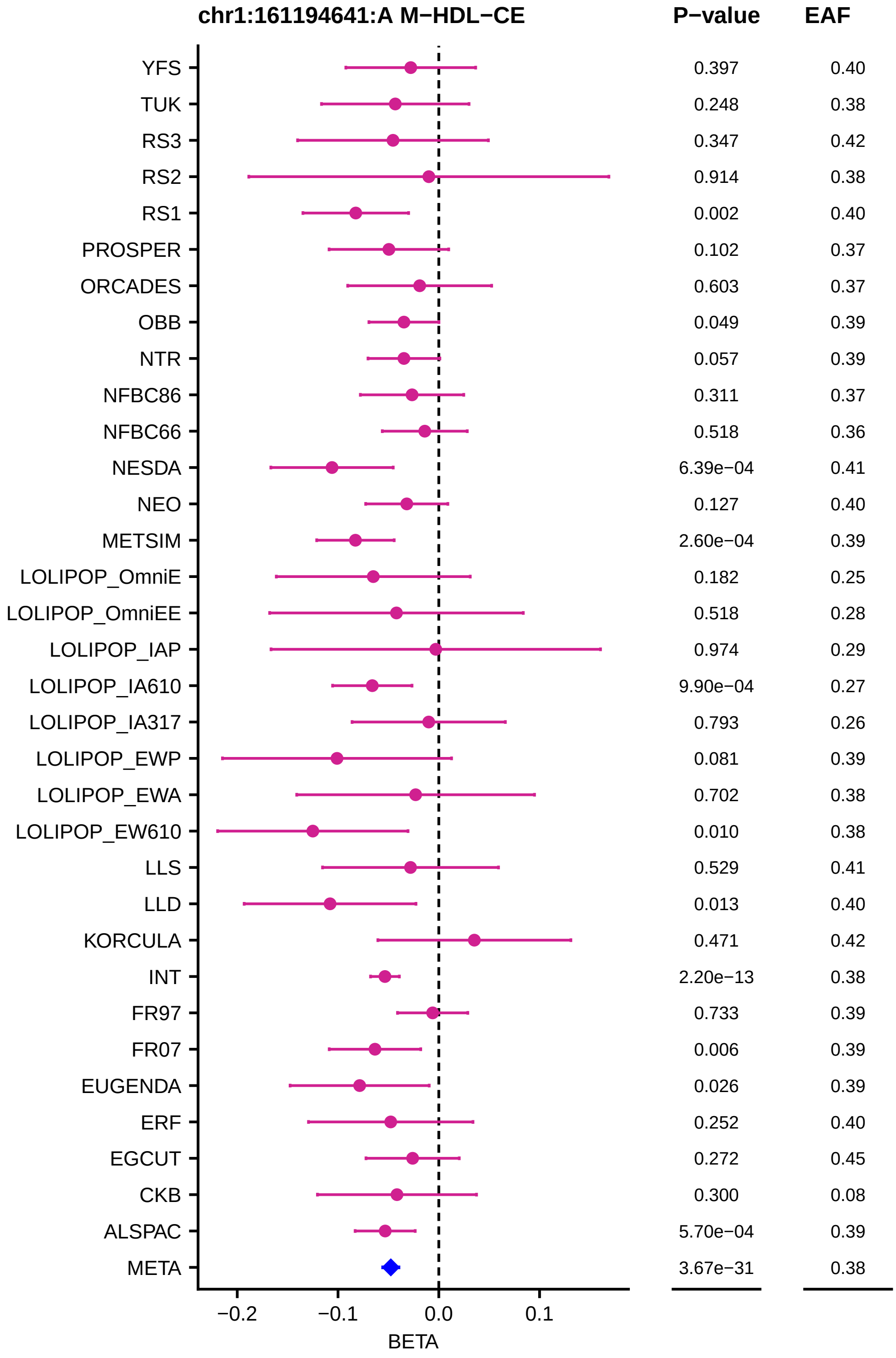
chr1:161194641:A M−HDL−CE
P−value
EAF
YFS 0.397 0.40
TUK 0.248 0.38
RS3 0.347 0.42
RS2 0.914 0.38
RS1 0.002 0.40
PROSPER 0.102 0.37
ORCADES 0.603 0.37
OBB 0.049 0.39
NTR 0.057 0.39
NFBC86 0.311 0.37
NFBC66 0.518 0.36
NESDA 6.39e−04 0.41
NEO 0.127 0.40
METSIM 2.60e−04 0.39
LOLIPOP_OmniE 0.182 0.25
LOLIPOP_OmniEE 0.518 0.28
LOLIPOP_IAP 0.974 0.29
LOLIPOP_IA610 9.90e−04 0.27
LOLIPOP_IA317 0.793 0.26
LOLIPOP_EWP 0.081 0.39
LOLIPOP_EWA 0.702 0.38
LOLIPOP_EW610 0.010 0.38
LLS 0.529 0.41
LLD 0.013 0.40
KORCULA 0.471 0.42
INT 2.20e−13 0.38
FR97 0.733 0.39
FR07 0.006 0.39
EUGENDA 0.026 0.39
ERF 0.252 0.40
EGCUT 0.272 0.45
CKB 0.300 0.08
ALSPAC 5.70e−04 0.39
META 3.67e−31 0.38
−0.2
−0.1
0.0
0.1
BETA

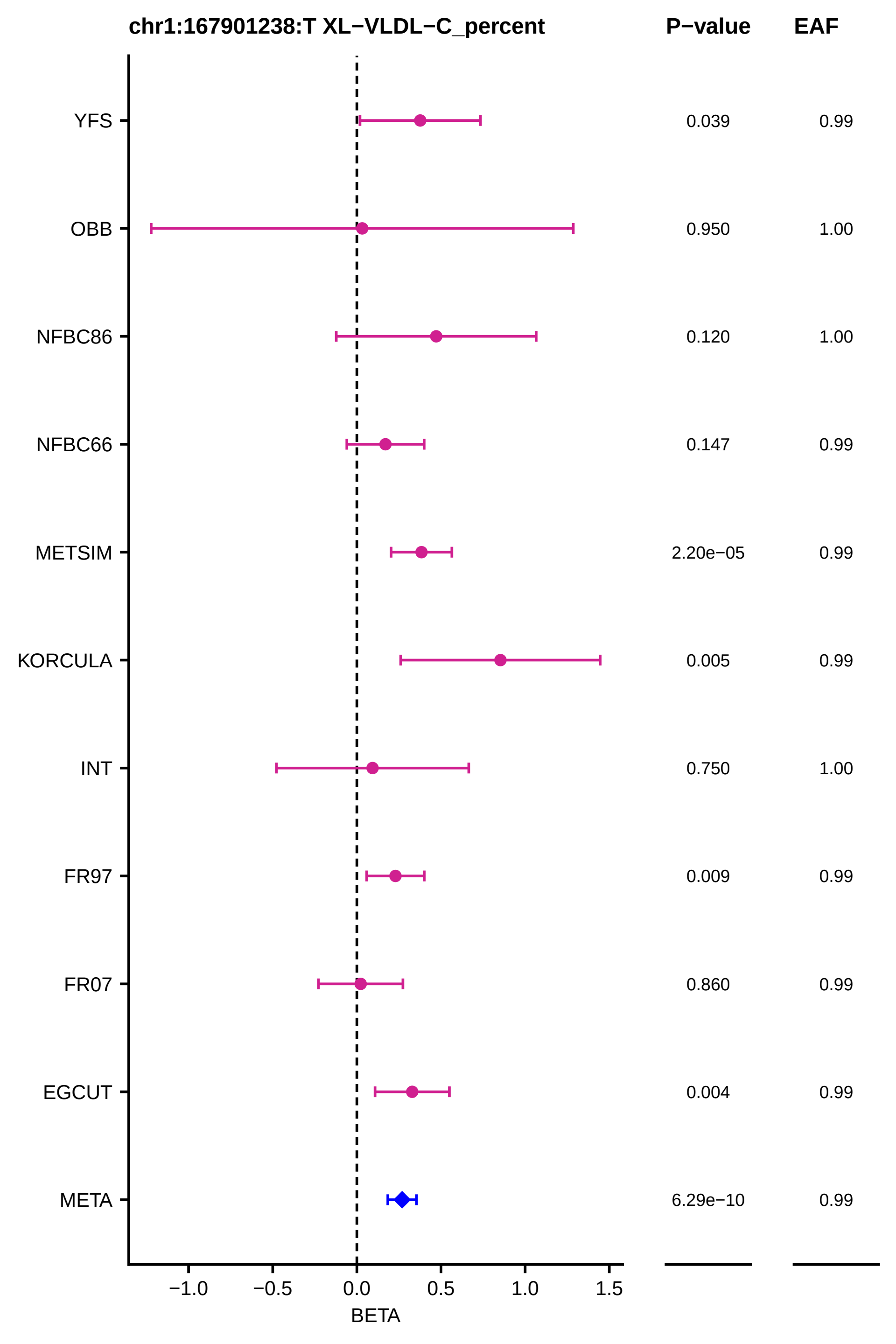
chr1:167901238:T XL−VLDL−C_percent
P−value
EAF
YFS
OBB
NFBC86
NFBC66
METSIM
KORCULA
INT
FR97
FR07
EGCUT
META
0.039
0.950
0.120
0.147
2.20e−05
0.005
0.750
0.009
0.860
0.004
6.29e−10
0.99
1.00
1.00
0.99
0.99
0.99
1.00
0.99
0.99
0.99
0.99
−1.0
−0.5
0.0
0.5
1.0
1.5
BETA

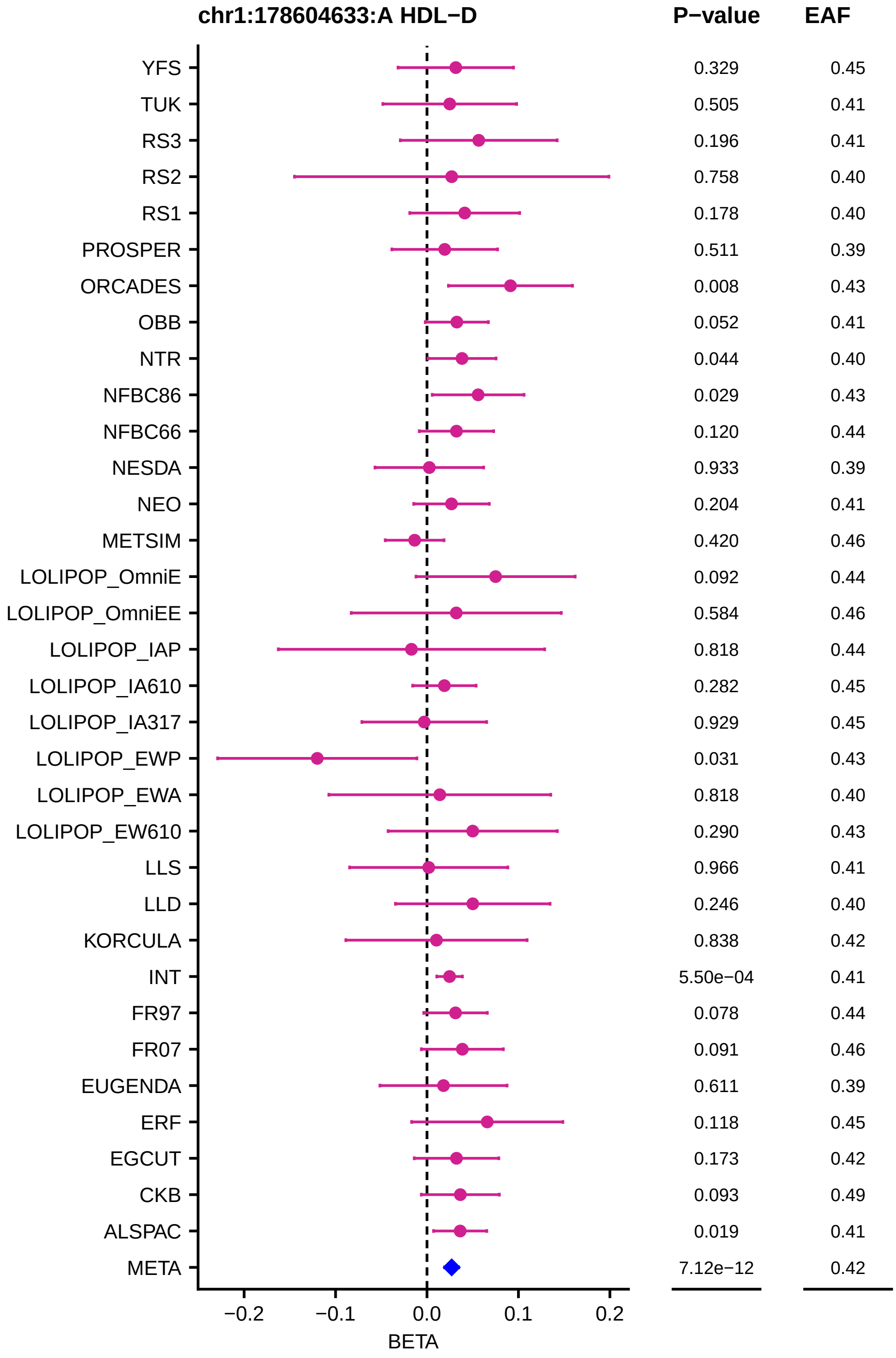
chr1:178604633:A HDL−D
P−value
EAF
YFS 0.329 0.45
TUK 0.505 0.41
RS3 0.196 0.41
RS2 0.758 0.40
RS1 0.178 0.40
PROSPER 0.511 0.39
ORCADES 0.008 0.43
OBB 0.052 0.41
NTR 0.044 0.40
NFBC86 0.029 0.43
NFBC66 0.120 0.44
NESDA 0.933 0.39
NEO 0.204 0.41
METSIM 0.420 0.46
LOLIPOP_OmniE 0.092 0.44
LOLIPOP_OmniEE 0.584 0.46
LOLIPOP_IAP 0.818 0.44
LOLIPOP_IA610 0.282 0.45
LOLIPOP_IA317 0.929 0.45
LOLIPOP_EWP 0.031 0.43
LOLIPOP_EWA 0.818 0.40
LOLIPOP_EW610 0.290 0.43
LLS 0.966 0.41
LLD 0.246 0.40
KORCULA 0.838 0.42
INT 5.50e−04 0.41
FR97 0.078 0.44
FR07 0.091 0.46
EUGENDA 0.611 0.39
ERF 0.118 0.45
EGCUT 0.173 0.42
CKB 0.093 0.49
ALSPAC 0.019 0.41
META 7.12e−12 0.42
−0.2
−0.1
0.0
0.1
0.2
BETA

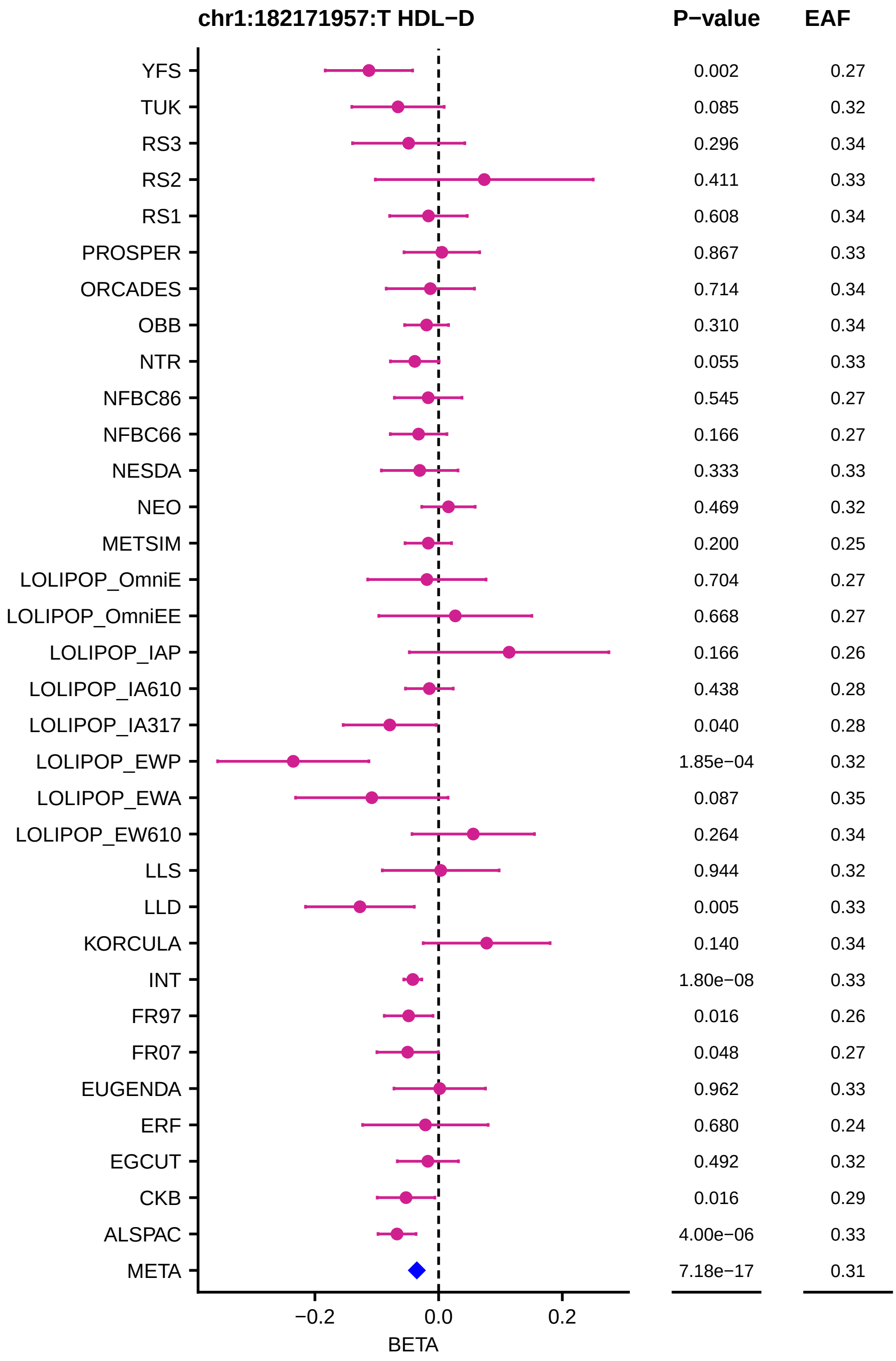

chr1:182171957:T HDL−D
P−value
EAF
YFS 0.002 0.27
TUK 0.085 0.32
RS3 0.296 0.34
RS2 0.411 0.33
RS1 0.608 0.34
PROSPER 0.867 0.33
ORCADES 0.714 0.34
OBB 0.310 0.34
NTR 0.055 0.33
NFBC86 0.545 0.27
NFBC66 0.166 0.27
NESDA 0.333 0.33
NEO 0.469 0.32
METSIM 0.200 0.25
LOLIPOP_OmniE 0.704 0.27
LOLIPOP_OmniEE 0.668 0.27
LOLIPOP_IAP 0.166 0.26
LOLIPOP_IA610 0.438 0.28
LOLIPOP_IA317 0.040 0.28
LOLIPOP_EWP 1.85e−04 0.32
LOLIPOP_EWA 0.087 0.35
LOLIPOP_EW610 0.264 0.34
LLS 0.944 0.32
LLD 0.005 0.33
KORCULA 0.140 0.34
INT 1.80e−08 0.33
FR97 0.016 0.26
FR07 0.048 0.27
EUGENDA 0.962 0.33
ERF 0.680 0.24
EGCUT 0.492 0.32
CKB 0.016 0.29
ALSPAC 4.00e−06 0.33
META 7.18e−17 0.31
−0.2
0.0
0.2
BETA

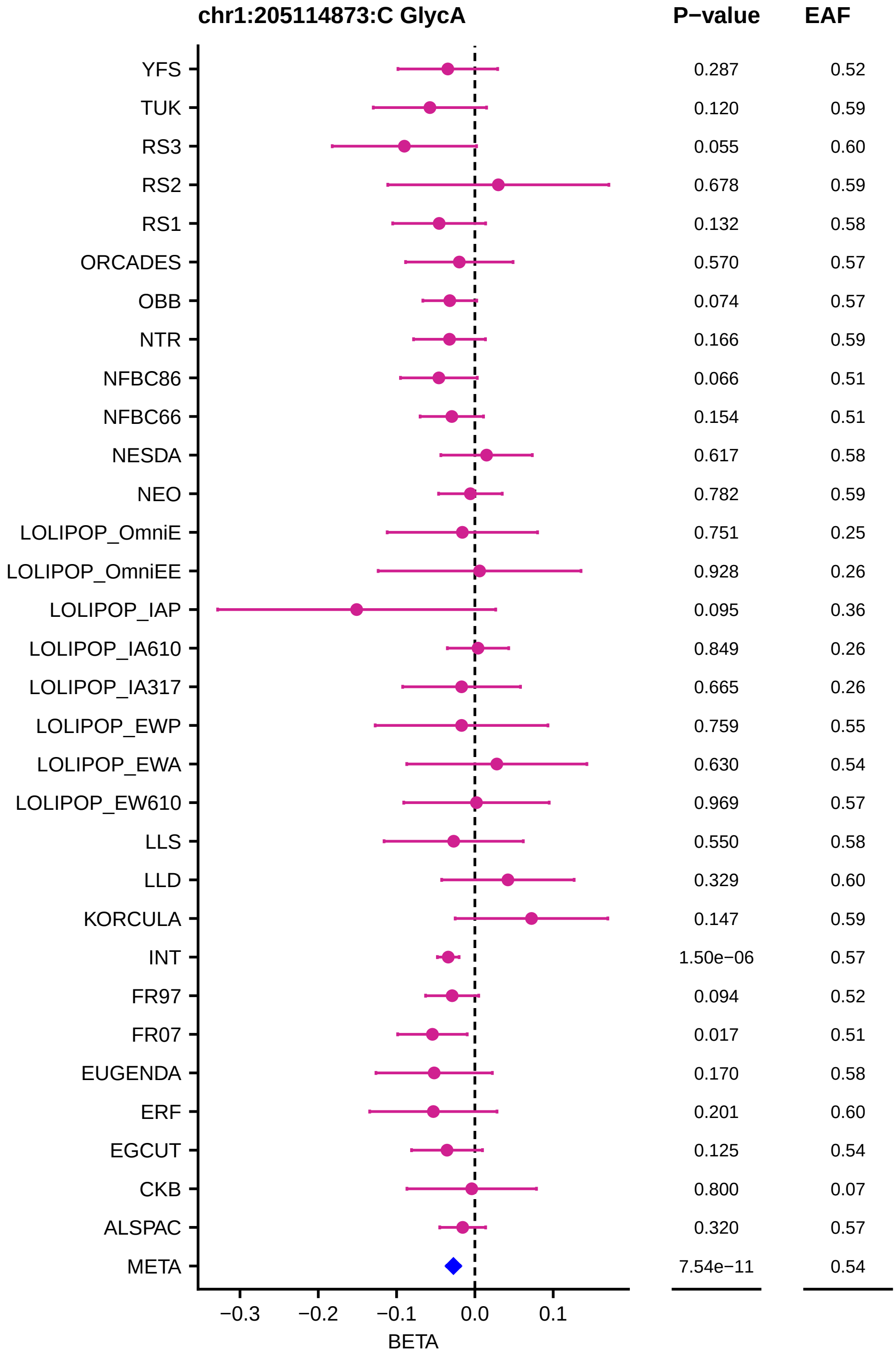
chr1:205114873:C GlycA
P−value
EAF
YFS 0.287 0.52
TUK 0.120 0.59
RS3 0.055 0.60
RS2 0.678 0.59
RS1 0.132 0.58
ORCADES 0.570 0.57
OBB 0.074 0.57
NTR 0.166 0.59
NFBC86 0.066 0.51
NFBC66 0.154 0.51
NESDA 0.617 0.58
NEO 0.782 0.59
LOLIPOP_OmniE 0.751 0.25
LOLIPOP_OmniEE 0.928 0.26
LOLIPOP_IAP 0.095 0.36
LOLIPOP_IA610 0.849 0.26
LOLIPOP_IA317 0.665 0.26
LOLIPOP_EWP 0.759 0.55
LOLIPOP_EWA 0.630 0.54
LOLIPOP_EW610 0.969 0.57
LLS 0.550 0.58
LLD 0.329 0.60
KORCULA 0.147 0.59
INT 1.50e−06 0.57
FR97 0.094 0.52
FR07 0.017 0.51
EUGENDA 0.170 0.58
ERF 0.201 0.60
EGCUT 0.125 0.54
CKB 0.800 0.07
ALSPAC 0.320 0.57
META 7.54e−11 0.54
−0.3
−0.2
−0.1
0.0
0.1
BETA

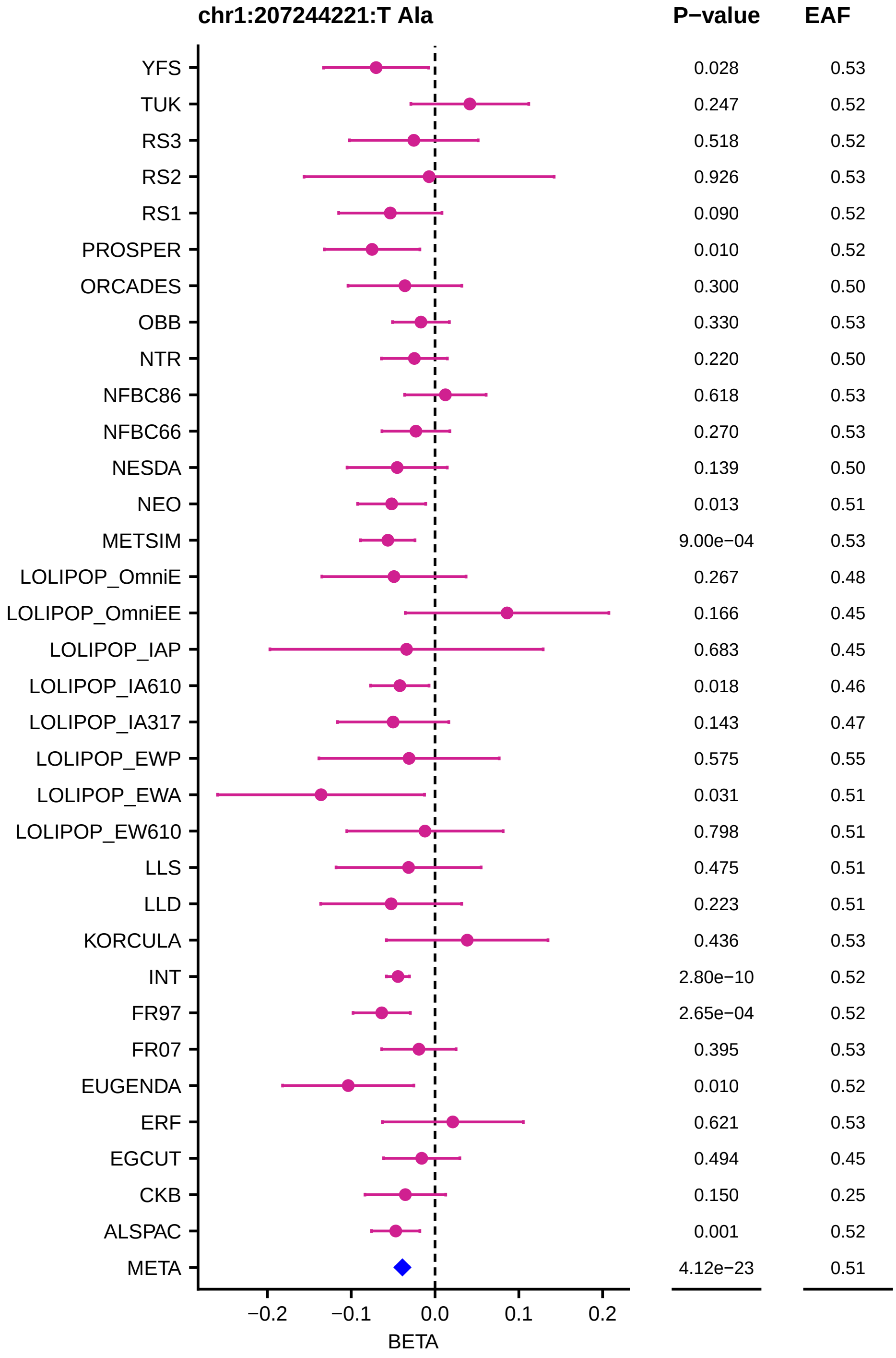

chr1:207244221:T Ala
P−value
EAF
YFS 0.028 0.53
TUK 0.247 0.52
RS3 0.518 0.52
RS2 0.926 0.53
RS1 0.090 0.52
PROSPER 0.010 0.52
ORCADES 0.300 0.50
OBB 0.330 0.53
NTR 0.220 0.50
NFBC86 0.618 0.53
NFBC66 0.270 0.53
NESDA 0.139 0.50
NEO 0.013 0.51
METSIM 9.00e−04 0.53
LOLIPOP_OmniE 0.267 0.48
LOLIPOP_OmniEE 0.166 0.45
LOLIPOP_IAP 0.683 0.45
LOLIPOP_IA610 0.018 0.46
LOLIPOP_IA317 0.143 0.47
LOLIPOP_EWP 0.575 0.55
LOLIPOP_EWA 0.031 0.51
LOLIPOP_EW610 0.798 0.51
LLS 0.475 0.51
LLD 0.223 0.51
KORCULA 0.436 0.53
INT 2.80e−10 0.52
FR97 2.65e−04 0.52
FR07 0.395 0.53
EUGENDA 0.010 0.52
ERF 0.621 0.53
EGCUT 0.494 0.45
CKB 0.150 0.25
ALSPAC 0.001 0.52
META 4.12e−23 0.51
−0.2
−0.1
0.0
0.1
0.2
BETA

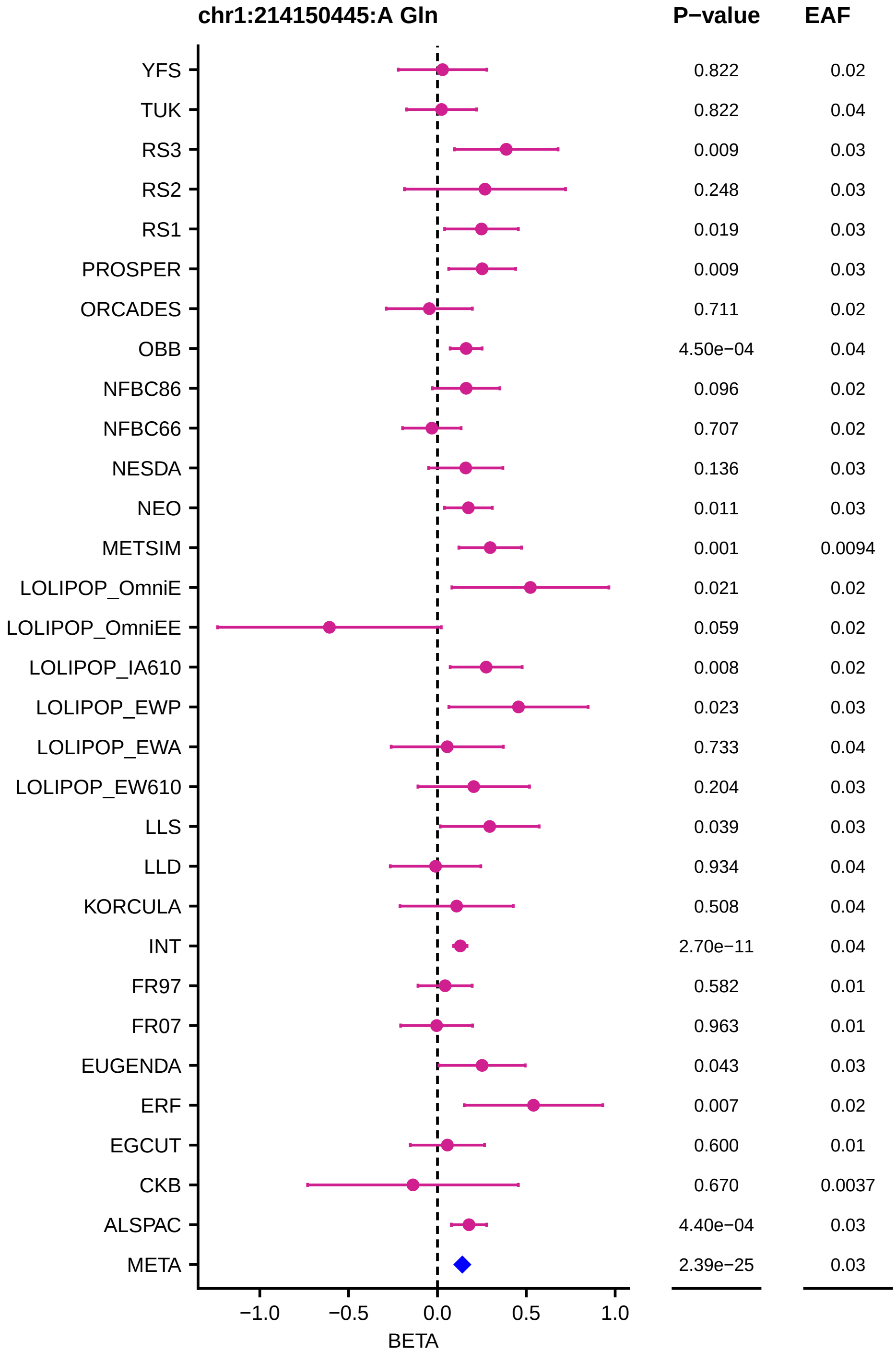

chr1:214150445:A Gln
P−value
EAF
YFS
TUK
RS3
RS2
RS1
PROSPER
ORCADES
OBB
NFBC86
NFBC66
NESDA
NEO
METSIM
LOLIPOP_OmniE
LOLIPOP_OmniEE
LOLIPOP_IA610
LOLIPOP_EWP
LOLIPOP_EWA
LOLIPOP_EW610
LLS
LLD
KORCULA
INT
FR97
FR07
EUGENDA
ERF
EGCUT
CKB
ALSPAC
META
0.822 0.02
0.822 0.04
0.009 0.03
0.248 0.03
0.019 0.03
0.009 0.03
0.711 0.02
4.50e−04 0.04
0.096 0.02
0.707 0.02
0.136 0.03
0.011 0.03
0.001 0.0094
0.021 0.02
0.059 0.02
0.008 0.02
0.023 0.03
0.733 0.04
0.204 0.03
0.039 0.03
0.934 0.04
0.508 0.04
2.70e−11 0.04
0.582 0.01
0.963 0.01
0.043 0.03
0.007 0.02
0.600 0.01
0.670 0.0037
4.40e−04 0.03
2.39e−25 0.03
−1.0
−0.5
0.0
0.5
1.0
BETA

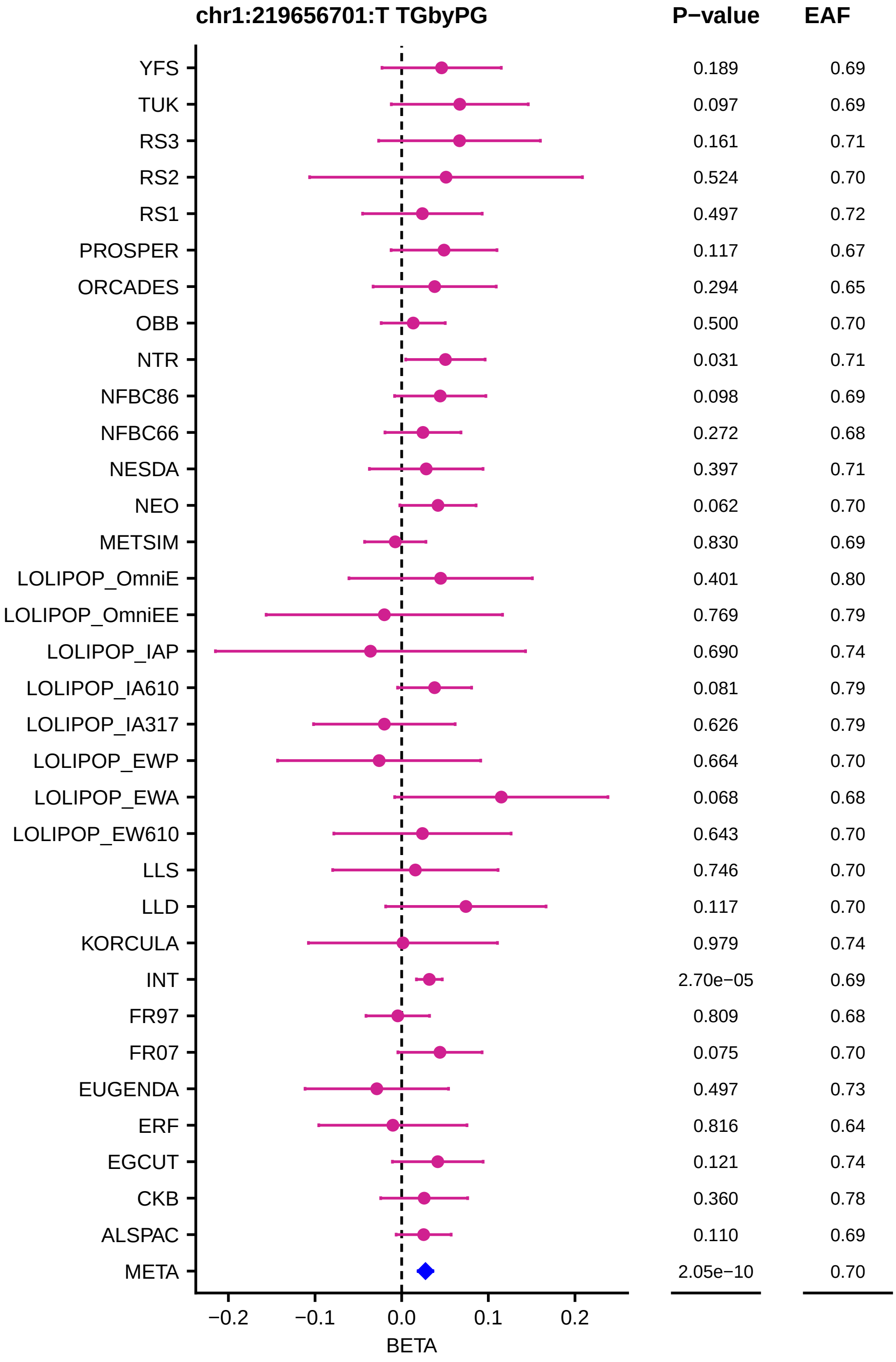

chr1:219656701:T TGbyPG
P−value
EAF
YFS 0.189 0.69
TUK 0.097 0.69
RS3 0.161 0.71
RS2 0.524 0.70
RS1 0.497 0.72
PROSPER 0.117 0.67
ORCADES 0.294 0.65
OBB 0.500 0.70
NTR 0.031 0.71
NFBC86 0.098 0.69
NFBC66 0.272 0.68
NESDA 0.397 0.71
NEO 0.062 0.70
METSIM 0.830 0.69
LOLIPOP_OmniE 0.401 0.80
LOLIPOP_OmniEE 0.769 0.79
LOLIPOP_IAP 0.690 0.74
LOLIPOP_IA610 0.081 0.79
LOLIPOP_IA317 0.626 0.79
LOLIPOP_EWP 0.664 0.70
LOLIPOP_EWA 0.068 0.68
LOLIPOP_EW610 0.643 0.70
LLS 0.746 0.70
LLD 0.117 0.70
KORCULA 0.979 0.74
INT 2.70e−05 0.69
FR97 0.809 0.68
FR07 0.075 0.70
EUGENDA 0.497 0.73
ERF 0.816 0.64
EGCUT 0.121 0.74
CKB 0.360 0.78
ALSPAC 0.110 0.69
META 2.05e−10 0.70
−0.2
−0.1
0.0
0.1
0.2
BETA

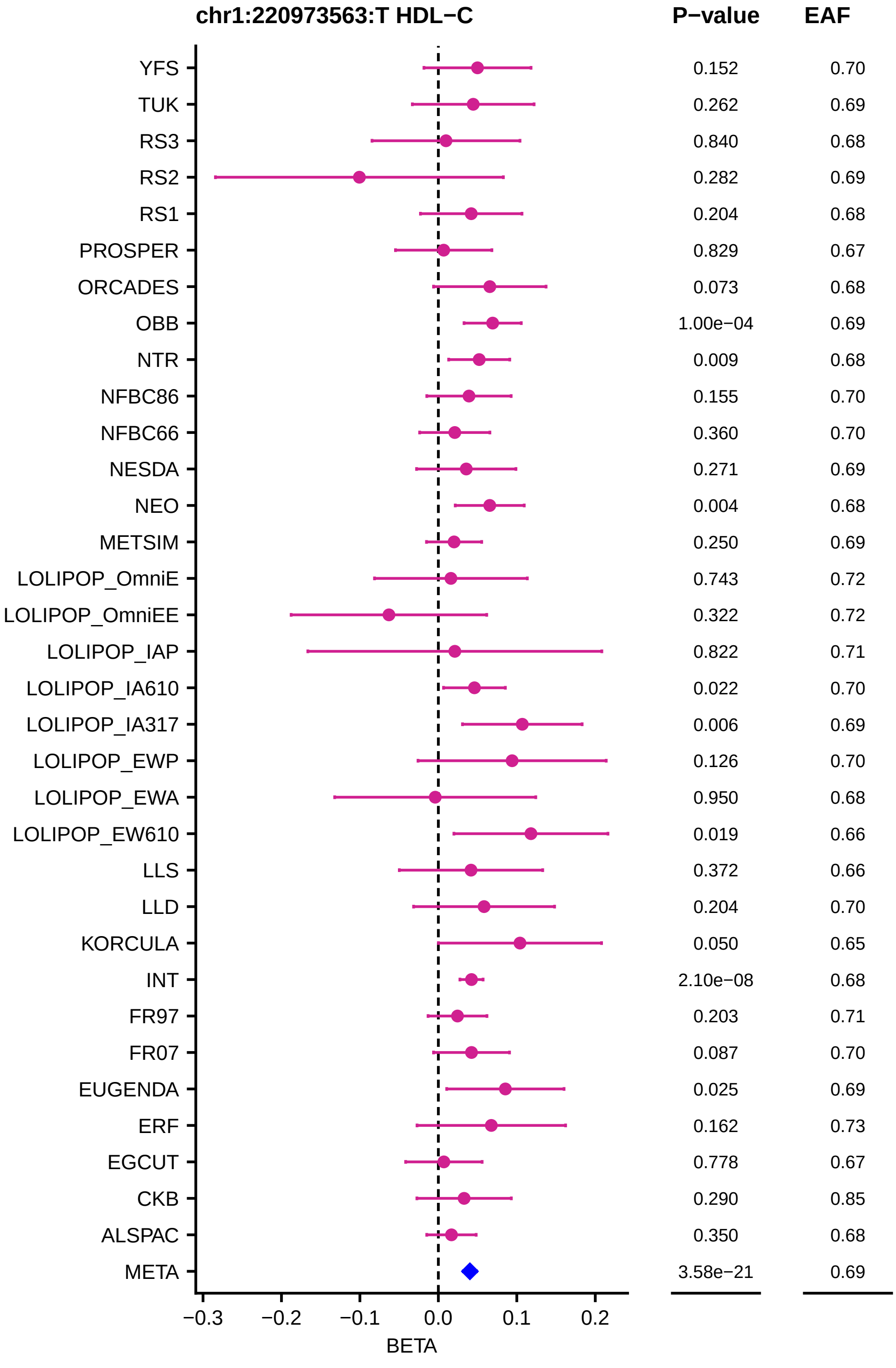
chr1:220973563:T HDL−C
P−value
EAF
YFS 0.152 0.70
TUK 0.262 0.69
RS3 0.840 0.68
RS2 0.282 0.69
RS1 0.204 0.68
PROSPER 0.829 0.67
ORCADES 0.073 0.68
OBB 1.00e−04 0.69
NTR 0.009 0.68
NFBC86 0.155 0.70
NFBC66 0.360 0.70
NESDA 0.271 0.69
NEO 0.004 0.68
METSIM 0.250 0.69
LOLIPOP_OmniE 0.743 0.72
LOLIPOP_OmniEE 0.322 0.72
LOLIPOP_IAP 0.822 0.71
LOLIPOP_IA610 0.022 0.70
LOLIPOP_IA317 0.006 0.69
LOLIPOP_EWP 0.126 0.70
LOLIPOP_EWA 0.950 0.68
LOLIPOP_EW610 0.019 0.66
LLS 0.372 0.66
LLD 0.204 0.70
KORCULA 0.050 0.65
INT 2.10e−08 0.68
FR97 0.203 0.71
FR07 0.087 0.70
EUGENDA 0.025 0.69
ERF 0.162 0.73
EGCUT 0.778 0.67
CKB 0.290 0.85
ALSPAC 0.350 0.68
META 3.58e−21 0.69
−0.3
−0.2
−0.1
0.0
0.1
0.2
BETA

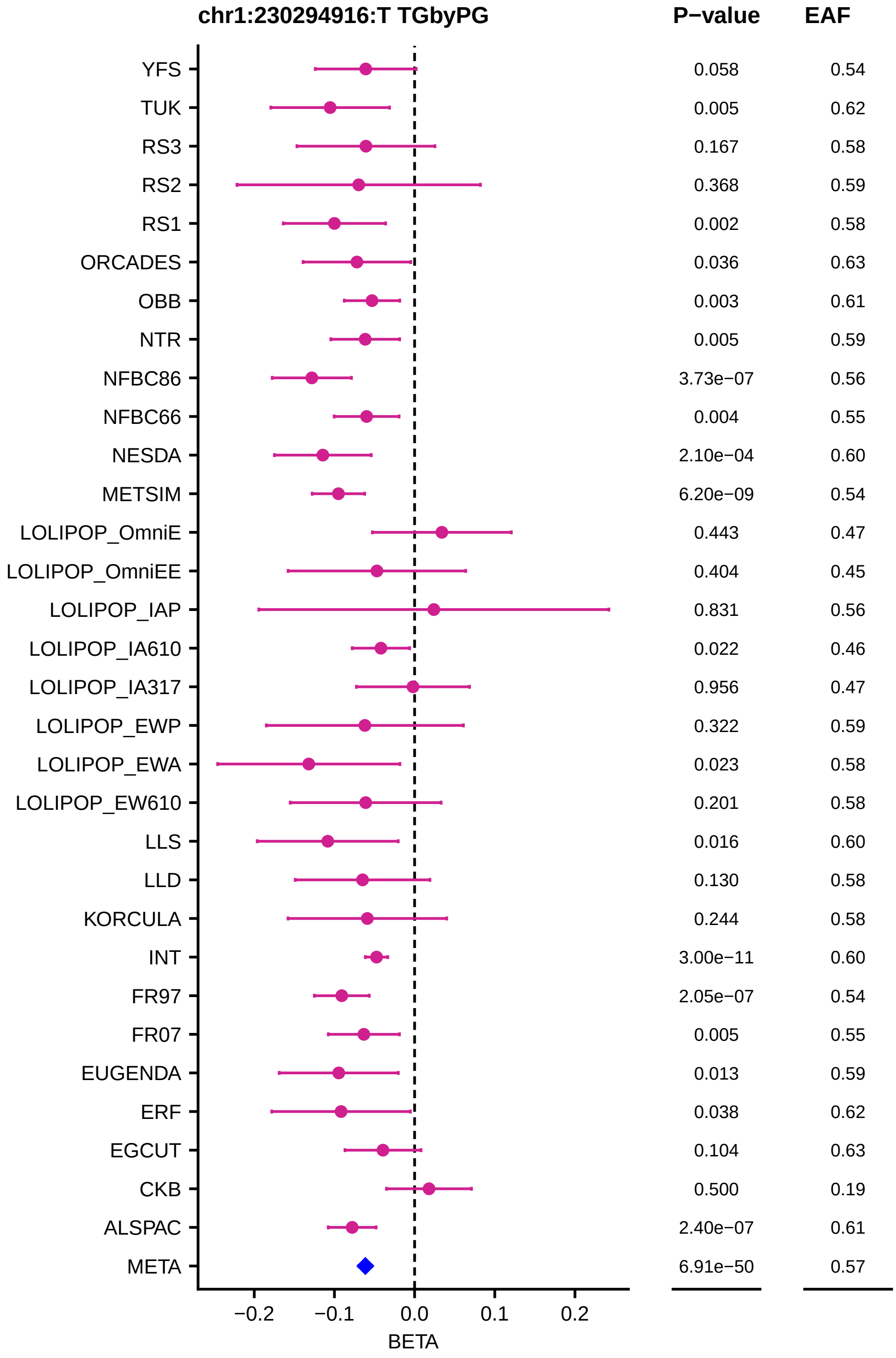
chr1:230294916:T TGbyPG
P−value
EAF
YFS 0.058 0.54
TUK 0.005 0.62
RS3 0.167 0.58
RS2 0.368 0.59
RS1 0.002 0.58
ORCADES 0.036 0.63
OBB 0.003 0.61
NTR 0.005 0.59
NFBC86 3.73e−07 0.56
NFBC66 0.004 0.55
NESDA 2.10e−04 0.60
METSIM 6.20e−09 0.54
LOLIPOP_OmniE 0.443 0.47
LOLIPOP_OmniEE 0.404 0.45
LOLIPOP_IAP 0.831 0.56
LOLIPOP_IA610 0.022 0.46
LOLIPOP_IA317 0.956 0.47
LOLIPOP_EWP 0.322 0.59
LOLIPOP_EWA 0.023 0.58
LOLIPOP_EW610 0.201 0.58
LLS 0.016 0.60
LLD 0.130 0.58
KORCULA 0.244 0.58
INT 3.00e−11 0.60
FR97 2.05e−07 0.54
FR07 0.005 0.55
EUGENDA 0.013 0.59
ERF 0.038 0.62
EGCUT 0.104 0.63
CKB 0.500 0.19
ALSPAC 2.40e−07 0.61
META 6.91e−50 0.57
−0.2
−0.1
0.0
0.1
0.2
BETA

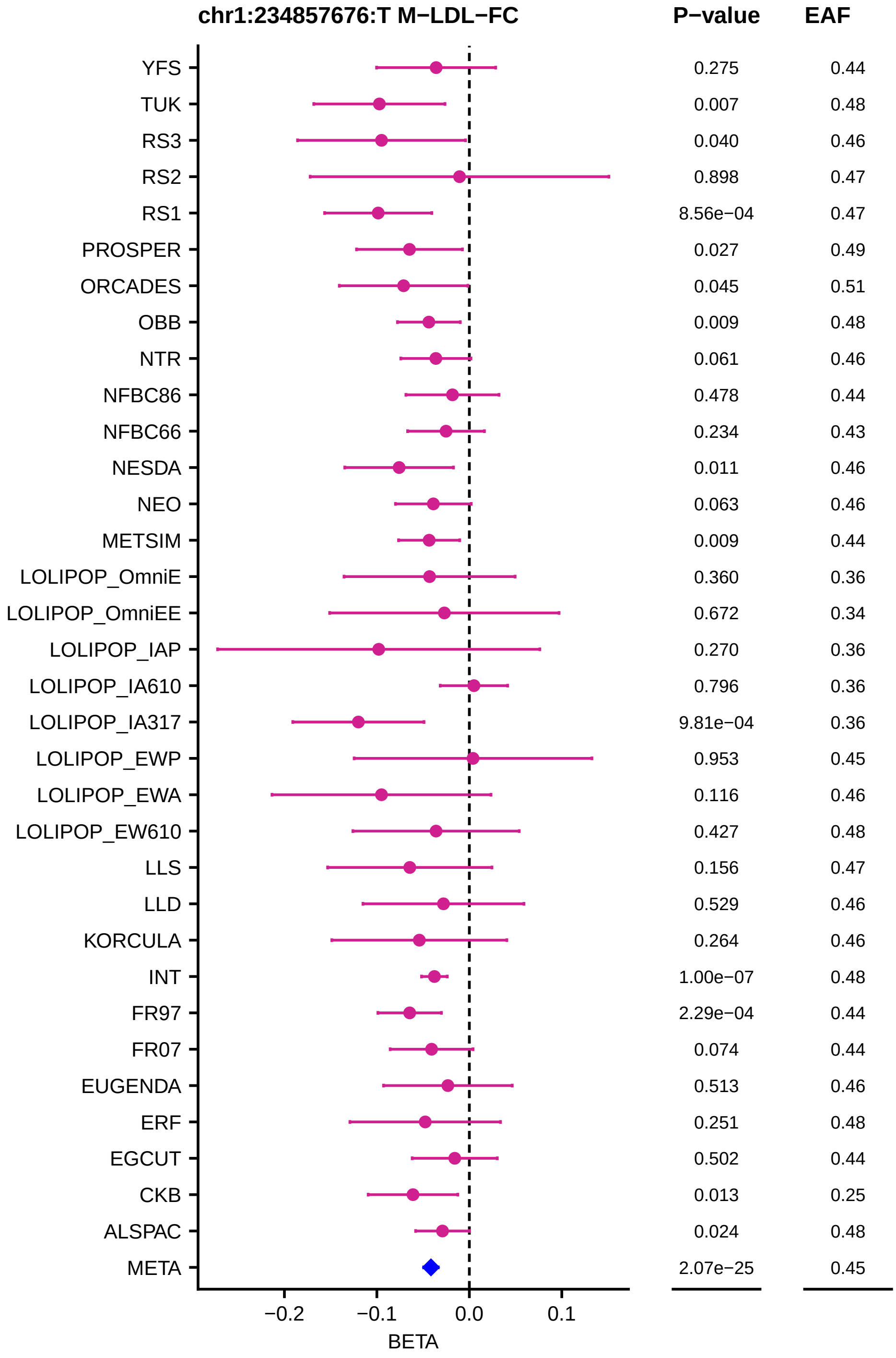
chr1:234857676:T M−LDL−FC
P−value
EAF
YFS 0.275 0.44
TUK 0.007 0.48
RS3 0.040 0.46
RS2 0.898 0.47
RS1 8.56e−04 0.47
PROSPER 0.027 0.49
ORCADES 0.045 0.51
OBB 0.009 0.48
NTR 0.061 0.46
NFBC86 0.478 0.44
NFBC66 0.234 0.43
NESDA 0.011 0.46
NEO 0.063 0.46
METSIM 0.009 0.44
LOLIPOP_OmniE 0.360 0.36
LOLIPOP_OmniEE 0.672 0.34
LOLIPOP_IAP 0.270 0.36
LOLIPOP_IA610 0.796 0.36
LOLIPOP_IA317 9.81e−04 0.36
LOLIPOP_EWP 0.953 0.45
LOLIPOP_EWA 0.116 0.46
LOLIPOP_EW610 0.427 0.48
LLS 0.156 0.47
LLD 0.529 0.46
KORCULA 0.264 0.46
INT 1.00e−07 0.48
FR97 2.29e−04 0.44
FR07 0.074 0.44
EUGENDA 0.513 0.46
ERF 0.251 0.48
EGCUT 0.502 0.44
CKB 0.013 0.25
ALSPAC 0.024 0.48
META 2.07e−25 0.45
−0.2
−0.1
0.0
0.1
BETA

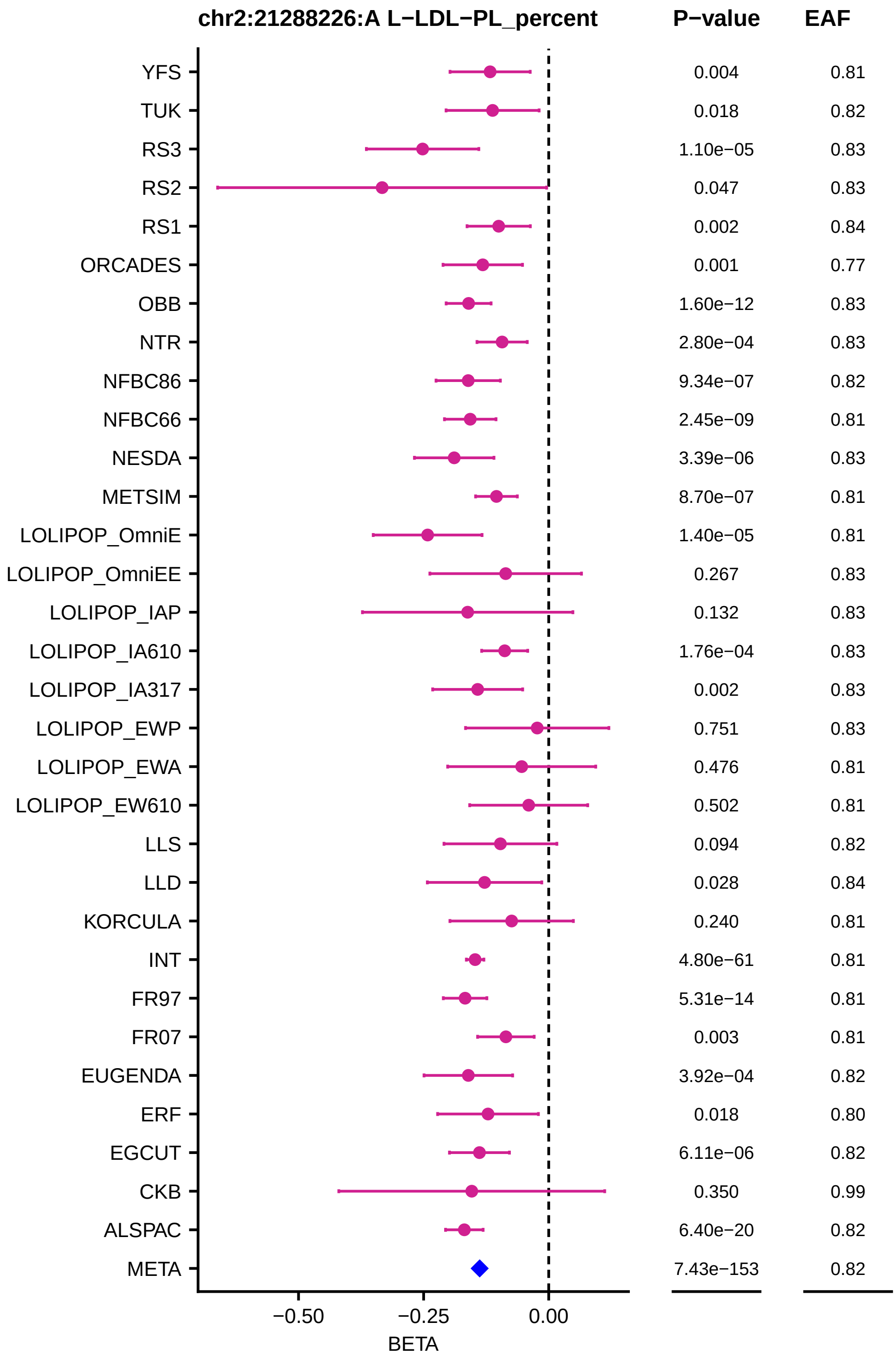
chr2:21288226:A L−LDL−PL_percent
P−value
EAF
YFS 0.004 0.81
TUK 0.018 0.82
RS3 1.10e−05 0.83
RS2 0.047 0.83
RS1 0.002 0.84
ORCADES 0.001 0.77
OBB 1.60e−12 0.83
NTR 2.80e−04 0.83
NFBC86 9.34e−07 0.82
NFBC66 2.45e−09 0.81
NESDA 3.39e−06 0.83
METSIM 8.70e−07 0.81
LOLIPOP_OmniE 1.40e−05 0.81
LOLIPOP_OmniEE 0.267 0.83
LOLIPOP_IAP 0.132 0.83
LOLIPOP_IA610 1.76e−04 0.83
LOLIPOP_IA317 0.002 0.83
LOLIPOP_EWP 0.751 0.83
LOLIPOP_EWA 0.476 0.81
LOLIPOP_EW610 0.502 0.81
LLS 0.094 0.82
LLD 0.028 0.84
KORCULA 0.240 0.81
INT 4.80e−61 0.81
FR97 5.31e−14 0.81
FR07 0.003 0.81
EUGENDA 3.92e−04 0.82
ERF 0.018 0.80
EGCUT 6.11e−06 0.82
CKB 0.350 0.99
ALSPAC 6.40e−20 0.82
META 7.43e−153 0.82
−0.50
−0.25
0.00
BETA

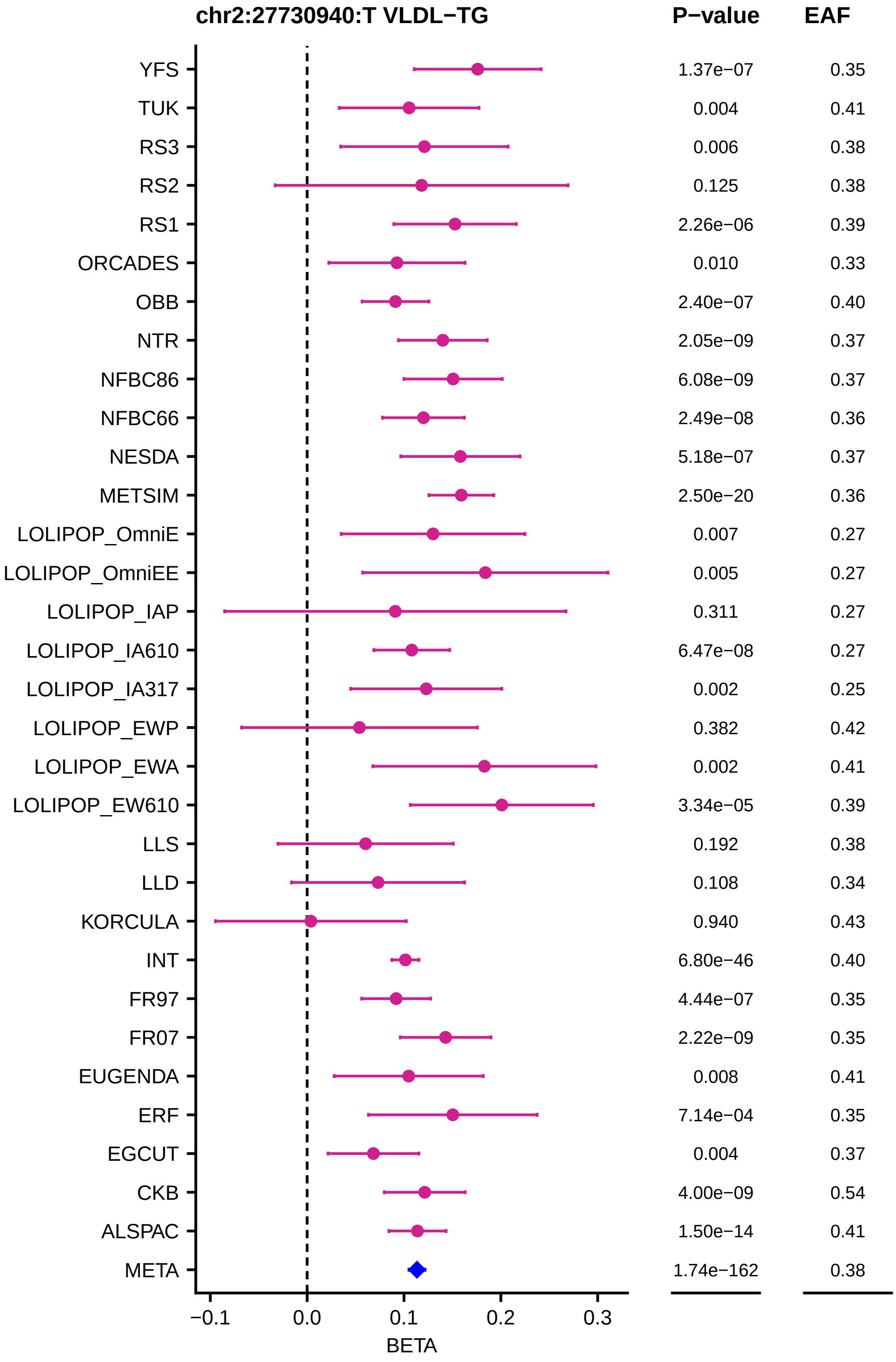
chr2:27730940:T VLDL−TG
P−value
EAF
YFS
1.37e−07
0.35
TUK
0.004
0.41
RS3
0.006
0.38
RS2
0.125
0.38
RS1
2.26e−06
0.39
ORCADES
0.010
0.33
OBB
2.40e−07
0.40
NTR
2.05e−09
0.37
NFBC86
6.08e−09
0.37
NFBC66
2.49e−08
0.36
NESDA
5.18e−07
0.37
METSIM
2.50e−20
0.36
LOLIPOP_OmniE
0.007
0.27
LOLIPOP_OmniEE
0.005
0.27
LOLIPOP_IAP
0.311
0.27
LOLIPOP_IA610
6.47e−08
0.27
LOLIPOP_IA317
0.002
0.25
LOLIPOP_EWP
0.382
0.42
LOLIPOP_EWA
0.002
0.41
LOLIPOP_EW610
3.34e−05
0.39
LLS
0.192
0.38
LLD
0.108
0.34
KORCULA
0.940
0.43
INT
6.80e−46
0.40
FR97
4.44e−07
0.35
FR07
2.22e−09
0.35
EUGENDA
0.008
0.41
ERF
7.14e−04
0.35
EGCUT
0.004
0.37
CKB
4.00e−09
0.54
ALSPAC
1.50e−14
0.41
META
1.74e−162
0.38
−0.1
0.0
0.1
0.2
0.3
BETA

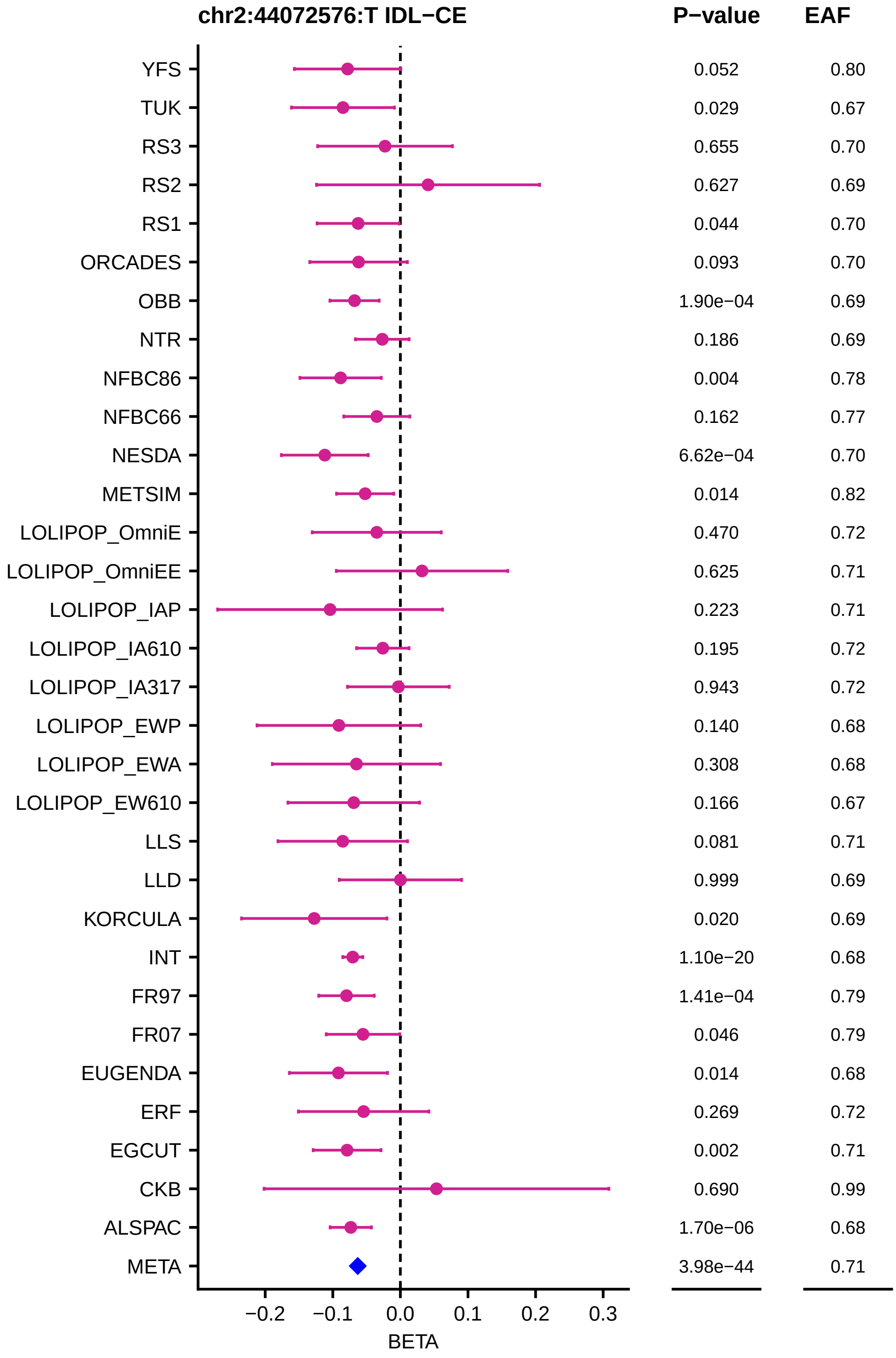
chr2:44072576:T IDL−CE
P−value
EAF
YFS 0.052 0.80
TUK 0.029 0.67
RS3 0.655 0.70
RS2 0.627 0.69
RS1 0.044 0.70
ORCADES 0.093 0.70
OBB 1.90e−04 0.69
NTR 0.186 0.69
NFBC86 0.004 0.78
NFBC66 0.162 0.77
NESDA 6.62e−04 0.70
METSIM 0.014 0.82
LOLIPOP_OmniE 0.470 0.72
LOLIPOP_OmniEE 0.625 0.71
LOLIPOP_IAP 0.223 0.71
LOLIPOP_IA610 0.195 0.72
LOLIPOP_IA317 0.943 0.72
LOLIPOP_EWP 0.140 0.68
LOLIPOP_EWA 0.308 0.68
LOLIPOP_EW610 0.166 0.67
LLS 0.081 0.71
LLD 0.999 0.69
KORCULA 0.020 0.69
INT 1.10e−20 0.68
FR97 1.41e−04 0.79
FR07 0.046 0.79
EUGENDA 0.014 0.68
ERF 0.269 0.72
EGCUT 0.002 0.71
CKB 0.690 0.99
ALSPAC 1.70e−06 0.68
META 3.98e−44 0.71
−0.2
−0.1
0.0
0.1
0.2
0.3
BETA

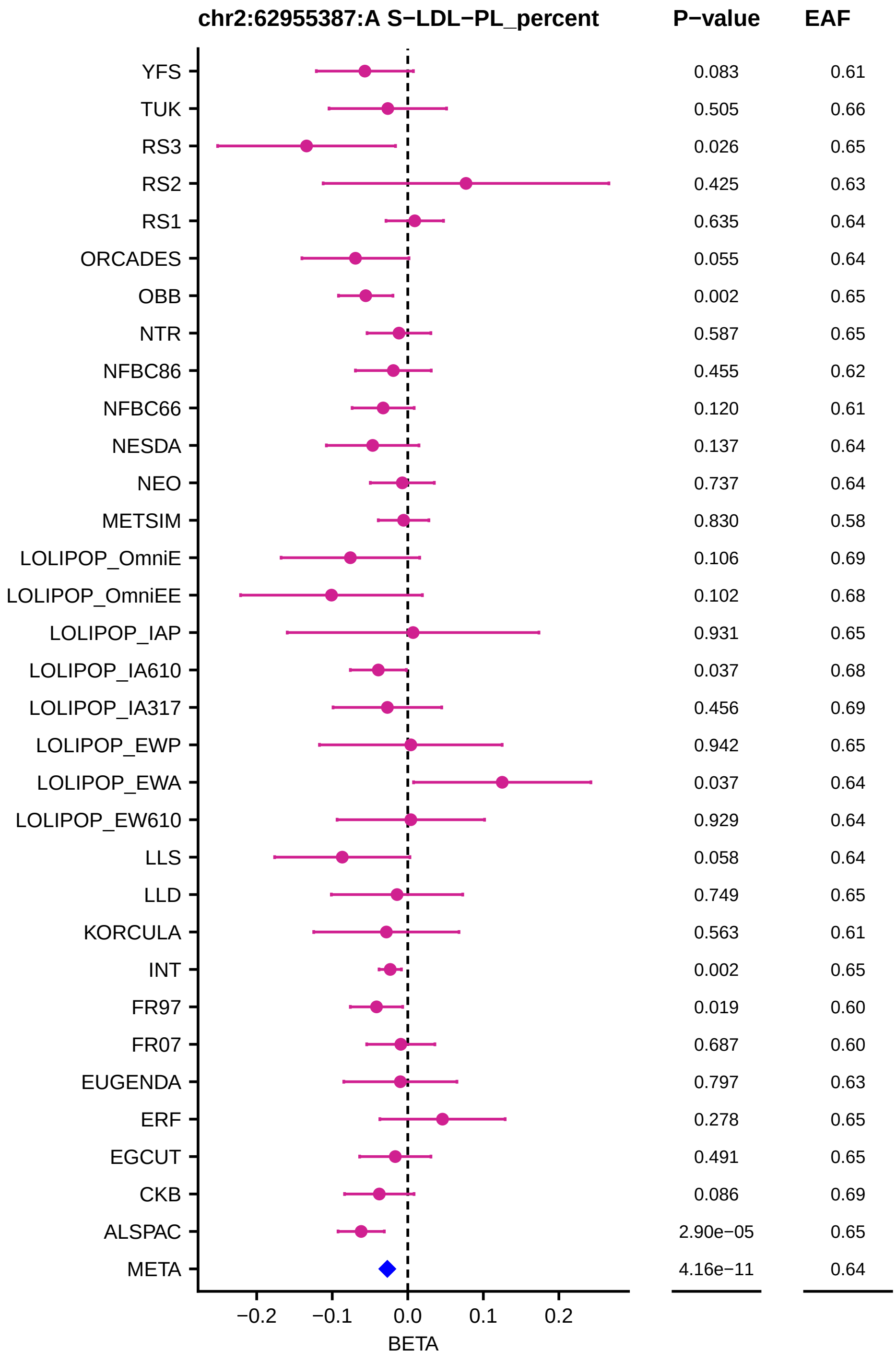
chr2:62955387:A S−LDL−PL_percent
P−value
EAF
YFS 0.083 0.61
TUK 0.505 0.66
RS3 0.026 0.65
RS2 0.425 0.63
RS1 0.635 0.64
ORCADES 0.055 0.64
OBB 0.002 0.65
NTR 0.587 0.65
NFBC86 0.455 0.62
NFBC66 0.120 0.61
NESDA 0.137 0.64
NEO 0.737 0.64
METSIM 0.830 0.58
LOLIPOP_OmniE 0.106 0.69
LOLIPOP_OmniEE 0.102 0.68
LOLIPOP_IAP 0.931 0.65
LOLIPOP_IA610 0.037 0.68
LOLIPOP_IA317 0.456 0.69
LOLIPOP_EWP 0.942 0.65
LOLIPOP_EWA 0.037 0.64
LOLIPOP_EW610 0.929 0.64
LLS 0.058 0.64
LLD 0.749 0.65
KORCULA 0.563 0.61
INT 0.002 0.65
FR97 0.019 0.60
FR07 0.687 0.60
EUGENDA 0.797 0.63
ERF 0.278 0.65
EGCUT 0.491 0.65
CKB 0.086 0.69
ALSPAC 2.90e−05 0.65
META 4.16e−11 0.64
−0.2 −0.1 0.0 0.1 0.2
BETA

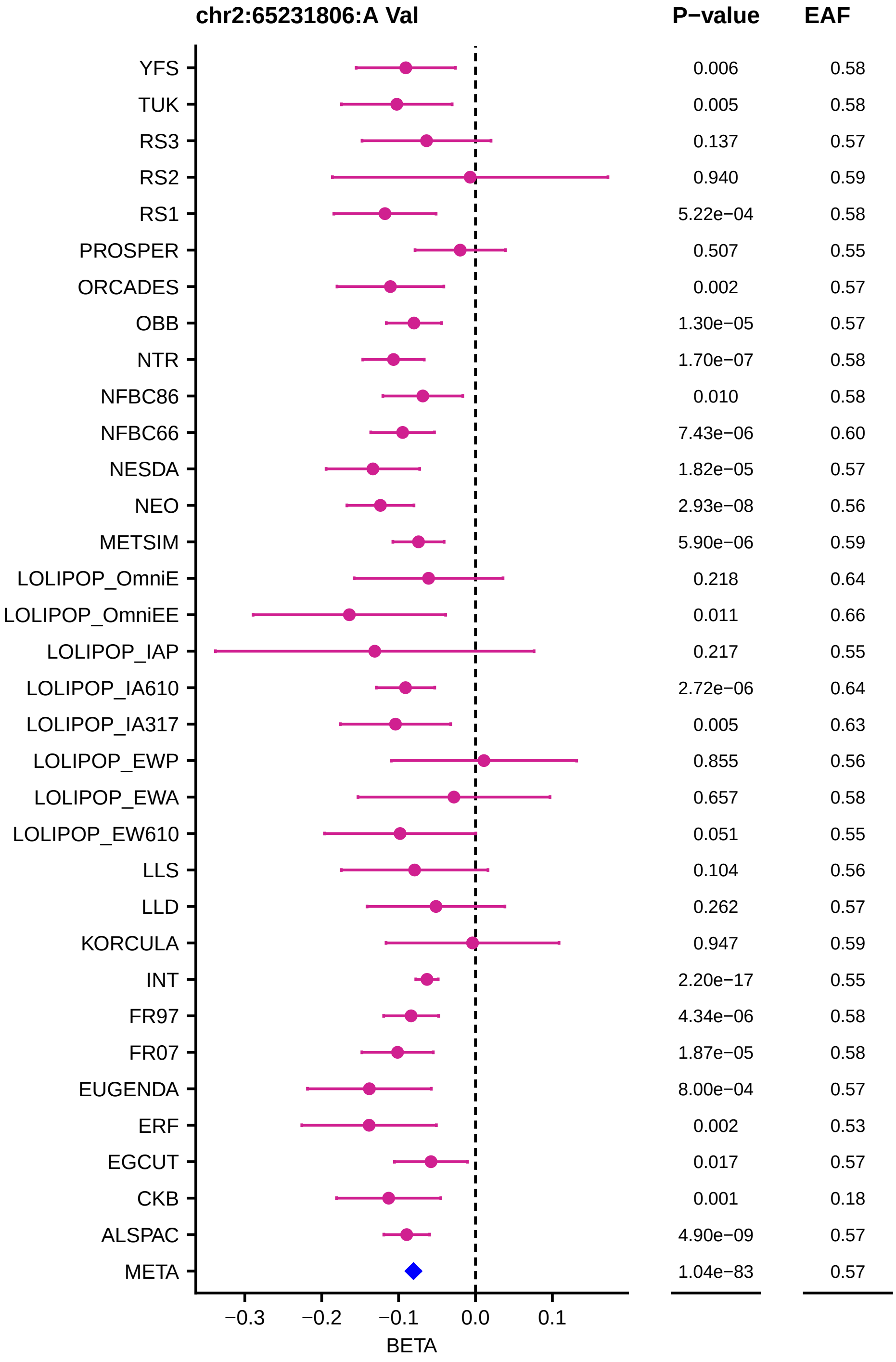

chr2:65231806:A Val
P−value
EAF
YFS
TUK
RS3
RS2
RS1
PROSPER
ORCADES
OBB
NTR
NFBC86
NFBC66
NESDA
NEO
METSIM
LOLIPOP_OmniE
LOLIPOP_OmniEE
LOLIPOP_IAP
LOLIPOP_IA610
LOLIPOP_IA317
LOLIPOP_EWP
LOLIPOP_EWA
LOLIPOP_EW610
LLS
LLD
KORCULA
INT
FR97
FR07
EUGENDA
ERF
EGCUT
CKB
ALSPAC
META
0.006 0.58
0.005 0.58
0.137 0.57
0.940 0.59
5.22e−04 0.58
0.507 0.55
0.002 0.57
1.30e−05 0.57
1.70e−07 0.58
0.010 0.58
7.43e−06 0.60
1.82e−05 0.57
2.93e−08 0.56
5.90e−06 0.59
0.218 0.64
0.011 0.66
0.217 0.55
2.72e−06 0.64
0.005 0.63
0.855 0.56
0.657 0.58
0.051 0.55
0.104 0.56
0.262 0.57
0.947 0.59
2.20e−17 0.55
4.34e−06 0.58
1.87e−05 0.58
8.00e−04 0.57
0.002 0.53
0.017 0.57
0.001 0.18
4.90e−09 0.57
1.04e−83 0.57
−0.3
−0.2
−0.1
0.0
0.1
BETA

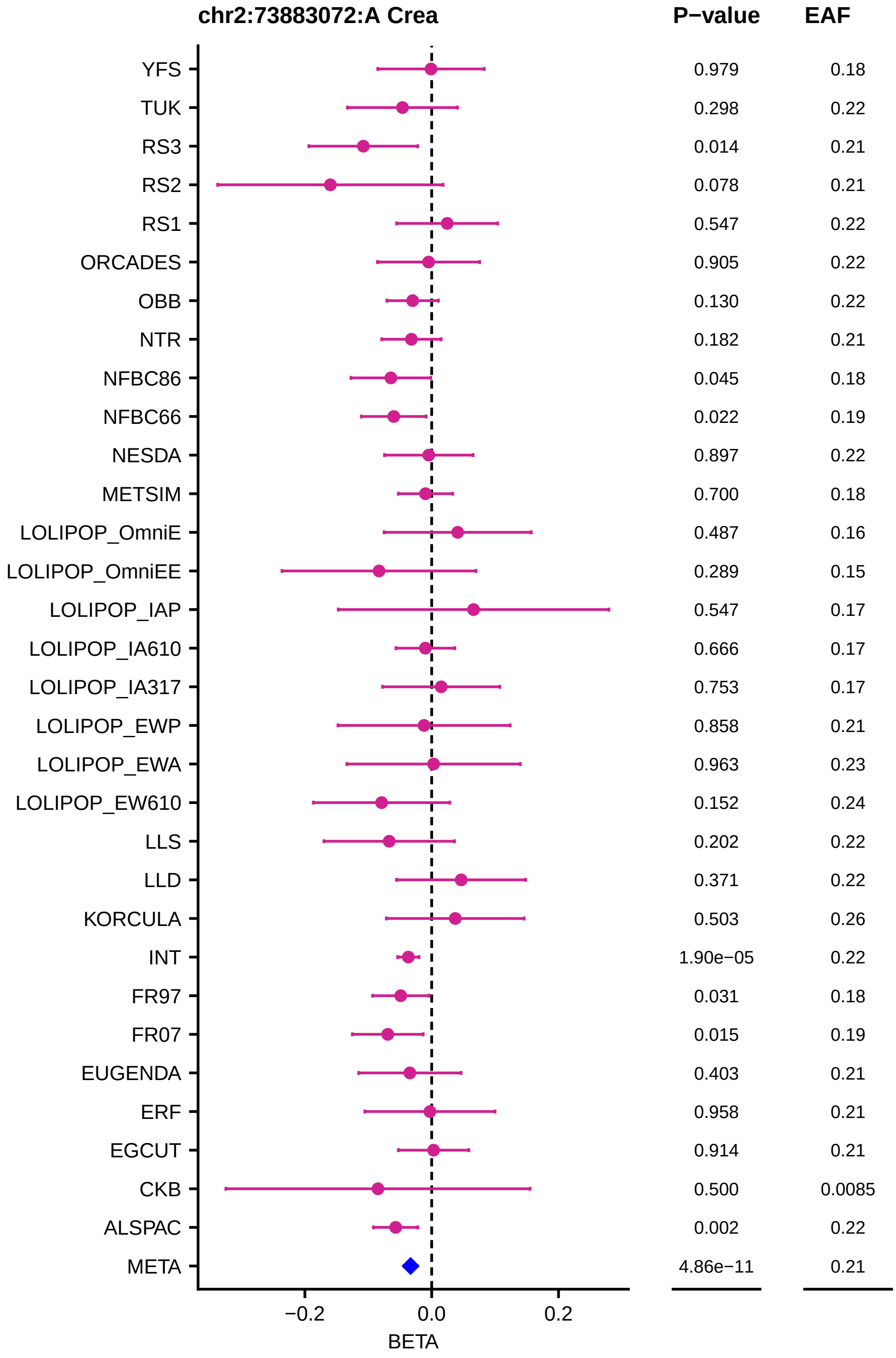
chr2:73883072:A Crea
P−value
EAF
YFS 0.979 0.18
TUK 0.298 0.22
RS3 0.014 0.21
RS2 0.078 0.21
RS1 0.547 0.22
ORCADES 0.905 0.22
OBB 0.130 0.22
NTR 0.182 0.21
NFBC86 0.045 0.18
NFBC66 0.022 0.19
NESDA 0.897 0.22
METSIM 0.700 0.18
LOLIPOP_OmniE 0.487 0.16
LOLIPOP_OmniEE 0.289 0.15
LOLIPOP_IAP 0.547 0.17
LOLIPOP_IA610 0.666 0.17
LOLIPOP_IA317 0.753 0.17
LOLIPOP_EWP 0.858 0.21
LOLIPOP_EWA 0.963 0.23
LOLIPOP_EW610 0.152 0.24
LLS 0.202 0.22
LLD 0.371 0.22
KORCULA 0.503 0.26
INT 1.90e−05 0.22
FR97 0.031 0.18
FR07 0.015 0.19
EUGENDA 0.403 0.21
ERF 0.958 0.21
EGCUT 0.914 0.21
CKB 0.500 0.0085
ALSPAC 0.002 0.22
META 4.86e−11 0.21
−0.2
0.0
0.2
BETA

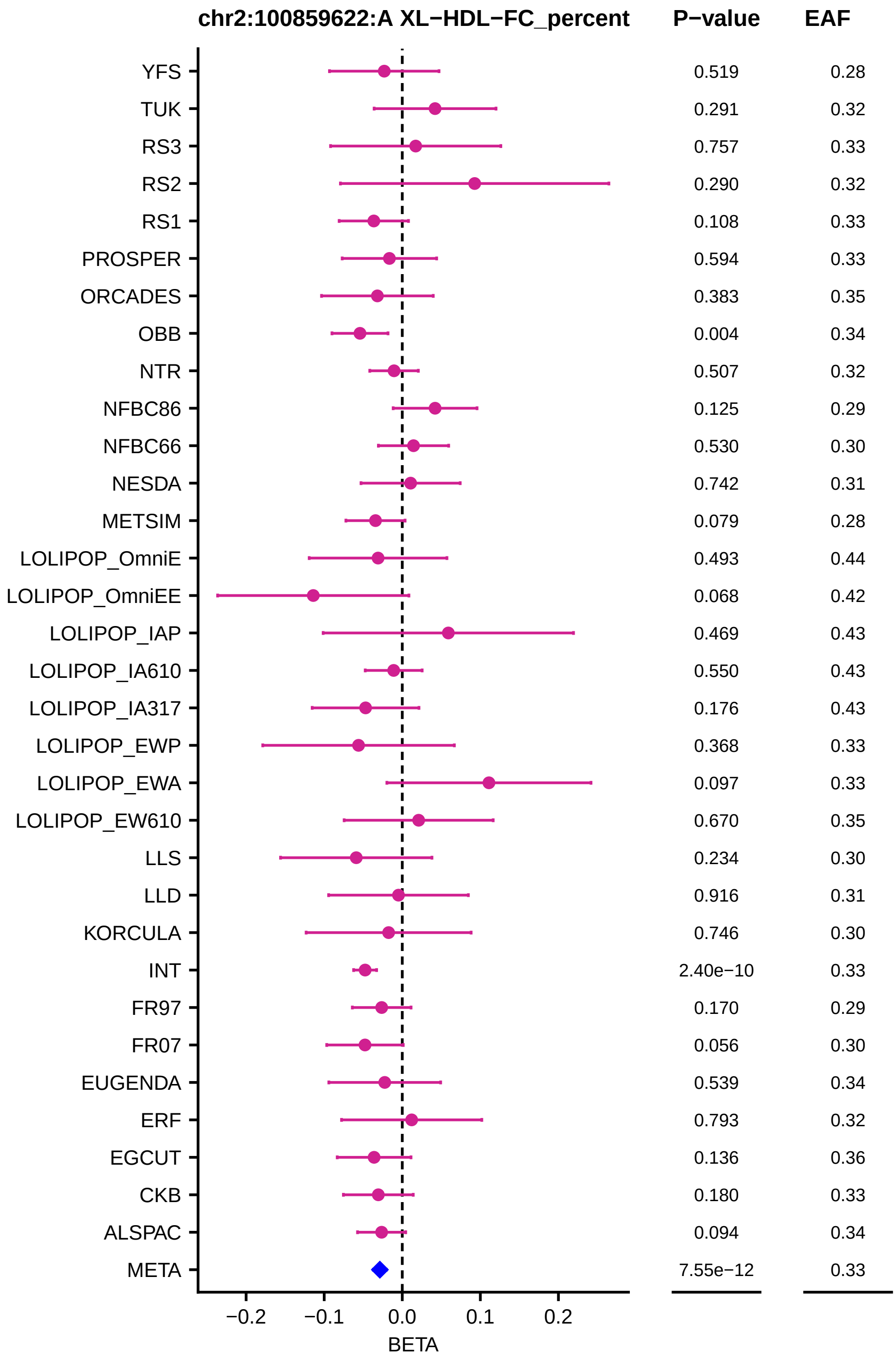
chr2:100859622:A XL−HDL−FC_percent
P−value
EAF
YFS 0.519 0.28
TUK 0.291 0.32
RS3 0.757 0.33
RS2 0.290 0.32
RS1 0.108 0.33
PROSPER 0.594 0.33
ORCADES 0.383 0.35
OBB 0.004 0.34
NTR 0.507 0.32
NFBC86 0.125 0.29
NFBC66 0.530 0.30
NESDA 0.742 0.31
METSIM 0.079 0.28
LOLIPOP_OmniE 0.493 0.44
LOLIPOP_OmniEE 0.068 0.42
LOLIPOP_IAP 0.469 0.43
LOLIPOP_IA610 0.550 0.43
LOLIPOP_IA317 0.176 0.43
LOLIPOP_EWP 0.368 0.33
LOLIPOP_EWA 0.097 0.33
LOLIPOP_EW610 0.670 0.35
LLS 0.234 0.30
LLD 0.916 0.31
KORCULA 0.746 0.30
INT 2.40e−10 0.33
FR97 0.170 0.29
FR07 0.056 0.30
EUGENDA 0.539 0.34
ERF 0.793 0.32
EGCUT 0.136 0.36
CKB 0.180 0.33
ALSPAC 0.094 0.34
META 7.55e−12 0.33
−0.2 −0.1 0.0 0.1 0.2
BETA

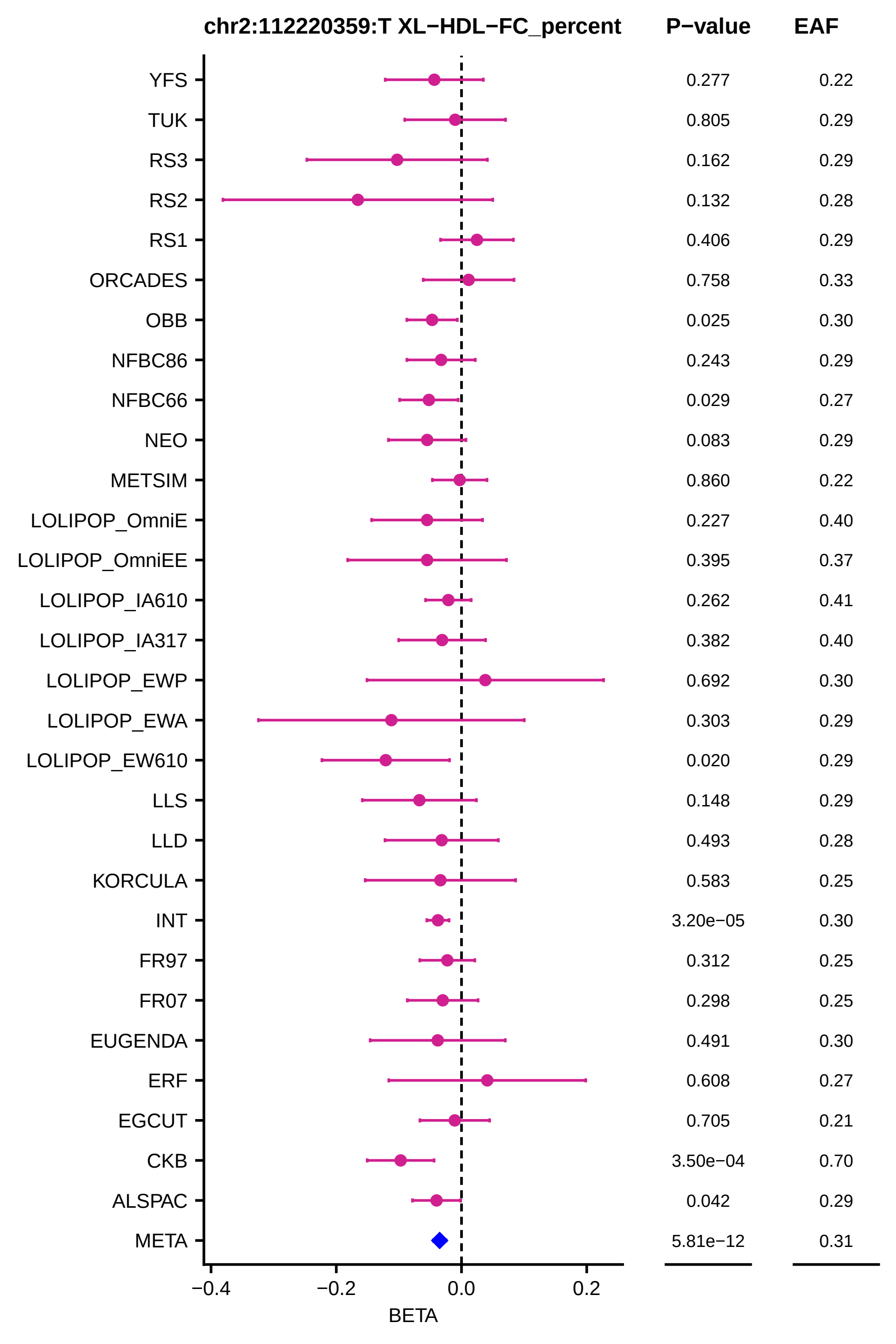
chr2:112220359:T XL−HDL−FC_percent
P−value
EAF
YFS 0.277 0.22
TUK 0.805 0.29
RS3 0.162 0.29
RS2 0.132 0.28
RS1 0.406 0.29
ORCADES 0.758 0.33
OBB 0.025 0.30
NFBC86 0.243 0.29
NFBC66 0.029 0.27
NEO 0.083 0.29
METSIM 0.860 0.22
LOLIPOP_OmniE 0.227 0.40
LOLIPOP_OmniEE 0.395 0.37
LOLIPOP_IA610 0.262 0.41
LOLIPOP_IA317 0.382 0.40
LOLIPOP_EWP 0.692 0.30
LOLIPOP_EWA 0.303 0.29
LOLIPOP_EW610 0.020 0.29
LLS 0.148 0.29
LLD 0.493 0.28
KORCULA 0.583 0.25
INT 3.20e−05 0.30
FR97 0.312 0.25
FR07 0.298 0.25
EUGENDA 0.491 0.30
ERF 0.608 0.27
EGCUT 0.705 0.21
CKB 3.50e−04 0.70
ALSPAC 0.042 0.29
META 5.81e−12 0.31
−0.4
−0.2
0.0
0.2
BETA

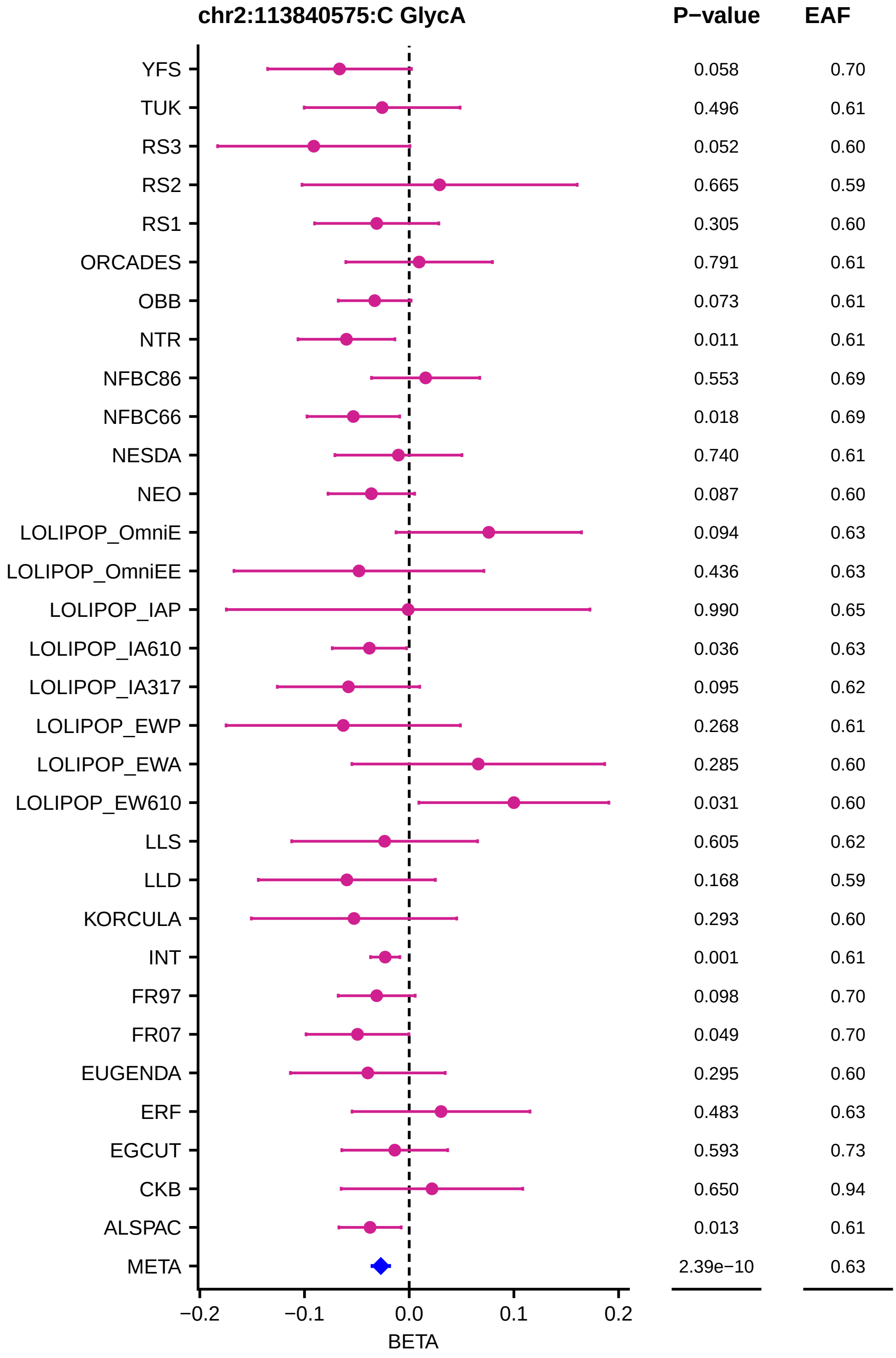
chr2:113840575:C GlycA
P−value
EAF
YFS 0.058 0.70
TUK 0.496 0.61
RS3 0.052 0.60
RS2 0.665 0.59
RS1 0.305 0.60
ORCADES 0.791 0.61
OBB 0.073 0.61
NTR 0.011 0.61
NFBC86 0.553 0.69
NFBC66 0.018 0.69
NESDA 0.740 0.61
NEO 0.087 0.60
LOLIPOP_OmniE 0.094 0.63
LOLIPOP_OmniEE 0.436 0.63
LOLIPOP_IAP 0.990 0.65
LOLIPOP_IA610 0.036 0.63
LOLIPOP_IA317 0.095 0.62
LOLIPOP_EWP 0.268 0.61
LOLIPOP_EWA 0.285 0.60
LOLIPOP_EW610 0.031 0.60
LLS 0.605 0.62
LLD 0.168 0.59
KORCULA 0.293 0.60
INT 0.001 0.61
FR97 0.098 0.70
FR07 0.049 0.70
EUGENDA 0.295 0.60
ERF 0.483 0.63
EGCUT 0.593 0.73
CKB 0.650 0.94
ALSPAC 0.013 0.61
META 2.39e−10 0.63
−0.2
−0.1
0.0
0.1
0.2
BETA

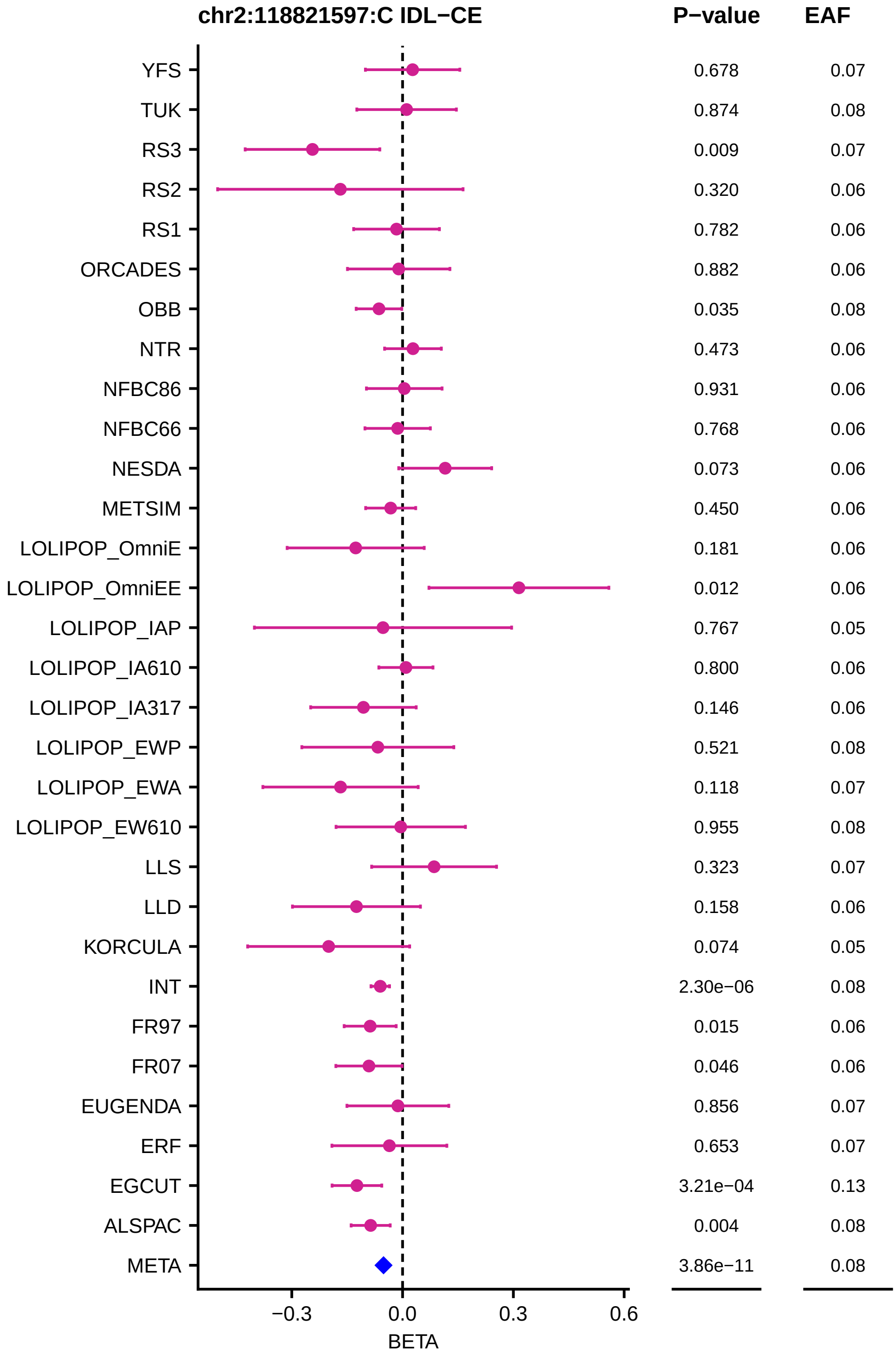
chr2:118821597:C IDL−CE
P−value
EAF
YFS 0.678 0.07
TUK 0.874 0.08
RS3 0.009 0.07
RS2 0.320 0.06
RS1 0.782 0.06
ORCADES 0.882 0.06
OBB 0.035 0.08
NTR 0.473 0.06
NFBC86 0.931 0.06
NFBC66 0.768 0.06
NESDA 0.073 0.06
METSIM 0.450 0.06
LOLIPOP_OmniE 0.181 0.06
LOLIPOP_OmniEE 0.012 0.06
LOLIPOP_IAP 0.767 0.05
LOLIPOP_IA610 0.800 0.06
LOLIPOP_IA317 0.146 0.06
LOLIPOP_EWP 0.521 0.08
LOLIPOP_EWA 0.118 0.07
LOLIPOP_EW610 0.955 0.08
LLS 0.323 0.07
LLD 0.158 0.06
KORCULA 0.074 0.05
INT 2.30e−06 0.08
FR97 0.015 0.06
FR07 0.046 0.06
EUGENDA 0.856 0.07
ERF 0.653 0.07
EGCUT 3.21e−04 0.13
ALSPAC 0.004 0.08
META 3.86e−11 0.08
−0.3
0.0
0.3
0.6
BETA

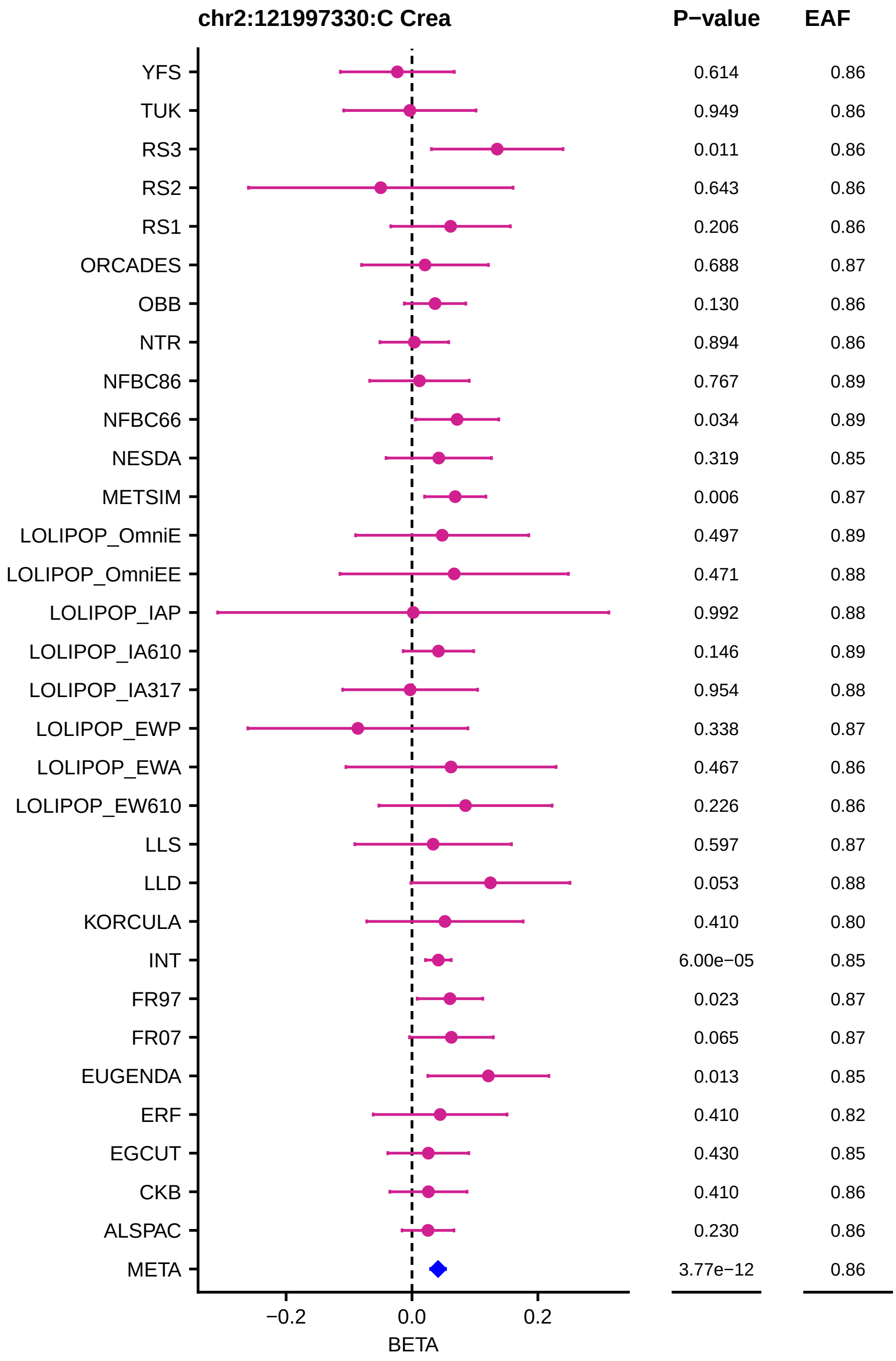

chr2:121997330:C Crea
P−value
EAF
YFS
TUK
RS3
RS2
RS1
ORCADES
OBB
NTR
NFBC86
NFBC66
NESDA
METSIM
LOLIPOP_OmniE
LOLIPOP_OmniEE
LOLIPOP_IAP
LOLIPOP_IA610
LOLIPOP_IA317
LOLIPOP_EWP
LOLIPOP_EWA
LOLIPOP_EW610
LLS
LLD
KORCULA
INT
FR97
FR07
EUGENDA
ERF
EGCUT
CKB
ALSPAC
META
0.614 0.86
0.949 0.86
0.011 0.86
0.643 0.86
0.206 0.86
0.688 0.87
0.130 0.86
0.894 0.86
0.767 0.89
0.034 0.89
0.319 0.85
0.006 0.87
0.497 0.89
0.471 0.88
0.992 0.88
0.146 0.89
0.954 0.88
0.338 0.87
0.467 0.86
0.226 0.86
0.597 0.87
0.053 0.88
0.410 0.80
6.00e−05 0.85
0.023 0.87
0.065 0.87
0.013 0.85
0.410 0.82
0.430 0.85
0.410 0.86
0.230 0.86
3.77e−12 0.86
−0.2
0.0
0.2
BETA

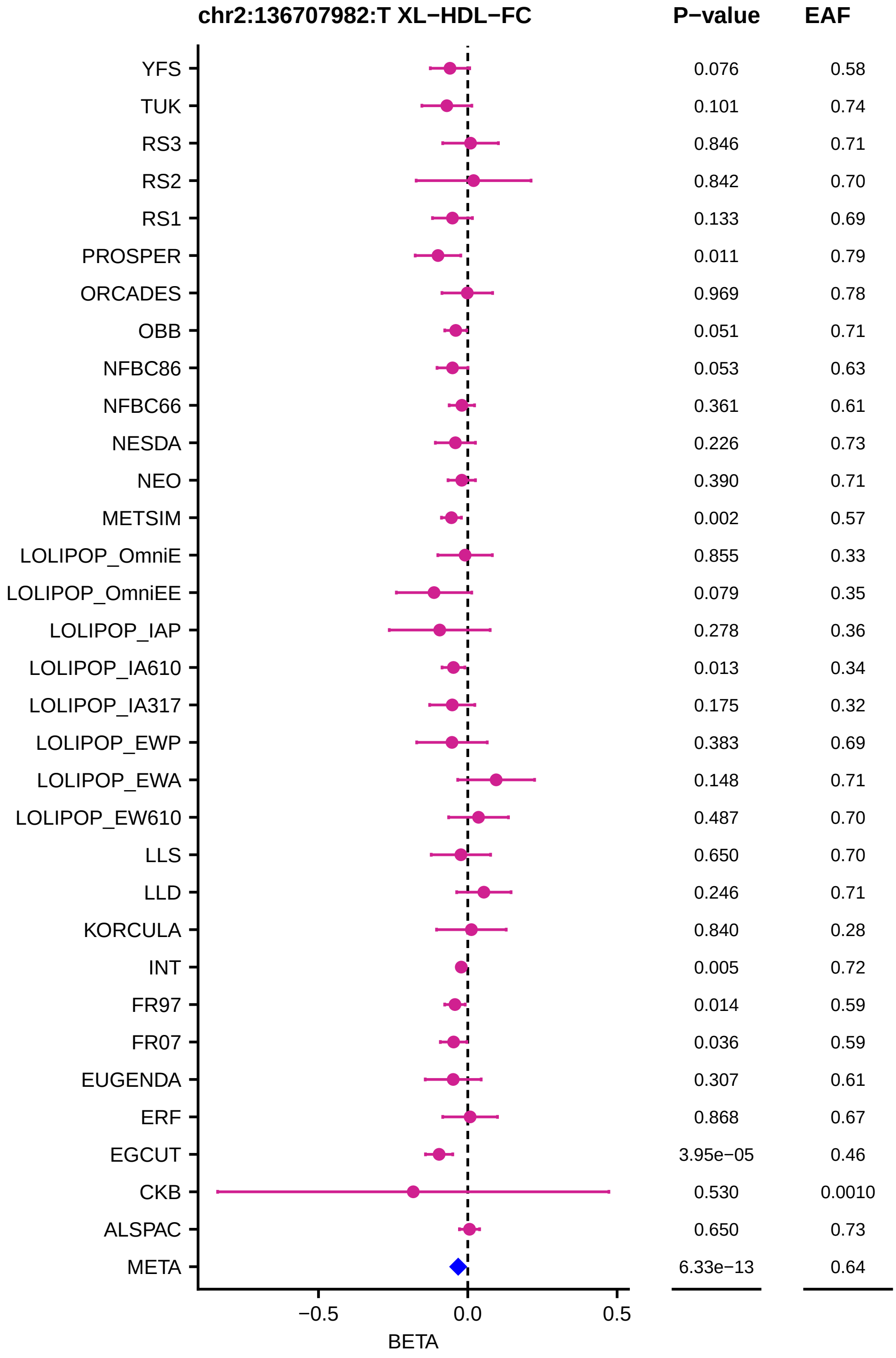
chr2:136707982:T XL−HDL−FC
P−value
EAF
YFS 0.076 0.58
TUK 0.101 0.74
RS3 0.846 0.71
RS2 0.842 0.70
RS1 0.133 0.69
PROSPER 0.011 0.79
ORCADES 0.969 0.78
OBB 0.051 0.71
NFBC86 0.053 0.63
NFBC66 0.361 0.61
NESDA 0.226 0.73
NEO 0.390 0.71
METSIM 0.002 0.57
LOLIPOP_OmniE 0.855 0.33
LOLIPOP_OmniEE 0.079 0.35
LOLIPOP_IAP 0.278 0.36
LOLIPOP_IA610 0.013 0.34
LOLIPOP_IA317 0.175 0.32
LOLIPOP_EWP 0.383 0.69
LOLIPOP_EWA 0.148 0.71
LOLIPOP_EW610 0.487 0.70
LLS 0.650 0.70
LLD 0.246 0.71
KORCULA 0.840 0.28
INT 0.005 0.72
FR97 0.014 0.59
FR07 0.036 0.59
EUGENDA 0.307 0.61
ERF 0.868 0.67
EGCUT 3.95e−05 0.46
CKB 0.530 0.0010
ALSPAC 0.650 0.73
META 6.33e−13 0.64
−0.5
0.0
0.5
BETA

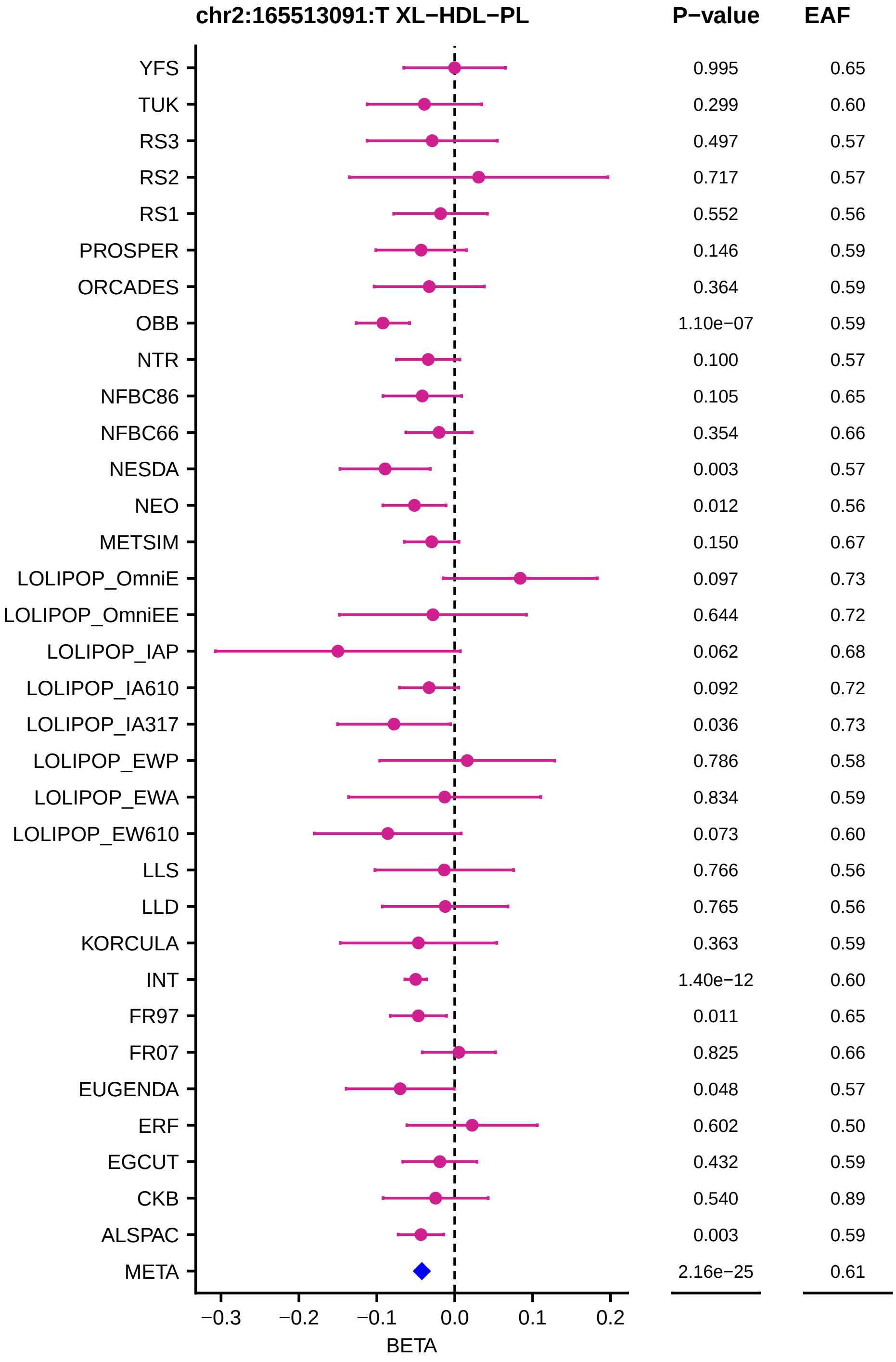
chr2:165513091:T XL−HDL−PL
P−value
EAF
YFS 0.995 0.65
TUK 0.299 0.60
RS3 0.497 0.57
RS2 0.717 0.57
RS1 0.552 0.56
PROSPER 0.146 0.59
ORCADES 0.364 0.59
OBB 1.10e−07 0.59
NTR 0.100 0.57
NFBC86 0.105 0.65
NFBC66 0.354 0.66
NESDA 0.003 0.57
NEO 0.012 0.56
METSIM 0.150 0.67
LOLIPOP_OmniE 0.097 0.73
LOLIPOP_OmniEE 0.644 0.72
LOLIPOP_IAP 0.062 0.68
LOLIPOP_IA610 0.092 0.72
LOLIPOP_IA317 0.036 0.73
LOLIPOP_EWP 0.786 0.58
LOLIPOP_EWA 0.834 0.59
LOLIPOP_EW610 0.073 0.60
LLS 0.766 0.56
LLD 0.765 0.56
KORCULA 0.363 0.59
INT 1.40e−12 0.60
FR97 0.011 0.65
FR07 0.825 0.66
EUGENDA 0.048 0.57
ERF 0.602 0.50
EGCUT 0.432 0.59
CKB 0.540 0.89
ALSPAC 0.003 0.59
META 2.16e−25 0.61
−0.3 −0.2 −0.1 0.0 0.1 0.2
BETA

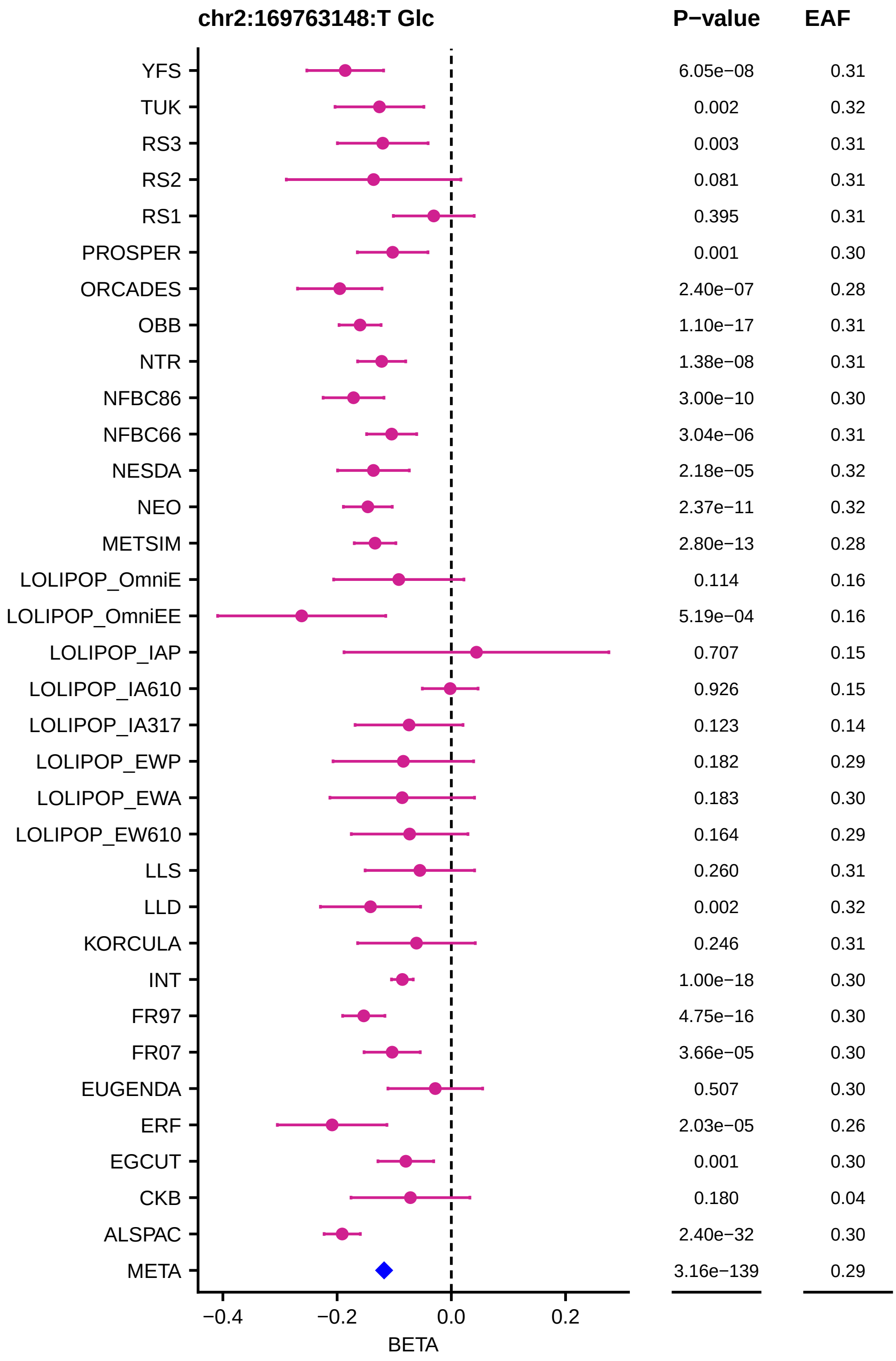
chr2:169763148:T Glc
P−value
EAF
YFS 6.05e−08 0.31
TUK 0.002 0.32
RS3 0.003 0.31
RS2 0.081 0.31
RS1 0.395 0.31
PROSPER 0.001 0.30
ORCADES 2.40e−07 0.28
OBB 1.10e−17 0.31
NTR 1.38e−08 0.31
NFBC86 3.00e−10 0.30
NFBC66 3.04e−06 0.31
NESDA 2.18e−05 0.32
NEO 2.37e−11 0.32
METSIM 2.80e−13 0.28
LOLIPOP_OmniE 0.114 0.16
LOLIPOP_OmniEE 5.19e−04 0.16
LOLIPOP_IAP 0.707 0.15
LOLIPOP_IA610 0.926 0.15
LOLIPOP_IA317 0.123 0.14
LOLIPOP_EWP 0.182 0.29
LOLIPOP_EWA 0.183 0.30
LOLIPOP_EW610 0.164 0.29
LLS 0.260 0.31
LLD 0.002 0.32
KORCULA 0.246 0.31
INT 1.00e−18 0.30
FR97 4.75e−16 0.30
FR07 3.66e−05 0.30
EUGENDA 0.507 0.30
ERF 2.03e−05 0.26
EGCUT 0.001 0.30
CKB 0.180 0.04
ALSPAC 2.40e−32 0.30
META 3.16e−139 0.29
−0.4
−0.2
0.0
0.2
BETA

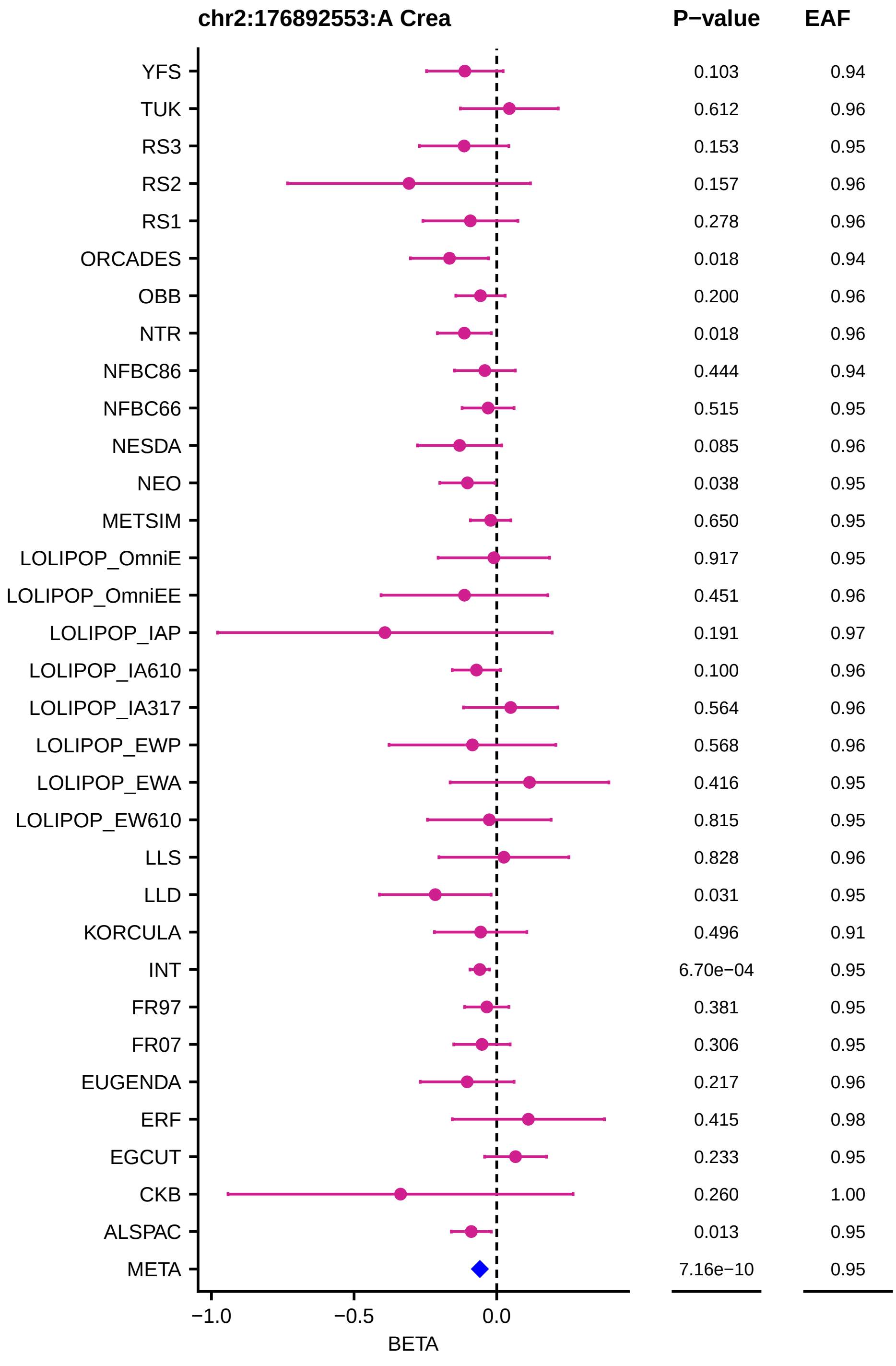
chr2:176892553:A Crea
P−value
EAF
YFS 0.103 0.94
TUK 0.612 0.96
RS3 0.153 0.95
RS2 0.157 0.96
RS1 0.278 0.96
ORCADES 0.018 0.94
OBB 0.200 0.96
NTR 0.018 0.96
NFBC86 0.444 0.94
NFBC66 0.515 0.95
NESDA 0.085 0.96
NEO 0.038 0.95
METSIM 0.650 0.95
LOLIPOP_OmniE 0.917 0.95
LOLIPOP_OmniEE 0.451 0.96
LOLIPOP_IAP 0.191 0.97
LOLIPOP_IA610 0.100 0.96
LOLIPOP_IA317 0.564 0.96
LOLIPOP_EWP 0.568 0.96
LOLIPOP_EWA 0.416 0.95
LOLIPOP_EW610 0.815 0.95
LLS 0.828 0.96
LLD 0.031 0.95
KORCULA 0.496 0.91
INT 6.70e−04 0.95
FR97 0.381 0.95
FR07 0.306 0.95
EUGENDA 0.217 0.96
ERF 0.415 0.98
EGCUT 0.233 0.95
CKB 0.260 1.00
ALSPAC 0.013 0.95
META 7.16e−10 0.95
−1.0
−0.5
0.0
BETA

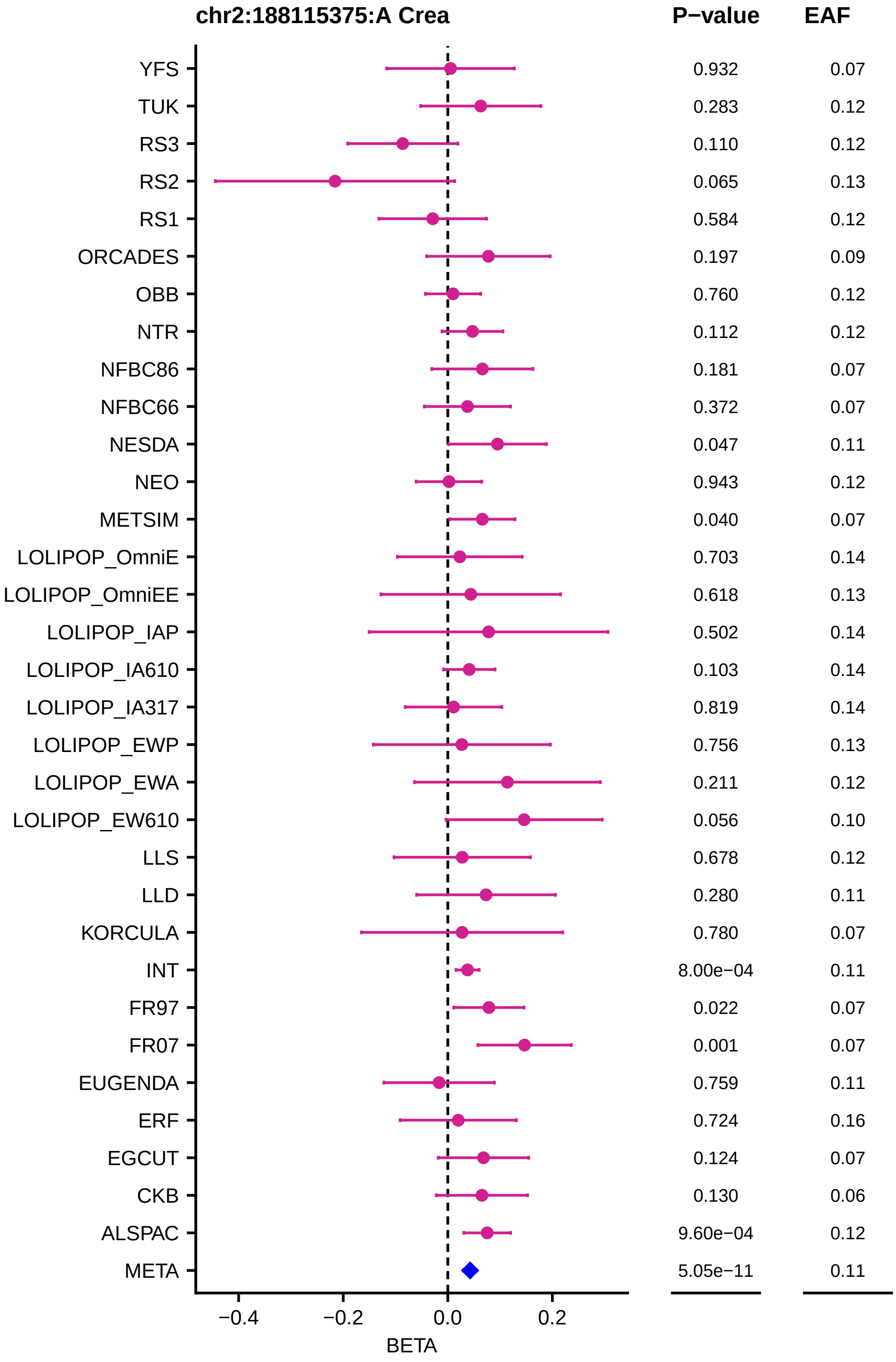
chr2:188115375:A Crea
P−value
EAF
YFS 0.932 0.07
TUK 0.283 0.12
RS3 0.110 0.12
RS2 0.065 0.13
RS1 0.584 0.12
ORCADES 0.197 0.09
OBB 0.760 0.12
NTR 0.112 0.12
NFBC86 0.181 0.07
NFBC66 0.372 0.07
NESDA 0.047 0.11
NEO 0.943 0.12
METSIM 0.040 0.07
LOLIPOP_OmniE 0.703 0.14
LOLIPOP_OmniEE 0.618 0.13
LOLIPOP_IAP 0.502 0.14
LOLIPOP_IA610 0.103 0.14
LOLIPOP_IA317 0.819 0.14
LOLIPOP_EWP 0.756 0.13
LOLIPOP_EWA 0.211 0.12
LOLIPOP_EW610 0.056 0.10
LLS 0.678 0.12
LLD 0.280 0.11
KORCULA 0.780 0.07
INT 8.00e−04 0.11
FR97 0.022 0.07
FR07 0.001 0.07
EUGENDA 0.759 0.11
ERF 0.724 0.16
EGCUT 0.124 0.07
CKB 0.130 0.06
ALSPAC 9.60e−04 0.12
META 5.05e−11 0.11
−0.4
−0.2
0.0
0.2
BETA

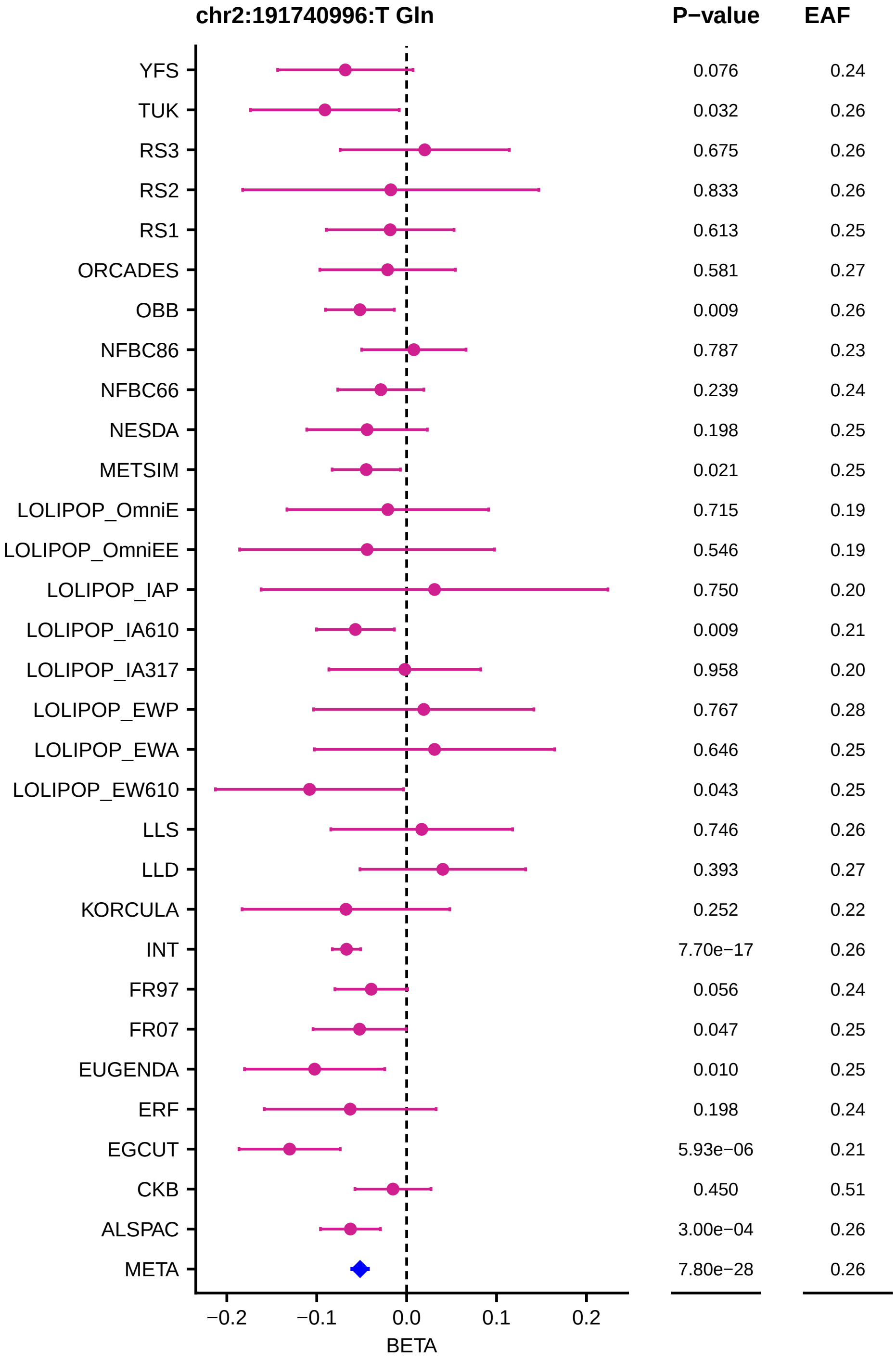
chr2:191740996:T Gln
P−value
EAF
YFS
TUK
RS3
RS2
RS1
ORCADES
OBB
NFBC86
NFBC66
NESDA
METSIM
LOLIPOP_OmniE
LOLIPOP_OmniEE
LOLIPOP_IAP
LOLIPOP_IA610
LOLIPOP_IA317
LOLIPOP_EWP
LOLIPOP_EWA
LOLIPOP_EW610
LLS
LLD
KORCULA
INT
FR97
FR07
EUGENDA
ERF
EGCUT
CKB
ALSPAC
META
0.076
0.032
0.675
0.833
0.613
0.581
0.009
0.787
0.239
0.198
0.021
0.715
0.546
0.750
0.009
0.958
0.767
0.646
0.043
0.746
0.393
0.252
7.70e−17
0.056
0.047
0.010
0.198
5.93e−06
0.450
3.00e−04
7.80e−28
0.24
0.26
0.26
0.26
0.25
0.27
0.26
0.23
0.24
0.25
0.25
0.19
0.19
0.20
0.21
0.20
0.28
0.25
0.25
0.26
0.27
0.22
0.26
0.24
0.25
0.25
0.24
0.21
0.51
0.26
0.26
−0.2
−0.1
0.0
0.1
0.2
BETA

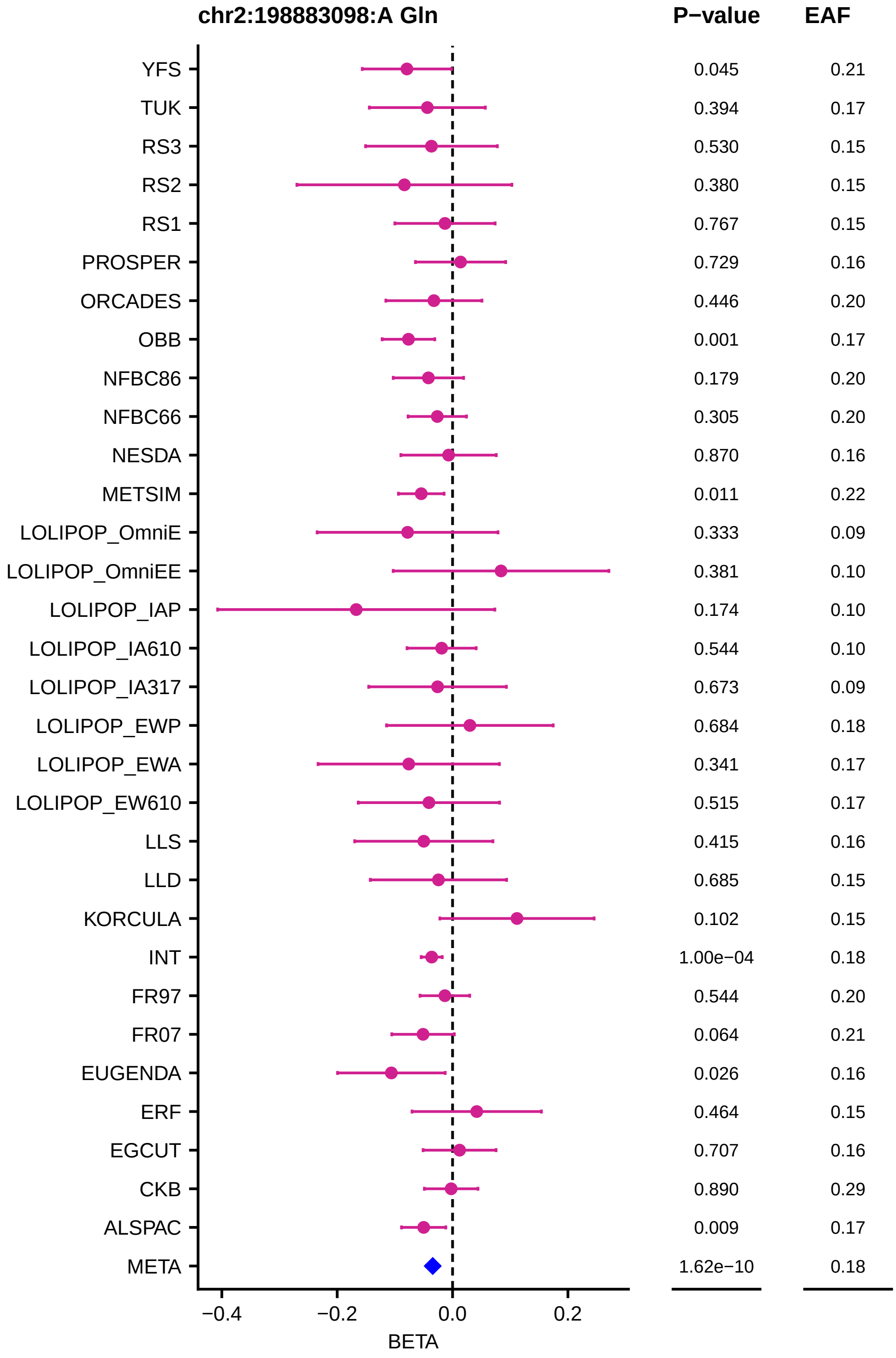
chr2:198883098:A Gln
P−value
EAF
YFS 0.045 0.21
TUK 0.394 0.17
RS3 0.530 0.15
RS2 0.380 0.15
RS1 0.767 0.15
PROSPER 0.729 0.16
ORCADES 0.446 0.20
OBB 0.001 0.17
NFBC86 0.179 0.20
NFBC66 0.305 0.20
NESDA 0.870 0.16
METSIM 0.011 0.22
LOLIPOP_OmniE 0.333 0.09
LOLIPOP_OmniEE 0.381 0.10
LOLIPOP_IAP 0.174 0.10
LOLIPOP_IA610 0.544 0.10
LOLIPOP_IA317 0.673 0.09
LOLIPOP_EWP 0.684 0.18
LOLIPOP_EWA 0.341 0.17
LOLIPOP_EW610 0.515 0.17
LLS 0.415 0.16
LLD 0.685 0.15
KORCULA 0.102 0.15
INT 1.00e−04 0.18
FR97 0.544 0.20
FR07 0.064 0.21
EUGENDA 0.026 0.16
ERF 0.464 0.15
EGCUT 0.707 0.16
CKB 0.890 0.29
ALSPAC 0.009 0.17
META 1.62e−10 0.18
−0.4
−0.2
0.0
0.2
BETA

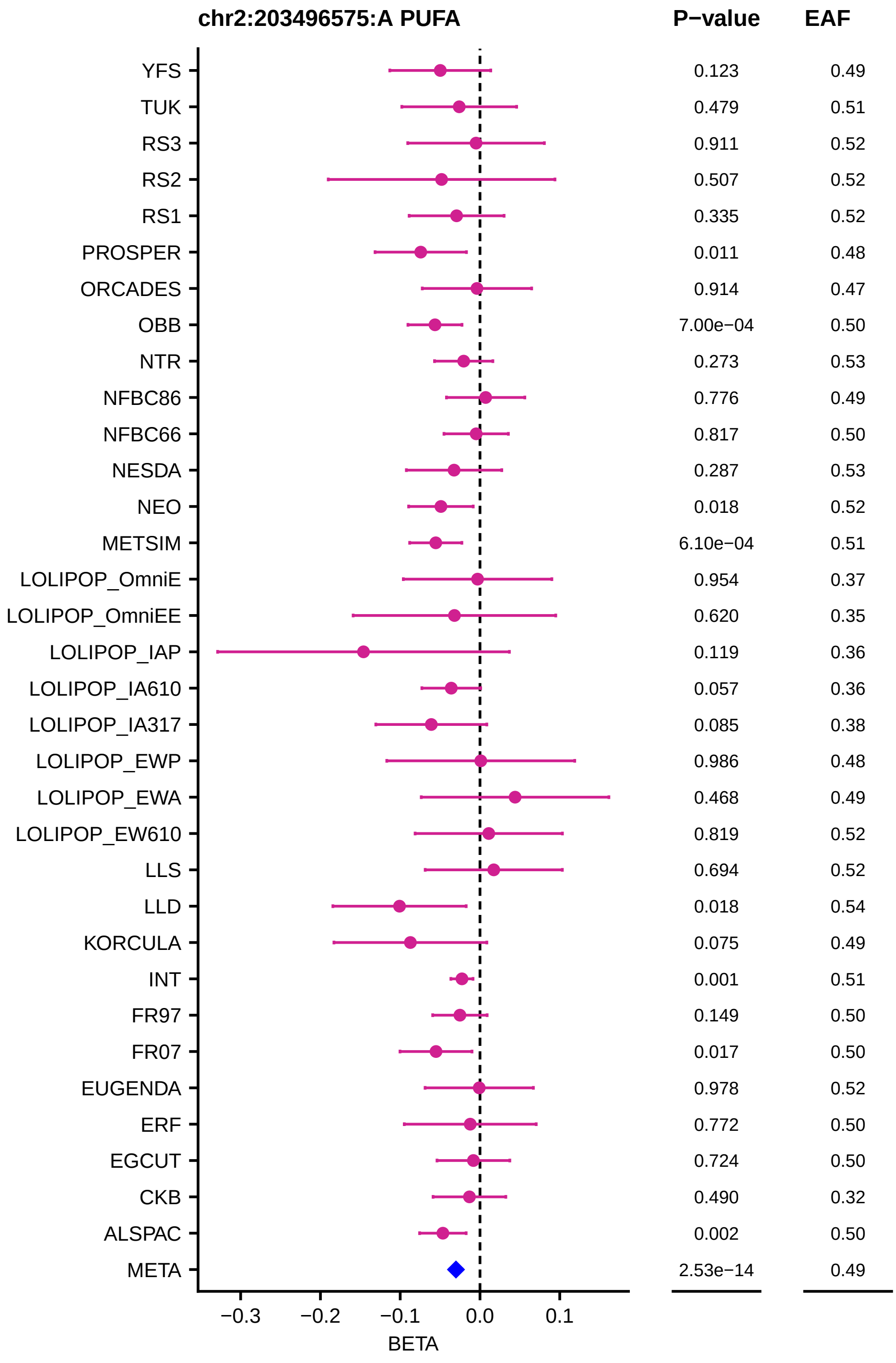
chr2:203496575:A PUFA
P−value
EAF
YFS 0.123 0.49
TUK 0.479 0.51
RS3 0.911 0.52
RS2 0.507 0.52
RS1 0.335 0.52
PROSPER 0.011 0.48
ORCADES 0.914 0.47
OBB 7.00e−04 0.50
NTR 0.273 0.53
NFBC86 0.776 0.49
NFBC66 0.817 0.50
NESDA 0.287 0.53
NEO 0.018 0.52
METSIM 6.10e−04 0.51
LOLIPOP_OmniE 0.954 0.37
LOLIPOP_OmniEE 0.620 0.35
LOLIPOP_IAP 0.119 0.36
LOLIPOP_IA610 0.057 0.36
LOLIPOP_IA317 0.085 0.38
LOLIPOP_EWP 0.986 0.48
LOLIPOP_EWA 0.468 0.49
LOLIPOP_EW610 0.819 0.52
LLS 0.694 0.52
LLD 0.018 0.54
KORCULA 0.075 0.49
INT 0.001 0.51
FR97 0.149 0.50
FR07 0.017 0.50
EUGENDA 0.978 0.52
ERF 0.772 0.50
EGCUT 0.724 0.50
CKB 0.490 0.32
ALSPAC 0.002 0.50
META 2.53e−14 0.49
−0.3
−0.2
−0.1
0.0
0.1
BETA

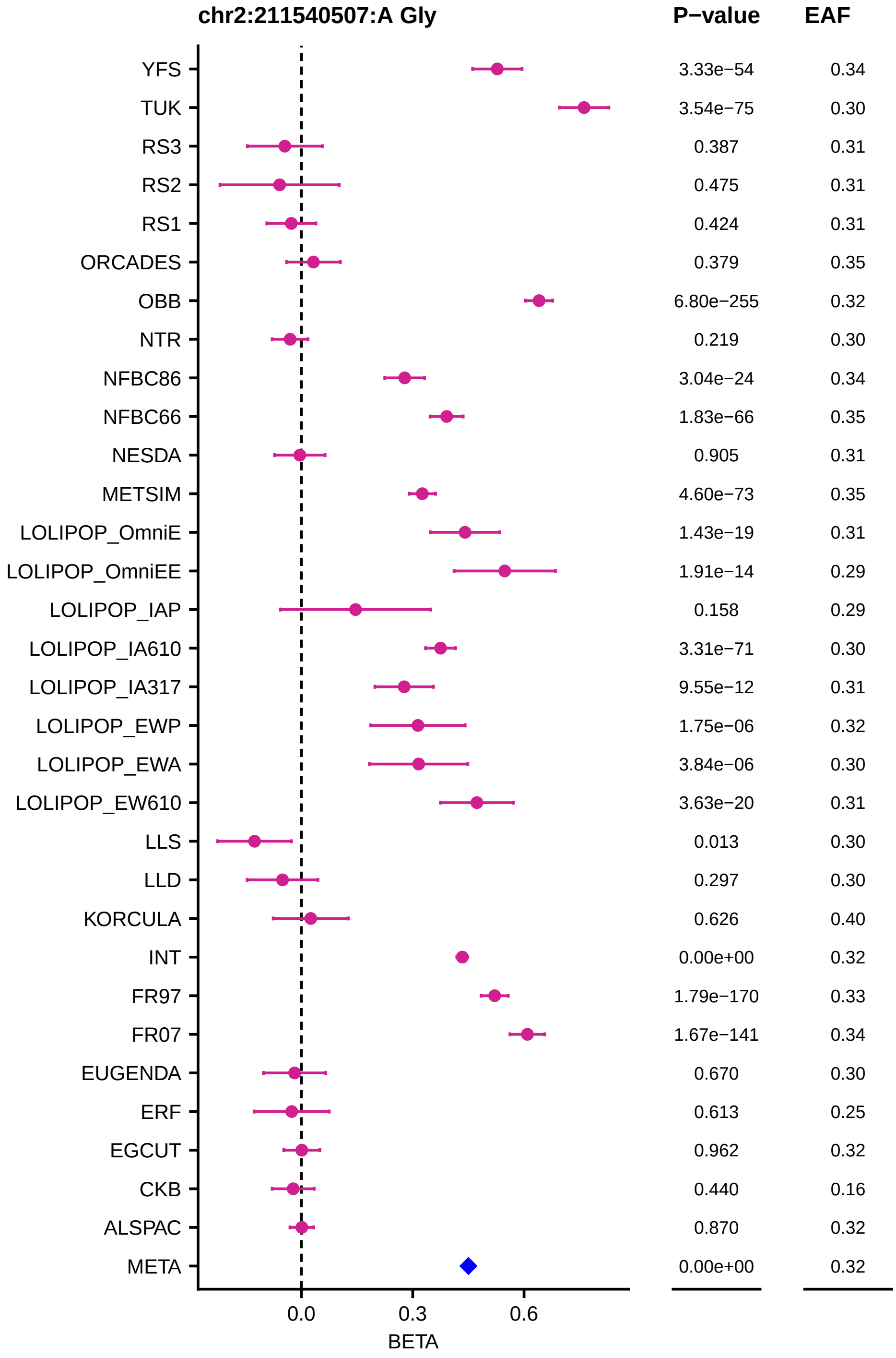
chr2:211540507:A Gly
P−value
EAF
YFS 3.33e−54 0.34
TUK 3.54e−75 0.30
RS3 0.387 0.31
RS2 0.475 0.31
RS1 0.424 0.31
ORCADES 0.379 0.35
OBB 6.80e−255 0.32
NTR 0.219 0.30
NFBC86 3.04e−24 0.34
NFBC66 1.83e−66 0.35
NESDA 0.905 0.31
METSIM 4.60e−73 0.35
LOLIPOP_OmniE 1.43e−19 0.31
LOLIPOP_OmniEE 1.91e−14 0.29
LOLIPOP_IAP 0.158 0.29
LOLIPOP_IA610 3.31e−71 0.30
LOLIPOP_IA317 9.55e−12 0.31
LOLIPOP_EWP 1.75e−06 0.32
LOLIPOP_EWA 3.84e−06 0.30
LOLIPOP_EW610 3.63e−20 0.31
LLS 0.013 0.30
LLD 0.297 0.30
KORCULA 0.626 0.40
INT 0.00e+00 0.32
FR97 1.79e−170 0.33
FR07 1.67e−141 0.34
EUGENDA 0.670 0.30
ERF 0.613 0.25
EGCUT 0.962 0.32
CKB 0.440 0.16
ALSPAC 0.870 0.32
META 0.00e+00 0.32
0.0
0.3
0.6
BETA

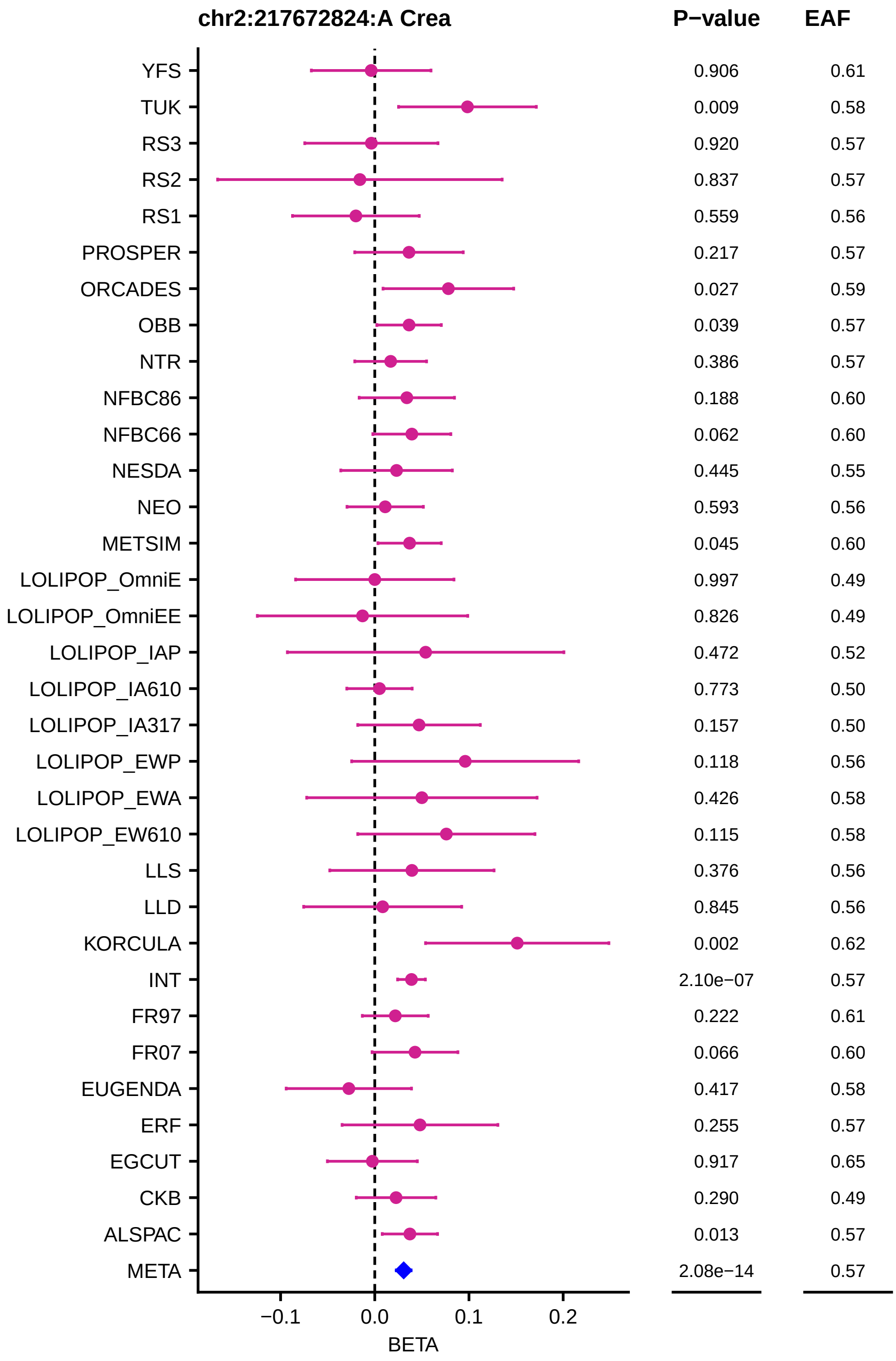

chr2:217672824:A Crea
P−value
EAF
YFS 0.906 0.61
TUK 0.009 0.58
RS3 0.920 0.57
RS2 0.837 0.57
RS1 0.559 0.56
PROSPER 0.217 0.57
ORCADES 0.027 0.59
OBB 0.039 0.57
NTR 0.386 0.57
NFBC86 0.188 0.60
NFBC66 0.062 0.60
NESDA 0.445 0.55
NEO 0.593 0.56
METSIM 0.045 0.60
LOLIPOP_OmniE 0.997 0.49
LOLIPOP_OmniEE 0.826 0.49
LOLIPOP_IAP 0.472 0.52
LOLIPOP_IA610 0.773 0.50
LOLIPOP_IA317 0.157 0.50
LOLIPOP_EWP 0.118 0.56
LOLIPOP_EWA 0.426 0.58
LOLIPOP_EW610 0.115 0.58
LLS 0.376 0.56
LLD 0.845 0.56
KORCULA 0.002 0.62
INT 2.10e−07 0.57
FR97 0.222 0.61
FR07 0.066 0.60
EUGENDA 0.417 0.58
ERF 0.255 0.57
EGCUT 0.917 0.65
CKB 0.290 0.49
ALSPAC 0.013 0.57
META 2.08e−14 0.57
−0.1
0.0
0.1
0.2
BETA

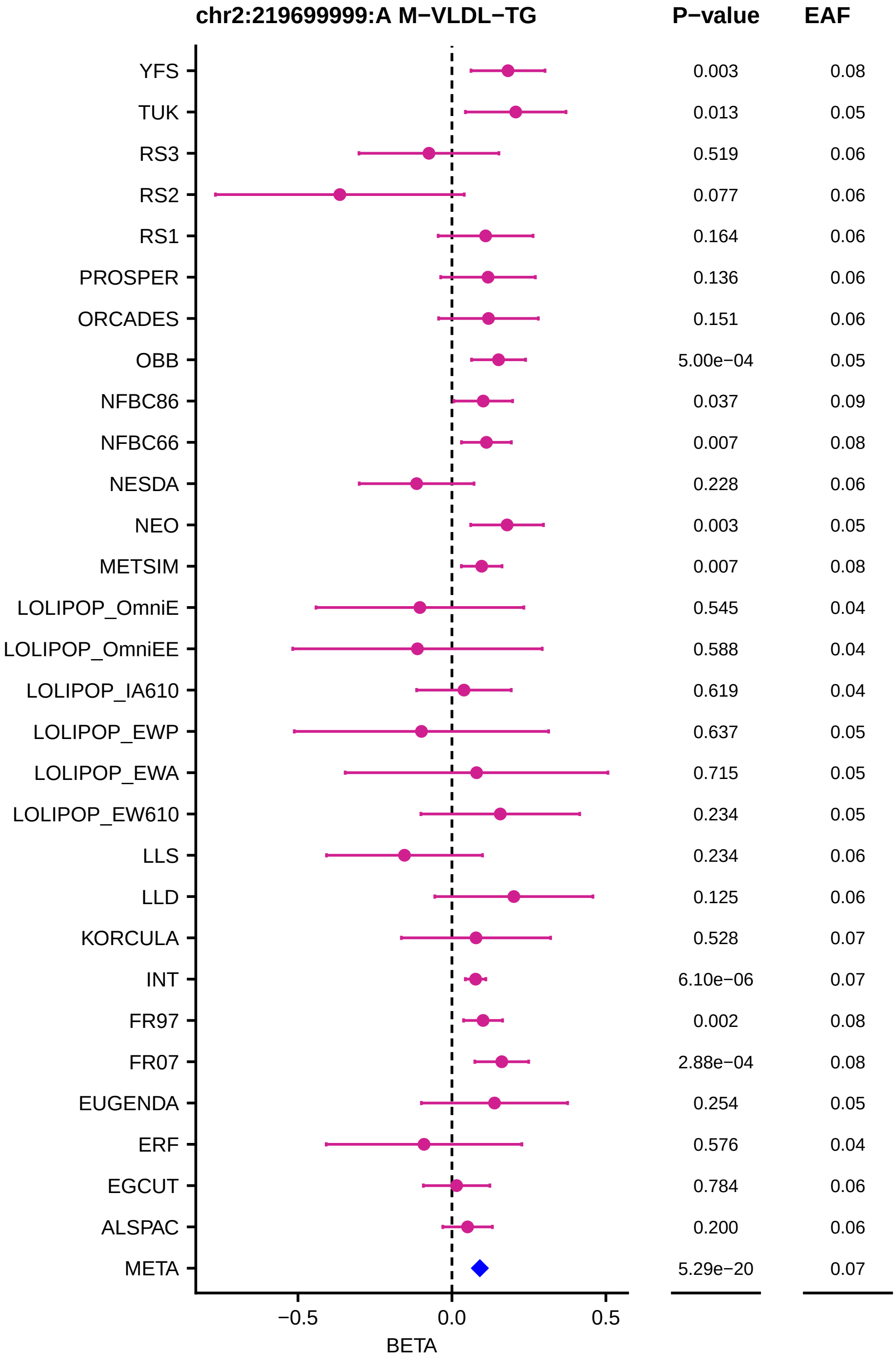
chr2:219699999:A M−VLDL−TG
P−value
EAF
YFS 0.003 0.08
TUK 0.013 0.05
RS3 0.519 0.06
RS2 0.077 0.06
RS1 0.164 0.06
PROSPER 0.136 0.06
ORCADES 0.151 0.06
OBB 5.00e−04 0.05
NFBC86 0.037 0.09
NFBC66 0.007 0.08
NESDA 0.228 0.06
NEO 0.003 0.05
METSIM 0.007 0.08
LOLIPOP_OmniE 0.545 0.04
LOLIPOP_OmniEE 0.588 0.04
LOLIPOP_IA610 0.619 0.04
LOLIPOP_EWP 0.637 0.05
LOLIPOP_EWA 0.715 0.05
LOLIPOP_EW610 0.234 0.05
LLS 0.234 0.06
LLD 0.125 0.06
KORCULA 0.528 0.07
INT 6.10e−06 0.07
FR97 0.002 0.08
FR07 2.88e−04 0.08
EUGENDA 0.254 0.05
ERF 0.576 0.04
EGCUT 0.784 0.06
ALSPAC 0.200 0.06
META 5.29e−20 0.07
−0.5
0.0
0.5
BETA

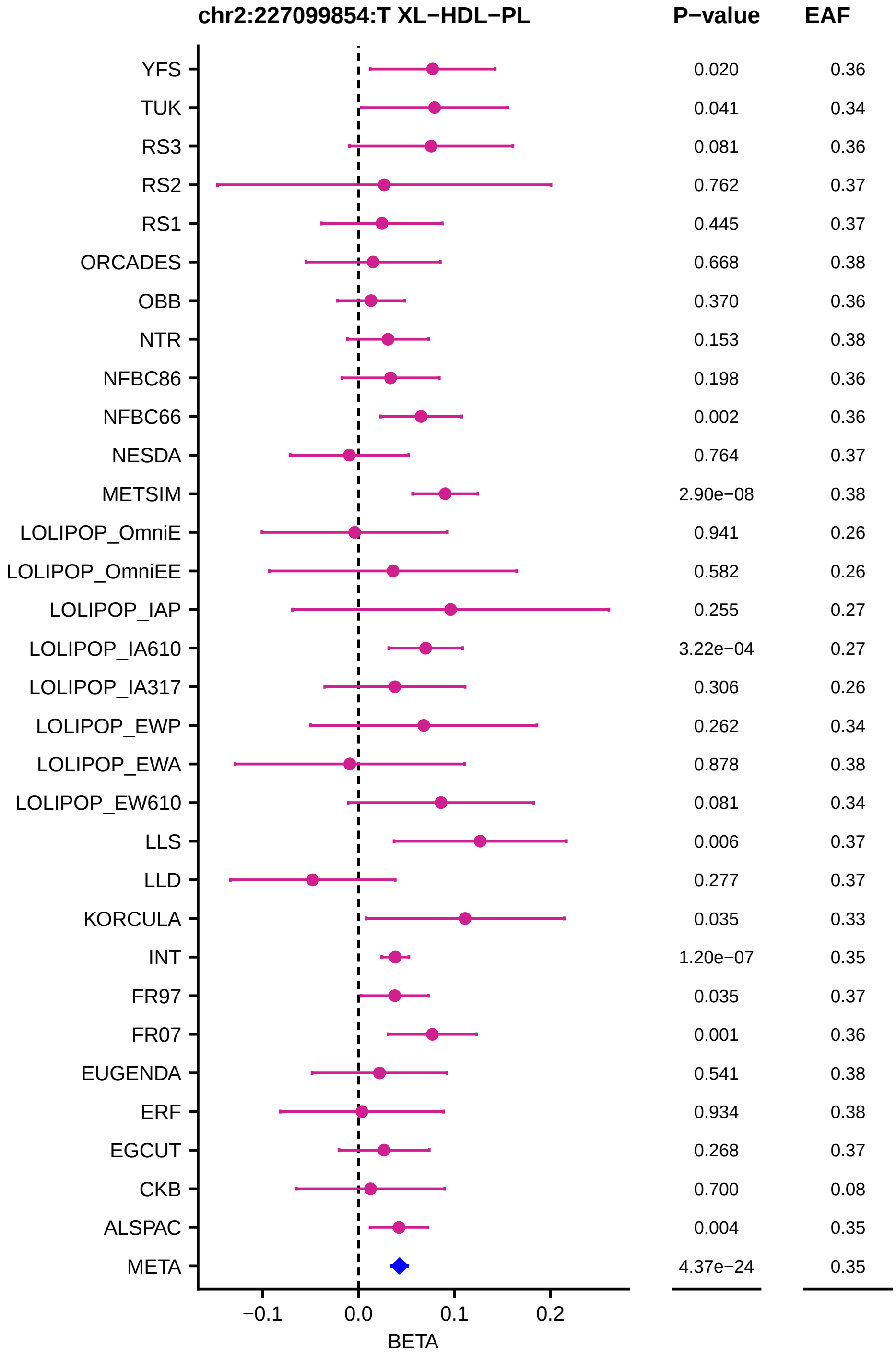
chr2:227099854:T XL−HDL−PL
P−value
EAF
YFS 0.020 0.36
TUK 0.041 0.34
RS3 0.081 0.36
RS2 0.762 0.37
RS1 0.445 0.37
ORCADES 0.668 0.38
OBB 0.370 0.36
NTR 0.153 0.38
NFBC86 0.198 0.36
NFBC66 0.002 0.36
NESDA 0.764 0.37
METSIM 2.90e−08 0.38
LOLIPOP_OmniE 0.941 0.26
LOLIPOP_OmniEE 0.582 0.26
LOLIPOP_IAP 0.255 0.27
LOLIPOP_IA610 3.22e−04 0.27
LOLIPOP_IA317 0.306 0.26
LOLIPOP_EWP 0.262 0.34
LOLIPOP_EWA 0.878 0.38
LOLIPOP_EW610 0.081 0.34
LLS 0.006 0.37
LLD 0.277 0.37
KORCULA 0.035 0.33
INT 1.20e−07 0.35
FR97 0.035 0.37
FR07 0.001 0.36
EUGENDA 0.541 0.38
ERF 0.934 0.38
EGCUT 0.268 0.37
CKB 0.700 0.08
ALSPAC 0.004 0.35
META 4.37e−24 0.35
−0.1
0.0
0.1
0.2
BETA

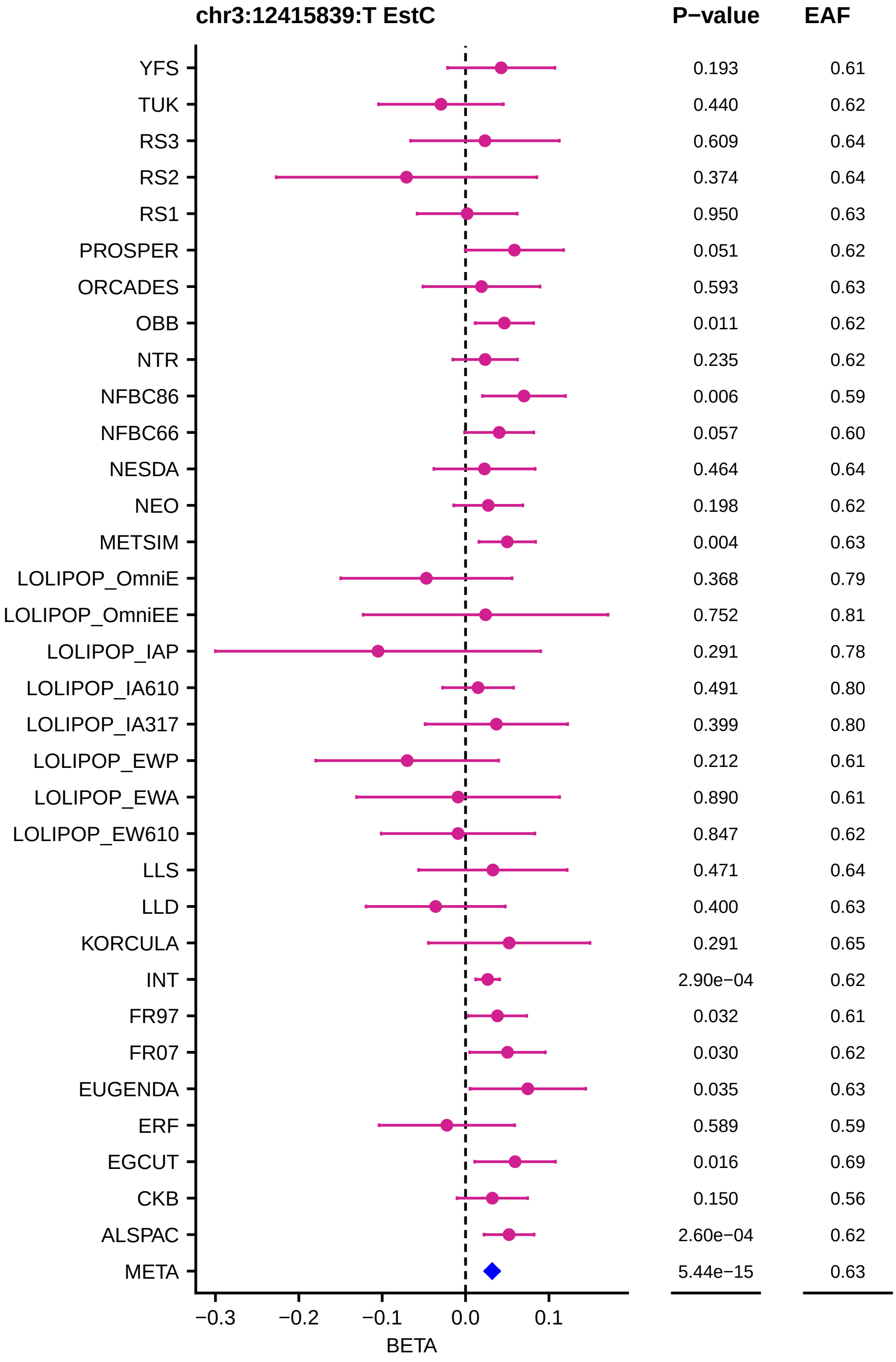
chr3:12415839:T EstC
P−value
EAF
YFS 0.193 0.61
TUK 0.440 0.62
RS3 0.609 0.64
RS2 0.374 0.64
RS1 0.950 0.63
PROSPER 0.051 0.62
ORCADES 0.593 0.63
OBB 0.011 0.62
NTR 0.235 0.62
NFBC86 0.006 0.59
NFBC66 0.057 0.60
NESDA 0.464 0.64
NEO 0.198 0.62
METSIM 0.004 0.63
LOLIPOP_OmniE 0.368 0.79
LOLIPOP_OmniEE 0.752 0.81
LOLIPOP_IAP 0.291 0.78
LOLIPOP_IA610 0.491 0.80
LOLIPOP_IA317 0.399 0.80
LOLIPOP_EWP 0.212 0.61
LOLIPOP_EWA 0.890 0.61
LOLIPOP_EW610 0.847 0.62
LLS 0.471 0.64
LLD 0.400 0.63
KORCULA 0.291 0.65
INT 2.90e−04 0.62
FR97 0.032 0.61
FR07 0.030 0.62
EUGENDA 0.035 0.63
ERF 0.589 0.59
EGCUT 0.016 0.69
CKB 0.150 0.56
ALSPAC 2.60e−04 0.62
META 5.44e−15 0.63
−0.3
−0.2
−0.1
0.0
0.1
BETA

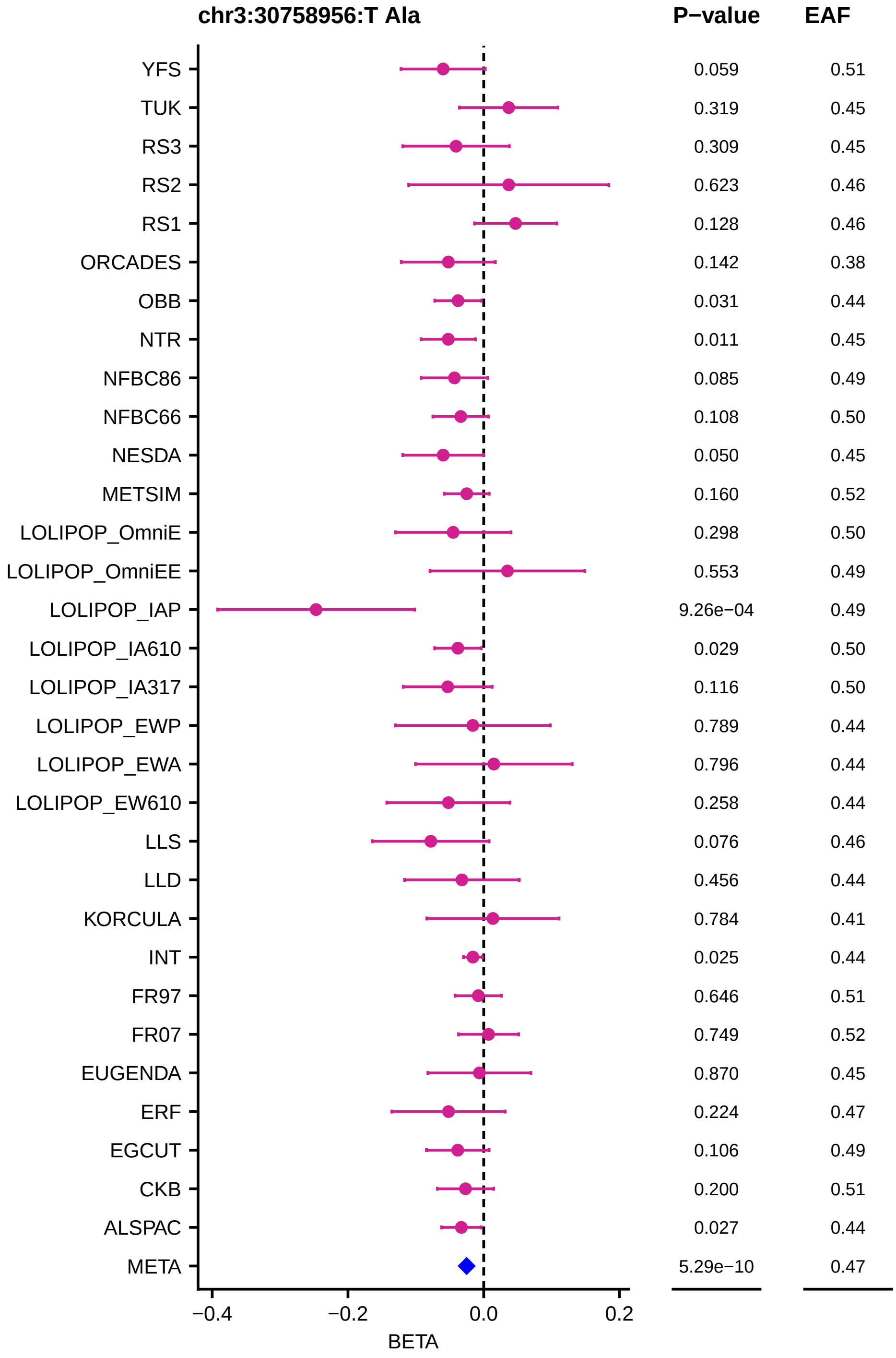
chr3:30758956:T Ala
P−value
EAF
YFS 0.059 0.51
TUK 0.319 0.45
RS3 0.309 0.45
RS2 0.623 0.46
RS1 0.128 0.46
ORCADES 0.142 0.38
OBB 0.031 0.44
NTR 0.011 0.45
NFBC86 0.085 0.49
NFBC66 0.108 0.50
NESDA 0.050 0.45
METSIM 0.160 0.52
LOLIPOP_OmniE 0.298 0.50
LOLIPOP_OmniEE 0.553 0.49
LOLIPOP_IAP 9.26e−04 0.49
LOLIPOP_IA610 0.029 0.50
LOLIPOP_IA317 0.116 0.50
LOLIPOP_EWP 0.789 0.44
LOLIPOP_EWA 0.796 0.44
LOLIPOP_EW610 0.258 0.44
LLS 0.076 0.46
LLD 0.456 0.44
KORCULA 0.784 0.41
INT 0.025 0.44
FR97 0.646 0.51
FR07 0.749 0.52
EUGENDA 0.870 0.45
ERF 0.224 0.47
EGCUT 0.106 0.49
CKB 0.200 0.51
ALSPAC 0.027 0.44
META 5.29e−10 0.47
−0.4
−0.2
0.0
0.2
BETA

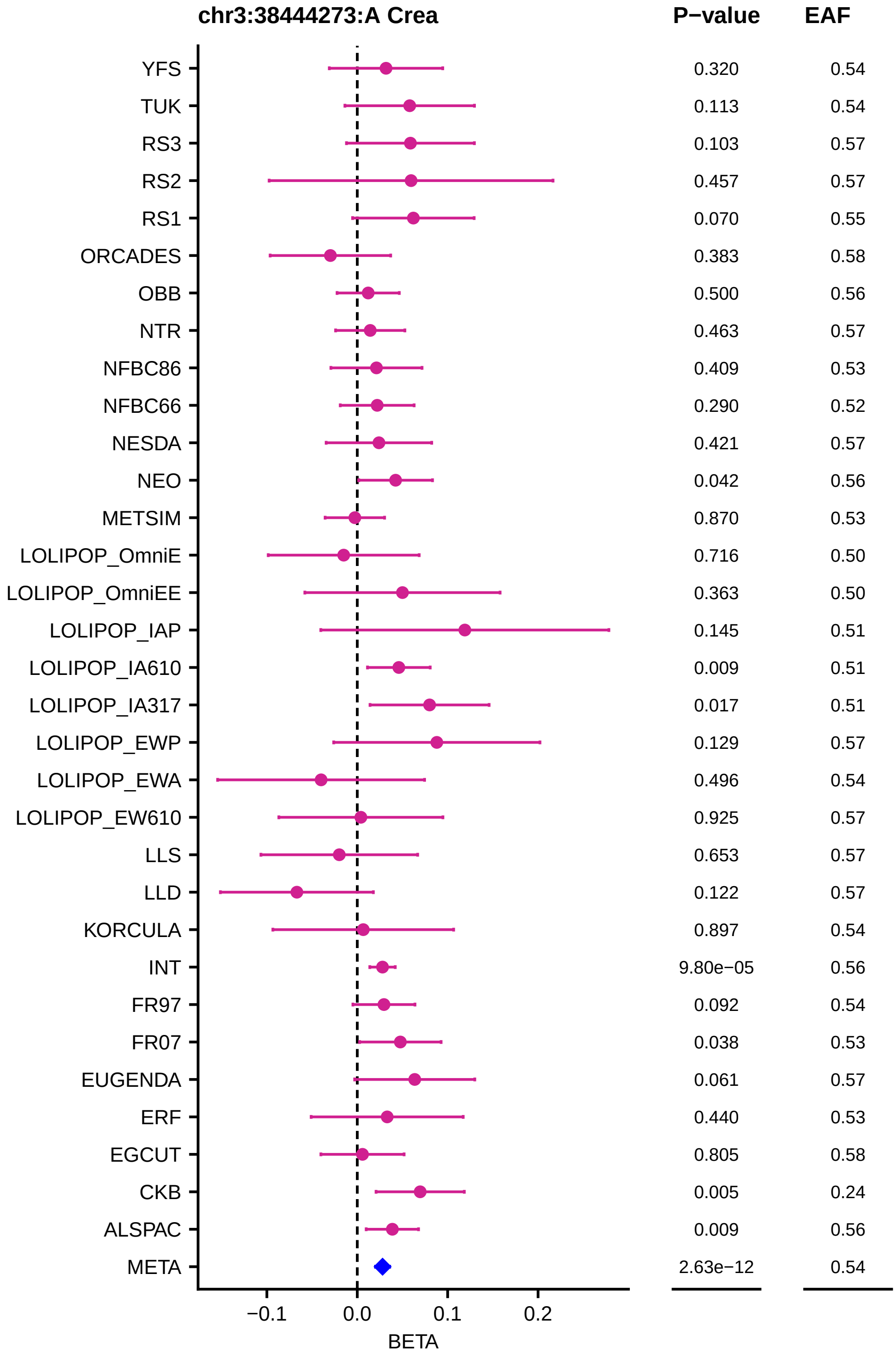
chr3:38444273:A Crea
P−value
EAF
YFS 0.320 0.54
TUK 0.113 0.54
RS3 0.103 0.57
RS2 0.457 0.57
RS1 0.070 0.55
ORCADES 0.383 0.58
OBB 0.500 0.56
NTR 0.463 0.57
NFBC86 0.409 0.53
NFBC66 0.290 0.52
NESDA 0.421 0.57
NEO 0.042 0.56
METSIM 0.870 0.53
LOLIPOP_OmniE 0.716 0.50
LOLIPOP_OmniEE 0.363 0.50
LOLIPOP_IAP 0.145 0.51
LOLIPOP_IA610 0.009 0.51
LOLIPOP_IA317 0.017 0.51
LOLIPOP_EWP 0.129 0.57
LOLIPOP_EWA 0.496 0.54
LOLIPOP_EW610 0.925 0.57
LLS 0.653 0.57
LLD 0.122 0.57
KORCULA 0.897 0.54
INT 9.80e−05 0.56
FR97 0.092 0.54
FR07 0.038 0.53
EUGENDA 0.061 0.57
ERF 0.440 0.53
EGCUT 0.805 0.58
CKB 0.005 0.24
ALSPAC 0.009 0.56
META 2.63e−12 0.54
−0.1
0.0
0.1
0.2
BETA

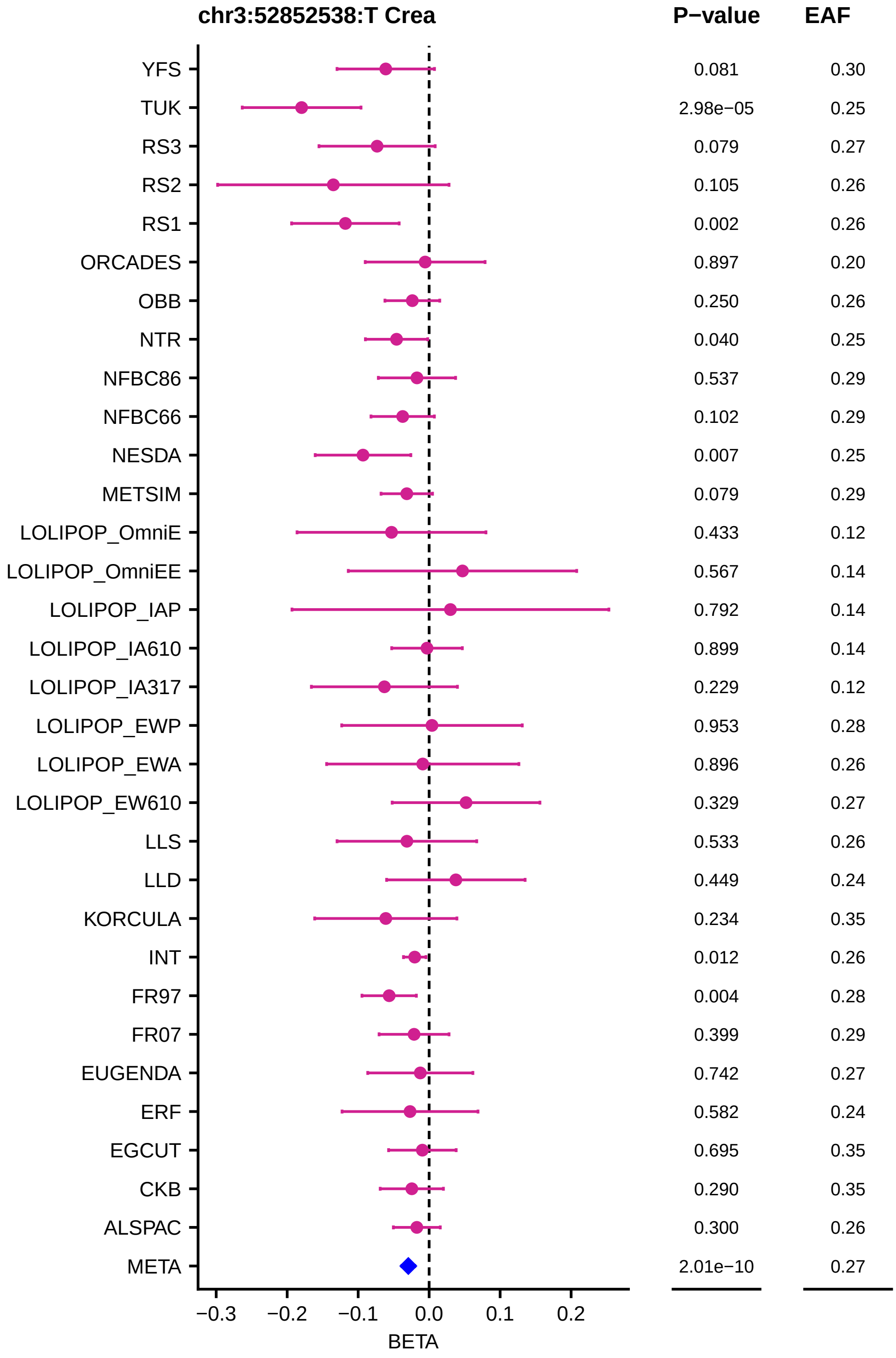

chr3:52852538:T Crea
P−value
EAF
YFS 0.081 0.30
TUK 2.98e−05 0.25
RS3 0.079 0.27
RS2 0.105 0.26
RS1 0.002 0.26
ORCADES 0.897 0.20
OBB 0.250 0.26
NTR 0.040 0.25
NFBC86 0.537 0.29
NFBC66 0.102 0.29
NESDA 0.007 0.25
METSIM 0.079 0.29
LOLIPOP_OmniE 0.433 0.12
LOLIPOP_OmniEE 0.567 0.14
LOLIPOP_IAP 0.792 0.14
LOLIPOP_IA610 0.899 0.14
LOLIPOP_IA317 0.229 0.12
LOLIPOP_EWP 0.953 0.28
LOLIPOP_EWA 0.896 0.26
LOLIPOP_EW610 0.329 0.27
LLS 0.533 0.26
LLD 0.449 0.24
KORCULA 0.234 0.35
INT 0.012 0.26
FR97 0.004 0.28
FR07 0.399 0.29
EUGENDA 0.742 0.27
ERF 0.582 0.24
EGCUT 0.695 0.35
CKB 0.290 0.35
ALSPAC 0.300 0.26
META 2.01e−10 0.27
−0.3
−0.2
−0.1
0.0
0.1
0.2
BETA

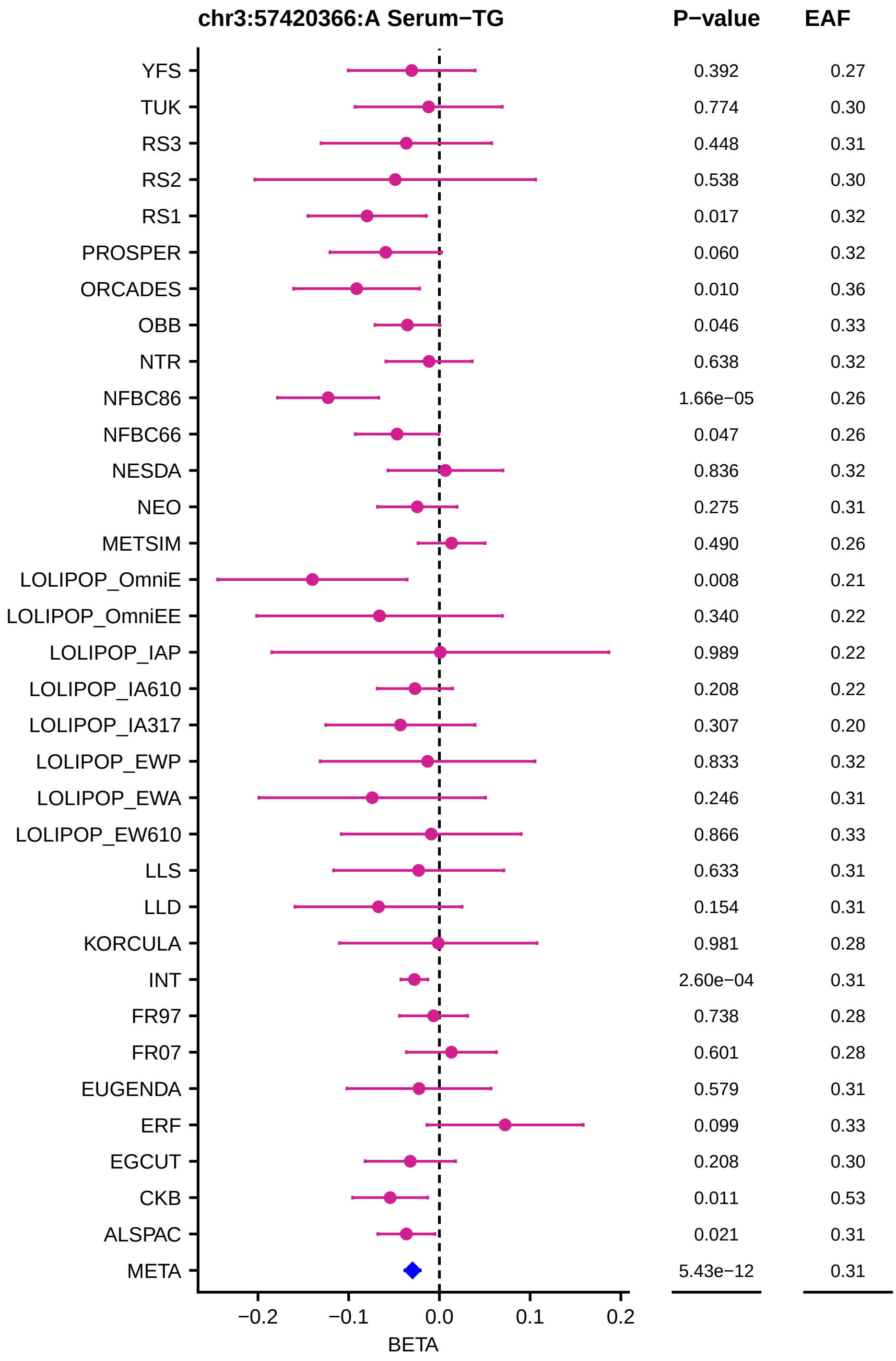
chr3:57420366:A Serum−TG
P−value
EAF
YFS 0.392 0.27
TUK 0.774 0.30
RS3 0.448 0.31
RS2 0.538 0.30
RS1 0.017 0.32
PROSPER 0.060 0.32
ORCADES 0.010 0.36
OBB 0.046 0.33
NTR 0.638 0.32
NFBC86 1.66e−05 0.26
NFBC66 0.047 0.26
NESDA 0.836 0.32
NEO 0.275 0.31
METSIM 0.490 0.26
LOLIPOP_OmniE 0.008 0.21
LOLIPOP_OmniEE 0.340 0.22
LOLIPOP_IAP 0.989 0.22
LOLIPOP_IA610 0.208 0.22
LOLIPOP_IA317 0.307 0.20
LOLIPOP_EWP 0.833 0.32
LOLIPOP_EWA 0.246 0.31
LOLIPOP_EW610 0.866 0.33
LLS 0.633 0.31
LLD 0.154 0.31
KORCULA 0.981 0.28
INT 2.60e−04 0.31
FR97 0.738 0.28
FR07 0.601 0.28
EUGENDA 0.579 0.31
ERF 0.099 0.33
EGCUT 0.208 0.30
CKB 0.011 0.53
ALSPAC 0.021 0.31
META 5.43e−12 0.31
−0.2
−0.1
0.0
0.1
0.2
BETA

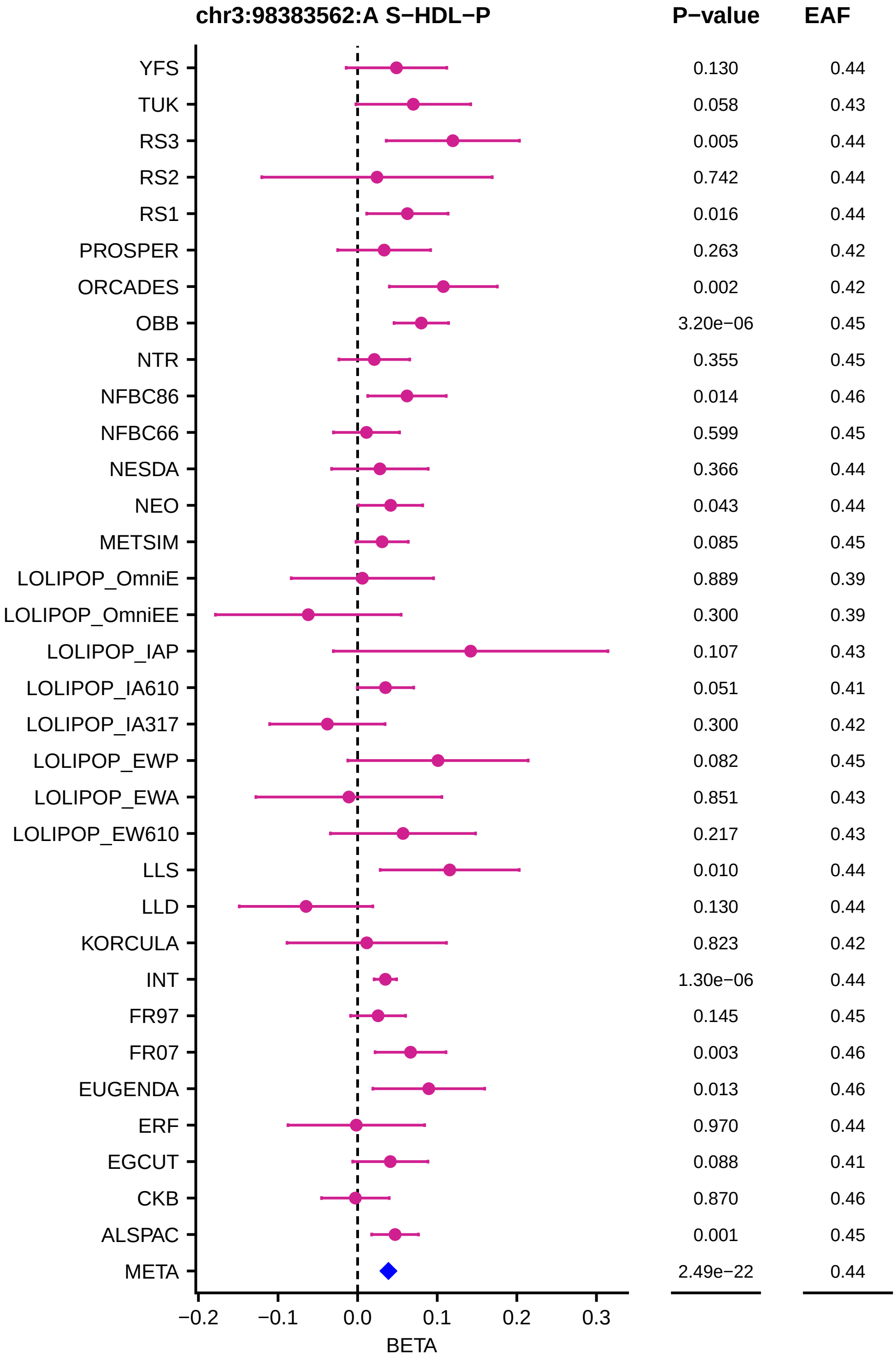
chr3:98383562:A S−HDL−P
P−value
EAF
YFS 0.130 0.44
TUK 0.058 0.43
RS3 0.005 0.44
RS2 0.742 0.44
RS1 0.016 0.44
PROSPER 0.263 0.42
ORCADES 0.002 0.42
OBB 3.20e−06 0.45
NTR 0.355 0.45
NFBC86 0.014 0.46
NFBC66 0.599 0.45
NESDA 0.366 0.44
NEO 0.043 0.44
METSIM 0.085 0.45
LOLIPOP_OmniE 0.889 0.39
LOLIPOP_OmniEE 0.300 0.39
LOLIPOP_IAP 0.107 0.43
LOLIPOP_IA610 0.051 0.41
LOLIPOP_IA317 0.300 0.42
LOLIPOP_EWP 0.082 0.45
LOLIPOP_EWA 0.851 0.43
LOLIPOP_EW610 0.217 0.43
LLS 0.010 0.44
LLD 0.130 0.44
KORCULA 0.823 0.42
INT 1.30e−06 0.44
FR97 0.145 0.45
FR07 0.003 0.46
EUGENDA 0.013 0.46
ERF 0.970 0.44
EGCUT 0.088 0.41
CKB 0.870 0.46
ALSPAC 0.001 0.45
META 2.49e−22 0.44
−0.2 −0.1 0.0 0.1 0.2 0.3
BETA

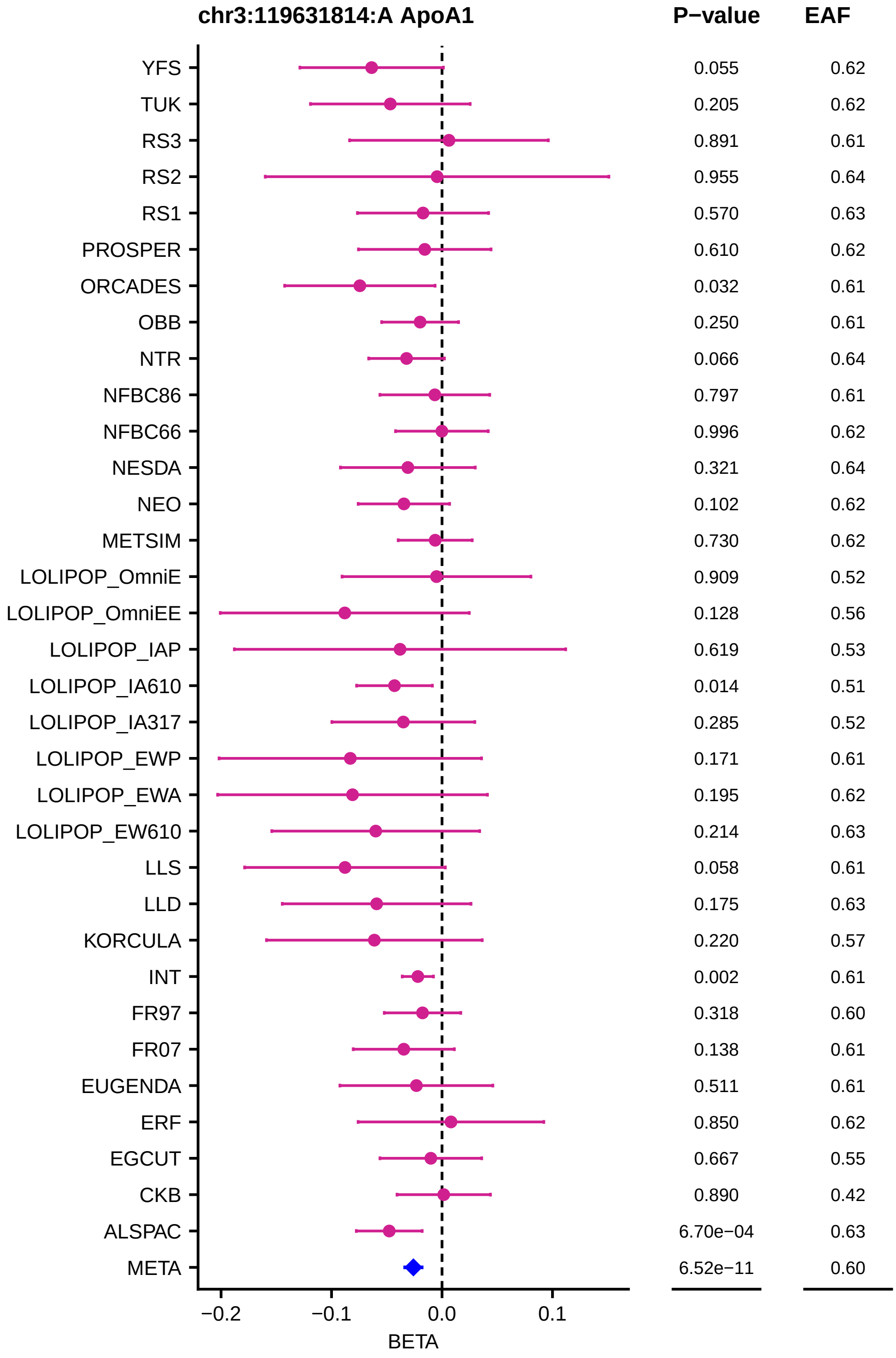
chr3:119631814:A ApoA1
P−value
EAF
YFS 0.055 0.62
TUK 0.205 0.62
RS3 0.891 0.61
RS2 0.955 0.64
RS1 0.570 0.63
PROSPER 0.610 0.62
ORCADES 0.032 0.61
OBB 0.250 0.61
NTR 0.066 0.64
NFBC86 0.797 0.61
NFBC66 0.996 0.62
NESDA 0.321 0.64
NEO 0.102 0.62
METSIM 0.730 0.62
LOLIPOP_OmniE 0.909 0.52
LOLIPOP_OmniEE 0.128 0.56
LOLIPOP_IAP 0.619 0.53
LOLIPOP_IA610 0.014 0.51
LOLIPOP_IA317 0.285 0.52
LOLIPOP_EWP 0.171 0.61
LOLIPOP_EWA 0.195 0.62
LOLIPOP_EW610 0.214 0.63
LLS 0.058 0.61
LLD 0.175 0.63
KORCULA 0.220 0.57
INT 0.002 0.61
FR97 0.318 0.60
FR07 0.138 0.61
EUGENDA 0.511 0.61
ERF 0.850 0.62
EGCUT 0.667 0.55
CKB 0.890 0.42
ALSPAC 6.70e−04 0.63
META 6.52e−11 0.60
−0.2
−0.1
0.0
0.1
BETA

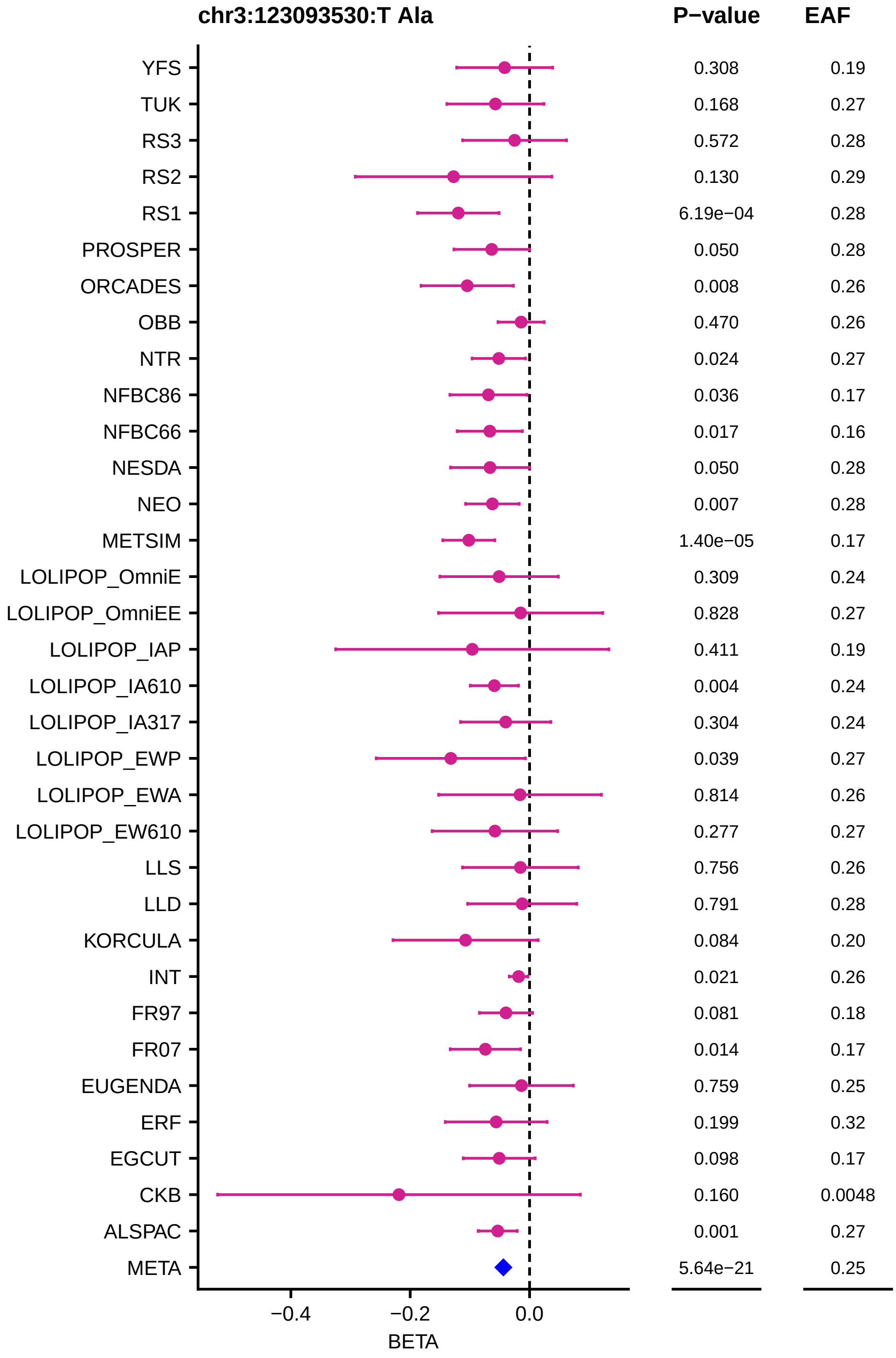
chr3:123093530:T Ala
P−value
EAF
YFS
0.308
0.19
TUK
0.168
0.27
RS3
0.572
0.28
RS2
0.130
0.29
RS1
6.19e−04
0.28
PROSPER
0.050
0.28
ORCADES
0.008
0.26
OBB
0.470
0.26
NTR
0.024
0.27
NFBC86
0.036
0.17
NFBC66
0.017
0.16
NESDA
0.050
0.28
NEO
0.007
0.28
METSIM
1.40e−05
0.17
LOLIPOP_OmniE
0.309
0.24
LOLIPOP_OmniEE
0.828
0.27
LOLIPOP_IAP
0.411
0.19
LOLIPOP_IA610
0.004
0.24
LOLIPOP_IA317
0.304
0.24
LOLIPOP_EWP
0.039
0.27
LOLIPOP_EWA
0.814
0.26
LOLIPOP_EW610
0.277
0.27
LLS
0.756
0.26
LLD
0.791
0.28
KORCULA
0.084
0.20
INT
0.021
0.26
FR97
0.081
0.18
FR07
0.014
0.17
EUGENDA
0.759
0.25
ERF
0.199
0.32
EGCUT
0.098
0.17
CKB
0.160
0.0048
ALSPAC
0.001
0.27
META
5.64e−21
0.25
−0.4
−0.2
0.0
BETA

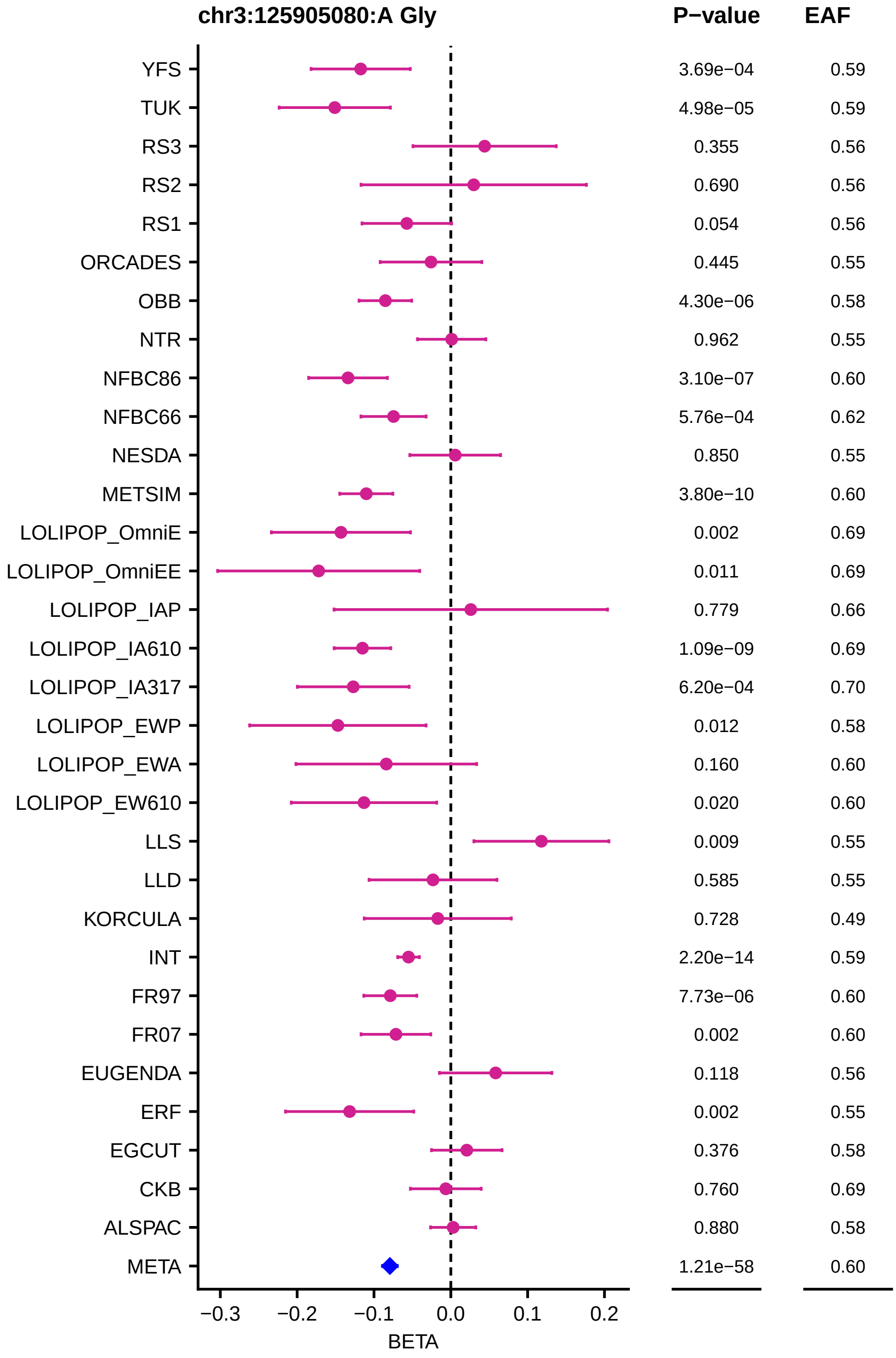
chr3:125905080:A Gly
P−value
EAF
YFS 3.69e−04 0.59
TUK 4.98e−05 0.59
RS3 0.355 0.56
RS2 0.690 0.56
RS1 0.054 0.56
ORCADES 0.445 0.55
OBB 4.30e−06 0.58
NTR 0.962 0.55
NFBC86 3.10e−07 0.60
NFBC66 5.76e−04 0.62
NESDA 0.850 0.55
METSIM 3.80e−10 0.60
LOLIPOP_OmniE 0.002 0.69
LOLIPOP_OmniEE 0.011 0.69
LOLIPOP_IAP 0.779 0.66
LOLIPOP_IA610 1.09e−09 0.69
LOLIPOP_IA317 6.20e−04 0.70
LOLIPOP_EWP 0.012 0.58
LOLIPOP_EWA 0.160 0.60
LOLIPOP_EW610 0.020 0.60
LLS 0.009 0.55
LLD 0.585 0.55
KORCULA 0.728 0.49
INT 2.20e−14 0.59
FR97 7.73e−06 0.60
FR07 0.002 0.60
EUGENDA 0.118 0.56
ERF 0.002 0.55
EGCUT 0.376 0.58
CKB 0.760 0.69
ALSPAC 0.880 0.58
META 1.21e−58 0.60
−0.3
−0.2
−0.1
0.0
0.1
0.2
BETA

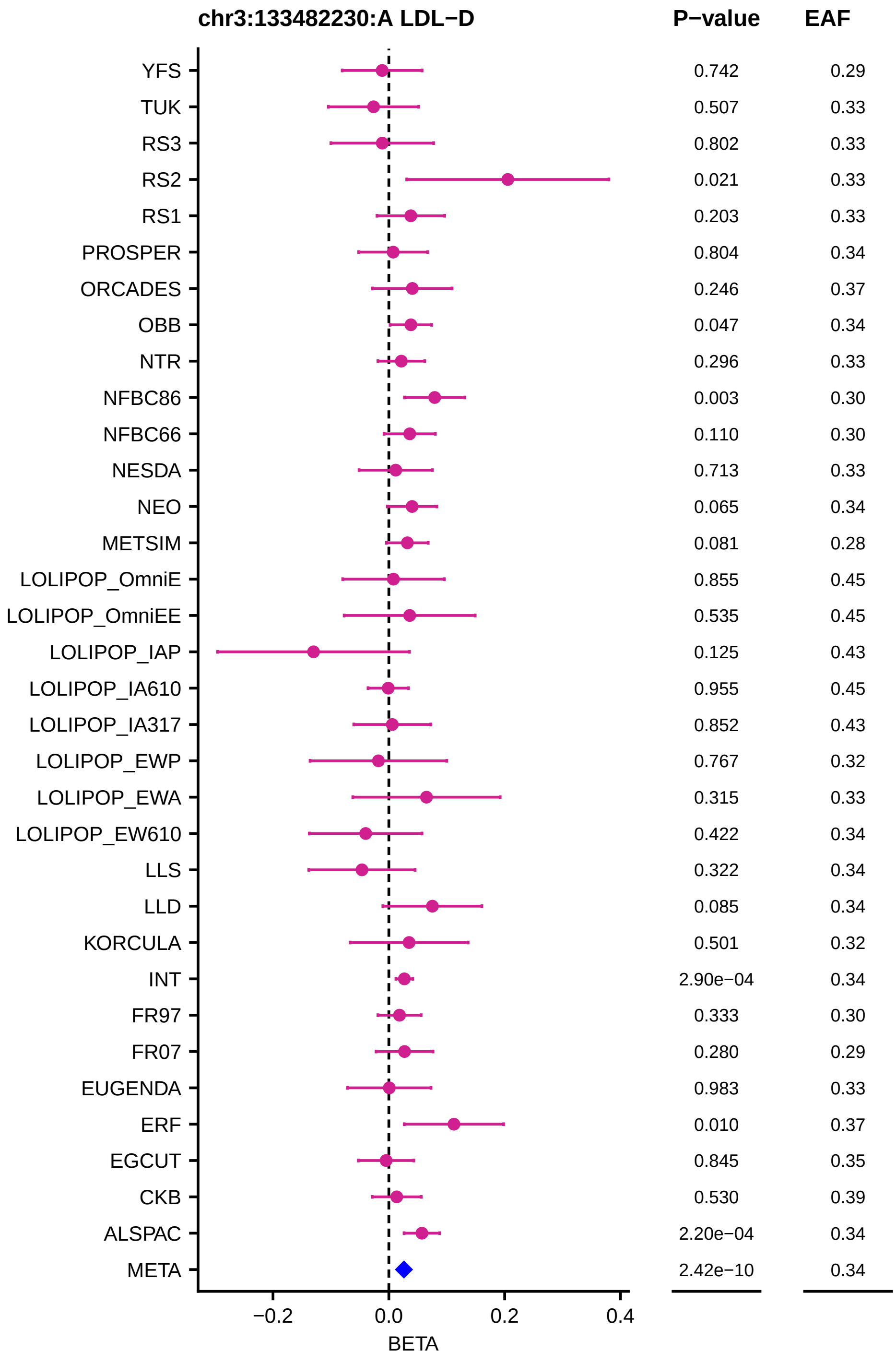
chr3:133482230:A LDL−D
P−value
EAF
YFS
TUK
RS3
RS2
RS1
PROSPER
ORCADES
OBB
NTR
NFBC86
NFBC66
NESDA
NEO
METSIM
LOLIPOP_OmniE
LOLIPOP_OmniEE
LOLIPOP_IAP
LOLIPOP_IA610
LOLIPOP_IA317
LOLIPOP_EWP
LOLIPOP_EWA
LOLIPOP_EW610
LLS
LLD
KORCULA
INT
FR97
FR07
EUGENDA
ERF
EGCUT
CKB
ALSPAC
META
0.742 0.29
0.507 0.33
0.802 0.33
0.021 0.33
0.203 0.33
0.804 0.34
0.246 0.37
0.047 0.34
0.296 0.33
0.003 0.30
0.110 0.30
0.713 0.33
0.065 0.34
0.081 0.28
0.855 0.45
0.535 0.45
0.125 0.43
0.955 0.45
0.852 0.43
0.767 0.32
0.315 0.33
0.422 0.34
0.322 0.34
0.085 0.34
0.501 0.32
2.90e−04 0.34
0.333 0.30
0.280 0.29
0.983 0.33
0.010 0.37
0.845 0.35
0.530 0.39
2.20e−04 0.34
2.42e−10 0.34
−0.2
0.0
0.2
0.4
BETA

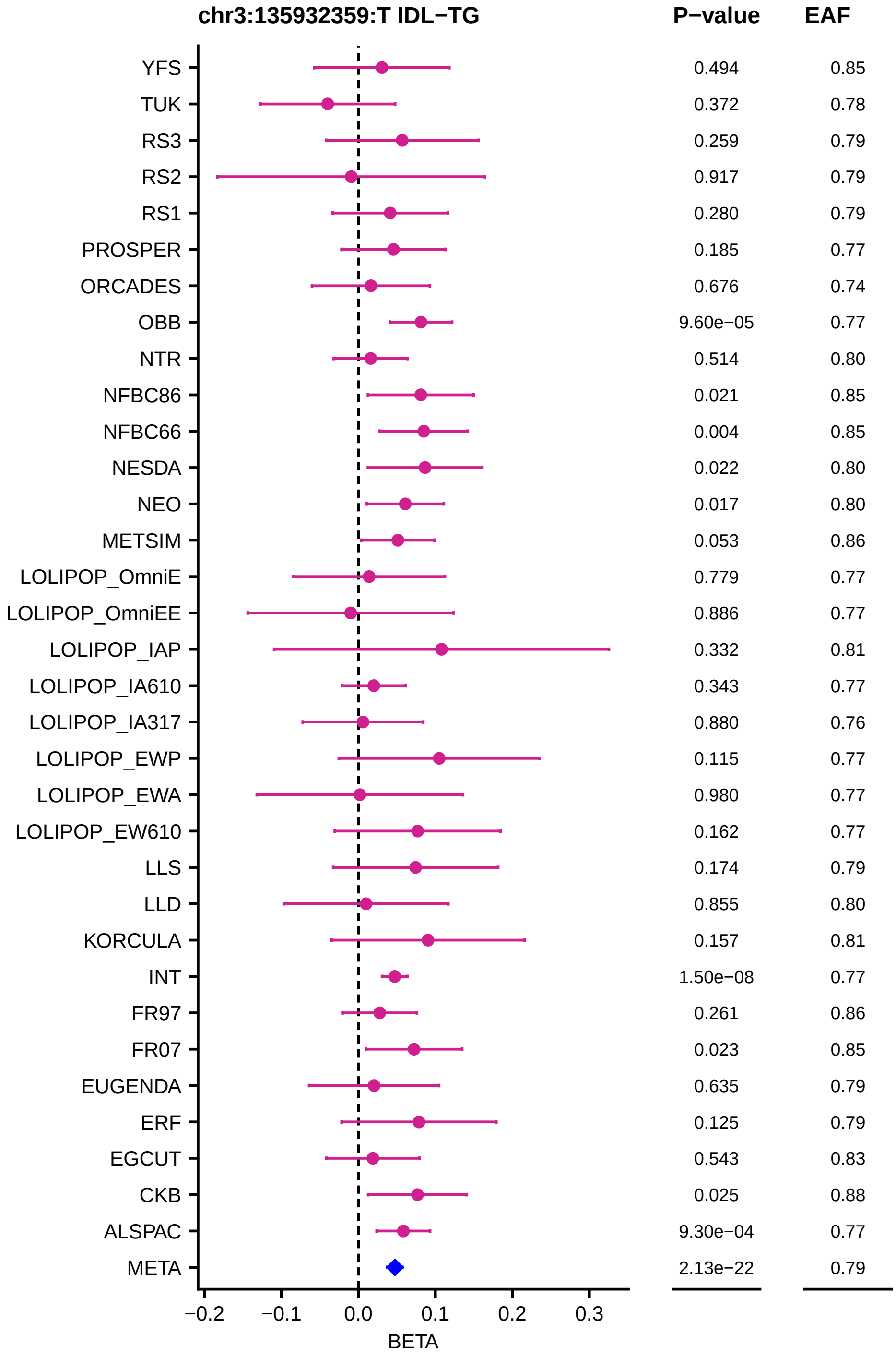
chr3:135932359:T IDL−TG
P−value
EAF
YFS 0.494 0.85
TUK 0.372 0.78
RS3 0.259 0.79
RS2 0.917 0.79
RS1 0.280 0.79
PROSPER 0.185 0.77
ORCADES 0.676 0.74
OBB 9.60e−05 0.77
NTR 0.514 0.80
NFBC86 0.021 0.85
NFBC66 0.004 0.85
NESDA 0.022 0.80
NEO 0.017 0.80
METSIM 0.053 0.86
LOLIPOP_OmniE 0.779 0.77
LOLIPOP_OmniEE 0.886 0.77
LOLIPOP_IAP 0.332 0.81
LOLIPOP_IA610 0.343 0.77
LOLIPOP_IA317 0.880 0.76
LOLIPOP_EWP 0.115 0.77
LOLIPOP_EWA 0.980 0.77
LOLIPOP_EW610 0.162 0.77
LLS 0.174 0.79
LLD 0.855 0.80
KORCULA 0.157 0.81
INT 1.50e−08 0.77
FR97 0.261 0.86
FR07 0.023 0.85
EUGENDA 0.635 0.79
ERF 0.125 0.79
EGCUT 0.543 0.83
CKB 0.025 0.88
ALSPAC 9.30e−04 0.77
META 2.13e−22 0.79
−0.2 −0.1 0.0 0.1 0.2 0.3
BETA

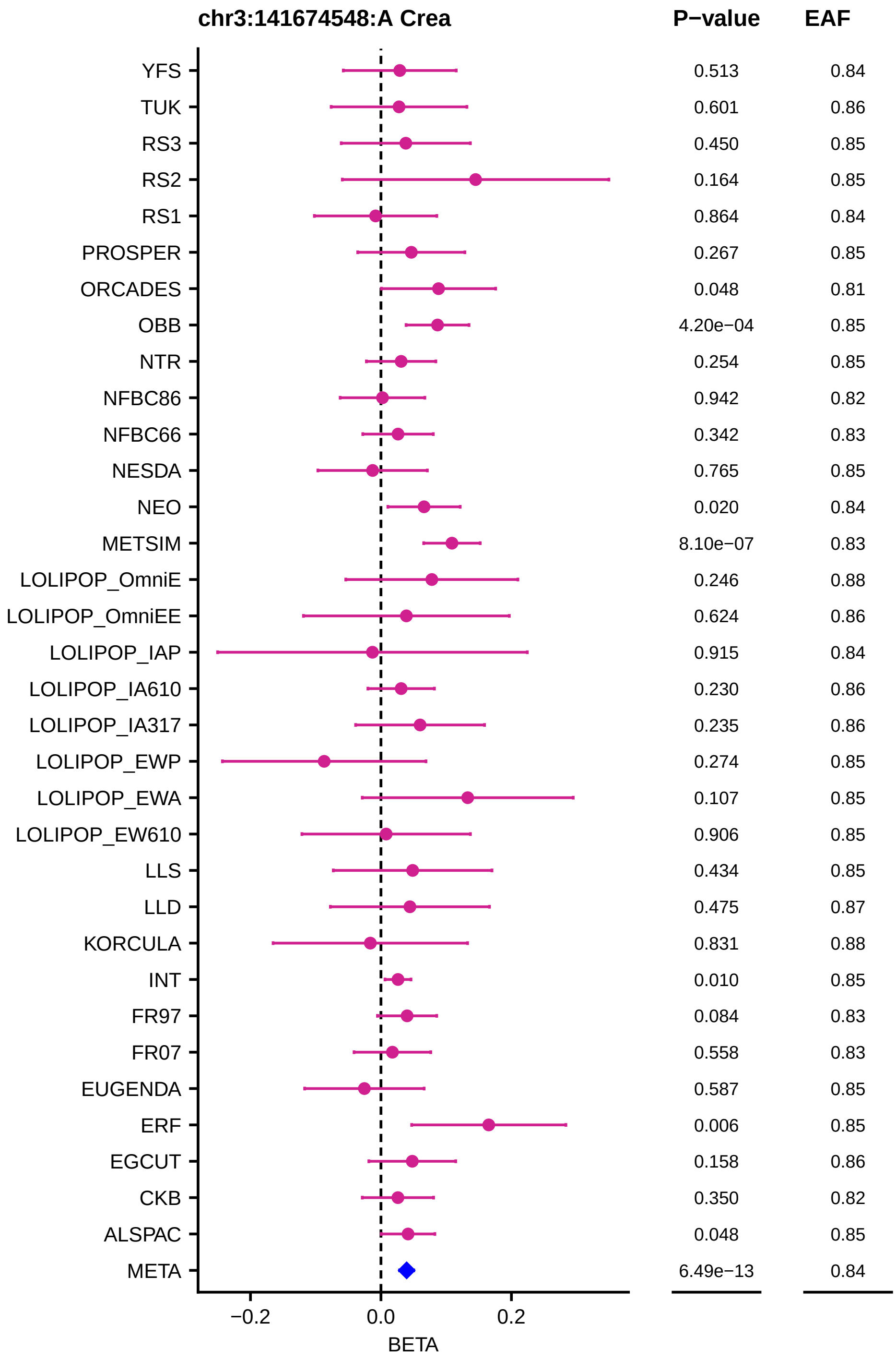
chr3:141674548:A Crea
P−value
EAF
YFS 0.513 0.84
TUK 0.601 0.86
RS3 0.450 0.85
RS2 0.164 0.85
RS1 0.864 0.84
PROSPER 0.267 0.85
ORCADES 0.048 0.81
OBB 4.20e−04 0.85
NTR 0.254 0.85
NFBC86 0.942 0.82
NFBC66 0.342 0.83
NESDA 0.765 0.85
NEO 0.020 0.84
METSIM 8.10e−07 0.83
LOLIPOP_OmniE 0.246 0.88
LOLIPOP_OmniEE 0.624 0.86
LOLIPOP_IAP 0.915 0.84
LOLIPOP_IA610 0.230 0.86
LOLIPOP_IA317 0.235 0.86
LOLIPOP_EWP 0.274 0.85
LOLIPOP_EWA 0.107 0.85
LOLIPOP_EW610 0.906 0.85
LLS 0.434 0.85
LLD 0.475 0.87
KORCULA 0.831 0.88
INT 0.010 0.85
FR97 0.084 0.83
FR07 0.558 0.83
EUGENDA 0.587 0.85
ERF 0.006 0.85
EGCUT 0.158 0.86
CKB 0.350 0.82
ALSPAC 0.048 0.85
META 6.49e−13 0.84
−0.2
0.0
0.2
BETA

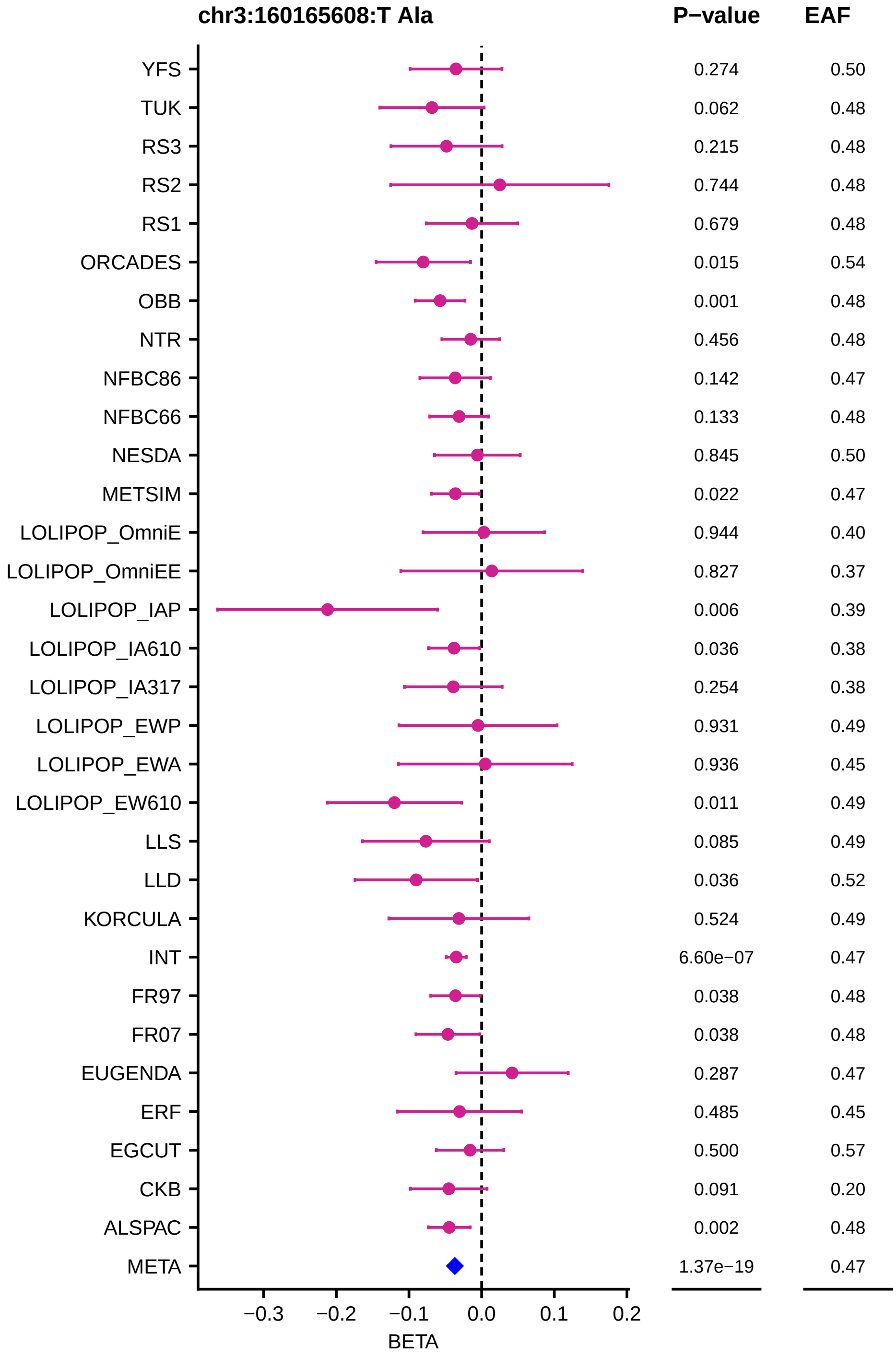
chr3:160165608:T Ala
P−value
EAF
YFS
TUK
RS3
RS2
RS1
ORCADES
OBB
NTR
NFBC86
NFBC66
NESDA
METSIM
LOLIPOP_OmniE
LOLIPOP_OmniEE
LOLIPOP_IAP
LOLIPOP_IA610
LOLIPOP_IA317
LOLIPOP_EWP
LOLIPOP_EWA
LOLIPOP_EW610
LLS
LLD
KORCULA
INT
FR97
FR07
EUGENDA
ERF
EGCUT
CKB
ALSPAC
META
0.274
0.062
0.215
0.744
0.679
0.015
0.001
0.456
0.142
0.133
0.845
0.022
0.944
0.827
0.006
0.036
0.254
0.931
0.936
0.011
0.085
0.036
0.524
6.60e−07
0.038
0.038
0.287
0.485
0.500
0.091
0.002
1.37e−19
0.50
0.48
0.48
0.48
0.48
0.54
0.48
0.48
0.47
0.48
0.50
0.47
0.40
0.37
0.39
0.38
0.38
0.49
0.45
0.49
0.49
0.52
0.49
0.47
0.48
0.48
0.47
0.45
0.57
0.20
0.48
0.47
−0.3
−0.2
−0.1
0.0
0.1
0.2
BETA

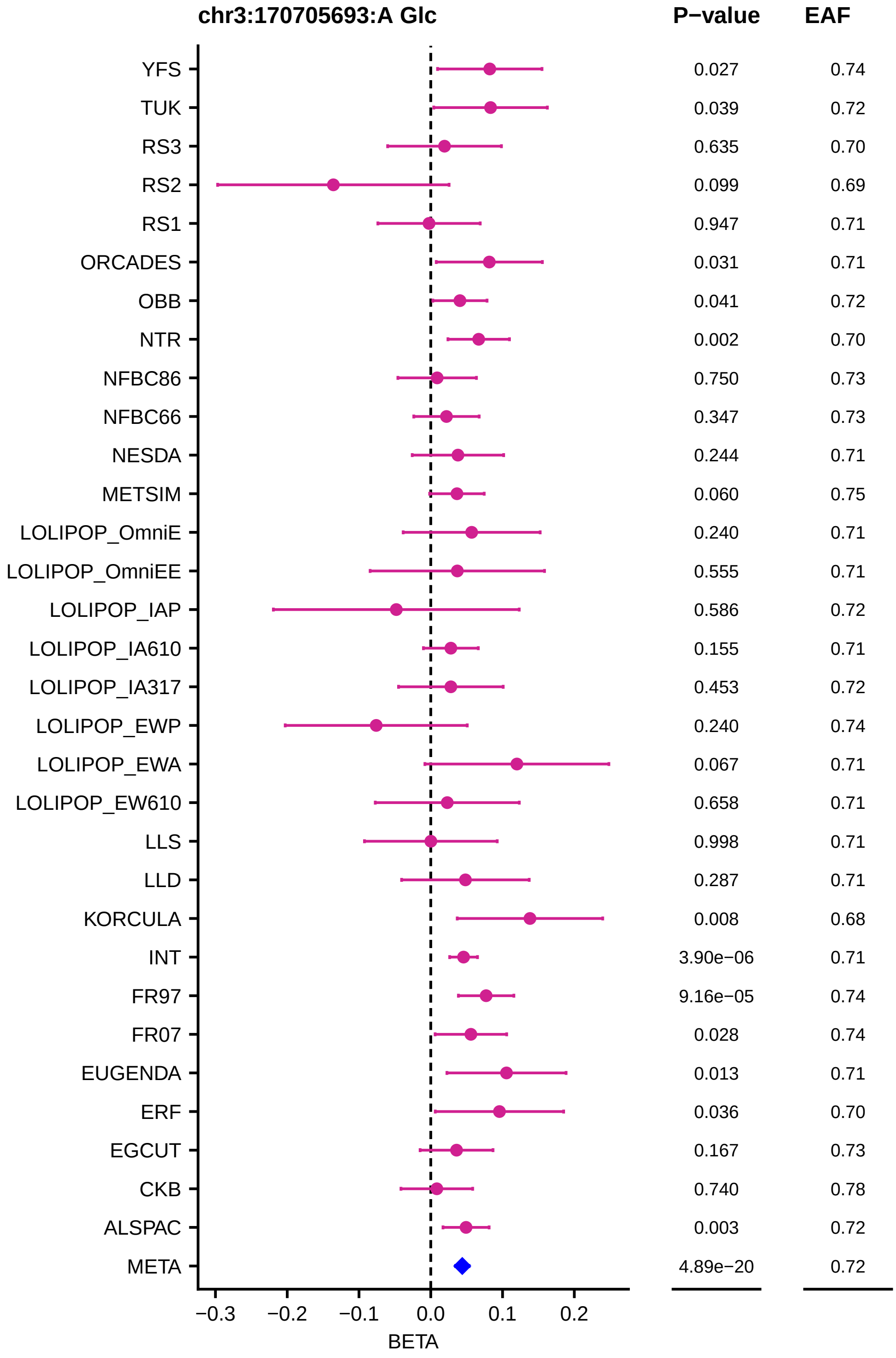

chr3:170705693:A Glc
P−value
EAF
YFS
0.027
0.74
TUK
0.039
0.72
RS3
0.635
0.70
RS2
0.099
0.69
RS1
0.947
0.71
ORCADES
0.031
0.71
OBB
0.041
0.72
NTR
0.002
0.70
NFBC86
0.750
0.73
NFBC66
0.347
0.73
NESDA
0.244
0.71
METSIM
0.060
0.75
LOLIPOP_OmniE
0.240
0.71
LOLIPOP_OmniEE
0.555
0.71
LOLIPOP_IAP
0.586
0.72
LOLIPOP_IA610
0.155
0.71
LOLIPOP_IA317
0.453
0.72
LOLIPOP_EWP
0.240
0.74
LOLIPOP_EWA
0.067
0.71
LOLIPOP_EW610
0.658
0.71
LLS
0.998
0.71
LLD
0.287
0.71
KORCULA
0.008
0.68
INT
3.90e−06
0.71
FR97
9.16e−05
0.74
FR07
0.028
0.74
EUGENDA
0.013
0.71
ERF
0.036
0.70
EGCUT
0.167
0.73
CKB
0.740
0.78
ALSPAC
0.003
0.72
META
4.89e−20
0.72
−0.3
−0.2
−0.1
0.0
0.1
0.2
BETA

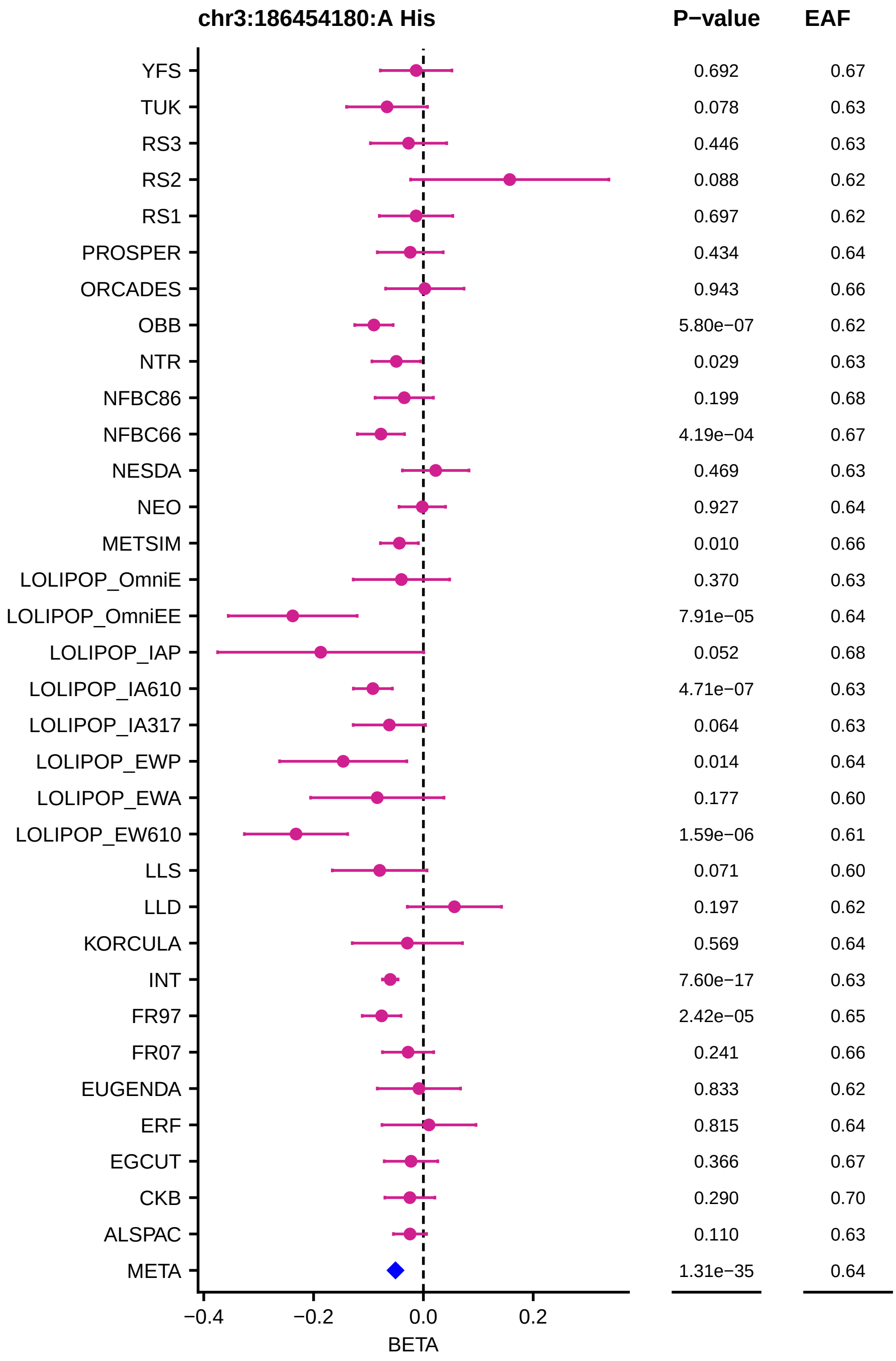
chr3:186454180:A His
P−value
EAF
YFS
TUK
RS3
RS2
RS1
PROSPER
ORCADES
OBB
NTR
NFBC86
NFBC66
NESDA
NEO
METSIM
LOLIPOP_OmniE
LOLIPOP_OmniEE
LOLIPOP_IAP
LOLIPOP_IA610
LOLIPOP_IA317
LOLIPOP_EWP
LOLIPOP_EWA
LOLIPOP_EW610
LLS
LLD
KORCULA
INT
FR97
FR07
EUGENDA
ERF
EGCUT
CKB
ALSPAC
META
0.692 0.67
0.078 0.63
0.446 0.63
0.088 0.62
0.697 0.62
0.434 0.64
0.943 0.66
5.80e−07 0.62
0.029 0.63
0.199 0.68
4.19e−04 0.67
0.469 0.63
0.927 0.64
0.010 0.66
0.370 0.63
7.91e−05 0.64
0.052 0.68
4.71e−07 0.63
0.064 0.63
0.014 0.64
0.177 0.60
1.59e−06 0.61
0.071 0.60
0.197 0.62
0.569 0.64
7.60e−17 0.63
2.42e−05 0.65
0.241 0.66
0.833 0.62
0.815 0.64
0.366 0.67
0.290 0.70
0.110 0.63
1.31e−35 0.64
−0.4
−0.2
0.0
0.2
BETA

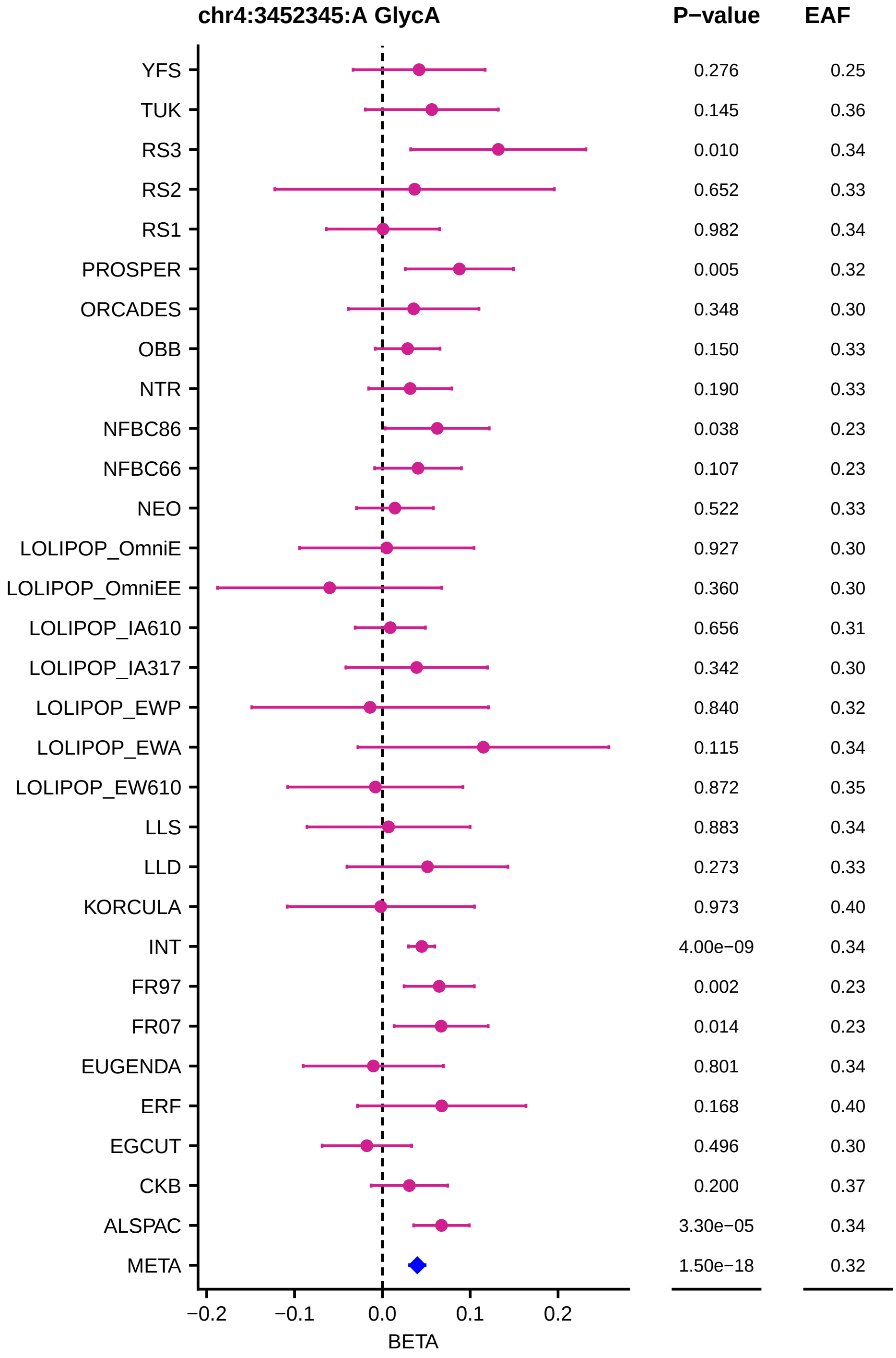

chr4:3452345:A GlycA
P−value
EAF
YFS 0.276 0.25
TUK 0.145 0.36
RS3 0.010 0.34
RS2 0.652 0.33
RS1 0.982 0.34
PROSPER 0.005 0.32
ORCADES 0.348 0.30
OBB 0.150 0.33
NTR 0.190 0.33
NFBC86 0.038 0.23
NFBC66 0.107 0.23
NEO 0.522 0.33
LOLIPOP_OmniE 0.927 0.30
LOLIPOP_OmniEE 0.360 0.30
LOLIPOP_IA610 0.656 0.31
LOLIPOP_IA317 0.342 0.30
LOLIPOP_EWP 0.840 0.32
LOLIPOP_EWA 0.115 0.34
LOLIPOP_EW610 0.872 0.35
LLS 0.883 0.34
LLD 0.273 0.33
KORCULA 0.973 0.40
INT 4.00e−09 0.34
FR97 0.002 0.23
FR07 0.014 0.23
EUGENDA 0.801 0.34
ERF 0.168 0.40
EGCUT 0.496 0.30
CKB 0.200 0.37
ALSPAC 3.30e−05 0.34
META 1.50e−18 0.32
−0.2
−0.1
0.0
0.1
0.2
BETA

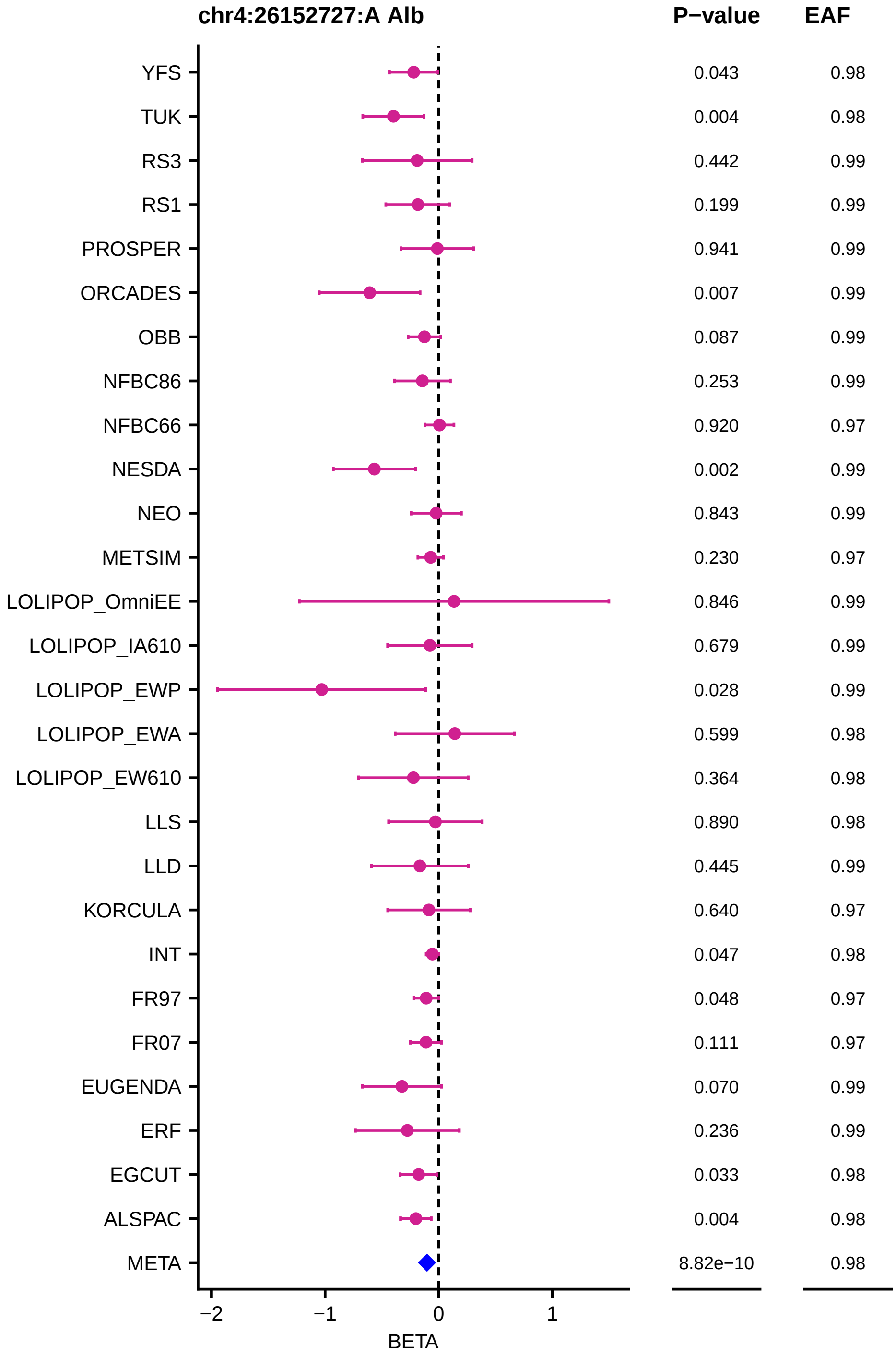
chr4:26152727:A Alb
P−value
EAF
YFS 0.043 0.98
TUK 0.004 0.98
RS3 0.442 0.99
RS1 0.199 0.99
PROSPER 0.941 0.99
ORCADES 0.007 0.99
OBB 0.087 0.99
NFBC86 0.253 0.99
NFBC66 0.920 0.97
NESDA 0.002 0.99
NEO 0.843 0.99
METSIM 0.230 0.97
LOLIPOP_OmniEE 0.846 0.99
LOLIPOP_IA610 0.679 0.99
LOLIPOP_EWP 0.028 0.99
LOLIPOP_EWA 0.599 0.98
LOLIPOP_EW610 0.364 0.98
LLS 0.890 0.98
LLD 0.445 0.99
KORCULA 0.640 0.97
INT 0.047 0.98
FR97 0.048 0.97
FR07 0.111 0.97
EUGENDA 0.070 0.99
ERF 0.236 0.99
EGCUT 0.033 0.98
ALSPAC 0.004 0.98
META 8.82e−10 0.98
−2
−1
0
1
BETA

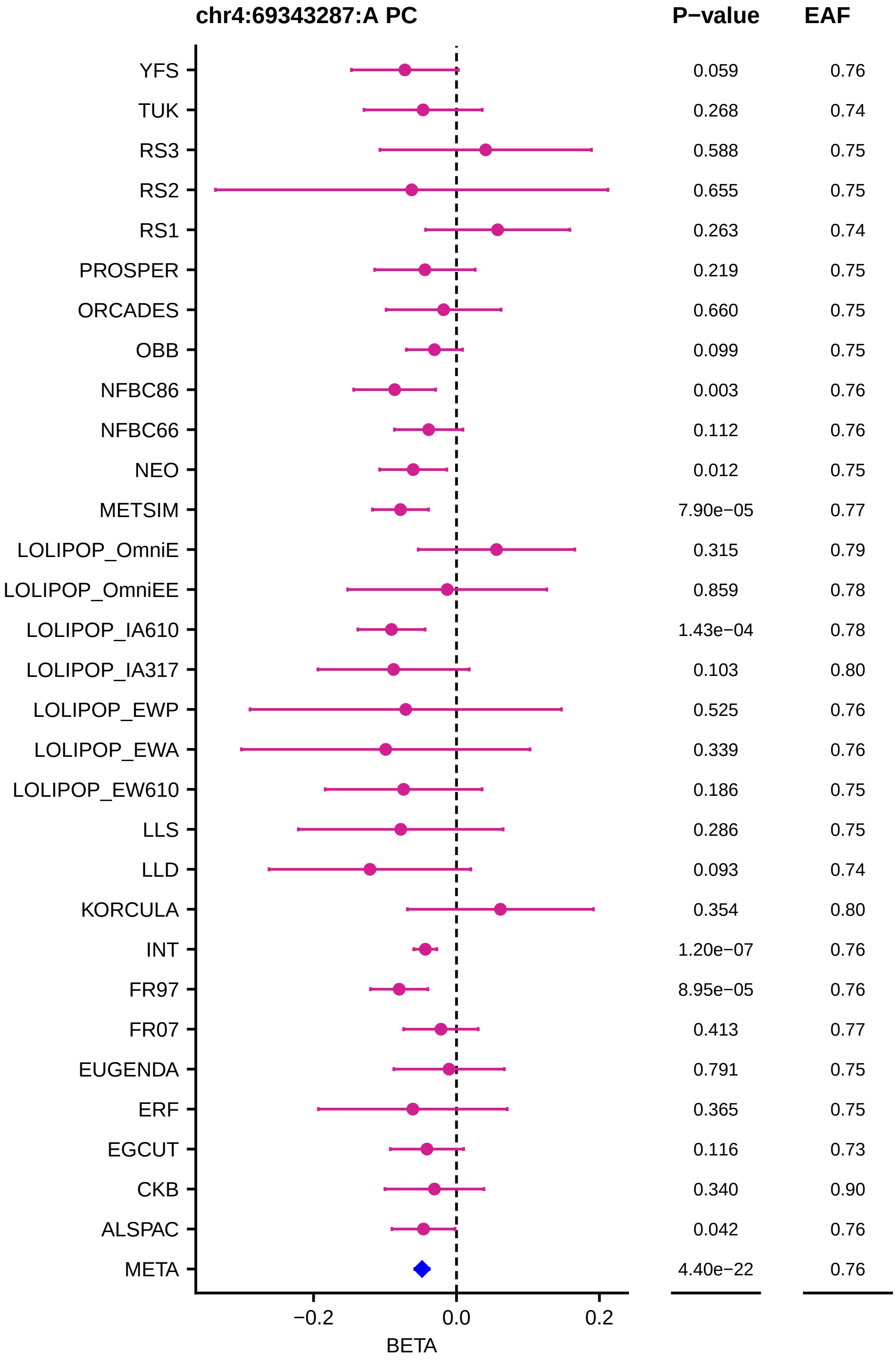
chr4:69343287:A PC
P−value
EAF
YFS 0.059 0.76
TUK 0.268 0.74
RS3 0.588 0.75
RS2 0.655 0.75
RS1 0.263 0.74
PROSPER 0.219 0.75
ORCADES 0.660 0.75
OBB 0.099 0.75
NFBC86 0.003 0.76
NFBC66 0.112 0.76
NEO 0.012 0.75
METSIM 7.90e−05 0.77
LOLIPOP_OmniE 0.315 0.79
LOLIPOP_OmniEE 0.859 0.78
LOLIPOP_IA610 1.43e−04 0.78
LOLIPOP_IA317 0.103 0.80
LOLIPOP_EWP 0.525 0.76
LOLIPOP_EWA 0.339 0.76
LOLIPOP_EW610 0.186 0.75
LLS 0.286 0.75
LLD 0.093 0.74
KORCULA 0.354 0.80
INT 1.20e−07 0.76
FR97 8.95e−05 0.76
FR07 0.413 0.77
EUGENDA 0.791 0.75
ERF 0.365 0.75
EGCUT 0.116 0.73
CKB 0.340 0.90
ALSPAC 0.042 0.76
META 4.40e−22 0.76
−0.2
0.0
0.2
BETA

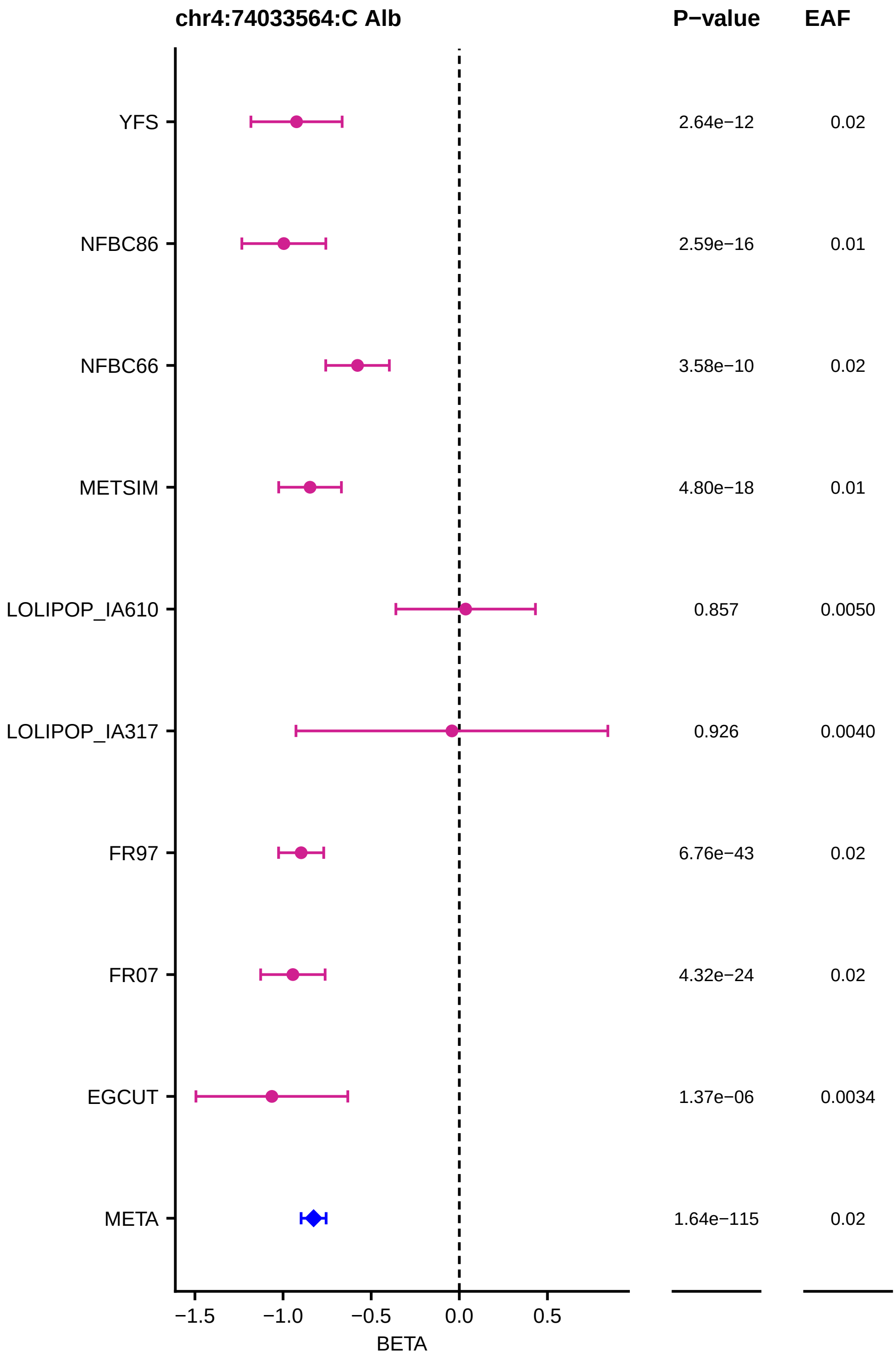
chr4:74033564:C Alb
P−value
EAF
YFS
2.64e−12
0.02
NFBC86
2.59e−16
0.01
NFBC66
3.58e−10
0.02
METSIM
4.80e−18
0.01
LOLIPOP_IA610
0.857
0.0050
LOLIPOP_IA317
0.926
0.0040
FR97
6.76e−43
0.02
FR07
4.32e−24
0.02
EGCUT
1.37e−06
0.0034
META
1.64e−115
0.02
−1.5
−1.0
−0.5
0.0
0.5
BETA

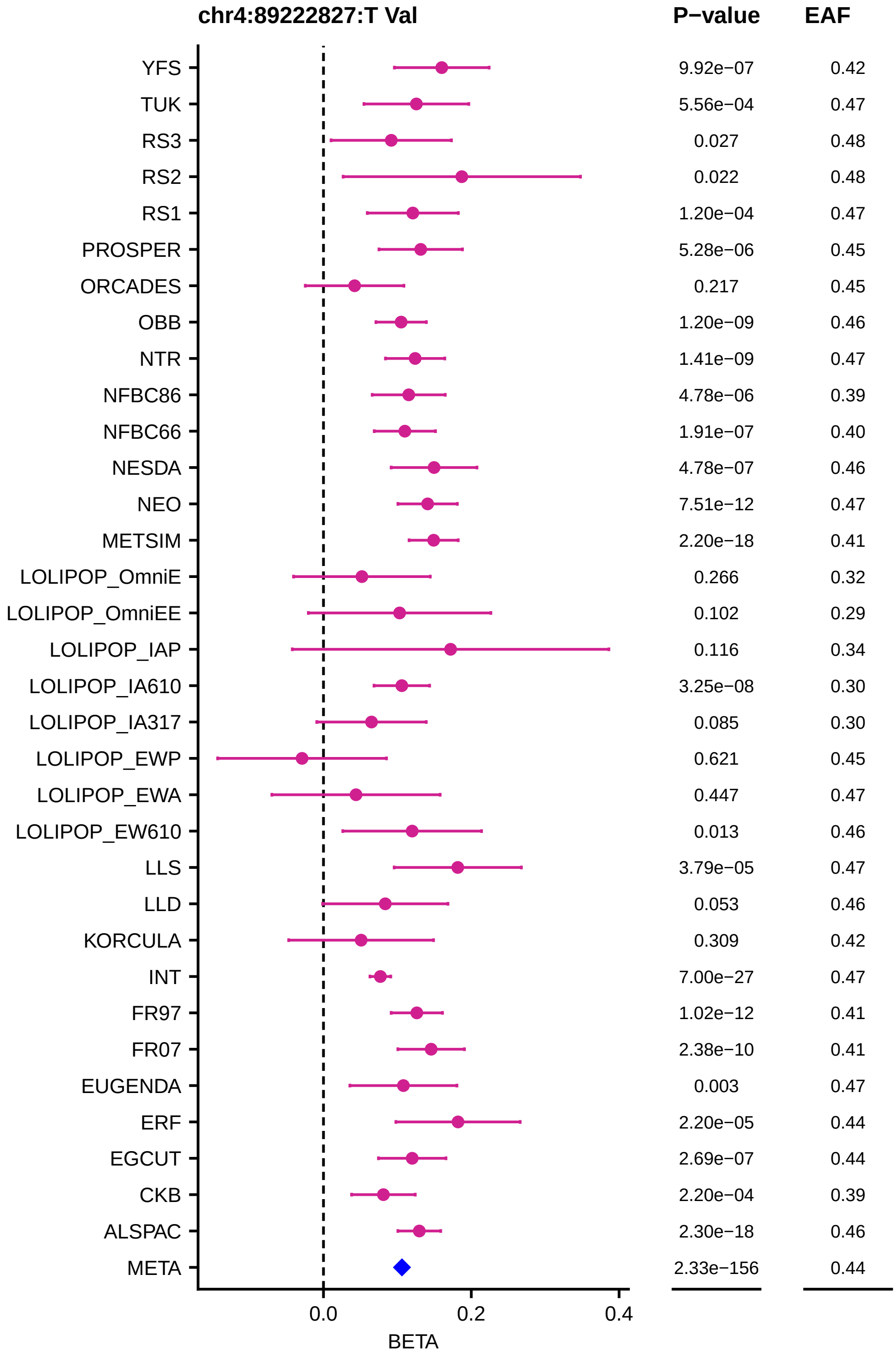
chr4:89222827:T Val
P−value
EAF
YFS 9.92e−07 0.42
TUK 5.56e−04 0.47
RS3 0.027 0.48
RS2 0.022 0.48
RS1 1.20e−04 0.47
PROSPER 5.28e−06 0.45
ORCADES 0.217 0.45
OBB 1.20e−09 0.46
NTR 1.41e−09 0.47
NFBC86 4.78e−06 0.39
NFBC66 1.91e−07 0.40
NESDA 4.78e−07 0.46
NEO 7.51e−12 0.47
METSIM 2.20e−18 0.41
LOLIPOP_OmniE 0.266 0.32
LOLIPOP_OmniEE 0.102 0.29
LOLIPOP_IAP 0.116 0.34
LOLIPOP_IA610 3.25e−08 0.30
LOLIPOP_IA317 0.085 0.30
LOLIPOP_EWP 0.621 0.45
LOLIPOP_EWA 0.447 0.47
LOLIPOP_EW610 0.013 0.46
LLS 3.79e−05 0.47
LLD 0.053 0.46
KORCULA 0.309 0.42
INT 7.00e−27 0.47
FR97 1.02e−12 0.41
FR07 2.38e−10 0.41
EUGENDA 0.003 0.47
ERF 2.20e−05 0.44
EGCUT 2.69e−07 0.44
CKB 2.20e−04 0.39
ALSPAC 2.30e−18 0.46
META 2.33e−156 0.44
0.0
0.2
0.4
BETA

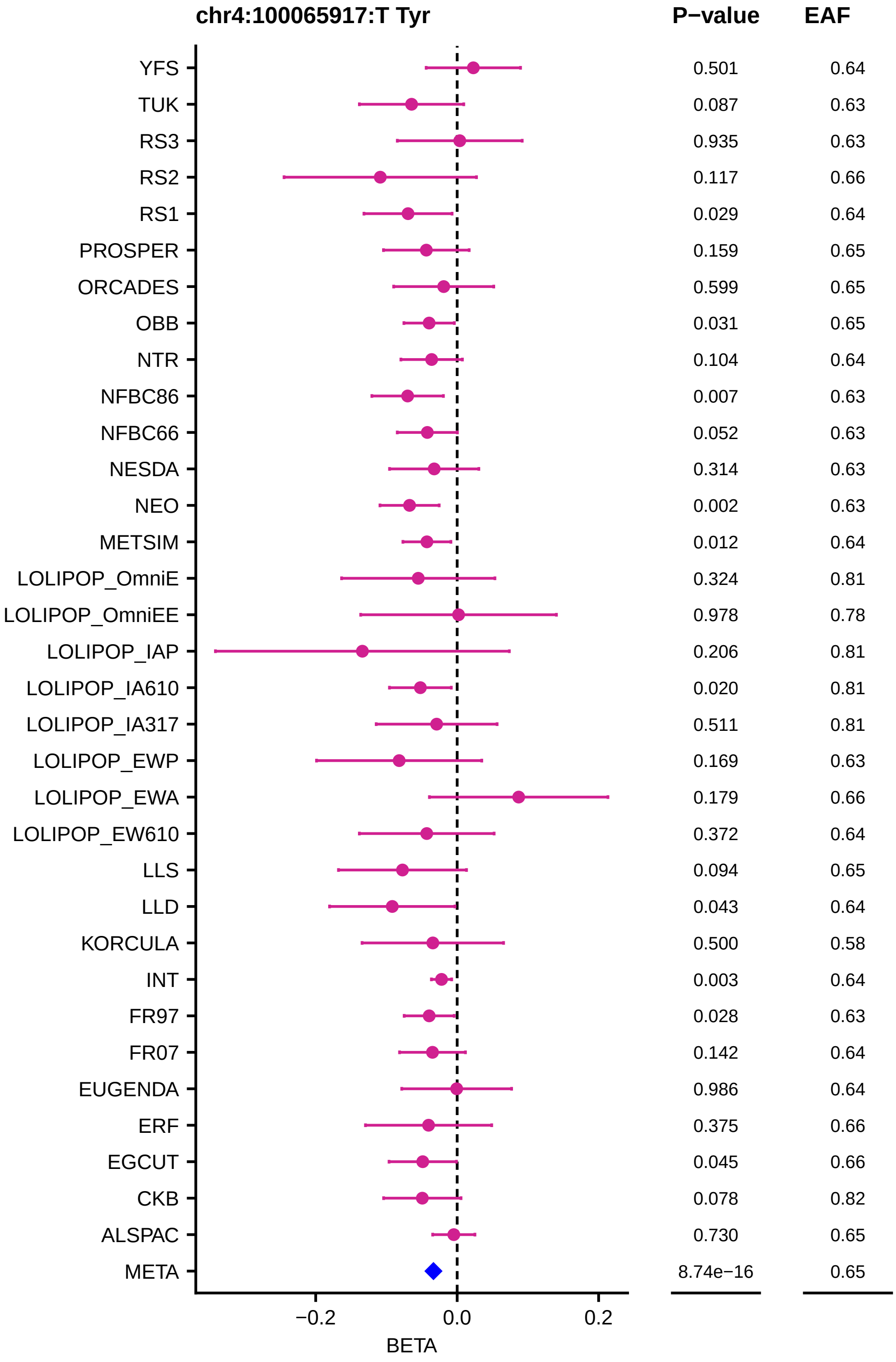
chr4:100065917:T Tyr
P−value
EAF
YFS 0.501 0.64
TUK 0.087 0.63
RS3 0.935 0.63
RS2 0.117 0.66
RS1 0.029 0.64
PROSPER 0.159 0.65
ORCADES 0.599 0.65
OBB 0.031 0.65
NTR 0.104 0.64
NFBC86 0.007 0.63
NFBC66 0.052 0.63
NESDA 0.314 0.63
NEO 0.002 0.63
METSIM 0.012 0.64
LOLIPOP_OmniE 0.324 0.81
LOLIPOP_OmniEE 0.978 0.78
LOLIPOP_IAP 0.206 0.81
LOLIPOP_IA610 0.020 0.81
LOLIPOP_IA317 0.511 0.81
LOLIPOP_EWP 0.169 0.63
LOLIPOP_EWA 0.179 0.66
LOLIPOP_EW610 0.372 0.64
LLS 0.094 0.65
LLD 0.043 0.64
KORCULA 0.500 0.58
INT 0.003 0.64
FR97 0.028 0.63
FR07 0.142 0.64
EUGENDA 0.986 0.64
ERF 0.375 0.66
EGCUT 0.045 0.66
CKB 0.078 0.82
ALSPAC 0.730 0.65
META 8.74e−16 0.65
−0.2
0.0
0.2
BETA

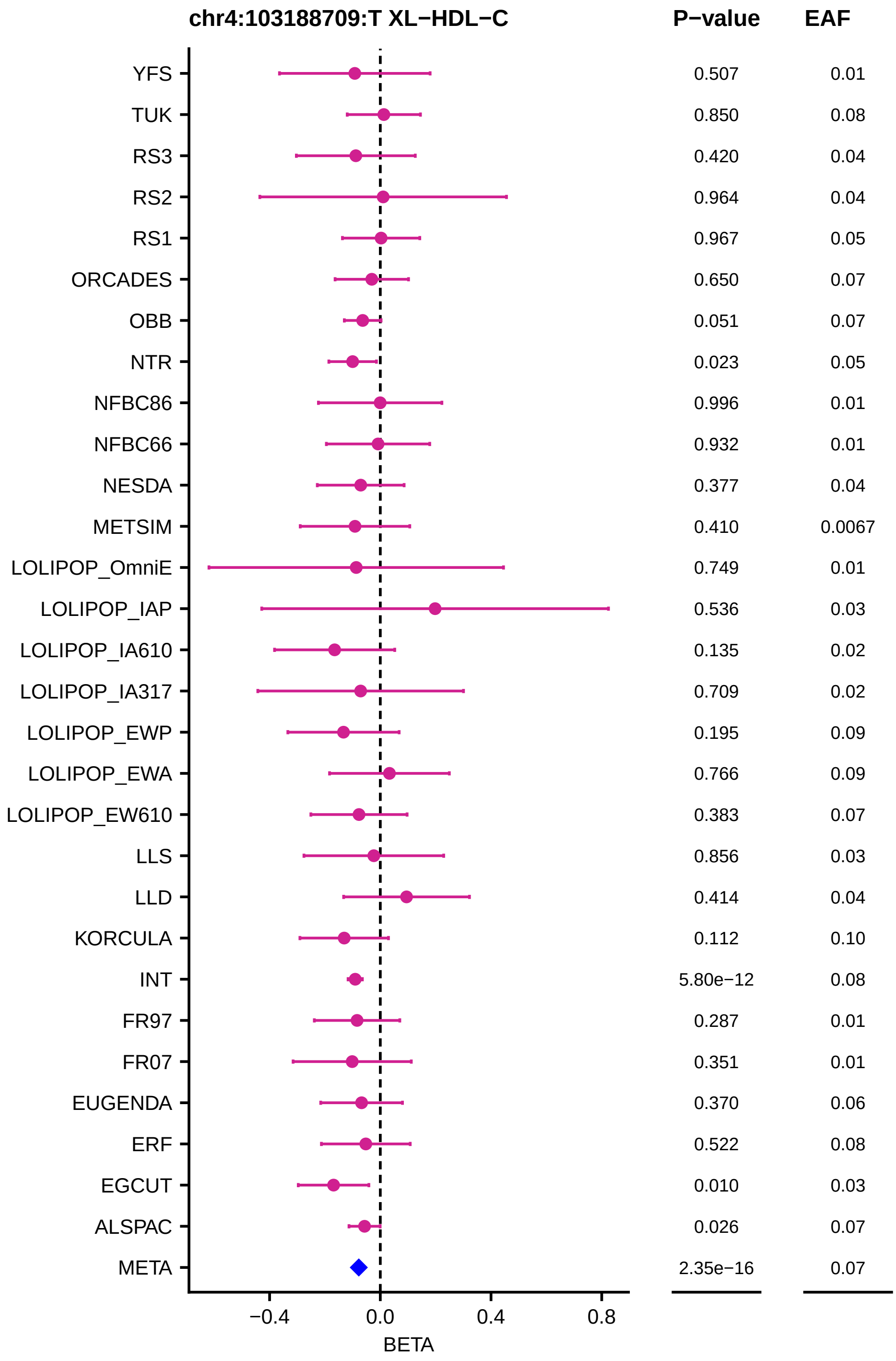
chr4:103188709:T XL−HDL−C
P−value
EAF
YFS 0.507 0.01
TUK 0.850 0.08
RS3 0.420 0.04
RS2 0.964 0.04
RS1 0.967 0.05
ORCADES 0.650 0.07
OBB 0.051 0.07
NTR 0.023 0.05
NFBC86 0.996 0.01
NFBC66 0.932 0.01
NESDA 0.377 0.04
METSIM 0.410 0.0067
LOLIPOP_OmniE 0.749 0.01
LOLIPOP_IAP 0.536 0.03
LOLIPOP_IA610 0.135 0.02
LOLIPOP_IA317 0.709 0.02
LOLIPOP_EWP 0.195 0.09
LOLIPOP_EWA 0.766 0.09
LOLIPOP_EW610 0.383 0.07
LLS 0.856 0.03
LLD 0.414 0.04
KORCULA 0.112 0.10
INT 5.80e−12 0.08
FR97 0.287 0.01
FR07 0.351 0.01
EUGENDA 0.370 0.06
ERF 0.522 0.08
EGCUT 0.010 0.03
ALSPAC 0.026 0.07
META 2.35e−16 0.07
−0.4
0.0
0.4
0.8
BETA

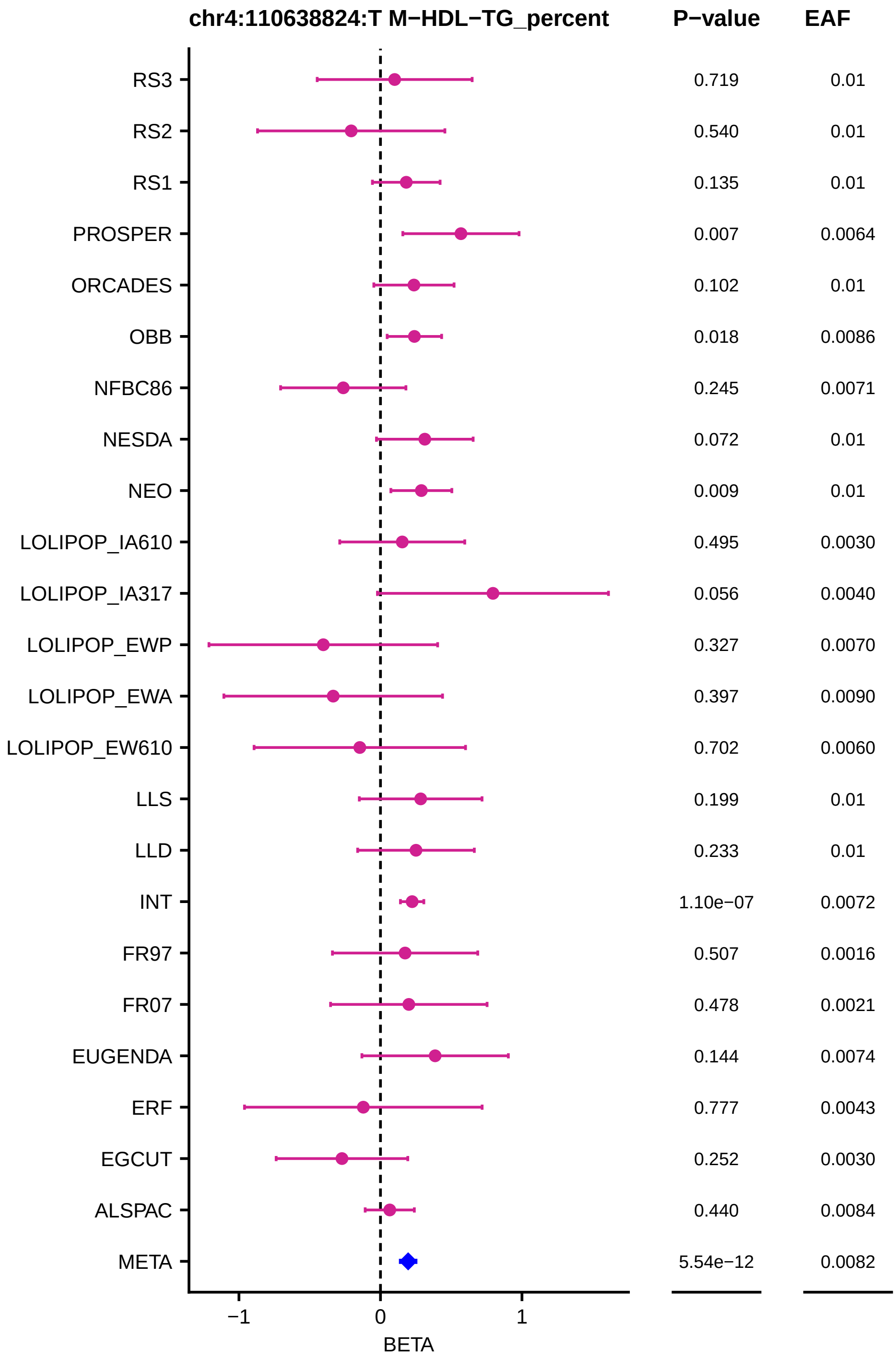
chr4:110638824:T M−HDL−TG_percent
P−value
EAF
RS3
0.719
0.01
RS2
0.540
0.01
RS1
0.135
0.01
PROSPER
0.007
0.0064
ORCADES
0.102
0.01
OBB
0.018
0.0086
NFBC86
0.245
0.0071
NESDA
0.072
0.01
NEO
0.009
0.01
LOLIPOP_IA610
0.495
0.0030
LOLIPOP_IA317
0.056
0.0040
LOLIPOP_EWP
0.327
0.0070
LOLIPOP_EWA
0.397
0.0090
LOLIPOP_EW610
0.702
0.0060
LLS
0.199
0.01
LLD
0.233
0.01
INT
1.10e−07
0.0072
FR97
0.507
0.0016
FR07
0.478
0.0021
EUGENDA
0.144
0.0074
ERF
0.777
0.0043
EGCUT
0.252
0.0030
ALSPAC
0.440
0.0084
META
5.54e−12
0.0082
−1
0
1
BETA

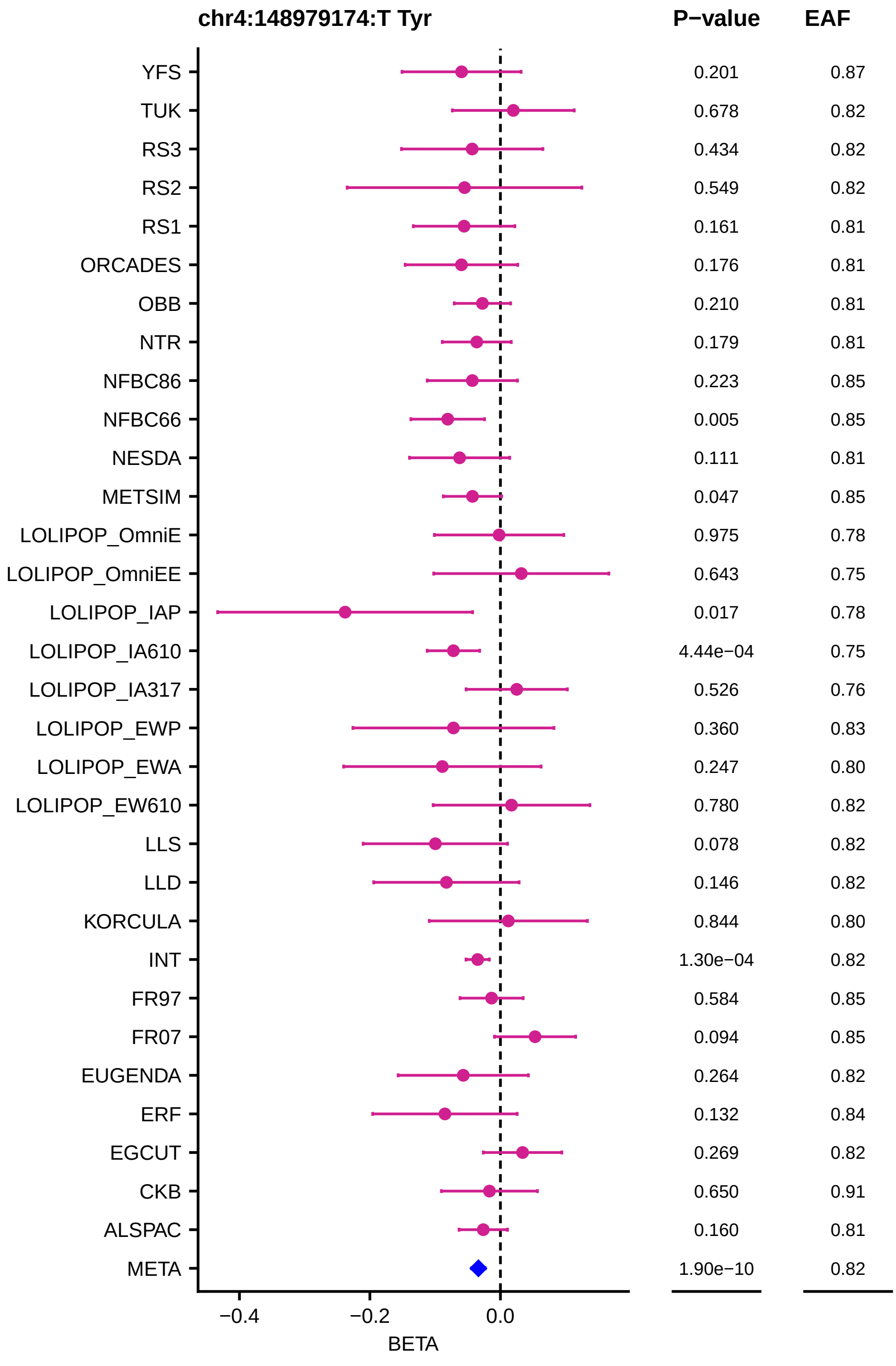
chr4:148979174:T Tyr
P−value
EAF
YFS 0.201 0.87
TUK 0.678 0.82
RS3 0.434 0.82
RS2 0.549 0.82
RS1 0.161 0.81
ORCADES 0.176 0.81
OBB 0.210 0.81
NTR 0.179 0.81
NFBC86 0.223 0.85
NFBC66 0.005 0.85
NESDA 0.111 0.81
METSIM 0.047 0.85
LOLIPOP_OmniE 0.975 0.78
LOLIPOP_OmniEE 0.643 0.75
LOLIPOP_IAP 0.017 0.78
LOLIPOP_IA610 4.44e−04 0.75
LOLIPOP_IA317 0.526 0.76
LOLIPOP_EWP 0.360 0.83
LOLIPOP_EWA 0.247 0.80
LOLIPOP_EW610 0.780 0.82
LLS 0.078 0.82
LLD 0.146 0.82
KORCULA 0.844 0.80
INT 1.30e−04 0.82
FR97 0.584 0.85
FR07 0.094 0.85
EUGENDA 0.264 0.82
ERF 0.132 0.84
EGCUT 0.269 0.82
CKB 0.650 0.91
ALSPAC 0.160 0.81
META 1.90e−10 0.82
−0.4
−0.2
0.0
BETA

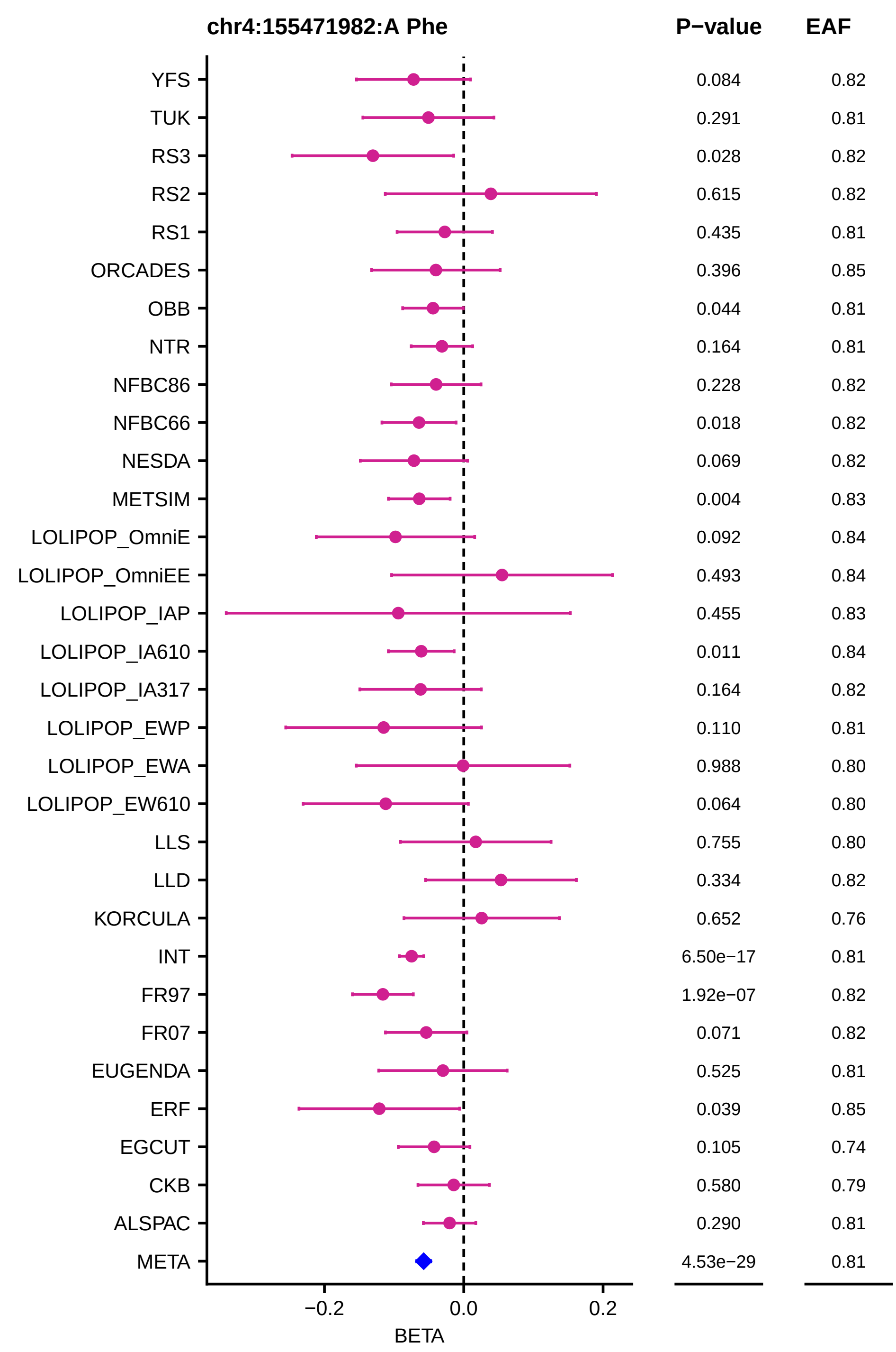
chr4:155471982:A Phe
P−value
EAF
YFS 0.084 0.82
TUK 0.291 0.81
RS3 0.028 0.82
RS2 0.615 0.82
RS1 0.435 0.81
ORCADES 0.396 0.85
OBB 0.044 0.81
NTR 0.164 0.81
NFBC86 0.228 0.82
NFBC66 0.018 0.82
NESDA 0.069 0.82
METSIM 0.004 0.83
LOLIPOP_OmniE 0.092 0.84
LOLIPOP_OmniEE 0.493 0.84
LOLIPOP_IAP 0.455 0.83
LOLIPOP_IA610 0.011 0.84
LOLIPOP_IA317 0.164 0.82
LOLIPOP_EWP 0.110 0.81
LOLIPOP_EWA 0.988 0.80
LOLIPOP_EW610 0.064 0.80
LLS 0.755 0.80
LLD 0.334 0.82
KORCULA 0.652 0.76
INT 6.50e−17 0.81
FR97 1.92e−07 0.82
FR07 0.071 0.82
EUGENDA 0.525 0.81
ERF 0.039 0.85
EGCUT 0.105 0.74
CKB 0.580 0.79
ALSPAC 0.290 0.81
META 4.53e−29 0.81
−0.2
0.0
0.2
BETA

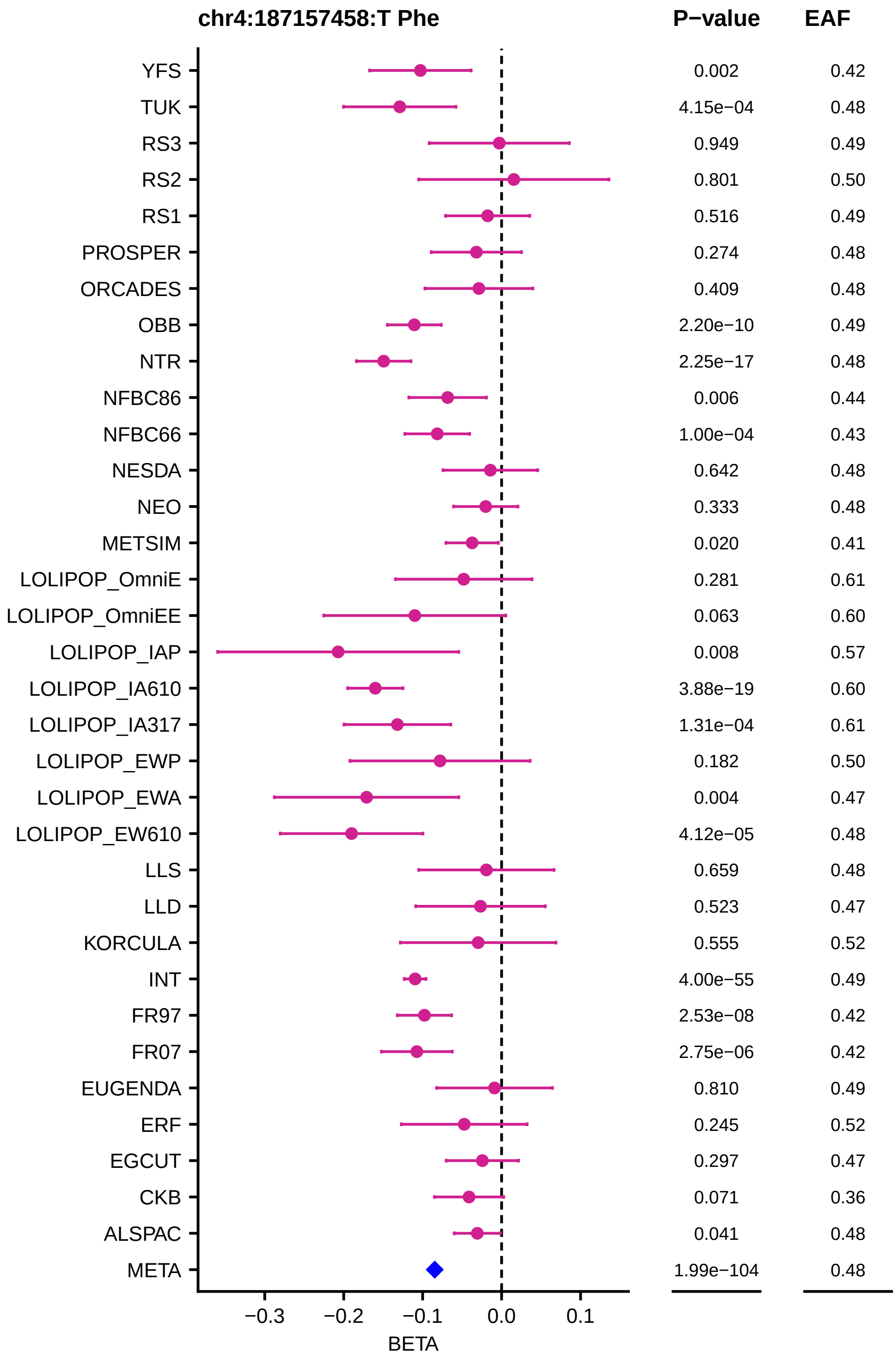
chr4:187157458:T Phe
P−value
EAF
YFS 0.002 0.42
TUK 4.15e−04 0.48
RS3 0.949 0.49
RS2 0.801 0.50
RS1 0.516 0.49
PROSPER 0.274 0.48
ORCADES 0.409 0.48
OBB 2.20e−10 0.49
NTR 2.25e−17 0.48
NFBC86 0.006 0.44
NFBC66 1.00e−04 0.43
NESDA 0.642 0.48
NEO 0.333 0.48
METSIM 0.020 0.41
LOLIPOP_OmniE 0.281 0.61
LOLIPOP_OmniEE 0.063 0.60
LOLIPOP_IAP 0.008 0.57
LOLIPOP_IA610 3.88e−19 0.60
LOLIPOP_IA317 1.31e−04 0.61
LOLIPOP_EWP 0.182 0.50
LOLIPOP_EWA 0.004 0.47
LOLIPOP_EW610 4.12e−05 0.48
LLS 0.659 0.48
LLD 0.523 0.47
KORCULA 0.555 0.52
INT 4.00e−55 0.49
FR97 2.53e−08 0.42
FR07 2.75e−06 0.42
EUGENDA 0.810 0.49
ERF 0.245 0.52
EGCUT 0.297 0.47
CKB 0.071 0.36
ALSPAC 0.041 0.48
META 1.99e−104 0.48
−0.3
−0.2
−0.1
0.0
0.1
BETA

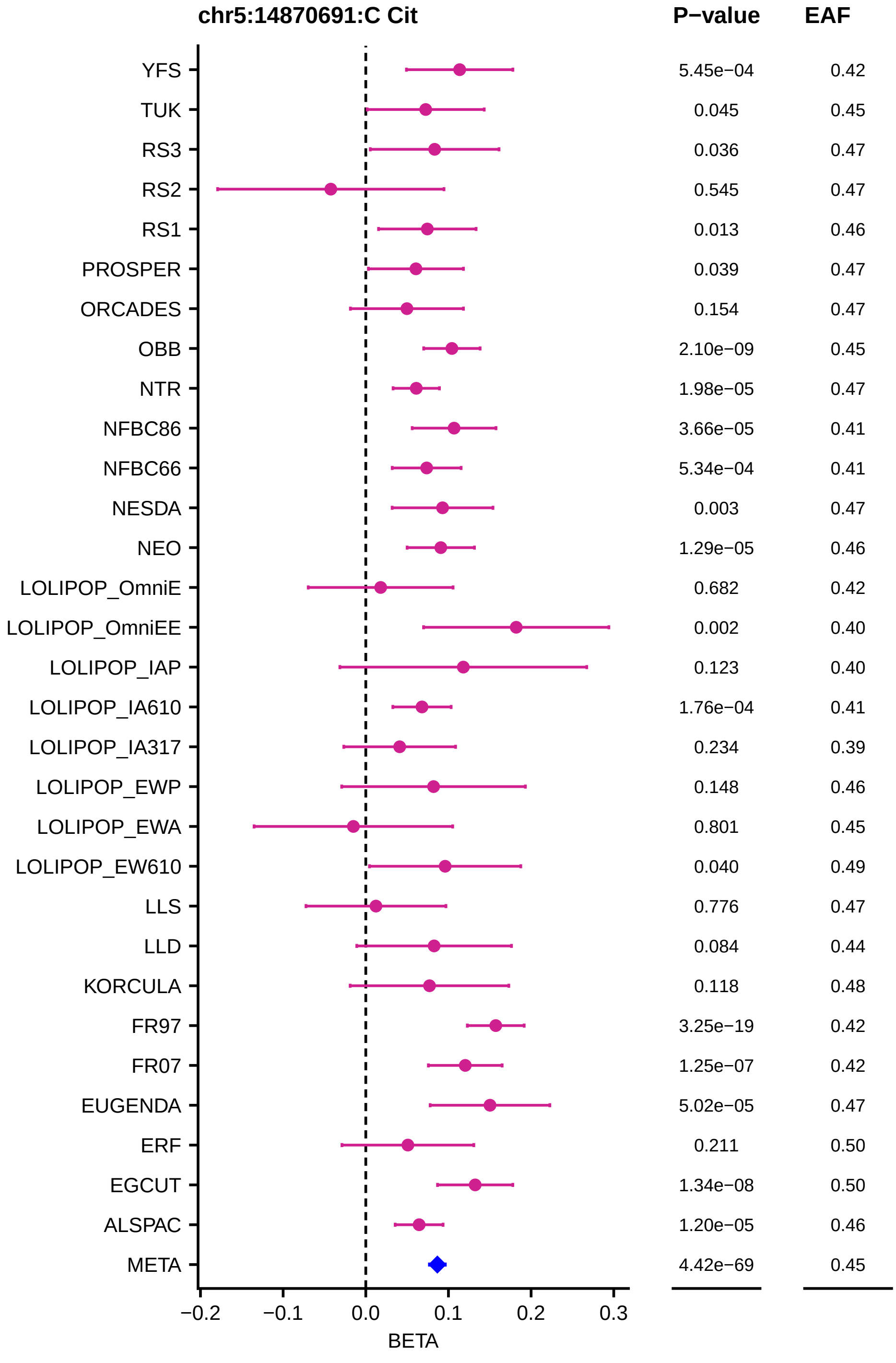

chr5:14870691:C Cit
P−value
EAF
YFS 5.45e−04 0.42
TUK 0.045 0.45
RS3 0.036 0.47
RS2 0.545 0.47
RS1 0.013 0.46
PROSPER 0.039 0.47
ORCADES 0.154 0.47
OBB 2.10e−09 0.45
NTR 1.98e−05 0.47
NFBC86 3.66e−05 0.41
NFBC66 5.34e−04 0.41
NESDA 0.003 0.47
NEO 1.29e−05 0.46
LOLIPOP_OmniE 0.682 0.42
LOLIPOP_OmniEE 0.002 0.40
LOLIPOP_IAP 0.123 0.40
LOLIPOP_IA610 1.76e−04 0.41
LOLIPOP_IA317 0.234 0.39
LOLIPOP_EWP 0.148 0.46
LOLIPOP_EWA 0.801 0.45
LOLIPOP_EW610 0.040 0.49
LLS 0.776 0.47
LLD 0.084 0.44
KORCULA 0.118 0.48
FR97 3.25e−19 0.42
FR07 1.25e−07 0.42
EUGENDA 5.02e−05 0.47
ERF 0.211 0.50
EGCUT 1.34e−08 0.50
ALSPAC 1.20e−05 0.46
META 4.42e−69 0.45
−0.2 −0.1 0.0 0.1 0.2 0.3
BETA

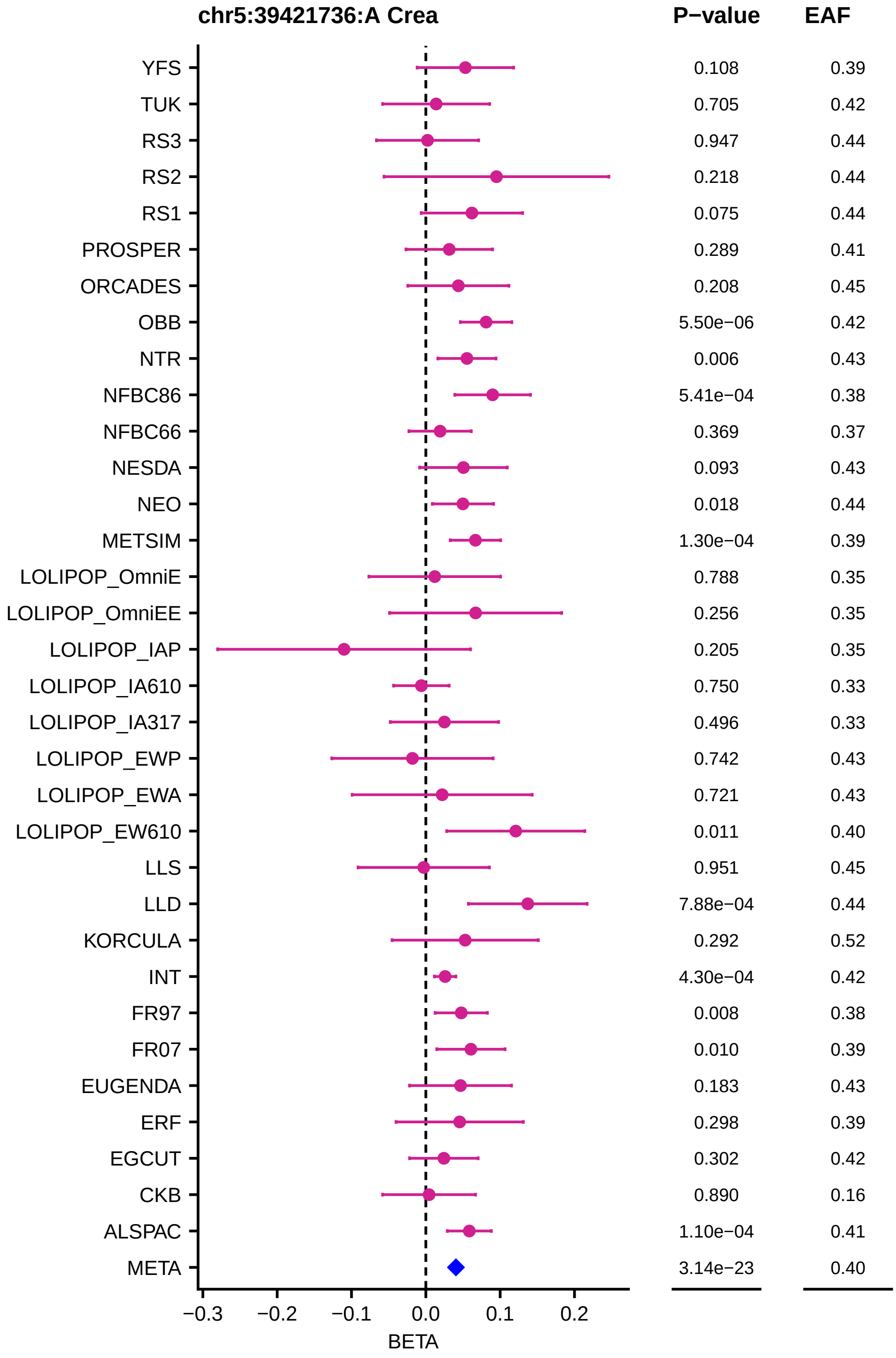
chr5:39421736:A Crea
P−value
EAF
YFS 0.108 0.39
TUK 0.705 0.42
RS3 0.947 0.44
RS2 0.218 0.44
RS1 0.075 0.44
PROSPER 0.289 0.41
ORCADES 0.208 0.45
OBB 5.50e−06 0.42
NTR 0.006 0.43
NFBC86 5.41e−04 0.38
NFBC66 0.369 0.37
NESDA 0.093 0.43
NEO 0.018 0.44
METSIM 1.30e−04 0.39
LOLIPOP_OmniE 0.788 0.35
LOLIPOP_OmniEE 0.256 0.35
LOLIPOP_IAP 0.205 0.35
LOLIPOP_IA610 0.750 0.33
LOLIPOP_IA317 0.496 0.33
LOLIPOP_EWP 0.742 0.43
LOLIPOP_EWA 0.721 0.43
LOLIPOP_EW610 0.011 0.40
LLS 0.951 0.45
LLD 7.88e−04 0.44
KORCULA 0.292 0.52
INT 4.30e−04 0.42
FR97 0.008 0.38
FR07 0.010 0.39
EUGENDA 0.183 0.43
ERF 0.298 0.39
EGCUT 0.302 0.42
CKB 0.890 0.16
ALSPAC 1.10e−04 0.41
META 3.14e−23 0.40
−0.3
−0.2
−0.1
0.0
0.1
0.2
BETA

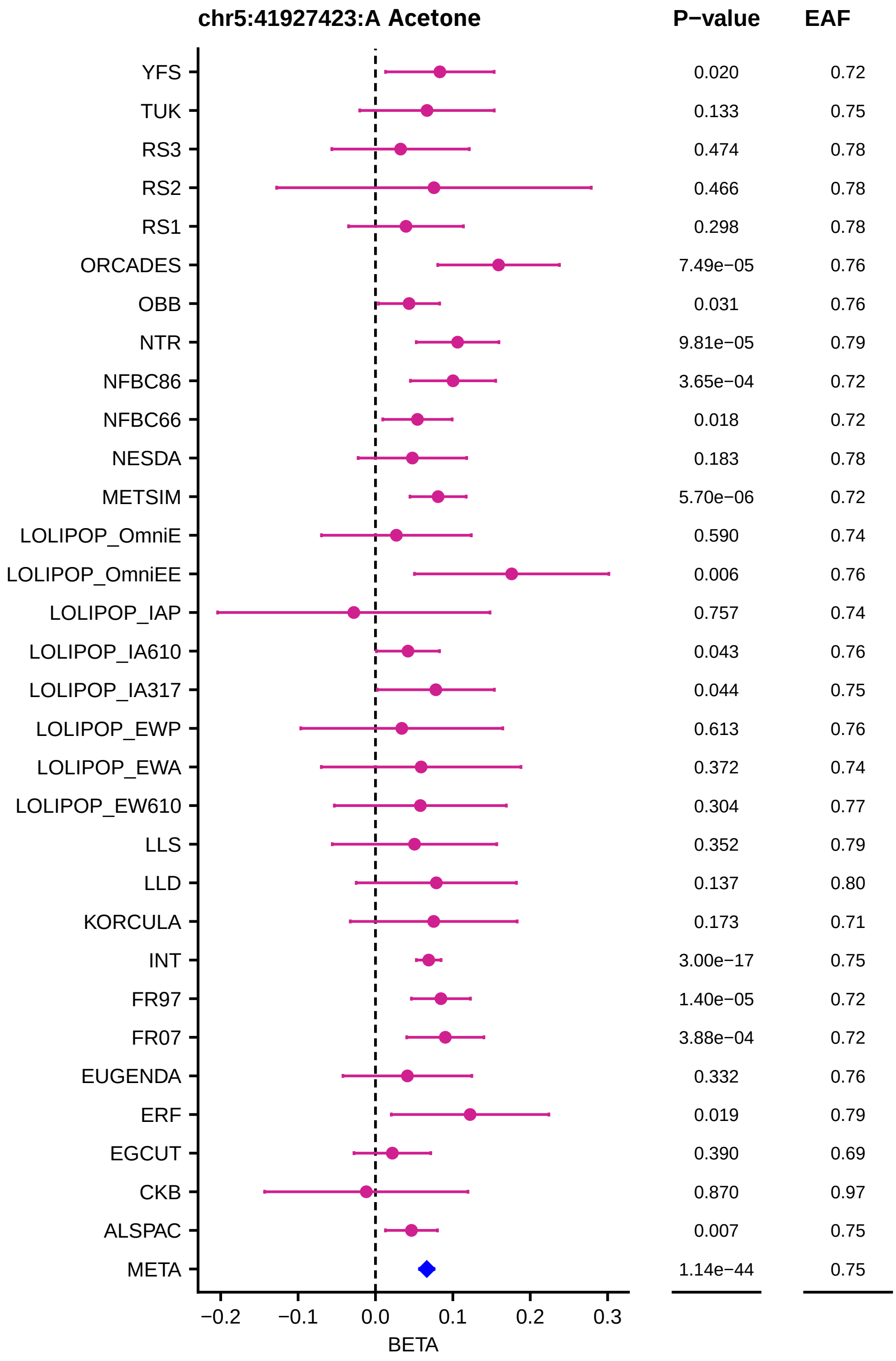
chr5:41927423:A Acetone
P−value
EAF
YFS 0.020 0.72
TUK 0.133 0.75
RS3 0.474 0.78
RS2 0.466 0.78
RS1 0.298 0.78
ORCADES 7.49e−05 0.76
OBB 0.031 0.76
NTR 9.81e−05 0.79
NFBC86 3.65e−04 0.72
NFBC66 0.018 0.72
NESDA 0.183 0.78
METSIM 5.70e−06 0.72
LOLIPOP_OmniE 0.590 0.74
LOLIPOP_OmniEE 0.006 0.76
LOLIPOP_IAP 0.757 0.74
LOLIPOP_IA610 0.043 0.76
LOLIPOP_IA317 0.044 0.75
LOLIPOP_EWP 0.613 0.76
LOLIPOP_EWA 0.372 0.74
LOLIPOP_EW610 0.304 0.77
LLS 0.352 0.79
LLD 0.137 0.80
KORCULA 0.173 0.71
INT 3.00e−17 0.75
FR97 1.40e−05 0.72
FR07 3.88e−04 0.72
EUGENDA 0.332 0.76
ERF 0.019 0.79
EGCUT 0.390 0.69
CKB 0.870 0.97
ALSPAC 0.007 0.75
META 1.14e−44 0.75
−0.2 −0.1 0.0 0.1 0.2 0.3
BETA

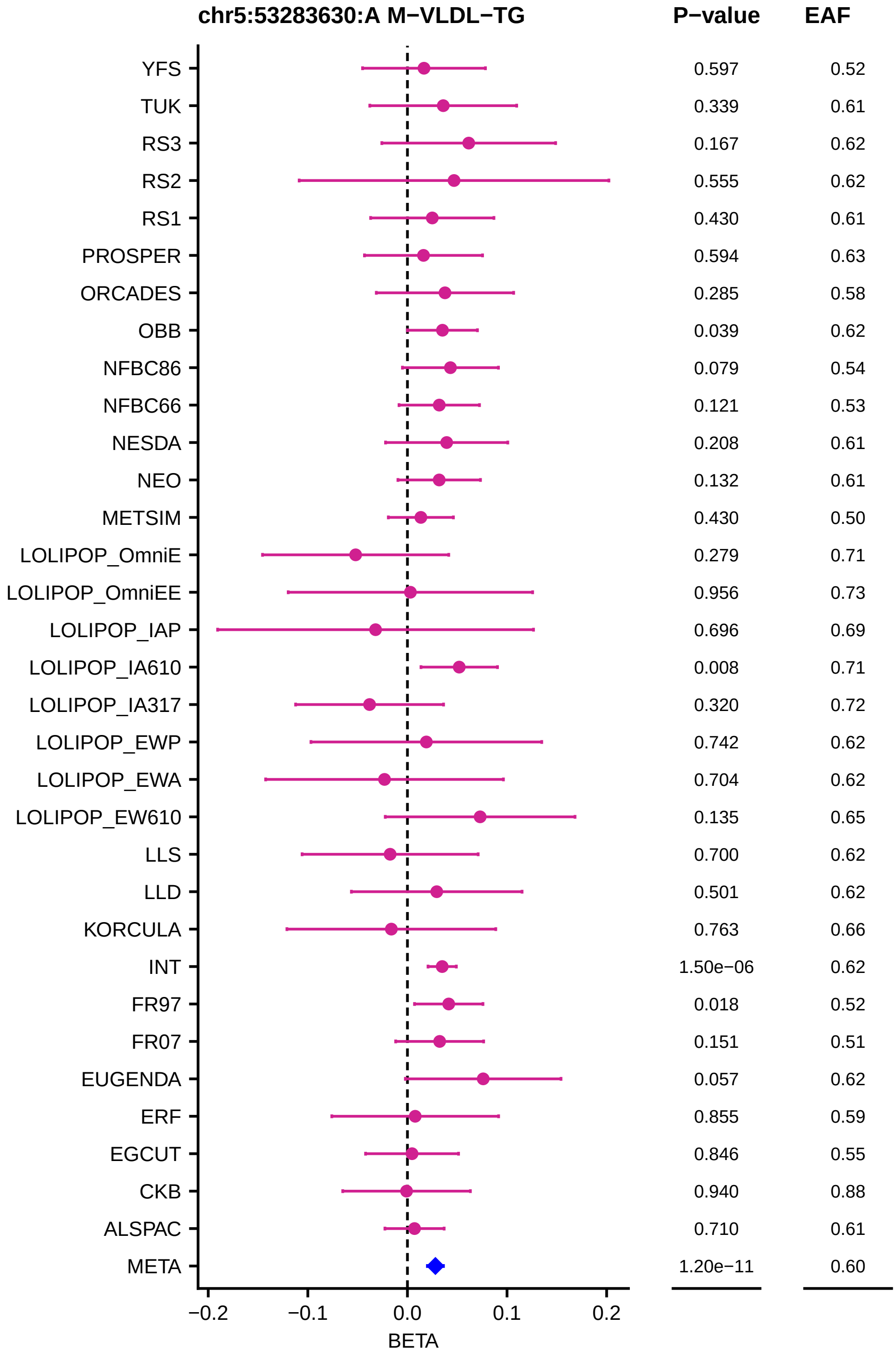
chr5:53283630:A M−VLDL−TG
P−value
EAF
YFS 0.597 0.52
TUK 0.339 0.61
RS3 0.167 0.62
RS2 0.555 0.62
RS1 0.430 0.61
PROSPER 0.594 0.63
ORCADES 0.285 0.58
OBB 0.039 0.62
NFBC86 0.079 0.54
NFBC66 0.121 0.53
NESDA 0.208 0.61
NEO 0.132 0.61
METSIM 0.430 0.50
LOLIPOP_OmniE 0.279 0.71
LOLIPOP_OmniEE 0.956 0.73
LOLIPOP_IAP 0.696 0.69
LOLIPOP_IA610 0.008 0.71
LOLIPOP_IA317 0.320 0.72
LOLIPOP_EWP 0.742 0.62
LOLIPOP_EWA 0.704 0.62
LOLIPOP_EW610 0.135 0.65
LLS 0.700 0.62
LLD 0.501 0.62
KORCULA 0.763 0.66
INT 1.50e−06 0.62
FR97 0.018 0.52
FR07 0.151 0.51
EUGENDA 0.057 0.62
ERF 0.855 0.59
EGCUT 0.846 0.55
CKB 0.940 0.88
ALSPAC 0.710 0.61
META 1.20e−11 0.60
−0.2
−0.1
0.0
0.1
0.2
BETA

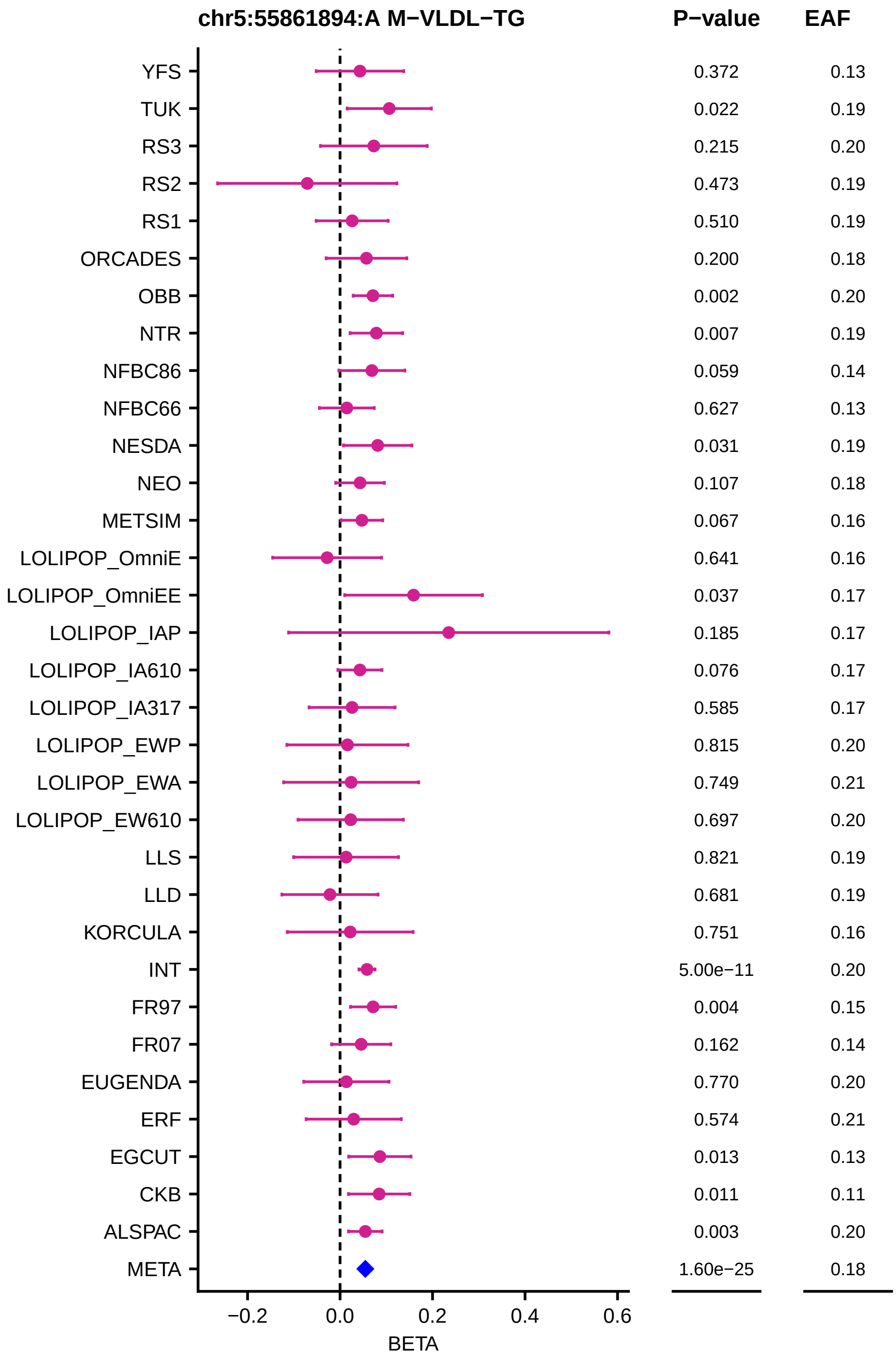
chr5:55861894:A M−VLDL−TG
P−value
EAF
YFS 0.372 0.13
TUK 0.022 0.19
RS3 0.215 0.20
RS2 0.473 0.19
RS1 0.510 0.19
ORCADES 0.200 0.18
OBB 0.002 0.20
NTR 0.007 0.19
NFBC86 0.059 0.14
NFBC66 0.627 0.13
NESDA 0.031 0.19
NEO 0.107 0.18
METSIM 0.067 0.16
LOLIPOP_OmniE 0.641 0.16
LOLIPOP_OmniEE 0.037 0.17
LOLIPOP_IAP 0.185 0.17
LOLIPOP_IA610 0.076 0.17
LOLIPOP_IA317 0.585 0.17
LOLIPOP_EWP 0.815 0.20
LOLIPOP_EWA 0.749 0.21
LOLIPOP_EW610 0.697 0.20
LLS 0.821 0.19
LLD 0.681 0.19
KORCULA 0.751 0.16
INT 5.00e−11 0.20
FR97 0.004 0.15
FR07 0.162 0.14
EUGENDA 0.770 0.20
ERF 0.574 0.21
EGCUT 0.013 0.13
CKB 0.011 0.11
ALSPAC 0.003 0.20
META 1.60e−25 0.18
−0.2
0.0
0.2
0.4
0.6
BETA

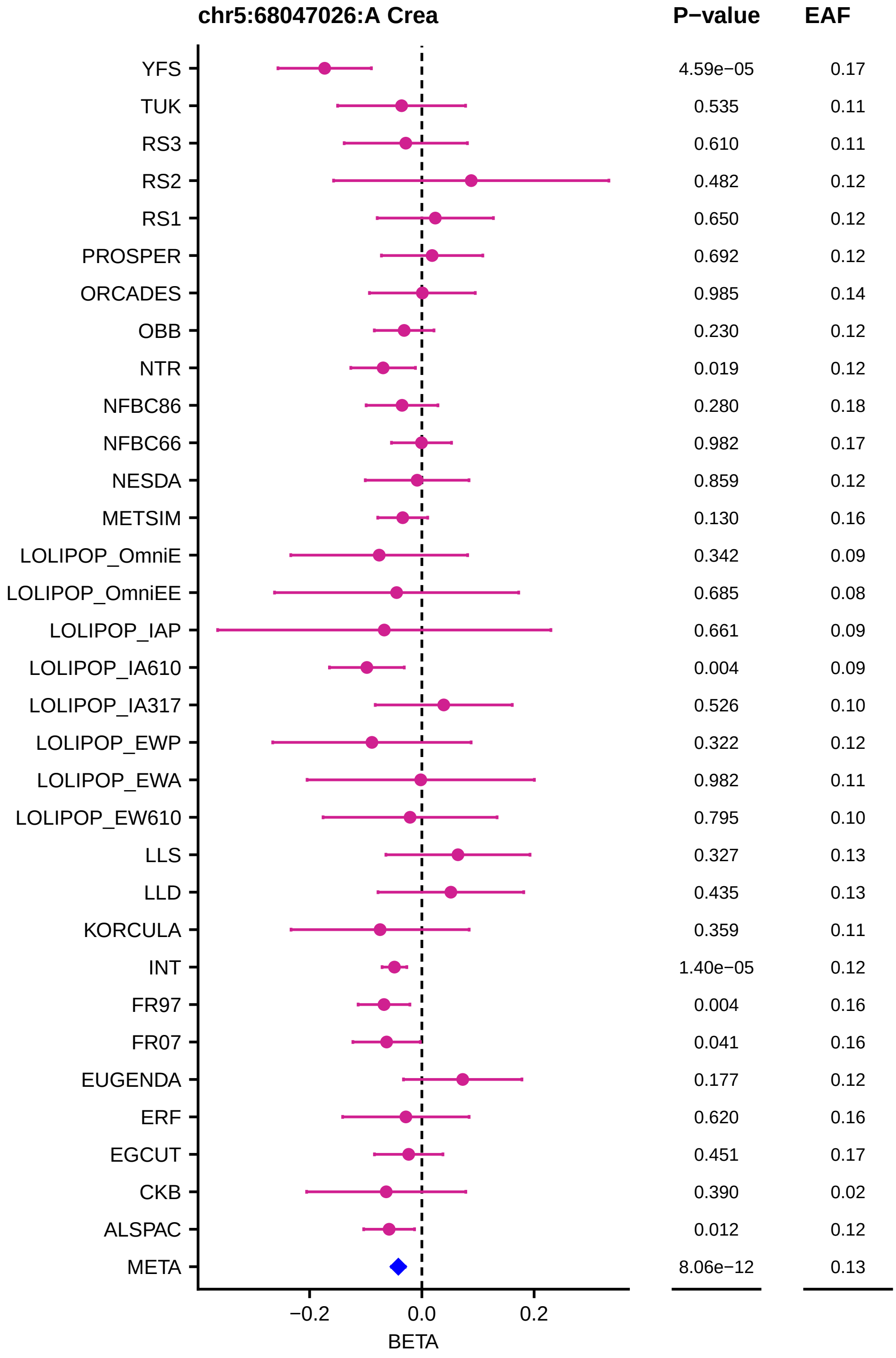

chr5:68047026:A Crea
P−value
EAF
YFS 4.59e−05 0.17
TUK 0.535 0.11
RS3 0.610 0.11
RS2 0.482 0.12
RS1 0.650 0.12
PROSPER 0.692 0.12
ORCADES 0.985 0.14
OBB 0.230 0.12
NTR 0.019 0.12
NFBC86 0.280 0.18
NFBC66 0.982 0.17
NESDA 0.859 0.12
METSIM 0.130 0.16
LOLIPOP_OmniE 0.342 0.09
LOLIPOP_OmniEE 0.685 0.08
LOLIPOP_IAP 0.661 0.09
LOLIPOP_IA610 0.004 0.09
LOLIPOP_IA317 0.526 0.10
LOLIPOP_EWP 0.322 0.12
LOLIPOP_EWA 0.982 0.11
LOLIPOP_EW610 0.795 0.10
LLS 0.327 0.13
LLD 0.435 0.13
KORCULA 0.359 0.11
INT 1.40e−05 0.12
FR97 0.004 0.16
FR07 0.041 0.16
EUGENDA 0.177 0.12
ERF 0.620 0.16
EGCUT 0.451 0.17
CKB 0.390 0.02
ALSPAC 0.012 0.12
META 8.06e−12 0.13
−0.2
0.0
0.2
BETA

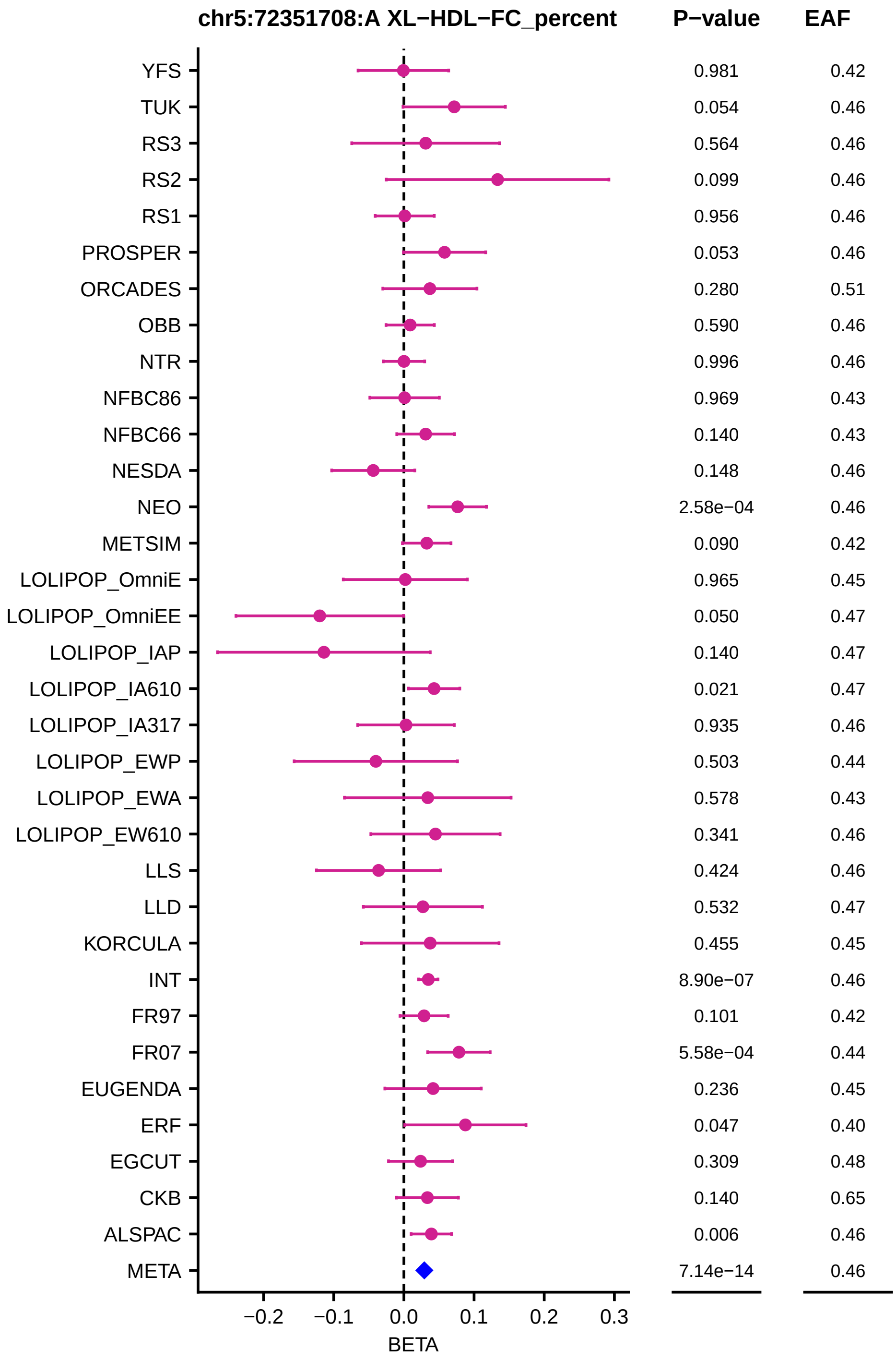

chr5:72351708:A XL−HDL−FC_percent
P−value
EAF
YFS 0.981 0.42
TUK 0.054 0.46
RS3 0.564 0.46
RS2 0.099 0.46
RS1 0.956 0.46
PROSPER 0.053 0.46
ORCADES 0.280 0.51
OBB 0.590 0.46
NTR 0.996 0.46
NFBC86 0.969 0.43
NFBC66 0.140 0.43
NESDA 0.148 0.46
NEO 2.58e−04 0.46
METSIM 0.090 0.42
LOLIPOP_OmniE 0.965 0.45
LOLIPOP_OmniEE 0.050 0.47
LOLIPOP_IAP 0.140 0.47
LOLIPOP_IA610 0.021 0.47
LOLIPOP_IA317 0.935 0.46
LOLIPOP_EWP 0.503 0.44
LOLIPOP_EWA 0.578 0.43
LOLIPOP_EW610 0.341 0.46
LLS 0.424 0.46
LLD 0.532 0.47
KORCULA 0.455 0.45
INT 8.90e−07 0.46
FR97 0.101 0.42
FR07 5.58e−04 0.44
EUGENDA 0.236 0.45
ERF 0.047 0.40
EGCUT 0.309 0.48
CKB 0.140 0.65
ALSPAC 0.006 0.46
META 7.14e−14 0.46
−0.2 −0.1 0.0 0.1 0.2 0.3
BETA

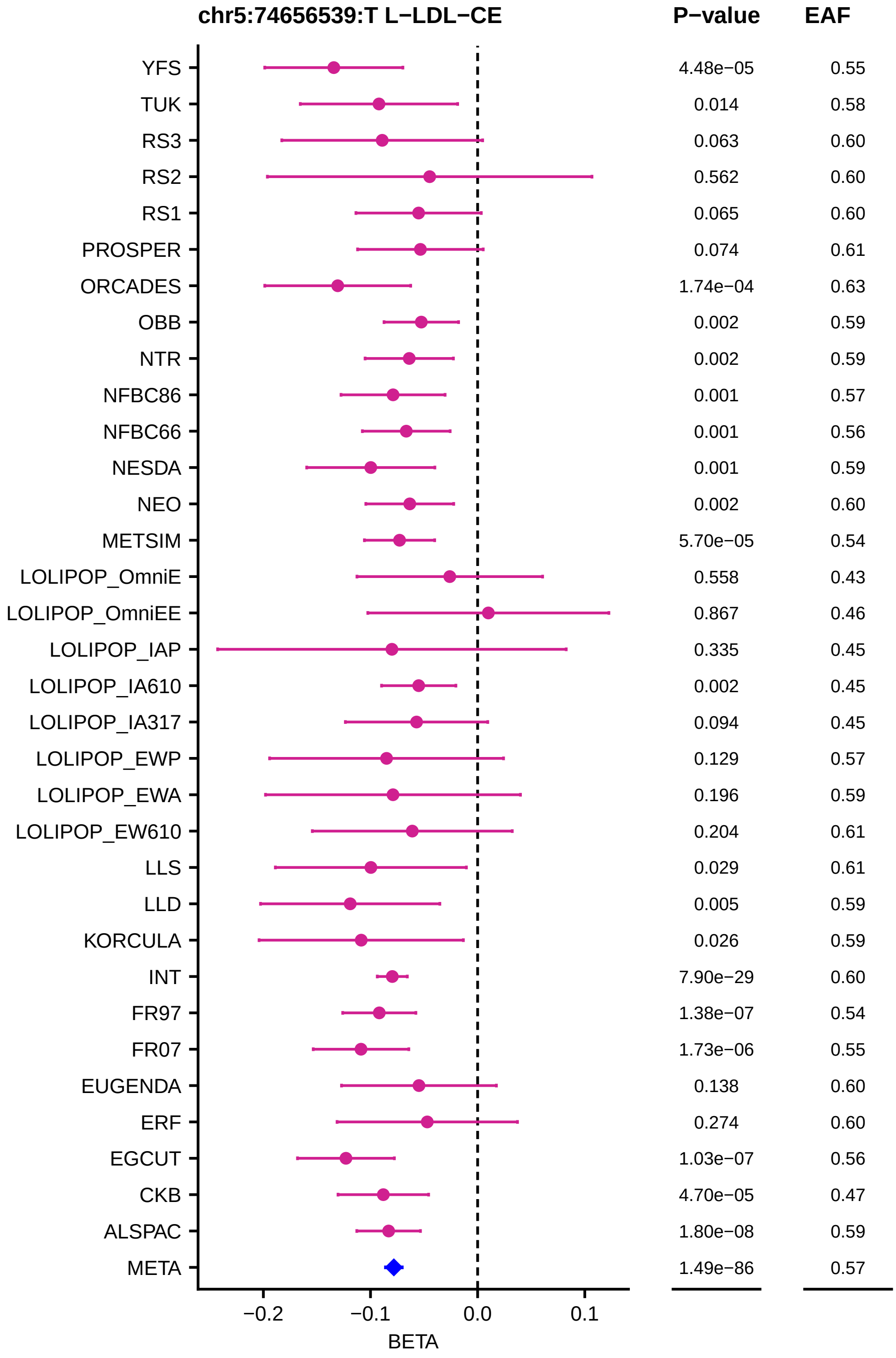

chr5:74656539:T L−LDL−CE
P−value
EAF
YFS 4.48e−05 0.55
TUK 0.014 0.58
RS3 0.063 0.60
RS2 0.562 0.60
RS1 0.065 0.60
PROSPER 0.074 0.61
ORCADES 1.74e−04 0.63
OBB 0.002 0.59
NTR 0.002 0.59
NFBC86 0.001 0.57
NFBC66 0.001 0.56
NESDA 0.001 0.59
NEO 0.002 0.60
METSIM 5.70e−05 0.54
LOLIPOP_OmniE 0.558 0.43
LOLIPOP_OmniEE 0.867 0.46
LOLIPOP_IAP 0.335 0.45
LOLIPOP_IA610 0.002 0.45
LOLIPOP_IA317 0.094 0.45
LOLIPOP_EWP 0.129 0.57
LOLIPOP_EWA 0.196 0.59
LOLIPOP_EW610 0.204 0.61
LLS 0.029 0.61
LLD 0.005 0.59
KORCULA 0.026 0.59
INT 7.90e−29 0.60
FR97 1.38e−07 0.54
FR07 1.73e−06 0.55
EUGENDA 0.138 0.60
ERF 0.274 0.60
EGCUT 1.03e−07 0.56
CKB 4.70e−05 0.47
ALSPAC 1.80e−08 0.59
META 1.49e−86 0.57
−0.2
−0.1
0.0
0.1
BETA

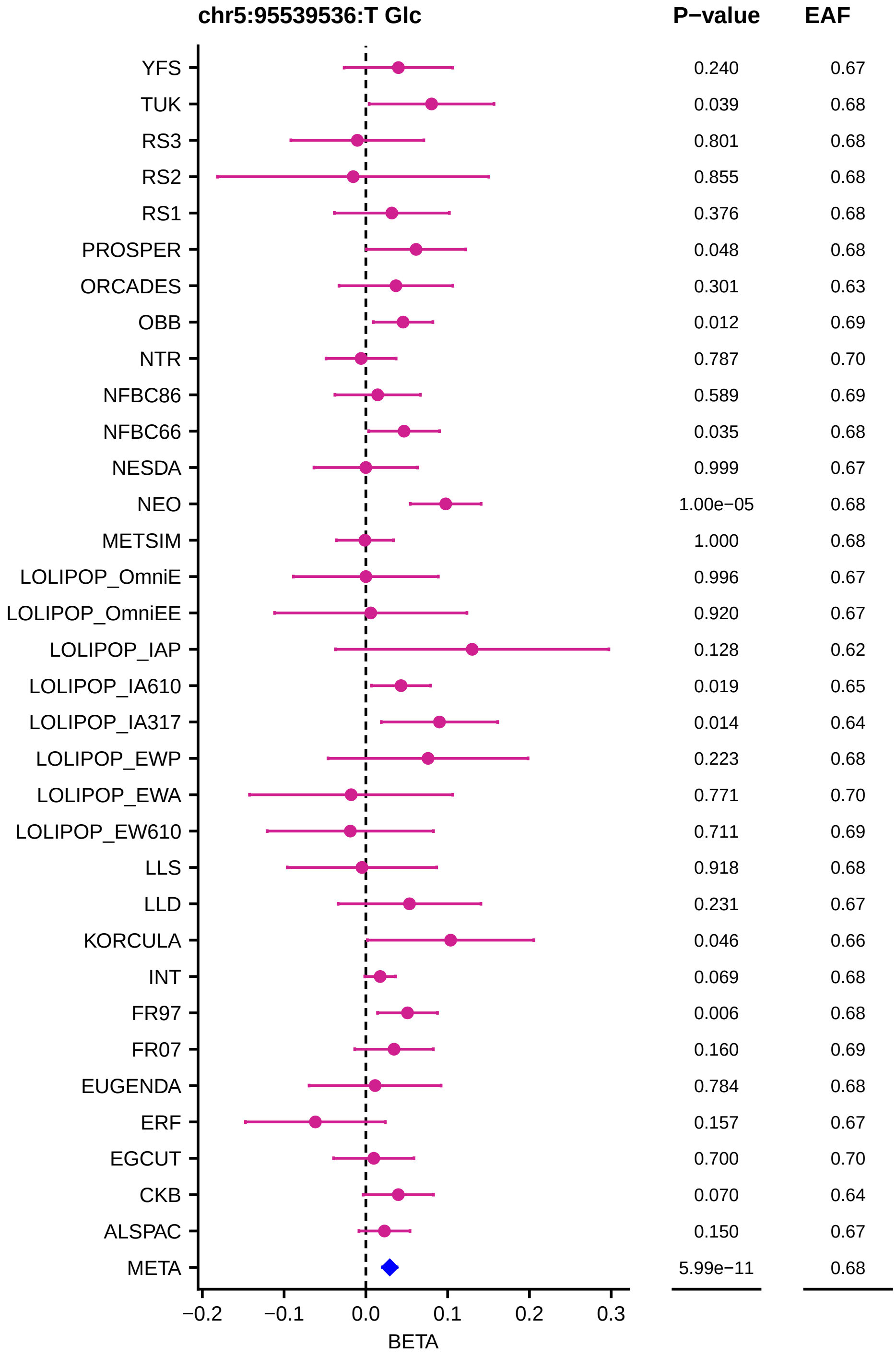
chr5:95539536:T Glc
P−value
EAF
YFS 0.240 0.67
TUK 0.039 0.68
RS3 0.801 0.68
RS2 0.855 0.68
RS1 0.376 0.68
PROSPER 0.048 0.68
ORCADES 0.301 0.63
OBB 0.012 0.69
NTR 0.787 0.70
NFBC86 0.589 0.69
NFBC66 0.035 0.68
NESDA 0.999 0.67
NEO 1.00e−05 0.68
METSIM 1.000 0.68
LOLIPOP_OmniE 0.996 0.67
LOLIPOP_OmniEE 0.920 0.67
LOLIPOP_IAP 0.128 0.62
LOLIPOP_IA610 0.019 0.65
LOLIPOP_IA317 0.014 0.64
LOLIPOP_EWP 0.223 0.68
LOLIPOP_EWA 0.771 0.70
LOLIPOP_EW610 0.711 0.69
LLS 0.918 0.68
LLD 0.231 0.67
KORCULA 0.046 0.66
INT 0.069 0.68
FR97 0.006 0.68
FR07 0.160 0.69
EUGENDA 0.784 0.68
ERF 0.157 0.67
EGCUT 0.700 0.70
CKB 0.070 0.64
ALSPAC 0.150 0.67
META 5.99e−11 0.68
−0.2 −0.1 0.0 0.1 0.2 0.3
BETA

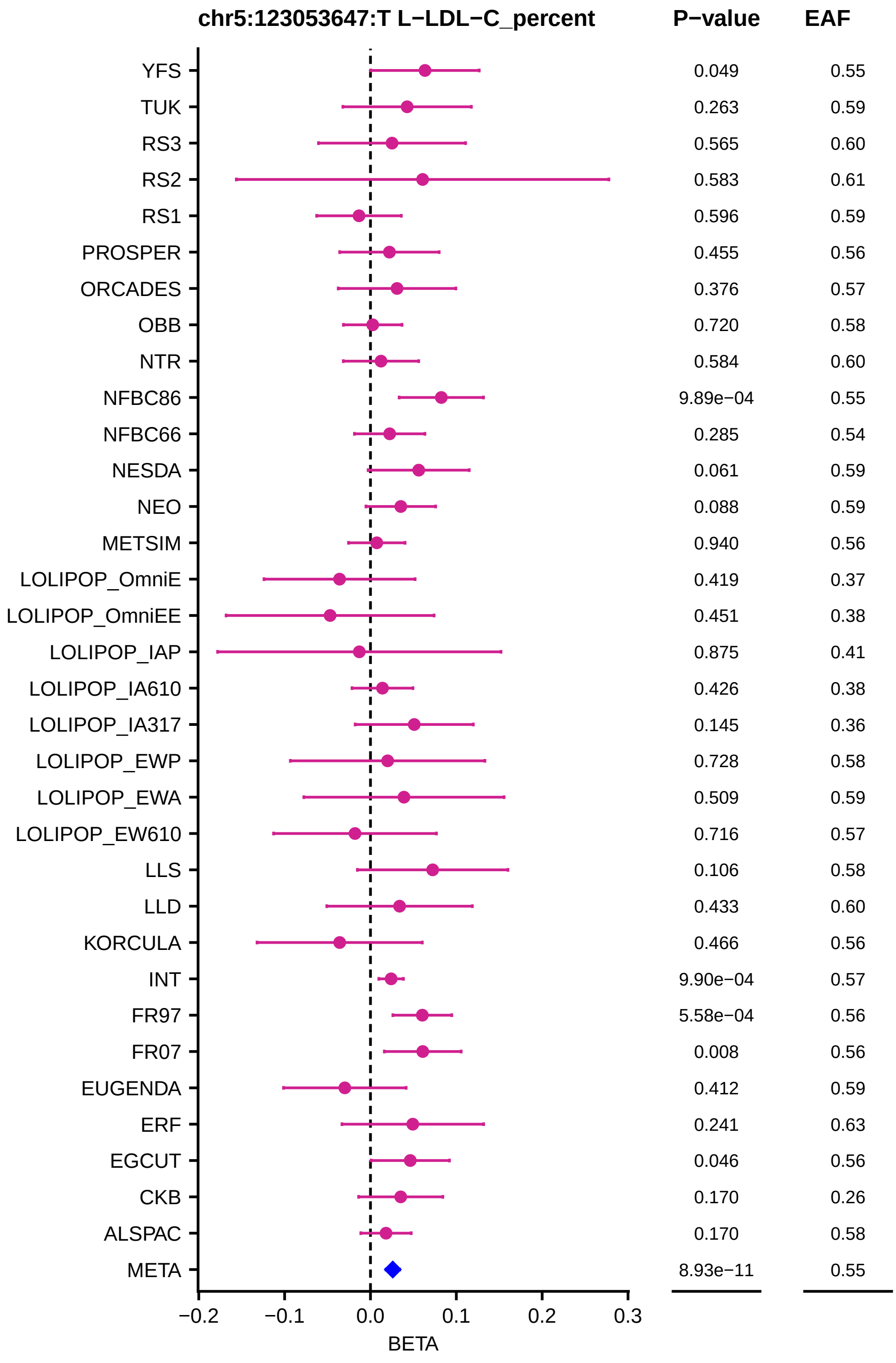
chr5:123053647:T L−LDL−C_percent
P−value
EAF
YFS 0.049 0.55
TUK 0.263 0.59
RS3 0.565 0.60
RS2 0.583 0.61
RS1 0.596 0.59
PROSPER 0.455 0.56
ORCADES 0.376 0.57
OBB 0.720 0.58
NTR 0.584 0.60
NFBC86 9.89e−04 0.55
NFBC66 0.285 0.54
NESDA 0.061 0.59
NEO 0.088 0.59
METSIM 0.940 0.56
LOLIPOP_OmniE 0.419 0.37
LOLIPOP_OmniEE 0.451 0.38
LOLIPOP_IAP 0.875 0.41
LOLIPOP_IA610 0.426 0.38
LOLIPOP_IA317 0.145 0.36
LOLIPOP_EWP 0.728 0.58
LOLIPOP_EWA 0.509 0.59
LOLIPOP_EW610 0.716 0.57
LLS 0.106 0.58
LLD 0.433 0.60
KORCULA 0.466 0.56
INT 9.90e−04 0.57
FR97 5.58e−04 0.56
FR07 0.008 0.56
EUGENDA 0.412 0.59
ERF 0.241 0.63
EGCUT 0.046 0.56
CKB 0.170 0.26
ALSPAC 0.170 0.58
META 8.93e−11 0.55
−0.2 −0.1 0.0 0.1 0.2 0.3
BETA

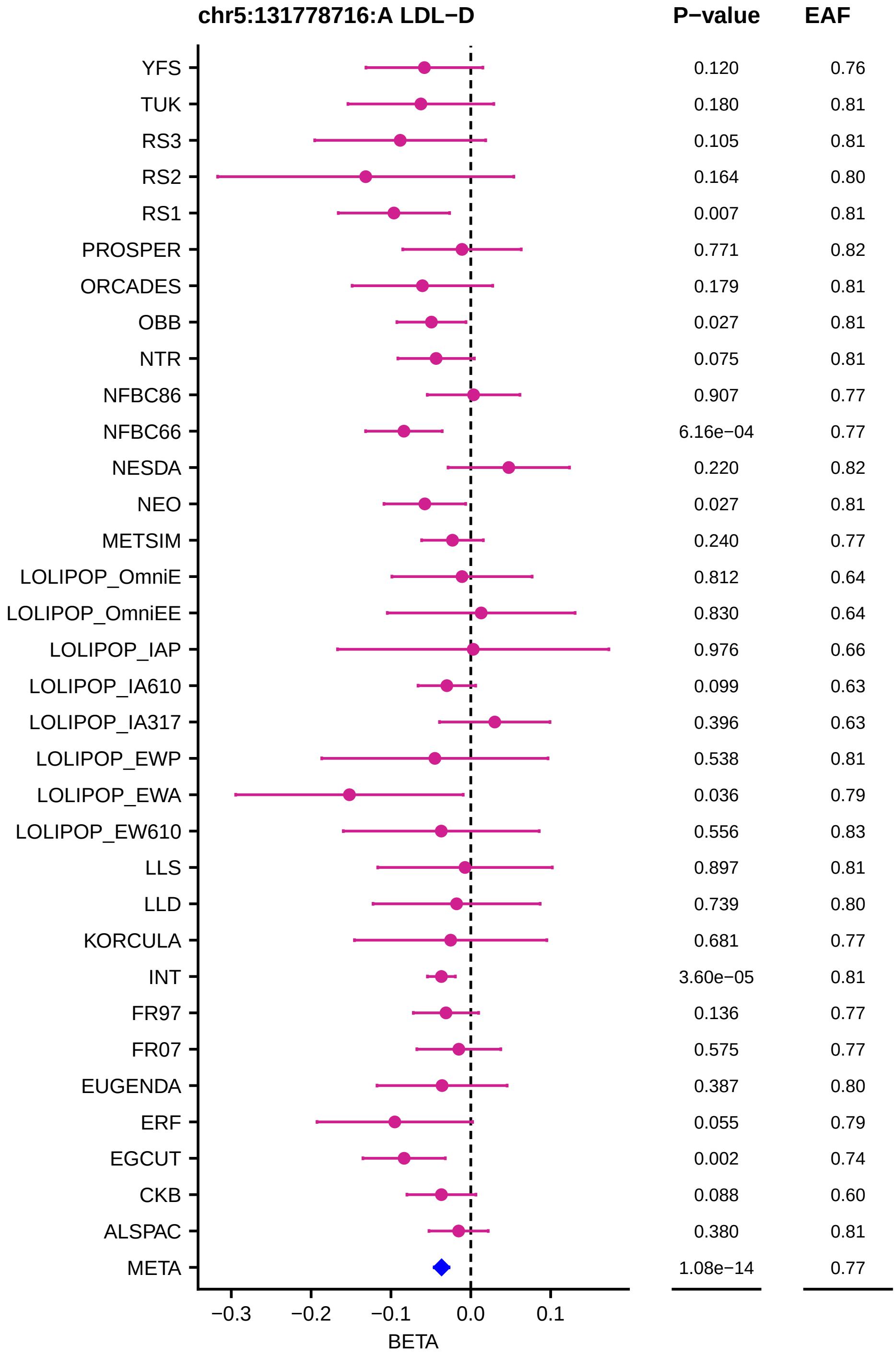
chr5:131778716:A LDL−D
P−value
EAF
YFS 0.120 0.76
TUK 0.180 0.81
RS3 0.105 0.81
RS2 0.164 0.80
RS1 0.007 0.81
PROSPER 0.771 0.82
ORCADES 0.179 0.81
OBB 0.027 0.81
NTR 0.075 0.81
NFBC86 0.907 0.77
NFBC66 6.16e−04 0.77
NESDA 0.220 0.82
NEO 0.027 0.81
METSIM 0.240 0.77
LOLIPOP_OmniE 0.812 0.64
LOLIPOP_OmniEE 0.830 0.64
LOLIPOP_IAP 0.976 0.66
LOLIPOP_IA610 0.099 0.63
LOLIPOP_IA317 0.396 0.63
LOLIPOP_EWP 0.538 0.81
LOLIPOP_EWA 0.036 0.79
LOLIPOP_EW610 0.556 0.83
LLS 0.897 0.81
LLD 0.739 0.80
KORCULA 0.681 0.77
INT 3.60e−05 0.81
FR97 0.136 0.77
FR07 0.575 0.77
EUGENDA 0.387 0.80
ERF 0.055 0.79
EGCUT 0.002 0.74
CKB 0.088 0.60
ALSPAC 0.380 0.81
META 1.08e−14 0.77
−0.3 −0.2 −0.1 0.0 0.1
BETA

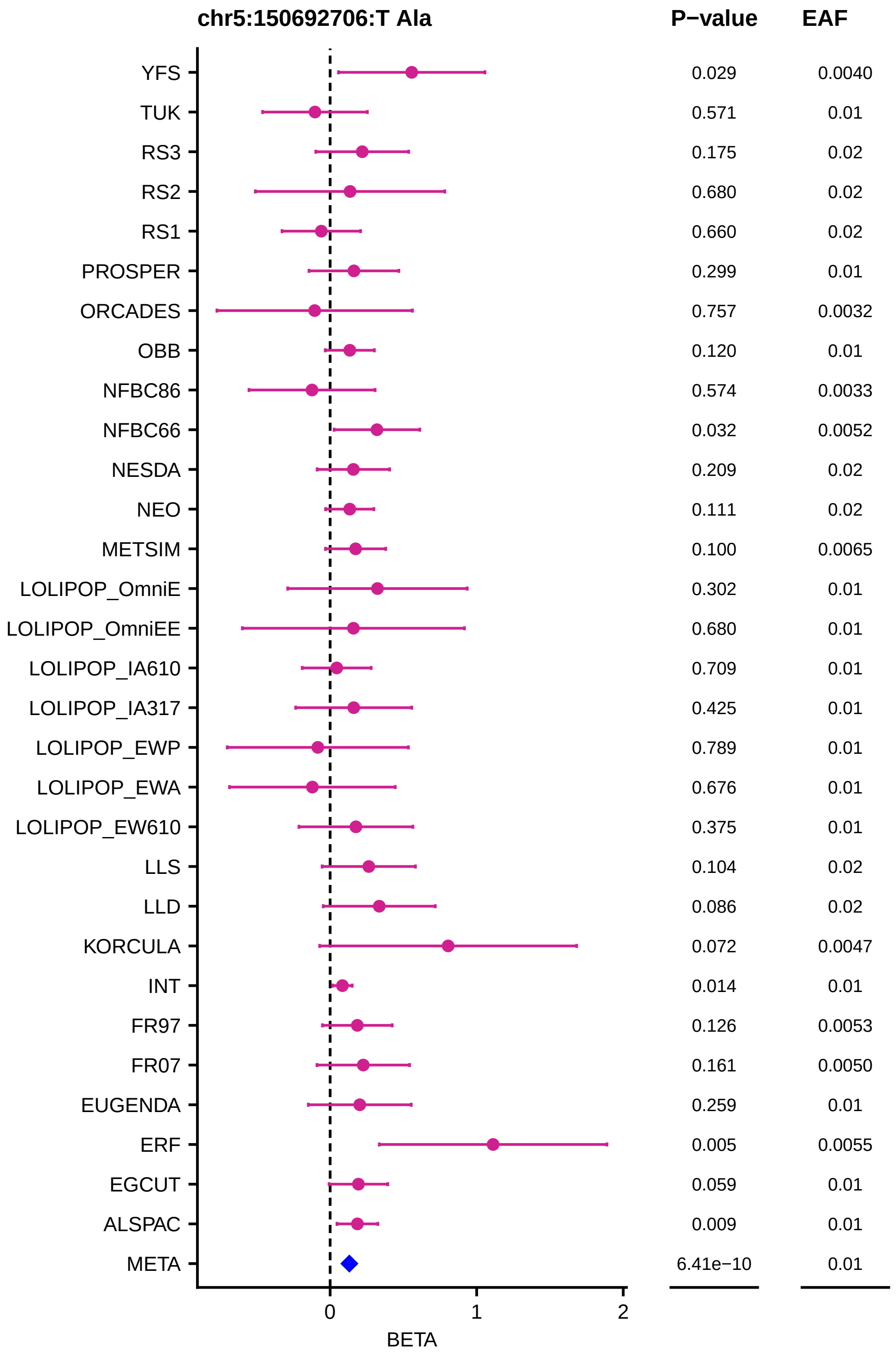
chr5:150692706:T Ala
P−value
EAF
YFS 0.029 0.0040
TUK 0.571 0.01
RS3 0.175 0.02
RS2 0.680 0.02
RS1 0.660 0.02
PROSPER 0.299 0.01
ORCADES 0.757 0.0032
OBB 0.120 0.01
NFBC86 0.574 0.0033
NFBC66 0.032 0.0052
NESDA 0.209 0.02
NEO 0.111 0.02
METSIM 0.100 0.0065
LOLIPOP_OmniE 0.302 0.01
LOLIPOP_OmniEE 0.680 0.01
LOLIPOP_IA610 0.709 0.01
LOLIPOP_IA317 0.425 0.01
LOLIPOP_EWP 0.789 0.01
LOLIPOP_EWA 0.676 0.01
LOLIPOP_EW610 0.375 0.01
LLS 0.104 0.02
LLD 0.086 0.02
KORCULA 0.072 0.0047
INT 0.014 0.01
FR97 0.126 0.0053
FR07 0.161 0.0050
EUGENDA 0.259 0.01
ERF 0.005 0.0055
EGCUT 0.059 0.01
ALSPAC 0.009 0.01
META 6.41e−10 0.01
0
1
2
BETA

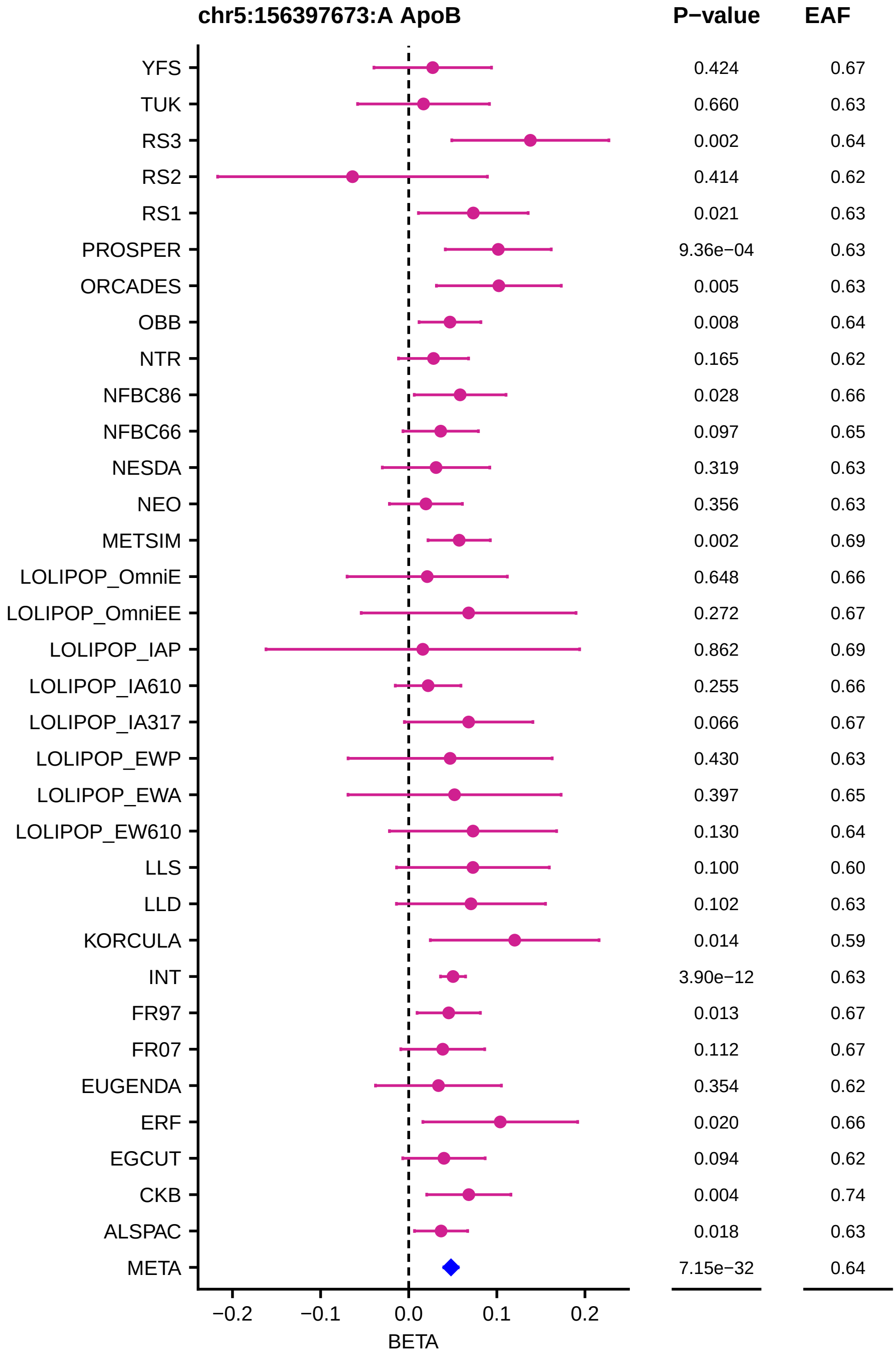

chr5:156397673:A ApoB
P−value
EAF
YFS 0.424 0.67
TUK 0.660 0.63
RS3 0.002 0.64
RS2 0.414 0.62
RS1 0.021 0.63
PROSPER 9.36e−04 0.63
ORCADES 0.005 0.63
OBB 0.008 0.64
NTR 0.165 0.62
NFBC86 0.028 0.66
NFBC66 0.097 0.65
NESDA 0.319 0.63
NEO 0.356 0.63
METSIM 0.002 0.69
LOLIPOP_OmniE 0.648 0.66
LOLIPOP_OmniEE 0.272 0.67
LOLIPOP_IAP 0.862 0.69
LOLIPOP_IA610 0.255 0.66
LOLIPOP_IA317 0.066 0.67
LOLIPOP_EWP 0.430 0.63
LOLIPOP_EWA 0.397 0.65
LOLIPOP_EW610 0.130 0.64
LLS 0.100 0.60
LLD 0.102 0.63
KORCULA 0.014 0.59
INT 3.90e−12 0.63
FR97 0.013 0.67
FR07 0.112 0.67
EUGENDA 0.354 0.62
ERF 0.020 0.66
EGCUT 0.094 0.62
CKB 0.004 0.74
ALSPAC 0.018 0.63
META 7.15e−32 0.64
−0.2
−0.1
0.0
0.1
0.2
BETA

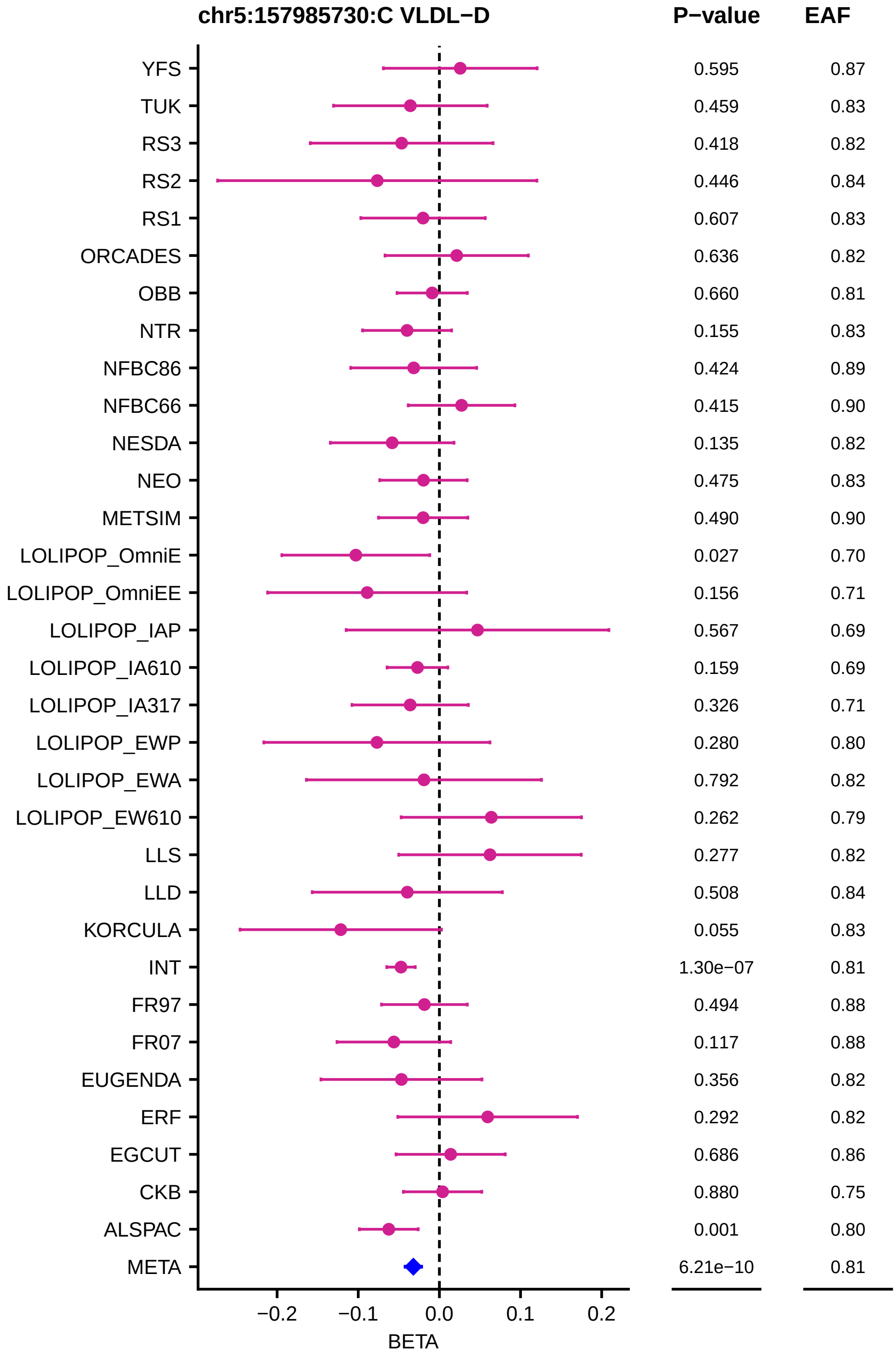
chr5:157985730:C VLDL−D
P−value
EAF
YFS 0.595 0.87
TUK 0.459 0.83
RS3 0.418 0.82
RS2 0.446 0.84
RS1 0.607 0.83
ORCADES 0.636 0.82
OBB 0.660 0.81
NTR 0.155 0.83
NFBC86 0.424 0.89
NFBC66 0.415 0.90
NESDA 0.135 0.82
NEO 0.475 0.83
METSIM 0.490 0.90
LOLIPOP_OmniE 0.027 0.70
LOLIPOP_OmniEE 0.156 0.71
LOLIPOP_IAP 0.567 0.69
LOLIPOP_IA610 0.159 0.69
LOLIPOP_IA317 0.326 0.71
LOLIPOP_EWP 0.280 0.80
LOLIPOP_EWA 0.792 0.82
LOLIPOP_EW610 0.262 0.79
LLS 0.277 0.82
LLD 0.508 0.84
KORCULA 0.055 0.83
INT 1.30e−07 0.81
FR97 0.494 0.88
FR07 0.117 0.88
EUGENDA 0.356 0.82
ERF 0.292 0.82
EGCUT 0.686 0.86
CKB 0.880 0.75
ALSPAC 0.001 0.80
META 6.21e−10 0.81
−0.2 −0.1 0.0 0.1 0.2
BETA

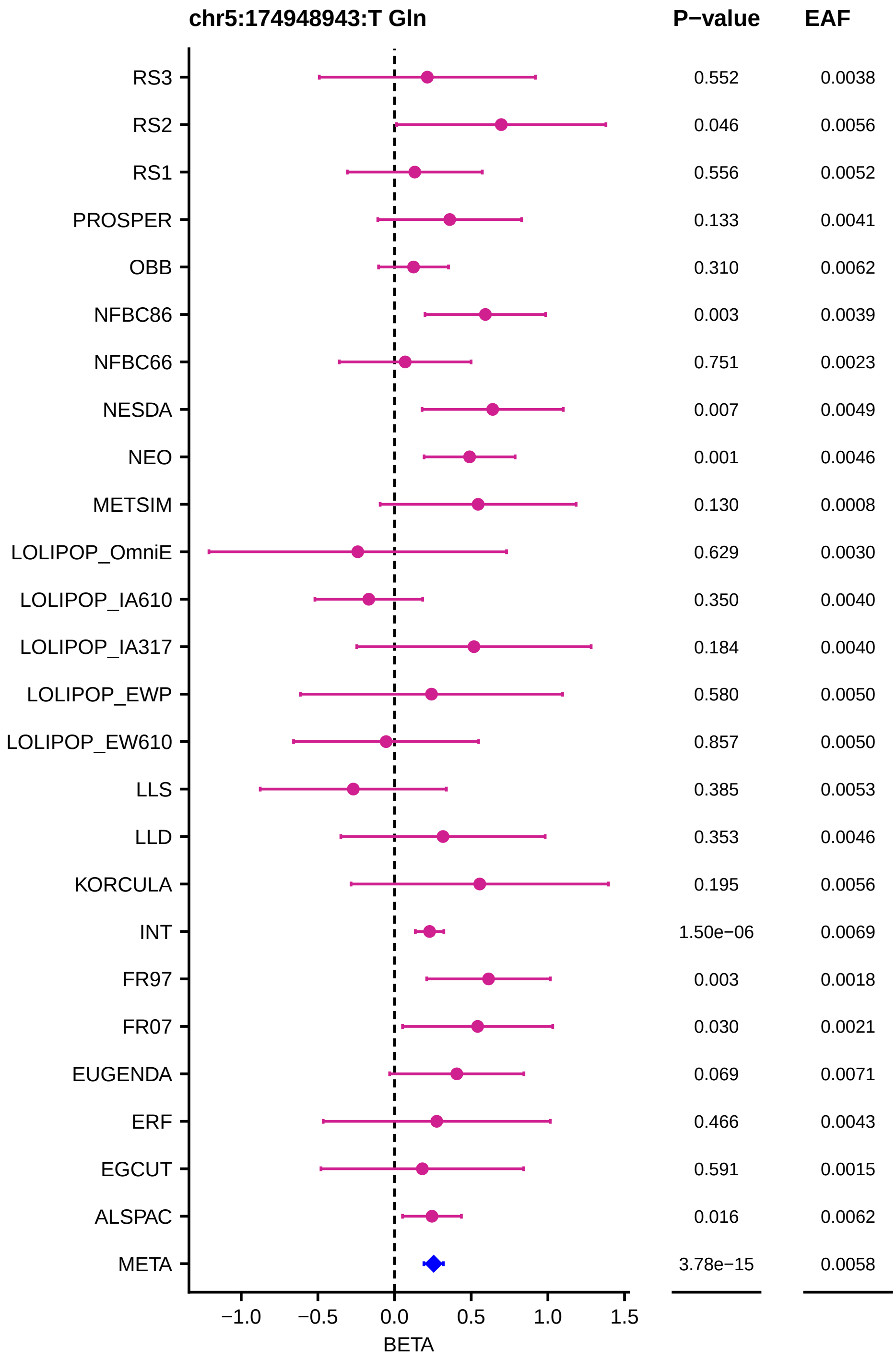
chr5:174948943:T Gln
P−value
EAF
RS3 0.552 0.0038
RS2 0.046 0.0056
RS1 0.556 0.0052
PROSPER 0.133 0.0041
OBB 0.310 0.0062
NFBC86 0.003 0.0039
NFBC66 0.751 0.0023
NESDA 0.007 0.0049
NEO 0.001 0.0046
METSIM 0.130 0.0008
LOLIPOP_OmniE 0.629 0.0030
LOLIPOP_IA610 0.350 0.0040
LOLIPOP_IA317 0.184 0.0040
LOLIPOP_EWP 0.580 0.0050
LOLIPOP_EW610 0.857 0.0050
LLS 0.385 0.0053
LLD 0.353 0.0046
KORCULA 0.195 0.0056
INT 1.50e−06 0.0069
FR97 0.003 0.0018
FR07 0.030 0.0021
EUGENDA 0.069 0.0071
ERF 0.466 0.0043
EGCUT 0.591 0.0015
ALSPAC 0.016 0.0062
META 3.78e−15 0.0058
−1.0
−0.5
0.0
0.5
1.0
1.5
BETA

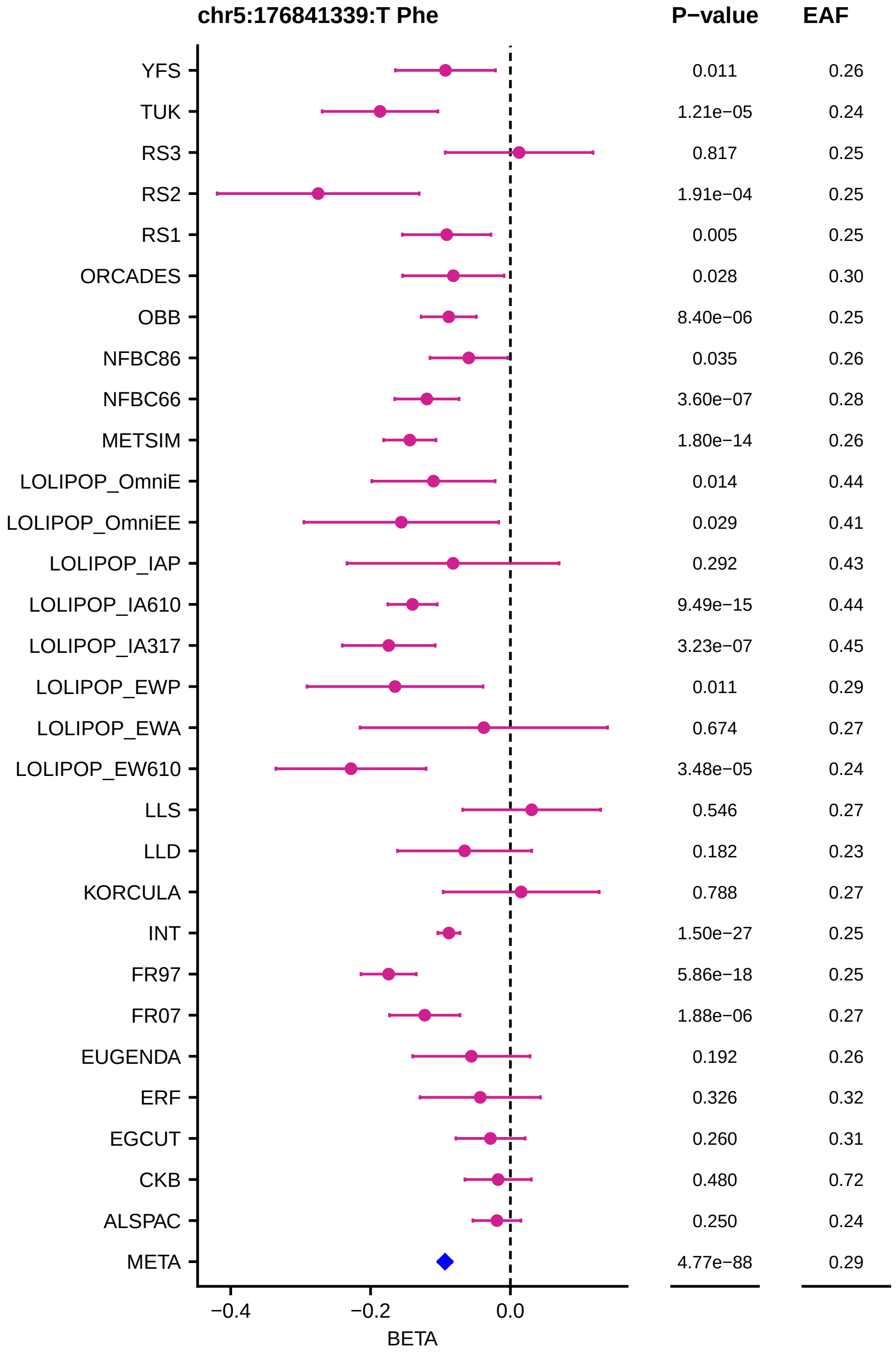

chr5:176841339:T Phe
P−value
EAF
YFS
TUK
RS3
RS2
RS1
ORCADES
OBB
NFBC86
NFBC66
METSIM
LOLIPOP_OmniE
LOLIPOP_OmniEE
LOLIPOP_IAP
LOLIPOP_IA610
LOLIPOP_IA317
LOLIPOP_EWP
LOLIPOP_EWA
LOLIPOP_EW610
LLS
LLD
KORCULA
INT
FR97
FR07
EUGENDA
ERF
EGCUT
CKB
ALSPAC
META
0.011 0.26
1.21e−05 0.24
0.817 0.25
1.91e−04 0.25
0.005 0.25
0.028 0.30
8.40e−06 0.25
0.035 0.26
3.60e−07 0.28
1.80e−14 0.26
0.014 0.44
0.029 0.41
0.292 0.43
9.49e−15 0.44
3.23e−07 0.45
0.011 0.29
0.674 0.27
3.48e−05 0.24
0.546 0.27
0.182 0.23
0.788 0.27
1.50e−27 0.25
5.86e−18 0.25
1.88e−06 0.27
0.192 0.26
0.326 0.32
0.260 0.31
0.480 0.72
0.250 0.24
4.77e−88 0.29
−0.4
−0.2
0.0
BETA

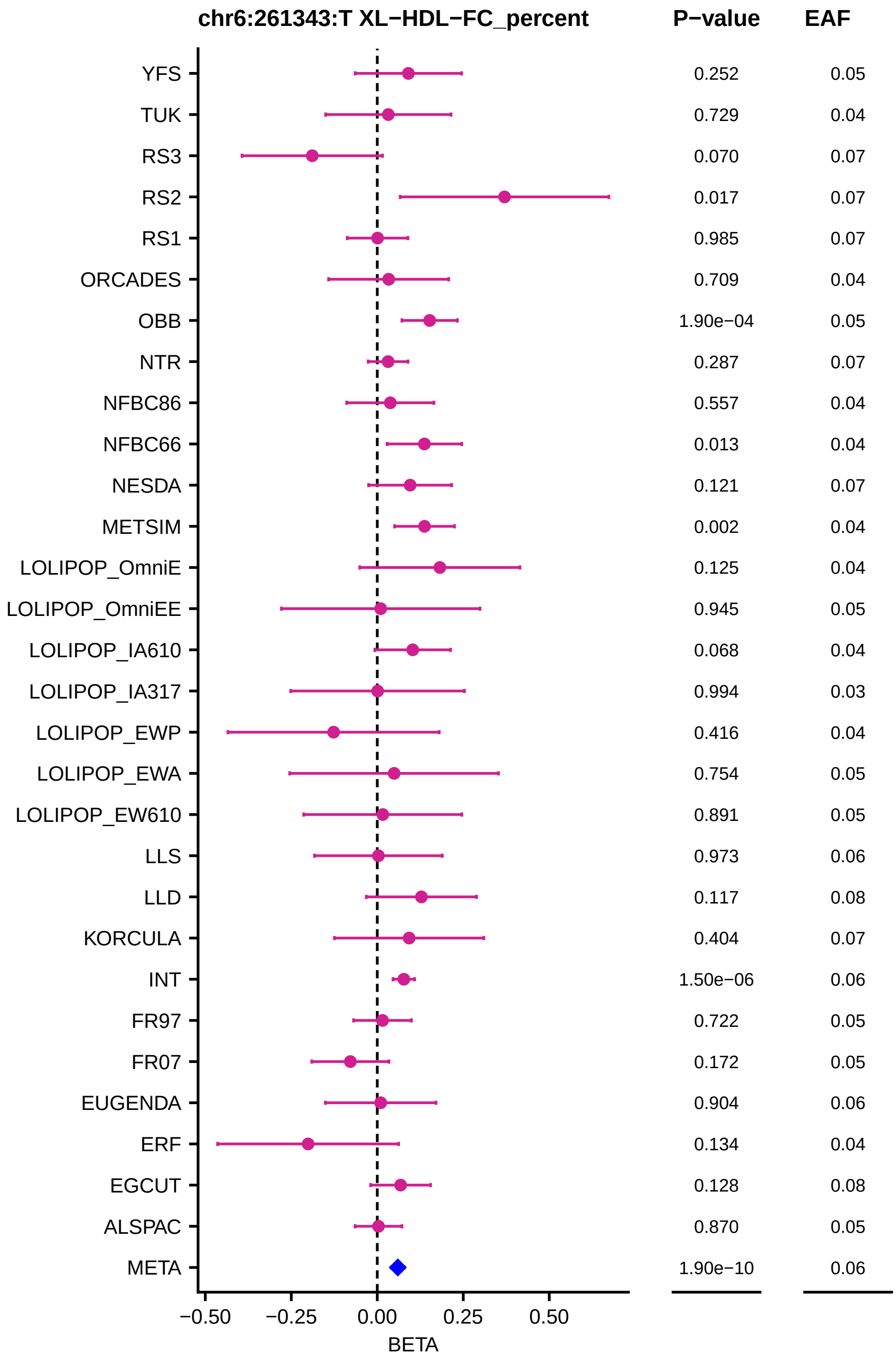
chr6:261343:T XL−HDL−FC_percent
P−value
EAF
YFS
TUK
RS3
RS2
RS1
ORCADES
OBB
NTR
NFBC86
NFBC66
NESDA
METSIM
LOLIPOP_OmniE
LOLIPOP_OmniEE
LOLIPOP_IA610
LOLIPOP_IA317
LOLIPOP_EWP
LOLIPOP_EWA
LOLIPOP_EW610
LLS
LLD
KORCULA
INT
FR97
FR07
EUGENDA
ERF
EGCUT
ALSPAC
META
0.252
0.729
0.070
0.017
0.985
0.709
1.90e−04
0.287
0.557
0.013
0.121
0.002
0.125
0.945
0.068
0.994
0.416
0.754
0.891
0.973
0.117
0.404
1.50e−06
0.722
0.172
0.904
0.134
0.128
0.870
1.90e−10
0.05
0.04
0.07
0.07
0.07
0.04
0.05
0.07
0.04
0.04
0.07
0.04
0.04
0.05
0.04
0.03
0.04
0.05
0.05
0.06
0.08
0.07
0.06
0.05
0.05
0.06
0.04
0.08
0.05
0.06
−0.50
−0.25
0.00
0.25
0.50
BETA

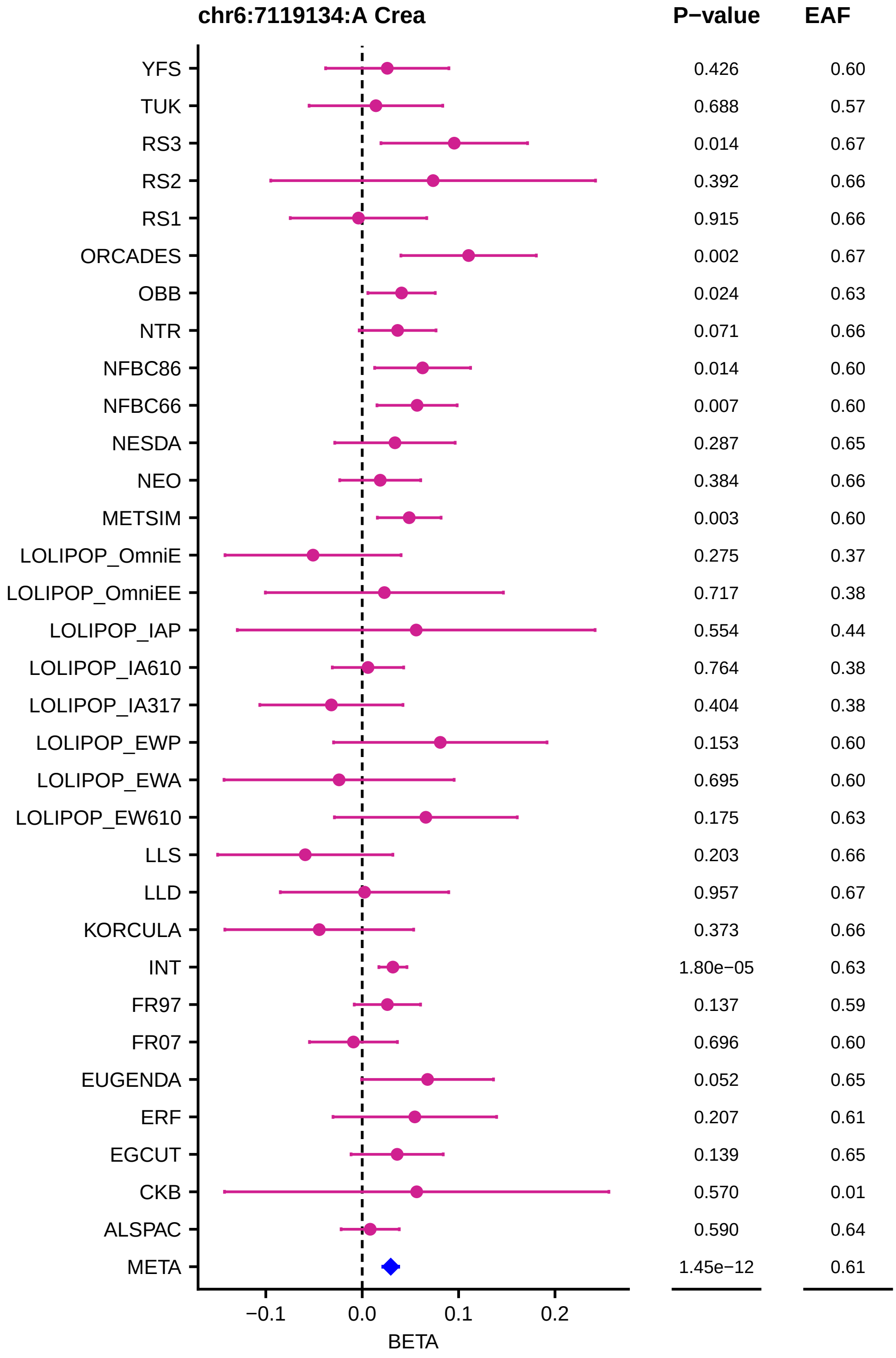
chr6:7119134:A Crea
P−value
EAF
YFS 0.426 0.60
TUK 0.688 0.57
RS3 0.014 0.67
RS2 0.392 0.66
RS1 0.915 0.66
ORCADES 0.002 0.67
OBB 0.024 0.63
NTR 0.071 0.66
NFBC86 0.014 0.60
NFBC66 0.007 0.60
NESDA 0.287 0.65
NEO 0.384 0.66
METSIM 0.003 0.60
LOLIPOP_OmniE 0.275 0.37
LOLIPOP_OmniEE 0.717 0.38
LOLIPOP_IAP 0.554 0.44
LOLIPOP_IA610 0.764 0.38
LOLIPOP_IA317 0.404 0.38
LOLIPOP_EWP 0.153 0.60
LOLIPOP_EWA 0.695 0.60
LOLIPOP_EW610 0.175 0.63
LLS 0.203 0.66
LLD 0.957 0.67
KORCULA 0.373 0.66
INT 1.80e−05 0.63
FR97 0.137 0.59
FR07 0.696 0.60
EUGENDA 0.052 0.65
ERF 0.207 0.61
EGCUT 0.139 0.65
CKB 0.570 0.01
ALSPAC 0.590 0.64
META 1.45e−12 0.61
−0.1
0.0
0.1
0.2
BETA

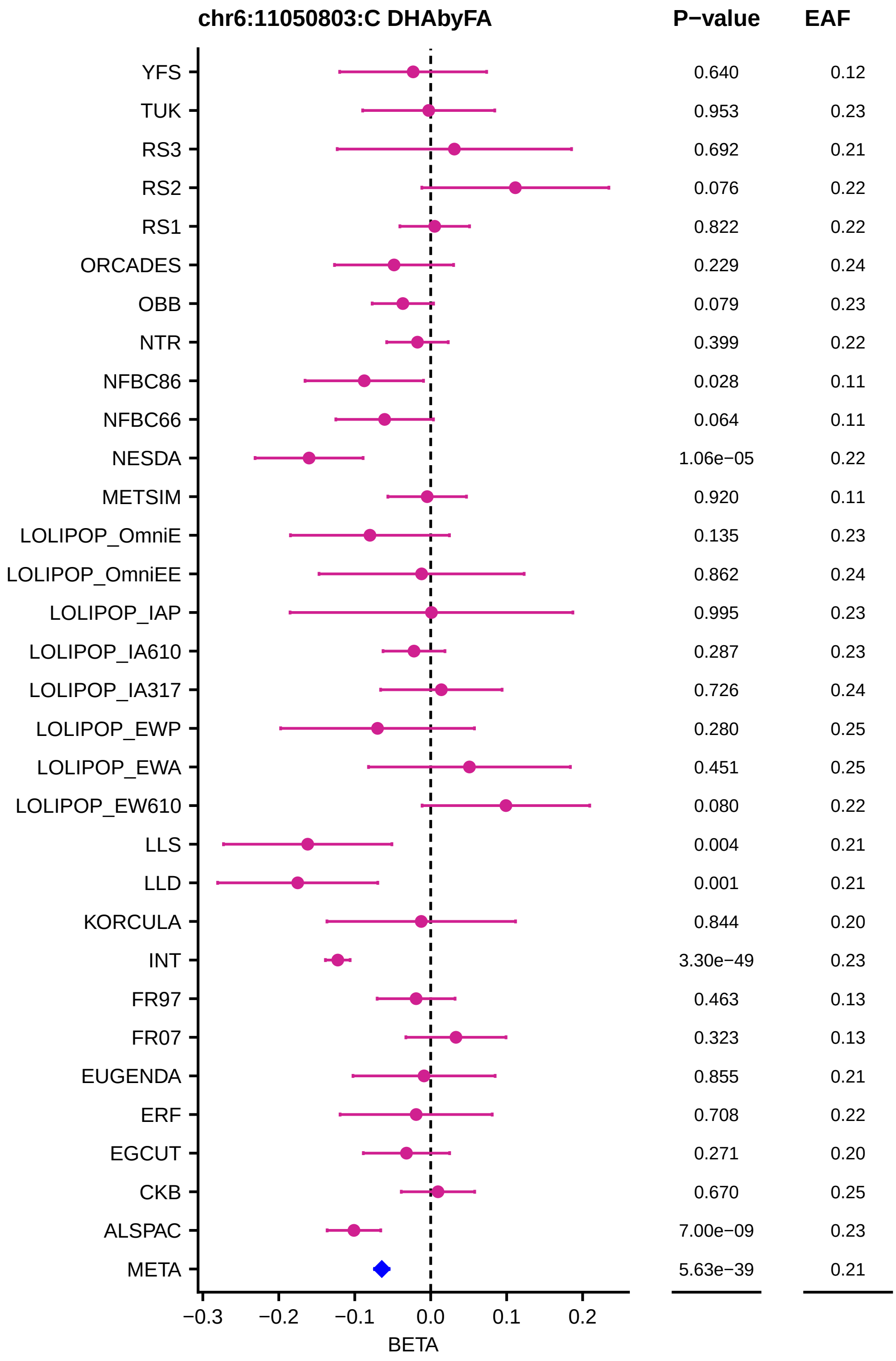

chr6:11050803:C DHAbyFA
P−value
EAF
YFS
TUK
RS3
RS2
RS1
ORCADES
OBB
NTR
NFBC86
NFBC66
NESDA
METSIM
LOLIPOP_OmniE
LOLIPOP_OmniEE
LOLIPOP_IAP
LOLIPOP_IA610
LOLIPOP_IA317
LOLIPOP_EWP
LOLIPOP_EWA
LOLIPOP_EW610
LLS
LLD
KORCULA
INT
FR97
FR07
EUGENDA
ERF
EGCUT
CKB
ALSPAC
META
0.640 0.12
0.953 0.23
0.692 0.21
0.076 0.22
0.822 0.22
0.229 0.24
0.079 0.23
0.399 0.22
0.028 0.11
0.064 0.11
1.06e−05 0.22
0.920 0.11
0.135 0.23
0.862 0.24
0.995 0.23
0.287 0.23
0.726 0.24
0.280 0.25
0.451 0.25
0.080 0.22
0.004 0.21
0.001 0.21
0.844 0.20
3.30e−49 0.23
0.463 0.13
0.323 0.13
0.855 0.21
0.708 0.22
0.271 0.20
0.670 0.25
7.00e−09 0.23
5.63e−39 0.21
−0.3
−0.2
−0.1
0.0
0.1
0.2
BETA

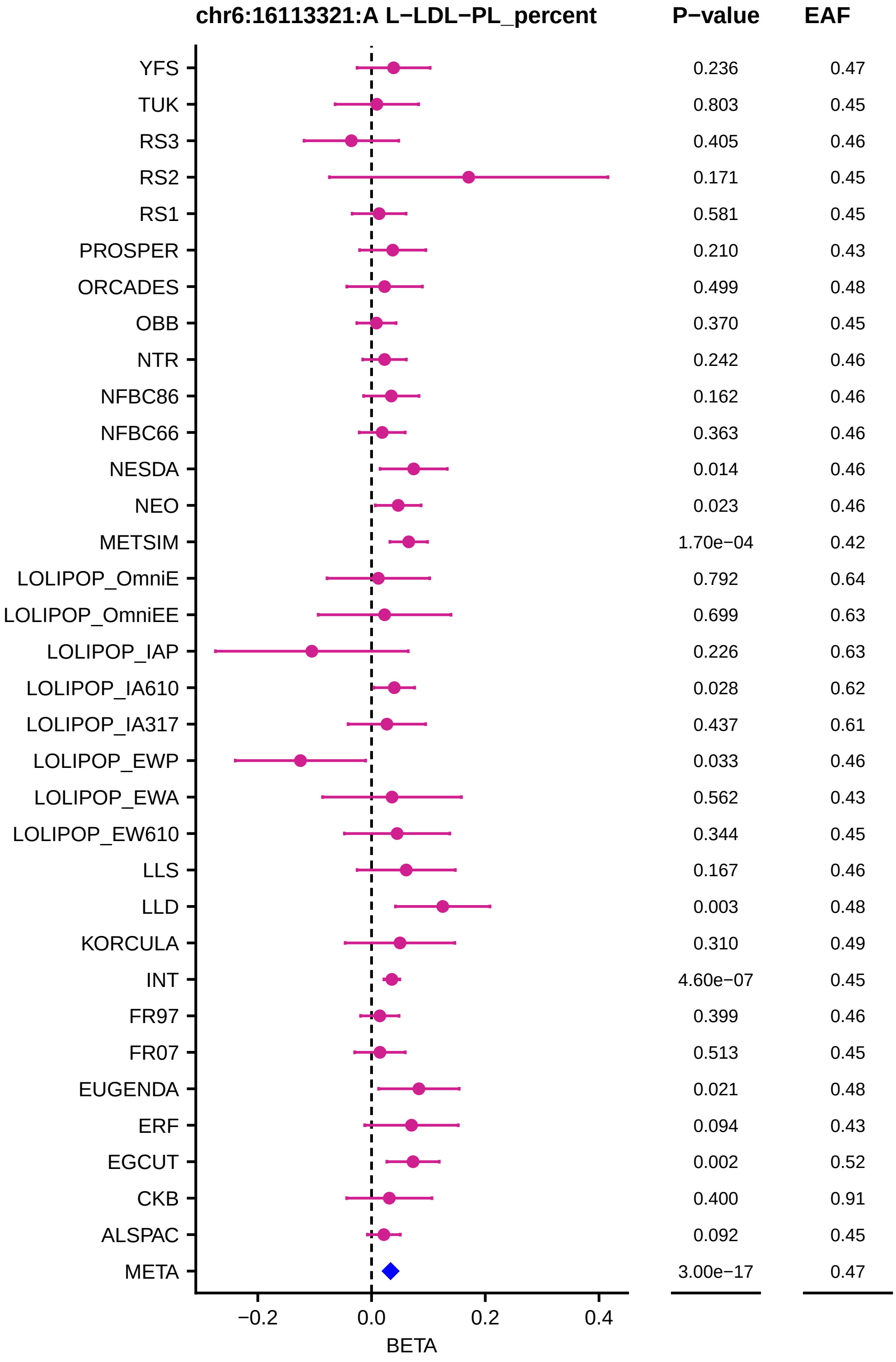

chr6:16113321:A L−LDL−PL_percent
P−value
EAF
YFS 0.236 0.47
TUK 0.803 0.45
RS3 0.405 0.46
RS2 0.171 0.45
RS1 0.581 0.45
PROSPER 0.210 0.43
ORCADES 0.499 0.48
OBB 0.370 0.45
NTR 0.242 0.46
NFBC86 0.162 0.46
NFBC66 0.363 0.46
NESDA 0.014 0.46
NEO 0.023 0.46
METSIM 1.70e−04 0.42
LOLIPOP_OmniE 0.792 0.64
LOLIPOP_OmniEE 0.699 0.63
LOLIPOP_IAP 0.226 0.63
LOLIPOP_IA610 0.028 0.62
LOLIPOP_IA317 0.437 0.61
LOLIPOP_EWP 0.033 0.46
LOLIPOP_EWA 0.562 0.43
LOLIPOP_EW610 0.344 0.45
LLS 0.167 0.46
LLD 0.003 0.48
KORCULA 0.310 0.49
INT 4.60e−07 0.45
FR97 0.399 0.46
FR07 0.513 0.45
EUGENDA 0.021 0.48
ERF 0.094 0.43
EGCUT 0.002 0.52
CKB 0.400 0.91
ALSPAC 0.092 0.45
META 3.00e−17 0.47
−0.2
0.0
0.2
0.4
BETA

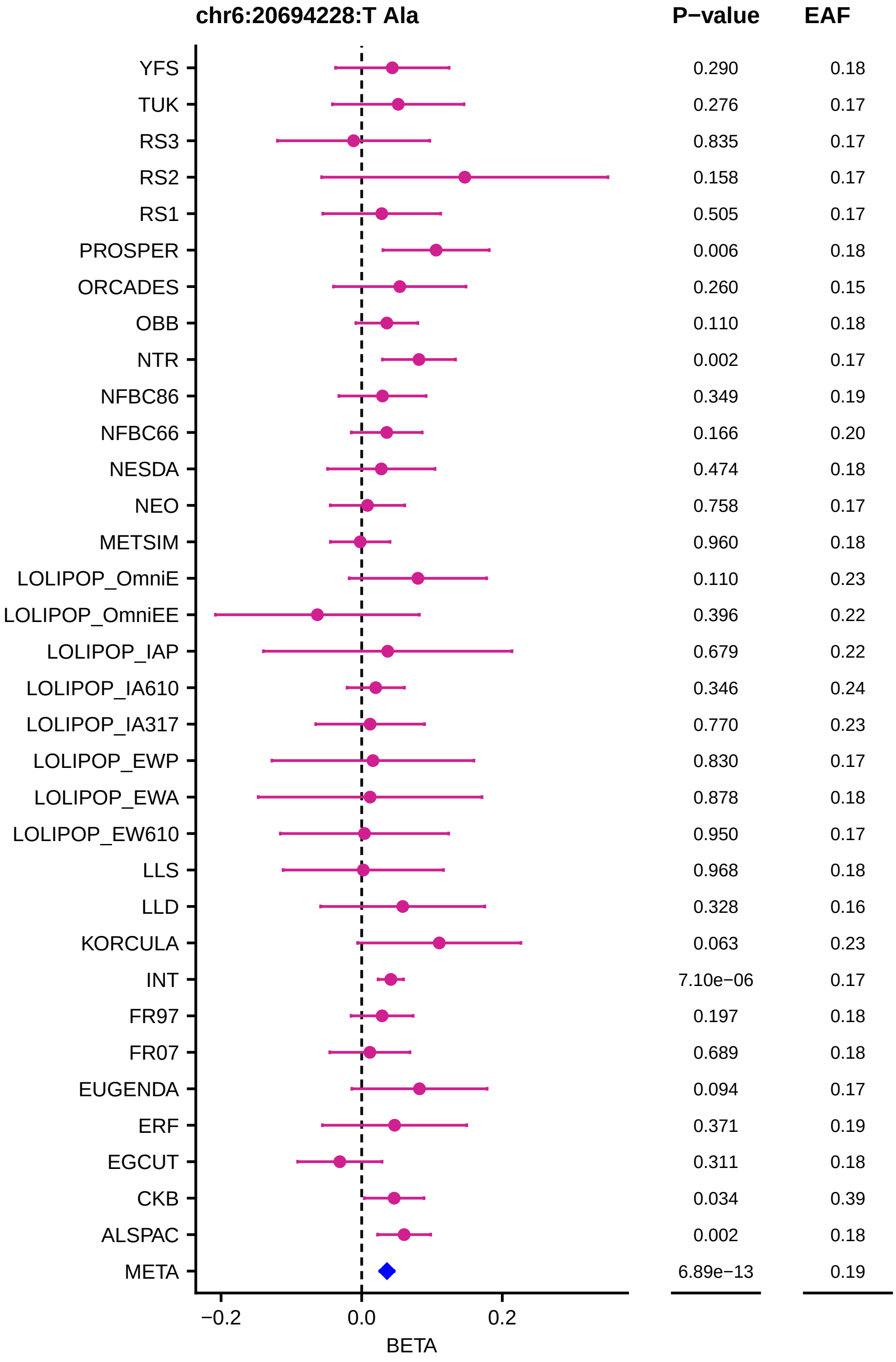
chr6:20694228:T Ala
P−value
EAF
YFS 0.290 0.18
TUK 0.276 0.17
RS3 0.835 0.17
RS2 0.158 0.17
RS1 0.505 0.17
PROSPER 0.006 0.18
ORCADES 0.260 0.15
OBB 0.110 0.18
NTR 0.002 0.17
NFBC86 0.349 0.19
NFBC66 0.166 0.20
NESDA 0.474 0.18
NEO 0.758 0.17
METSIM 0.960 0.18
LOLIPOP_OmniE 0.110 0.23
LOLIPOP_OmniEE 0.396 0.22
LOLIPOP_IAP 0.679 0.22
LOLIPOP_IA610 0.346 0.24
LOLIPOP_IA317 0.770 0.23
LOLIPOP_EWP 0.830 0.17
LOLIPOP_EWA 0.878 0.18
LOLIPOP_EW610 0.950 0.17
LLS 0.968 0.18
LLD 0.328 0.16
KORCULA 0.063 0.23
INT 7.10e−06 0.17
FR97 0.197 0.18
FR07 0.689 0.18
EUGENDA 0.094 0.17
ERF 0.371 0.19
EGCUT 0.311 0.18
CKB 0.034 0.39
ALSPAC 0.002 0.18
META 6.89e−13 0.19
−0.2
0.0
0.2
BETA

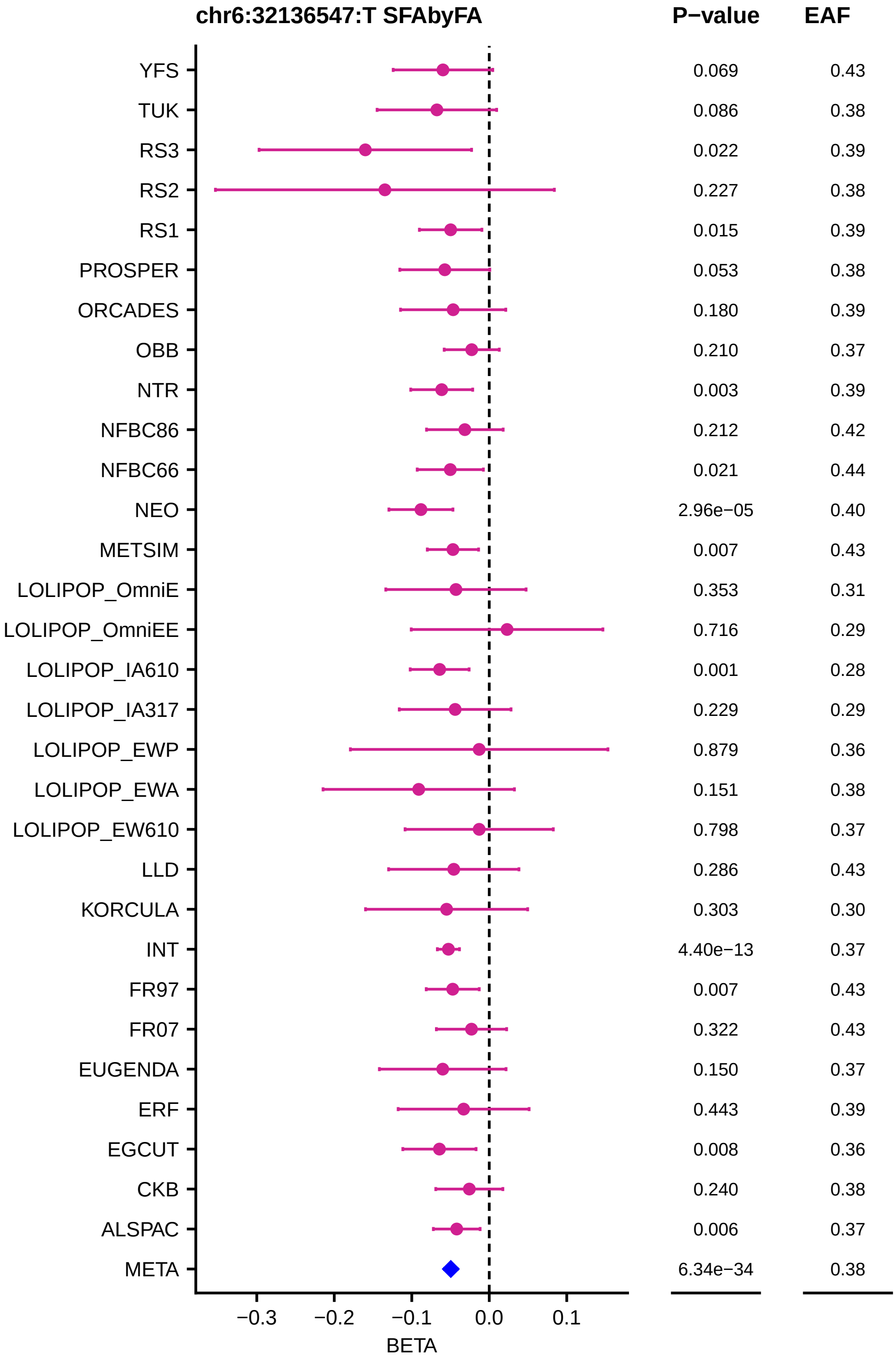
chr6:32136547:T SFAbyFA
P−value
EAF
YFS 0.069 0.43
TUK 0.086 0.38
RS3 0.022 0.39
RS2 0.227 0.38
RS1 0.015 0.39
PROSPER 0.053 0.38
ORCADES 0.180 0.39
OBB 0.210 0.37
NTR 0.003 0.39
NFBC86 0.212 0.42
NFBC66 0.021 0.44
NEO 2.96e−05 0.40
METSIM 0.007 0.43
LOLIPOP_OmniE 0.353 0.31
LOLIPOP_OmniEE 0.716 0.29
LOLIPOP_IA610 0.001 0.28
LOLIPOP_IA317 0.229 0.29
LOLIPOP_EWP 0.879 0.36
LOLIPOP_EWA 0.151 0.38
LOLIPOP_EW610 0.798 0.37
LLD 0.286 0.43
KORCULA 0.303 0.30
INT 4.40e−13 0.37
FR97 0.007 0.43
FR07 0.322 0.43
EUGENDA 0.150 0.37
ERF 0.443 0.39
EGCUT 0.008 0.36
CKB 0.240 0.38
ALSPAC 0.006 0.37
META 6.34e−34 0.38
−0.3
−0.2
−0.1
0.0
0.1
BETA

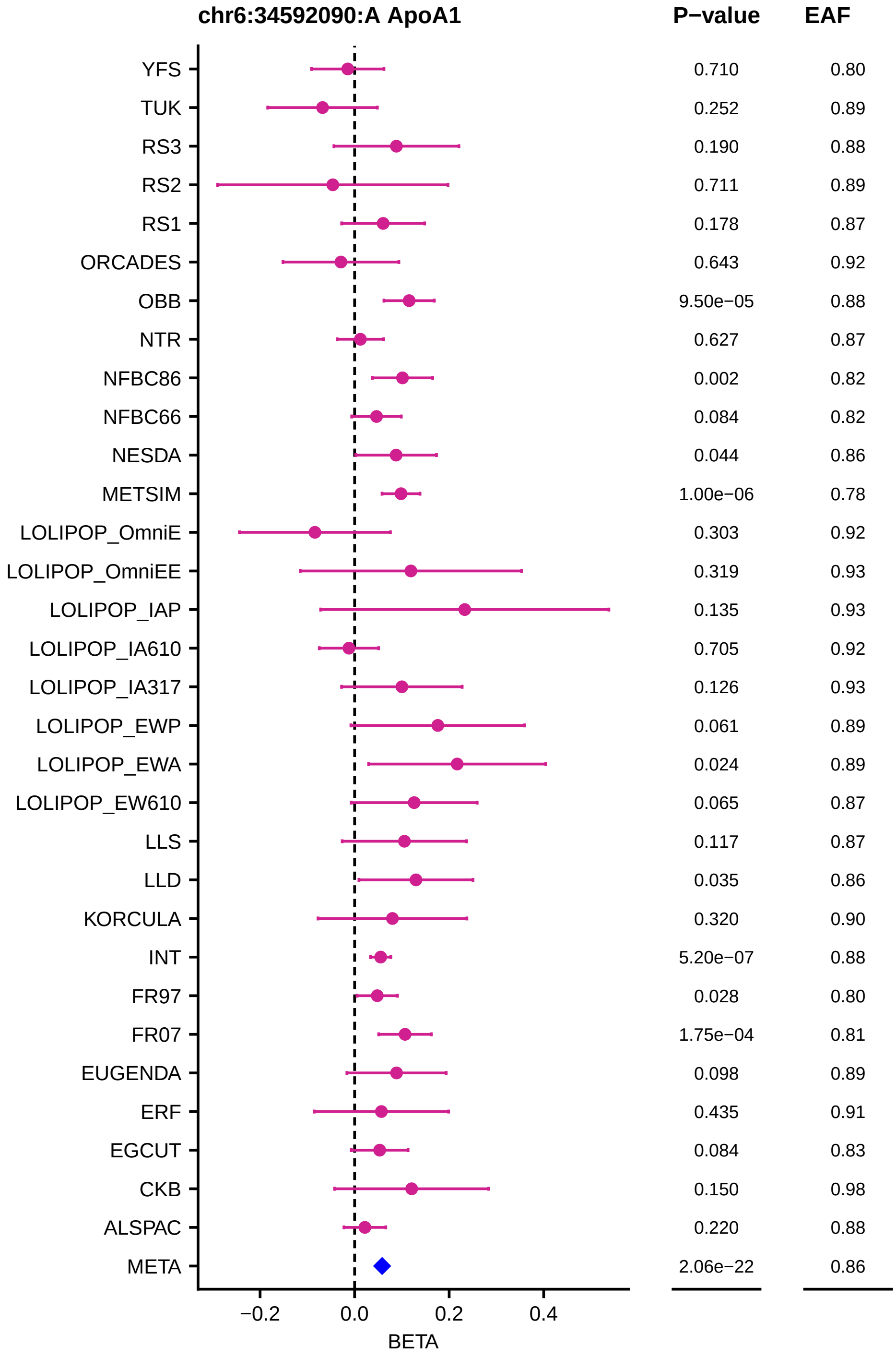
chr6:34592090:A ApoA1
P−value
EAF
YFS 0.710 0.80
TUK 0.252 0.89
RS3 0.190 0.88
RS2 0.711 0.89
RS1 0.178 0.87
ORCADES 0.643 0.92
OBB 9.50e−05 0.88
NTR 0.627 0.87
NFBC86 0.002 0.82
NFBC66 0.084 0.82
NESDA 0.044 0.86
METSIM 1.00e−06 0.78
LOLIPOP_OmniE 0.303 0.92
LOLIPOP_OmniEE 0.319 0.93
LOLIPOP_IAP 0.135 0.93
LOLIPOP_IA610 0.705 0.92
LOLIPOP_IA317 0.126 0.93
LOLIPOP_EWP 0.061 0.89
LOLIPOP_EWA 0.024 0.89
LOLIPOP_EW610 0.065 0.87
LLS 0.117 0.87
LLD 0.035 0.86
KORCULA 0.320 0.90
INT 5.20e−07 0.88
FR97 0.028 0.80
FR07 1.75e−04 0.81
EUGENDA 0.098 0.89
ERF 0.435 0.91
EGCUT 0.084 0.83
CKB 0.150 0.98
ALSPAC 0.220 0.88
META 2.06e−22 0.86
−0.2
0.0
0.2
0.4
BETA

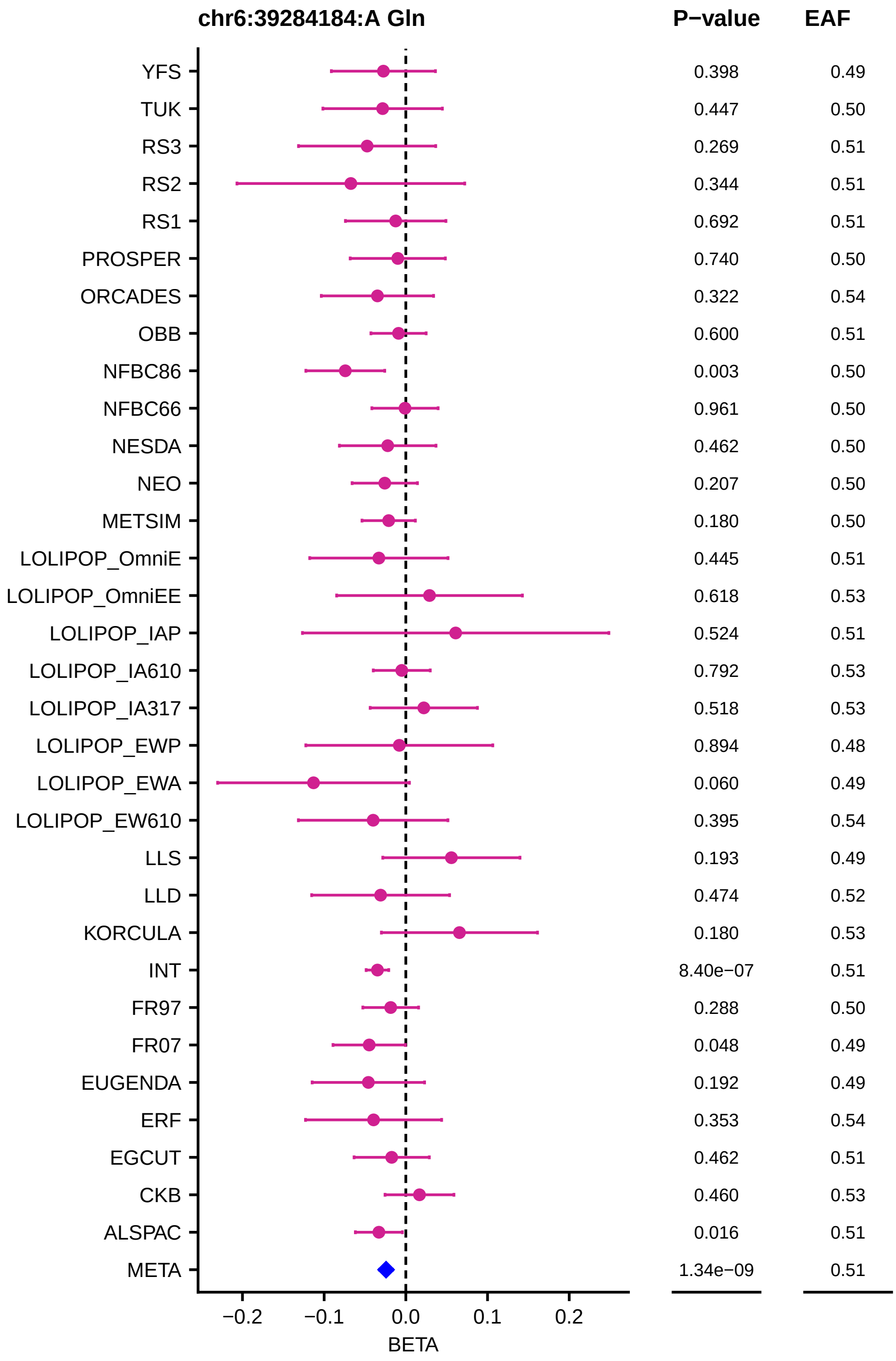
chr6:39284184:A Gln
P−value
EAF
YFS 0.398 0.49
TUK 0.447 0.50
RS3 0.269 0.51
RS2 0.344 0.51
RS1 0.692 0.51
PROSPER 0.740 0.50
ORCADES 0.322 0.54
OBB 0.600 0.51
NFBC86 0.003 0.50
NFBC66 0.961 0.50
NESDA 0.462 0.50
NEO 0.207 0.50
METSIM 0.180 0.50
LOLIPOP_OmniE 0.445 0.51
LOLIPOP_OmniEE 0.618 0.53
LOLIPOP_IAP 0.524 0.51
LOLIPOP_IA610 0.792 0.53
LOLIPOP_IA317 0.518 0.53
LOLIPOP_EWP 0.894 0.48
LOLIPOP_EWA 0.060 0.49
LOLIPOP_EW610 0.395 0.54
LLS 0.193 0.49
LLD 0.474 0.52
KORCULA 0.180 0.53
INT 8.40e−07 0.51
FR97 0.288 0.50
FR07 0.048 0.49
EUGENDA 0.192 0.49
ERF 0.353 0.54
EGCUT 0.462 0.51
CKB 0.460 0.53
ALSPAC 0.016 0.51
META 1.34e−09 0.51
−0.2
−0.1
0.0
0.1
0.2
BETA

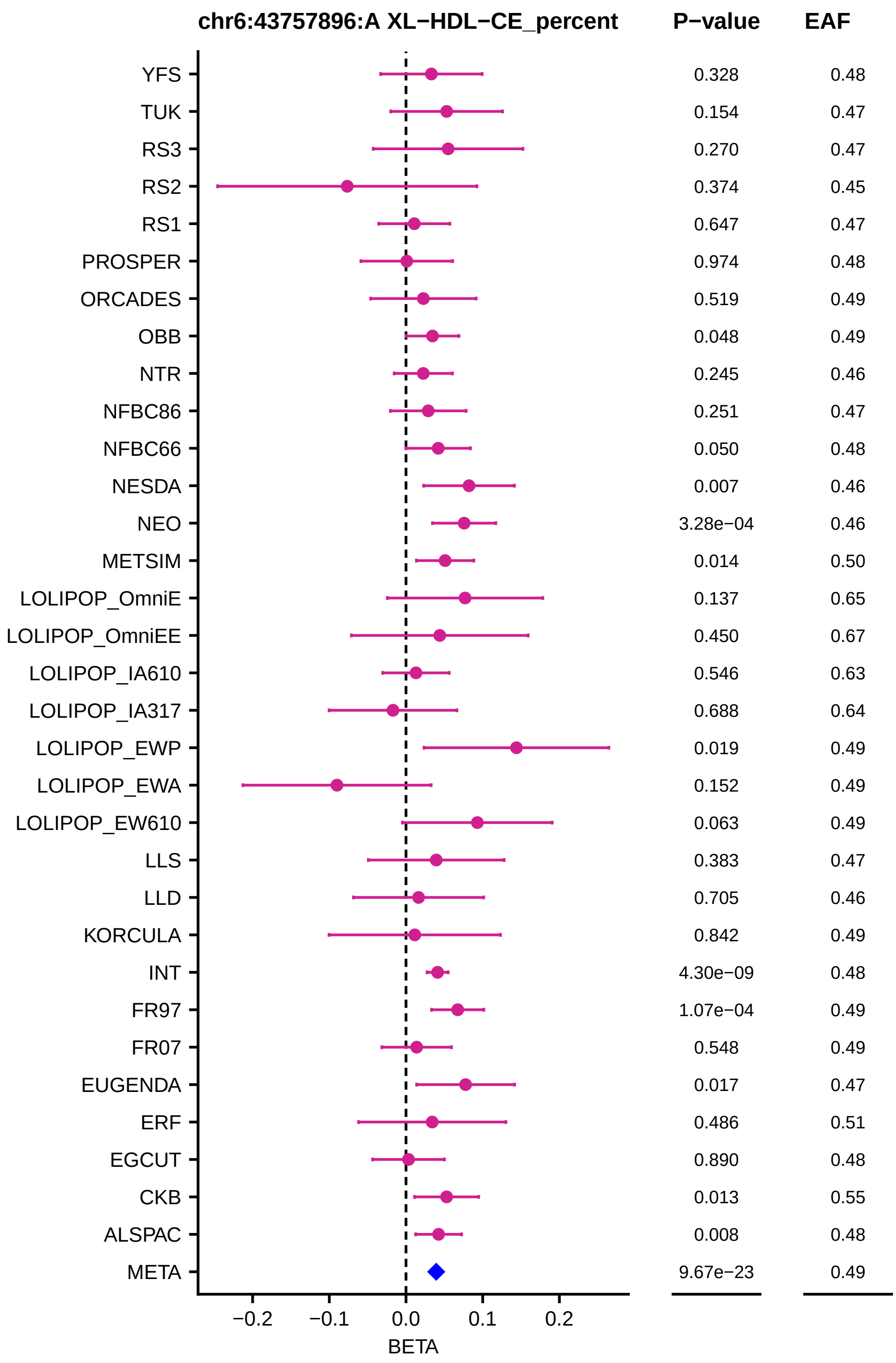
chr6:43757896:A XL−HDL−CE_percent
P−value
EAF
YFS
TUK
RS3
RS2
RS1
PROSPER
ORCADES
OBB
NTR
NFBC86
NFBC66
NESDA
NEO
METSIM
LOLIPOP_OmniE
LOLIPOP_OmniEE
LOLIPOP_IA610
LOLIPOP_IA317
LOLIPOP_EWP
LOLIPOP_EWA
LOLIPOP_EW610
LLS
LLD
KORCULA
INT
FR97
FR07
EUGENDA
ERF
EGCUT
CKB
ALSPAC
META
0.328 0.48
0.154 0.47
0.270 0.47
0.374 0.45
0.647 0.47
0.974 0.48
0.519 0.49
0.048 0.49
0.245 0.46
0.251 0.47
0.050 0.48
0.007 0.46
3.28e−04 0.46
0.014 0.50
0.137 0.65
0.450 0.67
0.546 0.63
0.688 0.64
0.019 0.49
0.152 0.49
0.063 0.49
0.383 0.47
0.705 0.46
0.842 0.49
4.30e−09 0.48
1.07e−04 0.49
0.548 0.49
0.017 0.47
0.486 0.51
0.890 0.48
0.013 0.55
0.008 0.48
9.67e−23 0.49
−0.2
−0.1
0.0
0.1
0.2
BETA

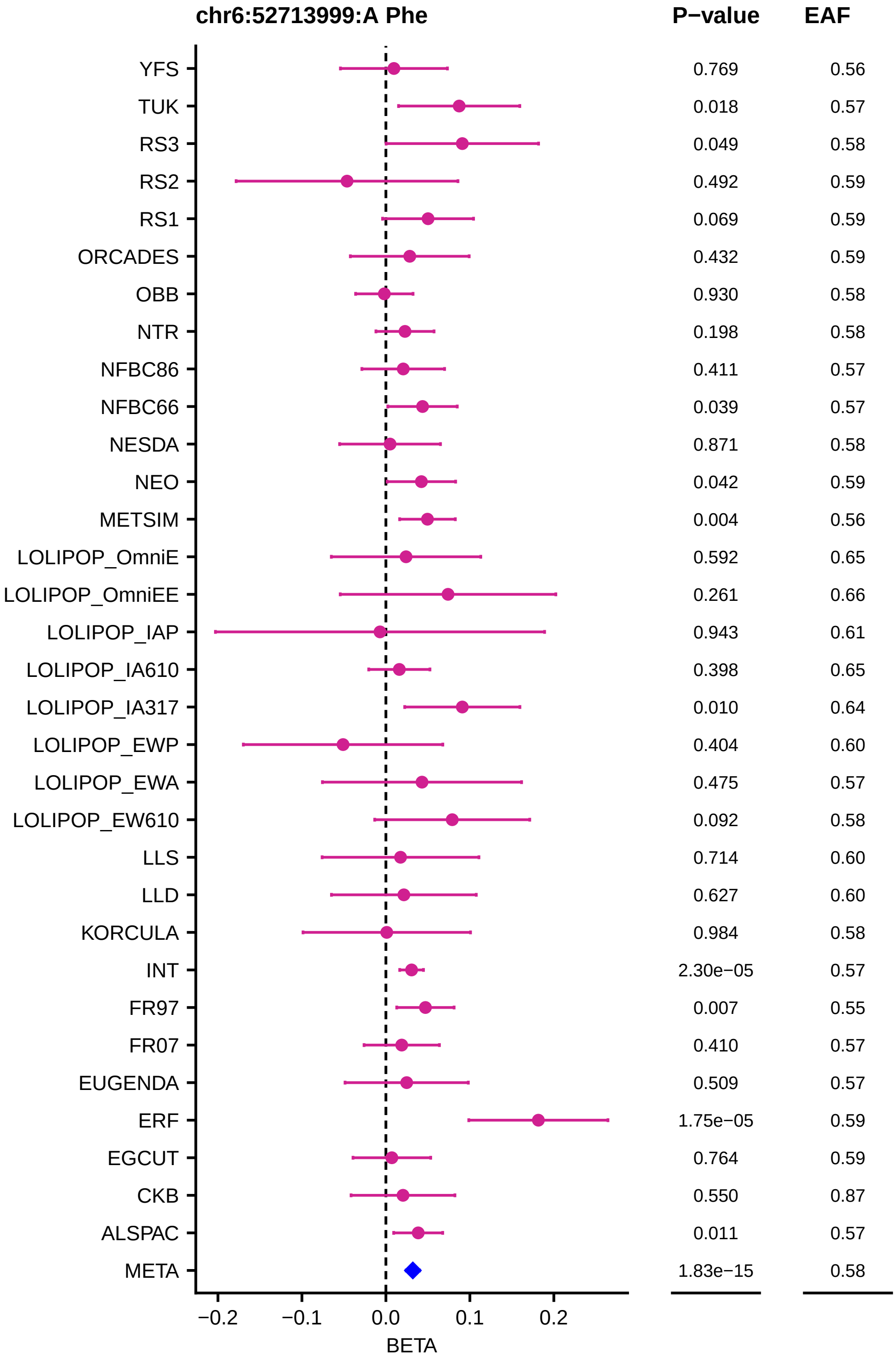
chr6:52713999:A Phe
P−value
EAF
YFS 0.769 0.56
TUK 0.018 0.57
RS3 0.049 0.58
RS2 0.492 0.59
RS1 0.069 0.59
ORCADES 0.432 0.59
OBB 0.930 0.58
NTR 0.198 0.58
NFBC86 0.411 0.57
NFBC66 0.039 0.57
NESDA 0.871 0.58
NEO 0.042 0.59
METSIM 0.004 0.56
LOLIPOP_OmniE 0.592 0.65
LOLIPOP_OmniEE 0.261 0.66
LOLIPOP_IAP 0.943 0.61
LOLIPOP_IA610 0.398 0.65
LOLIPOP_IA317 0.010 0.64
LOLIPOP_EWP 0.404 0.60
LOLIPOP_EWA 0.475 0.57
LOLIPOP_EW610 0.092 0.58
LLS 0.714 0.60
LLD 0.627 0.60
KORCULA 0.984 0.58
INT 2.30e−05 0.57
FR97 0.007 0.55
FR07 0.410 0.57
EUGENDA 0.509 0.57
ERF 1.75e−05 0.59
EGCUT 0.764 0.59
CKB 0.550 0.87
ALSPAC 0.011 0.57
META 1.83e−15 0.58
−0.2 −0.1 0.0 0.1 0.2
BETA

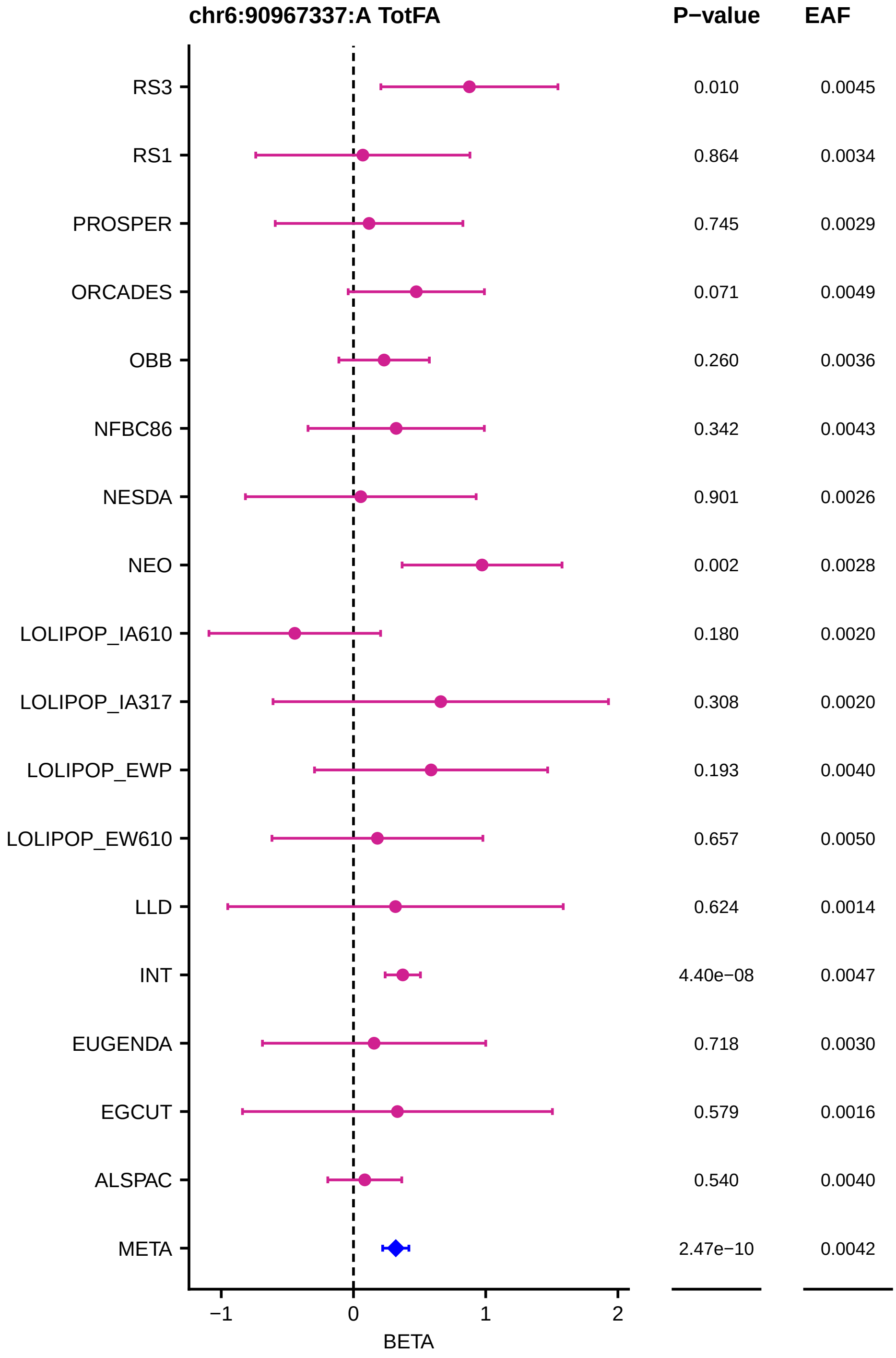
chr6:90967337:A TotFA
P−value
EAF
RS3 0.010 0.0045
RS1 0.864 0.0034
PROSPER 0.745 0.0029
ORCADES 0.071 0.0049
OBB 0.260 0.0036
NFBC86 0.342 0.0043
NESDA 0.901 0.0026
NEO 0.002 0.0028
LOLIPOP_IA610 0.180 0.0020
LOLIPOP_IA317 0.308 0.0020
LOLIPOP_EWP 0.193 0.0040
LOLIPOP_EW610 0.657 0.0050
LLD 0.624 0.0014
INT 4.40e−08 0.0047
EUGENDA 0.718 0.0030
EGCUT 0.579 0.0016
ALSPAC 0.540 0.0040
META 2.47e−10 0.0042
−1
0
1
2
BETA

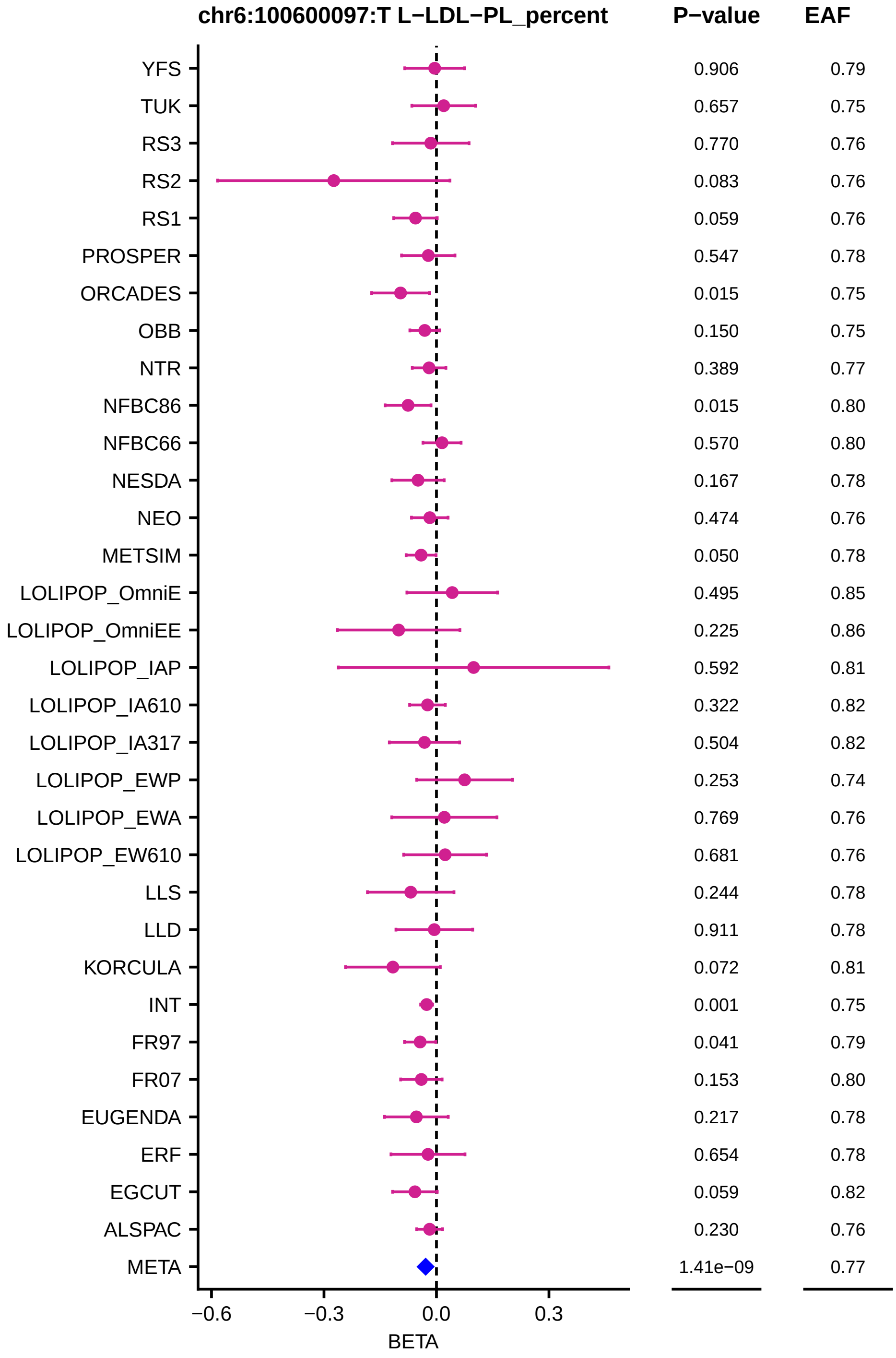
chr6:100600097:T L−LDL−PL_percent
P−value
EAF
YFS 0.906 0.79
TUK 0.657 0.75
RS3 0.770 0.76
RS2 0.083 0.76
RS1 0.059 0.76
PROSPER 0.547 0.78
ORCADES 0.015 0.75
OBB 0.150 0.75
NTR 0.389 0.77
NFBC86 0.015 0.80
NFBC66 0.570 0.80
NESDA 0.167 0.78
NEO 0.474 0.76
METSIM 0.050 0.78
LOLIPOP_OmniE 0.495 0.85
LOLIPOP_OmniEE 0.225 0.86
LOLIPOP_IAP 0.592 0.81
LOLIPOP_IA610 0.322 0.82
LOLIPOP_IA317 0.504 0.82
LOLIPOP_EWP 0.253 0.74
LOLIPOP_EWA 0.769 0.76
LOLIPOP_EW610 0.681 0.76
LLS 0.244 0.78
LLD 0.911 0.78
KORCULA 0.072 0.81
INT 0.001 0.75
FR97 0.041 0.79
FR07 0.153 0.80
EUGENDA 0.217 0.78
ERF 0.654 0.78
EGCUT 0.059 0.82
ALSPAC 0.230 0.76
META 1.41e−09 0.77
−0.6
−0.3
0.0
0.3
BETA

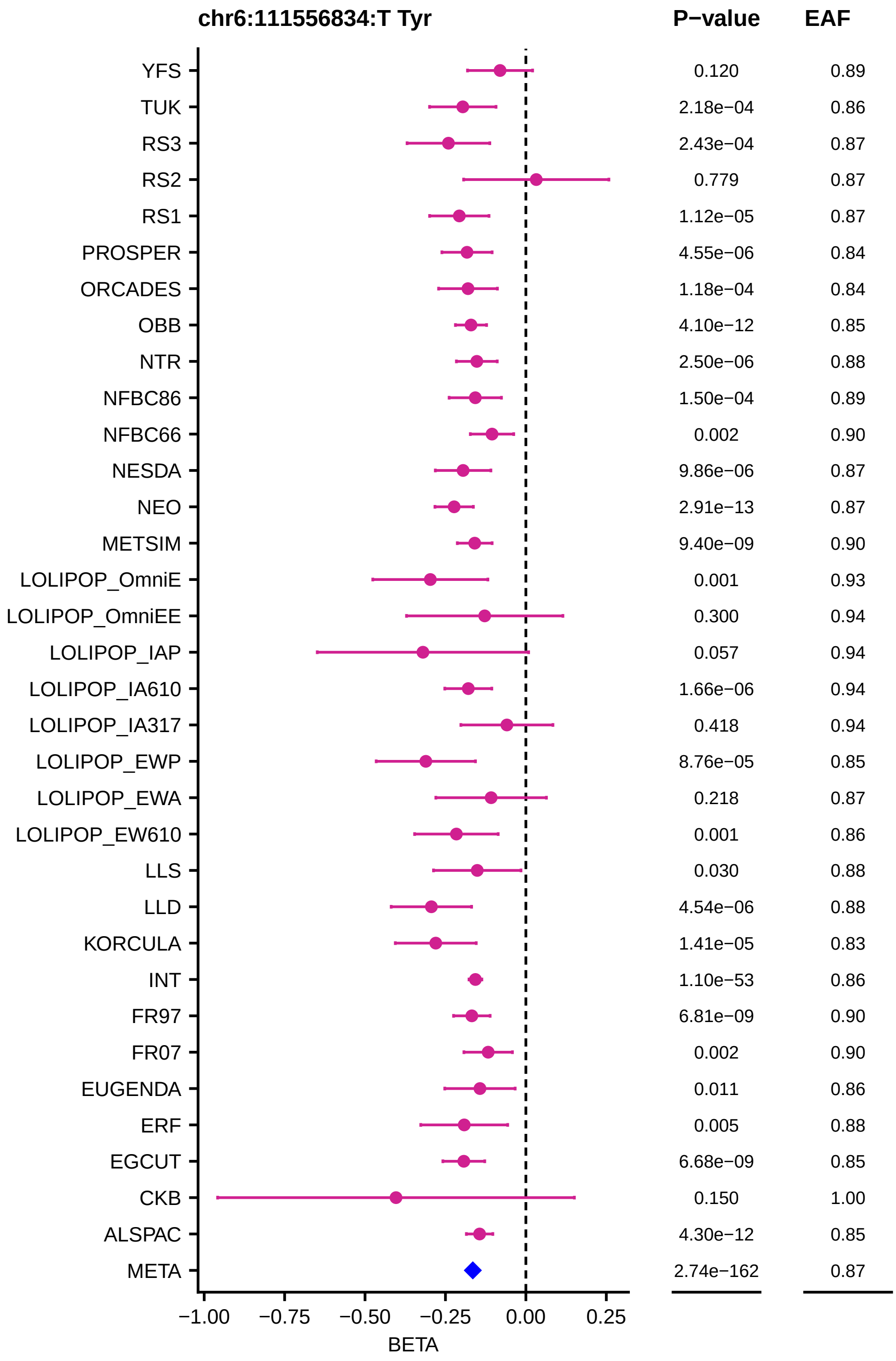

chr6:111556834:T Tyr
P−value
EAF
YFS 0.120 0.89
TUK 2.18e−04 0.86
RS3 2.43e−04 0.87
RS2 0.779 0.87
RS1 1.12e−05 0.87
PROSPER 4.55e−06 0.84
ORCADES 1.18e−04 0.84
OBB 4.10e−12 0.85
NTR 2.50e−06 0.88
NFBC86 1.50e−04 0.89
NFBC66 0.002 0.90
NESDA 9.86e−06 0.87
NEO 2.91e−13 0.87
METSIM 9.40e−09 0.90
LOLIPOP_OmniE 0.001 0.93
LOLIPOP_OmniEE 0.300 0.94
LOLIPOP_IAP 0.057 0.94
LOLIPOP_IA610 1.66e−06 0.94
LOLIPOP_IA317 0.418 0.94
LOLIPOP_EWP 8.76e−05 0.85
LOLIPOP_EWA 0.218 0.87
LOLIPOP_EW610 0.001 0.86
LLS 0.030 0.88
LLD 4.54e−06 0.88
KORCULA 1.41e−05 0.83
INT 1.10e−53 0.86
FR97 6.81e−09 0.90
FR07 0.002 0.90
EUGENDA 0.011 0.86
ERF 0.005 0.88
EGCUT 6.68e−09 0.85
CKB 0.150 1.00
ALSPAC 4.30e−12 0.85
META 2.74e−162 0.87
−1.00 −0.75 −0.50 −0.25 0.00 0.25
BETA

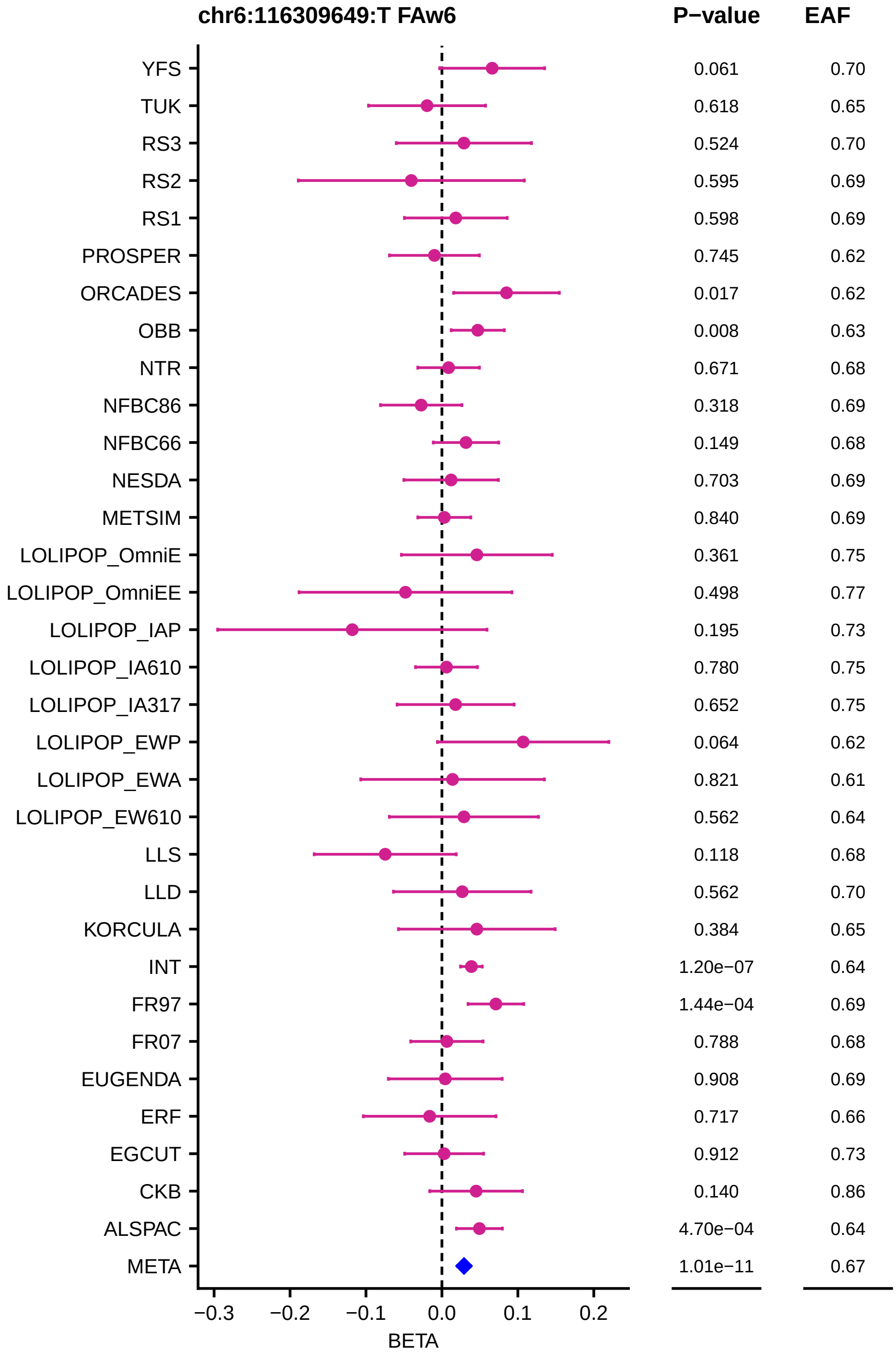
chr6:116309649:T FAw6
P−value
EAF
YFS 0.061 0.70
TUK 0.618 0.65
RS3 0.524 0.70
RS2 0.595 0.69
RS1 0.598 0.69
PROSPER 0.745 0.62
ORCADES 0.017 0.62
OBB 0.008 0.63
NTR 0.671 0.68
NFBC86 0.318 0.69
NFBC66 0.149 0.68
NESDA 0.703 0.69
METSIM 0.840 0.69
LOLIPOP_OmniE 0.361 0.75
LOLIPOP_OmniEE 0.498 0.77
LOLIPOP_IAP 0.195 0.73
LOLIPOP_IA610 0.780 0.75
LOLIPOP_IA317 0.652 0.75
LOLIPOP_EWP 0.064 0.62
LOLIPOP_EWA 0.821 0.61
LOLIPOP_EW610 0.562 0.64
LLS 0.118 0.68
LLD 0.562 0.70
KORCULA 0.384 0.65
INT 1.20e−07 0.64
FR97 1.44e−04 0.69
FR07 0.788 0.68
EUGENDA 0.908 0.69
ERF 0.717 0.66
EGCUT 0.912 0.73
CKB 0.140 0.86
ALSPAC 4.70e−04 0.64
META 1.01e−11 0.67
−0.3
−0.2
−0.1
0.0
0.1
0.2
BETA

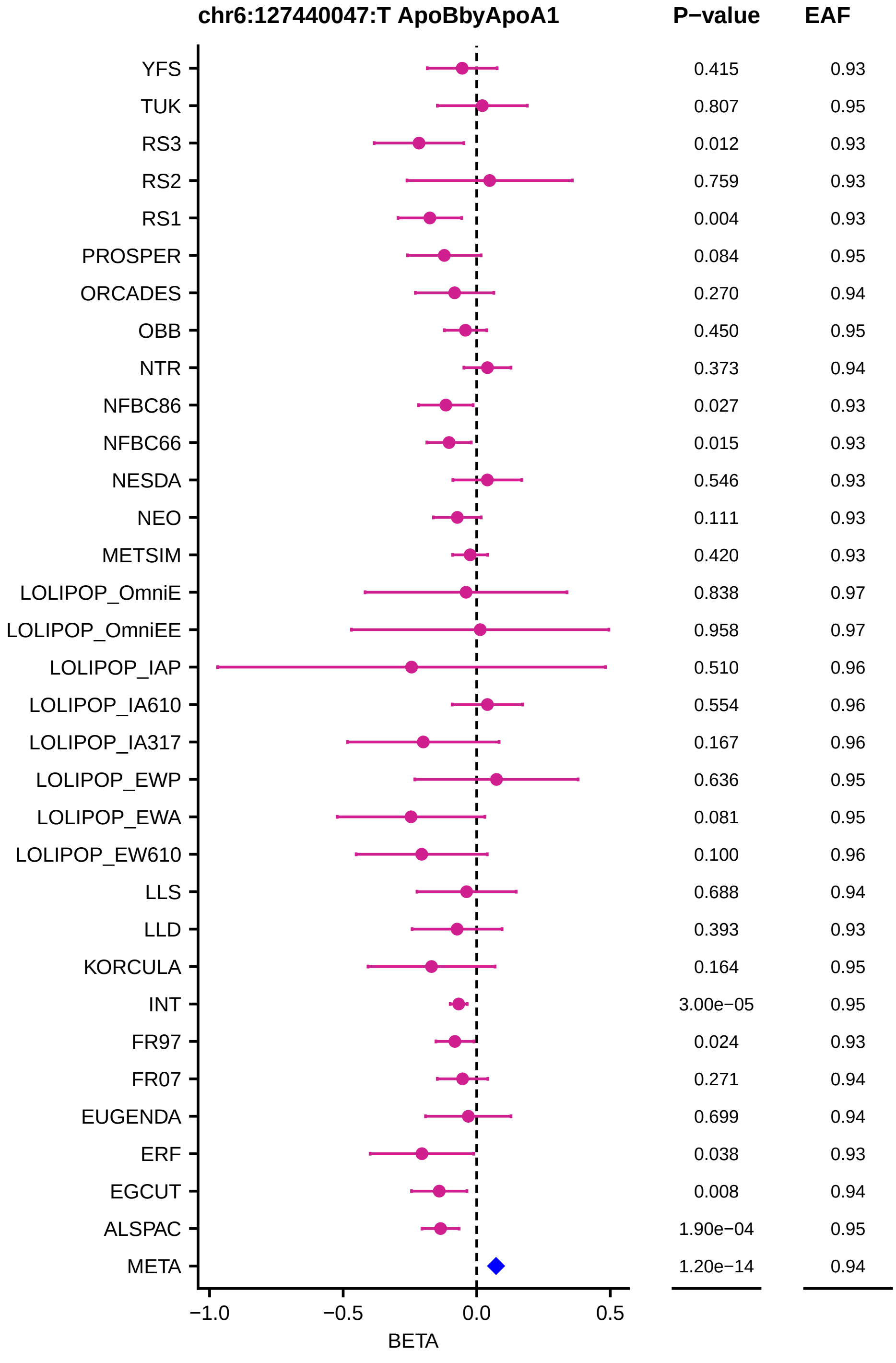

chr6:127440047:T ApoBbyApoA1
P−value
EAF
YFS 0.415 0.93
TUK 0.807 0.95
RS3 0.012 0.93
RS2 0.759 0.93
RS1 0.004 0.93
PROSPER 0.084 0.95
ORCADES 0.270 0.94
OBB 0.450 0.95
NTR 0.373 0.94
NFBC86 0.027 0.93
NFBC66 0.015 0.93
NESDA 0.546 0.93
NEO 0.111 0.93
METSIM 0.420 0.93
LOLIPOP_OmniE 0.838 0.97
LOLIPOP_OmniEE 0.958 0.97
LOLIPOP_IAP 0.510 0.96
LOLIPOP_IA610 0.554 0.96
LOLIPOP_IA317 0.167 0.96
LOLIPOP_EWP 0.636 0.95
LOLIPOP_EWA 0.081 0.95
LOLIPOP_EW610 0.100 0.96
LLS 0.688 0.94
LLD 0.393 0.93
KORCULA 0.164 0.95
INT 3.00e−05 0.95
FR97 0.024 0.93
FR07 0.271 0.94
EUGENDA 0.699 0.94
ERF 0.038 0.93
EGCUT 0.008 0.94
ALSPAC 1.90e−04 0.95
META 1.20e−14 0.94
−1.0
−0.5
0.0
0.5
BETA

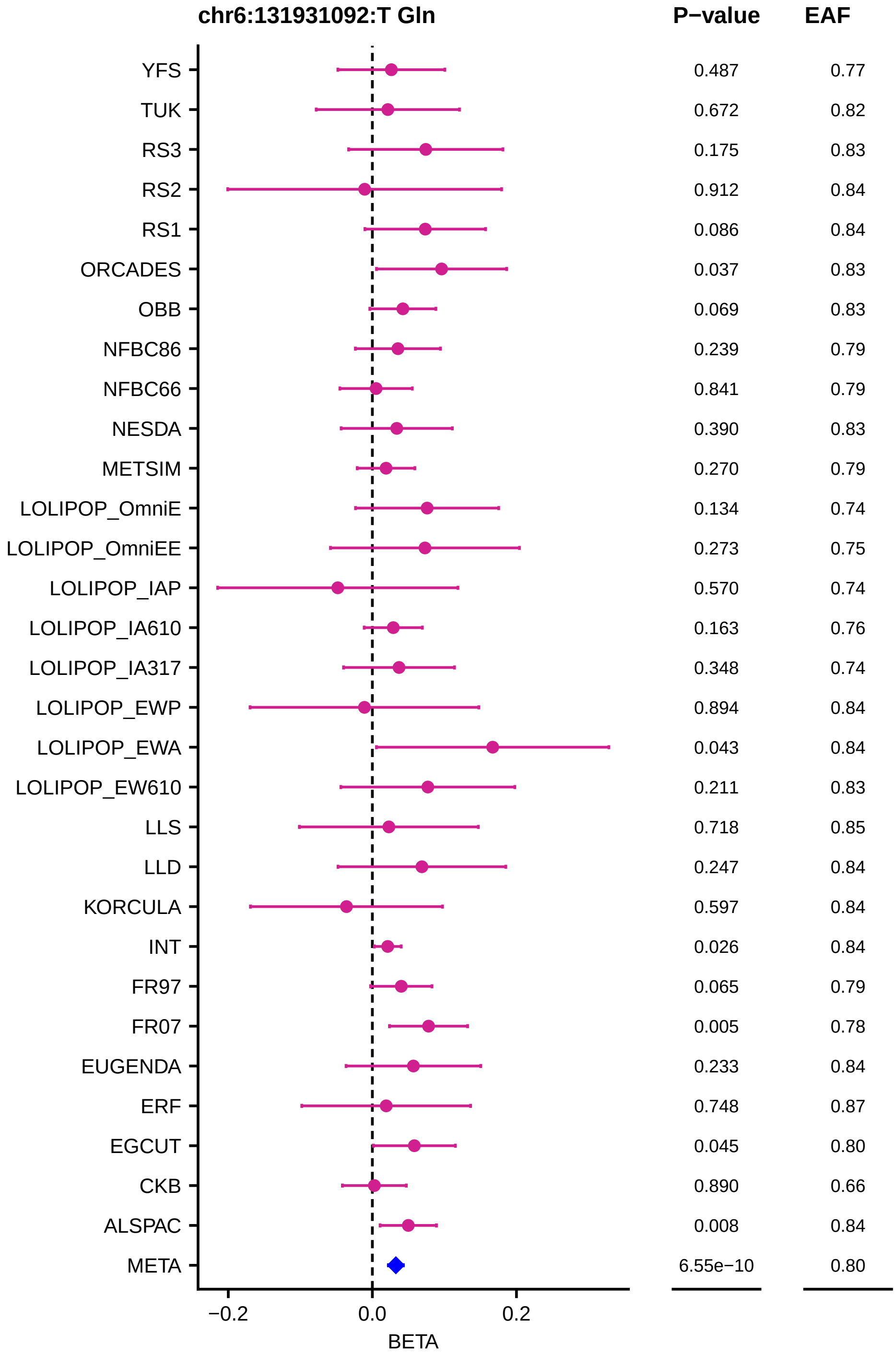
chr6:131931092:T Gln
P−value
EAF
YFS 0.487 0.77
TUK 0.672 0.82
RS3 0.175 0.83
RS2 0.912 0.84
RS1 0.086 0.84
ORCADES 0.037 0.83
OBB 0.069 0.83
NFBC86 0.239 0.79
NFBC66 0.841 0.79
NESDA 0.390 0.83
METSIM 0.270 0.79
LOLIPOP_OmniE 0.134 0.74
LOLIPOP_OmniEE 0.273 0.75
LOLIPOP_IAP 0.570 0.74
LOLIPOP_IA610 0.163 0.76
LOLIPOP_IA317 0.348 0.74
LOLIPOP_EWP 0.894 0.84
LOLIPOP_EWA 0.043 0.84
LOLIPOP_EW610 0.211 0.83
LLS 0.718 0.85
LLD 0.247 0.84
KORCULA 0.597 0.84
INT 0.026 0.84
FR97 0.065 0.79
FR07 0.005 0.78
EUGENDA 0.233 0.84
ERF 0.748 0.87
EGCUT 0.045 0.80
CKB 0.890 0.66
ALSPAC 0.008 0.84
META 6.55e−10 0.80
−0.2
0.0
0.2
BETA

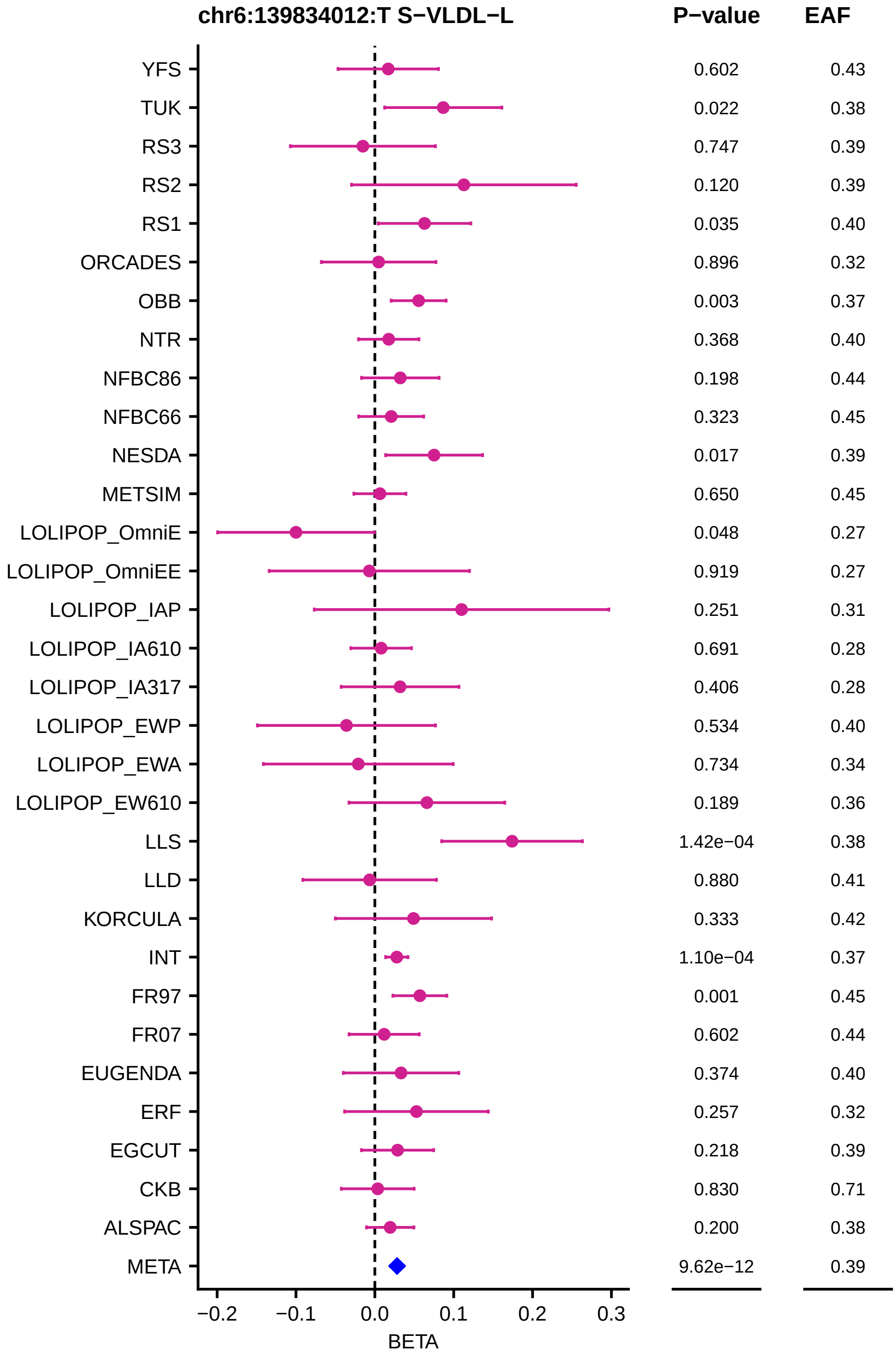
chr6:139834012:T S−VLDL−L
P−value
EAF
YFS 0.602 0.43
TUK 0.022 0.38
RS3 0.747 0.39
RS2 0.120 0.39
RS1 0.035 0.40
ORCADES 0.896 0.32
OBB 0.003 0.37
NTR 0.368 0.40
NFBC86 0.198 0.44
NFBC66 0.323 0.45
NESDA 0.017 0.39
METSIM 0.650 0.45
LOLIPOP_OmniE 0.048 0.27
LOLIPOP_OmniEE 0.919 0.27
LOLIPOP_IAP 0.251 0.31
LOLIPOP_IA610 0.691 0.28
LOLIPOP_IA317 0.406 0.28
LOLIPOP_EWP 0.534 0.40
LOLIPOP_EWA 0.734 0.34
LOLIPOP_EW610 0.189 0.36
LLS 1.42e−04 0.38
LLD 0.880 0.41
KORCULA 0.333 0.42
INT 1.10e−04 0.37
FR97 0.001 0.45
FR07 0.602 0.44
EUGENDA 0.374 0.40
ERF 0.257 0.32
EGCUT 0.218 0.39
CKB 0.830 0.71
ALSPAC 0.200 0.38
META 9.62e−12 0.39
−0.2 −0.1 0.0 0.1 0.2 0.3
BETA

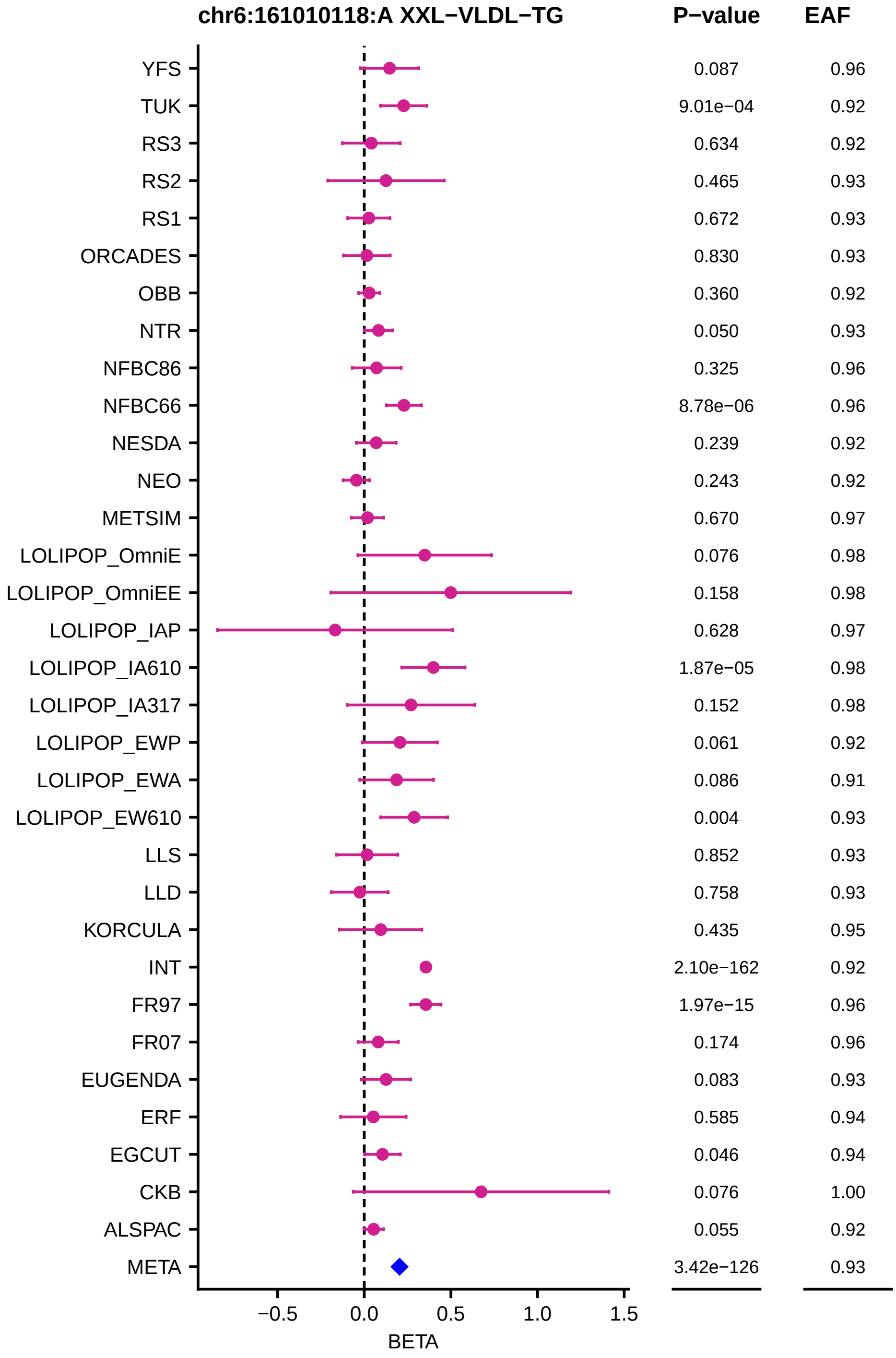
chr6:161010118:A XXL−VLDL−TG
P−value
EAF
YFS 0.087 0.96
TUK 9.01e−04 0.92
RS3 0.634 0.92
RS2 0.465 0.93
RS1 0.672 0.93
ORCADES 0.830 0.93
OBB 0.360 0.92
NTR 0.050 0.93
NFBC86 0.325 0.96
NFBC66 8.78e−06 0.96
NESDA 0.239 0.92
NEO 0.243 0.92
METSIM 0.670 0.97
LOLIPOP_OmniE 0.076 0.98
LOLIPOP_OmniEE 0.158 0.98
LOLIPOP_IAP 0.628 0.97
LOLIPOP_IA610 1.87e−05 0.98
LOLIPOP_IA317 0.152 0.98
LOLIPOP_EWP 0.061 0.92
LOLIPOP_EWA 0.086 0.91
LOLIPOP_EW610 0.004 0.93
LLS 0.852 0.93
LLD 0.758 0.93
KORCULA 0.435 0.95
INT 2.10e−162 0.92
FR97 1.97e−15 0.96
FR07 0.174 0.96
EUGENDA 0.083 0.93
ERF 0.585 0.94
EGCUT 0.046 0.94
CKB 0.076 1.00
ALSPAC 0.055 0.92
META 3.42e−126 0.93
−0.5
0.0
0.5
1.0
1.5
BETA

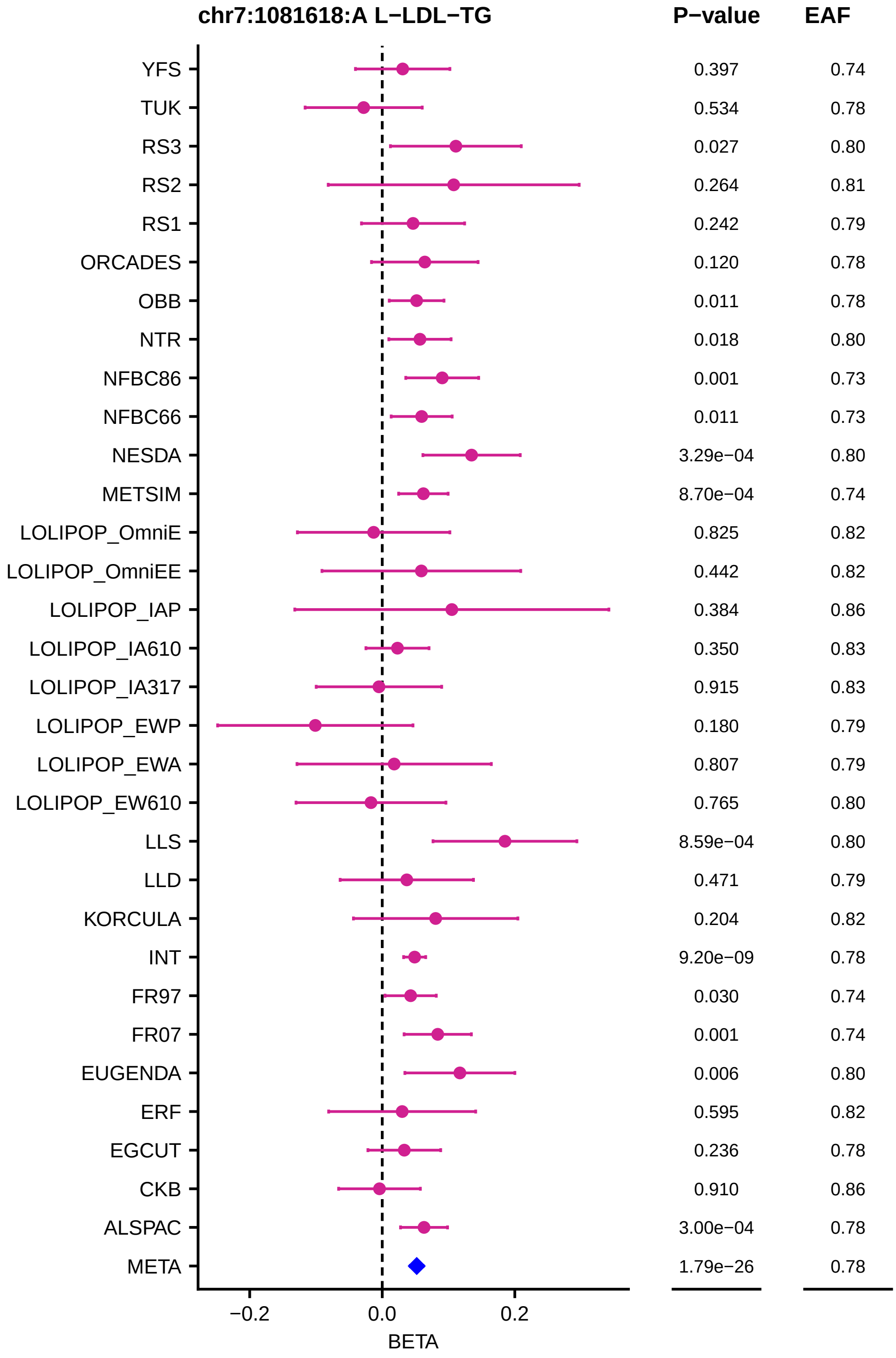
chr7:1081618:A L−LDL−TG
P−value
EAF
YFS 0.397 0.74
TUK 0.534 0.78
RS3 0.027 0.80
RS2 0.264 0.81
RS1 0.242 0.79
ORCADES 0.120 0.78
OBB 0.011 0.78
NTR 0.018 0.80
NFBC86 0.001 0.73
NFBC66 0.011 0.73
NESDA 3.29e−04 0.80
METSIM 8.70e−04 0.74
LOLIPOP_OmniE 0.825 0.82
LOLIPOP_OmniEE 0.442 0.82
LOLIPOP_IAP 0.384 0.86
LOLIPOP_IA610 0.350 0.83
LOLIPOP_IA317 0.915 0.83
LOLIPOP_EWP 0.180 0.79
LOLIPOP_EWA 0.807 0.79
LOLIPOP_EW610 0.765 0.80
LLS 8.59e−04 0.80
LLD 0.471 0.79
KORCULA 0.204 0.82
INT 9.20e−09 0.78
FR97 0.030 0.74
FR07 0.001 0.74
EUGENDA 0.006 0.80
ERF 0.595 0.82
EGCUT 0.236 0.78
CKB 0.910 0.86
ALSPAC 3.00e−04 0.78
META 1.79e−26 0.78
−0.2
0.0
0.2
BETA

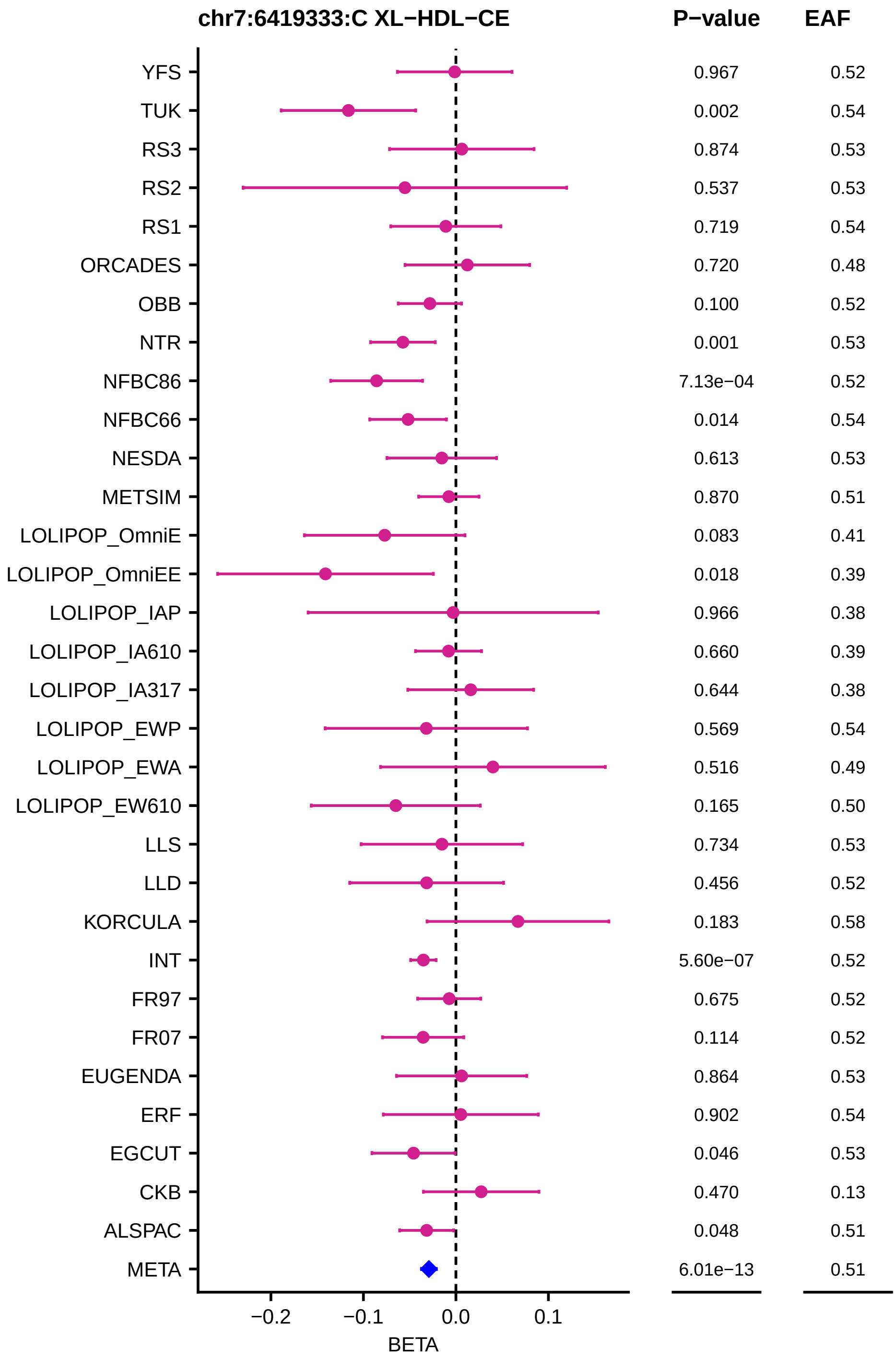
chr7:6419333:C XL−HDL−CE
P−value
EAF
YFS
TUK
RS3
RS2
RS1
ORCADES
OBB
NTR
NFBC86
NFBC66
NESDA
METSIM
LOLIPOP_OmniE
LOLIPOP_OmniEE
LOLIPOP_IAP
LOLIPOP_IA610
LOLIPOP_IA317
LOLIPOP_EWP
LOLIPOP_EWA
LOLIPOP_EW610
LLS
LLD
KORCULA
INT
FR97
FR07
EUGENDA
ERF
EGCUT
CKB
ALSPAC
META
0.967
0.002
0.874
0.537
0.719
0.720
0.100
0.001
7.13e−04
0.014
0.613
0.870
0.083
0.018
0.966
0.660
0.644
0.569
0.516
0.165
0.734
0.456
0.183
5.60e−07
0.675
0.114
0.864
0.902
0.046
0.470
0.048
6.01e−13
0.52
0.54
0.53
0.53
0.54
0.48
0.52
0.53
0.52
0.54
0.53
0.51
0.41
0.39
0.38
0.39
0.38
0.54
0.49
0.50
0.53
0.52
0.58
0.52
0.52
0.52
0.53
0.54
0.53
0.13
0.51
0.51
−0.2
−0.1
0.0
0.1
BETA

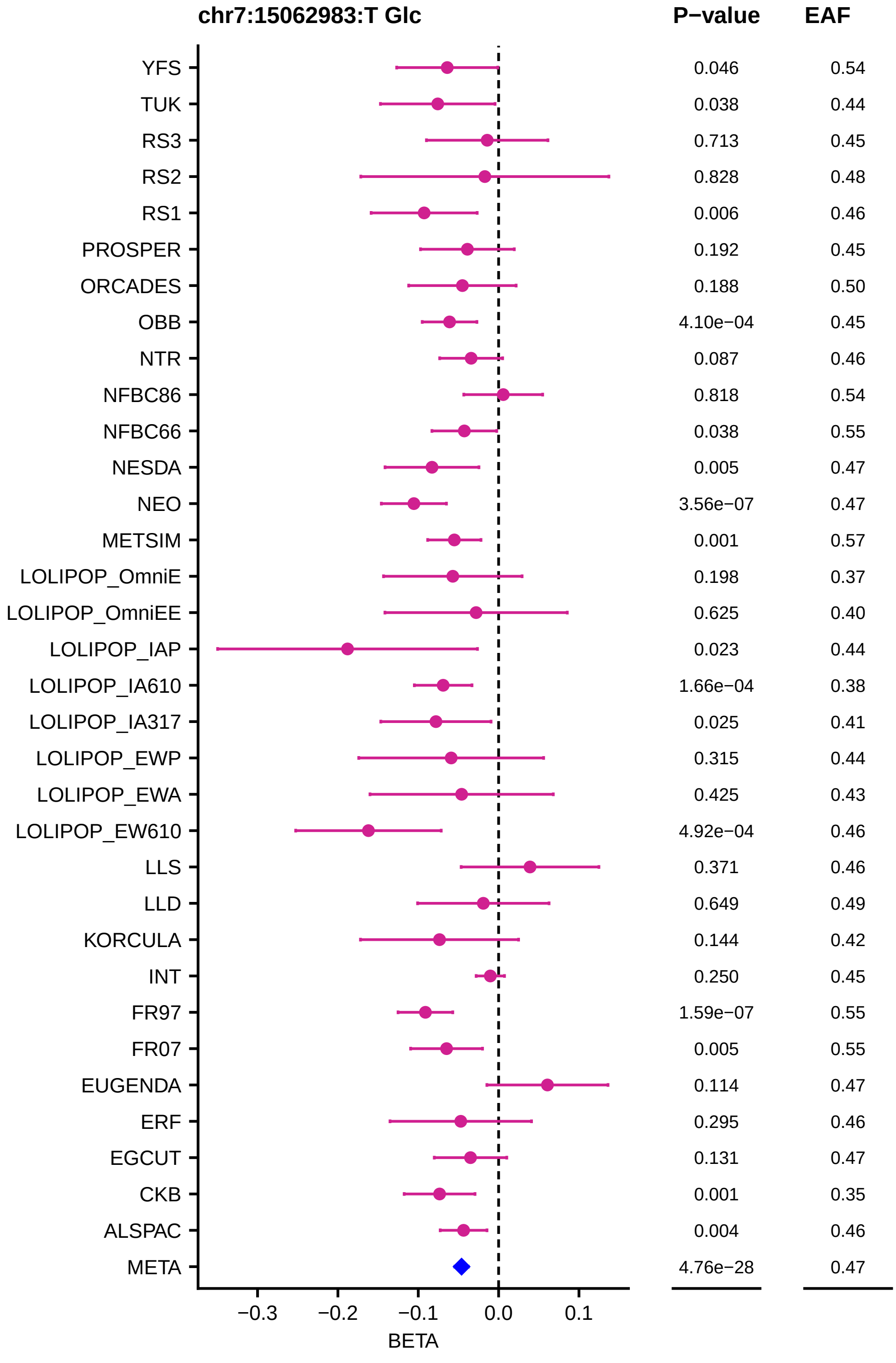
chr7:15062983:T Glc
P−value
EAF
YFS 0.046 0.54
TUK 0.038 0.44
RS3 0.713 0.45
RS2 0.828 0.48
RS1 0.006 0.46
PROSPER 0.192 0.45
ORCADES 0.188 0.50
OBB 4.10e−04 0.45
NTR 0.087 0.46
NFBC86 0.818 0.54
NFBC66 0.038 0.55
NESDA 0.005 0.47
NEO 3.56e−07 0.47
METSIM 0.001 0.57
LOLIPOP_OmniE 0.198 0.37
LOLIPOP_OmniEE 0.625 0.40
LOLIPOP_IAP 0.023 0.44
LOLIPOP_IA610 1.66e−04 0.38
LOLIPOP_IA317 0.025 0.41
LOLIPOP_EWP 0.315 0.44
LOLIPOP_EWA 0.425 0.43
LOLIPOP_EW610 4.92e−04 0.46
LLS 0.371 0.46
LLD 0.649 0.49
KORCULA 0.144 0.42
INT 0.250 0.45
FR97 1.59e−07 0.55
FR07 0.005 0.55
EUGENDA 0.114 0.47
ERF 0.295 0.46
EGCUT 0.131 0.47
CKB 0.001 0.35
ALSPAC 0.004 0.46
META 4.76e−28 0.47
−0.3
−0.2
−0.1
0.0
0.1
BETA

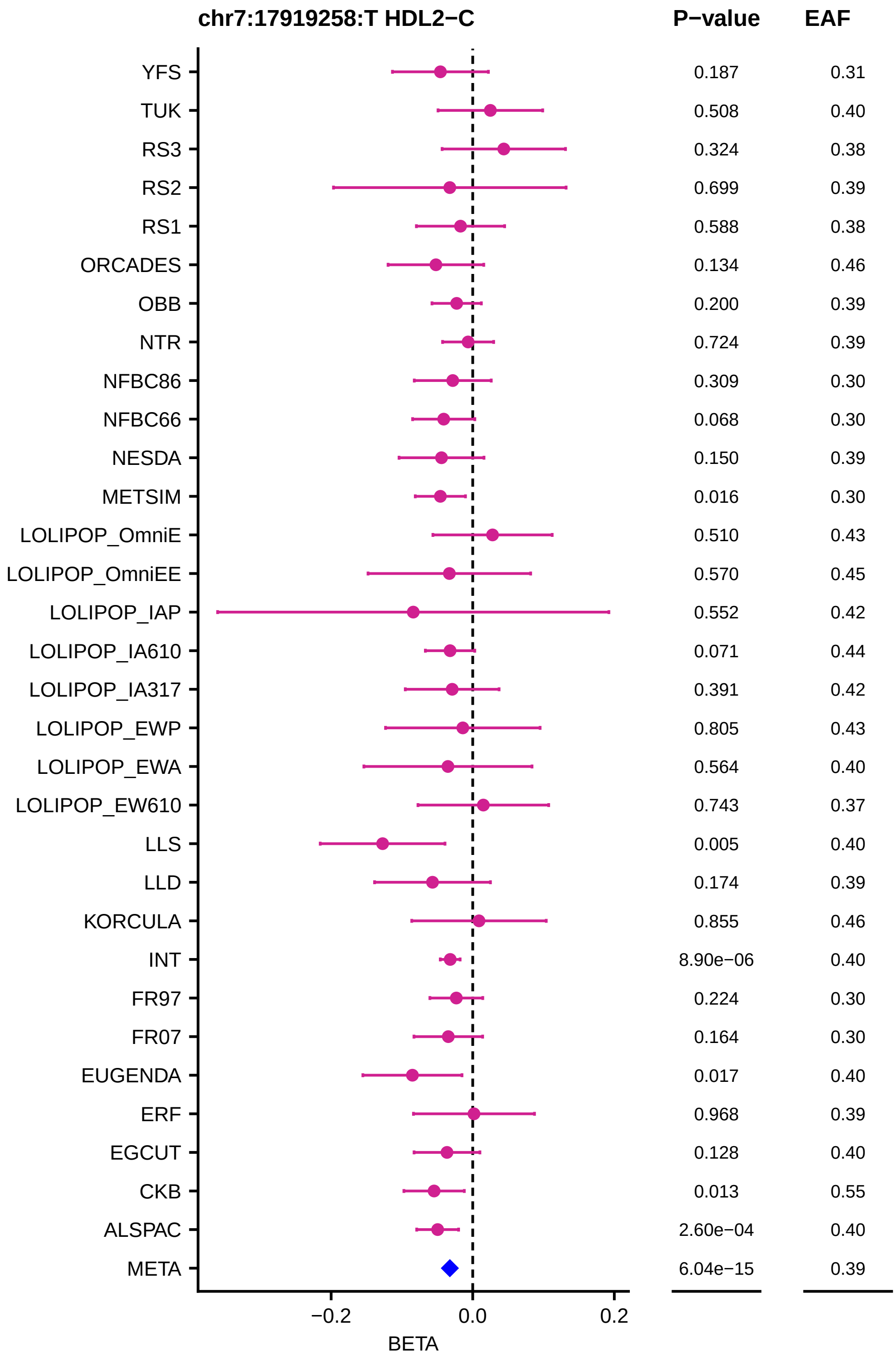

chr7:17919258:T HDL2−C
P−value
EAF
YFS 0.187 0.31
TUK 0.508 0.40
RS3 0.324 0.38
RS2 0.699 0.39
RS1 0.588 0.38
ORCADES 0.134 0.46
OBB 0.200 0.39
NTR 0.724 0.39
NFBC86 0.309 0.30
NFBC66 0.068 0.30
NESDA 0.150 0.39
METSIM 0.016 0.30
LOLIPOP_OmniE 0.510 0.43
LOLIPOP_OmniEE 0.570 0.45
LOLIPOP_IAP 0.552 0.42
LOLIPOP_IA610 0.071 0.44
LOLIPOP_IA317 0.391 0.42
LOLIPOP_EWP 0.805 0.43
LOLIPOP_EWA 0.564 0.40
LOLIPOP_EW610 0.743 0.37
LLS 0.005 0.40
LLD 0.174 0.39
KORCULA 0.855 0.46
INT 8.90e−06 0.40
FR97 0.224 0.30
FR07 0.164 0.30
EUGENDA 0.017 0.40
ERF 0.968 0.39
EGCUT 0.128 0.40
CKB 0.013 0.55
ALSPAC 2.60e−04 0.40
META 6.04e−15 0.39
−0.2
0.0
0.2
BETA

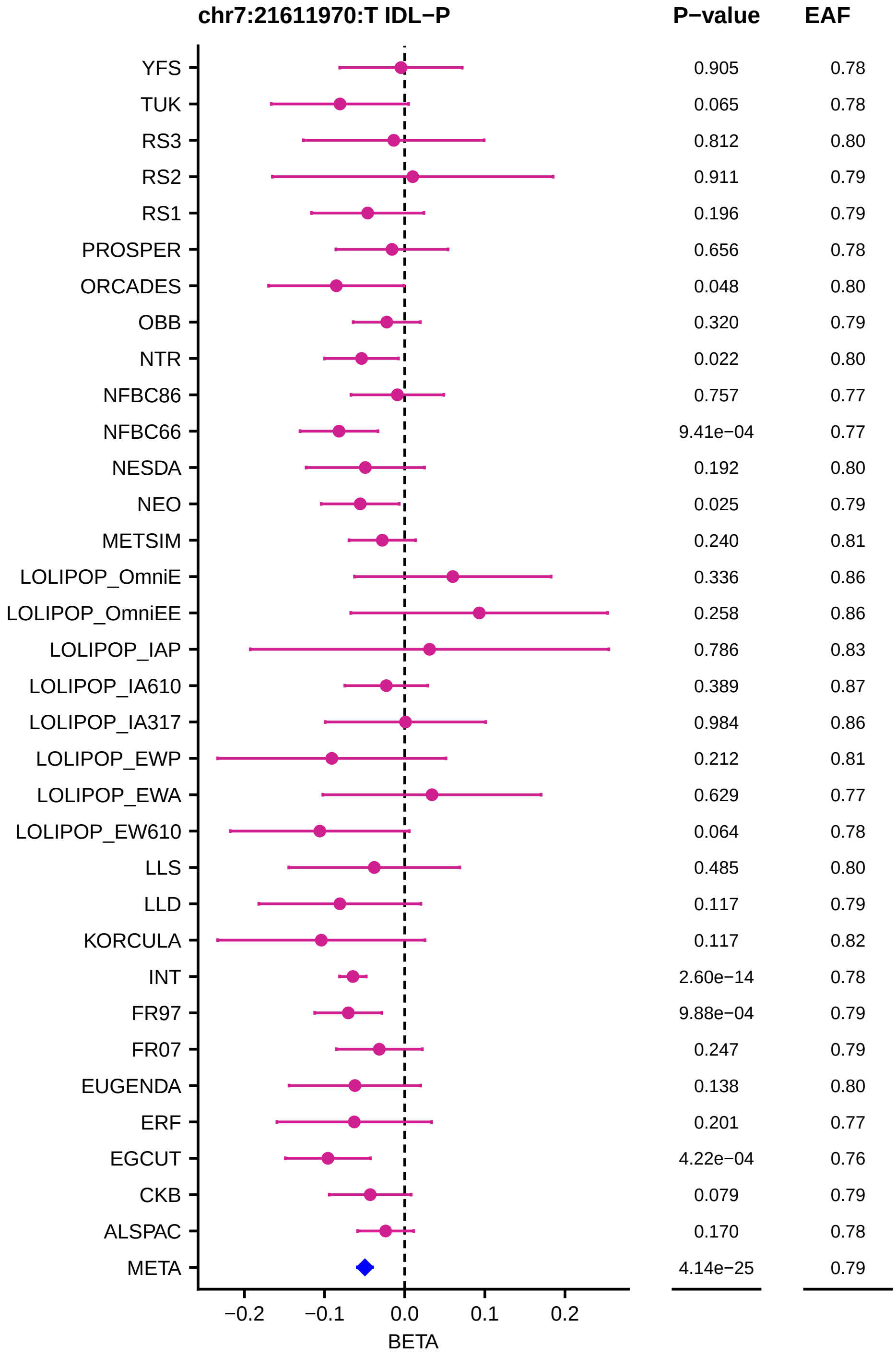
chr7:21611970:T IDL−P
P−value
EAF
YFS 0.905 0.78
TUK 0.065 0.78
RS3 0.812 0.80
RS2 0.911 0.79
RS1 0.196 0.79
PROSPER 0.656 0.78
ORCADES 0.048 0.80
OBB 0.320 0.79
NTR 0.022 0.80
NFBC86 0.757 0.77
NFBC66 9.41e−04 0.77
NESDA 0.192 0.80
NEO 0.025 0.79
METSIM 0.240 0.81
LOLIPOP_OmniE 0.336 0.86
LOLIPOP_OmniEE 0.258 0.86
LOLIPOP_IAP 0.786 0.83
LOLIPOP_IA610 0.389 0.87
LOLIPOP_IA317 0.984 0.86
LOLIPOP_EWP 0.212 0.81
LOLIPOP_EWA 0.629 0.77
LOLIPOP_EW610 0.064 0.78
LLS 0.485 0.80
LLD 0.117 0.79
KORCULA 0.117 0.82
INT 2.60e−14 0.78
FR97 9.88e−04 0.79
FR07 0.247 0.79
EUGENDA 0.138 0.80
ERF 0.201 0.77
EGCUT 4.22e−04 0.76
CKB 0.079 0.79
ALSPAC 0.170 0.78
META 4.14e−25 0.79
−0.2
−0.1
0.0
0.1
0.2
BETA

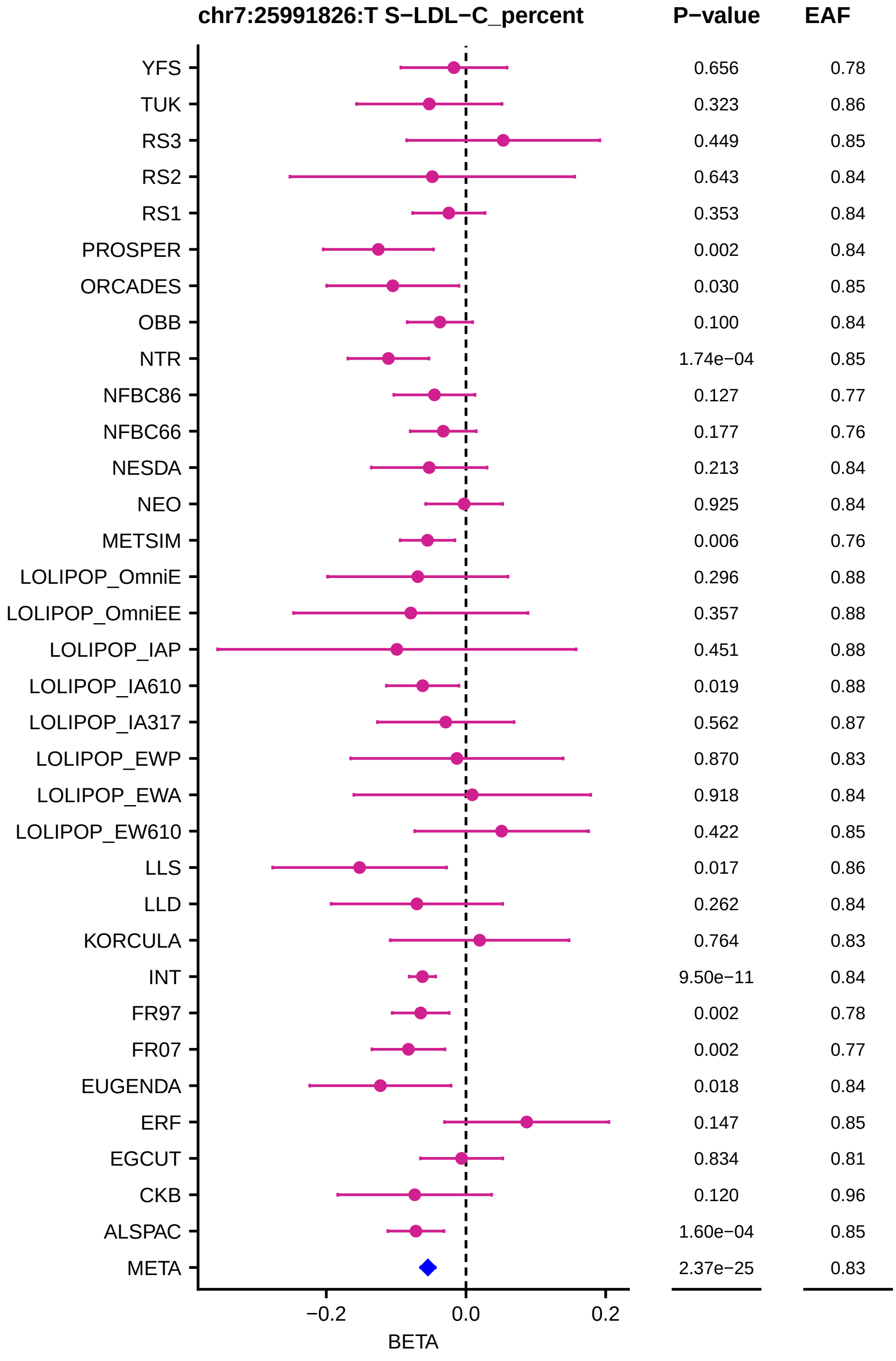
chr7:25991826:T S−LDL−C_percent
P−value
EAF
YFS 0.656 0.78
TUK 0.323 0.86
RS3 0.449 0.85
RS2 0.643 0.84
RS1 0.353 0.84
PROSPER 0.002 0.84
ORCADES 0.030 0.85
OBB 0.100 0.84
NTR 1.74e−04 0.85
NFBC86 0.127 0.77
NFBC66 0.177 0.76
NESDA 0.213 0.84
NEO 0.925 0.84
METSIM 0.006 0.76
LOLIPOP_OmniE 0.296 0.88
LOLIPOP_OmniEE 0.357 0.88
LOLIPOP_IAP 0.451 0.88
LOLIPOP_IA610 0.019 0.88
LOLIPOP_IA317 0.562 0.87
LOLIPOP_EWP 0.870 0.83
LOLIPOP_EWA 0.918 0.84
LOLIPOP_EW610 0.422 0.85
LLS 0.017 0.86
LLD 0.262 0.84
KORCULA 0.764 0.83
INT 9.50e−11 0.84
FR97 0.002 0.78
FR07 0.002 0.77
EUGENDA 0.018 0.84
ERF 0.147 0.85
EGCUT 0.834 0.81
CKB 0.120 0.96
ALSPAC 1.60e−04 0.85
META 2.37e−25 0.83
−0.2
0.0
0.2
BETA

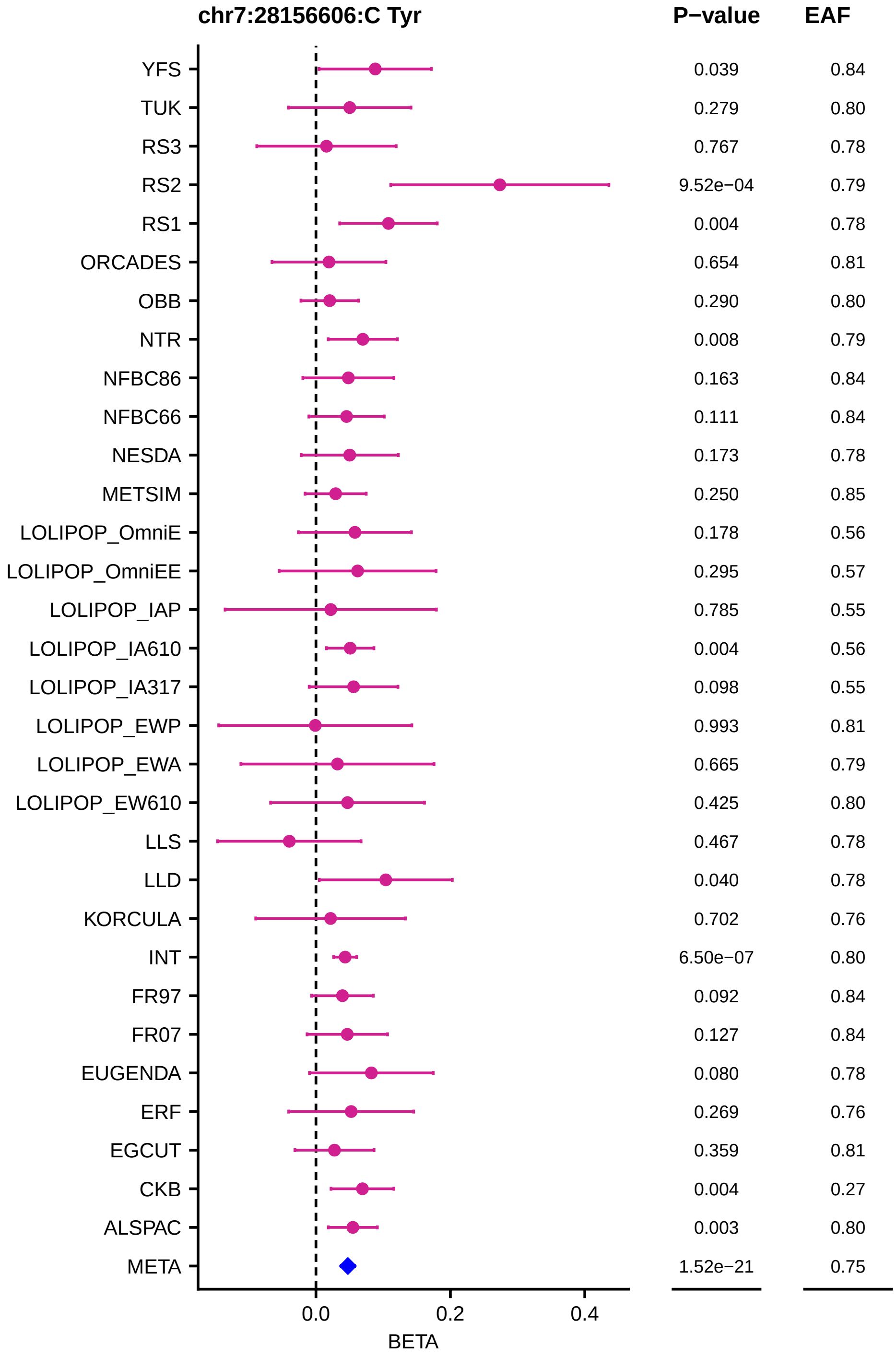
chr7:28156606:C Tyr
P−value
EAF
YFS 0.039 0.84
TUK 0.279 0.80
RS3 0.767 0.78
RS2 9.52e−04 0.79
RS1 0.004 0.78
ORCADES 0.654 0.81
OBB 0.290 0.80
NTR 0.008 0.79
NFBC86 0.163 0.84
NFBC66 0.111 0.84
NESDA 0.173 0.78
METSIM 0.250 0.85
LOLIPOP_OmniE 0.178 0.56
LOLIPOP_OmniEE 0.295 0.57
LOLIPOP_IAP 0.785 0.55
LOLIPOP_IA610 0.004 0.56
LOLIPOP_IA317 0.098 0.55
LOLIPOP_EWP 0.993 0.81
LOLIPOP_EWA 0.665 0.79
LOLIPOP_EW610 0.425 0.80
LLS 0.467 0.78
LLD 0.040 0.78
KORCULA 0.702 0.76
INT 6.50e−07 0.80
FR97 0.092 0.84
FR07 0.127 0.84
EUGENDA 0.080 0.78
ERF 0.269 0.76
EGCUT 0.359 0.81
CKB 0.004 0.27
ALSPAC 0.003 0.80
META 1.52e−21 0.75
0.0
0.2
0.4
BETA

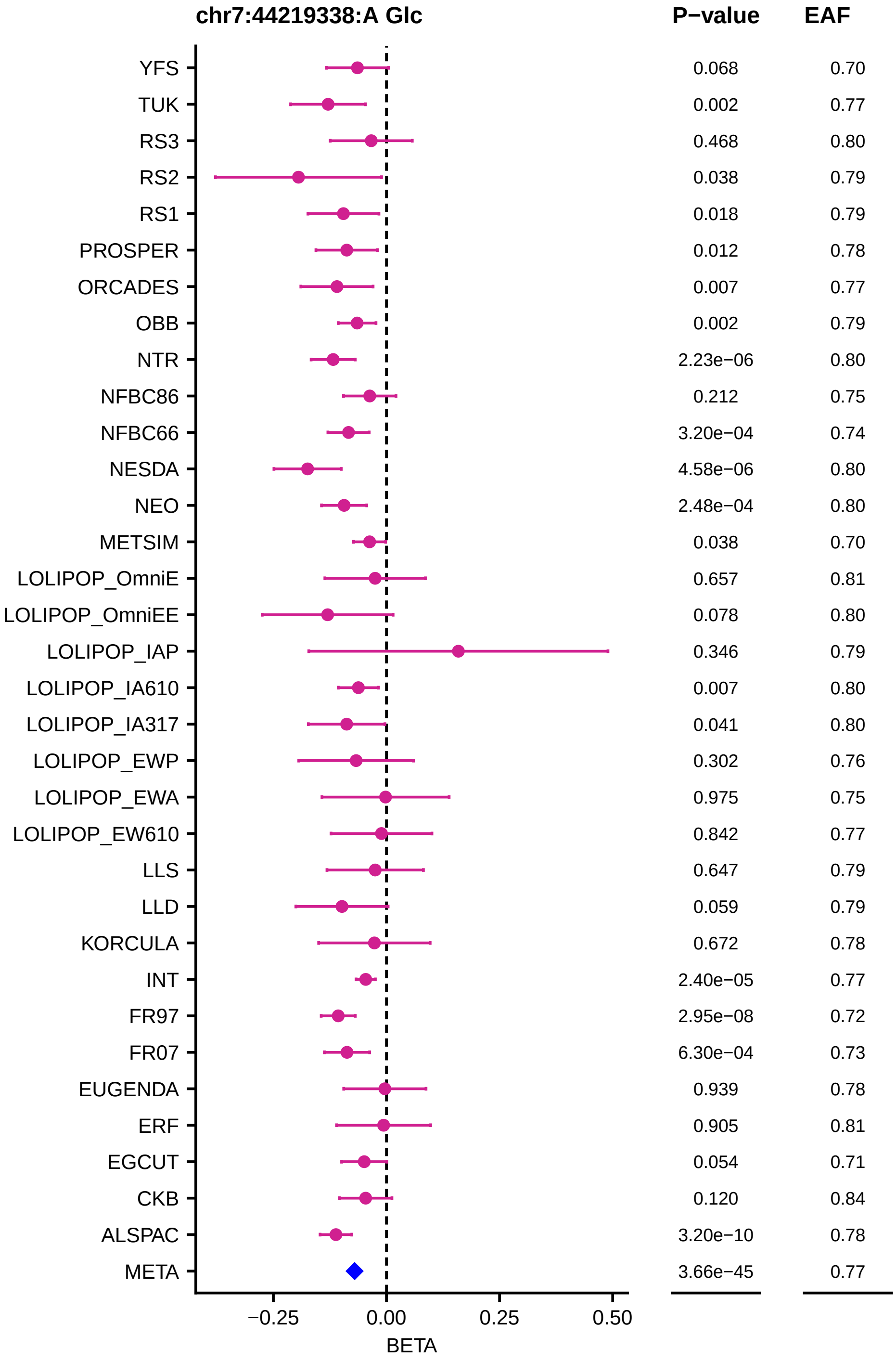
chr7:44219338:A Glc
P−value
EAF
YFS 0.068 0.70
TUK 0.002 0.77
RS3 0.468 0.80
RS2 0.038 0.79
RS1 0.018 0.79
PROSPER 0.012 0.78
ORCADES 0.007 0.77
OBB 0.002 0.79
NTR 2.23e−06 0.80
NFBC86 0.212 0.75
NFBC66 3.20e−04 0.74
NESDA 4.58e−06 0.80
NEO 2.48e−04 0.80
METSIM 0.038 0.70
LOLIPOP_OmniE 0.657 0.81
LOLIPOP_OmniEE 0.078 0.80
LOLIPOP_IAP 0.346 0.79
LOLIPOP_IA610 0.007 0.80
LOLIPOP_IA317 0.041 0.80
LOLIPOP_EWP 0.302 0.76
LOLIPOP_EWA 0.975 0.75
LOLIPOP_EW610 0.842 0.77
LLS 0.647 0.79
LLD 0.059 0.79
KORCULA 0.672 0.78
INT 2.40e−05 0.77
FR97 2.95e−08 0.72
FR07 6.30e−04 0.73
EUGENDA 0.939 0.78
ERF 0.905 0.81
EGCUT 0.054 0.71
CKB 0.120 0.84
ALSPAC 3.20e−10 0.78
META 3.66e−45 0.77
−0.25
0.00
0.25
0.50
BETA

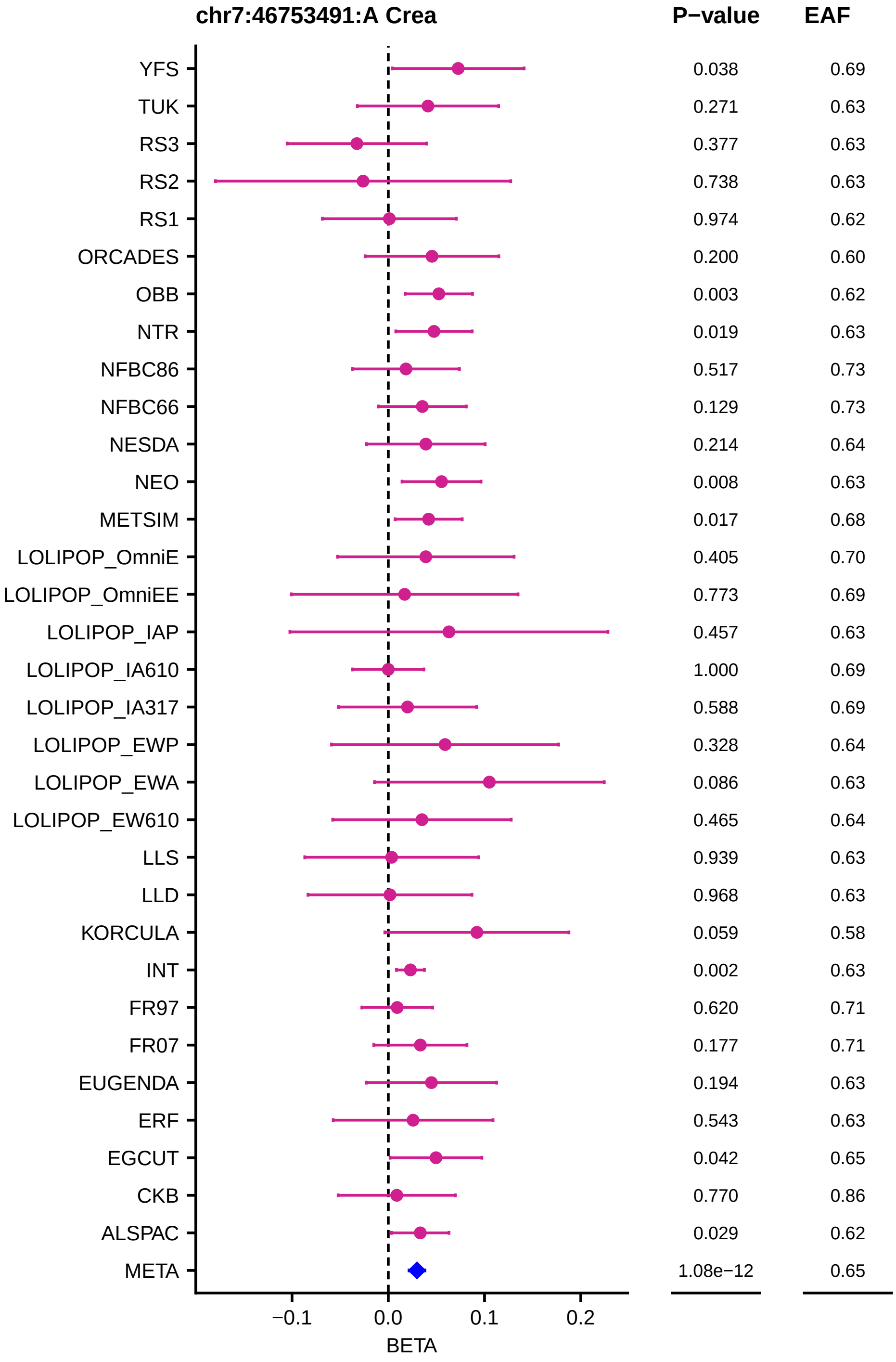
chr7:46753491:A Crea
P−value
EAF
YFS 0.038 0.69
TUK 0.271 0.63
RS3 0.377 0.63
RS2 0.738 0.63
RS1 0.974 0.62
ORCADES 0.200 0.60
OBB 0.003 0.62
NTR 0.019 0.63
NFBC86 0.517 0.73
NFBC66 0.129 0.73
NESDA 0.214 0.64
NEO 0.008 0.63
METSIM 0.017 0.68
LOLIPOP_OmniE 0.405 0.70
LOLIPOP_OmniEE 0.773 0.69
LOLIPOP_IAP 0.457 0.63
LOLIPOP_IA610 1.000 0.69
LOLIPOP_IA317 0.588 0.69
LOLIPOP_EWP 0.328 0.64
LOLIPOP_EWA 0.086 0.63
LOLIPOP_EW610 0.465 0.64
LLS 0.939 0.63
LLD 0.968 0.63
KORCULA 0.059 0.58
INT 0.002 0.63
FR97 0.620 0.71
FR07 0.177 0.71
EUGENDA 0.194 0.63
ERF 0.543 0.63
EGCUT 0.042 0.65
CKB 0.770 0.86
ALSPAC 0.029 0.62
META 1.08e−12 0.65
−0.1
0.0
0.1
0.2
BETA

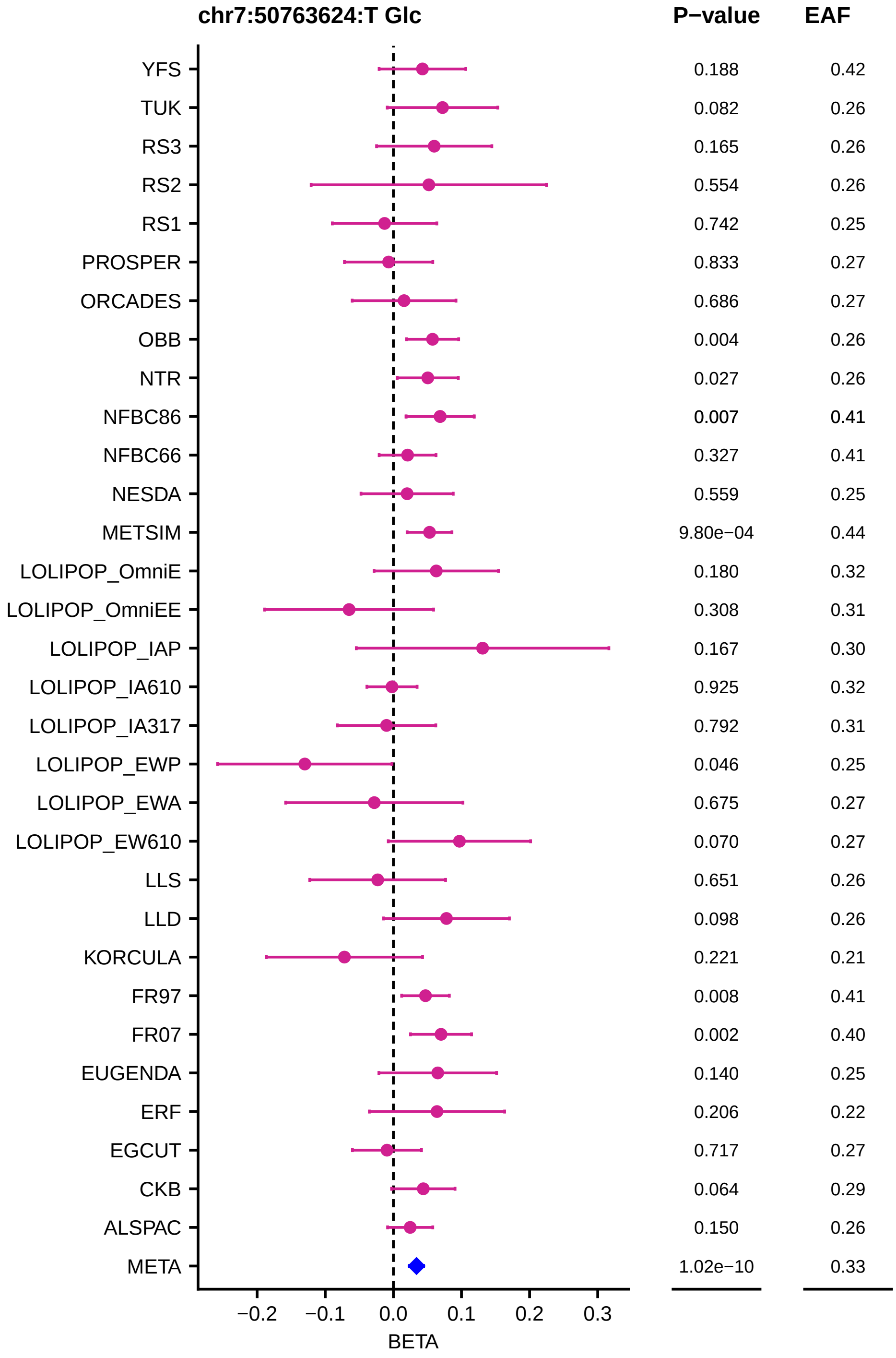
chr7:50763624:T Glc
P−value
EAF
YFS 0.188 0.42
TUK 0.082 0.26
RS3 0.165 0.26
RS2 0.554 0.26
RS1 0.742 0.25
PROSPER 0.833 0.27
ORCADES 0.686 0.27
OBB 0.004 0.26
NTR 0.027 0.26
NFBC86 0.007 0.41
NFBC66 0.327 0.41
NESDA 0.559 0.25
METSIM 9.80e−04 0.44
LOLIPOP_OmniE 0.180 0.32
LOLIPOP_OmniEE 0.308 0.31
LOLIPOP_IAP 0.167 0.30
LOLIPOP_IA610 0.925 0.32
LOLIPOP_IA317 0.792 0.31
LOLIPOP_EWP 0.046 0.25
LOLIPOP_EWA 0.675 0.27
LOLIPOP_EW610 0.070 0.27
LLS 0.651 0.26
LLD 0.098 0.26
KORCULA 0.221 0.21
FR97 0.008 0.41
FR07 0.002 0.40
EUGENDA 0.140 0.25
ERF 0.206 0.22
EGCUT 0.717 0.27
CKB 0.064 0.29
ALSPAC 0.150 0.26
META 1.02e−10 0.33
−0.2 −0.1 0.0 0.1 0.2 0.3
BETA

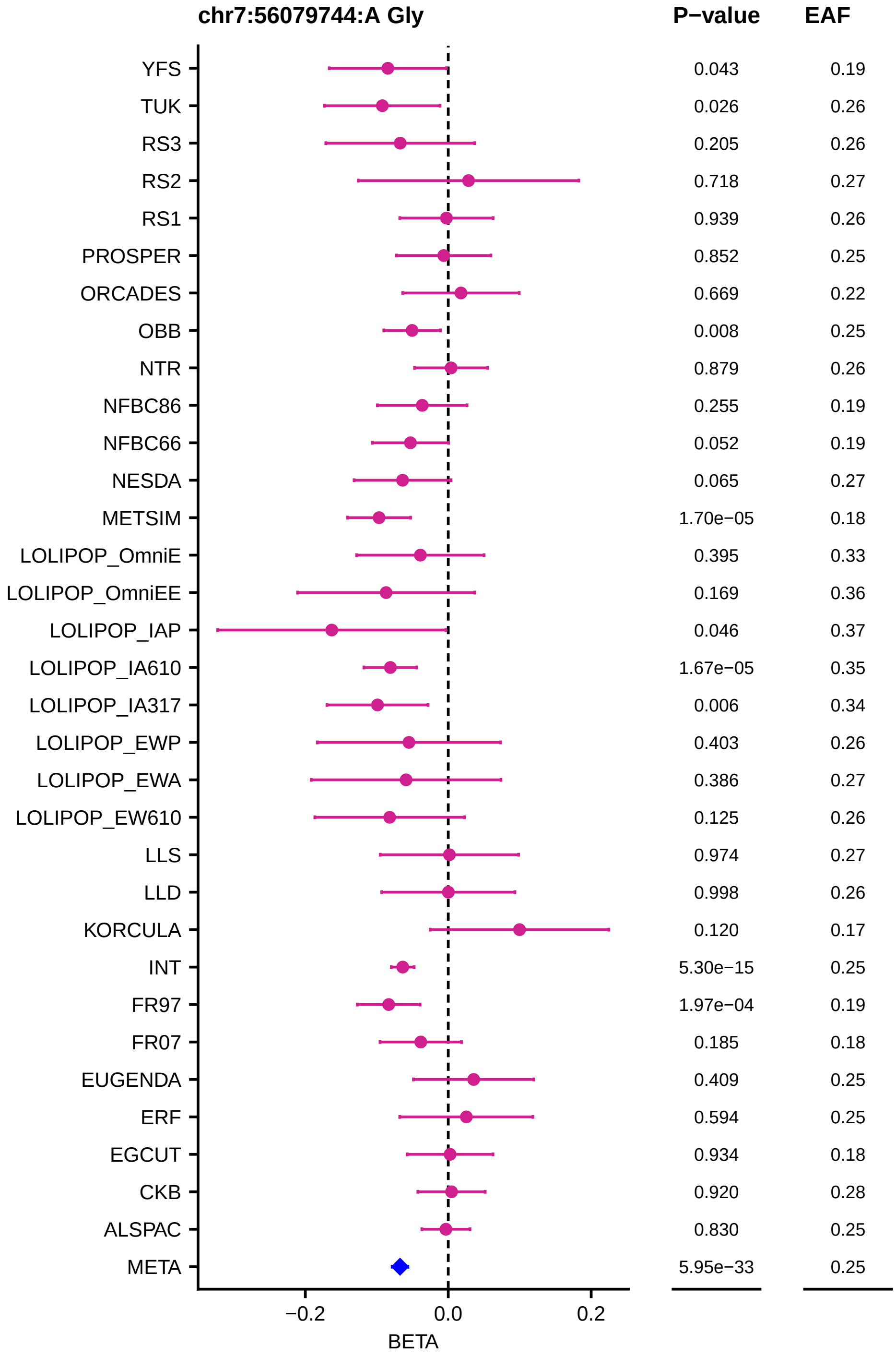

chr7:56079744:A Gly
P−value
EAF
YFS 0.043 0.19
TUK 0.026 0.26
RS3 0.205 0.26
RS2 0.718 0.27
RS1 0.939 0.26
PROSPER 0.852 0.25
ORCADES 0.669 0.22
OBB 0.008 0.25
NTR 0.879 0.26
NFBC86 0.255 0.19
NFBC66 0.052 0.19
NESDA 0.065 0.27
METSIM 1.70e−05 0.18
LOLIPOP_OmniE 0.395 0.33
LOLIPOP_OmniEE 0.169 0.36
LOLIPOP_IAP 0.046 0.37
LOLIPOP_IA610 1.67e−05 0.35
LOLIPOP_IA317 0.006 0.34
LOLIPOP_EWP 0.403 0.26
LOLIPOP_EWA 0.386 0.27
LOLIPOP_EW610 0.125 0.26
LLS 0.974 0.27
LLD 0.998 0.26
KORCULA 0.120 0.17
INT 5.30e−15 0.25
FR97 1.97e−04 0.19
FR07 0.185 0.18
EUGENDA 0.409 0.25
ERF 0.594 0.25
EGCUT 0.934 0.18
CKB 0.920 0.28
ALSPAC 0.830 0.25
META 5.95e−33 0.25
−0.2
0.0
0.2
BETA

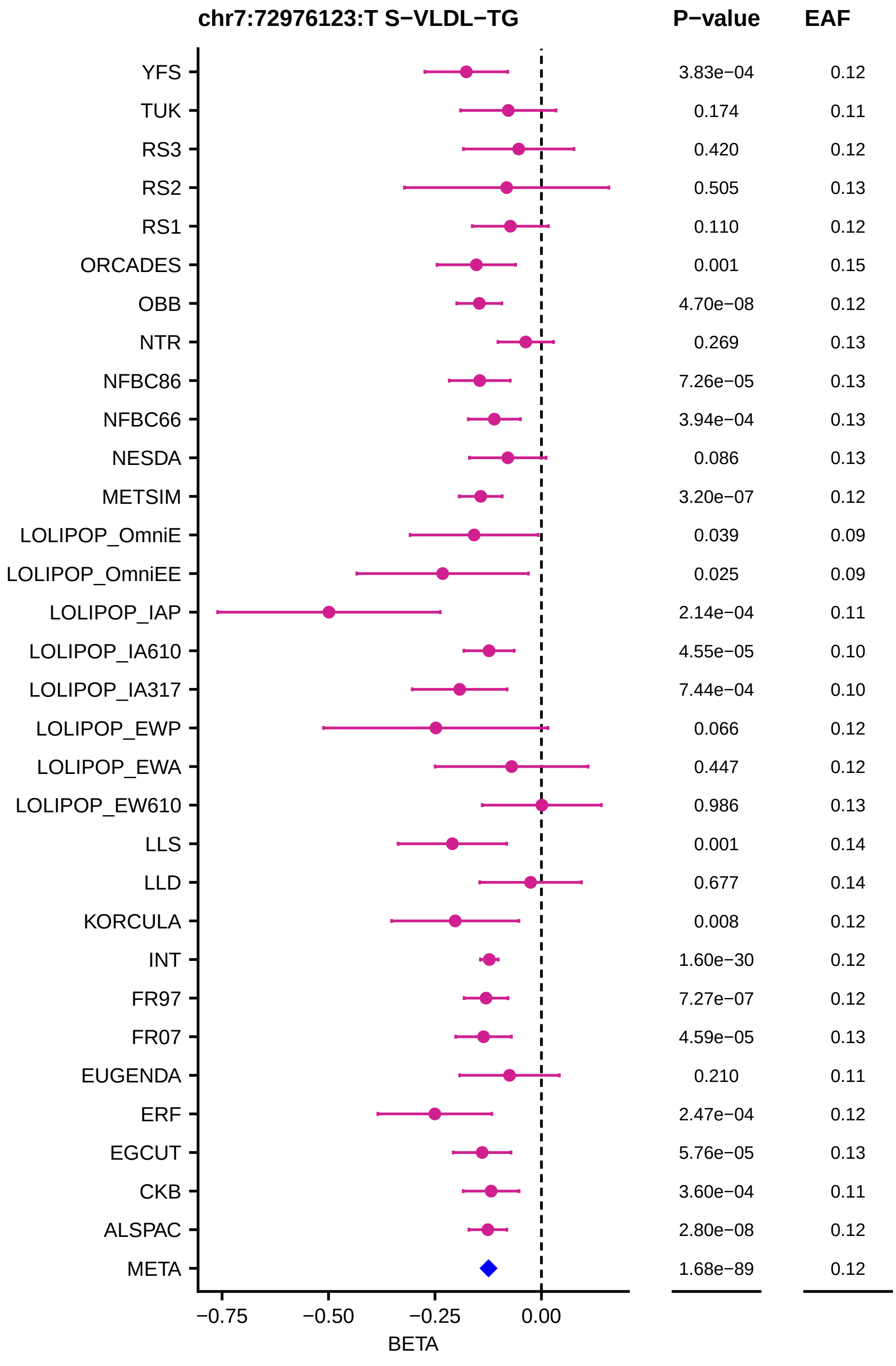
chr7:72976123:T S−VLDL−TG
P−value
EAF
YFS 3.83e−04 0.12
TUK 0.174 0.11
RS3 0.420 0.12
RS2 0.505 0.13
RS1 0.110 0.12
ORCADES 0.001 0.15
OBB 4.70e−08 0.12
NTR 0.269 0.13
NFBC86 7.26e−05 0.13
NFBC66 3.94e−04 0.13
NESDA 0.086 0.13
METSIM 3.20e−07 0.12
LOLIPOP_OmniE 0.039 0.09
LOLIPOP_OmniEE 0.025 0.09
LOLIPOP_IAP 2.14e−04 0.11
LOLIPOP_IA610 4.55e−05 0.10
LOLIPOP_IA317 7.44e−04 0.10
LOLIPOP_EWP 0.066 0.12
LOLIPOP_EWA 0.447 0.12
LOLIPOP_EW610 0.986 0.13
LLS 0.001 0.14
LLD 0.677 0.14
KORCULA 0.008 0.12
INT 1.60e−30 0.12
FR97 7.27e−07 0.12
FR07 4.59e−05 0.13
EUGENDA 0.210 0.11
ERF 2.47e−04 0.12
EGCUT 5.76e−05 0.13
CKB 3.60e−04 0.11
ALSPAC 2.80e−08 0.12
META 1.68e−89 0.12
−0.75
−0.50
−0.25
0.00
BETA

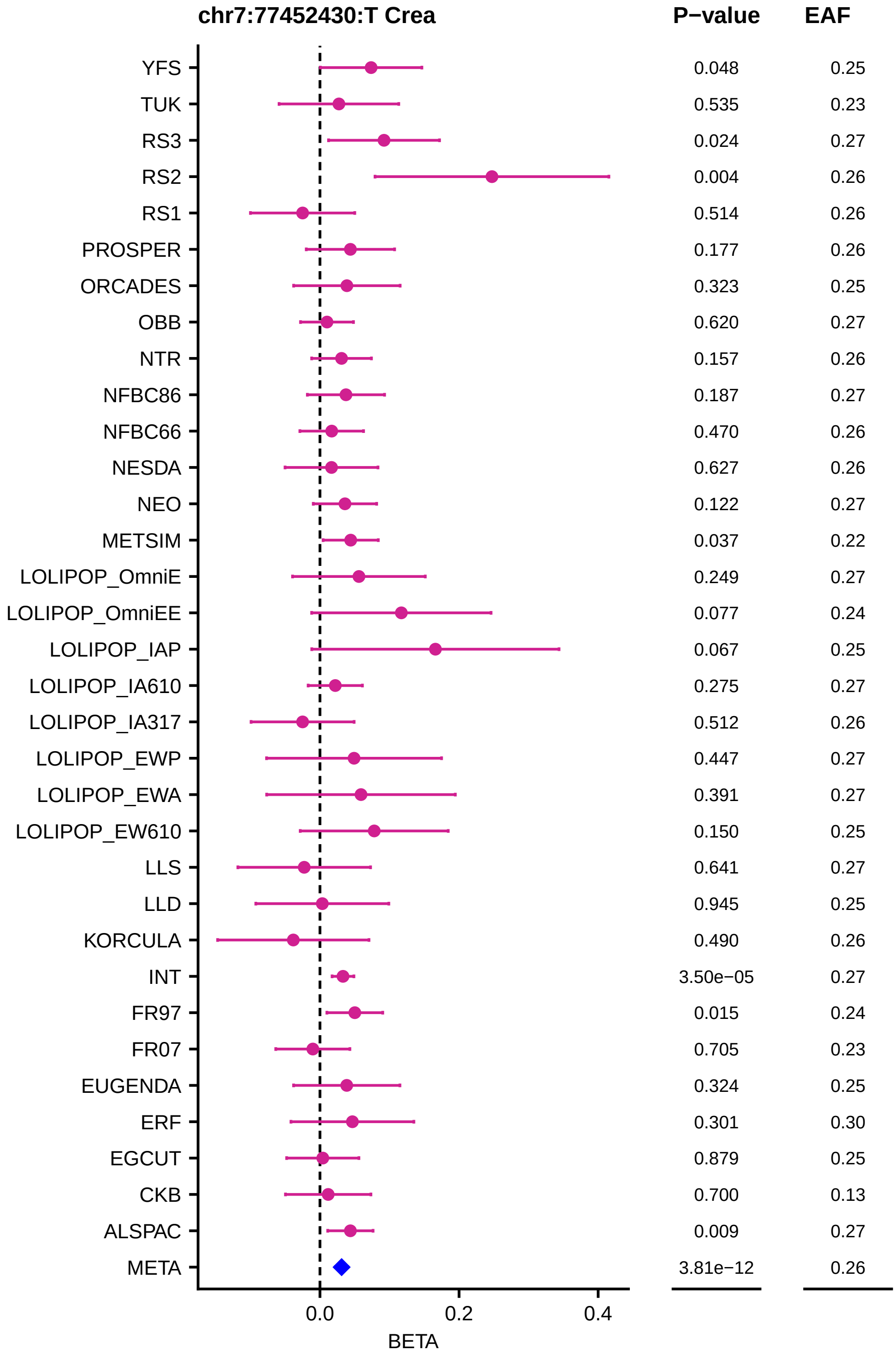

chr7:77452430:T Crea
P−value
EAF
YFS
0.048
0.25
TUK
0.535
0.23
RS3
0.024
0.27
RS2
0.004
0.26
RS1
0.514
0.26
PROSPER
0.177
0.26
ORCADES
0.323
0.25
OBB
0.620
0.27
NTR
0.157
0.26
NFBC86
0.187
0.27
NFBC66
0.470
0.26
NESDA
0.627
0.26
NEO
0.122
0.27
METSIM
0.037
0.22
LOLIPOP_OmniE
0.249
0.27
LOLIPOP_OmniEE
0.077
0.24
LOLIPOP_IAP
0.067
0.25
LOLIPOP_IA610
0.275
0.27
LOLIPOP_IA317
0.512
0.26
LOLIPOP_EWP
0.447
0.27
LOLIPOP_EWA
0.391
0.27
LOLIPOP_EW610
0.150
0.25
LLS
0.641
0.27
LLD
0.945
0.25
KORCULA
0.490
0.26
INT
3.50e−05
0.27
FR97
0.015
0.24
FR07
0.705
0.23
EUGENDA
0.324
0.25
ERF
0.301
0.30
EGCUT
0.879
0.25
CKB
0.700
0.13
ALSPAC
0.009
0.27
META
3.81e−12
0.26
0.0
0.2
0.4
BETA

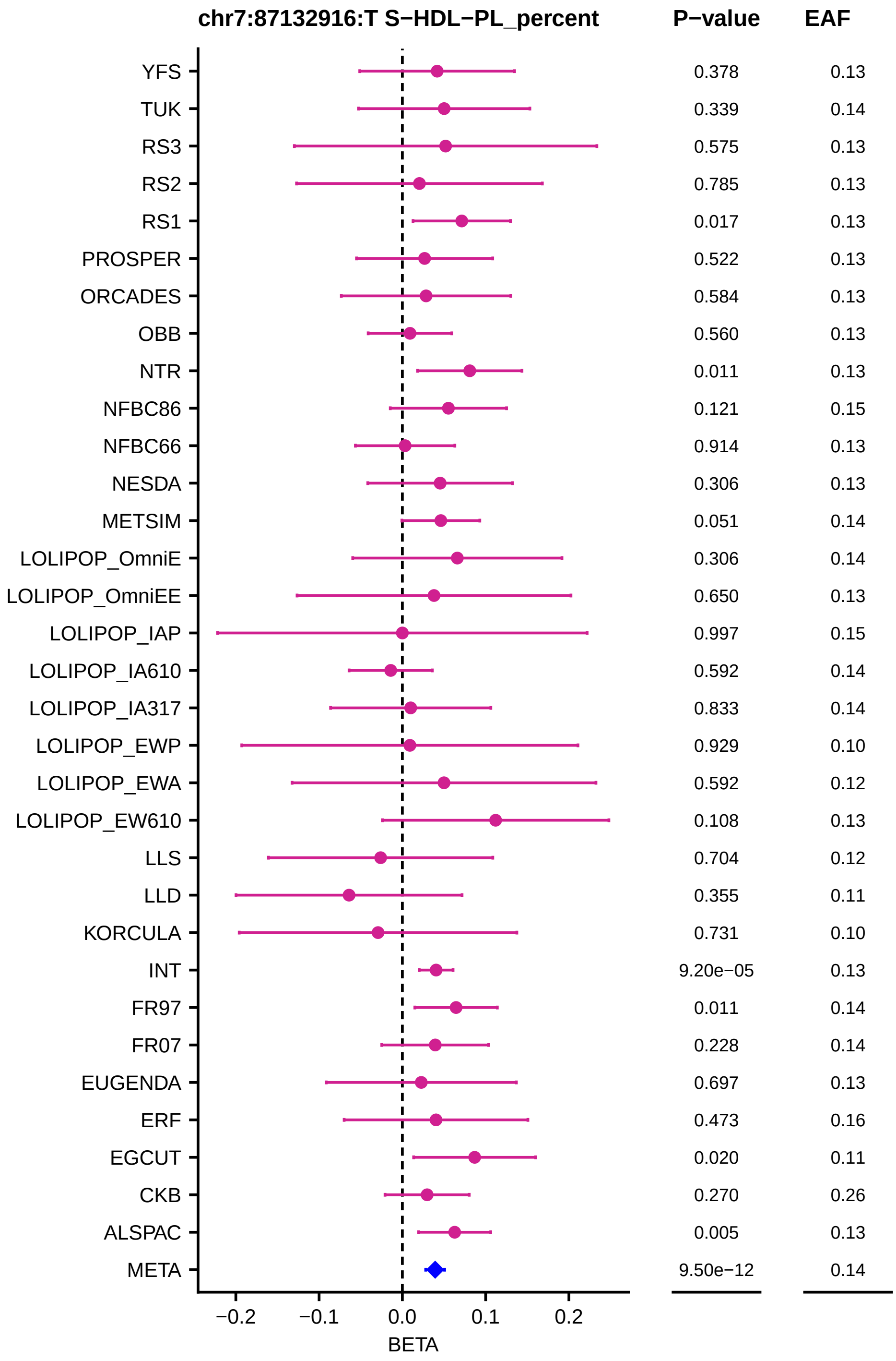
chr7:87132916:T S−HDL−PL_percent
P−value
EAF
YFS 0.378 0.13
TUK 0.339 0.14
RS3 0.575 0.13
RS2 0.785 0.13
RS1 0.017 0.13
PROSPER 0.522 0.13
ORCADES 0.584 0.13
OBB 0.560 0.13
NTR 0.011 0.13
NFBC86 0.121 0.15
NFBC66 0.914 0.13
NESDA 0.306 0.13
METSIM 0.051 0.14
LOLIPOP_OmniE 0.306 0.14
LOLIPOP_OmniEE 0.650 0.13
LOLIPOP_IAP 0.997 0.15
LOLIPOP_IA610 0.592 0.14
LOLIPOP_IA317 0.833 0.14
LOLIPOP_EWP 0.929 0.10
LOLIPOP_EWA 0.592 0.12
LOLIPOP_EW610 0.108 0.13
LLS 0.704 0.12
LLD 0.355 0.11
KORCULA 0.731 0.10
INT 9.20e−05 0.13
FR97 0.011 0.14
FR07 0.228 0.14
EUGENDA 0.697 0.13
ERF 0.473 0.16
EGCUT 0.020 0.11
CKB 0.270 0.26
ALSPAC 0.005 0.13
META 9.50e−12 0.14
−0.2
−0.1
0.0
0.1
0.2
BETA

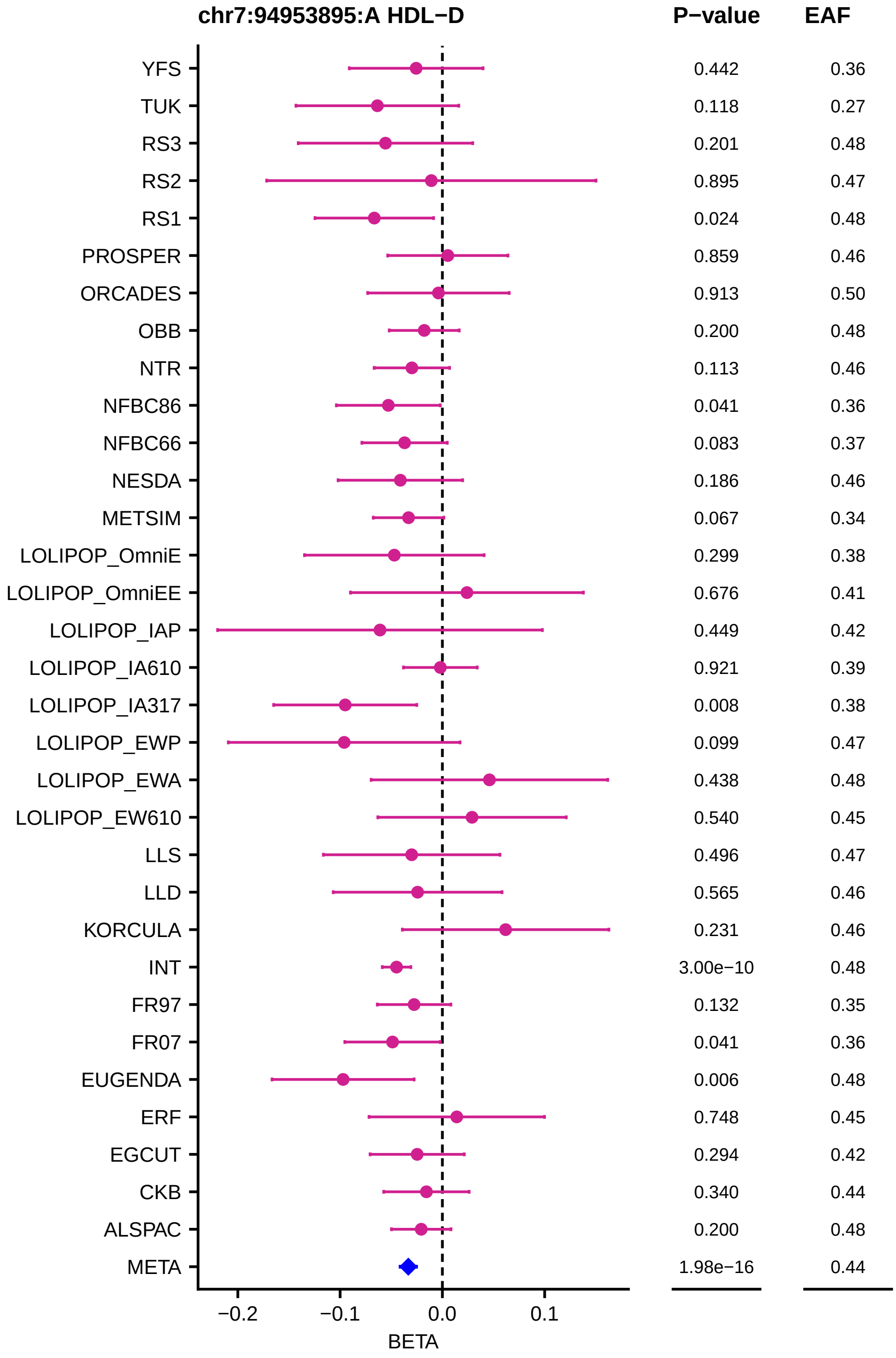

chr7:94953895:A HDL−D
P−value
EAF
YFS
0.442
0.36
TUK
0.118
0.27
RS3
0.201
0.48
RS2
0.895
0.47
RS1
0.024
0.48
PROSPER
0.859
0.46
ORCADES
0.913
0.50
OBB
0.200
0.48
NTR
0.113
0.46
NFBC86
0.041
0.36
NFBC66
0.083
0.37
NESDA
0.186
0.46
METSIM
0.067
0.34
LOLIPOP_OmniE
0.299
0.38
LOLIPOP_OmniEE
0.676
0.41
LOLIPOP_IAP
0.449
0.42
LOLIPOP_IA610
0.921
0.39
LOLIPOP_IA317
0.008
0.38
LOLIPOP_EWP
0.099
0.47
LOLIPOP_EWA
0.438
0.48
LOLIPOP_EW610
0.540
0.45
LLS
0.496
0.47
LLD
0.565
0.46
KORCULA
0.231
0.46
INT
3.00e−10
0.48
FR97
0.132
0.35
FR07
0.041
0.36
EUGENDA
0.006
0.48
ERF
0.748
0.45
EGCUT
0.294
0.42
CKB
0.340
0.44
ALSPAC
0.200
0.48
META
1.98e−16
0.44
−0.2
−0.1
0.0
0.1
BETA

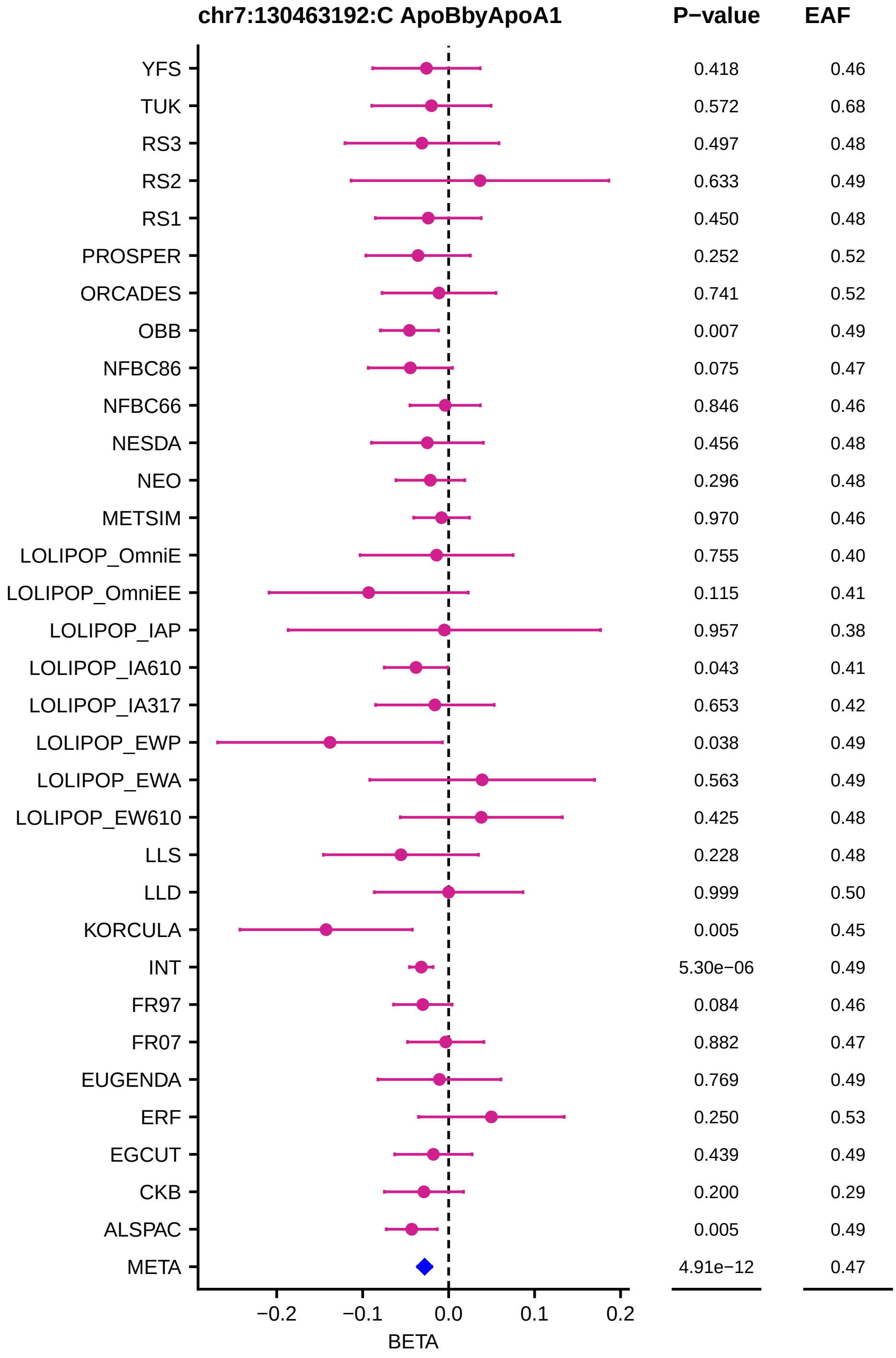
chr7:130463192:C ApoBbyApoA1
P−value
EAF
YFS 0.418 0.46
TUK 0.572 0.68
RS3 0.497 0.48
RS2 0.633 0.49
RS1 0.450 0.48
PROSPER 0.252 0.52
ORCADES 0.741 0.52
OBB 0.007 0.49
NFBC86 0.075 0.47
NFBC66 0.846 0.46
NESDA 0.456 0.48
NEO 0.296 0.48
METSIM 0.970 0.46
LOLIPOP_OmniE 0.755 0.40
LOLIPOP_OmniEE 0.115 0.41
LOLIPOP_IAP 0.957 0.38
LOLIPOP_IA610 0.043 0.41
LOLIPOP_IA317 0.653 0.42
LOLIPOP_EWP 0.038 0.49
LOLIPOP_EWA 0.563 0.49
LOLIPOP_EW610 0.425 0.48
LLS 0.228 0.48
LLD 0.999 0.50
KORCULA 0.005 0.45
INT 5.30e−06 0.49
FR97 0.084 0.46
FR07 0.882 0.47
EUGENDA 0.769 0.49
ERF 0.250 0.53
EGCUT 0.439 0.49
CKB 0.200 0.29
ALSPAC 0.005 0.49
META 4.91e−12 0.47
−0.2 −0.1 0.0 0.1 0.2
BETA

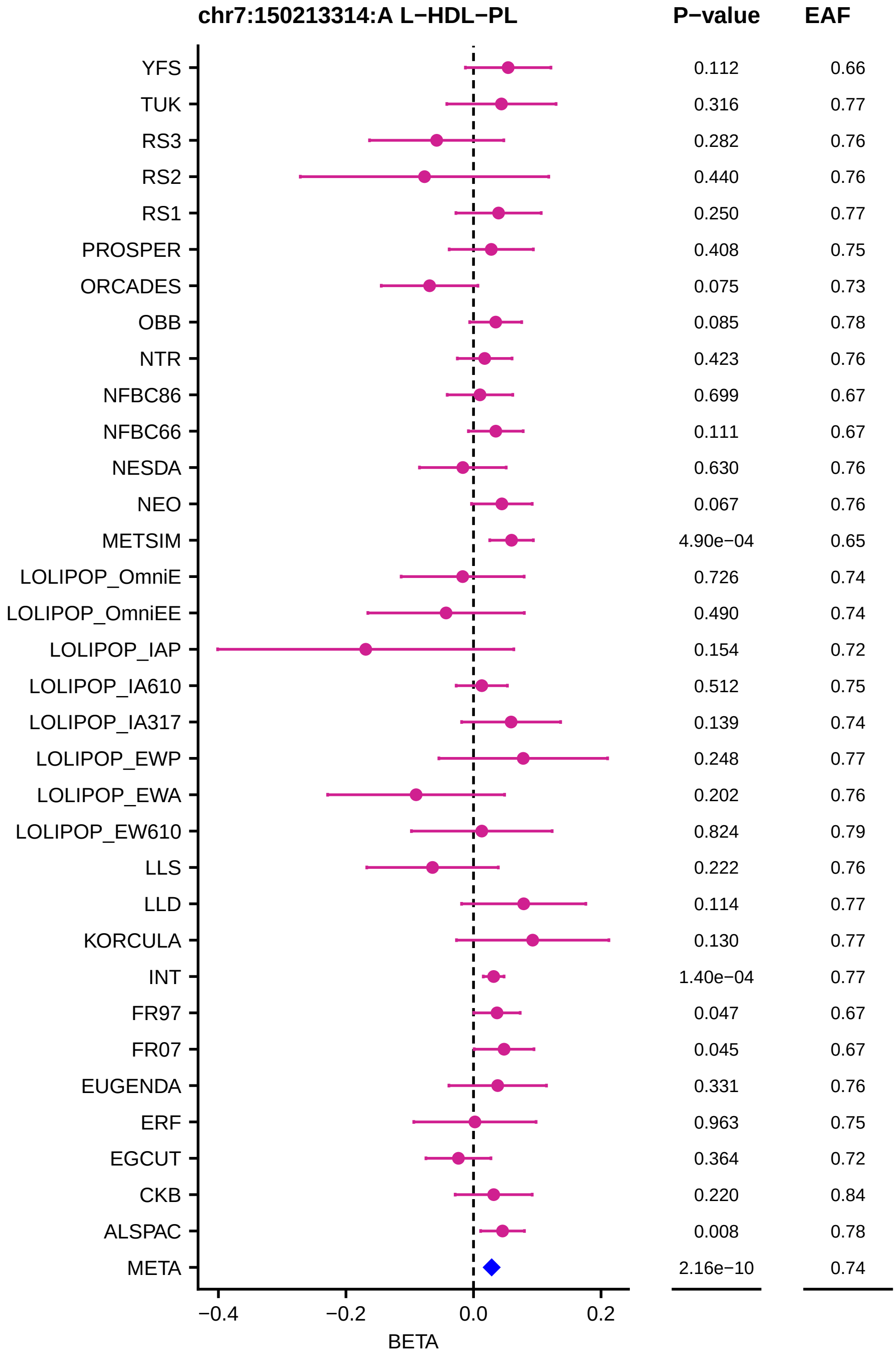
chr7:150213314:A L−HDL−PL
P−value
EAF
YFS 0.112 0.66
TUK 0.316 0.77
RS3 0.282 0.76
RS2 0.440 0.76
RS1 0.250 0.77
PROSPER 0.408 0.75
ORCADES 0.075 0.73
OBB 0.085 0.78
NTR 0.423 0.76
NFBC86 0.699 0.67
NFBC66 0.111 0.67
NESDA 0.630 0.76
NEO 0.067 0.76
METSIM 4.90e−04 0.65
LOLIPOP_OmniE 0.726 0.74
LOLIPOP_OmniEE 0.490 0.74
LOLIPOP_IAP 0.154 0.72
LOLIPOP_IA610 0.512 0.75
LOLIPOP_IA317 0.139 0.74
LOLIPOP_EWP 0.248 0.77
LOLIPOP_EWA 0.202 0.76
LOLIPOP_EW610 0.824 0.79
LLS 0.222 0.76
LLD 0.114 0.77
KORCULA 0.130 0.77
INT 1.40e−04 0.77
FR97 0.047 0.67
FR07 0.045 0.67
EUGENDA 0.331 0.76
ERF 0.963 0.75
EGCUT 0.364 0.72
CKB 0.220 0.84
ALSPAC 0.008 0.78
META 2.16e−10 0.74
−0.4
−0.2
0.0
0.2
BETA

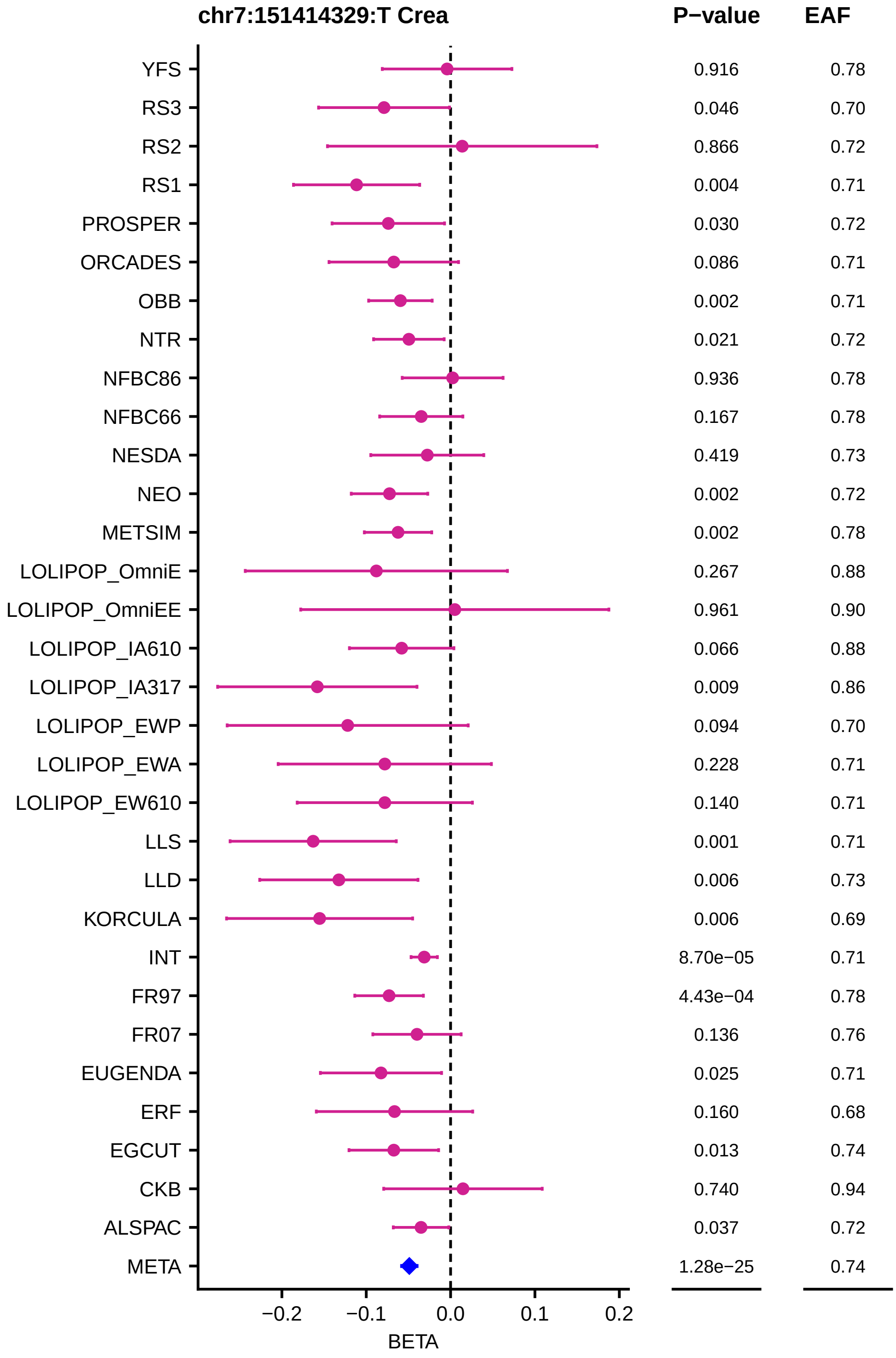
chr7:151414329:T Crea
P−value
EAF
YFS 0.916 0.78
RS3 0.046 0.70
RS2 0.866 0.72
RS1 0.004 0.71
PROSPER 0.030 0.72
ORCADES 0.086 0.71
OBB 0.002 0.71
NTR 0.021 0.72
NFBC86 0.936 0.78
NFBC66 0.167 0.78
NESDA 0.419 0.73
NEO 0.002 0.72
METSIM 0.002 0.78
LOLIPOP_OmniE 0.267 0.88
LOLIPOP_OmniEE 0.961 0.90
LOLIPOP_IA610 0.066 0.88
LOLIPOP_IA317 0.009 0.86
LOLIPOP_EWP 0.094 0.70
LOLIPOP_EWA 0.228 0.71
LOLIPOP_EW610 0.140 0.71
LLS 0.001 0.71
LLD 0.006 0.73
KORCULA 0.006 0.69
INT 8.70e−05 0.71
FR97 4.43e−04 0.78
FR07 0.136 0.76
EUGENDA 0.025 0.71
ERF 0.160 0.68
EGCUT 0.013 0.74
CKB 0.740 0.94
ALSPAC 0.037 0.72
META 1.28e−25 0.74
−0.2
−0.1
0.0
0.1
0.2
BETA

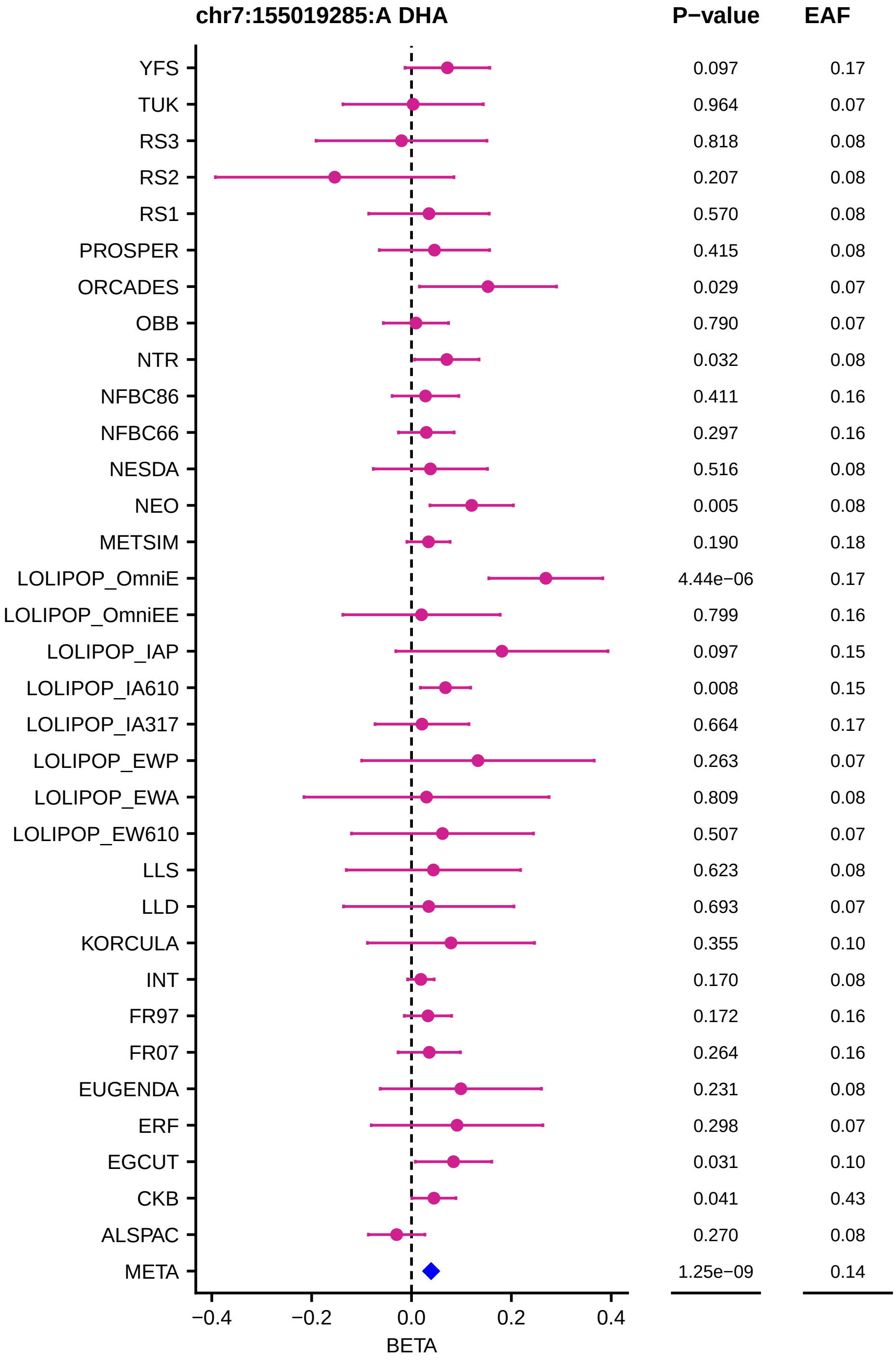
chr7:155019285:A DHA
P−value
EAF
YFS 0.097 0.17
TUK 0.964 0.07
RS3 0.818 0.08
RS2 0.207 0.08
RS1 0.570 0.08
PROSPER 0.415 0.08
ORCADES 0.029 0.07
OBB 0.790 0.07
NTR 0.032 0.08
NFBC86 0.411 0.16
NFBC66 0.297 0.16
NESDA 0.516 0.08
NEO 0.005 0.08
METSIM 0.190 0.18
LOLIPOP_OmniE 4.44e−06 0.17
LOLIPOP_OmniEE 0.799 0.16
LOLIPOP_IAP 0.097 0.15
LOLIPOP_IA610 0.008 0.15
LOLIPOP_IA317 0.664 0.17
LOLIPOP_EWP 0.263 0.07
LOLIPOP_EWA 0.809 0.08
LOLIPOP_EW610 0.507 0.07
LLS 0.623 0.08
LLD 0.693 0.07
KORCULA 0.355 0.10
INT 0.170 0.08
FR97 0.172 0.16
FR07 0.264 0.16
EUGENDA 0.231 0.08
ERF 0.298 0.07
EGCUT 0.031 0.10
CKB 0.041 0.43
ALSPAC 0.270 0.08
META 1.25e−09 0.14
−0.4
−0.2
0.0
0.2
0.4
BETA

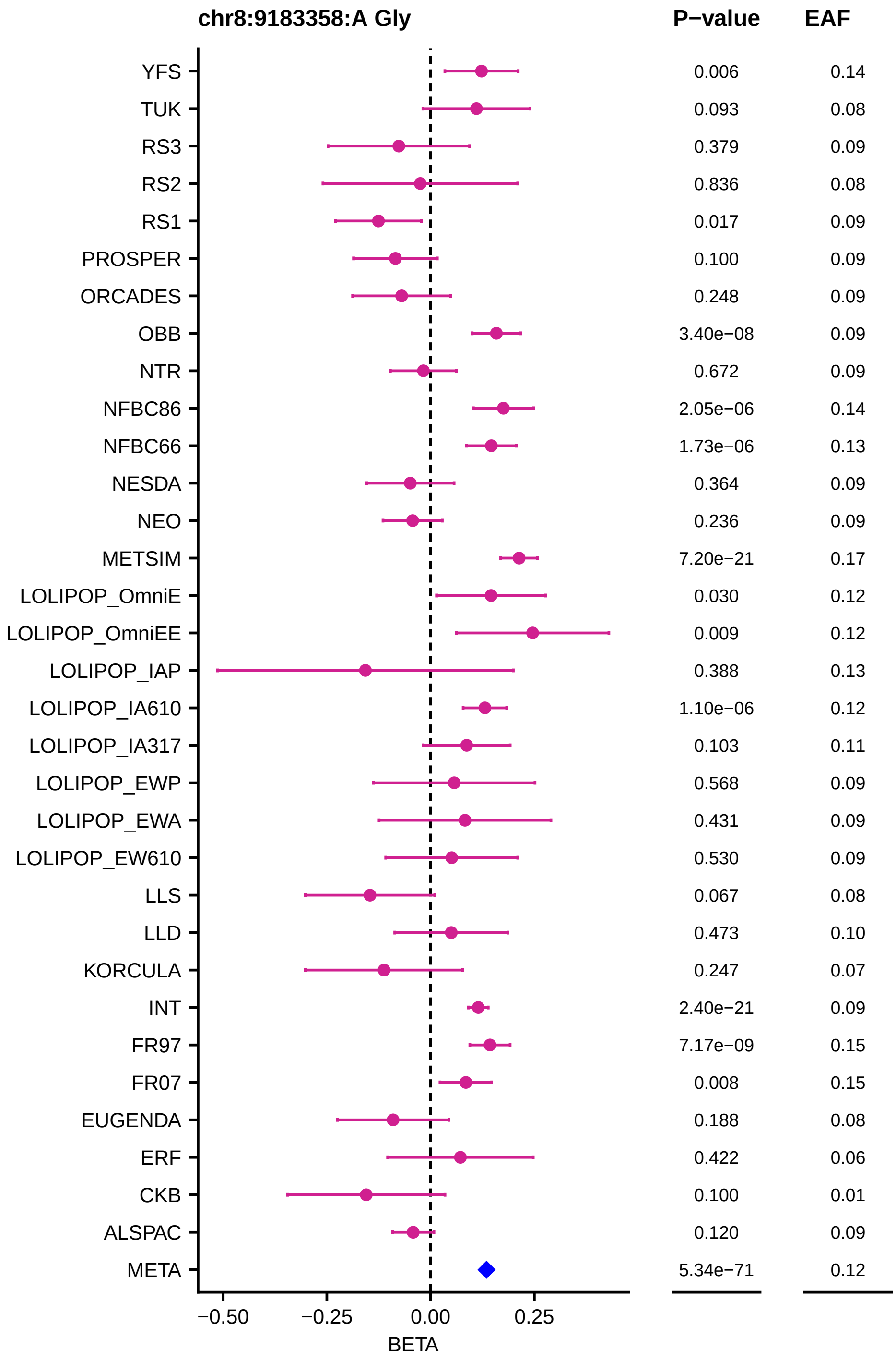
chr8:9183358:A Gly
P−value
EAF
YFS
TUK
RS3
RS2
RS1
PROSPER
ORCADES
OBB
NTR
NFBC86
NFBC66
NESDA
NEO
METSIM
LOLIPOP_OmniE
LOLIPOP_OmniEE
LOLIPOP_IAP
LOLIPOP_IA610
LOLIPOP_IA317
LOLIPOP_EWP
LOLIPOP_EWA
LOLIPOP_EW610
LLS
LLD
KORCULA
INT
FR97
FR07
EUGENDA
ERF
CKB
ALSPAC
META
0.006 0.14
0.093 0.08
0.379 0.09
0.836 0.08
0.017 0.09
0.100 0.09
0.248 0.09
3.40e−08 0.09
0.672 0.09
2.05e−06 0.14
1.73e−06 0.13
0.364 0.09
0.236 0.09
7.20e−21 0.17
0.030 0.12
0.009 0.12
0.388 0.13
1.10e−06 0.12
0.103 0.11
0.568 0.09
0.431 0.09
0.530 0.09
0.067 0.08
0.473 0.10
0.247 0.07
2.40e−21 0.09
7.17e−09 0.15
0.008 0.15
0.188 0.08
0.422 0.06
0.100 0.01
0.120 0.09
5.34e−71 0.12
−0.50
−0.25
0.00
0.25
BETA

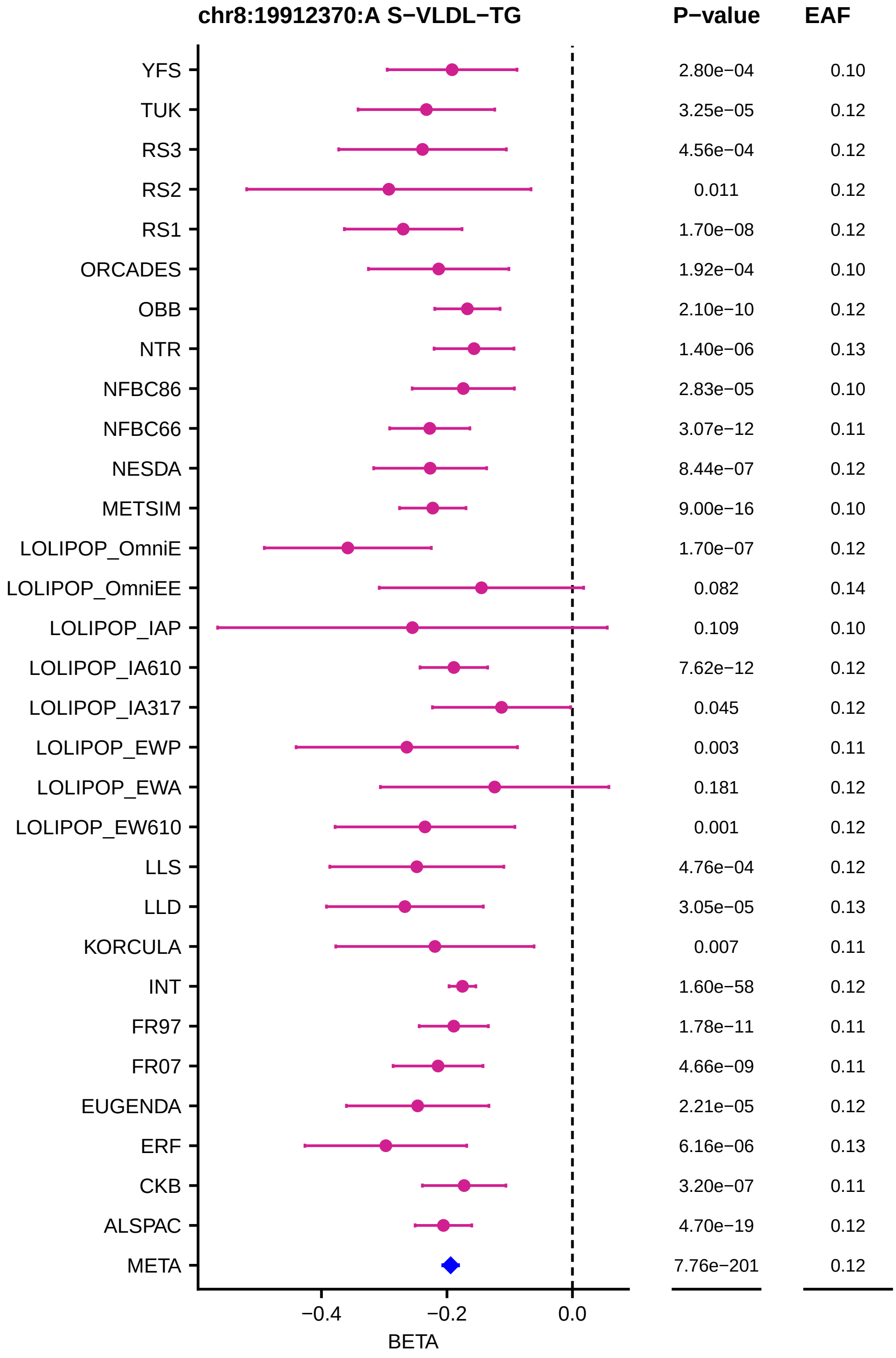
chr8:19912370:A S−VLDL−TG
P−value
EAF
YFS 2.80e−04 0.10
TUK 3.25e−05 0.12
RS3 4.56e−04 0.12
RS2 0.011 0.12
RS1 1.70e−08 0.12
ORCADES 1.92e−04 0.10
OBB 2.10e−10 0.12
NTR 1.40e−06 0.13
NFBC86 2.83e−05 0.10
NFBC66 3.07e−12 0.11
NESDA 8.44e−07 0.12
METSIM 9.00e−16 0.10
LOLIPOP_OmniE 1.70e−07 0.12
LOLIPOP_OmniEE 0.082 0.14
LOLIPOP_IAP 0.109 0.10
LOLIPOP_IA610 7.62e−12 0.12
LOLIPOP_IA317 0.045 0.12
LOLIPOP_EWP 0.003 0.11
LOLIPOP_EWA 0.181 0.12
LOLIPOP_EW610 0.001 0.12
LLS 4.76e−04 0.12
LLD 3.05e−05 0.13
KORCULA 0.007 0.11
INT 1.60e−58 0.12
FR97 1.78e−11 0.11
FR07 4.66e−09 0.11
EUGENDA 2.21e−05 0.12
ERF 6.16e−06 0.13
CKB 3.20e−07 0.11
ALSPAC 4.70e−19 0.12
META 7.76e−201 0.12
−0.4
−0.2
0.0
BETA

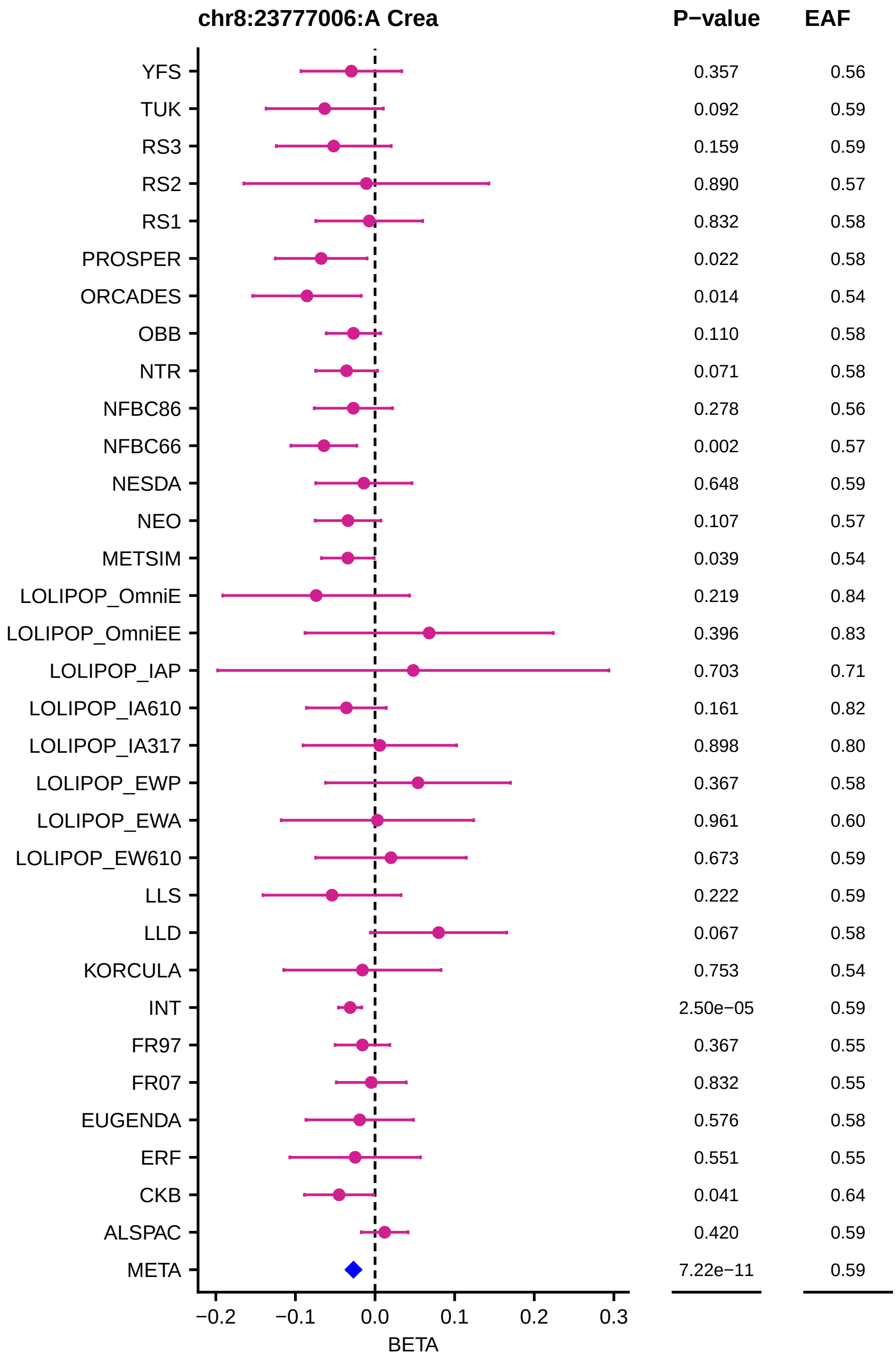
chr8:23777006:A Crea
P−value
EAF
YFS 0.357 0.56
TUK 0.092 0.59
RS3 0.159 0.59
RS2 0.890 0.57
RS1 0.832 0.58
PROSPER 0.022 0.58
ORCADES 0.014 0.54
OBB 0.110 0.58
NTR 0.071 0.58
NFBC86 0.278 0.56
NFBC66 0.002 0.57
NESDA 0.648 0.59
NEO 0.107 0.57
METSIM 0.039 0.54
LOLIPOP_OmniE 0.219 0.84
LOLIPOP_OmniEE 0.396 0.83
LOLIPOP_IAP 0.703 0.71
LOLIPOP_IA610 0.161 0.82
LOLIPOP_IA317 0.898 0.80
LOLIPOP_EWP 0.367 0.58
LOLIPOP_EWA 0.961 0.60
LOLIPOP_EW610 0.673 0.59
LLS 0.222 0.59
LLD 0.067 0.58
KORCULA 0.753 0.54
INT 2.50e−05 0.59
FR97 0.367 0.55
FR07 0.832 0.55
EUGENDA 0.576 0.58
ERF 0.551 0.55
CKB 0.041 0.64
ALSPAC 0.420 0.59
META 7.22e−11 0.59
−0.2 −0.1 0.0 0.1 0.2 0.3
BETA

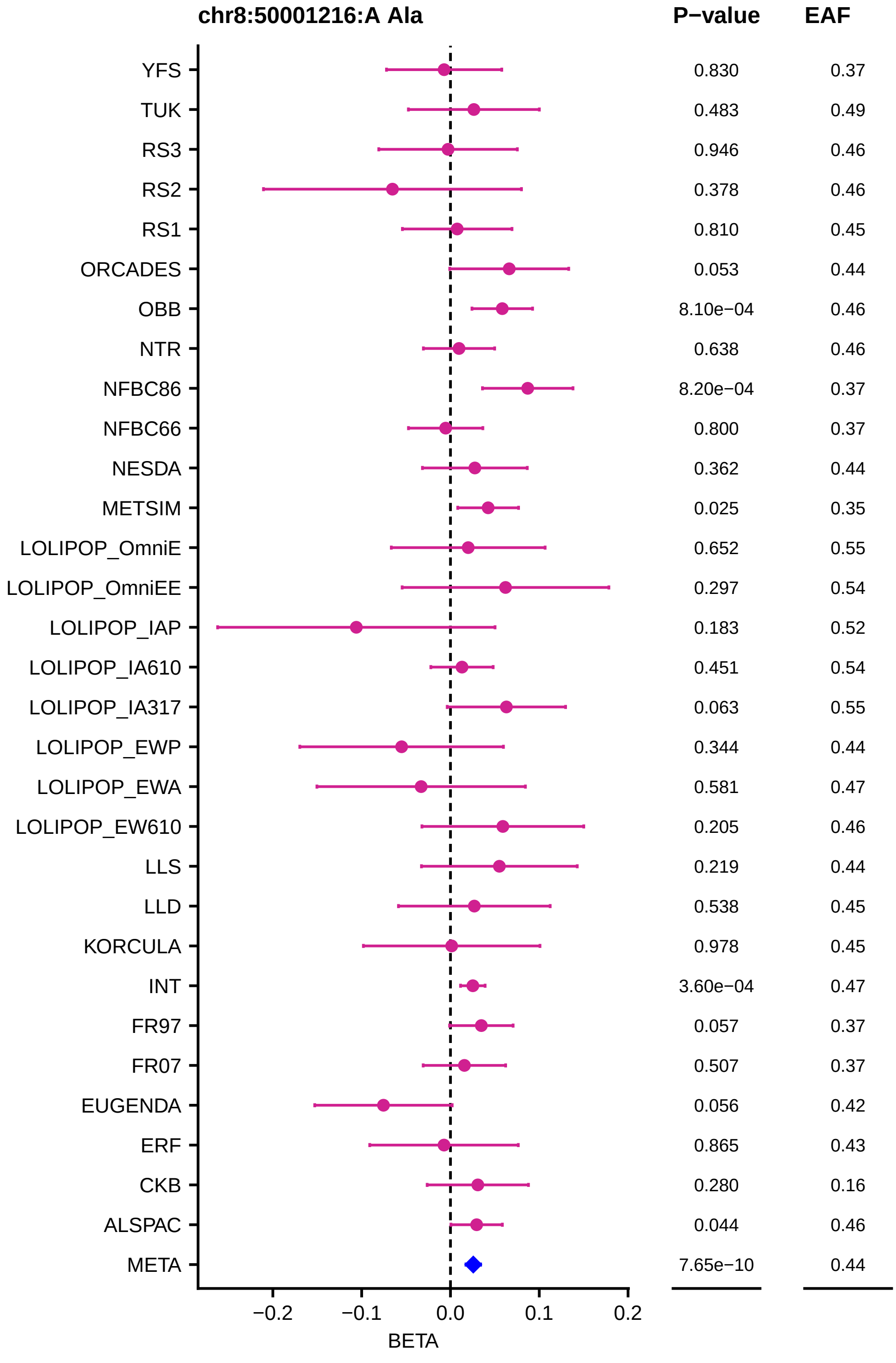
chr8:50001216:A Ala
P−value
EAF
YFS 0.830 0.37
TUK 0.483 0.49
RS3 0.946 0.46
RS2 0.378 0.46
RS1 0.810 0.45
ORCADES 0.053 0.44
OBB 8.10e−04 0.46
NTR 0.638 0.46
NFBC86 8.20e−04 0.37
NFBC66 0.800 0.37
NESDA 0.362 0.44
METSIM 0.025 0.35
LOLIPOP_OmniE 0.652 0.55
LOLIPOP_OmniEE 0.297 0.54
LOLIPOP_IAP 0.183 0.52
LOLIPOP_IA610 0.451 0.54
LOLIPOP_IA317 0.063 0.55
LOLIPOP_EWP 0.344 0.44
LOLIPOP_EWA 0.581 0.47
LOLIPOP_EW610 0.205 0.46
LLS 0.219 0.44
LLD 0.538 0.45
KORCULA 0.978 0.45
INT 3.60e−04 0.47
FR97 0.057 0.37
FR07 0.507 0.37
EUGENDA 0.056 0.42
ERF 0.865 0.43
CKB 0.280 0.16
ALSPAC 0.044 0.46
META 7.65e−10 0.44
−0.2
−0.1
0.0
0.1
0.2
BETA

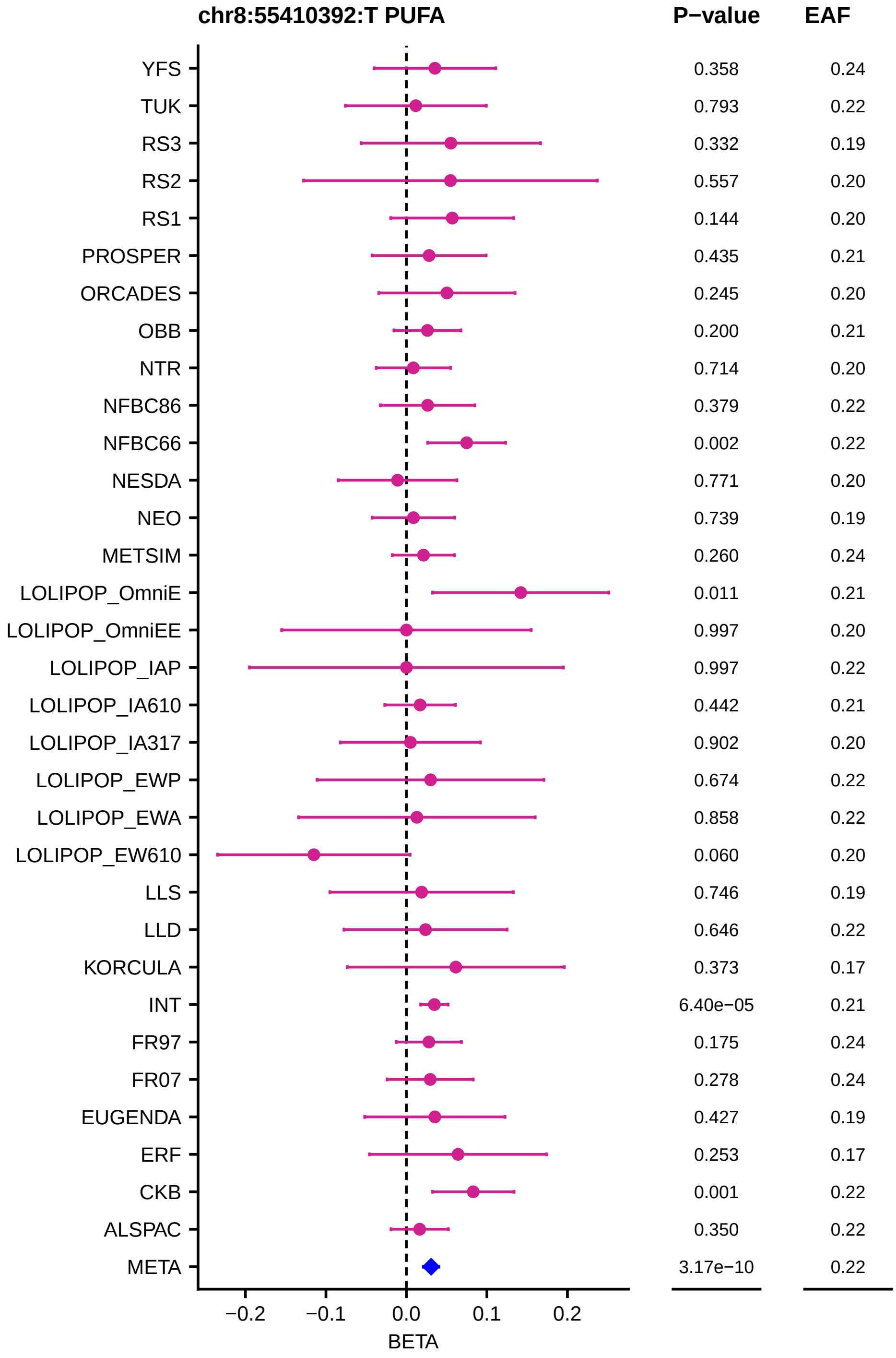
chr8:55410392:T PUFA
P−value
EAF
YFS
TUK
RS3
RS2
RS1
PROSPER
ORCADES
OBB
NTR
NFBC86
NFBC66
NESDA
NEO
METSIM
LOLIPOP_OmniE
LOLIPOP_OmniEE
LOLIPOP_IAP
LOLIPOP_IA610
LOLIPOP_IA317
LOLIPOP_EWP
LOLIPOP_EWA
LOLIPOP_EW610
LLS
LLD
KORCULA
INT
FR97
FR07
EUGENDA
ERF
CKB
ALSPAC
META
0.358 0.24
0.793 0.22
0.332 0.19
0.557 0.20
0.144 0.20
0.435 0.21
0.245 0.20
0.200 0.21
0.714 0.20
0.379 0.22
0.002 0.22
0.771 0.20
0.739 0.19
0.260 0.24
0.011 0.21
0.997 0.20
0.997 0.22
0.442 0.21
0.902 0.20
0.674 0.22
0.858 0.22
0.060 0.20
0.746 0.19
0.646 0.22
0.373 0.17
6.40e−05 0.21
0.175 0.24
0.278 0.24
0.427 0.19
0.253 0.17
0.001 0.22
0.350 0.22
3.17e−10 0.22
−0.2
−0.1
0.0
0.1
0.2
BETA

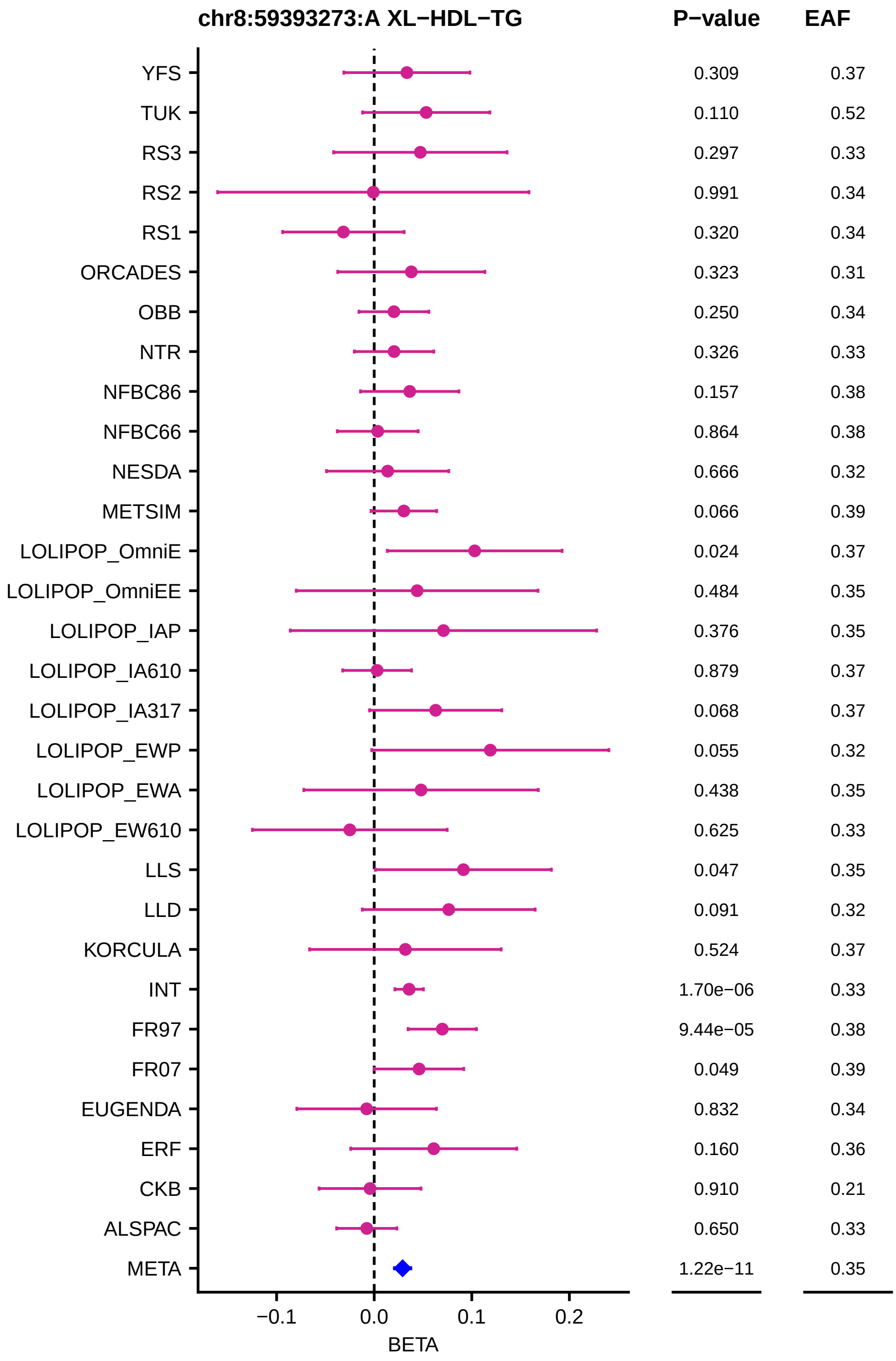
chr8:59393273:A XL−HDL−TG
P−value
EAF
YFS 0.309 0.37
TUK 0.110 0.52
RS3 0.297 0.33
RS2 0.991 0.34
RS1 0.320 0.34
ORCADES 0.323 0.31
OBB 0.250 0.34
NTR 0.326 0.33
NFBC86 0.157 0.38
NFBC66 0.864 0.38
NESDA 0.666 0.32
METSIM 0.066 0.39
LOLIPOP_OmniE 0.024 0.37
LOLIPOP_OmniEE 0.484 0.35
LOLIPOP_IAP 0.376 0.35
LOLIPOP_IA610 0.879 0.37
LOLIPOP_IA317 0.068 0.37
LOLIPOP_EWP 0.055 0.32
LOLIPOP_EWA 0.438 0.35
LOLIPOP_EW610 0.625 0.33
LLS 0.047 0.35
LLD 0.091 0.32
KORCULA 0.524 0.37
INT 1.70e−06 0.33
FR97 9.44e−05 0.38
FR07 0.049 0.39
EUGENDA 0.832 0.34
ERF 0.160 0.36
CKB 0.910 0.21
ALSPAC 0.650 0.33
META 1.22e−11 0.35
−0.1
0.0
0.1
0.2
BETA

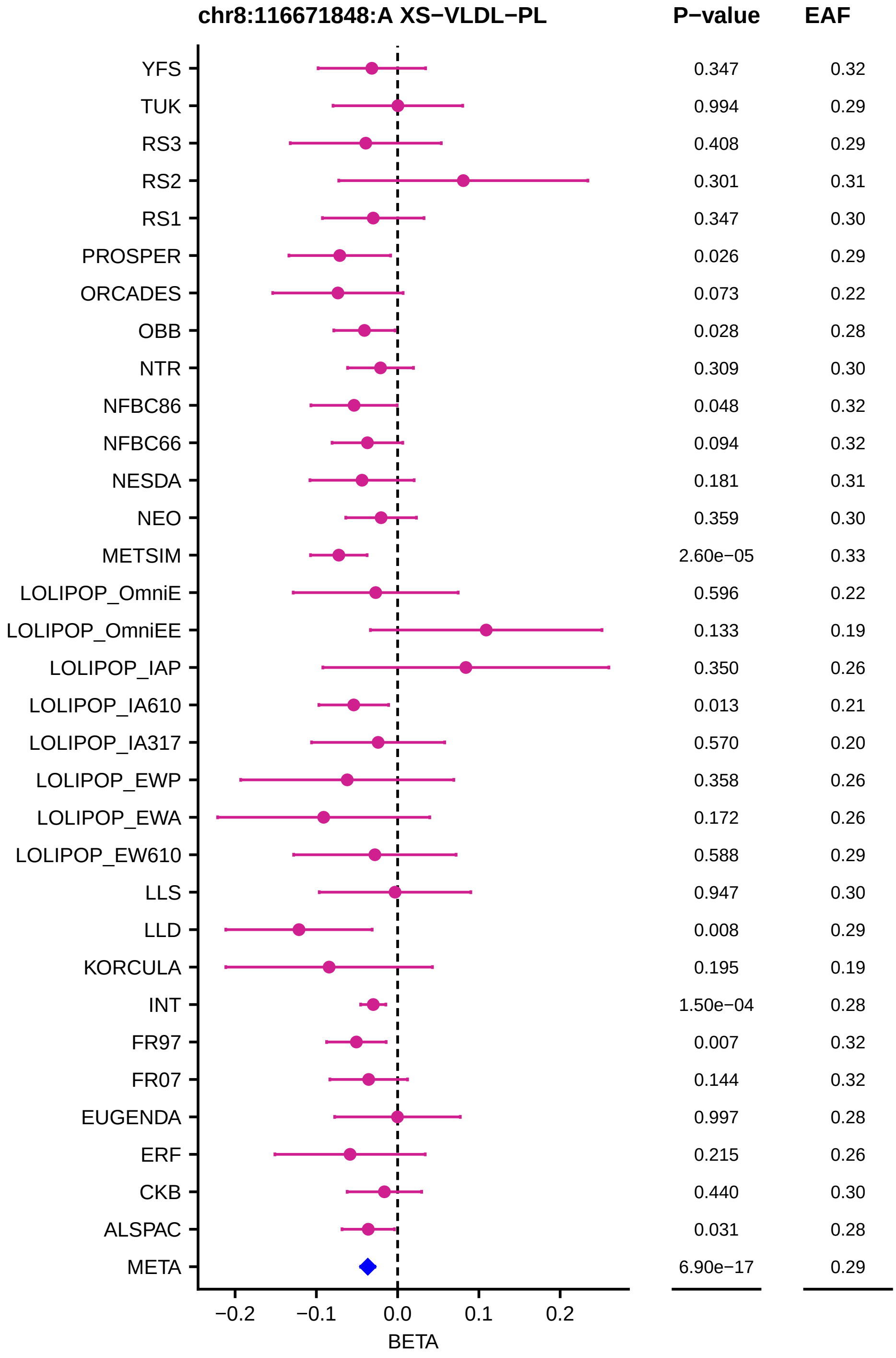

chr8:116671848:A XS−VLDL−PL
P−value
EAF
YFS 0.347 0.32
TUK 0.994 0.29
RS3 0.408 0.29
RS2 0.301 0.31
RS1 0.347 0.30
PROSPER 0.026 0.29
ORCADES 0.073 0.22
OBB 0.028 0.28
NTR 0.309 0.30
NFBC86 0.048 0.32
NFBC66 0.094 0.32
NESDA 0.181 0.31
NEO 0.359 0.30
METSIM 2.60e−05 0.33
LOLIPOP_OmniE 0.596 0.22
LOLIPOP_OmniEE 0.133 0.19
LOLIPOP_IAP 0.350 0.26
LOLIPOP_IA610 0.013 0.21
LOLIPOP_IA317 0.570 0.20
LOLIPOP_EWP 0.358 0.26
LOLIPOP_EWA 0.172 0.26
LOLIPOP_EW610 0.588 0.29
LLS 0.947 0.30
LLD 0.008 0.29
KORCULA 0.195 0.19
INT 1.50e−04 0.28
FR97 0.007 0.32
FR07 0.144 0.32
EUGENDA 0.997 0.28
ERF 0.215 0.26
CKB 0.440 0.30
ALSPAC 0.031 0.28
META 6.90e−17 0.29
−0.2
−0.1
0.0
0.1
0.2
BETA

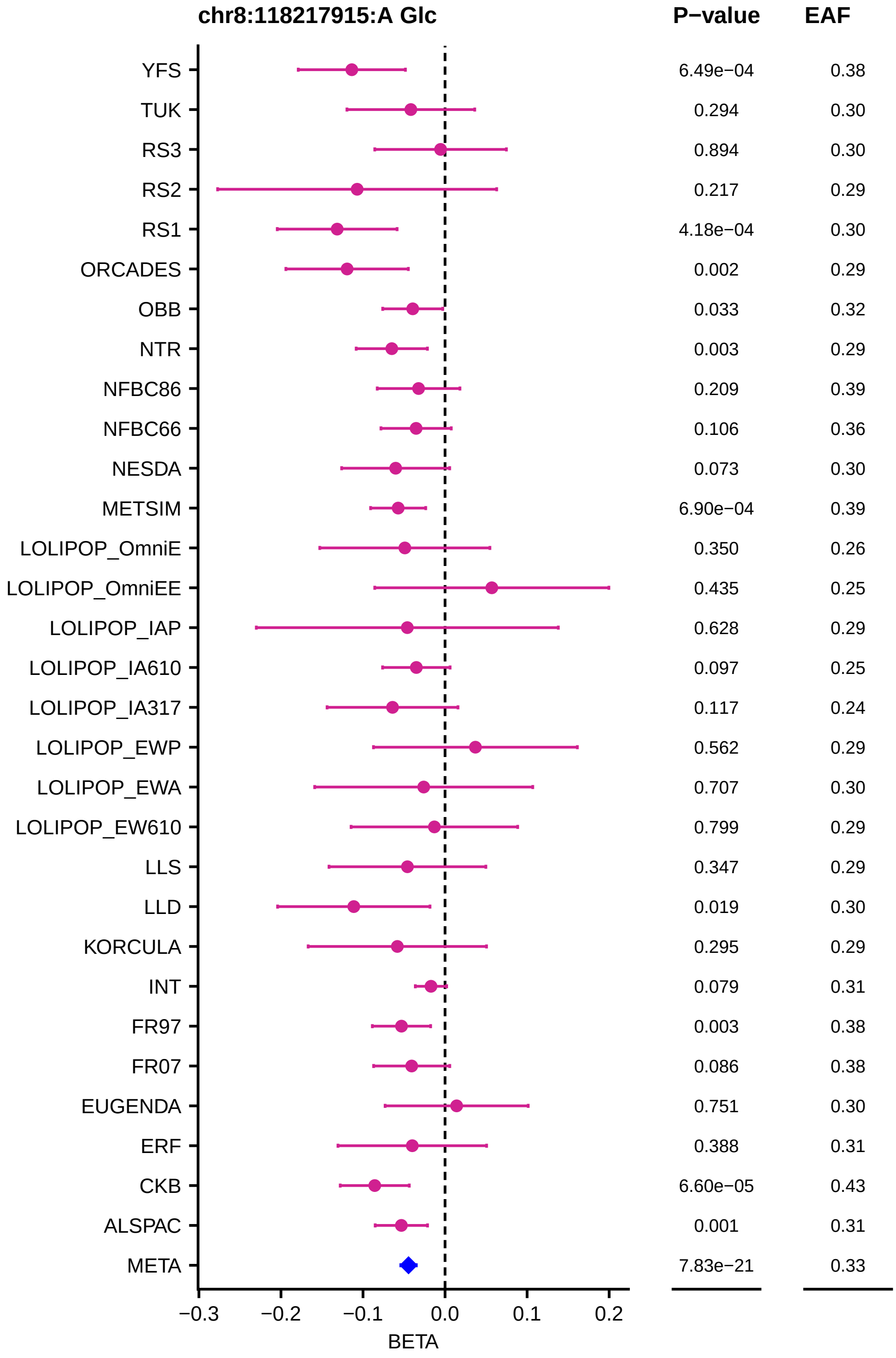

chr8:118217915:A Glc
P−value
EAF
YFS
6.49e−04
0.38
TUK
0.294
0.30
RS3
0.894
0.30
RS2
0.217
0.29
RS1
4.18e−04
0.30
ORCADES
0.002
0.29
OBB
0.033
0.32
NTR
0.003
0.29
NFBC86
0.209
0.39
NFBC66
0.106
0.36
NESDA
0.073
0.30
METSIM
6.90e−04
0.39
LOLIPOP_OmniE
0.350
0.26
LOLIPOP_OmniEE
0.435
0.25
LOLIPOP_IAP
0.628
0.29
LOLIPOP_IA610
0.097
0.25
LOLIPOP_IA317
0.117
0.24
LOLIPOP_EWP
0.562
0.29
LOLIPOP_EWA
0.707
0.30
LOLIPOP_EW610
0.799
0.29
LLS
0.347
0.29
LLD
0.019
0.30
KORCULA
0.295
0.29
INT
0.079
0.31
FR97
0.003
0.38
FR07
0.086
0.38
EUGENDA
0.751
0.30
ERF
0.388
0.31
CKB
6.60e−05
0.43
ALSPAC
0.001
0.31
META
7.83e−21
0.33
−0.3
−0.2
−0.1
0.0
0.1
0.2
BETA

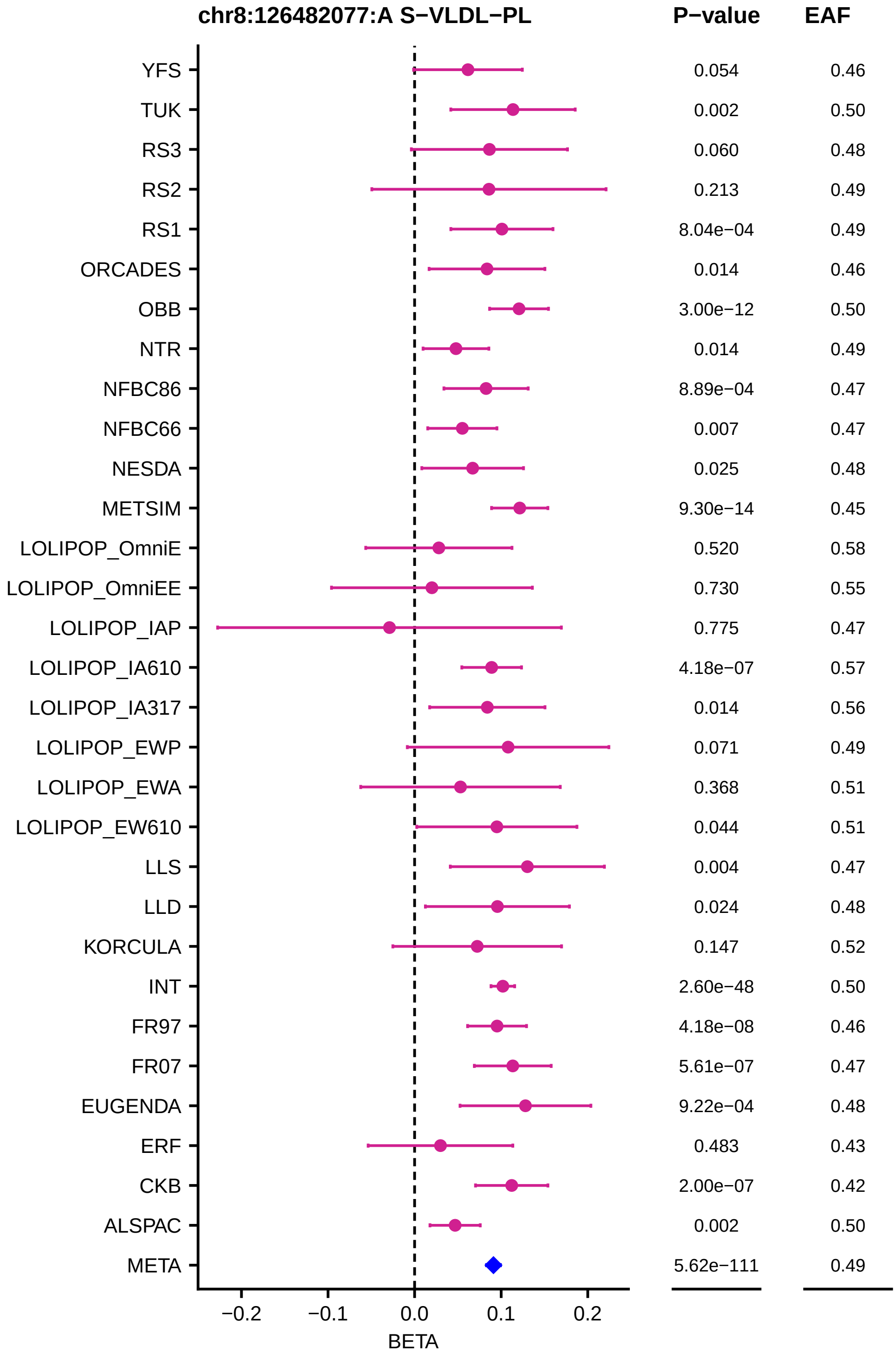
chr8:126482077:A S−VLDL−PL
P−value
EAF
YFS 0.054 0.46
TUK 0.002 0.50
RS3 0.060 0.48
RS2 0.213 0.49
RS1 8.04e−04 0.49
ORCADES 0.014 0.46
OBB 3.00e−12 0.50
NTR 0.014 0.49
NFBC86 8.89e−04 0.47
NFBC66 0.007 0.47
NESDA 0.025 0.48
METSIM 9.30e−14 0.45
LOLIPOP_OmniE 0.520 0.58
LOLIPOP_OmniEE 0.730 0.55
LOLIPOP_IAP 0.775 0.47
LOLIPOP_IA610 4.18e−07 0.57
LOLIPOP_IA317 0.014 0.56
LOLIPOP_EWP 0.071 0.49
LOLIPOP_EWA 0.368 0.51
LOLIPOP_EW610 0.044 0.51
LLS 0.004 0.47
LLD 0.024 0.48
KORCULA 0.147 0.52
INT 2.60e−48 0.50
FR97 4.18e−08 0.46
FR07 5.61e−07 0.47
EUGENDA 9.22e−04 0.48
ERF 0.483 0.43
CKB 2.00e−07 0.42
ALSPAC 0.002 0.50
META 5.62e−111 0.49
−0.2
−0.1
0.0
0.1
0.2
BETA

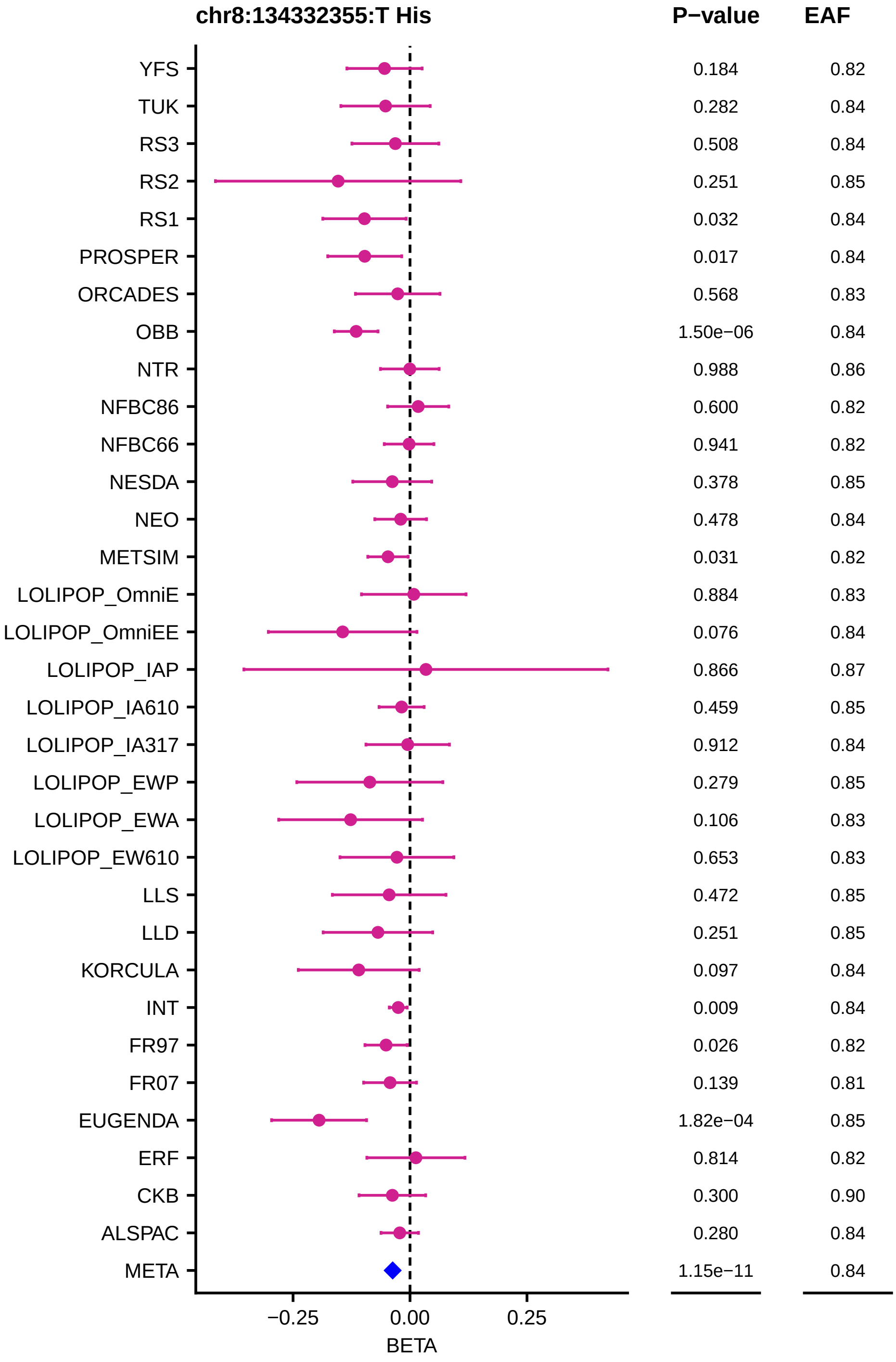
chr8:134332355:T His
P−value
EAF
YFS 0.184 0.82
TUK 0.282 0.84
RS3 0.508 0.84
RS2 0.251 0.85
RS1 0.032 0.84
PROSPER 0.017 0.84
ORCADES 0.568 0.83
OBB 1.50e−06 0.84
NTR 0.988 0.86
NFBC86 0.600 0.82
NFBC66 0.941 0.82
NESDA 0.378 0.85
NEO 0.478 0.84
METSIM 0.031 0.82
LOLIPOP_OmniE 0.884 0.83
LOLIPOP_OmniEE 0.076 0.84
LOLIPOP_IAP 0.866 0.87
LOLIPOP_IA610 0.459 0.85
LOLIPOP_IA317 0.912 0.84
LOLIPOP_EWP 0.279 0.85
LOLIPOP_EWA 0.106 0.83
LOLIPOP_EW610 0.653 0.83
LLS 0.472 0.85
LLD 0.251 0.85
KORCULA 0.097 0.84
INT 0.009 0.84
FR97 0.026 0.82
FR07 0.139 0.81
EUGENDA 1.82e−04 0.85
ERF 0.814 0.82
CKB 0.300 0.90
ALSPAC 0.280 0.84
META 1.15e−11 0.84
−0.25
0.00
0.25
BETA

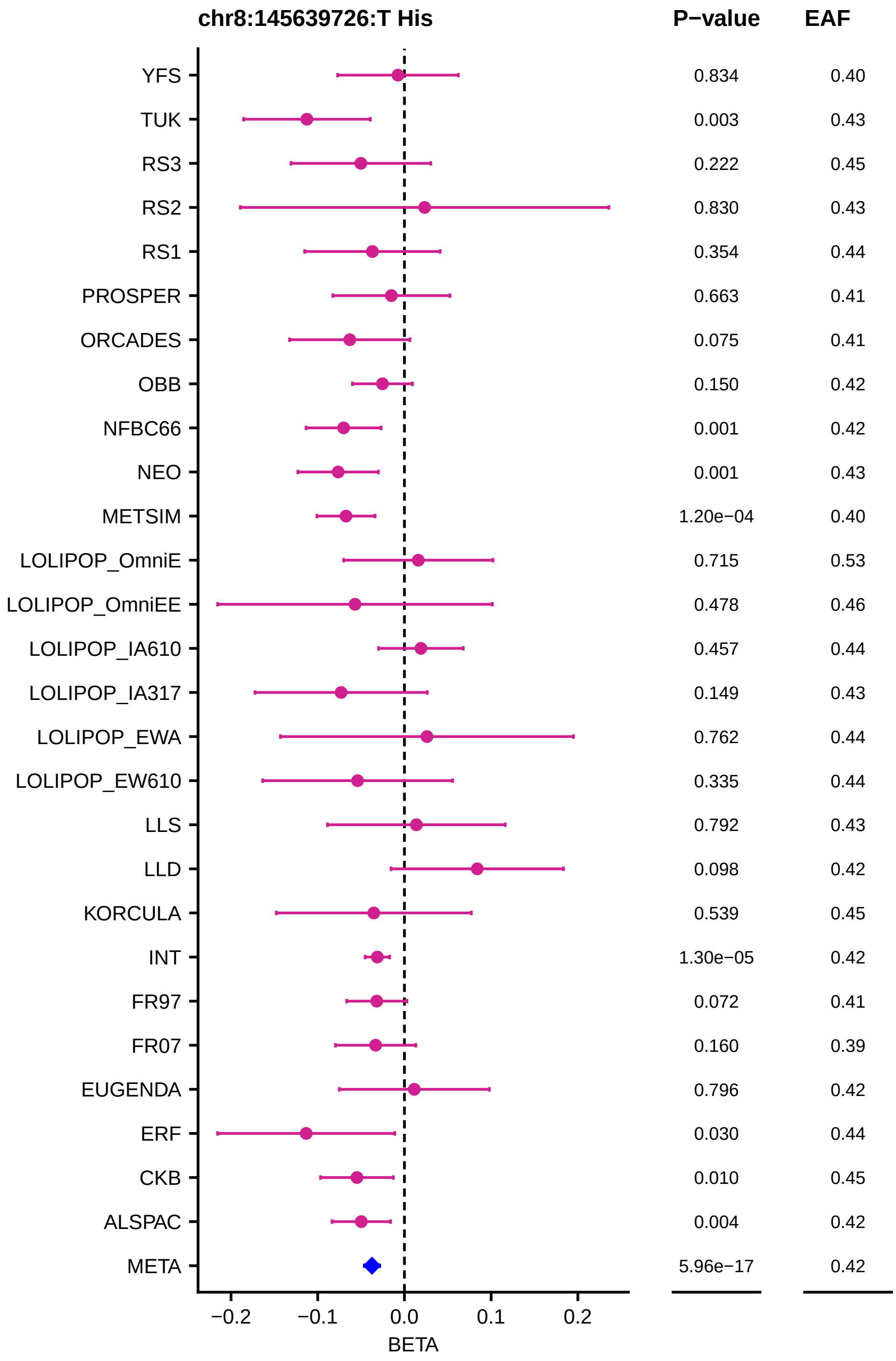

chr8:145639726:T His
P−value
EAF
YFS 0.834 0.40
TUK 0.003 0.43
RS3 0.222 0.45
RS2 0.830 0.43
RS1 0.354 0.44
PROSPER 0.663 0.41
ORCADES 0.075 0.41
OBB 0.150 0.42
NFBC66 0.001 0.42
NEO 0.001 0.43
METSIM 1.20e−04 0.40
LOLIPOP_OmniE 0.715 0.53
LOLIPOP_OmniEE 0.478 0.46
LOLIPOP_IA610 0.457 0.44
LOLIPOP_IA317 0.149 0.43
LOLIPOP_EWA 0.762 0.44
LOLIPOP_EW610 0.335 0.44
LLS 0.792 0.43
LLD 0.098 0.42
KORCULA 0.539 0.45
INT 1.30e−05 0.42
FR97 0.072 0.41
FR07 0.160 0.39
EUGENDA 0.796 0.42
ERF 0.030 0.44
CKB 0.010 0.45
ALSPAC 0.004 0.42
META 5.96e−17 0.42
−0.2
−0.1
0.0
0.1
0.2
BETA

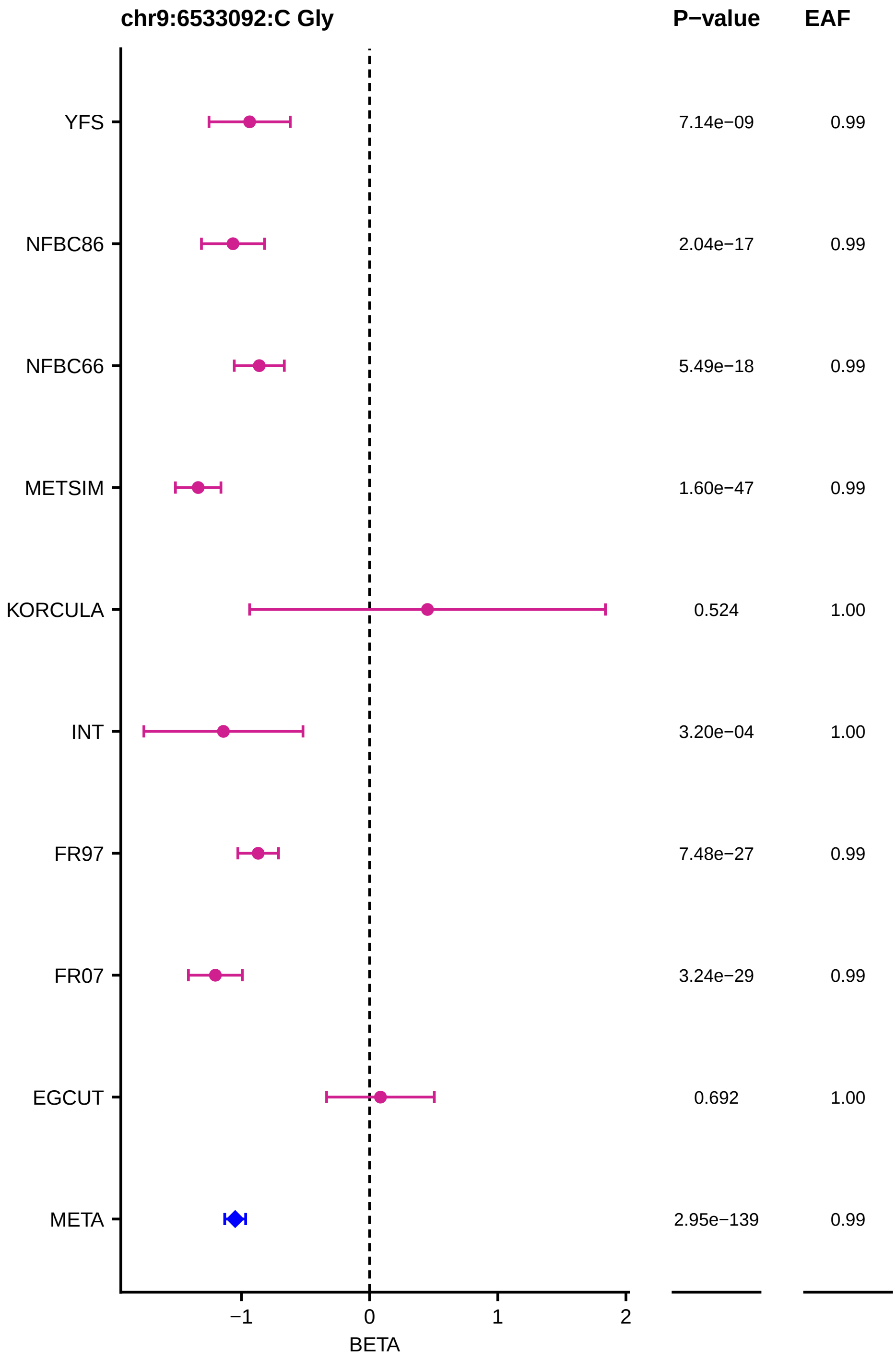

chr9:6533092:C Gly
P−value
EAF
YFS
7.14e−09
0.99
NFBC86
2.04e−17
0.99
NFBC66
5.49e−18
0.99
METSIM
1.60e−47
0.99
KORCULA
0.524
1.00
INT
3.20e−04
1.00
FR97
7.48e−27
0.99
FR07
3.24e−29
0.99
EGCUT
0.692
1.00
META
2.95e−139
0.99
−1
0
1
2
BETA

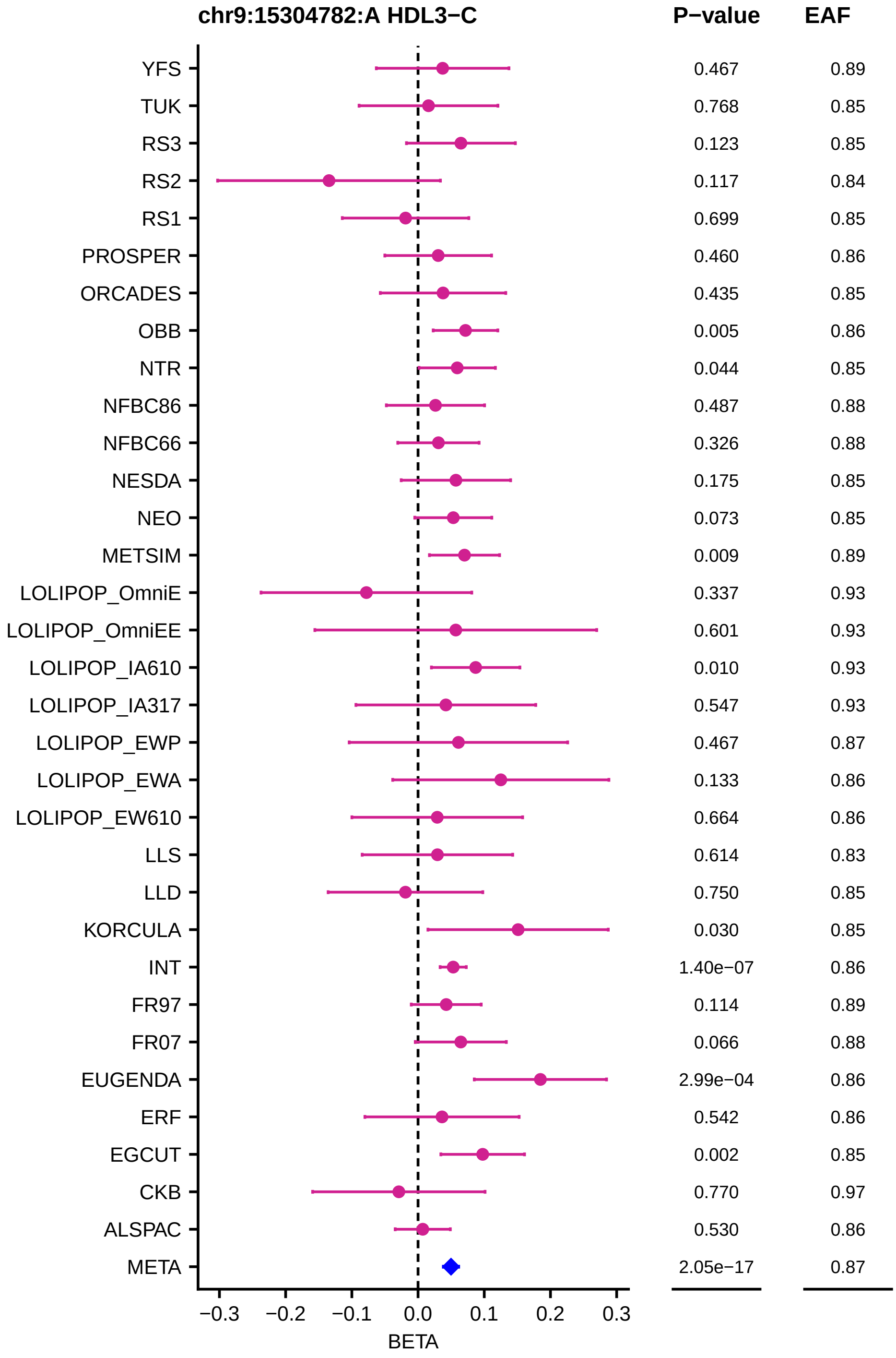
chr9:15304782:A HDL3−C
P−value
EAF
YFS 0.467 0.89
TUK 0.768 0.85
RS3 0.123 0.85
RS2 0.117 0.84
RS1 0.699 0.85
PROSPER 0.460 0.86
ORCADES 0.435 0.85
OBB 0.005 0.86
NTR 0.044 0.85
NFBC86 0.487 0.88
NFBC66 0.326 0.88
NESDA 0.175 0.85
NEO 0.073 0.85
METSIM 0.009 0.89
LOLIPOP_OmniE 0.337 0.93
LOLIPOP_OmniEE 0.601 0.93
LOLIPOP_IA610 0.010 0.93
LOLIPOP_IA317 0.547 0.93
LOLIPOP_EWP 0.467 0.87
LOLIPOP_EWA 0.133 0.86
LOLIPOP_EW610 0.664 0.86
LLS 0.614 0.83
LLD 0.750 0.85
KORCULA 0.030 0.85
INT 1.40e−07 0.86
FR97 0.114 0.89
FR07 0.066 0.88
EUGENDA 2.99e−04 0.86
ERF 0.542 0.86
EGCUT 0.002 0.85
CKB 0.770 0.97
ALSPAC 0.530 0.86
META 2.05e−17 0.87
−0.3 −0.2 −0.1 0.0 0.1 0.2 0.3
BETA

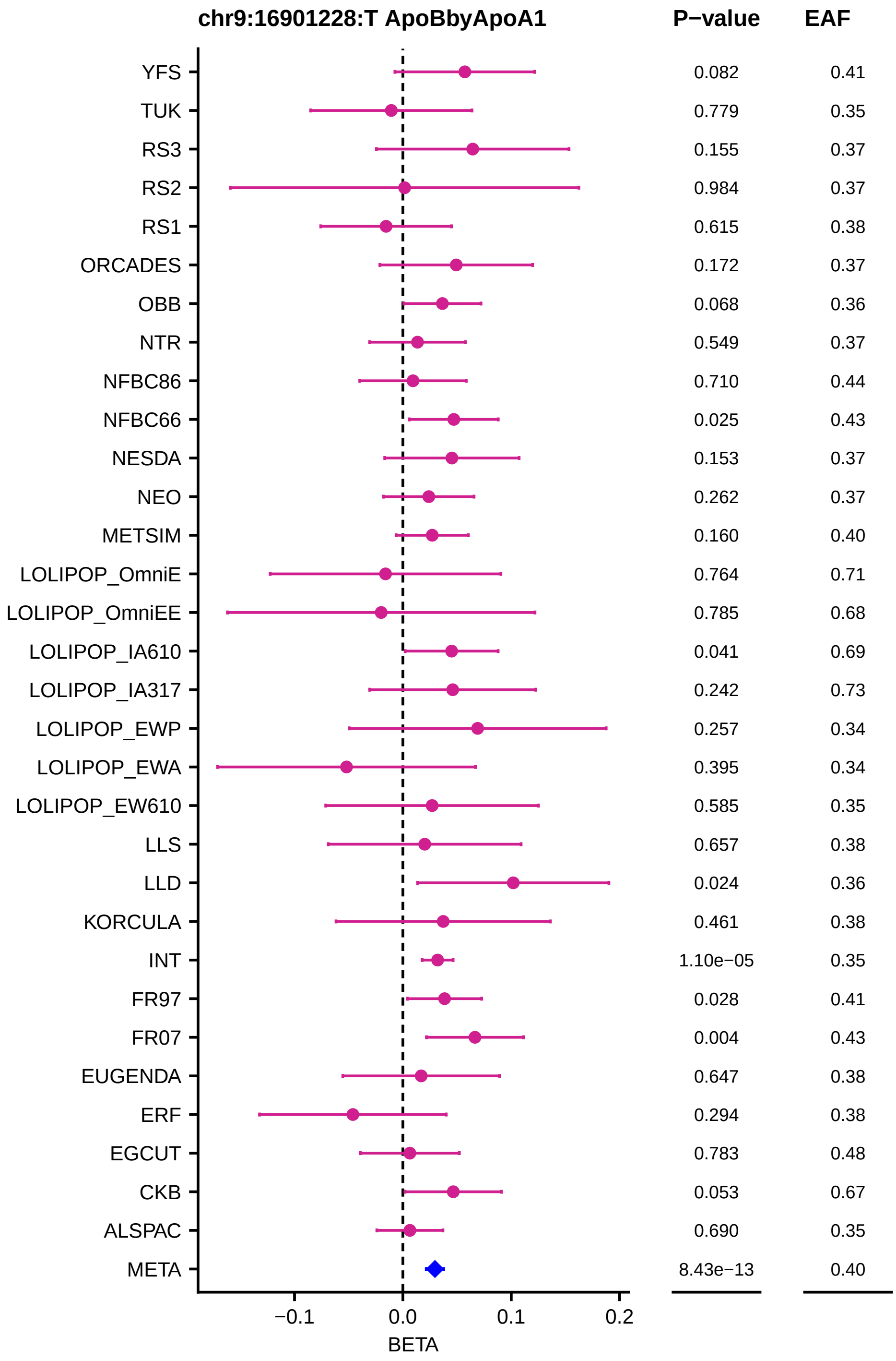
chr9:16901228:T ApoBbyApoA1
P−value
EAF
YFS 0.082 0.41
TUK 0.779 0.35
RS3 0.155 0.37
RS2 0.984 0.37
RS1 0.615 0.38
ORCADES 0.172 0.37
OBB 0.068 0.36
NTR 0.549 0.37
NFBC86 0.710 0.44
NFBC66 0.025 0.43
NESDA 0.153 0.37
NEO 0.262 0.37
METSIM 0.160 0.40
LOLIPOP_OmniE 0.764 0.71
LOLIPOP_OmniEE 0.785 0.68
LOLIPOP_IA610 0.041 0.69
LOLIPOP_IA317 0.242 0.73
LOLIPOP_EWP 0.257 0.34
LOLIPOP_EWA 0.395 0.34
LOLIPOP_EW610 0.585 0.35
LLS 0.657 0.38
LLD 0.024 0.36
KORCULA 0.461 0.38
INT 1.10e−05 0.35
FR97 0.028 0.41
FR07 0.004 0.43
EUGENDA 0.647 0.38
ERF 0.294 0.38
EGCUT 0.783 0.48
CKB 0.053 0.67
ALSPAC 0.690 0.35
META 8.43e−13 0.40
−0.1
0.0
0.1
0.2
BETA

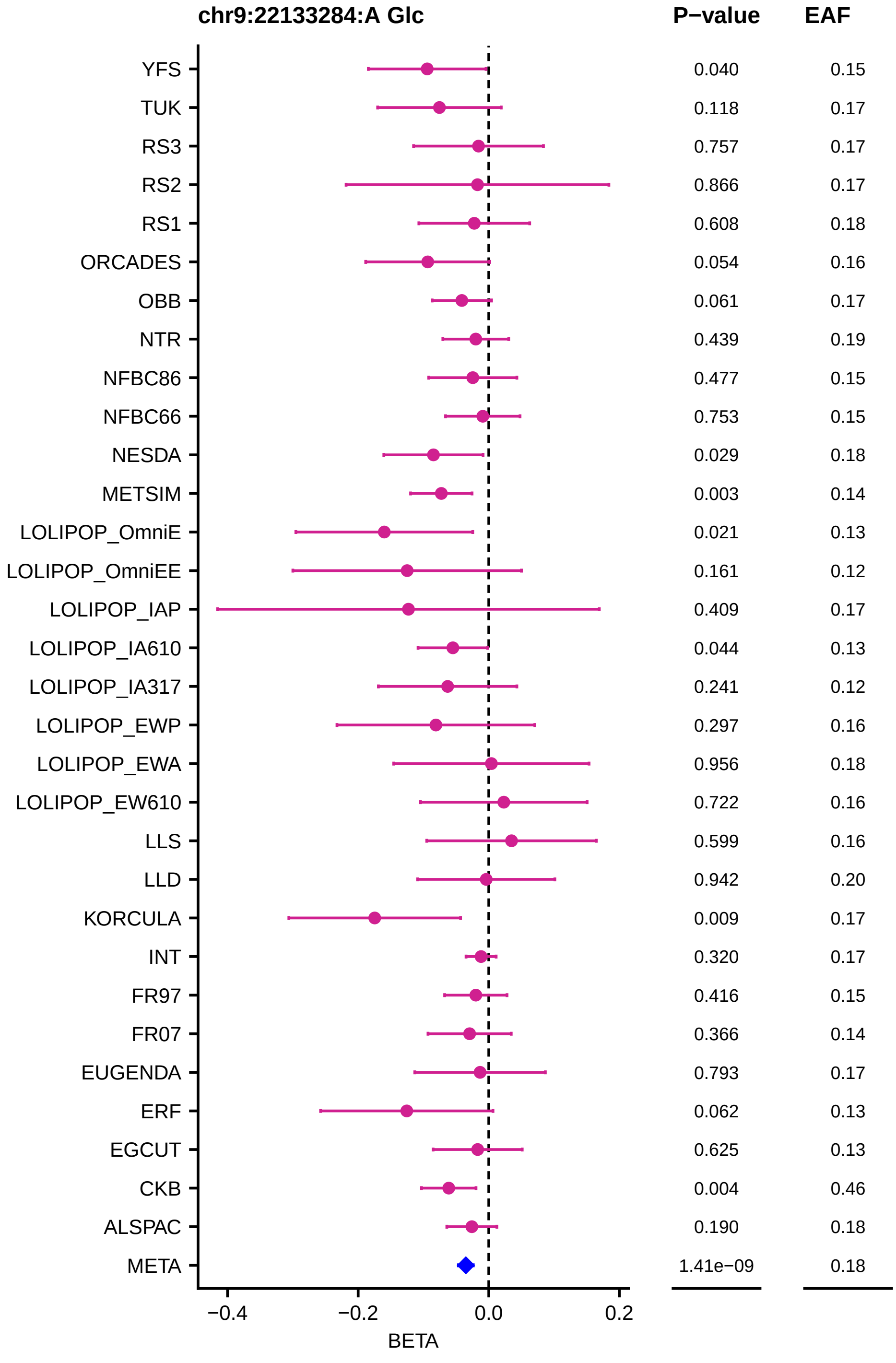
chr9:22133284:A Glc
P−value
EAF
YFS 0.040 0.15
TUK 0.118 0.17
RS3 0.757 0.17
RS2 0.866 0.17
RS1 0.608 0.18
ORCADES 0.054 0.16
OBB 0.061 0.17
NTR 0.439 0.19
NFBC86 0.477 0.15
NFBC66 0.753 0.15
NESDA 0.029 0.18
METSIM 0.003 0.14
LOLIPOP_OmniE 0.021 0.13
LOLIPOP_OmniEE 0.161 0.12
LOLIPOP_IAP 0.409 0.17
LOLIPOP_IA610 0.044 0.13
LOLIPOP_IA317 0.241 0.12
LOLIPOP_EWP 0.297 0.16
LOLIPOP_EWA 0.956 0.18
LOLIPOP_EW610 0.722 0.16
LLS 0.599 0.16
LLD 0.942 0.20
KORCULA 0.009 0.17
INT 0.320 0.17
FR97 0.416 0.15
FR07 0.366 0.14
EUGENDA 0.793 0.17
ERF 0.062 0.13
EGCUT 0.625 0.13
CKB 0.004 0.46
ALSPAC 0.190 0.18
META 1.41e−09 0.18
−0.4
−0.2
0.0
0.2
BETA

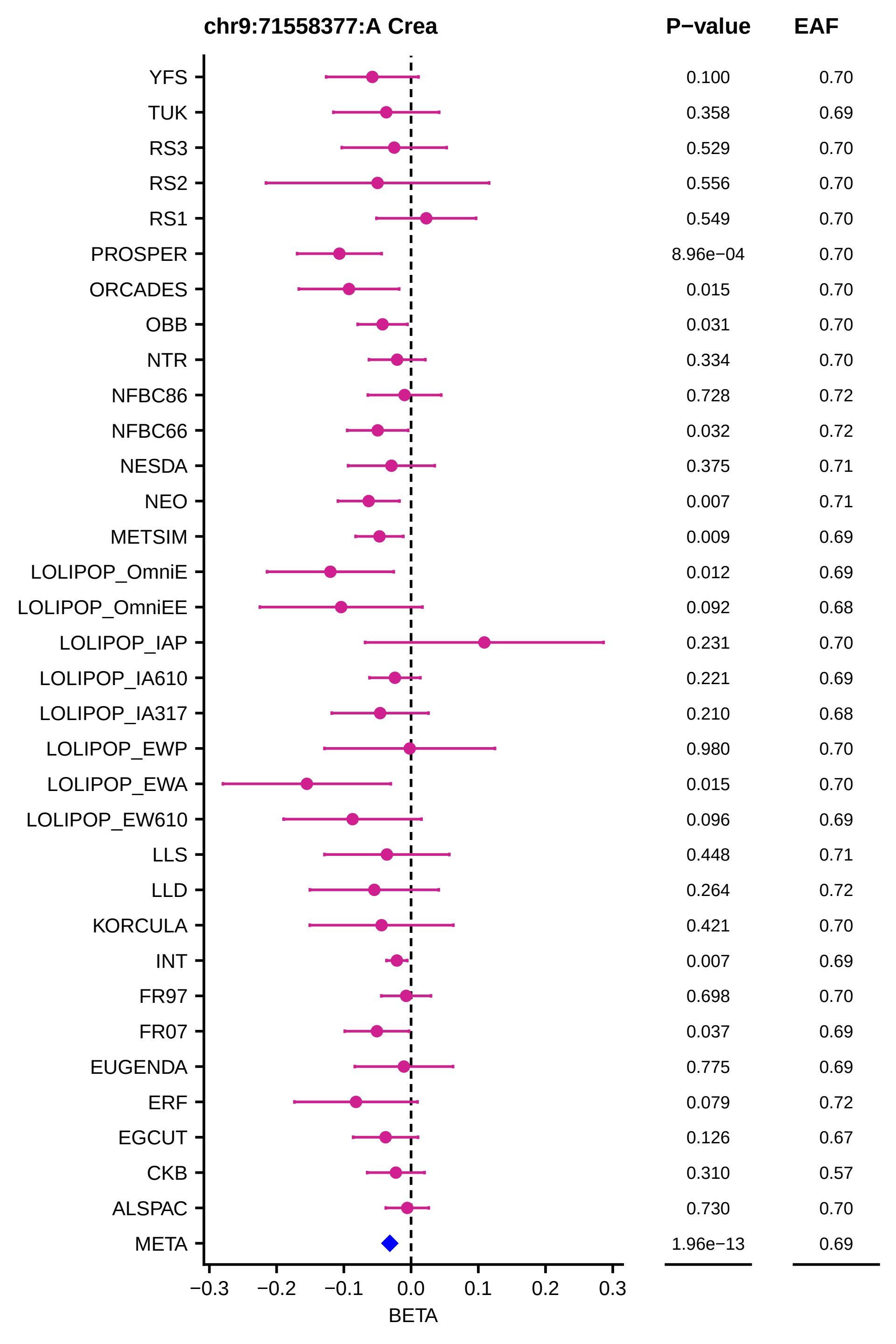
chr9:71558377:A Crea
P−value
EAF
YFS 0.100 0.70
TUK 0.358 0.69
RS3 0.529 0.70
RS2 0.556 0.70
RS1 0.549 0.70
PROSPER 8.96e−04 0.70
ORCADES 0.015 0.70
OBB 0.031 0.70
NTR 0.334 0.70
NFBC86 0.728 0.72
NFBC66 0.032 0.72
NESDA 0.375 0.71
NEO 0.007 0.71
METSIM 0.009 0.69
LOLIPOP_OmniE 0.012 0.69
LOLIPOP_OmniEE 0.092 0.68
LOLIPOP_IAP 0.231 0.70
LOLIPOP_IA610 0.221 0.69
LOLIPOP_IA317 0.210 0.68
LOLIPOP_EWP 0.980 0.70
LOLIPOP_EWA 0.015 0.70
LOLIPOP_EW610 0.096 0.69
LLS 0.448 0.71
LLD 0.264 0.72
KORCULA 0.421 0.70
INT 0.007 0.69
FR97 0.698 0.70
FR07 0.037 0.69
EUGENDA 0.775 0.69
ERF 0.079 0.72
EGCUT 0.126 0.67
CKB 0.310 0.57
ALSPAC 0.730 0.70
META 1.96e−13 0.69
−0.3 −0.2 −0.1 0.0 0.1 0.2 0.3
BETA

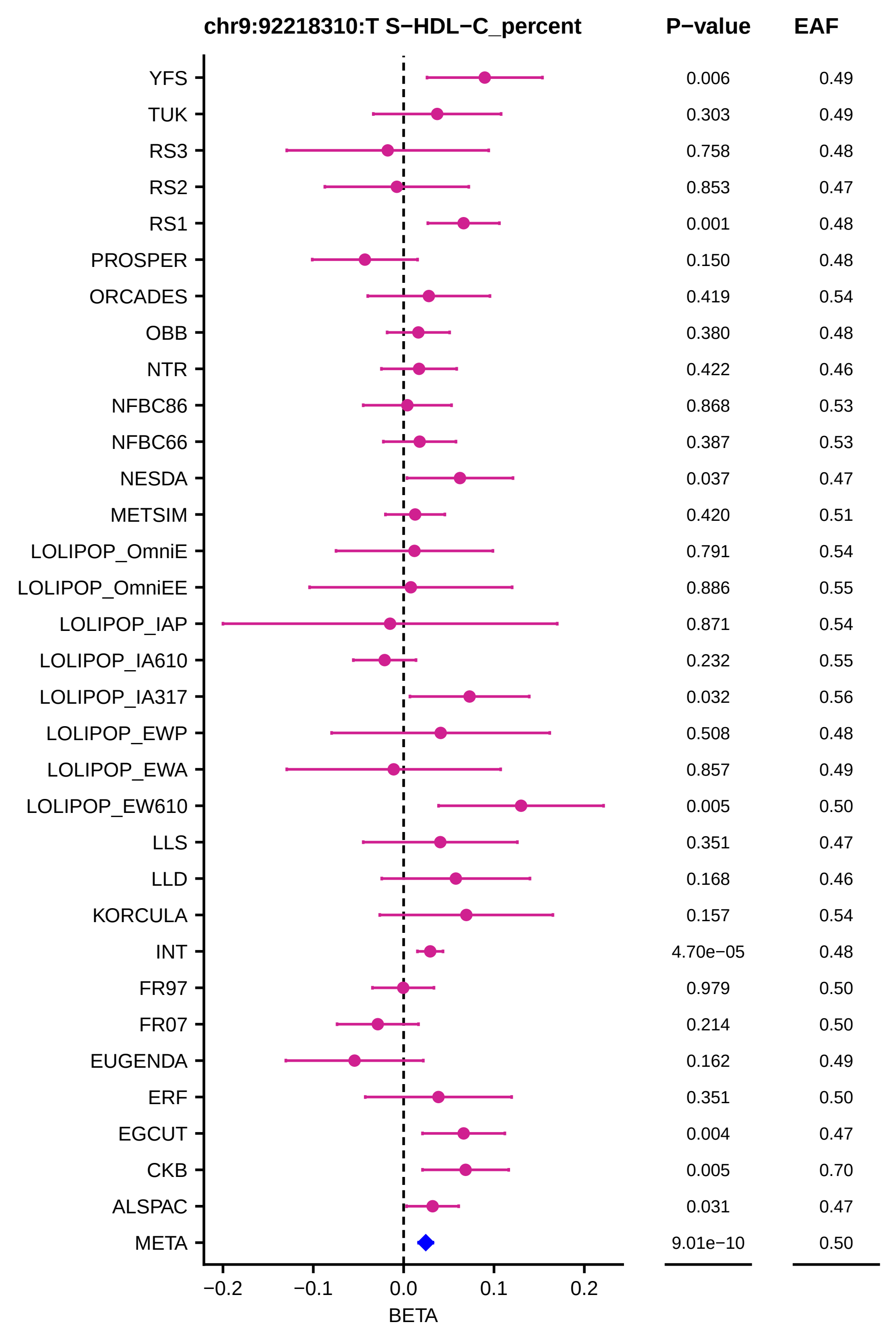
chr9:92218310:T S−HDL−C_percent
P−value
EAF
YFS 0.006 0.49
TUK 0.303 0.49
RS3 0.758 0.48
RS2 0.853 0.47
RS1 0.001 0.48
PROSPER 0.150 0.48
ORCADES 0.419 0.54
OBB 0.380 0.48
NTR 0.422 0.46
NFBC86 0.868 0.53
NFBC66 0.387 0.53
NESDA 0.037 0.47
METSIM 0.420 0.51
LOLIPOP_OmniE 0.791 0.54
LOLIPOP_OmniEE 0.886 0.55
LOLIPOP_IAP 0.871 0.54
LOLIPOP_IA610 0.232 0.55
LOLIPOP_IA317 0.032 0.56
LOLIPOP_EWP 0.508 0.48
LOLIPOP_EWA 0.857 0.49
LOLIPOP_EW610 0.005 0.50
LLS 0.351 0.47
LLD 0.168 0.46
KORCULA 0.157 0.54
INT 4.70e−05 0.48
FR97 0.979 0.50
FR07 0.214 0.50
EUGENDA 0.162 0.49
ERF 0.351 0.50
EGCUT 0.004 0.47
CKB 0.005 0.70
ALSPAC 0.031 0.47
META 9.01e−10 0.50
−0.2
−0.1
0.0
0.1
0.2
BETA

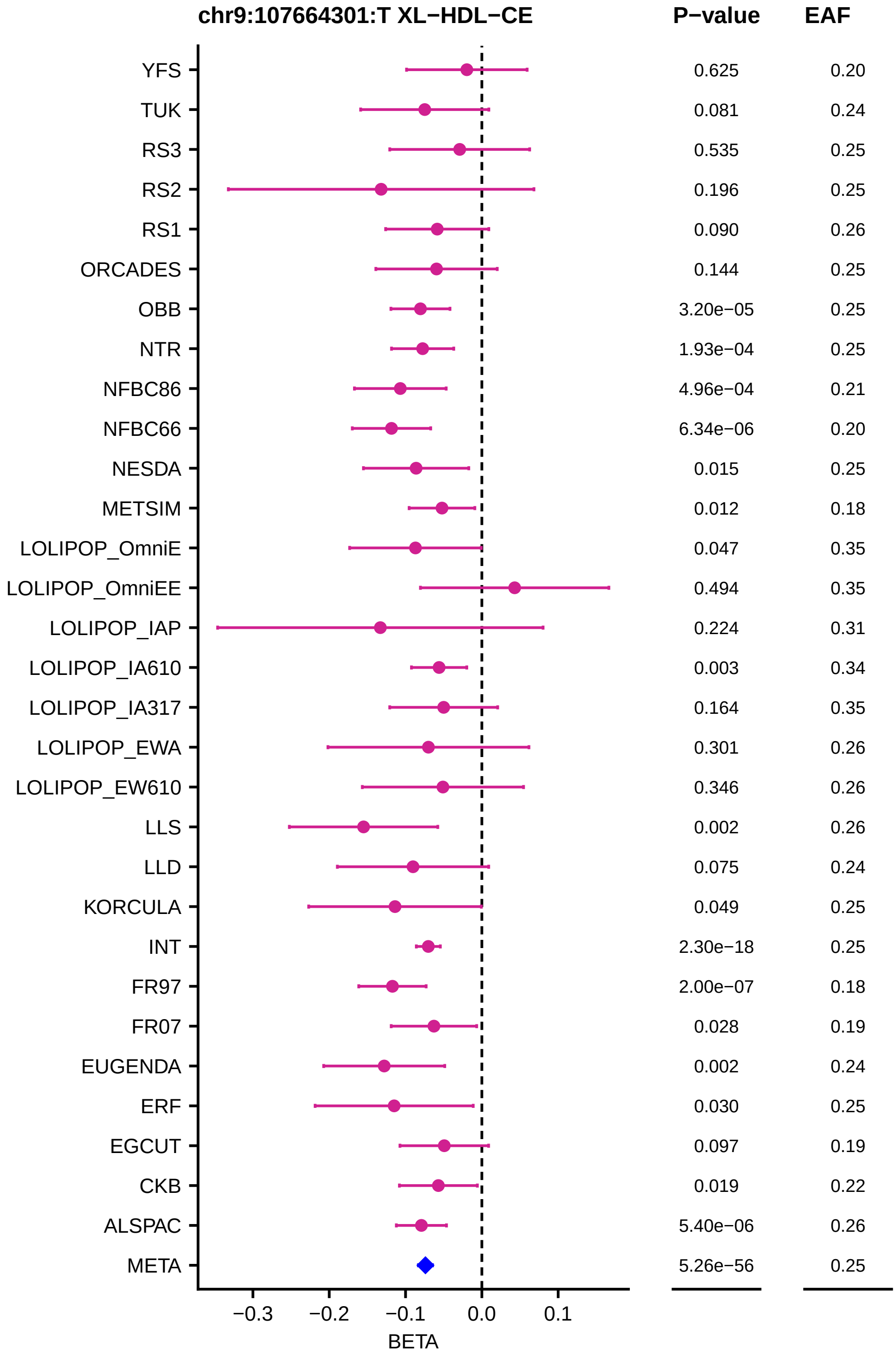
chr9:107664301:T XL−HDL−CE
P−value
EAF
YFS
TUK
RS3
RS2
RS1
ORCADES
OBB
NTR
NFBC86
NFBC66
NESDA
METSIM
LOLIPOP_OmniE
LOLIPOP_OmniEE
LOLIPOP_IAP
LOLIPOP_IA610
LOLIPOP_IA317
LOLIPOP_EWA
LOLIPOP_EW610
LLS
LLD
KORCULA
INT
FR97
FR07
EUGENDA
ERF
EGCUT
CKB
ALSPAC
META
0.625 0.20
0.081 0.24
0.535 0.25
0.196 0.25
0.090 0.26
0.144 0.25
3.20e−05 0.25
1.93e−04 0.25
4.96e−04 0.21
6.34e−06 0.20
0.015 0.25
0.012 0.18
0.047 0.35
0.494 0.35
0.224 0.31
0.003 0.34
0.164 0.35
0.301 0.26
0.346 0.26
0.002 0.26
0.075 0.24
0.049 0.25
2.30e−18 0.25
2.00e−07 0.18
0.028 0.19
0.002 0.24
0.030 0.25
0.097 0.19
0.019 0.22
5.40e−06 0.26
5.26e−56 0.25
−0.3
−0.2
−0.1
0.0
0.1
BETA

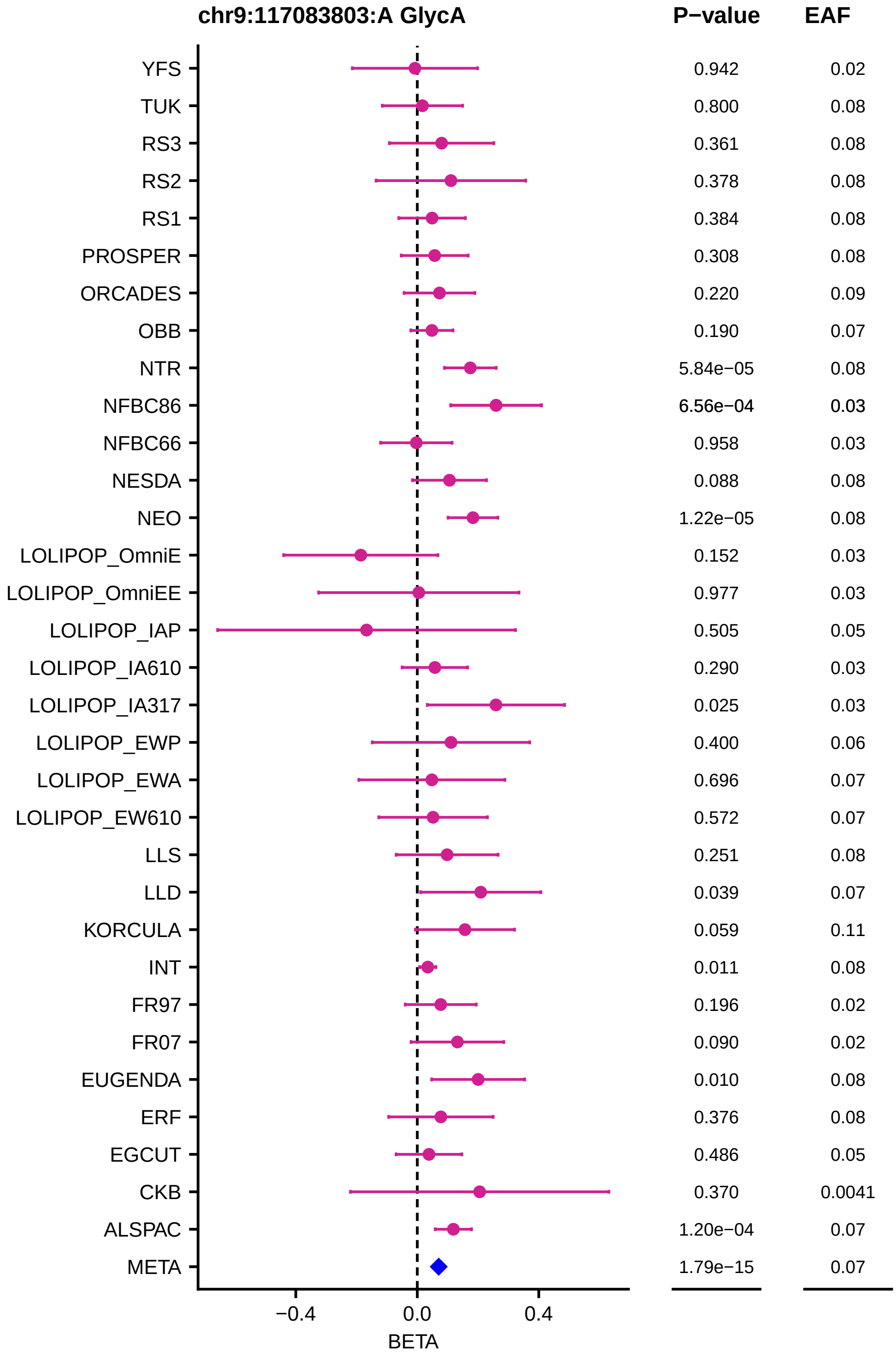

chr9:117083803:A GlycA
P−value
EAF
YFS 0.942 0.02
TUK 0.800 0.08
RS3 0.361 0.08
RS2 0.378 0.08
RS1 0.384 0.08
PROSPER 0.308 0.08
ORCADES 0.220 0.09
OBB 0.190 0.07
NTR 5.84e−05 0.08
NFBC86 6.56e−04 0.03
NFBC66 0.958 0.03
NESDA 0.088 0.08
NEO 1.22e−05 0.08
LOLIPOP_OmniE 0.152 0.03
LOLIPOP_OmniEE 0.977 0.03
LOLIPOP_IAP 0.505 0.05
LOLIPOP_IA610 0.290 0.03
LOLIPOP_IA317 0.025 0.03
LOLIPOP_EWP 0.400 0.06
LOLIPOP_EWA 0.696 0.07
LOLIPOP_EW610 0.572 0.07
LLS 0.251 0.08
LLD 0.039 0.07
KORCULA 0.059 0.11
INT 0.011 0.08
FR97 0.196 0.02
FR07 0.090 0.02
EUGENDA 0.010 0.08
ERF 0.376 0.08
EGCUT 0.486 0.05
CKB 0.370 0.0041
ALSPAC 1.20e−04 0.07
META 1.79e−15 0.07
−0.4
0.0
0.4
BETA

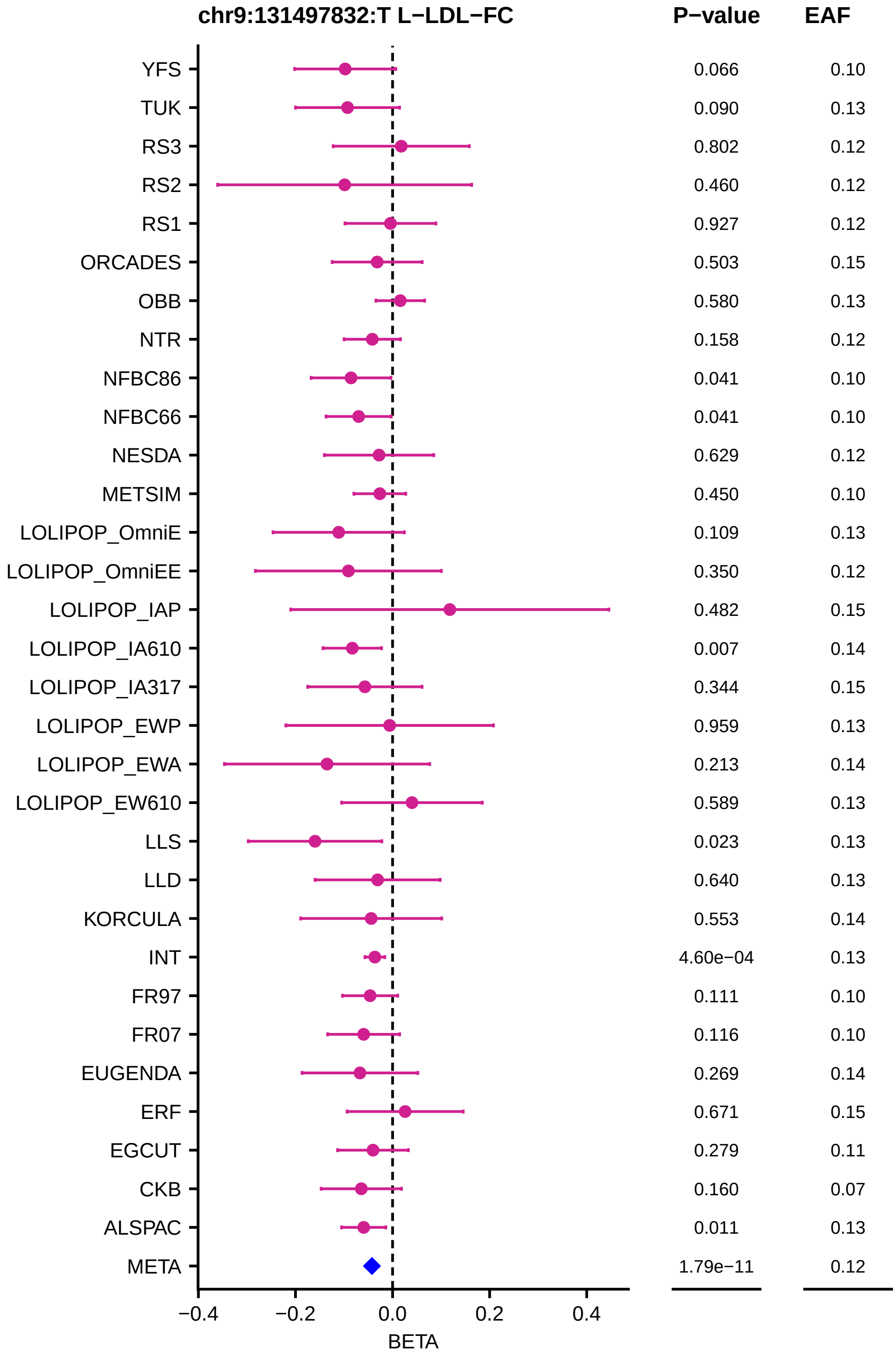
chr9:131497832:T L−LDL−FC
P−value
EAF
YFS 0.066 0.10
TUK 0.090 0.13
RS3 0.802 0.12
RS2 0.460 0.12
RS1 0.927 0.12
ORCADES 0.503 0.15
OBB 0.580 0.13
NTR 0.158 0.12
NFBC86 0.041 0.10
NFBC66 0.041 0.10
NESDA 0.629 0.12
METSIM 0.450 0.10
LOLIPOP_OmniE 0.109 0.13
LOLIPOP_OmniEE 0.350 0.12
LOLIPOP_IAP 0.482 0.15
LOLIPOP_IA610 0.007 0.14
LOLIPOP_IA317 0.344 0.15
LOLIPOP_EWP 0.959 0.13
LOLIPOP_EWA 0.213 0.14
LOLIPOP_EW610 0.589 0.13
LLS 0.023 0.13
LLD 0.640 0.13
KORCULA 0.553 0.14
INT 4.60e−04 0.13
FR97 0.111 0.10
FR07 0.116 0.10
EUGENDA 0.269 0.14
ERF 0.671 0.15
EGCUT 0.279 0.11
CKB 0.160 0.07
ALSPAC 0.011 0.13
META 1.79e−11 0.12
−0.4
−0.2
0.0
0.2
0.4
BETA

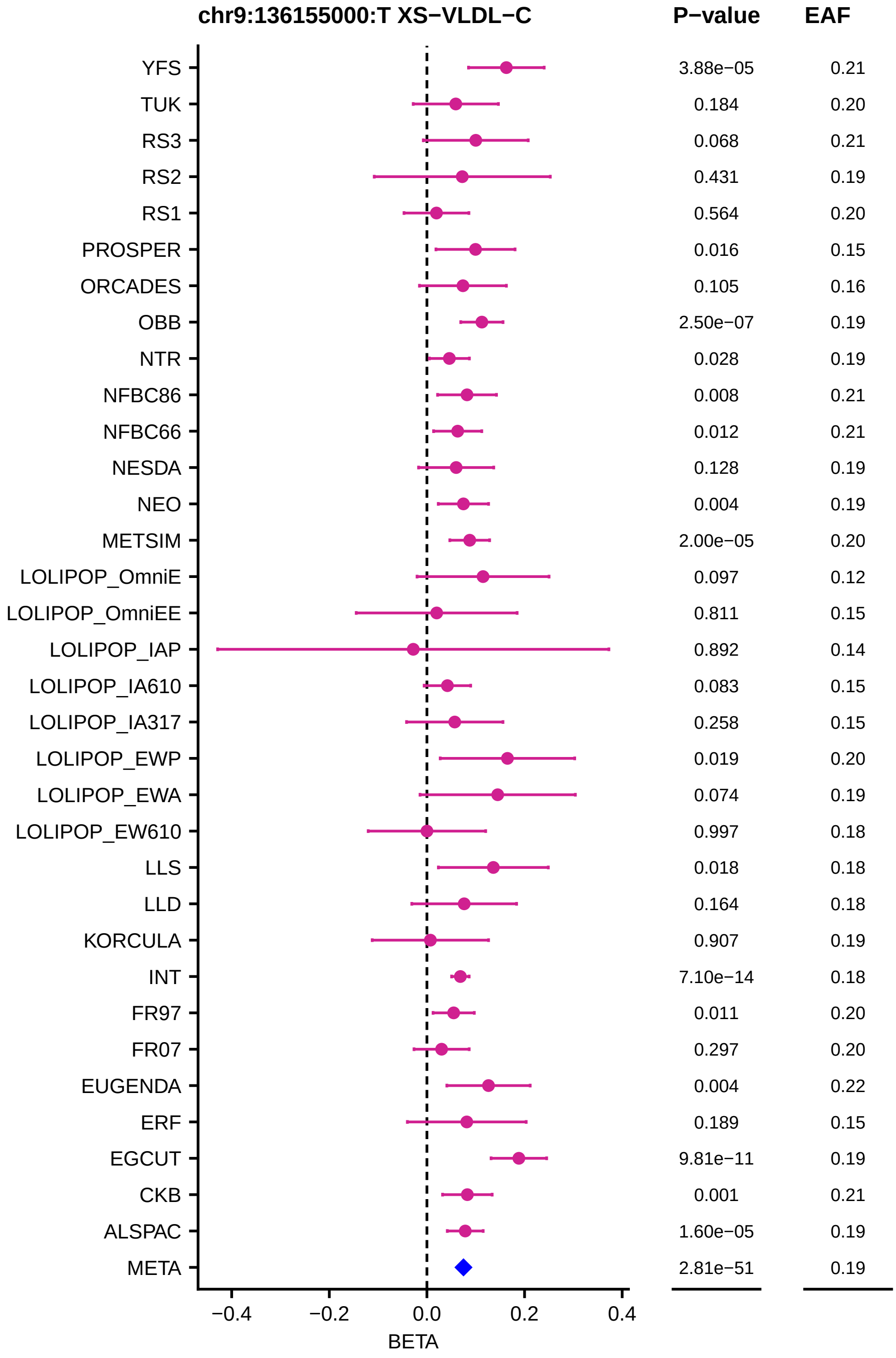
chr9:136155000:T XS−VLDL−C
P−value
EAF
YFS 3.88e−05 0.21
TUK 0.184 0.20
RS3 0.068 0.21
RS2 0.431 0.19
RS1 0.564 0.20
PROSPER 0.016 0.15
ORCADES 0.105 0.16
OBB 2.50e−07 0.19
NTR 0.028 0.19
NFBC86 0.008 0.21
NFBC66 0.012 0.21
NESDA 0.128 0.19
NEO 0.004 0.19
METSIM 2.00e−05 0.20
LOLIPOP_OmniE 0.097 0.12
LOLIPOP_OmniEE 0.811 0.15
LOLIPOP_IAP 0.892 0.14
LOLIPOP_IA610 0.083 0.15
LOLIPOP_IA317 0.258 0.15
LOLIPOP_EWP 0.019 0.20
LOLIPOP_EWA 0.074 0.19
LOLIPOP_EW610 0.997 0.18
LLS 0.018 0.18
LLD 0.164 0.18
KORCULA 0.907 0.19
INT 7.10e−14 0.18
FR97 0.011 0.20
FR07 0.297 0.20
EUGENDA 0.004 0.22
ERF 0.189 0.15
EGCUT 9.81e−11 0.19
CKB 0.001 0.21
ALSPAC 1.60e−05 0.19
META 2.81e−51 0.19
−0.4
−0.2
0.0
0.2
0.4
BETA

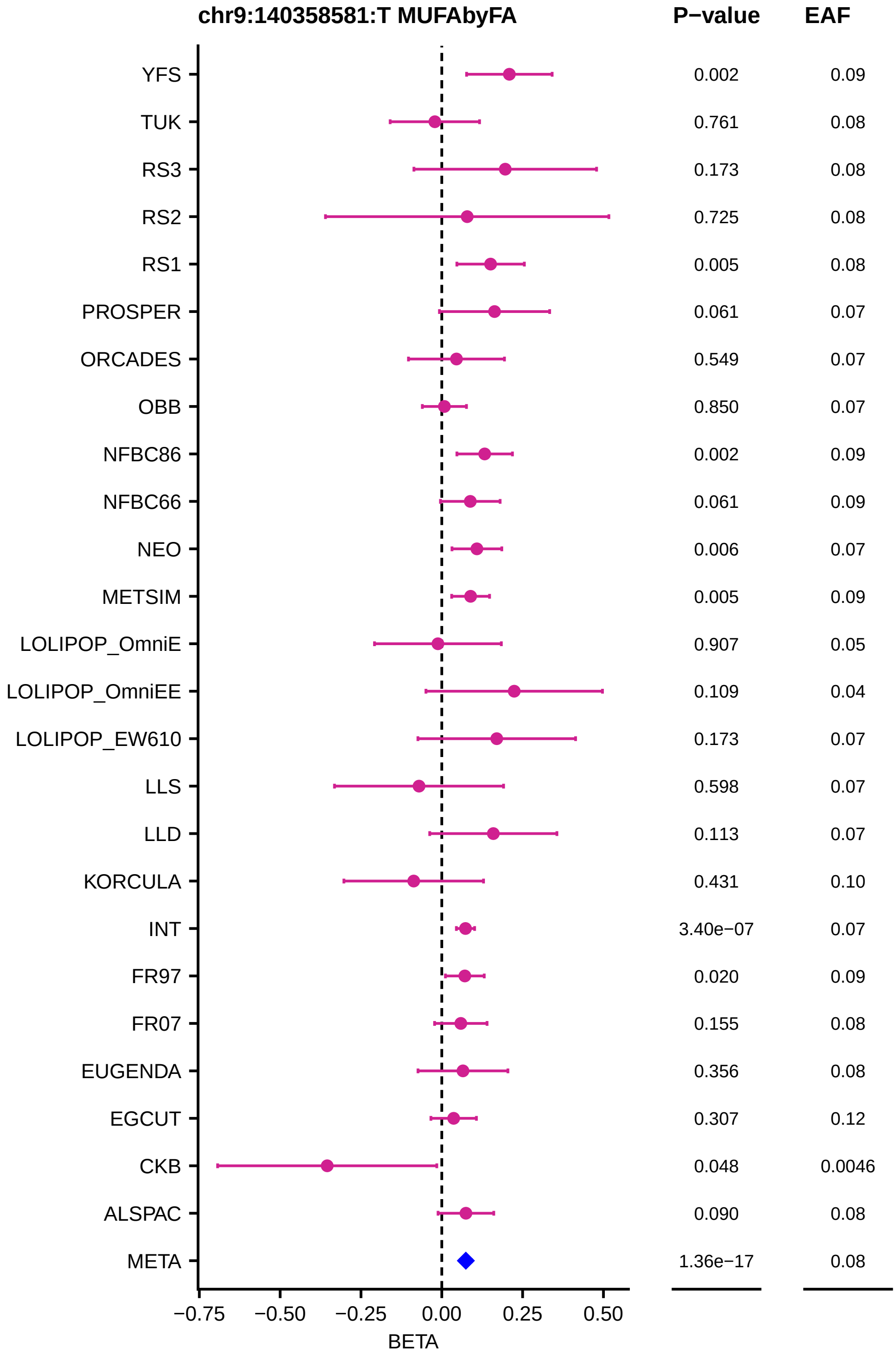
chr9:140358581:T MUFAbyFA
P−value
EAF
YFS 0.002 0.09
TUK 0.761 0.08
RS3 0.173 0.08
RS2 0.725 0.08
RS1 0.005 0.08
PROSPER 0.061 0.07
ORCADES 0.549 0.07
OBB 0.850 0.07
NFBC86 0.002 0.09
NFBC66 0.061 0.09
NEO 0.006 0.07
METSIM 0.005 0.09
LOLIPOP_OmniE 0.907 0.05
LOLIPOP_OmniEE 0.109 0.04
LOLIPOP_EW610 0.173 0.07
LLS 0.598 0.07
LLD 0.113 0.07
KORCULA 0.431 0.10
INT 3.40e−07 0.07
FR97 0.020 0.09
FR07 0.155 0.08
EUGENDA 0.356 0.08
EGCUT 0.307 0.12
CKB 0.048 0.0046
ALSPAC 0.090 0.08
META 1.36e−17 0.08
−0.75
−0.50
−0.25
0.00
0.25
0.50
BETA

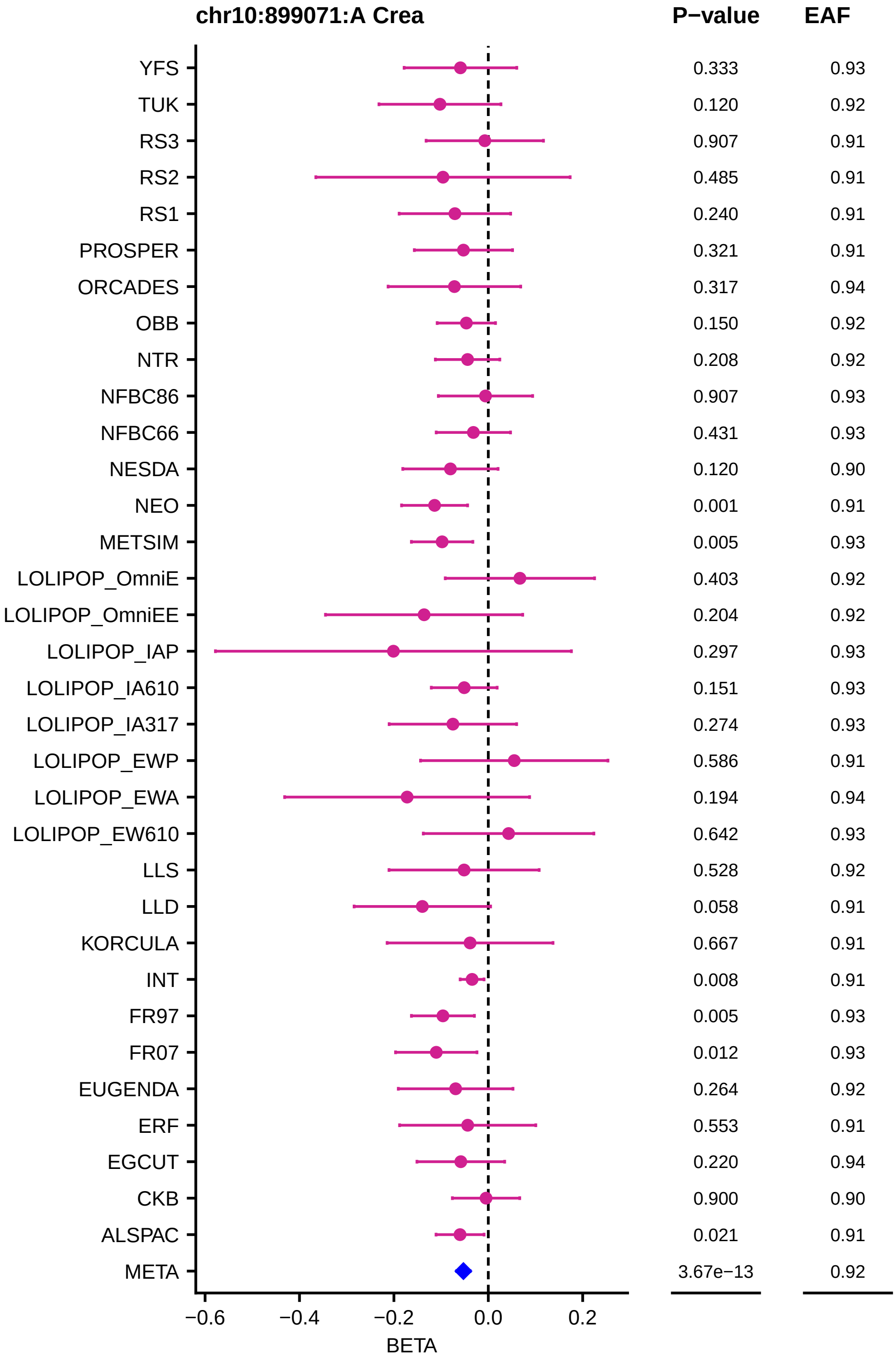
chr10:899071:A Crea
P−value
EAF
YFS 0.333 0.93
TUK 0.120 0.92
RS3 0.907 0.91
RS2 0.485 0.91
RS1 0.240 0.91
PROSPER 0.321 0.91
ORCADES 0.317 0.94
OBB 0.150 0.92
NTR 0.208 0.92
NFBC86 0.907 0.93
NFBC66 0.431 0.93
NESDA 0.120 0.90
NEO 0.001 0.91
METSIM 0.005 0.93
LOLIPOP_OmniE 0.403 0.92
LOLIPOP_OmniEE 0.204 0.92
LOLIPOP_IAP 0.297 0.93
LOLIPOP_IA610 0.151 0.93
LOLIPOP_IA317 0.274 0.93
LOLIPOP_EWP 0.586 0.91
LOLIPOP_EWA 0.194 0.94
LOLIPOP_EW610 0.642 0.93
LLS 0.528 0.92
LLD 0.058 0.91
KORCULA 0.667 0.91
INT 0.008 0.91
FR97 0.005 0.93
FR07 0.012 0.93
EUGENDA 0.264 0.92
ERF 0.553 0.91
EGCUT 0.220 0.94
CKB 0.900 0.90
ALSPAC 0.021 0.91
META 3.67e−13 0.92
−0.6
−0.4
−0.2
0.0
0.2
BETA

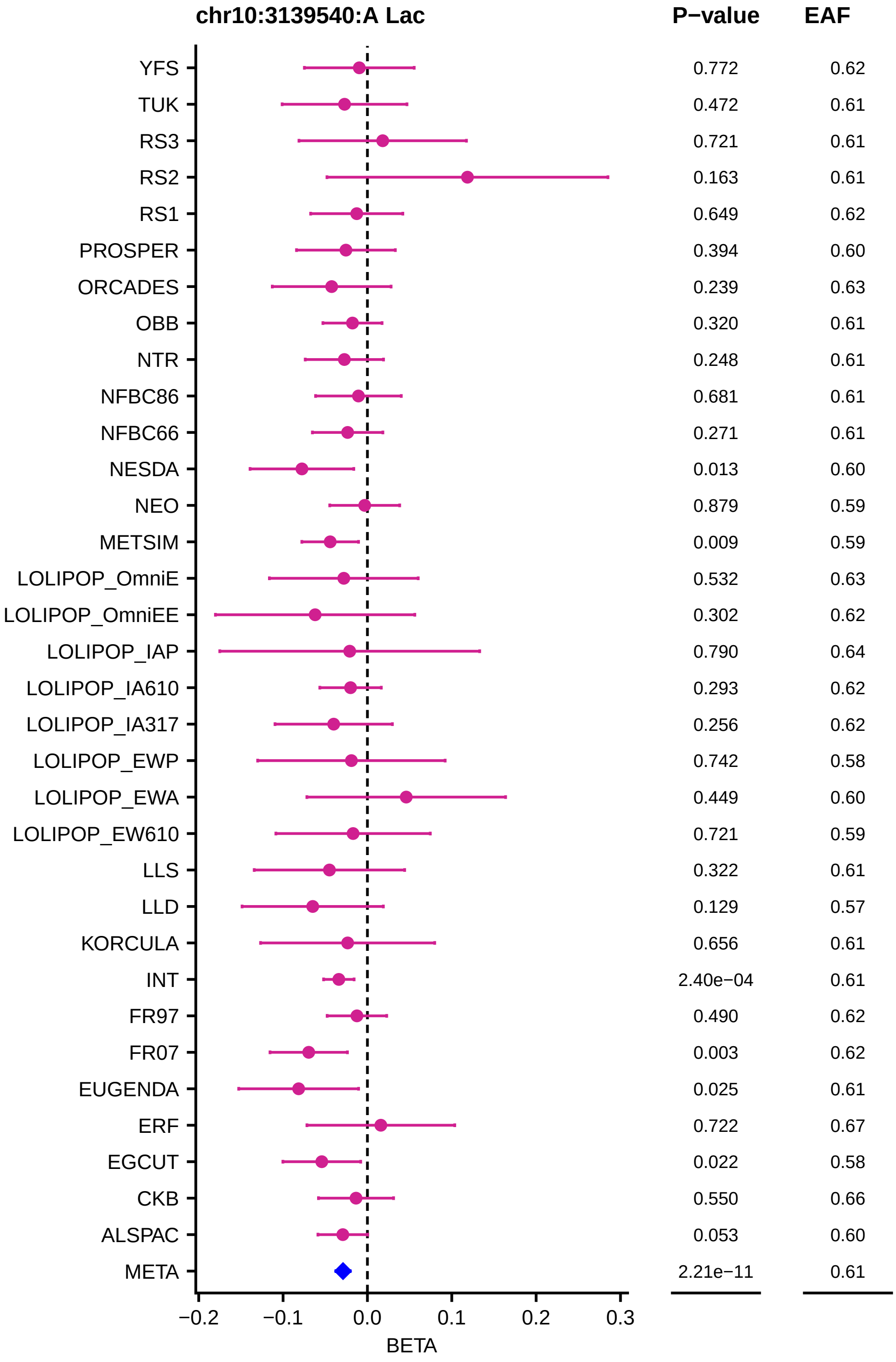
chr10:3139540:A Lac
P−value
EAF
YFS 0.772 0.62
TUK 0.472 0.61
RS3 0.721 0.61
RS2 0.163 0.61
RS1 0.649 0.62
PROSPER 0.394 0.60
ORCADES 0.239 0.63
OBB 0.320 0.61
NTR 0.248 0.61
NFBC86 0.681 0.61
NFBC66 0.271 0.61
NESDA 0.013 0.60
NEO 0.879 0.59
METSIM 0.009 0.59
LOLIPOP_OmniE 0.532 0.63
LOLIPOP_OmniEE 0.302 0.62
LOLIPOP_IAP 0.790 0.64
LOLIPOP_IA610 0.293 0.62
LOLIPOP_IA317 0.256 0.62
LOLIPOP_EWP 0.742 0.58
LOLIPOP_EWA 0.449 0.60
LOLIPOP_EW610 0.721 0.59
LLS 0.322 0.61
LLD 0.129 0.57
KORCULA 0.656 0.61
INT 2.40e−04 0.61
FR97 0.490 0.62
FR07 0.003 0.62
EUGENDA 0.025 0.61
ERF 0.722 0.67
EGCUT 0.022 0.58
CKB 0.550 0.66
ALSPAC 0.053 0.60
META 2.21e−11 0.61
−0.2
−0.1
0.0
0.1
0.2
0.3
BETA

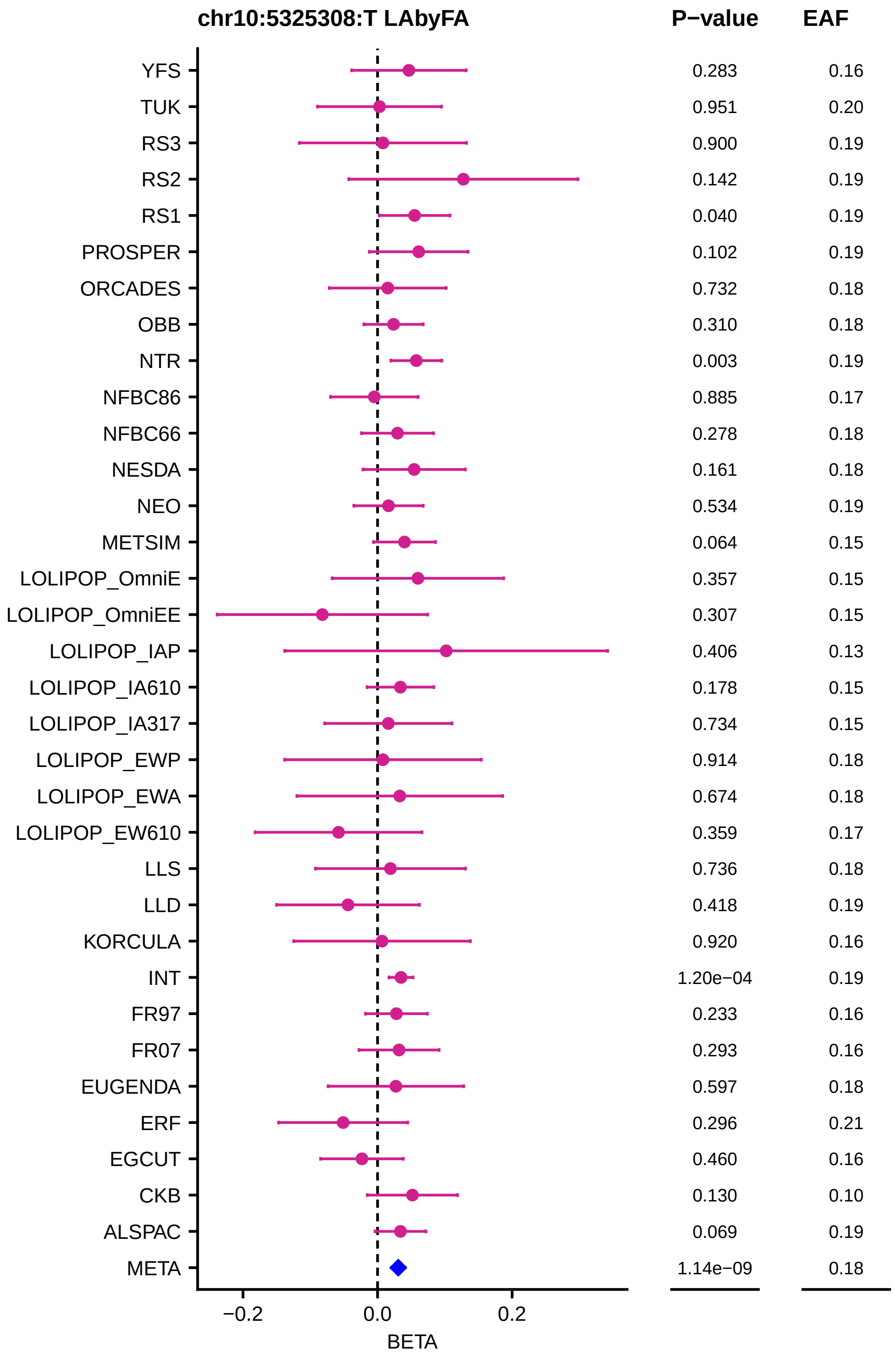
chr10:5325308:T LAbyFA
P−value
EAF
YFS 0.283 0.16
TUK 0.951 0.20
RS3 0.900 0.19
RS2 0.142 0.19
RS1 0.040 0.19
PROSPER 0.102 0.19
ORCADES 0.732 0.18
OBB 0.310 0.18
NTR 0.003 0.19
NFBC86 0.885 0.17
NFBC66 0.278 0.18
NESDA 0.161 0.18
NEO 0.534 0.19
METSIM 0.064 0.15
LOLIPOP_OmniE 0.357 0.15
LOLIPOP_OmniEE 0.307 0.15
LOLIPOP_IAP 0.406 0.13
LOLIPOP_IA610 0.178 0.15
LOLIPOP_IA317 0.734 0.15
LOLIPOP_EWP 0.914 0.18
LOLIPOP_EWA 0.674 0.18
LOLIPOP_EW610 0.359 0.17
LLS 0.736 0.18
LLD 0.418 0.19
KORCULA 0.920 0.16
INT 1.20e−04 0.19
FR97 0.233 0.16
FR07 0.293 0.16
EUGENDA 0.597 0.18
ERF 0.296 0.21
EGCUT 0.460 0.16
CKB 0.130 0.10
ALSPAC 0.069 0.19
META 1.14e−09 0.18
−0.2
0.0
0.2
BETA

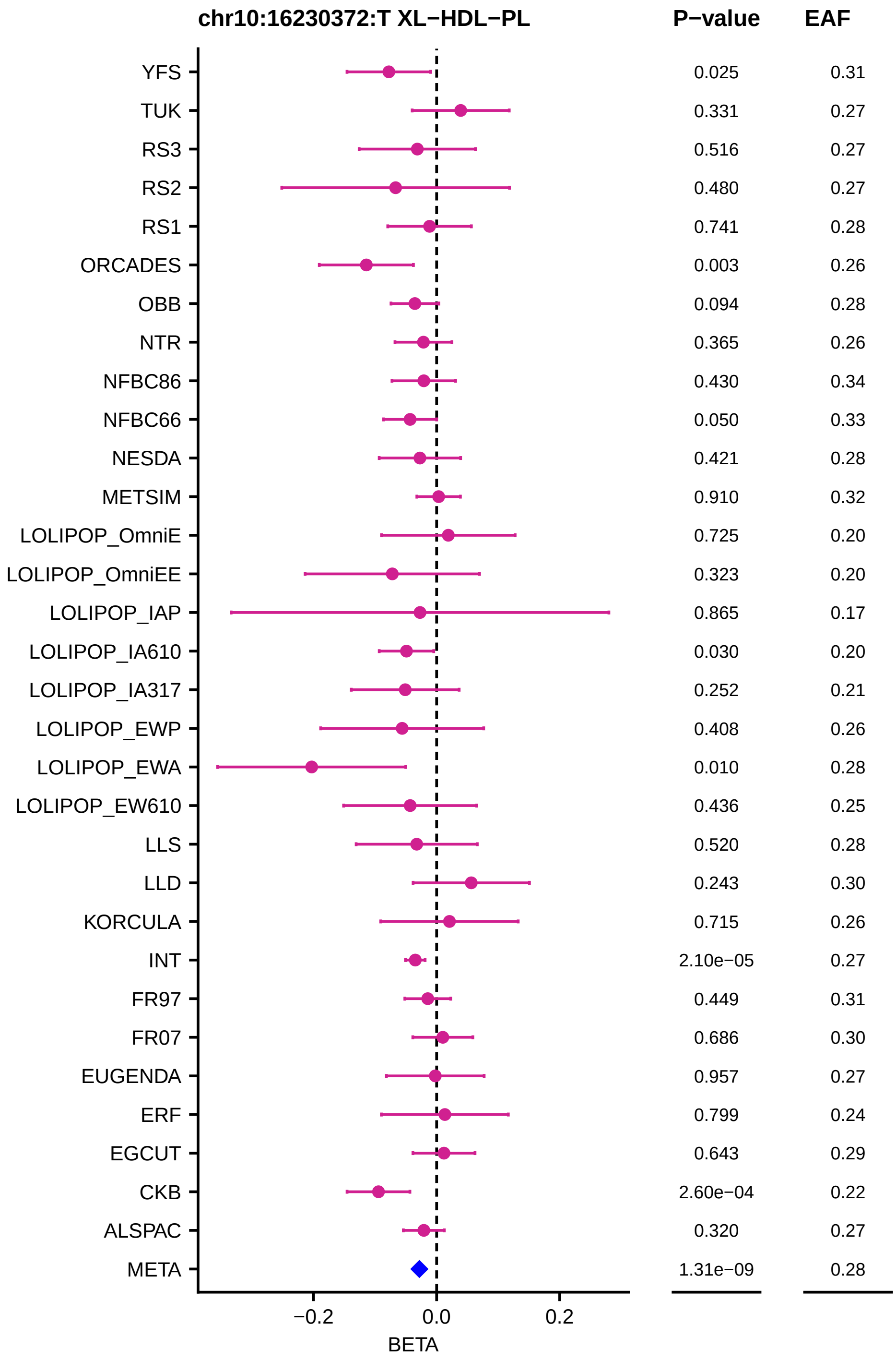
chr10:16230372:T XL−HDL−PL
P−value
EAF
YFS 0.025 0.31
TUK 0.331 0.27
RS3 0.516 0.27
RS2 0.480 0.27
RS1 0.741 0.28
ORCADES 0.003 0.26
OBB 0.094 0.28
NTR 0.365 0.26
NFBC86 0.430 0.34
NFBC66 0.050 0.33
NESDA 0.421 0.28
METSIM 0.910 0.32
LOLIPOP_OmniE 0.725 0.20
LOLIPOP_OmniEE 0.323 0.20
LOLIPOP_IAP 0.865 0.17
LOLIPOP_IA610 0.030 0.20
LOLIPOP_IA317 0.252 0.21
LOLIPOP_EWP 0.408 0.26
LOLIPOP_EWA 0.010 0.28
LOLIPOP_EW610 0.436 0.25
LLS 0.520 0.28
LLD 0.243 0.30
KORCULA 0.715 0.26
INT 2.10e−05 0.27
FR97 0.449 0.31
FR07 0.686 0.30
EUGENDA 0.957 0.27
ERF 0.799 0.24
EGCUT 0.643 0.29
CKB 2.60e−04 0.22
ALSPAC 0.320 0.27
META 1.31e−09 0.28
−0.2
0.0
0.2
BETA

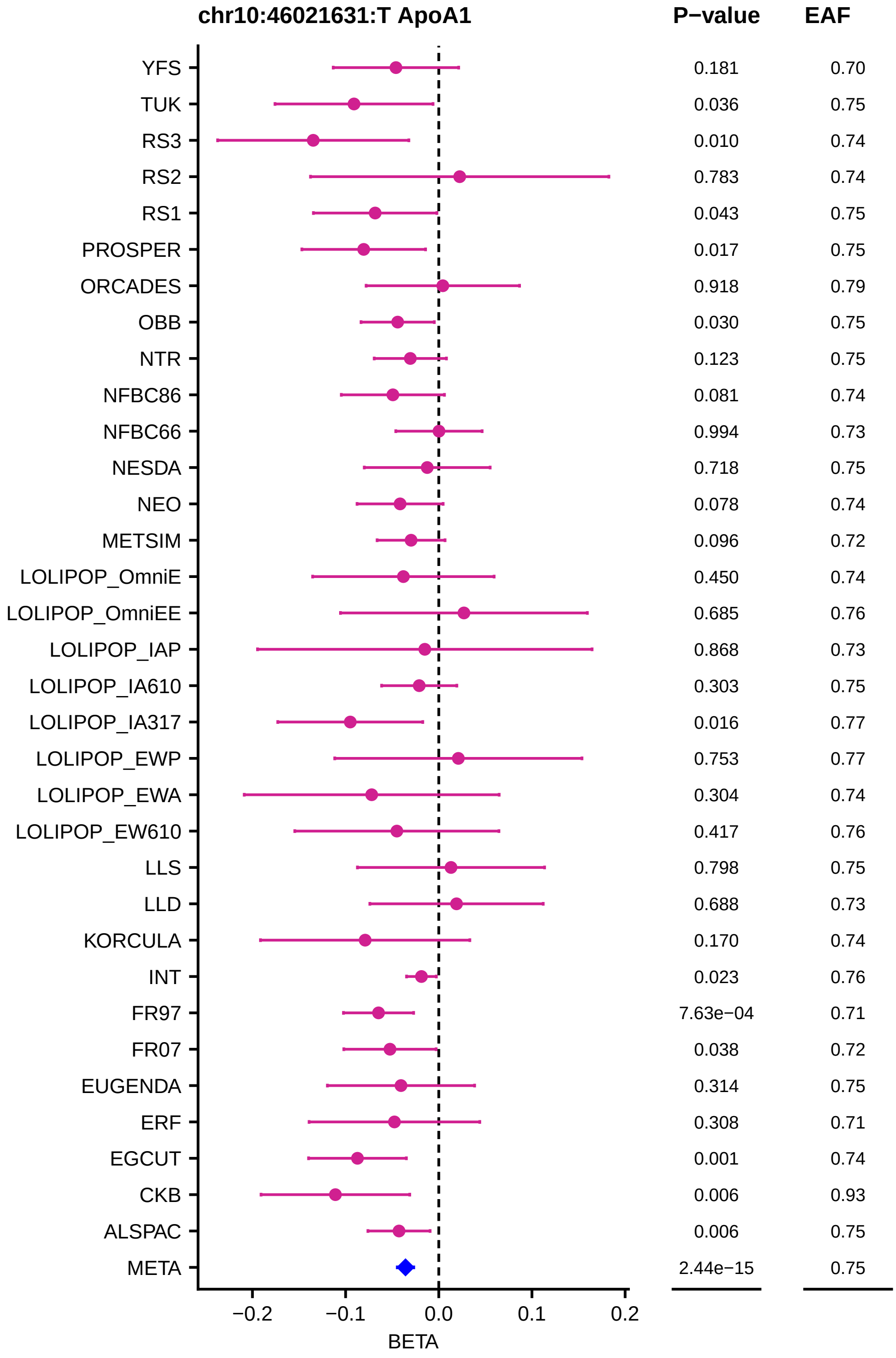
chr10:46021631:T ApoA1
P−value
EAF
YFS
0.181
0.70
TUK
0.036
0.75
RS3
0.010
0.74
RS2
0.783
0.74
RS1
0.043
0.75
PROSPER
0.017
0.75
ORCADES
0.918
0.79
OBB
0.030
0.75
NTR
0.123
0.75
NFBC86
0.081
0.74
NFBC66
0.994
0.73
NESDA
0.718
0.75
NEO
0.078
0.74
METSIM
0.096
0.72
LOLIPOP_OmniE
0.450
0.74
LOLIPOP_OmniEE
0.685
0.76
LOLIPOP_IAP
0.868
0.73
LOLIPOP_IA610
0.303
0.75
LOLIPOP_IA317
0.016
0.77
LOLIPOP_EWP
0.753
0.77
LOLIPOP_EWA
0.304
0.74
LOLIPOP_EW610
0.417
0.76
LLS
0.798
0.75
LLD
0.688
0.73
KORCULA
0.170
0.74
INT
0.023
0.76
FR97
7.63e−04
0.71
FR07
0.038
0.72
EUGENDA
0.314
0.75
ERF
0.308
0.71
EGCUT
0.001
0.74
CKB
0.006
0.93
ALSPAC
0.006
0.75
META
2.44e−15
0.75
−0.2
−0.1
0.0
0.1
0.2
BETA

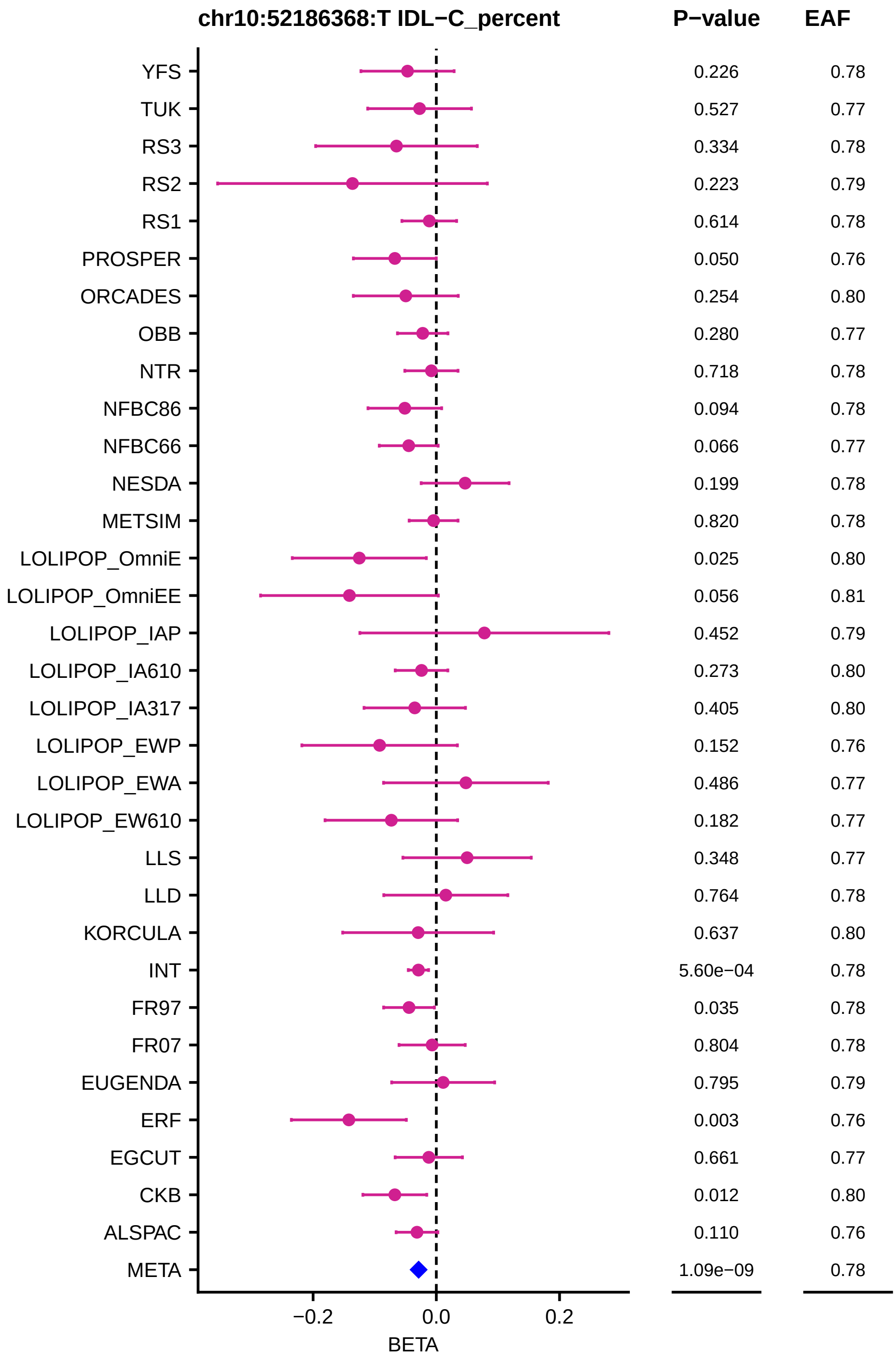
chr10:52186368:T IDL−C_percent
P−value
EAF
YFS
0.226
0.78
TUK
0.527
0.77
RS3
0.334
0.78
RS2
0.223
0.79
RS1
0.614
0.78
PROSPER
0.050
0.76
ORCADES
0.254
0.80
OBB
0.280
0.77
NTR
0.718
0.78
NFBC86
0.094
0.78
NFBC66
0.066
0.77
NESDA
0.199
0.78
METSIM
0.820
0.78
LOLIPOP_OmniE
0.025
0.80
LOLIPOP_OmniEE
0.056
0.81
LOLIPOP_IAP
0.452
0.79
LOLIPOP_IA610
0.273
0.80
LOLIPOP_IA317
0.405
0.80
LOLIPOP_EWP
0.152
0.76
LOLIPOP_EWA
0.486
0.77
LOLIPOP_EW610
0.182
0.77
LLS
0.348
0.77
LLD
0.764
0.78
KORCULA
0.637
0.80
INT
5.60e−04
0.78
FR97
0.035
0.78
FR07
0.804
0.78
EUGENDA
0.795
0.79
ERF
0.003
0.76
EGCUT
0.661
0.77
CKB
0.012
0.80
ALSPAC
0.110
0.76
META
1.09e−09
0.78
−0.2
0.0
0.2
BETA

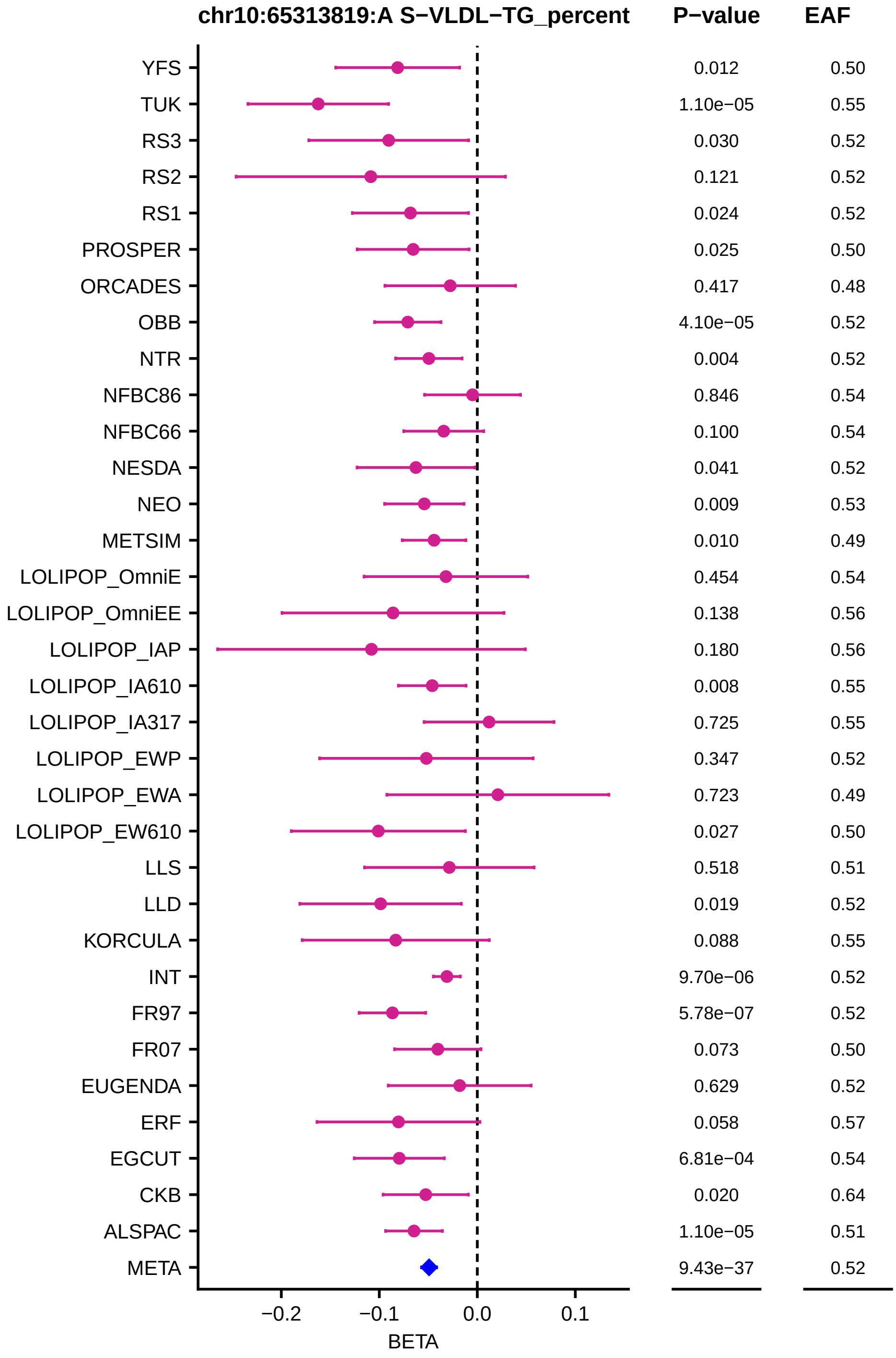
chr10:65313819:A S−VLDL−TG_percent
P−value
EAF
YFS 0.012 0.50
TUK 1.10e−05 0.55
RS3 0.030 0.52
RS2 0.121 0.52
RS1 0.024 0.52
PROSPER 0.025 0.50
ORCADES 0.417 0.48
OBB 4.10e−05 0.52
NTR 0.004 0.52
NFBC86 0.846 0.54
NFBC66 0.100 0.54
NESDA 0.041 0.52
NEO 0.009 0.53
METSIM 0.010 0.49
LOLIPOP_OmniE 0.454 0.54
LOLIPOP_OmniEE 0.138 0.56
LOLIPOP_IAP 0.180 0.56
LOLIPOP_IA610 0.008 0.55
LOLIPOP_IA317 0.725 0.55
LOLIPOP_EWP 0.347 0.52
LOLIPOP_EWA 0.723 0.49
LOLIPOP_EW610 0.027 0.50
LLS 0.518 0.51
LLD 0.019 0.52
KORCULA 0.088 0.55
INT 9.70e−06 0.52
FR97 5.78e−07 0.52
FR07 0.073 0.50
EUGENDA 0.629 0.52
ERF 0.058 0.57
EGCUT 6.81e−04 0.54
CKB 0.020 0.64
ALSPAC 1.10e−05 0.51
META 9.43e−37 0.52
−0.2
−0.1
0.0
0.1
BETA

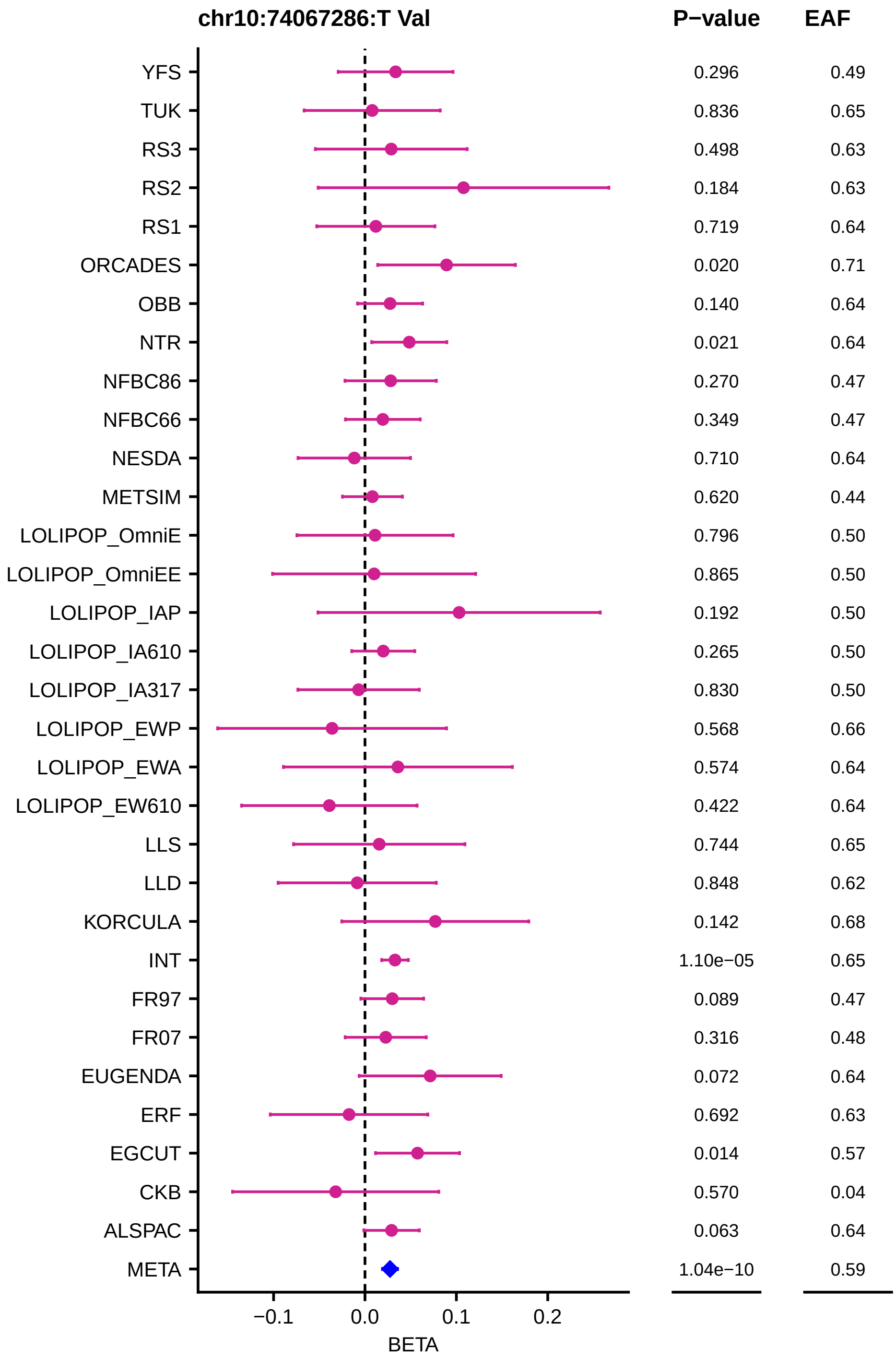

chr10:74067286:T Val
P−value
EAF
YFS 0.296 0.49
TUK 0.836 0.65
RS3 0.498 0.63
RS2 0.184 0.63
RS1 0.719 0.64
ORCADES 0.020 0.71
OBB 0.140 0.64
NTR 0.021 0.64
NFBC86 0.270 0.47
NFBC66 0.349 0.47
NESDA 0.710 0.64
METSIM 0.620 0.44
LOLIPOP_OmniE 0.796 0.50
LOLIPOP_OmniEE 0.865 0.50
LOLIPOP_IAP 0.192 0.50
LOLIPOP_IA610 0.265 0.50
LOLIPOP_IA317 0.830 0.50
LOLIPOP_EWP 0.568 0.66
LOLIPOP_EWA 0.574 0.64
LOLIPOP_EW610 0.422 0.64
LLS 0.744 0.65
LLD 0.848 0.62
KORCULA 0.142 0.68
INT 1.10e−05 0.65
FR97 0.089 0.47
FR07 0.316 0.48
EUGENDA 0.072 0.64
ERF 0.692 0.63
EGCUT 0.014 0.57
CKB 0.570 0.04
ALSPAC 0.063 0.64
META 1.04e−10 0.59
−0.1
0.0
0.1
0.2
BETA

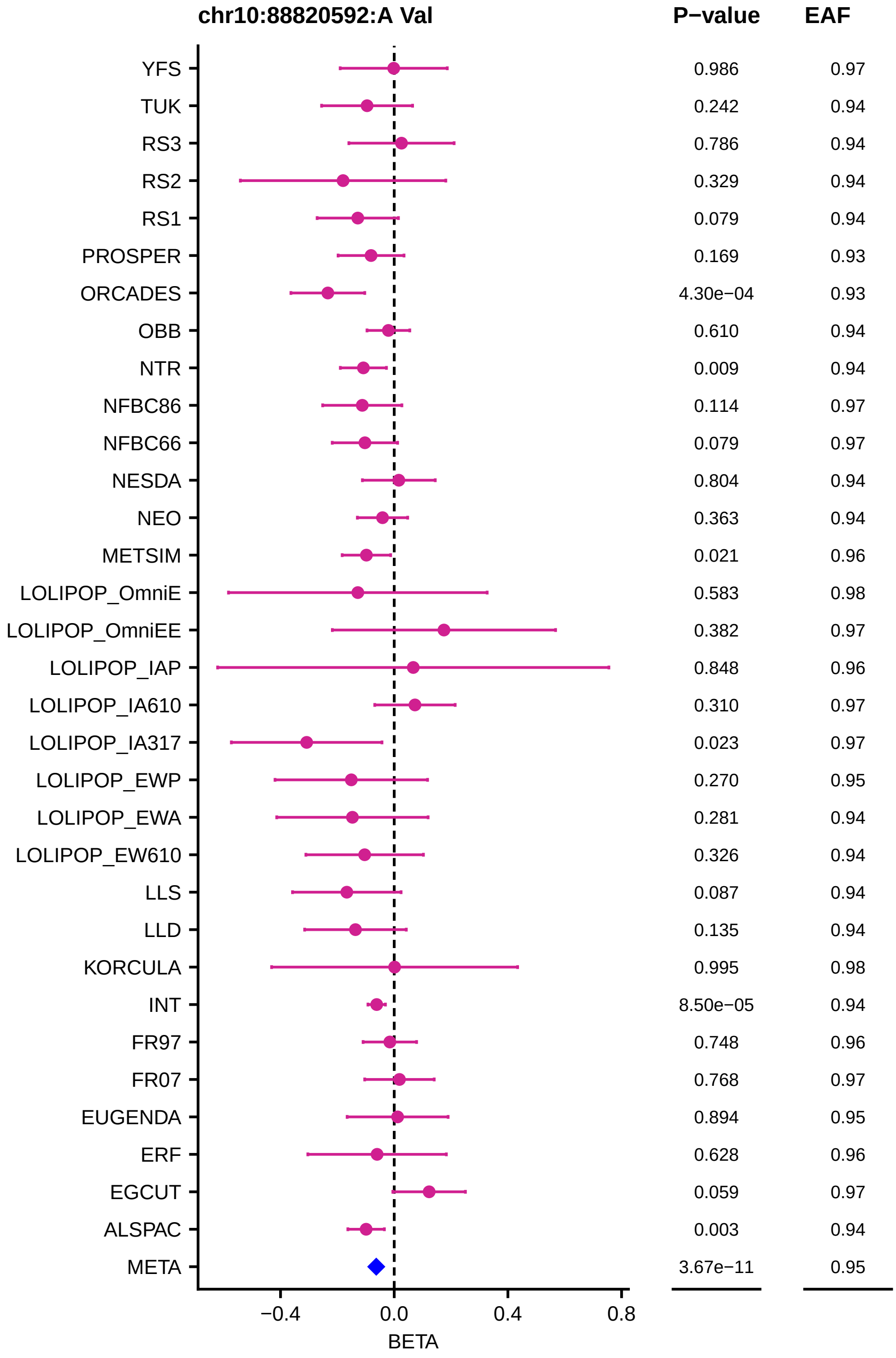
chr10:88820592:A Val
P−value
EAF
YFS 0.986 0.97
TUK 0.242 0.94
RS3 0.786 0.94
RS2 0.329 0.94
RS1 0.079 0.94
PROSPER 0.169 0.93
ORCADES 4.30e−04 0.93
OBB 0.610 0.94
NTR 0.009 0.94
NFBC86 0.114 0.97
NFBC66 0.079 0.97
NESDA 0.804 0.94
NEO 0.363 0.94
METSIM 0.021 0.96
LOLIPOP_OmniE 0.583 0.98
LOLIPOP_OmniEE 0.382 0.97
LOLIPOP_IAP 0.848 0.96
LOLIPOP_IA610 0.310 0.97
LOLIPOP_IA317 0.023 0.97
LOLIPOP_EWP 0.270 0.95
LOLIPOP_EWA 0.281 0.94
LOLIPOP_EW610 0.326 0.94
LLS 0.087 0.94
LLD 0.135 0.94
KORCULA 0.995 0.98
INT 8.50e−05 0.94
FR97 0.748 0.96
FR07 0.768 0.97
EUGENDA 0.894 0.95
ERF 0.628 0.96
EGCUT 0.059 0.97
ALSPAC 0.003 0.94
META 3.67e−11 0.95
−0.4
0.0
0.4
0.8
BETA

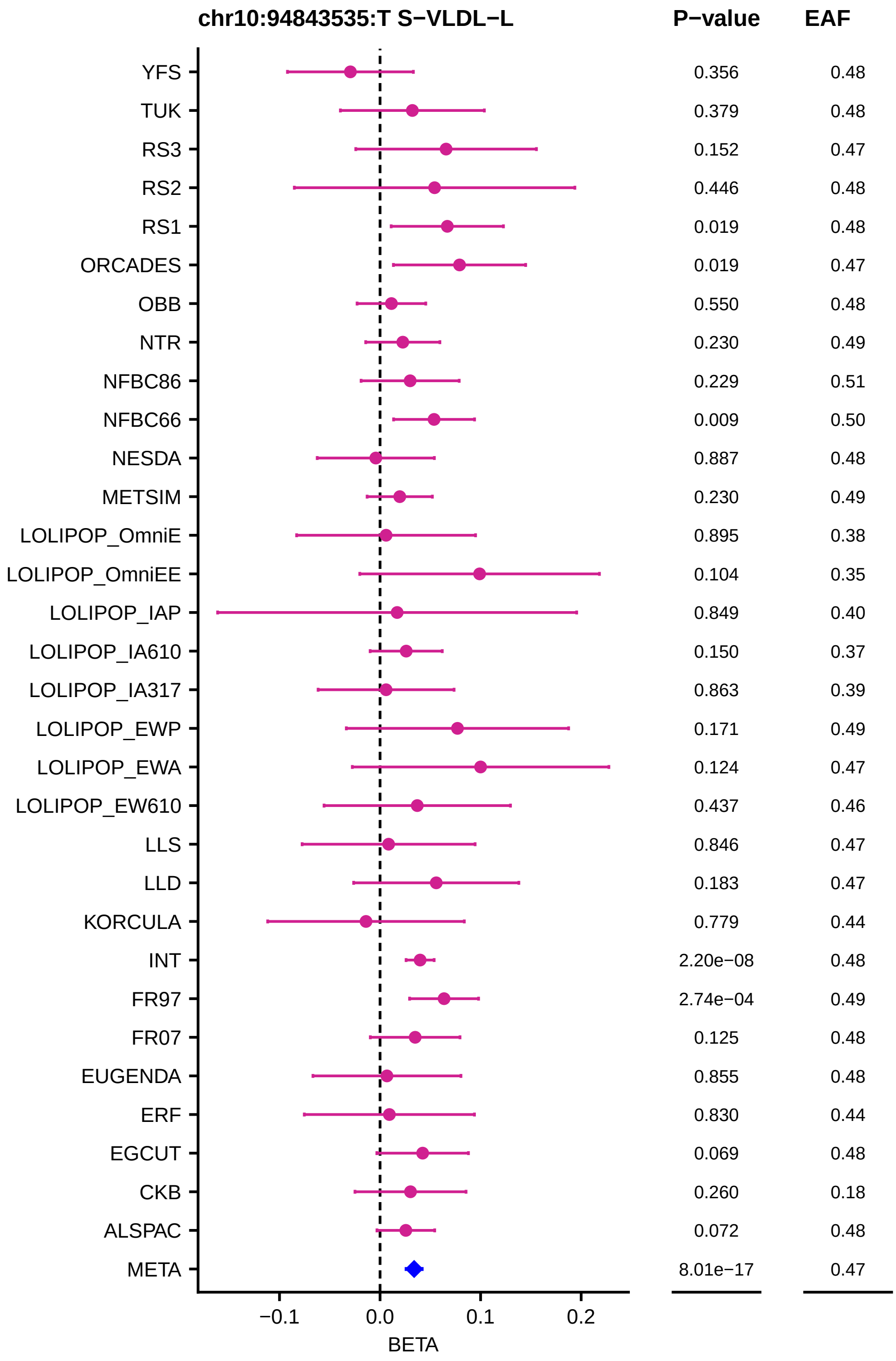
chr10:94843535:T S−VLDL−L
P−value
EAF
YFS 0.356 0.48
TUK 0.379 0.48
RS3 0.152 0.47
RS2 0.446 0.48
RS1 0.019 0.48
ORCADES 0.019 0.47
OBB 0.550 0.48
NTR 0.230 0.49
NFBC86 0.229 0.51
NFBC66 0.009 0.50
NESDA 0.887 0.48
METSIM 0.230 0.49
LOLIPOP_OmniE 0.895 0.38
LOLIPOP_OmniEE 0.104 0.35
LOLIPOP_IAP 0.849 0.40
LOLIPOP_IA610 0.150 0.37
LOLIPOP_IA317 0.863 0.39
LOLIPOP_EWP 0.171 0.49
LOLIPOP_EWA 0.124 0.47
LOLIPOP_EW610 0.437 0.46
LLS 0.846 0.47
LLD 0.183 0.47
KORCULA 0.779 0.44
INT 2.20e−08 0.48
FR97 2.74e−04 0.49
FR07 0.125 0.48
EUGENDA 0.855 0.48
ERF 0.830 0.44
EGCUT 0.069 0.48
CKB 0.260 0.18
ALSPAC 0.072 0.48
META 8.01e−17 0.47
−0.1
0.0
0.1
0.2
BETA

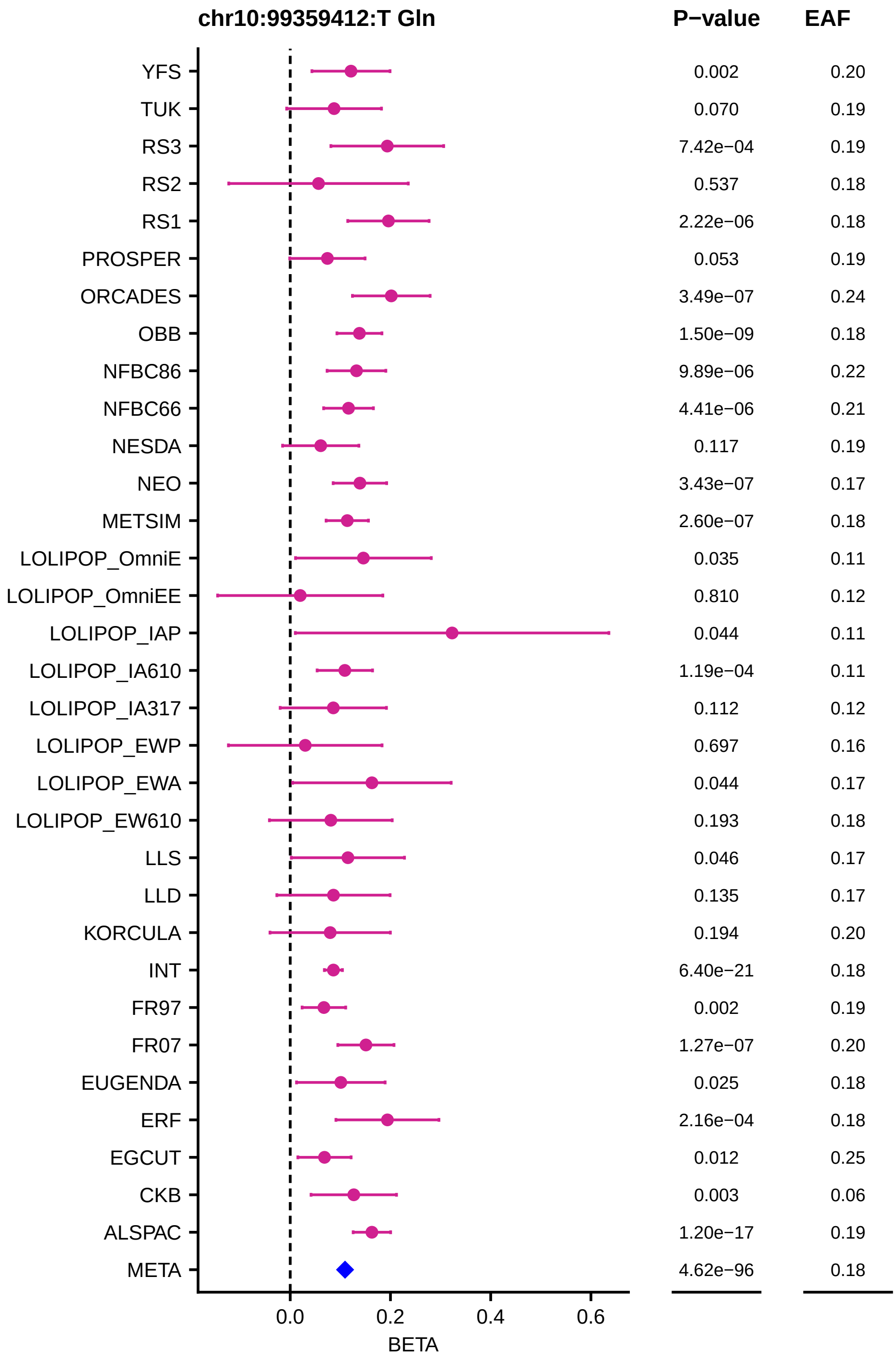
chr10:99359412:T Gln
P−value
EAF
YFS 0.002 0.20
TUK 0.070 0.19
RS3 7.42e−04 0.19
RS2 0.537 0.18
RS1 2.22e−06 0.18
PROSPER 0.053 0.19
ORCADES 3.49e−07 0.24
OBB 1.50e−09 0.18
NFBC86 9.89e−06 0.22
NFBC66 4.41e−06 0.21
NESDA 0.117 0.19
NEO 3.43e−07 0.17
METSIM 2.60e−07 0.18
LOLIPOP_OmniE 0.035 0.11
LOLIPOP_OmniEE 0.810 0.12
LOLIPOP_IAP 0.044 0.11
LOLIPOP_IA610 1.19e−04 0.11
LOLIPOP_IA317 0.112 0.12
LOLIPOP_EWP 0.697 0.16
LOLIPOP_EWA 0.044 0.17
LOLIPOP_EW610 0.193 0.18
LLS 0.046 0.17
LLD 0.135 0.17
KORCULA 0.194 0.20
INT 6.40e−21 0.18
FR97 0.002 0.19
FR07 1.27e−07 0.20
EUGENDA 0.025 0.18
ERF 2.16e−04 0.18
EGCUT 0.012 0.25
CKB 0.003 0.06
ALSPAC 1.20e−17 0.19
META 4.62e−96 0.18
0.0
0.2
0.4
0.6
BETA

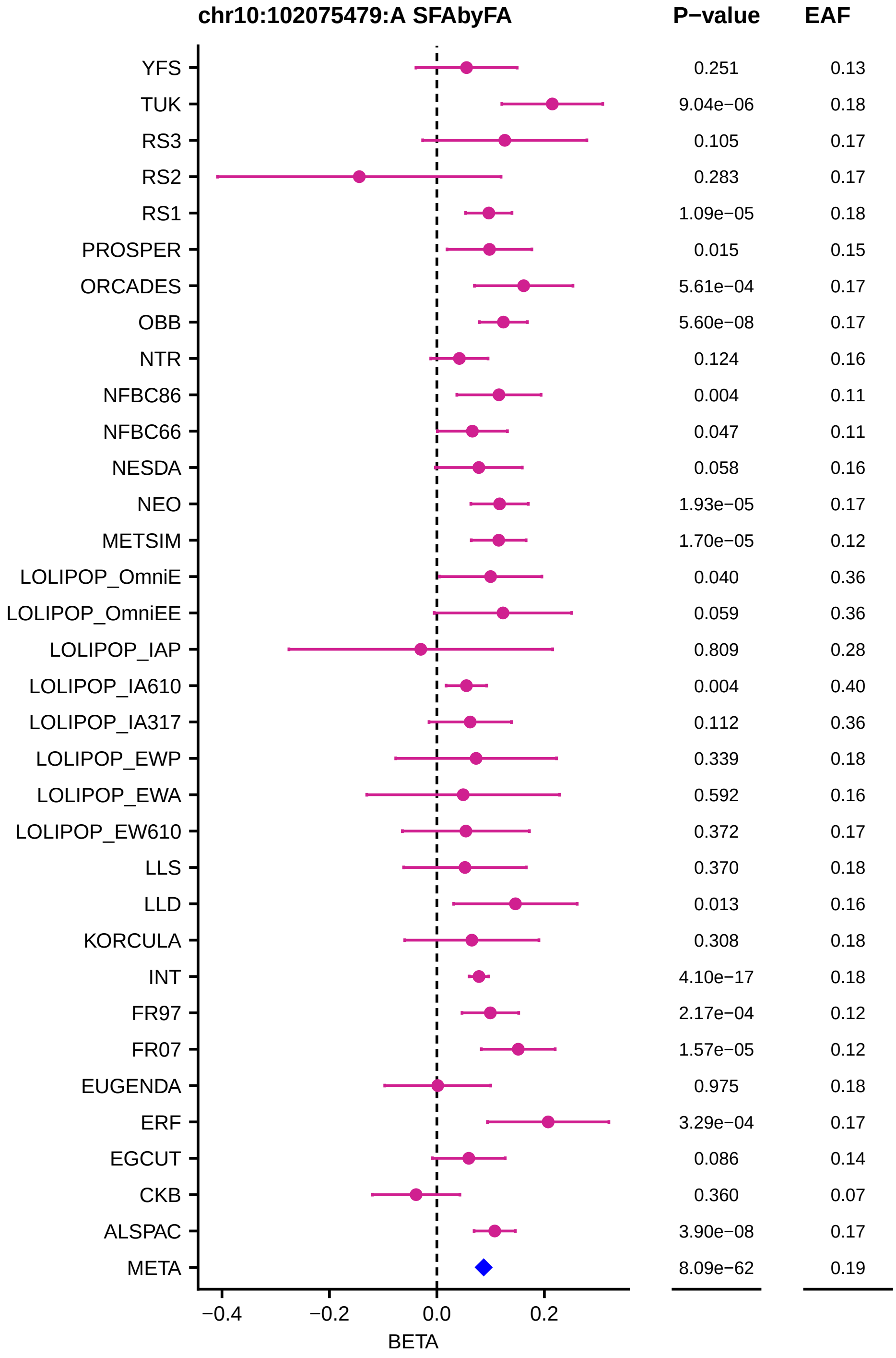

chr10:102075479:A SFAbyFA
P−value
EAF
YFS 0.251 0.13
TUK 9.04e−06 0.18
RS3 0.105 0.17
RS2 0.283 0.17
RS1 1.09e−05 0.18
PROSPER 0.015 0.15
ORCADES 5.61e−04 0.17
OBB 5.60e−08 0.17
NTR 0.124 0.16
NFBC86 0.004 0.11
NFBC66 0.047 0.11
NESDA 0.058 0.16
NEO 1.93e−05 0.17
METSIM 1.70e−05 0.12
LOLIPOP_OmniE 0.040 0.36
LOLIPOP_OmniEE 0.059 0.36
LOLIPOP_IAP 0.809 0.28
LOLIPOP_IA610 0.004 0.40
LOLIPOP_IA317 0.112 0.36
LOLIPOP_EWP 0.339 0.18
LOLIPOP_EWA 0.592 0.16
LOLIPOP_EW610 0.372 0.17
LLS 0.370 0.18
LLD 0.013 0.16
KORCULA 0.308 0.18
INT 4.10e−17 0.18
FR97 2.17e−04 0.12
FR07 1.57e−05 0.12
EUGENDA 0.975 0.18
ERF 3.29e−04 0.17
EGCUT 0.086 0.14
CKB 0.360 0.07
ALSPAC 3.90e−08 0.17
META 8.09e−62 0.19
−0.4
−0.2
0.0
0.2
BETA

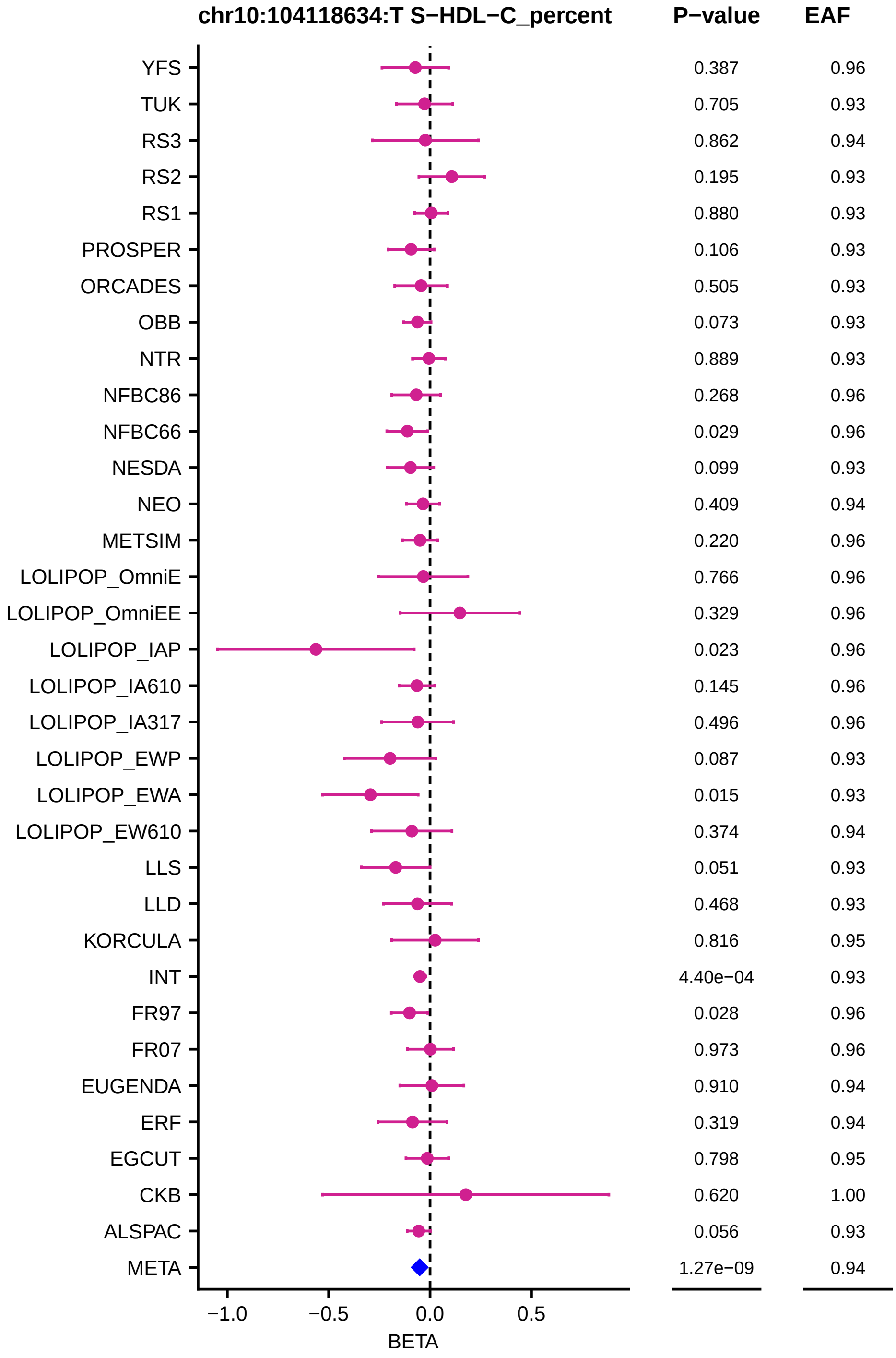
chr10:104118634:T S−HDL−C_percent
P−value
EAF
YFS
0.387
0.96
TUK
0.705
0.93
RS3
0.862
0.94
RS2
0.195
0.93
RS1
0.880
0.93
PROSPER
0.106
0.93
ORCADES
0.505
0.93
OBB
0.073
0.93
NTR
0.889
0.93
NFBC86
0.268
0.96
NFBC66
0.029
0.96
NESDA
0.099
0.93
NEO
0.409
0.94
METSIM
0.220
0.96
LOLIPOP_OmniE
0.766
0.96
LOLIPOP_OmniEE
0.329
0.96
LOLIPOP_IAP
0.023
0.96
LOLIPOP_IA610
0.145
0.96
LOLIPOP_IA317
0.496
0.96
LOLIPOP_EWP
0.087
0.93
LOLIPOP_EWA
0.015
0.93
LOLIPOP_EW610
0.374
0.94
LLS
0.051
0.93
LLD
0.468
0.93
KORCULA
0.816
0.95
INT
4.40e−04
0.93
FR97
0.028
0.96
FR07
0.973
0.96
EUGENDA
0.910
0.94
ERF
0.319
0.94
EGCUT
0.798
0.95
CKB
0.620
1.00
ALSPAC
0.056
0.93
META
1.27e−09
0.94
−1.0
−0.5
0.0
0.5
BETA

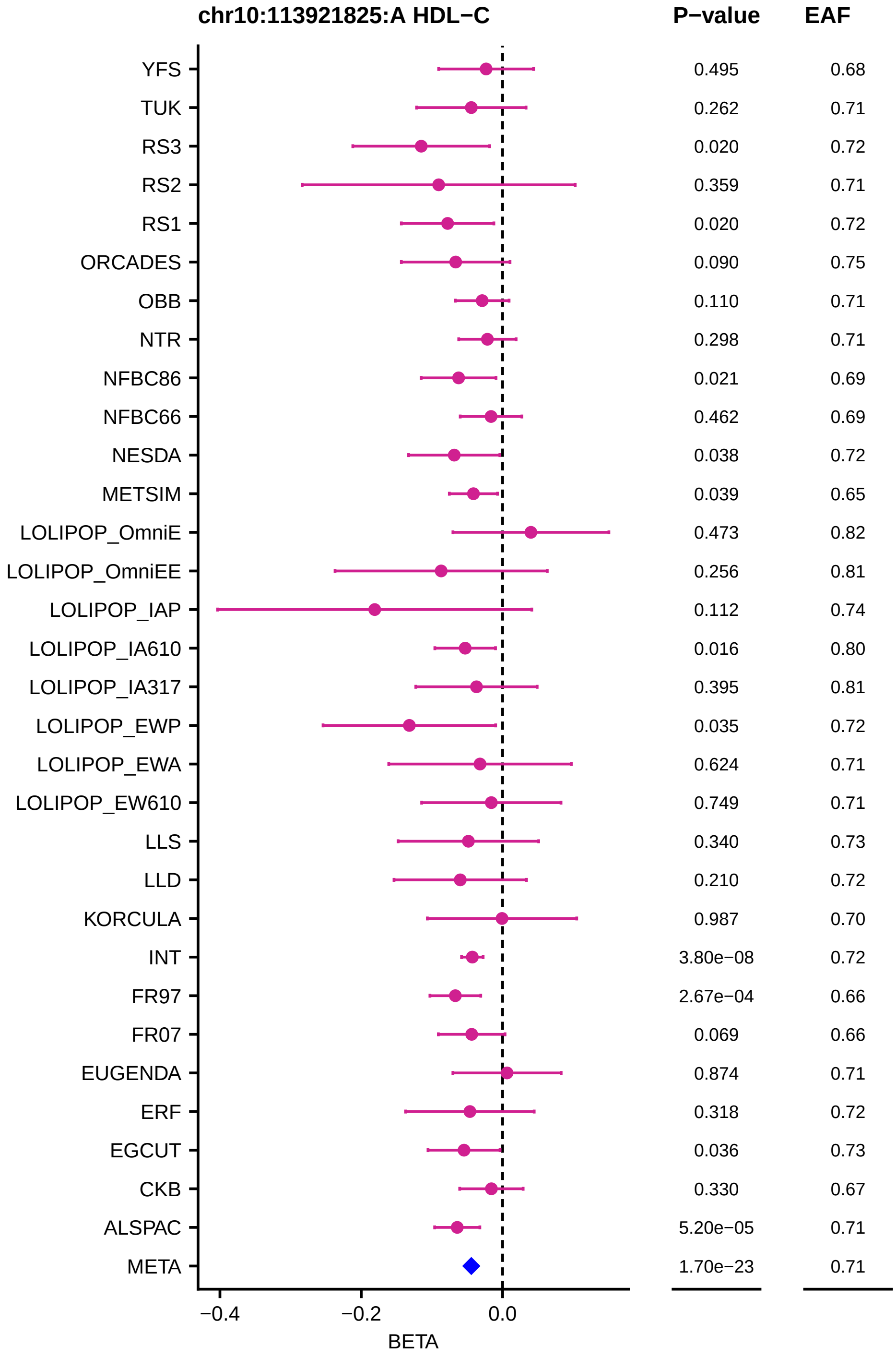
chr10:113921825:A HDL−C
P−value
EAF
YFS 0.495 0.68
TUK 0.262 0.71
RS3 0.020 0.72
RS2 0.359 0.71
RS1 0.020 0.72
ORCADES 0.090 0.75
OBB 0.110 0.71
NTR 0.298 0.71
NFBC86 0.021 0.69
NFBC66 0.462 0.69
NESDA 0.038 0.72
METSIM 0.039 0.65
LOLIPOP_OmniE 0.473 0.82
LOLIPOP_OmniEE 0.256 0.81
LOLIPOP_IAP 0.112 0.74
LOLIPOP_IA610 0.016 0.80
LOLIPOP_IA317 0.395 0.81
LOLIPOP_EWP 0.035 0.72
LOLIPOP_EWA 0.624 0.71
LOLIPOP_EW610 0.749 0.71
LLS 0.340 0.73
LLD 0.210 0.72
KORCULA 0.987 0.70
INT 3.80e−08 0.72
FR97 2.67e−04 0.66
FR07 0.069 0.66
EUGENDA 0.874 0.71
ERF 0.318 0.72
EGCUT 0.036 0.73
CKB 0.330 0.67
ALSPAC 5.20e−05 0.71
META 1.70e−23 0.71
−0.4
−0.2
0.0
BETA

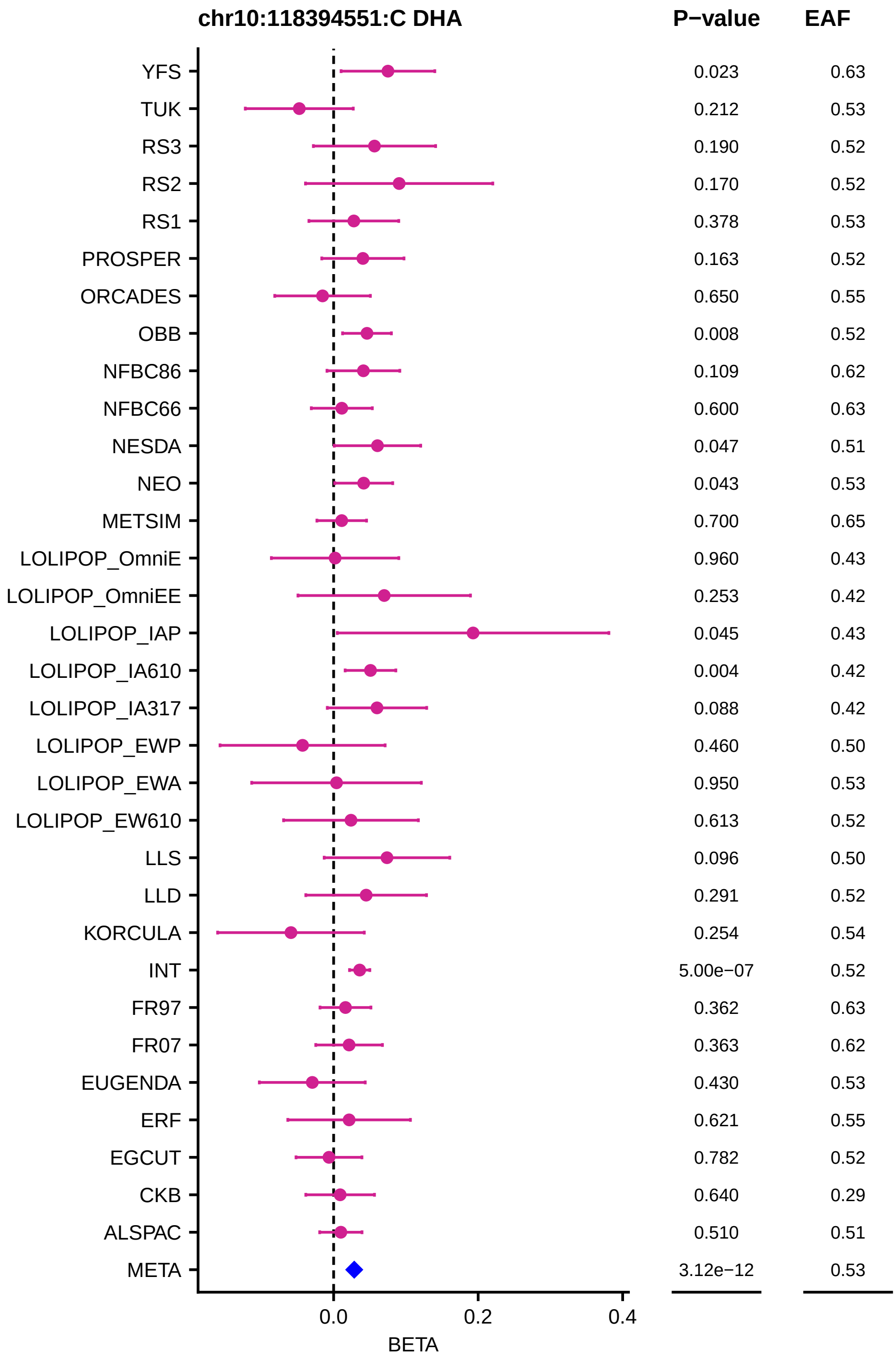

chr10:118394551:C DHA
P−value
EAF
YFS 0.023 0.63
TUK 0.212 0.53
RS3 0.190 0.52
RS2 0.170 0.52
RS1 0.378 0.53
PROSPER 0.163 0.52
ORCADES 0.650 0.55
OBB 0.008 0.52
NFBC86 0.109 0.62
NFBC66 0.600 0.63
NESDA 0.047 0.51
NEO 0.043 0.53
METSIM 0.700 0.65
LOLIPOP_OmniE 0.960 0.43
LOLIPOP_OmniEE 0.253 0.42
LOLIPOP_IAP 0.045 0.43
LOLIPOP_IA610 0.004 0.42
LOLIPOP_IA317 0.088 0.42
LOLIPOP_EWP 0.460 0.50
LOLIPOP_EWA 0.950 0.53
LOLIPOP_EW610 0.613 0.52
LLS 0.096 0.50
LLD 0.291 0.52
KORCULA 0.254 0.54
INT 5.00e−07 0.52
FR97 0.362 0.63
FR07 0.363 0.62
EUGENDA 0.430 0.53
ERF 0.621 0.55
EGCUT 0.782 0.52
CKB 0.640 0.29
ALSPAC 0.510 0.51
META 3.12e−12 0.53
0.0
0.2
0.4
BETA

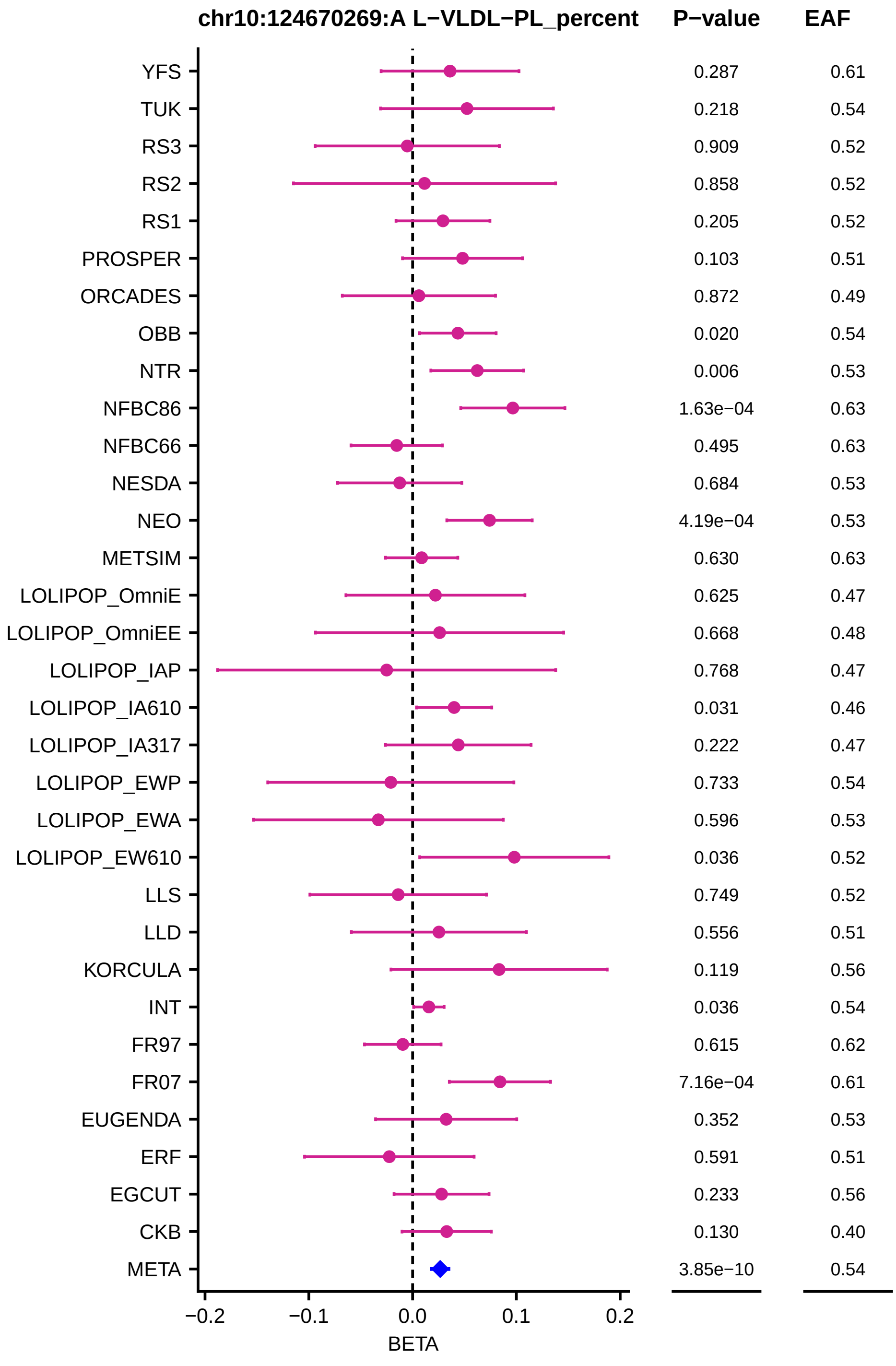

chr10:124670269:A L−VLDL−PL_percent
P−value
EAF
YFS 0.287 0.61
TUK 0.218 0.54
RS3 0.909 0.52
RS2 0.858 0.52
RS1 0.205 0.52
PROSPER 0.103 0.51
ORCADES 0.872 0.49
OBB 0.020 0.54
NTR 0.006 0.53
NFBC86 1.63e−04 0.63
NFBC66 0.495 0.63
NESDA 0.684 0.53
NEO 4.19e−04 0.53
METSIM 0.630 0.63
LOLIPOP_OmniE 0.625 0.47
LOLIPOP_OmniEE 0.668 0.48
LOLIPOP_IAP 0.768 0.47
LOLIPOP_IA610 0.031 0.46
LOLIPOP_IA317 0.222 0.47
LOLIPOP_EWP 0.733 0.54
LOLIPOP_EWA 0.596 0.53
LOLIPOP_EW610 0.036 0.52
LLS 0.749 0.52
LLD 0.556 0.51
KORCULA 0.119 0.56
INT 0.036 0.54
FR97 0.615 0.62
FR07 7.16e−04 0.61
EUGENDA 0.352 0.53
ERF 0.591 0.51
EGCUT 0.233 0.56
CKB 0.130 0.40
META 3.85e−10 0.54
−0.2
−0.1
0.0
0.1
0.2
BETA

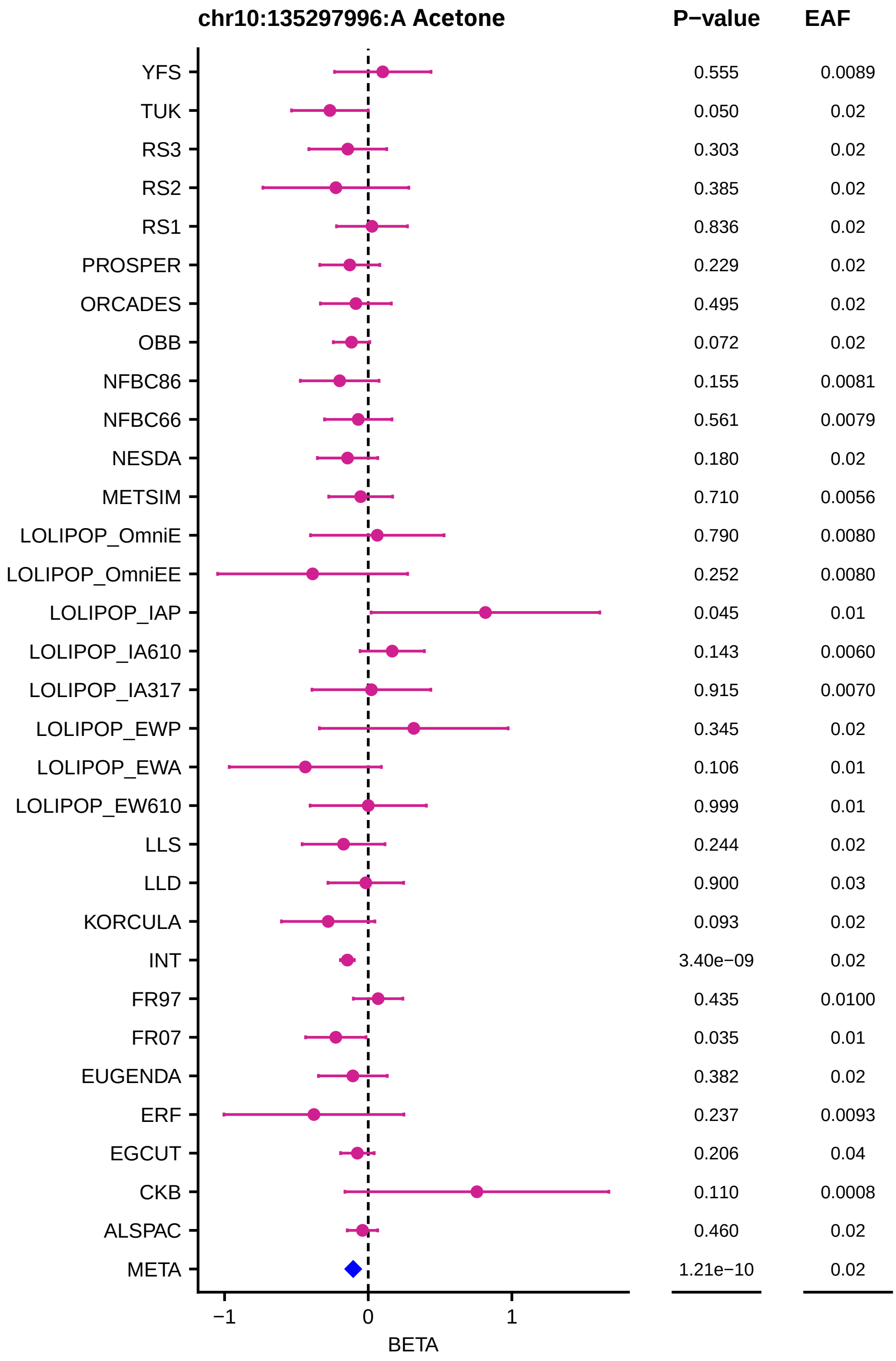
chr10:135297996:A Acetone
P−value
EAF
YFS 0.555 0.0089
TUK 0.050 0.02
RS3 0.303 0.02
RS2 0.385 0.02
RS1 0.836 0.02
PROSPER 0.229 0.02
ORCADES 0.495 0.02
OBB 0.072 0.02
NFBC86 0.155 0.0081
NFBC66 0.561 0.0079
NESDA 0.180 0.02
METSIM 0.710 0.0056
LOLIPOP_OmniE 0.790 0.0080
LOLIPOP_OmniEE 0.252 0.0080
LOLIPOP_IAP 0.045 0.01
LOLIPOP_IA610 0.143 0.0060
LOLIPOP_IA317 0.915 0.0070
LOLIPOP_EWP 0.345 0.02
LOLIPOP_EWA 0.106 0.01
LOLIPOP_EW610 0.999 0.01
LLS 0.244 0.02
LLD 0.900 0.03
KORCULA 0.093 0.02
INT 3.40e−09 0.02
FR97 0.435 0.0100
FR07 0.035 0.01
EUGENDA 0.382 0.02
ERF 0.237 0.0093
EGCUT 0.206 0.04
CKB 0.110 0.0008
ALSPAC 0.460 0.02
META 1.21e−10 0.02
−1
0
1
BETA

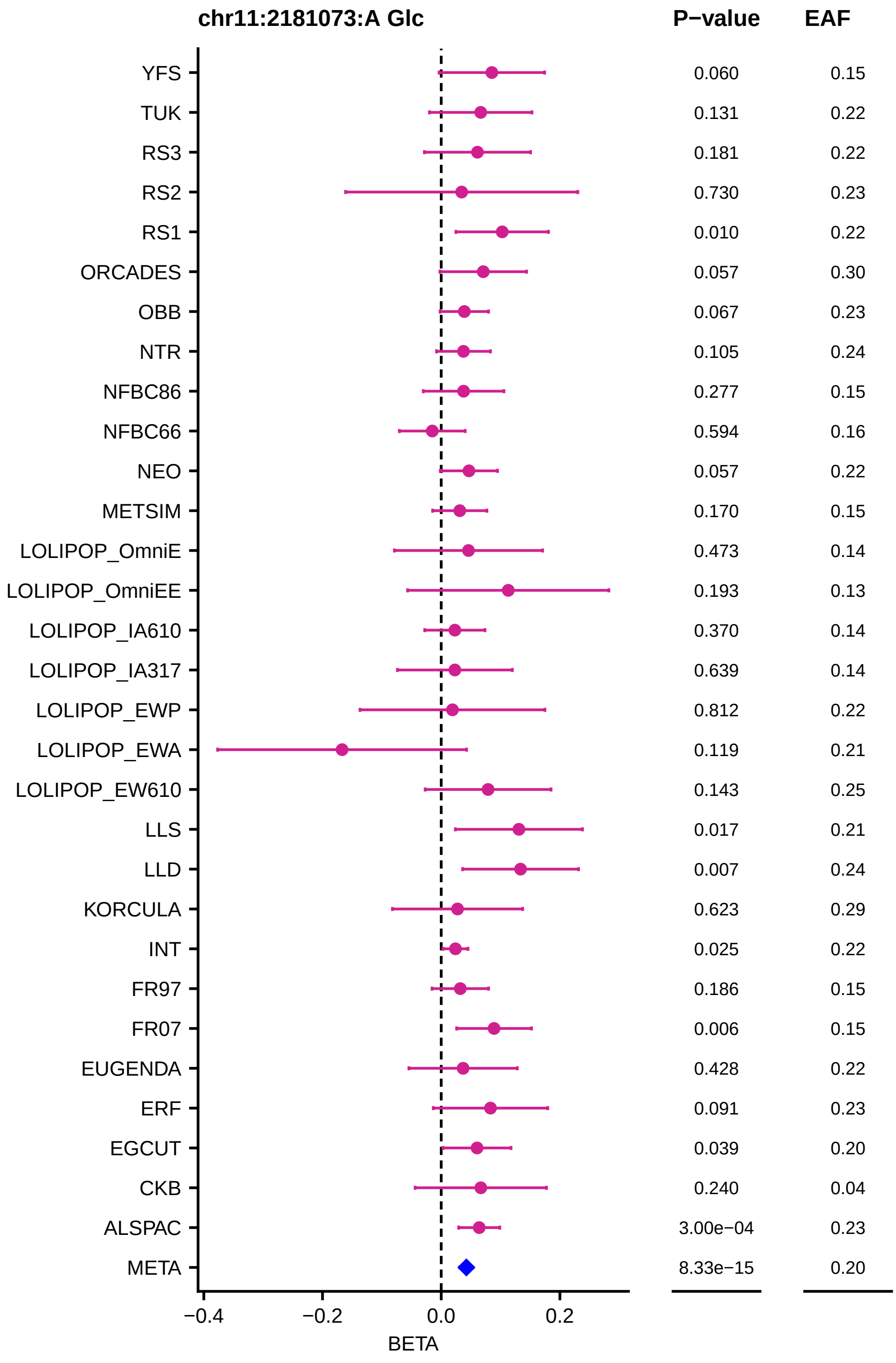
chr11:2181073:A Glc
P−value
EAF
YFS 0.060 0.15
TUK 0.131 0.22
RS3 0.181 0.22
RS2 0.730 0.23
RS1 0.010 0.22
ORCADES 0.057 0.30
OBB 0.067 0.23
NTR 0.105 0.24
NFBC86 0.277 0.15
NFBC66 0.594 0.16
NEO 0.057 0.22
METSIM 0.170 0.15
LOLIPOP_OmniE 0.473 0.14
LOLIPOP_OmniEE 0.193 0.13
LOLIPOP_IA610 0.370 0.14
LOLIPOP_IA317 0.639 0.14
LOLIPOP_EWP 0.812 0.22
LOLIPOP_EWA 0.119 0.21
LOLIPOP_EW610 0.143 0.25
LLS 0.017 0.21
LLD 0.007 0.24
KORCULA 0.623 0.29
INT 0.025 0.22
FR97 0.186 0.15
FR07 0.006 0.15
EUGENDA 0.428 0.22
ERF 0.091 0.23
EGCUT 0.039 0.20
CKB 0.240 0.04
ALSPAC 3.00e−04 0.23
META 8.33e−15 0.20
−0.4
−0.2
0.0
0.2
BETA

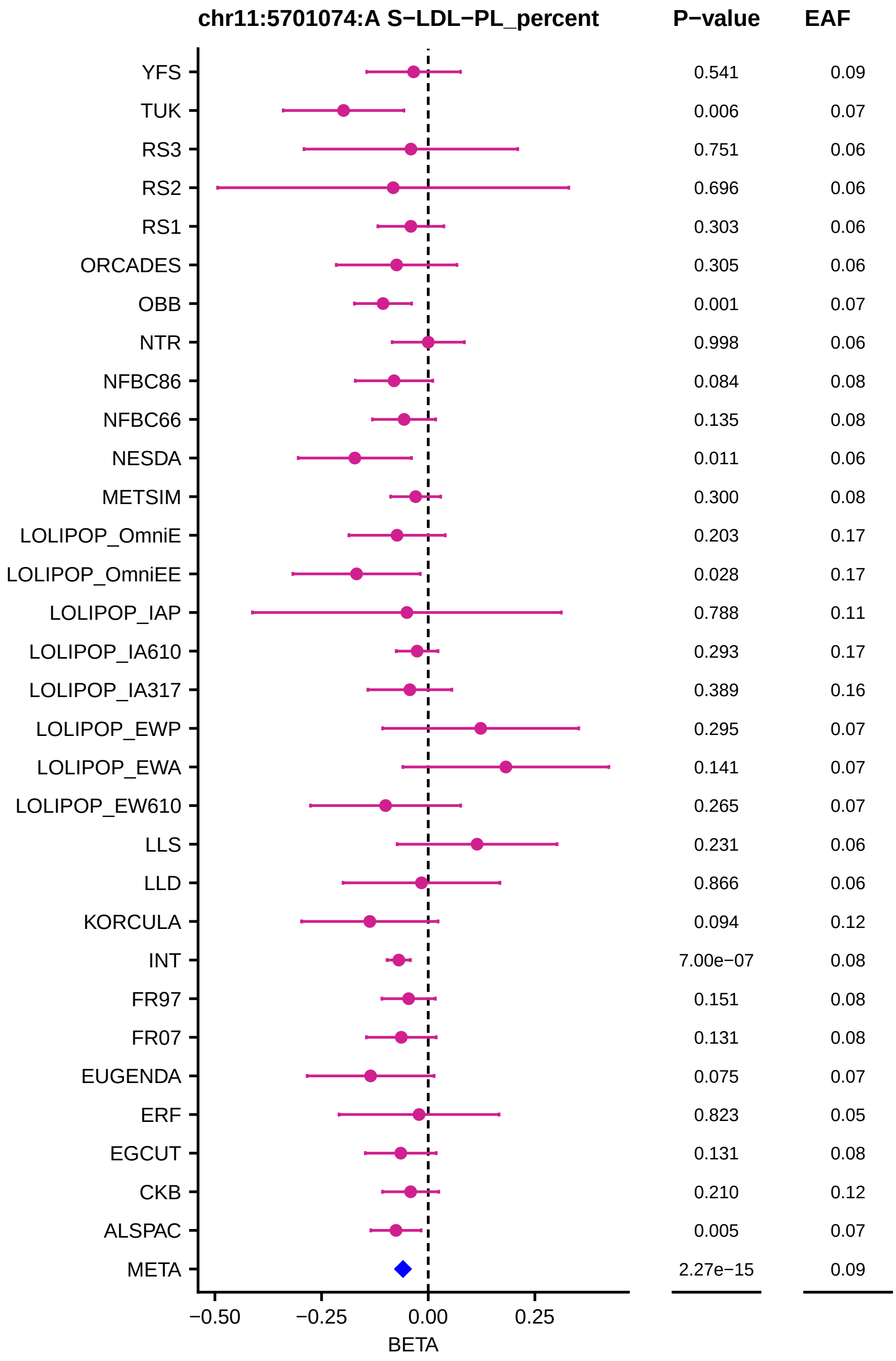
chr11:5701074:A S−LDL−PL_percent
P−value
EAF
YFS
TUK
RS3
RS2
RS1
ORCADES
OBB
NTR
NFBC86
NFBC66
NESDA
METSIM
LOLIPOP_OmniE
LOLIPOP_OmniEE
LOLIPOP_IAP
LOLIPOP_IA610
LOLIPOP_IA317
LOLIPOP_EWP
LOLIPOP_EWA
LOLIPOP_EW610
LLS
LLD
KORCULA
INT
FR97
FR07
EUGENDA
ERF
EGCUT
CKB
ALSPAC
META
0.541 0.09
0.006 0.07
0.751 0.06
0.696 0.06
0.303 0.06
0.305 0.06
0.001 0.07
0.998 0.06
0.084 0.08
0.135 0.08
0.011 0.06
0.300 0.08
0.203 0.17
0.028 0.17
0.788 0.11
0.293 0.17
0.389 0.16
0.295 0.07
0.141 0.07
0.265 0.07
0.231 0.06
0.866 0.06
0.094 0.12
7.00e−07 0.08
0.151 0.08
0.131 0.08
0.075 0.07
0.823 0.05
0.131 0.08
0.210 0.12
0.005 0.07
2.27e−15 0.09
−0.50
−0.25
0.00
0.25
BETA

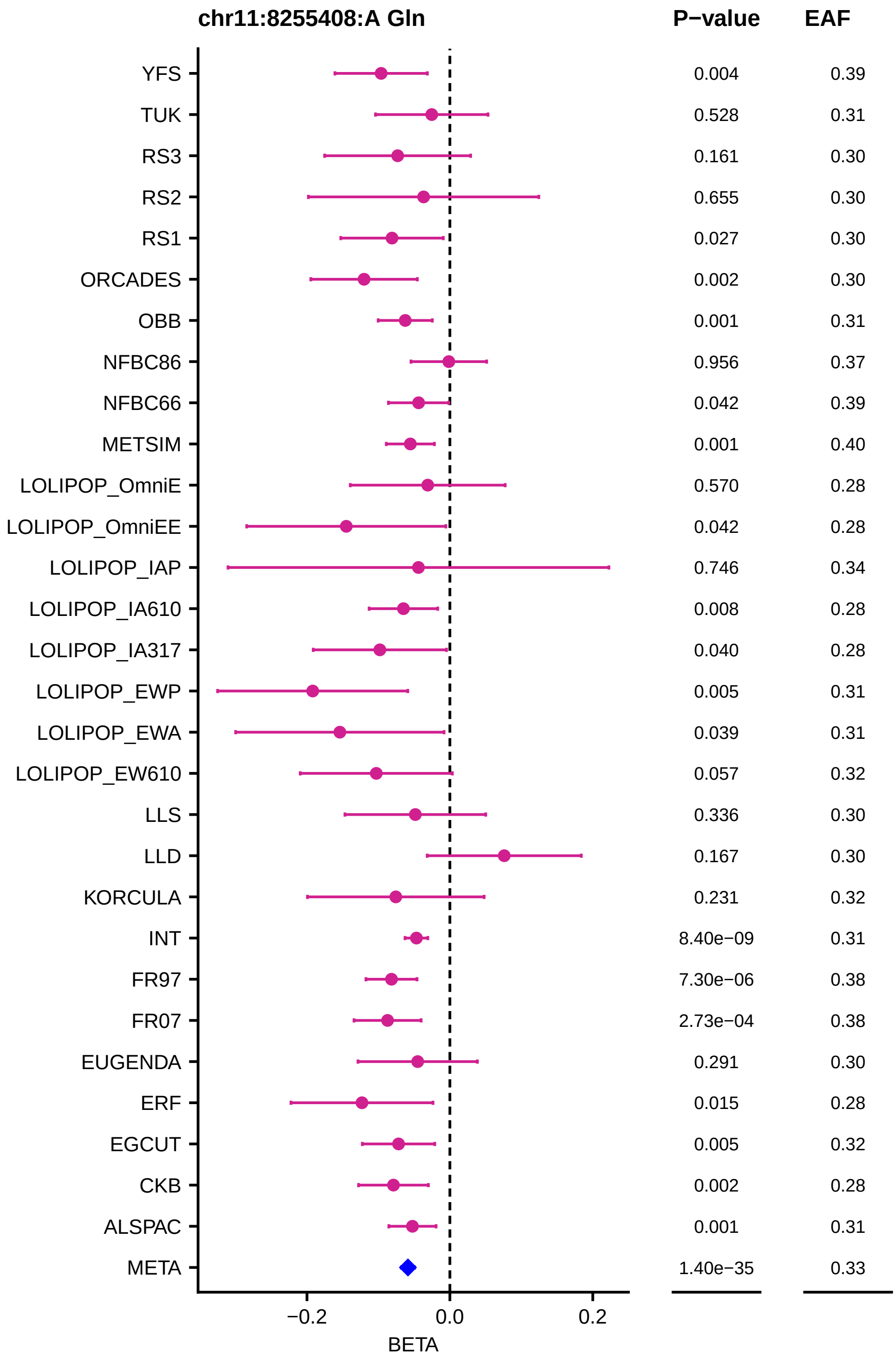

chr11:8255408:A Gln
P−value
EAF
YFS 0.004 0.39
TUK 0.528 0.31
RS3 0.161 0.30
RS2 0.655 0.30
RS1 0.027 0.30
ORCADES 0.002 0.30
OBB 0.001 0.31
NFBC86 0.956 0.37
NFBC66 0.042 0.39
METSIM 0.001 0.40
LOLIPOP_OmniE 0.570 0.28
LOLIPOP_OmniEE 0.042 0.28
LOLIPOP_IAP 0.746 0.34
LOLIPOP_IA610 0.008 0.28
LOLIPOP_IA317 0.040 0.28
LOLIPOP_EWP 0.005 0.31
LOLIPOP_EWA 0.039 0.31
LOLIPOP_EW610 0.057 0.32
LLS 0.336 0.30
LLD 0.167 0.30
KORCULA 0.231 0.32
INT 8.40e−09 0.31
FR97 7.30e−06 0.38
FR07 2.73e−04 0.38
EUGENDA 0.291 0.30
ERF 0.015 0.28
EGCUT 0.005 0.32
CKB 0.002 0.28
ALSPAC 0.001 0.31
META 1.40e−35 0.33
−0.2
0.0
0.2
BETA

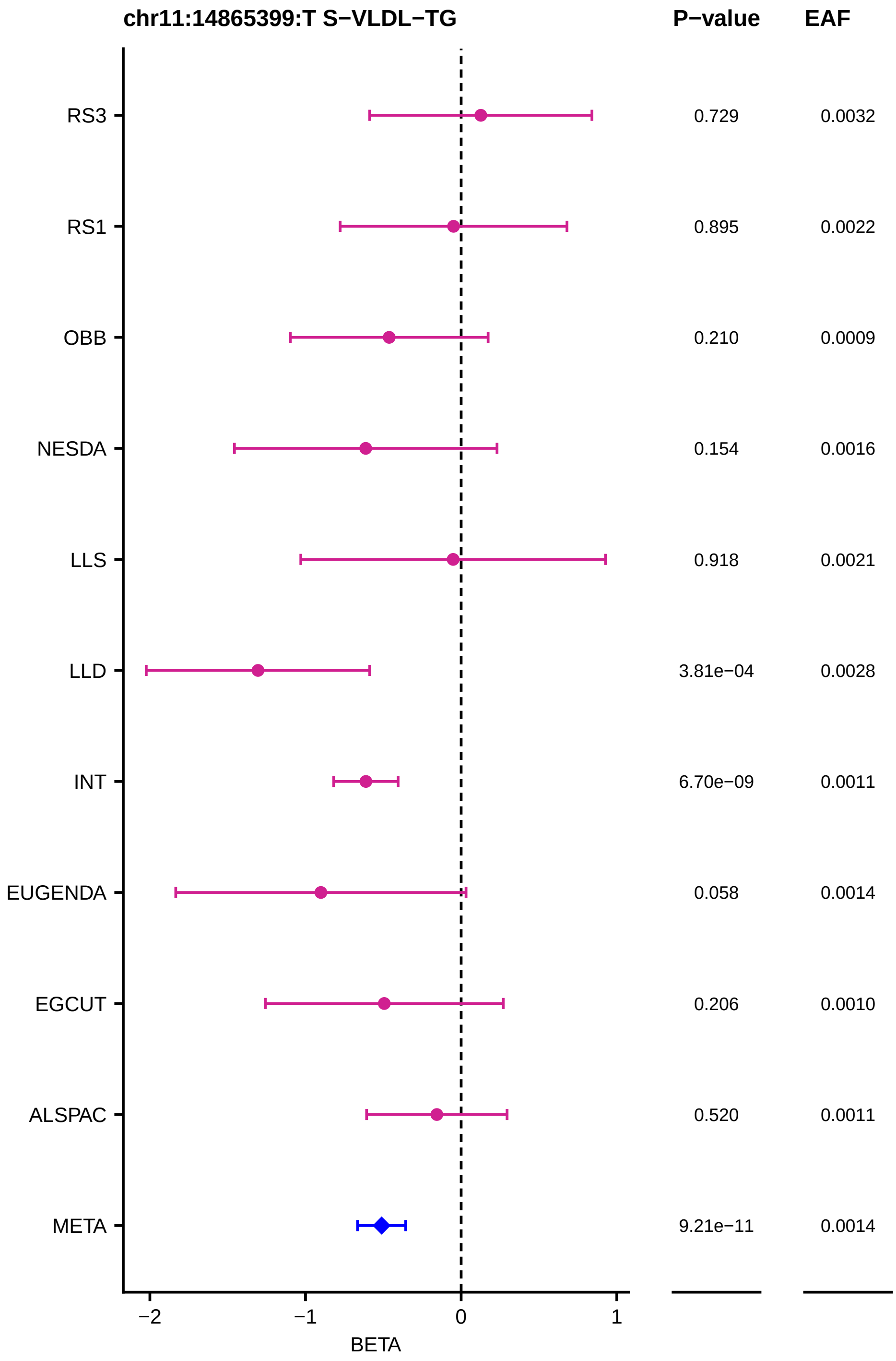

chr11:14865399:T S−VLDL−TG
P−value
EAF
RS3 0.729 0.0032
RS1 0.895 0.0022
OBB 0.210 0.0009
NESDA 0.154 0.0016
LLS 0.918 0.0021
LLD 3.81e−04 0.0028
INT 6.70e−09 0.0011
EUGENDA 0.058 0.0014
EGCUT 0.206 0.0010
ALSPAC 0.520 0.0011
META 9.21e−11 0.0014
−2
−1
0
1
BETA

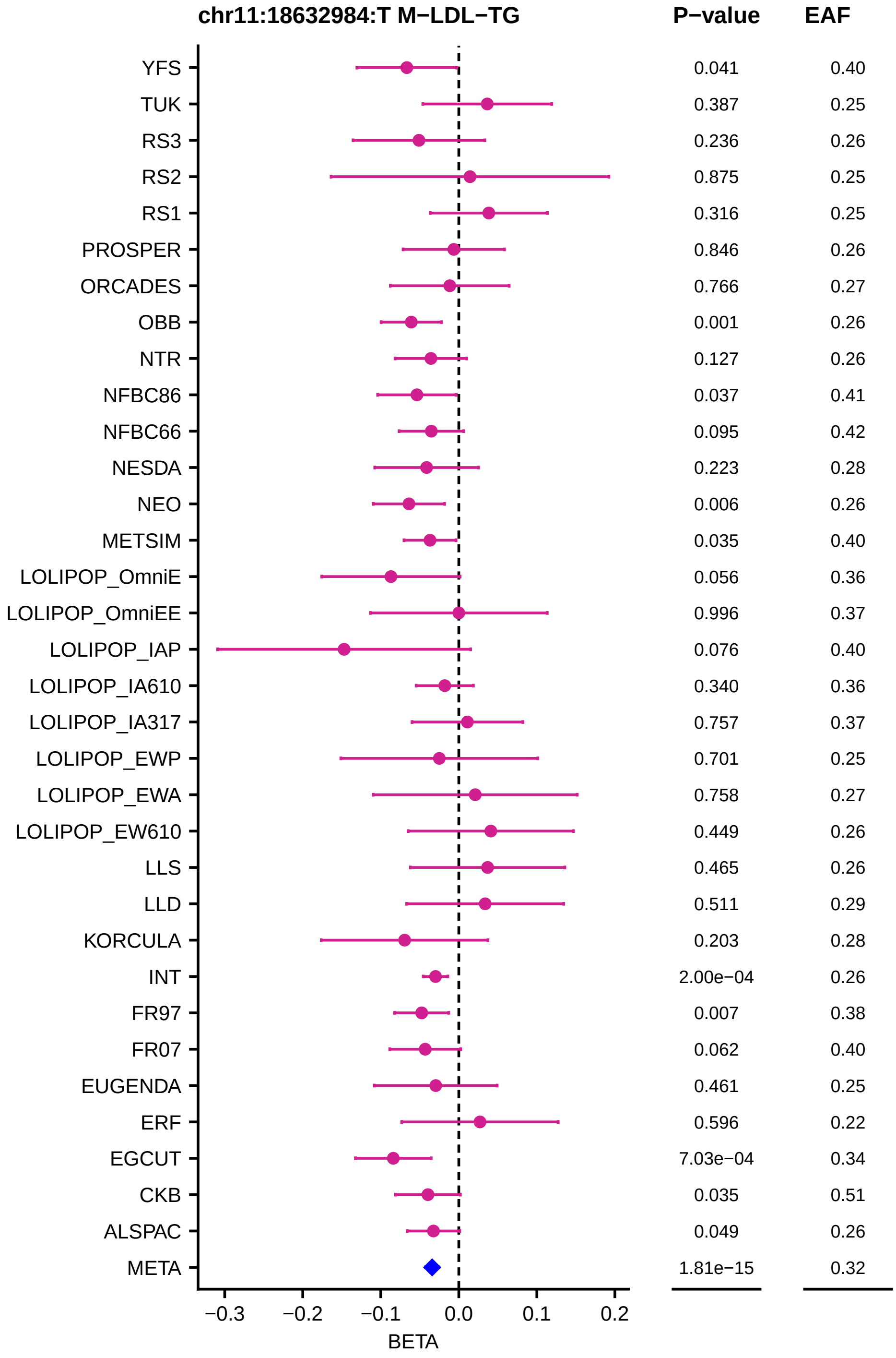
chr11:18632984:T M−LDL−TG
P−value
EAF
YFS 0.041 0.40
TUK 0.387 0.25
RS3 0.236 0.26
RS2 0.875 0.25
RS1 0.316 0.25
PROSPER 0.846 0.26
ORCADES 0.766 0.27
OBB 0.001 0.26
NTR 0.127 0.26
NFBC86 0.037 0.41
NFBC66 0.095 0.42
NESDA 0.223 0.28
NEO 0.006 0.26
METSIM 0.035 0.40
LOLIPOP_OmniE 0.056 0.36
LOLIPOP_OmniEE 0.996 0.37
LOLIPOP_IAP 0.076 0.40
LOLIPOP_IA610 0.340 0.36
LOLIPOP_IA317 0.757 0.37
LOLIPOP_EWP 0.701 0.25
LOLIPOP_EWA 0.758 0.27
LOLIPOP_EW610 0.449 0.26
LLS 0.465 0.26
LLD 0.511 0.29
KORCULA 0.203 0.28
INT 2.00e−04 0.26
FR97 0.007 0.38
FR07 0.062 0.40
EUGENDA 0.461 0.25
ERF 0.596 0.22
EGCUT 7.03e−04 0.34
CKB 0.035 0.51
ALSPAC 0.049 0.26
META 1.81e−15 0.32
−0.3
−0.2
−0.1
0.0
0.1
0.2
BETA

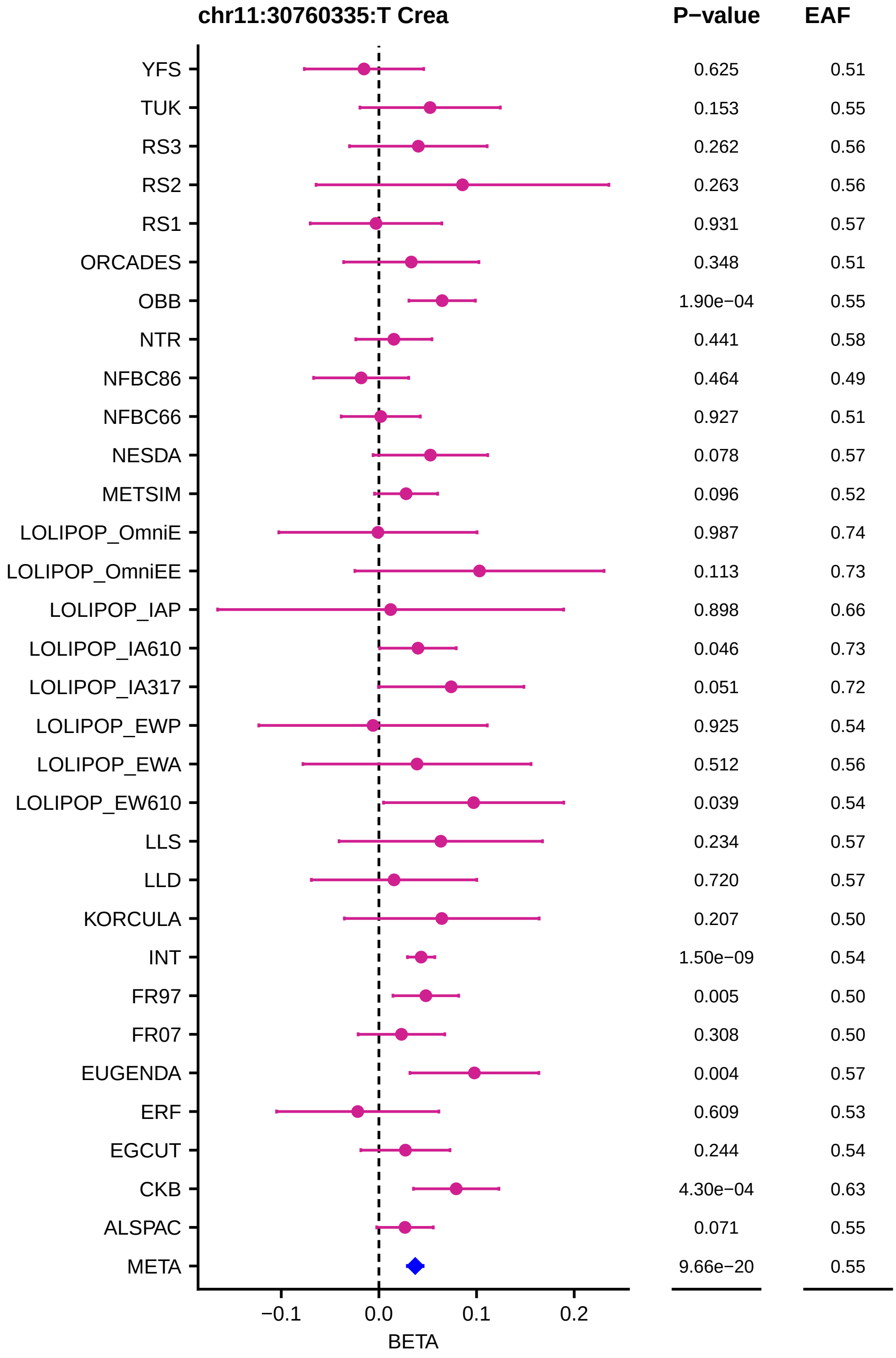

chr11:30760335:T Crea
P−value
EAF
YFS 0.625 0.51
TUK 0.153 0.55
RS3 0.262 0.56
RS2 0.263 0.56
RS1 0.931 0.57
ORCADES 0.348 0.51
OBB 1.90e−04 0.55
NTR 0.441 0.58
NFBC86 0.464 0.49
NFBC66 0.927 0.51
NESDA 0.078 0.57
METSIM 0.096 0.52
LOLIPOP_OmniE 0.987 0.74
LOLIPOP_OmniEE 0.113 0.73
LOLIPOP_IAP 0.898 0.66
LOLIPOP_IA610 0.046 0.73
LOLIPOP_IA317 0.051 0.72
LOLIPOP_EWP 0.925 0.54
LOLIPOP_EWA 0.512 0.56
LOLIPOP_EW610 0.039 0.54
LLS 0.234 0.57
LLD 0.720 0.57
KORCULA 0.207 0.50
INT 1.50e−09 0.54
FR97 0.005 0.50
FR07 0.308 0.50
EUGENDA 0.004 0.57
ERF 0.609 0.53
EGCUT 0.244 0.54
CKB 4.30e−04 0.63
ALSPAC 0.071 0.55
META 9.66e−20 0.55
−0.1
0.0
0.1
0.2
BETA

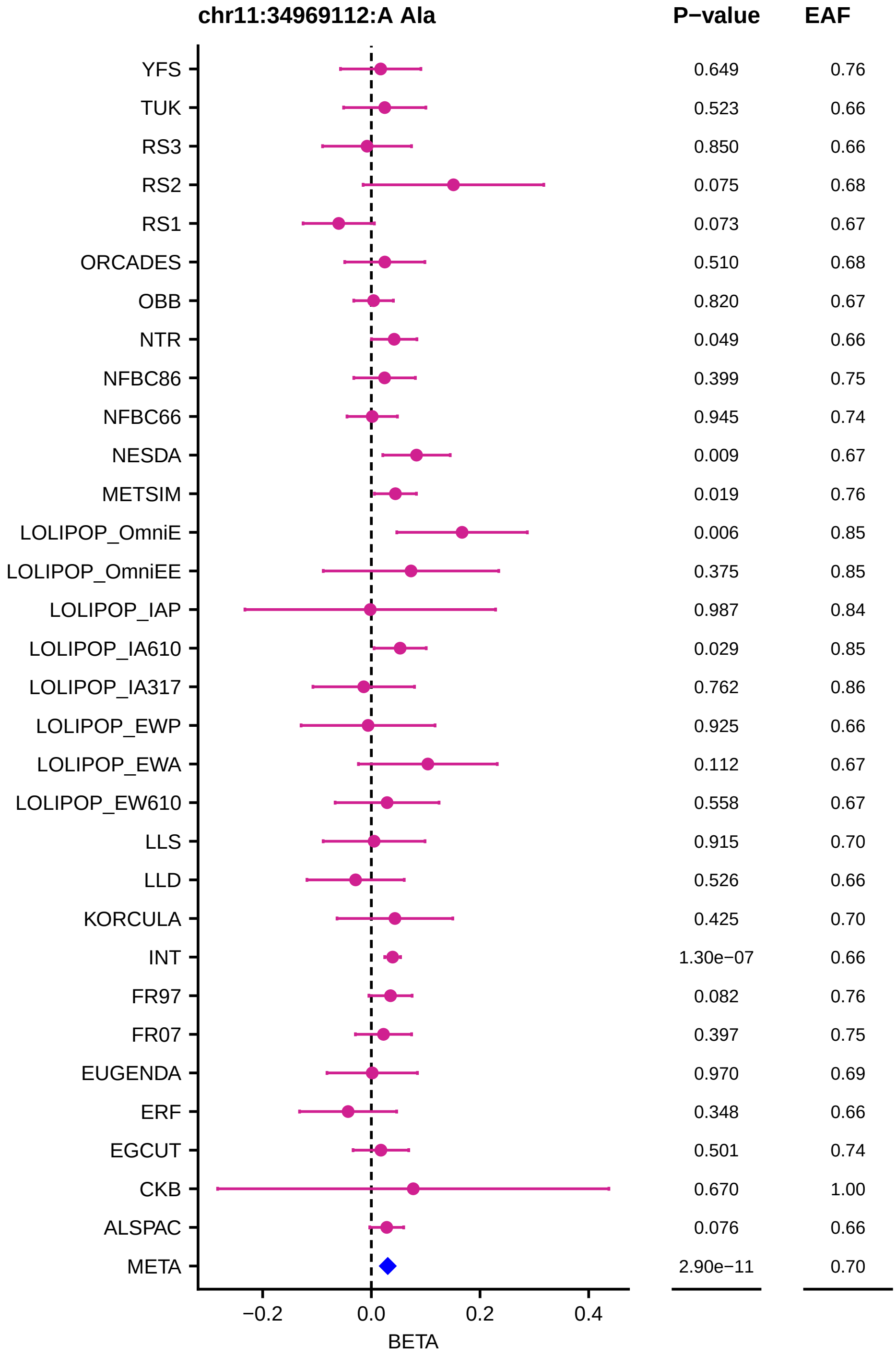
chr11:34969112:A Ala
P−value
EAF
YFS
TUK
RS3
RS2
RS1
ORCADES
OBB
NTR
NFBC86
NFBC66
NESDA
METSIM
LOLIPOP_OmniE
LOLIPOP_OmniEE
LOLIPOP_IAP
LOLIPOP_IA610
LOLIPOP_IA317
LOLIPOP_EWP
LOLIPOP_EWA
LOLIPOP_EW610
LLS
LLD
KORCULA
INT
FR97
FR07
EUGENDA
ERF
EGCUT
CKB
ALSPAC
META
0.649 0.76
0.523 0.66
0.850 0.66
0.075 0.68
0.073 0.67
0.510 0.68
0.820 0.67
0.049 0.66
0.399 0.75
0.945 0.74
0.009 0.67
0.019 0.76
0.006 0.85
0.375 0.85
0.987 0.84
0.029 0.85
0.762 0.86
0.925 0.66
0.112 0.67
0.558 0.67
0.915 0.70
0.526 0.66
0.425 0.70
1.30e−07 0.66
0.082 0.76
0.397 0.75
0.970 0.69
0.348 0.66
0.501 0.74
0.670 1.00
0.076 0.66
2.90e−11 0.70
−0.2
0.0
0.2
0.4
BETA

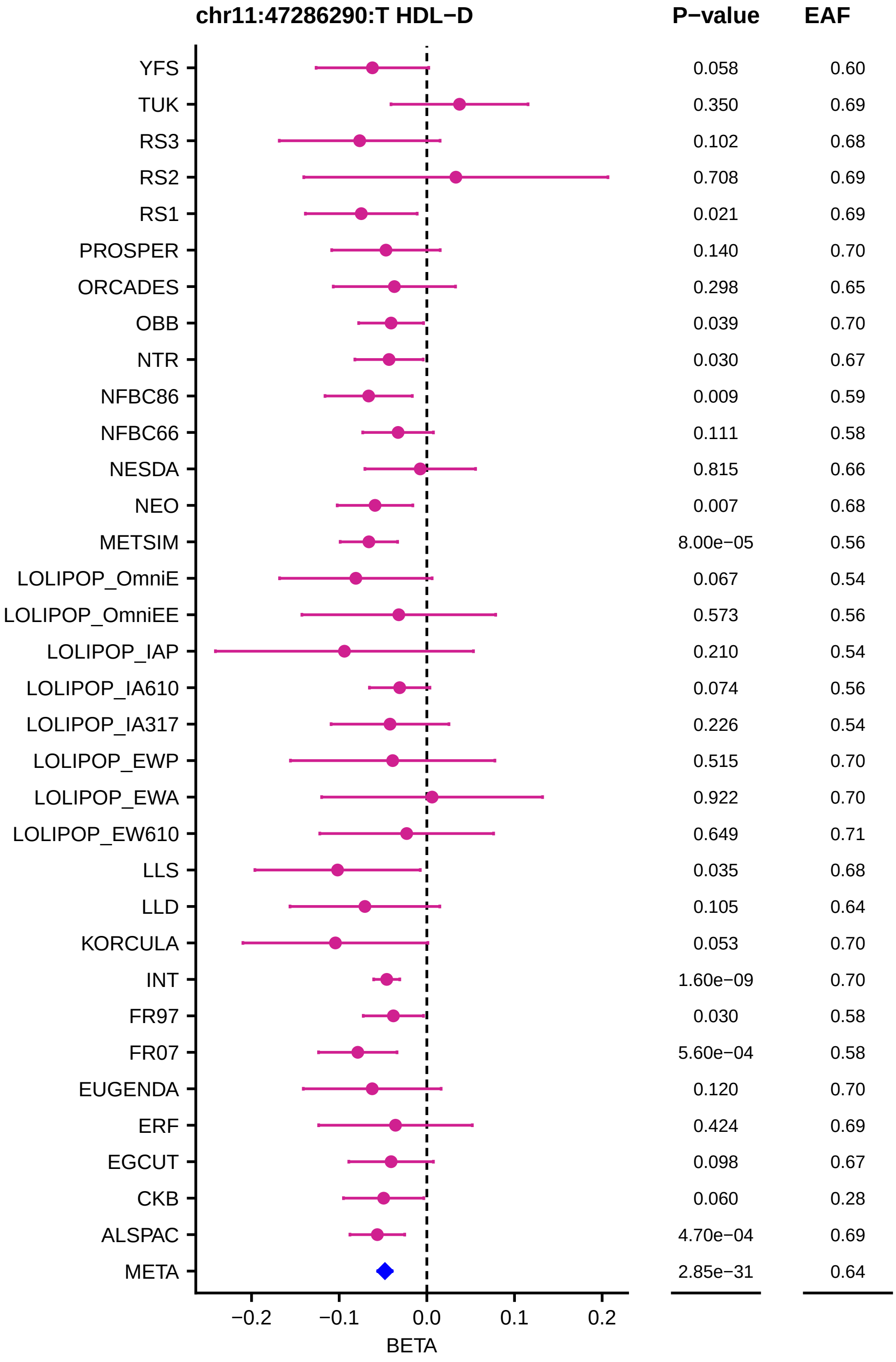
chr11:47286290:T HDL−D
P−value
EAF
YFS 0.058 0.60
TUK 0.350 0.69
RS3 0.102 0.68
RS2 0.708 0.69
RS1 0.021 0.69
PROSPER 0.140 0.70
ORCADES 0.298 0.65
OBB 0.039 0.70
NTR 0.030 0.67
NFBC86 0.009 0.59
NFBC66 0.111 0.58
NESDA 0.815 0.66
NEO 0.007 0.68
METSIM 8.00e−05 0.56
LOLIPOP_OmniE 0.067 0.54
LOLIPOP_OmniEE 0.573 0.56
LOLIPOP_IAP 0.210 0.54
LOLIPOP_IA610 0.074 0.56
LOLIPOP_IA317 0.226 0.54
LOLIPOP_EWP 0.515 0.70
LOLIPOP_EWA 0.922 0.70
LOLIPOP_EW610 0.649 0.71
LLS 0.035 0.68
LLD 0.105 0.64
KORCULA 0.053 0.70
INT 1.60e−09 0.70
FR97 0.030 0.58
FR07 5.60e−04 0.58
EUGENDA 0.120 0.70
ERF 0.424 0.69
EGCUT 0.098 0.67
CKB 0.060 0.28
ALSPAC 4.70e−04 0.69
META 2.85e−31 0.64
−0.2
−0.1
0.0
0.1
0.2
BETA

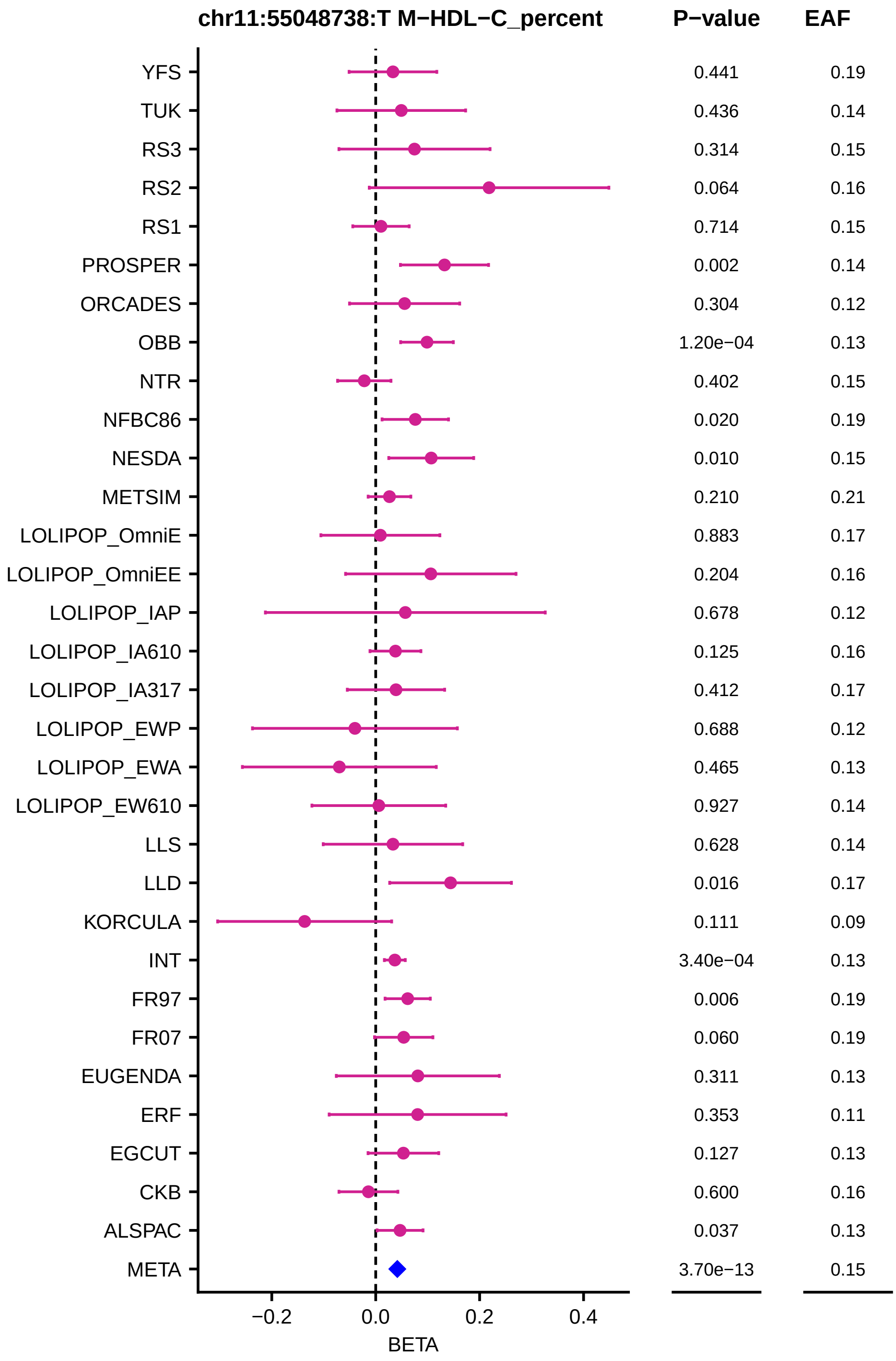
chr11:55048738:T M−HDL−C_percent
P−value
EAF
YFS 0.441 0.19
TUK 0.436 0.14
RS3 0.314 0.15
RS2 0.064 0.16
RS1 0.714 0.15
PROSPER 0.002 0.14
ORCADES 0.304 0.12
OBB 1.20e−04 0.13
NTR 0.402 0.15
NFBC86 0.020 0.19
NESDA 0.010 0.15
METSIM 0.210 0.21
LOLIPOP_OmniE 0.883 0.17
LOLIPOP_OmniEE 0.204 0.16
LOLIPOP_IAP 0.678 0.12
LOLIPOP_IA610 0.125 0.16
LOLIPOP_IA317 0.412 0.17
LOLIPOP_EWP 0.688 0.12
LOLIPOP_EWA 0.465 0.13
LOLIPOP_EW610 0.927 0.14
LLS 0.628 0.14
LLD 0.016 0.17
KORCULA 0.111 0.09
INT 3.40e−04 0.13
FR97 0.006 0.19
FR07 0.060 0.19
EUGENDA 0.311 0.13
ERF 0.353 0.11
EGCUT 0.127 0.13
CKB 0.600 0.16
ALSPAC 0.037 0.13
META 3.70e−13 0.15
−0.2
0.0
0.2
0.4
BETA

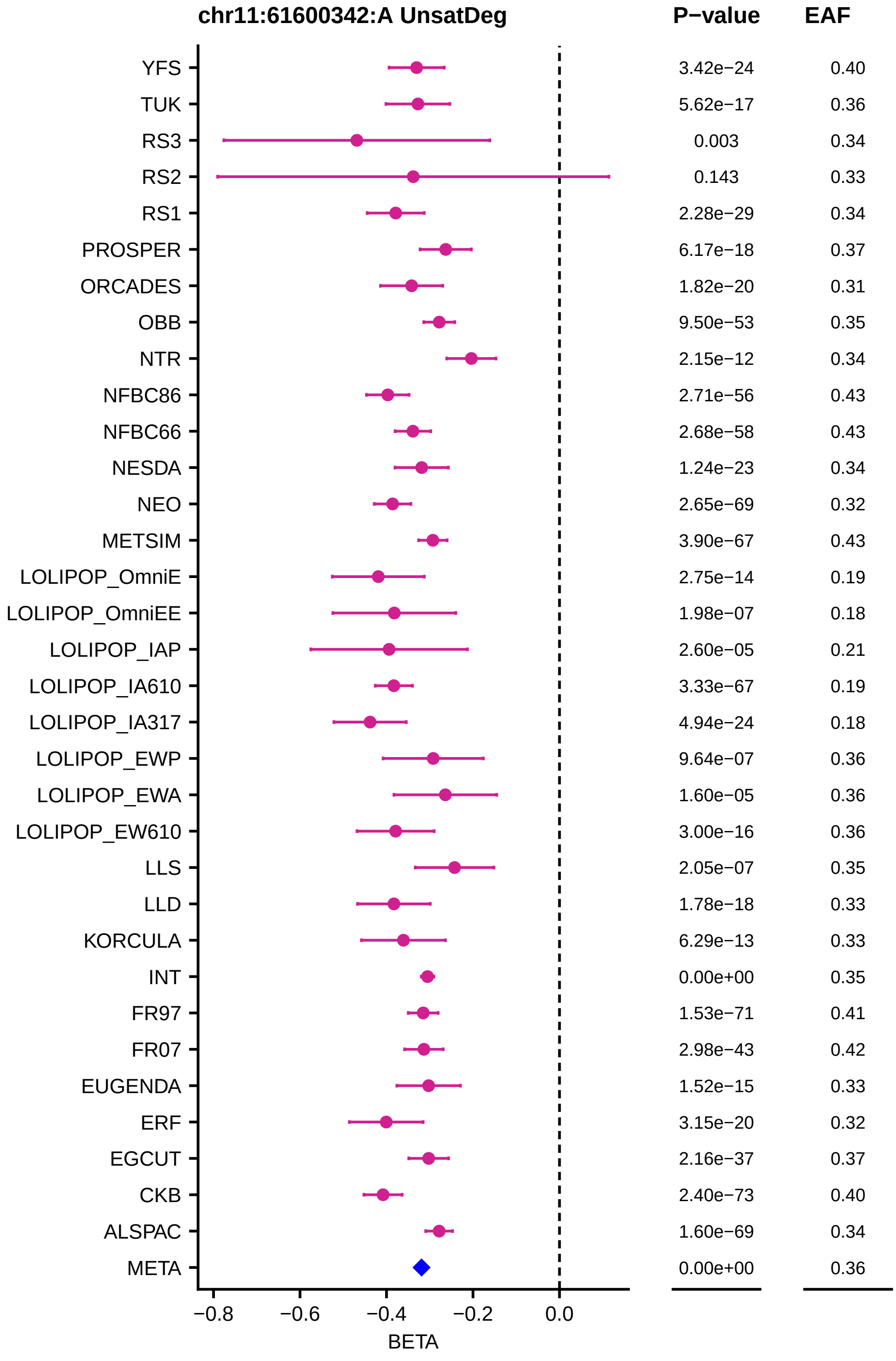
chr11:61600342:A UnsatDeg
P−value
EAF
YFS 3.42e−24 0.40
TUK 5.62e−17 0.36
RS3 0.003 0.34
RS2 0.143 0.33
RS1 2.28e−29 0.34
PROSPER 6.17e−18 0.37
ORCADES 1.82e−20 0.31
OBB 9.50e−53 0.35
NTR 2.15e−12 0.34
NFBC86 2.71e−56 0.43
NFBC66 2.68e−58 0.43
NESDA 1.24e−23 0.34
NEO 2.65e−69 0.32
METSIM 3.90e−67 0.43
LOLIPOP_OmniE 2.75e−14 0.19
LOLIPOP_OmniEE 1.98e−07 0.18
LOLIPOP_IAP 2.60e−05 0.21
LOLIPOP_IA610 3.33e−67 0.19
LOLIPOP_IA317 4.94e−24 0.18
LOLIPOP_EWP 9.64e−07 0.36
LOLIPOP_EWA 1.60e−05 0.36
LOLIPOP_EW610 3.00e−16 0.36
LLS 2.05e−07 0.35
LLD 1.78e−18 0.33
KORCULA 6.29e−13 0.33
INT 0.00e+00 0.35
FR97 1.53e−71 0.41
FR07 2.98e−43 0.42
EUGENDA 1.52e−15 0.33
ERF 3.15e−20 0.32
EGCUT 2.16e−37 0.37
CKB 2.40e−73 0.40
ALSPAC 1.60e−69 0.34
META 0.00e+00 0.36
−0.8
−0.6
−0.4
−0.2
0.0
BETA

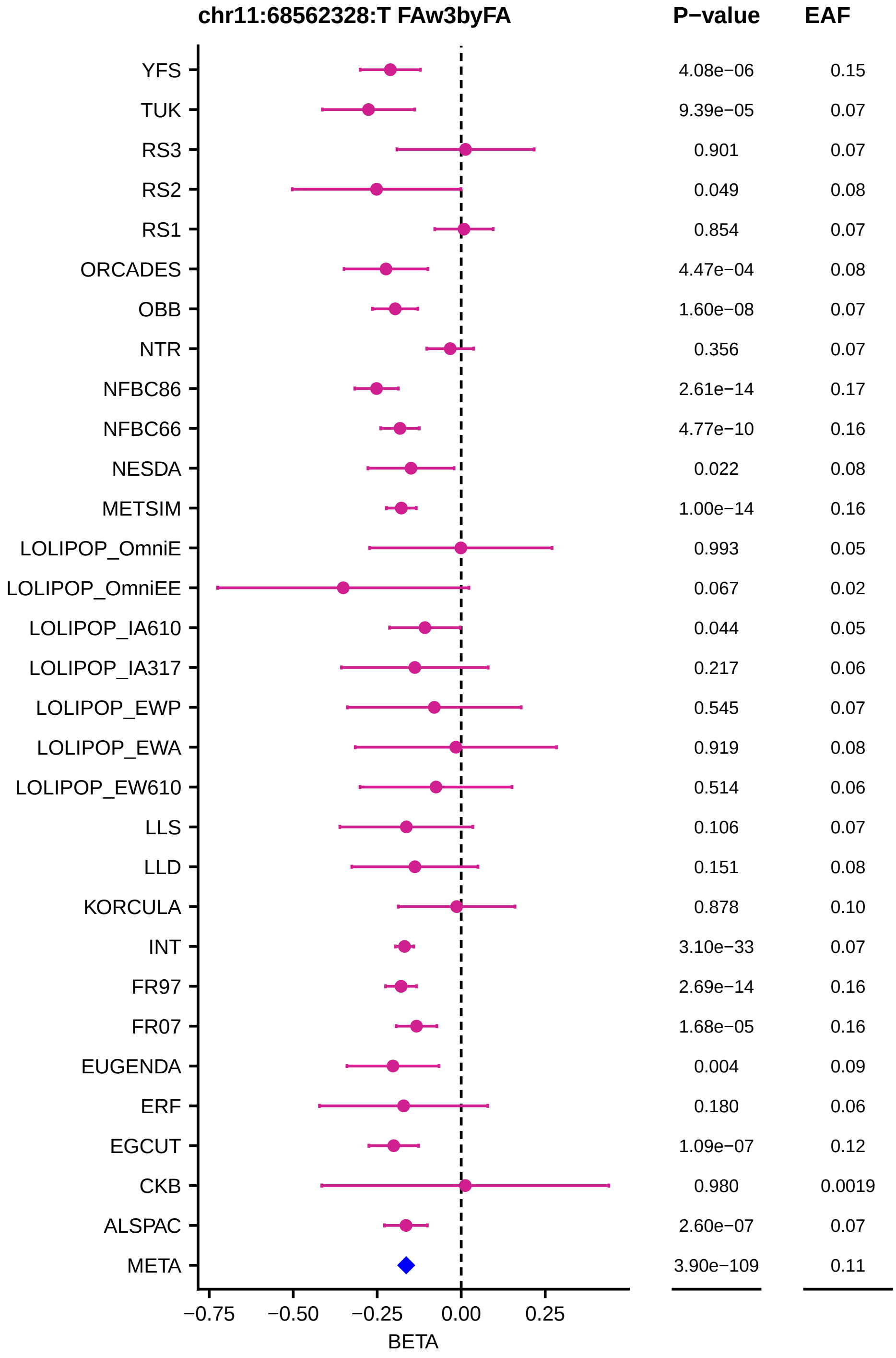
chr11:68562328:T FAw3byFA
P−value
EAF
YFS 4.08e−06 0.15
TUK 9.39e−05 0.07
RS3 0.901 0.07
RS2 0.049 0.08
RS1 0.854 0.07
ORCADES 4.47e−04 0.08
OBB 1.60e−08 0.07
NTR 0.356 0.07
NFBC86 2.61e−14 0.17
NFBC66 4.77e−10 0.16
NESDA 0.022 0.08
METSIM 1.00e−14 0.16
LOLIPOP_OmniE 0.993 0.05
LOLIPOP_OmniEE 0.067 0.02
LOLIPOP_IA610 0.044 0.05
LOLIPOP_IA317 0.217 0.06
LOLIPOP_EWP 0.545 0.07
LOLIPOP_EWA 0.919 0.08
LOLIPOP_EW610 0.514 0.06
LLS 0.106 0.07
LLD 0.151 0.08
KORCULA 0.878 0.10
INT 3.10e−33 0.07
FR97 2.69e−14 0.16
FR07 1.68e−05 0.16
EUGENDA 0.004 0.09
ERF 0.180 0.06
EGCUT 1.09e−07 0.12
CKB 0.980 0.0019
ALSPAC 2.60e−07 0.07
META 3.90e−109 0.11
−0.75
−0.50
−0.25
0.00
0.25
BETA

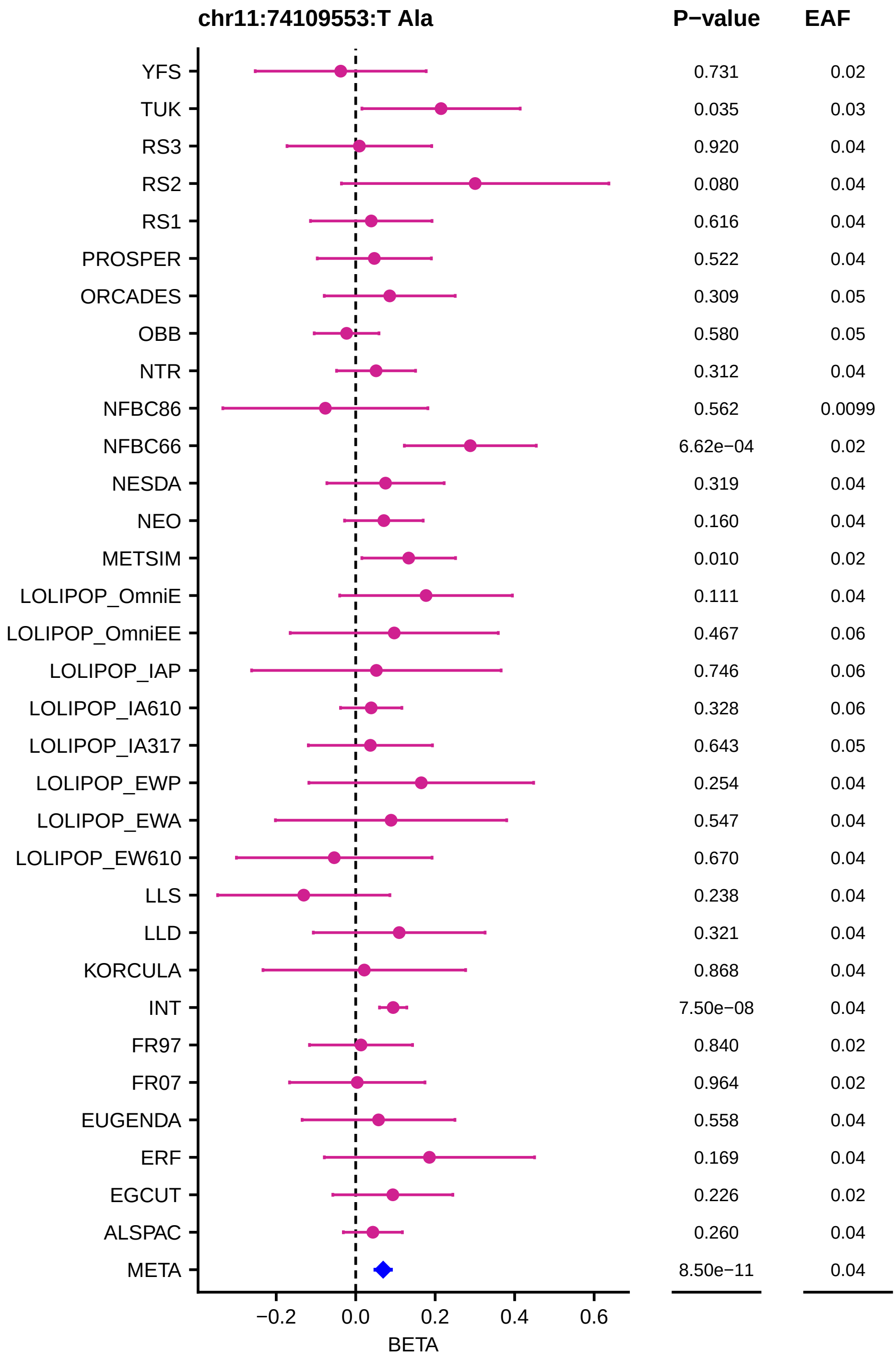
chr11:74109553:T Ala
P−value
EAF
YFS 0.731 0.02
TUK 0.035 0.03
RS3 0.920 0.04
RS2 0.080 0.04
RS1 0.616 0.04
PROSPER 0.522 0.04
ORCADES 0.309 0.05
OBB 0.580 0.05
NTR 0.312 0.04
NFBC86 0.562 0.0099
NFBC66 6.62e−04 0.02
NESDA 0.319 0.04
NEO 0.160 0.04
METSIM 0.010 0.02
LOLIPOP_OmniE 0.111 0.04
LOLIPOP_OmniEE 0.467 0.06
LOLIPOP_IAP 0.746 0.06
LOLIPOP_IA610 0.328 0.06
LOLIPOP_IA317 0.643 0.05
LOLIPOP_EWP 0.254 0.04
LOLIPOP_EWA 0.547 0.04
LOLIPOP_EW610 0.670 0.04
LLS 0.238 0.04
LLD 0.321 0.04
KORCULA 0.868 0.04
INT 7.50e−08 0.04
FR97 0.840 0.02
FR07 0.964 0.02
EUGENDA 0.558 0.04
ERF 0.169 0.04
EGCUT 0.226 0.02
ALSPAC 0.260 0.04
META 8.50e−11 0.04
−0.2 0.0 0.2 0.4 0.6
BETA

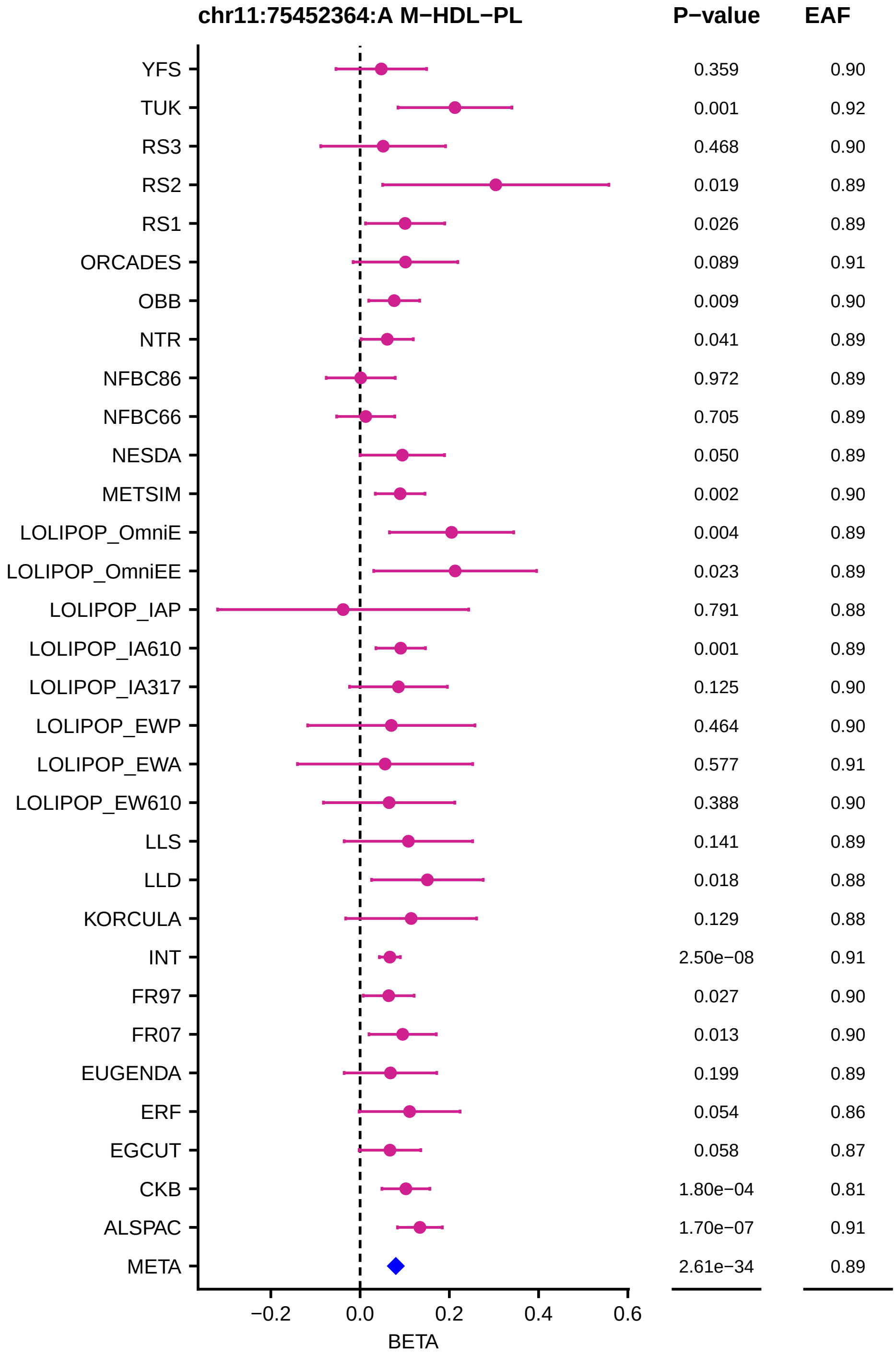
chr11:75452364:A M−HDL−PL
P−value
EAF
YFS 0.359 0.90
TUK 0.001 0.92
RS3 0.468 0.90
RS2 0.019 0.89
RS1 0.026 0.89
ORCADES 0.089 0.91
OBB 0.009 0.90
NTR 0.041 0.89
NFBC86 0.972 0.89
NFBC66 0.705 0.89
NESDA 0.050 0.89
METSIM 0.002 0.90
LOLIPOP_OmniE 0.004 0.89
LOLIPOP_OmniEE 0.023 0.89
LOLIPOP_IAP 0.791 0.88
LOLIPOP_IA610 0.001 0.89
LOLIPOP_IA317 0.125 0.90
LOLIPOP_EWP 0.464 0.90
LOLIPOP_EWA 0.577 0.91
LOLIPOP_EW610 0.388 0.90
LLS 0.141 0.89
LLD 0.018 0.88
KORCULA 0.129 0.88
INT 2.50e−08 0.91
FR97 0.027 0.90
FR07 0.013 0.90
EUGENDA 0.199 0.89
ERF 0.054 0.86
EGCUT 0.058 0.87
CKB 1.80e−04 0.81
ALSPAC 1.70e−07 0.91
META 2.61e−34 0.89
−0.2
0.0
0.2
0.4
0.6
BETA

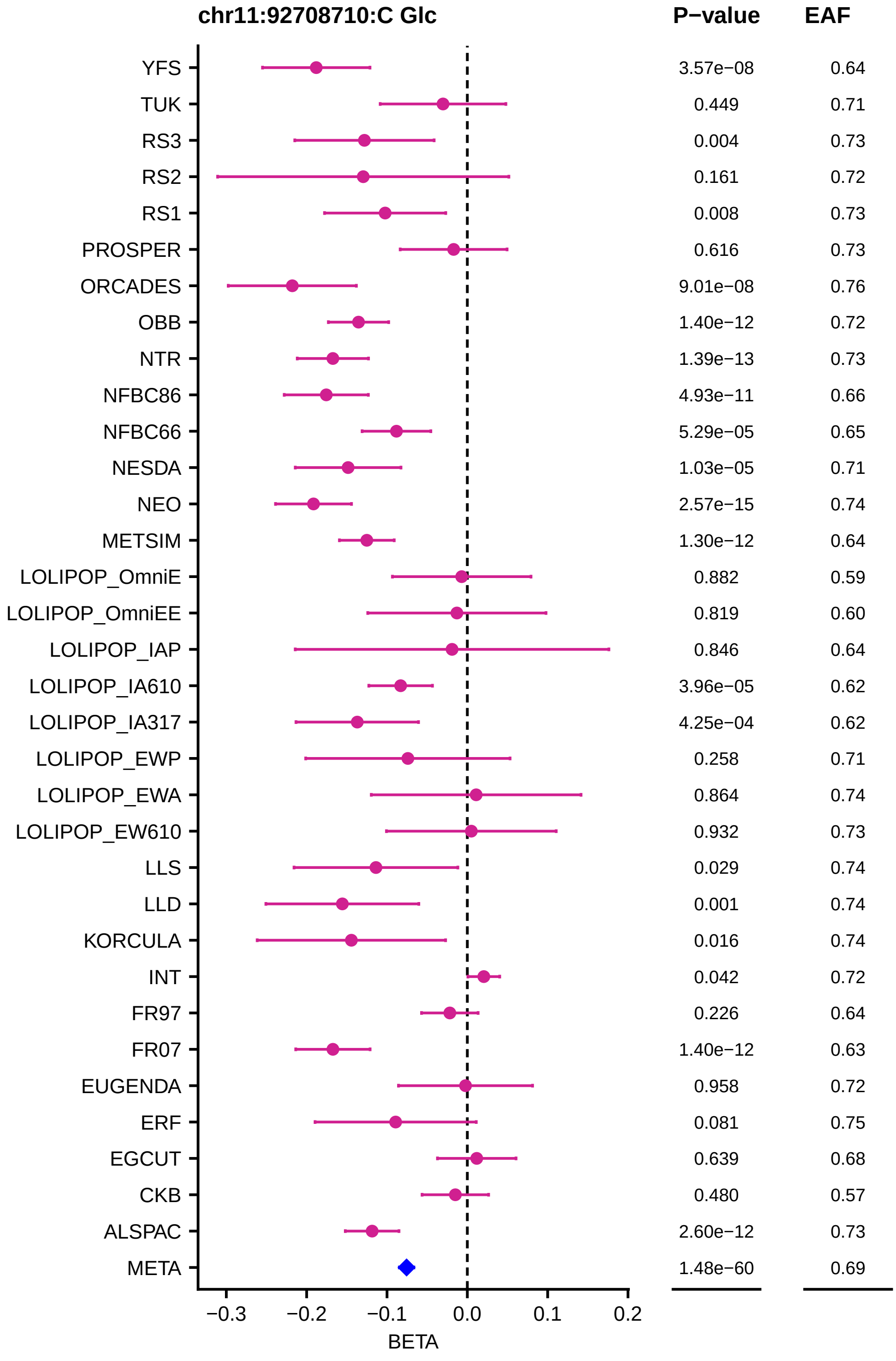
chr11:92708710:C Glc
P−value
EAF
YFS 3.57e−08 0.64
TUK 0.449 0.71
RS3 0.004 0.73
RS2 0.161 0.72
RS1 0.008 0.73
PROSPER 0.616 0.73
ORCADES 9.01e−08 0.76
OBB 1.40e−12 0.72
NTR 1.39e−13 0.73
NFBC86 4.93e−11 0.66
NFBC66 5.29e−05 0.65
NESDA 1.03e−05 0.71
NEO 2.57e−15 0.74
METSIM 1.30e−12 0.64
LOLIPOP_OmniE 0.882 0.59
LOLIPOP_OmniEE 0.819 0.60
LOLIPOP_IAP 0.846 0.64
LOLIPOP_IA610 3.96e−05 0.62
LOLIPOP_IA317 4.25e−04 0.62
LOLIPOP_EWP 0.258 0.71
LOLIPOP_EWA 0.864 0.74
LOLIPOP_EW610 0.932 0.73
LLS 0.029 0.74
LLD 0.001 0.74
KORCULA 0.016 0.74
INT 0.042 0.72
FR97 0.226 0.64
FR07 1.40e−12 0.63
EUGENDA 0.958 0.72
ERF 0.081 0.75
EGCUT 0.639 0.68
CKB 0.480 0.57
ALSPAC 2.60e−12 0.73
META 1.48e−60 0.69
−0.3 −0.2 −0.1 0.0 0.1 0.2
BETA

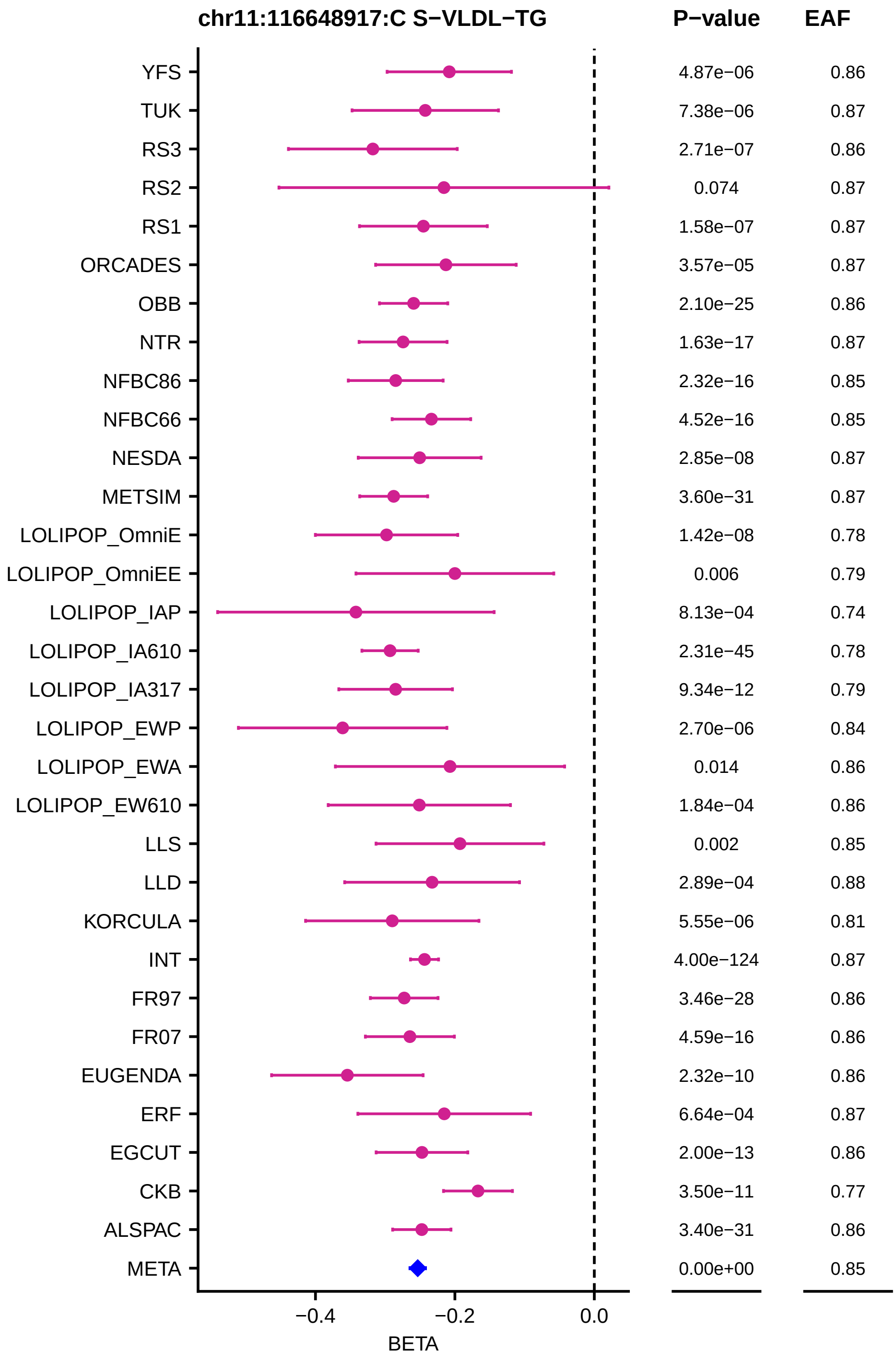
chr11:116648917:C S−VLDL−TG
P−value
EAF
YFS 4.87e−06 0.86
TUK 7.38e−06 0.87
RS3 2.71e−07 0.86
RS2 0.074 0.87
RS1 1.58e−07 0.87
ORCADES 3.57e−05 0.87
OBB 2.10e−25 0.86
NTR 1.63e−17 0.87
NFBC86 2.32e−16 0.85
NFBC66 4.52e−16 0.85
NESDA 2.85e−08 0.87
METSIM 3.60e−31 0.87
LOLIPOP_OmniE 1.42e−08 0.78
LOLIPOP_OmniEE 0.006 0.79
LOLIPOP_IAP 8.13e−04 0.74
LOLIPOP_IA610 2.31e−45 0.78
LOLIPOP_IA317 9.34e−12 0.79
LOLIPOP_EWP 2.70e−06 0.84
LOLIPOP_EWA 0.014 0.86
LOLIPOP_EW610 1.84e−04 0.86
LLS 0.002 0.85
LLD 2.89e−04 0.88
KORCULA 5.55e−06 0.81
INT 4.00e−124 0.87
FR97 3.46e−28 0.86
FR07 4.59e−16 0.86
EUGENDA 2.32e−10 0.86
ERF 6.64e−04 0.87
EGCUT 2.00e−13 0.86
CKB 3.50e−11 0.77
ALSPAC 3.40e−31 0.86
META 0.00e+00 0.85
−0.4
−0.2
0.0
BETA

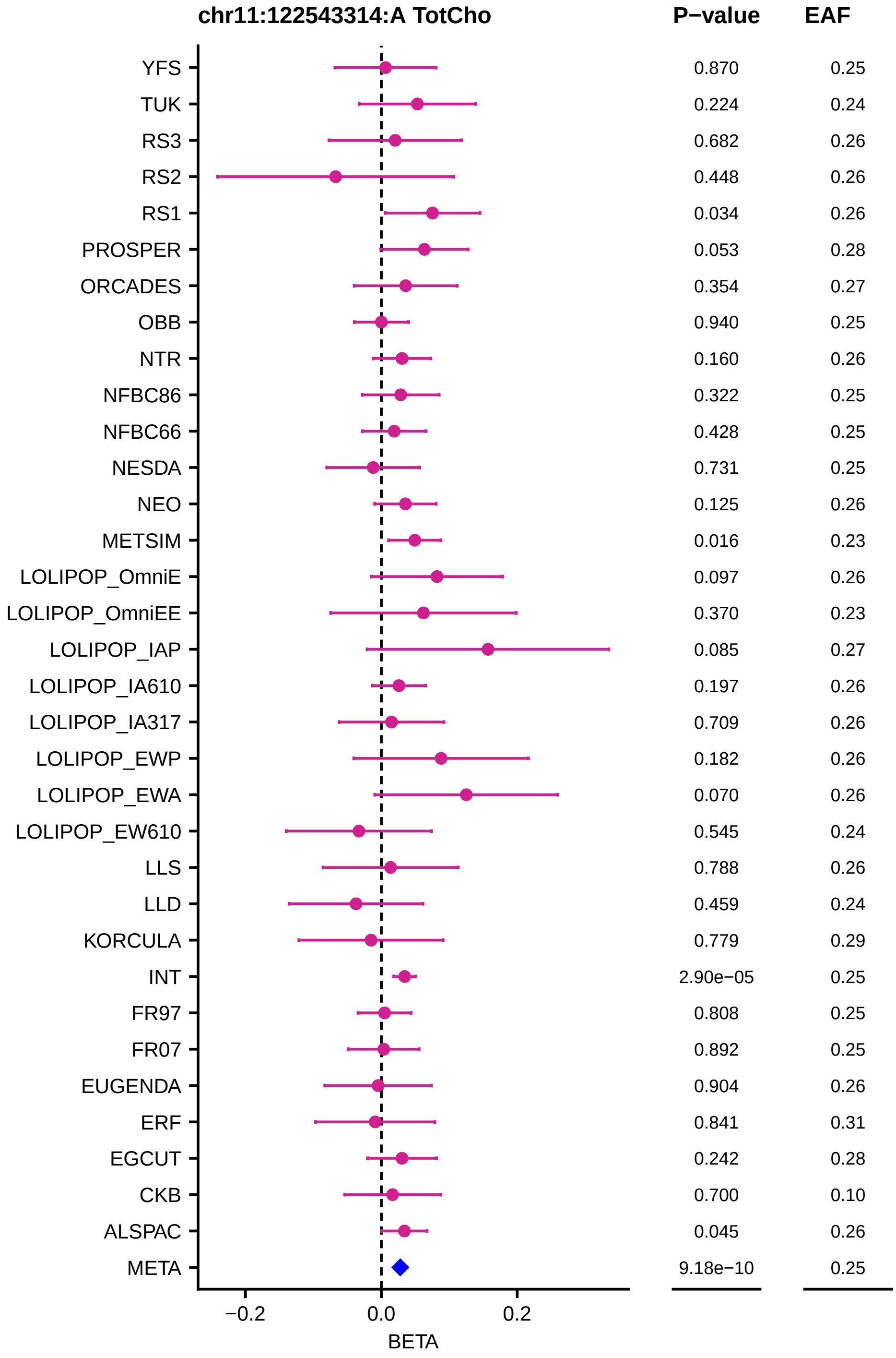
chr11:122543314:A TotCho
P−value
EAF
YFS 0.870 0.25
TUK 0.224 0.24
RS3 0.682 0.26
RS2 0.448 0.26
RS1 0.034 0.26
PROSPER 0.053 0.28
ORCADES 0.354 0.27
OBB 0.940 0.25
NTR 0.160 0.26
NFBC86 0.322 0.25
NFBC66 0.428 0.25
NESDA 0.731 0.25
NEO 0.125 0.26
METSIM 0.016 0.23
LOLIPOP_OmniE 0.097 0.26
LOLIPOP_OmniEE 0.370 0.23
LOLIPOP_IAP 0.085 0.27
LOLIPOP_IA610 0.197 0.26
LOLIPOP_IA317 0.709 0.26
LOLIPOP_EWP 0.182 0.26
LOLIPOP_EWA 0.070 0.26
LOLIPOP_EW610 0.545 0.24
LLS 0.788 0.26
LLD 0.459 0.24
KORCULA 0.779 0.29
INT 2.90e−05 0.25
FR97 0.808 0.25
FR07 0.892 0.25
EUGENDA 0.904 0.26
ERF 0.841 0.31
EGCUT 0.242 0.28
CKB 0.700 0.10
ALSPAC 0.045 0.26
META 9.18e−10 0.25
−0.2
0.0
0.2
BETA

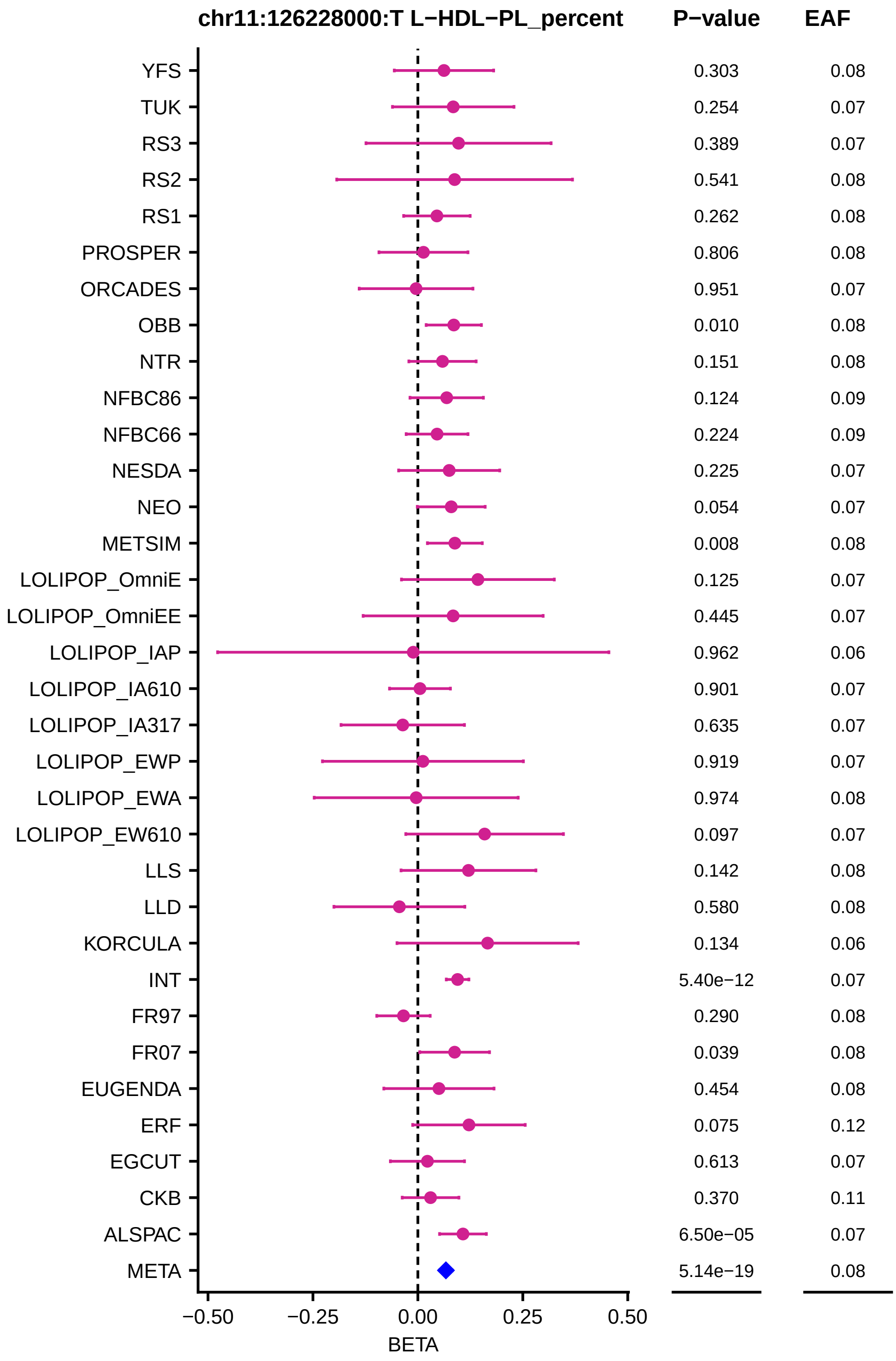
chr11:126228000:T L−HDL−PL_percent
P−value
EAF
YFS 0.303 0.08
TUK 0.254 0.07
RS3 0.389 0.07
RS2 0.541 0.08
RS1 0.262 0.08
PROSPER 0.806 0.08
ORCADES 0.951 0.07
OBB 0.010 0.08
NTR 0.151 0.08
NFBC86 0.124 0.09
NFBC66 0.224 0.09
NESDA 0.225 0.07
NEO 0.054 0.07
METSIM 0.008 0.08
LOLIPOP_OmniE 0.125 0.07
LOLIPOP_OmniEE 0.445 0.07
LOLIPOP_IAP 0.962 0.06
LOLIPOP_IA610 0.901 0.07
LOLIPOP_IA317 0.635 0.07
LOLIPOP_EWP 0.919 0.07
LOLIPOP_EWA 0.974 0.08
LOLIPOP_EW610 0.097 0.07
LLS 0.142 0.08
LLD 0.580 0.08
KORCULA 0.134 0.06
INT 5.40e−12 0.07
FR97 0.290 0.08
FR07 0.039 0.08
EUGENDA 0.454 0.08
ERF 0.075 0.12
EGCUT 0.613 0.07
CKB 0.370 0.11
ALSPAC 6.50e−05 0.07
META 5.14e−19 0.08
−0.50 −0.25 0.00 0.25 0.50
BETA

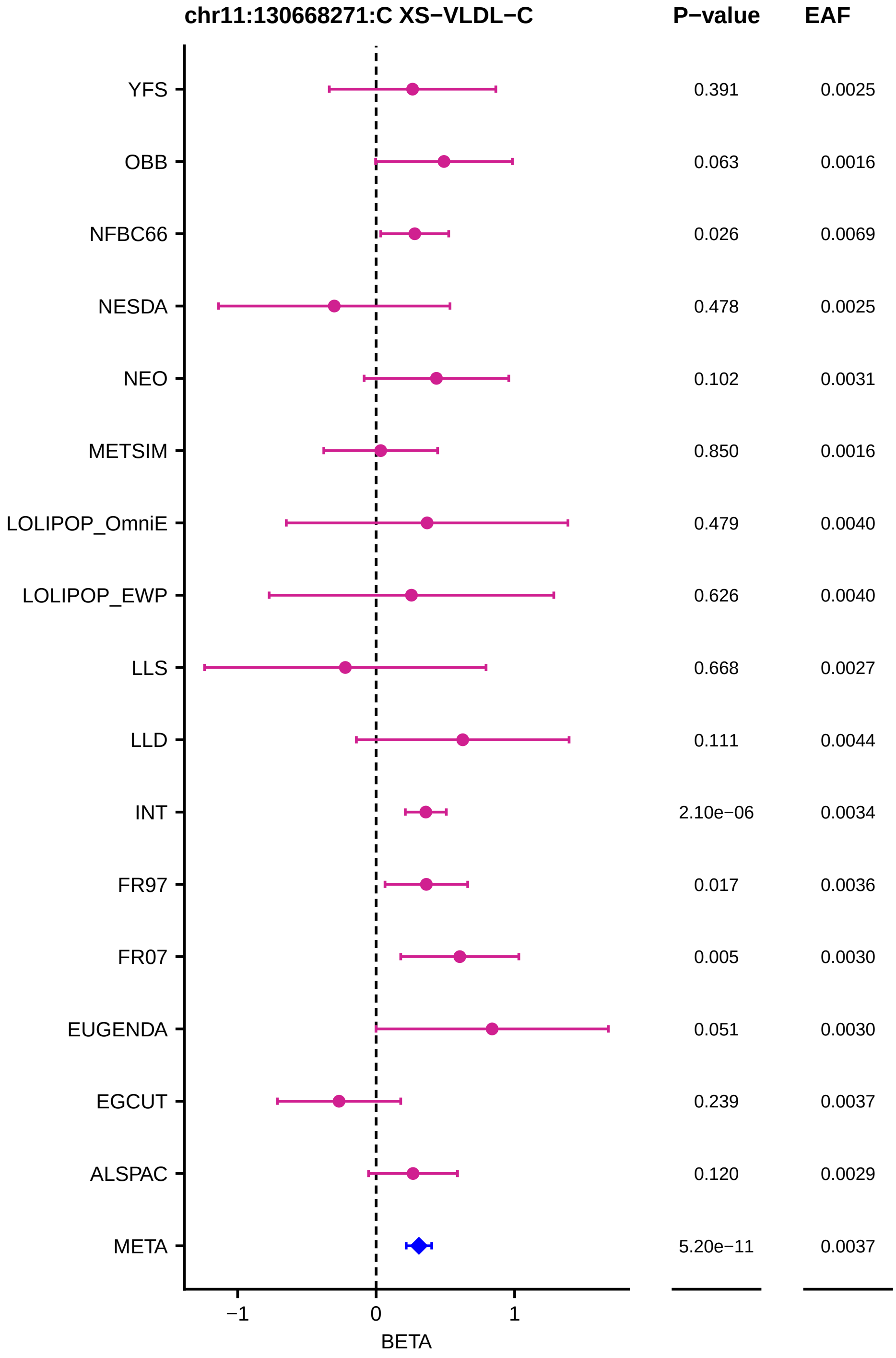
chr11:130668271:C XS−VLDL−C
P−value
EAF
YFS
OBB
NFBC66
NESDA
NEO
METSIM
LOLIPOP_OmniE
LOLIPOP_EWP
LLS
LLD
INT
FR97
FR07
EUGENDA
EGCUT
ALSPAC
META
0.391
0.063
0.026
0.478
0.102
0.850
0.479
0.626
0.668
0.111
2.10e−06
0.017
0.005
0.051
0.239
0.120
5.20e−11
0.0025
0.0016
0.0069
0.0025
0.0031
0.0016
0.0040
0.0040
0.0027
0.0044
0.0034
0.0036
0.0030
0.0030
0.0037
0.0029
0.0037
−1
0
1
BETA

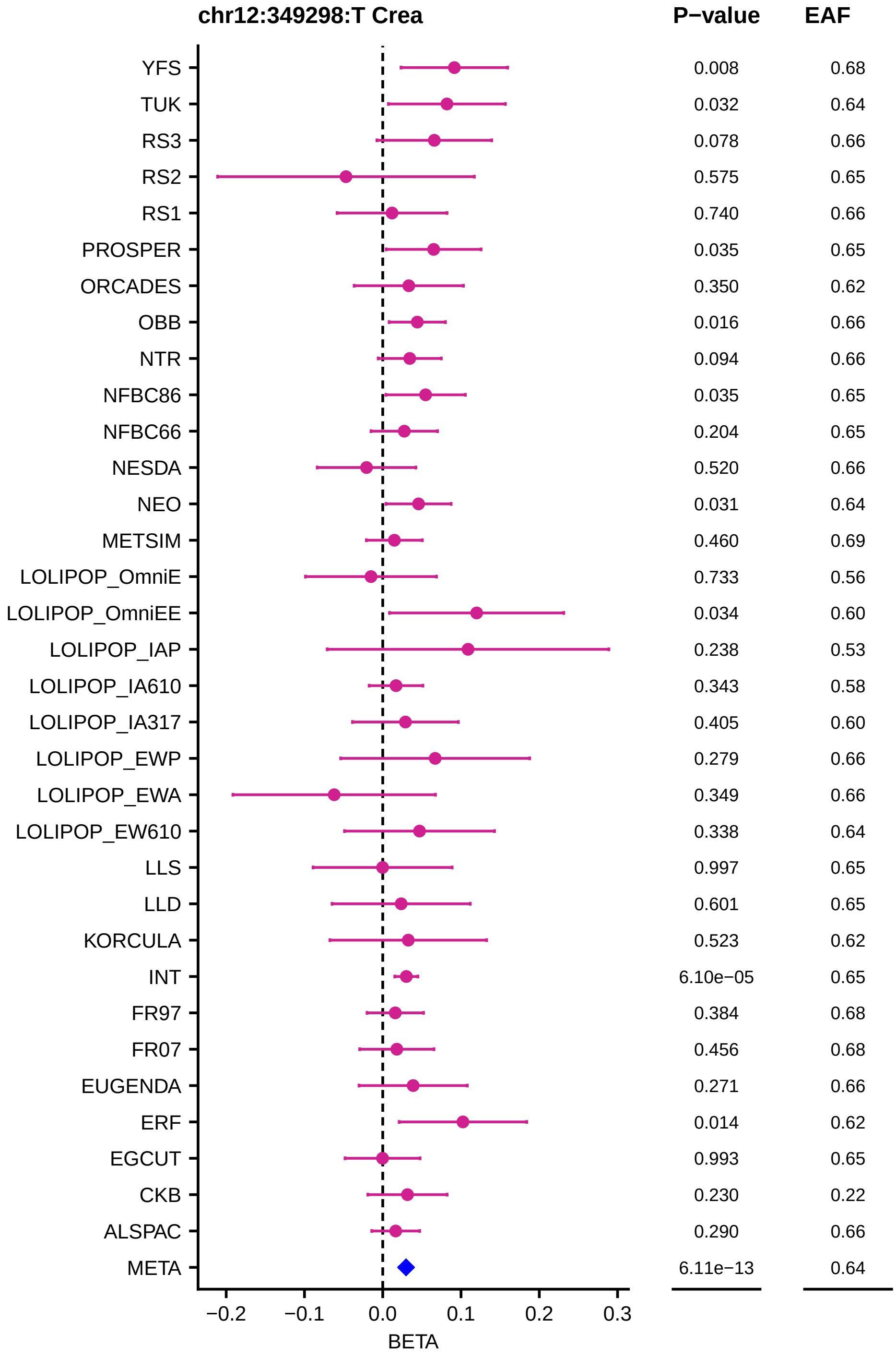

chr12:349298:T Crea
P−value
EAF
YFS 0.008 0.68
TUK 0.032 0.64
RS3 0.078 0.66
RS2 0.575 0.65
RS1 0.740 0.66
PROSPER 0.035 0.65
ORCADES 0.350 0.62
OBB 0.016 0.66
NTR 0.094 0.66
NFBC86 0.035 0.65
NFBC66 0.204 0.65
NESDA 0.520 0.66
NEO 0.031 0.64
METSIM 0.460 0.69
LOLIPOP_OmniE 0.733 0.56
LOLIPOP_OmniEE 0.034 0.60
LOLIPOP_IAP 0.238 0.53
LOLIPOP_IA610 0.343 0.58
LOLIPOP_IA317 0.405 0.60
LOLIPOP_EWP 0.279 0.66
LOLIPOP_EWA 0.349 0.66
LOLIPOP_EW610 0.338 0.64
LLS 0.997 0.65
LLD 0.601 0.65
KORCULA 0.523 0.62
INT 6.10e−05 0.65
FR97 0.384 0.68
FR07 0.456 0.68
EUGENDA 0.271 0.66
ERF 0.014 0.62
EGCUT 0.993 0.65
CKB 0.230 0.22
ALSPAC 0.290 0.66
META 6.11e−13 0.64
−0.2 −0.1 0.0 0.1 0.2 0.3
BETA

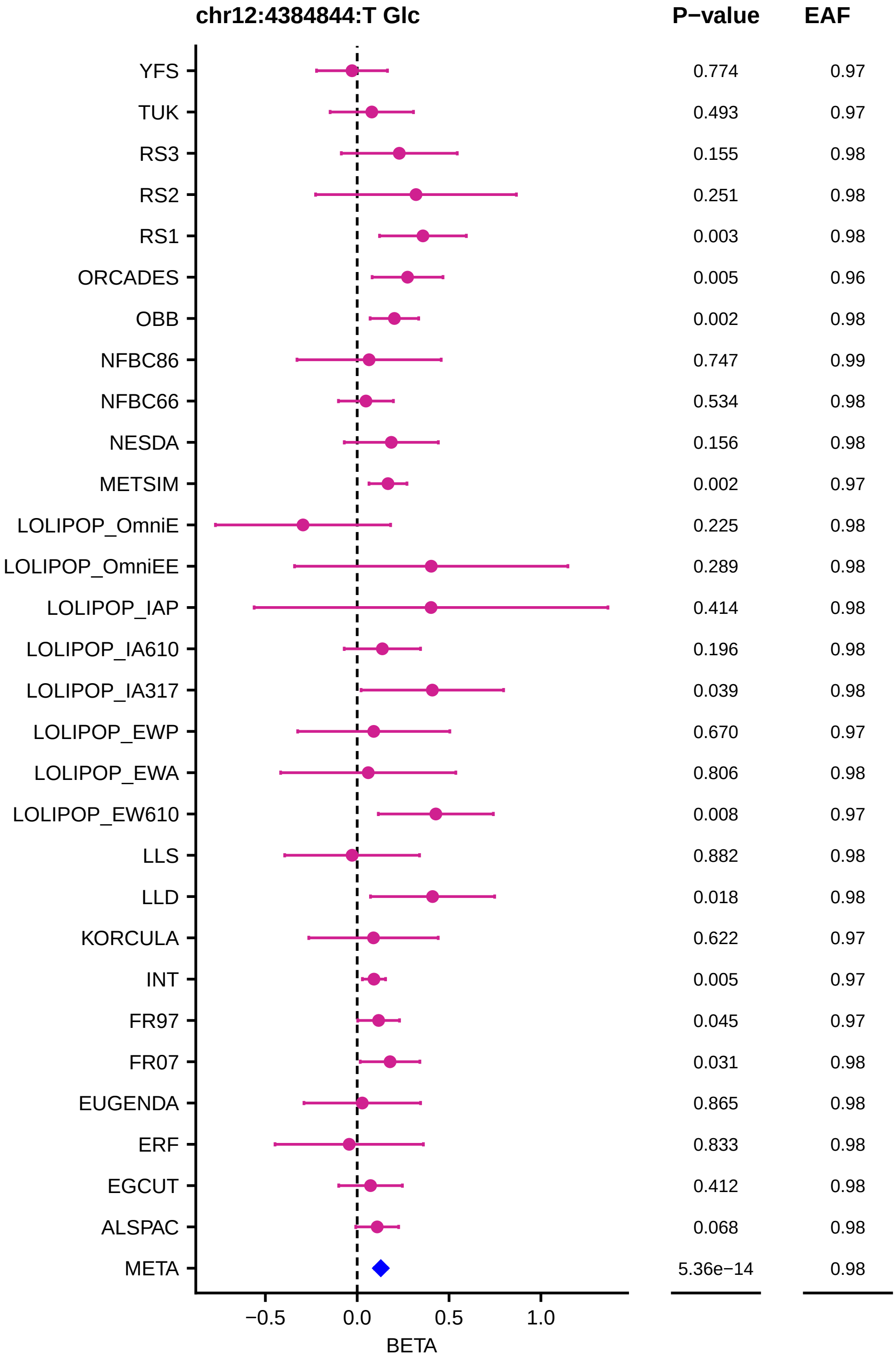
chr12:4384844:T Glc
P−value
EAF
YFS 0.774 0.97
TUK 0.493 0.97
RS3 0.155 0.98
RS2 0.251 0.98
RS1 0.003 0.98
ORCADES 0.005 0.96
OBB 0.002 0.98
NFBC86 0.747 0.99
NFBC66 0.534 0.98
NESDA 0.156 0.98
METSIM 0.002 0.97
LOLIPOP_OmniE 0.225 0.98
LOLIPOP_OmniEE 0.289 0.98
LOLIPOP_IAP 0.414 0.98
LOLIPOP_IA610 0.196 0.98
LOLIPOP_IA317 0.039 0.98
LOLIPOP_EWP 0.670 0.97
LOLIPOP_EWA 0.806 0.98
LOLIPOP_EW610 0.008 0.97
LLS 0.882 0.98
LLD 0.018 0.98
KORCULA 0.622 0.97
INT 0.005 0.97
FR97 0.045 0.97
FR07 0.031 0.98
EUGENDA 0.865 0.98
ERF 0.833 0.98
EGCUT 0.412 0.98
ALSPAC 0.068 0.98
META 5.36e−14 0.98
−0.5
0.0
0.5
1.0
BETA

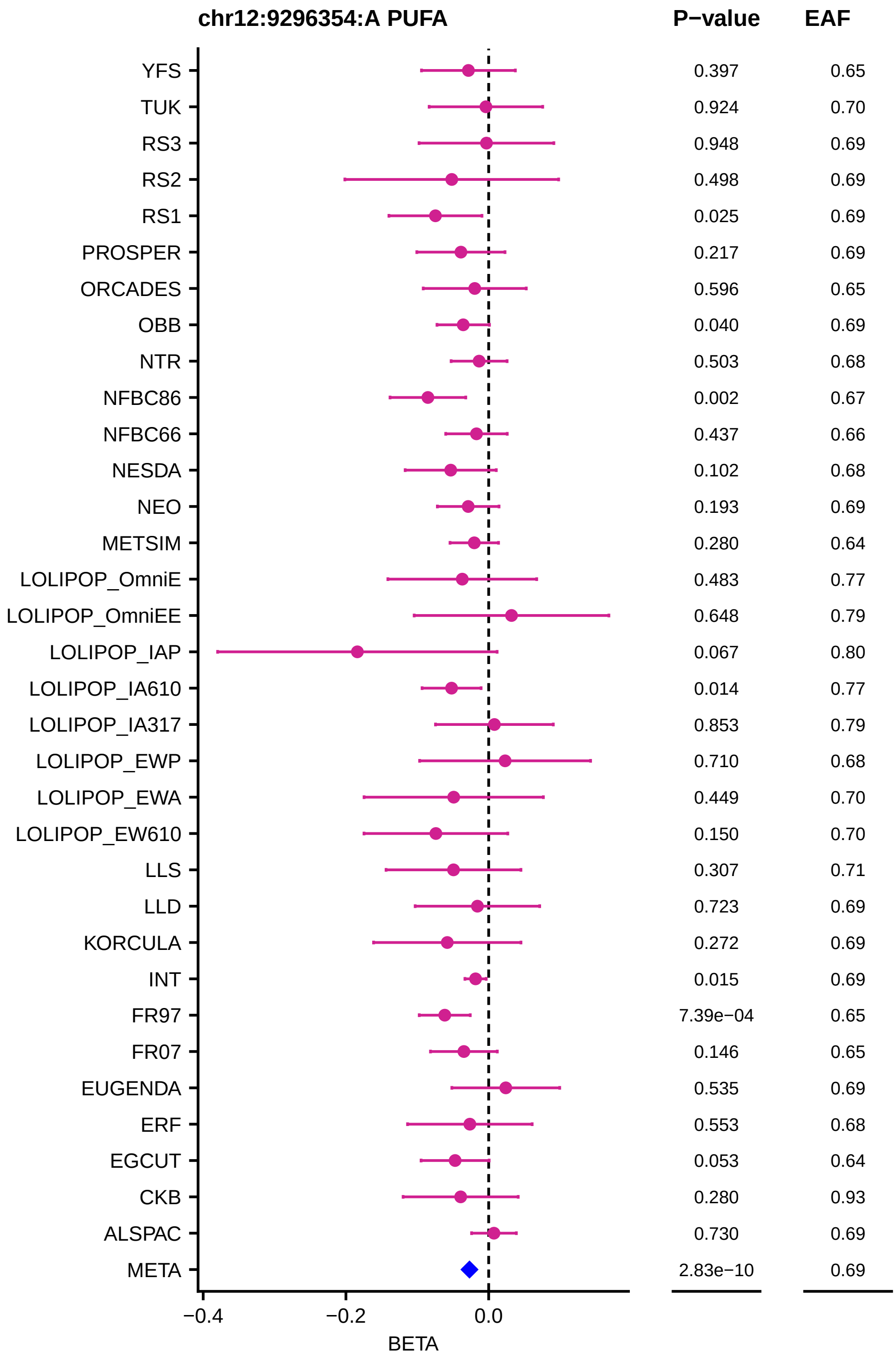

chr12:9296354:A PUFA
P−value
EAF
YFS
TUK
RS3
RS2
RS1
PROSPER
ORCADES
OBB
NTR
NFBC86
NFBC66
NESDA
NEO
METSIM
LOLIPOP_OmniE
LOLIPOP_OmniEE
LOLIPOP_IAP
LOLIPOP_IA610
LOLIPOP_IA317
LOLIPOP_EWP
LOLIPOP_EWA
LOLIPOP_EW610
LLS
LLD
KORCULA
INT
FR97
FR07
EUGENDA
ERF
EGCUT
CKB
ALSPAC
META
0.397 0.65
0.924 0.70
0.948 0.69
0.498 0.69
0.025 0.69
0.217 0.69
0.596 0.65
0.040 0.69
0.503 0.68
0.002 0.67
0.437 0.66
0.102 0.68
0.193 0.69
0.280 0.64
0.483 0.77
0.648 0.79
0.067 0.80
0.014 0.77
0.853 0.79
0.710 0.68
0.449 0.70
0.150 0.70
0.307 0.71
0.723 0.69
0.272 0.69
0.015 0.69
7.39e−04 0.65
0.146 0.65
0.535 0.69
0.553 0.68
0.053 0.64
0.280 0.93
0.730 0.69
2.83e−10 0.69
−0.4
−0.2
0.0
BETA

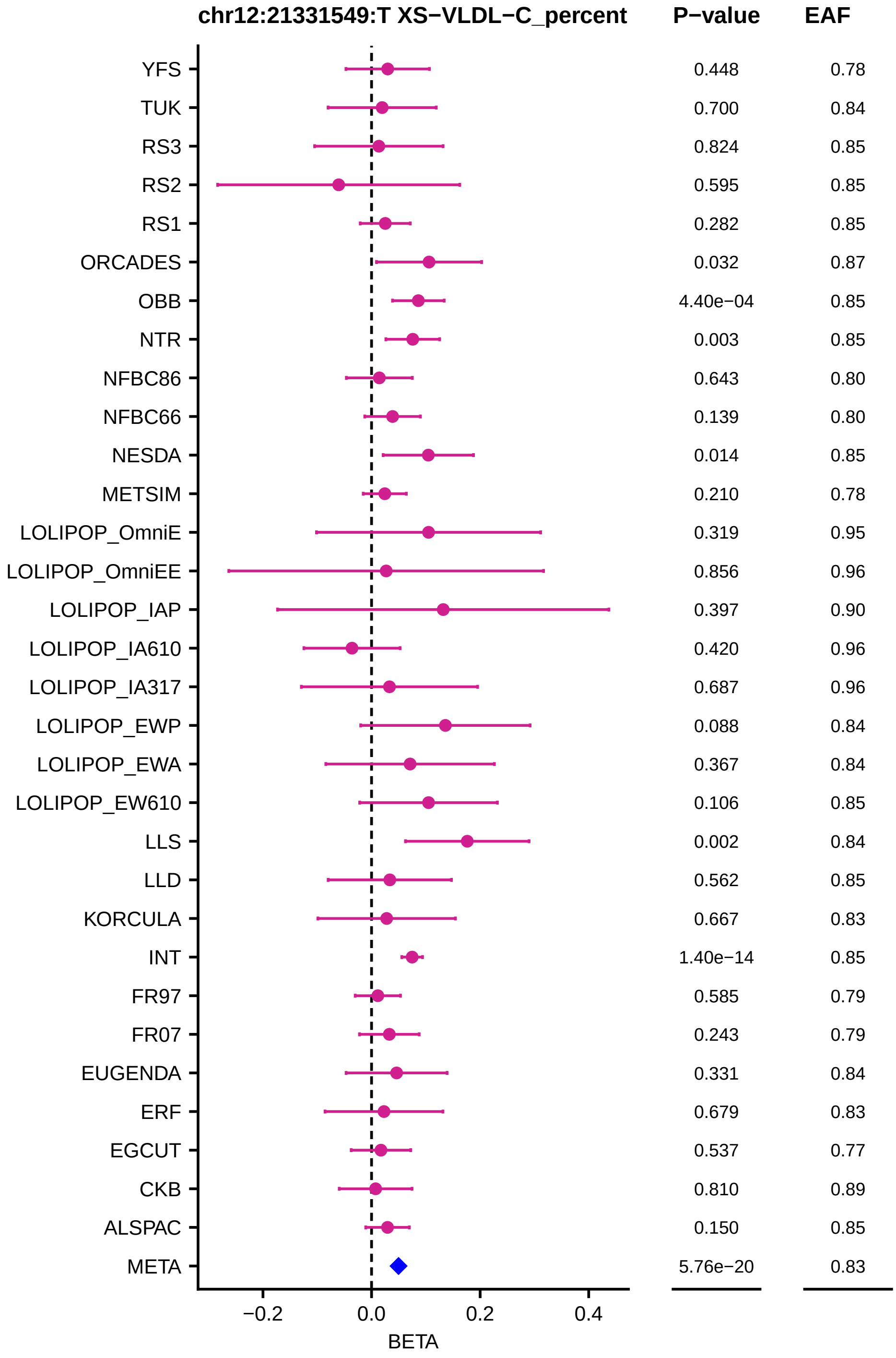
chr12:21331549:T XS−VLDL−C_percent
P−value
EAF
YFS 0.448 0.78
TUK 0.700 0.84
RS3 0.824 0.85
RS2 0.595 0.85
RS1 0.282 0.85
ORCADES 0.032 0.87
OBB 4.40e−04 0.85
NTR 0.003 0.85
NFBC86 0.643 0.80
NFBC66 0.139 0.80
NESDA 0.014 0.85
METSIM 0.210 0.78
LOLIPOP_OmniE 0.319 0.95
LOLIPOP_OmniEE 0.856 0.96
LOLIPOP_IAP 0.397 0.90
LOLIPOP_IA610 0.420 0.96
LOLIPOP_IA317 0.687 0.96
LOLIPOP_EWP 0.088 0.84
LOLIPOP_EWA 0.367 0.84
LOLIPOP_EW610 0.106 0.85
LLS 0.002 0.84
LLD 0.562 0.85
KORCULA 0.667 0.83
INT 1.40e−14 0.85
FR97 0.585 0.79
FR07 0.243 0.79
EUGENDA 0.331 0.84
ERF 0.679 0.83
EGCUT 0.537 0.77
CKB 0.810 0.89
ALSPAC 0.150 0.85
META 5.76e−20 0.83
−0.2
0.0
0.2
0.4
BETA

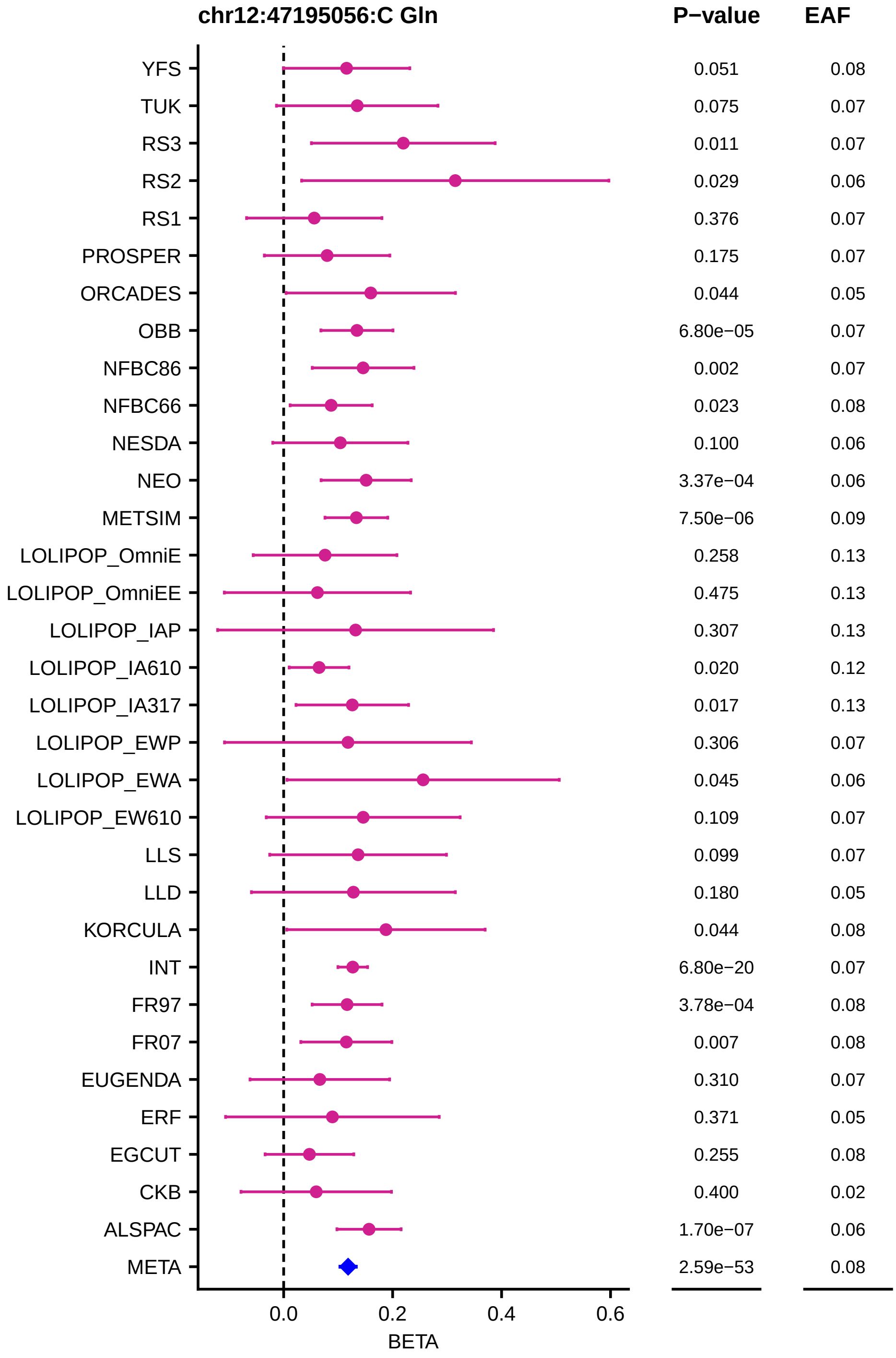

chr12:47195056:C Gln
P−value
EAF
YFS 0.051 0.08
TUK 0.075 0.07
RS3 0.011 0.07
RS2 0.029 0.06
RS1 0.376 0.07
PROSPER 0.175 0.07
ORCADES 0.044 0.05
OBB 6.80e−05 0.07
NFBC86 0.002 0.07
NFBC66 0.023 0.08
NESDA 0.100 0.06
NEO 3.37e−04 0.06
METSIM 7.50e−06 0.09
LOLIPOP_OmniE 0.258 0.13
LOLIPOP_OmniEE 0.475 0.13
LOLIPOP_IAP 0.307 0.13
LOLIPOP_IA610 0.020 0.12
LOLIPOP_IA317 0.017 0.13
LOLIPOP_EWP 0.306 0.07
LOLIPOP_EWA 0.045 0.06
LOLIPOP_EW610 0.109 0.07
LLS 0.099 0.07
LLD 0.180 0.05
KORCULA 0.044 0.08
INT 6.80e−20 0.07
FR97 3.78e−04 0.08
FR07 0.007 0.08
EUGENDA 0.310 0.07
ERF 0.371 0.05
EGCUT 0.255 0.08
CKB 0.400 0.02
ALSPAC 1.70e−07 0.06
META 2.59e−53 0.08
0.0
0.2
0.4
0.6
BETA

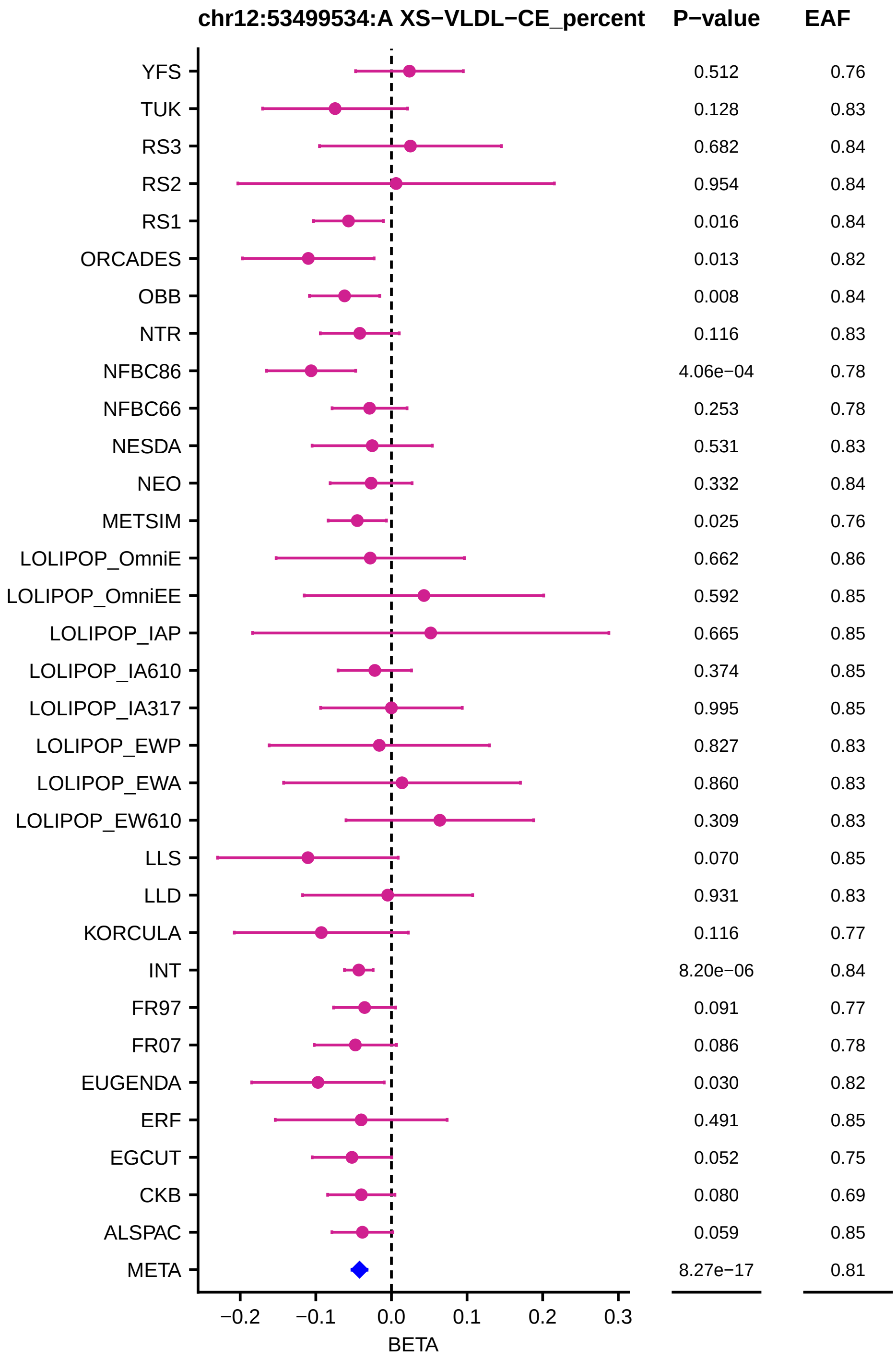
chr12:53499534:A XS−VLDL−CE_percent
P−value
EAF
YFS 0.512 0.76
TUK 0.128 0.83
RS3 0.682 0.84
RS2 0.954 0.84
RS1 0.016 0.84
ORCADES 0.013 0.82
OBB 0.008 0.84
NTR 0.116 0.83
NFBC86 4.06e−04 0.78
NFBC66 0.253 0.78
NESDA 0.531 0.83
NEO 0.332 0.84
METSIM 0.025 0.76
LOLIPOP_OmniE 0.662 0.86
LOLIPOP_OmniEE 0.592 0.85
LOLIPOP_IAP 0.665 0.85
LOLIPOP_IA610 0.374 0.85
LOLIPOP_IA317 0.995 0.85
LOLIPOP_EWP 0.827 0.83
LOLIPOP_EWA 0.860 0.83
LOLIPOP_EW610 0.309 0.83
LLS 0.070 0.85
LLD 0.931 0.83
KORCULA 0.116 0.77
INT 8.20e−06 0.84
FR97 0.091 0.77
FR07 0.086 0.78
EUGENDA 0.030 0.82
ERF 0.491 0.85
EGCUT 0.052 0.75
CKB 0.080 0.69
ALSPAC 0.059 0.85
META 8.27e−17 0.81
−0.2 −0.1 0.0 0.1 0.2 0.3
BETA

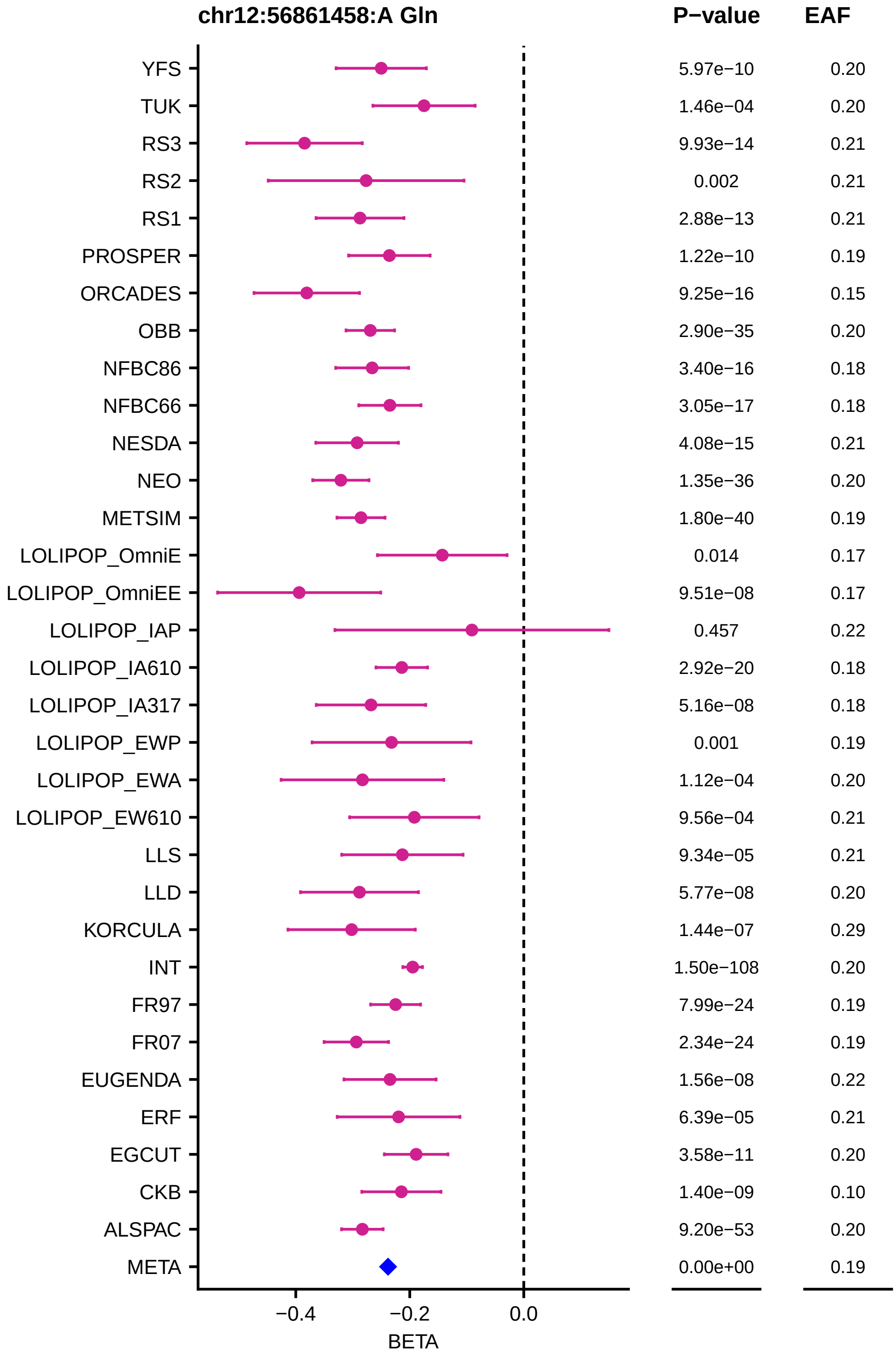
chr12:56861458:A Gln
P−value
EAF
YFS 5.97e−10 0.20
TUK 1.46e−04 0.20
RS3 9.93e−14 0.21
RS2 0.002 0.21
RS1 2.88e−13 0.21
PROSPER 1.22e−10 0.19
ORCADES 9.25e−16 0.15
OBB 2.90e−35 0.20
NFBC86 3.40e−16 0.18
NFBC66 3.05e−17 0.18
NESDA 4.08e−15 0.21
NEO 1.35e−36 0.20
METSIM 1.80e−40 0.19
LOLIPOP_OmniE 0.014 0.17
LOLIPOP_OmniEE 9.51e−08 0.17
LOLIPOP_IAP 0.457 0.22
LOLIPOP_IA610 2.92e−20 0.18
LOLIPOP_IA317 5.16e−08 0.18
LOLIPOP_EWP 0.001 0.19
LOLIPOP_EWA 1.12e−04 0.20
LOLIPOP_EW610 9.56e−04 0.21
LLS 9.34e−05 0.21
LLD 5.77e−08 0.20
KORCULA 1.44e−07 0.29
INT 1.50e−108 0.20
FR97 7.99e−24 0.19
FR07 2.34e−24 0.19
EUGENDA 1.56e−08 0.22
ERF 6.39e−05 0.21
EGCUT 3.58e−11 0.20
CKB 1.40e−09 0.10
ALSPAC 9.20e−53 0.20
META 0.00e+00 0.19
−0.4
−0.2
0.0
BETA

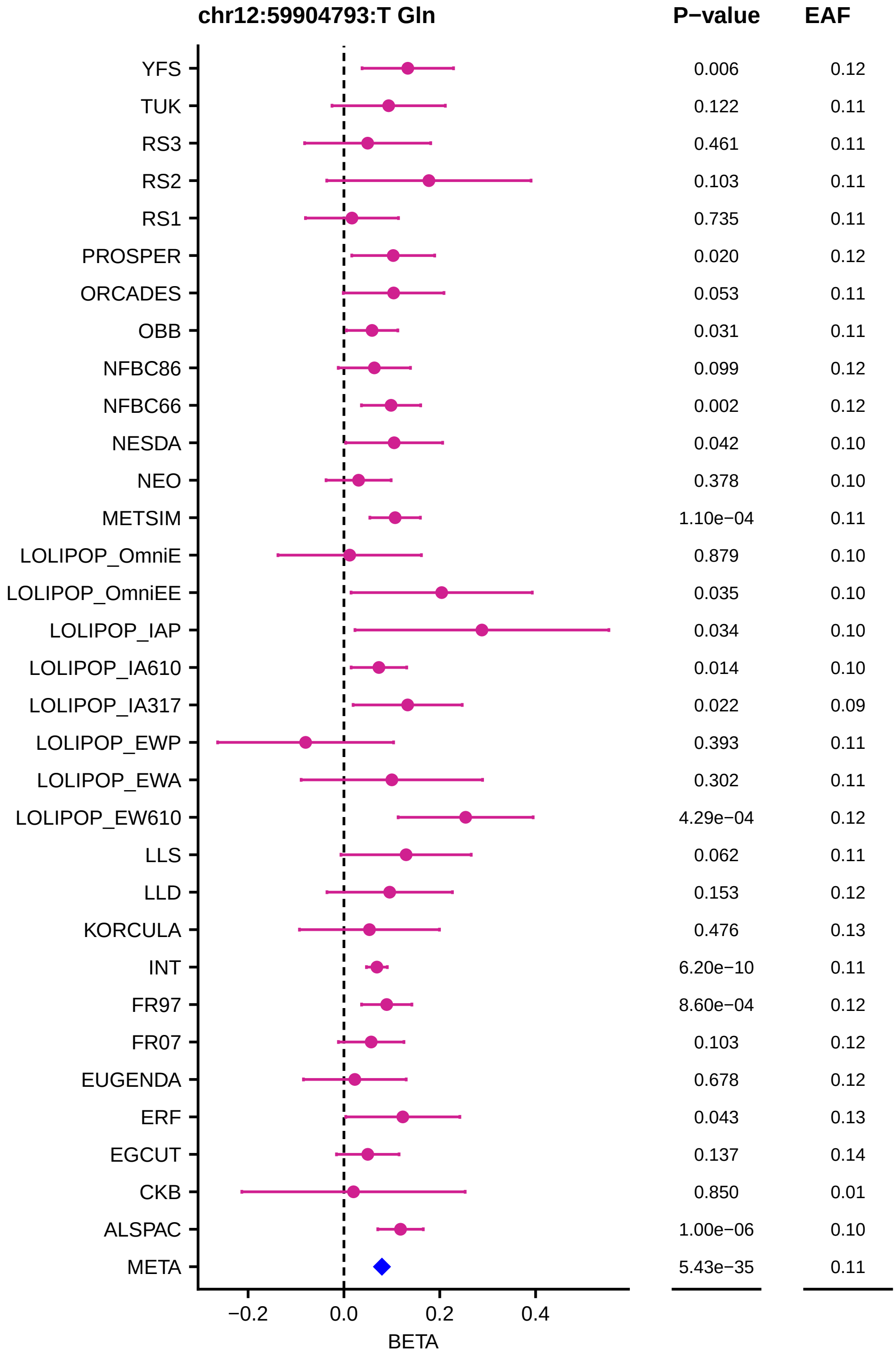
chr12:59904793:T Gln
P−value
EAF
YFS 0.006 0.12
TUK 0.122 0.11
RS3 0.461 0.11
RS2 0.103 0.11
RS1 0.735 0.11
PROSPER 0.020 0.12
ORCADES 0.053 0.11
OBB 0.031 0.11
NFBC86 0.099 0.12
NFBC66 0.002 0.12
NESDA 0.042 0.10
NEO 0.378 0.10
METSIM 1.10e−04 0.11
LOLIPOP_OmniE 0.879 0.10
LOLIPOP_OmniEE 0.035 0.10
LOLIPOP_IAP 0.034 0.10
LOLIPOP_IA610 0.014 0.10
LOLIPOP_IA317 0.022 0.09
LOLIPOP_EWP 0.393 0.11
LOLIPOP_EWA 0.302 0.11
LOLIPOP_EW610 4.29e−04 0.12
LLS 0.062 0.11
LLD 0.153 0.12
KORCULA 0.476 0.13
INT 6.20e−10 0.11
FR97 8.60e−04 0.12
FR07 0.103 0.12
EUGENDA 0.678 0.12
ERF 0.043 0.13
EGCUT 0.137 0.14
CKB 0.850 0.01
ALSPAC 1.00e−06 0.10
META 5.43e−35 0.11
−0.2
0.0
0.2
0.4
BETA

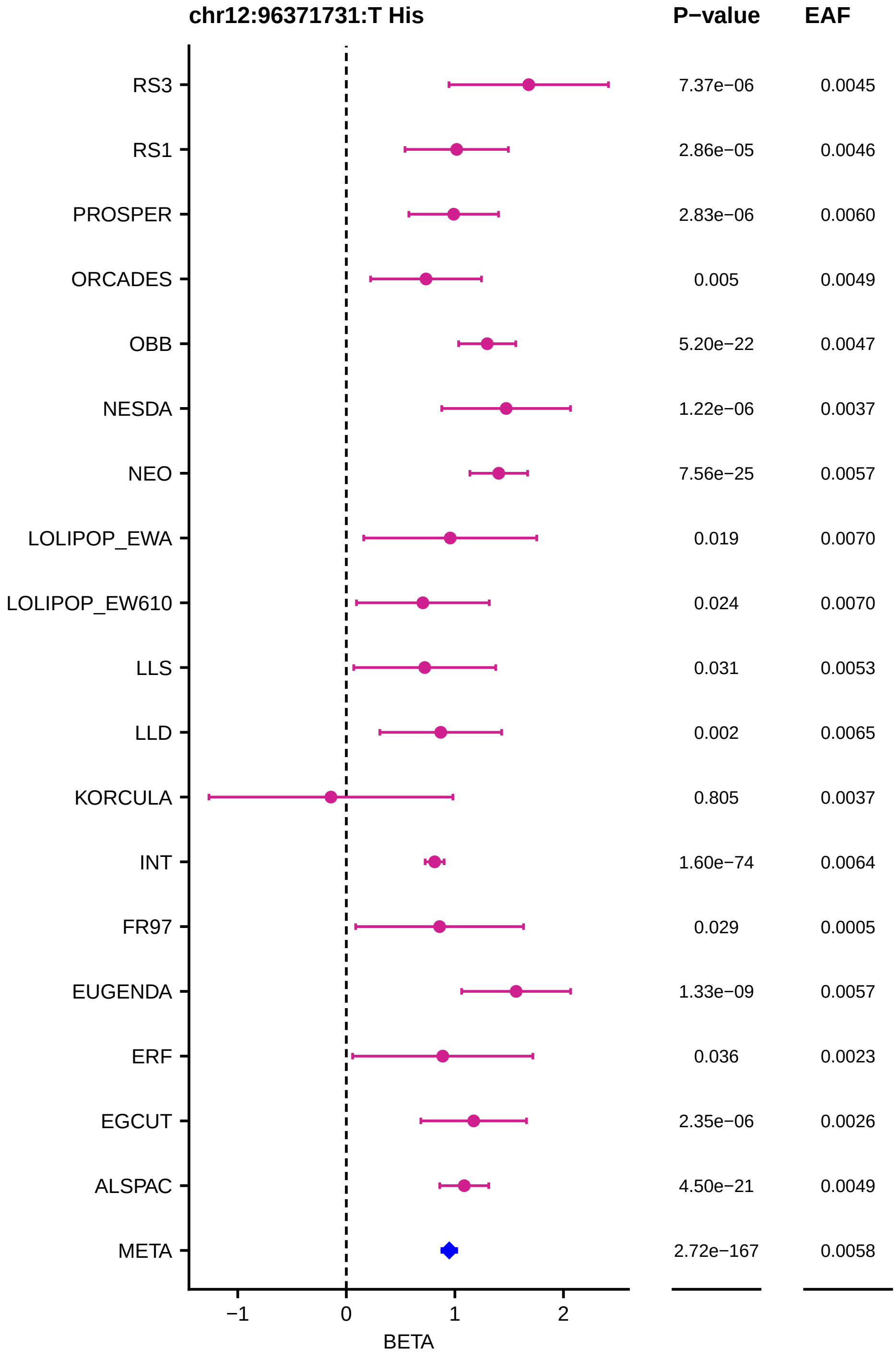
chr12:96371731:T His
P−value
EAF
RS3
RS1
PROSPER
ORCADES
OBB
NESDA
NEO
LOLIPOP_EWA
LOLIPOP_EW610
LLS
LLD
KORCULA
INT
FR97
EUGENDA
ERF
EGCUT
ALSPAC
META
7.37e−06 0.0045
2.86e−05 0.0046
2.83e−06 0.0060
0.005 0.0049
5.20e−22 0.0047
1.22e−06 0.0037
7.56e−25 0.0057
0.019 0.0070
0.024 0.0070
0.031 0.0053
0.002 0.0065
0.805 0.0037
1.60e−74 0.0064
0.029 0.0005
1.33e−09 0.0057
0.036 0.0023
2.35e−06 0.0026
4.50e−21 0.0049
2.72e−167 0.0058
−1
0
1
2
BETA

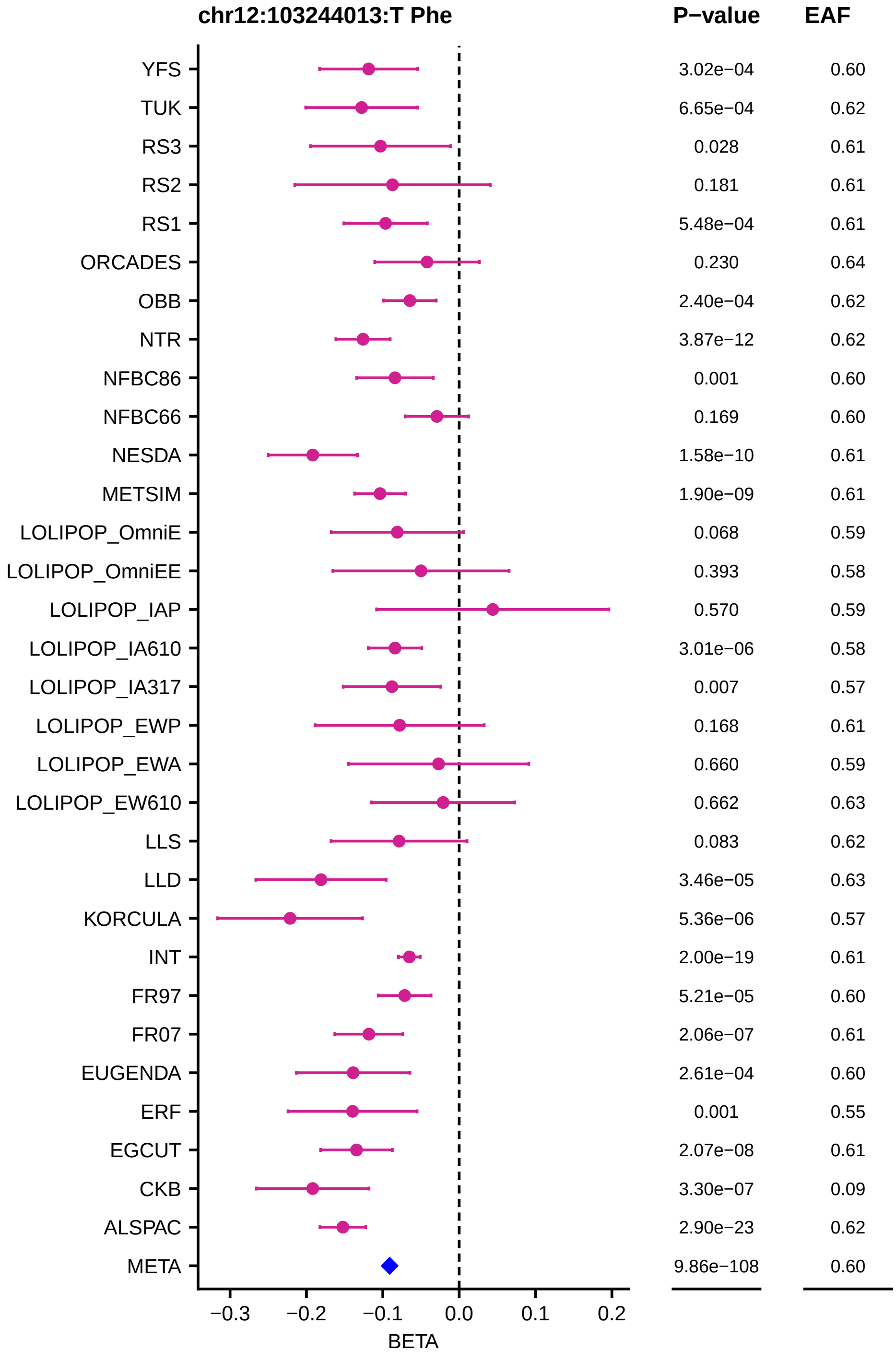
chr12:103244013:T Phe
P−value
EAF
YFS 3.02e−04 0.60
TUK 6.65e−04 0.62
RS3 0.028 0.61
RS2 0.181 0.61
RS1 5.48e−04 0.61
ORCADES 0.230 0.64
OBB 2.40e−04 0.62
NTR 3.87e−12 0.62
NFBC86 0.001 0.60
NFBC66 0.169 0.60
NESDA 1.58e−10 0.61
METSIM 1.90e−09 0.61
LOLIPOP_OmniE 0.068 0.59
LOLIPOP_OmniEE 0.393 0.58
LOLIPOP_IAP 0.570 0.59
LOLIPOP_IA610 3.01e−06 0.58
LOLIPOP_IA317 0.007 0.57
LOLIPOP_EWP 0.168 0.61
LOLIPOP_EWA 0.660 0.59
LOLIPOP_EW610 0.662 0.63
LLS 0.083 0.62
LLD 3.46e−05 0.63
KORCULA 5.36e−06 0.57
INT 2.00e−19 0.61
FR97 5.21e−05 0.60
FR07 2.06e−07 0.61
EUGENDA 2.61e−04 0.60
ERF 0.001 0.55
EGCUT 2.07e−08 0.61
CKB 3.30e−07 0.09
ALSPAC 2.90e−23 0.62
META 9.86e−108 0.60
−0.3 −0.2 −0.1 0.0 0.1 0.2
BETA

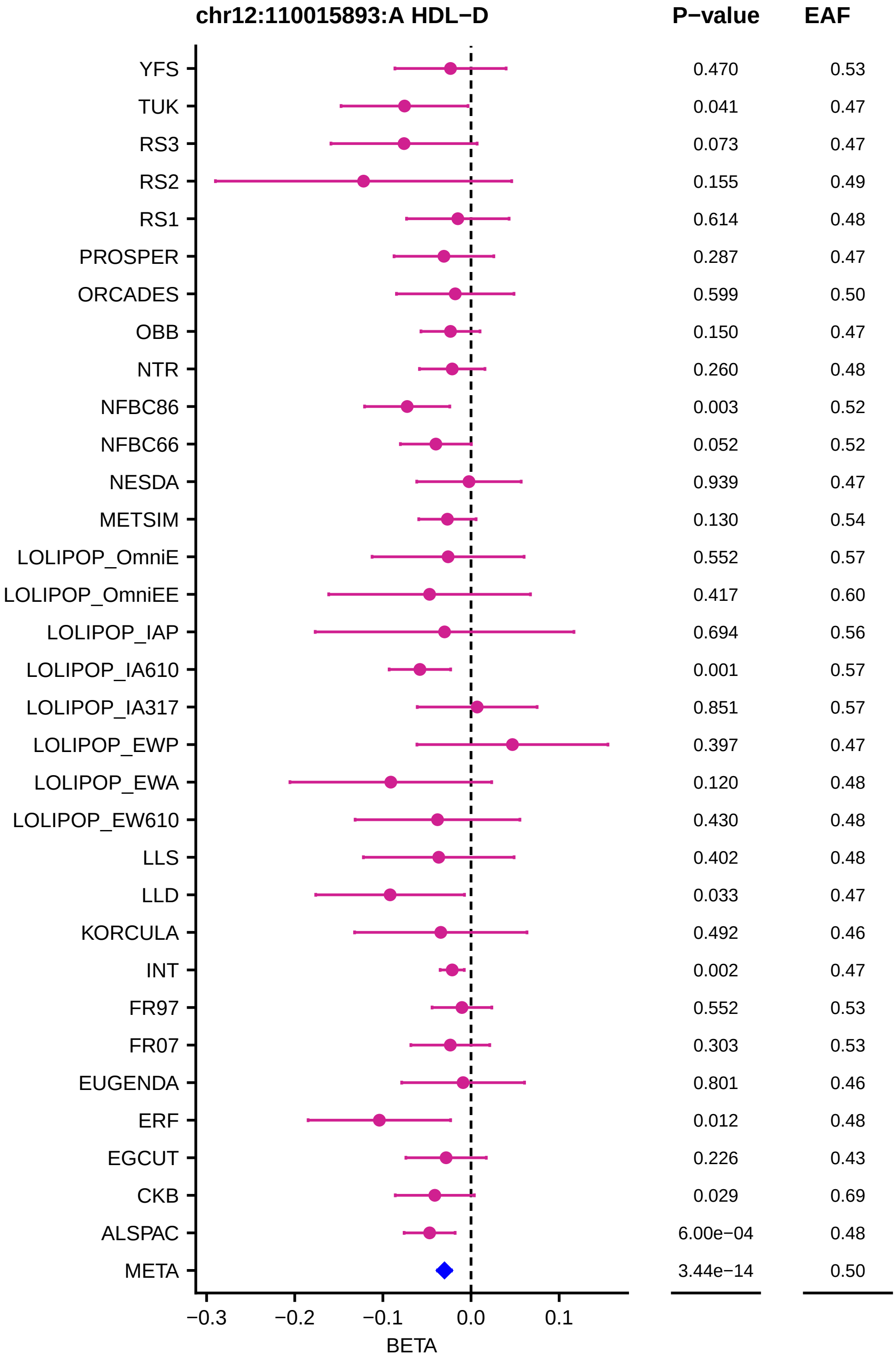

chr12:110015893:A HDL−D
P−value
EAF
YFS 0.470 0.53
TUK 0.041 0.47
RS3 0.073 0.47
RS2 0.155 0.49
RS1 0.614 0.48
PROSPER 0.287 0.47
ORCADES 0.599 0.50
OBB 0.150 0.47
NTR 0.260 0.48
NFBC86 0.003 0.52
NFBC66 0.052 0.52
NESDA 0.939 0.47
METSIM 0.130 0.54
LOLIPOP_OmniE 0.552 0.57
LOLIPOP_OmniEE 0.417 0.60
LOLIPOP_IAP 0.694 0.56
LOLIPOP_IA610 0.001 0.57
LOLIPOP_IA317 0.851 0.57
LOLIPOP_EWP 0.397 0.47
LOLIPOP_EWA 0.120 0.48
LOLIPOP_EW610 0.430 0.48
LLS 0.402 0.48
LLD 0.033 0.47
KORCULA 0.492 0.46
INT 0.002 0.47
FR97 0.552 0.53
FR07 0.303 0.53
EUGENDA 0.801 0.46
ERF 0.012 0.48
EGCUT 0.226 0.43
CKB 0.029 0.69
ALSPAC 6.00e−04 0.48
META 3.44e−14 0.50
−0.3
−0.2
−0.1
0.0
0.1
BETA

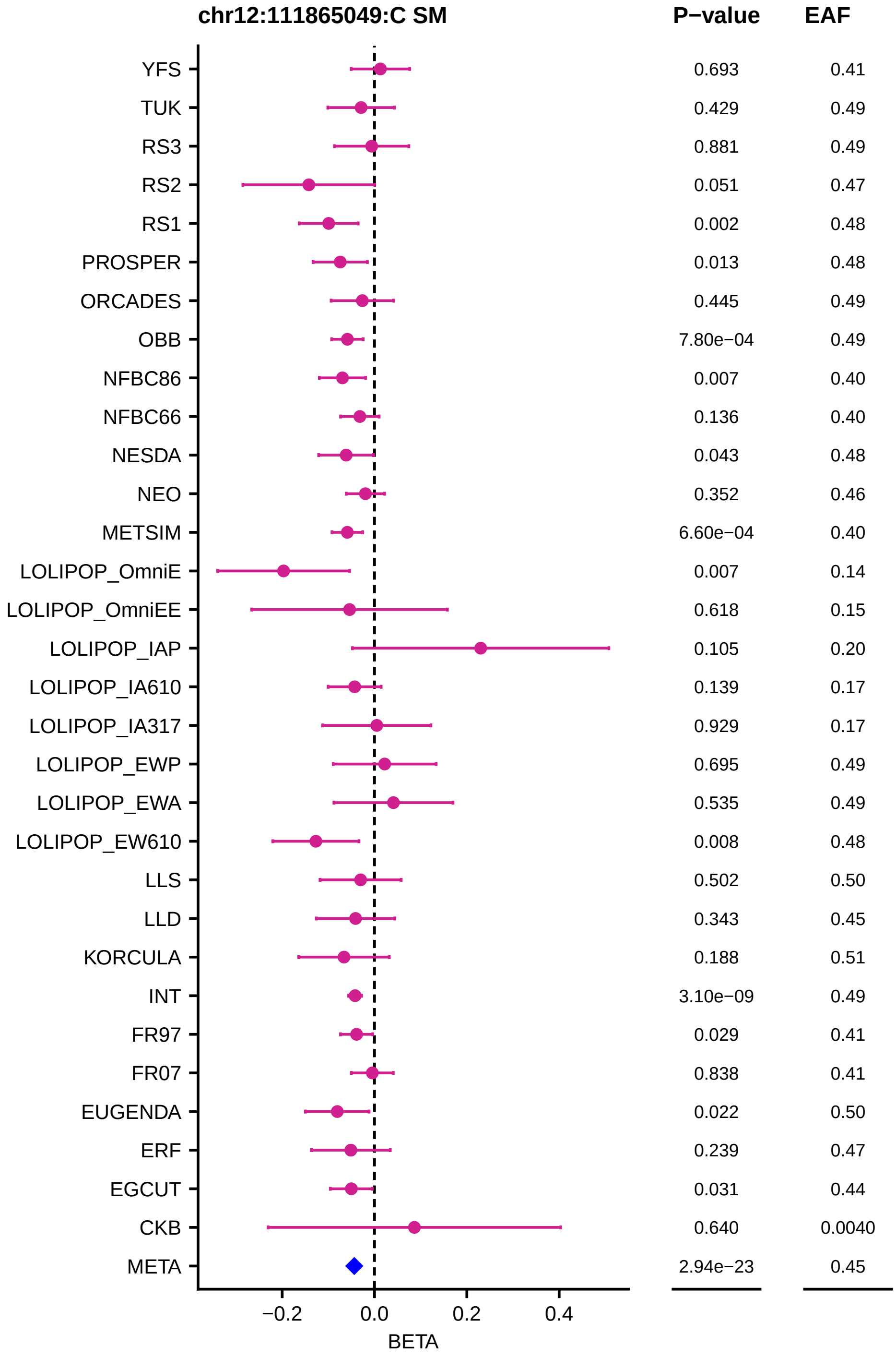
chr12:111865049:C SM
P−value
EAF
YFS 0.693 0.41
TUK 0.429 0.49
RS3 0.881 0.49
RS2 0.051 0.47
RS1 0.002 0.48
PROSPER 0.013 0.48
ORCADES 0.445 0.49
OBB 7.80e−04 0.49
NFBC86 0.007 0.40
NFBC66 0.136 0.40
NESDA 0.043 0.48
NEO 0.352 0.46
METSIM 6.60e−04 0.40
LOLIPOP_OmniE 0.007 0.14
LOLIPOP_OmniEE 0.618 0.15
LOLIPOP_IAP 0.105 0.20
LOLIPOP_IA610 0.139 0.17
LOLIPOP_IA317 0.929 0.17
LOLIPOP_EWP 0.695 0.49
LOLIPOP_EWA 0.535 0.49
LOLIPOP_EW610 0.008 0.48
LLS 0.502 0.50
LLD 0.343 0.45
KORCULA 0.188 0.51
INT 3.10e−09 0.49
FR97 0.029 0.41
FR07 0.838 0.41
EUGENDA 0.022 0.50
ERF 0.239 0.47
EGCUT 0.031 0.44
CKB 0.640 0.0040
META 2.94e−23 0.45
−0.2
0.0
0.2
0.4
BETA

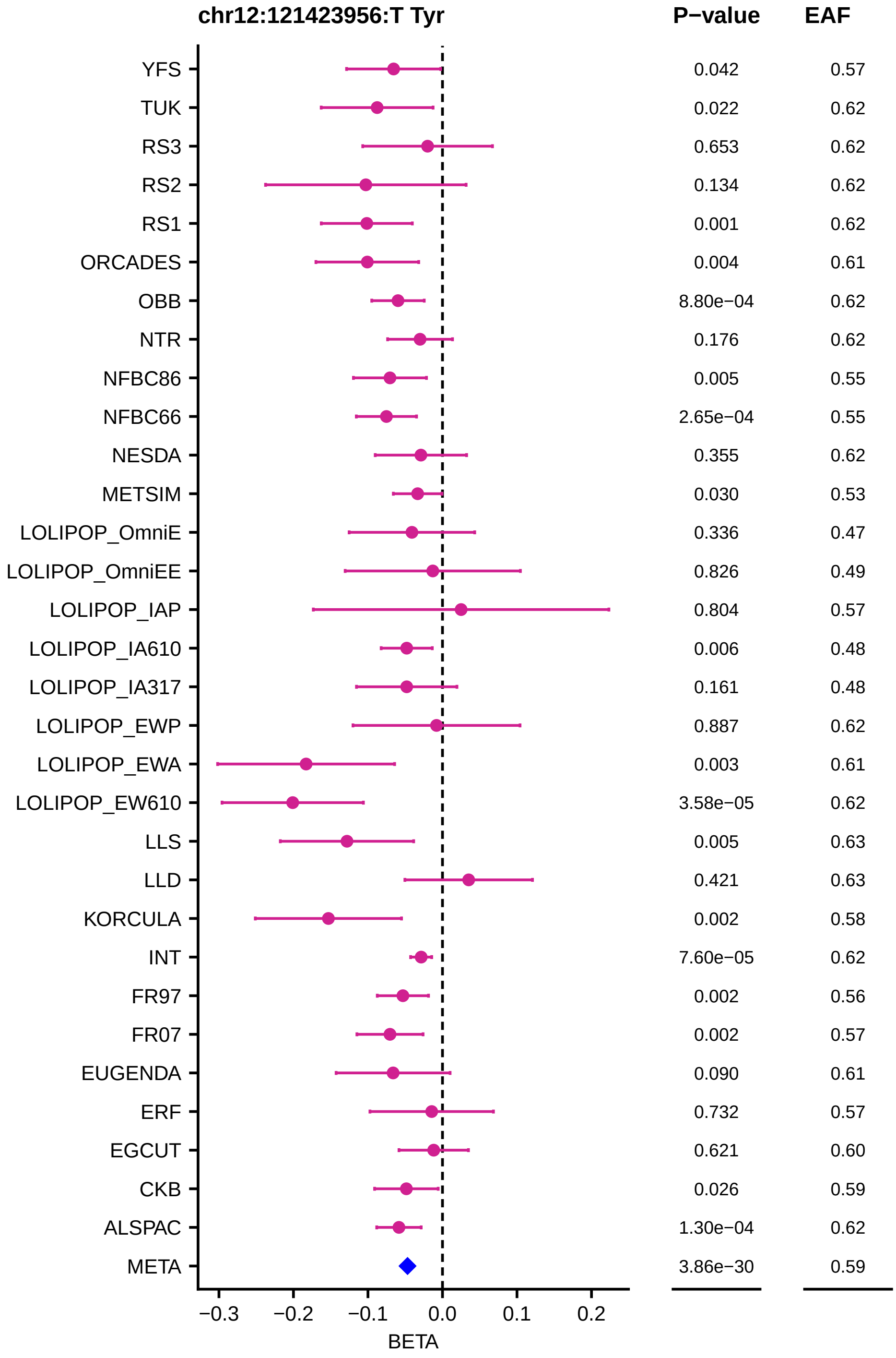
chr12:121423956:T Tyr
P−value
EAF
YFS 0.042 0.57
TUK 0.022 0.62
RS3 0.653 0.62
RS2 0.134 0.62
RS1 0.001 0.62
ORCADES 0.004 0.61
OBB 8.80e−04 0.62
NTR 0.176 0.62
NFBC86 0.005 0.55
NFBC66 2.65e−04 0.55
NESDA 0.355 0.62
METSIM 0.030 0.53
LOLIPOP_OmniE 0.336 0.47
LOLIPOP_OmniEE 0.826 0.49
LOLIPOP_IAP 0.804 0.57
LOLIPOP_IA610 0.006 0.48
LOLIPOP_IA317 0.161 0.48
LOLIPOP_EWP 0.887 0.62
LOLIPOP_EWA 0.003 0.61
LOLIPOP_EW610 3.58e−05 0.62
LLS 0.005 0.63
LLD 0.421 0.63
KORCULA 0.002 0.58
INT 7.60e−05 0.62
FR97 0.002 0.56
FR07 0.002 0.57
EUGENDA 0.090 0.61
ERF 0.732 0.57
EGCUT 0.621 0.60
CKB 0.026 0.59
ALSPAC 1.30e−04 0.62
META 3.86e−30 0.59
−0.3
−0.2
−0.1
0.0
0.1
0.2
BETA

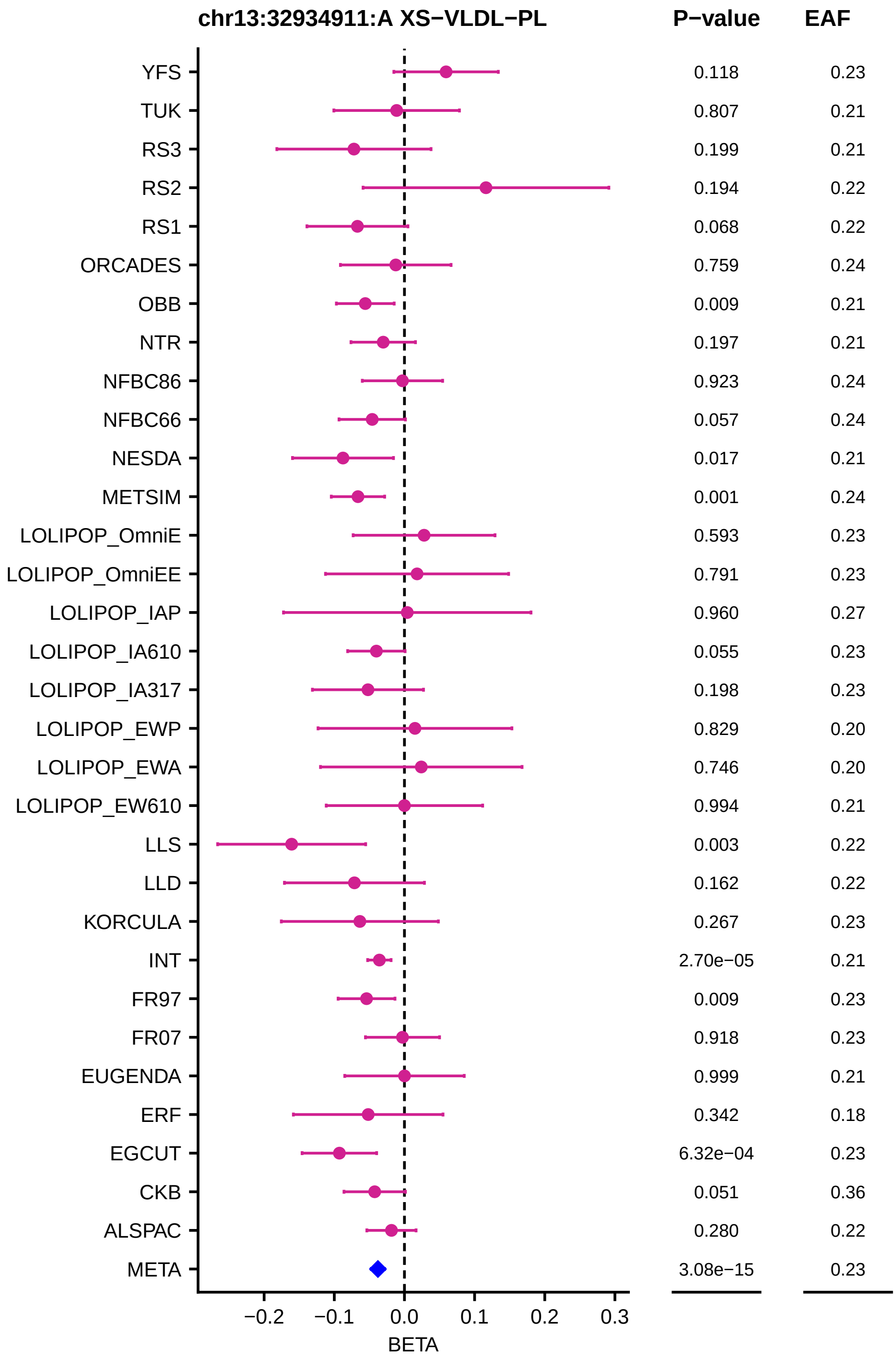
chr13:32934911:A XS−VLDL−PL
P−value
EAF
YFS 0.118 0.23
TUK 0.807 0.21
RS3 0.199 0.21
RS2 0.194 0.22
RS1 0.068 0.22
ORCADES 0.759 0.24
OBB 0.009 0.21
NTR 0.197 0.21
NFBC86 0.923 0.24
NFBC66 0.057 0.24
NESDA 0.017 0.21
METSIM 0.001 0.24
LOLIPOP_OmniE 0.593 0.23
LOLIPOP_OmniEE 0.791 0.23
LOLIPOP_IAP 0.960 0.27
LOLIPOP_IA610 0.055 0.23
LOLIPOP_IA317 0.198 0.23
LOLIPOP_EWP 0.829 0.20
LOLIPOP_EWA 0.746 0.20
LOLIPOP_EW610 0.994 0.21
LLS 0.003 0.22
LLD 0.162 0.22
KORCULA 0.267 0.23
INT 2.70e−05 0.21
FR97 0.009 0.23
FR07 0.918 0.23
EUGENDA 0.999 0.21
ERF 0.342 0.18
EGCUT 6.32e−04 0.23
CKB 0.051 0.36
ALSPAC 0.280 0.22
META 3.08e−15 0.23
−0.2 −0.1 0.0 0.1 0.2 0.3
BETA

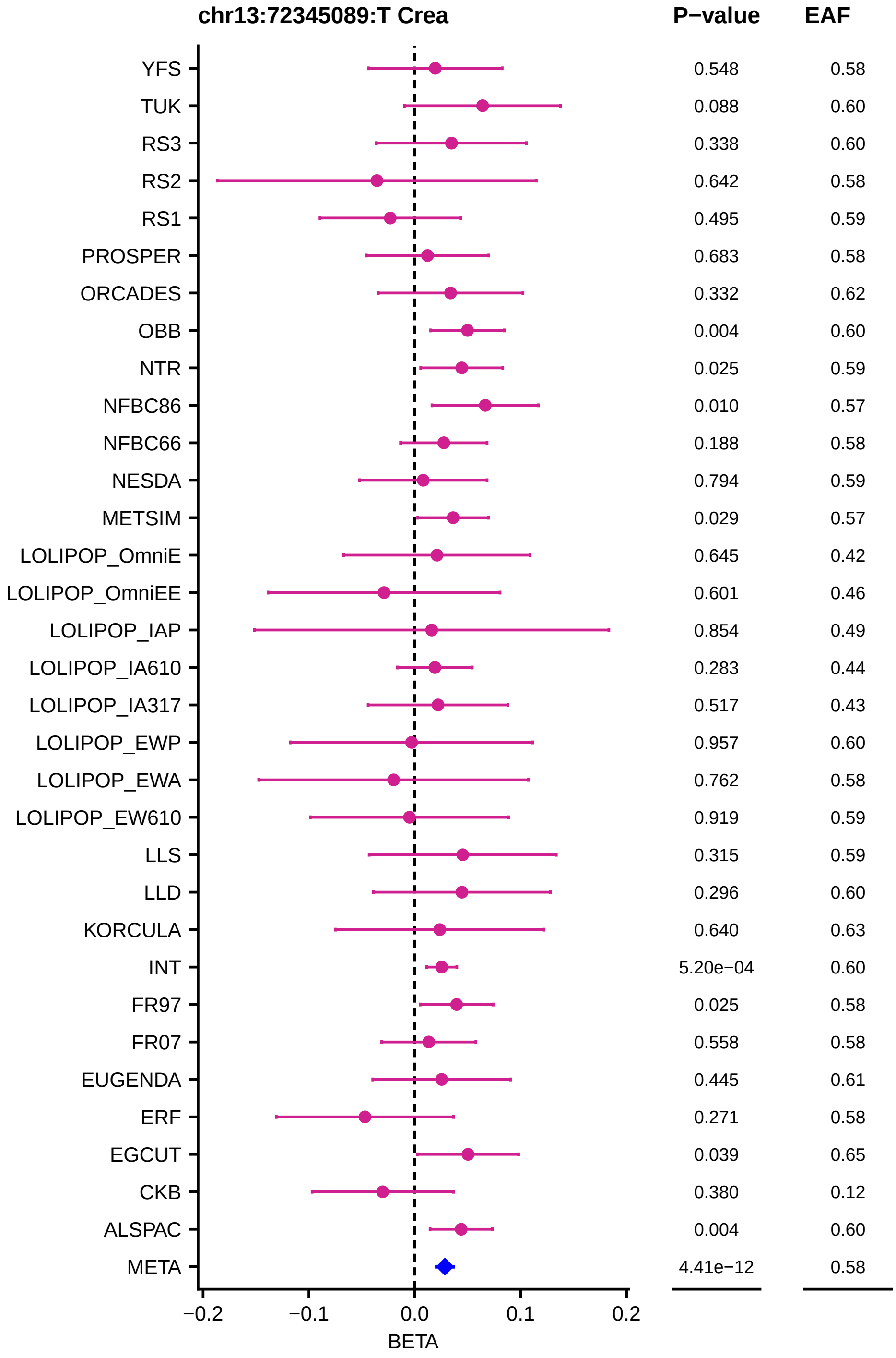
chr13:72345089:T Crea
P−value
EAF
YFS
0.548
0.58
TUK
0.088
0.60
RS3
0.338
0.60
RS2
0.642
0.58
RS1
0.495
0.59
PROSPER
0.683
0.58
ORCADES
0.332
0.62
OBB
0.004
0.60
NTR
0.025
0.59
NFBC86
0.010
0.57
NFBC66
0.188
0.58
NESDA
0.794
0.59
METSIM
0.029
0.57
LOLIPOP_OmniE
0.645
0.42
LOLIPOP_OmniEE
0.601
0.46
LOLIPOP_IAP
0.854
0.49
LOLIPOP_IA610
0.283
0.44
LOLIPOP_IA317
0.517
0.43
LOLIPOP_EWP
0.957
0.60
LOLIPOP_EWA
0.762
0.58
LOLIPOP_EW610
0.919
0.59
LLS
0.315
0.59
LLD
0.296
0.60
KORCULA
0.640
0.63
INT
5.20e−04
0.60
FR97
0.025
0.58
FR07
0.558
0.58
EUGENDA
0.445
0.61
ERF
0.271
0.58
EGCUT
0.039
0.65
CKB
0.380
0.12
ALSPAC
0.004
0.60
META
4.41e−12
0.58
−0.2
−0.1
0.0
0.1
0.2
BETA

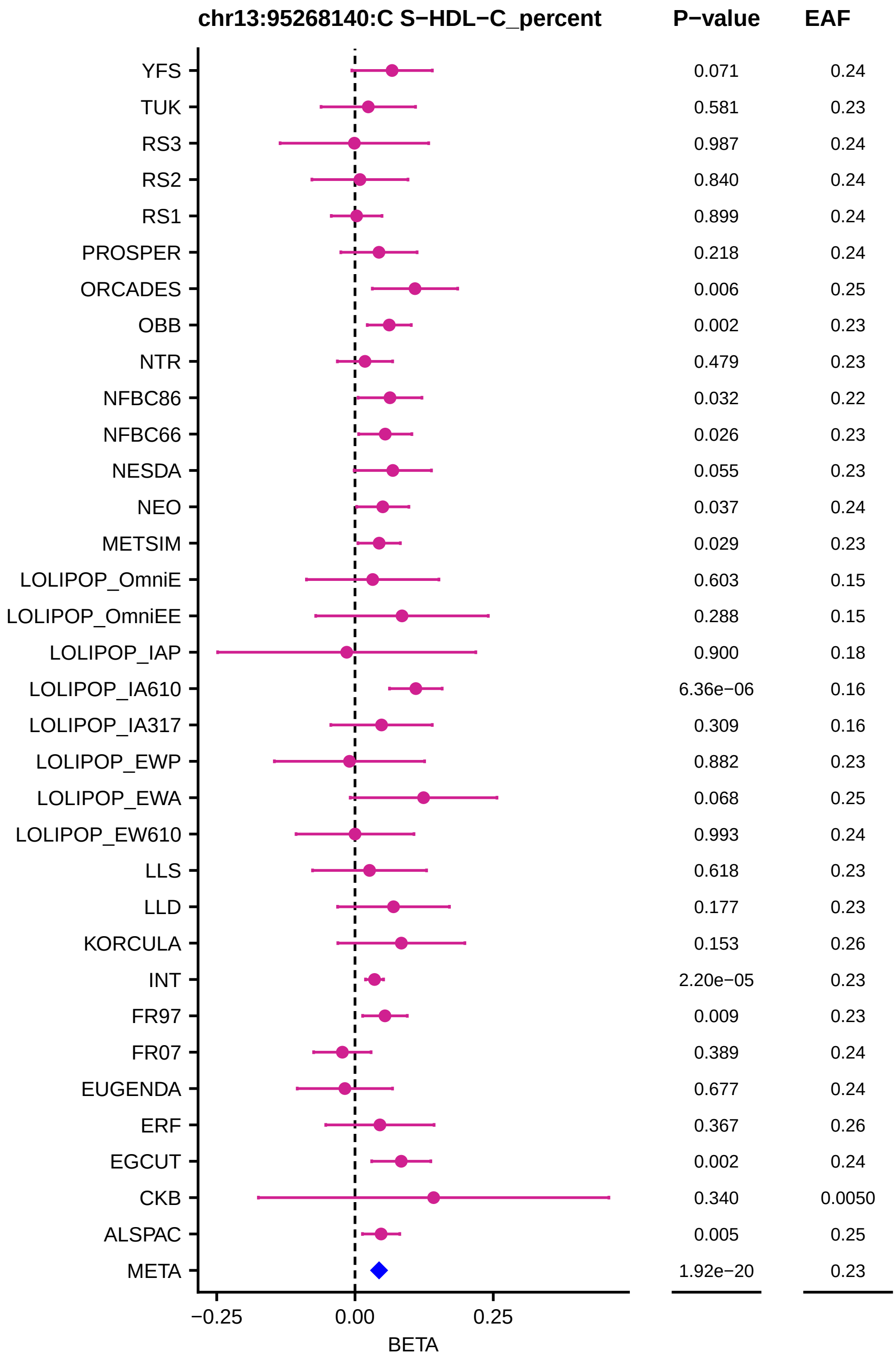

chr13:95268140:C S−HDL−C_percent
P−value
EAF
YFS 0.071 0.24
TUK 0.581 0.23
RS3 0.987 0.24
RS2 0.840 0.24
RS1 0.899 0.24
PROSPER 0.218 0.24
ORCADES 0.006 0.25
OBB 0.002 0.23
NTR 0.479 0.23
NFBC86 0.032 0.22
NFBC66 0.026 0.23
NESDA 0.055 0.23
NEO 0.037 0.24
METSIM 0.029 0.23
LOLIPOP_OmniE 0.603 0.15
LOLIPOP_OmniEE 0.288 0.15
LOLIPOP_IAP 0.900 0.18
LOLIPOP_IA610 6.36e−06 0.16
LOLIPOP_IA317 0.309 0.16
LOLIPOP_EWP 0.882 0.23
LOLIPOP_EWA 0.068 0.25
LOLIPOP_EW610 0.993 0.24
LLS 0.618 0.23
LLD 0.177 0.23
KORCULA 0.153 0.26
INT 2.20e−05 0.23
FR97 0.009 0.23
FR07 0.389 0.24
EUGENDA 0.677 0.24
ERF 0.367 0.26
EGCUT 0.002 0.24
CKB 0.340 0.0050
ALSPAC 0.005 0.25
META 1.92e−20 0.23
−0.25
0.00
0.25
BETA

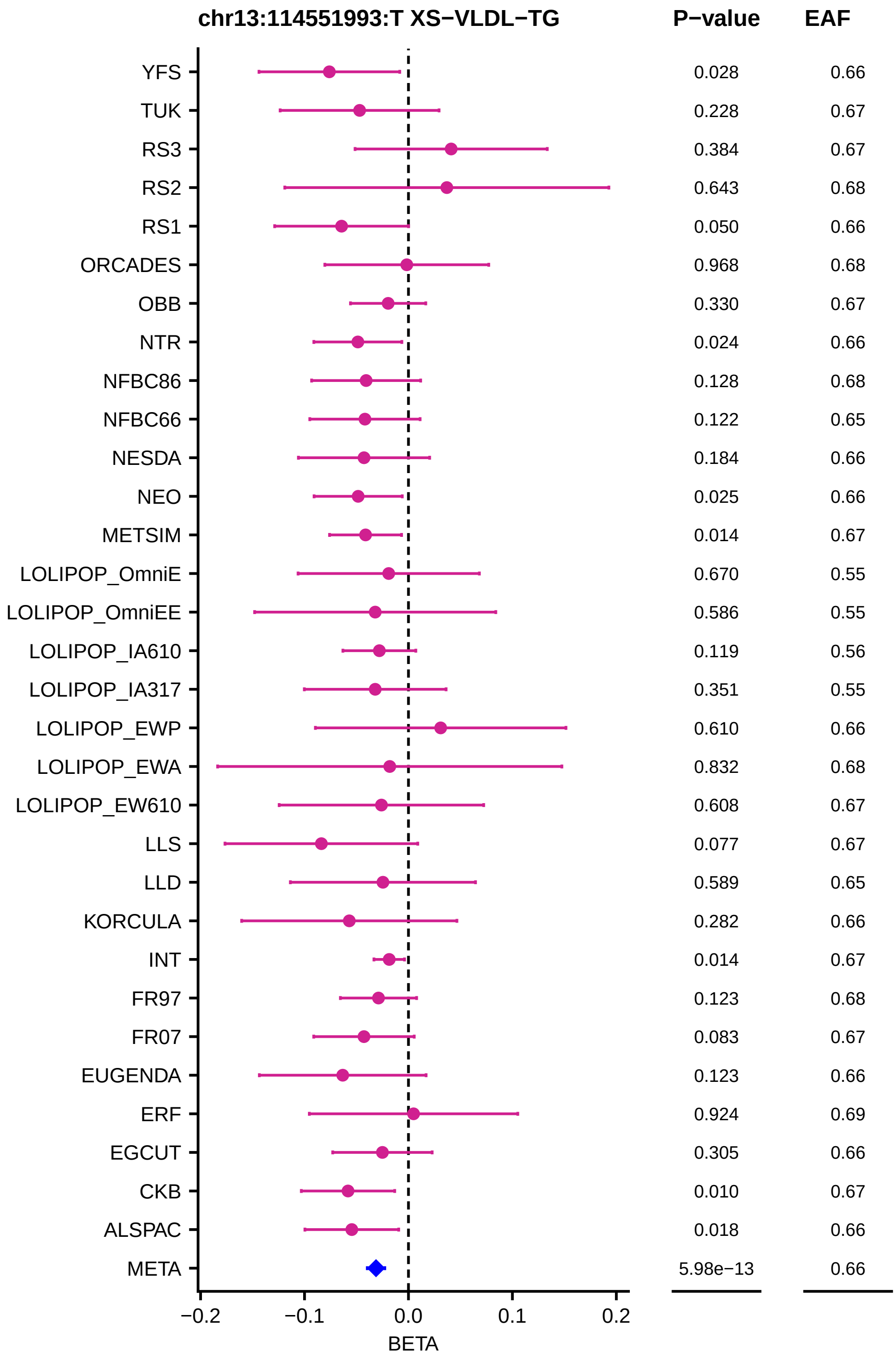
chr13:114551993:T XS−VLDL−TG
P−value
EAF
YFS 0.028 0.66
TUK 0.228 0.67
RS3 0.384 0.67
RS2 0.643 0.68
RS1 0.050 0.66
ORCADES 0.968 0.68
OBB 0.330 0.67
NTR 0.024 0.66
NFBC86 0.128 0.68
NFBC66 0.122 0.65
NESDA 0.184 0.66
NEO 0.025 0.66
METSIM 0.014 0.67
LOLIPOP_OmniE 0.670 0.55
LOLIPOP_OmniEE 0.586 0.55
LOLIPOP_IA610 0.119 0.56
LOLIPOP_IA317 0.351 0.55
LOLIPOP_EWP 0.610 0.66
LOLIPOP_EWA 0.832 0.68
LOLIPOP_EW610 0.608 0.67
LLS 0.077 0.67
LLD 0.589 0.65
KORCULA 0.282 0.66
INT 0.014 0.67
FR97 0.123 0.68
FR07 0.083 0.67
EUGENDA 0.123 0.66
ERF 0.924 0.69
EGCUT 0.305 0.66
CKB 0.010 0.67
ALSPAC 0.018 0.66
META 5.98e−13 0.66
−0.2 −0.1 0.0 0.1 0.2
BETA

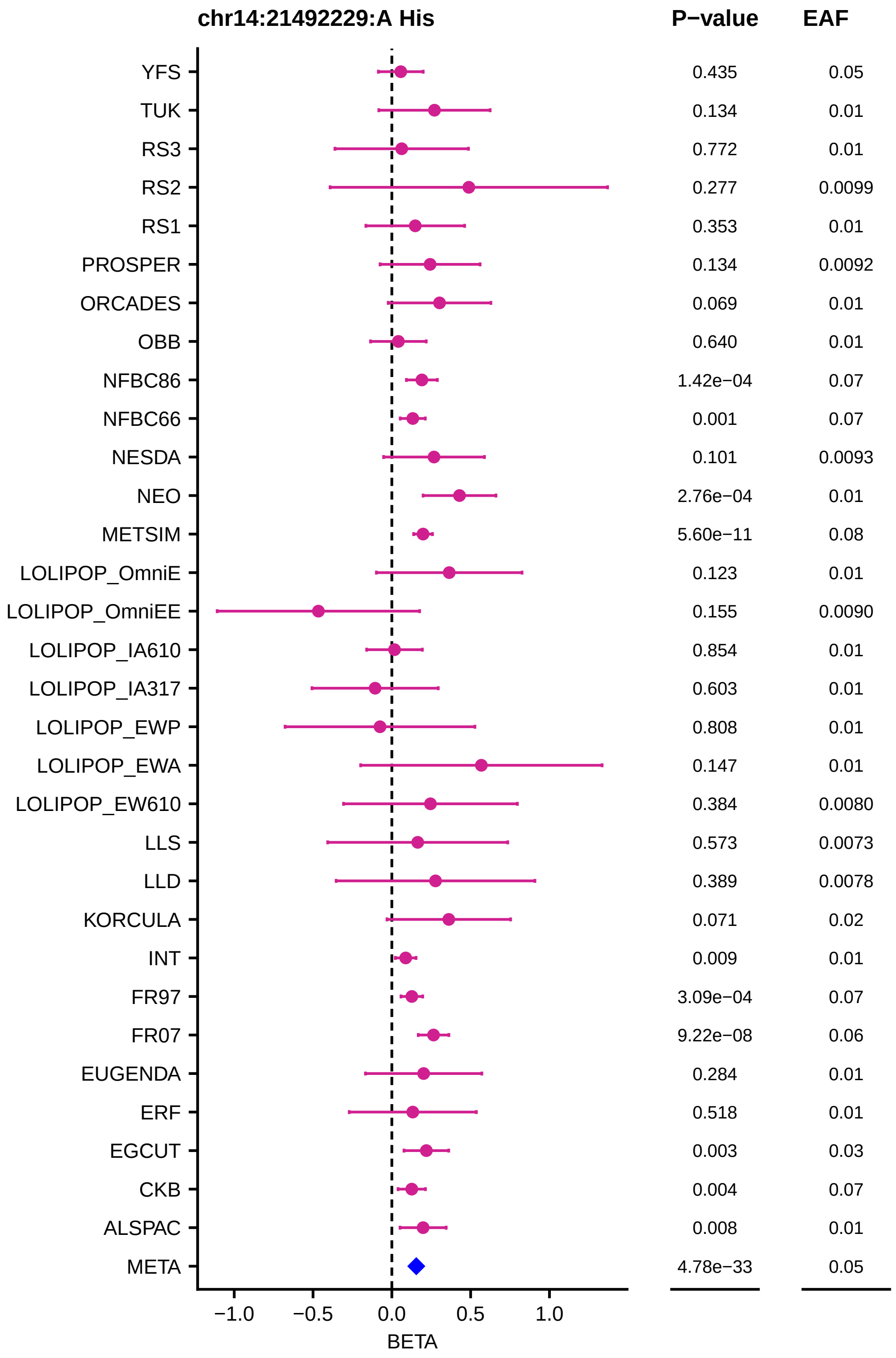
chr14:21492229:A His
P−value
EAF
YFS 0.435 0.05
TUK 0.134 0.01
RS3 0.772 0.01
RS2 0.277 0.0099
RS1 0.353 0.01
PROSPER 0.134 0.0092
ORCADES 0.069 0.01
OBB 0.640 0.01
NFBC86 1.42e−04 0.07
NFBC66 0.001 0.07
NESDA 0.101 0.0093
NEO 2.76e−04 0.01
METSIM 5.60e−11 0.08
LOLIPOP_OmniE 0.123 0.01
LOLIPOP_OmniEE 0.155 0.0090
LOLIPOP_IA610 0.854 0.01
LOLIPOP_IA317 0.603 0.01
LOLIPOP_EWP 0.808 0.01
LOLIPOP_EWA 0.147 0.01
LOLIPOP_EW610 0.384 0.0080
LLS 0.573 0.0073
LLD 0.389 0.0078
KORCULA 0.071 0.02
INT 0.009 0.01
FR97 3.09e−04 0.07
FR07 9.22e−08 0.06
EUGENDA 0.284 0.01
ERF 0.518 0.01
EGCUT 0.003 0.03
CKB 0.004 0.07
ALSPAC 0.008 0.01
META 4.78e−33 0.05
−1.0 −0.5 0.0 0.5 1.0
BETA

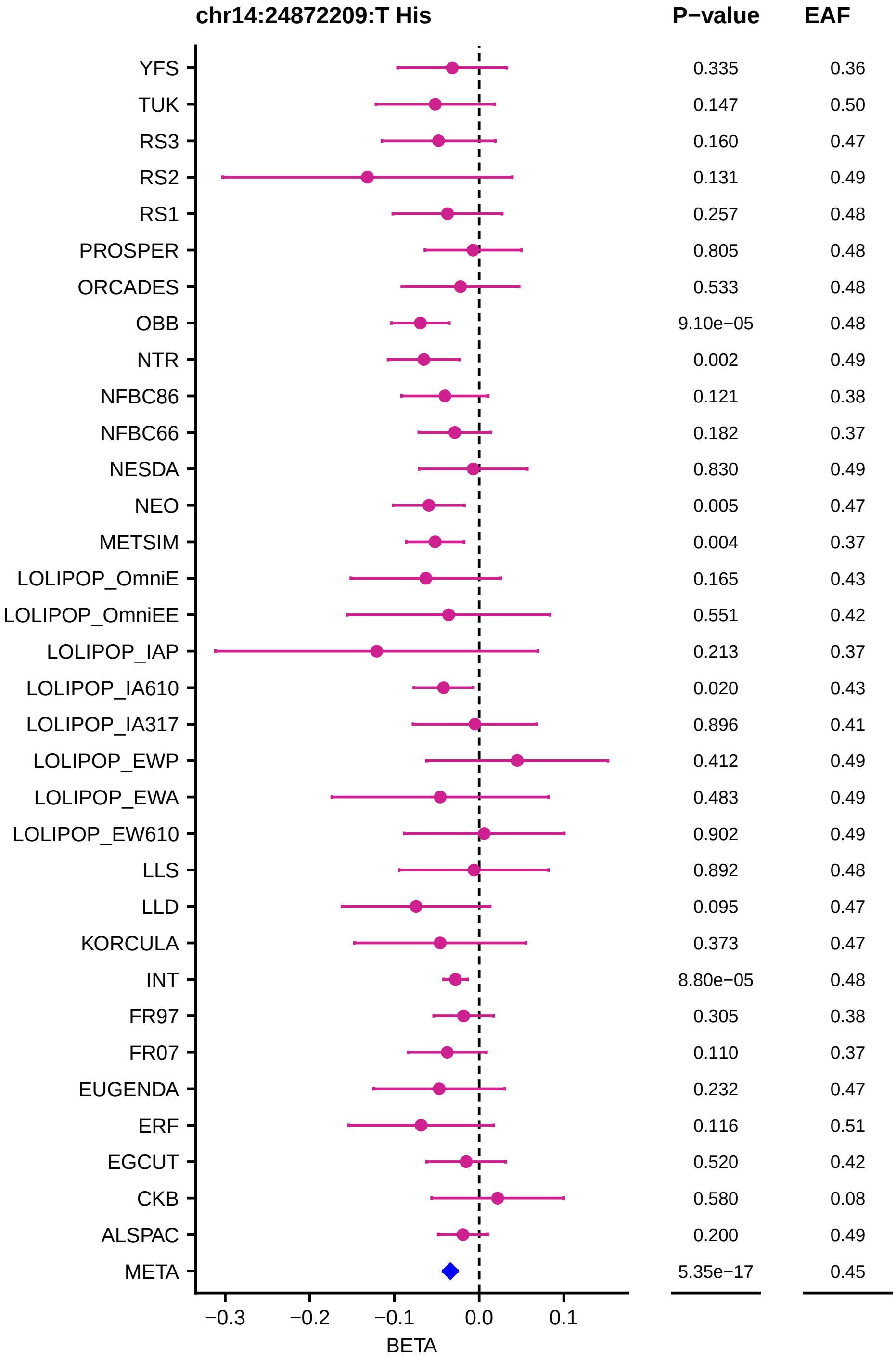
chr14:24872209:T His
P−value
EAF
YFS 0.335 0.36
TUK 0.147 0.50
RS3 0.160 0.47
RS2 0.131 0.49
RS1 0.257 0.48
PROSPER 0.805 0.48
ORCADES 0.533 0.48
OBB 9.10e−05 0.48
NTR 0.002 0.49
NFBC86 0.121 0.38
NFBC66 0.182 0.37
NESDA 0.830 0.49
NEO 0.005 0.47
METSIM 0.004 0.37
LOLIPOP_OmniE 0.165 0.43
LOLIPOP_OmniEE 0.551 0.42
LOLIPOP_IAP 0.213 0.37
LOLIPOP_IA610 0.020 0.43
LOLIPOP_IA317 0.896 0.41
LOLIPOP_EWP 0.412 0.49
LOLIPOP_EWA 0.483 0.49
LOLIPOP_EW610 0.902 0.49
LLS 0.892 0.48
LLD 0.095 0.47
KORCULA 0.373 0.47
INT 8.80e−05 0.48
FR97 0.305 0.38
FR07 0.110 0.37
EUGENDA 0.232 0.47
ERF 0.116 0.51
EGCUT 0.520 0.42
CKB 0.580 0.08
ALSPAC 0.200 0.49
META 5.35e−17 0.45
−0.3
−0.2
−0.1
0.0
0.1
BETA

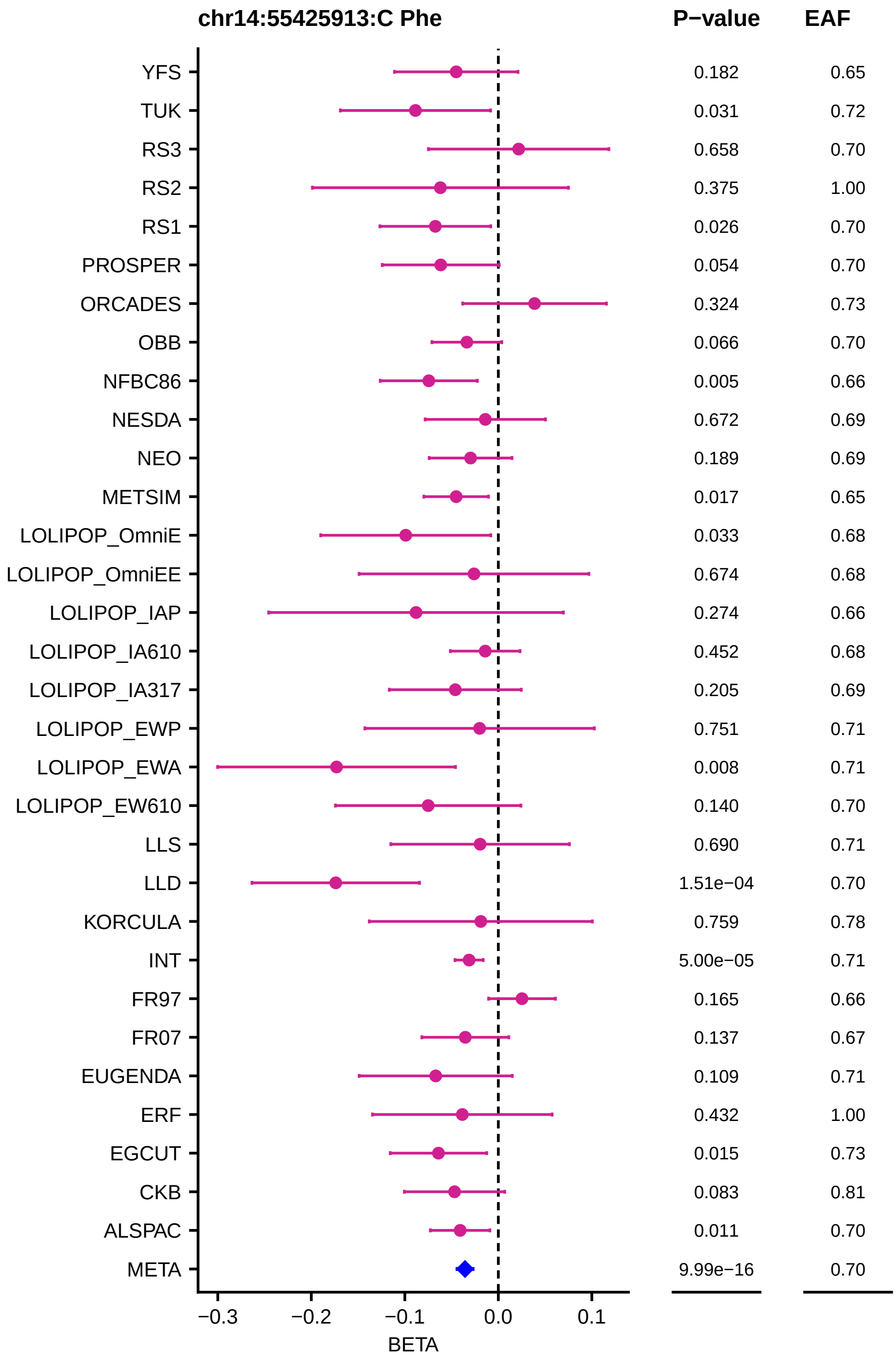
chr14:55425913:C Phe
P−value
EAF
YFS 0.182 0.65
TUK 0.031 0.72
RS3 0.658 0.70
RS2 0.375 1.00
RS1 0.026 0.70
PROSPER 0.054 0.70
ORCADES 0.324 0.73
OBB 0.066 0.70
NFBC86 0.005 0.66
NESDA 0.672 0.69
NEO 0.189 0.69
METSIM 0.017 0.65
LOLIPOP_OmniE 0.033 0.68
LOLIPOP_OmniEE 0.674 0.68
LOLIPOP_IAP 0.274 0.66
LOLIPOP_IA610 0.452 0.68
LOLIPOP_IA317 0.205 0.69
LOLIPOP_EWP 0.751 0.71
LOLIPOP_EWA 0.008 0.71
LOLIPOP_EW610 0.140 0.70
LLS 0.690 0.71
LLD 1.51e−04 0.70
KORCULA 0.759 0.78
INT 5.00e−05 0.71
FR97 0.165 0.66
FR07 0.137 0.67
EUGENDA 0.109 0.71
ERF 0.432 1.00
EGCUT 0.015 0.73
CKB 0.083 0.81
ALSPAC 0.011 0.70
META 9.99e−16 0.70
−0.3
−0.2
−0.1
0.0
0.1
BETA

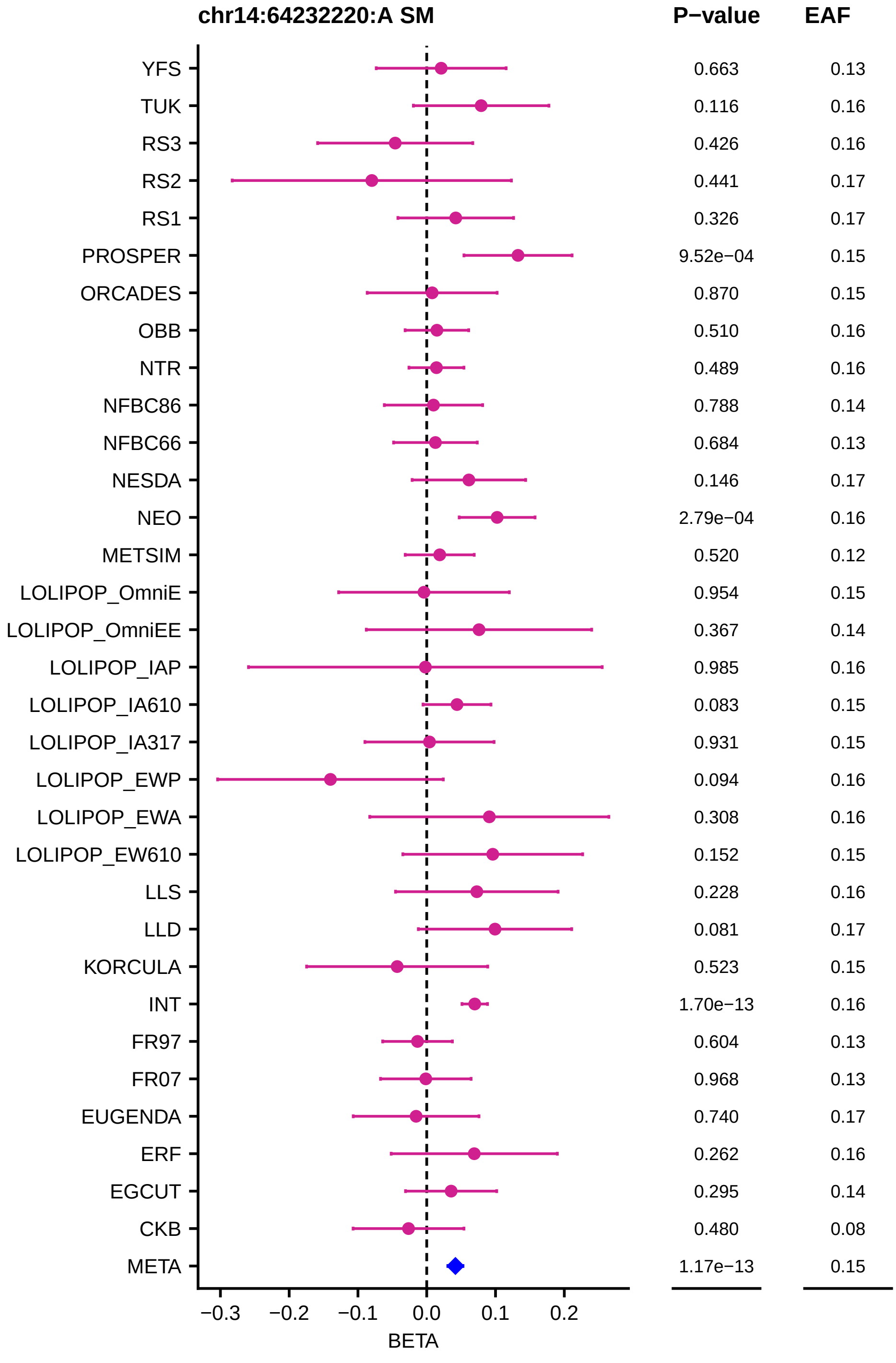
chr14:64232220:A SM
P−value
EAF
YFS 0.663 0.13
TUK 0.116 0.16
RS3 0.426 0.16
RS2 0.441 0.17
RS1 0.326 0.17
PROSPER 9.52e−04 0.15
ORCADES 0.870 0.15
OBB 0.510 0.16
NTR 0.489 0.16
NFBC86 0.788 0.14
NFBC66 0.684 0.13
NESDA 0.146 0.17
NEO 2.79e−04 0.16
METSIM 0.520 0.12
LOLIPOP_OmniE 0.954 0.15
LOLIPOP_OmniEE 0.367 0.14
LOLIPOP_IAP 0.985 0.16
LOLIPOP_IA610 0.083 0.15
LOLIPOP_IA317 0.931 0.15
LOLIPOP_EWP 0.094 0.16
LOLIPOP_EWA 0.308 0.16
LOLIPOP_EW610 0.152 0.15
LLS 0.228 0.16
LLD 0.081 0.17
KORCULA 0.523 0.15
INT 1.70e−13 0.16
FR97 0.604 0.13
FR07 0.968 0.13
EUGENDA 0.740 0.17
ERF 0.262 0.16
EGCUT 0.295 0.14
CKB 0.480 0.08
META 1.17e−13 0.15
−0.3 −0.2 −0.1 0.0 0.1 0.2
BETA

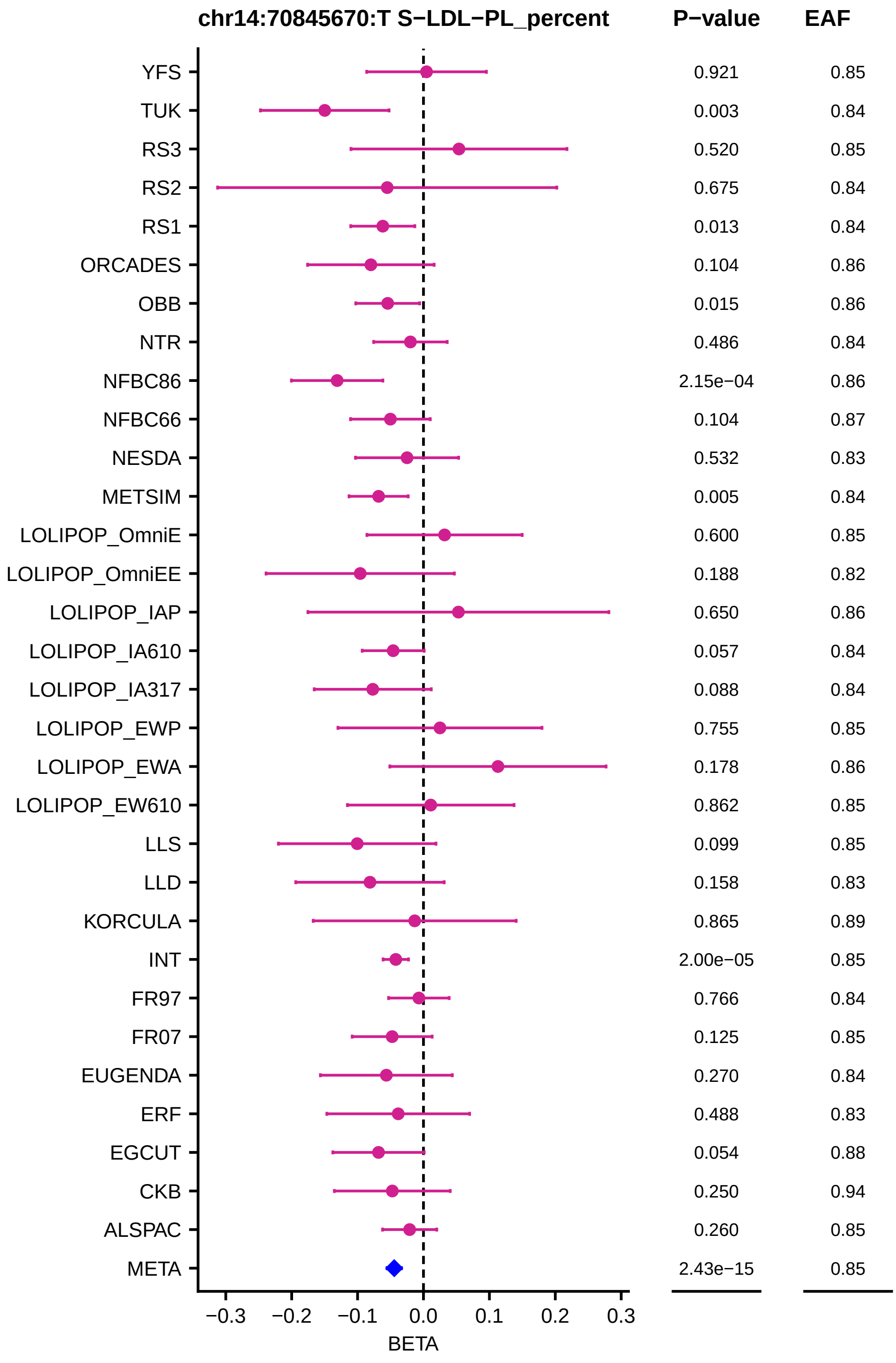

chr14:70845670:T S−LDL−PL_percent
P−value
EAF
YFS
TUK
RS3
RS2
RS1
ORCADES
OBB
NTR
NFBC86
NFBC66
NESDA
METSIM
LOLIPOP_OmniE
LOLIPOP_OmniEE
LOLIPOP_IAP
LOLIPOP_IA610
LOLIPOP_IA317
LOLIPOP_EWP
LOLIPOP_EWA
LOLIPOP_EW610
LLS
LLD
KORCULA
INT
FR97
FR07
EUGENDA
ERF
EGCUT
CKB
ALSPAC
META
0.921 0.85
0.003 0.84
0.520 0.85
0.675 0.84
0.013 0.84
0.104 0.86
0.015 0.86
0.486 0.84
2.15e−04 0.86
0.104 0.87
0.532 0.83
0.005 0.84
0.600 0.85
0.188 0.82
0.650 0.86
0.057 0.84
0.088 0.84
0.755 0.85
0.178 0.86
0.862 0.85
0.099 0.85
0.158 0.83
0.865 0.89
2.00e−05 0.85
0.766 0.84
0.125 0.85
0.270 0.84
0.488 0.83
0.054 0.88
0.250 0.94
0.260 0.85
2.43e−15 0.85
−0.3 −0.2 −0.1 0.0 0.1 0.2 0.3
BETA

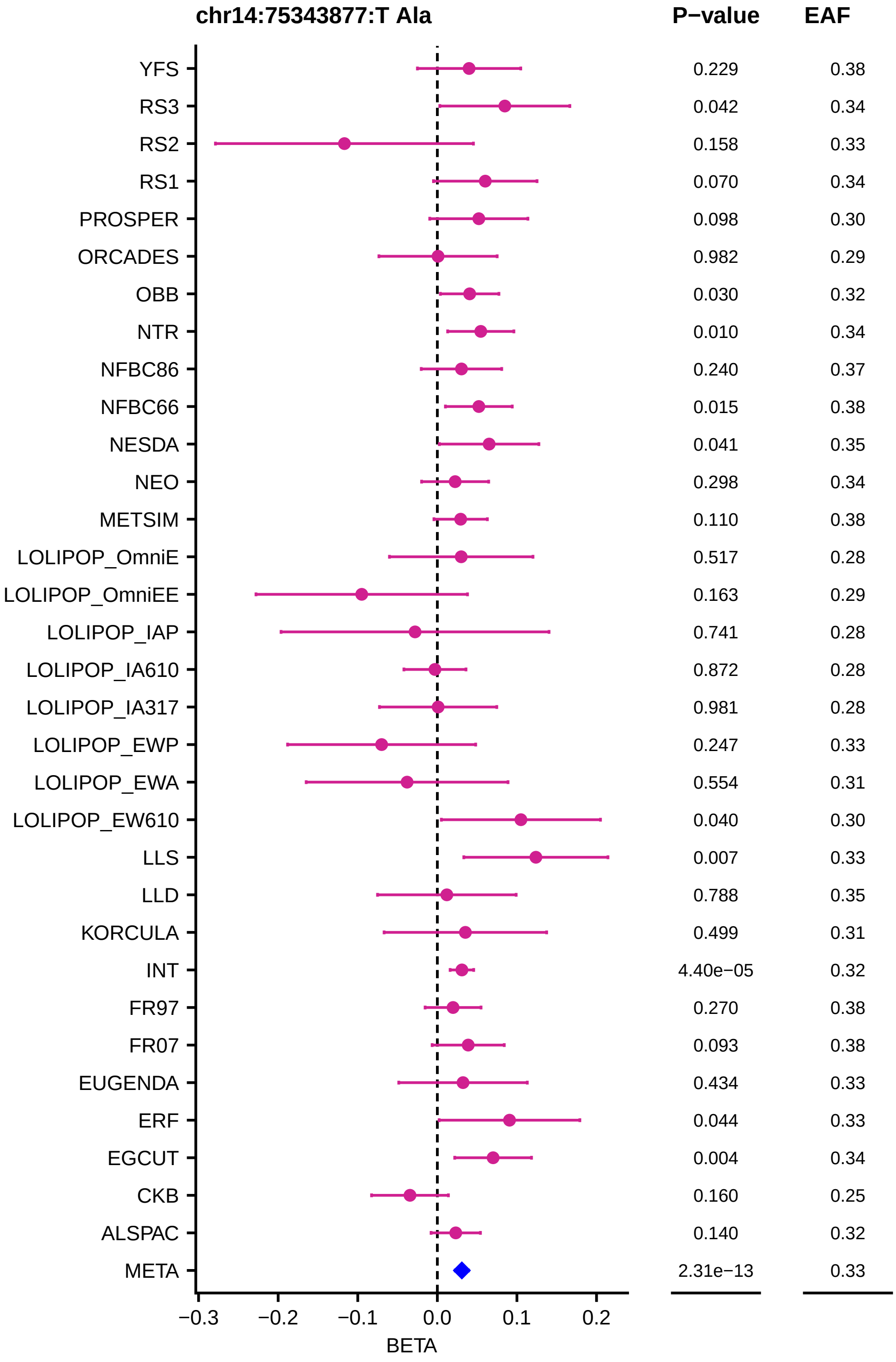
chr14:75343877:T Ala
P−value
EAF
YFS 0.229 0.38
RS3 0.042 0.34
RS2 0.158 0.33
RS1 0.070 0.34
PROSPER 0.098 0.30
ORCADES 0.982 0.29
OBB 0.030 0.32
NTR 0.010 0.34
NFBC86 0.240 0.37
NFBC66 0.015 0.38
NESDA 0.041 0.35
NEO 0.298 0.34
METSIM 0.110 0.38
LOLIPOP_OmniE 0.517 0.28
LOLIPOP_OmniEE 0.163 0.29
LOLIPOP_IAP 0.741 0.28
LOLIPOP_IA610 0.872 0.28
LOLIPOP_IA317 0.981 0.28
LOLIPOP_EWP 0.247 0.33
LOLIPOP_EWA 0.554 0.31
LOLIPOP_EW610 0.040 0.30
LLS 0.007 0.33
LLD 0.788 0.35
KORCULA 0.499 0.31
INT 4.40e−05 0.32
FR97 0.270 0.38
FR07 0.093 0.38
EUGENDA 0.434 0.33
ERF 0.044 0.33
EGCUT 0.004 0.34
CKB 0.160 0.25
ALSPAC 0.140 0.32
META 2.31e−13 0.33
−0.3
−0.2
−0.1
0.0
0.1
0.2
BETA

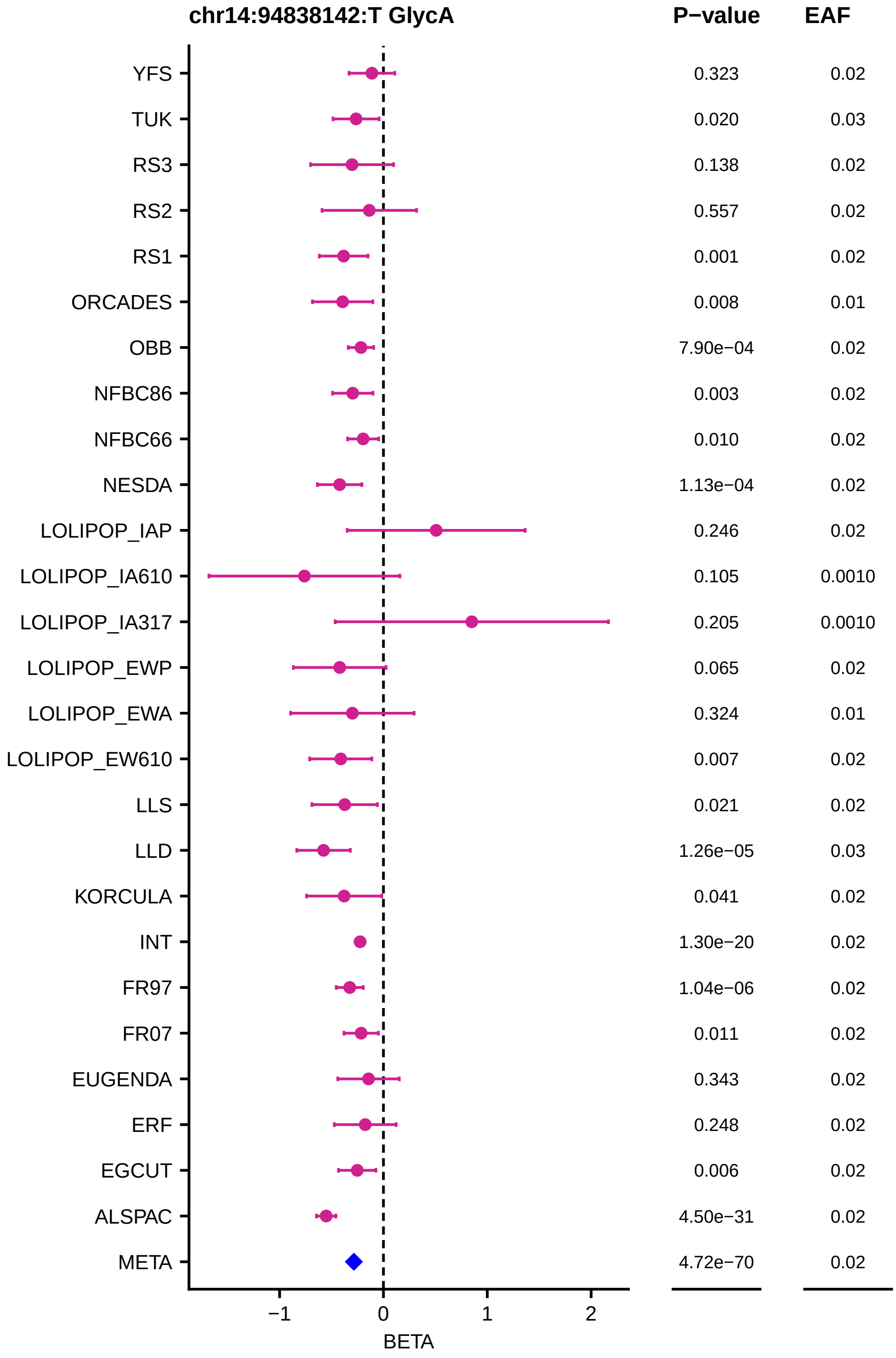

chr14:94838142:T GlycA
P−value
EAF
YFS 0.323 0.02
TUK 0.020 0.03
RS3 0.138 0.02
RS2 0.557 0.02
RS1 0.001 0.02
ORCADES 0.008 0.01
OBB 7.90e−04 0.02
NFBC86 0.003 0.02
NFBC66 0.010 0.02
NESDA 1.13e−04 0.02
LOLIPOP_IAP 0.246 0.02
LOLIPOP_IA610 0.105 0.0010
LOLIPOP_IA317 0.205 0.0010
LOLIPOP_EWP 0.065 0.02
LOLIPOP_EWA 0.324 0.01
LOLIPOP_EW610 0.007 0.02
LLS 0.021 0.02
LLD 1.26e−05 0.03
KORCULA 0.041 0.02
INT 1.30e−20 0.02
FR97 1.04e−06 0.02
FR07 0.011 0.02
EUGENDA 0.343 0.02
ERF 0.248 0.02
EGCUT 0.006 0.02
ALSPAC 4.50e−31 0.02
META 4.72e−70 0.02
−1
0
1
2
BETA

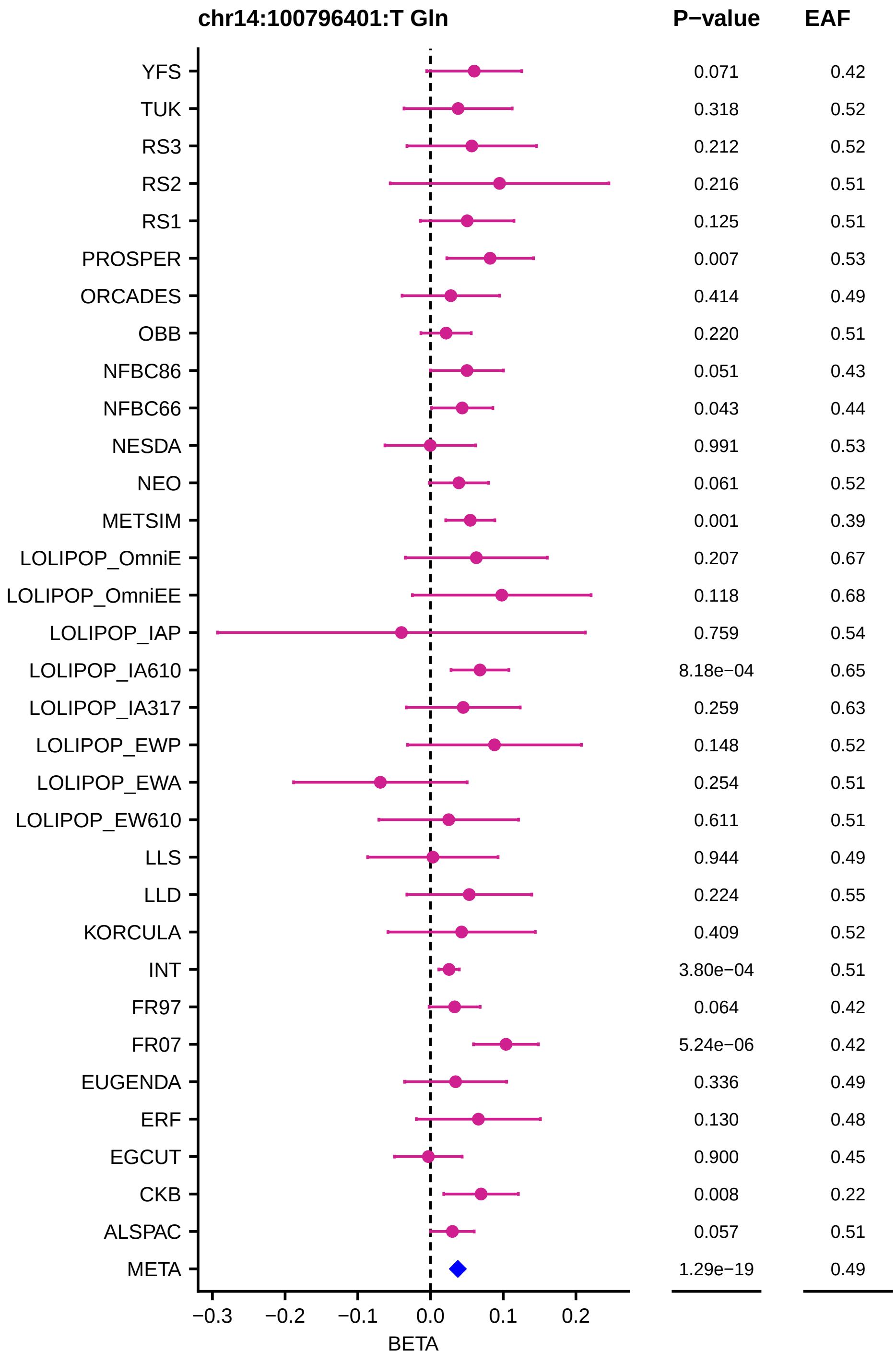

chr14:100796401:T Gln
P−value
EAF
YFS
TUK
RS3
RS2
RS1
PROSPER
ORCADES
OBB
NFBC86
NFBC66
NESDA
NEO
METSIM
LOLIPOP_OmniE
LOLIPOP_OmniEE
LOLIPOP_IAP
LOLIPOP_IA610
LOLIPOP_IA317
LOLIPOP_EWP
LOLIPOP_EWA
LOLIPOP_EW610
LLS
LLD
KORCULA
INT
FR97
FR07
EUGENDA
ERF
EGCUT
CKB
ALSPAC
META
0.071 0.42
0.318 0.52
0.212 0.52
0.216 0.51
0.125 0.51
0.007 0.53
0.414 0.49
0.220 0.51
0.051 0.43
0.043 0.44
0.991 0.53
0.061 0.52
0.001 0.39
0.207 0.67
0.118 0.68
0.759 0.54
8.18e−04 0.65
0.259 0.63
0.148 0.52
0.254 0.51
0.611 0.51
0.944 0.49
0.224 0.55
0.409 0.52
3.80e−04 0.51
0.064 0.42
5.24e−06 0.42
0.336 0.49
0.130 0.48
0.900 0.45
0.008 0.22
0.057 0.51
1.29e−19 0.49
−0.3 −0.2 −0.1 0.0 0.1 0.2
BETA

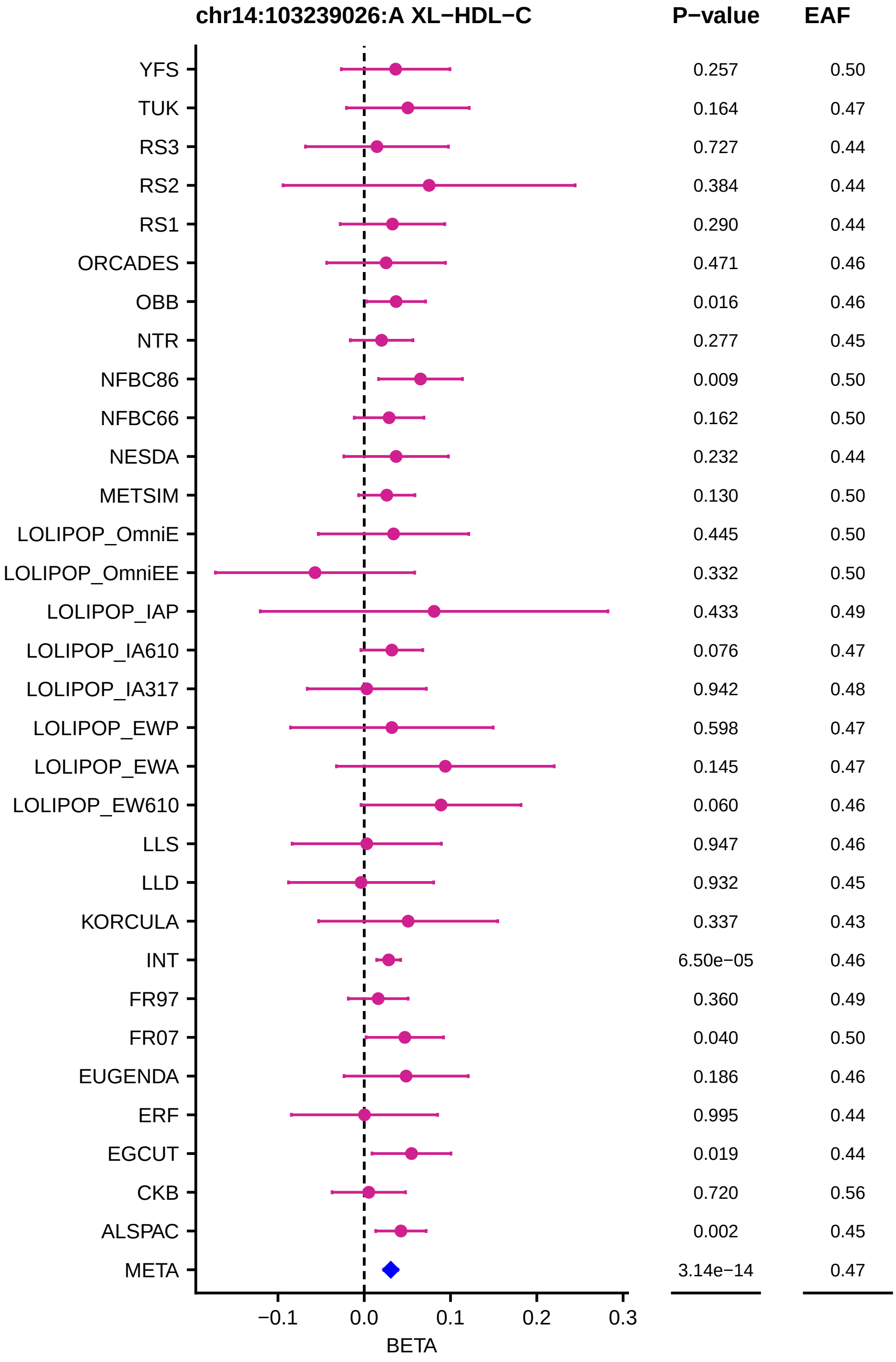
chr14:103239026:A XL−HDL−C
P−value
EAF
YFS 0.257 0.50
TUK 0.164 0.47
RS3 0.727 0.44
RS2 0.384 0.44
RS1 0.290 0.44
ORCADES 0.471 0.46
OBB 0.016 0.46
NTR 0.277 0.45
NFBC86 0.009 0.50
NFBC66 0.162 0.50
NESDA 0.232 0.44
METSIM 0.130 0.50
LOLIPOP_OmniE 0.445 0.50
LOLIPOP_OmniEE 0.332 0.50
LOLIPOP_IAP 0.433 0.49
LOLIPOP_IA610 0.076 0.47
LOLIPOP_IA317 0.942 0.48
LOLIPOP_EWP 0.598 0.47
LOLIPOP_EWA 0.145 0.47
LOLIPOP_EW610 0.060 0.46
LLS 0.947 0.46
LLD 0.932 0.45
KORCULA 0.337 0.43
INT 6.50e−05 0.46
FR97 0.360 0.49
FR07 0.040 0.50
EUGENDA 0.186 0.46
ERF 0.995 0.44
EGCUT 0.019 0.44
CKB 0.720 0.56
ALSPAC 0.002 0.45
META 3.14e−14 0.47
−0.1
0.0
0.1
0.2
0.3
BETA

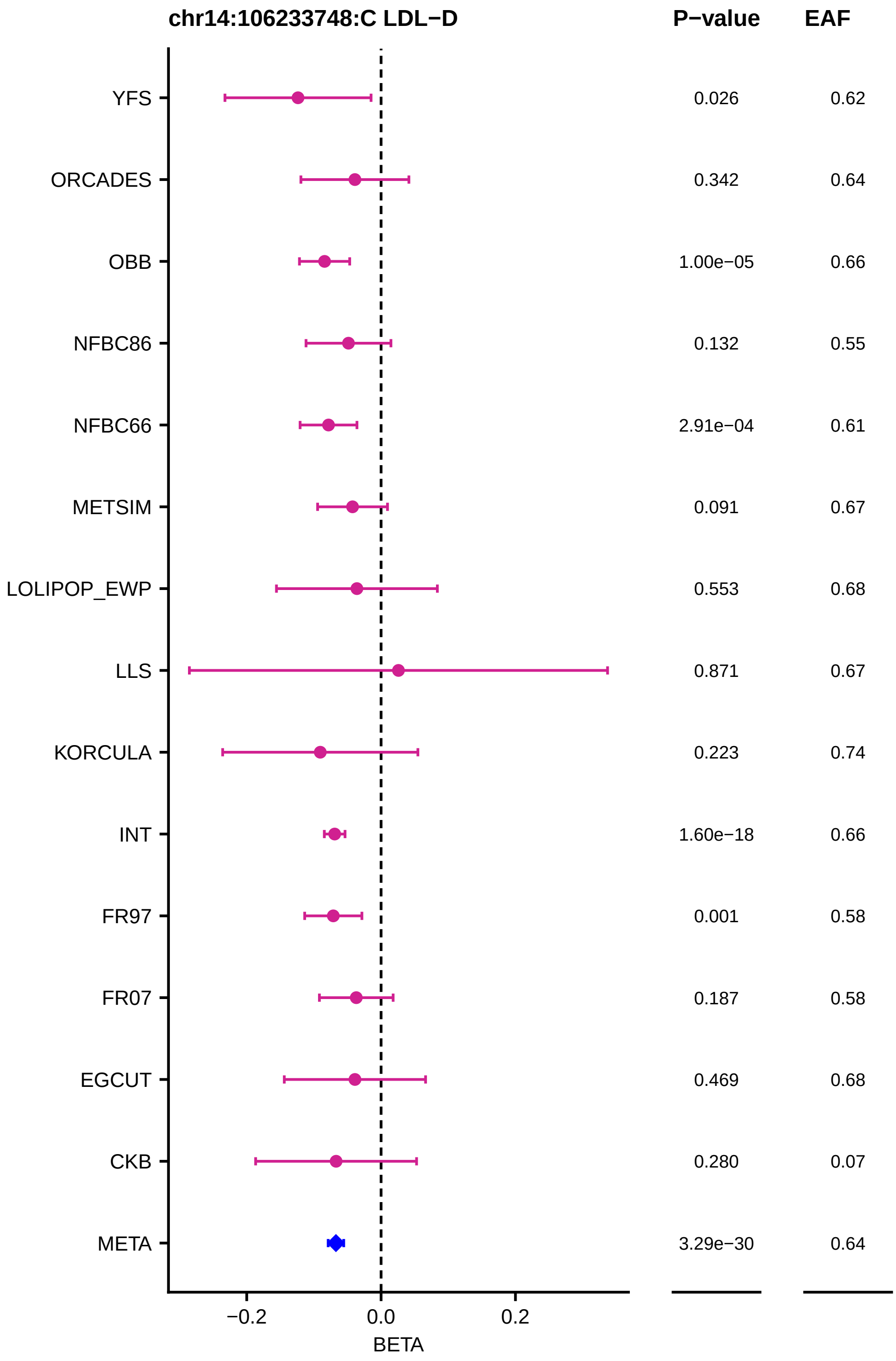
chr14:106233748:C LDL−D
P−value
EAF
YFS
ORCADES
OBB
NFBC86
NFBC66
METSIM
LOLIPOP_EWP
LLS
KORCULA
INT
FR97
FR07
EGCUT
CKB
META
0.026
0.342
1.00e−05
0.132
2.91e−04
0.091
0.553
0.871
0.223
1.60e−18
0.001
0.187
0.469
0.280
3.29e−30
0.62
0.64
0.66
0.55
0.61
0.67
0.68
0.67
0.74
0.66
0.58
0.58
0.68
0.07
0.64
−0.2
0.0
0.2
BETA

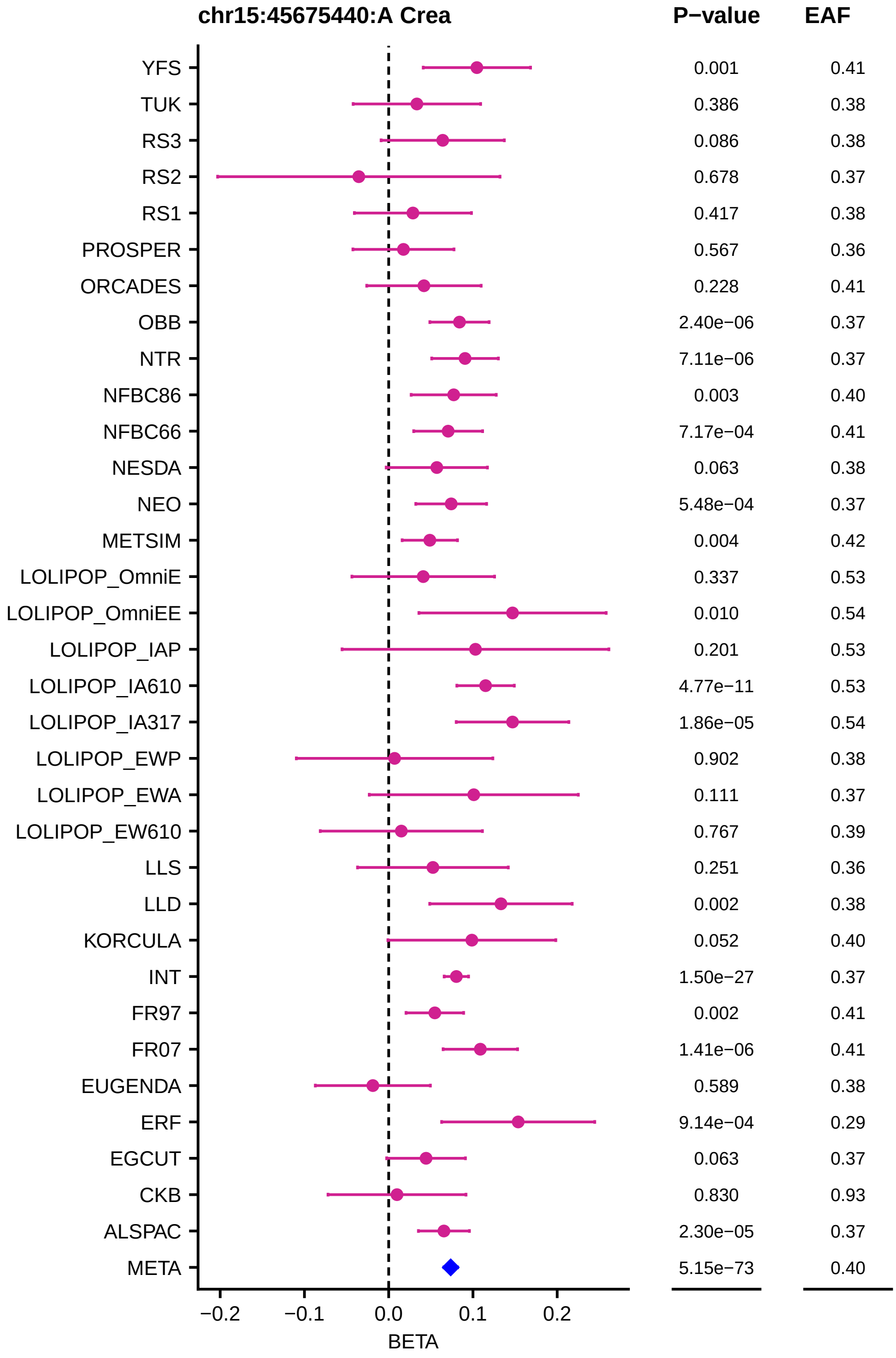

chr15:45675440:A Crea
P−value
EAF
YFS 0.001 0.41
TUK 0.386 0.38
RS3 0.086 0.38
RS2 0.678 0.37
RS1 0.417 0.38
PROSPER 0.567 0.36
ORCADES 0.228 0.41
OBB 2.40e−06 0.37
NTR 7.11e−06 0.37
NFBC86 0.003 0.40
NFBC66 7.17e−04 0.41
NESDA 0.063 0.38
NEO 5.48e−04 0.37
METSIM 0.004 0.42
LOLIPOP_OmniE 0.337 0.53
LOLIPOP_OmniEE 0.010 0.54
LOLIPOP_IAP 0.201 0.53
LOLIPOP_IA610 4.77e−11 0.53
LOLIPOP_IA317 1.86e−05 0.54
LOLIPOP_EWP 0.902 0.38
LOLIPOP_EWA 0.111 0.37
LOLIPOP_EW610 0.767 0.39
LLS 0.251 0.36
LLD 0.002 0.38
KORCULA 0.052 0.40
INT 1.50e−27 0.37
FR97 0.002 0.41
FR07 1.41e−06 0.41
EUGENDA 0.589 0.38
ERF 9.14e−04 0.29
EGCUT 0.063 0.37
CKB 0.830 0.93
ALSPAC 2.30e−05 0.37
META 5.15e−73 0.40
−0.2 −0.1 0.0 0.1 0.2
BETA

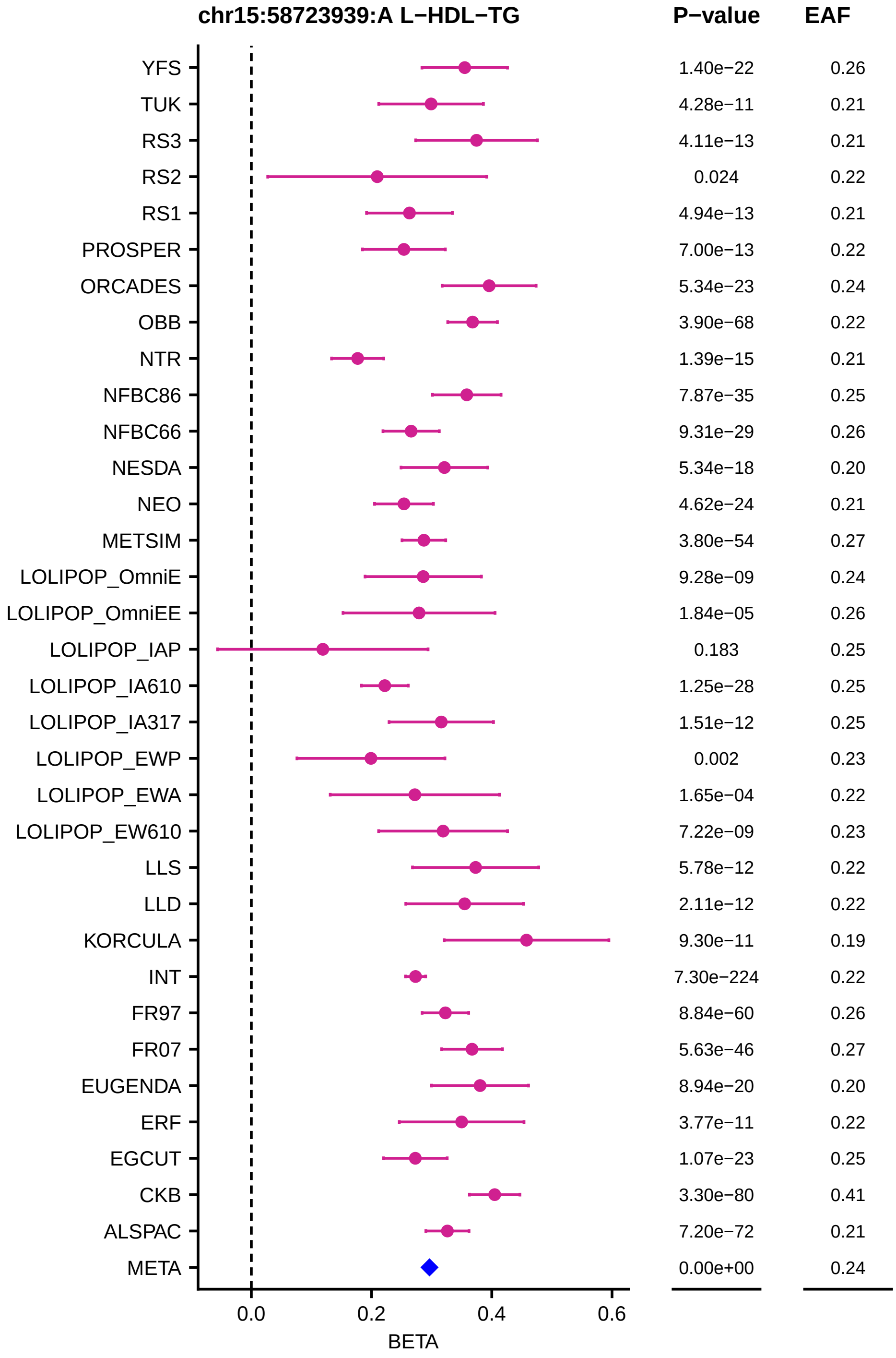

chr15:58723939:A L−HDL−TG
P−value
EAF
YFS 1.40e−22 0.26
TUK 4.28e−11 0.21
RS3 4.11e−13 0.21
RS2 0.024 0.22
RS1 4.94e−13 0.21
PROSPER 7.00e−13 0.22
ORCADES 5.34e−23 0.24
OBB 3.90e−68 0.22
NTR 1.39e−15 0.21
NFBC86 7.87e−35 0.25
NFBC66 9.31e−29 0.26
NESDA 5.34e−18 0.20
NEO 4.62e−24 0.21
METSIM 3.80e−54 0.27
LOLIPOP_OmniE 9.28e−09 0.24
LOLIPOP_OmniEE 1.84e−05 0.26
LOLIPOP_IAP 0.183 0.25
LOLIPOP_IA610 1.25e−28 0.25
LOLIPOP_IA317 1.51e−12 0.25
LOLIPOP_EWP 0.002 0.23
LOLIPOP_EWA 1.65e−04 0.22
LOLIPOP_EW610 7.22e−09 0.23
LLS 5.78e−12 0.22
LLD 2.11e−12 0.22
KORCULA 9.30e−11 0.19
INT 7.30e−224 0.22
FR97 8.84e−60 0.26
FR07 5.63e−46 0.27
EUGENDA 8.94e−20 0.20
ERF 3.77e−11 0.22
EGCUT 1.07e−23 0.25
CKB 3.30e−80 0.41
ALSPAC 7.20e−72 0.21
META 0.00e+00 0.24
0.0
0.2
0.4
0.6
BETA

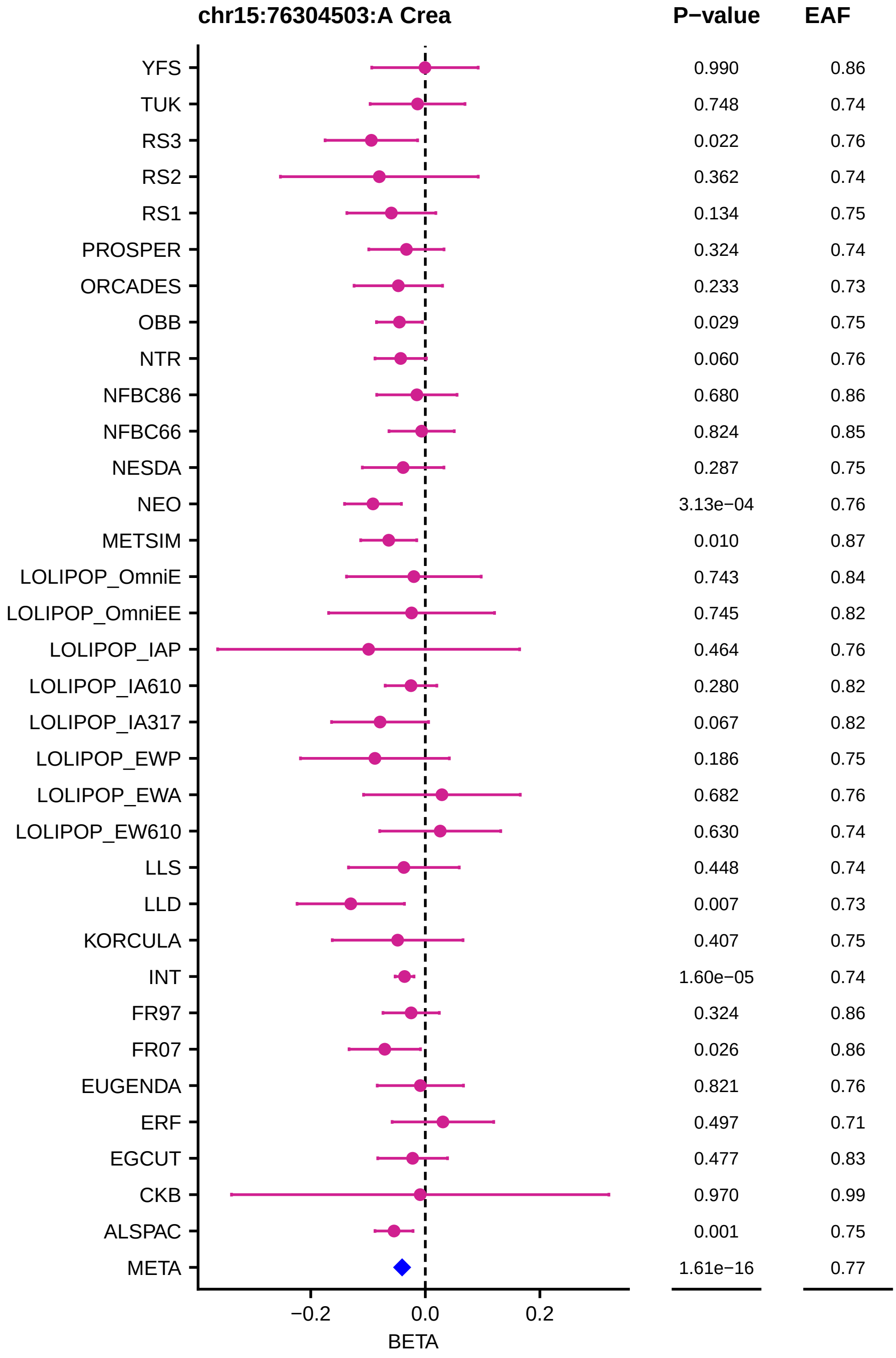
chr15:76304503:A Crea
P−value
EAF
YFS 0.990 0.86
TUK 0.748 0.74
RS3 0.022 0.76
RS2 0.362 0.74
RS1 0.134 0.75
PROSPER 0.324 0.74
ORCADES 0.233 0.73
OBB 0.029 0.75
NTR 0.060 0.76
NFBC86 0.680 0.86
NFBC66 0.824 0.85
NESDA 0.287 0.75
NEO 3.13e−04 0.76
METSIM 0.010 0.87
LOLIPOP_OmniE 0.743 0.84
LOLIPOP_OmniEE 0.745 0.82
LOLIPOP_IAP 0.464 0.76
LOLIPOP_IA610 0.280 0.82
LOLIPOP_IA317 0.067 0.82
LOLIPOP_EWP 0.186 0.75
LOLIPOP_EWA 0.682 0.76
LOLIPOP_EW610 0.630 0.74
LLS 0.448 0.74
LLD 0.007 0.73
KORCULA 0.407 0.75
INT 1.60e−05 0.74
FR97 0.324 0.86
FR07 0.026 0.86
EUGENDA 0.821 0.76
ERF 0.497 0.71
EGCUT 0.477 0.83
CKB 0.970 0.99
ALSPAC 0.001 0.75
META 1.61e−16 0.77
−0.2
0.0
0.2
BETA

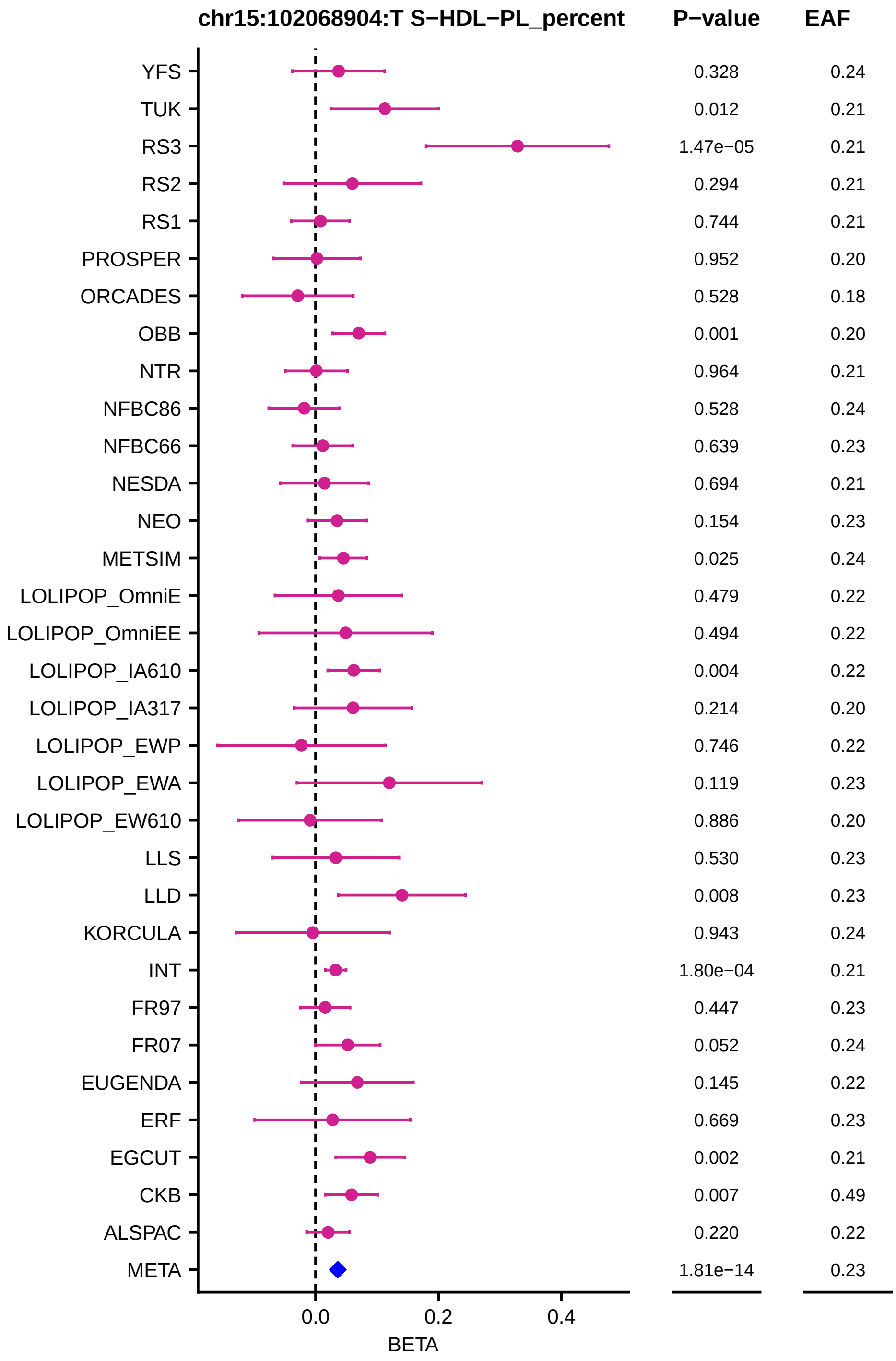
chr15:102068904:T S−HDL−PL_percent
P−value
EAF
YFS
TUK
RS3
RS2
RS1
PROSPER
ORCADES
OBB
NTR
NFBC86
NFBC66
NESDA
NEO
METSIM
LOLIPOP_OmniE
LOLIPOP_OmniEE
LOLIPOP_IA610
LOLIPOP_IA317
LOLIPOP_EWP
LOLIPOP_EWA
LOLIPOP_EW610
LLS
LLD
KORCULA
INT
FR97
FR07
EUGENDA
ERF
EGCUT
CKB
ALSPAC
META
0.328 0.24
0.012 0.21
1.47e−05 0.21
0.294 0.21
0.744 0.21
0.952 0.20
0.528 0.18
0.001 0.20
0.964 0.21
0.528 0.24
0.639 0.23
0.694 0.21
0.154 0.23
0.025 0.24
0.479 0.22
0.494 0.22
0.004 0.22
0.214 0.20
0.746 0.22
0.119 0.23
0.886 0.20
0.530 0.23
0.008 0.23
0.943 0.24
1.80e−04 0.21
0.447 0.23
0.052 0.24
0.145 0.22
0.669 0.23
0.002 0.21
0.007 0.49
0.220 0.22
1.81e−14 0.23
0.0
0.2
0.4
BETA

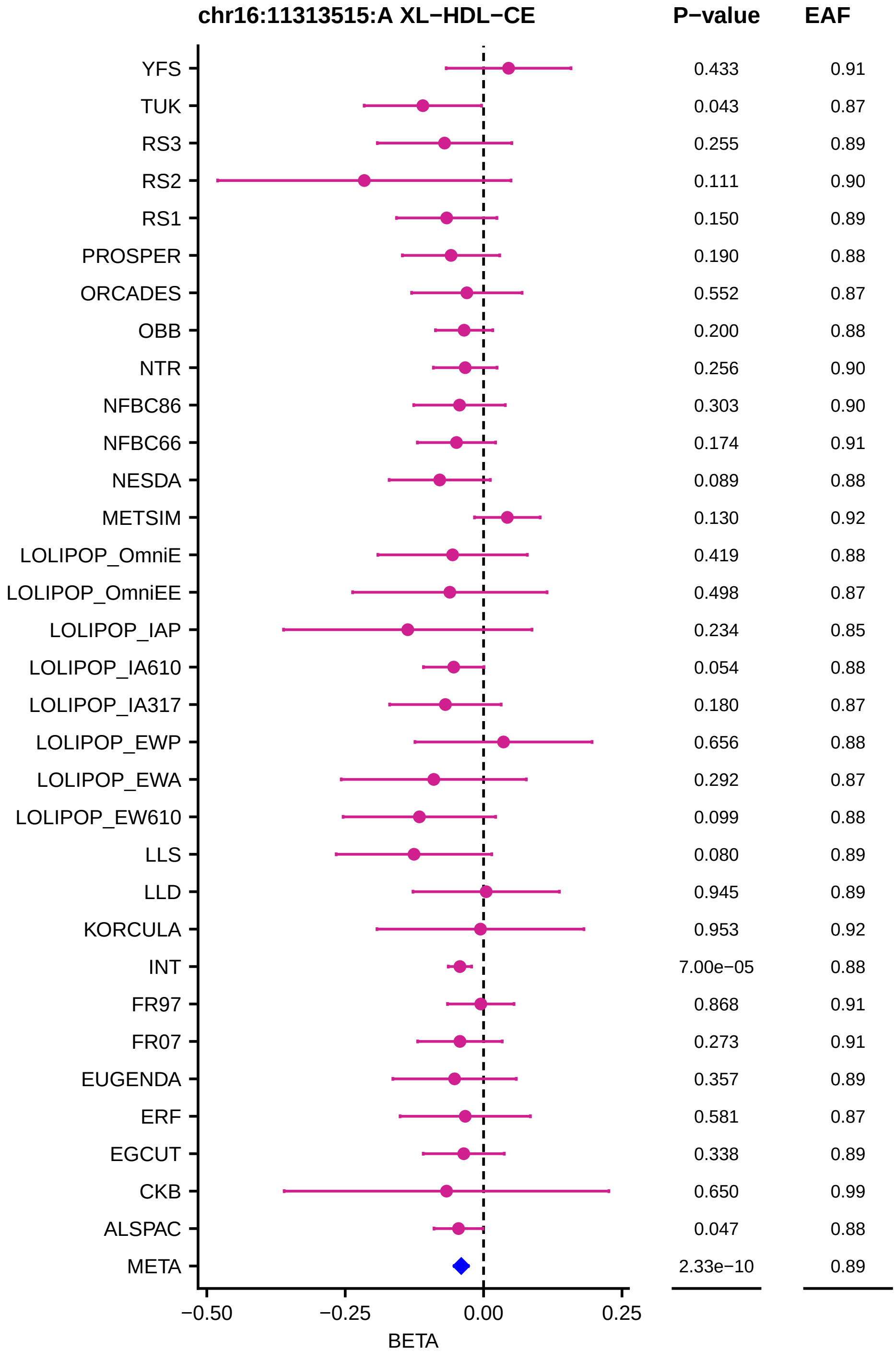
chr16:11313515:A XL−HDL−CE
P−value
EAF
YFS 0.433 0.91
TUK 0.043 0.87
RS3 0.255 0.89
RS2 0.111 0.90
RS1 0.150 0.89
PROSPER 0.190 0.88
ORCADES 0.552 0.87
OBB 0.200 0.88
NTR 0.256 0.90
NFBC86 0.303 0.90
NFBC66 0.174 0.91
NESDA 0.089 0.88
METSIM 0.130 0.92
LOLIPOP_OmniE 0.419 0.88
LOLIPOP_OmniEE 0.498 0.87
LOLIPOP_IAP 0.234 0.85
LOLIPOP_IA610 0.054 0.88
LOLIPOP_IA317 0.180 0.87
LOLIPOP_EWP 0.656 0.88
LOLIPOP_EWA 0.292 0.87
LOLIPOP_EW610 0.099 0.88
LLS 0.080 0.89
LLD 0.945 0.89
KORCULA 0.953 0.92
INT 7.00e−05 0.88
FR97 0.868 0.91
FR07 0.273 0.91
EUGENDA 0.357 0.89
ERF 0.581 0.87
EGCUT 0.338 0.89
CKB 0.650 0.99
ALSPAC 0.047 0.88
META 2.33e−10 0.89
−0.50
−0.25
0.00
0.25
BETA

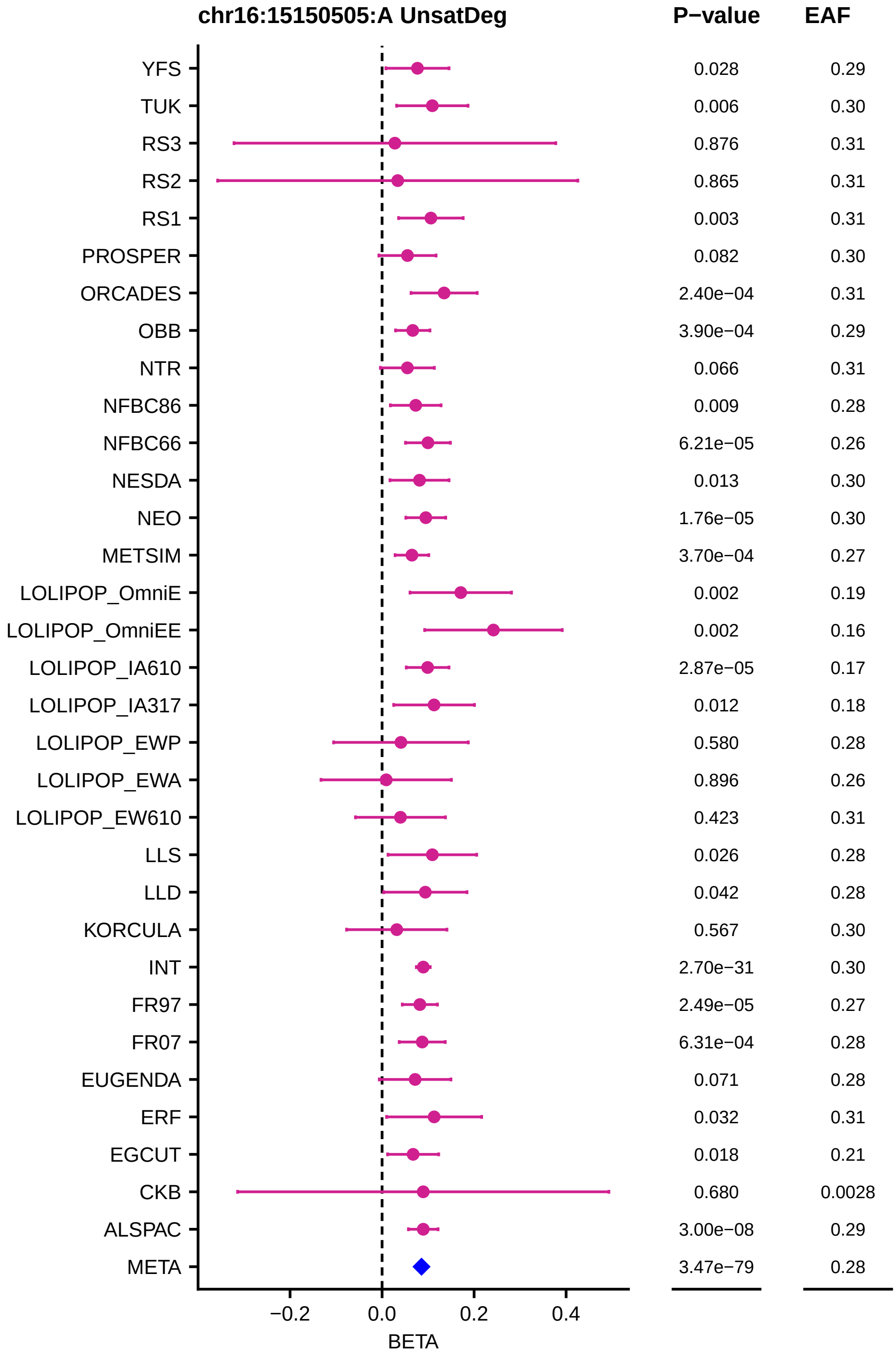

chr16:15150505:A UnsatDeg
P−value
EAF
YFS
0.028
0.29
TUK
0.006
0.30
RS3
0.876
0.31
RS2
0.865
0.31
RS1
0.003
0.31
PROSPER
0.082
0.30
ORCADES
2.40e−04
0.31
OBB
3.90e−04
0.29
NTR
0.066
0.31
NFBC86
0.009
0.28
NFBC66
6.21e−05
0.26
NESDA
0.013
0.30
NEO
1.76e−05
0.30
METSIM
3.70e−04
0.27
LOLIPOP_OmniE
0.002
0.19
LOLIPOP_OmniEE
0.002
0.16
LOLIPOP_IA610
2.87e−05
0.17
LOLIPOP_IA317
0.012
0.18
LOLIPOP_EWP
0.580
0.28
LOLIPOP_EWA
0.896
0.26
LOLIPOP_EW610
0.423
0.31
LLS
0.026
0.28
LLD
0.042
0.28
KORCULA
0.567
0.30
INT
2.70e−31
0.30
FR97
2.49e−05
0.27
FR07
6.31e−04
0.28
EUGENDA
0.071
0.28
ERF
0.032
0.31
EGCUT
0.018
0.21
CKB
0.680
0.0028
ALSPAC
3.00e−08
0.29
META
3.47e−79
0.28
−0.2
0.0
0.2
0.4
BETA

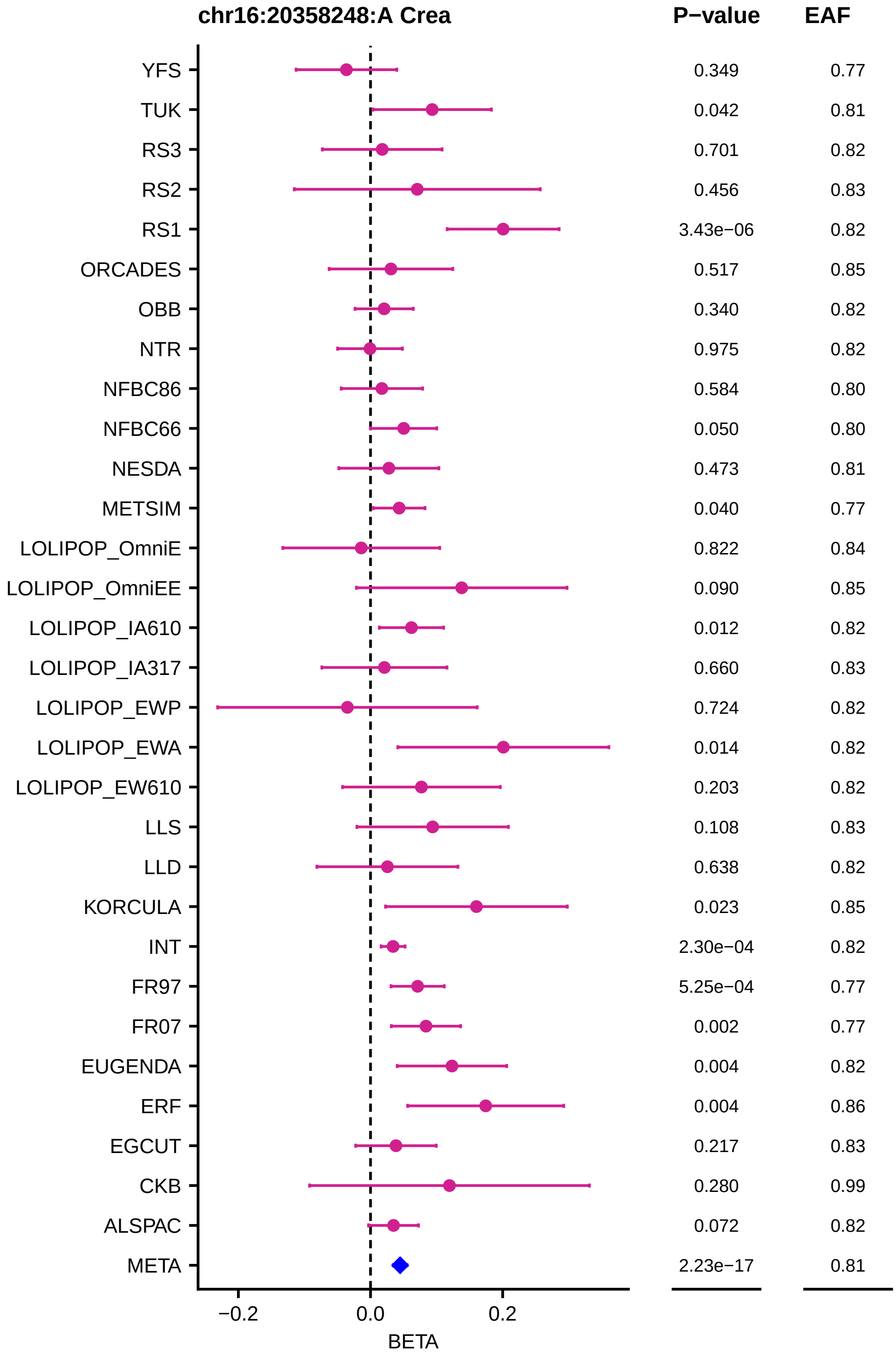

chr16:20358248:A Crea
P−value
EAF
YFS 0.349 0.77
TUK 0.042 0.81
RS3 0.701 0.82
RS2 0.456 0.83
RS1 3.43e−06 0.82
ORCADES 0.517 0.85
OBB 0.340 0.82
NTR 0.975 0.82
NFBC86 0.584 0.80
NFBC66 0.050 0.80
NESDA 0.473 0.81
METSIM 0.040 0.77
LOLIPOP_OmniE 0.822 0.84
LOLIPOP_OmniEE 0.090 0.85
LOLIPOP_IA610 0.012 0.82
LOLIPOP_IA317 0.660 0.83
LOLIPOP_EWP 0.724 0.82
LOLIPOP_EWA 0.014 0.82
LOLIPOP_EW610 0.203 0.82
LLS 0.108 0.83
LLD 0.638 0.82
KORCULA 0.023 0.85
INT 2.30e−04 0.82
FR97 5.25e−04 0.77
FR07 0.002 0.77
EUGENDA 0.004 0.82
ERF 0.004 0.86
EGCUT 0.217 0.83
CKB 0.280 0.99
ALSPAC 0.072 0.82
META 2.23e−17 0.81
−0.2
0.0
0.2
BETA

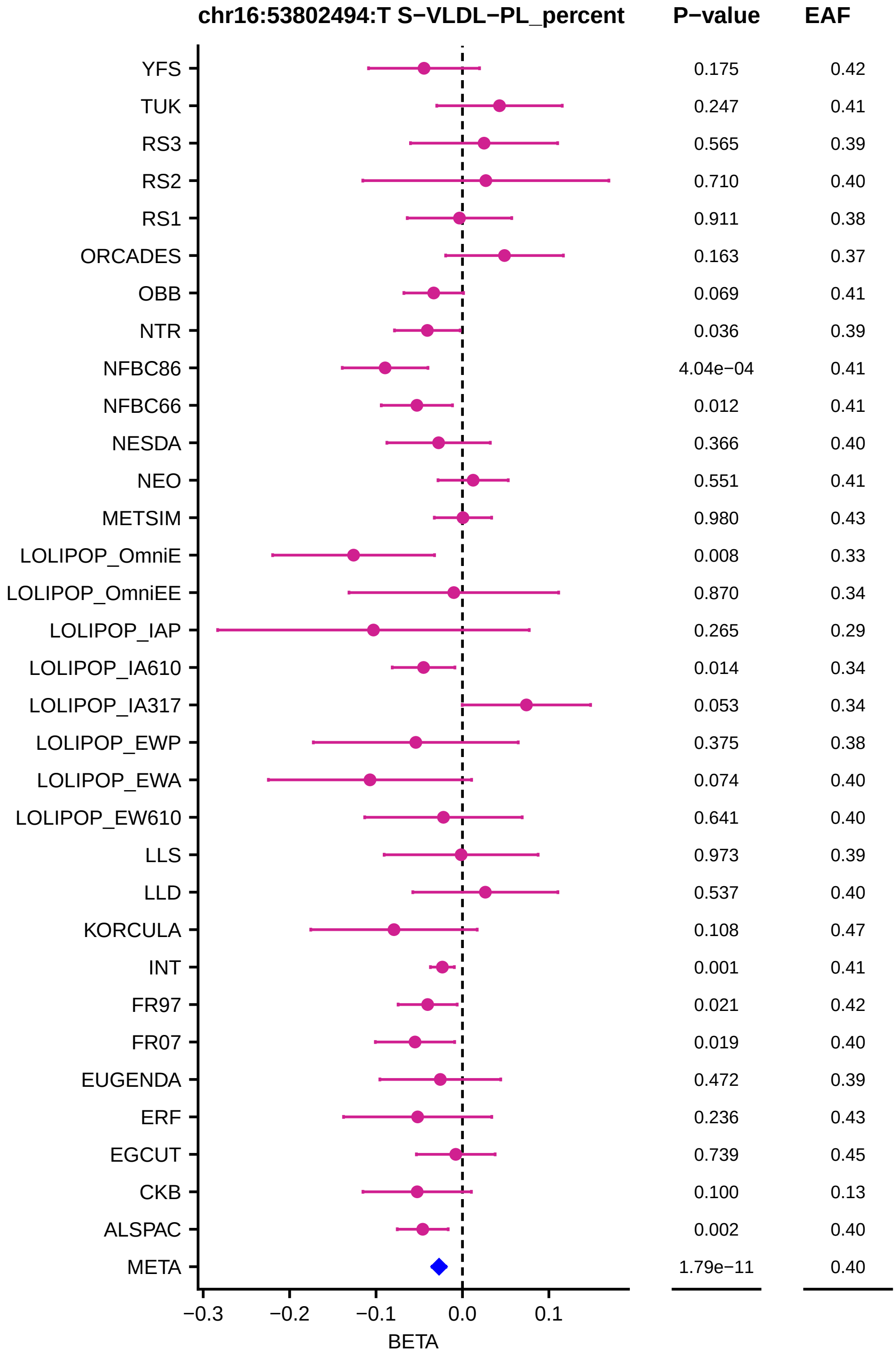
chr16:53802494:T S−VLDL−PL_percent
P−value
EAF
YFS 0.175 0.42
TUK 0.247 0.41
RS3 0.565 0.39
RS2 0.710 0.40
RS1 0.911 0.38
ORCADES 0.163 0.37
OBB 0.069 0.41
NTR 0.036 0.39
NFBC86 4.04e−04 0.41
NFBC66 0.012 0.41
NESDA 0.366 0.40
NEO 0.551 0.41
METSIM 0.980 0.43
LOLIPOP_OmniE 0.008 0.33
LOLIPOP_OmniEE 0.870 0.34
LOLIPOP_IAP 0.265 0.29
LOLIPOP_IA610 0.014 0.34
LOLIPOP_IA317 0.053 0.34
LOLIPOP_EWP 0.375 0.38
LOLIPOP_EWA 0.074 0.40
LOLIPOP_EW610 0.641 0.40
LLS 0.973 0.39
LLD 0.537 0.40
KORCULA 0.108 0.47
INT 0.001 0.41
FR97 0.021 0.42
FR07 0.019 0.40
EUGENDA 0.472 0.39
ERF 0.236 0.43
EGCUT 0.739 0.45
CKB 0.100 0.13
ALSPAC 0.002 0.40
META 1.79e−11 0.40
−0.3
−0.2
−0.1
0.0
0.1
BETA

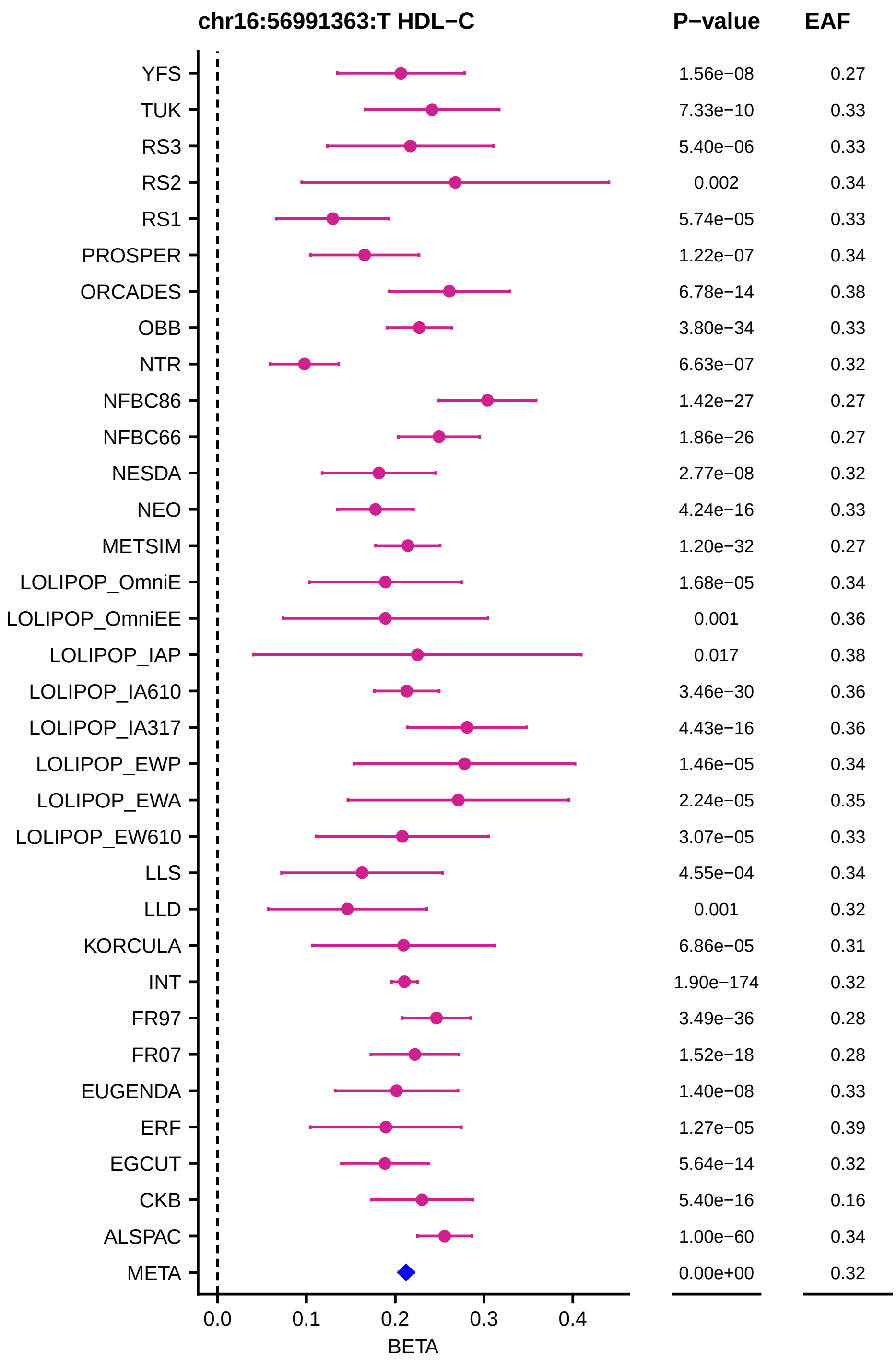
chr16:56991363:T HDL−C
P−value
EAF
YFS 1.56e−08 0.27
TUK 7.33e−10 0.33
RS3 5.40e−06 0.33
RS2 0.002 0.34
RS1 5.74e−05 0.33
PROSPER 1.22e−07 0.34
ORCADES 6.78e−14 0.38
OBB 3.80e−34 0.33
NTR 6.63e−07 0.32
NFBC86 1.42e−27 0.27
NFBC66 1.86e−26 0.27
NESDA 2.77e−08 0.32
NEO 4.24e−16 0.33
METSIM 1.20e−32 0.27
LOLIPOP_OmniE 1.68e−05 0.34
LOLIPOP_OmniEE 0.001 0.36
LOLIPOP_IAP 0.017 0.38
LOLIPOP_IA610 3.46e−30 0.36
LOLIPOP_IA317 4.43e−16 0.36
LOLIPOP_EWP 1.46e−05 0.34
LOLIPOP_EWA 2.24e−05 0.35
LOLIPOP_EW610 3.07e−05 0.33
LLS 4.55e−04 0.34
LLD 0.001 0.32
KORCULA 6.86e−05 0.31
INT 1.90e−174 0.32
FR97 3.49e−36 0.28
FR07 1.52e−18 0.28
EUGENDA 1.40e−08 0.33
ERF 1.27e−05 0.39
EGCUT 5.64e−14 0.32
CKB 5.40e−16 0.16
ALSPAC 1.00e−60 0.34
META 0.00e+00 0.32
0.0
0.1
0.2
0.3
0.4
BETA

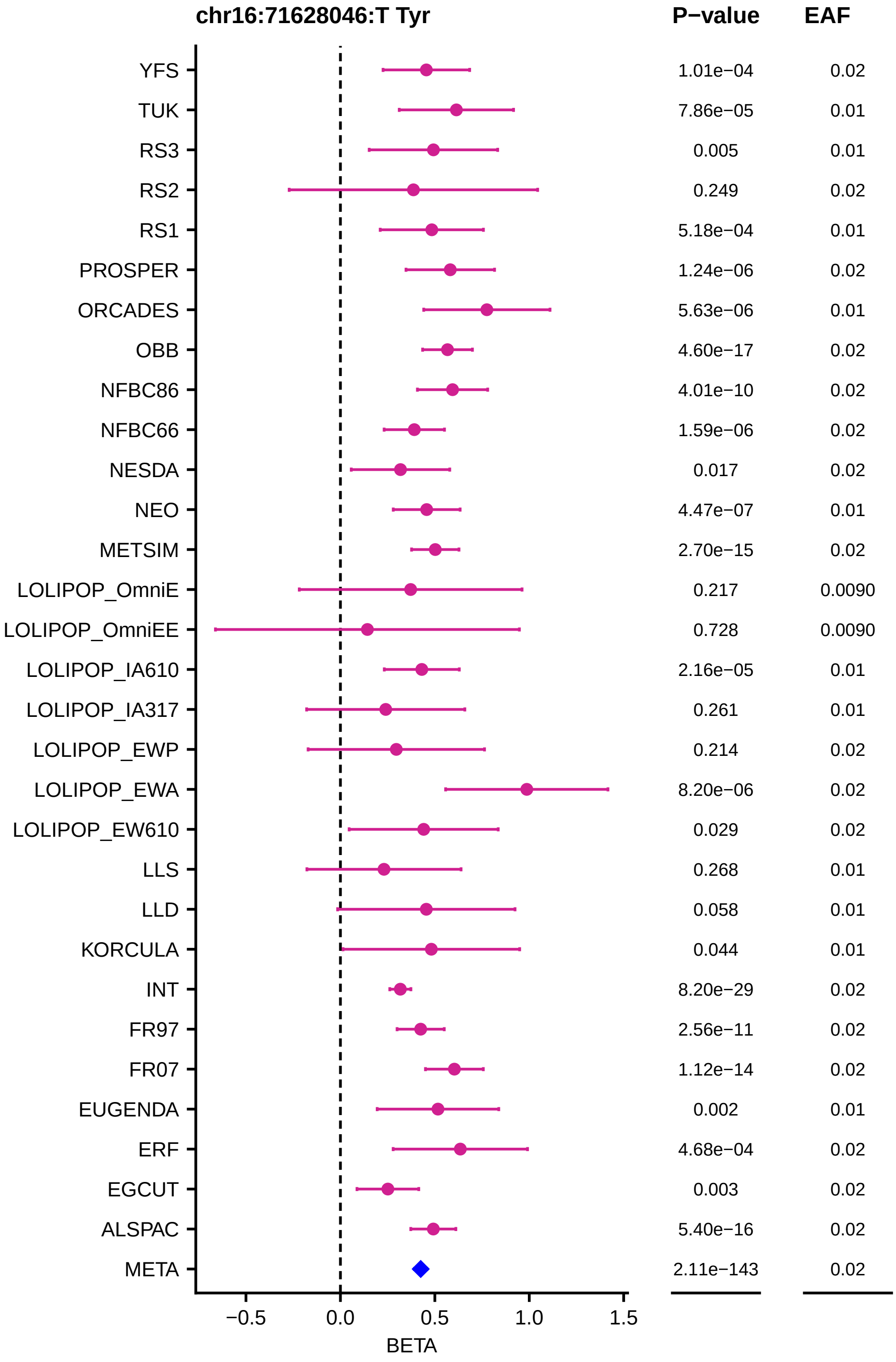

chr16:71628046:T Tyr
P−value
EAF
YFS 1.01e−04 0.02
TUK 7.86e−05 0.01
RS3 0.005 0.01
RS2 0.249 0.02
RS1 5.18e−04 0.01
PROSPER 1.24e−06 0.02
ORCADES 5.63e−06 0.01
OBB 4.60e−17 0.02
NFBC86 4.01e−10 0.02
NFBC66 1.59e−06 0.02
NESDA 0.017 0.02
NEO 4.47e−07 0.01
METSIM 2.70e−15 0.02
LOLIPOP_OmniE 0.217 0.0090
LOLIPOP_OmniEE 0.728 0.0090
LOLIPOP_IA610 2.16e−05 0.01
LOLIPOP_IA317 0.261 0.01
LOLIPOP_EWP 0.214 0.02
LOLIPOP_EWA 8.20e−06 0.02
LOLIPOP_EW610 0.029 0.02
LLS 0.268 0.01
LLD 0.058 0.01
KORCULA 0.044 0.01
INT 8.20e−29 0.02
FR97 2.56e−11 0.02
FR07 1.12e−14 0.02
EUGENDA 0.002 0.01
ERF 4.68e−04 0.02
EGCUT 0.003 0.02
ALSPAC 5.40e−16 0.02
META 2.11e−143 0.02
−0.5
0.0
0.5
1.0
1.5
BETA

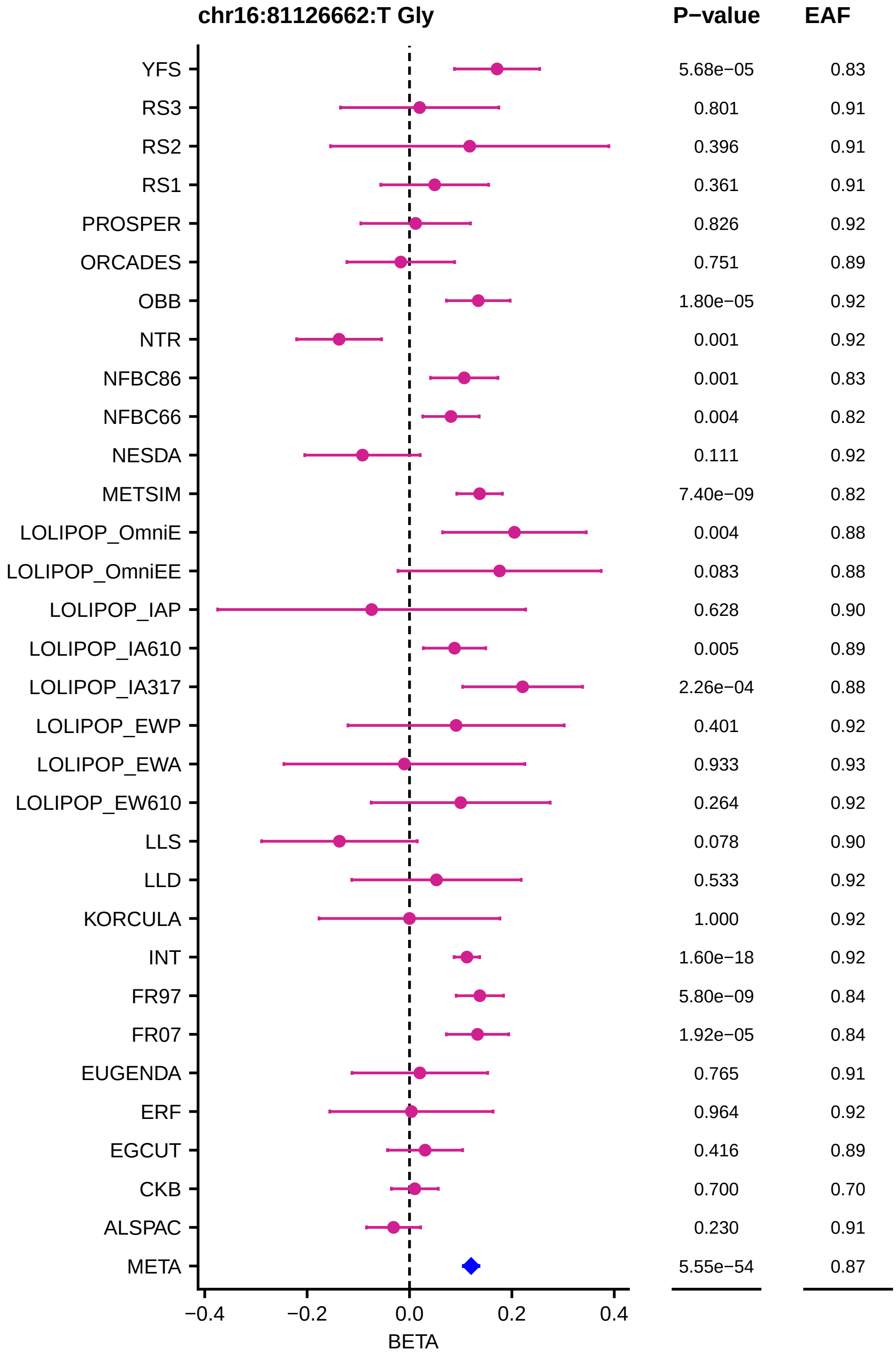
chr16:81126662:T Gly
P−value
EAF
YFS 5.68e−05 0.83
RS3 0.801 0.91
RS2 0.396 0.91
RS1 0.361 0.91
PROSPER 0.826 0.92
ORCADES 0.751 0.89
OBB 1.80e−05 0.92
NTR 0.001 0.92
NFBC86 0.001 0.83
NFBC66 0.004 0.82
NESDA 0.111 0.92
METSIM 7.40e−09 0.82
LOLIPOP_OmniE 0.004 0.88
LOLIPOP_OmniEE 0.083 0.88
LOLIPOP_IAP 0.628 0.90
LOLIPOP_IA610 0.005 0.89
LOLIPOP_IA317 2.26e−04 0.88
LOLIPOP_EWP 0.401 0.92
LOLIPOP_EWA 0.933 0.93
LOLIPOP_EW610 0.264 0.92
LLS 0.078 0.90
LLD 0.533 0.92
KORCULA 1.000 0.92
INT 1.60e−18 0.92
FR97 5.80e−09 0.84
FR07 1.92e−05 0.84
EUGENDA 0.765 0.91
ERF 0.964 0.92
EGCUT 0.416 0.89
CKB 0.700 0.70
ALSPAC 0.230 0.91
META 5.55e−54 0.87
−0.4
−0.2
0.0
0.2
0.4
BETA

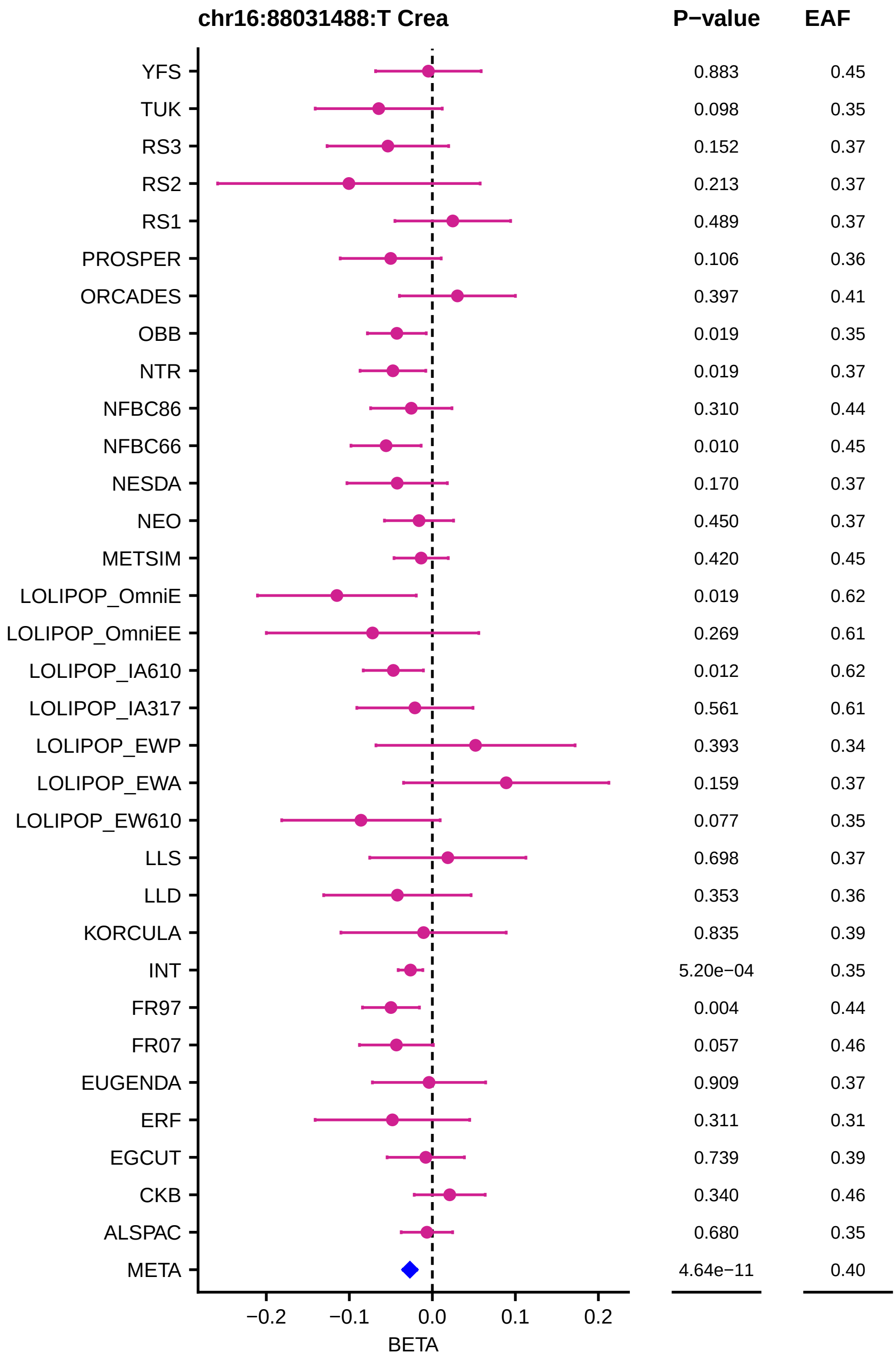
chr16:88031488:T Crea
P−value
EAF
YFS 0.883 0.45
TUK 0.098 0.35
RS3 0.152 0.37
RS2 0.213 0.37
RS1 0.489 0.37
PROSPER 0.106 0.36
ORCADES 0.397 0.41
OBB 0.019 0.35
NTR 0.019 0.37
NFBC86 0.310 0.44
NFBC66 0.010 0.45
NESDA 0.170 0.37
NEO 0.450 0.37
METSIM 0.420 0.45
LOLIPOP_OmniE 0.019 0.62
LOLIPOP_OmniEE 0.269 0.61
LOLIPOP_IA610 0.012 0.62
LOLIPOP_IA317 0.561 0.61
LOLIPOP_EWP 0.393 0.34
LOLIPOP_EWA 0.159 0.37
LOLIPOP_EW610 0.077 0.35
LLS 0.698 0.37
LLD 0.353 0.36
KORCULA 0.835 0.39
INT 5.20e−04 0.35
FR97 0.004 0.44
FR07 0.057 0.46
EUGENDA 0.909 0.37
ERF 0.311 0.31
EGCUT 0.739 0.39
CKB 0.340 0.46
ALSPAC 0.680 0.35
META 4.64e−11 0.40
−0.2 −0.1 0.0 0.1 0.2
BETA

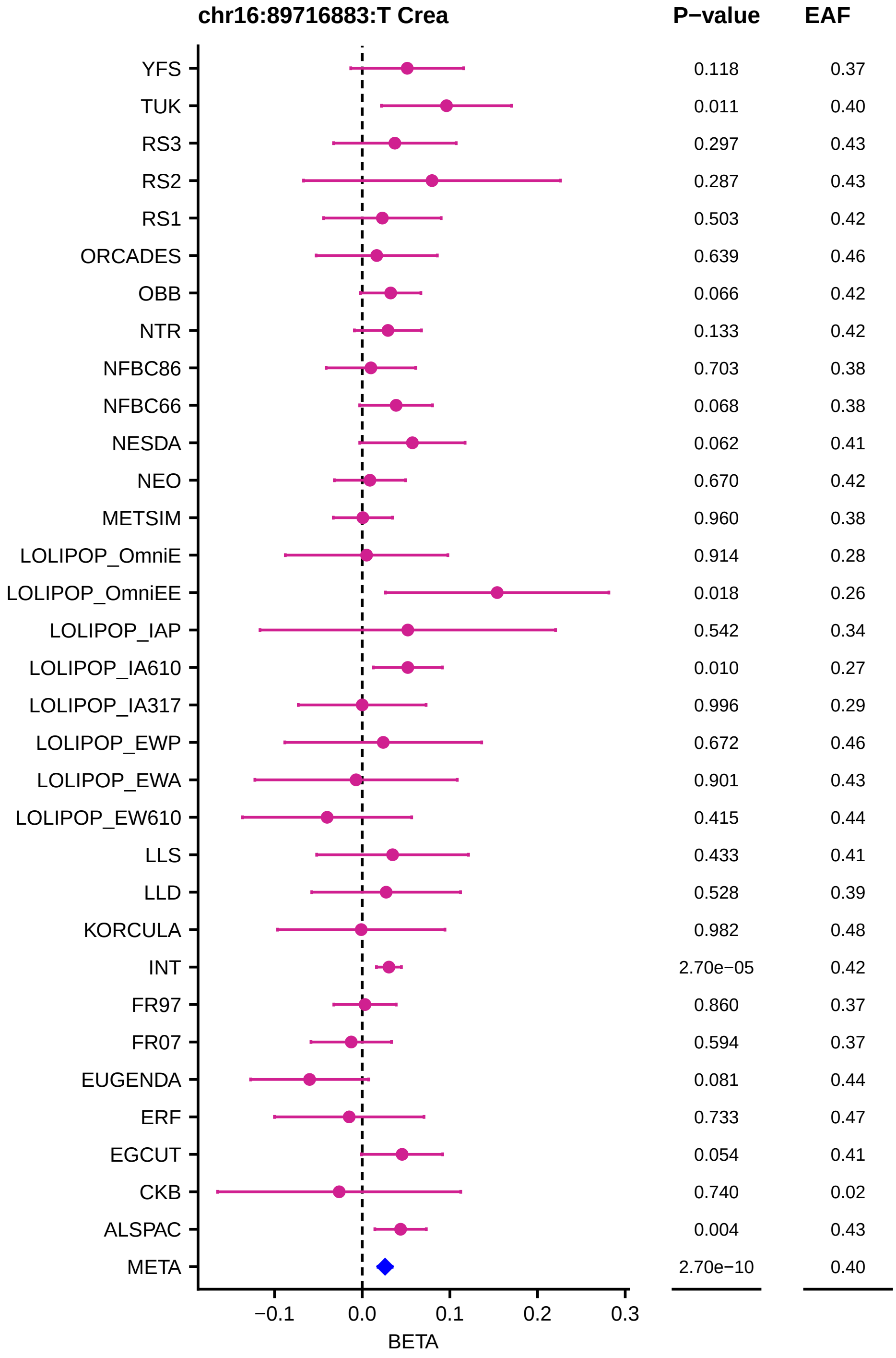

chr16:89716883:T Crea
P−value
EAF
YFS 0.118 0.37
TUK 0.011 0.40
RS3 0.297 0.43
RS2 0.287 0.43
RS1 0.503 0.42
ORCADES 0.639 0.46
OBB 0.066 0.42
NTR 0.133 0.42
NFBC86 0.703 0.38
NFBC66 0.068 0.38
NESDA 0.062 0.41
NEO 0.670 0.42
METSIM 0.960 0.38
LOLIPOP_OmniE 0.914 0.28
LOLIPOP_OmniEE 0.018 0.26
LOLIPOP_IAP 0.542 0.34
LOLIPOP_IA610 0.010 0.27
LOLIPOP_IA317 0.996 0.29
LOLIPOP_EWP 0.672 0.46
LOLIPOP_EWA 0.901 0.43
LOLIPOP_EW610 0.415 0.44
LLS 0.433 0.41
LLD 0.528 0.39
KORCULA 0.982 0.48
INT 2.70e−05 0.42
FR97 0.860 0.37
FR07 0.594 0.37
EUGENDA 0.081 0.44
ERF 0.733 0.47
EGCUT 0.054 0.41
CKB 0.740 0.02
ALSPAC 0.004 0.43
META 2.70e−10 0.40
−0.1
0.0
0.1
0.2
0.3
BETA

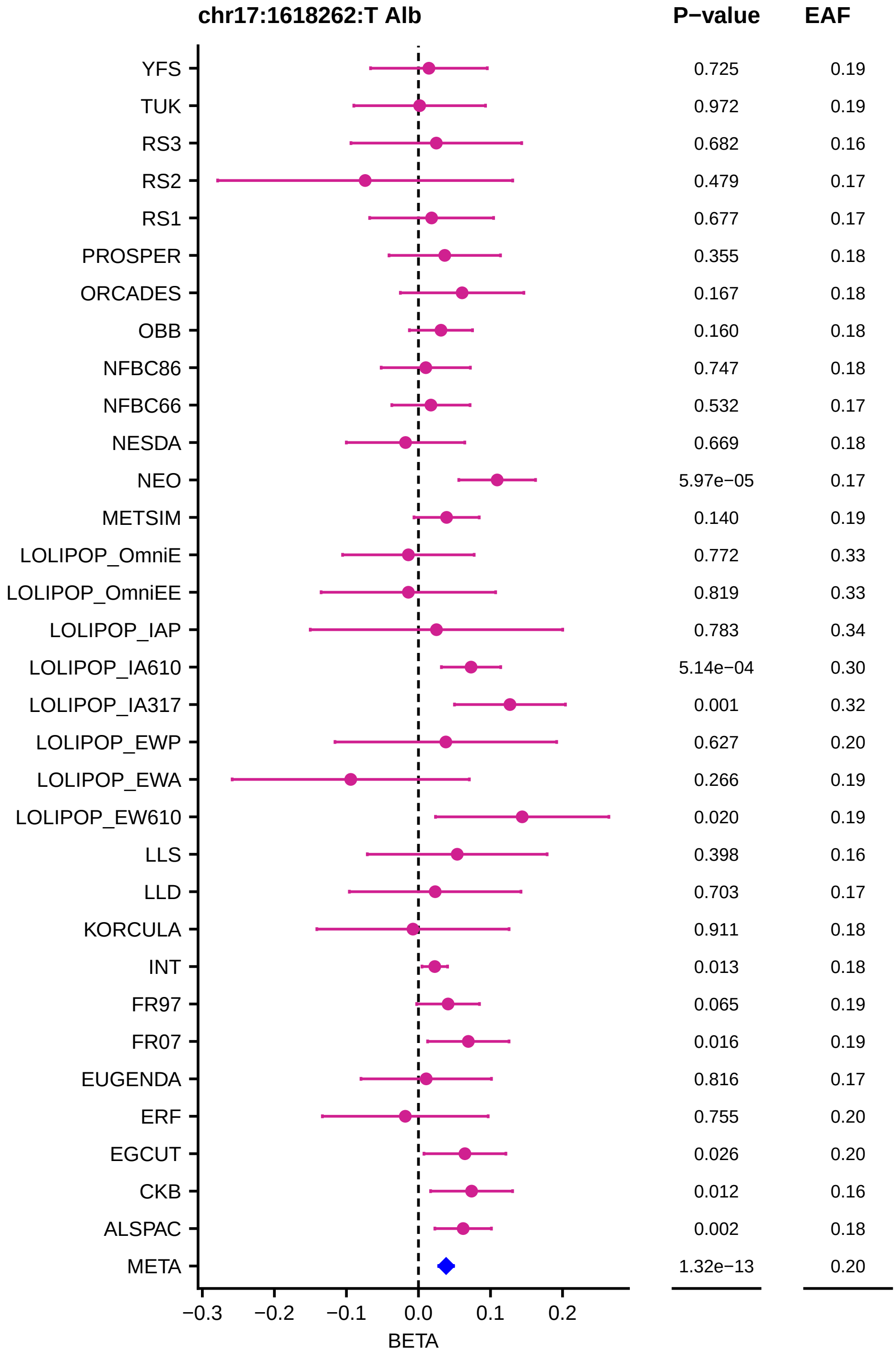
chr17:1618262:T Alb
P−value
EAF
YFS 0.725 0.19
TUK 0.972 0.19
RS3 0.682 0.16
RS2 0.479 0.17
RS1 0.677 0.17
PROSPER 0.355 0.18
ORCADES 0.167 0.18
OBB 0.160 0.18
NFBC86 0.747 0.18
NFBC66 0.532 0.17
NESDA 0.669 0.18
NEO 5.97e−05 0.17
METSIM 0.140 0.19
LOLIPOP_OmniE 0.772 0.33
LOLIPOP_OmniEE 0.819 0.33
LOLIPOP_IAP 0.783 0.34
LOLIPOP_IA610 5.14e−04 0.30
LOLIPOP_IA317 0.001 0.32
LOLIPOP_EWP 0.627 0.20
LOLIPOP_EWA 0.266 0.19
LOLIPOP_EW610 0.020 0.19
LLS 0.398 0.16
LLD 0.703 0.17
KORCULA 0.911 0.18
INT 0.013 0.18
FR97 0.065 0.19
FR07 0.016 0.19
EUGENDA 0.816 0.17
ERF 0.755 0.20
EGCUT 0.026 0.20
CKB 0.012 0.16
ALSPAC 0.002 0.18
META 1.32e−13 0.20
−0.3
−0.2
−0.1
0.0
0.1
0.2
BETA

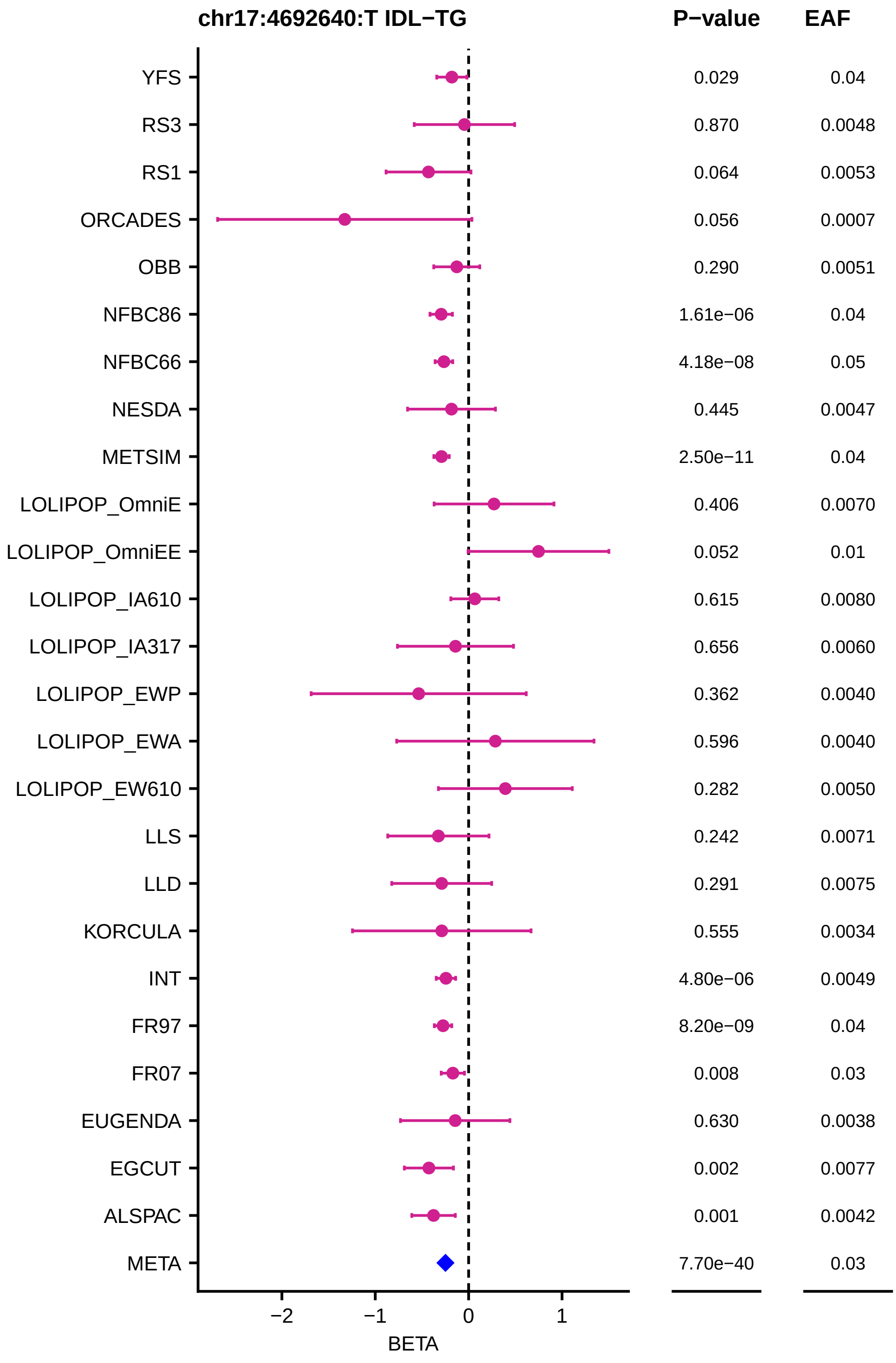

chr17:4692640:T IDL−TG
P−value
EAF
YFS
0.029
0.04
RS3
0.870
0.0048
RS1
0.064
0.0053
ORCADES
0.056
0.0007
OBB
0.290
0.0051
NFBC86
1.61e−06
0.04
NFBC66
4.18e−08
0.05
NESDA
0.445
0.0047
METSIM
2.50e−11
0.04
LOLIPOP_OmniE
0.406
0.0070
LOLIPOP_OmniEE
0.052
0.01
LOLIPOP_IA610
0.615
0.0080
LOLIPOP_IA317
0.656
0.0060
LOLIPOP_EWP
0.362
0.0040
LOLIPOP_EWA
0.596
0.0040
LOLIPOP_EW610
0.282
0.0050
LLS
0.242
0.0071
LLD
0.291
0.0075
KORCULA
0.555
0.0034
INT
4.80e−06
0.0049
FR97
8.20e−09
0.04
FR07
0.008
0.03
EUGENDA
0.630
0.0038
EGCUT
0.002
0.0077
ALSPAC
0.001
0.0042
META
7.70e−40
0.03
−2
−1
0
1
BETA

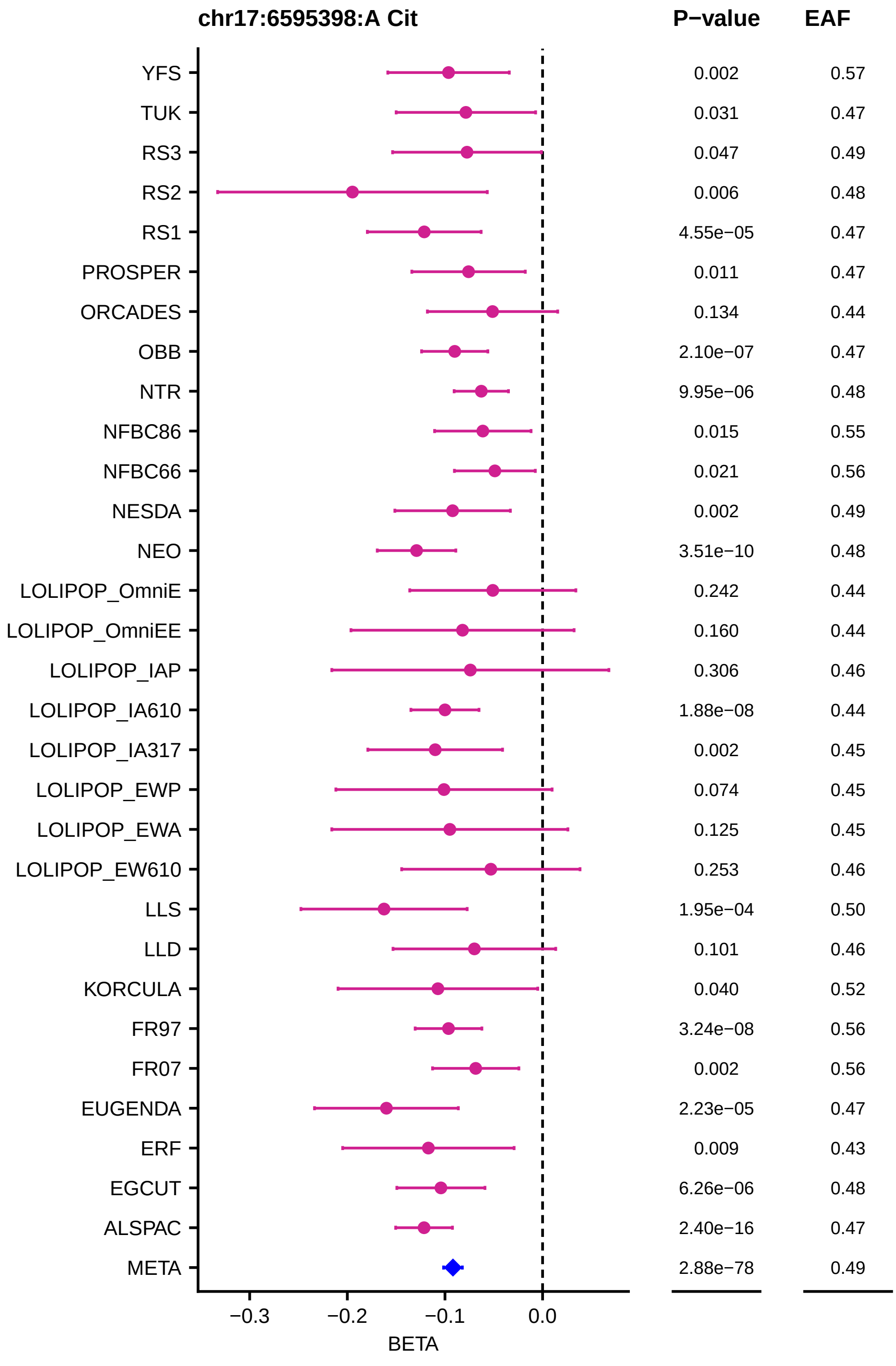
chr17:6595398:A Cit
P−value
EAF
YFS 0.002 0.57
TUK 0.031 0.47
RS3 0.047 0.49
RS2 0.006 0.48
RS1 4.55e−05 0.47
PROSPER 0.011 0.47
ORCADES 0.134 0.44
OBB 2.10e−07 0.47
NTR 9.95e−06 0.48
NFBC86 0.015 0.55
NFBC66 0.021 0.56
NESDA 0.002 0.49
NEO 3.51e−10 0.48
LOLIPOP_OmniE 0.242 0.44
LOLIPOP_OmniEE 0.160 0.44
LOLIPOP_IAP 0.306 0.46
LOLIPOP_IA610 1.88e−08 0.44
LOLIPOP_IA317 0.002 0.45
LOLIPOP_EWP 0.074 0.45
LOLIPOP_EWA 0.125 0.45
LOLIPOP_EW610 0.253 0.46
LLS 1.95e−04 0.50
LLD 0.101 0.46
KORCULA 0.040 0.52
FR97 3.24e−08 0.56
FR07 0.002 0.56
EUGENDA 2.23e−05 0.47
ERF 0.009 0.43
EGCUT 6.26e−06 0.48
ALSPAC 2.40e−16 0.47
META 2.88e−78 0.49
−0.3
−0.2
−0.1
0.0
BETA

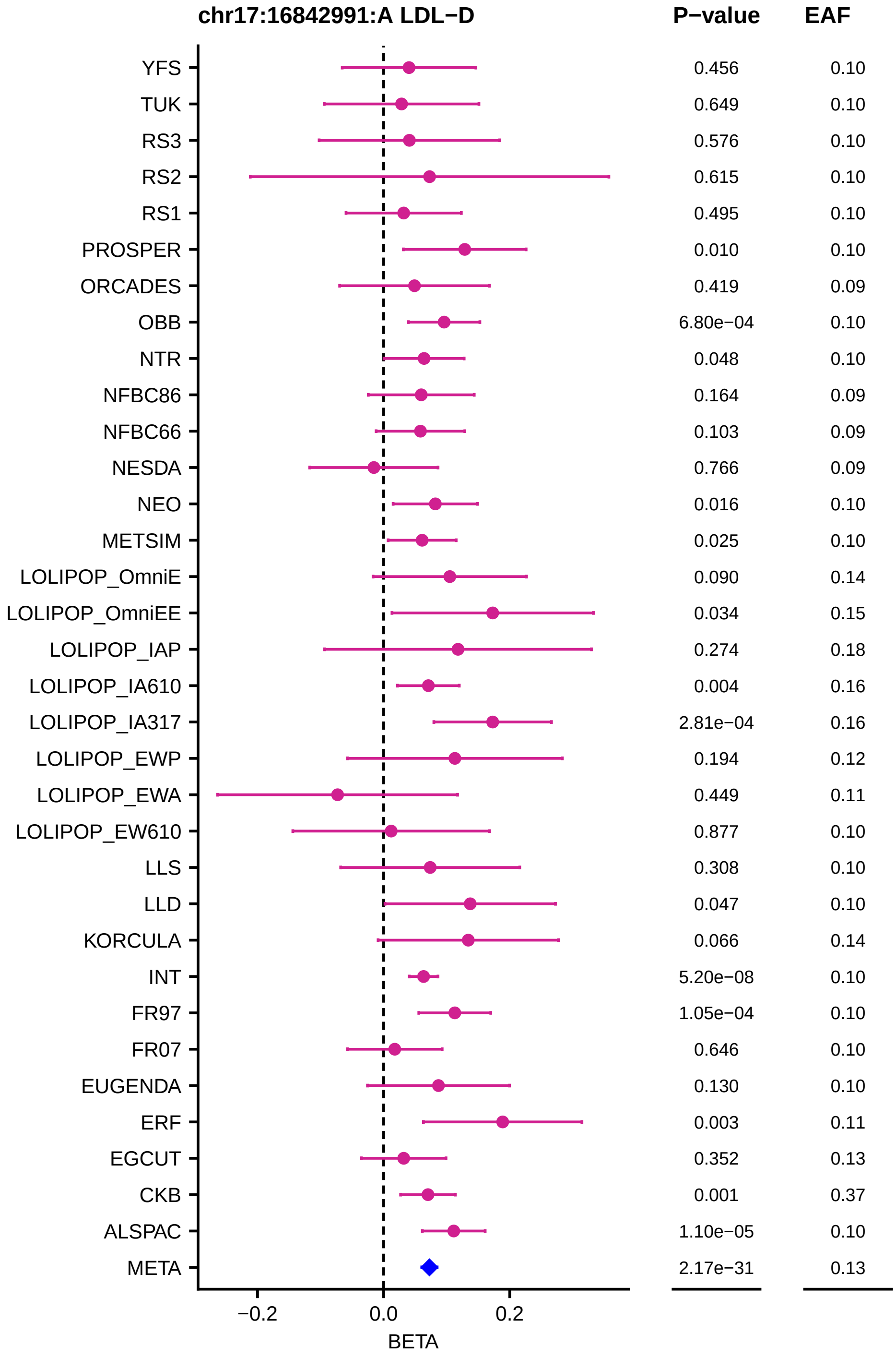
chr17:16842991:A LDL−D
P−value
EAF
YFS
TUK
RS3
RS2
RS1
PROSPER
ORCADES
OBB
NTR
NFBC86
NFBC66
NESDA
NEO
METSIM
LOLIPOP_OmniE
LOLIPOP_OmniEE
LOLIPOP_IAP
LOLIPOP_IA610
LOLIPOP_IA317
LOLIPOP_EWP
LOLIPOP_EWA
LOLIPOP_EW610
LLS
LLD
KORCULA
INT
FR97
FR07
EUGENDA
ERF
EGCUT
CKB
ALSPAC
META
0.456 0.10
0.649 0.10
0.576 0.10
0.615 0.10
0.495 0.10
0.010 0.10
0.419 0.09
6.80e−04 0.10
0.048 0.10
0.164 0.09
0.103 0.09
0.766 0.09
0.016 0.10
0.025 0.10
0.090 0.14
0.034 0.15
0.274 0.18
0.004 0.16
2.81e−04 0.16
0.194 0.12
0.449 0.11
0.877 0.10
0.308 0.10
0.047 0.10
0.066 0.14
5.20e−08 0.10
1.05e−04 0.10
0.646 0.10
0.130 0.10
0.003 0.11
0.352 0.13
0.001 0.37
1.10e−05 0.10
2.17e−31 0.13
−0.2
0.0
0.2
BETA

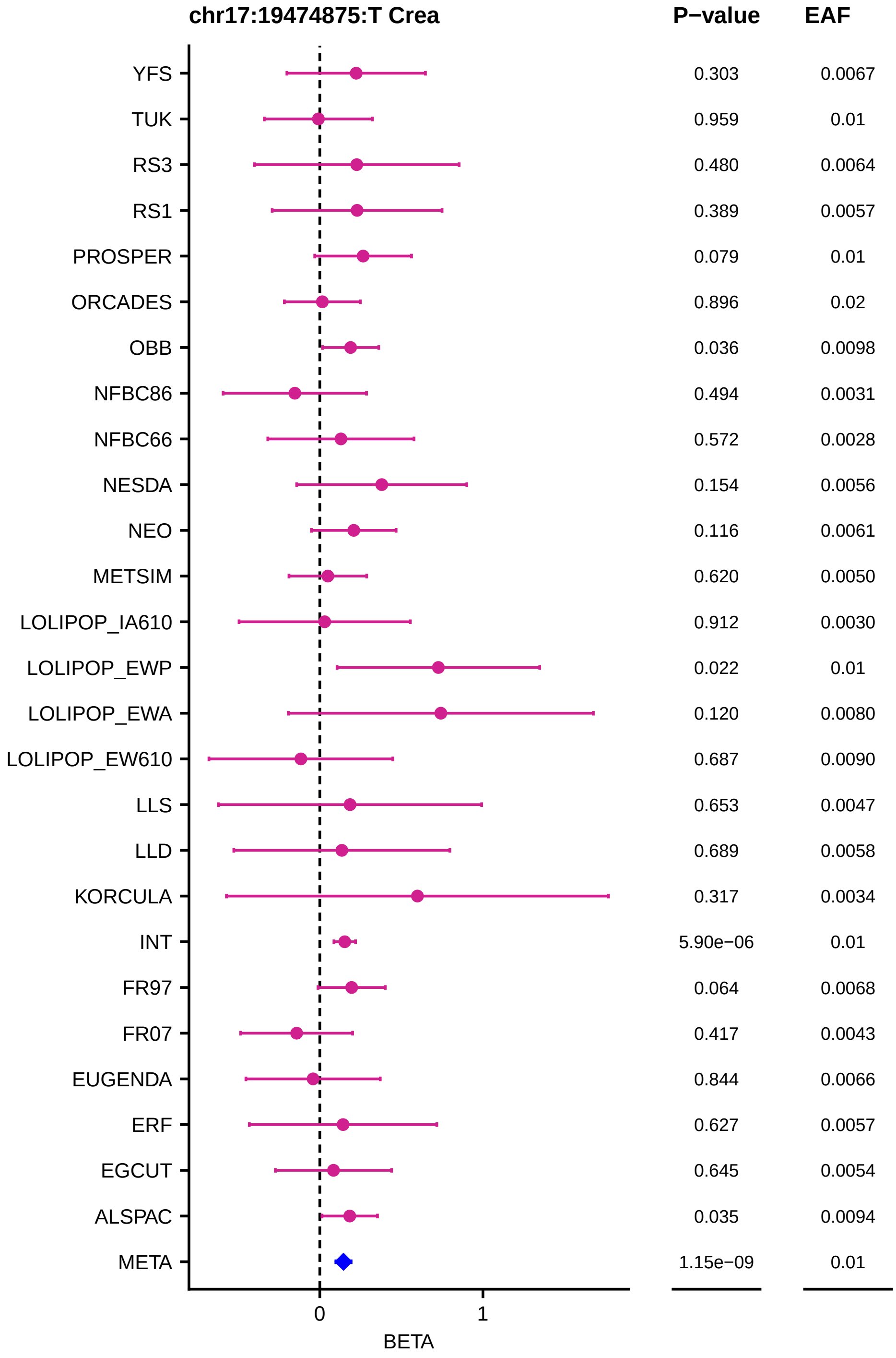

chr17:19474875:T Crea
P−value
EAF
YFS 0.303 0.0067
TUK 0.959 0.01
RS3 0.480 0.0064
RS1 0.389 0.0057
PROSPER 0.079 0.01
ORCADES 0.896 0.02
OBB 0.036 0.0098
NFBC86 0.494 0.0031
NFBC66 0.572 0.0028
NESDA 0.154 0.0056
NEO 0.116 0.0061
METSIM 0.620 0.0050
LOLIPOP_IA610 0.912 0.0030
LOLIPOP_EWP 0.022 0.01
LOLIPOP_EWA 0.120 0.0080
LOLIPOP_EW610 0.687 0.0090
LLS 0.653 0.0047
LLD 0.689 0.0058
KORCULA 0.317 0.0034
INT 5.90e−06 0.01
FR97 0.064 0.0068
FR07 0.417 0.0043
EUGENDA 0.844 0.0066
ERF 0.627 0.0057
EGCUT 0.645 0.0054
ALSPAC 0.035 0.0094
META 1.15e−09 0.01
0
1
BETA

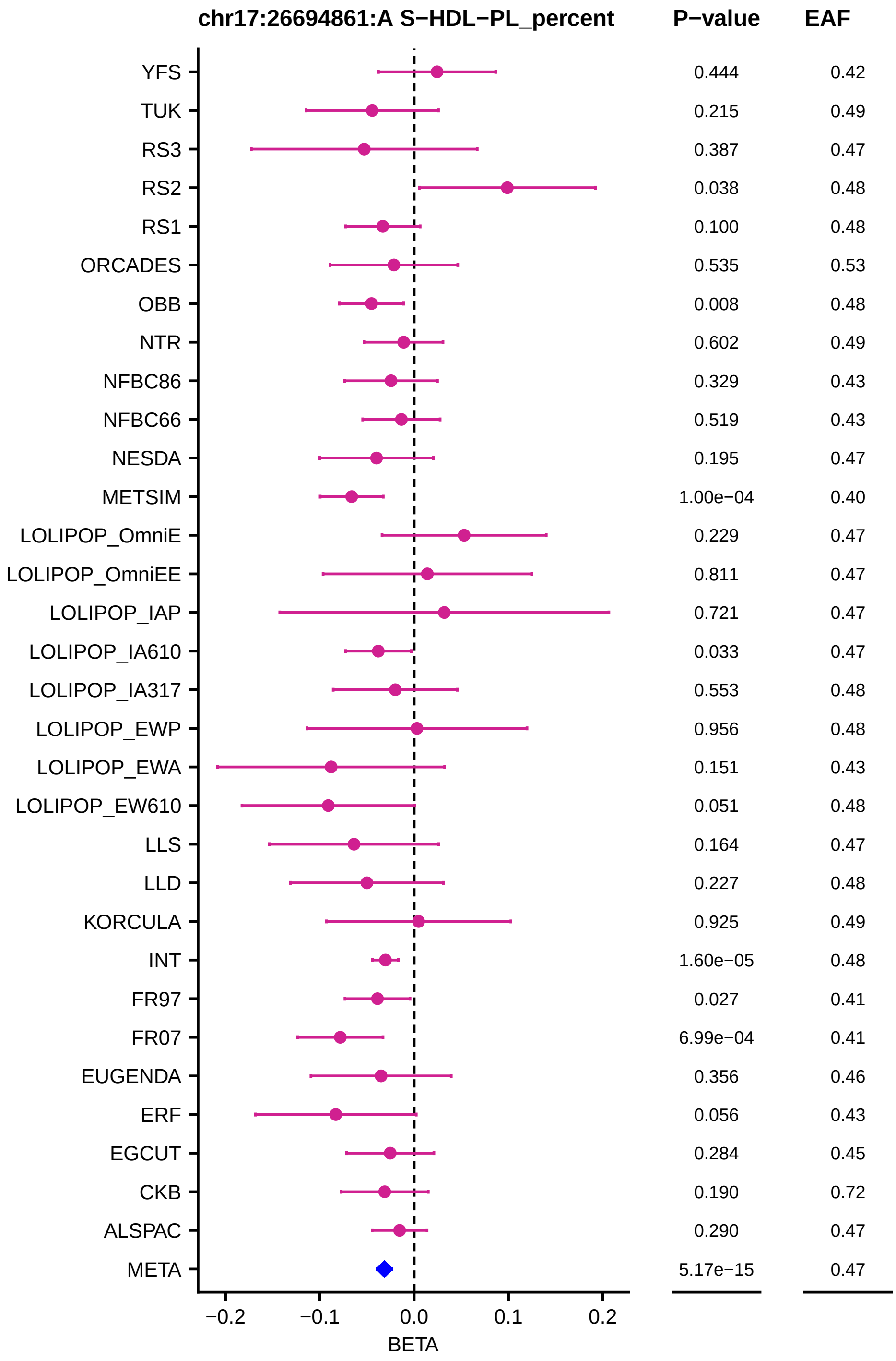

chr17:26694861:A S−HDL−PL_percent
P−value
EAF
YFS 0.444 0.42
TUK 0.215 0.49
RS3 0.387 0.47
RS2 0.038 0.48
RS1 0.100 0.48
ORCADES 0.535 0.53
OBB 0.008 0.48
NTR 0.602 0.49
NFBC86 0.329 0.43
NFBC66 0.519 0.43
NESDA 0.195 0.47
METSIM 1.00e−04 0.40
LOLIPOP_OmniE 0.229 0.47
LOLIPOP_OmniEE 0.811 0.47
LOLIPOP_IAP 0.721 0.47
LOLIPOP_IA610 0.033 0.47
LOLIPOP_IA317 0.553 0.48
LOLIPOP_EWP 0.956 0.48
LOLIPOP_EWA 0.151 0.43
LOLIPOP_EW610 0.051 0.48
LLS 0.164 0.47
LLD 0.227 0.48
KORCULA 0.925 0.49
INT 1.60e−05 0.48
FR97 0.027 0.41
FR07 6.99e−04 0.41
EUGENDA 0.356 0.46
ERF 0.056 0.43
EGCUT 0.284 0.45
CKB 0.190 0.72
ALSPAC 0.290 0.47
META 5.17e−15 0.47
−0.2
−0.1
0.0
0.1
0.2
BETA

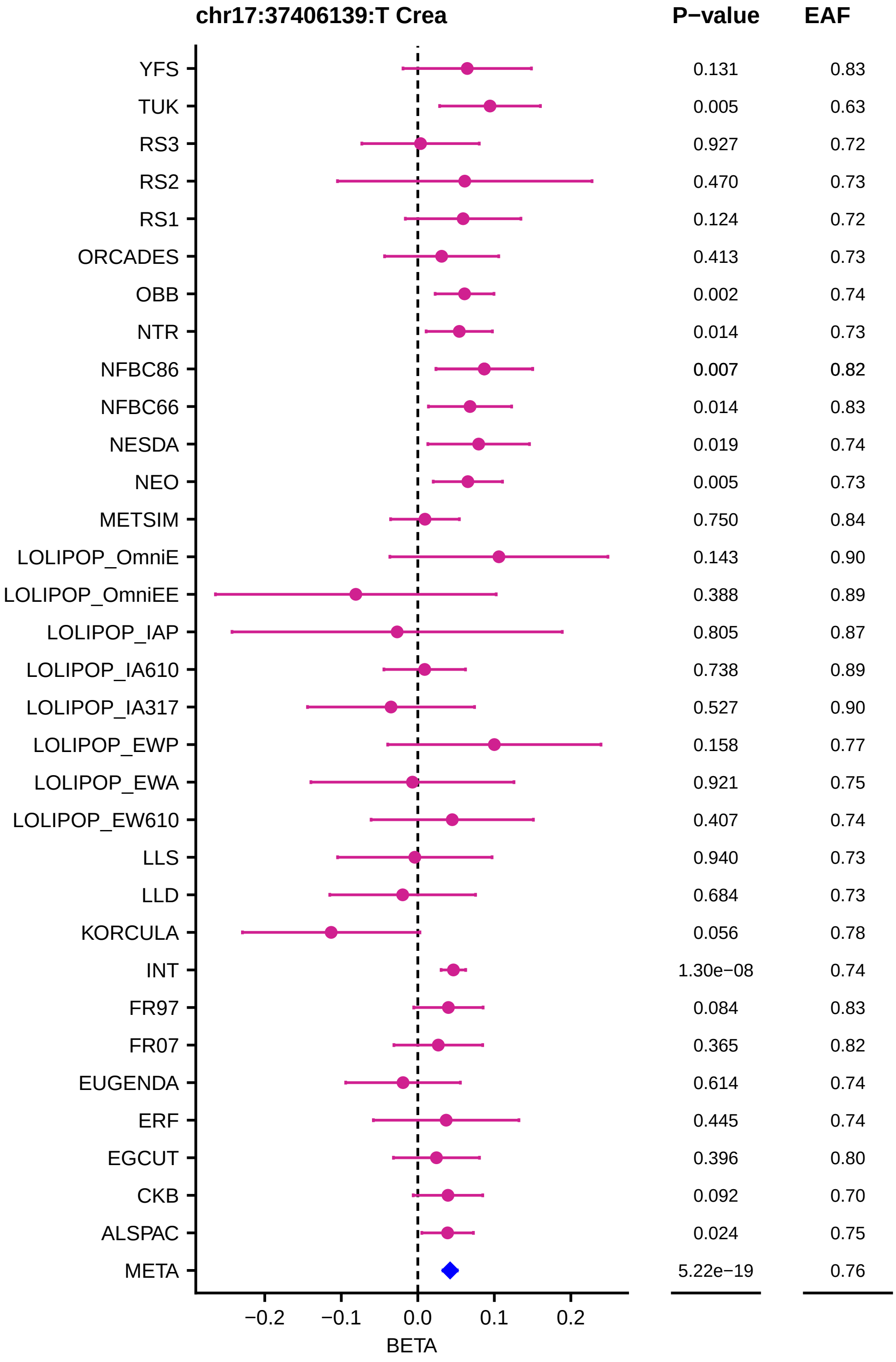
chr17:37406139:T Crea
P−value
EAF
YFS 0.131 0.83
TUK 0.005 0.63
RS3 0.927 0.72
RS2 0.470 0.73
RS1 0.124 0.72
ORCADES 0.413 0.73
OBB 0.002 0.74
NTR 0.014 0.73
NFBC86 0.007 0.82
NFBC66 0.014 0.83
NESDA 0.019 0.74
NEO 0.005 0.73
METSIM 0.750 0.84
LOLIPOP_OmniE 0.143 0.90
LOLIPOP_OmniEE 0.388 0.89
LOLIPOP_IAP 0.805 0.87
LOLIPOP_IA610 0.738 0.89
LOLIPOP_IA317 0.527 0.90
LOLIPOP_EWP 0.158 0.77
LOLIPOP_EWA 0.921 0.75
LOLIPOP_EW610 0.407 0.74
LLS 0.940 0.73
LLD 0.684 0.73
KORCULA 0.056 0.78
INT 1.30e−08 0.74
FR97 0.084 0.83
FR07 0.365 0.82
EUGENDA 0.614 0.74
ERF 0.445 0.74
EGCUT 0.396 0.80
CKB 0.092 0.70
ALSPAC 0.024 0.75
META 5.22e−19 0.76
−0.2
−0.1
0.0
0.1
0.2
BETA

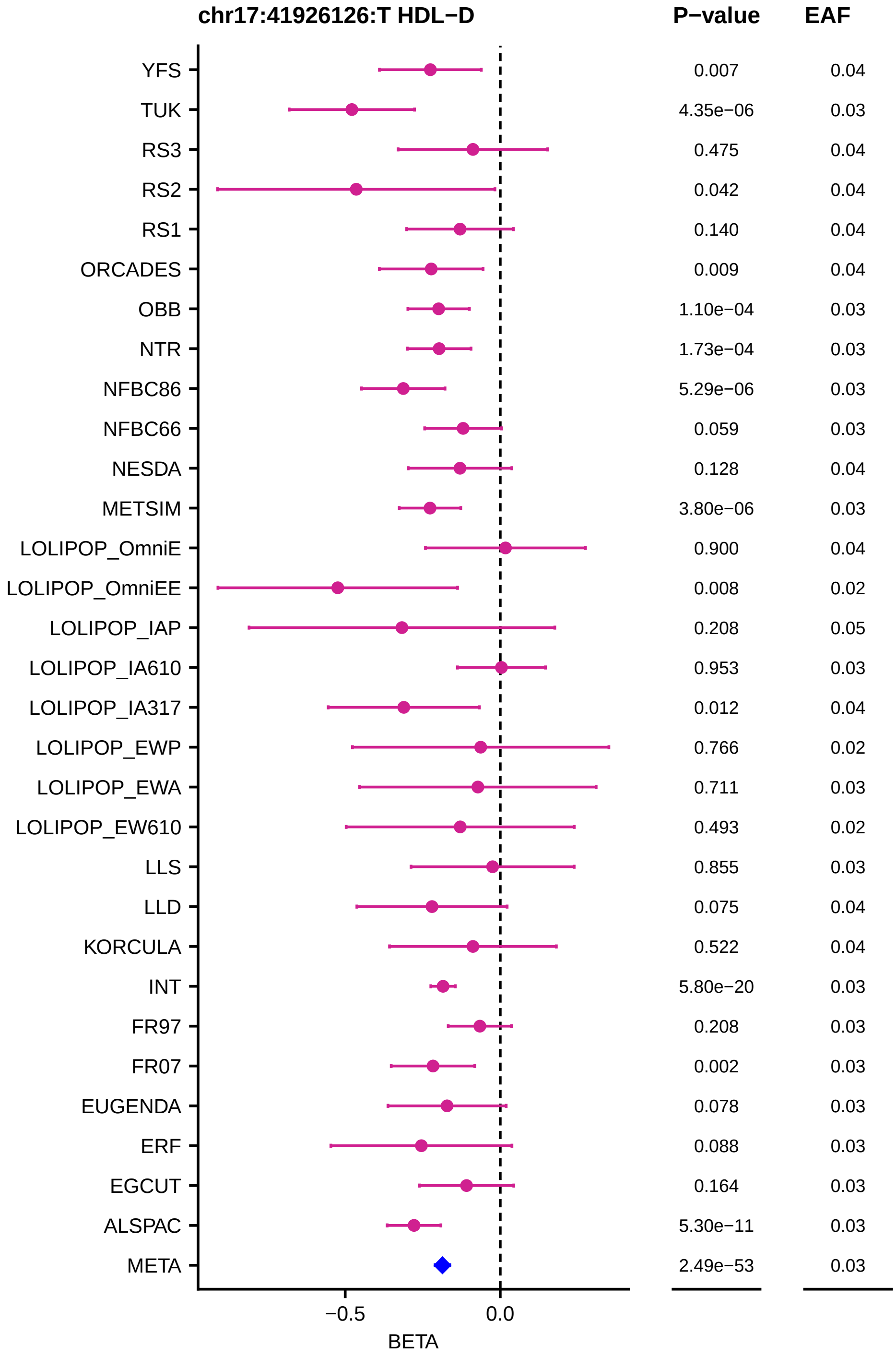
chr17:41926126:T HDL−D
P−value
EAF
YFS 0.007 0.04
TUK 4.35e−06 0.03
RS3 0.475 0.04
RS2 0.042 0.04
RS1 0.140 0.04
ORCADES 0.009 0.04
OBB 1.10e−04 0.03
NTR 1.73e−04 0.03
NFBC86 5.29e−06 0.03
NFBC66 0.059 0.03
NESDA 0.128 0.04
METSIM 3.80e−06 0.03
LOLIPOP_OmniE 0.900 0.04
LOLIPOP_OmniEE 0.008 0.02
LOLIPOP_IAP 0.208 0.05
LOLIPOP_IA610 0.953 0.03
LOLIPOP_IA317 0.012 0.04
LOLIPOP_EWP 0.766 0.02
LOLIPOP_EWA 0.711 0.03
LOLIPOP_EW610 0.493 0.02
LLS 0.855 0.03
LLD 0.075 0.04
KORCULA 0.522 0.04
INT 5.80e−20 0.03
FR97 0.208 0.03
FR07 0.002 0.03
EUGENDA 0.078 0.03
ERF 0.088 0.03
EGCUT 0.164 0.03
ALSPAC 5.30e−11 0.03
META 2.49e−53 0.03
−0.5
0.0
BETA

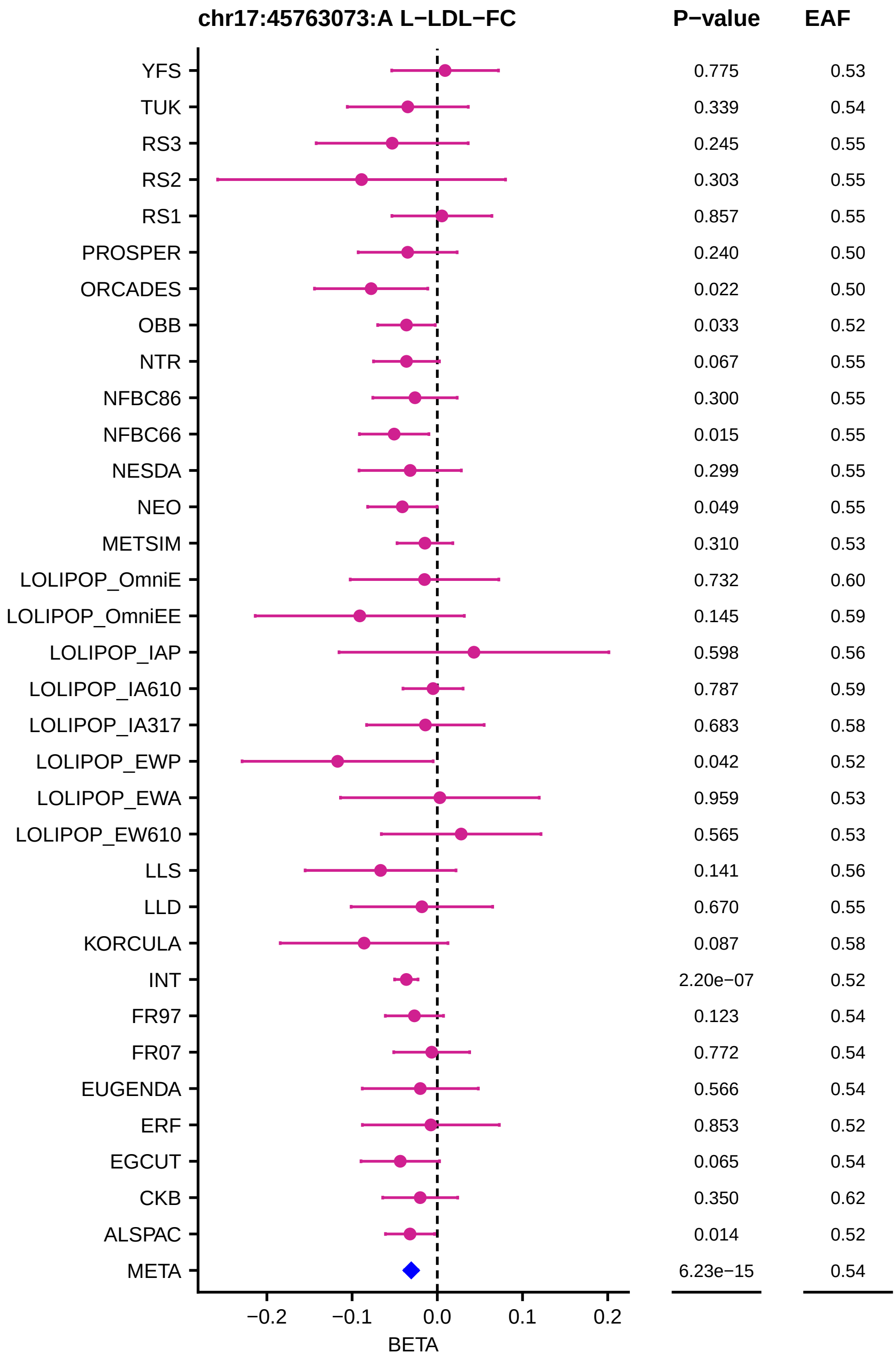

chr17:45763073:A L−LDL−FC
P−value
EAF
YFS 0.775 0.53
TUK 0.339 0.54
RS3 0.245 0.55
RS2 0.303 0.55
RS1 0.857 0.55
PROSPER 0.240 0.50
ORCADES 0.022 0.50
OBB 0.033 0.52
NTR 0.067 0.55
NFBC86 0.300 0.55
NFBC66 0.015 0.55
NESDA 0.299 0.55
NEO 0.049 0.55
METSIM 0.310 0.53
LOLIPOP_OmniE 0.732 0.60
LOLIPOP_OmniEE 0.145 0.59
LOLIPOP_IAP 0.598 0.56
LOLIPOP_IA610 0.787 0.59
LOLIPOP_IA317 0.683 0.58
LOLIPOP_EWP 0.042 0.52
LOLIPOP_EWA 0.959 0.53
LOLIPOP_EW610 0.565 0.53
LLS 0.141 0.56
LLD 0.670 0.55
KORCULA 0.087 0.58
INT 2.20e−07 0.52
FR97 0.123 0.54
FR07 0.772 0.54
EUGENDA 0.566 0.54
ERF 0.853 0.52
EGCUT 0.065 0.54
CKB 0.350 0.62
ALSPAC 0.014 0.52
META 6.23e−15 0.54
−0.2
−0.1
0.0
0.1
0.2
BETA

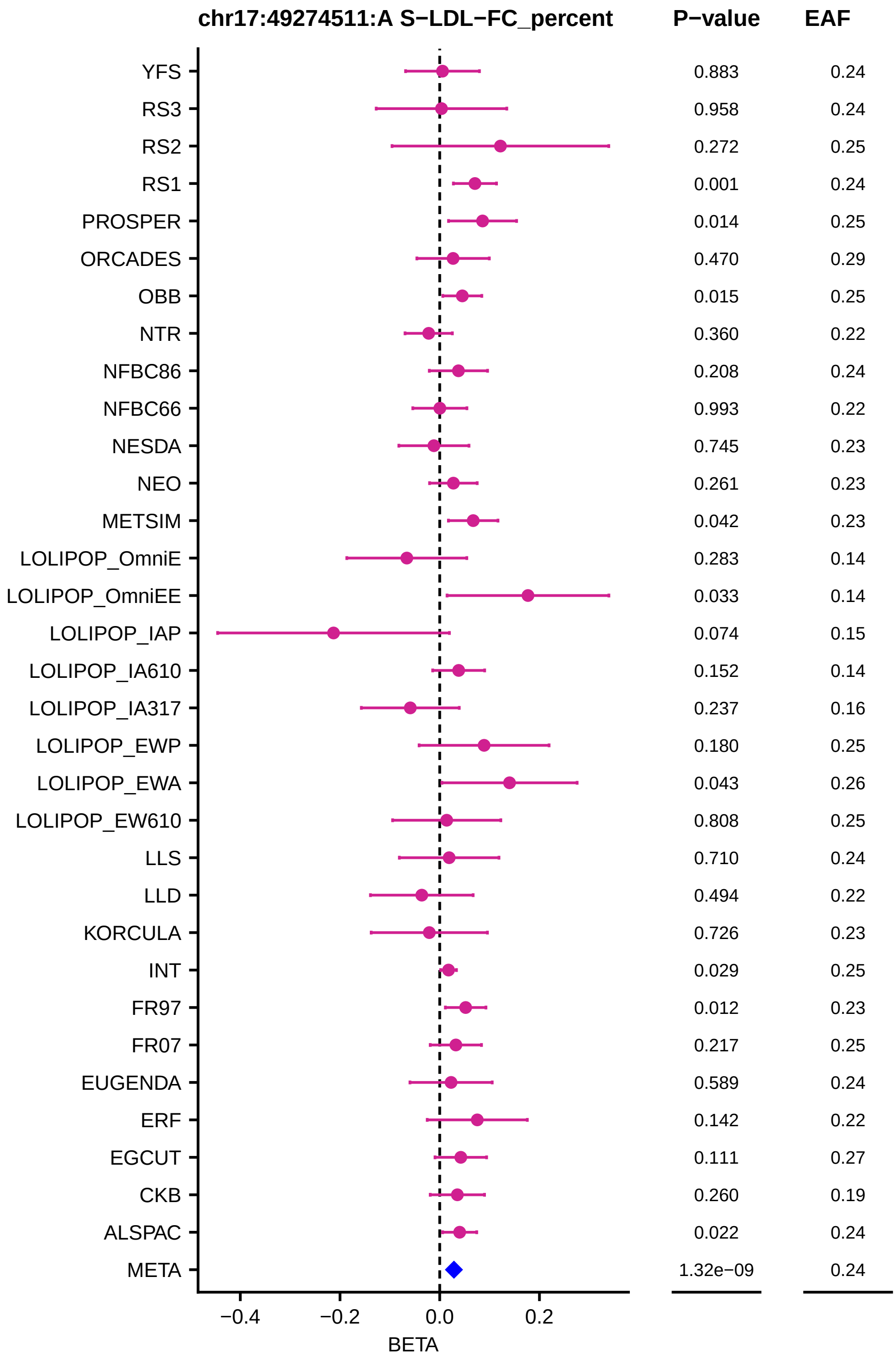

chr17:49274511:A S−LDL−FC_percent
P−value
EAF
YFS 0.883 0.24
RS3 0.958 0.24
RS2 0.272 0.25
RS1 0.001 0.24
PROSPER 0.014 0.25
ORCADES 0.470 0.29
OBB 0.015 0.25
NTR 0.360 0.22
NFBC86 0.208 0.24
NFBC66 0.993 0.22
NESDA 0.745 0.23
NEO 0.261 0.23
METSIM 0.042 0.23
LOLIPOP_OmniE 0.283 0.14
LOLIPOP_OmniEE 0.033 0.14
LOLIPOP_IAP 0.074 0.15
LOLIPOP_IA610 0.152 0.14
LOLIPOP_IA317 0.237 0.16
LOLIPOP_EWP 0.180 0.25
LOLIPOP_EWA 0.043 0.26
LOLIPOP_EW610 0.808 0.25
LLS 0.710 0.24
LLD 0.494 0.22
KORCULA 0.726 0.23
INT 0.029 0.25
FR97 0.012 0.23
FR07 0.217 0.25
EUGENDA 0.589 0.24
ERF 0.142 0.22
EGCUT 0.111 0.27
CKB 0.260 0.19
ALSPAC 0.022 0.24
META 1.32e−09 0.24
−0.4
−0.2
0.0
0.2
BETA

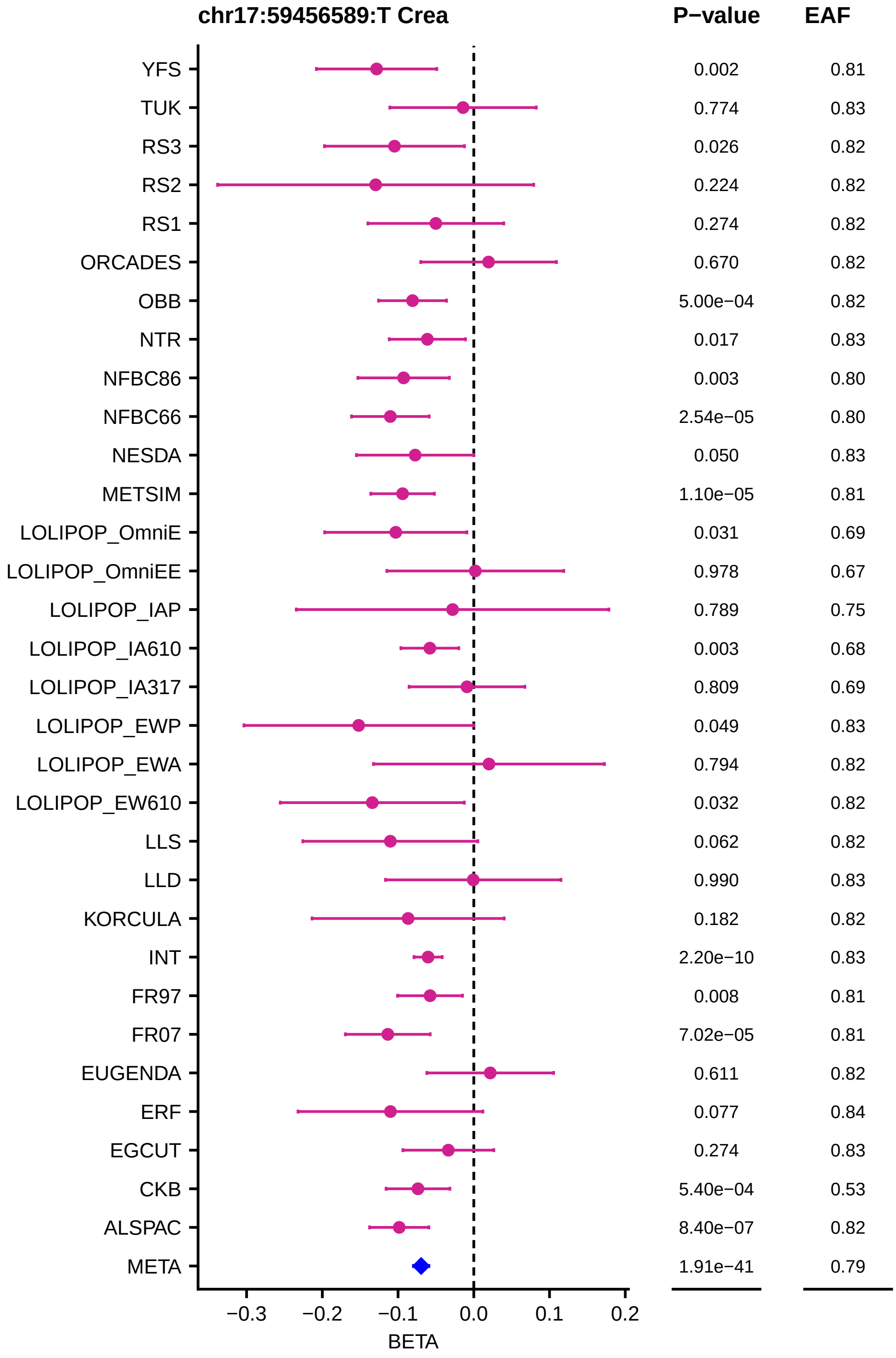
chr17:59456589:T Crea
P−value
EAF
YFS
TUK
RS3
RS2
RS1
ORCADES
OBB
NTR
NFBC86
NFBC66
NESDA
METSIM
LOLIPOP_OmniE
LOLIPOP_OmniEE
LOLIPOP_IAP
LOLIPOP_IA610
LOLIPOP_IA317
LOLIPOP_EWP
LOLIPOP_EWA
LOLIPOP_EW610
LLS
LLD
KORCULA
INT
FR97
FR07
EUGENDA
ERF
EGCUT
CKB
ALSPAC
META
0.002 0.81
0.774 0.83
0.026 0.82
0.224 0.82
0.274 0.82
0.670 0.82
5.00e−04 0.82
0.017 0.83
0.003 0.80
2.54e−05 0.80
0.050 0.83
1.10e−05 0.81
0.031 0.69
0.978 0.67
0.789 0.75
0.003 0.68
0.809 0.69
0.049 0.83
0.794 0.82
0.032 0.82
0.062 0.82
0.990 0.83
0.182 0.82
2.20e−10 0.83
0.008 0.81
7.02e−05 0.81
0.611 0.82
0.077 0.84
0.274 0.83
5.40e−04 0.53
8.40e−07 0.82
1.91e−41 0.79
−0.3
−0.2
−0.1
0.0
0.1
0.2
BETA

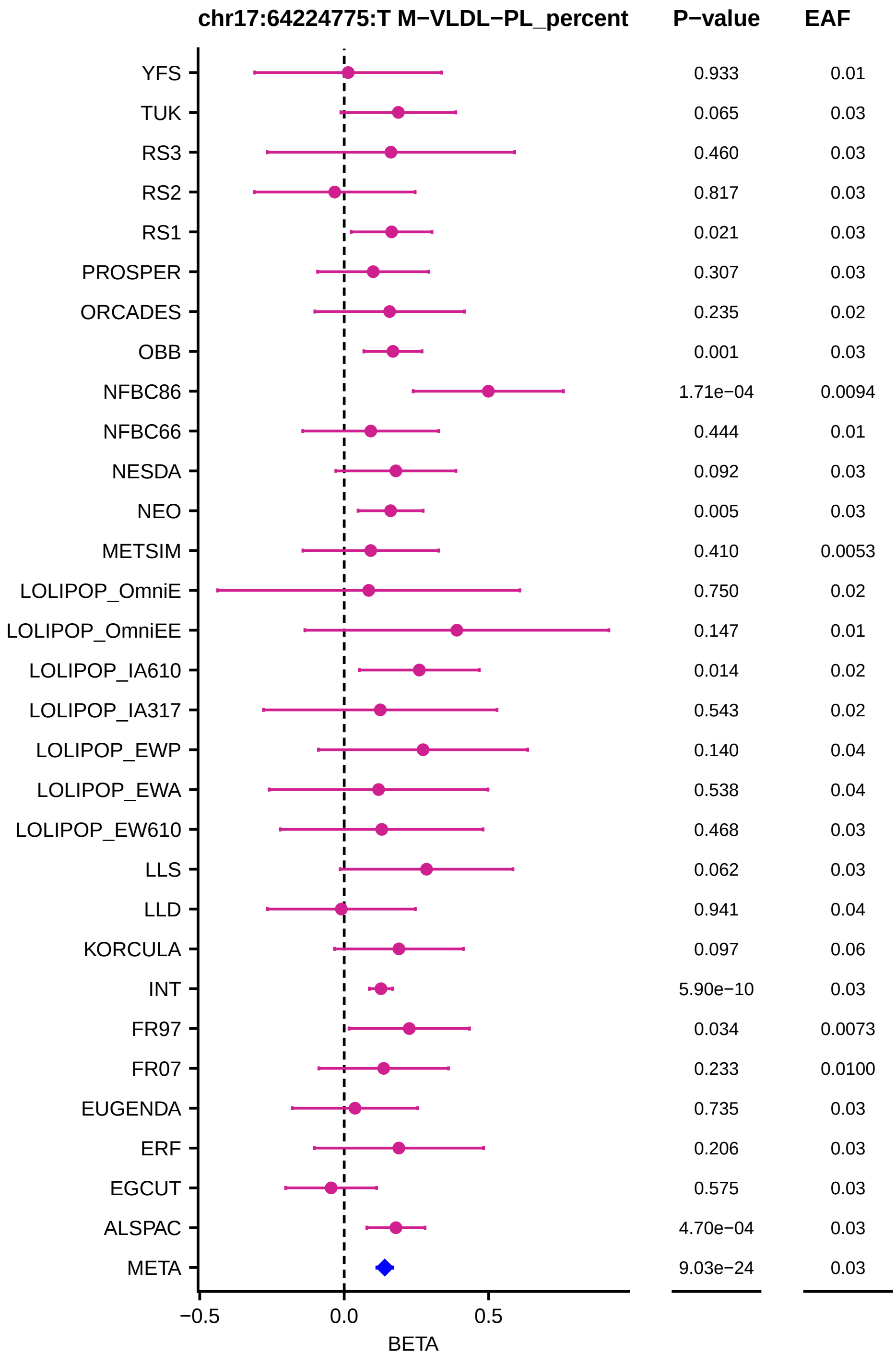
chr17:64224775:T M−VLDL−PL_percent
P−value
EAF
YFS 0.933 0.01
TUK 0.065 0.03
RS3 0.460 0.03
RS2 0.817 0.03
RS1 0.021 0.03
PROSPER 0.307 0.03
ORCADES 0.235 0.02
OBB 0.001 0.03
NFBC86 1.71e−04 0.0094
NFBC66 0.444 0.01
NESDA 0.092 0.03
NEO 0.005 0.03
METSIM 0.410 0.0053
LOLIPOP_OmniE 0.750 0.02
LOLIPOP_OmniEE 0.147 0.01
LOLIPOP_IA610 0.014 0.02
LOLIPOP_IA317 0.543 0.02
LOLIPOP_EWP 0.140 0.04
LOLIPOP_EWA 0.538 0.04
LOLIPOP_EW610 0.468 0.03
LLS 0.062 0.03
LLD 0.941 0.04
KORCULA 0.097 0.06
INT 5.90e−10 0.03
FR97 0.034 0.0073
FR07 0.233 0.0100
EUGENDA 0.735 0.03
ERF 0.206 0.03
EGCUT 0.575 0.03
ALSPAC 4.70e−04 0.03
META 9.03e−24 0.03
−0.5
0.0
0.5
BETA

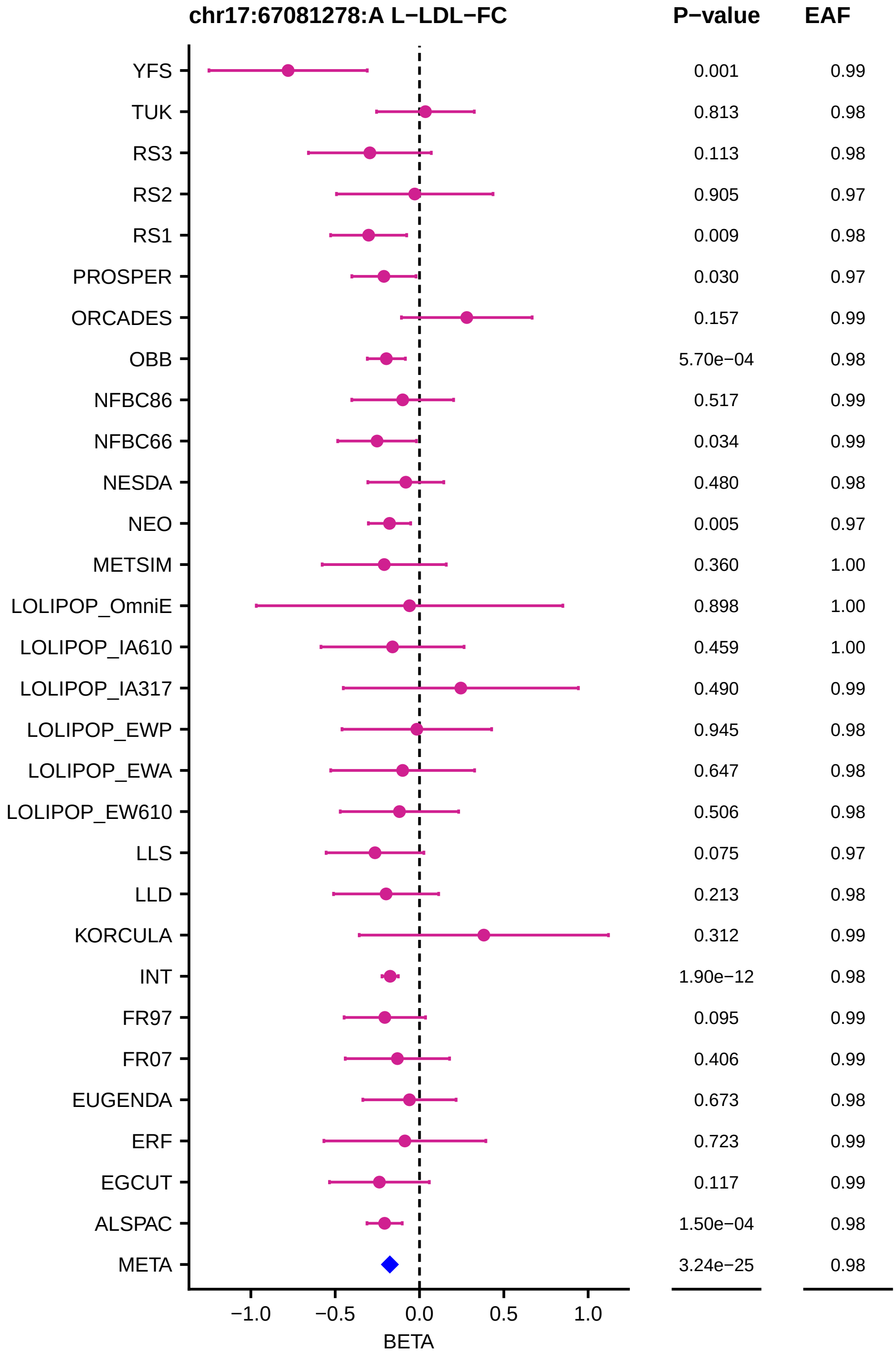
chr17:67081278:A L−LDL−FC
P−value
EAF
YFS 0.001 0.99
TUK 0.813 0.98
RS3 0.113 0.98
RS2 0.905 0.97
RS1 0.009 0.98
PROSPER 0.030 0.97
ORCADES 0.157 0.99
OBB 5.70e−04 0.98
NFBC86 0.517 0.99
NFBC66 0.034 0.99
NESDA 0.480 0.98
NEO 0.005 0.97
METSIM 0.360 1.00
LOLIPOP_OmniE 0.898 1.00
LOLIPOP_IA610 0.459 1.00
LOLIPOP_IA317 0.490 0.99
LOLIPOP_EWP 0.945 0.98
LOLIPOP_EWA 0.647 0.98
LOLIPOP_EW610 0.506 0.98
LLS 0.075 0.97
LLD 0.213 0.98
KORCULA 0.312 0.99
INT 1.90e−12 0.98
FR97 0.095 0.99
FR07 0.406 0.99
EUGENDA 0.673 0.98
ERF 0.723 0.99
EGCUT 0.117 0.99
ALSPAC 1.50e−04 0.98
META 3.24e−25 0.98
−1.0 −0.5 0.0 0.5 1.0
BETA

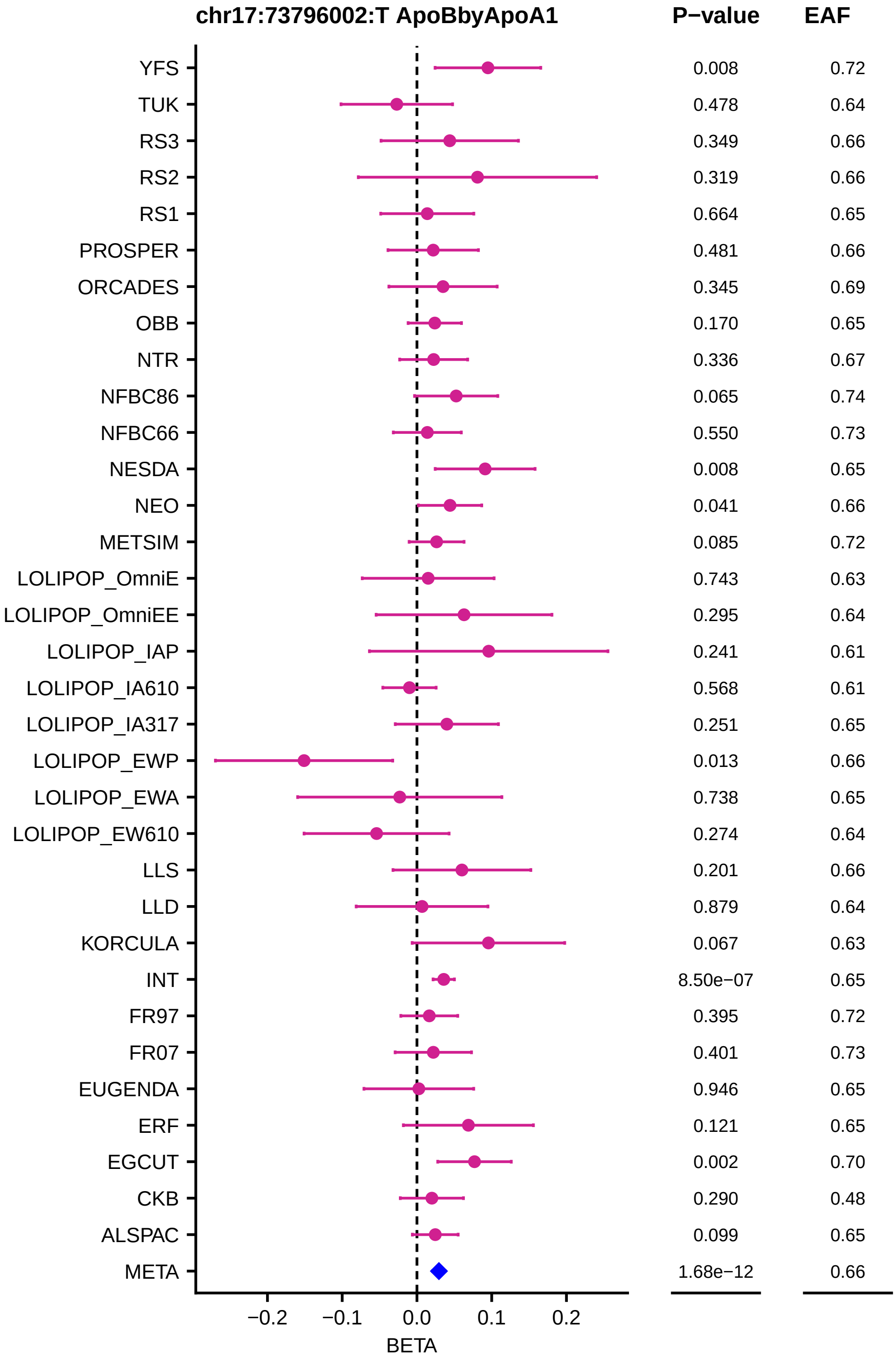
chr17:73796002:T ApoBbyApoA1
P−value
EAF
YFS 0.008 0.72
TUK 0.478 0.64
RS3 0.349 0.66
RS2 0.319 0.66
RS1 0.664 0.65
PROSPER 0.481 0.66
ORCADES 0.345 0.69
OBB 0.170 0.65
NTR 0.336 0.67
NFBC86 0.065 0.74
NFBC66 0.550 0.73
NESDA 0.008 0.65
NEO 0.041 0.66
METSIM 0.085 0.72
LOLIPOP_OmniE 0.743 0.63
LOLIPOP_OmniEE 0.295 0.64
LOLIPOP_IAP 0.241 0.61
LOLIPOP_IA610 0.568 0.61
LOLIPOP_IA317 0.251 0.65
LOLIPOP_EWP 0.013 0.66
LOLIPOP_EWA 0.738 0.65
LOLIPOP_EW610 0.274 0.64
LLS 0.201 0.66
LLD 0.879 0.64
KORCULA 0.067 0.63
INT 8.50e−07 0.65
FR97 0.395 0.72
FR07 0.401 0.73
EUGENDA 0.946 0.65
ERF 0.121 0.65
EGCUT 0.002 0.70
CKB 0.290 0.48
ALSPAC 0.099 0.65
META 1.68e−12 0.66
−0.2
−0.1
0.0
0.1
0.2
BETA

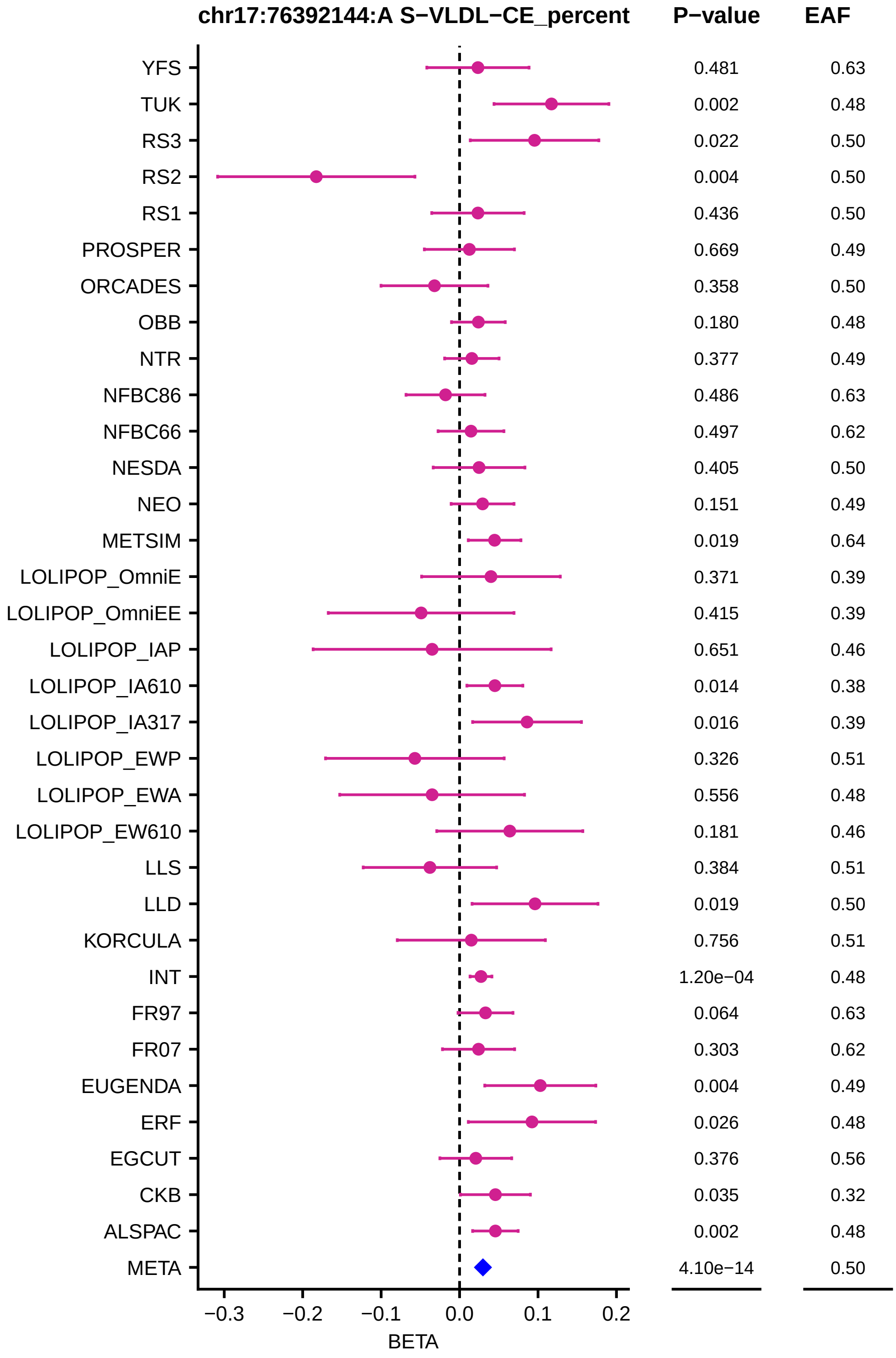
chr17:76392144:A S−VLDL−CE_percent
P−value
EAF
YFS 0.481 0.63
TUK 0.002 0.48
RS3 0.022 0.50
RS2 0.004 0.50
RS1 0.436 0.50
PROSPER 0.669 0.49
ORCADES 0.358 0.50
OBB 0.180 0.48
NTR 0.377 0.49
NFBC86 0.486 0.63
NFBC66 0.497 0.62
NESDA 0.405 0.50
NEO 0.151 0.49
METSIM 0.019 0.64
LOLIPOP_OmniE 0.371 0.39
LOLIPOP_OmniEE 0.415 0.39
LOLIPOP_IAP 0.651 0.46
LOLIPOP_IA610 0.014 0.38
LOLIPOP_IA317 0.016 0.39
LOLIPOP_EWP 0.326 0.51
LOLIPOP_EWA 0.556 0.48
LOLIPOP_EW610 0.181 0.46
LLS 0.384 0.51
LLD 0.019 0.50
KORCULA 0.756 0.51
INT 1.20e−04 0.48
FR97 0.064 0.63
FR07 0.303 0.62
EUGENDA 0.004 0.49
ERF 0.026 0.48
EGCUT 0.376 0.56
CKB 0.035 0.32
ALSPAC 0.002 0.48
META 4.10e−14 0.50
−0.3
−0.2
−0.1
0.0
0.1
0.2
BETA

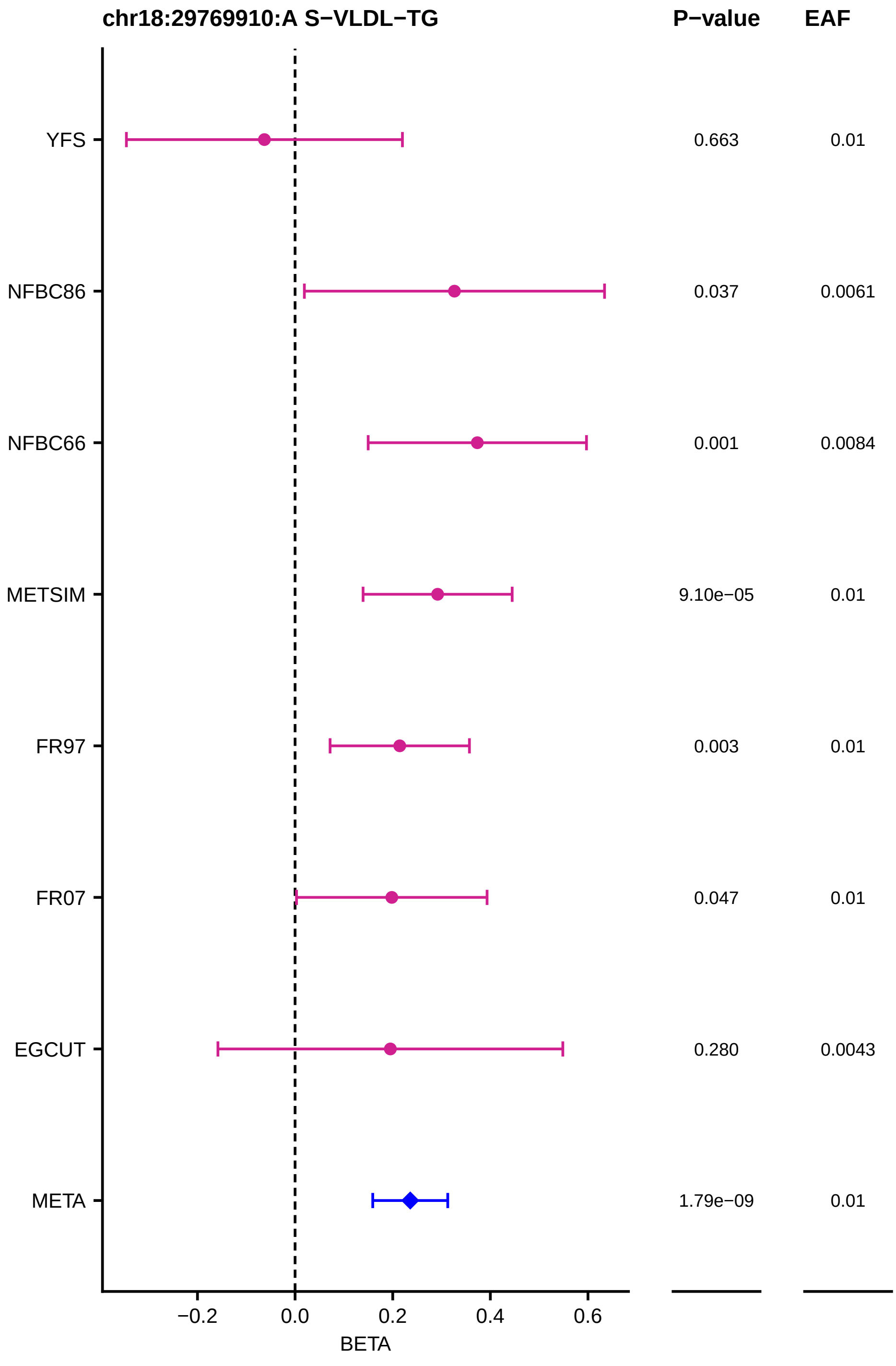

chr18:29769910:A S−VLDL−TG
P−value
EAF
YFS
NFBC86
NFBC66
METSIM
FR97
FR07
EGCUT
META
0.663
0.037
0.001
9.10e−05
0.003
0.047
0.280
1.79e−09
0.01
0.0061
0.0084
0.01
0.01
0.01
0.0043
0.01
−0.2
0.0
0.2
0.4
0.6
BETA

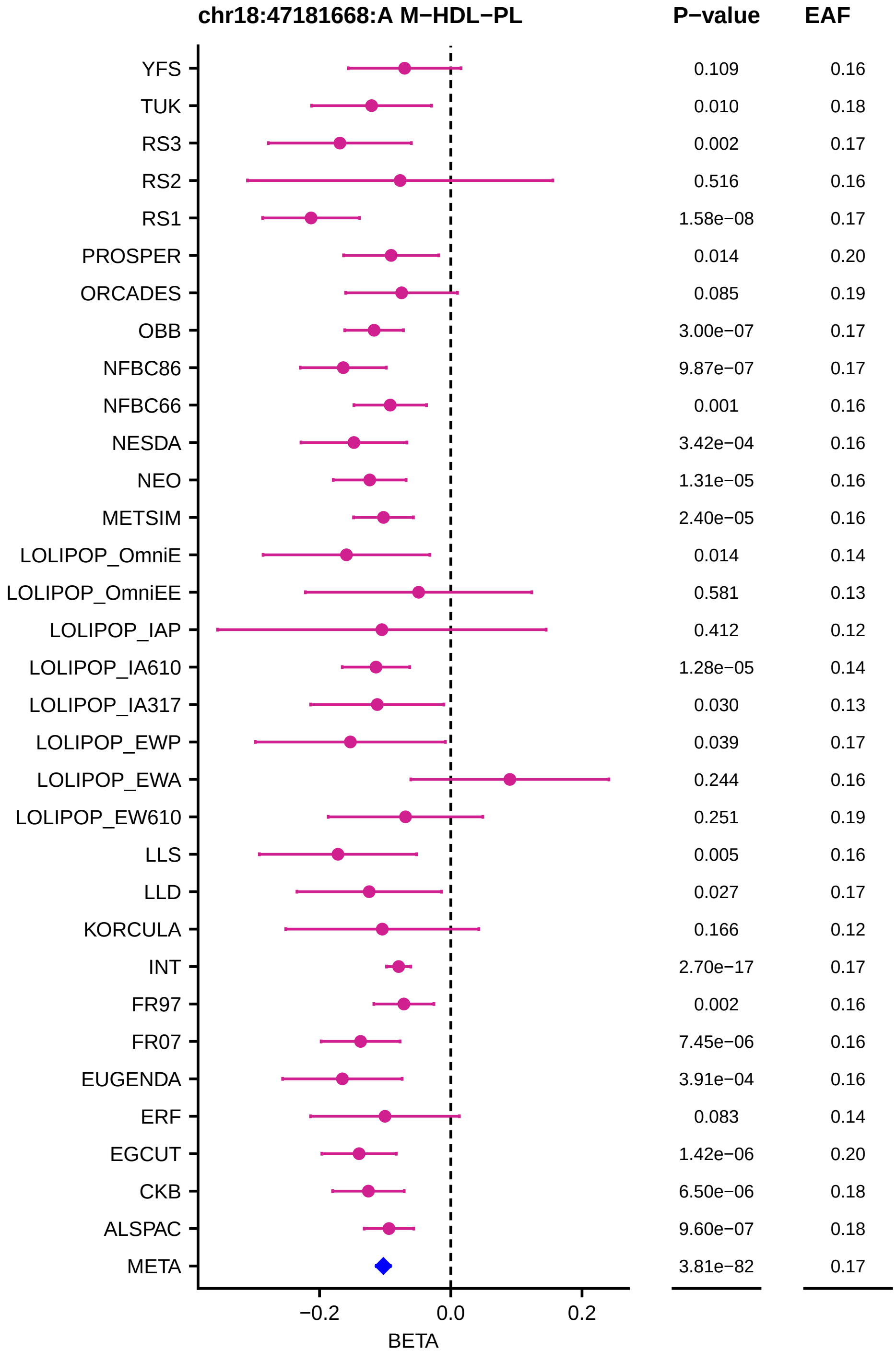
chr18:47181668:A M−HDL−PL
P−value
EAF
YFS 0.109 0.16
TUK 0.010 0.18
RS3 0.002 0.17
RS2 0.516 0.16
RS1 1.58e−08 0.17
PROSPER 0.014 0.20
ORCADES 0.085 0.19
OBB 3.00e−07 0.17
NFBC86 9.87e−07 0.17
NFBC66 0.001 0.16
NESDA 3.42e−04 0.16
NEO 1.31e−05 0.16
METSIM 2.40e−05 0.16
LOLIPOP_OmniE 0.014 0.14
LOLIPOP_OmniEE 0.581 0.13
LOLIPOP_IAP 0.412 0.12
LOLIPOP_IA610 1.28e−05 0.14
LOLIPOP_IA317 0.030 0.13
LOLIPOP_EWP 0.039 0.17
LOLIPOP_EWA 0.244 0.16
LOLIPOP_EW610 0.251 0.19
LLS 0.005 0.16
LLD 0.027 0.17
KORCULA 0.166 0.12
INT 2.70e−17 0.17
FR97 0.002 0.16
FR07 7.45e−06 0.16
EUGENDA 3.91e−04 0.16
ERF 0.083 0.14
EGCUT 1.42e−06 0.20
CKB 6.50e−06 0.18
ALSPAC 9.60e−07 0.18
META 3.81e−82 0.17
−0.2
0.0
0.2
BETA

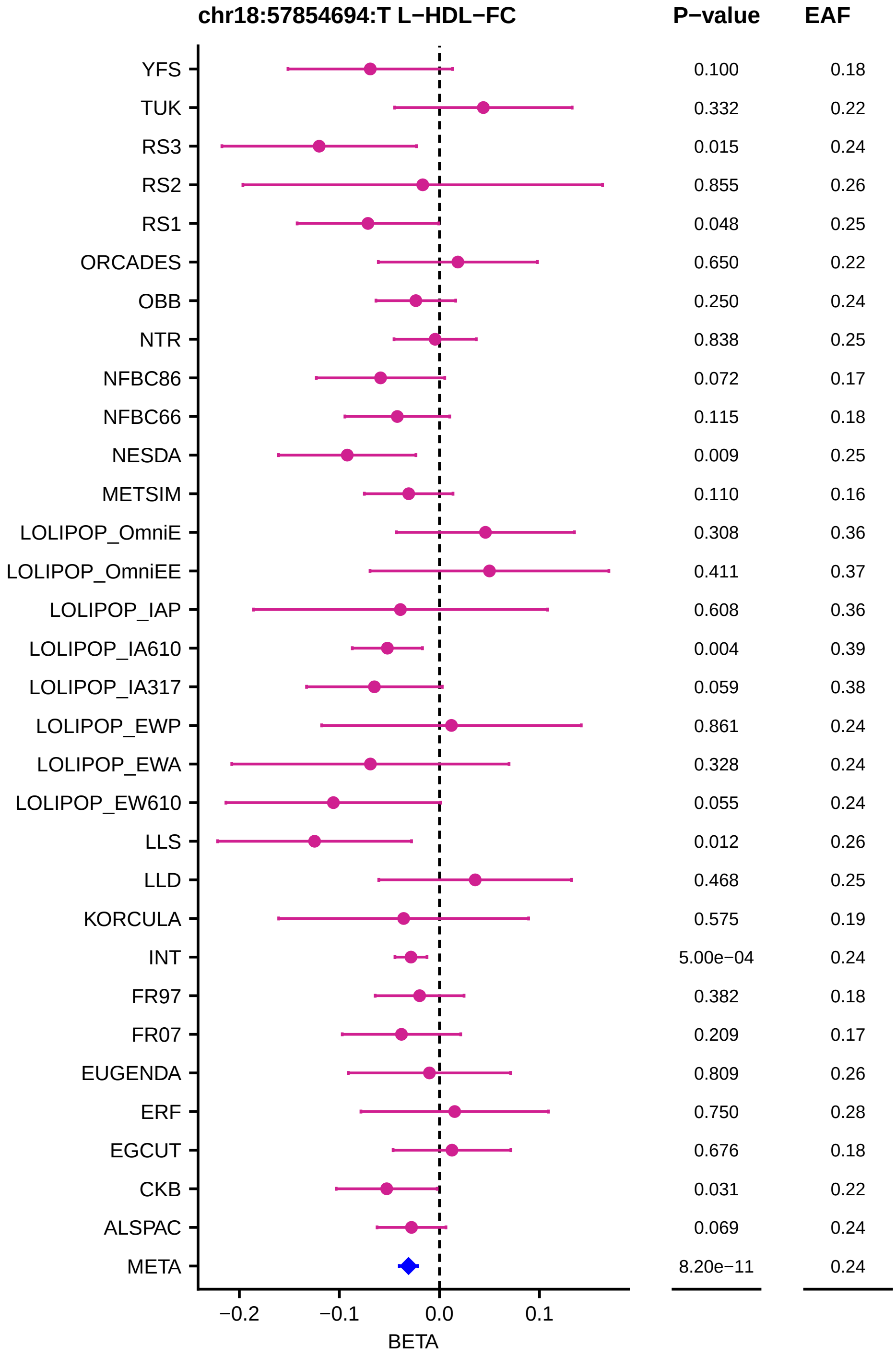
chr18:57854694:T L−HDL−FC
P−value
EAF
YFS 0.100 0.18
TUK 0.332 0.22
RS3 0.015 0.24
RS2 0.855 0.26
RS1 0.048 0.25
ORCADES 0.650 0.22
OBB 0.250 0.24
NTR 0.838 0.25
NFBC86 0.072 0.17
NFBC66 0.115 0.18
NESDA 0.009 0.25
METSIM 0.110 0.16
LOLIPOP_OmniE 0.308 0.36
LOLIPOP_OmniEE 0.411 0.37
LOLIPOP_IAP 0.608 0.36
LOLIPOP_IA610 0.004 0.39
LOLIPOP_IA317 0.059 0.38
LOLIPOP_EWP 0.861 0.24
LOLIPOP_EWA 0.328 0.24
LOLIPOP_EW610 0.055 0.24
LLS 0.012 0.26
LLD 0.468 0.25
KORCULA 0.575 0.19
INT 5.00e−04 0.24
FR97 0.382 0.18
FR07 0.209 0.17
EUGENDA 0.809 0.26
ERF 0.750 0.28
EGCUT 0.676 0.18
CKB 0.031 0.22
ALSPAC 0.069 0.24
META 8.20e−11 0.24
−0.2
−0.1
0.0
0.1
BETA

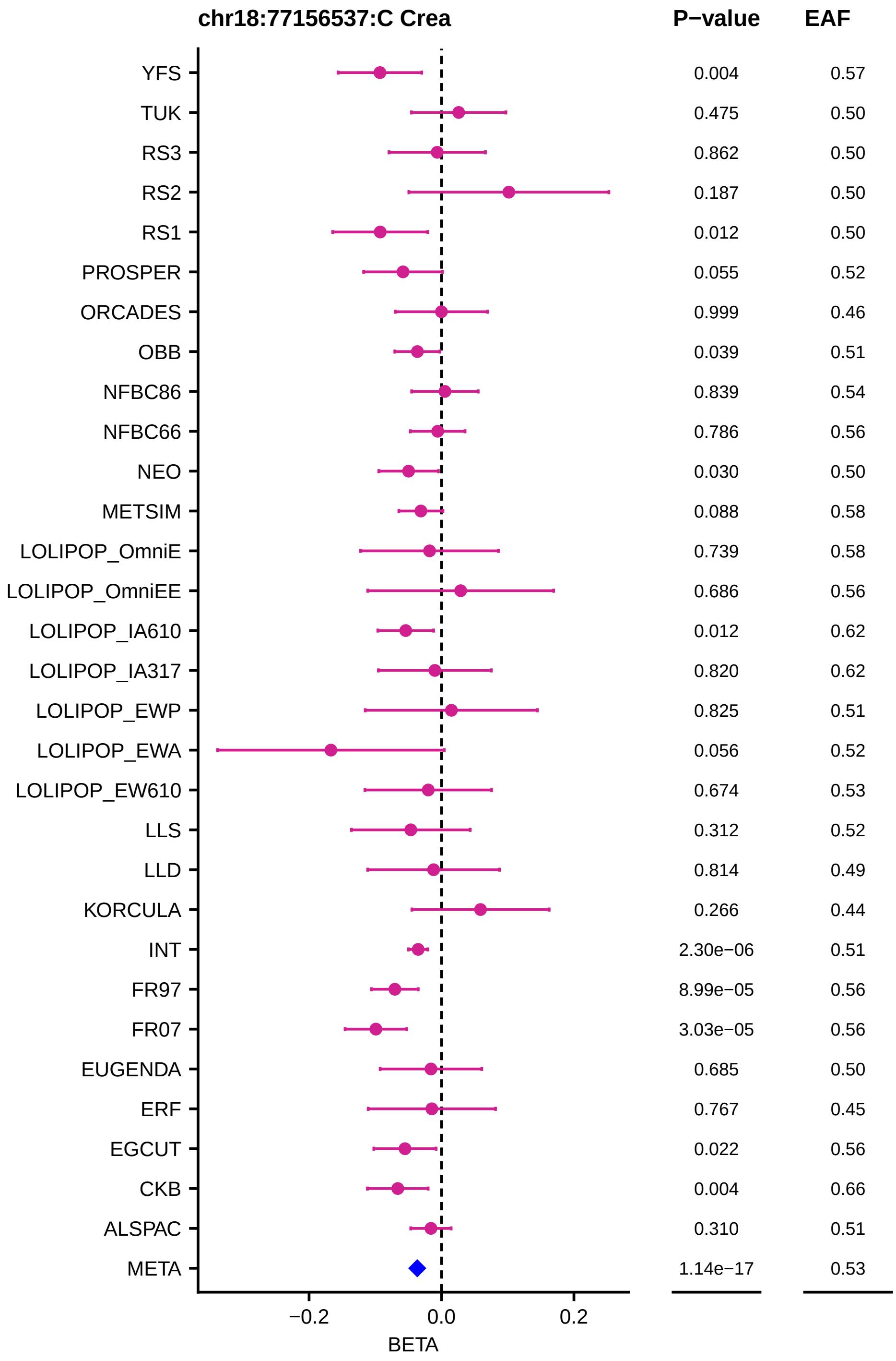
chr18:77156537:C Crea
P−value
EAF
YFS
TUK
RS3
RS2
RS1
PROSPER
ORCADES
OBB
NFBC86
NFBC66
NEO
METSIM
LOLIPOP_OmniE
LOLIPOP_OmniEE
LOLIPOP_IA610
LOLIPOP_IA317
LOLIPOP_EWP
LOLIPOP_EWA
LOLIPOP_EW610
LLS
LLD
KORCULA
INT
FR97
FR07
EUGENDA
ERF
EGCUT
CKB
ALSPAC
META
0.004 0.57
0.475 0.50
0.862 0.50
0.187 0.50
0.012 0.50
0.055 0.52
0.999 0.46
0.039 0.51
0.839 0.54
0.786 0.56
0.030 0.50
0.088 0.58
0.739 0.58
0.686 0.56
0.012 0.62
0.820 0.62
0.825 0.51
0.056 0.52
0.674 0.53
0.312 0.52
0.814 0.49
0.266 0.44
2.30e−06 0.51
8.99e−05 0.56
3.03e−05 0.56
0.685 0.50
0.767 0.45
0.022 0.56
0.004 0.66
0.310 0.51
1.14e−17 0.53
−0.2
0.0
0.2
BETA

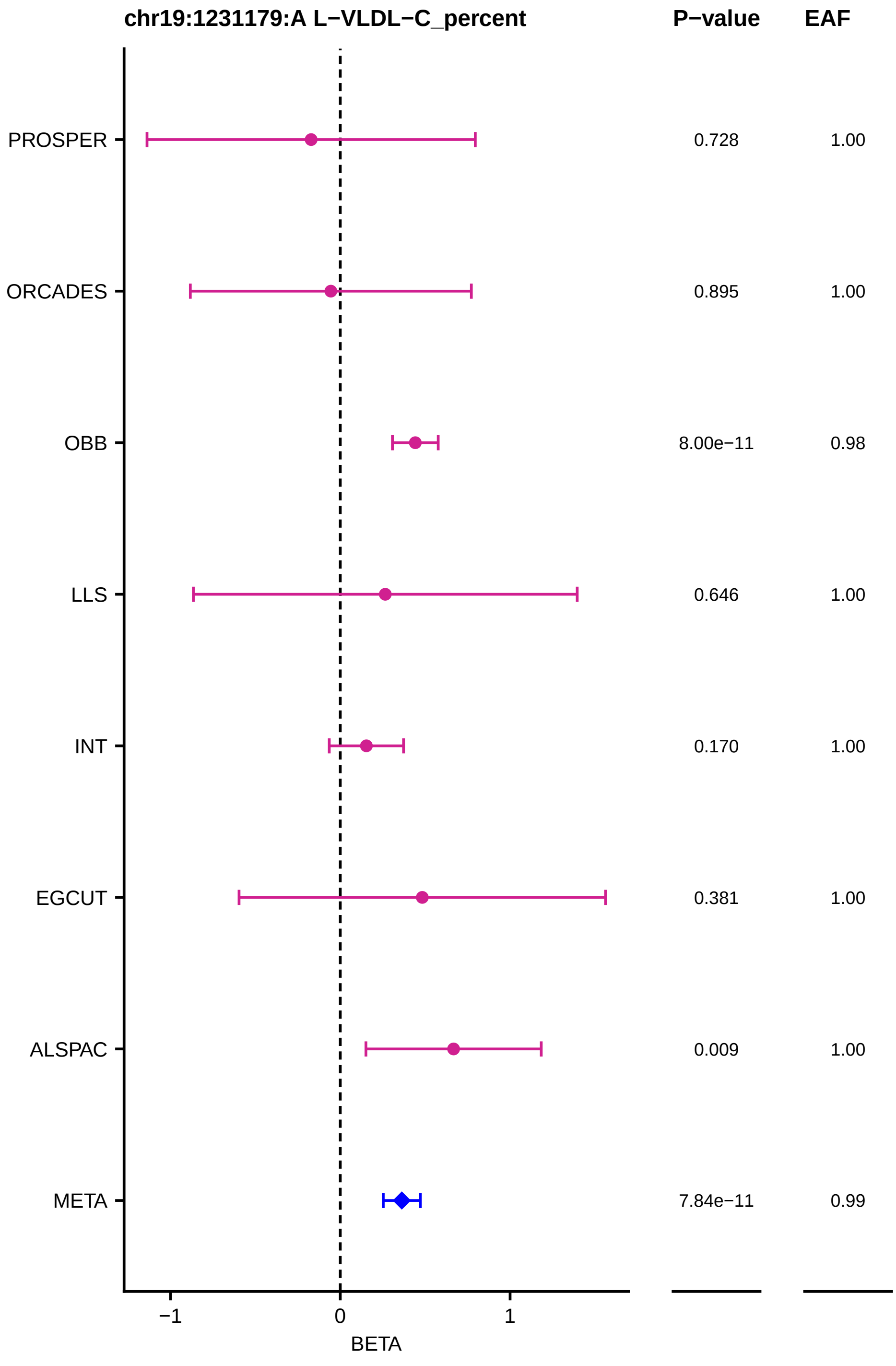

chr19:1231179:A L−VLDL−C_percent
P−value
EAF
PROSPER
0.728
1.00
ORCADES
0.895
1.00
OBB
8.00e−11
0.98
LLS
0.646
1.00
INT
0.170
1.00
EGCUT
0.381
1.00
ALSPAC
0.009
1.00
META
7.84e−11
0.99
−1
0
1
BETA

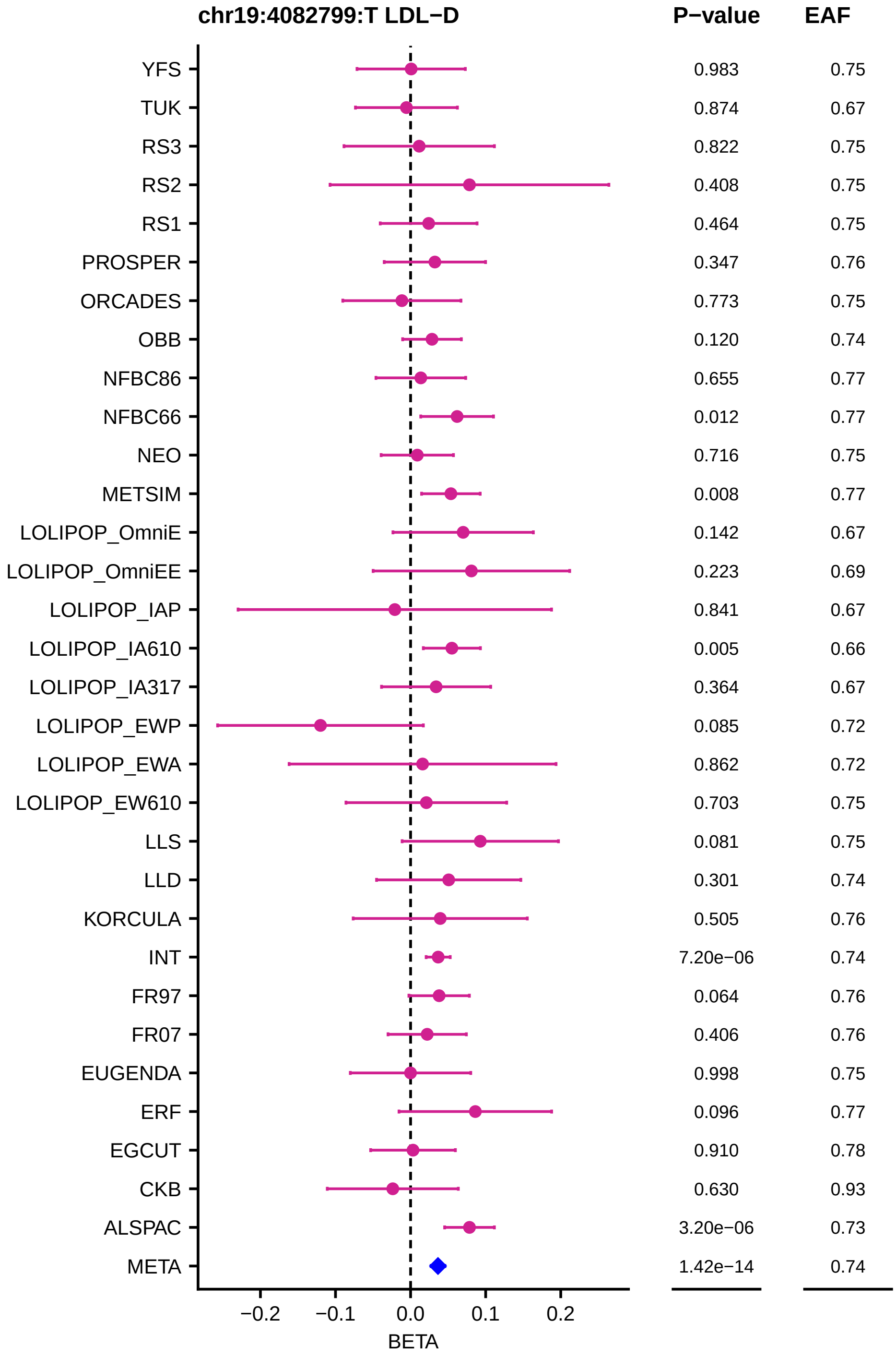
chr19:4082799:T LDL−D
P−value
EAF
YFS
0.983
0.75
TUK
0.874
0.67
RS3
0.822
0.75
RS2
0.408
0.75
RS1
0.464
0.75
PROSPER
0.347
0.76
ORCADES
0.773
0.75
OBB
0.120
0.74
NFBC86
0.655
0.77
NFBC66
0.012
0.77
NEO
0.716
0.75
METSIM
0.008
0.77
LOLIPOP_OmniE
0.142
0.67
LOLIPOP_OmniEE
0.223
0.69
LOLIPOP_IAP
0.841
0.67
LOLIPOP_IA610
0.005
0.66
LOLIPOP_IA317
0.364
0.67
LOLIPOP_EWP
0.085
0.72
LOLIPOP_EWA
0.862
0.72
LOLIPOP_EW610
0.703
0.75
LLS
0.081
0.75
LLD
0.301
0.74
KORCULA
0.505
0.76
INT
7.20e−06
0.74
FR97
0.064
0.76
FR07
0.406
0.76
EUGENDA
0.998
0.75
ERF
0.096
0.77
EGCUT
0.910
0.78
CKB
0.630
0.93
ALSPAC
3.20e−06
0.73
META
1.42e−14
0.74
−0.2
−0.1
0.0
0.1
0.2
BETA

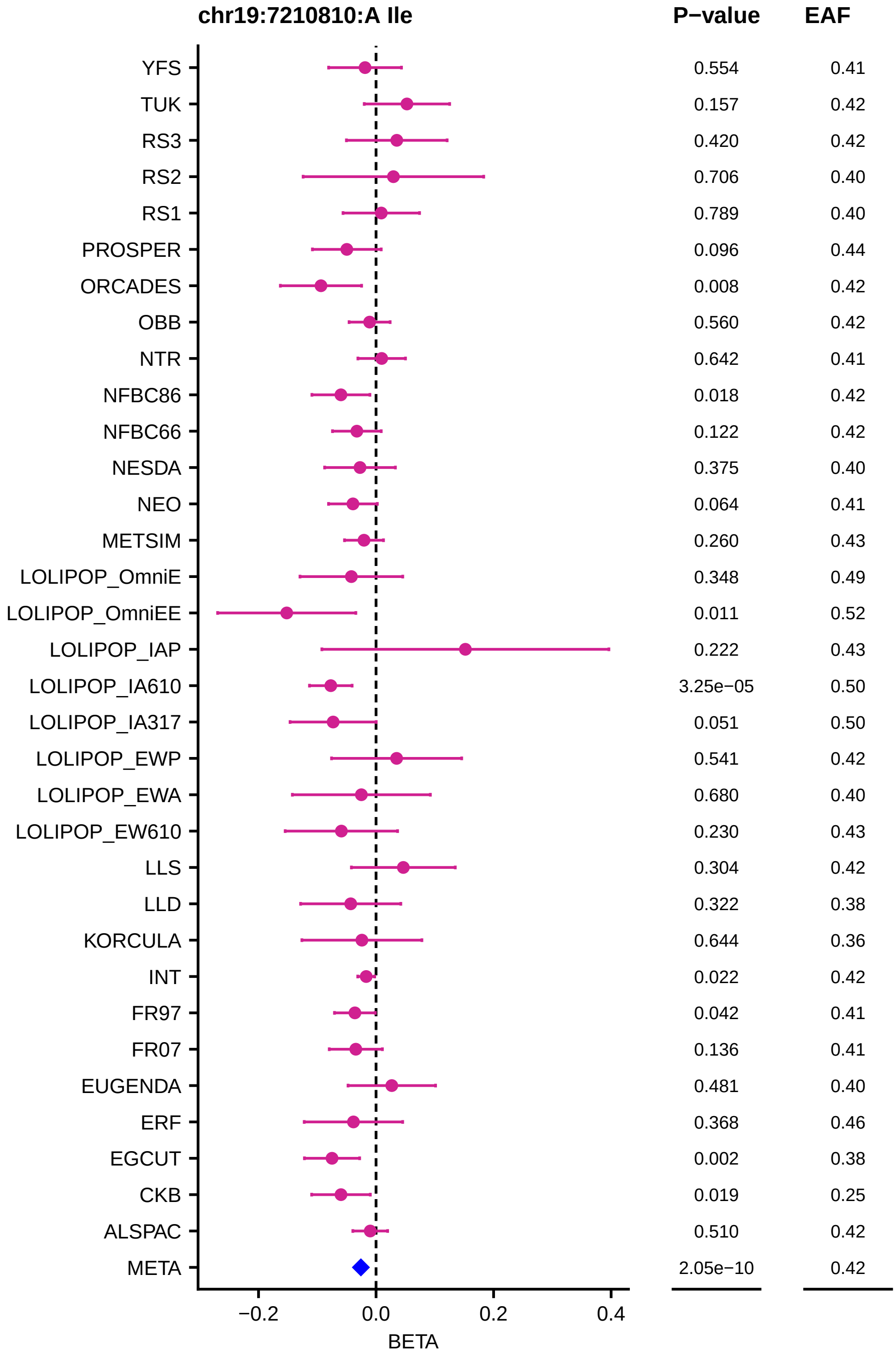

chr19:7210810:A Ile
P−value
EAF
YFS 0.554 0.41
TUK 0.157 0.42
RS3 0.420 0.42
RS2 0.706 0.40
RS1 0.789 0.40
PROSPER 0.096 0.44
ORCADES 0.008 0.42
OBB 0.560 0.42
NTR 0.642 0.41
NFBC86 0.018 0.42
NFBC66 0.122 0.42
NESDA 0.375 0.40
NEO 0.064 0.41
METSIM 0.260 0.43
LOLIPOP_OmniE 0.348 0.49
LOLIPOP_OmniEE 0.011 0.52
LOLIPOP_IAP 0.222 0.43
LOLIPOP_IA610 3.25e−05 0.50
LOLIPOP_IA317 0.051 0.50
LOLIPOP_EWP 0.541 0.42
LOLIPOP_EWA 0.680 0.40
LOLIPOP_EW610 0.230 0.43
LLS 0.304 0.42
LLD 0.322 0.38
KORCULA 0.644 0.36
INT 0.022 0.42
FR97 0.042 0.41
FR07 0.136 0.41
EUGENDA 0.481 0.40
ERF 0.368 0.46
EGCUT 0.002 0.38
CKB 0.019 0.25
ALSPAC 0.510 0.42
META 2.05e−10 0.42
−0.2
0.0
0.2
0.4
BETA

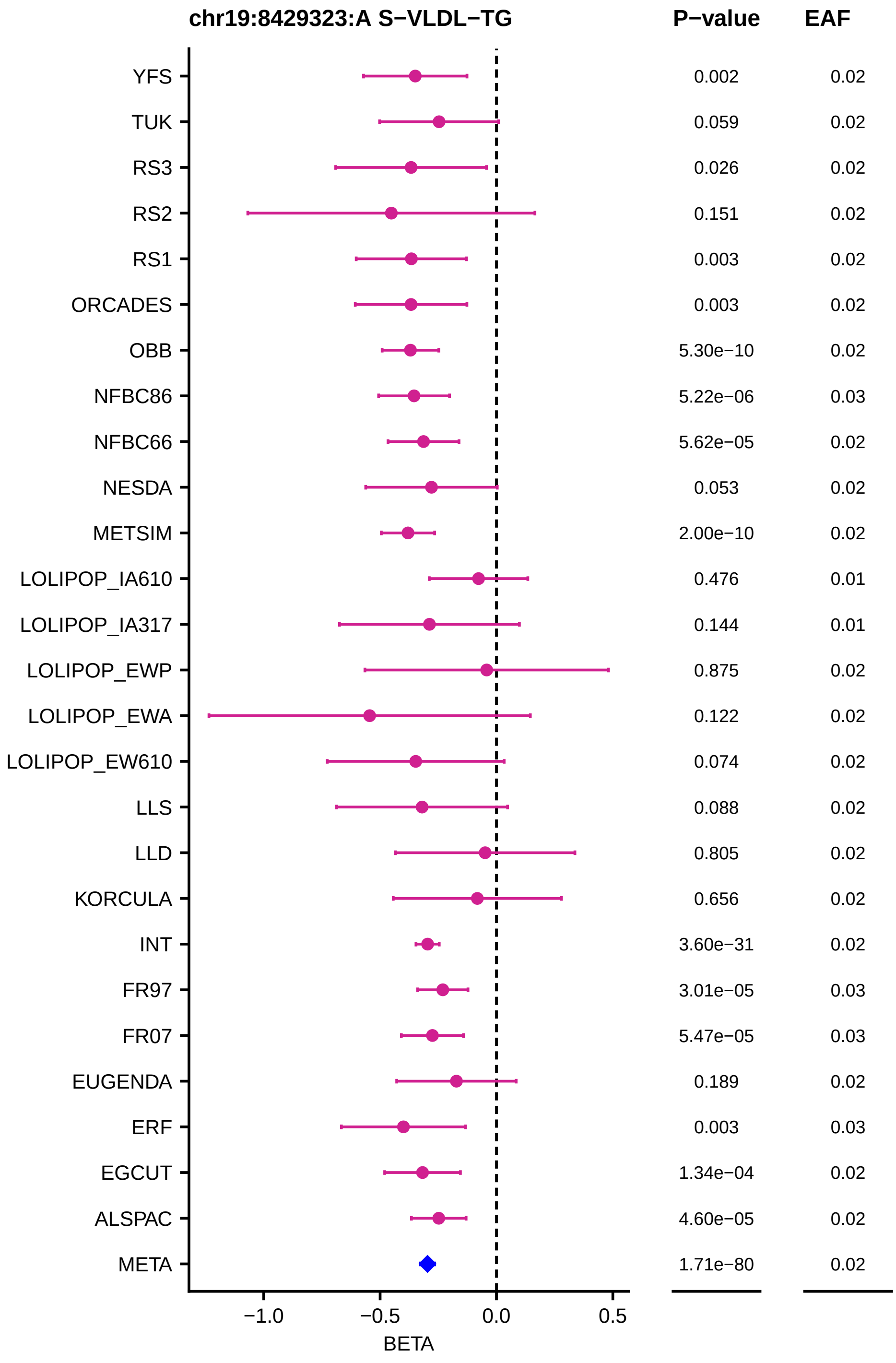
chr19:8429323:A S−VLDL−TG
P−value
EAF
YFS 0.002 0.02
TUK 0.059 0.02
RS3 0.026 0.02
RS2 0.151 0.02
RS1 0.003 0.02
ORCADES 0.003 0.02
OBB 5.30e−10 0.02
NFBC86 5.22e−06 0.03
NFBC66 5.62e−05 0.02
NESDA 0.053 0.02
METSIM 2.00e−10 0.02
LOLIPOP_IA610 0.476 0.01
LOLIPOP_IA317 0.144 0.01
LOLIPOP_EWP 0.875 0.02
LOLIPOP_EWA 0.122 0.02
LOLIPOP_EW610 0.074 0.02
LLS 0.088 0.02
LLD 0.805 0.02
KORCULA 0.656 0.02
INT 3.60e−31 0.02
FR97 3.01e−05 0.03
FR07 5.47e−05 0.03
EUGENDA 0.189 0.02
ERF 0.003 0.03
EGCUT 1.34e−04 0.02
ALSPAC 4.60e−05 0.02
META 1.71e−80 0.02
−1.0
−0.5
0.0
0.5
BETA

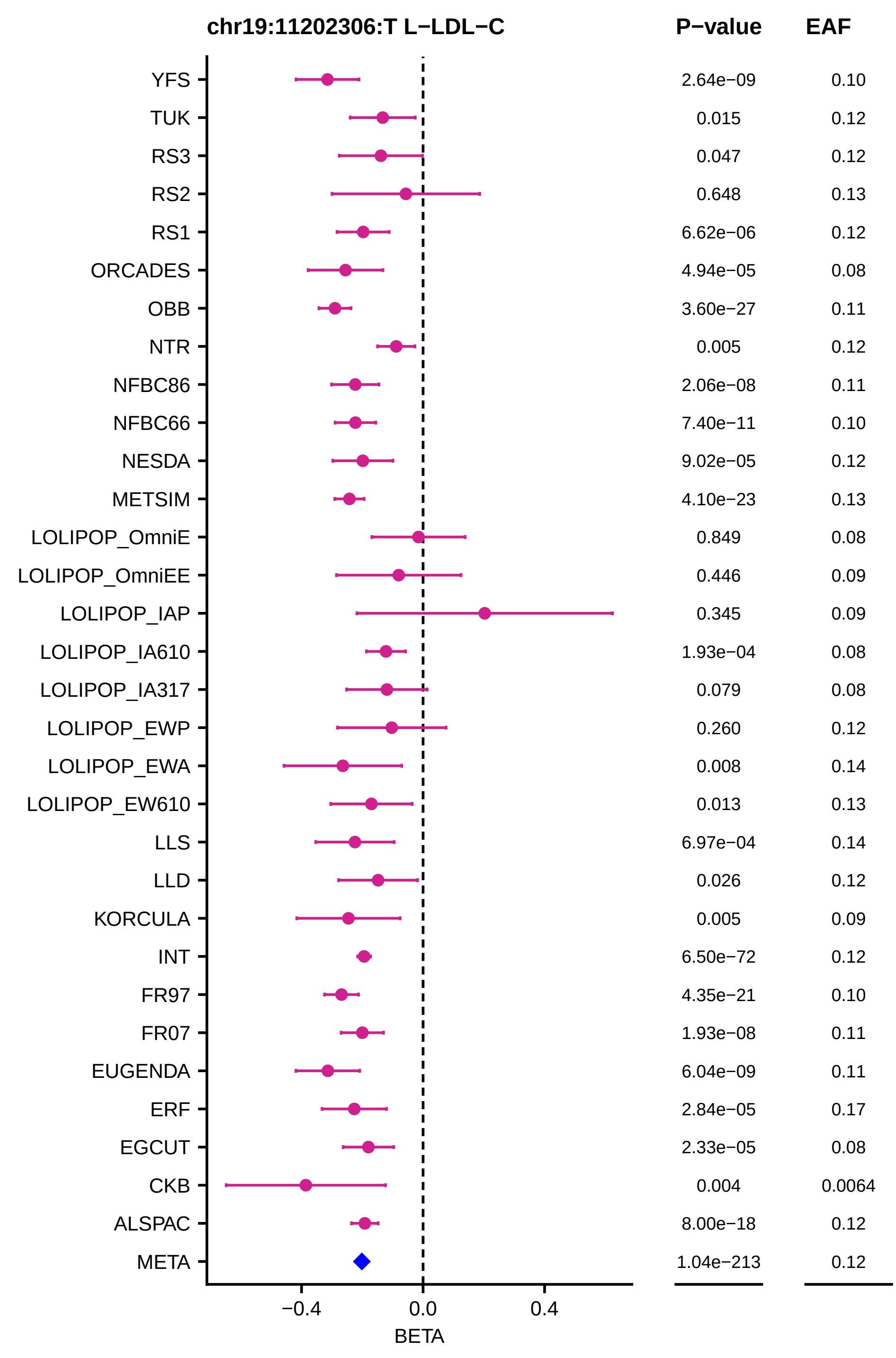
chr19:11202306:T L−LDL−C
P−value
EAF
YFS 2.64e−09 0.10
TUK 0.015 0.12
RS3 0.047 0.12
RS2 0.648 0.13
RS1 6.62e−06 0.12
ORCADES 4.94e−05 0.08
OBB 3.60e−27 0.11
NTR 0.005 0.12
NFBC86 2.06e−08 0.11
NFBC66 7.40e−11 0.10
NESDA 9.02e−05 0.12
METSIM 4.10e−23 0.13
LOLIPOP_OmniE 0.849 0.08
LOLIPOP_OmniEE 0.446 0.09
LOLIPOP_IAP 0.345 0.09
LOLIPOP_IA610 1.93e−04 0.08
LOLIPOP_IA317 0.079 0.08
LOLIPOP_EWP 0.260 0.12
LOLIPOP_EWA 0.008 0.14
LOLIPOP_EW610 0.013 0.13
LLS 6.97e−04 0.14
LLD 0.026 0.12
KORCULA 0.005 0.09
INT 6.50e−72 0.12
FR97 4.35e−21 0.10
FR07 1.93e−08 0.11
EUGENDA 6.04e−09 0.11
ERF 2.84e−05 0.17
EGCUT 2.33e−05 0.08
CKB 0.004 0.0064
ALSPAC 8.00e−18 0.12
META 1.04e−213 0.12
−0.4
0.0
0.4
BETA

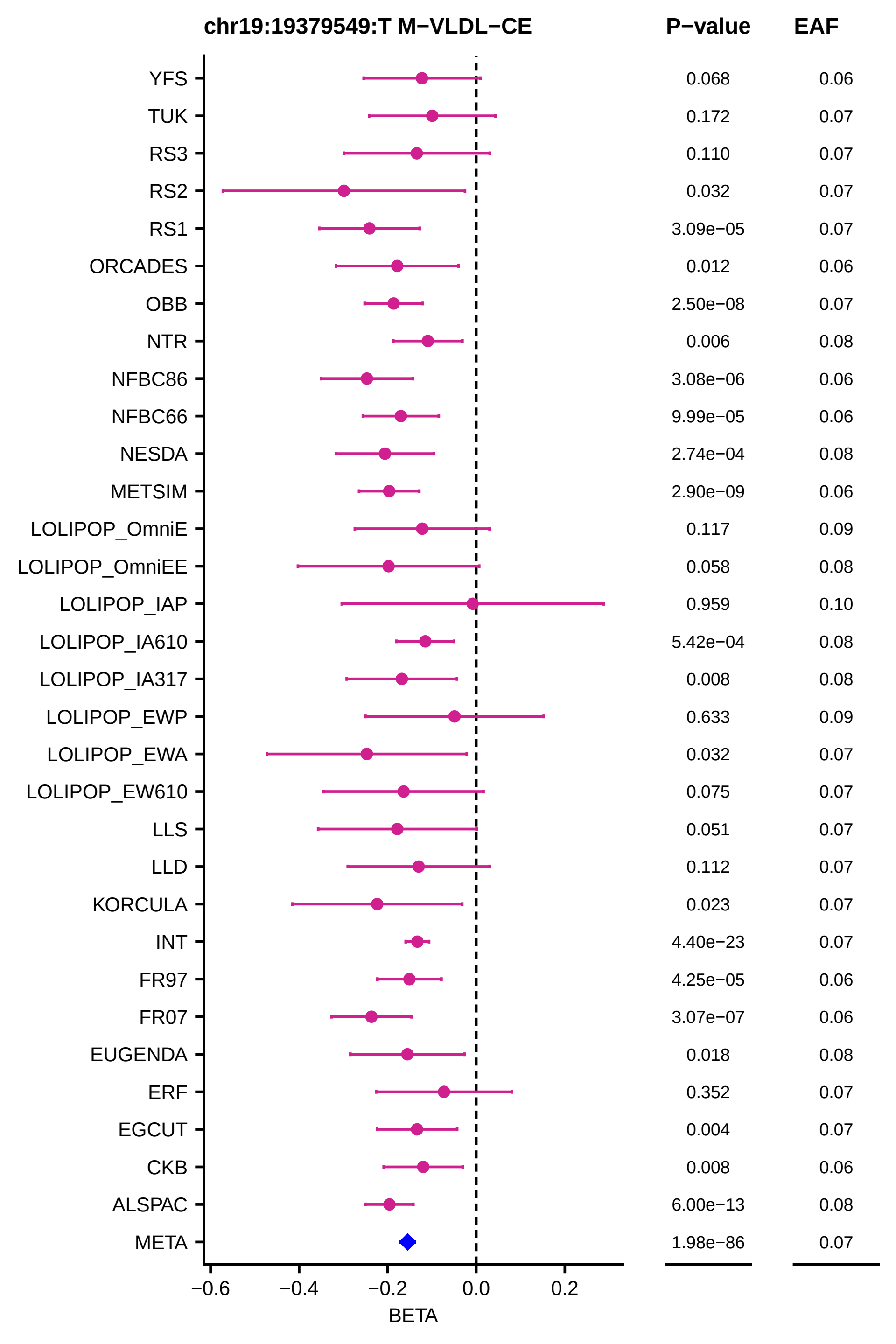

chr19:19379549:T M−VLDL−CE
P−value
EAF
YFS 0.068 0.06
TUK 0.172 0.07
RS3 0.110 0.07
RS2 0.032 0.07
RS1 3.09e−05 0.07
ORCADES 0.012 0.06
OBB 2.50e−08 0.07
NTR 0.006 0.08
NFBC86 3.08e−06 0.06
NFBC66 9.99e−05 0.06
NESDA 2.74e−04 0.08
METSIM 2.90e−09 0.06
LOLIPOP_OmniE 0.117 0.09
LOLIPOP_OmniEE 0.058 0.08
LOLIPOP_IAP 0.959 0.10
LOLIPOP_IA610 5.42e−04 0.08
LOLIPOP_IA317 0.008 0.08
LOLIPOP_EWP 0.633 0.09
LOLIPOP_EWA 0.032 0.07
LOLIPOP_EW610 0.075 0.07
LLS 0.051 0.07
LLD 0.112 0.07
KORCULA 0.023 0.07
INT 4.40e−23 0.07
FR97 4.25e−05 0.06
FR07 3.07e−07 0.06
EUGENDA 0.018 0.08
ERF 0.352 0.07
EGCUT 0.004 0.07
CKB 0.008 0.06
ALSPAC 6.00e−13 0.08
META 1.98e−86 0.07
−0.6
−0.4
−0.2
0.0
0.2
BETA

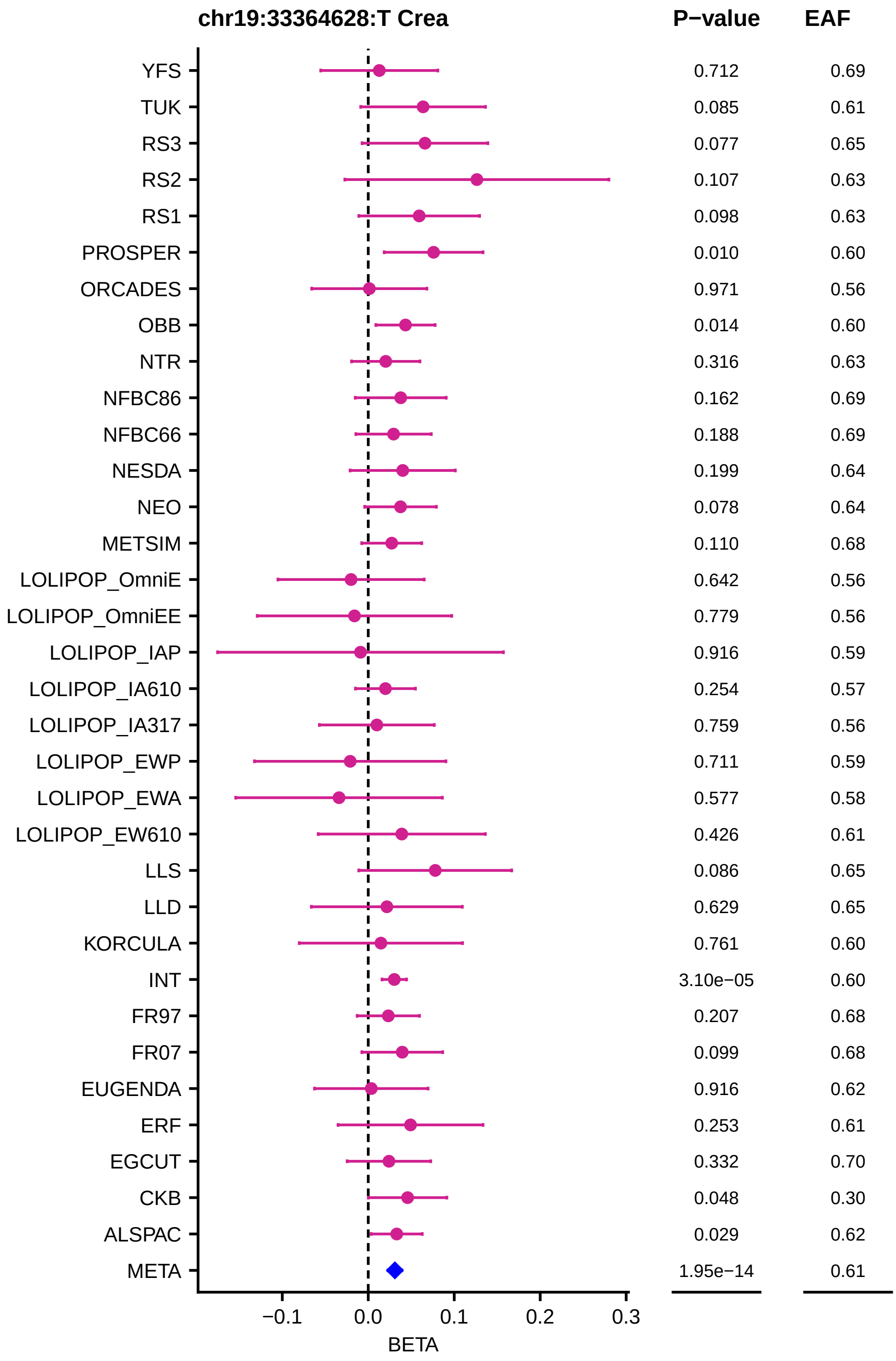
chr19:33364628:T Crea
P−value
EAF
YFS 0.712 0.69
TUK 0.085 0.61
RS3 0.077 0.65
RS2 0.107 0.63
RS1 0.098 0.63
PROSPER 0.010 0.60
ORCADES 0.971 0.56
OBB 0.014 0.60
NTR 0.316 0.63
NFBC86 0.162 0.69
NFBC66 0.188 0.69
NESDA 0.199 0.64
NEO 0.078 0.64
METSIM 0.110 0.68
LOLIPOP_OmniE 0.642 0.56
LOLIPOP_OmniEE 0.779 0.56
LOLIPOP_IAP 0.916 0.59
LOLIPOP_IA610 0.254 0.57
LOLIPOP_IA317 0.759 0.56
LOLIPOP_EWP 0.711 0.59
LOLIPOP_EWA 0.577 0.58
LOLIPOP_EW610 0.426 0.61
LLS 0.086 0.65
LLD 0.629 0.65
KORCULA 0.761 0.60
INT 3.10e−05 0.60
FR97 0.207 0.68
FR07 0.099 0.68
EUGENDA 0.916 0.62
ERF 0.253 0.61
EGCUT 0.332 0.70
CKB 0.048 0.30
ALSPAC 0.029 0.62
META 1.95e−14 0.61
−0.1
0.0
0.1
0.2
0.3
BETA

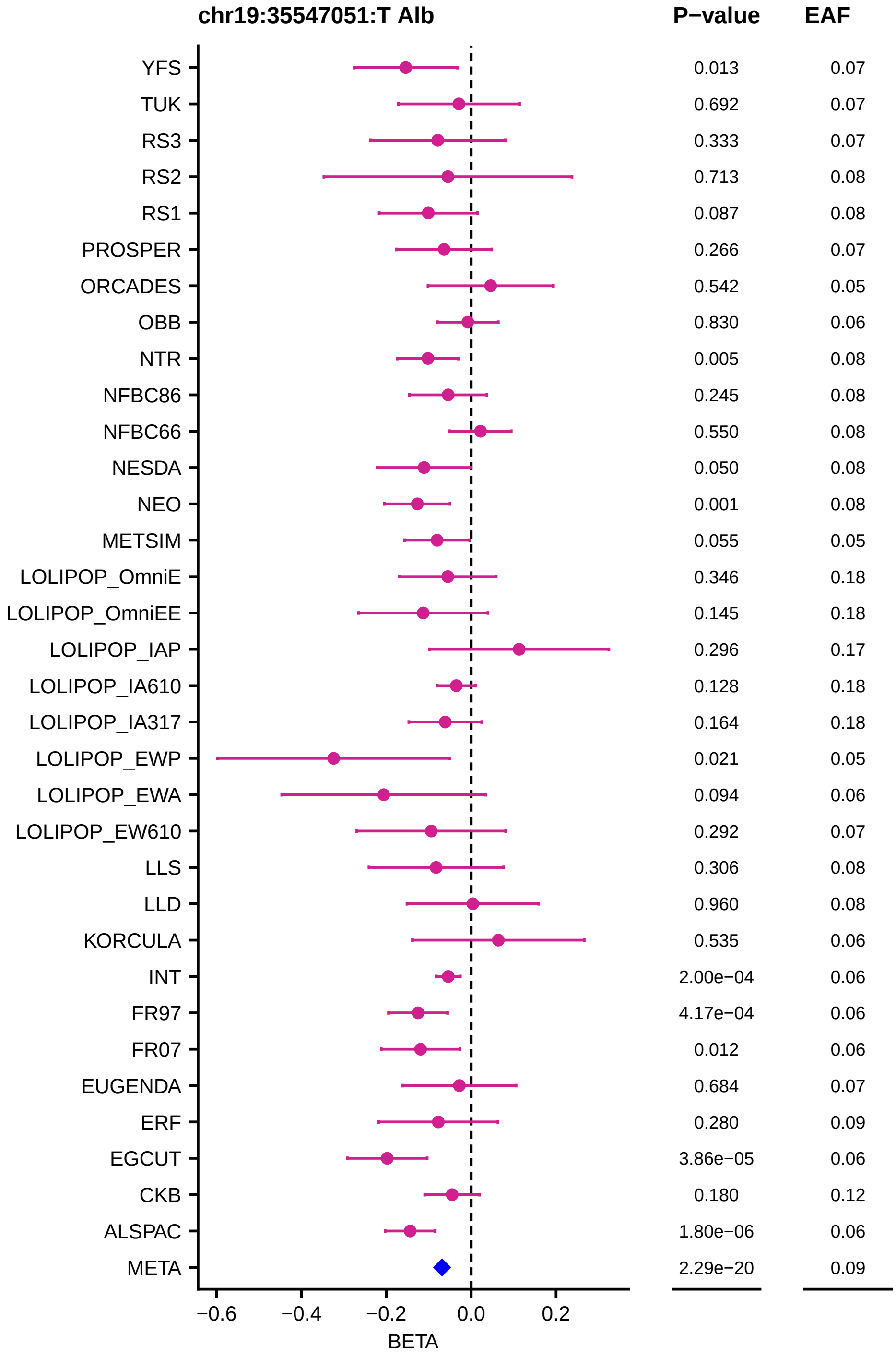
chr19:35547051:T Alb
P−value
EAF
YFS 0.013 0.07
TUK 0.692 0.07
RS3 0.333 0.07
RS2 0.713 0.08
RS1 0.087 0.08
PROSPER 0.266 0.07
ORCADES 0.542 0.05
OBB 0.830 0.06
NTR 0.005 0.08
NFBC86 0.245 0.08
NFBC66 0.550 0.08
NESDA 0.050 0.08
NEO 0.001 0.08
METSIM 0.055 0.05
LOLIPOP_OmniE 0.346 0.18
LOLIPOP_OmniEE 0.145 0.18
LOLIPOP_IAP 0.296 0.17
LOLIPOP_IA610 0.128 0.18
LOLIPOP_IA317 0.164 0.18
LOLIPOP_EWP 0.021 0.05
LOLIPOP_EWA 0.094 0.06
LOLIPOP_EW610 0.292 0.07
LLS 0.306 0.08
LLD 0.960 0.08
KORCULA 0.535 0.06
INT 2.00e−04 0.06
FR97 4.17e−04 0.06
FR07 0.012 0.06
EUGENDA 0.684 0.07
ERF 0.280 0.09
EGCUT 3.86e−05 0.06
CKB 0.180 0.12
ALSPAC 1.80e−06 0.06
META 2.29e−20 0.09
−0.6
−0.4
−0.2
0.0
0.2
BETA

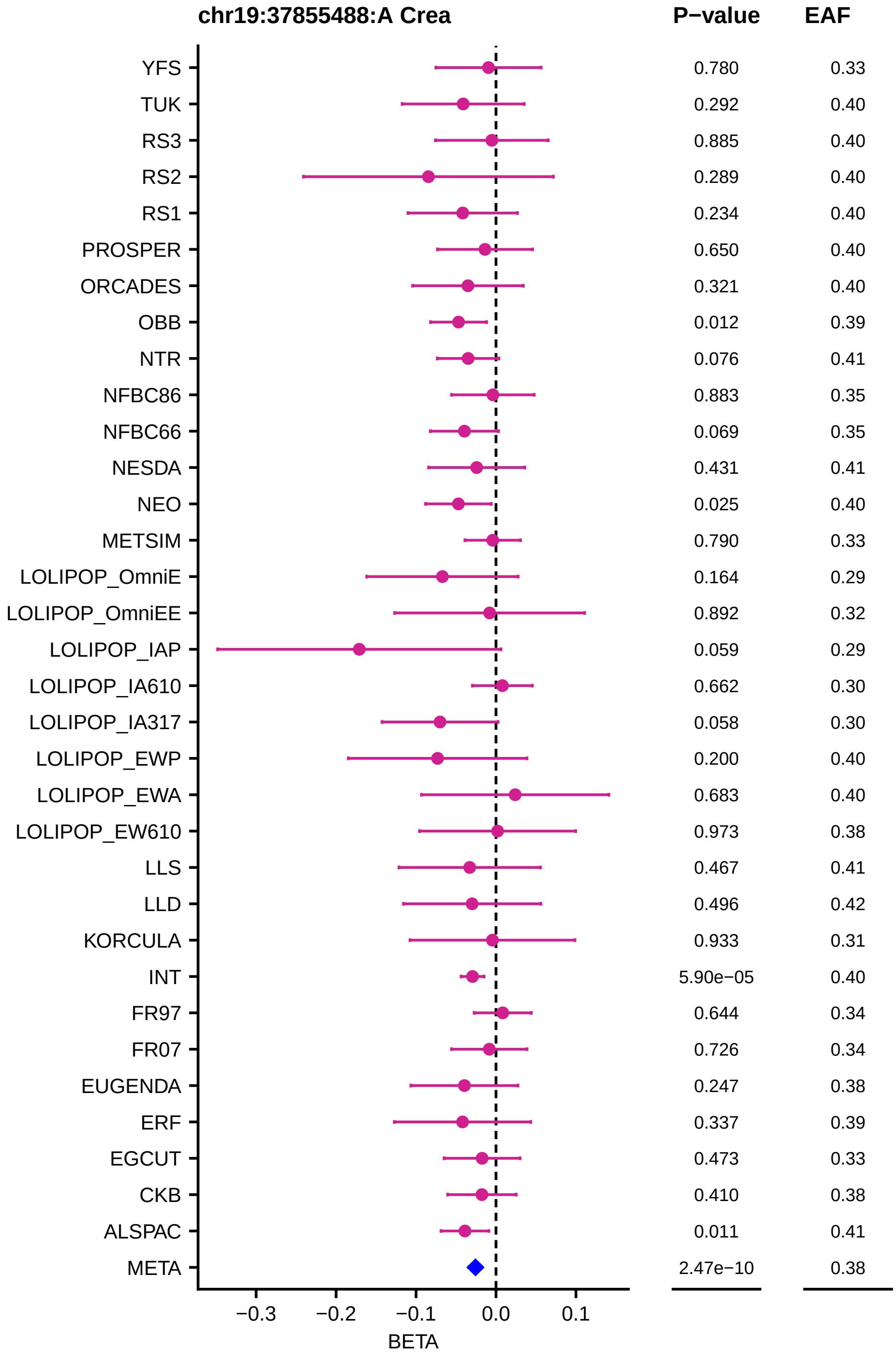

chr19:37855488:A Crea
P−value
EAF
YFS 0.780 0.33
TUK 0.292 0.40
RS3 0.885 0.40
RS2 0.289 0.40
RS1 0.234 0.40
PROSPER 0.650 0.40
ORCADES 0.321 0.40
OBB 0.012 0.39
NTR 0.076 0.41
NFBC86 0.883 0.35
NFBC66 0.069 0.35
NESDA 0.431 0.41
NEO 0.025 0.40
METSIM 0.790 0.33
LOLIPOP_OmniE 0.164 0.29
LOLIPOP_OmniEE 0.892 0.32
LOLIPOP_IAP 0.059 0.29
LOLIPOP_IA610 0.662 0.30
LOLIPOP_IA317 0.058 0.30
LOLIPOP_EWP 0.200 0.40
LOLIPOP_EWA 0.683 0.40
LOLIPOP_EW610 0.973 0.38
LLS 0.467 0.41
LLD 0.496 0.42
KORCULA 0.933 0.31
INT 5.90e−05 0.40
FR97 0.644 0.34
FR07 0.726 0.34
EUGENDA 0.247 0.38
ERF 0.337 0.39
EGCUT 0.473 0.33
CKB 0.410 0.38
ALSPAC 0.011 0.41
META 2.47e−10 0.38
−0.3
−0.2
−0.1
0.0
0.1
BETA

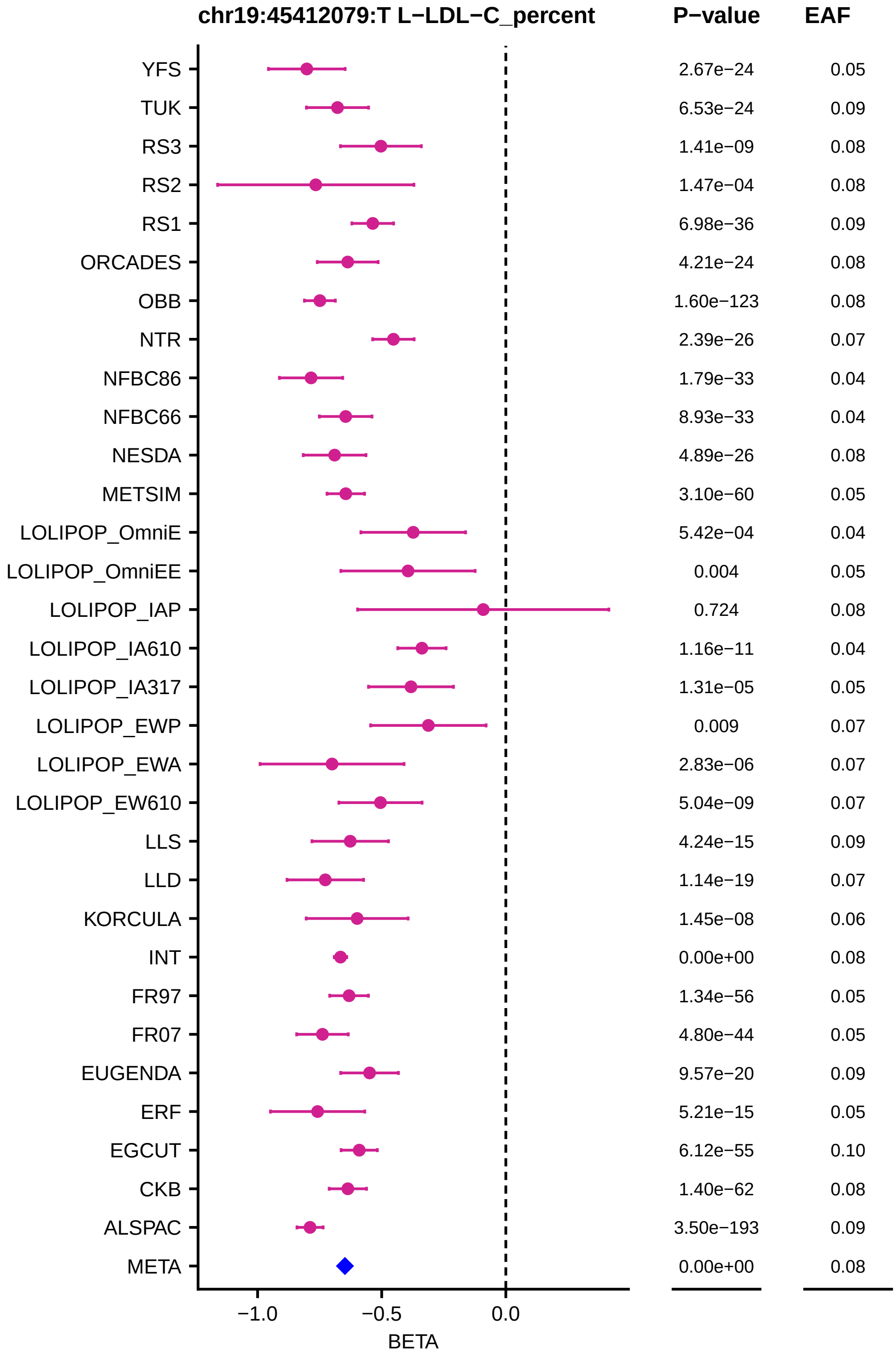

chr19:45412079:T L−LDL−C_percent
P−value
EAF
YFS 2.67e−24 0.05
TUK 6.53e−24 0.09
RS3 1.41e−09 0.08
RS2 1.47e−04 0.08
RS1 6.98e−36 0.09
ORCADES 4.21e−24 0.08
OBB 1.60e−123 0.08
NTR 2.39e−26 0.07
NFBC86 1.79e−33 0.04
NFBC66 8.93e−33 0.04
NESDA 4.89e−26 0.08
METSIM 3.10e−60 0.05
LOLIPOP_OmniE 5.42e−04 0.04
LOLIPOP_OmniEE 0.004 0.05
LOLIPOP_IAP 0.724 0.08
LOLIPOP_IA610 1.16e−11 0.04
LOLIPOP_IA317 1.31e−05 0.05
LOLIPOP_EWP 0.009 0.07
LOLIPOP_EWA 2.83e−06 0.07
LOLIPOP_EW610 5.04e−09 0.07
LLS 4.24e−15 0.09
LLD 1.14e−19 0.07
KORCULA 1.45e−08 0.06
INT 0.00e+00 0.08
FR97 1.34e−56 0.05
FR07 4.80e−44 0.05
EUGENDA 9.57e−20 0.09
ERF 5.21e−15 0.05
EGCUT 6.12e−55 0.10
CKB 1.40e−62 0.08
ALSPAC 3.50e−193 0.09
META 0.00e+00 0.08
−1.0
−0.5
0.0
BETA

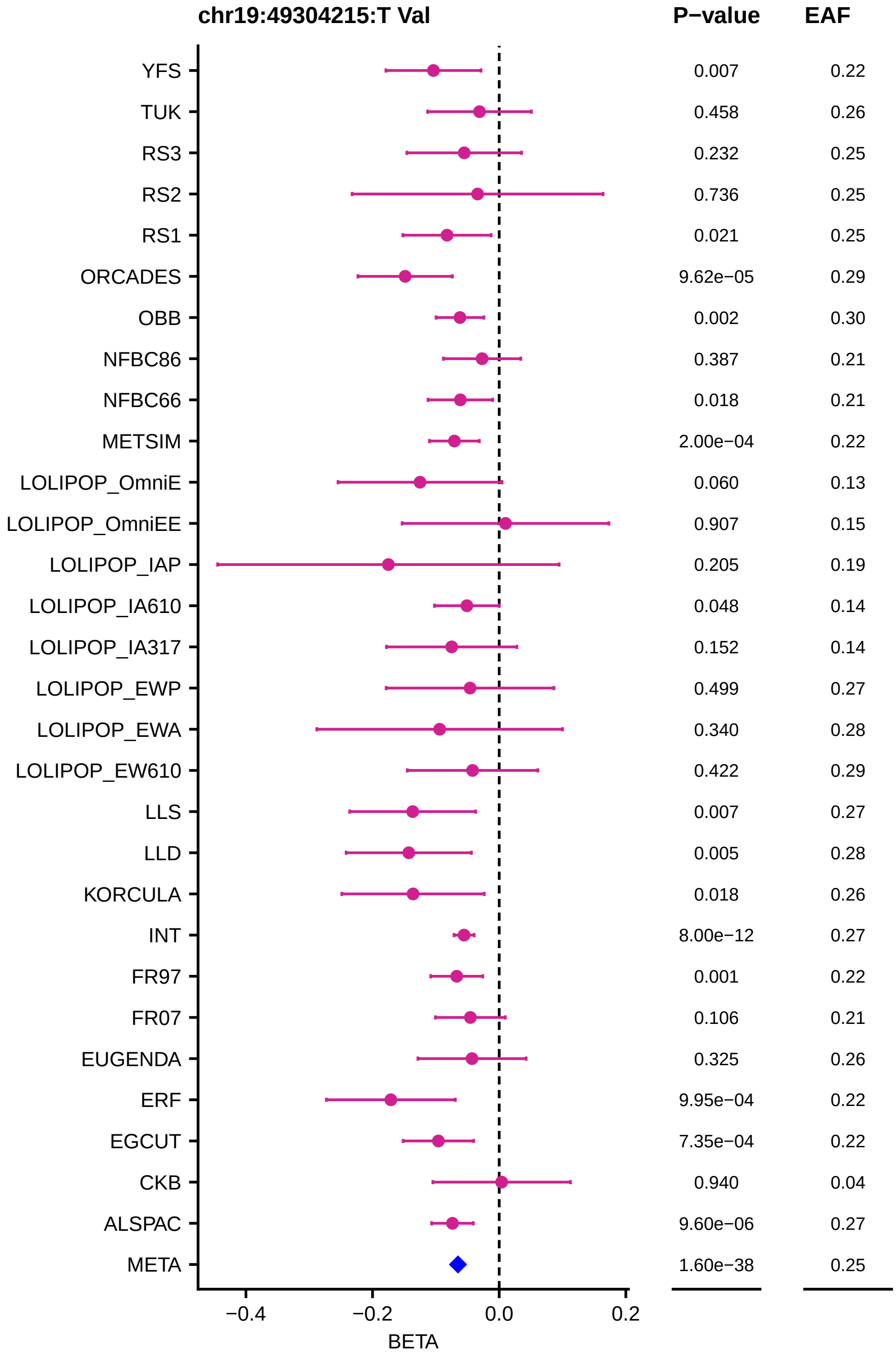

chr19:49304215:T Val
P−value
EAF
YFS
TUK
RS3
RS2
RS1
ORCADES
OBB
NFBC86
NFBC66
METSIM
LOLIPOP_OmniE
LOLIPOP_OmniEE
LOLIPOP_IAP
LOLIPOP_IA610
LOLIPOP_IA317
LOLIPOP_EWP
LOLIPOP_EWA
LOLIPOP_EW610
LLS
LLD
KORCULA
INT
FR97
FR07
EUGENDA
ERF
EGCUT
CKB
ALSPAC
META
0.007 0.22
0.458 0.26
0.232 0.25
0.736 0.25
0.021 0.25
9.62e−05 0.29
0.002 0.30
0.387 0.21
0.018 0.21
2.00e−04 0.22
0.060 0.13
0.907 0.15
0.205 0.19
0.048 0.14
0.152 0.14
0.499 0.27
0.340 0.28
0.422 0.29
0.007 0.27
0.005 0.28
0.018 0.26
8.00e−12 0.27
0.001 0.22
0.106 0.21
0.325 0.26
9.95e−04 0.22
7.35e−04 0.22
0.940 0.04
9.60e−06 0.27
1.60e−38 0.25
−0.4
−0.2
0.0
0.2
BETA

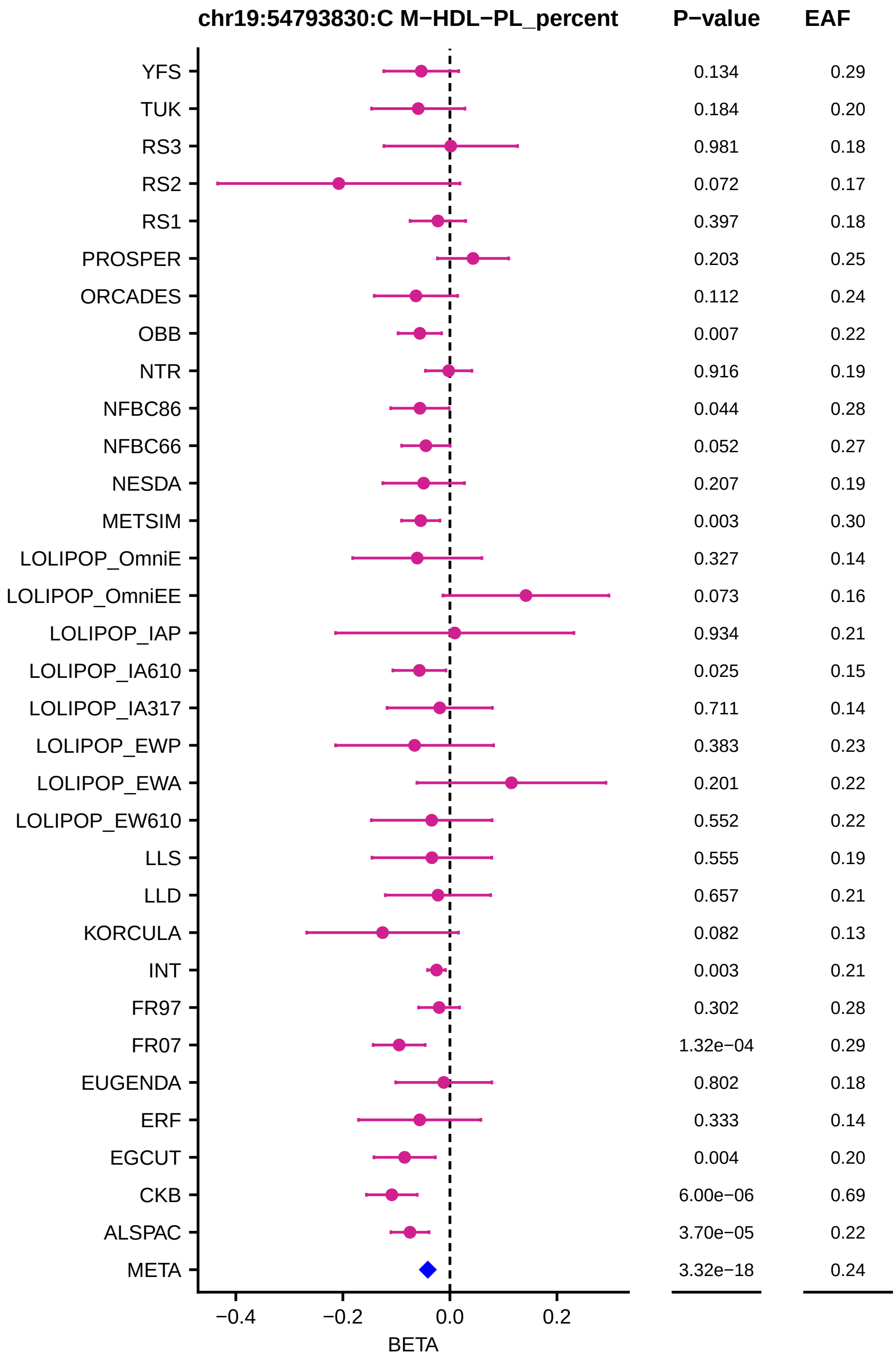
chr19:54793830:C M−HDL−PL_percent
P−value
EAF
YFS 0.134 0.29
TUK 0.184 0.20
RS3 0.981 0.18
RS2 0.072 0.17
RS1 0.397 0.18
PROSPER 0.203 0.25
ORCADES 0.112 0.24
OBB 0.007 0.22
NTR 0.916 0.19
NFBC86 0.044 0.28
NFBC66 0.052 0.27
NESDA 0.207 0.19
METSIM 0.003 0.30
LOLIPOP_OmniE 0.327 0.14
LOLIPOP_OmniEE 0.073 0.16
LOLIPOP_IAP 0.934 0.21
LOLIPOP_IA610 0.025 0.15
LOLIPOP_IA317 0.711 0.14
LOLIPOP_EWP 0.383 0.23
LOLIPOP_EWA 0.201 0.22
LOLIPOP_EW610 0.552 0.22
LLS 0.555 0.19
LLD 0.657 0.21
KORCULA 0.082 0.13
INT 0.003 0.21
FR97 0.302 0.28
FR07 1.32e−04 0.29
EUGENDA 0.802 0.18
ERF 0.333 0.14
EGCUT 0.004 0.20
CKB 6.00e−06 0.69
ALSPAC 3.70e−05 0.22
META 3.32e−18 0.24
−0.4
−0.2
0.0
0.2
BETA

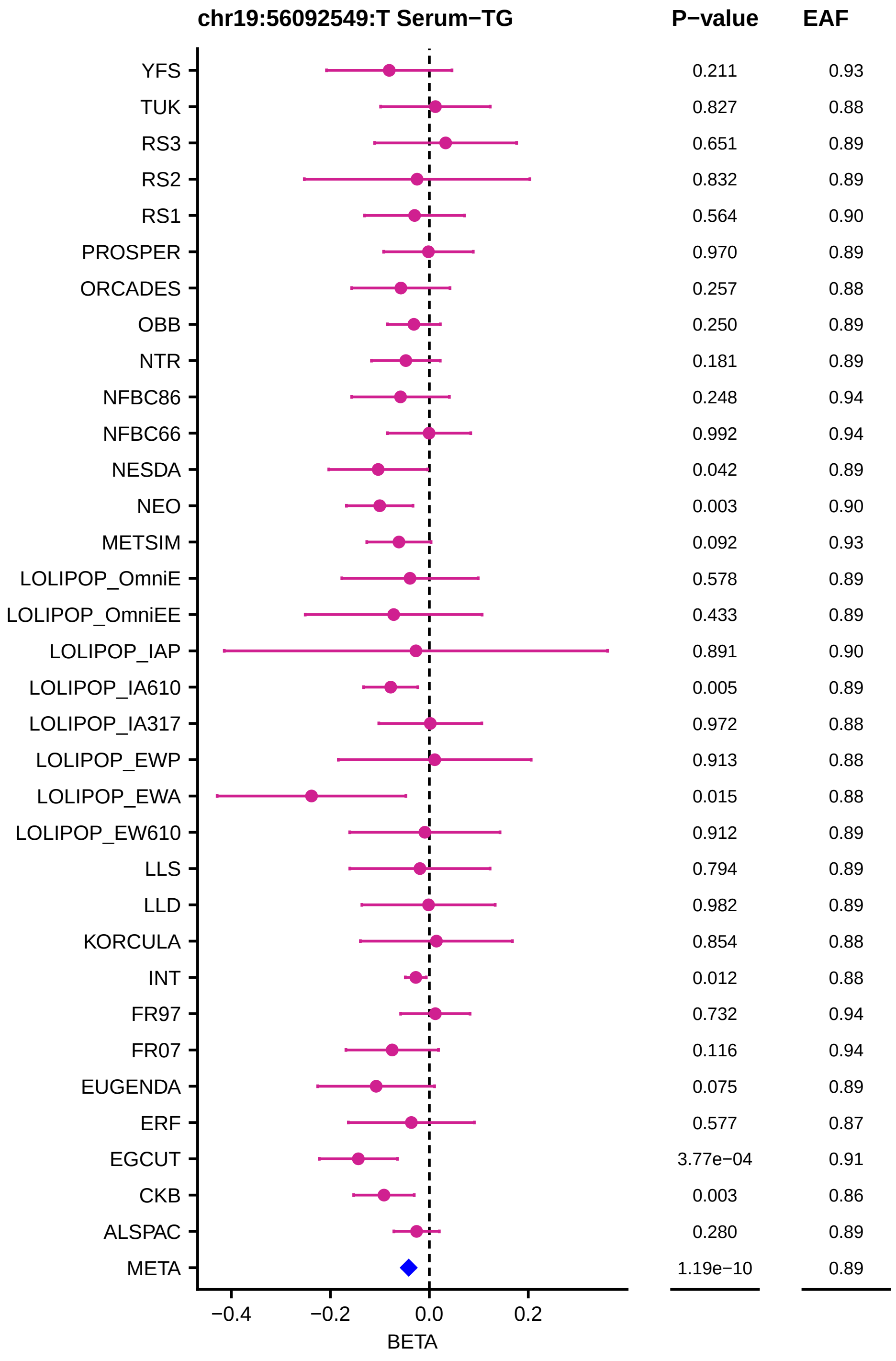
chr19:56092549:T Serum−TG
P−value
EAF
YFS 0.211 0.93
TUK 0.827 0.88
RS3 0.651 0.89
RS2 0.832 0.89
RS1 0.564 0.90
PROSPER 0.970 0.89
ORCADES 0.257 0.88
OBB 0.250 0.89
NTR 0.181 0.89
NFBC86 0.248 0.94
NFBC66 0.992 0.94
NESDA 0.042 0.89
NEO 0.003 0.90
METSIM 0.092 0.93
LOLIPOP_OmniE 0.578 0.89
LOLIPOP_OmniEE 0.433 0.89
LOLIPOP_IAP 0.891 0.90
LOLIPOP_IA610 0.005 0.89
LOLIPOP_IA317 0.972 0.88
LOLIPOP_EWP 0.913 0.88
LOLIPOP_EWA 0.015 0.88
LOLIPOP_EW610 0.912 0.89
LLS 0.794 0.89
LLD 0.982 0.89
KORCULA 0.854 0.88
INT 0.012 0.88
FR97 0.732 0.94
FR07 0.116 0.94
EUGENDA 0.075 0.89
ERF 0.577 0.87
EGCUT 3.77e−04 0.91
CKB 0.003 0.86
ALSPAC 0.280 0.89
META 1.19e−10 0.89
−0.4
−0.2
0.0
0.2
BETA

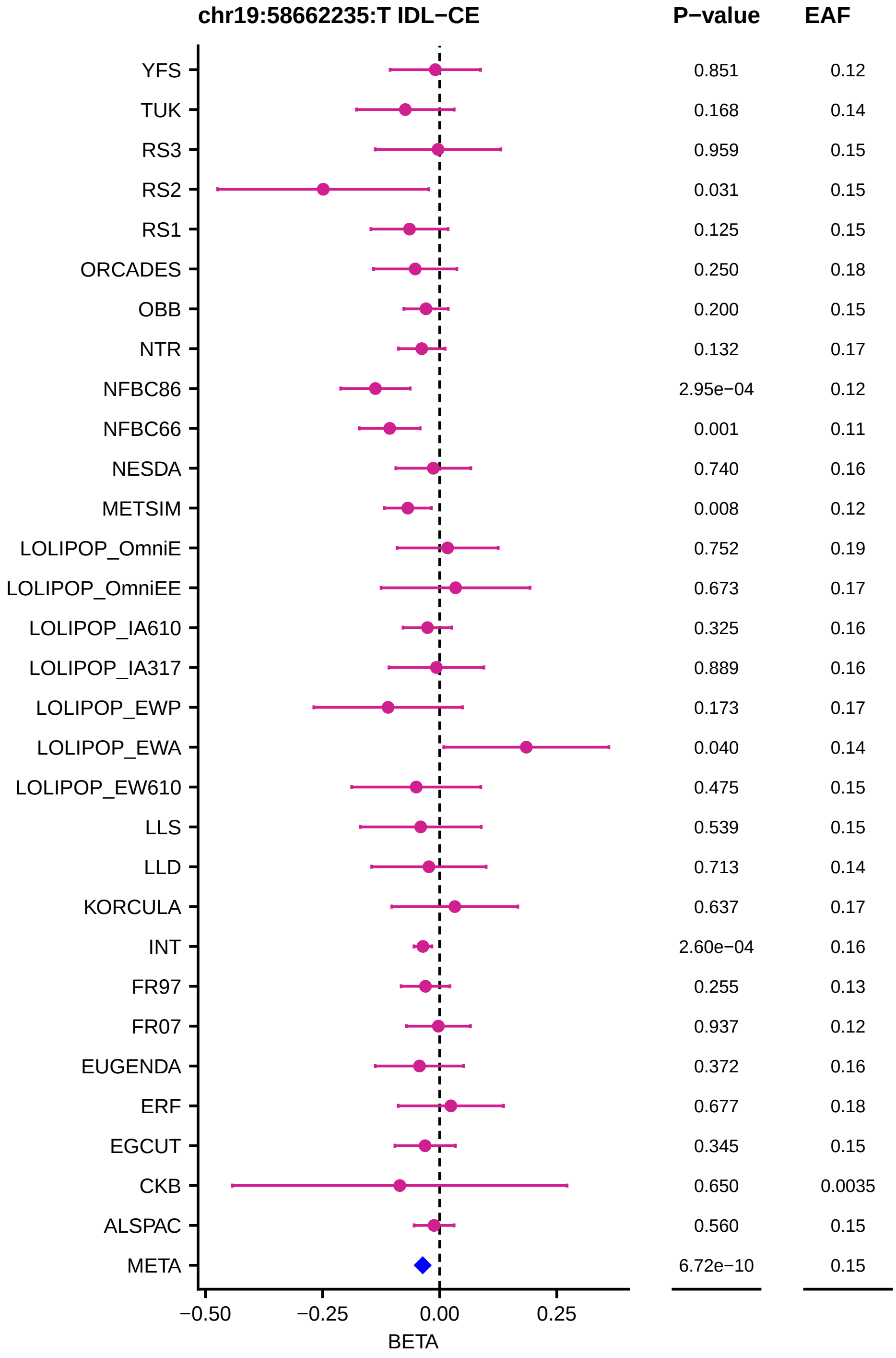
chr19:58662235:T IDL−CE
P−value
EAF
YFS 0.851 0.12
TUK 0.168 0.14
RS3 0.959 0.15
RS2 0.031 0.15
RS1 0.125 0.15
ORCADES 0.250 0.18
OBB 0.200 0.15
NTR 0.132 0.17
NFBC86 2.95e−04 0.12
NFBC66 0.001 0.11
NESDA 0.740 0.16
METSIM 0.008 0.12
LOLIPOP_OmniE 0.752 0.19
LOLIPOP_OmniEE 0.673 0.17
LOLIPOP_IA610 0.325 0.16
LOLIPOP_IA317 0.889 0.16
LOLIPOP_EWP 0.173 0.17
LOLIPOP_EWA 0.040 0.14
LOLIPOP_EW610 0.475 0.15
LLS 0.539 0.15
LLD 0.713 0.14
KORCULA 0.637 0.17
INT 2.60e−04 0.16
FR97 0.255 0.13
FR07 0.937 0.12
EUGENDA 0.372 0.16
ERF 0.677 0.18
EGCUT 0.345 0.15
CKB 0.650 0.0035
ALSPAC 0.560 0.15
META 6.72e−10 0.15
−0.50
−0.25
0.00
0.25
BETA

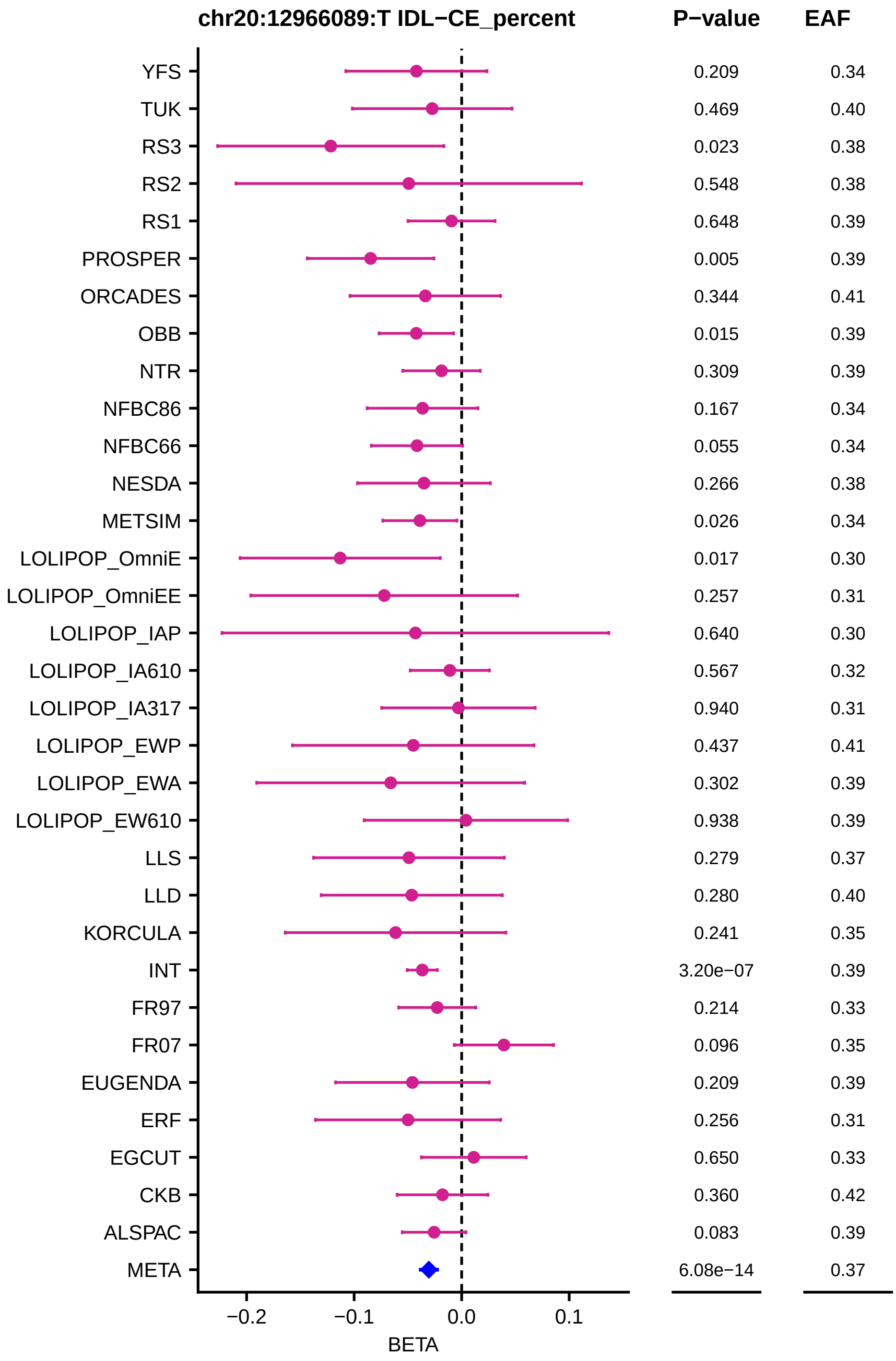

chr20:12966089:T IDL−CE_percent
P−value
EAF
YFS 0.209 0.34
TUK 0.469 0.40
RS3 0.023 0.38
RS2 0.548 0.38
RS1 0.648 0.39
PROSPER 0.005 0.39
ORCADES 0.344 0.41
OBB 0.015 0.39
NTR 0.309 0.39
NFBC86 0.167 0.34
NFBC66 0.055 0.34
NESDA 0.266 0.38
METSIM 0.026 0.34
LOLIPOP_OmniE 0.017 0.30
LOLIPOP_OmniEE 0.257 0.31
LOLIPOP_IAP 0.640 0.30
LOLIPOP_IA610 0.567 0.32
LOLIPOP_IA317 0.940 0.31
LOLIPOP_EWP 0.437 0.41
LOLIPOP_EWA 0.302 0.39
LOLIPOP_EW610 0.938 0.39
LLS 0.279 0.37
LLD 0.280 0.40
KORCULA 0.241 0.35
INT 3.20e−07 0.39
FR97 0.214 0.33
FR07 0.096 0.35
EUGENDA 0.209 0.39
ERF 0.256 0.31
EGCUT 0.650 0.33
CKB 0.360 0.42
ALSPAC 0.083 0.39
META 6.08e−14 0.37
−0.2
−0.1
0.0
0.1
BETA

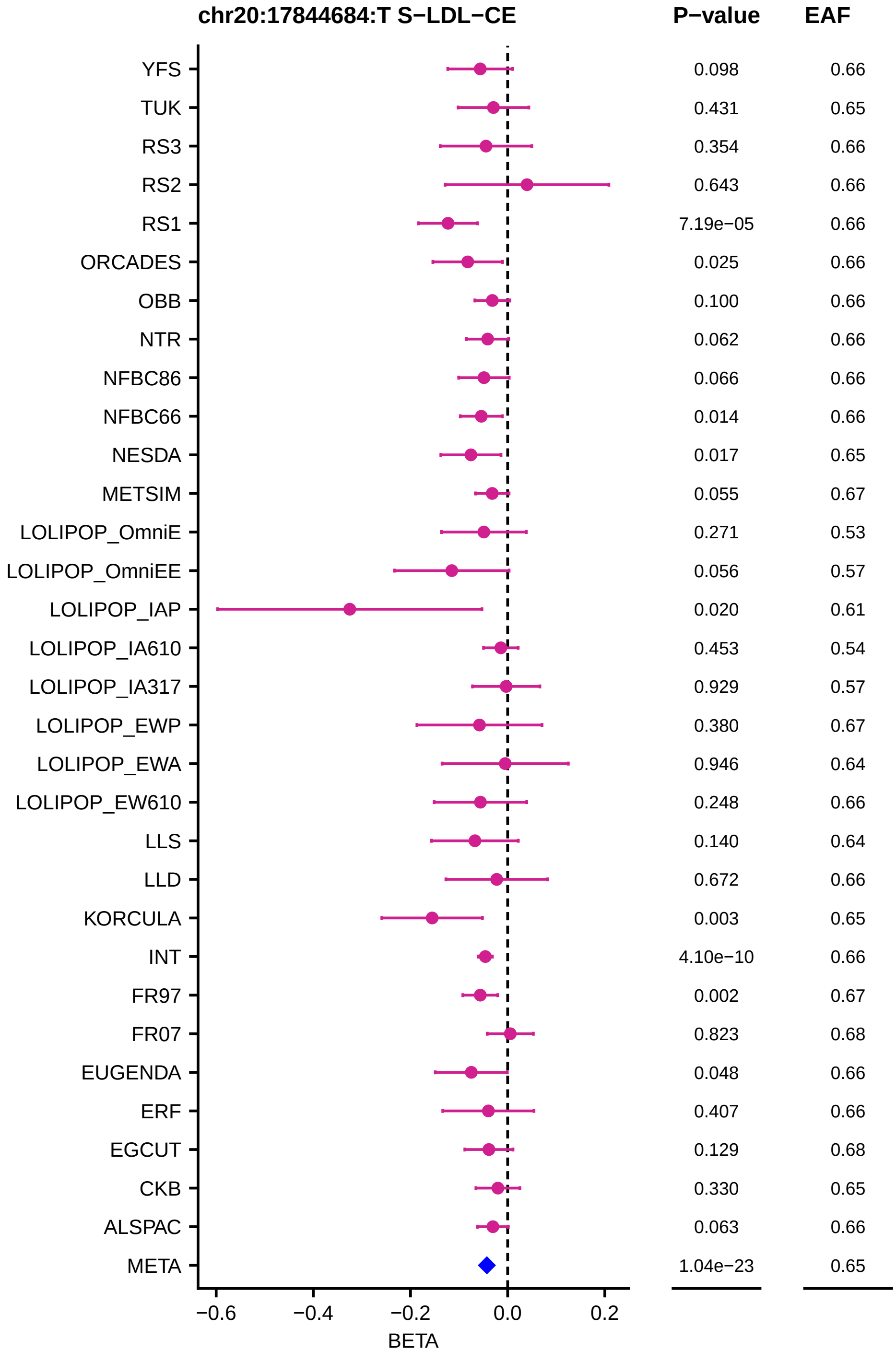
chr20:17844684:T S−LDL−CE
P−value
EAF
YFS 0.098 0.66
TUK 0.431 0.65
RS3 0.354 0.66
RS2 0.643 0.66
RS1 7.19e−05 0.66
ORCADES 0.025 0.66
OBB 0.100 0.66
NTR 0.062 0.66
NFBC86 0.066 0.66
NFBC66 0.014 0.66
NESDA 0.017 0.65
METSIM 0.055 0.67
LOLIPOP_OmniE 0.271 0.53
LOLIPOP_OmniEE 0.056 0.57
LOLIPOP_IAP 0.020 0.61
LOLIPOP_IA610 0.453 0.54
LOLIPOP_IA317 0.929 0.57
LOLIPOP_EWP 0.380 0.67
LOLIPOP_EWA 0.946 0.64
LOLIPOP_EW610 0.248 0.66
LLS 0.140 0.64
LLD 0.672 0.66
KORCULA 0.003 0.65
INT 4.10e−10 0.66
FR97 0.002 0.67
FR07 0.823 0.68
EUGENDA 0.048 0.66
ERF 0.407 0.66
EGCUT 0.129 0.68
CKB 0.330 0.65
ALSPAC 0.063 0.66
META 1.04e−23 0.65
−0.6
−0.4
−0.2
0.0
0.2
BETA

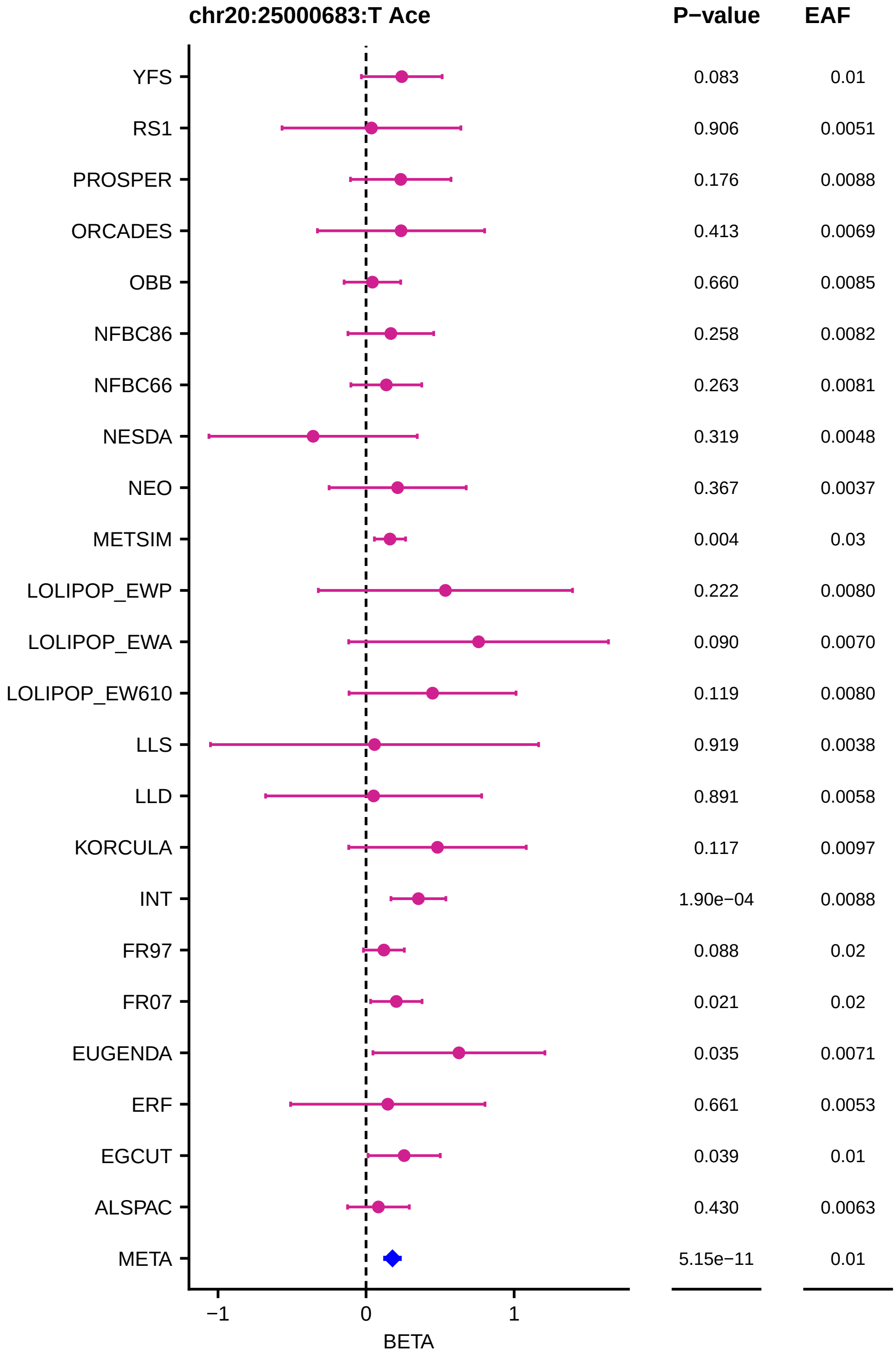
chr20:25000683:T Ace
P−value
EAF
YFS
0.083
0.01
RS1
0.906
0.0051
PROSPER
0.176
0.0088
ORCADES
0.413
0.0069
OBB
0.660
0.0085
NFBC86
0.258
0.0082
NFBC66
0.263
0.0081
NESDA
0.319
0.0048
NEO
0.367
0.0037
METSIM
0.004
0.03
LOLIPOP_EWP
0.222
0.0080
LOLIPOP_EWA
0.090
0.0070
LOLIPOP_EW610
0.119
0.0080
LLS
0.919
0.0038
LLD
0.891
0.0058
KORCULA
0.117
0.0097
INT
1.90e−04
0.0088
FR97
0.088
0.02
FR07
0.021
0.02
EUGENDA
0.035
0.0071
ERF
0.661
0.0053
EGCUT
0.039
0.01
ALSPAC
0.430
0.0063
META
5.15e−11
0.01
−1
0
1
BETA

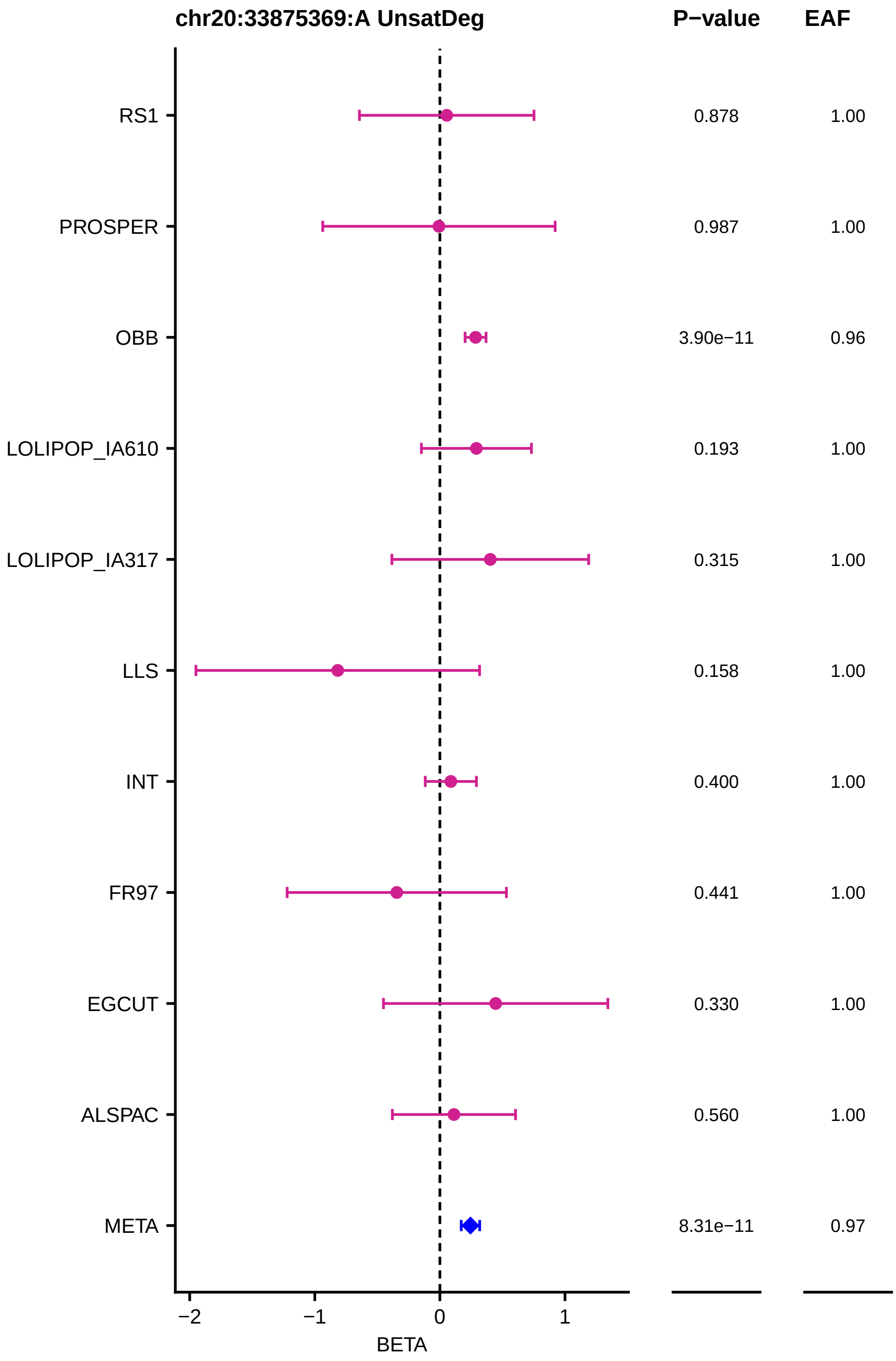
chr20:33875369:A UnsatDeg
P−value
EAF
RS1
PROSPER
OBB
LOLIPOP_IA610
LOLIPOP_IA317
LLS
INT
FR97
EGCUT
ALSPAC
META
0.878
0.987
3.90e−11
0.193
0.315
0.158
0.400
0.441
0.330
0.560
8.31e−11
1.00
1.00
0.96
1.00
1.00
1.00
1.00
1.00
1.00
1.00
0.97
−2
−1
0
1
BETA

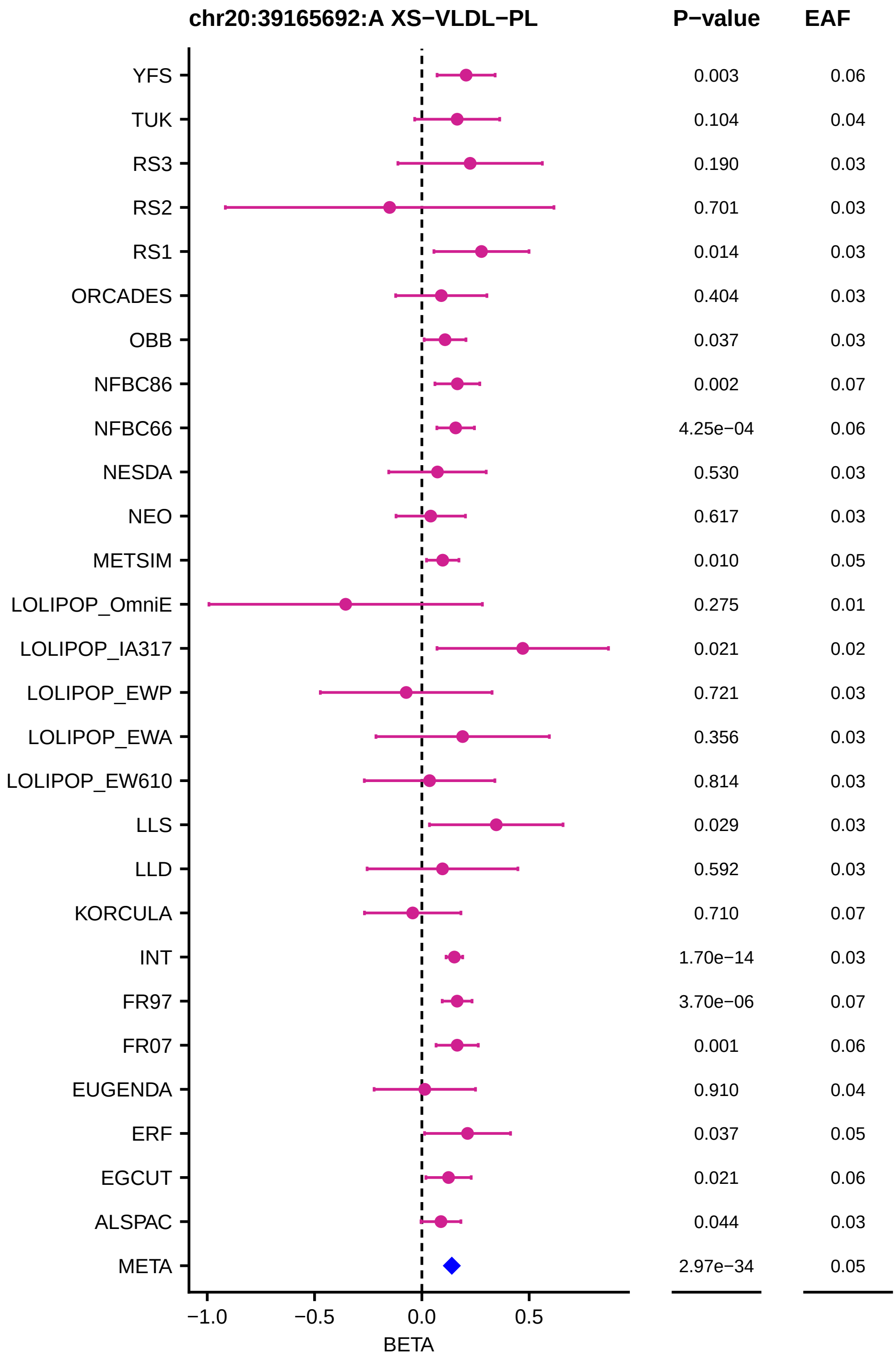

chr20:39165692:A XS−VLDL−PL
P−value
EAF
YFS
TUK
RS3
RS2
RS1
ORCADES
OBB
NFBC86
NFBC66
NESDA
NEO
METSIM
LOLIPOP_OmniE
LOLIPOP_IA317
LOLIPOP_EWP
LOLIPOP_EWA
LOLIPOP_EW610
LLS
LLD
KORCULA
INT
FR97
FR07
EUGENDA
ERF
EGCUT
ALSPAC
META
0.003 0.06
0.104 0.04
0.190 0.03
0.701 0.03
0.014 0.03
0.404 0.03
0.037 0.03
0.002 0.07
4.25e−04 0.06
0.530 0.03
0.617 0.03
0.010 0.05
0.275 0.01
0.021 0.02
0.721 0.03
0.356 0.03
0.814 0.03
0.029 0.03
0.592 0.03
0.710 0.07
1.70e−14 0.03
3.70e−06 0.07
0.001 0.06
0.910 0.04
0.037 0.05
0.021 0.06
0.044 0.03
2.97e−34 0.05
−1.0
−0.5
0.0
0.5
BETA

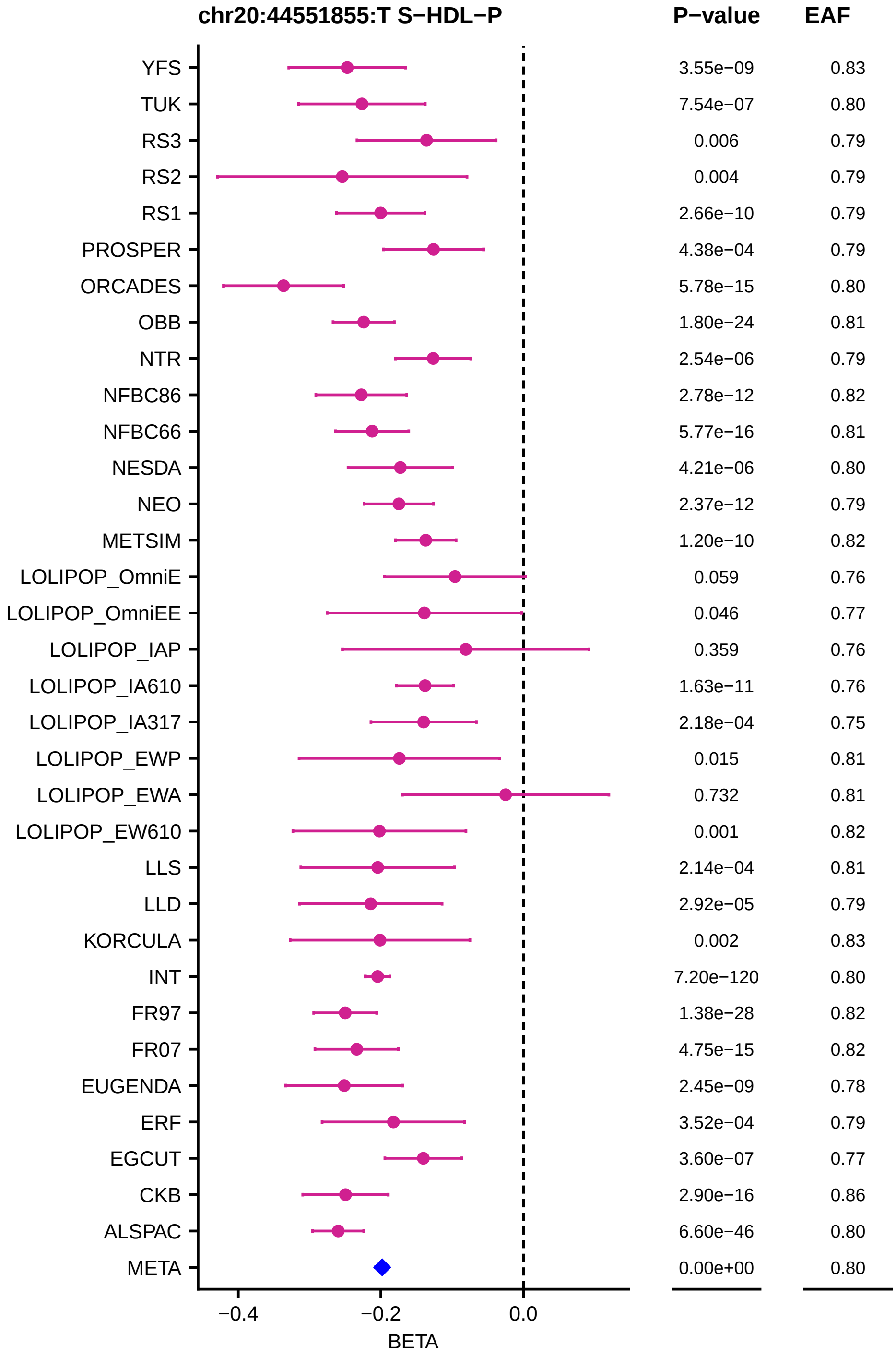

chr20:44551855:T S−HDL−P
P−value
EAF
YFS 3.55e−09 0.83
TUK 7.54e−07 0.80
RS3 0.006 0.79
RS2 0.004 0.79
RS1 2.66e−10 0.79
PROSPER 4.38e−04 0.79
ORCADES 5.78e−15 0.80
OBB 1.80e−24 0.81
NTR 2.54e−06 0.79
NFBC86 2.78e−12 0.82
NFBC66 5.77e−16 0.81
NESDA 4.21e−06 0.80
NEO 2.37e−12 0.79
METSIM 1.20e−10 0.82
LOLIPOP_OmniE 0.059 0.76
LOLIPOP_OmniEE 0.046 0.77
LOLIPOP_IAP 0.359 0.76
LOLIPOP_IA610 1.63e−11 0.76
LOLIPOP_IA317 2.18e−04 0.75
LOLIPOP_EWP 0.015 0.81
LOLIPOP_EWA 0.732 0.81
LOLIPOP_EW610 0.001 0.82
LLS 2.14e−04 0.81
LLD 2.92e−05 0.79
KORCULA 0.002 0.83
INT 7.20e−120 0.80
FR97 1.38e−28 0.82
FR07 4.75e−15 0.82
EUGENDA 2.45e−09 0.78
ERF 3.52e−04 0.79
EGCUT 3.60e−07 0.77
CKB 2.90e−16 0.86
ALSPAC 6.60e−46 0.80
META 0.00e+00 0.80
−0.4
−0.2
0.0
BETA

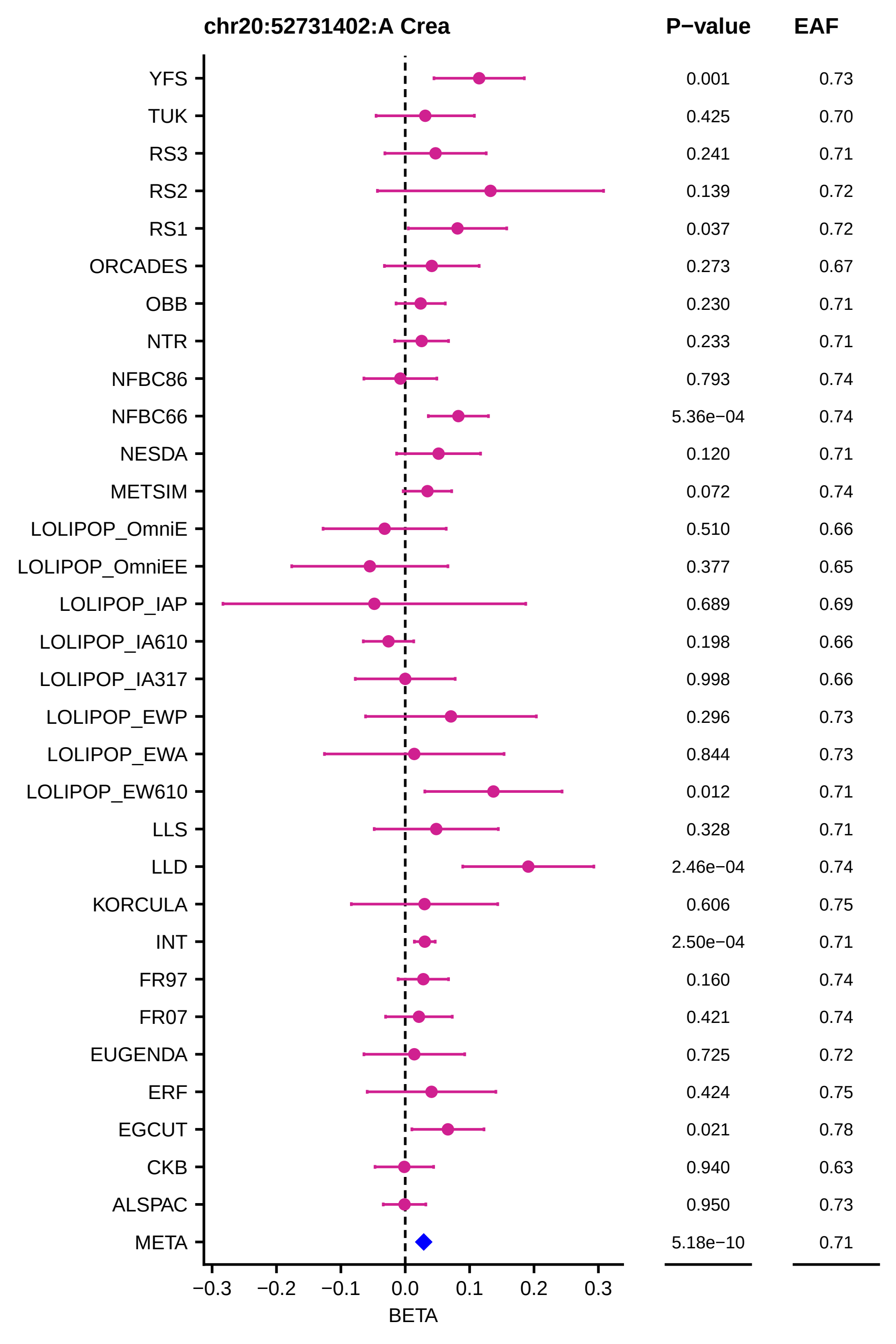
chr20:52731402:A Crea
P−value
EAF
YFS 0.001 0.73
TUK 0.425 0.70
RS3 0.241 0.71
RS2 0.139 0.72
RS1 0.037 0.72
ORCADES 0.273 0.67
OBB 0.230 0.71
NTR 0.233 0.71
NFBC86 0.793 0.74
NFBC66 5.36e−04 0.74
NESDA 0.120 0.71
METSIM 0.072 0.74
LOLIPOP_OmniE 0.510 0.66
LOLIPOP_OmniEE 0.377 0.65
LOLIPOP_IAP 0.689 0.69
LOLIPOP_IA610 0.198 0.66
LOLIPOP_IA317 0.998 0.66
LOLIPOP_EWP 0.296 0.73
LOLIPOP_EWA 0.844 0.73
LOLIPOP_EW610 0.012 0.71
LLS 0.328 0.71
LLD 2.46e−04 0.74
KORCULA 0.606 0.75
INT 2.50e−04 0.71
FR97 0.160 0.74
FR07 0.421 0.74
EUGENDA 0.725 0.72
ERF 0.424 0.75
EGCUT 0.021 0.78
CKB 0.940 0.63
ALSPAC 0.950 0.73
META 5.18e−10 0.71
−0.3
−0.2
−0.1
0.0
0.1
0.2
0.3
BETA

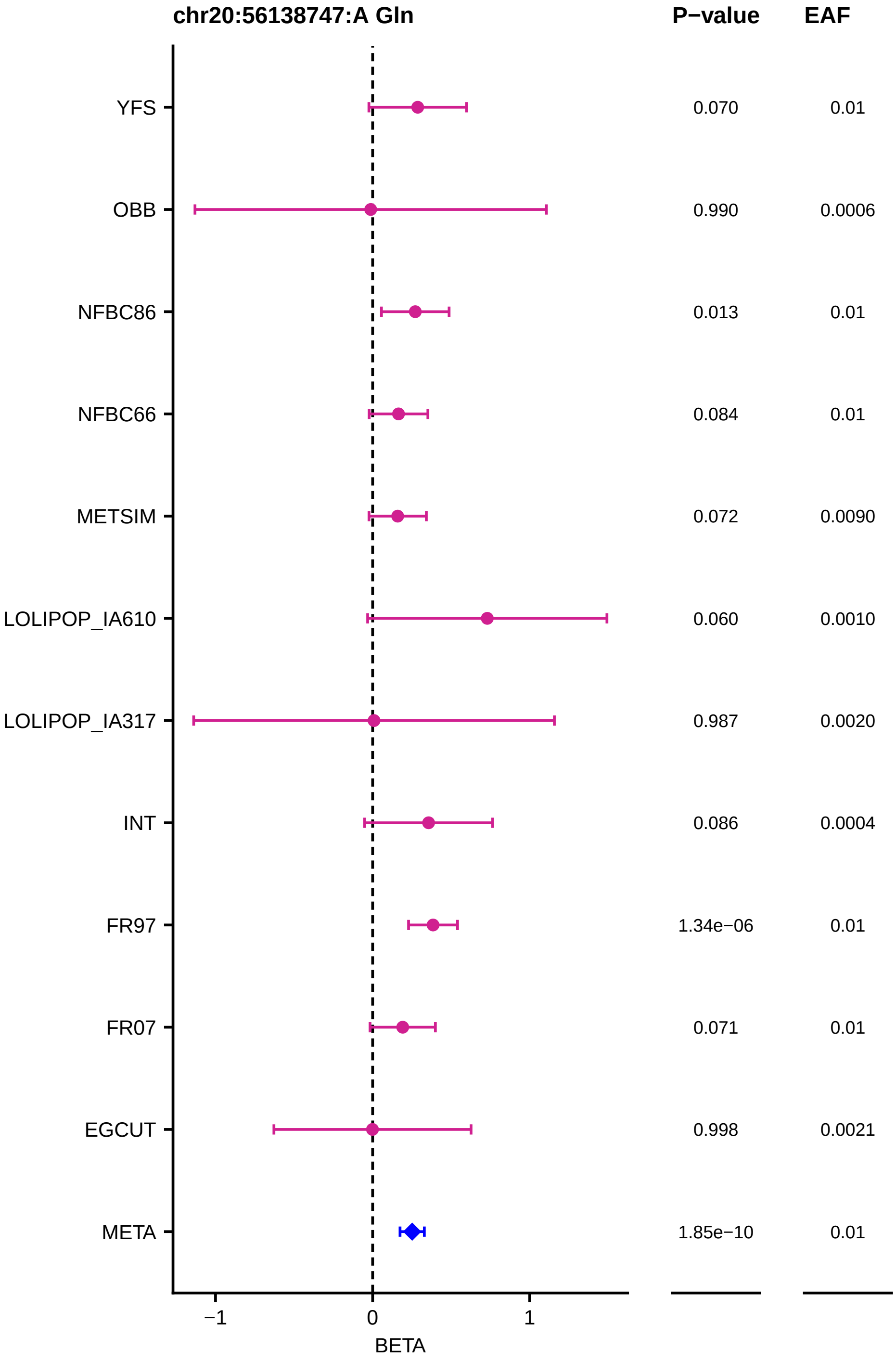
chr20:56138747:A Gln
P−value
EAF
YFS
OBB
NFBC86
NFBC66
METSIM
LOLIPOP_IA610
LOLIPOP_IA317
INT
FR97
FR07
EGCUT
META
0.070
0.990
0.013
0.084
0.072
0.060
0.987
0.086
1.34e−06
0.071
0.998
1.85e−10
0.01
0.0006
0.01
0.01
0.0090
0.0010
0.0020
0.0004
0.01
0.01
0.0021
0.01
−1
0
1
BETA

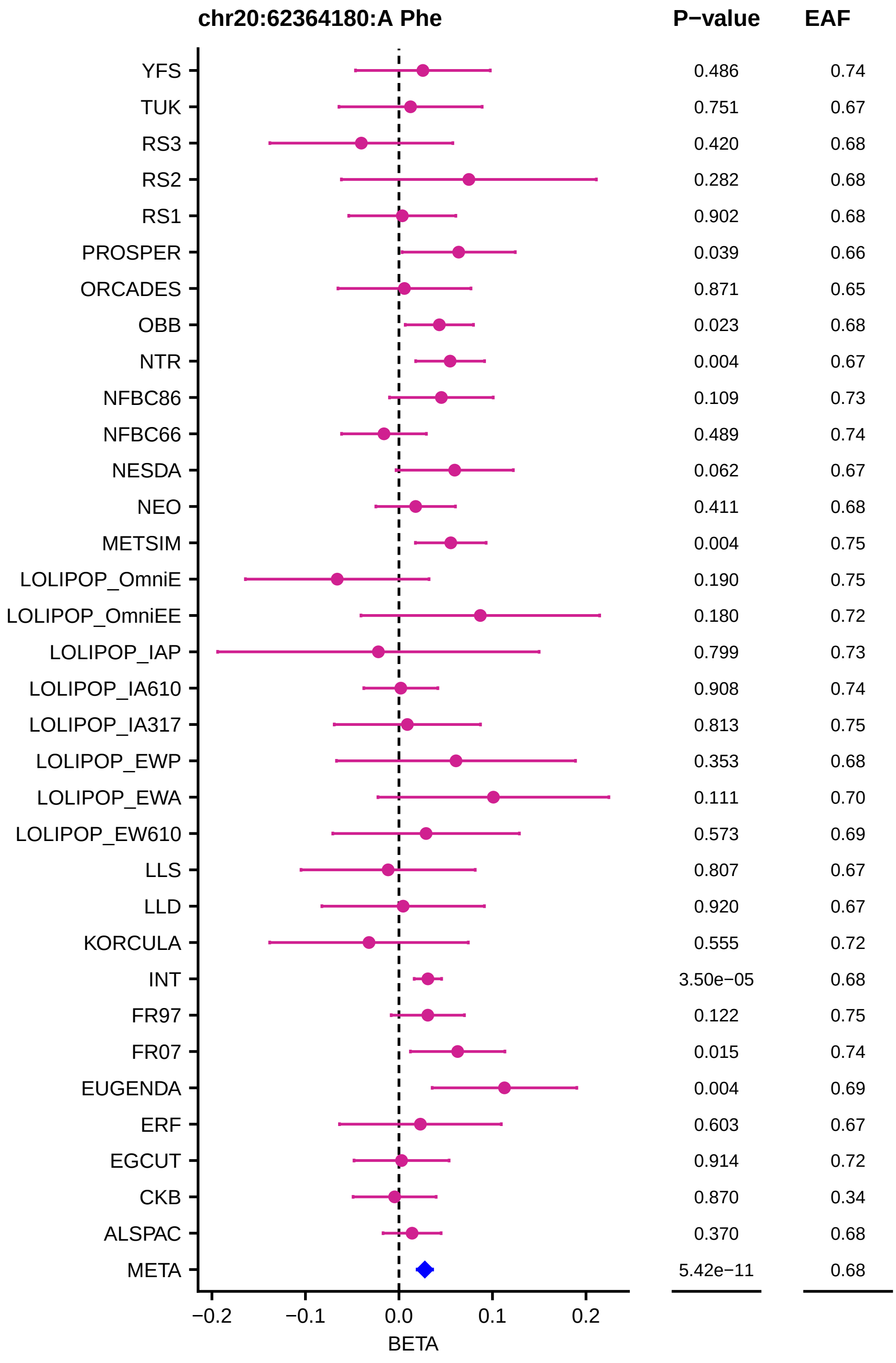

chr20:62364180:A Phe
P−value
EAF
YFS 0.486 0.74
TUK 0.751 0.67
RS3 0.420 0.68
RS2 0.282 0.68
RS1 0.902 0.68
PROSPER 0.039 0.66
ORCADES 0.871 0.65
OBB 0.023 0.68
NTR 0.004 0.67
NFBC86 0.109 0.73
NFBC66 0.489 0.74
NESDA 0.062 0.67
NEO 0.411 0.68
METSIM 0.004 0.75
LOLIPOP_OmniE 0.190 0.75
LOLIPOP_OmniEE 0.180 0.72
LOLIPOP_IAP 0.799 0.73
LOLIPOP_IA610 0.908 0.74
LOLIPOP_IA317 0.813 0.75
LOLIPOP_EWP 0.353 0.68
LOLIPOP_EWA 0.111 0.70
LOLIPOP_EW610 0.573 0.69
LLS 0.807 0.67
LLD 0.920 0.67
KORCULA 0.555 0.72
INT 3.50e−05 0.68
FR97 0.122 0.75
FR07 0.015 0.74
EUGENDA 0.004 0.69
ERF 0.603 0.67
EGCUT 0.914 0.72
CKB 0.870 0.34
ALSPAC 0.370 0.68
META 5.42e−11 0.68
−0.2 −0.1 0.0 0.1 0.2
BETA

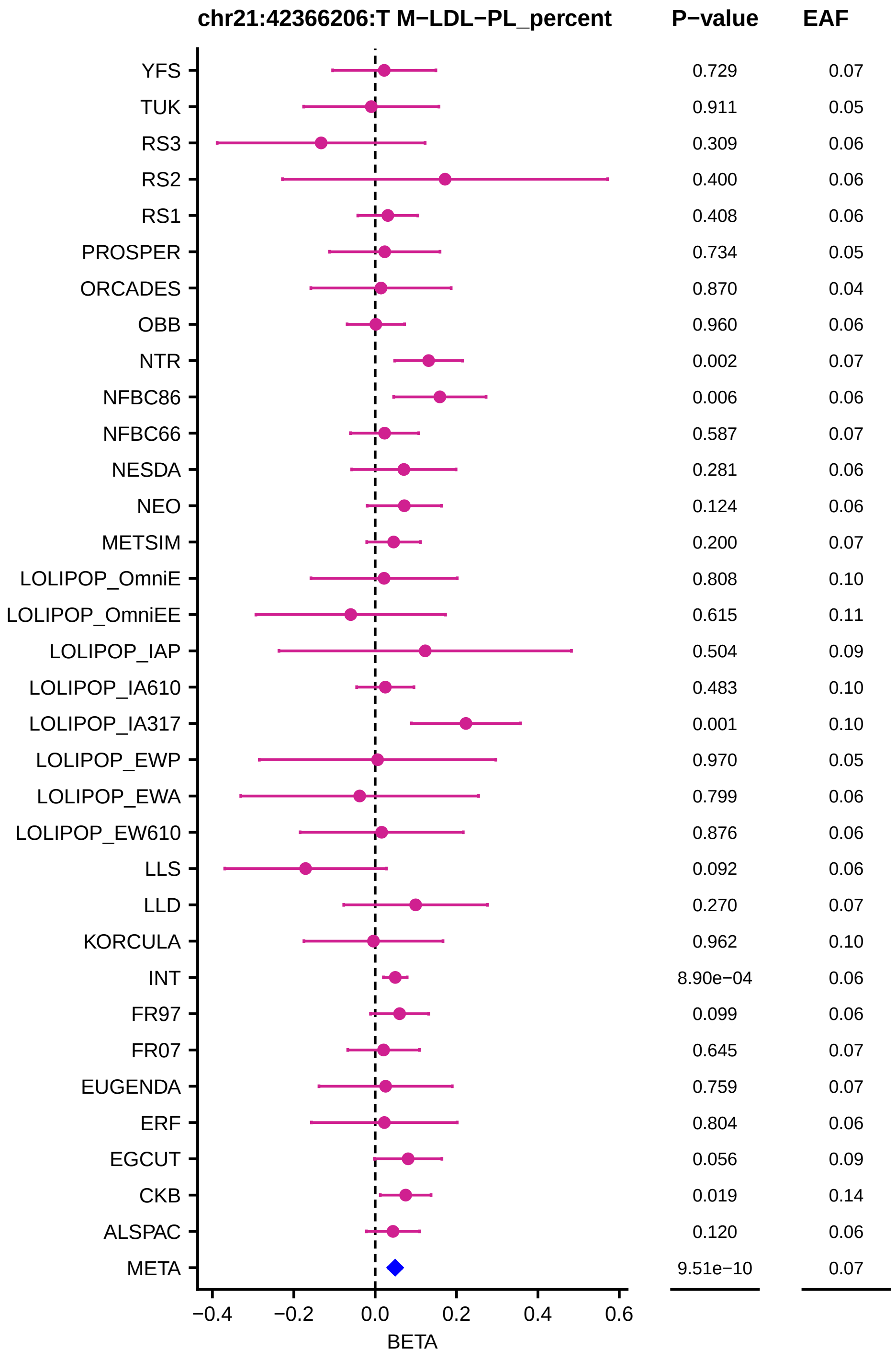
chr21:42366206:T M−LDL−PL_percent
P−value
EAF
YFS 0.729 0.07
TUK 0.911 0.05
RS3 0.309 0.06
RS2 0.400 0.06
RS1 0.408 0.06
PROSPER 0.734 0.05
ORCADES 0.870 0.04
OBB 0.960 0.06
NTR 0.002 0.07
NFBC86 0.006 0.06
NFBC66 0.587 0.07
NESDA 0.281 0.06
NEO 0.124 0.06
METSIM 0.200 0.07
LOLIPOP_OmniE 0.808 0.10
LOLIPOP_OmniEE 0.615 0.11
LOLIPOP_IAP 0.504 0.09
LOLIPOP_IA610 0.483 0.10
LOLIPOP_IA317 0.001 0.10
LOLIPOP_EWP 0.970 0.05
LOLIPOP_EWA 0.799 0.06
LOLIPOP_EW610 0.876 0.06
LLS 0.092 0.06
LLD 0.270 0.07
KORCULA 0.962 0.10
INT 8.90e−04 0.06
FR97 0.099 0.06
FR07 0.645 0.07
EUGENDA 0.759 0.07
ERF 0.804 0.06
EGCUT 0.056 0.09
CKB 0.019 0.14
ALSPAC 0.120 0.06
META 9.51e−10 0.07
−0.4
−0.2
0.0
0.2
0.4
0.6
BETA

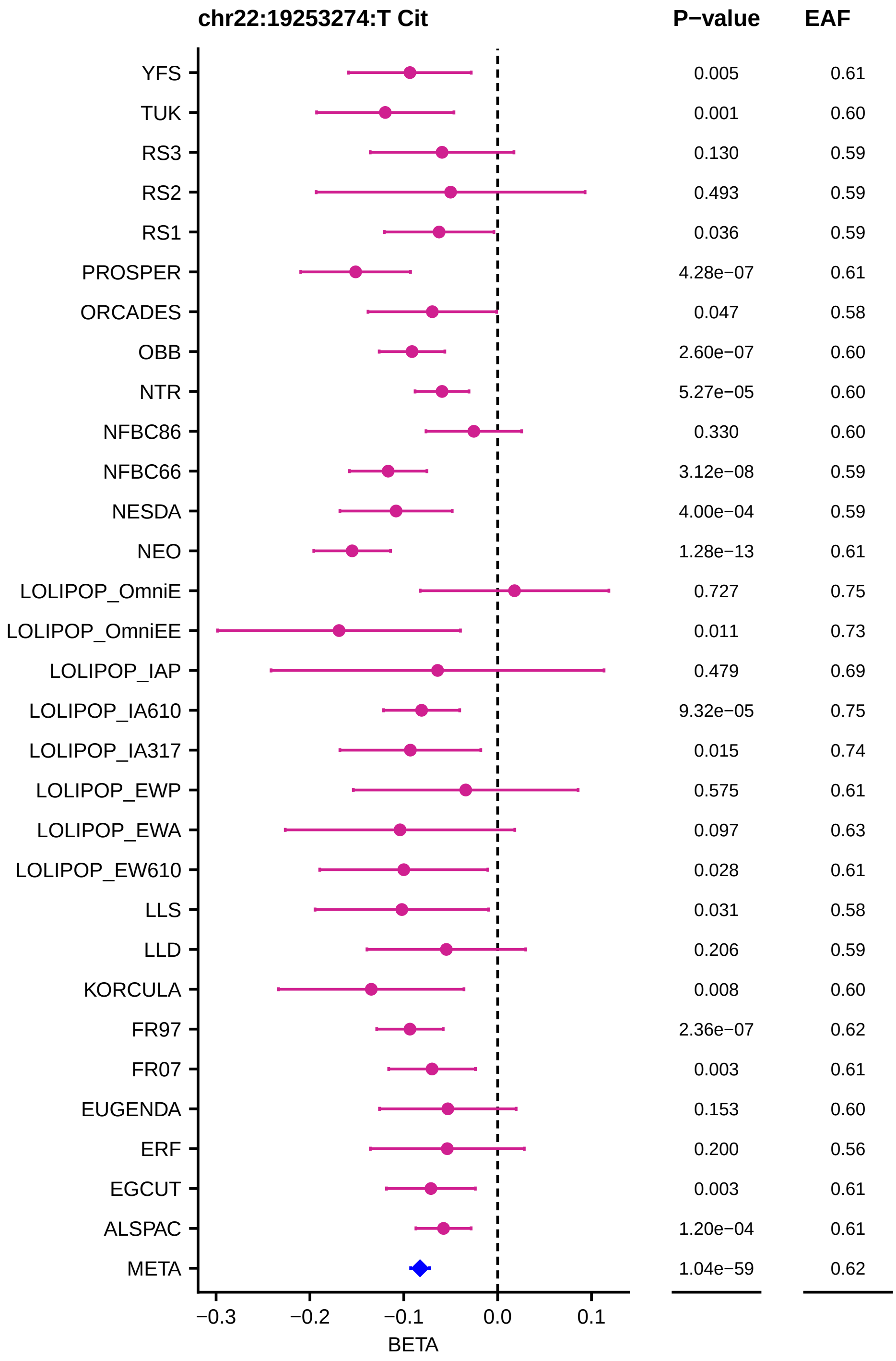
chr22:19253274:T Cit
P−value
EAF
YFS 0.005 0.61
TUK 0.001 0.60
RS3 0.130 0.59
RS2 0.493 0.59
RS1 0.036 0.59
PROSPER 4.28e−07 0.61
ORCADES 0.047 0.58
OBB 2.60e−07 0.60
NTR 5.27e−05 0.60
NFBC86 0.330 0.60
NFBC66 3.12e−08 0.59
NESDA 4.00e−04 0.59
NEO 1.28e−13 0.61
LOLIPOP_OmniE 0.727 0.75
LOLIPOP_OmniEE 0.011 0.73
LOLIPOP_IAP 0.479 0.69
LOLIPOP_IA610 9.32e−05 0.75
LOLIPOP_IA317 0.015 0.74
LOLIPOP_EWP 0.575 0.61
LOLIPOP_EWA 0.097 0.63
LOLIPOP_EW610 0.028 0.61
LLS 0.031 0.58
LLD 0.206 0.59
KORCULA 0.008 0.60
FR97 2.36e−07 0.62
FR07 0.003 0.61
EUGENDA 0.153 0.60
ERF 0.200 0.56
EGCUT 0.003 0.61
ALSPAC 1.20e−04 0.61
META 1.04e−59 0.62
−0.3
−0.2
−0.1
0.0
0.1
BETA

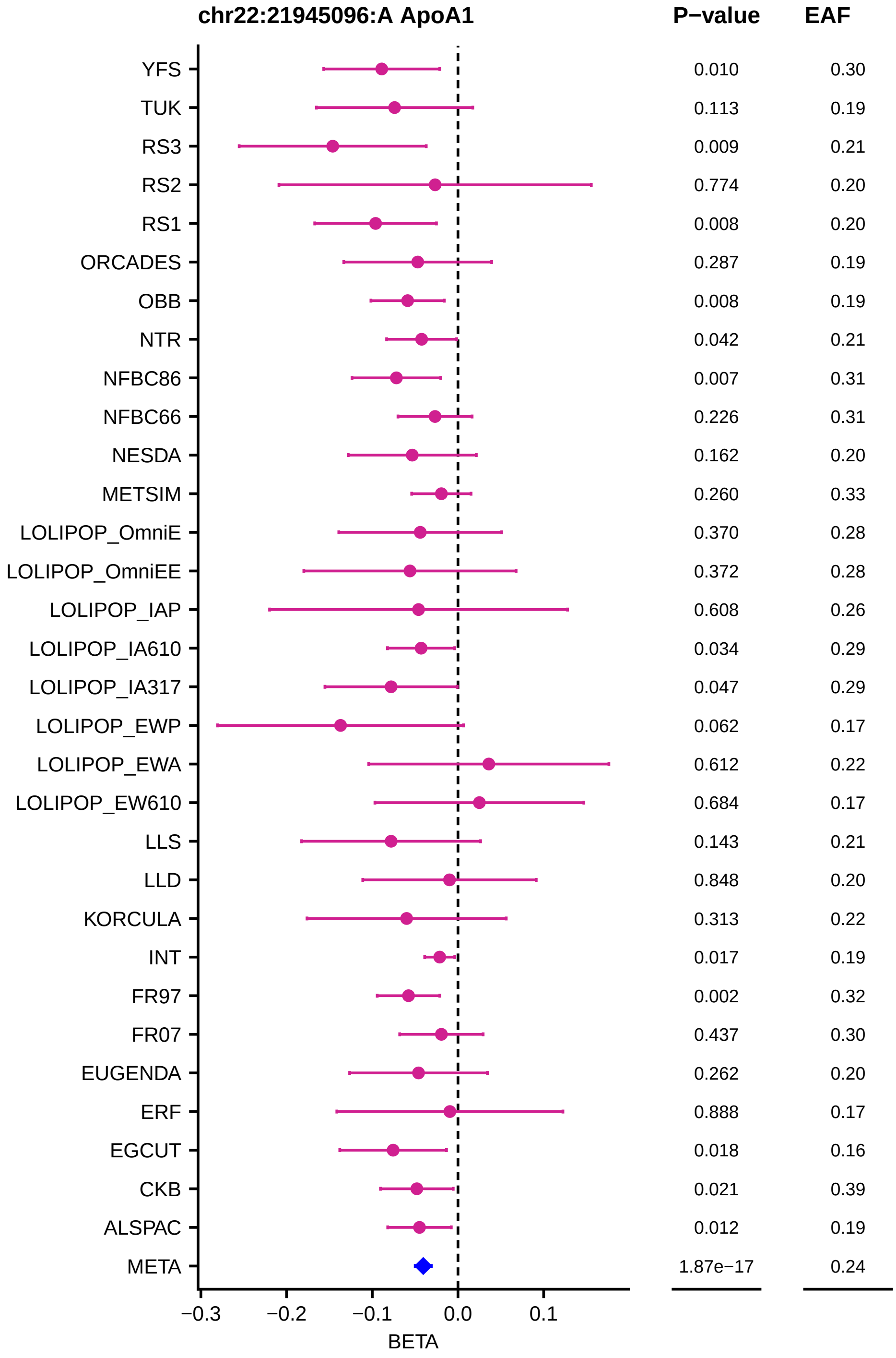
chr22:21945096:A ApoA1
P−value
EAF
YFS 0.010 0.30
TUK 0.113 0.19
RS3 0.009 0.21
RS2 0.774 0.20
RS1 0.008 0.20
ORCADES 0.287 0.19
OBB 0.008 0.19
NTR 0.042 0.21
NFBC86 0.007 0.31
NFBC66 0.226 0.31
NESDA 0.162 0.20
METSIM 0.260 0.33
LOLIPOP_OmniE 0.370 0.28
LOLIPOP_OmniEE 0.372 0.28
LOLIPOP_IAP 0.608 0.26
LOLIPOP_IA610 0.034 0.29
LOLIPOP_IA317 0.047 0.29
LOLIPOP_EWP 0.062 0.17
LOLIPOP_EWA 0.612 0.22
LOLIPOP_EW610 0.684 0.17
LLS 0.143 0.21
LLD 0.848 0.20
KORCULA 0.313 0.22
INT 0.017 0.19
FR97 0.002 0.32
FR07 0.437 0.30
EUGENDA 0.262 0.20
ERF 0.888 0.17
EGCUT 0.018 0.16
CKB 0.021 0.39
ALSPAC 0.012 0.19
META 1.87e−17 0.24
−0.3
−0.2
−0.1
0.0
0.1
BETA

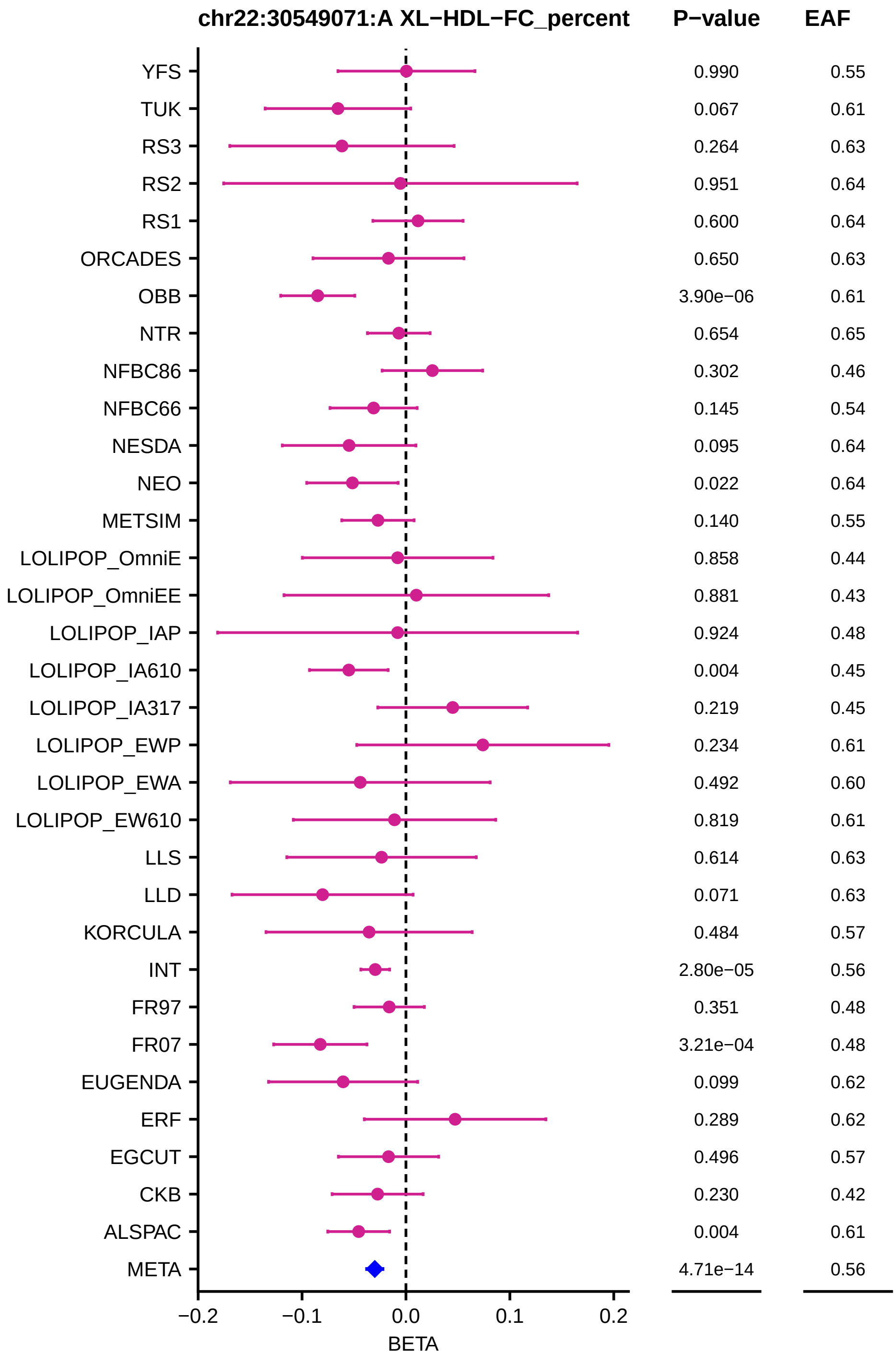
chr22:30549071:A XL−HDL−FC_percent
P−value
EAF
YFS 0.990 0.55
TUK 0.067 0.61
RS3 0.264 0.63
RS2 0.951 0.64
RS1 0.600 0.64
ORCADES 0.650 0.63
OBB 3.90e−06 0.61
NTR 0.654 0.65
NFBC86 0.302 0.46
NFBC66 0.145 0.54
NESDA 0.095 0.64
NEO 0.022 0.64
METSIM 0.140 0.55
LOLIPOP_OmniE 0.858 0.44
LOLIPOP_OmniEE 0.881 0.43
LOLIPOP_IAP 0.924 0.48
LOLIPOP_IA610 0.004 0.45
LOLIPOP_IA317 0.219 0.45
LOLIPOP_EWP 0.234 0.61
LOLIPOP_EWA 0.492 0.60
LOLIPOP_EW610 0.819 0.61
LLS 0.614 0.63
LLD 0.071 0.63
KORCULA 0.484 0.57
INT 2.80e−05 0.56
FR97 0.351 0.48
FR07 3.21e−04 0.48
EUGENDA 0.099 0.62
ERF 0.289 0.62
EGCUT 0.496 0.57
CKB 0.230 0.42
ALSPAC 0.004 0.61
META 4.71e−14 0.56
−0.2
−0.1
0.0
0.1
0.2
BETA

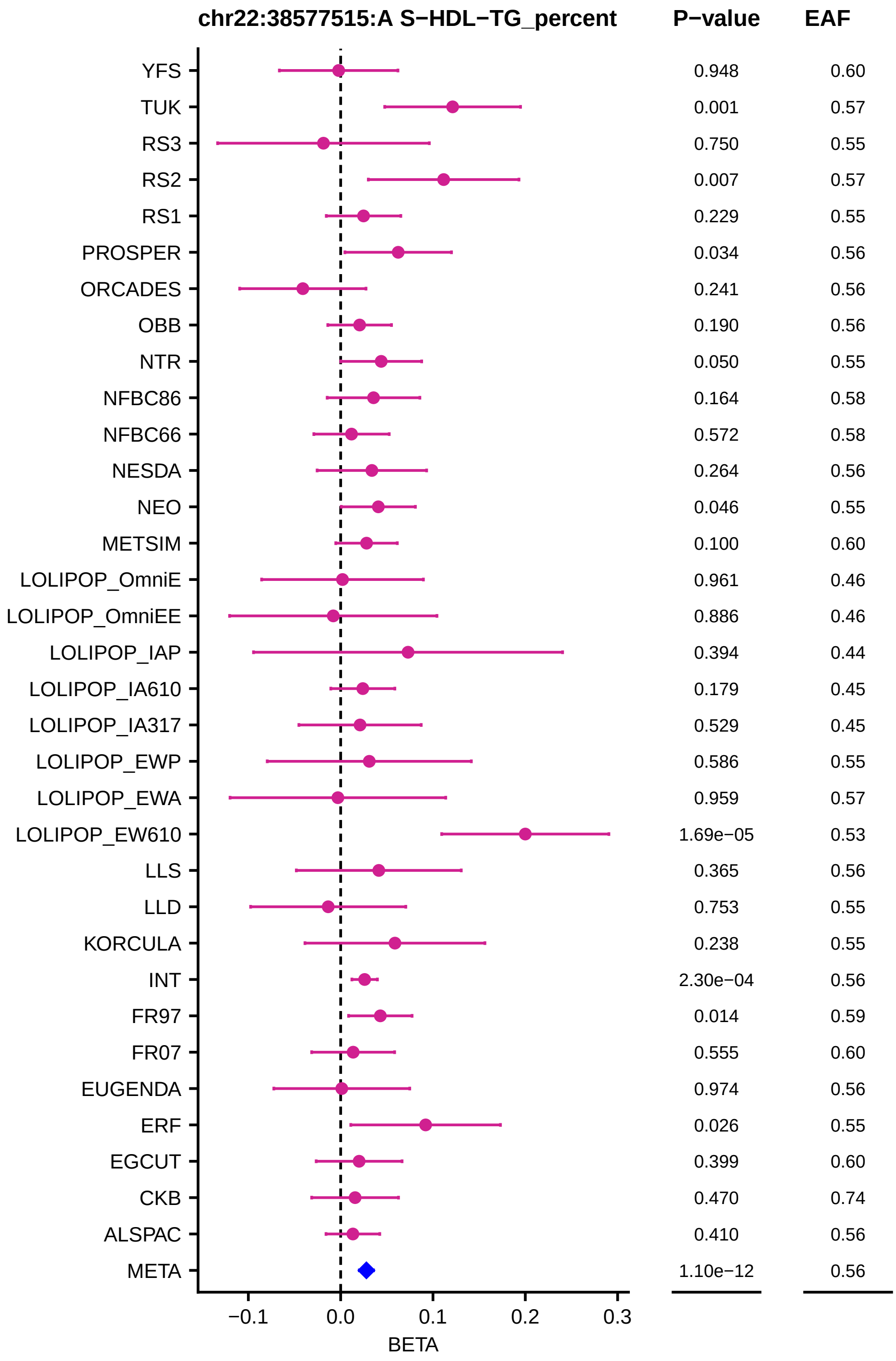

chr22:38577515:A S−HDL−TG_percent
P−value
EAF
YFS 0.948 0.60
TUK 0.001 0.57
RS3 0.750 0.55
RS2 0.007 0.57
RS1 0.229 0.55
PROSPER 0.034 0.56
ORCADES 0.241 0.56
OBB 0.190 0.56
NTR 0.050 0.55
NFBC86 0.164 0.58
NFBC66 0.572 0.58
NESDA 0.264 0.56
NEO 0.046 0.55
METSIM 0.100 0.60
LOLIPOP_OmniE 0.961 0.46
LOLIPOP_OmniEE 0.886 0.46
LOLIPOP_IAP 0.394 0.44
LOLIPOP_IA610 0.179 0.45
LOLIPOP_IA317 0.529 0.45
LOLIPOP_EWP 0.586 0.55
LOLIPOP_EWA 0.959 0.57
LOLIPOP_EW610 1.69e−05 0.53
LLS 0.365 0.56
LLD 0.753 0.55
KORCULA 0.238 0.55
INT 2.30e−04 0.56
FR97 0.014 0.59
FR07 0.555 0.60
EUGENDA 0.974 0.56
ERF 0.026 0.55
EGCUT 0.399 0.60
CKB 0.470 0.74
ALSPAC 0.410 0.56
META 1.10e−12 0.56
−0.1
0.0
0.1
0.2
0.3
BETA

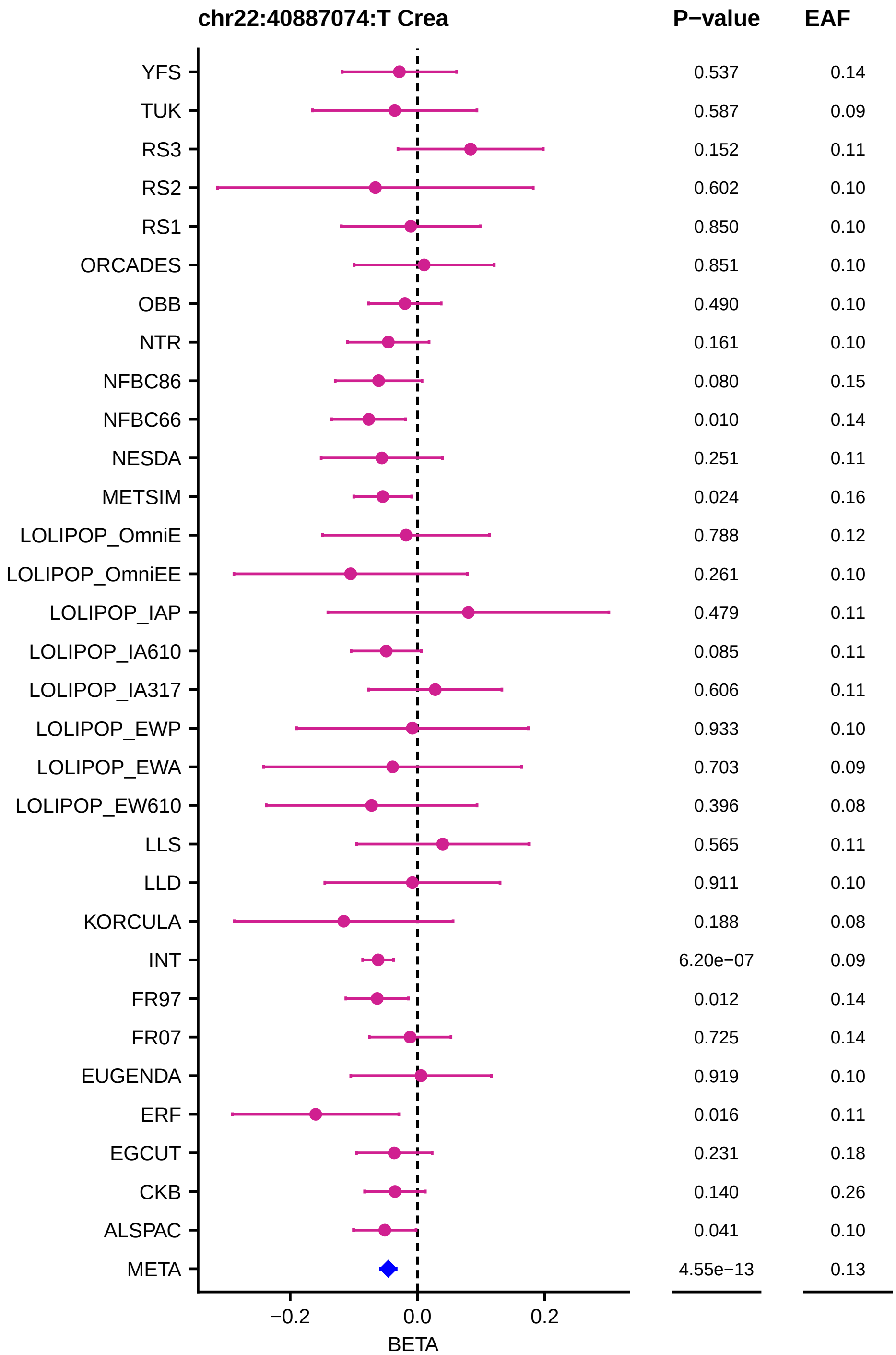
chr22:40887074:T Crea
P−value
EAF
YFS 0.537 0.14
TUK 0.587 0.09
RS3 0.152 0.11
RS2 0.602 0.10
RS1 0.850 0.10
ORCADES 0.851 0.10
OBB 0.490 0.10
NTR 0.161 0.10
NFBC86 0.080 0.15
NFBC66 0.010 0.14
NESDA 0.251 0.11
METSIM 0.024 0.16
LOLIPOP_OmniE 0.788 0.12
LOLIPOP_OmniEE 0.261 0.10
LOLIPOP_IAP 0.479 0.11
LOLIPOP_IA610 0.085 0.11
LOLIPOP_IA317 0.606 0.11
LOLIPOP_EWP 0.933 0.10
LOLIPOP_EWA 0.703 0.09
LOLIPOP_EW610 0.396 0.08
LLS 0.565 0.11
LLD 0.911 0.10
KORCULA 0.188 0.08
INT 6.20e−07 0.09
FR97 0.012 0.14
FR07 0.725 0.14
EUGENDA 0.919 0.10
ERF 0.016 0.11
EGCUT 0.231 0.18
CKB 0.140 0.26
ALSPAC 0.041 0.10
META 4.55e−13 0.13
−0.2
0.0
0.2
BETA

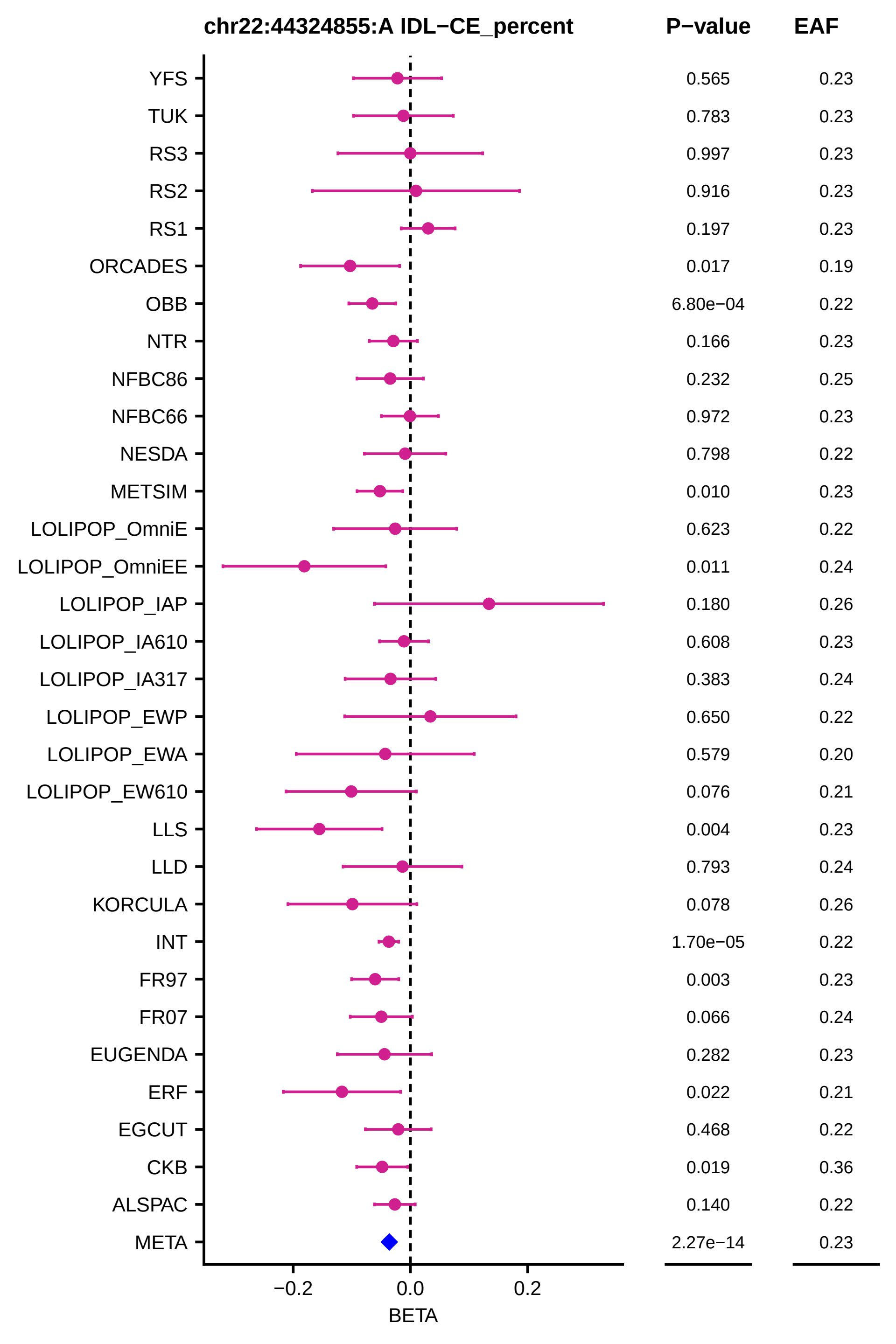

chr22:44324855:A IDL−CE_percent
P−value
EAF
YFS 0.565 0.23
TUK 0.783 0.23
RS3 0.997 0.23
RS2 0.916 0.23
RS1 0.197 0.23
ORCADES 0.017 0.19
OBB 6.80e−04 0.22
NTR 0.166 0.23
NFBC86 0.232 0.25
NFBC66 0.972 0.23
NESDA 0.798 0.22
METSIM 0.010 0.23
LOLIPOP_OmniE 0.623 0.22
LOLIPOP_OmniEE 0.011 0.24
LOLIPOP_IAP 0.180 0.26
LOLIPOP_IA610 0.608 0.23
LOLIPOP_IA317 0.383 0.24
LOLIPOP_EWP 0.650 0.22
LOLIPOP_EWA 0.579 0.20
LOLIPOP_EW610 0.076 0.21
LLS 0.004 0.23
LLD 0.793 0.24
KORCULA 0.078 0.26
INT 1.70e−05 0.22
FR97 0.003 0.23
FR07 0.066 0.24
EUGENDA 0.282 0.23
ERF 0.022 0.21
EGCUT 0.468 0.22
CKB 0.019 0.36
ALSPAC 0.140 0.22
META 2.27e−14 0.23
−0.2
0.0
0.2
BETA
